# Supplementary material for: Impact of COVID-19 on the mental health of public university hospital workers in Brazil: A cohort-based analysis of 32,691 workers
Source: PLoS One. 2022 Jun 16;17(6):e0269318. doi: 10.1371/journal.pone.0269318 (PMC9202958; doi:10.1371/journal.pone.0269318)
Supplement: S1 File — (PDF) [file pone.0269318.s001.pdf]

Supporting Information. Data Analysis. Part 1.

```
GET
  FILE='C:\Users\User\Documents\Pesquisa\Force II\BDForceII_AbsperInd_Horiz.sav'.
DATASET NAME DataSet2 WINDOW=FRONT.
DATASET ACTIVATE DataSet2.
DATASET CLOSE DataSet1.
EXAMINE VARIABLES=Age
  /PLOT HISTOGRAM NPPLOT
  /PERCENTILES(5,10,25,50,75,90,95) HAVERAGE
  /STATISTICS DESCRIPTIVES
  /CINTERVAL 95
  /MISSING LISTWISE
  /NOTOTAL.
```

General Features of the Sample

Explore

Case Processing Summary

	Cases					
	Valid		Missing		Total	
	N	Percent	N	Percent	N	Percent
Age (years)	32691	100,0%	0	0,0%	32691	100,0%

Supporting Information. Data Analysis. Part 1.

Descriptives

			Statistic	Std. Error
Age (years)	Mean		39,27	,042
	95% Confidence Interval for Mean	Lower Bound	39,19	
		Upper Bound	39,35	
	5% Trimmed Mean		38,85	
	Median		38,00	
	Variance		56,617	
	Std. Deviation		7,524	
	Minimum		20	
	Maximum		80	
	Range		60	
	Interquartile Range		9	
	Skewness		,914	,014
	Kurtosis		1,017	,027

Percentiles

		Percentiles						
		5	10	25	50	75	90	95
Weighted Average(Definition 1)	Age (years)	29,00	31,00	34,00	38,00	43,00	50,00	54,00
Tukey's Hinges	Age (years)			34,00	38,00	43,00		

Tests of Normality

	Kolmogorov-Smirnov[a]		
	Statistic	df	Sig.
Age (years)	,107	32691	,000

a. Lilliefors Significance Correction

Age (years)

Supporting Information. Data Analysis. Part 1.

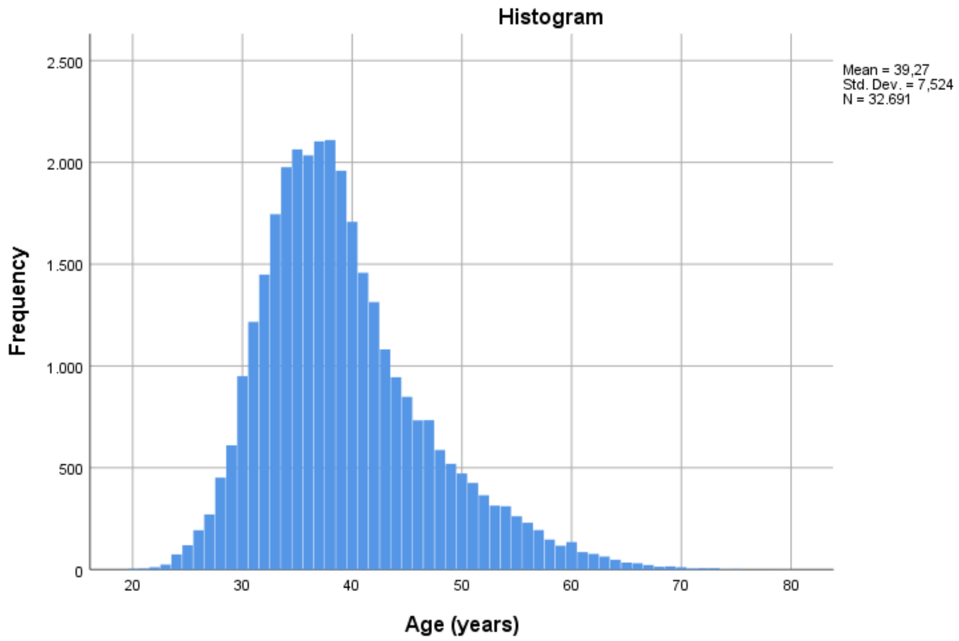

Supporting Information. Data Analysis. Part 1.

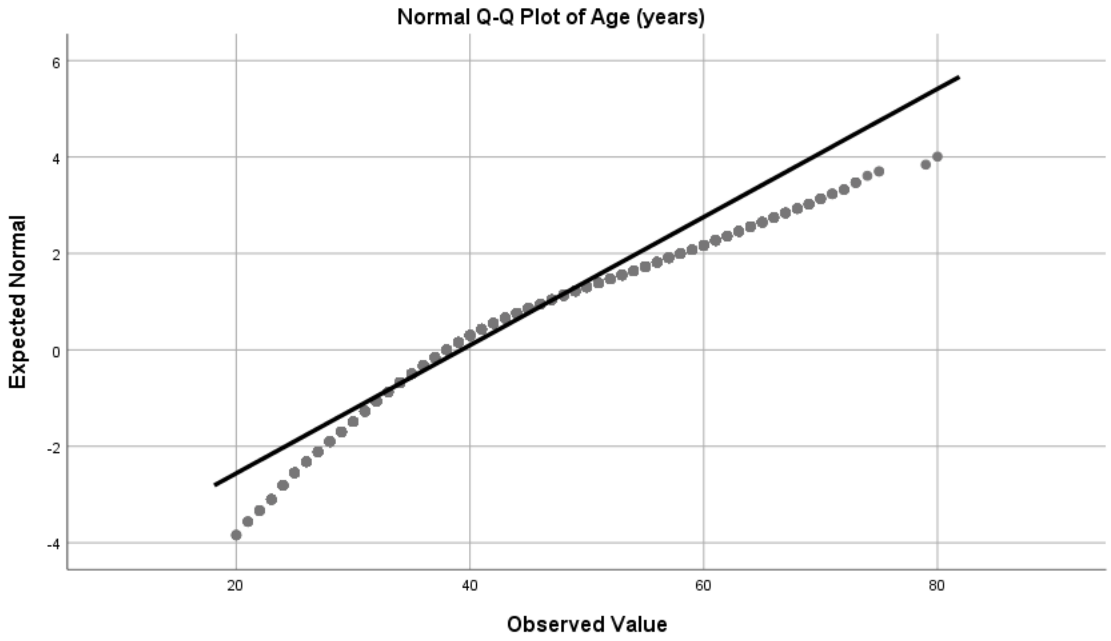


```
FREQUENCIES VARIABLES=Sex ProfCateg HCPcateg Profession Institution Region FedState City
  /FORMAT=DFREQ
  /ORDER=ANALYSIS.
```

Frequencies

Statistics

		Gender	Main type of professional activity	Healthcare provider category	Professional categories	Institution (workplace)	Country Region	Federal State	City
N	Valid	32691	31434	26956	32691	32691	32691	32691	32691
	Missing	0	1257	5735	0	0	0	0	0

Frequency Table

Gender

		Frequency	Percent	Valid Percent	Cumulative Percent
Valid	Female	22982	70,3	70,3	70,3
	Male	9709	29,7	29,7	100,0
	Total	32691	100,0	100,0	

Main type of professional activity

		Frequency	Percent	Valid Percent	Cumulative Percent

Valid	Healthcare provider (direct care for patients)	26956	82,5	85,8	85,8
	Support activities (no direct care for patients)	4478	13,7	14,2	100,0
	Total	31434	96,2	100,0	
Missing	999	1257	3,8		
Total		32691	100,0		

Healthcare provider category

		Frequency	Percent	Valid Percent	Cumulative Percent
Valid	Nurse assistants	10266	31,4	38,1	38,1
	Physicians	6871	21,0	25,5	63,6
	Nurses	5570	17,0	20,7	84,2
	Other HCP	3230	9,9	12,0	96,2
	Physiotherapists	1019	3,1	3,8	100,0
	Total	26956	82,5	100,0	
Missing	999	5735	17,5		
Total		32691	100,0		

Professional categories

		Frequency	Percent	Valid Percent	Cumulative Percent
Valid	TECNICO EM ENFERMAGEM	10266	31,40	31,40	31,40

	MEDICO	6871	21,02	21,02	52,42
	ENFERMEIRO	5570	17,04	17,04	69,46
	ASSISTENTE ADMINISTRATIVO	2440	7,46	7,46	76,92
	999	1257	3,85	3,85	80,77
	TECNICO EM SAUDE	1104	3,38	3,38	84,15
	FISIOTERAPEUTA	1019	3,12	3,12	87,26
	FARMACEUTICO	604	1,85	1,85	89,11
	TECNICO EM RADIOLOGIA	548	1,68	1,68	90,79
	ANALISTA ADMINISTRATIVO	512	1,57	1,57	92,35
	TECNICO ADMINISTRATIVO	387	1,18	1,18	93,54
	PSICOLOGO	264	,81	,81	94,34
	ASSISTENTE SOCIAL	263	,80	,80	95,15
	NUTRICIONISTA	258	,79	,79	95,94
	ANALISTA DE TECNOL DA INFORMACAO	204	,62	,62	96,56
	ENGENHEIRO	202	,62	,62	97,18
	FONOAUDIOLOGO	190	,58	,58	97,76
	TERAPEUTA OCUPACIONAL	136	,42	,42	98,18
	CIRURGIAO DENTISTA	126	,39	,39	98,56
	BIOMEDICO	107	,33	,33	98,89
	ADVOGADO	86	,26	,26	99,15
	BIOLOGO	66	,20	,20	99,35
	TECNOLOGO	50	,15	,15	99,51
	FISICO	49	,15	,15	99,66
	EDUCADOR FISICO	45	,14	,14	99,80

	PEDAGOGO	40	,12	,12	99,92
	JORNALISTA	14	,04	,04	99,96
	ARQUITETO	13	,04	,04	100,00
	Total	32691	100,0	100,0	

Institution (workplace)

		Frequency	Percent	Valid Percent	Cumulative Percent
Valid	HU da Universidade Federal do Maranhão - MA	1975	6,0	6,0	6,0
	CH da Universidade Federal do Ceará - CE	1953	6,0	6,0	12,0
	HC da Universidade Federal de Minas Gerais - MG	1715	5,2	5,2	17,3
	CHC da Universidade Federal do Paraná - PR	1658	5,1	5,1	22,3
	HU Professor Edgard Santos - UFBA - BA	1563	4,8	4,8	27,1
	HU de Brasília - UNB - DF	1440	4,4	4,4	31,5
	HU Onofre Lopes - UFRN - RN	1269	3,9	3,9	35,4
	HU da Universidade Federal do Piauí - PI	1258	3,8	3,8	39,2
	HU da Universidade Federal de Sergipe - SE	1230	3,8	3,8	43,0
	HU Lauro Wanderley - UFPB - PB	1217	3,7	3,7	46,7

	HU da Universidade Federal de Juiz de Fora - MG	1063	3,3	3,3	50,0
	HC da Universidade Federal de Pernambuco - PE	1061	3,2	3,2	53,2
	HU Cassiano Antonio de Moraes - UFES - ES	1049	3,2	3,2	56,4
	HC da Universidade Federal do Triângulo Mineiro - MG	1023	3,1	3,1	59,6
	HU Maria Aparecida Pedrossian - UFMS - MS	948	2,9	2,9	62,5
	HU da Universidade Federal de Santa Maria - RS	892	2,7	2,7	65,2
	HE da Universidade Federal de Pelotas - RS	855	2,6	2,6	67,8
	HU Professor Alberto Antunes - UFAL - AL	840	2,6	2,6	70,4
	CHU da Universidade Federal do Pará - UFPA - PA	785	2,4	2,4	72,8
	HU da Universidade Federal de Grande Dourados - MS	726	2,2	2,2	75,0
	HE Doutor Washigton Antônio de Barros - UNIVASF - PE	708	2,2	2,2	77,2
	HU Doutor Miguel Riet Corrêa Junior - FURG - RS	688	2,1	2,1	79,3

	HC da Universidade Federal de Goiás - GO	634	1,9	1,9	81,2
	HU de Lagarto - UFS - SE	617	1,9	1,9	83,1
	Maternidade Climério de Oliveira - UFBA - BA	594	1,8	1,8	84,9
	Maternidade Escola Januário Cicco - UFRN - RN	579	1,8	1,8	86,7
	HU da Universidade Federal de Santa Catarina - UFSC - SC	530	1,6	1,6	88,3
	HU Gaffrée e Guinle - UNIRIO - RJ	503	1,5	1,5	89,9
	HU Júlio Müller - UFMT - MT	455	1,4	1,4	91,2
	HU Antônio Pedro - UFF - RJ	439	1,3	1,3	92,6
	HU Getúlio Vargas - UFAM - AM	433	1,3	1,3	93,9
	HU Alcides Carneiro - UFCG - PB	425	1,3	1,3	95,2
	HU Ana Bezerra - UFRN - RN	385	1,2	1,2	96,4
	HU da Universidade Federal de São Carlos - SP	338	1,0	1,0	97,4
	Sede - DF	303	,9	,9	98,3
	Hospital de Doenças Tropicais - UFT - TO	271	,8	,8	99,2
	HU Júlio Bandeira - UFCG - PB	269	,8	,8	100,0
	Total	32691	100,0	100,0	

Country Region

		Frequency	Percent	Valid Percent	Cumulative Percent
Valid	Northeast	15942	48,8	48,8	48,8
	Southeast	6130	18,8	18,8	67,5
	South	4626	14,2	14,2	81,7
	Center-West	4504	13,8	13,8	95,4
	North	1489	4,6	4,6	100,0
	Total	32691	100,0	100,0	

Federal State

		Frequency	Percent	Valid Percent	Cumulative Percent
Valid	MG	3801	11,6	11,6	11,6
	RS	2436	7,5	7,5	19,1
	RN	2232	6,8	6,8	25,9
	BA	2157	6,6	6,6	32,5
	MA	1974	6,0	6,0	38,5
	CE	1954	6,0	6,0	44,5
	PB	1911	5,8	5,8	50,4
	SE	1847	5,6	5,6	56,0
	PE	1769	5,4	5,4	61,4
	DF	1743	5,3	5,3	66,8
	MS	1673	5,1	5,1	71,9
	PR	1659	5,1	5,1	77,0
	PI	1258	3,8	3,8	80,8

	ES	1049	3,2	3,2	84,0
	RJ	942	2,9	2,9	86,9
	AL	840	2,6	2,6	89,5
	PA	785	2,4	2,4	91,9
	GO	633	1,9	1,9	93,8
	SC	531	1,6	1,6	95,4
	MT	455	1,4	1,4	96,8
	AM	433	1,3	1,3	98,1
	SP	338	1,0	1,0	99,2
	TO	271	,8	,8	100,0
	Total	32691	100,0	100,0	

City

		Frequency	Percent	Valid Percent	Cumulative Percent
Valid	Salvador	2157	6,6	6,6	6,6
	Sao Luis	1974	6,0	6,0	12,6
	Fortaleza	1954	6,0	6,0	18,6
	Natal	1847	5,6	5,6	24,3
	Brasilia	1743	5,3	5,3	29,6
	Belo Horizonte	1715	5,2	5,2	34,8
	Curitiba	1659	5,1	5,1	39,9
	Teresina	1258	3,8	3,8	43,8
	Aracaju	1230	3,8	3,8	47,5

	Joao Pessoa	1217	3,7	3,7	51,2
	Juiz de Fora	1063	3,3	3,3	54,5
	Recife	1061	3,2	3,2	57,7
	Vitoria	1049	3,2	3,2	61,0
	Uberaba	1023	3,1	3,1	64,1
	Campo Grande	948	2,9	2,9	67,0
	Santa Maria	892	2,7	2,7	69,7
	Pelotas	855	2,6	2,6	72,3
	Maceio	840	2,6	2,6	74,9
	Belem	785	2,4	2,4	77,3
	Dourados	725	2,2	2,2	79,5
	Petrolina	708	2,2	2,2	81,7
	Rio Grande	689	2,1	2,1	83,8
	Goiania	633	1,9	1,9	85,7
	Lagarto	617	1,9	1,9	87,6
	Florianopolis	531	1,6	1,6	89,2
	Rio de Janeiro	503	1,5	1,5	90,8
	Cuiaba	455	1,4	1,4	92,2
	Niteroi	439	1,3	1,3	93,5
	Manaus	433	1,3	1,3	94,8
	Campina Grande	425	1,3	1,3	96,1
	Santa Cruz	385	1,2	1,2	97,3
	Sao Carlos	338	1,0	1,0	98,3
	Araguaina	271	,8	,8	99,2

	Cajazeiras	269	,8	,8	100,0
	Total	32691	100,0	100,0	

```
* Custom Tables.
CTABLES
  /VLABELS VARIABLES=City CityCovCases100k CityCovDeath100k DISPLAY=DEFAULT
  /TABLE City BY CityCovCases100k [MEAN] + CityCovDeath100k [MEAN]
  /CATEGORIES VARIABLES=City ORDER=A KEY=VALUE EMPTY=EXCLUDE
  /CRITERIA CILEVEL=95.
```

Custom Tables

		COVID cases / 100K inhab in the city	Deaths by COVID / 100K inhab in the city
		Mean	Mean
City	Aracaju	4318,79	81,73
	Araguaina	3970,19	53,75
	Belem	1701,23	135,99
	Belo Horizonte	807,14	21,02
	Brasilia	3107,15	44,21

	Cajazeiras	1998,61	46,78
	Campina Grande	2598,05	55,16
	Campo Grande	1145,34	15,07
	Cuiaba	1878,55	88,81
	Curitiba	921,11	28,71
	Dourados	1939,91	24,67
	Florianopolis	654,73	10,38
	Fortaleza	1571,70	138,24
	Goiania	1140,09	32,06
	Joao Pessoa	2636,54	78,00
	Juiz de Fora	601,89	18,98
	Lagarto	1580,34	43,10
	Maceio	2198,25	70,96
	Manaus	1630,59	92,22
	Natal	2224,35	93,77
	Niteroi	1767,77	60,17
	Pelotas	264,89	4,96
	Petrolina	679,09	15,75
	Recife	1622,69	129,24
	Rio de Janeiro	1061,51	123,68
	Rio Grande	408,52	29,38
	Salvador	1974,73	61,10
	Santa Cruz	821,70	17,64
	Santa Maria	393,80	9,22

	Sao Carlos	520,67	8,33
	Sao Luis	1466,58	103,73
	Teresina	2026,03	81,98
	Uberaba	503,02	16,48
	Vitoria	3107,45	97,21

```
* Custom Tables.
CTABLES
  /VLABELS VARIABLES=CoVAb CoVAb_N CoVAb_Day DISPLAY=DEFAULT
  /TABLE BY CoVAb [C][COUNT F40.0, ROWPCT.COUNT PCT40.1] + CoVAb_N [S][SUM] + CoVAb_Day [S][SUM]
  /CATEGORIES VARIABLES=CoVAb [1, 0, OTHERNM] EMPTY=INCLUDE TOTAL=YES POSITION=BEFORE
  /CRITERIA CILEVEL=95.
```

Custom Tables: absences due to suspected or confirmed COVID

Any work absence due to suspected or confirmed COVID in the period						Events of work absence due to suspected or confirmed COVID	Duration (days) of work absence due to suspected or confirmed COVID
Total		Yes		No			
Count	Row N %	Count	Row N %	Count	Row N %	Sum	Sum
32691	100,0%	10994	33,6%	21697	66,4%	21295	127551

Supporting Information. Data Analysis. Part 1.

```
* Custom Tables.
CTABLES
  /VLABELS VARIABLES=ExtWork ExtWrk_Day DISPLAY=DEFAULT
  /TABLE BY ExtWork [C][COUNT F40.0, ROWPCT.COUNT PCT40.1] + ExtWrk_Day [S][SUM]
  /CATEGORIES VARIABLES=ExtWork ORDER=A KEY=COUNT EMPTY=INCLUDE TOTAL=YES POSITION=BEFORE
  /CRITERIA CILEVEL=95.
```

Custom Tables: assignments to external (remote) work

Any assignment to external (remote) work during pandemia						Duration (days) of external (remote) work during pandemia
Total		Yes		No		
Count	Row N %	Count	Row N %	Count	Row N %	Sum
32691	100,0%	6504	19,9%	26187	80,1%	349016

```
FREQUENCIES VARIABLES=ExtWrk_5p
  /ORDER=ANALYSIS.
```

Frequencies

Supporting Information. Data Analysis. Part 1.

Statistics

Proportion of time assigned to external (remote) work during pandemia

N	Valid	32691
	Missing	0

Proportion of time assigned to external (remote) work during pandemia

		Frequency	Percent	Valid Percent	Cumulative Percent
Valid	No time at all	26187	80,1	80,1	80,1
	>0% and <=25%	3032	9,3	9,3	89,4
	>25% and <=50%	1394	4,3	4,3	93,6
	>50% and <=75%	1124	3,4	3,4	97,1
	>75%	954	2,9	2,9	100,0
	Total	32691	100,0	100,0	

```
* Custom Tables.
CTABLES
  /VLABELS VARIABLES=AbsType AsgnGrp DISPLAY=DEFAULT
  /TABLE AbsType [C][COUNT F40.0, COLPCT.COUNT PCT40.1] BY AsgnGrp [C]
  /CATEGORIES VARIABLES=AbsType [2, 1, 4, 3, 10, 8, 5] EMPTY=INCLUDE TOTAL=YES POSITION=AFTER
  /CATEGORIES VARIABLES=AsgnGrp ORDER=A KEY=VALUE EMPTY=INCLUDE
  /CRITERIA CILEVEL=95.
```

Comparisons between exposed and non-exposed individuals as to types (or causes) of work absence in the general sample

Custom Tables: events of work absence (non-COVID)

		Exposure to the pandemia environment			
		Non-Exposed (2019)		Exposed (2020)	
		Count	Column N %	Count	Column N %
Absence type (general classification)	Health-Related	29301	70,7%	20473	70,1%
	Administrative	8335	20,1%	5552	19,0%
	Wedding, maternity, paternity or adoption leaves	1819	4,4%	1636	5,6%
	Family member illness or death	934	2,3%	812	2,8%
	Blood donation	742	1,8%	522	1,8%
	Abortion (complete, failed or threat) and its complications	197	0,5%	147	0,5%
	Occupational accidents	141	0,3%	75	0,3%
	Total	41469	100,0%	29217	100,0%

```
SET TLook=None Small=0.0001 SUMMARY=None THREADS=AUTO TFit=Both DIGITGROUPING=No LEADZERO=No TABLERENDER=light.
CROSSTABS
```

```
  /TABLES=AsgnGrp BY AbAC AbADM AbHRel AbMnt AbFam AbAbor AbBLD AbAcd AbWMPA
  /FORMAT=AVALUE TABLES
  /STATISTICS=RISK
  /CELLS=COUNT ROW TOTAL
  /COUNT ROUND CELL.
```

Crosstabs

[DataSet4] C:\Users\trabalho\Documents\COVID-19\Centro Pesquisa HUB COVID19\BDForceII_AbsperInd_Vertic.sav

Case Processing Summary

	Cases					
	Valid		Missing		Total	
	N	Percent	N	Percent	N	Percent
Exposure to the pandemia environment * Any work absence due to ALL CAUSES occurred in the period	65382	100,0%	0	0,0%	65382	100,0%

Exposure to the pandemia environment * Any work absence due to ADMINISTRATIVE CAUSES occurred in the period	65382	100,0%	0	0,0%	65382	100,0%
Exposure to the pandemia environment * Any work absence due to HEALTH-RELATED CAUSES occurred in the period	65382	100,0%	0	0,0%	65382	100,0%
Exposure to the pandemia environment * Any work absence due to MENTAL DISEASE occurred in the period	65382	100,0%	0	0,0%	65382	100,0%
Exposure to the pandemia environment * Any work absence due to FAMILY member's illness or death in the period	65382	100,0%	0	0,0%	65382	100,0%
Exposure to the pandemia environment * Any work absence due to ABORTION and its complications occurred in the period	65382	100,0%	0	0,0%	65382	100,0%
Exposure to the pandemia environment * Any work absence due to BLOOD DONATION occurred in the period	65382	100,0%	0	0,0%	65382	100,0%

Exposure to the pandemia environment * Any work absence due to OCCUPATIONAL ACCIDENTS occurred in the period	65382	100,0%	0	0,0%	65382	100,0%
Exposure to the pandemia environment * Any work absence due to wedding, maternity, paternity or adoption leaves in the period - T1	65382	100,0%	0	0,0%	65382	100,0%

Exposure to the pandemia environment * Any work absence due to ALL CAUSES occurred in the period

Crosstab

			Any work absence due to ALL CAUSES occurred in the period		
			No	Yes	Total
Exposure to the pandemia environment	Non-Exposed (2019)	Count	15917	16774	32691
		% within Exposure to the pandemia environment	48,7%	51,3%	100,0%
		% of Total	24,3%	25,7%	50,0%
	Exposed (2020)	Count	18430	14261	32691

	% within Exposure to the pandemia environment	56,4%	43,6%	100,0%
	% of Total	28,2%	21,8%	50,0%
Total	Count	34347	31035	65382
	% within Exposure to the pandemia environment	52,5%	47,5%	100,0%
	% of Total	52,5%	47,5%	100,0%

Risk Estimate

	Value	95% Confidence Interval	
		Lower	Upper
Odds Ratio for Exposure to the pandemia environment (Non-Exposed (2019) / Exposed (2020))	,734	,712	,757
For cohort Any work absence due to ALL CAUSES occurred in the period = No	,864	,851	,876
For cohort Any work absence due to ALL CAUSES occurred in the period = Yes	1,176	1,157	1,195
N of Valid Cases	65382		

Exposure to the pandemia environment * Any work absence due to ADMINISTRATIVE CAUSES occurred in the period

Crosstab

			Any work absence due to ADMINISTRATIVE CAUSES occurred in the period		
			No	Yes	Total
Exposure to the pandemia environment	Non-Exposed (2019)	Count	28351	4340	32691
		% within Exposure to the pandemia environment	86,7%	13,3%	100,0%
		% of Total	43,4%	6,6%	50,0%
	Exposed (2020)	Count	29833	2858	32691
		% within Exposure to the pandemia environment	91,3%	8,7%	100,0%
		% of Total	45,6%	4,4%	50,0%
Total		Count	58184	7198	65382
		% within Exposure to the pandemia environment	89,0%	11,0%	100,0%
		% of Total	89,0%	11,0%	100,0%

Risk Estimate

	Value	95% Confidence Interval	
		Lower	Upper
Odds Ratio for Exposure to the pandemia environment (Non-Exposed (2019) / Exposed (2020))	,626	,595	,658
For cohort Any work absence due to ADMINISTRATIVE CAUSES occurred in the period = No	,950	,945	,955
For cohort Any work absence due to ADMINISTRATIVE CAUSES occurred in the period = Yes	1,519	1,452	1,588
N of Valid Cases	65382		

Exposure to the pandemia environment * Any work absence due to HEALTH-RELATED CAUSES occurred in the period

Crosstab

	Any work absence due to HEALTH-RELATED CAUSES occurred in the period		Total
	No	Yes	

Exposure to the pandemia environment	Non-Exposed (2019)	Count	19427	13264	32691
		% within Exposure to the pandemia environment	59,4%	40,6%	100,0%
		% of Total	29,7%	20,3%	50,0%
	Exposed (2020)	Count	21542	11149	32691
		% within Exposure to the pandemia environment	65,9%	34,1%	100,0%
		% of Total	32,9%	17,1%	50,0%
Total		Count	40969	24413	65382
		% within Exposure to the pandemia environment	62,7%	37,3%	100,0%
		% of Total	62,7%	37,3%	100,0%

Risk Estimate

	Value	95% Confidence Interval	
		Lower	Upper
Odds Ratio for Exposure to the pandemia environment (Non-Exposed (2019) / Exposed (2020))	,758	,734	,783
For cohort Any work absence due to HEALTH-RELATED CAUSES occurred in the period = No	,902	,891	,913

For cohort Any work absence due to HEALTH-RELATED CAUSES occurred in the period = Yes	1,190	1,166	1,214
N of Valid Cases	65382		

Exposure to the pandemia environment * Any work absence due to FAMILY member's illness or death in the period

Crosstab

			Any work absence due to FAMILY member's illness or death in the period		
			No	Yes	Total
Exposure to the pandemia environment	Non-Exposed (2019)	Count	31780	911	32691
		% within Exposure to the pandemia environment	97,2%	2,8%	100,0%
		% of Total	48,6%	1,4%	50,0%
	Exposed (2020)	Count	31905	786	32691
		% within Exposure to the pandemia environment	97,6%	2,4%	100,0%
		% of Total	48,8%	1,2%	50,0%
Total		Count	63685	1697	65382

	% within Exposure to the pandemia environment	97,4%	2,6%	100,0%
	% of Total	97,4%	2,6%	100,0%

Risk Estimate

	Value	95% Confidence Interval	
		Lower	Upper
Odds Ratio for Exposure to the pandemia environment (Non-Exposed (2019) / Exposed (2020))	,859	,780	,947
For cohort Any work absence due to FAMILY member's illness or death in the period = No	,996	,994	,999
For cohort Any work absence due to FAMILY member's illness or death in the period = Yes	1,159	1,055	1,273
N of Valid Cases	65382		

Exposure to the pandemia environment * Any work absence due to BLOOD DONATION occurred in the period

Crosstab

			Any work absence due to BLOOD DONATION occurred in the period		
			No	Yes	Total
Exposure to the pandemia environment	Non-Exposed (2019)	Count	31953	738	32691
		% within Exposure to the pandemia environment	97,7%	2,3%	100,0%
		% of Total	48,9%	1,1%	50,0%
	Exposed (2020)	Count	32176	515	32691
		% within Exposure to the pandemia environment	98,4%	1,6%	100,0%
		% of Total	49,2%	0,8%	50,0%
Total		Count	64129	1253	65382
		% within Exposure to the pandemia environment	98,1%	1,9%	100,0%
		% of Total	98,1%	1,9%	100,0%

Risk Estimate

	Value	95% Confidence Interval	
		Lower	Upper
Odds Ratio for Exposure to the pandemia environment (Non-Exposed (2019) / Exposed (2020))	,693	,619	,776

For cohort Any work absence due to BLOOD DONATION occurred in the period = No	,993	,991	,995
For cohort Any work absence due to BLOOD DONATION occurred in the period = Yes	1,433	1,282	1,602
N of Valid Cases	65382		

Exposure to the pandemia environment * Any work absence due to OCCUPATIONAL ACCIDENTS occurred in the period

Crosstab

			Any work absence due to OCCUPATIONAL ACCIDENTS occurred in the period		
			No	Yes	Total
Exposure to the pandemia environment	Non-Exposed (2019)	Count	32603	88	32691
		% within Exposure to the pandemia environment	99,7%	0,3%	100,0%
		% of Total	49,9%	0,1%	50,0%
	Exposed (2020)	Count	32645	46	32691

	% within Exposure to the pandemia environment	99,9%	0,1%	100,0%
	% of Total	49,9%	0,1%	50,0%
Total	Count	65248	134	65382
	% within Exposure to the pandemia environment	99,8%	0,2%	100,0%
	% of Total	99,8%	0,2%	100,0%

Risk Estimate

	Value	95% Confidence Interval Lower	95% Confidence Interval Upper
Odds Ratio for Exposure to the pandemia environment (Non-Exposed (2019) / Exposed (2020))	,522	,365	,746
For cohort Any work absence due to OCCUPATIONAL ACCIDENTS occurred in the period = No	,999	,998	,999
For cohort Any work absence due to OCCUPATIONAL ACCIDENTS occurred in the period = Yes	1,913	1,340	2,732
N of Valid Cases	65382		

Exposure to the pandemia environment * Any work absence due to wedding, maternity, paternity or adoption leaves in the period

Crosstab

			Any work absence due to wedding, maternity, paternity or adoption leaves in the period		
			No	Yes	Total
Exposure to the pandemia environment	Non-Exposed (2019)	Count	31218	1473	32691
		% within Exposure to the pandemia environment	95,5%	4,5%	100,0%
		% of Total	47,7%	2,3%	50,0%
	Exposed (2020)	Count	31343	1348	32691
		% within Exposure to the pandemia environment	95,9%	4,1%	100,0%
		% of Total	47,9%	2,1%	50,0%
Total		Count	62561	2821	65382
		% within Exposure to the pandemia environment	95,7%	4,3%	100,0%
		% of Total	95,7%	4,3%	100,0%

Risk Estimate

	Value	95% Confidence Interval	
		Lower	Upper
Odds Ratio for Exposure to the pandemia environment (Non-Exposed (2019) / Exposed (2020))	,911	,845	,983
For cohort Any work absence due to wedding, maternity, paternity or adoption leaves in the period = No	,996	,993	,999
For cohort Any work absence due to wedding, maternity, paternity or adoption leaves in the period = Yes	1,093	1,017	1,175
N of Valid Cases	65382		

```
CROSSTABS
  /TABLES=AsgnGrp BY AbAbor
  /FORMAT=AVALUE TABLES
  /STATISTICS=RISK
  /CELLS=COUNT ROW TOTAL
  /COUNT ROUND CELL.
```

Crosstabs: **Abortion -** only women under 45 years of age considered

[BDForceII_Under45]

Case Processing Summary

	Cases					
	Valid		Missing		Total	
	N	Percent	N	Percent	N	Percent
Exposure to the pandemia environment * Any work absence due to ABORTION and its complications occurred in the period	37678	100,0%	0	0,0%	37678	100,0%

Exposure to the pandemia environment * Any work absence due to ABORTION and its complications occurred in the period Crosstabulation

			Any work absence due to ABORTION and its complications occurred in the period		Total
			No	Yes	
Exposure to the pandemia environment	Non-Exposed (2019)	Count	18686	153	18839
		% within Exposure to the pandemia environment	99,19%	0,81%	100,0%
		% of Total	49,59%	0,41%	50,0%
	Exposed (2020)	Count	18721	118	18839
		% within Exposure to the pandemia environment	99,37%	0,63%	100,0%

	% of Total	49,69%	0,31%	50,0%
Total	Count	37407	271	37678
	% within Exposure to the pandemia environment	99,3%	0,7%	100,0%
	% of Total	99,3%	0,7%	100,0%

Risk Estimate

	Value	95% Confidence Interval Lower	95% Confidence Interval Upper
Odds Ratio for Exposure to the pandemia environment (Non-Exposed (2019) / Exposed (2020))	,770	,605	,980
For cohort Any work absence due to ABORTION and its complications occurred in the period = No	,998	,996	1,000
For cohort Any work absence due to ABORTION and its complications occurred in the period = Yes	1,297	1,021	1,647
N of Valid Cases	37678		

```
DATASET COPY  BDForceII_FemaleUnder45.
DATASET ACTIVATE  BDForceII_FemaleUnder45.
FILTER OFF.
USE ALL.
SELECT IF (Sex = 0).
```

```
EXECUTE.
DATASET ACTIVATE  BDForceII_Under45.
DATASET ACTIVATE BDForceII_FemaleUnder45.
USE ALL.
COMPUTE filter_$=(CoVAb = 1).
VARIABLE LABELS filter_$ 'CoVAb = 1 (FILTER)'.
VALUE LABELS filter_$ 0 'Not Selected' 1 'Selected'.
FORMATS filter_$ (f1.0).
FILTER BY filter_$.
EXECUTE.
CROSSTABS
  /TABLES=AsgnGrp BY AbAbor
  /FORMAT=AVALUE TABLES
  /STATISTICS=RISK
  /CELLS=COUNT ROW TOTAL
  /COUNT ROUND CELL.
```

Crosstabs: only women under 45 years of age who had at least one absence due to suspect or confirmed COVID

[BDForceII_FemaleUnder45]

Case Processing Summary

Cases					
Valid		Missing		Total	
N	Percent	N	Percent	N	Percent

Exposure to the pandemia environment * Any work absence due to ABORTION and its complications occurred in the period	13650	100,0%	0	0,0%	13650	100,0%

Exposure to the pandemia environment * Any work absence due to ABORTION and its complications occurred in the period Crosstabulation

			Any work absence due to ABORTION and its complications occurred in the period		
			No	Yes	Total
Exposure to the pandemia environment	Non-Exposed (2019)	Count	6785	40	6825
		% within Exposure to the pandemia environment	99,41%	0,59%	100,0%
		% of Total	49,71%	0,29%	50,0%
	Exposed (2020)	Count	6788	37	6825
		% within Exposure to the pandemia environment	99,46%	0,54%	100,0%
		% of Total	49,73%	0,27%	50,0%
Total		Count	13573	77	13650
		% within Exposure to the pandemia environment	99,4%	0,6%	100,0%
		% of Total	99,4%	0,6%	100,0%

Risk Estimate

	Value	95% Confidence Interval Lower	Upper
Odds Ratio for Exposure to the pandemia environment (Non-Exposed (2019) / Exposed (2020))	,925	,591	1,448
For cohort Any work absence due to ABORTION and its complications occurred in the period = No	1,000	,997	1,002
For cohort Any work absence due to ABORTION and its complications occurred in the period = Yes	1,081	,692	1,688
N of Valid Cases	13650		

```
DATASET ACTIVATE DataSet2.
FILTER OFF.
USE ALL.
EXECUTE.
DATASET ACTIVATE DataSet1.
DATASET COPY  BDForceII_Under45_Horiz.
DATASET ACTIVATE  BDForceII_Under45_Horiz.
FILTER OFF.
USE ALL.
SELECT IF (Age <= 45).
EXECUTE.
DATASET ACTIVATE  DataSet1.
DATASET ACTIVATE BDForceII_Under45_Horiz.
USE ALL.
COMPUTE filter_$=(Sex = 0).
```

Supporting Information. Data Analysis. Part 1.

```
VARIABLE LABELS filter_$ 'Sex = 0 (FILTER)'.
VALUE LABELS filter_$ 0 'Not Selected' 1 'Selected'.
FORMATS filter_$ (f1.0).
FILTER BY filter_$.
EXECUTE.
CROSSTABS
  /TABLES=AbAbor_T1 BY AbAbor_T2
  /FORMAT=AVALUE TABLES
  /STATISTICS=MCNEMAR
  /CELLS=COUNT
  /COUNT ROUND CELL.
```

Crosstabs: only women under 45 years of age

[BDForceII_Under45_Horiz]

Case Processing Summary

Cases					
Valid		Missing		Total	
N	Percent	N	Percent	N	Percent

Any work absence due to ABORTION and its complications occurred in the period - T1 * Any work absence due to ABORTION and its complications occurred in the period - T2	18839	100,0%	0	0,0%	18839	100,0%

Any work absence due to ABORTION and its complications occurred in the period - T1 * Any work absence due to ABORTION and its complications occurred in the period - T2 Crosstabulation

Count

		Any work absence due to ABORTION and its complications occurred in the period - T2		
		No	Yes	Total
Any work absence due to ABORTION and its complications occurred in the period - T1	No	18576	110	18686
	Yes	145	8	153
Total		18721	118	18839

Chi-Square Tests

	Value	Exact Sig. (2-sided)
McNemar Test		,033[a]

N of Valid Cases	18839

a. Binomial distribution used.

```
DATASET COPY  BDForceII_WomenUnder45_Horiz.
DATASET ACTIVATE  BDForceII_WomenUnder45_Horiz.
FILTER OFF.
USE ALL.
SELECT IF (Sex = 0).
EXECUTE.
DATASET ACTIVATE  BDForceII_Under45_Horiz.
DATASET ACTIVATE BDForceII_WomenUnder45_Horiz.
USE ALL.
COMPUTE filter_$=(CoVAb = 1).
VARIABLE LABELS filter_$ 'CoVAb = 1 (FILTER)'.
VALUE LABELS filter_$ 0 'Not Selected' 1 'Selected'.
FORMATS filter_$ (f1.0).
FILTER BY filter_$.
EXECUTE.
CROSSTABS
  /TABLES=AbAbor_T1 BY AbAbor_T2
  /FORMAT=AVALUE TABLES
  /STATISTICS=MCNEMAR
  /CELLS=COUNT
  /COUNT ROUND CELL.
```

Crosstabs: only women under 45 years of age who had at least 01 absence due to suspect or confirmed COVID

[BDForceII_WomenUnder45_Horiz]

Case Processing Summary

	Cases					
	Valid		Missing		Total	
	N	Percent	N	Percent	N	Percent
Any work absence due to ABORTION and its complications occurred in the period - T1 * Any work absence due to ABORTION and its complications occurred in the period - T2	6825	100,0%	0	0,0%	6825	100,0%

Any work absence due to ABORTION and its complications occurred in the period - T1 * Any work absence due to ABORTION and its complications occurred in the period - T2 Crosstabulation

Count

		Any work absence due to ABORTION and its complications occurred in the period - T2		
		No	Yes	Total
Any work absence due to ABORTION and its complications occurred in the period - T1	No	6749	36	6785
	Yes	39	1	40

Total	6788	37	6825

Chi-Square Tests

	Value	Exact Sig. (2-sided)
McNemar Test		,818[a]
N of Valid Cases	6825	

a. Binomial distribution used.

```
DATASET ACTIVATE DataSet1.
DATASET CLOSE BDForceII_WomenUnder45_Horiz.
DATASET CLOSE BDForceII_Under45_Horiz.
DATASET CLOSE BDForceII_FemaleUnder45.
DATASET CLOSE BDForceII_Under45.
FILTER OFF.
USE ALL.
EXECUTE.
FILTER OFF.
USE ALL.
EXECUTE.
DATASET ACTIVATE DataSet2.
FILTER OFF.
USE ALL.
EXECUTE.
DATASET ACTIVATE DataSet1.
DATASET ACTIVATE DataSet1.

SAVE OUTFILE='C:\Users\trabalho\Documents\COVID-19\Centro Pesquisa HUB '+
    'COVID19\BDForceII_AbsperInd_Horiz.sav'
  /COMPRESSED.
DATASET ACTIVATE DataSet2.
DATASET ACTIVATE DataSet2.
```

Supporting Information. Data Analysis. Part 1.

```
SAVE OUTFILE='C:\Users\trabalho\Documents\COVID-19\Centro Pesquisa HUB '+
    'COVID19\BDForceII_AbsperInd_Vertic.sav'
  /COMPRESSED.
DATASET ACTIVATE DataSet1.
CROSSTABS
  /TABLES=AbAC_T1 BY AbAC_T2
  /FORMAT=AVALUE TABLES
  /STATISTICS=MCNEMAR
  /CELLS=COUNT
  /COUNT ROUND CELL.
```

Crosstabs

Case Processing Summary

	Cases					
	Valid		Missing		Total	
	N	Percent	N	Percent	N	Percent
Any work absence due to ALL CAUSES occurred in the period - T1 * Any work absence due to ALL CAUSES occurred in the period - T2	32691	100,0%	0	0,0%	32691	100,0%

Any work absence due to ALL CAUSES occurred in the period - T1 * Any work absence due to ALL CAUSES occurred in the period - T2 Crosstabulation

Count

		Any work absence due to ALL CAUSES occurred in the period - T2		
		No	Yes	Total
Any work absence due to ALL CAUSES occurred in the period - T1	No	10819	5098	15917
	Yes	7611	9163	16774
Total		18430	14261	32691

Chi-Square Tests

	Value	Exact Sig. (2-sided)
McNemar Test		,000[a]
N of Valid Cases	32691	

a. Binomial distribution used.

```
CROSSTABS
  /TABLES=AbADM_T1 BY AbADM_T2
  /FORMAT=AVALUE TABLES
  /STATISTICS=MCNEMAR
  /CELLS=COUNT
  /COUNT ROUND CELL.
```

Crosstabs

Case Processing Summary

	Cases					
	Valid		Missing		Total	
	N	Percent	N	Percent	N	Percent
Any work absence due to ADMINISTRATIVE CAUSES occurred in the period - T1 * Any work absence due to ADMINISTRATIVE CAUSES occurred in the period - T2	32691	100,0%	0	0,0%	32691	100,0%

Any work absence due to ADMINISTRATIVE CAUSES occurred in the period - T1 * Any work absence due to ADMINISTRATIVE CAUSES occurred in the period - T2 Crosstabulation

Count

		Any work absence due to ADMINISTRATIVE CAUSES occurred in the period - T2		
		No	Yes	Total
Any work absence due to ADMINISTRATIVE CAUSES occurred in the period - T1	No	26292	2059	28351
	Yes	3541	799	4340
Total		29833	2858	32691

Chi-Square Tests

	Value	Exact Sig. (2-sided)
McNemar Test		,000[a]
N of Valid Cases	32691	

a. Binomial distribution used.

```
CROSSTABS
  /TABLES=AbHRel_T1 BY AbHRel_T2
  /FORMAT=AVALUE TABLES
  /STATISTICS=MCNEMAR
  /CELLS=COUNT
  /COUNT ROUND CELL.
```

Crosstabs

Case Processing Summary

	Cases					
	Valid		Missing		Total	
	N	Percent	N	Percent	N	Percent
Any work absence due to HEALTH-RELATED CAUSES occurred in the period - T1 * Any work absence due to HEALTH-RELATED CAUSES occurred in the period - T2	32691	100,0%	0	0,0%	32691	100,0%

Any work absence due to HEALTH-RELATED CAUSES occurred in the period - T1 * Any work absence due to HEALTH-RELATED CAUSES occurred in the period - T2 Crosstabulation

Count

		Any work absence due to HEALTH-RELATED CAUSES occurred in the period - T2		
		No	Yes	Total
Any work absence due to HEALTH-RELATED CAUSES occurred in the period - T1	No	14695	4732	19427
	Yes	6847	6417	13264

Total	21542	11149	32691

Chi-Square Tests

	Value	Exact Sig. (2-sided)
McNemar Test		,000[a]
N of Valid Cases	32691	

a. Binomial distribution used.

```
CROSSTABS
  /TABLES=AbFam_T1 BY AbFam_T2
  /FORMAT=AVALUE TABLES
  /STATISTICS=MCNEMAR
  /CELLS=COUNT
  /COUNT ROUND CELL.
```

Crosstabs

Case Processing Summary

	Cases	
Valid	Missing	Total

	N	Percent	N	Percent	N	Percent
Any work absence due to FAMILY member's illness or death in the period - T1 * Any work absence due to FAMILY member's illness or death in the period - T2	32691	100,0%	0	0,0%	32691	100,0%

Any work absence due to FAMILY member's illness or death in the period - T1 * Any work absence due to FAMILY member's illness or death in the period - T2 Crosstabulation

Count

		Any work absence due to FAMILY member's illness or death in the period - T2		
		No	Yes	Total
Any work absence due to FAMILY member's illness or death in the period - T1	No	31037	743	31780
	Yes	868	43	911
Total		31905	786	32691

Chi-Square Tests

	Value	Exact Sig. (2-sided)
McNemar Test		,002[a]

N of Valid Cases	32691	

a. Binomial distribution used.

```
CROSSTABS
  /TABLES=AbBLD_T1 BY AbBLD_T2
  /FORMAT=AVALUE TABLES
  /STATISTICS=MCNEMAR
  /CELLS=COUNT
  /COUNT ROUND CELL.
```

Crosstabs

Case Processing Summary

	Cases					
	Valid		Missing		Total	
	N	Percent	N	Percent	N	Percent
Any work absence due to BLOOD DONATION occurred in the period - T1 * Any work absence due to BLOOD DONATION occurred in the period - T2	32691	100,0%	0	0,0%	32691	100,0%

Any work absence due to BLOOD DONATION occurred in the period - T1 * Any work absence due to BLOOD DONATION occurred in the period - T2 Crosstabulation

Count

		Any work absence due to BLOOD DONATION occurred in the period - T2		
		No	Yes	Total
Any work absence due to BLOOD DONATION occurred in the period - T1	No	31567	386	31953
	Yes	609	129	738
Total		32176	515	32691

Chi-Square Tests

	Value	Exact Sig. (2-sided)
McNemar Test		,000[a]
N of Valid Cases	32691	

a. Binomial distribution used.

```
CROSSTABS
  /TABLES=AbWMPA_T1 BY AbWMPA_T2
  /FORMAT=AVALUE TABLES
  /STATISTICS=MCNEMAR
  /CELLS=COUNT
  /COUNT ROUND CELL.
```

Crosstabs

Case Processing Summary

	Cases					
	Valid		Missing		Total	
	N	Percent	N	Percent	N	Percent
Any work absence due to wedding, maternity, paternity or adoption leaves in the period - T1 * Any work absence due to wedding, maternity, paternity or adoption leaves in the period - T1	32691	100,0%	0	0,0%	32691	100,0%

Any work absence due to wedding, maternity, paternity or adoption leaves in the period - T1 * Any work absence due to wedding, maternity, paternity or adoption leaves in the period - T1 Crosstabulation

Count

		Any work absence due to wedding, maternity, paternity or adoption leaves in the period - T1		
		No	Yes	Total
Any work absence due to wedding, maternity, paternity or adoption leaves in the period - T1	No	29902	1316	31218
	Yes	1441	32	1473
Total		31343	1348	32691

Chi-Square Tests

	Value	Exact Sig. (2-sided)
McNemar Test		,018[a]
N of Valid Cases	32691	

a. Binomial distribution used.

```
CROSSTABS
  /TABLES=AbAcd_T1 BY AbAcd_T2
  /FORMAT=AVALUE TABLES
  /STATISTICS=MCNEMAR
  /CELLS=COUNT
  /COUNT ROUND CELL.
```

Crosstabs

Case Processing Summary

	Cases					
	Valid		Missing		Total	
	N	Percent	N	Percent	N	Percent
Any work absence due to OCCUPATIONAL ACCIDENTS occurred in the period - T1 * Any work absence due to OCCUPATIONAL ACCIDENTS occurred in the period - T2	32691	100,0%	0	0,0%	32691	100,0%

Any work absence due to OCCUPATIONAL ACCIDENTS occurred in the period - T1 * Any work absence due to OCCUPATIONAL ACCIDENTS occurred in the period - T2 Crosstabulation

Count

		Any work absence due to OCCUPATIONAL ACCIDENTS occurred in the period - T2		
		No	Yes	Total
Any work absence due to	No	32557	46	32603

OCCUPATIONAL ACCIDENTS occurred in the period - T1	Yes	88	0	88
Total		32645	46	32691

Chi-Square Tests

	Value	Exact Sig. (2-sided)
McNemar Test		,000[a]
N of Valid Cases	32691	

a. Binomial distribution used.

```
DATASET ACTIVATE DataSet2.
GET
  FILE='C:\Users\trabalho\Documents\COVID-19\Centro Pesquisa HUB COVID19\BDForceII_KM_ALLCause.sav'.
DATASET NAME DataSet3 WINDOW=FRONT.
DATASET ACTIVATE DataSet3.

SAVE OUTFILE='C:\Users\trabalho\Documents\COVID-19\Centro Pesquisa HUB '+
    'COVID19\BDForceII_KM_ALLCause.sav'
  /COMPRESSED.
KM TTE BY AssgnGrp
  /STATUS=AbsEvent(1)
  /PRINT MEAN
  /PLOT SURVIVAL HAZARD LOGSURV
  /TEST LOGRANK BRESLOW TARONE
  /COMPARE OVERALL POOLED
  /SAVE SURVIVAL CUMEVENT.
```

Indirect assessment of the uniformity (steadiness) of the occurrence of work absences

throughout the period of observation

Kaplan-Meier

[DataSet3] C:\Users\trabalho\Documents\COVID-19\Centro Pesquisa HUB COVID19\BDForceII_KM_ALLCause.sav

Case Processing Summary

Exposure status as to the pandemia environment	Total N	N of Events	Censored N	Censored Percent
Non-Exposed (2019)	32691	16775	15916	48,7%
Exposed (2020)	32691	14261	18430	56,4%
Overall	65382	31036	34346	52,5%

Means and Medians for Survival Time

Exposure status as to the pandemia environment	Mean[a]				Median			
	Estimate	Std. Error	95% Confidence Interval		Estimate	Std. Error	95% Confidence Interval	
			Lower Bound	Upper Bound			Lower Bound	Upper Bound

Non-Exposed (2019)	103,785	,309	103,179	104,391	145,000	.	.	.
Exposed (2020)	110,510	,313	109,896	111,123	.	.	.	.
Overall	107,147	,220	106,716	107,579	.	.	.	.

a. Estimation is limited to the largest survival time if it is censored.

Overall Comparisons

	Chi-Square	df	Sig.
Log Rank (Mantel-Cox)	336,910	1	,000
Breslow (Generalized Wilcoxon)	246,353	1	,000
Tarone-Ware	294,469	1	,000

Test of equality of survival distributions for the different levels of Exposure status as to the pandemia environment.

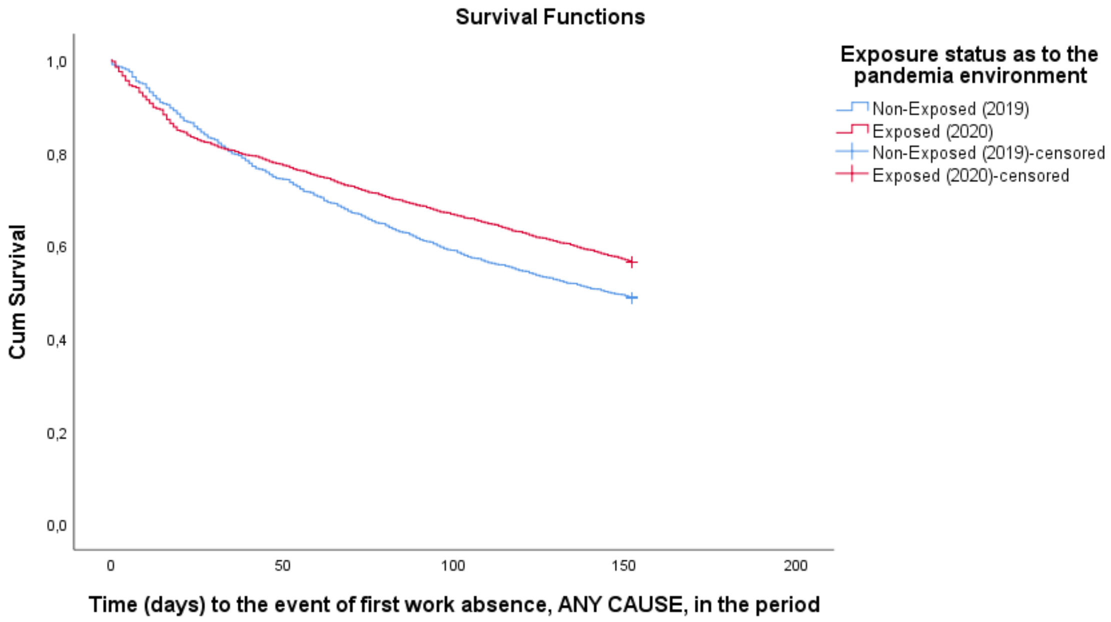
Survival Functions
Cum Survival
1,0
0,8
0,6
0,4
0,2
0,0
0
50
100
150
200
Time (days) to the event of first work absence, ANY CAUSE, in the period
Exposure status as to the pandemia environment
Non-Exposed (2019)
Exposed (2020)
Non-Exposed (2019)-censored
Exposed (2020)-censored

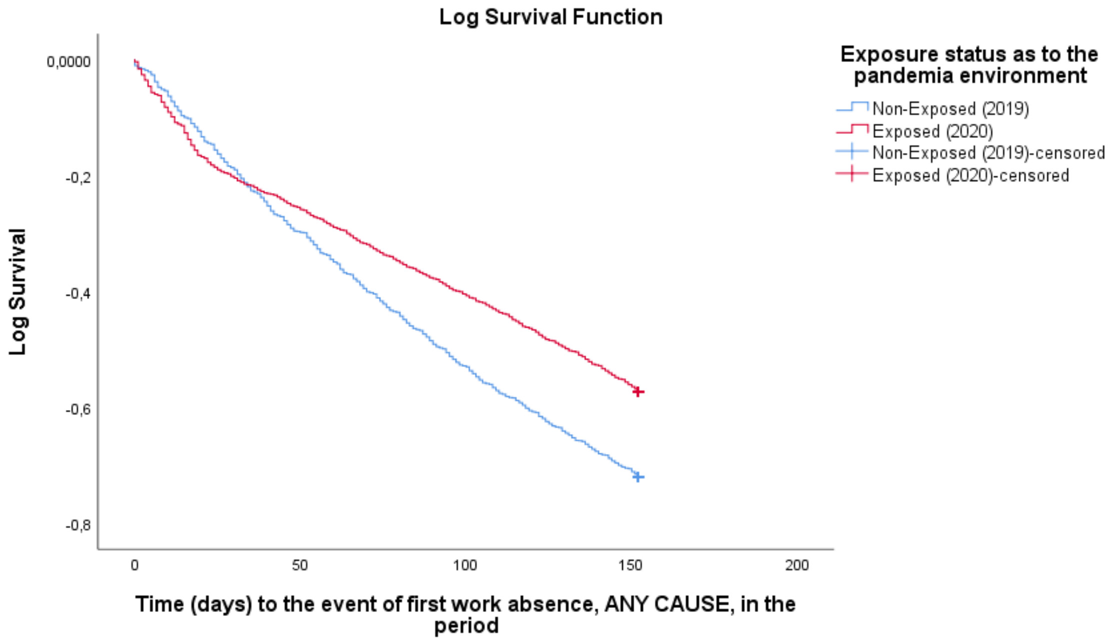
Log Survival Function
Log Survival
0,0000
-0,2
-0,4
-0,6
-0,8
0
50
100
150
200
Time (days) to the event of first work absence, ANY CAUSE, in the period
Exposure status as to the pandemia environment
Non-Exposed (2019)
Exposed (2020)
Non-Exposed (2019)-censored
Exposed (2020)-censored

Supporting Information. Data Analysis. Part 1.

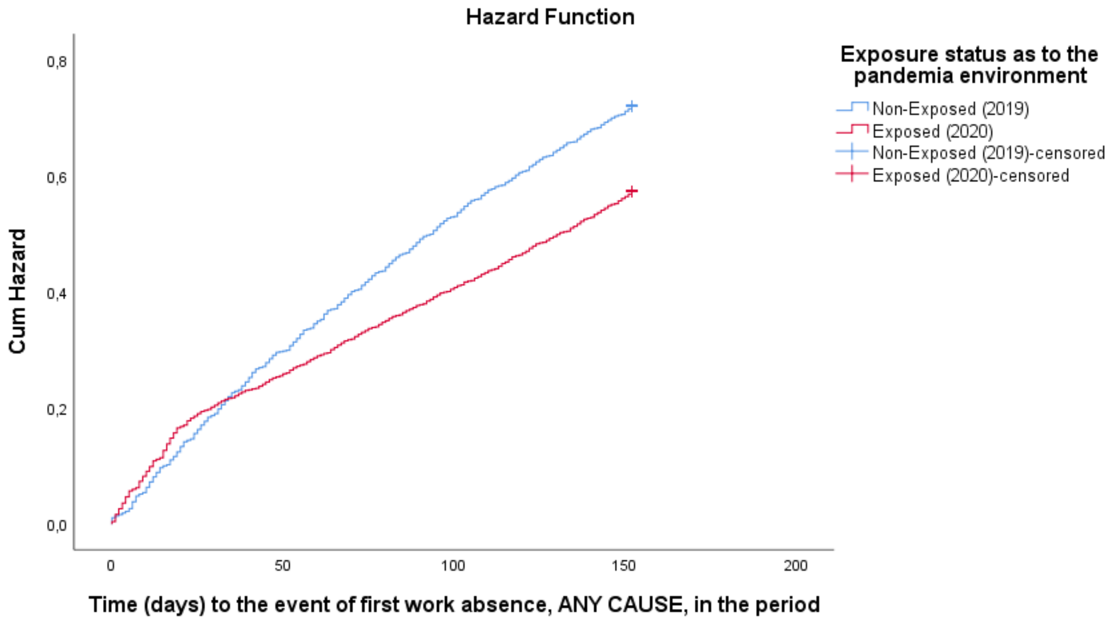


```
KM TTE BY AssgnGrp
  /STATUS=AbsEvent(1)
  /PRINT MEAN
  /PLOT SURVIVAL HAZARD LOGSURV
  /TEST LOGRANK BRESLOW TARONE
  /COMPARE OVERALL POOLED.
```

Kaplan-Meier

Case Processing Summary

Exposure status as to the pandemia environment	Total N	N of Events	Censored N	Censored Percent
Non-Exposed (2019)	32691	13343	19348	59,2%
Exposed (2020)	32691	11234	21457	65,6%
Overall	65382	24577	40805	62,4%

Means and Medians for Survival Time

Exposure status as to the pandemia environment	Mean[a] Estimate	Mean[a] Std. Error	Mean[a] 95% Confidence Interval Lower Bound	Mean[a] 95% Confidence Interval Upper Bound	Median Estimate	Median Std. Error	Median 95% Confidence Interval Lower Bound	Median 95% Confidence Interval Upper Bound
Non-Exposed (2019)	115,140	,289	114,574	115,705	.	.	.	.
Exposed (2020)	118,660	,296	118,080	119,240	.	.	.	.
Overall	116,900	,207	116,494	117,305	.	.	.	.

a. Estimation is limited to the largest survival time if it is censored.

Overall Comparisons

	Chi-Square	df	Sig.

Log Rank (Mantel-Cox)	220,825	1	,000
Breslow (Generalized Wilcoxon)	146,360	1	,000
Tarone-Ware	183,244	1	,000

Test of equality of survival distributions for the different levels of Exposure status as to the pandemia environment.

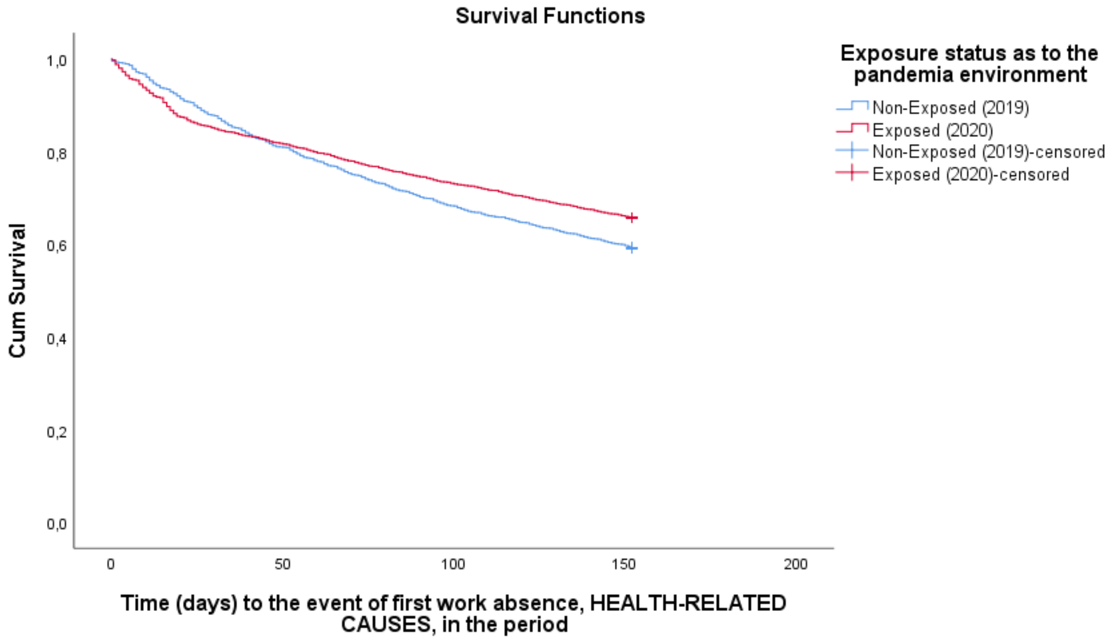

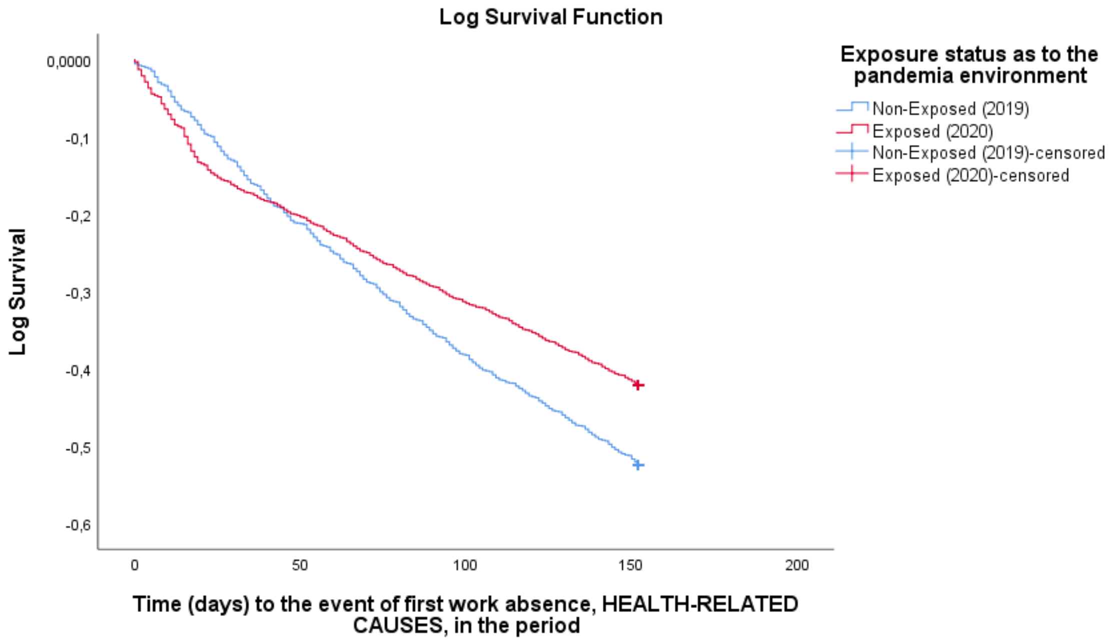
Log Survival Function
Exposure status as to the pandemia environment
Non-Exposed (2019)
Exposed (2020)
Non-Exposed (2019)-censored
Exposed (2020)-censored
Log Survival
0,0000
-0,1
-0,2
-0,3
-0,4
-0,5
-0,6
0
50
100
150
200
Time (days) to the event of first work absence, HEALTH-RELATED CAUSES, in the period

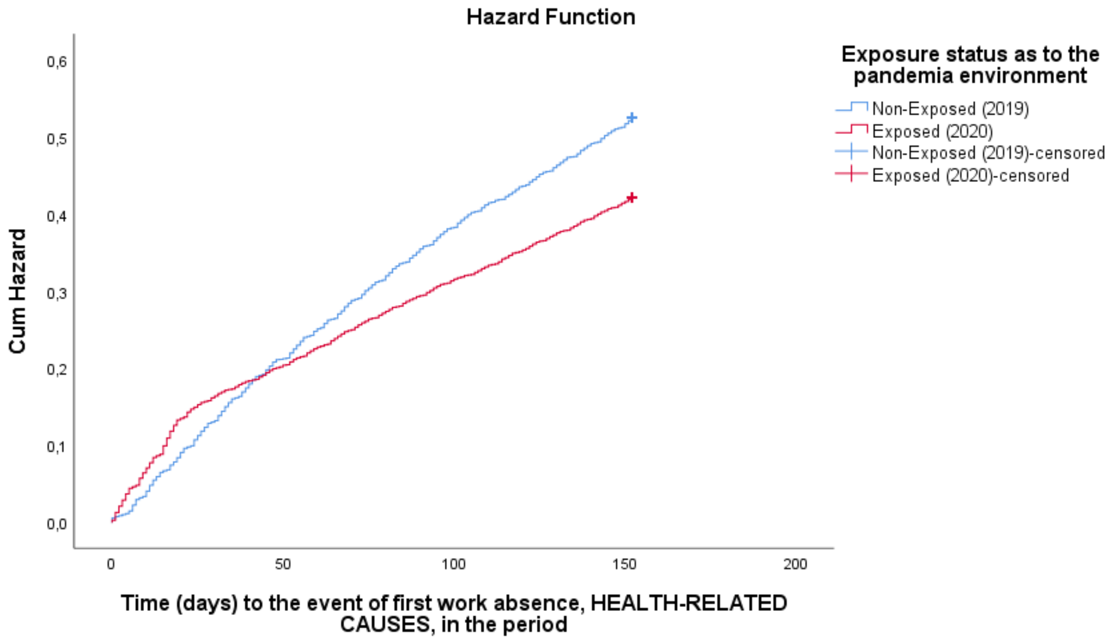


```
* Custom Tables.
CTABLES
  /VLABELS VARIABLES=DisGroup AsgnGrp DISPLAY=DEFAULT
  /TABLE DisGroup [C][COUNT F40.0, COLPCT.COUNT PCT40.1] BY AsgnGrp [C]
  /CATEGORIES VARIABLES=DisGroup ORDER=A KEY=VALUE EMPTY=INCLUDE TOTAL=YES POSITION=AFTER
  /CATEGORIES VARIABLES=AsgnGrp ORDER=A KEY=VALUE EMPTY=INCLUDE
  /CRITERIA CILEVEL=95.
```

Assessment of the occurence and relative distribution of specific groups of diseases as underlying causes of work absences between exposed and non-exposed subjects

Custom Tables

		Assignment group			
		Non-exposed (2019)		Exposed (2020)	
		Count	Column N %	Count	Column N %
General groups of diseases	Infectious	2507	12,1%	1364	8,9%
	Neoplasms	198	1,0%	134	0,9%
	Immunodeficiencies and blood discrasias	23	0,1%	22	0,1%
	Endocrine, metabolic and nutritional	108	0,5%	102	0,7%
	Mental and behavioral	1239	6,0%	1657	10,8%
	Neurological	935	4,5%	833	5,4%
	Ophtalmological	778	3,8%	459	3,0%
	Ear and mastoid	211	1,0%	223	1,4%
	Cardiovascular	479	2,3%	415	2,7%
	Upper and lower respiratory	3732	18,1%	2374	15,4%
	Digestive system	2180	10,6%	1445	9,4%
	Skin and subcutaneous	289	1,4%	256	1,7%

	Musculoskeletal and rheumatic	2754	13,3%	2288	14,9%
	Genitourinary	1040	5,0%	786	5,1%
	Pregnancy, childbirth and puerperium	1264	6,1%	647	4,2%
	Other diseases, symptoms and labs findings	393	1,9%	326	2,1%
	Trauma and external causes	1061	5,1%	831	5,4%
	Exams, proceedures and convalescence	1415	6,9%	1188	7,7%
	Allergy and related disorders	46	0,2%	45	0,3%
	Total	20652	100,0%	15395	100,0%

```
DATASET ACTIVATE DataSet11.
* Chart Builder.
GGRAPH
  /GRAPHDATASET NAME="graphdataset" VARIABLES=DisGroup COUNT()[name="COUNT"] AsgnGrp
    MISSING=LISTWISE REPORTMISSING=NO
  /GRAPHSPEC SOURCE=INLINE.
BEGIN GPL
  SOURCE: s=userSource(id("graphdataset"))
  DATA: DisGroup=col(source(s), name("DisGroup"), unit.category())
  DATA: COUNT=col(source(s), name("COUNT"))
  DATA: AsgnGrp=col(source(s), name("AsgnGrp"), unit.category())
  COORD: rect(dim(1,2), cluster(3,0))
  GUIDE: axis(dim(3), label("General groups of diseases"))
  GUIDE: axis(dim(2), label("Count"))
  GUIDE: legend(aesthetic(aesthetic.color.interior), label("Assignment group"))
  GUIDE: text.title(label("Clustered Bar Count of General groups of diseases by Assignment group"))
  SCALE: cat(dim(3), include("1", "2", "3", "4", "5", "6", "7", "8", "9", "10", "11", "12"
, "13", "14", "15", "16", "17", "18", "19"))
  SCALE: linear(dim(2), include(0))
```

```
  SCALE: cat(aesthetic(aesthetic.color.interior), include("0", "1"))
  SCALE: cat(dim(1), include("0", "1"))
  ELEMENT: interval(position(AsgnGrp*COUNT*DisGroup), color.interior(AsgnGrp),
    shape.interior(shape.square))
END GPL.
```

Supporting Information. Continues in the next file.
