# Supplementary material for: Impact of COVID-19 on the mental health of public university hospital workers in Brazil: A cohort-based analysis of 32,691 workers
Source: PLoS One. 2022 Jun 16;17(6):e0269318. doi: 10.1371/journal.pone.0269318 (PMC9202958; doi:10.1371/journal.pone.0269318)
Supplement: S2 File — (PDF) [file pone.0269318.s002.pdf]

Supporting Information. Data Analysis. Part2

GGraph

[DataSet11] C:\Users\trabalho\Documents\COVID-19\Centro Pesquisa HUB COVID19\BDForceII_OnlyEvents_ICDCompst_04102020.sav

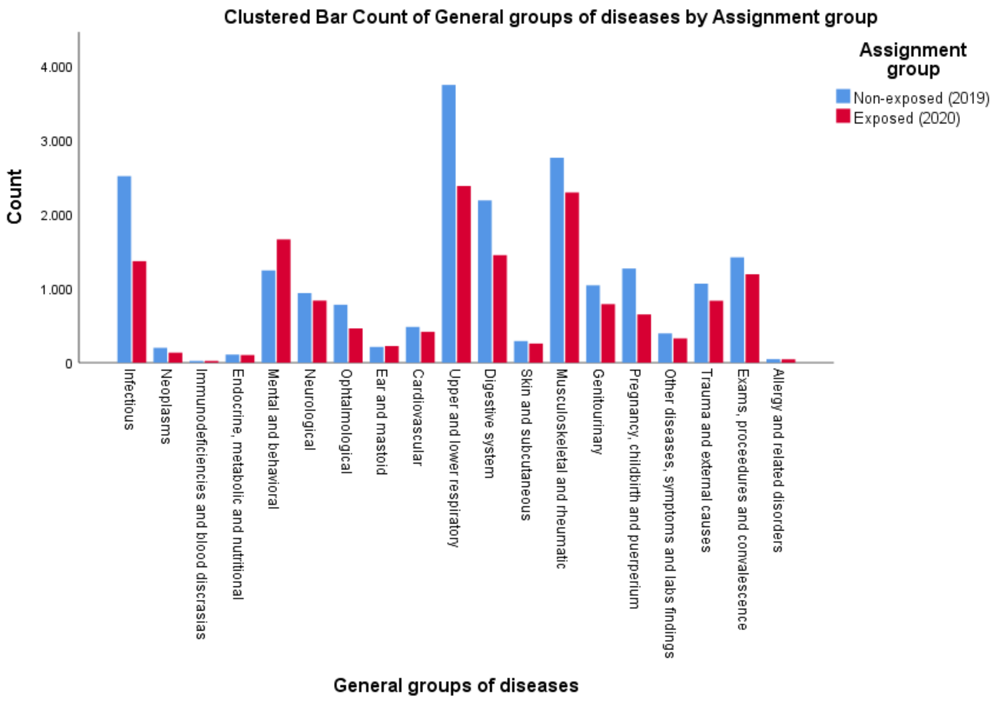

* Chart Builder.

```
GGRAPH
  /GRAPHDATASET NAME="graphdataset" VARIABLES=DisGroup COUNT()[name="COUNT"] AsgnGrp
    MISSING=LISTWISE REPORTMISSING=NO
  /GRAPHSPEC SOURCE=INLINE.
BEGIN GPL
  SOURCE: s=userSource(id("graphdataset"))
  DATA: DisGroup=col(source(s), name("DisGroup"), unit.category())
  DATA: COUNT=col(source(s), name("COUNT"))
  DATA: AsgnGrp=col(source(s), name("AsgnGrp"), unit.category())
  COORD: rect(dim(1,2), cluster(3,0))
  GUIDE: axis(dim(3), label("General groups of diseases"))
  GUIDE: axis(dim(2), label("Percent"))
  GUIDE: legend(aesthetic(aesthetic.color.interior), label("Assignment group"))
  GUIDE: text.title(label("Clustered Bar Percent of General groups of diseases by Assignment ",
    "group"))
  SCALE: cat(dim(3), include("1", "2", "3", "4", "5", "6", "7", "8", "9", "10", "11", "12"
, "13", "14", "15", "16", "17", "18", "19"))
  SCALE: linear(dim(2), include(0))
  SCALE: cat(aesthetic(aesthetic.color.interior), include("0", "1"))
  SCALE: cat(dim(1), include("0", "1"))
  ELEMENT: interval(position(summary.percent(AsgnGrp*COUNT*DisGroup,
    base.aesthetic(aesthetic(aesthetic.color.interior)))), color.interior(AsgnGrp),
    shape.interior(shape.square))
END GPL.
```

GGraph

Supporting Information. Data Analysis. Part2

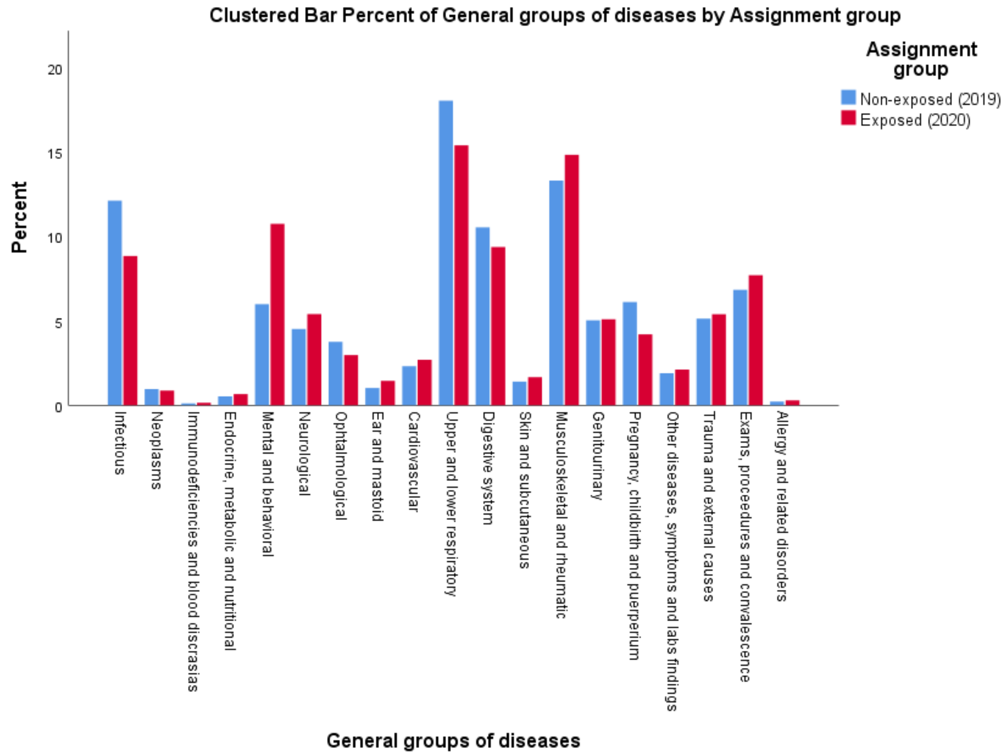

Assessment of the differences in work absences due to Mental Disease between exposed and non-exposed individuals

```
DATASET ACTIVATE DataSet1.
DATASET CLOSE DataSet11.
DATASET ACTIVATE DataSet1.
DATASET CLOSE DataSet10.
```

Supporting Information. Data Analysis. Part2

```
DATASET ACTIVATE DataSet2.
CROSSTABS
  /TABLES=AsgnGrp BY AbMnt
  /FORMAT=AVALUE TABLES
  /STATISTICS=RISK
  /CELLS=COUNT ROW TOTAL
  /COUNT ROUND CELL.
```

Crosstabs

[DataSet2] C:\Users\trabalho\Documents\COVID-19\Centro Pesquisa HUB COVID19\BDForceII_AbsperInd_Vertic.sav

Case Processing Summary

	Cases					
	Valid		Missing		Total	
	N	Percent	N	Percent	N	Percent
Exposure to the pandemia environment * Any work absence due to MENTAL DISEASE occurred in the period	65382	100,0%	0	0,0%	65382	100,0%

Exposure to the pandemia environment * Any work absence due to MENTAL DISEASE occurred in the period Crosstabulation

			Any work absence due to MENTAL DISEASE occurred in the period		
			No	Yes	Total
Exposure to the pandemia environment	Non-Exposed (2019)	Count	31875	816	32691
		% within Exposure to the pandemia environment	97,5%	2,5%	100,0%
		% of Total	48,8%	1,2%	50,0%
	Exposed (2020)	Count	31571	1120	32691
		% within Exposure to the pandemia environment	96,6%	3,4%	100,0%
		% of Total	48,3%	1,7%	50,0%
Total		Count	63446	1936	65382
		% within Exposure to the pandemia environment	97,0%	3,0%	100,0%
		% of Total	97,0%	3,0%	100,0%

Risk Estimate

	Value	95% Confidence Interval	
		Lower	Upper
Odds Ratio for Exposure to the pandemia environment (Non-Exposed (2019) / Exposed (2020))	1,386	1,265	1,519

For cohort Any work absence due to MENTAL DISEASE occurred in the period = No	1,010	1,007	1,012
For cohort Any work absence due to MENTAL DISEASE occurred in the period = Yes	,729	,667	,796
N of Valid Cases	65382		

```
DATASET ACTIVATE DataSet2.
DATASET CLOSE DataSet1.
DATASET ACTIVATE DataSet5.
DATASET CLOSE DataSet2.
```

```
GET
  FILE='C:\Users\trabalho\Documents\COVID-19\Centro Pesquisa HUB COVID19\BDForceII_AbsperInd_Horiz.sav'.
DATASET NAME DataSet2 WINDOW=FRONT.
CROSSTABS
  /TABLES=AbMnt_T1 BY AbMnt_T2
  /FORMAT=AVALUE TABLES
  /STATISTICS=MCNEMAR
  /CELLS=COUNT
  /COUNT ROUND CELL.
```

Crosstabs

[DataSet2] C:\Users\trabalho\Documents\COVID-19\Centro Pesquisa HUB COVID19\BDForceII_AbsperInd_Horiz.sav

Case Processing Summary

	Cases					
	Valid		Missing		Total	
	N	Percent	N	Percent	N	Percent
Any work absence due to MENTAL DISEASE occurred in the period - T1 * Any work absence due to MENTAL DISEASE occurred in the period - T2	32691	100,0%	0	0,0%	32691	100,0%

Any work absence due to MENTAL DISEASE occurred in the period - T1 * Any work absence due to MENTAL DISEASE occurred in the period - T2 Crosstabulation

Count

		Any work absence due to MENTAL DISEASE occurred in the period - T2		
		No	Yes	Total
Any work absence due to MENTAL	No	30939	936	31875

DISEASE occurred in the period - T1	Yes	632	184	816
Total		31571	1120	32691

Chi-Square Tests

	Value	Exact Sig. (2-sided)
McNemar Test		,000[a]
N of Valid Cases	32691	

a. Binomial distribution used.

```
DATASET ACTIVATE DataSet1.
EXAMINE VARIABLES=AbMnt_N BY AsgnGrp
  /PLOT HISTOGRAM NPPLOT
  /PERCENTILES(5,10,25,50,75,90,95) HAVERAGE
  /STATISTICS DESCRIPTIVES
  /CINTERVAL 95
  /MISSING LISTWISE
  /NOTOTAL.
```

Explore: events of work absence per individual due to Mental Diseases (counts)

[DataSet1] C:\Users\trabalho\Documents\COVID-19\Centro Pesquisa HUB COVID19\BDForceII_AbsperInd_Vertic.sav

Exposure to the pandemia environment

Case Processing Summary

		Cases					
	Exposure to the pandemia environment	Valid		Missing		Total	
		N	Percent	N	Percent	N	Percent
Events of work absence due to MENTAL DISEASE occurring in the period	Non-Exposed (2019)	32691	100,0%	0	0,0%	32691	100,0%
	Exposed (2020)	32691	100,0%	0	0,0%	32691	100,0%

Descriptives

	Exposure to the pandemia environment			Statistic	Std. Error
Events of work absence due to MENTAL DISEASE occurring in the period	Non-Exposed (2019)	Mean		,0379	,0015
		95% Confidence Interval for Mean	Lower Bound	,0349	
			Upper Bound	,0409	
		5% Trimmed Mean		,0000	
		Median		,0000	
		Variance		,0757	
		Std. Deviation		,2752	

		Minimum		0	
		Maximum		8	
		Range		8	
		Interquartile Range		0	
		Skewness		10,0935	,0135
		Kurtosis		137,0622	,0271
	Exposed (2020)	Mean		,0508	,0017
		95% Confidence Interval for Mean	Lower Bound	,0475	
			Upper Bound	,0542	
		5% Trimmed Mean		,0000	
		Median		,0000	
		Variance		,0971	
		Std. Deviation		,3117	
		Minimum		0	
		Maximum		7	
		Range		7	
		Interquartile Range		0	
		Skewness		8,3336	,0135
		Kurtosis		91,1899	,0271

Tests of Normality

	Exposure to the pandemia environment	Kolmogorov-Smirnov[a]		
		Statistic	df	Sig.

Events of work absence due to MENTAL DISEASE occurring in the period	Non-Exposed (2019)	,530	32691	,000
	Exposed (2020)	,531	32691	,000

a. Lilliefors Significance Correction

Events of work absence due to MENTAL DISEASE occurring in the period

Histograms

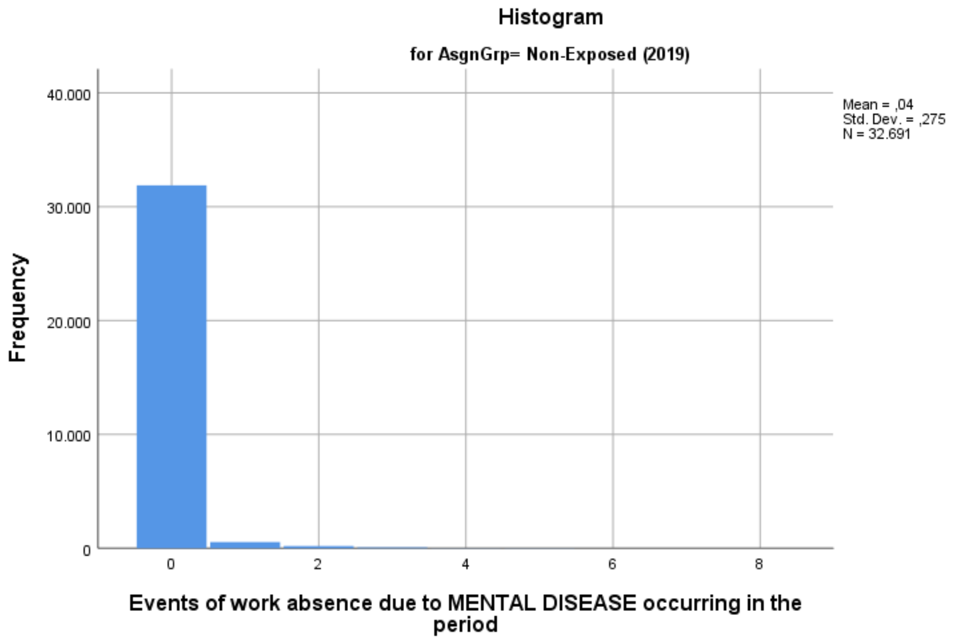
Histogram
for AsgnGrp= Non-Exposed (2019)
Frequency
40.000
30.000
20.000
10.000
0
0
2
4
6
8
Events of work absence due to MENTAL DISEASE occurring in the period
Mean = ,04
Std. Dev. = ,275
N = 32.691

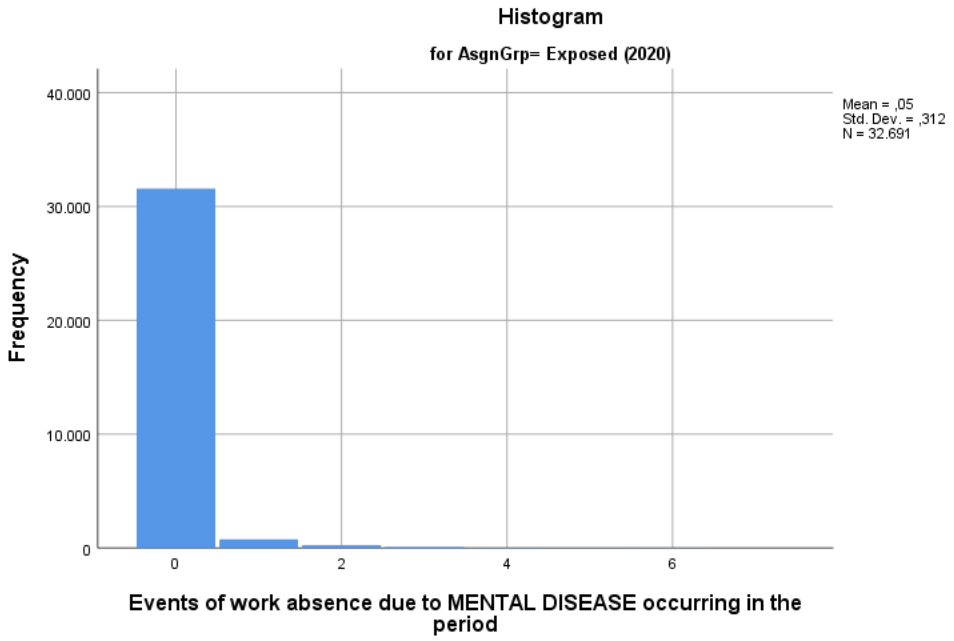

Normal Q-Q Plots

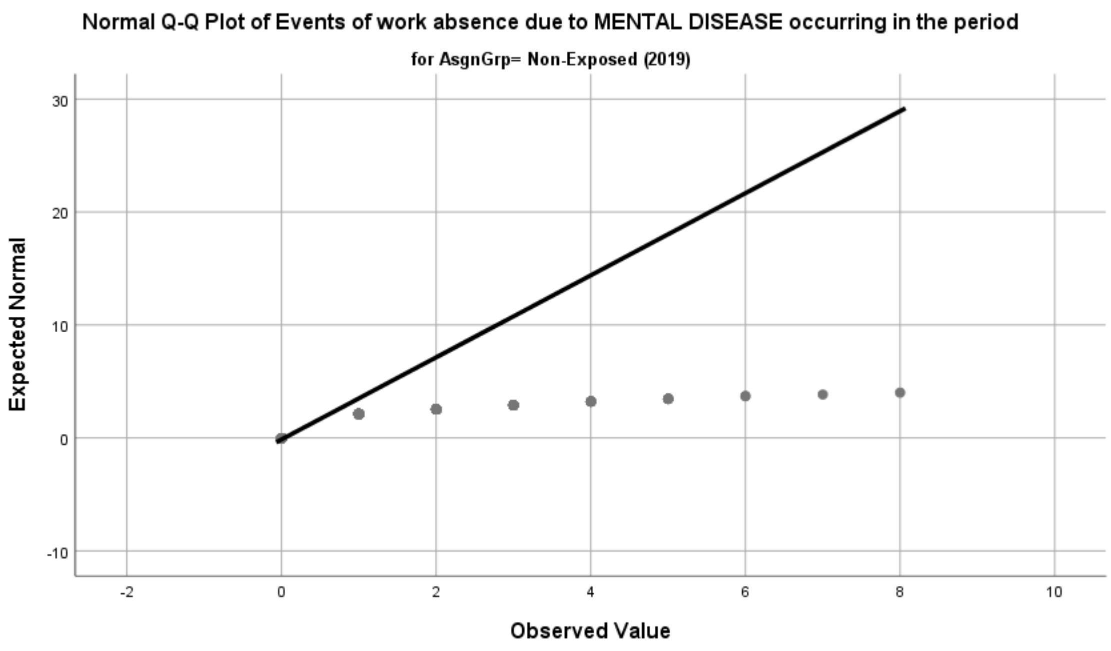
Normal Q-Q Plot of Events of work absence due to MENTAL DISEASE occurring in the period
for AsgnGrp= Non-Exposed (2019)
Expected Normal
30
20
10
0
-10
-2
0
2
4
6
8
10
Observed Value

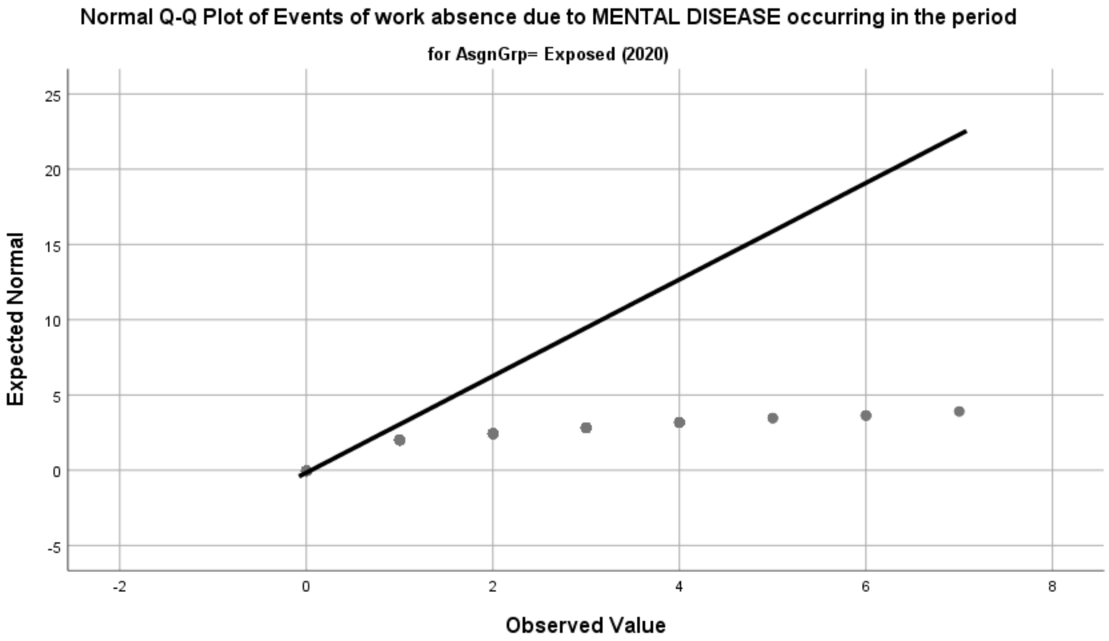

Detrended Normal Q-Q Plots

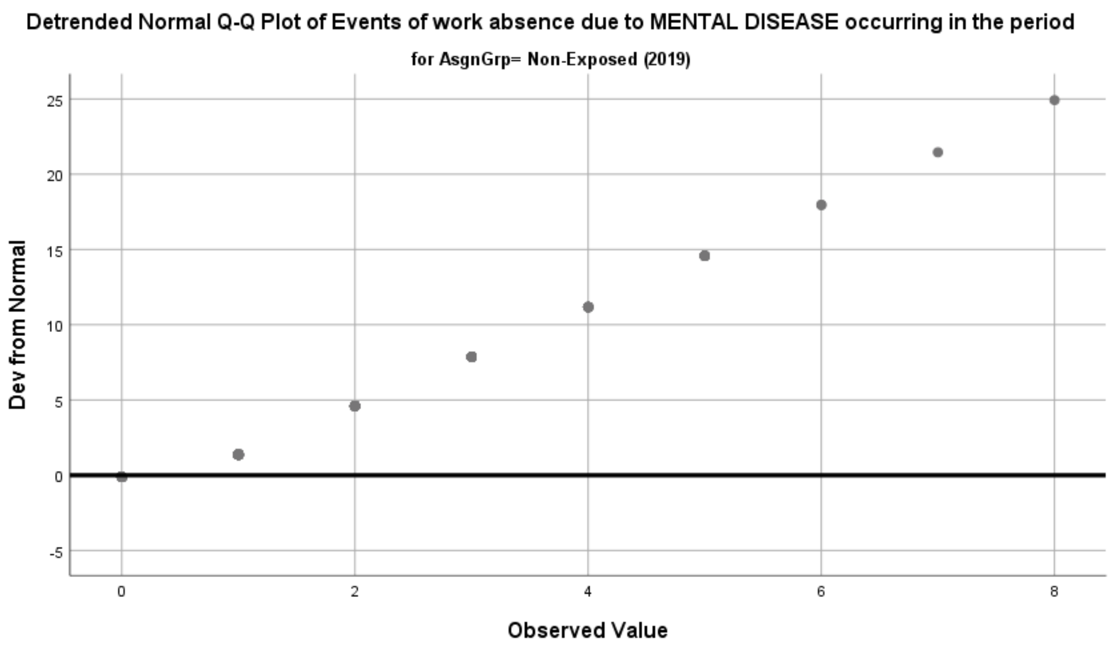
Detrended Normal Q-Q Plot of Events of work absence due to MENTAL DISEASE occurring in the period
for AsgnGrp= Non-Exposed (2019)
Dev from Normal
25
20
15
10
5
0
-5
0
2
4
6
8
Observed Value

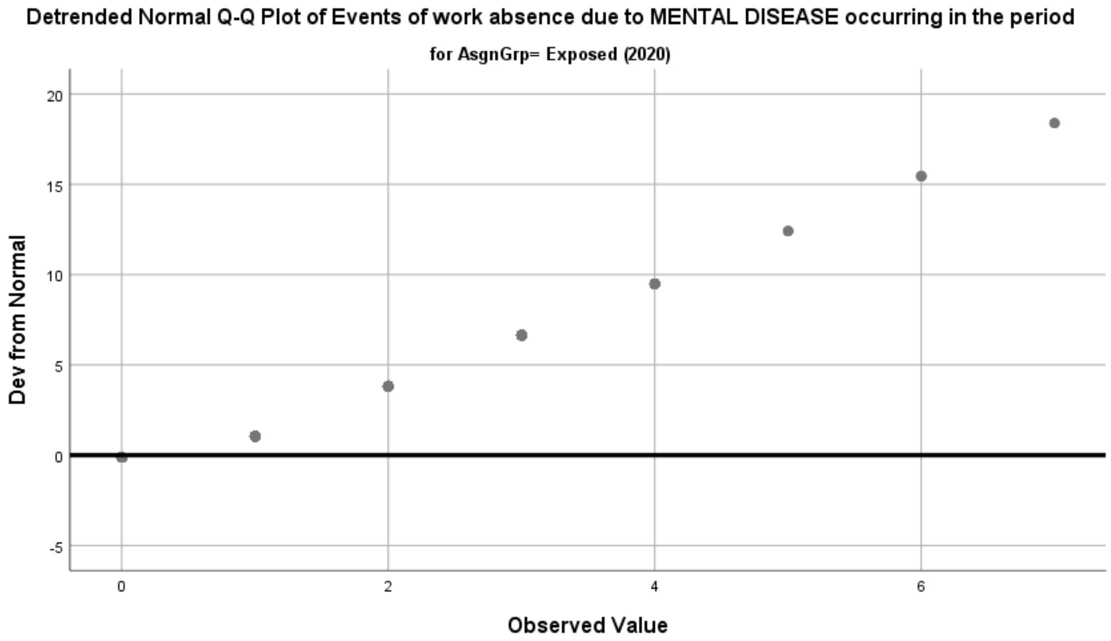


```
COMPUTE AbMnt_N100=AbMnt_N * 100.
EXECUTE.
* Generalized Estimating Equations.
GENLIN AbMnt_N100 BY AsgnGrp Sex (ORDER=DESCENDING)
  /MODEL AsgnGrp Sex INTERCEPT=YES
 DISTRIBUTION=NEGBIN(MLE) LINK=LOG
  /CRITERIA METHOD=FISHER(1) SCALE=1 MAXITERATIONS=100 MAXSTEPHALVING=5 PCONVERGE=1E-006(ABSOLUTE)
    SINGULAR=1E-012 ANALYSISTYPE=3(WALD) CILEVEL=95 LIKELIHOOD=FULL
  /EMMEANS TABLES=AsgnGrp SCALE=ORIGINAL
  /EMMEANS TABLES=Sex SCALE=ORIGINAL
  /REPEATED SUBJECT=ID WITHINSUBJECT=AsgnGrp SORT=YES CORRTYPE=UNSTRUCTURED ADJUSTCORR=YES
    COVB=ROBUST MAXITERATIONS=100 PCONVERGE=1e-006(ABSOLUTE) UPDATECORR=1
```

```
  /MISSING CLASSMISSING=EXCLUDE
  /PRINT CPS DESCRIPTIVES MODELINFO FIT SUMMARY SOLUTION (EXPONENTIATED).
```

Generalized Linear Models

Model Information

Dependent Variable		Events of work absence due to MENTAL DISEASE occurring in the period per 100 professionals
Probability Distribution		Negative binomial (MLE)
Link Function		Log
Subject Effect	1	ID
Within-Subject Effect	1	Exposure to the pandemia environment
Working Correlation Matrix Structure		Unstructured

Case Processing Summary

	N	Percent
Included	65382	100,0%
Excluded	0	0,0%

Total	65382	100,0%

Correlated Data Summary

Number of Levels	Subject Effect	ID	32691
	Within-Subject Effect	Exposure to the pandemia environment	2
Number of Subjects			32691
Number of Measurements per Subject	Minimum		2
	Maximum		2
Correlation Matrix Dimension			2

Categorical Variable Information

			N	Percent
Factor	Exposure to the pandemia environment	Exposed (2020)	32691	50,0%
		Non-Exposed (2019)	32691	50,0%
		Total	65382	100,0%
	Gender	Male	19418	29,7%
		Female	45964	70,3%
		Total	65382	100,0%

Continuous Variable Information

		N	Minimum	Maximum	Mean	Std. Deviation
Dependent Variable	Events of work absence due to MENTAL DISEASE occurring in the period per 100 professionals	65382	,00	800,00	4,4370	29,40783

Goodness of Fit[a]

	Value
Quasi Likelihood under Independence Model Criterion (QIC)[b]	4352,568
Corrected Quasi Likelihood under Independence Model Criterion (QICC)[b]	4356,937

Dependent Variable: Events of work absence due to MENTAL DISEASE occurring in the period per 100 professionals
Model: (Intercept), Exposure to the pandemia environment, Gender[a]
a. Information criteria are in smaller-is-better form.
b. Computed using the full log quasi-likelihood function.

Tests of Model Effects

		Type III	
Source	Wald Chi-Square	df	Sig.
(Intercept)	1058,402	1	,000
Exposure to the pandemia environment	32,060	1	,000
Gender	105,628	1	,000

Dependent Variable: Events of work absence due to MENTAL DISEASE occurring in the period per 100 professionals

Model: (Intercept), Exposure to the pandemia environment, Gender

Parameter Estimates

			95% Wald Confidence Interval		Hypothesis Test				95% Wald Confidence Interval for Exp	
Parameter	B	Std. Error	Lower	Upper	Wald Chi-Square	df	Sig.	Exp(B)	Lower	Upper
(Intercept)	1,526	,0426	1,443	1,610	1284,167	1	,000	4,600	4,232	[illegible]
[Exposure to the pandemia environment=1]	,274	,0505	,175	,373	29,424	1	,000	1,315	1,191	[illegible]
[Exposure to the pandemia environment=0]	0[a]	.	.	.	.	.	.	1	.	
[Gender=1]	-,796	,0774	-,947	-,644	105,628	1	,000	,451	,388	[illegible]
[Gender=0]	0[a]	.	.	.	.	.	.	1	.	
(Scale)	1									
(Negative binomial)	225,555[b]									

Dependent Variable: Events of work absence due to MENTAL DISEASE occurring in the period per 100 professionals

Model: (Intercept), Exposure to the pandemia environment, Gender

a. Set to zero because this parameter is redundant.

b. Fixed at the displayed value.

Estimated Marginal Means 1: Exposure to the pandemia environment

Estimates

Exposure to the pandemia environment	Mean	Std. Error	95% Wald Confidence Interval	
			Lower	Upper
Exposed (2020)	4,0650	,17595	3,7343	4,4249
Non-Exposed (2019)	3,0533	,14960	2,7737	3,3610

Estimated Marginal Means 2: Gender

Estimates

Gender	Mean	Std. Error	95% Wald Confidence Interval	
			Lower	Upper

Male	2,3667	,16752	2,0601	2,7189
Female	5,2442	,16441	4,9317	5,5766

```
DATASET ACTIVATE DataSet2.
T-TEST PAIRS=AbMnt_N_T2 WITH AbMnt_N_T1 (PAIRED)
  /CRITERIA=CI(.9500)
  /MISSING=ANALYSIS.
```

T-Test

[DataSet2] C:\Users\trabalho\Documents\COVID-19\Centro Pesquisa HUB COVID19\BDForceII_AbsperInd_Horiz.sav

Paired Samples Statistics

		Mean	N	Std. Deviation	Std. Error Mean
Pair 1	Events of work absence due to MENTAL DISEASE occurring in the period - T2	,0508	32691	,3117	,0017
	Events of work absence due to MENTAL DISEASE occurring in the period - T1	,0379	32691	,2752	,0015

Paired Samples Correlations

		N	Correlation	Sig.
Pair 1	Events of work absence due to MENTAL DISEASE occurring in the period - T2 & Events of work absence due to MENTAL DISEASE occurring in the period - T1	32691	,198	,000

Paired Samples Test

		Paired Differences					t	df	Sig. (2-tailed)
					95% Confidence Interval of the Difference				
		Mean	Std. Deviation	Std. Error Mean	Lower	Upper			
Pair 1	Events of work absence due to MENTAL DISEASE occurring in the period - T2 - Events of work absence due to MENTAL DISEASE occurring in the period - T1	,0129	,3727	,0021	,0089	,0170	6,277	32690	,0[illegible]

```
GET
  FILE='C:\Users\trabalho\Documents\COVID-19\Centro Pesquisa HUB COVID19\BDForceII_AbsperInd_Vertic.sav'.
```

Supporting Information. Data Analysis. Part2

```
DATASET NAME DataSet1 WINDOW=FRONT.
GET
  FILE='C:\Users\trabalho\Documents\COVID-19\Centro Pesquisa HUB COVID19\BDForceII_AbsperInd_Horiz.sav'.
DATASET NAME DataSet2 WINDOW=FRONT.
*Nonparametric Tests: Related Samples.
NPTESTS
  /RELATED TEST(AbMnt_N_T1 AbMnt_N_T2)
  /MISSING SCOPE=ANALYSIS USERMISSING=EXCLUDE
  /CRITERIA ALPHA=0.05  CILEVEL=95.
```

Nonparametric Tests

[DataSet2] C:\Users\trabalho\Documents\COVID-19\Centro Pesquisa HUB COVID19\BDForceII_AbsperInd_Horiz.sav

null : null

Hypothesis Test Summary

	Null Hypothesis	Test	Sig.	Decision
1	The median of differences between Events of work absence due to MENTAL DISEASE occurring in the period - T1 and Events of work absence due to MENTAL DISEASE occurring in the period - T2 equals 0.	Related-Samples Wilcoxon Signed Rank Test	,000	Reject the null hypothesis.

Asymptotic significances are displayed. The significance level is ,05.

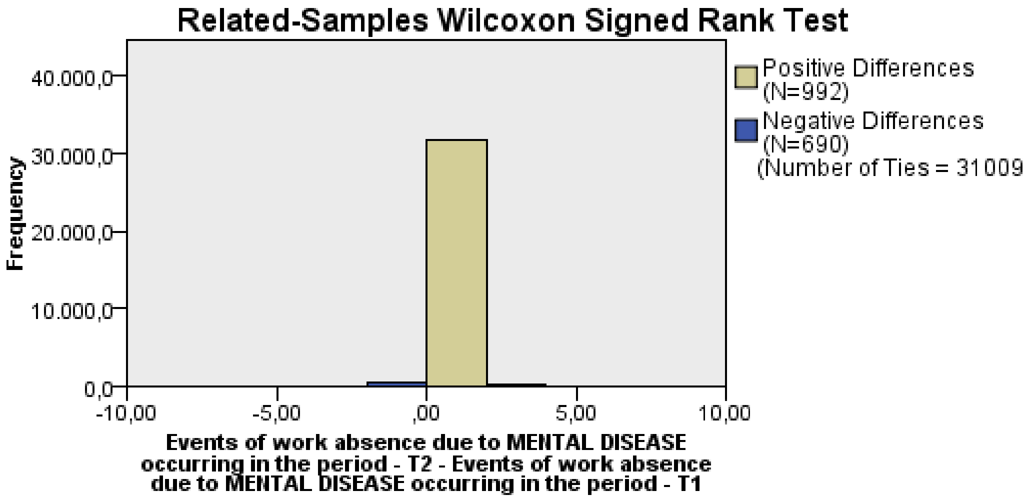

**Total N**	32.691
**Test Statistic**	834.347,500
**Standard Error**	19.069,593
**Standardized Test Statistic**	6,641
**Asymptotic Sig. (2-sided test)**	,000

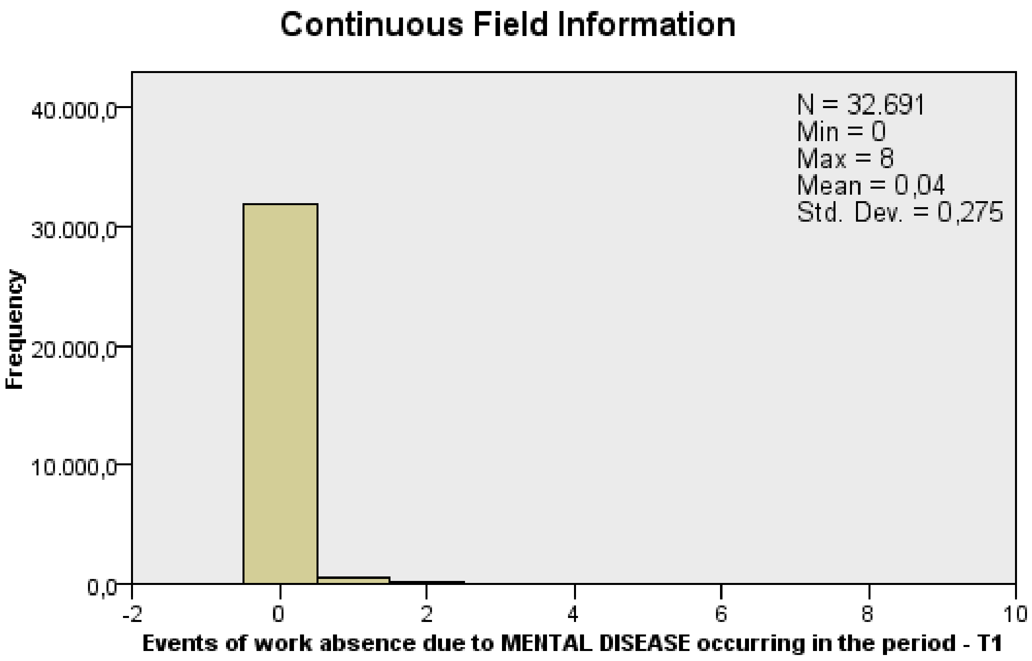
Continuous Field Information
N = 32.691
Min = 0
Max = 8
Mean = 0,04
Std. Dev. = 0,275
40.000,0
30.000,0
20.000,0
10.000,0
0,0
Frequency
-2
0
2
4
6
8
10
Events of work absence due to MENTAL DISEASE occurring in the period - T1

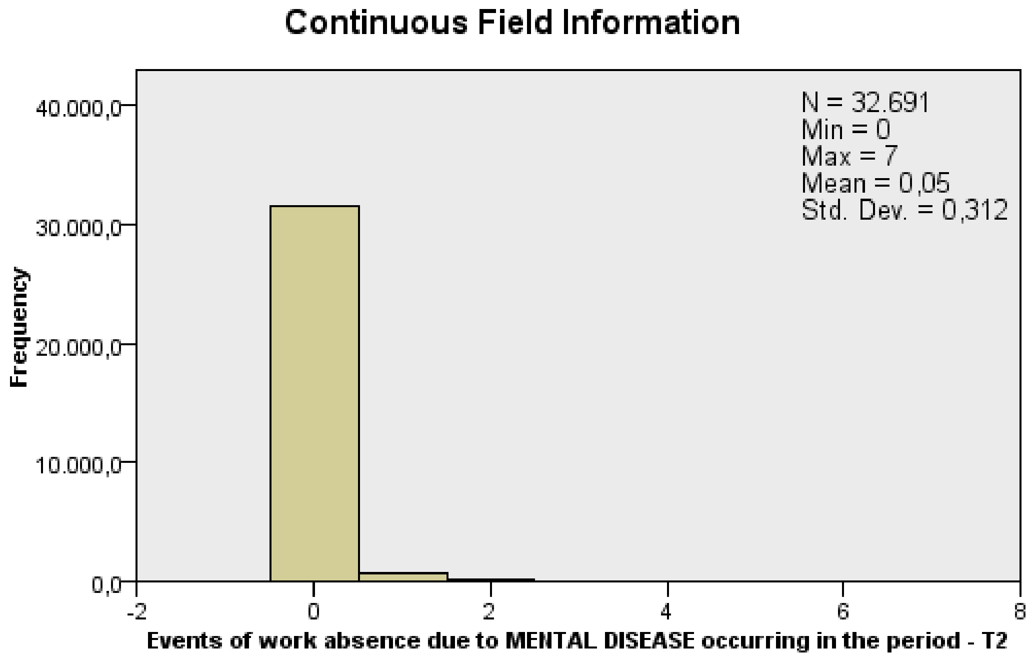

Supporting Information. Continues in the next file.
