# Supplementary material for: Impact of COVID-19 on the mental health of public university hospital workers in Brazil: A cohort-based analysis of 32,691 workers
Source: PLoS One. 2022 Jun 16;17(6):e0269318. doi: 10.1371/journal.pone.0269318 (PMC9202958; doi:10.1371/journal.pone.0269318)
Supplement: S3 File — (PDF) [file pone.0269318.s003.pdf]

Supporting Information. Part3

In the following table, the identification numbers of the healthcare workers were removed in order to preserve the data of the participants of the study.

Mixed Model Analysis: duration (days) work absence due to Mental Diseases

Label		Count	Marginal Percentage
ID		1	0,1%
		1	0,1%
		1	0,1%
		1	0,1%
		1	0,1%
		1	0,1%
		1	0,1%
		2	0,1%
		1	0,1%
		1	0,1%
		1	0,1%
		1	0,1%
		2	0,1%
		1	0,1%
		1	0,1%
		1	0,1%
		1	0,1%
		1	0,1%
		1	0,1%
		1	0,1%
		1	0,1%
		1	0,1%
		1	0,1%
		1	0,1%
		1	0,1%
		1	0,1%
		1	0,1%
		2	0,1%
		1	0,1%
		1	0,1%
		1	0,1%
		1	0,1%
		1	0,1%
		1	0,1%
		1	0,1%
		1	0,1%
		1	0,1%
		2	0,1%
		1	0,1%
		1	0,1%
		1	0,1%
		1	0,1%
		1	0,1%
		1	0,1%
		2	0,1%
		1	0,1%
		1	0,1%
		1	0,1%
		1	0,1%
		1	0,1%
		1	0,1%
		1	0,1%
		1	0,1%
		1	0,1%
		1	0,1%
		1	0,1%
		1	0,1%
		1	0,1%
		1	0,1%

		1	0,1%
		1	0,1%
		1	0,1%
		2	0,1%
		1	0,1%
		1	0,1%
		1	0,1%
		1	0,1%
		1	0,1%
		1	0,1%
		1	0,1%
		1	0,1%
		1	0,1%
		1	0,1%
		1	0,1%
		1	0,1%
		1	0,1%
		1	0,1%
		1	0,1%
		1	0,1%
		1	0,1%
		1	0,1%
		1	0,1%
		1	0,1%
		2	0,1%
		1	0,1%
		1	0,1%
		1	0,1%
		1	0,1%
		1	0,1%
		1	0,1%
		1	0,1%
		1	0,1%
		1	0,1%
		1	0,1%
		1	0,1%
		1	0,1%
		1	0,1%
		1	0,1%
		1	0,1%
		1	0,1%
		1	0,1%
		1	0,1%
		1	0,1%
		1	0,1%
		1	0,1%
		1	0,1%
		1	0,1%
		1	0,1%
		1	0,1%
		1	0,1%
		1	0,1%
		2	0,1%
		1	0,1%
		1	0,1%
		1	0,1%
		2	0,1%
		1	0,1%
		1	0,1%
		1	0,1%
		1	0,1%
		1	0,1%
		2	0,1%
		1	0,1%
		1	0,1%
		1	0,1%
		2	0,1%
		1	0,1%
		1	0,1%

		1	0,1%
		1	0,1%
		2	0,1%
		1	0,1%
		1	0,1%
		1	0,1%
		1	0,1%
		1	0,1%
		1	0,1%
		1	0,1%
		1	0,1%
		1	0,1%
		1	0,1%
		2	0,1%
		1	0,1%
		1	0,1%
		1	0,1%
		1	0,1%
		1	0,1%
		1	0,1%
		1	0,1%
		1	0,1%
		1	0,1%
		1	0,1%
		1	0,1%
		1	0,1%
		1	0,1%
		1	0,1%
		2	0,1%
		1	0,1%
		1	0,1%
		1	0,1%
		1	0,1%
		1	0,1%
		1	0,1%
		1	0,1%
		1	0,1%
		1	0,1%
		1	0,1%
		1	0,1%
		2	0,1%
		1	0,1%
		1	0,1%
		1	0,1%
		1	0,1%
		1	0,1%
		1	0,1%
		1	0,1%
		1	0,1%
		1	0,1%
		1	0,1%
		1	0,1%
		1	0,1%
		1	0,1%
		1	0,1%
		1	0,1%
		1	0,1%
		1	0,1%
		1	0,1%
		1	0,1%
		2	0,1%
		1	0,1%
		1	0,1%
		1	0,1%
		1	0,1%
		1	0,1%
		2	0,1%

		1	0,1%
		1	0,1%
		1	0,1%
		1	0,1%
		1	0,1%
		1	0,1%
		1	0,1%
		1	0,1%
		1	0,1%
		1	0,1%
		2	0,1%
		2	0,1%
		1	0,1%
		1	0,1%
		1	0,1%
		2	0,1%
		1	0,1%
		1	0,1%
		1	0,1%
		1	0,1%
		1	0,1%
		1	0,1%
		2	0,1%
		1	0,1%
		1	0,1%
		1	0,1%
		1	0,1%
		2	0,1%
		1	0,1%
		1	0,1%
		1	0,1%
		1	0,1%
		1	0,1%
		1	0,1%
		1	0,1%
		1	0,1%
		1	0,1%
		1	0,1%
		1	0,1%
		1	0,1%
		1	0,1%
		1	0,1%
		1	0,1%
		1	0,1%
		1	0,1%
		1	0,1%
		1	0,1%
		1	0,1%
		1	0,1%
		1	0,1%
		1	0,1%
		2	0,1%
		1	0,1%
		1	0,1%
		1	0,1%
		2	0,1%
		1	0,1%
		1	0,1%
		1	0,1%
		1	0,1%
		1	0,1%
		1	0,1%
		1	0,1%
		1	0,1%
		1	0,1%
		1	0,1%
		1	0,1%
		1	0,1%
		1	0,1%

		1	0,1%
		1	0,1%
		1	0,1%
		1	0,1%
		1	0,1%
		1	0,1%
		1	0,1%
		1	0,1%
		1	0,1%
		1	0,1%
		1	0,1%
		1	0,1%
		1	0,1%
		1	0,1%
		1	0,1%
		1	0,1%
		1	0,1%
		1	0,1%
		1	0,1%
		1	0,1%
		1	0,1%
		1	0,1%
		1	0,1%
		1	0,1%
		1	0,1%
		1	0,1%
		2	0,1%
		1	0,1%
		1	0,1%
		1	0,1%
		1	0,1%
		1	0,1%
		1	0,1%
		1	0,1%
		1	0,1%
		2	0,1%
		2	0,1%
		1	0,1%
		1	0,1%
		1	0,1%
		1	0,1%
		1	0,1%
		1	0,1%
		1	0,1%
		2	0,1%
		1	0,1%
		1	0,1%
		1	0,1%
		1	0,1%
		1	0,1%
		1	0,1%
		1	0,1%
		1	0,1%
		1	0,1%
		1	0,1%
		1	0,1%
		1	0,1%
		1	0,1%
		1	0,1%
		1	0,1%
		1	0,1%
		1	0,1%
		1	0,1%
		1	0,1%
		1	0,1%
		1	0,1%
		1	0,1%
		1	0,1%

		1	0,1%
		2	0,1%
		1	0,1%
		1	0,1%
		1	0,1%
		1	0,1%
		1	0,1%
		1	0,1%
		1	0,1%
		1	0,1%
		1	0,1%
		1	0,1%
		1	0,1%
		1	0,1%
		1	0,1%
		1	0,1%
		1	0,1%
		1	0,1%
		1	0,1%
		1	0,1%
		1	0,1%
		1	0,1%
		1	0,1%
		1	0,1%
		1	0,1%
		1	0,1%
		1	0,1%
		1	0,1%
		1	0,1%
		1	0,1%
		2	0,1%
		1	0,1%
		1	0,1%
		1	0,1%
		1	0,1%
		1	0,1%
		1	0,1%
		2	0,1%
		1	0,1%
		1	0,1%
		1	0,1%
		1	0,1%
		2	0,1%
		1	0,1%
		1	0,1%
		1	0,1%
		1	0,1%
		1	0,1%
		1	0,1%
		1	0,1%
		1	0,1%
		1	0,1%
		1	0,1%
		1	0,1%
		1	0,1%
		1	0,1%
		1	0,1%
		1	0,1%
		2	0,1%
		2	0,1%
		1	0,1%
		1	0,1%
		1	0,1%
		1	0,1%
		1	0,1%
		1	0,1%
		1	0,1%
		1	0,1%

		1	0,1%
		1	0,1%
		1	0,1%
		1	0,1%
		1	0,1%
		1	0,1%
		1	0,1%
		1	0,1%
		1	0,1%
		1	0,1%
		1	0,1%
		1	0,1%
		1	0,1%
		1	0,1%
		1	0,1%
		1	0,1%
		1	0,1%
		1	0,1%
		1	0,1%
		1	0,1%
		1	0,1%
		1	0,1%
		1	0,1%
		1	0,1%
		1	0,1%
		1	0,1%
		1	0,1%
		1	0,1%
		1	0,1%
		1	0,1%
		1	0,1%
		1	0,1%
		1	0,1%
		1	0,1%
		1	0,1%
		1	0,1%
		1	0,1%
		1	0,1%
		1	0,1%
		1	0,1%
		1	0,1%
		1	0,1%
		1	0,1%
		1	0,1%
		1	0,1%
		1	0,1%
		2	0,1%
		1	0,1%
		1	0,1%
		1	0,1%
		1	0,1%
		1	0,1%
		1	0,1%
		1	0,1%
		1	0,1%
		2	0,1%
		2	0,1%
		1	0,1%
		1	0,1%
		1	0,1%
		2	0,1%
		1	0,1%
		2	0,1%
		1	0,1%
		1	0,1%
		1	0,1%
		1	0,1%
		2	0,1%

		1	0,1%
		2	0,1%
		1	0,1%
		1	0,1%
		1	0,1%
		1	0,1%
		1	0,1%
		1	0,1%
		1	0,1%
		1	0,1%
		2	0,1%
		1	0,1%
		2	0,1%
		1	0,1%
		1	0,1%
		2	0,1%
		2	0,1%
		1	0,1%
		1	0,1%
		1	0,1%
		1	0,1%
		1	0,1%
		1	0,1%
		2	0,1%
		1	0,1%
		1	0,1%
		1	0,1%
		1	0,1%
		1	0,1%
		2	0,1%
		1	0,1%
		1	0,1%
		1	0,1%
		1	0,1%
		1	0,1%
		2	0,1%
		1	0,1%
		1	0,1%
		1	0,1%
		1	0,1%
		1	0,1%
		1	0,1%
		1	0,1%
		1	0,1%
		1	0,1%
		1	0,1%
		1	0,1%
		2	0,1%
		1	0,1%
		1	0,1%
		1	0,1%
		1	0,1%
		1	0,1%
		1	0,1%
		1	0,1%
		1	0,1%
		1	0,1%
		1	0,1%
		1	0,1%
		1	0,1%
		1	0,1%
		1	0,1%
		1	0,1%
		1	0,1%
		1	0,1%
		1	0,1%
		1	0,1%

		1	0,1%
		1	0,1%
		1	0,1%
		2	0,1%
		1	0,1%
		1	0,1%
		1	0,1%
		1	0,1%
		1	0,1%
		1	0,1%
		1	0,1%
		1	0,1%
		1	0,1%
		1	0,1%
		1	0,1%
		2	0,1%
		1	0,1%
		1	0,1%
		1	0,1%
		1	0,1%
		1	0,1%
		1	0,1%
		1	0,1%
		1	0,1%
		1	0,1%
		1	0,1%
		1	0,1%
		2	0,1%
		1	0,1%
		1	0,1%
		1	0,1%
		1	0,1%
		1	0,1%
		1	0,1%
		1	0,1%
		2	0,1%
		1	0,1%
		1	0,1%
		1	0,1%
		1	0,1%
		1	0,1%
		1	0,1%
		1	0,1%
		1	0,1%
		1	0,1%
		1	0,1%
		1	0,1%
		1	0,1%
		1	0,1%
		1	0,1%
		1	0,1%
		1	0,1%
		1	0,1%
		1	0,1%
		1	0,1%
		1	0,1%
		1	0,1%
		1	0,1%
		1	0,1%
		1	0,1%
		2	0,1%
		1	0,1%
		1	0,1%
		1	0,1%
		2	0,1%
		1	0,1%
		1	0,1%
		1	0,1%
		1	0,1%

		1	0,1%
		1	0,1%
		1	0,1%
		1	0,1%
		1	0,1%
		1	0,1%
		1	0,1%
		1	0,1%
		1	0,1%
		1	0,1%
		1	0,1%
		1	0,1%
		1	0,1%
		1	0,1%
		1	0,1%
		1	0,1%
		1	0,1%
		1	0,1%
		1	0,1%
		1	0,1%
		1	0,1%
		1	0,1%
		1	0,1%
		1	0,1%
		1	0,1%
		1	0,1%
		1	0,1%
		1	0,1%
		1	0,1%
		1	0,1%
		1	0,1%
		1	0,1%
		2	0,1%
		1	0,1%
		1	0,1%
		1	0,1%
		1	0,1%
		1	0,1%
		1	0,1%
		1	0,1%
		1	0,1%
		1	0,1%
		1	0,1%
		1	0,1%
		1	0,1%
		1	0,1%
		2	0,1%
		1	0,1%
		1	0,1%
		1	0,1%
		2	0,1%
		1	0,1%
		1	0,1%
		1	0,1%
		1	0,1%
		1	0,1%
		1	0,1%
		1	0,1%
		2	0,1%
		1	0,1%
		2	0,1%
		1	0,1%
		1	0,1%
		1	0,1%
		1	0,1%
		1	0,1%
		1	0,1%
		1	0,1%

		1	0,1%
		2	0,1%
		1	0,1%
		1	0,1%
		1	0,1%
		1	0,1%
		1	0,1%
		1	0,1%
		1	0,1%
		1	0,1%
		1	0,1%
		1	0,1%
		1	0,1%
		1	0,1%
		1	0,1%
		1	0,1%
		1	0,1%
		1	0,1%
		1	0,1%
		1	0,1%
		1	0,1%
		1	0,1%
		2	0,1%
		2	0,1%
		1	0,1%
		1	0,1%
		1	0,1%
		1	0,1%
		1	0,1%
		1	0,1%
		1	0,1%
		1	0,1%
		2	0,1%
		2	0,1%
		1	0,1%
		1	0,1%
		1	0,1%
		1	0,1%
		1	0,1%
		1	0,1%
		1	0,1%
		2	0,1%
		1	0,1%
		1	0,1%
		1	0,1%
		1	0,1%
		1	0,1%
		1	0,1%
		1	0,1%
		1	0,1%
		1	0,1%
		1	0,1%
		1	0,1%
		1	0,1%
		2	0,1%
		1	0,1%
		1	0,1%
		1	0,1%
		1	0,1%
		1	0,1%
		1	0,1%
		1	0,1%
		1	0,1%
		1	0,1%
		1	0,1%
		1	0,1%
		1	0,1%

		1	0,1%
		2	0,1%
		1	0,1%
		1	0,1%
		1	0,1%
		1	0,1%
		2	0,1%
		1	0,1%
		1	0,1%
		1	0,1%
		1	0,1%
		1	0,1%
		1	0,1%
		1	0,1%
		1	0,1%
		1	0,1%
		1	0,1%
		2	0,1%
		1	0,1%
		2	0,1%
		1	0,1%
		1	0,1%
		1	0,1%
		1	0,1%
		1	0,1%
		1	0,1%
		1	0,1%
		2	0,1%
		1	0,1%
		1	0,1%
		1	0,1%
		2	0,1%
		1	0,1%
		1	0,1%
		1	0,1%
		1	0,1%
		1	0,1%
		1	0,1%
		1	0,1%
		1	0,1%
		1	0,1%
		1	0,1%
		1	0,1%
		1	0,1%
		1	0,1%
		1	0,1%
		1	0,1%
		1	0,1%
		1	0,1%
		1	0,1%
		1	0,1%
		1	0,1%
		1	0,1%
		1	0,1%
		1	0,1%
		1	0,1%
		2	0,1%
		1	0,1%
		1	0,1%
		1	0,1%
		1	0,1%
		1	0,1%
		1	0,1%
		1	0,1%
		1	0,1%
		1	0,1%
		1	0,1%
		1	0,1%

		1	0,1%
		1	0,1%
		1	0,1%
		1	0,1%
		1	0,1%
		1	0,1%
		1	0,1%
		2	0,1%
		1	0,1%
		1	0,1%
		1	0,1%
		1	0,1%
		1	0,1%
		1	0,1%
		1	0,1%
		1	0,1%
		1	0,1%
		1	0,1%
		2	0,1%
		1	0,1%
		1	0,1%
		1	0,1%
		1	0,1%
		1	0,1%
		1	0,1%
		1	0,1%
		1	0,1%
		1	0,1%
		1	0,1%
		1	0,1%
		1	0,1%
		1	0,1%
		1	0,1%
		1	0,1%
		1	0,1%
		1	0,1%
		1	0,1%
		1	0,1%
		1	0,1%
		1	0,1%
		1	0,1%
		1	0,1%
		1	0,1%
		2	0,1%
		1	0,1%
		2	0,1%
		1	0,1%
		1	0,1%
		1	0,1%
		1	0,1%
		1	0,1%
		1	0,1%
		1	0,1%
		1	0,1%
		1	0,1%
		1	0,1%
		2	0,1%
		1	0,1%
		1	0,1%
		1	0,1%
		1	0,1%
		1	0,1%
		1	0,1%
		1	0,1%
		1	0,1%
		1	0,1%
		1	0,1%
		1	0,1%

		2	0,1%
		1	0,1%
		1	0,1%
		1	0,1%
		2	0,1%
		1	0,1%
		1	0,1%
		1	0,1%
		2	0,1%
		2	0,1%
		1	0,1%
		1	0,1%
		1	0,1%
		1	0,1%
		1	0,1%
		1	0,1%
		1	0,1%
		1	0,1%
		1	0,1%
		1	0,1%
		1	0,1%
		1	0,1%
		1	0,1%
		1	0,1%
		1	0,1%
		1	0,1%
		1	0,1%
		1	0,1%
		1	0,1%
		1	0,1%
		1	0,1%
		1	0,1%
		1	0,1%
		1	0,1%
		2	0,1%
		1	0,1%
		1	0,1%
		1	0,1%
		1	0,1%
		1	0,1%
		1	0,1%
		1	0,1%
		1	0,1%
		1	0,1%
		1	0,1%
		1	0,1%
		1	0,1%
		1	0,1%
		1	0,1%
		1	0,1%
		1	0,1%
		1	0,1%
		1	0,1%
		1	0,1%
		1	0,1%
		2	0,1%
		2	0,1%
		1	0,1%
		2	0,1%
		1	0,1%
		2	0,1%
		1	0,1%
		1	0,1%
		1	0,1%
		1	0,1%
		1	0,1%
		1	0,1%
		1	0,1%

		1	0,1%
		1	0,1%
		1	0,1%
		1	0,1%
		1	0,1%
		1	0,1%
		1	0,1%
		1	0,1%
		1	0,1%
		1	0,1%
		2	0,1%
		2	0,1%
		1	0,1%
		1	0,1%
		2	0,1%
		1	0,1%
		1	0,1%
		1	0,1%
		1	0,1%
		1	0,1%
		1	0,1%
		2	0,1%
		1	0,1%
		1	0,1%
		1	0,1%
		1	0,1%
		1	0,1%
		1	0,1%
		1	0,1%
		2	0,1%
		1	0,1%
		1	0,1%
		1	0,1%
		2	0,1%
		1	0,1%
		1	0,1%
		1	0,1%
		1	0,1%
		1	0,1%
		2	0,1%
		1	0,1%
		2	0,1%
		2	0,1%
		1	0,1%
		1	0,1%
		1	0,1%
		1	0,1%
		2	0,1%
		1	0,1%
		2	0,1%
		1	0,1%
		1	0,1%
		1	0,1%
		1	0,1%
		1	0,1%
		1	0,1%
		1	0,1%
		1	0,1%
		1	0,1%
		2	0,1%
		2	0,1%
		1	0,1%
		2	0,1%
		2	0,1%
		1	0,1%
		1	0,1%
		1	0,1%
		1	0,1%

		1	0,1%
		1	0,1%
		1	0,1%
		2	0,1%
		1	0,1%
		1	0,1%
		1	0,1%
		1	0,1%
		2	0,1%
		1	0,1%
		1	0,1%
		1	0,1%
		1	0,1%
		1	0,1%
		2	0,1%
		1	0,1%
		1	0,1%
		1	0,1%
		1	0,1%
		2	0,1%
		1	0,1%
		1	0,1%
		1	0,1%
		1	0,1%
		1	0,1%
		1	0,1%
		1	0,1%
		1	0,1%
		1	0,1%
		2	0,1%
		2	0,1%
		1	0,1%
		1	0,1%
		1	0,1%
		1	0,1%
		1	0,1%
		1	0,1%
		1	0,1%
		1	0,1%
		1	0,1%
		1	0,1%
		1	0,1%
		1	0,1%
		1	0,1%
		1	0,1%
		1	0,1%
		2	0,1%
		1	0,1%
		2	0,1%
		1	0,1%
		2	0,1%
		1	0,1%
		1	0,1%
		1	0,1%
		1	0,1%
		1	0,1%
		1	0,1%
		1	0,1%
		1	0,1%
		1	0,1%
		1	0,1%
		1	0,1%
		1	0,1%
		1	0,1%
		1	0,1%
		1	0,1%
		1	0,1%
		1	0,1%
		1	0,1%

		1	0,1%
		1	0,1%
		1	0,1%
		1	0,1%
		1	0,1%
		1	0,1%
		1	0,1%
		2	0,1%
		1	0,1%
		1	0,1%
		1	0,1%
		1	0,1%
		1	0,1%
		1	0,1%
		1	0,1%
		1	0,1%
		1	0,1%
		1	0,1%
		1	0,1%
		1	0,1%
		1	0,1%
		1	0,1%
		1	0,1%
		1	0,1%
		1	0,1%
		1	0,1%
		1	0,1%
		1	0,1%
		1	0,1%
		1	0,1%
		2	0,1%
		1	0,1%
		1	0,1%
		1	0,1%
		1	0,1%
		1	0,1%
		1	0,1%
		1	0,1%
		1	0,1%
		1	0,1%
		1	0,1%
		1	0,1%
		1	0,1%
		1	0,1%
		1	0,1%
		1	0,1%
		1	0,1%
		1	0,1%
		1	0,1%
		1	0,1%
		2	0,1%
		1	0,1%
		1	0,1%
		1	0,1%
		1	0,1%
		1	0,1%
		1	0,1%
		1	0,1%
		1	0,1%
		2	0,1%
		1	0,1%
		1	0,1%
		1	0,1%
		1	0,1%
		1	0,1%
		1	0,1%
		1	0,1%

		1	0,1%
		1	0,1%
		1	0,1%
		1	0,1%
		1	0,1%
		2	0,1%
		2	0,1%
		1	0,1%
		1	0,1%
		1	0,1%
		1	0,1%
		1	0,1%
		1	0,1%
		1	0,1%
		1	0,1%
		1	0,1%
		1	0,1%
		1	0,1%
		1	0,1%
		1	0,1%
		1	0,1%
		1	0,1%
		2	0,1%
		1	0,1%
		2	0,1%
		1	0,1%
		1	0,1%
		1	0,1%
		1	0,1%
		2	0,1%
		1	0,1%
		1	0,1%
		2	0,1%
		1	0,1%
		1	0,1%
		1	0,1%
		1	0,1%
		1	0,1%
		1	0,1%
		1	0,1%
		2	0,1%
		1	0,1%
		2	0,1%
		2	0,1%
		1	0,1%
		1	0,1%
		1	0,1%
		1	0,1%
		1	0,1%
		1	0,1%
		1	0,1%
		1	0,1%
		2	0,1%
		1	0,1%
		1	0,1%
		1	0,1%
		2	0,1%
		1	0,1%
		1	0,1%
		1	0,1%
		1	0,1%
		1	0,1%
		1	0,1%
		1	0,1%
		1	0,1%
		1	0,1%
		1	0,1%
		1	0,1%

		1	0,1%
		1	0,1%
		1	0,1%
		1	0,1%
		2	0,1%
		1	0,1%
		1	0,1%
		1	0,1%
		2	0,1%
		1	0,1%
		2	0,1%
		1	0,1%
		1	0,1%
		1	0,1%
		2	0,1%
		1	0,1%
		1	0,1%
		1	0,1%
		2	0,1%
		1	0,1%
		1	0,1%
		1	0,1%
		1	0,1%
		1	0,1%
		1	0,1%
		1	0,1%
		1	0,1%
		1	0,1%
		2	0,1%
		1	0,1%
		1	0,1%
		1	0,1%
		1	0,1%
		1	0,1%
		1	0,1%
		1	0,1%
		1	0,1%
		2	0,1%
		1	0,1%
		1	0,1%
		1	0,1%
		1	0,1%
		1	0,1%
		1	0,1%
		1	0,1%
		1	0,1%
		2	0,1%
		1	0,1%
		1	0,1%
		1	0,1%
		1	0,1%
		1	0,1%
		2	0,1%
		1	0,1%
		1	0,1%
		1	0,1%
		1	0,1%
		1	0,1%
		1	0,1%
		1	0,1%
		1	0,1%
		1	0,1%
		1	0,1%
		1	0,1%
		1	0,1%
		1	0,1%
		1	0,1%
		1	0,1%

		1	0,1%
		1	0,1%
		1	0,1%
		1	0,1%
		1	0,1%
		2	0,1%
		2	0,1%
		1	0,1%
		1	0,1%
		1	0,1%
		1	0,1%
		1	0,1%
		1	0,1%
		1	0,1%
		2	0,1%
		1	0,1%
		1	0,1%
		1	0,1%
		1	0,1%
		1	0,1%
		1	0,1%
		1	0,1%
		1	0,1%
		1	0,1%
		1	0,1%
		1	0,1%
		1	0,1%
		1	0,1%
		1	0,1%
		1	0,1%
		1	0,1%
		1	0,1%
		1	0,1%
		1	0,1%
		1	0,1%
		1	0,1%
		1	0,1%
		1	0,1%
		1	0,1%
		1	0,1%
		1	0,1%
		1	0,1%
		2	0,1%
		1	0,1%
		1	0,1%
		1	0,1%
		1	0,1%
		1	0,1%
		1	0,1%
		1	0,1%
		1	0,1%
		1	0,1%
		2	0,1%
		1	0,1%
		2	0,1%
		1	0,1%
		1	0,1%
		1	0,1%
		1	0,1%
		1	0,1%
		1	0,1%
		1	0,1%
		1	0,1%
		1	0,1%
		1	0,1%
		2	0,1%
		1	0,1%
		1	0,1%
		1	0,1%

		2	0,1%
		1	0,1%
		2	0,1%
		1	0,1%
		1	0,1%
		1	0,1%
		1	0,1%
		2	0,1%
		1	0,1%
		1	0,1%
		1	0,1%
		1	0,1%
		2	0,1%
		1	0,1%
		1	0,1%
		1	0,1%
		1	0,1%
		1	0,1%
		1	0,1%
		1	0,1%
		1	0,1%
		1	0,1%
		2	0,1%
		1	0,1%
		1	0,1%
		1	0,1%
		1	0,1%
		1	0,1%
		1	0,1%
		1	0,1%
		1	0,1%
		2	0,1%
		1	0,1%
		1	0,1%
		1	0,1%
		1	0,1%
		1	0,1%
		2	0,1%
		1	0,1%
		1	0,1%
		1	0,1%
		1	0,1%
		1	0,1%
		1	0,1%
		1	0,1%
		1	0,1%
		1	0,1%
		1	0,1%
		1	0,1%
		2	0,1%
		1	0,1%
		1	0,1%
		1	0,1%
		1	0,1%
		1	0,1%
		1	0,1%
		1	0,1%
		1	0,1%
		1	0,1%
		1	0,1%
		1	0,1%
		1	0,1%
		1	0,1%
		2	0,1%
		1	0,1%
		1	0,1%
		1	0,1%

		1	0,1%
		1	0,1%
		1	0,1%
		2	0,1%
		1	0,1%
		1	0,1%
		1	0,1%
		1	0,1%
		1	0,1%
		1	0,1%
		2	0,1%
		1	0,1%
		1	0,1%
		1	0,1%
		1	0,1%
		1	0,1%
		2	0,1%
		2	0,1%
		1	0,1%
		1	0,1%
		1	0,1%
		1	0,1%
		1	0,1%
		1	0,1%
		1	0,1%
		1	0,1%
		1	0,1%
		1	0,1%
		1	0,1%
		1	0,1%
		1	0,1%
		1	0,1%
		1	0,1%
		2	0,1%
		2	0,1%
		1	0,1%
		1	0,1%
		2	0,1%
		1	0,1%
		1	0,1%
		2	0,1%
		1	0,1%
		1	0,1%
		1	0,1%
		1	0,1%
		1	0,1%
		1	0,1%
		1	0,1%
		1	0,1%
		1	0,1%
		1	0,1%
		1	0,1%
		2	0,1%
		1	0,1%
		1	0,1%
		1	0,1%
		1	0,1%
		1	0,1%
		1	0,1%
		1	0,1%
		1	0,1%
		1	0,1%
		1	0,1%
		1	0,1%
		1	0,1%
		1	0,1%
		1	0,1%
		1	0,1%

		1	0,1%
		1	0,1%
		1	0,1%
		1	0,1%
		1	0,1%
		1	0,1%
		1	0,1%
		1	0,1%
		1	0,1%
		1	0,1%
		1	0,1%
		2	0,1%
		1	0,1%
		1	0,1%
		2	0,1%
		1	0,1%
		1	0,1%
		1	0,1%
		2	0,1%
		1	0,1%
		1	0,1%
		2	0,1%
		1	0,1%
		1	0,1%
		1	0,1%
		1	0,1%
		1	0,1%
		1	0,1%
		1	0,1%
		1	0,1%
		1	0,1%
		1	0,1%
		1	0,1%
		1	0,1%
		1	0,1%
		1	0,1%
		2	0,1%
		1	0,1%
		1	0,1%
		1	0,1%
		1	0,1%
		1	0,1%
		1	0,1%
		1	0,1%
		1	0,1%
		1	0,1%
		1	0,1%
		1	0,1%
		1	0,1%
		1	0,1%
		1	0,1%
		1	0,1%
		2	0,1%
		1	0,1%
		1	0,1%
		1	0,1%
		1	0,1%
		2	0,1%
		1	0,1%
		1	0,1%
		1	0,1%
		1	0,1%
		1	0,1%
		1	0,1%
		1	0,1%
		1	0,1%
		1	0,1%
		1	0,1%

		1	0,1%
		1	0,1%
		1	0,1%
		1	0,1%
		1	0,1%
		1	0,1%
		1	0,1%
		1	0,1%
		1	0,1%
		1	0,1%
		2	0,1%
		2	0,1%
		1	0,1%
		1	0,1%
		1	0,1%
		1	0,1%
		1	0,1%
		1	0,1%
		1	0,1%
		1	0,1%
		1	0,1%
		1	0,1%
		1	0,1%
		1	0,1%
		1	0,1%
		1	0,1%
		1	0,1%
		1	0,1%
		1	0,1%
		2	0,1%
		1	0,1%
		1	0,1%
		1	0,1%
		1	0,1%
		1	0,1%
		1	0,1%
		1	0,1%
		1	0,1%
		2	0,1%
		1	0,1%
		1	0,1%
		1	0,1%
		1	0,1%
		1	0,1%
		1	0,1%
		1	0,1%
		1	0,1%
		1	0,1%
		1	0,1%
		1	0,1%
		1	0,1%
		1	0,1%
		1	0,1%
		1	0,1%
		2	0,1%
		1	0,1%
		1	0,1%
		1	0,1%
		1	0,1%
		2	0,1%
		1	0,1%
		1	0,1%
		1	0,1%
		1	0,1%
		1	0,1%
		1	0,1%
		1	0,1%
		1	0,1%

		1	0,1%
		1	0,1%
		1	0,1%
		1	0,1%
		1	0,1%
		1	0,1%
		1	0,1%
		1	0,1%
		1	0,1%
		1	0,1%
		1	0,1%
		1	0,1%
		1	0,1%
		2	0,1%
		1	0,1%
		1	0,1%
		1	0,1%
		1	0,1%
		1	0,1%
		1	0,1%
		1	0,1%
		1	0,1%
		1	0,1%
		1	0,1%
		1	0,1%
		1	0,1%
		1	0,1%
		1	0,1%
		1	0,1%
		1	0,1%
		1	0,1%
		1	0,1%
		2	0,1%
		1	0,1%
		1	0,1%
		1	0,1%
		2	0,1%
		1	0,1%
		1	0,1%
		1	0,1%
		1	0,1%
		1	0,1%
		1	0,1%
		1	0,1%
		2	0,1%
		1	0,1%
		2	0,1%
		1	0,1%
		1	0,1%
		1	0,1%
		2	0,1%
		1	0,1%
		1	0,1%
		1	0,1%
		1	0,1%
		1	0,1%
		2	0,1%
		1	0,1%
		1	0,1%
		2	0,1%
		1	0,1%
		1	0,1%
		2	0,1%
		1	0,1%
		1	0,1%
		1	0,1%
		1	0,1%
		2	0,1%

		1	0,1%
		1	0,1%
		1	0,1%
		1	0,1%
		1	0,1%
		1	0,1%
		1	0,1%
		1	0,1%
		1	0,1%
		1	0,1%
		1	0,1%
		1	0,1%
		1	0,1%
		1	0,1%
		1	0,1%
		1	0,1%
		1	0,1%
		1	0,1%
		1	0,1%
		1	0,1%
		1	0,1%
		1	0,1%
		1	0,1%
		1	0,1%
		1	0,1%
		1	0,1%
		1	0,1%
		1	0,1%
		1	0,1%
		1	0,1%
		1	0,1%
		1	0,1%
		1	0,1%
		1	0,1%
		1	0,1%
		1	0,1%
		1	0,1%
		1	0,1%
		1	0,1%
		1	0,1%
		1	0,1%
		1	0,1%
		1	0,1%
		1	0,1%
		1	0,1%
		1	0,1%
		1	0,1%
		1	0,1%
		1	0,1%
		1	0,1%
		1	0,1%
		1	0,1%
		1	0,1%
		1	0,1%
		1	0,1%
		1	0,1%
		1	0,1%
		1	0,1%
		1	0,1%
		1	0,1%
		1	0,1%
		1	0,1%
Exposure to the pandemia environment	Non-Exposed (2019)	816	42,1%
	Exposed (2020)	1120	57,9%
Gender	Female	1626	84,0%
	Male	310	16,0%

	1936	100,0%
	63446	

Total		65382	

Model Dimension[a]					
		Number of Levels	Covariance Structure	Number of Parameters	Subject Variables
Fixed Effects	Intercept	1		1	
	AsgnGrp	2		1	
	Sex	2		1	
Random Effects	Intercept[b]	1	Variance Components	1	ID
Residual				1	
Total		6		5	

a. Dependent Variable: Duration (days) of work absence due to MENTAL DISEASE in the period.

b. As of version 11.5, the syntax rules for the RANDOM subcommand have changed. Your command syntax may yield results that differ from those produced by prior versions. If you are using version 11 syntax, please consult the current syntax reference guide for more information.

Information Criteria[a]	
-2 Restricted Log Likelihood	18343,973
Akaike's Information Criterion (AIC)	18347,973
Hurvich and Tsai's Criterion (AICC)	18347,979
Bozdogan's Criterion (CAIC)	18361,107
Schwarz's Bayesian Criterion (BIC)	18359,107

The information criteria are displayed in smaller-is-better form.

a. Dependent Variable: Duration (days) of work absence due to MENTAL DISEASE in the period.

Fixed Effects

Type III Tests of Fixed Effects[a]				
Source	Numerator df	Denominator df	F	Sig.
Intercept	1	1745,206	564,543	,000
AsgnGrp	1	1680,051	10,242	,001
Sex	1	1743,641	2,451	,118

a. Dependent Variable: Duration (days) of work absence due to MENTAL DISEASE in the period.

Estimates of Fixed Effects[a]							
Parameter	Estimate	Std. Error	df	t	Sig.	95% Confidence Interval	
						Lower Bound	Upper Bound
Intercept	24,060707	1,673694	1824,212	14,376	,000	20,778149	27,343266
[AsgnGrp=0]	-4,042402	1,263123	1680,051	-3,200	,001	-6,519862	-1,564942
[AsgnGrp=1]	0[b]	0	.	.	.	.	.
[Sex=0]	-2,708456	1,730011	1743,641	-1,566	,118	-6,101572	,684659
[Sex=1]	0[b]	0	.	.	.	.	.

a. Dependent Variable: Duration (days) of work absence due to MENTAL DISEASE in the period.

b. This parameter is set to zero because it is redundant.

Covariance Parameters

Estimates of Covariance Parameters[a]			
Parameter		Estimate	Std. Error
Residual		698,747452	50,320312
Intercept [subject = ID]	Variance	68,501479	45,860717

a. Dependent Variable: Duration (days) of work absence due to MENTAL DISEASE in the period.

Estimated Marginal Means

1. Exposure to the pandemia environment[a]					
Exposure to the pandemia environment	Mean	Std. Error	df	95% Confidence Interval	
				Lower Bound	Upper Bound
Non-Exposed (2019)	18,664	1,132	1924,946	16,444	20,884
Exposed (2020)	22,706	1,016	1913,271	20,714	24,699

a. Dependent Variable: Duration (days) of work absence due to MENTAL DISEASE in the period.

2. Gender[a]					
Gender	Mean	Std. Error	df	95% Confidence Interval	
				Lower Bound	Upper Bound
Female	19,331	,700	1741,223	17,959	20,704
Male	22,040	1,588	1745,049	18,924	25,155

a. Dependent Variable: Duration (days) of work absence due to MENTAL DISEASE in the period.

Gender differences regarding proportions of individuals with work absences due to mental disease

```
* Custom Tables.
CTABLES
 /VLABELS VARIABLES=AbMnt AsgnGrp Sex DISPLAY=DEFAULT
 /TABLE AbMnt [C] BY AsgnGrp [C] > Sex [C][COUNT F40.0, COLPCT.COUNT PCT40.1]
 /CATEGORIES VARIABLES=AbMnt Sex ORDER=A KEY=VALUE EMPTY=INCLUDE TOTAL=YES POSITION=AFTER
 /CATEGORIES VARIABLES=AsgnGrp ORDER=A KEY=VALUE EMPTY=INCLUDE
 /CRITERIA CILEVEL=95.
```

Custom Tables

		Exposure to the pandemia environment											
		Non-Exposed (2019)						Exposed (2020)					
		Gender						Gender					
		Female		Male		Total		Female		Male		Total	
		Count	Column N %	Count	Column N %	Count	Column N %	Count	Column N %	Count	Column N %	Count	Column N %
Any work absence due to MENTAL DISEASE occurred in the period	No	22298	97,02%	9577	98,64%	31875	97,50%	22040	95,90%	9531	98,17%	31571	96,57%
	Yes	684	2,98%	132	1,36%	816	2,50%	942	4,10%	178	1,83%	1120	3,43%
	Total	22982	100,00%	9709	100,00%	32691	100,00%	22982	100,00%	9709	100,00%	32691	100,00%

```
* Chart Builder.
GGRAPH
 /GRAPHDATASET NAME="graphdataset" VARIABLES=AbMnt COUNT()[name="COUNT"] Sex AsgnGrp
   MISSING=LISTWISE REPORTMISSING=NO
 /GRAPHSPEC SOURCE=INLINE.
BEGIN GPL
 SOURCE: s=userSource(id("graphdataset"))
 DATA: AbMnt=col(source(s), name("AbMnt"),
notIn("0"), unit.category())
 DATA: COUNT=col(source(s), name("COUNT"))
 DATA: Sex=col(source(s), name("Sex"), unit.category())
 DATA: AsgnGrp=col(source(s), name("AsgnGrp"), unit.category())
 COORD: rect(dim(1,2), cluster(3,0))
 GUIDE: axis(dim(3), label("Any work absence due to MENTAL DISEASE occurred in the period"))
 GUIDE: axis(dim(2), label("Count"))
 GUIDE: axis(dim(4), label("Exposure to the pandemia environment"), opposite())
 GUIDE: legend(aesthetic(aesthetic.color.interior), label("Gender"))
 GUIDE: text.title(label("Clustered Bar Count of Any work absence due to MENTAL DISEASE ",
   "occurred in the period by Gender by Exposure to the pandemia environment"))
 SCALE: cat(dim(3), include("1"))
 SCALE: linear(dim(2), include(0))
 SCALE: cat(dim(4), include("0", "1"))
 SCALE: cat(aesthetic(aesthetic.color.interior), include("0", "1"))
 SCALE: cat(dim(1), include("0", "1"))
 ELEMENT: interval(position(Sex*COUNT*AbMnt*AsgnGrp), color.interior(Sex),
   shape.interior(shape.square))
END GPL.
```

GGraph

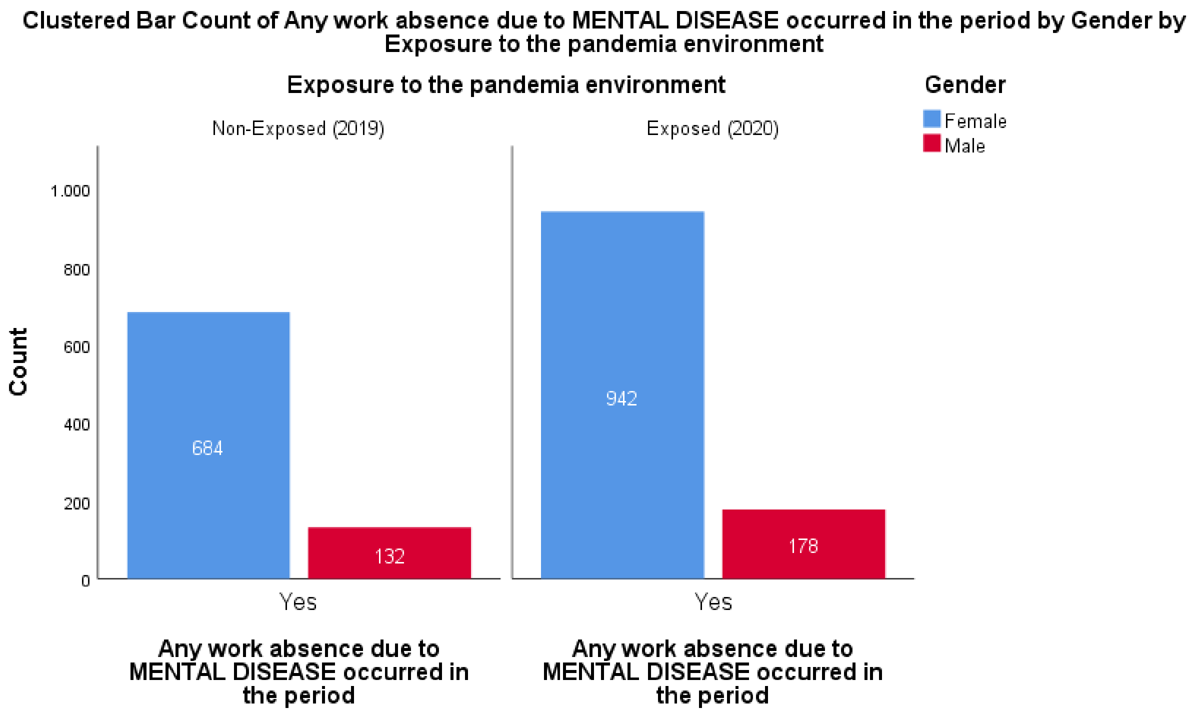


```
* Chart Builder.
GGRAPH
 /GRAPHDATASET NAME="graphdataset" VARIABLES=AbMnt COUNT()[name="COUNT"] Sex AsgnGrp
   MISSING=LISTWISE REPORTMISSING=NO
 /GRAPHSPEC SOURCE=INLINE.
BEGIN GPL
 SOURCE: s=userSource(id("graphdataset"))
 DATA: AbMnt=col(source(s), name("AbMnt"), unit.category())
 DATA: COUNT=col(source(s), name("COUNT"))
 DATA: Sex=col(source(s), name("Sex"), unit.category())
 DATA: AsgnGrp=col(source(s), name("AsgnGrp"), unit.category())
 COORD: rect(dim(1,2), cluster(3,0))
 GUIDE: axis(dim(3), label("Any work absence due to MENTAL DISEASE occurred in the period"))
 GUIDE: axis(dim(2), label("Percent"))
 GUIDE: axis(dim(4), label("Exposure to the pandemia environment"), opposite())
 GUIDE: legend(aesthetic(aesthetic.color.interior), label("Gender"))
 GUIDE: text.title(label("Clustered Bar Percent of Any work absence due to MENTAL DISEASE ",
   "occurred in the period by Gender by Exposure to the pandemia environment"))
 SCALE: cat(dim(3), include("1", "0"), sort.values("1", "0"))
 SCALE: linear(dim(2), include(0))
 SCALE: cat(dim(4), include("0", "1"))
 SCALE: cat(aesthetic(aesthetic.color.interior), include("0", "1"))
 SCALE: cat(dim(1), include("0", "1"))
 ELEMENT: interval(position(summary.percent(Sex*COUNT*AbMnt*AsgnGrp,
   base.aesthetic(aesthetic(aesthetic.color.interior)))), color.interior(Sex),
   shape.interior(shape.square))
END GPL.
```

GGraph

Clustered Bar Percent of Any work absence due to MENTAL DISEASE occurred in the period by Gender by Exposure to the pandemia environment

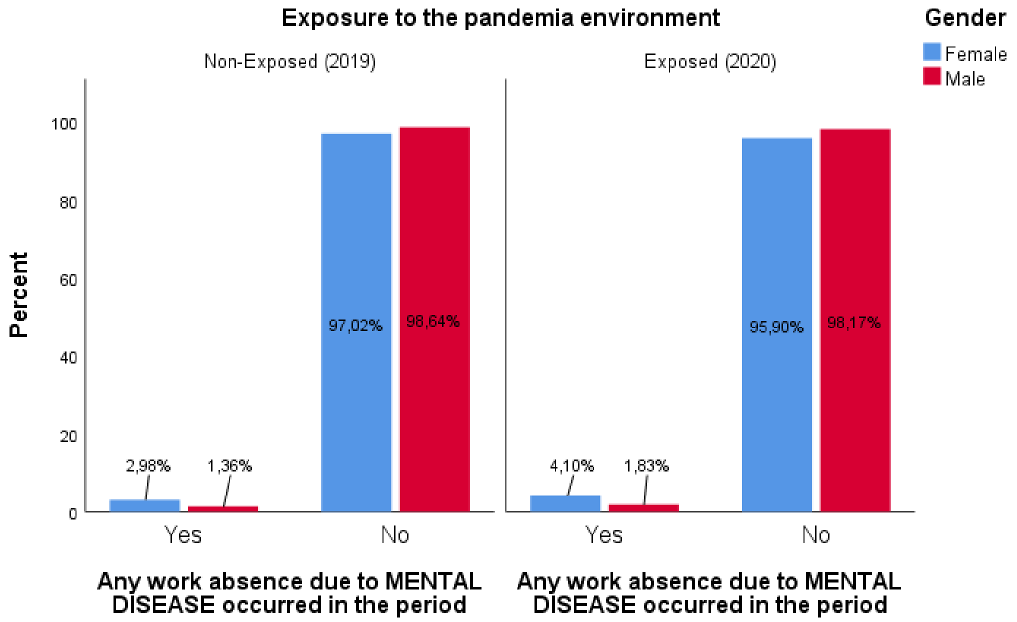


```
* Generalized Estimating Equations.
GENLIN AbMnt (REFERENCE=FIRST) BY AsgnGrp Sex (ORDER=DESCENDING)
 /MODEL AsgnGrp Sex AsgnGrp*Sex INTERCEPT=YES
DISTRIBUTION=BINOMIAL LINK=LOGIT
 /CRITERIA METHOD=FISHER(1) SCALE=1 MAXITERATIONS=100 MAXSTEPHALVING=5 PCONVERGE=1E-006(ABSOLUTE)
   SINGULAR=1E-012 ANALYSISTYPE=3(WALD) CILEVEL=95 LIKELIHOOD=FULL
 /REPEATED SUBJECT=ID WITHINSUBJECT=AsgnGrp SORT=YES CORRTYPE=UNSTRUCTURED ADJUSTCORR=YES
   COVB=ROBUST MAXITERATIONS=100 PCONVERGE=1e-006(ABSOLUTE) UPDATECORR=1
 /MISSING CLASSMISSING=EXCLUDE
 /PRINT CPS DESCRIPTIVES MODELINFO FIT SUMMARY SOLUTION (EXPONENTIATED).
```

Generalized Linear Models

Model Information

Dependent Variable		Any work absence due to MENTAL DISEASE occurred in the period[a]
Probability Distribution		Binomial
Link Function		Logit
Subject Effect	1	ID
Within-Subject Effect	1	Exposure to the pandemia environment
Working Correlation Matrix Structure		Unstructured

a. The procedure models Yes as the response, treating No as the reference category.

Case Processing Summary

	N	Percent
Included	65382	100,0%
Excluded	0	0,0%
Total	65382	100,0%

Correlated Data Summary

Number of Levels	Subject Effect	ID	32691
	Within-Subject Effect	Exposure to the pandemia environment	2
Number of Subjects			32691
Number of Measurements per Subject	Minimum		2
	Maximum		2
Correlation Matrix Dimension			2

Categorical Variable Information				
			N	Percent
Dependent Variable	Any work absence due to MENTAL DISEASE occurred in the period	No	63446	97,0%
		Yes	1936	3,0%
		Total	65382	100,0%
Factor	Exposure to the pandemia environment	Exposed (2020)	32691	50,0%
		Non-Exposed (2019)	32691	50,0%
		Total	65382	100,0%
	Gender	Male	19418	29,7%
		Female	45964	70,3%
		Total	65382	100,0%

Goodness of Fit[a]	
	Value
Quasi Likelihood under Independence Model Criterion (QIC)[b]	17199,741
Corrected Quasi Likelihood under Independence Model Criterion (QICC)[b]	17199,741

Dependent Variable: Any work absence due to MENTAL DISEASE occurred in the period
Model: (Intercept), Exposure to the pandemia environment, Gender, Exposure to the pandemia environment * Gender[a]

a. Information criteria are in smaller-is-better form.

b. Computed using the full log quasi-likelihood function.

Tests of Model Effects			
Source	Type III		
	Wald Chi-Square	df	Sig.
(Intercept)	11826,317	1	,000
Exposure to the pandemia environment	30,463	1	,000
Gender	141,140	1	,000
Exposure to the pandemia environment * Gender	,059	1	,809

Dependent Variable: Any work absence due to MENTAL DISEASE occurred in the period
Model: (Intercept), Exposure to the pandemia environment, Gender, Exposure to the pandemia environment * Gender

Parameter Estimates										
Parameter	B	Std. Error	95% Wald Confidence Interval		Hypothesis Test			Exp(B)	95% Wald Confidence Interval for Exp(B)	
			Lower	Upper	Wald Chi-Square	df	Sig.		Lower	Upper
(Intercept)	-3,484	,0388	-3,560	-3,408	8056,824	1	,000	,031	,028	,033
[Exposure to the pandemia environment=1]	,332	,0468	,240	,423	50,230	1	,000	1,393	1,271	1,527
[Exposure to the pandemia environment=0]	0[a]	.	.	.	.	.	.	1	.	.
[Gender=1]	-,800	,0958	-,988	-,612	69,668	1	,000	,449	,372	,542
[Gender=0]	0[a]	.	.	.	.	.	.	1	.	.
[Exposure to the pandemia environment=1] * [Gender=1]	-,028	,1151	-,254	,198	,059	1	,809	,972	,776	1,219
[Exposure to the pandemia environment=1] * [Gender=0]	0[a]	.	.	.	.	.	.	1	.	.
[Exposure to the pandemia environment=0] * [Gender=1]	0[a]	.	.	.	.	.	.	1	.	.
[Exposure to the pandemia environment=0] * [Gender=0]	0[a]	.	.	.	.	.	.	1	.	.
(Scale)	1									

Dependent Variable: Any work absence due to MENTAL DISEASE occurred in the period
Model: (Intercept), Exposure to the pandemia environment, Gender, Exposure to the pandemia environment * Gender

a. Set to zero because this parameter is redundant.

```
GET
 FILE='C:\Users\trabalho\Documents\COVID-19\Centro  Pesquisa  HUB  COVID19\BDForceII_AbsperInd_Horiz.sav'.
DATASET NAME DataSet1 WINDOW=FRONT.
GET
 FILE='C:\Users\trabalho\Documents\COVID-19\Centro  Pesquisa  HUB  COVID19\BDForceII_AbsperInd_Vertic.sav'.
DATASET NAME DataSet2 WINDOW=FRONT.
```

Supporting Information. Continues in the next file.
