# Supplementary material for: Impact of COVID-19 on the mental health of public university hospital workers in Brazil: A cohort-based analysis of 32,691 workers
Source: PLoS One. 2022 Jun 16;17(6):e0269318. doi: 10.1371/journal.pone.0269318 (PMC9202958; doi:10.1371/journal.pone.0269318)
Supplement: S1 Data — (ZIP) [file Raw study data 2020.pdf › Raw study data 2020.pdf]

EMPRESA BRASILEIRA DE SERVIÇOS HOSPITALARES

Cód. Afast.	Afastamento	Hospital
4	LICENÇA MATERNIDADE - 120 DIAS	CH da Universidade Federal do Ceará - CE
4	LICENÇA MATERNIDADE - 120 DIAS	CH da Universidade Federal do Ceará - CE
4	LICENÇA MATERNIDADE - 120 DIAS	CH da Universidade Federal do Ceará - CE
4	LICENÇA MATERNIDADE - 120 DIAS	CH da Universidade Federal do Ceará - CE
4	LICENÇA MATERNIDADE - 120 DIAS	CH da Universidade Federal do Ceará - CE
4	LICENÇA MATERNIDADE - 120 DIAS	CH da Universidade Federal do Ceará - CE
4	LICENÇA MATERNIDADE - 120 DIAS	CH da Universidade Federal do Ceará - CE
4	LICENÇA MATERNIDADE - 120 DIAS	CH da Universidade Federal do Ceará - CE
4	LICENÇA MATERNIDADE - 120 DIAS	CH da Universidade Federal do Ceará - CE
4	LICENÇA MATERNIDADE - 120 DIAS	CH da Universidade Federal do Ceará - CE
4	LICENÇA MATERNIDADE - 120 DIAS	CH da Universidade Federal do Ceará - CE
4	LICENÇA MATERNIDADE - 120 DIAS	CH da Universidade Federal do Ceará - CE
4	LICENÇA MATERNIDADE - 120 DIAS	CH da Universidade Federal do Ceará - CE
4	LICENÇA MATERNIDADE - 120 DIAS	CH da Universidade Federal do Ceará - CE
4	LICENÇA MATERNIDADE - 120 DIAS	CH da Universidade Federal do Ceará - CE
4	LICENÇA MATERNIDADE - 120 DIAS	CH da Universidade Federal do Ceará - CE
4	LICENÇA MATERNIDADE - 120 DIAS	CH da Universidade Federal do Ceará - CE
4	LICENÇA MATERNIDADE - 120 DIAS	CH da Universidade Federal do Ceará - CE
4	LICENÇA MATERNIDADE - 120 DIAS	CH da Universidade Federal do Ceará - CE
4	LICENÇA MATERNIDADE - 120 DIAS	CH da Universidade Federal do Ceará - CE
4	LICENÇA MATERNIDADE - 120 DIAS	CH da Universidade Federal do Ceará - CE
4	LICENÇA MATERNIDADE - 120 DIAS	CH da Universidade Federal do Ceará - CE
4	LICENÇA MATERNIDADE - 120 DIAS	CH da Universidade Federal do Ceará - CE
4	LICENÇA MATERNIDADE - 120 DIAS	CH da Universidade Federal do Ceará - CE
4	LICENÇA MATERNIDADE - 120 DIAS	CH da Universidade Federal do Ceará - CE
4	LICENÇA MATERNIDADE - 120 DIAS	CH da Universidade Federal do Ceará - CE
4	LICENÇA MATERNIDADE - 120 DIAS	CH da Universidade Federal do Ceará - CE
4	LICENÇA MATERNIDADE - 120 DIAS	CH da Universidade Federal do Ceará - CE
4	LICENÇA MATERNIDADE - 120 DIAS	CH da Universidade Federal do Ceará - CE
4	LICENÇA MATERNIDADE - 120 DIAS	CH da Universidade Federal do Ceará - CE
4	LICENÇA MATERNIDADE - 120 DIAS	CH da Universidade Federal do Ceará - CE
4	LICENÇA MATERNIDADE - 120 DIAS	CH da Universidade Federal do Ceará - CE
4	LICENÇA MATERNIDADE - 120 DIAS	CH da Universidade Federal do Ceará - CE
4	LICENÇA MATERNIDADE - 120 DIAS	CH da Universidade Federal do Ceará - CE
4	LICENÇA MATERNIDADE - 120 DIAS	CH da Universidade Federal do Ceará - CE
4	LICENÇA MATERNIDADE - 120 DIAS	CH da Universidade Federal do Ceará - CE
4	LICENÇA MATERNIDADE - 120 DIAS	CH da Universidade Federal do Ceará - CE
4	LICENÇA MATERNIDADE - 120 DIAS	CH da Universidade Federal do Ceará - CE
4	LICENÇA MATERNIDADE - 120 DIAS	CH da Universidade Federal do Ceará - CE
4	LICENÇA MATERNIDADE - 120 DIAS	CH da Universidade Federal do Ceará - CE
4	LICENÇA MATERNIDADE - 120 DIAS	CH da Universidade Federal do Ceará - CE
4	LICENÇA MATERNIDADE - 120 DIAS	CH da Universidade Federal do Ceará - CE
4	LICENÇA MATERNIDADE - 120 DIAS	CH da Universidade Federal do Ceará - CE
4	LICENÇA MATERNIDADE - 120 DIAS	CH da Universidade Federal do Ceará - CE

4	LICENÇA MATERNIDADE - 120 DIAS	CH da Universidade Federal do Ceará - CE
4	LICENÇA MATERNIDADE - 120 DIAS	CH da Universidade Federal do Ceará - CE
4	LICENÇA MATERNIDADE - 120 DIAS	CH da Universidade Federal do Ceará - CE
4	LICENÇA MATERNIDADE - 120 DIAS	CH da Universidade Federal do Ceará - CE
4	LICENÇA MATERNIDADE - 120 DIAS	CH da Universidade Federal do Ceará - CE
4	LICENÇA MATERNIDADE - 120 DIAS	CH da Universidade Federal do Ceará - CE
4	LICENÇA MATERNIDADE - 120 DIAS	CH da Universidade Federal do Ceará - CE
4	LICENÇA MATERNIDADE - 120 DIAS	CH da Universidade Federal do Ceará - CE
4	LICENÇA MATERNIDADE - 120 DIAS	CH da Universidade Federal do Ceará - CE
4	LICENÇA MATERNIDADE - 120 DIAS	CH da Universidade Federal do Ceará - CE
4	LICENÇA MATERNIDADE - 120 DIAS	CH da Universidade Federal do Ceará - CE
4	LICENÇA MATERNIDADE - 120 DIAS	CH da Universidade Federal do Ceará - CE
4	LICENÇA MATERNIDADE - 120 DIAS	CH da Universidade Federal do Ceará - CE
4	LICENÇA MATERNIDADE - 120 DIAS	CH da Universidade Federal do Ceará - CE
4	LICENÇA MATERNIDADE - 120 DIAS	CH da Universidade Federal do Ceará - CE
4	LICENÇA MATERNIDADE - 120 DIAS	CH da Universidade Federal do Ceará - CE
4	LICENÇA MATERNIDADE - 120 DIAS	CH da Universidade Federal do Ceará - CE
4	LICENÇA MATERNIDADE - 120 DIAS	CH da Universidade Federal do Ceará - CE
4	LICENÇA MATERNIDADE - 120 DIAS	CH da Universidade Federal do Ceará - CE
4	LICENÇA MATERNIDADE - 120 DIAS	CH da Universidade Federal do Ceará - CE
4	LICENÇA MATERNIDADE - 120 DIAS	CH da Universidade Federal do Ceará - CE
4	LICENÇA MATERNIDADE - 120 DIAS	CH da Universidade Federal do Ceará - CE
4	LICENÇA MATERNIDADE - 120 DIAS	CH da Universidade Federal do Ceará - CE
4	LICENÇA MATERNIDADE - 120 DIAS	CH da Universidade Federal do Ceará - CE
4	LICENÇA MATERNIDADE - 120 DIAS	CH da Universidade Federal do Ceará - CE
4	LICENÇA MATERNIDADE - 120 DIAS	CH da Universidade Federal do Ceará - CE
4	LICENÇA MATERNIDADE - 120 DIAS	CH da Universidade Federal do Ceará - CE
5	LICENÇA POR MORTE DE FAMILIAR - CLT E REGULAMENTO DE PESSOAL	CH da Universidade Federal do Ceará - CE
5	LICENÇA POR MORTE DE FAMILIAR - CLT E REGULAMENTO DE PESSOAL	CH da Universidade Federal do Ceará - CE
5	LICENÇA POR MORTE DE FAMILIAR - CLT E REGULAMENTO DE PESSOAL	CH da Universidade Federal do Ceará - CE
5	LICENÇA POR MORTE DE FAMILIAR - CLT E REGULAMENTO DE PESSOAL	CH da Universidade Federal do Ceará - CE
5	LICENÇA POR MORTE DE FAMILIAR - CLT E REGULAMENTO DE PESSOAL	CH da Universidade Federal do Ceará - CE
5	LICENÇA POR MORTE DE FAMILIAR - CLT E REGULAMENTO DE PESSOAL	CH da Universidade Federal do Ceará - CE
5	LICENÇA POR MORTE DE FAMILIAR - CLT E REGULAMENTO DE PESSOAL	CH da Universidade Federal do Ceará - CE
5	LICENÇA POR MORTE DE FAMILIAR - CLT E REGULAMENTO DE PESSOAL	CH da Universidade Federal do Ceará - CE
5	LICENÇA POR MORTE DE FAMILIAR - CLT E REGULAMENTO DE PESSOAL	CH da Universidade Federal do Ceará - CE
5	LICENÇA POR MORTE DE FAMILIAR - CLT E REGULAMENTO DE PESSOAL	CH da Universidade Federal do Ceará - CE

5	LICENÇA POR MORTE DE FAMILIAR - CLT E REGULAMENTO DE PESSOAL	CH da Universidade Federal do Ceará - CE
5	LICENÇA POR MORTE DE FAMILIAR - CLT E REGULAMENTO DE PESSOAL	CH da Universidade Federal do Ceará - CE
5	LICENÇA POR MORTE DE FAMILIAR - CLT E REGULAMENTO DE PESSOAL	CH da Universidade Federal do Ceará - CE
5	LICENÇA POR MORTE DE FAMILIAR - CLT E REGULAMENTO DE PESSOAL	CH da Universidade Federal do Ceará - CE
5	LICENÇA POR MORTE DE FAMILIAR - CLT E REGULAMENTO DE PESSOAL	CH da Universidade Federal do Ceará - CE
5	LICENÇA POR MORTE DE FAMILIAR - CLT E REGULAMENTO DE PESSOAL	CH da Universidade Federal do Ceará - CE
5	LICENÇA POR MORTE DE FAMILIAR - CLT E REGULAMENTO DE PESSOAL	CH da Universidade Federal do Ceará - CE
5	LICENÇA POR MORTE DE FAMILIAR - CLT E REGULAMENTO DE PESSOAL	CH da Universidade Federal do Ceará - CE
5	LICENÇA POR MORTE DE FAMILIAR - CLT E REGULAMENTO DE PESSOAL	CH da Universidade Federal do Ceará - CE
5	LICENÇA POR MORTE DE FAMILIAR - CLT E REGULAMENTO DE PESSOAL	CH da Universidade Federal do Ceará - CE
5	LICENÇA POR MORTE DE FAMILIAR - CLT E REGULAMENTO DE PESSOAL	CH da Universidade Federal do Ceará - CE
5	LICENÇA POR MORTE DE FAMILIAR - CLT E REGULAMENTO DE PESSOAL	CH da Universidade Federal do Ceará - CE
5	LICENÇA POR MORTE DE FAMILIAR - CLT E REGULAMENTO DE PESSOAL	CH da Universidade Federal do Ceará - CE
5	LICENÇA POR MORTE DE FAMILIAR - CLT E REGULAMENTO DE PESSOAL	CH da Universidade Federal do Ceará - CE
5	LICENÇA POR MORTE DE FAMILIAR - CLT E REGULAMENTO DE PESSOAL	CH da Universidade Federal do Ceará - CE
5	LICENÇA POR MORTE DE FAMILIAR - CLT E REGULAMENTO DE PESSOAL	CH da Universidade Federal do Ceará - CE
5	LICENÇA POR MORTE DE FAMILIAR - CLT E REGULAMENTO DE PESSOAL	CH da Universidade Federal do Ceará - CE
5	LICENÇA POR MORTE DE FAMILIAR - CLT E REGULAMENTO DE PESSOAL	CH da Universidade Federal do Ceará - CE
5	LICENÇA POR MORTE DE FAMILIAR - CLT E REGULAMENTO DE PESSOAL	CH da Universidade Federal do Ceará - CE
5	LICENÇA POR MORTE DE FAMILIAR - CLT E REGULAMENTO DE PESSOAL	CH da Universidade Federal do Ceará - CE
5	LICENÇA POR MORTE DE FAMILIAR - CLT E REGULAMENTO DE PESSOAL	CH da Universidade Federal do Ceará - CE
5	LICENÇA POR MORTE DE FAMILIAR - CLT E REGULAMENTO DE PESSOAL	CH da Universidade Federal do Ceará - CE
5	LICENÇA POR MORTE DE FAMILIAR - CLT E REGULAMENTO DE PESSOAL	CH da Universidade Federal do Ceará - CE
5	LICENÇA POR MORTE DE FAMILIAR - CLT E REGULAMENTO DE PESSOAL	CH da Universidade Federal do Ceará - CE
5	LICENÇA POR MORTE DE FAMILIAR - CLT E REGULAMENTO DE PESSOAL	CH da Universidade Federal do Ceará - CE
5	LICENÇA POR MORTE DE FAMILIAR - CLT E REGULAMENTO DE PESSOAL	CH da Universidade Federal do Ceará - CE
5	LICENÇA POR MORTE DE FAMILIAR - CLT E REGULAMENTO DE PESSOAL	CH da Universidade Federal do Ceará - CE
5	LICENÇA POR MORTE DE FAMILIAR - CLT E REGULAMENTO DE PESSOAL	CH da Universidade Federal do Ceará - CE
5	LICENÇA POR MORTE DE FAMILIAR - CLT E REGULAMENTO DE PESSOAL	CH da Universidade Federal do Ceará - CE
5	LICENÇA POR MORTE DE FAMILIAR - CLT E REGULAMENTO DE PESSOAL	CH da Universidade Federal do Ceará - CE
5	LICENÇA POR MORTE DE FAMILIAR - CLT E REGULAMENTO DE PESSOAL	CH da Universidade Federal do Ceará - CE
5	LICENÇA POR MORTE DE FAMILIAR - CLT E REGULAMENTO DE PESSOAL	CH da Universidade Federal do Ceará - CE

5	LICENÇA POR MORTE DE FAMILIAR - CLT E REGULAMENTO DE PESSOAL	CH da Universidade Federal do Ceará - CE
5	LICENÇA POR MORTE DE FAMILIAR - CLT E REGULAMENTO DE PESSOAL	CH da Universidade Federal do Ceará - CE
5	LICENÇA POR MORTE DE FAMILIAR - CLT E REGULAMENTO DE PESSOAL	CH da Universidade Federal do Ceará - CE
5	LICENÇA POR MORTE DE FAMILIAR - CLT E REGULAMENTO DE PESSOAL	CH da Universidade Federal do Ceará - CE
5	LICENÇA POR MORTE DE FAMILIAR - CLT E REGULAMENTO DE PESSOAL	CH da Universidade Federal do Ceará - CE
5	LICENÇA POR MORTE DE FAMILIAR - CLT E REGULAMENTO DE PESSOAL	CH da Universidade Federal do Ceará - CE
5	LICENÇA POR MORTE DE FAMILIAR - CLT E REGULAMENTO DE PESSOAL	CH da Universidade Federal do Ceará - CE
5	LICENÇA POR MORTE DE FAMILIAR - CLT E REGULAMENTO DE PESSOAL	CH da Universidade Federal do Ceará - CE
5	LICENÇA POR MORTE DE FAMILIAR - CLT E REGULAMENTO DE PESSOAL	CH da Universidade Federal do Ceará - CE
5	LICENÇA POR MORTE DE FAMILIAR - CLT E REGULAMENTO DE PESSOAL	CH da Universidade Federal do Ceará - CE
5	LICENÇA POR MORTE DE FAMILIAR - CLT E REGULAMENTO DE PESSOAL	CH da Universidade Federal do Ceará - CE
5	LICENÇA POR MORTE DE FAMILIAR - CLT E REGULAMENTO DE PESSOAL	CH da Universidade Federal do Ceará - CE
5	LICENÇA POR MORTE DE FAMILIAR - CLT E REGULAMENTO DE PESSOAL	CH da Universidade Federal do Ceará - CE
5	LICENÇA POR MORTE DE FAMILIAR - CLT E REGULAMENTO DE PESSOAL	CH da Universidade Federal do Ceará - CE
5	LICENÇA POR MORTE DE FAMILIAR - CLT E REGULAMENTO DE PESSOAL	CH da Universidade Federal do Ceará - CE
5	LICENÇA POR MORTE DE FAMILIAR - CLT E REGULAMENTO DE PESSOAL	CH da Universidade Federal do Ceará - CE
5	LICENÇA POR MORTE DE FAMILIAR - CLT E REGULAMENTO DE PESSOAL	CH da Universidade Federal do Ceará - CE
5	LICENÇA POR MORTE DE FAMILIAR - CLT E REGULAMENTO DE PESSOAL	CH da Universidade Federal do Ceará - CE
5	LICENÇA POR MORTE DE FAMILIAR - CLT E REGULAMENTO DE PESSOAL	CH da Universidade Federal do Ceará - CE
5	LICENÇA POR MORTE DE FAMILIAR - CLT E REGULAMENTO DE PESSOAL	CH da Universidade Federal do Ceará - CE
5	LICENÇA POR MORTE DE FAMILIAR - CLT E REGULAMENTO DE PESSOAL	CH da Universidade Federal do Ceará - CE
5	LICENÇA POR MORTE DE FAMILIAR - CLT E REGULAMENTO DE PESSOAL	CH da Universidade Federal do Ceará - CE
5	LICENÇA POR MORTE DE FAMILIAR - CLT E REGULAMENTO DE PESSOAL	CH da Universidade Federal do Ceará - CE
5	LICENÇA POR MORTE DE FAMILIAR - CLT E REGULAMENTO DE PESSOAL	CH da Universidade Federal do Ceará - CE
5	LICENÇA POR MORTE DE FAMILIAR - CLT E REGULAMENTO DE PESSOAL	CH da Universidade Federal do Ceará - CE
5	LICENÇA POR MORTE DE FAMILIAR - CLT E REGULAMENTO DE PESSOAL	CH da Universidade Federal do Ceará - CE
6	LICENÇA INTERESSE PARTICULAR - REGULAMENTO DE PESSOAL ART 35 VII	CH da Universidade Federal do Ceará - CE
6	LICENÇA INTERESSE PARTICULAR - REGULAMENTO DE PESSOAL ART 35 VII	CH da Universidade Federal do Ceará - CE
6	LICENÇA INTERESSE PARTICULAR - REGULAMENTO DE PESSOAL ART 35 VII	CH da Universidade Federal do Ceará - CE
6	LICENÇA INTERESSE PARTICULAR - REGULAMENTO DE PESSOAL ART 35 VII	CH da Universidade Federal do Ceará - CE
6	LICENÇA INTERESSE PARTICULAR - REGULAMENTO DE PESSOAL ART 35 VII	CH da Universidade Federal do Ceará - CE
6	LICENÇA INTERESSE PARTICULAR - REGULAMENTO DE PESSOAL ART 35 VII	CH da Universidade Federal do Ceará - CE

6	LICENÇA INTERESSE PARTICULAR - REGULAMENTO DE PESSOAL ART 35 VII	CH da Universidade Federal do Ceará - CE
6	LICENÇA INTERESSE PARTICULAR - REGULAMENTO DE PESSOAL ART 35 VII	CH da Universidade Federal do Ceará - CE
6	LICENÇA INTERESSE PARTICULAR - REGULAMENTO DE PESSOAL ART 35 VII	CH da Universidade Federal do Ceará - CE
6	LICENÇA INTERESSE PARTICULAR - REGULAMENTO DE PESSOAL ART 35 VII	CH da Universidade Federal do Ceará - CE
6	LICENÇA INTERESSE PARTICULAR - REGULAMENTO DE PESSOAL ART 35 VII	CH da Universidade Federal do Ceará - CE
6	LICENÇA INTERESSE PARTICULAR - REGULAMENTO DE PESSOAL ART 35 VII	CH da Universidade Federal do Ceará - CE
6	LICENÇA INTERESSE PARTICULAR - REGULAMENTO DE PESSOAL ART 35 VII	CH da Universidade Federal do Ceará - CE
6	LICENÇA INTERESSE PARTICULAR - REGULAMENTO DE PESSOAL ART 35 VII	CH da Universidade Federal do Ceará - CE
6	LICENÇA INTERESSE PARTICULAR - REGULAMENTO DE PESSOAL ART 35 VII	CH da Universidade Federal do Ceará - CE
6	LICENÇA INTERESSE PARTICULAR - REGULAMENTO DE PESSOAL ART 35 VII	CH da Universidade Federal do Ceará - CE
7	ALISTAMENTO ELEITORAL - CLT ART 473 V	CH da Universidade Federal do Ceará - CE
12	LICENÇA MATERNIDADE (COMPLEMENTAR 60 DIAS)	CH da Universidade Federal do Ceará - CE
12	LICENÇA MATERNIDADE (COMPLEMENTAR 60 DIAS)	CH da Universidade Federal do Ceará - CE
12	LICENÇA MATERNIDADE (COMPLEMENTAR 60 DIAS)	CH da Universidade Federal do Ceará - CE
12	LICENÇA MATERNIDADE (COMPLEMENTAR 60 DIAS)	CH da Universidade Federal do Ceará - CE
12	LICENÇA MATERNIDADE (COMPLEMENTAR 60 DIAS)	CH da Universidade Federal do Ceará - CE
12	LICENÇA MATERNIDADE (COMPLEMENTAR 60 DIAS)	CH da Universidade Federal do Ceará - CE
12	LICENÇA MATERNIDADE (COMPLEMENTAR 60 DIAS)	CH da Universidade Federal do Ceará - CE
12	LICENÇA MATERNIDADE (COMPLEMENTAR 60 DIAS)	CH da Universidade Federal do Ceará - CE
12	LICENÇA MATERNIDADE (COMPLEMENTAR 60 DIAS)	CH da Universidade Federal do Ceará - CE
12	LICENÇA MATERNIDADE (COMPLEMENTAR 60 DIAS)	CH da Universidade Federal do Ceará - CE
12	LICENÇA MATERNIDADE (COMPLEMENTAR 60 DIAS)	CH da Universidade Federal do Ceará - CE
12	LICENÇA MATERNIDADE (COMPLEMENTAR 60 DIAS)	CH da Universidade Federal do Ceará - CE
12	LICENÇA MATERNIDADE (COMPLEMENTAR 60 DIAS)	CH da Universidade Federal do Ceará - CE
12	LICENÇA MATERNIDADE (COMPLEMENTAR 60 DIAS)	CH da Universidade Federal do Ceará - CE
12	LICENÇA MATERNIDADE (COMPLEMENTAR 60 DIAS)	CH da Universidade Federal do Ceará - CE
12	LICENÇA MATERNIDADE (COMPLEMENTAR 60 DIAS)	CH da Universidade Federal do Ceará - CE
12	LICENÇA MATERNIDADE (COMPLEMENTAR 60 DIAS)	CH da Universidade Federal do Ceará - CE
12	LICENÇA MATERNIDADE (COMPLEMENTAR 60 DIAS)	CH da Universidade Federal do Ceará - CE
12	LICENÇA MATERNIDADE (COMPLEMENTAR 60 DIAS)	CH da Universidade Federal do Ceará - CE
12	LICENÇA MATERNIDADE (COMPLEMENTAR 60 DIAS)	CH da Universidade Federal do Ceará - CE
12	LICENÇA MATERNIDADE (COMPLEMENTAR 60 DIAS)	CH da Universidade Federal do Ceará - CE
12	LICENÇA MATERNIDADE (COMPLEMENTAR 60 DIAS)	CH da Universidade Federal do Ceará - CE
12	LICENÇA MATERNIDADE (COMPLEMENTAR 60 DIAS)	CH da Universidade Federal do Ceará - CE
12	LICENÇA MATERNIDADE (COMPLEMENTAR 60 DIAS)	CH da Universidade Federal do Ceará - CE
12	LICENÇA MATERNIDADE (COMPLEMENTAR 60 DIAS)	CH da Universidade Federal do Ceará - CE
12	LICENÇA MATERNIDADE (COMPLEMENTAR 60 DIAS)	CH da Universidade Federal do Ceará - CE
12	LICENÇA MATERNIDADE (COMPLEMENTAR 60 DIAS)	CH da Universidade Federal do Ceará - CE
12	LICENÇA MATERNIDADE (COMPLEMENTAR 60 DIAS)	CH da Universidade Federal do Ceará - CE
12	LICENÇA MATERNIDADE (COMPLEMENTAR 60 DIAS)	CH da Universidade Federal do Ceará - CE
12	LICENÇA MATERNIDADE (COMPLEMENTAR 60 DIAS)	CH da Universidade Federal do Ceará - CE
12	LICENÇA MATERNIDADE (COMPLEMENTAR 60 DIAS)	CH da Universidade Federal do Ceará - CE
12	LICENÇA MATERNIDADE (COMPLEMENTAR 60 DIAS)	CH da Universidade Federal do Ceará - CE

12	LICENÇA MATERNIDADE (COMPLEMENTAR 60 DIAS)	CH da Universidade Federal do Ceará - CE
12	LICENÇA MATERNIDADE (COMPLEMENTAR 60 DIAS)	CH da Universidade Federal do Ceará - CE
12	LICENÇA MATERNIDADE (COMPLEMENTAR 60 DIAS)	CH da Universidade Federal do Ceará - CE
12	LICENÇA MATERNIDADE (COMPLEMENTAR 60 DIAS)	CH da Universidade Federal do Ceará - CE
12	LICENÇA MATERNIDADE (COMPLEMENTAR 60 DIAS)	CH da Universidade Federal do Ceará - CE
12	LICENÇA MATERNIDADE (COMPLEMENTAR 60 DIAS)	CH da Universidade Federal do Ceará - CE
12	LICENÇA MATERNIDADE (COMPLEMENTAR 60 DIAS)	CH da Universidade Federal do Ceará - CE
12	LICENÇA MATERNIDADE (COMPLEMENTAR 60 DIAS)	CH da Universidade Federal do Ceará - CE
12	LICENÇA MATERNIDADE (COMPLEMENTAR 60 DIAS)	CH da Universidade Federal do Ceará - CE
12	LICENÇA MATERNIDADE (COMPLEMENTAR 60 DIAS)	CH da Universidade Federal do Ceará - CE
12	LICENÇA MATERNIDADE (COMPLEMENTAR 60 DIAS)	CH da Universidade Federal do Ceará - CE
12	LICENÇA MATERNIDADE (COMPLEMENTAR 60 DIAS)	CH da Universidade Federal do Ceará - CE
12	LICENÇA MATERNIDADE (COMPLEMENTAR 60 DIAS)	CH da Universidade Federal do Ceará - CE
12	LICENÇA MATERNIDADE (COMPLEMENTAR 60 DIAS)	CH da Universidade Federal do Ceará - CE
12	LICENÇA MATERNIDADE (COMPLEMENTAR 60 DIAS)	CH da Universidade Federal do Ceará - CE
12	LICENÇA MATERNIDADE (COMPLEMENTAR 60 DIAS)	CH da Universidade Federal do Ceará - CE
12	LICENÇA MATERNIDADE (COMPLEMENTAR 60 DIAS)	CH da Universidade Federal do Ceará - CE
12	LICENÇA MATERNIDADE (COMPLEMENTAR 60 DIAS)	CH da Universidade Federal do Ceará - CE
12	LICENÇA MATERNIDADE (COMPLEMENTAR 60 DIAS)	CH da Universidade Federal do Ceará - CE
12	LICENÇA MATERNIDADE (COMPLEMENTAR 60 DIAS)	CH da Universidade Federal do Ceará - CE
12	LICENÇA MATERNIDADE (COMPLEMENTAR 60 DIAS)	CH da Universidade Federal do Ceará - CE
12	LICENÇA MATERNIDADE (COMPLEMENTAR 60 DIAS)	CH da Universidade Federal do Ceará - CE
12	LICENÇA MATERNIDADE (COMPLEMENTAR 60 DIAS)	CH da Universidade Federal do Ceará - CE
12	LICENÇA MATERNIDADE (COMPLEMENTAR 60 DIAS)	CH da Universidade Federal do Ceará - CE
12	LICENÇA MATERNIDADE (COMPLEMENTAR 60 DIAS)	CH da Universidade Federal do Ceará - CE
12	LICENÇA MATERNIDADE (COMPLEMENTAR 60 DIAS)	CH da Universidade Federal do Ceará - CE
12	LICENÇA MATERNIDADE (COMPLEMENTAR 60 DIAS)	CH da Universidade Federal do Ceará - CE
14	LICENÇA GALA - CLT ART 473 II	CH da Universidade Federal do Ceará - CE
14	LICENÇA GALA - CLT ART 473 II	CH da Universidade Federal do Ceará - CE
14	LICENÇA GALA - CLT ART 473 II	CH da Universidade Federal do Ceará - CE
14	LICENÇA GALA - CLT ART 473 II	CH da Universidade Federal do Ceará - CE
14	LICENÇA GALA - CLT ART 473 II	CH da Universidade Federal do Ceará - CE
23	CAPACITAÇAO/EVENTO POR INTERESSE DA EMPRESA - ATE 30 DIAS	CH da Universidade Federal do Ceará - CE
23	CAPACITAÇAO/EVENTO POR INTERESSE DA EMPRESA - ATE 30 DIAS	CH da Universidade Federal do Ceará - CE
23	CAPACITAÇAO/EVENTO POR INTERESSE DA EMPRESA - ATE 30 DIAS	CH da Universidade Federal do Ceará - CE
23	CAPACITAÇAO/EVENTO POR INTERESSE DA EMPRESA - ATE 30 DIAS	CH da Universidade Federal do Ceará - CE
23	CAPACITAÇAO/EVENTO POR INTERESSE DA EMPRESA - ATE 30 DIAS	CH da Universidade Federal do Ceará - CE
23	CAPACITAÇAO/EVENTO POR INTERESSE DA EMPRESA - ATE 30 DIAS	CH da Universidade Federal do Ceará - CE
23	CAPACITAÇAO/EVENTO POR INTERESSE DA EMPRESA - ATE 30 DIAS	CH da Universidade Federal do Ceará - CE

23	CAPACITAÇAO/EVENTO POR INTERESSE DA EMPRESA - ATE 30 DIAS	CH da Universidade Federal do Ceará - CE
23	CAPACITAÇAO/EVENTO POR INTERESSE DA EMPRESA - ATE 30 DIAS	CH da Universidade Federal do Ceará - CE
23	CAPACITAÇAO/EVENTO POR INTERESSE DA EMPRESA - ATE 30 DIAS	CH da Universidade Federal do Ceará - CE
23	CAPACITAÇAO/EVENTO POR INTERESSE DA EMPRESA - ATE 30 DIAS	CH da Universidade Federal do Ceará - CE
23	CAPACITAÇAO/EVENTO POR INTERESSE DA EMPRESA - ATE 30 DIAS	CH da Universidade Federal do Ceará - CE
23	CAPACITAÇAO/EVENTO POR INTERESSE DA EMPRESA - ATE 30 DIAS	CH da Universidade Federal do Ceará - CE
23	CAPACITAÇAO/EVENTO POR INTERESSE DA EMPRESA - ATE 30 DIAS	CH da Universidade Federal do Ceará - CE
23	CAPACITAÇAO/EVENTO POR INTERESSE DA EMPRESA - ATE 30 DIAS	CH da Universidade Federal do Ceará - CE
23	CAPACITAÇAO/EVENTO POR INTERESSE DA EMPRESA - ATE 30 DIAS	CH da Universidade Federal do Ceará - CE
23	CAPACITAÇAO/EVENTO POR INTERESSE DA EMPRESA - ATE 30 DIAS	CH da Universidade Federal do Ceará - CE
23	CAPACITAÇAO/EVENTO POR INTERESSE DA EMPRESA - ATE 30 DIAS	CH da Universidade Federal do Ceará - CE
23	CAPACITAÇAO/EVENTO POR INTERESSE DA EMPRESA - ATE 30 DIAS	CH da Universidade Federal do Ceará - CE
23	CAPACITAÇAO/EVENTO POR INTERESSE DA EMPRESA - ATE 30 DIAS	CH da Universidade Federal do Ceará - CE
23	CAPACITAÇAO/EVENTO POR INTERESSE DA EMPRESA - ATE 30 DIAS	CH da Universidade Federal do Ceará - CE
23	CAPACITAÇAO/EVENTO POR INTERESSE DA EMPRESA - ATE 30 DIAS	CH da Universidade Federal do Ceará - CE
23	CAPACITAÇAO/EVENTO POR INTERESSE DA EMPRESA - ATE 30 DIAS	CH da Universidade Federal do Ceará - CE
23	CAPACITAÇAO/EVENTO POR INTERESSE DA EMPRESA - ATE 30 DIAS	CH da Universidade Federal do Ceará - CE
23	CAPACITAÇAO/EVENTO POR INTERESSE DA EMPRESA - ATE 30 DIAS	CH da Universidade Federal do Ceará - CE
23	CAPACITAÇAO/EVENTO POR INTERESSE DA EMPRESA - ATE 30 DIAS	CH da Universidade Federal do Ceará - CE
23	CAPACITAÇAO/EVENTO POR INTERESSE DA EMPRESA - ATE 30 DIAS	CH da Universidade Federal do Ceará - CE
23	CAPACITAÇAO/EVENTO POR INTERESSE DA EMPRESA - ATE 30 DIAS	CH da Universidade Federal do Ceará - CE
23	CAPACITAÇAO/EVENTO POR INTERESSE DA EMPRESA - ATE 30 DIAS	CH da Universidade Federal do Ceará - CE
23	CAPACITAÇAO/EVENTO POR INTERESSE DA EMPRESA - ATE 30 DIAS	CH da Universidade Federal do Ceará - CE
23	CAPACITAÇAO/EVENTO POR INTERESSE DA EMPRESA - ATE 30 DIAS	CH da Universidade Federal do Ceará - CE
23	CAPACITAÇAO/EVENTO POR INTERESSE DA EMPRESA - ATE 30 DIAS	CH da Universidade Federal do Ceará - CE
23	CAPACITAÇAO/EVENTO POR INTERESSE DA EMPRESA - ATE 30 DIAS	CH da Universidade Federal do Ceará - CE
23	CAPACITAÇAO/EVENTO POR INTERESSE DA EMPRESA - ATE 30 DIAS	CH da Universidade Federal do Ceará - CE
23	CAPACITAÇAO/EVENTO POR INTERESSE DA EMPRESA - ATE 30 DIAS	CH da Universidade Federal do Ceará - CE
23	CAPACITAÇAO/EVENTO POR INTERESSE DA EMPRESA - ATE 30 DIAS	CH da Universidade Federal do Ceará - CE
23	CAPACITAÇAO/EVENTO POR INTERESSE DA EMPRESA - ATE 30 DIAS	CH da Universidade Federal do Ceará - CE
23	CAPACITAÇAO/EVENTO POR INTERESSE DA EMPRESA - ATE 30 DIAS	CH da Universidade Federal do Ceará - CE
23	CAPACITAÇAO/EVENTO POR INTERESSE DA EMPRESA - ATE 30 DIAS	CH da Universidade Federal do Ceará - CE

23	CAPACITAÇAO/EVENTO POR INTERESSE DA EMPRESA - ATE 30 DIAS	CH da Universidade Federal do Ceará - CE
23	CAPACITAÇAO/EVENTO POR INTERESSE DA EMPRESA - ATE 30 DIAS	CH da Universidade Federal do Ceará - CE
23	CAPACITAÇAO/EVENTO POR INTERESSE DA EMPRESA - ATE 30 DIAS	CH da Universidade Federal do Ceará - CE
23	CAPACITAÇAO/EVENTO POR INTERESSE DA EMPRESA - ATE 30 DIAS	CH da Universidade Federal do Ceará - CE
23	CAPACITAÇAO/EVENTO POR INTERESSE DA EMPRESA - ATE 30 DIAS	CH da Universidade Federal do Ceará - CE
23	CAPACITAÇAO/EVENTO POR INTERESSE DA EMPRESA - ATE 30 DIAS	CH da Universidade Federal do Ceará - CE
23	CAPACITAÇAO/EVENTO POR INTERESSE DA EMPRESA - ATE 30 DIAS	CH da Universidade Federal do Ceará - CE
23	CAPACITAÇAO/EVENTO POR INTERESSE DA EMPRESA - ATE 30 DIAS	CH da Universidade Federal do Ceará - CE
23	CAPACITAÇAO/EVENTO POR INTERESSE DA EMPRESA - ATE 30 DIAS	CH da Universidade Federal do Ceará - CE
23	CAPACITAÇAO/EVENTO POR INTERESSE DA EMPRESA - ATE 30 DIAS	CH da Universidade Federal do Ceará - CE
23	CAPACITAÇAO/EVENTO POR INTERESSE DA EMPRESA - ATE 30 DIAS	CH da Universidade Federal do Ceará - CE
23	CAPACITAÇAO/EVENTO POR INTERESSE DA EMPRESA - ATE 30 DIAS	CH da Universidade Federal do Ceará - CE
23	CAPACITAÇAO/EVENTO POR INTERESSE DA EMPRESA - ATE 30 DIAS	CH da Universidade Federal do Ceará - CE
23	CAPACITAÇAO/EVENTO POR INTERESSE DA EMPRESA - ATE 30 DIAS	CH da Universidade Federal do Ceará - CE
23	CAPACITAÇAO/EVENTO POR INTERESSE DA EMPRESA - ATE 30 DIAS	CH da Universidade Federal do Ceará - CE
23	CAPACITAÇAO/EVENTO POR INTERESSE DA EMPRESA - ATE 30 DIAS	CH da Universidade Federal do Ceará - CE
23	CAPACITAÇAO/EVENTO POR INTERESSE DA EMPRESA - ATE 30 DIAS	CH da Universidade Federal do Ceará - CE
23	CAPACITAÇAO/EVENTO POR INTERESSE DA EMPRESA - ATE 30 DIAS	CH da Universidade Federal do Ceará - CE
23	CAPACITAÇAO/EVENTO POR INTERESSE DA EMPRESA - ATE 30 DIAS	CH da Universidade Federal do Ceará - CE
23	CAPACITAÇAO/EVENTO POR INTERESSE DA EMPRESA - ATE 30 DIAS	CH da Universidade Federal do Ceará - CE
23	CAPACITAÇAO/EVENTO POR INTERESSE DA EMPRESA - ATE 30 DIAS	CH da Universidade Federal do Ceará - CE
23	CAPACITAÇAO/EVENTO POR INTERESSE DA EMPRESA - ATE 30 DIAS	CH da Universidade Federal do Ceará - CE
23	CAPACITAÇAO/EVENTO POR INTERESSE DA EMPRESA - ATE 30 DIAS	CH da Universidade Federal do Ceará - CE
23	CAPACITAÇAO/EVENTO POR INTERESSE DA EMPRESA - ATE 30 DIAS	CH da Universidade Federal do Ceará - CE
23	CAPACITAÇAO/EVENTO POR INTERESSE DA EMPRESA - ATE 30 DIAS	CH da Universidade Federal do Ceará - CE
23	CAPACITAÇAO/EVENTO POR INTERESSE DA EMPRESA - ATE 30 DIAS	CH da Universidade Federal do Ceará - CE
23	CAPACITAÇAO/EVENTO POR INTERESSE DA EMPRESA - ATE 30 DIAS	CH da Universidade Federal do Ceará - CE
23	CAPACITAÇAO/EVENTO POR INTERESSE DA EMPRESA - ATE 30 DIAS	CH da Universidade Federal do Ceará - CE
23	CAPACITAÇAO/EVENTO POR INTERESSE DA EMPRESA - ATE 30 DIAS	CH da Universidade Federal do Ceará - CE
23	CAPACITAÇAO/EVENTO POR INTERESSE DA EMPRESA - ATE 30 DIAS	CH da Universidade Federal do Ceará - CE
23	CAPACITAÇAO/EVENTO POR INTERESSE DA EMPRESA - ATE 30 DIAS	CH da Universidade Federal do Ceará - CE
23	CAPACITAÇAO/EVENTO POR INTERESSE DA EMPRESA - ATE 30 DIAS	CH da Universidade Federal do Ceará - CE

23	CAPACITAÇAO/EVENTO POR INTERESSE DA EMPRESA - ATE 30 DIAS	CH da Universidade Federal do Ceará - CE
23	CAPACITAÇAO/EVENTO POR INTERESSE DA EMPRESA - ATE 30 DIAS	CH da Universidade Federal do Ceará - CE
23	CAPACITAÇAO/EVENTO POR INTERESSE DA EMPRESA - ATE 30 DIAS	CH da Universidade Federal do Ceará - CE
23	CAPACITAÇAO/EVENTO POR INTERESSE DA EMPRESA - ATE 30 DIAS	CH da Universidade Federal do Ceará - CE
23	CAPACITAÇAO/EVENTO POR INTERESSE DA EMPRESA - ATE 30 DIAS	CH da Universidade Federal do Ceará - CE
23	CAPACITAÇAO/EVENTO POR INTERESSE DA EMPRESA - ATE 30 DIAS	CH da Universidade Federal do Ceará - CE
23	CAPACITAÇAO/EVENTO POR INTERESSE DA EMPRESA - ATE 30 DIAS	CH da Universidade Federal do Ceará - CE
23	CAPACITAÇAO/EVENTO POR INTERESSE DA EMPRESA - ATE 30 DIAS	CH da Universidade Federal do Ceará - CE
23	CAPACITAÇAO/EVENTO POR INTERESSE DA EMPRESA - ATE 30 DIAS	CH da Universidade Federal do Ceará - CE
23	CAPACITAÇAO/EVENTO POR INTERESSE DA EMPRESA - ATE 30 DIAS	CH da Universidade Federal do Ceará - CE
23	CAPACITAÇAO/EVENTO POR INTERESSE DA EMPRESA - ATE 30 DIAS	CH da Universidade Federal do Ceará - CE
23	CAPACITAÇAO/EVENTO POR INTERESSE DA EMPRESA - ATE 30 DIAS	CH da Universidade Federal do Ceará - CE
23	CAPACITAÇAO/EVENTO POR INTERESSE DA EMPRESA - ATE 30 DIAS	CH da Universidade Federal do Ceará - CE
23	CAPACITAÇAO/EVENTO POR INTERESSE DA EMPRESA - ATE 30 DIAS	CH da Universidade Federal do Ceará - CE
23	CAPACITAÇAO/EVENTO POR INTERESSE DA EMPRESA - ATE 30 DIAS	CH da Universidade Federal do Ceará - CE
23	CAPACITAÇAO/EVENTO POR INTERESSE DA EMPRESA - ATE 30 DIAS	CH da Universidade Federal do Ceará - CE
23	CAPACITAÇAO/EVENTO POR INTERESSE DA EMPRESA - ATE 30 DIAS	CH da Universidade Federal do Ceará - CE
23	CAPACITAÇAO/EVENTO POR INTERESSE DA EMPRESA - ATE 30 DIAS	CH da Universidade Federal do Ceará - CE
23	CAPACITAÇAO/EVENTO POR INTERESSE DA EMPRESA - ATE 30 DIAS	CH da Universidade Federal do Ceará - CE
23	CAPACITAÇAO/EVENTO POR INTERESSE DA EMPRESA - ATE 30 DIAS	CH da Universidade Federal do Ceará - CE
23	CAPACITAÇAO/EVENTO POR INTERESSE DA EMPRESA - ATE 30 DIAS	CH da Universidade Federal do Ceará - CE
23	CAPACITAÇAO/EVENTO POR INTERESSE DA EMPRESA - ATE 30 DIAS	CH da Universidade Federal do Ceará - CE
23	CAPACITAÇAO/EVENTO POR INTERESSE DA EMPRESA - ATE 30 DIAS	CH da Universidade Federal do Ceará - CE
23	CAPACITAÇAO/EVENTO POR INTERESSE DA EMPRESA - ATE 30 DIAS	CH da Universidade Federal do Ceará - CE
23	CAPACITAÇAO/EVENTO POR INTERESSE DA EMPRESA - ATE 30 DIAS	CH da Universidade Federal do Ceará - CE
23	CAPACITAÇAO/EVENTO POR INTERESSE DA EMPRESA - ATE 30 DIAS	CH da Universidade Federal do Ceará - CE
23	CAPACITAÇAO/EVENTO POR INTERESSE DA EMPRESA - ATE 30 DIAS	CH da Universidade Federal do Ceará - CE
23	CAPACITAÇAO/EVENTO POR INTERESSE DA EMPRESA - ATE 30 DIAS	CH da Universidade Federal do Ceará - CE
23	CAPACITAÇAO/EVENTO POR INTERESSE DA EMPRESA - ATE 30 DIAS	CH da Universidade Federal do Ceará - CE
23	CAPACITAÇAO/EVENTO POR INTERESSE DA EMPRESA - ATE 30 DIAS	CH da Universidade Federal do Ceará - CE
23	CAPACITAÇAO/EVENTO POR INTERESSE DA EMPRESA - ATE 30 DIAS	CH da Universidade Federal do Ceará - CE
23	CAPACITAÇAO/EVENTO POR INTERESSE DA EMPRESA - ATE 30 DIAS	CH da Universidade Federal do Ceará - CE

23	CAPACITAÇAO/EVENTO POR INTERESSE DA EMPRESA - ATE 30 DIAS	CH da Universidade Federal do Ceará - CE
23	CAPACITAÇAO/EVENTO POR INTERESSE DA EMPRESA - ATE 30 DIAS	CH da Universidade Federal do Ceará - CE
23	CAPACITAÇAO/EVENTO POR INTERESSE DA EMPRESA - ATE 30 DIAS	CH da Universidade Federal do Ceará - CE
23	CAPACITAÇAO/EVENTO POR INTERESSE DA EMPRESA - ATE 30 DIAS	CH da Universidade Federal do Ceará - CE
23	CAPACITAÇAO/EVENTO POR INTERESSE DA EMPRESA - ATE 30 DIAS	CH da Universidade Federal do Ceará - CE
23	CAPACITAÇAO/EVENTO POR INTERESSE DA EMPRESA - ATE 30 DIAS	CH da Universidade Federal do Ceará - CE
23	CAPACITAÇAO/EVENTO POR INTERESSE DA EMPRESA - ATE 30 DIAS	CH da Universidade Federal do Ceará - CE
23	CAPACITAÇAO/EVENTO POR INTERESSE DA EMPRESA - ATE 30 DIAS	CH da Universidade Federal do Ceará - CE
23	CAPACITAÇAO/EVENTO POR INTERESSE DA EMPRESA - ATE 30 DIAS	CH da Universidade Federal do Ceará - CE
23	CAPACITAÇAO/EVENTO POR INTERESSE DA EMPRESA - ATE 30 DIAS	CH da Universidade Federal do Ceará - CE
23	CAPACITAÇAO/EVENTO POR INTERESSE DA EMPRESA - ATE 30 DIAS	CH da Universidade Federal do Ceará - CE
23	CAPACITAÇAO/EVENTO POR INTERESSE DA EMPRESA - ATE 30 DIAS	CH da Universidade Federal do Ceará - CE
23	CAPACITAÇAO/EVENTO POR INTERESSE DA EMPRESA - ATE 30 DIAS	CH da Universidade Federal do Ceará - CE
23	CAPACITAÇAO/EVENTO POR INTERESSE DA EMPRESA - ATE 30 DIAS	CH da Universidade Federal do Ceará - CE
23	CAPACITAÇAO/EVENTO POR INTERESSE DA EMPRESA - ATE 30 DIAS	CH da Universidade Federal do Ceará - CE
23	CAPACITAÇAO/EVENTO POR INTERESSE DA EMPRESA - ATE 30 DIAS	CH da Universidade Federal do Ceará - CE
23	CAPACITAÇAO/EVENTO POR INTERESSE DA EMPRESA - ATE 30 DIAS	CH da Universidade Federal do Ceará - CE
23	CAPACITAÇAO/EVENTO POR INTERESSE DA EMPRESA - ATE 30 DIAS	CH da Universidade Federal do Ceará - CE
23	CAPACITAÇAO/EVENTO POR INTERESSE DA EMPRESA - ATE 30 DIAS	CH da Universidade Federal do Ceará - CE
23	CAPACITAÇAO/EVENTO POR INTERESSE DA EMPRESA - ATE 30 DIAS	CH da Universidade Federal do Ceará - CE
23	CAPACITAÇAO/EVENTO POR INTERESSE DA EMPRESA - ATE 30 DIAS	CH da Universidade Federal do Ceará - CE
23	CAPACITAÇAO/EVENTO POR INTERESSE DA EMPRESA - ATE 30 DIAS	CH da Universidade Federal do Ceará - CE
23	CAPACITAÇAO/EVENTO POR INTERESSE DA EMPRESA - ATE 30 DIAS	CH da Universidade Federal do Ceará - CE
24	PARTICIPAÇAO EM REUNIOES DA COMISSAO DE NEGOCIAÇAO - REG. PESSOAL ART 36 VI	CH da Universidade Federal do Ceará - CE
24	PARTICIPAÇAO EM REUNIOES DA COMISSAO DE NEGOCIAÇAO - REG. PESSOAL ART 36 VI	CH da Universidade Federal do Ceará - CE
36	AUXILIO DOENÇA (INSS) - A PARTIR DO 16 DIA	CH da Universidade Federal do Ceará - CE
36	AUXILIO DOENÇA (INSS) - A PARTIR DO 16 DIA	CH da Universidade Federal do Ceará - CE
36	AUXILIO DOENÇA (INSS) - A PARTIR DO 16 DIA	CH da Universidade Federal do Ceará - CE
36	AUXILIO DOENÇA (INSS) - A PARTIR DO 16 DIA	CH da Universidade Federal do Ceará - CE
36	AUXILIO DOENÇA (INSS) - A PARTIR DO 16 DIA	CH da Universidade Federal do Ceará - CE
36	AUXILIO DOENÇA (INSS) - A PARTIR DO 16 DIA	CH da Universidade Federal do Ceará - CE
36	AUXILIO DOENÇA (INSS) - A PARTIR DO 16 DIA	CH da Universidade Federal do Ceará - CE
36	AUXILIO DOENÇA (INSS) - A PARTIR DO 16 DIA	CH da Universidade Federal do Ceará - CE
36	AUXILIO DOENÇA (INSS) - A PARTIR DO 16 DIA	CH da Universidade Federal do Ceará - CE
36	AUXILIO DOENÇA (INSS) - A PARTIR DO 16 DIA	CH da Universidade Federal do Ceará - CE

36	AUXILIO DOENÇA (INSS) - A PARTIR DO 16 DIA	CH da Universidade Federal do Ceará - CE
36	AUXILIO DOENÇA (INSS) - A PARTIR DO 16 DIA	CH da Universidade Federal do Ceará - CE
36	AUXILIO DOENÇA (INSS) - A PARTIR DO 16 DIA	CH da Universidade Federal do Ceará - CE
36	AUXILIO DOENÇA (INSS) - A PARTIR DO 16 DIA	CH da Universidade Federal do Ceará - CE
36	AUXILIO DOENÇA (INSS) - A PARTIR DO 16 DIA	CH da Universidade Federal do Ceará - CE
36	AUXILIO DOENÇA (INSS) - A PARTIR DO 16 DIA	CH da Universidade Federal do Ceará - CE
36	AUXILIO DOENÇA (INSS) - A PARTIR DO 16 DIA	CH da Universidade Federal do Ceará - CE
36	AUXILIO DOENÇA (INSS) - A PARTIR DO 16 DIA	CH da Universidade Federal do Ceará - CE
36	AUXILIO DOENÇA (INSS) - A PARTIR DO 16 DIA	CH da Universidade Federal do Ceará - CE
36	AUXILIO DOENÇA (INSS) - A PARTIR DO 16 DIA	CH da Universidade Federal do Ceará - CE
36	AUXILIO DOENÇA (INSS) - A PARTIR DO 16 DIA	CH da Universidade Federal do Ceará - CE
36	AUXILIO DOENÇA (INSS) - A PARTIR DO 16 DIA	CH da Universidade Federal do Ceará - CE
36	AUXILIO DOENÇA (INSS) - A PARTIR DO 16 DIA	CH da Universidade Federal do Ceará - CE
36	AUXILIO DOENÇA (INSS) - A PARTIR DO 16 DIA	CH da Universidade Federal do Ceará - CE
36	AUXILIO DOENÇA (INSS) - A PARTIR DO 16 DIA	CH da Universidade Federal do Ceará - CE
36	AUXILIO DOENÇA (INSS) - A PARTIR DO 16 DIA	CH da Universidade Federal do Ceará - CE
36	AUXILIO DOENÇA (INSS) - A PARTIR DO 16 DIA	CH da Universidade Federal do Ceará - CE
36	AUXILIO DOENÇA (INSS) - A PARTIR DO 16 DIA	CH da Universidade Federal do Ceará - CE
36	AUXILIO DOENÇA (INSS) - A PARTIR DO 16 DIA	CH da Universidade Federal do Ceará - CE
36	AUXILIO DOENÇA (INSS) - A PARTIR DO 16 DIA	CH da Universidade Federal do Ceará - CE
36	AUXILIO DOENÇA (INSS) - A PARTIR DO 16 DIA	CH da Universidade Federal do Ceará - CE
36	AUXILIO DOENÇA (INSS) - A PARTIR DO 16 DIA	CH da Universidade Federal do Ceará - CE
36	AUXILIO DOENÇA (INSS) - A PARTIR DO 16 DIA	CH da Universidade Federal do Ceará - CE
36	AUXILIO DOENÇA (INSS) - A PARTIR DO 16 DIA	CH da Universidade Federal do Ceará - CE
36	AUXILIO DOENÇA (INSS) - A PARTIR DO 16 DIA	CH da Universidade Federal do Ceará - CE
36	AUXILIO DOENÇA (INSS) - A PARTIR DO 16 DIA	CH da Universidade Federal do Ceará - CE
36	AUXILIO DOENÇA (INSS) - A PARTIR DO 16 DIA	CH da Universidade Federal do Ceará - CE
36	AUXILIO DOENÇA (INSS) - A PARTIR DO 16 DIA	CH da Universidade Federal do Ceará - CE
36	AUXILIO DOENÇA (INSS) - A PARTIR DO 16 DIA	CH da Universidade Federal do Ceará - CE
36	AUXILIO DOENÇA (INSS) - A PARTIR DO 16 DIA	CH da Universidade Federal do Ceará - CE
36	AUXILIO DOENÇA (INSS) - A PARTIR DO 16 DIA	CH da Universidade Federal do Ceará - CE
36	AUXILIO DOENÇA (INSS) - A PARTIR DO 16 DIA	CH da Universidade Federal do Ceará - CE
36	AUXILIO DOENÇA (INSS) - A PARTIR DO 16 DIA	CH da Universidade Federal do Ceará - CE
36	AUXILIO DOENÇA (INSS) - A PARTIR DO 16 DIA	CH da Universidade Federal do Ceará - CE
36	AUXILIO DOENÇA (INSS) - A PARTIR DO 16 DIA	CH da Universidade Federal do Ceará - CE
36	AUXILIO DOENÇA (INSS) - A PARTIR DO 16 DIA	CH da Universidade Federal do Ceará - CE
36	AUXILIO DOENÇA (INSS) - A PARTIR DO 16 DIA	CH da Universidade Federal do Ceará - CE
36	AUXILIO DOENÇA (INSS) - A PARTIR DO 16 DIA	CH da Universidade Federal do Ceará - CE
36	AUXILIO DOENÇA (INSS) - A PARTIR DO 16 DIA	CH da Universidade Federal do Ceará - CE
36	AUXILIO DOENÇA (INSS) - A PARTIR DO 16 DIA	CH da Universidade Federal do Ceará - CE
36	AUXILIO DOENÇA (INSS) - A PARTIR DO 16 DIA	CH da Universidade Federal do Ceará - CE
36	AUXILIO DOENÇA (INSS) - A PARTIR DO 16 DIA	CH da Universidade Federal do Ceará - CE
36	AUXILIO DOENÇA (INSS) - A PARTIR DO 16 DIA	CH da Universidade Federal do Ceará - CE
36	AUXILIO DOENÇA (INSS) - A PARTIR DO 16 DIA	CH da Universidade Federal do Ceará - CE
36	AUXILIO DOENÇA (INSS) - A PARTIR DO 16 DIA	CH da Universidade Federal do Ceará - CE
36	AUXILIO DOENÇA (INSS) - A PARTIR DO 16 DIA	CH da Universidade Federal do Ceará - CE
36	AUXILIO DOENÇA (INSS) - A PARTIR DO 16 DIA	CH da Universidade Federal do Ceará - CE
36	AUXILIO DOENÇA (INSS) - A PARTIR DO 16 DIA	CH da Universidade Federal do Ceará - CE

36	AUXILIO DOENÇA (INSS) - A PARTIR DO 16 DIA	CH da Universidade Federal do Ceará - CE
36	AUXILIO DOENÇA (INSS) - A PARTIR DO 16 DIA	CH da Universidade Federal do Ceará - CE
36	AUXILIO DOENÇA (INSS) - A PARTIR DO 16 DIA	CH da Universidade Federal do Ceará - CE
36	AUXILIO DOENÇA (INSS) - A PARTIR DO 16 DIA	CH da Universidade Federal do Ceará - CE
36	AUXILIO DOENÇA (INSS) - A PARTIR DO 16 DIA	CH da Universidade Federal do Ceará - CE
36	AUXILIO DOENÇA (INSS) - A PARTIR DO 16 DIA	CH da Universidade Federal do Ceará - CE
36	AUXILIO DOENÇA (INSS) - A PARTIR DO 16 DIA	CH da Universidade Federal do Ceará - CE
36	AUXILIO DOENÇA (INSS) - A PARTIR DO 16 DIA	CH da Universidade Federal do Ceará - CE
36	AUXILIO DOENÇA (INSS) - A PARTIR DO 16 DIA	CH da Universidade Federal do Ceará - CE
36	AUXILIO DOENÇA (INSS) - A PARTIR DO 16 DIA	CH da Universidade Federal do Ceará - CE
36	AUXILIO DOENÇA (INSS) - A PARTIR DO 16 DIA	CH da Universidade Federal do Ceará - CE
36	AUXILIO DOENÇA (INSS) - A PARTIR DO 16 DIA	CH da Universidade Federal do Ceará - CE
36	AUXILIO DOENÇA (INSS) - A PARTIR DO 16 DIA	CH da Universidade Federal do Ceará - CE
36	AUXILIO DOENÇA (INSS) - A PARTIR DO 16 DIA	CH da Universidade Federal do Ceará - CE
36	AUXILIO DOENÇA (INSS) - A PARTIR DO 16 DIA	CH da Universidade Federal do Ceará - CE
36	AUXILIO DOENÇA (INSS) - A PARTIR DO 16 DIA	CH da Universidade Federal do Ceará - CE
36	AUXILIO DOENÇA (INSS) - A PARTIR DO 16 DIA	CH da Universidade Federal do Ceará - CE
36	AUXILIO DOENÇA (INSS) - A PARTIR DO 16 DIA	CH da Universidade Federal do Ceará - CE
36	AUXILIO DOENÇA (INSS) - A PARTIR DO 16 DIA	CH da Universidade Federal do Ceará - CE
36	AUXILIO DOENÇA (INSS) - A PARTIR DO 16 DIA	CH da Universidade Federal do Ceará - CE
36	AUXILIO DOENÇA (INSS) - A PARTIR DO 16 DIA	CH da Universidade Federal do Ceará - CE
36	AUXILIO DOENÇA (INSS) - A PARTIR DO 16 DIA	CH da Universidade Federal do Ceará - CE
36	AUXILIO DOENÇA (INSS) - A PARTIR DO 16 DIA	CH da Universidade Federal do Ceará - CE
36	AUXILIO DOENÇA (INSS) - A PARTIR DO 16 DIA	CH da Universidade Federal do Ceará - CE
36	AUXILIO DOENÇA (INSS) - A PARTIR DO 16 DIA	CH da Universidade Federal do Ceará - CE
36	AUXILIO DOENÇA (INSS) - A PARTIR DO 16 DIA	CH da Universidade Federal do Ceará - CE
36	AUXILIO DOENÇA (INSS) - A PARTIR DO 16 DIA	CH da Universidade Federal do Ceará - CE
36	AUXILIO DOENÇA (INSS) - A PARTIR DO 16 DIA	CH da Universidade Federal do Ceará - CE
36	AUXILIO DOENÇA (INSS) - A PARTIR DO 16 DIA	CH da Universidade Federal do Ceará - CE
36	AUXILIO DOENÇA (INSS) - A PARTIR DO 16 DIA	CH da Universidade Federal do Ceará - CE
36	AUXILIO DOENÇA (INSS) - A PARTIR DO 16 DIA	CH da Universidade Federal do Ceará - CE
36	AUXILIO DOENÇA (INSS) - A PARTIR DO 16 DIA	CH da Universidade Federal do Ceará - CE
36	AUXILIO DOENÇA (INSS) - A PARTIR DO 16 DIA	CH da Universidade Federal do Ceará - CE
36	AUXILIO DOENÇA (INSS) - A PARTIR DO 16 DIA	CH da Universidade Federal do Ceará - CE
36	AUXILIO DOENÇA (INSS) - A PARTIR DO 16 DIA	CH da Universidade Federal do Ceará - CE
36	AUXILIO DOENÇA (INSS) - A PARTIR DO 16 DIA	CH da Universidade Federal do Ceará - CE
36	AUXILIO DOENÇA (INSS) - A PARTIR DO 16 DIA	CH da Universidade Federal do Ceará - CE
36	AUXILIO DOENÇA (INSS) - A PARTIR DO 16 DIA	CH da Universidade Federal do Ceará - CE
36	AUXILIO DOENÇA (INSS) - A PARTIR DO 16 DIA	CH da Universidade Federal do Ceará - CE
36	AUXILIO DOENÇA (INSS) - A PARTIR DO 16 DIA	CH da Universidade Federal do Ceará - CE
36	AUXILIO DOENÇA (INSS) - A PARTIR DO 16 DIA	CH da Universidade Federal do Ceará - CE
36	AUXILIO DOENÇA (INSS) - A PARTIR DO 16 DIA	CH da Universidade Federal do Ceará - CE
36	AUXILIO DOENÇA (INSS) - A PARTIR DO 16 DIA	CH da Universidade Federal do Ceará - CE
36	AUXILIO DOENÇA (INSS) - A PARTIR DO 16 DIA	CH da Universidade Federal do Ceará - CE
36	AUXILIO DOENÇA (INSS) - A PARTIR DO 16 DIA	CH da Universidade Federal do Ceará - CE

36	AUXILIO DOENÇA (INSS) - A PARTIR DO 16 DIA	CH da Universidade Federal do Ceará - CE
36	AUXILIO DOENÇA (INSS) - A PARTIR DO 16 DIA	CH da Universidade Federal do Ceará - CE
36	AUXILIO DOENÇA (INSS) - A PARTIR DO 16 DIA	CH da Universidade Federal do Ceará - CE
36	AUXILIO DOENÇA (INSS) - A PARTIR DO 16 DIA	CH da Universidade Federal do Ceará - CE
36	AUXILIO DOENÇA (INSS) - A PARTIR DO 16 DIA	CH da Universidade Federal do Ceará - CE
36	AUXILIO DOENÇA (INSS) - A PARTIR DO 16 DIA	CH da Universidade Federal do Ceará - CE
36	AUXILIO DOENÇA (INSS) - A PARTIR DO 16 DIA	CH da Universidade Federal do Ceará - CE
36	AUXILIO DOENÇA (INSS) - A PARTIR DO 16 DIA	CH da Universidade Federal do Ceará - CE
36	AUXILIO DOENÇA (INSS) - A PARTIR DO 16 DIA	CH da Universidade Federal do Ceará - CE
36	AUXILIO DOENÇA (INSS) - A PARTIR DO 16 DIA	CH da Universidade Federal do Ceará - CE
36	AUXILIO DOENÇA (INSS) - A PARTIR DO 16 DIA	CH da Universidade Federal do Ceará - CE
36	AUXILIO DOENÇA (INSS) - A PARTIR DO 16 DIA	CH da Universidade Federal do Ceará - CE
36	AUXILIO DOENÇA (INSS) - A PARTIR DO 16 DIA	CH da Universidade Federal do Ceará - CE
36	AUXILIO DOENÇA (INSS) - A PARTIR DO 16 DIA	CH da Universidade Federal do Ceará - CE
36	AUXILIO DOENÇA (INSS) - A PARTIR DO 16 DIA	CH da Universidade Federal do Ceará - CE
36	AUXILIO DOENÇA (INSS) - A PARTIR DO 16 DIA	CH da Universidade Federal do Ceará - CE
36	AUXILIO DOENÇA (INSS) - A PARTIR DO 16 DIA	CH da Universidade Federal do Ceará - CE
36	AUXILIO DOENÇA (INSS) - A PARTIR DO 16 DIA	CH da Universidade Federal do Ceará - CE
36	AUXILIO DOENÇA (INSS) - A PARTIR DO 16 DIA	CH da Universidade Federal do Ceará - CE
36	AUXILIO DOENÇA (INSS) - A PARTIR DO 16 DIA	CH da Universidade Federal do Ceará - CE
36	AUXILIO DOENÇA (INSS) - A PARTIR DO 16 DIA	CH da Universidade Federal do Ceará - CE
36	AUXILIO DOENÇA (INSS) - A PARTIR DO 16 DIA	CH da Universidade Federal do Ceará - CE
36	AUXILIO DOENÇA (INSS) - A PARTIR DO 16 DIA	CH da Universidade Federal do Ceará - CE
36	AUXILIO DOENÇA (INSS) - A PARTIR DO 16 DIA	CH da Universidade Federal do Ceará - CE
36	AUXILIO DOENÇA (INSS) - A PARTIR DO 16 DIA	CH da Universidade Federal do Ceará - CE
36	AUXILIO DOENÇA (INSS) - A PARTIR DO 16 DIA	CH da Universidade Federal do Ceará - CE
36	AUXILIO DOENÇA (INSS) - A PARTIR DO 16 DIA	CH da Universidade Federal do Ceará - CE
36	AUXILIO DOENÇA (INSS) - A PARTIR DO 16 DIA	CH da Universidade Federal do Ceará - CE
36	AUXILIO DOENÇA (INSS) - A PARTIR DO 16 DIA	CH da Universidade Federal do Ceará - CE
36	AUXILIO DOENÇA (INSS) - A PARTIR DO 16 DIA	CH da Universidade Federal do Ceará - CE
36	AUXILIO DOENÇA (INSS) - A PARTIR DO 16 DIA	CH da Universidade Federal do Ceará - CE
36	AUXILIO DOENÇA (INSS) - A PARTIR DO 16 DIA	CH da Universidade Federal do Ceará - CE
36	AUXILIO DOENÇA (INSS) - A PARTIR DO 16 DIA	CH da Universidade Federal do Ceará - CE
36	AUXILIO DOENÇA (INSS) - A PARTIR DO 16 DIA	CH da Universidade Federal do Ceará - CE
36	AUXILIO DOENÇA (INSS) - A PARTIR DO 16 DIA	CH da Universidade Federal do Ceará - CE
42	DOAÇÃO DE SANGUE - REGULAMENTO DE PESSOAL ART 36 I	CH da Universidade Federal do Ceará - CE
42	DOAÇÃO DE SANGUE - REGULAMENTO DE PESSOAL ART 36 I	CH da Universidade Federal do Ceará - CE
42	DOAÇÃO DE SANGUE - REGULAMENTO DE PESSOAL ART 36 I	CH da Universidade Federal do Ceará - CE
42	DOAÇÃO DE SANGUE - REGULAMENTO DE PESSOAL ART 36 I	CH da Universidade Federal do Ceará - CE
42	DOAÇÃO DE SANGUE - REGULAMENTO DE PESSOAL ART 36 I	CH da Universidade Federal do Ceará - CE
42	DOAÇÃO DE SANGUE - REGULAMENTO DE PESSOAL ART 36 I	CH da Universidade Federal do Ceará - CE
42	DOAÇÃO DE SANGUE - REGULAMENTO DE PESSOAL ART 36 I	CH da Universidade Federal do Ceará - CE
42	DOAÇÃO DE SANGUE - REGULAMENTO DE PESSOAL ART 36 I	CH da Universidade Federal do Ceará - CE

42	DOAÇÃO DE SANGUE - REGULAMENTO DE PESSOAL ART 36 I	CH da Universidade Federal do Ceará - CE
42	DOAÇÃO DE SANGUE - REGULAMENTO DE PESSOAL ART 36 I	CH da Universidade Federal do Ceará - CE
42	DOAÇÃO DE SANGUE - REGULAMENTO DE PESSOAL ART 36 I	CH da Universidade Federal do Ceará - CE
42	DOAÇÃO DE SANGUE - REGULAMENTO DE PESSOAL ART 36 I	CH da Universidade Federal do Ceará - CE
42	DOAÇÃO DE SANGUE - REGULAMENTO DE PESSOAL ART 36 I	CH da Universidade Federal do Ceará - CE
42	DOAÇÃO DE SANGUE - REGULAMENTO DE PESSOAL ART 36 I	CH da Universidade Federal do Ceará - CE
42	DOAÇÃO DE SANGUE - REGULAMENTO DE PESSOAL ART 36 I	CH da Universidade Federal do Ceará - CE
42	DOAÇÃO DE SANGUE - REGULAMENTO DE PESSOAL ART 36 I	CH da Universidade Federal do Ceará - CE
42	DOAÇÃO DE SANGUE - REGULAMENTO DE PESSOAL ART 36 I	CH da Universidade Federal do Ceará - CE
42	DOAÇÃO DE SANGUE - REGULAMENTO DE PESSOAL ART 36 I	CH da Universidade Federal do Ceará - CE
42	DOAÇÃO DE SANGUE - REGULAMENTO DE PESSOAL ART 36 I	CH da Universidade Federal do Ceará - CE
42	DOAÇÃO DE SANGUE - REGULAMENTO DE PESSOAL ART 36 I	CH da Universidade Federal do Ceará - CE
42	DOAÇÃO DE SANGUE - REGULAMENTO DE PESSOAL ART 36 I	CH da Universidade Federal do Ceará - CE
42	DOAÇÃO DE SANGUE - REGULAMENTO DE PESSOAL ART 36 I	CH da Universidade Federal do Ceará - CE
42	DOAÇÃO DE SANGUE - REGULAMENTO DE PESSOAL ART 36 I	CH da Universidade Federal do Ceará - CE
42	DOAÇÃO DE SANGUE - REGULAMENTO DE PESSOAL ART 36 I	CH da Universidade Federal do Ceará - CE
42	DOAÇÃO DE SANGUE - REGULAMENTO DE PESSOAL ART 36 I	CH da Universidade Federal do Ceará - CE
42	DOAÇÃO DE SANGUE - REGULAMENTO DE PESSOAL ART 36 I	CH da Universidade Federal do Ceará - CE
42	DOAÇÃO DE SANGUE - REGULAMENTO DE PESSOAL ART 36 I	CH da Universidade Federal do Ceará - CE
42	DOAÇÃO DE SANGUE - REGULAMENTO DE PESSOAL ART 36 I	CH da Universidade Federal do Ceará - CE
42	DOAÇÃO DE SANGUE - REGULAMENTO DE PESSOAL ART 36 I	CH da Universidade Federal do Ceará - CE
42	DOAÇÃO DE SANGUE - REGULAMENTO DE PESSOAL ART 36 I	CH da Universidade Federal do Ceará - CE
42	DOAÇÃO DE SANGUE - REGULAMENTO DE PESSOAL ART 36 I	CH da Universidade Federal do Ceará - CE
42	DOAÇÃO DE SANGUE - REGULAMENTO DE PESSOAL ART 36 I	CH da Universidade Federal do Ceará - CE
42	DOAÇÃO DE SANGUE - REGULAMENTO DE PESSOAL ART 36 I	CH da Universidade Federal do Ceará - CE
42	DOAÇÃO DE SANGUE - REGULAMENTO DE PESSOAL ART 36 I	CH da Universidade Federal do Ceará - CE
42	DOAÇÃO DE SANGUE - REGULAMENTO DE PESSOAL ART 36 I	CH da Universidade Federal do Ceará - CE
42	DOAÇÃO DE SANGUE - REGULAMENTO DE PESSOAL ART 36 I	CH da Universidade Federal do Ceará - CE
42	DOAÇÃO DE SANGUE - REGULAMENTO DE PESSOAL ART 36 I	CH da Universidade Federal do Ceará - CE
42	DOAÇÃO DE SANGUE - REGULAMENTO DE PESSOAL ART 36 I	CH da Universidade Federal do Ceará - CE
42	DOAÇÃO DE SANGUE - REGULAMENTO DE PESSOAL ART 36 I	CH da Universidade Federal do Ceará - CE
42	DOAÇÃO DE SANGUE - REGULAMENTO DE PESSOAL ART 36 I	CH da Universidade Federal do Ceará - CE
42	DOAÇÃO DE SANGUE - REGULAMENTO DE PESSOAL ART 36 I	CH da Universidade Federal do Ceará - CE
42	DOAÇÃO DE SANGUE - REGULAMENTO DE PESSOAL ART 36 I	CH da Universidade Federal do Ceará - CE
42	DOAÇÃO DE SANGUE - REGULAMENTO DE PESSOAL ART 36 I	CH da Universidade Federal do Ceará - CE
42	DOAÇÃO DE SANGUE - REGULAMENTO DE PESSOAL ART 36 I	CH da Universidade Federal do Ceará - CE
42	DOAÇÃO DE SANGUE - REGULAMENTO DE PESSOAL ART 36 I	CH da Universidade Federal do Ceará - CE
42	DOAÇÃO DE SANGUE - REGULAMENTO DE PESSOAL ART 36 I	CH da Universidade Federal do Ceará - CE
45	FALTA INJUSTIFICADA (GERA DESCONTO NA FOLHA)	CH da Universidade Federal do Ceará - CE
45	FALTA INJUSTIFICADA (GERA DESCONTO NA FOLHA)	CH da Universidade Federal do Ceará - CE
45	FALTA INJUSTIFICADA (GERA DESCONTO NA FOLHA)	CH da Universidade Federal do Ceará - CE
45	FALTA INJUSTIFICADA (GERA DESCONTO NA FOLHA)	CH da Universidade Federal do Ceará - CE
45	FALTA INJUSTIFICADA (GERA DESCONTO NA FOLHA)	CH da Universidade Federal do Ceará - CE
45	FALTA INJUSTIFICADA (GERA DESCONTO NA FOLHA)	CH da Universidade Federal do Ceará - CE
45	FALTA INJUSTIFICADA (GERA DESCONTO NA FOLHA)	CH da Universidade Federal do Ceará - CE

45	FALTA INJUSTIFICADA (GERA DESCONTO NA FOLHA)	CH da Universidade Federal do Ceará - CE
45	FALTA INJUSTIFICADA (GERA DESCONTO NA FOLHA)	CH da Universidade Federal do Ceará - CE
45	FALTA INJUSTIFICADA (GERA DESCONTO NA FOLHA)	CH da Universidade Federal do Ceará - CE
45	FALTA INJUSTIFICADA (GERA DESCONTO NA FOLHA)	CH da Universidade Federal do Ceará - CE
45	FALTA INJUSTIFICADA (GERA DESCONTO NA FOLHA)	CH da Universidade Federal do Ceará - CE
45	FALTA INJUSTIFICADA (GERA DESCONTO NA FOLHA)	CH da Universidade Federal do Ceará - CE
45	FALTA INJUSTIFICADA (GERA DESCONTO NA FOLHA)	CH da Universidade Federal do Ceará - CE
45	FALTA INJUSTIFICADA (GERA DESCONTO NA FOLHA)	CH da Universidade Federal do Ceará - CE
45	FALTA INJUSTIFICADA (GERA DESCONTO NA FOLHA)	CH da Universidade Federal do Ceará - CE
45	FALTA INJUSTIFICADA (GERA DESCONTO NA FOLHA)	CH da Universidade Federal do Ceará - CE
45	FALTA INJUSTIFICADA (GERA DESCONTO NA FOLHA)	CH da Universidade Federal do Ceará - CE
45	FALTA INJUSTIFICADA (GERA DESCONTO NA FOLHA)	CH da Universidade Federal do Ceará - CE
45	FALTA INJUSTIFICADA (GERA DESCONTO NA FOLHA)	CH da Universidade Federal do Ceará - CE
45	FALTA INJUSTIFICADA (GERA DESCONTO NA FOLHA)	CH da Universidade Federal do Ceará - CE
45	FALTA INJUSTIFICADA (GERA DESCONTO NA FOLHA)	CH da Universidade Federal do Ceará - CE
45	FALTA INJUSTIFICADA (GERA DESCONTO NA FOLHA)	CH da Universidade Federal do Ceará - CE
45	FALTA INJUSTIFICADA (GERA DESCONTO NA FOLHA)	CH da Universidade Federal do Ceará - CE
45	FALTA INJUSTIFICADA (GERA DESCONTO NA FOLHA)	CH da Universidade Federal do Ceará - CE
45	FALTA INJUSTIFICADA (GERA DESCONTO NA FOLHA)	CH da Universidade Federal do Ceará - CE
45	FALTA INJUSTIFICADA (GERA DESCONTO NA FOLHA)	CH da Universidade Federal do Ceará - CE
45	FALTA INJUSTIFICADA (GERA DESCONTO NA FOLHA)	CH da Universidade Federal do Ceará - CE
45	FALTA INJUSTIFICADA (GERA DESCONTO NA FOLHA)	CH da Universidade Federal do Ceará - CE
45	FALTA INJUSTIFICADA (GERA DESCONTO NA FOLHA)	CH da Universidade Federal do Ceará - CE
45	FALTA INJUSTIFICADA (GERA DESCONTO NA FOLHA)	CH da Universidade Federal do Ceará - CE
45	FALTA INJUSTIFICADA (GERA DESCONTO NA FOLHA)	CH da Universidade Federal do Ceará - CE
45	FALTA INJUSTIFICADA (GERA DESCONTO NA FOLHA)	CH da Universidade Federal do Ceará - CE
45	FALTA INJUSTIFICADA (GERA DESCONTO NA FOLHA)	CH da Universidade Federal do Ceará - CE
45	FALTA INJUSTIFICADA (GERA DESCONTO NA FOLHA)	CH da Universidade Federal do Ceará - CE
45	FALTA INJUSTIFICADA (GERA DESCONTO NA FOLHA)	CH da Universidade Federal do Ceará - CE
45	FALTA INJUSTIFICADA (GERA DESCONTO NA FOLHA)	CH da Universidade Federal do Ceará - CE
45	FALTA INJUSTIFICADA (GERA DESCONTO NA FOLHA)	CH da Universidade Federal do Ceará - CE
45	FALTA INJUSTIFICADA (GERA DESCONTO NA FOLHA)	CH da Universidade Federal do Ceará - CE
45	FALTA INJUSTIFICADA (GERA DESCONTO NA FOLHA)	CH da Universidade Federal do Ceará - CE
45	FALTA INJUSTIFICADA (GERA DESCONTO NA FOLHA)	CH da Universidade Federal do Ceará - CE
45	FALTA INJUSTIFICADA (GERA DESCONTO NA FOLHA)	CH da Universidade Federal do Ceará - CE
45	FALTA INJUSTIFICADA (GERA DESCONTO NA FOLHA)	CH da Universidade Federal do Ceará - CE
45	FALTA INJUSTIFICADA (GERA DESCONTO NA FOLHA)	CH da Universidade Federal do Ceará - CE
45	FALTA INJUSTIFICADA (GERA DESCONTO NA FOLHA)	CH da Universidade Federal do Ceará - CE
45	FALTA INJUSTIFICADA (GERA DESCONTO NA FOLHA)	CH da Universidade Federal do Ceará - CE
45	FALTA INJUSTIFICADA (GERA DESCONTO NA FOLHA)	CH da Universidade Federal do Ceará - CE
45	FALTA INJUSTIFICADA (GERA DESCONTO NA FOLHA)	CH da Universidade Federal do Ceará - CE
45	FALTA INJUSTIFICADA (GERA DESCONTO NA FOLHA)	CH da Universidade Federal do Ceará - CE
45	FALTA INJUSTIFICADA (GERA DESCONTO NA FOLHA)	CH da Universidade Federal do Ceará - CE
45	FALTA INJUSTIFICADA (GERA DESCONTO NA FOLHA)	CH da Universidade Federal do Ceará - CE
45	FALTA INJUSTIFICADA (GERA DESCONTO NA FOLHA)	CH da Universidade Federal do Ceará - CE
45	FALTA INJUSTIFICADA (GERA DESCONTO NA FOLHA)	CH da Universidade Federal do Ceará - CE
45	FALTA INJUSTIFICADA (GERA DESCONTO NA FOLHA)	CH da Universidade Federal do Ceará - CE
45	FALTA INJUSTIFICADA (GERA DESCONTO NA FOLHA)	CH da Universidade Federal do Ceará - CE

45	FALTA INJUSTIFICADA (GERA DESCONTO NA FOLHA)	CH da Universidade Federal do Ceará - CE
45	FALTA INJUSTIFICADA (GERA DESCONTO NA FOLHA)	CH da Universidade Federal do Ceará - CE
45	FALTA INJUSTIFICADA (GERA DESCONTO NA FOLHA)	CH da Universidade Federal do Ceará - CE
45	FALTA INJUSTIFICADA (GERA DESCONTO NA FOLHA)	CH da Universidade Federal do Ceará - CE
45	FALTA INJUSTIFICADA (GERA DESCONTO NA FOLHA)	CH da Universidade Federal do Ceará - CE
45	FALTA INJUSTIFICADA (GERA DESCONTO NA FOLHA)	CH da Universidade Federal do Ceará - CE
45	FALTA INJUSTIFICADA (GERA DESCONTO NA FOLHA)	CH da Universidade Federal do Ceará - CE
45	FALTA INJUSTIFICADA (GERA DESCONTO NA FOLHA)	CH da Universidade Federal do Ceará - CE
45	FALTA INJUSTIFICADA (GERA DESCONTO NA FOLHA)	CH da Universidade Federal do Ceará - CE
45	FALTA INJUSTIFICADA (GERA DESCONTO NA FOLHA)	CH da Universidade Federal do Ceará - CE
45	FALTA INJUSTIFICADA (GERA DESCONTO NA FOLHA)	CH da Universidade Federal do Ceará - CE
45	FALTA INJUSTIFICADA (GERA DESCONTO NA FOLHA)	CH da Universidade Federal do Ceará - CE
45	FALTA INJUSTIFICADA (GERA DESCONTO NA FOLHA)	CH da Universidade Federal do Ceará - CE
45	FALTA INJUSTIFICADA (GERA DESCONTO NA FOLHA)	CH da Universidade Federal do Ceará - CE
45	FALTA INJUSTIFICADA (GERA DESCONTO NA FOLHA)	CH da Universidade Federal do Ceará - CE
45	FALTA INJUSTIFICADA (GERA DESCONTO NA FOLHA)	CH da Universidade Federal do Ceará - CE
45	FALTA INJUSTIFICADA (GERA DESCONTO NA FOLHA)	CH da Universidade Federal do Ceará - CE
45	FALTA INJUSTIFICADA (GERA DESCONTO NA FOLHA)	CH da Universidade Federal do Ceará - CE
45	FALTA INJUSTIFICADA (GERA DESCONTO NA FOLHA)	CH da Universidade Federal do Ceará - CE
45	FALTA INJUSTIFICADA (GERA DESCONTO NA FOLHA)	CH da Universidade Federal do Ceará - CE
45	FALTA INJUSTIFICADA (GERA DESCONTO NA FOLHA)	CH da Universidade Federal do Ceará - CE
45	FALTA INJUSTIFICADA (GERA DESCONTO NA FOLHA)	CH da Universidade Federal do Ceará - CE
45	FALTA INJUSTIFICADA (GERA DESCONTO NA FOLHA)	CH da Universidade Federal do Ceará - CE
45	FALTA INJUSTIFICADA (GERA DESCONTO NA FOLHA)	CH da Universidade Federal do Ceará - CE
45	FALTA INJUSTIFICADA (GERA DESCONTO NA FOLHA)	CH da Universidade Federal do Ceará - CE
45	FALTA INJUSTIFICADA (GERA DESCONTO NA FOLHA)	CH da Universidade Federal do Ceará - CE
45	FALTA INJUSTIFICADA (GERA DESCONTO NA FOLHA)	CH da Universidade Federal do Ceará - CE
45	FALTA INJUSTIFICADA (GERA DESCONTO NA FOLHA)	CH da Universidade Federal do Ceará - CE
45	FALTA INJUSTIFICADA (GERA DESCONTO NA FOLHA)	CH da Universidade Federal do Ceará - CE
45	FALTA INJUSTIFICADA (GERA DESCONTO NA FOLHA)	CH da Universidade Federal do Ceará - CE
45	FALTA INJUSTIFICADA (GERA DESCONTO NA FOLHA)	CH da Universidade Federal do Ceará - CE
45	FALTA INJUSTIFICADA (GERA DESCONTO NA FOLHA)	CH da Universidade Federal do Ceará - CE
45	FALTA INJUSTIFICADA (GERA DESCONTO NA FOLHA)	CH da Universidade Federal do Ceará - CE
45	FALTA INJUSTIFICADA (GERA DESCONTO NA FOLHA)	CH da Universidade Federal do Ceará - CE
45	FALTA INJUSTIFICADA (GERA DESCONTO NA FOLHA)	CH da Universidade Federal do Ceará - CE
45	FALTA INJUSTIFICADA (GERA DESCONTO NA FOLHA)	CH da Universidade Federal do Ceará - CE
45	FALTA INJUSTIFICADA (GERA DESCONTO NA FOLHA)	CH da Universidade Federal do Ceará - CE
45	FALTA INJUSTIFICADA (GERA DESCONTO NA FOLHA)	CH da Universidade Federal do Ceará - CE
45	FALTA INJUSTIFICADA (GERA DESCONTO NA FOLHA)	CH da Universidade Federal do Ceará - CE
45	FALTA INJUSTIFICADA (GERA DESCONTO NA FOLHA)	CH da Universidade Federal do Ceará - CE
45	FALTA INJUSTIFICADA (GERA DESCONTO NA FOLHA)	CH da Universidade Federal do Ceará - CE
45	FALTA INJUSTIFICADA (GERA DESCONTO NA FOLHA)	CH da Universidade Federal do Ceará - CE
45	FALTA INJUSTIFICADA (GERA DESCONTO NA FOLHA)	CH da Universidade Federal do Ceará - CE
45	FALTA INJUSTIFICADA (GERA DESCONTO NA FOLHA)	CH da Universidade Federal do Ceará - CE
45	FALTA INJUSTIFICADA (GERA DESCONTO NA FOLHA)	CH da Universidade Federal do Ceará - CE
45	FALTA INJUSTIFICADA (GERA DESCONTO NA FOLHA)	CH da Universidade Federal do Ceará - CE
45	FALTA INJUSTIFICADA (GERA DESCONTO NA FOLHA)	CH da Universidade Federal do Ceará - CE
45	FALTA INJUSTIFICADA (GERA DESCONTO NA FOLHA)	CH da Universidade Federal do Ceará - CE

45	FALTA INJUSTIFICADA (GERA DESCONTO NA FOLHA)	CH da Universidade Federal do Ceará - CE
45	FALTA INJUSTIFICADA (GERA DESCONTO NA FOLHA)	CH da Universidade Federal do Ceará - CE
45	FALTA INJUSTIFICADA (GERA DESCONTO NA FOLHA)	CH da Universidade Federal do Ceará - CE
45	FALTA INJUSTIFICADA (GERA DESCONTO NA FOLHA)	CH da Universidade Federal do Ceará - CE
45	FALTA INJUSTIFICADA (GERA DESCONTO NA FOLHA)	CH da Universidade Federal do Ceará - CE
45	FALTA INJUSTIFICADA (GERA DESCONTO NA FOLHA)	CH da Universidade Federal do Ceará - CE
45	FALTA INJUSTIFICADA (GERA DESCONTO NA FOLHA)	CH da Universidade Federal do Ceará - CE
45	FALTA INJUSTIFICADA (GERA DESCONTO NA FOLHA)	CH da Universidade Federal do Ceará - CE
45	FALTA INJUSTIFICADA (GERA DESCONTO NA FOLHA)	CH da Universidade Federal do Ceará - CE
45	FALTA INJUSTIFICADA (GERA DESCONTO NA FOLHA)	CH da Universidade Federal do Ceará - CE
45	FALTA INJUSTIFICADA (GERA DESCONTO NA FOLHA)	CH da Universidade Federal do Ceará - CE
45	FALTA INJUSTIFICADA (GERA DESCONTO NA FOLHA)	CH da Universidade Federal do Ceará - CE
45	FALTA INJUSTIFICADA (GERA DESCONTO NA FOLHA)	CH da Universidade Federal do Ceará - CE
45	FALTA INJUSTIFICADA (GERA DESCONTO NA FOLHA)	CH da Universidade Federal do Ceará - CE
45	FALTA INJUSTIFICADA (GERA DESCONTO NA FOLHA)	CH da Universidade Federal do Ceará - CE
45	FALTA INJUSTIFICADA (GERA DESCONTO NA FOLHA)	CH da Universidade Federal do Ceará - CE
45	FALTA INJUSTIFICADA (GERA DESCONTO NA FOLHA)	CH da Universidade Federal do Ceará - CE
45	FALTA INJUSTIFICADA (GERA DESCONTO NA FOLHA)	CH da Universidade Federal do Ceará - CE
45	FALTA INJUSTIFICADA (GERA DESCONTO NA FOLHA)	CH da Universidade Federal do Ceará - CE
45	FALTA INJUSTIFICADA (GERA DESCONTO NA FOLHA)	CH da Universidade Federal do Ceará - CE
45	FALTA INJUSTIFICADA (GERA DESCONTO NA FOLHA)	CH da Universidade Federal do Ceará - CE
45	FALTA INJUSTIFICADA (GERA DESCONTO NA FOLHA)	CH da Universidade Federal do Ceará - CE
45	FALTA INJUSTIFICADA (GERA DESCONTO NA FOLHA)	CH da Universidade Federal do Ceará - CE
45	FALTA INJUSTIFICADA (GERA DESCONTO NA FOLHA)	CH da Universidade Federal do Ceará - CE
45	FALTA INJUSTIFICADA (GERA DESCONTO NA FOLHA)	CH da Universidade Federal do Ceará - CE
45	FALTA INJUSTIFICADA (GERA DESCONTO NA FOLHA)	CH da Universidade Federal do Ceará - CE
45	FALTA INJUSTIFICADA (GERA DESCONTO NA FOLHA)	CH da Universidade Federal do Ceará - CE
45	FALTA INJUSTIFICADA (GERA DESCONTO NA FOLHA)	CH da Universidade Federal do Ceará - CE
45	FALTA INJUSTIFICADA (GERA DESCONTO NA FOLHA)	CH da Universidade Federal do Ceará - CE
45	FALTA INJUSTIFICADA (GERA DESCONTO NA FOLHA)	CH da Universidade Federal do Ceará - CE
45	FALTA INJUSTIFICADA (GERA DESCONTO NA FOLHA)	CH da Universidade Federal do Ceará - CE
45	FALTA INJUSTIFICADA (GERA DESCONTO NA FOLHA)	CH da Universidade Federal do Ceará - CE
45	FALTA INJUSTIFICADA (GERA DESCONTO NA FOLHA)	CH da Universidade Federal do Ceará - CE
45	FALTA INJUSTIFICADA (GERA DESCONTO NA FOLHA)	CH da Universidade Federal do Ceará - CE
45	FALTA INJUSTIFICADA (GERA DESCONTO NA FOLHA)	CH da Universidade Federal do Ceará - CE
45	FALTA INJUSTIFICADA (GERA DESCONTO NA FOLHA)	CH da Universidade Federal do Ceará - CE
45	FALTA INJUSTIFICADA (GERA DESCONTO NA FOLHA)	CH da Universidade Federal do Ceará - CE
45	FALTA INJUSTIFICADA (GERA DESCONTO NA FOLHA)	CH da Universidade Federal do Ceará - CE
45	FALTA INJUSTIFICADA (GERA DESCONTO NA FOLHA)	CH da Universidade Federal do Ceará - CE
45	FALTA INJUSTIFICADA (GERA DESCONTO NA FOLHA)	CH da Universidade Federal do Ceará - CE
45	FALTA INJUSTIFICADA (GERA DESCONTO NA FOLHA)	CH da Universidade Federal do Ceará - CE
45	FALTA INJUSTIFICADA (GERA DESCONTO NA FOLHA)	CH da Universidade Federal do Ceará - CE
45	FALTA INJUSTIFICADA (GERA DESCONTO NA FOLHA)	CH da Universidade Federal do Ceará - CE
45	FALTA INJUSTIFICADA (GERA DESCONTO NA FOLHA)	CH da Universidade Federal do Ceará - CE
45	FALTA INJUSTIFICADA (GERA DESCONTO NA FOLHA)	CH da Universidade Federal do Ceará - CE
45	FALTA INJUSTIFICADA (GERA DESCONTO NA FOLHA)	CH da Universidade Federal do Ceará - CE
45	FALTA INJUSTIFICADA (GERA DESCONTO NA FOLHA)	CH da Universidade Federal do Ceará - CE
45	FALTA INJUSTIFICADA (GERA DESCONTO NA FOLHA)	CH da Universidade Federal do Ceará - CE

45	FALTA INJUSTIFICADA (GERA DESCONTO NA FOLHA)	CH da Universidade Federal do Ceará - CE
45	FALTA INJUSTIFICADA (GERA DESCONTO NA FOLHA)	CH da Universidade Federal do Ceará - CE
45	FALTA INJUSTIFICADA (GERA DESCONTO NA FOLHA)	CH da Universidade Federal do Ceará - CE
45	FALTA INJUSTIFICADA (GERA DESCONTO NA FOLHA)	CH da Universidade Federal do Ceará - CE
45	FALTA INJUSTIFICADA (GERA DESCONTO NA FOLHA)	CH da Universidade Federal do Ceará - CE
45	FALTA INJUSTIFICADA (GERA DESCONTO NA FOLHA)	CH da Universidade Federal do Ceará - CE
45	FALTA INJUSTIFICADA (GERA DESCONTO NA FOLHA)	CH da Universidade Federal do Ceará - CE
45	FALTA INJUSTIFICADA (GERA DESCONTO NA FOLHA)	CH da Universidade Federal do Ceará - CE
45	FALTA INJUSTIFICADA (GERA DESCONTO NA FOLHA)	CH da Universidade Federal do Ceará - CE
45	FALTA INJUSTIFICADA (GERA DESCONTO NA FOLHA)	CH da Universidade Federal do Ceará - CE
45	FALTA INJUSTIFICADA (GERA DESCONTO NA FOLHA)	CH da Universidade Federal do Ceará - CE
45	FALTA INJUSTIFICADA (GERA DESCONTO NA FOLHA)	CH da Universidade Federal do Ceará - CE
45	FALTA INJUSTIFICADA (GERA DESCONTO NA FOLHA)	CH da Universidade Federal do Ceará - CE
45	FALTA INJUSTIFICADA (GERA DESCONTO NA FOLHA)	CH da Universidade Federal do Ceará - CE
45	FALTA INJUSTIFICADA (GERA DESCONTO NA FOLHA)	CH da Universidade Federal do Ceará - CE
45	FALTA INJUSTIFICADA (GERA DESCONTO NA FOLHA)	CH da Universidade Federal do Ceará - CE
45	FALTA INJUSTIFICADA (GERA DESCONTO NA FOLHA)	CH da Universidade Federal do Ceará - CE
45	FALTA INJUSTIFICADA (GERA DESCONTO NA FOLHA)	CH da Universidade Federal do Ceará - CE
45	FALTA INJUSTIFICADA (GERA DESCONTO NA FOLHA)	CH da Universidade Federal do Ceará - CE
45	FALTA INJUSTIFICADA (GERA DESCONTO NA FOLHA)	CH da Universidade Federal do Ceará - CE
45	FALTA INJUSTIFICADA (GERA DESCONTO NA FOLHA)	CH da Universidade Federal do Ceará - CE
45	FALTA INJUSTIFICADA (GERA DESCONTO NA FOLHA)	CH da Universidade Federal do Ceará - CE
45	FALTA INJUSTIFICADA (GERA DESCONTO NA FOLHA)	CH da Universidade Federal do Ceará - CE
45	FALTA INJUSTIFICADA (GERA DESCONTO NA FOLHA)	CH da Universidade Federal do Ceará - CE
45	FALTA INJUSTIFICADA (GERA DESCONTO NA FOLHA)	CH da Universidade Federal do Ceará - CE
45	FALTA INJUSTIFICADA (GERA DESCONTO NA FOLHA)	CH da Universidade Federal do Ceará - CE
45	FALTA INJUSTIFICADA (GERA DESCONTO NA FOLHA)	CH da Universidade Federal do Ceará - CE
45	FALTA INJUSTIFICADA (GERA DESCONTO NA FOLHA)	CH da Universidade Federal do Ceará - CE
45	FALTA INJUSTIFICADA (GERA DESCONTO NA FOLHA)	CH da Universidade Federal do Ceará - CE
45	FALTA INJUSTIFICADA (GERA DESCONTO NA FOLHA)	CH da Universidade Federal do Ceará - CE
45	FALTA INJUSTIFICADA (GERA DESCONTO NA FOLHA)	CH da Universidade Federal do Ceará - CE
45	FALTA INJUSTIFICADA (GERA DESCONTO NA FOLHA)	CH da Universidade Federal do Ceará - CE
45	FALTA INJUSTIFICADA (GERA DESCONTO NA FOLHA)	CH da Universidade Federal do Ceará - CE
45	FALTA INJUSTIFICADA (GERA DESCONTO NA FOLHA)	CH da Universidade Federal do Ceará - CE
45	FALTA INJUSTIFICADA (GERA DESCONTO NA FOLHA)	CH da Universidade Federal do Ceará - CE
45	FALTA INJUSTIFICADA (GERA DESCONTO NA FOLHA)	CH da Universidade Federal do Ceará - CE
45	FALTA INJUSTIFICADA (GERA DESCONTO NA FOLHA)	CH da Universidade Federal do Ceará - CE
45	FALTA INJUSTIFICADA (GERA DESCONTO NA FOLHA)	CH da Universidade Federal do Ceará - CE
45	FALTA INJUSTIFICADA (GERA DESCONTO NA FOLHA)	CH da Universidade Federal do Ceará - CE
45	FALTA INJUSTIFICADA (GERA DESCONTO NA FOLHA)	CH da Universidade Federal do Ceará - CE
45	FALTA INJUSTIFICADA (GERA DESCONTO NA FOLHA)	CH da Universidade Federal do Ceará - CE
45	FALTA INJUSTIFICADA (GERA DESCONTO NA FOLHA)	CH da Universidade Federal do Ceará - CE
45	FALTA INJUSTIFICADA (GERA DESCONTO NA FOLHA)	CH da Universidade Federal do Ceará - CE

45	FALTA INJUSTIFICADA (GERA DESCONTO NA FOLHA)	CH da Universidade Federal do Ceará - CE
45	FALTA INJUSTIFICADA (GERA DESCONTO NA FOLHA)	CH da Universidade Federal do Ceará - CE
45	FALTA INJUSTIFICADA (GERA DESCONTO NA FOLHA)	CH da Universidade Federal do Ceará - CE
45	FALTA INJUSTIFICADA (GERA DESCONTO NA FOLHA)	CH da Universidade Federal do Ceará - CE
45	FALTA INJUSTIFICADA (GERA DESCONTO NA FOLHA)	CH da Universidade Federal do Ceará - CE
45	FALTA INJUSTIFICADA (GERA DESCONTO NA FOLHA)	CH da Universidade Federal do Ceará - CE
45	FALTA INJUSTIFICADA (GERA DESCONTO NA FOLHA)	CH da Universidade Federal do Ceará - CE
45	FALTA INJUSTIFICADA (GERA DESCONTO NA FOLHA)	CH da Universidade Federal do Ceará - CE
45	FALTA INJUSTIFICADA (GERA DESCONTO NA FOLHA)	CH da Universidade Federal do Ceará - CE
45	FALTA INJUSTIFICADA (GERA DESCONTO NA FOLHA)	CH da Universidade Federal do Ceará - CE
45	FALTA INJUSTIFICADA (GERA DESCONTO NA FOLHA)	CH da Universidade Federal do Ceará - CE
45	FALTA INJUSTIFICADA (GERA DESCONTO NA FOLHA)	CH da Universidade Federal do Ceará - CE
45	FALTA INJUSTIFICADA (GERA DESCONTO NA FOLHA)	CH da Universidade Federal do Ceará - CE
45	FALTA INJUSTIFICADA (GERA DESCONTO NA FOLHA)	CH da Universidade Federal do Ceará - CE
45	FALTA INJUSTIFICADA (GERA DESCONTO NA FOLHA)	CH da Universidade Federal do Ceará - CE
45	FALTA INJUSTIFICADA (GERA DESCONTO NA FOLHA)	CH da Universidade Federal do Ceará - CE
45	FALTA INJUSTIFICADA (GERA DESCONTO NA FOLHA)	CH da Universidade Federal do Ceará - CE
45	FALTA INJUSTIFICADA (GERA DESCONTO NA FOLHA)	CH da Universidade Federal do Ceará - CE
45	FALTA INJUSTIFICADA (GERA DESCONTO NA FOLHA)	CH da Universidade Federal do Ceará - CE
45	FALTA INJUSTIFICADA (GERA DESCONTO NA FOLHA)	CH da Universidade Federal do Ceará - CE
45	FALTA INJUSTIFICADA (GERA DESCONTO NA FOLHA)	CH da Universidade Federal do Ceará - CE
45	FALTA INJUSTIFICADA (GERA DESCONTO NA FOLHA)	CH da Universidade Federal do Ceará - CE
51	A SERVIÇO DA JUSTIÇA ELEITORAL - LEI 9.504 ART. 98	CH da Universidade Federal do Ceará - CE
51	A SERVIÇO DA JUSTIÇA ELEITORAL - LEI 9.504 ART. 98	CH da Universidade Federal do Ceará - CE
51	A SERVIÇO DA JUSTIÇA ELEITORAL - LEI 9.504 ART. 98	CH da Universidade Federal do Ceará - CE
51	A SERVIÇO DA JUSTIÇA ELEITORAL - LEI 9.504 ART. 98	CH da Universidade Federal do Ceará - CE
51	A SERVIÇO DA JUSTIÇA ELEITORAL - LEI 9.504 ART. 98	CH da Universidade Federal do Ceará - CE
51	A SERVIÇO DA JUSTIÇA ELEITORAL - LEI 9.504 ART. 98	CH da Universidade Federal do Ceará - CE
51	A SERVIÇO DA JUSTIÇA ELEITORAL - LEI 9.504 ART. 98	CH da Universidade Federal do Ceará - CE
51	A SERVIÇO DA JUSTIÇA ELEITORAL - LEI 9.504 ART. 98	CH da Universidade Federal do Ceará - CE
51	A SERVIÇO DA JUSTIÇA ELEITORAL - LEI 9.504 ART. 98	CH da Universidade Federal do Ceará - CE
51	A SERVIÇO DA JUSTIÇA ELEITORAL - LEI 9.504 ART. 98	CH da Universidade Federal do Ceará - CE
51	A SERVIÇO DA JUSTIÇA ELEITORAL - LEI 9.504 ART. 98	CH da Universidade Federal do Ceará - CE
51	A SERVIÇO DA JUSTIÇA ELEITORAL - LEI 9.504 ART. 98	CH da Universidade Federal do Ceará - CE
51	A SERVIÇO DA JUSTIÇA ELEITORAL - LEI 9.504 ART. 98	CH da Universidade Federal do Ceará - CE
51	A SERVIÇO DA JUSTIÇA ELEITORAL - LEI 9.504 ART. 98	CH da Universidade Federal do Ceará - CE
51	A SERVIÇO DA JUSTIÇA ELEITORAL - LEI 9.504 ART. 98	CH da Universidade Federal do Ceará - CE
51	A SERVIÇO DA JUSTIÇA ELEITORAL - LEI 9.504 ART. 98	CH da Universidade Federal do Ceará - CE
51	A SERVIÇO DA JUSTIÇA ELEITORAL - LEI 9.504 ART. 98	CH da Universidade Federal do Ceará - CE
51	A SERVIÇO DA JUSTIÇA ELEITORAL - LEI 9.504 ART. 98	CH da Universidade Federal do Ceará - CE
51	A SERVIÇO DA JUSTIÇA ELEITORAL - LEI 9.504 ART. 98	CH da Universidade Federal do Ceará - CE
51	A SERVIÇO DA JUSTIÇA ELEITORAL - LEI 9.504 ART. 98	CH da Universidade Federal do Ceará - CE
51	A SERVIÇO DA JUSTIÇA ELEITORAL - LEI 9.504 ART. 98	CH da Universidade Federal do Ceará - CE
51	A SERVIÇO DA JUSTIÇA ELEITORAL - LEI 9.504 ART. 98	CH da Universidade Federal do Ceará - CE
51	A SERVIÇO DA JUSTIÇA ELEITORAL - LEI 9.504 ART. 98	CH da Universidade Federal do Ceará - CE
51	A SERVIÇO DA JUSTIÇA ELEITORAL - LEI 9.504 ART. 98	CH da Universidade Federal do Ceará - CE

51	A SERVIÇO DA JUSTIÇA ELEITORAL - LEI 9.504 ART. 98	CH da Universidade Federal do Ceará - CE
51	A SERVIÇO DA JUSTIÇA ELEITORAL - LEI 9.504 ART. 98	CH da Universidade Federal do Ceará - CE
51	A SERVIÇO DA JUSTIÇA ELEITORAL - LEI 9.504 ART. 98	CH da Universidade Federal do Ceará - CE
51	A SERVIÇO DA JUSTIÇA ELEITORAL - LEI 9.504 ART. 98	CH da Universidade Federal do Ceará - CE
51	A SERVIÇO DA JUSTIÇA ELEITORAL - LEI 9.504 ART. 98	CH da Universidade Federal do Ceará - CE
51	A SERVIÇO DA JUSTIÇA ELEITORAL - LEI 9.504 ART. 98	CH da Universidade Federal do Ceará - CE
51	A SERVIÇO DA JUSTIÇA ELEITORAL - LEI 9.504 ART. 98	CH da Universidade Federal do Ceará - CE
51	A SERVIÇO DA JUSTIÇA ELEITORAL - LEI 9.504 ART. 98	CH da Universidade Federal do Ceará - CE
51	A SERVIÇO DA JUSTIÇA ELEITORAL - LEI 9.504 ART. 98	CH da Universidade Federal do Ceará - CE
51	A SERVIÇO DA JUSTIÇA ELEITORAL - LEI 9.504 ART. 98	CH da Universidade Federal do Ceará - CE
51	A SERVIÇO DA JUSTIÇA ELEITORAL - LEI 9.504 ART. 98	CH da Universidade Federal do Ceará - CE
51	A SERVIÇO DA JUSTIÇA ELEITORAL - LEI 9.504 ART. 98	CH da Universidade Federal do Ceará - CE
51	A SERVIÇO DA JUSTIÇA ELEITORAL - LEI 9.504 ART. 98	CH da Universidade Federal do Ceará - CE
51	A SERVIÇO DA JUSTIÇA ELEITORAL - LEI 9.504 ART. 98	CH da Universidade Federal do Ceará - CE
51	A SERVIÇO DA JUSTIÇA ELEITORAL - LEI 9.504 ART. 98	CH da Universidade Federal do Ceará - CE
51	A SERVIÇO DA JUSTIÇA ELEITORAL - LEI 9.504 ART. 98	CH da Universidade Federal do Ceará - CE
51	A SERVIÇO DA JUSTIÇA ELEITORAL - LEI 9.504 ART. 98	CH da Universidade Federal do Ceará - CE
51	A SERVIÇO DA JUSTIÇA ELEITORAL - LEI 9.504 ART. 98	CH da Universidade Federal do Ceará - CE
51	A SERVIÇO DA JUSTIÇA ELEITORAL - LEI 9.504 ART. 98	CH da Universidade Federal do Ceará - CE
51	A SERVIÇO DA JUSTIÇA ELEITORAL - LEI 9.504 ART. 98	CH da Universidade Federal do Ceará - CE
51	A SERVIÇO DA JUSTIÇA ELEITORAL - LEI 9.504 ART. 98	CH da Universidade Federal do Ceará - CE
51	A SERVIÇO DA JUSTIÇA ELEITORAL - LEI 9.504 ART. 98	CH da Universidade Federal do Ceará - CE
51	A SERVIÇO DA JUSTIÇA ELEITORAL - LEI 9.504 ART. 98	CH da Universidade Federal do Ceará - CE
51	A SERVIÇO DA JUSTIÇA ELEITORAL - LEI 9.504 ART. 98	CH da Universidade Federal do Ceará - CE
51	A SERVIÇO DA JUSTIÇA ELEITORAL - LEI 9.504 ART. 98	CH da Universidade Federal do Ceará - CE
51	A SERVIÇO DA JUSTIÇA ELEITORAL - LEI 9.504 ART. 98	CH da Universidade Federal do Ceará - CE
51	A SERVIÇO DA JUSTIÇA ELEITORAL - LEI 9.504 ART. 98	CH da Universidade Federal do Ceará - CE
51	A SERVIÇO DA JUSTIÇA ELEITORAL - LEI 9.504 ART. 98	CH da Universidade Federal do Ceará - CE
51	A SERVIÇO DA JUSTIÇA ELEITORAL - LEI 9.504 ART. 98	CH da Universidade Federal do Ceará - CE
51	A SERVIÇO DA JUSTIÇA ELEITORAL - LEI 9.504 ART. 98	CH da Universidade Federal do Ceará - CE
51	A SERVIÇO DA JUSTIÇA ELEITORAL - LEI 9.504 ART. 98	CH da Universidade Federal do Ceará - CE
51	A SERVIÇO DA JUSTIÇA ELEITORAL - LEI 9.504 ART. 98	CH da Universidade Federal do Ceará - CE
51	A SERVIÇO DA JUSTIÇA ELEITORAL - LEI 9.504 ART. 98	CH da Universidade Federal do Ceará - CE
51	A SERVIÇO DA JUSTIÇA ELEITORAL - LEI 9.504 ART. 98	CH da Universidade Federal do Ceará - CE
51	A SERVIÇO DA JUSTIÇA ELEITORAL - LEI 9.504 ART. 98	CH da Universidade Federal do Ceará - CE
51	A SERVIÇO DA JUSTIÇA ELEITORAL - LEI 9.504 ART. 98	CH da Universidade Federal do Ceará - CE
51	A SERVIÇO DA JUSTIÇA ELEITORAL - LEI 9.504 ART. 98	CH da Universidade Federal do Ceará - CE
51	A SERVIÇO DA JUSTIÇA ELEITORAL - LEI 9.504 ART. 98	CH da Universidade Federal do Ceará - CE
51	A SERVIÇO DA JUSTIÇA ELEITORAL - LEI 9.504 ART. 98	CH da Universidade Federal do Ceará - CE
51	A SERVIÇO DA JUSTIÇA ELEITORAL - LEI 9.504 ART. 98	CH da Universidade Federal do Ceará - CE
51	A SERVIÇO DA JUSTIÇA ELEITORAL - LEI 9.504 ART. 98	CH da Universidade Federal do Ceará - CE
51	A SERVIÇO DA JUSTIÇA ELEITORAL - LEI 9.504 ART. 98	CH da Universidade Federal do Ceará - CE
51	A SERVIÇO DA JUSTIÇA ELEITORAL - LEI 9.504 ART. 98	CH da Universidade Federal do Ceará - CE
51	A SERVIÇO DA JUSTIÇA ELEITORAL - LEI 9.504 ART. 98	CH da Universidade Federal do Ceará - CE
51	A SERVIÇO DA JUSTIÇA ELEITORAL - LEI 9.504 ART. 98	CH da Universidade Federal do Ceará - CE

51	A SERVIÇO DA JUSTIÇA ELEITORAL - LEI 9.504 ART. 98	CH da Universidade Federal do Ceará - CE
51	A SERVIÇO DA JUSTIÇA ELEITORAL - LEI 9.504 ART. 98	CH da Universidade Federal do Ceará - CE
51	A SERVIÇO DA JUSTIÇA ELEITORAL - LEI 9.504 ART. 98	CH da Universidade Federal do Ceará - CE
51	A SERVIÇO DA JUSTIÇA ELEITORAL - LEI 9.504 ART. 98	CH da Universidade Federal do Ceará - CE
51	A SERVIÇO DA JUSTIÇA ELEITORAL - LEI 9.504 ART. 98	CH da Universidade Federal do Ceará - CE
51	A SERVIÇO DA JUSTIÇA ELEITORAL - LEI 9.504 ART. 98	CH da Universidade Federal do Ceará - CE
51	A SERVIÇO DA JUSTIÇA ELEITORAL - LEI 9.504 ART. 98	CH da Universidade Federal do Ceará - CE
51	A SERVIÇO DA JUSTIÇA ELEITORAL - LEI 9.504 ART. 98	CH da Universidade Federal do Ceará - CE
51	A SERVIÇO DA JUSTIÇA ELEITORAL - LEI 9.504 ART. 98	CH da Universidade Federal do Ceará - CE
51	A SERVIÇO DA JUSTIÇA ELEITORAL - LEI 9.504 ART. 98	CH da Universidade Federal do Ceará - CE
51	A SERVIÇO DA JUSTIÇA ELEITORAL - LEI 9.504 ART. 98	CH da Universidade Federal do Ceará - CE
51	A SERVIÇO DA JUSTIÇA ELEITORAL - LEI 9.504 ART. 98	CH da Universidade Federal do Ceará - CE
51	A SERVIÇO DA JUSTIÇA ELEITORAL - LEI 9.504 ART. 98	CH da Universidade Federal do Ceará - CE
51	A SERVIÇO DA JUSTIÇA ELEITORAL - LEI 9.504 ART. 98	CH da Universidade Federal do Ceará - CE
51	A SERVIÇO DA JUSTIÇA ELEITORAL - LEI 9.504 ART. 98	CH da Universidade Federal do Ceará - CE
51	A SERVIÇO DA JUSTIÇA ELEITORAL - LEI 9.504 ART. 98	CH da Universidade Federal do Ceará - CE
51	A SERVIÇO DA JUSTIÇA ELEITORAL - LEI 9.504 ART. 98	CH da Universidade Federal do Ceará - CE
51	A SERVIÇO DA JUSTIÇA ELEITORAL - LEI 9.504 ART. 98	CH da Universidade Federal do Ceará - CE
51	A SERVIÇO DA JUSTIÇA ELEITORAL - LEI 9.504 ART. 98	CH da Universidade Federal do Ceará - CE
51	A SERVIÇO DA JUSTIÇA ELEITORAL - LEI 9.504 ART. 98	CH da Universidade Federal do Ceará - CE
51	A SERVIÇO DA JUSTIÇA ELEITORAL - LEI 9.504 ART. 98	CH da Universidade Federal do Ceará - CE
51	A SERVIÇO DA JUSTIÇA ELEITORAL - LEI 9.504 ART. 98	CH da Universidade Federal do Ceará - CE
51	A SERVIÇO DA JUSTIÇA ELEITORAL - LEI 9.504 ART. 98	CH da Universidade Federal do Ceará - CE
51	A SERVIÇO DA JUSTIÇA ELEITORAL - LEI 9.504 ART. 98	CH da Universidade Federal do Ceará - CE
51	A SERVIÇO DA JUSTIÇA ELEITORAL - LEI 9.504 ART. 98	CH da Universidade Federal do Ceará - CE
51	A SERVIÇO DA JUSTIÇA ELEITORAL - LEI 9.504 ART. 98	CH da Universidade Federal do Ceará - CE
51	A SERVIÇO DA JUSTIÇA ELEITORAL - LEI 9.504 ART. 98	CH da Universidade Federal do Ceará - CE
51	A SERVIÇO DA JUSTIÇA ELEITORAL - LEI 9.504 ART. 98	CH da Universidade Federal do Ceará - CE
51	A SERVIÇO DA JUSTIÇA ELEITORAL - LEI 9.504 ART. 98	CH da Universidade Federal do Ceará - CE
51	A SERVIÇO DA JUSTIÇA ELEITORAL - LEI 9.504 ART. 98	CH da Universidade Federal do Ceará - CE
51	A SERVIÇO DA JUSTIÇA ELEITORAL - LEI 9.504 ART. 98	CH da Universidade Federal do Ceará - CE
51	A SERVIÇO DA JUSTIÇA ELEITORAL - LEI 9.504 ART. 98	CH da Universidade Federal do Ceará - CE
51	A SERVIÇO DA JUSTIÇA ELEITORAL - LEI 9.504 ART. 98	CH da Universidade Federal do Ceará - CE
51	A SERVIÇO DA JUSTIÇA ELEITORAL - LEI 9.504 ART. 98	CH da Universidade Federal do Ceará - CE
51	A SERVIÇO DA JUSTIÇA ELEITORAL - LEI 9.504 ART. 98	CH da Universidade Federal do Ceará - CE
51	A SERVIÇO DA JUSTIÇA ELEITORAL - LEI 9.504 ART. 98	CH da Universidade Federal do Ceará - CE
51	A SERVIÇO DA JUSTIÇA ELEITORAL - LEI 9.504 ART. 98	CH da Universidade Federal do Ceará - CE
51	A SERVIÇO DA JUSTIÇA ELEITORAL - LEI 9.504 ART. 98	CH da Universidade Federal do Ceará - CE

51	A SERVIÇO DA JUSTIÇA ELEITORAL - LEI 9.504 ART. 98	CH da Universidade Federal do Ceará - CE
51	A SERVIÇO DA JUSTIÇA ELEITORAL - LEI 9.504 ART. 98	CH da Universidade Federal do Ceará - CE
51	A SERVIÇO DA JUSTIÇA ELEITORAL - LEI 9.504 ART. 98	CH da Universidade Federal do Ceará - CE
51	A SERVIÇO DA JUSTIÇA ELEITORAL - LEI 9.504 ART. 98	CH da Universidade Federal do Ceará - CE
51	A SERVIÇO DA JUSTIÇA ELEITORAL - LEI 9.504 ART. 98	CH da Universidade Federal do Ceará - CE
51	A SERVIÇO DA JUSTIÇA ELEITORAL - LEI 9.504 ART. 98	CH da Universidade Federal do Ceará - CE
51	A SERVIÇO DA JUSTIÇA ELEITORAL - LEI 9.504 ART. 98	CH da Universidade Federal do Ceará - CE
51	A SERVIÇO DA JUSTIÇA ELEITORAL - LEI 9.504 ART. 98	CH da Universidade Federal do Ceará - CE
51	A SERVIÇO DA JUSTIÇA ELEITORAL - LEI 9.504 ART. 98	CH da Universidade Federal do Ceará - CE
51	A SERVIÇO DA JUSTIÇA ELEITORAL - LEI 9.504 ART. 98	CH da Universidade Federal do Ceará - CE
51	A SERVIÇO DA JUSTIÇA ELEITORAL - LEI 9.504 ART. 98	CH da Universidade Federal do Ceará - CE
51	A SERVIÇO DA JUSTIÇA ELEITORAL - LEI 9.504 ART. 98	CH da Universidade Federal do Ceará - CE
51	A SERVIÇO DA JUSTIÇA ELEITORAL - LEI 9.504 ART. 98	CH da Universidade Federal do Ceará - CE
51	A SERVIÇO DA JUSTIÇA ELEITORAL - LEI 9.504 ART. 98	CH da Universidade Federal do Ceará - CE
51	A SERVIÇO DA JUSTIÇA ELEITORAL - LEI 9.504 ART. 98	CH da Universidade Federal do Ceará - CE
51	A SERVIÇO DA JUSTIÇA ELEITORAL - LEI 9.504 ART. 98	CH da Universidade Federal do Ceará - CE
51	A SERVIÇO DA JUSTIÇA ELEITORAL - LEI 9.504 ART. 98	CH da Universidade Federal do Ceará - CE
57	AFASTAMENTO POR ATESTADO MÉDICO - ATE 15 DIAS	CH da Universidade Federal do Ceará - CE
57	AFASTAMENTO POR ATESTADO MÉDICO - ATE 15 DIAS	CH da Universidade Federal do Ceará - CE
57	AFASTAMENTO POR ATESTADO MÉDICO - ATE 15 DIAS	CH da Universidade Federal do Ceará - CE
57	AFASTAMENTO POR ATESTADO MÉDICO - ATE 15 DIAS	CH da Universidade Federal do Ceará - CE
57	AFASTAMENTO POR ATESTADO MÉDICO - ATE 15 DIAS	CH da Universidade Federal do Ceará - CE
57	AFASTAMENTO POR ATESTADO MÉDICO - ATE 15 DIAS	CH da Universidade Federal do Ceará - CE
57	AFASTAMENTO POR ATESTADO MÉDICO - ATE 15 DIAS	CH da Universidade Federal do Ceará - CE
57	AFASTAMENTO POR ATESTADO MÉDICO - ATE 15 DIAS	CH da Universidade Federal do Ceará - CE
57	AFASTAMENTO POR ATESTADO MÉDICO - ATE 15 DIAS	CH da Universidade Federal do Ceará - CE
57	AFASTAMENTO POR ATESTADO MÉDICO - ATE 15 DIAS	CH da Universidade Federal do Ceará - CE
57	AFASTAMENTO POR ATESTADO MÉDICO - ATE 15 DIAS	CH da Universidade Federal do Ceará - CE
57	AFASTAMENTO POR ATESTADO MÉDICO - ATE 15 DIAS	CH da Universidade Federal do Ceará - CE
57	AFASTAMENTO POR ATESTADO MÉDICO - ATE 15 DIAS	CH da Universidade Federal do Ceará - CE
57	AFASTAMENTO POR ATESTADO MÉDICO - ATE 15 DIAS	CH da Universidade Federal do Ceará - CE
57	AFASTAMENTO POR ATESTADO MÉDICO - ATE 15 DIAS	CH da Universidade Federal do Ceará - CE
57	AFASTAMENTO POR ATESTADO MÉDICO - ATE 15 DIAS	CH da Universidade Federal do Ceará - CE
57	AFASTAMENTO POR ATESTADO MÉDICO - ATE 15 DIAS	CH da Universidade Federal do Ceará - CE
57	AFASTAMENTO POR ATESTADO MÉDICO - ATE 15 DIAS	CH da Universidade Federal do Ceará - CE
57	AFASTAMENTO POR ATESTADO MÉDICO - ATE 15 DIAS	CH da Universidade Federal do Ceará - CE
57	AFASTAMENTO POR ATESTADO MÉDICO - ATE 15 DIAS	CH da Universidade Federal do Ceará - CE
57	AFASTAMENTO POR ATESTADO MÉDICO - ATE 15 DIAS	CH da Universidade Federal do Ceará - CE
57	AFASTAMENTO POR ATESTADO MÉDICO - ATE 15 DIAS	CH da Universidade Federal do Ceará - CE
57	AFASTAMENTO POR ATESTADO MÉDICO - ATE 15 DIAS	CH da Universidade Federal do Ceará - CE
57	AFASTAMENTO POR ATESTADO MÉDICO - ATE 15 DIAS	CH da Universidade Federal do Ceará - CE
57	AFASTAMENTO POR ATESTADO MÉDICO - ATE 15 DIAS	CH da Universidade Federal do Ceará - CE
57	AFASTAMENTO POR ATESTADO MÉDICO - ATE 15 DIAS	CH da Universidade Federal do Ceará - CE
57	AFASTAMENTO POR ATESTADO MÉDICO - ATE 15 DIAS	CH da Universidade Federal do Ceará - CE

57	AFASTAMENTO POR ATESTADO MÉDICO - ATE 15 DIAS	CH da Universidade Federal do Ceará - CE
57	AFASTAMENTO POR ATESTADO MÉDICO - ATE 15 DIAS	CH da Universidade Federal do Ceará - CE
57	AFASTAMENTO POR ATESTADO MÉDICO - ATE 15 DIAS	CH da Universidade Federal do Ceará - CE
57	AFASTAMENTO POR ATESTADO MÉDICO - ATE 15 DIAS	CH da Universidade Federal do Ceará - CE
57	AFASTAMENTO POR ATESTADO MÉDICO - ATE 15 DIAS	CH da Universidade Federal do Ceará - CE
57	AFASTAMENTO POR ATESTADO MÉDICO - ATE 15 DIAS	CH da Universidade Federal do Ceará - CE
57	AFASTAMENTO POR ATESTADO MÉDICO - ATE 15 DIAS	CH da Universidade Federal do Ceará - CE
57	AFASTAMENTO POR ATESTADO MÉDICO - ATE 15 DIAS	CH da Universidade Federal do Ceará - CE
57	AFASTAMENTO POR ATESTADO MÉDICO - ATE 15 DIAS	CH da Universidade Federal do Ceará - CE
57	AFASTAMENTO POR ATESTADO MÉDICO - ATE 15 DIAS	CH da Universidade Federal do Ceará - CE
57	AFASTAMENTO POR ATESTADO MÉDICO - ATE 15 DIAS	CH da Universidade Federal do Ceará - CE
57	AFASTAMENTO POR ATESTADO MÉDICO - ATE 15 DIAS	CH da Universidade Federal do Ceará - CE
57	AFASTAMENTO POR ATESTADO MÉDICO - ATE 15 DIAS	CH da Universidade Federal do Ceará - CE
57	AFASTAMENTO POR ATESTADO MÉDICO - ATE 15 DIAS	CH da Universidade Federal do Ceará - CE
57	AFASTAMENTO POR ATESTADO MÉDICO - ATE 15 DIAS	CH da Universidade Federal do Ceará - CE
57	AFASTAMENTO POR ATESTADO MÉDICO - ATE 15 DIAS	CH da Universidade Federal do Ceará - CE
57	AFASTAMENTO POR ATESTADO MÉDICO - ATE 15 DIAS	CH da Universidade Federal do Ceará - CE
57	AFASTAMENTO POR ATESTADO MÉDICO - ATE 15 DIAS	CH da Universidade Federal do Ceará - CE
57	AFASTAMENTO POR ATESTADO MÉDICO - ATE 15 DIAS	CH da Universidade Federal do Ceará - CE
57	AFASTAMENTO POR ATESTADO MÉDICO - ATE 15 DIAS	CH da Universidade Federal do Ceará - CE
57	AFASTAMENTO POR ATESTADO MÉDICO - ATE 15 DIAS	CH da Universidade Federal do Ceará - CE
57	AFASTAMENTO POR ATESTADO MÉDICO - ATE 15 DIAS	CH da Universidade Federal do Ceará - CE
57	AFASTAMENTO POR ATESTADO MÉDICO - ATE 15 DIAS	CH da Universidade Federal do Ceará - CE
57	AFASTAMENTO POR ATESTADO MÉDICO - ATE 15 DIAS	CH da Universidade Federal do Ceará - CE
57	AFASTAMENTO POR ATESTADO MÉDICO - ATE 15 DIAS	CH da Universidade Federal do Ceará - CE
57	AFASTAMENTO POR ATESTADO MÉDICO - ATE 15 DIAS	CH da Universidade Federal do Ceará - CE
57	AFASTAMENTO POR ATESTADO MÉDICO - ATE 15 DIAS	CH da Universidade Federal do Ceará - CE
57	AFASTAMENTO POR ATESTADO MÉDICO - ATE 15 DIAS	CH da Universidade Federal do Ceará - CE
57	AFASTAMENTO POR ATESTADO MÉDICO - ATE 15 DIAS	CH da Universidade Federal do Ceará - CE
57	AFASTAMENTO POR ATESTADO MÉDICO - ATE 15 DIAS	CH da Universidade Federal do Ceará - CE
57	AFASTAMENTO POR ATESTADO MÉDICO - ATE 15 DIAS	CH da Universidade Federal do Ceará - CE
57	AFASTAMENTO POR ATESTADO MÉDICO - ATE 15 DIAS	CH da Universidade Federal do Ceará - CE
57	AFASTAMENTO POR ATESTADO MÉDICO - ATE 15 DIAS	CH da Universidade Federal do Ceará - CE
57	AFASTAMENTO POR ATESTADO MÉDICO - ATE 15 DIAS	CH da Universidade Federal do Ceará - CE
57	AFASTAMENTO POR ATESTADO MÉDICO - ATE 15 DIAS	CH da Universidade Federal do Ceará - CE
57	AFASTAMENTO POR ATESTADO MÉDICO - ATE 15 DIAS	CH da Universidade Federal do Ceará - CE
57	AFASTAMENTO POR ATESTADO MÉDICO - ATE 15 DIAS	CH da Universidade Federal do Ceará - CE
57	AFASTAMENTO POR ATESTADO MÉDICO - ATE 15 DIAS	CH da Universidade Federal do Ceará - CE
57	AFASTAMENTO POR ATESTADO MÉDICO - ATE 15 DIAS	CH da Universidade Federal do Ceará - CE
57	AFASTAMENTO POR ATESTADO MÉDICO - ATE 15 DIAS	CH da Universidade Federal do Ceará - CE
57	AFASTAMENTO POR ATESTADO MÉDICO - ATE 15 DIAS	CH da Universidade Federal do Ceará - CE
57	AFASTAMENTO POR ATESTADO MÉDICO - ATE 15 DIAS	CH da Universidade Federal do Ceará - CE
57	AFASTAMENTO POR ATESTADO MÉDICO - ATE 15 DIAS	CH da Universidade Federal do Ceará - CE
57	AFASTAMENTO POR ATESTADO MÉDICO - ATE 15 DIAS	CH da Universidade Federal do Ceará - CE
57	AFASTAMENTO POR ATESTADO MÉDICO - ATE 15 DIAS	CH da Universidade Federal do Ceará - CE
57	AFASTAMENTO POR ATESTADO MÉDICO - ATE 15 DIAS	CH da Universidade Federal do Ceará - CE
57	AFASTAMENTO POR ATESTADO MÉDICO - ATE 15 DIAS	CH da Universidade Federal do Ceará - CE
57	AFASTAMENTO POR ATESTADO MÉDICO - ATE 15 DIAS	CH da Universidade Federal do Ceará - CE

57	AFASTAMENTO POR ATESTADO MÉDICO - ATE 15 DIAS	CH da Universidade Federal do Ceará - CE
57	AFASTAMENTO POR ATESTADO MÉDICO - ATE 15 DIAS	CH da Universidade Federal do Ceará - CE
57	AFASTAMENTO POR ATESTADO MÉDICO - ATE 15 DIAS	CH da Universidade Federal do Ceará - CE
57	AFASTAMENTO POR ATESTADO MÉDICO - ATE 15 DIAS	CH da Universidade Federal do Ceará - CE
57	AFASTAMENTO POR ATESTADO MÉDICO - ATE 15 DIAS	CH da Universidade Federal do Ceará - CE
57	AFASTAMENTO POR ATESTADO MÉDICO - ATE 15 DIAS	CH da Universidade Federal do Ceará - CE
57	AFASTAMENTO POR ATESTADO MÉDICO - ATE 15 DIAS	CH da Universidade Federal do Ceará - CE
57	AFASTAMENTO POR ATESTADO MÉDICO - ATE 15 DIAS	CH da Universidade Federal do Ceará - CE
57	AFASTAMENTO POR ATESTADO MÉDICO - ATE 15 DIAS	CH da Universidade Federal do Ceará - CE
57	AFASTAMENTO POR ATESTADO MÉDICO - ATE 15 DIAS	CH da Universidade Federal do Ceará - CE
57	AFASTAMENTO POR ATESTADO MÉDICO - ATE 15 DIAS	CH da Universidade Federal do Ceará - CE
57	AFASTAMENTO POR ATESTADO MÉDICO - ATE 15 DIAS	CH da Universidade Federal do Ceará - CE
57	AFASTAMENTO POR ATESTADO MÉDICO - ATE 15 DIAS	CH da Universidade Federal do Ceará - CE
57	AFASTAMENTO POR ATESTADO MÉDICO - ATE 15 DIAS	CH da Universidade Federal do Ceará - CE
57	AFASTAMENTO POR ATESTADO MÉDICO - ATE 15 DIAS	CH da Universidade Federal do Ceará - CE
57	AFASTAMENTO POR ATESTADO MÉDICO - ATE 15 DIAS	CH da Universidade Federal do Ceará - CE
57	AFASTAMENTO POR ATESTADO MÉDICO - ATE 15 DIAS	CH da Universidade Federal do Ceará - CE
57	AFASTAMENTO POR ATESTADO MÉDICO - ATE 15 DIAS	CH da Universidade Federal do Ceará - CE
57	AFASTAMENTO POR ATESTADO MÉDICO - ATE 15 DIAS	CH da Universidade Federal do Ceará - CE
57	AFASTAMENTO POR ATESTADO MÉDICO - ATE 15 DIAS	CH da Universidade Federal do Ceará - CE
57	AFASTAMENTO POR ATESTADO MÉDICO - ATE 15 DIAS	CH da Universidade Federal do Ceará - CE
57	AFASTAMENTO POR ATESTADO MÉDICO - ATE 15 DIAS	CH da Universidade Federal do Ceará - CE
57	AFASTAMENTO POR ATESTADO MÉDICO - ATE 15 DIAS	CH da Universidade Federal do Ceará - CE
57	AFASTAMENTO POR ATESTADO MÉDICO - ATE 15 DIAS	CH da Universidade Federal do Ceará - CE
57	AFASTAMENTO POR ATESTADO MÉDICO - ATE 15 DIAS	CH da Universidade Federal do Ceará - CE
57	AFASTAMENTO POR ATESTADO MÉDICO - ATE 15 DIAS	CH da Universidade Federal do Ceará - CE
57	AFASTAMENTO POR ATESTADO MÉDICO - ATE 15 DIAS	CH da Universidade Federal do Ceará - CE
57	AFASTAMENTO POR ATESTADO MÉDICO - ATE 15 DIAS	CH da Universidade Federal do Ceará - CE
57	AFASTAMENTO POR ATESTADO MÉDICO - ATE 15 DIAS	CH da Universidade Federal do Ceará - CE
57	AFASTAMENTO POR ATESTADO MÉDICO - ATE 15 DIAS	CH da Universidade Federal do Ceará - CE
57	AFASTAMENTO POR ATESTADO MÉDICO - ATE 15 DIAS	CH da Universidade Federal do Ceará - CE
57	AFASTAMENTO POR ATESTADO MÉDICO - ATE 15 DIAS	CH da Universidade Federal do Ceará - CE
57	AFASTAMENTO POR ATESTADO MÉDICO - ATE 15 DIAS	CH da Universidade Federal do Ceará - CE
57	AFASTAMENTO POR ATESTADO MÉDICO - ATE 15 DIAS	CH da Universidade Federal do Ceará - CE
57	AFASTAMENTO POR ATESTADO MÉDICO - ATE 15 DIAS	CH da Universidade Federal do Ceará - CE
57	AFASTAMENTO POR ATESTADO MÉDICO - ATE 15 DIAS	CH da Universidade Federal do Ceará - CE
57	AFASTAMENTO POR ATESTADO MÉDICO - ATE 15 DIAS	CH da Universidade Federal do Ceará - CE
57	AFASTAMENTO POR ATESTADO MÉDICO - ATE 15 DIAS	CH da Universidade Federal do Ceará - CE
57	AFASTAMENTO POR ATESTADO MÉDICO - ATE 15 DIAS	CH da Universidade Federal do Ceará - CE
57	AFASTAMENTO POR ATESTADO MÉDICO - ATE 15 DIAS	CH da Universidade Federal do Ceará - CE
57	AFASTAMENTO POR ATESTADO MÉDICO - ATE 15 DIAS	CH da Universidade Federal do Ceará - CE
57	AFASTAMENTO POR ATESTADO MÉDICO - ATE 15 DIAS	CH da Universidade Federal do Ceará - CE
57	AFASTAMENTO POR ATESTADO MÉDICO - ATE 15 DIAS	CH da Universidade Federal do Ceará - CE
57	AFASTAMENTO POR ATESTADO MÉDICO - ATE 15 DIAS	CH da Universidade Federal do Ceará - CE
57	AFASTAMENTO POR ATESTADO MÉDICO - ATE 15 DIAS	CH da Universidade Federal do Ceará - CE
57	AFASTAMENTO POR ATESTADO MÉDICO - ATE 15 DIAS	CH da Universidade Federal do Ceará - CE
57	AFASTAMENTO POR ATESTADO MÉDICO - ATE 15 DIAS	CH da Universidade Federal do Ceará - CE
57	AFASTAMENTO POR ATESTADO MÉDICO - ATE 15 DIAS	CH da Universidade Federal do Ceará - CE

57	AFASTAMENTO POR ATESTADO MÉDICO - ATE 15 DIAS	CH da Universidade Federal do Ceará - CE
57	AFASTAMENTO POR ATESTADO MÉDICO - ATE 15 DIAS	CH da Universidade Federal do Ceará - CE
57	AFASTAMENTO POR ATESTADO MÉDICO - ATE 15 DIAS	CH da Universidade Federal do Ceará - CE
57	AFASTAMENTO POR ATESTADO MÉDICO - ATE 15 DIAS	CH da Universidade Federal do Ceará - CE
57	AFASTAMENTO POR ATESTADO MÉDICO - ATE 15 DIAS	CH da Universidade Federal do Ceará - CE
57	AFASTAMENTO POR ATESTADO MÉDICO - ATE 15 DIAS	CH da Universidade Federal do Ceará - CE
57	AFASTAMENTO POR ATESTADO MÉDICO - ATE 15 DIAS	CH da Universidade Federal do Ceará - CE
57	AFASTAMENTO POR ATESTADO MÉDICO - ATE 15 DIAS	CH da Universidade Federal do Ceará - CE
57	AFASTAMENTO POR ATESTADO MÉDICO - ATE 15 DIAS	CH da Universidade Federal do Ceará - CE
57	AFASTAMENTO POR ATESTADO MÉDICO - ATE 15 DIAS	CH da Universidade Federal do Ceará - CE
57	AFASTAMENTO POR ATESTADO MÉDICO - ATE 15 DIAS	CH da Universidade Federal do Ceará - CE
57	AFASTAMENTO POR ATESTADO MÉDICO - ATE 15 DIAS	CH da Universidade Federal do Ceará - CE
57	AFASTAMENTO POR ATESTADO MÉDICO - ATE 15 DIAS	CH da Universidade Federal do Ceará - CE
57	AFASTAMENTO POR ATESTADO MÉDICO - ATE 15 DIAS	CH da Universidade Federal do Ceará - CE
57	AFASTAMENTO POR ATESTADO MÉDICO - ATE 15 DIAS	CH da Universidade Federal do Ceará - CE
57	AFASTAMENTO POR ATESTADO MÉDICO - ATE 15 DIAS	CH da Universidade Federal do Ceará - CE
57	AFASTAMENTO POR ATESTADO MÉDICO - ATE 15 DIAS	CH da Universidade Federal do Ceará - CE
57	AFASTAMENTO POR ATESTADO MÉDICO - ATE 15 DIAS	CH da Universidade Federal do Ceará - CE
57	AFASTAMENTO POR ATESTADO MÉDICO - ATE 15 DIAS	CH da Universidade Federal do Ceará - CE
57	AFASTAMENTO POR ATESTADO MÉDICO - ATE 15 DIAS	CH da Universidade Federal do Ceará - CE
57	AFASTAMENTO POR ATESTADO MÉDICO - ATE 15 DIAS	CH da Universidade Federal do Ceará - CE
57	AFASTAMENTO POR ATESTADO MÉDICO - ATE 15 DIAS	CH da Universidade Federal do Ceará - CE
57	AFASTAMENTO POR ATESTADO MÉDICO - ATE 15 DIAS	CH da Universidade Federal do Ceará - CE
57	AFASTAMENTO POR ATESTADO MÉDICO - ATE 15 DIAS	CH da Universidade Federal do Ceará - CE
57	AFASTAMENTO POR ATESTADO MÉDICO - ATE 15 DIAS	CH da Universidade Federal do Ceará - CE
57	AFASTAMENTO POR ATESTADO MÉDICO - ATE 15 DIAS	CH da Universidade Federal do Ceará - CE
57	AFASTAMENTO POR ATESTADO MÉDICO - ATE 15 DIAS	CH da Universidade Federal do Ceará - CE
57	AFASTAMENTO POR ATESTADO MÉDICO - ATE 15 DIAS	CH da Universidade Federal do Ceará - CE
57	AFASTAMENTO POR ATESTADO MÉDICO - ATE 15 DIAS	CH da Universidade Federal do Ceará - CE
57	AFASTAMENTO POR ATESTADO MÉDICO - ATE 15 DIAS	CH da Universidade Federal do Ceará - CE
57	AFASTAMENTO POR ATESTADO MÉDICO - ATE 15 DIAS	CH da Universidade Federal do Ceará - CE
57	AFASTAMENTO POR ATESTADO MÉDICO - ATE 15 DIAS	CH da Universidade Federal do Ceará - CE
57	AFASTAMENTO POR ATESTADO MÉDICO - ATE 15 DIAS	CH da Universidade Federal do Ceará - CE
57	AFASTAMENTO POR ATESTADO MÉDICO - ATE 15 DIAS	CH da Universidade Federal do Ceará - CE
57	AFASTAMENTO POR ATESTADO MÉDICO - ATE 15 DIAS	CH da Universidade Federal do Ceará - CE
57	AFASTAMENTO POR ATESTADO MÉDICO - ATE 15 DIAS	CH da Universidade Federal do Ceará - CE
57	AFASTAMENTO POR ATESTADO MÉDICO - ATE 15 DIAS	CH da Universidade Federal do Ceará - CE
57	AFASTAMENTO POR ATESTADO MÉDICO - ATE 15 DIAS	CH da Universidade Federal do Ceará - CE
57	AFASTAMENTO POR ATESTADO MÉDICO - ATE 15 DIAS	CH da Universidade Federal do Ceará - CE
57	AFASTAMENTO POR ATESTADO MÉDICO - ATE 15 DIAS	CH da Universidade Federal do Ceará - CE
57	AFASTAMENTO POR ATESTADO MÉDICO - ATE 15 DIAS	CH da Universidade Federal do Ceará - CE
57	AFASTAMENTO POR ATESTADO MÉDICO - ATE 15 DIAS	CH da Universidade Federal do Ceará - CE
57	AFASTAMENTO POR ATESTADO MÉDICO - ATE 15 DIAS	CH da Universidade Federal do Ceará - CE
57	AFASTAMENTO POR ATESTADO MÉDICO - ATE 15 DIAS	CH da Universidade Federal do Ceará - CE
57	AFASTAMENTO POR ATESTADO MÉDICO - ATE 15 DIAS	CH da Universidade Federal do Ceará - CE
57	AFASTAMENTO POR ATESTADO MÉDICO - ATE 15 DIAS	CH da Universidade Federal do Ceará - CE
57	AFASTAMENTO POR ATESTADO MÉDICO - ATE 15 DIAS	CH da Universidade Federal do Ceará - CE
57	AFASTAMENTO POR ATESTADO MÉDICO - ATE 15 DIAS	CH da Universidade Federal do Ceará - CE

57	AFASTAMENTO POR ATESTADO MÉDICO - ATE 15 DIAS	CH da Universidade Federal do Ceará - CE
57	AFASTAMENTO POR ATESTADO MÉDICO - ATE 15 DIAS	CH da Universidade Federal do Ceará - CE
57	AFASTAMENTO POR ATESTADO MÉDICO - ATE 15 DIAS	CH da Universidade Federal do Ceará - CE
57	AFASTAMENTO POR ATESTADO MÉDICO - ATE 15 DIAS	CH da Universidade Federal do Ceará - CE
57	AFASTAMENTO POR ATESTADO MÉDICO - ATE 15 DIAS	CH da Universidade Federal do Ceará - CE
57	AFASTAMENTO POR ATESTADO MÉDICO - ATE 15 DIAS	CH da Universidade Federal do Ceará - CE
57	AFASTAMENTO POR ATESTADO MÉDICO - ATE 15 DIAS	CH da Universidade Federal do Ceará - CE
57	AFASTAMENTO POR ATESTADO MÉDICO - ATE 15 DIAS	CH da Universidade Federal do Ceará - CE
57	AFASTAMENTO POR ATESTADO MÉDICO - ATE 15 DIAS	CH da Universidade Federal do Ceará - CE
57	AFASTAMENTO POR ATESTADO MÉDICO - ATE 15 DIAS	CH da Universidade Federal do Ceará - CE
57	AFASTAMENTO POR ATESTADO MÉDICO - ATE 15 DIAS	CH da Universidade Federal do Ceará - CE
57	AFASTAMENTO POR ATESTADO MÉDICO - ATE 15 DIAS	CH da Universidade Federal do Ceará - CE
57	AFASTAMENTO POR ATESTADO MÉDICO - ATE 15 DIAS	CH da Universidade Federal do Ceará - CE
57	AFASTAMENTO POR ATESTADO MÉDICO - ATE 15 DIAS	CH da Universidade Federal do Ceará - CE
57	AFASTAMENTO POR ATESTADO MÉDICO - ATE 15 DIAS	CH da Universidade Federal do Ceará - CE
57	AFASTAMENTO POR ATESTADO MÉDICO - ATE 15 DIAS	CH da Universidade Federal do Ceará - CE
57	AFASTAMENTO POR ATESTADO MÉDICO - ATE 15 DIAS	CH da Universidade Federal do Ceará - CE
57	AFASTAMENTO POR ATESTADO MÉDICO - ATE 15 DIAS	CH da Universidade Federal do Ceará - CE
57	AFASTAMENTO POR ATESTADO MÉDICO - ATE 15 DIAS	CH da Universidade Federal do Ceará - CE
57	AFASTAMENTO POR ATESTADO MÉDICO - ATE 15 DIAS	CH da Universidade Federal do Ceará - CE
57	AFASTAMENTO POR ATESTADO MÉDICO - ATE 15 DIAS	CH da Universidade Federal do Ceará - CE
57	AFASTAMENTO POR ATESTADO MÉDICO - ATE 15 DIAS	CH da Universidade Federal do Ceará - CE
57	AFASTAMENTO POR ATESTADO MÉDICO - ATE 15 DIAS	CH da Universidade Federal do Ceará - CE
57	AFASTAMENTO POR ATESTADO MÉDICO - ATE 15 DIAS	CH da Universidade Federal do Ceará - CE
57	AFASTAMENTO POR ATESTADO MÉDICO - ATE 15 DIAS	CH da Universidade Federal do Ceará - CE
57	AFASTAMENTO POR ATESTADO MÉDICO - ATE 15 DIAS	CH da Universidade Federal do Ceará - CE
57	AFASTAMENTO POR ATESTADO MÉDICO - ATE 15 DIAS	CH da Universidade Federal do Ceará - CE
57	AFASTAMENTO POR ATESTADO MÉDICO - ATE 15 DIAS	CH da Universidade Federal do Ceará - CE
57	AFASTAMENTO POR ATESTADO MÉDICO - ATE 15 DIAS	CH da Universidade Federal do Ceará - CE
57	AFASTAMENTO POR ATESTADO MÉDICO - ATE 15 DIAS	CH da Universidade Federal do Ceará - CE
57	AFASTAMENTO POR ATESTADO MÉDICO - ATE 15 DIAS	CH da Universidade Federal do Ceará - CE
57	AFASTAMENTO POR ATESTADO MÉDICO - ATE 15 DIAS	CH da Universidade Federal do Ceará - CE
57	AFASTAMENTO POR ATESTADO MÉDICO - ATE 15 DIAS	CH da Universidade Federal do Ceará - CE
57	AFASTAMENTO POR ATESTADO MÉDICO - ATE 15 DIAS	CH da Universidade Federal do Ceará - CE
57	AFASTAMENTO POR ATESTADO MÉDICO - ATE 15 DIAS	CH da Universidade Federal do Ceará - CE
57	AFASTAMENTO POR ATESTADO MÉDICO - ATE 15 DIAS	CH da Universidade Federal do Ceará - CE
57	AFASTAMENTO POR ATESTADO MÉDICO - ATE 15 DIAS	CH da Universidade Federal do Ceará - CE
57	AFASTAMENTO POR ATESTADO MÉDICO - ATE 15 DIAS	CH da Universidade Federal do Ceará - CE
57	AFASTAMENTO POR ATESTADO MÉDICO - ATE 15 DIAS	CH da Universidade Federal do Ceará - CE
57	AFASTAMENTO POR ATESTADO MÉDICO - ATE 15 DIAS	CH da Universidade Federal do Ceará - CE
57	AFASTAMENTO POR ATESTADO MÉDICO - ATE 15 DIAS	CH da Universidade Federal do Ceará - CE
57	AFASTAMENTO POR ATESTADO MÉDICO - ATE 15 DIAS	CH da Universidade Federal do Ceará - CE
57	AFASTAMENTO POR ATESTADO MÉDICO - ATE 15 DIAS	CH da Universidade Federal do Ceará - CE
57	AFASTAMENTO POR ATESTADO MÉDICO - ATE 15 DIAS	CH da Universidade Federal do Ceará - CE
57	AFASTAMENTO POR ATESTADO MÉDICO - ATE 15 DIAS	CH da Universidade Federal do Ceará - CE
57	AFASTAMENTO POR ATESTADO MÉDICO - ATE 15 DIAS	CH da Universidade Federal do Ceará - CE
57	AFASTAMENTO POR ATESTADO MÉDICO - ATE 15 DIAS	CH da Universidade Federal do Ceará - CE
57	AFASTAMENTO POR ATESTADO MÉDICO - ATE 15 DIAS	CH da Universidade Federal do Ceará - CE

57	AFASTAMENTO POR ATESTADO MÉDICO - ATE 15 DIAS	CH da Universidade Federal do Ceará - CE
57	AFASTAMENTO POR ATESTADO MÉDICO - ATE 15 DIAS	CH da Universidade Federal do Ceará - CE
57	AFASTAMENTO POR ATESTADO MÉDICO - ATE 15 DIAS	CH da Universidade Federal do Ceará - CE
57	AFASTAMENTO POR ATESTADO MÉDICO - ATE 15 DIAS	CH da Universidade Federal do Ceará - CE
57	AFASTAMENTO POR ATESTADO MÉDICO - ATE 15 DIAS	CH da Universidade Federal do Ceará - CE
57	AFASTAMENTO POR ATESTADO MÉDICO - ATE 15 DIAS	CH da Universidade Federal do Ceará - CE
57	AFASTAMENTO POR ATESTADO MÉDICO - ATE 15 DIAS	CH da Universidade Federal do Ceará - CE
57	AFASTAMENTO POR ATESTADO MÉDICO - ATE 15 DIAS	CH da Universidade Federal do Ceará - CE
57	AFASTAMENTO POR ATESTADO MÉDICO - ATE 15 DIAS	CH da Universidade Federal do Ceará - CE
57	AFASTAMENTO POR ATESTADO MÉDICO - ATE 15 DIAS	CH da Universidade Federal do Ceará - CE
57	AFASTAMENTO POR ATESTADO MÉDICO - ATE 15 DIAS	CH da Universidade Federal do Ceará - CE
57	AFASTAMENTO POR ATESTADO MÉDICO - ATE 15 DIAS	CH da Universidade Federal do Ceará - CE
57	AFASTAMENTO POR ATESTADO MÉDICO - ATE 15 DIAS	CH da Universidade Federal do Ceará - CE
57	AFASTAMENTO POR ATESTADO MÉDICO - ATE 15 DIAS	CH da Universidade Federal do Ceará - CE
57	AFASTAMENTO POR ATESTADO MÉDICO - ATE 15 DIAS	CH da Universidade Federal do Ceará - CE
57	AFASTAMENTO POR ATESTADO MÉDICO - ATE 15 DIAS	CH da Universidade Federal do Ceará - CE
57	AFASTAMENTO POR ATESTADO MÉDICO - ATE 15 DIAS	CH da Universidade Federal do Ceará - CE
57	AFASTAMENTO POR ATESTADO MÉDICO - ATE 15 DIAS	CH da Universidade Federal do Ceará - CE
57	AFASTAMENTO POR ATESTADO MÉDICO - ATE 15 DIAS	CH da Universidade Federal do Ceará - CE
57	AFASTAMENTO POR ATESTADO MÉDICO - ATE 15 DIAS	CH da Universidade Federal do Ceará - CE
57	AFASTAMENTO POR ATESTADO MÉDICO - ATE 15 DIAS	CH da Universidade Federal do Ceará - CE
57	AFASTAMENTO POR ATESTADO MÉDICO - ATE 15 DIAS	CH da Universidade Federal do Ceará - CE
57	AFASTAMENTO POR ATESTADO MÉDICO - ATE 15 DIAS	CH da Universidade Federal do Ceará - CE
57	AFASTAMENTO POR ATESTADO MÉDICO - ATE 15 DIAS	CH da Universidade Federal do Ceará - CE
57	AFASTAMENTO POR ATESTADO MÉDICO - ATE 15 DIAS	CH da Universidade Federal do Ceará - CE
57	AFASTAMENTO POR ATESTADO MÉDICO - ATE 15 DIAS	CH da Universidade Federal do Ceará - CE
57	AFASTAMENTO POR ATESTADO MÉDICO - ATE 15 DIAS	CH da Universidade Federal do Ceará - CE
57	AFASTAMENTO POR ATESTADO MÉDICO - ATE 15 DIAS	CH da Universidade Federal do Ceará - CE
57	AFASTAMENTO POR ATESTADO MÉDICO - ATE 15 DIAS	CH da Universidade Federal do Ceará - CE
57	AFASTAMENTO POR ATESTADO MÉDICO - ATE 15 DIAS	CH da Universidade Federal do Ceará - CE
57	AFASTAMENTO POR ATESTADO MÉDICO - ATE 15 DIAS	CH da Universidade Federal do Ceará - CE
57	AFASTAMENTO POR ATESTADO MÉDICO - ATE 15 DIAS	CH da Universidade Federal do Ceará - CE
57	AFASTAMENTO POR ATESTADO MÉDICO - ATE 15 DIAS	CH da Universidade Federal do Ceará - CE
57	AFASTAMENTO POR ATESTADO MÉDICO - ATE 15 DIAS	CH da Universidade Federal do Ceará - CE
57	AFASTAMENTO POR ATESTADO MÉDICO - ATE 15 DIAS	CH da Universidade Federal do Ceará - CE
57	AFASTAMENTO POR ATESTADO MÉDICO - ATE 15 DIAS	CH da Universidade Federal do Ceará - CE
57	AFASTAMENTO POR ATESTADO MÉDICO - ATE 15 DIAS	CH da Universidade Federal do Ceará - CE
57	AFASTAMENTO POR ATESTADO MÉDICO - ATE 15 DIAS	CH da Universidade Federal do Ceará - CE
57	AFASTAMENTO POR ATESTADO MÉDICO - ATE 15 DIAS	CH da Universidade Federal do Ceará - CE
57	AFASTAMENTO POR ATESTADO MÉDICO - ATE 15 DIAS	CH da Universidade Federal do Ceará - CE
57	AFASTAMENTO POR ATESTADO MÉDICO - ATE 15 DIAS	CH da Universidade Federal do Ceará - CE
57	AFASTAMENTO POR ATESTADO MÉDICO - ATE 15 DIAS	CH da Universidade Federal do Ceará - CE
57	AFASTAMENTO POR ATESTADO MÉDICO - ATE 15 DIAS	CH da Universidade Federal do Ceará - CE
57	AFASTAMENTO POR ATESTADO MÉDICO - ATE 15 DIAS	CH da Universidade Federal do Ceará - CE
57	AFASTAMENTO POR ATESTADO MÉDICO - ATE 15 DIAS	CH da Universidade Federal do Ceará - CE
57	AFASTAMENTO POR ATESTADO MÉDICO - ATE 15 DIAS	CH da Universidade Federal do Ceará - CE
57	AFASTAMENTO POR ATESTADO MÉDICO - ATE 15 DIAS	CH da Universidade Federal do Ceará - CE
57	AFASTAMENTO POR ATESTADO MÉDICO - ATE 15 DIAS	CH da Universidade Federal do Ceará - CE

57	AFASTAMENTO POR ATESTADO MÉDICO - ATE 15 DIAS	CH da Universidade Federal do Ceará - CE
57	AFASTAMENTO POR ATESTADO MÉDICO - ATE 15 DIAS	CH da Universidade Federal do Ceará - CE
57	AFASTAMENTO POR ATESTADO MÉDICO - ATE 15 DIAS	CH da Universidade Federal do Ceará - CE
57	AFASTAMENTO POR ATESTADO MÉDICO - ATE 15 DIAS	CH da Universidade Federal do Ceará - CE
57	AFASTAMENTO POR ATESTADO MÉDICO - ATE 15 DIAS	CH da Universidade Federal do Ceará - CE
57	AFASTAMENTO POR ATESTADO MÉDICO - ATE 15 DIAS	CH da Universidade Federal do Ceará - CE
57	AFASTAMENTO POR ATESTADO MÉDICO - ATE 15 DIAS	CH da Universidade Federal do Ceará - CE
57	AFASTAMENTO POR ATESTADO MÉDICO - ATE 15 DIAS	CH da Universidade Federal do Ceará - CE
57	AFASTAMENTO POR ATESTADO MÉDICO - ATE 15 DIAS	CH da Universidade Federal do Ceará - CE
57	AFASTAMENTO POR ATESTADO MÉDICO - ATE 15 DIAS	CH da Universidade Federal do Ceará - CE
57	AFASTAMENTO POR ATESTADO MÉDICO - ATE 15 DIAS	CH da Universidade Federal do Ceará - CE
57	AFASTAMENTO POR ATESTADO MÉDICO - ATE 15 DIAS	CH da Universidade Federal do Ceará - CE
57	AFASTAMENTO POR ATESTADO MÉDICO - ATE 15 DIAS	CH da Universidade Federal do Ceará - CE
57	AFASTAMENTO POR ATESTADO MÉDICO - ATE 15 DIAS	CH da Universidade Federal do Ceará - CE
57	AFASTAMENTO POR ATESTADO MÉDICO - ATE 15 DIAS	CH da Universidade Federal do Ceará - CE
57	AFASTAMENTO POR ATESTADO MÉDICO - ATE 15 DIAS	CH da Universidade Federal do Ceará - CE
57	AFASTAMENTO POR ATESTADO MÉDICO - ATE 15 DIAS	CH da Universidade Federal do Ceará - CE
57	AFASTAMENTO POR ATESTADO MÉDICO - ATE 15 DIAS	CH da Universidade Federal do Ceará - CE
57	AFASTAMENTO POR ATESTADO MÉDICO - ATE 15 DIAS	CH da Universidade Federal do Ceará - CE
57	AFASTAMENTO POR ATESTADO MÉDICO - ATE 15 DIAS	CH da Universidade Federal do Ceará - CE
57	AFASTAMENTO POR ATESTADO MÉDICO - ATE 15 DIAS	CH da Universidade Federal do Ceará - CE
57	AFASTAMENTO POR ATESTADO MÉDICO - ATE 15 DIAS	CH da Universidade Federal do Ceará - CE
57	AFASTAMENTO POR ATESTADO MÉDICO - ATE 15 DIAS	CH da Universidade Federal do Ceará - CE
57	AFASTAMENTO POR ATESTADO MÉDICO - ATE 15 DIAS	CH da Universidade Federal do Ceará - CE
57	AFASTAMENTO POR ATESTADO MÉDICO - ATE 15 DIAS	CH da Universidade Federal do Ceará - CE
57	AFASTAMENTO POR ATESTADO MÉDICO - ATE 15 DIAS	CH da Universidade Federal do Ceará - CE
57	AFASTAMENTO POR ATESTADO MÉDICO - ATE 15 DIAS	CH da Universidade Federal do Ceará - CE
57	AFASTAMENTO POR ATESTADO MÉDICO - ATE 15 DIAS	CH da Universidade Federal do Ceará - CE
57	AFASTAMENTO POR ATESTADO MÉDICO - ATE 15 DIAS	CH da Universidade Federal do Ceará - CE
57	AFASTAMENTO POR ATESTADO MÉDICO - ATE 15 DIAS	CH da Universidade Federal do Ceará - CE
57	AFASTAMENTO POR ATESTADO MÉDICO - ATE 15 DIAS	CH da Universidade Federal do Ceará - CE
57	AFASTAMENTO POR ATESTADO MÉDICO - ATE 15 DIAS	CH da Universidade Federal do Ceará - CE
57	AFASTAMENTO POR ATESTADO MÉDICO - ATE 15 DIAS	CH da Universidade Federal do Ceará - CE
57	AFASTAMENTO POR ATESTADO MÉDICO - ATE 15 DIAS	CH da Universidade Federal do Ceará - CE
57	AFASTAMENTO POR ATESTADO MÉDICO - ATE 15 DIAS	CH da Universidade Federal do Ceará - CE
57	AFASTAMENTO POR ATESTADO MÉDICO - ATE 15 DIAS	CH da Universidade Federal do Ceará - CE
57	AFASTAMENTO POR ATESTADO MÉDICO - ATE 15 DIAS	CH da Universidade Federal do Ceará - CE
57	AFASTAMENTO POR ATESTADO MÉDICO - ATE 15 DIAS	CH da Universidade Federal do Ceará - CE
57	AFASTAMENTO POR ATESTADO MÉDICO - ATE 15 DIAS	CH da Universidade Federal do Ceará - CE
57	AFASTAMENTO POR ATESTADO MÉDICO - ATE 15 DIAS	CH da Universidade Federal do Ceará - CE
57	AFASTAMENTO POR ATESTADO MÉDICO - ATE 15 DIAS	CH da Universidade Federal do Ceará - CE
57	AFASTAMENTO POR ATESTADO MÉDICO - ATE 15 DIAS	CH da Universidade Federal do Ceará - CE
57	AFASTAMENTO POR ATESTADO MÉDICO - ATE 15 DIAS	CH da Universidade Federal do Ceará - CE
57	AFASTAMENTO POR ATESTADO MÉDICO - ATE 15 DIAS	CH da Universidade Federal do Ceará - CE
57	AFASTAMENTO POR ATESTADO MÉDICO - ATE 15 DIAS	CH da Universidade Federal do Ceará - CE
57	AFASTAMENTO POR ATESTADO MÉDICO - ATE 15 DIAS	CH da Universidade Federal do Ceará - CE
57	AFASTAMENTO POR ATESTADO MÉDICO - ATE 15 DIAS	CH da Universidade Federal do Ceará - CE
57	AFASTAMENTO POR ATESTADO MÉDICO - ATE 15 DIAS	CH da Universidade Federal do Ceará - CE

57	AFASTAMENTO POR ATESTADO MÉDICO - ATE 15 DIAS	CH da Universidade Federal do Ceará - CE
57	AFASTAMENTO POR ATESTADO MÉDICO - ATE 15 DIAS	CH da Universidade Federal do Ceará - CE
57	AFASTAMENTO POR ATESTADO MÉDICO - ATE 15 DIAS	CH da Universidade Federal do Ceará - CE
57	AFASTAMENTO POR ATESTADO MÉDICO - ATE 15 DIAS	CH da Universidade Federal do Ceará - CE
57	AFASTAMENTO POR ATESTADO MÉDICO - ATE 15 DIAS	CH da Universidade Federal do Ceará - CE
57	AFASTAMENTO POR ATESTADO MÉDICO - ATE 15 DIAS	CH da Universidade Federal do Ceará - CE
57	AFASTAMENTO POR ATESTADO MÉDICO - ATE 15 DIAS	CH da Universidade Federal do Ceará - CE
57	AFASTAMENTO POR ATESTADO MÉDICO - ATE 15 DIAS	CH da Universidade Federal do Ceará - CE
57	AFASTAMENTO POR ATESTADO MÉDICO - ATE 15 DIAS	CH da Universidade Federal do Ceará - CE
57	AFASTAMENTO POR ATESTADO MÉDICO - ATE 15 DIAS	CH da Universidade Federal do Ceará - CE
57	AFASTAMENTO POR ATESTADO MÉDICO - ATE 15 DIAS	CH da Universidade Federal do Ceará - CE
57	AFASTAMENTO POR ATESTADO MÉDICO - ATE 15 DIAS	CH da Universidade Federal do Ceará - CE
57	AFASTAMENTO POR ATESTADO MÉDICO - ATE 15 DIAS	CH da Universidade Federal do Ceará - CE
57	AFASTAMENTO POR ATESTADO MÉDICO - ATE 15 DIAS	CH da Universidade Federal do Ceará - CE
57	AFASTAMENTO POR ATESTADO MÉDICO - ATE 15 DIAS	CH da Universidade Federal do Ceará - CE
57	AFASTAMENTO POR ATESTADO MÉDICO - ATE 15 DIAS	CH da Universidade Federal do Ceará - CE
57	AFASTAMENTO POR ATESTADO MÉDICO - ATE 15 DIAS	CH da Universidade Federal do Ceará - CE
57	AFASTAMENTO POR ATESTADO MÉDICO - ATE 15 DIAS	CH da Universidade Federal do Ceará - CE
57	AFASTAMENTO POR ATESTADO MÉDICO - ATE 15 DIAS	CH da Universidade Federal do Ceará - CE
57	AFASTAMENTO POR ATESTADO MÉDICO - ATE 15 DIAS	CH da Universidade Federal do Ceará - CE
57	AFASTAMENTO POR ATESTADO MÉDICO - ATE 15 DIAS	CH da Universidade Federal do Ceará - CE
57	AFASTAMENTO POR ATESTADO MÉDICO - ATE 15 DIAS	CH da Universidade Federal do Ceará - CE
57	AFASTAMENTO POR ATESTADO MÉDICO - ATE 15 DIAS	CH da Universidade Federal do Ceará - CE
57	AFASTAMENTO POR ATESTADO MÉDICO - ATE 15 DIAS	CH da Universidade Federal do Ceará - CE
57	AFASTAMENTO POR ATESTADO MÉDICO - ATE 15 DIAS	CH da Universidade Federal do Ceará - CE
57	AFASTAMENTO POR ATESTADO MÉDICO - ATE 15 DIAS	CH da Universidade Federal do Ceará - CE
57	AFASTAMENTO POR ATESTADO MÉDICO - ATE 15 DIAS	CH da Universidade Federal do Ceará - CE
57	AFASTAMENTO POR ATESTADO MÉDICO - ATE 15 DIAS	CH da Universidade Federal do Ceará - CE
57	AFASTAMENTO POR ATESTADO MÉDICO - ATE 15 DIAS	CH da Universidade Federal do Ceará - CE
57	AFASTAMENTO POR ATESTADO MÉDICO - ATE 15 DIAS	CH da Universidade Federal do Ceará - CE
57	AFASTAMENTO POR ATESTADO MÉDICO - ATE 15 DIAS	CH da Universidade Federal do Ceará - CE
57	AFASTAMENTO POR ATESTADO MÉDICO - ATE 15 DIAS	CH da Universidade Federal do Ceará - CE
57	AFASTAMENTO POR ATESTADO MÉDICO - ATE 15 DIAS	CH da Universidade Federal do Ceará - CE
57	AFASTAMENTO POR ATESTADO MÉDICO - ATE 15 DIAS	CH da Universidade Federal do Ceará - CE
57	AFASTAMENTO POR ATESTADO MÉDICO - ATE 15 DIAS	CH da Universidade Federal do Ceará - CE
57	AFASTAMENTO POR ATESTADO MÉDICO - ATE 15 DIAS	CH da Universidade Federal do Ceará - CE
57	AFASTAMENTO POR ATESTADO MÉDICO - ATE 15 DIAS	CH da Universidade Federal do Ceará - CE
57	AFASTAMENTO POR ATESTADO MÉDICO - ATE 15 DIAS	CH da Universidade Federal do Ceará - CE
57	AFASTAMENTO POR ATESTADO MÉDICO - ATE 15 DIAS	CH da Universidade Federal do Ceará - CE
57	AFASTAMENTO POR ATESTADO MÉDICO - ATE 15 DIAS	CH da Universidade Federal do Ceará - CE
57	AFASTAMENTO POR ATESTADO MÉDICO - ATE 15 DIAS	CH da Universidade Federal do Ceará - CE
57	AFASTAMENTO POR ATESTADO MÉDICO - ATE 15 DIAS	CH da Universidade Federal do Ceará - CE
57	AFASTAMENTO POR ATESTADO MÉDICO - ATE 15 DIAS	CH da Universidade Federal do Ceará - CE
57	AFASTAMENTO POR ATESTADO MÉDICO - ATE 15 DIAS	CH da Universidade Federal do Ceará - CE
57	AFASTAMENTO POR ATESTADO MÉDICO - ATE 15 DIAS	CH da Universidade Federal do Ceará - CE
57	AFASTAMENTO POR ATESTADO MÉDICO - ATE 15 DIAS	CH da Universidade Federal do Ceará - CE
57	AFASTAMENTO POR ATESTADO MÉDICO - ATE 15 DIAS	CH da Universidade Federal do Ceará - CE
57	AFASTAMENTO POR ATESTADO MÉDICO - ATE 15 DIAS	CH da Universidade Federal do Ceará - CE

57	AFASTAMENTO POR ATESTADO MÉDICO - ATE 15 DIAS	CH da Universidade Federal do Ceará - CE
57	AFASTAMENTO POR ATESTADO MÉDICO - ATE 15 DIAS	CH da Universidade Federal do Ceará - CE
57	AFASTAMENTO POR ATESTADO MÉDICO - ATE 15 DIAS	CH da Universidade Federal do Ceará - CE
57	AFASTAMENTO POR ATESTADO MÉDICO - ATE 15 DIAS	CH da Universidade Federal do Ceará - CE
57	AFASTAMENTO POR ATESTADO MÉDICO - ATE 15 DIAS	CH da Universidade Federal do Ceará - CE
57	AFASTAMENTO POR ATESTADO MÉDICO - ATE 15 DIAS	CH da Universidade Federal do Ceará - CE
57	AFASTAMENTO POR ATESTADO MÉDICO - ATE 15 DIAS	CH da Universidade Federal do Ceará - CE
57	AFASTAMENTO POR ATESTADO MÉDICO - ATE 15 DIAS	CH da Universidade Federal do Ceará - CE
57	AFASTAMENTO POR ATESTADO MÉDICO - ATE 15 DIAS	CH da Universidade Federal do Ceará - CE
57	AFASTAMENTO POR ATESTADO MÉDICO - ATE 15 DIAS	CH da Universidade Federal do Ceará - CE
57	AFASTAMENTO POR ATESTADO MÉDICO - ATE 15 DIAS	CH da Universidade Federal do Ceará - CE
57	AFASTAMENTO POR ATESTADO MÉDICO - ATE 15 DIAS	CH da Universidade Federal do Ceará - CE
57	AFASTAMENTO POR ATESTADO MÉDICO - ATE 15 DIAS	CH da Universidade Federal do Ceará - CE
57	AFASTAMENTO POR ATESTADO MÉDICO - ATE 15 DIAS	CH da Universidade Federal do Ceará - CE
57	AFASTAMENTO POR ATESTADO MÉDICO - ATE 15 DIAS	CH da Universidade Federal do Ceará - CE
57	AFASTAMENTO POR ATESTADO MÉDICO - ATE 15 DIAS	CH da Universidade Federal do Ceará - CE
57	AFASTAMENTO POR ATESTADO MÉDICO - ATE 15 DIAS	CH da Universidade Federal do Ceará - CE
57	AFASTAMENTO POR ATESTADO MÉDICO - ATE 15 DIAS	CH da Universidade Federal do Ceará - CE
57	AFASTAMENTO POR ATESTADO MÉDICO - ATE 15 DIAS	CH da Universidade Federal do Ceará - CE
57	AFASTAMENTO POR ATESTADO MÉDICO - ATE 15 DIAS	CH da Universidade Federal do Ceará - CE
57	AFASTAMENTO POR ATESTADO MÉDICO - ATE 15 DIAS	CH da Universidade Federal do Ceará - CE
57	AFASTAMENTO POR ATESTADO MÉDICO - ATE 15 DIAS	CH da Universidade Federal do Ceará - CE
57	AFASTAMENTO POR ATESTADO MÉDICO - ATE 15 DIAS	CH da Universidade Federal do Ceará - CE
57	AFASTAMENTO POR ATESTADO MÉDICO - ATE 15 DIAS	CH da Universidade Federal do Ceará - CE
57	AFASTAMENTO POR ATESTADO MÉDICO - ATE 15 DIAS	CH da Universidade Federal do Ceará - CE
57	AFASTAMENTO POR ATESTADO MÉDICO - ATE 15 DIAS	CH da Universidade Federal do Ceará - CE
57	AFASTAMENTO POR ATESTADO MÉDICO - ATE 15 DIAS	CH da Universidade Federal do Ceará - CE
57	AFASTAMENTO POR ATESTADO MÉDICO - ATE 15 DIAS	CH da Universidade Federal do Ceará - CE
57	AFASTAMENTO POR ATESTADO MÉDICO - ATE 15 DIAS	CH da Universidade Federal do Ceará - CE
57	AFASTAMENTO POR ATESTADO MÉDICO - ATE 15 DIAS	CH da Universidade Federal do Ceará - CE
57	AFASTAMENTO POR ATESTADO MÉDICO - ATE 15 DIAS	CH da Universidade Federal do Ceará - CE
57	AFASTAMENTO POR ATESTADO MÉDICO - ATE 15 DIAS	CH da Universidade Federal do Ceará - CE
57	AFASTAMENTO POR ATESTADO MÉDICO - ATE 15 DIAS	CH da Universidade Federal do Ceará - CE
57	AFASTAMENTO POR ATESTADO MÉDICO - ATE 15 DIAS	CH da Universidade Federal do Ceará - CE
57	AFASTAMENTO POR ATESTADO MÉDICO - ATE 15 DIAS	CH da Universidade Federal do Ceará - CE
57	AFASTAMENTO POR ATESTADO MÉDICO - ATE 15 DIAS	CH da Universidade Federal do Ceará - CE
57	AFASTAMENTO POR ATESTADO MÉDICO - ATE 15 DIAS	CH da Universidade Federal do Ceará - CE
57	AFASTAMENTO POR ATESTADO MÉDICO - ATE 15 DIAS	CH da Universidade Federal do Ceará - CE
57	AFASTAMENTO POR ATESTADO MÉDICO - ATE 15 DIAS	CH da Universidade Federal do Ceará - CE
57	AFASTAMENTO POR ATESTADO MÉDICO - ATE 15 DIAS	CH da Universidade Federal do Ceará - CE
57	AFASTAMENTO POR ATESTADO MÉDICO - ATE 15 DIAS	CH da Universidade Federal do Ceará - CE
57	AFASTAMENTO POR ATESTADO MÉDICO - ATE 15 DIAS	CH da Universidade Federal do Ceará - CE
57	AFASTAMENTO POR ATESTADO MÉDICO - ATE 15 DIAS	CH da Universidade Federal do Ceará - CE
57	AFASTAMENTO POR ATESTADO MÉDICO - ATE 15 DIAS	CH da Universidade Federal do Ceará - CE
57	AFASTAMENTO POR ATESTADO MÉDICO - ATE 15 DIAS	CH da Universidade Federal do Ceará - CE
57	AFASTAMENTO POR ATESTADO MÉDICO - ATE 15 DIAS	CH da Universidade Federal do Ceará - CE
57	AFASTAMENTO POR ATESTADO MÉDICO - ATE 15 DIAS	CH da Universidade Federal do Ceará - CE
57	AFASTAMENTO POR ATESTADO MÉDICO - ATE 15 DIAS	CH da Universidade Federal do Ceará - CE

57	AFASTAMENTO POR ATESTADO MÉDICO - ATE 15 DIAS	CH da Universidade Federal do Ceará - CE
57	AFASTAMENTO POR ATESTADO MÉDICO - ATE 15 DIAS	CH da Universidade Federal do Ceará - CE
57	AFASTAMENTO POR ATESTADO MÉDICO - ATE 15 DIAS	CH da Universidade Federal do Ceará - CE
57	AFASTAMENTO POR ATESTADO MÉDICO - ATE 15 DIAS	CH da Universidade Federal do Ceará - CE
57	AFASTAMENTO POR ATESTADO MÉDICO - ATE 15 DIAS	CH da Universidade Federal do Ceará - CE
57	AFASTAMENTO POR ATESTADO MÉDICO - ATE 15 DIAS	CH da Universidade Federal do Ceará - CE
57	AFASTAMENTO POR ATESTADO MÉDICO - ATE 15 DIAS	CH da Universidade Federal do Ceará - CE
57	AFASTAMENTO POR ATESTADO MÉDICO - ATE 15 DIAS	CH da Universidade Federal do Ceará - CE
57	AFASTAMENTO POR ATESTADO MÉDICO - ATE 15 DIAS	CH da Universidade Federal do Ceará - CE
57	AFASTAMENTO POR ATESTADO MÉDICO - ATE 15 DIAS	CH da Universidade Federal do Ceará - CE
57	AFASTAMENTO POR ATESTADO MÉDICO - ATE 15 DIAS	CH da Universidade Federal do Ceará - CE
57	AFASTAMENTO POR ATESTADO MÉDICO - ATE 15 DIAS	CH da Universidade Federal do Ceará - CE
57	AFASTAMENTO POR ATESTADO MÉDICO - ATE 15 DIAS	CH da Universidade Federal do Ceará - CE
57	AFASTAMENTO POR ATESTADO MÉDICO - ATE 15 DIAS	CH da Universidade Federal do Ceará - CE
57	AFASTAMENTO POR ATESTADO MÉDICO - ATE 15 DIAS	CH da Universidade Federal do Ceará - CE
57	AFASTAMENTO POR ATESTADO MÉDICO - ATE 15 DIAS	CH da Universidade Federal do Ceará - CE
57	AFASTAMENTO POR ATESTADO MÉDICO - ATE 15 DIAS	CH da Universidade Federal do Ceará - CE
57	AFASTAMENTO POR ATESTADO MÉDICO - ATE 15 DIAS	CH da Universidade Federal do Ceará - CE
57	AFASTAMENTO POR ATESTADO MÉDICO - ATE 15 DIAS	CH da Universidade Federal do Ceará - CE
57	AFASTAMENTO POR ATESTADO MÉDICO - ATE 15 DIAS	CH da Universidade Federal do Ceará - CE
57	AFASTAMENTO POR ATESTADO MÉDICO - ATE 15 DIAS	CH da Universidade Federal do Ceará - CE
57	AFASTAMENTO POR ATESTADO MÉDICO - ATE 15 DIAS	CH da Universidade Federal do Ceará - CE
57	AFASTAMENTO POR ATESTADO MÉDICO - ATE 15 DIAS	CH da Universidade Federal do Ceará - CE
57	AFASTAMENTO POR ATESTADO MÉDICO - ATE 15 DIAS	CH da Universidade Federal do Ceará - CE
57	AFASTAMENTO POR ATESTADO MÉDICO - ATE 15 DIAS	CH da Universidade Federal do Ceará - CE
57	AFASTAMENTO POR ATESTADO MÉDICO - ATE 15 DIAS	CH da Universidade Federal do Ceará - CE
57	AFASTAMENTO POR ATESTADO MÉDICO - ATE 15 DIAS	CH da Universidade Federal do Ceará - CE
57	AFASTAMENTO POR ATESTADO MÉDICO - ATE 15 DIAS	CH da Universidade Federal do Ceará - CE
57	AFASTAMENTO POR ATESTADO MÉDICO - ATE 15 DIAS	CH da Universidade Federal do Ceará - CE
57	AFASTAMENTO POR ATESTADO MÉDICO - ATE 15 DIAS	CH da Universidade Federal do Ceará - CE
57	AFASTAMENTO POR ATESTADO MÉDICO - ATE 15 DIAS	CH da Universidade Federal do Ceará - CE
57	AFASTAMENTO POR ATESTADO MÉDICO - ATE 15 DIAS	CH da Universidade Federal do Ceará - CE
57	AFASTAMENTO POR ATESTADO MÉDICO - ATE 15 DIAS	CH da Universidade Federal do Ceará - CE
57	AFASTAMENTO POR ATESTADO MÉDICO - ATE 15 DIAS	CH da Universidade Federal do Ceará - CE
57	AFASTAMENTO POR ATESTADO MÉDICO - ATE 15 DIAS	CH da Universidade Federal do Ceará - CE
57	AFASTAMENTO POR ATESTADO MÉDICO - ATE 15 DIAS	CH da Universidade Federal do Ceará - CE
57	AFASTAMENTO POR ATESTADO MÉDICO - ATE 15 DIAS	CH da Universidade Federal do Ceará - CE
57	AFASTAMENTO POR ATESTADO MÉDICO - ATE 15 DIAS	CH da Universidade Federal do Ceará - CE
57	AFASTAMENTO POR ATESTADO MÉDICO - ATE 15 DIAS	CH da Universidade Federal do Ceará - CE
57	AFASTAMENTO POR ATESTADO MÉDICO - ATE 15 DIAS	CH da Universidade Federal do Ceará - CE
57	AFASTAMENTO POR ATESTADO MÉDICO - ATE 15 DIAS	CH da Universidade Federal do Ceará - CE
57	AFASTAMENTO POR ATESTADO MÉDICO - ATE 15 DIAS	CH da Universidade Federal do Ceará - CE
57	AFASTAMENTO POR ATESTADO MÉDICO - ATE 15 DIAS	CH da Universidade Federal do Ceará - CE
57	AFASTAMENTO POR ATESTADO MÉDICO - ATE 15 DIAS	CH da Universidade Federal do Ceará - CE
57	AFASTAMENTO POR ATESTADO MÉDICO - ATE 15 DIAS	CH da Universidade Federal do Ceará - CE
57	AFASTAMENTO POR ATESTADO MÉDICO - ATE 15 DIAS	CH da Universidade Federal do Ceará - CE
57	AFASTAMENTO POR ATESTADO MÉDICO - ATE 15 DIAS	CH da Universidade Federal do Ceará - CE
57	AFASTAMENTO POR ATESTADO MÉDICO - ATE 15 DIAS	CH da Universidade Federal do Ceará - CE

57	AFASTAMENTO POR ATESTADO MÉDICO - ATE 15 DIAS	CH da Universidade Federal do Ceará - CE
57	AFASTAMENTO POR ATESTADO MÉDICO - ATE 15 DIAS	CH da Universidade Federal do Ceará - CE
57	AFASTAMENTO POR ATESTADO MÉDICO - ATE 15 DIAS	CH da Universidade Federal do Ceará - CE
57	AFASTAMENTO POR ATESTADO MÉDICO - ATE 15 DIAS	CH da Universidade Federal do Ceará - CE
57	AFASTAMENTO POR ATESTADO MÉDICO - ATE 15 DIAS	CH da Universidade Federal do Ceará - CE
57	AFASTAMENTO POR ATESTADO MÉDICO - ATE 15 DIAS	CH da Universidade Federal do Ceará - CE
57	AFASTAMENTO POR ATESTADO MÉDICO - ATE 15 DIAS	CH da Universidade Federal do Ceará - CE
57	AFASTAMENTO POR ATESTADO MÉDICO - ATE 15 DIAS	CH da Universidade Federal do Ceará - CE
57	AFASTAMENTO POR ATESTADO MÉDICO - ATE 15 DIAS	CH da Universidade Federal do Ceará - CE
57	AFASTAMENTO POR ATESTADO MÉDICO - ATE 15 DIAS	CH da Universidade Federal do Ceará - CE
57	AFASTAMENTO POR ATESTADO MÉDICO - ATE 15 DIAS	CH da Universidade Federal do Ceará - CE
57	AFASTAMENTO POR ATESTADO MÉDICO - ATE 15 DIAS	CH da Universidade Federal do Ceará - CE
57	AFASTAMENTO POR ATESTADO MÉDICO - ATE 15 DIAS	CH da Universidade Federal do Ceará - CE
57	AFASTAMENTO POR ATESTADO MÉDICO - ATE 15 DIAS	CH da Universidade Federal do Ceará - CE
57	AFASTAMENTO POR ATESTADO MÉDICO - ATE 15 DIAS	CH da Universidade Federal do Ceará - CE
57	AFASTAMENTO POR ATESTADO MÉDICO - ATE 15 DIAS	CH da Universidade Federal do Ceará - CE
57	AFASTAMENTO POR ATESTADO MÉDICO - ATE 15 DIAS	CH da Universidade Federal do Ceará - CE
57	AFASTAMENTO POR ATESTADO MÉDICO - ATE 15 DIAS	CH da Universidade Federal do Ceará - CE
57	AFASTAMENTO POR ATESTADO MÉDICO - ATE 15 DIAS	CH da Universidade Federal do Ceará - CE
57	AFASTAMENTO POR ATESTADO MÉDICO - ATE 15 DIAS	CH da Universidade Federal do Ceará - CE
57	AFASTAMENTO POR ATESTADO MÉDICO - ATE 15 DIAS	CH da Universidade Federal do Ceará - CE
57	AFASTAMENTO POR ATESTADO MÉDICO - ATE 15 DIAS	CH da Universidade Federal do Ceará - CE
57	AFASTAMENTO POR ATESTADO MÉDICO - ATE 15 DIAS	CH da Universidade Federal do Ceará - CE
57	AFASTAMENTO POR ATESTADO MÉDICO - ATE 15 DIAS	CH da Universidade Federal do Ceará - CE
57	AFASTAMENTO POR ATESTADO MÉDICO - ATE 15 DIAS	CH da Universidade Federal do Ceará - CE
57	AFASTAMENTO POR ATESTADO MÉDICO - ATE 15 DIAS	CH da Universidade Federal do Ceará - CE
57	AFASTAMENTO POR ATESTADO MÉDICO - ATE 15 DIAS	CH da Universidade Federal do Ceará - CE
57	AFASTAMENTO POR ATESTADO MÉDICO - ATE 15 DIAS	CH da Universidade Federal do Ceará - CE
57	AFASTAMENTO POR ATESTADO MÉDICO - ATE 15 DIAS	CH da Universidade Federal do Ceará - CE
57	AFASTAMENTO POR ATESTADO MÉDICO - ATE 15 DIAS	CH da Universidade Federal do Ceará - CE
57	AFASTAMENTO POR ATESTADO MÉDICO - ATE 15 DIAS	CH da Universidade Federal do Ceará - CE
57	AFASTAMENTO POR ATESTADO MÉDICO - ATE 15 DIAS	CH da Universidade Federal do Ceará - CE
57	AFASTAMENTO POR ATESTADO MÉDICO - ATE 15 DIAS	CH da Universidade Federal do Ceará - CE
57	AFASTAMENTO POR ATESTADO MÉDICO - ATE 15 DIAS	CH da Universidade Federal do Ceará - CE
57	AFASTAMENTO POR ATESTADO MÉDICO - ATE 15 DIAS	CH da Universidade Federal do Ceará - CE
57	AFASTAMENTO POR ATESTADO MÉDICO - ATE 15 DIAS	CH da Universidade Federal do Ceará - CE
57	AFASTAMENTO POR ATESTADO MÉDICO - ATE 15 DIAS	CH da Universidade Federal do Ceará - CE
57	AFASTAMENTO POR ATESTADO MÉDICO - ATE 15 DIAS	CH da Universidade Federal do Ceará - CE
57	AFASTAMENTO POR ATESTADO MÉDICO - ATE 15 DIAS	CH da Universidade Federal do Ceará - CE
57	AFASTAMENTO POR ATESTADO MÉDICO - ATE 15 DIAS	CH da Universidade Federal do Ceará - CE
57	AFASTAMENTO POR ATESTADO MÉDICO - ATE 15 DIAS	CH da Universidade Federal do Ceará - CE
57	AFASTAMENTO POR ATESTADO MÉDICO - ATE 15 DIAS	CH da Universidade Federal do Ceará - CE
57	AFASTAMENTO POR ATESTADO MÉDICO - ATE 15 DIAS	CH da Universidade Federal do Ceará - CE
57	AFASTAMENTO POR ATESTADO MÉDICO - ATE 15 DIAS	CH da Universidade Federal do Ceará - CE
57	AFASTAMENTO POR ATESTADO MÉDICO - ATE 15 DIAS	CH da Universidade Federal do Ceará - CE
57	AFASTAMENTO POR ATESTADO MÉDICO - ATE 15 DIAS	CH da Universidade Federal do Ceará - CE
57	AFASTAMENTO POR ATESTADO MÉDICO - ATE 15 DIAS	CH da Universidade Federal do Ceará - CE
57	AFASTAMENTO POR ATESTADO MÉDICO - ATE 15 DIAS	CH da Universidade Federal do Ceará - CE

57	AFASTAMENTO POR ATESTADO MÉDICO - ATE 15 DIAS	CH da Universidade Federal do Ceará - CE
57	AFASTAMENTO POR ATESTADO MÉDICO - ATE 15 DIAS	CH da Universidade Federal do Ceará - CE
57	AFASTAMENTO POR ATESTADO MÉDICO - ATE 15 DIAS	CH da Universidade Federal do Ceará - CE
57	AFASTAMENTO POR ATESTADO MÉDICO - ATE 15 DIAS	CH da Universidade Federal do Ceará - CE
57	AFASTAMENTO POR ATESTADO MÉDICO - ATE 15 DIAS	CH da Universidade Federal do Ceará - CE
57	AFASTAMENTO POR ATESTADO MÉDICO - ATE 15 DIAS	CH da Universidade Federal do Ceará - CE
57	AFASTAMENTO POR ATESTADO MÉDICO - ATE 15 DIAS	CH da Universidade Federal do Ceará - CE
57	AFASTAMENTO POR ATESTADO MÉDICO - ATE 15 DIAS	CH da Universidade Federal do Ceará - CE
57	AFASTAMENTO POR ATESTADO MÉDICO - ATE 15 DIAS	CH da Universidade Federal do Ceará - CE
57	AFASTAMENTO POR ATESTADO MÉDICO - ATE 15 DIAS	CH da Universidade Federal do Ceará - CE
57	AFASTAMENTO POR ATESTADO MÉDICO - ATE 15 DIAS	CH da Universidade Federal do Ceará - CE
57	AFASTAMENTO POR ATESTADO MÉDICO - ATE 15 DIAS	CH da Universidade Federal do Ceará - CE
57	AFASTAMENTO POR ATESTADO MÉDICO - ATE 15 DIAS	CH da Universidade Federal do Ceará - CE
57	AFASTAMENTO POR ATESTADO MÉDICO - ATE 15 DIAS	CH da Universidade Federal do Ceará - CE
57	AFASTAMENTO POR ATESTADO MÉDICO - ATE 15 DIAS	CH da Universidade Federal do Ceará - CE
57	AFASTAMENTO POR ATESTADO MÉDICO - ATE 15 DIAS	CH da Universidade Federal do Ceará - CE
57	AFASTAMENTO POR ATESTADO MÉDICO - ATE 15 DIAS	CH da Universidade Federal do Ceará - CE
57	AFASTAMENTO POR ATESTADO MÉDICO - ATE 15 DIAS	CH da Universidade Federal do Ceará - CE
57	AFASTAMENTO POR ATESTADO MÉDICO - ATE 15 DIAS	CH da Universidade Federal do Ceará - CE
57	AFASTAMENTO POR ATESTADO MÉDICO - ATE 15 DIAS	CH da Universidade Federal do Ceará - CE
57	AFASTAMENTO POR ATESTADO MÉDICO - ATE 15 DIAS	CH da Universidade Federal do Ceará - CE
57	AFASTAMENTO POR ATESTADO MÉDICO - ATE 15 DIAS	CH da Universidade Federal do Ceará - CE
57	AFASTAMENTO POR ATESTADO MÉDICO - ATE 15 DIAS	CH da Universidade Federal do Ceará - CE
57	AFASTAMENTO POR ATESTADO MÉDICO - ATE 15 DIAS	CH da Universidade Federal do Ceará - CE
57	AFASTAMENTO POR ATESTADO MÉDICO - ATE 15 DIAS	CH da Universidade Federal do Ceará - CE
57	AFASTAMENTO POR ATESTADO MÉDICO - ATE 15 DIAS	CH da Universidade Federal do Ceará - CE
57	AFASTAMENTO POR ATESTADO MÉDICO - ATE 15 DIAS	CH da Universidade Federal do Ceará - CE
57	AFASTAMENTO POR ATESTADO MÉDICO - ATE 15 DIAS	CH da Universidade Federal do Ceará - CE
57	AFASTAMENTO POR ATESTADO MÉDICO - ATE 15 DIAS	CH da Universidade Federal do Ceará - CE
57	AFASTAMENTO POR ATESTADO MÉDICO - ATE 15 DIAS	CH da Universidade Federal do Ceará - CE
57	AFASTAMENTO POR ATESTADO MÉDICO - ATE 15 DIAS	CH da Universidade Federal do Ceará - CE
57	AFASTAMENTO POR ATESTADO MÉDICO - ATE 15 DIAS	CH da Universidade Federal do Ceará - CE
57	AFASTAMENTO POR ATESTADO MÉDICO - ATE 15 DIAS	CH da Universidade Federal do Ceará - CE
57	AFASTAMENTO POR ATESTADO MÉDICO - ATE 15 DIAS	CH da Universidade Federal do Ceará - CE
57	AFASTAMENTO POR ATESTADO MÉDICO - ATE 15 DIAS	CH da Universidade Federal do Ceará - CE
57	AFASTAMENTO POR ATESTADO MÉDICO - ATE 15 DIAS	CH da Universidade Federal do Ceará - CE
57	AFASTAMENTO POR ATESTADO MÉDICO - ATE 15 DIAS	CH da Universidade Federal do Ceará - CE
57	AFASTAMENTO POR ATESTADO MÉDICO - ATE 15 DIAS	CH da Universidade Federal do Ceará - CE
57	AFASTAMENTO POR ATESTADO MÉDICO - ATE 15 DIAS	CH da Universidade Federal do Ceará - CE
57	AFASTAMENTO POR ATESTADO MÉDICO - ATE 15 DIAS	CH da Universidade Federal do Ceará - CE
57	AFASTAMENTO POR ATESTADO MÉDICO - ATE 15 DIAS	CH da Universidade Federal do Ceará - CE
57	AFASTAMENTO POR ATESTADO MÉDICO - ATE 15 DIAS	CH da Universidade Federal do Ceará - CE
57	AFASTAMENTO POR ATESTADO MÉDICO - ATE 15 DIAS	CH da Universidade Federal do Ceará - CE
57	AFASTAMENTO POR ATESTADO MÉDICO - ATE 15 DIAS	CH da Universidade Federal do Ceará - CE
57	AFASTAMENTO POR ATESTADO MÉDICO - ATE 15 DIAS	CH da Universidade Federal do Ceará - CE
57	AFASTAMENTO POR ATESTADO MÉDICO - ATE 15 DIAS	CH da Universidade Federal do Ceará - CE
57	AFASTAMENTO POR ATESTADO MÉDICO - ATE 15 DIAS	CH da Universidade Federal do Ceará - CE
57	AFASTAMENTO POR ATESTADO MÉDICO - ATE 15 DIAS	CH da Universidade Federal do Ceará - CE

57	AFASTAMENTO POR ATESTADO MÉDICO - ATE 15 DIAS	CH da Universidade Federal do Ceará - CE
57	AFASTAMENTO POR ATESTADO MÉDICO - ATE 15 DIAS	CH da Universidade Federal do Ceará - CE
57	AFASTAMENTO POR ATESTADO MÉDICO - ATE 15 DIAS	CH da Universidade Federal do Ceará - CE
57	AFASTAMENTO POR ATESTADO MÉDICO - ATE 15 DIAS	CH da Universidade Federal do Ceará - CE
57	AFASTAMENTO POR ATESTADO MÉDICO - ATE 15 DIAS	CH da Universidade Federal do Ceará - CE
57	AFASTAMENTO POR ATESTADO MÉDICO - ATE 15 DIAS	CH da Universidade Federal do Ceará - CE
57	AFASTAMENTO POR ATESTADO MÉDICO - ATE 15 DIAS	CH da Universidade Federal do Ceará - CE
57	AFASTAMENTO POR ATESTADO MÉDICO - ATE 15 DIAS	CH da Universidade Federal do Ceará - CE
57	AFASTAMENTO POR ATESTADO MÉDICO - ATE 15 DIAS	CH da Universidade Federal do Ceará - CE
57	AFASTAMENTO POR ATESTADO MÉDICO - ATE 15 DIAS	CH da Universidade Federal do Ceará - CE
57	AFASTAMENTO POR ATESTADO MÉDICO - ATE 15 DIAS	CH da Universidade Federal do Ceará - CE
57	AFASTAMENTO POR ATESTADO MÉDICO - ATE 15 DIAS	CH da Universidade Federal do Ceará - CE
57	AFASTAMENTO POR ATESTADO MÉDICO - ATE 15 DIAS	CH da Universidade Federal do Ceará - CE
57	AFASTAMENTO POR ATESTADO MÉDICO - ATE 15 DIAS	CH da Universidade Federal do Ceará - CE
57	AFASTAMENTO POR ATESTADO MÉDICO - ATE 15 DIAS	CH da Universidade Federal do Ceará - CE
57	AFASTAMENTO POR ATESTADO MÉDICO - ATE 15 DIAS	CH da Universidade Federal do Ceará - CE
57	AFASTAMENTO POR ATESTADO MÉDICO - ATE 15 DIAS	CH da Universidade Federal do Ceará - CE
57	AFASTAMENTO POR ATESTADO MÉDICO - ATE 15 DIAS	CH da Universidade Federal do Ceará - CE
57	AFASTAMENTO POR ATESTADO MÉDICO - ATE 15 DIAS	CH da Universidade Federal do Ceará - CE
57	AFASTAMENTO POR ATESTADO MÉDICO - ATE 15 DIAS	CH da Universidade Federal do Ceará - CE
57	AFASTAMENTO POR ATESTADO MÉDICO - ATE 15 DIAS	CH da Universidade Federal do Ceará - CE
57	AFASTAMENTO POR ATESTADO MÉDICO - ATE 15 DIAS	CH da Universidade Federal do Ceará - CE
57	AFASTAMENTO POR ATESTADO MÉDICO - ATE 15 DIAS	CH da Universidade Federal do Ceará - CE
57	AFASTAMENTO POR ATESTADO MÉDICO - ATE 15 DIAS	CH da Universidade Federal do Ceará - CE
57	AFASTAMENTO POR ATESTADO MÉDICO - ATE 15 DIAS	CH da Universidade Federal do Ceará - CE
57	AFASTAMENTO POR ATESTADO MÉDICO - ATE 15 DIAS	CH da Universidade Federal do Ceará - CE
57	AFASTAMENTO POR ATESTADO MÉDICO - ATE 15 DIAS	CH da Universidade Federal do Ceará - CE
57	AFASTAMENTO POR ATESTADO MÉDICO - ATE 15 DIAS	CH da Universidade Federal do Ceará - CE
57	AFASTAMENTO POR ATESTADO MÉDICO - ATE 15 DIAS	CH da Universidade Federal do Ceará - CE
57	AFASTAMENTO POR ATESTADO MÉDICO - ATE 15 DIAS	CH da Universidade Federal do Ceará - CE
57	AFASTAMENTO POR ATESTADO MÉDICO - ATE 15 DIAS	CH da Universidade Federal do Ceará - CE
57	AFASTAMENTO POR ATESTADO MÉDICO - ATE 15 DIAS	CH da Universidade Federal do Ceará - CE
57	AFASTAMENTO POR ATESTADO MÉDICO - ATE 15 DIAS	CH da Universidade Federal do Ceará - CE
57	AFASTAMENTO POR ATESTADO MÉDICO - ATE 15 DIAS	CH da Universidade Federal do Ceará - CE
57	AFASTAMENTO POR ATESTADO MÉDICO - ATE 15 DIAS	CH da Universidade Federal do Ceará - CE
57	AFASTAMENTO POR ATESTADO MÉDICO - ATE 15 DIAS	CH da Universidade Federal do Ceará - CE
57	AFASTAMENTO POR ATESTADO MÉDICO - ATE 15 DIAS	CH da Universidade Federal do Ceará - CE
57	AFASTAMENTO POR ATESTADO MÉDICO - ATE 15 DIAS	CH da Universidade Federal do Ceará - CE
57	AFASTAMENTO POR ATESTADO MÉDICO - ATE 15 DIAS	CH da Universidade Federal do Ceará - CE
57	AFASTAMENTO POR ATESTADO MÉDICO - ATE 15 DIAS	CH da Universidade Federal do Ceará - CE
57	AFASTAMENTO POR ATESTADO MÉDICO - ATE 15 DIAS	CH da Universidade Federal do Ceará - CE
57	AFASTAMENTO POR ATESTADO MÉDICO - ATE 15 DIAS	CH da Universidade Federal do Ceará - CE
57	AFASTAMENTO POR ATESTADO MÉDICO - ATE 15 DIAS	CH da Universidade Federal do Ceará - CE
57	AFASTAMENTO POR ATESTADO MÉDICO - ATE 15 DIAS	CH da Universidade Federal do Ceará - CE
57	AFASTAMENTO POR ATESTADO MÉDICO - ATE 15 DIAS	CH da Universidade Federal do Ceará - CE
57	AFASTAMENTO POR ATESTADO MÉDICO - ATE 15 DIAS	CH da Universidade Federal do Ceará - CE
57	AFASTAMENTO POR ATESTADO MÉDICO - ATE 15 DIAS	CH da Universidade Federal do Ceará - CE
57	AFASTAMENTO POR ATESTADO MÉDICO - ATE 15 DIAS	CH da Universidade Federal do Ceará - CE

57	AFASTAMENTO POR ATESTADO MÉDICO - ATE 15 DIAS	CH da Universidade Federal do Ceará - CE
57	AFASTAMENTO POR ATESTADO MÉDICO - ATE 15 DIAS	CH da Universidade Federal do Ceará - CE
57	AFASTAMENTO POR ATESTADO MÉDICO - ATE 15 DIAS	CH da Universidade Federal do Ceará - CE
57	AFASTAMENTO POR ATESTADO MÉDICO - ATE 15 DIAS	CH da Universidade Federal do Ceará - CE
57	AFASTAMENTO POR ATESTADO MÉDICO - ATE 15 DIAS	CH da Universidade Federal do Ceará - CE
57	AFASTAMENTO POR ATESTADO MÉDICO - ATE 15 DIAS	CH da Universidade Federal do Ceará - CE
57	AFASTAMENTO POR ATESTADO MÉDICO - ATE 15 DIAS	CH da Universidade Federal do Ceará - CE
57	AFASTAMENTO POR ATESTADO MÉDICO - ATE 15 DIAS	CH da Universidade Federal do Ceará - CE
57	AFASTAMENTO POR ATESTADO MÉDICO - ATE 15 DIAS	CH da Universidade Federal do Ceará - CE
57	AFASTAMENTO POR ATESTADO MÉDICO - ATE 15 DIAS	CH da Universidade Federal do Ceará - CE
57	AFASTAMENTO POR ATESTADO MÉDICO - ATE 15 DIAS	CH da Universidade Federal do Ceará - CE
57	AFASTAMENTO POR ATESTADO MÉDICO - ATE 15 DIAS	CH da Universidade Federal do Ceará - CE
57	AFASTAMENTO POR ATESTADO MÉDICO - ATE 15 DIAS	CH da Universidade Federal do Ceará - CE
57	AFASTAMENTO POR ATESTADO MÉDICO - ATE 15 DIAS	CH da Universidade Federal do Ceará - CE
57	AFASTAMENTO POR ATESTADO MÉDICO - ATE 15 DIAS	CH da Universidade Federal do Ceará - CE
57	AFASTAMENTO POR ATESTADO MÉDICO - ATE 15 DIAS	CH da Universidade Federal do Ceará - CE
57	AFASTAMENTO POR ATESTADO MÉDICO - ATE 15 DIAS	CH da Universidade Federal do Ceará - CE
57	AFASTAMENTO POR ATESTADO MÉDICO - ATE 15 DIAS	CH da Universidade Federal do Ceará - CE
57	AFASTAMENTO POR ATESTADO MÉDICO - ATE 15 DIAS	CH da Universidade Federal do Ceará - CE
57	AFASTAMENTO POR ATESTADO MÉDICO - ATE 15 DIAS	CH da Universidade Federal do Ceará - CE
57	AFASTAMENTO POR ATESTADO MÉDICO - ATE 15 DIAS	CH da Universidade Federal do Ceará - CE
57	AFASTAMENTO POR ATESTADO MÉDICO - ATE 15 DIAS	CH da Universidade Federal do Ceará - CE
57	AFASTAMENTO POR ATESTADO MÉDICO - ATE 15 DIAS	CH da Universidade Federal do Ceará - CE
57	AFASTAMENTO POR ATESTADO MÉDICO - ATE 15 DIAS	CH da Universidade Federal do Ceará - CE
57	AFASTAMENTO POR ATESTADO MÉDICO - ATE 15 DIAS	CH da Universidade Federal do Ceará - CE
57	AFASTAMENTO POR ATESTADO MÉDICO - ATE 15 DIAS	CH da Universidade Federal do Ceará - CE
57	AFASTAMENTO POR ATESTADO MÉDICO - ATE 15 DIAS	CH da Universidade Federal do Ceará - CE
57	AFASTAMENTO POR ATESTADO MÉDICO - ATE 15 DIAS	CH da Universidade Federal do Ceará - CE
57	AFASTAMENTO POR ATESTADO MÉDICO - ATE 15 DIAS	CH da Universidade Federal do Ceará - CE
57	AFASTAMENTO POR ATESTADO MÉDICO - ATE 15 DIAS	CH da Universidade Federal do Ceará - CE
57	AFASTAMENTO POR ATESTADO MÉDICO - ATE 15 DIAS	CH da Universidade Federal do Ceará - CE
57	AFASTAMENTO POR ATESTADO MÉDICO - ATE 15 DIAS	CH da Universidade Federal do Ceará - CE
57	AFASTAMENTO POR ATESTADO MÉDICO - ATE 15 DIAS	CH da Universidade Federal do Ceará - CE
57	AFASTAMENTO POR ATESTADO MÉDICO - ATE 15 DIAS	CH da Universidade Federal do Ceará - CE
57	AFASTAMENTO POR ATESTADO MÉDICO - ATE 15 DIAS	CH da Universidade Federal do Ceará - CE
57	AFASTAMENTO POR ATESTADO MÉDICO - ATE 15 DIAS	CH da Universidade Federal do Ceará - CE
57	AFASTAMENTO POR ATESTADO MÉDICO - ATE 15 DIAS	CH da Universidade Federal do Ceará - CE
57	AFASTAMENTO POR ATESTADO MÉDICO - ATE 15 DIAS	CH da Universidade Federal do Ceará - CE
57	AFASTAMENTO POR ATESTADO MÉDICO - ATE 15 DIAS	CH da Universidade Federal do Ceará - CE
57	AFASTAMENTO POR ATESTADO MÉDICO - ATE 15 DIAS	CH da Universidade Federal do Ceará - CE
57	AFASTAMENTO POR ATESTADO MÉDICO - ATE 15 DIAS	CH da Universidade Federal do Ceará - CE
57	AFASTAMENTO POR ATESTADO MÉDICO - ATE 15 DIAS	CH da Universidade Federal do Ceará - CE
57	AFASTAMENTO POR ATESTADO MÉDICO - ATE 15 DIAS	CH da Universidade Federal do Ceará - CE
57	AFASTAMENTO POR ATESTADO MÉDICO - ATE 15 DIAS	CH da Universidade Federal do Ceará - CE
57	AFASTAMENTO POR ATESTADO MÉDICO - ATE 15 DIAS	CH da Universidade Federal do Ceará - CE
57	AFASTAMENTO POR ATESTADO MÉDICO - ATE 15 DIAS	CH da Universidade Federal do Ceará - CE
57	AFASTAMENTO POR ATESTADO MÉDICO - ATE 15 DIAS	CH da Universidade Federal do Ceará - CE
57	AFASTAMENTO POR ATESTADO MÉDICO - ATE 15 DIAS	CH da Universidade Federal do Ceará - CE

57	AFASTAMENTO POR ATESTADO MÉDICO - ATE 15 DIAS	CH da Universidade Federal do Ceará - CE
57	AFASTAMENTO POR ATESTADO MÉDICO - ATE 15 DIAS	CH da Universidade Federal do Ceará - CE
57	AFASTAMENTO POR ATESTADO MÉDICO - ATE 15 DIAS	CH da Universidade Federal do Ceará - CE
57	AFASTAMENTO POR ATESTADO MÉDICO - ATE 15 DIAS	CH da Universidade Federal do Ceará - CE
57	AFASTAMENTO POR ATESTADO MÉDICO - ATE 15 DIAS	CH da Universidade Federal do Ceará - CE
57	AFASTAMENTO POR ATESTADO MÉDICO - ATE 15 DIAS	CH da Universidade Federal do Ceará - CE
57	AFASTAMENTO POR ATESTADO MÉDICO - ATE 15 DIAS	CH da Universidade Federal do Ceará - CE
57	AFASTAMENTO POR ATESTADO MÉDICO - ATE 15 DIAS	CH da Universidade Federal do Ceará - CE
57	AFASTAMENTO POR ATESTADO MÉDICO - ATE 15 DIAS	CH da Universidade Federal do Ceará - CE
57	AFASTAMENTO POR ATESTADO MÉDICO - ATE 15 DIAS	CH da Universidade Federal do Ceará - CE
57	AFASTAMENTO POR ATESTADO MÉDICO - ATE 15 DIAS	CH da Universidade Federal do Ceará - CE
57	AFASTAMENTO POR ATESTADO MÉDICO - ATE 15 DIAS	CH da Universidade Federal do Ceará - CE
57	AFASTAMENTO POR ATESTADO MÉDICO - ATE 15 DIAS	CH da Universidade Federal do Ceará - CE
57	AFASTAMENTO POR ATESTADO MÉDICO - ATE 15 DIAS	CH da Universidade Federal do Ceará - CE
57	AFASTAMENTO POR ATESTADO MÉDICO - ATE 15 DIAS	CH da Universidade Federal do Ceará - CE
57	AFASTAMENTO POR ATESTADO MÉDICO - ATE 15 DIAS	CH da Universidade Federal do Ceará - CE
57	AFASTAMENTO POR ATESTADO MÉDICO - ATE 15 DIAS	CH da Universidade Federal do Ceará - CE
57	AFASTAMENTO POR ATESTADO MÉDICO - ATE 15 DIAS	CH da Universidade Federal do Ceará - CE
57	AFASTAMENTO POR ATESTADO MÉDICO - ATE 15 DIAS	CH da Universidade Federal do Ceará - CE
57	AFASTAMENTO POR ATESTADO MÉDICO - ATE 15 DIAS	CH da Universidade Federal do Ceará - CE
57	AFASTAMENTO POR ATESTADO MÉDICO - ATE 15 DIAS	CH da Universidade Federal do Ceará - CE
57	AFASTAMENTO POR ATESTADO MÉDICO - ATE 15 DIAS	CH da Universidade Federal do Ceará - CE
57	AFASTAMENTO POR ATESTADO MÉDICO - ATE 15 DIAS	CH da Universidade Federal do Ceará - CE
57	AFASTAMENTO POR ATESTADO MÉDICO - ATE 15 DIAS	CH da Universidade Federal do Ceará - CE
57	AFASTAMENTO POR ATESTADO MÉDICO - ATE 15 DIAS	CH da Universidade Federal do Ceará - CE
57	AFASTAMENTO POR ATESTADO MÉDICO - ATE 15 DIAS	CH da Universidade Federal do Ceará - CE
57	AFASTAMENTO POR ATESTADO MÉDICO - ATE 15 DIAS	CH da Universidade Federal do Ceará - CE
57	AFASTAMENTO POR ATESTADO MÉDICO - ATE 15 DIAS	CH da Universidade Federal do Ceará - CE
57	AFASTAMENTO POR ATESTADO MÉDICO - ATE 15 DIAS	CH da Universidade Federal do Ceará - CE
57	AFASTAMENTO POR ATESTADO MÉDICO - ATE 15 DIAS	CH da Universidade Federal do Ceará - CE
57	AFASTAMENTO POR ATESTADO MÉDICO - ATE 15 DIAS	CH da Universidade Federal do Ceará - CE
57	AFASTAMENTO POR ATESTADO MÉDICO - ATE 15 DIAS	CH da Universidade Federal do Ceará - CE
57	AFASTAMENTO POR ATESTADO MÉDICO - ATE 15 DIAS	CH da Universidade Federal do Ceará - CE
57	AFASTAMENTO POR ATESTADO MÉDICO - ATE 15 DIAS	CH da Universidade Federal do Ceará - CE
57	AFASTAMENTO POR ATESTADO MÉDICO - ATE 15 DIAS	CH da Universidade Federal do Ceará - CE
57	AFASTAMENTO POR ATESTADO MÉDICO - ATE 15 DIAS	CH da Universidade Federal do Ceará - CE
57	AFASTAMENTO POR ATESTADO MÉDICO - ATE 15 DIAS	CH da Universidade Federal do Ceará - CE
57	AFASTAMENTO POR ATESTADO MÉDICO - ATE 15 DIAS	CH da Universidade Federal do Ceará - CE
57	AFASTAMENTO POR ATESTADO MÉDICO - ATE 15 DIAS	CH da Universidade Federal do Ceará - CE
57	AFASTAMENTO POR ATESTADO MÉDICO - ATE 15 DIAS	CH da Universidade Federal do Ceará - CE
57	AFASTAMENTO POR ATESTADO MÉDICO - ATE 15 DIAS	CH da Universidade Federal do Ceará - CE
57	AFASTAMENTO POR ATESTADO MÉDICO - ATE 15 DIAS	CH da Universidade Federal do Ceará - CE
57	AFASTAMENTO POR ATESTADO MÉDICO - ATE 15 DIAS	CH da Universidade Federal do Ceará - CE
57	AFASTAMENTO POR ATESTADO MÉDICO - ATE 15 DIAS	CH da Universidade Federal do Ceará - CE
57	AFASTAMENTO POR ATESTADO MÉDICO - ATE 15 DIAS	CH da Universidade Federal do Ceará - CE
57	AFASTAMENTO POR ATESTADO MÉDICO - ATE 15 DIAS	CH da Universidade Federal do Ceará - CE
57	AFASTAMENTO POR ATESTADO MÉDICO - ATE 15 DIAS	CH da Universidade Federal do Ceará - CE
57	AFASTAMENTO POR ATESTADO MÉDICO - ATE 15 DIAS	CH da Universidade Federal do Ceará - CE

57	AFASTAMENTO POR ATESTADO MÉDICO - ATE 15 DIAS	CH da Universidade Federal do Ceará - CE
57	AFASTAMENTO POR ATESTADO MÉDICO - ATE 15 DIAS	CH da Universidade Federal do Ceará - CE
57	AFASTAMENTO POR ATESTADO MÉDICO - ATE 15 DIAS	CH da Universidade Federal do Ceará - CE
57	AFASTAMENTO POR ATESTADO MÉDICO - ATE 15 DIAS	CH da Universidade Federal do Ceará - CE
57	AFASTAMENTO POR ATESTADO MÉDICO - ATE 15 DIAS	CH da Universidade Federal do Ceará - CE
57	AFASTAMENTO POR ATESTADO MÉDICO - ATE 15 DIAS	CH da Universidade Federal do Ceará - CE
57	AFASTAMENTO POR ATESTADO MÉDICO - ATE 15 DIAS	CH da Universidade Federal do Ceará - CE
57	AFASTAMENTO POR ATESTADO MÉDICO - ATE 15 DIAS	CH da Universidade Federal do Ceará - CE
57	AFASTAMENTO POR ATESTADO MÉDICO - ATE 15 DIAS	CH da Universidade Federal do Ceará - CE
57	AFASTAMENTO POR ATESTADO MÉDICO - ATE 15 DIAS	CH da Universidade Federal do Ceará - CE
57	AFASTAMENTO POR ATESTADO MÉDICO - ATE 15 DIAS	CH da Universidade Federal do Ceará - CE
57	AFASTAMENTO POR ATESTADO MÉDICO - ATE 15 DIAS	CH da Universidade Federal do Ceará - CE
57	AFASTAMENTO POR ATESTADO MÉDICO - ATE 15 DIAS	CH da Universidade Federal do Ceará - CE
57	AFASTAMENTO POR ATESTADO MÉDICO - ATE 15 DIAS	CH da Universidade Federal do Ceará - CE
57	AFASTAMENTO POR ATESTADO MÉDICO - ATE 15 DIAS	CH da Universidade Federal do Ceará - CE
57	AFASTAMENTO POR ATESTADO MÉDICO - ATE 15 DIAS	CH da Universidade Federal do Ceará - CE
57	AFASTAMENTO POR ATESTADO MÉDICO - ATE 15 DIAS	CH da Universidade Federal do Ceará - CE
57	AFASTAMENTO POR ATESTADO MÉDICO - ATE 15 DIAS	CH da Universidade Federal do Ceará - CE
57	AFASTAMENTO POR ATESTADO MÉDICO - ATE 15 DIAS	CH da Universidade Federal do Ceará - CE
57	AFASTAMENTO POR ATESTADO MÉDICO - ATE 15 DIAS	CH da Universidade Federal do Ceará - CE
57	AFASTAMENTO POR ATESTADO MÉDICO - ATE 15 DIAS	CH da Universidade Federal do Ceará - CE
57	AFASTAMENTO POR ATESTADO MÉDICO - ATE 15 DIAS	CH da Universidade Federal do Ceará - CE
57	AFASTAMENTO POR ATESTADO MÉDICO - ATE 15 DIAS	CH da Universidade Federal do Ceará - CE
57	AFASTAMENTO POR ATESTADO MÉDICO - ATE 15 DIAS	CH da Universidade Federal do Ceará - CE
57	AFASTAMENTO POR ATESTADO MÉDICO - ATE 15 DIAS	CH da Universidade Federal do Ceará - CE
57	AFASTAMENTO POR ATESTADO MÉDICO - ATE 15 DIAS	CH da Universidade Federal do Ceará - CE
57	AFASTAMENTO POR ATESTADO MÉDICO - ATE 15 DIAS	CH da Universidade Federal do Ceará - CE
57	AFASTAMENTO POR ATESTADO MÉDICO - ATE 15 DIAS	CH da Universidade Federal do Ceará - CE
57	AFASTAMENTO POR ATESTADO MÉDICO - ATE 15 DIAS	CH da Universidade Federal do Ceará - CE
57	AFASTAMENTO POR ATESTADO MÉDICO - ATE 15 DIAS	CH da Universidade Federal do Ceará - CE
57	AFASTAMENTO POR ATESTADO MÉDICO - ATE 15 DIAS	CH da Universidade Federal do Ceará - CE
57	AFASTAMENTO POR ATESTADO MÉDICO - ATE 15 DIAS	CH da Universidade Federal do Ceará - CE
57	AFASTAMENTO POR ATESTADO MÉDICO - ATE 15 DIAS	CH da Universidade Federal do Ceará - CE
57	AFASTAMENTO POR ATESTADO MÉDICO - ATE 15 DIAS	CH da Universidade Federal do Ceará - CE
57	AFASTAMENTO POR ATESTADO MÉDICO - ATE 15 DIAS	CH da Universidade Federal do Ceará - CE
57	AFASTAMENTO POR ATESTADO MÉDICO - ATE 15 DIAS	CH da Universidade Federal do Ceará - CE
57	AFASTAMENTO POR ATESTADO MÉDICO - ATE 15 DIAS	CH da Universidade Federal do Ceará - CE
57	AFASTAMENTO POR ATESTADO MÉDICO - ATE 15 DIAS	CH da Universidade Federal do Ceará - CE
57	AFASTAMENTO POR ATESTADO MÉDICO - ATE 15 DIAS	CH da Universidade Federal do Ceará - CE
57	AFASTAMENTO POR ATESTADO MÉDICO - ATE 15 DIAS	CH da Universidade Federal do Ceará - CE
57	AFASTAMENTO POR ATESTADO MÉDICO - ATE 15 DIAS	CH da Universidade Federal do Ceará - CE
57	AFASTAMENTO POR ATESTADO MÉDICO - ATE 15 DIAS	CH da Universidade Federal do Ceará - CE
57	AFASTAMENTO POR ATESTADO MÉDICO - ATE 15 DIAS	CH da Universidade Federal do Ceará - CE
57	AFASTAMENTO POR ATESTADO MÉDICO - ATE 15 DIAS	CH da Universidade Federal do Ceará - CE
57	AFASTAMENTO POR ATESTADO MÉDICO - ATE 15 DIAS	CH da Universidade Federal do Ceará - CE
57	AFASTAMENTO POR ATESTADO MÉDICO - ATE 15 DIAS	CH da Universidade Federal do Ceará - CE
57	AFASTAMENTO POR ATESTADO MÉDICO - ATE 15 DIAS	CH da Universidade Federal do Ceará - CE
57	AFASTAMENTO POR ATESTADO MÉDICO - ATE 15 DIAS	CH da Universidade Federal do Ceará - CE

57	AFASTAMENTO POR ATESTADO MÉDICO - ATE 15 DIAS	CH da Universidade Federal do Ceará - CE
57	AFASTAMENTO POR ATESTADO MÉDICO - ATE 15 DIAS	CH da Universidade Federal do Ceará - CE
57	AFASTAMENTO POR ATESTADO MÉDICO - ATE 15 DIAS	CH da Universidade Federal do Ceará - CE
57	AFASTAMENTO POR ATESTADO MÉDICO - ATE 15 DIAS	CH da Universidade Federal do Ceará - CE
57	AFASTAMENTO POR ATESTADO MÉDICO - ATE 15 DIAS	CH da Universidade Federal do Ceará - CE
57	AFASTAMENTO POR ATESTADO MÉDICO - ATE 15 DIAS	CH da Universidade Federal do Ceará - CE
57	AFASTAMENTO POR ATESTADO MÉDICO - ATE 15 DIAS	CH da Universidade Federal do Ceará - CE
57	AFASTAMENTO POR ATESTADO MÉDICO - ATE 15 DIAS	CH da Universidade Federal do Ceará - CE
57	AFASTAMENTO POR ATESTADO MÉDICO - ATE 15 DIAS	CH da Universidade Federal do Ceará - CE
57	AFASTAMENTO POR ATESTADO MÉDICO - ATE 15 DIAS	CH da Universidade Federal do Ceará - CE
57	AFASTAMENTO POR ATESTADO MÉDICO - ATE 15 DIAS	CH da Universidade Federal do Ceará - CE
57	AFASTAMENTO POR ATESTADO MÉDICO - ATE 15 DIAS	CH da Universidade Federal do Ceará - CE
57	AFASTAMENTO POR ATESTADO MÉDICO - ATE 15 DIAS	CH da Universidade Federal do Ceará - CE
57	AFASTAMENTO POR ATESTADO MÉDICO - ATE 15 DIAS	CH da Universidade Federal do Ceará - CE
57	AFASTAMENTO POR ATESTADO MÉDICO - ATE 15 DIAS	CH da Universidade Federal do Ceará - CE
57	AFASTAMENTO POR ATESTADO MÉDICO - ATE 15 DIAS	CH da Universidade Federal do Ceará - CE
57	AFASTAMENTO POR ATESTADO MÉDICO - ATE 15 DIAS	CH da Universidade Federal do Ceará - CE
57	AFASTAMENTO POR ATESTADO MÉDICO - ATE 15 DIAS	CH da Universidade Federal do Ceará - CE
57	AFASTAMENTO POR ATESTADO MÉDICO - ATE 15 DIAS	CH da Universidade Federal do Ceará - CE
57	AFASTAMENTO POR ATESTADO MÉDICO - ATE 15 DIAS	CH da Universidade Federal do Ceará - CE
57	AFASTAMENTO POR ATESTADO MÉDICO - ATE 15 DIAS	CH da Universidade Federal do Ceará - CE
57	AFASTAMENTO POR ATESTADO MÉDICO - ATE 15 DIAS	CH da Universidade Federal do Ceará - CE
57	AFASTAMENTO POR ATESTADO MÉDICO - ATE 15 DIAS	CH da Universidade Federal do Ceará - CE
57	AFASTAMENTO POR ATESTADO MÉDICO - ATE 15 DIAS	CH da Universidade Federal do Ceará - CE
57	AFASTAMENTO POR ATESTADO MÉDICO - ATE 15 DIAS	CH da Universidade Federal do Ceará - CE
57	AFASTAMENTO POR ATESTADO MÉDICO - ATE 15 DIAS	CH da Universidade Federal do Ceará - CE
57	AFASTAMENTO POR ATESTADO MÉDICO - ATE 15 DIAS	CH da Universidade Federal do Ceará - CE
57	AFASTAMENTO POR ATESTADO MÉDICO - ATE 15 DIAS	CH da Universidade Federal do Ceará - CE
57	AFASTAMENTO POR ATESTADO MÉDICO - ATE 15 DIAS	CH da Universidade Federal do Ceará - CE
57	AFASTAMENTO POR ATESTADO MÉDICO - ATE 15 DIAS	CH da Universidade Federal do Ceará - CE
57	AFASTAMENTO POR ATESTADO MÉDICO - ATE 15 DIAS	CH da Universidade Federal do Ceará - CE
57	AFASTAMENTO POR ATESTADO MÉDICO - ATE 15 DIAS	CH da Universidade Federal do Ceará - CE
57	AFASTAMENTO POR ATESTADO MÉDICO - ATE 15 DIAS	CH da Universidade Federal do Ceará - CE
57	AFASTAMENTO POR ATESTADO MÉDICO - ATE 15 DIAS	CH da Universidade Federal do Ceará - CE
57	AFASTAMENTO POR ATESTADO MÉDICO - ATE 15 DIAS	CH da Universidade Federal do Ceará - CE
57	AFASTAMENTO POR ATESTADO MÉDICO - ATE 15 DIAS	CH da Universidade Federal do Ceará - CE
57	AFASTAMENTO POR ATESTADO MÉDICO - ATE 15 DIAS	CH da Universidade Federal do Ceará - CE
57	AFASTAMENTO POR ATESTADO MÉDICO - ATE 15 DIAS	CH da Universidade Federal do Ceará - CE
57	AFASTAMENTO POR ATESTADO MÉDICO - ATE 15 DIAS	CH da Universidade Federal do Ceará - CE
57	AFASTAMENTO POR ATESTADO MÉDICO - ATE 15 DIAS	CH da Universidade Federal do Ceará - CE
57	AFASTAMENTO POR ATESTADO MÉDICO - ATE 15 DIAS	CH da Universidade Federal do Ceará - CE
57	AFASTAMENTO POR ATESTADO MÉDICO - ATE 15 DIAS	CH da Universidade Federal do Ceará - CE
57	AFASTAMENTO POR ATESTADO MÉDICO - ATE 15 DIAS	CH da Universidade Federal do Ceará - CE
57	AFASTAMENTO POR ATESTADO MÉDICO - ATE 15 DIAS	CH da Universidade Federal do Ceará - CE
57	AFASTAMENTO POR ATESTADO MÉDICO - ATE 15 DIAS	CH da Universidade Federal do Ceará - CE
57	AFASTAMENTO POR ATESTADO MÉDICO - ATE 15 DIAS	CH da Universidade Federal do Ceará - CE
57	AFASTAMENTO POR ATESTADO MÉDICO - ATE 15 DIAS	CH da Universidade Federal do Ceará - CE
57	AFASTAMENTO POR ATESTADO MÉDICO - ATE 15 DIAS	CH da Universidade Federal do Ceará - CE

57	AFASTAMENTO POR ATESTADO MÉDICO - ATE 15 DIAS	CH da Universidade Federal do Ceará - CE
57	AFASTAMENTO POR ATESTADO MÉDICO - ATE 15 DIAS	CH da Universidade Federal do Ceará - CE
57	AFASTAMENTO POR ATESTADO MÉDICO - ATE 15 DIAS	CH da Universidade Federal do Ceará - CE
57	AFASTAMENTO POR ATESTADO MÉDICO - ATE 15 DIAS	CH da Universidade Federal do Ceará - CE
57	AFASTAMENTO POR ATESTADO MÉDICO - ATE 15 DIAS	CH da Universidade Federal do Ceará - CE
57	AFASTAMENTO POR ATESTADO MÉDICO - ATE 15 DIAS	CH da Universidade Federal do Ceará - CE
57	AFASTAMENTO POR ATESTADO MÉDICO - ATE 15 DIAS	CH da Universidade Federal do Ceará - CE
57	AFASTAMENTO POR ATESTADO MÉDICO - ATE 15 DIAS	CH da Universidade Federal do Ceará - CE
57	AFASTAMENTO POR ATESTADO MÉDICO - ATE 15 DIAS	CH da Universidade Federal do Ceará - CE
57	AFASTAMENTO POR ATESTADO MÉDICO - ATE 15 DIAS	CH da Universidade Federal do Ceará - CE
57	AFASTAMENTO POR ATESTADO MÉDICO - ATE 15 DIAS	CH da Universidade Federal do Ceará - CE
57	AFASTAMENTO POR ATESTADO MÉDICO - ATE 15 DIAS	CH da Universidade Federal do Ceará - CE
57	AFASTAMENTO POR ATESTADO MÉDICO - ATE 15 DIAS	CH da Universidade Federal do Ceará - CE
57	AFASTAMENTO POR ATESTADO MÉDICO - ATE 15 DIAS	CH da Universidade Federal do Ceará - CE
57	AFASTAMENTO POR ATESTADO MÉDICO - ATE 15 DIAS	CH da Universidade Federal do Ceará - CE
57	AFASTAMENTO POR ATESTADO MÉDICO - ATE 15 DIAS	CH da Universidade Federal do Ceará - CE
57	AFASTAMENTO POR ATESTADO MÉDICO - ATE 15 DIAS	CH da Universidade Federal do Ceará - CE
57	AFASTAMENTO POR ATESTADO MÉDICO - ATE 15 DIAS	CH da Universidade Federal do Ceará - CE
57	AFASTAMENTO POR ATESTADO MÉDICO - ATE 15 DIAS	CH da Universidade Federal do Ceará - CE
57	AFASTAMENTO POR ATESTADO MÉDICO - ATE 15 DIAS	CH da Universidade Federal do Ceará - CE
57	AFASTAMENTO POR ATESTADO MÉDICO - ATE 15 DIAS	CH da Universidade Federal do Ceará - CE
57	AFASTAMENTO POR ATESTADO MÉDICO - ATE 15 DIAS	CH da Universidade Federal do Ceará - CE
57	AFASTAMENTO POR ATESTADO MÉDICO - ATE 15 DIAS	CH da Universidade Federal do Ceará - CE
57	AFASTAMENTO POR ATESTADO MÉDICO - ATE 15 DIAS	CH da Universidade Federal do Ceará - CE
57	AFASTAMENTO POR ATESTADO MÉDICO - ATE 15 DIAS	CH da Universidade Federal do Ceará - CE
57	AFASTAMENTO POR ATESTADO MÉDICO - ATE 15 DIAS	CH da Universidade Federal do Ceará - CE
57	AFASTAMENTO POR ATESTADO MÉDICO - ATE 15 DIAS	CH da Universidade Federal do Ceará - CE
57	AFASTAMENTO POR ATESTADO MÉDICO - ATE 15 DIAS	CH da Universidade Federal do Ceará - CE
57	AFASTAMENTO POR ATESTADO MÉDICO - ATE 15 DIAS	CH da Universidade Federal do Ceará - CE
57	AFASTAMENTO POR ATESTADO MÉDICO - ATE 15 DIAS	CH da Universidade Federal do Ceará - CE
57	AFASTAMENTO POR ATESTADO MÉDICO - ATE 15 DIAS	CH da Universidade Federal do Ceará - CE
57	AFASTAMENTO POR ATESTADO MÉDICO - ATE 15 DIAS	CH da Universidade Federal do Ceará - CE
57	AFASTAMENTO POR ATESTADO MÉDICO - ATE 15 DIAS	CH da Universidade Federal do Ceará - CE
57	AFASTAMENTO POR ATESTADO MÉDICO - ATE 15 DIAS	CH da Universidade Federal do Ceará - CE
57	AFASTAMENTO POR ATESTADO MÉDICO - ATE 15 DIAS	CH da Universidade Federal do Ceará - CE
57	AFASTAMENTO POR ATESTADO MÉDICO - ATE 15 DIAS	CH da Universidade Federal do Ceará - CE
57	AFASTAMENTO POR ATESTADO MÉDICO - ATE 15 DIAS	CH da Universidade Federal do Ceará - CE
57	AFASTAMENTO POR ATESTADO MÉDICO - ATE 15 DIAS	CH da Universidade Federal do Ceará - CE
57	AFASTAMENTO POR ATESTADO MÉDICO - ATE 15 DIAS	CH da Universidade Federal do Ceará - CE
57	AFASTAMENTO POR ATESTADO MÉDICO - ATE 15 DIAS	CH da Universidade Federal do Ceará - CE
57	AFASTAMENTO POR ATESTADO MÉDICO - ATE 15 DIAS	CH da Universidade Federal do Ceará - CE
57	AFASTAMENTO POR ATESTADO MÉDICO - ATE 15 DIAS	CH da Universidade Federal do Ceará - CE
57	AFASTAMENTO POR ATESTADO MÉDICO - ATE 15 DIAS	CH da Universidade Federal do Ceará - CE
57	AFASTAMENTO POR ATESTADO MÉDICO - ATE 15 DIAS	CH da Universidade Federal do Ceará - CE
57	AFASTAMENTO POR ATESTADO MÉDICO - ATE 15 DIAS	CH da Universidade Federal do Ceará - CE
57	AFASTAMENTO POR ATESTADO MÉDICO - ATE 15 DIAS	CH da Universidade Federal do Ceará - CE
57	AFASTAMENTO POR ATESTADO MÉDICO - ATE 15 DIAS	CH da Universidade Federal do Ceará - CE
57	AFASTAMENTO POR ATESTADO MÉDICO - ATE 15 DIAS	CH da Universidade Federal do Ceará - CE

57	AFASTAMENTO POR ATESTADO MÉDICO - ATE 15 DIAS	CH da Universidade Federal do Ceará - CE
57	AFASTAMENTO POR ATESTADO MÉDICO - ATE 15 DIAS	CH da Universidade Federal do Ceará - CE
57	AFASTAMENTO POR ATESTADO MÉDICO - ATE 15 DIAS	CH da Universidade Federal do Ceará - CE
57	AFASTAMENTO POR ATESTADO MÉDICO - ATE 15 DIAS	CH da Universidade Federal do Ceará - CE
57	AFASTAMENTO POR ATESTADO MÉDICO - ATE 15 DIAS	CH da Universidade Federal do Ceará - CE
57	AFASTAMENTO POR ATESTADO MÉDICO - ATE 15 DIAS	CH da Universidade Federal do Ceará - CE
57	AFASTAMENTO POR ATESTADO MÉDICO - ATE 15 DIAS	CH da Universidade Federal do Ceará - CE
57	AFASTAMENTO POR ATESTADO MÉDICO - ATE 15 DIAS	CH da Universidade Federal do Ceará - CE
57	AFASTAMENTO POR ATESTADO MÉDICO - ATE 15 DIAS	CH da Universidade Federal do Ceará - CE
57	AFASTAMENTO POR ATESTADO MÉDICO - ATE 15 DIAS	CH da Universidade Federal do Ceará - CE
57	AFASTAMENTO POR ATESTADO MÉDICO - ATE 15 DIAS	CH da Universidade Federal do Ceará - CE
57	AFASTAMENTO POR ATESTADO MÉDICO - ATE 15 DIAS	CH da Universidade Federal do Ceará - CE
57	AFASTAMENTO POR ATESTADO MÉDICO - ATE 15 DIAS	CH da Universidade Federal do Ceará - CE
57	AFASTAMENTO POR ATESTADO MÉDICO - ATE 15 DIAS	CH da Universidade Federal do Ceará - CE
57	AFASTAMENTO POR ATESTADO MÉDICO - ATE 15 DIAS	CH da Universidade Federal do Ceará - CE
57	AFASTAMENTO POR ATESTADO MÉDICO - ATE 15 DIAS	CH da Universidade Federal do Ceará - CE
57	AFASTAMENTO POR ATESTADO MÉDICO - ATE 15 DIAS	CH da Universidade Federal do Ceará - CE
57	AFASTAMENTO POR ATESTADO MÉDICO - ATE 15 DIAS	CH da Universidade Federal do Ceará - CE
57	AFASTAMENTO POR ATESTADO MÉDICO - ATE 15 DIAS	CH da Universidade Federal do Ceará - CE
57	AFASTAMENTO POR ATESTADO MÉDICO - ATE 15 DIAS	CH da Universidade Federal do Ceará - CE
57	AFASTAMENTO POR ATESTADO MÉDICO - ATE 15 DIAS	CH da Universidade Federal do Ceará - CE
57	AFASTAMENTO POR ATESTADO MÉDICO - ATE 15 DIAS	CH da Universidade Federal do Ceará - CE
57	AFASTAMENTO POR ATESTADO MÉDICO - ATE 15 DIAS	CH da Universidade Federal do Ceará - CE
57	AFASTAMENTO POR ATESTADO MÉDICO - ATE 15 DIAS	CH da Universidade Federal do Ceará - CE
57	AFASTAMENTO POR ATESTADO MÉDICO - ATE 15 DIAS	CH da Universidade Federal do Ceará - CE
57	AFASTAMENTO POR ATESTADO MÉDICO - ATE 15 DIAS	CH da Universidade Federal do Ceará - CE
57	AFASTAMENTO POR ATESTADO MÉDICO - ATE 15 DIAS	CH da Universidade Federal do Ceará - CE
57	AFASTAMENTO POR ATESTADO MÉDICO - ATE 15 DIAS	CH da Universidade Federal do Ceará - CE
57	AFASTAMENTO POR ATESTADO MÉDICO - ATE 15 DIAS	CH da Universidade Federal do Ceará - CE
57	AFASTAMENTO POR ATESTADO MÉDICO - ATE 15 DIAS	CH da Universidade Federal do Ceará - CE
57	AFASTAMENTO POR ATESTADO MÉDICO - ATE 15 DIAS	CH da Universidade Federal do Ceará - CE
57	AFASTAMENTO POR ATESTADO MÉDICO - ATE 15 DIAS	CH da Universidade Federal do Ceará - CE
57	AFASTAMENTO POR ATESTADO MÉDICO - ATE 15 DIAS	CH da Universidade Federal do Ceará - CE
57	AFASTAMENTO POR ATESTADO MÉDICO - ATE 15 DIAS	CH da Universidade Federal do Ceará - CE
57	AFASTAMENTO POR ATESTADO MÉDICO - ATE 15 DIAS	CH da Universidade Federal do Ceará - CE
57	AFASTAMENTO POR ATESTADO MÉDICO - ATE 15 DIAS	CH da Universidade Federal do Ceará - CE
57	AFASTAMENTO POR ATESTADO MÉDICO - ATE 15 DIAS	CH da Universidade Federal do Ceará - CE
57	AFASTAMENTO POR ATESTADO MÉDICO - ATE 15 DIAS	CH da Universidade Federal do Ceará - CE
57	AFASTAMENTO POR ATESTADO MÉDICO - ATE 15 DIAS	CH da Universidade Federal do Ceará - CE
57	AFASTAMENTO POR ATESTADO MÉDICO - ATE 15 DIAS	CH da Universidade Federal do Ceará - CE
57	AFASTAMENTO POR ATESTADO MÉDICO - ATE 15 DIAS	CH da Universidade Federal do Ceará - CE
57	AFASTAMENTO POR ATESTADO MÉDICO - ATE 15 DIAS	CH da Universidade Federal do Ceará - CE
57	AFASTAMENTO POR ATESTADO MÉDICO - ATE 15 DIAS	CH da Universidade Federal do Ceará - CE
57	AFASTAMENTO POR ATESTADO MÉDICO - ATE 15 DIAS	CH da Universidade Federal do Ceará - CE
57	AFASTAMENTO POR ATESTADO MÉDICO - ATE 15 DIAS	CH da Universidade Federal do Ceará - CE
57	AFASTAMENTO POR ATESTADO MÉDICO - ATE 15 DIAS	CH da Universidade Federal do Ceará - CE
57	AFASTAMENTO POR ATESTADO MÉDICO - ATE 15 DIAS	CH da Universidade Federal do Ceará - CE
57	AFASTAMENTO POR ATESTADO MÉDICO - ATE 15 DIAS	CH da Universidade Federal do Ceará - CE

57	AFASTAMENTO POR ATESTADO MÉDICO - ATE 15 DIAS	CH da Universidade Federal do Ceará - CE
57	AFASTAMENTO POR ATESTADO MÉDICO - ATE 15 DIAS	CH da Universidade Federal do Ceará - CE
57	AFASTAMENTO POR ATESTADO MÉDICO - ATE 15 DIAS	CH da Universidade Federal do Ceará - CE
57	AFASTAMENTO POR ATESTADO MÉDICO - ATE 15 DIAS	CH da Universidade Federal do Ceará - CE
57	AFASTAMENTO POR ATESTADO MÉDICO - ATE 15 DIAS	CH da Universidade Federal do Ceará - CE
57	AFASTAMENTO POR ATESTADO MÉDICO - ATE 15 DIAS	CH da Universidade Federal do Ceará - CE
57	AFASTAMENTO POR ATESTADO MÉDICO - ATE 15 DIAS	CH da Universidade Federal do Ceará - CE
57	AFASTAMENTO POR ATESTADO MÉDICO - ATE 15 DIAS	CH da Universidade Federal do Ceará - CE
57	AFASTAMENTO POR ATESTADO MÉDICO - ATE 15 DIAS	CH da Universidade Federal do Ceará - CE
57	AFASTAMENTO POR ATESTADO MÉDICO - ATE 15 DIAS	CH da Universidade Federal do Ceará - CE
57	AFASTAMENTO POR ATESTADO MÉDICO - ATE 15 DIAS	CH da Universidade Federal do Ceará - CE
57	AFASTAMENTO POR ATESTADO MÉDICO - ATE 15 DIAS	CH da Universidade Federal do Ceará - CE
57	AFASTAMENTO POR ATESTADO MÉDICO - ATE 15 DIAS	CH da Universidade Federal do Ceará - CE
57	AFASTAMENTO POR ATESTADO MÉDICO - ATE 15 DIAS	CH da Universidade Federal do Ceará - CE
57	AFASTAMENTO POR ATESTADO MÉDICO - ATE 15 DIAS	CH da Universidade Federal do Ceará - CE
57	AFASTAMENTO POR ATESTADO MÉDICO - ATE 15 DIAS	CH da Universidade Federal do Ceará - CE
57	AFASTAMENTO POR ATESTADO MÉDICO - ATE 15 DIAS	CH da Universidade Federal do Ceará - CE
57	AFASTAMENTO POR ATESTADO MÉDICO - ATE 15 DIAS	CH da Universidade Federal do Ceará - CE
57	AFASTAMENTO POR ATESTADO MÉDICO - ATE 15 DIAS	CH da Universidade Federal do Ceará - CE
57	AFASTAMENTO POR ATESTADO MÉDICO - ATE 15 DIAS	CH da Universidade Federal do Ceará - CE
57	AFASTAMENTO POR ATESTADO MÉDICO - ATE 15 DIAS	CH da Universidade Federal do Ceará - CE
57	AFASTAMENTO POR ATESTADO MÉDICO - ATE 15 DIAS	CH da Universidade Federal do Ceará - CE
57	AFASTAMENTO POR ATESTADO MÉDICO - ATE 15 DIAS	CH da Universidade Federal do Ceará - CE
57	AFASTAMENTO POR ATESTADO MÉDICO - ATE 15 DIAS	CH da Universidade Federal do Ceará - CE
57	AFASTAMENTO POR ATESTADO MÉDICO - ATE 15 DIAS	CH da Universidade Federal do Ceará - CE
57	AFASTAMENTO POR ATESTADO MÉDICO - ATE 15 DIAS	CH da Universidade Federal do Ceará - CE
57	AFASTAMENTO POR ATESTADO MÉDICO - ATE 15 DIAS	CH da Universidade Federal do Ceará - CE
57	AFASTAMENTO POR ATESTADO MÉDICO - ATE 15 DIAS	CH da Universidade Federal do Ceará - CE
57	AFASTAMENTO POR ATESTADO MÉDICO - ATE 15 DIAS	CH da Universidade Federal do Ceará - CE
57	AFASTAMENTO POR ATESTADO MÉDICO - ATE 15 DIAS	CH da Universidade Federal do Ceará - CE
57	AFASTAMENTO POR ATESTADO MÉDICO - ATE 15 DIAS	CH da Universidade Federal do Ceará - CE
57	AFASTAMENTO POR ATESTADO MÉDICO - ATE 15 DIAS	CH da Universidade Federal do Ceará - CE
57	AFASTAMENTO POR ATESTADO MÉDICO - ATE 15 DIAS	CH da Universidade Federal do Ceará - CE
57	AFASTAMENTO POR ATESTADO MÉDICO - ATE 15 DIAS	CH da Universidade Federal do Ceará - CE
57	AFASTAMENTO POR ATESTADO MÉDICO - ATE 15 DIAS	CH da Universidade Federal do Ceará - CE
57	AFASTAMENTO POR ATESTADO MÉDICO - ATE 15 DIAS	CH da Universidade Federal do Ceará - CE
57	AFASTAMENTO POR ATESTADO MÉDICO - ATE 15 DIAS	CH da Universidade Federal do Ceará - CE
57	AFASTAMENTO POR ATESTADO MÉDICO - ATE 15 DIAS	CH da Universidade Federal do Ceará - CE
57	AFASTAMENTO POR ATESTADO MÉDICO - ATE 15 DIAS	CH da Universidade Federal do Ceará - CE
57	AFASTAMENTO POR ATESTADO MÉDICO - ATE 15 DIAS	CH da Universidade Federal do Ceará - CE
57	AFASTAMENTO POR ATESTADO MÉDICO - ATE 15 DIAS	CH da Universidade Federal do Ceará - CE
57	AFASTAMENTO POR ATESTADO MÉDICO - ATE 15 DIAS	CH da Universidade Federal do Ceará - CE
57	AFASTAMENTO POR ATESTADO MÉDICO - ATE 15 DIAS	CH da Universidade Federal do Ceará - CE
57	AFASTAMENTO POR ATESTADO MÉDICO - ATE 15 DIAS	CH da Universidade Federal do Ceará - CE
57	AFASTAMENTO POR ATESTADO MÉDICO - ATE 15 DIAS	CH da Universidade Federal do Ceará - CE
57	AFASTAMENTO POR ATESTADO MÉDICO - ATE 15 DIAS	CH da Universidade Federal do Ceará - CE
57	AFASTAMENTO POR ATESTADO MÉDICO - ATE 15 DIAS	CH da Universidade Federal do Ceará - CE
57	AFASTAMENTO POR ATESTADO MÉDICO - ATE 15 DIAS	CH da Universidade Federal do Ceará - CE

57	AFASTAMENTO POR ATESTADO MÉDICO - ATE 15 DIAS	CH da Universidade Federal do Ceará - CE
57	AFASTAMENTO POR ATESTADO MÉDICO - ATE 15 DIAS	CH da Universidade Federal do Ceará - CE
57	AFASTAMENTO POR ATESTADO MÉDICO - ATE 15 DIAS	CH da Universidade Federal do Ceará - CE
57	AFASTAMENTO POR ATESTADO MÉDICO - ATE 15 DIAS	CH da Universidade Federal do Ceará - CE
57	AFASTAMENTO POR ATESTADO MÉDICO - ATE 15 DIAS	CH da Universidade Federal do Ceará - CE
57	AFASTAMENTO POR ATESTADO MÉDICO - ATE 15 DIAS	CH da Universidade Federal do Ceará - CE
57	AFASTAMENTO POR ATESTADO MÉDICO - ATE 15 DIAS	CH da Universidade Federal do Ceará - CE
57	AFASTAMENTO POR ATESTADO MÉDICO - ATE 15 DIAS	CH da Universidade Federal do Ceará - CE
57	AFASTAMENTO POR ATESTADO MÉDICO - ATE 15 DIAS	CH da Universidade Federal do Ceará - CE
57	AFASTAMENTO POR ATESTADO MÉDICO - ATE 15 DIAS	CH da Universidade Federal do Ceará - CE
57	AFASTAMENTO POR ATESTADO MÉDICO - ATE 15 DIAS	CH da Universidade Federal do Ceará - CE
57	AFASTAMENTO POR ATESTADO MÉDICO - ATE 15 DIAS	CH da Universidade Federal do Ceará - CE
57	AFASTAMENTO POR ATESTADO MÉDICO - ATE 15 DIAS	CH da Universidade Federal do Ceará - CE
57	AFASTAMENTO POR ATESTADO MÉDICO - ATE 15 DIAS	CH da Universidade Federal do Ceará - CE
57	AFASTAMENTO POR ATESTADO MÉDICO - ATE 15 DIAS	CH da Universidade Federal do Ceará - CE
57	AFASTAMENTO POR ATESTADO MÉDICO - ATE 15 DIAS	CH da Universidade Federal do Ceará - CE
57	AFASTAMENTO POR ATESTADO MÉDICO - ATE 15 DIAS	CH da Universidade Federal do Ceará - CE
57	AFASTAMENTO POR ATESTADO MÉDICO - ATE 15 DIAS	CH da Universidade Federal do Ceará - CE
57	AFASTAMENTO POR ATESTADO MÉDICO - ATE 15 DIAS	CH da Universidade Federal do Ceará - CE
57	AFASTAMENTO POR ATESTADO MÉDICO - ATE 15 DIAS	CH da Universidade Federal do Ceará - CE
57	AFASTAMENTO POR ATESTADO MÉDICO - ATE 15 DIAS	CH da Universidade Federal do Ceará - CE
57	AFASTAMENTO POR ATESTADO MÉDICO - ATE 15 DIAS	CH da Universidade Federal do Ceará - CE
57	AFASTAMENTO POR ATESTADO MÉDICO - ATE 15 DIAS	CH da Universidade Federal do Ceará - CE
57	AFASTAMENTO POR ATESTADO MÉDICO - ATE 15 DIAS	CH da Universidade Federal do Ceará - CE
57	AFASTAMENTO POR ATESTADO MÉDICO - ATE 15 DIAS	CH da Universidade Federal do Ceará - CE
57	AFASTAMENTO POR ATESTADO MÉDICO - ATE 15 DIAS	CH da Universidade Federal do Ceará - CE
57	AFASTAMENTO POR ATESTADO MÉDICO - ATE 15 DIAS	CH da Universidade Federal do Ceará - CE
57	AFASTAMENTO POR ATESTADO MÉDICO - ATE 15 DIAS	CH da Universidade Federal do Ceará - CE
57	AFASTAMENTO POR ATESTADO MÉDICO - ATE 15 DIAS	CH da Universidade Federal do Ceará - CE
57	AFASTAMENTO POR ATESTADO MÉDICO - ATE 15 DIAS	CH da Universidade Federal do Ceará - CE
57	AFASTAMENTO POR ATESTADO MÉDICO - ATE 15 DIAS	CH da Universidade Federal do Ceará - CE
57	AFASTAMENTO POR ATESTADO MÉDICO - ATE 15 DIAS	CH da Universidade Federal do Ceará - CE
57	AFASTAMENTO POR ATESTADO MÉDICO - ATE 15 DIAS	CH da Universidade Federal do Ceará - CE
57	AFASTAMENTO POR ATESTADO MÉDICO - ATE 15 DIAS	CH da Universidade Federal do Ceará - CE
57	AFASTAMENTO POR ATESTADO MÉDICO - ATE 15 DIAS	CH da Universidade Federal do Ceará - CE
57	AFASTAMENTO POR ATESTADO MÉDICO - ATE 15 DIAS	CH da Universidade Federal do Ceará - CE
57	AFASTAMENTO POR ATESTADO MÉDICO - ATE 15 DIAS	CH da Universidade Federal do Ceará - CE
57	AFASTAMENTO POR ATESTADO MÉDICO - ATE 15 DIAS	CH da Universidade Federal do Ceará - CE
57	AFASTAMENTO POR ATESTADO MÉDICO - ATE 15 DIAS	CH da Universidade Federal do Ceará - CE
57	AFASTAMENTO POR ATESTADO MÉDICO - ATE 15 DIAS	CH da Universidade Federal do Ceará - CE
57	AFASTAMENTO POR ATESTADO MÉDICO - ATE 15 DIAS	CH da Universidade Federal do Ceará - CE
57	AFASTAMENTO POR ATESTADO MÉDICO - ATE 15 DIAS	CH da Universidade Federal do Ceará - CE
57	AFASTAMENTO POR ATESTADO MÉDICO - ATE 15 DIAS	CH da Universidade Federal do Ceará - CE
57	AFASTAMENTO POR ATESTADO MÉDICO - ATE 15 DIAS	CH da Universidade Federal do Ceará - CE
57	AFASTAMENTO POR ATESTADO MÉDICO - ATE 15 DIAS	CH da Universidade Federal do Ceará - CE
57	AFASTAMENTO POR ATESTADO MÉDICO - ATE 15 DIAS	CH da Universidade Federal do Ceará - CE
57	AFASTAMENTO POR ATESTADO MÉDICO - ATE 15 DIAS	CH da Universidade Federal do Ceará - CE
57	AFASTAMENTO POR ATESTADO MÉDICO - ATE 15 DIAS	CH da Universidade Federal do Ceará - CE

57	AFASTAMENTO POR ATESTADO MÉDICO - ATE 15 DIAS	CH da Universidade Federal do Ceará - CE
57	AFASTAMENTO POR ATESTADO MÉDICO - ATE 15 DIAS	CH da Universidade Federal do Ceará - CE
57	AFASTAMENTO POR ATESTADO MÉDICO - ATE 15 DIAS	CH da Universidade Federal do Ceará - CE
57	AFASTAMENTO POR ATESTADO MÉDICO - ATE 15 DIAS	CH da Universidade Federal do Ceará - CE
57	AFASTAMENTO POR ATESTADO MÉDICO - ATE 15 DIAS	CH da Universidade Federal do Ceará - CE
57	AFASTAMENTO POR ATESTADO MÉDICO - ATE 15 DIAS	CH da Universidade Federal do Ceará - CE
57	AFASTAMENTO POR ATESTADO MÉDICO - ATE 15 DIAS	CH da Universidade Federal do Ceará - CE
57	AFASTAMENTO POR ATESTADO MÉDICO - ATE 15 DIAS	CH da Universidade Federal do Ceará - CE
57	AFASTAMENTO POR ATESTADO MÉDICO - ATE 15 DIAS	CH da Universidade Federal do Ceará - CE
57	AFASTAMENTO POR ATESTADO MÉDICO - ATE 15 DIAS	CH da Universidade Federal do Ceará - CE
57	AFASTAMENTO POR ATESTADO MÉDICO - ATE 15 DIAS	CH da Universidade Federal do Ceará - CE
57	AFASTAMENTO POR ATESTADO MÉDICO - ATE 15 DIAS	CH da Universidade Federal do Ceará - CE
57	AFASTAMENTO POR ATESTADO MÉDICO - ATE 15 DIAS	CH da Universidade Federal do Ceará - CE
57	AFASTAMENTO POR ATESTADO MÉDICO - ATE 15 DIAS	CH da Universidade Federal do Ceará - CE
57	AFASTAMENTO POR ATESTADO MÉDICO - ATE 15 DIAS	CH da Universidade Federal do Ceará - CE
57	AFASTAMENTO POR ATESTADO MÉDICO - ATE 15 DIAS	CH da Universidade Federal do Ceará - CE
57	AFASTAMENTO POR ATESTADO MÉDICO - ATE 15 DIAS	CH da Universidade Federal do Ceará - CE
57	AFASTAMENTO POR ATESTADO MÉDICO - ATE 15 DIAS	CH da Universidade Federal do Ceará - CE
57	AFASTAMENTO POR ATESTADO MÉDICO - ATE 15 DIAS	CH da Universidade Federal do Ceará - CE
57	AFASTAMENTO POR ATESTADO MÉDICO - ATE 15 DIAS	CH da Universidade Federal do Ceará - CE
57	AFASTAMENTO POR ATESTADO MÉDICO - ATE 15 DIAS	CH da Universidade Federal do Ceará - CE
57	AFASTAMENTO POR ATESTADO MÉDICO - ATE 15 DIAS	CH da Universidade Federal do Ceará - CE
57	AFASTAMENTO POR ATESTADO MÉDICO - ATE 15 DIAS	CH da Universidade Federal do Ceará - CE
57	AFASTAMENTO POR ATESTADO MÉDICO - ATE 15 DIAS	CH da Universidade Federal do Ceará - CE
57	AFASTAMENTO POR ATESTADO MÉDICO - ATE 15 DIAS	CH da Universidade Federal do Ceará - CE
57	AFASTAMENTO POR ATESTADO MÉDICO - ATE 15 DIAS	CH da Universidade Federal do Ceará - CE
57	AFASTAMENTO POR ATESTADO MÉDICO - ATE 15 DIAS	CH da Universidade Federal do Ceará - CE
57	AFASTAMENTO POR ATESTADO MÉDICO - ATE 15 DIAS	CH da Universidade Federal do Ceará - CE
57	AFASTAMENTO POR ATESTADO MÉDICO - ATE 15 DIAS	CH da Universidade Federal do Ceará - CE
57	AFASTAMENTO POR ATESTADO MÉDICO - ATE 15 DIAS	CH da Universidade Federal do Ceará - CE
57	AFASTAMENTO POR ATESTADO MÉDICO - ATE 15 DIAS	CH da Universidade Federal do Ceará - CE
57	AFASTAMENTO POR ATESTADO MÉDICO - ATE 15 DIAS	CH da Universidade Federal do Ceará - CE
57	AFASTAMENTO POR ATESTADO MÉDICO - ATE 15 DIAS	CH da Universidade Federal do Ceará - CE
57	AFASTAMENTO POR ATESTADO MÉDICO - ATE 15 DIAS	CH da Universidade Federal do Ceará - CE
57	AFASTAMENTO POR ATESTADO MÉDICO - ATE 15 DIAS	CH da Universidade Federal do Ceará - CE
57	AFASTAMENTO POR ATESTADO MÉDICO - ATE 15 DIAS	CH da Universidade Federal do Ceará - CE
57	AFASTAMENTO POR ATESTADO MÉDICO - ATE 15 DIAS	CH da Universidade Federal do Ceará - CE
57	AFASTAMENTO POR ATESTADO MÉDICO - ATE 15 DIAS	CH da Universidade Federal do Ceará - CE
57	AFASTAMENTO POR ATESTADO MÉDICO - ATE 15 DIAS	CH da Universidade Federal do Ceará - CE
57	AFASTAMENTO POR ATESTADO MÉDICO - ATE 15 DIAS	CH da Universidade Federal do Ceará - CE
57	AFASTAMENTO POR ATESTADO MÉDICO - ATE 15 DIAS	CH da Universidade Federal do Ceará - CE
57	AFASTAMENTO POR ATESTADO MÉDICO - ATE 15 DIAS	CH da Universidade Federal do Ceará - CE
57	AFASTAMENTO POR ATESTADO MÉDICO - ATE 15 DIAS	CH da Universidade Federal do Ceará - CE
57	AFASTAMENTO POR ATESTADO MÉDICO - ATE 15 DIAS	CH da Universidade Federal do Ceará - CE
57	AFASTAMENTO POR ATESTADO MÉDICO - ATE 15 DIAS	CH da Universidade Federal do Ceará - CE
57	AFASTAMENTO POR ATESTADO MÉDICO - ATE 15 DIAS	CH da Universidade Federal do Ceará - CE
57	AFASTAMENTO POR ATESTADO MÉDICO - ATE 15 DIAS	CH da Universidade Federal do Ceará - CE
57	AFASTAMENTO POR ATESTADO MÉDICO - ATE 15 DIAS	CH da Universidade Federal do Ceará - CE

57	AFASTAMENTO POR ATESTADO MÉDICO - ATE 15 DIAS	CH da Universidade Federal do Ceará - CE
57	AFASTAMENTO POR ATESTADO MÉDICO - ATE 15 DIAS	CH da Universidade Federal do Ceará - CE
57	AFASTAMENTO POR ATESTADO MÉDICO - ATE 15 DIAS	CH da Universidade Federal do Ceará - CE
57	AFASTAMENTO POR ATESTADO MÉDICO - ATE 15 DIAS	CH da Universidade Federal do Ceará - CE
57	AFASTAMENTO POR ATESTADO MÉDICO - ATE 15 DIAS	CH da Universidade Federal do Ceará - CE
57	AFASTAMENTO POR ATESTADO MÉDICO - ATE 15 DIAS	CH da Universidade Federal do Ceará - CE
57	AFASTAMENTO POR ATESTADO MÉDICO - ATE 15 DIAS	CH da Universidade Federal do Ceará - CE
57	AFASTAMENTO POR ATESTADO MÉDICO - ATE 15 DIAS	CH da Universidade Federal do Ceará - CE
57	AFASTAMENTO POR ATESTADO MÉDICO - ATE 15 DIAS	CH da Universidade Federal do Ceará - CE
57	AFASTAMENTO POR ATESTADO MÉDICO - ATE 15 DIAS	CH da Universidade Federal do Ceará - CE
57	AFASTAMENTO POR ATESTADO MÉDICO - ATE 15 DIAS	CH da Universidade Federal do Ceará - CE
57	AFASTAMENTO POR ATESTADO MÉDICO - ATE 15 DIAS	CH da Universidade Federal do Ceará - CE
57	AFASTAMENTO POR ATESTADO MÉDICO - ATE 15 DIAS	CH da Universidade Federal do Ceará - CE
57	AFASTAMENTO POR ATESTADO MÉDICO - ATE 15 DIAS	CH da Universidade Federal do Ceará - CE
57	AFASTAMENTO POR ATESTADO MÉDICO - ATE 15 DIAS	CH da Universidade Federal do Ceará - CE
57	AFASTAMENTO POR ATESTADO MÉDICO - ATE 15 DIAS	CH da Universidade Federal do Ceará - CE
57	AFASTAMENTO POR ATESTADO MÉDICO - ATE 15 DIAS	CH da Universidade Federal do Ceará - CE
57	AFASTAMENTO POR ATESTADO MÉDICO - ATE 15 DIAS	CH da Universidade Federal do Ceará - CE
57	AFASTAMENTO POR ATESTADO MÉDICO - ATE 15 DIAS	CH da Universidade Federal do Ceará - CE
57	AFASTAMENTO POR ATESTADO MÉDICO - ATE 15 DIAS	CH da Universidade Federal do Ceará - CE
57	AFASTAMENTO POR ATESTADO MÉDICO - ATE 15 DIAS	CH da Universidade Federal do Ceará - CE
57	AFASTAMENTO POR ATESTADO MÉDICO - ATE 15 DIAS	CH da Universidade Federal do Ceará - CE
57	AFASTAMENTO POR ATESTADO MÉDICO - ATE 15 DIAS	CH da Universidade Federal do Ceará - CE
57	AFASTAMENTO POR ATESTADO MÉDICO - ATE 15 DIAS	CH da Universidade Federal do Ceará - CE
57	AFASTAMENTO POR ATESTADO MÉDICO - ATE 15 DIAS	CH da Universidade Federal do Ceará - CE
57	AFASTAMENTO POR ATESTADO MÉDICO - ATE 15 DIAS	CH da Universidade Federal do Ceará - CE
57	AFASTAMENTO POR ATESTADO MÉDICO - ATE 15 DIAS	CH da Universidade Federal do Ceará - CE
57	AFASTAMENTO POR ATESTADO MÉDICO - ATE 15 DIAS	CH da Universidade Federal do Ceará - CE
57	AFASTAMENTO POR ATESTADO MÉDICO - ATE 15 DIAS	CH da Universidade Federal do Ceará - CE
57	AFASTAMENTO POR ATESTADO MÉDICO - ATE 15 DIAS	CH da Universidade Federal do Ceará - CE
57	AFASTAMENTO POR ATESTADO MÉDICO - ATE 15 DIAS	CH da Universidade Federal do Ceará - CE
57	AFASTAMENTO POR ATESTADO MÉDICO - ATE 15 DIAS	CH da Universidade Federal do Ceará - CE
57	AFASTAMENTO POR ATESTADO MÉDICO - ATE 15 DIAS	CH da Universidade Federal do Ceará - CE
57	AFASTAMENTO POR ATESTADO MÉDICO - ATE 15 DIAS	CH da Universidade Federal do Ceará - CE
57	AFASTAMENTO POR ATESTADO MÉDICO - ATE 15 DIAS	CH da Universidade Federal do Ceará - CE
57	AFASTAMENTO POR ATESTADO MÉDICO - ATE 15 DIAS	CH da Universidade Federal do Ceará - CE
57	AFASTAMENTO POR ATESTADO MÉDICO - ATE 15 DIAS	CH da Universidade Federal do Ceará - CE
57	AFASTAMENTO POR ATESTADO MÉDICO - ATE 15 DIAS	CH da Universidade Federal do Ceará - CE
57	AFASTAMENTO POR ATESTADO MÉDICO - ATE 15 DIAS	CH da Universidade Federal do Ceará - CE
57	AFASTAMENTO POR ATESTADO MÉDICO - ATE 15 DIAS	CH da Universidade Federal do Ceará - CE
57	AFASTAMENTO POR ATESTADO MÉDICO - ATE 15 DIAS	CH da Universidade Federal do Ceará - CE
57	AFASTAMENTO POR ATESTADO MÉDICO - ATE 15 DIAS	CH da Universidade Federal do Ceará - CE
57	AFASTAMENTO POR ATESTADO MÉDICO - ATE 15 DIAS	CH da Universidade Federal do Ceará - CE
57	AFASTAMENTO POR ATESTADO MÉDICO - ATE 15 DIAS	CH da Universidade Federal do Ceará - CE
57	AFASTAMENTO POR ATESTADO MÉDICO - ATE 15 DIAS	CH da Universidade Federal do Ceará - CE

57	AFASTAMENTO POR ATESTADO MÉDICO - ATE 15 DIAS	CH da Universidade Federal do Ceará - CE
57	AFASTAMENTO POR ATESTADO MÉDICO - ATE 15 DIAS	CH da Universidade Federal do Ceará - CE
57	AFASTAMENTO POR ATESTADO MÉDICO - ATE 15 DIAS	CH da Universidade Federal do Ceará - CE
57	AFASTAMENTO POR ATESTADO MÉDICO - ATE 15 DIAS	CH da Universidade Federal do Ceará - CE
57	AFASTAMENTO POR ATESTADO MÉDICO - ATE 15 DIAS	CH da Universidade Federal do Ceará - CE
57	AFASTAMENTO POR ATESTADO MÉDICO - ATE 15 DIAS	CH da Universidade Federal do Ceará - CE
57	AFASTAMENTO POR ATESTADO MÉDICO - ATE 15 DIAS	CH da Universidade Federal do Ceará - CE
57	AFASTAMENTO POR ATESTADO MÉDICO - ATE 15 DIAS	CH da Universidade Federal do Ceará - CE
57	AFASTAMENTO POR ATESTADO MÉDICO - ATE 15 DIAS	CH da Universidade Federal do Ceará - CE
57	AFASTAMENTO POR ATESTADO MÉDICO - ATE 15 DIAS	CH da Universidade Federal do Ceará - CE
57	AFASTAMENTO POR ATESTADO MÉDICO - ATE 15 DIAS	CH da Universidade Federal do Ceará - CE
57	AFASTAMENTO POR ATESTADO MÉDICO - ATE 15 DIAS	CH da Universidade Federal do Ceará - CE
57	AFASTAMENTO POR ATESTADO MÉDICO - ATE 15 DIAS	CH da Universidade Federal do Ceará - CE
57	AFASTAMENTO POR ATESTADO MÉDICO - ATE 15 DIAS	CH da Universidade Federal do Ceará - CE
57	AFASTAMENTO POR ATESTADO MÉDICO - ATE 15 DIAS	CH da Universidade Federal do Ceará - CE
57	AFASTAMENTO POR ATESTADO MÉDICO - ATE 15 DIAS	CH da Universidade Federal do Ceará - CE
57	AFASTAMENTO POR ATESTADO MÉDICO - ATE 15 DIAS	CH da Universidade Federal do Ceará - CE
57	AFASTAMENTO POR ATESTADO MÉDICO - ATE 15 DIAS	CH da Universidade Federal do Ceará - CE
57	AFASTAMENTO POR ATESTADO MÉDICO - ATE 15 DIAS	CH da Universidade Federal do Ceará - CE
57	AFASTAMENTO POR ATESTADO MÉDICO - ATE 15 DIAS	CH da Universidade Federal do Ceará - CE
57	AFASTAMENTO POR ATESTADO MÉDICO - ATE 15 DIAS	CH da Universidade Federal do Ceará - CE
57	AFASTAMENTO POR ATESTADO MÉDICO - ATE 15 DIAS	CH da Universidade Federal do Ceará - CE
57	AFASTAMENTO POR ATESTADO MÉDICO - ATE 15 DIAS	CH da Universidade Federal do Ceará - CE
57	AFASTAMENTO POR ATESTADO MÉDICO - ATE 15 DIAS	CH da Universidade Federal do Ceará - CE
57	AFASTAMENTO POR ATESTADO MÉDICO - ATE 15 DIAS	CH da Universidade Federal do Ceará - CE
57	AFASTAMENTO POR ATESTADO MÉDICO - ATE 15 DIAS	CH da Universidade Federal do Ceará - CE
57	AFASTAMENTO POR ATESTADO MÉDICO - ATE 15 DIAS	CH da Universidade Federal do Ceará - CE
57	AFASTAMENTO POR ATESTADO MÉDICO - ATE 15 DIAS	CH da Universidade Federal do Ceará - CE
57	AFASTAMENTO POR ATESTADO MÉDICO - ATE 15 DIAS	CH da Universidade Federal do Ceará - CE
57	AFASTAMENTO POR ATESTADO MÉDICO - ATE 15 DIAS	CH da Universidade Federal do Ceará - CE
57	AFASTAMENTO POR ATESTADO MÉDICO - ATE 15 DIAS	CH da Universidade Federal do Ceará - CE
57	AFASTAMENTO POR ATESTADO MÉDICO - ATE 15 DIAS	CH da Universidade Federal do Ceará - CE
57	AFASTAMENTO POR ATESTADO MÉDICO - ATE 15 DIAS	CH da Universidade Federal do Ceará - CE
57	AFASTAMENTO POR ATESTADO MÉDICO - ATE 15 DIAS	CH da Universidade Federal do Ceará - CE
57	AFASTAMENTO POR ATESTADO MÉDICO - ATE 15 DIAS	CH da Universidade Federal do Ceará - CE
57	AFASTAMENTO POR ATESTADO MÉDICO - ATE 15 DIAS	CH da Universidade Federal do Ceará - CE
57	AFASTAMENTO POR ATESTADO MÉDICO - ATE 15 DIAS	CH da Universidade Federal do Ceará - CE
57	AFASTAMENTO POR ATESTADO MÉDICO - ATE 15 DIAS	CH da Universidade Federal do Ceará - CE
57	AFASTAMENTO POR ATESTADO MÉDICO - ATE 15 DIAS	CH da Universidade Federal do Ceará - CE
57	AFASTAMENTO POR ATESTADO MÉDICO - ATE 15 DIAS	CH da Universidade Federal do Ceará - CE
57	AFASTAMENTO POR ATESTADO MÉDICO - ATE 15 DIAS	CH da Universidade Federal do Ceará - CE
57	AFASTAMENTO POR ATESTADO MÉDICO - ATE 15 DIAS	CH da Universidade Federal do Ceará - CE
57	AFASTAMENTO POR ATESTADO MÉDICO - ATE 15 DIAS	CH da Universidade Federal do Ceará - CE
57	AFASTAMENTO POR ATESTADO MÉDICO - ATE 15 DIAS	CH da Universidade Federal do Ceará - CE
57	AFASTAMENTO POR ATESTADO MÉDICO - ATE 15 DIAS	CH da Universidade Federal do Ceará - CE
57	AFASTAMENTO POR ATESTADO MÉDICO - ATE 15 DIAS	CH da Universidade Federal do Ceará - CE
57	AFASTAMENTO POR ATESTADO MÉDICO - ATE 15 DIAS	CH da Universidade Federal do Ceará - CE
57	AFASTAMENTO POR ATESTADO MÉDICO - ATE 15 DIAS	CH da Universidade Federal do Ceará - CE

57	AFASTAMENTO POR ATESTADO MÉDICO - ATE 15 DIAS	CH da Universidade Federal do Ceará - CE
57	AFASTAMENTO POR ATESTADO MÉDICO - ATE 15 DIAS	CH da Universidade Federal do Ceará - CE
57	AFASTAMENTO POR ATESTADO MÉDICO - ATE 15 DIAS	CH da Universidade Federal do Ceará - CE
57	AFASTAMENTO POR ATESTADO MÉDICO - ATE 15 DIAS	CH da Universidade Federal do Ceará - CE
57	AFASTAMENTO POR ATESTADO MÉDICO - ATE 15 DIAS	CH da Universidade Federal do Ceará - CE
57	AFASTAMENTO POR ATESTADO MÉDICO - ATE 15 DIAS	CH da Universidade Federal do Ceará - CE
57	AFASTAMENTO POR ATESTADO MÉDICO - ATE 15 DIAS	CH da Universidade Federal do Ceará - CE
57	AFASTAMENTO POR ATESTADO MÉDICO - ATE 15 DIAS	CH da Universidade Federal do Ceará - CE
57	AFASTAMENTO POR ATESTADO MÉDICO - ATE 15 DIAS	CH da Universidade Federal do Ceará - CE
57	AFASTAMENTO POR ATESTADO MÉDICO - ATE 15 DIAS	CH da Universidade Federal do Ceará - CE
57	AFASTAMENTO POR ATESTADO MÉDICO - ATE 15 DIAS	CH da Universidade Federal do Ceará - CE
57	AFASTAMENTO POR ATESTADO MÉDICO - ATE 15 DIAS	CH da Universidade Federal do Ceará - CE
57	AFASTAMENTO POR ATESTADO MÉDICO - ATE 15 DIAS	CH da Universidade Federal do Ceará - CE
57	AFASTAMENTO POR ATESTADO MÉDICO - ATE 15 DIAS	CH da Universidade Federal do Ceará - CE
57	AFASTAMENTO POR ATESTADO MÉDICO - ATE 15 DIAS	CH da Universidade Federal do Ceará - CE
57	AFASTAMENTO POR ATESTADO MÉDICO - ATE 15 DIAS	CH da Universidade Federal do Ceará - CE
57	AFASTAMENTO POR ATESTADO MÉDICO - ATE 15 DIAS	CH da Universidade Federal do Ceará - CE
57	AFASTAMENTO POR ATESTADO MÉDICO - ATE 15 DIAS	CH da Universidade Federal do Ceará - CE
57	AFASTAMENTO POR ATESTADO MÉDICO - ATE 15 DIAS	CH da Universidade Federal do Ceará - CE
57	AFASTAMENTO POR ATESTADO MÉDICO - ATE 15 DIAS	CH da Universidade Federal do Ceará - CE
57	AFASTAMENTO POR ATESTADO MÉDICO - ATE 15 DIAS	CH da Universidade Federal do Ceará - CE
57	AFASTAMENTO POR ATESTADO MÉDICO - ATE 15 DIAS	CH da Universidade Federal do Ceará - CE
57	AFASTAMENTO POR ATESTADO MÉDICO - ATE 15 DIAS	CH da Universidade Federal do Ceará - CE
57	AFASTAMENTO POR ATESTADO MÉDICO - ATE 15 DIAS	CH da Universidade Federal do Ceará - CE
57	AFASTAMENTO POR ATESTADO MÉDICO - ATE 15 DIAS	CH da Universidade Federal do Ceará - CE
57	AFASTAMENTO POR ATESTADO MÉDICO - ATE 15 DIAS	CH da Universidade Federal do Ceará - CE
57	AFASTAMENTO POR ATESTADO MÉDICO - ATE 15 DIAS	CH da Universidade Federal do Ceará - CE
57	AFASTAMENTO POR ATESTADO MÉDICO - ATE 15 DIAS	CH da Universidade Federal do Ceará - CE
57	AFASTAMENTO POR ATESTADO MÉDICO - ATE 15 DIAS	CH da Universidade Federal do Ceará - CE
57	AFASTAMENTO POR ATESTADO MÉDICO - ATE 15 DIAS	CH da Universidade Federal do Ceará - CE
57	AFASTAMENTO POR ATESTADO MÉDICO - ATE 15 DIAS	CH da Universidade Federal do Ceará - CE
57	AFASTAMENTO POR ATESTADO MÉDICO - ATE 15 DIAS	CH da Universidade Federal do Ceará - CE
57	AFASTAMENTO POR ATESTADO MÉDICO - ATE 15 DIAS	CH da Universidade Federal do Ceará - CE
57	AFASTAMENTO POR ATESTADO MÉDICO - ATE 15 DIAS	CH da Universidade Federal do Ceará - CE
57	AFASTAMENTO POR ATESTADO MÉDICO - ATE 15 DIAS	CH da Universidade Federal do Ceará - CE
57	AFASTAMENTO POR ATESTADO MÉDICO - ATE 15 DIAS	CH da Universidade Federal do Ceará - CE
57	AFASTAMENTO POR ATESTADO MÉDICO - ATE 15 DIAS	CH da Universidade Federal do Ceará - CE
57	AFASTAMENTO POR ATESTADO MÉDICO - ATE 15 DIAS	CH da Universidade Federal do Ceará - CE
57	AFASTAMENTO POR ATESTADO MÉDICO - ATE 15 DIAS	CH da Universidade Federal do Ceará - CE

57	AFASTAMENTO POR ATESTADO MÉDICO - ATE 15 DIAS	CH da Universidade Federal do Ceará - CE
57	AFASTAMENTO POR ATESTADO MÉDICO - ATE 15 DIAS	CH da Universidade Federal do Ceará - CE
57	AFASTAMENTO POR ATESTADO MÉDICO - ATE 15 DIAS	CH da Universidade Federal do Ceará - CE
57	AFASTAMENTO POR ATESTADO MÉDICO - ATE 15 DIAS	CH da Universidade Federal do Ceará - CE
57	AFASTAMENTO POR ATESTADO MÉDICO - ATE 15 DIAS	CH da Universidade Federal do Ceará - CE
57	AFASTAMENTO POR ATESTADO MÉDICO - ATE 15 DIAS	CH da Universidade Federal do Ceará - CE
57	AFASTAMENTO POR ATESTADO MÉDICO - ATE 15 DIAS	CH da Universidade Federal do Ceará - CE
57	AFASTAMENTO POR ATESTADO MÉDICO - ATE 15 DIAS	CH da Universidade Federal do Ceará - CE
57	AFASTAMENTO POR ATESTADO MÉDICO - ATE 15 DIAS	CH da Universidade Federal do Ceará - CE
57	AFASTAMENTO POR ATESTADO MÉDICO - ATE 15 DIAS	CH da Universidade Federal do Ceará - CE
57	AFASTAMENTO POR ATESTADO MÉDICO - ATE 15 DIAS	CH da Universidade Federal do Ceará - CE
57	AFASTAMENTO POR ATESTADO MÉDICO - ATE 15 DIAS	CH da Universidade Federal do Ceará - CE
57	AFASTAMENTO POR ATESTADO MÉDICO - ATE 15 DIAS	CH da Universidade Federal do Ceará - CE
57	AFASTAMENTO POR ATESTADO MÉDICO - ATE 15 DIAS	CH da Universidade Federal do Ceará - CE
57	AFASTAMENTO POR ATESTADO MÉDICO - ATE 15 DIAS	CH da Universidade Federal do Ceará - CE
57	AFASTAMENTO POR ATESTADO MÉDICO - ATE 15 DIAS	CH da Universidade Federal do Ceará - CE
57	AFASTAMENTO POR ATESTADO MÉDICO - ATE 15 DIAS	CH da Universidade Federal do Ceará - CE
57	AFASTAMENTO POR ATESTADO MÉDICO - ATE 15 DIAS	CH da Universidade Federal do Ceará - CE
57	AFASTAMENTO POR ATESTADO MÉDICO - ATE 15 DIAS	CH da Universidade Federal do Ceará - CE
57	AFASTAMENTO POR ATESTADO MÉDICO - ATE 15 DIAS	CH da Universidade Federal do Ceará - CE
57	AFASTAMENTO POR ATESTADO MÉDICO - ATE 15 DIAS	CH da Universidade Federal do Ceará - CE
57	AFASTAMENTO POR ATESTADO MÉDICO - ATE 15 DIAS	CH da Universidade Federal do Ceará - CE
57	AFASTAMENTO POR ATESTADO MÉDICO - ATE 15 DIAS	CH da Universidade Federal do Ceará - CE
57	AFASTAMENTO POR ATESTADO MÉDICO - ATE 15 DIAS	CH da Universidade Federal do Ceará - CE
57	AFASTAMENTO POR ATESTADO MÉDICO - ATE 15 DIAS	CH da Universidade Federal do Ceará - CE
57	AFASTAMENTO POR ATESTADO MÉDICO - ATE 15 DIAS	CH da Universidade Federal do Ceará - CE
57	AFASTAMENTO POR ATESTADO MÉDICO - ATE 15 DIAS	CH da Universidade Federal do Ceará - CE
57	AFASTAMENTO POR ATESTADO MÉDICO - ATE 15 DIAS	CH da Universidade Federal do Ceará - CE
57	AFASTAMENTO POR ATESTADO MÉDICO - ATE 15 DIAS	CH da Universidade Federal do Ceará - CE
57	AFASTAMENTO POR ATESTADO MÉDICO - ATE 15 DIAS	CH da Universidade Federal do Ceará - CE
57	AFASTAMENTO POR ATESTADO MÉDICO - ATE 15 DIAS	CH da Universidade Federal do Ceará - CE
57	AFASTAMENTO POR ATESTADO MÉDICO - ATE 15 DIAS	CH da Universidade Federal do Ceará - CE
57	AFASTAMENTO POR ATESTADO MÉDICO - ATE 15 DIAS	CH da Universidade Federal do Ceará - CE
57	AFASTAMENTO POR ATESTADO MÉDICO - ATE 15 DIAS	CH da Universidade Federal do Ceará - CE
57	AFASTAMENTO POR ATESTADO MÉDICO - ATE 15 DIAS	CH da Universidade Federal do Ceará - CE
57	AFASTAMENTO POR ATESTADO MÉDICO - ATE 15 DIAS	CH da Universidade Federal do Ceará - CE
57	AFASTAMENTO POR ATESTADO MÉDICO - ATE 15 DIAS	CH da Universidade Federal do Ceará - CE
57	AFASTAMENTO POR ATESTADO MÉDICO - ATE 15 DIAS	CH da Universidade Federal do Ceará - CE
57	AFASTAMENTO POR ATESTADO MÉDICO - ATE 15 DIAS	CH da Universidade Federal do Ceará - CE
57	AFASTAMENTO POR ATESTADO MÉDICO - ATE 15 DIAS	CH da Universidade Federal do Ceará - CE
57	AFASTAMENTO POR ATESTADO MÉDICO - ATE 15 DIAS	CH da Universidade Federal do Ceará - CE
57	AFASTAMENTO POR ATESTADO MÉDICO - ATE 15 DIAS	CH da Universidade Federal do Ceará - CE
57	AFASTAMENTO POR ATESTADO MÉDICO - ATE 15 DIAS	CH da Universidade Federal do Ceará - CE
57	AFASTAMENTO POR ATESTADO MÉDICO - ATE 15 DIAS	CH da Universidade Federal do Ceará - CE
57	AFASTAMENTO POR ATESTADO MÉDICO - ATE 15 DIAS	CH da Universidade Federal do Ceará - CE
57	AFASTAMENTO POR ATESTADO MÉDICO - ATE 15 DIAS	CH da Universidade Federal do Ceará - CE
57	AFASTAMENTO POR ATESTADO MÉDICO - ATE 15 DIAS	CH da Universidade Federal do Ceará - CE
57	AFASTAMENTO POR ATESTADO MÉDICO - ATE 15 DIAS	CH da Universidade Federal do Ceará - CE

57	AFASTAMENTO POR ATESTADO MÉDICO - ATE 15 DIAS	CH da Universidade Federal do Ceará - CE
57	AFASTAMENTO POR ATESTADO MÉDICO - ATE 15 DIAS	CH da Universidade Federal do Ceará - CE
57	AFASTAMENTO POR ATESTADO MÉDICO - ATE 15 DIAS	CH da Universidade Federal do Ceará - CE
57	AFASTAMENTO POR ATESTADO MÉDICO - ATE 15 DIAS	CH da Universidade Federal do Ceará - CE
57	AFASTAMENTO POR ATESTADO MÉDICO - ATE 15 DIAS	CH da Universidade Federal do Ceará - CE
57	AFASTAMENTO POR ATESTADO MÉDICO - ATE 15 DIAS	CH da Universidade Federal do Ceará - CE
57	AFASTAMENTO POR ATESTADO MÉDICO - ATE 15 DIAS	CH da Universidade Federal do Ceará - CE
57	AFASTAMENTO POR ATESTADO MÉDICO - ATE 15 DIAS	CH da Universidade Federal do Ceará - CE
57	AFASTAMENTO POR ATESTADO MÉDICO - ATE 15 DIAS	CH da Universidade Federal do Ceará - CE
57	AFASTAMENTO POR ATESTADO MÉDICO - ATE 15 DIAS	CH da Universidade Federal do Ceará - CE
57	AFASTAMENTO POR ATESTADO MÉDICO - ATE 15 DIAS	CH da Universidade Federal do Ceará - CE
57	AFASTAMENTO POR ATESTADO MÉDICO - ATE 15 DIAS	CH da Universidade Federal do Ceará - CE
57	AFASTAMENTO POR ATESTADO MÉDICO - ATE 15 DIAS	CH da Universidade Federal do Ceará - CE
57	AFASTAMENTO POR ATESTADO MÉDICO - ATE 15 DIAS	CH da Universidade Federal do Ceará - CE
57	AFASTAMENTO POR ATESTADO MÉDICO - ATE 15 DIAS	CH da Universidade Federal do Ceará - CE
57	AFASTAMENTO POR ATESTADO MÉDICO - ATE 15 DIAS	CH da Universidade Federal do Ceará - CE
57	AFASTAMENTO POR ATESTADO MÉDICO - ATE 15 DIAS	CH da Universidade Federal do Ceará - CE
57	AFASTAMENTO POR ATESTADO MÉDICO - ATE 15 DIAS	CH da Universidade Federal do Ceará - CE
57	AFASTAMENTO POR ATESTADO MÉDICO - ATE 15 DIAS	CH da Universidade Federal do Ceará - CE
57	AFASTAMENTO POR ATESTADO MÉDICO - ATE 15 DIAS	CH da Universidade Federal do Ceará - CE
57	AFASTAMENTO POR ATESTADO MÉDICO - ATE 15 DIAS	CH da Universidade Federal do Ceará - CE
57	AFASTAMENTO POR ATESTADO MÉDICO - ATE 15 DIAS	CH da Universidade Federal do Ceará - CE
57	AFASTAMENTO POR ATESTADO MÉDICO - ATE 15 DIAS	CH da Universidade Federal do Ceará - CE
57	AFASTAMENTO POR ATESTADO MÉDICO - ATE 15 DIAS	CH da Universidade Federal do Ceará - CE
57	AFASTAMENTO POR ATESTADO MÉDICO - ATE 15 DIAS	CH da Universidade Federal do Ceará - CE
57	AFASTAMENTO POR ATESTADO MÉDICO - ATE 15 DIAS	CH da Universidade Federal do Ceará - CE
57	AFASTAMENTO POR ATESTADO MÉDICO - ATE 15 DIAS	CH da Universidade Federal do Ceará - CE
57	AFASTAMENTO POR ATESTADO MÉDICO - ATE 15 DIAS	CH da Universidade Federal do Ceará - CE
57	AFASTAMENTO POR ATESTADO MÉDICO - ATE 15 DIAS	CH da Universidade Federal do Ceará - CE
57	AFASTAMENTO POR ATESTADO MÉDICO - ATE 15 DIAS	CH da Universidade Federal do Ceará - CE
57	AFASTAMENTO POR ATESTADO MÉDICO - ATE 15 DIAS	CH da Universidade Federal do Ceará - CE
57	AFASTAMENTO POR ATESTADO MÉDICO - ATE 15 DIAS	CH da Universidade Federal do Ceará - CE
57	AFASTAMENTO POR ATESTADO MÉDICO - ATE 15 DIAS	CH da Universidade Federal do Ceará - CE
57	AFASTAMENTO POR ATESTADO MÉDICO - ATE 15 DIAS	CH da Universidade Federal do Ceará - CE
57	AFASTAMENTO POR ATESTADO MÉDICO - ATE 15 DIAS	CH da Universidade Federal do Ceará - CE
57	AFASTAMENTO POR ATESTADO MÉDICO - ATE 15 DIAS	CH da Universidade Federal do Ceará - CE
57	AFASTAMENTO POR ATESTADO MÉDICO - ATE 15 DIAS	CH da Universidade Federal do Ceará - CE
57	AFASTAMENTO POR ATESTADO MÉDICO - ATE 15 DIAS	CH da Universidade Federal do Ceará - CE
57	AFASTAMENTO POR ATESTADO MÉDICO - ATE 15 DIAS	CH da Universidade Federal do Ceará - CE
57	AFASTAMENTO POR ATESTADO MÉDICO - ATE 15 DIAS	CH da Universidade Federal do Ceará - CE
57	AFASTAMENTO POR ATESTADO MÉDICO - ATE 15 DIAS	CH da Universidade Federal do Ceará - CE
57	AFASTAMENTO POR ATESTADO MÉDICO - ATE 15 DIAS	CH da Universidade Federal do Ceará - CE
57	AFASTAMENTO POR ATESTADO MÉDICO - ATE 15 DIAS	CH da Universidade Federal do Ceará - CE
57	AFASTAMENTO POR ATESTADO MÉDICO - ATE 15 DIAS	CH da Universidade Federal do Ceará - CE
57	AFASTAMENTO POR ATESTADO MÉDICO - ATE 15 DIAS	CH da Universidade Federal do Ceará - CE
57	AFASTAMENTO POR ATESTADO MÉDICO - ATE 15 DIAS	CH da Universidade Federal do Ceará - CE
57	AFASTAMENTO POR ATESTADO MÉDICO - ATE 15 DIAS	CH da Universidade Federal do Ceará - CE
57	AFASTAMENTO POR ATESTADO MÉDICO - ATE 15 DIAS	CH da Universidade Federal do Ceará - CE

57	AFASTAMENTO POR ATESTADO MÉDICO - ATE 15 DIAS	CH da Universidade Federal do Ceará - CE
57	AFASTAMENTO POR ATESTADO MÉDICO - ATE 15 DIAS	CH da Universidade Federal do Ceará - CE
57	AFASTAMENTO POR ATESTADO MÉDICO - ATE 15 DIAS	CH da Universidade Federal do Ceará - CE
57	AFASTAMENTO POR ATESTADO MÉDICO - ATE 15 DIAS	CH da Universidade Federal do Ceará - CE
57	AFASTAMENTO POR ATESTADO MÉDICO - ATE 15 DIAS	CH da Universidade Federal do Ceará - CE
57	AFASTAMENTO POR ATESTADO MÉDICO - ATE 15 DIAS	CH da Universidade Federal do Ceará - CE
57	AFASTAMENTO POR ATESTADO MÉDICO - ATE 15 DIAS	CH da Universidade Federal do Ceará - CE
57	AFASTAMENTO POR ATESTADO MÉDICO - ATE 15 DIAS	CH da Universidade Federal do Ceará - CE
57	AFASTAMENTO POR ATESTADO MÉDICO - ATE 15 DIAS	CH da Universidade Federal do Ceará - CE
57	AFASTAMENTO POR ATESTADO MÉDICO - ATE 15 DIAS	CH da Universidade Federal do Ceará - CE
57	AFASTAMENTO POR ATESTADO MÉDICO - ATE 15 DIAS	CH da Universidade Federal do Ceará - CE
57	AFASTAMENTO POR ATESTADO MÉDICO - ATE 15 DIAS	CH da Universidade Federal do Ceará - CE
57	AFASTAMENTO POR ATESTADO MÉDICO - ATE 15 DIAS	CH da Universidade Federal do Ceará - CE
57	AFASTAMENTO POR ATESTADO MÉDICO - ATE 15 DIAS	CH da Universidade Federal do Ceará - CE
57	AFASTAMENTO POR ATESTADO MÉDICO - ATE 15 DIAS	CH da Universidade Federal do Ceará - CE
57	AFASTAMENTO POR ATESTADO MÉDICO - ATE 15 DIAS	CH da Universidade Federal do Ceará - CE
57	AFASTAMENTO POR ATESTADO MÉDICO - ATE 15 DIAS	CH da Universidade Federal do Ceará - CE
57	AFASTAMENTO POR ATESTADO MÉDICO - ATE 15 DIAS	CH da Universidade Federal do Ceará - CE
57	AFASTAMENTO POR ATESTADO MÉDICO - ATE 15 DIAS	CH da Universidade Federal do Ceará - CE
57	AFASTAMENTO POR ATESTADO MÉDICO - ATE 15 DIAS	CH da Universidade Federal do Ceará - CE
57	AFASTAMENTO POR ATESTADO MÉDICO - ATE 15 DIAS	CH da Universidade Federal do Ceará - CE
57	AFASTAMENTO POR ATESTADO MÉDICO - ATE 15 DIAS	CH da Universidade Federal do Ceará - CE
57	AFASTAMENTO POR ATESTADO MÉDICO - ATE 15 DIAS	CH da Universidade Federal do Ceará - CE
57	AFASTAMENTO POR ATESTADO MÉDICO - ATE 15 DIAS	CH da Universidade Federal do Ceará - CE
57	AFASTAMENTO POR ATESTADO MÉDICO - ATE 15 DIAS	CH da Universidade Federal do Ceará - CE
57	AFASTAMENTO POR ATESTADO MÉDICO - ATE 15 DIAS	CH da Universidade Federal do Ceará - CE
57	AFASTAMENTO POR ATESTADO MÉDICO - ATE 15 DIAS	CH da Universidade Federal do Ceará - CE
57	AFASTAMENTO POR ATESTADO MÉDICO - ATE 15 DIAS	CH da Universidade Federal do Ceará - CE
57	AFASTAMENTO POR ATESTADO MÉDICO - ATE 15 DIAS	CH da Universidade Federal do Ceará - CE
57	AFASTAMENTO POR ATESTADO MÉDICO - ATE 15 DIAS	CH da Universidade Federal do Ceará - CE
57	AFASTAMENTO POR ATESTADO MÉDICO - ATE 15 DIAS	CH da Universidade Federal do Ceará - CE
57	AFASTAMENTO POR ATESTADO MÉDICO - ATE 15 DIAS	CH da Universidade Federal do Ceará - CE
57	AFASTAMENTO POR ATESTADO MÉDICO - ATE 15 DIAS	CH da Universidade Federal do Ceará - CE
57	AFASTAMENTO POR ATESTADO MÉDICO - ATE 15 DIAS	CH da Universidade Federal do Ceará - CE
57	AFASTAMENTO POR ATESTADO MÉDICO - ATE 15 DIAS	CH da Universidade Federal do Ceará - CE
57	AFASTAMENTO POR ATESTADO MÉDICO - ATE 15 DIAS	CH da Universidade Federal do Ceará - CE
57	AFASTAMENTO POR ATESTADO MÉDICO - ATE 15 DIAS	CH da Universidade Federal do Ceará - CE
57	AFASTAMENTO POR ATESTADO MÉDICO - ATE 15 DIAS	CH da Universidade Federal do Ceará - CE
57	AFASTAMENTO POR ATESTADO MÉDICO - ATE 15 DIAS	CH da Universidade Federal do Ceará - CE
57	AFASTAMENTO POR ATESTADO MÉDICO - ATE 15 DIAS	CH da Universidade Federal do Ceará - CE
57	AFASTAMENTO POR ATESTADO MÉDICO - ATE 15 DIAS	CH da Universidade Federal do Ceará - CE
57	AFASTAMENTO POR ATESTADO MÉDICO - ATE 15 DIAS	CH da Universidade Federal do Ceará - CE
57	AFASTAMENTO POR ATESTADO MÉDICO - ATE 15 DIAS	CH da Universidade Federal do Ceará - CE
57	AFASTAMENTO POR ATESTADO MÉDICO - ATE 15 DIAS	CH da Universidade Federal do Ceará - CE
57	AFASTAMENTO POR ATESTADO MÉDICO - ATE 15 DIAS	CH da Universidade Federal do Ceará - CE
57	AFASTAMENTO POR ATESTADO MÉDICO - ATE 15 DIAS	CH da Universidade Federal do Ceará - CE

57	AFASTAMENTO POR ATESTADO MÉDICO - ATE 15 DIAS	CH da Universidade Federal do Ceará - CE
57	AFASTAMENTO POR ATESTADO MÉDICO - ATE 15 DIAS	CH da Universidade Federal do Ceará - CE
57	AFASTAMENTO POR ATESTADO MÉDICO - ATE 15 DIAS	CH da Universidade Federal do Ceará - CE
57	AFASTAMENTO POR ATESTADO MÉDICO - ATE 15 DIAS	CH da Universidade Federal do Ceará - CE
57	AFASTAMENTO POR ATESTADO MÉDICO - ATE 15 DIAS	CH da Universidade Federal do Ceará - CE
57	AFASTAMENTO POR ATESTADO MÉDICO - ATE 15 DIAS	CH da Universidade Federal do Ceará - CE
57	AFASTAMENTO POR ATESTADO MÉDICO - ATE 15 DIAS	CH da Universidade Federal do Ceará - CE
57	AFASTAMENTO POR ATESTADO MÉDICO - ATE 15 DIAS	CH da Universidade Federal do Ceará - CE
57	AFASTAMENTO POR ATESTADO MÉDICO - ATE 15 DIAS	CH da Universidade Federal do Ceará - CE
57	AFASTAMENTO POR ATESTADO MÉDICO - ATE 15 DIAS	CH da Universidade Federal do Ceará - CE
57	AFASTAMENTO POR ATESTADO MÉDICO - ATE 15 DIAS	CH da Universidade Federal do Ceará - CE
57	AFASTAMENTO POR ATESTADO MÉDICO - ATE 15 DIAS	CH da Universidade Federal do Ceará - CE
57	AFASTAMENTO POR ATESTADO MÉDICO - ATE 15 DIAS	CH da Universidade Federal do Ceará - CE
57	AFASTAMENTO POR ATESTADO MÉDICO - ATE 15 DIAS	CH da Universidade Federal do Ceará - CE
57	AFASTAMENTO POR ATESTADO MÉDICO - ATE 15 DIAS	CH da Universidade Federal do Ceará - CE
57	AFASTAMENTO POR ATESTADO MÉDICO - ATE 15 DIAS	CH da Universidade Federal do Ceará - CE
57	AFASTAMENTO POR ATESTADO MÉDICO - ATE 15 DIAS	CH da Universidade Federal do Ceará - CE
57	AFASTAMENTO POR ATESTADO MÉDICO - ATE 15 DIAS	CH da Universidade Federal do Ceará - CE
57	AFASTAMENTO POR ATESTADO MÉDICO - ATE 15 DIAS	CH da Universidade Federal do Ceará - CE
57	AFASTAMENTO POR ATESTADO MÉDICO - ATE 15 DIAS	CH da Universidade Federal do Ceará - CE
57	AFASTAMENTO POR ATESTADO MÉDICO - ATE 15 DIAS	CH da Universidade Federal do Ceará - CE
57	AFASTAMENTO POR ATESTADO MÉDICO - ATE 15 DIAS	CH da Universidade Federal do Ceará - CE
57	AFASTAMENTO POR ATESTADO MÉDICO - ATE 15 DIAS	CH da Universidade Federal do Ceará - CE
57	AFASTAMENTO POR ATESTADO MÉDICO - ATE 15 DIAS	CH da Universidade Federal do Ceará - CE
57	AFASTAMENTO POR ATESTADO MÉDICO - ATE 15 DIAS	CH da Universidade Federal do Ceará - CE
57	AFASTAMENTO POR ATESTADO MÉDICO - ATE 15 DIAS	CH da Universidade Federal do Ceará - CE
57	AFASTAMENTO POR ATESTADO MÉDICO - ATE 15 DIAS	CH da Universidade Federal do Ceará - CE
57	AFASTAMENTO POR ATESTADO MÉDICO - ATE 15 DIAS	CH da Universidade Federal do Ceará - CE
57	AFASTAMENTO POR ATESTADO MÉDICO - ATE 15 DIAS	CH da Universidade Federal do Ceará - CE
57	AFASTAMENTO POR ATESTADO MÉDICO - ATE 15 DIAS	CH da Universidade Federal do Ceará - CE
57	AFASTAMENTO POR ATESTADO MÉDICO - ATE 15 DIAS	CH da Universidade Federal do Ceará - CE
57	AFASTAMENTO POR ATESTADO MÉDICO - ATE 15 DIAS	CH da Universidade Federal do Ceará - CE
57	AFASTAMENTO POR ATESTADO MÉDICO - ATE 15 DIAS	CH da Universidade Federal do Ceará - CE
57	AFASTAMENTO POR ATESTADO MÉDICO - ATE 15 DIAS	CH da Universidade Federal do Ceará - CE
57	AFASTAMENTO POR ATESTADO MÉDICO - ATE 15 DIAS	CH da Universidade Federal do Ceará - CE
57	AFASTAMENTO POR ATESTADO MÉDICO - ATE 15 DIAS	CH da Universidade Federal do Ceará - CE
57	AFASTAMENTO POR ATESTADO MÉDICO - ATE 15 DIAS	CH da Universidade Federal do Ceará - CE
57	AFASTAMENTO POR ATESTADO MÉDICO - ATE 15 DIAS	CH da Universidade Federal do Ceará - CE
57	AFASTAMENTO POR ATESTADO MÉDICO - ATE 15 DIAS	CH da Universidade Federal do Ceará - CE
57	AFASTAMENTO POR ATESTADO MÉDICO - ATE 15 DIAS	CH da Universidade Federal do Ceará - CE

57	AFASTAMENTO POR ATESTADO MÉDICO - ATE 15 DIAS	CH da Universidade Federal do Ceará - CE
57	AFASTAMENTO POR ATESTADO MÉDICO - ATE 15 DIAS	CH da Universidade Federal do Ceará - CE
57	AFASTAMENTO POR ATESTADO MÉDICO - ATE 15 DIAS	CH da Universidade Federal do Ceará - CE
57	AFASTAMENTO POR ATESTADO MÉDICO - ATE 15 DIAS	CH da Universidade Federal do Ceará - CE
57	AFASTAMENTO POR ATESTADO MÉDICO - ATE 15 DIAS	CH da Universidade Federal do Ceará - CE
57	AFASTAMENTO POR ATESTADO MÉDICO - ATE 15 DIAS	CH da Universidade Federal do Ceará - CE
57	AFASTAMENTO POR ATESTADO MÉDICO - ATE 15 DIAS	CH da Universidade Federal do Ceará - CE
57	AFASTAMENTO POR ATESTADO MÉDICO - ATE 15 DIAS	CH da Universidade Federal do Ceará - CE
57	AFASTAMENTO POR ATESTADO MÉDICO - ATE 15 DIAS	CH da Universidade Federal do Ceará - CE
57	AFASTAMENTO POR ATESTADO MÉDICO - ATE 15 DIAS	CH da Universidade Federal do Ceará - CE
57	AFASTAMENTO POR ATESTADO MÉDICO - ATE 15 DIAS	CH da Universidade Federal do Ceará - CE
57	AFASTAMENTO POR ATESTADO MÉDICO - ATE 15 DIAS	CH da Universidade Federal do Ceará - CE
57	AFASTAMENTO POR ATESTADO MÉDICO - ATE 15 DIAS	CH da Universidade Federal do Ceará - CE
57	AFASTAMENTO POR ATESTADO MÉDICO - ATE 15 DIAS	CH da Universidade Federal do Ceará - CE
57	AFASTAMENTO POR ATESTADO MÉDICO - ATE 15 DIAS	CH da Universidade Federal do Ceará - CE
57	AFASTAMENTO POR ATESTADO MÉDICO - ATE 15 DIAS	CH da Universidade Federal do Ceará - CE
57	AFASTAMENTO POR ATESTADO MÉDICO - ATE 15 DIAS	CH da Universidade Federal do Ceará - CE
57	AFASTAMENTO POR ATESTADO MÉDICO - ATE 15 DIAS	CH da Universidade Federal do Ceará - CE
57	AFASTAMENTO POR ATESTADO MÉDICO - ATE 15 DIAS	CH da Universidade Federal do Ceará - CE
57	AFASTAMENTO POR ATESTADO MÉDICO - ATE 15 DIAS	CH da Universidade Federal do Ceará - CE
57	AFASTAMENTO POR ATESTADO MÉDICO - ATE 15 DIAS	CH da Universidade Federal do Ceará - CE
57	AFASTAMENTO POR ATESTADO MÉDICO - ATE 15 DIAS	CH da Universidade Federal do Ceará - CE
57	AFASTAMENTO POR ATESTADO MÉDICO - ATE 15 DIAS	CH da Universidade Federal do Ceará - CE
57	AFASTAMENTO POR ATESTADO MÉDICO - ATE 15 DIAS	CH da Universidade Federal do Ceará - CE
57	AFASTAMENTO POR ATESTADO MÉDICO - ATE 15 DIAS	CH da Universidade Federal do Ceará - CE
57	AFASTAMENTO POR ATESTADO MÉDICO - ATE 15 DIAS	CH da Universidade Federal do Ceará - CE
57	AFASTAMENTO POR ATESTADO MÉDICO - ATE 15 DIAS	CH da Universidade Federal do Ceará - CE
57	AFASTAMENTO POR ATESTADO MÉDICO - ATE 15 DIAS	CH da Universidade Federal do Ceará - CE
57	AFASTAMENTO POR ATESTADO MÉDICO - ATE 15 DIAS	CH da Universidade Federal do Ceará - CE
57	AFASTAMENTO POR ATESTADO MÉDICO - ATE 15 DIAS	CH da Universidade Federal do Ceará - CE
57	AFASTAMENTO POR ATESTADO MÉDICO - ATE 15 DIAS	CH da Universidade Federal do Ceará - CE
57	AFASTAMENTO POR ATESTADO MÉDICO - ATE 15 DIAS	CH da Universidade Federal do Ceará - CE
57	AFASTAMENTO POR ATESTADO MÉDICO - ATE 15 DIAS	CH da Universidade Federal do Ceará - CE
57	AFASTAMENTO POR ATESTADO MÉDICO - ATE 15 DIAS	CH da Universidade Federal do Ceará - CE
57	AFASTAMENTO POR ATESTADO MÉDICO - ATE 15 DIAS	CH da Universidade Federal do Ceará - CE
57	AFASTAMENTO POR ATESTADO MÉDICO - ATE 15 DIAS	CH da Universidade Federal do Ceará - CE
57	AFASTAMENTO POR ATESTADO MÉDICO - ATE 15 DIAS	CH da Universidade Federal do Ceará - CE
57	AFASTAMENTO POR ATESTADO MÉDICO - ATE 15 DIAS	CH da Universidade Federal do Ceará - CE
57	AFASTAMENTO POR ATESTADO MÉDICO - ATE 15 DIAS	CH da Universidade Federal do Ceará - CE
57	AFASTAMENTO POR ATESTADO MÉDICO - ATE 15 DIAS	CH da Universidade Federal do Ceará - CE
57	AFASTAMENTO POR ATESTADO MÉDICO - ATE 15 DIAS	CH da Universidade Federal do Ceará - CE
57	AFASTAMENTO POR ATESTADO MÉDICO - ATE 15 DIAS	CH da Universidade Federal do Ceará - CE
57	AFASTAMENTO POR ATESTADO MÉDICO - ATE 15 DIAS	CH da Universidade Federal do Ceará - CE
57	AFASTAMENTO POR ATESTADO MÉDICO - ATE 15 DIAS	CH da Universidade Federal do Ceará - CE
57	AFASTAMENTO POR ATESTADO MÉDICO - ATE 15 DIAS	CH da Universidade Federal do Ceará - CE
57	AFASTAMENTO POR ATESTADO MÉDICO - ATE 15 DIAS	CH da Universidade Federal do Ceará - CE
57	AFASTAMENTO POR ATESTADO MÉDICO - ATE 15 DIAS	CH da Universidade Federal do Ceará - CE
57	AFASTAMENTO POR ATESTADO MÉDICO - ATE 15 DIAS	CH da Universidade Federal do Ceará - CE

57	AFASTAMENTO POR ATESTADO MÉDICO - ATE 15 DIAS	CH da Universidade Federal do Ceará - CE
57	AFASTAMENTO POR ATESTADO MÉDICO - ATE 15 DIAS	CH da Universidade Federal do Ceará - CE
57	AFASTAMENTO POR ATESTADO MÉDICO - ATE 15 DIAS	CH da Universidade Federal do Ceará - CE
57	AFASTAMENTO POR ATESTADO MÉDICO - ATE 15 DIAS	CH da Universidade Federal do Ceará - CE
57	AFASTAMENTO POR ATESTADO MÉDICO - ATE 15 DIAS	CH da Universidade Federal do Ceará - CE
57	AFASTAMENTO POR ATESTADO MÉDICO - ATE 15 DIAS	CH da Universidade Federal do Ceará - CE
57	AFASTAMENTO POR ATESTADO MÉDICO - ATE 15 DIAS	CH da Universidade Federal do Ceará - CE
57	AFASTAMENTO POR ATESTADO MÉDICO - ATE 15 DIAS	CH da Universidade Federal do Ceará - CE
57	AFASTAMENTO POR ATESTADO MÉDICO - ATE 15 DIAS	CH da Universidade Federal do Ceará - CE
57	AFASTAMENTO POR ATESTADO MÉDICO - ATE 15 DIAS	CH da Universidade Federal do Ceará - CE
57	AFASTAMENTO POR ATESTADO MÉDICO - ATE 15 DIAS	CH da Universidade Federal do Ceará - CE
57	AFASTAMENTO POR ATESTADO MÉDICO - ATE 15 DIAS	CH da Universidade Federal do Ceará - CE
57	AFASTAMENTO POR ATESTADO MÉDICO - ATE 15 DIAS	CH da Universidade Federal do Ceará - CE
57	AFASTAMENTO POR ATESTADO MÉDICO - ATE 15 DIAS	CH da Universidade Federal do Ceará - CE
57	AFASTAMENTO POR ATESTADO MÉDICO - ATE 15 DIAS	CH da Universidade Federal do Ceará - CE
57	AFASTAMENTO POR ATESTADO MÉDICO - ATE 15 DIAS	CH da Universidade Federal do Ceará - CE
57	AFASTAMENTO POR ATESTADO MÉDICO - ATE 15 DIAS	CH da Universidade Federal do Ceará - CE
57	AFASTAMENTO POR ATESTADO MÉDICO - ATE 15 DIAS	CH da Universidade Federal do Ceará - CE
57	AFASTAMENTO POR ATESTADO MÉDICO - ATE 15 DIAS	CH da Universidade Federal do Ceará - CE
57	AFASTAMENTO POR ATESTADO MÉDICO - ATE 15 DIAS	CH da Universidade Federal do Ceará - CE
57	AFASTAMENTO POR ATESTADO MÉDICO - ATE 15 DIAS	CH da Universidade Federal do Ceará - CE
57	AFASTAMENTO POR ATESTADO MÉDICO - ATE 15 DIAS	CH da Universidade Federal do Ceará - CE
57	AFASTAMENTO POR ATESTADO MÉDICO - ATE 15 DIAS	CH da Universidade Federal do Ceará - CE
57	AFASTAMENTO POR ATESTADO MÉDICO - ATE 15 DIAS	CH da Universidade Federal do Ceará - CE
57	AFASTAMENTO POR ATESTADO MÉDICO - ATE 15 DIAS	CH da Universidade Federal do Ceará - CE
57	AFASTAMENTO POR ATESTADO MÉDICO - ATE 15 DIAS	CH da Universidade Federal do Ceará - CE
57	AFASTAMENTO POR ATESTADO MÉDICO - ATE 15 DIAS	CH da Universidade Federal do Ceará - CE
57	AFASTAMENTO POR ATESTADO MÉDICO - ATE 15 DIAS	CH da Universidade Federal do Ceará - CE
57	AFASTAMENTO POR ATESTADO MÉDICO - ATE 15 DIAS	CH da Universidade Federal do Ceará - CE
57	AFASTAMENTO POR ATESTADO MÉDICO - ATE 15 DIAS	CH da Universidade Federal do Ceará - CE
57	AFASTAMENTO POR ATESTADO MÉDICO - ATE 15 DIAS	CH da Universidade Federal do Ceará - CE
57	AFASTAMENTO POR ATESTADO MÉDICO - ATE 15 DIAS	CH da Universidade Federal do Ceará - CE
57	AFASTAMENTO POR ATESTADO MÉDICO - ATE 15 DIAS	CH da Universidade Federal do Ceará - CE
57	AFASTAMENTO POR ATESTADO MÉDICO - ATE 15 DIAS	CH da Universidade Federal do Ceará - CE
57	AFASTAMENTO POR ATESTADO MÉDICO - ATE 15 DIAS	CH da Universidade Federal do Ceará - CE
57	AFASTAMENTO POR ATESTADO MÉDICO - ATE 15 DIAS	CH da Universidade Federal do Ceará - CE
57	AFASTAMENTO POR ATESTADO MÉDICO - ATE 15 DIAS	CH da Universidade Federal do Ceará - CE
57	AFASTAMENTO POR ATESTADO MÉDICO - ATE 15 DIAS	CH da Universidade Federal do Ceará - CE
57	AFASTAMENTO POR ATESTADO MÉDICO - ATE 15 DIAS	CH da Universidade Federal do Ceará - CE
57	AFASTAMENTO POR ATESTADO MÉDICO - ATE 15 DIAS	CH da Universidade Federal do Ceará - CE
57	AFASTAMENTO POR ATESTADO MÉDICO - ATE 15 DIAS	CH da Universidade Federal do Ceará - CE
57	AFASTAMENTO POR ATESTADO MÉDICO - ATE 15 DIAS	CH da Universidade Federal do Ceará - CE
57	AFASTAMENTO POR ATESTADO MÉDICO - ATE 15 DIAS	CH da Universidade Federal do Ceará - CE
57	AFASTAMENTO POR ATESTADO MÉDICO - ATE 15 DIAS	CH da Universidade Federal do Ceará - CE
57	AFASTAMENTO POR ATESTADO MÉDICO - ATE 15 DIAS	CH da Universidade Federal do Ceará - CE
57	AFASTAMENTO POR ATESTADO MÉDICO - ATE 15 DIAS	CH da Universidade Federal do Ceará - CE
57	AFASTAMENTO POR ATESTADO MÉDICO - ATE 15 DIAS	CH da Universidade Federal do Ceará - CE
57	AFASTAMENTO POR ATESTADO MÉDICO - ATE 15 DIAS	CH da Universidade Federal do Ceará - CE

57	AFASTAMENTO POR ATESTADO MÉDICO - ATE 15 DIAS	CH da Universidade Federal do Ceará - CE
57	AFASTAMENTO POR ATESTADO MÉDICO - ATE 15 DIAS	CH da Universidade Federal do Ceará - CE
57	AFASTAMENTO POR ATESTADO MÉDICO - ATE 15 DIAS	CH da Universidade Federal do Ceará - CE
57	AFASTAMENTO POR ATESTADO MÉDICO - ATE 15 DIAS	CH da Universidade Federal do Ceará - CE
57	AFASTAMENTO POR ATESTADO MÉDICO - ATE 15 DIAS	CH da Universidade Federal do Ceará - CE
57	AFASTAMENTO POR ATESTADO MÉDICO - ATE 15 DIAS	CH da Universidade Federal do Ceará - CE
57	AFASTAMENTO POR ATESTADO MÉDICO - ATE 15 DIAS	CH da Universidade Federal do Ceará - CE
57	AFASTAMENTO POR ATESTADO MÉDICO - ATE 15 DIAS	CH da Universidade Federal do Ceará - CE
57	AFASTAMENTO POR ATESTADO MÉDICO - ATE 15 DIAS	CH da Universidade Federal do Ceará - CE
57	AFASTAMENTO POR ATESTADO MÉDICO - ATE 15 DIAS	CH da Universidade Federal do Ceará - CE
57	AFASTAMENTO POR ATESTADO MÉDICO - ATE 15 DIAS	CH da Universidade Federal do Ceará - CE
57	AFASTAMENTO POR ATESTADO MÉDICO - ATE 15 DIAS	CH da Universidade Federal do Ceará - CE
57	AFASTAMENTO POR ATESTADO MÉDICO - ATE 15 DIAS	CH da Universidade Federal do Ceará - CE
57	AFASTAMENTO POR ATESTADO MÉDICO - ATE 15 DIAS	CH da Universidade Federal do Ceará - CE
57	AFASTAMENTO POR ATESTADO MÉDICO - ATE 15 DIAS	CH da Universidade Federal do Ceará - CE
57	AFASTAMENTO POR ATESTADO MÉDICO - ATE 15 DIAS	CH da Universidade Federal do Ceará - CE
57	AFASTAMENTO POR ATESTADO MÉDICO - ATE 15 DIAS	CH da Universidade Federal do Ceará - CE
57	AFASTAMENTO POR ATESTADO MÉDICO - ATE 15 DIAS	CH da Universidade Federal do Ceará - CE
57	AFASTAMENTO POR ATESTADO MÉDICO - ATE 15 DIAS	CH da Universidade Federal do Ceará - CE
57	AFASTAMENTO POR ATESTADO MÉDICO - ATE 15 DIAS	CH da Universidade Federal do Ceará - CE
57	AFASTAMENTO POR ATESTADO MÉDICO - ATE 15 DIAS	CH da Universidade Federal do Ceará - CE
57	AFASTAMENTO POR ATESTADO MÉDICO - ATE 15 DIAS	CH da Universidade Federal do Ceará - CE
57	AFASTAMENTO POR ATESTADO MÉDICO - ATE 15 DIAS	CH da Universidade Federal do Ceará - CE
57	AFASTAMENTO POR ATESTADO MÉDICO - ATE 15 DIAS	CH da Universidade Federal do Ceará - CE
57	AFASTAMENTO POR ATESTADO MÉDICO - ATE 15 DIAS	CH da Universidade Federal do Ceará - CE
57	AFASTAMENTO POR ATESTADO MÉDICO - ATE 15 DIAS	CH da Universidade Federal do Ceará - CE
57	AFASTAMENTO POR ATESTADO MÉDICO - ATE 15 DIAS	CH da Universidade Federal do Ceará - CE
57	AFASTAMENTO POR ATESTADO MÉDICO - ATE 15 DIAS	CH da Universidade Federal do Ceará - CE
57	AFASTAMENTO POR ATESTADO MÉDICO - ATE 15 DIAS	CH da Universidade Federal do Ceará - CE
57	AFASTAMENTO POR ATESTADO MÉDICO - ATE 15 DIAS	CH da Universidade Federal do Ceará - CE
57	AFASTAMENTO POR ATESTADO MÉDICO - ATE 15 DIAS	CH da Universidade Federal do Ceará - CE
57	AFASTAMENTO POR ATESTADO MÉDICO - ATE 15 DIAS	CH da Universidade Federal do Ceará - CE
57	AFASTAMENTO POR ATESTADO MÉDICO - ATE 15 DIAS	CH da Universidade Federal do Ceará - CE
57	AFASTAMENTO POR ATESTADO MÉDICO - ATE 15 DIAS	CH da Universidade Federal do Ceará - CE
57	AFASTAMENTO POR ATESTADO MÉDICO - ATE 15 DIAS	CH da Universidade Federal do Ceará - CE
57	AFASTAMENTO POR ATESTADO MÉDICO - ATE 15 DIAS	CH da Universidade Federal do Ceará - CE
57	AFASTAMENTO POR ATESTADO MÉDICO - ATE 15 DIAS	CH da Universidade Federal do Ceará - CE
57	AFASTAMENTO POR ATESTADO MÉDICO - ATE 15 DIAS	CH da Universidade Federal do Ceará - CE
57	AFASTAMENTO POR ATESTADO MÉDICO - ATE 15 DIAS	CH da Universidade Federal do Ceará - CE
57	AFASTAMENTO POR ATESTADO MÉDICO - ATE 15 DIAS	CH da Universidade Federal do Ceará - CE
57	AFASTAMENTO POR ATESTADO MÉDICO - ATE 15 DIAS	CH da Universidade Federal do Ceará - CE
57	AFASTAMENTO POR ATESTADO MÉDICO - ATE 15 DIAS	CH da Universidade Federal do Ceará - CE
57	AFASTAMENTO POR ATESTADO MÉDICO - ATE 15 DIAS	CH da Universidade Federal do Ceará - CE
57	AFASTAMENTO POR ATESTADO MÉDICO - ATE 15 DIAS	CH da Universidade Federal do Ceará - CE
57	AFASTAMENTO POR ATESTADO MÉDICO - ATE 15 DIAS	CH da Universidade Federal do Ceará - CE
57	AFASTAMENTO POR ATESTADO MÉDICO - ATE 15 DIAS	CH da Universidade Federal do Ceará - CE
57	AFASTAMENTO POR ATESTADO MÉDICO - ATE 15 DIAS	CH da Universidade Federal do Ceará - CE
57	AFASTAMENTO POR ATESTADO MÉDICO - ATE 15 DIAS	CH da Universidade Federal do Ceará - CE

57	AFASTAMENTO POR ATESTADO MÉDICO - ATE 15 DIAS	CH da Universidade Federal do Ceará - CE
57	AFASTAMENTO POR ATESTADO MÉDICO - ATE 15 DIAS	CH da Universidade Federal do Ceará - CE
57	AFASTAMENTO POR ATESTADO MÉDICO - ATE 15 DIAS	CH da Universidade Federal do Ceará - CE
57	AFASTAMENTO POR ATESTADO MÉDICO - ATE 15 DIAS	CH da Universidade Federal do Ceará - CE
57	AFASTAMENTO POR ATESTADO MÉDICO - ATE 15 DIAS	CH da Universidade Federal do Ceará - CE
57	AFASTAMENTO POR ATESTADO MÉDICO - ATE 15 DIAS	CH da Universidade Federal do Ceará - CE
57	AFASTAMENTO POR ATESTADO MÉDICO - ATE 15 DIAS	CH da Universidade Federal do Ceará - CE
57	AFASTAMENTO POR ATESTADO MÉDICO - ATE 15 DIAS	CH da Universidade Federal do Ceará - CE
57	AFASTAMENTO POR ATESTADO MÉDICO - ATE 15 DIAS	CH da Universidade Federal do Ceará - CE
57	AFASTAMENTO POR ATESTADO MÉDICO - ATE 15 DIAS	CH da Universidade Federal do Ceará - CE
57	AFASTAMENTO POR ATESTADO MÉDICO - ATE 15 DIAS	CH da Universidade Federal do Ceará - CE
57	AFASTAMENTO POR ATESTADO MÉDICO - ATE 15 DIAS	CH da Universidade Federal do Ceará - CE
57	AFASTAMENTO POR ATESTADO MÉDICO - ATE 15 DIAS	CH da Universidade Federal do Ceará - CE
57	AFASTAMENTO POR ATESTADO MÉDICO - ATE 15 DIAS	CH da Universidade Federal do Ceará - CE
57	AFASTAMENTO POR ATESTADO MÉDICO - ATE 15 DIAS	CH da Universidade Federal do Ceará - CE
57	AFASTAMENTO POR ATESTADO MÉDICO - ATE 15 DIAS	CH da Universidade Federal do Ceará - CE
57	AFASTAMENTO POR ATESTADO MÉDICO - ATE 15 DIAS	CH da Universidade Federal do Ceará - CE
57	AFASTAMENTO POR ATESTADO MÉDICO - ATE 15 DIAS	CH da Universidade Federal do Ceará - CE
57	AFASTAMENTO POR ATESTADO MÉDICO - ATE 15 DIAS	CH da Universidade Federal do Ceará - CE
57	AFASTAMENTO POR ATESTADO MÉDICO - ATE 15 DIAS	CH da Universidade Federal do Ceará - CE
57	AFASTAMENTO POR ATESTADO MÉDICO - ATE 15 DIAS	CH da Universidade Federal do Ceará - CE
57	AFASTAMENTO POR ATESTADO MÉDICO - ATE 15 DIAS	CH da Universidade Federal do Ceará - CE
57	AFASTAMENTO POR ATESTADO MÉDICO - ATE 15 DIAS	CH da Universidade Federal do Ceará - CE
57	AFASTAMENTO POR ATESTADO MÉDICO - ATE 15 DIAS	CH da Universidade Federal do Ceará - CE
57	AFASTAMENTO POR ATESTADO MÉDICO - ATE 15 DIAS	CH da Universidade Federal do Ceará - CE
57	AFASTAMENTO POR ATESTADO MÉDICO - ATE 15 DIAS	CH da Universidade Federal do Ceará - CE
57	AFASTAMENTO POR ATESTADO MÉDICO - ATE 15 DIAS	CH da Universidade Federal do Ceará - CE
57	AFASTAMENTO POR ATESTADO MÉDICO - ATE 15 DIAS	CH da Universidade Federal do Ceará - CE
57	AFASTAMENTO POR ATESTADO MÉDICO - ATE 15 DIAS	CH da Universidade Federal do Ceará - CE
57	AFASTAMENTO POR ATESTADO MÉDICO - ATE 15 DIAS	CH da Universidade Federal do Ceará - CE
57	AFASTAMENTO POR ATESTADO MÉDICO - ATE 15 DIAS	CH da Universidade Federal do Ceará - CE
57	AFASTAMENTO POR ATESTADO MÉDICO - ATE 15 DIAS	CH da Universidade Federal do Ceará - CE
57	AFASTAMENTO POR ATESTADO MÉDICO - ATE 15 DIAS	CH da Universidade Federal do Ceará - CE
57	AFASTAMENTO POR ATESTADO MÉDICO - ATE 15 DIAS	CH da Universidade Federal do Ceará - CE
57	AFASTAMENTO POR ATESTADO MÉDICO - ATE 15 DIAS	CH da Universidade Federal do Ceará - CE
57	AFASTAMENTO POR ATESTADO MÉDICO - ATE 15 DIAS	CH da Universidade Federal do Ceará - CE
57	AFASTAMENTO POR ATESTADO MÉDICO - ATE 15 DIAS	CH da Universidade Federal do Ceará - CE
57	AFASTAMENTO POR ATESTADO MÉDICO - ATE 15 DIAS	CH da Universidade Federal do Ceará - CE
57	AFASTAMENTO POR ATESTADO MÉDICO - ATE 15 DIAS	CH da Universidade Federal do Ceará - CE
57	AFASTAMENTO POR ATESTADO MÉDICO - ATE 15 DIAS	CH da Universidade Federal do Ceará - CE
57	AFASTAMENTO POR ATESTADO MÉDICO - ATE 15 DIAS	CH da Universidade Federal do Ceará - CE
57	AFASTAMENTO POR ATESTADO MÉDICO - ATE 15 DIAS	CH da Universidade Federal do Ceará - CE
57	AFASTAMENTO POR ATESTADO MÉDICO - ATE 15 DIAS	CH da Universidade Federal do Ceará - CE
57	AFASTAMENTO POR ATESTADO MÉDICO - ATE 15 DIAS	CH da Universidade Federal do Ceará - CE
57	AFASTAMENTO POR ATESTADO MÉDICO - ATE 15 DIAS	CH da Universidade Federal do Ceará - CE
57	AFASTAMENTO POR ATESTADO MÉDICO - ATE 15 DIAS	CH da Universidade Federal do Ceará - CE
57	AFASTAMENTO POR ATESTADO MÉDICO - ATE 15 DIAS	CH da Universidade Federal do Ceará - CE
57	AFASTAMENTO POR ATESTADO MÉDICO - ATE 15 DIAS	CH da Universidade Federal do Ceará - CE

57	AFASTAMENTO POR ATESTADO MÉDICO - ATE 15 DIAS	CH da Universidade Federal do Ceará - CE
57	AFASTAMENTO POR ATESTADO MÉDICO - ATE 15 DIAS	CH da Universidade Federal do Ceará - CE
57	AFASTAMENTO POR ATESTADO MÉDICO - ATE 15 DIAS	CH da Universidade Federal do Ceará - CE
57	AFASTAMENTO POR ATESTADO MÉDICO - ATE 15 DIAS	CH da Universidade Federal do Ceará - CE
57	AFASTAMENTO POR ATESTADO MÉDICO - ATE 15 DIAS	CH da Universidade Federal do Ceará - CE
57	AFASTAMENTO POR ATESTADO MÉDICO - ATE 15 DIAS	CH da Universidade Federal do Ceará - CE
57	AFASTAMENTO POR ATESTADO MÉDICO - ATE 15 DIAS	CH da Universidade Federal do Ceará - CE
57	AFASTAMENTO POR ATESTADO MÉDICO - ATE 15 DIAS	CH da Universidade Federal do Ceará - CE
57	AFASTAMENTO POR ATESTADO MÉDICO - ATE 15 DIAS	CH da Universidade Federal do Ceará - CE
57	AFASTAMENTO POR ATESTADO MÉDICO - ATE 15 DIAS	CH da Universidade Federal do Ceará - CE
57	AFASTAMENTO POR ATESTADO MÉDICO - ATE 15 DIAS	CH da Universidade Federal do Ceará - CE
57	AFASTAMENTO POR ATESTADO MÉDICO - ATE 15 DIAS	CH da Universidade Federal do Ceará - CE
57	AFASTAMENTO POR ATESTADO MÉDICO - ATE 15 DIAS	CH da Universidade Federal do Ceará - CE
57	AFASTAMENTO POR ATESTADO MÉDICO - ATE 15 DIAS	CH da Universidade Federal do Ceará - CE
57	AFASTAMENTO POR ATESTADO MÉDICO - ATE 15 DIAS	CH da Universidade Federal do Ceará - CE
57	AFASTAMENTO POR ATESTADO MÉDICO - ATE 15 DIAS	CH da Universidade Federal do Ceará - CE
57	AFASTAMENTO POR ATESTADO MÉDICO - ATE 15 DIAS	CH da Universidade Federal do Ceará - CE
57	AFASTAMENTO POR ATESTADO MÉDICO - ATE 15 DIAS	CH da Universidade Federal do Ceará - CE
57	AFASTAMENTO POR ATESTADO MÉDICO - ATE 15 DIAS	CH da Universidade Federal do Ceará - CE
57	AFASTAMENTO POR ATESTADO MÉDICO - ATE 15 DIAS	CH da Universidade Federal do Ceará - CE
57	AFASTAMENTO POR ATESTADO MÉDICO - ATE 15 DIAS	CH da Universidade Federal do Ceará - CE
57	AFASTAMENTO POR ATESTADO MÉDICO - ATE 15 DIAS	CH da Universidade Federal do Ceará - CE
57	AFASTAMENTO POR ATESTADO MÉDICO - ATE 15 DIAS	CH da Universidade Federal do Ceará - CE
57	AFASTAMENTO POR ATESTADO MÉDICO - ATE 15 DIAS	CH da Universidade Federal do Ceará - CE
57	AFASTAMENTO POR ATESTADO MÉDICO - ATE 15 DIAS	CH da Universidade Federal do Ceará - CE
57	AFASTAMENTO POR ATESTADO MÉDICO - ATE 15 DIAS	CH da Universidade Federal do Ceará - CE
57	AFASTAMENTO POR ATESTADO MÉDICO - ATE 15 DIAS	CH da Universidade Federal do Ceará - CE
57	AFASTAMENTO POR ATESTADO MÉDICO - ATE 15 DIAS	CH da Universidade Federal do Ceará - CE
57	AFASTAMENTO POR ATESTADO MÉDICO - ATE 15 DIAS	CH da Universidade Federal do Ceará - CE
57	AFASTAMENTO POR ATESTADO MÉDICO - ATE 15 DIAS	CH da Universidade Federal do Ceará - CE
57	AFASTAMENTO POR ATESTADO MÉDICO - ATE 15 DIAS	CH da Universidade Federal do Ceará - CE
57	AFASTAMENTO POR ATESTADO MÉDICO - ATE 15 DIAS	CH da Universidade Federal do Ceará - CE
57	AFASTAMENTO POR ATESTADO MÉDICO - ATE 15 DIAS	CH da Universidade Federal do Ceará - CE
57	AFASTAMENTO POR ATESTADO MÉDICO - ATE 15 DIAS	CH da Universidade Federal do Ceará - CE
57	AFASTAMENTO POR ATESTADO MÉDICO - ATE 15 DIAS	CH da Universidade Federal do Ceará - CE
57	AFASTAMENTO POR ATESTADO MÉDICO - ATE 15 DIAS	CH da Universidade Federal do Ceará - CE
57	AFASTAMENTO POR ATESTADO MÉDICO - ATE 15 DIAS	CH da Universidade Federal do Ceará - CE
57	AFASTAMENTO POR ATESTADO MÉDICO - ATE 15 DIAS	CH da Universidade Federal do Ceará - CE
57	AFASTAMENTO POR ATESTADO MÉDICO - ATE 15 DIAS	CH da Universidade Federal do Ceará - CE
57	AFASTAMENTO POR ATESTADO MÉDICO - ATE 15 DIAS	CH da Universidade Federal do Ceará - CE
57	AFASTAMENTO POR ATESTADO MÉDICO - ATE 15 DIAS	CH da Universidade Federal do Ceará - CE
57	AFASTAMENTO POR ATESTADO MÉDICO - ATE 15 DIAS	CH da Universidade Federal do Ceará - CE
57	AFASTAMENTO POR ATESTADO MÉDICO - ATE 15 DIAS	CH da Universidade Federal do Ceará - CE
57	AFASTAMENTO POR ATESTADO MÉDICO - ATE 15 DIAS	CH da Universidade Federal do Ceará - CE
57	AFASTAMENTO POR ATESTADO MÉDICO - ATE 15 DIAS	CH da Universidade Federal do Ceará - CE
57	AFASTAMENTO POR ATESTADO MÉDICO - ATE 15 DIAS	CH da Universidade Federal do Ceará - CE
57	AFASTAMENTO POR ATESTADO MÉDICO - ATE 15 DIAS	CH da Universidade Federal do Ceará - CE
57	AFASTAMENTO POR ATESTADO MÉDICO - ATE 15 DIAS	CH da Universidade Federal do Ceará - CE

57	AFASTAMENTO POR ATESTADO MÉDICO - ATE 15 DIAS	CH da Universidade Federal do Ceará - CE
57	AFASTAMENTO POR ATESTADO MÉDICO - ATE 15 DIAS	CH da Universidade Federal do Ceará - CE
57	AFASTAMENTO POR ATESTADO MÉDICO - ATE 15 DIAS	CH da Universidade Federal do Ceará - CE
57	AFASTAMENTO POR ATESTADO MÉDICO - ATE 15 DIAS	CH da Universidade Federal do Ceará - CE
57	AFASTAMENTO POR ATESTADO MÉDICO - ATE 15 DIAS	CH da Universidade Federal do Ceará - CE
57	AFASTAMENTO POR ATESTADO MÉDICO - ATE 15 DIAS	CH da Universidade Federal do Ceará - CE
57	AFASTAMENTO POR ATESTADO MÉDICO - ATE 15 DIAS	CH da Universidade Federal do Ceará - CE
57	AFASTAMENTO POR ATESTADO MÉDICO - ATE 15 DIAS	CH da Universidade Federal do Ceará - CE
57	AFASTAMENTO POR ATESTADO MÉDICO - ATE 15 DIAS	CH da Universidade Federal do Ceará - CE
57	AFASTAMENTO POR ATESTADO MÉDICO - ATE 15 DIAS	CH da Universidade Federal do Ceará - CE
57	AFASTAMENTO POR ATESTADO MÉDICO - ATE 15 DIAS	CH da Universidade Federal do Ceará - CE
57	AFASTAMENTO POR ATESTADO MÉDICO - ATE 15 DIAS	CH da Universidade Federal do Ceará - CE
57	AFASTAMENTO POR ATESTADO MÉDICO - ATE 15 DIAS	CH da Universidade Federal do Ceará - CE
57	AFASTAMENTO POR ATESTADO MÉDICO - ATE 15 DIAS	CH da Universidade Federal do Ceará - CE
57	AFASTAMENTO POR ATESTADO MÉDICO - ATE 15 DIAS	CH da Universidade Federal do Ceará - CE
57	AFASTAMENTO POR ATESTADO MÉDICO - ATE 15 DIAS	CH da Universidade Federal do Ceará - CE
57	AFASTAMENTO POR ATESTADO MÉDICO - ATE 15 DIAS	CH da Universidade Federal do Ceará - CE
57	AFASTAMENTO POR ATESTADO MÉDICO - ATE 15 DIAS	CH da Universidade Federal do Ceará - CE
57	AFASTAMENTO POR ATESTADO MÉDICO - ATE 15 DIAS	CH da Universidade Federal do Ceará - CE
57	AFASTAMENTO POR ATESTADO MÉDICO - ATE 15 DIAS	CH da Universidade Federal do Ceará - CE
57	AFASTAMENTO POR ATESTADO MÉDICO - ATE 15 DIAS	CH da Universidade Federal do Ceará - CE
57	AFASTAMENTO POR ATESTADO MÉDICO - ATE 15 DIAS	CH da Universidade Federal do Ceará - CE
57	AFASTAMENTO POR ATESTADO MÉDICO - ATE 15 DIAS	CH da Universidade Federal do Ceará - CE
57	AFASTAMENTO POR ATESTADO MÉDICO - ATE 15 DIAS	CH da Universidade Federal do Ceará - CE
57	AFASTAMENTO POR ATESTADO MÉDICO - ATE 15 DIAS	CH da Universidade Federal do Ceará - CE
57	AFASTAMENTO POR ATESTADO MÉDICO - ATE 15 DIAS	CH da Universidade Federal do Ceará - CE
57	AFASTAMENTO POR ATESTADO MÉDICO - ATE 15 DIAS	CH da Universidade Federal do Ceará - CE
57	AFASTAMENTO POR ATESTADO MÉDICO - ATE 15 DIAS	CH da Universidade Federal do Ceará - CE
57	AFASTAMENTO POR ATESTADO MÉDICO - ATE 15 DIAS	CH da Universidade Federal do Ceará - CE
57	AFASTAMENTO POR ATESTADO MÉDICO - ATE 15 DIAS	CH da Universidade Federal do Ceará - CE
57	AFASTAMENTO POR ATESTADO MÉDICO - ATE 15 DIAS	CH da Universidade Federal do Ceará - CE
57	AFASTAMENTO POR ATESTADO MÉDICO - ATE 15 DIAS	CH da Universidade Federal do Ceará - CE
57	AFASTAMENTO POR ATESTADO MÉDICO - ATE 15 DIAS	CH da Universidade Federal do Ceará - CE
57	AFASTAMENTO POR ATESTADO MÉDICO - ATE 15 DIAS	CH da Universidade Federal do Ceará - CE
57	AFASTAMENTO POR ATESTADO MÉDICO - ATE 15 DIAS	CH da Universidade Federal do Ceará - CE
57	AFASTAMENTO POR ATESTADO MÉDICO - ATE 15 DIAS	CH da Universidade Federal do Ceará - CE
57	AFASTAMENTO POR ATESTADO MÉDICO - ATE 15 DIAS	CH da Universidade Federal do Ceará - CE
57	AFASTAMENTO POR ATESTADO MÉDICO - ATE 15 DIAS	CH da Universidade Federal do Ceará - CE
57	AFASTAMENTO POR ATESTADO MÉDICO - ATE 15 DIAS	CH da Universidade Federal do Ceará - CE
57	AFASTAMENTO POR ATESTADO MÉDICO - ATE 15 DIAS	CH da Universidade Federal do Ceará - CE
57	AFASTAMENTO POR ATESTADO MÉDICO - ATE 15 DIAS	CH da Universidade Federal do Ceará - CE
57	AFASTAMENTO POR ATESTADO MÉDICO - ATE 15 DIAS	CH da Universidade Federal do Ceará - CE
57	AFASTAMENTO POR ATESTADO MÉDICO - ATE 15 DIAS	CH da Universidade Federal do Ceará - CE
57	AFASTAMENTO POR ATESTADO MÉDICO - ATE 15 DIAS	CH da Universidade Federal do Ceará - CE
57	AFASTAMENTO POR ATESTADO MÉDICO - ATE 15 DIAS	CH da Universidade Federal do Ceará - CE
57	AFASTAMENTO POR ATESTADO MÉDICO - ATE 15 DIAS	CH da Universidade Federal do Ceará - CE
57	AFASTAMENTO POR ATESTADO MÉDICO - ATE 15 DIAS	CH da Universidade Federal do Ceará - CE
57	AFASTAMENTO POR ATESTADO MÉDICO - ATE 15 DIAS	CH da Universidade Federal do Ceará - CE

57	AFASTAMENTO POR ATESTADO MÉDICO - ATE 15 DIAS	CH da Universidade Federal do Ceará - CE
57	AFASTAMENTO POR ATESTADO MÉDICO - ATE 15 DIAS	CH da Universidade Federal do Ceará - CE
57	AFASTAMENTO POR ATESTADO MÉDICO - ATE 15 DIAS	CH da Universidade Federal do Ceará - CE
57	AFASTAMENTO POR ATESTADO MÉDICO - ATE 15 DIAS	CH da Universidade Federal do Ceará - CE
57	AFASTAMENTO POR ATESTADO MÉDICO - ATE 15 DIAS	CH da Universidade Federal do Ceará - CE
57	AFASTAMENTO POR ATESTADO MÉDICO - ATE 15 DIAS	CH da Universidade Federal do Ceará - CE
57	AFASTAMENTO POR ATESTADO MÉDICO - ATE 15 DIAS	CH da Universidade Federal do Ceará - CE
57	AFASTAMENTO POR ATESTADO MÉDICO - ATE 15 DIAS	CH da Universidade Federal do Ceará - CE
57	AFASTAMENTO POR ATESTADO MÉDICO - ATE 15 DIAS	CH da Universidade Federal do Ceará - CE
57	AFASTAMENTO POR ATESTADO MÉDICO - ATE 15 DIAS	CH da Universidade Federal do Ceará - CE
57	AFASTAMENTO POR ATESTADO MÉDICO - ATE 15 DIAS	CH da Universidade Federal do Ceará - CE
57	AFASTAMENTO POR ATESTADO MÉDICO - ATE 15 DIAS	CH da Universidade Federal do Ceará - CE
57	AFASTAMENTO POR ATESTADO MÉDICO - ATE 15 DIAS	CH da Universidade Federal do Ceará - CE
57	AFASTAMENTO POR ATESTADO MÉDICO - ATE 15 DIAS	CH da Universidade Federal do Ceará - CE
57	AFASTAMENTO POR ATESTADO MÉDICO - ATE 15 DIAS	CH da Universidade Federal do Ceará - CE
57	AFASTAMENTO POR ATESTADO MÉDICO - ATE 15 DIAS	CH da Universidade Federal do Ceará - CE
57	AFASTAMENTO POR ATESTADO MÉDICO - ATE 15 DIAS	CH da Universidade Federal do Ceará - CE
57	AFASTAMENTO POR ATESTADO MÉDICO - ATE 15 DIAS	CH da Universidade Federal do Ceará - CE
57	AFASTAMENTO POR ATESTADO MÉDICO - ATE 15 DIAS	CH da Universidade Federal do Ceará - CE
57	AFASTAMENTO POR ATESTADO MÉDICO - ATE 15 DIAS	CH da Universidade Federal do Ceará - CE
57	AFASTAMENTO POR ATESTADO MÉDICO - ATE 15 DIAS	CH da Universidade Federal do Ceará - CE
57	AFASTAMENTO POR ATESTADO MÉDICO - ATE 15 DIAS	CH da Universidade Federal do Ceará - CE
57	AFASTAMENTO POR ATESTADO MÉDICO - ATE 15 DIAS	CH da Universidade Federal do Ceará - CE
57	AFASTAMENTO POR ATESTADO MÉDICO - ATE 15 DIAS	CH da Universidade Federal do Ceará - CE
57	AFASTAMENTO POR ATESTADO MÉDICO - ATE 15 DIAS	CH da Universidade Federal do Ceará - CE
57	AFASTAMENTO POR ATESTADO MÉDICO - ATE 15 DIAS	CH da Universidade Federal do Ceará - CE
57	AFASTAMENTO POR ATESTADO MÉDICO - ATE 15 DIAS	CH da Universidade Federal do Ceará - CE
57	AFASTAMENTO POR ATESTADO MÉDICO - ATE 15 DIAS	CH da Universidade Federal do Ceará - CE
57	AFASTAMENTO POR ATESTADO MÉDICO - ATE 15 DIAS	CH da Universidade Federal do Ceará - CE
57	AFASTAMENTO POR ATESTADO MÉDICO - ATE 15 DIAS	CH da Universidade Federal do Ceará - CE
57	AFASTAMENTO POR ATESTADO MÉDICO - ATE 15 DIAS	CH da Universidade Federal do Ceará - CE
57	AFASTAMENTO POR ATESTADO MÉDICO - ATE 15 DIAS	CH da Universidade Federal do Ceará - CE
57	AFASTAMENTO POR ATESTADO MÉDICO - ATE 15 DIAS	CH da Universidade Federal do Ceará - CE
57	AFASTAMENTO POR ATESTADO MÉDICO - ATE 15 DIAS	CH da Universidade Federal do Ceará - CE
57	AFASTAMENTO POR ATESTADO MÉDICO - ATE 15 DIAS	CH da Universidade Federal do Ceará - CE
57	AFASTAMENTO POR ATESTADO MÉDICO - ATE 15 DIAS	CH da Universidade Federal do Ceará - CE
57	AFASTAMENTO POR ATESTADO MÉDICO - ATE 15 DIAS	CH da Universidade Federal do Ceará - CE
57	AFASTAMENTO POR ATESTADO MÉDICO - ATE 15 DIAS	CH da Universidade Federal do Ceará - CE
57	AFASTAMENTO POR ATESTADO MÉDICO - ATE 15 DIAS	CH da Universidade Federal do Ceará - CE
57	AFASTAMENTO POR ATESTADO MÉDICO - ATE 15 DIAS	CH da Universidade Federal do Ceará - CE
57	AFASTAMENTO POR ATESTADO MÉDICO - ATE 15 DIAS	CH da Universidade Federal do Ceará - CE
57	AFASTAMENTO POR ATESTADO MÉDICO - ATE 15 DIAS	CH da Universidade Federal do Ceará - CE
57	AFASTAMENTO POR ATESTADO MÉDICO - ATE 15 DIAS	CH da Universidade Federal do Ceará - CE
57	AFASTAMENTO POR ATESTADO MÉDICO - ATE 15 DIAS	CH da Universidade Federal do Ceará - CE
57	AFASTAMENTO POR ATESTADO MÉDICO - ATE 15 DIAS	CH da Universidade Federal do Ceará - CE
57	AFASTAMENTO POR ATESTADO MÉDICO - ATE 15 DIAS	CH da Universidade Federal do Ceará - CE
57	AFASTAMENTO POR ATESTADO MÉDICO - ATE 15 DIAS	CH da Universidade Federal do Ceará - CE
57	AFASTAMENTO POR ATESTADO MÉDICO - ATE 15 DIAS	CH da Universidade Federal do Ceará - CE

57	AFASTAMENTO POR ATESTADO MÉDICO - ATE 15 DIAS	CH da Universidade Federal do Ceará - CE
57	AFASTAMENTO POR ATESTADO MÉDICO - ATE 15 DIAS	CH da Universidade Federal do Ceará - CE
57	AFASTAMENTO POR ATESTADO MÉDICO - ATE 15 DIAS	CH da Universidade Federal do Ceará - CE
57	AFASTAMENTO POR ATESTADO MÉDICO - ATE 15 DIAS	CH da Universidade Federal do Ceará - CE
57	AFASTAMENTO POR ATESTADO MÉDICO - ATE 15 DIAS	CH da Universidade Federal do Ceará - CE
57	AFASTAMENTO POR ATESTADO MÉDICO - ATE 15 DIAS	CH da Universidade Federal do Ceará - CE
57	AFASTAMENTO POR ATESTADO MÉDICO - ATE 15 DIAS	CH da Universidade Federal do Ceará - CE
57	AFASTAMENTO POR ATESTADO MÉDICO - ATE 15 DIAS	CH da Universidade Federal do Ceará - CE
57	AFASTAMENTO POR ATESTADO MÉDICO - ATE 15 DIAS	CH da Universidade Federal do Ceará - CE
57	AFASTAMENTO POR ATESTADO MÉDICO - ATE 15 DIAS	CH da Universidade Federal do Ceará - CE
57	AFASTAMENTO POR ATESTADO MÉDICO - ATE 15 DIAS	CH da Universidade Federal do Ceará - CE
57	AFASTAMENTO POR ATESTADO MÉDICO - ATE 15 DIAS	CH da Universidade Federal do Ceará - CE
57	AFASTAMENTO POR ATESTADO MÉDICO - ATE 15 DIAS	CH da Universidade Federal do Ceará - CE
57	AFASTAMENTO POR ATESTADO MÉDICO - ATE 15 DIAS	CH da Universidade Federal do Ceará - CE
57	AFASTAMENTO POR ATESTADO MÉDICO - ATE 15 DIAS	CH da Universidade Federal do Ceará - CE
57	AFASTAMENTO POR ATESTADO MÉDICO - ATE 15 DIAS	CH da Universidade Federal do Ceará - CE
57	AFASTAMENTO POR ATESTADO MÉDICO - ATE 15 DIAS	CH da Universidade Federal do Ceará - CE
57	AFASTAMENTO POR ATESTADO MÉDICO - ATE 15 DIAS	CH da Universidade Federal do Ceará - CE
57	AFASTAMENTO POR ATESTADO MÉDICO - ATE 15 DIAS	CH da Universidade Federal do Ceará - CE
57	AFASTAMENTO POR ATESTADO MÉDICO - ATE 15 DIAS	CH da Universidade Federal do Ceará - CE
57	AFASTAMENTO POR ATESTADO MÉDICO - ATE 15 DIAS	CH da Universidade Federal do Ceará - CE
57	AFASTAMENTO POR ATESTADO MÉDICO - ATE 15 DIAS	CH da Universidade Federal do Ceará - CE
57	AFASTAMENTO POR ATESTADO MÉDICO - ATE 15 DIAS	CH da Universidade Federal do Ceará - CE
57	AFASTAMENTO POR ATESTADO MÉDICO - ATE 15 DIAS	CH da Universidade Federal do Ceará - CE
57	AFASTAMENTO POR ATESTADO MÉDICO - ATE 15 DIAS	CH da Universidade Federal do Ceará - CE
57	AFASTAMENTO POR ATESTADO MÉDICO - ATE 15 DIAS	CH da Universidade Federal do Ceará - CE
57	AFASTAMENTO POR ATESTADO MÉDICO - ATE 15 DIAS	CH da Universidade Federal do Ceará - CE
57	AFASTAMENTO POR ATESTADO MÉDICO - ATE 15 DIAS	CH da Universidade Federal do Ceará - CE
57	AFASTAMENTO POR ATESTADO MÉDICO - ATE 15 DIAS	CH da Universidade Federal do Ceará - CE
57	AFASTAMENTO POR ATESTADO MÉDICO - ATE 15 DIAS	CH da Universidade Federal do Ceará - CE
57	AFASTAMENTO POR ATESTADO MÉDICO - ATE 15 DIAS	CH da Universidade Federal do Ceará - CE
57	AFASTAMENTO POR ATESTADO MÉDICO - ATE 15 DIAS	CH da Universidade Federal do Ceará - CE
57	AFASTAMENTO POR ATESTADO MÉDICO - ATE 15 DIAS	CH da Universidade Federal do Ceará - CE
57	AFASTAMENTO POR ATESTADO MÉDICO - ATE 15 DIAS	CH da Universidade Federal do Ceará - CE
57	AFASTAMENTO POR ATESTADO MÉDICO - ATE 15 DIAS	CH da Universidade Federal do Ceará - CE
57	AFASTAMENTO POR ATESTADO MÉDICO - ATE 15 DIAS	CH da Universidade Federal do Ceará - CE
57	AFASTAMENTO POR ATESTADO MÉDICO - ATE 15 DIAS	CH da Universidade Federal do Ceará - CE
57	AFASTAMENTO POR ATESTADO MÉDICO - ATE 15 DIAS	CH da Universidade Federal do Ceará - CE
57	AFASTAMENTO POR ATESTADO MÉDICO - ATE 15 DIAS	CH da Universidade Federal do Ceará - CE
57	AFASTAMENTO POR ATESTADO MÉDICO - ATE 15 DIAS	CH da Universidade Federal do Ceará - CE
57	AFASTAMENTO POR ATESTADO MÉDICO - ATE 15 DIAS	CH da Universidade Federal do Ceará - CE
57	AFASTAMENTO POR ATESTADO MÉDICO - ATE 15 DIAS	CH da Universidade Federal do Ceará - CE
57	AFASTAMENTO POR ATESTADO MÉDICO - ATE 15 DIAS	CH da Universidade Federal do Ceará - CE
57	AFASTAMENTO POR ATESTADO MÉDICO - ATE 15 DIAS	CH da Universidade Federal do Ceará - CE
57	AFASTAMENTO POR ATESTADO MÉDICO - ATE 15 DIAS	CH da Universidade Federal do Ceará - CE
57	AFASTAMENTO POR ATESTADO MÉDICO - ATE 15 DIAS	CH da Universidade Federal do Ceará - CE
57	AFASTAMENTO POR ATESTADO MÉDICO - ATE 15 DIAS	CH da Universidade Federal do Ceará - CE
57	AFASTAMENTO POR ATESTADO MÉDICO - ATE 15 DIAS	CH da Universidade Federal do Ceará - CE

57	AFASTAMENTO POR ATESTADO MÉDICO - ATE 15 DIAS	CH da Universidade Federal do Ceará - CE
57	AFASTAMENTO POR ATESTADO MÉDICO - ATE 15 DIAS	CH da Universidade Federal do Ceará - CE
57	AFASTAMENTO POR ATESTADO MÉDICO - ATE 15 DIAS	CH da Universidade Federal do Ceará - CE
57	AFASTAMENTO POR ATESTADO MÉDICO - ATE 15 DIAS	CH da Universidade Federal do Ceará - CE
57	AFASTAMENTO POR ATESTADO MÉDICO - ATE 15 DIAS	CH da Universidade Federal do Ceará - CE
57	AFASTAMENTO POR ATESTADO MÉDICO - ATE 15 DIAS	CH da Universidade Federal do Ceará - CE
57	AFASTAMENTO POR ATESTADO MÉDICO - ATE 15 DIAS	CH da Universidade Federal do Ceará - CE
57	AFASTAMENTO POR ATESTADO MÉDICO - ATE 15 DIAS	CH da Universidade Federal do Ceará - CE
57	AFASTAMENTO POR ATESTADO MÉDICO - ATE 15 DIAS	CH da Universidade Federal do Ceará - CE
57	AFASTAMENTO POR ATESTADO MÉDICO - ATE 15 DIAS	CH da Universidade Federal do Ceará - CE
57	AFASTAMENTO POR ATESTADO MÉDICO - ATE 15 DIAS	CH da Universidade Federal do Ceará - CE
57	AFASTAMENTO POR ATESTADO MÉDICO - ATE 15 DIAS	CH da Universidade Federal do Ceará - CE
57	AFASTAMENTO POR ATESTADO MÉDICO - ATE 15 DIAS	CH da Universidade Federal do Ceará - CE
57	AFASTAMENTO POR ATESTADO MÉDICO - ATE 15 DIAS	CH da Universidade Federal do Ceará - CE
57	AFASTAMENTO POR ATESTADO MÉDICO - ATE 15 DIAS	CH da Universidade Federal do Ceará - CE
57	AFASTAMENTO POR ATESTADO MÉDICO - ATE 15 DIAS	CH da Universidade Federal do Ceará - CE
57	AFASTAMENTO POR ATESTADO MÉDICO - ATE 15 DIAS	CH da Universidade Federal do Ceará - CE
57	AFASTAMENTO POR ATESTADO MÉDICO - ATE 15 DIAS	CH da Universidade Federal do Ceará - CE
57	AFASTAMENTO POR ATESTADO MÉDICO - ATE 15 DIAS	CH da Universidade Federal do Ceará - CE
57	AFASTAMENTO POR ATESTADO MÉDICO - ATE 15 DIAS	CH da Universidade Federal do Ceará - CE
57	AFASTAMENTO POR ATESTADO MÉDICO - ATE 15 DIAS	CH da Universidade Federal do Ceará - CE
57	AFASTAMENTO POR ATESTADO MÉDICO - ATE 15 DIAS	CH da Universidade Federal do Ceará - CE
57	AFASTAMENTO POR ATESTADO MÉDICO - ATE 15 DIAS	CH da Universidade Federal do Ceará - CE
57	AFASTAMENTO POR ATESTADO MÉDICO - ATE 15 DIAS	CH da Universidade Federal do Ceará - CE
57	AFASTAMENTO POR ATESTADO MÉDICO - ATE 15 DIAS	CH da Universidade Federal do Ceará - CE
57	AFASTAMENTO POR ATESTADO MÉDICO - ATE 15 DIAS	CH da Universidade Federal do Ceará - CE
57	AFASTAMENTO POR ATESTADO MÉDICO - ATE 15 DIAS	CH da Universidade Federal do Ceará - CE
57	AFASTAMENTO POR ATESTADO MÉDICO - ATE 15 DIAS	CH da Universidade Federal do Ceará - CE
57	AFASTAMENTO POR ATESTADO MÉDICO - ATE 15 DIAS	CH da Universidade Federal do Ceará - CE
57	AFASTAMENTO POR ATESTADO MÉDICO - ATE 15 DIAS	CH da Universidade Federal do Ceará - CE
57	AFASTAMENTO POR ATESTADO MÉDICO - ATE 15 DIAS	CH da Universidade Federal do Ceará - CE
57	AFASTAMENTO POR ATESTADO MÉDICO - ATE 15 DIAS	CH da Universidade Federal do Ceará - CE
57	AFASTAMENTO POR ATESTADO MÉDICO - ATE 15 DIAS	CH da Universidade Federal do Ceará - CE
57	AFASTAMENTO POR ATESTADO MÉDICO - ATE 15 DIAS	CH da Universidade Federal do Ceará - CE
57	AFASTAMENTO POR ATESTADO MÉDICO - ATE 15 DIAS	CH da Universidade Federal do Ceará - CE
57	AFASTAMENTO POR ATESTADO MÉDICO - ATE 15 DIAS	CH da Universidade Federal do Ceará - CE
57	AFASTAMENTO POR ATESTADO MÉDICO - ATE 15 DIAS	CH da Universidade Federal do Ceará - CE
57	AFASTAMENTO POR ATESTADO MÉDICO - ATE 15 DIAS	CH da Universidade Federal do Ceará - CE
57	AFASTAMENTO POR ATESTADO MÉDICO - ATE 15 DIAS	CH da Universidade Federal do Ceará - CE

57	AFASTAMENTO POR ATESTADO MÉDICO - ATE 15 DIAS	CH da Universidade Federal do Ceará - CE
57	AFASTAMENTO POR ATESTADO MÉDICO - ATE 15 DIAS	CH da Universidade Federal do Ceará - CE
57	AFASTAMENTO POR ATESTADO MÉDICO - ATE 15 DIAS	CH da Universidade Federal do Ceará - CE
57	AFASTAMENTO POR ATESTADO MÉDICO - ATE 15 DIAS	CH da Universidade Federal do Ceará - CE
57	AFASTAMENTO POR ATESTADO MÉDICO - ATE 15 DIAS	CH da Universidade Federal do Ceará - CE
57	AFASTAMENTO POR ATESTADO MÉDICO - ATE 15 DIAS	CH da Universidade Federal do Ceará - CE
57	AFASTAMENTO POR ATESTADO MÉDICO - ATE 15 DIAS	CH da Universidade Federal do Ceará - CE
57	AFASTAMENTO POR ATESTADO MÉDICO - ATE 15 DIAS	CH da Universidade Federal do Ceará - CE
57	AFASTAMENTO POR ATESTADO MÉDICO - ATE 15 DIAS	CH da Universidade Federal do Ceará - CE
57	AFASTAMENTO POR ATESTADO MÉDICO - ATE 15 DIAS	CH da Universidade Federal do Ceará - CE
57	AFASTAMENTO POR ATESTADO MÉDICO - ATE 15 DIAS	CH da Universidade Federal do Ceará - CE
57	AFASTAMENTO POR ATESTADO MÉDICO - ATE 15 DIAS	CH da Universidade Federal do Ceará - CE
57	AFASTAMENTO POR ATESTADO MÉDICO - ATE 15 DIAS	CH da Universidade Federal do Ceará - CE
57	AFASTAMENTO POR ATESTADO MÉDICO - ATE 15 DIAS	CH da Universidade Federal do Ceará - CE
57	AFASTAMENTO POR ATESTADO MÉDICO - ATE 15 DIAS	CH da Universidade Federal do Ceará - CE
57	AFASTAMENTO POR ATESTADO MÉDICO - ATE 15 DIAS	CH da Universidade Federal do Ceará - CE
57	AFASTAMENTO POR ATESTADO MÉDICO - ATE 15 DIAS	CH da Universidade Federal do Ceará - CE
57	AFASTAMENTO POR ATESTADO MÉDICO - ATE 15 DIAS	CH da Universidade Federal do Ceará - CE
57	AFASTAMENTO POR ATESTADO MÉDICO - ATE 15 DIAS	CH da Universidade Federal do Ceará - CE
57	AFASTAMENTO POR ATESTADO MÉDICO - ATE 15 DIAS	CH da Universidade Federal do Ceará - CE
57	AFASTAMENTO POR ATESTADO MÉDICO - ATE 15 DIAS	CH da Universidade Federal do Ceará - CE
57	AFASTAMENTO POR ATESTADO MÉDICO - ATE 15 DIAS	CH da Universidade Federal do Ceará - CE
57	AFASTAMENTO POR ATESTADO MÉDICO - ATE 15 DIAS	CH da Universidade Federal do Ceará - CE
57	AFASTAMENTO POR ATESTADO MÉDICO - ATE 15 DIAS	CH da Universidade Federal do Ceará - CE
57	AFASTAMENTO POR ATESTADO MÉDICO - ATE 15 DIAS	CH da Universidade Federal do Ceará - CE
57	AFASTAMENTO POR ATESTADO MÉDICO - ATE 15 DIAS	CH da Universidade Federal do Ceará - CE
57	AFASTAMENTO POR ATESTADO MÉDICO - ATE 15 DIAS	CH da Universidade Federal do Ceará - CE
57	AFASTAMENTO POR ATESTADO MÉDICO - ATE 15 DIAS	CH da Universidade Federal do Ceará - CE
57	AFASTAMENTO POR ATESTADO MÉDICO - ATE 15 DIAS	CH da Universidade Federal do Ceará - CE
57	AFASTAMENTO POR ATESTADO MÉDICO - ATE 15 DIAS	CH da Universidade Federal do Ceará - CE
57	AFASTAMENTO POR ATESTADO MÉDICO - ATE 15 DIAS	CH da Universidade Federal do Ceará - CE
57	AFASTAMENTO POR ATESTADO MÉDICO - ATE 15 DIAS	CH da Universidade Federal do Ceará - CE
57	AFASTAMENTO POR ATESTADO MÉDICO - ATE 15 DIAS	CH da Universidade Federal do Ceará - CE
57	AFASTAMENTO POR ATESTADO MÉDICO - ATE 15 DIAS	CH da Universidade Federal do Ceará - CE
57	AFASTAMENTO POR ATESTADO MÉDICO - ATE 15 DIAS	CH da Universidade Federal do Ceará - CE
57	AFASTAMENTO POR ATESTADO MÉDICO - ATE 15 DIAS	CH da Universidade Federal do Ceará - CE
57	AFASTAMENTO POR ATESTADO MÉDICO - ATE 15 DIAS	CH da Universidade Federal do Ceará - CE
57	AFASTAMENTO POR ATESTADO MÉDICO - ATE 15 DIAS	CH da Universidade Federal do Ceará - CE
57	AFASTAMENTO POR ATESTADO MÉDICO - ATE 15 DIAS	CH da Universidade Federal do Ceará - CE
57	AFASTAMENTO POR ATESTADO MÉDICO - ATE 15 DIAS	CH da Universidade Federal do Ceará - CE
57	AFASTAMENTO POR ATESTADO MÉDICO - ATE 15 DIAS	CH da Universidade Federal do Ceará - CE
57	AFASTAMENTO POR ATESTADO MÉDICO - ATE 15 DIAS	CH da Universidade Federal do Ceará - CE
57	AFASTAMENTO POR ATESTADO MÉDICO - ATE 15 DIAS	CH da Universidade Federal do Ceará - CE

57	AFASTAMENTO POR ATESTADO MÉDICO - ATE 15 DIAS	CH da Universidade Federal do Ceará - CE
57	AFASTAMENTO POR ATESTADO MÉDICO - ATE 15 DIAS	CH da Universidade Federal do Ceará - CE
57	AFASTAMENTO POR ATESTADO MÉDICO - ATE 15 DIAS	CH da Universidade Federal do Ceará - CE
57	AFASTAMENTO POR ATESTADO MÉDICO - ATE 15 DIAS	CH da Universidade Federal do Ceará - CE
57	AFASTAMENTO POR ATESTADO MÉDICO - ATE 15 DIAS	CH da Universidade Federal do Ceará - CE
57	AFASTAMENTO POR ATESTADO MÉDICO - ATE 15 DIAS	CH da Universidade Federal do Ceará - CE
57	AFASTAMENTO POR ATESTADO MÉDICO - ATE 15 DIAS	CH da Universidade Federal do Ceará - CE
57	AFASTAMENTO POR ATESTADO MÉDICO - ATE 15 DIAS	CH da Universidade Federal do Ceará - CE
57	AFASTAMENTO POR ATESTADO MÉDICO - ATE 15 DIAS	CH da Universidade Federal do Ceará - CE
57	AFASTAMENTO POR ATESTADO MÉDICO - ATE 15 DIAS	CH da Universidade Federal do Ceará - CE
57	AFASTAMENTO POR ATESTADO MÉDICO - ATE 15 DIAS	CH da Universidade Federal do Ceará - CE
57	AFASTAMENTO POR ATESTADO MÉDICO - ATE 15 DIAS	CH da Universidade Federal do Ceará - CE
57	AFASTAMENTO POR ATESTADO MÉDICO - ATE 15 DIAS	CH da Universidade Federal do Ceará - CE
57	AFASTAMENTO POR ATESTADO MÉDICO - ATE 15 DIAS	CH da Universidade Federal do Ceará - CE
57	AFASTAMENTO POR ATESTADO MÉDICO - ATE 15 DIAS	CH da Universidade Federal do Ceará - CE
57	AFASTAMENTO POR ATESTADO MÉDICO - ATE 15 DIAS	CH da Universidade Federal do Ceará - CE
57	AFASTAMENTO POR ATESTADO MÉDICO - ATE 15 DIAS	CH da Universidade Federal do Ceará - CE
57	AFASTAMENTO POR ATESTADO MÉDICO - ATE 15 DIAS	CH da Universidade Federal do Ceará - CE
57	AFASTAMENTO POR ATESTADO MÉDICO - ATE 15 DIAS	CH da Universidade Federal do Ceará - CE
57	AFASTAMENTO POR ATESTADO MÉDICO - ATE 15 DIAS	CH da Universidade Federal do Ceará - CE
57	AFASTAMENTO POR ATESTADO MÉDICO - ATE 15 DIAS	CH da Universidade Federal do Ceará - CE
57	AFASTAMENTO POR ATESTADO MÉDICO - ATE 15 DIAS	CH da Universidade Federal do Ceará - CE
57	AFASTAMENTO POR ATESTADO MÉDICO - ATE 15 DIAS	CH da Universidade Federal do Ceará - CE
57	AFASTAMENTO POR ATESTADO MÉDICO - ATE 15 DIAS	CH da Universidade Federal do Ceará - CE
57	AFASTAMENTO POR ATESTADO MÉDICO - ATE 15 DIAS	CH da Universidade Federal do Ceará - CE
57	AFASTAMENTO POR ATESTADO MÉDICO - ATE 15 DIAS	CH da Universidade Federal do Ceará - CE
57	AFASTAMENTO POR ATESTADO MÉDICO - ATE 15 DIAS	CH da Universidade Federal do Ceará - CE
57	AFASTAMENTO POR ATESTADO MÉDICO - ATE 15 DIAS	CH da Universidade Federal do Ceará - CE
57	AFASTAMENTO POR ATESTADO MÉDICO - ATE 15 DIAS	CH da Universidade Federal do Ceará - CE
57	AFASTAMENTO POR ATESTADO MÉDICO - ATE 15 DIAS	CH da Universidade Federal do Ceará - CE
57	AFASTAMENTO POR ATESTADO MÉDICO - ATE 15 DIAS	CH da Universidade Federal do Ceará - CE
57	AFASTAMENTO POR ATESTADO MÉDICO - ATE 15 DIAS	CH da Universidade Federal do Ceará - CE
57	AFASTAMENTO POR ATESTADO MÉDICO - ATE 15 DIAS	CH da Universidade Federal do Ceará - CE
57	AFASTAMENTO POR ATESTADO MÉDICO - ATE 15 DIAS	CH da Universidade Federal do Ceará - CE
57	AFASTAMENTO POR ATESTADO MÉDICO - ATE 15 DIAS	CH da Universidade Federal do Ceará - CE
57	AFASTAMENTO POR ATESTADO MÉDICO - ATE 15 DIAS	CH da Universidade Federal do Ceará - CE
57	AFASTAMENTO POR ATESTADO MÉDICO - ATE 15 DIAS	CH da Universidade Federal do Ceará - CE

57	AFASTAMENTO POR ATESTADO MÉDICO - ATE 15 DIAS	CH da Universidade Federal do Ceará - CE
57	AFASTAMENTO POR ATESTADO MÉDICO - ATE 15 DIAS	CH da Universidade Federal do Ceará - CE
57	AFASTAMENTO POR ATESTADO MÉDICO - ATE 15 DIAS	CH da Universidade Federal do Ceará - CE
57	AFASTAMENTO POR ATESTADO MÉDICO - ATE 15 DIAS	CH da Universidade Federal do Ceará - CE
57	AFASTAMENTO POR ATESTADO MÉDICO - ATE 15 DIAS	CH da Universidade Federal do Ceará - CE
57	AFASTAMENTO POR ATESTADO MÉDICO - ATE 15 DIAS	CH da Universidade Federal do Ceará - CE
57	AFASTAMENTO POR ATESTADO MÉDICO - ATE 15 DIAS	CH da Universidade Federal do Ceará - CE
57	AFASTAMENTO POR ATESTADO MÉDICO - ATE 15 DIAS	CH da Universidade Federal do Ceará - CE
57	AFASTAMENTO POR ATESTADO MÉDICO - ATE 15 DIAS	CH da Universidade Federal do Ceará - CE
57	AFASTAMENTO POR ATESTADO MÉDICO - ATE 15 DIAS	CH da Universidade Federal do Ceará - CE
57	AFASTAMENTO POR ATESTADO MÉDICO - ATE 15 DIAS	CH da Universidade Federal do Ceará - CE
57	AFASTAMENTO POR ATESTADO MÉDICO - ATE 15 DIAS	CH da Universidade Federal do Ceará - CE
57	AFASTAMENTO POR ATESTADO MÉDICO - ATE 15 DIAS	CH da Universidade Federal do Ceará - CE
57	AFASTAMENTO POR ATESTADO MÉDICO - ATE 15 DIAS	CH da Universidade Federal do Ceará - CE
57	AFASTAMENTO POR ATESTADO MÉDICO - ATE 15 DIAS	CH da Universidade Federal do Ceará - CE
57	AFASTAMENTO POR ATESTADO MÉDICO - ATE 15 DIAS	CH da Universidade Federal do Ceará - CE
57	AFASTAMENTO POR ATESTADO MÉDICO - ATE 15 DIAS	CH da Universidade Federal do Ceará - CE
57	AFASTAMENTO POR ATESTADO MÉDICO - ATE 15 DIAS	CH da Universidade Federal do Ceará - CE
57	AFASTAMENTO POR ATESTADO MÉDICO - ATE 15 DIAS	CH da Universidade Federal do Ceará - CE
57	AFASTAMENTO POR ATESTADO MÉDICO - ATE 15 DIAS	CH da Universidade Federal do Ceará - CE
57	AFASTAMENTO POR ATESTADO MÉDICO - ATE 15 DIAS	CH da Universidade Federal do Ceará - CE
57	AFASTAMENTO POR ATESTADO MÉDICO - ATE 15 DIAS	CH da Universidade Federal do Ceará - CE
57	AFASTAMENTO POR ATESTADO MÉDICO - ATE 15 DIAS	CH da Universidade Federal do Ceará - CE
57	AFASTAMENTO POR ATESTADO MÉDICO - ATE 15 DIAS	CH da Universidade Federal do Ceará - CE
57	AFASTAMENTO POR ATESTADO MÉDICO - ATE 15 DIAS	CH da Universidade Federal do Ceará - CE
57	AFASTAMENTO POR ATESTADO MÉDICO - ATE 15 DIAS	CH da Universidade Federal do Ceará - CE
57	AFASTAMENTO POR ATESTADO MÉDICO - ATE 15 DIAS	CH da Universidade Federal do Ceará - CE
57	AFASTAMENTO POR ATESTADO MÉDICO - ATE 15 DIAS	CH da Universidade Federal do Ceará - CE
57	AFASTAMENTO POR ATESTADO MÉDICO - ATE 15 DIAS	CH da Universidade Federal do Ceará - CE
57	AFASTAMENTO POR ATESTADO MÉDICO - ATE 15 DIAS	CH da Universidade Federal do Ceará - CE
57	AFASTAMENTO POR ATESTADO MÉDICO - ATE 15 DIAS	CH da Universidade Federal do Ceará - CE
57	AFASTAMENTO POR ATESTADO MÉDICO - ATE 15 DIAS	CH da Universidade Federal do Ceará - CE
57	AFASTAMENTO POR ATESTADO MÉDICO - ATE 15 DIAS	CH da Universidade Federal do Ceará - CE
57	AFASTAMENTO POR ATESTADO MÉDICO - ATE 15 DIAS	CH da Universidade Federal do Ceará - CE
57	AFASTAMENTO POR ATESTADO MÉDICO - ATE 15 DIAS	CH da Universidade Federal do Ceará - CE
57	AFASTAMENTO POR ATESTADO MÉDICO - ATE 15 DIAS	CH da Universidade Federal do Ceará - CE
57	AFASTAMENTO POR ATESTADO MÉDICO - ATE 15 DIAS	CH da Universidade Federal do Ceará - CE
57	AFASTAMENTO POR ATESTADO MÉDICO - ATE 15 DIAS	CH da Universidade Federal do Ceará - CE
57	AFASTAMENTO POR ATESTADO MÉDICO - ATE 15 DIAS	CH da Universidade Federal do Ceará - CE
57	AFASTAMENTO POR ATESTADO MÉDICO - ATE 15 DIAS	CH da Universidade Federal do Ceará - CE
57	AFASTAMENTO POR ATESTADO MÉDICO - ATE 15 DIAS	CH da Universidade Federal do Ceará - CE
57	AFASTAMENTO POR ATESTADO MÉDICO - ATE 15 DIAS	CH da Universidade Federal do Ceará - CE
57	AFASTAMENTO POR ATESTADO MÉDICO - ATE 15 DIAS	CH da Universidade Federal do Ceará - CE
57	AFASTAMENTO POR ATESTADO MÉDICO - ATE 15 DIAS	CH da Universidade Federal do Ceará - CE
57	AFASTAMENTO POR ATESTADO MÉDICO - ATE 15 DIAS	CH da Universidade Federal do Ceará - CE
57	AFASTAMENTO POR ATESTADO MÉDICO - ATE 15 DIAS	CH da Universidade Federal do Ceará - CE
57	AFASTAMENTO POR ATESTADO MÉDICO - ATE 15 DIAS	CH da Universidade Federal do Ceará - CE
57	AFASTAMENTO POR ATESTADO MÉDICO - ATE 15 DIAS	CH da Universidade Federal do Ceará - CE

57	AFASTAMENTO POR ATESTADO MÉDICO - ATE 15 DIAS	CH da Universidade Federal do Ceará - CE
57	AFASTAMENTO POR ATESTADO MÉDICO - ATE 15 DIAS	CH da Universidade Federal do Ceará - CE
57	AFASTAMENTO POR ATESTADO MÉDICO - ATE 15 DIAS	CH da Universidade Federal do Ceará - CE
57	AFASTAMENTO POR ATESTADO MÉDICO - ATE 15 DIAS	CH da Universidade Federal do Ceará - CE
57	AFASTAMENTO POR ATESTADO MÉDICO - ATE 15 DIAS	CH da Universidade Federal do Ceará - CE
57	AFASTAMENTO POR ATESTADO MÉDICO - ATE 15 DIAS	CH da Universidade Federal do Ceará - CE
57	AFASTAMENTO POR ATESTADO MÉDICO - ATE 15 DIAS	CH da Universidade Federal do Ceará - CE
57	AFASTAMENTO POR ATESTADO MÉDICO - ATE 15 DIAS	CH da Universidade Federal do Ceará - CE
57	AFASTAMENTO POR ATESTADO MÉDICO - ATE 15 DIAS	CH da Universidade Federal do Ceará - CE
57	AFASTAMENTO POR ATESTADO MÉDICO - ATE 15 DIAS	CH da Universidade Federal do Ceará - CE
57	AFASTAMENTO POR ATESTADO MÉDICO - ATE 15 DIAS	CH da Universidade Federal do Ceará - CE
57	AFASTAMENTO POR ATESTADO MÉDICO - ATE 15 DIAS	CH da Universidade Federal do Ceará - CE
57	AFASTAMENTO POR ATESTADO MÉDICO - ATE 15 DIAS	CH da Universidade Federal do Ceará - CE
57	AFASTAMENTO POR ATESTADO MÉDICO - ATE 15 DIAS	CH da Universidade Federal do Ceará - CE
57	AFASTAMENTO POR ATESTADO MÉDICO - ATE 15 DIAS	CH da Universidade Federal do Ceará - CE
57	AFASTAMENTO POR ATESTADO MÉDICO - ATE 15 DIAS	CH da Universidade Federal do Ceará - CE
57	AFASTAMENTO POR ATESTADO MÉDICO - ATE 15 DIAS	CH da Universidade Federal do Ceará - CE
57	AFASTAMENTO POR ATESTADO MÉDICO - ATE 15 DIAS	CH da Universidade Federal do Ceará - CE
57	AFASTAMENTO POR ATESTADO MÉDICO - ATE 15 DIAS	CH da Universidade Federal do Ceará - CE
57	AFASTAMENTO POR ATESTADO MÉDICO - ATE 15 DIAS	CH da Universidade Federal do Ceará - CE
57	AFASTAMENTO POR ATESTADO MÉDICO - ATE 15 DIAS	CH da Universidade Federal do Ceará - CE
57	AFASTAMENTO POR ATESTADO MÉDICO - ATE 15 DIAS	CH da Universidade Federal do Ceará - CE
57	AFASTAMENTO POR ATESTADO MÉDICO - ATE 15 DIAS	CH da Universidade Federal do Ceará - CE
57	AFASTAMENTO POR ATESTADO MÉDICO - ATE 15 DIAS	CH da Universidade Federal do Ceará - CE
57	AFASTAMENTO POR ATESTADO MÉDICO - ATE 15 DIAS	CH da Universidade Federal do Ceará - CE
57	AFASTAMENTO POR ATESTADO MÉDICO - ATE 15 DIAS	CH da Universidade Federal do Ceará - CE
57	AFASTAMENTO POR ATESTADO MÉDICO - ATE 15 DIAS	CH da Universidade Federal do Ceará - CE
57	AFASTAMENTO POR ATESTADO MÉDICO - ATE 15 DIAS	CH da Universidade Federal do Ceará - CE
57	AFASTAMENTO POR ATESTADO MÉDICO - ATE 15 DIAS	CH da Universidade Federal do Ceará - CE
57	AFASTAMENTO POR ATESTADO MÉDICO - ATE 15 DIAS	CH da Universidade Federal do Ceará - CE
57	AFASTAMENTO POR ATESTADO MÉDICO - ATE 15 DIAS	CH da Universidade Federal do Ceará - CE
57	AFASTAMENTO POR ATESTADO MÉDICO - ATE 15 DIAS	CH da Universidade Federal do Ceará - CE
57	AFASTAMENTO POR ATESTADO MÉDICO - ATE 15 DIAS	CH da Universidade Federal do Ceará - CE
57	AFASTAMENTO POR ATESTADO MÉDICO - ATE 15 DIAS	CH da Universidade Federal do Ceará - CE
57	AFASTAMENTO POR ATESTADO MÉDICO - ATE 15 DIAS	CH da Universidade Federal do Ceará - CE
57	AFASTAMENTO POR ATESTADO MÉDICO - ATE 15 DIAS	CH da Universidade Federal do Ceará - CE
57	AFASTAMENTO POR ATESTADO MÉDICO - ATE 15 DIAS	CH da Universidade Federal do Ceará - CE
57	AFASTAMENTO POR ATESTADO MÉDICO - ATE 15 DIAS	CH da Universidade Federal do Ceará - CE
57	AFASTAMENTO POR ATESTADO MÉDICO - ATE 15 DIAS	CH da Universidade Federal do Ceará - CE

57	AFASTAMENTO POR ATESTADO MÉDICO - ATE 15 DIAS	CH da Universidade Federal do Ceará - CE
57	AFASTAMENTO POR ATESTADO MÉDICO - ATE 15 DIAS	CH da Universidade Federal do Ceará - CE
57	AFASTAMENTO POR ATESTADO MÉDICO - ATE 15 DIAS	CH da Universidade Federal do Ceará - CE
57	AFASTAMENTO POR ATESTADO MÉDICO - ATE 15 DIAS	CH da Universidade Federal do Ceará - CE
57	AFASTAMENTO POR ATESTADO MÉDICO - ATE 15 DIAS	CH da Universidade Federal do Ceará - CE
57	AFASTAMENTO POR ATESTADO MÉDICO - ATE 15 DIAS	CH da Universidade Federal do Ceará - CE
57	AFASTAMENTO POR ATESTADO MÉDICO - ATE 15 DIAS	CH da Universidade Federal do Ceará - CE
57	AFASTAMENTO POR ATESTADO MÉDICO - ATE 15 DIAS	CH da Universidade Federal do Ceará - CE
57	AFASTAMENTO POR ATESTADO MÉDICO - ATE 15 DIAS	CH da Universidade Federal do Ceará - CE
57	AFASTAMENTO POR ATESTADO MÉDICO - ATE 15 DIAS	CH da Universidade Federal do Ceará - CE
57	AFASTAMENTO POR ATESTADO MÉDICO - ATE 15 DIAS	CH da Universidade Federal do Ceará - CE
57	AFASTAMENTO POR ATESTADO MÉDICO - ATE 15 DIAS	CH da Universidade Federal do Ceará - CE
57	AFASTAMENTO POR ATESTADO MÉDICO - ATE 15 DIAS	CH da Universidade Federal do Ceará - CE
57	AFASTAMENTO POR ATESTADO MÉDICO - ATE 15 DIAS	CH da Universidade Federal do Ceará - CE
57	AFASTAMENTO POR ATESTADO MÉDICO - ATE 15 DIAS	CH da Universidade Federal do Ceará - CE
57	AFASTAMENTO POR ATESTADO MÉDICO - ATE 15 DIAS	CH da Universidade Federal do Ceará - CE
57	AFASTAMENTO POR ATESTADO MÉDICO - ATE 15 DIAS	CH da Universidade Federal do Ceará - CE
57	AFASTAMENTO POR ATESTADO MÉDICO - ATE 15 DIAS	CH da Universidade Federal do Ceará - CE
57	AFASTAMENTO POR ATESTADO MÉDICO - ATE 15 DIAS	CH da Universidade Federal do Ceará - CE
57	AFASTAMENTO POR ATESTADO MÉDICO - ATE 15 DIAS	CH da Universidade Federal do Ceará - CE
57	AFASTAMENTO POR ATESTADO MÉDICO - ATE 15 DIAS	CH da Universidade Federal do Ceará - CE
57	AFASTAMENTO POR ATESTADO MÉDICO - ATE 15 DIAS	CH da Universidade Federal do Ceará - CE
57	AFASTAMENTO POR ATESTADO MÉDICO - ATE 15 DIAS	CH da Universidade Federal do Ceará - CE
57	AFASTAMENTO POR ATESTADO MÉDICO - ATE 15 DIAS	CH da Universidade Federal do Ceará - CE
57	AFASTAMENTO POR ATESTADO MÉDICO - ATE 15 DIAS	CH da Universidade Federal do Ceará - CE
57	AFASTAMENTO POR ATESTADO MÉDICO - ATE 15 DIAS	CH da Universidade Federal do Ceará - CE
57	AFASTAMENTO POR ATESTADO MÉDICO - ATE 15 DIAS	CH da Universidade Federal do Ceará - CE
57	AFASTAMENTO POR ATESTADO MÉDICO - ATE 15 DIAS	CH da Universidade Federal do Ceará - CE
57	AFASTAMENTO POR ATESTADO MÉDICO - ATE 15 DIAS	CH da Universidade Federal do Ceará - CE
57	AFASTAMENTO POR ATESTADO MÉDICO - ATE 15 DIAS	CH da Universidade Federal do Ceará - CE
57	AFASTAMENTO POR ATESTADO MÉDICO - ATE 15 DIAS	CH da Universidade Federal do Ceará - CE
57	AFASTAMENTO POR ATESTADO MÉDICO - ATE 15 DIAS	CH da Universidade Federal do Ceará - CE

57	AFASTAMENTO POR ATESTADO MÉDICO - ATE 15 DIAS	CH da Universidade Federal do Ceará - CE
57	AFASTAMENTO POR ATESTADO MÉDICO - ATE 15 DIAS	CH da Universidade Federal do Ceará - CE
57	AFASTAMENTO POR ATESTADO MÉDICO - ATE 15 DIAS	CH da Universidade Federal do Ceará - CE
57	AFASTAMENTO POR ATESTADO MÉDICO - ATE 15 DIAS	CH da Universidade Federal do Ceará - CE
57	AFASTAMENTO POR ATESTADO MÉDICO - ATE 15 DIAS	CH da Universidade Federal do Ceará - CE
57	AFASTAMENTO POR ATESTADO MÉDICO - ATE 15 DIAS	CH da Universidade Federal do Ceará - CE
57	AFASTAMENTO POR ATESTADO MÉDICO - ATE 15 DIAS	CH da Universidade Federal do Ceará - CE
57	AFASTAMENTO POR ATESTADO MÉDICO - ATE 15 DIAS	CH da Universidade Federal do Ceará - CE
57	AFASTAMENTO POR ATESTADO MÉDICO - ATE 15 DIAS	CH da Universidade Federal do Ceará - CE
57	AFASTAMENTO POR ATESTADO MÉDICO - ATE 15 DIAS	CH da Universidade Federal do Ceará - CE
57	AFASTAMENTO POR ATESTADO MÉDICO - ATE 15 DIAS	CH da Universidade Federal do Ceará - CE
57	AFASTAMENTO POR ATESTADO MÉDICO - ATE 15 DIAS	CH da Universidade Federal do Ceará - CE
57	AFASTAMENTO POR ATESTADO MÉDICO - ATE 15 DIAS	CH da Universidade Federal do Ceará - CE
57	AFASTAMENTO POR ATESTADO MÉDICO - ATE 15 DIAS	CH da Universidade Federal do Ceará - CE
57	AFASTAMENTO POR ATESTADO MÉDICO - ATE 15 DIAS	CH da Universidade Federal do Ceará - CE
57	AFASTAMENTO POR ATESTADO MÉDICO - ATE 15 DIAS	CH da Universidade Federal do Ceará - CE
57	AFASTAMENTO POR ATESTADO MÉDICO - ATE 15 DIAS	CH da Universidade Federal do Ceará - CE
57	AFASTAMENTO POR ATESTADO MÉDICO - ATE 15 DIAS	CH da Universidade Federal do Ceará - CE
57	AFASTAMENTO POR ATESTADO MÉDICO - ATE 15 DIAS	CH da Universidade Federal do Ceará - CE
57	AFASTAMENTO POR ATESTADO MÉDICO - ATE 15 DIAS	CH da Universidade Federal do Ceará - CE
57	AFASTAMENTO POR ATESTADO MÉDICO - ATE 15 DIAS	CH da Universidade Federal do Ceará - CE
57	AFASTAMENTO POR ATESTADO MÉDICO - ATE 15 DIAS	CH da Universidade Federal do Ceará - CE
57	AFASTAMENTO POR ATESTADO MÉDICO - ATE 15 DIAS	CH da Universidade Federal do Ceará - CE
57	AFASTAMENTO POR ATESTADO MÉDICO - ATE 15 DIAS	CH da Universidade Federal do Ceará - CE
57	AFASTAMENTO POR ATESTADO MÉDICO - ATE 15 DIAS	CH da Universidade Federal do Ceará - CE
57	AFASTAMENTO POR ATESTADO MÉDICO - ATE 15 DIAS	CH da Universidade Federal do Ceará - CE
57	AFASTAMENTO POR ATESTADO MÉDICO - ATE 15 DIAS	CH da Universidade Federal do Ceará - CE
57	AFASTAMENTO POR ATESTADO MÉDICO - ATE 15 DIAS	CH da Universidade Federal do Ceará - CE
57	AFASTAMENTO POR ATESTADO MÉDICO - ATE 15 DIAS	CH da Universidade Federal do Ceará - CE
57	AFASTAMENTO POR ATESTADO MÉDICO - ATE 15 DIAS	CH da Universidade Federal do Ceará - CE
57	AFASTAMENTO POR ATESTADO MÉDICO - ATE 15 DIAS	CH da Universidade Federal do Ceará - CE
57	AFASTAMENTO POR ATESTADO MÉDICO - ATE 15 DIAS	CH da Universidade Federal do Ceará - CE
57	AFASTAMENTO POR ATESTADO MÉDICO - ATE 15 DIAS	CH da Universidade Federal do Ceará - CE
57	AFASTAMENTO POR ATESTADO MÉDICO - ATE 15 DIAS	CH da Universidade Federal do Ceará - CE
57	AFASTAMENTO POR ATESTADO MÉDICO - ATE 15 DIAS	CH da Universidade Federal do Ceará - CE
57	AFASTAMENTO POR ATESTADO MÉDICO - ATE 15 DIAS	CH da Universidade Federal do Ceará - CE
57	AFASTAMENTO POR ATESTADO MÉDICO - ATE 15 DIAS	CH da Universidade Federal do Ceará - CE
57	AFASTAMENTO POR ATESTADO MÉDICO - ATE 15 DIAS	CH da Universidade Federal do Ceará - CE
57	AFASTAMENTO POR ATESTADO MÉDICO - ATE 15 DIAS	CH da Universidade Federal do Ceará - CE
57	AFASTAMENTO POR ATESTADO MÉDICO - ATE 15 DIAS	CH da Universidade Federal do Ceará - CE
57	AFASTAMENTO POR ATESTADO MÉDICO - ATE 15 DIAS	CH da Universidade Federal do Ceará - CE
57	AFASTAMENTO POR ATESTADO MÉDICO - ATE 15 DIAS	CH da Universidade Federal do Ceará - CE
57	AFASTAMENTO POR ATESTADO MÉDICO - ATE 15 DIAS	CH da Universidade Federal do Ceará - CE
57	AFASTAMENTO POR ATESTADO MÉDICO - ATE 15 DIAS	CH da Universidade Federal do Ceará - CE
57	AFASTAMENTO POR ATESTADO MÉDICO - ATE 15 DIAS	CH da Universidade Federal do Ceará - CE
57	AFASTAMENTO POR ATESTADO MÉDICO - ATE 15 DIAS	CH da Universidade Federal do Ceará - CE
57	AFASTAMENTO POR ATESTADO MÉDICO - ATE 15 DIAS	CH da Universidade Federal do Ceará - CE

57	AFASTAMENTO POR ATESTADO MÉDICO - ATE 15 DIAS	CH da Universidade Federal do Ceará - CE
57	AFASTAMENTO POR ATESTADO MÉDICO - ATE 15 DIAS	CH da Universidade Federal do Ceará - CE
57	AFASTAMENTO POR ATESTADO MÉDICO - ATE 15 DIAS	CH da Universidade Federal do Ceará - CE
57	AFASTAMENTO POR ATESTADO MÉDICO - ATE 15 DIAS	CH da Universidade Federal do Ceará - CE
57	AFASTAMENTO POR ATESTADO MÉDICO - ATE 15 DIAS	CH da Universidade Federal do Ceará - CE
57	AFASTAMENTO POR ATESTADO MÉDICO - ATE 15 DIAS	CH da Universidade Federal do Ceará - CE
57	AFASTAMENTO POR ATESTADO MÉDICO - ATE 15 DIAS	CH da Universidade Federal do Ceará - CE
57	AFASTAMENTO POR ATESTADO MÉDICO - ATE 15 DIAS	CH da Universidade Federal do Ceará - CE
57	AFASTAMENTO POR ATESTADO MÉDICO - ATE 15 DIAS	CH da Universidade Federal do Ceará - CE
57	AFASTAMENTO POR ATESTADO MÉDICO - ATE 15 DIAS	CH da Universidade Federal do Ceará - CE
57	AFASTAMENTO POR ATESTADO MÉDICO - ATE 15 DIAS	CH da Universidade Federal do Ceará - CE
57	AFASTAMENTO POR ATESTADO MÉDICO - ATE 15 DIAS	CH da Universidade Federal do Ceará - CE
57	AFASTAMENTO POR ATESTADO MÉDICO - ATE 15 DIAS	CH da Universidade Federal do Ceará - CE
57	AFASTAMENTO POR ATESTADO MÉDICO - ATE 15 DIAS	CH da Universidade Federal do Ceará - CE
57	AFASTAMENTO POR ATESTADO MÉDICO - ATE 15 DIAS	CH da Universidade Federal do Ceará - CE
57	AFASTAMENTO POR ATESTADO MÉDICO - ATE 15 DIAS	CH da Universidade Federal do Ceará - CE
57	AFASTAMENTO POR ATESTADO MÉDICO - ATE 15 DIAS	CH da Universidade Federal do Ceará - CE
57	AFASTAMENTO POR ATESTADO MÉDICO - ATE 15 DIAS	CH da Universidade Federal do Ceará - CE
57	AFASTAMENTO POR ATESTADO MÉDICO - ATE 15 DIAS	CH da Universidade Federal do Ceará - CE
57	AFASTAMENTO POR ATESTADO MÉDICO - ATE 15 DIAS	CH da Universidade Federal do Ceará - CE
57	AFASTAMENTO POR ATESTADO MÉDICO - ATE 15 DIAS	CH da Universidade Federal do Ceará - CE
57	AFASTAMENTO POR ATESTADO MÉDICO - ATE 15 DIAS	CH da Universidade Federal do Ceará - CE
57	AFASTAMENTO POR ATESTADO MÉDICO - ATE 15 DIAS	CH da Universidade Federal do Ceará - CE
57	AFASTAMENTO POR ATESTADO MÉDICO - ATE 15 DIAS	CH da Universidade Federal do Ceará - CE
57	AFASTAMENTO POR ATESTADO MÉDICO - ATE 15 DIAS	CH da Universidade Federal do Ceará - CE
57	AFASTAMENTO POR ATESTADO MÉDICO - ATE 15 DIAS	CH da Universidade Federal do Ceará - CE
57	AFASTAMENTO POR ATESTADO MÉDICO - ATE 15 DIAS	CH da Universidade Federal do Ceará - CE
57	AFASTAMENTO POR ATESTADO MÉDICO - ATE 15 DIAS	CH da Universidade Federal do Ceará - CE
57	AFASTAMENTO POR ATESTADO MÉDICO - ATE 15 DIAS	CH da Universidade Federal do Ceará - CE
57	AFASTAMENTO POR ATESTADO MÉDICO - ATE 15 DIAS	CH da Universidade Federal do Ceará - CE
57	AFASTAMENTO POR ATESTADO MÉDICO - ATE 15 DIAS	CH da Universidade Federal do Ceará - CE
57	AFASTAMENTO POR ATESTADO MÉDICO - ATE 15 DIAS	CH da Universidade Federal do Ceará - CE
57	AFASTAMENTO POR ATESTADO MÉDICO - ATE 15 DIAS	CH da Universidade Federal do Ceará - CE
57	AFASTAMENTO POR ATESTADO MÉDICO - ATE 15 DIAS	CH da Universidade Federal do Ceará - CE
57	AFASTAMENTO POR ATESTADO MÉDICO - ATE 15 DIAS	CH da Universidade Federal do Ceará - CE
57	AFASTAMENTO POR ATESTADO MÉDICO - ATE 15 DIAS	CH da Universidade Federal do Ceará - CE
57	AFASTAMENTO POR ATESTADO MÉDICO - ATE 15 DIAS	CH da Universidade Federal do Ceará - CE
57	AFASTAMENTO POR ATESTADO MÉDICO - ATE 15 DIAS	CH da Universidade Federal do Ceará - CE
57	AFASTAMENTO POR ATESTADO MÉDICO - ATE 15 DIAS	CH da Universidade Federal do Ceará - CE
57	AFASTAMENTO POR ATESTADO MÉDICO - ATE 15 DIAS	CH da Universidade Federal do Ceará - CE
57	AFASTAMENTO POR ATESTADO MÉDICO - ATE 15 DIAS	CH da Universidade Federal do Ceará - CE
57	AFASTAMENTO POR ATESTADO MÉDICO - ATE 15 DIAS	CH da Universidade Federal do Ceará - CE
57	AFASTAMENTO POR ATESTADO MÉDICO - ATE 15 DIAS	CH da Universidade Federal do Ceará - CE

57	AFASTAMENTO POR ATESTADO MÉDICO - ATE 15 DIAS	CH da Universidade Federal do Ceará - CE
57	AFASTAMENTO POR ATESTADO MÉDICO - ATE 15 DIAS	CH da Universidade Federal do Ceará - CE
57	AFASTAMENTO POR ATESTADO MÉDICO - ATE 15 DIAS	CH da Universidade Federal do Ceará - CE
57	AFASTAMENTO POR ATESTADO MÉDICO - ATE 15 DIAS	CH da Universidade Federal do Ceará - CE
57	AFASTAMENTO POR ATESTADO MÉDICO - ATE 15 DIAS	CH da Universidade Federal do Ceará - CE
57	AFASTAMENTO POR ATESTADO MÉDICO - ATE 15 DIAS	CH da Universidade Federal do Ceará - CE
57	AFASTAMENTO POR ATESTADO MÉDICO - ATE 15 DIAS	CH da Universidade Federal do Ceará - CE
57	AFASTAMENTO POR ATESTADO MÉDICO - ATE 15 DIAS	CH da Universidade Federal do Ceará - CE
57	AFASTAMENTO POR ATESTADO MÉDICO - ATE 15 DIAS	CH da Universidade Federal do Ceará - CE
57	AFASTAMENTO POR ATESTADO MÉDICO - ATE 15 DIAS	CH da Universidade Federal do Ceará - CE
57	AFASTAMENTO POR ATESTADO MÉDICO - ATE 15 DIAS	CH da Universidade Federal do Ceará - CE
57	AFASTAMENTO POR ATESTADO MÉDICO - ATE 15 DIAS	CH da Universidade Federal do Ceará - CE
57	AFASTAMENTO POR ATESTADO MÉDICO - ATE 15 DIAS	CH da Universidade Federal do Ceará - CE
57	AFASTAMENTO POR ATESTADO MÉDICO - ATE 15 DIAS	CH da Universidade Federal do Ceará - CE
57	AFASTAMENTO POR ATESTADO MÉDICO - ATE 15 DIAS	CH da Universidade Federal do Ceará - CE
57	AFASTAMENTO POR ATESTADO MÉDICO - ATE 15 DIAS	CH da Universidade Federal do Ceará - CE
57	AFASTAMENTO POR ATESTADO MÉDICO - ATE 15 DIAS	CH da Universidade Federal do Ceará - CE
57	AFASTAMENTO POR ATESTADO MÉDICO - ATE 15 DIAS	CH da Universidade Federal do Ceará - CE
57	AFASTAMENTO POR ATESTADO MÉDICO - ATE 15 DIAS	CH da Universidade Federal do Ceará - CE
57	AFASTAMENTO POR ATESTADO MÉDICO - ATE 15 DIAS	CH da Universidade Federal do Ceará - CE
57	AFASTAMENTO POR ATESTADO MÉDICO - ATE 15 DIAS	CH da Universidade Federal do Ceará - CE
57	AFASTAMENTO POR ATESTADO MÉDICO - ATE 15 DIAS	CH da Universidade Federal do Ceará - CE
57	AFASTAMENTO POR ATESTADO MÉDICO - ATE 15 DIAS	CH da Universidade Federal do Ceará - CE
57	AFASTAMENTO POR ATESTADO MÉDICO - ATE 15 DIAS	CH da Universidade Federal do Ceará - CE
57	AFASTAMENTO POR ATESTADO MÉDICO - ATE 15 DIAS	CH da Universidade Federal do Ceará - CE
57	AFASTAMENTO POR ATESTADO MÉDICO - ATE 15 DIAS	CH da Universidade Federal do Ceará - CE
57	AFASTAMENTO POR ATESTADO MÉDICO - ATE 15 DIAS	CH da Universidade Federal do Ceará - CE
57	AFASTAMENTO POR ATESTADO MÉDICO - ATE 15 DIAS	CH da Universidade Federal do Ceará - CE
57	AFASTAMENTO POR ATESTADO MÉDICO - ATE 15 DIAS	CH da Universidade Federal do Ceará - CE
57	AFASTAMENTO POR ATESTADO MÉDICO - ATE 15 DIAS	CH da Universidade Federal do Ceará - CE
57	AFASTAMENTO POR ATESTADO MÉDICO - ATE 15 DIAS	CH da Universidade Federal do Ceará - CE
57	AFASTAMENTO POR ATESTADO MÉDICO - ATE 15 DIAS	CH da Universidade Federal do Ceará - CE
57	AFASTAMENTO POR ATESTADO MÉDICO - ATE 15 DIAS	CH da Universidade Federal do Ceará - CE
57	AFASTAMENTO POR ATESTADO MÉDICO - ATE 15 DIAS	CH da Universidade Federal do Ceará - CE
57	AFASTAMENTO POR ATESTADO MÉDICO - ATE 15 DIAS	CH da Universidade Federal do Ceará - CE
57	AFASTAMENTO POR ATESTADO MÉDICO - ATE 15 DIAS	CH da Universidade Federal do Ceará - CE
57	AFASTAMENTO POR ATESTADO MÉDICO - ATE 15 DIAS	CH da Universidade Federal do Ceará - CE
57	AFASTAMENTO POR ATESTADO MÉDICO - ATE 15 DIAS	CH da Universidade Federal do Ceará - CE
57	AFASTAMENTO POR ATESTADO MÉDICO - ATE 15 DIAS	CH da Universidade Federal do Ceará - CE
57	AFASTAMENTO POR ATESTADO MÉDICO - ATE 15 DIAS	CH da Universidade Federal do Ceará - CE
57	AFASTAMENTO POR ATESTADO MÉDICO - ATE 15 DIAS	CH da Universidade Federal do Ceará - CE
57	AFASTAMENTO POR ATESTADO MÉDICO - ATE 15 DIAS	CH da Universidade Federal do Ceará - CE
57	AFASTAMENTO POR ATESTADO MÉDICO - ATE 15 DIAS	CH da Universidade Federal do Ceará - CE
57	AFASTAMENTO POR ATESTADO MÉDICO - ATE 15 DIAS	CH da Universidade Federal do Ceará - CE
57	AFASTAMENTO POR ATESTADO MÉDICO - ATE 15 DIAS	CH da Universidade Federal do Ceará - CE
57	AFASTAMENTO POR ATESTADO MÉDICO - ATE 15 DIAS	CH da Universidade Federal do Ceará - CE
57	AFASTAMENTO POR ATESTADO MÉDICO - ATE 15 DIAS	CH da Universidade Federal do Ceará - CE

57	AFASTAMENTO POR ATESTADO MÉDICO - ATE 15 DIAS	CH da Universidade Federal do Ceará - CE
57	AFASTAMENTO POR ATESTADO MÉDICO - ATE 15 DIAS	CH da Universidade Federal do Ceará - CE
57	AFASTAMENTO POR ATESTADO MÉDICO - ATE 15 DIAS	CH da Universidade Federal do Ceará - CE
57	AFASTAMENTO POR ATESTADO MÉDICO - ATE 15 DIAS	CH da Universidade Federal do Ceará - CE
57	AFASTAMENTO POR ATESTADO MÉDICO - ATE 15 DIAS	CH da Universidade Federal do Ceará - CE
57	AFASTAMENTO POR ATESTADO MÉDICO - ATE 15 DIAS	CH da Universidade Federal do Ceará - CE
57	AFASTAMENTO POR ATESTADO MÉDICO - ATE 15 DIAS	CH da Universidade Federal do Ceará - CE
57	AFASTAMENTO POR ATESTADO MÉDICO - ATE 15 DIAS	CH da Universidade Federal do Ceará - CE
57	AFASTAMENTO POR ATESTADO MÉDICO - ATE 15 DIAS	CH da Universidade Federal do Ceará - CE
57	AFASTAMENTO POR ATESTADO MÉDICO - ATE 15 DIAS	CH da Universidade Federal do Ceará - CE
57	AFASTAMENTO POR ATESTADO MÉDICO - ATE 15 DIAS	CH da Universidade Federal do Ceará - CE
57	AFASTAMENTO POR ATESTADO MÉDICO - ATE 15 DIAS	CH da Universidade Federal do Ceará - CE
57	AFASTAMENTO POR ATESTADO MÉDICO - ATE 15 DIAS	CH da Universidade Federal do Ceará - CE
57	AFASTAMENTO POR ATESTADO MÉDICO - ATE 15 DIAS	CH da Universidade Federal do Ceará - CE
57	AFASTAMENTO POR ATESTADO MÉDICO - ATE 15 DIAS	CH da Universidade Federal do Ceará - CE
57	AFASTAMENTO POR ATESTADO MÉDICO - ATE 15 DIAS	CH da Universidade Federal do Ceará - CE
57	AFASTAMENTO POR ATESTADO MÉDICO - ATE 15 DIAS	CH da Universidade Federal do Ceará - CE
57	AFASTAMENTO POR ATESTADO MÉDICO - ATE 15 DIAS	CH da Universidade Federal do Ceará - CE
57	AFASTAMENTO POR ATESTADO MÉDICO - ATE 15 DIAS	CH da Universidade Federal do Ceará - CE
57	AFASTAMENTO POR ATESTADO MÉDICO - ATE 15 DIAS	CH da Universidade Federal do Ceará - CE
57	AFASTAMENTO POR ATESTADO MÉDICO - ATE 15 DIAS	CH da Universidade Federal do Ceará - CE
57	AFASTAMENTO POR ATESTADO MÉDICO - ATE 15 DIAS	CH da Universidade Federal do Ceará - CE
57	AFASTAMENTO POR ATESTADO MÉDICO - ATE 15 DIAS	CH da Universidade Federal do Ceará - CE
57	AFASTAMENTO POR ATESTADO MÉDICO - ATE 15 DIAS	CH da Universidade Federal do Ceará - CE
57	AFASTAMENTO POR ATESTADO MÉDICO - ATE 15 DIAS	CH da Universidade Federal do Ceará - CE
57	AFASTAMENTO POR ATESTADO MÉDICO - ATE 15 DIAS	CH da Universidade Federal do Ceará - CE
57	AFASTAMENTO POR ATESTADO MÉDICO - ATE 15 DIAS	CH da Universidade Federal do Ceará - CE
57	AFASTAMENTO POR ATESTADO MÉDICO - ATE 15 DIAS	CH da Universidade Federal do Ceará - CE
57	AFASTAMENTO POR ATESTADO MÉDICO - ATE 15 DIAS	CH da Universidade Federal do Ceará - CE
57	AFASTAMENTO POR ATESTADO MÉDICO - ATE 15 DIAS	CH da Universidade Federal do Ceará - CE
57	AFASTAMENTO POR ATESTADO MÉDICO - ATE 15 DIAS	CH da Universidade Federal do Ceará - CE
57	AFASTAMENTO POR ATESTADO MÉDICO - ATE 15 DIAS	CH da Universidade Federal do Ceará - CE
57	AFASTAMENTO POR ATESTADO MÉDICO - ATE 15 DIAS	CH da Universidade Federal do Ceará - CE
57	AFASTAMENTO POR ATESTADO MÉDICO - ATE 15 DIAS	CH da Universidade Federal do Ceará - CE
57	AFASTAMENTO POR ATESTADO MÉDICO - ATE 15 DIAS	CH da Universidade Federal do Ceará - CE
57	AFASTAMENTO POR ATESTADO MÉDICO - ATE 15 DIAS	CH da Universidade Federal do Ceará - CE
57	AFASTAMENTO POR ATESTADO MÉDICO - ATE 15 DIAS	CH da Universidade Federal do Ceará - CE
57	AFASTAMENTO POR ATESTADO MÉDICO - ATE 15 DIAS	CH da Universidade Federal do Ceará - CE
57	AFASTAMENTO POR ATESTADO MÉDICO - ATE 15 DIAS	CH da Universidade Federal do Ceará - CE
57	AFASTAMENTO POR ATESTADO MÉDICO - ATE 15 DIAS	CH da Universidade Federal do Ceará - CE
57	AFASTAMENTO POR ATESTADO MÉDICO - ATE 15 DIAS	CH da Universidade Federal do Ceará - CE
57	AFASTAMENTO POR ATESTADO MÉDICO - ATE 15 DIAS	CH da Universidade Federal do Ceará - CE
57	AFASTAMENTO POR ATESTADO MÉDICO - ATE 15 DIAS	CH da Universidade Federal do Ceará - CE
57	AFASTAMENTO POR ATESTADO MÉDICO - ATE 15 DIAS	CH da Universidade Federal do Ceará - CE

57	AFASTAMENTO POR ATESTADO MÉDICO - ATE 15 DIAS	CH da Universidade Federal do Ceará - CE
57	AFASTAMENTO POR ATESTADO MÉDICO - ATE 15 DIAS	CH da Universidade Federal do Ceará - CE
57	AFASTAMENTO POR ATESTADO MÉDICO - ATE 15 DIAS	CH da Universidade Federal do Ceará - CE
57	AFASTAMENTO POR ATESTADO MÉDICO - ATE 15 DIAS	CH da Universidade Federal do Ceará - CE
57	AFASTAMENTO POR ATESTADO MÉDICO - ATE 15 DIAS	CH da Universidade Federal do Ceará - CE
57	AFASTAMENTO POR ATESTADO MÉDICO - ATE 15 DIAS	CH da Universidade Federal do Ceará - CE
57	AFASTAMENTO POR ATESTADO MÉDICO - ATE 15 DIAS	CH da Universidade Federal do Ceará - CE
57	AFASTAMENTO POR ATESTADO MÉDICO - ATE 15 DIAS	CH da Universidade Federal do Ceará - CE
57	AFASTAMENTO POR ATESTADO MÉDICO - ATE 15 DIAS	CH da Universidade Federal do Ceará - CE
57	AFASTAMENTO POR ATESTADO MÉDICO - ATE 15 DIAS	CH da Universidade Federal do Ceará - CE
57	AFASTAMENTO POR ATESTADO MÉDICO - ATE 15 DIAS	CH da Universidade Federal do Ceará - CE
57	AFASTAMENTO POR ATESTADO MÉDICO - ATE 15 DIAS	CH da Universidade Federal do Ceará - CE
57	AFASTAMENTO POR ATESTADO MÉDICO - ATE 15 DIAS	CH da Universidade Federal do Ceará - CE
57	AFASTAMENTO POR ATESTADO MÉDICO - ATE 15 DIAS	CH da Universidade Federal do Ceará - CE
57	AFASTAMENTO POR ATESTADO MÉDICO - ATE 15 DIAS	CH da Universidade Federal do Ceará - CE
57	AFASTAMENTO POR ATESTADO MÉDICO - ATE 15 DIAS	CH da Universidade Federal do Ceará - CE
57	AFASTAMENTO POR ATESTADO MÉDICO - ATE 15 DIAS	CH da Universidade Federal do Ceará - CE
57	AFASTAMENTO POR ATESTADO MÉDICO - ATE 15 DIAS	CH da Universidade Federal do Ceará - CE
57	AFASTAMENTO POR ATESTADO MÉDICO - ATE 15 DIAS	CH da Universidade Federal do Ceará - CE
57	AFASTAMENTO POR ATESTADO MÉDICO - ATE 15 DIAS	CH da Universidade Federal do Ceará - CE
57	AFASTAMENTO POR ATESTADO MÉDICO - ATE 15 DIAS	CH da Universidade Federal do Ceará - CE
57	AFASTAMENTO POR ATESTADO MÉDICO - ATE 15 DIAS	CH da Universidade Federal do Ceará - CE
57	AFASTAMENTO POR ATESTADO MÉDICO - ATE 15 DIAS	CH da Universidade Federal do Ceará - CE
57	AFASTAMENTO POR ATESTADO MÉDICO - ATE 15 DIAS	CH da Universidade Federal do Ceará - CE
57	AFASTAMENTO POR ATESTADO MÉDICO - ATE 15 DIAS	CH da Universidade Federal do Ceará - CE
57	AFASTAMENTO POR ATESTADO MÉDICO - ATE 15 DIAS	CH da Universidade Federal do Ceará - CE
57	AFASTAMENTO POR ATESTADO MÉDICO - ATE 15 DIAS	CH da Universidade Federal do Ceará - CE
57	AFASTAMENTO POR ATESTADO MÉDICO - ATE 15 DIAS	CH da Universidade Federal do Ceará - CE
57	AFASTAMENTO POR ATESTADO MÉDICO - ATE 15 DIAS	CH da Universidade Federal do Ceará - CE
57	AFASTAMENTO POR ATESTADO MÉDICO - ATE 15 DIAS	CH da Universidade Federal do Ceará - CE
57	AFASTAMENTO POR ATESTADO MÉDICO - ATE 15 DIAS	CH da Universidade Federal do Ceará - CE
57	AFASTAMENTO POR ATESTADO MÉDICO - ATE 15 DIAS	CH da Universidade Federal do Ceará - CE

57	AFASTAMENTO POR ATESTADO MÉDICO - ATE 15 DIAS	CH da Universidade Federal do Ceará - CE
57	AFASTAMENTO POR ATESTADO MÉDICO - ATE 15 DIAS	CH da Universidade Federal do Ceará - CE
57	AFASTAMENTO POR ATESTADO MÉDICO - ATE 15 DIAS	CH da Universidade Federal do Ceará - CE
57	AFASTAMENTO POR ATESTADO MÉDICO - ATE 15 DIAS	CH da Universidade Federal do Ceará - CE
57	AFASTAMENTO POR ATESTADO MÉDICO - ATE 15 DIAS	CH da Universidade Federal do Ceará - CE
57	AFASTAMENTO POR ATESTADO MÉDICO - ATE 15 DIAS	CH da Universidade Federal do Ceará - CE
57	AFASTAMENTO POR ATESTADO MÉDICO - ATE 15 DIAS	CH da Universidade Federal do Ceará - CE
57	AFASTAMENTO POR ATESTADO MÉDICO - ATE 15 DIAS	CH da Universidade Federal do Ceará - CE
57	AFASTAMENTO POR ATESTADO MÉDICO - ATE 15 DIAS	CH da Universidade Federal do Ceará - CE
57	AFASTAMENTO POR ATESTADO MÉDICO - ATE 15 DIAS	CH da Universidade Federal do Ceará - CE
57	AFASTAMENTO POR ATESTADO MÉDICO - ATE 15 DIAS	CH da Universidade Federal do Ceará - CE
57	AFASTAMENTO POR ATESTADO MÉDICO - ATE 15 DIAS	CH da Universidade Federal do Ceará - CE
57	AFASTAMENTO POR ATESTADO MÉDICO - ATE 15 DIAS	CH da Universidade Federal do Ceará - CE
57	AFASTAMENTO POR ATESTADO MÉDICO - ATE 15 DIAS	CH da Universidade Federal do Ceará - CE
57	AFASTAMENTO POR ATESTADO MÉDICO - ATE 15 DIAS	CH da Universidade Federal do Ceará - CE
57	AFASTAMENTO POR ATESTADO MÉDICO - ATE 15 DIAS	CH da Universidade Federal do Ceará - CE
57	AFASTAMENTO POR ATESTADO MÉDICO - ATE 15 DIAS	CH da Universidade Federal do Ceará - CE
57	AFASTAMENTO POR ATESTADO MÉDICO - ATE 15 DIAS	CH da Universidade Federal do Ceará - CE
57	AFASTAMENTO POR ATESTADO MÉDICO - ATE 15 DIAS	CH da Universidade Federal do Ceará - CE
57	AFASTAMENTO POR ATESTADO MÉDICO - ATE 15 DIAS	CH da Universidade Federal do Ceará - CE
57	AFASTAMENTO POR ATESTADO MÉDICO - ATE 15 DIAS	CH da Universidade Federal do Ceará - CE
57	AFASTAMENTO POR ATESTADO MÉDICO - ATE 15 DIAS	CH da Universidade Federal do Ceará - CE
57	AFASTAMENTO POR ATESTADO MÉDICO - ATE 15 DIAS	CH da Universidade Federal do Ceará - CE
57	AFASTAMENTO POR ATESTADO MÉDICO - ATE 15 DIAS	CH da Universidade Federal do Ceará - CE
57	AFASTAMENTO POR ATESTADO MÉDICO - ATE 15 DIAS	CH da Universidade Federal do Ceará - CE
57	AFASTAMENTO POR ATESTADO MÉDICO - ATE 15 DIAS	CH da Universidade Federal do Ceará - CE
57	AFASTAMENTO POR ATESTADO MÉDICO - ATE 15 DIAS	CH da Universidade Federal do Ceará - CE
57	AFASTAMENTO POR ATESTADO MÉDICO - ATE 15 DIAS	CH da Universidade Federal do Ceará - CE
57	AFASTAMENTO POR ATESTADO MÉDICO - ATE 15 DIAS	CH da Universidade Federal do Ceará - CE
57	AFASTAMENTO POR ATESTADO MÉDICO - ATE 15 DIAS	CH da Universidade Federal do Ceará - CE
57	AFASTAMENTO POR ATESTADO MÉDICO - ATE 15 DIAS	CH da Universidade Federal do Ceará - CE
57	AFASTAMENTO POR ATESTADO MÉDICO - ATE 15 DIAS	CH da Universidade Federal do Ceará - CE
57	AFASTAMENTO POR ATESTADO MÉDICO - ATE 15 DIAS	CH da Universidade Federal do Ceará - CE
57	AFASTAMENTO POR ATESTADO MÉDICO - ATE 15 DIAS	CH da Universidade Federal do Ceará - CE
57	AFASTAMENTO POR ATESTADO MÉDICO - ATE 15 DIAS	CH da Universidade Federal do Ceará - CE

57	AFASTAMENTO POR ATESTADO MÉDICO - ATE 15 DIAS	CH da Universidade Federal do Ceará - CE
57	AFASTAMENTO POR ATESTADO MÉDICO - ATE 15 DIAS	CH da Universidade Federal do Ceará - CE
57	AFASTAMENTO POR ATESTADO MÉDICO - ATE 15 DIAS	CH da Universidade Federal do Ceará - CE
57	AFASTAMENTO POR ATESTADO MÉDICO - ATE 15 DIAS	CH da Universidade Federal do Ceará - CE
57	AFASTAMENTO POR ATESTADO MÉDICO - ATE 15 DIAS	CH da Universidade Federal do Ceará - CE
57	AFASTAMENTO POR ATESTADO MÉDICO - ATE 15 DIAS	CH da Universidade Federal do Ceará - CE
57	AFASTAMENTO POR ATESTADO MÉDICO - ATE 15 DIAS	CH da Universidade Federal do Ceará - CE
57	AFASTAMENTO POR ATESTADO MÉDICO - ATE 15 DIAS	CH da Universidade Federal do Ceará - CE
57	AFASTAMENTO POR ATESTADO MÉDICO - ATE 15 DIAS	CH da Universidade Federal do Ceará - CE
57	AFASTAMENTO POR ATESTADO MÉDICO - ATE 15 DIAS	CH da Universidade Federal do Ceará - CE
57	AFASTAMENTO POR ATESTADO MÉDICO - ATE 15 DIAS	CH da Universidade Federal do Ceará - CE
57	AFASTAMENTO POR ATESTADO MÉDICO - ATE 15 DIAS	CH da Universidade Federal do Ceará - CE
57	AFASTAMENTO POR ATESTADO MÉDICO - ATE 15 DIAS	CH da Universidade Federal do Ceará - CE
57	AFASTAMENTO POR ATESTADO MÉDICO - ATE 15 DIAS	CH da Universidade Federal do Ceará - CE
57	AFASTAMENTO POR ATESTADO MÉDICO - ATE 15 DIAS	CH da Universidade Federal do Ceará - CE
57	AFASTAMENTO POR ATESTADO MÉDICO - ATE 15 DIAS	CH da Universidade Federal do Ceará - CE
57	AFASTAMENTO POR ATESTADO MÉDICO - ATE 15 DIAS	CH da Universidade Federal do Ceará - CE
57	AFASTAMENTO POR ATESTADO MÉDICO - ATE 15 DIAS	CH da Universidade Federal do Ceará - CE
57	AFASTAMENTO POR ATESTADO MÉDICO - ATE 15 DIAS	CH da Universidade Federal do Ceará - CE
57	AFASTAMENTO POR ATESTADO MÉDICO - ATE 15 DIAS	CH da Universidade Federal do Ceará - CE
57	AFASTAMENTO POR ATESTADO MÉDICO - ATE 15 DIAS	CH da Universidade Federal do Ceará - CE
57	AFASTAMENTO POR ATESTADO MÉDICO - ATE 15 DIAS	CH da Universidade Federal do Ceará - CE
57	AFASTAMENTO POR ATESTADO MÉDICO - ATE 15 DIAS	CH da Universidade Federal do Ceará - CE
57	AFASTAMENTO POR ATESTADO MÉDICO - ATE 15 DIAS	CH da Universidade Federal do Ceará - CE
57	AFASTAMENTO POR ATESTADO MÉDICO - ATE 15 DIAS	CH da Universidade Federal do Ceará - CE
57	AFASTAMENTO POR ATESTADO MÉDICO - ATE 15 DIAS	CH da Universidade Federal do Ceará - CE
57	AFASTAMENTO POR ATESTADO MÉDICO - ATE 15 DIAS	CH da Universidade Federal do Ceará - CE
57	AFASTAMENTO POR ATESTADO MÉDICO - ATE 15 DIAS	CH da Universidade Federal do Ceará - CE
57	AFASTAMENTO POR ATESTADO MÉDICO - ATE 15 DIAS	CH da Universidade Federal do Ceará - CE
57	AFASTAMENTO POR ATESTADO MÉDICO - ATE 15 DIAS	CH da Universidade Federal do Ceará - CE
57	AFASTAMENTO POR ATESTADO MÉDICO - ATE 15 DIAS	CH da Universidade Federal do Ceará - CE
57	AFASTAMENTO POR ATESTADO MÉDICO - ATE 15 DIAS	CH da Universidade Federal do Ceará - CE
57	AFASTAMENTO POR ATESTADO MÉDICO - ATE 15 DIAS	CH da Universidade Federal do Ceará - CE
57	AFASTAMENTO POR ATESTADO MÉDICO - ATE 15 DIAS	CH da Universidade Federal do Ceará - CE
57	AFASTAMENTO POR ATESTADO MÉDICO - ATE 15 DIAS	CH da Universidade Federal do Ceará - CE
57	AFASTAMENTO POR ATESTADO MÉDICO - ATE 15 DIAS	CH da Universidade Federal do Ceará - CE

57	AFASTAMENTO POR ATESTADO MÉDICO - ATE 15 DIAS	CH da Universidade Federal do Ceará - CE
57	AFASTAMENTO POR ATESTADO MÉDICO - ATE 15 DIAS	CH da Universidade Federal do Ceará - CE
57	AFASTAMENTO POR ATESTADO MÉDICO - ATE 15 DIAS	CH da Universidade Federal do Ceará - CE
57	AFASTAMENTO POR ATESTADO MÉDICO - ATE 15 DIAS	CH da Universidade Federal do Ceará - CE
57	AFASTAMENTO POR ATESTADO MÉDICO - ATE 15 DIAS	CH da Universidade Federal do Ceará - CE
57	AFASTAMENTO POR ATESTADO MÉDICO - ATE 15 DIAS	CH da Universidade Federal do Ceará - CE
57	AFASTAMENTO POR ATESTADO MÉDICO - ATE 15 DIAS	CH da Universidade Federal do Ceará - CE
57	AFASTAMENTO POR ATESTADO MÉDICO - ATE 15 DIAS	CH da Universidade Federal do Ceará - CE
57	AFASTAMENTO POR ATESTADO MÉDICO - ATE 15 DIAS	CH da Universidade Federal do Ceará - CE
57	AFASTAMENTO POR ATESTADO MÉDICO - ATE 15 DIAS	CH da Universidade Federal do Ceará - CE
57	AFASTAMENTO POR ATESTADO MÉDICO - ATE 15 DIAS	CH da Universidade Federal do Ceará - CE
57	AFASTAMENTO POR ATESTADO MÉDICO - ATE 15 DIAS	CH da Universidade Federal do Ceará - CE
57	AFASTAMENTO POR ATESTADO MÉDICO - ATE 15 DIAS	CH da Universidade Federal do Ceará - CE
57	AFASTAMENTO POR ATESTADO MÉDICO - ATE 15 DIAS	CH da Universidade Federal do Ceará - CE
57	AFASTAMENTO POR ATESTADO MÉDICO - ATE 15 DIAS	CH da Universidade Federal do Ceará - CE
57	AFASTAMENTO POR ATESTADO MÉDICO - ATE 15 DIAS	CH da Universidade Federal do Ceará - CE
57	AFASTAMENTO POR ATESTADO MÉDICO - ATE 15 DIAS	CH da Universidade Federal do Ceará - CE
57	AFASTAMENTO POR ATESTADO MÉDICO - ATE 15 DIAS	CH da Universidade Federal do Ceará - CE
57	AFASTAMENTO POR ATESTADO MÉDICO - ATE 15 DIAS	CH da Universidade Federal do Ceará - CE
57	AFASTAMENTO POR ATESTADO MÉDICO - ATE 15 DIAS	CH da Universidade Federal do Ceará - CE
57	AFASTAMENTO POR ATESTADO MÉDICO - ATE 15 DIAS	CH da Universidade Federal do Ceará - CE
57	AFASTAMENTO POR ATESTADO MÉDICO - ATE 15 DIAS	CH da Universidade Federal do Ceará - CE
57	AFASTAMENTO POR ATESTADO MÉDICO - ATE 15 DIAS	CH da Universidade Federal do Ceará - CE
57	AFASTAMENTO POR ATESTADO MÉDICO - ATE 15 DIAS	CH da Universidade Federal do Ceará - CE
57	AFASTAMENTO POR ATESTADO MÉDICO - ATE 15 DIAS	CH da Universidade Federal do Ceará - CE
57	AFASTAMENTO POR ATESTADO MÉDICO - ATE 15 DIAS	CH da Universidade Federal do Ceará - CE
57	AFASTAMENTO POR ATESTADO MÉDICO - ATE 15 DIAS	CH da Universidade Federal do Ceará - CE
57	AFASTAMENTO POR ATESTADO MÉDICO - ATE 15 DIAS	CH da Universidade Federal do Ceará - CE
57	AFASTAMENTO POR ATESTADO MÉDICO - ATE 15 DIAS	CH da Universidade Federal do Ceará - CE
57	AFASTAMENTO POR ATESTADO MÉDICO - ATE 15 DIAS	CH da Universidade Federal do Ceará - CE
57	AFASTAMENTO POR ATESTADO MÉDICO - ATE 15 DIAS	CH da Universidade Federal do Ceará - CE
57	AFASTAMENTO POR ATESTADO MÉDICO - ATE 15 DIAS	CH da Universidade Federal do Ceará - CE
57	AFASTAMENTO POR ATESTADO MÉDICO - ATE 15 DIAS	CH da Universidade Federal do Ceará - CE
57	AFASTAMENTO POR ATESTADO MÉDICO - ATE 15 DIAS	CH da Universidade Federal do Ceará - CE
57	AFASTAMENTO POR ATESTADO MÉDICO - ATE 15 DIAS	CH da Universidade Federal do Ceará - CE
57	AFASTAMENTO POR ATESTADO MÉDICO - ATE 15 DIAS	CH da Universidade Federal do Ceará - CE
57	AFASTAMENTO POR ATESTADO MÉDICO - ATE 15 DIAS	CH da Universidade Federal do Ceará - CE
57	AFASTAMENTO POR ATESTADO MÉDICO - ATE 15 DIAS	CH da Universidade Federal do Ceará - CE
57	AFASTAMENTO POR ATESTADO MÉDICO - ATE 15 DIAS	CH da Universidade Federal do Ceará - CE
57	AFASTAMENTO POR ATESTADO MÉDICO - ATE 15 DIAS	CH da Universidade Federal do Ceará - CE
57	AFASTAMENTO POR ATESTADO MÉDICO - ATE 15 DIAS	CH da Universidade Federal do Ceará - CE
57	AFASTAMENTO POR ATESTADO MÉDICO - ATE 15 DIAS	CH da Universidade Federal do Ceará - CE
57	AFASTAMENTO POR ATESTADO MÉDICO - ATE 15 DIAS	CH da Universidade Federal do Ceará - CE
57	AFASTAMENTO POR ATESTADO MÉDICO - ATE 15 DIAS	CH da Universidade Federal do Ceará - CE
57	AFASTAMENTO POR ATESTADO MÉDICO - ATE 15 DIAS	CH da Universidade Federal do Ceará - CE
57	AFASTAMENTO POR ATESTADO MÉDICO - ATE 15 DIAS	CH da Universidade Federal do Ceará - CE
57	AFASTAMENTO POR ATESTADO MÉDICO - ATE 15 DIAS	CH da Universidade Federal do Ceará - CE
57	AFASTAMENTO POR ATESTADO MÉDICO - ATE 15 DIAS	CH da Universidade Federal do Ceará - CE

57	AFASTAMENTO POR ATESTADO MÉDICO - ATE 15 DIAS	CH da Universidade Federal do Ceará - CE
57	AFASTAMENTO POR ATESTADO MÉDICO - ATE 15 DIAS	CH da Universidade Federal do Ceará - CE
57	AFASTAMENTO POR ATESTADO MÉDICO - ATE 15 DIAS	CH da Universidade Federal do Ceará - CE
57	AFASTAMENTO POR ATESTADO MÉDICO - ATE 15 DIAS	CH da Universidade Federal do Ceará - CE
57	AFASTAMENTO POR ATESTADO MÉDICO - ATE 15 DIAS	CH da Universidade Federal do Ceará - CE
57	AFASTAMENTO POR ATESTADO MÉDICO - ATE 15 DIAS	CH da Universidade Federal do Ceará - CE
57	AFASTAMENTO POR ATESTADO MÉDICO - ATE 15 DIAS	CH da Universidade Federal do Ceará - CE
57	AFASTAMENTO POR ATESTADO MÉDICO - ATE 15 DIAS	CH da Universidade Federal do Ceará - CE
57	AFASTAMENTO POR ATESTADO MÉDICO - ATE 15 DIAS	CH da Universidade Federal do Ceará - CE
57	AFASTAMENTO POR ATESTADO MÉDICO - ATE 15 DIAS	CH da Universidade Federal do Ceará - CE
57	AFASTAMENTO POR ATESTADO MÉDICO - ATE 15 DIAS	CH da Universidade Federal do Ceará - CE
57	AFASTAMENTO POR ATESTADO MÉDICO - ATE 15 DIAS	CH da Universidade Federal do Ceará - CE
57	AFASTAMENTO POR ATESTADO MÉDICO - ATE 15 DIAS	CH da Universidade Federal do Ceará - CE
57	AFASTAMENTO POR ATESTADO MÉDICO - ATE 15 DIAS	CH da Universidade Federal do Ceará - CE
57	AFASTAMENTO POR ATESTADO MÉDICO - ATE 15 DIAS	CH da Universidade Federal do Ceará - CE
57	AFASTAMENTO POR ATESTADO MÉDICO - ATE 15 DIAS	CH da Universidade Federal do Ceará - CE
57	AFASTAMENTO POR ATESTADO MÉDICO - ATE 15 DIAS	CH da Universidade Federal do Ceará - CE
57	AFASTAMENTO POR ATESTADO MÉDICO - ATE 15 DIAS	CH da Universidade Federal do Ceará - CE
57	AFASTAMENTO POR ATESTADO MÉDICO - ATE 15 DIAS	CH da Universidade Federal do Ceará - CE
57	AFASTAMENTO POR ATESTADO MÉDICO - ATE 15 DIAS	CH da Universidade Federal do Ceará - CE
57	AFASTAMENTO POR ATESTADO MÉDICO - ATE 15 DIAS	CH da Universidade Federal do Ceará - CE
57	AFASTAMENTO POR ATESTADO MÉDICO - ATE 15 DIAS	CH da Universidade Federal do Ceará - CE
57	AFASTAMENTO POR ATESTADO MÉDICO - ATE 15 DIAS	CH da Universidade Federal do Ceará - CE
57	AFASTAMENTO POR ATESTADO MÉDICO - ATE 15 DIAS	CH da Universidade Federal do Ceará - CE
57	AFASTAMENTO POR ATESTADO MÉDICO - ATE 15 DIAS	CH da Universidade Federal do Ceará - CE
57	AFASTAMENTO POR ATESTADO MÉDICO - ATE 15 DIAS	CH da Universidade Federal do Ceará - CE
57	AFASTAMENTO POR ATESTADO MÉDICO - ATE 15 DIAS	CH da Universidade Federal do Ceará - CE
57	AFASTAMENTO POR ATESTADO MÉDICO - ATE 15 DIAS	CH da Universidade Federal do Ceará - CE
57	AFASTAMENTO POR ATESTADO MÉDICO - ATE 15 DIAS	CH da Universidade Federal do Ceará - CE
57	AFASTAMENTO POR ATESTADO MÉDICO - ATE 15 DIAS	CH da Universidade Federal do Ceará - CE
57	AFASTAMENTO POR ATESTADO MÉDICO - ATE 15 DIAS	CH da Universidade Federal do Ceará - CE
57	AFASTAMENTO POR ATESTADO MÉDICO - ATE 15 DIAS	CH da Universidade Federal do Ceará - CE
57	AFASTAMENTO POR ATESTADO MÉDICO - ATE 15 DIAS	CH da Universidade Federal do Ceará - CE
57	AFASTAMENTO POR ATESTADO MÉDICO - ATE 15 DIAS	CH da Universidade Federal do Ceará - CE
57	AFASTAMENTO POR ATESTADO MÉDICO - ATE 15 DIAS	CH da Universidade Federal do Ceará - CE
57	AFASTAMENTO POR ATESTADO MÉDICO - ATE 15 DIAS	CH da Universidade Federal do Ceará - CE
57	AFASTAMENTO POR ATESTADO MÉDICO - ATE 15 DIAS	CH da Universidade Federal do Ceará - CE
57	AFASTAMENTO POR ATESTADO MÉDICO - ATE 15 DIAS	CH da Universidade Federal do Ceará - CE
57	AFASTAMENTO POR ATESTADO MÉDICO - ATE 15 DIAS	CH da Universidade Federal do Ceará - CE
57	AFASTAMENTO POR ATESTADO MÉDICO - ATE 15 DIAS	CH da Universidade Federal do Ceará - CE
57	AFASTAMENTO POR ATESTADO MÉDICO - ATE 15 DIAS	CH da Universidade Federal do Ceará - CE
57	AFASTAMENTO POR ATESTADO MÉDICO - ATE 15 DIAS	CH da Universidade Federal do Ceará - CE
57	AFASTAMENTO POR ATESTADO MÉDICO - ATE 15 DIAS	CH da Universidade Federal do Ceará - CE
57	AFASTAMENTO POR ATESTADO MÉDICO - ATE 15 DIAS	CH da Universidade Federal do Ceará - CE
57	AFASTAMENTO POR ATESTADO MÉDICO - ATE 15 DIAS	CH da Universidade Federal do Ceará - CE
57	AFASTAMENTO POR ATESTADO MÉDICO - ATE 15 DIAS	CH da Universidade Federal do Ceará - CE
57	AFASTAMENTO POR ATESTADO MÉDICO - ATE 15 DIAS	CH da Universidade Federal do Ceará - CE
57	AFASTAMENTO POR ATESTADO MÉDICO - ATE 15 DIAS	CH da Universidade Federal do Ceará - CE

57	AFASTAMENTO POR ATESTADO MÉDICO - ATE 15 DIAS	CH da Universidade Federal do Ceará - CE
57	AFASTAMENTO POR ATESTADO MÉDICO - ATE 15 DIAS	CH da Universidade Federal do Ceará - CE
57	AFASTAMENTO POR ATESTADO MÉDICO - ATE 15 DIAS	CH da Universidade Federal do Ceará - CE
57	AFASTAMENTO POR ATESTADO MÉDICO - ATE 15 DIAS	CH da Universidade Federal do Ceará - CE
57	AFASTAMENTO POR ATESTADO MÉDICO - ATE 15 DIAS	CH da Universidade Federal do Ceará - CE
57	AFASTAMENTO POR ATESTADO MÉDICO - ATE 15 DIAS	CH da Universidade Federal do Ceará - CE
57	AFASTAMENTO POR ATESTADO MÉDICO - ATE 15 DIAS	CH da Universidade Federal do Ceará - CE
57	AFASTAMENTO POR ATESTADO MÉDICO - ATE 15 DIAS	CH da Universidade Federal do Ceará - CE
57	AFASTAMENTO POR ATESTADO MÉDICO - ATE 15 DIAS	CH da Universidade Federal do Ceará - CE
57	AFASTAMENTO POR ATESTADO MÉDICO - ATE 15 DIAS	CH da Universidade Federal do Ceará - CE
57	AFASTAMENTO POR ATESTADO MÉDICO - ATE 15 DIAS	CH da Universidade Federal do Ceará - CE
57	AFASTAMENTO POR ATESTADO MÉDICO - ATE 15 DIAS	CH da Universidade Federal do Ceará - CE
57	AFASTAMENTO POR ATESTADO MÉDICO - ATE 15 DIAS	CH da Universidade Federal do Ceará - CE
57	AFASTAMENTO POR ATESTADO MÉDICO - ATE 15 DIAS	CH da Universidade Federal do Ceará - CE
57	AFASTAMENTO POR ATESTADO MÉDICO - ATE 15 DIAS	CH da Universidade Federal do Ceará - CE
57	AFASTAMENTO POR ATESTADO MÉDICO - ATE 15 DIAS	CH da Universidade Federal do Ceará - CE
57	AFASTAMENTO POR ATESTADO MÉDICO - ATE 15 DIAS	CH da Universidade Federal do Ceará - CE
57	AFASTAMENTO POR ATESTADO MÉDICO - ATE 15 DIAS	CH da Universidade Federal do Ceará - CE
57	AFASTAMENTO POR ATESTADO MÉDICO - ATE 15 DIAS	CH da Universidade Federal do Ceará - CE
57	AFASTAMENTO POR ATESTADO MÉDICO - ATE 15 DIAS	CH da Universidade Federal do Ceará - CE
57	AFASTAMENTO POR ATESTADO MÉDICO - ATE 15 DIAS	CH da Universidade Federal do Ceará - CE
57	AFASTAMENTO POR ATESTADO MÉDICO - ATE 15 DIAS	CH da Universidade Federal do Ceará - CE
57	AFASTAMENTO POR ATESTADO MÉDICO - ATE 15 DIAS	CH da Universidade Federal do Ceará - CE
57	AFASTAMENTO POR ATESTADO MÉDICO - ATE 15 DIAS	CH da Universidade Federal do Ceará - CE
57	AFASTAMENTO POR ATESTADO MÉDICO - ATE 15 DIAS	CH da Universidade Federal do Ceará - CE
57	AFASTAMENTO POR ATESTADO MÉDICO - ATE 15 DIAS	CH da Universidade Federal do Ceará - CE
57	AFASTAMENTO POR ATESTADO MÉDICO - ATE 15 DIAS	CH da Universidade Federal do Ceará - CE
57	AFASTAMENTO POR ATESTADO MÉDICO - ATE 15 DIAS	CH da Universidade Federal do Ceará - CE
57	AFASTAMENTO POR ATESTADO MÉDICO - ATE 15 DIAS	CH da Universidade Federal do Ceará - CE
57	AFASTAMENTO POR ATESTADO MÉDICO - ATE 15 DIAS	CH da Universidade Federal do Ceará - CE
57	AFASTAMENTO POR ATESTADO MÉDICO - ATE 15 DIAS	CH da Universidade Federal do Ceará - CE
57	AFASTAMENTO POR ATESTADO MÉDICO - ATE 15 DIAS	CH da Universidade Federal do Ceará - CE
57	AFASTAMENTO POR ATESTADO MÉDICO - ATE 15 DIAS	CH da Universidade Federal do Ceará - CE
57	AFASTAMENTO POR ATESTADO MÉDICO - ATE 15 DIAS	CH da Universidade Federal do Ceará - CE
57	AFASTAMENTO POR ATESTADO MÉDICO - ATE 15 DIAS	CH da Universidade Federal do Ceará - CE
57	AFASTAMENTO POR ATESTADO MÉDICO - ATE 15 DIAS	CH da Universidade Federal do Ceará - CE
57	AFASTAMENTO POR ATESTADO MÉDICO - ATE 15 DIAS	CH da Universidade Federal do Ceará - CE
57	AFASTAMENTO POR ATESTADO MÉDICO - ATE 15 DIAS	CH da Universidade Federal do Ceará - CE
57	AFASTAMENTO POR ATESTADO MÉDICO - ATE 15 DIAS	CH da Universidade Federal do Ceará - CE
57	AFASTAMENTO POR ATESTADO MÉDICO - ATE 15 DIAS	CH da Universidade Federal do Ceará - CE
57	AFASTAMENTO POR ATESTADO MÉDICO - ATE 15 DIAS	CH da Universidade Federal do Ceará - CE
57	AFASTAMENTO POR ATESTADO MÉDICO - ATE 15 DIAS	CH da Universidade Federal do Ceará - CE
57	AFASTAMENTO POR ATESTADO MÉDICO - ATE 15 DIAS	CH da Universidade Federal do Ceará - CE
57	AFASTAMENTO POR ATESTADO MÉDICO - ATE 15 DIAS	CH da Universidade Federal do Ceará - CE
57	AFASTAMENTO POR ATESTADO MÉDICO - ATE 15 DIAS	CH da Universidade Federal do Ceará - CE
57	AFASTAMENTO POR ATESTADO MÉDICO - ATE 15 DIAS	CH da Universidade Federal do Ceará - CE

57	AFASTAMENTO POR ATESTADO MÉDICO - ATE 15 DIAS	CH da Universidade Federal do Ceará - CE
57	AFASTAMENTO POR ATESTADO MÉDICO - ATE 15 DIAS	CH da Universidade Federal do Ceará - CE
57	AFASTAMENTO POR ATESTADO MÉDICO - ATE 15 DIAS	CH da Universidade Federal do Ceará - CE
57	AFASTAMENTO POR ATESTADO MÉDICO - ATE 15 DIAS	CH da Universidade Federal do Ceará - CE
57	AFASTAMENTO POR ATESTADO MÉDICO - ATE 15 DIAS	CH da Universidade Federal do Ceará - CE
57	AFASTAMENTO POR ATESTADO MÉDICO - ATE 15 DIAS	CH da Universidade Federal do Ceará - CE
57	AFASTAMENTO POR ATESTADO MÉDICO - ATE 15 DIAS	CH da Universidade Federal do Ceará - CE
57	AFASTAMENTO POR ATESTADO MÉDICO - ATE 15 DIAS	CH da Universidade Federal do Ceará - CE
57	AFASTAMENTO POR ATESTADO MÉDICO - ATE 15 DIAS	CH da Universidade Federal do Ceará - CE
57	AFASTAMENTO POR ATESTADO MÉDICO - ATE 15 DIAS	CH da Universidade Federal do Ceará - CE
57	AFASTAMENTO POR ATESTADO MÉDICO - ATE 15 DIAS	CH da Universidade Federal do Ceará - CE
57	AFASTAMENTO POR ATESTADO MÉDICO - ATE 15 DIAS	CH da Universidade Federal do Ceará - CE
57	AFASTAMENTO POR ATESTADO MÉDICO - ATE 15 DIAS	CH da Universidade Federal do Ceará - CE
57	AFASTAMENTO POR ATESTADO MÉDICO - ATE 15 DIAS	CH da Universidade Federal do Ceará - CE
57	AFASTAMENTO POR ATESTADO MÉDICO - ATE 15 DIAS	CH da Universidade Federal do Ceará - CE
57	AFASTAMENTO POR ATESTADO MÉDICO - ATE 15 DIAS	CH da Universidade Federal do Ceará - CE
57	AFASTAMENTO POR ATESTADO MÉDICO - ATE 15 DIAS	CH da Universidade Federal do Ceará - CE
57	AFASTAMENTO POR ATESTADO MÉDICO - ATE 15 DIAS	CH da Universidade Federal do Ceará - CE
57	AFASTAMENTO POR ATESTADO MÉDICO - ATE 15 DIAS	CH da Universidade Federal do Ceará - CE
57	AFASTAMENTO POR ATESTADO MÉDICO - ATE 15 DIAS	CH da Universidade Federal do Ceará - CE
57	AFASTAMENTO POR ATESTADO MÉDICO - ATE 15 DIAS	CH da Universidade Federal do Ceará - CE
57	AFASTAMENTO POR ATESTADO MÉDICO - ATE 15 DIAS	CH da Universidade Federal do Ceará - CE
57	AFASTAMENTO POR ATESTADO MÉDICO - ATE 15 DIAS	CH da Universidade Federal do Ceará - CE
57	AFASTAMENTO POR ATESTADO MÉDICO - ATE 15 DIAS	CH da Universidade Federal do Ceará - CE
57	AFASTAMENTO POR ATESTADO MÉDICO - ATE 15 DIAS	CH da Universidade Federal do Ceará - CE
57	AFASTAMENTO POR ATESTADO MÉDICO - ATE 15 DIAS	CH da Universidade Federal do Ceará - CE
57	AFASTAMENTO POR ATESTADO MÉDICO - ATE 15 DIAS	CH da Universidade Federal do Ceará - CE
57	AFASTAMENTO POR ATESTADO MÉDICO - ATE 15 DIAS	CH da Universidade Federal do Ceará - CE
57	AFASTAMENTO POR ATESTADO MÉDICO - ATE 15 DIAS	CH da Universidade Federal do Ceará - CE
57	AFASTAMENTO POR ATESTADO MÉDICO - ATE 15 DIAS	CH da Universidade Federal do Ceará - CE
57	AFASTAMENTO POR ATESTADO MÉDICO - ATE 15 DIAS	CH da Universidade Federal do Ceará - CE
57	AFASTAMENTO POR ATESTADO MÉDICO - ATE 15 DIAS	CH da Universidade Federal do Ceará - CE

57	AFASTAMENTO POR ATESTADO MÉDICO - ATE 15 DIAS	CH da Universidade Federal do Ceará - CE
57	AFASTAMENTO POR ATESTADO MÉDICO - ATE 15 DIAS	CH da Universidade Federal do Ceará - CE
57	AFASTAMENTO POR ATESTADO MÉDICO - ATE 15 DIAS	CH da Universidade Federal do Ceará - CE
57	AFASTAMENTO POR ATESTADO MÉDICO - ATE 15 DIAS	CH da Universidade Federal do Ceará - CE
57	AFASTAMENTO POR ATESTADO MÉDICO - ATE 15 DIAS	CH da Universidade Federal do Ceará - CE
57	AFASTAMENTO POR ATESTADO MÉDICO - ATE 15 DIAS	CH da Universidade Federal do Ceará - CE
57	AFASTAMENTO POR ATESTADO MÉDICO - ATE 15 DIAS	CH da Universidade Federal do Ceará - CE
57	AFASTAMENTO POR ATESTADO MÉDICO - ATE 15 DIAS	CH da Universidade Federal do Ceará - CE
57	AFASTAMENTO POR ATESTADO MÉDICO - ATE 15 DIAS	CH da Universidade Federal do Ceará - CE
57	AFASTAMENTO POR ATESTADO MÉDICO - ATE 15 DIAS	CH da Universidade Federal do Ceará - CE
57	AFASTAMENTO POR ATESTADO MÉDICO - ATE 15 DIAS	CH da Universidade Federal do Ceará - CE
57	AFASTAMENTO POR ATESTADO MÉDICO - ATE 15 DIAS	CH da Universidade Federal do Ceará - CE
57	AFASTAMENTO POR ATESTADO MÉDICO - ATE 15 DIAS	CH da Universidade Federal do Ceará - CE
57	AFASTAMENTO POR ATESTADO MÉDICO - ATE 15 DIAS	CH da Universidade Federal do Ceará - CE
57	AFASTAMENTO POR ATESTADO MÉDICO - ATE 15 DIAS	CH da Universidade Federal do Ceará - CE
57	AFASTAMENTO POR ATESTADO MÉDICO - ATE 15 DIAS	CH da Universidade Federal do Ceará - CE
57	AFASTAMENTO POR ATESTADO MÉDICO - ATE 15 DIAS	CH da Universidade Federal do Ceará - CE
57	AFASTAMENTO POR ATESTADO MÉDICO - ATE 15 DIAS	CH da Universidade Federal do Ceará - CE
57	AFASTAMENTO POR ATESTADO MÉDICO - ATE 15 DIAS	CH da Universidade Federal do Ceará - CE
57	AFASTAMENTO POR ATESTADO MÉDICO - ATE 15 DIAS	CH da Universidade Federal do Ceará - CE
57	AFASTAMENTO POR ATESTADO MÉDICO - ATE 15 DIAS	CH da Universidade Federal do Ceará - CE
57	AFASTAMENTO POR ATESTADO MÉDICO - ATE 15 DIAS	CH da Universidade Federal do Ceará - CE
57	AFASTAMENTO POR ATESTADO MÉDICO - ATE 15 DIAS	CH da Universidade Federal do Ceará - CE
57	AFASTAMENTO POR ATESTADO MÉDICO - ATE 15 DIAS	CH da Universidade Federal do Ceará - CE
57	AFASTAMENTO POR ATESTADO MÉDICO - ATE 15 DIAS	CH da Universidade Federal do Ceará - CE
57	AFASTAMENTO POR ATESTADO MÉDICO - ATE 15 DIAS	CH da Universidade Federal do Ceará - CE
57	AFASTAMENTO POR ATESTADO MÉDICO - ATE 15 DIAS	CH da Universidade Federal do Ceará - CE
57	AFASTAMENTO POR ATESTADO MÉDICO - ATE 15 DIAS	CH da Universidade Federal do Ceará - CE
57	AFASTAMENTO POR ATESTADO MÉDICO - ATE 15 DIAS	CH da Universidade Federal do Ceará - CE
57	AFASTAMENTO POR ATESTADO MÉDICO - ATE 15 DIAS	CH da Universidade Federal do Ceará - CE
57	AFASTAMENTO POR ATESTADO MÉDICO - ATE 15 DIAS	CH da Universidade Federal do Ceará - CE
57	AFASTAMENTO POR ATESTADO MÉDICO - ATE 15 DIAS	CH da Universidade Federal do Ceará - CE
57	AFASTAMENTO POR ATESTADO MÉDICO - ATE 15 DIAS	CH da Universidade Federal do Ceará - CE
57	AFASTAMENTO POR ATESTADO MÉDICO - ATE 15 DIAS	CH da Universidade Federal do Ceará - CE
57	AFASTAMENTO POR ATESTADO MÉDICO - ATE 15 DIAS	CH da Universidade Federal do Ceará - CE
57	AFASTAMENTO POR ATESTADO MÉDICO - ATE 15 DIAS	CH da Universidade Federal do Ceará - CE
57	AFASTAMENTO POR ATESTADO MÉDICO - ATE 15 DIAS	CH da Universidade Federal do Ceará - CE
57	AFASTAMENTO POR ATESTADO MÉDICO - ATE 15 DIAS	CH da Universidade Federal do Ceará - CE
57	AFASTAMENTO POR ATESTADO MÉDICO - ATE 15 DIAS	CH da Universidade Federal do Ceará - CE
57	AFASTAMENTO POR ATESTADO MÉDICO - ATE 15 DIAS	CH da Universidade Federal do Ceará - CE
57	AFASTAMENTO POR ATESTADO MÉDICO - ATE 15 DIAS	CH da Universidade Federal do Ceará - CE

57	AFASTAMENTO POR ATESTADO MÉDICO - ATE 15 DIAS	CH da Universidade Federal do Ceará - CE
57	AFASTAMENTO POR ATESTADO MÉDICO - ATE 15 DIAS	CH da Universidade Federal do Ceará - CE
57	AFASTAMENTO POR ATESTADO MÉDICO - ATE 15 DIAS	CH da Universidade Federal do Ceará - CE
57	AFASTAMENTO POR ATESTADO MÉDICO - ATE 15 DIAS	CH da Universidade Federal do Ceará - CE
57	AFASTAMENTO POR ATESTADO MÉDICO - ATE 15 DIAS	CH da Universidade Federal do Ceará - CE
57	AFASTAMENTO POR ATESTADO MÉDICO - ATE 15 DIAS	CH da Universidade Federal do Ceará - CE
57	AFASTAMENTO POR ATESTADO MÉDICO - ATE 15 DIAS	CH da Universidade Federal do Ceará - CE
57	AFASTAMENTO POR ATESTADO MÉDICO - ATE 15 DIAS	CH da Universidade Federal do Ceará - CE
57	AFASTAMENTO POR ATESTADO MÉDICO - ATE 15 DIAS	CH da Universidade Federal do Ceará - CE
57	AFASTAMENTO POR ATESTADO MÉDICO - ATE 15 DIAS	CH da Universidade Federal do Ceará - CE
57	AFASTAMENTO POR ATESTADO MÉDICO - ATE 15 DIAS	CH da Universidade Federal do Ceará - CE
57	AFASTAMENTO POR ATESTADO MÉDICO - ATE 15 DIAS	CH da Universidade Federal do Ceará - CE
57	AFASTAMENTO POR ATESTADO MÉDICO - ATE 15 DIAS	CH da Universidade Federal do Ceará - CE
57	AFASTAMENTO POR ATESTADO MÉDICO - ATE 15 DIAS	CH da Universidade Federal do Ceará - CE
57	AFASTAMENTO POR ATESTADO MÉDICO - ATE 15 DIAS	CH da Universidade Federal do Ceará - CE
57	AFASTAMENTO POR ATESTADO MÉDICO - ATE 15 DIAS	CH da Universidade Federal do Ceará - CE
57	AFASTAMENTO POR ATESTADO MÉDICO - ATE 15 DIAS	CH da Universidade Federal do Ceará - CE
57	AFASTAMENTO POR ATESTADO MÉDICO - ATE 15 DIAS	CH da Universidade Federal do Ceará - CE
57	AFASTAMENTO POR ATESTADO MÉDICO - ATE 15 DIAS	CH da Universidade Federal do Ceará - CE
57	AFASTAMENTO POR ATESTADO MÉDICO - ATE 15 DIAS	CH da Universidade Federal do Ceará - CE
57	AFASTAMENTO POR ATESTADO MÉDICO - ATE 15 DIAS	CH da Universidade Federal do Ceará - CE
57	AFASTAMENTO POR ATESTADO MÉDICO - ATE 15 DIAS	CH da Universidade Federal do Ceará - CE
57	AFASTAMENTO POR ATESTADO MÉDICO - ATE 15 DIAS	CH da Universidade Federal do Ceará - CE
57	AFASTAMENTO POR ATESTADO MÉDICO - ATE 15 DIAS	CH da Universidade Federal do Ceará - CE
57	AFASTAMENTO POR ATESTADO MÉDICO - ATE 15 DIAS	CH da Universidade Federal do Ceará - CE
57	AFASTAMENTO POR ATESTADO MÉDICO - ATE 15 DIAS	CH da Universidade Federal do Ceará - CE
57	AFASTAMENTO POR ATESTADO MÉDICO - ATE 15 DIAS	CH da Universidade Federal do Ceará - CE
57	AFASTAMENTO POR ATESTADO MÉDICO - ATE 15 DIAS	CH da Universidade Federal do Ceará - CE
57	AFASTAMENTO POR ATESTADO MÉDICO - ATE 15 DIAS	CH da Universidade Federal do Ceará - CE
57	AFASTAMENTO POR ATESTADO MÉDICO - ATE 15 DIAS	CH da Universidade Federal do Ceará - CE
57	AFASTAMENTO POR ATESTADO MÉDICO - ATE 15 DIAS	CH da Universidade Federal do Ceará - CE
57	AFASTAMENTO POR ATESTADO MÉDICO - ATE 15 DIAS	CH da Universidade Federal do Ceará - CE
57	AFASTAMENTO POR ATESTADO MÉDICO - ATE 15 DIAS	CH da Universidade Federal do Ceará - CE
57	AFASTAMENTO POR ATESTADO MÉDICO - ATE 15 DIAS	CH da Universidade Federal do Ceará - CE
57	AFASTAMENTO POR ATESTADO MÉDICO - ATE 15 DIAS	CH da Universidade Federal do Ceará - CE
57	AFASTAMENTO POR ATESTADO MÉDICO - ATE 15 DIAS	CH da Universidade Federal do Ceará - CE
57	AFASTAMENTO POR ATESTADO MÉDICO - ATE 15 DIAS	CH da Universidade Federal do Ceará - CE
57	AFASTAMENTO POR ATESTADO MÉDICO - ATE 15 DIAS	CH da Universidade Federal do Ceará - CE
57	AFASTAMENTO POR ATESTADO MÉDICO - ATE 15 DIAS	CH da Universidade Federal do Ceará - CE
57	AFASTAMENTO POR ATESTADO MÉDICO - ATE 15 DIAS	CH da Universidade Federal do Ceará - CE
57	AFASTAMENTO POR ATESTADO MÉDICO - ATE 15 DIAS	CH da Universidade Federal do Ceará - CE

57	AFASTAMENTO POR ATESTADO MÉDICO - ATE 15 DIAS	CH da Universidade Federal do Ceará - CE
57	AFASTAMENTO POR ATESTADO MÉDICO - ATE 15 DIAS	CH da Universidade Federal do Ceará - CE
57	AFASTAMENTO POR ATESTADO MÉDICO - ATE 15 DIAS	CH da Universidade Federal do Ceará - CE
57	AFASTAMENTO POR ATESTADO MÉDICO - ATE 15 DIAS	CH da Universidade Federal do Ceará - CE
57	AFASTAMENTO POR ATESTADO MÉDICO - ATE 15 DIAS	CH da Universidade Federal do Ceará - CE
57	AFASTAMENTO POR ATESTADO MÉDICO - ATE 15 DIAS	CH da Universidade Federal do Ceará - CE
57	AFASTAMENTO POR ATESTADO MÉDICO - ATE 15 DIAS	CH da Universidade Federal do Ceará - CE
57	AFASTAMENTO POR ATESTADO MÉDICO - ATE 15 DIAS	CH da Universidade Federal do Ceará - CE
57	AFASTAMENTO POR ATESTADO MÉDICO - ATE 15 DIAS	CH da Universidade Federal do Ceará - CE
57	AFASTAMENTO POR ATESTADO MÉDICO - ATE 15 DIAS	CH da Universidade Federal do Ceará - CE
57	AFASTAMENTO POR ATESTADO MÉDICO - ATE 15 DIAS	CH da Universidade Federal do Ceará - CE
57	AFASTAMENTO POR ATESTADO MÉDICO - ATE 15 DIAS	CH da Universidade Federal do Ceará - CE
57	AFASTAMENTO POR ATESTADO MÉDICO - ATE 15 DIAS	CH da Universidade Federal do Ceará - CE
57	AFASTAMENTO POR ATESTADO MÉDICO - ATE 15 DIAS	CH da Universidade Federal do Ceará - CE
57	AFASTAMENTO POR ATESTADO MÉDICO - ATE 15 DIAS	CH da Universidade Federal do Ceará - CE
57	AFASTAMENTO POR ATESTADO MÉDICO - ATE 15 DIAS	CH da Universidade Federal do Ceará - CE
57	AFASTAMENTO POR ATESTADO MÉDICO - ATE 15 DIAS	CH da Universidade Federal do Ceará - CE
57	AFASTAMENTO POR ATESTADO MÉDICO - ATE 15 DIAS	CH da Universidade Federal do Ceará - CE
57	AFASTAMENTO POR ATESTADO MÉDICO - ATE 15 DIAS	CH da Universidade Federal do Ceará - CE
57	AFASTAMENTO POR ATESTADO MÉDICO - ATE 15 DIAS	CH da Universidade Federal do Ceará - CE
57	AFASTAMENTO POR ATESTADO MÉDICO - ATE 15 DIAS	CH da Universidade Federal do Ceará - CE
57	AFASTAMENTO POR ATESTADO MÉDICO - ATE 15 DIAS	CH da Universidade Federal do Ceará - CE
57	AFASTAMENTO POR ATESTADO MÉDICO - ATE 15 DIAS	CH da Universidade Federal do Ceará - CE
57	AFASTAMENTO POR ATESTADO MÉDICO - ATE 15 DIAS	CH da Universidade Federal do Ceará - CE
57	AFASTAMENTO POR ATESTADO MÉDICO - ATE 15 DIAS	CH da Universidade Federal do Ceará - CE
57	AFASTAMENTO POR ATESTADO MÉDICO - ATE 15 DIAS	CH da Universidade Federal do Ceará - CE
57	AFASTAMENTO POR ATESTADO MÉDICO - ATE 15 DIAS	CH da Universidade Federal do Ceará - CE
57	AFASTAMENTO POR ATESTADO MÉDICO - ATE 15 DIAS	CH da Universidade Federal do Ceará - CE
57	AFASTAMENTO POR ATESTADO MÉDICO - ATE 15 DIAS	CH da Universidade Federal do Ceará - CE
57	AFASTAMENTO POR ATESTADO MÉDICO - ATE 15 DIAS	CH da Universidade Federal do Ceará - CE
57	AFASTAMENTO POR ATESTADO MÉDICO - ATE 15 DIAS	CH da Universidade Federal do Ceará - CE
57	AFASTAMENTO POR ATESTADO MÉDICO - ATE 15 DIAS	CH da Universidade Federal do Ceará - CE

57	AFASTAMENTO POR ATESTADO MÉDICO - ATE 15 DIAS	CH da Universidade Federal do Ceará - CE
57	AFASTAMENTO POR ATESTADO MÉDICO - ATE 15 DIAS	CH da Universidade Federal do Ceará - CE
57	AFASTAMENTO POR ATESTADO MÉDICO - ATE 15 DIAS	CH da Universidade Federal do Ceará - CE
57	AFASTAMENTO POR ATESTADO MÉDICO - ATE 15 DIAS	CH da Universidade Federal do Ceará - CE
57	AFASTAMENTO POR ATESTADO MÉDICO - ATE 15 DIAS	CH da Universidade Federal do Ceará - CE
57	AFASTAMENTO POR ATESTADO MÉDICO - ATE 15 DIAS	CH da Universidade Federal do Ceará - CE
57	AFASTAMENTO POR ATESTADO MÉDICO - ATE 15 DIAS	CH da Universidade Federal do Ceará - CE
57	AFASTAMENTO POR ATESTADO MÉDICO - ATE 15 DIAS	CH da Universidade Federal do Ceará - CE
57	AFASTAMENTO POR ATESTADO MÉDICO - ATE 15 DIAS	CH da Universidade Federal do Ceará - CE
57	AFASTAMENTO POR ATESTADO MÉDICO - ATE 15 DIAS	CH da Universidade Federal do Ceará - CE
57	AFASTAMENTO POR ATESTADO MÉDICO - ATE 15 DIAS	CH da Universidade Federal do Ceará - CE
57	AFASTAMENTO POR ATESTADO MÉDICO - ATE 15 DIAS	CH da Universidade Federal do Ceará - CE
57	AFASTAMENTO POR ATESTADO MÉDICO - ATE 15 DIAS	CH da Universidade Federal do Ceará - CE
57	AFASTAMENTO POR ATESTADO MÉDICO - ATE 15 DIAS	CH da Universidade Federal do Ceará - CE
57	AFASTAMENTO POR ATESTADO MÉDICO - ATE 15 DIAS	CH da Universidade Federal do Ceará - CE
57	AFASTAMENTO POR ATESTADO MÉDICO - ATE 15 DIAS	CH da Universidade Federal do Ceará - CE
57	AFASTAMENTO POR ATESTADO MÉDICO - ATE 15 DIAS	CH da Universidade Federal do Ceará - CE
57	AFASTAMENTO POR ATESTADO MÉDICO - ATE 15 DIAS	CH da Universidade Federal do Ceará - CE
57	AFASTAMENTO POR ATESTADO MÉDICO - ATE 15 DIAS	CH da Universidade Federal do Ceará - CE
57	AFASTAMENTO POR ATESTADO MÉDICO - ATE 15 DIAS	CH da Universidade Federal do Ceará - CE
57	AFASTAMENTO POR ATESTADO MÉDICO - ATE 15 DIAS	CH da Universidade Federal do Ceará - CE
57	AFASTAMENTO POR ATESTADO MÉDICO - ATE 15 DIAS	CH da Universidade Federal do Ceará - CE
57	AFASTAMENTO POR ATESTADO MÉDICO - ATE 15 DIAS	CH da Universidade Federal do Ceará - CE
57	AFASTAMENTO POR ATESTADO MÉDICO - ATE 15 DIAS	CH da Universidade Federal do Ceará - CE
57	AFASTAMENTO POR ATESTADO MÉDICO - ATE 15 DIAS	CH da Universidade Federal do Ceará - CE
57	AFASTAMENTO POR ATESTADO MÉDICO - ATE 15 DIAS	CH da Universidade Federal do Ceará - CE
57	AFASTAMENTO POR ATESTADO MÉDICO - ATE 15 DIAS	CH da Universidade Federal do Ceará - CE
57	AFASTAMENTO POR ATESTADO MÉDICO - ATE 15 DIAS	CH da Universidade Federal do Ceará - CE
57	AFASTAMENTO POR ATESTADO MÉDICO - ATE 15 DIAS	CH da Universidade Federal do Ceará - CE
57	AFASTAMENTO POR ATESTADO MÉDICO - ATE 15 DIAS	CH da Universidade Federal do Ceará - CE
57	AFASTAMENTO POR ATESTADO MÉDICO - ATE 15 DIAS	CH da Universidade Federal do Ceará - CE
57	AFASTAMENTO POR ATESTADO MÉDICO - ATE 15 DIAS	CH da Universidade Federal do Ceará - CE

57	AFASTAMENTO POR ATESTADO MÉDICO - ATE 15 DIAS	CH da Universidade Federal do Ceará - CE
57	AFASTAMENTO POR ATESTADO MÉDICO - ATE 15 DIAS	CH da Universidade Federal do Ceará - CE
57	AFASTAMENTO POR ATESTADO MÉDICO - ATE 15 DIAS	CH da Universidade Federal do Ceará - CE
57	AFASTAMENTO POR ATESTADO MÉDICO - ATE 15 DIAS	CH da Universidade Federal do Ceará - CE
57	AFASTAMENTO POR ATESTADO MÉDICO - ATE 15 DIAS	CH da Universidade Federal do Ceará - CE
57	AFASTAMENTO POR ATESTADO MÉDICO - ATE 15 DIAS	CH da Universidade Federal do Ceará - CE
57	AFASTAMENTO POR ATESTADO MÉDICO - ATE 15 DIAS	CH da Universidade Federal do Ceará - CE
57	AFASTAMENTO POR ATESTADO MÉDICO - ATE 15 DIAS	CH da Universidade Federal do Ceará - CE
57	AFASTAMENTO POR ATESTADO MÉDICO - ATE 15 DIAS	CH da Universidade Federal do Ceará - CE
57	AFASTAMENTO POR ATESTADO MÉDICO - ATE 15 DIAS	CH da Universidade Federal do Ceará - CE
57	AFASTAMENTO POR ATESTADO MÉDICO - ATE 15 DIAS	CH da Universidade Federal do Ceará - CE
57	AFASTAMENTO POR ATESTADO MÉDICO - ATE 15 DIAS	CH da Universidade Federal do Ceará - CE
57	AFASTAMENTO POR ATESTADO MÉDICO - ATE 15 DIAS	CH da Universidade Federal do Ceará - CE
57	AFASTAMENTO POR ATESTADO MÉDICO - ATE 15 DIAS	CH da Universidade Federal do Ceará - CE
57	AFASTAMENTO POR ATESTADO MÉDICO - ATE 15 DIAS	CH da Universidade Federal do Ceará - CE
57	AFASTAMENTO POR ATESTADO MÉDICO - ATE 15 DIAS	CH da Universidade Federal do Ceará - CE
57	AFASTAMENTO POR ATESTADO MÉDICO - ATE 15 DIAS	CH da Universidade Federal do Ceará - CE
57	AFASTAMENTO POR ATESTADO MÉDICO - ATE 15 DIAS	CH da Universidade Federal do Ceará - CE
57	AFASTAMENTO POR ATESTADO MÉDICO - ATE 15 DIAS	CH da Universidade Federal do Ceará - CE
57	AFASTAMENTO POR ATESTADO MÉDICO - ATE 15 DIAS	CH da Universidade Federal do Ceará - CE
57	AFASTAMENTO POR ATESTADO MÉDICO - ATE 15 DIAS	CH da Universidade Federal do Ceará - CE
57	AFASTAMENTO POR ATESTADO MÉDICO - ATE 15 DIAS	CH da Universidade Federal do Ceará - CE
57	AFASTAMENTO POR ATESTADO MÉDICO - ATE 15 DIAS	CH da Universidade Federal do Ceará - CE
58	AFASTAMENTO POR ATESTADO ODONTOLOGICO - ATE 15 DIAS	CH da Universidade Federal do Ceará - CE
58	AFASTAMENTO POR ATESTADO ODONTOLOGICO - ATE 15 DIAS	CH da Universidade Federal do Ceará - CE
58	AFASTAMENTO POR ATESTADO ODONTOLOGICO - ATE 15 DIAS	CH da Universidade Federal do Ceará - CE
58	AFASTAMENTO POR ATESTADO ODONTOLOGICO - ATE 15 DIAS	CH da Universidade Federal do Ceará - CE
58	AFASTAMENTO POR ATESTADO ODONTOLOGICO - ATE 15 DIAS	CH da Universidade Federal do Ceará - CE
58	AFASTAMENTO POR ATESTADO ODONTOLOGICO - ATE 15 DIAS	CH da Universidade Federal do Ceará - CE
58	AFASTAMENTO POR ATESTADO ODONTOLOGICO - ATE 15 DIAS	CH da Universidade Federal do Ceará - CE
58	AFASTAMENTO POR ATESTADO ODONTOLOGICO - ATE 15 DIAS	CH da Universidade Federal do Ceará - CE
58	AFASTAMENTO POR ATESTADO ODONTOLOGICO - ATE 15 DIAS	CH da Universidade Federal do Ceará - CE
58	AFASTAMENTO POR ATESTADO ODONTOLOGICO - ATE 15 DIAS	CH da Universidade Federal do Ceará - CE
58	AFASTAMENTO POR ATESTADO ODONTOLOGICO - ATE 15 DIAS	CH da Universidade Federal do Ceará - CE
58	AFASTAMENTO POR ATESTADO ODONTOLOGICO - ATE 15 DIAS	CH da Universidade Federal do Ceará - CE
58	AFASTAMENTO POR ATESTADO ODONTOLOGICO - ATE 15 DIAS	CH da Universidade Federal do Ceará - CE

58	AFASTAMENTO POR ATESTADO ODONTOLOGICO - ATE 15 DIAS	CH da Universidade Federal do Ceará - CE
58	AFASTAMENTO POR ATESTADO ODONTOLOGICO - ATE 15 DIAS	CH da Universidade Federal do Ceará - CE
58	AFASTAMENTO POR ATESTADO ODONTOLOGICO - ATE 15 DIAS	CH da Universidade Federal do Ceará - CE
58	AFASTAMENTO POR ATESTADO ODONTOLOGICO - ATE 15 DIAS	CH da Universidade Federal do Ceará - CE
58	AFASTAMENTO POR ATESTADO ODONTOLOGICO - ATE 15 DIAS	CH da Universidade Federal do Ceará - CE
58	AFASTAMENTO POR ATESTADO ODONTOLOGICO - ATE 15 DIAS	CH da Universidade Federal do Ceará - CE
58	AFASTAMENTO POR ATESTADO ODONTOLOGICO - ATE 15 DIAS	CH da Universidade Federal do Ceará - CE
58	AFASTAMENTO POR ATESTADO ODONTOLOGICO - ATE 15 DIAS	CH da Universidade Federal do Ceará - CE
58	AFASTAMENTO POR ATESTADO ODONTOLOGICO - ATE 15 DIAS	CH da Universidade Federal do Ceará - CE
58	AFASTAMENTO POR ATESTADO ODONTOLOGICO - ATE 15 DIAS	CH da Universidade Federal do Ceará - CE
58	AFASTAMENTO POR ATESTADO ODONTOLOGICO - ATE 15 DIAS	CH da Universidade Federal do Ceará - CE
58	AFASTAMENTO POR ATESTADO ODONTOLOGICO - ATE 15 DIAS	CH da Universidade Federal do Ceará - CE
58	AFASTAMENTO POR ATESTADO ODONTOLOGICO - ATE 15 DIAS	CH da Universidade Federal do Ceará - CE
58	AFASTAMENTO POR ATESTADO ODONTOLOGICO - ATE 15 DIAS	CH da Universidade Federal do Ceará - CE
58	AFASTAMENTO POR ATESTADO ODONTOLOGICO - ATE 15 DIAS	CH da Universidade Federal do Ceará - CE
58	AFASTAMENTO POR ATESTADO ODONTOLOGICO - ATE 15 DIAS	CH da Universidade Federal do Ceará - CE
58	AFASTAMENTO POR ATESTADO ODONTOLOGICO - ATE 15 DIAS	CH da Universidade Federal do Ceará - CE
58	AFASTAMENTO POR ATESTADO ODONTOLOGICO - ATE 15 DIAS	CH da Universidade Federal do Ceará - CE
58	AFASTAMENTO POR ATESTADO ODONTOLOGICO - ATE 15 DIAS	CH da Universidade Federal do Ceará - CE
58	AFASTAMENTO POR ATESTADO ODONTOLOGICO - ATE 15 DIAS	CH da Universidade Federal do Ceará - CE
58	AFASTAMENTO POR ATESTADO ODONTOLOGICO - ATE 15 DIAS	CH da Universidade Federal do Ceará - CE
58	AFASTAMENTO POR ATESTADO ODONTOLOGICO - ATE 15 DIAS	CH da Universidade Federal do Ceará - CE
58	AFASTAMENTO POR ATESTADO ODONTOLOGICO - ATE 15 DIAS	CH da Universidade Federal do Ceará - CE
58	AFASTAMENTO POR ATESTADO ODONTOLOGICO - ATE 15 DIAS	CH da Universidade Federal do Ceará - CE
58	AFASTAMENTO POR ATESTADO ODONTOLOGICO - ATE 15 DIAS	CH da Universidade Federal do Ceará - CE
58	AFASTAMENTO POR ATESTADO ODONTOLOGICO - ATE 15 DIAS	CH da Universidade Federal do Ceará - CE
58	AFASTAMENTO POR ATESTADO ODONTOLOGICO - ATE 15 DIAS	CH da Universidade Federal do Ceará - CE
58	AFASTAMENTO POR ATESTADO ODONTOLOGICO - ATE 15 DIAS	CH da Universidade Federal do Ceará - CE
58	AFASTAMENTO POR ATESTADO ODONTOLOGICO - ATE 15 DIAS	CH da Universidade Federal do Ceará - CE
58	AFASTAMENTO POR ATESTADO ODONTOLOGICO - ATE 15 DIAS	CH da Universidade Federal do Ceará - CE
58	AFASTAMENTO POR ATESTADO ODONTOLOGICO - ATE 15 DIAS	CH da Universidade Federal do Ceará - CE
58	AFASTAMENTO POR ATESTADO ODONTOLOGICO - ATE 15 DIAS	CH da Universidade Federal do Ceará - CE

58	AFASTAMENTO POR ATESTADO ODONTOLOGICO - ATE 15 DIAS	CH da Universidade Federal do Ceará - CE
58	AFASTAMENTO POR ATESTADO ODONTOLOGICO - ATE 15 DIAS	CH da Universidade Federal do Ceará - CE
58	AFASTAMENTO POR ATESTADO ODONTOLOGICO - ATE 15 DIAS	CH da Universidade Federal do Ceará - CE
58	AFASTAMENTO POR ATESTADO ODONTOLOGICO - ATE 15 DIAS	CH da Universidade Federal do Ceará - CE
58	AFASTAMENTO POR ATESTADO ODONTOLOGICO - ATE 15 DIAS	CH da Universidade Federal do Ceará - CE
58	AFASTAMENTO POR ATESTADO ODONTOLOGICO - ATE 15 DIAS	CH da Universidade Federal do Ceará - CE
58	AFASTAMENTO POR ATESTADO ODONTOLOGICO - ATE 15 DIAS	CH da Universidade Federal do Ceará - CE
58	AFASTAMENTO POR ATESTADO ODONTOLOGICO - ATE 15 DIAS	CH da Universidade Federal do Ceará - CE
58	AFASTAMENTO POR ATESTADO ODONTOLOGICO - ATE 15 DIAS	CH da Universidade Federal do Ceará - CE
58	AFASTAMENTO POR ATESTADO ODONTOLOGICO - ATE 15 DIAS	CH da Universidade Federal do Ceará - CE
58	AFASTAMENTO POR ATESTADO ODONTOLOGICO - ATE 15 DIAS	CH da Universidade Federal do Ceará - CE
58	AFASTAMENTO POR ATESTADO ODONTOLOGICO - ATE 15 DIAS	CH da Universidade Federal do Ceará - CE
58	AFASTAMENTO POR ATESTADO ODONTOLOGICO - ATE 15 DIAS	CH da Universidade Federal do Ceará - CE
58	AFASTAMENTO POR ATESTADO ODONTOLOGICO - ATE 15 DIAS	CH da Universidade Federal do Ceará - CE
58	AFASTAMENTO POR ATESTADO ODONTOLOGICO - ATE 15 DIAS	CH da Universidade Federal do Ceará - CE
58	AFASTAMENTO POR ATESTADO ODONTOLOGICO - ATE 15 DIAS	CH da Universidade Federal do Ceará - CE
58	AFASTAMENTO POR ATESTADO ODONTOLOGICO - ATE 15 DIAS	CH da Universidade Federal do Ceará - CE
58	AFASTAMENTO POR ATESTADO ODONTOLOGICO - ATE 15 DIAS	CH da Universidade Federal do Ceará - CE
58	AFASTAMENTO POR ATESTADO ODONTOLOGICO - ATE 15 DIAS	CH da Universidade Federal do Ceará - CE
58	AFASTAMENTO POR ATESTADO ODONTOLOGICO - ATE 15 DIAS	CH da Universidade Federal do Ceará - CE
58	AFASTAMENTO POR ATESTADO ODONTOLOGICO - ATE 15 DIAS	CH da Universidade Federal do Ceará - CE
58	AFASTAMENTO POR ATESTADO ODONTOLOGICO - ATE 15 DIAS	CH da Universidade Federal do Ceará - CE
58	AFASTAMENTO POR ATESTADO ODONTOLOGICO - ATE 15 DIAS	CH da Universidade Federal do Ceará - CE
58	AFASTAMENTO POR ATESTADO ODONTOLOGICO - ATE 15 DIAS	CH da Universidade Federal do Ceará - CE
58	AFASTAMENTO POR ATESTADO ODONTOLOGICO - ATE 15 DIAS	CH da Universidade Federal do Ceará - CE
58	AFASTAMENTO POR ATESTADO ODONTOLOGICO - ATE 15 DIAS	CH da Universidade Federal do Ceará - CE
58	AFASTAMENTO POR ATESTADO ODONTOLOGICO - ATE 15 DIAS	CH da Universidade Federal do Ceará - CE
58	AFASTAMENTO POR ATESTADO ODONTOLOGICO - ATE 15 DIAS	CH da Universidade Federal do Ceará - CE
58	AFASTAMENTO POR ATESTADO ODONTOLOGICO - ATE 15 DIAS	CH da Universidade Federal do Ceará - CE
58	AFASTAMENTO POR ATESTADO ODONTOLOGICO - ATE 15 DIAS	CH da Universidade Federal do Ceará - CE
58	AFASTAMENTO POR ATESTADO ODONTOLOGICO - ATE 15 DIAS	CH da Universidade Federal do Ceará - CE
58	AFASTAMENTO POR ATESTADO ODONTOLOGICO - ATE 15 DIAS	CH da Universidade Federal do Ceará - CE

58	AFASTAMENTO POR ATESTADO ODONTOLOGICO - ATE 15 DIAS	CH da Universidade Federal do Ceará - CE
58	AFASTAMENTO POR ATESTADO ODONTOLOGICO - ATE 15 DIAS	CH da Universidade Federal do Ceará - CE
58	AFASTAMENTO POR ATESTADO ODONTOLOGICO - ATE 15 DIAS	CH da Universidade Federal do Ceará - CE
58	AFASTAMENTO POR ATESTADO ODONTOLOGICO - ATE 15 DIAS	CH da Universidade Federal do Ceará - CE
62	LICENÇA PATERNIDADE CELETISTA - Até 5 DIAS - ADCT CF ART 10	CH da Universidade Federal do Ceará - CE
62	LICENÇA PATERNIDADE CELETISTA - Até 5 DIAS - ADCT CF ART 10	CH da Universidade Federal do Ceará - CE
62	LICENÇA PATERNIDADE CELETISTA - Até 5 DIAS - ADCT CF ART 10	CH da Universidade Federal do Ceará - CE
62	LICENÇA PATERNIDADE CELETISTA - Até 5 DIAS - ADCT CF ART 10	CH da Universidade Federal do Ceará - CE
62	LICENÇA PATERNIDADE CELETISTA - Até 5 DIAS - ADCT CF ART 10	CH da Universidade Federal do Ceará - CE
62	LICENÇA PATERNIDADE CELETISTA - Até 5 DIAS - ADCT CF ART 10	CH da Universidade Federal do Ceará - CE
62	LICENÇA PATERNIDADE CELETISTA - Até 5 DIAS - ADCT CF ART 10	CH da Universidade Federal do Ceará - CE
62	LICENÇA PATERNIDADE CELETISTA - Até 5 DIAS - ADCT CF ART 10	CH da Universidade Federal do Ceará - CE
62	LICENÇA PATERNIDADE CELETISTA - Até 5 DIAS - ADCT CF ART 10	CH da Universidade Federal do Ceará - CE
62	LICENÇA PATERNIDADE CELETISTA - Até 5 DIAS - ADCT CF ART 10	CH da Universidade Federal do Ceará - CE
62	LICENÇA PATERNIDADE CELETISTA - Até 5 DIAS - ADCT CF ART 10	CH da Universidade Federal do Ceará - CE
62	LICENÇA PATERNIDADE CELETISTA - Até 5 DIAS - ADCT CF ART 10	CH da Universidade Federal do Ceará - CE
62	LICENÇA PATERNIDADE CELETISTA - Até 5 DIAS - ADCT CF ART 10	CH da Universidade Federal do Ceará - CE
62	LICENÇA PATERNIDADE CELETISTA - Até 5 DIAS - ADCT CF ART 10	CH da Universidade Federal do Ceará - CE
62	LICENÇA PATERNIDADE CELETISTA - Até 5 DIAS - ADCT CF ART 10	CH da Universidade Federal do Ceará - CE
62	LICENÇA PATERNIDADE CELETISTA - Até 5 DIAS - ADCT CF ART 10	CH da Universidade Federal do Ceará - CE
62	LICENÇA PATERNIDADE CELETISTA - Até 5 DIAS - ADCT CF ART 10	CH da Universidade Federal do Ceará - CE
62	LICENÇA PATERNIDADE CELETISTA - Até 5 DIAS - ADCT CF ART 10	CH da Universidade Federal do Ceará - CE
62	LICENÇA PATERNIDADE CELETISTA - Até 5 DIAS - ADCT CF ART 10	CH da Universidade Federal do Ceará - CE
62	LICENÇA PATERNIDADE CELETISTA - Até 5 DIAS - ADCT CF ART 10	CH da Universidade Federal do Ceará - CE
62	LICENÇA PATERNIDADE CELETISTA - Até 5 DIAS - ADCT CF ART 10	CH da Universidade Federal do Ceará - CE
65	AFASTAMENTO DO PAÍS (AUTORIZADO PELA EMPRESA)	CH da Universidade Federal do Ceará - CE
65	AFASTAMENTO DO PAÍS (AUTORIZADO PELA EMPRESA)	CH da Universidade Federal do Ceará - CE
68	COMPARECIMENTO A JUIZO (JURI, INQUERITO POLICIAL, PROC. JUDICIAL) - CLT ART. 473 VIII	CH da Universidade Federal do Ceará - CE
68	COMPARECIMENTO A JUIZO (JURI, INQUERITO POLICIAL, PROC. JUDICIAL) - CLT ART. 473 VIII	CH da Universidade Federal do Ceará - CE
68	COMPARECIMENTO A JUIZO (JURI, INQUERITO POLICIAL, PROC. JUDICIAL) - CLT ART. 473 VIII	CH da Universidade Federal do Ceará - CE
68	COMPARECIMENTO A JUIZO (JURI, INQUERITO POLICIAL, PROC. JUDICIAL) - CLT ART. 473 VIII	CH da Universidade Federal do Ceará - CE
68	COMPARECIMENTO A JUIZO (JURI, INQUERITO POLICIAL, PROC. JUDICIAL) - CLT ART. 473 VIII	CH da Universidade Federal do Ceará - CE

68	COMPARECIMENTO A JUIZO (JURI, INQUERITO POLICIAL, PROC. JUDICIAL) - CLT ART. 473 VIII	CH da Universidade Federal do Ceará - CE
68	COMPARECIMENTO A JUIZO (JURI, INQUERITO POLICIAL, PROC. JUDICIAL) - CLT ART. 473 VIII	CH da Universidade Federal do Ceará - CE
68	COMPARECIMENTO A JUIZO (JURI, INQUERITO POLICIAL, PROC. JUDICIAL) - CLT ART. 473 VIII	CH da Universidade Federal do Ceará - CE
68	COMPARECIMENTO A JUIZO (JURI, INQUERITO POLICIAL, PROC. JUDICIAL) - CLT ART. 473 VIII	CH da Universidade Federal do Ceará - CE
68	COMPARECIMENTO A JUIZO (JURI, INQUERITO POLICIAL, PROC. JUDICIAL) - CLT ART. 473 VIII	CH da Universidade Federal do Ceará - CE
68	COMPARECIMENTO A JUIZO (JURI, INQUERITO POLICIAL, PROC. JUDICIAL) - CLT ART. 473 VIII	CH da Universidade Federal do Ceará - CE
68	COMPARECIMENTO A JUIZO (JURI, INQUERITO POLICIAL, PROC. JUDICIAL) - CLT ART. 473 VIII	CH da Universidade Federal do Ceará - CE
68	COMPARECIMENTO A JUIZO (JURI, INQUERITO POLICIAL, PROC. JUDICIAL) - CLT ART. 473 VIII	CH da Universidade Federal do Ceará - CE
68	COMPARECIMENTO A JUIZO (JURI, INQUERITO POLICIAL, PROC. JUDICIAL) - CLT ART. 473 VIII	CH da Universidade Federal do Ceará - CE
69	ABONO (ACT)	CH da Universidade Federal do Ceará - CE
69	ABONO (ACT)	CH da Universidade Federal do Ceará - CE
69	ABONO (ACT)	CH da Universidade Federal do Ceará - CE
69	ABONO (ACT)	CH da Universidade Federal do Ceará - CE
69	ABONO (ACT)	CH da Universidade Federal do Ceará - CE
69	ABONO (ACT)	CH da Universidade Federal do Ceará - CE
69	ABONO (ACT)	CH da Universidade Federal do Ceará - CE
69	ABONO (ACT)	CH da Universidade Federal do Ceará - CE
69	ABONO (ACT)	CH da Universidade Federal do Ceará - CE
69	ABONO (ACT)	CH da Universidade Federal do Ceará - CE
69	ABONO (ACT)	CH da Universidade Federal do Ceará - CE
69	ABONO (ACT)	CH da Universidade Federal do Ceará - CE
69	ABONO (ACT)	CH da Universidade Federal do Ceará - CE
69	ABONO (ACT)	CH da Universidade Federal do Ceará - CE
69	ABONO (ACT)	CH da Universidade Federal do Ceará - CE
69	ABONO (ACT)	CH da Universidade Federal do Ceará - CE
69	ABONO (ACT)	CH da Universidade Federal do Ceará - CE
69	ABONO (ACT)	CH da Universidade Federal do Ceará - CE
69	ABONO (ACT)	CH da Universidade Federal do Ceará - CE
69	ABONO (ACT)	CH da Universidade Federal do Ceará - CE
69	ABONO (ACT)	CH da Universidade Federal do Ceará - CE
69	ABONO (ACT)	CH da Universidade Federal do Ceará - CE
69	ABONO (ACT)	CH da Universidade Federal do Ceará - CE
69	ABONO (ACT)	CH da Universidade Federal do Ceará - CE
69	ABONO (ACT)	CH da Universidade Federal do Ceará - CE
69	ABONO (ACT)	CH da Universidade Federal do Ceará - CE
69	ABONO (ACT)	CH da Universidade Federal do Ceará - CE
69	ABONO (ACT)	CH da Universidade Federal do Ceará - CE
69	ABONO (ACT)	CH da Universidade Federal do Ceará - CE
69	ABONO (ACT)	CH da Universidade Federal do Ceará - CE
69	ABONO (ACT)	CH da Universidade Federal do Ceará - CE
69	ABONO (ACT)	CH da Universidade Federal do Ceará - CE
69	ABONO (ACT)	CH da Universidade Federal do Ceará - CE

69	ABONO (ACT)	CH da Universidade Federal do Ceará - CE
69	ABONO (ACT)	CH da Universidade Federal do Ceará - CE
69	ABONO (ACT)	CH da Universidade Federal do Ceará - CE
69	ABONO (ACT)	CH da Universidade Federal do Ceará - CE
69	ABONO (ACT)	CH da Universidade Federal do Ceará - CE
69	ABONO (ACT)	CH da Universidade Federal do Ceará - CE
69	ABONO (ACT)	CH da Universidade Federal do Ceará - CE
69	ABONO (ACT)	CH da Universidade Federal do Ceará - CE
69	ABONO (ACT)	CH da Universidade Federal do Ceará - CE
69	ABONO (ACT)	CH da Universidade Federal do Ceará - CE
69	ABONO (ACT)	CH da Universidade Federal do Ceará - CE
69	ABONO (ACT)	CH da Universidade Federal do Ceará - CE
69	ABONO (ACT)	CH da Universidade Federal do Ceará - CE
69	ABONO (ACT)	CH da Universidade Federal do Ceará - CE
69	ABONO (ACT)	CH da Universidade Federal do Ceará - CE
69	ABONO (ACT)	CH da Universidade Federal do Ceará - CE
69	ABONO (ACT)	CH da Universidade Federal do Ceará - CE
69	ABONO (ACT)	CH da Universidade Federal do Ceará - CE
69	ABONO (ACT)	CH da Universidade Federal do Ceará - CE
69	ABONO (ACT)	CH da Universidade Federal do Ceará - CE
69	ABONO (ACT)	CH da Universidade Federal do Ceará - CE
69	ABONO (ACT)	CH da Universidade Federal do Ceará - CE
69	ABONO (ACT)	CH da Universidade Federal do Ceará - CE
69	ABONO (ACT)	CH da Universidade Federal do Ceará - CE
69	ABONO (ACT)	CH da Universidade Federal do Ceará - CE
69	ABONO (ACT)	CH da Universidade Federal do Ceará - CE
69	ABONO (ACT)	CH da Universidade Federal do Ceará - CE
69	ABONO (ACT)	CH da Universidade Federal do Ceará - CE
69	ABONO (ACT)	CH da Universidade Federal do Ceará - CE
69	ABONO (ACT)	CH da Universidade Federal do Ceará - CE
69	ABONO (ACT)	CH da Universidade Federal do Ceará - CE
69	ABONO (ACT)	CH da Universidade Federal do Ceará - CE
69	ABONO (ACT)	CH da Universidade Federal do Ceará - CE
69	ABONO (ACT)	CH da Universidade Federal do Ceará - CE
69	ABONO (ACT)	CH da Universidade Federal do Ceará - CE
69	ABONO (ACT)	CH da Universidade Federal do Ceará - CE
69	ABONO (ACT)	CH da Universidade Federal do Ceará - CE
69	ABONO (ACT)	CH da Universidade Federal do Ceará - CE
69	ABONO (ACT)	CH da Universidade Federal do Ceará - CE
69	ABONO (ACT)	CH da Universidade Federal do Ceará - CE
69	ABONO (ACT)	CH da Universidade Federal do Ceará - CE
69	ABONO (ACT)	CH da Universidade Federal do Ceará - CE
69	ABONO (ACT)	CH da Universidade Federal do Ceará - CE
69	ABONO (ACT)	CH da Universidade Federal do Ceará - CE
69	ABONO (ACT)	CH da Universidade Federal do Ceará - CE
69	ABONO (ACT)	CH da Universidade Federal do Ceará - CE
69	ABONO (ACT)	CH da Universidade Federal do Ceará - CE
69	ABONO (ACT)	CH da Universidade Federal do Ceará - CE

69	ABONO (ACT)	CH da Universidade Federal do Ceará - CE
69	ABONO (ACT)	CH da Universidade Federal do Ceará - CE
69	ABONO (ACT)	CH da Universidade Federal do Ceará - CE
69	ABONO (ACT)	CH da Universidade Federal do Ceará - CE
69	ABONO (ACT)	CH da Universidade Federal do Ceará - CE
69	ABONO (ACT)	CH da Universidade Federal do Ceará - CE
69	ABONO (ACT)	CH da Universidade Federal do Ceará - CE
69	ABONO (ACT)	CH da Universidade Federal do Ceará - CE
69	ABONO (ACT)	CH da Universidade Federal do Ceará - CE
69	ABONO (ACT)	CH da Universidade Federal do Ceará - CE
69	ABONO (ACT)	CH da Universidade Federal do Ceará - CE
69	ABONO (ACT)	CH da Universidade Federal do Ceará - CE
69	ABONO (ACT)	CH da Universidade Federal do Ceará - CE
69	ABONO (ACT)	CH da Universidade Federal do Ceará - CE
69	ABONO (ACT)	CH da Universidade Federal do Ceará - CE
69	ABONO (ACT)	CH da Universidade Federal do Ceará - CE
69	ABONO (ACT)	CH da Universidade Federal do Ceará - CE
69	ABONO (ACT)	CH da Universidade Federal do Ceará - CE
69	ABONO (ACT)	CH da Universidade Federal do Ceará - CE
69	ABONO (ACT)	CH da Universidade Federal do Ceará - CE
69	ABONO (ACT)	CH da Universidade Federal do Ceará - CE
69	ABONO (ACT)	CH da Universidade Federal do Ceará - CE
69	ABONO (ACT)	CH da Universidade Federal do Ceará - CE
69	ABONO (ACT)	CH da Universidade Federal do Ceará - CE
69	ABONO (ACT)	CH da Universidade Federal do Ceará - CE
69	ABONO (ACT)	CH da Universidade Federal do Ceará - CE
69	ABONO (ACT)	CH da Universidade Federal do Ceará - CE
69	ABONO (ACT)	CH da Universidade Federal do Ceará - CE
69	ABONO (ACT)	CH da Universidade Federal do Ceará - CE
69	ABONO (ACT)	CH da Universidade Federal do Ceará - CE
69	ABONO (ACT)	CH da Universidade Federal do Ceará - CE
69	ABONO (ACT)	CH da Universidade Federal do Ceará - CE
69	ABONO (ACT)	CH da Universidade Federal do Ceará - CE
69	ABONO (ACT)	CH da Universidade Federal do Ceará - CE
69	ABONO (ACT)	CH da Universidade Federal do Ceará - CE
69	ABONO (ACT)	CH da Universidade Federal do Ceará - CE
69	ABONO (ACT)	CH da Universidade Federal do Ceará - CE
69	ABONO (ACT)	CH da Universidade Federal do Ceará - CE
69	ABONO (ACT)	CH da Universidade Federal do Ceará - CE
69	ABONO (ACT)	CH da Universidade Federal do Ceará - CE
69	ABONO (ACT)	CH da Universidade Federal do Ceará - CE
69	ABONO (ACT)	CH da Universidade Federal do Ceará - CE
69	ABONO (ACT)	CH da Universidade Federal do Ceará - CE
69	ABONO (ACT)	CH da Universidade Federal do Ceará - CE
69	ABONO (ACT)	CH da Universidade Federal do Ceará - CE
69	ABONO (ACT)	CH da Universidade Federal do Ceará - CE
69	ABONO (ACT)	CH da Universidade Federal do Ceará - CE
69	ABONO (ACT)	CH da Universidade Federal do Ceará - CE

69	ABONO (ACT)	CH da Universidade Federal do Ceará - CE
69	ABONO (ACT)	CH da Universidade Federal do Ceará - CE
69	ABONO (ACT)	CH da Universidade Federal do Ceará - CE
69	ABONO (ACT)	CH da Universidade Federal do Ceará - CE
69	ABONO (ACT)	CH da Universidade Federal do Ceará - CE
69	ABONO (ACT)	CH da Universidade Federal do Ceará - CE
69	ABONO (ACT)	CH da Universidade Federal do Ceará - CE
69	ABONO (ACT)	CH da Universidade Federal do Ceará - CE
69	ABONO (ACT)	CH da Universidade Federal do Ceará - CE
69	ABONO (ACT)	CH da Universidade Federal do Ceará - CE
69	ABONO (ACT)	CH da Universidade Federal do Ceará - CE
69	ABONO (ACT)	CH da Universidade Federal do Ceará - CE
69	ABONO (ACT)	CH da Universidade Federal do Ceará - CE
69	ABONO (ACT)	CH da Universidade Federal do Ceará - CE
69	ABONO (ACT)	CH da Universidade Federal do Ceará - CE
69	ABONO (ACT)	CH da Universidade Federal do Ceará - CE
69	ABONO (ACT)	CH da Universidade Federal do Ceará - CE
69	ABONO (ACT)	CH da Universidade Federal do Ceará - CE
69	ABONO (ACT)	CH da Universidade Federal do Ceará - CE
69	ABONO (ACT)	CH da Universidade Federal do Ceará - CE
69	ABONO (ACT)	CH da Universidade Federal do Ceará - CE
69	ABONO (ACT)	CH da Universidade Federal do Ceará - CE
69	ABONO (ACT)	CH da Universidade Federal do Ceará - CE
69	ABONO (ACT)	CH da Universidade Federal do Ceará - CE
69	ABONO (ACT)	CH da Universidade Federal do Ceará - CE
69	ABONO (ACT)	CH da Universidade Federal do Ceará - CE
69	ABONO (ACT)	CH da Universidade Federal do Ceará - CE
69	ABONO (ACT)	CH da Universidade Federal do Ceará - CE
69	ABONO (ACT)	CH da Universidade Federal do Ceará - CE
69	ABONO (ACT)	CH da Universidade Federal do Ceará - CE
69	ABONO (ACT)	CH da Universidade Federal do Ceará - CE
69	ABONO (ACT)	CH da Universidade Federal do Ceará - CE
69	ABONO (ACT)	CH da Universidade Federal do Ceará - CE
69	ABONO (ACT)	CH da Universidade Federal do Ceará - CE
69	ABONO (ACT)	CH da Universidade Federal do Ceará - CE
69	ABONO (ACT)	CH da Universidade Federal do Ceará - CE
69	ABONO (ACT)	CH da Universidade Federal do Ceará - CE
69	ABONO (ACT)	CH da Universidade Federal do Ceará - CE
69	ABONO (ACT)	CH da Universidade Federal do Ceará - CE
69	ABONO (ACT)	CH da Universidade Federal do Ceará - CE
69	ABONO (ACT)	CH da Universidade Federal do Ceará - CE
69	ABONO (ACT)	CH da Universidade Federal do Ceará - CE
69	ABONO (ACT)	CH da Universidade Federal do Ceará - CE
69	ABONO (ACT)	CH da Universidade Federal do Ceará - CE
69	ABONO (ACT)	CH da Universidade Federal do Ceará - CE
69	ABONO (ACT)	CH da Universidade Federal do Ceará - CE
69	ABONO (ACT)	CH da Universidade Federal do Ceará - CE
69	ABONO (ACT)	CH da Universidade Federal do Ceará - CE

69	ABONO (ACT)	CH da Universidade Federal do Ceará - CE
69	ABONO (ACT)	CH da Universidade Federal do Ceará - CE
69	ABONO (ACT)	CH da Universidade Federal do Ceará - CE
69	ABONO (ACT)	CH da Universidade Federal do Ceará - CE
69	ABONO (ACT)	CH da Universidade Federal do Ceará - CE
69	ABONO (ACT)	CH da Universidade Federal do Ceará - CE
69	ABONO (ACT)	CH da Universidade Federal do Ceará - CE
69	ABONO (ACT)	CH da Universidade Federal do Ceará - CE
69	ABONO (ACT)	CH da Universidade Federal do Ceará - CE
69	ABONO (ACT)	CH da Universidade Federal do Ceará - CE
69	ABONO (ACT)	CH da Universidade Federal do Ceará - CE
69	ABONO (ACT)	CH da Universidade Federal do Ceará - CE
69	ABONO (ACT)	CH da Universidade Federal do Ceará - CE
69	ABONO (ACT)	CH da Universidade Federal do Ceará - CE
69	ABONO (ACT)	CH da Universidade Federal do Ceará - CE
69	ABONO (ACT)	CH da Universidade Federal do Ceará - CE
69	ABONO (ACT)	CH da Universidade Federal do Ceará - CE
69	ABONO (ACT)	CH da Universidade Federal do Ceará - CE
69	ABONO (ACT)	CH da Universidade Federal do Ceará - CE
69	ABONO (ACT)	CH da Universidade Federal do Ceará - CE
69	ABONO (ACT)	CH da Universidade Federal do Ceará - CE
69	ABONO (ACT)	CH da Universidade Federal do Ceará - CE
69	ABONO (ACT)	CH da Universidade Federal do Ceará - CE
69	ABONO (ACT)	CH da Universidade Federal do Ceará - CE
69	ABONO (ACT)	CH da Universidade Federal do Ceará - CE
69	ABONO (ACT)	CH da Universidade Federal do Ceará - CE
69	ABONO (ACT)	CH da Universidade Federal do Ceará - CE
69	ABONO (ACT)	CH da Universidade Federal do Ceará - CE
69	ABONO (ACT)	CH da Universidade Federal do Ceará - CE
69	ABONO (ACT)	CH da Universidade Federal do Ceará - CE
69	ABONO (ACT)	CH da Universidade Federal do Ceará - CE
69	ABONO (ACT)	CH da Universidade Federal do Ceará - CE
69	ABONO (ACT)	CH da Universidade Federal do Ceará - CE
69	ABONO (ACT)	CH da Universidade Federal do Ceará - CE
69	ABONO (ACT)	CH da Universidade Federal do Ceará - CE
69	ABONO (ACT)	CH da Universidade Federal do Ceará - CE
69	ABONO (ACT)	CH da Universidade Federal do Ceará - CE
69	ABONO (ACT)	CH da Universidade Federal do Ceará - CE
69	ABONO (ACT)	CH da Universidade Federal do Ceará - CE
69	ABONO (ACT)	CH da Universidade Federal do Ceará - CE
69	ABONO (ACT)	CH da Universidade Federal do Ceará - CE
69	ABONO (ACT)	CH da Universidade Federal do Ceará - CE
69	ABONO (ACT)	CH da Universidade Federal do Ceará - CE
69	ABONO (ACT)	CH da Universidade Federal do Ceará - CE
69	ABONO (ACT)	CH da Universidade Federal do Ceará - CE
69	ABONO (ACT)	CH da Universidade Federal do Ceará - CE
69	ABONO (ACT)	CH da Universidade Federal do Ceará - CE
69	ABONO (ACT)	CH da Universidade Federal do Ceará - CE

69	ABONO (ACT)	CH da Universidade Federal do Ceará - CE
69	ABONO (ACT)	CH da Universidade Federal do Ceará - CE
69	ABONO (ACT)	CH da Universidade Federal do Ceará - CE
69	ABONO (ACT)	CH da Universidade Federal do Ceará - CE
69	ABONO (ACT)	CH da Universidade Federal do Ceará - CE
69	ABONO (ACT)	CH da Universidade Federal do Ceará - CE
69	ABONO (ACT)	CH da Universidade Federal do Ceará - CE
69	ABONO (ACT)	CH da Universidade Federal do Ceará - CE
69	ABONO (ACT)	CH da Universidade Federal do Ceará - CE
69	ABONO (ACT)	CH da Universidade Federal do Ceará - CE
69	ABONO (ACT)	CH da Universidade Federal do Ceará - CE
69	ABONO (ACT)	CH da Universidade Federal do Ceará - CE
69	ABONO (ACT)	CH da Universidade Federal do Ceará - CE
69	ABONO (ACT)	CH da Universidade Federal do Ceará - CE
69	ABONO (ACT)	CH da Universidade Federal do Ceará - CE
69	ABONO (ACT)	CH da Universidade Federal do Ceará - CE
69	ABONO (ACT)	CH da Universidade Federal do Ceará - CE
69	ABONO (ACT)	CH da Universidade Federal do Ceará - CE
69	ABONO (ACT)	CH da Universidade Federal do Ceará - CE
69	ABONO (ACT)	CH da Universidade Federal do Ceará - CE
69	ABONO (ACT)	CH da Universidade Federal do Ceará - CE
69	ABONO (ACT)	CH da Universidade Federal do Ceará - CE
69	ABONO (ACT)	CH da Universidade Federal do Ceará - CE
69	ABONO (ACT)	CH da Universidade Federal do Ceará - CE
69	ABONO (ACT)	CH da Universidade Federal do Ceará - CE
69	ABONO (ACT)	CH da Universidade Federal do Ceará - CE
69	ABONO (ACT)	CH da Universidade Federal do Ceará - CE
69	ABONO (ACT)	CH da Universidade Federal do Ceará - CE
69	ABONO (ACT)	CH da Universidade Federal do Ceará - CE
69	ABONO (ACT)	CH da Universidade Federal do Ceará - CE
69	ABONO (ACT)	CH da Universidade Federal do Ceará - CE
69	ABONO (ACT)	CH da Universidade Federal do Ceará - CE
69	ABONO (ACT)	CH da Universidade Federal do Ceará - CE
69	ABONO (ACT)	CH da Universidade Federal do Ceará - CE
69	ABONO (ACT)	CH da Universidade Federal do Ceará - CE
69	ABONO (ACT)	CH da Universidade Federal do Ceará - CE
69	ABONO (ACT)	CH da Universidade Federal do Ceará - CE
69	ABONO (ACT)	CH da Universidade Federal do Ceará - CE
69	ABONO (ACT)	CH da Universidade Federal do Ceará - CE
69	ABONO (ACT)	CH da Universidade Federal do Ceará - CE
69	ABONO (ACT)	CH da Universidade Federal do Ceará - CE
69	ABONO (ACT)	CH da Universidade Federal do Ceará - CE
69	ABONO (ACT)	CH da Universidade Federal do Ceará - CE
69	ABONO (ACT)	CH da Universidade Federal do Ceará - CE
69	ABONO (ACT)	CH da Universidade Federal do Ceará - CE
69	ABONO (ACT)	CH da Universidade Federal do Ceará - CE
69	ABONO (ACT)	CH da Universidade Federal do Ceará - CE
69	ABONO (ACT)	CH da Universidade Federal do Ceará - CE

69	ABONO (ACT)	CH da Universidade Federal do Ceará - CE
69	ABONO (ACT)	CH da Universidade Federal do Ceará - CE
69	ABONO (ACT)	CH da Universidade Federal do Ceará - CE
69	ABONO (ACT)	CH da Universidade Federal do Ceará - CE
69	ABONO (ACT)	CH da Universidade Federal do Ceará - CE
69	ABONO (ACT)	CH da Universidade Federal do Ceará - CE
69	ABONO (ACT)	CH da Universidade Federal do Ceará - CE
69	ABONO (ACT)	CH da Universidade Federal do Ceará - CE
69	ABONO (ACT)	CH da Universidade Federal do Ceará - CE
69	ABONO (ACT)	CH da Universidade Federal do Ceará - CE
69	ABONO (ACT)	CH da Universidade Federal do Ceará - CE
69	ABONO (ACT)	CH da Universidade Federal do Ceará - CE
69	ABONO (ACT)	CH da Universidade Federal do Ceará - CE
69	ABONO (ACT)	CH da Universidade Federal do Ceará - CE
69	ABONO (ACT)	CH da Universidade Federal do Ceará - CE
69	ABONO (ACT)	CH da Universidade Federal do Ceará - CE
69	ABONO (ACT)	CH da Universidade Federal do Ceará - CE
69	ABONO (ACT)	CH da Universidade Federal do Ceará - CE
69	ABONO (ACT)	CH da Universidade Federal do Ceará - CE
69	ABONO (ACT)	CH da Universidade Federal do Ceará - CE
69	ABONO (ACT)	CH da Universidade Federal do Ceará - CE
69	ABONO (ACT)	CH da Universidade Federal do Ceará - CE
69	ABONO (ACT)	CH da Universidade Federal do Ceará - CE
69	ABONO (ACT)	CH da Universidade Federal do Ceará - CE
69	ABONO (ACT)	CH da Universidade Federal do Ceará - CE
69	ABONO (ACT)	CH da Universidade Federal do Ceará - CE
69	ABONO (ACT)	CH da Universidade Federal do Ceará - CE
69	ABONO (ACT)	CH da Universidade Federal do Ceará - CE
69	ABONO (ACT)	CH da Universidade Federal do Ceará - CE
69	ABONO (ACT)	CH da Universidade Federal do Ceará - CE
69	ABONO (ACT)	CH da Universidade Federal do Ceará - CE
69	ABONO (ACT)	CH da Universidade Federal do Ceará - CE
69	ABONO (ACT)	CH da Universidade Federal do Ceará - CE
69	ABONO (ACT)	CH da Universidade Federal do Ceará - CE
69	ABONO (ACT)	CH da Universidade Federal do Ceará - CE
69	ABONO (ACT)	CH da Universidade Federal do Ceará - CE
69	ABONO (ACT)	CH da Universidade Federal do Ceará - CE
69	ABONO (ACT)	CH da Universidade Federal do Ceará - CE
69	ABONO (ACT)	CH da Universidade Federal do Ceará - CE
69	ABONO (ACT)	CH da Universidade Federal do Ceará - CE
69	ABONO (ACT)	CH da Universidade Federal do Ceará - CE
69	ABONO (ACT)	CH da Universidade Federal do Ceará - CE
69	ABONO (ACT)	CH da Universidade Federal do Ceará - CE
69	ABONO (ACT)	CH da Universidade Federal do Ceará - CE
69	ABONO (ACT)	CH da Universidade Federal do Ceará - CE
69	ABONO (ACT)	CH da Universidade Federal do Ceará - CE
69	ABONO (ACT)	CH da Universidade Federal do Ceará - CE
69	ABONO (ACT)	CH da Universidade Federal do Ceará - CE

69	ABONO (ACT)	CH da Universidade Federal do Ceará - CE
69	ABONO (ACT)	CH da Universidade Federal do Ceará - CE
69	ABONO (ACT)	CH da Universidade Federal do Ceará - CE
69	ABONO (ACT)	CH da Universidade Federal do Ceará - CE
69	ABONO (ACT)	CH da Universidade Federal do Ceará - CE
69	ABONO (ACT)	CH da Universidade Federal do Ceará - CE
69	ABONO (ACT)	CH da Universidade Federal do Ceará - CE
69	ABONO (ACT)	CH da Universidade Federal do Ceará - CE
69	ABONO (ACT)	CH da Universidade Federal do Ceará - CE
69	ABONO (ACT)	CH da Universidade Federal do Ceará - CE
69	ABONO (ACT)	CH da Universidade Federal do Ceará - CE
69	ABONO (ACT)	CH da Universidade Federal do Ceará - CE
69	ABONO (ACT)	CH da Universidade Federal do Ceará - CE
69	ABONO (ACT)	CH da Universidade Federal do Ceará - CE
69	ABONO (ACT)	CH da Universidade Federal do Ceará - CE
69	ABONO (ACT)	CH da Universidade Federal do Ceará - CE
69	ABONO (ACT)	CH da Universidade Federal do Ceará - CE
69	ABONO (ACT)	CH da Universidade Federal do Ceará - CE
69	ABONO (ACT)	CH da Universidade Federal do Ceará - CE
69	ABONO (ACT)	CH da Universidade Federal do Ceará - CE
69	ABONO (ACT)	CH da Universidade Federal do Ceará - CE
69	ABONO (ACT)	CH da Universidade Federal do Ceará - CE
69	ABONO (ACT)	CH da Universidade Federal do Ceará - CE
69	ABONO (ACT)	CH da Universidade Federal do Ceará - CE
69	ABONO (ACT)	CH da Universidade Federal do Ceará - CE
69	ABONO (ACT)	CH da Universidade Federal do Ceará - CE
69	ABONO (ACT)	CH da Universidade Federal do Ceará - CE
69	ABONO (ACT)	CH da Universidade Federal do Ceará - CE
69	ABONO (ACT)	CH da Universidade Federal do Ceará - CE
69	ABONO (ACT)	CH da Universidade Federal do Ceará - CE
69	ABONO (ACT)	CH da Universidade Federal do Ceará - CE
69	ABONO (ACT)	CH da Universidade Federal do Ceará - CE
69	ABONO (ACT)	CH da Universidade Federal do Ceará - CE
69	ABONO (ACT)	CH da Universidade Federal do Ceará - CE
69	ABONO (ACT)	CH da Universidade Federal do Ceará - CE
69	ABONO (ACT)	CH da Universidade Federal do Ceará - CE
69	ABONO (ACT)	CH da Universidade Federal do Ceará - CE
69	ABONO (ACT)	CH da Universidade Federal do Ceará - CE
69	ABONO (ACT)	CH da Universidade Federal do Ceará - CE
69	ABONO (ACT)	CH da Universidade Federal do Ceará - CE
69	ABONO (ACT)	CH da Universidade Federal do Ceará - CE
69	ABONO (ACT)	CH da Universidade Federal do Ceará - CE
69	ABONO (ACT)	CH da Universidade Federal do Ceará - CE
69	ABONO (ACT)	CH da Universidade Federal do Ceará - CE
69	ABONO (ACT)	CH da Universidade Federal do Ceará - CE
69	ABONO (ACT)	CH da Universidade Federal do Ceará - CE
69	ABONO (ACT)	CH da Universidade Federal do Ceará - CE
69	ABONO (ACT)	CH da Universidade Federal do Ceará - CE

69	ABONO (ACT)	CH da Universidade Federal do Ceará - CE
69	ABONO (ACT)	CH da Universidade Federal do Ceará - CE
69	ABONO (ACT)	CH da Universidade Federal do Ceará - CE
69	ABONO (ACT)	CH da Universidade Federal do Ceará - CE
69	ABONO (ACT)	CH da Universidade Federal do Ceará - CE
69	ABONO (ACT)	CH da Universidade Federal do Ceará - CE
69	ABONO (ACT)	CH da Universidade Federal do Ceará - CE
69	ABONO (ACT)	CH da Universidade Federal do Ceará - CE
69	ABONO (ACT)	CH da Universidade Federal do Ceará - CE
69	ABONO (ACT)	CH da Universidade Federal do Ceará - CE
69	ABONO (ACT)	CH da Universidade Federal do Ceará - CE
69	ABONO (ACT)	CH da Universidade Federal do Ceará - CE
69	ABONO (ACT)	CH da Universidade Federal do Ceará - CE
69	ABONO (ACT)	CH da Universidade Federal do Ceará - CE
69	ABONO (ACT)	CH da Universidade Federal do Ceará - CE
69	ABONO (ACT)	CH da Universidade Federal do Ceará - CE
69	ABONO (ACT)	CH da Universidade Federal do Ceará - CE
69	ABONO (ACT)	CH da Universidade Federal do Ceará - CE
69	ABONO (ACT)	CH da Universidade Federal do Ceará - CE
69	ABONO (ACT)	CH da Universidade Federal do Ceará - CE
69	ABONO (ACT)	CH da Universidade Federal do Ceará - CE
69	ABONO (ACT)	CH da Universidade Federal do Ceará - CE
69	ABONO (ACT)	CH da Universidade Federal do Ceará - CE
69	ABONO (ACT)	CH da Universidade Federal do Ceará - CE
69	ABONO (ACT)	CH da Universidade Federal do Ceará - CE
69	ABONO (ACT)	CH da Universidade Federal do Ceará - CE
69	ABONO (ACT)	CH da Universidade Federal do Ceará - CE
69	ABONO (ACT)	CH da Universidade Federal do Ceará - CE
69	ABONO (ACT)	CH da Universidade Federal do Ceará - CE
69	ABONO (ACT)	CH da Universidade Federal do Ceará - CE
69	ABONO (ACT)	CH da Universidade Federal do Ceará - CE
69	ABONO (ACT)	CH da Universidade Federal do Ceará - CE
69	ABONO (ACT)	CH da Universidade Federal do Ceará - CE
69	ABONO (ACT)	CH da Universidade Federal do Ceará - CE
69	ABONO (ACT)	CH da Universidade Federal do Ceará - CE
69	ABONO (ACT)	CH da Universidade Federal do Ceará - CE
69	ABONO (ACT)	CH da Universidade Federal do Ceará - CE
69	ABONO (ACT)	CH da Universidade Federal do Ceará - CE
69	ABONO (ACT)	CH da Universidade Federal do Ceará - CE
69	ABONO (ACT)	CH da Universidade Federal do Ceará - CE
69	ABONO (ACT)	CH da Universidade Federal do Ceará - CE
69	ABONO (ACT)	CH da Universidade Federal do Ceará - CE
69	ABONO (ACT)	CH da Universidade Federal do Ceará - CE
69	ABONO (ACT)	CH da Universidade Federal do Ceará - CE
69	ABONO (ACT)	CH da Universidade Federal do Ceará - CE
69	ABONO (ACT)	CH da Universidade Federal do Ceará - CE
69	ABONO (ACT)	CH da Universidade Federal do Ceará - CE
69	ABONO (ACT)	CH da Universidade Federal do Ceará - CE

69	ABONO (ACT)	CH da Universidade Federal do Ceará - CE
69	ABONO (ACT)	CH da Universidade Federal do Ceará - CE
69	ABONO (ACT)	CH da Universidade Federal do Ceará - CE
69	ABONO (ACT)	CH da Universidade Federal do Ceará - CE
69	ABONO (ACT)	CH da Universidade Federal do Ceará - CE
69	ABONO (ACT)	CH da Universidade Federal do Ceará - CE
69	ABONO (ACT)	CH da Universidade Federal do Ceará - CE
69	ABONO (ACT)	CH da Universidade Federal do Ceará - CE
69	ABONO (ACT)	CH da Universidade Federal do Ceará - CE
69	ABONO (ACT)	CH da Universidade Federal do Ceará - CE
69	ABONO (ACT)	CH da Universidade Federal do Ceará - CE
69	ABONO (ACT)	CH da Universidade Federal do Ceará - CE
69	ABONO (ACT)	CH da Universidade Federal do Ceará - CE
69	ABONO (ACT)	CH da Universidade Federal do Ceará - CE
69	ABONO (ACT)	CH da Universidade Federal do Ceará - CE
69	ABONO (ACT)	CH da Universidade Federal do Ceará - CE
69	ABONO (ACT)	CH da Universidade Federal do Ceará - CE
69	ABONO (ACT)	CH da Universidade Federal do Ceará - CE
69	ABONO (ACT)	CH da Universidade Federal do Ceará - CE
69	ABONO (ACT)	CH da Universidade Federal do Ceará - CE
69	ABONO (ACT)	CH da Universidade Federal do Ceará - CE
69	ABONO (ACT)	CH da Universidade Federal do Ceará - CE
69	ABONO (ACT)	CH da Universidade Federal do Ceará - CE
69	ABONO (ACT)	CH da Universidade Federal do Ceará - CE
69	ABONO (ACT)	CH da Universidade Federal do Ceará - CE
69	ABONO (ACT)	CH da Universidade Federal do Ceará - CE
69	ABONO (ACT)	CH da Universidade Federal do Ceará - CE
69	ABONO (ACT)	CH da Universidade Federal do Ceará - CE
69	ABONO (ACT)	CH da Universidade Federal do Ceará - CE
69	ABONO (ACT)	CH da Universidade Federal do Ceará - CE
69	ABONO (ACT)	CH da Universidade Federal do Ceará - CE
69	ABONO (ACT)	CH da Universidade Federal do Ceará - CE
69	ABONO (ACT)	CH da Universidade Federal do Ceará - CE
69	ABONO (ACT)	CH da Universidade Federal do Ceará - CE
69	ABONO (ACT)	CH da Universidade Federal do Ceará - CE
69	ABONO (ACT)	CH da Universidade Federal do Ceará - CE
69	ABONO (ACT)	CH da Universidade Federal do Ceará - CE
69	ABONO (ACT)	CH da Universidade Federal do Ceará - CE
69	ABONO (ACT)	CH da Universidade Federal do Ceará - CE
69	ABONO (ACT)	CH da Universidade Federal do Ceará - CE
69	ABONO (ACT)	CH da Universidade Federal do Ceará - CE
69	ABONO (ACT)	CH da Universidade Federal do Ceará - CE
69	ABONO (ACT)	CH da Universidade Federal do Ceará - CE
69	ABONO (ACT)	CH da Universidade Federal do Ceará - CE
69	ABONO (ACT)	CH da Universidade Federal do Ceará - CE
69	ABONO (ACT)	CH da Universidade Federal do Ceará - CE
69	ABONO (ACT)	CH da Universidade Federal do Ceará - CE
69	ABONO (ACT)	CH da Universidade Federal do Ceará - CE

69	ABONO (ACT)	CH da Universidade Federal do Ceará - CE
69	ABONO (ACT)	CH da Universidade Federal do Ceará - CE
69	ABONO (ACT)	CH da Universidade Federal do Ceará - CE
69	ABONO (ACT)	CH da Universidade Federal do Ceará - CE
69	ABONO (ACT)	CH da Universidade Federal do Ceará - CE
69	ABONO (ACT)	CH da Universidade Federal do Ceará - CE
69	ABONO (ACT)	CH da Universidade Federal do Ceará - CE
69	ABONO (ACT)	CH da Universidade Federal do Ceará - CE
69	ABONO (ACT)	CH da Universidade Federal do Ceará - CE
69	ABONO (ACT)	CH da Universidade Federal do Ceará - CE
69	ABONO (ACT)	CH da Universidade Federal do Ceará - CE
69	ABONO (ACT)	CH da Universidade Federal do Ceará - CE
69	ABONO (ACT)	CH da Universidade Federal do Ceará - CE
69	ABONO (ACT)	CH da Universidade Federal do Ceará - CE
69	ABONO (ACT)	CH da Universidade Federal do Ceará - CE
69	ABONO (ACT)	CH da Universidade Federal do Ceará - CE
69	ABONO (ACT)	CH da Universidade Federal do Ceará - CE
69	ABONO (ACT)	CH da Universidade Federal do Ceará - CE
69	ABONO (ACT)	CH da Universidade Federal do Ceará - CE
69	ABONO (ACT)	CH da Universidade Federal do Ceará - CE
69	ABONO (ACT)	CH da Universidade Federal do Ceará - CE
69	ABONO (ACT)	CH da Universidade Federal do Ceará - CE
69	ABONO (ACT)	CH da Universidade Federal do Ceará - CE
69	ABONO (ACT)	CH da Universidade Federal do Ceará - CE
69	ABONO (ACT)	CH da Universidade Federal do Ceará - CE
69	ABONO (ACT)	CH da Universidade Federal do Ceará - CE
69	ABONO (ACT)	CH da Universidade Federal do Ceará - CE
69	ABONO (ACT)	CH da Universidade Federal do Ceará - CE
69	ABONO (ACT)	CH da Universidade Federal do Ceará - CE
69	ABONO (ACT)	CH da Universidade Federal do Ceará - CE
69	ABONO (ACT)	CH da Universidade Federal do Ceará - CE
69	ABONO (ACT)	CH da Universidade Federal do Ceará - CE
69	ABONO (ACT)	CH da Universidade Federal do Ceará - CE
69	ABONO (ACT)	CH da Universidade Federal do Ceará - CE
69	ABONO (ACT)	CH da Universidade Federal do Ceará - CE
69	ABONO (ACT)	CH da Universidade Federal do Ceará - CE
69	ABONO (ACT)	CH da Universidade Federal do Ceará - CE
69	ABONO (ACT)	CH da Universidade Federal do Ceará - CE
69	ABONO (ACT)	CH da Universidade Federal do Ceará - CE
69	ABONO (ACT)	CH da Universidade Federal do Ceará - CE
69	ABONO (ACT)	CH da Universidade Federal do Ceará - CE
69	ABONO (ACT)	CH da Universidade Federal do Ceará - CE
69	ABONO (ACT)	CH da Universidade Federal do Ceará - CE
69	ABONO (ACT)	CH da Universidade Federal do Ceará - CE
69	ABONO (ACT)	CH da Universidade Federal do Ceará - CE
69	ABONO (ACT)	CH da Universidade Federal do Ceará - CE
69	ABONO (ACT)	CH da Universidade Federal do Ceará - CE
69	ABONO (ACT)	CH da Universidade Federal do Ceará - CE

69	ABONO (ACT)	CH da Universidade Federal do Ceará - CE
69	ABONO (ACT)	CH da Universidade Federal do Ceará - CE
69	ABONO (ACT)	CH da Universidade Federal do Ceará - CE
69	ABONO (ACT)	CH da Universidade Federal do Ceará - CE
69	ABONO (ACT)	CH da Universidade Federal do Ceará - CE
69	ABONO (ACT)	CH da Universidade Federal do Ceará - CE
69	ABONO (ACT)	CH da Universidade Federal do Ceará - CE
69	ABONO (ACT)	CH da Universidade Federal do Ceará - CE
69	ABONO (ACT)	CH da Universidade Federal do Ceará - CE
69	ABONO (ACT)	CH da Universidade Federal do Ceará - CE
69	ABONO (ACT)	CH da Universidade Federal do Ceará - CE
69	ABONO (ACT)	CH da Universidade Federal do Ceará - CE
69	ABONO (ACT)	CH da Universidade Federal do Ceará - CE
69	ABONO (ACT)	CH da Universidade Federal do Ceará - CE
69	ABONO (ACT)	CH da Universidade Federal do Ceará - CE
69	ABONO (ACT)	CH da Universidade Federal do Ceará - CE
69	ABONO (ACT)	CH da Universidade Federal do Ceará - CE
69	ABONO (ACT)	CH da Universidade Federal do Ceará - CE
69	ABONO (ACT)	CH da Universidade Federal do Ceará - CE
69	ABONO (ACT)	CH da Universidade Federal do Ceará - CE
69	ABONO (ACT)	CH da Universidade Federal do Ceará - CE
69	ABONO (ACT)	CH da Universidade Federal do Ceará - CE
69	ABONO (ACT)	CH da Universidade Federal do Ceará - CE
69	ABONO (ACT)	CH da Universidade Federal do Ceará - CE
69	ABONO (ACT)	CH da Universidade Federal do Ceará - CE
69	ABONO (ACT)	CH da Universidade Federal do Ceará - CE
69	ABONO (ACT)	CH da Universidade Federal do Ceará - CE
69	ABONO (ACT)	CH da Universidade Federal do Ceará - CE
69	ABONO (ACT)	CH da Universidade Federal do Ceará - CE
69	ABONO (ACT)	CH da Universidade Federal do Ceará - CE
69	ABONO (ACT)	CH da Universidade Federal do Ceará - CE
69	ABONO (ACT)	CH da Universidade Federal do Ceará - CE
69	ABONO (ACT)	CH da Universidade Federal do Ceará - CE
69	ABONO (ACT)	CH da Universidade Federal do Ceará - CE
69	ABONO (ACT)	CH da Universidade Federal do Ceará - CE
69	ABONO (ACT)	CH da Universidade Federal do Ceará - CE
69	ABONO (ACT)	CH da Universidade Federal do Ceará - CE
69	ABONO (ACT)	CH da Universidade Federal do Ceará - CE
69	ABONO (ACT)	CH da Universidade Federal do Ceará - CE
69	ABONO (ACT)	CH da Universidade Federal do Ceará - CE
69	ABONO (ACT)	CH da Universidade Federal do Ceará - CE
69	ABONO (ACT)	CH da Universidade Federal do Ceará - CE
69	ABONO (ACT)	CH da Universidade Federal do Ceará - CE
69	ABONO (ACT)	CH da Universidade Federal do Ceará - CE
69	ABONO (ACT)	CH da Universidade Federal do Ceará - CE
69	ABONO (ACT)	CH da Universidade Federal do Ceará - CE
69	ABONO (ACT)	CH da Universidade Federal do Ceará - CE
69	ABONO (ACT)	CH da Universidade Federal do Ceará - CE

69	ABONO (ACT)	CH da Universidade Federal do Ceará - CE
69	ABONO (ACT)	CH da Universidade Federal do Ceará - CE
69	ABONO (ACT)	CH da Universidade Federal do Ceará - CE
69	ABONO (ACT)	CH da Universidade Federal do Ceará - CE
69	ABONO (ACT)	CH da Universidade Federal do Ceará - CE
69	ABONO (ACT)	CH da Universidade Federal do Ceará - CE
69	ABONO (ACT)	CH da Universidade Federal do Ceará - CE
69	ABONO (ACT)	CH da Universidade Federal do Ceará - CE
69	ABONO (ACT)	CH da Universidade Federal do Ceará - CE
69	ABONO (ACT)	CH da Universidade Federal do Ceará - CE
69	ABONO (ACT)	CH da Universidade Federal do Ceará - CE
69	ABONO (ACT)	CH da Universidade Federal do Ceará - CE
69	ABONO (ACT)	CH da Universidade Federal do Ceará - CE
69	ABONO (ACT)	CH da Universidade Federal do Ceará - CE
69	ABONO (ACT)	CH da Universidade Federal do Ceará - CE
69	ABONO (ACT)	CH da Universidade Federal do Ceará - CE
69	ABONO (ACT)	CH da Universidade Federal do Ceará - CE
69	ABONO (ACT)	CH da Universidade Federal do Ceará - CE
69	ABONO (ACT)	CH da Universidade Federal do Ceará - CE
69	ABONO (ACT)	CH da Universidade Federal do Ceará - CE
69	ABONO (ACT)	CH da Universidade Federal do Ceará - CE
69	ABONO (ACT)	CH da Universidade Federal do Ceará - CE
69	ABONO (ACT)	CH da Universidade Federal do Ceará - CE
69	ABONO (ACT)	CH da Universidade Federal do Ceará - CE
69	ABONO (ACT)	CH da Universidade Federal do Ceará - CE
69	ABONO (ACT)	CH da Universidade Federal do Ceará - CE
69	ABONO (ACT)	CH da Universidade Federal do Ceará - CE
69	ABONO (ACT)	CH da Universidade Federal do Ceará - CE
69	ABONO (ACT)	CH da Universidade Federal do Ceará - CE
69	ABONO (ACT)	CH da Universidade Federal do Ceará - CE
69	ABONO (ACT)	CH da Universidade Federal do Ceará - CE
69	ABONO (ACT)	CH da Universidade Federal do Ceará - CE
69	ABONO (ACT)	CH da Universidade Federal do Ceará - CE
69	ABONO (ACT)	CH da Universidade Federal do Ceará - CE
69	ABONO (ACT)	CH da Universidade Federal do Ceará - CE
69	ABONO (ACT)	CH da Universidade Federal do Ceará - CE
69	ABONO (ACT)	CH da Universidade Federal do Ceará - CE
69	ABONO (ACT)	CH da Universidade Federal do Ceará - CE
69	ABONO (ACT)	CH da Universidade Federal do Ceará - CE
69	ABONO (ACT)	CH da Universidade Federal do Ceará - CE
69	ABONO (ACT)	CH da Universidade Federal do Ceará - CE
69	ABONO (ACT)	CH da Universidade Federal do Ceará - CE
69	ABONO (ACT)	CH da Universidade Federal do Ceará - CE
69	ABONO (ACT)	CH da Universidade Federal do Ceará - CE
69	ABONO (ACT)	CH da Universidade Federal do Ceará - CE
69	ABONO (ACT)	CH da Universidade Federal do Ceará - CE
69	ABONO (ACT)	CH da Universidade Federal do Ceará - CE
69	ABONO (ACT)	CH da Universidade Federal do Ceará - CE

69	ABONO (ACT)	CH da Universidade Federal do Ceará - CE
69	ABONO (ACT)	CH da Universidade Federal do Ceará - CE
69	ABONO (ACT)	CH da Universidade Federal do Ceará - CE
69	ABONO (ACT)	CH da Universidade Federal do Ceará - CE
69	ABONO (ACT)	CH da Universidade Federal do Ceará - CE
69	ABONO (ACT)	CH da Universidade Federal do Ceará - CE
69	ABONO (ACT)	CH da Universidade Federal do Ceará - CE
69	ABONO (ACT)	CH da Universidade Federal do Ceará - CE
69	ABONO (ACT)	CH da Universidade Federal do Ceará - CE
69	ABONO (ACT)	CH da Universidade Federal do Ceará - CE
69	ABONO (ACT)	CH da Universidade Federal do Ceará - CE
69	ABONO (ACT)	CH da Universidade Federal do Ceará - CE
69	ABONO (ACT)	CH da Universidade Federal do Ceará - CE
69	ABONO (ACT)	CH da Universidade Federal do Ceará - CE
69	ABONO (ACT)	CH da Universidade Federal do Ceará - CE
69	ABONO (ACT)	CH da Universidade Federal do Ceará - CE
69	ABONO (ACT)	CH da Universidade Federal do Ceará - CE
69	ABONO (ACT)	CH da Universidade Federal do Ceará - CE
69	ABONO (ACT)	CH da Universidade Federal do Ceará - CE
69	ABONO (ACT)	CH da Universidade Federal do Ceará - CE
69	ABONO (ACT)	CH da Universidade Federal do Ceará - CE
69	ABONO (ACT)	CH da Universidade Federal do Ceará - CE
69	ABONO (ACT)	CH da Universidade Federal do Ceará - CE
69	ABONO (ACT)	CH da Universidade Federal do Ceará - CE
69	ABONO (ACT)	CH da Universidade Federal do Ceará - CE
69	ABONO (ACT)	CH da Universidade Federal do Ceará - CE
69	ABONO (ACT)	CH da Universidade Federal do Ceará - CE
69	ABONO (ACT)	CH da Universidade Federal do Ceará - CE
69	ABONO (ACT)	CH da Universidade Federal do Ceará - CE
69	ABONO (ACT)	CH da Universidade Federal do Ceará - CE
69	ABONO (ACT)	CH da Universidade Federal do Ceará - CE
69	ABONO (ACT)	CH da Universidade Federal do Ceará - CE
69	ABONO (ACT)	CH da Universidade Federal do Ceará - CE
69	ABONO (ACT)	CH da Universidade Federal do Ceará - CE
69	ABONO (ACT)	CH da Universidade Federal do Ceará - CE
69	ABONO (ACT)	CH da Universidade Federal do Ceará - CE
69	ABONO (ACT)	CH da Universidade Federal do Ceará - CE
69	ABONO (ACT)	CH da Universidade Federal do Ceará - CE
69	ABONO (ACT)	CH da Universidade Federal do Ceará - CE
69	ABONO (ACT)	CH da Universidade Federal do Ceará - CE
69	ABONO (ACT)	CH da Universidade Federal do Ceará - CE
69	ABONO (ACT)	CH da Universidade Federal do Ceará - CE
69	ABONO (ACT)	CH da Universidade Federal do Ceará - CE
69	ABONO (ACT)	CH da Universidade Federal do Ceará - CE
69	ABONO (ACT)	CH da Universidade Federal do Ceará - CE
69	ABONO (ACT)	CH da Universidade Federal do Ceará - CE
69	ABONO (ACT)	CH da Universidade Federal do Ceará - CE
69	ABONO (ACT)	CH da Universidade Federal do Ceará - CE

69	ABONO (ACT)	CH da Universidade Federal do Ceará - CE
69	ABONO (ACT)	CH da Universidade Federal do Ceará - CE
69	ABONO (ACT)	CH da Universidade Federal do Ceará - CE
69	ABONO (ACT)	CH da Universidade Federal do Ceará - CE
69	ABONO (ACT)	CH da Universidade Federal do Ceará - CE
69	ABONO (ACT)	CH da Universidade Federal do Ceará - CE
69	ABONO (ACT)	CH da Universidade Federal do Ceará - CE
69	ABONO (ACT)	CH da Universidade Federal do Ceará - CE
69	ABONO (ACT)	CH da Universidade Federal do Ceará - CE
69	ABONO (ACT)	CH da Universidade Federal do Ceará - CE
69	ABONO (ACT)	CH da Universidade Federal do Ceará - CE
69	ABONO (ACT)	CH da Universidade Federal do Ceará - CE
69	ABONO (ACT)	CH da Universidade Federal do Ceará - CE
69	ABONO (ACT)	CH da Universidade Federal do Ceará - CE
69	ABONO (ACT)	CH da Universidade Federal do Ceará - CE
69	ABONO (ACT)	CH da Universidade Federal do Ceará - CE
69	ABONO (ACT)	CH da Universidade Federal do Ceará - CE
69	ABONO (ACT)	CH da Universidade Federal do Ceará - CE
69	ABONO (ACT)	CH da Universidade Federal do Ceará - CE
69	ABONO (ACT)	CH da Universidade Federal do Ceará - CE
69	ABONO (ACT)	CH da Universidade Federal do Ceará - CE
69	ABONO (ACT)	CH da Universidade Federal do Ceará - CE
69	ABONO (ACT)	CH da Universidade Federal do Ceará - CE
69	ABONO (ACT)	CH da Universidade Federal do Ceará - CE
69	ABONO (ACT)	CH da Universidade Federal do Ceará - CE
69	ABONO (ACT)	CH da Universidade Federal do Ceará - CE
69	ABONO (ACT)	CH da Universidade Federal do Ceará - CE
69	ABONO (ACT)	CH da Universidade Federal do Ceará - CE
69	ABONO (ACT)	CH da Universidade Federal do Ceará - CE
69	ABONO (ACT)	CH da Universidade Federal do Ceará - CE
69	ABONO (ACT)	CH da Universidade Federal do Ceará - CE
69	ABONO (ACT)	CH da Universidade Federal do Ceará - CE
69	ABONO (ACT)	CH da Universidade Federal do Ceará - CE
69	ABONO (ACT)	CH da Universidade Federal do Ceará - CE
69	ABONO (ACT)	CH da Universidade Federal do Ceará - CE
69	ABONO (ACT)	CH da Universidade Federal do Ceará - CE
69	ABONO (ACT)	CH da Universidade Federal do Ceará - CE
69	ABONO (ACT)	CH da Universidade Federal do Ceará - CE
69	ABONO (ACT)	CH da Universidade Federal do Ceará - CE
69	ABONO (ACT)	CH da Universidade Federal do Ceará - CE
69	ABONO (ACT)	CH da Universidade Federal do Ceará - CE
69	ABONO (ACT)	CH da Universidade Federal do Ceará - CE
69	ABONO (ACT)	CH da Universidade Federal do Ceará - CE
69	ABONO (ACT)	CH da Universidade Federal do Ceará - CE
69	ABONO (ACT)	CH da Universidade Federal do Ceará - CE
69	ABONO (ACT)	CH da Universidade Federal do Ceará - CE
69	ABONO (ACT)	CH da Universidade Federal do Ceará - CE
69	ABONO (ACT)	CH da Universidade Federal do Ceará - CE

69	ABONO (ACT)	CH da Universidade Federal do Ceará - CE
69	ABONO (ACT)	CH da Universidade Federal do Ceará - CE
69	ABONO (ACT)	CH da Universidade Federal do Ceará - CE
69	ABONO (ACT)	CH da Universidade Federal do Ceará - CE
69	ABONO (ACT)	CH da Universidade Federal do Ceará - CE
69	ABONO (ACT)	CH da Universidade Federal do Ceará - CE
69	ABONO (ACT)	CH da Universidade Federal do Ceará - CE
69	ABONO (ACT)	CH da Universidade Federal do Ceará - CE
69	ABONO (ACT)	CH da Universidade Federal do Ceará - CE
69	ABONO (ACT)	CH da Universidade Federal do Ceará - CE
69	ABONO (ACT)	CH da Universidade Federal do Ceará - CE
69	ABONO (ACT)	CH da Universidade Federal do Ceará - CE
69	ABONO (ACT)	CH da Universidade Federal do Ceará - CE
69	ABONO (ACT)	CH da Universidade Federal do Ceará - CE
69	ABONO (ACT)	CH da Universidade Federal do Ceará - CE
69	ABONO (ACT)	CH da Universidade Federal do Ceará - CE
69	ABONO (ACT)	CH da Universidade Federal do Ceará - CE
69	ABONO (ACT)	CH da Universidade Federal do Ceará - CE
69	ABONO (ACT)	CH da Universidade Federal do Ceará - CE
69	ABONO (ACT)	CH da Universidade Federal do Ceará - CE
69	ABONO (ACT)	CH da Universidade Federal do Ceará - CE
69	ABONO (ACT)	CH da Universidade Federal do Ceará - CE
69	ABONO (ACT)	CH da Universidade Federal do Ceará - CE
69	ABONO (ACT)	CH da Universidade Federal do Ceará - CE
69	ABONO (ACT)	CH da Universidade Federal do Ceará - CE
69	ABONO (ACT)	CH da Universidade Federal do Ceará - CE
69	ABONO (ACT)	CH da Universidade Federal do Ceará - CE
69	ABONO (ACT)	CH da Universidade Federal do Ceará - CE
69	ABONO (ACT)	CH da Universidade Federal do Ceará - CE
69	ABONO (ACT)	CH da Universidade Federal do Ceará - CE
69	ABONO (ACT)	CH da Universidade Federal do Ceará - CE
69	ABONO (ACT)	CH da Universidade Federal do Ceará - CE
69	ABONO (ACT)	CH da Universidade Federal do Ceará - CE
69	ABONO (ACT)	CH da Universidade Federal do Ceará - CE
69	ABONO (ACT)	CH da Universidade Federal do Ceará - CE
69	ABONO (ACT)	CH da Universidade Federal do Ceará - CE
69	ABONO (ACT)	CH da Universidade Federal do Ceará - CE
69	ABONO (ACT)	CH da Universidade Federal do Ceará - CE
69	ABONO (ACT)	CH da Universidade Federal do Ceará - CE
69	ABONO (ACT)	CH da Universidade Federal do Ceará - CE
69	ABONO (ACT)	CH da Universidade Federal do Ceará - CE
69	ABONO (ACT)	CH da Universidade Federal do Ceará - CE
69	ABONO (ACT)	CH da Universidade Federal do Ceará - CE
69	ABONO (ACT)	CH da Universidade Federal do Ceará - CE
69	ABONO (ACT)	CH da Universidade Federal do Ceará - CE
69	ABONO (ACT)	CH da Universidade Federal do Ceará - CE
69	ABONO (ACT)	CH da Universidade Federal do Ceará - CE
69	ABONO (ACT)	CH da Universidade Federal do Ceará - CE

69	ABONO (ACT)	CH da Universidade Federal do Ceará - CE
69	ABONO (ACT)	CH da Universidade Federal do Ceará - CE
69	ABONO (ACT)	CH da Universidade Federal do Ceará - CE
69	ABONO (ACT)	CH da Universidade Federal do Ceará - CE
69	ABONO (ACT)	CH da Universidade Federal do Ceará - CE
69	ABONO (ACT)	CH da Universidade Federal do Ceará - CE
69	ABONO (ACT)	CH da Universidade Federal do Ceará - CE
69	ABONO (ACT)	CH da Universidade Federal do Ceará - CE
69	ABONO (ACT)	CH da Universidade Federal do Ceará - CE
69	ABONO (ACT)	CH da Universidade Federal do Ceará - CE
69	ABONO (ACT)	CH da Universidade Federal do Ceará - CE
69	ABONO (ACT)	CH da Universidade Federal do Ceará - CE
69	ABONO (ACT)	CH da Universidade Federal do Ceará - CE
69	ABONO (ACT)	CH da Universidade Federal do Ceará - CE
69	ABONO (ACT)	CH da Universidade Federal do Ceará - CE
69	ABONO (ACT)	CH da Universidade Federal do Ceará - CE
69	ABONO (ACT)	CH da Universidade Federal do Ceará - CE
69	ABONO (ACT)	CH da Universidade Federal do Ceará - CE
69	ABONO (ACT)	CH da Universidade Federal do Ceará - CE
69	ABONO (ACT)	CH da Universidade Federal do Ceará - CE
69	ABONO (ACT)	CH da Universidade Federal do Ceará - CE
69	ABONO (ACT)	CH da Universidade Federal do Ceará - CE
69	ABONO (ACT)	CH da Universidade Federal do Ceará - CE
69	ABONO (ACT)	CH da Universidade Federal do Ceará - CE
69	ABONO (ACT)	CH da Universidade Federal do Ceará - CE
69	ABONO (ACT)	CH da Universidade Federal do Ceará - CE
69	ABONO (ACT)	CH da Universidade Federal do Ceará - CE
69	ABONO (ACT)	CH da Universidade Federal do Ceará - CE
69	ABONO (ACT)	CH da Universidade Federal do Ceará - CE
69	ABONO (ACT)	CH da Universidade Federal do Ceará - CE
69	ABONO (ACT)	CH da Universidade Federal do Ceará - CE
69	ABONO (ACT)	CH da Universidade Federal do Ceará - CE
69	ABONO (ACT)	CH da Universidade Federal do Ceará - CE
69	ABONO (ACT)	CH da Universidade Federal do Ceará - CE
69	ABONO (ACT)	CH da Universidade Federal do Ceará - CE
69	ABONO (ACT)	CH da Universidade Federal do Ceará - CE
69	ABONO (ACT)	CH da Universidade Federal do Ceará - CE
69	ABONO (ACT)	CH da Universidade Federal do Ceará - CE
69	ABONO (ACT)	CH da Universidade Federal do Ceará - CE
69	ABONO (ACT)	CH da Universidade Federal do Ceará - CE
69	ABONO (ACT)	CH da Universidade Federal do Ceará - CE
69	ABONO (ACT)	CH da Universidade Federal do Ceará - CE
69	ABONO (ACT)	CH da Universidade Federal do Ceará - CE
69	ABONO (ACT)	CH da Universidade Federal do Ceará - CE
69	ABONO (ACT)	CH da Universidade Federal do Ceará - CE
69	ABONO (ACT)	CH da Universidade Federal do Ceará - CE
69	ABONO (ACT)	CH da Universidade Federal do Ceará - CE
69	ABONO (ACT)	CH da Universidade Federal do Ceará - CE

69	ABONO (ACT)	CH da Universidade Federal do Ceará - CE
69	ABONO (ACT)	CH da Universidade Federal do Ceará - CE
69	ABONO (ACT)	CH da Universidade Federal do Ceará - CE
69	ABONO (ACT)	CH da Universidade Federal do Ceará - CE
69	ABONO (ACT)	CH da Universidade Federal do Ceará - CE
69	ABONO (ACT)	CH da Universidade Federal do Ceará - CE
69	ABONO (ACT)	CH da Universidade Federal do Ceará - CE
69	ABONO (ACT)	CH da Universidade Federal do Ceará - CE
69	ABONO (ACT)	CH da Universidade Federal do Ceará - CE
69	ABONO (ACT)	CH da Universidade Federal do Ceará - CE
69	ABONO (ACT)	CH da Universidade Federal do Ceará - CE
69	ABONO (ACT)	CH da Universidade Federal do Ceará - CE
69	ABONO (ACT)	CH da Universidade Federal do Ceará - CE
69	ABONO (ACT)	CH da Universidade Federal do Ceará - CE
69	ABONO (ACT)	CH da Universidade Federal do Ceará - CE
69	ABONO (ACT)	CH da Universidade Federal do Ceará - CE
69	ABONO (ACT)	CH da Universidade Federal do Ceará - CE
69	ABONO (ACT)	CH da Universidade Federal do Ceará - CE
69	ABONO (ACT)	CH da Universidade Federal do Ceará - CE
69	ABONO (ACT)	CH da Universidade Federal do Ceará - CE
69	ABONO (ACT)	CH da Universidade Federal do Ceará - CE
69	ABONO (ACT)	CH da Universidade Federal do Ceará - CE
69	ABONO (ACT)	CH da Universidade Federal do Ceará - CE
69	ABONO (ACT)	CH da Universidade Federal do Ceará - CE
69	ABONO (ACT)	CH da Universidade Federal do Ceará - CE
69	ABONO (ACT)	CH da Universidade Federal do Ceará - CE
69	ABONO (ACT)	CH da Universidade Federal do Ceará - CE
69	ABONO (ACT)	CH da Universidade Federal do Ceará - CE
69	ABONO (ACT)	CH da Universidade Federal do Ceará - CE
69	ABONO (ACT)	CH da Universidade Federal do Ceará - CE
69	ABONO (ACT)	CH da Universidade Federal do Ceará - CE
69	ABONO (ACT)	CH da Universidade Federal do Ceará - CE
69	ABONO (ACT)	CH da Universidade Federal do Ceará - CE
69	ABONO (ACT)	CH da Universidade Federal do Ceará - CE
69	ABONO (ACT)	CH da Universidade Federal do Ceará - CE
69	ABONO (ACT)	CH da Universidade Federal do Ceará - CE
69	ABONO (ACT)	CH da Universidade Federal do Ceará - CE
69	ABONO (ACT)	CH da Universidade Federal do Ceará - CE
69	ABONO (ACT)	CH da Universidade Federal do Ceará - CE
69	ABONO (ACT)	CH da Universidade Federal do Ceará - CE
69	ABONO (ACT)	CH da Universidade Federal do Ceará - CE
69	ABONO (ACT)	CH da Universidade Federal do Ceará - CE
69	ABONO (ACT)	CH da Universidade Federal do Ceará - CE
69	ABONO (ACT)	CH da Universidade Federal do Ceará - CE
69	ABONO (ACT)	CH da Universidade Federal do Ceará - CE
69	ABONO (ACT)	CH da Universidade Federal do Ceará - CE
69	ABONO (ACT)	CH da Universidade Federal do Ceará - CE

69	ABONO (ACT)	CH da Universidade Federal do Ceará - CE
69	ABONO (ACT)	CH da Universidade Federal do Ceará - CE
69	ABONO (ACT)	CH da Universidade Federal do Ceará - CE
69	ABONO (ACT)	CH da Universidade Federal do Ceará - CE
69	ABONO (ACT)	CH da Universidade Federal do Ceará - CE
69	ABONO (ACT)	CH da Universidade Federal do Ceará - CE
69	ABONO (ACT)	CH da Universidade Federal do Ceará - CE
69	ABONO (ACT)	CH da Universidade Federal do Ceará - CE
69	ABONO (ACT)	CH da Universidade Federal do Ceará - CE
69	ABONO (ACT)	CH da Universidade Federal do Ceará - CE
69	ABONO (ACT)	CH da Universidade Federal do Ceará - CE
69	ABONO (ACT)	CH da Universidade Federal do Ceará - CE
69	ABONO (ACT)	CH da Universidade Federal do Ceará - CE
69	ABONO (ACT)	CH da Universidade Federal do Ceará - CE
69	ABONO (ACT)	CH da Universidade Federal do Ceará - CE
69	ABONO (ACT)	CH da Universidade Federal do Ceará - CE
69	ABONO (ACT)	CH da Universidade Federal do Ceará - CE
69	ABONO (ACT)	CH da Universidade Federal do Ceará - CE
69	ABONO (ACT)	CH da Universidade Federal do Ceará - CE
69	ABONO (ACT)	CH da Universidade Federal do Ceará - CE
69	ABONO (ACT)	CH da Universidade Federal do Ceará - CE
69	ABONO (ACT)	CH da Universidade Federal do Ceará - CE
69	ABONO (ACT)	CH da Universidade Federal do Ceará - CE
69	ABONO (ACT)	CH da Universidade Federal do Ceará - CE
69	ABONO (ACT)	CH da Universidade Federal do Ceará - CE
69	ABONO (ACT)	CH da Universidade Federal do Ceará - CE
69	ABONO (ACT)	CH da Universidade Federal do Ceará - CE
69	ABONO (ACT)	CH da Universidade Federal do Ceará - CE
69	ABONO (ACT)	CH da Universidade Federal do Ceará - CE
69	ABONO (ACT)	CH da Universidade Federal do Ceará - CE
69	ABONO (ACT)	CH da Universidade Federal do Ceará - CE
69	ABONO (ACT)	CH da Universidade Federal do Ceará - CE
69	ABONO (ACT)	CH da Universidade Federal do Ceará - CE
69	ABONO (ACT)	CH da Universidade Federal do Ceará - CE
69	ABONO (ACT)	CH da Universidade Federal do Ceará - CE
69	ABONO (ACT)	CH da Universidade Federal do Ceará - CE
69	ABONO (ACT)	CH da Universidade Federal do Ceará - CE
69	ABONO (ACT)	CH da Universidade Federal do Ceará - CE
69	ABONO (ACT)	CH da Universidade Federal do Ceará - CE
69	ABONO (ACT)	CH da Universidade Federal do Ceará - CE
69	ABONO (ACT)	CH da Universidade Federal do Ceará - CE
69	ABONO (ACT)	CH da Universidade Federal do Ceará - CE
69	ABONO (ACT)	CH da Universidade Federal do Ceará - CE
69	ABONO (ACT)	CH da Universidade Federal do Ceará - CE
69	ABONO (ACT)	CH da Universidade Federal do Ceará - CE

69	ABONO (ACT)	CH da Universidade Federal do Ceará - CE
69	ABONO (ACT)	CH da Universidade Federal do Ceará - CE
69	ABONO (ACT)	CH da Universidade Federal do Ceará - CE
69	ABONO (ACT)	CH da Universidade Federal do Ceará - CE
69	ABONO (ACT)	CH da Universidade Federal do Ceará - CE
69	ABONO (ACT)	CH da Universidade Federal do Ceará - CE
69	ABONO (ACT)	CH da Universidade Federal do Ceará - CE
69	ABONO (ACT)	CH da Universidade Federal do Ceará - CE
69	ABONO (ACT)	CH da Universidade Federal do Ceará - CE
69	ABONO (ACT)	CH da Universidade Federal do Ceará - CE
69	ABONO (ACT)	CH da Universidade Federal do Ceará - CE
69	ABONO (ACT)	CH da Universidade Federal do Ceará - CE
69	ABONO (ACT)	CH da Universidade Federal do Ceará - CE
69	ABONO (ACT)	CH da Universidade Federal do Ceará - CE
69	ABONO (ACT)	CH da Universidade Federal do Ceará - CE
69	ABONO (ACT)	CH da Universidade Federal do Ceará - CE
69	ABONO (ACT)	CH da Universidade Federal do Ceará - CE
69	ABONO (ACT)	CH da Universidade Federal do Ceará - CE
69	ABONO (ACT)	CH da Universidade Federal do Ceará - CE
69	ABONO (ACT)	CH da Universidade Federal do Ceará - CE
69	ABONO (ACT)	CH da Universidade Federal do Ceará - CE
69	ABONO (ACT)	CH da Universidade Federal do Ceará - CE
69	ABONO (ACT)	CH da Universidade Federal do Ceará - CE
69	ABONO (ACT)	CH da Universidade Federal do Ceará - CE
69	ABONO (ACT)	CH da Universidade Federal do Ceará - CE
69	ABONO (ACT)	CH da Universidade Federal do Ceará - CE
69	ABONO (ACT)	CH da Universidade Federal do Ceará - CE
69	ABONO (ACT)	CH da Universidade Federal do Ceará - CE
69	ABONO (ACT)	CH da Universidade Federal do Ceará - CE
69	ABONO (ACT)	CH da Universidade Federal do Ceará - CE
69	ABONO (ACT)	CH da Universidade Federal do Ceará - CE
69	ABONO (ACT)	CH da Universidade Federal do Ceará - CE
69	ABONO (ACT)	CH da Universidade Federal do Ceará - CE
69	ABONO (ACT)	CH da Universidade Federal do Ceará - CE
69	ABONO (ACT)	CH da Universidade Federal do Ceará - CE
69	ABONO (ACT)	CH da Universidade Federal do Ceará - CE
69	ABONO (ACT)	CH da Universidade Federal do Ceará - CE
69	ABONO (ACT)	CH da Universidade Federal do Ceará - CE
69	ABONO (ACT)	CH da Universidade Federal do Ceará - CE
69	ABONO (ACT)	CH da Universidade Federal do Ceará - CE

69	ABONO (ACT)	CH da Universidade Federal do Ceará - CE
69	ABONO (ACT)	CH da Universidade Federal do Ceará - CE
69	ABONO (ACT)	CH da Universidade Federal do Ceará - CE
69	ABONO (ACT)	CH da Universidade Federal do Ceará - CE
69	ABONO (ACT)	CH da Universidade Federal do Ceará - CE
69	ABONO (ACT)	CH da Universidade Federal do Ceará - CE
69	ABONO (ACT)	CH da Universidade Federal do Ceará - CE
69	ABONO (ACT)	CH da Universidade Federal do Ceará - CE
69	ABONO (ACT)	CH da Universidade Federal do Ceará - CE
69	ABONO (ACT)	CH da Universidade Federal do Ceará - CE
69	ABONO (ACT)	CH da Universidade Federal do Ceará - CE
69	ABONO (ACT)	CH da Universidade Federal do Ceará - CE
69	ABONO (ACT)	CH da Universidade Federal do Ceará - CE
69	ABONO (ACT)	CH da Universidade Federal do Ceará - CE
69	ABONO (ACT)	CH da Universidade Federal do Ceará - CE
69	ABONO (ACT)	CH da Universidade Federal do Ceará - CE
69	ABONO (ACT)	CH da Universidade Federal do Ceará - CE
69	ABONO (ACT)	CH da Universidade Federal do Ceará - CE
69	ABONO (ACT)	CH da Universidade Federal do Ceará - CE
69	ABONO (ACT)	CH da Universidade Federal do Ceará - CE
69	ABONO (ACT)	CH da Universidade Federal do Ceará - CE
69	ABONO (ACT)	CH da Universidade Federal do Ceará - CE
69	ABONO (ACT)	CH da Universidade Federal do Ceará - CE
69	ABONO (ACT)	CH da Universidade Federal do Ceará - CE
69	ABONO (ACT)	CH da Universidade Federal do Ceará - CE
69	ABONO (ACT)	CH da Universidade Federal do Ceará - CE
69	ABONO (ACT)	CH da Universidade Federal do Ceará - CE
69	ABONO (ACT)	CH da Universidade Federal do Ceará - CE
69	ABONO (ACT)	CH da Universidade Federal do Ceará - CE
69	ABONO (ACT)	CH da Universidade Federal do Ceará - CE
69	ABONO (ACT)	CH da Universidade Federal do Ceará - CE
69	ABONO (ACT)	CH da Universidade Federal do Ceará - CE
69	ABONO (ACT)	CH da Universidade Federal do Ceará - CE
69	ABONO (ACT)	CH da Universidade Federal do Ceará - CE
69	ABONO (ACT)	CH da Universidade Federal do Ceará - CE
69	ABONO (ACT)	CH da Universidade Federal do Ceará - CE
69	ABONO (ACT)	CH da Universidade Federal do Ceará - CE
69	ABONO (ACT)	CH da Universidade Federal do Ceará - CE
69	ABONO (ACT)	CH da Universidade Federal do Ceará - CE
69	ABONO (ACT)	CH da Universidade Federal do Ceará - CE
69	ABONO (ACT)	CH da Universidade Federal do Ceará - CE
69	ABONO (ACT)	CH da Universidade Federal do Ceará - CE
69	ABONO (ACT)	CH da Universidade Federal do Ceará - CE
69	ABONO (ACT)	CH da Universidade Federal do Ceará - CE
69	ABONO (ACT)	CH da Universidade Federal do Ceará - CE
69	ABONO (ACT)	CH da Universidade Federal do Ceará - CE
69	ABONO (ACT)	CH da Universidade Federal do Ceará - CE
69	ABONO (ACT)	CH da Universidade Federal do Ceará - CE

69	ABONO (ACT)	CH da Universidade Federal do Ceará - CE
69	ABONO (ACT)	CH da Universidade Federal do Ceará - CE
69	ABONO (ACT)	CH da Universidade Federal do Ceará - CE
69	ABONO (ACT)	CH da Universidade Federal do Ceará - CE
69	ABONO (ACT)	CH da Universidade Federal do Ceará - CE
69	ABONO (ACT)	CH da Universidade Federal do Ceará - CE
69	ABONO (ACT)	CH da Universidade Federal do Ceará - CE
69	ABONO (ACT)	CH da Universidade Federal do Ceará - CE
69	ABONO (ACT)	CH da Universidade Federal do Ceará - CE
69	ABONO (ACT)	CH da Universidade Federal do Ceará - CE
69	ABONO (ACT)	CH da Universidade Federal do Ceará - CE
69	ABONO (ACT)	CH da Universidade Federal do Ceará - CE
69	ABONO (ACT)	CH da Universidade Federal do Ceará - CE
69	ABONO (ACT)	CH da Universidade Federal do Ceará - CE
69	ABONO (ACT)	CH da Universidade Federal do Ceará - CE
69	ABONO (ACT)	CH da Universidade Federal do Ceará - CE
69	ABONO (ACT)	CH da Universidade Federal do Ceará - CE
69	ABONO (ACT)	CH da Universidade Federal do Ceará - CE
69	ABONO (ACT)	CH da Universidade Federal do Ceará - CE
69	ABONO (ACT)	CH da Universidade Federal do Ceará - CE
69	ABONO (ACT)	CH da Universidade Federal do Ceará - CE
69	ABONO (ACT)	CH da Universidade Federal do Ceará - CE
69	ABONO (ACT)	CH da Universidade Federal do Ceará - CE
69	ABONO (ACT)	CH da Universidade Federal do Ceará - CE
69	ABONO (ACT)	CH da Universidade Federal do Ceará - CE
69	ABONO (ACT)	CH da Universidade Federal do Ceará - CE
69	ABONO (ACT)	CH da Universidade Federal do Ceará - CE
69	ABONO (ACT)	CH da Universidade Federal do Ceará - CE
69	ABONO (ACT)	CH da Universidade Federal do Ceará - CE
69	ABONO (ACT)	CH da Universidade Federal do Ceará - CE
69	ABONO (ACT)	CH da Universidade Federal do Ceará - CE
69	ABONO (ACT)	CH da Universidade Federal do Ceará - CE
69	ABONO (ACT)	CH da Universidade Federal do Ceará - CE
69	ABONO (ACT)	CH da Universidade Federal do Ceará - CE
69	ABONO (ACT)	CH da Universidade Federal do Ceará - CE
69	ABONO (ACT)	CH da Universidade Federal do Ceará - CE
69	ABONO (ACT)	CH da Universidade Federal do Ceará - CE
69	ABONO (ACT)	CH da Universidade Federal do Ceará - CE
69	ABONO (ACT)	CH da Universidade Federal do Ceará - CE
69	ABONO (ACT)	CH da Universidade Federal do Ceará - CE
69	ABONO (ACT)	CH da Universidade Federal do Ceará - CE
69	ABONO (ACT)	CH da Universidade Federal do Ceará - CE
69	ABONO (ACT)	CH da Universidade Federal do Ceará - CE
69	ABONO (ACT)	CH da Universidade Federal do Ceará - CE
69	ABONO (ACT)	CH da Universidade Federal do Ceará - CE
69	ABONO (ACT)	CH da Universidade Federal do Ceará - CE
69	ABONO (ACT)	CH da Universidade Federal do Ceará - CE

69	ABONO (ACT)	CH da Universidade Federal do Ceará - CE
69	ABONO (ACT)	CH da Universidade Federal do Ceará - CE
69	ABONO (ACT)	CH da Universidade Federal do Ceará - CE
69	ABONO (ACT)	CH da Universidade Federal do Ceará - CE
69	ABONO (ACT)	CH da Universidade Federal do Ceará - CE
69	ABONO (ACT)	CH da Universidade Federal do Ceará - CE
69	ABONO (ACT)	CH da Universidade Federal do Ceará - CE
69	ABONO (ACT)	CH da Universidade Federal do Ceará - CE
69	ABONO (ACT)	CH da Universidade Federal do Ceará - CE
69	ABONO (ACT)	CH da Universidade Federal do Ceará - CE
69	ABONO (ACT)	CH da Universidade Federal do Ceará - CE
69	ABONO (ACT)	CH da Universidade Federal do Ceará - CE
69	ABONO (ACT)	CH da Universidade Federal do Ceará - CE
69	ABONO (ACT)	CH da Universidade Federal do Ceará - CE
69	ABONO (ACT)	CH da Universidade Federal do Ceará - CE
69	ABONO (ACT)	CH da Universidade Federal do Ceará - CE
69	ABONO (ACT)	CH da Universidade Federal do Ceará - CE
69	ABONO (ACT)	CH da Universidade Federal do Ceará - CE
69	ABONO (ACT)	CH da Universidade Federal do Ceará - CE
69	ABONO (ACT)	CH da Universidade Federal do Ceará - CE
69	ABONO (ACT)	CH da Universidade Federal do Ceará - CE
69	ABONO (ACT)	CH da Universidade Federal do Ceará - CE
69	ABONO (ACT)	CH da Universidade Federal do Ceará - CE
69	ABONO (ACT)	CH da Universidade Federal do Ceará - CE
69	ABONO (ACT)	CH da Universidade Federal do Ceará - CE
69	ABONO (ACT)	CH da Universidade Federal do Ceará - CE
69	ABONO (ACT)	CH da Universidade Federal do Ceará - CE
69	ABONO (ACT)	CH da Universidade Federal do Ceará - CE
69	ABONO (ACT)	CH da Universidade Federal do Ceará - CE
69	ABONO (ACT)	CH da Universidade Federal do Ceará - CE
69	ABONO (ACT)	CH da Universidade Federal do Ceará - CE
69	ABONO (ACT)	CH da Universidade Federal do Ceará - CE
69	ABONO (ACT)	CH da Universidade Federal do Ceará - CE
69	ABONO (ACT)	CH da Universidade Federal do Ceará - CE
69	ABONO (ACT)	CH da Universidade Federal do Ceará - CE
69	ABONO (ACT)	CH da Universidade Federal do Ceará - CE
69	ABONO (ACT)	CH da Universidade Federal do Ceará - CE
69	ABONO (ACT)	CH da Universidade Federal do Ceará - CE

69	ABONO (ACT)	CH da Universidade Federal do Ceará - CE
69	ABONO (ACT)	CH da Universidade Federal do Ceará - CE
69	ABONO (ACT)	CH da Universidade Federal do Ceará - CE
69	ABONO (ACT)	CH da Universidade Federal do Ceará - CE
69	ABONO (ACT)	CH da Universidade Federal do Ceará - CE
69	ABONO (ACT)	CH da Universidade Federal do Ceará - CE
69	ABONO (ACT)	CH da Universidade Federal do Ceará - CE
69	ABONO (ACT)	CH da Universidade Federal do Ceará - CE
69	ABONO (ACT)	CH da Universidade Federal do Ceará - CE
69	ABONO (ACT)	CH da Universidade Federal do Ceará - CE
69	ABONO (ACT)	CH da Universidade Federal do Ceará - CE
69	ABONO (ACT)	CH da Universidade Federal do Ceará - CE
69	ABONO (ACT)	CH da Universidade Federal do Ceará - CE
69	ABONO (ACT)	CH da Universidade Federal do Ceará - CE
69	ABONO (ACT)	CH da Universidade Federal do Ceará - CE
69	ABONO (ACT)	CH da Universidade Federal do Ceará - CE
69	ABONO (ACT)	CH da Universidade Federal do Ceará - CE
69	ABONO (ACT)	CH da Universidade Federal do Ceará - CE
69	ABONO (ACT)	CH da Universidade Federal do Ceará - CE
69	ABONO (ACT)	CH da Universidade Federal do Ceará - CE
69	ABONO (ACT)	CH da Universidade Federal do Ceará - CE
69	ABONO (ACT)	CH da Universidade Federal do Ceará - CE
69	ABONO (ACT)	CH da Universidade Federal do Ceará - CE
69	ABONO (ACT)	CH da Universidade Federal do Ceará - CE
69	ABONO (ACT)	CH da Universidade Federal do Ceará - CE
69	ABONO (ACT)	CH da Universidade Federal do Ceará - CE
69	ABONO (ACT)	CH da Universidade Federal do Ceará - CE
69	ABONO (ACT)	CH da Universidade Federal do Ceará - CE
69	ABONO (ACT)	CH da Universidade Federal do Ceará - CE
69	ABONO (ACT)	CH da Universidade Federal do Ceará - CE
69	ABONO (ACT)	CH da Universidade Federal do Ceará - CE
69	ABONO (ACT)	CH da Universidade Federal do Ceará - CE
69	ABONO (ACT)	CH da Universidade Federal do Ceará - CE
69	ABONO (ACT)	CH da Universidade Federal do Ceará - CE
69	ABONO (ACT)	CH da Universidade Federal do Ceará - CE
69	ABONO (ACT)	CH da Universidade Federal do Ceará - CE
69	ABONO (ACT)	CH da Universidade Federal do Ceará - CE
69	ABONO (ACT)	CH da Universidade Federal do Ceará - CE
69	ABONO (ACT)	CH da Universidade Federal do Ceará - CE
69	ABONO (ACT)	CH da Universidade Federal do Ceará - CE
69	ABONO (ACT)	CH da Universidade Federal do Ceará - CE
69	ABONO (ACT)	CH da Universidade Federal do Ceará - CE
69	ABONO (ACT)	CH da Universidade Federal do Ceará - CE
69	ABONO (ACT)	CH da Universidade Federal do Ceará - CE
69	ABONO (ACT)	CH da Universidade Federal do Ceará - CE
69	ABONO (ACT)	CH da Universidade Federal do Ceará - CE
69	ABONO (ACT)	CH da Universidade Federal do Ceará - CE
69	ABONO (ACT)	CH da Universidade Federal do Ceará - CE

69	ABONO (ACT)	CH da Universidade Federal do Ceará - CE
69	ABONO (ACT)	CH da Universidade Federal do Ceará - CE
69	ABONO (ACT)	CH da Universidade Federal do Ceará - CE
69	ABONO (ACT)	CH da Universidade Federal do Ceará - CE
69	ABONO (ACT)	CH da Universidade Federal do Ceará - CE
69	ABONO (ACT)	CH da Universidade Federal do Ceará - CE
69	ABONO (ACT)	CH da Universidade Federal do Ceará - CE
69	ABONO (ACT)	CH da Universidade Federal do Ceará - CE
69	ABONO (ACT)	CH da Universidade Federal do Ceará - CE
69	ABONO (ACT)	CH da Universidade Federal do Ceará - CE
69	ABONO (ACT)	CH da Universidade Federal do Ceará - CE
69	ABONO (ACT)	CH da Universidade Federal do Ceará - CE
69	ABONO (ACT)	CH da Universidade Federal do Ceará - CE
69	ABONO (ACT)	CH da Universidade Federal do Ceará - CE
69	ABONO (ACT)	CH da Universidade Federal do Ceará - CE
69	ABONO (ACT)	CH da Universidade Federal do Ceará - CE
69	ABONO (ACT)	CH da Universidade Federal do Ceará - CE
69	ABONO (ACT)	CH da Universidade Federal do Ceará - CE
69	ABONO (ACT)	CH da Universidade Federal do Ceará - CE
69	ABONO (ACT)	CH da Universidade Federal do Ceará - CE
69	ABONO (ACT)	CH da Universidade Federal do Ceará - CE
69	ABONO (ACT)	CH da Universidade Federal do Ceará - CE
69	ABONO (ACT)	CH da Universidade Federal do Ceará - CE
69	ABONO (ACT)	CH da Universidade Federal do Ceará - CE
69	ABONO (ACT)	CH da Universidade Federal do Ceará - CE
69	ABONO (ACT)	CH da Universidade Federal do Ceará - CE
69	ABONO (ACT)	CH da Universidade Federal do Ceará - CE
69	ABONO (ACT)	CH da Universidade Federal do Ceará - CE
69	ABONO (ACT)	CH da Universidade Federal do Ceará - CE
69	ABONO (ACT)	CH da Universidade Federal do Ceará - CE
69	ABONO (ACT)	CH da Universidade Federal do Ceará - CE
69	ABONO (ACT)	CH da Universidade Federal do Ceará - CE
69	ABONO (ACT)	CH da Universidade Federal do Ceará - CE
69	ABONO (ACT)	CH da Universidade Federal do Ceará - CE
69	ABONO (ACT)	CH da Universidade Federal do Ceará - CE
69	ABONO (ACT)	CH da Universidade Federal do Ceará - CE
69	ABONO (ACT)	CH da Universidade Federal do Ceará - CE
69	ABONO (ACT)	CH da Universidade Federal do Ceará - CE
69	ABONO (ACT)	CH da Universidade Federal do Ceará - CE
69	ABONO (ACT)	CH da Universidade Federal do Ceará - CE
69	ABONO (ACT)	CH da Universidade Federal do Ceará - CE
69	ABONO (ACT)	CH da Universidade Federal do Ceará - CE
69	ABONO (ACT)	CH da Universidade Federal do Ceará - CE
69	ABONO (ACT)	CH da Universidade Federal do Ceará - CE
69	ABONO (ACT)	CH da Universidade Federal do Ceará - CE
69	ABONO (ACT)	CH da Universidade Federal do Ceará - CE
69	ABONO (ACT)	CH da Universidade Federal do Ceará - CE
69	ABONO (ACT)	CH da Universidade Federal do Ceará - CE

69	ABONO (ACT)	CH da Universidade Federal do Ceará - CE
69	ABONO (ACT)	CH da Universidade Federal do Ceará - CE
69	ABONO (ACT)	CH da Universidade Federal do Ceará - CE
69	ABONO (ACT)	CH da Universidade Federal do Ceará - CE
69	ABONO (ACT)	CH da Universidade Federal do Ceará - CE
69	ABONO (ACT)	CH da Universidade Federal do Ceará - CE
69	ABONO (ACT)	CH da Universidade Federal do Ceará - CE
69	ABONO (ACT)	CH da Universidade Federal do Ceará - CE
69	ABONO (ACT)	CH da Universidade Federal do Ceará - CE
69	ABONO (ACT)	CH da Universidade Federal do Ceará - CE
69	ABONO (ACT)	CH da Universidade Federal do Ceará - CE
69	ABONO (ACT)	CH da Universidade Federal do Ceará - CE
69	ABONO (ACT)	CH da Universidade Federal do Ceará - CE
69	ABONO (ACT)	CH da Universidade Federal do Ceará - CE
69	ABONO (ACT)	CH da Universidade Federal do Ceará - CE
69	ABONO (ACT)	CH da Universidade Federal do Ceará - CE
69	ABONO (ACT)	CH da Universidade Federal do Ceará - CE
69	ABONO (ACT)	CH da Universidade Federal do Ceará - CE
69	ABONO (ACT)	CH da Universidade Federal do Ceará - CE
69	ABONO (ACT)	CH da Universidade Federal do Ceará - CE
69	ABONO (ACT)	CH da Universidade Federal do Ceará - CE
69	ABONO (ACT)	CH da Universidade Federal do Ceará - CE
69	ABONO (ACT)	CH da Universidade Federal do Ceará - CE
69	ABONO (ACT)	CH da Universidade Federal do Ceará - CE
69	ABONO (ACT)	CH da Universidade Federal do Ceará - CE
69	ABONO (ACT)	CH da Universidade Federal do Ceará - CE
69	ABONO (ACT)	CH da Universidade Federal do Ceará - CE
69	ABONO (ACT)	CH da Universidade Federal do Ceará - CE
69	ABONO (ACT)	CH da Universidade Federal do Ceará - CE
69	ABONO (ACT)	CH da Universidade Federal do Ceará - CE
69	ABONO (ACT)	CH da Universidade Federal do Ceará - CE
69	ABONO (ACT)	CH da Universidade Federal do Ceará - CE
69	ABONO (ACT)	CH da Universidade Federal do Ceará - CE
69	ABONO (ACT)	CH da Universidade Federal do Ceará - CE
69	ABONO (ACT)	CH da Universidade Federal do Ceará - CE
69	ABONO (ACT)	CH da Universidade Federal do Ceará - CE
69	ABONO (ACT)	CH da Universidade Federal do Ceará - CE
69	ABONO (ACT)	CH da Universidade Federal do Ceará - CE
69	ABONO (ACT)	CH da Universidade Federal do Ceará - CE
69	ABONO (ACT)	CH da Universidade Federal do Ceará - CE
69	ABONO (ACT)	CH da Universidade Federal do Ceará - CE
69	ABONO (ACT)	CH da Universidade Federal do Ceará - CE
69	ABONO (ACT)	CH da Universidade Federal do Ceará - CE
69	ABONO (ACT)	CH da Universidade Federal do Ceará - CE
69	ABONO (ACT)	CH da Universidade Federal do Ceará - CE
69	ABONO (ACT)	CH da Universidade Federal do Ceará - CE
69	ABONO (ACT)	CH da Universidade Federal do Ceará - CE
69	ABONO (ACT)	CH da Universidade Federal do Ceará - CE

69	ABONO (ACT)	CH da Universidade Federal do Ceará - CE
69	ABONO (ACT)	CH da Universidade Federal do Ceará - CE
69	ABONO (ACT)	CH da Universidade Federal do Ceará - CE
69	ABONO (ACT)	CH da Universidade Federal do Ceará - CE
69	ABONO (ACT)	CH da Universidade Federal do Ceará - CE
69	ABONO (ACT)	CH da Universidade Federal do Ceará - CE
69	ABONO (ACT)	CH da Universidade Federal do Ceará - CE
69	ABONO (ACT)	CH da Universidade Federal do Ceará - CE
69	ABONO (ACT)	CH da Universidade Federal do Ceará - CE
69	ABONO (ACT)	CH da Universidade Federal do Ceará - CE
69	ABONO (ACT)	CH da Universidade Federal do Ceará - CE
69	ABONO (ACT)	CH da Universidade Federal do Ceará - CE
69	ABONO (ACT)	CH da Universidade Federal do Ceará - CE
69	ABONO (ACT)	CH da Universidade Federal do Ceará - CE
69	ABONO (ACT)	CH da Universidade Federal do Ceará - CE
69	ABONO (ACT)	CH da Universidade Federal do Ceará - CE
69	ABONO (ACT)	CH da Universidade Federal do Ceará - CE
69	ABONO (ACT)	CH da Universidade Federal do Ceará - CE
69	ABONO (ACT)	CH da Universidade Federal do Ceará - CE
69	ABONO (ACT)	CH da Universidade Federal do Ceará - CE
69	ABONO (ACT)	CH da Universidade Federal do Ceará - CE
69	ABONO (ACT)	CH da Universidade Federal do Ceará - CE
69	ABONO (ACT)	CH da Universidade Federal do Ceará - CE
69	ABONO (ACT)	CH da Universidade Federal do Ceará - CE
69	ABONO (ACT)	CH da Universidade Federal do Ceará - CE
69	ABONO (ACT)	CH da Universidade Federal do Ceará - CE
69	ABONO (ACT)	CH da Universidade Federal do Ceará - CE
69	ABONO (ACT)	CH da Universidade Federal do Ceará - CE
69	ABONO (ACT)	CH da Universidade Federal do Ceará - CE
69	ABONO (ACT)	CH da Universidade Federal do Ceará - CE
69	ABONO (ACT)	CH da Universidade Federal do Ceará - CE
69	ABONO (ACT)	CH da Universidade Federal do Ceará - CE
69	ABONO (ACT)	CH da Universidade Federal do Ceará - CE
69	ABONO (ACT)	CH da Universidade Federal do Ceará - CE
69	ABONO (ACT)	CH da Universidade Federal do Ceará - CE
69	ABONO (ACT)	CH da Universidade Federal do Ceará - CE
69	ABONO (ACT)	CH da Universidade Federal do Ceará - CE
69	ABONO (ACT)	CH da Universidade Federal do Ceará - CE
69	ABONO (ACT)	CH da Universidade Federal do Ceará - CE
69	ABONO (ACT)	CH da Universidade Federal do Ceará - CE
69	ABONO (ACT)	CH da Universidade Federal do Ceará - CE
69	ABONO (ACT)	CH da Universidade Federal do Ceará - CE
69	ABONO (ACT)	CH da Universidade Federal do Ceará - CE
69	ABONO (ACT)	CH da Universidade Federal do Ceará - CE
69	ABONO (ACT)	CH da Universidade Federal do Ceará - CE
69	ABONO (ACT)	CH da Universidade Federal do Ceará - CE
69	ABONO (ACT)	CH da Universidade Federal do Ceará - CE
69	ABONO (ACT)	CH da Universidade Federal do Ceará - CE

69	ABONO (ACT)	CH da Universidade Federal do Ceará - CE
69	ABONO (ACT)	CH da Universidade Federal do Ceará - CE
69	ABONO (ACT)	CH da Universidade Federal do Ceará - CE
69	ABONO (ACT)	CH da Universidade Federal do Ceará - CE
69	ABONO (ACT)	CH da Universidade Federal do Ceará - CE
69	ABONO (ACT)	CH da Universidade Federal do Ceará - CE
69	ABONO (ACT)	CH da Universidade Federal do Ceará - CE
69	ABONO (ACT)	CH da Universidade Federal do Ceará - CE
69	ABONO (ACT)	CH da Universidade Federal do Ceará - CE
69	ABONO (ACT)	CH da Universidade Federal do Ceará - CE
69	ABONO (ACT)	CH da Universidade Federal do Ceará - CE
69	ABONO (ACT)	CH da Universidade Federal do Ceará - CE
69	ABONO (ACT)	CH da Universidade Federal do Ceará - CE
69	ABONO (ACT)	CH da Universidade Federal do Ceará - CE
69	ABONO (ACT)	CH da Universidade Federal do Ceará - CE
69	ABONO (ACT)	CH da Universidade Federal do Ceará - CE
69	ABONO (ACT)	CH da Universidade Federal do Ceará - CE
69	ABONO (ACT)	CH da Universidade Federal do Ceará - CE
69	ABONO (ACT)	CH da Universidade Federal do Ceará - CE
69	ABONO (ACT)	CH da Universidade Federal do Ceará - CE
69	ABONO (ACT)	CH da Universidade Federal do Ceará - CE
69	ABONO (ACT)	CH da Universidade Federal do Ceará - CE
69	ABONO (ACT)	CH da Universidade Federal do Ceará - CE
69	ABONO (ACT)	CH da Universidade Federal do Ceará - CE
69	ABONO (ACT)	CH da Universidade Federal do Ceará - CE
69	ABONO (ACT)	CH da Universidade Federal do Ceará - CE
69	ABONO (ACT)	CH da Universidade Federal do Ceará - CE
69	ABONO (ACT)	CH da Universidade Federal do Ceará - CE
69	ABONO (ACT)	CH da Universidade Federal do Ceará - CE
69	ABONO (ACT)	CH da Universidade Federal do Ceará - CE
69	ABONO (ACT)	CH da Universidade Federal do Ceará - CE
69	ABONO (ACT)	CH da Universidade Federal do Ceará - CE
69	ABONO (ACT)	CH da Universidade Federal do Ceará - CE
69	ABONO (ACT)	CH da Universidade Federal do Ceará - CE
69	ABONO (ACT)	CH da Universidade Federal do Ceará - CE
69	ABONO (ACT)	CH da Universidade Federal do Ceará - CE
69	ABONO (ACT)	CH da Universidade Federal do Ceará - CE
69	ABONO (ACT)	CH da Universidade Federal do Ceará - CE
69	ABONO (ACT)	CH da Universidade Federal do Ceará - CE
69	ABONO (ACT)	CH da Universidade Federal do Ceará - CE
69	ABONO (ACT)	CH da Universidade Federal do Ceará - CE
69	ABONO (ACT)	CH da Universidade Federal do Ceará - CE
69	ABONO (ACT)	CH da Universidade Federal do Ceará - CE
69	ABONO (ACT)	CH da Universidade Federal do Ceará - CE
69	ABONO (ACT)	CH da Universidade Federal do Ceará - CE
69	ABONO (ACT)	CH da Universidade Federal do Ceará - CE
69	ABONO (ACT)	CH da Universidade Federal do Ceará - CE
69	ABONO (ACT)	CH da Universidade Federal do Ceará - CE

69	ABONO (ACT)	CH da Universidade Federal do Ceará - CE
69	ABONO (ACT)	CH da Universidade Federal do Ceará - CE
69	ABONO (ACT)	CH da Universidade Federal do Ceará - CE
69	ABONO (ACT)	CH da Universidade Federal do Ceará - CE
69	ABONO (ACT)	CH da Universidade Federal do Ceará - CE
69	ABONO (ACT)	CH da Universidade Federal do Ceará - CE
69	ABONO (ACT)	CH da Universidade Federal do Ceará - CE
69	ABONO (ACT)	CH da Universidade Federal do Ceará - CE
69	ABONO (ACT)	CH da Universidade Federal do Ceará - CE
69	ABONO (ACT)	CH da Universidade Federal do Ceará - CE
69	ABONO (ACT)	CH da Universidade Federal do Ceará - CE
69	ABONO (ACT)	CH da Universidade Federal do Ceará - CE
69	ABONO (ACT)	CH da Universidade Federal do Ceará - CE
69	ABONO (ACT)	CH da Universidade Federal do Ceará - CE
69	ABONO (ACT)	CH da Universidade Federal do Ceará - CE
69	ABONO (ACT)	CH da Universidade Federal do Ceará - CE
69	ABONO (ACT)	CH da Universidade Federal do Ceará - CE
69	ABONO (ACT)	CH da Universidade Federal do Ceará - CE
69	ABONO (ACT)	CH da Universidade Federal do Ceará - CE
69	ABONO (ACT)	CH da Universidade Federal do Ceará - CE
69	ABONO (ACT)	CH da Universidade Federal do Ceará - CE
69	ABONO (ACT)	CH da Universidade Federal do Ceará - CE
69	ABONO (ACT)	CH da Universidade Federal do Ceará - CE
69	ABONO (ACT)	CH da Universidade Federal do Ceará - CE
69	ABONO (ACT)	CH da Universidade Federal do Ceará - CE
69	ABONO (ACT)	CH da Universidade Federal do Ceará - CE
69	ABONO (ACT)	CH da Universidade Federal do Ceará - CE
69	ABONO (ACT)	CH da Universidade Federal do Ceará - CE
69	ABONO (ACT)	CH da Universidade Federal do Ceará - CE
69	ABONO (ACT)	CH da Universidade Federal do Ceará - CE
69	ABONO (ACT)	CH da Universidade Federal do Ceará - CE
69	ABONO (ACT)	CH da Universidade Federal do Ceará - CE
69	ABONO (ACT)	CH da Universidade Federal do Ceará - CE
69	ABONO (ACT)	CH da Universidade Federal do Ceará - CE
69	ABONO (ACT)	CH da Universidade Federal do Ceará - CE
69	ABONO (ACT)	CH da Universidade Federal do Ceará - CE
69	ABONO (ACT)	CH da Universidade Federal do Ceará - CE
69	ABONO (ACT)	CH da Universidade Federal do Ceará - CE
69	ABONO (ACT)	CH da Universidade Federal do Ceará - CE
69	ABONO (ACT)	CH da Universidade Federal do Ceará - CE
69	ABONO (ACT)	CH da Universidade Federal do Ceará - CE
69	ABONO (ACT)	CH da Universidade Federal do Ceará - CE
69	ABONO (ACT)	CH da Universidade Federal do Ceará - CE
69	ABONO (ACT)	CH da Universidade Federal do Ceará - CE
69	ABONO (ACT)	CH da Universidade Federal do Ceará - CE
69	ABONO (ACT)	CH da Universidade Federal do Ceará - CE
69	ABONO (ACT)	CH da Universidade Federal do Ceará - CE
69	ABONO (ACT)	CH da Universidade Federal do Ceará - CE

69	ABONO (ACT)	CH da Universidade Federal do Ceará - CE
69	ABONO (ACT)	CH da Universidade Federal do Ceará - CE
69	ABONO (ACT)	CH da Universidade Federal do Ceará - CE
69	ABONO (ACT)	CH da Universidade Federal do Ceará - CE
69	ABONO (ACT)	CH da Universidade Federal do Ceará - CE
69	ABONO (ACT)	CH da Universidade Federal do Ceará - CE
69	ABONO (ACT)	CH da Universidade Federal do Ceará - CE
69	ABONO (ACT)	CH da Universidade Federal do Ceará - CE
69	ABONO (ACT)	CH da Universidade Federal do Ceará - CE
69	ABONO (ACT)	CH da Universidade Federal do Ceará - CE
69	ABONO (ACT)	CH da Universidade Federal do Ceará - CE
69	ABONO (ACT)	CH da Universidade Federal do Ceará - CE
69	ABONO (ACT)	CH da Universidade Federal do Ceará - CE
69	ABONO (ACT)	CH da Universidade Federal do Ceará - CE
69	ABONO (ACT)	CH da Universidade Federal do Ceará - CE
69	ABONO (ACT)	CH da Universidade Federal do Ceará - CE
69	ABONO (ACT)	CH da Universidade Federal do Ceará - CE
69	ABONO (ACT)	CH da Universidade Federal do Ceará - CE
69	ABONO (ACT)	CH da Universidade Federal do Ceará - CE
69	ABONO (ACT)	CH da Universidade Federal do Ceará - CE
69	ABONO (ACT)	CH da Universidade Federal do Ceará - CE
69	ABONO (ACT)	CH da Universidade Federal do Ceará - CE
69	ABONO (ACT)	CH da Universidade Federal do Ceará - CE
69	ABONO (ACT)	CH da Universidade Federal do Ceará - CE
69	ABONO (ACT)	CH da Universidade Federal do Ceará - CE
69	ABONO (ACT)	CH da Universidade Federal do Ceará - CE
69	ABONO (ACT)	CH da Universidade Federal do Ceará - CE
69	ABONO (ACT)	CH da Universidade Federal do Ceará - CE
69	ABONO (ACT)	CH da Universidade Federal do Ceará - CE
69	ABONO (ACT)	CH da Universidade Federal do Ceará - CE
69	ABONO (ACT)	CH da Universidade Federal do Ceará - CE
69	ABONO (ACT)	CH da Universidade Federal do Ceará - CE
69	ABONO (ACT)	CH da Universidade Federal do Ceará - CE
69	ABONO (ACT)	CH da Universidade Federal do Ceará - CE
69	ABONO (ACT)	CH da Universidade Federal do Ceará - CE
69	ABONO (ACT)	CH da Universidade Federal do Ceará - CE
69	ABONO (ACT)	CH da Universidade Federal do Ceará - CE
69	ABONO (ACT)	CH da Universidade Federal do Ceará - CE
69	ABONO (ACT)	CH da Universidade Federal do Ceará - CE
69	ABONO (ACT)	CH da Universidade Federal do Ceará - CE
69	ABONO (ACT)	CH da Universidade Federal do Ceará - CE
69	ABONO (ACT)	CH da Universidade Federal do Ceará - CE
69	ABONO (ACT)	CH da Universidade Federal do Ceará - CE
69	ABONO (ACT)	CH da Universidade Federal do Ceará - CE
69	ABONO (ACT)	CH da Universidade Federal do Ceará - CE
69	ABONO (ACT)	CH da Universidade Federal do Ceará - CE
69	ABONO (ACT)	CH da Universidade Federal do Ceará - CE

69	ABONO (ACT)	CH da Universidade Federal do Ceará - CE
69	ABONO (ACT)	CH da Universidade Federal do Ceará - CE
69	ABONO (ACT)	CH da Universidade Federal do Ceará - CE
69	ABONO (ACT)	CH da Universidade Federal do Ceará - CE
69	ABONO (ACT)	CH da Universidade Federal do Ceará - CE
69	ABONO (ACT)	CH da Universidade Federal do Ceará - CE
69	ABONO (ACT)	CH da Universidade Federal do Ceará - CE
69	ABONO (ACT)	CH da Universidade Federal do Ceará - CE
69	ABONO (ACT)	CH da Universidade Federal do Ceará - CE
69	ABONO (ACT)	CH da Universidade Federal do Ceará - CE
69	ABONO (ACT)	CH da Universidade Federal do Ceará - CE
69	ABONO (ACT)	CH da Universidade Federal do Ceará - CE
69	ABONO (ACT)	CH da Universidade Federal do Ceará - CE
69	ABONO (ACT)	CH da Universidade Federal do Ceará - CE
69	ABONO (ACT)	CH da Universidade Federal do Ceará - CE
69	ABONO (ACT)	CH da Universidade Federal do Ceará - CE
69	ABONO (ACT)	CH da Universidade Federal do Ceará - CE
69	ABONO (ACT)	CH da Universidade Federal do Ceará - CE
69	ABONO (ACT)	CH da Universidade Federal do Ceará - CE
69	ABONO (ACT)	CH da Universidade Federal do Ceará - CE
69	ABONO (ACT)	CH da Universidade Federal do Ceará - CE
69	ABONO (ACT)	CH da Universidade Federal do Ceará - CE
69	ABONO (ACT)	CH da Universidade Federal do Ceará - CE
69	ABONO (ACT)	CH da Universidade Federal do Ceará - CE
69	ABONO (ACT)	CH da Universidade Federal do Ceará - CE
69	ABONO (ACT)	CH da Universidade Federal do Ceará - CE
69	ABONO (ACT)	CH da Universidade Federal do Ceará - CE
69	ABONO (ACT)	CH da Universidade Federal do Ceará - CE
69	ABONO (ACT)	CH da Universidade Federal do Ceará - CE
69	ABONO (ACT)	CH da Universidade Federal do Ceará - CE
69	ABONO (ACT)	CH da Universidade Federal do Ceará - CE
69	ABONO (ACT)	CH da Universidade Federal do Ceará - CE

69	ABONO (ACT)	CH da Universidade Federal do Ceará - CE
69	ABONO (ACT)	CH da Universidade Federal do Ceará - CE
69	ABONO (ACT)	CH da Universidade Federal do Ceará - CE
69	ABONO (ACT)	CH da Universidade Federal do Ceará - CE
69	ABONO (ACT)	CH da Universidade Federal do Ceará - CE
69	ABONO (ACT)	CH da Universidade Federal do Ceará - CE
69	ABONO (ACT)	CH da Universidade Federal do Ceará - CE
69	ABONO (ACT)	CH da Universidade Federal do Ceará - CE
69	ABONO (ACT)	CH da Universidade Federal do Ceará - CE
69	ABONO (ACT)	CH da Universidade Federal do Ceará - CE
69	ABONO (ACT)	CH da Universidade Federal do Ceará - CE
69	ABONO (ACT)	CH da Universidade Federal do Ceará - CE
69	ABONO (ACT)	CH da Universidade Federal do Ceará - CE
69	ABONO (ACT)	CH da Universidade Federal do Ceará - CE
69	ABONO (ACT)	CH da Universidade Federal do Ceará - CE
69	ABONO (ACT)	CH da Universidade Federal do Ceará - CE
69	ABONO (ACT)	CH da Universidade Federal do Ceará - CE
69	ABONO (ACT)	CH da Universidade Federal do Ceará - CE
69	ABONO (ACT)	CH da Universidade Federal do Ceará - CE
69	ABONO (ACT)	CH da Universidade Federal do Ceará - CE
69	ABONO (ACT)	CH da Universidade Federal do Ceará - CE
69	ABONO (ACT)	CH da Universidade Federal do Ceará - CE
69	ABONO (ACT)	CH da Universidade Federal do Ceará - CE
69	ABONO (ACT)	CH da Universidade Federal do Ceará - CE
69	ABONO (ACT)	CH da Universidade Federal do Ceará - CE
69	ABONO (ACT)	CH da Universidade Federal do Ceará - CE
69	ABONO (ACT)	CH da Universidade Federal do Ceará - CE
69	ABONO (ACT)	CH da Universidade Federal do Ceará - CE
69	ABONO (ACT)	CH da Universidade Federal do Ceará - CE
69	ABONO (ACT)	CH da Universidade Federal do Ceará - CE
69	ABONO (ACT)	CH da Universidade Federal do Ceará - CE
69	ABONO (ACT)	CH da Universidade Federal do Ceará - CE
69	ABONO (ACT)	CH da Universidade Federal do Ceará - CE
69	ABONO (ACT)	CH da Universidade Federal do Ceará - CE
69	ABONO (ACT)	CH da Universidade Federal do Ceará - CE
69	ABONO (ACT)	CH da Universidade Federal do Ceará - CE
69	ABONO (ACT)	CH da Universidade Federal do Ceará - CE
69	ABONO (ACT)	CH da Universidade Federal do Ceará - CE
69	ABONO (ACT)	CH da Universidade Federal do Ceará - CE
69	ABONO (ACT)	CH da Universidade Federal do Ceará - CE
69	ABONO (ACT)	CH da Universidade Federal do Ceará - CE
69	ABONO (ACT)	CH da Universidade Federal do Ceará - CE

69	ABONO (ACT)	CH da Universidade Federal do Ceará - CE
69	ABONO (ACT)	CH da Universidade Federal do Ceará - CE
69	ABONO (ACT)	CH da Universidade Federal do Ceará - CE
69	ABONO (ACT)	CH da Universidade Federal do Ceará - CE
69	ABONO (ACT)	CH da Universidade Federal do Ceará - CE
69	ABONO (ACT)	CH da Universidade Federal do Ceará - CE
69	ABONO (ACT)	CH da Universidade Federal do Ceará - CE
69	ABONO (ACT)	CH da Universidade Federal do Ceará - CE
69	ABONO (ACT)	CH da Universidade Federal do Ceará - CE
69	ABONO (ACT)	CH da Universidade Federal do Ceará - CE
69	ABONO (ACT)	CH da Universidade Federal do Ceará - CE
69	ABONO (ACT)	CH da Universidade Federal do Ceará - CE
69	ABONO (ACT)	CH da Universidade Federal do Ceará - CE
69	ABONO (ACT)	CH da Universidade Federal do Ceará - CE
69	ABONO (ACT)	CH da Universidade Federal do Ceará - CE
69	ABONO (ACT)	CH da Universidade Federal do Ceará - CE
69	ABONO (ACT)	CH da Universidade Federal do Ceará - CE
69	ABONO (ACT)	CH da Universidade Federal do Ceará - CE
69	ABONO (ACT)	CH da Universidade Federal do Ceará - CE
69	ABONO (ACT)	CH da Universidade Federal do Ceará - CE
69	ABONO (ACT)	CH da Universidade Federal do Ceará - CE
69	ABONO (ACT)	CH da Universidade Federal do Ceará - CE
69	ABONO (ACT)	CH da Universidade Federal do Ceará - CE
69	ABONO (ACT)	CH da Universidade Federal do Ceará - CE
69	ABONO (ACT)	CH da Universidade Federal do Ceará - CE
69	ABONO (ACT)	CH da Universidade Federal do Ceará - CE
69	ABONO (ACT)	CH da Universidade Federal do Ceará - CE
69	ABONO (ACT)	CH da Universidade Federal do Ceará - CE
69	ABONO (ACT)	CH da Universidade Federal do Ceará - CE
69	ABONO (ACT)	CH da Universidade Federal do Ceará - CE
69	ABONO (ACT)	CH da Universidade Federal do Ceará - CE
69	ABONO (ACT)	CH da Universidade Federal do Ceará - CE
69	ABONO (ACT)	CH da Universidade Federal do Ceará - CE
69	ABONO (ACT)	CH da Universidade Federal do Ceará - CE
69	ABONO (ACT)	CH da Universidade Federal do Ceará - CE
69	ABONO (ACT)	CH da Universidade Federal do Ceará - CE
69	ABONO (ACT)	CH da Universidade Federal do Ceará - CE
69	ABONO (ACT)	CH da Universidade Federal do Ceará - CE
69	ABONO (ACT)	CH da Universidade Federal do Ceará - CE
69	ABONO (ACT)	CH da Universidade Federal do Ceará - CE
69	ABONO (ACT)	CH da Universidade Federal do Ceará - CE
69	ABONO (ACT)	CH da Universidade Federal do Ceará - CE
69	ABONO (ACT)	CH da Universidade Federal do Ceará - CE
69	ABONO (ACT)	CH da Universidade Federal do Ceará - CE
69	ABONO (ACT)	CH da Universidade Federal do Ceará - CE
69	ABONO (ACT)	CH da Universidade Federal do Ceará - CE
69	ABONO (ACT)	CH da Universidade Federal do Ceará - CE

69	ABONO (ACT)	CH da Universidade Federal do Ceará - CE
69	ABONO (ACT)	CH da Universidade Federal do Ceará - CE
69	ABONO (ACT)	CH da Universidade Federal do Ceará - CE
69	ABONO (ACT)	CH da Universidade Federal do Ceará - CE
69	ABONO (ACT)	CH da Universidade Federal do Ceará - CE
69	ABONO (ACT)	CH da Universidade Federal do Ceará - CE
69	ABONO (ACT)	CH da Universidade Federal do Ceará - CE
69	ABONO (ACT)	CH da Universidade Federal do Ceará - CE
69	ABONO (ACT)	CH da Universidade Federal do Ceará - CE
69	ABONO (ACT)	CH da Universidade Federal do Ceará - CE
69	ABONO (ACT)	CH da Universidade Federal do Ceará - CE
69	ABONO (ACT)	CH da Universidade Federal do Ceará - CE
69	ABONO (ACT)	CH da Universidade Federal do Ceará - CE
69	ABONO (ACT)	CH da Universidade Federal do Ceará - CE
69	ABONO (ACT)	CH da Universidade Federal do Ceará - CE
69	ABONO (ACT)	CH da Universidade Federal do Ceará - CE
69	ABONO (ACT)	CH da Universidade Federal do Ceará - CE
69	ABONO (ACT)	CH da Universidade Federal do Ceará - CE
69	ABONO (ACT)	CH da Universidade Federal do Ceará - CE
69	ABONO (ACT)	CH da Universidade Federal do Ceará - CE
69	ABONO (ACT)	CH da Universidade Federal do Ceará - CE
69	ABONO (ACT)	CH da Universidade Federal do Ceará - CE
69	ABONO (ACT)	CH da Universidade Federal do Ceará - CE
69	ABONO (ACT)	CH da Universidade Federal do Ceará - CE
69	ABONO (ACT)	CH da Universidade Federal do Ceará - CE
69	ABONO (ACT)	CH da Universidade Federal do Ceará - CE
69	ABONO (ACT)	CH da Universidade Federal do Ceará - CE
69	ABONO (ACT)	CH da Universidade Federal do Ceará - CE
69	ABONO (ACT)	CH da Universidade Federal do Ceará - CE
69	ABONO (ACT)	CH da Universidade Federal do Ceará - CE
69	ABONO (ACT)	CH da Universidade Federal do Ceará - CE
69	ABONO (ACT)	CH da Universidade Federal do Ceará - CE
69	ABONO (ACT)	CH da Universidade Federal do Ceará - CE
69	ABONO (ACT)	CH da Universidade Federal do Ceará - CE
69	ABONO (ACT)	CH da Universidade Federal do Ceará - CE
69	ABONO (ACT)	CH da Universidade Federal do Ceará - CE
69	ABONO (ACT)	CH da Universidade Federal do Ceará - CE
69	ABONO (ACT)	CH da Universidade Federal do Ceará - CE
69	ABONO (ACT)	CH da Universidade Federal do Ceará - CE
69	ABONO (ACT)	CH da Universidade Federal do Ceará - CE
69	ABONO (ACT)	CH da Universidade Federal do Ceará - CE

69	ABONO (ACT)	CH da Universidade Federal do Ceará - CE
69	ABONO (ACT)	CH da Universidade Federal do Ceará - CE
69	ABONO (ACT)	CH da Universidade Federal do Ceará - CE
69	ABONO (ACT)	CH da Universidade Federal do Ceará - CE
69	ABONO (ACT)	CH da Universidade Federal do Ceará - CE
69	ABONO (ACT)	CH da Universidade Federal do Ceará - CE
69	ABONO (ACT)	CH da Universidade Federal do Ceará - CE
69	ABONO (ACT)	CH da Universidade Federal do Ceará - CE
69	ABONO (ACT)	CH da Universidade Federal do Ceará - CE
69	ABONO (ACT)	CH da Universidade Federal do Ceará - CE
69	ABONO (ACT)	CH da Universidade Federal do Ceará - CE
69	ABONO (ACT)	CH da Universidade Federal do Ceará - CE
69	ABONO (ACT)	CH da Universidade Federal do Ceará - CE
69	ABONO (ACT)	CH da Universidade Federal do Ceará - CE
69	ABONO (ACT)	CH da Universidade Federal do Ceará - CE
69	ABONO (ACT)	CH da Universidade Federal do Ceará - CE
69	ABONO (ACT)	CH da Universidade Federal do Ceará - CE
69	ABONO (ACT)	CH da Universidade Federal do Ceará - CE
69	ABONO (ACT)	CH da Universidade Federal do Ceará - CE
69	ABONO (ACT)	CH da Universidade Federal do Ceará - CE
69	ABONO (ACT)	CH da Universidade Federal do Ceará - CE
69	ABONO (ACT)	CH da Universidade Federal do Ceará - CE
69	ABONO (ACT)	CH da Universidade Federal do Ceará - CE
69	ABONO (ACT)	CH da Universidade Federal do Ceará - CE
69	ABONO (ACT)	CH da Universidade Federal do Ceará - CE
69	ABONO (ACT)	CH da Universidade Federal do Ceará - CE
69	ABONO (ACT)	CH da Universidade Federal do Ceará - CE
69	ABONO (ACT)	CH da Universidade Federal do Ceará - CE
69	ABONO (ACT)	CH da Universidade Federal do Ceará - CE
69	ABONO (ACT)	CH da Universidade Federal do Ceará - CE
69	ABONO (ACT)	CH da Universidade Federal do Ceará - CE
69	ABONO (ACT)	CH da Universidade Federal do Ceará - CE
69	ABONO (ACT)	CH da Universidade Federal do Ceará - CE
69	ABONO (ACT)	CH da Universidade Federal do Ceará - CE
69	ABONO (ACT)	CH da Universidade Federal do Ceará - CE
69	ABONO (ACT)	CH da Universidade Federal do Ceará - CE
69	ABONO (ACT)	CH da Universidade Federal do Ceará - CE
69	ABONO (ACT)	CH da Universidade Federal do Ceará - CE
69	ABONO (ACT)	CH da Universidade Federal do Ceará - CE
69	ABONO (ACT)	CH da Universidade Federal do Ceará - CE
69	ABONO (ACT)	CH da Universidade Federal do Ceará - CE
69	ABONO (ACT)	CH da Universidade Federal do Ceará - CE
69	ABONO (ACT)	CH da Universidade Federal do Ceará - CE

69	ABONO (ACT)	CH da Universidade Federal do Ceará - CE
69	ABONO (ACT)	CH da Universidade Federal do Ceará - CE
69	ABONO (ACT)	CH da Universidade Federal do Ceará - CE
69	ABONO (ACT)	CH da Universidade Federal do Ceará - CE
69	ABONO (ACT)	CH da Universidade Federal do Ceará - CE
69	ABONO (ACT)	CH da Universidade Federal do Ceará - CE
69	ABONO (ACT)	CH da Universidade Federal do Ceará - CE
69	ABONO (ACT)	CH da Universidade Federal do Ceará - CE
69	ABONO (ACT)	CH da Universidade Federal do Ceará - CE
69	ABONO (ACT)	CH da Universidade Federal do Ceará - CE
69	ABONO (ACT)	CH da Universidade Federal do Ceará - CE
69	ABONO (ACT)	CH da Universidade Federal do Ceará - CE
69	ABONO (ACT)	CH da Universidade Federal do Ceará - CE
69	ABONO (ACT)	CH da Universidade Federal do Ceará - CE
69	ABONO (ACT)	CH da Universidade Federal do Ceará - CE
69	ABONO (ACT)	CH da Universidade Federal do Ceará - CE
69	ABONO (ACT)	CH da Universidade Federal do Ceará - CE
69	ABONO (ACT)	CH da Universidade Federal do Ceará - CE
69	ABONO (ACT)	CH da Universidade Federal do Ceará - CE
69	ABONO (ACT)	CH da Universidade Federal do Ceará - CE
69	ABONO (ACT)	CH da Universidade Federal do Ceará - CE
69	ABONO (ACT)	CH da Universidade Federal do Ceará - CE
69	ABONO (ACT)	CH da Universidade Federal do Ceará - CE
69	ABONO (ACT)	CH da Universidade Federal do Ceará - CE
69	ABONO (ACT)	CH da Universidade Federal do Ceará - CE
69	ABONO (ACT)	CH da Universidade Federal do Ceará - CE
69	ABONO (ACT)	CH da Universidade Federal do Ceará - CE
69	ABONO (ACT)	CH da Universidade Federal do Ceará - CE
69	ABONO (ACT)	CH da Universidade Federal do Ceará - CE
69	ABONO (ACT)	CH da Universidade Federal do Ceará - CE
69	ABONO (ACT)	CH da Universidade Federal do Ceará - CE
69	ABONO (ACT)	CH da Universidade Federal do Ceará - CE
69	ABONO (ACT)	CH da Universidade Federal do Ceará - CE
69	ABONO (ACT)	CH da Universidade Federal do Ceará - CE
69	ABONO (ACT)	CH da Universidade Federal do Ceará - CE
69	ABONO (ACT)	CH da Universidade Federal do Ceará - CE
69	ABONO (ACT)	CH da Universidade Federal do Ceará - CE
69	ABONO (ACT)	CH da Universidade Federal do Ceará - CE
69	ABONO (ACT)	CH da Universidade Federal do Ceará - CE
69	ABONO (ACT)	CH da Universidade Federal do Ceará - CE
69	ABONO (ACT)	CH da Universidade Federal do Ceará - CE
69	ABONO (ACT)	CH da Universidade Federal do Ceará - CE
69	ABONO (ACT)	CH da Universidade Federal do Ceará - CE
69	ABONO (ACT)	CH da Universidade Federal do Ceará - CE
69	ABONO (ACT)	CH da Universidade Federal do Ceará - CE

69	ABONO (ACT)	CH da Universidade Federal do Ceará - CE
69	ABONO (ACT)	CH da Universidade Federal do Ceará - CE
69	ABONO (ACT)	CH da Universidade Federal do Ceará - CE
69	ABONO (ACT)	CH da Universidade Federal do Ceará - CE
69	ABONO (ACT)	CH da Universidade Federal do Ceará - CE
69	ABONO (ACT)	CH da Universidade Federal do Ceará - CE
69	ABONO (ACT)	CH da Universidade Federal do Ceará - CE
69	ABONO (ACT)	CH da Universidade Federal do Ceará - CE
69	ABONO (ACT)	CH da Universidade Federal do Ceará - CE
69	ABONO (ACT)	CH da Universidade Federal do Ceará - CE
69	ABONO (ACT)	CH da Universidade Federal do Ceará - CE
69	ABONO (ACT)	CH da Universidade Federal do Ceará - CE
69	ABONO (ACT)	CH da Universidade Federal do Ceará - CE
69	ABONO (ACT)	CH da Universidade Federal do Ceará - CE
69	ABONO (ACT)	CH da Universidade Federal do Ceará - CE
69	ABONO (ACT)	CH da Universidade Federal do Ceará - CE
69	ABONO (ACT)	CH da Universidade Federal do Ceará - CE
69	ABONO (ACT)	CH da Universidade Federal do Ceará - CE
69	ABONO (ACT)	CH da Universidade Federal do Ceará - CE
69	ABONO (ACT)	CH da Universidade Federal do Ceará - CE
69	ABONO (ACT)	CH da Universidade Federal do Ceará - CE
69	ABONO (ACT)	CH da Universidade Federal do Ceará - CE
69	ABONO (ACT)	CH da Universidade Federal do Ceará - CE
69	ABONO (ACT)	CH da Universidade Federal do Ceará - CE
69	ABONO (ACT)	CH da Universidade Federal do Ceará - CE
69	ABONO (ACT)	CH da Universidade Federal do Ceará - CE
69	ABONO (ACT)	CH da Universidade Federal do Ceará - CE
69	ABONO (ACT)	CH da Universidade Federal do Ceará - CE
69	ABONO (ACT)	CH da Universidade Federal do Ceará - CE
69	ABONO (ACT)	CH da Universidade Federal do Ceará - CE
69	ABONO (ACT)	CH da Universidade Federal do Ceará - CE
69	ABONO (ACT)	CH da Universidade Federal do Ceará - CE
69	ABONO (ACT)	CH da Universidade Federal do Ceará - CE
69	ABONO (ACT)	CH da Universidade Federal do Ceará - CE
69	ABONO (ACT)	CH da Universidade Federal do Ceará - CE
69	ABONO (ACT)	CH da Universidade Federal do Ceará - CE
69	ABONO (ACT)	CH da Universidade Federal do Ceará - CE
69	ABONO (ACT)	CH da Universidade Federal do Ceará - CE
69	ABONO (ACT)	CH da Universidade Federal do Ceará - CE
69	ABONO (ACT)	CH da Universidade Federal do Ceará - CE
69	ABONO (ACT)	CH da Universidade Federal do Ceará - CE
69	ABONO (ACT)	CH da Universidade Federal do Ceará - CE
69	ABONO (ACT)	CH da Universidade Federal do Ceará - CE
69	ABONO (ACT)	CH da Universidade Federal do Ceará - CE
69	ABONO (ACT)	CH da Universidade Federal do Ceará - CE
69	ABONO (ACT)	CH da Universidade Federal do Ceará - CE
69	ABONO (ACT)	CH da Universidade Federal do Ceará - CE
69	ABONO (ACT)	CH da Universidade Federal do Ceará - CE

69	ABONO (ACT)	CH da Universidade Federal do Ceará - CE
69	ABONO (ACT)	CH da Universidade Federal do Ceará - CE
69	ABONO (ACT)	CH da Universidade Federal do Ceará - CE
69	ABONO (ACT)	CH da Universidade Federal do Ceará - CE
69	ABONO (ACT)	CH da Universidade Federal do Ceará - CE
69	ABONO (ACT)	CH da Universidade Federal do Ceará - CE
69	ABONO (ACT)	CH da Universidade Federal do Ceará - CE
69	ABONO (ACT)	CH da Universidade Federal do Ceará - CE
69	ABONO (ACT)	CH da Universidade Federal do Ceará - CE
69	ABONO (ACT)	CH da Universidade Federal do Ceará - CE
69	ABONO (ACT)	CH da Universidade Federal do Ceará - CE
69	ABONO (ACT)	CH da Universidade Federal do Ceará - CE
69	ABONO (ACT)	CH da Universidade Federal do Ceará - CE
69	ABONO (ACT)	CH da Universidade Federal do Ceará - CE
69	ABONO (ACT)	CH da Universidade Federal do Ceará - CE
69	ABONO (ACT)	CH da Universidade Federal do Ceará - CE
69	ABONO (ACT)	CH da Universidade Federal do Ceará - CE
69	ABONO (ACT)	CH da Universidade Federal do Ceará - CE
69	ABONO (ACT)	CH da Universidade Federal do Ceará - CE
69	ABONO (ACT)	CH da Universidade Federal do Ceará - CE
69	ABONO (ACT)	CH da Universidade Federal do Ceará - CE
69	ABONO (ACT)	CH da Universidade Federal do Ceará - CE
69	ABONO (ACT)	CH da Universidade Federal do Ceará - CE
69	ABONO (ACT)	CH da Universidade Federal do Ceará - CE
69	ABONO (ACT)	CH da Universidade Federal do Ceará - CE
69	ABONO (ACT)	CH da Universidade Federal do Ceará - CE
69	ABONO (ACT)	CH da Universidade Federal do Ceará - CE
69	ABONO (ACT)	CH da Universidade Federal do Ceará - CE
69	ABONO (ACT)	CH da Universidade Federal do Ceará - CE
69	ABONO (ACT)	CH da Universidade Federal do Ceará - CE
69	ABONO (ACT)	CH da Universidade Federal do Ceará - CE
69	ABONO (ACT)	CH da Universidade Federal do Ceará - CE
69	ABONO (ACT)	CH da Universidade Federal do Ceará - CE
69	ABONO (ACT)	CH da Universidade Federal do Ceará - CE
69	ABONO (ACT)	CH da Universidade Federal do Ceará - CE
69	ABONO (ACT)	CH da Universidade Federal do Ceará - CE
69	ABONO (ACT)	CH da Universidade Federal do Ceará - CE

69	ABONO (ACT)	CH da Universidade Federal do Ceará - CE
69	ABONO (ACT)	CH da Universidade Federal do Ceará - CE
69	ABONO (ACT)	CH da Universidade Federal do Ceará - CE
69	ABONO (ACT)	CH da Universidade Federal do Ceará - CE
69	ABONO (ACT)	CH da Universidade Federal do Ceará - CE
69	ABONO (ACT)	CH da Universidade Federal do Ceará - CE
69	ABONO (ACT)	CH da Universidade Federal do Ceará - CE
69	ABONO (ACT)	CH da Universidade Federal do Ceará - CE
69	ABONO (ACT)	CH da Universidade Federal do Ceará - CE
69	ABONO (ACT)	CH da Universidade Federal do Ceará - CE
69	ABONO (ACT)	CH da Universidade Federal do Ceará - CE
69	ABONO (ACT)	CH da Universidade Federal do Ceará - CE
69	ABONO (ACT)	CH da Universidade Federal do Ceará - CE
69	ABONO (ACT)	CH da Universidade Federal do Ceará - CE
69	ABONO (ACT)	CH da Universidade Federal do Ceará - CE
69	ABONO (ACT)	CH da Universidade Federal do Ceará - CE
69	ABONO (ACT)	CH da Universidade Federal do Ceará - CE
69	ABONO (ACT)	CH da Universidade Federal do Ceará - CE
69	ABONO (ACT)	CH da Universidade Federal do Ceará - CE
69	ABONO (ACT)	CH da Universidade Federal do Ceará - CE
69	ABONO (ACT)	CH da Universidade Federal do Ceará - CE
69	ABONO (ACT)	CH da Universidade Federal do Ceará - CE
69	ABONO (ACT)	CH da Universidade Federal do Ceará - CE
69	ABONO (ACT)	CH da Universidade Federal do Ceará - CE
69	ABONO (ACT)	CH da Universidade Federal do Ceará - CE
69	ABONO (ACT)	CH da Universidade Federal do Ceará - CE
69	ABONO (ACT)	CH da Universidade Federal do Ceará - CE
69	ABONO (ACT)	CH da Universidade Federal do Ceará - CE
69	ABONO (ACT)	CH da Universidade Federal do Ceará - CE
69	ABONO (ACT)	CH da Universidade Federal do Ceará - CE
69	ABONO (ACT)	CH da Universidade Federal do Ceará - CE
69	ABONO (ACT)	CH da Universidade Federal do Ceará - CE

69	ABONO (ACT)	CH da Universidade Federal do Ceará - CE
69	ABONO (ACT)	CH da Universidade Federal do Ceará - CE
69	ABONO (ACT)	CH da Universidade Federal do Ceará - CE
69	ABONO (ACT)	CH da Universidade Federal do Ceará - CE
69	ABONO (ACT)	CH da Universidade Federal do Ceará - CE
69	ABONO (ACT)	CH da Universidade Federal do Ceará - CE
69	ABONO (ACT)	CH da Universidade Federal do Ceará - CE
69	ABONO (ACT)	CH da Universidade Federal do Ceará - CE
69	ABONO (ACT)	CH da Universidade Federal do Ceará - CE
69	ABONO (ACT)	CH da Universidade Federal do Ceará - CE
69	ABONO (ACT)	CH da Universidade Federal do Ceará - CE
69	ABONO (ACT)	CH da Universidade Federal do Ceará - CE
69	ABONO (ACT)	CH da Universidade Federal do Ceará - CE
69	ABONO (ACT)	CH da Universidade Federal do Ceará - CE
69	ABONO (ACT)	CH da Universidade Federal do Ceará - CE
69	ABONO (ACT)	CH da Universidade Federal do Ceará - CE
69	ABONO (ACT)	CH da Universidade Federal do Ceará - CE
69	ABONO (ACT)	CH da Universidade Federal do Ceará - CE
69	ABONO (ACT)	CH da Universidade Federal do Ceará - CE
69	ABONO (ACT)	CH da Universidade Federal do Ceará - CE
69	ABONO (ACT)	CH da Universidade Federal do Ceará - CE
69	ABONO (ACT)	CH da Universidade Federal do Ceará - CE
69	ABONO (ACT)	CH da Universidade Federal do Ceará - CE
69	ABONO (ACT)	CH da Universidade Federal do Ceará - CE
69	ABONO (ACT)	CH da Universidade Federal do Ceará - CE
69	ABONO (ACT)	CH da Universidade Federal do Ceará - CE
69	ABONO (ACT)	CH da Universidade Federal do Ceará - CE
69	ABONO (ACT)	CH da Universidade Federal do Ceará - CE
69	ABONO (ACT)	CH da Universidade Federal do Ceará - CE
69	ABONO (ACT)	CH da Universidade Federal do Ceará - CE
69	ABONO (ACT)	CH da Universidade Federal do Ceará - CE
69	ABONO (ACT)	CH da Universidade Federal do Ceará - CE
69	ABONO (ACT)	CH da Universidade Federal do Ceará - CE

69	ABONO (ACT)	CH da Universidade Federal do Ceará - CE
69	ABONO (ACT)	CH da Universidade Federal do Ceará - CE
69	ABONO (ACT)	CH da Universidade Federal do Ceará - CE
69	ABONO (ACT)	CH da Universidade Federal do Ceará - CE
69	ABONO (ACT)	CH da Universidade Federal do Ceará - CE
69	ABONO (ACT)	CH da Universidade Federal do Ceará - CE
69	ABONO (ACT)	CH da Universidade Federal do Ceará - CE
69	ABONO (ACT)	CH da Universidade Federal do Ceará - CE
69	ABONO (ACT)	CH da Universidade Federal do Ceará - CE
69	ABONO (ACT)	CH da Universidade Federal do Ceará - CE
69	ABONO (ACT)	CH da Universidade Federal do Ceará - CE
69	ABONO (ACT)	CH da Universidade Federal do Ceará - CE
69	ABONO (ACT)	CH da Universidade Federal do Ceará - CE
69	ABONO (ACT)	CH da Universidade Federal do Ceará - CE
69	ABONO (ACT)	CH da Universidade Federal do Ceará - CE
69	ABONO (ACT)	CH da Universidade Federal do Ceará - CE
69	ABONO (ACT)	CH da Universidade Federal do Ceará - CE
69	ABONO (ACT)	CH da Universidade Federal do Ceará - CE
69	ABONO (ACT)	CH da Universidade Federal do Ceará - CE
69	ABONO (ACT)	CH da Universidade Federal do Ceará - CE
69	ABONO (ACT)	CH da Universidade Federal do Ceará - CE
69	ABONO (ACT)	CH da Universidade Federal do Ceará - CE
69	ABONO (ACT)	CH da Universidade Federal do Ceará - CE
69	ABONO (ACT)	CH da Universidade Federal do Ceará - CE
69	ABONO (ACT)	CH da Universidade Federal do Ceará - CE
69	ABONO (ACT)	CH da Universidade Federal do Ceará - CE
69	ABONO (ACT)	CH da Universidade Federal do Ceará - CE
69	ABONO (ACT)	CH da Universidade Federal do Ceará - CE
69	ABONO (ACT)	CH da Universidade Federal do Ceará - CE
69	ABONO (ACT)	CH da Universidade Federal do Ceará - CE
69	ABONO (ACT)	CH da Universidade Federal do Ceará - CE
69	ABONO (ACT)	CH da Universidade Federal do Ceará - CE
69	ABONO (ACT)	CH da Universidade Federal do Ceará - CE
69	ABONO (ACT)	CH da Universidade Federal do Ceará - CE
69	ABONO (ACT)	CH da Universidade Federal do Ceará - CE
69	ABONO (ACT)	CH da Universidade Federal do Ceará - CE
69	ABONO (ACT)	CH da Universidade Federal do Ceará - CE
69	ABONO (ACT)	CH da Universidade Federal do Ceará - CE
69	ABONO (ACT)	CH da Universidade Federal do Ceará - CE
69	ABONO (ACT)	CH da Universidade Federal do Ceará - CE
69	ABONO (ACT)	CH da Universidade Federal do Ceará - CE
69	ABONO (ACT)	CH da Universidade Federal do Ceará - CE
69	ABONO (ACT)	CH da Universidade Federal do Ceará - CE

69	ABONO (ACT)	CH da Universidade Federal do Ceará - CE
69	ABONO (ACT)	CH da Universidade Federal do Ceará - CE
69	ABONO (ACT)	CH da Universidade Federal do Ceará - CE
69	ABONO (ACT)	CH da Universidade Federal do Ceará - CE
69	ABONO (ACT)	CH da Universidade Federal do Ceará - CE
69	ABONO (ACT)	CH da Universidade Federal do Ceará - CE
69	ABONO (ACT)	CH da Universidade Federal do Ceará - CE
69	ABONO (ACT)	CH da Universidade Federal do Ceará - CE
69	ABONO (ACT)	CH da Universidade Federal do Ceará - CE
69	ABONO (ACT)	CH da Universidade Federal do Ceará - CE
69	ABONO (ACT)	CH da Universidade Federal do Ceará - CE
69	ABONO (ACT)	CH da Universidade Federal do Ceará - CE
69	ABONO (ACT)	CH da Universidade Federal do Ceará - CE
69	ABONO (ACT)	CH da Universidade Federal do Ceará - CE
69	ABONO (ACT)	CH da Universidade Federal do Ceará - CE
69	ABONO (ACT)	CH da Universidade Federal do Ceará - CE
69	ABONO (ACT)	CH da Universidade Federal do Ceará - CE
69	ABONO (ACT)	CH da Universidade Federal do Ceará - CE
69	ABONO (ACT)	CH da Universidade Federal do Ceará - CE
69	ABONO (ACT)	CH da Universidade Federal do Ceará - CE
69	ABONO (ACT)	CH da Universidade Federal do Ceará - CE
69	ABONO (ACT)	CH da Universidade Federal do Ceará - CE
69	ABONO (ACT)	CH da Universidade Federal do Ceará - CE
69	ABONO (ACT)	CH da Universidade Federal do Ceará - CE
69	ABONO (ACT)	CH da Universidade Federal do Ceará - CE
69	ABONO (ACT)	CH da Universidade Federal do Ceará - CE
69	ABONO (ACT)	CH da Universidade Federal do Ceará - CE
69	ABONO (ACT)	CH da Universidade Federal do Ceará - CE
69	ABONO (ACT)	CH da Universidade Federal do Ceará - CE
69	ABONO (ACT)	CH da Universidade Federal do Ceará - CE
69	ABONO (ACT)	CH da Universidade Federal do Ceará - CE
69	ABONO (ACT)	CH da Universidade Federal do Ceará - CE
69	ABONO (ACT)	CH da Universidade Federal do Ceará - CE
69	ABONO (ACT)	CH da Universidade Federal do Ceará - CE
69	ABONO (ACT)	CH da Universidade Federal do Ceará - CE
69	ABONO (ACT)	CH da Universidade Federal do Ceará - CE
69	ABONO (ACT)	CH da Universidade Federal do Ceará - CE
69	ABONO (ACT)	CH da Universidade Federal do Ceará - CE
69	ABONO (ACT)	CH da Universidade Federal do Ceará - CE
69	ABONO (ACT)	CH da Universidade Federal do Ceará - CE
69	ABONO (ACT)	CH da Universidade Federal do Ceará - CE
69	ABONO (ACT)	CH da Universidade Federal do Ceará - CE
69	ABONO (ACT)	CH da Universidade Federal do Ceará - CE
69	ABONO (ACT)	CH da Universidade Federal do Ceará - CE
69	ABONO (ACT)	CH da Universidade Federal do Ceará - CE
69	ABONO (ACT)	CH da Universidade Federal do Ceará - CE
69	ABONO (ACT)	CH da Universidade Federal do Ceará - CE
69	ABONO (ACT)	CH da Universidade Federal do Ceará - CE

69	ABONO (ACT)	CH da Universidade Federal do Ceará - CE
69	ABONO (ACT)	CH da Universidade Federal do Ceará - CE
69	ABONO (ACT)	CH da Universidade Federal do Ceará - CE
69	ABONO (ACT)	CH da Universidade Federal do Ceará - CE
69	ABONO (ACT)	CH da Universidade Federal do Ceará - CE
69	ABONO (ACT)	CH da Universidade Federal do Ceará - CE
69	ABONO (ACT)	CH da Universidade Federal do Ceará - CE
69	ABONO (ACT)	CH da Universidade Federal do Ceará - CE
69	ABONO (ACT)	CH da Universidade Federal do Ceará - CE
69	ABONO (ACT)	CH da Universidade Federal do Ceará - CE
69	ABONO (ACT)	CH da Universidade Federal do Ceará - CE
69	ABONO (ACT)	CH da Universidade Federal do Ceará - CE
69	ABONO (ACT)	CH da Universidade Federal do Ceará - CE
69	ABONO (ACT)	CH da Universidade Federal do Ceará - CE
69	ABONO (ACT)	CH da Universidade Federal do Ceará - CE
69	ABONO (ACT)	CH da Universidade Federal do Ceará - CE
69	ABONO (ACT)	CH da Universidade Federal do Ceará - CE
69	ABONO (ACT)	CH da Universidade Federal do Ceará - CE
69	ABONO (ACT)	CH da Universidade Federal do Ceará - CE
69	ABONO (ACT)	CH da Universidade Federal do Ceará - CE
69	ABONO (ACT)	CH da Universidade Federal do Ceará - CE
69	ABONO (ACT)	CH da Universidade Federal do Ceará - CE
69	ABONO (ACT)	CH da Universidade Federal do Ceará - CE
69	ABONO (ACT)	CH da Universidade Federal do Ceará - CE
69	ABONO (ACT)	CH da Universidade Federal do Ceará - CE
69	ABONO (ACT)	CH da Universidade Federal do Ceará - CE
69	ABONO (ACT)	CH da Universidade Federal do Ceará - CE
69	ABONO (ACT)	CH da Universidade Federal do Ceará - CE
69	ABONO (ACT)	CH da Universidade Federal do Ceará - CE
69	ABONO (ACT)	CH da Universidade Federal do Ceará - CE
69	ABONO (ACT)	CH da Universidade Federal do Ceará - CE
69	ABONO (ACT)	CH da Universidade Federal do Ceará - CE
69	ABONO (ACT)	CH da Universidade Federal do Ceará - CE
69	ABONO (ACT)	CH da Universidade Federal do Ceará - CE
69	ABONO (ACT)	CH da Universidade Federal do Ceará - CE
69	ABONO (ACT)	CH da Universidade Federal do Ceará - CE
69	ABONO (ACT)	CH da Universidade Federal do Ceará - CE
69	ABONO (ACT)	CH da Universidade Federal do Ceará - CE
69	ABONO (ACT)	CH da Universidade Federal do Ceará - CE
69	ABONO (ACT)	CH da Universidade Federal do Ceará - CE
69	ABONO (ACT)	CH da Universidade Federal do Ceará - CE
69	ABONO (ACT)	CH da Universidade Federal do Ceará - CE
69	ABONO (ACT)	CH da Universidade Federal do Ceará - CE

69	ABONO (ACT)	CH da Universidade Federal do Ceará - CE
69	ABONO (ACT)	CH da Universidade Federal do Ceará - CE
69	ABONO (ACT)	CH da Universidade Federal do Ceará - CE
69	ABONO (ACT)	CH da Universidade Federal do Ceará - CE
69	ABONO (ACT)	CH da Universidade Federal do Ceará - CE
69	ABONO (ACT)	CH da Universidade Federal do Ceará - CE
69	ABONO (ACT)	CH da Universidade Federal do Ceará - CE
69	ABONO (ACT)	CH da Universidade Federal do Ceará - CE
69	ABONO (ACT)	CH da Universidade Federal do Ceará - CE
69	ABONO (ACT)	CH da Universidade Federal do Ceará - CE
69	ABONO (ACT)	CH da Universidade Federal do Ceará - CE
69	ABONO (ACT)	CH da Universidade Federal do Ceará - CE
69	ABONO (ACT)	CH da Universidade Federal do Ceará - CE
69	ABONO (ACT)	CH da Universidade Federal do Ceará - CE
69	ABONO (ACT)	CH da Universidade Federal do Ceará - CE
69	ABONO (ACT)	CH da Universidade Federal do Ceará - CE
69	ABONO (ACT)	CH da Universidade Federal do Ceará - CE
69	ABONO (ACT)	CH da Universidade Federal do Ceará - CE
69	ABONO (ACT)	CH da Universidade Federal do Ceará - CE
69	ABONO (ACT)	CH da Universidade Federal do Ceará - CE
69	ABONO (ACT)	CH da Universidade Federal do Ceará - CE
69	ABONO (ACT)	CH da Universidade Federal do Ceará - CE
69	ABONO (ACT)	CH da Universidade Federal do Ceará - CE
69	ABONO (ACT)	CH da Universidade Federal do Ceará - CE
69	ABONO (ACT)	CH da Universidade Federal do Ceará - CE
69	ABONO (ACT)	CH da Universidade Federal do Ceará - CE
69	ABONO (ACT)	CH da Universidade Federal do Ceará - CE
69	ABONO (ACT)	CH da Universidade Federal do Ceará - CE
69	ABONO (ACT)	CH da Universidade Federal do Ceará - CE
69	ABONO (ACT)	CH da Universidade Federal do Ceará - CE
69	ABONO (ACT)	CH da Universidade Federal do Ceará - CE
69	ABONO (ACT)	CH da Universidade Federal do Ceará - CE
69	ABONO (ACT)	CH da Universidade Federal do Ceará - CE
69	ABONO (ACT)	CH da Universidade Federal do Ceará - CE
69	ABONO (ACT)	CH da Universidade Federal do Ceará - CE
69	ABONO (ACT)	CH da Universidade Federal do Ceará - CE
69	ABONO (ACT)	CH da Universidade Federal do Ceará - CE
69	ABONO (ACT)	CH da Universidade Federal do Ceará - CE
69	ABONO (ACT)	CH da Universidade Federal do Ceará - CE
69	ABONO (ACT)	CH da Universidade Federal do Ceará - CE
69	ABONO (ACT)	CH da Universidade Federal do Ceará - CE
69	ABONO (ACT)	CH da Universidade Federal do Ceará - CE
69	ABONO (ACT)	CH da Universidade Federal do Ceará - CE
69	ABONO (ACT)	CH da Universidade Federal do Ceará - CE
69	ABONO (ACT)	CH da Universidade Federal do Ceará - CE
69	ABONO (ACT)	CH da Universidade Federal do Ceará - CE

69	ABONO (ACT)	CH da Universidade Federal do Ceará - CE
69	ABONO (ACT)	CH da Universidade Federal do Ceará - CE
69	ABONO (ACT)	CH da Universidade Federal do Ceará - CE
69	ABONO (ACT)	CH da Universidade Federal do Ceará - CE
69	ABONO (ACT)	CH da Universidade Federal do Ceará - CE
69	ABONO (ACT)	CH da Universidade Federal do Ceará - CE
69	ABONO (ACT)	CH da Universidade Federal do Ceará - CE
69	ABONO (ACT)	CH da Universidade Federal do Ceará - CE
69	ABONO (ACT)	CH da Universidade Federal do Ceará - CE
69	ABONO (ACT)	CH da Universidade Federal do Ceará - CE
69	ABONO (ACT)	CH da Universidade Federal do Ceará - CE
69	ABONO (ACT)	CH da Universidade Federal do Ceará - CE
69	ABONO (ACT)	CH da Universidade Federal do Ceará - CE
69	ABONO (ACT)	CH da Universidade Federal do Ceará - CE
69	ABONO (ACT)	CH da Universidade Federal do Ceará - CE
69	ABONO (ACT)	CH da Universidade Federal do Ceará - CE
69	ABONO (ACT)	CH da Universidade Federal do Ceará - CE
69	ABONO (ACT)	CH da Universidade Federal do Ceará - CE
69	ABONO (ACT)	CH da Universidade Federal do Ceará - CE
69	ABONO (ACT)	CH da Universidade Federal do Ceará - CE
69	ABONO (ACT)	CH da Universidade Federal do Ceará - CE
69	ABONO (ACT)	CH da Universidade Federal do Ceará - CE
69	ABONO (ACT)	CH da Universidade Federal do Ceará - CE
69	ABONO (ACT)	CH da Universidade Federal do Ceará - CE
69	ABONO (ACT)	CH da Universidade Federal do Ceará - CE
69	ABONO (ACT)	CH da Universidade Federal do Ceará - CE
69	ABONO (ACT)	CH da Universidade Federal do Ceará - CE
69	ABONO (ACT)	CH da Universidade Federal do Ceará - CE
69	ABONO (ACT)	CH da Universidade Federal do Ceará - CE
69	ABONO (ACT)	CH da Universidade Federal do Ceará - CE
69	ABONO (ACT)	CH da Universidade Federal do Ceará - CE
69	ABONO (ACT)	CH da Universidade Federal do Ceará - CE
69	ABONO (ACT)	CH da Universidade Federal do Ceará - CE
69	ABONO (ACT)	CH da Universidade Federal do Ceará - CE
69	ABONO (ACT)	CH da Universidade Federal do Ceará - CE
69	ABONO (ACT)	CH da Universidade Federal do Ceará - CE

69	ABONO (ACT)	CH da Universidade Federal do Ceará - CE
69	ABONO (ACT)	CH da Universidade Federal do Ceará - CE
69	ABONO (ACT)	CH da Universidade Federal do Ceará - CE
69	ABONO (ACT)	CH da Universidade Federal do Ceará - CE
69	ABONO (ACT)	CH da Universidade Federal do Ceará - CE
69	ABONO (ACT)	CH da Universidade Federal do Ceará - CE
69	ABONO (ACT)	CH da Universidade Federal do Ceará - CE
69	ABONO (ACT)	CH da Universidade Federal do Ceará - CE
69	ABONO (ACT)	CH da Universidade Federal do Ceará - CE
69	ABONO (ACT)	CH da Universidade Federal do Ceará - CE
69	ABONO (ACT)	CH da Universidade Federal do Ceará - CE
69	ABONO (ACT)	CH da Universidade Federal do Ceará - CE
69	ABONO (ACT)	CH da Universidade Federal do Ceará - CE
69	ABONO (ACT)	CH da Universidade Federal do Ceará - CE
69	ABONO (ACT)	CH da Universidade Federal do Ceará - CE
69	ABONO (ACT)	CH da Universidade Federal do Ceará - CE
69	ABONO (ACT)	CH da Universidade Federal do Ceará - CE
69	ABONO (ACT)	CH da Universidade Federal do Ceará - CE
69	ABONO (ACT)	CH da Universidade Federal do Ceará - CE
69	ABONO (ACT)	CH da Universidade Federal do Ceará - CE
69	ABONO (ACT)	CH da Universidade Federal do Ceará - CE
69	ABONO (ACT)	CH da Universidade Federal do Ceará - CE
69	ABONO (ACT)	CH da Universidade Federal do Ceará - CE
69	ABONO (ACT)	CH da Universidade Federal do Ceará - CE
69	ABONO (ACT)	CH da Universidade Federal do Ceará - CE
69	ABONO (ACT)	CH da Universidade Federal do Ceará - CE
69	ABONO (ACT)	CH da Universidade Federal do Ceará - CE
69	ABONO (ACT)	CH da Universidade Federal do Ceará - CE
69	ABONO (ACT)	CH da Universidade Federal do Ceará - CE
69	ABONO (ACT)	CH da Universidade Federal do Ceará - CE
69	ABONO (ACT)	CH da Universidade Federal do Ceará - CE
69	ABONO (ACT)	CH da Universidade Federal do Ceará - CE
69	ABONO (ACT)	CH da Universidade Federal do Ceará - CE
69	ABONO (ACT)	CH da Universidade Federal do Ceará - CE
69	ABONO (ACT)	CH da Universidade Federal do Ceará - CE
69	ABONO (ACT)	CH da Universidade Federal do Ceará - CE
69	ABONO (ACT)	CH da Universidade Federal do Ceará - CE

69	ABONO (ACT)	CH da Universidade Federal do Ceará - CE
69	ABONO (ACT)	CH da Universidade Federal do Ceará - CE
69	ABONO (ACT)	CH da Universidade Federal do Ceará - CE
69	ABONO (ACT)	CH da Universidade Federal do Ceará - CE
69	ABONO (ACT)	CH da Universidade Federal do Ceará - CE
69	ABONO (ACT)	CH da Universidade Federal do Ceará - CE
70	AUSÊNCIA COM COMPENSAÇÃO	CH da Universidade Federal do Ceará - CE
70	AUSÊNCIA COM COMPENSAÇÃO	CH da Universidade Federal do Ceará - CE
70	AUSÊNCIA COM COMPENSAÇÃO	CH da Universidade Federal do Ceará - CE
70	AUSÊNCIA COM COMPENSAÇÃO	CH da Universidade Federal do Ceará - CE
70	AUSÊNCIA COM COMPENSAÇÃO	CH da Universidade Federal do Ceará - CE
70	AUSÊNCIA COM COMPENSAÇÃO	CH da Universidade Federal do Ceará - CE
70	AUSÊNCIA COM COMPENSAÇÃO	CH da Universidade Federal do Ceará - CE
70	AUSÊNCIA COM COMPENSAÇÃO	CH da Universidade Federal do Ceará - CE
70	AUSÊNCIA COM COMPENSAÇÃO	CH da Universidade Federal do Ceará - CE
70	AUSÊNCIA COM COMPENSAÇÃO	CH da Universidade Federal do Ceará - CE
70	AUSÊNCIA COM COMPENSAÇÃO	CH da Universidade Federal do Ceará - CE
70	AUSÊNCIA COM COMPENSAÇÃO	CH da Universidade Federal do Ceará - CE
70	AUSÊNCIA COM COMPENSAÇÃO	CH da Universidade Federal do Ceará - CE
70	AUSÊNCIA COM COMPENSAÇÃO	CH da Universidade Federal do Ceará - CE
70	AUSÊNCIA COM COMPENSAÇÃO	CH da Universidade Federal do Ceará - CE
70	AUSÊNCIA COM COMPENSAÇÃO	CH da Universidade Federal do Ceará - CE
70	AUSÊNCIA COM COMPENSAÇÃO	CH da Universidade Federal do Ceará - CE
70	AUSÊNCIA COM COMPENSAÇÃO	CH da Universidade Federal do Ceará - CE
70	AUSÊNCIA COM COMPENSAÇÃO	CH da Universidade Federal do Ceará - CE
70	AUSÊNCIA COM COMPENSAÇÃO	CH da Universidade Federal do Ceará - CE
70	AUSÊNCIA COM COMPENSAÇÃO	CH da Universidade Federal do Ceará - CE
70	AUSÊNCIA COM COMPENSAÇÃO	CH da Universidade Federal do Ceará - CE
70	AUSÊNCIA COM COMPENSAÇÃO	CH da Universidade Federal do Ceará - CE
70	AUSÊNCIA COM COMPENSAÇÃO	CH da Universidade Federal do Ceará - CE
70	AUSÊNCIA COM COMPENSAÇÃO	CH da Universidade Federal do Ceará - CE
70	AUSÊNCIA COM COMPENSAÇÃO	CH da Universidade Federal do Ceará - CE
70	AUSÊNCIA COM COMPENSAÇÃO	CH da Universidade Federal do Ceará - CE
70	AUSÊNCIA COM COMPENSAÇÃO	CH da Universidade Federal do Ceará - CE
70	AUSÊNCIA COM COMPENSAÇÃO	CH da Universidade Federal do Ceará - CE
70	AUSÊNCIA COM COMPENSAÇÃO	CH da Universidade Federal do Ceará - CE
70	AUSÊNCIA COM COMPENSAÇÃO	CH da Universidade Federal do Ceará - CE
70	AUSÊNCIA COM COMPENSAÇÃO	CH da Universidade Federal do Ceará - CE
70	AUSÊNCIA COM COMPENSAÇÃO	CH da Universidade Federal do Ceará - CE
70	AUSÊNCIA COM COMPENSAÇÃO	CH da Universidade Federal do Ceará - CE
70	AUSÊNCIA COM COMPENSAÇÃO	CH da Universidade Federal do Ceará - CE
70	AUSÊNCIA COM COMPENSAÇÃO	CH da Universidade Federal do Ceará - CE
70	AUSÊNCIA COM COMPENSAÇÃO	CH da Universidade Federal do Ceará - CE
70	AUSÊNCIA COM COMPENSAÇÃO	CH da Universidade Federal do Ceará - CE
70	AUSÊNCIA COM COMPENSAÇÃO	CH da Universidade Federal do Ceará - CE
70	AUSÊNCIA COM COMPENSAÇÃO	CH da Universidade Federal do Ceará - CE
70	AUSÊNCIA COM COMPENSAÇÃO	CH da Universidade Federal do Ceará - CE
70	AUSÊNCIA COM COMPENSAÇÃO	CH da Universidade Federal do Ceará - CE

70	AUSÊNCIA COM COMPENSAÇÃO	CH da Universidade Federal do Ceará - CE
70	AUSÊNCIA COM COMPENSAÇÃO	CH da Universidade Federal do Ceará - CE
70	AUSÊNCIA COM COMPENSAÇÃO	CH da Universidade Federal do Ceará - CE
70	AUSÊNCIA COM COMPENSAÇÃO	CH da Universidade Federal do Ceará - CE
70	AUSÊNCIA COM COMPENSAÇÃO	CH da Universidade Federal do Ceará - CE
70	AUSÊNCIA COM COMPENSAÇÃO	CH da Universidade Federal do Ceará - CE
70	AUSÊNCIA COM COMPENSAÇÃO	CH da Universidade Federal do Ceará - CE
70	AUSÊNCIA COM COMPENSAÇÃO	CH da Universidade Federal do Ceará - CE
70	AUSÊNCIA COM COMPENSAÇÃO	CH da Universidade Federal do Ceará - CE
70	AUSÊNCIA COM COMPENSAÇÃO	CH da Universidade Federal do Ceará - CE
70	AUSÊNCIA COM COMPENSAÇÃO	CH da Universidade Federal do Ceará - CE
70	AUSÊNCIA COM COMPENSAÇÃO	CH da Universidade Federal do Ceará - CE
70	AUSÊNCIA COM COMPENSAÇÃO	CH da Universidade Federal do Ceará - CE
70	AUSÊNCIA COM COMPENSAÇÃO	CH da Universidade Federal do Ceará - CE
70	AUSÊNCIA COM COMPENSAÇÃO	CH da Universidade Federal do Ceará - CE
70	AUSÊNCIA COM COMPENSAÇÃO	CH da Universidade Federal do Ceará - CE
70	AUSÊNCIA COM COMPENSAÇÃO	CH da Universidade Federal do Ceará - CE
70	AUSÊNCIA COM COMPENSAÇÃO	CH da Universidade Federal do Ceará - CE
70	AUSÊNCIA COM COMPENSAÇÃO	CH da Universidade Federal do Ceará - CE
70	AUSÊNCIA COM COMPENSAÇÃO	CH da Universidade Federal do Ceará - CE
70	AUSÊNCIA COM COMPENSAÇÃO	CH da Universidade Federal do Ceará - CE
70	AUSÊNCIA COM COMPENSAÇÃO	CH da Universidade Federal do Ceará - CE
70	AUSÊNCIA COM COMPENSAÇÃO	CH da Universidade Federal do Ceará - CE
70	AUSÊNCIA COM COMPENSAÇÃO	CH da Universidade Federal do Ceará - CE
70	AUSÊNCIA COM COMPENSAÇÃO	CH da Universidade Federal do Ceará - CE
70	AUSÊNCIA COM COMPENSAÇÃO	CH da Universidade Federal do Ceará - CE
70	AUSÊNCIA COM COMPENSAÇÃO	CH da Universidade Federal do Ceará - CE
70	AUSÊNCIA COM COMPENSAÇÃO	CH da Universidade Federal do Ceará - CE
70	AUSÊNCIA COM COMPENSAÇÃO	CH da Universidade Federal do Ceará - CE
70	AUSÊNCIA COM COMPENSAÇÃO	CH da Universidade Federal do Ceará - CE
70	AUSÊNCIA COM COMPENSAÇÃO	CH da Universidade Federal do Ceará - CE
70	AUSÊNCIA COM COMPENSAÇÃO	CH da Universidade Federal do Ceará - CE
70	AUSÊNCIA COM COMPENSAÇÃO	CH da Universidade Federal do Ceará - CE
70	AUSÊNCIA COM COMPENSAÇÃO	CH da Universidade Federal do Ceará - CE
70	AUSÊNCIA COM COMPENSAÇÃO	CH da Universidade Federal do Ceará - CE
70	AUSÊNCIA COM COMPENSAÇÃO	CH da Universidade Federal do Ceará - CE
70	AUSÊNCIA COM COMPENSAÇÃO	CH da Universidade Federal do Ceará - CE
70	AUSÊNCIA COM COMPENSAÇÃO	CH da Universidade Federal do Ceará - CE
70	AUSÊNCIA COM COMPENSAÇÃO	CH da Universidade Federal do Ceará - CE
70	AUSÊNCIA COM COMPENSAÇÃO	CH da Universidade Federal do Ceará - CE
70	AUSÊNCIA COM COMPENSAÇÃO	CH da Universidade Federal do Ceará - CE
70	AUSÊNCIA COM COMPENSAÇÃO	CH da Universidade Federal do Ceará - CE
70	AUSÊNCIA COM COMPENSAÇÃO	CH da Universidade Federal do Ceará - CE
70	AUSÊNCIA COM COMPENSAÇÃO	CH da Universidade Federal do Ceará - CE
70	AUSÊNCIA COM COMPENSAÇÃO	CH da Universidade Federal do Ceará - CE
70	AUSÊNCIA COM COMPENSAÇÃO	CH da Universidade Federal do Ceará - CE
70	AUSÊNCIA COM COMPENSAÇÃO	CH da Universidade Federal do Ceará - CE
70	AUSÊNCIA COM COMPENSAÇÃO	CH da Universidade Federal do Ceará - CE

70	AUSÊNCIA COM COMPENSAÇÃO	CH da Universidade Federal do Ceará - CE
70	AUSÊNCIA COM COMPENSAÇÃO	CH da Universidade Federal do Ceará - CE
70	AUSÊNCIA COM COMPENSAÇÃO	CH da Universidade Federal do Ceará - CE
70	AUSÊNCIA COM COMPENSAÇÃO	CH da Universidade Federal do Ceará - CE
70	AUSÊNCIA COM COMPENSAÇÃO	CH da Universidade Federal do Ceará - CE
70	AUSÊNCIA COM COMPENSAÇÃO	CH da Universidade Federal do Ceará - CE
70	AUSÊNCIA COM COMPENSAÇÃO	CH da Universidade Federal do Ceará - CE
70	AUSÊNCIA COM COMPENSAÇÃO	CH da Universidade Federal do Ceará - CE
70	AUSÊNCIA COM COMPENSAÇÃO	CH da Universidade Federal do Ceará - CE
70	AUSÊNCIA COM COMPENSAÇÃO	CH da Universidade Federal do Ceará - CE
70	AUSÊNCIA COM COMPENSAÇÃO	CH da Universidade Federal do Ceará - CE
70	AUSÊNCIA COM COMPENSAÇÃO	CH da Universidade Federal do Ceará - CE
70	AUSÊNCIA COM COMPENSAÇÃO	CH da Universidade Federal do Ceará - CE
70	AUSÊNCIA COM COMPENSAÇÃO	CH da Universidade Federal do Ceará - CE
70	AUSÊNCIA COM COMPENSAÇÃO	CH da Universidade Federal do Ceará - CE
70	AUSÊNCIA COM COMPENSAÇÃO	CH da Universidade Federal do Ceará - CE
70	AUSÊNCIA COM COMPENSAÇÃO	CH da Universidade Federal do Ceará - CE
70	AUSÊNCIA COM COMPENSAÇÃO	CH da Universidade Federal do Ceará - CE
70	AUSÊNCIA COM COMPENSAÇÃO	CH da Universidade Federal do Ceará - CE
70	AUSÊNCIA COM COMPENSAÇÃO	CH da Universidade Federal do Ceará - CE
70	AUSÊNCIA COM COMPENSAÇÃO	CH da Universidade Federal do Ceará - CE
70	AUSÊNCIA COM COMPENSAÇÃO	CH da Universidade Federal do Ceará - CE
70	AUSÊNCIA COM COMPENSAÇÃO	CH da Universidade Federal do Ceará - CE
70	AUSÊNCIA COM COMPENSAÇÃO	CH da Universidade Federal do Ceará - CE
70	AUSÊNCIA COM COMPENSAÇÃO	CH da Universidade Federal do Ceará - CE
70	AUSÊNCIA COM COMPENSAÇÃO	CH da Universidade Federal do Ceará - CE
70	AUSÊNCIA COM COMPENSAÇÃO	CH da Universidade Federal do Ceará - CE
70	AUSÊNCIA COM COMPENSAÇÃO	CH da Universidade Federal do Ceará - CE
70	AUSÊNCIA COM COMPENSAÇÃO	CH da Universidade Federal do Ceará - CE
70	AUSÊNCIA COM COMPENSAÇÃO	CH da Universidade Federal do Ceará - CE
70	AUSÊNCIA COM COMPENSAÇÃO	CH da Universidade Federal do Ceará - CE
70	AUSÊNCIA COM COMPENSAÇÃO	CH da Universidade Federal do Ceará - CE
70	AUSÊNCIA COM COMPENSAÇÃO	CH da Universidade Federal do Ceará - CE
70	AUSÊNCIA COM COMPENSAÇÃO	CH da Universidade Federal do Ceará - CE
70	AUSÊNCIA COM COMPENSAÇÃO	CH da Universidade Federal do Ceará - CE
70	AUSÊNCIA COM COMPENSAÇÃO	CH da Universidade Federal do Ceará - CE
70	AUSÊNCIA COM COMPENSAÇÃO	CH da Universidade Federal do Ceará - CE
70	AUSÊNCIA COM COMPENSAÇÃO	CH da Universidade Federal do Ceará - CE
70	AUSÊNCIA COM COMPENSAÇÃO	CH da Universidade Federal do Ceará - CE
70	AUSÊNCIA COM COMPENSAÇÃO	CH da Universidade Federal do Ceará - CE
70	AUSÊNCIA COM COMPENSAÇÃO	CH da Universidade Federal do Ceará - CE
70	AUSÊNCIA COM COMPENSAÇÃO	CH da Universidade Federal do Ceará - CE
70	AUSÊNCIA COM COMPENSAÇÃO	CH da Universidade Federal do Ceará - CE
70	AUSÊNCIA COM COMPENSAÇÃO	CH da Universidade Federal do Ceará - CE
70	AUSÊNCIA COM COMPENSAÇÃO	CH da Universidade Federal do Ceará - CE
70	AUSÊNCIA COM COMPENSAÇÃO	CH da Universidade Federal do Ceará - CE
70	AUSÊNCIA COM COMPENSAÇÃO	CH da Universidade Federal do Ceará - CE
70	AUSÊNCIA COM COMPENSAÇÃO	CH da Universidade Federal do Ceará - CE

70	AUSÊNCIA COM COMPENSAÇÃO	CH da Universidade Federal do Ceará - CE
70	AUSÊNCIA COM COMPENSAÇÃO	CH da Universidade Federal do Ceará - CE
70	AUSÊNCIA COM COMPENSAÇÃO	CH da Universidade Federal do Ceará - CE
70	AUSÊNCIA COM COMPENSAÇÃO	CH da Universidade Federal do Ceará - CE
70	AUSÊNCIA COM COMPENSAÇÃO	CH da Universidade Federal do Ceará - CE
70	AUSÊNCIA COM COMPENSAÇÃO	CH da Universidade Federal do Ceará - CE
70	AUSÊNCIA COM COMPENSAÇÃO	CH da Universidade Federal do Ceará - CE
70	AUSÊNCIA COM COMPENSAÇÃO	CH da Universidade Federal do Ceará - CE
70	AUSÊNCIA COM COMPENSAÇÃO	CH da Universidade Federal do Ceará - CE
70	AUSÊNCIA COM COMPENSAÇÃO	CH da Universidade Federal do Ceará - CE
70	AUSÊNCIA COM COMPENSAÇÃO	CH da Universidade Federal do Ceará - CE
70	AUSÊNCIA COM COMPENSAÇÃO	CH da Universidade Federal do Ceará - CE
70	AUSÊNCIA COM COMPENSAÇÃO	CH da Universidade Federal do Ceará - CE
70	AUSÊNCIA COM COMPENSAÇÃO	CH da Universidade Federal do Ceará - CE
70	AUSÊNCIA COM COMPENSAÇÃO	CH da Universidade Federal do Ceará - CE
70	AUSÊNCIA COM COMPENSAÇÃO	CH da Universidade Federal do Ceará - CE
70	AUSÊNCIA COM COMPENSAÇÃO	CH da Universidade Federal do Ceará - CE
70	AUSÊNCIA COM COMPENSAÇÃO	CH da Universidade Federal do Ceará - CE
70	AUSÊNCIA COM COMPENSAÇÃO	CH da Universidade Federal do Ceará - CE
70	AUSÊNCIA COM COMPENSAÇÃO	CH da Universidade Federal do Ceará - CE
70	AUSÊNCIA COM COMPENSAÇÃO	CH da Universidade Federal do Ceará - CE
70	AUSÊNCIA COM COMPENSAÇÃO	CH da Universidade Federal do Ceará - CE
70	AUSÊNCIA COM COMPENSAÇÃO	CH da Universidade Federal do Ceará - CE
70	AUSÊNCIA COM COMPENSAÇÃO	CH da Universidade Federal do Ceará - CE
70	AUSÊNCIA COM COMPENSAÇÃO	CH da Universidade Federal do Ceará - CE
70	AUSÊNCIA COM COMPENSAÇÃO	CH da Universidade Federal do Ceará - CE
70	AUSÊNCIA COM COMPENSAÇÃO	CH da Universidade Federal do Ceará - CE
70	AUSÊNCIA COM COMPENSAÇÃO	CH da Universidade Federal do Ceará - CE
70	AUSÊNCIA COM COMPENSAÇÃO	CH da Universidade Federal do Ceará - CE
70	AUSÊNCIA COM COMPENSAÇÃO	CH da Universidade Federal do Ceará - CE
70	AUSÊNCIA COM COMPENSAÇÃO	CH da Universidade Federal do Ceará - CE
70	AUSÊNCIA COM COMPENSAÇÃO	CH da Universidade Federal do Ceará - CE
70	AUSÊNCIA COM COMPENSAÇÃO	CH da Universidade Federal do Ceará - CE
70	AUSÊNCIA COM COMPENSAÇÃO	CH da Universidade Federal do Ceará - CE
70	AUSÊNCIA COM COMPENSAÇÃO	CH da Universidade Federal do Ceará - CE
70	AUSÊNCIA COM COMPENSAÇÃO	CH da Universidade Federal do Ceará - CE
70	AUSÊNCIA COM COMPENSAÇÃO	CH da Universidade Federal do Ceará - CE
70	AUSÊNCIA COM COMPENSAÇÃO	CH da Universidade Federal do Ceará - CE
70	AUSÊNCIA COM COMPENSAÇÃO	CH da Universidade Federal do Ceará - CE
70	AUSÊNCIA COM COMPENSAÇÃO	CH da Universidade Federal do Ceará - CE
70	AUSÊNCIA COM COMPENSAÇÃO	CH da Universidade Federal do Ceará - CE
70	AUSÊNCIA COM COMPENSAÇÃO	CH da Universidade Federal do Ceará - CE
70	AUSÊNCIA COM COMPENSAÇÃO	CH da Universidade Federal do Ceará - CE
70	AUSÊNCIA COM COMPENSAÇÃO	CH da Universidade Federal do Ceará - CE
70	AUSÊNCIA COM COMPENSAÇÃO	CH da Universidade Federal do Ceará - CE
70	AUSÊNCIA COM COMPENSAÇÃO	CH da Universidade Federal do Ceará - CE
70	AUSÊNCIA COM COMPENSAÇÃO	CH da Universidade Federal do Ceará - CE
70	AUSÊNCIA COM COMPENSAÇÃO	CH da Universidade Federal do Ceará - CE

70	AUSÊNCIA COM COMPENSAÇÃO	CH da Universidade Federal do Ceará - CE
70	AUSÊNCIA COM COMPENSAÇÃO	CH da Universidade Federal do Ceará - CE
70	AUSÊNCIA COM COMPENSAÇÃO	CH da Universidade Federal do Ceará - CE
70	AUSÊNCIA COM COMPENSAÇÃO	CH da Universidade Federal do Ceará - CE
70	AUSÊNCIA COM COMPENSAÇÃO	CH da Universidade Federal do Ceará - CE
70	AUSÊNCIA COM COMPENSAÇÃO	CH da Universidade Federal do Ceará - CE
70	AUSÊNCIA COM COMPENSAÇÃO	CH da Universidade Federal do Ceará - CE
70	AUSÊNCIA COM COMPENSAÇÃO	CH da Universidade Federal do Ceará - CE
70	AUSÊNCIA COM COMPENSAÇÃO	CH da Universidade Federal do Ceará - CE
70	AUSÊNCIA COM COMPENSAÇÃO	CH da Universidade Federal do Ceará - CE
70	AUSÊNCIA COM COMPENSAÇÃO	CH da Universidade Federal do Ceará - CE
70	AUSÊNCIA COM COMPENSAÇÃO	CH da Universidade Federal do Ceará - CE
70	AUSÊNCIA COM COMPENSAÇÃO	CH da Universidade Federal do Ceará - CE
70	AUSÊNCIA COM COMPENSAÇÃO	CH da Universidade Federal do Ceará - CE
70	AUSÊNCIA COM COMPENSAÇÃO	CH da Universidade Federal do Ceará - CE
70	AUSÊNCIA COM COMPENSAÇÃO	CH da Universidade Federal do Ceará - CE
70	AUSÊNCIA COM COMPENSAÇÃO	CH da Universidade Federal do Ceará - CE
70	AUSÊNCIA COM COMPENSAÇÃO	CH da Universidade Federal do Ceará - CE
70	AUSÊNCIA COM COMPENSAÇÃO	CH da Universidade Federal do Ceará - CE
70	AUSÊNCIA COM COMPENSAÇÃO	CH da Universidade Federal do Ceará - CE
70	AUSÊNCIA COM COMPENSAÇÃO	CH da Universidade Federal do Ceará - CE
70	AUSÊNCIA COM COMPENSAÇÃO	CH da Universidade Federal do Ceará - CE
70	AUSÊNCIA COM COMPENSAÇÃO	CH da Universidade Federal do Ceará - CE
70	AUSÊNCIA COM COMPENSAÇÃO	CH da Universidade Federal do Ceará - CE
70	AUSÊNCIA COM COMPENSAÇÃO	CH da Universidade Federal do Ceará - CE
70	AUSÊNCIA COM COMPENSAÇÃO	CH da Universidade Federal do Ceará - CE
70	AUSÊNCIA COM COMPENSAÇÃO	CH da Universidade Federal do Ceará - CE
70	AUSÊNCIA COM COMPENSAÇÃO	CH da Universidade Federal do Ceará - CE
70	AUSÊNCIA COM COMPENSAÇÃO	CH da Universidade Federal do Ceará - CE
70	AUSÊNCIA COM COMPENSAÇÃO	CH da Universidade Federal do Ceará - CE
70	AUSÊNCIA COM COMPENSAÇÃO	CH da Universidade Federal do Ceará - CE
70	AUSÊNCIA COM COMPENSAÇÃO	CH da Universidade Federal do Ceará - CE
70	AUSÊNCIA COM COMPENSAÇÃO	CH da Universidade Federal do Ceará - CE
70	AUSÊNCIA COM COMPENSAÇÃO	CH da Universidade Federal do Ceará - CE
70	AUSÊNCIA COM COMPENSAÇÃO	CH da Universidade Federal do Ceará - CE
70	AUSÊNCIA COM COMPENSAÇÃO	CH da Universidade Federal do Ceará - CE
70	AUSÊNCIA COM COMPENSAÇÃO	CH da Universidade Federal do Ceará - CE
70	AUSÊNCIA COM COMPENSAÇÃO	CH da Universidade Federal do Ceará - CE
70	AUSÊNCIA COM COMPENSAÇÃO	CH da Universidade Federal do Ceará - CE
70	AUSÊNCIA COM COMPENSAÇÃO	CH da Universidade Federal do Ceará - CE
70	AUSÊNCIA COM COMPENSAÇÃO	CH da Universidade Federal do Ceará - CE
70	AUSÊNCIA COM COMPENSAÇÃO	CH da Universidade Federal do Ceará - CE
70	AUSÊNCIA COM COMPENSAÇÃO	CH da Universidade Federal do Ceará - CE
70	AUSÊNCIA COM COMPENSAÇÃO	CH da Universidade Federal do Ceará - CE
70	AUSÊNCIA COM COMPENSAÇÃO	CH da Universidade Federal do Ceará - CE
70	AUSÊNCIA COM COMPENSAÇÃO	CH da Universidade Federal do Ceará - CE
70	AUSÊNCIA COM COMPENSAÇÃO	CH da Universidade Federal do Ceará - CE
70	AUSÊNCIA COM COMPENSAÇÃO	CH da Universidade Federal do Ceará - CE

70	AUSÊNCIA COM COMPENSAÇÃO	CH da Universidade Federal do Ceará - CE
70	AUSÊNCIA COM COMPENSAÇÃO	CH da Universidade Federal do Ceará - CE
70	AUSÊNCIA COM COMPENSAÇÃO	CH da Universidade Federal do Ceará - CE
70	AUSÊNCIA COM COMPENSAÇÃO	CH da Universidade Federal do Ceará - CE
70	AUSÊNCIA COM COMPENSAÇÃO	CH da Universidade Federal do Ceará - CE
70	AUSÊNCIA COM COMPENSAÇÃO	CH da Universidade Federal do Ceará - CE
70	AUSÊNCIA COM COMPENSAÇÃO	CH da Universidade Federal do Ceará - CE
70	AUSÊNCIA COM COMPENSAÇÃO	CH da Universidade Federal do Ceará - CE
70	AUSÊNCIA COM COMPENSAÇÃO	CH da Universidade Federal do Ceará - CE
70	AUSÊNCIA COM COMPENSAÇÃO	CH da Universidade Federal do Ceará - CE
70	AUSÊNCIA COM COMPENSAÇÃO	CH da Universidade Federal do Ceará - CE
70	AUSÊNCIA COM COMPENSAÇÃO	CH da Universidade Federal do Ceará - CE
70	AUSÊNCIA COM COMPENSAÇÃO	CH da Universidade Federal do Ceará - CE
70	AUSÊNCIA COM COMPENSAÇÃO	CH da Universidade Federal do Ceará - CE
70	AUSÊNCIA COM COMPENSAÇÃO	CH da Universidade Federal do Ceará - CE
70	AUSÊNCIA COM COMPENSAÇÃO	CH da Universidade Federal do Ceará - CE
70	AUSÊNCIA COM COMPENSAÇÃO	CH da Universidade Federal do Ceará - CE
70	AUSÊNCIA COM COMPENSAÇÃO	CH da Universidade Federal do Ceará - CE
70	AUSÊNCIA COM COMPENSAÇÃO	CH da Universidade Federal do Ceará - CE
70	AUSÊNCIA COM COMPENSAÇÃO	CH da Universidade Federal do Ceará - CE
70	AUSÊNCIA COM COMPENSAÇÃO	CH da Universidade Federal do Ceará - CE
70	AUSÊNCIA COM COMPENSAÇÃO	CH da Universidade Federal do Ceará - CE
70	AUSÊNCIA COM COMPENSAÇÃO	CH da Universidade Federal do Ceará - CE
70	AUSÊNCIA COM COMPENSAÇÃO	CH da Universidade Federal do Ceará - CE
70	AUSÊNCIA COM COMPENSAÇÃO	CH da Universidade Federal do Ceará - CE
70	AUSÊNCIA COM COMPENSAÇÃO	CH da Universidade Federal do Ceará - CE
70	AUSÊNCIA COM COMPENSAÇÃO	CH da Universidade Federal do Ceará - CE
70	AUSÊNCIA COM COMPENSAÇÃO	CH da Universidade Federal do Ceará - CE
70	AUSÊNCIA COM COMPENSAÇÃO	CH da Universidade Federal do Ceará - CE
70	AUSÊNCIA COM COMPENSAÇÃO	CH da Universidade Federal do Ceará - CE
70	AUSÊNCIA COM COMPENSAÇÃO	CH da Universidade Federal do Ceará - CE
70	AUSÊNCIA COM COMPENSAÇÃO	CH da Universidade Federal do Ceará - CE
70	AUSÊNCIA COM COMPENSAÇÃO	CH da Universidade Federal do Ceará - CE
70	AUSÊNCIA COM COMPENSAÇÃO	CH da Universidade Federal do Ceará - CE
70	AUSÊNCIA COM COMPENSAÇÃO	CH da Universidade Federal do Ceará - CE
70	AUSÊNCIA COM COMPENSAÇÃO	CH da Universidade Federal do Ceará - CE
70	AUSÊNCIA COM COMPENSAÇÃO	CH da Universidade Federal do Ceará - CE
70	AUSÊNCIA COM COMPENSAÇÃO	CH da Universidade Federal do Ceará - CE
70	AUSÊNCIA COM COMPENSAÇÃO	CH da Universidade Federal do Ceará - CE
70	AUSÊNCIA COM COMPENSAÇÃO	CH da Universidade Federal do Ceará - CE
70	AUSÊNCIA COM COMPENSAÇÃO	CH da Universidade Federal do Ceará - CE
70	AUSÊNCIA COM COMPENSAÇÃO	CH da Universidade Federal do Ceará - CE
70	AUSÊNCIA COM COMPENSAÇÃO	CH da Universidade Federal do Ceará - CE
70	AUSÊNCIA COM COMPENSAÇÃO	CH da Universidade Federal do Ceará - CE
70	AUSÊNCIA COM COMPENSAÇÃO	CH da Universidade Federal do Ceará - CE
70	AUSÊNCIA COM COMPENSAÇÃO	CH da Universidade Federal do Ceará - CE
70	AUSÊNCIA COM COMPENSAÇÃO	CH da Universidade Federal do Ceará - CE
70	AUSÊNCIA COM COMPENSAÇÃO	CH da Universidade Federal do Ceará - CE

70	AUSÊNCIA COM COMPENSAÇÃO	CH da Universidade Federal do Ceará - CE
70	AUSÊNCIA COM COMPENSAÇÃO	CH da Universidade Federal do Ceará - CE
70	AUSÊNCIA COM COMPENSAÇÃO	CH da Universidade Federal do Ceará - CE
70	AUSÊNCIA COM COMPENSAÇÃO	CH da Universidade Federal do Ceará - CE
70	AUSÊNCIA COM COMPENSAÇÃO	CH da Universidade Federal do Ceará - CE
70	AUSÊNCIA COM COMPENSAÇÃO	CH da Universidade Federal do Ceará - CE
70	AUSÊNCIA COM COMPENSAÇÃO	CH da Universidade Federal do Ceará - CE
70	AUSÊNCIA COM COMPENSAÇÃO	CH da Universidade Federal do Ceará - CE
70	AUSÊNCIA COM COMPENSAÇÃO	CH da Universidade Federal do Ceará - CE
70	AUSÊNCIA COM COMPENSAÇÃO	CH da Universidade Federal do Ceará - CE
70	AUSÊNCIA COM COMPENSAÇÃO	CH da Universidade Federal do Ceará - CE
70	AUSÊNCIA COM COMPENSAÇÃO	CH da Universidade Federal do Ceará - CE
70	AUSÊNCIA COM COMPENSAÇÃO	CH da Universidade Federal do Ceará - CE
70	AUSÊNCIA COM COMPENSAÇÃO	CH da Universidade Federal do Ceará - CE
70	AUSÊNCIA COM COMPENSAÇÃO	CH da Universidade Federal do Ceará - CE
70	AUSÊNCIA COM COMPENSAÇÃO	CH da Universidade Federal do Ceará - CE
70	AUSÊNCIA COM COMPENSAÇÃO	CH da Universidade Federal do Ceará - CE
70	AUSÊNCIA COM COMPENSAÇÃO	CH da Universidade Federal do Ceará - CE
70	AUSÊNCIA COM COMPENSAÇÃO	CH da Universidade Federal do Ceará - CE
70	AUSÊNCIA COM COMPENSAÇÃO	CH da Universidade Federal do Ceará - CE
70	AUSÊNCIA COM COMPENSAÇÃO	CH da Universidade Federal do Ceará - CE
70	AUSÊNCIA COM COMPENSAÇÃO	CH da Universidade Federal do Ceará - CE
70	AUSÊNCIA COM COMPENSAÇÃO	CH da Universidade Federal do Ceará - CE
70	AUSÊNCIA COM COMPENSAÇÃO	CH da Universidade Federal do Ceará - CE
70	AUSÊNCIA COM COMPENSAÇÃO	CH da Universidade Federal do Ceará - CE
70	AUSÊNCIA COM COMPENSAÇÃO	CH da Universidade Federal do Ceará - CE
70	AUSÊNCIA COM COMPENSAÇÃO	CH da Universidade Federal do Ceará - CE
70	AUSÊNCIA COM COMPENSAÇÃO	CH da Universidade Federal do Ceará - CE
70	AUSÊNCIA COM COMPENSAÇÃO	CH da Universidade Federal do Ceará - CE
70	AUSÊNCIA COM COMPENSAÇÃO	CH da Universidade Federal do Ceará - CE
70	AUSÊNCIA COM COMPENSAÇÃO	CH da Universidade Federal do Ceará - CE
70	AUSÊNCIA COM COMPENSAÇÃO	CH da Universidade Federal do Ceará - CE
70	AUSÊNCIA COM COMPENSAÇÃO	CH da Universidade Federal do Ceará - CE
70	AUSÊNCIA COM COMPENSAÇÃO	CH da Universidade Federal do Ceará - CE
70	AUSÊNCIA COM COMPENSAÇÃO	CH da Universidade Federal do Ceará - CE
70	AUSÊNCIA COM COMPENSAÇÃO	CH da Universidade Federal do Ceará - CE
70	AUSÊNCIA COM COMPENSAÇÃO	CH da Universidade Federal do Ceará - CE
70	AUSÊNCIA COM COMPENSAÇÃO	CH da Universidade Federal do Ceará - CE
70	AUSÊNCIA COM COMPENSAÇÃO	CH da Universidade Federal do Ceará - CE
70	AUSÊNCIA COM COMPENSAÇÃO	CH da Universidade Federal do Ceará - CE
70	AUSÊNCIA COM COMPENSAÇÃO	CH da Universidade Federal do Ceará - CE
70	AUSÊNCIA COM COMPENSAÇÃO	CH da Universidade Federal do Ceará - CE
70	AUSÊNCIA COM COMPENSAÇÃO	CH da Universidade Federal do Ceará - CE
70	AUSÊNCIA COM COMPENSAÇÃO	CH da Universidade Federal do Ceará - CE
70	AUSÊNCIA COM COMPENSAÇÃO	CH da Universidade Federal do Ceará - CE
70	AUSÊNCIA COM COMPENSAÇÃO	CH da Universidade Federal do Ceará - CE
70	AUSÊNCIA COM COMPENSAÇÃO	CH da Universidade Federal do Ceará - CE
70	AUSÊNCIA COM COMPENSAÇÃO	CH da Universidade Federal do Ceará - CE

70	AUSÊNCIA COM COMPENSAÇÃO	CH da Universidade Federal do Ceará - CE
70	AUSÊNCIA COM COMPENSAÇÃO	CH da Universidade Federal do Ceará - CE
70	AUSÊNCIA COM COMPENSAÇÃO	CH da Universidade Federal do Ceará - CE
70	AUSÊNCIA COM COMPENSAÇÃO	CH da Universidade Federal do Ceará - CE
70	AUSÊNCIA COM COMPENSAÇÃO	CH da Universidade Federal do Ceará - CE
70	AUSÊNCIA COM COMPENSAÇÃO	CH da Universidade Federal do Ceará - CE
70	AUSÊNCIA COM COMPENSAÇÃO	CH da Universidade Federal do Ceará - CE
70	AUSÊNCIA COM COMPENSAÇÃO	CH da Universidade Federal do Ceará - CE
70	AUSÊNCIA COM COMPENSAÇÃO	CH da Universidade Federal do Ceará - CE
70	AUSÊNCIA COM COMPENSAÇÃO	CH da Universidade Federal do Ceará - CE
70	AUSÊNCIA COM COMPENSAÇÃO	CH da Universidade Federal do Ceará - CE
70	AUSÊNCIA COM COMPENSAÇÃO	CH da Universidade Federal do Ceará - CE
70	AUSÊNCIA COM COMPENSAÇÃO	CH da Universidade Federal do Ceará - CE
70	AUSÊNCIA COM COMPENSAÇÃO	CH da Universidade Federal do Ceará - CE
70	AUSÊNCIA COM COMPENSAÇÃO	CH da Universidade Federal do Ceará - CE
70	AUSÊNCIA COM COMPENSAÇÃO	CH da Universidade Federal do Ceará - CE
70	AUSÊNCIA COM COMPENSAÇÃO	CH da Universidade Federal do Ceará - CE
70	AUSÊNCIA COM COMPENSAÇÃO	CH da Universidade Federal do Ceará - CE
70	AUSÊNCIA COM COMPENSAÇÃO	CH da Universidade Federal do Ceará - CE
70	AUSÊNCIA COM COMPENSAÇÃO	CH da Universidade Federal do Ceará - CE
70	AUSÊNCIA COM COMPENSAÇÃO	CH da Universidade Federal do Ceará - CE
70	AUSÊNCIA COM COMPENSAÇÃO	CH da Universidade Federal do Ceará - CE
70	AUSÊNCIA COM COMPENSAÇÃO	CH da Universidade Federal do Ceará - CE
70	AUSÊNCIA COM COMPENSAÇÃO	CH da Universidade Federal do Ceará - CE
70	AUSÊNCIA COM COMPENSAÇÃO	CH da Universidade Federal do Ceará - CE
70	AUSÊNCIA COM COMPENSAÇÃO	CH da Universidade Federal do Ceará - CE
70	AUSÊNCIA COM COMPENSAÇÃO	CH da Universidade Federal do Ceará - CE
70	AUSÊNCIA COM COMPENSAÇÃO	CH da Universidade Federal do Ceará - CE
70	AUSÊNCIA COM COMPENSAÇÃO	CH da Universidade Federal do Ceará - CE
70	AUSÊNCIA COM COMPENSAÇÃO	CH da Universidade Federal do Ceará - CE
70	AUSÊNCIA COM COMPENSAÇÃO	CH da Universidade Federal do Ceará - CE
70	AUSÊNCIA COM COMPENSAÇÃO	CH da Universidade Federal do Ceará - CE
70	AUSÊNCIA COM COMPENSAÇÃO	CH da Universidade Federal do Ceará - CE
70	AUSÊNCIA COM COMPENSAÇÃO	CH da Universidade Federal do Ceará - CE
70	AUSÊNCIA COM COMPENSAÇÃO	CH da Universidade Federal do Ceará - CE
70	AUSÊNCIA COM COMPENSAÇÃO	CH da Universidade Federal do Ceará - CE
70	AUSÊNCIA COM COMPENSAÇÃO	CH da Universidade Federal do Ceará - CE
70	AUSÊNCIA COM COMPENSAÇÃO	CH da Universidade Federal do Ceará - CE
70	AUSÊNCIA COM COMPENSAÇÃO	CH da Universidade Federal do Ceará - CE
70	AUSÊNCIA COM COMPENSAÇÃO	CH da Universidade Federal do Ceará - CE
70	AUSÊNCIA COM COMPENSAÇÃO	CH da Universidade Federal do Ceará - CE
70	AUSÊNCIA COM COMPENSAÇÃO	CH da Universidade Federal do Ceará - CE
70	AUSÊNCIA COM COMPENSAÇÃO	CH da Universidade Federal do Ceará - CE
70	AUSÊNCIA COM COMPENSAÇÃO	CH da Universidade Federal do Ceará - CE
70	AUSÊNCIA COM COMPENSAÇÃO	CH da Universidade Federal do Ceará - CE

70	AUSÊNCIA COM COMPENSAÇÃO	CH da Universidade Federal do Ceará - CE
70	AUSÊNCIA COM COMPENSAÇÃO	CH da Universidade Federal do Ceará - CE
70	AUSÊNCIA COM COMPENSAÇÃO	CH da Universidade Federal do Ceará - CE
70	AUSÊNCIA COM COMPENSAÇÃO	CH da Universidade Federal do Ceará - CE
70	AUSÊNCIA COM COMPENSAÇÃO	CH da Universidade Federal do Ceará - CE
70	AUSÊNCIA COM COMPENSAÇÃO	CH da Universidade Federal do Ceará - CE
70	AUSÊNCIA COM COMPENSAÇÃO	CH da Universidade Federal do Ceará - CE
70	AUSÊNCIA COM COMPENSAÇÃO	CH da Universidade Federal do Ceará - CE
70	AUSÊNCIA COM COMPENSAÇÃO	CH da Universidade Federal do Ceará - CE
70	AUSÊNCIA COM COMPENSAÇÃO	CH da Universidade Federal do Ceará - CE
70	AUSÊNCIA COM COMPENSAÇÃO	CH da Universidade Federal do Ceará - CE
70	AUSÊNCIA COM COMPENSAÇÃO	CH da Universidade Federal do Ceará - CE
70	AUSÊNCIA COM COMPENSAÇÃO	CH da Universidade Federal do Ceará - CE
70	AUSÊNCIA COM COMPENSAÇÃO	CH da Universidade Federal do Ceará - CE
70	AUSÊNCIA COM COMPENSAÇÃO	CH da Universidade Federal do Ceará - CE
70	AUSÊNCIA COM COMPENSAÇÃO	CH da Universidade Federal do Ceará - CE
70	AUSÊNCIA COM COMPENSAÇÃO	CH da Universidade Federal do Ceará - CE
70	AUSÊNCIA COM COMPENSAÇÃO	CH da Universidade Federal do Ceará - CE
70	AUSÊNCIA COM COMPENSAÇÃO	CH da Universidade Federal do Ceará - CE
70	AUSÊNCIA COM COMPENSAÇÃO	CH da Universidade Federal do Ceará - CE
70	AUSÊNCIA COM COMPENSAÇÃO	CH da Universidade Federal do Ceará - CE
70	AUSÊNCIA COM COMPENSAÇÃO	CH da Universidade Federal do Ceará - CE
70	AUSÊNCIA COM COMPENSAÇÃO	CH da Universidade Federal do Ceará - CE
70	AUSÊNCIA COM COMPENSAÇÃO	CH da Universidade Federal do Ceará - CE
70	AUSÊNCIA COM COMPENSAÇÃO	CH da Universidade Federal do Ceará - CE
70	AUSÊNCIA COM COMPENSAÇÃO	CH da Universidade Federal do Ceará - CE
70	AUSÊNCIA COM COMPENSAÇÃO	CH da Universidade Federal do Ceará - CE
70	AUSÊNCIA COM COMPENSAÇÃO	CH da Universidade Federal do Ceará - CE
70	AUSÊNCIA COM COMPENSAÇÃO	CH da Universidade Federal do Ceará - CE
70	AUSÊNCIA COM COMPENSAÇÃO	CH da Universidade Federal do Ceará - CE
70	AUSÊNCIA COM COMPENSAÇÃO	CH da Universidade Federal do Ceará - CE
70	AUSÊNCIA COM COMPENSAÇÃO	CH da Universidade Federal do Ceará - CE
70	AUSÊNCIA COM COMPENSAÇÃO	CH da Universidade Federal do Ceará - CE
70	AUSÊNCIA COM COMPENSAÇÃO	CH da Universidade Federal do Ceará - CE
70	AUSÊNCIA COM COMPENSAÇÃO	CH da Universidade Federal do Ceará - CE
70	AUSÊNCIA COM COMPENSAÇÃO	CH da Universidade Federal do Ceará - CE
70	AUSÊNCIA COM COMPENSAÇÃO	CH da Universidade Federal do Ceará - CE
70	AUSÊNCIA COM COMPENSAÇÃO	CH da Universidade Federal do Ceará - CE
70	AUSÊNCIA COM COMPENSAÇÃO	CH da Universidade Federal do Ceará - CE
70	AUSÊNCIA COM COMPENSAÇÃO	CH da Universidade Federal do Ceará - CE
70	AUSÊNCIA COM COMPENSAÇÃO	CH da Universidade Federal do Ceará - CE
70	AUSÊNCIA COM COMPENSAÇÃO	CH da Universidade Federal do Ceará - CE
70	AUSÊNCIA COM COMPENSAÇÃO	CH da Universidade Federal do Ceará - CE
70	AUSÊNCIA COM COMPENSAÇÃO	CH da Universidade Federal do Ceará - CE
70	AUSÊNCIA COM COMPENSAÇÃO	CH da Universidade Federal do Ceará - CE
70	AUSÊNCIA COM COMPENSAÇÃO	CH da Universidade Federal do Ceará - CE
70	AUSÊNCIA COM COMPENSAÇÃO	CH da Universidade Federal do Ceará - CE
70	AUSÊNCIA COM COMPENSAÇÃO	CH da Universidade Federal do Ceará - CE

70	AUSÊNCIA COM COMPENSAÇÃO	CH da Universidade Federal do Ceará - CE
70	AUSÊNCIA COM COMPENSAÇÃO	CH da Universidade Federal do Ceará - CE
70	AUSÊNCIA COM COMPENSAÇÃO	CH da Universidade Federal do Ceará - CE
70	AUSÊNCIA COM COMPENSAÇÃO	CH da Universidade Federal do Ceará - CE
70	AUSÊNCIA COM COMPENSAÇÃO	CH da Universidade Federal do Ceará - CE
70	AUSÊNCIA COM COMPENSAÇÃO	CH da Universidade Federal do Ceará - CE
70	AUSÊNCIA COM COMPENSAÇÃO	CH da Universidade Federal do Ceará - CE
70	AUSÊNCIA COM COMPENSAÇÃO	CH da Universidade Federal do Ceará - CE
70	AUSÊNCIA COM COMPENSAÇÃO	CH da Universidade Federal do Ceará - CE
70	AUSÊNCIA COM COMPENSAÇÃO	CH da Universidade Federal do Ceará - CE
70	AUSÊNCIA COM COMPENSAÇÃO	CH da Universidade Federal do Ceará - CE
70	AUSÊNCIA COM COMPENSAÇÃO	CH da Universidade Federal do Ceará - CE
70	AUSÊNCIA COM COMPENSAÇÃO	CH da Universidade Federal do Ceará - CE
70	AUSÊNCIA COM COMPENSAÇÃO	CH da Universidade Federal do Ceará - CE
70	AUSÊNCIA COM COMPENSAÇÃO	CH da Universidade Federal do Ceará - CE
70	AUSÊNCIA COM COMPENSAÇÃO	CH da Universidade Federal do Ceará - CE
70	AUSÊNCIA COM COMPENSAÇÃO	CH da Universidade Federal do Ceará - CE
70	AUSÊNCIA COM COMPENSAÇÃO	CH da Universidade Federal do Ceará - CE
70	AUSÊNCIA COM COMPENSAÇÃO	CH da Universidade Federal do Ceará - CE
70	AUSÊNCIA COM COMPENSAÇÃO	CH da Universidade Federal do Ceará - CE
70	AUSÊNCIA COM COMPENSAÇÃO	CH da Universidade Federal do Ceará - CE
70	AUSÊNCIA COM COMPENSAÇÃO	CH da Universidade Federal do Ceará - CE
70	AUSÊNCIA COM COMPENSAÇÃO	CH da Universidade Federal do Ceará - CE
70	AUSÊNCIA COM COMPENSAÇÃO	CH da Universidade Federal do Ceará - CE
70	AUSÊNCIA COM COMPENSAÇÃO	CH da Universidade Federal do Ceará - CE
70	AUSÊNCIA COM COMPENSAÇÃO	CH da Universidade Federal do Ceará - CE
70	AUSÊNCIA COM COMPENSAÇÃO	CH da Universidade Federal do Ceará - CE
70	AUSÊNCIA COM COMPENSAÇÃO	CH da Universidade Federal do Ceará - CE
70	AUSÊNCIA COM COMPENSAÇÃO	CH da Universidade Federal do Ceará - CE
70	AUSÊNCIA COM COMPENSAÇÃO	CH da Universidade Federal do Ceará - CE
70	AUSÊNCIA COM COMPENSAÇÃO	CH da Universidade Federal do Ceará - CE
70	AUSÊNCIA COM COMPENSAÇÃO	CH da Universidade Federal do Ceará - CE
70	AUSÊNCIA COM COMPENSAÇÃO	CH da Universidade Federal do Ceará - CE
70	AUSÊNCIA COM COMPENSAÇÃO	CH da Universidade Federal do Ceará - CE
70	AUSÊNCIA COM COMPENSAÇÃO	CH da Universidade Federal do Ceará - CE
70	AUSÊNCIA COM COMPENSAÇÃO	CH da Universidade Federal do Ceará - CE
70	AUSÊNCIA COM COMPENSAÇÃO	CH da Universidade Federal do Ceará - CE
70	AUSÊNCIA COM COMPENSAÇÃO	CH da Universidade Federal do Ceará - CE
70	AUSÊNCIA COM COMPENSAÇÃO	CH da Universidade Federal do Ceará - CE
70	AUSÊNCIA COM COMPENSAÇÃO	CH da Universidade Federal do Ceará - CE
70	AUSÊNCIA COM COMPENSAÇÃO	CH da Universidade Federal do Ceará - CE
70	AUSÊNCIA COM COMPENSAÇÃO	CH da Universidade Federal do Ceará - CE
70	AUSÊNCIA COM COMPENSAÇÃO	CH da Universidade Federal do Ceará - CE
70	AUSÊNCIA COM COMPENSAÇÃO	CH da Universidade Federal do Ceará - CE
70	AUSÊNCIA COM COMPENSAÇÃO	CH da Universidade Federal do Ceará - CE

70	AUSÊNCIA COM COMPENSAÇÃO	CH da Universidade Federal do Ceará - CE
70	AUSÊNCIA COM COMPENSAÇÃO	CH da Universidade Federal do Ceará - CE
70	AUSÊNCIA COM COMPENSAÇÃO	CH da Universidade Federal do Ceará - CE
70	AUSÊNCIA COM COMPENSAÇÃO	CH da Universidade Federal do Ceará - CE
70	AUSÊNCIA COM COMPENSAÇÃO	CH da Universidade Federal do Ceará - CE
70	AUSÊNCIA COM COMPENSAÇÃO	CH da Universidade Federal do Ceará - CE
70	AUSÊNCIA COM COMPENSAÇÃO	CH da Universidade Federal do Ceará - CE
70	AUSÊNCIA COM COMPENSAÇÃO	CH da Universidade Federal do Ceará - CE
70	AUSÊNCIA COM COMPENSAÇÃO	CH da Universidade Federal do Ceará - CE
70	AUSÊNCIA COM COMPENSAÇÃO	CH da Universidade Federal do Ceará - CE
70	AUSÊNCIA COM COMPENSAÇÃO	CH da Universidade Federal do Ceará - CE
70	AUSÊNCIA COM COMPENSAÇÃO	CH da Universidade Federal do Ceará - CE
70	AUSÊNCIA COM COMPENSAÇÃO	CH da Universidade Federal do Ceará - CE
70	AUSÊNCIA COM COMPENSAÇÃO	CH da Universidade Federal do Ceará - CE
70	AUSÊNCIA COM COMPENSAÇÃO	CH da Universidade Federal do Ceará - CE
70	AUSÊNCIA COM COMPENSAÇÃO	CH da Universidade Federal do Ceará - CE
70	AUSÊNCIA COM COMPENSAÇÃO	CH da Universidade Federal do Ceará - CE
70	AUSÊNCIA COM COMPENSAÇÃO	CH da Universidade Federal do Ceará - CE
70	AUSÊNCIA COM COMPENSAÇÃO	CH da Universidade Federal do Ceará - CE
70	AUSÊNCIA COM COMPENSAÇÃO	CH da Universidade Federal do Ceará - CE
70	AUSÊNCIA COM COMPENSAÇÃO	CH da Universidade Federal do Ceará - CE
70	AUSÊNCIA COM COMPENSAÇÃO	CH da Universidade Federal do Ceará - CE
70	AUSÊNCIA COM COMPENSAÇÃO	CH da Universidade Federal do Ceará - CE
70	AUSÊNCIA COM COMPENSAÇÃO	CH da Universidade Federal do Ceará - CE
70	AUSÊNCIA COM COMPENSAÇÃO	CH da Universidade Federal do Ceará - CE
70	AUSÊNCIA COM COMPENSAÇÃO	CH da Universidade Federal do Ceará - CE
70	AUSÊNCIA COM COMPENSAÇÃO	CH da Universidade Federal do Ceará - CE
70	AUSÊNCIA COM COMPENSAÇÃO	CH da Universidade Federal do Ceará - CE
70	AUSÊNCIA COM COMPENSAÇÃO	CH da Universidade Federal do Ceará - CE
70	AUSÊNCIA COM COMPENSAÇÃO	CH da Universidade Federal do Ceará - CE
70	AUSÊNCIA COM COMPENSAÇÃO	CH da Universidade Federal do Ceará - CE
70	AUSÊNCIA COM COMPENSAÇÃO	CH da Universidade Federal do Ceará - CE
70	AUSÊNCIA COM COMPENSAÇÃO	CH da Universidade Federal do Ceará - CE
70	AUSÊNCIA COM COMPENSAÇÃO	CH da Universidade Federal do Ceará - CE
70	AUSÊNCIA COM COMPENSAÇÃO	CH da Universidade Federal do Ceará - CE
70	AUSÊNCIA COM COMPENSAÇÃO	CH da Universidade Federal do Ceará - CE
70	AUSÊNCIA COM COMPENSAÇÃO	CH da Universidade Federal do Ceará - CE
70	AUSÊNCIA COM COMPENSAÇÃO	CH da Universidade Federal do Ceará - CE
70	AUSÊNCIA COM COMPENSAÇÃO	CH da Universidade Federal do Ceará - CE
70	AUSÊNCIA COM COMPENSAÇÃO	CH da Universidade Federal do Ceará - CE
70	AUSÊNCIA COM COMPENSAÇÃO	CH da Universidade Federal do Ceará - CE

70	AUSÊNCIA COM COMPENSAÇÃO	CH da Universidade Federal do Ceará - CE
70	AUSÊNCIA COM COMPENSAÇÃO	CH da Universidade Federal do Ceará - CE
70	AUSÊNCIA COM COMPENSAÇÃO	CH da Universidade Federal do Ceará - CE
70	AUSÊNCIA COM COMPENSAÇÃO	CH da Universidade Federal do Ceará - CE
70	AUSÊNCIA COM COMPENSAÇÃO	CH da Universidade Federal do Ceará - CE
70	AUSÊNCIA COM COMPENSAÇÃO	CH da Universidade Federal do Ceará - CE
70	AUSÊNCIA COM COMPENSAÇÃO	CH da Universidade Federal do Ceará - CE
70	AUSÊNCIA COM COMPENSAÇÃO	CH da Universidade Federal do Ceará - CE
70	AUSÊNCIA COM COMPENSAÇÃO	CH da Universidade Federal do Ceará - CE
70	AUSÊNCIA COM COMPENSAÇÃO	CH da Universidade Federal do Ceará - CE
70	AUSÊNCIA COM COMPENSAÇÃO	CH da Universidade Federal do Ceará - CE
70	AUSÊNCIA COM COMPENSAÇÃO	CH da Universidade Federal do Ceará - CE
70	AUSÊNCIA COM COMPENSAÇÃO	CH da Universidade Federal do Ceará - CE
70	AUSÊNCIA COM COMPENSAÇÃO	CH da Universidade Federal do Ceará - CE
70	AUSÊNCIA COM COMPENSAÇÃO	CH da Universidade Federal do Ceará - CE
70	AUSÊNCIA COM COMPENSAÇÃO	CH da Universidade Federal do Ceará - CE
70	AUSÊNCIA COM COMPENSAÇÃO	CH da Universidade Federal do Ceará - CE
70	AUSÊNCIA COM COMPENSAÇÃO	CH da Universidade Federal do Ceará - CE
70	AUSÊNCIA COM COMPENSAÇÃO	CH da Universidade Federal do Ceará - CE
70	AUSÊNCIA COM COMPENSAÇÃO	CH da Universidade Federal do Ceará - CE
70	AUSÊNCIA COM COMPENSAÇÃO	CH da Universidade Federal do Ceará - CE
70	AUSÊNCIA COM COMPENSAÇÃO	CH da Universidade Federal do Ceará - CE
70	AUSÊNCIA COM COMPENSAÇÃO	CH da Universidade Federal do Ceará - CE
70	AUSÊNCIA COM COMPENSAÇÃO	CH da Universidade Federal do Ceará - CE
70	AUSÊNCIA COM COMPENSAÇÃO	CH da Universidade Federal do Ceará - CE
70	AUSÊNCIA COM COMPENSAÇÃO	CH da Universidade Federal do Ceará - CE
70	AUSÊNCIA COM COMPENSAÇÃO	CH da Universidade Federal do Ceará - CE
70	AUSÊNCIA COM COMPENSAÇÃO	CH da Universidade Federal do Ceará - CE
70	AUSÊNCIA COM COMPENSAÇÃO	CH da Universidade Federal do Ceará - CE
70	AUSÊNCIA COM COMPENSAÇÃO	CH da Universidade Federal do Ceará - CE
70	AUSÊNCIA COM COMPENSAÇÃO	CH da Universidade Federal do Ceará - CE
70	AUSÊNCIA COM COMPENSAÇÃO	CH da Universidade Federal do Ceará - CE
70	AUSÊNCIA COM COMPENSAÇÃO	CH da Universidade Federal do Ceará - CE
70	AUSÊNCIA COM COMPENSAÇÃO	CH da Universidade Federal do Ceará - CE
70	AUSÊNCIA COM COMPENSAÇÃO	CH da Universidade Federal do Ceará - CE
70	AUSÊNCIA COM COMPENSAÇÃO	CH da Universidade Federal do Ceará - CE
70	AUSÊNCIA COM COMPENSAÇÃO	CH da Universidade Federal do Ceará - CE
70	AUSÊNCIA COM COMPENSAÇÃO	CH da Universidade Federal do Ceará - CE
70	AUSÊNCIA COM COMPENSAÇÃO	CH da Universidade Federal do Ceará - CE
70	AUSÊNCIA COM COMPENSAÇÃO	CH da Universidade Federal do Ceará - CE
70	AUSÊNCIA COM COMPENSAÇÃO	CH da Universidade Federal do Ceará - CE
70	AUSÊNCIA COM COMPENSAÇÃO	CH da Universidade Federal do Ceará - CE
70	AUSÊNCIA COM COMPENSAÇÃO	CH da Universidade Federal do Ceará - CE
70	AUSÊNCIA COM COMPENSAÇÃO	CH da Universidade Federal do Ceará - CE
70	AUSÊNCIA COM COMPENSAÇÃO	CH da Universidade Federal do Ceará - CE
70	AUSÊNCIA COM COMPENSAÇÃO	CH da Universidade Federal do Ceará - CE
70	AUSÊNCIA COM COMPENSAÇÃO	CH da Universidade Federal do Ceará - CE
70	AUSÊNCIA COM COMPENSAÇÃO	CH da Universidade Federal do Ceará - CE

70	AUSÊNCIA COM COMPENSAÇÃO	CH da Universidade Federal do Ceará - CE
70	AUSÊNCIA COM COMPENSAÇÃO	CH da Universidade Federal do Ceará - CE
70	AUSÊNCIA COM COMPENSAÇÃO	CH da Universidade Federal do Ceará - CE
70	AUSÊNCIA COM COMPENSAÇÃO	CH da Universidade Federal do Ceará - CE
70	AUSÊNCIA COM COMPENSAÇÃO	CH da Universidade Federal do Ceará - CE
70	AUSÊNCIA COM COMPENSAÇÃO	CH da Universidade Federal do Ceará - CE
70	AUSÊNCIA COM COMPENSAÇÃO	CH da Universidade Federal do Ceará - CE
70	AUSÊNCIA COM COMPENSAÇÃO	CH da Universidade Federal do Ceará - CE
70	AUSÊNCIA COM COMPENSAÇÃO	CH da Universidade Federal do Ceará - CE
70	AUSÊNCIA COM COMPENSAÇÃO	CH da Universidade Federal do Ceará - CE
70	AUSÊNCIA COM COMPENSAÇÃO	CH da Universidade Federal do Ceará - CE
70	AUSÊNCIA COM COMPENSAÇÃO	CH da Universidade Federal do Ceará - CE
70	AUSÊNCIA COM COMPENSAÇÃO	CH da Universidade Federal do Ceará - CE
70	AUSÊNCIA COM COMPENSAÇÃO	CH da Universidade Federal do Ceará - CE
70	AUSÊNCIA COM COMPENSAÇÃO	CH da Universidade Federal do Ceará - CE
70	AUSÊNCIA COM COMPENSAÇÃO	CH da Universidade Federal do Ceará - CE
70	AUSÊNCIA COM COMPENSAÇÃO	CH da Universidade Federal do Ceará - CE
70	AUSÊNCIA COM COMPENSAÇÃO	CH da Universidade Federal do Ceará - CE
70	AUSÊNCIA COM COMPENSAÇÃO	CH da Universidade Federal do Ceará - CE
70	AUSÊNCIA COM COMPENSAÇÃO	CH da Universidade Federal do Ceará - CE
70	AUSÊNCIA COM COMPENSAÇÃO	CH da Universidade Federal do Ceará - CE
70	AUSÊNCIA COM COMPENSAÇÃO	CH da Universidade Federal do Ceará - CE
70	AUSÊNCIA COM COMPENSAÇÃO	CH da Universidade Federal do Ceará - CE
70	AUSÊNCIA COM COMPENSAÇÃO	CH da Universidade Federal do Ceará - CE
70	AUSÊNCIA COM COMPENSAÇÃO	CH da Universidade Federal do Ceará - CE
70	AUSÊNCIA COM COMPENSAÇÃO	CH da Universidade Federal do Ceará - CE
70	AUSÊNCIA COM COMPENSAÇÃO	CH da Universidade Federal do Ceará - CE
70	AUSÊNCIA COM COMPENSAÇÃO	CH da Universidade Federal do Ceará - CE
70	AUSÊNCIA COM COMPENSAÇÃO	CH da Universidade Federal do Ceará - CE
70	AUSÊNCIA COM COMPENSAÇÃO	CH da Universidade Federal do Ceará - CE
70	AUSÊNCIA COM COMPENSAÇÃO	CH da Universidade Federal do Ceará - CE
70	AUSÊNCIA COM COMPENSAÇÃO	CH da Universidade Federal do Ceará - CE
70	AUSÊNCIA COM COMPENSAÇÃO	CH da Universidade Federal do Ceará - CE
70	AUSÊNCIA COM COMPENSAÇÃO	CH da Universidade Federal do Ceará - CE
70	AUSÊNCIA COM COMPENSAÇÃO	CH da Universidade Federal do Ceará - CE
70	AUSÊNCIA COM COMPENSAÇÃO	CH da Universidade Federal do Ceará - CE
70	AUSÊNCIA COM COMPENSAÇÃO	CH da Universidade Federal do Ceará - CE
70	AUSÊNCIA COM COMPENSAÇÃO	CH da Universidade Federal do Ceará - CE
70	AUSÊNCIA COM COMPENSAÇÃO	CH da Universidade Federal do Ceará - CE
70	AUSÊNCIA COM COMPENSAÇÃO	CH da Universidade Federal do Ceará - CE
70	AUSÊNCIA COM COMPENSAÇÃO	CH da Universidade Federal do Ceará - CE
70	AUSÊNCIA COM COMPENSAÇÃO	CH da Universidade Federal do Ceará - CE
70	AUSÊNCIA COM COMPENSAÇÃO	CH da Universidade Federal do Ceará - CE
70	AUSÊNCIA COM COMPENSAÇÃO	CH da Universidade Federal do Ceará - CE
70	AUSÊNCIA COM COMPENSAÇÃO	CH da Universidade Federal do Ceará - CE
70	AUSÊNCIA COM COMPENSAÇÃO	CH da Universidade Federal do Ceará - CE
70	AUSÊNCIA COM COMPENSAÇÃO	CH da Universidade Federal do Ceará - CE
70	AUSÊNCIA COM COMPENSAÇÃO	CH da Universidade Federal do Ceará - CE

70	AUSÊNCIA COM COMPENSAÇÃO	CH da Universidade Federal do Ceará - CE
70	AUSÊNCIA COM COMPENSAÇÃO	CH da Universidade Federal do Ceará - CE
70	AUSÊNCIA COM COMPENSAÇÃO	CH da Universidade Federal do Ceará - CE
70	AUSÊNCIA COM COMPENSAÇÃO	CH da Universidade Federal do Ceará - CE
70	AUSÊNCIA COM COMPENSAÇÃO	CH da Universidade Federal do Ceará - CE
70	AUSÊNCIA COM COMPENSAÇÃO	CH da Universidade Federal do Ceará - CE
70	AUSÊNCIA COM COMPENSAÇÃO	CH da Universidade Federal do Ceará - CE
70	AUSÊNCIA COM COMPENSAÇÃO	CH da Universidade Federal do Ceará - CE
70	AUSÊNCIA COM COMPENSAÇÃO	CH da Universidade Federal do Ceará - CE
70	AUSÊNCIA COM COMPENSAÇÃO	CH da Universidade Federal do Ceará - CE
70	AUSÊNCIA COM COMPENSAÇÃO	CH da Universidade Federal do Ceará - CE
70	AUSÊNCIA COM COMPENSAÇÃO	CH da Universidade Federal do Ceará - CE
70	AUSÊNCIA COM COMPENSAÇÃO	CH da Universidade Federal do Ceará - CE
70	AUSÊNCIA COM COMPENSAÇÃO	CH da Universidade Federal do Ceará - CE
70	AUSÊNCIA COM COMPENSAÇÃO	CH da Universidade Federal do Ceará - CE
70	AUSÊNCIA COM COMPENSAÇÃO	CH da Universidade Federal do Ceará - CE
70	AUSÊNCIA COM COMPENSAÇÃO	CH da Universidade Federal do Ceará - CE
70	AUSÊNCIA COM COMPENSAÇÃO	CH da Universidade Federal do Ceará - CE
70	AUSÊNCIA COM COMPENSAÇÃO	CH da Universidade Federal do Ceará - CE
70	AUSÊNCIA COM COMPENSAÇÃO	CH da Universidade Federal do Ceará - CE
70	AUSÊNCIA COM COMPENSAÇÃO	CH da Universidade Federal do Ceará - CE
70	AUSÊNCIA COM COMPENSAÇÃO	CH da Universidade Federal do Ceará - CE
70	AUSÊNCIA COM COMPENSAÇÃO	CH da Universidade Federal do Ceará - CE
70	AUSÊNCIA COM COMPENSAÇÃO	CH da Universidade Federal do Ceará - CE
70	AUSÊNCIA COM COMPENSAÇÃO	CH da Universidade Federal do Ceará - CE
70	AUSÊNCIA COM COMPENSAÇÃO	CH da Universidade Federal do Ceará - CE
70	AUSÊNCIA COM COMPENSAÇÃO	CH da Universidade Federal do Ceará - CE
70	AUSÊNCIA COM COMPENSAÇÃO	CH da Universidade Federal do Ceará - CE
70	AUSÊNCIA COM COMPENSAÇÃO	CH da Universidade Federal do Ceará - CE
70	AUSÊNCIA COM COMPENSAÇÃO	CH da Universidade Federal do Ceará - CE
70	AUSÊNCIA COM COMPENSAÇÃO	CH da Universidade Federal do Ceará - CE
70	AUSÊNCIA COM COMPENSAÇÃO	CH da Universidade Federal do Ceará - CE
70	AUSÊNCIA COM COMPENSAÇÃO	CH da Universidade Federal do Ceará - CE
70	AUSÊNCIA COM COMPENSAÇÃO	CH da Universidade Federal do Ceará - CE
70	AUSÊNCIA COM COMPENSAÇÃO	CH da Universidade Federal do Ceará - CE
70	AUSÊNCIA COM COMPENSAÇÃO	CH da Universidade Federal do Ceará - CE
70	AUSÊNCIA COM COMPENSAÇÃO	CH da Universidade Federal do Ceará - CE
70	AUSÊNCIA COM COMPENSAÇÃO	CH da Universidade Federal do Ceará - CE
70	AUSÊNCIA COM COMPENSAÇÃO	CH da Universidade Federal do Ceará - CE
70	AUSÊNCIA COM COMPENSAÇÃO	CH da Universidade Federal do Ceará - CE
70	AUSÊNCIA COM COMPENSAÇÃO	CH da Universidade Federal do Ceará - CE
70	AUSÊNCIA COM COMPENSAÇÃO	CH da Universidade Federal do Ceará - CE
70	AUSÊNCIA COM COMPENSAÇÃO	CH da Universidade Federal do Ceará - CE
70	AUSÊNCIA COM COMPENSAÇÃO	CH da Universidade Federal do Ceará - CE
70	AUSÊNCIA COM COMPENSAÇÃO	CH da Universidade Federal do Ceará - CE
70	AUSÊNCIA COM COMPENSAÇÃO	CH da Universidade Federal do Ceará - CE
70	AUSÊNCIA COM COMPENSAÇÃO	CH da Universidade Federal do Ceará - CE
70	AUSÊNCIA COM COMPENSAÇÃO	CH da Universidade Federal do Ceará - CE

70	AUSÊNCIA COM COMPENSAÇÃO	CH da Universidade Federal do Ceará - CE
70	AUSÊNCIA COM COMPENSAÇÃO	CH da Universidade Federal do Ceará - CE
70	AUSÊNCIA COM COMPENSAÇÃO	CH da Universidade Federal do Ceará - CE
70	AUSÊNCIA COM COMPENSAÇÃO	CH da Universidade Federal do Ceará - CE
70	AUSÊNCIA COM COMPENSAÇÃO	CH da Universidade Federal do Ceará - CE
70	AUSÊNCIA COM COMPENSAÇÃO	CH da Universidade Federal do Ceará - CE
70	AUSÊNCIA COM COMPENSAÇÃO	CH da Universidade Federal do Ceará - CE
70	AUSÊNCIA COM COMPENSAÇÃO	CH da Universidade Federal do Ceará - CE
70	AUSÊNCIA COM COMPENSAÇÃO	CH da Universidade Federal do Ceará - CE
70	AUSÊNCIA COM COMPENSAÇÃO	CH da Universidade Federal do Ceará - CE
70	AUSÊNCIA COM COMPENSAÇÃO	CH da Universidade Federal do Ceará - CE
70	AUSÊNCIA COM COMPENSAÇÃO	CH da Universidade Federal do Ceará - CE
70	AUSÊNCIA COM COMPENSAÇÃO	CH da Universidade Federal do Ceará - CE
70	AUSÊNCIA COM COMPENSAÇÃO	CH da Universidade Federal do Ceará - CE
70	AUSÊNCIA COM COMPENSAÇÃO	CH da Universidade Federal do Ceará - CE
70	AUSÊNCIA COM COMPENSAÇÃO	CH da Universidade Federal do Ceará - CE
70	AUSÊNCIA COM COMPENSAÇÃO	CH da Universidade Federal do Ceará - CE
70	AUSÊNCIA COM COMPENSAÇÃO	CH da Universidade Federal do Ceará - CE
70	AUSÊNCIA COM COMPENSAÇÃO	CH da Universidade Federal do Ceará - CE
70	AUSÊNCIA COM COMPENSAÇÃO	CH da Universidade Federal do Ceará - CE
70	AUSÊNCIA COM COMPENSAÇÃO	CH da Universidade Federal do Ceará - CE
70	AUSÊNCIA COM COMPENSAÇÃO	CH da Universidade Federal do Ceará - CE
70	AUSÊNCIA COM COMPENSAÇÃO	CH da Universidade Federal do Ceará - CE
70	AUSÊNCIA COM COMPENSAÇÃO	CH da Universidade Federal do Ceará - CE
70	AUSÊNCIA COM COMPENSAÇÃO	CH da Universidade Federal do Ceará - CE
70	AUSÊNCIA COM COMPENSAÇÃO	CH da Universidade Federal do Ceará - CE
70	AUSÊNCIA COM COMPENSAÇÃO	CH da Universidade Federal do Ceará - CE
70	AUSÊNCIA COM COMPENSAÇÃO	CH da Universidade Federal do Ceará - CE
70	AUSÊNCIA COM COMPENSAÇÃO	CH da Universidade Federal do Ceará - CE
70	AUSÊNCIA COM COMPENSAÇÃO	CH da Universidade Federal do Ceará - CE
70	AUSÊNCIA COM COMPENSAÇÃO	CH da Universidade Federal do Ceará - CE
70	AUSÊNCIA COM COMPENSAÇÃO	CH da Universidade Federal do Ceará - CE
70	AUSÊNCIA COM COMPENSAÇÃO	CH da Universidade Federal do Ceará - CE
70	AUSÊNCIA COM COMPENSAÇÃO	CH da Universidade Federal do Ceará - CE
70	AUSÊNCIA COM COMPENSAÇÃO	CH da Universidade Federal do Ceará - CE
70	AUSÊNCIA COM COMPENSAÇÃO	CH da Universidade Federal do Ceará - CE
70	AUSÊNCIA COM COMPENSAÇÃO	CH da Universidade Federal do Ceará - CE
70	AUSÊNCIA COM COMPENSAÇÃO	CH da Universidade Federal do Ceará - CE
70	AUSÊNCIA COM COMPENSAÇÃO	CH da Universidade Federal do Ceará - CE
70	AUSÊNCIA COM COMPENSAÇÃO	CH da Universidade Federal do Ceará - CE
70	AUSÊNCIA COM COMPENSAÇÃO	CH da Universidade Federal do Ceará - CE
70	AUSÊNCIA COM COMPENSAÇÃO	CH da Universidade Federal do Ceará - CE
70	AUSÊNCIA COM COMPENSAÇÃO	CH da Universidade Federal do Ceará - CE
70	AUSÊNCIA COM COMPENSAÇÃO	CH da Universidade Federal do Ceará - CE
70	AUSÊNCIA COM COMPENSAÇÃO	CH da Universidade Federal do Ceará - CE
70	AUSÊNCIA COM COMPENSAÇÃO	CH da Universidade Federal do Ceará - CE
70	AUSÊNCIA COM COMPENSAÇÃO	CH da Universidade Federal do Ceará - CE
70	AUSÊNCIA COM COMPENSAÇÃO	CH da Universidade Federal do Ceará - CE

70	AUSÊNCIA COM COMPENSAÇÃO	CH da Universidade Federal do Ceará - CE
70	AUSÊNCIA COM COMPENSAÇÃO	CH da Universidade Federal do Ceará - CE
70	AUSÊNCIA COM COMPENSAÇÃO	CH da Universidade Federal do Ceará - CE
70	AUSÊNCIA COM COMPENSAÇÃO	CH da Universidade Federal do Ceará - CE
70	AUSÊNCIA COM COMPENSAÇÃO	CH da Universidade Federal do Ceará - CE
70	AUSÊNCIA COM COMPENSAÇÃO	CH da Universidade Federal do Ceará - CE
70	AUSÊNCIA COM COMPENSAÇÃO	CH da Universidade Federal do Ceará - CE
70	AUSÊNCIA COM COMPENSAÇÃO	CH da Universidade Federal do Ceará - CE
70	AUSÊNCIA COM COMPENSAÇÃO	CH da Universidade Federal do Ceará - CE
70	AUSÊNCIA COM COMPENSAÇÃO	CH da Universidade Federal do Ceará - CE
70	AUSÊNCIA COM COMPENSAÇÃO	CH da Universidade Federal do Ceará - CE
70	AUSÊNCIA COM COMPENSAÇÃO	CH da Universidade Federal do Ceará - CE
70	AUSÊNCIA COM COMPENSAÇÃO	CH da Universidade Federal do Ceará - CE
70	AUSÊNCIA COM COMPENSAÇÃO	CH da Universidade Federal do Ceará - CE
70	AUSÊNCIA COM COMPENSAÇÃO	CH da Universidade Federal do Ceará - CE
70	AUSÊNCIA COM COMPENSAÇÃO	CH da Universidade Federal do Ceará - CE
70	AUSÊNCIA COM COMPENSAÇÃO	CH da Universidade Federal do Ceará - CE
70	AUSÊNCIA COM COMPENSAÇÃO	CH da Universidade Federal do Ceará - CE
70	AUSÊNCIA COM COMPENSAÇÃO	CH da Universidade Federal do Ceará - CE
70	AUSÊNCIA COM COMPENSAÇÃO	CH da Universidade Federal do Ceará - CE
70	AUSÊNCIA COM COMPENSAÇÃO	CH da Universidade Federal do Ceará - CE
70	AUSÊNCIA COM COMPENSAÇÃO	CH da Universidade Federal do Ceará - CE
70	AUSÊNCIA COM COMPENSAÇÃO	CH da Universidade Federal do Ceará - CE
70	AUSÊNCIA COM COMPENSAÇÃO	CH da Universidade Federal do Ceará - CE
70	AUSÊNCIA COM COMPENSAÇÃO	CH da Universidade Federal do Ceará - CE
70	AUSÊNCIA COM COMPENSAÇÃO	CH da Universidade Federal do Ceará - CE
70	AUSÊNCIA COM COMPENSAÇÃO	CH da Universidade Federal do Ceará - CE
70	AUSÊNCIA COM COMPENSAÇÃO	CH da Universidade Federal do Ceará - CE
70	AUSÊNCIA COM COMPENSAÇÃO	CH da Universidade Federal do Ceará - CE
70	AUSÊNCIA COM COMPENSAÇÃO	CH da Universidade Federal do Ceará - CE
70	AUSÊNCIA COM COMPENSAÇÃO	CH da Universidade Federal do Ceará - CE
70	AUSÊNCIA COM COMPENSAÇÃO	CH da Universidade Federal do Ceará - CE
70	AUSÊNCIA COM COMPENSAÇÃO	CH da Universidade Federal do Ceará - CE
70	AUSÊNCIA COM COMPENSAÇÃO	CH da Universidade Federal do Ceará - CE
70	AUSÊNCIA COM COMPENSAÇÃO	CH da Universidade Federal do Ceará - CE
70	AUSÊNCIA COM COMPENSAÇÃO	CH da Universidade Federal do Ceará - CE
70	AUSÊNCIA COM COMPENSAÇÃO	CH da Universidade Federal do Ceará - CE
70	AUSÊNCIA COM COMPENSAÇÃO	CH da Universidade Federal do Ceará - CE
70	AUSÊNCIA COM COMPENSAÇÃO	CH da Universidade Federal do Ceará - CE
70	AUSÊNCIA COM COMPENSAÇÃO	CH da Universidade Federal do Ceará - CE

70	AUSÊNCIA COM COMPENSAÇÃO	CH da Universidade Federal do Ceará - CE
70	AUSÊNCIA COM COMPENSAÇÃO	CH da Universidade Federal do Ceará - CE
70	AUSÊNCIA COM COMPENSAÇÃO	CH da Universidade Federal do Ceará - CE
70	AUSÊNCIA COM COMPENSAÇÃO	CH da Universidade Federal do Ceará - CE
70	AUSÊNCIA COM COMPENSAÇÃO	CH da Universidade Federal do Ceará - CE
70	AUSÊNCIA COM COMPENSAÇÃO	CH da Universidade Federal do Ceará - CE
70	AUSÊNCIA COM COMPENSAÇÃO	CH da Universidade Federal do Ceará - CE
70	AUSÊNCIA COM COMPENSAÇÃO	CH da Universidade Federal do Ceará - CE
70	AUSÊNCIA COM COMPENSAÇÃO	CH da Universidade Federal do Ceará - CE
70	AUSÊNCIA COM COMPENSAÇÃO	CH da Universidade Federal do Ceará - CE
70	AUSÊNCIA COM COMPENSAÇÃO	CH da Universidade Federal do Ceará - CE
70	AUSÊNCIA COM COMPENSAÇÃO	CH da Universidade Federal do Ceará - CE
70	AUSÊNCIA COM COMPENSAÇÃO	CH da Universidade Federal do Ceará - CE
70	AUSÊNCIA COM COMPENSAÇÃO	CH da Universidade Federal do Ceará - CE
70	AUSÊNCIA COM COMPENSAÇÃO	CH da Universidade Federal do Ceará - CE
70	AUSÊNCIA COM COMPENSAÇÃO	CH da Universidade Federal do Ceará - CE
70	AUSÊNCIA COM COMPENSAÇÃO	CH da Universidade Federal do Ceará - CE
70	AUSÊNCIA COM COMPENSAÇÃO	CH da Universidade Federal do Ceará - CE
70	AUSÊNCIA COM COMPENSAÇÃO	CH da Universidade Federal do Ceará - CE
70	AUSÊNCIA COM COMPENSAÇÃO	CH da Universidade Federal do Ceará - CE
70	AUSÊNCIA COM COMPENSAÇÃO	CH da Universidade Federal do Ceará - CE
70	AUSÊNCIA COM COMPENSAÇÃO	CH da Universidade Federal do Ceará - CE
70	AUSÊNCIA COM COMPENSAÇÃO	CH da Universidade Federal do Ceará - CE
70	AUSÊNCIA COM COMPENSAÇÃO	CH da Universidade Federal do Ceará - CE
70	AUSÊNCIA COM COMPENSAÇÃO	CH da Universidade Federal do Ceará - CE
70	AUSÊNCIA COM COMPENSAÇÃO	CH da Universidade Federal do Ceará - CE
70	AUSÊNCIA COM COMPENSAÇÃO	CH da Universidade Federal do Ceará - CE
70	AUSÊNCIA COM COMPENSAÇÃO	CH da Universidade Federal do Ceará - CE
70	AUSÊNCIA COM COMPENSAÇÃO	CH da Universidade Federal do Ceará - CE
70	AUSÊNCIA COM COMPENSAÇÃO	CH da Universidade Federal do Ceará - CE
70	AUSÊNCIA COM COMPENSAÇÃO	CH da Universidade Federal do Ceará - CE
70	AUSÊNCIA COM COMPENSAÇÃO	CH da Universidade Federal do Ceará - CE
70	AUSÊNCIA COM COMPENSAÇÃO	CH da Universidade Federal do Ceará - CE
70	AUSÊNCIA COM COMPENSAÇÃO	CH da Universidade Federal do Ceará - CE
70	AUSÊNCIA COM COMPENSAÇÃO	CH da Universidade Federal do Ceará - CE
70	AUSÊNCIA COM COMPENSAÇÃO	CH da Universidade Federal do Ceará - CE
70	AUSÊNCIA COM COMPENSAÇÃO	CH da Universidade Federal do Ceará - CE
70	AUSÊNCIA COM COMPENSAÇÃO	CH da Universidade Federal do Ceará - CE
70	AUSÊNCIA COM COMPENSAÇÃO	CH da Universidade Federal do Ceará - CE
70	AUSÊNCIA COM COMPENSAÇÃO	CH da Universidade Federal do Ceará - CE
70	AUSÊNCIA COM COMPENSAÇÃO	CH da Universidade Federal do Ceará - CE
70	AUSÊNCIA COM COMPENSAÇÃO	CH da Universidade Federal do Ceará - CE
70	AUSÊNCIA COM COMPENSAÇÃO	CH da Universidade Federal do Ceará - CE
70	AUSÊNCIA COM COMPENSAÇÃO	CH da Universidade Federal do Ceará - CE
70	AUSÊNCIA COM COMPENSAÇÃO	CH da Universidade Federal do Ceará - CE

70	AUSÊNCIA COM COMPENSAÇÃO	CH da Universidade Federal do Ceará - CE
70	AUSÊNCIA COM COMPENSAÇÃO	CH da Universidade Federal do Ceará - CE
70	AUSÊNCIA COM COMPENSAÇÃO	CH da Universidade Federal do Ceará - CE
70	AUSÊNCIA COM COMPENSAÇÃO	CH da Universidade Federal do Ceará - CE
70	AUSÊNCIA COM COMPENSAÇÃO	CH da Universidade Federal do Ceará - CE
70	AUSÊNCIA COM COMPENSAÇÃO	CH da Universidade Federal do Ceará - CE
70	AUSÊNCIA COM COMPENSAÇÃO	CH da Universidade Federal do Ceará - CE
70	AUSÊNCIA COM COMPENSAÇÃO	CH da Universidade Federal do Ceará - CE
70	AUSÊNCIA COM COMPENSAÇÃO	CH da Universidade Federal do Ceará - CE
70	AUSÊNCIA COM COMPENSAÇÃO	CH da Universidade Federal do Ceará - CE
70	AUSÊNCIA COM COMPENSAÇÃO	CH da Universidade Federal do Ceará - CE
70	AUSÊNCIA COM COMPENSAÇÃO	CH da Universidade Federal do Ceará - CE
70	AUSÊNCIA COM COMPENSAÇÃO	CH da Universidade Federal do Ceará - CE
70	AUSÊNCIA COM COMPENSAÇÃO	CH da Universidade Federal do Ceará - CE
70	AUSÊNCIA COM COMPENSAÇÃO	CH da Universidade Federal do Ceará - CE
70	AUSÊNCIA COM COMPENSAÇÃO	CH da Universidade Federal do Ceará - CE
70	AUSÊNCIA COM COMPENSAÇÃO	CH da Universidade Federal do Ceará - CE
70	AUSÊNCIA COM COMPENSAÇÃO	CH da Universidade Federal do Ceará - CE
70	AUSÊNCIA COM COMPENSAÇÃO	CH da Universidade Federal do Ceará - CE
70	AUSÊNCIA COM COMPENSAÇÃO	CH da Universidade Federal do Ceará - CE
70	AUSÊNCIA COM COMPENSAÇÃO	CH da Universidade Federal do Ceará - CE
70	AUSÊNCIA COM COMPENSAÇÃO	CH da Universidade Federal do Ceará - CE
70	AUSÊNCIA COM COMPENSAÇÃO	CH da Universidade Federal do Ceará - CE
70	AUSÊNCIA COM COMPENSAÇÃO	CH da Universidade Federal do Ceará - CE
70	AUSÊNCIA COM COMPENSAÇÃO	CH da Universidade Federal do Ceará - CE
70	AUSÊNCIA COM COMPENSAÇÃO	CH da Universidade Federal do Ceará - CE
70	AUSÊNCIA COM COMPENSAÇÃO	CH da Universidade Federal do Ceará - CE
70	AUSÊNCIA COM COMPENSAÇÃO	CH da Universidade Federal do Ceará - CE
70	AUSÊNCIA COM COMPENSAÇÃO	CH da Universidade Federal do Ceará - CE
70	AUSÊNCIA COM COMPENSAÇÃO	CH da Universidade Federal do Ceará - CE
70	AUSÊNCIA COM COMPENSAÇÃO	CH da Universidade Federal do Ceará - CE
70	AUSÊNCIA COM COMPENSAÇÃO	CH da Universidade Federal do Ceará - CE
70	AUSÊNCIA COM COMPENSAÇÃO	CH da Universidade Federal do Ceará - CE
70	AUSÊNCIA COM COMPENSAÇÃO	CH da Universidade Federal do Ceará - CE
70	AUSÊNCIA COM COMPENSAÇÃO	CH da Universidade Federal do Ceará - CE
70	AUSÊNCIA COM COMPENSAÇÃO	CH da Universidade Federal do Ceará - CE
70	AUSÊNCIA COM COMPENSAÇÃO	CH da Universidade Federal do Ceará - CE
70	AUSÊNCIA COM COMPENSAÇÃO	CH da Universidade Federal do Ceará - CE
70	AUSÊNCIA COM COMPENSAÇÃO	CH da Universidade Federal do Ceará - CE
70	AUSÊNCIA COM COMPENSAÇÃO	CH da Universidade Federal do Ceará - CE

70	AUSÊNCIA COM COMPENSAÇÃO	CH da Universidade Federal do Ceará - CE
70	AUSÊNCIA COM COMPENSAÇÃO	CH da Universidade Federal do Ceará - CE
70	AUSÊNCIA COM COMPENSAÇÃO	CH da Universidade Federal do Ceará - CE
70	AUSÊNCIA COM COMPENSAÇÃO	CH da Universidade Federal do Ceará - CE
70	AUSÊNCIA COM COMPENSAÇÃO	CH da Universidade Federal do Ceará - CE
70	AUSÊNCIA COM COMPENSAÇÃO	CH da Universidade Federal do Ceará - CE
70	AUSÊNCIA COM COMPENSAÇÃO	CH da Universidade Federal do Ceará - CE
70	AUSÊNCIA COM COMPENSAÇÃO	CH da Universidade Federal do Ceará - CE
70	AUSÊNCIA COM COMPENSAÇÃO	CH da Universidade Federal do Ceará - CE
70	AUSÊNCIA COM COMPENSAÇÃO	CH da Universidade Federal do Ceará - CE
70	AUSÊNCIA COM COMPENSAÇÃO	CH da Universidade Federal do Ceará - CE
70	AUSÊNCIA COM COMPENSAÇÃO	CH da Universidade Federal do Ceará - CE
70	AUSÊNCIA COM COMPENSAÇÃO	CH da Universidade Federal do Ceará - CE
70	AUSÊNCIA COM COMPENSAÇÃO	CH da Universidade Federal do Ceará - CE
70	AUSÊNCIA COM COMPENSAÇÃO	CH da Universidade Federal do Ceará - CE
70	AUSÊNCIA COM COMPENSAÇÃO	CH da Universidade Federal do Ceará - CE
70	AUSÊNCIA COM COMPENSAÇÃO	CH da Universidade Federal do Ceará - CE
70	AUSÊNCIA COM COMPENSAÇÃO	CH da Universidade Federal do Ceará - CE
70	AUSÊNCIA COM COMPENSAÇÃO	CH da Universidade Federal do Ceará - CE
70	AUSÊNCIA COM COMPENSAÇÃO	CH da Universidade Federal do Ceará - CE
70	AUSÊNCIA COM COMPENSAÇÃO	CH da Universidade Federal do Ceará - CE
70	AUSÊNCIA COM COMPENSAÇÃO	CH da Universidade Federal do Ceará - CE
70	AUSÊNCIA COM COMPENSAÇÃO	CH da Universidade Federal do Ceará - CE
70	AUSÊNCIA COM COMPENSAÇÃO	CH da Universidade Federal do Ceará - CE
70	AUSÊNCIA COM COMPENSAÇÃO	CH da Universidade Federal do Ceará - CE
70	AUSÊNCIA COM COMPENSAÇÃO	CH da Universidade Federal do Ceará - CE
70	AUSÊNCIA COM COMPENSAÇÃO	CH da Universidade Federal do Ceará - CE
70	AUSÊNCIA COM COMPENSAÇÃO	CH da Universidade Federal do Ceará - CE
70	AUSÊNCIA COM COMPENSAÇÃO	CH da Universidade Federal do Ceará - CE
70	AUSÊNCIA COM COMPENSAÇÃO	CH da Universidade Federal do Ceará - CE
70	AUSÊNCIA COM COMPENSAÇÃO	CH da Universidade Federal do Ceará - CE
70	AUSÊNCIA COM COMPENSAÇÃO	CH da Universidade Federal do Ceará - CE
70	AUSÊNCIA COM COMPENSAÇÃO	CH da Universidade Federal do Ceará - CE
70	AUSÊNCIA COM COMPENSAÇÃO	CH da Universidade Federal do Ceará - CE
70	AUSÊNCIA COM COMPENSAÇÃO	CH da Universidade Federal do Ceará - CE
70	AUSÊNCIA COM COMPENSAÇÃO	CH da Universidade Federal do Ceará - CE
70	AUSÊNCIA COM COMPENSAÇÃO	CH da Universidade Federal do Ceará - CE
70	AUSÊNCIA COM COMPENSAÇÃO	CH da Universidade Federal do Ceará - CE
70	AUSÊNCIA COM COMPENSAÇÃO	CH da Universidade Federal do Ceará - CE
70	AUSÊNCIA COM COMPENSAÇÃO	CH da Universidade Federal do Ceará - CE
70	AUSÊNCIA COM COMPENSAÇÃO	CH da Universidade Federal do Ceará - CE
70	AUSÊNCIA COM COMPENSAÇÃO	CH da Universidade Federal do Ceará - CE
70	AUSÊNCIA COM COMPENSAÇÃO	CH da Universidade Federal do Ceará - CE
70	AUSÊNCIA COM COMPENSAÇÃO	CH da Universidade Federal do Ceará - CE

70	AUSÊNCIA COM COMPENSAÇÃO	CH da Universidade Federal do Ceará - CE
70	AUSÊNCIA COM COMPENSAÇÃO	CH da Universidade Federal do Ceará - CE
70	AUSÊNCIA COM COMPENSAÇÃO	CH da Universidade Federal do Ceará - CE
70	AUSÊNCIA COM COMPENSAÇÃO	CH da Universidade Federal do Ceará - CE
70	AUSÊNCIA COM COMPENSAÇÃO	CH da Universidade Federal do Ceará - CE
70	AUSÊNCIA COM COMPENSAÇÃO	CH da Universidade Federal do Ceará - CE
70	AUSÊNCIA COM COMPENSAÇÃO	CH da Universidade Federal do Ceará - CE
70	AUSÊNCIA COM COMPENSAÇÃO	CH da Universidade Federal do Ceará - CE
70	AUSÊNCIA COM COMPENSAÇÃO	CH da Universidade Federal do Ceará - CE
70	AUSÊNCIA COM COMPENSAÇÃO	CH da Universidade Federal do Ceará - CE
70	AUSÊNCIA COM COMPENSAÇÃO	CH da Universidade Federal do Ceará - CE
70	AUSÊNCIA COM COMPENSAÇÃO	CH da Universidade Federal do Ceará - CE
70	AUSÊNCIA COM COMPENSAÇÃO	CH da Universidade Federal do Ceará - CE
70	AUSÊNCIA COM COMPENSAÇÃO	CH da Universidade Federal do Ceará - CE
70	AUSÊNCIA COM COMPENSAÇÃO	CH da Universidade Federal do Ceará - CE
70	AUSÊNCIA COM COMPENSAÇÃO	CH da Universidade Federal do Ceará - CE
70	AUSÊNCIA COM COMPENSAÇÃO	CH da Universidade Federal do Ceará - CE
70	AUSÊNCIA COM COMPENSAÇÃO	CH da Universidade Federal do Ceará - CE
70	AUSÊNCIA COM COMPENSAÇÃO	CH da Universidade Federal do Ceará - CE
70	AUSÊNCIA COM COMPENSAÇÃO	CH da Universidade Federal do Ceará - CE
70	AUSÊNCIA COM COMPENSAÇÃO	CH da Universidade Federal do Ceará - CE
70	AUSÊNCIA COM COMPENSAÇÃO	CH da Universidade Federal do Ceará - CE
70	AUSÊNCIA COM COMPENSAÇÃO	CH da Universidade Federal do Ceará - CE
70	AUSÊNCIA COM COMPENSAÇÃO	CH da Universidade Federal do Ceará - CE
70	AUSÊNCIA COM COMPENSAÇÃO	CH da Universidade Federal do Ceará - CE
70	AUSÊNCIA COM COMPENSAÇÃO	CH da Universidade Federal do Ceará - CE
70	AUSÊNCIA COM COMPENSAÇÃO	CH da Universidade Federal do Ceará - CE
70	AUSÊNCIA COM COMPENSAÇÃO	CH da Universidade Federal do Ceará - CE
70	AUSÊNCIA COM COMPENSAÇÃO	CH da Universidade Federal do Ceará - CE
70	AUSÊNCIA COM COMPENSAÇÃO	CH da Universidade Federal do Ceará - CE
70	AUSÊNCIA COM COMPENSAÇÃO	CH da Universidade Federal do Ceará - CE
70	AUSÊNCIA COM COMPENSAÇÃO	CH da Universidade Federal do Ceará - CE
70	AUSÊNCIA COM COMPENSAÇÃO	CH da Universidade Federal do Ceará - CE
70	AUSÊNCIA COM COMPENSAÇÃO	CH da Universidade Federal do Ceará - CE
70	AUSÊNCIA COM COMPENSAÇÃO	CH da Universidade Federal do Ceará - CE
70	AUSÊNCIA COM COMPENSAÇÃO	CH da Universidade Federal do Ceará - CE
70	AUSÊNCIA COM COMPENSAÇÃO	CH da Universidade Federal do Ceará - CE

70	AUSÊNCIA COM COMPENSAÇÃO	CH da Universidade Federal do Ceará - CE
70	AUSÊNCIA COM COMPENSAÇÃO	CH da Universidade Federal do Ceará - CE
70	AUSÊNCIA COM COMPENSAÇÃO	CH da Universidade Federal do Ceará - CE
70	AUSÊNCIA COM COMPENSAÇÃO	CH da Universidade Federal do Ceará - CE
70	AUSÊNCIA COM COMPENSAÇÃO	CH da Universidade Federal do Ceará - CE
70	AUSÊNCIA COM COMPENSAÇÃO	CH da Universidade Federal do Ceará - CE
70	AUSÊNCIA COM COMPENSAÇÃO	CH da Universidade Federal do Ceará - CE
70	AUSÊNCIA COM COMPENSAÇÃO	CH da Universidade Federal do Ceará - CE
70	AUSÊNCIA COM COMPENSAÇÃO	CH da Universidade Federal do Ceará - CE
70	AUSÊNCIA COM COMPENSAÇÃO	CH da Universidade Federal do Ceará - CE
70	AUSÊNCIA COM COMPENSAÇÃO	CH da Universidade Federal do Ceará - CE
70	AUSÊNCIA COM COMPENSAÇÃO	CH da Universidade Federal do Ceará - CE
70	AUSÊNCIA COM COMPENSAÇÃO	CH da Universidade Federal do Ceará - CE
70	AUSÊNCIA COM COMPENSAÇÃO	CH da Universidade Federal do Ceará - CE
70	AUSÊNCIA COM COMPENSAÇÃO	CH da Universidade Federal do Ceará - CE
70	AUSÊNCIA COM COMPENSAÇÃO	CH da Universidade Federal do Ceará - CE
70	AUSÊNCIA COM COMPENSAÇÃO	CH da Universidade Federal do Ceará - CE
70	AUSÊNCIA COM COMPENSAÇÃO	CH da Universidade Federal do Ceará - CE
70	AUSÊNCIA COM COMPENSAÇÃO	CH da Universidade Federal do Ceará - CE
70	AUSÊNCIA COM COMPENSAÇÃO	CH da Universidade Federal do Ceará - CE
70	AUSÊNCIA COM COMPENSAÇÃO	CH da Universidade Federal do Ceará - CE
70	AUSÊNCIA COM COMPENSAÇÃO	CH da Universidade Federal do Ceará - CE
70	AUSÊNCIA COM COMPENSAÇÃO	CH da Universidade Federal do Ceará - CE
70	AUSÊNCIA COM COMPENSAÇÃO	CH da Universidade Federal do Ceará - CE
70	AUSÊNCIA COM COMPENSAÇÃO	CH da Universidade Federal do Ceará - CE
70	AUSÊNCIA COM COMPENSAÇÃO	CH da Universidade Federal do Ceará - CE
70	AUSÊNCIA COM COMPENSAÇÃO	CH da Universidade Federal do Ceará - CE
70	AUSÊNCIA COM COMPENSAÇÃO	CH da Universidade Federal do Ceará - CE
70	AUSÊNCIA COM COMPENSAÇÃO	CH da Universidade Federal do Ceará - CE
70	AUSÊNCIA COM COMPENSAÇÃO	CH da Universidade Federal do Ceará - CE
70	AUSÊNCIA COM COMPENSAÇÃO	CH da Universidade Federal do Ceará - CE
70	AUSÊNCIA COM COMPENSAÇÃO	CH da Universidade Federal do Ceará - CE
70	AUSÊNCIA COM COMPENSAÇÃO	CH da Universidade Federal do Ceará - CE
70	AUSÊNCIA COM COMPENSAÇÃO	CH da Universidade Federal do Ceará - CE
70	AUSÊNCIA COM COMPENSAÇÃO	CH da Universidade Federal do Ceará - CE
70	AUSÊNCIA COM COMPENSAÇÃO	CH da Universidade Federal do Ceará - CE
70	AUSÊNCIA COM COMPENSAÇÃO	CH da Universidade Federal do Ceará - CE
70	AUSÊNCIA COM COMPENSAÇÃO	CH da Universidade Federal do Ceará - CE
70	AUSÊNCIA COM COMPENSAÇÃO	CH da Universidade Federal do Ceará - CE
70	AUSÊNCIA COM COMPENSAÇÃO	CH da Universidade Federal do Ceará - CE
70	AUSÊNCIA COM COMPENSAÇÃO	CH da Universidade Federal do Ceará - CE
70	AUSÊNCIA COM COMPENSAÇÃO	CH da Universidade Federal do Ceará - CE
70	AUSÊNCIA COM COMPENSAÇÃO	CH da Universidade Federal do Ceará - CE
70	AUSÊNCIA COM COMPENSAÇÃO	CH da Universidade Federal do Ceará - CE

70	AUSÊNCIA COM COMPENSAÇÃO	CH da Universidade Federal do Ceará - CE
70	AUSÊNCIA COM COMPENSAÇÃO	CH da Universidade Federal do Ceará - CE
70	AUSÊNCIA COM COMPENSAÇÃO	CH da Universidade Federal do Ceará - CE
70	AUSÊNCIA COM COMPENSAÇÃO	CH da Universidade Federal do Ceará - CE
70	AUSÊNCIA COM COMPENSAÇÃO	CH da Universidade Federal do Ceará - CE
70	AUSÊNCIA COM COMPENSAÇÃO	CH da Universidade Federal do Ceará - CE
70	AUSÊNCIA COM COMPENSAÇÃO	CH da Universidade Federal do Ceará - CE
70	AUSÊNCIA COM COMPENSAÇÃO	CH da Universidade Federal do Ceará - CE
70	AUSÊNCIA COM COMPENSAÇÃO	CH da Universidade Federal do Ceará - CE
70	AUSÊNCIA COM COMPENSAÇÃO	CH da Universidade Federal do Ceará - CE
70	AUSÊNCIA COM COMPENSAÇÃO	CH da Universidade Federal do Ceará - CE
70	AUSÊNCIA COM COMPENSAÇÃO	CH da Universidade Federal do Ceará - CE
70	AUSÊNCIA COM COMPENSAÇÃO	CH da Universidade Federal do Ceará - CE
70	AUSÊNCIA COM COMPENSAÇÃO	CH da Universidade Federal do Ceará - CE
70	AUSÊNCIA COM COMPENSAÇÃO	CH da Universidade Federal do Ceará - CE
70	AUSÊNCIA COM COMPENSAÇÃO	CH da Universidade Federal do Ceará - CE
70	AUSÊNCIA COM COMPENSAÇÃO	CH da Universidade Federal do Ceará - CE
70	AUSÊNCIA COM COMPENSAÇÃO	CH da Universidade Federal do Ceará - CE
70	AUSÊNCIA COM COMPENSAÇÃO	CH da Universidade Federal do Ceará - CE
70	AUSÊNCIA COM COMPENSAÇÃO	CH da Universidade Federal do Ceará - CE
70	AUSÊNCIA COM COMPENSAÇÃO	CH da Universidade Federal do Ceará - CE
70	AUSÊNCIA COM COMPENSAÇÃO	CH da Universidade Federal do Ceará - CE
70	AUSÊNCIA COM COMPENSAÇÃO	CH da Universidade Federal do Ceará - CE
70	AUSÊNCIA COM COMPENSAÇÃO	CH da Universidade Federal do Ceará - CE
70	AUSÊNCIA COM COMPENSAÇÃO	CH da Universidade Federal do Ceará - CE
70	AUSÊNCIA COM COMPENSAÇÃO	CH da Universidade Federal do Ceará - CE
70	AUSÊNCIA COM COMPENSAÇÃO	CH da Universidade Federal do Ceará - CE
70	AUSÊNCIA COM COMPENSAÇÃO	CH da Universidade Federal do Ceará - CE
70	AUSÊNCIA COM COMPENSAÇÃO	CH da Universidade Federal do Ceará - CE
70	AUSÊNCIA COM COMPENSAÇÃO	CH da Universidade Federal do Ceará - CE
70	AUSÊNCIA COM COMPENSAÇÃO	CH da Universidade Federal do Ceará - CE
70	AUSÊNCIA COM COMPENSAÇÃO	CH da Universidade Federal do Ceará - CE
70	AUSÊNCIA COM COMPENSAÇÃO	CH da Universidade Federal do Ceará - CE
70	AUSÊNCIA COM COMPENSAÇÃO	CH da Universidade Federal do Ceará - CE
70	AUSÊNCIA COM COMPENSAÇÃO	CH da Universidade Federal do Ceará - CE
70	AUSÊNCIA COM COMPENSAÇÃO	CH da Universidade Federal do Ceará - CE
70	AUSÊNCIA COM COMPENSAÇÃO	CH da Universidade Federal do Ceará - CE
70	AUSÊNCIA COM COMPENSAÇÃO	CH da Universidade Federal do Ceará - CE
70	AUSÊNCIA COM COMPENSAÇÃO	CH da Universidade Federal do Ceará - CE
70	AUSÊNCIA COM COMPENSAÇÃO	CH da Universidade Federal do Ceará - CE
70	AUSÊNCIA COM COMPENSAÇÃO	CH da Universidade Federal do Ceará - CE
70	AUSÊNCIA COM COMPENSAÇÃO	CH da Universidade Federal do Ceará - CE
70	AUSÊNCIA COM COMPENSAÇÃO	CH da Universidade Federal do Ceará - CE
70	AUSÊNCIA COM COMPENSAÇÃO	CH da Universidade Federal do Ceará - CE
70	AUSÊNCIA COM COMPENSAÇÃO	CH da Universidade Federal do Ceará - CE
70	AUSÊNCIA COM COMPENSAÇÃO	CH da Universidade Federal do Ceará - CE
70	AUSÊNCIA COM COMPENSAÇÃO	CH da Universidade Federal do Ceará - CE
70	AUSÊNCIA COM COMPENSAÇÃO	CH da Universidade Federal do Ceará - CE

70	AUSÊNCIA COM COMPENSAÇÃO	CH da Universidade Federal do Ceará - CE
70	AUSÊNCIA COM COMPENSAÇÃO	CH da Universidade Federal do Ceará - CE
70	AUSÊNCIA COM COMPENSAÇÃO	CH da Universidade Federal do Ceará - CE
70	AUSÊNCIA COM COMPENSAÇÃO	CH da Universidade Federal do Ceará - CE
70	AUSÊNCIA COM COMPENSAÇÃO	CH da Universidade Federal do Ceará - CE
70	AUSÊNCIA COM COMPENSAÇÃO	CH da Universidade Federal do Ceará - CE
70	AUSÊNCIA COM COMPENSAÇÃO	CH da Universidade Federal do Ceará - CE
70	AUSÊNCIA COM COMPENSAÇÃO	CH da Universidade Federal do Ceará - CE
70	AUSÊNCIA COM COMPENSAÇÃO	CH da Universidade Federal do Ceará - CE
70	AUSÊNCIA COM COMPENSAÇÃO	CH da Universidade Federal do Ceará - CE
70	AUSÊNCIA COM COMPENSAÇÃO	CH da Universidade Federal do Ceará - CE
70	AUSÊNCIA COM COMPENSAÇÃO	CH da Universidade Federal do Ceará - CE
70	AUSÊNCIA COM COMPENSAÇÃO	CH da Universidade Federal do Ceará - CE
70	AUSÊNCIA COM COMPENSAÇÃO	CH da Universidade Federal do Ceará - CE
70	AUSÊNCIA COM COMPENSAÇÃO	CH da Universidade Federal do Ceará - CE
70	AUSÊNCIA COM COMPENSAÇÃO	CH da Universidade Federal do Ceará - CE
70	AUSÊNCIA COM COMPENSAÇÃO	CH da Universidade Federal do Ceará - CE
70	AUSÊNCIA COM COMPENSAÇÃO	CH da Universidade Federal do Ceará - CE
70	AUSÊNCIA COM COMPENSAÇÃO	CH da Universidade Federal do Ceará - CE
70	AUSÊNCIA COM COMPENSAÇÃO	CH da Universidade Federal do Ceará - CE
70	AUSÊNCIA COM COMPENSAÇÃO	CH da Universidade Federal do Ceará - CE
70	AUSÊNCIA COM COMPENSAÇÃO	CH da Universidade Federal do Ceará - CE
70	AUSÊNCIA COM COMPENSAÇÃO	CH da Universidade Federal do Ceará - CE
71	Repouso Remunerado - Artigo 395 CLT - 14 dias	CH da Universidade Federal do Ceará - CE
71	Repouso Remunerado - Artigo 395 CLT - 14 dias	CH da Universidade Federal do Ceará - CE
71	Repouso Remunerado - Artigo 395 CLT - 14 dias	CH da Universidade Federal do Ceará - CE
71	Repouso Remunerado - Artigo 395 CLT - 14 dias	CH da Universidade Federal do Ceará - CE
72	CONSULTA PRE NATAL - ATESTADO MEDICO	CH da Universidade Federal do Ceará - CE
72	CONSULTA PRE NATAL - ATESTADO MEDICO	CH da Universidade Federal do Ceará - CE
72	CONSULTA PRE NATAL - ATESTADO MEDICO	CH da Universidade Federal do Ceará - CE
72	CONSULTA PRE NATAL - ATESTADO MEDICO	CH da Universidade Federal do Ceará - CE
72	CONSULTA PRE NATAL - ATESTADO MEDICO	CH da Universidade Federal do Ceará - CE
72	CONSULTA PRE NATAL - ATESTADO MEDICO	CH da Universidade Federal do Ceará - CE
72	CONSULTA PRE NATAL - ATESTADO MEDICO	CH da Universidade Federal do Ceará - CE
72	CONSULTA PRE NATAL - ATESTADO MEDICO	CH da Universidade Federal do Ceará - CE
72	CONSULTA PRE NATAL - ATESTADO MEDICO	CH da Universidade Federal do Ceará - CE
72	CONSULTA PRE NATAL - ATESTADO MEDICO	CH da Universidade Federal do Ceará - CE
72	CONSULTA PRE NATAL - ATESTADO MEDICO	CH da Universidade Federal do Ceará - CE
72	CONSULTA PRE NATAL - ATESTADO MEDICO	CH da Universidade Federal do Ceará - CE
72	CONSULTA PRE NATAL - ATESTADO MEDICO	CH da Universidade Federal do Ceará - CE
72	CONSULTA PRE NATAL - ATESTADO MEDICO	CH da Universidade Federal do Ceará - CE
72	CONSULTA PRE NATAL - ATESTADO MEDICO	CH da Universidade Federal do Ceará - CE
72	CONSULTA PRE NATAL - ATESTADO MEDICO	CH da Universidade Federal do Ceará - CE
72	CONSULTA PRE NATAL - ATESTADO MEDICO	CH da Universidade Federal do Ceará - CE
72	CONSULTA PRE NATAL - ATESTADO MEDICO	CH da Universidade Federal do Ceará - CE
72	CONSULTA PRE NATAL - ATESTADO MEDICO	CH da Universidade Federal do Ceará - CE
72	CONSULTA PRE NATAL - ATESTADO MEDICO	CH da Universidade Federal do Ceará - CE

72	CONSULTA PRE NATAL - ATESTADO MEDICO	CH da Universidade Federal do Ceará - CE
72	CONSULTA PRE NATAL - ATESTADO MEDICO	CH da Universidade Federal do Ceará - CE
72	CONSULTA PRE NATAL - ATESTADO MEDICO	CH da Universidade Federal do Ceará - CE
72	CONSULTA PRE NATAL - ATESTADO MEDICO	CH da Universidade Federal do Ceará - CE
72	CONSULTA PRE NATAL - ATESTADO MEDICO	CH da Universidade Federal do Ceará - CE
72	CONSULTA PRE NATAL - ATESTADO MEDICO	CH da Universidade Federal do Ceará - CE
72	CONSULTA PRE NATAL - ATESTADO MEDICO	CH da Universidade Federal do Ceará - CE
72	CONSULTA PRE NATAL - ATESTADO MEDICO	CH da Universidade Federal do Ceará - CE
72	CONSULTA PRE NATAL - ATESTADO MEDICO	CH da Universidade Federal do Ceará - CE
72	CONSULTA PRE NATAL - ATESTADO MEDICO	CH da Universidade Federal do Ceará - CE
72	CONSULTA PRE NATAL - ATESTADO MEDICO	CH da Universidade Federal do Ceará - CE
72	CONSULTA PRE NATAL - ATESTADO MEDICO	CH da Universidade Federal do Ceará - CE
72	CONSULTA PRE NATAL - ATESTADO MEDICO	CH da Universidade Federal do Ceará - CE
72	CONSULTA PRE NATAL - ATESTADO MEDICO	CH da Universidade Federal do Ceará - CE
72	CONSULTA PRE NATAL - ATESTADO MEDICO	CH da Universidade Federal do Ceará - CE
72	CONSULTA PRE NATAL - ATESTADO MEDICO	CH da Universidade Federal do Ceará - CE
72	CONSULTA PRE NATAL - ATESTADO MEDICO	CH da Universidade Federal do Ceará - CE
72	CONSULTA PRE NATAL - ATESTADO MEDICO	CH da Universidade Federal do Ceará - CE
72	CONSULTA PRE NATAL - ATESTADO MEDICO	CH da Universidade Federal do Ceará - CE
72	CONSULTA PRE NATAL - ATESTADO MEDICO	CH da Universidade Federal do Ceará - CE
72	CONSULTA PRE NATAL - ATESTADO MEDICO	CH da Universidade Federal do Ceará - CE
72	CONSULTA PRE NATAL - ATESTADO MEDICO	CH da Universidade Federal do Ceará - CE
72	CONSULTA PRE NATAL - ATESTADO MEDICO	CH da Universidade Federal do Ceará - CE
72	CONSULTA PRE NATAL - ATESTADO MEDICO	CH da Universidade Federal do Ceará - CE
72	CONSULTA PRE NATAL - ATESTADO MEDICO	CH da Universidade Federal do Ceará - CE
72	CONSULTA PRE NATAL - ATESTADO MEDICO	CH da Universidade Federal do Ceará - CE
72	CONSULTA PRE NATAL - ATESTADO MEDICO	CH da Universidade Federal do Ceará - CE
72	CONSULTA PRE NATAL - ATESTADO MEDICO	CH da Universidade Federal do Ceará - CE
72	CONSULTA PRE NATAL - ATESTADO MEDICO	CH da Universidade Federal do Ceará - CE
72	CONSULTA PRE NATAL - ATESTADO MEDICO	CH da Universidade Federal do Ceará - CE
72	CONSULTA PRE NATAL - ATESTADO MEDICO	CH da Universidade Federal do Ceará - CE
72	CONSULTA PRE NATAL - ATESTADO MEDICO	CH da Universidade Federal do Ceará - CE
72	CONSULTA PRE NATAL - ATESTADO MEDICO	CH da Universidade Federal do Ceará - CE
72	CONSULTA PRE NATAL - ATESTADO MEDICO	CH da Universidade Federal do Ceará - CE
72	CONSULTA PRE NATAL - ATESTADO MEDICO	CH da Universidade Federal do Ceará - CE
72	CONSULTA PRE NATAL - ATESTADO MEDICO	CH da Universidade Federal do Ceará - CE
72	CONSULTA PRE NATAL - ATESTADO MEDICO	CH da Universidade Federal do Ceará - CE
72	CONSULTA PRE NATAL - ATESTADO MEDICO	CH da Universidade Federal do Ceará - CE
72	CONSULTA PRE NATAL - ATESTADO MEDICO	CH da Universidade Federal do Ceará - CE
72	CONSULTA PRE NATAL - ATESTADO MEDICO	CH da Universidade Federal do Ceará - CE
72	CONSULTA PRE NATAL - ATESTADO MEDICO	CH da Universidade Federal do Ceará - CE
72	CONSULTA PRE NATAL - ATESTADO MEDICO	CH da Universidade Federal do Ceará - CE
72	CONSULTA PRE NATAL - ATESTADO MEDICO	CH da Universidade Federal do Ceará - CE
72	CONSULTA PRE NATAL - ATESTADO MEDICO	CH da Universidade Federal do Ceará - CE
72	CONSULTA PRE NATAL - ATESTADO MEDICO	CH da Universidade Federal do Ceará - CE
72	CONSULTA PRE NATAL - ATESTADO MEDICO	CH da Universidade Federal do Ceará - CE
72	CONSULTA PRE NATAL - ATESTADO MEDICO	CH da Universidade Federal do Ceará - CE

72	CONSULTA PRE NATAL - ATESTADO MEDICO	CH da Universidade Federal do Ceará - CE
72	CONSULTA PRE NATAL - ATESTADO MEDICO	CH da Universidade Federal do Ceará - CE
72	CONSULTA PRE NATAL - ATESTADO MEDICO	CH da Universidade Federal do Ceará - CE
72	CONSULTA PRE NATAL - ATESTADO MEDICO	CH da Universidade Federal do Ceará - CE
72	CONSULTA PRE NATAL - ATESTADO MEDICO	CH da Universidade Federal do Ceará - CE
72	CONSULTA PRE NATAL - ATESTADO MEDICO	CH da Universidade Federal do Ceará - CE
72	CONSULTA PRE NATAL - ATESTADO MEDICO	CH da Universidade Federal do Ceará - CE
72	CONSULTA PRE NATAL - ATESTADO MEDICO	CH da Universidade Federal do Ceará - CE
72	CONSULTA PRE NATAL - ATESTADO MEDICO	CH da Universidade Federal do Ceará - CE
72	CONSULTA PRE NATAL - ATESTADO MEDICO	CH da Universidade Federal do Ceará - CE
72	CONSULTA PRE NATAL - ATESTADO MEDICO	CH da Universidade Federal do Ceará - CE
72	CONSULTA PRE NATAL - ATESTADO MEDICO	CH da Universidade Federal do Ceará - CE
72	CONSULTA PRE NATAL - ATESTADO MEDICO	CH da Universidade Federal do Ceará - CE
72	CONSULTA PRE NATAL - ATESTADO MEDICO	CH da Universidade Federal do Ceará - CE
72	CONSULTA PRE NATAL - ATESTADO MEDICO	CH da Universidade Federal do Ceará - CE
72	CONSULTA PRE NATAL - ATESTADO MEDICO	CH da Universidade Federal do Ceará - CE
72	CONSULTA PRE NATAL - ATESTADO MEDICO	CH da Universidade Federal do Ceará - CE
72	CONSULTA PRE NATAL - ATESTADO MEDICO	CH da Universidade Federal do Ceará - CE
72	CONSULTA PRE NATAL - ATESTADO MEDICO	CH da Universidade Federal do Ceará - CE
72	CONSULTA PRE NATAL - ATESTADO MEDICO	CH da Universidade Federal do Ceará - CE
72	CONSULTA PRE NATAL - ATESTADO MEDICO	CH da Universidade Federal do Ceará - CE
72	CONSULTA PRE NATAL - ATESTADO MEDICO	CH da Universidade Federal do Ceará - CE
72	CONSULTA PRE NATAL - ATESTADO MEDICO	CH da Universidade Federal do Ceará - CE
72	CONSULTA PRE NATAL - ATESTADO MEDICO	CH da Universidade Federal do Ceará - CE
72	CONSULTA PRE NATAL - ATESTADO MEDICO	CH da Universidade Federal do Ceará - CE
72	CONSULTA PRE NATAL - ATESTADO MEDICO	CH da Universidade Federal do Ceará - CE
72	CONSULTA PRE NATAL - ATESTADO MEDICO	CH da Universidade Federal do Ceará - CE
72	CONSULTA PRE NATAL - ATESTADO MEDICO	CH da Universidade Federal do Ceará - CE
72	CONSULTA PRE NATAL - ATESTADO MEDICO	CH da Universidade Federal do Ceará - CE
72	CONSULTA PRE NATAL - ATESTADO MEDICO	CH da Universidade Federal do Ceará - CE
72	CONSULTA PRE NATAL - ATESTADO MEDICO	CH da Universidade Federal do Ceará - CE
72	CONSULTA PRE NATAL - ATESTADO MEDICO	CH da Universidade Federal do Ceará - CE
72	CONSULTA PRE NATAL - ATESTADO MEDICO	CH da Universidade Federal do Ceará - CE
72	CONSULTA PRE NATAL - ATESTADO MEDICO	CH da Universidade Federal do Ceará - CE
72	CONSULTA PRE NATAL - ATESTADO MEDICO	CH da Universidade Federal do Ceará - CE
72	CONSULTA PRE NATAL - ATESTADO MEDICO	CH da Universidade Federal do Ceará - CE
72	CONSULTA PRE NATAL - ATESTADO MEDICO	CH da Universidade Federal do Ceará - CE
72	CONSULTA PRE NATAL - ATESTADO MEDICO	CH da Universidade Federal do Ceará - CE

72	CONSULTA PRE NATAL - ATESTADO MEDICO	CH da Universidade Federal do Ceará - CE
72	CONSULTA PRE NATAL - ATESTADO MEDICO	CH da Universidade Federal do Ceará - CE
72	CONSULTA PRE NATAL - ATESTADO MEDICO	CH da Universidade Federal do Ceará - CE
72	CONSULTA PRE NATAL - ATESTADO MEDICO	CH da Universidade Federal do Ceará - CE
72	CONSULTA PRE NATAL - ATESTADO MEDICO	CH da Universidade Federal do Ceará - CE
72	CONSULTA PRE NATAL - ATESTADO MEDICO	CH da Universidade Federal do Ceará - CE
72	CONSULTA PRE NATAL - ATESTADO MEDICO	CH da Universidade Federal do Ceará - CE
72	CONSULTA PRE NATAL - ATESTADO MEDICO	CH da Universidade Federal do Ceará - CE
72	CONSULTA PRE NATAL - ATESTADO MEDICO	CH da Universidade Federal do Ceará - CE
72	CONSULTA PRE NATAL - ATESTADO MEDICO	CH da Universidade Federal do Ceará - CE
72	CONSULTA PRE NATAL - ATESTADO MEDICO	CH da Universidade Federal do Ceará - CE
72	CONSULTA PRE NATAL - ATESTADO MEDICO	CH da Universidade Federal do Ceará - CE
75	LICENÇA PATERNIDADE CELETISTA - 20 Dias (c/ prorrogação) - LEI 11.770/08 ART 1 II	CH da Universidade Federal do Ceará - CE
75	LICENÇA PATERNIDADE CELETISTA - 20 Dias (c/ prorrogação) - LEI 11.770/08 ART 1 II	CH da Universidade Federal do Ceará - CE
75	LICENÇA PATERNIDADE CELETISTA - 20 Dias (c/ prorrogação) - LEI 11.770/08 ART 1 II	CH da Universidade Federal do Ceará - CE
75	LICENÇA PATERNIDADE CELETISTA - 20 Dias (c/ prorrogação) - LEI 11.770/08 ART 1 II	CH da Universidade Federal do Ceará - CE
75	LICENÇA PATERNIDADE CELETISTA - 20 Dias (c/ prorrogação) - LEI 11.770/08 ART 1 II	CH da Universidade Federal do Ceará - CE
75	LICENÇA PATERNIDADE CELETISTA - 20 Dias (c/ prorrogação) - LEI 11.770/08 ART 1 II	CH da Universidade Federal do Ceará - CE
75	LICENÇA PATERNIDADE CELETISTA - 20 Dias (c/ prorrogação) - LEI 11.770/08 ART 1 II	CH da Universidade Federal do Ceará - CE
75	LICENÇA PATERNIDADE CELETISTA - 20 Dias (c/ prorrogação) - LEI 11.770/08 ART 1 II	CH da Universidade Federal do Ceará - CE
75	LICENÇA PATERNIDADE CELETISTA - 20 Dias (c/ prorrogação) - LEI 11.770/08 ART 1 II	CH da Universidade Federal do Ceará - CE
75	LICENÇA PATERNIDADE CELETISTA - 20 Dias (c/ prorrogação) - LEI 11.770/08 ART 1 II	CH da Universidade Federal do Ceará - CE
75	LICENÇA PATERNIDADE CELETISTA - 20 Dias (c/ prorrogação) - LEI 11.770/08 ART 1 II	CH da Universidade Federal do Ceará - CE
75	LICENÇA PATERNIDADE CELETISTA - 20 Dias (c/ prorrogação) - LEI 11.770/08 ART 1 II	CH da Universidade Federal do Ceará - CE
75	LICENÇA PATERNIDADE CELETISTA - 20 Dias (c/ prorrogação) - LEI 11.770/08 ART 1 II	CH da Universidade Federal do Ceará - CE
75	LICENÇA PATERNIDADE CELETISTA - 20 Dias (c/ prorrogação) - LEI 11.770/08 ART 1 II	CH da Universidade Federal do Ceará - CE
75	LICENÇA PATERNIDADE CELETISTA - 20 Dias (c/ prorrogação) - LEI 11.770/08 ART 1 II	CH da Universidade Federal do Ceará - CE
75	LICENÇA PATERNIDADE CELETISTA - 20 Dias (c/ prorrogação) - LEI 11.770/08 ART 1 II	CH da Universidade Federal do Ceará - CE
75	LICENÇA PATERNIDADE CELETISTA - 20 Dias (c/ prorrogação) - LEI 11.770/08 ART 1 II	CH da Universidade Federal do Ceará - CE
75	LICENÇA PATERNIDADE CELETISTA - 20 Dias (c/ prorrogação) - LEI 11.770/08 ART 1 II	CH da Universidade Federal do Ceará - CE
75	LICENÇA PATERNIDADE CELETISTA - 20 Dias (c/ prorrogação) - LEI 11.770/08 ART 1 II	CH da Universidade Federal do Ceará - CE
97	RECESSO FINAL DE ANO	CH da Universidade Federal do Ceará - CE
97	RECESSO FINAL DE ANO	CH da Universidade Federal do Ceará - CE

97	RECESSO FINAL DE ANO	CH da Universidade Federal do Ceará - CE
97	RECESSO FINAL DE ANO	CH da Universidade Federal do Ceará - CE
97	RECESSO FINAL DE ANO	CH da Universidade Federal do Ceará - CE
97	RECESSO FINAL DE ANO	CH da Universidade Federal do Ceará - CE
97	RECESSO FINAL DE ANO	CH da Universidade Federal do Ceará - CE
97	RECESSO FINAL DE ANO	CH da Universidade Federal do Ceará - CE
97	RECESSO FINAL DE ANO	CH da Universidade Federal do Ceará - CE
97	RECESSO FINAL DE ANO	CH da Universidade Federal do Ceará - CE
97	RECESSO FINAL DE ANO	CH da Universidade Federal do Ceará - CE
97	RECESSO FINAL DE ANO	CH da Universidade Federal do Ceará - CE
97	RECESSO FINAL DE ANO	CH da Universidade Federal do Ceará - CE
97	RECESSO FINAL DE ANO	CH da Universidade Federal do Ceará - CE
97	RECESSO FINAL DE ANO	CH da Universidade Federal do Ceará - CE
97	RECESSO FINAL DE ANO	CH da Universidade Federal do Ceará - CE
97	RECESSO FINAL DE ANO	CH da Universidade Federal do Ceará - CE
97	RECESSO FINAL DE ANO	CH da Universidade Federal do Ceará - CE
97	RECESSO FINAL DE ANO	CH da Universidade Federal do Ceará - CE
97	RECESSO FINAL DE ANO	CH da Universidade Federal do Ceará - CE
97	RECESSO FINAL DE ANO	CH da Universidade Federal do Ceará - CE
97	RECESSO FINAL DE ANO	CH da Universidade Federal do Ceará - CE
97	RECESSO FINAL DE ANO	CH da Universidade Federal do Ceará - CE
97	RECESSO FINAL DE ANO	CH da Universidade Federal do Ceará - CE
97	RECESSO FINAL DE ANO	CH da Universidade Federal do Ceará - CE
97	RECESSO FINAL DE ANO	CH da Universidade Federal do Ceará - CE
97	RECESSO FINAL DE ANO	CH da Universidade Federal do Ceará - CE
97	RECESSO FINAL DE ANO	CH da Universidade Federal do Ceará - CE
97	RECESSO FINAL DE ANO	CH da Universidade Federal do Ceará - CE
97	RECESSO FINAL DE ANO	CH da Universidade Federal do Ceará - CE
97	RECESSO FINAL DE ANO	CH da Universidade Federal do Ceará - CE
97	RECESSO FINAL DE ANO	CH da Universidade Federal do Ceará - CE
97	RECESSO FINAL DE ANO	CH da Universidade Federal do Ceará - CE
97	RECESSO FINAL DE ANO	CH da Universidade Federal do Ceará - CE
97	RECESSO FINAL DE ANO	CH da Universidade Federal do Ceará - CE
97	RECESSO FINAL DE ANO	CH da Universidade Federal do Ceará - CE
97	RECESSO FINAL DE ANO	CH da Universidade Federal do Ceará - CE
97	RECESSO FINAL DE ANO	CH da Universidade Federal do Ceará - CE
97	RECESSO FINAL DE ANO	CH da Universidade Federal do Ceará - CE
97	RECESSO FINAL DE ANO	CH da Universidade Federal do Ceará - CE
97	RECESSO FINAL DE ANO	CH da Universidade Federal do Ceará - CE
97	RECESSO FINAL DE ANO	CH da Universidade Federal do Ceará - CE
97	RECESSO FINAL DE ANO	CH da Universidade Federal do Ceará - CE
97	RECESSO FINAL DE ANO	CH da Universidade Federal do Ceará - CE
97	RECESSO FINAL DE ANO	CH da Universidade Federal do Ceará - CE

97	RECESSO FINAL DE ANO	CH da Universidade Federal do Ceará - CE
97	RECESSO FINAL DE ANO	CH da Universidade Federal do Ceará - CE
97	RECESSO FINAL DE ANO	CH da Universidade Federal do Ceará - CE
97	RECESSO FINAL DE ANO	CH da Universidade Federal do Ceará - CE
97	RECESSO FINAL DE ANO	CH da Universidade Federal do Ceará - CE
97	RECESSO FINAL DE ANO	CH da Universidade Federal do Ceará - CE
97	RECESSO FINAL DE ANO	CH da Universidade Federal do Ceará - CE
97	RECESSO FINAL DE ANO	CH da Universidade Federal do Ceará - CE
97	RECESSO FINAL DE ANO	CH da Universidade Federal do Ceará - CE
97	RECESSO FINAL DE ANO	CH da Universidade Federal do Ceará - CE
97	RECESSO FINAL DE ANO	CH da Universidade Federal do Ceará - CE
98	DESLOCAMENTO PARA NOVA UNIDADE (TRANSITO) - NORMA OPERACIONAL DGP N 01/2017	CH da Universidade Federal do Ceará - CE
98	DESLOCAMENTO PARA NOVA UNIDADE (TRANSITO) - NORMA OPERACIONAL DGP N 01/2017	CH da Universidade Federal do Ceará - CE
98	DESLOCAMENTO PARA NOVA UNIDADE (TRANSITO) - NORMA OPERACIONAL DGP N 01/2017	CH da Universidade Federal do Ceará - CE
98	DESLOCAMENTO PARA NOVA UNIDADE (TRANSITO) - NORMA OPERACIONAL DGP N 01/2017	CH da Universidade Federal do Ceará - CE
98	DESLOCAMENTO PARA NOVA UNIDADE (TRANSITO) - NORMA OPERACIONAL DGP N 01/2017	CH da Universidade Federal do Ceará - CE
104	Acompanhamento filho menor 6 anos - Art. 473, XI, CLT	CH da Universidade Federal do Ceará - CE
104	Acompanhamento filho menor 6 anos - Art. 473, XI, CLT	CH da Universidade Federal do Ceará - CE
104	Acompanhamento filho menor 6 anos - Art. 473, XI, CLT	CH da Universidade Federal do Ceará - CE
104	Acompanhamento filho menor 6 anos - Art. 473, XI, CLT	CH da Universidade Federal do Ceará - CE
104	Acompanhamento filho menor 6 anos - Art. 473, XI, CLT	CH da Universidade Federal do Ceará - CE
104	Acompanhamento filho menor 6 anos - Art. 473, XI, CLT	CH da Universidade Federal do Ceará - CE
104	Acompanhamento filho menor 6 anos - Art. 473, XI, CLT	CH da Universidade Federal do Ceará - CE
104	Acompanhamento filho menor 6 anos - Art. 473, XI, CLT	CH da Universidade Federal do Ceará - CE
104	Acompanhamento filho menor 6 anos - Art. 473, XI, CLT	CH da Universidade Federal do Ceará - CE
104	Acompanhamento filho menor 6 anos - Art. 473, XI, CLT	CH da Universidade Federal do Ceará - CE
104	Acompanhamento filho menor 6 anos - Art. 473, XI, CLT	CH da Universidade Federal do Ceará - CE
104	Acompanhamento filho menor 6 anos - Art. 473, XI, CLT	CH da Universidade Federal do Ceará - CE
104	Acompanhamento filho menor 6 anos - Art. 473, XI, CLT	CH da Universidade Federal do Ceará - CE
104	Acompanhamento filho menor 6 anos - Art. 473, XI, CLT	CH da Universidade Federal do Ceará - CE
104	Acompanhamento filho menor 6 anos - Art. 473, XI, CLT	CH da Universidade Federal do Ceará - CE
104	Acompanhamento filho menor 6 anos - Art. 473, XI, CLT	CH da Universidade Federal do Ceará - CE
104	Acompanhamento filho menor 6 anos - Art. 473, XI, CLT	CH da Universidade Federal do Ceará - CE
104	Acompanhamento filho menor 6 anos - Art. 473, XI, CLT	CH da Universidade Federal do Ceará - CE
104	Acompanhamento filho menor 6 anos - Art. 473, XI, CLT	CH da Universidade Federal do Ceará - CE
104	Acompanhamento filho menor 6 anos - Art. 473, XI, CLT	CH da Universidade Federal do Ceará - CE
104	Acompanhamento filho menor 6 anos - Art. 473, XI, CLT	CH da Universidade Federal do Ceará - CE
104	Acompanhamento filho menor 6 anos - Art. 473, XI, CLT	CH da Universidade Federal do Ceará - CE
104	Acompanhamento filho menor 6 anos - Art. 473, XI, CLT	CH da Universidade Federal do Ceará - CE
104	Acompanhamento filho menor 6 anos - Art. 473, XI, CLT	CH da Universidade Federal do Ceará - CE
104	Acompanhamento filho menor 6 anos - Art. 473, XI, CLT	CH da Universidade Federal do Ceará - CE

104	Acompanhamento filho menor 6 anos - Art. 473, XI, CLT	CH da Universidade Federal do Ceará - CE
104	Acompanhamento filho menor 6 anos - Art. 473, XI, CLT	CH da Universidade Federal do Ceará - CE
104	Acompanhamento filho menor 6 anos - Art. 473, XI, CLT	CH da Universidade Federal do Ceará - CE
104	Acompanhamento filho menor 6 anos - Art. 473, XI, CLT	CH da Universidade Federal do Ceará - CE
104	Acompanhamento filho menor 6 anos - Art. 473, XI, CLT	CH da Universidade Federal do Ceará - CE
104	Acompanhamento filho menor 6 anos - Art. 473, XI, CLT	CH da Universidade Federal do Ceará - CE
104	Acompanhamento filho menor 6 anos - Art. 473, XI, CLT	CH da Universidade Federal do Ceará - CE
104	Acompanhamento filho menor 6 anos - Art. 473, XI, CLT	CH da Universidade Federal do Ceará - CE
106	Afastamento por Atestado Medico/Acompanhamento - RJU	CH da Universidade Federal do Ceará - CE
106	Afastamento por Atestado Medico/Acompanhamento - RJU	CH da Universidade Federal do Ceará - CE
107	LICENÇA MATERNIDADE/ADOÇÃO - RJU	CH da Universidade Federal do Ceará - CE
224	REALIZAÇÃO DE EXAME PREVENTIVO DE CÂNCER. LEI 13.767	CH da Universidade Federal do Ceará - CE
224	REALIZAÇÃO DE EXAME PREVENTIVO DE CÂNCER. LEI 13.767	CH da Universidade Federal do Ceará - CE
224	REALIZAÇÃO DE EXAME PREVENTIVO DE CÂNCER. LEI 13.767	CH da Universidade Federal do Ceará - CE
224	REALIZAÇÃO DE EXAME PREVENTIVO DE CÂNCER. LEI 13.767	CH da Universidade Federal do Ceará - CE
224	REALIZAÇÃO DE EXAME PREVENTIVO DE CÂNCER. LEI 13.767	CH da Universidade Federal do Ceará - CE
224	REALIZAÇÃO DE EXAME PREVENTIVO DE CÂNCER. LEI 13.767	CH da Universidade Federal do Ceará - CE
224	REALIZAÇÃO DE EXAME PREVENTIVO DE CÂNCER. LEI 13.767	CH da Universidade Federal do Ceará - CE
224	REALIZAÇÃO DE EXAME PREVENTIVO DE CÂNCER. LEI 13.767	CH da Universidade Federal do Ceará - CE
224	REALIZAÇÃO DE EXAME PREVENTIVO DE CÂNCER. LEI 13.767	CH da Universidade Federal do Ceará - CE
224	REALIZAÇÃO DE EXAME PREVENTIVO DE CÂNCER. LEI 13.767	CH da Universidade Federal do Ceará - CE
224	REALIZAÇÃO DE EXAME PREVENTIVO DE CÂNCER. LEI 13.767	CH da Universidade Federal do Ceará - CE
224	REALIZAÇÃO DE EXAME PREVENTIVO DE CÂNCER. LEI 13.767	CH da Universidade Federal do Ceará - CE
224	REALIZAÇÃO DE EXAME PREVENTIVO DE CÂNCER. LEI 13.767	CH da Universidade Federal do Ceará - CE
224	REALIZAÇÃO DE EXAME PREVENTIVO DE CÂNCER. LEI 13.767	CH da Universidade Federal do Ceará - CE
224	REALIZAÇÃO DE EXAME PREVENTIVO DE CÂNCER. LEI 13.767	CH da Universidade Federal do Ceará - CE
224	REALIZAÇÃO DE EXAME PREVENTIVO DE CÂNCER. LEI 13.767	CH da Universidade Federal do Ceará - CE
224	REALIZAÇÃO DE EXAME PREVENTIVO DE CÂNCER. LEI 13.767	CH da Universidade Federal do Ceará - CE
224	REALIZAÇÃO DE EXAME PREVENTIVO DE CÂNCER. LEI 13.767	CH da Universidade Federal do Ceará - CE
224	REALIZAÇÃO DE EXAME PREVENTIVO DE CÂNCER. LEI 13.767	CH da Universidade Federal do Ceará - CE
224	REALIZAÇÃO DE EXAME PREVENTIVO DE CÂNCER. LEI 13.767	CH da Universidade Federal do Ceará - CE
224	REALIZAÇÃO DE EXAME PREVENTIVO DE CÂNCER. LEI 13.767	CH da Universidade Federal do Ceará - CE
224	REALIZAÇÃO DE EXAME PREVENTIVO DE CÂNCER. LEI 13.767	CH da Universidade Federal do Ceará - CE
224	REALIZAÇÃO DE EXAME PREVENTIVO DE CÂNCER. LEI 13.767	CH da Universidade Federal do Ceará - CE
224	REALIZAÇÃO DE EXAME PREVENTIVO DE CÂNCER. LEI 13.767	CH da Universidade Federal do Ceará - CE
224	REALIZAÇÃO DE EXAME PREVENTIVO DE CÂNCER. LEI 13.767	CH da Universidade Federal do Ceará - CE
224	REALIZAÇÃO DE EXAME PREVENTIVO DE CÂNCER. LEI 13.767	CH da Universidade Federal do Ceará - CE
224	REALIZAÇÃO DE EXAME PREVENTIVO DE CÂNCER. LEI 13.767	CH da Universidade Federal do Ceará - CE
224	REALIZAÇÃO DE EXAME PREVENTIVO DE CÂNCER. LEI 13.767	CH da Universidade Federal do Ceará - CE
224	REALIZAÇÃO DE EXAME PREVENTIVO DE CÂNCER. LEI 13.767	CH da Universidade Federal do Ceará - CE
224	REALIZAÇÃO DE EXAME PREVENTIVO DE CÂNCER. LEI 13.767	CH da Universidade Federal do Ceará - CE
224	REALIZAÇÃO DE EXAME PREVENTIVO DE CÂNCER. LEI 13.767	CH da Universidade Federal do Ceará - CE
224	REALIZAÇÃO DE EXAME PREVENTIVO DE CÂNCER. LEI 13.767	CH da Universidade Federal do Ceará - CE
224	REALIZAÇÃO DE EXAME PREVENTIVO DE CÂNCER. LEI 13.767	CH da Universidade Federal do Ceará - CE
224	REALIZAÇÃO DE EXAME PREVENTIVO DE CÂNCER. LEI 13.767	CH da Universidade Federal do Ceará - CE

224	REALIZAÇÃO DE EXAME PREVENTIVO DE CÂNCER. LEI 13.767	CH da Universidade Federal do Ceará - CE
224	REALIZAÇÃO DE EXAME PREVENTIVO DE CÂNCER. LEI 13.767	CH da Universidade Federal do Ceará - CE
224	REALIZAÇÃO DE EXAME PREVENTIVO DE CÂNCER. LEI 13.767	CH da Universidade Federal do Ceará - CE
224	REALIZAÇÃO DE EXAME PREVENTIVO DE CÂNCER. LEI 13.767	CH da Universidade Federal do Ceará - CE
224	REALIZAÇÃO DE EXAME PREVENTIVO DE CÂNCER. LEI 13.767	CH da Universidade Federal do Ceará - CE
224	REALIZAÇÃO DE EXAME PREVENTIVO DE CÂNCER. LEI 13.767	CH da Universidade Federal do Ceará - CE
224	REALIZAÇÃO DE EXAME PREVENTIVO DE CÂNCER. LEI 13.767	CH da Universidade Federal do Ceará - CE
224	REALIZAÇÃO DE EXAME PREVENTIVO DE CÂNCER. LEI 13.767	CH da Universidade Federal do Ceará - CE
224	REALIZAÇÃO DE EXAME PREVENTIVO DE CÂNCER. LEI 13.767	CH da Universidade Federal do Ceará - CE
224	REALIZAÇÃO DE EXAME PREVENTIVO DE CÂNCER. LEI 13.767	CH da Universidade Federal do Ceará - CE
224	REALIZAÇÃO DE EXAME PREVENTIVO DE CÂNCER. LEI 13.767	CH da Universidade Federal do Ceará - CE
224	REALIZAÇÃO DE EXAME PREVENTIVO DE CÂNCER. LEI 13.767	CH da Universidade Federal do Ceará - CE
224	REALIZAÇÃO DE EXAME PREVENTIVO DE CÂNCER. LEI 13.767	CH da Universidade Federal do Ceará - CE
224	REALIZAÇÃO DE EXAME PREVENTIVO DE CÂNCER. LEI 13.767	CH da Universidade Federal do Ceará - CE
224	REALIZAÇÃO DE EXAME PREVENTIVO DE CÂNCER. LEI 13.767	CH da Universidade Federal do Ceará - CE
224	REALIZAÇÃO DE EXAME PREVENTIVO DE CÂNCER. LEI 13.767	CH da Universidade Federal do Ceará - CE
224	REALIZAÇÃO DE EXAME PREVENTIVO DE CÂNCER. LEI 13.767	CH da Universidade Federal do Ceará - CE
224	REALIZAÇÃO DE EXAME PREVENTIVO DE CÂNCER. LEI 13.767	CH da Universidade Federal do Ceará - CE
224	REALIZAÇÃO DE EXAME PREVENTIVO DE CÂNCER. LEI 13.767	CH da Universidade Federal do Ceará - CE
224	REALIZAÇÃO DE EXAME PREVENTIVO DE CÂNCER. LEI 13.767	CH da Universidade Federal do Ceará - CE
224	REALIZAÇÃO DE EXAME PREVENTIVO DE CÂNCER. LEI 13.767	CH da Universidade Federal do Ceará - CE
224	REALIZAÇÃO DE EXAME PREVENTIVO DE CÂNCER. LEI 13.767	CH da Universidade Federal do Ceará - CE
224	REALIZAÇÃO DE EXAME PREVENTIVO DE CÂNCER. LEI 13.767	CH da Universidade Federal do Ceará - CE
224	REALIZAÇÃO DE EXAME PREVENTIVO DE CÂNCER. LEI 13.767	CH da Universidade Federal do Ceará - CE
224	REALIZAÇÃO DE EXAME PREVENTIVO DE CÂNCER. LEI 13.767	CH da Universidade Federal do Ceará - CE
224	REALIZAÇÃO DE EXAME PREVENTIVO DE CÂNCER. LEI 13.767	CH da Universidade Federal do Ceará - CE
224	REALIZAÇÃO DE EXAME PREVENTIVO DE CÂNCER. LEI 13.767	CH da Universidade Federal do Ceará - CE
224	REALIZAÇÃO DE EXAME PREVENTIVO DE CÂNCER. LEI 13.767	CH da Universidade Federal do Ceará - CE
224	REALIZAÇÃO DE EXAME PREVENTIVO DE CÂNCER. LEI 13.767	CH da Universidade Federal do Ceará - CE
224	REALIZAÇÃO DE EXAME PREVENTIVO DE CÂNCER. LEI 13.767	CH da Universidade Federal do Ceará - CE
224	REALIZAÇÃO DE EXAME PREVENTIVO DE CÂNCER. LEI 13.767	CH da Universidade Federal do Ceará - CE
224	REALIZAÇÃO DE EXAME PREVENTIVO DE CÂNCER. LEI 13.767	CH da Universidade Federal do Ceará - CE
224	REALIZAÇÃO DE EXAME PREVENTIVO DE CÂNCER. LEI 13.767	CH da Universidade Federal do Ceará - CE
224	REALIZAÇÃO DE EXAME PREVENTIVO DE CÂNCER. LEI 13.767	CH da Universidade Federal do Ceará - CE
224	REALIZAÇÃO DE EXAME PREVENTIVO DE CÂNCER. LEI 13.767	CH da Universidade Federal do Ceará - CE
224	REALIZAÇÃO DE EXAME PREVENTIVO DE CÂNCER. LEI 13.767	CH da Universidade Federal do Ceará - CE
224	REALIZAÇÃO DE EXAME PREVENTIVO DE CÂNCER. LEI 13.767	CH da Universidade Federal do Ceará - CE
224	REALIZAÇÃO DE EXAME PREVENTIVO DE CÂNCER. LEI 13.767	CH da Universidade Federal do Ceará - CE
224	REALIZAÇÃO DE EXAME PREVENTIVO DE CÂNCER. LEI 13.767	CH da Universidade Federal do Ceará - CE
224	REALIZAÇÃO DE EXAME PREVENTIVO DE CÂNCER. LEI 13.767	CH da Universidade Federal do Ceará - CE
224	REALIZAÇÃO DE EXAME PREVENTIVO DE CÂNCER. LEI 13.767	CH da Universidade Federal do Ceará - CE
224	REALIZAÇÃO DE EXAME PREVENTIVO DE CÂNCER. LEI 13.767	CH da Universidade Federal do Ceará - CE
224	REALIZAÇÃO DE EXAME PREVENTIVO DE CÂNCER. LEI 13.767	CH da Universidade Federal do Ceará - CE
224	REALIZAÇÃO DE EXAME PREVENTIVO DE CÂNCER. LEI 13.767	CH da Universidade Federal do Ceará - CE
224	REALIZAÇÃO DE EXAME PREVENTIVO DE CÂNCER. LEI 13.767	CH da Universidade Federal do Ceará - CE
224	REALIZAÇÃO DE EXAME PREVENTIVO DE CÂNCER. LEI 13.767	CH da Universidade Federal do Ceará - CE

224	REALIZAÇÃO DE EXAME PREVENTIVO DE CÂNCER. LEI 13.767	CH da Universidade Federal do Ceará - CE
224	REALIZAÇÃO DE EXAME PREVENTIVO DE CÂNCER. LEI 13.767	CH da Universidade Federal do Ceará - CE
224	REALIZAÇÃO DE EXAME PREVENTIVO DE CÂNCER. LEI 13.767	CH da Universidade Federal do Ceará - CE
224	REALIZAÇÃO DE EXAME PREVENTIVO DE CÂNCER. LEI 13.767	CH da Universidade Federal do Ceará - CE
224	REALIZAÇÃO DE EXAME PREVENTIVO DE CÂNCER. LEI 13.767	CH da Universidade Federal do Ceará - CE
224	REALIZAÇÃO DE EXAME PREVENTIVO DE CÂNCER. LEI 13.767	CH da Universidade Federal do Ceará - CE
224	REALIZAÇÃO DE EXAME PREVENTIVO DE CÂNCER. LEI 13.767	CH da Universidade Federal do Ceará - CE
224	REALIZAÇÃO DE EXAME PREVENTIVO DE CÂNCER. LEI 13.767	CH da Universidade Federal do Ceará - CE
224	REALIZAÇÃO DE EXAME PREVENTIVO DE CÂNCER. LEI 13.767	CH da Universidade Federal do Ceará - CE
224	REALIZAÇÃO DE EXAME PREVENTIVO DE CÂNCER. LEI 13.767	CH da Universidade Federal do Ceará - CE
224	REALIZAÇÃO DE EXAME PREVENTIVO DE CÂNCER. LEI 13.767	CH da Universidade Federal do Ceará - CE
224	REALIZAÇÃO DE EXAME PREVENTIVO DE CÂNCER. LEI 13.767	CH da Universidade Federal do Ceará - CE
224	REALIZAÇÃO DE EXAME PREVENTIVO DE CÂNCER. LEI 13.767	CH da Universidade Federal do Ceará - CE
224	REALIZAÇÃO DE EXAME PREVENTIVO DE CÂNCER. LEI 13.767	CH da Universidade Federal do Ceará - CE
224	REALIZAÇÃO DE EXAME PREVENTIVO DE CÂNCER. LEI 13.767	CH da Universidade Federal do Ceará - CE
224	REALIZAÇÃO DE EXAME PREVENTIVO DE CÂNCER. LEI 13.767	CH da Universidade Federal do Ceará - CE
224	REALIZAÇÃO DE EXAME PREVENTIVO DE CÂNCER. LEI 13.767	CH da Universidade Federal do Ceará - CE
224	REALIZAÇÃO DE EXAME PREVENTIVO DE CÂNCER. LEI 13.767	CH da Universidade Federal do Ceará - CE
224	REALIZAÇÃO DE EXAME PREVENTIVO DE CÂNCER. LEI 13.767	CH da Universidade Federal do Ceará - CE
224	REALIZAÇÃO DE EXAME PREVENTIVO DE CÂNCER. LEI 13.767	CH da Universidade Federal do Ceará - CE
224	REALIZAÇÃO DE EXAME PREVENTIVO DE CÂNCER. LEI 13.767	CH da Universidade Federal do Ceará - CE
224	REALIZAÇÃO DE EXAME PREVENTIVO DE CÂNCER. LEI 13.767	CH da Universidade Federal do Ceará - CE
224	REALIZAÇÃO DE EXAME PREVENTIVO DE CÂNCER. LEI 13.767	CH da Universidade Federal do Ceará - CE
224	REALIZAÇÃO DE EXAME PREVENTIVO DE CÂNCER. LEI 13.767	CH da Universidade Federal do Ceará - CE
224	REALIZAÇÃO DE EXAME PREVENTIVO DE CÂNCER. LEI 13.767	CH da Universidade Federal do Ceará - CE
224	REALIZAÇÃO DE EXAME PREVENTIVO DE CÂNCER. LEI 13.767	CH da Universidade Federal do Ceará - CE
224	REALIZAÇÃO DE EXAME PREVENTIVO DE CÂNCER. LEI 13.767	CH da Universidade Federal do Ceará - CE
224	REALIZAÇÃO DE EXAME PREVENTIVO DE CÂNCER. LEI 13.767	CH da Universidade Federal do Ceará - CE
224	REALIZAÇÃO DE EXAME PREVENTIVO DE CÂNCER. LEI 13.767	CH da Universidade Federal do Ceará - CE
224	REALIZAÇÃO DE EXAME PREVENTIVO DE CÂNCER. LEI 13.767	CH da Universidade Federal do Ceará - CE
224	REALIZAÇÃO DE EXAME PREVENTIVO DE CÂNCER. LEI 13.767	CH da Universidade Federal do Ceará - CE
224	REALIZAÇÃO DE EXAME PREVENTIVO DE CÂNCER. LEI 13.767	CH da Universidade Federal do Ceará - CE
224	REALIZAÇÃO DE EXAME PREVENTIVO DE CÂNCER. LEI 13.767	CH da Universidade Federal do Ceará - CE
224	REALIZAÇÃO DE EXAME PREVENTIVO DE CÂNCER. LEI 13.767	CH da Universidade Federal do Ceará - CE
224	REALIZAÇÃO DE EXAME PREVENTIVO DE CÂNCER. LEI 13.767	CH da Universidade Federal do Ceará - CE
224	REALIZAÇÃO DE EXAME PREVENTIVO DE CÂNCER. LEI 13.767	CH da Universidade Federal do Ceará - CE
224	REALIZAÇÃO DE EXAME PREVENTIVO DE CÂNCER. LEI 13.767	CH da Universidade Federal do Ceará - CE
224	REALIZAÇÃO DE EXAME PREVENTIVO DE CÂNCER. LEI 13.767	CH da Universidade Federal do Ceará - CE
224	REALIZAÇÃO DE EXAME PREVENTIVO DE CÂNCER. LEI 13.767	CH da Universidade Federal do Ceará - CE
224	REALIZAÇÃO DE EXAME PREVENTIVO DE CÂNCER. LEI 13.767	CH da Universidade Federal do Ceará - CE

224	REALIZAÇÃO DE EXAME PREVENTIVO DE CÂNCER. LEI 13.767	CH da Universidade Federal do Ceará - CE
227	ATIVIDADE EXTERNA (ESPII COVID19)	CH da Universidade Federal do Ceará - CE
227	ATIVIDADE EXTERNA (ESPII COVID19)	CH da Universidade Federal do Ceará - CE
227	ATIVIDADE EXTERNA (ESPII COVID19)	CH da Universidade Federal do Ceará - CE
227	ATIVIDADE EXTERNA (ESPII COVID19)	CH da Universidade Federal do Ceará - CE
227	ATIVIDADE EXTERNA (ESPII COVID19)	CH da Universidade Federal do Ceará - CE
227	ATIVIDADE EXTERNA (ESPII COVID19)	CH da Universidade Federal do Ceará - CE
227	ATIVIDADE EXTERNA (ESPII COVID19)	CH da Universidade Federal do Ceará - CE
227	ATIVIDADE EXTERNA (ESPII COVID19)	CH da Universidade Federal do Ceará - CE
227	ATIVIDADE EXTERNA (ESPII COVID19)	CH da Universidade Federal do Ceará - CE
227	ATIVIDADE EXTERNA (ESPII COVID19)	CH da Universidade Federal do Ceará - CE
227	ATIVIDADE EXTERNA (ESPII COVID19)	CH da Universidade Federal do Ceará - CE
227	ATIVIDADE EXTERNA (ESPII COVID19)	CH da Universidade Federal do Ceará - CE
227	ATIVIDADE EXTERNA (ESPII COVID19)	CH da Universidade Federal do Ceará - CE
227	ATIVIDADE EXTERNA (ESPII COVID19)	CH da Universidade Federal do Ceará - CE
227	ATIVIDADE EXTERNA (ESPII COVID19)	CH da Universidade Federal do Ceará - CE
227	ATIVIDADE EXTERNA (ESPII COVID19)	CH da Universidade Federal do Ceará - CE
227	ATIVIDADE EXTERNA (ESPII COVID19)	CH da Universidade Federal do Ceará - CE
227	ATIVIDADE EXTERNA (ESPII COVID19)	CH da Universidade Federal do Ceará - CE
227	ATIVIDADE EXTERNA (ESPII COVID19)	CH da Universidade Federal do Ceará - CE
227	ATIVIDADE EXTERNA (ESPII COVID19)	CH da Universidade Federal do Ceará - CE
227	ATIVIDADE EXTERNA (ESPII COVID19)	CH da Universidade Federal do Ceará - CE
227	ATIVIDADE EXTERNA (ESPII COVID19)	CH da Universidade Federal do Ceará - CE
227	ATIVIDADE EXTERNA (ESPII COVID19)	CH da Universidade Federal do Ceará - CE
227	ATIVIDADE EXTERNA (ESPII COVID19)	CH da Universidade Federal do Ceará - CE
227	ATIVIDADE EXTERNA (ESPII COVID19)	CH da Universidade Federal do Ceará - CE
227	ATIVIDADE EXTERNA (ESPII COVID19)	CH da Universidade Federal do Ceará - CE
227	ATIVIDADE EXTERNA (ESPII COVID19)	CH da Universidade Federal do Ceará - CE
227	ATIVIDADE EXTERNA (ESPII COVID19)	CH da Universidade Federal do Ceará - CE
227	ATIVIDADE EXTERNA (ESPII COVID19)	CH da Universidade Federal do Ceará - CE
227	ATIVIDADE EXTERNA (ESPII COVID19)	CH da Universidade Federal do Ceará - CE
227	ATIVIDADE EXTERNA (ESPII COVID19)	CH da Universidade Federal do Ceará - CE
227	ATIVIDADE EXTERNA (ESPII COVID19)	CH da Universidade Federal do Ceará - CE
228	ATIVIDADE EXTERNA (ESPII COVID19) - 7 Dias. DESATIVADO DE ACORDO COM A IN 02 - DGP de 26/03/2020.	CH da Universidade Federal do Ceará - CE
228	ATIVIDADE EXTERNA (ESPII COVID19) - 7 Dias. DESATIVADO DE ACORDO COM A IN 02 - DGP de 26/03/2020.	CH da Universidade Federal do Ceará - CE
228	ATIVIDADE EXTERNA (ESPII COVID19) - 7 Dias. DESATIVADO DE ACORDO COM A IN 02 - DGP de 26/03/2020.	CH da Universidade Federal do Ceará - CE
228	ATIVIDADE EXTERNA (ESPII COVID19) - 7 Dias. DESATIVADO DE ACORDO COM A IN 02 - DGP de 26/03/2020.	CH da Universidade Federal do Ceará - CE
228	ATIVIDADE EXTERNA (ESPII COVID19) - 7 Dias. DESATIVADO DE ACORDO COM A IN 02 - DGP de 26/03/2020.	CH da Universidade Federal do Ceará - CE
228	ATIVIDADE EXTERNA (ESPII COVID19) - 7 Dias. DESATIVADO DE ACORDO COM A IN 02 - DGP de 26/03/2020.	CH da Universidade Federal do Ceará - CE

228	ATIVIDADE EXTERNA (ESPII COVID19) - 7 Dias. DESATIVADO DE ACORDO COM A IN 02 - DGP de 26/03/2020.	CH da Universidade Federal do Ceará - CE
228	ATIVIDADE EXTERNA (ESPII COVID19) - 7 Dias. DESATIVADO DE ACORDO COM A IN 02 - DGP de 26/03/2020.	CH da Universidade Federal do Ceará - CE
228	ATIVIDADE EXTERNA (ESPII COVID19) - 7 Dias. DESATIVADO DE ACORDO COM A IN 02 - DGP de 26/03/2020.	CH da Universidade Federal do Ceará - CE
228	ATIVIDADE EXTERNA (ESPII COVID19) - 7 Dias. DESATIVADO DE ACORDO COM A IN 02 - DGP de 26/03/2020.	CH da Universidade Federal do Ceará - CE
228	ATIVIDADE EXTERNA (ESPII COVID19) - 7 Dias. DESATIVADO DE ACORDO COM A IN 02 - DGP de 26/03/2020.	CH da Universidade Federal do Ceará - CE
229	ATIVIDADE EXTERNA (ESPII COVID19) - 14 dias.	CH da Universidade Federal do Ceará - CE
229	ATIVIDADE EXTERNA (ESPII COVID19) - 14 dias.	CH da Universidade Federal do Ceará - CE
229	ATIVIDADE EXTERNA (ESPII COVID19) - 14 dias.	CH da Universidade Federal do Ceará - CE
231	Estado gripal (autodeclaratório) COVID-19. DESATIVADO DE ACORDO COM A IN 02 - DGP de 26/03/2020.	CH da Universidade Federal do Ceará - CE
232	ABONO ADMINISTRATIVO - COVID 19.	CH da Universidade Federal do Ceará - CE
232	ABONO ADMINISTRATIVO - COVID 19.	CH da Universidade Federal do Ceará - CE
233	ATIVIDADE EXTERNA ESPII - RODÍZIO	CH da Universidade Federal do Ceará - CE
233	ATIVIDADE EXTERNA ESPII - RODÍZIO	CH da Universidade Federal do Ceará - CE
233	ATIVIDADE EXTERNA ESPII - RODÍZIO	CH da Universidade Federal do Ceará - CE
233	ATIVIDADE EXTERNA ESPII - RODÍZIO	CH da Universidade Federal do Ceará - CE
233	ATIVIDADE EXTERNA ESPII - RODÍZIO	CH da Universidade Federal do Ceará - CE
233	ATIVIDADE EXTERNA ESPII - RODÍZIO	CH da Universidade Federal do Ceará - CE
233	ATIVIDADE EXTERNA ESPII - RODÍZIO	CH da Universidade Federal do Ceará - CE
233	ATIVIDADE EXTERNA ESPII - RODÍZIO	CH da Universidade Federal do Ceará - CE
233	ATIVIDADE EXTERNA ESPII - RODÍZIO	CH da Universidade Federal do Ceará - CE
233	ATIVIDADE EXTERNA ESPII - RODÍZIO	CH da Universidade Federal do Ceará - CE
233	ATIVIDADE EXTERNA ESPII - RODÍZIO	CH da Universidade Federal do Ceará - CE
233	ATIVIDADE EXTERNA ESPII - RODÍZIO	CH da Universidade Federal do Ceará - CE
233	ATIVIDADE EXTERNA ESPII - RODÍZIO	CH da Universidade Federal do Ceará - CE
233	ATIVIDADE EXTERNA ESPII - RODÍZIO	CH da Universidade Federal do Ceará - CE
233	ATIVIDADE EXTERNA ESPII - RODÍZIO	CH da Universidade Federal do Ceará - CE
233	ATIVIDADE EXTERNA ESPII - RODÍZIO	CH da Universidade Federal do Ceará - CE
233	ATIVIDADE EXTERNA ESPII - RODÍZIO	CH da Universidade Federal do Ceará - CE
233	ATIVIDADE EXTERNA ESPII - RODÍZIO	CH da Universidade Federal do Ceará - CE
233	ATIVIDADE EXTERNA ESPII - RODÍZIO	CH da Universidade Federal do Ceará - CE
233	ATIVIDADE EXTERNA ESPII - RODÍZIO	CH da Universidade Federal do Ceará - CE
233	ATIVIDADE EXTERNA ESPII - RODÍZIO	CH da Universidade Federal do Ceará - CE
233	ATIVIDADE EXTERNA ESPII - RODÍZIO	CH da Universidade Federal do Ceará - CE
233	ATIVIDADE EXTERNA ESPII - RODÍZIO	CH da Universidade Federal do Ceará - CE
233	ATIVIDADE EXTERNA ESPII - RODÍZIO	CH da Universidade Federal do Ceará - CE
233	ATIVIDADE EXTERNA ESPII - RODÍZIO	CH da Universidade Federal do Ceará - CE
233	ATIVIDADE EXTERNA ESPII - RODÍZIO	CH da Universidade Federal do Ceará - CE
233	ATIVIDADE EXTERNA ESPII - RODÍZIO	CH da Universidade Federal do Ceará - CE
233	ATIVIDADE EXTERNA ESPII - RODÍZIO	CH da Universidade Federal do Ceará - CE
233	ATIVIDADE EXTERNA ESPII - RODÍZIO	CH da Universidade Federal do Ceará - CE
233	ATIVIDADE EXTERNA ESPII - RODÍZIO	CH da Universidade Federal do Ceará - CE
233	ATIVIDADE EXTERNA ESPII - RODÍZIO	CH da Universidade Federal do Ceará - CE
233	ATIVIDADE EXTERNA ESPII - RODÍZIO	CH da Universidade Federal do Ceará - CE
233	ATIVIDADE EXTERNA ESPII - RODÍZIO	CH da Universidade Federal do Ceará - CE
233	ATIVIDADE EXTERNA ESPII - RODÍZIO	CH da Universidade Federal do Ceará - CE

233	ATIVIDADE EXTERNA ESPII - RODÍZIO	CH da Universidade Federal do Ceará - CE
233	ATIVIDADE EXTERNA ESPII - RODÍZIO	CH da Universidade Federal do Ceará - CE
233	ATIVIDADE EXTERNA ESPII - RODÍZIO	CH da Universidade Federal do Ceará - CE
233	ATIVIDADE EXTERNA ESPII - RODÍZIO	CH da Universidade Federal do Ceará - CE
233	ATIVIDADE EXTERNA ESPII - RODÍZIO	CH da Universidade Federal do Ceará - CE
233	ATIVIDADE EXTERNA ESPII - RODÍZIO	CH da Universidade Federal do Ceará - CE
233	ATIVIDADE EXTERNA ESPII - RODÍZIO	CH da Universidade Federal do Ceará - CE
233	ATIVIDADE EXTERNA ESPII - RODÍZIO	CH da Universidade Federal do Ceará - CE
233	ATIVIDADE EXTERNA ESPII - RODÍZIO	CH da Universidade Federal do Ceará - CE
233	ATIVIDADE EXTERNA ESPII - RODÍZIO	CH da Universidade Federal do Ceará - CE
233	ATIVIDADE EXTERNA ESPII - RODÍZIO	CH da Universidade Federal do Ceará - CE
233	ATIVIDADE EXTERNA ESPII - RODÍZIO	CH da Universidade Federal do Ceará - CE
233	ATIVIDADE EXTERNA ESPII - RODÍZIO	CH da Universidade Federal do Ceará - CE
233	ATIVIDADE EXTERNA ESPII - RODÍZIO	CH da Universidade Federal do Ceará - CE
233	ATIVIDADE EXTERNA ESPII - RODÍZIO	CH da Universidade Federal do Ceará - CE
233	ATIVIDADE EXTERNA ESPII - RODÍZIO	CH da Universidade Federal do Ceará - CE
233	ATIVIDADE EXTERNA ESPII - RODÍZIO	CH da Universidade Federal do Ceará - CE
233	ATIVIDADE EXTERNA ESPII - RODÍZIO	CH da Universidade Federal do Ceará - CE
233	ATIVIDADE EXTERNA ESPII - RODÍZIO	CH da Universidade Federal do Ceará - CE
233	ATIVIDADE EXTERNA ESPII - RODÍZIO	CH da Universidade Federal do Ceará - CE
233	ATIVIDADE EXTERNA ESPII - RODÍZIO	CH da Universidade Federal do Ceará - CE
233	ATIVIDADE EXTERNA ESPII - RODÍZIO	CH da Universidade Federal do Ceará - CE
233	ATIVIDADE EXTERNA ESPII - RODÍZIO	CH da Universidade Federal do Ceará - CE
233	ATIVIDADE EXTERNA ESPII - RODÍZIO	CH da Universidade Federal do Ceará - CE
233	ATIVIDADE EXTERNA ESPII - RODÍZIO	CH da Universidade Federal do Ceará - CE
233	ATIVIDADE EXTERNA ESPII - RODÍZIO	CH da Universidade Federal do Ceará - CE
233	ATIVIDADE EXTERNA ESPII - RODÍZIO	CH da Universidade Federal do Ceará - CE
233	ATIVIDADE EXTERNA ESPII - RODÍZIO	CH da Universidade Federal do Ceará - CE
233	ATIVIDADE EXTERNA ESPII - RODÍZIO	CH da Universidade Federal do Ceará - CE
233	ATIVIDADE EXTERNA ESPII - RODÍZIO	CH da Universidade Federal do Ceará - CE
233	ATIVIDADE EXTERNA ESPII - RODÍZIO	CH da Universidade Federal do Ceará - CE
233	ATIVIDADE EXTERNA ESPII - RODÍZIO	CH da Universidade Federal do Ceará - CE
233	ATIVIDADE EXTERNA ESPII - RODÍZIO	CH da Universidade Federal do Ceará - CE
233	ATIVIDADE EXTERNA ESPII - RODÍZIO	CH da Universidade Federal do Ceará - CE
233	ATIVIDADE EXTERNA ESPII - RODÍZIO	CH da Universidade Federal do Ceará - CE
233	ATIVIDADE EXTERNA ESPII - RODÍZIO	CH da Universidade Federal do Ceará - CE
233	ATIVIDADE EXTERNA ESPII - RODÍZIO	CH da Universidade Federal do Ceará - CE
233	ATIVIDADE EXTERNA ESPII - RODÍZIO	CH da Universidade Federal do Ceará - CE
233	ATIVIDADE EXTERNA ESPII - RODÍZIO	CH da Universidade Federal do Ceará - CE
233	ATIVIDADE EXTERNA ESPII - RODÍZIO	CH da Universidade Federal do Ceará - CE
233	ATIVIDADE EXTERNA ESPII - RODÍZIO	CH da Universidade Federal do Ceará - CE
233	ATIVIDADE EXTERNA ESPII - RODÍZIO	CH da Universidade Federal do Ceará - CE
233	ATIVIDADE EXTERNA ESPII - RODÍZIO	CH da Universidade Federal do Ceará - CE
233	ATIVIDADE EXTERNA ESPII - RODÍZIO	CH da Universidade Federal do Ceará - CE
233	ATIVIDADE EXTERNA ESPII - RODÍZIO	CH da Universidade Federal do Ceará - CE
233	ATIVIDADE EXTERNA ESPII - RODÍZIO	CH da Universidade Federal do Ceará - CE
233	ATIVIDADE EXTERNA ESPII - RODÍZIO	CH da Universidade Federal do Ceará - CE
233	ATIVIDADE EXTERNA ESPII - RODÍZIO	CH da Universidade Federal do Ceará - CE

233	ATIVIDADE EXTERNA ESPII - RODÍZIO	CH da Universidade Federal do Ceará - CE
233	ATIVIDADE EXTERNA ESPII - RODÍZIO	CH da Universidade Federal do Ceará - CE
233	ATIVIDADE EXTERNA ESPII - RODÍZIO	CH da Universidade Federal do Ceará - CE
233	ATIVIDADE EXTERNA ESPII - RODÍZIO	CH da Universidade Federal do Ceará - CE
233	ATIVIDADE EXTERNA ESPII - RODÍZIO	CH da Universidade Federal do Ceará - CE
233	ATIVIDADE EXTERNA ESPII - RODÍZIO	CH da Universidade Federal do Ceará - CE
233	ATIVIDADE EXTERNA ESPII - RODÍZIO	CH da Universidade Federal do Ceará - CE
233	ATIVIDADE EXTERNA ESPII - RODÍZIO	CH da Universidade Federal do Ceará - CE
233	ATIVIDADE EXTERNA ESPII - RODÍZIO	CH da Universidade Federal do Ceará - CE
233	ATIVIDADE EXTERNA ESPII - RODÍZIO	CH da Universidade Federal do Ceará - CE
233	ATIVIDADE EXTERNA ESPII - RODÍZIO	CH da Universidade Federal do Ceará - CE
233	ATIVIDADE EXTERNA ESPII - RODÍZIO	CH da Universidade Federal do Ceará - CE
233	ATIVIDADE EXTERNA ESPII - RODÍZIO	CH da Universidade Federal do Ceará - CE
233	ATIVIDADE EXTERNA ESPII - RODÍZIO	CH da Universidade Federal do Ceará - CE
233	ATIVIDADE EXTERNA ESPII - RODÍZIO	CH da Universidade Federal do Ceará - CE
233	ATIVIDADE EXTERNA ESPII - RODÍZIO	CH da Universidade Federal do Ceará - CE
233	ATIVIDADE EXTERNA ESPII - RODÍZIO	CH da Universidade Federal do Ceará - CE
233	ATIVIDADE EXTERNA ESPII - RODÍZIO	CH da Universidade Federal do Ceará - CE
233	ATIVIDADE EXTERNA ESPII - RODÍZIO	CH da Universidade Federal do Ceará - CE
233	ATIVIDADE EXTERNA ESPII - RODÍZIO	CH da Universidade Federal do Ceará - CE
233	ATIVIDADE EXTERNA ESPII - RODÍZIO	CH da Universidade Federal do Ceará - CE
233	ATIVIDADE EXTERNA ESPII - RODÍZIO	CH da Universidade Federal do Ceará - CE
233	ATIVIDADE EXTERNA ESPII - RODÍZIO	CH da Universidade Federal do Ceará - CE
233	ATIVIDADE EXTERNA ESPII - RODÍZIO	CH da Universidade Federal do Ceará - CE
233	ATIVIDADE EXTERNA ESPII - RODÍZIO	CH da Universidade Federal do Ceará - CE
233	ATIVIDADE EXTERNA ESPII - RODÍZIO	CH da Universidade Federal do Ceará - CE
233	ATIVIDADE EXTERNA ESPII - RODÍZIO	CH da Universidade Federal do Ceará - CE
233	ATIVIDADE EXTERNA ESPII - RODÍZIO	CH da Universidade Federal do Ceará - CE
233	ATIVIDADE EXTERNA ESPII - RODÍZIO	CH da Universidade Federal do Ceará - CE
233	ATIVIDADE EXTERNA ESPII - RODÍZIO	CH da Universidade Federal do Ceará - CE
233	ATIVIDADE EXTERNA ESPII - RODÍZIO	CH da Universidade Federal do Ceará - CE
233	ATIVIDADE EXTERNA ESPII - RODÍZIO	CH da Universidade Federal do Ceará - CE
233	ATIVIDADE EXTERNA ESPII - RODÍZIO	CH da Universidade Federal do Ceará - CE
233	ATIVIDADE EXTERNA ESPII - RODÍZIO	CH da Universidade Federal do Ceará - CE
233	ATIVIDADE EXTERNA ESPII - RODÍZIO	CH da Universidade Federal do Ceará - CE
233	ATIVIDADE EXTERNA ESPII - RODÍZIO	CH da Universidade Federal do Ceará - CE
233	ATIVIDADE EXTERNA ESPII - RODÍZIO	CH da Universidade Federal do Ceará - CE
233	ATIVIDADE EXTERNA ESPII - RODÍZIO	CH da Universidade Federal do Ceará - CE
233	ATIVIDADE EXTERNA ESPII - RODÍZIO	CH da Universidade Federal do Ceará - CE
233	ATIVIDADE EXTERNA ESPII - RODÍZIO	CH da Universidade Federal do Ceará - CE
233	ATIVIDADE EXTERNA ESPII - RODÍZIO	CH da Universidade Federal do Ceará - CE
233	ATIVIDADE EXTERNA ESPII - RODÍZIO	CH da Universidade Federal do Ceará - CE
233	ATIVIDADE EXTERNA ESPII - RODÍZIO	CH da Universidade Federal do Ceará - CE
233	ATIVIDADE EXTERNA ESPII - RODÍZIO	CH da Universidade Federal do Ceará - CE
233	ATIVIDADE EXTERNA ESPII - RODÍZIO	CH da Universidade Federal do Ceará - CE
233	ATIVIDADE EXTERNA ESPII - RODÍZIO	CH da Universidade Federal do Ceará - CE
233	ATIVIDADE EXTERNA ESPII - RODÍZIO	CH da Universidade Federal do Ceará - CE
233	ATIVIDADE EXTERNA ESPII - RODÍZIO	CH da Universidade Federal do Ceará - CE

233	ATIVIDADE EXTERNA ESPII - RODÍZIO	CH da Universidade Federal do Ceará - CE
233	ATIVIDADE EXTERNA ESPII - RODÍZIO	CH da Universidade Federal do Ceará - CE
233	ATIVIDADE EXTERNA ESPII - RODÍZIO	CH da Universidade Federal do Ceará - CE
233	ATIVIDADE EXTERNA ESPII - RODÍZIO	CH da Universidade Federal do Ceará - CE
233	ATIVIDADE EXTERNA ESPII - RODÍZIO	CH da Universidade Federal do Ceará - CE
233	ATIVIDADE EXTERNA ESPII - RODÍZIO	CH da Universidade Federal do Ceará - CE
233	ATIVIDADE EXTERNA ESPII - RODÍZIO	CH da Universidade Federal do Ceará - CE
233	ATIVIDADE EXTERNA ESPII - RODÍZIO	CH da Universidade Federal do Ceará - CE
233	ATIVIDADE EXTERNA ESPII - RODÍZIO	CH da Universidade Federal do Ceará - CE
233	ATIVIDADE EXTERNA ESPII - RODÍZIO	CH da Universidade Federal do Ceará - CE
233	ATIVIDADE EXTERNA ESPII - RODÍZIO	CH da Universidade Federal do Ceará - CE
233	ATIVIDADE EXTERNA ESPII - RODÍZIO	CH da Universidade Federal do Ceará - CE
233	ATIVIDADE EXTERNA ESPII - RODÍZIO	CH da Universidade Federal do Ceará - CE
233	ATIVIDADE EXTERNA ESPII - RODÍZIO	CH da Universidade Federal do Ceará - CE
233	ATIVIDADE EXTERNA ESPII - RODÍZIO	CH da Universidade Federal do Ceará - CE
233	ATIVIDADE EXTERNA ESPII - RODÍZIO	CH da Universidade Federal do Ceará - CE
233	ATIVIDADE EXTERNA ESPII - RODÍZIO	CH da Universidade Federal do Ceará - CE
233	ATIVIDADE EXTERNA ESPII - RODÍZIO	CH da Universidade Federal do Ceará - CE
233	ATIVIDADE EXTERNA ESPII - RODÍZIO	CH da Universidade Federal do Ceará - CE
233	ATIVIDADE EXTERNA ESPII - RODÍZIO	CH da Universidade Federal do Ceará - CE
233	ATIVIDADE EXTERNA ESPII - RODÍZIO	CH da Universidade Federal do Ceará - CE
233	ATIVIDADE EXTERNA ESPII - RODÍZIO	CH da Universidade Federal do Ceará - CE
233	ATIVIDADE EXTERNA ESPII - RODÍZIO	CH da Universidade Federal do Ceará - CE
233	ATIVIDADE EXTERNA ESPII - RODÍZIO	CH da Universidade Federal do Ceará - CE
233	ATIVIDADE EXTERNA ESPII - RODÍZIO	CH da Universidade Federal do Ceará - CE
233	ATIVIDADE EXTERNA ESPII - RODÍZIO	CH da Universidade Federal do Ceará - CE
233	ATIVIDADE EXTERNA ESPII - RODÍZIO	CH da Universidade Federal do Ceará - CE
233	ATIVIDADE EXTERNA ESPII - RODÍZIO	CH da Universidade Federal do Ceará - CE
233	ATIVIDADE EXTERNA ESPII - RODÍZIO	CH da Universidade Federal do Ceará - CE
233	ATIVIDADE EXTERNA ESPII - RODÍZIO	CH da Universidade Federal do Ceará - CE
233	ATIVIDADE EXTERNA ESPII - RODÍZIO	CH da Universidade Federal do Ceará - CE
233	ATIVIDADE EXTERNA ESPII - RODÍZIO	CH da Universidade Federal do Ceará - CE
233	ATIVIDADE EXTERNA ESPII - RODÍZIO	CH da Universidade Federal do Ceará - CE
233	ATIVIDADE EXTERNA ESPII - RODÍZIO	CH da Universidade Federal do Ceará - CE
233	ATIVIDADE EXTERNA ESPII - RODÍZIO	CH da Universidade Federal do Ceará - CE
233	ATIVIDADE EXTERNA ESPII - RODÍZIO	CH da Universidade Federal do Ceará - CE
233	ATIVIDADE EXTERNA ESPII - RODÍZIO	CH da Universidade Federal do Ceará - CE
233	ATIVIDADE EXTERNA ESPII - RODÍZIO	CH da Universidade Federal do Ceará - CE
233	ATIVIDADE EXTERNA ESPII - RODÍZIO	CH da Universidade Federal do Ceará - CE
233	ATIVIDADE EXTERNA ESPII - RODÍZIO	CH da Universidade Federal do Ceará - CE
233	ATIVIDADE EXTERNA ESPII - RODÍZIO	CH da Universidade Federal do Ceará - CE
233	ATIVIDADE EXTERNA ESPII - RODÍZIO	CH da Universidade Federal do Ceará - CE
233	ATIVIDADE EXTERNA ESPII - RODÍZIO	CH da Universidade Federal do Ceará - CE
233	ATIVIDADE EXTERNA ESPII - RODÍZIO	CH da Universidade Federal do Ceará - CE
233	ATIVIDADE EXTERNA ESPII - RODÍZIO	CH da Universidade Federal do Ceará - CE
233	ATIVIDADE EXTERNA ESPII - RODÍZIO	CH da Universidade Federal do Ceará - CE
233	ATIVIDADE EXTERNA ESPII - RODÍZIO	CH da Universidade Federal do Ceará - CE
233	ATIVIDADE EXTERNA ESPII - RODÍZIO	CH da Universidade Federal do Ceará - CE

233	ATIVIDADE EXTERNA ESPII - RODÍZIO	CH da Universidade Federal do Ceará - CE
233	ATIVIDADE EXTERNA ESPII - RODÍZIO	CH da Universidade Federal do Ceará - CE
233	ATIVIDADE EXTERNA ESPII - RODÍZIO	CH da Universidade Federal do Ceará - CE
233	ATIVIDADE EXTERNA ESPII - RODÍZIO	CH da Universidade Federal do Ceará - CE
233	ATIVIDADE EXTERNA ESPII - RODÍZIO	CH da Universidade Federal do Ceará - CE
233	ATIVIDADE EXTERNA ESPII - RODÍZIO	CH da Universidade Federal do Ceará - CE
233	ATIVIDADE EXTERNA ESPII - RODÍZIO	CH da Universidade Federal do Ceará - CE
233	ATIVIDADE EXTERNA ESPII - RODÍZIO	CH da Universidade Federal do Ceará - CE
233	ATIVIDADE EXTERNA ESPII - RODÍZIO	CH da Universidade Federal do Ceará - CE
233	ATIVIDADE EXTERNA ESPII - RODÍZIO	CH da Universidade Federal do Ceará - CE
233	ATIVIDADE EXTERNA ESPII - RODÍZIO	CH da Universidade Federal do Ceará - CE
233	ATIVIDADE EXTERNA ESPII - RODÍZIO	CH da Universidade Federal do Ceará - CE
233	ATIVIDADE EXTERNA ESPII - RODÍZIO	CH da Universidade Federal do Ceará - CE
233	ATIVIDADE EXTERNA ESPII - RODÍZIO	CH da Universidade Federal do Ceará - CE
233	ATIVIDADE EXTERNA ESPII - RODÍZIO	CH da Universidade Federal do Ceará - CE
233	ATIVIDADE EXTERNA ESPII - RODÍZIO	CH da Universidade Federal do Ceará - CE
233	ATIVIDADE EXTERNA ESPII - RODÍZIO	CH da Universidade Federal do Ceará - CE
233	ATIVIDADE EXTERNA ESPII - RODÍZIO	CH da Universidade Federal do Ceará - CE
233	ATIVIDADE EXTERNA ESPII - RODÍZIO	CH da Universidade Federal do Ceará - CE
233	ATIVIDADE EXTERNA ESPII - RODÍZIO	CH da Universidade Federal do Ceará - CE
233	ATIVIDADE EXTERNA ESPII - RODÍZIO	CH da Universidade Federal do Ceará - CE
233	ATIVIDADE EXTERNA ESPII - RODÍZIO	CH da Universidade Federal do Ceará - CE
233	ATIVIDADE EXTERNA ESPII - RODÍZIO	CH da Universidade Federal do Ceará - CE
233	ATIVIDADE EXTERNA ESPII - RODÍZIO	CH da Universidade Federal do Ceará - CE
233	ATIVIDADE EXTERNA ESPII - RODÍZIO	CH da Universidade Federal do Ceará - CE
233	ATIVIDADE EXTERNA ESPII - RODÍZIO	CH da Universidade Federal do Ceará - CE
233	ATIVIDADE EXTERNA ESPII - RODÍZIO	CH da Universidade Federal do Ceará - CE
233	ATIVIDADE EXTERNA ESPII - RODÍZIO	CH da Universidade Federal do Ceará - CE
233	ATIVIDADE EXTERNA ESPII - RODÍZIO	CH da Universidade Federal do Ceará - CE
233	ATIVIDADE EXTERNA ESPII - RODÍZIO	CH da Universidade Federal do Ceará - CE
233	ATIVIDADE EXTERNA ESPII - RODÍZIO	CH da Universidade Federal do Ceará - CE
233	ATIVIDADE EXTERNA ESPII - RODÍZIO	CH da Universidade Federal do Ceará - CE
233	ATIVIDADE EXTERNA ESPII - RODÍZIO	CH da Universidade Federal do Ceará - CE
233	ATIVIDADE EXTERNA ESPII - RODÍZIO	CH da Universidade Federal do Ceará - CE
233	ATIVIDADE EXTERNA ESPII - RODÍZIO	CH da Universidade Federal do Ceará - CE
233	ATIVIDADE EXTERNA ESPII - RODÍZIO	CH da Universidade Federal do Ceará - CE
233	ATIVIDADE EXTERNA ESPII - RODÍZIO	CH da Universidade Federal do Ceará - CE
233	ATIVIDADE EXTERNA ESPII - RODÍZIO	CH da Universidade Federal do Ceará - CE
233	ATIVIDADE EXTERNA ESPII - RODÍZIO	CH da Universidade Federal do Ceará - CE
233	ATIVIDADE EXTERNA ESPII - RODÍZIO	CH da Universidade Federal do Ceará - CE
233	ATIVIDADE EXTERNA ESPII - RODÍZIO	CH da Universidade Federal do Ceará - CE
233	ATIVIDADE EXTERNA ESPII - RODÍZIO	CH da Universidade Federal do Ceará - CE
233	ATIVIDADE EXTERNA ESPII - RODÍZIO	CH da Universidade Federal do Ceará - CE

233	ATIVIDADE EXTERNA ESPII - RODÍZIO	CH da Universidade Federal do Ceará - CE
233	ATIVIDADE EXTERNA ESPII - RODÍZIO	CH da Universidade Federal do Ceará - CE
233	ATIVIDADE EXTERNA ESPII - RODÍZIO	CH da Universidade Federal do Ceará - CE
233	ATIVIDADE EXTERNA ESPII - RODÍZIO	CH da Universidade Federal do Ceará - CE
233	ATIVIDADE EXTERNA ESPII - RODÍZIO	CH da Universidade Federal do Ceará - CE
233	ATIVIDADE EXTERNA ESPII - RODÍZIO	CH da Universidade Federal do Ceará - CE
233	ATIVIDADE EXTERNA ESPII - RODÍZIO	CH da Universidade Federal do Ceará - CE
233	ATIVIDADE EXTERNA ESPII - RODÍZIO	CH da Universidade Federal do Ceará - CE
233	ATIVIDADE EXTERNA ESPII - RODÍZIO	CH da Universidade Federal do Ceará - CE
233	ATIVIDADE EXTERNA ESPII - RODÍZIO	CH da Universidade Federal do Ceará - CE
233	ATIVIDADE EXTERNA ESPII - RODÍZIO	CH da Universidade Federal do Ceará - CE
233	ATIVIDADE EXTERNA ESPII - RODÍZIO	CH da Universidade Federal do Ceará - CE
233	ATIVIDADE EXTERNA ESPII - RODÍZIO	CH da Universidade Federal do Ceará - CE
233	ATIVIDADE EXTERNA ESPII - RODÍZIO	CH da Universidade Federal do Ceará - CE
233	ATIVIDADE EXTERNA ESPII - RODÍZIO	CH da Universidade Federal do Ceará - CE
233	ATIVIDADE EXTERNA ESPII - RODÍZIO	CH da Universidade Federal do Ceará - CE
233	ATIVIDADE EXTERNA ESPII - RODÍZIO	CH da Universidade Federal do Ceará - CE
233	ATIVIDADE EXTERNA ESPII - RODÍZIO	CH da Universidade Federal do Ceará - CE
233	ATIVIDADE EXTERNA ESPII - RODÍZIO	CH da Universidade Federal do Ceará - CE
233	ATIVIDADE EXTERNA ESPII - RODÍZIO	CH da Universidade Federal do Ceará - CE
233	ATIVIDADE EXTERNA ESPII - RODÍZIO	CH da Universidade Federal do Ceará - CE
233	ATIVIDADE EXTERNA ESPII - RODÍZIO	CH da Universidade Federal do Ceará - CE
233	ATIVIDADE EXTERNA ESPII - RODÍZIO	CH da Universidade Federal do Ceará - CE
233	ATIVIDADE EXTERNA ESPII - RODÍZIO	CH da Universidade Federal do Ceará - CE
233	ATIVIDADE EXTERNA ESPII - RODÍZIO	CH da Universidade Federal do Ceará - CE
233	ATIVIDADE EXTERNA ESPII - RODÍZIO	CH da Universidade Federal do Ceará - CE
233	ATIVIDADE EXTERNA ESPII - RODÍZIO	CH da Universidade Federal do Ceará - CE
233	ATIVIDADE EXTERNA ESPII - RODÍZIO	CH da Universidade Federal do Ceará - CE
233	ATIVIDADE EXTERNA ESPII - RODÍZIO	CH da Universidade Federal do Ceará - CE
233	ATIVIDADE EXTERNA ESPII - RODÍZIO	CH da Universidade Federal do Ceará - CE
233	ATIVIDADE EXTERNA ESPII - RODÍZIO	CH da Universidade Federal do Ceará - CE
233	ATIVIDADE EXTERNA ESPII - RODÍZIO	CH da Universidade Federal do Ceará - CE
233	ATIVIDADE EXTERNA ESPII - RODÍZIO	CH da Universidade Federal do Ceará - CE
233	ATIVIDADE EXTERNA ESPII - RODÍZIO	CH da Universidade Federal do Ceará - CE
233	ATIVIDADE EXTERNA ESPII - RODÍZIO	CH da Universidade Federal do Ceará - CE
233	ATIVIDADE EXTERNA ESPII - RODÍZIO	CH da Universidade Federal do Ceará - CE
233	ATIVIDADE EXTERNA ESPII - RODÍZIO	CH da Universidade Federal do Ceará - CE
233	ATIVIDADE EXTERNA ESPII - RODÍZIO	CH da Universidade Federal do Ceará - CE
233	ATIVIDADE EXTERNA ESPII - RODÍZIO	CH da Universidade Federal do Ceará - CE
233	ATIVIDADE EXTERNA ESPII - RODÍZIO	CH da Universidade Federal do Ceará - CE
233	ATIVIDADE EXTERNA ESPII - RODÍZIO	CH da Universidade Federal do Ceará - CE
233	ATIVIDADE EXTERNA ESPII - RODÍZIO	CH da Universidade Federal do Ceará - CE
233	ATIVIDADE EXTERNA ESPII - RODÍZIO	CH da Universidade Federal do Ceará - CE
233	ATIVIDADE EXTERNA ESPII - RODÍZIO	CH da Universidade Federal do Ceará - CE
233	ATIVIDADE EXTERNA ESPII - RODÍZIO	CH da Universidade Federal do Ceará - CE
233	ATIVIDADE EXTERNA ESPII - RODÍZIO	CH da Universidade Federal do Ceará - CE
233	ATIVIDADE EXTERNA ESPII - RODÍZIO	CH da Universidade Federal do Ceará - CE
233	ATIVIDADE EXTERNA ESPII - RODÍZIO	CH da Universidade Federal do Ceará - CE

233	ATIVIDADE EXTERNA ESPII - RODÍZIO	CH da Universidade Federal do Ceará - CE
233	ATIVIDADE EXTERNA ESPII - RODÍZIO	CH da Universidade Federal do Ceará - CE
233	ATIVIDADE EXTERNA ESPII - RODÍZIO	CH da Universidade Federal do Ceará - CE
233	ATIVIDADE EXTERNA ESPII - RODÍZIO	CH da Universidade Federal do Ceará - CE
233	ATIVIDADE EXTERNA ESPII - RODÍZIO	CH da Universidade Federal do Ceará - CE
233	ATIVIDADE EXTERNA ESPII - RODÍZIO	CH da Universidade Federal do Ceará - CE
233	ATIVIDADE EXTERNA ESPII - RODÍZIO	CH da Universidade Federal do Ceará - CE
233	ATIVIDADE EXTERNA ESPII - RODÍZIO	CH da Universidade Federal do Ceará - CE
233	ATIVIDADE EXTERNA ESPII - RODÍZIO	CH da Universidade Federal do Ceará - CE
233	ATIVIDADE EXTERNA ESPII - RODÍZIO	CH da Universidade Federal do Ceará - CE
233	ATIVIDADE EXTERNA ESPII - RODÍZIO	CH da Universidade Federal do Ceará - CE
233	ATIVIDADE EXTERNA ESPII - RODÍZIO	CH da Universidade Federal do Ceará - CE
233	ATIVIDADE EXTERNA ESPII - RODÍZIO	CH da Universidade Federal do Ceará - CE
233	ATIVIDADE EXTERNA ESPII - RODÍZIO	CH da Universidade Federal do Ceará - CE
233	ATIVIDADE EXTERNA ESPII - RODÍZIO	CH da Universidade Federal do Ceará - CE
233	ATIVIDADE EXTERNA ESPII - RODÍZIO	CH da Universidade Federal do Ceará - CE
233	ATIVIDADE EXTERNA ESPII - RODÍZIO	CH da Universidade Federal do Ceará - CE
233	ATIVIDADE EXTERNA ESPII - RODÍZIO	CH da Universidade Federal do Ceará - CE
233	ATIVIDADE EXTERNA ESPII - RODÍZIO	CH da Universidade Federal do Ceará - CE
233	ATIVIDADE EXTERNA ESPII - RODÍZIO	CH da Universidade Federal do Ceará - CE
233	ATIVIDADE EXTERNA ESPII - RODÍZIO	CH da Universidade Federal do Ceará - CE
233	ATIVIDADE EXTERNA ESPII - RODÍZIO	CH da Universidade Federal do Ceará - CE
233	ATIVIDADE EXTERNA ESPII - RODÍZIO	CH da Universidade Federal do Ceará - CE
233	ATIVIDADE EXTERNA ESPII - RODÍZIO	CH da Universidade Federal do Ceará - CE
233	ATIVIDADE EXTERNA ESPII - RODÍZIO	CH da Universidade Federal do Ceará - CE
233	ATIVIDADE EXTERNA ESPII - RODÍZIO	CH da Universidade Federal do Ceará - CE
233	ATIVIDADE EXTERNA ESPII - RODÍZIO	CH da Universidade Federal do Ceará - CE
233	ATIVIDADE EXTERNA ESPII - RODÍZIO	CH da Universidade Federal do Ceará - CE
233	ATIVIDADE EXTERNA ESPII - RODÍZIO	CH da Universidade Federal do Ceará - CE
233	ATIVIDADE EXTERNA ESPII - RODÍZIO	CH da Universidade Federal do Ceará - CE
233	ATIVIDADE EXTERNA ESPII - RODÍZIO	CH da Universidade Federal do Ceará - CE
233	ATIVIDADE EXTERNA ESPII - RODÍZIO	CH da Universidade Federal do Ceará - CE
233	ATIVIDADE EXTERNA ESPII - RODÍZIO	CH da Universidade Federal do Ceará - CE
233	ATIVIDADE EXTERNA ESPII - RODÍZIO	CH da Universidade Federal do Ceará - CE
233	ATIVIDADE EXTERNA ESPII - RODÍZIO	CH da Universidade Federal do Ceará - CE
233	ATIVIDADE EXTERNA ESPII - RODÍZIO	CH da Universidade Federal do Ceará - CE
233	ATIVIDADE EXTERNA ESPII - RODÍZIO	CH da Universidade Federal do Ceará - CE
233	ATIVIDADE EXTERNA ESPII - RODÍZIO	CH da Universidade Federal do Ceará - CE
233	ATIVIDADE EXTERNA ESPII - RODÍZIO	CH da Universidade Federal do Ceará - CE
233	ATIVIDADE EXTERNA ESPII - RODÍZIO	CH da Universidade Federal do Ceará - CE
233	ATIVIDADE EXTERNA ESPII - RODÍZIO	CH da Universidade Federal do Ceará - CE
233	ATIVIDADE EXTERNA ESPII - RODÍZIO	CH da Universidade Federal do Ceará - CE
233	ATIVIDADE EXTERNA ESPII - RODÍZIO	CH da Universidade Federal do Ceará - CE
233	ATIVIDADE EXTERNA ESPII - RODÍZIO	CH da Universidade Federal do Ceará - CE
233	ATIVIDADE EXTERNA ESPII - RODÍZIO	CH da Universidade Federal do Ceará - CE
233	ATIVIDADE EXTERNA ESPII - RODÍZIO	CH da Universidade Federal do Ceará - CE
233	ATIVIDADE EXTERNA ESPII - RODÍZIO	CH da Universidade Federal do Ceará - CE
233	ATIVIDADE EXTERNA ESPII - RODÍZIO	CH da Universidade Federal do Ceará - CE

233	ATIVIDADE EXTERNA ESPII - RODÍZIO	CH da Universidade Federal do Ceará - CE
233	ATIVIDADE EXTERNA ESPII - RODÍZIO	CH da Universidade Federal do Ceará - CE
233	ATIVIDADE EXTERNA ESPII - RODÍZIO	CH da Universidade Federal do Ceará - CE
233	ATIVIDADE EXTERNA ESPII - RODÍZIO	CH da Universidade Federal do Ceará - CE
233	ATIVIDADE EXTERNA ESPII - RODÍZIO	CH da Universidade Federal do Ceará - CE
233	ATIVIDADE EXTERNA ESPII - RODÍZIO	CH da Universidade Federal do Ceará - CE
233	ATIVIDADE EXTERNA ESPII - RODÍZIO	CH da Universidade Federal do Ceará - CE
233	ATIVIDADE EXTERNA ESPII - RODÍZIO	CH da Universidade Federal do Ceará - CE
233	ATIVIDADE EXTERNA ESPII - RODÍZIO	CH da Universidade Federal do Ceará - CE
233	ATIVIDADE EXTERNA ESPII - RODÍZIO	CH da Universidade Federal do Ceará - CE
233	ATIVIDADE EXTERNA ESPII - RODÍZIO	CH da Universidade Federal do Ceará - CE
233	ATIVIDADE EXTERNA ESPII - RODÍZIO	CH da Universidade Federal do Ceará - CE
233	ATIVIDADE EXTERNA ESPII - RODÍZIO	CH da Universidade Federal do Ceará - CE
233	ATIVIDADE EXTERNA ESPII - RODÍZIO	CH da Universidade Federal do Ceará - CE
233	ATIVIDADE EXTERNA ESPII - RODÍZIO	CH da Universidade Federal do Ceará - CE
233	ATIVIDADE EXTERNA ESPII - RODÍZIO	CH da Universidade Federal do Ceará - CE
233	ATIVIDADE EXTERNA ESPII - RODÍZIO	CH da Universidade Federal do Ceará - CE
233	ATIVIDADE EXTERNA ESPII - RODÍZIO	CH da Universidade Federal do Ceará - CE
233	ATIVIDADE EXTERNA ESPII - RODÍZIO	CH da Universidade Federal do Ceará - CE
233	ATIVIDADE EXTERNA ESPII - RODÍZIO	CH da Universidade Federal do Ceará - CE
233	ATIVIDADE EXTERNA ESPII - RODÍZIO	CH da Universidade Federal do Ceará - CE
233	ATIVIDADE EXTERNA ESPII - RODÍZIO	CH da Universidade Federal do Ceará - CE
233	ATIVIDADE EXTERNA ESPII - RODÍZIO	CH da Universidade Federal do Ceará - CE
233	ATIVIDADE EXTERNA ESPII - RODÍZIO	CH da Universidade Federal do Ceará - CE
233	ATIVIDADE EXTERNA ESPII - RODÍZIO	CH da Universidade Federal do Ceará - CE
233	ATIVIDADE EXTERNA ESPII - RODÍZIO	CH da Universidade Federal do Ceará - CE
233	ATIVIDADE EXTERNA ESPII - RODÍZIO	CH da Universidade Federal do Ceará - CE
233	ATIVIDADE EXTERNA ESPII - RODÍZIO	CH da Universidade Federal do Ceará - CE
233	ATIVIDADE EXTERNA ESPII - RODÍZIO	CH da Universidade Federal do Ceará - CE
233	ATIVIDADE EXTERNA ESPII - RODÍZIO	CH da Universidade Federal do Ceará - CE
233	ATIVIDADE EXTERNA ESPII - RODÍZIO	CH da Universidade Federal do Ceará - CE
233	ATIVIDADE EXTERNA ESPII - RODÍZIO	CH da Universidade Federal do Ceará - CE
233	ATIVIDADE EXTERNA ESPII - RODÍZIO	CH da Universidade Federal do Ceará - CE
233	ATIVIDADE EXTERNA ESPII - RODÍZIO	CH da Universidade Federal do Ceará - CE
233	ATIVIDADE EXTERNA ESPII - RODÍZIO	CH da Universidade Federal do Ceará - CE
233	ATIVIDADE EXTERNA ESPII - RODÍZIO	CH da Universidade Federal do Ceará - CE
233	ATIVIDADE EXTERNA ESPII - RODÍZIO	CH da Universidade Federal do Ceará - CE
233	ATIVIDADE EXTERNA ESPII - RODÍZIO	CH da Universidade Federal do Ceará - CE
233	ATIVIDADE EXTERNA ESPII - RODÍZIO	CH da Universidade Federal do Ceará - CE
233	ATIVIDADE EXTERNA ESPII - RODÍZIO	CH da Universidade Federal do Ceará - CE
233	ATIVIDADE EXTERNA ESPII - RODÍZIO	CH da Universidade Federal do Ceará - CE
233	ATIVIDADE EXTERNA ESPII - RODÍZIO	CH da Universidade Federal do Ceará - CE
233	ATIVIDADE EXTERNA ESPII - RODÍZIO	CH da Universidade Federal do Ceará - CE
233	ATIVIDADE EXTERNA ESPII - RODÍZIO	CH da Universidade Federal do Ceará - CE
233	ATIVIDADE EXTERNA ESPII - RODÍZIO	CH da Universidade Federal do Ceará - CE
233	ATIVIDADE EXTERNA ESPII - RODÍZIO	CH da Universidade Federal do Ceará - CE
233	ATIVIDADE EXTERNA ESPII - RODÍZIO	CH da Universidade Federal do Ceará - CE
233	ATIVIDADE EXTERNA ESPII - RODÍZIO	CH da Universidade Federal do Ceará - CE

233	ATIVIDADE EXTERNA ESPII - RODÍZIO	CH da Universidade Federal do Ceará - CE
233	ATIVIDADE EXTERNA ESPII - RODÍZIO	CH da Universidade Federal do Ceará - CE
233	ATIVIDADE EXTERNA ESPII - RODÍZIO	CH da Universidade Federal do Ceará - CE
233	ATIVIDADE EXTERNA ESPII - RODÍZIO	CH da Universidade Federal do Ceará - CE
233	ATIVIDADE EXTERNA ESPII - RODÍZIO	CH da Universidade Federal do Ceará - CE
233	ATIVIDADE EXTERNA ESPII - RODÍZIO	CH da Universidade Federal do Ceará - CE
233	ATIVIDADE EXTERNA ESPII - RODÍZIO	CH da Universidade Federal do Ceará - CE
233	ATIVIDADE EXTERNA ESPII - RODÍZIO	CH da Universidade Federal do Ceará - CE
233	ATIVIDADE EXTERNA ESPII - RODÍZIO	CH da Universidade Federal do Ceará - CE
233	ATIVIDADE EXTERNA ESPII - RODÍZIO	CH da Universidade Federal do Ceará - CE
233	ATIVIDADE EXTERNA ESPII - RODÍZIO	CH da Universidade Federal do Ceará - CE
233	ATIVIDADE EXTERNA ESPII - RODÍZIO	CH da Universidade Federal do Ceará - CE
233	ATIVIDADE EXTERNA ESPII - RODÍZIO	CH da Universidade Federal do Ceará - CE
233	ATIVIDADE EXTERNA ESPII - RODÍZIO	CH da Universidade Federal do Ceará - CE
233	ATIVIDADE EXTERNA ESPII - RODÍZIO	CH da Universidade Federal do Ceará - CE
233	ATIVIDADE EXTERNA ESPII - RODÍZIO	CH da Universidade Federal do Ceará - CE
233	ATIVIDADE EXTERNA ESPII - RODÍZIO	CH da Universidade Federal do Ceará - CE
233	ATIVIDADE EXTERNA ESPII - RODÍZIO	CH da Universidade Federal do Ceará - CE
233	ATIVIDADE EXTERNA ESPII - RODÍZIO	CH da Universidade Federal do Ceará - CE
233	ATIVIDADE EXTERNA ESPII - RODÍZIO	CH da Universidade Federal do Ceará - CE
233	ATIVIDADE EXTERNA ESPII - RODÍZIO	CH da Universidade Federal do Ceará - CE
233	ATIVIDADE EXTERNA ESPII - RODÍZIO	CH da Universidade Federal do Ceará - CE
233	ATIVIDADE EXTERNA ESPII - RODÍZIO	CH da Universidade Federal do Ceará - CE
233	ATIVIDADE EXTERNA ESPII - RODÍZIO	CH da Universidade Federal do Ceará - CE
233	ATIVIDADE EXTERNA ESPII - RODÍZIO	CH da Universidade Federal do Ceará - CE
233	ATIVIDADE EXTERNA ESPII - RODÍZIO	CH da Universidade Federal do Ceará - CE
233	ATIVIDADE EXTERNA ESPII - RODÍZIO	CH da Universidade Federal do Ceará - CE
233	ATIVIDADE EXTERNA ESPII - RODÍZIO	CH da Universidade Federal do Ceará - CE
233	ATIVIDADE EXTERNA ESPII - RODÍZIO	CH da Universidade Federal do Ceará - CE
233	ATIVIDADE EXTERNA ESPII - RODÍZIO	CH da Universidade Federal do Ceará - CE
233	ATIVIDADE EXTERNA ESPII - RODÍZIO	CH da Universidade Federal do Ceará - CE
233	ATIVIDADE EXTERNA ESPII - RODÍZIO	CH da Universidade Federal do Ceará - CE
233	ATIVIDADE EXTERNA ESPII - RODÍZIO	CH da Universidade Federal do Ceará - CE
233	ATIVIDADE EXTERNA ESPII - RODÍZIO	CH da Universidade Federal do Ceará - CE
233	ATIVIDADE EXTERNA ESPII - RODÍZIO	CH da Universidade Federal do Ceará - CE
233	ATIVIDADE EXTERNA ESPII - RODÍZIO	CH da Universidade Federal do Ceará - CE
233	ATIVIDADE EXTERNA ESPII - RODÍZIO	CH da Universidade Federal do Ceará - CE
233	ATIVIDADE EXTERNA ESPII - RODÍZIO	CH da Universidade Federal do Ceará - CE
233	ATIVIDADE EXTERNA ESPII - RODÍZIO	CH da Universidade Federal do Ceará - CE
233	ATIVIDADE EXTERNA ESPII - RODÍZIO	CH da Universidade Federal do Ceará - CE
233	ATIVIDADE EXTERNA ESPII - RODÍZIO	CH da Universidade Federal do Ceará - CE
233	ATIVIDADE EXTERNA ESPII - RODÍZIO	CH da Universidade Federal do Ceará - CE
233	ATIVIDADE EXTERNA ESPII - RODÍZIO	CH da Universidade Federal do Ceará - CE
233	ATIVIDADE EXTERNA ESPII - RODÍZIO	CH da Universidade Federal do Ceará - CE
233	ATIVIDADE EXTERNA ESPII - RODÍZIO	CH da Universidade Federal do Ceará - CE
233	ATIVIDADE EXTERNA ESPII - RODÍZIO	CH da Universidade Federal do Ceará - CE
233	ATIVIDADE EXTERNA ESPII - RODÍZIO	CH da Universidade Federal do Ceará - CE

233	ATIVIDADE EXTERNA ESPII - RODÍZIO	CH da Universidade Federal do Ceará - CE
233	ATIVIDADE EXTERNA ESPII - RODÍZIO	CH da Universidade Federal do Ceará - CE
233	ATIVIDADE EXTERNA ESPII - RODÍZIO	CH da Universidade Federal do Ceará - CE
233	ATIVIDADE EXTERNA ESPII - RODÍZIO	CH da Universidade Federal do Ceará - CE
233	ATIVIDADE EXTERNA ESPII - RODÍZIO	CH da Universidade Federal do Ceará - CE
233	ATIVIDADE EXTERNA ESPII - RODÍZIO	CH da Universidade Federal do Ceará - CE
233	ATIVIDADE EXTERNA ESPII - RODÍZIO	CH da Universidade Federal do Ceará - CE
233	ATIVIDADE EXTERNA ESPII - RODÍZIO	CH da Universidade Federal do Ceará - CE
233	ATIVIDADE EXTERNA ESPII - RODÍZIO	CH da Universidade Federal do Ceará - CE
233	ATIVIDADE EXTERNA ESPII - RODÍZIO	CH da Universidade Federal do Ceará - CE
233	ATIVIDADE EXTERNA ESPII - RODÍZIO	CH da Universidade Federal do Ceará - CE
233	ATIVIDADE EXTERNA ESPII - RODÍZIO	CH da Universidade Federal do Ceará - CE
233	ATIVIDADE EXTERNA ESPII - RODÍZIO	CH da Universidade Federal do Ceará - CE
233	ATIVIDADE EXTERNA ESPII - RODÍZIO	CH da Universidade Federal do Ceará - CE
233	ATIVIDADE EXTERNA ESPII - RODÍZIO	CH da Universidade Federal do Ceará - CE
233	ATIVIDADE EXTERNA ESPII - RODÍZIO	CH da Universidade Federal do Ceará - CE
233	ATIVIDADE EXTERNA ESPII - RODÍZIO	CH da Universidade Federal do Ceará - CE
233	ATIVIDADE EXTERNA ESPII - RODÍZIO	CH da Universidade Federal do Ceará - CE
233	ATIVIDADE EXTERNA ESPII - RODÍZIO	CH da Universidade Federal do Ceará - CE
233	ATIVIDADE EXTERNA ESPII - RODÍZIO	CH da Universidade Federal do Ceará - CE
233	ATIVIDADE EXTERNA ESPII - RODÍZIO	CH da Universidade Federal do Ceará - CE
233	ATIVIDADE EXTERNA ESPII - RODÍZIO	CH da Universidade Federal do Ceará - CE
233	ATIVIDADE EXTERNA ESPII - RODÍZIO	CH da Universidade Federal do Ceará - CE
233	ATIVIDADE EXTERNA ESPII - RODÍZIO	CH da Universidade Federal do Ceará - CE
233	ATIVIDADE EXTERNA ESPII - RODÍZIO	CH da Universidade Federal do Ceará - CE
233	ATIVIDADE EXTERNA ESPII - RODÍZIO	CH da Universidade Federal do Ceará - CE
233	ATIVIDADE EXTERNA ESPII - RODÍZIO	CH da Universidade Federal do Ceará - CE
233	ATIVIDADE EXTERNA ESPII - RODÍZIO	CH da Universidade Federal do Ceará - CE
233	ATIVIDADE EXTERNA ESPII - RODÍZIO	CH da Universidade Federal do Ceará - CE
233	ATIVIDADE EXTERNA ESPII - RODÍZIO	CH da Universidade Federal do Ceará - CE
233	ATIVIDADE EXTERNA ESPII - RODÍZIO	CH da Universidade Federal do Ceará - CE
233	ATIVIDADE EXTERNA ESPII - RODÍZIO	CH da Universidade Federal do Ceará - CE
233	ATIVIDADE EXTERNA ESPII - RODÍZIO	CH da Universidade Federal do Ceará - CE
233	ATIVIDADE EXTERNA ESPII - RODÍZIO	CH da Universidade Federal do Ceará - CE
233	ATIVIDADE EXTERNA ESPII - RODÍZIO	CH da Universidade Federal do Ceará - CE
233	ATIVIDADE EXTERNA ESPII - RODÍZIO	CH da Universidade Federal do Ceará - CE
233	ATIVIDADE EXTERNA ESPII - RODÍZIO	CH da Universidade Federal do Ceará - CE
233	ATIVIDADE EXTERNA ESPII - RODÍZIO	CH da Universidade Federal do Ceará - CE
233	ATIVIDADE EXTERNA ESPII - RODÍZIO	CH da Universidade Federal do Ceará - CE
233	ATIVIDADE EXTERNA ESPII - RODÍZIO	CH da Universidade Federal do Ceará - CE
233	ATIVIDADE EXTERNA ESPII - RODÍZIO	CH da Universidade Federal do Ceará - CE
233	ATIVIDADE EXTERNA ESPII - RODÍZIO	CH da Universidade Federal do Ceará - CE
233	ATIVIDADE EXTERNA ESPII - RODÍZIO	CH da Universidade Federal do Ceará - CE
233	ATIVIDADE EXTERNA ESPII - RODÍZIO	CH da Universidade Federal do Ceará - CE
233	ATIVIDADE EXTERNA ESPII - RODÍZIO	CH da Universidade Federal do Ceará - CE
233	ATIVIDADE EXTERNA ESPII - RODÍZIO	CH da Universidade Federal do Ceará - CE
233	ATIVIDADE EXTERNA ESPII - RODÍZIO	CH da Universidade Federal do Ceará - CE
233	ATIVIDADE EXTERNA ESPII - RODÍZIO	CH da Universidade Federal do Ceará - CE

233	ATIVIDADE EXTERNA ESPII - RODÍZIO	CH da Universidade Federal do Ceará - CE
233	ATIVIDADE EXTERNA ESPII - RODÍZIO	CH da Universidade Federal do Ceará - CE
233	ATIVIDADE EXTERNA ESPII - RODÍZIO	CH da Universidade Federal do Ceará - CE
233	ATIVIDADE EXTERNA ESPII - RODÍZIO	CH da Universidade Federal do Ceará - CE
233	ATIVIDADE EXTERNA ESPII - RODÍZIO	CH da Universidade Federal do Ceará - CE
233	ATIVIDADE EXTERNA ESPII - RODÍZIO	CH da Universidade Federal do Ceará - CE
233	ATIVIDADE EXTERNA ESPII - RODÍZIO	CH da Universidade Federal do Ceará - CE
233	ATIVIDADE EXTERNA ESPII - RODÍZIO	CH da Universidade Federal do Ceará - CE
233	ATIVIDADE EXTERNA ESPII - RODÍZIO	CH da Universidade Federal do Ceará - CE
233	ATIVIDADE EXTERNA ESPII - RODÍZIO	CH da Universidade Federal do Ceará - CE
233	ATIVIDADE EXTERNA ESPII - RODÍZIO	CH da Universidade Federal do Ceará - CE
233	ATIVIDADE EXTERNA ESPII - RODÍZIO	CH da Universidade Federal do Ceará - CE
233	ATIVIDADE EXTERNA ESPII - RODÍZIO	CH da Universidade Federal do Ceará - CE
233	ATIVIDADE EXTERNA ESPII - RODÍZIO	CH da Universidade Federal do Ceará - CE
233	ATIVIDADE EXTERNA ESPII - RODÍZIO	CH da Universidade Federal do Ceará - CE
233	ATIVIDADE EXTERNA ESPII - RODÍZIO	CH da Universidade Federal do Ceará - CE
233	ATIVIDADE EXTERNA ESPII - RODÍZIO	CH da Universidade Federal do Ceará - CE
233	ATIVIDADE EXTERNA ESPII - RODÍZIO	CH da Universidade Federal do Ceará - CE
233	ATIVIDADE EXTERNA ESPII - RODÍZIO	CH da Universidade Federal do Ceará - CE
233	ATIVIDADE EXTERNA ESPII - RODÍZIO	CH da Universidade Federal do Ceará - CE
233	ATIVIDADE EXTERNA ESPII - RODÍZIO	CH da Universidade Federal do Ceará - CE
233	ATIVIDADE EXTERNA ESPII - RODÍZIO	CH da Universidade Federal do Ceará - CE
233	ATIVIDADE EXTERNA ESPII - RODÍZIO	CH da Universidade Federal do Ceará - CE
233	ATIVIDADE EXTERNA ESPII - RODÍZIO	CH da Universidade Federal do Ceará - CE
233	ATIVIDADE EXTERNA ESPII - RODÍZIO	CH da Universidade Federal do Ceará - CE
233	ATIVIDADE EXTERNA ESPII - RODÍZIO	CH da Universidade Federal do Ceará - CE
233	ATIVIDADE EXTERNA ESPII - RODÍZIO	CH da Universidade Federal do Ceará - CE
233	ATIVIDADE EXTERNA ESPII - RODÍZIO	CH da Universidade Federal do Ceará - CE
233	ATIVIDADE EXTERNA ESPII - RODÍZIO	CH da Universidade Federal do Ceará - CE
233	ATIVIDADE EXTERNA ESPII - RODÍZIO	CH da Universidade Federal do Ceará - CE
233	ATIVIDADE EXTERNA ESPII - RODÍZIO	CH da Universidade Federal do Ceará - CE
233	ATIVIDADE EXTERNA ESPII - RODÍZIO	CH da Universidade Federal do Ceará - CE
233	ATIVIDADE EXTERNA ESPII - RODÍZIO	CH da Universidade Federal do Ceará - CE
233	ATIVIDADE EXTERNA ESPII - RODÍZIO	CH da Universidade Federal do Ceará - CE
233	ATIVIDADE EXTERNA ESPII - RODÍZIO	CH da Universidade Federal do Ceará - CE
233	ATIVIDADE EXTERNA ESPII - RODÍZIO	CH da Universidade Federal do Ceará - CE
233	ATIVIDADE EXTERNA ESPII - RODÍZIO	CH da Universidade Federal do Ceará - CE
233	ATIVIDADE EXTERNA ESPII - RODÍZIO	CH da Universidade Federal do Ceará - CE
233	ATIVIDADE EXTERNA ESPII - RODÍZIO	CH da Universidade Federal do Ceará - CE
233	ATIVIDADE EXTERNA ESPII - RODÍZIO	CH da Universidade Federal do Ceará - CE
233	ATIVIDADE EXTERNA ESPII - RODÍZIO	CH da Universidade Federal do Ceará - CE
233	ATIVIDADE EXTERNA ESPII - RODÍZIO	CH da Universidade Federal do Ceará - CE
233	ATIVIDADE EXTERNA ESPII - RODÍZIO	CH da Universidade Federal do Ceará - CE
233	ATIVIDADE EXTERNA ESPII - RODÍZIO	CH da Universidade Federal do Ceará - CE
233	ATIVIDADE EXTERNA ESPII - RODÍZIO	CH da Universidade Federal do Ceará - CE
233	ATIVIDADE EXTERNA ESPII - RODÍZIO	CH da Universidade Federal do Ceará - CE
233	ATIVIDADE EXTERNA ESPII - RODÍZIO	CH da Universidade Federal do Ceará - CE

233	ATIVIDADE EXTERNA ESPII - RODÍZIO	CH da Universidade Federal do Ceará - CE
233	ATIVIDADE EXTERNA ESPII - RODÍZIO	CH da Universidade Federal do Ceará - CE
233	ATIVIDADE EXTERNA ESPII - RODÍZIO	CH da Universidade Federal do Ceará - CE
233	ATIVIDADE EXTERNA ESPII - RODÍZIO	CH da Universidade Federal do Ceará - CE
233	ATIVIDADE EXTERNA ESPII - RODÍZIO	CH da Universidade Federal do Ceará - CE
233	ATIVIDADE EXTERNA ESPII - RODÍZIO	CH da Universidade Federal do Ceará - CE
233	ATIVIDADE EXTERNA ESPII - RODÍZIO	CH da Universidade Federal do Ceará - CE
233	ATIVIDADE EXTERNA ESPII - RODÍZIO	CH da Universidade Federal do Ceará - CE
233	ATIVIDADE EXTERNA ESPII - RODÍZIO	CH da Universidade Federal do Ceará - CE
233	ATIVIDADE EXTERNA ESPII - RODÍZIO	CH da Universidade Federal do Ceará - CE
233	ATIVIDADE EXTERNA ESPII - RODÍZIO	CH da Universidade Federal do Ceará - CE
233	ATIVIDADE EXTERNA ESPII - RODÍZIO	CH da Universidade Federal do Ceará - CE
233	ATIVIDADE EXTERNA ESPII - RODÍZIO	CH da Universidade Federal do Ceará - CE
233	ATIVIDADE EXTERNA ESPII - RODÍZIO	CH da Universidade Federal do Ceará - CE
233	ATIVIDADE EXTERNA ESPII - RODÍZIO	CH da Universidade Federal do Ceará - CE
233	ATIVIDADE EXTERNA ESPII - RODÍZIO	CH da Universidade Federal do Ceará - CE
233	ATIVIDADE EXTERNA ESPII - RODÍZIO	CH da Universidade Federal do Ceará - CE
233	ATIVIDADE EXTERNA ESPII - RODÍZIO	CH da Universidade Federal do Ceará - CE
233	ATIVIDADE EXTERNA ESPII - RODÍZIO	CH da Universidade Federal do Ceará - CE
233	ATIVIDADE EXTERNA ESPII - RODÍZIO	CH da Universidade Federal do Ceará - CE
233	ATIVIDADE EXTERNA ESPII - RODÍZIO	CH da Universidade Federal do Ceará - CE
233	ATIVIDADE EXTERNA ESPII - RODÍZIO	CH da Universidade Federal do Ceará - CE
233	ATIVIDADE EXTERNA ESPII - RODÍZIO	CH da Universidade Federal do Ceará - CE
233	ATIVIDADE EXTERNA ESPII - RODÍZIO	CH da Universidade Federal do Ceará - CE
233	ATIVIDADE EXTERNA ESPII - RODÍZIO	CH da Universidade Federal do Ceará - CE
233	ATIVIDADE EXTERNA ESPII - RODÍZIO	CH da Universidade Federal do Ceará - CE
233	ATIVIDADE EXTERNA ESPII - RODÍZIO	CH da Universidade Federal do Ceará - CE
233	ATIVIDADE EXTERNA ESPII - RODÍZIO	CH da Universidade Federal do Ceará - CE
233	ATIVIDADE EXTERNA ESPII - RODÍZIO	CH da Universidade Federal do Ceará - CE
233	ATIVIDADE EXTERNA ESPII - RODÍZIO	CH da Universidade Federal do Ceará - CE
233	ATIVIDADE EXTERNA ESPII - RODÍZIO	CH da Universidade Federal do Ceará - CE
233	ATIVIDADE EXTERNA ESPII - RODÍZIO	CH da Universidade Federal do Ceará - CE
233	ATIVIDADE EXTERNA ESPII - RODÍZIO	CH da Universidade Federal do Ceará - CE
233	ATIVIDADE EXTERNA ESPII - RODÍZIO	CH da Universidade Federal do Ceará - CE
233	ATIVIDADE EXTERNA ESPII - RODÍZIO	CH da Universidade Federal do Ceará - CE
233	ATIVIDADE EXTERNA ESPII - RODÍZIO	CH da Universidade Federal do Ceará - CE
233	ATIVIDADE EXTERNA ESPII - RODÍZIO	CH da Universidade Federal do Ceará - CE
233	ATIVIDADE EXTERNA ESPII - RODÍZIO	CH da Universidade Federal do Ceará - CE
233	ATIVIDADE EXTERNA ESPII - RODÍZIO	CH da Universidade Federal do Ceará - CE
233	ATIVIDADE EXTERNA ESPII - RODÍZIO	CH da Universidade Federal do Ceará - CE
233	ATIVIDADE EXTERNA ESPII - RODÍZIO	CH da Universidade Federal do Ceará - CE
233	ATIVIDADE EXTERNA ESPII - RODÍZIO	CH da Universidade Federal do Ceará - CE
233	ATIVIDADE EXTERNA ESPII - RODÍZIO	CH da Universidade Federal do Ceará - CE
233	ATIVIDADE EXTERNA ESPII - RODÍZIO	CH da Universidade Federal do Ceará - CE
233	ATIVIDADE EXTERNA ESPII - RODÍZIO	CH da Universidade Federal do Ceará - CE
233	ATIVIDADE EXTERNA ESPII - RODÍZIO	CH da Universidade Federal do Ceará - CE
233	ATIVIDADE EXTERNA ESPII - RODÍZIO	CH da Universidade Federal do Ceará - CE
233	ATIVIDADE EXTERNA ESPII - RODÍZIO	CH da Universidade Federal do Ceará - CE

233	ATIVIDADE EXTERNA ESPII - RODÍZIO	CH da Universidade Federal do Ceará - CE
233	ATIVIDADE EXTERNA ESPII - RODÍZIO	CH da Universidade Federal do Ceará - CE
233	ATIVIDADE EXTERNA ESPII - RODÍZIO	CH da Universidade Federal do Ceará - CE
233	ATIVIDADE EXTERNA ESPII - RODÍZIO	CH da Universidade Federal do Ceará - CE
233	ATIVIDADE EXTERNA ESPII - RODÍZIO	CH da Universidade Federal do Ceará - CE
233	ATIVIDADE EXTERNA ESPII - RODÍZIO	CH da Universidade Federal do Ceará - CE
233	ATIVIDADE EXTERNA ESPII - RODÍZIO	CH da Universidade Federal do Ceará - CE
233	ATIVIDADE EXTERNA ESPII - RODÍZIO	CH da Universidade Federal do Ceará - CE
233	ATIVIDADE EXTERNA ESPII - RODÍZIO	CH da Universidade Federal do Ceará - CE
233	ATIVIDADE EXTERNA ESPII - RODÍZIO	CH da Universidade Federal do Ceará - CE
233	ATIVIDADE EXTERNA ESPII - RODÍZIO	CH da Universidade Federal do Ceará - CE
233	ATIVIDADE EXTERNA ESPII - RODÍZIO	CH da Universidade Federal do Ceará - CE
233	ATIVIDADE EXTERNA ESPII - RODÍZIO	CH da Universidade Federal do Ceará - CE
233	ATIVIDADE EXTERNA ESPII - RODÍZIO	CH da Universidade Federal do Ceará - CE
233	ATIVIDADE EXTERNA ESPII - RODÍZIO	CH da Universidade Federal do Ceará - CE
233	ATIVIDADE EXTERNA ESPII - RODÍZIO	CH da Universidade Federal do Ceará - CE
233	ATIVIDADE EXTERNA ESPII - RODÍZIO	CH da Universidade Federal do Ceará - CE
233	ATIVIDADE EXTERNA ESPII - RODÍZIO	CH da Universidade Federal do Ceará - CE
233	ATIVIDADE EXTERNA ESPII - RODÍZIO	CH da Universidade Federal do Ceará - CE
233	ATIVIDADE EXTERNA ESPII - RODÍZIO	CH da Universidade Federal do Ceará - CE
233	ATIVIDADE EXTERNA ESPII - RODÍZIO	CH da Universidade Federal do Ceará - CE
233	ATIVIDADE EXTERNA ESPII - RODÍZIO	CH da Universidade Federal do Ceará - CE
233	ATIVIDADE EXTERNA ESPII - RODÍZIO	CH da Universidade Federal do Ceará - CE
233	ATIVIDADE EXTERNA ESPII - RODÍZIO	CH da Universidade Federal do Ceará - CE
233	ATIVIDADE EXTERNA ESPII - RODÍZIO	CH da Universidade Federal do Ceará - CE
233	ATIVIDADE EXTERNA ESPII - RODÍZIO	CH da Universidade Federal do Ceará - CE
233	ATIVIDADE EXTERNA ESPII - RODÍZIO	CH da Universidade Federal do Ceará - CE
233	ATIVIDADE EXTERNA ESPII - RODÍZIO	CH da Universidade Federal do Ceará - CE
233	ATIVIDADE EXTERNA ESPII - RODÍZIO	CH da Universidade Federal do Ceará - CE
233	ATIVIDADE EXTERNA ESPII - RODÍZIO	CH da Universidade Federal do Ceará - CE
233	ATIVIDADE EXTERNA ESPII - RODÍZIO	CH da Universidade Federal do Ceará - CE
233	ATIVIDADE EXTERNA ESPII - RODÍZIO	CH da Universidade Federal do Ceará - CE
233	ATIVIDADE EXTERNA ESPII - RODÍZIO	CH da Universidade Federal do Ceará - CE
233	ATIVIDADE EXTERNA ESPII - RODÍZIO	CH da Universidade Federal do Ceará - CE
233	ATIVIDADE EXTERNA ESPII - RODÍZIO	CH da Universidade Federal do Ceará - CE
233	ATIVIDADE EXTERNA ESPII - RODÍZIO	CH da Universidade Federal do Ceará - CE
233	ATIVIDADE EXTERNA ESPII - RODÍZIO	CH da Universidade Federal do Ceará - CE

233	ATIVIDADE EXTERNA ESPII - RODÍZIO	CH da Universidade Federal do Ceará - CE
233	ATIVIDADE EXTERNA ESPII - RODÍZIO	CH da Universidade Federal do Ceará - CE
233	ATIVIDADE EXTERNA ESPII - RODÍZIO	CH da Universidade Federal do Ceará - CE
233	ATIVIDADE EXTERNA ESPII - RODÍZIO	CH da Universidade Federal do Ceará - CE
233	ATIVIDADE EXTERNA ESPII - RODÍZIO	CH da Universidade Federal do Ceará - CE
233	ATIVIDADE EXTERNA ESPII - RODÍZIO	CH da Universidade Federal do Ceará - CE
233	ATIVIDADE EXTERNA ESPII - RODÍZIO	CH da Universidade Federal do Ceará - CE
233	ATIVIDADE EXTERNA ESPII - RODÍZIO	CH da Universidade Federal do Ceará - CE
233	ATIVIDADE EXTERNA ESPII - RODÍZIO	CH da Universidade Federal do Ceará - CE
233	ATIVIDADE EXTERNA ESPII - RODÍZIO	CH da Universidade Federal do Ceará - CE
233	ATIVIDADE EXTERNA ESPII - RODÍZIO	CH da Universidade Federal do Ceará - CE
233	ATIVIDADE EXTERNA ESPII - RODÍZIO	CH da Universidade Federal do Ceará - CE
233	ATIVIDADE EXTERNA ESPII - RODÍZIO	CH da Universidade Federal do Ceará - CE
233	ATIVIDADE EXTERNA ESPII - RODÍZIO	CH da Universidade Federal do Ceará - CE
233	ATIVIDADE EXTERNA ESPII - RODÍZIO	CH da Universidade Federal do Ceará - CE
233	ATIVIDADE EXTERNA ESPII - RODÍZIO	CH da Universidade Federal do Ceará - CE
233	ATIVIDADE EXTERNA ESPII - RODÍZIO	CH da Universidade Federal do Ceará - CE
233	ATIVIDADE EXTERNA ESPII - RODÍZIO	CH da Universidade Federal do Ceará - CE
233	ATIVIDADE EXTERNA ESPII - RODÍZIO	CH da Universidade Federal do Ceará - CE
233	ATIVIDADE EXTERNA ESPII - RODÍZIO	CH da Universidade Federal do Ceará - CE
233	ATIVIDADE EXTERNA ESPII - RODÍZIO	CH da Universidade Federal do Ceará - CE
233	ATIVIDADE EXTERNA ESPII - RODÍZIO	CH da Universidade Federal do Ceará - CE
233	ATIVIDADE EXTERNA ESPII - RODÍZIO	CH da Universidade Federal do Ceará - CE
233	ATIVIDADE EXTERNA ESPII - RODÍZIO	CH da Universidade Federal do Ceará - CE
233	ATIVIDADE EXTERNA ESPII - RODÍZIO	CH da Universidade Federal do Ceará - CE
233	ATIVIDADE EXTERNA ESPII - RODÍZIO	CH da Universidade Federal do Ceará - CE
233	ATIVIDADE EXTERNA ESPII - RODÍZIO	CH da Universidade Federal do Ceará - CE
233	ATIVIDADE EXTERNA ESPII - RODÍZIO	CH da Universidade Federal do Ceará - CE
233	ATIVIDADE EXTERNA ESPII - RODÍZIO	CH da Universidade Federal do Ceará - CE
233	ATIVIDADE EXTERNA ESPII - RODÍZIO	CH da Universidade Federal do Ceará - CE
233	ATIVIDADE EXTERNA ESPII - RODÍZIO	CH da Universidade Federal do Ceará - CE
233	ATIVIDADE EXTERNA ESPII - RODÍZIO	CH da Universidade Federal do Ceará - CE

233	ATIVIDADE EXTERNA ESPII - RODÍZIO	CH da Universidade Federal do Ceará - CE
233	ATIVIDADE EXTERNA ESPII - RODÍZIO	CH da Universidade Federal do Ceará - CE
233	ATIVIDADE EXTERNA ESPII - RODÍZIO	CH da Universidade Federal do Ceará - CE
233	ATIVIDADE EXTERNA ESPII - RODÍZIO	CH da Universidade Federal do Ceará - CE
233	ATIVIDADE EXTERNA ESPII - RODÍZIO	CH da Universidade Federal do Ceará - CE
233	ATIVIDADE EXTERNA ESPII - RODÍZIO	CH da Universidade Federal do Ceará - CE
233	ATIVIDADE EXTERNA ESPII - RODÍZIO	CH da Universidade Federal do Ceará - CE
233	ATIVIDADE EXTERNA ESPII - RODÍZIO	CH da Universidade Federal do Ceará - CE
233	ATIVIDADE EXTERNA ESPII - RODÍZIO	CH da Universidade Federal do Ceará - CE
233	ATIVIDADE EXTERNA ESPII - RODÍZIO	CH da Universidade Federal do Ceará - CE
233	ATIVIDADE EXTERNA ESPII - RODÍZIO	CH da Universidade Federal do Ceará - CE
233	ATIVIDADE EXTERNA ESPII - RODÍZIO	CH da Universidade Federal do Ceará - CE
233	ATIVIDADE EXTERNA ESPII - RODÍZIO	CH da Universidade Federal do Ceará - CE
233	ATIVIDADE EXTERNA ESPII - RODÍZIO	CH da Universidade Federal do Ceará - CE
233	ATIVIDADE EXTERNA ESPII - RODÍZIO	CH da Universidade Federal do Ceará - CE
233	ATIVIDADE EXTERNA ESPII - RODÍZIO	CH da Universidade Federal do Ceará - CE
233	ATIVIDADE EXTERNA ESPII - RODÍZIO	CH da Universidade Federal do Ceará - CE
233	ATIVIDADE EXTERNA ESPII - RODÍZIO	CH da Universidade Federal do Ceará - CE
233	ATIVIDADE EXTERNA ESPII - RODÍZIO	CH da Universidade Federal do Ceará - CE
233	ATIVIDADE EXTERNA ESPII - RODÍZIO	CH da Universidade Federal do Ceará - CE
233	ATIVIDADE EXTERNA ESPII - RODÍZIO	CH da Universidade Federal do Ceará - CE
233	ATIVIDADE EXTERNA ESPII - RODÍZIO	CH da Universidade Federal do Ceará - CE
233	ATIVIDADE EXTERNA ESPII - RODÍZIO	CH da Universidade Federal do Ceará - CE
233	ATIVIDADE EXTERNA ESPII - RODÍZIO	CH da Universidade Federal do Ceará - CE
233	ATIVIDADE EXTERNA ESPII - RODÍZIO	CH da Universidade Federal do Ceará - CE
233	ATIVIDADE EXTERNA ESPII - RODÍZIO	CH da Universidade Federal do Ceará - CE
233	ATIVIDADE EXTERNA ESPII - RODÍZIO	CH da Universidade Federal do Ceará - CE
233	ATIVIDADE EXTERNA ESPII - RODÍZIO	CH da Universidade Federal do Ceará - CE
233	ATIVIDADE EXTERNA ESPII - RODÍZIO	CH da Universidade Federal do Ceará - CE
233	ATIVIDADE EXTERNA ESPII - RODÍZIO	CH da Universidade Federal do Ceará - CE
233	ATIVIDADE EXTERNA ESPII - RODÍZIO	CH da Universidade Federal do Ceará - CE
233	ATIVIDADE EXTERNA ESPII - RODÍZIO	CH da Universidade Federal do Ceará - CE
233	ATIVIDADE EXTERNA ESPII - RODÍZIO	CH da Universidade Federal do Ceará - CE
233	ATIVIDADE EXTERNA ESPII - RODÍZIO	CH da Universidade Federal do Ceará - CE
233	ATIVIDADE EXTERNA ESPII - RODÍZIO	CH da Universidade Federal do Ceará - CE
233	ATIVIDADE EXTERNA ESPII - RODÍZIO	CH da Universidade Federal do Ceará - CE
233	ATIVIDADE EXTERNA ESPII - RODÍZIO	CH da Universidade Federal do Ceará - CE
233	ATIVIDADE EXTERNA ESPII - RODÍZIO	CH da Universidade Federal do Ceará - CE

233	ATIVIDADE EXTERNA ESPII - RODÍZIO	CH da Universidade Federal do Ceará - CE
233	ATIVIDADE EXTERNA ESPII - RODÍZIO	CH da Universidade Federal do Ceará - CE
233	ATIVIDADE EXTERNA ESPII - RODÍZIO	CH da Universidade Federal do Ceará - CE
233	ATIVIDADE EXTERNA ESPII - RODÍZIO	CH da Universidade Federal do Ceará - CE
233	ATIVIDADE EXTERNA ESPII - RODÍZIO	CH da Universidade Federal do Ceará - CE
233	ATIVIDADE EXTERNA ESPII - RODÍZIO	CH da Universidade Federal do Ceará - CE
233	ATIVIDADE EXTERNA ESPII - RODÍZIO	CH da Universidade Federal do Ceará - CE
233	ATIVIDADE EXTERNA ESPII - RODÍZIO	CH da Universidade Federal do Ceará - CE
233	ATIVIDADE EXTERNA ESPII - RODÍZIO	CH da Universidade Federal do Ceará - CE
233	ATIVIDADE EXTERNA ESPII - RODÍZIO	CH da Universidade Federal do Ceará - CE
233	ATIVIDADE EXTERNA ESPII - RODÍZIO	CH da Universidade Federal do Ceará - CE
233	ATIVIDADE EXTERNA ESPII - RODÍZIO	CH da Universidade Federal do Ceará - CE
233	ATIVIDADE EXTERNA ESPII - RODÍZIO	CH da Universidade Federal do Ceará - CE
233	ATIVIDADE EXTERNA ESPII - RODÍZIO	CH da Universidade Federal do Ceará - CE
233	ATIVIDADE EXTERNA ESPII - RODÍZIO	CH da Universidade Federal do Ceará - CE
233	ATIVIDADE EXTERNA ESPII - RODÍZIO	CH da Universidade Federal do Ceará - CE
233	ATIVIDADE EXTERNA ESPII - RODÍZIO	CH da Universidade Federal do Ceará - CE
233	ATIVIDADE EXTERNA ESPII - RODÍZIO	CH da Universidade Federal do Ceará - CE
233	ATIVIDADE EXTERNA ESPII - RODÍZIO	CH da Universidade Federal do Ceará - CE
233	ATIVIDADE EXTERNA ESPII - RODÍZIO	CH da Universidade Federal do Ceará - CE
233	ATIVIDADE EXTERNA ESPII - RODÍZIO	CH da Universidade Federal do Ceará - CE
233	ATIVIDADE EXTERNA ESPII - RODÍZIO	CH da Universidade Federal do Ceará - CE
233	ATIVIDADE EXTERNA ESPII - RODÍZIO	CH da Universidade Federal do Ceará - CE
233	ATIVIDADE EXTERNA ESPII - RODÍZIO	CH da Universidade Federal do Ceará - CE
233	ATIVIDADE EXTERNA ESPII - RODÍZIO	CH da Universidade Federal do Ceará - CE
233	ATIVIDADE EXTERNA ESPII - RODÍZIO	CH da Universidade Federal do Ceará - CE
233	ATIVIDADE EXTERNA ESPII - RODÍZIO	CH da Universidade Federal do Ceará - CE
233	ATIVIDADE EXTERNA ESPII - RODÍZIO	CH da Universidade Federal do Ceará - CE
233	ATIVIDADE EXTERNA ESPII - RODÍZIO	CH da Universidade Federal do Ceará - CE
233	ATIVIDADE EXTERNA ESPII - RODÍZIO	CH da Universidade Federal do Ceará - CE
233	ATIVIDADE EXTERNA ESPII - RODÍZIO	CH da Universidade Federal do Ceará - CE
233	ATIVIDADE EXTERNA ESPII - RODÍZIO	CH da Universidade Federal do Ceará - CE
233	ATIVIDADE EXTERNA ESPII - RODÍZIO	CH da Universidade Federal do Ceará - CE
233	ATIVIDADE EXTERNA ESPII - RODÍZIO	CH da Universidade Federal do Ceará - CE
233	ATIVIDADE EXTERNA ESPII - RODÍZIO	CH da Universidade Federal do Ceará - CE
233	ATIVIDADE EXTERNA ESPII - RODÍZIO	CH da Universidade Federal do Ceará - CE
233	ATIVIDADE EXTERNA ESPII - RODÍZIO	CH da Universidade Federal do Ceará - CE
233	ATIVIDADE EXTERNA ESPII - RODÍZIO	CH da Universidade Federal do Ceará - CE
233	ATIVIDADE EXTERNA ESPII - RODÍZIO	CH da Universidade Federal do Ceará - CE
233	ATIVIDADE EXTERNA ESPII - RODÍZIO	CH da Universidade Federal do Ceará - CE
233	ATIVIDADE EXTERNA ESPII - RODÍZIO	CH da Universidade Federal do Ceará - CE
233	ATIVIDADE EXTERNA ESPII - RODÍZIO	CH da Universidade Federal do Ceará - CE
233	ATIVIDADE EXTERNA ESPII - RODÍZIO	CH da Universidade Federal do Ceará - CE
233	ATIVIDADE EXTERNA ESPII - RODÍZIO	CH da Universidade Federal do Ceará - CE
233	ATIVIDADE EXTERNA ESPII - RODÍZIO	CH da Universidade Federal do Ceará - CE
233	ATIVIDADE EXTERNA ESPII - RODÍZIO	CH da Universidade Federal do Ceará - CE
233	ATIVIDADE EXTERNA ESPII - RODÍZIO	CH da Universidade Federal do Ceará - CE

233	ATIVIDADE EXTERNA ESPII - RODÍZIO	CH da Universidade Federal do Ceará - CE
233	ATIVIDADE EXTERNA ESPII - RODÍZIO	CH da Universidade Federal do Ceará - CE
233	ATIVIDADE EXTERNA ESPII - RODÍZIO	CH da Universidade Federal do Ceará - CE
233	ATIVIDADE EXTERNA ESPII - RODÍZIO	CH da Universidade Federal do Ceará - CE
233	ATIVIDADE EXTERNA ESPII - RODÍZIO	CH da Universidade Federal do Ceará - CE
233	ATIVIDADE EXTERNA ESPII - RODÍZIO	CH da Universidade Federal do Ceará - CE
233	ATIVIDADE EXTERNA ESPII - RODÍZIO	CH da Universidade Federal do Ceará - CE
233	ATIVIDADE EXTERNA ESPII - RODÍZIO	CH da Universidade Federal do Ceará - CE
233	ATIVIDADE EXTERNA ESPII - RODÍZIO	CH da Universidade Federal do Ceará - CE
233	ATIVIDADE EXTERNA ESPII - RODÍZIO	CH da Universidade Federal do Ceará - CE
233	ATIVIDADE EXTERNA ESPII - RODÍZIO	CH da Universidade Federal do Ceará - CE
233	ATIVIDADE EXTERNA ESPII - RODÍZIO	CH da Universidade Federal do Ceará - CE
233	ATIVIDADE EXTERNA ESPII - RODÍZIO	CH da Universidade Federal do Ceará - CE
233	ATIVIDADE EXTERNA ESPII - RODÍZIO	CH da Universidade Federal do Ceará - CE
233	ATIVIDADE EXTERNA ESPII - RODÍZIO	CH da Universidade Federal do Ceará - CE
233	ATIVIDADE EXTERNA ESPII - RODÍZIO	CH da Universidade Federal do Ceará - CE
233	ATIVIDADE EXTERNA ESPII - RODÍZIO	CH da Universidade Federal do Ceará - CE
233	ATIVIDADE EXTERNA ESPII - RODÍZIO	CH da Universidade Federal do Ceará - CE
233	ATIVIDADE EXTERNA ESPII - RODÍZIO	CH da Universidade Federal do Ceará - CE
235	Afastamento Portaria n 454 art 3	CH da Universidade Federal do Ceará - CE
235	Afastamento Portaria n 454 art 3	CH da Universidade Federal do Ceará - CE
235	Afastamento Portaria n 454 art 3	CH da Universidade Federal do Ceará - CE
235	Afastamento Portaria n 454 art 3	CH da Universidade Federal do Ceará - CE
235	Afastamento Portaria n 454 art 3	CH da Universidade Federal do Ceará - CE
235	Afastamento Portaria n 454 art 3	CH da Universidade Federal do Ceará - CE
235	Afastamento Portaria n 454 art 3	CH da Universidade Federal do Ceará - CE
235	Afastamento Portaria n 454 art 3	CH da Universidade Federal do Ceará - CE
235	Afastamento Portaria n 454 art 3	CH da Universidade Federal do Ceará - CE
235	Afastamento Portaria n 454 art 3	CH da Universidade Federal do Ceará - CE
235	Afastamento Portaria n 454 art 3	CH da Universidade Federal do Ceará - CE
235	Afastamento Portaria n 454 art 3	CH da Universidade Federal do Ceará - CE
235	Afastamento Portaria n 454 art 3	CH da Universidade Federal do Ceará - CE
235	Afastamento Portaria n 454 art 3	CH da Universidade Federal do Ceará - CE
235	Afastamento Portaria n 454 art 3	CH da Universidade Federal do Ceará - CE
235	Afastamento Portaria n 454 art 3	CH da Universidade Federal do Ceará - CE
235	Afastamento Portaria n 454 art 3	CH da Universidade Federal do Ceará - CE
235	Afastamento Portaria n 454 art 3	CH da Universidade Federal do Ceará - CE
235	Afastamento Portaria n 454 art 3	CH da Universidade Federal do Ceará - CE
235	Afastamento Portaria n 454 art 3	CH da Universidade Federal do Ceará - CE
235	Afastamento Portaria n 454 art 3	CH da Universidade Federal do Ceará - CE
235	Afastamento Portaria n 454 art 3	CH da Universidade Federal do Ceará - CE
235	Afastamento Portaria n 454 art 3	CH da Universidade Federal do Ceará - CE
235	Afastamento Portaria n 454 art 3	CH da Universidade Federal do Ceará - CE

235	Afastamento Portaria n 454 art 3	CH da Universidade Federal do Ceará - CE
235	Afastamento Portaria n 454 art 3	CH da Universidade Federal do Ceará - CE
235	Afastamento Portaria n 454 art 3	CH da Universidade Federal do Ceará - CE
235	Afastamento Portaria n 454 art 3	CH da Universidade Federal do Ceará - CE
235	Afastamento Portaria n 454 art 3	CH da Universidade Federal do Ceará - CE
235	Afastamento Portaria n 454 art 3	CH da Universidade Federal do Ceará - CE
235	Afastamento Portaria n 454 art 3	CH da Universidade Federal do Ceará - CE
235	Afastamento Portaria n 454 art 3	CH da Universidade Federal do Ceará - CE
235	Afastamento Portaria n 454 art 3	CH da Universidade Federal do Ceará - CE
235	Afastamento Portaria n 454 art 3	CH da Universidade Federal do Ceará - CE
235	Afastamento Portaria n 454 art 3	CH da Universidade Federal do Ceará - CE
235	Afastamento Portaria n 454 art 3	CH da Universidade Federal do Ceará - CE
235	Afastamento Portaria n 454 art 3	CH da Universidade Federal do Ceará - CE
235	Afastamento Portaria n 454 art 3	CH da Universidade Federal do Ceará - CE
235	Afastamento Portaria n 454 art 3	CH da Universidade Federal do Ceará - CE
235	Afastamento Portaria n 454 art 3	CH da Universidade Federal do Ceará - CE
235	Afastamento Portaria n 454 art 3	CH da Universidade Federal do Ceará - CE
235	Afastamento Portaria n 454 art 3	CH da Universidade Federal do Ceará - CE
235	Afastamento Portaria n 454 art 3	CH da Universidade Federal do Ceará - CE
235	Afastamento Portaria n 454 art 3	CH da Universidade Federal do Ceará - CE
235	Afastamento Portaria n 454 art 3	CH da Universidade Federal do Ceará - CE
235	Afastamento Portaria n 454 art 3	CH da Universidade Federal do Ceará - CE
235	Afastamento Portaria n 454 art 3	CH da Universidade Federal do Ceará - CE
235	Afastamento Portaria n 454 art 3	CH da Universidade Federal do Ceará - CE
235	Afastamento Portaria n 454 art 3	CH da Universidade Federal do Ceará - CE
235	Afastamento Portaria n 454 art 3	CH da Universidade Federal do Ceará - CE
235	Afastamento Portaria n 454 art 3	CH da Universidade Federal do Ceará - CE
235	Afastamento Portaria n 454 art 3	CH da Universidade Federal do Ceará - CE
235	Afastamento Portaria n 454 art 3	CH da Universidade Federal do Ceará - CE
235	Afastamento Portaria n 454 art 3	CH da Universidade Federal do Ceará - CE
235	Afastamento Portaria n 454 art 3	CH da Universidade Federal do Ceará - CE
235	Afastamento Portaria n 454 art 3	CH da Universidade Federal do Ceará - CE
235	Afastamento Portaria n 454 art 3	CH da Universidade Federal do Ceará - CE
235	Afastamento Portaria n 454 art 3	CH da Universidade Federal do Ceará - CE
235	Afastamento Portaria n 454 art 3	CH da Universidade Federal do Ceará - CE
235	Afastamento Portaria n 454 art 3	CH da Universidade Federal do Ceará - CE
235	Afastamento Portaria n 454 art 3	CH da Universidade Federal do Ceará - CE
235	Afastamento Portaria n 454 art 3	CH da Universidade Federal do Ceará - CE
235	Afastamento Portaria n 454 art 3	CH da Universidade Federal do Ceará - CE
235	Afastamento Portaria n 454 art 3	CH da Universidade Federal do Ceará - CE
235	Afastamento Portaria n 454 art 3	CH da Universidade Federal do Ceará - CE
235	Afastamento Portaria n 454 art 3	CH da Universidade Federal do Ceará - CE
235	Afastamento Portaria n 454 art 3	CH da Universidade Federal do Ceará - CE
235	Afastamento Portaria n 454 art 3	CH da Universidade Federal do Ceará - CE
235	Afastamento Portaria n 454 art 3	CH da Universidade Federal do Ceará - CE
235	Afastamento Portaria n 454 art 3	CH da Universidade Federal do Ceará - CE

235	Afastamento Portaria n 454 art 3	CH da Universidade Federal do Ceará - CE
235	Afastamento Portaria n 454 art 3	CH da Universidade Federal do Ceará - CE
235	Afastamento Portaria n 454 art 3	CH da Universidade Federal do Ceará - CE
235	Afastamento Portaria n 454 art 3	CH da Universidade Federal do Ceará - CE
235	Afastamento Portaria n 454 art 3	CH da Universidade Federal do Ceará - CE
235	Afastamento Portaria n 454 art 3	CH da Universidade Federal do Ceará - CE
235	Afastamento Portaria n 454 art 3	CH da Universidade Federal do Ceará - CE
235	Afastamento Portaria n 454 art 3	CH da Universidade Federal do Ceará - CE
235	Afastamento Portaria n 454 art 3	CH da Universidade Federal do Ceará - CE
235	Afastamento Portaria n 454 art 3	CH da Universidade Federal do Ceará - CE
235	Afastamento Portaria n 454 art 3	CH da Universidade Federal do Ceará - CE
235	Afastamento Portaria n 454 art 3	CH da Universidade Federal do Ceará - CE
235	Afastamento Portaria n 454 art 3	CH da Universidade Federal do Ceará - CE
235	Afastamento Portaria n 454 art 3	CH da Universidade Federal do Ceará - CE
235	Afastamento Portaria n 454 art 3	CH da Universidade Federal do Ceará - CE
235	Afastamento Portaria n 454 art 3	CH da Universidade Federal do Ceará - CE
235	Afastamento Portaria n 454 art 3	CH da Universidade Federal do Ceará - CE
235	Afastamento Portaria n 454 art 3	CH da Universidade Federal do Ceará - CE
235	Afastamento Portaria n 454 art 3	CH da Universidade Federal do Ceará - CE
235	Afastamento Portaria n 454 art 3	CH da Universidade Federal do Ceará - CE
235	Afastamento Portaria n 454 art 3	CH da Universidade Federal do Ceará - CE
235	Afastamento Portaria n 454 art 3	CH da Universidade Federal do Ceará - CE
235	Afastamento Portaria n 454 art 3	CH da Universidade Federal do Ceará - CE
235	Afastamento Portaria n 454 art 3	CH da Universidade Federal do Ceará - CE
235	Afastamento Portaria n 454 art 3	CH da Universidade Federal do Ceará - CE
235	Afastamento Portaria n 454 art 3	CH da Universidade Federal do Ceará - CE
235	Afastamento Portaria n 454 art 3	CH da Universidade Federal do Ceará - CE
235	Afastamento Portaria n 454 art 3	CH da Universidade Federal do Ceará - CE
235	Afastamento Portaria n 454 art 3	CH da Universidade Federal do Ceará - CE
235	Afastamento Portaria n 454 art 3	CH da Universidade Federal do Ceará - CE
235	Afastamento Portaria n 454 art 3	CH da Universidade Federal do Ceará - CE
235	Afastamento Portaria n 454 art 3	CH da Universidade Federal do Ceará - CE
235	Afastamento Portaria n 454 art 3	CH da Universidade Federal do Ceará - CE
235	Afastamento Portaria n 454 art 3	CH da Universidade Federal do Ceará - CE
235	Afastamento Portaria n 454 art 3	CH da Universidade Federal do Ceará - CE
235	Afastamento Portaria n 454 art 3	CH da Universidade Federal do Ceará - CE
235	Afastamento Portaria n 454 art 3	CH da Universidade Federal do Ceará - CE
235	Afastamento Portaria n 454 art 3	CH da Universidade Federal do Ceará - CE
235	Afastamento Portaria n 454 art 3	CH da Universidade Federal do Ceará - CE
235	Afastamento Portaria n 454 art 3	CH da Universidade Federal do Ceará - CE
235	Afastamento Portaria n 454 art 3	CH da Universidade Federal do Ceará - CE
235	Afastamento Portaria n 454 art 3	CH da Universidade Federal do Ceará - CE
235	Afastamento Portaria n 454 art 3	CH da Universidade Federal do Ceará - CE
235	Afastamento Portaria n 454 art 3	CH da Universidade Federal do Ceará - CE

235	Afastamento Portaria n 454 art 3	CH da Universidade Federal do Ceará - CE
235	Afastamento Portaria n 454 art 3	CH da Universidade Federal do Ceará - CE
235	Afastamento Portaria n 454 art 3	CH da Universidade Federal do Ceará - CE
235	Afastamento Portaria n 454 art 3	CH da Universidade Federal do Ceará - CE
235	Afastamento Portaria n 454 art 3	CH da Universidade Federal do Ceará - CE
235	Afastamento Portaria n 454 art 3	CH da Universidade Federal do Ceará - CE
235	Afastamento Portaria n 454 art 3	CH da Universidade Federal do Ceará - CE
235	Afastamento Portaria n 454 art 3	CH da Universidade Federal do Ceará - CE
235	Afastamento Portaria n 454 art 3	CH da Universidade Federal do Ceará - CE
235	Afastamento Portaria n 454 art 3	CH da Universidade Federal do Ceará - CE
235	Afastamento Portaria n 454 art 3	CH da Universidade Federal do Ceará - CE
235	Afastamento Portaria n 454 art 3	CH da Universidade Federal do Ceará - CE
235	Afastamento Portaria n 454 art 3	CH da Universidade Federal do Ceará - CE
235	Afastamento Portaria n 454 art 3	CH da Universidade Federal do Ceará - CE
235	Afastamento Portaria n 454 art 3	CH da Universidade Federal do Ceará - CE
235	Afastamento Portaria n 454 art 3	CH da Universidade Federal do Ceará - CE
235	Afastamento Portaria n 454 art 3	CH da Universidade Federal do Ceará - CE
235	Afastamento Portaria n 454 art 3	CH da Universidade Federal do Ceará - CE
235	Afastamento Portaria n 454 art 3	CH da Universidade Federal do Ceará - CE
235	Afastamento Portaria n 454 art 3	CH da Universidade Federal do Ceará - CE
235	Afastamento Portaria n 454 art 3	CH da Universidade Federal do Ceará - CE
235	Afastamento Portaria n 454 art 3	CH da Universidade Federal do Ceará - CE
235	Afastamento Portaria n 454 art 3	CH da Universidade Federal do Ceará - CE
235	Afastamento Portaria n 454 art 3	CH da Universidade Federal do Ceará - CE
235	Afastamento Portaria n 454 art 3	CH da Universidade Federal do Ceará - CE
235	Afastamento Portaria n 454 art 3	CH da Universidade Federal do Ceará - CE
235	Afastamento Portaria n 454 art 3	CH da Universidade Federal do Ceará - CE
235	Afastamento Portaria n 454 art 3	CH da Universidade Federal do Ceará - CE
235	Afastamento Portaria n 454 art 3	CH da Universidade Federal do Ceará - CE
235	Afastamento Portaria n 454 art 3	CH da Universidade Federal do Ceará - CE
235	Afastamento Portaria n 454 art 3	CH da Universidade Federal do Ceará - CE
235	Afastamento Portaria n 454 art 3	CH da Universidade Federal do Ceará - CE
235	Afastamento Portaria n 454 art 3	CH da Universidade Federal do Ceará - CE
235	Afastamento Portaria n 454 art 3	CH da Universidade Federal do Ceará - CE
235	Afastamento Portaria n 454 art 3	CH da Universidade Federal do Ceará - CE
235	Afastamento Portaria n 454 art 3	CH da Universidade Federal do Ceará - CE
235	Afastamento Portaria n 454 art 3	CH da Universidade Federal do Ceará - CE
235	Afastamento Portaria n 454 art 3	CH da Universidade Federal do Ceará - CE
235	Afastamento Portaria n 454 art 3	CH da Universidade Federal do Ceará - CE
235	Afastamento Portaria n 454 art 3	CH da Universidade Federal do Ceará - CE
235	Afastamento Portaria n 454 art 3	CH da Universidade Federal do Ceará - CE

236	Afastamento Portaria n 454 art. 2 Único	CH da Universidade Federal do Ceará - CE
236	Afastamento Portaria n 454 art. 2 Único	CH da Universidade Federal do Ceará - CE
236	Afastamento Portaria n 454 art. 2 Único	CH da Universidade Federal do Ceará - CE
236	Afastamento Portaria n 454 art. 2 Único	CH da Universidade Federal do Ceará - CE
236	Afastamento Portaria n 454 art. 2 Único	CH da Universidade Federal do Ceará - CE
236	Afastamento Portaria n 454 art. 2 Único	CH da Universidade Federal do Ceará - CE
236	Afastamento Portaria n 454 art. 2 Único	CH da Universidade Federal do Ceará - CE
236	Afastamento Portaria n 454 art. 2 Único	CH da Universidade Federal do Ceará - CE
236	Afastamento Portaria n 454 art. 2 Único	CH da Universidade Federal do Ceará - CE
236	Afastamento Portaria n 454 art. 2 Único	CH da Universidade Federal do Ceará - CE
236	Afastamento Portaria n 454 art. 2 Único	CH da Universidade Federal do Ceará - CE
236	Afastamento Portaria n 454 art. 2 Único	CH da Universidade Federal do Ceará - CE
236	Afastamento Portaria n 454 art. 2 Único	CH da Universidade Federal do Ceará - CE
236	Afastamento Portaria n 454 art. 2 Único	CH da Universidade Federal do Ceará - CE
236	Afastamento Portaria n 454 art. 2 Único	CH da Universidade Federal do Ceará - CE
236	Afastamento Portaria n 454 art. 2 Único	CH da Universidade Federal do Ceará - CE
236	Afastamento Portaria n 454 art. 2 Único	CH da Universidade Federal do Ceará - CE
236	Afastamento Portaria n 454 art. 2 Único	CH da Universidade Federal do Ceará - CE
236	Afastamento Portaria n 454 art. 2 Único	CH da Universidade Federal do Ceará - CE
236	Afastamento Portaria n 454 art. 2 Único	CH da Universidade Federal do Ceará - CE
236	Afastamento Portaria n 454 art. 2 Único	CH da Universidade Federal do Ceará - CE
236	Afastamento Portaria n 454 art. 2 Único	CH da Universidade Federal do Ceará - CE
236	Afastamento Portaria n 454 art. 2 Único	CH da Universidade Federal do Ceará - CE
236	Afastamento Portaria n 454 art. 2 Único	CH da Universidade Federal do Ceará - CE
236	Afastamento Portaria n 454 art. 2 Único	CH da Universidade Federal do Ceará - CE
236	Afastamento Portaria n 454 art. 2 Único	CH da Universidade Federal do Ceará - CE
236	Afastamento Portaria n 454 art. 2 Único	CH da Universidade Federal do Ceará - CE
236	Afastamento Portaria n 454 art. 2 Único	CH da Universidade Federal do Ceará - CE
236	Afastamento Portaria n 454 art. 2 Único	CH da Universidade Federal do Ceará - CE
236	Afastamento Portaria n 454 art. 2 Único	CH da Universidade Federal do Ceará - CE
236	Afastamento Portaria n 454 art. 2 Único	CH da Universidade Federal do Ceará - CE
236	Afastamento Portaria n 454 art. 2 Único	CH da Universidade Federal do Ceará - CE
236	Afastamento Portaria n 454 art. 2 Único	CH da Universidade Federal do Ceará - CE
236	Afastamento Portaria n 454 art. 2 Único	CH da Universidade Federal do Ceará - CE
236	Afastamento Portaria n 454 art. 2 Único	CH da Universidade Federal do Ceará - CE
236	Afastamento Portaria n 454 art. 2 Único	CH da Universidade Federal do Ceará - CE
236	Afastamento Portaria n 454 art. 2 Único	CH da Universidade Federal do Ceará - CE
236	Afastamento Portaria n 454 art. 2 Único	CH da Universidade Federal do Ceará - CE
236	Afastamento Portaria n 454 art. 2 Único	CH da Universidade Federal do Ceará - CE
236	Afastamento Portaria n 454 art. 2 Único	CH da Universidade Federal do Ceará - CE
236	Afastamento Portaria n 454 art. 2 Único	CH da Universidade Federal do Ceará - CE
236	Afastamento Portaria n 454 art. 2 Único	CH da Universidade Federal do Ceará - CE
236	Afastamento Portaria n 454 art. 2 Único	CH da Universidade Federal do Ceará - CE
236	Afastamento Portaria n 454 art. 2 Único	CH da Universidade Federal do Ceará - CE
236	Afastamento Portaria n 454 art. 2 Único	CH da Universidade Federal do Ceará - CE
236	Afastamento Portaria n 454 art. 2 Único	CH da Universidade Federal do Ceará - CE
236	Afastamento Portaria n 454 art. 2 Único	CH da Universidade Federal do Ceará - CE
236	Afastamento Portaria n 454 art. 2 Único	CH da Universidade Federal do Ceará - CE

236	Afastamento Portaria n 454 art. 2 Único	CH da Universidade Federal do Ceará - CE
236	Afastamento Portaria n 454 art. 2 Único	CH da Universidade Federal do Ceará - CE
236	Afastamento Portaria n 454 art. 2 Único	CH da Universidade Federal do Ceará - CE
236	Afastamento Portaria n 454 art. 2 Único	CH da Universidade Federal do Ceará - CE
236	Afastamento Portaria n 454 art. 2 Único	CH da Universidade Federal do Ceará - CE
236	Afastamento Portaria n 454 art. 2 Único	CH da Universidade Federal do Ceará - CE
236	Afastamento Portaria n 454 art. 2 Único	CH da Universidade Federal do Ceará - CE
236	Afastamento Portaria n 454 art. 2 Único	CH da Universidade Federal do Ceará - CE
236	Afastamento Portaria n 454 art. 2 Único	CH da Universidade Federal do Ceará - CE
236	Afastamento Portaria n 454 art. 2 Único	CH da Universidade Federal do Ceará - CE
236	Afastamento Portaria n 454 art. 2 Único	CH da Universidade Federal do Ceará - CE
236	Afastamento Portaria n 454 art. 2 Único	CH da Universidade Federal do Ceará - CE
236	Afastamento Portaria n 454 art. 2 Único	CH da Universidade Federal do Ceará - CE
236	Afastamento Portaria n 454 art. 2 Único	CH da Universidade Federal do Ceará - CE
236	Afastamento Portaria n 454 art. 2 Único	CH da Universidade Federal do Ceará - CE
236	Afastamento Portaria n 454 art. 2 Único	CH da Universidade Federal do Ceará - CE
236	Afastamento Portaria n 454 art. 2 Único	CH da Universidade Federal do Ceará - CE
236	Afastamento Portaria n 454 art. 2 Único	CH da Universidade Federal do Ceará - CE
236	Afastamento Portaria n 454 art. 2 Único	CH da Universidade Federal do Ceará - CE
236	Afastamento Portaria n 454 art. 2 Único	CH da Universidade Federal do Ceará - CE
236	Afastamento Portaria n 454 art. 2 Único	CH da Universidade Federal do Ceará - CE
236	Afastamento Portaria n 454 art. 2 Único	CH da Universidade Federal do Ceará - CE
236	Afastamento Portaria n 454 art. 2 Único	CH da Universidade Federal do Ceará - CE
236	Afastamento Portaria n 454 art. 2 Único	CH da Universidade Federal do Ceará - CE
236	Afastamento Portaria n 454 art. 2 Único	CH da Universidade Federal do Ceará - CE
236	Afastamento Portaria n 454 art. 2 Único	CH da Universidade Federal do Ceará - CE
236	Afastamento Portaria n 454 art. 2 Único	CH da Universidade Federal do Ceará - CE
236	Afastamento Portaria n 454 art. 2 Único	CH da Universidade Federal do Ceará - CE
236	Afastamento Portaria n 454 art. 2 Único	CH da Universidade Federal do Ceará - CE
236	Afastamento Portaria n 454 art. 2 Único	CH da Universidade Federal do Ceará - CE
236	Afastamento Portaria n 454 art. 2 Único	CH da Universidade Federal do Ceará - CE
236	Afastamento Portaria n 454 art. 2 Único	CH da Universidade Federal do Ceará - CE
236	Afastamento Portaria n 454 art. 2 Único	CH da Universidade Federal do Ceará - CE
236	Afastamento Portaria n 454 art. 2 Único	CH da Universidade Federal do Ceará - CE
236	Afastamento Portaria n 454 art. 2 Único	CH da Universidade Federal do Ceará - CE
236	Afastamento Portaria n 454 art. 2 Único	CH da Universidade Federal do Ceará - CE
236	Afastamento Portaria n 454 art. 2 Único	CH da Universidade Federal do Ceará - CE
236	Afastamento Portaria n 454 art. 2 Único	CH da Universidade Federal do Ceará - CE
236	Afastamento Portaria n 454 art. 2 Único	CH da Universidade Federal do Ceará - CE
236	Afastamento Portaria n 454 art. 2 Único	CH da Universidade Federal do Ceará - CE
236	Afastamento Portaria n 454 art. 2 Único	CH da Universidade Federal do Ceará - CE
236	Afastamento Portaria n 454 art. 2 Único	CH da Universidade Federal do Ceará - CE
236	Afastamento Portaria n 454 art. 2 Único	CH da Universidade Federal do Ceará - CE
236	Afastamento Portaria n 454 art. 2 Único	CH da Universidade Federal do Ceará - CE
236	Afastamento Portaria n 454 art. 2 Único	CH da Universidade Federal do Ceará - CE
236	Afastamento Portaria n 454 art. 2 Único	CH da Universidade Federal do Ceará - CE
236	Afastamento Portaria n 454 art. 2 Único	CH da Universidade Federal do Ceará - CE
236	Afastamento Portaria n 454 art. 2 Único	CH da Universidade Federal do Ceará - CE

236	Afastamento Portaria n 454 art. 2 Único	CH da Universidade Federal do Ceará - CE
236	Afastamento Portaria n 454 art. 2 Único	CH da Universidade Federal do Ceará - CE
236	Afastamento Portaria n 454 art. 2 Único	CH da Universidade Federal do Ceará - CE
236	Afastamento Portaria n 454 art. 2 Único	CH da Universidade Federal do Ceará - CE
236	Afastamento Portaria n 454 art. 2 Único	CH da Universidade Federal do Ceará - CE
236	Afastamento Portaria n 454 art. 2 Único	CH da Universidade Federal do Ceará - CE
236	Afastamento Portaria n 454 art. 2 Único	CH da Universidade Federal do Ceará - CE
236	Afastamento Portaria n 454 art. 2 Único	CH da Universidade Federal do Ceará - CE
236	Afastamento Portaria n 454 art. 2 Único	CH da Universidade Federal do Ceará - CE
236	Afastamento Portaria n 454 art. 2 Único	CH da Universidade Federal do Ceará - CE
236	Afastamento Portaria n 454 art. 2 Único	CH da Universidade Federal do Ceará - CE
236	Afastamento Portaria n 454 art. 2 Único	CH da Universidade Federal do Ceará - CE
236	Afastamento Portaria n 454 art. 2 Único	CH da Universidade Federal do Ceará - CE
236	Afastamento Portaria n 454 art. 2 Único	CH da Universidade Federal do Ceará - CE
236	Afastamento Portaria n 454 art. 2 Único	CH da Universidade Federal do Ceará - CE
236	Afastamento Portaria n 454 art. 2 Único	CH da Universidade Federal do Ceará - CE
236	Afastamento Portaria n 454 art. 2 Único	CH da Universidade Federal do Ceará - CE
236	Afastamento Portaria n 454 art. 2 Único	CH da Universidade Federal do Ceará - CE
236	Afastamento Portaria n 454 art. 2 Único	CH da Universidade Federal do Ceará - CE
236	Afastamento Portaria n 454 art. 2 Único	CH da Universidade Federal do Ceará - CE
236	Afastamento Portaria n 454 art. 2 Único	CH da Universidade Federal do Ceará - CE
236	Afastamento Portaria n 454 art. 2 Único	CH da Universidade Federal do Ceará - CE
236	Afastamento Portaria n 454 art. 2 Único	CH da Universidade Federal do Ceará - CE
236	Afastamento Portaria n 454 art. 2 Único	CH da Universidade Federal do Ceará - CE
236	Afastamento Portaria n 454 art. 2 Único	CH da Universidade Federal do Ceará - CE
236	Afastamento Portaria n 454 art. 2 Único	CH da Universidade Federal do Ceará - CE
236	Afastamento Portaria n 454 art. 2 Único	CH da Universidade Federal do Ceará - CE
236	Afastamento Portaria n 454 art. 2 Único	CH da Universidade Federal do Ceará - CE
236	Afastamento Portaria n 454 art. 2 Único	CH da Universidade Federal do Ceará - CE
236	Afastamento Portaria n 454 art. 2 Único	CH da Universidade Federal do Ceará - CE
236	Afastamento Portaria n 454 art. 2 Único	CH da Universidade Federal do Ceará - CE
236	Afastamento Portaria n 454 art. 2 Único	CH da Universidade Federal do Ceará - CE
236	Afastamento Portaria n 454 art. 2 Único	CH da Universidade Federal do Ceará - CE
236	Afastamento Portaria n 454 art. 2 Único	CH da Universidade Federal do Ceará - CE
236	Afastamento Portaria n 454 art. 2 Único	CH da Universidade Federal do Ceará - CE
236	Afastamento Portaria n 454 art. 2 Único	CH da Universidade Federal do Ceará - CE
236	Afastamento Portaria n 454 art. 2 Único	CH da Universidade Federal do Ceará - CE
236	Afastamento Portaria n 454 art. 2 Único	CH da Universidade Federal do Ceará - CE
236	Afastamento Portaria n 454 art. 2 Único	CH da Universidade Federal do Ceará - CE
236	Afastamento Portaria n 454 art. 2 Único	CH da Universidade Federal do Ceará - CE
236	Afastamento Portaria n 454 art. 2 Único	CH da Universidade Federal do Ceará - CE
236	Afastamento Portaria n 454 art. 2 Único	CH da Universidade Federal do Ceará - CE
236	Afastamento Portaria n 454 art. 2 Único	CH da Universidade Federal do Ceará - CE
236	Afastamento Portaria n 454 art. 2 Único	CH da Universidade Federal do Ceará - CE
236	Afastamento Portaria n 454 art. 2 Único	CH da Universidade Federal do Ceará - CE
236	Afastamento Portaria n 454 art. 2 Único	CH da Universidade Federal do Ceará - CE

236	Afastamento Portaria n 454 art. 2 Único	CH da Universidade Federal do Ceará - CE
236	Afastamento Portaria n 454 art. 2 Único	CH da Universidade Federal do Ceará - CE
236	Afastamento Portaria n 454 art. 2 Único	CH da Universidade Federal do Ceará - CE
236	Afastamento Portaria n 454 art. 2 Único	CH da Universidade Federal do Ceará - CE
236	Afastamento Portaria n 454 art. 2 Único	CH da Universidade Federal do Ceará - CE
236	Afastamento Portaria n 454 art. 2 Único	CH da Universidade Federal do Ceará - CE
236	Afastamento Portaria n 454 art. 2 Único	CH da Universidade Federal do Ceará - CE
236	Afastamento Portaria n 454 art. 2 Único	CH da Universidade Federal do Ceará - CE
236	Afastamento Portaria n 454 art. 2 Único	CH da Universidade Federal do Ceará - CE
236	Afastamento Portaria n 454 art. 2 Único	CH da Universidade Federal do Ceará - CE
236	Afastamento Portaria n 454 art. 2 Único	CH da Universidade Federal do Ceará - CE
236	Afastamento Portaria n 454 art. 2 Único	CH da Universidade Federal do Ceará - CE
236	Afastamento Portaria n 454 art. 2 Único	CH da Universidade Federal do Ceará - CE
236	Afastamento Portaria n 454 art. 2 Único	CH da Universidade Federal do Ceará - CE
236	Afastamento Portaria n 454 art. 2 Único	CH da Universidade Federal do Ceará - CE
236	Afastamento Portaria n 454 art. 2 Único	CH da Universidade Federal do Ceará - CE
236	Afastamento Portaria n 454 art. 2 Único	CH da Universidade Federal do Ceará - CE
236	Afastamento Portaria n 454 art. 2 Único	CH da Universidade Federal do Ceará - CE
236	Afastamento Portaria n 454 art. 2 Único	CH da Universidade Federal do Ceará - CE
236	Afastamento Portaria n 454 art. 2 Único	CH da Universidade Federal do Ceará - CE
236	Afastamento Portaria n 454 art. 2 Único	CH da Universidade Federal do Ceará - CE
236	Afastamento Portaria n 454 art. 2 Único	CH da Universidade Federal do Ceará - CE
236	Afastamento Portaria n 454 art. 2 Único	CH da Universidade Federal do Ceará - CE
236	Afastamento Portaria n 454 art. 2 Único	CH da Universidade Federal do Ceará - CE
236	Afastamento Portaria n 454 art. 2 Único	CH da Universidade Federal do Ceará - CE
236	Afastamento Portaria n 454 art. 2 Único	CH da Universidade Federal do Ceará - CE
236	Afastamento Portaria n 454 art. 2 Único	CH da Universidade Federal do Ceará - CE
236	Afastamento Portaria n 454 art. 2 Único	CH da Universidade Federal do Ceará - CE
236	Afastamento Portaria n 454 art. 2 Único	CH da Universidade Federal do Ceará - CE
236	Afastamento Portaria n 454 art. 2 Único	CH da Universidade Federal do Ceará - CE
236	Afastamento Portaria n 454 art. 2 Único	CH da Universidade Federal do Ceará - CE
236	Afastamento Portaria n 454 art. 2 Único	CH da Universidade Federal do Ceará - CE
236	Afastamento Portaria n 454 art. 2 Único	CH da Universidade Federal do Ceará - CE
236	Afastamento Portaria n 454 art. 2 Único	CH da Universidade Federal do Ceará - CE
236	Afastamento Portaria n 454 art. 2 Único	CH da Universidade Federal do Ceará - CE
236	Afastamento Portaria n 454 art. 2 Único	CH da Universidade Federal do Ceará - CE
236	Afastamento Portaria n 454 art. 2 Único	CH da Universidade Federal do Ceará - CE
236	Afastamento Portaria n 454 art. 2 Único	CH da Universidade Federal do Ceará - CE
236	Afastamento Portaria n 454 art. 2 Único	CH da Universidade Federal do Ceará - CE
236	Afastamento Portaria n 454 art. 2 Único	CH da Universidade Federal do Ceará - CE
236	Afastamento Portaria n 454 art. 2 Único	CH da Universidade Federal do Ceará - CE
236	Afastamento Portaria n 454 art. 2 Único	CH da Universidade Federal do Ceará - CE
236	Afastamento Portaria n 454 art. 2 Único	CH da Universidade Federal do Ceará - CE
236	Afastamento Portaria n 454 art. 2 Único	CH da Universidade Federal do Ceará - CE
236	Afastamento Portaria n 454 art. 2 Único	CH da Universidade Federal do Ceará - CE
236	Afastamento Portaria n 454 art. 2 Único	CH da Universidade Federal do Ceará - CE
236	Afastamento Portaria n 454 art. 2 Único	CH da Universidade Federal do Ceará - CE

236	Afastamento Portaria n 454 art. 2 Único	CH da Universidade Federal do Ceará - CE
236	Afastamento Portaria n 454 art. 2 Único	CH da Universidade Federal do Ceará - CE
236	Afastamento Portaria n 454 art. 2 Único	CH da Universidade Federal do Ceará - CE
236	Afastamento Portaria n 454 art. 2 Único	CH da Universidade Federal do Ceará - CE
236	Afastamento Portaria n 454 art. 2 Único	CH da Universidade Federal do Ceará - CE
236	Afastamento Portaria n 454 art. 2 Único	CH da Universidade Federal do Ceará - CE
236	Afastamento Portaria n 454 art. 2 Único	CH da Universidade Federal do Ceará - CE
236	Afastamento Portaria n 454 art. 2 Único	CH da Universidade Federal do Ceará - CE
236	Afastamento Portaria n 454 art. 2 Único	CH da Universidade Federal do Ceará - CE
236	Afastamento Portaria n 454 art. 2 Único	CH da Universidade Federal do Ceará - CE
236	Afastamento Portaria n 454 art. 2 Único	CH da Universidade Federal do Ceará - CE
236	Afastamento Portaria n 454 art. 2 Único	CH da Universidade Federal do Ceará - CE
236	Afastamento Portaria n 454 art. 2 Único	CH da Universidade Federal do Ceará - CE
236	Afastamento Portaria n 454 art. 2 Único	CH da Universidade Federal do Ceará - CE
236	Afastamento Portaria n 454 art. 2 Único	CH da Universidade Federal do Ceará - CE
236	Afastamento Portaria n 454 art. 2 Único	CH da Universidade Federal do Ceará - CE
236	Afastamento Portaria n 454 art. 2 Único	CH da Universidade Federal do Ceará - CE
236	Afastamento Portaria n 454 art. 2 Único	CH da Universidade Federal do Ceará - CE
236	Afastamento Portaria n 454 art. 2 Único	CH da Universidade Federal do Ceará - CE
236	Afastamento Portaria n 454 art. 2 Único	CH da Universidade Federal do Ceará - CE
236	Afastamento Portaria n 454 art. 2 Único	CH da Universidade Federal do Ceará - CE
236	Afastamento Portaria n 454 art. 2 Único	CH da Universidade Federal do Ceará - CE
236	Afastamento Portaria n 454 art. 2 Único	CH da Universidade Federal do Ceará - CE
236	Afastamento Portaria n 454 art. 2 Único	CH da Universidade Federal do Ceará - CE
236	Afastamento Portaria n 454 art. 2 Único	CH da Universidade Federal do Ceará - CE
236	Afastamento Portaria n 454 art. 2 Único	CH da Universidade Federal do Ceará - CE
236	Afastamento Portaria n 454 art. 2 Único	CH da Universidade Federal do Ceará - CE
236	Afastamento Portaria n 454 art. 2 Único	CH da Universidade Federal do Ceará - CE
236	Afastamento Portaria n 454 art. 2 Único	CH da Universidade Federal do Ceará - CE
236	Afastamento Portaria n 454 art. 2 Único	CH da Universidade Federal do Ceará - CE
236	Afastamento Portaria n 454 art. 2 Único	CH da Universidade Federal do Ceará - CE
236	Afastamento Portaria n 454 art. 2 Único	CH da Universidade Federal do Ceará - CE
236	Afastamento Portaria n 454 art. 2 Único	CH da Universidade Federal do Ceará - CE
236	Afastamento Portaria n 454 art. 2 Único	CH da Universidade Federal do Ceará - CE
236	Afastamento Portaria n 454 art. 2 Único	CH da Universidade Federal do Ceará - CE
236	Afastamento Portaria n 454 art. 2 Único	CH da Universidade Federal do Ceará - CE
236	Afastamento Portaria n 454 art. 2 Único	CH da Universidade Federal do Ceará - CE
236	Afastamento Portaria n 454 art. 2 Único	CH da Universidade Federal do Ceará - CE
236	Afastamento Portaria n 454 art. 2 Único	CH da Universidade Federal do Ceará - CE
236	Afastamento Portaria n 454 art. 2 Único	CH da Universidade Federal do Ceará - CE
236	Afastamento Portaria n 454 art. 2 Único	CH da Universidade Federal do Ceará - CE
236	Afastamento Portaria n 454 art. 2 Único	CH da Universidade Federal do Ceará - CE
236	Afastamento Portaria n 454 art. 2 Único	CH da Universidade Federal do Ceará - CE

236	Afastamento Portaria n 454 art. 2 Único	CH da Universidade Federal do Ceará - CE
236	Afastamento Portaria n 454 art. 2 Único	CH da Universidade Federal do Ceará - CE
236	Afastamento Portaria n 454 art. 2 Único	CH da Universidade Federal do Ceará - CE
236	Afastamento Portaria n 454 art. 2 Único	CH da Universidade Federal do Ceará - CE
236	Afastamento Portaria n 454 art. 2 Único	CH da Universidade Federal do Ceará - CE
236	Afastamento Portaria n 454 art. 2 Único	CH da Universidade Federal do Ceará - CE
236	Afastamento Portaria n 454 art. 2 Único	CH da Universidade Federal do Ceará - CE
236	Afastamento Portaria n 454 art. 2 Único	CH da Universidade Federal do Ceará - CE
236	Afastamento Portaria n 454 art. 2 Único	CH da Universidade Federal do Ceará - CE
236	Afastamento Portaria n 454 art. 2 Único	CH da Universidade Federal do Ceará - CE
236	Afastamento Portaria n 454 art. 2 Único	CH da Universidade Federal do Ceará - CE
236	Afastamento Portaria n 454 art. 2 Único	CH da Universidade Federal do Ceará - CE
236	Afastamento Portaria n 454 art. 2 Único	CH da Universidade Federal do Ceará - CE
236	Afastamento Portaria n 454 art. 2 Único	CH da Universidade Federal do Ceará - CE
236	Afastamento Portaria n 454 art. 2 Único	CH da Universidade Federal do Ceará - CE
236	Afastamento Portaria n 454 art. 2 Único	CH da Universidade Federal do Ceará - CE
236	Afastamento Portaria n 454 art. 2 Único	CH da Universidade Federal do Ceará - CE
236	Afastamento Portaria n 454 art. 2 Único	CH da Universidade Federal do Ceará - CE
236	Afastamento Portaria n 454 art. 2 Único	CH da Universidade Federal do Ceará - CE
236	Afastamento Portaria n 454 art. 2 Único	CH da Universidade Federal do Ceará - CE
236	Afastamento Portaria n 454 art. 2 Único	CH da Universidade Federal do Ceará - CE
236	Afastamento Portaria n 454 art. 2 Único	CH da Universidade Federal do Ceará - CE
236	Afastamento Portaria n 454 art. 2 Único	CH da Universidade Federal do Ceará - CE
236	Afastamento Portaria n 454 art. 2 Único	CH da Universidade Federal do Ceará - CE
236	Afastamento Portaria n 454 art. 2 Único	CH da Universidade Federal do Ceará - CE
236	Afastamento Portaria n 454 art. 2 Único	CH da Universidade Federal do Ceará - CE
236	Afastamento Portaria n 454 art. 2 Único	CH da Universidade Federal do Ceará - CE
236	Afastamento Portaria n 454 art. 2 Único	CH da Universidade Federal do Ceará - CE
236	Afastamento Portaria n 454 art. 2 Único	CH da Universidade Federal do Ceará - CE
236	Afastamento Portaria n 454 art. 2 Único	CH da Universidade Federal do Ceará - CE
236	Afastamento Portaria n 454 art. 2 Único	CH da Universidade Federal do Ceará - CE
236	Afastamento Portaria n 454 art. 2 Único	CH da Universidade Federal do Ceará - CE
236	Afastamento Portaria n 454 art. 2 Único	CH da Universidade Federal do Ceará - CE
236	Afastamento Portaria n 454 art. 2 Único	CH da Universidade Federal do Ceará - CE
236	Afastamento Portaria n 454 art. 2 Único	CH da Universidade Federal do Ceará - CE
236	Afastamento Portaria n 454 art. 2 Único	CH da Universidade Federal do Ceará - CE
236	Afastamento Portaria n 454 art. 2 Único	CH da Universidade Federal do Ceará - CE
236	Afastamento Portaria n 454 art. 2 Único	CH da Universidade Federal do Ceará - CE
236	Afastamento Portaria n 454 art. 2 Único	CH da Universidade Federal do Ceará - CE
236	Afastamento Portaria n 454 art. 2 Único	CH da Universidade Federal do Ceará - CE
236	Afastamento Portaria n 454 art. 2 Único	CH da Universidade Federal do Ceará - CE

236	Afastamento Portaria n 454 art. 2 Único	CH da Universidade Federal do Ceará - CE
236	Afastamento Portaria n 454 art. 2 Único	CH da Universidade Federal do Ceará - CE
237	Trabalho Remoto Instrução Normativa DGP 03/2020 art 6, IX.	CH da Universidade Federal do Ceará - CE
237	Trabalho Remoto Instrução Normativa DGP 03/2020 art 6, IX.	CH da Universidade Federal do Ceará - CE
237	Trabalho Remoto Instrução Normativa DGP 03/2020 art 6, IX.	CH da Universidade Federal do Ceará - CE
237	Trabalho Remoto Instrução Normativa DGP 03/2020 art 6, IX.	CH da Universidade Federal do Ceará - CE
237	Trabalho Remoto Instrução Normativa DGP 03/2020 art 6, IX.	CH da Universidade Federal do Ceará - CE
237	Trabalho Remoto Instrução Normativa DGP 03/2020 art 6, IX.	CH da Universidade Federal do Ceará - CE
237	Trabalho Remoto Instrução Normativa DGP 03/2020 art 6, IX.	CH da Universidade Federal do Ceará - CE
237	Trabalho Remoto Instrução Normativa DGP 03/2020 art 6, IX.	CH da Universidade Federal do Ceará - CE
237	Trabalho Remoto Instrução Normativa DGP 03/2020 art 6, IX.	CH da Universidade Federal do Ceará - CE
237	Trabalho Remoto Instrução Normativa DGP 03/2020 art 6, IX.	CH da Universidade Federal do Ceará - CE
237	Trabalho Remoto Instrução Normativa DGP 03/2020 art 6, IX.	CH da Universidade Federal do Ceará - CE
237	Trabalho Remoto Instrução Normativa DGP 03/2020 art 6, IX.	CH da Universidade Federal do Ceará - CE
237	Trabalho Remoto Instrução Normativa DGP 03/2020 art 6, IX.	CH da Universidade Federal do Ceará - CE
237	Trabalho Remoto Instrução Normativa DGP 03/2020 art 6, IX.	CH da Universidade Federal do Ceará - CE
237	Trabalho Remoto Instrução Normativa DGP 03/2020 art 6, IX.	CH da Universidade Federal do Ceará - CE
237	Trabalho Remoto Instrução Normativa DGP 03/2020 art 6, IX.	CH da Universidade Federal do Ceará - CE
237	Trabalho Remoto Instrução Normativa DGP 03/2020 art 6, IX.	CH da Universidade Federal do Ceará - CE
237	Trabalho Remoto Instrução Normativa DGP 03/2020 art 6, IX.	CH da Universidade Federal do Ceará - CE
237	Trabalho Remoto Instrução Normativa DGP 03/2020 art 6, IX.	CH da Universidade Federal do Ceará - CE
237	Trabalho Remoto Instrução Normativa DGP 03/2020 art 6, IX.	CH da Universidade Federal do Ceará - CE
237	Trabalho Remoto Instrução Normativa DGP 03/2020 art 6, IX.	CH da Universidade Federal do Ceará - CE
237	Trabalho Remoto Instrução Normativa DGP 03/2020 art 6, IX.	CH da Universidade Federal do Ceará - CE
237	Trabalho Remoto Instrução Normativa DGP 03/2020 art 6, IX.	CH da Universidade Federal do Ceará - CE
237	Trabalho Remoto Instrução Normativa DGP 03/2020 art 6, IX.	CH da Universidade Federal do Ceará - CE
237	Trabalho Remoto Instrução Normativa DGP 03/2020 art 6, IX.	CH da Universidade Federal do Ceará - CE
237	Trabalho Remoto Instrução Normativa DGP 03/2020 art 6, IX.	CH da Universidade Federal do Ceará - CE
237	Trabalho Remoto Instrução Normativa DGP 03/2020 art 6, IX.	CH da Universidade Federal do Ceará - CE
237	Trabalho Remoto Instrução Normativa DGP 03/2020 art 6, IX.	CH da Universidade Federal do Ceará - CE
237	Trabalho Remoto Instrução Normativa DGP 03/2020 art 6, IX.	CH da Universidade Federal do Ceará - CE
237	Trabalho Remoto Instrução Normativa DGP 03/2020 art 6, IX.	CH da Universidade Federal do Ceará - CE

237	Trabalho Remoto Instrução Normativa DGP 03/2020 art 6, IX.	CH da Universidade Federal do Ceará - CE
237	Trabalho Remoto Instrução Normativa DGP 03/2020 art 6, IX.	CH da Universidade Federal do Ceará - CE
237	Trabalho Remoto Instrução Normativa DGP 03/2020 art 6, IX.	CH da Universidade Federal do Ceará - CE
237	Trabalho Remoto Instrução Normativa DGP 03/2020 art 6, IX.	CH da Universidade Federal do Ceará - CE
237	Trabalho Remoto Instrução Normativa DGP 03/2020 art 6, IX.	CH da Universidade Federal do Ceará - CE
237	Trabalho Remoto Instrução Normativa DGP 03/2020 art 6, IX.	CH da Universidade Federal do Ceará - CE
237	Trabalho Remoto Instrução Normativa DGP 03/2020 art 6, IX.	CH da Universidade Federal do Ceará - CE
237	Trabalho Remoto Instrução Normativa DGP 03/2020 art 6, IX.	CH da Universidade Federal do Ceará - CE
237	Trabalho Remoto Instrução Normativa DGP 03/2020 art 6, IX.	CH da Universidade Federal do Ceará - CE
237	Trabalho Remoto Instrução Normativa DGP 03/2020 art 6, IX.	CH da Universidade Federal do Ceará - CE
237	Trabalho Remoto Instrução Normativa DGP 03/2020 art 6, IX.	CH da Universidade Federal do Ceará - CE
237	Trabalho Remoto Instrução Normativa DGP 03/2020 art 6, IX.	CH da Universidade Federal do Ceará - CE
237	Trabalho Remoto Instrução Normativa DGP 03/2020 art 6, IX.	CH da Universidade Federal do Ceará - CE
237	Trabalho Remoto Instrução Normativa DGP 03/2020 art 6, IX.	CH da Universidade Federal do Ceará - CE
237	Trabalho Remoto Instrução Normativa DGP 03/2020 art 6, IX.	CH da Universidade Federal do Ceará - CE
237	Trabalho Remoto Instrução Normativa DGP 03/2020 art 6, IX.	CH da Universidade Federal do Ceará - CE
237	Trabalho Remoto Instrução Normativa DGP 03/2020 art 6, IX.	CH da Universidade Federal do Ceará - CE
237	Trabalho Remoto Instrução Normativa DGP 03/2020 art 6, IX.	CH da Universidade Federal do Ceará - CE
237	Trabalho Remoto Instrução Normativa DGP 03/2020 art 6, IX.	CH da Universidade Federal do Ceará - CE
237	Trabalho Remoto Instrução Normativa DGP 03/2020 art 6, IX.	CH da Universidade Federal do Ceará - CE
237	Trabalho Remoto Instrução Normativa DGP 03/2020 art 6, IX.	CH da Universidade Federal do Ceará - CE
237	Trabalho Remoto Instrução Normativa DGP 03/2020 art 6, IX.	CH da Universidade Federal do Ceará - CE
237	Trabalho Remoto Instrução Normativa DGP 03/2020 art 6, IX.	CH da Universidade Federal do Ceará - CE
237	Trabalho Remoto Instrução Normativa DGP 03/2020 art 6, IX.	CH da Universidade Federal do Ceará - CE
237	Trabalho Remoto Instrução Normativa DGP 03/2020 art 6, IX.	CH da Universidade Federal do Ceará - CE
237	Trabalho Remoto Instrução Normativa DGP 03/2020 art 6, IX.	CH da Universidade Federal do Ceará - CE
237	Trabalho Remoto Instrução Normativa DGP 03/2020 art 6, IX.	CH da Universidade Federal do Ceará - CE
237	Trabalho Remoto Instrução Normativa DGP 03/2020 art 6, IX.	CH da Universidade Federal do Ceará - CE
237	Trabalho Remoto Instrução Normativa DGP 03/2020 art 6, IX.	CH da Universidade Federal do Ceará - CE
237	Trabalho Remoto Instrução Normativa DGP 03/2020 art 6, IX.	CH da Universidade Federal do Ceará - CE
237	Trabalho Remoto Instrução Normativa DGP 03/2020 art 6, IX.	CH da Universidade Federal do Ceará - CE
237	Trabalho Remoto Instrução Normativa DGP 03/2020 art 6, IX.	CH da Universidade Federal do Ceará - CE

237	Trabalho Remoto Instrução Normativa DGP 03/2020 art 6, IX.	CH da Universidade Federal do Ceará - CE
237	Trabalho Remoto Instrução Normativa DGP 03/2020 art 6, IX.	CH da Universidade Federal do Ceará - CE
237	Trabalho Remoto Instrução Normativa DGP 03/2020 art 6, IX.	CH da Universidade Federal do Ceará - CE
237	Trabalho Remoto Instrução Normativa DGP 03/2020 art 6, IX.	CH da Universidade Federal do Ceará - CE
237	Trabalho Remoto Instrução Normativa DGP 03/2020 art 6, IX.	CH da Universidade Federal do Ceará - CE
237	Trabalho Remoto Instrução Normativa DGP 03/2020 art 6, IX.	CH da Universidade Federal do Ceará - CE
237	Trabalho Remoto Instrução Normativa DGP 03/2020 art 6, IX.	CH da Universidade Federal do Ceará - CE
237	Trabalho Remoto Instrução Normativa DGP 03/2020 art 6, IX.	CH da Universidade Federal do Ceará - CE
237	Trabalho Remoto Instrução Normativa DGP 03/2020 art 6, IX.	CH da Universidade Federal do Ceará - CE
237	Trabalho Remoto Instrução Normativa DGP 03/2020 art 6, IX.	CH da Universidade Federal do Ceará - CE
237	Trabalho Remoto Instrução Normativa DGP 03/2020 art 6, IX.	CH da Universidade Federal do Ceará - CE
237	Trabalho Remoto Instrução Normativa DGP 03/2020 art 6, IX.	CH da Universidade Federal do Ceará - CE
237	Trabalho Remoto Instrução Normativa DGP 03/2020 art 6, IX.	CH da Universidade Federal do Ceará - CE
237	Trabalho Remoto Instrução Normativa DGP 03/2020 art 6, IX.	CH da Universidade Federal do Ceará - CE
237	Trabalho Remoto Instrução Normativa DGP 03/2020 art 6, IX.	CH da Universidade Federal do Ceará - CE
237	Trabalho Remoto Instrução Normativa DGP 03/2020 art 6, IX.	CH da Universidade Federal do Ceará - CE
237	Trabalho Remoto Instrução Normativa DGP 03/2020 art 6, IX.	CH da Universidade Federal do Ceará - CE
237	Trabalho Remoto Instrução Normativa DGP 03/2020 art 6, IX.	CH da Universidade Federal do Ceará - CE
237	Trabalho Remoto Instrução Normativa DGP 03/2020 art 6, IX.	CH da Universidade Federal do Ceará - CE
237	Trabalho Remoto Instrução Normativa DGP 03/2020 art 6, IX.	CH da Universidade Federal do Ceará - CE
237	Trabalho Remoto Instrução Normativa DGP 03/2020 art 6, IX.	CH da Universidade Federal do Ceará - CE
237	Trabalho Remoto Instrução Normativa DGP 03/2020 art 6, IX.	CH da Universidade Federal do Ceará - CE
237	Trabalho Remoto Instrução Normativa DGP 03/2020 art 6, IX.	CH da Universidade Federal do Ceará - CE
237	Trabalho Remoto Instrução Normativa DGP 03/2020 art 6, IX.	CH da Universidade Federal do Ceará - CE
237	Trabalho Remoto Instrução Normativa DGP 03/2020 art 6, IX.	CH da Universidade Federal do Ceará - CE
237	Trabalho Remoto Instrução Normativa DGP 03/2020 art 6, IX.	CH da Universidade Federal do Ceará - CE
237	Trabalho Remoto Instrução Normativa DGP 03/2020 art 6, IX.	CH da Universidade Federal do Ceará - CE
237	Trabalho Remoto Instrução Normativa DGP 03/2020 art 6, IX.	CH da Universidade Federal do Ceará - CE
237	Trabalho Remoto Instrução Normativa DGP 03/2020 art 6, IX.	CH da Universidade Federal do Ceará - CE
237	Trabalho Remoto Instrução Normativa DGP 03/2020 art 6, IX.	CH da Universidade Federal do Ceará - CE
237	Trabalho Remoto Instrução Normativa DGP 03/2020 art 6, IX.	CH da Universidade Federal do Ceará - CE
237	Trabalho Remoto Instrução Normativa DGP 03/2020 art 6, IX.	CH da Universidade Federal do Ceará - CE

237	Trabalho Remoto Instrução Normativa DGP 03/2020 art 6, IX.	CH da Universidade Federal do Ceará - CE
237	Trabalho Remoto Instrução Normativa DGP 03/2020 art 6, IX.	CH da Universidade Federal do Ceará - CE
237	Trabalho Remoto Instrução Normativa DGP 03/2020 art 6, IX.	CH da Universidade Federal do Ceará - CE
237	Trabalho Remoto Instrução Normativa DGP 03/2020 art 6, IX.	CH da Universidade Federal do Ceará - CE
237	Trabalho Remoto Instrução Normativa DGP 03/2020 art 6, IX.	CH da Universidade Federal do Ceará - CE
237	Trabalho Remoto Instrução Normativa DGP 03/2020 art 6, IX.	CH da Universidade Federal do Ceará - CE
237	Trabalho Remoto Instrução Normativa DGP 03/2020 art 6, IX.	CH da Universidade Federal do Ceará - CE
237	Trabalho Remoto Instrução Normativa DGP 03/2020 art 6, IX.	CH da Universidade Federal do Ceará - CE
237	Trabalho Remoto Instrução Normativa DGP 03/2020 art 6, IX.	CH da Universidade Federal do Ceará - CE
237	Trabalho Remoto Instrução Normativa DGP 03/2020 art 6, IX.	CH da Universidade Federal do Ceará - CE
237	Trabalho Remoto Instrução Normativa DGP 03/2020 art 6, IX.	CH da Universidade Federal do Ceará - CE
237	Trabalho Remoto Instrução Normativa DGP 03/2020 art 6, IX.	CH da Universidade Federal do Ceará - CE
237	Trabalho Remoto Instrução Normativa DGP 03/2020 art 6, IX.	CH da Universidade Federal do Ceará - CE
237	Trabalho Remoto Instrução Normativa DGP 03/2020 art 6, IX.	CH da Universidade Federal do Ceará - CE
237	Trabalho Remoto Instrução Normativa DGP 03/2020 art 6, IX.	CH da Universidade Federal do Ceará - CE
237	Trabalho Remoto Instrução Normativa DGP 03/2020 art 6, IX.	CH da Universidade Federal do Ceará - CE
237	Trabalho Remoto Instrução Normativa DGP 03/2020 art 6, IX.	CH da Universidade Federal do Ceará - CE
237	Trabalho Remoto Instrução Normativa DGP 03/2020 art 6, IX.	CH da Universidade Federal do Ceará - CE
237	Trabalho Remoto Instrução Normativa DGP 03/2020 art 6, IX.	CH da Universidade Federal do Ceará - CE
237	Trabalho Remoto Instrução Normativa DGP 03/2020 art 6, IX.	CH da Universidade Federal do Ceará - CE
237	Trabalho Remoto Instrução Normativa DGP 03/2020 art 6, IX.	CH da Universidade Federal do Ceará - CE
237	Trabalho Remoto Instrução Normativa DGP 03/2020 art 6, IX.	CH da Universidade Federal do Ceará - CE
237	Trabalho Remoto Instrução Normativa DGP 03/2020 art 6, IX.	CH da Universidade Federal do Ceará - CE
237	Trabalho Remoto Instrução Normativa DGP 03/2020 art 6, IX.	CH da Universidade Federal do Ceará - CE
237	Trabalho Remoto Instrução Normativa DGP 03/2020 art 6, IX.	CH da Universidade Federal do Ceará - CE
237	Trabalho Remoto Instrução Normativa DGP 03/2020 art 6, IX.	CH da Universidade Federal do Ceará - CE
237	Trabalho Remoto Instrução Normativa DGP 03/2020 art 6, IX.	CH da Universidade Federal do Ceará - CE
237	Trabalho Remoto Instrução Normativa DGP 03/2020 art 6, IX.	CH da Universidade Federal do Ceará - CE
237	Trabalho Remoto Instrução Normativa DGP 03/2020 art 6, IX.	CH da Universidade Federal do Ceará - CE
237	Trabalho Remoto Instrução Normativa DGP 03/2020 art 6, IX.	CH da Universidade Federal do Ceará - CE
237	Trabalho Remoto Instrução Normativa DGP 03/2020 art 6, IX.	CH da Universidade Federal do Ceará - CE
237	Trabalho Remoto Instrução Normativa DGP 03/2020 art 6, IX.	CH da Universidade Federal do Ceará - CE

237	Trabalho Remoto Instrução Normativa DGP 03/2020 art 6, IX.	CH da Universidade Federal do Ceará - CE
237	Trabalho Remoto Instrução Normativa DGP 03/2020 art 6, IX.	CH da Universidade Federal do Ceará - CE
237	Trabalho Remoto Instrução Normativa DGP 03/2020 art 6, IX.	CH da Universidade Federal do Ceará - CE
4	LICENÇA MATERNIDADE - 120 DIAS	CHC da Universidade Federal do Paraná - PR
4	LICENÇA MATERNIDADE - 120 DIAS	CHC da Universidade Federal do Paraná - PR
4	LICENÇA MATERNIDADE - 120 DIAS	CHC da Universidade Federal do Paraná - PR
4	LICENÇA MATERNIDADE - 120 DIAS	CHC da Universidade Federal do Paraná - PR
4	LICENÇA MATERNIDADE - 120 DIAS	CHC da Universidade Federal do Paraná - PR
4	LICENÇA MATERNIDADE - 120 DIAS	CHC da Universidade Federal do Paraná - PR
4	LICENÇA MATERNIDADE - 120 DIAS	CHC da Universidade Federal do Paraná - PR
4	LICENÇA MATERNIDADE - 120 DIAS	CHC da Universidade Federal do Paraná - PR
4	LICENÇA MATERNIDADE - 120 DIAS	CHC da Universidade Federal do Paraná - PR
4	LICENÇA MATERNIDADE - 120 DIAS	CHC da Universidade Federal do Paraná - PR
4	LICENÇA MATERNIDADE - 120 DIAS	CHC da Universidade Federal do Paraná - PR
4	LICENÇA MATERNIDADE - 120 DIAS	CHC da Universidade Federal do Paraná - PR
4	LICENÇA MATERNIDADE - 120 DIAS	CHC da Universidade Federal do Paraná - PR
4	LICENÇA MATERNIDADE - 120 DIAS	CHC da Universidade Federal do Paraná - PR
4	LICENÇA MATERNIDADE - 120 DIAS	CHC da Universidade Federal do Paraná - PR
4	LICENÇA MATERNIDADE - 120 DIAS	CHC da Universidade Federal do Paraná - PR
4	LICENÇA MATERNIDADE - 120 DIAS	CHC da Universidade Federal do Paraná - PR
4	LICENÇA MATERNIDADE - 120 DIAS	CHC da Universidade Federal do Paraná - PR
4	LICENÇA MATERNIDADE - 120 DIAS	CHC da Universidade Federal do Paraná - PR
4	LICENÇA MATERNIDADE - 120 DIAS	CHC da Universidade Federal do Paraná - PR
4	LICENÇA MATERNIDADE - 120 DIAS	CHC da Universidade Federal do Paraná - PR
4	LICENÇA MATERNIDADE - 120 DIAS	CHC da Universidade Federal do Paraná - PR
4	LICENÇA MATERNIDADE - 120 DIAS	CHC da Universidade Federal do Paraná - PR
4	LICENÇA MATERNIDADE - 120 DIAS	CHC da Universidade Federal do Paraná - PR
4	LICENÇA MATERNIDADE - 120 DIAS	CHC da Universidade Federal do Paraná - PR
4	LICENÇA MATERNIDADE - 120 DIAS	CHC da Universidade Federal do Paraná - PR
4	LICENÇA MATERNIDADE - 120 DIAS	CHC da Universidade Federal do Paraná - PR
4	LICENÇA MATERNIDADE - 120 DIAS	CHC da Universidade Federal do Paraná - PR
4	LICENÇA MATERNIDADE - 120 DIAS	CHC da Universidade Federal do Paraná - PR
4	LICENÇA MATERNIDADE - 120 DIAS	CHC da Universidade Federal do Paraná - PR
4	LICENÇA MATERNIDADE - 120 DIAS	CHC da Universidade Federal do Paraná - PR
4	LICENÇA MATERNIDADE - 120 DIAS	CHC da Universidade Federal do Paraná - PR
4	LICENÇA MATERNIDADE - 120 DIAS	CHC da Universidade Federal do Paraná - PR
4	LICENÇA MATERNIDADE - 120 DIAS	CHC da Universidade Federal do Paraná - PR
4	LICENÇA MATERNIDADE - 120 DIAS	CHC da Universidade Federal do Paraná - PR
4	LICENÇA MATERNIDADE - 120 DIAS	CHC da Universidade Federal do Paraná - PR
4	LICENÇA MATERNIDADE - 120 DIAS	CHC da Universidade Federal do Paraná - PR
4	LICENÇA MATERNIDADE - 120 DIAS	CHC da Universidade Federal do Paraná - PR
4	LICENÇA MATERNIDADE - 120 DIAS	CHC da Universidade Federal do Paraná - PR
4	LICENÇA MATERNIDADE - 120 DIAS	CHC da Universidade Federal do Paraná - PR
4	LICENÇA MATERNIDADE - 120 DIAS	CHC da Universidade Federal do Paraná - PR
4	LICENÇA MATERNIDADE - 120 DIAS	CHC da Universidade Federal do Paraná - PR
4	LICENÇA MATERNIDADE - 120 DIAS	CHC da Universidade Federal do Paraná - PR

4	LICENÇA MATERNIDADE - 120 DIAS	CHC da Universidade Federal do Paraná - PR
4	LICENÇA MATERNIDADE - 120 DIAS	CHC da Universidade Federal do Paraná - PR
4	LICENÇA MATERNIDADE - 120 DIAS	CHC da Universidade Federal do Paraná - PR
4	LICENÇA MATERNIDADE - 120 DIAS	CHC da Universidade Federal do Paraná - PR
4	LICENÇA MATERNIDADE - 120 DIAS	CHC da Universidade Federal do Paraná - PR
4	LICENÇA MATERNIDADE - 120 DIAS	CHC da Universidade Federal do Paraná - PR
4	LICENÇA MATERNIDADE - 120 DIAS	CHC da Universidade Federal do Paraná - PR
4	LICENÇA MATERNIDADE - 120 DIAS	CHC da Universidade Federal do Paraná - PR
4	LICENÇA MATERNIDADE - 120 DIAS	CHC da Universidade Federal do Paraná - PR
4	LICENÇA MATERNIDADE - 120 DIAS	CHC da Universidade Federal do Paraná - PR
4	LICENÇA MATERNIDADE - 120 DIAS	CHC da Universidade Federal do Paraná - PR
4	LICENÇA MATERNIDADE - 120 DIAS	CHC da Universidade Federal do Paraná - PR
4	LICENÇA MATERNIDADE - 120 DIAS	CHC da Universidade Federal do Paraná - PR
4	LICENÇA MATERNIDADE - 120 DIAS	CHC da Universidade Federal do Paraná - PR
4	LICENÇA MATERNIDADE - 120 DIAS	CHC da Universidade Federal do Paraná - PR
4	LICENÇA MATERNIDADE - 120 DIAS	CHC da Universidade Federal do Paraná - PR
4	LICENÇA MATERNIDADE - 120 DIAS	CHC da Universidade Federal do Paraná - PR
4	LICENÇA MATERNIDADE - 120 DIAS	CHC da Universidade Federal do Paraná - PR
4	LICENÇA MATERNIDADE - 120 DIAS	CHC da Universidade Federal do Paraná - PR
4	LICENÇA MATERNIDADE - 120 DIAS	CHC da Universidade Federal do Paraná - PR
5	LICENÇA POR MORTE DE FAMILIAR - CLT E REGULAMENTO DE PESSOAL	CHC da Universidade Federal do Paraná - PR
5	LICENÇA POR MORTE DE FAMILIAR - CLT E REGULAMENTO DE PESSOAL	CHC da Universidade Federal do Paraná - PR
5	LICENÇA POR MORTE DE FAMILIAR - CLT E REGULAMENTO DE PESSOAL	CHC da Universidade Federal do Paraná - PR
5	LICENÇA POR MORTE DE FAMILIAR - CLT E REGULAMENTO DE PESSOAL	CHC da Universidade Federal do Paraná - PR
5	LICENÇA POR MORTE DE FAMILIAR - CLT E REGULAMENTO DE PESSOAL	CHC da Universidade Federal do Paraná - PR
5	LICENÇA POR MORTE DE FAMILIAR - CLT E REGULAMENTO DE PESSOAL	CHC da Universidade Federal do Paraná - PR
5	LICENÇA POR MORTE DE FAMILIAR - CLT E REGULAMENTO DE PESSOAL	CHC da Universidade Federal do Paraná - PR
5	LICENÇA POR MORTE DE FAMILIAR - CLT E REGULAMENTO DE PESSOAL	CHC da Universidade Federal do Paraná - PR
5	LICENÇA POR MORTE DE FAMILIAR - CLT E REGULAMENTO DE PESSOAL	CHC da Universidade Federal do Paraná - PR
5	LICENÇA POR MORTE DE FAMILIAR - CLT E REGULAMENTO DE PESSOAL	CHC da Universidade Federal do Paraná - PR
5	LICENÇA POR MORTE DE FAMILIAR - CLT E REGULAMENTO DE PESSOAL	CHC da Universidade Federal do Paraná - PR
5	LICENÇA POR MORTE DE FAMILIAR - CLT E REGULAMENTO DE PESSOAL	CHC da Universidade Federal do Paraná - PR
5	LICENÇA POR MORTE DE FAMILIAR - CLT E REGULAMENTO DE PESSOAL	CHC da Universidade Federal do Paraná - PR
5	LICENÇA POR MORTE DE FAMILIAR - CLT E REGULAMENTO DE PESSOAL	CHC da Universidade Federal do Paraná - PR
5	LICENÇA POR MORTE DE FAMILIAR - CLT E REGULAMENTO DE PESSOAL	CHC da Universidade Federal do Paraná - PR
5	LICENÇA POR MORTE DE FAMILIAR - CLT E REGULAMENTO DE PESSOAL	CHC da Universidade Federal do Paraná - PR
5	LICENÇA POR MORTE DE FAMILIAR - CLT E REGULAMENTO DE PESSOAL	CHC da Universidade Federal do Paraná - PR
5	LICENÇA POR MORTE DE FAMILIAR - CLT E REGULAMENTO DE PESSOAL	CHC da Universidade Federal do Paraná - PR

5	LICENÇA POR MORTE DE FAMILIAR - CLT E REGULAMENTO DE PESSOAL	CHC da Universidade Federal do Paraná - PR
5	LICENÇA POR MORTE DE FAMILIAR - CLT E REGULAMENTO DE PESSOAL	CHC da Universidade Federal do Paraná - PR
5	LICENÇA POR MORTE DE FAMILIAR - CLT E REGULAMENTO DE PESSOAL	CHC da Universidade Federal do Paraná - PR
5	LICENÇA POR MORTE DE FAMILIAR - CLT E REGULAMENTO DE PESSOAL	CHC da Universidade Federal do Paraná - PR
5	LICENÇA POR MORTE DE FAMILIAR - CLT E REGULAMENTO DE PESSOAL	CHC da Universidade Federal do Paraná - PR
5	LICENÇA POR MORTE DE FAMILIAR - CLT E REGULAMENTO DE PESSOAL	CHC da Universidade Federal do Paraná - PR
5	LICENÇA POR MORTE DE FAMILIAR - CLT E REGULAMENTO DE PESSOAL	CHC da Universidade Federal do Paraná - PR
5	LICENÇA POR MORTE DE FAMILIAR - CLT E REGULAMENTO DE PESSOAL	CHC da Universidade Federal do Paraná - PR
5	LICENÇA POR MORTE DE FAMILIAR - CLT E REGULAMENTO DE PESSOAL	CHC da Universidade Federal do Paraná - PR
5	LICENÇA POR MORTE DE FAMILIAR - CLT E REGULAMENTO DE PESSOAL	CHC da Universidade Federal do Paraná - PR
5	LICENÇA POR MORTE DE FAMILIAR - CLT E REGULAMENTO DE PESSOAL	CHC da Universidade Federal do Paraná - PR
5	LICENÇA POR MORTE DE FAMILIAR - CLT E REGULAMENTO DE PESSOAL	CHC da Universidade Federal do Paraná - PR
5	LICENÇA POR MORTE DE FAMILIAR - CLT E REGULAMENTO DE PESSOAL	CHC da Universidade Federal do Paraná - PR
5	LICENÇA POR MORTE DE FAMILIAR - CLT E REGULAMENTO DE PESSOAL	CHC da Universidade Federal do Paraná - PR
5	LICENÇA POR MORTE DE FAMILIAR - CLT E REGULAMENTO DE PESSOAL	CHC da Universidade Federal do Paraná - PR
5	LICENÇA POR MORTE DE FAMILIAR - CLT E REGULAMENTO DE PESSOAL	CHC da Universidade Federal do Paraná - PR
5	LICENÇA POR MORTE DE FAMILIAR - CLT E REGULAMENTO DE PESSOAL	CHC da Universidade Federal do Paraná - PR
5	LICENÇA POR MORTE DE FAMILIAR - CLT E REGULAMENTO DE PESSOAL	CHC da Universidade Federal do Paraná - PR
5	LICENÇA POR MORTE DE FAMILIAR - CLT E REGULAMENTO DE PESSOAL	CHC da Universidade Federal do Paraná - PR
5	LICENÇA POR MORTE DE FAMILIAR - CLT E REGULAMENTO DE PESSOAL	CHC da Universidade Federal do Paraná - PR
5	LICENÇA POR MORTE DE FAMILIAR - CLT E REGULAMENTO DE PESSOAL	CHC da Universidade Federal do Paraná - PR
5	LICENÇA POR MORTE DE FAMILIAR - CLT E REGULAMENTO DE PESSOAL	CHC da Universidade Federal do Paraná - PR
5	LICENÇA POR MORTE DE FAMILIAR - CLT E REGULAMENTO DE PESSOAL	CHC da Universidade Federal do Paraná - PR
5	LICENÇA POR MORTE DE FAMILIAR - CLT E REGULAMENTO DE PESSOAL	CHC da Universidade Federal do Paraná - PR
5	LICENÇA POR MORTE DE FAMILIAR - CLT E REGULAMENTO DE PESSOAL	CHC da Universidade Federal do Paraná - PR
6	LICENÇA INTERESSE PARTICULAR - REGULAMENTO DE PESSOAL ART 35 VII	CHC da Universidade Federal do Paraná - PR
6	LICENÇA INTERESSE PARTICULAR - REGULAMENTO DE PESSOAL ART 35 VII	CHC da Universidade Federal do Paraná - PR
12	LICENÇA MATERNIDADE (COMPLEMENTAR 60 DIAS)	CHC da Universidade Federal do Paraná - PR
12	LICENÇA MATERNIDADE (COMPLEMENTAR 60 DIAS)	CHC da Universidade Federal do Paraná - PR
12	LICENÇA MATERNIDADE (COMPLEMENTAR 60 DIAS)	CHC da Universidade Federal do Paraná - PR
12	LICENÇA MATERNIDADE (COMPLEMENTAR 60 DIAS)	CHC da Universidade Federal do Paraná - PR
12	LICENÇA MATERNIDADE (COMPLEMENTAR 60 DIAS)	CHC da Universidade Federal do Paraná - PR
12	LICENÇA MATERNIDADE (COMPLEMENTAR 60 DIAS)	CHC da Universidade Federal do Paraná - PR
12	LICENÇA MATERNIDADE (COMPLEMENTAR 60 DIAS)	CHC da Universidade Federal do Paraná - PR

12	LICENÇA MATERNIDADE (COMPLEMENTAR 60 DIAS)	CHC da Universidade Federal do Paraná - PR
12	LICENÇA MATERNIDADE (COMPLEMENTAR 60 DIAS)	CHC da Universidade Federal do Paraná - PR
12	LICENÇA MATERNIDADE (COMPLEMENTAR 60 DIAS)	CHC da Universidade Federal do Paraná - PR
12	LICENÇA MATERNIDADE (COMPLEMENTAR 60 DIAS)	CHC da Universidade Federal do Paraná - PR
12	LICENÇA MATERNIDADE (COMPLEMENTAR 60 DIAS)	CHC da Universidade Federal do Paraná - PR
12	LICENÇA MATERNIDADE (COMPLEMENTAR 60 DIAS)	CHC da Universidade Federal do Paraná - PR
12	LICENÇA MATERNIDADE (COMPLEMENTAR 60 DIAS)	CHC da Universidade Federal do Paraná - PR
12	LICENÇA MATERNIDADE (COMPLEMENTAR 60 DIAS)	CHC da Universidade Federal do Paraná - PR
12	LICENÇA MATERNIDADE (COMPLEMENTAR 60 DIAS)	CHC da Universidade Federal do Paraná - PR
12	LICENÇA MATERNIDADE (COMPLEMENTAR 60 DIAS)	CHC da Universidade Federal do Paraná - PR
12	LICENÇA MATERNIDADE (COMPLEMENTAR 60 DIAS)	CHC da Universidade Federal do Paraná - PR
12	LICENÇA MATERNIDADE (COMPLEMENTAR 60 DIAS)	CHC da Universidade Federal do Paraná - PR
12	LICENÇA MATERNIDADE (COMPLEMENTAR 60 DIAS)	CHC da Universidade Federal do Paraná - PR
12	LICENÇA MATERNIDADE (COMPLEMENTAR 60 DIAS)	CHC da Universidade Federal do Paraná - PR
12	LICENÇA MATERNIDADE (COMPLEMENTAR 60 DIAS)	CHC da Universidade Federal do Paraná - PR
12	LICENÇA MATERNIDADE (COMPLEMENTAR 60 DIAS)	CHC da Universidade Federal do Paraná - PR
12	LICENÇA MATERNIDADE (COMPLEMENTAR 60 DIAS)	CHC da Universidade Federal do Paraná - PR
12	LICENÇA MATERNIDADE (COMPLEMENTAR 60 DIAS)	CHC da Universidade Federal do Paraná - PR
12	LICENÇA MATERNIDADE (COMPLEMENTAR 60 DIAS)	CHC da Universidade Federal do Paraná - PR
12	LICENÇA MATERNIDADE (COMPLEMENTAR 60 DIAS)	CHC da Universidade Federal do Paraná - PR
12	LICENÇA MATERNIDADE (COMPLEMENTAR 60 DIAS)	CHC da Universidade Federal do Paraná - PR
12	LICENÇA MATERNIDADE (COMPLEMENTAR 60 DIAS)	CHC da Universidade Federal do Paraná - PR
12	LICENÇA MATERNIDADE (COMPLEMENTAR 60 DIAS)	CHC da Universidade Federal do Paraná - PR
12	LICENÇA MATERNIDADE (COMPLEMENTAR 60 DIAS)	CHC da Universidade Federal do Paraná - PR
12	LICENÇA MATERNIDADE (COMPLEMENTAR 60 DIAS)	CHC da Universidade Federal do Paraná - PR
12	LICENÇA MATERNIDADE (COMPLEMENTAR 60 DIAS)	CHC da Universidade Federal do Paraná - PR
12	LICENÇA MATERNIDADE (COMPLEMENTAR 60 DIAS)	CHC da Universidade Federal do Paraná - PR
12	LICENÇA MATERNIDADE (COMPLEMENTAR 60 DIAS)	CHC da Universidade Federal do Paraná - PR
12	LICENÇA MATERNIDADE (COMPLEMENTAR 60 DIAS)	CHC da Universidade Federal do Paraná - PR
12	LICENÇA MATERNIDADE (COMPLEMENTAR 60 DIAS)	CHC da Universidade Federal do Paraná - PR
12	LICENÇA MATERNIDADE (COMPLEMENTAR 60 DIAS)	CHC da Universidade Federal do Paraná - PR
12	LICENÇA MATERNIDADE (COMPLEMENTAR 60 DIAS)	CHC da Universidade Federal do Paraná - PR
12	LICENÇA MATERNIDADE (COMPLEMENTAR 60 DIAS)	CHC da Universidade Federal do Paraná - PR
12	LICENÇA MATERNIDADE (COMPLEMENTAR 60 DIAS)	CHC da Universidade Federal do Paraná - PR
12	LICENÇA MATERNIDADE (COMPLEMENTAR 60 DIAS)	CHC da Universidade Federal do Paraná - PR
12	LICENÇA MATERNIDADE (COMPLEMENTAR 60 DIAS)	CHC da Universidade Federal do Paraná - PR
12	LICENÇA MATERNIDADE (COMPLEMENTAR 60 DIAS)	CHC da Universidade Federal do Paraná - PR
12	LICENÇA MATERNIDADE (COMPLEMENTAR 60 DIAS)	CHC da Universidade Federal do Paraná - PR
12	LICENÇA MATERNIDADE (COMPLEMENTAR 60 DIAS)	CHC da Universidade Federal do Paraná - PR
12	LICENÇA MATERNIDADE (COMPLEMENTAR 60 DIAS)	CHC da Universidade Federal do Paraná - PR
14	LICENÇA GALA - CLT ART 473 II	CHC da Universidade Federal do Paraná - PR
14	LICENÇA GALA - CLT ART 473 II	CHC da Universidade Federal do Paraná - PR
14	LICENÇA GALA - CLT ART 473 II	CHC da Universidade Federal do Paraná - PR
14	LICENÇA GALA - CLT ART 473 II	CHC da Universidade Federal do Paraná - PR
14	LICENÇA GALA - CLT ART 473 II	CHC da Universidade Federal do Paraná - PR
14	LICENÇA GALA - CLT ART 473 II	CHC da Universidade Federal do Paraná - PR
14	LICENÇA GALA - CLT ART 473 II	CHC da Universidade Federal do Paraná - PR

14	LICENÇA GALA - CLT ART 473 II	CHC da Universidade Federal do Paraná - PR
14	LICENÇA GALA - CLT ART 473 II	CHC da Universidade Federal do Paraná - PR
14	LICENÇA GALA - CLT ART 473 II	CHC da Universidade Federal do Paraná - PR
23	CAPACITAÇAO/EVENTO POR INTERESSE DA EMPRESA - ATE 30 DIAS	CHC da Universidade Federal do Paraná - PR
36	AUXILIO DOENÇA (INSS) - A PARTIR DO 16 DIA	CHC da Universidade Federal do Paraná - PR
36	AUXILIO DOENÇA (INSS) - A PARTIR DO 16 DIA	CHC da Universidade Federal do Paraná - PR
36	AUXILIO DOENÇA (INSS) - A PARTIR DO 16 DIA	CHC da Universidade Federal do Paraná - PR
36	AUXILIO DOENÇA (INSS) - A PARTIR DO 16 DIA	CHC da Universidade Federal do Paraná - PR
36	AUXILIO DOENÇA (INSS) - A PARTIR DO 16 DIA	CHC da Universidade Federal do Paraná - PR
36	AUXILIO DOENÇA (INSS) - A PARTIR DO 16 DIA	CHC da Universidade Federal do Paraná - PR
36	AUXILIO DOENÇA (INSS) - A PARTIR DO 16 DIA	CHC da Universidade Federal do Paraná - PR
36	AUXILIO DOENÇA (INSS) - A PARTIR DO 16 DIA	CHC da Universidade Federal do Paraná - PR
36	AUXILIO DOENÇA (INSS) - A PARTIR DO 16 DIA	CHC da Universidade Federal do Paraná - PR
36	AUXILIO DOENÇA (INSS) - A PARTIR DO 16 DIA	CHC da Universidade Federal do Paraná - PR
36	AUXILIO DOENÇA (INSS) - A PARTIR DO 16 DIA	CHC da Universidade Federal do Paraná - PR
36	AUXILIO DOENÇA (INSS) - A PARTIR DO 16 DIA	CHC da Universidade Federal do Paraná - PR
36	AUXILIO DOENÇA (INSS) - A PARTIR DO 16 DIA	CHC da Universidade Federal do Paraná - PR
36	AUXILIO DOENÇA (INSS) - A PARTIR DO 16 DIA	CHC da Universidade Federal do Paraná - PR
36	AUXILIO DOENÇA (INSS) - A PARTIR DO 16 DIA	CHC da Universidade Federal do Paraná - PR
36	AUXILIO DOENÇA (INSS) - A PARTIR DO 16 DIA	CHC da Universidade Federal do Paraná - PR
36	AUXILIO DOENÇA (INSS) - A PARTIR DO 16 DIA	CHC da Universidade Federal do Paraná - PR
36	AUXILIO DOENÇA (INSS) - A PARTIR DO 16 DIA	CHC da Universidade Federal do Paraná - PR
36	AUXILIO DOENÇA (INSS) - A PARTIR DO 16 DIA	CHC da Universidade Federal do Paraná - PR
36	AUXILIO DOENÇA (INSS) - A PARTIR DO 16 DIA	CHC da Universidade Federal do Paraná - PR
36	AUXILIO DOENÇA (INSS) - A PARTIR DO 16 DIA	CHC da Universidade Federal do Paraná - PR
36	AUXILIO DOENÇA (INSS) - A PARTIR DO 16 DIA	CHC da Universidade Federal do Paraná - PR
36	AUXILIO DOENÇA (INSS) - A PARTIR DO 16 DIA	CHC da Universidade Federal do Paraná - PR
36	AUXILIO DOENÇA (INSS) - A PARTIR DO 16 DIA	CHC da Universidade Federal do Paraná - PR
36	AUXILIO DOENÇA (INSS) - A PARTIR DO 16 DIA	CHC da Universidade Federal do Paraná - PR
36	AUXILIO DOENÇA (INSS) - A PARTIR DO 16 DIA	CHC da Universidade Federal do Paraná - PR
36	AUXILIO DOENÇA (INSS) - A PARTIR DO 16 DIA	CHC da Universidade Federal do Paraná - PR
36	AUXILIO DOENÇA (INSS) - A PARTIR DO 16 DIA	CHC da Universidade Federal do Paraná - PR
36	AUXILIO DOENÇA (INSS) - A PARTIR DO 16 DIA	CHC da Universidade Federal do Paraná - PR
36	AUXILIO DOENÇA (INSS) - A PARTIR DO 16 DIA	CHC da Universidade Federal do Paraná - PR
36	AUXILIO DOENÇA (INSS) - A PARTIR DO 16 DIA	CHC da Universidade Federal do Paraná - PR
36	AUXILIO DOENÇA (INSS) - A PARTIR DO 16 DIA	CHC da Universidade Federal do Paraná - PR
36	AUXILIO DOENÇA (INSS) - A PARTIR DO 16 DIA	CHC da Universidade Federal do Paraná - PR
36	AUXILIO DOENÇA (INSS) - A PARTIR DO 16 DIA	CHC da Universidade Federal do Paraná - PR
36	AUXILIO DOENÇA (INSS) - A PARTIR DO 16 DIA	CHC da Universidade Federal do Paraná - PR
36	AUXILIO DOENÇA (INSS) - A PARTIR DO 16 DIA	CHC da Universidade Federal do Paraná - PR
36	AUXILIO DOENÇA (INSS) - A PARTIR DO 16 DIA	CHC da Universidade Federal do Paraná - PR
36	AUXILIO DOENÇA (INSS) - A PARTIR DO 16 DIA	CHC da Universidade Federal do Paraná - PR
36	AUXILIO DOENÇA (INSS) - A PARTIR DO 16 DIA	CHC da Universidade Federal do Paraná - PR
36	AUXILIO DOENÇA (INSS) - A PARTIR DO 16 DIA	CHC da Universidade Federal do Paraná - PR
36	AUXILIO DOENÇA (INSS) - A PARTIR DO 16 DIA	CHC da Universidade Federal do Paraná - PR
36	AUXILIO DOENÇA (INSS) - A PARTIR DO 16 DIA	CHC da Universidade Federal do Paraná - PR
36	AUXILIO DOENÇA (INSS) - A PARTIR DO 16 DIA	CHC da Universidade Federal do Paraná - PR

36	AUXILIO DOENÇA (INSS) - A PARTIR DO 16 DIA	CHC da Universidade Federal do Paraná - PR
36	AUXILIO DOENÇA (INSS) - A PARTIR DO 16 DIA	CHC da Universidade Federal do Paraná - PR
36	AUXILIO DOENÇA (INSS) - A PARTIR DO 16 DIA	CHC da Universidade Federal do Paraná - PR
36	AUXILIO DOENÇA (INSS) - A PARTIR DO 16 DIA	CHC da Universidade Federal do Paraná - PR
36	AUXILIO DOENÇA (INSS) - A PARTIR DO 16 DIA	CHC da Universidade Federal do Paraná - PR
36	AUXILIO DOENÇA (INSS) - A PARTIR DO 16 DIA	CHC da Universidade Federal do Paraná - PR
36	AUXILIO DOENÇA (INSS) - A PARTIR DO 16 DIA	CHC da Universidade Federal do Paraná - PR
36	AUXILIO DOENÇA (INSS) - A PARTIR DO 16 DIA	CHC da Universidade Federal do Paraná - PR
36	AUXILIO DOENÇA (INSS) - A PARTIR DO 16 DIA	CHC da Universidade Federal do Paraná - PR
36	AUXILIO DOENÇA (INSS) - A PARTIR DO 16 DIA	CHC da Universidade Federal do Paraná - PR
36	AUXILIO DOENÇA (INSS) - A PARTIR DO 16 DIA	CHC da Universidade Federal do Paraná - PR
36	AUXILIO DOENÇA (INSS) - A PARTIR DO 16 DIA	CHC da Universidade Federal do Paraná - PR
36	AUXILIO DOENÇA (INSS) - A PARTIR DO 16 DIA	CHC da Universidade Federal do Paraná - PR
36	AUXILIO DOENÇA (INSS) - A PARTIR DO 16 DIA	CHC da Universidade Federal do Paraná - PR
36	AUXILIO DOENÇA (INSS) - A PARTIR DO 16 DIA	CHC da Universidade Federal do Paraná - PR
36	AUXILIO DOENÇA (INSS) - A PARTIR DO 16 DIA	CHC da Universidade Federal do Paraná - PR
36	AUXILIO DOENÇA (INSS) - A PARTIR DO 16 DIA	CHC da Universidade Federal do Paraná - PR
36	AUXILIO DOENÇA (INSS) - A PARTIR DO 16 DIA	CHC da Universidade Federal do Paraná - PR
36	AUXILIO DOENÇA (INSS) - A PARTIR DO 16 DIA	CHC da Universidade Federal do Paraná - PR
36	AUXILIO DOENÇA (INSS) - A PARTIR DO 16 DIA	CHC da Universidade Federal do Paraná - PR
36	AUXILIO DOENÇA (INSS) - A PARTIR DO 16 DIA	CHC da Universidade Federal do Paraná - PR
36	AUXILIO DOENÇA (INSS) - A PARTIR DO 16 DIA	CHC da Universidade Federal do Paraná - PR
36	AUXILIO DOENÇA (INSS) - A PARTIR DO 16 DIA	CHC da Universidade Federal do Paraná - PR
36	AUXILIO DOENÇA (INSS) - A PARTIR DO 16 DIA	CHC da Universidade Federal do Paraná - PR
36	AUXILIO DOENÇA (INSS) - A PARTIR DO 16 DIA	CHC da Universidade Federal do Paraná - PR
36	AUXILIO DOENÇA (INSS) - A PARTIR DO 16 DIA	CHC da Universidade Federal do Paraná - PR
36	AUXILIO DOENÇA (INSS) - A PARTIR DO 16 DIA	CHC da Universidade Federal do Paraná - PR
36	AUXILIO DOENÇA (INSS) - A PARTIR DO 16 DIA	CHC da Universidade Federal do Paraná - PR
36	AUXILIO DOENÇA (INSS) - A PARTIR DO 16 DIA	CHC da Universidade Federal do Paraná - PR
36	AUXILIO DOENÇA (INSS) - A PARTIR DO 16 DIA	CHC da Universidade Federal do Paraná - PR
36	AUXILIO DOENÇA (INSS) - A PARTIR DO 16 DIA	CHC da Universidade Federal do Paraná - PR
36	AUXILIO DOENÇA (INSS) - A PARTIR DO 16 DIA	CHC da Universidade Federal do Paraná - PR
36	AUXILIO DOENÇA (INSS) - A PARTIR DO 16 DIA	CHC da Universidade Federal do Paraná - PR
36	AUXILIO DOENÇA (INSS) - A PARTIR DO 16 DIA	CHC da Universidade Federal do Paraná - PR
36	AUXILIO DOENÇA (INSS) - A PARTIR DO 16 DIA	CHC da Universidade Federal do Paraná - PR
36	AUXILIO DOENÇA (INSS) - A PARTIR DO 16 DIA	CHC da Universidade Federal do Paraná - PR
36	AUXILIO DOENÇA (INSS) - A PARTIR DO 16 DIA	CHC da Universidade Federal do Paraná - PR
36	AUXILIO DOENÇA (INSS) - A PARTIR DO 16 DIA	CHC da Universidade Federal do Paraná - PR
36	AUXILIO DOENÇA (INSS) - A PARTIR DO 16 DIA	CHC da Universidade Federal do Paraná - PR
36	AUXILIO DOENÇA (INSS) - A PARTIR DO 16 DIA	CHC da Universidade Federal do Paraná - PR
36	AUXILIO DOENÇA (INSS) - A PARTIR DO 16 DIA	CHC da Universidade Federal do Paraná - PR
36	AUXILIO DOENÇA (INSS) - A PARTIR DO 16 DIA	CHC da Universidade Federal do Paraná - PR
36	AUXILIO DOENÇA (INSS) - A PARTIR DO 16 DIA	CHC da Universidade Federal do Paraná - PR
36	AUXILIO DOENÇA (INSS) - A PARTIR DO 16 DIA	CHC da Universidade Federal do Paraná - PR

36	AUXILIO DOENÇA (INSS) - A PARTIR DO 16 DIA	CHC da Universidade Federal do Paraná - PR
36	AUXILIO DOENÇA (INSS) - A PARTIR DO 16 DIA	CHC da Universidade Federal do Paraná - PR
36	AUXILIO DOENÇA (INSS) - A PARTIR DO 16 DIA	CHC da Universidade Federal do Paraná - PR
36	AUXILIO DOENÇA (INSS) - A PARTIR DO 16 DIA	CHC da Universidade Federal do Paraná - PR
36	AUXILIO DOENÇA (INSS) - A PARTIR DO 16 DIA	CHC da Universidade Federal do Paraná - PR
36	AUXILIO DOENÇA (INSS) - A PARTIR DO 16 DIA	CHC da Universidade Federal do Paraná - PR
36	AUXILIO DOENÇA (INSS) - A PARTIR DO 16 DIA	CHC da Universidade Federal do Paraná - PR
36	AUXILIO DOENÇA (INSS) - A PARTIR DO 16 DIA	CHC da Universidade Federal do Paraná - PR
36	AUXILIO DOENÇA (INSS) - A PARTIR DO 16 DIA	CHC da Universidade Federal do Paraná - PR
42	DOAÇÃO DE SANGUE - REGULAMENTO DE PESSOAL ART 36 I	CHC da Universidade Federal do Paraná - PR
42	DOAÇÃO DE SANGUE - REGULAMENTO DE PESSOAL ART 36 I	CHC da Universidade Federal do Paraná - PR
42	DOAÇÃO DE SANGUE - REGULAMENTO DE PESSOAL ART 36 I	CHC da Universidade Federal do Paraná - PR
42	DOAÇÃO DE SANGUE - REGULAMENTO DE PESSOAL ART 36 I	CHC da Universidade Federal do Paraná - PR
42	DOAÇÃO DE SANGUE - REGULAMENTO DE PESSOAL ART 36 I	CHC da Universidade Federal do Paraná - PR
42	DOAÇÃO DE SANGUE - REGULAMENTO DE PESSOAL ART 36 I	CHC da Universidade Federal do Paraná - PR
42	DOAÇÃO DE SANGUE - REGULAMENTO DE PESSOAL ART 36 I	CHC da Universidade Federal do Paraná - PR
42	DOAÇÃO DE SANGUE - REGULAMENTO DE PESSOAL ART 36 I	CHC da Universidade Federal do Paraná - PR
42	DOAÇÃO DE SANGUE - REGULAMENTO DE PESSOAL ART 36 I	CHC da Universidade Federal do Paraná - PR
42	DOAÇÃO DE SANGUE - REGULAMENTO DE PESSOAL ART 36 I	CHC da Universidade Federal do Paraná - PR
42	DOAÇÃO DE SANGUE - REGULAMENTO DE PESSOAL ART 36 I	CHC da Universidade Federal do Paraná - PR
42	DOAÇÃO DE SANGUE - REGULAMENTO DE PESSOAL ART 36 I	CHC da Universidade Federal do Paraná - PR
42	DOAÇÃO DE SANGUE - REGULAMENTO DE PESSOAL ART 36 I	CHC da Universidade Federal do Paraná - PR
42	DOAÇÃO DE SANGUE - REGULAMENTO DE PESSOAL ART 36 I	CHC da Universidade Federal do Paraná - PR
42	DOAÇÃO DE SANGUE - REGULAMENTO DE PESSOAL ART 36 I	CHC da Universidade Federal do Paraná - PR
42	DOAÇÃO DE SANGUE - REGULAMENTO DE PESSOAL ART 36 I	CHC da Universidade Federal do Paraná - PR
42	DOAÇÃO DE SANGUE - REGULAMENTO DE PESSOAL ART 36 I	CHC da Universidade Federal do Paraná - PR
42	DOAÇÃO DE SANGUE - REGULAMENTO DE PESSOAL ART 36 I	CHC da Universidade Federal do Paraná - PR
42	DOAÇÃO DE SANGUE - REGULAMENTO DE PESSOAL ART 36 I	CHC da Universidade Federal do Paraná - PR
42	DOAÇÃO DE SANGUE - REGULAMENTO DE PESSOAL ART 36 I	CHC da Universidade Federal do Paraná - PR
42	DOAÇÃO DE SANGUE - REGULAMENTO DE PESSOAL ART 36 I	CHC da Universidade Federal do Paraná - PR
42	DOAÇÃO DE SANGUE - REGULAMENTO DE PESSOAL ART 36 I	CHC da Universidade Federal do Paraná - PR
42	DOAÇÃO DE SANGUE - REGULAMENTO DE PESSOAL ART 36 I	CHC da Universidade Federal do Paraná - PR
42	DOAÇÃO DE SANGUE - REGULAMENTO DE PESSOAL ART 36 I	CHC da Universidade Federal do Paraná - PR
42	DOAÇÃO DE SANGUE - REGULAMENTO DE PESSOAL ART 36 I	CHC da Universidade Federal do Paraná - PR
42	DOAÇÃO DE SANGUE - REGULAMENTO DE PESSOAL ART 36 I	CHC da Universidade Federal do Paraná - PR
42	DOAÇÃO DE SANGUE - REGULAMENTO DE PESSOAL ART 36 I	CHC da Universidade Federal do Paraná - PR
42	DOAÇÃO DE SANGUE - REGULAMENTO DE PESSOAL ART 36 I	CHC da Universidade Federal do Paraná - PR
42	DOAÇÃO DE SANGUE - REGULAMENTO DE PESSOAL ART 36 I	CHC da Universidade Federal do Paraná - PR
42	DOAÇÃO DE SANGUE - REGULAMENTO DE PESSOAL ART 36 I	CHC da Universidade Federal do Paraná - PR
42	DOAÇÃO DE SANGUE - REGULAMENTO DE PESSOAL ART 36 I	CHC da Universidade Federal do Paraná - PR
42	DOAÇÃO DE SANGUE - REGULAMENTO DE PESSOAL ART 36 I	CHC da Universidade Federal do Paraná - PR
42	DOAÇÃO DE SANGUE - REGULAMENTO DE PESSOAL ART 36 I	CHC da Universidade Federal do Paraná - PR
42	DOAÇÃO DE SANGUE - REGULAMENTO DE PESSOAL ART 36 I	CHC da Universidade Federal do Paraná - PR
42	DOAÇÃO DE SANGUE - REGULAMENTO DE PESSOAL ART 36 I	CHC da Universidade Federal do Paraná - PR
42	DOAÇÃO DE SANGUE - REGULAMENTO DE PESSOAL ART 36 I	CHC da Universidade Federal do Paraná - PR
42	DOAÇÃO DE SANGUE - REGULAMENTO DE PESSOAL ART 36 I	CHC da Universidade Federal do Paraná - PR
42	DOAÇÃO DE SANGUE - REGULAMENTO DE PESSOAL ART 36 I	CHC da Universidade Federal do Paraná - PR

42	DOAÇÃO DE SANGUE - REGULAMENTO DE PESSOAL ART 36 I	CHC da Universidade Federal do Paraná - PR
42	DOAÇÃO DE SANGUE - REGULAMENTO DE PESSOAL ART 36 I	CHC da Universidade Federal do Paraná - PR
42	DOAÇÃO DE SANGUE - REGULAMENTO DE PESSOAL ART 36 I	CHC da Universidade Federal do Paraná - PR
42	DOAÇÃO DE SANGUE - REGULAMENTO DE PESSOAL ART 36 I	CHC da Universidade Federal do Paraná - PR
42	DOAÇÃO DE SANGUE - REGULAMENTO DE PESSOAL ART 36 I	CHC da Universidade Federal do Paraná - PR
42	DOAÇÃO DE SANGUE - REGULAMENTO DE PESSOAL ART 36 I	CHC da Universidade Federal do Paraná - PR
42	DOAÇÃO DE SANGUE - REGULAMENTO DE PESSOAL ART 36 I	CHC da Universidade Federal do Paraná - PR
42	DOAÇÃO DE SANGUE - REGULAMENTO DE PESSOAL ART 36 I	CHC da Universidade Federal do Paraná - PR
42	DOAÇÃO DE SANGUE - REGULAMENTO DE PESSOAL ART 36 I	CHC da Universidade Federal do Paraná - PR
45	FALTA INJUSTIFICADA (GERA DESCONTO NA FOLHA)	CHC da Universidade Federal do Paraná - PR
45	FALTA INJUSTIFICADA (GERA DESCONTO NA FOLHA)	CHC da Universidade Federal do Paraná - PR
45	FALTA INJUSTIFICADA (GERA DESCONTO NA FOLHA)	CHC da Universidade Federal do Paraná - PR
45	FALTA INJUSTIFICADA (GERA DESCONTO NA FOLHA)	CHC da Universidade Federal do Paraná - PR
45	FALTA INJUSTIFICADA (GERA DESCONTO NA FOLHA)	CHC da Universidade Federal do Paraná - PR
45	FALTA INJUSTIFICADA (GERA DESCONTO NA FOLHA)	CHC da Universidade Federal do Paraná - PR
45	FALTA INJUSTIFICADA (GERA DESCONTO NA FOLHA)	CHC da Universidade Federal do Paraná - PR
45	FALTA INJUSTIFICADA (GERA DESCONTO NA FOLHA)	CHC da Universidade Federal do Paraná - PR
45	FALTA INJUSTIFICADA (GERA DESCONTO NA FOLHA)	CHC da Universidade Federal do Paraná - PR
45	FALTA INJUSTIFICADA (GERA DESCONTO NA FOLHA)	CHC da Universidade Federal do Paraná - PR
45	FALTA INJUSTIFICADA (GERA DESCONTO NA FOLHA)	CHC da Universidade Federal do Paraná - PR
45	FALTA INJUSTIFICADA (GERA DESCONTO NA FOLHA)	CHC da Universidade Federal do Paraná - PR
45	FALTA INJUSTIFICADA (GERA DESCONTO NA FOLHA)	CHC da Universidade Federal do Paraná - PR
45	FALTA INJUSTIFICADA (GERA DESCONTO NA FOLHA)	CHC da Universidade Federal do Paraná - PR
45	FALTA INJUSTIFICADA (GERA DESCONTO NA FOLHA)	CHC da Universidade Federal do Paraná - PR
45	FALTA INJUSTIFICADA (GERA DESCONTO NA FOLHA)	CHC da Universidade Federal do Paraná - PR
45	FALTA INJUSTIFICADA (GERA DESCONTO NA FOLHA)	CHC da Universidade Federal do Paraná - PR
45	FALTA INJUSTIFICADA (GERA DESCONTO NA FOLHA)	CHC da Universidade Federal do Paraná - PR
45	FALTA INJUSTIFICADA (GERA DESCONTO NA FOLHA)	CHC da Universidade Federal do Paraná - PR
45	FALTA INJUSTIFICADA (GERA DESCONTO NA FOLHA)	CHC da Universidade Federal do Paraná - PR
45	FALTA INJUSTIFICADA (GERA DESCONTO NA FOLHA)	CHC da Universidade Federal do Paraná - PR
45	FALTA INJUSTIFICADA (GERA DESCONTO NA FOLHA)	CHC da Universidade Federal do Paraná - PR
45	FALTA INJUSTIFICADA (GERA DESCONTO NA FOLHA)	CHC da Universidade Federal do Paraná - PR
45	FALTA INJUSTIFICADA (GERA DESCONTO NA FOLHA)	CHC da Universidade Federal do Paraná - PR
45	FALTA INJUSTIFICADA (GERA DESCONTO NA FOLHA)	CHC da Universidade Federal do Paraná - PR
45	FALTA INJUSTIFICADA (GERA DESCONTO NA FOLHA)	CHC da Universidade Federal do Paraná - PR
45	FALTA INJUSTIFICADA (GERA DESCONTO NA FOLHA)	CHC da Universidade Federal do Paraná - PR
45	FALTA INJUSTIFICADA (GERA DESCONTO NA FOLHA)	CHC da Universidade Federal do Paraná - PR
45	FALTA INJUSTIFICADA (GERA DESCONTO NA FOLHA)	CHC da Universidade Federal do Paraná - PR
45	FALTA INJUSTIFICADA (GERA DESCONTO NA FOLHA)	CHC da Universidade Federal do Paraná - PR
45	FALTA INJUSTIFICADA (GERA DESCONTO NA FOLHA)	CHC da Universidade Federal do Paraná - PR
45	FALTA INJUSTIFICADA (GERA DESCONTO NA FOLHA)	CHC da Universidade Federal do Paraná - PR
45	FALTA INJUSTIFICADA (GERA DESCONTO NA FOLHA)	CHC da Universidade Federal do Paraná - PR
45	FALTA INJUSTIFICADA (GERA DESCONTO NA FOLHA)	CHC da Universidade Federal do Paraná - PR
45	FALTA INJUSTIFICADA (GERA DESCONTO NA FOLHA)	CHC da Universidade Federal do Paraná - PR
45	FALTA INJUSTIFICADA (GERA DESCONTO NA FOLHA)	CHC da Universidade Federal do Paraná - PR
45	FALTA INJUSTIFICADA (GERA DESCONTO NA FOLHA)	CHC da Universidade Federal do Paraná - PR
45	FALTA INJUSTIFICADA (GERA DESCONTO NA FOLHA)	CHC da Universidade Federal do Paraná - PR
45	FALTA INJUSTIFICADA (GERA DESCONTO NA FOLHA)	CHC da Universidade Federal do Paraná - PR

45	FALTA INJUSTIFICADA (GERA DESCONTO NA FOLHA)	CHC da Universidade Federal do Paraná - PR
45	FALTA INJUSTIFICADA (GERA DESCONTO NA FOLHA)	CHC da Universidade Federal do Paraná - PR
45	FALTA INJUSTIFICADA (GERA DESCONTO NA FOLHA)	CHC da Universidade Federal do Paraná - PR
45	FALTA INJUSTIFICADA (GERA DESCONTO NA FOLHA)	CHC da Universidade Federal do Paraná - PR
45	FALTA INJUSTIFICADA (GERA DESCONTO NA FOLHA)	CHC da Universidade Federal do Paraná - PR
45	FALTA INJUSTIFICADA (GERA DESCONTO NA FOLHA)	CHC da Universidade Federal do Paraná - PR
45	FALTA INJUSTIFICADA (GERA DESCONTO NA FOLHA)	CHC da Universidade Federal do Paraná - PR
45	FALTA INJUSTIFICADA (GERA DESCONTO NA FOLHA)	CHC da Universidade Federal do Paraná - PR
45	FALTA INJUSTIFICADA (GERA DESCONTO NA FOLHA)	CHC da Universidade Federal do Paraná - PR
45	FALTA INJUSTIFICADA (GERA DESCONTO NA FOLHA)	CHC da Universidade Federal do Paraná - PR
45	FALTA INJUSTIFICADA (GERA DESCONTO NA FOLHA)	CHC da Universidade Federal do Paraná - PR
45	FALTA INJUSTIFICADA (GERA DESCONTO NA FOLHA)	CHC da Universidade Federal do Paraná - PR
45	FALTA INJUSTIFICADA (GERA DESCONTO NA FOLHA)	CHC da Universidade Federal do Paraná - PR
45	FALTA INJUSTIFICADA (GERA DESCONTO NA FOLHA)	CHC da Universidade Federal do Paraná - PR
45	FALTA INJUSTIFICADA (GERA DESCONTO NA FOLHA)	CHC da Universidade Federal do Paraná - PR
45	FALTA INJUSTIFICADA (GERA DESCONTO NA FOLHA)	CHC da Universidade Federal do Paraná - PR
45	FALTA INJUSTIFICADA (GERA DESCONTO NA FOLHA)	CHC da Universidade Federal do Paraná - PR
45	FALTA INJUSTIFICADA (GERA DESCONTO NA FOLHA)	CHC da Universidade Federal do Paraná - PR
45	FALTA INJUSTIFICADA (GERA DESCONTO NA FOLHA)	CHC da Universidade Federal do Paraná - PR
45	FALTA INJUSTIFICADA (GERA DESCONTO NA FOLHA)	CHC da Universidade Federal do Paraná - PR
45	FALTA INJUSTIFICADA (GERA DESCONTO NA FOLHA)	CHC da Universidade Federal do Paraná - PR
45	FALTA INJUSTIFICADA (GERA DESCONTO NA FOLHA)	CHC da Universidade Federal do Paraná - PR
45	FALTA INJUSTIFICADA (GERA DESCONTO NA FOLHA)	CHC da Universidade Federal do Paraná - PR
45	FALTA INJUSTIFICADA (GERA DESCONTO NA FOLHA)	CHC da Universidade Federal do Paraná - PR
45	FALTA INJUSTIFICADA (GERA DESCONTO NA FOLHA)	CHC da Universidade Federal do Paraná - PR
45	FALTA INJUSTIFICADA (GERA DESCONTO NA FOLHA)	CHC da Universidade Federal do Paraná - PR
45	FALTA INJUSTIFICADA (GERA DESCONTO NA FOLHA)	CHC da Universidade Federal do Paraná - PR
45	FALTA INJUSTIFICADA (GERA DESCONTO NA FOLHA)	CHC da Universidade Federal do Paraná - PR
45	FALTA INJUSTIFICADA (GERA DESCONTO NA FOLHA)	CHC da Universidade Federal do Paraná - PR
45	FALTA INJUSTIFICADA (GERA DESCONTO NA FOLHA)	CHC da Universidade Federal do Paraná - PR
45	FALTA INJUSTIFICADA (GERA DESCONTO NA FOLHA)	CHC da Universidade Federal do Paraná - PR
45	FALTA INJUSTIFICADA (GERA DESCONTO NA FOLHA)	CHC da Universidade Federal do Paraná - PR
45	FALTA INJUSTIFICADA (GERA DESCONTO NA FOLHA)	CHC da Universidade Federal do Paraná - PR
45	FALTA INJUSTIFICADA (GERA DESCONTO NA FOLHA)	CHC da Universidade Federal do Paraná - PR
45	FALTA INJUSTIFICADA (GERA DESCONTO NA FOLHA)	CHC da Universidade Federal do Paraná - PR
45	FALTA INJUSTIFICADA (GERA DESCONTO NA FOLHA)	CHC da Universidade Federal do Paraná - PR
45	FALTA INJUSTIFICADA (GERA DESCONTO NA FOLHA)	CHC da Universidade Federal do Paraná - PR
45	FALTA INJUSTIFICADA (GERA DESCONTO NA FOLHA)	CHC da Universidade Federal do Paraná - PR
45	FALTA INJUSTIFICADA (GERA DESCONTO NA FOLHA)	CHC da Universidade Federal do Paraná - PR
45	FALTA INJUSTIFICADA (GERA DESCONTO NA FOLHA)	CHC da Universidade Federal do Paraná - PR
45	FALTA INJUSTIFICADA (GERA DESCONTO NA FOLHA)	CHC da Universidade Federal do Paraná - PR
45	FALTA INJUSTIFICADA (GERA DESCONTO NA FOLHA)	CHC da Universidade Federal do Paraná - PR
45	FALTA INJUSTIFICADA (GERA DESCONTO NA FOLHA)	CHC da Universidade Federal do Paraná - PR
45	FALTA INJUSTIFICADA (GERA DESCONTO NA FOLHA)	CHC da Universidade Federal do Paraná - PR
45	FALTA INJUSTIFICADA (GERA DESCONTO NA FOLHA)	CHC da Universidade Federal do Paraná - PR
45	FALTA INJUSTIFICADA (GERA DESCONTO NA FOLHA)	CHC da Universidade Federal do Paraná - PR
45	FALTA INJUSTIFICADA (GERA DESCONTO NA FOLHA)	CHC da Universidade Federal do Paraná - PR
45	FALTA INJUSTIFICADA (GERA DESCONTO NA FOLHA)	CHC da Universidade Federal do Paraná - PR

45	FALTA INJUSTIFICADA (GERA DESCONTO NA FOLHA)	CHC da Universidade Federal do Paraná - PR
45	FALTA INJUSTIFICADA (GERA DESCONTO NA FOLHA)	CHC da Universidade Federal do Paraná - PR
45	FALTA INJUSTIFICADA (GERA DESCONTO NA FOLHA)	CHC da Universidade Federal do Paraná - PR
45	FALTA INJUSTIFICADA (GERA DESCONTO NA FOLHA)	CHC da Universidade Federal do Paraná - PR
45	FALTA INJUSTIFICADA (GERA DESCONTO NA FOLHA)	CHC da Universidade Federal do Paraná - PR
45	FALTA INJUSTIFICADA (GERA DESCONTO NA FOLHA)	CHC da Universidade Federal do Paraná - PR
45	FALTA INJUSTIFICADA (GERA DESCONTO NA FOLHA)	CHC da Universidade Federal do Paraná - PR
45	FALTA INJUSTIFICADA (GERA DESCONTO NA FOLHA)	CHC da Universidade Federal do Paraná - PR
45	FALTA INJUSTIFICADA (GERA DESCONTO NA FOLHA)	CHC da Universidade Federal do Paraná - PR
45	FALTA INJUSTIFICADA (GERA DESCONTO NA FOLHA)	CHC da Universidade Federal do Paraná - PR
45	FALTA INJUSTIFICADA (GERA DESCONTO NA FOLHA)	CHC da Universidade Federal do Paraná - PR
45	FALTA INJUSTIFICADA (GERA DESCONTO NA FOLHA)	CHC da Universidade Federal do Paraná - PR
45	FALTA INJUSTIFICADA (GERA DESCONTO NA FOLHA)	CHC da Universidade Federal do Paraná - PR
45	FALTA INJUSTIFICADA (GERA DESCONTO NA FOLHA)	CHC da Universidade Federal do Paraná - PR
45	FALTA INJUSTIFICADA (GERA DESCONTO NA FOLHA)	CHC da Universidade Federal do Paraná - PR
45	FALTA INJUSTIFICADA (GERA DESCONTO NA FOLHA)	CHC da Universidade Federal do Paraná - PR
45	FALTA INJUSTIFICADA (GERA DESCONTO NA FOLHA)	CHC da Universidade Federal do Paraná - PR
45	FALTA INJUSTIFICADA (GERA DESCONTO NA FOLHA)	CHC da Universidade Federal do Paraná - PR
45	FALTA INJUSTIFICADA (GERA DESCONTO NA FOLHA)	CHC da Universidade Federal do Paraná - PR
45	FALTA INJUSTIFICADA (GERA DESCONTO NA FOLHA)	CHC da Universidade Federal do Paraná - PR
45	FALTA INJUSTIFICADA (GERA DESCONTO NA FOLHA)	CHC da Universidade Federal do Paraná - PR
45	FALTA INJUSTIFICADA (GERA DESCONTO NA FOLHA)	CHC da Universidade Federal do Paraná - PR
45	FALTA INJUSTIFICADA (GERA DESCONTO NA FOLHA)	CHC da Universidade Federal do Paraná - PR
45	FALTA INJUSTIFICADA (GERA DESCONTO NA FOLHA)	CHC da Universidade Federal do Paraná - PR
45	FALTA INJUSTIFICADA (GERA DESCONTO NA FOLHA)	CHC da Universidade Federal do Paraná - PR
45	FALTA INJUSTIFICADA (GERA DESCONTO NA FOLHA)	CHC da Universidade Federal do Paraná - PR
45	FALTA INJUSTIFICADA (GERA DESCONTO NA FOLHA)	CHC da Universidade Federal do Paraná - PR
45	FALTA INJUSTIFICADA (GERA DESCONTO NA FOLHA)	CHC da Universidade Federal do Paraná - PR
45	FALTA INJUSTIFICADA (GERA DESCONTO NA FOLHA)	CHC da Universidade Federal do Paraná - PR
45	FALTA INJUSTIFICADA (GERA DESCONTO NA FOLHA)	CHC da Universidade Federal do Paraná - PR
45	FALTA INJUSTIFICADA (GERA DESCONTO NA FOLHA)	CHC da Universidade Federal do Paraná - PR
45	FALTA INJUSTIFICADA (GERA DESCONTO NA FOLHA)	CHC da Universidade Federal do Paraná - PR
45	FALTA INJUSTIFICADA (GERA DESCONTO NA FOLHA)	CHC da Universidade Federal do Paraná - PR
45	FALTA INJUSTIFICADA (GERA DESCONTO NA FOLHA)	CHC da Universidade Federal do Paraná - PR
45	FALTA INJUSTIFICADA (GERA DESCONTO NA FOLHA)	CHC da Universidade Federal do Paraná - PR
45	FALTA INJUSTIFICADA (GERA DESCONTO NA FOLHA)	CHC da Universidade Federal do Paraná - PR
45	FALTA INJUSTIFICADA (GERA DESCONTO NA FOLHA)	CHC da Universidade Federal do Paraná - PR
45	FALTA INJUSTIFICADA (GERA DESCONTO NA FOLHA)	CHC da Universidade Federal do Paraná - PR
45	FALTA INJUSTIFICADA (GERA DESCONTO NA FOLHA)	CHC da Universidade Federal do Paraná - PR
45	FALTA INJUSTIFICADA (GERA DESCONTO NA FOLHA)	CHC da Universidade Federal do Paraná - PR
45	FALTA INJUSTIFICADA (GERA DESCONTO NA FOLHA)	CHC da Universidade Federal do Paraná - PR
45	FALTA INJUSTIFICADA (GERA DESCONTO NA FOLHA)	CHC da Universidade Federal do Paraná - PR
47	AFASTAMENTO ACIDENTE DO TRABALHO - ATE 15 DIA	CHC da Universidade Federal do Paraná - PR
47	AFASTAMENTO ACIDENTE DO TRABALHO - ATE 15 DIA	CHC da Universidade Federal do Paraná - PR
47	AFASTAMENTO ACIDENTE DO TRABALHO - ATE 15 DIA	CHC da Universidade Federal do Paraná - PR
47	AFASTAMENTO ACIDENTE DO TRABALHO - ATE 15 DIA	CHC da Universidade Federal do Paraná - PR

47	AFASTAMENTO ACIDENTE DO TRABALHO - ATE 15 DIA	CHC da Universidade Federal do Paraná - PR
47	AFASTAMENTO ACIDENTE DO TRABALHO - ATE 15 DIA	CHC da Universidade Federal do Paraná - PR
47	AFASTAMENTO ACIDENTE DO TRABALHO - ATE 15 DIA	CHC da Universidade Federal do Paraná - PR
47	AFASTAMENTO ACIDENTE DO TRABALHO - ATE 15 DIA	CHC da Universidade Federal do Paraná - PR
47	AFASTAMENTO ACIDENTE DO TRABALHO - ATE 15 DIA	CHC da Universidade Federal do Paraná - PR
47	AFASTAMENTO ACIDENTE DO TRABALHO - ATE 15 DIA	CHC da Universidade Federal do Paraná - PR
48	AFASTAMENTO ACIDENTE DO TRABALHO A PARTIR 16 DIA (INSS)	CHC da Universidade Federal do Paraná - PR
48	AFASTAMENTO ACIDENTE DO TRABALHO A PARTIR 16 DIA (INSS)	CHC da Universidade Federal do Paraná - PR
52	AFASTAMENTO ACIDENTE DO TRABALHO (TRAJETO) ATE 15 DIA	CHC da Universidade Federal do Paraná - PR
52	AFASTAMENTO ACIDENTE DO TRABALHO (TRAJETO) ATE 15 DIA	CHC da Universidade Federal do Paraná - PR
52	AFASTAMENTO ACIDENTE DO TRABALHO (TRAJETO) ATE 15 DIA	CHC da Universidade Federal do Paraná - PR
53	APOSENTADORIA POR INVALIDEZ - CLT ART. 475	CHC da Universidade Federal do Paraná - PR
57	AFASTAMENTO POR ATESTADO MÉDICO - ATE 15 DIAS	CHC da Universidade Federal do Paraná - PR
57	AFASTAMENTO POR ATESTADO MÉDICO - ATE 15 DIAS	CHC da Universidade Federal do Paraná - PR
57	AFASTAMENTO POR ATESTADO MÉDICO - ATE 15 DIAS	CHC da Universidade Federal do Paraná - PR
57	AFASTAMENTO POR ATESTADO MÉDICO - ATE 15 DIAS	CHC da Universidade Federal do Paraná - PR
57	AFASTAMENTO POR ATESTADO MÉDICO - ATE 15 DIAS	CHC da Universidade Federal do Paraná - PR
57	AFASTAMENTO POR ATESTADO MÉDICO - ATE 15 DIAS	CHC da Universidade Federal do Paraná - PR
57	AFASTAMENTO POR ATESTADO MÉDICO - ATE 15 DIAS	CHC da Universidade Federal do Paraná - PR
57	AFASTAMENTO POR ATESTADO MÉDICO - ATE 15 DIAS	CHC da Universidade Federal do Paraná - PR
57	AFASTAMENTO POR ATESTADO MÉDICO - ATE 15 DIAS	CHC da Universidade Federal do Paraná - PR
57	AFASTAMENTO POR ATESTADO MÉDICO - ATE 15 DIAS	CHC da Universidade Federal do Paraná - PR
57	AFASTAMENTO POR ATESTADO MÉDICO - ATE 15 DIAS	CHC da Universidade Federal do Paraná - PR
57	AFASTAMENTO POR ATESTADO MÉDICO - ATE 15 DIAS	CHC da Universidade Federal do Paraná - PR
57	AFASTAMENTO POR ATESTADO MÉDICO - ATE 15 DIAS	CHC da Universidade Federal do Paraná - PR
57	AFASTAMENTO POR ATESTADO MÉDICO - ATE 15 DIAS	CHC da Universidade Federal do Paraná - PR
57	AFASTAMENTO POR ATESTADO MÉDICO - ATE 15 DIAS	CHC da Universidade Federal do Paraná - PR
57	AFASTAMENTO POR ATESTADO MÉDICO - ATE 15 DIAS	CHC da Universidade Federal do Paraná - PR
57	AFASTAMENTO POR ATESTADO MÉDICO - ATE 15 DIAS	CHC da Universidade Federal do Paraná - PR
57	AFASTAMENTO POR ATESTADO MÉDICO - ATE 15 DIAS	CHC da Universidade Federal do Paraná - PR
57	AFASTAMENTO POR ATESTADO MÉDICO - ATE 15 DIAS	CHC da Universidade Federal do Paraná - PR
57	AFASTAMENTO POR ATESTADO MÉDICO - ATE 15 DIAS	CHC da Universidade Federal do Paraná - PR
57	AFASTAMENTO POR ATESTADO MÉDICO - ATE 15 DIAS	CHC da Universidade Federal do Paraná - PR
57	AFASTAMENTO POR ATESTADO MÉDICO - ATE 15 DIAS	CHC da Universidade Federal do Paraná - PR
57	AFASTAMENTO POR ATESTADO MÉDICO - ATE 15 DIAS	CHC da Universidade Federal do Paraná - PR
57	AFASTAMENTO POR ATESTADO MÉDICO - ATE 15 DIAS	CHC da Universidade Federal do Paraná - PR
57	AFASTAMENTO POR ATESTADO MÉDICO - ATE 15 DIAS	CHC da Universidade Federal do Paraná - PR
57	AFASTAMENTO POR ATESTADO MÉDICO - ATE 15 DIAS	CHC da Universidade Federal do Paraná - PR
57	AFASTAMENTO POR ATESTADO MÉDICO - ATE 15 DIAS	CHC da Universidade Federal do Paraná - PR
57	AFASTAMENTO POR ATESTADO MÉDICO - ATE 15 DIAS	CHC da Universidade Federal do Paraná - PR
57	AFASTAMENTO POR ATESTADO MÉDICO - ATE 15 DIAS	CHC da Universidade Federal do Paraná - PR
57	AFASTAMENTO POR ATESTADO MÉDICO - ATE 15 DIAS	CHC da Universidade Federal do Paraná - PR
57	AFASTAMENTO POR ATESTADO MÉDICO - ATE 15 DIAS	CHC da Universidade Federal do Paraná - PR
57	AFASTAMENTO POR ATESTADO MÉDICO - ATE 15 DIAS	CHC da Universidade Federal do Paraná - PR
57	AFASTAMENTO POR ATESTADO MÉDICO - ATE 15 DIAS	CHC da Universidade Federal do Paraná - PR
57	AFASTAMENTO POR ATESTADO MÉDICO - ATE 15 DIAS	CHC da Universidade Federal do Paraná - PR
57	AFASTAMENTO POR ATESTADO MÉDICO - ATE 15 DIAS	CHC da Universidade Federal do Paraná - PR

57	AFASTAMENTO POR ATESTADO MÉDICO - ATE 15 DIAS	CHC da Universidade Federal do Paraná - PR
57	AFASTAMENTO POR ATESTADO MÉDICO - ATE 15 DIAS	CHC da Universidade Federal do Paraná - PR
57	AFASTAMENTO POR ATESTADO MÉDICO - ATE 15 DIAS	CHC da Universidade Federal do Paraná - PR
57	AFASTAMENTO POR ATESTADO MÉDICO - ATE 15 DIAS	CHC da Universidade Federal do Paraná - PR
57	AFASTAMENTO POR ATESTADO MÉDICO - ATE 15 DIAS	CHC da Universidade Federal do Paraná - PR
57	AFASTAMENTO POR ATESTADO MÉDICO - ATE 15 DIAS	CHC da Universidade Federal do Paraná - PR
57	AFASTAMENTO POR ATESTADO MÉDICO - ATE 15 DIAS	CHC da Universidade Federal do Paraná - PR
57	AFASTAMENTO POR ATESTADO MÉDICO - ATE 15 DIAS	CHC da Universidade Federal do Paraná - PR
57	AFASTAMENTO POR ATESTADO MÉDICO - ATE 15 DIAS	CHC da Universidade Federal do Paraná - PR
57	AFASTAMENTO POR ATESTADO MÉDICO - ATE 15 DIAS	CHC da Universidade Federal do Paraná - PR
57	AFASTAMENTO POR ATESTADO MÉDICO - ATE 15 DIAS	CHC da Universidade Federal do Paraná - PR
57	AFASTAMENTO POR ATESTADO MÉDICO - ATE 15 DIAS	CHC da Universidade Federal do Paraná - PR
57	AFASTAMENTO POR ATESTADO MÉDICO - ATE 15 DIAS	CHC da Universidade Federal do Paraná - PR
57	AFASTAMENTO POR ATESTADO MÉDICO - ATE 15 DIAS	CHC da Universidade Federal do Paraná - PR
57	AFASTAMENTO POR ATESTADO MÉDICO - ATE 15 DIAS	CHC da Universidade Federal do Paraná - PR
57	AFASTAMENTO POR ATESTADO MÉDICO - ATE 15 DIAS	CHC da Universidade Federal do Paraná - PR
57	AFASTAMENTO POR ATESTADO MÉDICO - ATE 15 DIAS	CHC da Universidade Federal do Paraná - PR
57	AFASTAMENTO POR ATESTADO MÉDICO - ATE 15 DIAS	CHC da Universidade Federal do Paraná - PR
57	AFASTAMENTO POR ATESTADO MÉDICO - ATE 15 DIAS	CHC da Universidade Federal do Paraná - PR
57	AFASTAMENTO POR ATESTADO MÉDICO - ATE 15 DIAS	CHC da Universidade Federal do Paraná - PR
57	AFASTAMENTO POR ATESTADO MÉDICO - ATE 15 DIAS	CHC da Universidade Federal do Paraná - PR
57	AFASTAMENTO POR ATESTADO MÉDICO - ATE 15 DIAS	CHC da Universidade Federal do Paraná - PR
57	AFASTAMENTO POR ATESTADO MÉDICO - ATE 15 DIAS	CHC da Universidade Federal do Paraná - PR
57	AFASTAMENTO POR ATESTADO MÉDICO - ATE 15 DIAS	CHC da Universidade Federal do Paraná - PR
57	AFASTAMENTO POR ATESTADO MÉDICO - ATE 15 DIAS	CHC da Universidade Federal do Paraná - PR
57	AFASTAMENTO POR ATESTADO MÉDICO - ATE 15 DIAS	CHC da Universidade Federal do Paraná - PR
57	AFASTAMENTO POR ATESTADO MÉDICO - ATE 15 DIAS	CHC da Universidade Federal do Paraná - PR
57	AFASTAMENTO POR ATESTADO MÉDICO - ATE 15 DIAS	CHC da Universidade Federal do Paraná - PR
57	AFASTAMENTO POR ATESTADO MÉDICO - ATE 15 DIAS	CHC da Universidade Federal do Paraná - PR
57	AFASTAMENTO POR ATESTADO MÉDICO - ATE 15 DIAS	CHC da Universidade Federal do Paraná - PR
57	AFASTAMENTO POR ATESTADO MÉDICO - ATE 15 DIAS	CHC da Universidade Federal do Paraná - PR
57	AFASTAMENTO POR ATESTADO MÉDICO - ATE 15 DIAS	CHC da Universidade Federal do Paraná - PR
57	AFASTAMENTO POR ATESTADO MÉDICO - ATE 15 DIAS	CHC da Universidade Federal do Paraná - PR
57	AFASTAMENTO POR ATESTADO MÉDICO - ATE 15 DIAS	CHC da Universidade Federal do Paraná - PR
57	AFASTAMENTO POR ATESTADO MÉDICO - ATE 15 DIAS	CHC da Universidade Federal do Paraná - PR
57	AFASTAMENTO POR ATESTADO MÉDICO - ATE 15 DIAS	CHC da Universidade Federal do Paraná - PR
57	AFASTAMENTO POR ATESTADO MÉDICO - ATE 15 DIAS	CHC da Universidade Federal do Paraná - PR
57	AFASTAMENTO POR ATESTADO MÉDICO - ATE 15 DIAS	CHC da Universidade Federal do Paraná - PR
57	AFASTAMENTO POR ATESTADO MÉDICO - ATE 15 DIAS	CHC da Universidade Federal do Paraná - PR
57	AFASTAMENTO POR ATESTADO MÉDICO - ATE 15 DIAS	CHC da Universidade Federal do Paraná - PR
57	AFASTAMENTO POR ATESTADO MÉDICO - ATE 15 DIAS	CHC da Universidade Federal do Paraná - PR
57	AFASTAMENTO POR ATESTADO MÉDICO - ATE 15 DIAS	CHC da Universidade Federal do Paraná - PR
57	AFASTAMENTO POR ATESTADO MÉDICO - ATE 15 DIAS	CHC da Universidade Federal do Paraná - PR
57	AFASTAMENTO POR ATESTADO MÉDICO - ATE 15 DIAS	CHC da Universidade Federal do Paraná - PR
57	AFASTAMENTO POR ATESTADO MÉDICO - ATE 15 DIAS	CHC da Universidade Federal do Paraná - PR
57	AFASTAMENTO POR ATESTADO MÉDICO - ATE 15 DIAS	CHC da Universidade Federal do Paraná - PR
57	AFASTAMENTO POR ATESTADO MÉDICO - ATE 15 DIAS	CHC da Universidade Federal do Paraná - PR
57	AFASTAMENTO POR ATESTADO MÉDICO - ATE 15 DIAS	CHC da Universidade Federal do Paraná - PR

57	AFASTAMENTO POR ATESTADO MÉDICO - ATE 15 DIAS	CHC da Universidade Federal do Paraná - PR
57	AFASTAMENTO POR ATESTADO MÉDICO - ATE 15 DIAS	CHC da Universidade Federal do Paraná - PR
57	AFASTAMENTO POR ATESTADO MÉDICO - ATE 15 DIAS	CHC da Universidade Federal do Paraná - PR
57	AFASTAMENTO POR ATESTADO MÉDICO - ATE 15 DIAS	CHC da Universidade Federal do Paraná - PR
57	AFASTAMENTO POR ATESTADO MÉDICO - ATE 15 DIAS	CHC da Universidade Federal do Paraná - PR
57	AFASTAMENTO POR ATESTADO MÉDICO - ATE 15 DIAS	CHC da Universidade Federal do Paraná - PR
57	AFASTAMENTO POR ATESTADO MÉDICO - ATE 15 DIAS	CHC da Universidade Federal do Paraná - PR
57	AFASTAMENTO POR ATESTADO MÉDICO - ATE 15 DIAS	CHC da Universidade Federal do Paraná - PR
57	AFASTAMENTO POR ATESTADO MÉDICO - ATE 15 DIAS	CHC da Universidade Federal do Paraná - PR
57	AFASTAMENTO POR ATESTADO MÉDICO - ATE 15 DIAS	CHC da Universidade Federal do Paraná - PR
57	AFASTAMENTO POR ATESTADO MÉDICO - ATE 15 DIAS	CHC da Universidade Federal do Paraná - PR
57	AFASTAMENTO POR ATESTADO MÉDICO - ATE 15 DIAS	CHC da Universidade Federal do Paraná - PR
57	AFASTAMENTO POR ATESTADO MÉDICO - ATE 15 DIAS	CHC da Universidade Federal do Paraná - PR
57	AFASTAMENTO POR ATESTADO MÉDICO - ATE 15 DIAS	CHC da Universidade Federal do Paraná - PR
57	AFASTAMENTO POR ATESTADO MÉDICO - ATE 15 DIAS	CHC da Universidade Federal do Paraná - PR
57	AFASTAMENTO POR ATESTADO MÉDICO - ATE 15 DIAS	CHC da Universidade Federal do Paraná - PR
57	AFASTAMENTO POR ATESTADO MÉDICO - ATE 15 DIAS	CHC da Universidade Federal do Paraná - PR
57	AFASTAMENTO POR ATESTADO MÉDICO - ATE 15 DIAS	CHC da Universidade Federal do Paraná - PR
57	AFASTAMENTO POR ATESTADO MÉDICO - ATE 15 DIAS	CHC da Universidade Federal do Paraná - PR
57	AFASTAMENTO POR ATESTADO MÉDICO - ATE 15 DIAS	CHC da Universidade Federal do Paraná - PR
57	AFASTAMENTO POR ATESTADO MÉDICO - ATE 15 DIAS	CHC da Universidade Federal do Paraná - PR
57	AFASTAMENTO POR ATESTADO MÉDICO - ATE 15 DIAS	CHC da Universidade Federal do Paraná - PR
57	AFASTAMENTO POR ATESTADO MÉDICO - ATE 15 DIAS	CHC da Universidade Federal do Paraná - PR
57	AFASTAMENTO POR ATESTADO MÉDICO - ATE 15 DIAS	CHC da Universidade Federal do Paraná - PR
57	AFASTAMENTO POR ATESTADO MÉDICO - ATE 15 DIAS	CHC da Universidade Federal do Paraná - PR
57	AFASTAMENTO POR ATESTADO MÉDICO - ATE 15 DIAS	CHC da Universidade Federal do Paraná - PR
57	AFASTAMENTO POR ATESTADO MÉDICO - ATE 15 DIAS	CHC da Universidade Federal do Paraná - PR
57	AFASTAMENTO POR ATESTADO MÉDICO - ATE 15 DIAS	CHC da Universidade Federal do Paraná - PR
57	AFASTAMENTO POR ATESTADO MÉDICO - ATE 15 DIAS	CHC da Universidade Federal do Paraná - PR
57	AFASTAMENTO POR ATESTADO MÉDICO - ATE 15 DIAS	CHC da Universidade Federal do Paraná - PR
57	AFASTAMENTO POR ATESTADO MÉDICO - ATE 15 DIAS	CHC da Universidade Federal do Paraná - PR
57	AFASTAMENTO POR ATESTADO MÉDICO - ATE 15 DIAS	CHC da Universidade Federal do Paraná - PR
57	AFASTAMENTO POR ATESTADO MÉDICO - ATE 15 DIAS	CHC da Universidade Federal do Paraná - PR
57	AFASTAMENTO POR ATESTADO MÉDICO - ATE 15 DIAS	CHC da Universidade Federal do Paraná - PR
57	AFASTAMENTO POR ATESTADO MÉDICO - ATE 15 DIAS	CHC da Universidade Federal do Paraná - PR
57	AFASTAMENTO POR ATESTADO MÉDICO - ATE 15 DIAS	CHC da Universidade Federal do Paraná - PR
57	AFASTAMENTO POR ATESTADO MÉDICO - ATE 15 DIAS	CHC da Universidade Federal do Paraná - PR
57	AFASTAMENTO POR ATESTADO MÉDICO - ATE 15 DIAS	CHC da Universidade Federal do Paraná - PR
57	AFASTAMENTO POR ATESTADO MÉDICO - ATE 15 DIAS	CHC da Universidade Federal do Paraná - PR
57	AFASTAMENTO POR ATESTADO MÉDICO - ATE 15 DIAS	CHC da Universidade Federal do Paraná - PR
57	AFASTAMENTO POR ATESTADO MÉDICO - ATE 15 DIAS	CHC da Universidade Federal do Paraná - PR
57	AFASTAMENTO POR ATESTADO MÉDICO - ATE 15 DIAS	CHC da Universidade Federal do Paraná - PR
57	AFASTAMENTO POR ATESTADO MÉDICO - ATE 15 DIAS	CHC da Universidade Federal do Paraná - PR
57	AFASTAMENTO POR ATESTADO MÉDICO - ATE 15 DIAS	CHC da Universidade Federal do Paraná - PR
57	AFASTAMENTO POR ATESTADO MÉDICO - ATE 15 DIAS	CHC da Universidade Federal do Paraná - PR
57	AFASTAMENTO POR ATESTADO MÉDICO - ATE 15 DIAS	CHC da Universidade Federal do Paraná - PR
57	AFASTAMENTO POR ATESTADO MÉDICO - ATE 15 DIAS	CHC da Universidade Federal do Paraná - PR
57	AFASTAMENTO POR ATESTADO MÉDICO - ATE 15 DIAS	CHC da Universidade Federal do Paraná - PR

57	AFASTAMENTO POR ATESTADO MÉDICO - ATE 15 DIAS	CHC da Universidade Federal do Paraná - PR
57	AFASTAMENTO POR ATESTADO MÉDICO - ATE 15 DIAS	CHC da Universidade Federal do Paraná - PR
57	AFASTAMENTO POR ATESTADO MÉDICO - ATE 15 DIAS	CHC da Universidade Federal do Paraná - PR
57	AFASTAMENTO POR ATESTADO MÉDICO - ATE 15 DIAS	CHC da Universidade Federal do Paraná - PR
57	AFASTAMENTO POR ATESTADO MÉDICO - ATE 15 DIAS	CHC da Universidade Federal do Paraná - PR
57	AFASTAMENTO POR ATESTADO MÉDICO - ATE 15 DIAS	CHC da Universidade Federal do Paraná - PR
57	AFASTAMENTO POR ATESTADO MÉDICO - ATE 15 DIAS	CHC da Universidade Federal do Paraná - PR
57	AFASTAMENTO POR ATESTADO MÉDICO - ATE 15 DIAS	CHC da Universidade Federal do Paraná - PR
57	AFASTAMENTO POR ATESTADO MÉDICO - ATE 15 DIAS	CHC da Universidade Federal do Paraná - PR
57	AFASTAMENTO POR ATESTADO MÉDICO - ATE 15 DIAS	CHC da Universidade Federal do Paraná - PR
57	AFASTAMENTO POR ATESTADO MÉDICO - ATE 15 DIAS	CHC da Universidade Federal do Paraná - PR
57	AFASTAMENTO POR ATESTADO MÉDICO - ATE 15 DIAS	CHC da Universidade Federal do Paraná - PR
57	AFASTAMENTO POR ATESTADO MÉDICO - ATE 15 DIAS	CHC da Universidade Federal do Paraná - PR
57	AFASTAMENTO POR ATESTADO MÉDICO - ATE 15 DIAS	CHC da Universidade Federal do Paraná - PR
57	AFASTAMENTO POR ATESTADO MÉDICO - ATE 15 DIAS	CHC da Universidade Federal do Paraná - PR
57	AFASTAMENTO POR ATESTADO MÉDICO - ATE 15 DIAS	CHC da Universidade Federal do Paraná - PR
57	AFASTAMENTO POR ATESTADO MÉDICO - ATE 15 DIAS	CHC da Universidade Federal do Paraná - PR
57	AFASTAMENTO POR ATESTADO MÉDICO - ATE 15 DIAS	CHC da Universidade Federal do Paraná - PR
57	AFASTAMENTO POR ATESTADO MÉDICO - ATE 15 DIAS	CHC da Universidade Federal do Paraná - PR
57	AFASTAMENTO POR ATESTADO MÉDICO - ATE 15 DIAS	CHC da Universidade Federal do Paraná - PR
57	AFASTAMENTO POR ATESTADO MÉDICO - ATE 15 DIAS	CHC da Universidade Federal do Paraná - PR
57	AFASTAMENTO POR ATESTADO MÉDICO - ATE 15 DIAS	CHC da Universidade Federal do Paraná - PR
57	AFASTAMENTO POR ATESTADO MÉDICO - ATE 15 DIAS	CHC da Universidade Federal do Paraná - PR
57	AFASTAMENTO POR ATESTADO MÉDICO - ATE 15 DIAS	CHC da Universidade Federal do Paraná - PR
57	AFASTAMENTO POR ATESTADO MÉDICO - ATE 15 DIAS	CHC da Universidade Federal do Paraná - PR
57	AFASTAMENTO POR ATESTADO MÉDICO - ATE 15 DIAS	CHC da Universidade Federal do Paraná - PR
57	AFASTAMENTO POR ATESTADO MÉDICO - ATE 15 DIAS	CHC da Universidade Federal do Paraná - PR
57	AFASTAMENTO POR ATESTADO MÉDICO - ATE 15 DIAS	CHC da Universidade Federal do Paraná - PR
57	AFASTAMENTO POR ATESTADO MÉDICO - ATE 15 DIAS	CHC da Universidade Federal do Paraná - PR
57	AFASTAMENTO POR ATESTADO MÉDICO - ATE 15 DIAS	CHC da Universidade Federal do Paraná - PR
57	AFASTAMENTO POR ATESTADO MÉDICO - ATE 15 DIAS	CHC da Universidade Federal do Paraná - PR
57	AFASTAMENTO POR ATESTADO MÉDICO - ATE 15 DIAS	CHC da Universidade Federal do Paraná - PR
57	AFASTAMENTO POR ATESTADO MÉDICO - ATE 15 DIAS	CHC da Universidade Federal do Paraná - PR
57	AFASTAMENTO POR ATESTADO MÉDICO - ATE 15 DIAS	CHC da Universidade Federal do Paraná - PR
57	AFASTAMENTO POR ATESTADO MÉDICO - ATE 15 DIAS	CHC da Universidade Federal do Paraná - PR
57	AFASTAMENTO POR ATESTADO MÉDICO - ATE 15 DIAS	CHC da Universidade Federal do Paraná - PR
57	AFASTAMENTO POR ATESTADO MÉDICO - ATE 15 DIAS	CHC da Universidade Federal do Paraná - PR
57	AFASTAMENTO POR ATESTADO MÉDICO - ATE 15 DIAS	CHC da Universidade Federal do Paraná - PR
57	AFASTAMENTO POR ATESTADO MÉDICO - ATE 15 DIAS	CHC da Universidade Federal do Paraná - PR
57	AFASTAMENTO POR ATESTADO MÉDICO - ATE 15 DIAS	CHC da Universidade Federal do Paraná - PR
57	AFASTAMENTO POR ATESTADO MÉDICO - ATE 15 DIAS	CHC da Universidade Federal do Paraná - PR
57	AFASTAMENTO POR ATESTADO MÉDICO - ATE 15 DIAS	CHC da Universidade Federal do Paraná - PR
57	AFASTAMENTO POR ATESTADO MÉDICO - ATE 15 DIAS	CHC da Universidade Federal do Paraná - PR
57	AFASTAMENTO POR ATESTADO MÉDICO - ATE 15 DIAS	CHC da Universidade Federal do Paraná - PR
57	AFASTAMENTO POR ATESTADO MÉDICO - ATE 15 DIAS	CHC da Universidade Federal do Paraná - PR
57	AFASTAMENTO POR ATESTADO MÉDICO - ATE 15 DIAS	CHC da Universidade Federal do Paraná - PR
57	AFASTAMENTO POR ATESTADO MÉDICO - ATE 15 DIAS	CHC da Universidade Federal do Paraná - PR
57	AFASTAMENTO POR ATESTADO MÉDICO - ATE 15 DIAS	CHC da Universidade Federal do Paraná - PR

57	AFASTAMENTO POR ATESTADO MÉDICO - ATE 15 DIAS	CHC da Universidade Federal do Paraná - PR
57	AFASTAMENTO POR ATESTADO MÉDICO - ATE 15 DIAS	CHC da Universidade Federal do Paraná - PR
57	AFASTAMENTO POR ATESTADO MÉDICO - ATE 15 DIAS	CHC da Universidade Federal do Paraná - PR
57	AFASTAMENTO POR ATESTADO MÉDICO - ATE 15 DIAS	CHC da Universidade Federal do Paraná - PR
57	AFASTAMENTO POR ATESTADO MÉDICO - ATE 15 DIAS	CHC da Universidade Federal do Paraná - PR
57	AFASTAMENTO POR ATESTADO MÉDICO - ATE 15 DIAS	CHC da Universidade Federal do Paraná - PR
57	AFASTAMENTO POR ATESTADO MÉDICO - ATE 15 DIAS	CHC da Universidade Federal do Paraná - PR
57	AFASTAMENTO POR ATESTADO MÉDICO - ATE 15 DIAS	CHC da Universidade Federal do Paraná - PR
57	AFASTAMENTO POR ATESTADO MÉDICO - ATE 15 DIAS	CHC da Universidade Federal do Paraná - PR
57	AFASTAMENTO POR ATESTADO MÉDICO - ATE 15 DIAS	CHC da Universidade Federal do Paraná - PR
57	AFASTAMENTO POR ATESTADO MÉDICO - ATE 15 DIAS	CHC da Universidade Federal do Paraná - PR
57	AFASTAMENTO POR ATESTADO MÉDICO - ATE 15 DIAS	CHC da Universidade Federal do Paraná - PR
57	AFASTAMENTO POR ATESTADO MÉDICO - ATE 15 DIAS	CHC da Universidade Federal do Paraná - PR
57	AFASTAMENTO POR ATESTADO MÉDICO - ATE 15 DIAS	CHC da Universidade Federal do Paraná - PR
57	AFASTAMENTO POR ATESTADO MÉDICO - ATE 15 DIAS	CHC da Universidade Federal do Paraná - PR
57	AFASTAMENTO POR ATESTADO MÉDICO - ATE 15 DIAS	CHC da Universidade Federal do Paraná - PR
57	AFASTAMENTO POR ATESTADO MÉDICO - ATE 15 DIAS	CHC da Universidade Federal do Paraná - PR
57	AFASTAMENTO POR ATESTADO MÉDICO - ATE 15 DIAS	CHC da Universidade Federal do Paraná - PR
57	AFASTAMENTO POR ATESTADO MÉDICO - ATE 15 DIAS	CHC da Universidade Federal do Paraná - PR
57	AFASTAMENTO POR ATESTADO MÉDICO - ATE 15 DIAS	CHC da Universidade Federal do Paraná - PR
57	AFASTAMENTO POR ATESTADO MÉDICO - ATE 15 DIAS	CHC da Universidade Federal do Paraná - PR
57	AFASTAMENTO POR ATESTADO MÉDICO - ATE 15 DIAS	CHC da Universidade Federal do Paraná - PR
57	AFASTAMENTO POR ATESTADO MÉDICO - ATE 15 DIAS	CHC da Universidade Federal do Paraná - PR
57	AFASTAMENTO POR ATESTADO MÉDICO - ATE 15 DIAS	CHC da Universidade Federal do Paraná - PR
57	AFASTAMENTO POR ATESTADO MÉDICO - ATE 15 DIAS	CHC da Universidade Federal do Paraná - PR
57	AFASTAMENTO POR ATESTADO MÉDICO - ATE 15 DIAS	CHC da Universidade Federal do Paraná - PR
57	AFASTAMENTO POR ATESTADO MÉDICO - ATE 15 DIAS	CHC da Universidade Federal do Paraná - PR
57	AFASTAMENTO POR ATESTADO MÉDICO - ATE 15 DIAS	CHC da Universidade Federal do Paraná - PR
57	AFASTAMENTO POR ATESTADO MÉDICO - ATE 15 DIAS	CHC da Universidade Federal do Paraná - PR
57	AFASTAMENTO POR ATESTADO MÉDICO - ATE 15 DIAS	CHC da Universidade Federal do Paraná - PR
57	AFASTAMENTO POR ATESTADO MÉDICO - ATE 15 DIAS	CHC da Universidade Federal do Paraná - PR
57	AFASTAMENTO POR ATESTADO MÉDICO - ATE 15 DIAS	CHC da Universidade Federal do Paraná - PR
57	AFASTAMENTO POR ATESTADO MÉDICO - ATE 15 DIAS	CHC da Universidade Federal do Paraná - PR
57	AFASTAMENTO POR ATESTADO MÉDICO - ATE 15 DIAS	CHC da Universidade Federal do Paraná - PR
57	AFASTAMENTO POR ATESTADO MÉDICO - ATE 15 DIAS	CHC da Universidade Federal do Paraná - PR
57	AFASTAMENTO POR ATESTADO MÉDICO - ATE 15 DIAS	CHC da Universidade Federal do Paraná - PR
57	AFASTAMENTO POR ATESTADO MÉDICO - ATE 15 DIAS	CHC da Universidade Federal do Paraná - PR
57	AFASTAMENTO POR ATESTADO MÉDICO - ATE 15 DIAS	CHC da Universidade Federal do Paraná - PR
57	AFASTAMENTO POR ATESTADO MÉDICO - ATE 15 DIAS	CHC da Universidade Federal do Paraná - PR
57	AFASTAMENTO POR ATESTADO MÉDICO - ATE 15 DIAS	CHC da Universidade Federal do Paraná - PR
57	AFASTAMENTO POR ATESTADO MÉDICO - ATE 15 DIAS	CHC da Universidade Federal do Paraná - PR
57	AFASTAMENTO POR ATESTADO MÉDICO - ATE 15 DIAS	CHC da Universidade Federal do Paraná - PR
57	AFASTAMENTO POR ATESTADO MÉDICO - ATE 15 DIAS	CHC da Universidade Federal do Paraná - PR
57	AFASTAMENTO POR ATESTADO MÉDICO - ATE 15 DIAS	CHC da Universidade Federal do Paraná - PR
57	AFASTAMENTO POR ATESTADO MÉDICO - ATE 15 DIAS	CHC da Universidade Federal do Paraná - PR
57	AFASTAMENTO POR ATESTADO MÉDICO - ATE 15 DIAS	CHC da Universidade Federal do Paraná - PR
57	AFASTAMENTO POR ATESTADO MÉDICO - ATE 15 DIAS	CHC da Universidade Federal do Paraná - PR
57	AFASTAMENTO POR ATESTADO MÉDICO - ATE 15 DIAS	CHC da Universidade Federal do Paraná - PR

57	AFASTAMENTO POR ATESTADO MÉDICO - ATE 15 DIAS	CHC da Universidade Federal do Paraná - PR
57	AFASTAMENTO POR ATESTADO MÉDICO - ATE 15 DIAS	CHC da Universidade Federal do Paraná - PR
57	AFASTAMENTO POR ATESTADO MÉDICO - ATE 15 DIAS	CHC da Universidade Federal do Paraná - PR
57	AFASTAMENTO POR ATESTADO MÉDICO - ATE 15 DIAS	CHC da Universidade Federal do Paraná - PR
57	AFASTAMENTO POR ATESTADO MÉDICO - ATE 15 DIAS	CHC da Universidade Federal do Paraná - PR
57	AFASTAMENTO POR ATESTADO MÉDICO - ATE 15 DIAS	CHC da Universidade Federal do Paraná - PR
57	AFASTAMENTO POR ATESTADO MÉDICO - ATE 15 DIAS	CHC da Universidade Federal do Paraná - PR
57	AFASTAMENTO POR ATESTADO MÉDICO - ATE 15 DIAS	CHC da Universidade Federal do Paraná - PR
57	AFASTAMENTO POR ATESTADO MÉDICO - ATE 15 DIAS	CHC da Universidade Federal do Paraná - PR
57	AFASTAMENTO POR ATESTADO MÉDICO - ATE 15 DIAS	CHC da Universidade Federal do Paraná - PR
57	AFASTAMENTO POR ATESTADO MÉDICO - ATE 15 DIAS	CHC da Universidade Federal do Paraná - PR
57	AFASTAMENTO POR ATESTADO MÉDICO - ATE 15 DIAS	CHC da Universidade Federal do Paraná - PR
57	AFASTAMENTO POR ATESTADO MÉDICO - ATE 15 DIAS	CHC da Universidade Federal do Paraná - PR
57	AFASTAMENTO POR ATESTADO MÉDICO - ATE 15 DIAS	CHC da Universidade Federal do Paraná - PR
57	AFASTAMENTO POR ATESTADO MÉDICO - ATE 15 DIAS	CHC da Universidade Federal do Paraná - PR
57	AFASTAMENTO POR ATESTADO MÉDICO - ATE 15 DIAS	CHC da Universidade Federal do Paraná - PR
57	AFASTAMENTO POR ATESTADO MÉDICO - ATE 15 DIAS	CHC da Universidade Federal do Paraná - PR
57	AFASTAMENTO POR ATESTADO MÉDICO - ATE 15 DIAS	CHC da Universidade Federal do Paraná - PR
57	AFASTAMENTO POR ATESTADO MÉDICO - ATE 15 DIAS	CHC da Universidade Federal do Paraná - PR
57	AFASTAMENTO POR ATESTADO MÉDICO - ATE 15 DIAS	CHC da Universidade Federal do Paraná - PR
57	AFASTAMENTO POR ATESTADO MÉDICO - ATE 15 DIAS	CHC da Universidade Federal do Paraná - PR
57	AFASTAMENTO POR ATESTADO MÉDICO - ATE 15 DIAS	CHC da Universidade Federal do Paraná - PR
57	AFASTAMENTO POR ATESTADO MÉDICO - ATE 15 DIAS	CHC da Universidade Federal do Paraná - PR
57	AFASTAMENTO POR ATESTADO MÉDICO - ATE 15 DIAS	CHC da Universidade Federal do Paraná - PR
57	AFASTAMENTO POR ATESTADO MÉDICO - ATE 15 DIAS	CHC da Universidade Federal do Paraná - PR
57	AFASTAMENTO POR ATESTADO MÉDICO - ATE 15 DIAS	CHC da Universidade Federal do Paraná - PR
57	AFASTAMENTO POR ATESTADO MÉDICO - ATE 15 DIAS	CHC da Universidade Federal do Paraná - PR
57	AFASTAMENTO POR ATESTADO MÉDICO - ATE 15 DIAS	CHC da Universidade Federal do Paraná - PR
57	AFASTAMENTO POR ATESTADO MÉDICO - ATE 15 DIAS	CHC da Universidade Federal do Paraná - PR
57	AFASTAMENTO POR ATESTADO MÉDICO - ATE 15 DIAS	CHC da Universidade Federal do Paraná - PR
57	AFASTAMENTO POR ATESTADO MÉDICO - ATE 15 DIAS	CHC da Universidade Federal do Paraná - PR
57	AFASTAMENTO POR ATESTADO MÉDICO - ATE 15 DIAS	CHC da Universidade Federal do Paraná - PR
57	AFASTAMENTO POR ATESTADO MÉDICO - ATE 15 DIAS	CHC da Universidade Federal do Paraná - PR
57	AFASTAMENTO POR ATESTADO MÉDICO - ATE 15 DIAS	CHC da Universidade Federal do Paraná - PR
57	AFASTAMENTO POR ATESTADO MÉDICO - ATE 15 DIAS	CHC da Universidade Federal do Paraná - PR
57	AFASTAMENTO POR ATESTADO MÉDICO - ATE 15 DIAS	CHC da Universidade Federal do Paraná - PR
57	AFASTAMENTO POR ATESTADO MÉDICO - ATE 15 DIAS	CHC da Universidade Federal do Paraná - PR
57	AFASTAMENTO POR ATESTADO MÉDICO - ATE 15 DIAS	CHC da Universidade Federal do Paraná - PR
57	AFASTAMENTO POR ATESTADO MÉDICO - ATE 15 DIAS	CHC da Universidade Federal do Paraná - PR
57	AFASTAMENTO POR ATESTADO MÉDICO - ATE 15 DIAS	CHC da Universidade Federal do Paraná - PR
57	AFASTAMENTO POR ATESTADO MÉDICO - ATE 15 DIAS	CHC da Universidade Federal do Paraná - PR
57	AFASTAMENTO POR ATESTADO MÉDICO - ATE 15 DIAS	CHC da Universidade Federal do Paraná - PR
57	AFASTAMENTO POR ATESTADO MÉDICO - ATE 15 DIAS	CHC da Universidade Federal do Paraná - PR
57	AFASTAMENTO POR ATESTADO MÉDICO - ATE 15 DIAS	CHC da Universidade Federal do Paraná - PR
57	AFASTAMENTO POR ATESTADO MÉDICO - ATE 15 DIAS	CHC da Universidade Federal do Paraná - PR
57	AFASTAMENTO POR ATESTADO MÉDICO - ATE 15 DIAS	CHC da Universidade Federal do Paraná - PR
57	AFASTAMENTO POR ATESTADO MÉDICO - ATE 15 DIAS	CHC da Universidade Federal do Paraná - PR
57	AFASTAMENTO POR ATESTADO MÉDICO - ATE 15 DIAS	CHC da Universidade Federal do Paraná - PR

57	AFASTAMENTO POR ATESTADO MÉDICO - ATE 15 DIAS	CHC da Universidade Federal do Paraná - PR
57	AFASTAMENTO POR ATESTADO MÉDICO - ATE 15 DIAS	CHC da Universidade Federal do Paraná - PR
57	AFASTAMENTO POR ATESTADO MÉDICO - ATE 15 DIAS	CHC da Universidade Federal do Paraná - PR
57	AFASTAMENTO POR ATESTADO MÉDICO - ATE 15 DIAS	CHC da Universidade Federal do Paraná - PR
57	AFASTAMENTO POR ATESTADO MÉDICO - ATE 15 DIAS	CHC da Universidade Federal do Paraná - PR
57	AFASTAMENTO POR ATESTADO MÉDICO - ATE 15 DIAS	CHC da Universidade Federal do Paraná - PR
57	AFASTAMENTO POR ATESTADO MÉDICO - ATE 15 DIAS	CHC da Universidade Federal do Paraná - PR
57	AFASTAMENTO POR ATESTADO MÉDICO - ATE 15 DIAS	CHC da Universidade Federal do Paraná - PR
57	AFASTAMENTO POR ATESTADO MÉDICO - ATE 15 DIAS	CHC da Universidade Federal do Paraná - PR
57	AFASTAMENTO POR ATESTADO MÉDICO - ATE 15 DIAS	CHC da Universidade Federal do Paraná - PR
57	AFASTAMENTO POR ATESTADO MÉDICO - ATE 15 DIAS	CHC da Universidade Federal do Paraná - PR
57	AFASTAMENTO POR ATESTADO MÉDICO - ATE 15 DIAS	CHC da Universidade Federal do Paraná - PR
57	AFASTAMENTO POR ATESTADO MÉDICO - ATE 15 DIAS	CHC da Universidade Federal do Paraná - PR
57	AFASTAMENTO POR ATESTADO MÉDICO - ATE 15 DIAS	CHC da Universidade Federal do Paraná - PR
57	AFASTAMENTO POR ATESTADO MÉDICO - ATE 15 DIAS	CHC da Universidade Federal do Paraná - PR
57	AFASTAMENTO POR ATESTADO MÉDICO - ATE 15 DIAS	CHC da Universidade Federal do Paraná - PR
57	AFASTAMENTO POR ATESTADO MÉDICO - ATE 15 DIAS	CHC da Universidade Federal do Paraná - PR
57	AFASTAMENTO POR ATESTADO MÉDICO - ATE 15 DIAS	CHC da Universidade Federal do Paraná - PR
57	AFASTAMENTO POR ATESTADO MÉDICO - ATE 15 DIAS	CHC da Universidade Federal do Paraná - PR
57	AFASTAMENTO POR ATESTADO MÉDICO - ATE 15 DIAS	CHC da Universidade Federal do Paraná - PR
57	AFASTAMENTO POR ATESTADO MÉDICO - ATE 15 DIAS	CHC da Universidade Federal do Paraná - PR
57	AFASTAMENTO POR ATESTADO MÉDICO - ATE 15 DIAS	CHC da Universidade Federal do Paraná - PR
57	AFASTAMENTO POR ATESTADO MÉDICO - ATE 15 DIAS	CHC da Universidade Federal do Paraná - PR
57	AFASTAMENTO POR ATESTADO MÉDICO - ATE 15 DIAS	CHC da Universidade Federal do Paraná - PR
57	AFASTAMENTO POR ATESTADO MÉDICO - ATE 15 DIAS	CHC da Universidade Federal do Paraná - PR
57	AFASTAMENTO POR ATESTADO MÉDICO - ATE 15 DIAS	CHC da Universidade Federal do Paraná - PR
57	AFASTAMENTO POR ATESTADO MÉDICO - ATE 15 DIAS	CHC da Universidade Federal do Paraná - PR
57	AFASTAMENTO POR ATESTADO MÉDICO - ATE 15 DIAS	CHC da Universidade Federal do Paraná - PR
57	AFASTAMENTO POR ATESTADO MÉDICO - ATE 15 DIAS	CHC da Universidade Federal do Paraná - PR
57	AFASTAMENTO POR ATESTADO MÉDICO - ATE 15 DIAS	CHC da Universidade Federal do Paraná - PR
57	AFASTAMENTO POR ATESTADO MÉDICO - ATE 15 DIAS	CHC da Universidade Federal do Paraná - PR
57	AFASTAMENTO POR ATESTADO MÉDICO - ATE 15 DIAS	CHC da Universidade Federal do Paraná - PR
57	AFASTAMENTO POR ATESTADO MÉDICO - ATE 15 DIAS	CHC da Universidade Federal do Paraná - PR
57	AFASTAMENTO POR ATESTADO MÉDICO - ATE 15 DIAS	CHC da Universidade Federal do Paraná - PR
57	AFASTAMENTO POR ATESTADO MÉDICO - ATE 15 DIAS	CHC da Universidade Federal do Paraná - PR
57	AFASTAMENTO POR ATESTADO MÉDICO - ATE 15 DIAS	CHC da Universidade Federal do Paraná - PR
57	AFASTAMENTO POR ATESTADO MÉDICO - ATE 15 DIAS	CHC da Universidade Federal do Paraná - PR
57	AFASTAMENTO POR ATESTADO MÉDICO - ATE 15 DIAS	CHC da Universidade Federal do Paraná - PR
57	AFASTAMENTO POR ATESTADO MÉDICO - ATE 15 DIAS	CHC da Universidade Federal do Paraná - PR
57	AFASTAMENTO POR ATESTADO MÉDICO - ATE 15 DIAS	CHC da Universidade Federal do Paraná - PR
57	AFASTAMENTO POR ATESTADO MÉDICO - ATE 15 DIAS	CHC da Universidade Federal do Paraná - PR
57	AFASTAMENTO POR ATESTADO MÉDICO - ATE 15 DIAS	CHC da Universidade Federal do Paraná - PR
57	AFASTAMENTO POR ATESTADO MÉDICO - ATE 15 DIAS	CHC da Universidade Federal do Paraná - PR
57	AFASTAMENTO POR ATESTADO MÉDICO - ATE 15 DIAS	CHC da Universidade Federal do Paraná - PR
57	AFASTAMENTO POR ATESTADO MÉDICO - ATE 15 DIAS	CHC da Universidade Federal do Paraná - PR
57	AFASTAMENTO POR ATESTADO MÉDICO - ATE 15 DIAS	CHC da Universidade Federal do Paraná - PR
57	AFASTAMENTO POR ATESTADO MÉDICO - ATE 15 DIAS	CHC da Universidade Federal do Paraná - PR
57	AFASTAMENTO POR ATESTADO MÉDICO - ATE 15 DIAS	CHC da Universidade Federal do Paraná - PR

57	AFASTAMENTO POR ATESTADO MÉDICO - ATE 15 DIAS	CHC da Universidade Federal do Paraná - PR
57	AFASTAMENTO POR ATESTADO MÉDICO - ATE 15 DIAS	CHC da Universidade Federal do Paraná - PR
57	AFASTAMENTO POR ATESTADO MÉDICO - ATE 15 DIAS	CHC da Universidade Federal do Paraná - PR
57	AFASTAMENTO POR ATESTADO MÉDICO - ATE 15 DIAS	CHC da Universidade Federal do Paraná - PR
57	AFASTAMENTO POR ATESTADO MÉDICO - ATE 15 DIAS	CHC da Universidade Federal do Paraná - PR
57	AFASTAMENTO POR ATESTADO MÉDICO - ATE 15 DIAS	CHC da Universidade Federal do Paraná - PR
57	AFASTAMENTO POR ATESTADO MÉDICO - ATE 15 DIAS	CHC da Universidade Federal do Paraná - PR
57	AFASTAMENTO POR ATESTADO MÉDICO - ATE 15 DIAS	CHC da Universidade Federal do Paraná - PR
57	AFASTAMENTO POR ATESTADO MÉDICO - ATE 15 DIAS	CHC da Universidade Federal do Paraná - PR
57	AFASTAMENTO POR ATESTADO MÉDICO - ATE 15 DIAS	CHC da Universidade Federal do Paraná - PR
57	AFASTAMENTO POR ATESTADO MÉDICO - ATE 15 DIAS	CHC da Universidade Federal do Paraná - PR
57	AFASTAMENTO POR ATESTADO MÉDICO - ATE 15 DIAS	CHC da Universidade Federal do Paraná - PR
57	AFASTAMENTO POR ATESTADO MÉDICO - ATE 15 DIAS	CHC da Universidade Federal do Paraná - PR
57	AFASTAMENTO POR ATESTADO MÉDICO - ATE 15 DIAS	CHC da Universidade Federal do Paraná - PR
57	AFASTAMENTO POR ATESTADO MÉDICO - ATE 15 DIAS	CHC da Universidade Federal do Paraná - PR
57	AFASTAMENTO POR ATESTADO MÉDICO - ATE 15 DIAS	CHC da Universidade Federal do Paraná - PR
57	AFASTAMENTO POR ATESTADO MÉDICO - ATE 15 DIAS	CHC da Universidade Federal do Paraná - PR
57	AFASTAMENTO POR ATESTADO MÉDICO - ATE 15 DIAS	CHC da Universidade Federal do Paraná - PR
57	AFASTAMENTO POR ATESTADO MÉDICO - ATE 15 DIAS	CHC da Universidade Federal do Paraná - PR
57	AFASTAMENTO POR ATESTADO MÉDICO - ATE 15 DIAS	CHC da Universidade Federal do Paraná - PR
57	AFASTAMENTO POR ATESTADO MÉDICO - ATE 15 DIAS	CHC da Universidade Federal do Paraná - PR
57	AFASTAMENTO POR ATESTADO MÉDICO - ATE 15 DIAS	CHC da Universidade Federal do Paraná - PR
57	AFASTAMENTO POR ATESTADO MÉDICO - ATE 15 DIAS	CHC da Universidade Federal do Paraná - PR
57	AFASTAMENTO POR ATESTADO MÉDICO - ATE 15 DIAS	CHC da Universidade Federal do Paraná - PR
57	AFASTAMENTO POR ATESTADO MÉDICO - ATE 15 DIAS	CHC da Universidade Federal do Paraná - PR
57	AFASTAMENTO POR ATESTADO MÉDICO - ATE 15 DIAS	CHC da Universidade Federal do Paraná - PR
57	AFASTAMENTO POR ATESTADO MÉDICO - ATE 15 DIAS	CHC da Universidade Federal do Paraná - PR
57	AFASTAMENTO POR ATESTADO MÉDICO - ATE 15 DIAS	CHC da Universidade Federal do Paraná - PR
57	AFASTAMENTO POR ATESTADO MÉDICO - ATE 15 DIAS	CHC da Universidade Federal do Paraná - PR
57	AFASTAMENTO POR ATESTADO MÉDICO - ATE 15 DIAS	CHC da Universidade Federal do Paraná - PR
57	AFASTAMENTO POR ATESTADO MÉDICO - ATE 15 DIAS	CHC da Universidade Federal do Paraná - PR
57	AFASTAMENTO POR ATESTADO MÉDICO - ATE 15 DIAS	CHC da Universidade Federal do Paraná - PR
57	AFASTAMENTO POR ATESTADO MÉDICO - ATE 15 DIAS	CHC da Universidade Federal do Paraná - PR
57	AFASTAMENTO POR ATESTADO MÉDICO - ATE 15 DIAS	CHC da Universidade Federal do Paraná - PR
57	AFASTAMENTO POR ATESTADO MÉDICO - ATE 15 DIAS	CHC da Universidade Federal do Paraná - PR
57	AFASTAMENTO POR ATESTADO MÉDICO - ATE 15 DIAS	CHC da Universidade Federal do Paraná - PR
57	AFASTAMENTO POR ATESTADO MÉDICO - ATE 15 DIAS	CHC da Universidade Federal do Paraná - PR
57	AFASTAMENTO POR ATESTADO MÉDICO - ATE 15 DIAS	CHC da Universidade Federal do Paraná - PR
57	AFASTAMENTO POR ATESTADO MÉDICO - ATE 15 DIAS	CHC da Universidade Federal do Paraná - PR
57	AFASTAMENTO POR ATESTADO MÉDICO - ATE 15 DIAS	CHC da Universidade Federal do Paraná - PR
57	AFASTAMENTO POR ATESTADO MÉDICO - ATE 15 DIAS	CHC da Universidade Federal do Paraná - PR
57	AFASTAMENTO POR ATESTADO MÉDICO - ATE 15 DIAS	CHC da Universidade Federal do Paraná - PR
57	AFASTAMENTO POR ATESTADO MÉDICO - ATE 15 DIAS	CHC da Universidade Federal do Paraná - PR
57	AFASTAMENTO POR ATESTADO MÉDICO - ATE 15 DIAS	CHC da Universidade Federal do Paraná - PR
57	AFASTAMENTO POR ATESTADO MÉDICO - ATE 15 DIAS	CHC da Universidade Federal do Paraná - PR
57	AFASTAMENTO POR ATESTADO MÉDICO - ATE 15 DIAS	CHC da Universidade Federal do Paraná - PR
57	AFASTAMENTO POR ATESTADO MÉDICO - ATE 15 DIAS	CHC da Universidade Federal do Paraná - PR
57	AFASTAMENTO POR ATESTADO MÉDICO - ATE 15 DIAS	CHC da Universidade Federal do Paraná - PR

57	AFASTAMENTO POR ATESTADO MÉDICO - ATE 15 DIAS	CHC da Universidade Federal do Paraná - PR
57	AFASTAMENTO POR ATESTADO MÉDICO - ATE 15 DIAS	CHC da Universidade Federal do Paraná - PR
57	AFASTAMENTO POR ATESTADO MÉDICO - ATE 15 DIAS	CHC da Universidade Federal do Paraná - PR
57	AFASTAMENTO POR ATESTADO MÉDICO - ATE 15 DIAS	CHC da Universidade Federal do Paraná - PR
57	AFASTAMENTO POR ATESTADO MÉDICO - ATE 15 DIAS	CHC da Universidade Federal do Paraná - PR
57	AFASTAMENTO POR ATESTADO MÉDICO - ATE 15 DIAS	CHC da Universidade Federal do Paraná - PR
57	AFASTAMENTO POR ATESTADO MÉDICO - ATE 15 DIAS	CHC da Universidade Federal do Paraná - PR
57	AFASTAMENTO POR ATESTADO MÉDICO - ATE 15 DIAS	CHC da Universidade Federal do Paraná - PR
57	AFASTAMENTO POR ATESTADO MÉDICO - ATE 15 DIAS	CHC da Universidade Federal do Paraná - PR
57	AFASTAMENTO POR ATESTADO MÉDICO - ATE 15 DIAS	CHC da Universidade Federal do Paraná - PR
57	AFASTAMENTO POR ATESTADO MÉDICO - ATE 15 DIAS	CHC da Universidade Federal do Paraná - PR
57	AFASTAMENTO POR ATESTADO MÉDICO - ATE 15 DIAS	CHC da Universidade Federal do Paraná - PR
57	AFASTAMENTO POR ATESTADO MÉDICO - ATE 15 DIAS	CHC da Universidade Federal do Paraná - PR
57	AFASTAMENTO POR ATESTADO MÉDICO - ATE 15 DIAS	CHC da Universidade Federal do Paraná - PR
57	AFASTAMENTO POR ATESTADO MÉDICO - ATE 15 DIAS	CHC da Universidade Federal do Paraná - PR
57	AFASTAMENTO POR ATESTADO MÉDICO - ATE 15 DIAS	CHC da Universidade Federal do Paraná - PR
57	AFASTAMENTO POR ATESTADO MÉDICO - ATE 15 DIAS	CHC da Universidade Federal do Paraná - PR
57	AFASTAMENTO POR ATESTADO MÉDICO - ATE 15 DIAS	CHC da Universidade Federal do Paraná - PR
57	AFASTAMENTO POR ATESTADO MÉDICO - ATE 15 DIAS	CHC da Universidade Federal do Paraná - PR
57	AFASTAMENTO POR ATESTADO MÉDICO - ATE 15 DIAS	CHC da Universidade Federal do Paraná - PR
57	AFASTAMENTO POR ATESTADO MÉDICO - ATE 15 DIAS	CHC da Universidade Federal do Paraná - PR
57	AFASTAMENTO POR ATESTADO MÉDICO - ATE 15 DIAS	CHC da Universidade Federal do Paraná - PR
57	AFASTAMENTO POR ATESTADO MÉDICO - ATE 15 DIAS	CHC da Universidade Federal do Paraná - PR
57	AFASTAMENTO POR ATESTADO MÉDICO - ATE 15 DIAS	CHC da Universidade Federal do Paraná - PR
57	AFASTAMENTO POR ATESTADO MÉDICO - ATE 15 DIAS	CHC da Universidade Federal do Paraná - PR
57	AFASTAMENTO POR ATESTADO MÉDICO - ATE 15 DIAS	CHC da Universidade Federal do Paraná - PR
57	AFASTAMENTO POR ATESTADO MÉDICO - ATE 15 DIAS	CHC da Universidade Federal do Paraná - PR
57	AFASTAMENTO POR ATESTADO MÉDICO - ATE 15 DIAS	CHC da Universidade Federal do Paraná - PR
57	AFASTAMENTO POR ATESTADO MÉDICO - ATE 15 DIAS	CHC da Universidade Federal do Paraná - PR
57	AFASTAMENTO POR ATESTADO MÉDICO - ATE 15 DIAS	CHC da Universidade Federal do Paraná - PR
57	AFASTAMENTO POR ATESTADO MÉDICO - ATE 15 DIAS	CHC da Universidade Federal do Paraná - PR
57	AFASTAMENTO POR ATESTADO MÉDICO - ATE 15 DIAS	CHC da Universidade Federal do Paraná - PR
57	AFASTAMENTO POR ATESTADO MÉDICO - ATE 15 DIAS	CHC da Universidade Federal do Paraná - PR
57	AFASTAMENTO POR ATESTADO MÉDICO - ATE 15 DIAS	CHC da Universidade Federal do Paraná - PR
57	AFASTAMENTO POR ATESTADO MÉDICO - ATE 15 DIAS	CHC da Universidade Federal do Paraná - PR
57	AFASTAMENTO POR ATESTADO MÉDICO - ATE 15 DIAS	CHC da Universidade Federal do Paraná - PR
57	AFASTAMENTO POR ATESTADO MÉDICO - ATE 15 DIAS	CHC da Universidade Federal do Paraná - PR
57	AFASTAMENTO POR ATESTADO MÉDICO - ATE 15 DIAS	CHC da Universidade Federal do Paraná - PR
57	AFASTAMENTO POR ATESTADO MÉDICO - ATE 15 DIAS	CHC da Universidade Federal do Paraná - PR
57	AFASTAMENTO POR ATESTADO MÉDICO - ATE 15 DIAS	CHC da Universidade Federal do Paraná - PR
57	AFASTAMENTO POR ATESTADO MÉDICO - ATE 15 DIAS	CHC da Universidade Federal do Paraná - PR
57	AFASTAMENTO POR ATESTADO MÉDICO - ATE 15 DIAS	CHC da Universidade Federal do Paraná - PR
57	AFASTAMENTO POR ATESTADO MÉDICO - ATE 15 DIAS	CHC da Universidade Federal do Paraná - PR
57	AFASTAMENTO POR ATESTADO MÉDICO - ATE 15 DIAS	CHC da Universidade Federal do Paraná - PR
57	AFASTAMENTO POR ATESTADO MÉDICO - ATE 15 DIAS	CHC da Universidade Federal do Paraná - PR
57	AFASTAMENTO POR ATESTADO MÉDICO - ATE 15 DIAS	CHC da Universidade Federal do Paraná - PR
57	AFASTAMENTO POR ATESTADO MÉDICO - ATE 15 DIAS	CHC da Universidade Federal do Paraná - PR
57	AFASTAMENTO POR ATESTADO MÉDICO - ATE 15 DIAS	CHC da Universidade Federal do Paraná - PR

57	AFASTAMENTO POR ATESTADO MÉDICO - ATE 15 DIAS	CHC da Universidade Federal do Paraná - PR
57	AFASTAMENTO POR ATESTADO MÉDICO - ATE 15 DIAS	CHC da Universidade Federal do Paraná - PR
57	AFASTAMENTO POR ATESTADO MÉDICO - ATE 15 DIAS	CHC da Universidade Federal do Paraná - PR
57	AFASTAMENTO POR ATESTADO MÉDICO - ATE 15 DIAS	CHC da Universidade Federal do Paraná - PR
57	AFASTAMENTO POR ATESTADO MÉDICO - ATE 15 DIAS	CHC da Universidade Federal do Paraná - PR
57	AFASTAMENTO POR ATESTADO MÉDICO - ATE 15 DIAS	CHC da Universidade Federal do Paraná - PR
57	AFASTAMENTO POR ATESTADO MÉDICO - ATE 15 DIAS	CHC da Universidade Federal do Paraná - PR
57	AFASTAMENTO POR ATESTADO MÉDICO - ATE 15 DIAS	CHC da Universidade Federal do Paraná - PR
57	AFASTAMENTO POR ATESTADO MÉDICO - ATE 15 DIAS	CHC da Universidade Federal do Paraná - PR
57	AFASTAMENTO POR ATESTADO MÉDICO - ATE 15 DIAS	CHC da Universidade Federal do Paraná - PR
57	AFASTAMENTO POR ATESTADO MÉDICO - ATE 15 DIAS	CHC da Universidade Federal do Paraná - PR
57	AFASTAMENTO POR ATESTADO MÉDICO - ATE 15 DIAS	CHC da Universidade Federal do Paraná - PR
57	AFASTAMENTO POR ATESTADO MÉDICO - ATE 15 DIAS	CHC da Universidade Federal do Paraná - PR
57	AFASTAMENTO POR ATESTADO MÉDICO - ATE 15 DIAS	CHC da Universidade Federal do Paraná - PR
57	AFASTAMENTO POR ATESTADO MÉDICO - ATE 15 DIAS	CHC da Universidade Federal do Paraná - PR
57	AFASTAMENTO POR ATESTADO MÉDICO - ATE 15 DIAS	CHC da Universidade Federal do Paraná - PR
57	AFASTAMENTO POR ATESTADO MÉDICO - ATE 15 DIAS	CHC da Universidade Federal do Paraná - PR
57	AFASTAMENTO POR ATESTADO MÉDICO - ATE 15 DIAS	CHC da Universidade Federal do Paraná - PR
57	AFASTAMENTO POR ATESTADO MÉDICO - ATE 15 DIAS	CHC da Universidade Federal do Paraná - PR
57	AFASTAMENTO POR ATESTADO MÉDICO - ATE 15 DIAS	CHC da Universidade Federal do Paraná - PR
57	AFASTAMENTO POR ATESTADO MÉDICO - ATE 15 DIAS	CHC da Universidade Federal do Paraná - PR
57	AFASTAMENTO POR ATESTADO MÉDICO - ATE 15 DIAS	CHC da Universidade Federal do Paraná - PR
57	AFASTAMENTO POR ATESTADO MÉDICO - ATE 15 DIAS	CHC da Universidade Federal do Paraná - PR
57	AFASTAMENTO POR ATESTADO MÉDICO - ATE 15 DIAS	CHC da Universidade Federal do Paraná - PR
57	AFASTAMENTO POR ATESTADO MÉDICO - ATE 15 DIAS	CHC da Universidade Federal do Paraná - PR
57	AFASTAMENTO POR ATESTADO MÉDICO - ATE 15 DIAS	CHC da Universidade Federal do Paraná - PR
57	AFASTAMENTO POR ATESTADO MÉDICO - ATE 15 DIAS	CHC da Universidade Federal do Paraná - PR
57	AFASTAMENTO POR ATESTADO MÉDICO - ATE 15 DIAS	CHC da Universidade Federal do Paraná - PR
57	AFASTAMENTO POR ATESTADO MÉDICO - ATE 15 DIAS	CHC da Universidade Federal do Paraná - PR
57	AFASTAMENTO POR ATESTADO MÉDICO - ATE 15 DIAS	CHC da Universidade Federal do Paraná - PR
57	AFASTAMENTO POR ATESTADO MÉDICO - ATE 15 DIAS	CHC da Universidade Federal do Paraná - PR
57	AFASTAMENTO POR ATESTADO MÉDICO - ATE 15 DIAS	CHC da Universidade Federal do Paraná - PR
57	AFASTAMENTO POR ATESTADO MÉDICO - ATE 15 DIAS	CHC da Universidade Federal do Paraná - PR
57	AFASTAMENTO POR ATESTADO MÉDICO - ATE 15 DIAS	CHC da Universidade Federal do Paraná - PR
57	AFASTAMENTO POR ATESTADO MÉDICO - ATE 15 DIAS	CHC da Universidade Federal do Paraná - PR
57	AFASTAMENTO POR ATESTADO MÉDICO - ATE 15 DIAS	CHC da Universidade Federal do Paraná - PR
57	AFASTAMENTO POR ATESTADO MÉDICO - ATE 15 DIAS	CHC da Universidade Federal do Paraná - PR
57	AFASTAMENTO POR ATESTADO MÉDICO - ATE 15 DIAS	CHC da Universidade Federal do Paraná - PR
57	AFASTAMENTO POR ATESTADO MÉDICO - ATE 15 DIAS	CHC da Universidade Federal do Paraná - PR
57	AFASTAMENTO POR ATESTADO MÉDICO - ATE 15 DIAS	CHC da Universidade Federal do Paraná - PR
57	AFASTAMENTO POR ATESTADO MÉDICO - ATE 15 DIAS	CHC da Universidade Federal do Paraná - PR
57	AFASTAMENTO POR ATESTADO MÉDICO - ATE 15 DIAS	CHC da Universidade Federal do Paraná - PR
57	AFASTAMENTO POR ATESTADO MÉDICO - ATE 15 DIAS	CHC da Universidade Federal do Paraná - PR
57	AFASTAMENTO POR ATESTADO MÉDICO - ATE 15 DIAS	CHC da Universidade Federal do Paraná - PR
57	AFASTAMENTO POR ATESTADO MÉDICO - ATE 15 DIAS	CHC da Universidade Federal do Paraná - PR
57	AFASTAMENTO POR ATESTADO MÉDICO - ATE 15 DIAS	CHC da Universidade Federal do Paraná - PR
57	AFASTAMENTO POR ATESTADO MÉDICO - ATE 15 DIAS	CHC da Universidade Federal do Paraná - PR
57	AFASTAMENTO POR ATESTADO MÉDICO - ATE 15 DIAS	CHC da Universidade Federal do Paraná - PR

57	AFASTAMENTO POR ATESTADO MÉDICO - ATE 15 DIAS	CHC da Universidade Federal do Paraná - PR
57	AFASTAMENTO POR ATESTADO MÉDICO - ATE 15 DIAS	CHC da Universidade Federal do Paraná - PR
57	AFASTAMENTO POR ATESTADO MÉDICO - ATE 15 DIAS	CHC da Universidade Federal do Paraná - PR
57	AFASTAMENTO POR ATESTADO MÉDICO - ATE 15 DIAS	CHC da Universidade Federal do Paraná - PR
57	AFASTAMENTO POR ATESTADO MÉDICO - ATE 15 DIAS	CHC da Universidade Federal do Paraná - PR
57	AFASTAMENTO POR ATESTADO MÉDICO - ATE 15 DIAS	CHC da Universidade Federal do Paraná - PR
57	AFASTAMENTO POR ATESTADO MÉDICO - ATE 15 DIAS	CHC da Universidade Federal do Paraná - PR
57	AFASTAMENTO POR ATESTADO MÉDICO - ATE 15 DIAS	CHC da Universidade Federal do Paraná - PR
57	AFASTAMENTO POR ATESTADO MÉDICO - ATE 15 DIAS	CHC da Universidade Federal do Paraná - PR
57	AFASTAMENTO POR ATESTADO MÉDICO - ATE 15 DIAS	CHC da Universidade Federal do Paraná - PR
57	AFASTAMENTO POR ATESTADO MÉDICO - ATE 15 DIAS	CHC da Universidade Federal do Paraná - PR
57	AFASTAMENTO POR ATESTADO MÉDICO - ATE 15 DIAS	CHC da Universidade Federal do Paraná - PR
57	AFASTAMENTO POR ATESTADO MÉDICO - ATE 15 DIAS	CHC da Universidade Federal do Paraná - PR
57	AFASTAMENTO POR ATESTADO MÉDICO - ATE 15 DIAS	CHC da Universidade Federal do Paraná - PR
57	AFASTAMENTO POR ATESTADO MÉDICO - ATE 15 DIAS	CHC da Universidade Federal do Paraná - PR
57	AFASTAMENTO POR ATESTADO MÉDICO - ATE 15 DIAS	CHC da Universidade Federal do Paraná - PR
57	AFASTAMENTO POR ATESTADO MÉDICO - ATE 15 DIAS	CHC da Universidade Federal do Paraná - PR
57	AFASTAMENTO POR ATESTADO MÉDICO - ATE 15 DIAS	CHC da Universidade Federal do Paraná - PR
57	AFASTAMENTO POR ATESTADO MÉDICO - ATE 15 DIAS	CHC da Universidade Federal do Paraná - PR
57	AFASTAMENTO POR ATESTADO MÉDICO - ATE 15 DIAS	CHC da Universidade Federal do Paraná - PR
57	AFASTAMENTO POR ATESTADO MÉDICO - ATE 15 DIAS	CHC da Universidade Federal do Paraná - PR
57	AFASTAMENTO POR ATESTADO MÉDICO - ATE 15 DIAS	CHC da Universidade Federal do Paraná - PR
57	AFASTAMENTO POR ATESTADO MÉDICO - ATE 15 DIAS	CHC da Universidade Federal do Paraná - PR
57	AFASTAMENTO POR ATESTADO MÉDICO - ATE 15 DIAS	CHC da Universidade Federal do Paraná - PR
57	AFASTAMENTO POR ATESTADO MÉDICO - ATE 15 DIAS	CHC da Universidade Federal do Paraná - PR
57	AFASTAMENTO POR ATESTADO MÉDICO - ATE 15 DIAS	CHC da Universidade Federal do Paraná - PR
57	AFASTAMENTO POR ATESTADO MÉDICO - ATE 15 DIAS	CHC da Universidade Federal do Paraná - PR
57	AFASTAMENTO POR ATESTADO MÉDICO - ATE 15 DIAS	CHC da Universidade Federal do Paraná - PR
57	AFASTAMENTO POR ATESTADO MÉDICO - ATE 15 DIAS	CHC da Universidade Federal do Paraná - PR
57	AFASTAMENTO POR ATESTADO MÉDICO - ATE 15 DIAS	CHC da Universidade Federal do Paraná - PR
57	AFASTAMENTO POR ATESTADO MÉDICO - ATE 15 DIAS	CHC da Universidade Federal do Paraná - PR
57	AFASTAMENTO POR ATESTADO MÉDICO - ATE 15 DIAS	CHC da Universidade Federal do Paraná - PR
57	AFASTAMENTO POR ATESTADO MÉDICO - ATE 15 DIAS	CHC da Universidade Federal do Paraná - PR
57	AFASTAMENTO POR ATESTADO MÉDICO - ATE 15 DIAS	CHC da Universidade Federal do Paraná - PR
57	AFASTAMENTO POR ATESTADO MÉDICO - ATE 15 DIAS	CHC da Universidade Federal do Paraná - PR
57	AFASTAMENTO POR ATESTADO MÉDICO - ATE 15 DIAS	CHC da Universidade Federal do Paraná - PR
57	AFASTAMENTO POR ATESTADO MÉDICO - ATE 15 DIAS	CHC da Universidade Federal do Paraná - PR
57	AFASTAMENTO POR ATESTADO MÉDICO - ATE 15 DIAS	CHC da Universidade Federal do Paraná - PR
57	AFASTAMENTO POR ATESTADO MÉDICO - ATE 15 DIAS	CHC da Universidade Federal do Paraná - PR
57	AFASTAMENTO POR ATESTADO MÉDICO - ATE 15 DIAS	CHC da Universidade Federal do Paraná - PR
57	AFASTAMENTO POR ATESTADO MÉDICO - ATE 15 DIAS	CHC da Universidade Federal do Paraná - PR
57	AFASTAMENTO POR ATESTADO MÉDICO - ATE 15 DIAS	CHC da Universidade Federal do Paraná - PR
57	AFASTAMENTO POR ATESTADO MÉDICO - ATE 15 DIAS	CHC da Universidade Federal do Paraná - PR
57	AFASTAMENTO POR ATESTADO MÉDICO - ATE 15 DIAS	CHC da Universidade Federal do Paraná - PR
57	AFASTAMENTO POR ATESTADO MÉDICO - ATE 15 DIAS	CHC da Universidade Federal do Paraná - PR
57	AFASTAMENTO POR ATESTADO MÉDICO - ATE 15 DIAS	CHC da Universidade Federal do Paraná - PR
57	AFASTAMENTO POR ATESTADO MÉDICO - ATE 15 DIAS	CHC da Universidade Federal do Paraná - PR
57	AFASTAMENTO POR ATESTADO MÉDICO - ATE 15 DIAS	CHC da Universidade Federal do Paraná - PR

57	AFASTAMENTO POR ATESTADO MÉDICO - ATE 15 DIAS	CHC da Universidade Federal do Paraná - PR
57	AFASTAMENTO POR ATESTADO MÉDICO - ATE 15 DIAS	CHC da Universidade Federal do Paraná - PR
57	AFASTAMENTO POR ATESTADO MÉDICO - ATE 15 DIAS	CHC da Universidade Federal do Paraná - PR
57	AFASTAMENTO POR ATESTADO MÉDICO - ATE 15 DIAS	CHC da Universidade Federal do Paraná - PR
57	AFASTAMENTO POR ATESTADO MÉDICO - ATE 15 DIAS	CHC da Universidade Federal do Paraná - PR
57	AFASTAMENTO POR ATESTADO MÉDICO - ATE 15 DIAS	CHC da Universidade Federal do Paraná - PR
57	AFASTAMENTO POR ATESTADO MÉDICO - ATE 15 DIAS	CHC da Universidade Federal do Paraná - PR
57	AFASTAMENTO POR ATESTADO MÉDICO - ATE 15 DIAS	CHC da Universidade Federal do Paraná - PR
57	AFASTAMENTO POR ATESTADO MÉDICO - ATE 15 DIAS	CHC da Universidade Federal do Paraná - PR
57	AFASTAMENTO POR ATESTADO MÉDICO - ATE 15 DIAS	CHC da Universidade Federal do Paraná - PR
57	AFASTAMENTO POR ATESTADO MÉDICO - ATE 15 DIAS	CHC da Universidade Federal do Paraná - PR
57	AFASTAMENTO POR ATESTADO MÉDICO - ATE 15 DIAS	CHC da Universidade Federal do Paraná - PR
57	AFASTAMENTO POR ATESTADO MÉDICO - ATE 15 DIAS	CHC da Universidade Federal do Paraná - PR
57	AFASTAMENTO POR ATESTADO MÉDICO - ATE 15 DIAS	CHC da Universidade Federal do Paraná - PR
57	AFASTAMENTO POR ATESTADO MÉDICO - ATE 15 DIAS	CHC da Universidade Federal do Paraná - PR
57	AFASTAMENTO POR ATESTADO MÉDICO - ATE 15 DIAS	CHC da Universidade Federal do Paraná - PR
57	AFASTAMENTO POR ATESTADO MÉDICO - ATE 15 DIAS	CHC da Universidade Federal do Paraná - PR
57	AFASTAMENTO POR ATESTADO MÉDICO - ATE 15 DIAS	CHC da Universidade Federal do Paraná - PR
57	AFASTAMENTO POR ATESTADO MÉDICO - ATE 15 DIAS	CHC da Universidade Federal do Paraná - PR
57	AFASTAMENTO POR ATESTADO MÉDICO - ATE 15 DIAS	CHC da Universidade Federal do Paraná - PR
57	AFASTAMENTO POR ATESTADO MÉDICO - ATE 15 DIAS	CHC da Universidade Federal do Paraná - PR
57	AFASTAMENTO POR ATESTADO MÉDICO - ATE 15 DIAS	CHC da Universidade Federal do Paraná - PR
57	AFASTAMENTO POR ATESTADO MÉDICO - ATE 15 DIAS	CHC da Universidade Federal do Paraná - PR
57	AFASTAMENTO POR ATESTADO MÉDICO - ATE 15 DIAS	CHC da Universidade Federal do Paraná - PR
57	AFASTAMENTO POR ATESTADO MÉDICO - ATE 15 DIAS	CHC da Universidade Federal do Paraná - PR
57	AFASTAMENTO POR ATESTADO MÉDICO - ATE 15 DIAS	CHC da Universidade Federal do Paraná - PR
57	AFASTAMENTO POR ATESTADO MÉDICO - ATE 15 DIAS	CHC da Universidade Federal do Paraná - PR
57	AFASTAMENTO POR ATESTADO MÉDICO - ATE 15 DIAS	CHC da Universidade Federal do Paraná - PR
57	AFASTAMENTO POR ATESTADO MÉDICO - ATE 15 DIAS	CHC da Universidade Federal do Paraná - PR
57	AFASTAMENTO POR ATESTADO MÉDICO - ATE 15 DIAS	CHC da Universidade Federal do Paraná - PR
57	AFASTAMENTO POR ATESTADO MÉDICO - ATE 15 DIAS	CHC da Universidade Federal do Paraná - PR
57	AFASTAMENTO POR ATESTADO MÉDICO - ATE 15 DIAS	CHC da Universidade Federal do Paraná - PR
57	AFASTAMENTO POR ATESTADO MÉDICO - ATE 15 DIAS	CHC da Universidade Federal do Paraná - PR
57	AFASTAMENTO POR ATESTADO MÉDICO - ATE 15 DIAS	CHC da Universidade Federal do Paraná - PR
57	AFASTAMENTO POR ATESTADO MÉDICO - ATE 15 DIAS	CHC da Universidade Federal do Paraná - PR
57	AFASTAMENTO POR ATESTADO MÉDICO - ATE 15 DIAS	CHC da Universidade Federal do Paraná - PR
57	AFASTAMENTO POR ATESTADO MÉDICO - ATE 15 DIAS	CHC da Universidade Federal do Paraná - PR
57	AFASTAMENTO POR ATESTADO MÉDICO - ATE 15 DIAS	CHC da Universidade Federal do Paraná - PR
57	AFASTAMENTO POR ATESTADO MÉDICO - ATE 15 DIAS	CHC da Universidade Federal do Paraná - PR
57	AFASTAMENTO POR ATESTADO MÉDICO - ATE 15 DIAS	CHC da Universidade Federal do Paraná - PR
57	AFASTAMENTO POR ATESTADO MÉDICO - ATE 15 DIAS	CHC da Universidade Federal do Paraná - PR
57	AFASTAMENTO POR ATESTADO MÉDICO - ATE 15 DIAS	CHC da Universidade Federal do Paraná - PR
57	AFASTAMENTO POR ATESTADO MÉDICO - ATE 15 DIAS	CHC da Universidade Federal do Paraná - PR
57	AFASTAMENTO POR ATESTADO MÉDICO - ATE 15 DIAS	CHC da Universidade Federal do Paraná - PR
57	AFASTAMENTO POR ATESTADO MÉDICO - ATE 15 DIAS	CHC da Universidade Federal do Paraná - PR
57	AFASTAMENTO POR ATESTADO MÉDICO - ATE 15 DIAS	CHC da Universidade Federal do Paraná - PR
57	AFASTAMENTO POR ATESTADO MÉDICO - ATE 15 DIAS	CHC da Universidade Federal do Paraná - PR
57	AFASTAMENTO POR ATESTADO MÉDICO - ATE 15 DIAS	CHC da Universidade Federal do Paraná - PR

57	AFASTAMENTO POR ATESTADO MÉDICO - ATE 15 DIAS	CHC da Universidade Federal do Paraná - PR
57	AFASTAMENTO POR ATESTADO MÉDICO - ATE 15 DIAS	CHC da Universidade Federal do Paraná - PR
57	AFASTAMENTO POR ATESTADO MÉDICO - ATE 15 DIAS	CHC da Universidade Federal do Paraná - PR
57	AFASTAMENTO POR ATESTADO MÉDICO - ATE 15 DIAS	CHC da Universidade Federal do Paraná - PR
57	AFASTAMENTO POR ATESTADO MÉDICO - ATE 15 DIAS	CHC da Universidade Federal do Paraná - PR
57	AFASTAMENTO POR ATESTADO MÉDICO - ATE 15 DIAS	CHC da Universidade Federal do Paraná - PR
57	AFASTAMENTO POR ATESTADO MÉDICO - ATE 15 DIAS	CHC da Universidade Federal do Paraná - PR
57	AFASTAMENTO POR ATESTADO MÉDICO - ATE 15 DIAS	CHC da Universidade Federal do Paraná - PR
57	AFASTAMENTO POR ATESTADO MÉDICO - ATE 15 DIAS	CHC da Universidade Federal do Paraná - PR
57	AFASTAMENTO POR ATESTADO MÉDICO - ATE 15 DIAS	CHC da Universidade Federal do Paraná - PR
57	AFASTAMENTO POR ATESTADO MÉDICO - ATE 15 DIAS	CHC da Universidade Federal do Paraná - PR
57	AFASTAMENTO POR ATESTADO MÉDICO - ATE 15 DIAS	CHC da Universidade Federal do Paraná - PR
57	AFASTAMENTO POR ATESTADO MÉDICO - ATE 15 DIAS	CHC da Universidade Federal do Paraná - PR
57	AFASTAMENTO POR ATESTADO MÉDICO - ATE 15 DIAS	CHC da Universidade Federal do Paraná - PR
57	AFASTAMENTO POR ATESTADO MÉDICO - ATE 15 DIAS	CHC da Universidade Federal do Paraná - PR
57	AFASTAMENTO POR ATESTADO MÉDICO - ATE 15 DIAS	CHC da Universidade Federal do Paraná - PR
57	AFASTAMENTO POR ATESTADO MÉDICO - ATE 15 DIAS	CHC da Universidade Federal do Paraná - PR
57	AFASTAMENTO POR ATESTADO MÉDICO - ATE 15 DIAS	CHC da Universidade Federal do Paraná - PR
57	AFASTAMENTO POR ATESTADO MÉDICO - ATE 15 DIAS	CHC da Universidade Federal do Paraná - PR
57	AFASTAMENTO POR ATESTADO MÉDICO - ATE 15 DIAS	CHC da Universidade Federal do Paraná - PR
57	AFASTAMENTO POR ATESTADO MÉDICO - ATE 15 DIAS	CHC da Universidade Federal do Paraná - PR
57	AFASTAMENTO POR ATESTADO MÉDICO - ATE 15 DIAS	CHC da Universidade Federal do Paraná - PR
57	AFASTAMENTO POR ATESTADO MÉDICO - ATE 15 DIAS	CHC da Universidade Federal do Paraná - PR
57	AFASTAMENTO POR ATESTADO MÉDICO - ATE 15 DIAS	CHC da Universidade Federal do Paraná - PR
57	AFASTAMENTO POR ATESTADO MÉDICO - ATE 15 DIAS	CHC da Universidade Federal do Paraná - PR
57	AFASTAMENTO POR ATESTADO MÉDICO - ATE 15 DIAS	CHC da Universidade Federal do Paraná - PR
57	AFASTAMENTO POR ATESTADO MÉDICO - ATE 15 DIAS	CHC da Universidade Federal do Paraná - PR
57	AFASTAMENTO POR ATESTADO MÉDICO - ATE 15 DIAS	CHC da Universidade Federal do Paraná - PR
57	AFASTAMENTO POR ATESTADO MÉDICO - ATE 15 DIAS	CHC da Universidade Federal do Paraná - PR
57	AFASTAMENTO POR ATESTADO MÉDICO - ATE 15 DIAS	CHC da Universidade Federal do Paraná - PR
57	AFASTAMENTO POR ATESTADO MÉDICO - ATE 15 DIAS	CHC da Universidade Federal do Paraná - PR
57	AFASTAMENTO POR ATESTADO MÉDICO - ATE 15 DIAS	CHC da Universidade Federal do Paraná - PR
57	AFASTAMENTO POR ATESTADO MÉDICO - ATE 15 DIAS	CHC da Universidade Federal do Paraná - PR
57	AFASTAMENTO POR ATESTADO MÉDICO - ATE 15 DIAS	CHC da Universidade Federal do Paraná - PR
57	AFASTAMENTO POR ATESTADO MÉDICO - ATE 15 DIAS	CHC da Universidade Federal do Paraná - PR
57	AFASTAMENTO POR ATESTADO MÉDICO - ATE 15 DIAS	CHC da Universidade Federal do Paraná - PR
57	AFASTAMENTO POR ATESTADO MÉDICO - ATE 15 DIAS	CHC da Universidade Federal do Paraná - PR
57	AFASTAMENTO POR ATESTADO MÉDICO - ATE 15 DIAS	CHC da Universidade Federal do Paraná - PR
57	AFASTAMENTO POR ATESTADO MÉDICO - ATE 15 DIAS	CHC da Universidade Federal do Paraná - PR
57	AFASTAMENTO POR ATESTADO MÉDICO - ATE 15 DIAS	CHC da Universidade Federal do Paraná - PR
57	AFASTAMENTO POR ATESTADO MÉDICO - ATE 15 DIAS	CHC da Universidade Federal do Paraná - PR
57	AFASTAMENTO POR ATESTADO MÉDICO - ATE 15 DIAS	CHC da Universidade Federal do Paraná - PR
57	AFASTAMENTO POR ATESTADO MÉDICO - ATE 15 DIAS	CHC da Universidade Federal do Paraná - PR
57	AFASTAMENTO POR ATESTADO MÉDICO - ATE 15 DIAS	CHC da Universidade Federal do Paraná - PR
57	AFASTAMENTO POR ATESTADO MÉDICO - ATE 15 DIAS	CHC da Universidade Federal do Paraná - PR
57	AFASTAMENTO POR ATESTADO MÉDICO - ATE 15 DIAS	CHC da Universidade Federal do Paraná - PR
57	AFASTAMENTO POR ATESTADO MÉDICO - ATE 15 DIAS	CHC da Universidade Federal do Paraná - PR
57	AFASTAMENTO POR ATESTADO MÉDICO - ATE 15 DIAS	CHC da Universidade Federal do Paraná - PR

57	AFASTAMENTO POR ATESTADO MÉDICO - ATE 15 DIAS	CHC da Universidade Federal do Paraná - PR
57	AFASTAMENTO POR ATESTADO MÉDICO - ATE 15 DIAS	CHC da Universidade Federal do Paraná - PR
57	AFASTAMENTO POR ATESTADO MÉDICO - ATE 15 DIAS	CHC da Universidade Federal do Paraná - PR
57	AFASTAMENTO POR ATESTADO MÉDICO - ATE 15 DIAS	CHC da Universidade Federal do Paraná - PR
57	AFASTAMENTO POR ATESTADO MÉDICO - ATE 15 DIAS	CHC da Universidade Federal do Paraná - PR
57	AFASTAMENTO POR ATESTADO MÉDICO - ATE 15 DIAS	CHC da Universidade Federal do Paraná - PR
57	AFASTAMENTO POR ATESTADO MÉDICO - ATE 15 DIAS	CHC da Universidade Federal do Paraná - PR
57	AFASTAMENTO POR ATESTADO MÉDICO - ATE 15 DIAS	CHC da Universidade Federal do Paraná - PR
57	AFASTAMENTO POR ATESTADO MÉDICO - ATE 15 DIAS	CHC da Universidade Federal do Paraná - PR
57	AFASTAMENTO POR ATESTADO MÉDICO - ATE 15 DIAS	CHC da Universidade Federal do Paraná - PR
57	AFASTAMENTO POR ATESTADO MÉDICO - ATE 15 DIAS	CHC da Universidade Federal do Paraná - PR
57	AFASTAMENTO POR ATESTADO MÉDICO - ATE 15 DIAS	CHC da Universidade Federal do Paraná - PR
57	AFASTAMENTO POR ATESTADO MÉDICO - ATE 15 DIAS	CHC da Universidade Federal do Paraná - PR
57	AFASTAMENTO POR ATESTADO MÉDICO - ATE 15 DIAS	CHC da Universidade Federal do Paraná - PR
57	AFASTAMENTO POR ATESTADO MÉDICO - ATE 15 DIAS	CHC da Universidade Federal do Paraná - PR
57	AFASTAMENTO POR ATESTADO MÉDICO - ATE 15 DIAS	CHC da Universidade Federal do Paraná - PR
57	AFASTAMENTO POR ATESTADO MÉDICO - ATE 15 DIAS	CHC da Universidade Federal do Paraná - PR
57	AFASTAMENTO POR ATESTADO MÉDICO - ATE 15 DIAS	CHC da Universidade Federal do Paraná - PR
57	AFASTAMENTO POR ATESTADO MÉDICO - ATE 15 DIAS	CHC da Universidade Federal do Paraná - PR
57	AFASTAMENTO POR ATESTADO MÉDICO - ATE 15 DIAS	CHC da Universidade Federal do Paraná - PR
57	AFASTAMENTO POR ATESTADO MÉDICO - ATE 15 DIAS	CHC da Universidade Federal do Paraná - PR
57	AFASTAMENTO POR ATESTADO MÉDICO - ATE 15 DIAS	CHC da Universidade Federal do Paraná - PR
57	AFASTAMENTO POR ATESTADO MÉDICO - ATE 15 DIAS	CHC da Universidade Federal do Paraná - PR
57	AFASTAMENTO POR ATESTADO MÉDICO - ATE 15 DIAS	CHC da Universidade Federal do Paraná - PR
57	AFASTAMENTO POR ATESTADO MÉDICO - ATE 15 DIAS	CHC da Universidade Federal do Paraná - PR
57	AFASTAMENTO POR ATESTADO MÉDICO - ATE 15 DIAS	CHC da Universidade Federal do Paraná - PR
57	AFASTAMENTO POR ATESTADO MÉDICO - ATE 15 DIAS	CHC da Universidade Federal do Paraná - PR
57	AFASTAMENTO POR ATESTADO MÉDICO - ATE 15 DIAS	CHC da Universidade Federal do Paraná - PR
57	AFASTAMENTO POR ATESTADO MÉDICO - ATE 15 DIAS	CHC da Universidade Federal do Paraná - PR
57	AFASTAMENTO POR ATESTADO MÉDICO - ATE 15 DIAS	CHC da Universidade Federal do Paraná - PR
57	AFASTAMENTO POR ATESTADO MÉDICO - ATE 15 DIAS	CHC da Universidade Federal do Paraná - PR
57	AFASTAMENTO POR ATESTADO MÉDICO - ATE 15 DIAS	CHC da Universidade Federal do Paraná - PR
57	AFASTAMENTO POR ATESTADO MÉDICO - ATE 15 DIAS	CHC da Universidade Federal do Paraná - PR
57	AFASTAMENTO POR ATESTADO MÉDICO - ATE 15 DIAS	CHC da Universidade Federal do Paraná - PR
57	AFASTAMENTO POR ATESTADO MÉDICO - ATE 15 DIAS	CHC da Universidade Federal do Paraná - PR
57	AFASTAMENTO POR ATESTADO MÉDICO - ATE 15 DIAS	CHC da Universidade Federal do Paraná - PR
57	AFASTAMENTO POR ATESTADO MÉDICO - ATE 15 DIAS	CHC da Universidade Federal do Paraná - PR
57	AFASTAMENTO POR ATESTADO MÉDICO - ATE 15 DIAS	CHC da Universidade Federal do Paraná - PR
57	AFASTAMENTO POR ATESTADO MÉDICO - ATE 15 DIAS	CHC da Universidade Federal do Paraná - PR
57	AFASTAMENTO POR ATESTADO MÉDICO - ATE 15 DIAS	CHC da Universidade Federal do Paraná - PR
57	AFASTAMENTO POR ATESTADO MÉDICO - ATE 15 DIAS	CHC da Universidade Federal do Paraná - PR
57	AFASTAMENTO POR ATESTADO MÉDICO - ATE 15 DIAS	CHC da Universidade Federal do Paraná - PR
57	AFASTAMENTO POR ATESTADO MÉDICO - ATE 15 DIAS	CHC da Universidade Federal do Paraná - PR
57	AFASTAMENTO POR ATESTADO MÉDICO - ATE 15 DIAS	CHC da Universidade Federal do Paraná - PR
57	AFASTAMENTO POR ATESTADO MÉDICO - ATE 15 DIAS	CHC da Universidade Federal do Paraná - PR
57	AFASTAMENTO POR ATESTADO MÉDICO - ATE 15 DIAS	CHC da Universidade Federal do Paraná - PR
57	AFASTAMENTO POR ATESTADO MÉDICO - ATE 15 DIAS	CHC da Universidade Federal do Paraná - PR
57	AFASTAMENTO POR ATESTADO MÉDICO - ATE 15 DIAS	CHC da Universidade Federal do Paraná - PR

57	AFASTAMENTO POR ATESTADO MÉDICO - ATE 15 DIAS	CHC da Universidade Federal do Paraná - PR
57	AFASTAMENTO POR ATESTADO MÉDICO - ATE 15 DIAS	CHC da Universidade Federal do Paraná - PR
57	AFASTAMENTO POR ATESTADO MÉDICO - ATE 15 DIAS	CHC da Universidade Federal do Paraná - PR
57	AFASTAMENTO POR ATESTADO MÉDICO - ATE 15 DIAS	CHC da Universidade Federal do Paraná - PR
57	AFASTAMENTO POR ATESTADO MÉDICO - ATE 15 DIAS	CHC da Universidade Federal do Paraná - PR
57	AFASTAMENTO POR ATESTADO MÉDICO - ATE 15 DIAS	CHC da Universidade Federal do Paraná - PR
57	AFASTAMENTO POR ATESTADO MÉDICO - ATE 15 DIAS	CHC da Universidade Federal do Paraná - PR
57	AFASTAMENTO POR ATESTADO MÉDICO - ATE 15 DIAS	CHC da Universidade Federal do Paraná - PR
57	AFASTAMENTO POR ATESTADO MÉDICO - ATE 15 DIAS	CHC da Universidade Federal do Paraná - PR
57	AFASTAMENTO POR ATESTADO MÉDICO - ATE 15 DIAS	CHC da Universidade Federal do Paraná - PR
57	AFASTAMENTO POR ATESTADO MÉDICO - ATE 15 DIAS	CHC da Universidade Federal do Paraná - PR
57	AFASTAMENTO POR ATESTADO MÉDICO - ATE 15 DIAS	CHC da Universidade Federal do Paraná - PR
57	AFASTAMENTO POR ATESTADO MÉDICO - ATE 15 DIAS	CHC da Universidade Federal do Paraná - PR
57	AFASTAMENTO POR ATESTADO MÉDICO - ATE 15 DIAS	CHC da Universidade Federal do Paraná - PR
57	AFASTAMENTO POR ATESTADO MÉDICO - ATE 15 DIAS	CHC da Universidade Federal do Paraná - PR
57	AFASTAMENTO POR ATESTADO MÉDICO - ATE 15 DIAS	CHC da Universidade Federal do Paraná - PR
57	AFASTAMENTO POR ATESTADO MÉDICO - ATE 15 DIAS	CHC da Universidade Federal do Paraná - PR
57	AFASTAMENTO POR ATESTADO MÉDICO - ATE 15 DIAS	CHC da Universidade Federal do Paraná - PR
57	AFASTAMENTO POR ATESTADO MÉDICO - ATE 15 DIAS	CHC da Universidade Federal do Paraná - PR
57	AFASTAMENTO POR ATESTADO MÉDICO - ATE 15 DIAS	CHC da Universidade Federal do Paraná - PR
57	AFASTAMENTO POR ATESTADO MÉDICO - ATE 15 DIAS	CHC da Universidade Federal do Paraná - PR
57	AFASTAMENTO POR ATESTADO MÉDICO - ATE 15 DIAS	CHC da Universidade Federal do Paraná - PR
57	AFASTAMENTO POR ATESTADO MÉDICO - ATE 15 DIAS	CHC da Universidade Federal do Paraná - PR
57	AFASTAMENTO POR ATESTADO MÉDICO - ATE 15 DIAS	CHC da Universidade Federal do Paraná - PR
57	AFASTAMENTO POR ATESTADO MÉDICO - ATE 15 DIAS	CHC da Universidade Federal do Paraná - PR
57	AFASTAMENTO POR ATESTADO MÉDICO - ATE 15 DIAS	CHC da Universidade Federal do Paraná - PR
57	AFASTAMENTO POR ATESTADO MÉDICO - ATE 15 DIAS	CHC da Universidade Federal do Paraná - PR
57	AFASTAMENTO POR ATESTADO MÉDICO - ATE 15 DIAS	CHC da Universidade Federal do Paraná - PR
57	AFASTAMENTO POR ATESTADO MÉDICO - ATE 15 DIAS	CHC da Universidade Federal do Paraná - PR
57	AFASTAMENTO POR ATESTADO MÉDICO - ATE 15 DIAS	CHC da Universidade Federal do Paraná - PR
57	AFASTAMENTO POR ATESTADO MÉDICO - ATE 15 DIAS	CHC da Universidade Federal do Paraná - PR
57	AFASTAMENTO POR ATESTADO MÉDICO - ATE 15 DIAS	CHC da Universidade Federal do Paraná - PR
57	AFASTAMENTO POR ATESTADO MÉDICO - ATE 15 DIAS	CHC da Universidade Federal do Paraná - PR
57	AFASTAMENTO POR ATESTADO MÉDICO - ATE 15 DIAS	CHC da Universidade Federal do Paraná - PR
57	AFASTAMENTO POR ATESTADO MÉDICO - ATE 15 DIAS	CHC da Universidade Federal do Paraná - PR
57	AFASTAMENTO POR ATESTADO MÉDICO - ATE 15 DIAS	CHC da Universidade Federal do Paraná - PR
57	AFASTAMENTO POR ATESTADO MÉDICO - ATE 15 DIAS	CHC da Universidade Federal do Paraná - PR
57	AFASTAMENTO POR ATESTADO MÉDICO - ATE 15 DIAS	CHC da Universidade Federal do Paraná - PR
57	AFASTAMENTO POR ATESTADO MÉDICO - ATE 15 DIAS	CHC da Universidade Federal do Paraná - PR
57	AFASTAMENTO POR ATESTADO MÉDICO - ATE 15 DIAS	CHC da Universidade Federal do Paraná - PR
57	AFASTAMENTO POR ATESTADO MÉDICO - ATE 15 DIAS	CHC da Universidade Federal do Paraná - PR
57	AFASTAMENTO POR ATESTADO MÉDICO - ATE 15 DIAS	CHC da Universidade Federal do Paraná - PR
57	AFASTAMENTO POR ATESTADO MÉDICO - ATE 15 DIAS	CHC da Universidade Federal do Paraná - PR
57	AFASTAMENTO POR ATESTADO MÉDICO - ATE 15 DIAS	CHC da Universidade Federal do Paraná - PR
57	AFASTAMENTO POR ATESTADO MÉDICO - ATE 15 DIAS	CHC da Universidade Federal do Paraná - PR
57	AFASTAMENTO POR ATESTADO MÉDICO - ATE 15 DIAS	CHC da Universidade Federal do Paraná - PR
57	AFASTAMENTO POR ATESTADO MÉDICO - ATE 15 DIAS	CHC da Universidade Federal do Paraná - PR
57	AFASTAMENTO POR ATESTADO MÉDICO - ATE 15 DIAS	CHC da Universidade Federal do Paraná - PR

57	AFASTAMENTO POR ATESTADO MÉDICO - ATE 15 DIAS	CHC da Universidade Federal do Paraná - PR
57	AFASTAMENTO POR ATESTADO MÉDICO - ATE 15 DIAS	CHC da Universidade Federal do Paraná - PR
57	AFASTAMENTO POR ATESTADO MÉDICO - ATE 15 DIAS	CHC da Universidade Federal do Paraná - PR
57	AFASTAMENTO POR ATESTADO MÉDICO - ATE 15 DIAS	CHC da Universidade Federal do Paraná - PR
57	AFASTAMENTO POR ATESTADO MÉDICO - ATE 15 DIAS	CHC da Universidade Federal do Paraná - PR
57	AFASTAMENTO POR ATESTADO MÉDICO - ATE 15 DIAS	CHC da Universidade Federal do Paraná - PR
57	AFASTAMENTO POR ATESTADO MÉDICO - ATE 15 DIAS	CHC da Universidade Federal do Paraná - PR
57	AFASTAMENTO POR ATESTADO MÉDICO - ATE 15 DIAS	CHC da Universidade Federal do Paraná - PR
57	AFASTAMENTO POR ATESTADO MÉDICO - ATE 15 DIAS	CHC da Universidade Federal do Paraná - PR
57	AFASTAMENTO POR ATESTADO MÉDICO - ATE 15 DIAS	CHC da Universidade Federal do Paraná - PR
57	AFASTAMENTO POR ATESTADO MÉDICO - ATE 15 DIAS	CHC da Universidade Federal do Paraná - PR
57	AFASTAMENTO POR ATESTADO MÉDICO - ATE 15 DIAS	CHC da Universidade Federal do Paraná - PR
57	AFASTAMENTO POR ATESTADO MÉDICO - ATE 15 DIAS	CHC da Universidade Federal do Paraná - PR
57	AFASTAMENTO POR ATESTADO MÉDICO - ATE 15 DIAS	CHC da Universidade Federal do Paraná - PR
57	AFASTAMENTO POR ATESTADO MÉDICO - ATE 15 DIAS	CHC da Universidade Federal do Paraná - PR
57	AFASTAMENTO POR ATESTADO MÉDICO - ATE 15 DIAS	CHC da Universidade Federal do Paraná - PR
57	AFASTAMENTO POR ATESTADO MÉDICO - ATE 15 DIAS	CHC da Universidade Federal do Paraná - PR
57	AFASTAMENTO POR ATESTADO MÉDICO - ATE 15 DIAS	CHC da Universidade Federal do Paraná - PR
57	AFASTAMENTO POR ATESTADO MÉDICO - ATE 15 DIAS	CHC da Universidade Federal do Paraná - PR
57	AFASTAMENTO POR ATESTADO MÉDICO - ATE 15 DIAS	CHC da Universidade Federal do Paraná - PR
57	AFASTAMENTO POR ATESTADO MÉDICO - ATE 15 DIAS	CHC da Universidade Federal do Paraná - PR
57	AFASTAMENTO POR ATESTADO MÉDICO - ATE 15 DIAS	CHC da Universidade Federal do Paraná - PR
57	AFASTAMENTO POR ATESTADO MÉDICO - ATE 15 DIAS	CHC da Universidade Federal do Paraná - PR
57	AFASTAMENTO POR ATESTADO MÉDICO - ATE 15 DIAS	CHC da Universidade Federal do Paraná - PR
57	AFASTAMENTO POR ATESTADO MÉDICO - ATE 15 DIAS	CHC da Universidade Federal do Paraná - PR
57	AFASTAMENTO POR ATESTADO MÉDICO - ATE 15 DIAS	CHC da Universidade Federal do Paraná - PR
57	AFASTAMENTO POR ATESTADO MÉDICO - ATE 15 DIAS	CHC da Universidade Federal do Paraná - PR
57	AFASTAMENTO POR ATESTADO MÉDICO - ATE 15 DIAS	CHC da Universidade Federal do Paraná - PR
57	AFASTAMENTO POR ATESTADO MÉDICO - ATE 15 DIAS	CHC da Universidade Federal do Paraná - PR
57	AFASTAMENTO POR ATESTADO MÉDICO - ATE 15 DIAS	CHC da Universidade Federal do Paraná - PR
57	AFASTAMENTO POR ATESTADO MÉDICO - ATE 15 DIAS	CHC da Universidade Federal do Paraná - PR
57	AFASTAMENTO POR ATESTADO MÉDICO - ATE 15 DIAS	CHC da Universidade Federal do Paraná - PR
57	AFASTAMENTO POR ATESTADO MÉDICO - ATE 15 DIAS	CHC da Universidade Federal do Paraná - PR
57	AFASTAMENTO POR ATESTADO MÉDICO - ATE 15 DIAS	CHC da Universidade Federal do Paraná - PR
57	AFASTAMENTO POR ATESTADO MÉDICO - ATE 15 DIAS	CHC da Universidade Federal do Paraná - PR
57	AFASTAMENTO POR ATESTADO MÉDICO - ATE 15 DIAS	CHC da Universidade Federal do Paraná - PR
57	AFASTAMENTO POR ATESTADO MÉDICO - ATE 15 DIAS	CHC da Universidade Federal do Paraná - PR
57	AFASTAMENTO POR ATESTADO MÉDICO - ATE 15 DIAS	CHC da Universidade Federal do Paraná - PR
57	AFASTAMENTO POR ATESTADO MÉDICO - ATE 15 DIAS	CHC da Universidade Federal do Paraná - PR
57	AFASTAMENTO POR ATESTADO MÉDICO - ATE 15 DIAS	CHC da Universidade Federal do Paraná - PR
57	AFASTAMENTO POR ATESTADO MÉDICO - ATE 15 DIAS	CHC da Universidade Federal do Paraná - PR
57	AFASTAMENTO POR ATESTADO MÉDICO - ATE 15 DIAS	CHC da Universidade Federal do Paraná - PR
57	AFASTAMENTO POR ATESTADO MÉDICO - ATE 15 DIAS	CHC da Universidade Federal do Paraná - PR
57	AFASTAMENTO POR ATESTADO MÉDICO - ATE 15 DIAS	CHC da Universidade Federal do Paraná - PR
57	AFASTAMENTO POR ATESTADO MÉDICO - ATE 15 DIAS	CHC da Universidade Federal do Paraná - PR
57	AFASTAMENTO POR ATESTADO MÉDICO - ATE 15 DIAS	CHC da Universidade Federal do Paraná - PR
57	AFASTAMENTO POR ATESTADO MÉDICO - ATE 15 DIAS	CHC da Universidade Federal do Paraná - PR
57	AFASTAMENTO POR ATESTADO MÉDICO - ATE 15 DIAS	CHC da Universidade Federal do Paraná - PR

57	AFASTAMENTO POR ATESTADO MÉDICO - ATE 15 DIAS	CHC da Universidade Federal do Paraná - PR
57	AFASTAMENTO POR ATESTADO MÉDICO - ATE 15 DIAS	CHC da Universidade Federal do Paraná - PR
57	AFASTAMENTO POR ATESTADO MÉDICO - ATE 15 DIAS	CHC da Universidade Federal do Paraná - PR
57	AFASTAMENTO POR ATESTADO MÉDICO - ATE 15 DIAS	CHC da Universidade Federal do Paraná - PR
57	AFASTAMENTO POR ATESTADO MÉDICO - ATE 15 DIAS	CHC da Universidade Federal do Paraná - PR
57	AFASTAMENTO POR ATESTADO MÉDICO - ATE 15 DIAS	CHC da Universidade Federal do Paraná - PR
57	AFASTAMENTO POR ATESTADO MÉDICO - ATE 15 DIAS	CHC da Universidade Federal do Paraná - PR
57	AFASTAMENTO POR ATESTADO MÉDICO - ATE 15 DIAS	CHC da Universidade Federal do Paraná - PR
57	AFASTAMENTO POR ATESTADO MÉDICO - ATE 15 DIAS	CHC da Universidade Federal do Paraná - PR
57	AFASTAMENTO POR ATESTADO MÉDICO - ATE 15 DIAS	CHC da Universidade Federal do Paraná - PR
57	AFASTAMENTO POR ATESTADO MÉDICO - ATE 15 DIAS	CHC da Universidade Federal do Paraná - PR
57	AFASTAMENTO POR ATESTADO MÉDICO - ATE 15 DIAS	CHC da Universidade Federal do Paraná - PR
57	AFASTAMENTO POR ATESTADO MÉDICO - ATE 15 DIAS	CHC da Universidade Federal do Paraná - PR
57	AFASTAMENTO POR ATESTADO MÉDICO - ATE 15 DIAS	CHC da Universidade Federal do Paraná - PR
57	AFASTAMENTO POR ATESTADO MÉDICO - ATE 15 DIAS	CHC da Universidade Federal do Paraná - PR
57	AFASTAMENTO POR ATESTADO MÉDICO - ATE 15 DIAS	CHC da Universidade Federal do Paraná - PR
57	AFASTAMENTO POR ATESTADO MÉDICO - ATE 15 DIAS	CHC da Universidade Federal do Paraná - PR
57	AFASTAMENTO POR ATESTADO MÉDICO - ATE 15 DIAS	CHC da Universidade Federal do Paraná - PR
57	AFASTAMENTO POR ATESTADO MÉDICO - ATE 15 DIAS	CHC da Universidade Federal do Paraná - PR
57	AFASTAMENTO POR ATESTADO MÉDICO - ATE 15 DIAS	CHC da Universidade Federal do Paraná - PR
57	AFASTAMENTO POR ATESTADO MÉDICO - ATE 15 DIAS	CHC da Universidade Federal do Paraná - PR
57	AFASTAMENTO POR ATESTADO MÉDICO - ATE 15 DIAS	CHC da Universidade Federal do Paraná - PR
57	AFASTAMENTO POR ATESTADO MÉDICO - ATE 15 DIAS	CHC da Universidade Federal do Paraná - PR
57	AFASTAMENTO POR ATESTADO MÉDICO - ATE 15 DIAS	CHC da Universidade Federal do Paraná - PR
57	AFASTAMENTO POR ATESTADO MÉDICO - ATE 15 DIAS	CHC da Universidade Federal do Paraná - PR
57	AFASTAMENTO POR ATESTADO MÉDICO - ATE 15 DIAS	CHC da Universidade Federal do Paraná - PR
57	AFASTAMENTO POR ATESTADO MÉDICO - ATE 15 DIAS	CHC da Universidade Federal do Paraná - PR
57	AFASTAMENTO POR ATESTADO MÉDICO - ATE 15 DIAS	CHC da Universidade Federal do Paraná - PR
57	AFASTAMENTO POR ATESTADO MÉDICO - ATE 15 DIAS	CHC da Universidade Federal do Paraná - PR
57	AFASTAMENTO POR ATESTADO MÉDICO - ATE 15 DIAS	CHC da Universidade Federal do Paraná - PR
57	AFASTAMENTO POR ATESTADO MÉDICO - ATE 15 DIAS	CHC da Universidade Federal do Paraná - PR
57	AFASTAMENTO POR ATESTADO MÉDICO - ATE 15 DIAS	CHC da Universidade Federal do Paraná - PR
57	AFASTAMENTO POR ATESTADO MÉDICO - ATE 15 DIAS	CHC da Universidade Federal do Paraná - PR
57	AFASTAMENTO POR ATESTADO MÉDICO - ATE 15 DIAS	CHC da Universidade Federal do Paraná - PR
57	AFASTAMENTO POR ATESTADO MÉDICO - ATE 15 DIAS	CHC da Universidade Federal do Paraná - PR
57	AFASTAMENTO POR ATESTADO MÉDICO - ATE 15 DIAS	CHC da Universidade Federal do Paraná - PR
57	AFASTAMENTO POR ATESTADO MÉDICO - ATE 15 DIAS	CHC da Universidade Federal do Paraná - PR
57	AFASTAMENTO POR ATESTADO MÉDICO - ATE 15 DIAS	CHC da Universidade Federal do Paraná - PR
57	AFASTAMENTO POR ATESTADO MÉDICO - ATE 15 DIAS	CHC da Universidade Federal do Paraná - PR
57	AFASTAMENTO POR ATESTADO MÉDICO - ATE 15 DIAS	CHC da Universidade Federal do Paraná - PR
57	AFASTAMENTO POR ATESTADO MÉDICO - ATE 15 DIAS	CHC da Universidade Federal do Paraná - PR
57	AFASTAMENTO POR ATESTADO MÉDICO - ATE 15 DIAS	CHC da Universidade Federal do Paraná - PR
57	AFASTAMENTO POR ATESTADO MÉDICO - ATE 15 DIAS	CHC da Universidade Federal do Paraná - PR
57	AFASTAMENTO POR ATESTADO MÉDICO - ATE 15 DIAS	CHC da Universidade Federal do Paraná - PR
57	AFASTAMENTO POR ATESTADO MÉDICO - ATE 15 DIAS	CHC da Universidade Federal do Paraná - PR
57	AFASTAMENTO POR ATESTADO MÉDICO - ATE 15 DIAS	CHC da Universidade Federal do Paraná - PR
57	AFASTAMENTO POR ATESTADO MÉDICO - ATE 15 DIAS	CHC da Universidade Federal do Paraná - PR
57	AFASTAMENTO POR ATESTADO MÉDICO - ATE 15 DIAS	CHC da Universidade Federal do Paraná - PR

57	AFASTAMENTO POR ATESTADO MÉDICO - ATE 15 DIAS	CHC da Universidade Federal do Paraná - PR
57	AFASTAMENTO POR ATESTADO MÉDICO - ATE 15 DIAS	CHC da Universidade Federal do Paraná - PR
57	AFASTAMENTO POR ATESTADO MÉDICO - ATE 15 DIAS	CHC da Universidade Federal do Paraná - PR
57	AFASTAMENTO POR ATESTADO MÉDICO - ATE 15 DIAS	CHC da Universidade Federal do Paraná - PR
57	AFASTAMENTO POR ATESTADO MÉDICO - ATE 15 DIAS	CHC da Universidade Federal do Paraná - PR
57	AFASTAMENTO POR ATESTADO MÉDICO - ATE 15 DIAS	CHC da Universidade Federal do Paraná - PR
57	AFASTAMENTO POR ATESTADO MÉDICO - ATE 15 DIAS	CHC da Universidade Federal do Paraná - PR
57	AFASTAMENTO POR ATESTADO MÉDICO - ATE 15 DIAS	CHC da Universidade Federal do Paraná - PR
57	AFASTAMENTO POR ATESTADO MÉDICO - ATE 15 DIAS	CHC da Universidade Federal do Paraná - PR
57	AFASTAMENTO POR ATESTADO MÉDICO - ATE 15 DIAS	CHC da Universidade Federal do Paraná - PR
57	AFASTAMENTO POR ATESTADO MÉDICO - ATE 15 DIAS	CHC da Universidade Federal do Paraná - PR
57	AFASTAMENTO POR ATESTADO MÉDICO - ATE 15 DIAS	CHC da Universidade Federal do Paraná - PR
57	AFASTAMENTO POR ATESTADO MÉDICO - ATE 15 DIAS	CHC da Universidade Federal do Paraná - PR
57	AFASTAMENTO POR ATESTADO MÉDICO - ATE 15 DIAS	CHC da Universidade Federal do Paraná - PR
57	AFASTAMENTO POR ATESTADO MÉDICO - ATE 15 DIAS	CHC da Universidade Federal do Paraná - PR
57	AFASTAMENTO POR ATESTADO MÉDICO - ATE 15 DIAS	CHC da Universidade Federal do Paraná - PR
57	AFASTAMENTO POR ATESTADO MÉDICO - ATE 15 DIAS	CHC da Universidade Federal do Paraná - PR
57	AFASTAMENTO POR ATESTADO MÉDICO - ATE 15 DIAS	CHC da Universidade Federal do Paraná - PR
57	AFASTAMENTO POR ATESTADO MÉDICO - ATE 15 DIAS	CHC da Universidade Federal do Paraná - PR
57	AFASTAMENTO POR ATESTADO MÉDICO - ATE 15 DIAS	CHC da Universidade Federal do Paraná - PR
57	AFASTAMENTO POR ATESTADO MÉDICO - ATE 15 DIAS	CHC da Universidade Federal do Paraná - PR
57	AFASTAMENTO POR ATESTADO MÉDICO - ATE 15 DIAS	CHC da Universidade Federal do Paraná - PR
57	AFASTAMENTO POR ATESTADO MÉDICO - ATE 15 DIAS	CHC da Universidade Federal do Paraná - PR
57	AFASTAMENTO POR ATESTADO MÉDICO - ATE 15 DIAS	CHC da Universidade Federal do Paraná - PR
57	AFASTAMENTO POR ATESTADO MÉDICO - ATE 15 DIAS	CHC da Universidade Federal do Paraná - PR
57	AFASTAMENTO POR ATESTADO MÉDICO - ATE 15 DIAS	CHC da Universidade Federal do Paraná - PR
57	AFASTAMENTO POR ATESTADO MÉDICO - ATE 15 DIAS	CHC da Universidade Federal do Paraná - PR
57	AFASTAMENTO POR ATESTADO MÉDICO - ATE 15 DIAS	CHC da Universidade Federal do Paraná - PR
57	AFASTAMENTO POR ATESTADO MÉDICO - ATE 15 DIAS	CHC da Universidade Federal do Paraná - PR
57	AFASTAMENTO POR ATESTADO MÉDICO - ATE 15 DIAS	CHC da Universidade Federal do Paraná - PR
57	AFASTAMENTO POR ATESTADO MÉDICO - ATE 15 DIAS	CHC da Universidade Federal do Paraná - PR
57	AFASTAMENTO POR ATESTADO MÉDICO - ATE 15 DIAS	CHC da Universidade Federal do Paraná - PR
57	AFASTAMENTO POR ATESTADO MÉDICO - ATE 15 DIAS	CHC da Universidade Federal do Paraná - PR
57	AFASTAMENTO POR ATESTADO MÉDICO - ATE 15 DIAS	CHC da Universidade Federal do Paraná - PR
57	AFASTAMENTO POR ATESTADO MÉDICO - ATE 15 DIAS	CHC da Universidade Federal do Paraná - PR
57	AFASTAMENTO POR ATESTADO MÉDICO - ATE 15 DIAS	CHC da Universidade Federal do Paraná - PR
57	AFASTAMENTO POR ATESTADO MÉDICO - ATE 15 DIAS	CHC da Universidade Federal do Paraná - PR
57	AFASTAMENTO POR ATESTADO MÉDICO - ATE 15 DIAS	CHC da Universidade Federal do Paraná - PR
57	AFASTAMENTO POR ATESTADO MÉDICO - ATE 15 DIAS	CHC da Universidade Federal do Paraná - PR
57	AFASTAMENTO POR ATESTADO MÉDICO - ATE 15 DIAS	CHC da Universidade Federal do Paraná - PR
57	AFASTAMENTO POR ATESTADO MÉDICO - ATE 15 DIAS	CHC da Universidade Federal do Paraná - PR
57	AFASTAMENTO POR ATESTADO MÉDICO - ATE 15 DIAS	CHC da Universidade Federal do Paraná - PR
57	AFASTAMENTO POR ATESTADO MÉDICO - ATE 15 DIAS	CHC da Universidade Federal do Paraná - PR
57	AFASTAMENTO POR ATESTADO MÉDICO - ATE 15 DIAS	CHC da Universidade Federal do Paraná - PR
57	AFASTAMENTO POR ATESTADO MÉDICO - ATE 15 DIAS	CHC da Universidade Federal do Paraná - PR
57	AFASTAMENTO POR ATESTADO MÉDICO - ATE 15 DIAS	CHC da Universidade Federal do Paraná - PR
57	AFASTAMENTO POR ATESTADO MÉDICO - ATE 15 DIAS	CHC da Universidade Federal do Paraná - PR
57	AFASTAMENTO POR ATESTADO MÉDICO - ATE 15 DIAS	CHC da Universidade Federal do Paraná - PR

57	AFASTAMENTO POR ATESTADO MÉDICO - ATE 15 DIAS	CHC da Universidade Federal do Paraná - PR
57	AFASTAMENTO POR ATESTADO MÉDICO - ATE 15 DIAS	CHC da Universidade Federal do Paraná - PR
57	AFASTAMENTO POR ATESTADO MÉDICO - ATE 15 DIAS	CHC da Universidade Federal do Paraná - PR
57	AFASTAMENTO POR ATESTADO MÉDICO - ATE 15 DIAS	CHC da Universidade Federal do Paraná - PR
57	AFASTAMENTO POR ATESTADO MÉDICO - ATE 15 DIAS	CHC da Universidade Federal do Paraná - PR
57	AFASTAMENTO POR ATESTADO MÉDICO - ATE 15 DIAS	CHC da Universidade Federal do Paraná - PR
57	AFASTAMENTO POR ATESTADO MÉDICO - ATE 15 DIAS	CHC da Universidade Federal do Paraná - PR
57	AFASTAMENTO POR ATESTADO MÉDICO - ATE 15 DIAS	CHC da Universidade Federal do Paraná - PR
57	AFASTAMENTO POR ATESTADO MÉDICO - ATE 15 DIAS	CHC da Universidade Federal do Paraná - PR
57	AFASTAMENTO POR ATESTADO MÉDICO - ATE 15 DIAS	CHC da Universidade Federal do Paraná - PR
57	AFASTAMENTO POR ATESTADO MÉDICO - ATE 15 DIAS	CHC da Universidade Federal do Paraná - PR
57	AFASTAMENTO POR ATESTADO MÉDICO - ATE 15 DIAS	CHC da Universidade Federal do Paraná - PR
57	AFASTAMENTO POR ATESTADO MÉDICO - ATE 15 DIAS	CHC da Universidade Federal do Paraná - PR
57	AFASTAMENTO POR ATESTADO MÉDICO - ATE 15 DIAS	CHC da Universidade Federal do Paraná - PR
57	AFASTAMENTO POR ATESTADO MÉDICO - ATE 15 DIAS	CHC da Universidade Federal do Paraná - PR
57	AFASTAMENTO POR ATESTADO MÉDICO - ATE 15 DIAS	CHC da Universidade Federal do Paraná - PR
57	AFASTAMENTO POR ATESTADO MÉDICO - ATE 15 DIAS	CHC da Universidade Federal do Paraná - PR
57	AFASTAMENTO POR ATESTADO MÉDICO - ATE 15 DIAS	CHC da Universidade Federal do Paraná - PR
57	AFASTAMENTO POR ATESTADO MÉDICO - ATE 15 DIAS	CHC da Universidade Federal do Paraná - PR
57	AFASTAMENTO POR ATESTADO MÉDICO - ATE 15 DIAS	CHC da Universidade Federal do Paraná - PR
57	AFASTAMENTO POR ATESTADO MÉDICO - ATE 15 DIAS	CHC da Universidade Federal do Paraná - PR
57	AFASTAMENTO POR ATESTADO MÉDICO - ATE 15 DIAS	CHC da Universidade Federal do Paraná - PR
57	AFASTAMENTO POR ATESTADO MÉDICO - ATE 15 DIAS	CHC da Universidade Federal do Paraná - PR
57	AFASTAMENTO POR ATESTADO MÉDICO - ATE 15 DIAS	CHC da Universidade Federal do Paraná - PR
57	AFASTAMENTO POR ATESTADO MÉDICO - ATE 15 DIAS	CHC da Universidade Federal do Paraná - PR
57	AFASTAMENTO POR ATESTADO MÉDICO - ATE 15 DIAS	CHC da Universidade Federal do Paraná - PR
57	AFASTAMENTO POR ATESTADO MÉDICO - ATE 15 DIAS	CHC da Universidade Federal do Paraná - PR
57	AFASTAMENTO POR ATESTADO MÉDICO - ATE 15 DIAS	CHC da Universidade Federal do Paraná - PR
57	AFASTAMENTO POR ATESTADO MÉDICO - ATE 15 DIAS	CHC da Universidade Federal do Paraná - PR
57	AFASTAMENTO POR ATESTADO MÉDICO - ATE 15 DIAS	CHC da Universidade Federal do Paraná - PR
57	AFASTAMENTO POR ATESTADO MÉDICO - ATE 15 DIAS	CHC da Universidade Federal do Paraná - PR
57	AFASTAMENTO POR ATESTADO MÉDICO - ATE 15 DIAS	CHC da Universidade Federal do Paraná - PR
57	AFASTAMENTO POR ATESTADO MÉDICO - ATE 15 DIAS	CHC da Universidade Federal do Paraná - PR
57	AFASTAMENTO POR ATESTADO MÉDICO - ATE 15 DIAS	CHC da Universidade Federal do Paraná - PR
57	AFASTAMENTO POR ATESTADO MÉDICO - ATE 15 DIAS	CHC da Universidade Federal do Paraná - PR
57	AFASTAMENTO POR ATESTADO MÉDICO - ATE 15 DIAS	CHC da Universidade Federal do Paraná - PR
57	AFASTAMENTO POR ATESTADO MÉDICO - ATE 15 DIAS	CHC da Universidade Federal do Paraná - PR
57	AFASTAMENTO POR ATESTADO MÉDICO - ATE 15 DIAS	CHC da Universidade Federal do Paraná - PR
57	AFASTAMENTO POR ATESTADO MÉDICO - ATE 15 DIAS	CHC da Universidade Federal do Paraná - PR
57	AFASTAMENTO POR ATESTADO MÉDICO - ATE 15 DIAS	CHC da Universidade Federal do Paraná - PR
57	AFASTAMENTO POR ATESTADO MÉDICO - ATE 15 DIAS	CHC da Universidade Federal do Paraná - PR
57	AFASTAMENTO POR ATESTADO MÉDICO - ATE 15 DIAS	CHC da Universidade Federal do Paraná - PR
57	AFASTAMENTO POR ATESTADO MÉDICO - ATE 15 DIAS	CHC da Universidade Federal do Paraná - PR
57	AFASTAMENTO POR ATESTADO MÉDICO - ATE 15 DIAS	CHC da Universidade Federal do Paraná - PR
57	AFASTAMENTO POR ATESTADO MÉDICO - ATE 15 DIAS	CHC da Universidade Federal do Paraná - PR
57	AFASTAMENTO POR ATESTADO MÉDICO - ATE 15 DIAS	CHC da Universidade Federal do Paraná - PR
57	AFASTAMENTO POR ATESTADO MÉDICO - ATE 15 DIAS	CHC da Universidade Federal do Paraná - PR
57	AFASTAMENTO POR ATESTADO MÉDICO - ATE 15 DIAS	CHC da Universidade Federal do Paraná - PR

57	AFASTAMENTO POR ATESTADO MÉDICO - ATE 15 DIAS	CHC da Universidade Federal do Paraná - PR
57	AFASTAMENTO POR ATESTADO MÉDICO - ATE 15 DIAS	CHC da Universidade Federal do Paraná - PR
57	AFASTAMENTO POR ATESTADO MÉDICO - ATE 15 DIAS	CHC da Universidade Federal do Paraná - PR
57	AFASTAMENTO POR ATESTADO MÉDICO - ATE 15 DIAS	CHC da Universidade Federal do Paraná - PR
57	AFASTAMENTO POR ATESTADO MÉDICO - ATE 15 DIAS	CHC da Universidade Federal do Paraná - PR
57	AFASTAMENTO POR ATESTADO MÉDICO - ATE 15 DIAS	CHC da Universidade Federal do Paraná - PR
57	AFASTAMENTO POR ATESTADO MÉDICO - ATE 15 DIAS	CHC da Universidade Federal do Paraná - PR
57	AFASTAMENTO POR ATESTADO MÉDICO - ATE 15 DIAS	CHC da Universidade Federal do Paraná - PR
57	AFASTAMENTO POR ATESTADO MÉDICO - ATE 15 DIAS	CHC da Universidade Federal do Paraná - PR
57	AFASTAMENTO POR ATESTADO MÉDICO - ATE 15 DIAS	CHC da Universidade Federal do Paraná - PR
57	AFASTAMENTO POR ATESTADO MÉDICO - ATE 15 DIAS	CHC da Universidade Federal do Paraná - PR
57	AFASTAMENTO POR ATESTADO MÉDICO - ATE 15 DIAS	CHC da Universidade Federal do Paraná - PR
57	AFASTAMENTO POR ATESTADO MÉDICO - ATE 15 DIAS	CHC da Universidade Federal do Paraná - PR
57	AFASTAMENTO POR ATESTADO MÉDICO - ATE 15 DIAS	CHC da Universidade Federal do Paraná - PR
57	AFASTAMENTO POR ATESTADO MÉDICO - ATE 15 DIAS	CHC da Universidade Federal do Paraná - PR
57	AFASTAMENTO POR ATESTADO MÉDICO - ATE 15 DIAS	CHC da Universidade Federal do Paraná - PR
57	AFASTAMENTO POR ATESTADO MÉDICO - ATE 15 DIAS	CHC da Universidade Federal do Paraná - PR
57	AFASTAMENTO POR ATESTADO MÉDICO - ATE 15 DIAS	CHC da Universidade Federal do Paraná - PR
57	AFASTAMENTO POR ATESTADO MÉDICO - ATE 15 DIAS	CHC da Universidade Federal do Paraná - PR
57	AFASTAMENTO POR ATESTADO MÉDICO - ATE 15 DIAS	CHC da Universidade Federal do Paraná - PR
57	AFASTAMENTO POR ATESTADO MÉDICO - ATE 15 DIAS	CHC da Universidade Federal do Paraná - PR
57	AFASTAMENTO POR ATESTADO MÉDICO - ATE 15 DIAS	CHC da Universidade Federal do Paraná - PR
57	AFASTAMENTO POR ATESTADO MÉDICO - ATE 15 DIAS	CHC da Universidade Federal do Paraná - PR
57	AFASTAMENTO POR ATESTADO MÉDICO - ATE 15 DIAS	CHC da Universidade Federal do Paraná - PR
57	AFASTAMENTO POR ATESTADO MÉDICO - ATE 15 DIAS	CHC da Universidade Federal do Paraná - PR
57	AFASTAMENTO POR ATESTADO MÉDICO - ATE 15 DIAS	CHC da Universidade Federal do Paraná - PR
57	AFASTAMENTO POR ATESTADO MÉDICO - ATE 15 DIAS	CHC da Universidade Federal do Paraná - PR
57	AFASTAMENTO POR ATESTADO MÉDICO - ATE 15 DIAS	CHC da Universidade Federal do Paraná - PR
57	AFASTAMENTO POR ATESTADO MÉDICO - ATE 15 DIAS	CHC da Universidade Federal do Paraná - PR
57	AFASTAMENTO POR ATESTADO MÉDICO - ATE 15 DIAS	CHC da Universidade Federal do Paraná - PR
57	AFASTAMENTO POR ATESTADO MÉDICO - ATE 15 DIAS	CHC da Universidade Federal do Paraná - PR
57	AFASTAMENTO POR ATESTADO MÉDICO - ATE 15 DIAS	CHC da Universidade Federal do Paraná - PR
57	AFASTAMENTO POR ATESTADO MÉDICO - ATE 15 DIAS	CHC da Universidade Federal do Paraná - PR
57	AFASTAMENTO POR ATESTADO MÉDICO - ATE 15 DIAS	CHC da Universidade Federal do Paraná - PR
57	AFASTAMENTO POR ATESTADO MÉDICO - ATE 15 DIAS	CHC da Universidade Federal do Paraná - PR
57	AFASTAMENTO POR ATESTADO MÉDICO - ATE 15 DIAS	CHC da Universidade Federal do Paraná - PR
57	AFASTAMENTO POR ATESTADO MÉDICO - ATE 15 DIAS	CHC da Universidade Federal do Paraná - PR
57	AFASTAMENTO POR ATESTADO MÉDICO - ATE 15 DIAS	CHC da Universidade Federal do Paraná - PR
57	AFASTAMENTO POR ATESTADO MÉDICO - ATE 15 DIAS	CHC da Universidade Federal do Paraná - PR
57	AFASTAMENTO POR ATESTADO MÉDICO - ATE 15 DIAS	CHC da Universidade Federal do Paraná - PR
57	AFASTAMENTO POR ATESTADO MÉDICO - ATE 15 DIAS	CHC da Universidade Federal do Paraná - PR
57	AFASTAMENTO POR ATESTADO MÉDICO - ATE 15 DIAS	CHC da Universidade Federal do Paraná - PR
57	AFASTAMENTO POR ATESTADO MÉDICO - ATE 15 DIAS	CHC da Universidade Federal do Paraná - PR
57	AFASTAMENTO POR ATESTADO MÉDICO - ATE 15 DIAS	CHC da Universidade Federal do Paraná - PR
57	AFASTAMENTO POR ATESTADO MÉDICO - ATE 15 DIAS	CHC da Universidade Federal do Paraná - PR
57	AFASTAMENTO POR ATESTADO MÉDICO - ATE 15 DIAS	CHC da Universidade Federal do Paraná - PR
57	AFASTAMENTO POR ATESTADO MÉDICO - ATE 15 DIAS	CHC da Universidade Federal do Paraná - PR
57	AFASTAMENTO POR ATESTADO MÉDICO - ATE 15 DIAS	CHC da Universidade Federal do Paraná - PR

57	AFASTAMENTO POR ATESTADO MÉDICO - ATE 15 DIAS	CHC da Universidade Federal do Paraná - PR
57	AFASTAMENTO POR ATESTADO MÉDICO - ATE 15 DIAS	CHC da Universidade Federal do Paraná - PR
57	AFASTAMENTO POR ATESTADO MÉDICO - ATE 15 DIAS	CHC da Universidade Federal do Paraná - PR
57	AFASTAMENTO POR ATESTADO MÉDICO - ATE 15 DIAS	CHC da Universidade Federal do Paraná - PR
57	AFASTAMENTO POR ATESTADO MÉDICO - ATE 15 DIAS	CHC da Universidade Federal do Paraná - PR
57	AFASTAMENTO POR ATESTADO MÉDICO - ATE 15 DIAS	CHC da Universidade Federal do Paraná - PR
57	AFASTAMENTO POR ATESTADO MÉDICO - ATE 15 DIAS	CHC da Universidade Federal do Paraná - PR
57	AFASTAMENTO POR ATESTADO MÉDICO - ATE 15 DIAS	CHC da Universidade Federal do Paraná - PR
57	AFASTAMENTO POR ATESTADO MÉDICO - ATE 15 DIAS	CHC da Universidade Federal do Paraná - PR
57	AFASTAMENTO POR ATESTADO MÉDICO - ATE 15 DIAS	CHC da Universidade Federal do Paraná - PR
57	AFASTAMENTO POR ATESTADO MÉDICO - ATE 15 DIAS	CHC da Universidade Federal do Paraná - PR
57	AFASTAMENTO POR ATESTADO MÉDICO - ATE 15 DIAS	CHC da Universidade Federal do Paraná - PR
57	AFASTAMENTO POR ATESTADO MÉDICO - ATE 15 DIAS	CHC da Universidade Federal do Paraná - PR
57	AFASTAMENTO POR ATESTADO MÉDICO - ATE 15 DIAS	CHC da Universidade Federal do Paraná - PR
57	AFASTAMENTO POR ATESTADO MÉDICO - ATE 15 DIAS	CHC da Universidade Federal do Paraná - PR
57	AFASTAMENTO POR ATESTADO MÉDICO - ATE 15 DIAS	CHC da Universidade Federal do Paraná - PR
57	AFASTAMENTO POR ATESTADO MÉDICO - ATE 15 DIAS	CHC da Universidade Federal do Paraná - PR
57	AFASTAMENTO POR ATESTADO MÉDICO - ATE 15 DIAS	CHC da Universidade Federal do Paraná - PR
57	AFASTAMENTO POR ATESTADO MÉDICO - ATE 15 DIAS	CHC da Universidade Federal do Paraná - PR
57	AFASTAMENTO POR ATESTADO MÉDICO - ATE 15 DIAS	CHC da Universidade Federal do Paraná - PR
57	AFASTAMENTO POR ATESTADO MÉDICO - ATE 15 DIAS	CHC da Universidade Federal do Paraná - PR
57	AFASTAMENTO POR ATESTADO MÉDICO - ATE 15 DIAS	CHC da Universidade Federal do Paraná - PR
57	AFASTAMENTO POR ATESTADO MÉDICO - ATE 15 DIAS	CHC da Universidade Federal do Paraná - PR
57	AFASTAMENTO POR ATESTADO MÉDICO - ATE 15 DIAS	CHC da Universidade Federal do Paraná - PR
57	AFASTAMENTO POR ATESTADO MÉDICO - ATE 15 DIAS	CHC da Universidade Federal do Paraná - PR
57	AFASTAMENTO POR ATESTADO MÉDICO - ATE 15 DIAS	CHC da Universidade Federal do Paraná - PR
57	AFASTAMENTO POR ATESTADO MÉDICO - ATE 15 DIAS	CHC da Universidade Federal do Paraná - PR
57	AFASTAMENTO POR ATESTADO MÉDICO - ATE 15 DIAS	CHC da Universidade Federal do Paraná - PR
57	AFASTAMENTO POR ATESTADO MÉDICO - ATE 15 DIAS	CHC da Universidade Federal do Paraná - PR
57	AFASTAMENTO POR ATESTADO MÉDICO - ATE 15 DIAS	CHC da Universidade Federal do Paraná - PR
57	AFASTAMENTO POR ATESTADO MÉDICO - ATE 15 DIAS	CHC da Universidade Federal do Paraná - PR
57	AFASTAMENTO POR ATESTADO MÉDICO - ATE 15 DIAS	CHC da Universidade Federal do Paraná - PR
57	AFASTAMENTO POR ATESTADO MÉDICO - ATE 15 DIAS	CHC da Universidade Federal do Paraná - PR
57	AFASTAMENTO POR ATESTADO MÉDICO - ATE 15 DIAS	CHC da Universidade Federal do Paraná - PR

57	AFASTAMENTO POR ATESTADO MÉDICO - ATE 15 DIAS	CHC da Universidade Federal do Paraná - PR
57	AFASTAMENTO POR ATESTADO MÉDICO - ATE 15 DIAS	CHC da Universidade Federal do Paraná - PR
57	AFASTAMENTO POR ATESTADO MÉDICO - ATE 15 DIAS	CHC da Universidade Federal do Paraná - PR
57	AFASTAMENTO POR ATESTADO MÉDICO - ATE 15 DIAS	CHC da Universidade Federal do Paraná - PR
57	AFASTAMENTO POR ATESTADO MÉDICO - ATE 15 DIAS	CHC da Universidade Federal do Paraná - PR
57	AFASTAMENTO POR ATESTADO MÉDICO - ATE 15 DIAS	CHC da Universidade Federal do Paraná - PR
57	AFASTAMENTO POR ATESTADO MÉDICO - ATE 15 DIAS	CHC da Universidade Federal do Paraná - PR
57	AFASTAMENTO POR ATESTADO MÉDICO - ATE 15 DIAS	CHC da Universidade Federal do Paraná - PR
57	AFASTAMENTO POR ATESTADO MÉDICO - ATE 15 DIAS	CHC da Universidade Federal do Paraná - PR
57	AFASTAMENTO POR ATESTADO MÉDICO - ATE 15 DIAS	CHC da Universidade Federal do Paraná - PR
57	AFASTAMENTO POR ATESTADO MÉDICO - ATE 15 DIAS	CHC da Universidade Federal do Paraná - PR
57	AFASTAMENTO POR ATESTADO MÉDICO - ATE 15 DIAS	CHC da Universidade Federal do Paraná - PR
57	AFASTAMENTO POR ATESTADO MÉDICO - ATE 15 DIAS	CHC da Universidade Federal do Paraná - PR
57	AFASTAMENTO POR ATESTADO MÉDICO - ATE 15 DIAS	CHC da Universidade Federal do Paraná - PR
57	AFASTAMENTO POR ATESTADO MÉDICO - ATE 15 DIAS	CHC da Universidade Federal do Paraná - PR
57	AFASTAMENTO POR ATESTADO MÉDICO - ATE 15 DIAS	CHC da Universidade Federal do Paraná - PR
57	AFASTAMENTO POR ATESTADO MÉDICO - ATE 15 DIAS	CHC da Universidade Federal do Paraná - PR
57	AFASTAMENTO POR ATESTADO MÉDICO - ATE 15 DIAS	CHC da Universidade Federal do Paraná - PR
57	AFASTAMENTO POR ATESTADO MÉDICO - ATE 15 DIAS	CHC da Universidade Federal do Paraná - PR
57	AFASTAMENTO POR ATESTADO MÉDICO - ATE 15 DIAS	CHC da Universidade Federal do Paraná - PR
57	AFASTAMENTO POR ATESTADO MÉDICO - ATE 15 DIAS	CHC da Universidade Federal do Paraná - PR
57	AFASTAMENTO POR ATESTADO MÉDICO - ATE 15 DIAS	CHC da Universidade Federal do Paraná - PR
57	AFASTAMENTO POR ATESTADO MÉDICO - ATE 15 DIAS	CHC da Universidade Federal do Paraná - PR
57	AFASTAMENTO POR ATESTADO MÉDICO - ATE 15 DIAS	CHC da Universidade Federal do Paraná - PR
57	AFASTAMENTO POR ATESTADO MÉDICO - ATE 15 DIAS	CHC da Universidade Federal do Paraná - PR
57	AFASTAMENTO POR ATESTADO MÉDICO - ATE 15 DIAS	CHC da Universidade Federal do Paraná - PR
57	AFASTAMENTO POR ATESTADO MÉDICO - ATE 15 DIAS	CHC da Universidade Federal do Paraná - PR
57	AFASTAMENTO POR ATESTADO MÉDICO - ATE 15 DIAS	CHC da Universidade Federal do Paraná - PR
57	AFASTAMENTO POR ATESTADO MÉDICO - ATE 15 DIAS	CHC da Universidade Federal do Paraná - PR
57	AFASTAMENTO POR ATESTADO MÉDICO - ATE 15 DIAS	CHC da Universidade Federal do Paraná - PR
57	AFASTAMENTO POR ATESTADO MÉDICO - ATE 15 DIAS	CHC da Universidade Federal do Paraná - PR
57	AFASTAMENTO POR ATESTADO MÉDICO - ATE 15 DIAS	CHC da Universidade Federal do Paraná - PR

57	AFASTAMENTO POR ATESTADO MÉDICO - ATE 15 DIAS	CHC da Universidade Federal do Paraná - PR
57	AFASTAMENTO POR ATESTADO MÉDICO - ATE 15 DIAS	CHC da Universidade Federal do Paraná - PR
57	AFASTAMENTO POR ATESTADO MÉDICO - ATE 15 DIAS	CHC da Universidade Federal do Paraná - PR
57	AFASTAMENTO POR ATESTADO MÉDICO - ATE 15 DIAS	CHC da Universidade Federal do Paraná - PR
57	AFASTAMENTO POR ATESTADO MÉDICO - ATE 15 DIAS	CHC da Universidade Federal do Paraná - PR
57	AFASTAMENTO POR ATESTADO MÉDICO - ATE 15 DIAS	CHC da Universidade Federal do Paraná - PR
57	AFASTAMENTO POR ATESTADO MÉDICO - ATE 15 DIAS	CHC da Universidade Federal do Paraná - PR
57	AFASTAMENTO POR ATESTADO MÉDICO - ATE 15 DIAS	CHC da Universidade Federal do Paraná - PR
57	AFASTAMENTO POR ATESTADO MÉDICO - ATE 15 DIAS	CHC da Universidade Federal do Paraná - PR
57	AFASTAMENTO POR ATESTADO MÉDICO - ATE 15 DIAS	CHC da Universidade Federal do Paraná - PR
57	AFASTAMENTO POR ATESTADO MÉDICO - ATE 15 DIAS	CHC da Universidade Federal do Paraná - PR
57	AFASTAMENTO POR ATESTADO MÉDICO - ATE 15 DIAS	CHC da Universidade Federal do Paraná - PR
57	AFASTAMENTO POR ATESTADO MÉDICO - ATE 15 DIAS	CHC da Universidade Federal do Paraná - PR
57	AFASTAMENTO POR ATESTADO MÉDICO - ATE 15 DIAS	CHC da Universidade Federal do Paraná - PR
57	AFASTAMENTO POR ATESTADO MÉDICO - ATE 15 DIAS	CHC da Universidade Federal do Paraná - PR
57	AFASTAMENTO POR ATESTADO MÉDICO - ATE 15 DIAS	CHC da Universidade Federal do Paraná - PR
57	AFASTAMENTO POR ATESTADO MÉDICO - ATE 15 DIAS	CHC da Universidade Federal do Paraná - PR
57	AFASTAMENTO POR ATESTADO MÉDICO - ATE 15 DIAS	CHC da Universidade Federal do Paraná - PR
57	AFASTAMENTO POR ATESTADO MÉDICO - ATE 15 DIAS	CHC da Universidade Federal do Paraná - PR
57	AFASTAMENTO POR ATESTADO MÉDICO - ATE 15 DIAS	CHC da Universidade Federal do Paraná - PR
57	AFASTAMENTO POR ATESTADO MÉDICO - ATE 15 DIAS	CHC da Universidade Federal do Paraná - PR
57	AFASTAMENTO POR ATESTADO MÉDICO - ATE 15 DIAS	CHC da Universidade Federal do Paraná - PR
57	AFASTAMENTO POR ATESTADO MÉDICO - ATE 15 DIAS	CHC da Universidade Federal do Paraná - PR
57	AFASTAMENTO POR ATESTADO MÉDICO - ATE 15 DIAS	CHC da Universidade Federal do Paraná - PR
57	AFASTAMENTO POR ATESTADO MÉDICO - ATE 15 DIAS	CHC da Universidade Federal do Paraná - PR
57	AFASTAMENTO POR ATESTADO MÉDICO - ATE 15 DIAS	CHC da Universidade Federal do Paraná - PR
57	AFASTAMENTO POR ATESTADO MÉDICO - ATE 15 DIAS	CHC da Universidade Federal do Paraná - PR
57	AFASTAMENTO POR ATESTADO MÉDICO - ATE 15 DIAS	CHC da Universidade Federal do Paraná - PR
57	AFASTAMENTO POR ATESTADO MÉDICO - ATE 15 DIAS	CHC da Universidade Federal do Paraná - PR
57	AFASTAMENTO POR ATESTADO MÉDICO - ATE 15 DIAS	CHC da Universidade Federal do Paraná - PR
57	AFASTAMENTO POR ATESTADO MÉDICO - ATE 15 DIAS	CHC da Universidade Federal do Paraná - PR
57	AFASTAMENTO POR ATESTADO MÉDICO - ATE 15 DIAS	CHC da Universidade Federal do Paraná - PR
57	AFASTAMENTO POR ATESTADO MÉDICO - ATE 15 DIAS	CHC da Universidade Federal do Paraná - PR
57	AFASTAMENTO POR ATESTADO MÉDICO - ATE 15 DIAS	CHC da Universidade Federal do Paraná - PR
57	AFASTAMENTO POR ATESTADO MÉDICO - ATE 15 DIAS	CHC da Universidade Federal do Paraná - PR
57	AFASTAMENTO POR ATESTADO MÉDICO - ATE 15 DIAS	CHC da Universidade Federal do Paraná - PR
57	AFASTAMENTO POR ATESTADO MÉDICO - ATE 15 DIAS	CHC da Universidade Federal do Paraná - PR
57	AFASTAMENTO POR ATESTADO MÉDICO - ATE 15 DIAS	CHC da Universidade Federal do Paraná - PR
57	AFASTAMENTO POR ATESTADO MÉDICO - ATE 15 DIAS	CHC da Universidade Federal do Paraná - PR

57	AFASTAMENTO POR ATESTADO MÉDICO - ATE 15 DIAS	CHC da Universidade Federal do Paraná - PR
57	AFASTAMENTO POR ATESTADO MÉDICO - ATE 15 DIAS	CHC da Universidade Federal do Paraná - PR
57	AFASTAMENTO POR ATESTADO MÉDICO - ATE 15 DIAS	CHC da Universidade Federal do Paraná - PR
57	AFASTAMENTO POR ATESTADO MÉDICO - ATE 15 DIAS	CHC da Universidade Federal do Paraná - PR
57	AFASTAMENTO POR ATESTADO MÉDICO - ATE 15 DIAS	CHC da Universidade Federal do Paraná - PR
57	AFASTAMENTO POR ATESTADO MÉDICO - ATE 15 DIAS	CHC da Universidade Federal do Paraná - PR
57	AFASTAMENTO POR ATESTADO MÉDICO - ATE 15 DIAS	CHC da Universidade Federal do Paraná - PR
57	AFASTAMENTO POR ATESTADO MÉDICO - ATE 15 DIAS	CHC da Universidade Federal do Paraná - PR
57	AFASTAMENTO POR ATESTADO MÉDICO - ATE 15 DIAS	CHC da Universidade Federal do Paraná - PR
57	AFASTAMENTO POR ATESTADO MÉDICO - ATE 15 DIAS	CHC da Universidade Federal do Paraná - PR
57	AFASTAMENTO POR ATESTADO MÉDICO - ATE 15 DIAS	CHC da Universidade Federal do Paraná - PR
57	AFASTAMENTO POR ATESTADO MÉDICO - ATE 15 DIAS	CHC da Universidade Federal do Paraná - PR
57	AFASTAMENTO POR ATESTADO MÉDICO - ATE 15 DIAS	CHC da Universidade Federal do Paraná - PR
57	AFASTAMENTO POR ATESTADO MÉDICO - ATE 15 DIAS	CHC da Universidade Federal do Paraná - PR
57	AFASTAMENTO POR ATESTADO MÉDICO - ATE 15 DIAS	CHC da Universidade Federal do Paraná - PR
57	AFASTAMENTO POR ATESTADO MÉDICO - ATE 15 DIAS	CHC da Universidade Federal do Paraná - PR
57	AFASTAMENTO POR ATESTADO MÉDICO - ATE 15 DIAS	CHC da Universidade Federal do Paraná - PR
57	AFASTAMENTO POR ATESTADO MÉDICO - ATE 15 DIAS	CHC da Universidade Federal do Paraná - PR
57	AFASTAMENTO POR ATESTADO MÉDICO - ATE 15 DIAS	CHC da Universidade Federal do Paraná - PR
57	AFASTAMENTO POR ATESTADO MÉDICO - ATE 15 DIAS	CHC da Universidade Federal do Paraná - PR
57	AFASTAMENTO POR ATESTADO MÉDICO - ATE 15 DIAS	CHC da Universidade Federal do Paraná - PR
57	AFASTAMENTO POR ATESTADO MÉDICO - ATE 15 DIAS	CHC da Universidade Federal do Paraná - PR
57	AFASTAMENTO POR ATESTADO MÉDICO - ATE 15 DIAS	CHC da Universidade Federal do Paraná - PR
57	AFASTAMENTO POR ATESTADO MÉDICO - ATE 15 DIAS	CHC da Universidade Federal do Paraná - PR
57	AFASTAMENTO POR ATESTADO MÉDICO - ATE 15 DIAS	CHC da Universidade Federal do Paraná - PR
57	AFASTAMENTO POR ATESTADO MÉDICO - ATE 15 DIAS	CHC da Universidade Federal do Paraná - PR
57	AFASTAMENTO POR ATESTADO MÉDICO - ATE 15 DIAS	CHC da Universidade Federal do Paraná - PR
57	AFASTAMENTO POR ATESTADO MÉDICO - ATE 15 DIAS	CHC da Universidade Federal do Paraná - PR
57	AFASTAMENTO POR ATESTADO MÉDICO - ATE 15 DIAS	CHC da Universidade Federal do Paraná - PR
57	AFASTAMENTO POR ATESTADO MÉDICO - ATE 15 DIAS	CHC da Universidade Federal do Paraná - PR
57	AFASTAMENTO POR ATESTADO MÉDICO - ATE 15 DIAS	CHC da Universidade Federal do Paraná - PR
57	AFASTAMENTO POR ATESTADO MÉDICO - ATE 15 DIAS	CHC da Universidade Federal do Paraná - PR
57	AFASTAMENTO POR ATESTADO MÉDICO - ATE 15 DIAS	CHC da Universidade Federal do Paraná - PR
57	AFASTAMENTO POR ATESTADO MÉDICO - ATE 15 DIAS	CHC da Universidade Federal do Paraná - PR
57	AFASTAMENTO POR ATESTADO MÉDICO - ATE 15 DIAS	CHC da Universidade Federal do Paraná - PR
57	AFASTAMENTO POR ATESTADO MÉDICO - ATE 15 DIAS	CHC da Universidade Federal do Paraná - PR
57	AFASTAMENTO POR ATESTADO MÉDICO - ATE 15 DIAS	CHC da Universidade Federal do Paraná - PR
57	AFASTAMENTO POR ATESTADO MÉDICO - ATE 15 DIAS	CHC da Universidade Federal do Paraná - PR
57	AFASTAMENTO POR ATESTADO MÉDICO - ATE 15 DIAS	CHC da Universidade Federal do Paraná - PR
57	AFASTAMENTO POR ATESTADO MÉDICO - ATE 15 DIAS	CHC da Universidade Federal do Paraná - PR
57	AFASTAMENTO POR ATESTADO MÉDICO - ATE 15 DIAS	CHC da Universidade Federal do Paraná - PR
57	AFASTAMENTO POR ATESTADO MÉDICO - ATE 15 DIAS	CHC da Universidade Federal do Paraná - PR
57	AFASTAMENTO POR ATESTADO MÉDICO - ATE 15 DIAS	CHC da Universidade Federal do Paraná - PR
57	AFASTAMENTO POR ATESTADO MÉDICO - ATE 15 DIAS	CHC da Universidade Federal do Paraná - PR
57	AFASTAMENTO POR ATESTADO MÉDICO - ATE 15 DIAS	CHC da Universidade Federal do Paraná - PR
57	AFASTAMENTO POR ATESTADO MÉDICO - ATE 15 DIAS	CHC da Universidade Federal do Paraná - PR
57	AFASTAMENTO POR ATESTADO MÉDICO - ATE 15 DIAS	CHC da Universidade Federal do Paraná - PR
57	AFASTAMENTO POR ATESTADO MÉDICO - ATE 15 DIAS	CHC da Universidade Federal do Paraná - PR

57	AFASTAMENTO POR ATESTADO MÉDICO - ATE 15 DIAS	CHC da Universidade Federal do Paraná - PR
57	AFASTAMENTO POR ATESTADO MÉDICO - ATE 15 DIAS	CHC da Universidade Federal do Paraná - PR
57	AFASTAMENTO POR ATESTADO MÉDICO - ATE 15 DIAS	CHC da Universidade Federal do Paraná - PR
57	AFASTAMENTO POR ATESTADO MÉDICO - ATE 15 DIAS	CHC da Universidade Federal do Paraná - PR
57	AFASTAMENTO POR ATESTADO MÉDICO - ATE 15 DIAS	CHC da Universidade Federal do Paraná - PR
57	AFASTAMENTO POR ATESTADO MÉDICO - ATE 15 DIAS	CHC da Universidade Federal do Paraná - PR
57	AFASTAMENTO POR ATESTADO MÉDICO - ATE 15 DIAS	CHC da Universidade Federal do Paraná - PR
57	AFASTAMENTO POR ATESTADO MÉDICO - ATE 15 DIAS	CHC da Universidade Federal do Paraná - PR
57	AFASTAMENTO POR ATESTADO MÉDICO - ATE 15 DIAS	CHC da Universidade Federal do Paraná - PR
57	AFASTAMENTO POR ATESTADO MÉDICO - ATE 15 DIAS	CHC da Universidade Federal do Paraná - PR
57	AFASTAMENTO POR ATESTADO MÉDICO - ATE 15 DIAS	CHC da Universidade Federal do Paraná - PR
57	AFASTAMENTO POR ATESTADO MÉDICO - ATE 15 DIAS	CHC da Universidade Federal do Paraná - PR
57	AFASTAMENTO POR ATESTADO MÉDICO - ATE 15 DIAS	CHC da Universidade Federal do Paraná - PR
57	AFASTAMENTO POR ATESTADO MÉDICO - ATE 15 DIAS	CHC da Universidade Federal do Paraná - PR
57	AFASTAMENTO POR ATESTADO MÉDICO - ATE 15 DIAS	CHC da Universidade Federal do Paraná - PR
57	AFASTAMENTO POR ATESTADO MÉDICO - ATE 15 DIAS	CHC da Universidade Federal do Paraná - PR
57	AFASTAMENTO POR ATESTADO MÉDICO - ATE 15 DIAS	CHC da Universidade Federal do Paraná - PR
57	AFASTAMENTO POR ATESTADO MÉDICO - ATE 15 DIAS	CHC da Universidade Federal do Paraná - PR
57	AFASTAMENTO POR ATESTADO MÉDICO - ATE 15 DIAS	CHC da Universidade Federal do Paraná - PR
57	AFASTAMENTO POR ATESTADO MÉDICO - ATE 15 DIAS	CHC da Universidade Federal do Paraná - PR
57	AFASTAMENTO POR ATESTADO MÉDICO - ATE 15 DIAS	CHC da Universidade Federal do Paraná - PR
57	AFASTAMENTO POR ATESTADO MÉDICO - ATE 15 DIAS	CHC da Universidade Federal do Paraná - PR
57	AFASTAMENTO POR ATESTADO MÉDICO - ATE 15 DIAS	CHC da Universidade Federal do Paraná - PR
57	AFASTAMENTO POR ATESTADO MÉDICO - ATE 15 DIAS	CHC da Universidade Federal do Paraná - PR
57	AFASTAMENTO POR ATESTADO MÉDICO - ATE 15 DIAS	CHC da Universidade Federal do Paraná - PR
57	AFASTAMENTO POR ATESTADO MÉDICO - ATE 15 DIAS	CHC da Universidade Federal do Paraná - PR
57	AFASTAMENTO POR ATESTADO MÉDICO - ATE 15 DIAS	CHC da Universidade Federal do Paraná - PR
57	AFASTAMENTO POR ATESTADO MÉDICO - ATE 15 DIAS	CHC da Universidade Federal do Paraná - PR
57	AFASTAMENTO POR ATESTADO MÉDICO - ATE 15 DIAS	CHC da Universidade Federal do Paraná - PR
57	AFASTAMENTO POR ATESTADO MÉDICO - ATE 15 DIAS	CHC da Universidade Federal do Paraná - PR
57	AFASTAMENTO POR ATESTADO MÉDICO - ATE 15 DIAS	CHC da Universidade Federal do Paraná - PR
57	AFASTAMENTO POR ATESTADO MÉDICO - ATE 15 DIAS	CHC da Universidade Federal do Paraná - PR
57	AFASTAMENTO POR ATESTADO MÉDICO - ATE 15 DIAS	CHC da Universidade Federal do Paraná - PR
57	AFASTAMENTO POR ATESTADO MÉDICO - ATE 15 DIAS	CHC da Universidade Federal do Paraná - PR
57	AFASTAMENTO POR ATESTADO MÉDICO - ATE 15 DIAS	CHC da Universidade Federal do Paraná - PR
57	AFASTAMENTO POR ATESTADO MÉDICO - ATE 15 DIAS	CHC da Universidade Federal do Paraná - PR
57	AFASTAMENTO POR ATESTADO MÉDICO - ATE 15 DIAS	CHC da Universidade Federal do Paraná - PR
57	AFASTAMENTO POR ATESTADO MÉDICO - ATE 15 DIAS	CHC da Universidade Federal do Paraná - PR
57	AFASTAMENTO POR ATESTADO MÉDICO - ATE 15 DIAS	CHC da Universidade Federal do Paraná - PR
57	AFASTAMENTO POR ATESTADO MÉDICO - ATE 15 DIAS	CHC da Universidade Federal do Paraná - PR
57	AFASTAMENTO POR ATESTADO MÉDICO - ATE 15 DIAS	CHC da Universidade Federal do Paraná - PR
57	AFASTAMENTO POR ATESTADO MÉDICO - ATE 15 DIAS	CHC da Universidade Federal do Paraná - PR
57	AFASTAMENTO POR ATESTADO MÉDICO - ATE 15 DIAS	CHC da Universidade Federal do Paraná - PR
57	AFASTAMENTO POR ATESTADO MÉDICO - ATE 15 DIAS	CHC da Universidade Federal do Paraná - PR
57	AFASTAMENTO POR ATESTADO MÉDICO - ATE 15 DIAS	CHC da Universidade Federal do Paraná - PR
57	AFASTAMENTO POR ATESTADO MÉDICO - ATE 15 DIAS	CHC da Universidade Federal do Paraná - PR
57	AFASTAMENTO POR ATESTADO MÉDICO - ATE 15 DIAS	CHC da Universidade Federal do Paraná - PR
57	AFASTAMENTO POR ATESTADO MÉDICO - ATE 15 DIAS	CHC da Universidade Federal do Paraná - PR

57	AFASTAMENTO POR ATESTADO MÉDICO - ATE 15 DIAS	CHC da Universidade Federal do Paraná - PR
57	AFASTAMENTO POR ATESTADO MÉDICO - ATE 15 DIAS	CHC da Universidade Federal do Paraná - PR
57	AFASTAMENTO POR ATESTADO MÉDICO - ATE 15 DIAS	CHC da Universidade Federal do Paraná - PR
57	AFASTAMENTO POR ATESTADO MÉDICO - ATE 15 DIAS	CHC da Universidade Federal do Paraná - PR
57	AFASTAMENTO POR ATESTADO MÉDICO - ATE 15 DIAS	CHC da Universidade Federal do Paraná - PR
57	AFASTAMENTO POR ATESTADO MÉDICO - ATE 15 DIAS	CHC da Universidade Federal do Paraná - PR
57	AFASTAMENTO POR ATESTADO MÉDICO - ATE 15 DIAS	CHC da Universidade Federal do Paraná - PR
57	AFASTAMENTO POR ATESTADO MÉDICO - ATE 15 DIAS	CHC da Universidade Federal do Paraná - PR
57	AFASTAMENTO POR ATESTADO MÉDICO - ATE 15 DIAS	CHC da Universidade Federal do Paraná - PR
57	AFASTAMENTO POR ATESTADO MÉDICO - ATE 15 DIAS	CHC da Universidade Federal do Paraná - PR
57	AFASTAMENTO POR ATESTADO MÉDICO - ATE 15 DIAS	CHC da Universidade Federal do Paraná - PR
57	AFASTAMENTO POR ATESTADO MÉDICO - ATE 15 DIAS	CHC da Universidade Federal do Paraná - PR
57	AFASTAMENTO POR ATESTADO MÉDICO - ATE 15 DIAS	CHC da Universidade Federal do Paraná - PR
57	AFASTAMENTO POR ATESTADO MÉDICO - ATE 15 DIAS	CHC da Universidade Federal do Paraná - PR
57	AFASTAMENTO POR ATESTADO MÉDICO - ATE 15 DIAS	CHC da Universidade Federal do Paraná - PR
57	AFASTAMENTO POR ATESTADO MÉDICO - ATE 15 DIAS	CHC da Universidade Federal do Paraná - PR
57	AFASTAMENTO POR ATESTADO MÉDICO - ATE 15 DIAS	CHC da Universidade Federal do Paraná - PR
57	AFASTAMENTO POR ATESTADO MÉDICO - ATE 15 DIAS	CHC da Universidade Federal do Paraná - PR
57	AFASTAMENTO POR ATESTADO MÉDICO - ATE 15 DIAS	CHC da Universidade Federal do Paraná - PR
57	AFASTAMENTO POR ATESTADO MÉDICO - ATE 15 DIAS	CHC da Universidade Federal do Paraná - PR
57	AFASTAMENTO POR ATESTADO MÉDICO - ATE 15 DIAS	CHC da Universidade Federal do Paraná - PR
57	AFASTAMENTO POR ATESTADO MÉDICO - ATE 15 DIAS	CHC da Universidade Federal do Paraná - PR
57	AFASTAMENTO POR ATESTADO MÉDICO - ATE 15 DIAS	CHC da Universidade Federal do Paraná - PR
57	AFASTAMENTO POR ATESTADO MÉDICO - ATE 15 DIAS	CHC da Universidade Federal do Paraná - PR
57	AFASTAMENTO POR ATESTADO MÉDICO - ATE 15 DIAS	CHC da Universidade Federal do Paraná - PR
57	AFASTAMENTO POR ATESTADO MÉDICO - ATE 15 DIAS	CHC da Universidade Federal do Paraná - PR
57	AFASTAMENTO POR ATESTADO MÉDICO - ATE 15 DIAS	CHC da Universidade Federal do Paraná - PR
57	AFASTAMENTO POR ATESTADO MÉDICO - ATE 15 DIAS	CHC da Universidade Federal do Paraná - PR
57	AFASTAMENTO POR ATESTADO MÉDICO - ATE 15 DIAS	CHC da Universidade Federal do Paraná - PR
57	AFASTAMENTO POR ATESTADO MÉDICO - ATE 15 DIAS	CHC da Universidade Federal do Paraná - PR
57	AFASTAMENTO POR ATESTADO MÉDICO - ATE 15 DIAS	CHC da Universidade Federal do Paraná - PR
57	AFASTAMENTO POR ATESTADO MÉDICO - ATE 15 DIAS	CHC da Universidade Federal do Paraná - PR
57	AFASTAMENTO POR ATESTADO MÉDICO - ATE 15 DIAS	CHC da Universidade Federal do Paraná - PR
57	AFASTAMENTO POR ATESTADO MÉDICO - ATE 15 DIAS	CHC da Universidade Federal do Paraná - PR
57	AFASTAMENTO POR ATESTADO MÉDICO - ATE 15 DIAS	CHC da Universidade Federal do Paraná - PR
57	AFASTAMENTO POR ATESTADO MÉDICO - ATE 15 DIAS	CHC da Universidade Federal do Paraná - PR
57	AFASTAMENTO POR ATESTADO MÉDICO - ATE 15 DIAS	CHC da Universidade Federal do Paraná - PR
57	AFASTAMENTO POR ATESTADO MÉDICO - ATE 15 DIAS	CHC da Universidade Federal do Paraná - PR
57	AFASTAMENTO POR ATESTADO MÉDICO - ATE 15 DIAS	CHC da Universidade Federal do Paraná - PR
57	AFASTAMENTO POR ATESTADO MÉDICO - ATE 15 DIAS	CHC da Universidade Federal do Paraná - PR
57	AFASTAMENTO POR ATESTADO MÉDICO - ATE 15 DIAS	CHC da Universidade Federal do Paraná - PR
57	AFASTAMENTO POR ATESTADO MÉDICO - ATE 15 DIAS	CHC da Universidade Federal do Paraná - PR
57	AFASTAMENTO POR ATESTADO MÉDICO - ATE 15 DIAS	CHC da Universidade Federal do Paraná - PR
57	AFASTAMENTO POR ATESTADO MÉDICO - ATE 15 DIAS	CHC da Universidade Federal do Paraná - PR
57	AFASTAMENTO POR ATESTADO MÉDICO - ATE 15 DIAS	CHC da Universidade Federal do Paraná - PR
57	AFASTAMENTO POR ATESTADO MÉDICO - ATE 15 DIAS	CHC da Universidade Federal do Paraná - PR
57	AFASTAMENTO POR ATESTADO MÉDICO - ATE 15 DIAS	CHC da Universidade Federal do Paraná - PR
57	AFASTAMENTO POR ATESTADO MÉDICO - ATE 15 DIAS	CHC da Universidade Federal do Paraná - PR

57	AFASTAMENTO POR ATESTADO MÉDICO - ATE 15 DIAS	CHC da Universidade Federal do Paraná - PR
57	AFASTAMENTO POR ATESTADO MÉDICO - ATE 15 DIAS	CHC da Universidade Federal do Paraná - PR
57	AFASTAMENTO POR ATESTADO MÉDICO - ATE 15 DIAS	CHC da Universidade Federal do Paraná - PR
57	AFASTAMENTO POR ATESTADO MÉDICO - ATE 15 DIAS	CHC da Universidade Federal do Paraná - PR
57	AFASTAMENTO POR ATESTADO MÉDICO - ATE 15 DIAS	CHC da Universidade Federal do Paraná - PR
57	AFASTAMENTO POR ATESTADO MÉDICO - ATE 15 DIAS	CHC da Universidade Federal do Paraná - PR
57	AFASTAMENTO POR ATESTADO MÉDICO - ATE 15 DIAS	CHC da Universidade Federal do Paraná - PR
57	AFASTAMENTO POR ATESTADO MÉDICO - ATE 15 DIAS	CHC da Universidade Federal do Paraná - PR
57	AFASTAMENTO POR ATESTADO MÉDICO - ATE 15 DIAS	CHC da Universidade Federal do Paraná - PR
57	AFASTAMENTO POR ATESTADO MÉDICO - ATE 15 DIAS	CHC da Universidade Federal do Paraná - PR
57	AFASTAMENTO POR ATESTADO MÉDICO - ATE 15 DIAS	CHC da Universidade Federal do Paraná - PR
57	AFASTAMENTO POR ATESTADO MÉDICO - ATE 15 DIAS	CHC da Universidade Federal do Paraná - PR
57	AFASTAMENTO POR ATESTADO MÉDICO - ATE 15 DIAS	CHC da Universidade Federal do Paraná - PR
57	AFASTAMENTO POR ATESTADO MÉDICO - ATE 15 DIAS	CHC da Universidade Federal do Paraná - PR
57	AFASTAMENTO POR ATESTADO MÉDICO - ATE 15 DIAS	CHC da Universidade Federal do Paraná - PR
57	AFASTAMENTO POR ATESTADO MÉDICO - ATE 15 DIAS	CHC da Universidade Federal do Paraná - PR
57	AFASTAMENTO POR ATESTADO MÉDICO - ATE 15 DIAS	CHC da Universidade Federal do Paraná - PR
57	AFASTAMENTO POR ATESTADO MÉDICO - ATE 15 DIAS	CHC da Universidade Federal do Paraná - PR
57	AFASTAMENTO POR ATESTADO MÉDICO - ATE 15 DIAS	CHC da Universidade Federal do Paraná - PR
57	AFASTAMENTO POR ATESTADO MÉDICO - ATE 15 DIAS	CHC da Universidade Federal do Paraná - PR
57	AFASTAMENTO POR ATESTADO MÉDICO - ATE 15 DIAS	CHC da Universidade Federal do Paraná - PR
57	AFASTAMENTO POR ATESTADO MÉDICO - ATE 15 DIAS	CHC da Universidade Federal do Paraná - PR
57	AFASTAMENTO POR ATESTADO MÉDICO - ATE 15 DIAS	CHC da Universidade Federal do Paraná - PR
57	AFASTAMENTO POR ATESTADO MÉDICO - ATE 15 DIAS	CHC da Universidade Federal do Paraná - PR
57	AFASTAMENTO POR ATESTADO MÉDICO - ATE 15 DIAS	CHC da Universidade Federal do Paraná - PR
57	AFASTAMENTO POR ATESTADO MÉDICO - ATE 15 DIAS	CHC da Universidade Federal do Paraná - PR
57	AFASTAMENTO POR ATESTADO MÉDICO - ATE 15 DIAS	CHC da Universidade Federal do Paraná - PR
57	AFASTAMENTO POR ATESTADO MÉDICO - ATE 15 DIAS	CHC da Universidade Federal do Paraná - PR
57	AFASTAMENTO POR ATESTADO MÉDICO - ATE 15 DIAS	CHC da Universidade Federal do Paraná - PR
57	AFASTAMENTO POR ATESTADO MÉDICO - ATE 15 DIAS	CHC da Universidade Federal do Paraná - PR
57	AFASTAMENTO POR ATESTADO MÉDICO - ATE 15 DIAS	CHC da Universidade Federal do Paraná - PR
57	AFASTAMENTO POR ATESTADO MÉDICO - ATE 15 DIAS	CHC da Universidade Federal do Paraná - PR
57	AFASTAMENTO POR ATESTADO MÉDICO - ATE 15 DIAS	CHC da Universidade Federal do Paraná - PR
57	AFASTAMENTO POR ATESTADO MÉDICO - ATE 15 DIAS	CHC da Universidade Federal do Paraná - PR
57	AFASTAMENTO POR ATESTADO MÉDICO - ATE 15 DIAS	CHC da Universidade Federal do Paraná - PR
57	AFASTAMENTO POR ATESTADO MÉDICO - ATE 15 DIAS	CHC da Universidade Federal do Paraná - PR
57	AFASTAMENTO POR ATESTADO MÉDICO - ATE 15 DIAS	CHC da Universidade Federal do Paraná - PR
57	AFASTAMENTO POR ATESTADO MÉDICO - ATE 15 DIAS	CHC da Universidade Federal do Paraná - PR
57	AFASTAMENTO POR ATESTADO MÉDICO - ATE 15 DIAS	CHC da Universidade Federal do Paraná - PR
57	AFASTAMENTO POR ATESTADO MÉDICO - ATE 15 DIAS	CHC da Universidade Federal do Paraná - PR
57	AFASTAMENTO POR ATESTADO MÉDICO - ATE 15 DIAS	CHC da Universidade Federal do Paraná - PR
57	AFASTAMENTO POR ATESTADO MÉDICO - ATE 15 DIAS	CHC da Universidade Federal do Paraná - PR
57	AFASTAMENTO POR ATESTADO MÉDICO - ATE 15 DIAS	CHC da Universidade Federal do Paraná - PR
57	AFASTAMENTO POR ATESTADO MÉDICO - ATE 15 DIAS	CHC da Universidade Federal do Paraná - PR
57	AFASTAMENTO POR ATESTADO MÉDICO - ATE 15 DIAS	CHC da Universidade Federal do Paraná - PR
57	AFASTAMENTO POR ATESTADO MÉDICO - ATE 15 DIAS	CHC da Universidade Federal do Paraná - PR
57	AFASTAMENTO POR ATESTADO MÉDICO - ATE 15 DIAS	CHC da Universidade Federal do Paraná - PR
57	AFASTAMENTO POR ATESTADO MÉDICO - ATE 15 DIAS	CHC da Universidade Federal do Paraná - PR

57	AFASTAMENTO POR ATESTADO MÉDICO - ATE 15 DIAS	CHC da Universidade Federal do Paraná - PR
57	AFASTAMENTO POR ATESTADO MÉDICO - ATE 15 DIAS	CHC da Universidade Federal do Paraná - PR
57	AFASTAMENTO POR ATESTADO MÉDICO - ATE 15 DIAS	CHC da Universidade Federal do Paraná - PR
57	AFASTAMENTO POR ATESTADO MÉDICO - ATE 15 DIAS	CHC da Universidade Federal do Paraná - PR
57	AFASTAMENTO POR ATESTADO MÉDICO - ATE 15 DIAS	CHC da Universidade Federal do Paraná - PR
57	AFASTAMENTO POR ATESTADO MÉDICO - ATE 15 DIAS	CHC da Universidade Federal do Paraná - PR
57	AFASTAMENTO POR ATESTADO MÉDICO - ATE 15 DIAS	CHC da Universidade Federal do Paraná - PR
57	AFASTAMENTO POR ATESTADO MÉDICO - ATE 15 DIAS	CHC da Universidade Federal do Paraná - PR
57	AFASTAMENTO POR ATESTADO MÉDICO - ATE 15 DIAS	CHC da Universidade Federal do Paraná - PR
57	AFASTAMENTO POR ATESTADO MÉDICO - ATE 15 DIAS	CHC da Universidade Federal do Paraná - PR
57	AFASTAMENTO POR ATESTADO MÉDICO - ATE 15 DIAS	CHC da Universidade Federal do Paraná - PR
57	AFASTAMENTO POR ATESTADO MÉDICO - ATE 15 DIAS	CHC da Universidade Federal do Paraná - PR
57	AFASTAMENTO POR ATESTADO MÉDICO - ATE 15 DIAS	CHC da Universidade Federal do Paraná - PR
57	AFASTAMENTO POR ATESTADO MÉDICO - ATE 15 DIAS	CHC da Universidade Federal do Paraná - PR
57	AFASTAMENTO POR ATESTADO MÉDICO - ATE 15 DIAS	CHC da Universidade Federal do Paraná - PR
57	AFASTAMENTO POR ATESTADO MÉDICO - ATE 15 DIAS	CHC da Universidade Federal do Paraná - PR
57	AFASTAMENTO POR ATESTADO MÉDICO - ATE 15 DIAS	CHC da Universidade Federal do Paraná - PR
57	AFASTAMENTO POR ATESTADO MÉDICO - ATE 15 DIAS	CHC da Universidade Federal do Paraná - PR
57	AFASTAMENTO POR ATESTADO MÉDICO - ATE 15 DIAS	CHC da Universidade Federal do Paraná - PR
57	AFASTAMENTO POR ATESTADO MÉDICO - ATE 15 DIAS	CHC da Universidade Federal do Paraná - PR
57	AFASTAMENTO POR ATESTADO MÉDICO - ATE 15 DIAS	CHC da Universidade Federal do Paraná - PR
57	AFASTAMENTO POR ATESTADO MÉDICO - ATE 15 DIAS	CHC da Universidade Federal do Paraná - PR
57	AFASTAMENTO POR ATESTADO MÉDICO - ATE 15 DIAS	CHC da Universidade Federal do Paraná - PR
57	AFASTAMENTO POR ATESTADO MÉDICO - ATE 15 DIAS	CHC da Universidade Federal do Paraná - PR
57	AFASTAMENTO POR ATESTADO MÉDICO - ATE 15 DIAS	CHC da Universidade Federal do Paraná - PR
57	AFASTAMENTO POR ATESTADO MÉDICO - ATE 15 DIAS	CHC da Universidade Federal do Paraná - PR
57	AFASTAMENTO POR ATESTADO MÉDICO - ATE 15 DIAS	CHC da Universidade Federal do Paraná - PR
57	AFASTAMENTO POR ATESTADO MÉDICO - ATE 15 DIAS	CHC da Universidade Federal do Paraná - PR
57	AFASTAMENTO POR ATESTADO MÉDICO - ATE 15 DIAS	CHC da Universidade Federal do Paraná - PR
57	AFASTAMENTO POR ATESTADO MÉDICO - ATE 15 DIAS	CHC da Universidade Federal do Paraná - PR
57	AFASTAMENTO POR ATESTADO MÉDICO - ATE 15 DIAS	CHC da Universidade Federal do Paraná - PR
57	AFASTAMENTO POR ATESTADO MÉDICO - ATE 15 DIAS	CHC da Universidade Federal do Paraná - PR
57	AFASTAMENTO POR ATESTADO MÉDICO - ATE 15 DIAS	CHC da Universidade Federal do Paraná - PR
57	AFASTAMENTO POR ATESTADO MÉDICO - ATE 15 DIAS	CHC da Universidade Federal do Paraná - PR
57	AFASTAMENTO POR ATESTADO MÉDICO - ATE 15 DIAS	CHC da Universidade Federal do Paraná - PR
57	AFASTAMENTO POR ATESTADO MÉDICO - ATE 15 DIAS	CHC da Universidade Federal do Paraná - PR
57	AFASTAMENTO POR ATESTADO MÉDICO - ATE 15 DIAS	CHC da Universidade Federal do Paraná - PR
57	AFASTAMENTO POR ATESTADO MÉDICO - ATE 15 DIAS	CHC da Universidade Federal do Paraná - PR
57	AFASTAMENTO POR ATESTADO MÉDICO - ATE 15 DIAS	CHC da Universidade Federal do Paraná - PR
57	AFASTAMENTO POR ATESTADO MÉDICO - ATE 15 DIAS	CHC da Universidade Federal do Paraná - PR
57	AFASTAMENTO POR ATESTADO MÉDICO - ATE 15 DIAS	CHC da Universidade Federal do Paraná - PR
57	AFASTAMENTO POR ATESTADO MÉDICO - ATE 15 DIAS	CHC da Universidade Federal do Paraná - PR
57	AFASTAMENTO POR ATESTADO MÉDICO - ATE 15 DIAS	CHC da Universidade Federal do Paraná - PR
57	AFASTAMENTO POR ATESTADO MÉDICO - ATE 15 DIAS	CHC da Universidade Federal do Paraná - PR
57	AFASTAMENTO POR ATESTADO MÉDICO - ATE 15 DIAS	CHC da Universidade Federal do Paraná - PR
57	AFASTAMENTO POR ATESTADO MÉDICO - ATE 15 DIAS	CHC da Universidade Federal do Paraná - PR
57	AFASTAMENTO POR ATESTADO MÉDICO - ATE 15 DIAS	CHC da Universidade Federal do Paraná - PR
57	AFASTAMENTO POR ATESTADO MÉDICO - ATE 15 DIAS	CHC da Universidade Federal do Paraná - PR

57	AFASTAMENTO POR ATESTADO MÉDICO - ATE 15 DIAS	CHC da Universidade Federal do Paraná - PR
57	AFASTAMENTO POR ATESTADO MÉDICO - ATE 15 DIAS	CHC da Universidade Federal do Paraná - PR
57	AFASTAMENTO POR ATESTADO MÉDICO - ATE 15 DIAS	CHC da Universidade Federal do Paraná - PR
57	AFASTAMENTO POR ATESTADO MÉDICO - ATE 15 DIAS	CHC da Universidade Federal do Paraná - PR
57	AFASTAMENTO POR ATESTADO MÉDICO - ATE 15 DIAS	CHC da Universidade Federal do Paraná - PR
57	AFASTAMENTO POR ATESTADO MÉDICO - ATE 15 DIAS	CHC da Universidade Federal do Paraná - PR
57	AFASTAMENTO POR ATESTADO MÉDICO - ATE 15 DIAS	CHC da Universidade Federal do Paraná - PR
57	AFASTAMENTO POR ATESTADO MÉDICO - ATE 15 DIAS	CHC da Universidade Federal do Paraná - PR
57	AFASTAMENTO POR ATESTADO MÉDICO - ATE 15 DIAS	CHC da Universidade Federal do Paraná - PR
57	AFASTAMENTO POR ATESTADO MÉDICO - ATE 15 DIAS	CHC da Universidade Federal do Paraná - PR
57	AFASTAMENTO POR ATESTADO MÉDICO - ATE 15 DIAS	CHC da Universidade Federal do Paraná - PR
57	AFASTAMENTO POR ATESTADO MÉDICO - ATE 15 DIAS	CHC da Universidade Federal do Paraná - PR
57	AFASTAMENTO POR ATESTADO MÉDICO - ATE 15 DIAS	CHC da Universidade Federal do Paraná - PR
57	AFASTAMENTO POR ATESTADO MÉDICO - ATE 15 DIAS	CHC da Universidade Federal do Paraná - PR
57	AFASTAMENTO POR ATESTADO MÉDICO - ATE 15 DIAS	CHC da Universidade Federal do Paraná - PR
57	AFASTAMENTO POR ATESTADO MÉDICO - ATE 15 DIAS	CHC da Universidade Federal do Paraná - PR
57	AFASTAMENTO POR ATESTADO MÉDICO - ATE 15 DIAS	CHC da Universidade Federal do Paraná - PR
57	AFASTAMENTO POR ATESTADO MÉDICO - ATE 15 DIAS	CHC da Universidade Federal do Paraná - PR
57	AFASTAMENTO POR ATESTADO MÉDICO - ATE 15 DIAS	CHC da Universidade Federal do Paraná - PR
57	AFASTAMENTO POR ATESTADO MÉDICO - ATE 15 DIAS	CHC da Universidade Federal do Paraná - PR
57	AFASTAMENTO POR ATESTADO MÉDICO - ATE 15 DIAS	CHC da Universidade Federal do Paraná - PR
57	AFASTAMENTO POR ATESTADO MÉDICO - ATE 15 DIAS	CHC da Universidade Federal do Paraná - PR
57	AFASTAMENTO POR ATESTADO MÉDICO - ATE 15 DIAS	CHC da Universidade Federal do Paraná - PR
57	AFASTAMENTO POR ATESTADO MÉDICO - ATE 15 DIAS	CHC da Universidade Federal do Paraná - PR
57	AFASTAMENTO POR ATESTADO MÉDICO - ATE 15 DIAS	CHC da Universidade Federal do Paraná - PR
57	AFASTAMENTO POR ATESTADO MÉDICO - ATE 15 DIAS	CHC da Universidade Federal do Paraná - PR
57	AFASTAMENTO POR ATESTADO MÉDICO - ATE 15 DIAS	CHC da Universidade Federal do Paraná - PR
57	AFASTAMENTO POR ATESTADO MÉDICO - ATE 15 DIAS	CHC da Universidade Federal do Paraná - PR
57	AFASTAMENTO POR ATESTADO MÉDICO - ATE 15 DIAS	CHC da Universidade Federal do Paraná - PR
57	AFASTAMENTO POR ATESTADO MÉDICO - ATE 15 DIAS	CHC da Universidade Federal do Paraná - PR
57	AFASTAMENTO POR ATESTADO MÉDICO - ATE 15 DIAS	CHC da Universidade Federal do Paraná - PR
57	AFASTAMENTO POR ATESTADO MÉDICO - ATE 15 DIAS	CHC da Universidade Federal do Paraná - PR
57	AFASTAMENTO POR ATESTADO MÉDICO - ATE 15 DIAS	CHC da Universidade Federal do Paraná - PR
57	AFASTAMENTO POR ATESTADO MÉDICO - ATE 15 DIAS	CHC da Universidade Federal do Paraná - PR
57	AFASTAMENTO POR ATESTADO MÉDICO - ATE 15 DIAS	CHC da Universidade Federal do Paraná - PR
57	AFASTAMENTO POR ATESTADO MÉDICO - ATE 15 DIAS	CHC da Universidade Federal do Paraná - PR
57	AFASTAMENTO POR ATESTADO MÉDICO - ATE 15 DIAS	CHC da Universidade Federal do Paraná - PR
57	AFASTAMENTO POR ATESTADO MÉDICO - ATE 15 DIAS	CHC da Universidade Federal do Paraná - PR
57	AFASTAMENTO POR ATESTADO MÉDICO - ATE 15 DIAS	CHC da Universidade Federal do Paraná - PR
57	AFASTAMENTO POR ATESTADO MÉDICO - ATE 15 DIAS	CHC da Universidade Federal do Paraná - PR
57	AFASTAMENTO POR ATESTADO MÉDICO - ATE 15 DIAS	CHC da Universidade Federal do Paraná - PR
57	AFASTAMENTO POR ATESTADO MÉDICO - ATE 15 DIAS	CHC da Universidade Federal do Paraná - PR
57	AFASTAMENTO POR ATESTADO MÉDICO - ATE 15 DIAS	CHC da Universidade Federal do Paraná - PR
57	AFASTAMENTO POR ATESTADO MÉDICO - ATE 15 DIAS	CHC da Universidade Federal do Paraná - PR
57	AFASTAMENTO POR ATESTADO MÉDICO - ATE 15 DIAS	CHC da Universidade Federal do Paraná - PR
57	AFASTAMENTO POR ATESTADO MÉDICO - ATE 15 DIAS	CHC da Universidade Federal do Paraná - PR
57	AFASTAMENTO POR ATESTADO MÉDICO - ATE 15 DIAS	CHC da Universidade Federal do Paraná - PR
57	AFASTAMENTO POR ATESTADO MÉDICO - ATE 15 DIAS	CHC da Universidade Federal do Paraná - PR

57	AFASTAMENTO POR ATESTADO MÉDICO - ATE 15 DIAS	CHC da Universidade Federal do Paraná - PR
57	AFASTAMENTO POR ATESTADO MÉDICO - ATE 15 DIAS	CHC da Universidade Federal do Paraná - PR
57	AFASTAMENTO POR ATESTADO MÉDICO - ATE 15 DIAS	CHC da Universidade Federal do Paraná - PR
57	AFASTAMENTO POR ATESTADO MÉDICO - ATE 15 DIAS	CHC da Universidade Federal do Paraná - PR
57	AFASTAMENTO POR ATESTADO MÉDICO - ATE 15 DIAS	CHC da Universidade Federal do Paraná - PR
57	AFASTAMENTO POR ATESTADO MÉDICO - ATE 15 DIAS	CHC da Universidade Federal do Paraná - PR
57	AFASTAMENTO POR ATESTADO MÉDICO - ATE 15 DIAS	CHC da Universidade Federal do Paraná - PR
57	AFASTAMENTO POR ATESTADO MÉDICO - ATE 15 DIAS	CHC da Universidade Federal do Paraná - PR
57	AFASTAMENTO POR ATESTADO MÉDICO - ATE 15 DIAS	CHC da Universidade Federal do Paraná - PR
57	AFASTAMENTO POR ATESTADO MÉDICO - ATE 15 DIAS	CHC da Universidade Federal do Paraná - PR
57	AFASTAMENTO POR ATESTADO MÉDICO - ATE 15 DIAS	CHC da Universidade Federal do Paraná - PR
57	AFASTAMENTO POR ATESTADO MÉDICO - ATE 15 DIAS	CHC da Universidade Federal do Paraná - PR
57	AFASTAMENTO POR ATESTADO MÉDICO - ATE 15 DIAS	CHC da Universidade Federal do Paraná - PR
57	AFASTAMENTO POR ATESTADO MÉDICO - ATE 15 DIAS	CHC da Universidade Federal do Paraná - PR
57	AFASTAMENTO POR ATESTADO MÉDICO - ATE 15 DIAS	CHC da Universidade Federal do Paraná - PR
57	AFASTAMENTO POR ATESTADO MÉDICO - ATE 15 DIAS	CHC da Universidade Federal do Paraná - PR
57	AFASTAMENTO POR ATESTADO MÉDICO - ATE 15 DIAS	CHC da Universidade Federal do Paraná - PR
57	AFASTAMENTO POR ATESTADO MÉDICO - ATE 15 DIAS	CHC da Universidade Federal do Paraná - PR
57	AFASTAMENTO POR ATESTADO MÉDICO - ATE 15 DIAS	CHC da Universidade Federal do Paraná - PR
57	AFASTAMENTO POR ATESTADO MÉDICO - ATE 15 DIAS	CHC da Universidade Federal do Paraná - PR
57	AFASTAMENTO POR ATESTADO MÉDICO - ATE 15 DIAS	CHC da Universidade Federal do Paraná - PR
57	AFASTAMENTO POR ATESTADO MÉDICO - ATE 15 DIAS	CHC da Universidade Federal do Paraná - PR
57	AFASTAMENTO POR ATESTADO MÉDICO - ATE 15 DIAS	CHC da Universidade Federal do Paraná - PR
57	AFASTAMENTO POR ATESTADO MÉDICO - ATE 15 DIAS	CHC da Universidade Federal do Paraná - PR
57	AFASTAMENTO POR ATESTADO MÉDICO - ATE 15 DIAS	CHC da Universidade Federal do Paraná - PR
57	AFASTAMENTO POR ATESTADO MÉDICO - ATE 15 DIAS	CHC da Universidade Federal do Paraná - PR
57	AFASTAMENTO POR ATESTADO MÉDICO - ATE 15 DIAS	CHC da Universidade Federal do Paraná - PR
57	AFASTAMENTO POR ATESTADO MÉDICO - ATE 15 DIAS	CHC da Universidade Federal do Paraná - PR
57	AFASTAMENTO POR ATESTADO MÉDICO - ATE 15 DIAS	CHC da Universidade Federal do Paraná - PR
57	AFASTAMENTO POR ATESTADO MÉDICO - ATE 15 DIAS	CHC da Universidade Federal do Paraná - PR
57	AFASTAMENTO POR ATESTADO MÉDICO - ATE 15 DIAS	CHC da Universidade Federal do Paraná - PR
57	AFASTAMENTO POR ATESTADO MÉDICO - ATE 15 DIAS	CHC da Universidade Federal do Paraná - PR
57	AFASTAMENTO POR ATESTADO MÉDICO - ATE 15 DIAS	CHC da Universidade Federal do Paraná - PR
57	AFASTAMENTO POR ATESTADO MÉDICO - ATE 15 DIAS	CHC da Universidade Federal do Paraná - PR
57	AFASTAMENTO POR ATESTADO MÉDICO - ATE 15 DIAS	CHC da Universidade Federal do Paraná - PR
57	AFASTAMENTO POR ATESTADO MÉDICO - ATE 15 DIAS	CHC da Universidade Federal do Paraná - PR
57	AFASTAMENTO POR ATESTADO MÉDICO - ATE 15 DIAS	CHC da Universidade Federal do Paraná - PR
57	AFASTAMENTO POR ATESTADO MÉDICO - ATE 15 DIAS	CHC da Universidade Federal do Paraná - PR
57	AFASTAMENTO POR ATESTADO MÉDICO - ATE 15 DIAS	CHC da Universidade Federal do Paraná - PR
57	AFASTAMENTO POR ATESTADO MÉDICO - ATE 15 DIAS	CHC da Universidade Federal do Paraná - PR
57	AFASTAMENTO POR ATESTADO MÉDICO - ATE 15 DIAS	CHC da Universidade Federal do Paraná - PR
57	AFASTAMENTO POR ATESTADO MÉDICO - ATE 15 DIAS	CHC da Universidade Federal do Paraná - PR
57	AFASTAMENTO POR ATESTADO MÉDICO - ATE 15 DIAS	CHC da Universidade Federal do Paraná - PR
57	AFASTAMENTO POR ATESTADO MÉDICO - ATE 15 DIAS	CHC da Universidade Federal do Paraná - PR
57	AFASTAMENTO POR ATESTADO MÉDICO - ATE 15 DIAS	CHC da Universidade Federal do Paraná - PR
57	AFASTAMENTO POR ATESTADO MÉDICO - ATE 15 DIAS	CHC da Universidade Federal do Paraná - PR
57	AFASTAMENTO POR ATESTADO MÉDICO - ATE 15 DIAS	CHC da Universidade Federal do Paraná - PR
57	AFASTAMENTO POR ATESTADO MÉDICO - ATE 15 DIAS	CHC da Universidade Federal do Paraná - PR

57	AFASTAMENTO POR ATESTADO MÉDICO - ATE 15 DIAS	CHC da Universidade Federal do Paraná - PR
57	AFASTAMENTO POR ATESTADO MÉDICO - ATE 15 DIAS	CHC da Universidade Federal do Paraná - PR
57	AFASTAMENTO POR ATESTADO MÉDICO - ATE 15 DIAS	CHC da Universidade Federal do Paraná - PR
58	AFASTAMENTO POR ATESTADO ODONTOLOGICO - ATE 15 DIAS	CHC da Universidade Federal do Paraná - PR
58	AFASTAMENTO POR ATESTADO ODONTOLOGICO - ATE 15 DIAS	CHC da Universidade Federal do Paraná - PR
58	AFASTAMENTO POR ATESTADO ODONTOLOGICO - ATE 15 DIAS	CHC da Universidade Federal do Paraná - PR
58	AFASTAMENTO POR ATESTADO ODONTOLOGICO - ATE 15 DIAS	CHC da Universidade Federal do Paraná - PR
58	AFASTAMENTO POR ATESTADO ODONTOLOGICO - ATE 15 DIAS	CHC da Universidade Federal do Paraná - PR
58	AFASTAMENTO POR ATESTADO ODONTOLOGICO - ATE 15 DIAS	CHC da Universidade Federal do Paraná - PR
58	AFASTAMENTO POR ATESTADO ODONTOLOGICO - ATE 15 DIAS	CHC da Universidade Federal do Paraná - PR
58	AFASTAMENTO POR ATESTADO ODONTOLOGICO - ATE 15 DIAS	CHC da Universidade Federal do Paraná - PR
58	AFASTAMENTO POR ATESTADO ODONTOLOGICO - ATE 15 DIAS	CHC da Universidade Federal do Paraná - PR
58	AFASTAMENTO POR ATESTADO ODONTOLOGICO - ATE 15 DIAS	CHC da Universidade Federal do Paraná - PR
58	AFASTAMENTO POR ATESTADO ODONTOLOGICO - ATE 15 DIAS	CHC da Universidade Federal do Paraná - PR
58	AFASTAMENTO POR ATESTADO ODONTOLOGICO - ATE 15 DIAS	CHC da Universidade Federal do Paraná - PR
58	AFASTAMENTO POR ATESTADO ODONTOLOGICO - ATE 15 DIAS	CHC da Universidade Federal do Paraná - PR
58	AFASTAMENTO POR ATESTADO ODONTOLOGICO - ATE 15 DIAS	CHC da Universidade Federal do Paraná - PR
58	AFASTAMENTO POR ATESTADO ODONTOLOGICO - ATE 15 DIAS	CHC da Universidade Federal do Paraná - PR
58	AFASTAMENTO POR ATESTADO ODONTOLOGICO - ATE 15 DIAS	CHC da Universidade Federal do Paraná - PR
58	AFASTAMENTO POR ATESTADO ODONTOLOGICO - ATE 15 DIAS	CHC da Universidade Federal do Paraná - PR
58	AFASTAMENTO POR ATESTADO ODONTOLOGICO - ATE 15 DIAS	CHC da Universidade Federal do Paraná - PR
58	AFASTAMENTO POR ATESTADO ODONTOLOGICO - ATE 15 DIAS	CHC da Universidade Federal do Paraná - PR
58	AFASTAMENTO POR ATESTADO ODONTOLOGICO - ATE 15 DIAS	CHC da Universidade Federal do Paraná - PR
58	AFASTAMENTO POR ATESTADO ODONTOLOGICO - ATE 15 DIAS	CHC da Universidade Federal do Paraná - PR
58	AFASTAMENTO POR ATESTADO ODONTOLOGICO - ATE 15 DIAS	CHC da Universidade Federal do Paraná - PR
58	AFASTAMENTO POR ATESTADO ODONTOLOGICO - ATE 15 DIAS	CHC da Universidade Federal do Paraná - PR
58	AFASTAMENTO POR ATESTADO ODONTOLOGICO - ATE 15 DIAS	CHC da Universidade Federal do Paraná - PR
58	AFASTAMENTO POR ATESTADO ODONTOLOGICO - ATE 15 DIAS	CHC da Universidade Federal do Paraná - PR
58	AFASTAMENTO POR ATESTADO ODONTOLOGICO - ATE 15 DIAS	CHC da Universidade Federal do Paraná - PR
58	AFASTAMENTO POR ATESTADO ODONTOLOGICO - ATE 15 DIAS	CHC da Universidade Federal do Paraná - PR
58	AFASTAMENTO POR ATESTADO ODONTOLOGICO - ATE 15 DIAS	CHC da Universidade Federal do Paraná - PR
58	AFASTAMENTO POR ATESTADO ODONTOLOGICO - ATE 15 DIAS	CHC da Universidade Federal do Paraná - PR

58	AFASTAMENTO POR ATESTADO ODONTOLOGICO - ATE 15 DIAS	CHC da Universidade Federal do Paraná - PR
58	AFASTAMENTO POR ATESTADO ODONTOLOGICO - ATE 15 DIAS	CHC da Universidade Federal do Paraná - PR
58	AFASTAMENTO POR ATESTADO ODONTOLOGICO - ATE 15 DIAS	CHC da Universidade Federal do Paraná - PR
58	AFASTAMENTO POR ATESTADO ODONTOLOGICO - ATE 15 DIAS	CHC da Universidade Federal do Paraná - PR
58	AFASTAMENTO POR ATESTADO ODONTOLOGICO - ATE 15 DIAS	CHC da Universidade Federal do Paraná - PR
58	AFASTAMENTO POR ATESTADO ODONTOLOGICO - ATE 15 DIAS	CHC da Universidade Federal do Paraná - PR
58	AFASTAMENTO POR ATESTADO ODONTOLOGICO - ATE 15 DIAS	CHC da Universidade Federal do Paraná - PR
58	AFASTAMENTO POR ATESTADO ODONTOLOGICO - ATE 15 DIAS	CHC da Universidade Federal do Paraná - PR
58	AFASTAMENTO POR ATESTADO ODONTOLOGICO - ATE 15 DIAS	CHC da Universidade Federal do Paraná - PR
58	AFASTAMENTO POR ATESTADO ODONTOLOGICO - ATE 15 DIAS	CHC da Universidade Federal do Paraná - PR
58	AFASTAMENTO POR ATESTADO ODONTOLOGICO - ATE 15 DIAS	CHC da Universidade Federal do Paraná - PR
58	AFASTAMENTO POR ATESTADO ODONTOLOGICO - ATE 15 DIAS	CHC da Universidade Federal do Paraná - PR
58	AFASTAMENTO POR ATESTADO ODONTOLOGICO - ATE 15 DIAS	CHC da Universidade Federal do Paraná - PR
58	AFASTAMENTO POR ATESTADO ODONTOLOGICO - ATE 15 DIAS	CHC da Universidade Federal do Paraná - PR
58	AFASTAMENTO POR ATESTADO ODONTOLOGICO - ATE 15 DIAS	CHC da Universidade Federal do Paraná - PR
58	AFASTAMENTO POR ATESTADO ODONTOLOGICO - ATE 15 DIAS	CHC da Universidade Federal do Paraná - PR
58	AFASTAMENTO POR ATESTADO ODONTOLOGICO - ATE 15 DIAS	CHC da Universidade Federal do Paraná - PR
58	AFASTAMENTO POR ATESTADO ODONTOLOGICO - ATE 15 DIAS	CHC da Universidade Federal do Paraná - PR
58	AFASTAMENTO POR ATESTADO ODONTOLOGICO - ATE 15 DIAS	CHC da Universidade Federal do Paraná - PR
58	AFASTAMENTO POR ATESTADO ODONTOLOGICO - ATE 15 DIAS	CHC da Universidade Federal do Paraná - PR
58	AFASTAMENTO POR ATESTADO ODONTOLOGICO - ATE 15 DIAS	CHC da Universidade Federal do Paraná - PR
58	AFASTAMENTO POR ATESTADO ODONTOLOGICO - ATE 15 DIAS	CHC da Universidade Federal do Paraná - PR
58	AFASTAMENTO POR ATESTADO ODONTOLOGICO - ATE 15 DIAS	CHC da Universidade Federal do Paraná - PR
58	AFASTAMENTO POR ATESTADO ODONTOLOGICO - ATE 15 DIAS	CHC da Universidade Federal do Paraná - PR
58	AFASTAMENTO POR ATESTADO ODONTOLOGICO - ATE 15 DIAS	CHC da Universidade Federal do Paraná - PR
58	AFASTAMENTO POR ATESTADO ODONTOLOGICO - ATE 15 DIAS	CHC da Universidade Federal do Paraná - PR
58	AFASTAMENTO POR ATESTADO ODONTOLOGICO - ATE 15 DIAS	CHC da Universidade Federal do Paraná - PR
58	AFASTAMENTO POR ATESTADO ODONTOLOGICO - ATE 15 DIAS	CHC da Universidade Federal do Paraná - PR
58	AFASTAMENTO POR ATESTADO ODONTOLOGICO - ATE 15 DIAS	CHC da Universidade Federal do Paraná - PR
58	AFASTAMENTO POR ATESTADO ODONTOLOGICO - ATE 15 DIAS	CHC da Universidade Federal do Paraná - PR
58	AFASTAMENTO POR ATESTADO ODONTOLOGICO - ATE 15 DIAS	CHC da Universidade Federal do Paraná - PR
58	AFASTAMENTO POR ATESTADO ODONTOLOGICO - ATE 15 DIAS	CHC da Universidade Federal do Paraná - PR

58	AFASTAMENTO POR ATESTADO ODONTOLOGICO - ATE 15 DIAS	CHC da Universidade Federal do Paraná - PR
58	AFASTAMENTO POR ATESTADO ODONTOLOGICO - ATE 15 DIAS	CHC da Universidade Federal do Paraná - PR
58	AFASTAMENTO POR ATESTADO ODONTOLOGICO - ATE 15 DIAS	CHC da Universidade Federal do Paraná - PR
58	AFASTAMENTO POR ATESTADO ODONTOLOGICO - ATE 15 DIAS	CHC da Universidade Federal do Paraná - PR
58	AFASTAMENTO POR ATESTADO ODONTOLOGICO - ATE 15 DIAS	CHC da Universidade Federal do Paraná - PR
58	AFASTAMENTO POR ATESTADO ODONTOLOGICO - ATE 15 DIAS	CHC da Universidade Federal do Paraná - PR
58	AFASTAMENTO POR ATESTADO ODONTOLOGICO - ATE 15 DIAS	CHC da Universidade Federal do Paraná - PR
58	AFASTAMENTO POR ATESTADO ODONTOLOGICO - ATE 15 DIAS	CHC da Universidade Federal do Paraná - PR
58	AFASTAMENTO POR ATESTADO ODONTOLOGICO - ATE 15 DIAS	CHC da Universidade Federal do Paraná - PR
58	AFASTAMENTO POR ATESTADO ODONTOLOGICO - ATE 15 DIAS	CHC da Universidade Federal do Paraná - PR
58	AFASTAMENTO POR ATESTADO ODONTOLOGICO - ATE 15 DIAS	CHC da Universidade Federal do Paraná - PR
58	AFASTAMENTO POR ATESTADO ODONTOLOGICO - ATE 15 DIAS	CHC da Universidade Federal do Paraná - PR
58	AFASTAMENTO POR ATESTADO ODONTOLOGICO - ATE 15 DIAS	CHC da Universidade Federal do Paraná - PR
58	AFASTAMENTO POR ATESTADO ODONTOLOGICO - ATE 15 DIAS	CHC da Universidade Federal do Paraná - PR
58	AFASTAMENTO POR ATESTADO ODONTOLOGICO - ATE 15 DIAS	CHC da Universidade Federal do Paraná - PR
58	AFASTAMENTO POR ATESTADO ODONTOLOGICO - ATE 15 DIAS	CHC da Universidade Federal do Paraná - PR
58	AFASTAMENTO POR ATESTADO ODONTOLOGICO - ATE 15 DIAS	CHC da Universidade Federal do Paraná - PR
58	AFASTAMENTO POR ATESTADO ODONTOLOGICO - ATE 15 DIAS	CHC da Universidade Federal do Paraná - PR
58	AFASTAMENTO POR ATESTADO ODONTOLOGICO - ATE 15 DIAS	CHC da Universidade Federal do Paraná - PR
58	AFASTAMENTO POR ATESTADO ODONTOLOGICO - ATE 15 DIAS	CHC da Universidade Federal do Paraná - PR
58	AFASTAMENTO POR ATESTADO ODONTOLOGICO - ATE 15 DIAS	CHC da Universidade Federal do Paraná - PR
58	AFASTAMENTO POR ATESTADO ODONTOLOGICO - ATE 15 DIAS	CHC da Universidade Federal do Paraná - PR
58	AFASTAMENTO POR ATESTADO ODONTOLOGICO - ATE 15 DIAS	CHC da Universidade Federal do Paraná - PR
62	LICENÇA PATERNIDADE CELETISTA - Até 5 DIAS - ADCT CF ART 10	CHC da Universidade Federal do Paraná - PR
62	LICENÇA PATERNIDADE CELETISTA - Até 5 DIAS - ADCT CF ART 10	CHC da Universidade Federal do Paraná - PR
62	LICENÇA PATERNIDADE CELETISTA - Até 5 DIAS - ADCT CF ART 10	CHC da Universidade Federal do Paraná - PR
62	LICENÇA PATERNIDADE CELETISTA - Até 5 DIAS - ADCT CF ART 10	CHC da Universidade Federal do Paraná - PR
62	LICENÇA PATERNIDADE CELETISTA - Até 5 DIAS - ADCT CF ART 10	CHC da Universidade Federal do Paraná - PR
62	LICENÇA PATERNIDADE CELETISTA - Até 5 DIAS - ADCT CF ART 10	CHC da Universidade Federal do Paraná - PR
62	LICENÇA PATERNIDADE CELETISTA - Até 5 DIAS - ADCT CF ART 10	CHC da Universidade Federal do Paraná - PR
62	LICENÇA PATERNIDADE CELETISTA - Até 5 DIAS - ADCT CF ART 10	CHC da Universidade Federal do Paraná - PR
67	SUSPENSÃO DE CONTRATO DE TRABALHO	CHC da Universidade Federal do Paraná - PR

68	COMPARECIMENTO A JUIZO (JURI, INQUERITO POLICIAL, PROC. JUDICIAL) - CLT ART. 473 VIII	CHC da Universidade Federal do Paraná - PR
68	COMPARECIMENTO A JUIZO (JURI, INQUERITO POLICIAL, PROC. JUDICIAL) - CLT ART. 473 VIII	CHC da Universidade Federal do Paraná - PR
69	ABONO (ACT)	CHC da Universidade Federal do Paraná - PR
69	ABONO (ACT)	CHC da Universidade Federal do Paraná - PR
69	ABONO (ACT)	CHC da Universidade Federal do Paraná - PR
69	ABONO (ACT)	CHC da Universidade Federal do Paraná - PR
69	ABONO (ACT)	CHC da Universidade Federal do Paraná - PR
69	ABONO (ACT)	CHC da Universidade Federal do Paraná - PR
69	ABONO (ACT)	CHC da Universidade Federal do Paraná - PR
69	ABONO (ACT)	CHC da Universidade Federal do Paraná - PR
69	ABONO (ACT)	CHC da Universidade Federal do Paraná - PR
69	ABONO (ACT)	CHC da Universidade Federal do Paraná - PR
69	ABONO (ACT)	CHC da Universidade Federal do Paraná - PR
69	ABONO (ACT)	CHC da Universidade Federal do Paraná - PR
69	ABONO (ACT)	CHC da Universidade Federal do Paraná - PR
69	ABONO (ACT)	CHC da Universidade Federal do Paraná - PR
69	ABONO (ACT)	CHC da Universidade Federal do Paraná - PR
69	ABONO (ACT)	CHC da Universidade Federal do Paraná - PR
69	ABONO (ACT)	CHC da Universidade Federal do Paraná - PR
69	ABONO (ACT)	CHC da Universidade Federal do Paraná - PR
69	ABONO (ACT)	CHC da Universidade Federal do Paraná - PR
69	ABONO (ACT)	CHC da Universidade Federal do Paraná - PR
69	ABONO (ACT)	CHC da Universidade Federal do Paraná - PR
69	ABONO (ACT)	CHC da Universidade Federal do Paraná - PR
69	ABONO (ACT)	CHC da Universidade Federal do Paraná - PR
69	ABONO (ACT)	CHC da Universidade Federal do Paraná - PR
69	ABONO (ACT)	CHC da Universidade Federal do Paraná - PR
69	ABONO (ACT)	CHC da Universidade Federal do Paraná - PR
69	ABONO (ACT)	CHC da Universidade Federal do Paraná - PR
69	ABONO (ACT)	CHC da Universidade Federal do Paraná - PR
69	ABONO (ACT)	CHC da Universidade Federal do Paraná - PR
69	ABONO (ACT)	CHC da Universidade Federal do Paraná - PR
69	ABONO (ACT)	CHC da Universidade Federal do Paraná - PR
69	ABONO (ACT)	CHC da Universidade Federal do Paraná - PR
69	ABONO (ACT)	CHC da Universidade Federal do Paraná - PR
69	ABONO (ACT)	CHC da Universidade Federal do Paraná - PR
69	ABONO (ACT)	CHC da Universidade Federal do Paraná - PR
69	ABONO (ACT)	CHC da Universidade Federal do Paraná - PR
69	ABONO (ACT)	CHC da Universidade Federal do Paraná - PR
69	ABONO (ACT)	CHC da Universidade Federal do Paraná - PR
69	ABONO (ACT)	CHC da Universidade Federal do Paraná - PR
69	ABONO (ACT)	CHC da Universidade Federal do Paraná - PR
69	ABONO (ACT)	CHC da Universidade Federal do Paraná - PR
69	ABONO (ACT)	CHC da Universidade Federal do Paraná - PR
69	ABONO (ACT)	CHC da Universidade Federal do Paraná - PR

69	ABONO (ACT)	CHC da Universidade Federal do Paraná - PR
69	ABONO (ACT)	CHC da Universidade Federal do Paraná - PR
69	ABONO (ACT)	CHC da Universidade Federal do Paraná - PR
69	ABONO (ACT)	CHC da Universidade Federal do Paraná - PR
69	ABONO (ACT)	CHC da Universidade Federal do Paraná - PR
69	ABONO (ACT)	CHC da Universidade Federal do Paraná - PR
69	ABONO (ACT)	CHC da Universidade Federal do Paraná - PR
69	ABONO (ACT)	CHC da Universidade Federal do Paraná - PR
69	ABONO (ACT)	CHC da Universidade Federal do Paraná - PR
69	ABONO (ACT)	CHC da Universidade Federal do Paraná - PR
69	ABONO (ACT)	CHC da Universidade Federal do Paraná - PR
69	ABONO (ACT)	CHC da Universidade Federal do Paraná - PR
69	ABONO (ACT)	CHC da Universidade Federal do Paraná - PR
69	ABONO (ACT)	CHC da Universidade Federal do Paraná - PR
69	ABONO (ACT)	CHC da Universidade Federal do Paraná - PR
69	ABONO (ACT)	CHC da Universidade Federal do Paraná - PR
69	ABONO (ACT)	CHC da Universidade Federal do Paraná - PR
69	ABONO (ACT)	CHC da Universidade Federal do Paraná - PR
69	ABONO (ACT)	CHC da Universidade Federal do Paraná - PR
69	ABONO (ACT)	CHC da Universidade Federal do Paraná - PR
69	ABONO (ACT)	CHC da Universidade Federal do Paraná - PR
69	ABONO (ACT)	CHC da Universidade Federal do Paraná - PR
69	ABONO (ACT)	CHC da Universidade Federal do Paraná - PR
69	ABONO (ACT)	CHC da Universidade Federal do Paraná - PR
69	ABONO (ACT)	CHC da Universidade Federal do Paraná - PR
69	ABONO (ACT)	CHC da Universidade Federal do Paraná - PR
69	ABONO (ACT)	CHC da Universidade Federal do Paraná - PR
69	ABONO (ACT)	CHC da Universidade Federal do Paraná - PR
69	ABONO (ACT)	CHC da Universidade Federal do Paraná - PR
69	ABONO (ACT)	CHC da Universidade Federal do Paraná - PR
69	ABONO (ACT)	CHC da Universidade Federal do Paraná - PR
69	ABONO (ACT)	CHC da Universidade Federal do Paraná - PR
69	ABONO (ACT)	CHC da Universidade Federal do Paraná - PR
69	ABONO (ACT)	CHC da Universidade Federal do Paraná - PR
69	ABONO (ACT)	CHC da Universidade Federal do Paraná - PR
69	ABONO (ACT)	CHC da Universidade Federal do Paraná - PR
69	ABONO (ACT)	CHC da Universidade Federal do Paraná - PR
69	ABONO (ACT)	CHC da Universidade Federal do Paraná - PR
69	ABONO (ACT)	CHC da Universidade Federal do Paraná - PR
69	ABONO (ACT)	CHC da Universidade Federal do Paraná - PR
69	ABONO (ACT)	CHC da Universidade Federal do Paraná - PR
69	ABONO (ACT)	CHC da Universidade Federal do Paraná - PR
69	ABONO (ACT)	CHC da Universidade Federal do Paraná - PR
69	ABONO (ACT)	CHC da Universidade Federal do Paraná - PR
69	ABONO (ACT)	CHC da Universidade Federal do Paraná - PR
69	ABONO (ACT)	CHC da Universidade Federal do Paraná - PR
69	ABONO (ACT)	CHC da Universidade Federal do Paraná - PR
69	ABONO (ACT)	CHC da Universidade Federal do Paraná - PR

69	ABONO (ACT)	CHC da Universidade Federal do Paraná - PR
69	ABONO (ACT)	CHC da Universidade Federal do Paraná - PR
69	ABONO (ACT)	CHC da Universidade Federal do Paraná - PR
69	ABONO (ACT)	CHC da Universidade Federal do Paraná - PR
69	ABONO (ACT)	CHC da Universidade Federal do Paraná - PR
69	ABONO (ACT)	CHC da Universidade Federal do Paraná - PR
69	ABONO (ACT)	CHC da Universidade Federal do Paraná - PR
69	ABONO (ACT)	CHC da Universidade Federal do Paraná - PR
69	ABONO (ACT)	CHC da Universidade Federal do Paraná - PR
69	ABONO (ACT)	CHC da Universidade Federal do Paraná - PR
69	ABONO (ACT)	CHC da Universidade Federal do Paraná - PR
69	ABONO (ACT)	CHC da Universidade Federal do Paraná - PR
69	ABONO (ACT)	CHC da Universidade Federal do Paraná - PR
69	ABONO (ACT)	CHC da Universidade Federal do Paraná - PR
69	ABONO (ACT)	CHC da Universidade Federal do Paraná - PR
69	ABONO (ACT)	CHC da Universidade Federal do Paraná - PR
69	ABONO (ACT)	CHC da Universidade Federal do Paraná - PR
69	ABONO (ACT)	CHC da Universidade Federal do Paraná - PR
69	ABONO (ACT)	CHC da Universidade Federal do Paraná - PR
69	ABONO (ACT)	CHC da Universidade Federal do Paraná - PR
69	ABONO (ACT)	CHC da Universidade Federal do Paraná - PR
69	ABONO (ACT)	CHC da Universidade Federal do Paraná - PR
69	ABONO (ACT)	CHC da Universidade Federal do Paraná - PR
69	ABONO (ACT)	CHC da Universidade Federal do Paraná - PR
69	ABONO (ACT)	CHC da Universidade Federal do Paraná - PR
69	ABONO (ACT)	CHC da Universidade Federal do Paraná - PR
69	ABONO (ACT)	CHC da Universidade Federal do Paraná - PR
69	ABONO (ACT)	CHC da Universidade Federal do Paraná - PR
69	ABONO (ACT)	CHC da Universidade Federal do Paraná - PR
69	ABONO (ACT)	CHC da Universidade Federal do Paraná - PR
69	ABONO (ACT)	CHC da Universidade Federal do Paraná - PR
69	ABONO (ACT)	CHC da Universidade Federal do Paraná - PR
69	ABONO (ACT)	CHC da Universidade Federal do Paraná - PR
69	ABONO (ACT)	CHC da Universidade Federal do Paraná - PR
69	ABONO (ACT)	CHC da Universidade Federal do Paraná - PR
69	ABONO (ACT)	CHC da Universidade Federal do Paraná - PR
69	ABONO (ACT)	CHC da Universidade Federal do Paraná - PR
69	ABONO (ACT)	CHC da Universidade Federal do Paraná - PR
69	ABONO (ACT)	CHC da Universidade Federal do Paraná - PR
69	ABONO (ACT)	CHC da Universidade Federal do Paraná - PR
69	ABONO (ACT)	CHC da Universidade Federal do Paraná - PR
69	ABONO (ACT)	CHC da Universidade Federal do Paraná - PR
69	ABONO (ACT)	CHC da Universidade Federal do Paraná - PR
69	ABONO (ACT)	CHC da Universidade Federal do Paraná - PR
69	ABONO (ACT)	CHC da Universidade Federal do Paraná - PR
69	ABONO (ACT)	CHC da Universidade Federal do Paraná - PR
69	ABONO (ACT)	CHC da Universidade Federal do Paraná - PR
69	ABONO (ACT)	CHC da Universidade Federal do Paraná - PR

69	ABONO (ACT)	CHC da Universidade Federal do Paraná - PR
69	ABONO (ACT)	CHC da Universidade Federal do Paraná - PR
69	ABONO (ACT)	CHC da Universidade Federal do Paraná - PR
69	ABONO (ACT)	CHC da Universidade Federal do Paraná - PR
69	ABONO (ACT)	CHC da Universidade Federal do Paraná - PR
69	ABONO (ACT)	CHC da Universidade Federal do Paraná - PR
69	ABONO (ACT)	CHC da Universidade Federal do Paraná - PR
69	ABONO (ACT)	CHC da Universidade Federal do Paraná - PR
69	ABONO (ACT)	CHC da Universidade Federal do Paraná - PR
69	ABONO (ACT)	CHC da Universidade Federal do Paraná - PR
69	ABONO (ACT)	CHC da Universidade Federal do Paraná - PR
69	ABONO (ACT)	CHC da Universidade Federal do Paraná - PR
69	ABONO (ACT)	CHC da Universidade Federal do Paraná - PR
69	ABONO (ACT)	CHC da Universidade Federal do Paraná - PR
69	ABONO (ACT)	CHC da Universidade Federal do Paraná - PR
69	ABONO (ACT)	CHC da Universidade Federal do Paraná - PR
69	ABONO (ACT)	CHC da Universidade Federal do Paraná - PR
69	ABONO (ACT)	CHC da Universidade Federal do Paraná - PR
69	ABONO (ACT)	CHC da Universidade Federal do Paraná - PR
69	ABONO (ACT)	CHC da Universidade Federal do Paraná - PR
69	ABONO (ACT)	CHC da Universidade Federal do Paraná - PR
69	ABONO (ACT)	CHC da Universidade Federal do Paraná - PR
69	ABONO (ACT)	CHC da Universidade Federal do Paraná - PR
69	ABONO (ACT)	CHC da Universidade Federal do Paraná - PR
69	ABONO (ACT)	CHC da Universidade Federal do Paraná - PR
69	ABONO (ACT)	CHC da Universidade Federal do Paraná - PR
69	ABONO (ACT)	CHC da Universidade Federal do Paraná - PR
69	ABONO (ACT)	CHC da Universidade Federal do Paraná - PR
69	ABONO (ACT)	CHC da Universidade Federal do Paraná - PR
69	ABONO (ACT)	CHC da Universidade Federal do Paraná - PR
69	ABONO (ACT)	CHC da Universidade Federal do Paraná - PR
69	ABONO (ACT)	CHC da Universidade Federal do Paraná - PR
69	ABONO (ACT)	CHC da Universidade Federal do Paraná - PR
69	ABONO (ACT)	CHC da Universidade Federal do Paraná - PR
69	ABONO (ACT)	CHC da Universidade Federal do Paraná - PR
69	ABONO (ACT)	CHC da Universidade Federal do Paraná - PR
69	ABONO (ACT)	CHC da Universidade Federal do Paraná - PR
69	ABONO (ACT)	CHC da Universidade Federal do Paraná - PR
69	ABONO (ACT)	CHC da Universidade Federal do Paraná - PR
69	ABONO (ACT)	CHC da Universidade Federal do Paraná - PR
69	ABONO (ACT)	CHC da Universidade Federal do Paraná - PR
69	ABONO (ACT)	CHC da Universidade Federal do Paraná - PR
69	ABONO (ACT)	CHC da Universidade Federal do Paraná - PR
69	ABONO (ACT)	CHC da Universidade Federal do Paraná - PR
69	ABONO (ACT)	CHC da Universidade Federal do Paraná - PR
69	ABONO (ACT)	CHC da Universidade Federal do Paraná - PR
69	ABONO (ACT)	CHC da Universidade Federal do Paraná - PR
69	ABONO (ACT)	CHC da Universidade Federal do Paraná - PR

69	ABONO (ACT)	CHC da Universidade Federal do Paraná - PR
69	ABONO (ACT)	CHC da Universidade Federal do Paraná - PR
69	ABONO (ACT)	CHC da Universidade Federal do Paraná - PR
69	ABONO (ACT)	CHC da Universidade Federal do Paraná - PR
69	ABONO (ACT)	CHC da Universidade Federal do Paraná - PR
69	ABONO (ACT)	CHC da Universidade Federal do Paraná - PR
69	ABONO (ACT)	CHC da Universidade Federal do Paraná - PR
69	ABONO (ACT)	CHC da Universidade Federal do Paraná - PR
69	ABONO (ACT)	CHC da Universidade Federal do Paraná - PR
69	ABONO (ACT)	CHC da Universidade Federal do Paraná - PR
69	ABONO (ACT)	CHC da Universidade Federal do Paraná - PR
69	ABONO (ACT)	CHC da Universidade Federal do Paraná - PR
69	ABONO (ACT)	CHC da Universidade Federal do Paraná - PR
69	ABONO (ACT)	CHC da Universidade Federal do Paraná - PR
69	ABONO (ACT)	CHC da Universidade Federal do Paraná - PR
69	ABONO (ACT)	CHC da Universidade Federal do Paraná - PR
69	ABONO (ACT)	CHC da Universidade Federal do Paraná - PR
69	ABONO (ACT)	CHC da Universidade Federal do Paraná - PR
69	ABONO (ACT)	CHC da Universidade Federal do Paraná - PR
69	ABONO (ACT)	CHC da Universidade Federal do Paraná - PR
69	ABONO (ACT)	CHC da Universidade Federal do Paraná - PR
69	ABONO (ACT)	CHC da Universidade Federal do Paraná - PR
69	ABONO (ACT)	CHC da Universidade Federal do Paraná - PR
69	ABONO (ACT)	CHC da Universidade Federal do Paraná - PR
69	ABONO (ACT)	CHC da Universidade Federal do Paraná - PR
69	ABONO (ACT)	CHC da Universidade Federal do Paraná - PR
69	ABONO (ACT)	CHC da Universidade Federal do Paraná - PR
69	ABONO (ACT)	CHC da Universidade Federal do Paraná - PR
69	ABONO (ACT)	CHC da Universidade Federal do Paraná - PR
69	ABONO (ACT)	CHC da Universidade Federal do Paraná - PR
69	ABONO (ACT)	CHC da Universidade Federal do Paraná - PR
69	ABONO (ACT)	CHC da Universidade Federal do Paraná - PR
69	ABONO (ACT)	CHC da Universidade Federal do Paraná - PR
69	ABONO (ACT)	CHC da Universidade Federal do Paraná - PR
69	ABONO (ACT)	CHC da Universidade Federal do Paraná - PR
69	ABONO (ACT)	CHC da Universidade Federal do Paraná - PR
69	ABONO (ACT)	CHC da Universidade Federal do Paraná - PR
69	ABONO (ACT)	CHC da Universidade Federal do Paraná - PR
69	ABONO (ACT)	CHC da Universidade Federal do Paraná - PR
69	ABONO (ACT)	CHC da Universidade Federal do Paraná - PR
69	ABONO (ACT)	CHC da Universidade Federal do Paraná - PR
69	ABONO (ACT)	CHC da Universidade Federal do Paraná - PR
69	ABONO (ACT)	CHC da Universidade Federal do Paraná - PR
69	ABONO (ACT)	CHC da Universidade Federal do Paraná - PR
69	ABONO (ACT)	CHC da Universidade Federal do Paraná - PR
69	ABONO (ACT)	CHC da Universidade Federal do Paraná - PR
69	ABONO (ACT)	CHC da Universidade Federal do Paraná - PR
69	ABONO (ACT)	CHC da Universidade Federal do Paraná - PR

69	ABONO (ACT)	CHC da Universidade Federal do Paraná - PR
69	ABONO (ACT)	CHC da Universidade Federal do Paraná - PR
69	ABONO (ACT)	CHC da Universidade Federal do Paraná - PR
69	ABONO (ACT)	CHC da Universidade Federal do Paraná - PR
69	ABONO (ACT)	CHC da Universidade Federal do Paraná - PR
69	ABONO (ACT)	CHC da Universidade Federal do Paraná - PR
69	ABONO (ACT)	CHC da Universidade Federal do Paraná - PR
69	ABONO (ACT)	CHC da Universidade Federal do Paraná - PR
69	ABONO (ACT)	CHC da Universidade Federal do Paraná - PR
69	ABONO (ACT)	CHC da Universidade Federal do Paraná - PR
69	ABONO (ACT)	CHC da Universidade Federal do Paraná - PR
69	ABONO (ACT)	CHC da Universidade Federal do Paraná - PR
69	ABONO (ACT)	CHC da Universidade Federal do Paraná - PR
69	ABONO (ACT)	CHC da Universidade Federal do Paraná - PR
69	ABONO (ACT)	CHC da Universidade Federal do Paraná - PR
69	ABONO (ACT)	CHC da Universidade Federal do Paraná - PR
69	ABONO (ACT)	CHC da Universidade Federal do Paraná - PR
69	ABONO (ACT)	CHC da Universidade Federal do Paraná - PR
69	ABONO (ACT)	CHC da Universidade Federal do Paraná - PR
69	ABONO (ACT)	CHC da Universidade Federal do Paraná - PR
69	ABONO (ACT)	CHC da Universidade Federal do Paraná - PR
69	ABONO (ACT)	CHC da Universidade Federal do Paraná - PR
69	ABONO (ACT)	CHC da Universidade Federal do Paraná - PR
69	ABONO (ACT)	CHC da Universidade Federal do Paraná - PR
69	ABONO (ACT)	CHC da Universidade Federal do Paraná - PR
69	ABONO (ACT)	CHC da Universidade Federal do Paraná - PR
69	ABONO (ACT)	CHC da Universidade Federal do Paraná - PR
69	ABONO (ACT)	CHC da Universidade Federal do Paraná - PR
69	ABONO (ACT)	CHC da Universidade Federal do Paraná - PR
69	ABONO (ACT)	CHC da Universidade Federal do Paraná - PR
69	ABONO (ACT)	CHC da Universidade Federal do Paraná - PR
69	ABONO (ACT)	CHC da Universidade Federal do Paraná - PR
69	ABONO (ACT)	CHC da Universidade Federal do Paraná - PR
69	ABONO (ACT)	CHC da Universidade Federal do Paraná - PR
69	ABONO (ACT)	CHC da Universidade Federal do Paraná - PR
69	ABONO (ACT)	CHC da Universidade Federal do Paraná - PR
69	ABONO (ACT)	CHC da Universidade Federal do Paraná - PR
69	ABONO (ACT)	CHC da Universidade Federal do Paraná - PR
69	ABONO (ACT)	CHC da Universidade Federal do Paraná - PR
69	ABONO (ACT)	CHC da Universidade Federal do Paraná - PR
69	ABONO (ACT)	CHC da Universidade Federal do Paraná - PR
69	ABONO (ACT)	CHC da Universidade Federal do Paraná - PR
69	ABONO (ACT)	CHC da Universidade Federal do Paraná - PR
69	ABONO (ACT)	CHC da Universidade Federal do Paraná - PR
69	ABONO (ACT)	CHC da Universidade Federal do Paraná - PR
69	ABONO (ACT)	CHC da Universidade Federal do Paraná - PR
69	ABONO (ACT)	CHC da Universidade Federal do Paraná - PR
69	ABONO (ACT)	CHC da Universidade Federal do Paraná - PR

69	ABONO (ACT)	CHC da Universidade Federal do Paraná - PR
69	ABONO (ACT)	CHC da Universidade Federal do Paraná - PR
69	ABONO (ACT)	CHC da Universidade Federal do Paraná - PR
69	ABONO (ACT)	CHC da Universidade Federal do Paraná - PR
69	ABONO (ACT)	CHC da Universidade Federal do Paraná - PR
69	ABONO (ACT)	CHC da Universidade Federal do Paraná - PR
69	ABONO (ACT)	CHC da Universidade Federal do Paraná - PR
69	ABONO (ACT)	CHC da Universidade Federal do Paraná - PR
69	ABONO (ACT)	CHC da Universidade Federal do Paraná - PR
69	ABONO (ACT)	CHC da Universidade Federal do Paraná - PR
69	ABONO (ACT)	CHC da Universidade Federal do Paraná - PR
69	ABONO (ACT)	CHC da Universidade Federal do Paraná - PR
69	ABONO (ACT)	CHC da Universidade Federal do Paraná - PR
69	ABONO (ACT)	CHC da Universidade Federal do Paraná - PR
69	ABONO (ACT)	CHC da Universidade Federal do Paraná - PR
69	ABONO (ACT)	CHC da Universidade Federal do Paraná - PR
69	ABONO (ACT)	CHC da Universidade Federal do Paraná - PR
69	ABONO (ACT)	CHC da Universidade Federal do Paraná - PR
69	ABONO (ACT)	CHC da Universidade Federal do Paraná - PR
69	ABONO (ACT)	CHC da Universidade Federal do Paraná - PR
69	ABONO (ACT)	CHC da Universidade Federal do Paraná - PR
69	ABONO (ACT)	CHC da Universidade Federal do Paraná - PR
69	ABONO (ACT)	CHC da Universidade Federal do Paraná - PR
69	ABONO (ACT)	CHC da Universidade Federal do Paraná - PR
69	ABONO (ACT)	CHC da Universidade Federal do Paraná - PR
69	ABONO (ACT)	CHC da Universidade Federal do Paraná - PR
69	ABONO (ACT)	CHC da Universidade Federal do Paraná - PR
69	ABONO (ACT)	CHC da Universidade Federal do Paraná - PR
69	ABONO (ACT)	CHC da Universidade Federal do Paraná - PR
69	ABONO (ACT)	CHC da Universidade Federal do Paraná - PR
69	ABONO (ACT)	CHC da Universidade Federal do Paraná - PR
69	ABONO (ACT)	CHC da Universidade Federal do Paraná - PR
69	ABONO (ACT)	CHC da Universidade Federal do Paraná - PR
69	ABONO (ACT)	CHC da Universidade Federal do Paraná - PR
69	ABONO (ACT)	CHC da Universidade Federal do Paraná - PR
69	ABONO (ACT)	CHC da Universidade Federal do Paraná - PR
69	ABONO (ACT)	CHC da Universidade Federal do Paraná - PR
69	ABONO (ACT)	CHC da Universidade Federal do Paraná - PR
69	ABONO (ACT)	CHC da Universidade Federal do Paraná - PR
69	ABONO (ACT)	CHC da Universidade Federal do Paraná - PR
69	ABONO (ACT)	CHC da Universidade Federal do Paraná - PR
69	ABONO (ACT)	CHC da Universidade Federal do Paraná - PR
69	ABONO (ACT)	CHC da Universidade Federal do Paraná - PR
69	ABONO (ACT)	CHC da Universidade Federal do Paraná - PR
69	ABONO (ACT)	CHC da Universidade Federal do Paraná - PR
69	ABONO (ACT)	CHC da Universidade Federal do Paraná - PR
69	ABONO (ACT)	CHC da Universidade Federal do Paraná - PR
69	ABONO (ACT)	CHC da Universidade Federal do Paraná - PR

69	ABONO (ACT)	CHC da Universidade Federal do Paraná - PR
69	ABONO (ACT)	CHC da Universidade Federal do Paraná - PR
69	ABONO (ACT)	CHC da Universidade Federal do Paraná - PR
69	ABONO (ACT)	CHC da Universidade Federal do Paraná - PR
69	ABONO (ACT)	CHC da Universidade Federal do Paraná - PR
69	ABONO (ACT)	CHC da Universidade Federal do Paraná - PR
69	ABONO (ACT)	CHC da Universidade Federal do Paraná - PR
69	ABONO (ACT)	CHC da Universidade Federal do Paraná - PR
69	ABONO (ACT)	CHC da Universidade Federal do Paraná - PR
69	ABONO (ACT)	CHC da Universidade Federal do Paraná - PR
69	ABONO (ACT)	CHC da Universidade Federal do Paraná - PR
69	ABONO (ACT)	CHC da Universidade Federal do Paraná - PR
69	ABONO (ACT)	CHC da Universidade Federal do Paraná - PR
69	ABONO (ACT)	CHC da Universidade Federal do Paraná - PR
69	ABONO (ACT)	CHC da Universidade Federal do Paraná - PR
69	ABONO (ACT)	CHC da Universidade Federal do Paraná - PR
69	ABONO (ACT)	CHC da Universidade Federal do Paraná - PR
69	ABONO (ACT)	CHC da Universidade Federal do Paraná - PR
69	ABONO (ACT)	CHC da Universidade Federal do Paraná - PR
69	ABONO (ACT)	CHC da Universidade Federal do Paraná - PR
69	ABONO (ACT)	CHC da Universidade Federal do Paraná - PR
69	ABONO (ACT)	CHC da Universidade Federal do Paraná - PR
69	ABONO (ACT)	CHC da Universidade Federal do Paraná - PR
69	ABONO (ACT)	CHC da Universidade Federal do Paraná - PR
69	ABONO (ACT)	CHC da Universidade Federal do Paraná - PR
69	ABONO (ACT)	CHC da Universidade Federal do Paraná - PR
69	ABONO (ACT)	CHC da Universidade Federal do Paraná - PR
69	ABONO (ACT)	CHC da Universidade Federal do Paraná - PR
69	ABONO (ACT)	CHC da Universidade Federal do Paraná - PR
69	ABONO (ACT)	CHC da Universidade Federal do Paraná - PR
69	ABONO (ACT)	CHC da Universidade Federal do Paraná - PR
69	ABONO (ACT)	CHC da Universidade Federal do Paraná - PR
69	ABONO (ACT)	CHC da Universidade Federal do Paraná - PR
69	ABONO (ACT)	CHC da Universidade Federal do Paraná - PR
69	ABONO (ACT)	CHC da Universidade Federal do Paraná - PR
69	ABONO (ACT)	CHC da Universidade Federal do Paraná - PR
69	ABONO (ACT)	CHC da Universidade Federal do Paraná - PR
69	ABONO (ACT)	CHC da Universidade Federal do Paraná - PR
69	ABONO (ACT)	CHC da Universidade Federal do Paraná - PR
69	ABONO (ACT)	CHC da Universidade Federal do Paraná - PR
69	ABONO (ACT)	CHC da Universidade Federal do Paraná - PR
69	ABONO (ACT)	CHC da Universidade Federal do Paraná - PR
69	ABONO (ACT)	CHC da Universidade Federal do Paraná - PR
69	ABONO (ACT)	CHC da Universidade Federal do Paraná - PR
69	ABONO (ACT)	CHC da Universidade Federal do Paraná - PR
69	ABONO (ACT)	CHC da Universidade Federal do Paraná - PR
69	ABONO (ACT)	CHC da Universidade Federal do Paraná - PR
69	ABONO (ACT)	CHC da Universidade Federal do Paraná - PR

69	ABONO (ACT)	CHC da Universidade Federal do Paraná - PR
69	ABONO (ACT)	CHC da Universidade Federal do Paraná - PR
69	ABONO (ACT)	CHC da Universidade Federal do Paraná - PR
69	ABONO (ACT)	CHC da Universidade Federal do Paraná - PR
69	ABONO (ACT)	CHC da Universidade Federal do Paraná - PR
69	ABONO (ACT)	CHC da Universidade Federal do Paraná - PR
69	ABONO (ACT)	CHC da Universidade Federal do Paraná - PR
69	ABONO (ACT)	CHC da Universidade Federal do Paraná - PR
69	ABONO (ACT)	CHC da Universidade Federal do Paraná - PR
69	ABONO (ACT)	CHC da Universidade Federal do Paraná - PR
69	ABONO (ACT)	CHC da Universidade Federal do Paraná - PR
69	ABONO (ACT)	CHC da Universidade Federal do Paraná - PR
69	ABONO (ACT)	CHC da Universidade Federal do Paraná - PR
69	ABONO (ACT)	CHC da Universidade Federal do Paraná - PR
69	ABONO (ACT)	CHC da Universidade Federal do Paraná - PR
69	ABONO (ACT)	CHC da Universidade Federal do Paraná - PR
69	ABONO (ACT)	CHC da Universidade Federal do Paraná - PR
69	ABONO (ACT)	CHC da Universidade Federal do Paraná - PR
69	ABONO (ACT)	CHC da Universidade Federal do Paraná - PR
69	ABONO (ACT)	CHC da Universidade Federal do Paraná - PR
69	ABONO (ACT)	CHC da Universidade Federal do Paraná - PR
69	ABONO (ACT)	CHC da Universidade Federal do Paraná - PR
69	ABONO (ACT)	CHC da Universidade Federal do Paraná - PR
69	ABONO (ACT)	CHC da Universidade Federal do Paraná - PR
69	ABONO (ACT)	CHC da Universidade Federal do Paraná - PR
69	ABONO (ACT)	CHC da Universidade Federal do Paraná - PR
69	ABONO (ACT)	CHC da Universidade Federal do Paraná - PR
69	ABONO (ACT)	CHC da Universidade Federal do Paraná - PR
69	ABONO (ACT)	CHC da Universidade Federal do Paraná - PR
69	ABONO (ACT)	CHC da Universidade Federal do Paraná - PR
69	ABONO (ACT)	CHC da Universidade Federal do Paraná - PR
69	ABONO (ACT)	CHC da Universidade Federal do Paraná - PR
69	ABONO (ACT)	CHC da Universidade Federal do Paraná - PR
69	ABONO (ACT)	CHC da Universidade Federal do Paraná - PR
69	ABONO (ACT)	CHC da Universidade Federal do Paraná - PR
69	ABONO (ACT)	CHC da Universidade Federal do Paraná - PR
69	ABONO (ACT)	CHC da Universidade Federal do Paraná - PR
69	ABONO (ACT)	CHC da Universidade Federal do Paraná - PR
69	ABONO (ACT)	CHC da Universidade Federal do Paraná - PR
69	ABONO (ACT)	CHC da Universidade Federal do Paraná - PR
69	ABONO (ACT)	CHC da Universidade Federal do Paraná - PR
69	ABONO (ACT)	CHC da Universidade Federal do Paraná - PR
69	ABONO (ACT)	CHC da Universidade Federal do Paraná - PR
69	ABONO (ACT)	CHC da Universidade Federal do Paraná - PR
69	ABONO (ACT)	CHC da Universidade Federal do Paraná - PR
69	ABONO (ACT)	CHC da Universidade Federal do Paraná - PR
69	ABONO (ACT)	CHC da Universidade Federal do Paraná - PR
69	ABONO (ACT)	CHC da Universidade Federal do Paraná - PR

69	ABONO (ACT)	CHC da Universidade Federal do Paraná - PR
69	ABONO (ACT)	CHC da Universidade Federal do Paraná - PR
69	ABONO (ACT)	CHC da Universidade Federal do Paraná - PR
69	ABONO (ACT)	CHC da Universidade Federal do Paraná - PR
69	ABONO (ACT)	CHC da Universidade Federal do Paraná - PR
69	ABONO (ACT)	CHC da Universidade Federal do Paraná - PR
69	ABONO (ACT)	CHC da Universidade Federal do Paraná - PR
69	ABONO (ACT)	CHC da Universidade Federal do Paraná - PR
69	ABONO (ACT)	CHC da Universidade Federal do Paraná - PR
69	ABONO (ACT)	CHC da Universidade Federal do Paraná - PR
69	ABONO (ACT)	CHC da Universidade Federal do Paraná - PR
69	ABONO (ACT)	CHC da Universidade Federal do Paraná - PR
69	ABONO (ACT)	CHC da Universidade Federal do Paraná - PR
69	ABONO (ACT)	CHC da Universidade Federal do Paraná - PR
69	ABONO (ACT)	CHC da Universidade Federal do Paraná - PR
69	ABONO (ACT)	CHC da Universidade Federal do Paraná - PR
69	ABONO (ACT)	CHC da Universidade Federal do Paraná - PR
69	ABONO (ACT)	CHC da Universidade Federal do Paraná - PR
69	ABONO (ACT)	CHC da Universidade Federal do Paraná - PR
69	ABONO (ACT)	CHC da Universidade Federal do Paraná - PR
69	ABONO (ACT)	CHC da Universidade Federal do Paraná - PR
69	ABONO (ACT)	CHC da Universidade Federal do Paraná - PR
69	ABONO (ACT)	CHC da Universidade Federal do Paraná - PR
69	ABONO (ACT)	CHC da Universidade Federal do Paraná - PR
69	ABONO (ACT)	CHC da Universidade Federal do Paraná - PR
69	ABONO (ACT)	CHC da Universidade Federal do Paraná - PR
69	ABONO (ACT)	CHC da Universidade Federal do Paraná - PR
69	ABONO (ACT)	CHC da Universidade Federal do Paraná - PR
69	ABONO (ACT)	CHC da Universidade Federal do Paraná - PR
69	ABONO (ACT)	CHC da Universidade Federal do Paraná - PR
69	ABONO (ACT)	CHC da Universidade Federal do Paraná - PR
69	ABONO (ACT)	CHC da Universidade Federal do Paraná - PR
69	ABONO (ACT)	CHC da Universidade Federal do Paraná - PR
69	ABONO (ACT)	CHC da Universidade Federal do Paraná - PR
69	ABONO (ACT)	CHC da Universidade Federal do Paraná - PR
69	ABONO (ACT)	CHC da Universidade Federal do Paraná - PR
69	ABONO (ACT)	CHC da Universidade Federal do Paraná - PR
69	ABONO (ACT)	CHC da Universidade Federal do Paraná - PR
69	ABONO (ACT)	CHC da Universidade Federal do Paraná - PR
69	ABONO (ACT)	CHC da Universidade Federal do Paraná - PR
69	ABONO (ACT)	CHC da Universidade Federal do Paraná - PR
69	ABONO (ACT)	CHC da Universidade Federal do Paraná - PR
69	ABONO (ACT)	CHC da Universidade Federal do Paraná - PR
69	ABONO (ACT)	CHC da Universidade Federal do Paraná - PR
69	ABONO (ACT)	CHC da Universidade Federal do Paraná - PR
69	ABONO (ACT)	CHC da Universidade Federal do Paraná - PR
69	ABONO (ACT)	CHC da Universidade Federal do Paraná - PR
69	ABONO (ACT)	CHC da Universidade Federal do Paraná - PR

69	ABONO (ACT)	CHC da Universidade Federal do Paraná - PR
69	ABONO (ACT)	CHC da Universidade Federal do Paraná - PR
69	ABONO (ACT)	CHC da Universidade Federal do Paraná - PR
69	ABONO (ACT)	CHC da Universidade Federal do Paraná - PR
69	ABONO (ACT)	CHC da Universidade Federal do Paraná - PR
69	ABONO (ACT)	CHC da Universidade Federal do Paraná - PR
69	ABONO (ACT)	CHC da Universidade Federal do Paraná - PR
69	ABONO (ACT)	CHC da Universidade Federal do Paraná - PR
69	ABONO (ACT)	CHC da Universidade Federal do Paraná - PR
69	ABONO (ACT)	CHC da Universidade Federal do Paraná - PR
69	ABONO (ACT)	CHC da Universidade Federal do Paraná - PR
69	ABONO (ACT)	CHC da Universidade Federal do Paraná - PR
69	ABONO (ACT)	CHC da Universidade Federal do Paraná - PR
69	ABONO (ACT)	CHC da Universidade Federal do Paraná - PR
69	ABONO (ACT)	CHC da Universidade Federal do Paraná - PR
69	ABONO (ACT)	CHC da Universidade Federal do Paraná - PR
69	ABONO (ACT)	CHC da Universidade Federal do Paraná - PR
69	ABONO (ACT)	CHC da Universidade Federal do Paraná - PR
69	ABONO (ACT)	CHC da Universidade Federal do Paraná - PR
69	ABONO (ACT)	CHC da Universidade Federal do Paraná - PR
69	ABONO (ACT)	CHC da Universidade Federal do Paraná - PR
69	ABONO (ACT)	CHC da Universidade Federal do Paraná - PR
69	ABONO (ACT)	CHC da Universidade Federal do Paraná - PR
69	ABONO (ACT)	CHC da Universidade Federal do Paraná - PR
69	ABONO (ACT)	CHC da Universidade Federal do Paraná - PR
69	ABONO (ACT)	CHC da Universidade Federal do Paraná - PR
69	ABONO (ACT)	CHC da Universidade Federal do Paraná - PR
69	ABONO (ACT)	CHC da Universidade Federal do Paraná - PR
69	ABONO (ACT)	CHC da Universidade Federal do Paraná - PR
69	ABONO (ACT)	CHC da Universidade Federal do Paraná - PR
69	ABONO (ACT)	CHC da Universidade Federal do Paraná - PR
69	ABONO (ACT)	CHC da Universidade Federal do Paraná - PR
69	ABONO (ACT)	CHC da Universidade Federal do Paraná - PR
69	ABONO (ACT)	CHC da Universidade Federal do Paraná - PR
69	ABONO (ACT)	CHC da Universidade Federal do Paraná - PR
69	ABONO (ACT)	CHC da Universidade Federal do Paraná - PR
69	ABONO (ACT)	CHC da Universidade Federal do Paraná - PR
69	ABONO (ACT)	CHC da Universidade Federal do Paraná - PR
69	ABONO (ACT)	CHC da Universidade Federal do Paraná - PR
69	ABONO (ACT)	CHC da Universidade Federal do Paraná - PR
69	ABONO (ACT)	CHC da Universidade Federal do Paraná - PR
69	ABONO (ACT)	CHC da Universidade Federal do Paraná - PR
69	ABONO (ACT)	CHC da Universidade Federal do Paraná - PR
69	ABONO (ACT)	CHC da Universidade Federal do Paraná - PR
69	ABONO (ACT)	CHC da Universidade Federal do Paraná - PR
69	ABONO (ACT)	CHC da Universidade Federal do Paraná - PR
69	ABONO (ACT)	CHC da Universidade Federal do Paraná - PR
69	ABONO (ACT)	CHC da Universidade Federal do Paraná - PR

69	ABONO (ACT)	CHC da Universidade Federal do Paraná - PR
69	ABONO (ACT)	CHC da Universidade Federal do Paraná - PR
69	ABONO (ACT)	CHC da Universidade Federal do Paraná - PR
69	ABONO (ACT)	CHC da Universidade Federal do Paraná - PR
69	ABONO (ACT)	CHC da Universidade Federal do Paraná - PR
69	ABONO (ACT)	CHC da Universidade Federal do Paraná - PR
69	ABONO (ACT)	CHC da Universidade Federal do Paraná - PR
69	ABONO (ACT)	CHC da Universidade Federal do Paraná - PR
69	ABONO (ACT)	CHC da Universidade Federal do Paraná - PR
69	ABONO (ACT)	CHC da Universidade Federal do Paraná - PR
69	ABONO (ACT)	CHC da Universidade Federal do Paraná - PR
69	ABONO (ACT)	CHC da Universidade Federal do Paraná - PR
69	ABONO (ACT)	CHC da Universidade Federal do Paraná - PR
69	ABONO (ACT)	CHC da Universidade Federal do Paraná - PR
69	ABONO (ACT)	CHC da Universidade Federal do Paraná - PR
69	ABONO (ACT)	CHC da Universidade Federal do Paraná - PR
69	ABONO (ACT)	CHC da Universidade Federal do Paraná - PR
69	ABONO (ACT)	CHC da Universidade Federal do Paraná - PR
69	ABONO (ACT)	CHC da Universidade Federal do Paraná - PR
69	ABONO (ACT)	CHC da Universidade Federal do Paraná - PR
69	ABONO (ACT)	CHC da Universidade Federal do Paraná - PR
69	ABONO (ACT)	CHC da Universidade Federal do Paraná - PR
69	ABONO (ACT)	CHC da Universidade Federal do Paraná - PR
69	ABONO (ACT)	CHC da Universidade Federal do Paraná - PR
69	ABONO (ACT)	CHC da Universidade Federal do Paraná - PR
69	ABONO (ACT)	CHC da Universidade Federal do Paraná - PR
69	ABONO (ACT)	CHC da Universidade Federal do Paraná - PR
69	ABONO (ACT)	CHC da Universidade Federal do Paraná - PR
69	ABONO (ACT)	CHC da Universidade Federal do Paraná - PR
69	ABONO (ACT)	CHC da Universidade Federal do Paraná - PR
69	ABONO (ACT)	CHC da Universidade Federal do Paraná - PR
69	ABONO (ACT)	CHC da Universidade Federal do Paraná - PR
69	ABONO (ACT)	CHC da Universidade Federal do Paraná - PR
69	ABONO (ACT)	CHC da Universidade Federal do Paraná - PR
69	ABONO (ACT)	CHC da Universidade Federal do Paraná - PR
69	ABONO (ACT)	CHC da Universidade Federal do Paraná - PR
69	ABONO (ACT)	CHC da Universidade Federal do Paraná - PR
69	ABONO (ACT)	CHC da Universidade Federal do Paraná - PR
69	ABONO (ACT)	CHC da Universidade Federal do Paraná - PR
69	ABONO (ACT)	CHC da Universidade Federal do Paraná - PR
69	ABONO (ACT)	CHC da Universidade Federal do Paraná - PR
69	ABONO (ACT)	CHC da Universidade Federal do Paraná - PR
69	ABONO (ACT)	CHC da Universidade Federal do Paraná - PR
69	ABONO (ACT)	CHC da Universidade Federal do Paraná - PR
69	ABONO (ACT)	CHC da Universidade Federal do Paraná - PR
69	ABONO (ACT)	CHC da Universidade Federal do Paraná - PR
69	ABONO (ACT)	CHC da Universidade Federal do Paraná - PR
69	ABONO (ACT)	CHC da Universidade Federal do Paraná - PR

69	ABONO (ACT)	CHC da Universidade Federal do Paraná - PR
69	ABONO (ACT)	CHC da Universidade Federal do Paraná - PR
69	ABONO (ACT)	CHC da Universidade Federal do Paraná - PR
69	ABONO (ACT)	CHC da Universidade Federal do Paraná - PR
69	ABONO (ACT)	CHC da Universidade Federal do Paraná - PR
69	ABONO (ACT)	CHC da Universidade Federal do Paraná - PR
69	ABONO (ACT)	CHC da Universidade Federal do Paraná - PR
69	ABONO (ACT)	CHC da Universidade Federal do Paraná - PR
69	ABONO (ACT)	CHC da Universidade Federal do Paraná - PR
69	ABONO (ACT)	CHC da Universidade Federal do Paraná - PR
69	ABONO (ACT)	CHC da Universidade Federal do Paraná - PR
69	ABONO (ACT)	CHC da Universidade Federal do Paraná - PR
69	ABONO (ACT)	CHC da Universidade Federal do Paraná - PR
69	ABONO (ACT)	CHC da Universidade Federal do Paraná - PR
69	ABONO (ACT)	CHC da Universidade Federal do Paraná - PR
69	ABONO (ACT)	CHC da Universidade Federal do Paraná - PR
69	ABONO (ACT)	CHC da Universidade Federal do Paraná - PR
69	ABONO (ACT)	CHC da Universidade Federal do Paraná - PR
69	ABONO (ACT)	CHC da Universidade Federal do Paraná - PR
69	ABONO (ACT)	CHC da Universidade Federal do Paraná - PR
69	ABONO (ACT)	CHC da Universidade Federal do Paraná - PR
69	ABONO (ACT)	CHC da Universidade Federal do Paraná - PR
69	ABONO (ACT)	CHC da Universidade Federal do Paraná - PR
69	ABONO (ACT)	CHC da Universidade Federal do Paraná - PR
69	ABONO (ACT)	CHC da Universidade Federal do Paraná - PR
69	ABONO (ACT)	CHC da Universidade Federal do Paraná - PR
69	ABONO (ACT)	CHC da Universidade Federal do Paraná - PR
69	ABONO (ACT)	CHC da Universidade Federal do Paraná - PR
69	ABONO (ACT)	CHC da Universidade Federal do Paraná - PR
69	ABONO (ACT)	CHC da Universidade Federal do Paraná - PR
69	ABONO (ACT)	CHC da Universidade Federal do Paraná - PR
69	ABONO (ACT)	CHC da Universidade Federal do Paraná - PR
69	ABONO (ACT)	CHC da Universidade Federal do Paraná - PR
69	ABONO (ACT)	CHC da Universidade Federal do Paraná - PR
69	ABONO (ACT)	CHC da Universidade Federal do Paraná - PR
69	ABONO (ACT)	CHC da Universidade Federal do Paraná - PR
69	ABONO (ACT)	CHC da Universidade Federal do Paraná - PR
69	ABONO (ACT)	CHC da Universidade Federal do Paraná - PR
69	ABONO (ACT)	CHC da Universidade Federal do Paraná - PR
69	ABONO (ACT)	CHC da Universidade Federal do Paraná - PR
69	ABONO (ACT)	CHC da Universidade Federal do Paraná - PR
69	ABONO (ACT)	CHC da Universidade Federal do Paraná - PR
69	ABONO (ACT)	CHC da Universidade Federal do Paraná - PR
69	ABONO (ACT)	CHC da Universidade Federal do Paraná - PR
69	ABONO (ACT)	CHC da Universidade Federal do Paraná - PR
69	ABONO (ACT)	CHC da Universidade Federal do Paraná - PR
69	ABONO (ACT)	CHC da Universidade Federal do Paraná - PR
69	ABONO (ACT)	CHC da Universidade Federal do Paraná - PR

69	ABONO (ACT)	CHC da Universidade Federal do Paraná - PR
69	ABONO (ACT)	CHC da Universidade Federal do Paraná - PR
69	ABONO (ACT)	CHC da Universidade Federal do Paraná - PR
69	ABONO (ACT)	CHC da Universidade Federal do Paraná - PR
69	ABONO (ACT)	CHC da Universidade Federal do Paraná - PR
69	ABONO (ACT)	CHC da Universidade Federal do Paraná - PR
69	ABONO (ACT)	CHC da Universidade Federal do Paraná - PR
69	ABONO (ACT)	CHC da Universidade Federal do Paraná - PR
69	ABONO (ACT)	CHC da Universidade Federal do Paraná - PR
69	ABONO (ACT)	CHC da Universidade Federal do Paraná - PR
69	ABONO (ACT)	CHC da Universidade Federal do Paraná - PR
69	ABONO (ACT)	CHC da Universidade Federal do Paraná - PR
69	ABONO (ACT)	CHC da Universidade Federal do Paraná - PR
69	ABONO (ACT)	CHC da Universidade Federal do Paraná - PR
69	ABONO (ACT)	CHC da Universidade Federal do Paraná - PR
69	ABONO (ACT)	CHC da Universidade Federal do Paraná - PR
69	ABONO (ACT)	CHC da Universidade Federal do Paraná - PR
69	ABONO (ACT)	CHC da Universidade Federal do Paraná - PR
69	ABONO (ACT)	CHC da Universidade Federal do Paraná - PR
69	ABONO (ACT)	CHC da Universidade Federal do Paraná - PR
69	ABONO (ACT)	CHC da Universidade Federal do Paraná - PR
69	ABONO (ACT)	CHC da Universidade Federal do Paraná - PR
69	ABONO (ACT)	CHC da Universidade Federal do Paraná - PR
69	ABONO (ACT)	CHC da Universidade Federal do Paraná - PR
69	ABONO (ACT)	CHC da Universidade Federal do Paraná - PR
69	ABONO (ACT)	CHC da Universidade Federal do Paraná - PR
69	ABONO (ACT)	CHC da Universidade Federal do Paraná - PR
69	ABONO (ACT)	CHC da Universidade Federal do Paraná - PR
69	ABONO (ACT)	CHC da Universidade Federal do Paraná - PR
69	ABONO (ACT)	CHC da Universidade Federal do Paraná - PR
69	ABONO (ACT)	CHC da Universidade Federal do Paraná - PR
69	ABONO (ACT)	CHC da Universidade Federal do Paraná - PR
69	ABONO (ACT)	CHC da Universidade Federal do Paraná - PR
69	ABONO (ACT)	CHC da Universidade Federal do Paraná - PR
69	ABONO (ACT)	CHC da Universidade Federal do Paraná - PR
69	ABONO (ACT)	CHC da Universidade Federal do Paraná - PR
69	ABONO (ACT)	CHC da Universidade Federal do Paraná - PR
69	ABONO (ACT)	CHC da Universidade Federal do Paraná - PR
69	ABONO (ACT)	CHC da Universidade Federal do Paraná - PR
69	ABONO (ACT)	CHC da Universidade Federal do Paraná - PR
69	ABONO (ACT)	CHC da Universidade Federal do Paraná - PR
69	ABONO (ACT)	CHC da Universidade Federal do Paraná - PR
69	ABONO (ACT)	CHC da Universidade Federal do Paraná - PR
69	ABONO (ACT)	CHC da Universidade Federal do Paraná - PR
69	ABONO (ACT)	CHC da Universidade Federal do Paraná - PR
69	ABONO (ACT)	CHC da Universidade Federal do Paraná - PR
69	ABONO (ACT)	CHC da Universidade Federal do Paraná - PR
69	ABONO (ACT)	CHC da Universidade Federal do Paraná - PR

69	ABONO (ACT)	CHC da Universidade Federal do Paraná - PR
69	ABONO (ACT)	CHC da Universidade Federal do Paraná - PR
69	ABONO (ACT)	CHC da Universidade Federal do Paraná - PR
69	ABONO (ACT)	CHC da Universidade Federal do Paraná - PR
69	ABONO (ACT)	CHC da Universidade Federal do Paraná - PR
69	ABONO (ACT)	CHC da Universidade Federal do Paraná - PR
69	ABONO (ACT)	CHC da Universidade Federal do Paraná - PR
69	ABONO (ACT)	CHC da Universidade Federal do Paraná - PR
69	ABONO (ACT)	CHC da Universidade Federal do Paraná - PR
69	ABONO (ACT)	CHC da Universidade Federal do Paraná - PR
69	ABONO (ACT)	CHC da Universidade Federal do Paraná - PR
69	ABONO (ACT)	CHC da Universidade Federal do Paraná - PR
69	ABONO (ACT)	CHC da Universidade Federal do Paraná - PR
69	ABONO (ACT)	CHC da Universidade Federal do Paraná - PR
69	ABONO (ACT)	CHC da Universidade Federal do Paraná - PR
69	ABONO (ACT)	CHC da Universidade Federal do Paraná - PR
69	ABONO (ACT)	CHC da Universidade Federal do Paraná - PR
69	ABONO (ACT)	CHC da Universidade Federal do Paraná - PR
69	ABONO (ACT)	CHC da Universidade Federal do Paraná - PR
69	ABONO (ACT)	CHC da Universidade Federal do Paraná - PR
69	ABONO (ACT)	CHC da Universidade Federal do Paraná - PR
69	ABONO (ACT)	CHC da Universidade Federal do Paraná - PR
69	ABONO (ACT)	CHC da Universidade Federal do Paraná - PR
69	ABONO (ACT)	CHC da Universidade Federal do Paraná - PR
69	ABONO (ACT)	CHC da Universidade Federal do Paraná - PR
69	ABONO (ACT)	CHC da Universidade Federal do Paraná - PR
69	ABONO (ACT)	CHC da Universidade Federal do Paraná - PR
69	ABONO (ACT)	CHC da Universidade Federal do Paraná - PR
69	ABONO (ACT)	CHC da Universidade Federal do Paraná - PR
69	ABONO (ACT)	CHC da Universidade Federal do Paraná - PR
69	ABONO (ACT)	CHC da Universidade Federal do Paraná - PR
69	ABONO (ACT)	CHC da Universidade Federal do Paraná - PR
69	ABONO (ACT)	CHC da Universidade Federal do Paraná - PR
69	ABONO (ACT)	CHC da Universidade Federal do Paraná - PR
69	ABONO (ACT)	CHC da Universidade Federal do Paraná - PR
69	ABONO (ACT)	CHC da Universidade Federal do Paraná - PR
69	ABONO (ACT)	CHC da Universidade Federal do Paraná - PR
69	ABONO (ACT)	CHC da Universidade Federal do Paraná - PR
69	ABONO (ACT)	CHC da Universidade Federal do Paraná - PR
69	ABONO (ACT)	CHC da Universidade Federal do Paraná - PR
69	ABONO (ACT)	CHC da Universidade Federal do Paraná - PR
69	ABONO (ACT)	CHC da Universidade Federal do Paraná - PR
69	ABONO (ACT)	CHC da Universidade Federal do Paraná - PR
69	ABONO (ACT)	CHC da Universidade Federal do Paraná - PR
69	ABONO (ACT)	CHC da Universidade Federal do Paraná - PR
69	ABONO (ACT)	CHC da Universidade Federal do Paraná - PR
69	ABONO (ACT)	CHC da Universidade Federal do Paraná - PR
69	ABONO (ACT)	CHC da Universidade Federal do Paraná - PR

69	ABONO (ACT)	CHC da Universidade Federal do Paraná - PR
69	ABONO (ACT)	CHC da Universidade Federal do Paraná - PR
69	ABONO (ACT)	CHC da Universidade Federal do Paraná - PR
69	ABONO (ACT)	CHC da Universidade Federal do Paraná - PR
69	ABONO (ACT)	CHC da Universidade Federal do Paraná - PR
69	ABONO (ACT)	CHC da Universidade Federal do Paraná - PR
69	ABONO (ACT)	CHC da Universidade Federal do Paraná - PR
69	ABONO (ACT)	CHC da Universidade Federal do Paraná - PR
69	ABONO (ACT)	CHC da Universidade Federal do Paraná - PR
69	ABONO (ACT)	CHC da Universidade Federal do Paraná - PR
69	ABONO (ACT)	CHC da Universidade Federal do Paraná - PR
69	ABONO (ACT)	CHC da Universidade Federal do Paraná - PR
69	ABONO (ACT)	CHC da Universidade Federal do Paraná - PR
69	ABONO (ACT)	CHC da Universidade Federal do Paraná - PR
69	ABONO (ACT)	CHC da Universidade Federal do Paraná - PR
69	ABONO (ACT)	CHC da Universidade Federal do Paraná - PR
69	ABONO (ACT)	CHC da Universidade Federal do Paraná - PR
69	ABONO (ACT)	CHC da Universidade Federal do Paraná - PR
69	ABONO (ACT)	CHC da Universidade Federal do Paraná - PR
69	ABONO (ACT)	CHC da Universidade Federal do Paraná - PR
69	ABONO (ACT)	CHC da Universidade Federal do Paraná - PR
69	ABONO (ACT)	CHC da Universidade Federal do Paraná - PR
69	ABONO (ACT)	CHC da Universidade Federal do Paraná - PR
69	ABONO (ACT)	CHC da Universidade Federal do Paraná - PR
69	ABONO (ACT)	CHC da Universidade Federal do Paraná - PR
69	ABONO (ACT)	CHC da Universidade Federal do Paraná - PR
69	ABONO (ACT)	CHC da Universidade Federal do Paraná - PR
69	ABONO (ACT)	CHC da Universidade Federal do Paraná - PR
69	ABONO (ACT)	CHC da Universidade Federal do Paraná - PR
69	ABONO (ACT)	CHC da Universidade Federal do Paraná - PR
69	ABONO (ACT)	CHC da Universidade Federal do Paraná - PR
69	ABONO (ACT)	CHC da Universidade Federal do Paraná - PR
69	ABONO (ACT)	CHC da Universidade Federal do Paraná - PR
69	ABONO (ACT)	CHC da Universidade Federal do Paraná - PR
69	ABONO (ACT)	CHC da Universidade Federal do Paraná - PR
69	ABONO (ACT)	CHC da Universidade Federal do Paraná - PR
69	ABONO (ACT)	CHC da Universidade Federal do Paraná - PR
69	ABONO (ACT)	CHC da Universidade Federal do Paraná - PR
69	ABONO (ACT)	CHC da Universidade Federal do Paraná - PR
69	ABONO (ACT)	CHC da Universidade Federal do Paraná - PR
69	ABONO (ACT)	CHC da Universidade Federal do Paraná - PR
69	ABONO (ACT)	CHC da Universidade Federal do Paraná - PR
69	ABONO (ACT)	CHC da Universidade Federal do Paraná - PR
69	ABONO (ACT)	CHC da Universidade Federal do Paraná - PR
69	ABONO (ACT)	CHC da Universidade Federal do Paraná - PR
69	ABONO (ACT)	CHC da Universidade Federal do Paraná - PR
69	ABONO (ACT)	CHC da Universidade Federal do Paraná - PR
69	ABONO (ACT)	CHC da Universidade Federal do Paraná - PR

69	ABONO (ACT)	CHC da Universidade Federal do Paraná - PR
69	ABONO (ACT)	CHC da Universidade Federal do Paraná - PR
69	ABONO (ACT)	CHC da Universidade Federal do Paraná - PR
69	ABONO (ACT)	CHC da Universidade Federal do Paraná - PR
69	ABONO (ACT)	CHC da Universidade Federal do Paraná - PR
69	ABONO (ACT)	CHC da Universidade Federal do Paraná - PR
69	ABONO (ACT)	CHC da Universidade Federal do Paraná - PR
69	ABONO (ACT)	CHC da Universidade Federal do Paraná - PR
69	ABONO (ACT)	CHC da Universidade Federal do Paraná - PR
69	ABONO (ACT)	CHC da Universidade Federal do Paraná - PR
69	ABONO (ACT)	CHC da Universidade Federal do Paraná - PR
69	ABONO (ACT)	CHC da Universidade Federal do Paraná - PR
69	ABONO (ACT)	CHC da Universidade Federal do Paraná - PR
69	ABONO (ACT)	CHC da Universidade Federal do Paraná - PR
69	ABONO (ACT)	CHC da Universidade Federal do Paraná - PR
69	ABONO (ACT)	CHC da Universidade Federal do Paraná - PR
69	ABONO (ACT)	CHC da Universidade Federal do Paraná - PR
69	ABONO (ACT)	CHC da Universidade Federal do Paraná - PR
69	ABONO (ACT)	CHC da Universidade Federal do Paraná - PR
69	ABONO (ACT)	CHC da Universidade Federal do Paraná - PR
69	ABONO (ACT)	CHC da Universidade Federal do Paraná - PR
69	ABONO (ACT)	CHC da Universidade Federal do Paraná - PR
69	ABONO (ACT)	CHC da Universidade Federal do Paraná - PR
69	ABONO (ACT)	CHC da Universidade Federal do Paraná - PR
69	ABONO (ACT)	CHC da Universidade Federal do Paraná - PR
69	ABONO (ACT)	CHC da Universidade Federal do Paraná - PR
69	ABONO (ACT)	CHC da Universidade Federal do Paraná - PR
69	ABONO (ACT)	CHC da Universidade Federal do Paraná - PR
69	ABONO (ACT)	CHC da Universidade Federal do Paraná - PR
69	ABONO (ACT)	CHC da Universidade Federal do Paraná - PR
69	ABONO (ACT)	CHC da Universidade Federal do Paraná - PR
69	ABONO (ACT)	CHC da Universidade Federal do Paraná - PR
69	ABONO (ACT)	CHC da Universidade Federal do Paraná - PR
69	ABONO (ACT)	CHC da Universidade Federal do Paraná - PR
69	ABONO (ACT)	CHC da Universidade Federal do Paraná - PR
69	ABONO (ACT)	CHC da Universidade Federal do Paraná - PR
69	ABONO (ACT)	CHC da Universidade Federal do Paraná - PR
69	ABONO (ACT)	CHC da Universidade Federal do Paraná - PR
69	ABONO (ACT)	CHC da Universidade Federal do Paraná - PR
69	ABONO (ACT)	CHC da Universidade Federal do Paraná - PR
69	ABONO (ACT)	CHC da Universidade Federal do Paraná - PR
69	ABONO (ACT)	CHC da Universidade Federal do Paraná - PR
69	ABONO (ACT)	CHC da Universidade Federal do Paraná - PR
69	ABONO (ACT)	CHC da Universidade Federal do Paraná - PR
69	ABONO (ACT)	CHC da Universidade Federal do Paraná - PR
69	ABONO (ACT)	CHC da Universidade Federal do Paraná - PR
69	ABONO (ACT)	CHC da Universidade Federal do Paraná - PR
69	ABONO (ACT)	CHC da Universidade Federal do Paraná - PR

69	ABONO (ACT)	CHC da Universidade Federal do Paraná - PR
69	ABONO (ACT)	CHC da Universidade Federal do Paraná - PR
69	ABONO (ACT)	CHC da Universidade Federal do Paraná - PR
69	ABONO (ACT)	CHC da Universidade Federal do Paraná - PR
69	ABONO (ACT)	CHC da Universidade Federal do Paraná - PR
69	ABONO (ACT)	CHC da Universidade Federal do Paraná - PR
69	ABONO (ACT)	CHC da Universidade Federal do Paraná - PR
69	ABONO (ACT)	CHC da Universidade Federal do Paraná - PR
69	ABONO (ACT)	CHC da Universidade Federal do Paraná - PR
69	ABONO (ACT)	CHC da Universidade Federal do Paraná - PR
69	ABONO (ACT)	CHC da Universidade Federal do Paraná - PR
69	ABONO (ACT)	CHC da Universidade Federal do Paraná - PR
69	ABONO (ACT)	CHC da Universidade Federal do Paraná - PR
69	ABONO (ACT)	CHC da Universidade Federal do Paraná - PR
69	ABONO (ACT)	CHC da Universidade Federal do Paraná - PR
69	ABONO (ACT)	CHC da Universidade Federal do Paraná - PR
69	ABONO (ACT)	CHC da Universidade Federal do Paraná - PR
69	ABONO (ACT)	CHC da Universidade Federal do Paraná - PR
69	ABONO (ACT)	CHC da Universidade Federal do Paraná - PR
69	ABONO (ACT)	CHC da Universidade Federal do Paraná - PR
69	ABONO (ACT)	CHC da Universidade Federal do Paraná - PR
69	ABONO (ACT)	CHC da Universidade Federal do Paraná - PR
69	ABONO (ACT)	CHC da Universidade Federal do Paraná - PR
69	ABONO (ACT)	CHC da Universidade Federal do Paraná - PR
69	ABONO (ACT)	CHC da Universidade Federal do Paraná - PR
69	ABONO (ACT)	CHC da Universidade Federal do Paraná - PR
69	ABONO (ACT)	CHC da Universidade Federal do Paraná - PR
69	ABONO (ACT)	CHC da Universidade Federal do Paraná - PR
69	ABONO (ACT)	CHC da Universidade Federal do Paraná - PR
69	ABONO (ACT)	CHC da Universidade Federal do Paraná - PR
69	ABONO (ACT)	CHC da Universidade Federal do Paraná - PR
69	ABONO (ACT)	CHC da Universidade Federal do Paraná - PR
69	ABONO (ACT)	CHC da Universidade Federal do Paraná - PR
69	ABONO (ACT)	CHC da Universidade Federal do Paraná - PR
69	ABONO (ACT)	CHC da Universidade Federal do Paraná - PR
69	ABONO (ACT)	CHC da Universidade Federal do Paraná - PR
69	ABONO (ACT)	CHC da Universidade Federal do Paraná - PR
69	ABONO (ACT)	CHC da Universidade Federal do Paraná - PR
69	ABONO (ACT)	CHC da Universidade Federal do Paraná - PR
69	ABONO (ACT)	CHC da Universidade Federal do Paraná - PR
69	ABONO (ACT)	CHC da Universidade Federal do Paraná - PR
69	ABONO (ACT)	CHC da Universidade Federal do Paraná - PR
69	ABONO (ACT)	CHC da Universidade Federal do Paraná - PR
69	ABONO (ACT)	CHC da Universidade Federal do Paraná - PR
69	ABONO (ACT)	CHC da Universidade Federal do Paraná - PR
69	ABONO (ACT)	CHC da Universidade Federal do Paraná - PR
69	ABONO (ACT)	CHC da Universidade Federal do Paraná - PR
69	ABONO (ACT)	CHC da Universidade Federal do Paraná - PR

69	ABONO (ACT)	CHC da Universidade Federal do Paraná - PR
69	ABONO (ACT)	CHC da Universidade Federal do Paraná - PR
69	ABONO (ACT)	CHC da Universidade Federal do Paraná - PR
69	ABONO (ACT)	CHC da Universidade Federal do Paraná - PR
69	ABONO (ACT)	CHC da Universidade Federal do Paraná - PR
69	ABONO (ACT)	CHC da Universidade Federal do Paraná - PR
69	ABONO (ACT)	CHC da Universidade Federal do Paraná - PR
69	ABONO (ACT)	CHC da Universidade Federal do Paraná - PR
69	ABONO (ACT)	CHC da Universidade Federal do Paraná - PR
69	ABONO (ACT)	CHC da Universidade Federal do Paraná - PR
69	ABONO (ACT)	CHC da Universidade Federal do Paraná - PR
69	ABONO (ACT)	CHC da Universidade Federal do Paraná - PR
69	ABONO (ACT)	CHC da Universidade Federal do Paraná - PR
69	ABONO (ACT)	CHC da Universidade Federal do Paraná - PR
69	ABONO (ACT)	CHC da Universidade Federal do Paraná - PR
69	ABONO (ACT)	CHC da Universidade Federal do Paraná - PR
69	ABONO (ACT)	CHC da Universidade Federal do Paraná - PR
69	ABONO (ACT)	CHC da Universidade Federal do Paraná - PR
69	ABONO (ACT)	CHC da Universidade Federal do Paraná - PR
69	ABONO (ACT)	CHC da Universidade Federal do Paraná - PR
69	ABONO (ACT)	CHC da Universidade Federal do Paraná - PR
69	ABONO (ACT)	CHC da Universidade Federal do Paraná - PR
69	ABONO (ACT)	CHC da Universidade Federal do Paraná - PR
69	ABONO (ACT)	CHC da Universidade Federal do Paraná - PR
69	ABONO (ACT)	CHC da Universidade Federal do Paraná - PR
69	ABONO (ACT)	CHC da Universidade Federal do Paraná - PR
69	ABONO (ACT)	CHC da Universidade Federal do Paraná - PR
69	ABONO (ACT)	CHC da Universidade Federal do Paraná - PR
69	ABONO (ACT)	CHC da Universidade Federal do Paraná - PR
69	ABONO (ACT)	CHC da Universidade Federal do Paraná - PR
69	ABONO (ACT)	CHC da Universidade Federal do Paraná - PR
69	ABONO (ACT)	CHC da Universidade Federal do Paraná - PR
69	ABONO (ACT)	CHC da Universidade Federal do Paraná - PR
69	ABONO (ACT)	CHC da Universidade Federal do Paraná - PR
69	ABONO (ACT)	CHC da Universidade Federal do Paraná - PR
69	ABONO (ACT)	CHC da Universidade Federal do Paraná - PR
69	ABONO (ACT)	CHC da Universidade Federal do Paraná - PR
69	ABONO (ACT)	CHC da Universidade Federal do Paraná - PR
69	ABONO (ACT)	CHC da Universidade Federal do Paraná - PR
69	ABONO (ACT)	CHC da Universidade Federal do Paraná - PR
69	ABONO (ACT)	CHC da Universidade Federal do Paraná - PR
69	ABONO (ACT)	CHC da Universidade Federal do Paraná - PR
69	ABONO (ACT)	CHC da Universidade Federal do Paraná - PR
69	ABONO (ACT)	CHC da Universidade Federal do Paraná - PR
69	ABONO (ACT)	CHC da Universidade Federal do Paraná - PR
69	ABONO (ACT)	CHC da Universidade Federal do Paraná - PR
69	ABONO (ACT)	CHC da Universidade Federal do Paraná - PR
69	ABONO (ACT)	CHC da Universidade Federal do Paraná - PR

69	ABONO (ACT)	CHC da Universidade Federal do Paraná - PR
69	ABONO (ACT)	CHC da Universidade Federal do Paraná - PR
69	ABONO (ACT)	CHC da Universidade Federal do Paraná - PR
69	ABONO (ACT)	CHC da Universidade Federal do Paraná - PR
69	ABONO (ACT)	CHC da Universidade Federal do Paraná - PR
69	ABONO (ACT)	CHC da Universidade Federal do Paraná - PR
69	ABONO (ACT)	CHC da Universidade Federal do Paraná - PR
69	ABONO (ACT)	CHC da Universidade Federal do Paraná - PR
69	ABONO (ACT)	CHC da Universidade Federal do Paraná - PR
69	ABONO (ACT)	CHC da Universidade Federal do Paraná - PR
69	ABONO (ACT)	CHC da Universidade Federal do Paraná - PR
69	ABONO (ACT)	CHC da Universidade Federal do Paraná - PR
69	ABONO (ACT)	CHC da Universidade Federal do Paraná - PR
69	ABONO (ACT)	CHC da Universidade Federal do Paraná - PR
69	ABONO (ACT)	CHC da Universidade Federal do Paraná - PR
69	ABONO (ACT)	CHC da Universidade Federal do Paraná - PR
69	ABONO (ACT)	CHC da Universidade Federal do Paraná - PR
69	ABONO (ACT)	CHC da Universidade Federal do Paraná - PR
69	ABONO (ACT)	CHC da Universidade Federal do Paraná - PR
69	ABONO (ACT)	CHC da Universidade Federal do Paraná - PR
69	ABONO (ACT)	CHC da Universidade Federal do Paraná - PR
69	ABONO (ACT)	CHC da Universidade Federal do Paraná - PR
69	ABONO (ACT)	CHC da Universidade Federal do Paraná - PR
69	ABONO (ACT)	CHC da Universidade Federal do Paraná - PR
69	ABONO (ACT)	CHC da Universidade Federal do Paraná - PR
69	ABONO (ACT)	CHC da Universidade Federal do Paraná - PR
69	ABONO (ACT)	CHC da Universidade Federal do Paraná - PR
69	ABONO (ACT)	CHC da Universidade Federal do Paraná - PR
69	ABONO (ACT)	CHC da Universidade Federal do Paraná - PR
69	ABONO (ACT)	CHC da Universidade Federal do Paraná - PR
69	ABONO (ACT)	CHC da Universidade Federal do Paraná - PR
69	ABONO (ACT)	CHC da Universidade Federal do Paraná - PR
69	ABONO (ACT)	CHC da Universidade Federal do Paraná - PR
69	ABONO (ACT)	CHC da Universidade Federal do Paraná - PR
69	ABONO (ACT)	CHC da Universidade Federal do Paraná - PR
69	ABONO (ACT)	CHC da Universidade Federal do Paraná - PR
69	ABONO (ACT)	CHC da Universidade Federal do Paraná - PR
69	ABONO (ACT)	CHC da Universidade Federal do Paraná - PR
69	ABONO (ACT)	CHC da Universidade Federal do Paraná - PR
69	ABONO (ACT)	CHC da Universidade Federal do Paraná - PR
69	ABONO (ACT)	CHC da Universidade Federal do Paraná - PR
69	ABONO (ACT)	CHC da Universidade Federal do Paraná - PR
69	ABONO (ACT)	CHC da Universidade Federal do Paraná - PR
69	ABONO (ACT)	CHC da Universidade Federal do Paraná - PR
69	ABONO (ACT)	CHC da Universidade Federal do Paraná - PR
69	ABONO (ACT)	CHC da Universidade Federal do Paraná - PR
69	ABONO (ACT)	CHC da Universidade Federal do Paraná - PR
69	ABONO (ACT)	CHC da Universidade Federal do Paraná - PR

69	ABONO (ACT)	CHC da Universidade Federal do Paraná - PR
69	ABONO (ACT)	CHC da Universidade Federal do Paraná - PR
69	ABONO (ACT)	CHC da Universidade Federal do Paraná - PR
69	ABONO (ACT)	CHC da Universidade Federal do Paraná - PR
69	ABONO (ACT)	CHC da Universidade Federal do Paraná - PR
69	ABONO (ACT)	CHC da Universidade Federal do Paraná - PR
69	ABONO (ACT)	CHC da Universidade Federal do Paraná - PR
69	ABONO (ACT)	CHC da Universidade Federal do Paraná - PR
69	ABONO (ACT)	CHC da Universidade Federal do Paraná - PR
69	ABONO (ACT)	CHC da Universidade Federal do Paraná - PR
69	ABONO (ACT)	CHC da Universidade Federal do Paraná - PR
69	ABONO (ACT)	CHC da Universidade Federal do Paraná - PR
69	ABONO (ACT)	CHC da Universidade Federal do Paraná - PR
69	ABONO (ACT)	CHC da Universidade Federal do Paraná - PR
69	ABONO (ACT)	CHC da Universidade Federal do Paraná - PR
69	ABONO (ACT)	CHC da Universidade Federal do Paraná - PR
69	ABONO (ACT)	CHC da Universidade Federal do Paraná - PR
69	ABONO (ACT)	CHC da Universidade Federal do Paraná - PR
69	ABONO (ACT)	CHC da Universidade Federal do Paraná - PR
69	ABONO (ACT)	CHC da Universidade Federal do Paraná - PR
69	ABONO (ACT)	CHC da Universidade Federal do Paraná - PR
69	ABONO (ACT)	CHC da Universidade Federal do Paraná - PR
69	ABONO (ACT)	CHC da Universidade Federal do Paraná - PR
69	ABONO (ACT)	CHC da Universidade Federal do Paraná - PR
69	ABONO (ACT)	CHC da Universidade Federal do Paraná - PR
69	ABONO (ACT)	CHC da Universidade Federal do Paraná - PR
69	ABONO (ACT)	CHC da Universidade Federal do Paraná - PR
69	ABONO (ACT)	CHC da Universidade Federal do Paraná - PR
69	ABONO (ACT)	CHC da Universidade Federal do Paraná - PR
69	ABONO (ACT)	CHC da Universidade Federal do Paraná - PR
69	ABONO (ACT)	CHC da Universidade Federal do Paraná - PR
69	ABONO (ACT)	CHC da Universidade Federal do Paraná - PR
69	ABONO (ACT)	CHC da Universidade Federal do Paraná - PR
69	ABONO (ACT)	CHC da Universidade Federal do Paraná - PR
69	ABONO (ACT)	CHC da Universidade Federal do Paraná - PR
69	ABONO (ACT)	CHC da Universidade Federal do Paraná - PR
69	ABONO (ACT)	CHC da Universidade Federal do Paraná - PR
69	ABONO (ACT)	CHC da Universidade Federal do Paraná - PR
69	ABONO (ACT)	CHC da Universidade Federal do Paraná - PR
69	ABONO (ACT)	CHC da Universidade Federal do Paraná - PR
69	ABONO (ACT)	CHC da Universidade Federal do Paraná - PR
69	ABONO (ACT)	CHC da Universidade Federal do Paraná - PR
69	ABONO (ACT)	CHC da Universidade Federal do Paraná - PR
69	ABONO (ACT)	CHC da Universidade Federal do Paraná - PR
69	ABONO (ACT)	CHC da Universidade Federal do Paraná - PR
69	ABONO (ACT)	CHC da Universidade Federal do Paraná - PR
69	ABONO (ACT)	CHC da Universidade Federal do Paraná - PR
69	ABONO (ACT)	CHC da Universidade Federal do Paraná - PR

69	ABONO (ACT)	CHC da Universidade Federal do Paraná - PR
69	ABONO (ACT)	CHC da Universidade Federal do Paraná - PR
69	ABONO (ACT)	CHC da Universidade Federal do Paraná - PR
69	ABONO (ACT)	CHC da Universidade Federal do Paraná - PR
69	ABONO (ACT)	CHC da Universidade Federal do Paraná - PR
69	ABONO (ACT)	CHC da Universidade Federal do Paraná - PR
69	ABONO (ACT)	CHC da Universidade Federal do Paraná - PR
69	ABONO (ACT)	CHC da Universidade Federal do Paraná - PR
69	ABONO (ACT)	CHC da Universidade Federal do Paraná - PR
69	ABONO (ACT)	CHC da Universidade Federal do Paraná - PR
69	ABONO (ACT)	CHC da Universidade Federal do Paraná - PR
69	ABONO (ACT)	CHC da Universidade Federal do Paraná - PR
69	ABONO (ACT)	CHC da Universidade Federal do Paraná - PR
69	ABONO (ACT)	CHC da Universidade Federal do Paraná - PR
69	ABONO (ACT)	CHC da Universidade Federal do Paraná - PR
69	ABONO (ACT)	CHC da Universidade Federal do Paraná - PR
69	ABONO (ACT)	CHC da Universidade Federal do Paraná - PR
69	ABONO (ACT)	CHC da Universidade Federal do Paraná - PR
69	ABONO (ACT)	CHC da Universidade Federal do Paraná - PR
69	ABONO (ACT)	CHC da Universidade Federal do Paraná - PR
69	ABONO (ACT)	CHC da Universidade Federal do Paraná - PR
69	ABONO (ACT)	CHC da Universidade Federal do Paraná - PR
69	ABONO (ACT)	CHC da Universidade Federal do Paraná - PR
69	ABONO (ACT)	CHC da Universidade Federal do Paraná - PR
69	ABONO (ACT)	CHC da Universidade Federal do Paraná - PR
69	ABONO (ACT)	CHC da Universidade Federal do Paraná - PR
69	ABONO (ACT)	CHC da Universidade Federal do Paraná - PR
69	ABONO (ACT)	CHC da Universidade Federal do Paraná - PR
69	ABONO (ACT)	CHC da Universidade Federal do Paraná - PR
69	ABONO (ACT)	CHC da Universidade Federal do Paraná - PR
69	ABONO (ACT)	CHC da Universidade Federal do Paraná - PR
69	ABONO (ACT)	CHC da Universidade Federal do Paraná - PR
69	ABONO (ACT)	CHC da Universidade Federal do Paraná - PR
69	ABONO (ACT)	CHC da Universidade Federal do Paraná - PR
69	ABONO (ACT)	CHC da Universidade Federal do Paraná - PR
69	ABONO (ACT)	CHC da Universidade Federal do Paraná - PR
69	ABONO (ACT)	CHC da Universidade Federal do Paraná - PR
69	ABONO (ACT)	CHC da Universidade Federal do Paraná - PR
69	ABONO (ACT)	CHC da Universidade Federal do Paraná - PR
69	ABONO (ACT)	CHC da Universidade Federal do Paraná - PR
69	ABONO (ACT)	CHC da Universidade Federal do Paraná - PR
69	ABONO (ACT)	CHC da Universidade Federal do Paraná - PR
69	ABONO (ACT)	CHC da Universidade Federal do Paraná - PR

69	ABONO (ACT)	CHC da Universidade Federal do Paraná - PR
69	ABONO (ACT)	CHC da Universidade Federal do Paraná - PR
69	ABONO (ACT)	CHC da Universidade Federal do Paraná - PR
69	ABONO (ACT)	CHC da Universidade Federal do Paraná - PR
69	ABONO (ACT)	CHC da Universidade Federal do Paraná - PR
69	ABONO (ACT)	CHC da Universidade Federal do Paraná - PR
69	ABONO (ACT)	CHC da Universidade Federal do Paraná - PR
69	ABONO (ACT)	CHC da Universidade Federal do Paraná - PR
69	ABONO (ACT)	CHC da Universidade Federal do Paraná - PR
69	ABONO (ACT)	CHC da Universidade Federal do Paraná - PR
69	ABONO (ACT)	CHC da Universidade Federal do Paraná - PR
69	ABONO (ACT)	CHC da Universidade Federal do Paraná - PR
69	ABONO (ACT)	CHC da Universidade Federal do Paraná - PR
69	ABONO (ACT)	CHC da Universidade Federal do Paraná - PR
69	ABONO (ACT)	CHC da Universidade Federal do Paraná - PR
69	ABONO (ACT)	CHC da Universidade Federal do Paraná - PR
69	ABONO (ACT)	CHC da Universidade Federal do Paraná - PR
69	ABONO (ACT)	CHC da Universidade Federal do Paraná - PR
69	ABONO (ACT)	CHC da Universidade Federal do Paraná - PR
69	ABONO (ACT)	CHC da Universidade Federal do Paraná - PR
69	ABONO (ACT)	CHC da Universidade Federal do Paraná - PR
69	ABONO (ACT)	CHC da Universidade Federal do Paraná - PR
69	ABONO (ACT)	CHC da Universidade Federal do Paraná - PR
69	ABONO (ACT)	CHC da Universidade Federal do Paraná - PR
69	ABONO (ACT)	CHC da Universidade Federal do Paraná - PR
69	ABONO (ACT)	CHC da Universidade Federal do Paraná - PR
69	ABONO (ACT)	CHC da Universidade Federal do Paraná - PR
69	ABONO (ACT)	CHC da Universidade Federal do Paraná - PR
69	ABONO (ACT)	CHC da Universidade Federal do Paraná - PR
69	ABONO (ACT)	CHC da Universidade Federal do Paraná - PR
69	ABONO (ACT)	CHC da Universidade Federal do Paraná - PR
69	ABONO (ACT)	CHC da Universidade Federal do Paraná - PR

69	ABONO (ACT)	CHC da Universidade Federal do Paraná - PR
69	ABONO (ACT)	CHC da Universidade Federal do Paraná - PR
69	ABONO (ACT)	CHC da Universidade Federal do Paraná - PR
69	ABONO (ACT)	CHC da Universidade Federal do Paraná - PR
69	ABONO (ACT)	CHC da Universidade Federal do Paraná - PR
69	ABONO (ACT)	CHC da Universidade Federal do Paraná - PR
69	ABONO (ACT)	CHC da Universidade Federal do Paraná - PR
69	ABONO (ACT)	CHC da Universidade Federal do Paraná - PR
69	ABONO (ACT)	CHC da Universidade Federal do Paraná - PR
69	ABONO (ACT)	CHC da Universidade Federal do Paraná - PR
69	ABONO (ACT)	CHC da Universidade Federal do Paraná - PR
69	ABONO (ACT)	CHC da Universidade Federal do Paraná - PR
69	ABONO (ACT)	CHC da Universidade Federal do Paraná - PR
69	ABONO (ACT)	CHC da Universidade Federal do Paraná - PR
69	ABONO (ACT)	CHC da Universidade Federal do Paraná - PR
69	ABONO (ACT)	CHC da Universidade Federal do Paraná - PR
69	ABONO (ACT)	CHC da Universidade Federal do Paraná - PR
69	ABONO (ACT)	CHC da Universidade Federal do Paraná - PR
69	ABONO (ACT)	CHC da Universidade Federal do Paraná - PR
69	ABONO (ACT)	CHC da Universidade Federal do Paraná - PR
69	ABONO (ACT)	CHC da Universidade Federal do Paraná - PR
69	ABONO (ACT)	CHC da Universidade Federal do Paraná - PR
69	ABONO (ACT)	CHC da Universidade Federal do Paraná - PR
69	ABONO (ACT)	CHC da Universidade Federal do Paraná - PR
69	ABONO (ACT)	CHC da Universidade Federal do Paraná - PR
69	ABONO (ACT)	CHC da Universidade Federal do Paraná - PR
69	ABONO (ACT)	CHC da Universidade Federal do Paraná - PR
69	ABONO (ACT)	CHC da Universidade Federal do Paraná - PR
69	ABONO (ACT)	CHC da Universidade Federal do Paraná - PR
69	ABONO (ACT)	CHC da Universidade Federal do Paraná - PR
69	ABONO (ACT)	CHC da Universidade Federal do Paraná - PR
69	ABONO (ACT)	CHC da Universidade Federal do Paraná - PR
69	ABONO (ACT)	CHC da Universidade Federal do Paraná - PR
69	ABONO (ACT)	CHC da Universidade Federal do Paraná - PR
69	ABONO (ACT)	CHC da Universidade Federal do Paraná - PR
69	ABONO (ACT)	CHC da Universidade Federal do Paraná - PR
69	ABONO (ACT)	CHC da Universidade Federal do Paraná - PR
69	ABONO (ACT)	CHC da Universidade Federal do Paraná - PR
69	ABONO (ACT)	CHC da Universidade Federal do Paraná - PR
69	ABONO (ACT)	CHC da Universidade Federal do Paraná - PR
69	ABONO (ACT)	CHC da Universidade Federal do Paraná - PR
69	ABONO (ACT)	CHC da Universidade Federal do Paraná - PR
69	ABONO (ACT)	CHC da Universidade Federal do Paraná - PR
69	ABONO (ACT)	CHC da Universidade Federal do Paraná - PR
69	ABONO (ACT)	CHC da Universidade Federal do Paraná - PR
69	ABONO (ACT)	CHC da Universidade Federal do Paraná - PR
69	ABONO (ACT)	CHC da Universidade Federal do Paraná - PR

69	ABONO (ACT)	CHC da Universidade Federal do Paraná - PR
69	ABONO (ACT)	CHC da Universidade Federal do Paraná - PR
69	ABONO (ACT)	CHC da Universidade Federal do Paraná - PR
69	ABONO (ACT)	CHC da Universidade Federal do Paraná - PR
69	ABONO (ACT)	CHC da Universidade Federal do Paraná - PR
69	ABONO (ACT)	CHC da Universidade Federal do Paraná - PR
69	ABONO (ACT)	CHC da Universidade Federal do Paraná - PR
69	ABONO (ACT)	CHC da Universidade Federal do Paraná - PR
69	ABONO (ACT)	CHC da Universidade Federal do Paraná - PR
69	ABONO (ACT)	CHC da Universidade Federal do Paraná - PR
69	ABONO (ACT)	CHC da Universidade Federal do Paraná - PR
69	ABONO (ACT)	CHC da Universidade Federal do Paraná - PR
69	ABONO (ACT)	CHC da Universidade Federal do Paraná - PR
69	ABONO (ACT)	CHC da Universidade Federal do Paraná - PR
69	ABONO (ACT)	CHC da Universidade Federal do Paraná - PR
69	ABONO (ACT)	CHC da Universidade Federal do Paraná - PR
69	ABONO (ACT)	CHC da Universidade Federal do Paraná - PR
69	ABONO (ACT)	CHC da Universidade Federal do Paraná - PR
69	ABONO (ACT)	CHC da Universidade Federal do Paraná - PR
69	ABONO (ACT)	CHC da Universidade Federal do Paraná - PR
69	ABONO (ACT)	CHC da Universidade Federal do Paraná - PR
69	ABONO (ACT)	CHC da Universidade Federal do Paraná - PR
69	ABONO (ACT)	CHC da Universidade Federal do Paraná - PR
69	ABONO (ACT)	CHC da Universidade Federal do Paraná - PR
69	ABONO (ACT)	CHC da Universidade Federal do Paraná - PR
69	ABONO (ACT)	CHC da Universidade Federal do Paraná - PR
69	ABONO (ACT)	CHC da Universidade Federal do Paraná - PR
69	ABONO (ACT)	CHC da Universidade Federal do Paraná - PR
69	ABONO (ACT)	CHC da Universidade Federal do Paraná - PR
69	ABONO (ACT)	CHC da Universidade Federal do Paraná - PR
69	ABONO (ACT)	CHC da Universidade Federal do Paraná - PR
69	ABONO (ACT)	CHC da Universidade Federal do Paraná - PR
69	ABONO (ACT)	CHC da Universidade Federal do Paraná - PR
69	ABONO (ACT)	CHC da Universidade Federal do Paraná - PR
69	ABONO (ACT)	CHC da Universidade Federal do Paraná - PR
69	ABONO (ACT)	CHC da Universidade Federal do Paraná - PR
69	ABONO (ACT)	CHC da Universidade Federal do Paraná - PR
69	ABONO (ACT)	CHC da Universidade Federal do Paraná - PR
69	ABONO (ACT)	CHC da Universidade Federal do Paraná - PR
69	ABONO (ACT)	CHC da Universidade Federal do Paraná - PR
69	ABONO (ACT)	CHC da Universidade Federal do Paraná - PR
69	ABONO (ACT)	CHC da Universidade Federal do Paraná - PR
69	ABONO (ACT)	CHC da Universidade Federal do Paraná - PR
69	ABONO (ACT)	CHC da Universidade Federal do Paraná - PR
69	ABONO (ACT)	CHC da Universidade Federal do Paraná - PR
69	ABONO (ACT)	CHC da Universidade Federal do Paraná - PR
69	ABONO (ACT)	CHC da Universidade Federal do Paraná - PR
69	ABONO (ACT)	CHC da Universidade Federal do Paraná - PR

69	ABONO (ACT)	CHC da Universidade Federal do Paraná - PR
69	ABONO (ACT)	CHC da Universidade Federal do Paraná - PR
69	ABONO (ACT)	CHC da Universidade Federal do Paraná - PR
69	ABONO (ACT)	CHC da Universidade Federal do Paraná - PR
69	ABONO (ACT)	CHC da Universidade Federal do Paraná - PR
69	ABONO (ACT)	CHC da Universidade Federal do Paraná - PR
69	ABONO (ACT)	CHC da Universidade Federal do Paraná - PR
69	ABONO (ACT)	CHC da Universidade Federal do Paraná - PR
69	ABONO (ACT)	CHC da Universidade Federal do Paraná - PR
69	ABONO (ACT)	CHC da Universidade Federal do Paraná - PR
69	ABONO (ACT)	CHC da Universidade Federal do Paraná - PR
69	ABONO (ACT)	CHC da Universidade Federal do Paraná - PR
69	ABONO (ACT)	CHC da Universidade Federal do Paraná - PR
69	ABONO (ACT)	CHC da Universidade Federal do Paraná - PR
69	ABONO (ACT)	CHC da Universidade Federal do Paraná - PR
69	ABONO (ACT)	CHC da Universidade Federal do Paraná - PR
69	ABONO (ACT)	CHC da Universidade Federal do Paraná - PR
69	ABONO (ACT)	CHC da Universidade Federal do Paraná - PR
69	ABONO (ACT)	CHC da Universidade Federal do Paraná - PR
69	ABONO (ACT)	CHC da Universidade Federal do Paraná - PR
69	ABONO (ACT)	CHC da Universidade Federal do Paraná - PR
69	ABONO (ACT)	CHC da Universidade Federal do Paraná - PR
69	ABONO (ACT)	CHC da Universidade Federal do Paraná - PR
69	ABONO (ACT)	CHC da Universidade Federal do Paraná - PR
69	ABONO (ACT)	CHC da Universidade Federal do Paraná - PR
69	ABONO (ACT)	CHC da Universidade Federal do Paraná - PR
69	ABONO (ACT)	CHC da Universidade Federal do Paraná - PR
69	ABONO (ACT)	CHC da Universidade Federal do Paraná - PR
69	ABONO (ACT)	CHC da Universidade Federal do Paraná - PR
69	ABONO (ACT)	CHC da Universidade Federal do Paraná - PR
69	ABONO (ACT)	CHC da Universidade Federal do Paraná - PR
69	ABONO (ACT)	CHC da Universidade Federal do Paraná - PR
69	ABONO (ACT)	CHC da Universidade Federal do Paraná - PR
69	ABONO (ACT)	CHC da Universidade Federal do Paraná - PR
69	ABONO (ACT)	CHC da Universidade Federal do Paraná - PR
69	ABONO (ACT)	CHC da Universidade Federal do Paraná - PR
69	ABONO (ACT)	CHC da Universidade Federal do Paraná - PR
69	ABONO (ACT)	CHC da Universidade Federal do Paraná - PR
69	ABONO (ACT)	CHC da Universidade Federal do Paraná - PR
69	ABONO (ACT)	CHC da Universidade Federal do Paraná - PR
69	ABONO (ACT)	CHC da Universidade Federal do Paraná - PR
69	ABONO (ACT)	CHC da Universidade Federal do Paraná - PR
69	ABONO (ACT)	CHC da Universidade Federal do Paraná - PR
69	ABONO (ACT)	CHC da Universidade Federal do Paraná - PR
69	ABONO (ACT)	CHC da Universidade Federal do Paraná - PR
69	ABONO (ACT)	CHC da Universidade Federal do Paraná - PR
69	ABONO (ACT)	CHC da Universidade Federal do Paraná - PR
69	ABONO (ACT)	CHC da Universidade Federal do Paraná - PR

69	ABONO (ACT)	CHC da Universidade Federal do Paraná - PR
69	ABONO (ACT)	CHC da Universidade Federal do Paraná - PR
69	ABONO (ACT)	CHC da Universidade Federal do Paraná - PR
69	ABONO (ACT)	CHC da Universidade Federal do Paraná - PR
69	ABONO (ACT)	CHC da Universidade Federal do Paraná - PR
69	ABONO (ACT)	CHC da Universidade Federal do Paraná - PR
69	ABONO (ACT)	CHC da Universidade Federal do Paraná - PR
69	ABONO (ACT)	CHC da Universidade Federal do Paraná - PR
69	ABONO (ACT)	CHC da Universidade Federal do Paraná - PR
69	ABONO (ACT)	CHC da Universidade Federal do Paraná - PR
69	ABONO (ACT)	CHC da Universidade Federal do Paraná - PR
69	ABONO (ACT)	CHC da Universidade Federal do Paraná - PR
69	ABONO (ACT)	CHC da Universidade Federal do Paraná - PR
69	ABONO (ACT)	CHC da Universidade Federal do Paraná - PR
69	ABONO (ACT)	CHC da Universidade Federal do Paraná - PR
69	ABONO (ACT)	CHC da Universidade Federal do Paraná - PR
69	ABONO (ACT)	CHC da Universidade Federal do Paraná - PR
69	ABONO (ACT)	CHC da Universidade Federal do Paraná - PR
69	ABONO (ACT)	CHC da Universidade Federal do Paraná - PR
69	ABONO (ACT)	CHC da Universidade Federal do Paraná - PR
69	ABONO (ACT)	CHC da Universidade Federal do Paraná - PR
69	ABONO (ACT)	CHC da Universidade Federal do Paraná - PR
69	ABONO (ACT)	CHC da Universidade Federal do Paraná - PR
69	ABONO (ACT)	CHC da Universidade Federal do Paraná - PR
69	ABONO (ACT)	CHC da Universidade Federal do Paraná - PR
69	ABONO (ACT)	CHC da Universidade Federal do Paraná - PR
69	ABONO (ACT)	CHC da Universidade Federal do Paraná - PR
69	ABONO (ACT)	CHC da Universidade Federal do Paraná - PR
69	ABONO (ACT)	CHC da Universidade Federal do Paraná - PR
69	ABONO (ACT)	CHC da Universidade Federal do Paraná - PR
69	ABONO (ACT)	CHC da Universidade Federal do Paraná - PR
69	ABONO (ACT)	CHC da Universidade Federal do Paraná - PR
69	ABONO (ACT)	CHC da Universidade Federal do Paraná - PR
69	ABONO (ACT)	CHC da Universidade Federal do Paraná - PR
69	ABONO (ACT)	CHC da Universidade Federal do Paraná - PR
69	ABONO (ACT)	CHC da Universidade Federal do Paraná - PR
69	ABONO (ACT)	CHC da Universidade Federal do Paraná - PR
69	ABONO (ACT)	CHC da Universidade Federal do Paraná - PR
69	ABONO (ACT)	CHC da Universidade Federal do Paraná - PR
69	ABONO (ACT)	CHC da Universidade Federal do Paraná - PR
69	ABONO (ACT)	CHC da Universidade Federal do Paraná - PR
69	ABONO (ACT)	CHC da Universidade Federal do Paraná - PR
69	ABONO (ACT)	CHC da Universidade Federal do Paraná - PR
69	ABONO (ACT)	CHC da Universidade Federal do Paraná - PR
69	ABONO (ACT)	CHC da Universidade Federal do Paraná - PR
69	ABONO (ACT)	CHC da Universidade Federal do Paraná - PR
69	ABONO (ACT)	CHC da Universidade Federal do Paraná - PR
69	ABONO (ACT)	CHC da Universidade Federal do Paraná - PR

69	ABONO (ACT)	CHC da Universidade Federal do Paraná - PR
69	ABONO (ACT)	CHC da Universidade Federal do Paraná - PR
69	ABONO (ACT)	CHC da Universidade Federal do Paraná - PR
69	ABONO (ACT)	CHC da Universidade Federal do Paraná - PR
69	ABONO (ACT)	CHC da Universidade Federal do Paraná - PR
69	ABONO (ACT)	CHC da Universidade Federal do Paraná - PR
69	ABONO (ACT)	CHC da Universidade Federal do Paraná - PR
69	ABONO (ACT)	CHC da Universidade Federal do Paraná - PR
69	ABONO (ACT)	CHC da Universidade Federal do Paraná - PR
69	ABONO (ACT)	CHC da Universidade Federal do Paraná - PR
69	ABONO (ACT)	CHC da Universidade Federal do Paraná - PR
69	ABONO (ACT)	CHC da Universidade Federal do Paraná - PR
69	ABONO (ACT)	CHC da Universidade Federal do Paraná - PR
69	ABONO (ACT)	CHC da Universidade Federal do Paraná - PR
69	ABONO (ACT)	CHC da Universidade Federal do Paraná - PR
69	ABONO (ACT)	CHC da Universidade Federal do Paraná - PR
69	ABONO (ACT)	CHC da Universidade Federal do Paraná - PR
69	ABONO (ACT)	CHC da Universidade Federal do Paraná - PR
69	ABONO (ACT)	CHC da Universidade Federal do Paraná - PR
69	ABONO (ACT)	CHC da Universidade Federal do Paraná - PR
69	ABONO (ACT)	CHC da Universidade Federal do Paraná - PR
69	ABONO (ACT)	CHC da Universidade Federal do Paraná - PR
69	ABONO (ACT)	CHC da Universidade Federal do Paraná - PR
69	ABONO (ACT)	CHC da Universidade Federal do Paraná - PR
69	ABONO (ACT)	CHC da Universidade Federal do Paraná - PR
69	ABONO (ACT)	CHC da Universidade Federal do Paraná - PR
69	ABONO (ACT)	CHC da Universidade Federal do Paraná - PR
69	ABONO (ACT)	CHC da Universidade Federal do Paraná - PR
69	ABONO (ACT)	CHC da Universidade Federal do Paraná - PR
69	ABONO (ACT)	CHC da Universidade Federal do Paraná - PR
69	ABONO (ACT)	CHC da Universidade Federal do Paraná - PR
69	ABONO (ACT)	CHC da Universidade Federal do Paraná - PR
69	ABONO (ACT)	CHC da Universidade Federal do Paraná - PR
69	ABONO (ACT)	CHC da Universidade Federal do Paraná - PR
69	ABONO (ACT)	CHC da Universidade Federal do Paraná - PR
69	ABONO (ACT)	CHC da Universidade Federal do Paraná - PR
69	ABONO (ACT)	CHC da Universidade Federal do Paraná - PR
69	ABONO (ACT)	CHC da Universidade Federal do Paraná - PR
69	ABONO (ACT)	CHC da Universidade Federal do Paraná - PR
69	ABONO (ACT)	CHC da Universidade Federal do Paraná - PR
69	ABONO (ACT)	CHC da Universidade Federal do Paraná - PR
69	ABONO (ACT)	CHC da Universidade Federal do Paraná - PR
69	ABONO (ACT)	CHC da Universidade Federal do Paraná - PR
69	ABONO (ACT)	CHC da Universidade Federal do Paraná - PR
69	ABONO (ACT)	CHC da Universidade Federal do Paraná - PR
69	ABONO (ACT)	CHC da Universidade Federal do Paraná - PR
69	ABONO (ACT)	CHC da Universidade Federal do Paraná - PR
69	ABONO (ACT)	CHC da Universidade Federal do Paraná - PR

69	ABONO (ACT)	CHC da Universidade Federal do Paraná - PR
69	ABONO (ACT)	CHC da Universidade Federal do Paraná - PR
69	ABONO (ACT)	CHC da Universidade Federal do Paraná - PR
69	ABONO (ACT)	CHC da Universidade Federal do Paraná - PR
69	ABONO (ACT)	CHC da Universidade Federal do Paraná - PR
69	ABONO (ACT)	CHC da Universidade Federal do Paraná - PR
69	ABONO (ACT)	CHC da Universidade Federal do Paraná - PR
69	ABONO (ACT)	CHC da Universidade Federal do Paraná - PR
69	ABONO (ACT)	CHC da Universidade Federal do Paraná - PR
69	ABONO (ACT)	CHC da Universidade Federal do Paraná - PR
69	ABONO (ACT)	CHC da Universidade Federal do Paraná - PR
69	ABONO (ACT)	CHC da Universidade Federal do Paraná - PR
69	ABONO (ACT)	CHC da Universidade Federal do Paraná - PR
69	ABONO (ACT)	CHC da Universidade Federal do Paraná - PR
69	ABONO (ACT)	CHC da Universidade Federal do Paraná - PR
69	ABONO (ACT)	CHC da Universidade Federal do Paraná - PR
69	ABONO (ACT)	CHC da Universidade Federal do Paraná - PR
69	ABONO (ACT)	CHC da Universidade Federal do Paraná - PR
69	ABONO (ACT)	CHC da Universidade Federal do Paraná - PR
69	ABONO (ACT)	CHC da Universidade Federal do Paraná - PR
69	ABONO (ACT)	CHC da Universidade Federal do Paraná - PR
69	ABONO (ACT)	CHC da Universidade Federal do Paraná - PR
69	ABONO (ACT)	CHC da Universidade Federal do Paraná - PR
69	ABONO (ACT)	CHC da Universidade Federal do Paraná - PR
69	ABONO (ACT)	CHC da Universidade Federal do Paraná - PR
69	ABONO (ACT)	CHC da Universidade Federal do Paraná - PR
69	ABONO (ACT)	CHC da Universidade Federal do Paraná - PR
69	ABONO (ACT)	CHC da Universidade Federal do Paraná - PR
69	ABONO (ACT)	CHC da Universidade Federal do Paraná - PR
69	ABONO (ACT)	CHC da Universidade Federal do Paraná - PR
69	ABONO (ACT)	CHC da Universidade Federal do Paraná - PR
69	ABONO (ACT)	CHC da Universidade Federal do Paraná - PR
69	ABONO (ACT)	CHC da Universidade Federal do Paraná - PR
69	ABONO (ACT)	CHC da Universidade Federal do Paraná - PR
69	ABONO (ACT)	CHC da Universidade Federal do Paraná - PR
69	ABONO (ACT)	CHC da Universidade Federal do Paraná - PR
69	ABONO (ACT)	CHC da Universidade Federal do Paraná - PR
69	ABONO (ACT)	CHC da Universidade Federal do Paraná - PR
69	ABONO (ACT)	CHC da Universidade Federal do Paraná - PR
69	ABONO (ACT)	CHC da Universidade Federal do Paraná - PR
69	ABONO (ACT)	CHC da Universidade Federal do Paraná - PR
69	ABONO (ACT)	CHC da Universidade Federal do Paraná - PR
69	ABONO (ACT)	CHC da Universidade Federal do Paraná - PR
69	ABONO (ACT)	CHC da Universidade Federal do Paraná - PR
69	ABONO (ACT)	CHC da Universidade Federal do Paraná - PR
69	ABONO (ACT)	CHC da Universidade Federal do Paraná - PR
69	ABONO (ACT)	CHC da Universidade Federal do Paraná - PR
69	ABONO (ACT)	CHC da Universidade Federal do Paraná - PR

69	ABONO (ACT)	CHC da Universidade Federal do Paraná - PR
69	ABONO (ACT)	CHC da Universidade Federal do Paraná - PR
69	ABONO (ACT)	CHC da Universidade Federal do Paraná - PR
69	ABONO (ACT)	CHC da Universidade Federal do Paraná - PR
69	ABONO (ACT)	CHC da Universidade Federal do Paraná - PR
69	ABONO (ACT)	CHC da Universidade Federal do Paraná - PR
69	ABONO (ACT)	CHC da Universidade Federal do Paraná - PR
69	ABONO (ACT)	CHC da Universidade Federal do Paraná - PR
69	ABONO (ACT)	CHC da Universidade Federal do Paraná - PR
69	ABONO (ACT)	CHC da Universidade Federal do Paraná - PR
69	ABONO (ACT)	CHC da Universidade Federal do Paraná - PR
69	ABONO (ACT)	CHC da Universidade Federal do Paraná - PR
69	ABONO (ACT)	CHC da Universidade Federal do Paraná - PR
69	ABONO (ACT)	CHC da Universidade Federal do Paraná - PR
69	ABONO (ACT)	CHC da Universidade Federal do Paraná - PR
69	ABONO (ACT)	CHC da Universidade Federal do Paraná - PR
69	ABONO (ACT)	CHC da Universidade Federal do Paraná - PR
69	ABONO (ACT)	CHC da Universidade Federal do Paraná - PR
69	ABONO (ACT)	CHC da Universidade Federal do Paraná - PR
70	AUSÊNCIA COM COMPENSAÇÃO	CHC da Universidade Federal do Paraná - PR
71	Repouso Remunerado - Artigo 395 CLT - 14 dias	CHC da Universidade Federal do Paraná - PR
71	Repouso Remunerado - Artigo 395 CLT - 14 dias	CHC da Universidade Federal do Paraná - PR
71	Repouso Remunerado - Artigo 395 CLT - 14 dias	CHC da Universidade Federal do Paraná - PR
71	Repouso Remunerado - Artigo 395 CLT - 14 dias	CHC da Universidade Federal do Paraná - PR
72	CONSULTA PRE NATAL - ATESTADO MEDICO	CHC da Universidade Federal do Paraná - PR
72	CONSULTA PRE NATAL - ATESTADO MEDICO	CHC da Universidade Federal do Paraná - PR
72	CONSULTA PRE NATAL - ATESTADO MEDICO	CHC da Universidade Federal do Paraná - PR
72	CONSULTA PRE NATAL - ATESTADO MEDICO	CHC da Universidade Federal do Paraná - PR
72	CONSULTA PRE NATAL - ATESTADO MEDICO	CHC da Universidade Federal do Paraná - PR
72	CONSULTA PRE NATAL - ATESTADO MEDICO	CHC da Universidade Federal do Paraná - PR
72	CONSULTA PRE NATAL - ATESTADO MEDICO	CHC da Universidade Federal do Paraná - PR
72	CONSULTA PRE NATAL - ATESTADO MEDICO	CHC da Universidade Federal do Paraná - PR
72	CONSULTA PRE NATAL - ATESTADO MEDICO	CHC da Universidade Federal do Paraná - PR
72	CONSULTA PRE NATAL - ATESTADO MEDICO	CHC da Universidade Federal do Paraná - PR
72	CONSULTA PRE NATAL - ATESTADO MEDICO	CHC da Universidade Federal do Paraná - PR
72	CONSULTA PRE NATAL - ATESTADO MEDICO	CHC da Universidade Federal do Paraná - PR
72	CONSULTA PRE NATAL - ATESTADO MEDICO	CHC da Universidade Federal do Paraná - PR
72	CONSULTA PRE NATAL - ATESTADO MEDICO	CHC da Universidade Federal do Paraná - PR
72	CONSULTA PRE NATAL - ATESTADO MEDICO	CHC da Universidade Federal do Paraná - PR
72	CONSULTA PRE NATAL - ATESTADO MEDICO	CHC da Universidade Federal do Paraná - PR
72	CONSULTA PRE NATAL - ATESTADO MEDICO	CHC da Universidade Federal do Paraná - PR
72	CONSULTA PRE NATAL - ATESTADO MEDICO	CHC da Universidade Federal do Paraná - PR
72	CONSULTA PRE NATAL - ATESTADO MEDICO	CHC da Universidade Federal do Paraná - PR
72	CONSULTA PRE NATAL - ATESTADO MEDICO	CHC da Universidade Federal do Paraná - PR
72	CONSULTA PRE NATAL - ATESTADO MEDICO	CHC da Universidade Federal do Paraná - PR
72	CONSULTA PRE NATAL - ATESTADO MEDICO	CHC da Universidade Federal do Paraná - PR
72	CONSULTA PRE NATAL - ATESTADO MEDICO	CHC da Universidade Federal do Paraná - PR

72	CONSULTA PRE NATAL - ATESTADO MEDICO	CHC da Universidade Federal do Paraná - PR
72	CONSULTA PRE NATAL - ATESTADO MEDICO	CHC da Universidade Federal do Paraná - PR
72	CONSULTA PRE NATAL - ATESTADO MEDICO	CHC da Universidade Federal do Paraná - PR
72	CONSULTA PRE NATAL - ATESTADO MEDICO	CHC da Universidade Federal do Paraná - PR
72	CONSULTA PRE NATAL - ATESTADO MEDICO	CHC da Universidade Federal do Paraná - PR
72	CONSULTA PRE NATAL - ATESTADO MEDICO	CHC da Universidade Federal do Paraná - PR
72	CONSULTA PRE NATAL - ATESTADO MEDICO	CHC da Universidade Federal do Paraná - PR
72	CONSULTA PRE NATAL - ATESTADO MEDICO	CHC da Universidade Federal do Paraná - PR
72	CONSULTA PRE NATAL - ATESTADO MEDICO	CHC da Universidade Federal do Paraná - PR
72	CONSULTA PRE NATAL - ATESTADO MEDICO	CHC da Universidade Federal do Paraná - PR
72	CONSULTA PRE NATAL - ATESTADO MEDICO	CHC da Universidade Federal do Paraná - PR
72	CONSULTA PRE NATAL - ATESTADO MEDICO	CHC da Universidade Federal do Paraná - PR
72	CONSULTA PRE NATAL - ATESTADO MEDICO	CHC da Universidade Federal do Paraná - PR
72	CONSULTA PRE NATAL - ATESTADO MEDICO	CHC da Universidade Federal do Paraná - PR
72	CONSULTA PRE NATAL - ATESTADO MEDICO	CHC da Universidade Federal do Paraná - PR
72	CONSULTA PRE NATAL - ATESTADO MEDICO	CHC da Universidade Federal do Paraná - PR
72	CONSULTA PRE NATAL - ATESTADO MEDICO	CHC da Universidade Federal do Paraná - PR
72	CONSULTA PRE NATAL - ATESTADO MEDICO	CHC da Universidade Federal do Paraná - PR
72	CONSULTA PRE NATAL - ATESTADO MEDICO	CHC da Universidade Federal do Paraná - PR
72	CONSULTA PRE NATAL - ATESTADO MEDICO	CHC da Universidade Federal do Paraná - PR
72	CONSULTA PRE NATAL - ATESTADO MEDICO	CHC da Universidade Federal do Paraná - PR
72	CONSULTA PRE NATAL - ATESTADO MEDICO	CHC da Universidade Federal do Paraná - PR
72	CONSULTA PRE NATAL - ATESTADO MEDICO	CHC da Universidade Federal do Paraná - PR
72	CONSULTA PRE NATAL - ATESTADO MEDICO	CHC da Universidade Federal do Paraná - PR
72	CONSULTA PRE NATAL - ATESTADO MEDICO	CHC da Universidade Federal do Paraná - PR
72	CONSULTA PRE NATAL - ATESTADO MEDICO	CHC da Universidade Federal do Paraná - PR
72	CONSULTA PRE NATAL - ATESTADO MEDICO	CHC da Universidade Federal do Paraná - PR
72	CONSULTA PRE NATAL - ATESTADO MEDICO	CHC da Universidade Federal do Paraná - PR
72	CONSULTA PRE NATAL - ATESTADO MEDICO	CHC da Universidade Federal do Paraná - PR
72	CONSULTA PRE NATAL - ATESTADO MEDICO	CHC da Universidade Federal do Paraná - PR
72	CONSULTA PRE NATAL - ATESTADO MEDICO	CHC da Universidade Federal do Paraná - PR
72	CONSULTA PRE NATAL - ATESTADO MEDICO	CHC da Universidade Federal do Paraná - PR
72	CONSULTA PRE NATAL - ATESTADO MEDICO	CHC da Universidade Federal do Paraná - PR
72	CONSULTA PRE NATAL - ATESTADO MEDICO	CHC da Universidade Federal do Paraná - PR
72	CONSULTA PRE NATAL - ATESTADO MEDICO	CHC da Universidade Federal do Paraná - PR
72	CONSULTA PRE NATAL - ATESTADO MEDICO	CHC da Universidade Federal do Paraná - PR
72	CONSULTA PRE NATAL - ATESTADO MEDICO	CHC da Universidade Federal do Paraná - PR
72	CONSULTA PRE NATAL - ATESTADO MEDICO	CHC da Universidade Federal do Paraná - PR
72	CONSULTA PRE NATAL - ATESTADO MEDICO	CHC da Universidade Federal do Paraná - PR
72	CONSULTA PRE NATAL - ATESTADO MEDICO	CHC da Universidade Federal do Paraná - PR
72	CONSULTA PRE NATAL - ATESTADO MEDICO	CHC da Universidade Federal do Paraná - PR
72	CONSULTA PRE NATAL - ATESTADO MEDICO	CHC da Universidade Federal do Paraná - PR
72	CONSULTA PRE NATAL - ATESTADO MEDICO	CHC da Universidade Federal do Paraná - PR
72	CONSULTA PRE NATAL - ATESTADO MEDICO	CHC da Universidade Federal do Paraná - PR
72	CONSULTA PRE NATAL - ATESTADO MEDICO	CHC da Universidade Federal do Paraná - PR
72	CONSULTA PRE NATAL - ATESTADO MEDICO	CHC da Universidade Federal do Paraná - PR
72	CONSULTA PRE NATAL - ATESTADO MEDICO	CHC da Universidade Federal do Paraná - PR
72	CONSULTA PRE NATAL - ATESTADO MEDICO	CHC da Universidade Federal do Paraná - PR

72	CONSULTA PRE NATAL - ATESTADO MEDICO	CHC da Universidade Federal do Paraná - PR
72	CONSULTA PRE NATAL - ATESTADO MEDICO	CHC da Universidade Federal do Paraná - PR
72	CONSULTA PRE NATAL - ATESTADO MEDICO	CHC da Universidade Federal do Paraná - PR
72	CONSULTA PRE NATAL - ATESTADO MEDICO	CHC da Universidade Federal do Paraná - PR
72	CONSULTA PRE NATAL - ATESTADO MEDICO	CHC da Universidade Federal do Paraná - PR
72	CONSULTA PRE NATAL - ATESTADO MEDICO	CHC da Universidade Federal do Paraná - PR
72	CONSULTA PRE NATAL - ATESTADO MEDICO	CHC da Universidade Federal do Paraná - PR
72	CONSULTA PRE NATAL - ATESTADO MEDICO	CHC da Universidade Federal do Paraná - PR
72	CONSULTA PRE NATAL - ATESTADO MEDICO	CHC da Universidade Federal do Paraná - PR
72	CONSULTA PRE NATAL - ATESTADO MEDICO	CHC da Universidade Federal do Paraná - PR
72	CONSULTA PRE NATAL - ATESTADO MEDICO	CHC da Universidade Federal do Paraná - PR
72	CONSULTA PRE NATAL - ATESTADO MEDICO	CHC da Universidade Federal do Paraná - PR
72	CONSULTA PRE NATAL - ATESTADO MEDICO	CHC da Universidade Federal do Paraná - PR
72	CONSULTA PRE NATAL - ATESTADO MEDICO	CHC da Universidade Federal do Paraná - PR
72	CONSULTA PRE NATAL - ATESTADO MEDICO	CHC da Universidade Federal do Paraná - PR
72	CONSULTA PRE NATAL - ATESTADO MEDICO	CHC da Universidade Federal do Paraná - PR
72	CONSULTA PRE NATAL - ATESTADO MEDICO	CHC da Universidade Federal do Paraná - PR
72	CONSULTA PRE NATAL - ATESTADO MEDICO	CHC da Universidade Federal do Paraná - PR
72	CONSULTA PRE NATAL - ATESTADO MEDICO	CHC da Universidade Federal do Paraná - PR
72	CONSULTA PRE NATAL - ATESTADO MEDICO	CHC da Universidade Federal do Paraná - PR
72	CONSULTA PRE NATAL - ATESTADO MEDICO	CHC da Universidade Federal do Paraná - PR
72	CONSULTA PRE NATAL - ATESTADO MEDICO	CHC da Universidade Federal do Paraná - PR
72	CONSULTA PRE NATAL - ATESTADO MEDICO	CHC da Universidade Federal do Paraná - PR
72	CONSULTA PRE NATAL - ATESTADO MEDICO	CHC da Universidade Federal do Paraná - PR
72	CONSULTA PRE NATAL - ATESTADO MEDICO	CHC da Universidade Federal do Paraná - PR
72	CONSULTA PRE NATAL - ATESTADO MEDICO	CHC da Universidade Federal do Paraná - PR
72	CONSULTA PRE NATAL - ATESTADO MEDICO	CHC da Universidade Federal do Paraná - PR
72	CONSULTA PRE NATAL - ATESTADO MEDICO	CHC da Universidade Federal do Paraná - PR
72	CONSULTA PRE NATAL - ATESTADO MEDICO	CHC da Universidade Federal do Paraná - PR
72	CONSULTA PRE NATAL - ATESTADO MEDICO	CHC da Universidade Federal do Paraná - PR
72	CONSULTA PRE NATAL - ATESTADO MEDICO	CHC da Universidade Federal do Paraná - PR
72	CONSULTA PRE NATAL - ATESTADO MEDICO	CHC da Universidade Federal do Paraná - PR
72	CONSULTA PRE NATAL - ATESTADO MEDICO	CHC da Universidade Federal do Paraná - PR
72	CONSULTA PRE NATAL - ATESTADO MEDICO	CHC da Universidade Federal do Paraná - PR
72	CONSULTA PRE NATAL - ATESTADO MEDICO	CHC da Universidade Federal do Paraná - PR
72	CONSULTA PRE NATAL - ATESTADO MEDICO	CHC da Universidade Federal do Paraná - PR
72	CONSULTA PRE NATAL - ATESTADO MEDICO	CHC da Universidade Federal do Paraná - PR
72	CONSULTA PRE NATAL - ATESTADO MEDICO	CHC da Universidade Federal do Paraná - PR
72	CONSULTA PRE NATAL - ATESTADO MEDICO	CHC da Universidade Federal do Paraná - PR
72	CONSULTA PRE NATAL - ATESTADO MEDICO	CHC da Universidade Federal do Paraná - PR
72	CONSULTA PRE NATAL - ATESTADO MEDICO	CHC da Universidade Federal do Paraná - PR
72	CONSULTA PRE NATAL - ATESTADO MEDICO	CHC da Universidade Federal do Paraná - PR
72	CONSULTA PRE NATAL - ATESTADO MEDICO	CHC da Universidade Federal do Paraná - PR
72	CONSULTA PRE NATAL - ATESTADO MEDICO	CHC da Universidade Federal do Paraná - PR
72	CONSULTA PRE NATAL - ATESTADO MEDICO	CHC da Universidade Federal do Paraná - PR
72	CONSULTA PRE NATAL - ATESTADO MEDICO	CHC da Universidade Federal do Paraná - PR
72	CONSULTA PRE NATAL - ATESTADO MEDICO	CHC da Universidade Federal do Paraná - PR
72	CONSULTA PRE NATAL - ATESTADO MEDICO	CHC da Universidade Federal do Paraná - PR

72	CONSULTA PRE NATAL - ATESTADO MEDICO	CHC da Universidade Federal do Paraná - PR
72	CONSULTA PRE NATAL - ATESTADO MEDICO	CHC da Universidade Federal do Paraná - PR
72	CONSULTA PRE NATAL - ATESTADO MEDICO	CHC da Universidade Federal do Paraná - PR
72	CONSULTA PRE NATAL - ATESTADO MEDICO	CHC da Universidade Federal do Paraná - PR
72	CONSULTA PRE NATAL - ATESTADO MEDICO	CHC da Universidade Federal do Paraná - PR
72	CONSULTA PRE NATAL - ATESTADO MEDICO	CHC da Universidade Federal do Paraná - PR
72	CONSULTA PRE NATAL - ATESTADO MEDICO	CHC da Universidade Federal do Paraná - PR
75	LICENÇA PATERNIDADE CELETISTA - 20 Dias (c/ prorrogação) - LEI 11.770/08 ART 1 II	CHC da Universidade Federal do Paraná - PR
75	LICENÇA PATERNIDADE CELETISTA - 20 Dias (c/ prorrogação) - LEI 11.770/08 ART 1 II	CHC da Universidade Federal do Paraná - PR
75	LICENÇA PATERNIDADE CELETISTA - 20 Dias (c/ prorrogação) - LEI 11.770/08 ART 1 II	CHC da Universidade Federal do Paraná - PR
75	LICENÇA PATERNIDADE CELETISTA - 20 Dias (c/ prorrogação) - LEI 11.770/08 ART 1 II	CHC da Universidade Federal do Paraná - PR
75	LICENÇA PATERNIDADE CELETISTA - 20 Dias (c/ prorrogação) - LEI 11.770/08 ART 1 II	CHC da Universidade Federal do Paraná - PR
75	LICENÇA PATERNIDADE CELETISTA - 20 Dias (c/ prorrogação) - LEI 11.770/08 ART 1 II	CHC da Universidade Federal do Paraná - PR
75	LICENÇA PATERNIDADE CELETISTA - 20 Dias (c/ prorrogação) - LEI 11.770/08 ART 1 II	CHC da Universidade Federal do Paraná - PR
75	LICENÇA PATERNIDADE CELETISTA - 20 Dias (c/ prorrogação) - LEI 11.770/08 ART 1 II	CHC da Universidade Federal do Paraná - PR
75	LICENÇA PATERNIDADE CELETISTA - 20 Dias (c/ prorrogação) - LEI 11.770/08 ART 1 II	CHC da Universidade Federal do Paraná - PR
75	LICENÇA PATERNIDADE CELETISTA - 20 Dias (c/ prorrogação) - LEI 11.770/08 ART 1 II	CHC da Universidade Federal do Paraná - PR
75	LICENÇA PATERNIDADE CELETISTA - 20 Dias (c/ prorrogação) - LEI 11.770/08 ART 1 II	CHC da Universidade Federal do Paraná - PR
75	LICENÇA PATERNIDADE CELETISTA - 20 Dias (c/ prorrogação) - LEI 11.770/08 ART 1 II	CHC da Universidade Federal do Paraná - PR
75	LICENÇA PATERNIDADE CELETISTA - 20 Dias (c/ prorrogação) - LEI 11.770/08 ART 1 II	CHC da Universidade Federal do Paraná - PR
75	LICENÇA PATERNIDADE CELETISTA - 20 Dias (c/ prorrogação) - LEI 11.770/08 ART 1 II	CHC da Universidade Federal do Paraná - PR
90	HORAS EXTRAS/SOBREAVISO PAGO(AS)	CHC da Universidade Federal do Paraná - PR
90	HORAS EXTRAS/SOBREAVISO PAGO(AS)	CHC da Universidade Federal do Paraná - PR
90	HORAS EXTRAS/SOBREAVISO PAGO(AS)	CHC da Universidade Federal do Paraná - PR
90	HORAS EXTRAS/SOBREAVISO PAGO(AS)	CHC da Universidade Federal do Paraná - PR
90	HORAS EXTRAS/SOBREAVISO PAGO(AS)	CHC da Universidade Federal do Paraná - PR
90	HORAS EXTRAS/SOBREAVISO PAGO(AS)	CHC da Universidade Federal do Paraná - PR
90	HORAS EXTRAS/SOBREAVISO PAGO(AS)	CHC da Universidade Federal do Paraná - PR
90	HORAS EXTRAS/SOBREAVISO PAGO(AS)	CHC da Universidade Federal do Paraná - PR
90	HORAS EXTRAS/SOBREAVISO PAGO(AS)	CHC da Universidade Federal do Paraná - PR
90	HORAS EXTRAS/SOBREAVISO PAGO(AS)	CHC da Universidade Federal do Paraná - PR
90	HORAS EXTRAS/SOBREAVISO PAGO(AS)	CHC da Universidade Federal do Paraná - PR
90	HORAS EXTRAS/SOBREAVISO PAGO(AS)	CHC da Universidade Federal do Paraná - PR
90	HORAS EXTRAS/SOBREAVISO PAGO(AS)	CHC da Universidade Federal do Paraná - PR
90	HORAS EXTRAS/SOBREAVISO PAGO(AS)	CHC da Universidade Federal do Paraná - PR
90	HORAS EXTRAS/SOBREAVISO PAGO(AS)	CHC da Universidade Federal do Paraná - PR
90	HORAS EXTRAS/SOBREAVISO PAGO(AS)	CHC da Universidade Federal do Paraná - PR
90	HORAS EXTRAS/SOBREAVISO PAGO(AS)	CHC da Universidade Federal do Paraná - PR
90	HORAS EXTRAS/SOBREAVISO PAGO(AS)	CHC da Universidade Federal do Paraná - PR
90	HORAS EXTRAS/SOBREAVISO PAGO(AS)	CHC da Universidade Federal do Paraná - PR
90	HORAS EXTRAS/SOBREAVISO PAGO(AS)	CHC da Universidade Federal do Paraná - PR

90	HORAS EXTRAS/SOBREAVISO PAGO(AS)	CHC da Universidade Federal do Paraná - PR
90	HORAS EXTRAS/SOBREAVISO PAGO(AS)	CHC da Universidade Federal do Paraná - PR
90	HORAS EXTRAS/SOBREAVISO PAGO(AS)	CHC da Universidade Federal do Paraná - PR
90	HORAS EXTRAS/SOBREAVISO PAGO(AS)	CHC da Universidade Federal do Paraná - PR
90	HORAS EXTRAS/SOBREAVISO PAGO(AS)	CHC da Universidade Federal do Paraná - PR
90	HORAS EXTRAS/SOBREAVISO PAGO(AS)	CHC da Universidade Federal do Paraná - PR
90	HORAS EXTRAS/SOBREAVISO PAGO(AS)	CHC da Universidade Federal do Paraná - PR
90	HORAS EXTRAS/SOBREAVISO PAGO(AS)	CHC da Universidade Federal do Paraná - PR
90	HORAS EXTRAS/SOBREAVISO PAGO(AS)	CHC da Universidade Federal do Paraná - PR
90	HORAS EXTRAS/SOBREAVISO PAGO(AS)	CHC da Universidade Federal do Paraná - PR
90	HORAS EXTRAS/SOBREAVISO PAGO(AS)	CHC da Universidade Federal do Paraná - PR
90	HORAS EXTRAS/SOBREAVISO PAGO(AS)	CHC da Universidade Federal do Paraná - PR
90	HORAS EXTRAS/SOBREAVISO PAGO(AS)	CHC da Universidade Federal do Paraná - PR
90	HORAS EXTRAS/SOBREAVISO PAGO(AS)	CHC da Universidade Federal do Paraná - PR
90	HORAS EXTRAS/SOBREAVISO PAGO(AS)	CHC da Universidade Federal do Paraná - PR
90	HORAS EXTRAS/SOBREAVISO PAGO(AS)	CHC da Universidade Federal do Paraná - PR
90	HORAS EXTRAS/SOBREAVISO PAGO(AS)	CHC da Universidade Federal do Paraná - PR
90	HORAS EXTRAS/SOBREAVISO PAGO(AS)	CHC da Universidade Federal do Paraná - PR
90	HORAS EXTRAS/SOBREAVISO PAGO(AS)	CHC da Universidade Federal do Paraná - PR
90	HORAS EXTRAS/SOBREAVISO PAGO(AS)	CHC da Universidade Federal do Paraná - PR
90	HORAS EXTRAS/SOBREAVISO PAGO(AS)	CHC da Universidade Federal do Paraná - PR
90	HORAS EXTRAS/SOBREAVISO PAGO(AS)	CHC da Universidade Federal do Paraná - PR
90	HORAS EXTRAS/SOBREAVISO PAGO(AS)	CHC da Universidade Federal do Paraná - PR
90	HORAS EXTRAS/SOBREAVISO PAGO(AS)	CHC da Universidade Federal do Paraná - PR
90	HORAS EXTRAS/SOBREAVISO PAGO(AS)	CHC da Universidade Federal do Paraná - PR
90	HORAS EXTRAS/SOBREAVISO PAGO(AS)	CHC da Universidade Federal do Paraná - PR
90	HORAS EXTRAS/SOBREAVISO PAGO(AS)	CHC da Universidade Federal do Paraná - PR
90	HORAS EXTRAS/SOBREAVISO PAGO(AS)	CHC da Universidade Federal do Paraná - PR
90	HORAS EXTRAS/SOBREAVISO PAGO(AS)	CHC da Universidade Federal do Paraná - PR
90	HORAS EXTRAS/SOBREAVISO PAGO(AS)	CHC da Universidade Federal do Paraná - PR
90	HORAS EXTRAS/SOBREAVISO PAGO(AS)	CHC da Universidade Federal do Paraná - PR
90	HORAS EXTRAS/SOBREAVISO PAGO(AS)	CHC da Universidade Federal do Paraná - PR
90	HORAS EXTRAS/SOBREAVISO PAGO(AS)	CHC da Universidade Federal do Paraná - PR
90	HORAS EXTRAS/SOBREAVISO PAGO(AS)	CHC da Universidade Federal do Paraná - PR
90	HORAS EXTRAS/SOBREAVISO PAGO(AS)	CHC da Universidade Federal do Paraná - PR
90	HORAS EXTRAS/SOBREAVISO PAGO(AS)	CHC da Universidade Federal do Paraná - PR
90	HORAS EXTRAS/SOBREAVISO PAGO(AS)	CHC da Universidade Federal do Paraná - PR
90	HORAS EXTRAS/SOBREAVISO PAGO(AS)	CHC da Universidade Federal do Paraná - PR
90	HORAS EXTRAS/SOBREAVISO PAGO(AS)	CHC da Universidade Federal do Paraná - PR
90	HORAS EXTRAS/SOBREAVISO PAGO(AS)	CHC da Universidade Federal do Paraná - PR
90	HORAS EXTRAS/SOBREAVISO PAGO(AS)	CHC da Universidade Federal do Paraná - PR
90	HORAS EXTRAS/SOBREAVISO PAGO(AS)	CHC da Universidade Federal do Paraná - PR
90	HORAS EXTRAS/SOBREAVISO PAGO(AS)	CHC da Universidade Federal do Paraná - PR
90	HORAS EXTRAS/SOBREAVISO PAGO(AS)	CHC da Universidade Federal do Paraná - PR
90	HORAS EXTRAS/SOBREAVISO PAGO(AS)	CHC da Universidade Federal do Paraná - PR
90	HORAS EXTRAS/SOBREAVISO PAGO(AS)	CHC da Universidade Federal do Paraná - PR
90	HORAS EXTRAS/SOBREAVISO PAGO(AS)	CHC da Universidade Federal do Paraná - PR
90	HORAS EXTRAS/SOBREAVISO PAGO(AS)	CHC da Universidade Federal do Paraná - PR

90	HORAS EXTRAS/SOBREAVISO PAGO(AS)	CHC da Universidade Federal do Paraná - PR
90	HORAS EXTRAS/SOBREAVISO PAGO(AS)	CHC da Universidade Federal do Paraná - PR
90	HORAS EXTRAS/SOBREAVISO PAGO(AS)	CHC da Universidade Federal do Paraná - PR
90	HORAS EXTRAS/SOBREAVISO PAGO(AS)	CHC da Universidade Federal do Paraná - PR
90	HORAS EXTRAS/SOBREAVISO PAGO(AS)	CHC da Universidade Federal do Paraná - PR
90	HORAS EXTRAS/SOBREAVISO PAGO(AS)	CHC da Universidade Federal do Paraná - PR
90	HORAS EXTRAS/SOBREAVISO PAGO(AS)	CHC da Universidade Federal do Paraná - PR
90	HORAS EXTRAS/SOBREAVISO PAGO(AS)	CHC da Universidade Federal do Paraná - PR
90	HORAS EXTRAS/SOBREAVISO PAGO(AS)	CHC da Universidade Federal do Paraná - PR
90	HORAS EXTRAS/SOBREAVISO PAGO(AS)	CHC da Universidade Federal do Paraná - PR
90	HORAS EXTRAS/SOBREAVISO PAGO(AS)	CHC da Universidade Federal do Paraná - PR
90	HORAS EXTRAS/SOBREAVISO PAGO(AS)	CHC da Universidade Federal do Paraná - PR
90	HORAS EXTRAS/SOBREAVISO PAGO(AS)	CHC da Universidade Federal do Paraná - PR
90	HORAS EXTRAS/SOBREAVISO PAGO(AS)	CHC da Universidade Federal do Paraná - PR
90	HORAS EXTRAS/SOBREAVISO PAGO(AS)	CHC da Universidade Federal do Paraná - PR
90	HORAS EXTRAS/SOBREAVISO PAGO(AS)	CHC da Universidade Federal do Paraná - PR
90	HORAS EXTRAS/SOBREAVISO PAGO(AS)	CHC da Universidade Federal do Paraná - PR
90	HORAS EXTRAS/SOBREAVISO PAGO(AS)	CHC da Universidade Federal do Paraná - PR
90	HORAS EXTRAS/SOBREAVISO PAGO(AS)	CHC da Universidade Federal do Paraná - PR
90	HORAS EXTRAS/SOBREAVISO PAGO(AS)	CHC da Universidade Federal do Paraná - PR
90	HORAS EXTRAS/SOBREAVISO PAGO(AS)	CHC da Universidade Federal do Paraná - PR
90	HORAS EXTRAS/SOBREAVISO PAGO(AS)	CHC da Universidade Federal do Paraná - PR
90	HORAS EXTRAS/SOBREAVISO PAGO(AS)	CHC da Universidade Federal do Paraná - PR
90	HORAS EXTRAS/SOBREAVISO PAGO(AS)	CHC da Universidade Federal do Paraná - PR
90	HORAS EXTRAS/SOBREAVISO PAGO(AS)	CHC da Universidade Federal do Paraná - PR
90	HORAS EXTRAS/SOBREAVISO PAGO(AS)	CHC da Universidade Federal do Paraná - PR
90	HORAS EXTRAS/SOBREAVISO PAGO(AS)	CHC da Universidade Federal do Paraná - PR
90	HORAS EXTRAS/SOBREAVISO PAGO(AS)	CHC da Universidade Federal do Paraná - PR
90	HORAS EXTRAS/SOBREAVISO PAGO(AS)	CHC da Universidade Federal do Paraná - PR
90	HORAS EXTRAS/SOBREAVISO PAGO(AS)	CHC da Universidade Federal do Paraná - PR
90	HORAS EXTRAS/SOBREAVISO PAGO(AS)	CHC da Universidade Federal do Paraná - PR
90	HORAS EXTRAS/SOBREAVISO PAGO(AS)	CHC da Universidade Federal do Paraná - PR
90	HORAS EXTRAS/SOBREAVISO PAGO(AS)	CHC da Universidade Federal do Paraná - PR
90	HORAS EXTRAS/SOBREAVISO PAGO(AS)	CHC da Universidade Federal do Paraná - PR
90	HORAS EXTRAS/SOBREAVISO PAGO(AS)	CHC da Universidade Federal do Paraná - PR
90	HORAS EXTRAS/SOBREAVISO PAGO(AS)	CHC da Universidade Federal do Paraná - PR
90	HORAS EXTRAS/SOBREAVISO PAGO(AS)	CHC da Universidade Federal do Paraná - PR
90	HORAS EXTRAS/SOBREAVISO PAGO(AS)	CHC da Universidade Federal do Paraná - PR
90	HORAS EXTRAS/SOBREAVISO PAGO(AS)	CHC da Universidade Federal do Paraná - PR
90	HORAS EXTRAS/SOBREAVISO PAGO(AS)	CHC da Universidade Federal do Paraná - PR
90	HORAS EXTRAS/SOBREAVISO PAGO(AS)	CHC da Universidade Federal do Paraná - PR
90	HORAS EXTRAS/SOBREAVISO PAGO(AS)	CHC da Universidade Federal do Paraná - PR
90	HORAS EXTRAS/SOBREAVISO PAGO(AS)	CHC da Universidade Federal do Paraná - PR
90	HORAS EXTRAS/SOBREAVISO PAGO(AS)	CHC da Universidade Federal do Paraná - PR
90	HORAS EXTRAS/SOBREAVISO PAGO(AS)	CHC da Universidade Federal do Paraná - PR
90	HORAS EXTRAS/SOBREAVISO PAGO(AS)	CHC da Universidade Federal do Paraná - PR
90	HORAS EXTRAS/SOBREAVISO PAGO(AS)	CHC da Universidade Federal do Paraná - PR
90	HORAS EXTRAS/SOBREAVISO PAGO(AS)	CHC da Universidade Federal do Paraná - PR

90	HORAS EXTRAS/SOBREAVISO PAGO(AS)	CHC da Universidade Federal do Paraná - PR
90	HORAS EXTRAS/SOBREAVISO PAGO(AS)	CHC da Universidade Federal do Paraná - PR
90	HORAS EXTRAS/SOBREAVISO PAGO(AS)	CHC da Universidade Federal do Paraná - PR
90	HORAS EXTRAS/SOBREAVISO PAGO(AS)	CHC da Universidade Federal do Paraná - PR
90	HORAS EXTRAS/SOBREAVISO PAGO(AS)	CHC da Universidade Federal do Paraná - PR
90	HORAS EXTRAS/SOBREAVISO PAGO(AS)	CHC da Universidade Federal do Paraná - PR
90	HORAS EXTRAS/SOBREAVISO PAGO(AS)	CHC da Universidade Federal do Paraná - PR
90	HORAS EXTRAS/SOBREAVISO PAGO(AS)	CHC da Universidade Federal do Paraná - PR
90	HORAS EXTRAS/SOBREAVISO PAGO(AS)	CHC da Universidade Federal do Paraná - PR
90	HORAS EXTRAS/SOBREAVISO PAGO(AS)	CHC da Universidade Federal do Paraná - PR
90	HORAS EXTRAS/SOBREAVISO PAGO(AS)	CHC da Universidade Federal do Paraná - PR
90	HORAS EXTRAS/SOBREAVISO PAGO(AS)	CHC da Universidade Federal do Paraná - PR
90	HORAS EXTRAS/SOBREAVISO PAGO(AS)	CHC da Universidade Federal do Paraná - PR
90	HORAS EXTRAS/SOBREAVISO PAGO(AS)	CHC da Universidade Federal do Paraná - PR
90	HORAS EXTRAS/SOBREAVISO PAGO(AS)	CHC da Universidade Federal do Paraná - PR
90	HORAS EXTRAS/SOBREAVISO PAGO(AS)	CHC da Universidade Federal do Paraná - PR
90	HORAS EXTRAS/SOBREAVISO PAGO(AS)	CHC da Universidade Federal do Paraná - PR
90	HORAS EXTRAS/SOBREAVISO PAGO(AS)	CHC da Universidade Federal do Paraná - PR
90	HORAS EXTRAS/SOBREAVISO PAGO(AS)	CHC da Universidade Federal do Paraná - PR
90	HORAS EXTRAS/SOBREAVISO PAGO(AS)	CHC da Universidade Federal do Paraná - PR
90	HORAS EXTRAS/SOBREAVISO PAGO(AS)	CHC da Universidade Federal do Paraná - PR
90	HORAS EXTRAS/SOBREAVISO PAGO(AS)	CHC da Universidade Federal do Paraná - PR
90	HORAS EXTRAS/SOBREAVISO PAGO(AS)	CHC da Universidade Federal do Paraná - PR
90	HORAS EXTRAS/SOBREAVISO PAGO(AS)	CHC da Universidade Federal do Paraná - PR
90	HORAS EXTRAS/SOBREAVISO PAGO(AS)	CHC da Universidade Federal do Paraná - PR
90	HORAS EXTRAS/SOBREAVISO PAGO(AS)	CHC da Universidade Federal do Paraná - PR
90	HORAS EXTRAS/SOBREAVISO PAGO(AS)	CHC da Universidade Federal do Paraná - PR
90	HORAS EXTRAS/SOBREAVISO PAGO(AS)	CHC da Universidade Federal do Paraná - PR
90	HORAS EXTRAS/SOBREAVISO PAGO(AS)	CHC da Universidade Federal do Paraná - PR
90	HORAS EXTRAS/SOBREAVISO PAGO(AS)	CHC da Universidade Federal do Paraná - PR
90	HORAS EXTRAS/SOBREAVISO PAGO(AS)	CHC da Universidade Federal do Paraná - PR
90	HORAS EXTRAS/SOBREAVISO PAGO(AS)	CHC da Universidade Federal do Paraná - PR
90	HORAS EXTRAS/SOBREAVISO PAGO(AS)	CHC da Universidade Federal do Paraná - PR
90	HORAS EXTRAS/SOBREAVISO PAGO(AS)	CHC da Universidade Federal do Paraná - PR
90	HORAS EXTRAS/SOBREAVISO PAGO(AS)	CHC da Universidade Federal do Paraná - PR
90	HORAS EXTRAS/SOBREAVISO PAGO(AS)	CHC da Universidade Federal do Paraná - PR
90	HORAS EXTRAS/SOBREAVISO PAGO(AS)	CHC da Universidade Federal do Paraná - PR
90	HORAS EXTRAS/SOBREAVISO PAGO(AS)	CHC da Universidade Federal do Paraná - PR
90	HORAS EXTRAS/SOBREAVISO PAGO(AS)	CHC da Universidade Federal do Paraná - PR
90	HORAS EXTRAS/SOBREAVISO PAGO(AS)	CHC da Universidade Federal do Paraná - PR
90	HORAS EXTRAS/SOBREAVISO PAGO(AS)	CHC da Universidade Federal do Paraná - PR
90	HORAS EXTRAS/SOBREAVISO PAGO(AS)	CHC da Universidade Federal do Paraná - PR
90	HORAS EXTRAS/SOBREAVISO PAGO(AS)	CHC da Universidade Federal do Paraná - PR
90	HORAS EXTRAS/SOBREAVISO PAGO(AS)	CHC da Universidade Federal do Paraná - PR
90	HORAS EXTRAS/SOBREAVISO PAGO(AS)	CHC da Universidade Federal do Paraná - PR
90	HORAS EXTRAS/SOBREAVISO PAGO(AS)	CHC da Universidade Federal do Paraná - PR
90	HORAS EXTRAS/SOBREAVISO PAGO(AS)	CHC da Universidade Federal do Paraná - PR
90	HORAS EXTRAS/SOBREAVISO PAGO(AS)	CHC da Universidade Federal do Paraná - PR

90	HORAS EXTRAS/SOBREAVISO PAGO(AS)	CHC da Universidade Federal do Paraná - PR
90	HORAS EXTRAS/SOBREAVISO PAGO(AS)	CHC da Universidade Federal do Paraná - PR
90	HORAS EXTRAS/SOBREAVISO PAGO(AS)	CHC da Universidade Federal do Paraná - PR
90	HORAS EXTRAS/SOBREAVISO PAGO(AS)	CHC da Universidade Federal do Paraná - PR
90	HORAS EXTRAS/SOBREAVISO PAGO(AS)	CHC da Universidade Federal do Paraná - PR
90	HORAS EXTRAS/SOBREAVISO PAGO(AS)	CHC da Universidade Federal do Paraná - PR
90	HORAS EXTRAS/SOBREAVISO PAGO(AS)	CHC da Universidade Federal do Paraná - PR
90	HORAS EXTRAS/SOBREAVISO PAGO(AS)	CHC da Universidade Federal do Paraná - PR
90	HORAS EXTRAS/SOBREAVISO PAGO(AS)	CHC da Universidade Federal do Paraná - PR
90	HORAS EXTRAS/SOBREAVISO PAGO(AS)	CHC da Universidade Federal do Paraná - PR
90	HORAS EXTRAS/SOBREAVISO PAGO(AS)	CHC da Universidade Federal do Paraná - PR
90	HORAS EXTRAS/SOBREAVISO PAGO(AS)	CHC da Universidade Federal do Paraná - PR
90	HORAS EXTRAS/SOBREAVISO PAGO(AS)	CHC da Universidade Federal do Paraná - PR
90	HORAS EXTRAS/SOBREAVISO PAGO(AS)	CHC da Universidade Federal do Paraná - PR
90	HORAS EXTRAS/SOBREAVISO PAGO(AS)	CHC da Universidade Federal do Paraná - PR
90	HORAS EXTRAS/SOBREAVISO PAGO(AS)	CHC da Universidade Federal do Paraná - PR
90	HORAS EXTRAS/SOBREAVISO PAGO(AS)	CHC da Universidade Federal do Paraná - PR
90	HORAS EXTRAS/SOBREAVISO PAGO(AS)	CHC da Universidade Federal do Paraná - PR
90	HORAS EXTRAS/SOBREAVISO PAGO(AS)	CHC da Universidade Federal do Paraná - PR
90	HORAS EXTRAS/SOBREAVISO PAGO(AS)	CHC da Universidade Federal do Paraná - PR
90	HORAS EXTRAS/SOBREAVISO PAGO(AS)	CHC da Universidade Federal do Paraná - PR
90	HORAS EXTRAS/SOBREAVISO PAGO(AS)	CHC da Universidade Federal do Paraná - PR
90	HORAS EXTRAS/SOBREAVISO PAGO(AS)	CHC da Universidade Federal do Paraná - PR
90	HORAS EXTRAS/SOBREAVISO PAGO(AS)	CHC da Universidade Federal do Paraná - PR
90	HORAS EXTRAS/SOBREAVISO PAGO(AS)	CHC da Universidade Federal do Paraná - PR
90	HORAS EXTRAS/SOBREAVISO PAGO(AS)	CHC da Universidade Federal do Paraná - PR
90	HORAS EXTRAS/SOBREAVISO PAGO(AS)	CHC da Universidade Federal do Paraná - PR
90	HORAS EXTRAS/SOBREAVISO PAGO(AS)	CHC da Universidade Federal do Paraná - PR
90	HORAS EXTRAS/SOBREAVISO PAGO(AS)	CHC da Universidade Federal do Paraná - PR
90	HORAS EXTRAS/SOBREAVISO PAGO(AS)	CHC da Universidade Federal do Paraná - PR
90	HORAS EXTRAS/SOBREAVISO PAGO(AS)	CHC da Universidade Federal do Paraná - PR
90	HORAS EXTRAS/SOBREAVISO PAGO(AS)	CHC da Universidade Federal do Paraná - PR
90	HORAS EXTRAS/SOBREAVISO PAGO(AS)	CHC da Universidade Federal do Paraná - PR
90	HORAS EXTRAS/SOBREAVISO PAGO(AS)	CHC da Universidade Federal do Paraná - PR
90	HORAS EXTRAS/SOBREAVISO PAGO(AS)	CHC da Universidade Federal do Paraná - PR
90	HORAS EXTRAS/SOBREAVISO PAGO(AS)	CHC da Universidade Federal do Paraná - PR
90	HORAS EXTRAS/SOBREAVISO PAGO(AS)	CHC da Universidade Federal do Paraná - PR
90	HORAS EXTRAS/SOBREAVISO PAGO(AS)	CHC da Universidade Federal do Paraná - PR
90	HORAS EXTRAS/SOBREAVISO PAGO(AS)	CHC da Universidade Federal do Paraná - PR
90	HORAS EXTRAS/SOBREAVISO PAGO(AS)	CHC da Universidade Federal do Paraná - PR
90	HORAS EXTRAS/SOBREAVISO PAGO(AS)	CHC da Universidade Federal do Paraná - PR
90	HORAS EXTRAS/SOBREAVISO PAGO(AS)	CHC da Universidade Federal do Paraná - PR
90	HORAS EXTRAS/SOBREAVISO PAGO(AS)	CHC da Universidade Federal do Paraná - PR
90	HORAS EXTRAS/SOBREAVISO PAGO(AS)	CHC da Universidade Federal do Paraná - PR
90	HORAS EXTRAS/SOBREAVISO PAGO(AS)	CHC da Universidade Federal do Paraná - PR
90	HORAS EXTRAS/SOBREAVISO PAGO(AS)	CHC da Universidade Federal do Paraná - PR
90	HORAS EXTRAS/SOBREAVISO PAGO(AS)	CHC da Universidade Federal do Paraná - PR
90	HORAS EXTRAS/SOBREAVISO PAGO(AS)	CHC da Universidade Federal do Paraná - PR

90	HORAS EXTRAS/SOBREAVISO PAGO(AS)	CHC da Universidade Federal do Paraná - PR
90	HORAS EXTRAS/SOBREAVISO PAGO(AS)	CHC da Universidade Federal do Paraná - PR
90	HORAS EXTRAS/SOBREAVISO PAGO(AS)	CHC da Universidade Federal do Paraná - PR
90	HORAS EXTRAS/SOBREAVISO PAGO(AS)	CHC da Universidade Federal do Paraná - PR
90	HORAS EXTRAS/SOBREAVISO PAGO(AS)	CHC da Universidade Federal do Paraná - PR
90	HORAS EXTRAS/SOBREAVISO PAGO(AS)	CHC da Universidade Federal do Paraná - PR
90	HORAS EXTRAS/SOBREAVISO PAGO(AS)	CHC da Universidade Federal do Paraná - PR
90	HORAS EXTRAS/SOBREAVISO PAGO(AS)	CHC da Universidade Federal do Paraná - PR
90	HORAS EXTRAS/SOBREAVISO PAGO(AS)	CHC da Universidade Federal do Paraná - PR
90	HORAS EXTRAS/SOBREAVISO PAGO(AS)	CHC da Universidade Federal do Paraná - PR
90	HORAS EXTRAS/SOBREAVISO PAGO(AS)	CHC da Universidade Federal do Paraná - PR
90	HORAS EXTRAS/SOBREAVISO PAGO(AS)	CHC da Universidade Federal do Paraná - PR
90	HORAS EXTRAS/SOBREAVISO PAGO(AS)	CHC da Universidade Federal do Paraná - PR
90	HORAS EXTRAS/SOBREAVISO PAGO(AS)	CHC da Universidade Federal do Paraná - PR
90	HORAS EXTRAS/SOBREAVISO PAGO(AS)	CHC da Universidade Federal do Paraná - PR
90	HORAS EXTRAS/SOBREAVISO PAGO(AS)	CHC da Universidade Federal do Paraná - PR
90	HORAS EXTRAS/SOBREAVISO PAGO(AS)	CHC da Universidade Federal do Paraná - PR
90	HORAS EXTRAS/SOBREAVISO PAGO(AS)	CHC da Universidade Federal do Paraná - PR
90	HORAS EXTRAS/SOBREAVISO PAGO(AS)	CHC da Universidade Federal do Paraná - PR
90	HORAS EXTRAS/SOBREAVISO PAGO(AS)	CHC da Universidade Federal do Paraná - PR
90	HORAS EXTRAS/SOBREAVISO PAGO(AS)	CHC da Universidade Federal do Paraná - PR
90	HORAS EXTRAS/SOBREAVISO PAGO(AS)	CHC da Universidade Federal do Paraná - PR
90	HORAS EXTRAS/SOBREAVISO PAGO(AS)	CHC da Universidade Federal do Paraná - PR
90	HORAS EXTRAS/SOBREAVISO PAGO(AS)	CHC da Universidade Federal do Paraná - PR
90	HORAS EXTRAS/SOBREAVISO PAGO(AS)	CHC da Universidade Federal do Paraná - PR
90	HORAS EXTRAS/SOBREAVISO PAGO(AS)	CHC da Universidade Federal do Paraná - PR
90	HORAS EXTRAS/SOBREAVISO PAGO(AS)	CHC da Universidade Federal do Paraná - PR
90	HORAS EXTRAS/SOBREAVISO PAGO(AS)	CHC da Universidade Federal do Paraná - PR
90	HORAS EXTRAS/SOBREAVISO PAGO(AS)	CHC da Universidade Federal do Paraná - PR
90	HORAS EXTRAS/SOBREAVISO PAGO(AS)	CHC da Universidade Federal do Paraná - PR
90	HORAS EXTRAS/SOBREAVISO PAGO(AS)	CHC da Universidade Federal do Paraná - PR
90	HORAS EXTRAS/SOBREAVISO PAGO(AS)	CHC da Universidade Federal do Paraná - PR
90	HORAS EXTRAS/SOBREAVISO PAGO(AS)	CHC da Universidade Federal do Paraná - PR
90	HORAS EXTRAS/SOBREAVISO PAGO(AS)	CHC da Universidade Federal do Paraná - PR
90	HORAS EXTRAS/SOBREAVISO PAGO(AS)	CHC da Universidade Federal do Paraná - PR
90	HORAS EXTRAS/SOBREAVISO PAGO(AS)	CHC da Universidade Federal do Paraná - PR
90	HORAS EXTRAS/SOBREAVISO PAGO(AS)	CHC da Universidade Federal do Paraná - PR
90	HORAS EXTRAS/SOBREAVISO PAGO(AS)	CHC da Universidade Federal do Paraná - PR
90	HORAS EXTRAS/SOBREAVISO PAGO(AS)	CHC da Universidade Federal do Paraná - PR
90	HORAS EXTRAS/SOBREAVISO PAGO(AS)	CHC da Universidade Federal do Paraná - PR
90	HORAS EXTRAS/SOBREAVISO PAGO(AS)	CHC da Universidade Federal do Paraná - PR
90	HORAS EXTRAS/SOBREAVISO PAGO(AS)	CHC da Universidade Federal do Paraná - PR
90	HORAS EXTRAS/SOBREAVISO PAGO(AS)	CHC da Universidade Federal do Paraná - PR
90	HORAS EXTRAS/SOBREAVISO PAGO(AS)	CHC da Universidade Federal do Paraná - PR
90	HORAS EXTRAS/SOBREAVISO PAGO(AS)	CHC da Universidade Federal do Paraná - PR
90	HORAS EXTRAS/SOBREAVISO PAGO(AS)	CHC da Universidade Federal do Paraná - PR
90	HORAS EXTRAS/SOBREAVISO PAGO(AS)	CHC da Universidade Federal do Paraná - PR
90	HORAS EXTRAS/SOBREAVISO PAGO(AS)	CHC da Universidade Federal do Paraná - PR

90	HORAS EXTRAS/SOBREAVISO PAGO(AS)	CHC da Universidade Federal do Paraná - PR
90	HORAS EXTRAS/SOBREAVISO PAGO(AS)	CHC da Universidade Federal do Paraná - PR
90	HORAS EXTRAS/SOBREAVISO PAGO(AS)	CHC da Universidade Federal do Paraná - PR
90	HORAS EXTRAS/SOBREAVISO PAGO(AS)	CHC da Universidade Federal do Paraná - PR
90	HORAS EXTRAS/SOBREAVISO PAGO(AS)	CHC da Universidade Federal do Paraná - PR
90	HORAS EXTRAS/SOBREAVISO PAGO(AS)	CHC da Universidade Federal do Paraná - PR
90	HORAS EXTRAS/SOBREAVISO PAGO(AS)	CHC da Universidade Federal do Paraná - PR
90	HORAS EXTRAS/SOBREAVISO PAGO(AS)	CHC da Universidade Federal do Paraná - PR
90	HORAS EXTRAS/SOBREAVISO PAGO(AS)	CHC da Universidade Federal do Paraná - PR
90	HORAS EXTRAS/SOBREAVISO PAGO(AS)	CHC da Universidade Federal do Paraná - PR
90	HORAS EXTRAS/SOBREAVISO PAGO(AS)	CHC da Universidade Federal do Paraná - PR
90	HORAS EXTRAS/SOBREAVISO PAGO(AS)	CHC da Universidade Federal do Paraná - PR
90	HORAS EXTRAS/SOBREAVISO PAGO(AS)	CHC da Universidade Federal do Paraná - PR
90	HORAS EXTRAS/SOBREAVISO PAGO(AS)	CHC da Universidade Federal do Paraná - PR
90	HORAS EXTRAS/SOBREAVISO PAGO(AS)	CHC da Universidade Federal do Paraná - PR
90	HORAS EXTRAS/SOBREAVISO PAGO(AS)	CHC da Universidade Federal do Paraná - PR
90	HORAS EXTRAS/SOBREAVISO PAGO(AS)	CHC da Universidade Federal do Paraná - PR
90	HORAS EXTRAS/SOBREAVISO PAGO(AS)	CHC da Universidade Federal do Paraná - PR
90	HORAS EXTRAS/SOBREAVISO PAGO(AS)	CHC da Universidade Federal do Paraná - PR
90	HORAS EXTRAS/SOBREAVISO PAGO(AS)	CHC da Universidade Federal do Paraná - PR
90	HORAS EXTRAS/SOBREAVISO PAGO(AS)	CHC da Universidade Federal do Paraná - PR
90	HORAS EXTRAS/SOBREAVISO PAGO(AS)	CHC da Universidade Federal do Paraná - PR
90	HORAS EXTRAS/SOBREAVISO PAGO(AS)	CHC da Universidade Federal do Paraná - PR
90	HORAS EXTRAS/SOBREAVISO PAGO(AS)	CHC da Universidade Federal do Paraná - PR
97	RECESSO FINAL DE ANO	CHC da Universidade Federal do Paraná - PR
98	DESLOCAMENTO PARA NOVA UNIDADE (TRANSITO) - NORMA OPERACIONAL DGP N 01/2017	CHC da Universidade Federal do Paraná - PR
102	ACOMPANHAMENTO DE ESPOSA GESTANTE - CLT ART. 473 X	CHC da Universidade Federal do Paraná - PR
102	ACOMPANHAMENTO DE ESPOSA GESTANTE - CLT ART. 473 X	CHC da Universidade Federal do Paraná - PR
102	ACOMPANHAMENTO DE ESPOSA GESTANTE - CLT ART. 473 X	CHC da Universidade Federal do Paraná - PR
102	ACOMPANHAMENTO DE ESPOSA GESTANTE - CLT ART. 473 X	CHC da Universidade Federal do Paraná - PR
104	Acompanhamento filho menor 6 anos - Art. 473, XI, CLT	CHC da Universidade Federal do Paraná - PR
104	Acompanhamento filho menor 6 anos - Art. 473, XI, CLT	CHC da Universidade Federal do Paraná - PR
104	Acompanhamento filho menor 6 anos - Art. 473, XI, CLT	CHC da Universidade Federal do Paraná - PR
104	Acompanhamento filho menor 6 anos - Art. 473, XI, CLT	CHC da Universidade Federal do Paraná - PR
104	Acompanhamento filho menor 6 anos - Art. 473, XI, CLT	CHC da Universidade Federal do Paraná - PR
104	Acompanhamento filho menor 6 anos - Art. 473, XI, CLT	CHC da Universidade Federal do Paraná - PR
104	Acompanhamento filho menor 6 anos - Art. 473, XI, CLT	CHC da Universidade Federal do Paraná - PR
104	Acompanhamento filho menor 6 anos - Art. 473, XI, CLT	CHC da Universidade Federal do Paraná - PR
104	Acompanhamento filho menor 6 anos - Art. 473, XI, CLT	CHC da Universidade Federal do Paraná - PR
104	Acompanhamento filho menor 6 anos - Art. 473, XI, CLT	CHC da Universidade Federal do Paraná - PR
104	Acompanhamento filho menor 6 anos - Art. 473, XI, CLT	CHC da Universidade Federal do Paraná - PR
106	Afastamento por Atestado Medico/Acompanhamento - RJU	CHC da Universidade Federal do Paraná - PR
106	Afastamento por Atestado Medico/Acompanhamento - RJU	CHC da Universidade Federal do Paraná - PR
106	Afastamento por Atestado Medico/Acompanhamento - RJU	CHC da Universidade Federal do Paraná - PR
221	Afastamento Saúde Ocupacional	CHC da Universidade Federal do Paraná - PR

223	APOSENTADO INSS EFETIVO EBSERH	CHC da Universidade Federal do Paraná - PR
223	APOSENTADO INSS EFETIVO EBSERH	CHC da Universidade Federal do Paraná - PR
223	APOSENTADO INSS EFETIVO EBSERH	CHC da Universidade Federal do Paraná - PR
223	APOSENTADO INSS EFETIVO EBSERH	CHC da Universidade Federal do Paraná - PR
224	REALIZAÇÃO DE EXAME PREVENTIVO DE CÂNCER. LEI 13.767	CHC da Universidade Federal do Paraná - PR
224	REALIZAÇÃO DE EXAME PREVENTIVO DE CÂNCER. LEI 13.767	CHC da Universidade Federal do Paraná - PR
224	REALIZAÇÃO DE EXAME PREVENTIVO DE CÂNCER. LEI 13.767	CHC da Universidade Federal do Paraná - PR
224	REALIZAÇÃO DE EXAME PREVENTIVO DE CÂNCER. LEI 13.767	CHC da Universidade Federal do Paraná - PR
224	REALIZAÇÃO DE EXAME PREVENTIVO DE CÂNCER. LEI 13.767	CHC da Universidade Federal do Paraná - PR
224	REALIZAÇÃO DE EXAME PREVENTIVO DE CÂNCER. LEI 13.767	CHC da Universidade Federal do Paraná - PR
224	REALIZAÇÃO DE EXAME PREVENTIVO DE CÂNCER. LEI 13.767	CHC da Universidade Federal do Paraná - PR
224	REALIZAÇÃO DE EXAME PREVENTIVO DE CÂNCER. LEI 13.767	CHC da Universidade Federal do Paraná - PR
224	REALIZAÇÃO DE EXAME PREVENTIVO DE CÂNCER. LEI 13.767	CHC da Universidade Federal do Paraná - PR
224	REALIZAÇÃO DE EXAME PREVENTIVO DE CÂNCER. LEI 13.767	CHC da Universidade Federal do Paraná - PR
224	REALIZAÇÃO DE EXAME PREVENTIVO DE CÂNCER. LEI 13.767	CHC da Universidade Federal do Paraná - PR
224	REALIZAÇÃO DE EXAME PREVENTIVO DE CÂNCER. LEI 13.767	CHC da Universidade Federal do Paraná - PR
224	REALIZAÇÃO DE EXAME PREVENTIVO DE CÂNCER. LEI 13.767	CHC da Universidade Federal do Paraná - PR
224	REALIZAÇÃO DE EXAME PREVENTIVO DE CÂNCER. LEI 13.767	CHC da Universidade Federal do Paraná - PR
224	REALIZAÇÃO DE EXAME PREVENTIVO DE CÂNCER. LEI 13.767	CHC da Universidade Federal do Paraná - PR
227	ATIVIDADE EXTERNA (ESPII COVID19)	CHC da Universidade Federal do Paraná - PR
227	ATIVIDADE EXTERNA (ESPII COVID19)	CHC da Universidade Federal do Paraná - PR
227	ATIVIDADE EXTERNA (ESPII COVID19)	CHC da Universidade Federal do Paraná - PR
227	ATIVIDADE EXTERNA (ESPII COVID19)	CHC da Universidade Federal do Paraná - PR
227	ATIVIDADE EXTERNA (ESPII COVID19)	CHC da Universidade Federal do Paraná - PR
227	ATIVIDADE EXTERNA (ESPII COVID19)	CHC da Universidade Federal do Paraná - PR
227	ATIVIDADE EXTERNA (ESPII COVID19)	CHC da Universidade Federal do Paraná - PR
227	ATIVIDADE EXTERNA (ESPII COVID19)	CHC da Universidade Federal do Paraná - PR
227	ATIVIDADE EXTERNA (ESPII COVID19)	CHC da Universidade Federal do Paraná - PR
227	ATIVIDADE EXTERNA (ESPII COVID19)	CHC da Universidade Federal do Paraná - PR
227	ATIVIDADE EXTERNA (ESPII COVID19)	CHC da Universidade Federal do Paraná - PR
227	ATIVIDADE EXTERNA (ESPII COVID19)	CHC da Universidade Federal do Paraná - PR
227	ATIVIDADE EXTERNA (ESPII COVID19)	CHC da Universidade Federal do Paraná - PR
227	ATIVIDADE EXTERNA (ESPII COVID19)	CHC da Universidade Federal do Paraná - PR
227	ATIVIDADE EXTERNA (ESPII COVID19)	CHC da Universidade Federal do Paraná - PR
227	ATIVIDADE EXTERNA (ESPII COVID19)	CHC da Universidade Federal do Paraná - PR
227	ATIVIDADE EXTERNA (ESPII COVID19)	CHC da Universidade Federal do Paraná - PR
227	ATIVIDADE EXTERNA (ESPII COVID19)	CHC da Universidade Federal do Paraná - PR
227	ATIVIDADE EXTERNA (ESPII COVID19)	CHC da Universidade Federal do Paraná - PR
227	ATIVIDADE EXTERNA (ESPII COVID19)	CHC da Universidade Federal do Paraná - PR
227	ATIVIDADE EXTERNA (ESPII COVID19)	CHC da Universidade Federal do Paraná - PR
227	ATIVIDADE EXTERNA (ESPII COVID19)	CHC da Universidade Federal do Paraná - PR
227	ATIVIDADE EXTERNA (ESPII COVID19)	CHC da Universidade Federal do Paraná - PR
227	ATIVIDADE EXTERNA (ESPII COVID19)	CHC da Universidade Federal do Paraná - PR
227	ATIVIDADE EXTERNA (ESPII COVID19)	CHC da Universidade Federal do Paraná - PR
227	ATIVIDADE EXTERNA (ESPII COVID19)	CHC da Universidade Federal do Paraná - PR
227	ATIVIDADE EXTERNA (ESPII COVID19)	CHC da Universidade Federal do Paraná - PR
227	ATIVIDADE EXTERNA (ESPII COVID19)	CHC da Universidade Federal do Paraná - PR

227	ATIVIDADE EXTERNA (ESPII COVID19)	CHC da Universidade Federal do Paraná - PR
227	ATIVIDADE EXTERNA (ESPII COVID19)	CHC da Universidade Federal do Paraná - PR
227	ATIVIDADE EXTERNA (ESPII COVID19)	CHC da Universidade Federal do Paraná - PR
227	ATIVIDADE EXTERNA (ESPII COVID19)	CHC da Universidade Federal do Paraná - PR
227	ATIVIDADE EXTERNA (ESPII COVID19)	CHC da Universidade Federal do Paraná - PR
227	ATIVIDADE EXTERNA (ESPII COVID19)	CHC da Universidade Federal do Paraná - PR
227	ATIVIDADE EXTERNA (ESPII COVID19)	CHC da Universidade Federal do Paraná - PR
227	ATIVIDADE EXTERNA (ESPII COVID19)	CHC da Universidade Federal do Paraná - PR
227	ATIVIDADE EXTERNA (ESPII COVID19)	CHC da Universidade Federal do Paraná - PR
227	ATIVIDADE EXTERNA (ESPII COVID19)	CHC da Universidade Federal do Paraná - PR
227	ATIVIDADE EXTERNA (ESPII COVID19)	CHC da Universidade Federal do Paraná - PR
227	ATIVIDADE EXTERNA (ESPII COVID19)	CHC da Universidade Federal do Paraná - PR
227	ATIVIDADE EXTERNA (ESPII COVID19)	CHC da Universidade Federal do Paraná - PR
227	ATIVIDADE EXTERNA (ESPII COVID19)	CHC da Universidade Federal do Paraná - PR
227	ATIVIDADE EXTERNA (ESPII COVID19)	CHC da Universidade Federal do Paraná - PR
227	ATIVIDADE EXTERNA (ESPII COVID19)	CHC da Universidade Federal do Paraná - PR
227	ATIVIDADE EXTERNA (ESPII COVID19)	CHC da Universidade Federal do Paraná - PR
227	ATIVIDADE EXTERNA (ESPII COVID19)	CHC da Universidade Federal do Paraná - PR
227	ATIVIDADE EXTERNA (ESPII COVID19)	CHC da Universidade Federal do Paraná - PR
227	ATIVIDADE EXTERNA (ESPII COVID19)	CHC da Universidade Federal do Paraná - PR
227	ATIVIDADE EXTERNA (ESPII COVID19)	CHC da Universidade Federal do Paraná - PR
227	ATIVIDADE EXTERNA (ESPII COVID19)	CHC da Universidade Federal do Paraná - PR
227	ATIVIDADE EXTERNA (ESPII COVID19)	CHC da Universidade Federal do Paraná - PR
227	ATIVIDADE EXTERNA (ESPII COVID19)	CHC da Universidade Federal do Paraná - PR
227	ATIVIDADE EXTERNA (ESPII COVID19)	CHC da Universidade Federal do Paraná - PR
227	ATIVIDADE EXTERNA (ESPII COVID19)	CHC da Universidade Federal do Paraná - PR
227	ATIVIDADE EXTERNA (ESPII COVID19)	CHC da Universidade Federal do Paraná - PR
227	ATIVIDADE EXTERNA (ESPII COVID19)	CHC da Universidade Federal do Paraná - PR
227	ATIVIDADE EXTERNA (ESPII COVID19)	CHC da Universidade Federal do Paraná - PR
227	ATIVIDADE EXTERNA (ESPII COVID19)	CHC da Universidade Federal do Paraná - PR
227	ATIVIDADE EXTERNA (ESPII COVID19)	CHC da Universidade Federal do Paraná - PR
227	ATIVIDADE EXTERNA (ESPII COVID19)	CHC da Universidade Federal do Paraná - PR
227	ATIVIDADE EXTERNA (ESPII COVID19)	CHC da Universidade Federal do Paraná - PR
227	ATIVIDADE EXTERNA (ESPII COVID19)	CHC da Universidade Federal do Paraná - PR
227	ATIVIDADE EXTERNA (ESPII COVID19)	CHC da Universidade Federal do Paraná - PR
227	ATIVIDADE EXTERNA (ESPII COVID19)	CHC da Universidade Federal do Paraná - PR
227	ATIVIDADE EXTERNA (ESPII COVID19)	CHC da Universidade Federal do Paraná - PR
227	ATIVIDADE EXTERNA (ESPII COVID19)	CHC da Universidade Federal do Paraná - PR
227	ATIVIDADE EXTERNA (ESPII COVID19)	CHC da Universidade Federal do Paraná - PR
227	ATIVIDADE EXTERNA (ESPII COVID19)	CHC da Universidade Federal do Paraná - PR
227	ATIVIDADE EXTERNA (ESPII COVID19)	CHC da Universidade Federal do Paraná - PR
227	ATIVIDADE EXTERNA (ESPII COVID19)	CHC da Universidade Federal do Paraná - PR
227	ATIVIDADE EXTERNA (ESPII COVID19)	CHC da Universidade Federal do Paraná - PR
227	ATIVIDADE EXTERNA (ESPII COVID19)	CHC da Universidade Federal do Paraná - PR
227	ATIVIDADE EXTERNA (ESPII COVID19)	CHC da Universidade Federal do Paraná - PR
227	ATIVIDADE EXTERNA (ESPII COVID19)	CHC da Universidade Federal do Paraná - PR
227	ATIVIDADE EXTERNA (ESPII COVID19)	CHC da Universidade Federal do Paraná - PR
227	ATIVIDADE EXTERNA (ESPII COVID19)	CHC da Universidade Federal do Paraná - PR

227	ATIVIDADE EXTERNA (ESPII COVID19)	CHC da Universidade Federal do Paraná - PR
227	ATIVIDADE EXTERNA (ESPII COVID19)	CHC da Universidade Federal do Paraná - PR
227	ATIVIDADE EXTERNA (ESPII COVID19)	CHC da Universidade Federal do Paraná - PR
227	ATIVIDADE EXTERNA (ESPII COVID19)	CHC da Universidade Federal do Paraná - PR
227	ATIVIDADE EXTERNA (ESPII COVID19)	CHC da Universidade Federal do Paraná - PR
227	ATIVIDADE EXTERNA (ESPII COVID19)	CHC da Universidade Federal do Paraná - PR
227	ATIVIDADE EXTERNA (ESPII COVID19)	CHC da Universidade Federal do Paraná - PR
227	ATIVIDADE EXTERNA (ESPII COVID19)	CHC da Universidade Federal do Paraná - PR
227	ATIVIDADE EXTERNA (ESPII COVID19)	CHC da Universidade Federal do Paraná - PR
227	ATIVIDADE EXTERNA (ESPII COVID19)	CHC da Universidade Federal do Paraná - PR
227	ATIVIDADE EXTERNA (ESPII COVID19)	CHC da Universidade Federal do Paraná - PR
227	ATIVIDADE EXTERNA (ESPII COVID19)	CHC da Universidade Federal do Paraná - PR
227	ATIVIDADE EXTERNA (ESPII COVID19)	CHC da Universidade Federal do Paraná - PR
227	ATIVIDADE EXTERNA (ESPII COVID19)	CHC da Universidade Federal do Paraná - PR
227	ATIVIDADE EXTERNA (ESPII COVID19)	CHC da Universidade Federal do Paraná - PR
227	ATIVIDADE EXTERNA (ESPII COVID19)	CHC da Universidade Federal do Paraná - PR
227	ATIVIDADE EXTERNA (ESPII COVID19)	CHC da Universidade Federal do Paraná - PR
227	ATIVIDADE EXTERNA (ESPII COVID19)	CHC da Universidade Federal do Paraná - PR
227	ATIVIDADE EXTERNA (ESPII COVID19)	CHC da Universidade Federal do Paraná - PR
227	ATIVIDADE EXTERNA (ESPII COVID19)	CHC da Universidade Federal do Paraná - PR
227	ATIVIDADE EXTERNA (ESPII COVID19)	CHC da Universidade Federal do Paraná - PR
227	ATIVIDADE EXTERNA (ESPII COVID19)	CHC da Universidade Federal do Paraná - PR
227	ATIVIDADE EXTERNA (ESPII COVID19)	CHC da Universidade Federal do Paraná - PR
227	ATIVIDADE EXTERNA (ESPII COVID19)	CHC da Universidade Federal do Paraná - PR
227	ATIVIDADE EXTERNA (ESPII COVID19)	CHC da Universidade Federal do Paraná - PR
227	ATIVIDADE EXTERNA (ESPII COVID19)	CHC da Universidade Federal do Paraná - PR
227	ATIVIDADE EXTERNA (ESPII COVID19)	CHC da Universidade Federal do Paraná - PR
227	ATIVIDADE EXTERNA (ESPII COVID19)	CHC da Universidade Federal do Paraná - PR
227	ATIVIDADE EXTERNA (ESPII COVID19)	CHC da Universidade Federal do Paraná - PR
227	ATIVIDADE EXTERNA (ESPII COVID19)	CHC da Universidade Federal do Paraná - PR
227	ATIVIDADE EXTERNA (ESPII COVID19)	CHC da Universidade Federal do Paraná - PR
227	ATIVIDADE EXTERNA (ESPII COVID19)	CHC da Universidade Federal do Paraná - PR
227	ATIVIDADE EXTERNA (ESPII COVID19)	CHC da Universidade Federal do Paraná - PR
227	ATIVIDADE EXTERNA (ESPII COVID19)	CHC da Universidade Federal do Paraná - PR
227	ATIVIDADE EXTERNA (ESPII COVID19)	CHC da Universidade Federal do Paraná - PR
227	ATIVIDADE EXTERNA (ESPII COVID19)	CHC da Universidade Federal do Paraná - PR
227	ATIVIDADE EXTERNA (ESPII COVID19)	CHC da Universidade Federal do Paraná - PR
227	ATIVIDADE EXTERNA (ESPII COVID19)	CHC da Universidade Federal do Paraná - PR
227	ATIVIDADE EXTERNA (ESPII COVID19)	CHC da Universidade Federal do Paraná - PR
227	ATIVIDADE EXTERNA (ESPII COVID19)	CHC da Universidade Federal do Paraná - PR
227	ATIVIDADE EXTERNA (ESPII COVID19)	CHC da Universidade Federal do Paraná - PR
227	ATIVIDADE EXTERNA (ESPII COVID19)	CHC da Universidade Federal do Paraná - PR
227	ATIVIDADE EXTERNA (ESPII COVID19)	CHC da Universidade Federal do Paraná - PR
227	ATIVIDADE EXTERNA (ESPII COVID19)	CHC da Universidade Federal do Paraná - PR
227	ATIVIDADE EXTERNA (ESPII COVID19)	CHC da Universidade Federal do Paraná - PR
227	ATIVIDADE EXTERNA (ESPII COVID19)	CHC da Universidade Federal do Paraná - PR
227	ATIVIDADE EXTERNA (ESPII COVID19)	CHC da Universidade Federal do Paraná - PR
227	ATIVIDADE EXTERNA (ESPII COVID19)	CHC da Universidade Federal do Paraná - PR

227	ATIVIDADE EXTERNA (ESPII COVID19)	CHC da Universidade Federal do Paraná - PR
227	ATIVIDADE EXTERNA (ESPII COVID19)	CHC da Universidade Federal do Paraná - PR
227	ATIVIDADE EXTERNA (ESPII COVID19)	CHC da Universidade Federal do Paraná - PR
227	ATIVIDADE EXTERNA (ESPII COVID19)	CHC da Universidade Federal do Paraná - PR
227	ATIVIDADE EXTERNA (ESPII COVID19)	CHC da Universidade Federal do Paraná - PR
227	ATIVIDADE EXTERNA (ESPII COVID19)	CHC da Universidade Federal do Paraná - PR
227	ATIVIDADE EXTERNA (ESPII COVID19)	CHC da Universidade Federal do Paraná - PR
227	ATIVIDADE EXTERNA (ESPII COVID19)	CHC da Universidade Federal do Paraná - PR
227	ATIVIDADE EXTERNA (ESPII COVID19)	CHC da Universidade Federal do Paraná - PR
227	ATIVIDADE EXTERNA (ESPII COVID19)	CHC da Universidade Federal do Paraná - PR
227	ATIVIDADE EXTERNA (ESPII COVID19)	CHC da Universidade Federal do Paraná - PR
227	ATIVIDADE EXTERNA (ESPII COVID19)	CHC da Universidade Federal do Paraná - PR
227	ATIVIDADE EXTERNA (ESPII COVID19)	CHC da Universidade Federal do Paraná - PR
227	ATIVIDADE EXTERNA (ESPII COVID19)	CHC da Universidade Federal do Paraná - PR
227	ATIVIDADE EXTERNA (ESPII COVID19)	CHC da Universidade Federal do Paraná - PR
227	ATIVIDADE EXTERNA (ESPII COVID19)	CHC da Universidade Federal do Paraná - PR
227	ATIVIDADE EXTERNA (ESPII COVID19)	CHC da Universidade Federal do Paraná - PR
227	ATIVIDADE EXTERNA (ESPII COVID19)	CHC da Universidade Federal do Paraná - PR
227	ATIVIDADE EXTERNA (ESPII COVID19)	CHC da Universidade Federal do Paraná - PR
227	ATIVIDADE EXTERNA (ESPII COVID19)	CHC da Universidade Federal do Paraná - PR
227	ATIVIDADE EXTERNA (ESPII COVID19)	CHC da Universidade Federal do Paraná - PR
227	ATIVIDADE EXTERNA (ESPII COVID19)	CHC da Universidade Federal do Paraná - PR
227	ATIVIDADE EXTERNA (ESPII COVID19)	CHC da Universidade Federal do Paraná - PR
227	ATIVIDADE EXTERNA (ESPII COVID19)	CHC da Universidade Federal do Paraná - PR
227	ATIVIDADE EXTERNA (ESPII COVID19)	CHC da Universidade Federal do Paraná - PR
227	ATIVIDADE EXTERNA (ESPII COVID19)	CHC da Universidade Federal do Paraná - PR
227	ATIVIDADE EXTERNA (ESPII COVID19)	CHC da Universidade Federal do Paraná - PR
227	ATIVIDADE EXTERNA (ESPII COVID19)	CHC da Universidade Federal do Paraná - PR
227	ATIVIDADE EXTERNA (ESPII COVID19)	CHC da Universidade Federal do Paraná - PR
227	ATIVIDADE EXTERNA (ESPII COVID19)	CHC da Universidade Federal do Paraná - PR
227	ATIVIDADE EXTERNA (ESPII COVID19)	CHC da Universidade Federal do Paraná - PR
227	ATIVIDADE EXTERNA (ESPII COVID19)	CHC da Universidade Federal do Paraná - PR
227	ATIVIDADE EXTERNA (ESPII COVID19)	CHC da Universidade Federal do Paraná - PR
227	ATIVIDADE EXTERNA (ESPII COVID19)	CHC da Universidade Federal do Paraná - PR
227	ATIVIDADE EXTERNA (ESPII COVID19)	CHC da Universidade Federal do Paraná - PR
227	ATIVIDADE EXTERNA (ESPII COVID19)	CHC da Universidade Federal do Paraná - PR
227	ATIVIDADE EXTERNA (ESPII COVID19)	CHC da Universidade Federal do Paraná - PR
227	ATIVIDADE EXTERNA (ESPII COVID19)	CHC da Universidade Federal do Paraná - PR
227	ATIVIDADE EXTERNA (ESPII COVID19)	CHC da Universidade Federal do Paraná - PR
227	ATIVIDADE EXTERNA (ESPII COVID19)	CHC da Universidade Federal do Paraná - PR
227	ATIVIDADE EXTERNA (ESPII COVID19)	CHC da Universidade Federal do Paraná - PR
227	ATIVIDADE EXTERNA (ESPII COVID19)	CHC da Universidade Federal do Paraná - PR
227	ATIVIDADE EXTERNA (ESPII COVID19)	CHC da Universidade Federal do Paraná - PR
227	ATIVIDADE EXTERNA (ESPII COVID19)	CHC da Universidade Federal do Paraná - PR
227	ATIVIDADE EXTERNA (ESPII COVID19)	CHC da Universidade Federal do Paraná - PR
227	ATIVIDADE EXTERNA (ESPII COVID19)	CHC da Universidade Federal do Paraná - PR
227	ATIVIDADE EXTERNA (ESPII COVID19)	CHC da Universidade Federal do Paraná - PR
227	ATIVIDADE EXTERNA (ESPII COVID19)	CHC da Universidade Federal do Paraná - PR

227	ATIVIDADE EXTERNA (ESPII COVID19)	CHC da Universidade Federal do Paraná - PR
227	ATIVIDADE EXTERNA (ESPII COVID19)	CHC da Universidade Federal do Paraná - PR
227	ATIVIDADE EXTERNA (ESPII COVID19)	CHC da Universidade Federal do Paraná - PR
227	ATIVIDADE EXTERNA (ESPII COVID19)	CHC da Universidade Federal do Paraná - PR
227	ATIVIDADE EXTERNA (ESPII COVID19)	CHC da Universidade Federal do Paraná - PR
227	ATIVIDADE EXTERNA (ESPII COVID19)	CHC da Universidade Federal do Paraná - PR
227	ATIVIDADE EXTERNA (ESPII COVID19)	CHC da Universidade Federal do Paraná - PR
227	ATIVIDADE EXTERNA (ESPII COVID19)	CHC da Universidade Federal do Paraná - PR
227	ATIVIDADE EXTERNA (ESPII COVID19)	CHC da Universidade Federal do Paraná - PR
227	ATIVIDADE EXTERNA (ESPII COVID19)	CHC da Universidade Federal do Paraná - PR
227	ATIVIDADE EXTERNA (ESPII COVID19)	CHC da Universidade Federal do Paraná - PR
227	ATIVIDADE EXTERNA (ESPII COVID19)	CHC da Universidade Federal do Paraná - PR
227	ATIVIDADE EXTERNA (ESPII COVID19)	CHC da Universidade Federal do Paraná - PR
227	ATIVIDADE EXTERNA (ESPII COVID19)	CHC da Universidade Federal do Paraná - PR
227	ATIVIDADE EXTERNA (ESPII COVID19)	CHC da Universidade Federal do Paraná - PR
227	ATIVIDADE EXTERNA (ESPII COVID19)	CHC da Universidade Federal do Paraná - PR
227	ATIVIDADE EXTERNA (ESPII COVID19)	CHC da Universidade Federal do Paraná - PR
227	ATIVIDADE EXTERNA (ESPII COVID19)	CHC da Universidade Federal do Paraná - PR
227	ATIVIDADE EXTERNA (ESPII COVID19)	CHC da Universidade Federal do Paraná - PR
227	ATIVIDADE EXTERNA (ESPII COVID19)	CHC da Universidade Federal do Paraná - PR
227	ATIVIDADE EXTERNA (ESPII COVID19)	CHC da Universidade Federal do Paraná - PR
227	ATIVIDADE EXTERNA (ESPII COVID19)	CHC da Universidade Federal do Paraná - PR
227	ATIVIDADE EXTERNA (ESPII COVID19)	CHC da Universidade Federal do Paraná - PR
227	ATIVIDADE EXTERNA (ESPII COVID19)	CHC da Universidade Federal do Paraná - PR
227	ATIVIDADE EXTERNA (ESPII COVID19)	CHC da Universidade Federal do Paraná - PR
227	ATIVIDADE EXTERNA (ESPII COVID19)	CHC da Universidade Federal do Paraná - PR
227	ATIVIDADE EXTERNA (ESPII COVID19)	CHC da Universidade Federal do Paraná - PR
227	ATIVIDADE EXTERNA (ESPII COVID19)	CHC da Universidade Federal do Paraná - PR
227	ATIVIDADE EXTERNA (ESPII COVID19)	CHC da Universidade Federal do Paraná - PR
227	ATIVIDADE EXTERNA (ESPII COVID19)	CHC da Universidade Federal do Paraná - PR
227	ATIVIDADE EXTERNA (ESPII COVID19)	CHC da Universidade Federal do Paraná - PR
227	ATIVIDADE EXTERNA (ESPII COVID19)	CHC da Universidade Federal do Paraná - PR
227	ATIVIDADE EXTERNA (ESPII COVID19)	CHC da Universidade Federal do Paraná - PR
227	ATIVIDADE EXTERNA (ESPII COVID19)	CHC da Universidade Federal do Paraná - PR
227	ATIVIDADE EXTERNA (ESPII COVID19)	CHC da Universidade Federal do Paraná - PR
227	ATIVIDADE EXTERNA (ESPII COVID19)	CHC da Universidade Federal do Paraná - PR
227	ATIVIDADE EXTERNA (ESPII COVID19)	CHC da Universidade Federal do Paraná - PR
227	ATIVIDADE EXTERNA (ESPII COVID19)	CHC da Universidade Federal do Paraná - PR
227	ATIVIDADE EXTERNA (ESPII COVID19)	CHC da Universidade Federal do Paraná - PR
227	ATIVIDADE EXTERNA (ESPII COVID19)	CHC da Universidade Federal do Paraná - PR
227	ATIVIDADE EXTERNA (ESPII COVID19)	CHC da Universidade Federal do Paraná - PR
227	ATIVIDADE EXTERNA (ESPII COVID19)	CHC da Universidade Federal do Paraná - PR
227	ATIVIDADE EXTERNA (ESPII COVID19)	CHC da Universidade Federal do Paraná - PR
227	ATIVIDADE EXTERNA (ESPII COVID19)	CHC da Universidade Federal do Paraná - PR
227	ATIVIDADE EXTERNA (ESPII COVID19)	CHC da Universidade Federal do Paraná - PR
227	ATIVIDADE EXTERNA (ESPII COVID19)	CHC da Universidade Federal do Paraná - PR
227	ATIVIDADE EXTERNA (ESPII COVID19)	CHC da Universidade Federal do Paraná - PR
227	ATIVIDADE EXTERNA (ESPII COVID19)	CHC da Universidade Federal do Paraná - PR

227	ATIVIDADE EXTERNA (ESPII COVID19)	CHC da Universidade Federal do Paraná - PR
227	ATIVIDADE EXTERNA (ESPII COVID19)	CHC da Universidade Federal do Paraná - PR
227	ATIVIDADE EXTERNA (ESPII COVID19)	CHC da Universidade Federal do Paraná - PR
227	ATIVIDADE EXTERNA (ESPII COVID19)	CHC da Universidade Federal do Paraná - PR
227	ATIVIDADE EXTERNA (ESPII COVID19)	CHC da Universidade Federal do Paraná - PR
227	ATIVIDADE EXTERNA (ESPII COVID19)	CHC da Universidade Federal do Paraná - PR
227	ATIVIDADE EXTERNA (ESPII COVID19)	CHC da Universidade Federal do Paraná - PR
227	ATIVIDADE EXTERNA (ESPII COVID19)	CHC da Universidade Federal do Paraná - PR
227	ATIVIDADE EXTERNA (ESPII COVID19)	CHC da Universidade Federal do Paraná - PR
227	ATIVIDADE EXTERNA (ESPII COVID19)	CHC da Universidade Federal do Paraná - PR
227	ATIVIDADE EXTERNA (ESPII COVID19)	CHC da Universidade Federal do Paraná - PR
227	ATIVIDADE EXTERNA (ESPII COVID19)	CHC da Universidade Federal do Paraná - PR
227	ATIVIDADE EXTERNA (ESPII COVID19)	CHC da Universidade Federal do Paraná - PR
227	ATIVIDADE EXTERNA (ESPII COVID19)	CHC da Universidade Federal do Paraná - PR
227	ATIVIDADE EXTERNA (ESPII COVID19)	CHC da Universidade Federal do Paraná - PR
227	ATIVIDADE EXTERNA (ESPII COVID19)	CHC da Universidade Federal do Paraná - PR
227	ATIVIDADE EXTERNA (ESPII COVID19)	CHC da Universidade Federal do Paraná - PR
227	ATIVIDADE EXTERNA (ESPII COVID19)	CHC da Universidade Federal do Paraná - PR
227	ATIVIDADE EXTERNA (ESPII COVID19)	CHC da Universidade Federal do Paraná - PR
227	ATIVIDADE EXTERNA (ESPII COVID19)	CHC da Universidade Federal do Paraná - PR
227	ATIVIDADE EXTERNA (ESPII COVID19)	CHC da Universidade Federal do Paraná - PR
227	ATIVIDADE EXTERNA (ESPII COVID19)	CHC da Universidade Federal do Paraná - PR
227	ATIVIDADE EXTERNA (ESPII COVID19)	CHC da Universidade Federal do Paraná - PR
227	ATIVIDADE EXTERNA (ESPII COVID19)	CHC da Universidade Federal do Paraná - PR
227	ATIVIDADE EXTERNA (ESPII COVID19)	CHC da Universidade Federal do Paraná - PR
227	ATIVIDADE EXTERNA (ESPII COVID19)	CHC da Universidade Federal do Paraná - PR
227	ATIVIDADE EXTERNA (ESPII COVID19)	CHC da Universidade Federal do Paraná - PR
227	ATIVIDADE EXTERNA (ESPII COVID19)	CHC da Universidade Federal do Paraná - PR
227	ATIVIDADE EXTERNA (ESPII COVID19)	CHC da Universidade Federal do Paraná - PR
227	ATIVIDADE EXTERNA (ESPII COVID19)	CHC da Universidade Federal do Paraná - PR
227	ATIVIDADE EXTERNA (ESPII COVID19)	CHC da Universidade Federal do Paraná - PR
227	ATIVIDADE EXTERNA (ESPII COVID19)	CHC da Universidade Federal do Paraná - PR
227	ATIVIDADE EXTERNA (ESPII COVID19)	CHC da Universidade Federal do Paraná - PR
227	ATIVIDADE EXTERNA (ESPII COVID19)	CHC da Universidade Federal do Paraná - PR
227	ATIVIDADE EXTERNA (ESPII COVID19)	CHC da Universidade Federal do Paraná - PR
227	ATIVIDADE EXTERNA (ESPII COVID19)	CHC da Universidade Federal do Paraná - PR
227	ATIVIDADE EXTERNA (ESPII COVID19)	CHC da Universidade Federal do Paraná - PR
227	ATIVIDADE EXTERNA (ESPII COVID19)	CHC da Universidade Federal do Paraná - PR
227	ATIVIDADE EXTERNA (ESPII COVID19)	CHC da Universidade Federal do Paraná - PR
227	ATIVIDADE EXTERNA (ESPII COVID19)	CHC da Universidade Federal do Paraná - PR
227	ATIVIDADE EXTERNA (ESPII COVID19)	CHC da Universidade Federal do Paraná - PR
227	ATIVIDADE EXTERNA (ESPII COVID19)	CHC da Universidade Federal do Paraná - PR
227	ATIVIDADE EXTERNA (ESPII COVID19)	CHC da Universidade Federal do Paraná - PR
227	ATIVIDADE EXTERNA (ESPII COVID19)	CHC da Universidade Federal do Paraná - PR
227	ATIVIDADE EXTERNA (ESPII COVID19)	CHC da Universidade Federal do Paraná - PR
227	ATIVIDADE EXTERNA (ESPII COVID19)	CHC da Universidade Federal do Paraná - PR
227	ATIVIDADE EXTERNA (ESPII COVID19)	CHC da Universidade Federal do Paraná - PR
227	ATIVIDADE EXTERNA (ESPII COVID19)	CHC da Universidade Federal do Paraná - PR

227	ATIVIDADE EXTERNA (ESPII COVID19)	CHC da Universidade Federal do Paraná - PR
227	ATIVIDADE EXTERNA (ESPII COVID19)	CHC da Universidade Federal do Paraná - PR
227	ATIVIDADE EXTERNA (ESPII COVID19)	CHC da Universidade Federal do Paraná - PR
227	ATIVIDADE EXTERNA (ESPII COVID19)	CHC da Universidade Federal do Paraná - PR
227	ATIVIDADE EXTERNA (ESPII COVID19)	CHC da Universidade Federal do Paraná - PR
227	ATIVIDADE EXTERNA (ESPII COVID19)	CHC da Universidade Federal do Paraná - PR
227	ATIVIDADE EXTERNA (ESPII COVID19)	CHC da Universidade Federal do Paraná - PR
227	ATIVIDADE EXTERNA (ESPII COVID19)	CHC da Universidade Federal do Paraná - PR
227	ATIVIDADE EXTERNA (ESPII COVID19)	CHC da Universidade Federal do Paraná - PR
227	ATIVIDADE EXTERNA (ESPII COVID19)	CHC da Universidade Federal do Paraná - PR
227	ATIVIDADE EXTERNA (ESPII COVID19)	CHC da Universidade Federal do Paraná - PR
227	ATIVIDADE EXTERNA (ESPII COVID19)	CHC da Universidade Federal do Paraná - PR
227	ATIVIDADE EXTERNA (ESPII COVID19)	CHC da Universidade Federal do Paraná - PR
227	ATIVIDADE EXTERNA (ESPII COVID19)	CHC da Universidade Federal do Paraná - PR
227	ATIVIDADE EXTERNA (ESPII COVID19)	CHC da Universidade Federal do Paraná - PR
227	ATIVIDADE EXTERNA (ESPII COVID19)	CHC da Universidade Federal do Paraná - PR
227	ATIVIDADE EXTERNA (ESPII COVID19)	CHC da Universidade Federal do Paraná - PR
227	ATIVIDADE EXTERNA (ESPII COVID19)	CHC da Universidade Federal do Paraná - PR
227	ATIVIDADE EXTERNA (ESPII COVID19)	CHC da Universidade Federal do Paraná - PR
227	ATIVIDADE EXTERNA (ESPII COVID19)	CHC da Universidade Federal do Paraná - PR
227	ATIVIDADE EXTERNA (ESPII COVID19)	CHC da Universidade Federal do Paraná - PR
227	ATIVIDADE EXTERNA (ESPII COVID19)	CHC da Universidade Federal do Paraná - PR
227	ATIVIDADE EXTERNA (ESPII COVID19)	CHC da Universidade Federal do Paraná - PR
227	ATIVIDADE EXTERNA (ESPII COVID19)	CHC da Universidade Federal do Paraná - PR
227	ATIVIDADE EXTERNA (ESPII COVID19)	CHC da Universidade Federal do Paraná - PR
227	ATIVIDADE EXTERNA (ESPII COVID19)	CHC da Universidade Federal do Paraná - PR
227	ATIVIDADE EXTERNA (ESPII COVID19)	CHC da Universidade Federal do Paraná - PR
227	ATIVIDADE EXTERNA (ESPII COVID19)	CHC da Universidade Federal do Paraná - PR
227	ATIVIDADE EXTERNA (ESPII COVID19)	CHC da Universidade Federal do Paraná - PR
227	ATIVIDADE EXTERNA (ESPII COVID19)	CHC da Universidade Federal do Paraná - PR
227	ATIVIDADE EXTERNA (ESPII COVID19)	CHC da Universidade Federal do Paraná - PR
227	ATIVIDADE EXTERNA (ESPII COVID19)	CHC da Universidade Federal do Paraná - PR
227	ATIVIDADE EXTERNA (ESPII COVID19)	CHC da Universidade Federal do Paraná - PR
227	ATIVIDADE EXTERNA (ESPII COVID19)	CHC da Universidade Federal do Paraná - PR
227	ATIVIDADE EXTERNA (ESPII COVID19)	CHC da Universidade Federal do Paraná - PR
227	ATIVIDADE EXTERNA (ESPII COVID19)	CHC da Universidade Federal do Paraná - PR
227	ATIVIDADE EXTERNA (ESPII COVID19)	CHC da Universidade Federal do Paraná - PR
227	ATIVIDADE EXTERNA (ESPII COVID19)	CHC da Universidade Federal do Paraná - PR
227	ATIVIDADE EXTERNA (ESPII COVID19)	CHC da Universidade Federal do Paraná - PR
227	ATIVIDADE EXTERNA (ESPII COVID19)	CHC da Universidade Federal do Paraná - PR
227	ATIVIDADE EXTERNA (ESPII COVID19)	CHC da Universidade Federal do Paraná - PR
227	ATIVIDADE EXTERNA (ESPII COVID19)	CHC da Universidade Federal do Paraná - PR
227	ATIVIDADE EXTERNA (ESPII COVID19)	CHC da Universidade Federal do Paraná - PR
227	ATIVIDADE EXTERNA (ESPII COVID19)	CHC da Universidade Federal do Paraná - PR
227	ATIVIDADE EXTERNA (ESPII COVID19)	CHC da Universidade Federal do Paraná - PR
227	ATIVIDADE EXTERNA (ESPII COVID19)	CHC da Universidade Federal do Paraná - PR
227	ATIVIDADE EXTERNA (ESPII COVID19)	CHC da Universidade Federal do Paraná - PR
227	ATIVIDADE EXTERNA (ESPII COVID19)	CHC da Universidade Federal do Paraná - PR

227	ATIVIDADE EXTERNA (ESPII COVID19)	CHC da Universidade Federal do Paraná - PR
227	ATIVIDADE EXTERNA (ESPII COVID19)	CHC da Universidade Federal do Paraná - PR
227	ATIVIDADE EXTERNA (ESPII COVID19)	CHC da Universidade Federal do Paraná - PR
227	ATIVIDADE EXTERNA (ESPII COVID19)	CHC da Universidade Federal do Paraná - PR
227	ATIVIDADE EXTERNA (ESPII COVID19)	CHC da Universidade Federal do Paraná - PR
227	ATIVIDADE EXTERNA (ESPII COVID19)	CHC da Universidade Federal do Paraná - PR
227	ATIVIDADE EXTERNA (ESPII COVID19)	CHC da Universidade Federal do Paraná - PR
227	ATIVIDADE EXTERNA (ESPII COVID19)	CHC da Universidade Federal do Paraná - PR
227	ATIVIDADE EXTERNA (ESPII COVID19)	CHC da Universidade Federal do Paraná - PR
227	ATIVIDADE EXTERNA (ESPII COVID19)	CHC da Universidade Federal do Paraná - PR
227	ATIVIDADE EXTERNA (ESPII COVID19)	CHC da Universidade Federal do Paraná - PR
227	ATIVIDADE EXTERNA (ESPII COVID19)	CHC da Universidade Federal do Paraná - PR
227	ATIVIDADE EXTERNA (ESPII COVID19)	CHC da Universidade Federal do Paraná - PR
227	ATIVIDADE EXTERNA (ESPII COVID19)	CHC da Universidade Federal do Paraná - PR
227	ATIVIDADE EXTERNA (ESPII COVID19)	CHC da Universidade Federal do Paraná - PR
227	ATIVIDADE EXTERNA (ESPII COVID19)	CHC da Universidade Federal do Paraná - PR
227	ATIVIDADE EXTERNA (ESPII COVID19)	CHC da Universidade Federal do Paraná - PR
227	ATIVIDADE EXTERNA (ESPII COVID19)	CHC da Universidade Federal do Paraná - PR
227	ATIVIDADE EXTERNA (ESPII COVID19)	CHC da Universidade Federal do Paraná - PR
227	ATIVIDADE EXTERNA (ESPII COVID19)	CHC da Universidade Federal do Paraná - PR
227	ATIVIDADE EXTERNA (ESPII COVID19)	CHC da Universidade Federal do Paraná - PR
227	ATIVIDADE EXTERNA (ESPII COVID19)	CHC da Universidade Federal do Paraná - PR
227	ATIVIDADE EXTERNA (ESPII COVID19)	CHC da Universidade Federal do Paraná - PR
227	ATIVIDADE EXTERNA (ESPII COVID19)	CHC da Universidade Federal do Paraná - PR
227	ATIVIDADE EXTERNA (ESPII COVID19)	CHC da Universidade Federal do Paraná - PR
227	ATIVIDADE EXTERNA (ESPII COVID19)	CHC da Universidade Federal do Paraná - PR
227	ATIVIDADE EXTERNA (ESPII COVID19)	CHC da Universidade Federal do Paraná - PR
227	ATIVIDADE EXTERNA (ESPII COVID19)	CHC da Universidade Federal do Paraná - PR
227	ATIVIDADE EXTERNA (ESPII COVID19)	CHC da Universidade Federal do Paraná - PR
227	ATIVIDADE EXTERNA (ESPII COVID19)	CHC da Universidade Federal do Paraná - PR
227	ATIVIDADE EXTERNA (ESPII COVID19)	CHC da Universidade Federal do Paraná - PR
227	ATIVIDADE EXTERNA (ESPII COVID19)	CHC da Universidade Federal do Paraná - PR
227	ATIVIDADE EXTERNA (ESPII COVID19)	CHC da Universidade Federal do Paraná - PR
227	ATIVIDADE EXTERNA (ESPII COVID19)	CHC da Universidade Federal do Paraná - PR
227	ATIVIDADE EXTERNA (ESPII COVID19)	CHC da Universidade Federal do Paraná - PR
227	ATIVIDADE EXTERNA (ESPII COVID19)	CHC da Universidade Federal do Paraná - PR
227	ATIVIDADE EXTERNA (ESPII COVID19)	CHC da Universidade Federal do Paraná - PR
227	ATIVIDADE EXTERNA (ESPII COVID19)	CHC da Universidade Federal do Paraná - PR
227	ATIVIDADE EXTERNA (ESPII COVID19)	CHC da Universidade Federal do Paraná - PR
227	ATIVIDADE EXTERNA (ESPII COVID19)	CHC da Universidade Federal do Paraná - PR
227	ATIVIDADE EXTERNA (ESPII COVID19)	CHC da Universidade Federal do Paraná - PR
227	ATIVIDADE EXTERNA (ESPII COVID19)	CHC da Universidade Federal do Paraná - PR
227	ATIVIDADE EXTERNA (ESPII COVID19)	CHC da Universidade Federal do Paraná - PR
227	ATIVIDADE EXTERNA (ESPII COVID19)	CHC da Universidade Federal do Paraná - PR
227	ATIVIDADE EXTERNA (ESPII COVID19)	CHC da Universidade Federal do Paraná - PR
227	ATIVIDADE EXTERNA (ESPII COVID19)	CHC da Universidade Federal do Paraná - PR
227	ATIVIDADE EXTERNA (ESPII COVID19)	CHC da Universidade Federal do Paraná - PR
227	ATIVIDADE EXTERNA (ESPII COVID19)	CHC da Universidade Federal do Paraná - PR

227	ATIVIDADE EXTERNA (ESPII COVID19)	CHC da Universidade Federal do Paraná - PR
227	ATIVIDADE EXTERNA (ESPII COVID19)	CHC da Universidade Federal do Paraná - PR
227	ATIVIDADE EXTERNA (ESPII COVID19)	CHC da Universidade Federal do Paraná - PR
227	ATIVIDADE EXTERNA (ESPII COVID19)	CHC da Universidade Federal do Paraná - PR
227	ATIVIDADE EXTERNA (ESPII COVID19)	CHC da Universidade Federal do Paraná - PR
227	ATIVIDADE EXTERNA (ESPII COVID19)	CHC da Universidade Federal do Paraná - PR
227	ATIVIDADE EXTERNA (ESPII COVID19)	CHC da Universidade Federal do Paraná - PR
227	ATIVIDADE EXTERNA (ESPII COVID19)	CHC da Universidade Federal do Paraná - PR
227	ATIVIDADE EXTERNA (ESPII COVID19)	CHC da Universidade Federal do Paraná - PR
227	ATIVIDADE EXTERNA (ESPII COVID19)	CHC da Universidade Federal do Paraná - PR
227	ATIVIDADE EXTERNA (ESPII COVID19)	CHC da Universidade Federal do Paraná - PR
227	ATIVIDADE EXTERNA (ESPII COVID19)	CHC da Universidade Federal do Paraná - PR
227	ATIVIDADE EXTERNA (ESPII COVID19)	CHC da Universidade Federal do Paraná - PR
227	ATIVIDADE EXTERNA (ESPII COVID19)	CHC da Universidade Federal do Paraná - PR
227	ATIVIDADE EXTERNA (ESPII COVID19)	CHC da Universidade Federal do Paraná - PR
227	ATIVIDADE EXTERNA (ESPII COVID19)	CHC da Universidade Federal do Paraná - PR
227	ATIVIDADE EXTERNA (ESPII COVID19)	CHC da Universidade Federal do Paraná - PR
227	ATIVIDADE EXTERNA (ESPII COVID19)	CHC da Universidade Federal do Paraná - PR
227	ATIVIDADE EXTERNA (ESPII COVID19)	CHC da Universidade Federal do Paraná - PR
227	ATIVIDADE EXTERNA (ESPII COVID19)	CHC da Universidade Federal do Paraná - PR
227	ATIVIDADE EXTERNA (ESPII COVID19)	CHC da Universidade Federal do Paraná - PR
227	ATIVIDADE EXTERNA (ESPII COVID19)	CHC da Universidade Federal do Paraná - PR
227	ATIVIDADE EXTERNA (ESPII COVID19)	CHC da Universidade Federal do Paraná - PR
227	ATIVIDADE EXTERNA (ESPII COVID19)	CHC da Universidade Federal do Paraná - PR
227	ATIVIDADE EXTERNA (ESPII COVID19)	CHC da Universidade Federal do Paraná - PR
227	ATIVIDADE EXTERNA (ESPII COVID19)	CHC da Universidade Federal do Paraná - PR
227	ATIVIDADE EXTERNA (ESPII COVID19)	CHC da Universidade Federal do Paraná - PR
227	ATIVIDADE EXTERNA (ESPII COVID19)	CHC da Universidade Federal do Paraná - PR
227	ATIVIDADE EXTERNA (ESPII COVID19)	CHC da Universidade Federal do Paraná - PR
227	ATIVIDADE EXTERNA (ESPII COVID19)	CHC da Universidade Federal do Paraná - PR
227	ATIVIDADE EXTERNA (ESPII COVID19)	CHC da Universidade Federal do Paraná - PR
227	ATIVIDADE EXTERNA (ESPII COVID19)	CHC da Universidade Federal do Paraná - PR
227	ATIVIDADE EXTERNA (ESPII COVID19)	CHC da Universidade Federal do Paraná - PR
227	ATIVIDADE EXTERNA (ESPII COVID19)	CHC da Universidade Federal do Paraná - PR
227	ATIVIDADE EXTERNA (ESPII COVID19)	CHC da Universidade Federal do Paraná - PR
227	ATIVIDADE EXTERNA (ESPII COVID19)	CHC da Universidade Federal do Paraná - PR
227	ATIVIDADE EXTERNA (ESPII COVID19)	CHC da Universidade Federal do Paraná - PR
227	ATIVIDADE EXTERNA (ESPII COVID19)	CHC da Universidade Federal do Paraná - PR
227	ATIVIDADE EXTERNA (ESPII COVID19)	CHC da Universidade Federal do Paraná - PR
227	ATIVIDADE EXTERNA (ESPII COVID19)	CHC da Universidade Federal do Paraná - PR
227	ATIVIDADE EXTERNA (ESPII COVID19)	CHC da Universidade Federal do Paraná - PR
227	ATIVIDADE EXTERNA (ESPII COVID19)	CHC da Universidade Federal do Paraná - PR
227	ATIVIDADE EXTERNA (ESPII COVID19)	CHC da Universidade Federal do Paraná - PR
227	ATIVIDADE EXTERNA (ESPII COVID19)	CHC da Universidade Federal do Paraná - PR
227	ATIVIDADE EXTERNA (ESPII COVID19)	CHC da Universidade Federal do Paraná - PR
227	ATIVIDADE EXTERNA (ESPII COVID19)	CHC da Universidade Federal do Paraná - PR
227	ATIVIDADE EXTERNA (ESPII COVID19)	CHC da Universidade Federal do Paraná - PR
227	ATIVIDADE EXTERNA (ESPII COVID19)	CHC da Universidade Federal do Paraná - PR

227	ATIVIDADE EXTERNA (ESPII COVID19)	CHC da Universidade Federal do Paraná - PR
227	ATIVIDADE EXTERNA (ESPII COVID19)	CHC da Universidade Federal do Paraná - PR
227	ATIVIDADE EXTERNA (ESPII COVID19)	CHC da Universidade Federal do Paraná - PR
227	ATIVIDADE EXTERNA (ESPII COVID19)	CHC da Universidade Federal do Paraná - PR
227	ATIVIDADE EXTERNA (ESPII COVID19)	CHC da Universidade Federal do Paraná - PR
227	ATIVIDADE EXTERNA (ESPII COVID19)	CHC da Universidade Federal do Paraná - PR
227	ATIVIDADE EXTERNA (ESPII COVID19)	CHC da Universidade Federal do Paraná - PR
227	ATIVIDADE EXTERNA (ESPII COVID19)	CHC da Universidade Federal do Paraná - PR
227	ATIVIDADE EXTERNA (ESPII COVID19)	CHC da Universidade Federal do Paraná - PR
227	ATIVIDADE EXTERNA (ESPII COVID19)	CHC da Universidade Federal do Paraná - PR
227	ATIVIDADE EXTERNA (ESPII COVID19)	CHC da Universidade Federal do Paraná - PR
227	ATIVIDADE EXTERNA (ESPII COVID19)	CHC da Universidade Federal do Paraná - PR
227	ATIVIDADE EXTERNA (ESPII COVID19)	CHC da Universidade Federal do Paraná - PR
227	ATIVIDADE EXTERNA (ESPII COVID19)	CHC da Universidade Federal do Paraná - PR
227	ATIVIDADE EXTERNA (ESPII COVID19)	CHC da Universidade Federal do Paraná - PR
227	ATIVIDADE EXTERNA (ESPII COVID19)	CHC da Universidade Federal do Paraná - PR
227	ATIVIDADE EXTERNA (ESPII COVID19)	CHC da Universidade Federal do Paraná - PR
227	ATIVIDADE EXTERNA (ESPII COVID19)	CHC da Universidade Federal do Paraná - PR
227	ATIVIDADE EXTERNA (ESPII COVID19)	CHC da Universidade Federal do Paraná - PR
227	ATIVIDADE EXTERNA (ESPII COVID19)	CHC da Universidade Federal do Paraná - PR
227	ATIVIDADE EXTERNA (ESPII COVID19)	CHC da Universidade Federal do Paraná - PR
227	ATIVIDADE EXTERNA (ESPII COVID19)	CHC da Universidade Federal do Paraná - PR
227	ATIVIDADE EXTERNA (ESPII COVID19)	CHC da Universidade Federal do Paraná - PR
227	ATIVIDADE EXTERNA (ESPII COVID19)	CHC da Universidade Federal do Paraná - PR
227	ATIVIDADE EXTERNA (ESPII COVID19)	CHC da Universidade Federal do Paraná - PR
227	ATIVIDADE EXTERNA (ESPII COVID19)	CHC da Universidade Federal do Paraná - PR
227	ATIVIDADE EXTERNA (ESPII COVID19)	CHC da Universidade Federal do Paraná - PR
227	ATIVIDADE EXTERNA (ESPII COVID19)	CHC da Universidade Federal do Paraná - PR
227	ATIVIDADE EXTERNA (ESPII COVID19)	CHC da Universidade Federal do Paraná - PR
227	ATIVIDADE EXTERNA (ESPII COVID19)	CHC da Universidade Federal do Paraná - PR
227	ATIVIDADE EXTERNA (ESPII COVID19)	CHC da Universidade Federal do Paraná - PR
227	ATIVIDADE EXTERNA (ESPII COVID19)	CHC da Universidade Federal do Paraná - PR
227	ATIVIDADE EXTERNA (ESPII COVID19)	CHC da Universidade Federal do Paraná - PR
227	ATIVIDADE EXTERNA (ESPII COVID19)	CHC da Universidade Federal do Paraná - PR
227	ATIVIDADE EXTERNA (ESPII COVID19)	CHC da Universidade Federal do Paraná - PR
227	ATIVIDADE EXTERNA (ESPII COVID19)	CHC da Universidade Federal do Paraná - PR
227	ATIVIDADE EXTERNA (ESPII COVID19)	CHC da Universidade Federal do Paraná - PR
227	ATIVIDADE EXTERNA (ESPII COVID19)	CHC da Universidade Federal do Paraná - PR
227	ATIVIDADE EXTERNA (ESPII COVID19)	CHC da Universidade Federal do Paraná - PR
227	ATIVIDADE EXTERNA (ESPII COVID19)	CHC da Universidade Federal do Paraná - PR
227	ATIVIDADE EXTERNA (ESPII COVID19)	CHC da Universidade Federal do Paraná - PR
227	ATIVIDADE EXTERNA (ESPII COVID19)	CHC da Universidade Federal do Paraná - PR
227	ATIVIDADE EXTERNA (ESPII COVID19)	CHC da Universidade Federal do Paraná - PR
227	ATIVIDADE EXTERNA (ESPII COVID19)	CHC da Universidade Federal do Paraná - PR
227	ATIVIDADE EXTERNA (ESPII COVID19)	CHC da Universidade Federal do Paraná - PR
227	ATIVIDADE EXTERNA (ESPII COVID19)	CHC da Universidade Federal do Paraná - PR
227	ATIVIDADE EXTERNA (ESPII COVID19)	CHC da Universidade Federal do Paraná - PR
227	ATIVIDADE EXTERNA (ESPII COVID19)	CHC da Universidade Federal do Paraná - PR

227	ATIVIDADE EXTERNA (ESPII COVID19)	CHC da Universidade Federal do Paraná - PR
227	ATIVIDADE EXTERNA (ESPII COVID19)	CHC da Universidade Federal do Paraná - PR
227	ATIVIDADE EXTERNA (ESPII COVID19)	CHC da Universidade Federal do Paraná - PR
227	ATIVIDADE EXTERNA (ESPII COVID19)	CHC da Universidade Federal do Paraná - PR
227	ATIVIDADE EXTERNA (ESPII COVID19)	CHC da Universidade Federal do Paraná - PR
227	ATIVIDADE EXTERNA (ESPII COVID19)	CHC da Universidade Federal do Paraná - PR
227	ATIVIDADE EXTERNA (ESPII COVID19)	CHC da Universidade Federal do Paraná - PR
227	ATIVIDADE EXTERNA (ESPII COVID19)	CHC da Universidade Federal do Paraná - PR
227	ATIVIDADE EXTERNA (ESPII COVID19)	CHC da Universidade Federal do Paraná - PR
227	ATIVIDADE EXTERNA (ESPII COVID19)	CHC da Universidade Federal do Paraná - PR
227	ATIVIDADE EXTERNA (ESPII COVID19)	CHC da Universidade Federal do Paraná - PR
227	ATIVIDADE EXTERNA (ESPII COVID19)	CHC da Universidade Federal do Paraná - PR
227	ATIVIDADE EXTERNA (ESPII COVID19)	CHC da Universidade Federal do Paraná - PR
227	ATIVIDADE EXTERNA (ESPII COVID19)	CHC da Universidade Federal do Paraná - PR
227	ATIVIDADE EXTERNA (ESPII COVID19)	CHC da Universidade Federal do Paraná - PR
227	ATIVIDADE EXTERNA (ESPII COVID19)	CHC da Universidade Federal do Paraná - PR
227	ATIVIDADE EXTERNA (ESPII COVID19)	CHC da Universidade Federal do Paraná - PR
227	ATIVIDADE EXTERNA (ESPII COVID19)	CHC da Universidade Federal do Paraná - PR
227	ATIVIDADE EXTERNA (ESPII COVID19)	CHC da Universidade Federal do Paraná - PR
227	ATIVIDADE EXTERNA (ESPII COVID19)	CHC da Universidade Federal do Paraná - PR
227	ATIVIDADE EXTERNA (ESPII COVID19)	CHC da Universidade Federal do Paraná - PR
227	ATIVIDADE EXTERNA (ESPII COVID19)	CHC da Universidade Federal do Paraná - PR
227	ATIVIDADE EXTERNA (ESPII COVID19)	CHC da Universidade Federal do Paraná - PR
227	ATIVIDADE EXTERNA (ESPII COVID19)	CHC da Universidade Federal do Paraná - PR
227	ATIVIDADE EXTERNA (ESPII COVID19)	CHC da Universidade Federal do Paraná - PR
227	ATIVIDADE EXTERNA (ESPII COVID19)	CHC da Universidade Federal do Paraná - PR
227	ATIVIDADE EXTERNA (ESPII COVID19)	CHC da Universidade Federal do Paraná - PR
227	ATIVIDADE EXTERNA (ESPII COVID19)	CHC da Universidade Federal do Paraná - PR
227	ATIVIDADE EXTERNA (ESPII COVID19)	CHC da Universidade Federal do Paraná - PR
227	ATIVIDADE EXTERNA (ESPII COVID19)	CHC da Universidade Federal do Paraná - PR
227	ATIVIDADE EXTERNA (ESPII COVID19)	CHC da Universidade Federal do Paraná - PR
227	ATIVIDADE EXTERNA (ESPII COVID19)	CHC da Universidade Federal do Paraná - PR
227	ATIVIDADE EXTERNA (ESPII COVID19)	CHC da Universidade Federal do Paraná - PR
227	ATIVIDADE EXTERNA (ESPII COVID19)	CHC da Universidade Federal do Paraná - PR
227	ATIVIDADE EXTERNA (ESPII COVID19)	CHC da Universidade Federal do Paraná - PR
227	ATIVIDADE EXTERNA (ESPII COVID19)	CHC da Universidade Federal do Paraná - PR
227	ATIVIDADE EXTERNA (ESPII COVID19)	CHC da Universidade Federal do Paraná - PR
227	ATIVIDADE EXTERNA (ESPII COVID19)	CHC da Universidade Federal do Paraná - PR
227	ATIVIDADE EXTERNA (ESPII COVID19)	CHC da Universidade Federal do Paraná - PR
227	ATIVIDADE EXTERNA (ESPII COVID19)	CHC da Universidade Federal do Paraná - PR
227	ATIVIDADE EXTERNA (ESPII COVID19)	CHC da Universidade Federal do Paraná - PR
227	ATIVIDADE EXTERNA (ESPII COVID19)	CHC da Universidade Federal do Paraná - PR
227	ATIVIDADE EXTERNA (ESPII COVID19)	CHC da Universidade Federal do Paraná - PR
227	ATIVIDADE EXTERNA (ESPII COVID19)	CHC da Universidade Federal do Paraná - PR
227	ATIVIDADE EXTERNA (ESPII COVID19)	CHC da Universidade Federal do Paraná - PR
227	ATIVIDADE EXTERNA (ESPII COVID19)	CHC da Universidade Federal do Paraná - PR
227	ATIVIDADE EXTERNA (ESPII COVID19)	CHC da Universidade Federal do Paraná - PR
227	ATIVIDADE EXTERNA (ESPII COVID19)	CHC da Universidade Federal do Paraná - PR

227	ATIVIDADE EXTERNA (ESPII COVID19)	CHC da Universidade Federal do Paraná - PR
227	ATIVIDADE EXTERNA (ESPII COVID19)	CHC da Universidade Federal do Paraná - PR
227	ATIVIDADE EXTERNA (ESPII COVID19)	CHC da Universidade Federal do Paraná - PR
227	ATIVIDADE EXTERNA (ESPII COVID19)	CHC da Universidade Federal do Paraná - PR
227	ATIVIDADE EXTERNA (ESPII COVID19)	CHC da Universidade Federal do Paraná - PR
227	ATIVIDADE EXTERNA (ESPII COVID19)	CHC da Universidade Federal do Paraná - PR
227	ATIVIDADE EXTERNA (ESPII COVID19)	CHC da Universidade Federal do Paraná - PR
227	ATIVIDADE EXTERNA (ESPII COVID19)	CHC da Universidade Federal do Paraná - PR
227	ATIVIDADE EXTERNA (ESPII COVID19)	CHC da Universidade Federal do Paraná - PR
227	ATIVIDADE EXTERNA (ESPII COVID19)	CHC da Universidade Federal do Paraná - PR
227	ATIVIDADE EXTERNA (ESPII COVID19)	CHC da Universidade Federal do Paraná - PR
227	ATIVIDADE EXTERNA (ESPII COVID19)	CHC da Universidade Federal do Paraná - PR
227	ATIVIDADE EXTERNA (ESPII COVID19)	CHC da Universidade Federal do Paraná - PR
227	ATIVIDADE EXTERNA (ESPII COVID19)	CHC da Universidade Federal do Paraná - PR
227	ATIVIDADE EXTERNA (ESPII COVID19)	CHC da Universidade Federal do Paraná - PR
227	ATIVIDADE EXTERNA (ESPII COVID19)	CHC da Universidade Federal do Paraná - PR
227	ATIVIDADE EXTERNA (ESPII COVID19)	CHC da Universidade Federal do Paraná - PR
227	ATIVIDADE EXTERNA (ESPII COVID19)	CHC da Universidade Federal do Paraná - PR
227	ATIVIDADE EXTERNA (ESPII COVID19)	CHC da Universidade Federal do Paraná - PR
227	ATIVIDADE EXTERNA (ESPII COVID19)	CHC da Universidade Federal do Paraná - PR
227	ATIVIDADE EXTERNA (ESPII COVID19)	CHC da Universidade Federal do Paraná - PR
227	ATIVIDADE EXTERNA (ESPII COVID19)	CHC da Universidade Federal do Paraná - PR
227	ATIVIDADE EXTERNA (ESPII COVID19)	CHC da Universidade Federal do Paraná - PR
227	ATIVIDADE EXTERNA (ESPII COVID19)	CHC da Universidade Federal do Paraná - PR
227	ATIVIDADE EXTERNA (ESPII COVID19)	CHC da Universidade Federal do Paraná - PR
227	ATIVIDADE EXTERNA (ESPII COVID19)	CHC da Universidade Federal do Paraná - PR
227	ATIVIDADE EXTERNA (ESPII COVID19)	CHC da Universidade Federal do Paraná - PR
227	ATIVIDADE EXTERNA (ESPII COVID19)	CHC da Universidade Federal do Paraná - PR
227	ATIVIDADE EXTERNA (ESPII COVID19)	CHC da Universidade Federal do Paraná - PR
227	ATIVIDADE EXTERNA (ESPII COVID19)	CHC da Universidade Federal do Paraná - PR
227	ATIVIDADE EXTERNA (ESPII COVID19)	CHC da Universidade Federal do Paraná - PR
227	ATIVIDADE EXTERNA (ESPII COVID19)	CHC da Universidade Federal do Paraná - PR
227	ATIVIDADE EXTERNA (ESPII COVID19)	CHC da Universidade Federal do Paraná - PR
227	ATIVIDADE EXTERNA (ESPII COVID19)	CHC da Universidade Federal do Paraná - PR
227	ATIVIDADE EXTERNA (ESPII COVID19)	CHC da Universidade Federal do Paraná - PR
227	ATIVIDADE EXTERNA (ESPII COVID19)	CHC da Universidade Federal do Paraná - PR
227	ATIVIDADE EXTERNA (ESPII COVID19)	CHC da Universidade Federal do Paraná - PR
227	ATIVIDADE EXTERNA (ESPII COVID19)	CHC da Universidade Federal do Paraná - PR
227	ATIVIDADE EXTERNA (ESPII COVID19)	CHC da Universidade Federal do Paraná - PR
227	ATIVIDADE EXTERNA (ESPII COVID19)	CHC da Universidade Federal do Paraná - PR
227	ATIVIDADE EXTERNA (ESPII COVID19)	CHC da Universidade Federal do Paraná - PR
227	ATIVIDADE EXTERNA (ESPII COVID19)	CHC da Universidade Federal do Paraná - PR
227	ATIVIDADE EXTERNA (ESPII COVID19)	CHC da Universidade Federal do Paraná - PR
227	ATIVIDADE EXTERNA (ESPII COVID19)	CHC da Universidade Federal do Paraná - PR
227	ATIVIDADE EXTERNA (ESPII COVID19)	CHC da Universidade Federal do Paraná - PR
227	ATIVIDADE EXTERNA (ESPII COVID19)	CHC da Universidade Federal do Paraná - PR
227	ATIVIDADE EXTERNA (ESPII COVID19)	CHC da Universidade Federal do Paraná - PR
227	ATIVIDADE EXTERNA (ESPII COVID19)	CHC da Universidade Federal do Paraná - PR

227	ATIVIDADE EXTERNA (ESPII COVID19)	CHC da Universidade Federal do Paraná - PR
227	ATIVIDADE EXTERNA (ESPII COVID19)	CHC da Universidade Federal do Paraná - PR
227	ATIVIDADE EXTERNA (ESPII COVID19)	CHC da Universidade Federal do Paraná - PR
227	ATIVIDADE EXTERNA (ESPII COVID19)	CHC da Universidade Federal do Paraná - PR
227	ATIVIDADE EXTERNA (ESPII COVID19)	CHC da Universidade Federal do Paraná - PR
227	ATIVIDADE EXTERNA (ESPII COVID19)	CHC da Universidade Federal do Paraná - PR
227	ATIVIDADE EXTERNA (ESPII COVID19)	CHC da Universidade Federal do Paraná - PR
227	ATIVIDADE EXTERNA (ESPII COVID19)	CHC da Universidade Federal do Paraná - PR
227	ATIVIDADE EXTERNA (ESPII COVID19)	CHC da Universidade Federal do Paraná - PR
227	ATIVIDADE EXTERNA (ESPII COVID19)	CHC da Universidade Federal do Paraná - PR
227	ATIVIDADE EXTERNA (ESPII COVID19)	CHC da Universidade Federal do Paraná - PR
227	ATIVIDADE EXTERNA (ESPII COVID19)	CHC da Universidade Federal do Paraná - PR
227	ATIVIDADE EXTERNA (ESPII COVID19)	CHC da Universidade Federal do Paraná - PR
227	ATIVIDADE EXTERNA (ESPII COVID19)	CHC da Universidade Federal do Paraná - PR
227	ATIVIDADE EXTERNA (ESPII COVID19)	CHC da Universidade Federal do Paraná - PR
227	ATIVIDADE EXTERNA (ESPII COVID19)	CHC da Universidade Federal do Paraná - PR
227	ATIVIDADE EXTERNA (ESPII COVID19)	CHC da Universidade Federal do Paraná - PR
227	ATIVIDADE EXTERNA (ESPII COVID19)	CHC da Universidade Federal do Paraná - PR
227	ATIVIDADE EXTERNA (ESPII COVID19)	CHC da Universidade Federal do Paraná - PR
227	ATIVIDADE EXTERNA (ESPII COVID19)	CHC da Universidade Federal do Paraná - PR
227	ATIVIDADE EXTERNA (ESPII COVID19)	CHC da Universidade Federal do Paraná - PR
227	ATIVIDADE EXTERNA (ESPII COVID19)	CHC da Universidade Federal do Paraná - PR
227	ATIVIDADE EXTERNA (ESPII COVID19)	CHC da Universidade Federal do Paraná - PR
227	ATIVIDADE EXTERNA (ESPII COVID19)	CHC da Universidade Federal do Paraná - PR
227	ATIVIDADE EXTERNA (ESPII COVID19)	CHC da Universidade Federal do Paraná - PR
227	ATIVIDADE EXTERNA (ESPII COVID19)	CHC da Universidade Federal do Paraná - PR
227	ATIVIDADE EXTERNA (ESPII COVID19)	CHC da Universidade Federal do Paraná - PR
227	ATIVIDADE EXTERNA (ESPII COVID19)	CHC da Universidade Federal do Paraná - PR
227	ATIVIDADE EXTERNA (ESPII COVID19)	CHC da Universidade Federal do Paraná - PR
227	ATIVIDADE EXTERNA (ESPII COVID19)	CHC da Universidade Federal do Paraná - PR
227	ATIVIDADE EXTERNA (ESPII COVID19)	CHC da Universidade Federal do Paraná - PR
227	ATIVIDADE EXTERNA (ESPII COVID19)	CHC da Universidade Federal do Paraná - PR
227	ATIVIDADE EXTERNA (ESPII COVID19)	CHC da Universidade Federal do Paraná - PR
227	ATIVIDADE EXTERNA (ESPII COVID19)	CHC da Universidade Federal do Paraná - PR
227	ATIVIDADE EXTERNA (ESPII COVID19)	CHC da Universidade Federal do Paraná - PR
227	ATIVIDADE EXTERNA (ESPII COVID19)	CHC da Universidade Federal do Paraná - PR
227	ATIVIDADE EXTERNA (ESPII COVID19)	CHC da Universidade Federal do Paraná - PR
227	ATIVIDADE EXTERNA (ESPII COVID19)	CHC da Universidade Federal do Paraná - PR
227	ATIVIDADE EXTERNA (ESPII COVID19)	CHC da Universidade Federal do Paraná - PR
227	ATIVIDADE EXTERNA (ESPII COVID19)	CHC da Universidade Federal do Paraná - PR
227	ATIVIDADE EXTERNA (ESPII COVID19)	CHC da Universidade Federal do Paraná - PR
227	ATIVIDADE EXTERNA (ESPII COVID19)	CHC da Universidade Federal do Paraná - PR
227	ATIVIDADE EXTERNA (ESPII COVID19)	CHC da Universidade Federal do Paraná - PR
227	ATIVIDADE EXTERNA (ESPII COVID19)	CHC da Universidade Federal do Paraná - PR
227	ATIVIDADE EXTERNA (ESPII COVID19)	CHC da Universidade Federal do Paraná - PR
227	ATIVIDADE EXTERNA (ESPII COVID19)	CHC da Universidade Federal do Paraná - PR
227	ATIVIDADE EXTERNA (ESPII COVID19)	CHC da Universidade Federal do Paraná - PR
227	ATIVIDADE EXTERNA (ESPII COVID19)	CHC da Universidade Federal do Paraná - PR

227	ATIVIDADE EXTERNA (ESPII COVID19)	CHC da Universidade Federal do Paraná - PR
227	ATIVIDADE EXTERNA (ESPII COVID19)	CHC da Universidade Federal do Paraná - PR
227	ATIVIDADE EXTERNA (ESPII COVID19)	CHC da Universidade Federal do Paraná - PR
227	ATIVIDADE EXTERNA (ESPII COVID19)	CHC da Universidade Federal do Paraná - PR
227	ATIVIDADE EXTERNA (ESPII COVID19)	CHC da Universidade Federal do Paraná - PR
227	ATIVIDADE EXTERNA (ESPII COVID19)	CHC da Universidade Federal do Paraná - PR
227	ATIVIDADE EXTERNA (ESPII COVID19)	CHC da Universidade Federal do Paraná - PR
227	ATIVIDADE EXTERNA (ESPII COVID19)	CHC da Universidade Federal do Paraná - PR
227	ATIVIDADE EXTERNA (ESPII COVID19)	CHC da Universidade Federal do Paraná - PR
227	ATIVIDADE EXTERNA (ESPII COVID19)	CHC da Universidade Federal do Paraná - PR
227	ATIVIDADE EXTERNA (ESPII COVID19)	CHC da Universidade Federal do Paraná - PR
227	ATIVIDADE EXTERNA (ESPII COVID19)	CHC da Universidade Federal do Paraná - PR
227	ATIVIDADE EXTERNA (ESPII COVID19)	CHC da Universidade Federal do Paraná - PR
227	ATIVIDADE EXTERNA (ESPII COVID19)	CHC da Universidade Federal do Paraná - PR
227	ATIVIDADE EXTERNA (ESPII COVID19)	CHC da Universidade Federal do Paraná - PR
227	ATIVIDADE EXTERNA (ESPII COVID19)	CHC da Universidade Federal do Paraná - PR
227	ATIVIDADE EXTERNA (ESPII COVID19)	CHC da Universidade Federal do Paraná - PR
227	ATIVIDADE EXTERNA (ESPII COVID19)	CHC da Universidade Federal do Paraná - PR
227	ATIVIDADE EXTERNA (ESPII COVID19)	CHC da Universidade Federal do Paraná - PR
227	ATIVIDADE EXTERNA (ESPII COVID19)	CHC da Universidade Federal do Paraná - PR
227	ATIVIDADE EXTERNA (ESPII COVID19)	CHC da Universidade Federal do Paraná - PR
227	ATIVIDADE EXTERNA (ESPII COVID19)	CHC da Universidade Federal do Paraná - PR
227	ATIVIDADE EXTERNA (ESPII COVID19)	CHC da Universidade Federal do Paraná - PR
227	ATIVIDADE EXTERNA (ESPII COVID19)	CHC da Universidade Federal do Paraná - PR
227	ATIVIDADE EXTERNA (ESPII COVID19)	CHC da Universidade Federal do Paraná - PR
227	ATIVIDADE EXTERNA (ESPII COVID19)	CHC da Universidade Federal do Paraná - PR
227	ATIVIDADE EXTERNA (ESPII COVID19)	CHC da Universidade Federal do Paraná - PR
227	ATIVIDADE EXTERNA (ESPII COVID19)	CHC da Universidade Federal do Paraná - PR
227	ATIVIDADE EXTERNA (ESPII COVID19)	CHC da Universidade Federal do Paraná - PR
227	ATIVIDADE EXTERNA (ESPII COVID19)	CHC da Universidade Federal do Paraná - PR
227	ATIVIDADE EXTERNA (ESPII COVID19)	CHC da Universidade Federal do Paraná - PR
227	ATIVIDADE EXTERNA (ESPII COVID19)	CHC da Universidade Federal do Paraná - PR
227	ATIVIDADE EXTERNA (ESPII COVID19)	CHC da Universidade Federal do Paraná - PR
227	ATIVIDADE EXTERNA (ESPII COVID19)	CHC da Universidade Federal do Paraná - PR
227	ATIVIDADE EXTERNA (ESPII COVID19)	CHC da Universidade Federal do Paraná - PR
227	ATIVIDADE EXTERNA (ESPII COVID19)	CHC da Universidade Federal do Paraná - PR
227	ATIVIDADE EXTERNA (ESPII COVID19)	CHC da Universidade Federal do Paraná - PR
227	ATIVIDADE EXTERNA (ESPII COVID19)	CHC da Universidade Federal do Paraná - PR
227	ATIVIDADE EXTERNA (ESPII COVID19)	CHC da Universidade Federal do Paraná - PR
227	ATIVIDADE EXTERNA (ESPII COVID19)	CHC da Universidade Federal do Paraná - PR
227	ATIVIDADE EXTERNA (ESPII COVID19)	CHC da Universidade Federal do Paraná - PR
227	ATIVIDADE EXTERNA (ESPII COVID19)	CHC da Universidade Federal do Paraná - PR
227	ATIVIDADE EXTERNA (ESPII COVID19)	CHC da Universidade Federal do Paraná - PR

227	ATIVIDADE EXTERNA (ESPII COVID19)	CHC da Universidade Federal do Paraná - PR
227	ATIVIDADE EXTERNA (ESPII COVID19)	CHC da Universidade Federal do Paraná - PR
227	ATIVIDADE EXTERNA (ESPII COVID19)	CHC da Universidade Federal do Paraná - PR
227	ATIVIDADE EXTERNA (ESPII COVID19)	CHC da Universidade Federal do Paraná - PR
227	ATIVIDADE EXTERNA (ESPII COVID19)	CHC da Universidade Federal do Paraná - PR
227	ATIVIDADE EXTERNA (ESPII COVID19)	CHC da Universidade Federal do Paraná - PR
227	ATIVIDADE EXTERNA (ESPII COVID19)	CHC da Universidade Federal do Paraná - PR
227	ATIVIDADE EXTERNA (ESPII COVID19)	CHC da Universidade Federal do Paraná - PR
227	ATIVIDADE EXTERNA (ESPII COVID19)	CHC da Universidade Federal do Paraná - PR
227	ATIVIDADE EXTERNA (ESPII COVID19)	CHC da Universidade Federal do Paraná - PR
227	ATIVIDADE EXTERNA (ESPII COVID19)	CHC da Universidade Federal do Paraná - PR
227	ATIVIDADE EXTERNA (ESPII COVID19)	CHC da Universidade Federal do Paraná - PR
227	ATIVIDADE EXTERNA (ESPII COVID19)	CHC da Universidade Federal do Paraná - PR
227	ATIVIDADE EXTERNA (ESPII COVID19)	CHC da Universidade Federal do Paraná - PR
227	ATIVIDADE EXTERNA (ESPII COVID19)	CHC da Universidade Federal do Paraná - PR
227	ATIVIDADE EXTERNA (ESPII COVID19)	CHC da Universidade Federal do Paraná - PR
227	ATIVIDADE EXTERNA (ESPII COVID19)	CHC da Universidade Federal do Paraná - PR
227	ATIVIDADE EXTERNA (ESPII COVID19)	CHC da Universidade Federal do Paraná - PR
227	ATIVIDADE EXTERNA (ESPII COVID19)	CHC da Universidade Federal do Paraná - PR
227	ATIVIDADE EXTERNA (ESPII COVID19)	CHC da Universidade Federal do Paraná - PR
227	ATIVIDADE EXTERNA (ESPII COVID19)	CHC da Universidade Federal do Paraná - PR
227	ATIVIDADE EXTERNA (ESPII COVID19)	CHC da Universidade Federal do Paraná - PR
227	ATIVIDADE EXTERNA (ESPII COVID19)	CHC da Universidade Federal do Paraná - PR
227	ATIVIDADE EXTERNA (ESPII COVID19)	CHC da Universidade Federal do Paraná - PR
227	ATIVIDADE EXTERNA (ESPII COVID19)	CHC da Universidade Federal do Paraná - PR
227	ATIVIDADE EXTERNA (ESPII COVID19)	CHC da Universidade Federal do Paraná - PR
227	ATIVIDADE EXTERNA (ESPII COVID19)	CHC da Universidade Federal do Paraná - PR
227	ATIVIDADE EXTERNA (ESPII COVID19)	CHC da Universidade Federal do Paraná - PR
227	ATIVIDADE EXTERNA (ESPII COVID19)	CHC da Universidade Federal do Paraná - PR
227	ATIVIDADE EXTERNA (ESPII COVID19)	CHC da Universidade Federal do Paraná - PR
227	ATIVIDADE EXTERNA (ESPII COVID19)	CHC da Universidade Federal do Paraná - PR
227	ATIVIDADE EXTERNA (ESPII COVID19)	CHC da Universidade Federal do Paraná - PR
227	ATIVIDADE EXTERNA (ESPII COVID19)	CHC da Universidade Federal do Paraná - PR
227	ATIVIDADE EXTERNA (ESPII COVID19)	CHC da Universidade Federal do Paraná - PR
227	ATIVIDADE EXTERNA (ESPII COVID19)	CHC da Universidade Federal do Paraná - PR
227	ATIVIDADE EXTERNA (ESPII COVID19)	CHC da Universidade Federal do Paraná - PR
227	ATIVIDADE EXTERNA (ESPII COVID19)	CHC da Universidade Federal do Paraná - PR
227	ATIVIDADE EXTERNA (ESPII COVID19)	CHC da Universidade Federal do Paraná - PR
227	ATIVIDADE EXTERNA (ESPII COVID19)	CHC da Universidade Federal do Paraná - PR
227	ATIVIDADE EXTERNA (ESPII COVID19)	CHC da Universidade Federal do Paraná - PR
227	ATIVIDADE EXTERNA (ESPII COVID19)	CHC da Universidade Federal do Paraná - PR
227	ATIVIDADE EXTERNA (ESPII COVID19)	CHC da Universidade Federal do Paraná - PR
227	ATIVIDADE EXTERNA (ESPII COVID19)	CHC da Universidade Federal do Paraná - PR
227	ATIVIDADE EXTERNA (ESPII COVID19)	CHC da Universidade Federal do Paraná - PR
227	ATIVIDADE EXTERNA (ESPII COVID19)	CHC da Universidade Federal do Paraná - PR
227	ATIVIDADE EXTERNA (ESPII COVID19)	CHC da Universidade Federal do Paraná - PR
227	ATIVIDADE EXTERNA (ESPII COVID19)	CHC da Universidade Federal do Paraná - PR
227	ATIVIDADE EXTERNA (ESPII COVID19)	CHC da Universidade Federal do Paraná - PR

227	ATIVIDADE EXTERNA (ESPII COVID19)	CHC da Universidade Federal do Paraná - PR
227	ATIVIDADE EXTERNA (ESPII COVID19)	CHC da Universidade Federal do Paraná - PR
227	ATIVIDADE EXTERNA (ESPII COVID19)	CHC da Universidade Federal do Paraná - PR
227	ATIVIDADE EXTERNA (ESPII COVID19)	CHC da Universidade Federal do Paraná - PR
227	ATIVIDADE EXTERNA (ESPII COVID19)	CHC da Universidade Federal do Paraná - PR
227	ATIVIDADE EXTERNA (ESPII COVID19)	CHC da Universidade Federal do Paraná - PR
227	ATIVIDADE EXTERNA (ESPII COVID19)	CHC da Universidade Federal do Paraná - PR
227	ATIVIDADE EXTERNA (ESPII COVID19)	CHC da Universidade Federal do Paraná - PR
227	ATIVIDADE EXTERNA (ESPII COVID19)	CHC da Universidade Federal do Paraná - PR
227	ATIVIDADE EXTERNA (ESPII COVID19)	CHC da Universidade Federal do Paraná - PR
227	ATIVIDADE EXTERNA (ESPII COVID19)	CHC da Universidade Federal do Paraná - PR
227	ATIVIDADE EXTERNA (ESPII COVID19)	CHC da Universidade Federal do Paraná - PR
227	ATIVIDADE EXTERNA (ESPII COVID19)	CHC da Universidade Federal do Paraná - PR
227	ATIVIDADE EXTERNA (ESPII COVID19)	CHC da Universidade Federal do Paraná - PR
227	ATIVIDADE EXTERNA (ESPII COVID19)	CHC da Universidade Federal do Paraná - PR
227	ATIVIDADE EXTERNA (ESPII COVID19)	CHC da Universidade Federal do Paraná - PR
227	ATIVIDADE EXTERNA (ESPII COVID19)	CHC da Universidade Federal do Paraná - PR
227	ATIVIDADE EXTERNA (ESPII COVID19)	CHC da Universidade Federal do Paraná - PR
227	ATIVIDADE EXTERNA (ESPII COVID19)	CHC da Universidade Federal do Paraná - PR
227	ATIVIDADE EXTERNA (ESPII COVID19)	CHC da Universidade Federal do Paraná - PR
227	ATIVIDADE EXTERNA (ESPII COVID19)	CHC da Universidade Federal do Paraná - PR
227	ATIVIDADE EXTERNA (ESPII COVID19)	CHC da Universidade Federal do Paraná - PR
227	ATIVIDADE EXTERNA (ESPII COVID19)	CHC da Universidade Federal do Paraná - PR
227	ATIVIDADE EXTERNA (ESPII COVID19)	CHC da Universidade Federal do Paraná - PR
227	ATIVIDADE EXTERNA (ESPII COVID19)	CHC da Universidade Federal do Paraná - PR
227	ATIVIDADE EXTERNA (ESPII COVID19)	CHC da Universidade Federal do Paraná - PR
227	ATIVIDADE EXTERNA (ESPII COVID19)	CHC da Universidade Federal do Paraná - PR
227	ATIVIDADE EXTERNA (ESPII COVID19)	CHC da Universidade Federal do Paraná - PR
227	ATIVIDADE EXTERNA (ESPII COVID19)	CHC da Universidade Federal do Paraná - PR
227	ATIVIDADE EXTERNA (ESPII COVID19)	CHC da Universidade Federal do Paraná - PR
227	ATIVIDADE EXTERNA (ESPII COVID19)	CHC da Universidade Federal do Paraná - PR
227	ATIVIDADE EXTERNA (ESPII COVID19)	CHC da Universidade Federal do Paraná - PR
227	ATIVIDADE EXTERNA (ESPII COVID19)	CHC da Universidade Federal do Paraná - PR
227	ATIVIDADE EXTERNA (ESPII COVID19)	CHC da Universidade Federal do Paraná - PR
227	ATIVIDADE EXTERNA (ESPII COVID19)	CHC da Universidade Federal do Paraná - PR
227	ATIVIDADE EXTERNA (ESPII COVID19)	CHC da Universidade Federal do Paraná - PR
227	ATIVIDADE EXTERNA (ESPII COVID19)	CHC da Universidade Federal do Paraná - PR
227	ATIVIDADE EXTERNA (ESPII COVID19)	CHC da Universidade Federal do Paraná - PR
227	ATIVIDADE EXTERNA (ESPII COVID19)	CHC da Universidade Federal do Paraná - PR
227	ATIVIDADE EXTERNA (ESPII COVID19)	CHC da Universidade Federal do Paraná - PR
227	ATIVIDADE EXTERNA (ESPII COVID19)	CHC da Universidade Federal do Paraná - PR
227	ATIVIDADE EXTERNA (ESPII COVID19)	CHC da Universidade Federal do Paraná - PR
227	ATIVIDADE EXTERNA (ESPII COVID19)	CHC da Universidade Federal do Paraná - PR
227	ATIVIDADE EXTERNA (ESPII COVID19)	CHC da Universidade Federal do Paraná - PR
227	ATIVIDADE EXTERNA (ESPII COVID19)	CHC da Universidade Federal do Paraná - PR
227	ATIVIDADE EXTERNA (ESPII COVID19)	CHC da Universidade Federal do Paraná - PR
227	ATIVIDADE EXTERNA (ESPII COVID19)	CHC da Universidade Federal do Paraná - PR
227	ATIVIDADE EXTERNA (ESPII COVID19)	CHC da Universidade Federal do Paraná - PR

227	ATIVIDADE EXTERNA (ESPII COVID19)	CHC da Universidade Federal do Paraná - PR
227	ATIVIDADE EXTERNA (ESPII COVID19)	CHC da Universidade Federal do Paraná - PR
227	ATIVIDADE EXTERNA (ESPII COVID19)	CHC da Universidade Federal do Paraná - PR
227	ATIVIDADE EXTERNA (ESPII COVID19)	CHC da Universidade Federal do Paraná - PR
227	ATIVIDADE EXTERNA (ESPII COVID19)	CHC da Universidade Federal do Paraná - PR
227	ATIVIDADE EXTERNA (ESPII COVID19)	CHC da Universidade Federal do Paraná - PR
227	ATIVIDADE EXTERNA (ESPII COVID19)	CHC da Universidade Federal do Paraná - PR
227	ATIVIDADE EXTERNA (ESPII COVID19)	CHC da Universidade Federal do Paraná - PR
227	ATIVIDADE EXTERNA (ESPII COVID19)	CHC da Universidade Federal do Paraná - PR
227	ATIVIDADE EXTERNA (ESPII COVID19)	CHC da Universidade Federal do Paraná - PR
227	ATIVIDADE EXTERNA (ESPII COVID19)	CHC da Universidade Federal do Paraná - PR
227	ATIVIDADE EXTERNA (ESPII COVID19)	CHC da Universidade Federal do Paraná - PR
227	ATIVIDADE EXTERNA (ESPII COVID19)	CHC da Universidade Federal do Paraná - PR
227	ATIVIDADE EXTERNA (ESPII COVID19)	CHC da Universidade Federal do Paraná - PR
227	ATIVIDADE EXTERNA (ESPII COVID19)	CHC da Universidade Federal do Paraná - PR
227	ATIVIDADE EXTERNA (ESPII COVID19)	CHC da Universidade Federal do Paraná - PR
227	ATIVIDADE EXTERNA (ESPII COVID19)	CHC da Universidade Federal do Paraná - PR
227	ATIVIDADE EXTERNA (ESPII COVID19)	CHC da Universidade Federal do Paraná - PR
227	ATIVIDADE EXTERNA (ESPII COVID19)	CHC da Universidade Federal do Paraná - PR
227	ATIVIDADE EXTERNA (ESPII COVID19)	CHC da Universidade Federal do Paraná - PR
227	ATIVIDADE EXTERNA (ESPII COVID19)	CHC da Universidade Federal do Paraná - PR
227	ATIVIDADE EXTERNA (ESPII COVID19)	CHC da Universidade Federal do Paraná - PR
227	ATIVIDADE EXTERNA (ESPII COVID19)	CHC da Universidade Federal do Paraná - PR
227	ATIVIDADE EXTERNA (ESPII COVID19)	CHC da Universidade Federal do Paraná - PR
227	ATIVIDADE EXTERNA (ESPII COVID19)	CHC da Universidade Federal do Paraná - PR
227	ATIVIDADE EXTERNA (ESPII COVID19)	CHC da Universidade Federal do Paraná - PR
227	ATIVIDADE EXTERNA (ESPII COVID19)	CHC da Universidade Federal do Paraná - PR
227	ATIVIDADE EXTERNA (ESPII COVID19)	CHC da Universidade Federal do Paraná - PR
227	ATIVIDADE EXTERNA (ESPII COVID19)	CHC da Universidade Federal do Paraná - PR
227	ATIVIDADE EXTERNA (ESPII COVID19)	CHC da Universidade Federal do Paraná - PR
227	ATIVIDADE EXTERNA (ESPII COVID19)	CHC da Universidade Federal do Paraná - PR
227	ATIVIDADE EXTERNA (ESPII COVID19)	CHC da Universidade Federal do Paraná - PR
227	ATIVIDADE EXTERNA (ESPII COVID19)	CHC da Universidade Federal do Paraná - PR
227	ATIVIDADE EXTERNA (ESPII COVID19)	CHC da Universidade Federal do Paraná - PR
227	ATIVIDADE EXTERNA (ESPII COVID19)	CHC da Universidade Federal do Paraná - PR
227	ATIVIDADE EXTERNA (ESPII COVID19)	CHC da Universidade Federal do Paraná - PR
227	ATIVIDADE EXTERNA (ESPII COVID19)	CHC da Universidade Federal do Paraná - PR
227	ATIVIDADE EXTERNA (ESPII COVID19)	CHC da Universidade Federal do Paraná - PR
227	ATIVIDADE EXTERNA (ESPII COVID19)	CHC da Universidade Federal do Paraná - PR
227	ATIVIDADE EXTERNA (ESPII COVID19)	CHC da Universidade Federal do Paraná - PR
227	ATIVIDADE EXTERNA (ESPII COVID19)	CHC da Universidade Federal do Paraná - PR
227	ATIVIDADE EXTERNA (ESPII COVID19)	CHC da Universidade Federal do Paraná - PR
227	ATIVIDADE EXTERNA (ESPII COVID19)	CHC da Universidade Federal do Paraná - PR
227	ATIVIDADE EXTERNA (ESPII COVID19)	CHC da Universidade Federal do Paraná - PR
227	ATIVIDADE EXTERNA (ESPII COVID19)	CHC da Universidade Federal do Paraná - PR
227	ATIVIDADE EXTERNA (ESPII COVID19)	CHC da Universidade Federal do Paraná - PR
227	ATIVIDADE EXTERNA (ESPII COVID19)	CHC da Universidade Federal do Paraná - PR
227	ATIVIDADE EXTERNA (ESPII COVID19)	CHC da Universidade Federal do Paraná - PR

227	ATIVIDADE EXTERNA (ESPII COVID19)	CHC da Universidade Federal do Paraná - PR
227	ATIVIDADE EXTERNA (ESPII COVID19)	CHC da Universidade Federal do Paraná - PR
227	ATIVIDADE EXTERNA (ESPII COVID19)	CHC da Universidade Federal do Paraná - PR
227	ATIVIDADE EXTERNA (ESPII COVID19)	CHC da Universidade Federal do Paraná - PR
227	ATIVIDADE EXTERNA (ESPII COVID19)	CHC da Universidade Federal do Paraná - PR
227	ATIVIDADE EXTERNA (ESPII COVID19)	CHC da Universidade Federal do Paraná - PR
227	ATIVIDADE EXTERNA (ESPII COVID19)	CHC da Universidade Federal do Paraná - PR
227	ATIVIDADE EXTERNA (ESPII COVID19)	CHC da Universidade Federal do Paraná - PR
227	ATIVIDADE EXTERNA (ESPII COVID19)	CHC da Universidade Federal do Paraná - PR
227	ATIVIDADE EXTERNA (ESPII COVID19)	CHC da Universidade Federal do Paraná - PR
227	ATIVIDADE EXTERNA (ESPII COVID19)	CHC da Universidade Federal do Paraná - PR
227	ATIVIDADE EXTERNA (ESPII COVID19)	CHC da Universidade Federal do Paraná - PR
227	ATIVIDADE EXTERNA (ESPII COVID19)	CHC da Universidade Federal do Paraná - PR
227	ATIVIDADE EXTERNA (ESPII COVID19)	CHC da Universidade Federal do Paraná - PR
227	ATIVIDADE EXTERNA (ESPII COVID19)	CHC da Universidade Federal do Paraná - PR
227	ATIVIDADE EXTERNA (ESPII COVID19)	CHC da Universidade Federal do Paraná - PR
227	ATIVIDADE EXTERNA (ESPII COVID19)	CHC da Universidade Federal do Paraná - PR
227	ATIVIDADE EXTERNA (ESPII COVID19)	CHC da Universidade Federal do Paraná - PR
227	ATIVIDADE EXTERNA (ESPII COVID19)	CHC da Universidade Federal do Paraná - PR
227	ATIVIDADE EXTERNA (ESPII COVID19)	CHC da Universidade Federal do Paraná - PR
227	ATIVIDADE EXTERNA (ESPII COVID19)	CHC da Universidade Federal do Paraná - PR
227	ATIVIDADE EXTERNA (ESPII COVID19)	CHC da Universidade Federal do Paraná - PR
227	ATIVIDADE EXTERNA (ESPII COVID19)	CHC da Universidade Federal do Paraná - PR
227	ATIVIDADE EXTERNA (ESPII COVID19)	CHC da Universidade Federal do Paraná - PR
227	ATIVIDADE EXTERNA (ESPII COVID19)	CHC da Universidade Federal do Paraná - PR
227	ATIVIDADE EXTERNA (ESPII COVID19)	CHC da Universidade Federal do Paraná - PR
227	ATIVIDADE EXTERNA (ESPII COVID19)	CHC da Universidade Federal do Paraná - PR
227	ATIVIDADE EXTERNA (ESPII COVID19)	CHC da Universidade Federal do Paraná - PR
227	ATIVIDADE EXTERNA (ESPII COVID19)	CHC da Universidade Federal do Paraná - PR
227	ATIVIDADE EXTERNA (ESPII COVID19)	CHC da Universidade Federal do Paraná - PR
227	ATIVIDADE EXTERNA (ESPII COVID19)	CHC da Universidade Federal do Paraná - PR
227	ATIVIDADE EXTERNA (ESPII COVID19)	CHC da Universidade Federal do Paraná - PR
227	ATIVIDADE EXTERNA (ESPII COVID19)	CHC da Universidade Federal do Paraná - PR
227	ATIVIDADE EXTERNA (ESPII COVID19)	CHC da Universidade Federal do Paraná - PR
227	ATIVIDADE EXTERNA (ESPII COVID19)	CHC da Universidade Federal do Paraná - PR
227	ATIVIDADE EXTERNA (ESPII COVID19)	CHC da Universidade Federal do Paraná - PR
227	ATIVIDADE EXTERNA (ESPII COVID19)	CHC da Universidade Federal do Paraná - PR
227	ATIVIDADE EXTERNA (ESPII COVID19)	CHC da Universidade Federal do Paraná - PR
227	ATIVIDADE EXTERNA (ESPII COVID19)	CHC da Universidade Federal do Paraná - PR
227	ATIVIDADE EXTERNA (ESPII COVID19)	CHC da Universidade Federal do Paraná - PR
227	ATIVIDADE EXTERNA (ESPII COVID19)	CHC da Universidade Federal do Paraná - PR
227	ATIVIDADE EXTERNA (ESPII COVID19)	CHC da Universidade Federal do Paraná - PR
227	ATIVIDADE EXTERNA (ESPII COVID19)	CHC da Universidade Federal do Paraná - PR
227	ATIVIDADE EXTERNA (ESPII COVID19)	CHC da Universidade Federal do Paraná - PR
227	ATIVIDADE EXTERNA (ESPII COVID19)	CHC da Universidade Federal do Paraná - PR
227	ATIVIDADE EXTERNA (ESPII COVID19)	CHC da Universidade Federal do Paraná - PR
227	ATIVIDADE EXTERNA (ESPII COVID19)	CHC da Universidade Federal do Paraná - PR
227	ATIVIDADE EXTERNA (ESPII COVID19)	CHC da Universidade Federal do Paraná - PR

227	ATIVIDADE EXTERNA (ESPII COVID19)	CHC da Universidade Federal do Paraná - PR
227	ATIVIDADE EXTERNA (ESPII COVID19)	CHC da Universidade Federal do Paraná - PR
227	ATIVIDADE EXTERNA (ESPII COVID19)	CHC da Universidade Federal do Paraná - PR
227	ATIVIDADE EXTERNA (ESPII COVID19)	CHC da Universidade Federal do Paraná - PR
227	ATIVIDADE EXTERNA (ESPII COVID19)	CHC da Universidade Federal do Paraná - PR
227	ATIVIDADE EXTERNA (ESPII COVID19)	CHC da Universidade Federal do Paraná - PR
227	ATIVIDADE EXTERNA (ESPII COVID19)	CHC da Universidade Federal do Paraná - PR
227	ATIVIDADE EXTERNA (ESPII COVID19)	CHC da Universidade Federal do Paraná - PR
227	ATIVIDADE EXTERNA (ESPII COVID19)	CHC da Universidade Federal do Paraná - PR
227	ATIVIDADE EXTERNA (ESPII COVID19)	CHC da Universidade Federal do Paraná - PR
227	ATIVIDADE EXTERNA (ESPII COVID19)	CHC da Universidade Federal do Paraná - PR
227	ATIVIDADE EXTERNA (ESPII COVID19)	CHC da Universidade Federal do Paraná - PR
227	ATIVIDADE EXTERNA (ESPII COVID19)	CHC da Universidade Federal do Paraná - PR
227	ATIVIDADE EXTERNA (ESPII COVID19)	CHC da Universidade Federal do Paraná - PR
227	ATIVIDADE EXTERNA (ESPII COVID19)	CHC da Universidade Federal do Paraná - PR
227	ATIVIDADE EXTERNA (ESPII COVID19)	CHC da Universidade Federal do Paraná - PR
227	ATIVIDADE EXTERNA (ESPII COVID19)	CHC da Universidade Federal do Paraná - PR
227	ATIVIDADE EXTERNA (ESPII COVID19)	CHC da Universidade Federal do Paraná - PR
227	ATIVIDADE EXTERNA (ESPII COVID19)	CHC da Universidade Federal do Paraná - PR
227	ATIVIDADE EXTERNA (ESPII COVID19)	CHC da Universidade Federal do Paraná - PR
227	ATIVIDADE EXTERNA (ESPII COVID19)	CHC da Universidade Federal do Paraná - PR
227	ATIVIDADE EXTERNA (ESPII COVID19)	CHC da Universidade Federal do Paraná - PR
227	ATIVIDADE EXTERNA (ESPII COVID19)	CHC da Universidade Federal do Paraná - PR
227	ATIVIDADE EXTERNA (ESPII COVID19)	CHC da Universidade Federal do Paraná - PR
227	ATIVIDADE EXTERNA (ESPII COVID19)	CHC da Universidade Federal do Paraná - PR
227	ATIVIDADE EXTERNA (ESPII COVID19)	CHC da Universidade Federal do Paraná - PR
227	ATIVIDADE EXTERNA (ESPII COVID19)	CHC da Universidade Federal do Paraná - PR
227	ATIVIDADE EXTERNA (ESPII COVID19)	CHC da Universidade Federal do Paraná - PR
227	ATIVIDADE EXTERNA (ESPII COVID19)	CHC da Universidade Federal do Paraná - PR
227	ATIVIDADE EXTERNA (ESPII COVID19)	CHC da Universidade Federal do Paraná - PR
227	ATIVIDADE EXTERNA (ESPII COVID19)	CHC da Universidade Federal do Paraná - PR
227	ATIVIDADE EXTERNA (ESPII COVID19)	CHC da Universidade Federal do Paraná - PR
227	ATIVIDADE EXTERNA (ESPII COVID19)	CHC da Universidade Federal do Paraná - PR
227	ATIVIDADE EXTERNA (ESPII COVID19)	CHC da Universidade Federal do Paraná - PR
227	ATIVIDADE EXTERNA (ESPII COVID19)	CHC da Universidade Federal do Paraná - PR
227	ATIVIDADE EXTERNA (ESPII COVID19)	CHC da Universidade Federal do Paraná - PR
227	ATIVIDADE EXTERNA (ESPII COVID19)	CHC da Universidade Federal do Paraná - PR
227	ATIVIDADE EXTERNA (ESPII COVID19)	CHC da Universidade Federal do Paraná - PR
227	ATIVIDADE EXTERNA (ESPII COVID19)	CHC da Universidade Federal do Paraná - PR
227	ATIVIDADE EXTERNA (ESPII COVID19)	CHC da Universidade Federal do Paraná - PR
227	ATIVIDADE EXTERNA (ESPII COVID19)	CHC da Universidade Federal do Paraná - PR
227	ATIVIDADE EXTERNA (ESPII COVID19)	CHC da Universidade Federal do Paraná - PR
227	ATIVIDADE EXTERNA (ESPII COVID19)	CHC da Universidade Federal do Paraná - PR
227	ATIVIDADE EXTERNA (ESPII COVID19)	CHC da Universidade Federal do Paraná - PR
227	ATIVIDADE EXTERNA (ESPII COVID19)	CHC da Universidade Federal do Paraná - PR
227	ATIVIDADE EXTERNA (ESPII COVID19)	CHC da Universidade Federal do Paraná - PR
227	ATIVIDADE EXTERNA (ESPII COVID19)	CHC da Universidade Federal do Paraná - PR
227	ATIVIDADE EXTERNA (ESPII COVID19)	CHC da Universidade Federal do Paraná - PR

227	ATIVIDADE EXTERNA (ESPII COVID19)	CHC da Universidade Federal do Paraná - PR
227	ATIVIDADE EXTERNA (ESPII COVID19)	CHC da Universidade Federal do Paraná - PR
227	ATIVIDADE EXTERNA (ESPII COVID19)	CHC da Universidade Federal do Paraná - PR
227	ATIVIDADE EXTERNA (ESPII COVID19)	CHC da Universidade Federal do Paraná - PR
227	ATIVIDADE EXTERNA (ESPII COVID19)	CHC da Universidade Federal do Paraná - PR
227	ATIVIDADE EXTERNA (ESPII COVID19)	CHC da Universidade Federal do Paraná - PR
227	ATIVIDADE EXTERNA (ESPII COVID19)	CHC da Universidade Federal do Paraná - PR
227	ATIVIDADE EXTERNA (ESPII COVID19)	CHC da Universidade Federal do Paraná - PR
227	ATIVIDADE EXTERNA (ESPII COVID19)	CHC da Universidade Federal do Paraná - PR
227	ATIVIDADE EXTERNA (ESPII COVID19)	CHC da Universidade Federal do Paraná - PR
227	ATIVIDADE EXTERNA (ESPII COVID19)	CHC da Universidade Federal do Paraná - PR
227	ATIVIDADE EXTERNA (ESPII COVID19)	CHC da Universidade Federal do Paraná - PR
227	ATIVIDADE EXTERNA (ESPII COVID19)	CHC da Universidade Federal do Paraná - PR
227	ATIVIDADE EXTERNA (ESPII COVID19)	CHC da Universidade Federal do Paraná - PR
227	ATIVIDADE EXTERNA (ESPII COVID19)	CHC da Universidade Federal do Paraná - PR
227	ATIVIDADE EXTERNA (ESPII COVID19)	CHC da Universidade Federal do Paraná - PR
227	ATIVIDADE EXTERNA (ESPII COVID19)	CHC da Universidade Federal do Paraná - PR
227	ATIVIDADE EXTERNA (ESPII COVID19)	CHC da Universidade Federal do Paraná - PR
227	ATIVIDADE EXTERNA (ESPII COVID19)	CHC da Universidade Federal do Paraná - PR
227	ATIVIDADE EXTERNA (ESPII COVID19)	CHC da Universidade Federal do Paraná - PR
227	ATIVIDADE EXTERNA (ESPII COVID19)	CHC da Universidade Federal do Paraná - PR
227	ATIVIDADE EXTERNA (ESPII COVID19)	CHC da Universidade Federal do Paraná - PR
227	ATIVIDADE EXTERNA (ESPII COVID19)	CHC da Universidade Federal do Paraná - PR
227	ATIVIDADE EXTERNA (ESPII COVID19)	CHC da Universidade Federal do Paraná - PR
227	ATIVIDADE EXTERNA (ESPII COVID19)	CHC da Universidade Federal do Paraná - PR
227	ATIVIDADE EXTERNA (ESPII COVID19)	CHC da Universidade Federal do Paraná - PR
227	ATIVIDADE EXTERNA (ESPII COVID19)	CHC da Universidade Federal do Paraná - PR
227	ATIVIDADE EXTERNA (ESPII COVID19)	CHC da Universidade Federal do Paraná - PR
227	ATIVIDADE EXTERNA (ESPII COVID19)	CHC da Universidade Federal do Paraná - PR
227	ATIVIDADE EXTERNA (ESPII COVID19)	CHC da Universidade Federal do Paraná - PR
227	ATIVIDADE EXTERNA (ESPII COVID19)	CHC da Universidade Federal do Paraná - PR
227	ATIVIDADE EXTERNA (ESPII COVID19)	CHC da Universidade Federal do Paraná - PR
227	ATIVIDADE EXTERNA (ESPII COVID19)	CHC da Universidade Federal do Paraná - PR
227	ATIVIDADE EXTERNA (ESPII COVID19)	CHC da Universidade Federal do Paraná - PR
227	ATIVIDADE EXTERNA (ESPII COVID19)	CHC da Universidade Federal do Paraná - PR
227	ATIVIDADE EXTERNA (ESPII COVID19)	CHC da Universidade Federal do Paraná - PR
227	ATIVIDADE EXTERNA (ESPII COVID19)	CHC da Universidade Federal do Paraná - PR
227	ATIVIDADE EXTERNA (ESPII COVID19)	CHC da Universidade Federal do Paraná - PR
227	ATIVIDADE EXTERNA (ESPII COVID19)	CHC da Universidade Federal do Paraná - PR
227	ATIVIDADE EXTERNA (ESPII COVID19)	CHC da Universidade Federal do Paraná - PR
227	ATIVIDADE EXTERNA (ESPII COVID19)	CHC da Universidade Federal do Paraná - PR
227	ATIVIDADE EXTERNA (ESPII COVID19)	CHC da Universidade Federal do Paraná - PR
227	ATIVIDADE EXTERNA (ESPII COVID19)	CHC da Universidade Federal do Paraná - PR
227	ATIVIDADE EXTERNA (ESPII COVID19)	CHC da Universidade Federal do Paraná - PR
227	ATIVIDADE EXTERNA (ESPII COVID19)	CHC da Universidade Federal do Paraná - PR
227	ATIVIDADE EXTERNA (ESPII COVID19)	CHC da Universidade Federal do Paraná - PR
227	ATIVIDADE EXTERNA (ESPII COVID19)	CHC da Universidade Federal do Paraná - PR
227	ATIVIDADE EXTERNA (ESPII COVID19)	CHC da Universidade Federal do Paraná - PR

227	ATIVIDADE EXTERNA (ESPII COVID19)	CHC da Universidade Federal do Paraná - PR
227	ATIVIDADE EXTERNA (ESPII COVID19)	CHC da Universidade Federal do Paraná - PR
227	ATIVIDADE EXTERNA (ESPII COVID19)	CHC da Universidade Federal do Paraná - PR
227	ATIVIDADE EXTERNA (ESPII COVID19)	CHC da Universidade Federal do Paraná - PR
227	ATIVIDADE EXTERNA (ESPII COVID19)	CHC da Universidade Federal do Paraná - PR
227	ATIVIDADE EXTERNA (ESPII COVID19)	CHC da Universidade Federal do Paraná - PR
227	ATIVIDADE EXTERNA (ESPII COVID19)	CHC da Universidade Federal do Paraná - PR
227	ATIVIDADE EXTERNA (ESPII COVID19)	CHC da Universidade Federal do Paraná - PR
227	ATIVIDADE EXTERNA (ESPII COVID19)	CHC da Universidade Federal do Paraná - PR
227	ATIVIDADE EXTERNA (ESPII COVID19)	CHC da Universidade Federal do Paraná - PR
227	ATIVIDADE EXTERNA (ESPII COVID19)	CHC da Universidade Federal do Paraná - PR
227	ATIVIDADE EXTERNA (ESPII COVID19)	CHC da Universidade Federal do Paraná - PR
227	ATIVIDADE EXTERNA (ESPII COVID19)	CHC da Universidade Federal do Paraná - PR
227	ATIVIDADE EXTERNA (ESPII COVID19)	CHC da Universidade Federal do Paraná - PR
227	ATIVIDADE EXTERNA (ESPII COVID19)	CHC da Universidade Federal do Paraná - PR
228	ATIVIDADE EXTERNA (ESPII COVID19) - 7 Dias. DESATIVADO DE ACORDO COM A IN 02 - DGP de 26/03/2020.	CHC da Universidade Federal do Paraná - PR
228	ATIVIDADE EXTERNA (ESPII COVID19) - 7 Dias. DESATIVADO DE ACORDO COM A IN 02 - DGP de 26/03/2020.	CHC da Universidade Federal do Paraná - PR
228	ATIVIDADE EXTERNA (ESPII COVID19) - 7 Dias. DESATIVADO DE ACORDO COM A IN 02 - DGP de 26/03/2020.	CHC da Universidade Federal do Paraná - PR
228	ATIVIDADE EXTERNA (ESPII COVID19) - 7 Dias. DESATIVADO DE ACORDO COM A IN 02 - DGP de 26/03/2020.	CHC da Universidade Federal do Paraná - PR
228	ATIVIDADE EXTERNA (ESPII COVID19) - 7 Dias. DESATIVADO DE ACORDO COM A IN 02 - DGP de 26/03/2020.	CHC da Universidade Federal do Paraná - PR
228	ATIVIDADE EXTERNA (ESPII COVID19) - 7 Dias. DESATIVADO DE ACORDO COM A IN 02 - DGP de 26/03/2020.	CHC da Universidade Federal do Paraná - PR
228	ATIVIDADE EXTERNA (ESPII COVID19) - 7 Dias. DESATIVADO DE ACORDO COM A IN 02 - DGP de 26/03/2020.	CHC da Universidade Federal do Paraná - PR
228	ATIVIDADE EXTERNA (ESPII COVID19) - 7 Dias. DESATIVADO DE ACORDO COM A IN 02 - DGP de 26/03/2020.	CHC da Universidade Federal do Paraná - PR
228	ATIVIDADE EXTERNA (ESPII COVID19) - 7 Dias. DESATIVADO DE ACORDO COM A IN 02 - DGP de 26/03/2020.	CHC da Universidade Federal do Paraná - PR
228	ATIVIDADE EXTERNA (ESPII COVID19) - 7 Dias. DESATIVADO DE ACORDO COM A IN 02 - DGP de 26/03/2020.	CHC da Universidade Federal do Paraná - PR
228	ATIVIDADE EXTERNA (ESPII COVID19) - 7 Dias. DESATIVADO DE ACORDO COM A IN 02 - DGP de 26/03/2020.	CHC da Universidade Federal do Paraná - PR
228	ATIVIDADE EXTERNA (ESPII COVID19) - 7 Dias. DESATIVADO DE ACORDO COM A IN 02 - DGP de 26/03/2020.	CHC da Universidade Federal do Paraná - PR
228	ATIVIDADE EXTERNA (ESPII COVID19) - 7 Dias. DESATIVADO DE ACORDO COM A IN 02 - DGP de 26/03/2020.	CHC da Universidade Federal do Paraná - PR
228	ATIVIDADE EXTERNA (ESPII COVID19) - 7 Dias. DESATIVADO DE ACORDO COM A IN 02 - DGP de 26/03/2020.	CHC da Universidade Federal do Paraná - PR
228	ATIVIDADE EXTERNA (ESPII COVID19) - 7 Dias. DESATIVADO DE ACORDO COM A IN 02 - DGP de 26/03/2020.	CHC da Universidade Federal do Paraná - PR
228	ATIVIDADE EXTERNA (ESPII COVID19) - 7 Dias. DESATIVADO DE ACORDO COM A IN 02 - DGP de 26/03/2020.	CHC da Universidade Federal do Paraná - PR
228	ATIVIDADE EXTERNA (ESPII COVID19) - 7 Dias. DESATIVADO DE ACORDO COM A IN 02 - DGP de 26/03/2020.	CHC da Universidade Federal do Paraná - PR
228	ATIVIDADE EXTERNA (ESPII COVID19) - 7 Dias. DESATIVADO DE ACORDO COM A IN 02 - DGP de 26/03/2020.	CHC da Universidade Federal do Paraná - PR
228	ATIVIDADE EXTERNA (ESPII COVID19) - 7 Dias. DESATIVADO DE ACORDO COM A IN 02 - DGP de 26/03/2020.	CHC da Universidade Federal do Paraná - PR
228	ATIVIDADE EXTERNA (ESPII COVID19) - 7 Dias. DESATIVADO DE ACORDO COM A IN 02 - DGP de 26/03/2020.	CHC da Universidade Federal do Paraná - PR
228	ATIVIDADE EXTERNA (ESPII COVID19) - 7 Dias. DESATIVADO DE ACORDO COM A IN 02 - DGP de 26/03/2020.	CHC da Universidade Federal do Paraná - PR

228	ATIVIDADE EXTERNA (ESPII COVID19) - 7 Dias. DESATIVADO DE ACORDO COM A IN 02 - DGP de 26/03/2020.	CHC da Universidade Federal do Paraná - PR
228	ATIVIDADE EXTERNA (ESPII COVID19) - 7 Dias. DESATIVADO DE ACORDO COM A IN 02 - DGP de 26/03/2020.	CHC da Universidade Federal do Paraná - PR
228	ATIVIDADE EXTERNA (ESPII COVID19) - 7 Dias. DESATIVADO DE ACORDO COM A IN 02 - DGP de 26/03/2020.	CHC da Universidade Federal do Paraná - PR
228	ATIVIDADE EXTERNA (ESPII COVID19) - 7 Dias. DESATIVADO DE ACORDO COM A IN 02 - DGP de 26/03/2020.	CHC da Universidade Federal do Paraná - PR
228	ATIVIDADE EXTERNA (ESPII COVID19) - 7 Dias. DESATIVADO DE ACORDO COM A IN 02 - DGP de 26/03/2020.	CHC da Universidade Federal do Paraná - PR
228	ATIVIDADE EXTERNA (ESPII COVID19) - 7 Dias. DESATIVADO DE ACORDO COM A IN 02 - DGP de 26/03/2020.	CHC da Universidade Federal do Paraná - PR
228	ATIVIDADE EXTERNA (ESPII COVID19) - 7 Dias. DESATIVADO DE ACORDO COM A IN 02 - DGP de 26/03/2020.	CHC da Universidade Federal do Paraná - PR
228	ATIVIDADE EXTERNA (ESPII COVID19) - 7 Dias. DESATIVADO DE ACORDO COM A IN 02 - DGP de 26/03/2020.	CHC da Universidade Federal do Paraná - PR
228	ATIVIDADE EXTERNA (ESPII COVID19) - 7 Dias. DESATIVADO DE ACORDO COM A IN 02 - DGP de 26/03/2020.	CHC da Universidade Federal do Paraná - PR
228	ATIVIDADE EXTERNA (ESPII COVID19) - 7 Dias. DESATIVADO DE ACORDO COM A IN 02 - DGP de 26/03/2020.	CHC da Universidade Federal do Paraná - PR
228	ATIVIDADE EXTERNA (ESPII COVID19) - 7 Dias. DESATIVADO DE ACORDO COM A IN 02 - DGP de 26/03/2020.	CHC da Universidade Federal do Paraná - PR
228	ATIVIDADE EXTERNA (ESPII COVID19) - 7 Dias. DESATIVADO DE ACORDO COM A IN 02 - DGP de 26/03/2020.	CHC da Universidade Federal do Paraná - PR
228	ATIVIDADE EXTERNA (ESPII COVID19) - 7 Dias. DESATIVADO DE ACORDO COM A IN 02 - DGP de 26/03/2020.	CHC da Universidade Federal do Paraná - PR
228	ATIVIDADE EXTERNA (ESPII COVID19) - 7 Dias. DESATIVADO DE ACORDO COM A IN 02 - DGP de 26/03/2020.	CHC da Universidade Federal do Paraná - PR
228	ATIVIDADE EXTERNA (ESPII COVID19) - 7 Dias. DESATIVADO DE ACORDO COM A IN 02 - DGP de 26/03/2020.	CHC da Universidade Federal do Paraná - PR
228	ATIVIDADE EXTERNA (ESPII COVID19) - 7 Dias. DESATIVADO DE ACORDO COM A IN 02 - DGP de 26/03/2020.	CHC da Universidade Federal do Paraná - PR
228	ATIVIDADE EXTERNA (ESPII COVID19) - 7 Dias. DESATIVADO DE ACORDO COM A IN 02 - DGP de 26/03/2020.	CHC da Universidade Federal do Paraná - PR
228	ATIVIDADE EXTERNA (ESPII COVID19) - 7 Dias. DESATIVADO DE ACORDO COM A IN 02 - DGP de 26/03/2020.	CHC da Universidade Federal do Paraná - PR
228	ATIVIDADE EXTERNA (ESPII COVID19) - 7 Dias. DESATIVADO DE ACORDO COM A IN 02 - DGP de 26/03/2020.	CHC da Universidade Federal do Paraná - PR
229	ATIVIDADE EXTERNA (ESPII COVID19) - 14 dias.	CHC da Universidade Federal do Paraná - PR
229	ATIVIDADE EXTERNA (ESPII COVID19) - 14 dias.	CHC da Universidade Federal do Paraná - PR
229	ATIVIDADE EXTERNA (ESPII COVID19) - 14 dias.	CHC da Universidade Federal do Paraná - PR
229	ATIVIDADE EXTERNA (ESPII COVID19) - 14 dias.	CHC da Universidade Federal do Paraná - PR
229	ATIVIDADE EXTERNA (ESPII COVID19) - 14 dias.	CHC da Universidade Federal do Paraná - PR
229	ATIVIDADE EXTERNA (ESPII COVID19) - 14 dias.	CHC da Universidade Federal do Paraná - PR
229	ATIVIDADE EXTERNA (ESPII COVID19) - 14 dias.	CHC da Universidade Federal do Paraná - PR
229	ATIVIDADE EXTERNA (ESPII COVID19) - 14 dias.	CHC da Universidade Federal do Paraná - PR
229	ATIVIDADE EXTERNA (ESPII COVID19) - 14 dias.	CHC da Universidade Federal do Paraná - PR
229	ATIVIDADE EXTERNA (ESPII COVID19) - 14 dias.	CHC da Universidade Federal do Paraná - PR
229	ATIVIDADE EXTERNA (ESPII COVID19) - 14 dias.	CHC da Universidade Federal do Paraná - PR
229	ATIVIDADE EXTERNA (ESPII COVID19) - 14 dias.	CHC da Universidade Federal do Paraná - PR
229	ATIVIDADE EXTERNA (ESPII COVID19) - 14 dias.	CHC da Universidade Federal do Paraná - PR
231	Estado gripal (autodeclaratório) COVID-19. DESATIVADO DE ACORDO COM A IN 02 - DGP de 26/03/2020.	CHC da Universidade Federal do Paraná - PR
231	Estado gripal (autodeclaratório) COVID-19. DESATIVADO DE ACORDO COM A IN 02 - DGP de 26/03/2020.	CHC da Universidade Federal do Paraná - PR
231	Estado gripal (autodeclaratório) COVID-19. DESATIVADO DE ACORDO COM A IN 02 - DGP de 26/03/2020.	CHC da Universidade Federal do Paraná - PR
231	Estado gripal (autodeclaratório) COVID-19. DESATIVADO DE ACORDO COM A IN 02 - DGP de 26/03/2020.	CHC da Universidade Federal do Paraná - PR

231	Estado gripal (autodeclaratório) COVID-19. DESATIVADO DE ACORDO COM A IN 02 - DGP de 26/03/2020.	CHC da Universidade Federal do Paraná - PR
231	Estado gripal (autodeclaratório) COVID-19. DESATIVADO DE ACORDO COM A IN 02 - DGP de 26/03/2020.	CHC da Universidade Federal do Paraná - PR
231	Estado gripal (autodeclaratório) COVID-19. DESATIVADO DE ACORDO COM A IN 02 - DGP de 26/03/2020.	CHC da Universidade Federal do Paraná - PR
231	Estado gripal (autodeclaratório) COVID-19. DESATIVADO DE ACORDO COM A IN 02 - DGP de 26/03/2020.	CHC da Universidade Federal do Paraná - PR
231	Estado gripal (autodeclaratório) COVID-19. DESATIVADO DE ACORDO COM A IN 02 - DGP de 26/03/2020.	CHC da Universidade Federal do Paraná - PR
231	Estado gripal (autodeclaratório) COVID-19. DESATIVADO DE ACORDO COM A IN 02 - DGP de 26/03/2020.	CHC da Universidade Federal do Paraná - PR
231	Estado gripal (autodeclaratório) COVID-19. DESATIVADO DE ACORDO COM A IN 02 - DGP de 26/03/2020.	CHC da Universidade Federal do Paraná - PR
231	Estado gripal (autodeclaratório) COVID-19. DESATIVADO DE ACORDO COM A IN 02 - DGP de 26/03/2020.	CHC da Universidade Federal do Paraná - PR
231	Estado gripal (autodeclaratório) COVID-19. DESATIVADO DE ACORDO COM A IN 02 - DGP de 26/03/2020.	CHC da Universidade Federal do Paraná - PR
231	Estado gripal (autodeclaratório) COVID-19. DESATIVADO DE ACORDO COM A IN 02 - DGP de 26/03/2020.	CHC da Universidade Federal do Paraná - PR
231	Estado gripal (autodeclaratório) COVID-19. DESATIVADO DE ACORDO COM A IN 02 - DGP de 26/03/2020.	CHC da Universidade Federal do Paraná - PR
231	Estado gripal (autodeclaratório) COVID-19. DESATIVADO DE ACORDO COM A IN 02 - DGP de 26/03/2020.	CHC da Universidade Federal do Paraná - PR
231	Estado gripal (autodeclaratório) COVID-19. DESATIVADO DE ACORDO COM A IN 02 - DGP de 26/03/2020.	CHC da Universidade Federal do Paraná - PR
231	Estado gripal (autodeclaratório) COVID-19. DESATIVADO DE ACORDO COM A IN 02 - DGP de 26/03/2020.	CHC da Universidade Federal do Paraná - PR
231	Estado gripal (autodeclaratório) COVID-19. DESATIVADO DE ACORDO COM A IN 02 - DGP de 26/03/2020.	CHC da Universidade Federal do Paraná - PR
231	Estado gripal (autodeclaratório) COVID-19. DESATIVADO DE ACORDO COM A IN 02 - DGP de 26/03/2020.	CHC da Universidade Federal do Paraná - PR
231	Estado gripal (autodeclaratório) COVID-19. DESATIVADO DE ACORDO COM A IN 02 - DGP de 26/03/2020.	CHC da Universidade Federal do Paraná - PR
231	Estado gripal (autodeclaratório) COVID-19. DESATIVADO DE ACORDO COM A IN 02 - DGP de 26/03/2020.	CHC da Universidade Federal do Paraná - PR
231	Estado gripal (autodeclaratório) COVID-19. DESATIVADO DE ACORDO COM A IN 02 - DGP de 26/03/2020.	CHC da Universidade Federal do Paraná - PR
231	Estado gripal (autodeclaratório) COVID-19. DESATIVADO DE ACORDO COM A IN 02 - DGP de 26/03/2020.	CHC da Universidade Federal do Paraná - PR
231	Estado gripal (autodeclaratório) COVID-19. DESATIVADO DE ACORDO COM A IN 02 - DGP de 26/03/2020.	CHC da Universidade Federal do Paraná - PR
231	Estado gripal (autodeclaratório) COVID-19. DESATIVADO DE ACORDO COM A IN 02 - DGP de 26/03/2020.	CHC da Universidade Federal do Paraná - PR
231	Estado gripal (autodeclaratório) COVID-19. DESATIVADO DE ACORDO COM A IN 02 - DGP de 26/03/2020.	CHC da Universidade Federal do Paraná - PR
231	Estado gripal (autodeclaratório) COVID-19. DESATIVADO DE ACORDO COM A IN 02 - DGP de 26/03/2020.	CHC da Universidade Federal do Paraná - PR
231	Estado gripal (autodeclaratório) COVID-19. DESATIVADO DE ACORDO COM A IN 02 - DGP de 26/03/2020.	CHC da Universidade Federal do Paraná - PR
231	Estado gripal (autodeclaratório) COVID-19. DESATIVADO DE ACORDO COM A IN 02 - DGP de 26/03/2020.	CHC da Universidade Federal do Paraná - PR
231	Estado gripal (autodeclaratório) COVID-19. DESATIVADO DE ACORDO COM A IN 02 - DGP de 26/03/2020.	CHC da Universidade Federal do Paraná - PR
231	Estado gripal (autodeclaratório) COVID-19. DESATIVADO DE ACORDO COM A IN 02 - DGP de 26/03/2020.	CHC da Universidade Federal do Paraná - PR
231	Estado gripal (autodeclaratório) COVID-19. DESATIVADO DE ACORDO COM A IN 02 - DGP de 26/03/2020.	CHC da Universidade Federal do Paraná - PR
231	Estado gripal (autodeclaratório) COVID-19. DESATIVADO DE ACORDO COM A IN 02 - DGP de 26/03/2020.	CHC da Universidade Federal do Paraná - PR
231	Estado gripal (autodeclaratório) COVID-19. DESATIVADO DE ACORDO COM A IN 02 - DGP de 26/03/2020.	CHC da Universidade Federal do Paraná - PR
231	Estado gripal (autodeclaratório) COVID-19. DESATIVADO DE ACORDO COM A IN 02 - DGP de 26/03/2020.	CHC da Universidade Federal do Paraná - PR

231	Estado gripal (autodeclaratório) COVID-19. DESATIVADO DE ACORDO COM A IN 02 - DGP de 26/03/2020.	CHC da Universidade Federal do Paraná - PR
231	Estado gripal (autodeclaratório) COVID-19. DESATIVADO DE ACORDO COM A IN 02 - DGP de 26/03/2020.	CHC da Universidade Federal do Paraná - PR
231	Estado gripal (autodeclaratório) COVID-19. DESATIVADO DE ACORDO COM A IN 02 - DGP de 26/03/2020.	CHC da Universidade Federal do Paraná - PR
232	ABONO ADMINISTRATIVO - COVID 19.	CHC da Universidade Federal do Paraná - PR
232	ABONO ADMINISTRATIVO - COVID 19.	CHC da Universidade Federal do Paraná - PR
232	ABONO ADMINISTRATIVO - COVID 19.	CHC da Universidade Federal do Paraná - PR
232	ABONO ADMINISTRATIVO - COVID 19.	CHC da Universidade Federal do Paraná - PR
232	ABONO ADMINISTRATIVO - COVID 19.	CHC da Universidade Federal do Paraná - PR
232	ABONO ADMINISTRATIVO - COVID 19.	CHC da Universidade Federal do Paraná - PR
232	ABONO ADMINISTRATIVO - COVID 19.	CHC da Universidade Federal do Paraná - PR
232	ABONO ADMINISTRATIVO - COVID 19.	CHC da Universidade Federal do Paraná - PR
232	ABONO ADMINISTRATIVO - COVID 19.	CHC da Universidade Federal do Paraná - PR
232	ABONO ADMINISTRATIVO - COVID 19.	CHC da Universidade Federal do Paraná - PR
232	ABONO ADMINISTRATIVO - COVID 19.	CHC da Universidade Federal do Paraná - PR
232	ABONO ADMINISTRATIVO - COVID 19.	CHC da Universidade Federal do Paraná - PR
232	ABONO ADMINISTRATIVO - COVID 19.	CHC da Universidade Federal do Paraná - PR
232	ABONO ADMINISTRATIVO - COVID 19.	CHC da Universidade Federal do Paraná - PR
232	ABONO ADMINISTRATIVO - COVID 19.	CHC da Universidade Federal do Paraná - PR
232	ABONO ADMINISTRATIVO - COVID 19.	CHC da Universidade Federal do Paraná - PR
232	ABONO ADMINISTRATIVO - COVID 19.	CHC da Universidade Federal do Paraná - PR
232	ABONO ADMINISTRATIVO - COVID 19.	CHC da Universidade Federal do Paraná - PR
232	ABONO ADMINISTRATIVO - COVID 19.	CHC da Universidade Federal do Paraná - PR
232	ABONO ADMINISTRATIVO - COVID 19.	CHC da Universidade Federal do Paraná - PR
232	ABONO ADMINISTRATIVO - COVID 19.	CHC da Universidade Federal do Paraná - PR
232	ABONO ADMINISTRATIVO - COVID 19.	CHC da Universidade Federal do Paraná - PR
232	ABONO ADMINISTRATIVO - COVID 19.	CHC da Universidade Federal do Paraná - PR
232	ABONO ADMINISTRATIVO - COVID 19.	CHC da Universidade Federal do Paraná - PR
232	ABONO ADMINISTRATIVO - COVID 19.	CHC da Universidade Federal do Paraná - PR
232	ABONO ADMINISTRATIVO - COVID 19.	CHC da Universidade Federal do Paraná - PR
232	ABONO ADMINISTRATIVO - COVID 19.	CHC da Universidade Federal do Paraná - PR
232	ABONO ADMINISTRATIVO - COVID 19.	CHC da Universidade Federal do Paraná - PR
232	ABONO ADMINISTRATIVO - COVID 19.	CHC da Universidade Federal do Paraná - PR
232	ABONO ADMINISTRATIVO - COVID 19.	CHC da Universidade Federal do Paraná - PR
232	ABONO ADMINISTRATIVO - COVID 19.	CHC da Universidade Federal do Paraná - PR
232	ABONO ADMINISTRATIVO - COVID 19.	CHC da Universidade Federal do Paraná - PR
232	ABONO ADMINISTRATIVO - COVID 19.	CHC da Universidade Federal do Paraná - PR
232	ABONO ADMINISTRATIVO - COVID 19.	CHC da Universidade Federal do Paraná - PR
232	ABONO ADMINISTRATIVO - COVID 19.	CHC da Universidade Federal do Paraná - PR
232	ABONO ADMINISTRATIVO - COVID 19.	CHC da Universidade Federal do Paraná - PR
232	ABONO ADMINISTRATIVO - COVID 19.	CHC da Universidade Federal do Paraná - PR
232	ABONO ADMINISTRATIVO - COVID 19.	CHC da Universidade Federal do Paraná - PR
232	ABONO ADMINISTRATIVO - COVID 19.	CHC da Universidade Federal do Paraná - PR
232	ABONO ADMINISTRATIVO - COVID 19.	CHC da Universidade Federal do Paraná - PR
232	ABONO ADMINISTRATIVO - COVID 19.	CHC da Universidade Federal do Paraná - PR
232	ABONO ADMINISTRATIVO - COVID 19.	CHC da Universidade Federal do Paraná - PR
232	ABONO ADMINISTRATIVO - COVID 19.	CHC da Universidade Federal do Paraná - PR

232	ABONO ADMINISTRATIVO - COVID 19.	CHC da Universidade Federal do Paraná - PR
232	ABONO ADMINISTRATIVO - COVID 19.	CHC da Universidade Federal do Paraná - PR
232	ABONO ADMINISTRATIVO - COVID 19.	CHC da Universidade Federal do Paraná - PR
232	ABONO ADMINISTRATIVO - COVID 19.	CHC da Universidade Federal do Paraná - PR
232	ABONO ADMINISTRATIVO - COVID 19.	CHC da Universidade Federal do Paraná - PR
232	ABONO ADMINISTRATIVO - COVID 19.	CHC da Universidade Federal do Paraná - PR
232	ABONO ADMINISTRATIVO - COVID 19.	CHC da Universidade Federal do Paraná - PR
232	ABONO ADMINISTRATIVO - COVID 19.	CHC da Universidade Federal do Paraná - PR
232	ABONO ADMINISTRATIVO - COVID 19.	CHC da Universidade Federal do Paraná - PR
232	ABONO ADMINISTRATIVO - COVID 19.	CHC da Universidade Federal do Paraná - PR
232	ABONO ADMINISTRATIVO - COVID 19.	CHC da Universidade Federal do Paraná - PR
232	ABONO ADMINISTRATIVO - COVID 19.	CHC da Universidade Federal do Paraná - PR
232	ABONO ADMINISTRATIVO - COVID 19.	CHC da Universidade Federal do Paraná - PR
232	ABONO ADMINISTRATIVO - COVID 19.	CHC da Universidade Federal do Paraná - PR
232	ABONO ADMINISTRATIVO - COVID 19.	CHC da Universidade Federal do Paraná - PR
232	ABONO ADMINISTRATIVO - COVID 19.	CHC da Universidade Federal do Paraná - PR
232	ABONO ADMINISTRATIVO - COVID 19.	CHC da Universidade Federal do Paraná - PR
232	ABONO ADMINISTRATIVO - COVID 19.	CHC da Universidade Federal do Paraná - PR
232	ABONO ADMINISTRATIVO - COVID 19.	CHC da Universidade Federal do Paraná - PR
232	ABONO ADMINISTRATIVO - COVID 19.	CHC da Universidade Federal do Paraná - PR
232	ABONO ADMINISTRATIVO - COVID 19.	CHC da Universidade Federal do Paraná - PR
232	ABONO ADMINISTRATIVO - COVID 19.	CHC da Universidade Federal do Paraná - PR
232	ABONO ADMINISTRATIVO - COVID 19.	CHC da Universidade Federal do Paraná - PR
233	ATIVIDADE EXTERNA ESPII - RODÍZIO	CHC da Universidade Federal do Paraná - PR
233	ATIVIDADE EXTERNA ESPII - RODÍZIO	CHC da Universidade Federal do Paraná - PR
233	ATIVIDADE EXTERNA ESPII - RODÍZIO	CHC da Universidade Federal do Paraná - PR
233	ATIVIDADE EXTERNA ESPII - RODÍZIO	CHC da Universidade Federal do Paraná - PR
233	ATIVIDADE EXTERNA ESPII - RODÍZIO	CHC da Universidade Federal do Paraná - PR
233	ATIVIDADE EXTERNA ESPII - RODÍZIO	CHC da Universidade Federal do Paraná - PR
233	ATIVIDADE EXTERNA ESPII - RODÍZIO	CHC da Universidade Federal do Paraná - PR
233	ATIVIDADE EXTERNA ESPII - RODÍZIO	CHC da Universidade Federal do Paraná - PR
233	ATIVIDADE EXTERNA ESPII - RODÍZIO	CHC da Universidade Federal do Paraná - PR
233	ATIVIDADE EXTERNA ESPII - RODÍZIO	CHC da Universidade Federal do Paraná - PR
233	ATIVIDADE EXTERNA ESPII - RODÍZIO	CHC da Universidade Federal do Paraná - PR
233	ATIVIDADE EXTERNA ESPII - RODÍZIO	CHC da Universidade Federal do Paraná - PR
233	ATIVIDADE EXTERNA ESPII - RODÍZIO	CHC da Universidade Federal do Paraná - PR
233	ATIVIDADE EXTERNA ESPII - RODÍZIO	CHC da Universidade Federal do Paraná - PR
233	ATIVIDADE EXTERNA ESPII - RODÍZIO	CHC da Universidade Federal do Paraná - PR
233	ATIVIDADE EXTERNA ESPII - RODÍZIO	CHC da Universidade Federal do Paraná - PR
233	ATIVIDADE EXTERNA ESPII - RODÍZIO	CHC da Universidade Federal do Paraná - PR
233	ATIVIDADE EXTERNA ESPII - RODÍZIO	CHC da Universidade Federal do Paraná - PR
233	ATIVIDADE EXTERNA ESPII - RODÍZIO	CHC da Universidade Federal do Paraná - PR
233	ATIVIDADE EXTERNA ESPII - RODÍZIO	CHC da Universidade Federal do Paraná - PR
233	ATIVIDADE EXTERNA ESPII - RODÍZIO	CHC da Universidade Federal do Paraná - PR
233	ATIVIDADE EXTERNA ESPII - RODÍZIO	CHC da Universidade Federal do Paraná - PR
233	ATIVIDADE EXTERNA ESPII - RODÍZIO	CHC da Universidade Federal do Paraná - PR
233	ATIVIDADE EXTERNA ESPII - RODÍZIO	CHC da Universidade Federal do Paraná - PR

233	ATIVIDADE EXTERNA ESPII - RODÍZIO	CHC da Universidade Federal do Paraná - PR
233	ATIVIDADE EXTERNA ESPII - RODÍZIO	CHC da Universidade Federal do Paraná - PR
233	ATIVIDADE EXTERNA ESPII - RODÍZIO	CHC da Universidade Federal do Paraná - PR
233	ATIVIDADE EXTERNA ESPII - RODÍZIO	CHC da Universidade Federal do Paraná - PR
233	ATIVIDADE EXTERNA ESPII - RODÍZIO	CHC da Universidade Federal do Paraná - PR
233	ATIVIDADE EXTERNA ESPII - RODÍZIO	CHC da Universidade Federal do Paraná - PR
233	ATIVIDADE EXTERNA ESPII - RODÍZIO	CHC da Universidade Federal do Paraná - PR
233	ATIVIDADE EXTERNA ESPII - RODÍZIO	CHC da Universidade Federal do Paraná - PR
233	ATIVIDADE EXTERNA ESPII - RODÍZIO	CHC da Universidade Federal do Paraná - PR
233	ATIVIDADE EXTERNA ESPII - RODÍZIO	CHC da Universidade Federal do Paraná - PR
233	ATIVIDADE EXTERNA ESPII - RODÍZIO	CHC da Universidade Federal do Paraná - PR
233	ATIVIDADE EXTERNA ESPII - RODÍZIO	CHC da Universidade Federal do Paraná - PR
233	ATIVIDADE EXTERNA ESPII - RODÍZIO	CHC da Universidade Federal do Paraná - PR
233	ATIVIDADE EXTERNA ESPII - RODÍZIO	CHC da Universidade Federal do Paraná - PR
233	ATIVIDADE EXTERNA ESPII - RODÍZIO	CHC da Universidade Federal do Paraná - PR
233	ATIVIDADE EXTERNA ESPII - RODÍZIO	CHC da Universidade Federal do Paraná - PR
233	ATIVIDADE EXTERNA ESPII - RODÍZIO	CHC da Universidade Federal do Paraná - PR
233	ATIVIDADE EXTERNA ESPII - RODÍZIO	CHC da Universidade Federal do Paraná - PR
233	ATIVIDADE EXTERNA ESPII - RODÍZIO	CHC da Universidade Federal do Paraná - PR
233	ATIVIDADE EXTERNA ESPII - RODÍZIO	CHC da Universidade Federal do Paraná - PR
233	ATIVIDADE EXTERNA ESPII - RODÍZIO	CHC da Universidade Federal do Paraná - PR
233	ATIVIDADE EXTERNA ESPII - RODÍZIO	CHC da Universidade Federal do Paraná - PR
233	ATIVIDADE EXTERNA ESPII - RODÍZIO	CHC da Universidade Federal do Paraná - PR
233	ATIVIDADE EXTERNA ESPII - RODÍZIO	CHC da Universidade Federal do Paraná - PR
233	ATIVIDADE EXTERNA ESPII - RODÍZIO	CHC da Universidade Federal do Paraná - PR
233	ATIVIDADE EXTERNA ESPII - RODÍZIO	CHC da Universidade Federal do Paraná - PR
233	ATIVIDADE EXTERNA ESPII - RODÍZIO	CHC da Universidade Federal do Paraná - PR
233	ATIVIDADE EXTERNA ESPII - RODÍZIO	CHC da Universidade Federal do Paraná - PR
233	ATIVIDADE EXTERNA ESPII - RODÍZIO	CHC da Universidade Federal do Paraná - PR
233	ATIVIDADE EXTERNA ESPII - RODÍZIO	CHC da Universidade Federal do Paraná - PR
233	ATIVIDADE EXTERNA ESPII - RODÍZIO	CHC da Universidade Federal do Paraná - PR
233	ATIVIDADE EXTERNA ESPII - RODÍZIO	CHC da Universidade Federal do Paraná - PR
233	ATIVIDADE EXTERNA ESPII - RODÍZIO	CHC da Universidade Federal do Paraná - PR
233	ATIVIDADE EXTERNA ESPII - RODÍZIO	CHC da Universidade Federal do Paraná - PR
233	ATIVIDADE EXTERNA ESPII - RODÍZIO	CHC da Universidade Federal do Paraná - PR
233	ATIVIDADE EXTERNA ESPII - RODÍZIO	CHC da Universidade Federal do Paraná - PR
233	ATIVIDADE EXTERNA ESPII - RODÍZIO	CHC da Universidade Federal do Paraná - PR
233	ATIVIDADE EXTERNA ESPII - RODÍZIO	CHC da Universidade Federal do Paraná - PR
233	ATIVIDADE EXTERNA ESPII - RODÍZIO	CHC da Universidade Federal do Paraná - PR
233	ATIVIDADE EXTERNA ESPII - RODÍZIO	CHC da Universidade Federal do Paraná - PR
233	ATIVIDADE EXTERNA ESPII - RODÍZIO	CHC da Universidade Federal do Paraná - PR
233	ATIVIDADE EXTERNA ESPII - RODÍZIO	CHC da Universidade Federal do Paraná - PR
233	ATIVIDADE EXTERNA ESPII - RODÍZIO	CHC da Universidade Federal do Paraná - PR
233	ATIVIDADE EXTERNA ESPII - RODÍZIO	CHC da Universidade Federal do Paraná - PR
233	ATIVIDADE EXTERNA ESPII - RODÍZIO	CHC da Universidade Federal do Paraná - PR
233	ATIVIDADE EXTERNA ESPII - RODÍZIO	CHC da Universidade Federal do Paraná - PR
233	ATIVIDADE EXTERNA ESPII - RODÍZIO	CHC da Universidade Federal do Paraná - PR
233	ATIVIDADE EXTERNA ESPII - RODÍZIO	CHC da Universidade Federal do Paraná - PR

233	ATIVIDADE EXTERNA ESPII - RODÍZIO	CHC da Universidade Federal do Paraná - PR
233	ATIVIDADE EXTERNA ESPII - RODÍZIO	CHC da Universidade Federal do Paraná - PR
233	ATIVIDADE EXTERNA ESPII - RODÍZIO	CHC da Universidade Federal do Paraná - PR
233	ATIVIDADE EXTERNA ESPII - RODÍZIO	CHC da Universidade Federal do Paraná - PR
233	ATIVIDADE EXTERNA ESPII - RODÍZIO	CHC da Universidade Federal do Paraná - PR
233	ATIVIDADE EXTERNA ESPII - RODÍZIO	CHC da Universidade Federal do Paraná - PR
233	ATIVIDADE EXTERNA ESPII - RODÍZIO	CHC da Universidade Federal do Paraná - PR
233	ATIVIDADE EXTERNA ESPII - RODÍZIO	CHC da Universidade Federal do Paraná - PR
233	ATIVIDADE EXTERNA ESPII - RODÍZIO	CHC da Universidade Federal do Paraná - PR
233	ATIVIDADE EXTERNA ESPII - RODÍZIO	CHC da Universidade Federal do Paraná - PR
233	ATIVIDADE EXTERNA ESPII - RODÍZIO	CHC da Universidade Federal do Paraná - PR
233	ATIVIDADE EXTERNA ESPII - RODÍZIO	CHC da Universidade Federal do Paraná - PR
233	ATIVIDADE EXTERNA ESPII - RODÍZIO	CHC da Universidade Federal do Paraná - PR
233	ATIVIDADE EXTERNA ESPII - RODÍZIO	CHC da Universidade Federal do Paraná - PR
233	ATIVIDADE EXTERNA ESPII - RODÍZIO	CHC da Universidade Federal do Paraná - PR
233	ATIVIDADE EXTERNA ESPII - RODÍZIO	CHC da Universidade Federal do Paraná - PR
233	ATIVIDADE EXTERNA ESPII - RODÍZIO	CHC da Universidade Federal do Paraná - PR
233	ATIVIDADE EXTERNA ESPII - RODÍZIO	CHC da Universidade Federal do Paraná - PR
233	ATIVIDADE EXTERNA ESPII - RODÍZIO	CHC da Universidade Federal do Paraná - PR
233	ATIVIDADE EXTERNA ESPII - RODÍZIO	CHC da Universidade Federal do Paraná - PR
233	ATIVIDADE EXTERNA ESPII - RODÍZIO	CHC da Universidade Federal do Paraná - PR
233	ATIVIDADE EXTERNA ESPII - RODÍZIO	CHC da Universidade Federal do Paraná - PR
233	ATIVIDADE EXTERNA ESPII - RODÍZIO	CHC da Universidade Federal do Paraná - PR
233	ATIVIDADE EXTERNA ESPII - RODÍZIO	CHC da Universidade Federal do Paraná - PR
233	ATIVIDADE EXTERNA ESPII - RODÍZIO	CHC da Universidade Federal do Paraná - PR
233	ATIVIDADE EXTERNA ESPII - RODÍZIO	CHC da Universidade Federal do Paraná - PR
233	ATIVIDADE EXTERNA ESPII - RODÍZIO	CHC da Universidade Federal do Paraná - PR
233	ATIVIDADE EXTERNA ESPII - RODÍZIO	CHC da Universidade Federal do Paraná - PR
233	ATIVIDADE EXTERNA ESPII - RODÍZIO	CHC da Universidade Federal do Paraná - PR
233	ATIVIDADE EXTERNA ESPII - RODÍZIO	CHC da Universidade Federal do Paraná - PR
233	ATIVIDADE EXTERNA ESPII - RODÍZIO	CHC da Universidade Federal do Paraná - PR
233	ATIVIDADE EXTERNA ESPII - RODÍZIO	CHC da Universidade Federal do Paraná - PR
233	ATIVIDADE EXTERNA ESPII - RODÍZIO	CHC da Universidade Federal do Paraná - PR
233	ATIVIDADE EXTERNA ESPII - RODÍZIO	CHC da Universidade Federal do Paraná - PR
233	ATIVIDADE EXTERNA ESPII - RODÍZIO	CHC da Universidade Federal do Paraná - PR
233	ATIVIDADE EXTERNA ESPII - RODÍZIO	CHC da Universidade Federal do Paraná - PR
233	ATIVIDADE EXTERNA ESPII - RODÍZIO	CHC da Universidade Federal do Paraná - PR
233	ATIVIDADE EXTERNA ESPII - RODÍZIO	CHC da Universidade Federal do Paraná - PR
233	ATIVIDADE EXTERNA ESPII - RODÍZIO	CHC da Universidade Federal do Paraná - PR
233	ATIVIDADE EXTERNA ESPII - RODÍZIO	CHC da Universidade Federal do Paraná - PR
233	ATIVIDADE EXTERNA ESPII - RODÍZIO	CHC da Universidade Federal do Paraná - PR
233	ATIVIDADE EXTERNA ESPII - RODÍZIO	CHC da Universidade Federal do Paraná - PR
233	ATIVIDADE EXTERNA ESPII - RODÍZIO	CHC da Universidade Federal do Paraná - PR
233	ATIVIDADE EXTERNA ESPII - RODÍZIO	CHC da Universidade Federal do Paraná - PR
233	ATIVIDADE EXTERNA ESPII - RODÍZIO	CHC da Universidade Federal do Paraná - PR
233	ATIVIDADE EXTERNA ESPII - RODÍZIO	CHC da Universidade Federal do Paraná - PR
233	ATIVIDADE EXTERNA ESPII - RODÍZIO	CHC da Universidade Federal do Paraná - PR
233	ATIVIDADE EXTERNA ESPII - RODÍZIO	CHC da Universidade Federal do Paraná - PR

233	ATIVIDADE EXTERNA ESPII - RODÍZIO	CHC da Universidade Federal do Paraná - PR
233	ATIVIDADE EXTERNA ESPII - RODÍZIO	CHC da Universidade Federal do Paraná - PR
233	ATIVIDADE EXTERNA ESPII - RODÍZIO	CHC da Universidade Federal do Paraná - PR
233	ATIVIDADE EXTERNA ESPII - RODÍZIO	CHC da Universidade Federal do Paraná - PR
233	ATIVIDADE EXTERNA ESPII - RODÍZIO	CHC da Universidade Federal do Paraná - PR
233	ATIVIDADE EXTERNA ESPII - RODÍZIO	CHC da Universidade Federal do Paraná - PR
233	ATIVIDADE EXTERNA ESPII - RODÍZIO	CHC da Universidade Federal do Paraná - PR
233	ATIVIDADE EXTERNA ESPII - RODÍZIO	CHC da Universidade Federal do Paraná - PR
233	ATIVIDADE EXTERNA ESPII - RODÍZIO	CHC da Universidade Federal do Paraná - PR
233	ATIVIDADE EXTERNA ESPII - RODÍZIO	CHC da Universidade Federal do Paraná - PR
233	ATIVIDADE EXTERNA ESPII - RODÍZIO	CHC da Universidade Federal do Paraná - PR
233	ATIVIDADE EXTERNA ESPII - RODÍZIO	CHC da Universidade Federal do Paraná - PR
233	ATIVIDADE EXTERNA ESPII - RODÍZIO	CHC da Universidade Federal do Paraná - PR
233	ATIVIDADE EXTERNA ESPII - RODÍZIO	CHC da Universidade Federal do Paraná - PR
233	ATIVIDADE EXTERNA ESPII - RODÍZIO	CHC da Universidade Federal do Paraná - PR
233	ATIVIDADE EXTERNA ESPII - RODÍZIO	CHC da Universidade Federal do Paraná - PR
233	ATIVIDADE EXTERNA ESPII - RODÍZIO	CHC da Universidade Federal do Paraná - PR
233	ATIVIDADE EXTERNA ESPII - RODÍZIO	CHC da Universidade Federal do Paraná - PR
233	ATIVIDADE EXTERNA ESPII - RODÍZIO	CHC da Universidade Federal do Paraná - PR
233	ATIVIDADE EXTERNA ESPII - RODÍZIO	CHC da Universidade Federal do Paraná - PR
233	ATIVIDADE EXTERNA ESPII - RODÍZIO	CHC da Universidade Federal do Paraná - PR
233	ATIVIDADE EXTERNA ESPII - RODÍZIO	CHC da Universidade Federal do Paraná - PR
233	ATIVIDADE EXTERNA ESPII - RODÍZIO	CHC da Universidade Federal do Paraná - PR
233	ATIVIDADE EXTERNA ESPII - RODÍZIO	CHC da Universidade Federal do Paraná - PR
233	ATIVIDADE EXTERNA ESPII - RODÍZIO	CHC da Universidade Federal do Paraná - PR
233	ATIVIDADE EXTERNA ESPII - RODÍZIO	CHC da Universidade Federal do Paraná - PR
233	ATIVIDADE EXTERNA ESPII - RODÍZIO	CHC da Universidade Federal do Paraná - PR
233	ATIVIDADE EXTERNA ESPII - RODÍZIO	CHC da Universidade Federal do Paraná - PR
233	ATIVIDADE EXTERNA ESPII - RODÍZIO	CHC da Universidade Federal do Paraná - PR
233	ATIVIDADE EXTERNA ESPII - RODÍZIO	CHC da Universidade Federal do Paraná - PR
233	ATIVIDADE EXTERNA ESPII - RODÍZIO	CHC da Universidade Federal do Paraná - PR
233	ATIVIDADE EXTERNA ESPII - RODÍZIO	CHC da Universidade Federal do Paraná - PR
233	ATIVIDADE EXTERNA ESPII - RODÍZIO	CHC da Universidade Federal do Paraná - PR
233	ATIVIDADE EXTERNA ESPII - RODÍZIO	CHC da Universidade Federal do Paraná - PR
233	ATIVIDADE EXTERNA ESPII - RODÍZIO	CHC da Universidade Federal do Paraná - PR
233	ATIVIDADE EXTERNA ESPII - RODÍZIO	CHC da Universidade Federal do Paraná - PR
233	ATIVIDADE EXTERNA ESPII - RODÍZIO	CHC da Universidade Federal do Paraná - PR
233	ATIVIDADE EXTERNA ESPII - RODÍZIO	CHC da Universidade Federal do Paraná - PR
233	ATIVIDADE EXTERNA ESPII - RODÍZIO	CHC da Universidade Federal do Paraná - PR
233	ATIVIDADE EXTERNA ESPII - RODÍZIO	CHC da Universidade Federal do Paraná - PR
233	ATIVIDADE EXTERNA ESPII - RODÍZIO	CHC da Universidade Federal do Paraná - PR
233	ATIVIDADE EXTERNA ESPII - RODÍZIO	CHC da Universidade Federal do Paraná - PR
233	ATIVIDADE EXTERNA ESPII - RODÍZIO	CHC da Universidade Federal do Paraná - PR
233	ATIVIDADE EXTERNA ESPII - RODÍZIO	CHC da Universidade Federal do Paraná - PR
233	ATIVIDADE EXTERNA ESPII - RODÍZIO	CHC da Universidade Federal do Paraná - PR
233	ATIVIDADE EXTERNA ESPII - RODÍZIO	CHC da Universidade Federal do Paraná - PR
233	ATIVIDADE EXTERNA ESPII - RODÍZIO	CHC da Universidade Federal do Paraná - PR
233	ATIVIDADE EXTERNA ESPII - RODÍZIO	CHC da Universidade Federal do Paraná - PR

233	ATIVIDADE EXTERNA ESPII - RODÍZIO	CHC da Universidade Federal do Paraná - PR
233	ATIVIDADE EXTERNA ESPII - RODÍZIO	CHC da Universidade Federal do Paraná - PR
233	ATIVIDADE EXTERNA ESPII - RODÍZIO	CHC da Universidade Federal do Paraná - PR
233	ATIVIDADE EXTERNA ESPII - RODÍZIO	CHC da Universidade Federal do Paraná - PR
233	ATIVIDADE EXTERNA ESPII - RODÍZIO	CHC da Universidade Federal do Paraná - PR
233	ATIVIDADE EXTERNA ESPII - RODÍZIO	CHC da Universidade Federal do Paraná - PR
233	ATIVIDADE EXTERNA ESPII - RODÍZIO	CHC da Universidade Federal do Paraná - PR
233	ATIVIDADE EXTERNA ESPII - RODÍZIO	CHC da Universidade Federal do Paraná - PR
233	ATIVIDADE EXTERNA ESPII - RODÍZIO	CHC da Universidade Federal do Paraná - PR
233	ATIVIDADE EXTERNA ESPII - RODÍZIO	CHC da Universidade Federal do Paraná - PR
233	ATIVIDADE EXTERNA ESPII - RODÍZIO	CHC da Universidade Federal do Paraná - PR
233	ATIVIDADE EXTERNA ESPII - RODÍZIO	CHC da Universidade Federal do Paraná - PR
233	ATIVIDADE EXTERNA ESPII - RODÍZIO	CHC da Universidade Federal do Paraná - PR
233	ATIVIDADE EXTERNA ESPII - RODÍZIO	CHC da Universidade Federal do Paraná - PR
233	ATIVIDADE EXTERNA ESPII - RODÍZIO	CHC da Universidade Federal do Paraná - PR
233	ATIVIDADE EXTERNA ESPII - RODÍZIO	CHC da Universidade Federal do Paraná - PR
233	ATIVIDADE EXTERNA ESPII - RODÍZIO	CHC da Universidade Federal do Paraná - PR
233	ATIVIDADE EXTERNA ESPII - RODÍZIO	CHC da Universidade Federal do Paraná - PR
233	ATIVIDADE EXTERNA ESPII - RODÍZIO	CHC da Universidade Federal do Paraná - PR
233	ATIVIDADE EXTERNA ESPII - RODÍZIO	CHC da Universidade Federal do Paraná - PR
233	ATIVIDADE EXTERNA ESPII - RODÍZIO	CHC da Universidade Federal do Paraná - PR
233	ATIVIDADE EXTERNA ESPII - RODÍZIO	CHC da Universidade Federal do Paraná - PR
233	ATIVIDADE EXTERNA ESPII - RODÍZIO	CHC da Universidade Federal do Paraná - PR
233	ATIVIDADE EXTERNA ESPII - RODÍZIO	CHC da Universidade Federal do Paraná - PR
233	ATIVIDADE EXTERNA ESPII - RODÍZIO	CHC da Universidade Federal do Paraná - PR
233	ATIVIDADE EXTERNA ESPII - RODÍZIO	CHC da Universidade Federal do Paraná - PR
233	ATIVIDADE EXTERNA ESPII - RODÍZIO	CHC da Universidade Federal do Paraná - PR
233	ATIVIDADE EXTERNA ESPII - RODÍZIO	CHC da Universidade Federal do Paraná - PR
233	ATIVIDADE EXTERNA ESPII - RODÍZIO	CHC da Universidade Federal do Paraná - PR
233	ATIVIDADE EXTERNA ESPII - RODÍZIO	CHC da Universidade Federal do Paraná - PR
233	ATIVIDADE EXTERNA ESPII - RODÍZIO	CHC da Universidade Federal do Paraná - PR
233	ATIVIDADE EXTERNA ESPII - RODÍZIO	CHC da Universidade Federal do Paraná - PR
233	ATIVIDADE EXTERNA ESPII - RODÍZIO	CHC da Universidade Federal do Paraná - PR
233	ATIVIDADE EXTERNA ESPII - RODÍZIO	CHC da Universidade Federal do Paraná - PR
233	ATIVIDADE EXTERNA ESPII - RODÍZIO	CHC da Universidade Federal do Paraná - PR
233	ATIVIDADE EXTERNA ESPII - RODÍZIO	CHC da Universidade Federal do Paraná - PR
233	ATIVIDADE EXTERNA ESPII - RODÍZIO	CHC da Universidade Federal do Paraná - PR
233	ATIVIDADE EXTERNA ESPII - RODÍZIO	CHC da Universidade Federal do Paraná - PR
233	ATIVIDADE EXTERNA ESPII - RODÍZIO	CHC da Universidade Federal do Paraná - PR
233	ATIVIDADE EXTERNA ESPII - RODÍZIO	CHC da Universidade Federal do Paraná - PR
233	ATIVIDADE EXTERNA ESPII - RODÍZIO	CHC da Universidade Federal do Paraná - PR
233	ATIVIDADE EXTERNA ESPII - RODÍZIO	CHC da Universidade Federal do Paraná - PR
233	ATIVIDADE EXTERNA ESPII - RODÍZIO	CHC da Universidade Federal do Paraná - PR
233	ATIVIDADE EXTERNA ESPII - RODÍZIO	CHC da Universidade Federal do Paraná - PR
233	ATIVIDADE EXTERNA ESPII - RODÍZIO	CHC da Universidade Federal do Paraná - PR
233	ATIVIDADE EXTERNA ESPII - RODÍZIO	CHC da Universidade Federal do Paraná - PR
233	ATIVIDADE EXTERNA ESPII - RODÍZIO	CHC da Universidade Federal do Paraná - PR
233	ATIVIDADE EXTERNA ESPII - RODÍZIO	CHC da Universidade Federal do Paraná - PR

233	ATIVIDADE EXTERNA ESPII - RODÍZIO	CHC da Universidade Federal do Paraná - PR
233	ATIVIDADE EXTERNA ESPII - RODÍZIO	CHC da Universidade Federal do Paraná - PR
233	ATIVIDADE EXTERNA ESPII - RODÍZIO	CHC da Universidade Federal do Paraná - PR
233	ATIVIDADE EXTERNA ESPII - RODÍZIO	CHC da Universidade Federal do Paraná - PR
233	ATIVIDADE EXTERNA ESPII - RODÍZIO	CHC da Universidade Federal do Paraná - PR
233	ATIVIDADE EXTERNA ESPII - RODÍZIO	CHC da Universidade Federal do Paraná - PR
233	ATIVIDADE EXTERNA ESPII - RODÍZIO	CHC da Universidade Federal do Paraná - PR
233	ATIVIDADE EXTERNA ESPII - RODÍZIO	CHC da Universidade Federal do Paraná - PR
233	ATIVIDADE EXTERNA ESPII - RODÍZIO	CHC da Universidade Federal do Paraná - PR
233	ATIVIDADE EXTERNA ESPII - RODÍZIO	CHC da Universidade Federal do Paraná - PR
233	ATIVIDADE EXTERNA ESPII - RODÍZIO	CHC da Universidade Federal do Paraná - PR
233	ATIVIDADE EXTERNA ESPII - RODÍZIO	CHC da Universidade Federal do Paraná - PR
233	ATIVIDADE EXTERNA ESPII - RODÍZIO	CHC da Universidade Federal do Paraná - PR
233	ATIVIDADE EXTERNA ESPII - RODÍZIO	CHC da Universidade Federal do Paraná - PR
233	ATIVIDADE EXTERNA ESPII - RODÍZIO	CHC da Universidade Federal do Paraná - PR
233	ATIVIDADE EXTERNA ESPII - RODÍZIO	CHC da Universidade Federal do Paraná - PR
233	ATIVIDADE EXTERNA ESPII - RODÍZIO	CHC da Universidade Federal do Paraná - PR
233	ATIVIDADE EXTERNA ESPII - RODÍZIO	CHC da Universidade Federal do Paraná - PR
233	ATIVIDADE EXTERNA ESPII - RODÍZIO	CHC da Universidade Federal do Paraná - PR
233	ATIVIDADE EXTERNA ESPII - RODÍZIO	CHC da Universidade Federal do Paraná - PR
233	ATIVIDADE EXTERNA ESPII - RODÍZIO	CHC da Universidade Federal do Paraná - PR
233	ATIVIDADE EXTERNA ESPII - RODÍZIO	CHC da Universidade Federal do Paraná - PR
233	ATIVIDADE EXTERNA ESPII - RODÍZIO	CHC da Universidade Federal do Paraná - PR
233	ATIVIDADE EXTERNA ESPII - RODÍZIO	CHC da Universidade Federal do Paraná - PR
233	ATIVIDADE EXTERNA ESPII - RODÍZIO	CHC da Universidade Federal do Paraná - PR
233	ATIVIDADE EXTERNA ESPII - RODÍZIO	CHC da Universidade Federal do Paraná - PR
233	ATIVIDADE EXTERNA ESPII - RODÍZIO	CHC da Universidade Federal do Paraná - PR
233	ATIVIDADE EXTERNA ESPII - RODÍZIO	CHC da Universidade Federal do Paraná - PR
233	ATIVIDADE EXTERNA ESPII - RODÍZIO	CHC da Universidade Federal do Paraná - PR
233	ATIVIDADE EXTERNA ESPII - RODÍZIO	CHC da Universidade Federal do Paraná - PR
233	ATIVIDADE EXTERNA ESPII - RODÍZIO	CHC da Universidade Federal do Paraná - PR
233	ATIVIDADE EXTERNA ESPII - RODÍZIO	CHC da Universidade Federal do Paraná - PR
233	ATIVIDADE EXTERNA ESPII - RODÍZIO	CHC da Universidade Federal do Paraná - PR
233	ATIVIDADE EXTERNA ESPII - RODÍZIO	CHC da Universidade Federal do Paraná - PR
233	ATIVIDADE EXTERNA ESPII - RODÍZIO	CHC da Universidade Federal do Paraná - PR
233	ATIVIDADE EXTERNA ESPII - RODÍZIO	CHC da Universidade Federal do Paraná - PR
233	ATIVIDADE EXTERNA ESPII - RODÍZIO	CHC da Universidade Federal do Paraná - PR
233	ATIVIDADE EXTERNA ESPII - RODÍZIO	CHC da Universidade Federal do Paraná - PR
233	ATIVIDADE EXTERNA ESPII - RODÍZIO	CHC da Universidade Federal do Paraná - PR
233	ATIVIDADE EXTERNA ESPII - RODÍZIO	CHC da Universidade Federal do Paraná - PR
233	ATIVIDADE EXTERNA ESPII - RODÍZIO	CHC da Universidade Federal do Paraná - PR
233	ATIVIDADE EXTERNA ESPII - RODÍZIO	CHC da Universidade Federal do Paraná - PR
233	ATIVIDADE EXTERNA ESPII - RODÍZIO	CHC da Universidade Federal do Paraná - PR
233	ATIVIDADE EXTERNA ESPII - RODÍZIO	CHC da Universidade Federal do Paraná - PR
233	ATIVIDADE EXTERNA ESPII - RODÍZIO	CHC da Universidade Federal do Paraná - PR
233	ATIVIDADE EXTERNA ESPII - RODÍZIO	CHC da Universidade Federal do Paraná - PR
233	ATIVIDADE EXTERNA ESPII - RODÍZIO	CHC da Universidade Federal do Paraná - PR
233	ATIVIDADE EXTERNA ESPII - RODÍZIO	CHC da Universidade Federal do Paraná - PR

233	ATIVIDADE EXTERNA ESPII - RODÍZIO	CHC da Universidade Federal do Paraná - PR
233	ATIVIDADE EXTERNA ESPII - RODÍZIO	CHC da Universidade Federal do Paraná - PR
233	ATIVIDADE EXTERNA ESPII - RODÍZIO	CHC da Universidade Federal do Paraná - PR
233	ATIVIDADE EXTERNA ESPII - RODÍZIO	CHC da Universidade Federal do Paraná - PR
233	ATIVIDADE EXTERNA ESPII - RODÍZIO	CHC da Universidade Federal do Paraná - PR
233	ATIVIDADE EXTERNA ESPII - RODÍZIO	CHC da Universidade Federal do Paraná - PR
233	ATIVIDADE EXTERNA ESPII - RODÍZIO	CHC da Universidade Federal do Paraná - PR
233	ATIVIDADE EXTERNA ESPII - RODÍZIO	CHC da Universidade Federal do Paraná - PR
233	ATIVIDADE EXTERNA ESPII - RODÍZIO	CHC da Universidade Federal do Paraná - PR
233	ATIVIDADE EXTERNA ESPII - RODÍZIO	CHC da Universidade Federal do Paraná - PR
233	ATIVIDADE EXTERNA ESPII - RODÍZIO	CHC da Universidade Federal do Paraná - PR
233	ATIVIDADE EXTERNA ESPII - RODÍZIO	CHC da Universidade Federal do Paraná - PR
233	ATIVIDADE EXTERNA ESPII - RODÍZIO	CHC da Universidade Federal do Paraná - PR
233	ATIVIDADE EXTERNA ESPII - RODÍZIO	CHC da Universidade Federal do Paraná - PR
233	ATIVIDADE EXTERNA ESPII - RODÍZIO	CHC da Universidade Federal do Paraná - PR
233	ATIVIDADE EXTERNA ESPII - RODÍZIO	CHC da Universidade Federal do Paraná - PR
233	ATIVIDADE EXTERNA ESPII - RODÍZIO	CHC da Universidade Federal do Paraná - PR
233	ATIVIDADE EXTERNA ESPII - RODÍZIO	CHC da Universidade Federal do Paraná - PR
233	ATIVIDADE EXTERNA ESPII - RODÍZIO	CHC da Universidade Federal do Paraná - PR
233	ATIVIDADE EXTERNA ESPII - RODÍZIO	CHC da Universidade Federal do Paraná - PR
233	ATIVIDADE EXTERNA ESPII - RODÍZIO	CHC da Universidade Federal do Paraná - PR
233	ATIVIDADE EXTERNA ESPII - RODÍZIO	CHC da Universidade Federal do Paraná - PR
233	ATIVIDADE EXTERNA ESPII - RODÍZIO	CHC da Universidade Federal do Paraná - PR
233	ATIVIDADE EXTERNA ESPII - RODÍZIO	CHC da Universidade Federal do Paraná - PR
233	ATIVIDADE EXTERNA ESPII - RODÍZIO	CHC da Universidade Federal do Paraná - PR
233	ATIVIDADE EXTERNA ESPII - RODÍZIO	CHC da Universidade Federal do Paraná - PR
233	ATIVIDADE EXTERNA ESPII - RODÍZIO	CHC da Universidade Federal do Paraná - PR
233	ATIVIDADE EXTERNA ESPII - RODÍZIO	CHC da Universidade Federal do Paraná - PR
233	ATIVIDADE EXTERNA ESPII - RODÍZIO	CHC da Universidade Federal do Paraná - PR
233	ATIVIDADE EXTERNA ESPII - RODÍZIO	CHC da Universidade Federal do Paraná - PR
233	ATIVIDADE EXTERNA ESPII - RODÍZIO	CHC da Universidade Federal do Paraná - PR
233	ATIVIDADE EXTERNA ESPII - RODÍZIO	CHC da Universidade Federal do Paraná - PR
233	ATIVIDADE EXTERNA ESPII - RODÍZIO	CHC da Universidade Federal do Paraná - PR
233	ATIVIDADE EXTERNA ESPII - RODÍZIO	CHC da Universidade Federal do Paraná - PR
233	ATIVIDADE EXTERNA ESPII - RODÍZIO	CHC da Universidade Federal do Paraná - PR
233	ATIVIDADE EXTERNA ESPII - RODÍZIO	CHC da Universidade Federal do Paraná - PR
233	ATIVIDADE EXTERNA ESPII - RODÍZIO	CHC da Universidade Federal do Paraná - PR
233	ATIVIDADE EXTERNA ESPII - RODÍZIO	CHC da Universidade Federal do Paraná - PR
233	ATIVIDADE EXTERNA ESPII - RODÍZIO	CHC da Universidade Federal do Paraná - PR
233	ATIVIDADE EXTERNA ESPII - RODÍZIO	CHC da Universidade Federal do Paraná - PR
233	ATIVIDADE EXTERNA ESPII - RODÍZIO	CHC da Universidade Federal do Paraná - PR
233	ATIVIDADE EXTERNA ESPII - RODÍZIO	CHC da Universidade Federal do Paraná - PR
233	ATIVIDADE EXTERNA ESPII - RODÍZIO	CHC da Universidade Federal do Paraná - PR
233	ATIVIDADE EXTERNA ESPII - RODÍZIO	CHC da Universidade Federal do Paraná - PR
233	ATIVIDADE EXTERNA ESPII - RODÍZIO	CHC da Universidade Federal do Paraná - PR
233	ATIVIDADE EXTERNA ESPII - RODÍZIO	CHC da Universidade Federal do Paraná - PR
233	ATIVIDADE EXTERNA ESPII - RODÍZIO	CHC da Universidade Federal do Paraná - PR
233	ATIVIDADE EXTERNA ESPII - RODÍZIO	CHC da Universidade Federal do Paraná - PR

233	ATIVIDADE EXTERNA ESPII - RODÍZIO	CHC da Universidade Federal do Paraná - PR
233	ATIVIDADE EXTERNA ESPII - RODÍZIO	CHC da Universidade Federal do Paraná - PR
233	ATIVIDADE EXTERNA ESPII - RODÍZIO	CHC da Universidade Federal do Paraná - PR
233	ATIVIDADE EXTERNA ESPII - RODÍZIO	CHC da Universidade Federal do Paraná - PR
233	ATIVIDADE EXTERNA ESPII - RODÍZIO	CHC da Universidade Federal do Paraná - PR
233	ATIVIDADE EXTERNA ESPII - RODÍZIO	CHC da Universidade Federal do Paraná - PR
233	ATIVIDADE EXTERNA ESPII - RODÍZIO	CHC da Universidade Federal do Paraná - PR
233	ATIVIDADE EXTERNA ESPII - RODÍZIO	CHC da Universidade Federal do Paraná - PR
233	ATIVIDADE EXTERNA ESPII - RODÍZIO	CHC da Universidade Federal do Paraná - PR
233	ATIVIDADE EXTERNA ESPII - RODÍZIO	CHC da Universidade Federal do Paraná - PR
233	ATIVIDADE EXTERNA ESPII - RODÍZIO	CHC da Universidade Federal do Paraná - PR
233	ATIVIDADE EXTERNA ESPII - RODÍZIO	CHC da Universidade Federal do Paraná - PR
233	ATIVIDADE EXTERNA ESPII - RODÍZIO	CHC da Universidade Federal do Paraná - PR
233	ATIVIDADE EXTERNA ESPII - RODÍZIO	CHC da Universidade Federal do Paraná - PR
233	ATIVIDADE EXTERNA ESPII - RODÍZIO	CHC da Universidade Federal do Paraná - PR
233	ATIVIDADE EXTERNA ESPII - RODÍZIO	CHC da Universidade Federal do Paraná - PR
233	ATIVIDADE EXTERNA ESPII - RODÍZIO	CHC da Universidade Federal do Paraná - PR
233	ATIVIDADE EXTERNA ESPII - RODÍZIO	CHC da Universidade Federal do Paraná - PR
233	ATIVIDADE EXTERNA ESPII - RODÍZIO	CHC da Universidade Federal do Paraná - PR
233	ATIVIDADE EXTERNA ESPII - RODÍZIO	CHC da Universidade Federal do Paraná - PR
233	ATIVIDADE EXTERNA ESPII - RODÍZIO	CHC da Universidade Federal do Paraná - PR
233	ATIVIDADE EXTERNA ESPII - RODÍZIO	CHC da Universidade Federal do Paraná - PR
233	ATIVIDADE EXTERNA ESPII - RODÍZIO	CHC da Universidade Federal do Paraná - PR
233	ATIVIDADE EXTERNA ESPII - RODÍZIO	CHC da Universidade Federal do Paraná - PR
233	ATIVIDADE EXTERNA ESPII - RODÍZIO	CHC da Universidade Federal do Paraná - PR
233	ATIVIDADE EXTERNA ESPII - RODÍZIO	CHC da Universidade Federal do Paraná - PR
233	ATIVIDADE EXTERNA ESPII - RODÍZIO	CHC da Universidade Federal do Paraná - PR
233	ATIVIDADE EXTERNA ESPII - RODÍZIO	CHC da Universidade Federal do Paraná - PR
233	ATIVIDADE EXTERNA ESPII - RODÍZIO	CHC da Universidade Federal do Paraná - PR
233	ATIVIDADE EXTERNA ESPII - RODÍZIO	CHC da Universidade Federal do Paraná - PR
233	ATIVIDADE EXTERNA ESPII - RODÍZIO	CHC da Universidade Federal do Paraná - PR
233	ATIVIDADE EXTERNA ESPII - RODÍZIO	CHC da Universidade Federal do Paraná - PR
233	ATIVIDADE EXTERNA ESPII - RODÍZIO	CHC da Universidade Federal do Paraná - PR
233	ATIVIDADE EXTERNA ESPII - RODÍZIO	CHC da Universidade Federal do Paraná - PR
233	ATIVIDADE EXTERNA ESPII - RODÍZIO	CHC da Universidade Federal do Paraná - PR
233	ATIVIDADE EXTERNA ESPII - RODÍZIO	CHC da Universidade Federal do Paraná - PR
233	ATIVIDADE EXTERNA ESPII - RODÍZIO	CHC da Universidade Federal do Paraná - PR
233	ATIVIDADE EXTERNA ESPII - RODÍZIO	CHC da Universidade Federal do Paraná - PR
233	ATIVIDADE EXTERNA ESPII - RODÍZIO	CHC da Universidade Federal do Paraná - PR
233	ATIVIDADE EXTERNA ESPII - RODÍZIO	CHC da Universidade Federal do Paraná - PR
233	ATIVIDADE EXTERNA ESPII - RODÍZIO	CHC da Universidade Federal do Paraná - PR
233	ATIVIDADE EXTERNA ESPII - RODÍZIO	CHC da Universidade Federal do Paraná - PR
233	ATIVIDADE EXTERNA ESPII - RODÍZIO	CHC da Universidade Federal do Paraná - PR
233	ATIVIDADE EXTERNA ESPII - RODÍZIO	CHC da Universidade Federal do Paraná - PR
233	ATIVIDADE EXTERNA ESPII - RODÍZIO	CHC da Universidade Federal do Paraná - PR
233	ATIVIDADE EXTERNA ESPII - RODÍZIO	CHC da Universidade Federal do Paraná - PR
233	ATIVIDADE EXTERNA ESPII - RODÍZIO	CHC da Universidade Federal do Paraná - PR
233	ATIVIDADE EXTERNA ESPII - RODÍZIO	CHC da Universidade Federal do Paraná - PR

233	ATIVIDADE EXTERNA ESPII - RODÍZIO	CHC da Universidade Federal do Paraná - PR
233	ATIVIDADE EXTERNA ESPII - RODÍZIO	CHC da Universidade Federal do Paraná - PR
233	ATIVIDADE EXTERNA ESPII - RODÍZIO	CHC da Universidade Federal do Paraná - PR
233	ATIVIDADE EXTERNA ESPII - RODÍZIO	CHC da Universidade Federal do Paraná - PR
233	ATIVIDADE EXTERNA ESPII - RODÍZIO	CHC da Universidade Federal do Paraná - PR
233	ATIVIDADE EXTERNA ESPII - RODÍZIO	CHC da Universidade Federal do Paraná - PR
233	ATIVIDADE EXTERNA ESPII - RODÍZIO	CHC da Universidade Federal do Paraná - PR
233	ATIVIDADE EXTERNA ESPII - RODÍZIO	CHC da Universidade Federal do Paraná - PR
233	ATIVIDADE EXTERNA ESPII - RODÍZIO	CHC da Universidade Federal do Paraná - PR
233	ATIVIDADE EXTERNA ESPII - RODÍZIO	CHC da Universidade Federal do Paraná - PR
233	ATIVIDADE EXTERNA ESPII - RODÍZIO	CHC da Universidade Federal do Paraná - PR
233	ATIVIDADE EXTERNA ESPII - RODÍZIO	CHC da Universidade Federal do Paraná - PR
233	ATIVIDADE EXTERNA ESPII - RODÍZIO	CHC da Universidade Federal do Paraná - PR
233	ATIVIDADE EXTERNA ESPII - RODÍZIO	CHC da Universidade Federal do Paraná - PR
233	ATIVIDADE EXTERNA ESPII - RODÍZIO	CHC da Universidade Federal do Paraná - PR
233	ATIVIDADE EXTERNA ESPII - RODÍZIO	CHC da Universidade Federal do Paraná - PR
233	ATIVIDADE EXTERNA ESPII - RODÍZIO	CHC da Universidade Federal do Paraná - PR
233	ATIVIDADE EXTERNA ESPII - RODÍZIO	CHC da Universidade Federal do Paraná - PR
233	ATIVIDADE EXTERNA ESPII - RODÍZIO	CHC da Universidade Federal do Paraná - PR
233	ATIVIDADE EXTERNA ESPII - RODÍZIO	CHC da Universidade Federal do Paraná - PR
233	ATIVIDADE EXTERNA ESPII - RODÍZIO	CHC da Universidade Federal do Paraná - PR
233	ATIVIDADE EXTERNA ESPII - RODÍZIO	CHC da Universidade Federal do Paraná - PR
233	ATIVIDADE EXTERNA ESPII - RODÍZIO	CHC da Universidade Federal do Paraná - PR
233	ATIVIDADE EXTERNA ESPII - RODÍZIO	CHC da Universidade Federal do Paraná - PR
233	ATIVIDADE EXTERNA ESPII - RODÍZIO	CHC da Universidade Federal do Paraná - PR
233	ATIVIDADE EXTERNA ESPII - RODÍZIO	CHC da Universidade Federal do Paraná - PR
233	ATIVIDADE EXTERNA ESPII - RODÍZIO	CHC da Universidade Federal do Paraná - PR
233	ATIVIDADE EXTERNA ESPII - RODÍZIO	CHC da Universidade Federal do Paraná - PR
233	ATIVIDADE EXTERNA ESPII - RODÍZIO	CHC da Universidade Federal do Paraná - PR
233	ATIVIDADE EXTERNA ESPII - RODÍZIO	CHC da Universidade Federal do Paraná - PR
233	ATIVIDADE EXTERNA ESPII - RODÍZIO	CHC da Universidade Federal do Paraná - PR
233	ATIVIDADE EXTERNA ESPII - RODÍZIO	CHC da Universidade Federal do Paraná - PR
233	ATIVIDADE EXTERNA ESPII - RODÍZIO	CHC da Universidade Federal do Paraná - PR
233	ATIVIDADE EXTERNA ESPII - RODÍZIO	CHC da Universidade Federal do Paraná - PR
233	ATIVIDADE EXTERNA ESPII - RODÍZIO	CHC da Universidade Federal do Paraná - PR
233	ATIVIDADE EXTERNA ESPII - RODÍZIO	CHC da Universidade Federal do Paraná - PR
233	ATIVIDADE EXTERNA ESPII - RODÍZIO	CHC da Universidade Federal do Paraná - PR
233	ATIVIDADE EXTERNA ESPII - RODÍZIO	CHC da Universidade Federal do Paraná - PR
233	ATIVIDADE EXTERNA ESPII - RODÍZIO	CHC da Universidade Federal do Paraná - PR
233	ATIVIDADE EXTERNA ESPII - RODÍZIO	CHC da Universidade Federal do Paraná - PR
233	ATIVIDADE EXTERNA ESPII - RODÍZIO	CHC da Universidade Federal do Paraná - PR
233	ATIVIDADE EXTERNA ESPII - RODÍZIO	CHC da Universidade Federal do Paraná - PR
233	ATIVIDADE EXTERNA ESPII - RODÍZIO	CHC da Universidade Federal do Paraná - PR
233	ATIVIDADE EXTERNA ESPII - RODÍZIO	CHC da Universidade Federal do Paraná - PR
233	ATIVIDADE EXTERNA ESPII - RODÍZIO	CHC da Universidade Federal do Paraná - PR
233	ATIVIDADE EXTERNA ESPII - RODÍZIO	CHC da Universidade Federal do Paraná - PR
233	ATIVIDADE EXTERNA ESPII - RODÍZIO	CHC da Universidade Federal do Paraná - PR
233	ATIVIDADE EXTERNA ESPII - RODÍZIO	CHC da Universidade Federal do Paraná - PR

233	ATIVIDADE EXTERNA ESPII - RODÍZIO	CHC da Universidade Federal do Paraná - PR
233	ATIVIDADE EXTERNA ESPII - RODÍZIO	CHC da Universidade Federal do Paraná - PR
233	ATIVIDADE EXTERNA ESPII - RODÍZIO	CHC da Universidade Federal do Paraná - PR
233	ATIVIDADE EXTERNA ESPII - RODÍZIO	CHC da Universidade Federal do Paraná - PR
233	ATIVIDADE EXTERNA ESPII - RODÍZIO	CHC da Universidade Federal do Paraná - PR
233	ATIVIDADE EXTERNA ESPII - RODÍZIO	CHC da Universidade Federal do Paraná - PR
233	ATIVIDADE EXTERNA ESPII - RODÍZIO	CHC da Universidade Federal do Paraná - PR
233	ATIVIDADE EXTERNA ESPII - RODÍZIO	CHC da Universidade Federal do Paraná - PR
233	ATIVIDADE EXTERNA ESPII - RODÍZIO	CHC da Universidade Federal do Paraná - PR
233	ATIVIDADE EXTERNA ESPII - RODÍZIO	CHC da Universidade Federal do Paraná - PR
233	ATIVIDADE EXTERNA ESPII - RODÍZIO	CHC da Universidade Federal do Paraná - PR
233	ATIVIDADE EXTERNA ESPII - RODÍZIO	CHC da Universidade Federal do Paraná - PR
233	ATIVIDADE EXTERNA ESPII - RODÍZIO	CHC da Universidade Federal do Paraná - PR
233	ATIVIDADE EXTERNA ESPII - RODÍZIO	CHC da Universidade Federal do Paraná - PR
233	ATIVIDADE EXTERNA ESPII - RODÍZIO	CHC da Universidade Federal do Paraná - PR
233	ATIVIDADE EXTERNA ESPII - RODÍZIO	CHC da Universidade Federal do Paraná - PR
233	ATIVIDADE EXTERNA ESPII - RODÍZIO	CHC da Universidade Federal do Paraná - PR
233	ATIVIDADE EXTERNA ESPII - RODÍZIO	CHC da Universidade Federal do Paraná - PR
233	ATIVIDADE EXTERNA ESPII - RODÍZIO	CHC da Universidade Federal do Paraná - PR
233	ATIVIDADE EXTERNA ESPII - RODÍZIO	CHC da Universidade Federal do Paraná - PR
233	ATIVIDADE EXTERNA ESPII - RODÍZIO	CHC da Universidade Federal do Paraná - PR
233	ATIVIDADE EXTERNA ESPII - RODÍZIO	CHC da Universidade Federal do Paraná - PR
233	ATIVIDADE EXTERNA ESPII - RODÍZIO	CHC da Universidade Federal do Paraná - PR
233	ATIVIDADE EXTERNA ESPII - RODÍZIO	CHC da Universidade Federal do Paraná - PR
233	ATIVIDADE EXTERNA ESPII - RODÍZIO	CHC da Universidade Federal do Paraná - PR
233	ATIVIDADE EXTERNA ESPII - RODÍZIO	CHC da Universidade Federal do Paraná - PR
233	ATIVIDADE EXTERNA ESPII - RODÍZIO	CHC da Universidade Federal do Paraná - PR
233	ATIVIDADE EXTERNA ESPII - RODÍZIO	CHC da Universidade Federal do Paraná - PR
233	ATIVIDADE EXTERNA ESPII - RODÍZIO	CHC da Universidade Federal do Paraná - PR
233	ATIVIDADE EXTERNA ESPII - RODÍZIO	CHC da Universidade Federal do Paraná - PR
233	ATIVIDADE EXTERNA ESPII - RODÍZIO	CHC da Universidade Federal do Paraná - PR
233	ATIVIDADE EXTERNA ESPII - RODÍZIO	CHC da Universidade Federal do Paraná - PR
233	ATIVIDADE EXTERNA ESPII - RODÍZIO	CHC da Universidade Federal do Paraná - PR
233	ATIVIDADE EXTERNA ESPII - RODÍZIO	CHC da Universidade Federal do Paraná - PR
233	ATIVIDADE EXTERNA ESPII - RODÍZIO	CHC da Universidade Federal do Paraná - PR
233	ATIVIDADE EXTERNA ESPII - RODÍZIO	CHC da Universidade Federal do Paraná - PR
233	ATIVIDADE EXTERNA ESPII - RODÍZIO	CHC da Universidade Federal do Paraná - PR
233	ATIVIDADE EXTERNA ESPII - RODÍZIO	CHC da Universidade Federal do Paraná - PR
233	ATIVIDADE EXTERNA ESPII - RODÍZIO	CHC da Universidade Federal do Paraná - PR
233	ATIVIDADE EXTERNA ESPII - RODÍZIO	CHC da Universidade Federal do Paraná - PR
233	ATIVIDADE EXTERNA ESPII - RODÍZIO	CHC da Universidade Federal do Paraná - PR
233	ATIVIDADE EXTERNA ESPII - RODÍZIO	CHC da Universidade Federal do Paraná - PR
233	ATIVIDADE EXTERNA ESPII - RODÍZIO	CHC da Universidade Federal do Paraná - PR
233	ATIVIDADE EXTERNA ESPII - RODÍZIO	CHC da Universidade Federal do Paraná - PR
233	ATIVIDADE EXTERNA ESPII - RODÍZIO	CHC da Universidade Federal do Paraná - PR
233	ATIVIDADE EXTERNA ESPII - RODÍZIO	CHC da Universidade Federal do Paraná - PR
233	ATIVIDADE EXTERNA ESPII - RODÍZIO	CHC da Universidade Federal do Paraná - PR
233	ATIVIDADE EXTERNA ESPII - RODÍZIO	CHC da Universidade Federal do Paraná - PR

233	ATIVIDADE EXTERNA ESPII - RODÍZIO	CHC da Universidade Federal do Paraná - PR
233	ATIVIDADE EXTERNA ESPII - RODÍZIO	CHC da Universidade Federal do Paraná - PR
233	ATIVIDADE EXTERNA ESPII - RODÍZIO	CHC da Universidade Federal do Paraná - PR
233	ATIVIDADE EXTERNA ESPII - RODÍZIO	CHC da Universidade Federal do Paraná - PR
233	ATIVIDADE EXTERNA ESPII - RODÍZIO	CHC da Universidade Federal do Paraná - PR
233	ATIVIDADE EXTERNA ESPII - RODÍZIO	CHC da Universidade Federal do Paraná - PR
233	ATIVIDADE EXTERNA ESPII - RODÍZIO	CHC da Universidade Federal do Paraná - PR
233	ATIVIDADE EXTERNA ESPII - RODÍZIO	CHC da Universidade Federal do Paraná - PR
233	ATIVIDADE EXTERNA ESPII - RODÍZIO	CHC da Universidade Federal do Paraná - PR
233	ATIVIDADE EXTERNA ESPII - RODÍZIO	CHC da Universidade Federal do Paraná - PR
233	ATIVIDADE EXTERNA ESPII - RODÍZIO	CHC da Universidade Federal do Paraná - PR
233	ATIVIDADE EXTERNA ESPII - RODÍZIO	CHC da Universidade Federal do Paraná - PR
233	ATIVIDADE EXTERNA ESPII - RODÍZIO	CHC da Universidade Federal do Paraná - PR
233	ATIVIDADE EXTERNA ESPII - RODÍZIO	CHC da Universidade Federal do Paraná - PR
233	ATIVIDADE EXTERNA ESPII - RODÍZIO	CHC da Universidade Federal do Paraná - PR
233	ATIVIDADE EXTERNA ESPII - RODÍZIO	CHC da Universidade Federal do Paraná - PR
233	ATIVIDADE EXTERNA ESPII - RODÍZIO	CHC da Universidade Federal do Paraná - PR
233	ATIVIDADE EXTERNA ESPII - RODÍZIO	CHC da Universidade Federal do Paraná - PR
233	ATIVIDADE EXTERNA ESPII - RODÍZIO	CHC da Universidade Federal do Paraná - PR
233	ATIVIDADE EXTERNA ESPII - RODÍZIO	CHC da Universidade Federal do Paraná - PR
233	ATIVIDADE EXTERNA ESPII - RODÍZIO	CHC da Universidade Federal do Paraná - PR
233	ATIVIDADE EXTERNA ESPII - RODÍZIO	CHC da Universidade Federal do Paraná - PR
233	ATIVIDADE EXTERNA ESPII - RODÍZIO	CHC da Universidade Federal do Paraná - PR
233	ATIVIDADE EXTERNA ESPII - RODÍZIO	CHC da Universidade Federal do Paraná - PR
233	ATIVIDADE EXTERNA ESPII - RODÍZIO	CHC da Universidade Federal do Paraná - PR
233	ATIVIDADE EXTERNA ESPII - RODÍZIO	CHC da Universidade Federal do Paraná - PR
233	ATIVIDADE EXTERNA ESPII - RODÍZIO	CHC da Universidade Federal do Paraná - PR
233	ATIVIDADE EXTERNA ESPII - RODÍZIO	CHC da Universidade Federal do Paraná - PR
233	ATIVIDADE EXTERNA ESPII - RODÍZIO	CHC da Universidade Federal do Paraná - PR
233	ATIVIDADE EXTERNA ESPII - RODÍZIO	CHC da Universidade Federal do Paraná - PR
233	ATIVIDADE EXTERNA ESPII - RODÍZIO	CHC da Universidade Federal do Paraná - PR
233	ATIVIDADE EXTERNA ESPII - RODÍZIO	CHC da Universidade Federal do Paraná - PR
233	ATIVIDADE EXTERNA ESPII - RODÍZIO	CHC da Universidade Federal do Paraná - PR
233	ATIVIDADE EXTERNA ESPII - RODÍZIO	CHC da Universidade Federal do Paraná - PR
233	ATIVIDADE EXTERNA ESPII - RODÍZIO	CHC da Universidade Federal do Paraná - PR
233	ATIVIDADE EXTERNA ESPII - RODÍZIO	CHC da Universidade Federal do Paraná - PR
233	ATIVIDADE EXTERNA ESPII - RODÍZIO	CHC da Universidade Federal do Paraná - PR
233	ATIVIDADE EXTERNA ESPII - RODÍZIO	CHC da Universidade Federal do Paraná - PR
233	ATIVIDADE EXTERNA ESPII - RODÍZIO	CHC da Universidade Federal do Paraná - PR
233	ATIVIDADE EXTERNA ESPII - RODÍZIO	CHC da Universidade Federal do Paraná - PR
233	ATIVIDADE EXTERNA ESPII - RODÍZIO	CHC da Universidade Federal do Paraná - PR
233	ATIVIDADE EXTERNA ESPII - RODÍZIO	CHC da Universidade Federal do Paraná - PR
233	ATIVIDADE EXTERNA ESPII - RODÍZIO	CHC da Universidade Federal do Paraná - PR
233	ATIVIDADE EXTERNA ESPII - RODÍZIO	CHC da Universidade Federal do Paraná - PR
233	ATIVIDADE EXTERNA ESPII - RODÍZIO	CHC da Universidade Federal do Paraná - PR
233	ATIVIDADE EXTERNA ESPII - RODÍZIO	CHC da Universidade Federal do Paraná - PR
233	ATIVIDADE EXTERNA ESPII - RODÍZIO	CHC da Universidade Federal do Paraná - PR
233	ATIVIDADE EXTERNA ESPII - RODÍZIO	CHC da Universidade Federal do Paraná - PR

233	ATIVIDADE EXTERNA ESPII - RODÍZIO	CHC da Universidade Federal do Paraná - PR
233	ATIVIDADE EXTERNA ESPII - RODÍZIO	CHC da Universidade Federal do Paraná - PR
233	ATIVIDADE EXTERNA ESPII - RODÍZIO	CHC da Universidade Federal do Paraná - PR
233	ATIVIDADE EXTERNA ESPII - RODÍZIO	CHC da Universidade Federal do Paraná - PR
233	ATIVIDADE EXTERNA ESPII - RODÍZIO	CHC da Universidade Federal do Paraná - PR
233	ATIVIDADE EXTERNA ESPII - RODÍZIO	CHC da Universidade Federal do Paraná - PR
233	ATIVIDADE EXTERNA ESPII - RODÍZIO	CHC da Universidade Federal do Paraná - PR
233	ATIVIDADE EXTERNA ESPII - RODÍZIO	CHC da Universidade Federal do Paraná - PR
233	ATIVIDADE EXTERNA ESPII - RODÍZIO	CHC da Universidade Federal do Paraná - PR
233	ATIVIDADE EXTERNA ESPII - RODÍZIO	CHC da Universidade Federal do Paraná - PR
233	ATIVIDADE EXTERNA ESPII - RODÍZIO	CHC da Universidade Federal do Paraná - PR
233	ATIVIDADE EXTERNA ESPII - RODÍZIO	CHC da Universidade Federal do Paraná - PR
233	ATIVIDADE EXTERNA ESPII - RODÍZIO	CHC da Universidade Federal do Paraná - PR
233	ATIVIDADE EXTERNA ESPII - RODÍZIO	CHC da Universidade Federal do Paraná - PR
233	ATIVIDADE EXTERNA ESPII - RODÍZIO	CHC da Universidade Federal do Paraná - PR
233	ATIVIDADE EXTERNA ESPII - RODÍZIO	CHC da Universidade Federal do Paraná - PR
233	ATIVIDADE EXTERNA ESPII - RODÍZIO	CHC da Universidade Federal do Paraná - PR
233	ATIVIDADE EXTERNA ESPII - RODÍZIO	CHC da Universidade Federal do Paraná - PR
233	ATIVIDADE EXTERNA ESPII - RODÍZIO	CHC da Universidade Federal do Paraná - PR
233	ATIVIDADE EXTERNA ESPII - RODÍZIO	CHC da Universidade Federal do Paraná - PR
233	ATIVIDADE EXTERNA ESPII - RODÍZIO	CHC da Universidade Federal do Paraná - PR
233	ATIVIDADE EXTERNA ESPII - RODÍZIO	CHC da Universidade Federal do Paraná - PR
233	ATIVIDADE EXTERNA ESPII - RODÍZIO	CHC da Universidade Federal do Paraná - PR
233	ATIVIDADE EXTERNA ESPII - RODÍZIO	CHC da Universidade Federal do Paraná - PR
233	ATIVIDADE EXTERNA ESPII - RODÍZIO	CHC da Universidade Federal do Paraná - PR
233	ATIVIDADE EXTERNA ESPII - RODÍZIO	CHC da Universidade Federal do Paraná - PR
233	ATIVIDADE EXTERNA ESPII - RODÍZIO	CHC da Universidade Federal do Paraná - PR
233	ATIVIDADE EXTERNA ESPII - RODÍZIO	CHC da Universidade Federal do Paraná - PR
233	ATIVIDADE EXTERNA ESPII - RODÍZIO	CHC da Universidade Federal do Paraná - PR
233	ATIVIDADE EXTERNA ESPII - RODÍZIO	CHC da Universidade Federal do Paraná - PR
233	ATIVIDADE EXTERNA ESPII - RODÍZIO	CHC da Universidade Federal do Paraná - PR
233	ATIVIDADE EXTERNA ESPII - RODÍZIO	CHC da Universidade Federal do Paraná - PR
233	ATIVIDADE EXTERNA ESPII - RODÍZIO	CHC da Universidade Federal do Paraná - PR
233	ATIVIDADE EXTERNA ESPII - RODÍZIO	CHC da Universidade Federal do Paraná - PR
233	ATIVIDADE EXTERNA ESPII - RODÍZIO	CHC da Universidade Federal do Paraná - PR
233	ATIVIDADE EXTERNA ESPII - RODÍZIO	CHC da Universidade Federal do Paraná - PR
233	ATIVIDADE EXTERNA ESPII - RODÍZIO	CHC da Universidade Federal do Paraná - PR
233	ATIVIDADE EXTERNA ESPII - RODÍZIO	CHC da Universidade Federal do Paraná - PR
233	ATIVIDADE EXTERNA ESPII - RODÍZIO	CHC da Universidade Federal do Paraná - PR
233	ATIVIDADE EXTERNA ESPII - RODÍZIO	CHC da Universidade Federal do Paraná - PR
233	ATIVIDADE EXTERNA ESPII - RODÍZIO	CHC da Universidade Federal do Paraná - PR
233	ATIVIDADE EXTERNA ESPII - RODÍZIO	CHC da Universidade Federal do Paraná - PR
233	ATIVIDADE EXTERNA ESPII - RODÍZIO	CHC da Universidade Federal do Paraná - PR
233	ATIVIDADE EXTERNA ESPII - RODÍZIO	CHC da Universidade Federal do Paraná - PR
233	ATIVIDADE EXTERNA ESPII - RODÍZIO	CHC da Universidade Federal do Paraná - PR
233	ATIVIDADE EXTERNA ESPII - RODÍZIO	CHC da Universidade Federal do Paraná - PR
233	ATIVIDADE EXTERNA ESPII - RODÍZIO	CHC da Universidade Federal do Paraná - PR
233	ATIVIDADE EXTERNA ESPII - RODÍZIO	CHC da Universidade Federal do Paraná - PR

233	ATIVIDADE EXTERNA ESPII - RODÍZIO	CHC da Universidade Federal do Paraná - PR
233	ATIVIDADE EXTERNA ESPII - RODÍZIO	CHC da Universidade Federal do Paraná - PR
233	ATIVIDADE EXTERNA ESPII - RODÍZIO	CHC da Universidade Federal do Paraná - PR
233	ATIVIDADE EXTERNA ESPII - RODÍZIO	CHC da Universidade Federal do Paraná - PR
233	ATIVIDADE EXTERNA ESPII - RODÍZIO	CHC da Universidade Federal do Paraná - PR
233	ATIVIDADE EXTERNA ESPII - RODÍZIO	CHC da Universidade Federal do Paraná - PR
233	ATIVIDADE EXTERNA ESPII - RODÍZIO	CHC da Universidade Federal do Paraná - PR
233	ATIVIDADE EXTERNA ESPII - RODÍZIO	CHC da Universidade Federal do Paraná - PR
233	ATIVIDADE EXTERNA ESPII - RODÍZIO	CHC da Universidade Federal do Paraná - PR
233	ATIVIDADE EXTERNA ESPII - RODÍZIO	CHC da Universidade Federal do Paraná - PR
233	ATIVIDADE EXTERNA ESPII - RODÍZIO	CHC da Universidade Federal do Paraná - PR
233	ATIVIDADE EXTERNA ESPII - RODÍZIO	CHC da Universidade Federal do Paraná - PR
233	ATIVIDADE EXTERNA ESPII - RODÍZIO	CHC da Universidade Federal do Paraná - PR
233	ATIVIDADE EXTERNA ESPII - RODÍZIO	CHC da Universidade Federal do Paraná - PR
233	ATIVIDADE EXTERNA ESPII - RODÍZIO	CHC da Universidade Federal do Paraná - PR
233	ATIVIDADE EXTERNA ESPII - RODÍZIO	CHC da Universidade Federal do Paraná - PR
233	ATIVIDADE EXTERNA ESPII - RODÍZIO	CHC da Universidade Federal do Paraná - PR
233	ATIVIDADE EXTERNA ESPII - RODÍZIO	CHC da Universidade Federal do Paraná - PR
233	ATIVIDADE EXTERNA ESPII - RODÍZIO	CHC da Universidade Federal do Paraná - PR
233	ATIVIDADE EXTERNA ESPII - RODÍZIO	CHC da Universidade Federal do Paraná - PR
233	ATIVIDADE EXTERNA ESPII - RODÍZIO	CHC da Universidade Federal do Paraná - PR
233	ATIVIDADE EXTERNA ESPII - RODÍZIO	CHC da Universidade Federal do Paraná - PR
233	ATIVIDADE EXTERNA ESPII - RODÍZIO	CHC da Universidade Federal do Paraná - PR
233	ATIVIDADE EXTERNA ESPII - RODÍZIO	CHC da Universidade Federal do Paraná - PR
233	ATIVIDADE EXTERNA ESPII - RODÍZIO	CHC da Universidade Federal do Paraná - PR
233	ATIVIDADE EXTERNA ESPII - RODÍZIO	CHC da Universidade Federal do Paraná - PR
233	ATIVIDADE EXTERNA ESPII - RODÍZIO	CHC da Universidade Federal do Paraná - PR
233	ATIVIDADE EXTERNA ESPII - RODÍZIO	CHC da Universidade Federal do Paraná - PR
233	ATIVIDADE EXTERNA ESPII - RODÍZIO	CHC da Universidade Federal do Paraná - PR
233	ATIVIDADE EXTERNA ESPII - RODÍZIO	CHC da Universidade Federal do Paraná - PR
233	ATIVIDADE EXTERNA ESPII - RODÍZIO	CHC da Universidade Federal do Paraná - PR
233	ATIVIDADE EXTERNA ESPII - RODÍZIO	CHC da Universidade Federal do Paraná - PR
233	ATIVIDADE EXTERNA ESPII - RODÍZIO	CHC da Universidade Federal do Paraná - PR
233	ATIVIDADE EXTERNA ESPII - RODÍZIO	CHC da Universidade Federal do Paraná - PR
233	ATIVIDADE EXTERNA ESPII - RODÍZIO	CHC da Universidade Federal do Paraná - PR
233	ATIVIDADE EXTERNA ESPII - RODÍZIO	CHC da Universidade Federal do Paraná - PR
233	ATIVIDADE EXTERNA ESPII - RODÍZIO	CHC da Universidade Federal do Paraná - PR
233	ATIVIDADE EXTERNA ESPII - RODÍZIO	CHC da Universidade Federal do Paraná - PR
233	ATIVIDADE EXTERNA ESPII - RODÍZIO	CHC da Universidade Federal do Paraná - PR
233	ATIVIDADE EXTERNA ESPII - RODÍZIO	CHC da Universidade Federal do Paraná - PR
233	ATIVIDADE EXTERNA ESPII - RODÍZIO	CHC da Universidade Federal do Paraná - PR
233	ATIVIDADE EXTERNA ESPII - RODÍZIO	CHC da Universidade Federal do Paraná - PR
233	ATIVIDADE EXTERNA ESPII - RODÍZIO	CHC da Universidade Federal do Paraná - PR
233	ATIVIDADE EXTERNA ESPII - RODÍZIO	CHC da Universidade Federal do Paraná - PR
233	ATIVIDADE EXTERNA ESPII - RODÍZIO	CHC da Universidade Federal do Paraná - PR
233	ATIVIDADE EXTERNA ESPII - RODÍZIO	CHC da Universidade Federal do Paraná - PR
233	ATIVIDADE EXTERNA ESPII - RODÍZIO	CHC da Universidade Federal do Paraná - PR
233	ATIVIDADE EXTERNA ESPII - RODÍZIO	CHC da Universidade Federal do Paraná - PR

233	ATIVIDADE EXTERNA ESPII - RODÍZIO	CHC da Universidade Federal do Paraná - PR
233	ATIVIDADE EXTERNA ESPII - RODÍZIO	CHC da Universidade Federal do Paraná - PR
233	ATIVIDADE EXTERNA ESPII - RODÍZIO	CHC da Universidade Federal do Paraná - PR
233	ATIVIDADE EXTERNA ESPII - RODÍZIO	CHC da Universidade Federal do Paraná - PR
233	ATIVIDADE EXTERNA ESPII - RODÍZIO	CHC da Universidade Federal do Paraná - PR
233	ATIVIDADE EXTERNA ESPII - RODÍZIO	CHC da Universidade Federal do Paraná - PR
233	ATIVIDADE EXTERNA ESPII - RODÍZIO	CHC da Universidade Federal do Paraná - PR
233	ATIVIDADE EXTERNA ESPII - RODÍZIO	CHC da Universidade Federal do Paraná - PR
233	ATIVIDADE EXTERNA ESPII - RODÍZIO	CHC da Universidade Federal do Paraná - PR
233	ATIVIDADE EXTERNA ESPII - RODÍZIO	CHC da Universidade Federal do Paraná - PR
233	ATIVIDADE EXTERNA ESPII - RODÍZIO	CHC da Universidade Federal do Paraná - PR
233	ATIVIDADE EXTERNA ESPII - RODÍZIO	CHC da Universidade Federal do Paraná - PR
233	ATIVIDADE EXTERNA ESPII - RODÍZIO	CHC da Universidade Federal do Paraná - PR
233	ATIVIDADE EXTERNA ESPII - RODÍZIO	CHC da Universidade Federal do Paraná - PR
233	ATIVIDADE EXTERNA ESPII - RODÍZIO	CHC da Universidade Federal do Paraná - PR
233	ATIVIDADE EXTERNA ESPII - RODÍZIO	CHC da Universidade Federal do Paraná - PR
233	ATIVIDADE EXTERNA ESPII - RODÍZIO	CHC da Universidade Federal do Paraná - PR
233	ATIVIDADE EXTERNA ESPII - RODÍZIO	CHC da Universidade Federal do Paraná - PR
233	ATIVIDADE EXTERNA ESPII - RODÍZIO	CHC da Universidade Federal do Paraná - PR
233	ATIVIDADE EXTERNA ESPII - RODÍZIO	CHC da Universidade Federal do Paraná - PR
233	ATIVIDADE EXTERNA ESPII - RODÍZIO	CHC da Universidade Federal do Paraná - PR
233	ATIVIDADE EXTERNA ESPII - RODÍZIO	CHC da Universidade Federal do Paraná - PR
233	ATIVIDADE EXTERNA ESPII - RODÍZIO	CHC da Universidade Federal do Paraná - PR
233	ATIVIDADE EXTERNA ESPII - RODÍZIO	CHC da Universidade Federal do Paraná - PR
233	ATIVIDADE EXTERNA ESPII - RODÍZIO	CHC da Universidade Federal do Paraná - PR
233	ATIVIDADE EXTERNA ESPII - RODÍZIO	CHC da Universidade Federal do Paraná - PR
233	ATIVIDADE EXTERNA ESPII - RODÍZIO	CHC da Universidade Federal do Paraná - PR
233	ATIVIDADE EXTERNA ESPII - RODÍZIO	CHC da Universidade Federal do Paraná - PR
233	ATIVIDADE EXTERNA ESPII - RODÍZIO	CHC da Universidade Federal do Paraná - PR
233	ATIVIDADE EXTERNA ESPII - RODÍZIO	CHC da Universidade Federal do Paraná - PR
233	ATIVIDADE EXTERNA ESPII - RODÍZIO	CHC da Universidade Federal do Paraná - PR
233	ATIVIDADE EXTERNA ESPII - RODÍZIO	CHC da Universidade Federal do Paraná - PR
233	ATIVIDADE EXTERNA ESPII - RODÍZIO	CHC da Universidade Federal do Paraná - PR
233	ATIVIDADE EXTERNA ESPII - RODÍZIO	CHC da Universidade Federal do Paraná - PR
233	ATIVIDADE EXTERNA ESPII - RODÍZIO	CHC da Universidade Federal do Paraná - PR
233	ATIVIDADE EXTERNA ESPII - RODÍZIO	CHC da Universidade Federal do Paraná - PR
233	ATIVIDADE EXTERNA ESPII - RODÍZIO	CHC da Universidade Federal do Paraná - PR
233	ATIVIDADE EXTERNA ESPII - RODÍZIO	CHC da Universidade Federal do Paraná - PR
233	ATIVIDADE EXTERNA ESPII - RODÍZIO	CHC da Universidade Federal do Paraná - PR
233	ATIVIDADE EXTERNA ESPII - RODÍZIO	CHC da Universidade Federal do Paraná - PR
233	ATIVIDADE EXTERNA ESPII - RODÍZIO	CHC da Universidade Federal do Paraná - PR
233	ATIVIDADE EXTERNA ESPII - RODÍZIO	CHC da Universidade Federal do Paraná - PR
233	ATIVIDADE EXTERNA ESPII - RODÍZIO	CHC da Universidade Federal do Paraná - PR
233	ATIVIDADE EXTERNA ESPII - RODÍZIO	CHC da Universidade Federal do Paraná - PR
233	ATIVIDADE EXTERNA ESPII - RODÍZIO	CHC da Universidade Federal do Paraná - PR
233	ATIVIDADE EXTERNA ESPII - RODÍZIO	CHC da Universidade Federal do Paraná - PR
233	ATIVIDADE EXTERNA ESPII - RODÍZIO	CHC da Universidade Federal do Paraná - PR
233	ATIVIDADE EXTERNA ESPII - RODÍZIO	CHC da Universidade Federal do Paraná - PR

233	ATIVIDADE EXTERNA ESPII - RODÍZIO	CHC da Universidade Federal do Paraná - PR
233	ATIVIDADE EXTERNA ESPII - RODÍZIO	CHC da Universidade Federal do Paraná - PR
233	ATIVIDADE EXTERNA ESPII - RODÍZIO	CHC da Universidade Federal do Paraná - PR
233	ATIVIDADE EXTERNA ESPII - RODÍZIO	CHC da Universidade Federal do Paraná - PR
233	ATIVIDADE EXTERNA ESPII - RODÍZIO	CHC da Universidade Federal do Paraná - PR
233	ATIVIDADE EXTERNA ESPII - RODÍZIO	CHC da Universidade Federal do Paraná - PR
233	ATIVIDADE EXTERNA ESPII - RODÍZIO	CHC da Universidade Federal do Paraná - PR
233	ATIVIDADE EXTERNA ESPII - RODÍZIO	CHC da Universidade Federal do Paraná - PR
233	ATIVIDADE EXTERNA ESPII - RODÍZIO	CHC da Universidade Federal do Paraná - PR
233	ATIVIDADE EXTERNA ESPII - RODÍZIO	CHC da Universidade Federal do Paraná - PR
233	ATIVIDADE EXTERNA ESPII - RODÍZIO	CHC da Universidade Federal do Paraná - PR
233	ATIVIDADE EXTERNA ESPII - RODÍZIO	CHC da Universidade Federal do Paraná - PR
233	ATIVIDADE EXTERNA ESPII - RODÍZIO	CHC da Universidade Federal do Paraná - PR
233	ATIVIDADE EXTERNA ESPII - RODÍZIO	CHC da Universidade Federal do Paraná - PR
233	ATIVIDADE EXTERNA ESPII - RODÍZIO	CHC da Universidade Federal do Paraná - PR
233	ATIVIDADE EXTERNA ESPII - RODÍZIO	CHC da Universidade Federal do Paraná - PR
233	ATIVIDADE EXTERNA ESPII - RODÍZIO	CHC da Universidade Federal do Paraná - PR
233	ATIVIDADE EXTERNA ESPII - RODÍZIO	CHC da Universidade Federal do Paraná - PR
233	ATIVIDADE EXTERNA ESPII - RODÍZIO	CHC da Universidade Federal do Paraná - PR
233	ATIVIDADE EXTERNA ESPII - RODÍZIO	CHC da Universidade Federal do Paraná - PR
233	ATIVIDADE EXTERNA ESPII - RODÍZIO	CHC da Universidade Federal do Paraná - PR
233	ATIVIDADE EXTERNA ESPII - RODÍZIO	CHC da Universidade Federal do Paraná - PR
233	ATIVIDADE EXTERNA ESPII - RODÍZIO	CHC da Universidade Federal do Paraná - PR
233	ATIVIDADE EXTERNA ESPII - RODÍZIO	CHC da Universidade Federal do Paraná - PR
233	ATIVIDADE EXTERNA ESPII - RODÍZIO	CHC da Universidade Federal do Paraná - PR
233	ATIVIDADE EXTERNA ESPII - RODÍZIO	CHC da Universidade Federal do Paraná - PR
233	ATIVIDADE EXTERNA ESPII - RODÍZIO	CHC da Universidade Federal do Paraná - PR
233	ATIVIDADE EXTERNA ESPII - RODÍZIO	CHC da Universidade Federal do Paraná - PR
233	ATIVIDADE EXTERNA ESPII - RODÍZIO	CHC da Universidade Federal do Paraná - PR
233	ATIVIDADE EXTERNA ESPII - RODÍZIO	CHC da Universidade Federal do Paraná - PR
233	ATIVIDADE EXTERNA ESPII - RODÍZIO	CHC da Universidade Federal do Paraná - PR
233	ATIVIDADE EXTERNA ESPII - RODÍZIO	CHC da Universidade Federal do Paraná - PR
233	ATIVIDADE EXTERNA ESPII - RODÍZIO	CHC da Universidade Federal do Paraná - PR
233	ATIVIDADE EXTERNA ESPII - RODÍZIO	CHC da Universidade Federal do Paraná - PR
233	ATIVIDADE EXTERNA ESPII - RODÍZIO	CHC da Universidade Federal do Paraná - PR
233	ATIVIDADE EXTERNA ESPII - RODÍZIO	CHC da Universidade Federal do Paraná - PR
233	ATIVIDADE EXTERNA ESPII - RODÍZIO	CHC da Universidade Federal do Paraná - PR
233	ATIVIDADE EXTERNA ESPII - RODÍZIO	CHC da Universidade Federal do Paraná - PR
233	ATIVIDADE EXTERNA ESPII - RODÍZIO	CHC da Universidade Federal do Paraná - PR
233	ATIVIDADE EXTERNA ESPII - RODÍZIO	CHC da Universidade Federal do Paraná - PR
233	ATIVIDADE EXTERNA ESPII - RODÍZIO	CHC da Universidade Federal do Paraná - PR
233	ATIVIDADE EXTERNA ESPII - RODÍZIO	CHC da Universidade Federal do Paraná - PR
233	ATIVIDADE EXTERNA ESPII - RODÍZIO	CHC da Universidade Federal do Paraná - PR
233	ATIVIDADE EXTERNA ESPII - RODÍZIO	CHC da Universidade Federal do Paraná - PR
233	ATIVIDADE EXTERNA ESPII - RODÍZIO	CHC da Universidade Federal do Paraná - PR
233	ATIVIDADE EXTERNA ESPII - RODÍZIO	CHC da Universidade Federal do Paraná - PR
233	ATIVIDADE EXTERNA ESPII - RODÍZIO	CHC da Universidade Federal do Paraná - PR
233	ATIVIDADE EXTERNA ESPII - RODÍZIO	CHC da Universidade Federal do Paraná - PR

233	ATIVIDADE EXTERNA ESPII - RODÍZIO	CHC da Universidade Federal do Paraná - PR
233	ATIVIDADE EXTERNA ESPII - RODÍZIO	CHC da Universidade Federal do Paraná - PR
233	ATIVIDADE EXTERNA ESPII - RODÍZIO	CHC da Universidade Federal do Paraná - PR
233	ATIVIDADE EXTERNA ESPII - RODÍZIO	CHC da Universidade Federal do Paraná - PR
233	ATIVIDADE EXTERNA ESPII - RODÍZIO	CHC da Universidade Federal do Paraná - PR
233	ATIVIDADE EXTERNA ESPII - RODÍZIO	CHC da Universidade Federal do Paraná - PR
233	ATIVIDADE EXTERNA ESPII - RODÍZIO	CHC da Universidade Federal do Paraná - PR
233	ATIVIDADE EXTERNA ESPII - RODÍZIO	CHC da Universidade Federal do Paraná - PR
233	ATIVIDADE EXTERNA ESPII - RODÍZIO	CHC da Universidade Federal do Paraná - PR
233	ATIVIDADE EXTERNA ESPII - RODÍZIO	CHC da Universidade Federal do Paraná - PR
233	ATIVIDADE EXTERNA ESPII - RODÍZIO	CHC da Universidade Federal do Paraná - PR
233	ATIVIDADE EXTERNA ESPII - RODÍZIO	CHC da Universidade Federal do Paraná - PR
233	ATIVIDADE EXTERNA ESPII - RODÍZIO	CHC da Universidade Federal do Paraná - PR
233	ATIVIDADE EXTERNA ESPII - RODÍZIO	CHC da Universidade Federal do Paraná - PR
233	ATIVIDADE EXTERNA ESPII - RODÍZIO	CHC da Universidade Federal do Paraná - PR
233	ATIVIDADE EXTERNA ESPII - RODÍZIO	CHC da Universidade Federal do Paraná - PR
233	ATIVIDADE EXTERNA ESPII - RODÍZIO	CHC da Universidade Federal do Paraná - PR
233	ATIVIDADE EXTERNA ESPII - RODÍZIO	CHC da Universidade Federal do Paraná - PR
233	ATIVIDADE EXTERNA ESPII - RODÍZIO	CHC da Universidade Federal do Paraná - PR
233	ATIVIDADE EXTERNA ESPII - RODÍZIO	CHC da Universidade Federal do Paraná - PR
233	ATIVIDADE EXTERNA ESPII - RODÍZIO	CHC da Universidade Federal do Paraná - PR
233	ATIVIDADE EXTERNA ESPII - RODÍZIO	CHC da Universidade Federal do Paraná - PR
233	ATIVIDADE EXTERNA ESPII - RODÍZIO	CHC da Universidade Federal do Paraná - PR
233	ATIVIDADE EXTERNA ESPII - RODÍZIO	CHC da Universidade Federal do Paraná - PR
233	ATIVIDADE EXTERNA ESPII - RODÍZIO	CHC da Universidade Federal do Paraná - PR
233	ATIVIDADE EXTERNA ESPII - RODÍZIO	CHC da Universidade Federal do Paraná - PR
233	ATIVIDADE EXTERNA ESPII - RODÍZIO	CHC da Universidade Federal do Paraná - PR
233	ATIVIDADE EXTERNA ESPII - RODÍZIO	CHC da Universidade Federal do Paraná - PR
233	ATIVIDADE EXTERNA ESPII - RODÍZIO	CHC da Universidade Federal do Paraná - PR
233	ATIVIDADE EXTERNA ESPII - RODÍZIO	CHC da Universidade Federal do Paraná - PR
233	ATIVIDADE EXTERNA ESPII - RODÍZIO	CHC da Universidade Federal do Paraná - PR
233	ATIVIDADE EXTERNA ESPII - RODÍZIO	CHC da Universidade Federal do Paraná - PR
233	ATIVIDADE EXTERNA ESPII - RODÍZIO	CHC da Universidade Federal do Paraná - PR
233	ATIVIDADE EXTERNA ESPII - RODÍZIO	CHC da Universidade Federal do Paraná - PR
233	ATIVIDADE EXTERNA ESPII - RODÍZIO	CHC da Universidade Federal do Paraná - PR
233	ATIVIDADE EXTERNA ESPII - RODÍZIO	CHC da Universidade Federal do Paraná - PR
233	ATIVIDADE EXTERNA ESPII - RODÍZIO	CHC da Universidade Federal do Paraná - PR
233	ATIVIDADE EXTERNA ESPII - RODÍZIO	CHC da Universidade Federal do Paraná - PR
233	ATIVIDADE EXTERNA ESPII - RODÍZIO	CHC da Universidade Federal do Paraná - PR
233	ATIVIDADE EXTERNA ESPII - RODÍZIO	CHC da Universidade Federal do Paraná - PR
233	ATIVIDADE EXTERNA ESPII - RODÍZIO	CHC da Universidade Federal do Paraná - PR
233	ATIVIDADE EXTERNA ESPII - RODÍZIO	CHC da Universidade Federal do Paraná - PR
233	ATIVIDADE EXTERNA ESPII - RODÍZIO	CHC da Universidade Federal do Paraná - PR
233	ATIVIDADE EXTERNA ESPII - RODÍZIO	CHC da Universidade Federal do Paraná - PR
233	ATIVIDADE EXTERNA ESPII - RODÍZIO	CHC da Universidade Federal do Paraná - PR
233	ATIVIDADE EXTERNA ESPII - RODÍZIO	CHC da Universidade Federal do Paraná - PR
233	ATIVIDADE EXTERNA ESPII - RODÍZIO	CHC da Universidade Federal do Paraná - PR
233	ATIVIDADE EXTERNA ESPII - RODÍZIO	CHC da Universidade Federal do Paraná - PR

233	ATIVIDADE EXTERNA ESPII - RODÍZIO	CHC da Universidade Federal do Paraná - PR
233	ATIVIDADE EXTERNA ESPII - RODÍZIO	CHC da Universidade Federal do Paraná - PR
233	ATIVIDADE EXTERNA ESPII - RODÍZIO	CHC da Universidade Federal do Paraná - PR
233	ATIVIDADE EXTERNA ESPII - RODÍZIO	CHC da Universidade Federal do Paraná - PR
233	ATIVIDADE EXTERNA ESPII - RODÍZIO	CHC da Universidade Federal do Paraná - PR
233	ATIVIDADE EXTERNA ESPII - RODÍZIO	CHC da Universidade Federal do Paraná - PR
233	ATIVIDADE EXTERNA ESPII - RODÍZIO	CHC da Universidade Federal do Paraná - PR
233	ATIVIDADE EXTERNA ESPII - RODÍZIO	CHC da Universidade Federal do Paraná - PR
233	ATIVIDADE EXTERNA ESPII - RODÍZIO	CHC da Universidade Federal do Paraná - PR
233	ATIVIDADE EXTERNA ESPII - RODÍZIO	CHC da Universidade Federal do Paraná - PR
233	ATIVIDADE EXTERNA ESPII - RODÍZIO	CHC da Universidade Federal do Paraná - PR
233	ATIVIDADE EXTERNA ESPII - RODÍZIO	CHC da Universidade Federal do Paraná - PR
233	ATIVIDADE EXTERNA ESPII - RODÍZIO	CHC da Universidade Federal do Paraná - PR
233	ATIVIDADE EXTERNA ESPII - RODÍZIO	CHC da Universidade Federal do Paraná - PR
233	ATIVIDADE EXTERNA ESPII - RODÍZIO	CHC da Universidade Federal do Paraná - PR
233	ATIVIDADE EXTERNA ESPII - RODÍZIO	CHC da Universidade Federal do Paraná - PR
233	ATIVIDADE EXTERNA ESPII - RODÍZIO	CHC da Universidade Federal do Paraná - PR
233	ATIVIDADE EXTERNA ESPII - RODÍZIO	CHC da Universidade Federal do Paraná - PR
233	ATIVIDADE EXTERNA ESPII - RODÍZIO	CHC da Universidade Federal do Paraná - PR
233	ATIVIDADE EXTERNA ESPII - RODÍZIO	CHC da Universidade Federal do Paraná - PR
233	ATIVIDADE EXTERNA ESPII - RODÍZIO	CHC da Universidade Federal do Paraná - PR
233	ATIVIDADE EXTERNA ESPII - RODÍZIO	CHC da Universidade Federal do Paraná - PR
233	ATIVIDADE EXTERNA ESPII - RODÍZIO	CHC da Universidade Federal do Paraná - PR
233	ATIVIDADE EXTERNA ESPII - RODÍZIO	CHC da Universidade Federal do Paraná - PR
233	ATIVIDADE EXTERNA ESPII - RODÍZIO	CHC da Universidade Federal do Paraná - PR
233	ATIVIDADE EXTERNA ESPII - RODÍZIO	CHC da Universidade Federal do Paraná - PR
233	ATIVIDADE EXTERNA ESPII - RODÍZIO	CHC da Universidade Federal do Paraná - PR
233	ATIVIDADE EXTERNA ESPII - RODÍZIO	CHC da Universidade Federal do Paraná - PR
233	ATIVIDADE EXTERNA ESPII - RODÍZIO	CHC da Universidade Federal do Paraná - PR
233	ATIVIDADE EXTERNA ESPII - RODÍZIO	CHC da Universidade Federal do Paraná - PR
233	ATIVIDADE EXTERNA ESPII - RODÍZIO	CHC da Universidade Federal do Paraná - PR
233	ATIVIDADE EXTERNA ESPII - RODÍZIO	CHC da Universidade Federal do Paraná - PR
233	ATIVIDADE EXTERNA ESPII - RODÍZIO	CHC da Universidade Federal do Paraná - PR
233	ATIVIDADE EXTERNA ESPII - RODÍZIO	CHC da Universidade Federal do Paraná - PR
233	ATIVIDADE EXTERNA ESPII - RODÍZIO	CHC da Universidade Federal do Paraná - PR
233	ATIVIDADE EXTERNA ESPII - RODÍZIO	CHC da Universidade Federal do Paraná - PR
233	ATIVIDADE EXTERNA ESPII - RODÍZIO	CHC da Universidade Federal do Paraná - PR
233	ATIVIDADE EXTERNA ESPII - RODÍZIO	CHC da Universidade Federal do Paraná - PR
233	ATIVIDADE EXTERNA ESPII - RODÍZIO	CHC da Universidade Federal do Paraná - PR
233	ATIVIDADE EXTERNA ESPII - RODÍZIO	CHC da Universidade Federal do Paraná - PR
233	ATIVIDADE EXTERNA ESPII - RODÍZIO	CHC da Universidade Federal do Paraná - PR
233	ATIVIDADE EXTERNA ESPII - RODÍZIO	CHC da Universidade Federal do Paraná - PR
233	ATIVIDADE EXTERNA ESPII - RODÍZIO	CHC da Universidade Federal do Paraná - PR
233	ATIVIDADE EXTERNA ESPII - RODÍZIO	CHC da Universidade Federal do Paraná - PR
233	ATIVIDADE EXTERNA ESPII - RODÍZIO	CHC da Universidade Federal do Paraná - PR
233	ATIVIDADE EXTERNA ESPII - RODÍZIO	CHC da Universidade Federal do Paraná - PR
233	ATIVIDADE EXTERNA ESPII - RODÍZIO	CHC da Universidade Federal do Paraná - PR
233	ATIVIDADE EXTERNA ESPII - RODÍZIO	CHC da Universidade Federal do Paraná - PR

233	ATIVIDADE EXTERNA ESPII - RODÍZIO	CHC da Universidade Federal do Paraná - PR
233	ATIVIDADE EXTERNA ESPII - RODÍZIO	CHC da Universidade Federal do Paraná - PR
233	ATIVIDADE EXTERNA ESPII - RODÍZIO	CHC da Universidade Federal do Paraná - PR
233	ATIVIDADE EXTERNA ESPII - RODÍZIO	CHC da Universidade Federal do Paraná - PR
233	ATIVIDADE EXTERNA ESPII - RODÍZIO	CHC da Universidade Federal do Paraná - PR
233	ATIVIDADE EXTERNA ESPII - RODÍZIO	CHC da Universidade Federal do Paraná - PR
233	ATIVIDADE EXTERNA ESPII - RODÍZIO	CHC da Universidade Federal do Paraná - PR
233	ATIVIDADE EXTERNA ESPII - RODÍZIO	CHC da Universidade Federal do Paraná - PR
233	ATIVIDADE EXTERNA ESPII - RODÍZIO	CHC da Universidade Federal do Paraná - PR
233	ATIVIDADE EXTERNA ESPII - RODÍZIO	CHC da Universidade Federal do Paraná - PR
233	ATIVIDADE EXTERNA ESPII - RODÍZIO	CHC da Universidade Federal do Paraná - PR
233	ATIVIDADE EXTERNA ESPII - RODÍZIO	CHC da Universidade Federal do Paraná - PR
233	ATIVIDADE EXTERNA ESPII - RODÍZIO	CHC da Universidade Federal do Paraná - PR
233	ATIVIDADE EXTERNA ESPII - RODÍZIO	CHC da Universidade Federal do Paraná - PR
233	ATIVIDADE EXTERNA ESPII - RODÍZIO	CHC da Universidade Federal do Paraná - PR
233	ATIVIDADE EXTERNA ESPII - RODÍZIO	CHC da Universidade Federal do Paraná - PR
233	ATIVIDADE EXTERNA ESPII - RODÍZIO	CHC da Universidade Federal do Paraná - PR
233	ATIVIDADE EXTERNA ESPII - RODÍZIO	CHC da Universidade Federal do Paraná - PR
233	ATIVIDADE EXTERNA ESPII - RODÍZIO	CHC da Universidade Federal do Paraná - PR
233	ATIVIDADE EXTERNA ESPII - RODÍZIO	CHC da Universidade Federal do Paraná - PR
233	ATIVIDADE EXTERNA ESPII - RODÍZIO	CHC da Universidade Federal do Paraná - PR
233	ATIVIDADE EXTERNA ESPII - RODÍZIO	CHC da Universidade Federal do Paraná - PR
233	ATIVIDADE EXTERNA ESPII - RODÍZIO	CHC da Universidade Federal do Paraná - PR
233	ATIVIDADE EXTERNA ESPII - RODÍZIO	CHC da Universidade Federal do Paraná - PR
233	ATIVIDADE EXTERNA ESPII - RODÍZIO	CHC da Universidade Federal do Paraná - PR
233	ATIVIDADE EXTERNA ESPII - RODÍZIO	CHC da Universidade Federal do Paraná - PR
233	ATIVIDADE EXTERNA ESPII - RODÍZIO	CHC da Universidade Federal do Paraná - PR
233	ATIVIDADE EXTERNA ESPII - RODÍZIO	CHC da Universidade Federal do Paraná - PR
233	ATIVIDADE EXTERNA ESPII - RODÍZIO	CHC da Universidade Federal do Paraná - PR
233	ATIVIDADE EXTERNA ESPII - RODÍZIO	CHC da Universidade Federal do Paraná - PR
233	ATIVIDADE EXTERNA ESPII - RODÍZIO	CHC da Universidade Federal do Paraná - PR
233	ATIVIDADE EXTERNA ESPII - RODÍZIO	CHC da Universidade Federal do Paraná - PR
233	ATIVIDADE EXTERNA ESPII - RODÍZIO	CHC da Universidade Federal do Paraná - PR
233	ATIVIDADE EXTERNA ESPII - RODÍZIO	CHC da Universidade Federal do Paraná - PR
233	ATIVIDADE EXTERNA ESPII - RODÍZIO	CHC da Universidade Federal do Paraná - PR
233	ATIVIDADE EXTERNA ESPII - RODÍZIO	CHC da Universidade Federal do Paraná - PR
233	ATIVIDADE EXTERNA ESPII - RODÍZIO	CHC da Universidade Federal do Paraná - PR
233	ATIVIDADE EXTERNA ESPII - RODÍZIO	CHC da Universidade Federal do Paraná - PR
233	ATIVIDADE EXTERNA ESPII - RODÍZIO	CHC da Universidade Federal do Paraná - PR
233	ATIVIDADE EXTERNA ESPII - RODÍZIO	CHC da Universidade Federal do Paraná - PR
233	ATIVIDADE EXTERNA ESPII - RODÍZIO	CHC da Universidade Federal do Paraná - PR
233	ATIVIDADE EXTERNA ESPII - RODÍZIO	CHC da Universidade Federal do Paraná - PR
233	ATIVIDADE EXTERNA ESPII - RODÍZIO	CHC da Universidade Federal do Paraná - PR
233	ATIVIDADE EXTERNA ESPII - RODÍZIO	CHC da Universidade Federal do Paraná - PR
233	ATIVIDADE EXTERNA ESPII - RODÍZIO	CHC da Universidade Federal do Paraná - PR
233	ATIVIDADE EXTERNA ESPII - RODÍZIO	CHC da Universidade Federal do Paraná - PR
233	ATIVIDADE EXTERNA ESPII - RODÍZIO	CHC da Universidade Federal do Paraná - PR
233	ATIVIDADE EXTERNA ESPII - RODÍZIO	CHC da Universidade Federal do Paraná - PR

233	ATIVIDADE EXTERNA ESPII - RODÍZIO	CHC da Universidade Federal do Paraná - PR
233	ATIVIDADE EXTERNA ESPII - RODÍZIO	CHC da Universidade Federal do Paraná - PR
233	ATIVIDADE EXTERNA ESPII - RODÍZIO	CHC da Universidade Federal do Paraná - PR
233	ATIVIDADE EXTERNA ESPII - RODÍZIO	CHC da Universidade Federal do Paraná - PR
233	ATIVIDADE EXTERNA ESPII - RODÍZIO	CHC da Universidade Federal do Paraná - PR
233	ATIVIDADE EXTERNA ESPII - RODÍZIO	CHC da Universidade Federal do Paraná - PR
233	ATIVIDADE EXTERNA ESPII - RODÍZIO	CHC da Universidade Federal do Paraná - PR
233	ATIVIDADE EXTERNA ESPII - RODÍZIO	CHC da Universidade Federal do Paraná - PR
233	ATIVIDADE EXTERNA ESPII - RODÍZIO	CHC da Universidade Federal do Paraná - PR
233	ATIVIDADE EXTERNA ESPII - RODÍZIO	CHC da Universidade Federal do Paraná - PR
233	ATIVIDADE EXTERNA ESPII - RODÍZIO	CHC da Universidade Federal do Paraná - PR
233	ATIVIDADE EXTERNA ESPII - RODÍZIO	CHC da Universidade Federal do Paraná - PR
233	ATIVIDADE EXTERNA ESPII - RODÍZIO	CHC da Universidade Federal do Paraná - PR
233	ATIVIDADE EXTERNA ESPII - RODÍZIO	CHC da Universidade Federal do Paraná - PR
233	ATIVIDADE EXTERNA ESPII - RODÍZIO	CHC da Universidade Federal do Paraná - PR
233	ATIVIDADE EXTERNA ESPII - RODÍZIO	CHC da Universidade Federal do Paraná - PR
233	ATIVIDADE EXTERNA ESPII - RODÍZIO	CHC da Universidade Federal do Paraná - PR
233	ATIVIDADE EXTERNA ESPII - RODÍZIO	CHC da Universidade Federal do Paraná - PR
233	ATIVIDADE EXTERNA ESPII - RODÍZIO	CHC da Universidade Federal do Paraná - PR
233	ATIVIDADE EXTERNA ESPII - RODÍZIO	CHC da Universidade Federal do Paraná - PR
233	ATIVIDADE EXTERNA ESPII - RODÍZIO	CHC da Universidade Federal do Paraná - PR
233	ATIVIDADE EXTERNA ESPII - RODÍZIO	CHC da Universidade Federal do Paraná - PR
233	ATIVIDADE EXTERNA ESPII - RODÍZIO	CHC da Universidade Federal do Paraná - PR
233	ATIVIDADE EXTERNA ESPII - RODÍZIO	CHC da Universidade Federal do Paraná - PR
233	ATIVIDADE EXTERNA ESPII - RODÍZIO	CHC da Universidade Federal do Paraná - PR
233	ATIVIDADE EXTERNA ESPII - RODÍZIO	CHC da Universidade Federal do Paraná - PR
233	ATIVIDADE EXTERNA ESPII - RODÍZIO	CHC da Universidade Federal do Paraná - PR
233	ATIVIDADE EXTERNA ESPII - RODÍZIO	CHC da Universidade Federal do Paraná - PR
233	ATIVIDADE EXTERNA ESPII - RODÍZIO	CHC da Universidade Federal do Paraná - PR
233	ATIVIDADE EXTERNA ESPII - RODÍZIO	CHC da Universidade Federal do Paraná - PR
233	ATIVIDADE EXTERNA ESPII - RODÍZIO	CHC da Universidade Federal do Paraná - PR
233	ATIVIDADE EXTERNA ESPII - RODÍZIO	CHC da Universidade Federal do Paraná - PR
233	ATIVIDADE EXTERNA ESPII - RODÍZIO	CHC da Universidade Federal do Paraná - PR
233	ATIVIDADE EXTERNA ESPII - RODÍZIO	CHC da Universidade Federal do Paraná - PR
233	ATIVIDADE EXTERNA ESPII - RODÍZIO	CHC da Universidade Federal do Paraná - PR
233	ATIVIDADE EXTERNA ESPII - RODÍZIO	CHC da Universidade Federal do Paraná - PR
233	ATIVIDADE EXTERNA ESPII - RODÍZIO	CHC da Universidade Federal do Paraná - PR
233	ATIVIDADE EXTERNA ESPII - RODÍZIO	CHC da Universidade Federal do Paraná - PR
233	ATIVIDADE EXTERNA ESPII - RODÍZIO	CHC da Universidade Federal do Paraná - PR
233	ATIVIDADE EXTERNA ESPII - RODÍZIO	CHC da Universidade Federal do Paraná - PR
233	ATIVIDADE EXTERNA ESPII - RODÍZIO	CHC da Universidade Federal do Paraná - PR
233	ATIVIDADE EXTERNA ESPII - RODÍZIO	CHC da Universidade Federal do Paraná - PR
233	ATIVIDADE EXTERNA ESPII - RODÍZIO	CHC da Universidade Federal do Paraná - PR
233	ATIVIDADE EXTERNA ESPII - RODÍZIO	CHC da Universidade Federal do Paraná - PR
233	ATIVIDADE EXTERNA ESPII - RODÍZIO	CHC da Universidade Federal do Paraná - PR
233	ATIVIDADE EXTERNA ESPII - RODÍZIO	CHC da Universidade Federal do Paraná - PR
233	ATIVIDADE EXTERNA ESPII - RODÍZIO	CHC da Universidade Federal do Paraná - PR
233	ATIVIDADE EXTERNA ESPII - RODÍZIO	CHC da Universidade Federal do Paraná - PR

233	ATIVIDADE EXTERNA ESPII - RODÍZIO	CHC da Universidade Federal do Paraná - PR
233	ATIVIDADE EXTERNA ESPII - RODÍZIO	CHC da Universidade Federal do Paraná - PR
233	ATIVIDADE EXTERNA ESPII - RODÍZIO	CHC da Universidade Federal do Paraná - PR
233	ATIVIDADE EXTERNA ESPII - RODÍZIO	CHC da Universidade Federal do Paraná - PR
233	ATIVIDADE EXTERNA ESPII - RODÍZIO	CHC da Universidade Federal do Paraná - PR
233	ATIVIDADE EXTERNA ESPII - RODÍZIO	CHC da Universidade Federal do Paraná - PR
233	ATIVIDADE EXTERNA ESPII - RODÍZIO	CHC da Universidade Federal do Paraná - PR
233	ATIVIDADE EXTERNA ESPII - RODÍZIO	CHC da Universidade Federal do Paraná - PR
233	ATIVIDADE EXTERNA ESPII - RODÍZIO	CHC da Universidade Federal do Paraná - PR
233	ATIVIDADE EXTERNA ESPII - RODÍZIO	CHC da Universidade Federal do Paraná - PR
233	ATIVIDADE EXTERNA ESPII - RODÍZIO	CHC da Universidade Federal do Paraná - PR
233	ATIVIDADE EXTERNA ESPII - RODÍZIO	CHC da Universidade Federal do Paraná - PR
233	ATIVIDADE EXTERNA ESPII - RODÍZIO	CHC da Universidade Federal do Paraná - PR
233	ATIVIDADE EXTERNA ESPII - RODÍZIO	CHC da Universidade Federal do Paraná - PR
233	ATIVIDADE EXTERNA ESPII - RODÍZIO	CHC da Universidade Federal do Paraná - PR
233	ATIVIDADE EXTERNA ESPII - RODÍZIO	CHC da Universidade Federal do Paraná - PR
233	ATIVIDADE EXTERNA ESPII - RODÍZIO	CHC da Universidade Federal do Paraná - PR
233	ATIVIDADE EXTERNA ESPII - RODÍZIO	CHC da Universidade Federal do Paraná - PR
233	ATIVIDADE EXTERNA ESPII - RODÍZIO	CHC da Universidade Federal do Paraná - PR
233	ATIVIDADE EXTERNA ESPII - RODÍZIO	CHC da Universidade Federal do Paraná - PR
233	ATIVIDADE EXTERNA ESPII - RODÍZIO	CHC da Universidade Federal do Paraná - PR
233	ATIVIDADE EXTERNA ESPII - RODÍZIO	CHC da Universidade Federal do Paraná - PR
233	ATIVIDADE EXTERNA ESPII - RODÍZIO	CHC da Universidade Federal do Paraná - PR
233	ATIVIDADE EXTERNA ESPII - RODÍZIO	CHC da Universidade Federal do Paraná - PR
233	ATIVIDADE EXTERNA ESPII - RODÍZIO	CHC da Universidade Federal do Paraná - PR
233	ATIVIDADE EXTERNA ESPII - RODÍZIO	CHC da Universidade Federal do Paraná - PR
233	ATIVIDADE EXTERNA ESPII - RODÍZIO	CHC da Universidade Federal do Paraná - PR
233	ATIVIDADE EXTERNA ESPII - RODÍZIO	CHC da Universidade Federal do Paraná - PR
233	ATIVIDADE EXTERNA ESPII - RODÍZIO	CHC da Universidade Federal do Paraná - PR
233	ATIVIDADE EXTERNA ESPII - RODÍZIO	CHC da Universidade Federal do Paraná - PR
233	ATIVIDADE EXTERNA ESPII - RODÍZIO	CHC da Universidade Federal do Paraná - PR
233	ATIVIDADE EXTERNA ESPII - RODÍZIO	CHC da Universidade Federal do Paraná - PR
233	ATIVIDADE EXTERNA ESPII - RODÍZIO	CHC da Universidade Federal do Paraná - PR
233	ATIVIDADE EXTERNA ESPII - RODÍZIO	CHC da Universidade Federal do Paraná - PR
233	ATIVIDADE EXTERNA ESPII - RODÍZIO	CHC da Universidade Federal do Paraná - PR
233	ATIVIDADE EXTERNA ESPII - RODÍZIO	CHC da Universidade Federal do Paraná - PR
233	ATIVIDADE EXTERNA ESPII - RODÍZIO	CHC da Universidade Federal do Paraná - PR
233	ATIVIDADE EXTERNA ESPII - RODÍZIO	CHC da Universidade Federal do Paraná - PR
233	ATIVIDADE EXTERNA ESPII - RODÍZIO	CHC da Universidade Federal do Paraná - PR
233	ATIVIDADE EXTERNA ESPII - RODÍZIO	CHC da Universidade Federal do Paraná - PR
233	ATIVIDADE EXTERNA ESPII - RODÍZIO	CHC da Universidade Federal do Paraná - PR
233	ATIVIDADE EXTERNA ESPII - RODÍZIO	CHC da Universidade Federal do Paraná - PR
233	ATIVIDADE EXTERNA ESPII - RODÍZIO	CHC da Universidade Federal do Paraná - PR
233	ATIVIDADE EXTERNA ESPII - RODÍZIO	CHC da Universidade Federal do Paraná - PR
233	ATIVIDADE EXTERNA ESPII - RODÍZIO	CHC da Universidade Federal do Paraná - PR
233	ATIVIDADE EXTERNA ESPII - RODÍZIO	CHC da Universidade Federal do Paraná - PR
233	ATIVIDADE EXTERNA ESPII - RODÍZIO	CHC da Universidade Federal do Paraná - PR
233	ATIVIDADE EXTERNA ESPII - RODÍZIO	CHC da Universidade Federal do Paraná - PR

233	ATIVIDADE EXTERNA ESPII - RODÍZIO	CHC da Universidade Federal do Paraná - PR
233	ATIVIDADE EXTERNA ESPII - RODÍZIO	CHC da Universidade Federal do Paraná - PR
233	ATIVIDADE EXTERNA ESPII - RODÍZIO	CHC da Universidade Federal do Paraná - PR
233	ATIVIDADE EXTERNA ESPII - RODÍZIO	CHC da Universidade Federal do Paraná - PR
233	ATIVIDADE EXTERNA ESPII - RODÍZIO	CHC da Universidade Federal do Paraná - PR
233	ATIVIDADE EXTERNA ESPII - RODÍZIO	CHC da Universidade Federal do Paraná - PR
233	ATIVIDADE EXTERNA ESPII - RODÍZIO	CHC da Universidade Federal do Paraná - PR
233	ATIVIDADE EXTERNA ESPII - RODÍZIO	CHC da Universidade Federal do Paraná - PR
233	ATIVIDADE EXTERNA ESPII - RODÍZIO	CHC da Universidade Federal do Paraná - PR
233	ATIVIDADE EXTERNA ESPII - RODÍZIO	CHC da Universidade Federal do Paraná - PR
233	ATIVIDADE EXTERNA ESPII - RODÍZIO	CHC da Universidade Federal do Paraná - PR
233	ATIVIDADE EXTERNA ESPII - RODÍZIO	CHC da Universidade Federal do Paraná - PR
233	ATIVIDADE EXTERNA ESPII - RODÍZIO	CHC da Universidade Federal do Paraná - PR
233	ATIVIDADE EXTERNA ESPII - RODÍZIO	CHC da Universidade Federal do Paraná - PR
233	ATIVIDADE EXTERNA ESPII - RODÍZIO	CHC da Universidade Federal do Paraná - PR
233	ATIVIDADE EXTERNA ESPII - RODÍZIO	CHC da Universidade Federal do Paraná - PR
233	ATIVIDADE EXTERNA ESPII - RODÍZIO	CHC da Universidade Federal do Paraná - PR
233	ATIVIDADE EXTERNA ESPII - RODÍZIO	CHC da Universidade Federal do Paraná - PR
233	ATIVIDADE EXTERNA ESPII - RODÍZIO	CHC da Universidade Federal do Paraná - PR
233	ATIVIDADE EXTERNA ESPII - RODÍZIO	CHC da Universidade Federal do Paraná - PR
233	ATIVIDADE EXTERNA ESPII - RODÍZIO	CHC da Universidade Federal do Paraná - PR
233	ATIVIDADE EXTERNA ESPII - RODÍZIO	CHC da Universidade Federal do Paraná - PR
233	ATIVIDADE EXTERNA ESPII - RODÍZIO	CHC da Universidade Federal do Paraná - PR
233	ATIVIDADE EXTERNA ESPII - RODÍZIO	CHC da Universidade Federal do Paraná - PR
233	ATIVIDADE EXTERNA ESPII - RODÍZIO	CHC da Universidade Federal do Paraná - PR
233	ATIVIDADE EXTERNA ESPII - RODÍZIO	CHC da Universidade Federal do Paraná - PR
233	ATIVIDADE EXTERNA ESPII - RODÍZIO	CHC da Universidade Federal do Paraná - PR
233	ATIVIDADE EXTERNA ESPII - RODÍZIO	CHC da Universidade Federal do Paraná - PR
233	ATIVIDADE EXTERNA ESPII - RODÍZIO	CHC da Universidade Federal do Paraná - PR
233	ATIVIDADE EXTERNA ESPII - RODÍZIO	CHC da Universidade Federal do Paraná - PR
233	ATIVIDADE EXTERNA ESPII - RODÍZIO	CHC da Universidade Federal do Paraná - PR
233	ATIVIDADE EXTERNA ESPII - RODÍZIO	CHC da Universidade Federal do Paraná - PR
233	ATIVIDADE EXTERNA ESPII - RODÍZIO	CHC da Universidade Federal do Paraná - PR
233	ATIVIDADE EXTERNA ESPII - RODÍZIO	CHC da Universidade Federal do Paraná - PR
233	ATIVIDADE EXTERNA ESPII - RODÍZIO	CHC da Universidade Federal do Paraná - PR
233	ATIVIDADE EXTERNA ESPII - RODÍZIO	CHC da Universidade Federal do Paraná - PR
233	ATIVIDADE EXTERNA ESPII - RODÍZIO	CHC da Universidade Federal do Paraná - PR
233	ATIVIDADE EXTERNA ESPII - RODÍZIO	CHC da Universidade Federal do Paraná - PR
233	ATIVIDADE EXTERNA ESPII - RODÍZIO	CHC da Universidade Federal do Paraná - PR
233	ATIVIDADE EXTERNA ESPII - RODÍZIO	CHC da Universidade Federal do Paraná - PR
233	ATIVIDADE EXTERNA ESPII - RODÍZIO	CHC da Universidade Federal do Paraná - PR
233	ATIVIDADE EXTERNA ESPII - RODÍZIO	CHC da Universidade Federal do Paraná - PR
233	ATIVIDADE EXTERNA ESPII - RODÍZIO	CHC da Universidade Federal do Paraná - PR
233	ATIVIDADE EXTERNA ESPII - RODÍZIO	CHC da Universidade Federal do Paraná - PR
233	ATIVIDADE EXTERNA ESPII - RODÍZIO	CHC da Universidade Federal do Paraná - PR
233	ATIVIDADE EXTERNA ESPII - RODÍZIO	CHC da Universidade Federal do Paraná - PR
233	ATIVIDADE EXTERNA ESPII - RODÍZIO	CHC da Universidade Federal do Paraná - PR
233	ATIVIDADE EXTERNA ESPII - RODÍZIO	CHC da Universidade Federal do Paraná - PR

233	ATIVIDADE EXTERNA ESPII - RODÍZIO	CHC da Universidade Federal do Paraná - PR
233	ATIVIDADE EXTERNA ESPII - RODÍZIO	CHC da Universidade Federal do Paraná - PR
233	ATIVIDADE EXTERNA ESPII - RODÍZIO	CHC da Universidade Federal do Paraná - PR
233	ATIVIDADE EXTERNA ESPII - RODÍZIO	CHC da Universidade Federal do Paraná - PR
233	ATIVIDADE EXTERNA ESPII - RODÍZIO	CHC da Universidade Federal do Paraná - PR
233	ATIVIDADE EXTERNA ESPII - RODÍZIO	CHC da Universidade Federal do Paraná - PR
233	ATIVIDADE EXTERNA ESPII - RODÍZIO	CHC da Universidade Federal do Paraná - PR
233	ATIVIDADE EXTERNA ESPII - RODÍZIO	CHC da Universidade Federal do Paraná - PR
233	ATIVIDADE EXTERNA ESPII - RODÍZIO	CHC da Universidade Federal do Paraná - PR
233	ATIVIDADE EXTERNA ESPII - RODÍZIO	CHC da Universidade Federal do Paraná - PR
233	ATIVIDADE EXTERNA ESPII - RODÍZIO	CHC da Universidade Federal do Paraná - PR
233	ATIVIDADE EXTERNA ESPII - RODÍZIO	CHC da Universidade Federal do Paraná - PR
233	ATIVIDADE EXTERNA ESPII - RODÍZIO	CHC da Universidade Federal do Paraná - PR
233	ATIVIDADE EXTERNA ESPII - RODÍZIO	CHC da Universidade Federal do Paraná - PR
233	ATIVIDADE EXTERNA ESPII - RODÍZIO	CHC da Universidade Federal do Paraná - PR
233	ATIVIDADE EXTERNA ESPII - RODÍZIO	CHC da Universidade Federal do Paraná - PR
233	ATIVIDADE EXTERNA ESPII - RODÍZIO	CHC da Universidade Federal do Paraná - PR
233	ATIVIDADE EXTERNA ESPII - RODÍZIO	CHC da Universidade Federal do Paraná - PR
233	ATIVIDADE EXTERNA ESPII - RODÍZIO	CHC da Universidade Federal do Paraná - PR
233	ATIVIDADE EXTERNA ESPII - RODÍZIO	CHC da Universidade Federal do Paraná - PR
233	ATIVIDADE EXTERNA ESPII - RODÍZIO	CHC da Universidade Federal do Paraná - PR
233	ATIVIDADE EXTERNA ESPII - RODÍZIO	CHC da Universidade Federal do Paraná - PR
233	ATIVIDADE EXTERNA ESPII - RODÍZIO	CHC da Universidade Federal do Paraná - PR
233	ATIVIDADE EXTERNA ESPII - RODÍZIO	CHC da Universidade Federal do Paraná - PR
233	ATIVIDADE EXTERNA ESPII - RODÍZIO	CHC da Universidade Federal do Paraná - PR
233	ATIVIDADE EXTERNA ESPII - RODÍZIO	CHC da Universidade Federal do Paraná - PR
233	ATIVIDADE EXTERNA ESPII - RODÍZIO	CHC da Universidade Federal do Paraná - PR
233	ATIVIDADE EXTERNA ESPII - RODÍZIO	CHC da Universidade Federal do Paraná - PR
233	ATIVIDADE EXTERNA ESPII - RODÍZIO	CHC da Universidade Federal do Paraná - PR
233	ATIVIDADE EXTERNA ESPII - RODÍZIO	CHC da Universidade Federal do Paraná - PR
233	ATIVIDADE EXTERNA ESPII - RODÍZIO	CHC da Universidade Federal do Paraná - PR
233	ATIVIDADE EXTERNA ESPII - RODÍZIO	CHC da Universidade Federal do Paraná - PR
233	ATIVIDADE EXTERNA ESPII - RODÍZIO	CHC da Universidade Federal do Paraná - PR
233	ATIVIDADE EXTERNA ESPII - RODÍZIO	CHC da Universidade Federal do Paraná - PR
233	ATIVIDADE EXTERNA ESPII - RODÍZIO	CHC da Universidade Federal do Paraná - PR
233	ATIVIDADE EXTERNA ESPII - RODÍZIO	CHC da Universidade Federal do Paraná - PR
233	ATIVIDADE EXTERNA ESPII - RODÍZIO	CHC da Universidade Federal do Paraná - PR
233	ATIVIDADE EXTERNA ESPII - RODÍZIO	CHC da Universidade Federal do Paraná - PR
233	ATIVIDADE EXTERNA ESPII - RODÍZIO	CHC da Universidade Federal do Paraná - PR
233	ATIVIDADE EXTERNA ESPII - RODÍZIO	CHC da Universidade Federal do Paraná - PR
233	ATIVIDADE EXTERNA ESPII - RODÍZIO	CHC da Universidade Federal do Paraná - PR
233	ATIVIDADE EXTERNA ESPII - RODÍZIO	CHC da Universidade Federal do Paraná - PR
233	ATIVIDADE EXTERNA ESPII - RODÍZIO	CHC da Universidade Federal do Paraná - PR
233	ATIVIDADE EXTERNA ESPII - RODÍZIO	CHC da Universidade Federal do Paraná - PR
233	ATIVIDADE EXTERNA ESPII - RODÍZIO	CHC da Universidade Federal do Paraná - PR
233	ATIVIDADE EXTERNA ESPII - RODÍZIO	CHC da Universidade Federal do Paraná - PR
233	ATIVIDADE EXTERNA ESPII - RODÍZIO	CHC da Universidade Federal do Paraná - PR
233	ATIVIDADE EXTERNA ESPII - RODÍZIO	CHC da Universidade Federal do Paraná - PR

233	ATIVIDADE EXTERNA ESPII - RODÍZIO	CHC da Universidade Federal do Paraná - PR
233	ATIVIDADE EXTERNA ESPII - RODÍZIO	CHC da Universidade Federal do Paraná - PR
233	ATIVIDADE EXTERNA ESPII - RODÍZIO	CHC da Universidade Federal do Paraná - PR
233	ATIVIDADE EXTERNA ESPII - RODÍZIO	CHC da Universidade Federal do Paraná - PR
233	ATIVIDADE EXTERNA ESPII - RODÍZIO	CHC da Universidade Federal do Paraná - PR
233	ATIVIDADE EXTERNA ESPII - RODÍZIO	CHC da Universidade Federal do Paraná - PR
233	ATIVIDADE EXTERNA ESPII - RODÍZIO	CHC da Universidade Federal do Paraná - PR
233	ATIVIDADE EXTERNA ESPII - RODÍZIO	CHC da Universidade Federal do Paraná - PR
233	ATIVIDADE EXTERNA ESPII - RODÍZIO	CHC da Universidade Federal do Paraná - PR
233	ATIVIDADE EXTERNA ESPII - RODÍZIO	CHC da Universidade Federal do Paraná - PR
233	ATIVIDADE EXTERNA ESPII - RODÍZIO	CHC da Universidade Federal do Paraná - PR
233	ATIVIDADE EXTERNA ESPII - RODÍZIO	CHC da Universidade Federal do Paraná - PR
233	ATIVIDADE EXTERNA ESPII - RODÍZIO	CHC da Universidade Federal do Paraná - PR
233	ATIVIDADE EXTERNA ESPII - RODÍZIO	CHC da Universidade Federal do Paraná - PR
233	ATIVIDADE EXTERNA ESPII - RODÍZIO	CHC da Universidade Federal do Paraná - PR
233	ATIVIDADE EXTERNA ESPII - RODÍZIO	CHC da Universidade Federal do Paraná - PR
233	ATIVIDADE EXTERNA ESPII - RODÍZIO	CHC da Universidade Federal do Paraná - PR
233	ATIVIDADE EXTERNA ESPII - RODÍZIO	CHC da Universidade Federal do Paraná - PR
233	ATIVIDADE EXTERNA ESPII - RODÍZIO	CHC da Universidade Federal do Paraná - PR
233	ATIVIDADE EXTERNA ESPII - RODÍZIO	CHC da Universidade Federal do Paraná - PR
233	ATIVIDADE EXTERNA ESPII - RODÍZIO	CHC da Universidade Federal do Paraná - PR
233	ATIVIDADE EXTERNA ESPII - RODÍZIO	CHC da Universidade Federal do Paraná - PR
233	ATIVIDADE EXTERNA ESPII - RODÍZIO	CHC da Universidade Federal do Paraná - PR
233	ATIVIDADE EXTERNA ESPII - RODÍZIO	CHC da Universidade Federal do Paraná - PR
233	ATIVIDADE EXTERNA ESPII - RODÍZIO	CHC da Universidade Federal do Paraná - PR
233	ATIVIDADE EXTERNA ESPII - RODÍZIO	CHC da Universidade Federal do Paraná - PR
233	ATIVIDADE EXTERNA ESPII - RODÍZIO	CHC da Universidade Federal do Paraná - PR
233	ATIVIDADE EXTERNA ESPII - RODÍZIO	CHC da Universidade Federal do Paraná - PR
233	ATIVIDADE EXTERNA ESPII - RODÍZIO	CHC da Universidade Federal do Paraná - PR
233	ATIVIDADE EXTERNA ESPII - RODÍZIO	CHC da Universidade Federal do Paraná - PR
233	ATIVIDADE EXTERNA ESPII - RODÍZIO	CHC da Universidade Federal do Paraná - PR
233	ATIVIDADE EXTERNA ESPII - RODÍZIO	CHC da Universidade Federal do Paraná - PR
233	ATIVIDADE EXTERNA ESPII - RODÍZIO	CHC da Universidade Federal do Paraná - PR
233	ATIVIDADE EXTERNA ESPII - RODÍZIO	CHC da Universidade Federal do Paraná - PR
233	ATIVIDADE EXTERNA ESPII - RODÍZIO	CHC da Universidade Federal do Paraná - PR
233	ATIVIDADE EXTERNA ESPII - RODÍZIO	CHC da Universidade Federal do Paraná - PR
233	ATIVIDADE EXTERNA ESPII - RODÍZIO	CHC da Universidade Federal do Paraná - PR
233	ATIVIDADE EXTERNA ESPII - RODÍZIO	CHC da Universidade Federal do Paraná - PR
233	ATIVIDADE EXTERNA ESPII - RODÍZIO	CHC da Universidade Federal do Paraná - PR
233	ATIVIDADE EXTERNA ESPII - RODÍZIO	CHC da Universidade Federal do Paraná - PR
233	ATIVIDADE EXTERNA ESPII - RODÍZIO	CHC da Universidade Federal do Paraná - PR
233	ATIVIDADE EXTERNA ESPII - RODÍZIO	CHC da Universidade Federal do Paraná - PR
233	ATIVIDADE EXTERNA ESPII - RODÍZIO	CHC da Universidade Federal do Paraná - PR
233	ATIVIDADE EXTERNA ESPII - RODÍZIO	CHC da Universidade Federal do Paraná - PR
233	ATIVIDADE EXTERNA ESPII - RODÍZIO	CHC da Universidade Federal do Paraná - PR
233	ATIVIDADE EXTERNA ESPII - RODÍZIO	CHC da Universidade Federal do Paraná - PR
233	ATIVIDADE EXTERNA ESPII - RODÍZIO	CHC da Universidade Federal do Paraná - PR
233	ATIVIDADE EXTERNA ESPII - RODÍZIO	CHC da Universidade Federal do Paraná - PR

233	ATIVIDADE EXTERNA ESPII - RODÍZIO	CHC da Universidade Federal do Paraná - PR
233	ATIVIDADE EXTERNA ESPII - RODÍZIO	CHC da Universidade Federal do Paraná - PR
233	ATIVIDADE EXTERNA ESPII - RODÍZIO	CHC da Universidade Federal do Paraná - PR
233	ATIVIDADE EXTERNA ESPII - RODÍZIO	CHC da Universidade Federal do Paraná - PR
233	ATIVIDADE EXTERNA ESPII - RODÍZIO	CHC da Universidade Federal do Paraná - PR
233	ATIVIDADE EXTERNA ESPII - RODÍZIO	CHC da Universidade Federal do Paraná - PR
233	ATIVIDADE EXTERNA ESPII - RODÍZIO	CHC da Universidade Federal do Paraná - PR
233	ATIVIDADE EXTERNA ESPII - RODÍZIO	CHC da Universidade Federal do Paraná - PR
233	ATIVIDADE EXTERNA ESPII - RODÍZIO	CHC da Universidade Federal do Paraná - PR
233	ATIVIDADE EXTERNA ESPII - RODÍZIO	CHC da Universidade Federal do Paraná - PR
233	ATIVIDADE EXTERNA ESPII - RODÍZIO	CHC da Universidade Federal do Paraná - PR
233	ATIVIDADE EXTERNA ESPII - RODÍZIO	CHC da Universidade Federal do Paraná - PR
233	ATIVIDADE EXTERNA ESPII - RODÍZIO	CHC da Universidade Federal do Paraná - PR
233	ATIVIDADE EXTERNA ESPII - RODÍZIO	CHC da Universidade Federal do Paraná - PR
233	ATIVIDADE EXTERNA ESPII - RODÍZIO	CHC da Universidade Federal do Paraná - PR
233	ATIVIDADE EXTERNA ESPII - RODÍZIO	CHC da Universidade Federal do Paraná - PR
233	ATIVIDADE EXTERNA ESPII - RODÍZIO	CHC da Universidade Federal do Paraná - PR
233	ATIVIDADE EXTERNA ESPII - RODÍZIO	CHC da Universidade Federal do Paraná - PR
233	ATIVIDADE EXTERNA ESPII - RODÍZIO	CHC da Universidade Federal do Paraná - PR
233	ATIVIDADE EXTERNA ESPII - RODÍZIO	CHC da Universidade Federal do Paraná - PR
233	ATIVIDADE EXTERNA ESPII - RODÍZIO	CHC da Universidade Federal do Paraná - PR
233	ATIVIDADE EXTERNA ESPII - RODÍZIO	CHC da Universidade Federal do Paraná - PR
233	ATIVIDADE EXTERNA ESPII - RODÍZIO	CHC da Universidade Federal do Paraná - PR
233	ATIVIDADE EXTERNA ESPII - RODÍZIO	CHC da Universidade Federal do Paraná - PR
233	ATIVIDADE EXTERNA ESPII - RODÍZIO	CHC da Universidade Federal do Paraná - PR
233	ATIVIDADE EXTERNA ESPII - RODÍZIO	CHC da Universidade Federal do Paraná - PR
233	ATIVIDADE EXTERNA ESPII - RODÍZIO	CHC da Universidade Federal do Paraná - PR
233	ATIVIDADE EXTERNA ESPII - RODÍZIO	CHC da Universidade Federal do Paraná - PR
233	ATIVIDADE EXTERNA ESPII - RODÍZIO	CHC da Universidade Federal do Paraná - PR
233	ATIVIDADE EXTERNA ESPII - RODÍZIO	CHC da Universidade Federal do Paraná - PR
233	ATIVIDADE EXTERNA ESPII - RODÍZIO	CHC da Universidade Federal do Paraná - PR
233	ATIVIDADE EXTERNA ESPII - RODÍZIO	CHC da Universidade Federal do Paraná - PR
233	ATIVIDADE EXTERNA ESPII - RODÍZIO	CHC da Universidade Federal do Paraná - PR
233	ATIVIDADE EXTERNA ESPII - RODÍZIO	CHC da Universidade Federal do Paraná - PR
233	ATIVIDADE EXTERNA ESPII - RODÍZIO	CHC da Universidade Federal do Paraná - PR
233	ATIVIDADE EXTERNA ESPII - RODÍZIO	CHC da Universidade Federal do Paraná - PR
233	ATIVIDADE EXTERNA ESPII - RODÍZIO	CHC da Universidade Federal do Paraná - PR
233	ATIVIDADE EXTERNA ESPII - RODÍZIO	CHC da Universidade Federal do Paraná - PR
233	ATIVIDADE EXTERNA ESPII - RODÍZIO	CHC da Universidade Federal do Paraná - PR
233	ATIVIDADE EXTERNA ESPII - RODÍZIO	CHC da Universidade Federal do Paraná - PR
233	ATIVIDADE EXTERNA ESPII - RODÍZIO	CHC da Universidade Federal do Paraná - PR
233	ATIVIDADE EXTERNA ESPII - RODÍZIO	CHC da Universidade Federal do Paraná - PR
233	ATIVIDADE EXTERNA ESPII - RODÍZIO	CHC da Universidade Federal do Paraná - PR
233	ATIVIDADE EXTERNA ESPII - RODÍZIO	CHC da Universidade Federal do Paraná - PR
233	ATIVIDADE EXTERNA ESPII - RODÍZIO	CHC da Universidade Federal do Paraná - PR
233	ATIVIDADE EXTERNA ESPII - RODÍZIO	CHC da Universidade Federal do Paraná - PR
233	ATIVIDADE EXTERNA ESPII - RODÍZIO	CHC da Universidade Federal do Paraná - PR
233	ATIVIDADE EXTERNA ESPII - RODÍZIO	CHC da Universidade Federal do Paraná - PR

233	ATIVIDADE EXTERNA ESPII - RODÍZIO	CHC da Universidade Federal do Paraná - PR
233	ATIVIDADE EXTERNA ESPII - RODÍZIO	CHC da Universidade Federal do Paraná - PR
233	ATIVIDADE EXTERNA ESPII - RODÍZIO	CHC da Universidade Federal do Paraná - PR
233	ATIVIDADE EXTERNA ESPII - RODÍZIO	CHC da Universidade Federal do Paraná - PR
233	ATIVIDADE EXTERNA ESPII - RODÍZIO	CHC da Universidade Federal do Paraná - PR
233	ATIVIDADE EXTERNA ESPII - RODÍZIO	CHC da Universidade Federal do Paraná - PR
233	ATIVIDADE EXTERNA ESPII - RODÍZIO	CHC da Universidade Federal do Paraná - PR
233	ATIVIDADE EXTERNA ESPII - RODÍZIO	CHC da Universidade Federal do Paraná - PR
233	ATIVIDADE EXTERNA ESPII - RODÍZIO	CHC da Universidade Federal do Paraná - PR
233	ATIVIDADE EXTERNA ESPII - RODÍZIO	CHC da Universidade Federal do Paraná - PR
233	ATIVIDADE EXTERNA ESPII - RODÍZIO	CHC da Universidade Federal do Paraná - PR
233	ATIVIDADE EXTERNA ESPII - RODÍZIO	CHC da Universidade Federal do Paraná - PR
233	ATIVIDADE EXTERNA ESPII - RODÍZIO	CHC da Universidade Federal do Paraná - PR
233	ATIVIDADE EXTERNA ESPII - RODÍZIO	CHC da Universidade Federal do Paraná - PR
233	ATIVIDADE EXTERNA ESPII - RODÍZIO	CHC da Universidade Federal do Paraná - PR
233	ATIVIDADE EXTERNA ESPII - RODÍZIO	CHC da Universidade Federal do Paraná - PR
233	ATIVIDADE EXTERNA ESPII - RODÍZIO	CHC da Universidade Federal do Paraná - PR
233	ATIVIDADE EXTERNA ESPII - RODÍZIO	CHC da Universidade Federal do Paraná - PR
233	ATIVIDADE EXTERNA ESPII - RODÍZIO	CHC da Universidade Federal do Paraná - PR
233	ATIVIDADE EXTERNA ESPII - RODÍZIO	CHC da Universidade Federal do Paraná - PR
233	ATIVIDADE EXTERNA ESPII - RODÍZIO	CHC da Universidade Federal do Paraná - PR
233	ATIVIDADE EXTERNA ESPII - RODÍZIO	CHC da Universidade Federal do Paraná - PR
233	ATIVIDADE EXTERNA ESPII - RODÍZIO	CHC da Universidade Federal do Paraná - PR
233	ATIVIDADE EXTERNA ESPII - RODÍZIO	CHC da Universidade Federal do Paraná - PR
233	ATIVIDADE EXTERNA ESPII - RODÍZIO	CHC da Universidade Federal do Paraná - PR
233	ATIVIDADE EXTERNA ESPII - RODÍZIO	CHC da Universidade Federal do Paraná - PR
233	ATIVIDADE EXTERNA ESPII - RODÍZIO	CHC da Universidade Federal do Paraná - PR
233	ATIVIDADE EXTERNA ESPII - RODÍZIO	CHC da Universidade Federal do Paraná - PR
233	ATIVIDADE EXTERNA ESPII - RODÍZIO	CHC da Universidade Federal do Paraná - PR
233	ATIVIDADE EXTERNA ESPII - RODÍZIO	CHC da Universidade Federal do Paraná - PR
233	ATIVIDADE EXTERNA ESPII - RODÍZIO	CHC da Universidade Federal do Paraná - PR
233	ATIVIDADE EXTERNA ESPII - RODÍZIO	CHC da Universidade Federal do Paraná - PR
233	ATIVIDADE EXTERNA ESPII - RODÍZIO	CHC da Universidade Federal do Paraná - PR
233	ATIVIDADE EXTERNA ESPII - RODÍZIO	CHC da Universidade Federal do Paraná - PR
233	ATIVIDADE EXTERNA ESPII - RODÍZIO	CHC da Universidade Federal do Paraná - PR
233	ATIVIDADE EXTERNA ESPII - RODÍZIO	CHC da Universidade Federal do Paraná - PR
233	ATIVIDADE EXTERNA ESPII - RODÍZIO	CHC da Universidade Federal do Paraná - PR
233	ATIVIDADE EXTERNA ESPII - RODÍZIO	CHC da Universidade Federal do Paraná - PR
233	ATIVIDADE EXTERNA ESPII - RODÍZIO	CHC da Universidade Federal do Paraná - PR
233	ATIVIDADE EXTERNA ESPII - RODÍZIO	CHC da Universidade Federal do Paraná - PR
233	ATIVIDADE EXTERNA ESPII - RODÍZIO	CHC da Universidade Federal do Paraná - PR
233	ATIVIDADE EXTERNA ESPII - RODÍZIO	CHC da Universidade Federal do Paraná - PR
233	ATIVIDADE EXTERNA ESPII - RODÍZIO	CHC da Universidade Federal do Paraná - PR
233	ATIVIDADE EXTERNA ESPII - RODÍZIO	CHC da Universidade Federal do Paraná - PR
233	ATIVIDADE EXTERNA ESPII - RODÍZIO	CHC da Universidade Federal do Paraná - PR
233	ATIVIDADE EXTERNA ESPII - RODÍZIO	CHC da Universidade Federal do Paraná - PR
233	ATIVIDADE EXTERNA ESPII - RODÍZIO	CHC da Universidade Federal do Paraná - PR
233	ATIVIDADE EXTERNA ESPII - RODÍZIO	CHC da Universidade Federal do Paraná - PR

233	ATIVIDADE EXTERNA ESPII - RODÍZIO	CHC da Universidade Federal do Paraná - PR
233	ATIVIDADE EXTERNA ESPII - RODÍZIO	CHC da Universidade Federal do Paraná - PR
233	ATIVIDADE EXTERNA ESPII - RODÍZIO	CHC da Universidade Federal do Paraná - PR
233	ATIVIDADE EXTERNA ESPII - RODÍZIO	CHC da Universidade Federal do Paraná - PR
233	ATIVIDADE EXTERNA ESPII - RODÍZIO	CHC da Universidade Federal do Paraná - PR
233	ATIVIDADE EXTERNA ESPII - RODÍZIO	CHC da Universidade Federal do Paraná - PR
233	ATIVIDADE EXTERNA ESPII - RODÍZIO	CHC da Universidade Federal do Paraná - PR
233	ATIVIDADE EXTERNA ESPII - RODÍZIO	CHC da Universidade Federal do Paraná - PR
233	ATIVIDADE EXTERNA ESPII - RODÍZIO	CHC da Universidade Federal do Paraná - PR
233	ATIVIDADE EXTERNA ESPII - RODÍZIO	CHC da Universidade Federal do Paraná - PR
233	ATIVIDADE EXTERNA ESPII - RODÍZIO	CHC da Universidade Federal do Paraná - PR
233	ATIVIDADE EXTERNA ESPII - RODÍZIO	CHC da Universidade Federal do Paraná - PR
233	ATIVIDADE EXTERNA ESPII - RODÍZIO	CHC da Universidade Federal do Paraná - PR
233	ATIVIDADE EXTERNA ESPII - RODÍZIO	CHC da Universidade Federal do Paraná - PR
233	ATIVIDADE EXTERNA ESPII - RODÍZIO	CHC da Universidade Federal do Paraná - PR
233	ATIVIDADE EXTERNA ESPII - RODÍZIO	CHC da Universidade Federal do Paraná - PR
233	ATIVIDADE EXTERNA ESPII - RODÍZIO	CHC da Universidade Federal do Paraná - PR
233	ATIVIDADE EXTERNA ESPII - RODÍZIO	CHC da Universidade Federal do Paraná - PR
233	ATIVIDADE EXTERNA ESPII - RODÍZIO	CHC da Universidade Federal do Paraná - PR
233	ATIVIDADE EXTERNA ESPII - RODÍZIO	CHC da Universidade Federal do Paraná - PR
233	ATIVIDADE EXTERNA ESPII - RODÍZIO	CHC da Universidade Federal do Paraná - PR
233	ATIVIDADE EXTERNA ESPII - RODÍZIO	CHC da Universidade Federal do Paraná - PR
233	ATIVIDADE EXTERNA ESPII - RODÍZIO	CHC da Universidade Federal do Paraná - PR
233	ATIVIDADE EXTERNA ESPII - RODÍZIO	CHC da Universidade Federal do Paraná - PR
233	ATIVIDADE EXTERNA ESPII - RODÍZIO	CHC da Universidade Federal do Paraná - PR
233	ATIVIDADE EXTERNA ESPII - RODÍZIO	CHC da Universidade Federal do Paraná - PR
233	ATIVIDADE EXTERNA ESPII - RODÍZIO	CHC da Universidade Federal do Paraná - PR
233	ATIVIDADE EXTERNA ESPII - RODÍZIO	CHC da Universidade Federal do Paraná - PR
233	ATIVIDADE EXTERNA ESPII - RODÍZIO	CHC da Universidade Federal do Paraná - PR
233	ATIVIDADE EXTERNA ESPII - RODÍZIO	CHC da Universidade Federal do Paraná - PR
233	ATIVIDADE EXTERNA ESPII - RODÍZIO	CHC da Universidade Federal do Paraná - PR
233	ATIVIDADE EXTERNA ESPII - RODÍZIO	CHC da Universidade Federal do Paraná - PR
233	ATIVIDADE EXTERNA ESPII - RODÍZIO	CHC da Universidade Federal do Paraná - PR
233	ATIVIDADE EXTERNA ESPII - RODÍZIO	CHC da Universidade Federal do Paraná - PR
233	ATIVIDADE EXTERNA ESPII - RODÍZIO	CHC da Universidade Federal do Paraná - PR
233	ATIVIDADE EXTERNA ESPII - RODÍZIO	CHC da Universidade Federal do Paraná - PR
233	ATIVIDADE EXTERNA ESPII - RODÍZIO	CHC da Universidade Federal do Paraná - PR
233	ATIVIDADE EXTERNA ESPII - RODÍZIO	CHC da Universidade Federal do Paraná - PR
233	ATIVIDADE EXTERNA ESPII - RODÍZIO	CHC da Universidade Federal do Paraná - PR
233	ATIVIDADE EXTERNA ESPII - RODÍZIO	CHC da Universidade Federal do Paraná - PR
233	ATIVIDADE EXTERNA ESPII - RODÍZIO	CHC da Universidade Federal do Paraná - PR
233	ATIVIDADE EXTERNA ESPII - RODÍZIO	CHC da Universidade Federal do Paraná - PR
233	ATIVIDADE EXTERNA ESPII - RODÍZIO	CHC da Universidade Federal do Paraná - PR
233	ATIVIDADE EXTERNA ESPII - RODÍZIO	CHC da Universidade Federal do Paraná - PR
233	ATIVIDADE EXTERNA ESPII - RODÍZIO	CHC da Universidade Federal do Paraná - PR
233	ATIVIDADE EXTERNA ESPII - RODÍZIO	CHC da Universidade Federal do Paraná - PR
233	ATIVIDADE EXTERNA ESPII - RODÍZIO	CHC da Universidade Federal do Paraná - PR
233	ATIVIDADE EXTERNA ESPII - RODÍZIO	CHC da Universidade Federal do Paraná - PR

233	ATIVIDADE EXTERNA ESPII - RODÍZIO	CHC da Universidade Federal do Paraná - PR
233	ATIVIDADE EXTERNA ESPII - RODÍZIO	CHC da Universidade Federal do Paraná - PR
233	ATIVIDADE EXTERNA ESPII - RODÍZIO	CHC da Universidade Federal do Paraná - PR
233	ATIVIDADE EXTERNA ESPII - RODÍZIO	CHC da Universidade Federal do Paraná - PR
233	ATIVIDADE EXTERNA ESPII - RODÍZIO	CHC da Universidade Federal do Paraná - PR
233	ATIVIDADE EXTERNA ESPII - RODÍZIO	CHC da Universidade Federal do Paraná - PR
233	ATIVIDADE EXTERNA ESPII - RODÍZIO	CHC da Universidade Federal do Paraná - PR
233	ATIVIDADE EXTERNA ESPII - RODÍZIO	CHC da Universidade Federal do Paraná - PR
233	ATIVIDADE EXTERNA ESPII - RODÍZIO	CHC da Universidade Federal do Paraná - PR
233	ATIVIDADE EXTERNA ESPII - RODÍZIO	CHC da Universidade Federal do Paraná - PR
233	ATIVIDADE EXTERNA ESPII - RODÍZIO	CHC da Universidade Federal do Paraná - PR
233	ATIVIDADE EXTERNA ESPII - RODÍZIO	CHC da Universidade Federal do Paraná - PR
233	ATIVIDADE EXTERNA ESPII - RODÍZIO	CHC da Universidade Federal do Paraná - PR
233	ATIVIDADE EXTERNA ESPII - RODÍZIO	CHC da Universidade Federal do Paraná - PR
233	ATIVIDADE EXTERNA ESPII - RODÍZIO	CHC da Universidade Federal do Paraná - PR
233	ATIVIDADE EXTERNA ESPII - RODÍZIO	CHC da Universidade Federal do Paraná - PR
233	ATIVIDADE EXTERNA ESPII - RODÍZIO	CHC da Universidade Federal do Paraná - PR
233	ATIVIDADE EXTERNA ESPII - RODÍZIO	CHC da Universidade Federal do Paraná - PR
233	ATIVIDADE EXTERNA ESPII - RODÍZIO	CHC da Universidade Federal do Paraná - PR
233	ATIVIDADE EXTERNA ESPII - RODÍZIO	CHC da Universidade Federal do Paraná - PR
233	ATIVIDADE EXTERNA ESPII - RODÍZIO	CHC da Universidade Federal do Paraná - PR
233	ATIVIDADE EXTERNA ESPII - RODÍZIO	CHC da Universidade Federal do Paraná - PR
233	ATIVIDADE EXTERNA ESPII - RODÍZIO	CHC da Universidade Federal do Paraná - PR
233	ATIVIDADE EXTERNA ESPII - RODÍZIO	CHC da Universidade Federal do Paraná - PR
233	ATIVIDADE EXTERNA ESPII - RODÍZIO	CHC da Universidade Federal do Paraná - PR
233	ATIVIDADE EXTERNA ESPII - RODÍZIO	CHC da Universidade Federal do Paraná - PR
233	ATIVIDADE EXTERNA ESPII - RODÍZIO	CHC da Universidade Federal do Paraná - PR
233	ATIVIDADE EXTERNA ESPII - RODÍZIO	CHC da Universidade Federal do Paraná - PR
233	ATIVIDADE EXTERNA ESPII - RODÍZIO	CHC da Universidade Federal do Paraná - PR
233	ATIVIDADE EXTERNA ESPII - RODÍZIO	CHC da Universidade Federal do Paraná - PR
233	ATIVIDADE EXTERNA ESPII - RODÍZIO	CHC da Universidade Federal do Paraná - PR
233	ATIVIDADE EXTERNA ESPII - RODÍZIO	CHC da Universidade Federal do Paraná - PR
233	ATIVIDADE EXTERNA ESPII - RODÍZIO	CHC da Universidade Federal do Paraná - PR
233	ATIVIDADE EXTERNA ESPII - RODÍZIO	CHC da Universidade Federal do Paraná - PR
233	ATIVIDADE EXTERNA ESPII - RODÍZIO	CHC da Universidade Federal do Paraná - PR
233	ATIVIDADE EXTERNA ESPII - RODÍZIO	CHC da Universidade Federal do Paraná - PR
233	ATIVIDADE EXTERNA ESPII - RODÍZIO	CHC da Universidade Federal do Paraná - PR
233	ATIVIDADE EXTERNA ESPII - RODÍZIO	CHC da Universidade Federal do Paraná - PR
233	ATIVIDADE EXTERNA ESPII - RODÍZIO	CHC da Universidade Federal do Paraná - PR
233	ATIVIDADE EXTERNA ESPII - RODÍZIO	CHC da Universidade Federal do Paraná - PR
233	ATIVIDADE EXTERNA ESPII - RODÍZIO	CHC da Universidade Federal do Paraná - PR
233	ATIVIDADE EXTERNA ESPII - RODÍZIO	CHC da Universidade Federal do Paraná - PR
233	ATIVIDADE EXTERNA ESPII - RODÍZIO	CHC da Universidade Federal do Paraná - PR
233	ATIVIDADE EXTERNA ESPII - RODÍZIO	CHC da Universidade Federal do Paraná - PR
233	ATIVIDADE EXTERNA ESPII - RODÍZIO	CHC da Universidade Federal do Paraná - PR
233	ATIVIDADE EXTERNA ESPII - RODÍZIO	CHC da Universidade Federal do Paraná - PR
233	ATIVIDADE EXTERNA ESPII - RODÍZIO	CHC da Universidade Federal do Paraná - PR
233	ATIVIDADE EXTERNA ESPII - RODÍZIO	CHC da Universidade Federal do Paraná - PR

233	ATIVIDADE EXTERNA ESPII - RODÍZIO	CHC da Universidade Federal do Paraná - PR
233	ATIVIDADE EXTERNA ESPII - RODÍZIO	CHC da Universidade Federal do Paraná - PR
233	ATIVIDADE EXTERNA ESPII - RODÍZIO	CHC da Universidade Federal do Paraná - PR
233	ATIVIDADE EXTERNA ESPII - RODÍZIO	CHC da Universidade Federal do Paraná - PR
233	ATIVIDADE EXTERNA ESPII - RODÍZIO	CHC da Universidade Federal do Paraná - PR
233	ATIVIDADE EXTERNA ESPII - RODÍZIO	CHC da Universidade Federal do Paraná - PR
233	ATIVIDADE EXTERNA ESPII - RODÍZIO	CHC da Universidade Federal do Paraná - PR
233	ATIVIDADE EXTERNA ESPII - RODÍZIO	CHC da Universidade Federal do Paraná - PR
233	ATIVIDADE EXTERNA ESPII - RODÍZIO	CHC da Universidade Federal do Paraná - PR
233	ATIVIDADE EXTERNA ESPII - RODÍZIO	CHC da Universidade Federal do Paraná - PR
233	ATIVIDADE EXTERNA ESPII - RODÍZIO	CHC da Universidade Federal do Paraná - PR
233	ATIVIDADE EXTERNA ESPII - RODÍZIO	CHC da Universidade Federal do Paraná - PR
233	ATIVIDADE EXTERNA ESPII - RODÍZIO	CHC da Universidade Federal do Paraná - PR
233	ATIVIDADE EXTERNA ESPII - RODÍZIO	CHC da Universidade Federal do Paraná - PR
233	ATIVIDADE EXTERNA ESPII - RODÍZIO	CHC da Universidade Federal do Paraná - PR
233	ATIVIDADE EXTERNA ESPII - RODÍZIO	CHC da Universidade Federal do Paraná - PR
233	ATIVIDADE EXTERNA ESPII - RODÍZIO	CHC da Universidade Federal do Paraná - PR
233	ATIVIDADE EXTERNA ESPII - RODÍZIO	CHC da Universidade Federal do Paraná - PR
233	ATIVIDADE EXTERNA ESPII - RODÍZIO	CHC da Universidade Federal do Paraná - PR
233	ATIVIDADE EXTERNA ESPII - RODÍZIO	CHC da Universidade Federal do Paraná - PR
233	ATIVIDADE EXTERNA ESPII - RODÍZIO	CHC da Universidade Federal do Paraná - PR
233	ATIVIDADE EXTERNA ESPII - RODÍZIO	CHC da Universidade Federal do Paraná - PR
233	ATIVIDADE EXTERNA ESPII - RODÍZIO	CHC da Universidade Federal do Paraná - PR
233	ATIVIDADE EXTERNA ESPII - RODÍZIO	CHC da Universidade Federal do Paraná - PR
233	ATIVIDADE EXTERNA ESPII - RODÍZIO	CHC da Universidade Federal do Paraná - PR
233	ATIVIDADE EXTERNA ESPII - RODÍZIO	CHC da Universidade Federal do Paraná - PR
233	ATIVIDADE EXTERNA ESPII - RODÍZIO	CHC da Universidade Federal do Paraná - PR
233	ATIVIDADE EXTERNA ESPII - RODÍZIO	CHC da Universidade Federal do Paraná - PR
233	ATIVIDADE EXTERNA ESPII - RODÍZIO	CHC da Universidade Federal do Paraná - PR
233	ATIVIDADE EXTERNA ESPII - RODÍZIO	CHC da Universidade Federal do Paraná - PR
233	ATIVIDADE EXTERNA ESPII - RODÍZIO	CHC da Universidade Federal do Paraná - PR
233	ATIVIDADE EXTERNA ESPII - RODÍZIO	CHC da Universidade Federal do Paraná - PR
233	ATIVIDADE EXTERNA ESPII - RODÍZIO	CHC da Universidade Federal do Paraná - PR
233	ATIVIDADE EXTERNA ESPII - RODÍZIO	CHC da Universidade Federal do Paraná - PR
233	ATIVIDADE EXTERNA ESPII - RODÍZIO	CHC da Universidade Federal do Paraná - PR
233	ATIVIDADE EXTERNA ESPII - RODÍZIO	CHC da Universidade Federal do Paraná - PR
233	ATIVIDADE EXTERNA ESPII - RODÍZIO	CHC da Universidade Federal do Paraná - PR
233	ATIVIDADE EXTERNA ESPII - RODÍZIO	CHC da Universidade Federal do Paraná - PR
233	ATIVIDADE EXTERNA ESPII - RODÍZIO	CHC da Universidade Federal do Paraná - PR
233	ATIVIDADE EXTERNA ESPII - RODÍZIO	CHC da Universidade Federal do Paraná - PR
233	ATIVIDADE EXTERNA ESPII - RODÍZIO	CHC da Universidade Federal do Paraná - PR
233	ATIVIDADE EXTERNA ESPII - RODÍZIO	CHC da Universidade Federal do Paraná - PR
233	ATIVIDADE EXTERNA ESPII - RODÍZIO	CHC da Universidade Federal do Paraná - PR
233	ATIVIDADE EXTERNA ESPII - RODÍZIO	CHC da Universidade Federal do Paraná - PR
233	ATIVIDADE EXTERNA ESPII - RODÍZIO	CHC da Universidade Federal do Paraná - PR
233	ATIVIDADE EXTERNA ESPII - RODÍZIO	CHC da Universidade Federal do Paraná - PR
233	ATIVIDADE EXTERNA ESPII - RODÍZIO	CHC da Universidade Federal do Paraná - PR
233	ATIVIDADE EXTERNA ESPII - RODÍZIO	CHC da Universidade Federal do Paraná - PR

233	ATIVIDADE EXTERNA ESPII - RODÍZIO	CHC da Universidade Federal do Paraná - PR
233	ATIVIDADE EXTERNA ESPII - RODÍZIO	CHC da Universidade Federal do Paraná - PR
233	ATIVIDADE EXTERNA ESPII - RODÍZIO	CHC da Universidade Federal do Paraná - PR
233	ATIVIDADE EXTERNA ESPII - RODÍZIO	CHC da Universidade Federal do Paraná - PR
233	ATIVIDADE EXTERNA ESPII - RODÍZIO	CHC da Universidade Federal do Paraná - PR
233	ATIVIDADE EXTERNA ESPII - RODÍZIO	CHC da Universidade Federal do Paraná - PR
233	ATIVIDADE EXTERNA ESPII - RODÍZIO	CHC da Universidade Federal do Paraná - PR
233	ATIVIDADE EXTERNA ESPII - RODÍZIO	CHC da Universidade Federal do Paraná - PR
233	ATIVIDADE EXTERNA ESPII - RODÍZIO	CHC da Universidade Federal do Paraná - PR
233	ATIVIDADE EXTERNA ESPII - RODÍZIO	CHC da Universidade Federal do Paraná - PR
233	ATIVIDADE EXTERNA ESPII - RODÍZIO	CHC da Universidade Federal do Paraná - PR
233	ATIVIDADE EXTERNA ESPII - RODÍZIO	CHC da Universidade Federal do Paraná - PR
233	ATIVIDADE EXTERNA ESPII - RODÍZIO	CHC da Universidade Federal do Paraná - PR
233	ATIVIDADE EXTERNA ESPII - RODÍZIO	CHC da Universidade Federal do Paraná - PR
233	ATIVIDADE EXTERNA ESPII - RODÍZIO	CHC da Universidade Federal do Paraná - PR
233	ATIVIDADE EXTERNA ESPII - RODÍZIO	CHC da Universidade Federal do Paraná - PR
233	ATIVIDADE EXTERNA ESPII - RODÍZIO	CHC da Universidade Federal do Paraná - PR
233	ATIVIDADE EXTERNA ESPII - RODÍZIO	CHC da Universidade Federal do Paraná - PR
233	ATIVIDADE EXTERNA ESPII - RODÍZIO	CHC da Universidade Federal do Paraná - PR
233	ATIVIDADE EXTERNA ESPII - RODÍZIO	CHC da Universidade Federal do Paraná - PR
233	ATIVIDADE EXTERNA ESPII - RODÍZIO	CHC da Universidade Federal do Paraná - PR
233	ATIVIDADE EXTERNA ESPII - RODÍZIO	CHC da Universidade Federal do Paraná - PR
233	ATIVIDADE EXTERNA ESPII - RODÍZIO	CHC da Universidade Federal do Paraná - PR
233	ATIVIDADE EXTERNA ESPII - RODÍZIO	CHC da Universidade Federal do Paraná - PR
233	ATIVIDADE EXTERNA ESPII - RODÍZIO	CHC da Universidade Federal do Paraná - PR
233	ATIVIDADE EXTERNA ESPII - RODÍZIO	CHC da Universidade Federal do Paraná - PR
233	ATIVIDADE EXTERNA ESPII - RODÍZIO	CHC da Universidade Federal do Paraná - PR
233	ATIVIDADE EXTERNA ESPII - RODÍZIO	CHC da Universidade Federal do Paraná - PR
233	ATIVIDADE EXTERNA ESPII - RODÍZIO	CHC da Universidade Federal do Paraná - PR
233	ATIVIDADE EXTERNA ESPII - RODÍZIO	CHC da Universidade Federal do Paraná - PR
233	ATIVIDADE EXTERNA ESPII - RODÍZIO	CHC da Universidade Federal do Paraná - PR
233	ATIVIDADE EXTERNA ESPII - RODÍZIO	CHC da Universidade Federal do Paraná - PR
233	ATIVIDADE EXTERNA ESPII - RODÍZIO	CHC da Universidade Federal do Paraná - PR
233	ATIVIDADE EXTERNA ESPII - RODÍZIO	CHC da Universidade Federal do Paraná - PR
233	ATIVIDADE EXTERNA ESPII - RODÍZIO	CHC da Universidade Federal do Paraná - PR
233	ATIVIDADE EXTERNA ESPII - RODÍZIO	CHC da Universidade Federal do Paraná - PR
233	ATIVIDADE EXTERNA ESPII - RODÍZIO	CHC da Universidade Federal do Paraná - PR
233	ATIVIDADE EXTERNA ESPII - RODÍZIO	CHC da Universidade Federal do Paraná - PR
233	ATIVIDADE EXTERNA ESPII - RODÍZIO	CHC da Universidade Federal do Paraná - PR
233	ATIVIDADE EXTERNA ESPII - RODÍZIO	CHC da Universidade Federal do Paraná - PR
233	ATIVIDADE EXTERNA ESPII - RODÍZIO	CHC da Universidade Federal do Paraná - PR
233	ATIVIDADE EXTERNA ESPII - RODÍZIO	CHC da Universidade Federal do Paraná - PR
233	ATIVIDADE EXTERNA ESPII - RODÍZIO	CHC da Universidade Federal do Paraná - PR
233	ATIVIDADE EXTERNA ESPII - RODÍZIO	CHC da Universidade Federal do Paraná - PR
233	ATIVIDADE EXTERNA ESPII - RODÍZIO	CHC da Universidade Federal do Paraná - PR
233	ATIVIDADE EXTERNA ESPII - RODÍZIO	CHC da Universidade Federal do Paraná - PR
233	ATIVIDADE EXTERNA ESPII - RODÍZIO	CHC da Universidade Federal do Paraná - PR
233	ATIVIDADE EXTERNA ESPII - RODÍZIO	CHC da Universidade Federal do Paraná - PR

233	ATIVIDADE EXTERNA ESPII - RODÍZIO	CHC da Universidade Federal do Paraná - PR
233	ATIVIDADE EXTERNA ESPII - RODÍZIO	CHC da Universidade Federal do Paraná - PR
233	ATIVIDADE EXTERNA ESPII - RODÍZIO	CHC da Universidade Federal do Paraná - PR
233	ATIVIDADE EXTERNA ESPII - RODÍZIO	CHC da Universidade Federal do Paraná - PR
233	ATIVIDADE EXTERNA ESPII - RODÍZIO	CHC da Universidade Federal do Paraná - PR
233	ATIVIDADE EXTERNA ESPII - RODÍZIO	CHC da Universidade Federal do Paraná - PR
233	ATIVIDADE EXTERNA ESPII - RODÍZIO	CHC da Universidade Federal do Paraná - PR
233	ATIVIDADE EXTERNA ESPII - RODÍZIO	CHC da Universidade Federal do Paraná - PR
233	ATIVIDADE EXTERNA ESPII - RODÍZIO	CHC da Universidade Federal do Paraná - PR
233	ATIVIDADE EXTERNA ESPII - RODÍZIO	CHC da Universidade Federal do Paraná - PR
233	ATIVIDADE EXTERNA ESPII - RODÍZIO	CHC da Universidade Federal do Paraná - PR
233	ATIVIDADE EXTERNA ESPII - RODÍZIO	CHC da Universidade Federal do Paraná - PR
233	ATIVIDADE EXTERNA ESPII - RODÍZIO	CHC da Universidade Federal do Paraná - PR
233	ATIVIDADE EXTERNA ESPII - RODÍZIO	CHC da Universidade Federal do Paraná - PR
233	ATIVIDADE EXTERNA ESPII - RODÍZIO	CHC da Universidade Federal do Paraná - PR
233	ATIVIDADE EXTERNA ESPII - RODÍZIO	CHC da Universidade Federal do Paraná - PR
233	ATIVIDADE EXTERNA ESPII - RODÍZIO	CHC da Universidade Federal do Paraná - PR
233	ATIVIDADE EXTERNA ESPII - RODÍZIO	CHC da Universidade Federal do Paraná - PR
233	ATIVIDADE EXTERNA ESPII - RODÍZIO	CHC da Universidade Federal do Paraná - PR
233	ATIVIDADE EXTERNA ESPII - RODÍZIO	CHC da Universidade Federal do Paraná - PR
233	ATIVIDADE EXTERNA ESPII - RODÍZIO	CHC da Universidade Federal do Paraná - PR
233	ATIVIDADE EXTERNA ESPII - RODÍZIO	CHC da Universidade Federal do Paraná - PR
233	ATIVIDADE EXTERNA ESPII - RODÍZIO	CHC da Universidade Federal do Paraná - PR
233	ATIVIDADE EXTERNA ESPII - RODÍZIO	CHC da Universidade Federal do Paraná - PR
233	ATIVIDADE EXTERNA ESPII - RODÍZIO	CHC da Universidade Federal do Paraná - PR
233	ATIVIDADE EXTERNA ESPII - RODÍZIO	CHC da Universidade Federal do Paraná - PR
233	ATIVIDADE EXTERNA ESPII - RODÍZIO	CHC da Universidade Federal do Paraná - PR
233	ATIVIDADE EXTERNA ESPII - RODÍZIO	CHC da Universidade Federal do Paraná - PR
233	ATIVIDADE EXTERNA ESPII - RODÍZIO	CHC da Universidade Federal do Paraná - PR
233	ATIVIDADE EXTERNA ESPII - RODÍZIO	CHC da Universidade Federal do Paraná - PR
233	ATIVIDADE EXTERNA ESPII - RODÍZIO	CHC da Universidade Federal do Paraná - PR
233	ATIVIDADE EXTERNA ESPII - RODÍZIO	CHC da Universidade Federal do Paraná - PR
233	ATIVIDADE EXTERNA ESPII - RODÍZIO	CHC da Universidade Federal do Paraná - PR
233	ATIVIDADE EXTERNA ESPII - RODÍZIO	CHC da Universidade Federal do Paraná - PR
233	ATIVIDADE EXTERNA ESPII - RODÍZIO	CHC da Universidade Federal do Paraná - PR
233	ATIVIDADE EXTERNA ESPII - RODÍZIO	CHC da Universidade Federal do Paraná - PR
233	ATIVIDADE EXTERNA ESPII - RODÍZIO	CHC da Universidade Federal do Paraná - PR
233	ATIVIDADE EXTERNA ESPII - RODÍZIO	CHC da Universidade Federal do Paraná - PR
233	ATIVIDADE EXTERNA ESPII - RODÍZIO	CHC da Universidade Federal do Paraná - PR
233	ATIVIDADE EXTERNA ESPII - RODÍZIO	CHC da Universidade Federal do Paraná - PR
233	ATIVIDADE EXTERNA ESPII - RODÍZIO	CHC da Universidade Federal do Paraná - PR
233	ATIVIDADE EXTERNA ESPII - RODÍZIO	CHC da Universidade Federal do Paraná - PR
233	ATIVIDADE EXTERNA ESPII - RODÍZIO	CHC da Universidade Federal do Paraná - PR
233	ATIVIDADE EXTERNA ESPII - RODÍZIO	CHC da Universidade Federal do Paraná - PR
233	ATIVIDADE EXTERNA ESPII - RODÍZIO	CHC da Universidade Federal do Paraná - PR
233	ATIVIDADE EXTERNA ESPII - RODÍZIO	CHC da Universidade Federal do Paraná - PR
233	ATIVIDADE EXTERNA ESPII - RODÍZIO	CHC da Universidade Federal do Paraná - PR
233	ATIVIDADE EXTERNA ESPII - RODÍZIO	CHC da Universidade Federal do Paraná - PR

233	ATIVIDADE EXTERNA ESPII - RODÍZIO	CHC da Universidade Federal do Paraná - PR
233	ATIVIDADE EXTERNA ESPII - RODÍZIO	CHC da Universidade Federal do Paraná - PR
233	ATIVIDADE EXTERNA ESPII - RODÍZIO	CHC da Universidade Federal do Paraná - PR
233	ATIVIDADE EXTERNA ESPII - RODÍZIO	CHC da Universidade Federal do Paraná - PR
233	ATIVIDADE EXTERNA ESPII - RODÍZIO	CHC da Universidade Federal do Paraná - PR
233	ATIVIDADE EXTERNA ESPII - RODÍZIO	CHC da Universidade Federal do Paraná - PR
233	ATIVIDADE EXTERNA ESPII - RODÍZIO	CHC da Universidade Federal do Paraná - PR
233	ATIVIDADE EXTERNA ESPII - RODÍZIO	CHC da Universidade Federal do Paraná - PR
233	ATIVIDADE EXTERNA ESPII - RODÍZIO	CHC da Universidade Federal do Paraná - PR
233	ATIVIDADE EXTERNA ESPII - RODÍZIO	CHC da Universidade Federal do Paraná - PR
233	ATIVIDADE EXTERNA ESPII - RODÍZIO	CHC da Universidade Federal do Paraná - PR
233	ATIVIDADE EXTERNA ESPII - RODÍZIO	CHC da Universidade Federal do Paraná - PR
233	ATIVIDADE EXTERNA ESPII - RODÍZIO	CHC da Universidade Federal do Paraná - PR
233	ATIVIDADE EXTERNA ESPII - RODÍZIO	CHC da Universidade Federal do Paraná - PR
233	ATIVIDADE EXTERNA ESPII - RODÍZIO	CHC da Universidade Federal do Paraná - PR
233	ATIVIDADE EXTERNA ESPII - RODÍZIO	CHC da Universidade Federal do Paraná - PR
233	ATIVIDADE EXTERNA ESPII - RODÍZIO	CHC da Universidade Federal do Paraná - PR
233	ATIVIDADE EXTERNA ESPII - RODÍZIO	CHC da Universidade Federal do Paraná - PR
233	ATIVIDADE EXTERNA ESPII - RODÍZIO	CHC da Universidade Federal do Paraná - PR
233	ATIVIDADE EXTERNA ESPII - RODÍZIO	CHC da Universidade Federal do Paraná - PR
233	ATIVIDADE EXTERNA ESPII - RODÍZIO	CHC da Universidade Federal do Paraná - PR
233	ATIVIDADE EXTERNA ESPII - RODÍZIO	CHC da Universidade Federal do Paraná - PR
233	ATIVIDADE EXTERNA ESPII - RODÍZIO	CHC da Universidade Federal do Paraná - PR
233	ATIVIDADE EXTERNA ESPII - RODÍZIO	CHC da Universidade Federal do Paraná - PR
233	ATIVIDADE EXTERNA ESPII - RODÍZIO	CHC da Universidade Federal do Paraná - PR
233	ATIVIDADE EXTERNA ESPII - RODÍZIO	CHC da Universidade Federal do Paraná - PR
233	ATIVIDADE EXTERNA ESPII - RODÍZIO	CHC da Universidade Federal do Paraná - PR
233	ATIVIDADE EXTERNA ESPII - RODÍZIO	CHC da Universidade Federal do Paraná - PR
233	ATIVIDADE EXTERNA ESPII - RODÍZIO	CHC da Universidade Federal do Paraná - PR
233	ATIVIDADE EXTERNA ESPII - RODÍZIO	CHC da Universidade Federal do Paraná - PR
233	ATIVIDADE EXTERNA ESPII - RODÍZIO	CHC da Universidade Federal do Paraná - PR
233	ATIVIDADE EXTERNA ESPII - RODÍZIO	CHC da Universidade Federal do Paraná - PR
233	ATIVIDADE EXTERNA ESPII - RODÍZIO	CHC da Universidade Federal do Paraná - PR
233	ATIVIDADE EXTERNA ESPII - RODÍZIO	CHC da Universidade Federal do Paraná - PR
233	ATIVIDADE EXTERNA ESPII - RODÍZIO	CHC da Universidade Federal do Paraná - PR
233	ATIVIDADE EXTERNA ESPII - RODÍZIO	CHC da Universidade Federal do Paraná - PR
233	ATIVIDADE EXTERNA ESPII - RODÍZIO	CHC da Universidade Federal do Paraná - PR
233	ATIVIDADE EXTERNA ESPII - RODÍZIO	CHC da Universidade Federal do Paraná - PR
233	ATIVIDADE EXTERNA ESPII - RODÍZIO	CHC da Universidade Federal do Paraná - PR
233	ATIVIDADE EXTERNA ESPII - RODÍZIO	CHC da Universidade Federal do Paraná - PR
233	ATIVIDADE EXTERNA ESPII - RODÍZIO	CHC da Universidade Federal do Paraná - PR
233	ATIVIDADE EXTERNA ESPII - RODÍZIO	CHC da Universidade Federal do Paraná - PR
233	ATIVIDADE EXTERNA ESPII - RODÍZIO	CHC da Universidade Federal do Paraná - PR

233	ATIVIDADE EXTERNA ESPII - RODÍZIO	CHC da Universidade Federal do Paraná - PR
233	ATIVIDADE EXTERNA ESPII - RODÍZIO	CHC da Universidade Federal do Paraná - PR
233	ATIVIDADE EXTERNA ESPII - RODÍZIO	CHC da Universidade Federal do Paraná - PR
233	ATIVIDADE EXTERNA ESPII - RODÍZIO	CHC da Universidade Federal do Paraná - PR
233	ATIVIDADE EXTERNA ESPII - RODÍZIO	CHC da Universidade Federal do Paraná - PR
233	ATIVIDADE EXTERNA ESPII - RODÍZIO	CHC da Universidade Federal do Paraná - PR
233	ATIVIDADE EXTERNA ESPII - RODÍZIO	CHC da Universidade Federal do Paraná - PR
233	ATIVIDADE EXTERNA ESPII - RODÍZIO	CHC da Universidade Federal do Paraná - PR
233	ATIVIDADE EXTERNA ESPII - RODÍZIO	CHC da Universidade Federal do Paraná - PR
233	ATIVIDADE EXTERNA ESPII - RODÍZIO	CHC da Universidade Federal do Paraná - PR
233	ATIVIDADE EXTERNA ESPII - RODÍZIO	CHC da Universidade Federal do Paraná - PR
233	ATIVIDADE EXTERNA ESPII - RODÍZIO	CHC da Universidade Federal do Paraná - PR
233	ATIVIDADE EXTERNA ESPII - RODÍZIO	CHC da Universidade Federal do Paraná - PR
233	ATIVIDADE EXTERNA ESPII - RODÍZIO	CHC da Universidade Federal do Paraná - PR
233	ATIVIDADE EXTERNA ESPII - RODÍZIO	CHC da Universidade Federal do Paraná - PR
233	ATIVIDADE EXTERNA ESPII - RODÍZIO	CHC da Universidade Federal do Paraná - PR
233	ATIVIDADE EXTERNA ESPII - RODÍZIO	CHC da Universidade Federal do Paraná - PR
233	ATIVIDADE EXTERNA ESPII - RODÍZIO	CHC da Universidade Federal do Paraná - PR
233	ATIVIDADE EXTERNA ESPII - RODÍZIO	CHC da Universidade Federal do Paraná - PR
233	ATIVIDADE EXTERNA ESPII - RODÍZIO	CHC da Universidade Federal do Paraná - PR
233	ATIVIDADE EXTERNA ESPII - RODÍZIO	CHC da Universidade Federal do Paraná - PR
233	ATIVIDADE EXTERNA ESPII - RODÍZIO	CHC da Universidade Federal do Paraná - PR
233	ATIVIDADE EXTERNA ESPII - RODÍZIO	CHC da Universidade Federal do Paraná - PR
233	ATIVIDADE EXTERNA ESPII - RODÍZIO	CHC da Universidade Federal do Paraná - PR
233	ATIVIDADE EXTERNA ESPII - RODÍZIO	CHC da Universidade Federal do Paraná - PR
233	ATIVIDADE EXTERNA ESPII - RODÍZIO	CHC da Universidade Federal do Paraná - PR
233	ATIVIDADE EXTERNA ESPII - RODÍZIO	CHC da Universidade Federal do Paraná - PR
233	ATIVIDADE EXTERNA ESPII - RODÍZIO	CHC da Universidade Federal do Paraná - PR
233	ATIVIDADE EXTERNA ESPII - RODÍZIO	CHC da Universidade Federal do Paraná - PR
233	ATIVIDADE EXTERNA ESPII - RODÍZIO	CHC da Universidade Federal do Paraná - PR
233	ATIVIDADE EXTERNA ESPII - RODÍZIO	CHC da Universidade Federal do Paraná - PR
233	ATIVIDADE EXTERNA ESPII - RODÍZIO	CHC da Universidade Federal do Paraná - PR
233	ATIVIDADE EXTERNA ESPII - RODÍZIO	CHC da Universidade Federal do Paraná - PR
233	ATIVIDADE EXTERNA ESPII - RODÍZIO	CHC da Universidade Federal do Paraná - PR
233	ATIVIDADE EXTERNA ESPII - RODÍZIO	CHC da Universidade Federal do Paraná - PR
233	ATIVIDADE EXTERNA ESPII - RODÍZIO	CHC da Universidade Federal do Paraná - PR
233	ATIVIDADE EXTERNA ESPII - RODÍZIO	CHC da Universidade Federal do Paraná - PR
233	ATIVIDADE EXTERNA ESPII - RODÍZIO	CHC da Universidade Federal do Paraná - PR
233	ATIVIDADE EXTERNA ESPII - RODÍZIO	CHC da Universidade Federal do Paraná - PR
233	ATIVIDADE EXTERNA ESPII - RODÍZIO	CHC da Universidade Federal do Paraná - PR
233	ATIVIDADE EXTERNA ESPII - RODÍZIO	CHC da Universidade Federal do Paraná - PR
233	ATIVIDADE EXTERNA ESPII - RODÍZIO	CHC da Universidade Federal do Paraná - PR
233	ATIVIDADE EXTERNA ESPII - RODÍZIO	CHC da Universidade Federal do Paraná - PR
233	ATIVIDADE EXTERNA ESPII - RODÍZIO	CHC da Universidade Federal do Paraná - PR
233	ATIVIDADE EXTERNA ESPII - RODÍZIO	CHC da Universidade Federal do Paraná - PR
233	ATIVIDADE EXTERNA ESPII - RODÍZIO	CHC da Universidade Federal do Paraná - PR
233	ATIVIDADE EXTERNA ESPII - RODÍZIO	CHC da Universidade Federal do Paraná - PR

233	ATIVIDADE EXTERNA ESPII - RODÍZIO	CHC da Universidade Federal do Paraná - PR
233	ATIVIDADE EXTERNA ESPII - RODÍZIO	CHC da Universidade Federal do Paraná - PR
233	ATIVIDADE EXTERNA ESPII - RODÍZIO	CHC da Universidade Federal do Paraná - PR
233	ATIVIDADE EXTERNA ESPII - RODÍZIO	CHC da Universidade Federal do Paraná - PR
233	ATIVIDADE EXTERNA ESPII - RODÍZIO	CHC da Universidade Federal do Paraná - PR
233	ATIVIDADE EXTERNA ESPII - RODÍZIO	CHC da Universidade Federal do Paraná - PR
233	ATIVIDADE EXTERNA ESPII - RODÍZIO	CHC da Universidade Federal do Paraná - PR
233	ATIVIDADE EXTERNA ESPII - RODÍZIO	CHC da Universidade Federal do Paraná - PR
233	ATIVIDADE EXTERNA ESPII - RODÍZIO	CHC da Universidade Federal do Paraná - PR
233	ATIVIDADE EXTERNA ESPII - RODÍZIO	CHC da Universidade Federal do Paraná - PR
233	ATIVIDADE EXTERNA ESPII - RODÍZIO	CHC da Universidade Federal do Paraná - PR
233	ATIVIDADE EXTERNA ESPII - RODÍZIO	CHC da Universidade Federal do Paraná - PR
233	ATIVIDADE EXTERNA ESPII - RODÍZIO	CHC da Universidade Federal do Paraná - PR
233	ATIVIDADE EXTERNA ESPII - RODÍZIO	CHC da Universidade Federal do Paraná - PR
233	ATIVIDADE EXTERNA ESPII - RODÍZIO	CHC da Universidade Federal do Paraná - PR
233	ATIVIDADE EXTERNA ESPII - RODÍZIO	CHC da Universidade Federal do Paraná - PR
233	ATIVIDADE EXTERNA ESPII - RODÍZIO	CHC da Universidade Federal do Paraná - PR
233	ATIVIDADE EXTERNA ESPII - RODÍZIO	CHC da Universidade Federal do Paraná - PR
233	ATIVIDADE EXTERNA ESPII - RODÍZIO	CHC da Universidade Federal do Paraná - PR
233	ATIVIDADE EXTERNA ESPII - RODÍZIO	CHC da Universidade Federal do Paraná - PR
233	ATIVIDADE EXTERNA ESPII - RODÍZIO	CHC da Universidade Federal do Paraná - PR
233	ATIVIDADE EXTERNA ESPII - RODÍZIO	CHC da Universidade Federal do Paraná - PR
233	ATIVIDADE EXTERNA ESPII - RODÍZIO	CHC da Universidade Federal do Paraná - PR
233	ATIVIDADE EXTERNA ESPII - RODÍZIO	CHC da Universidade Federal do Paraná - PR
233	ATIVIDADE EXTERNA ESPII - RODÍZIO	CHC da Universidade Federal do Paraná - PR
233	ATIVIDADE EXTERNA ESPII - RODÍZIO	CHC da Universidade Federal do Paraná - PR
233	ATIVIDADE EXTERNA ESPII - RODÍZIO	CHC da Universidade Federal do Paraná - PR
233	ATIVIDADE EXTERNA ESPII - RODÍZIO	CHC da Universidade Federal do Paraná - PR
233	ATIVIDADE EXTERNA ESPII - RODÍZIO	CHC da Universidade Federal do Paraná - PR
233	ATIVIDADE EXTERNA ESPII - RODÍZIO	CHC da Universidade Federal do Paraná - PR
233	ATIVIDADE EXTERNA ESPII - RODÍZIO	CHC da Universidade Federal do Paraná - PR
233	ATIVIDADE EXTERNA ESPII - RODÍZIO	CHC da Universidade Federal do Paraná - PR
233	ATIVIDADE EXTERNA ESPII - RODÍZIO	CHC da Universidade Federal do Paraná - PR
233	ATIVIDADE EXTERNA ESPII - RODÍZIO	CHC da Universidade Federal do Paraná - PR
233	ATIVIDADE EXTERNA ESPII - RODÍZIO	CHC da Universidade Federal do Paraná - PR
233	ATIVIDADE EXTERNA ESPII - RODÍZIO	CHC da Universidade Federal do Paraná - PR
233	ATIVIDADE EXTERNA ESPII - RODÍZIO	CHC da Universidade Federal do Paraná - PR
233	ATIVIDADE EXTERNA ESPII - RODÍZIO	CHC da Universidade Federal do Paraná - PR
233	ATIVIDADE EXTERNA ESPII - RODÍZIO	CHC da Universidade Federal do Paraná - PR
233	ATIVIDADE EXTERNA ESPII - RODÍZIO	CHC da Universidade Federal do Paraná - PR
233	ATIVIDADE EXTERNA ESPII - RODÍZIO	CHC da Universidade Federal do Paraná - PR
233	ATIVIDADE EXTERNA ESPII - RODÍZIO	CHC da Universidade Federal do Paraná - PR
233	ATIVIDADE EXTERNA ESPII - RODÍZIO	CHC da Universidade Federal do Paraná - PR
233	ATIVIDADE EXTERNA ESPII - RODÍZIO	CHC da Universidade Federal do Paraná - PR
233	ATIVIDADE EXTERNA ESPII - RODÍZIO	CHC da Universidade Federal do Paraná - PR
233	ATIVIDADE EXTERNA ESPII - RODÍZIO	CHC da Universidade Federal do Paraná - PR
233	ATIVIDADE EXTERNA ESPII - RODÍZIO	CHC da Universidade Federal do Paraná - PR
233	ATIVIDADE EXTERNA ESPII - RODÍZIO	CHC da Universidade Federal do Paraná - PR

233	ATIVIDADE EXTERNA ESPII - RODÍZIO	CHC da Universidade Federal do Paraná - PR
233	ATIVIDADE EXTERNA ESPII - RODÍZIO	CHC da Universidade Federal do Paraná - PR
233	ATIVIDADE EXTERNA ESPII - RODÍZIO	CHC da Universidade Federal do Paraná - PR
233	ATIVIDADE EXTERNA ESPII - RODÍZIO	CHC da Universidade Federal do Paraná - PR
233	ATIVIDADE EXTERNA ESPII - RODÍZIO	CHC da Universidade Federal do Paraná - PR
233	ATIVIDADE EXTERNA ESPII - RODÍZIO	CHC da Universidade Federal do Paraná - PR
233	ATIVIDADE EXTERNA ESPII - RODÍZIO	CHC da Universidade Federal do Paraná - PR
233	ATIVIDADE EXTERNA ESPII - RODÍZIO	CHC da Universidade Federal do Paraná - PR
233	ATIVIDADE EXTERNA ESPII - RODÍZIO	CHC da Universidade Federal do Paraná - PR
233	ATIVIDADE EXTERNA ESPII - RODÍZIO	CHC da Universidade Federal do Paraná - PR
233	ATIVIDADE EXTERNA ESPII - RODÍZIO	CHC da Universidade Federal do Paraná - PR
233	ATIVIDADE EXTERNA ESPII - RODÍZIO	CHC da Universidade Federal do Paraná - PR
233	ATIVIDADE EXTERNA ESPII - RODÍZIO	CHC da Universidade Federal do Paraná - PR
233	ATIVIDADE EXTERNA ESPII - RODÍZIO	CHC da Universidade Federal do Paraná - PR
233	ATIVIDADE EXTERNA ESPII - RODÍZIO	CHC da Universidade Federal do Paraná - PR
233	ATIVIDADE EXTERNA ESPII - RODÍZIO	CHC da Universidade Federal do Paraná - PR
233	ATIVIDADE EXTERNA ESPII - RODÍZIO	CHC da Universidade Federal do Paraná - PR
233	ATIVIDADE EXTERNA ESPII - RODÍZIO	CHC da Universidade Federal do Paraná - PR
233	ATIVIDADE EXTERNA ESPII - RODÍZIO	CHC da Universidade Federal do Paraná - PR
233	ATIVIDADE EXTERNA ESPII - RODÍZIO	CHC da Universidade Federal do Paraná - PR
233	ATIVIDADE EXTERNA ESPII - RODÍZIO	CHC da Universidade Federal do Paraná - PR
233	ATIVIDADE EXTERNA ESPII - RODÍZIO	CHC da Universidade Federal do Paraná - PR
233	ATIVIDADE EXTERNA ESPII - RODÍZIO	CHC da Universidade Federal do Paraná - PR
233	ATIVIDADE EXTERNA ESPII - RODÍZIO	CHC da Universidade Federal do Paraná - PR
233	ATIVIDADE EXTERNA ESPII - RODÍZIO	CHC da Universidade Federal do Paraná - PR
233	ATIVIDADE EXTERNA ESPII - RODÍZIO	CHC da Universidade Federal do Paraná - PR
233	ATIVIDADE EXTERNA ESPII - RODÍZIO	CHC da Universidade Federal do Paraná - PR
233	ATIVIDADE EXTERNA ESPII - RODÍZIO	CHC da Universidade Federal do Paraná - PR
233	ATIVIDADE EXTERNA ESPII - RODÍZIO	CHC da Universidade Federal do Paraná - PR
233	ATIVIDADE EXTERNA ESPII - RODÍZIO	CHC da Universidade Federal do Paraná - PR
233	ATIVIDADE EXTERNA ESPII - RODÍZIO	CHC da Universidade Federal do Paraná - PR
233	ATIVIDADE EXTERNA ESPII - RODÍZIO	CHC da Universidade Federal do Paraná - PR
233	ATIVIDADE EXTERNA ESPII - RODÍZIO	CHC da Universidade Federal do Paraná - PR
233	ATIVIDADE EXTERNA ESPII - RODÍZIO	CHC da Universidade Federal do Paraná - PR
233	ATIVIDADE EXTERNA ESPII - RODÍZIO	CHC da Universidade Federal do Paraná - PR
233	ATIVIDADE EXTERNA ESPII - RODÍZIO	CHC da Universidade Federal do Paraná - PR
233	ATIVIDADE EXTERNA ESPII - RODÍZIO	CHC da Universidade Federal do Paraná - PR
233	ATIVIDADE EXTERNA ESPII - RODÍZIO	CHC da Universidade Federal do Paraná - PR
233	ATIVIDADE EXTERNA ESPII - RODÍZIO	CHC da Universidade Federal do Paraná - PR
233	ATIVIDADE EXTERNA ESPII - RODÍZIO	CHC da Universidade Federal do Paraná - PR
233	ATIVIDADE EXTERNA ESPII - RODÍZIO	CHC da Universidade Federal do Paraná - PR
233	ATIVIDADE EXTERNA ESPII - RODÍZIO	CHC da Universidade Federal do Paraná - PR
233	ATIVIDADE EXTERNA ESPII - RODÍZIO	CHC da Universidade Federal do Paraná - PR
233	ATIVIDADE EXTERNA ESPII - RODÍZIO	CHC da Universidade Federal do Paraná - PR
233	ATIVIDADE EXTERNA ESPII - RODÍZIO	CHC da Universidade Federal do Paraná - PR
233	ATIVIDADE EXTERNA ESPII - RODÍZIO	CHC da Universidade Federal do Paraná - PR
233	ATIVIDADE EXTERNA ESPII - RODÍZIO	CHC da Universidade Federal do Paraná - PR
233	ATIVIDADE EXTERNA ESPII - RODÍZIO	CHC da Universidade Federal do Paraná - PR

233	ATIVIDADE EXTERNA ESPII - RODÍZIO	CHC da Universidade Federal do Paraná - PR
233	ATIVIDADE EXTERNA ESPII - RODÍZIO	CHC da Universidade Federal do Paraná - PR
233	ATIVIDADE EXTERNA ESPII - RODÍZIO	CHC da Universidade Federal do Paraná - PR
233	ATIVIDADE EXTERNA ESPII - RODÍZIO	CHC da Universidade Federal do Paraná - PR
233	ATIVIDADE EXTERNA ESPII - RODÍZIO	CHC da Universidade Federal do Paraná - PR
233	ATIVIDADE EXTERNA ESPII - RODÍZIO	CHC da Universidade Federal do Paraná - PR
233	ATIVIDADE EXTERNA ESPII - RODÍZIO	CHC da Universidade Federal do Paraná - PR
233	ATIVIDADE EXTERNA ESPII - RODÍZIO	CHC da Universidade Federal do Paraná - PR
233	ATIVIDADE EXTERNA ESPII - RODÍZIO	CHC da Universidade Federal do Paraná - PR
233	ATIVIDADE EXTERNA ESPII - RODÍZIO	CHC da Universidade Federal do Paraná - PR
233	ATIVIDADE EXTERNA ESPII - RODÍZIO	CHC da Universidade Federal do Paraná - PR
233	ATIVIDADE EXTERNA ESPII - RODÍZIO	CHC da Universidade Federal do Paraná - PR
233	ATIVIDADE EXTERNA ESPII - RODÍZIO	CHC da Universidade Federal do Paraná - PR
233	ATIVIDADE EXTERNA ESPII - RODÍZIO	CHC da Universidade Federal do Paraná - PR
233	ATIVIDADE EXTERNA ESPII - RODÍZIO	CHC da Universidade Federal do Paraná - PR
233	ATIVIDADE EXTERNA ESPII - RODÍZIO	CHC da Universidade Federal do Paraná - PR
233	ATIVIDADE EXTERNA ESPII - RODÍZIO	CHC da Universidade Federal do Paraná - PR
233	ATIVIDADE EXTERNA ESPII - RODÍZIO	CHC da Universidade Federal do Paraná - PR
233	ATIVIDADE EXTERNA ESPII - RODÍZIO	CHC da Universidade Federal do Paraná - PR
233	ATIVIDADE EXTERNA ESPII - RODÍZIO	CHC da Universidade Federal do Paraná - PR
233	ATIVIDADE EXTERNA ESPII - RODÍZIO	CHC da Universidade Federal do Paraná - PR
233	ATIVIDADE EXTERNA ESPII - RODÍZIO	CHC da Universidade Federal do Paraná - PR
233	ATIVIDADE EXTERNA ESPII - RODÍZIO	CHC da Universidade Federal do Paraná - PR
233	ATIVIDADE EXTERNA ESPII - RODÍZIO	CHC da Universidade Federal do Paraná - PR
233	ATIVIDADE EXTERNA ESPII - RODÍZIO	CHC da Universidade Federal do Paraná - PR
233	ATIVIDADE EXTERNA ESPII - RODÍZIO	CHC da Universidade Federal do Paraná - PR
233	ATIVIDADE EXTERNA ESPII - RODÍZIO	CHC da Universidade Federal do Paraná - PR
233	ATIVIDADE EXTERNA ESPII - RODÍZIO	CHC da Universidade Federal do Paraná - PR
233	ATIVIDADE EXTERNA ESPII - RODÍZIO	CHC da Universidade Federal do Paraná - PR
233	ATIVIDADE EXTERNA ESPII - RODÍZIO	CHC da Universidade Federal do Paraná - PR
233	ATIVIDADE EXTERNA ESPII - RODÍZIO	CHC da Universidade Federal do Paraná - PR
233	ATIVIDADE EXTERNA ESPII - RODÍZIO	CHC da Universidade Federal do Paraná - PR
233	ATIVIDADE EXTERNA ESPII - RODÍZIO	CHC da Universidade Federal do Paraná - PR
233	ATIVIDADE EXTERNA ESPII - RODÍZIO	CHC da Universidade Federal do Paraná - PR
233	ATIVIDADE EXTERNA ESPII - RODÍZIO	CHC da Universidade Federal do Paraná - PR
233	ATIVIDADE EXTERNA ESPII - RODÍZIO	CHC da Universidade Federal do Paraná - PR
233	ATIVIDADE EXTERNA ESPII - RODÍZIO	CHC da Universidade Federal do Paraná - PR
233	ATIVIDADE EXTERNA ESPII - RODÍZIO	CHC da Universidade Federal do Paraná - PR
233	ATIVIDADE EXTERNA ESPII - RODÍZIO	CHC da Universidade Federal do Paraná - PR
233	ATIVIDADE EXTERNA ESPII - RODÍZIO	CHC da Universidade Federal do Paraná - PR
233	ATIVIDADE EXTERNA ESPII - RODÍZIO	CHC da Universidade Federal do Paraná - PR
233	ATIVIDADE EXTERNA ESPII - RODÍZIO	CHC da Universidade Federal do Paraná - PR
233	ATIVIDADE EXTERNA ESPII - RODÍZIO	CHC da Universidade Federal do Paraná - PR
233	ATIVIDADE EXTERNA ESPII - RODÍZIO	CHC da Universidade Federal do Paraná - PR
233	ATIVIDADE EXTERNA ESPII - RODÍZIO	CHC da Universidade Federal do Paraná - PR
233	ATIVIDADE EXTERNA ESPII - RODÍZIO	CHC da Universidade Federal do Paraná - PR
233	ATIVIDADE EXTERNA ESPII - RODÍZIO	CHC da Universidade Federal do Paraná - PR
233	ATIVIDADE EXTERNA ESPII - RODÍZIO	CHC da Universidade Federal do Paraná - PR

233	ATIVIDADE EXTERNA ESPII - RODÍZIO	CHC da Universidade Federal do Paraná - PR
233	ATIVIDADE EXTERNA ESPII - RODÍZIO	CHC da Universidade Federal do Paraná - PR
233	ATIVIDADE EXTERNA ESPII - RODÍZIO	CHC da Universidade Federal do Paraná - PR
233	ATIVIDADE EXTERNA ESPII - RODÍZIO	CHC da Universidade Federal do Paraná - PR
233	ATIVIDADE EXTERNA ESPII - RODÍZIO	CHC da Universidade Federal do Paraná - PR
233	ATIVIDADE EXTERNA ESPII - RODÍZIO	CHC da Universidade Federal do Paraná - PR
233	ATIVIDADE EXTERNA ESPII - RODÍZIO	CHC da Universidade Federal do Paraná - PR
233	ATIVIDADE EXTERNA ESPII - RODÍZIO	CHC da Universidade Federal do Paraná - PR
234	Participação em atividades de representação sindical	CHC da Universidade Federal do Paraná - PR
235	Afastamento Portaria n 454 art 3	CHC da Universidade Federal do Paraná - PR
235	Afastamento Portaria n 454 art 3	CHC da Universidade Federal do Paraná - PR
235	Afastamento Portaria n 454 art 3	CHC da Universidade Federal do Paraná - PR
235	Afastamento Portaria n 454 art 3	CHC da Universidade Federal do Paraná - PR
235	Afastamento Portaria n 454 art 3	CHC da Universidade Federal do Paraná - PR
235	Afastamento Portaria n 454 art 3	CHC da Universidade Federal do Paraná - PR
235	Afastamento Portaria n 454 art 3	CHC da Universidade Federal do Paraná - PR
235	Afastamento Portaria n 454 art 3	CHC da Universidade Federal do Paraná - PR
235	Afastamento Portaria n 454 art 3	CHC da Universidade Federal do Paraná - PR
235	Afastamento Portaria n 454 art 3	CHC da Universidade Federal do Paraná - PR
235	Afastamento Portaria n 454 art 3	CHC da Universidade Federal do Paraná - PR
235	Afastamento Portaria n 454 art 3	CHC da Universidade Federal do Paraná - PR
235	Afastamento Portaria n 454 art 3	CHC da Universidade Federal do Paraná - PR
235	Afastamento Portaria n 454 art 3	CHC da Universidade Federal do Paraná - PR
235	Afastamento Portaria n 454 art 3	CHC da Universidade Federal do Paraná - PR
235	Afastamento Portaria n 454 art 3	CHC da Universidade Federal do Paraná - PR
235	Afastamento Portaria n 454 art 3	CHC da Universidade Federal do Paraná - PR
235	Afastamento Portaria n 454 art 3	CHC da Universidade Federal do Paraná - PR
235	Afastamento Portaria n 454 art 3	CHC da Universidade Federal do Paraná - PR
235	Afastamento Portaria n 454 art 3	CHC da Universidade Federal do Paraná - PR
235	Afastamento Portaria n 454 art 3	CHC da Universidade Federal do Paraná - PR
235	Afastamento Portaria n 454 art 3	CHC da Universidade Federal do Paraná - PR
235	Afastamento Portaria n 454 art 3	CHC da Universidade Federal do Paraná - PR
235	Afastamento Portaria n 454 art 3	CHC da Universidade Federal do Paraná - PR
235	Afastamento Portaria n 454 art 3	CHC da Universidade Federal do Paraná - PR
235	Afastamento Portaria n 454 art 3	CHC da Universidade Federal do Paraná - PR
235	Afastamento Portaria n 454 art 3	CHC da Universidade Federal do Paraná - PR
235	Afastamento Portaria n 454 art 3	CHC da Universidade Federal do Paraná - PR
235	Afastamento Portaria n 454 art 3	CHC da Universidade Federal do Paraná - PR
235	Afastamento Portaria n 454 art 3	CHC da Universidade Federal do Paraná - PR
235	Afastamento Portaria n 454 art 3	CHC da Universidade Federal do Paraná - PR
235	Afastamento Portaria n 454 art 3	CHC da Universidade Federal do Paraná - PR
235	Afastamento Portaria n 454 art 3	CHC da Universidade Federal do Paraná - PR
235	Afastamento Portaria n 454 art 3	CHC da Universidade Federal do Paraná - PR
235	Afastamento Portaria n 454 art 3	CHC da Universidade Federal do Paraná - PR
235	Afastamento Portaria n 454 art 3	CHC da Universidade Federal do Paraná - PR
235	Afastamento Portaria n 454 art 3	CHC da Universidade Federal do Paraná - PR
235	Afastamento Portaria n 454 art 3	CHC da Universidade Federal do Paraná - PR

235	Afastamento Portaria n 454 art 3	CHC da Universidade Federal do Paraná - PR
235	Afastamento Portaria n 454 art 3	CHC da Universidade Federal do Paraná - PR
235	Afastamento Portaria n 454 art 3	CHC da Universidade Federal do Paraná - PR
235	Afastamento Portaria n 454 art 3	CHC da Universidade Federal do Paraná - PR
235	Afastamento Portaria n 454 art 3	CHC da Universidade Federal do Paraná - PR
235	Afastamento Portaria n 454 art 3	CHC da Universidade Federal do Paraná - PR
235	Afastamento Portaria n 454 art 3	CHC da Universidade Federal do Paraná - PR
235	Afastamento Portaria n 454 art 3	CHC da Universidade Federal do Paraná - PR
235	Afastamento Portaria n 454 art 3	CHC da Universidade Federal do Paraná - PR
235	Afastamento Portaria n 454 art 3	CHC da Universidade Federal do Paraná - PR
235	Afastamento Portaria n 454 art 3	CHC da Universidade Federal do Paraná - PR
235	Afastamento Portaria n 454 art 3	CHC da Universidade Federal do Paraná - PR
235	Afastamento Portaria n 454 art 3	CHC da Universidade Federal do Paraná - PR
235	Afastamento Portaria n 454 art 3	CHC da Universidade Federal do Paraná - PR
235	Afastamento Portaria n 454 art 3	CHC da Universidade Federal do Paraná - PR
235	Afastamento Portaria n 454 art 3	CHC da Universidade Federal do Paraná - PR
235	Afastamento Portaria n 454 art 3	CHC da Universidade Federal do Paraná - PR
235	Afastamento Portaria n 454 art 3	CHC da Universidade Federal do Paraná - PR
235	Afastamento Portaria n 454 art 3	CHC da Universidade Federal do Paraná - PR
235	Afastamento Portaria n 454 art 3	CHC da Universidade Federal do Paraná - PR
235	Afastamento Portaria n 454 art 3	CHC da Universidade Federal do Paraná - PR
235	Afastamento Portaria n 454 art 3	CHC da Universidade Federal do Paraná - PR
235	Afastamento Portaria n 454 art 3	CHC da Universidade Federal do Paraná - PR
235	Afastamento Portaria n 454 art 3	CHC da Universidade Federal do Paraná - PR
235	Afastamento Portaria n 454 art 3	CHC da Universidade Federal do Paraná - PR
235	Afastamento Portaria n 454 art 3	CHC da Universidade Federal do Paraná - PR
235	Afastamento Portaria n 454 art 3	CHC da Universidade Federal do Paraná - PR
235	Afastamento Portaria n 454 art 3	CHC da Universidade Federal do Paraná - PR
235	Afastamento Portaria n 454 art 3	CHC da Universidade Federal do Paraná - PR
235	Afastamento Portaria n 454 art 3	CHC da Universidade Federal do Paraná - PR
235	Afastamento Portaria n 454 art 3	CHC da Universidade Federal do Paraná - PR
235	Afastamento Portaria n 454 art 3	CHC da Universidade Federal do Paraná - PR
235	Afastamento Portaria n 454 art 3	CHC da Universidade Federal do Paraná - PR
235	Afastamento Portaria n 454 art 3	CHC da Universidade Federal do Paraná - PR
235	Afastamento Portaria n 454 art 3	CHC da Universidade Federal do Paraná - PR
235	Afastamento Portaria n 454 art 3	CHC da Universidade Federal do Paraná - PR
235	Afastamento Portaria n 454 art 3	CHC da Universidade Federal do Paraná - PR
235	Afastamento Portaria n 454 art 3	CHC da Universidade Federal do Paraná - PR
235	Afastamento Portaria n 454 art 3	CHC da Universidade Federal do Paraná - PR
235	Afastamento Portaria n 454 art 3	CHC da Universidade Federal do Paraná - PR
235	Afastamento Portaria n 454 art 3	CHC da Universidade Federal do Paraná - PR
235	Afastamento Portaria n 454 art 3	CHC da Universidade Federal do Paraná - PR
235	Afastamento Portaria n 454 art 3	CHC da Universidade Federal do Paraná - PR
235	Afastamento Portaria n 454 art 3	CHC da Universidade Federal do Paraná - PR
235	Afastamento Portaria n 454 art 3	CHC da Universidade Federal do Paraná - PR
235	Afastamento Portaria n 454 art 3	CHC da Universidade Federal do Paraná - PR
235	Afastamento Portaria n 454 art 3	CHC da Universidade Federal do Paraná - PR
235	Afastamento Portaria n 454 art 3	CHC da Universidade Federal do Paraná - PR

235	Afastamento Portaria n 454 art 3	CHC da Universidade Federal do Paraná - PR
235	Afastamento Portaria n 454 art 3	CHC da Universidade Federal do Paraná - PR
235	Afastamento Portaria n 454 art 3	CHC da Universidade Federal do Paraná - PR
235	Afastamento Portaria n 454 art 3	CHC da Universidade Federal do Paraná - PR
235	Afastamento Portaria n 454 art 3	CHC da Universidade Federal do Paraná - PR
235	Afastamento Portaria n 454 art 3	CHC da Universidade Federal do Paraná - PR
235	Afastamento Portaria n 454 art 3	CHC da Universidade Federal do Paraná - PR
235	Afastamento Portaria n 454 art 3	CHC da Universidade Federal do Paraná - PR
235	Afastamento Portaria n 454 art 3	CHC da Universidade Federal do Paraná - PR
235	Afastamento Portaria n 454 art 3	CHC da Universidade Federal do Paraná - PR
235	Afastamento Portaria n 454 art 3	CHC da Universidade Federal do Paraná - PR
235	Afastamento Portaria n 454 art 3	CHC da Universidade Federal do Paraná - PR
235	Afastamento Portaria n 454 art 3	CHC da Universidade Federal do Paraná - PR
235	Afastamento Portaria n 454 art 3	CHC da Universidade Federal do Paraná - PR
235	Afastamento Portaria n 454 art 3	CHC da Universidade Federal do Paraná - PR
235	Afastamento Portaria n 454 art 3	CHC da Universidade Federal do Paraná - PR
235	Afastamento Portaria n 454 art 3	CHC da Universidade Federal do Paraná - PR
235	Afastamento Portaria n 454 art 3	CHC da Universidade Federal do Paraná - PR
235	Afastamento Portaria n 454 art 3	CHC da Universidade Federal do Paraná - PR
235	Afastamento Portaria n 454 art 3	CHC da Universidade Federal do Paraná - PR
235	Afastamento Portaria n 454 art 3	CHC da Universidade Federal do Paraná - PR
235	Afastamento Portaria n 454 art 3	CHC da Universidade Federal do Paraná - PR
235	Afastamento Portaria n 454 art 3	CHC da Universidade Federal do Paraná - PR
235	Afastamento Portaria n 454 art 3	CHC da Universidade Federal do Paraná - PR
235	Afastamento Portaria n 454 art 3	CHC da Universidade Federal do Paraná - PR
235	Afastamento Portaria n 454 art 3	CHC da Universidade Federal do Paraná - PR
235	Afastamento Portaria n 454 art 3	CHC da Universidade Federal do Paraná - PR
235	Afastamento Portaria n 454 art 3	CHC da Universidade Federal do Paraná - PR
235	Afastamento Portaria n 454 art 3	CHC da Universidade Federal do Paraná - PR
235	Afastamento Portaria n 454 art 3	CHC da Universidade Federal do Paraná - PR
235	Afastamento Portaria n 454 art 3	CHC da Universidade Federal do Paraná - PR
235	Afastamento Portaria n 454 art 3	CHC da Universidade Federal do Paraná - PR
235	Afastamento Portaria n 454 art 3	CHC da Universidade Federal do Paraná - PR
235	Afastamento Portaria n 454 art 3	CHC da Universidade Federal do Paraná - PR
235	Afastamento Portaria n 454 art 3	CHC da Universidade Federal do Paraná - PR
235	Afastamento Portaria n 454 art 3	CHC da Universidade Federal do Paraná - PR
235	Afastamento Portaria n 454 art 3	CHC da Universidade Federal do Paraná - PR
235	Afastamento Portaria n 454 art 3	CHC da Universidade Federal do Paraná - PR
235	Afastamento Portaria n 454 art 3	CHC da Universidade Federal do Paraná - PR
235	Afastamento Portaria n 454 art 3	CHC da Universidade Federal do Paraná - PR
235	Afastamento Portaria n 454 art 3	CHC da Universidade Federal do Paraná - PR
235	Afastamento Portaria n 454 art 3	CHC da Universidade Federal do Paraná - PR
235	Afastamento Portaria n 454 art 3	CHC da Universidade Federal do Paraná - PR
235	Afastamento Portaria n 454 art 3	CHC da Universidade Federal do Paraná - PR
235	Afastamento Portaria n 454 art 3	CHC da Universidade Federal do Paraná - PR
235	Afastamento Portaria n 454 art 3	CHC da Universidade Federal do Paraná - PR
235	Afastamento Portaria n 454 art 3	CHC da Universidade Federal do Paraná - PR

235	Afastamento Portaria n 454 art 3	CHC da Universidade Federal do Paraná - PR
235	Afastamento Portaria n 454 art 3	CHC da Universidade Federal do Paraná - PR
235	Afastamento Portaria n 454 art 3	CHC da Universidade Federal do Paraná - PR
235	Afastamento Portaria n 454 art 3	CHC da Universidade Federal do Paraná - PR
235	Afastamento Portaria n 454 art 3	CHC da Universidade Federal do Paraná - PR
235	Afastamento Portaria n 454 art 3	CHC da Universidade Federal do Paraná - PR
235	Afastamento Portaria n 454 art 3	CHC da Universidade Federal do Paraná - PR
235	Afastamento Portaria n 454 art 3	CHC da Universidade Federal do Paraná - PR
235	Afastamento Portaria n 454 art 3	CHC da Universidade Federal do Paraná - PR
235	Afastamento Portaria n 454 art 3	CHC da Universidade Federal do Paraná - PR
235	Afastamento Portaria n 454 art 3	CHC da Universidade Federal do Paraná - PR
235	Afastamento Portaria n 454 art 3	CHC da Universidade Federal do Paraná - PR
235	Afastamento Portaria n 454 art 3	CHC da Universidade Federal do Paraná - PR
235	Afastamento Portaria n 454 art 3	CHC da Universidade Federal do Paraná - PR
235	Afastamento Portaria n 454 art 3	CHC da Universidade Federal do Paraná - PR
235	Afastamento Portaria n 454 art 3	CHC da Universidade Federal do Paraná - PR
235	Afastamento Portaria n 454 art 3	CHC da Universidade Federal do Paraná - PR
235	Afastamento Portaria n 454 art 3	CHC da Universidade Federal do Paraná - PR
235	Afastamento Portaria n 454 art 3	CHC da Universidade Federal do Paraná - PR
235	Afastamento Portaria n 454 art 3	CHC da Universidade Federal do Paraná - PR
235	Afastamento Portaria n 454 art 3	CHC da Universidade Federal do Paraná - PR
235	Afastamento Portaria n 454 art 3	CHC da Universidade Federal do Paraná - PR
235	Afastamento Portaria n 454 art 3	CHC da Universidade Federal do Paraná - PR
235	Afastamento Portaria n 454 art 3	CHC da Universidade Federal do Paraná - PR
235	Afastamento Portaria n 454 art 3	CHC da Universidade Federal do Paraná - PR
235	Afastamento Portaria n 454 art 3	CHC da Universidade Federal do Paraná - PR
235	Afastamento Portaria n 454 art 3	CHC da Universidade Federal do Paraná - PR
235	Afastamento Portaria n 454 art 3	CHC da Universidade Federal do Paraná - PR
236	Afastamento Portaria n 454 art. 2 Único	CHC da Universidade Federal do Paraná - PR
236	Afastamento Portaria n 454 art. 2 Único	CHC da Universidade Federal do Paraná - PR
236	Afastamento Portaria n 454 art. 2 Único	CHC da Universidade Federal do Paraná - PR
236	Afastamento Portaria n 454 art. 2 Único	CHC da Universidade Federal do Paraná - PR
236	Afastamento Portaria n 454 art. 2 Único	CHC da Universidade Federal do Paraná - PR
236	Afastamento Portaria n 454 art. 2 Único	CHC da Universidade Federal do Paraná - PR
236	Afastamento Portaria n 454 art. 2 Único	CHC da Universidade Federal do Paraná - PR
236	Afastamento Portaria n 454 art. 2 Único	CHC da Universidade Federal do Paraná - PR
236	Afastamento Portaria n 454 art. 2 Único	CHC da Universidade Federal do Paraná - PR
236	Afastamento Portaria n 454 art. 2 Único	CHC da Universidade Federal do Paraná - PR
236	Afastamento Portaria n 454 art. 2 Único	CHC da Universidade Federal do Paraná - PR
236	Afastamento Portaria n 454 art. 2 Único	CHC da Universidade Federal do Paraná - PR
236	Afastamento Portaria n 454 art. 2 Único	CHC da Universidade Federal do Paraná - PR
236	Afastamento Portaria n 454 art. 2 Único	CHC da Universidade Federal do Paraná - PR
236	Afastamento Portaria n 454 art. 2 Único	CHC da Universidade Federal do Paraná - PR
236	Afastamento Portaria n 454 art. 2 Único	CHC da Universidade Federal do Paraná - PR
236	Afastamento Portaria n 454 art. 2 Único	CHC da Universidade Federal do Paraná - PR
236	Afastamento Portaria n 454 art. 2 Único	CHC da Universidade Federal do Paraná - PR
236	Afastamento Portaria n 454 art. 2 Único	CHC da Universidade Federal do Paraná - PR
236	Afastamento Portaria n 454 art. 2 Único	CHC da Universidade Federal do Paraná - PR

236	Afastamento Portaria n 454 art. 2 Único	CHC da Universidade Federal do Paraná - PR
236	Afastamento Portaria n 454 art. 2 Único	CHC da Universidade Federal do Paraná - PR
236	Afastamento Portaria n 454 art. 2 Único	CHC da Universidade Federal do Paraná - PR
236	Afastamento Portaria n 454 art. 2 Único	CHC da Universidade Federal do Paraná - PR
236	Afastamento Portaria n 454 art. 2 Único	CHC da Universidade Federal do Paraná - PR
236	Afastamento Portaria n 454 art. 2 Único	CHC da Universidade Federal do Paraná - PR
236	Afastamento Portaria n 454 art. 2 Único	CHC da Universidade Federal do Paraná - PR
236	Afastamento Portaria n 454 art. 2 Único	CHC da Universidade Federal do Paraná - PR
236	Afastamento Portaria n 454 art. 2 Único	CHC da Universidade Federal do Paraná - PR
236	Afastamento Portaria n 454 art. 2 Único	CHC da Universidade Federal do Paraná - PR
236	Afastamento Portaria n 454 art. 2 Único	CHC da Universidade Federal do Paraná - PR
236	Afastamento Portaria n 454 art. 2 Único	CHC da Universidade Federal do Paraná - PR
236	Afastamento Portaria n 454 art. 2 Único	CHC da Universidade Federal do Paraná - PR
236	Afastamento Portaria n 454 art. 2 Único	CHC da Universidade Federal do Paraná - PR
236	Afastamento Portaria n 454 art. 2 Único	CHC da Universidade Federal do Paraná - PR
236	Afastamento Portaria n 454 art. 2 Único	CHC da Universidade Federal do Paraná - PR
236	Afastamento Portaria n 454 art. 2 Único	CHC da Universidade Federal do Paraná - PR
236	Afastamento Portaria n 454 art. 2 Único	CHC da Universidade Federal do Paraná - PR
236	Afastamento Portaria n 454 art. 2 Único	CHC da Universidade Federal do Paraná - PR
236	Afastamento Portaria n 454 art. 2 Único	CHC da Universidade Federal do Paraná - PR
236	Afastamento Portaria n 454 art. 2 Único	CHC da Universidade Federal do Paraná - PR
236	Afastamento Portaria n 454 art. 2 Único	CHC da Universidade Federal do Paraná - PR
236	Afastamento Portaria n 454 art. 2 Único	CHC da Universidade Federal do Paraná - PR
236	Afastamento Portaria n 454 art. 2 Único	CHC da Universidade Federal do Paraná - PR
236	Afastamento Portaria n 454 art. 2 Único	CHC da Universidade Federal do Paraná - PR
236	Afastamento Portaria n 454 art. 2 Único	CHC da Universidade Federal do Paraná - PR
236	Afastamento Portaria n 454 art. 2 Único	CHC da Universidade Federal do Paraná - PR
236	Afastamento Portaria n 454 art. 2 Único	CHC da Universidade Federal do Paraná - PR
236	Afastamento Portaria n 454 art. 2 Único	CHC da Universidade Federal do Paraná - PR
236	Afastamento Portaria n 454 art. 2 Único	CHC da Universidade Federal do Paraná - PR
236	Afastamento Portaria n 454 art. 2 Único	CHC da Universidade Federal do Paraná - PR
236	Afastamento Portaria n 454 art. 2 Único	CHC da Universidade Federal do Paraná - PR
236	Afastamento Portaria n 454 art. 2 Único	CHC da Universidade Federal do Paraná - PR
236	Afastamento Portaria n 454 art. 2 Único	CHC da Universidade Federal do Paraná - PR
236	Afastamento Portaria n 454 art. 2 Único	CHC da Universidade Federal do Paraná - PR
236	Afastamento Portaria n 454 art. 2 Único	CHC da Universidade Federal do Paraná - PR
236	Afastamento Portaria n 454 art. 2 Único	CHC da Universidade Federal do Paraná - PR
236	Afastamento Portaria n 454 art. 2 Único	CHC da Universidade Federal do Paraná - PR
236	Afastamento Portaria n 454 art. 2 Único	CHC da Universidade Federal do Paraná - PR
236	Afastamento Portaria n 454 art. 2 Único	CHC da Universidade Federal do Paraná - PR
236	Afastamento Portaria n 454 art. 2 Único	CHC da Universidade Federal do Paraná - PR
236	Afastamento Portaria n 454 art. 2 Único	CHC da Universidade Federal do Paraná - PR
236	Afastamento Portaria n 454 art. 2 Único	CHC da Universidade Federal do Paraná - PR
236	Afastamento Portaria n 454 art. 2 Único	CHC da Universidade Federal do Paraná - PR
236	Afastamento Portaria n 454 art. 2 Único	CHC da Universidade Federal do Paraná - PR
236	Afastamento Portaria n 454 art. 2 Único	CHC da Universidade Federal do Paraná - PR
236	Afastamento Portaria n 454 art. 2 Único	CHC da Universidade Federal do Paraná - PR
236	Afastamento Portaria n 454 art. 2 Único	CHC da Universidade Federal do Paraná - PR

236	Afastamento Portaria n 454 art. 2 Único	CHC da Universidade Federal do Paraná - PR
236	Afastamento Portaria n 454 art. 2 Único	CHC da Universidade Federal do Paraná - PR
236	Afastamento Portaria n 454 art. 2 Único	CHC da Universidade Federal do Paraná - PR
236	Afastamento Portaria n 454 art. 2 Único	CHC da Universidade Federal do Paraná - PR
236	Afastamento Portaria n 454 art. 2 Único	CHC da Universidade Federal do Paraná - PR
236	Afastamento Portaria n 454 art. 2 Único	CHC da Universidade Federal do Paraná - PR
236	Afastamento Portaria n 454 art. 2 Único	CHC da Universidade Federal do Paraná - PR
236	Afastamento Portaria n 454 art. 2 Único	CHC da Universidade Federal do Paraná - PR
236	Afastamento Portaria n 454 art. 2 Único	CHC da Universidade Federal do Paraná - PR
236	Afastamento Portaria n 454 art. 2 Único	CHC da Universidade Federal do Paraná - PR
236	Afastamento Portaria n 454 art. 2 Único	CHC da Universidade Federal do Paraná - PR
236	Afastamento Portaria n 454 art. 2 Único	CHC da Universidade Federal do Paraná - PR
236	Afastamento Portaria n 454 art. 2 Único	CHC da Universidade Federal do Paraná - PR
236	Afastamento Portaria n 454 art. 2 Único	CHC da Universidade Federal do Paraná - PR
236	Afastamento Portaria n 454 art. 2 Único	CHC da Universidade Federal do Paraná - PR
236	Afastamento Portaria n 454 art. 2 Único	CHC da Universidade Federal do Paraná - PR
236	Afastamento Portaria n 454 art. 2 Único	CHC da Universidade Federal do Paraná - PR
236	Afastamento Portaria n 454 art. 2 Único	CHC da Universidade Federal do Paraná - PR
236	Afastamento Portaria n 454 art. 2 Único	CHC da Universidade Federal do Paraná - PR
236	Afastamento Portaria n 454 art. 2 Único	CHC da Universidade Federal do Paraná - PR
236	Afastamento Portaria n 454 art. 2 Único	CHC da Universidade Federal do Paraná - PR
236	Afastamento Portaria n 454 art. 2 Único	CHC da Universidade Federal do Paraná - PR
236	Afastamento Portaria n 454 art. 2 Único	CHC da Universidade Federal do Paraná - PR
236	Afastamento Portaria n 454 art. 2 Único	CHC da Universidade Federal do Paraná - PR
236	Afastamento Portaria n 454 art. 2 Único	CHC da Universidade Federal do Paraná - PR
236	Afastamento Portaria n 454 art. 2 Único	CHC da Universidade Federal do Paraná - PR
236	Afastamento Portaria n 454 art. 2 Único	CHC da Universidade Federal do Paraná - PR
236	Afastamento Portaria n 454 art. 2 Único	CHC da Universidade Federal do Paraná - PR
236	Afastamento Portaria n 454 art. 2 Único	CHC da Universidade Federal do Paraná - PR
236	Afastamento Portaria n 454 art. 2 Único	CHC da Universidade Federal do Paraná - PR
236	Afastamento Portaria n 454 art. 2 Único	CHC da Universidade Federal do Paraná - PR
236	Afastamento Portaria n 454 art. 2 Único	CHC da Universidade Federal do Paraná - PR
236	Afastamento Portaria n 454 art. 2 Único	CHC da Universidade Federal do Paraná - PR
236	Afastamento Portaria n 454 art. 2 Único	CHC da Universidade Federal do Paraná - PR
236	Afastamento Portaria n 454 art. 2 Único	CHC da Universidade Federal do Paraná - PR
236	Afastamento Portaria n 454 art. 2 Único	CHC da Universidade Federal do Paraná - PR
236	Afastamento Portaria n 454 art. 2 Único	CHC da Universidade Federal do Paraná - PR
236	Afastamento Portaria n 454 art. 2 Único	CHC da Universidade Federal do Paraná - PR
236	Afastamento Portaria n 454 art. 2 Único	CHC da Universidade Federal do Paraná - PR
236	Afastamento Portaria n 454 art. 2 Único	CHC da Universidade Federal do Paraná - PR
236	Afastamento Portaria n 454 art. 2 Único	CHC da Universidade Federal do Paraná - PR
236	Afastamento Portaria n 454 art. 2 Único	CHC da Universidade Federal do Paraná - PR
236	Afastamento Portaria n 454 art. 2 Único	CHC da Universidade Federal do Paraná - PR
236	Afastamento Portaria n 454 art. 2 Único	CHC da Universidade Federal do Paraná - PR
236	Afastamento Portaria n 454 art. 2 Único	CHC da Universidade Federal do Paraná - PR
236	Afastamento Portaria n 454 art. 2 Único	CHC da Universidade Federal do Paraná - PR
236	Afastamento Portaria n 454 art. 2 Único	CHC da Universidade Federal do Paraná - PR
236	Afastamento Portaria n 454 art. 2 Único	CHC da Universidade Federal do Paraná - PR

236	Afastamento Portaria n 454 art. 2 Único	CHC da Universidade Federal do Paraná - PR
236	Afastamento Portaria n 454 art. 2 Único	CHC da Universidade Federal do Paraná - PR
236	Afastamento Portaria n 454 art. 2 Único	CHC da Universidade Federal do Paraná - PR
236	Afastamento Portaria n 454 art. 2 Único	CHC da Universidade Federal do Paraná - PR
236	Afastamento Portaria n 454 art. 2 Único	CHC da Universidade Federal do Paraná - PR
236	Afastamento Portaria n 454 art. 2 Único	CHC da Universidade Federal do Paraná - PR
236	Afastamento Portaria n 454 art. 2 Único	CHC da Universidade Federal do Paraná - PR
236	Afastamento Portaria n 454 art. 2 Único	CHC da Universidade Federal do Paraná - PR
236	Afastamento Portaria n 454 art. 2 Único	CHC da Universidade Federal do Paraná - PR
236	Afastamento Portaria n 454 art. 2 Único	CHC da Universidade Federal do Paraná - PR
236	Afastamento Portaria n 454 art. 2 Único	CHC da Universidade Federal do Paraná - PR
236	Afastamento Portaria n 454 art. 2 Único	CHC da Universidade Federal do Paraná - PR
236	Afastamento Portaria n 454 art. 2 Único	CHC da Universidade Federal do Paraná - PR
236	Afastamento Portaria n 454 art. 2 Único	CHC da Universidade Federal do Paraná - PR
236	Afastamento Portaria n 454 art. 2 Único	CHC da Universidade Federal do Paraná - PR
236	Afastamento Portaria n 454 art. 2 Único	CHC da Universidade Federal do Paraná - PR
236	Afastamento Portaria n 454 art. 2 Único	CHC da Universidade Federal do Paraná - PR
236	Afastamento Portaria n 454 art. 2 Único	CHC da Universidade Federal do Paraná - PR
236	Afastamento Portaria n 454 art. 2 Único	CHC da Universidade Federal do Paraná - PR
236	Afastamento Portaria n 454 art. 2 Único	CHC da Universidade Federal do Paraná - PR
236	Afastamento Portaria n 454 art. 2 Único	CHC da Universidade Federal do Paraná - PR
236	Afastamento Portaria n 454 art. 2 Único	CHC da Universidade Federal do Paraná - PR
236	Afastamento Portaria n 454 art. 2 Único	CHC da Universidade Federal do Paraná - PR
236	Afastamento Portaria n 454 art. 2 Único	CHC da Universidade Federal do Paraná - PR
236	Afastamento Portaria n 454 art. 2 Único	CHC da Universidade Federal do Paraná - PR
236	Afastamento Portaria n 454 art. 2 Único	CHC da Universidade Federal do Paraná - PR
236	Afastamento Portaria n 454 art. 2 Único	CHC da Universidade Federal do Paraná - PR
236	Afastamento Portaria n 454 art. 2 Único	CHC da Universidade Federal do Paraná - PR
236	Afastamento Portaria n 454 art. 2 Único	CHC da Universidade Federal do Paraná - PR
236	Afastamento Portaria n 454 art. 2 Único	CHC da Universidade Federal do Paraná - PR
236	Afastamento Portaria n 454 art. 2 Único	CHC da Universidade Federal do Paraná - PR
236	Afastamento Portaria n 454 art. 2 Único	CHC da Universidade Federal do Paraná - PR
236	Afastamento Portaria n 454 art. 2 Único	CHC da Universidade Federal do Paraná - PR
236	Afastamento Portaria n 454 art. 2 Único	CHC da Universidade Federal do Paraná - PR
236	Afastamento Portaria n 454 art. 2 Único	CHC da Universidade Federal do Paraná - PR
236	Afastamento Portaria n 454 art. 2 Único	CHC da Universidade Federal do Paraná - PR
236	Afastamento Portaria n 454 art. 2 Único	CHC da Universidade Federal do Paraná - PR
236	Afastamento Portaria n 454 art. 2 Único	CHC da Universidade Federal do Paraná - PR
236	Afastamento Portaria n 454 art. 2 Único	CHC da Universidade Federal do Paraná - PR
236	Afastamento Portaria n 454 art. 2 Único	CHC da Universidade Federal do Paraná - PR
236	Afastamento Portaria n 454 art. 2 Único	CHC da Universidade Federal do Paraná - PR
236	Afastamento Portaria n 454 art. 2 Único	CHC da Universidade Federal do Paraná - PR
236	Afastamento Portaria n 454 art. 2 Único	CHC da Universidade Federal do Paraná - PR
236	Afastamento Portaria n 454 art. 2 Único	CHC da Universidade Federal do Paraná - PR
236	Afastamento Portaria n 454 art. 2 Único	CHC da Universidade Federal do Paraná - PR
236	Afastamento Portaria n 454 art. 2 Único	CHC da Universidade Federal do Paraná - PR
236	Afastamento Portaria n 454 art. 2 Único	CHC da Universidade Federal do Paraná - PR
236	Afastamento Portaria n 454 art. 2 Único	CHC da Universidade Federal do Paraná - PR

236	Afastamento Portaria n 454 art. 2 Único	CHC da Universidade Federal do Paraná - PR
236	Afastamento Portaria n 454 art. 2 Único	CHC da Universidade Federal do Paraná - PR
236	Afastamento Portaria n 454 art. 2 Único	CHC da Universidade Federal do Paraná - PR
236	Afastamento Portaria n 454 art. 2 Único	CHC da Universidade Federal do Paraná - PR
236	Afastamento Portaria n 454 art. 2 Único	CHC da Universidade Federal do Paraná - PR
236	Afastamento Portaria n 454 art. 2 Único	CHC da Universidade Federal do Paraná - PR
236	Afastamento Portaria n 454 art. 2 Único	CHC da Universidade Federal do Paraná - PR
236	Afastamento Portaria n 454 art. 2 Único	CHC da Universidade Federal do Paraná - PR
236	Afastamento Portaria n 454 art. 2 Único	CHC da Universidade Federal do Paraná - PR
236	Afastamento Portaria n 454 art. 2 Único	CHC da Universidade Federal do Paraná - PR
236	Afastamento Portaria n 454 art. 2 Único	CHC da Universidade Federal do Paraná - PR
236	Afastamento Portaria n 454 art. 2 Único	CHC da Universidade Federal do Paraná - PR
236	Afastamento Portaria n 454 art. 2 Único	CHC da Universidade Federal do Paraná - PR
236	Afastamento Portaria n 454 art. 2 Único	CHC da Universidade Federal do Paraná - PR
236	Afastamento Portaria n 454 art. 2 Único	CHC da Universidade Federal do Paraná - PR
236	Afastamento Portaria n 454 art. 2 Único	CHC da Universidade Federal do Paraná - PR
236	Afastamento Portaria n 454 art. 2 Único	CHC da Universidade Federal do Paraná - PR
236	Afastamento Portaria n 454 art. 2 Único	CHC da Universidade Federal do Paraná - PR
236	Afastamento Portaria n 454 art. 2 Único	CHC da Universidade Federal do Paraná - PR
236	Afastamento Portaria n 454 art. 2 Único	CHC da Universidade Federal do Paraná - PR
236	Afastamento Portaria n 454 art. 2 Único	CHC da Universidade Federal do Paraná - PR
236	Afastamento Portaria n 454 art. 2 Único	CHC da Universidade Federal do Paraná - PR
236	Afastamento Portaria n 454 art. 2 Único	CHC da Universidade Federal do Paraná - PR
236	Afastamento Portaria n 454 art. 2 Único	CHC da Universidade Federal do Paraná - PR
236	Afastamento Portaria n 454 art. 2 Único	CHC da Universidade Federal do Paraná - PR
236	Afastamento Portaria n 454 art. 2 Único	CHC da Universidade Federal do Paraná - PR
236	Afastamento Portaria n 454 art. 2 Único	CHC da Universidade Federal do Paraná - PR
236	Afastamento Portaria n 454 art. 2 Único	CHC da Universidade Federal do Paraná - PR
236	Afastamento Portaria n 454 art. 2 Único	CHC da Universidade Federal do Paraná - PR
236	Afastamento Portaria n 454 art. 2 Único	CHC da Universidade Federal do Paraná - PR
236	Afastamento Portaria n 454 art. 2 Único	CHC da Universidade Federal do Paraná - PR
236	Afastamento Portaria n 454 art. 2 Único	CHC da Universidade Federal do Paraná - PR
236	Afastamento Portaria n 454 art. 2 Único	CHC da Universidade Federal do Paraná - PR
236	Afastamento Portaria n 454 art. 2 Único	CHC da Universidade Federal do Paraná - PR
236	Afastamento Portaria n 454 art. 2 Único	CHC da Universidade Federal do Paraná - PR
236	Afastamento Portaria n 454 art. 2 Único	CHC da Universidade Federal do Paraná - PR
236	Afastamento Portaria n 454 art. 2 Único	CHC da Universidade Federal do Paraná - PR
236	Afastamento Portaria n 454 art. 2 Único	CHC da Universidade Federal do Paraná - PR
236	Afastamento Portaria n 454 art. 2 Único	CHC da Universidade Federal do Paraná - PR
236	Afastamento Portaria n 454 art. 2 Único	CHC da Universidade Federal do Paraná - PR
236	Afastamento Portaria n 454 art. 2 Único	CHC da Universidade Federal do Paraná - PR
236	Afastamento Portaria n 454 art. 2 Único	CHC da Universidade Federal do Paraná - PR
236	Afastamento Portaria n 454 art. 2 Único	CHC da Universidade Federal do Paraná - PR
236	Afastamento Portaria n 454 art. 2 Único	CHC da Universidade Federal do Paraná - PR
236	Afastamento Portaria n 454 art. 2 Único	CHC da Universidade Federal do Paraná - PR
236	Afastamento Portaria n 454 art. 2 Único	CHC da Universidade Federal do Paraná - PR
236	Afastamento Portaria n 454 art. 2 Único	CHC da Universidade Federal do Paraná - PR
236	Afastamento Portaria n 454 art. 2 Único	CHC da Universidade Federal do Paraná - PR

236	Afastamento Portaria n 454 art. 2 Único	CHC da Universidade Federal do Paraná - PR
236	Afastamento Portaria n 454 art. 2 Único	CHC da Universidade Federal do Paraná - PR
236	Afastamento Portaria n 454 art. 2 Único	CHC da Universidade Federal do Paraná - PR
236	Afastamento Portaria n 454 art. 2 Único	CHC da Universidade Federal do Paraná - PR
236	Afastamento Portaria n 454 art. 2 Único	CHC da Universidade Federal do Paraná - PR
236	Afastamento Portaria n 454 art. 2 Único	CHC da Universidade Federal do Paraná - PR
236	Afastamento Portaria n 454 art. 2 Único	CHC da Universidade Federal do Paraná - PR
236	Afastamento Portaria n 454 art. 2 Único	CHC da Universidade Federal do Paraná - PR
236	Afastamento Portaria n 454 art. 2 Único	CHC da Universidade Federal do Paraná - PR
236	Afastamento Portaria n 454 art. 2 Único	CHC da Universidade Federal do Paraná - PR
236	Afastamento Portaria n 454 art. 2 Único	CHC da Universidade Federal do Paraná - PR
236	Afastamento Portaria n 454 art. 2 Único	CHC da Universidade Federal do Paraná - PR
236	Afastamento Portaria n 454 art. 2 Único	CHC da Universidade Federal do Paraná - PR
236	Afastamento Portaria n 454 art. 2 Único	CHC da Universidade Federal do Paraná - PR
236	Afastamento Portaria n 454 art. 2 Único	CHC da Universidade Federal do Paraná - PR
236	Afastamento Portaria n 454 art. 2 Único	CHC da Universidade Federal do Paraná - PR
236	Afastamento Portaria n 454 art. 2 Único	CHC da Universidade Federal do Paraná - PR
236	Afastamento Portaria n 454 art. 2 Único	CHC da Universidade Federal do Paraná - PR
236	Afastamento Portaria n 454 art. 2 Único	CHC da Universidade Federal do Paraná - PR
236	Afastamento Portaria n 454 art. 2 Único	CHC da Universidade Federal do Paraná - PR
236	Afastamento Portaria n 454 art. 2 Único	CHC da Universidade Federal do Paraná - PR
236	Afastamento Portaria n 454 art. 2 Único	CHC da Universidade Federal do Paraná - PR
236	Afastamento Portaria n 454 art. 2 Único	CHC da Universidade Federal do Paraná - PR
236	Afastamento Portaria n 454 art. 2 Único	CHC da Universidade Federal do Paraná - PR
236	Afastamento Portaria n 454 art. 2 Único	CHC da Universidade Federal do Paraná - PR
236	Afastamento Portaria n 454 art. 2 Único	CHC da Universidade Federal do Paraná - PR
236	Afastamento Portaria n 454 art. 2 Único	CHC da Universidade Federal do Paraná - PR
236	Afastamento Portaria n 454 art. 2 Único	CHC da Universidade Federal do Paraná - PR
236	Afastamento Portaria n 454 art. 2 Único	CHC da Universidade Federal do Paraná - PR
236	Afastamento Portaria n 454 art. 2 Único	CHC da Universidade Federal do Paraná - PR
236	Afastamento Portaria n 454 art. 2 Único	CHC da Universidade Federal do Paraná - PR
236	Afastamento Portaria n 454 art. 2 Único	CHC da Universidade Federal do Paraná - PR
236	Afastamento Portaria n 454 art. 2 Único	CHC da Universidade Federal do Paraná - PR
236	Afastamento Portaria n 454 art. 2 Único	CHC da Universidade Federal do Paraná - PR
236	Afastamento Portaria n 454 art. 2 Único	CHC da Universidade Federal do Paraná - PR
236	Afastamento Portaria n 454 art. 2 Único	CHC da Universidade Federal do Paraná - PR
236	Afastamento Portaria n 454 art. 2 Único	CHC da Universidade Federal do Paraná - PR
236	Afastamento Portaria n 454 art. 2 Único	CHC da Universidade Federal do Paraná - PR
236	Afastamento Portaria n 454 art. 2 Único	CHC da Universidade Federal do Paraná - PR
236	Afastamento Portaria n 454 art. 2 Único	CHC da Universidade Federal do Paraná - PR
236	Afastamento Portaria n 454 art. 2 Único	CHC da Universidade Federal do Paraná - PR
236	Afastamento Portaria n 454 art. 2 Único	CHC da Universidade Federal do Paraná - PR
236	Afastamento Portaria n 454 art. 2 Único	CHC da Universidade Federal do Paraná - PR
236	Afastamento Portaria n 454 art. 2 Único	CHC da Universidade Federal do Paraná - PR
236	Afastamento Portaria n 454 art. 2 Único	CHC da Universidade Federal do Paraná - PR
236	Afastamento Portaria n 454 art. 2 Único	CHC da Universidade Federal do Paraná - PR
236	Afastamento Portaria n 454 art. 2 Único	CHC da Universidade Federal do Paraná - PR
236	Afastamento Portaria n 454 art. 2 Único	CHC da Universidade Federal do Paraná - PR

236	Afastamento Portaria n 454 art. 2 Único	CHC da Universidade Federal do Paraná - PR
236	Afastamento Portaria n 454 art. 2 Único	CHC da Universidade Federal do Paraná - PR
236	Afastamento Portaria n 454 art. 2 Único	CHC da Universidade Federal do Paraná - PR
236	Afastamento Portaria n 454 art. 2 Único	CHC da Universidade Federal do Paraná - PR
236	Afastamento Portaria n 454 art. 2 Único	CHC da Universidade Federal do Paraná - PR
236	Afastamento Portaria n 454 art. 2 Único	CHC da Universidade Federal do Paraná - PR
236	Afastamento Portaria n 454 art. 2 Único	CHC da Universidade Federal do Paraná - PR
236	Afastamento Portaria n 454 art. 2 Único	CHC da Universidade Federal do Paraná - PR
236	Afastamento Portaria n 454 art. 2 Único	CHC da Universidade Federal do Paraná - PR
236	Afastamento Portaria n 454 art. 2 Único	CHC da Universidade Federal do Paraná - PR
236	Afastamento Portaria n 454 art. 2 Único	CHC da Universidade Federal do Paraná - PR
236	Afastamento Portaria n 454 art. 2 Único	CHC da Universidade Federal do Paraná - PR
236	Afastamento Portaria n 454 art. 2 Único	CHC da Universidade Federal do Paraná - PR
236	Afastamento Portaria n 454 art. 2 Único	CHC da Universidade Federal do Paraná - PR
236	Afastamento Portaria n 454 art. 2 Único	CHC da Universidade Federal do Paraná - PR
236	Afastamento Portaria n 454 art. 2 Único	CHC da Universidade Federal do Paraná - PR
236	Afastamento Portaria n 454 art. 2 Único	CHC da Universidade Federal do Paraná - PR
236	Afastamento Portaria n 454 art. 2 Único	CHC da Universidade Federal do Paraná - PR
236	Afastamento Portaria n 454 art. 2 Único	CHC da Universidade Federal do Paraná - PR
236	Afastamento Portaria n 454 art. 2 Único	CHC da Universidade Federal do Paraná - PR
236	Afastamento Portaria n 454 art. 2 Único	CHC da Universidade Federal do Paraná - PR
236	Afastamento Portaria n 454 art. 2 Único	CHC da Universidade Federal do Paraná - PR
236	Afastamento Portaria n 454 art. 2 Único	CHC da Universidade Federal do Paraná - PR
236	Afastamento Portaria n 454 art. 2 Único	CHC da Universidade Federal do Paraná - PR
236	Afastamento Portaria n 454 art. 2 Único	CHC da Universidade Federal do Paraná - PR
236	Afastamento Portaria n 454 art. 2 Único	CHC da Universidade Federal do Paraná - PR
236	Afastamento Portaria n 454 art. 2 Único	CHC da Universidade Federal do Paraná - PR
236	Afastamento Portaria n 454 art. 2 Único	CHC da Universidade Federal do Paraná - PR
236	Afastamento Portaria n 454 art. 2 Único	CHC da Universidade Federal do Paraná - PR
236	Afastamento Portaria n 454 art. 2 Único	CHC da Universidade Federal do Paraná - PR
236	Afastamento Portaria n 454 art. 2 Único	CHC da Universidade Federal do Paraná - PR
236	Afastamento Portaria n 454 art. 2 Único	CHC da Universidade Federal do Paraná - PR
236	Afastamento Portaria n 454 art. 2 Único	CHC da Universidade Federal do Paraná - PR
236	Afastamento Portaria n 454 art. 2 Único	CHC da Universidade Federal do Paraná - PR
236	Afastamento Portaria n 454 art. 2 Único	CHC da Universidade Federal do Paraná - PR
236	Afastamento Portaria n 454 art. 2 Único	CHC da Universidade Federal do Paraná - PR
236	Afastamento Portaria n 454 art. 2 Único	CHC da Universidade Federal do Paraná - PR
236	Afastamento Portaria n 454 art. 2 Único	CHC da Universidade Federal do Paraná - PR
236	Afastamento Portaria n 454 art. 2 Único	CHC da Universidade Federal do Paraná - PR
236	Afastamento Portaria n 454 art. 2 Único	CHC da Universidade Federal do Paraná - PR
236	Afastamento Portaria n 454 art. 2 Único	CHC da Universidade Federal do Paraná - PR
236	Afastamento Portaria n 454 art. 2 Único	CHC da Universidade Federal do Paraná - PR
236	Afastamento Portaria n 454 art. 2 Único	CHC da Universidade Federal do Paraná - PR
236	Afastamento Portaria n 454 art. 2 Único	CHC da Universidade Federal do Paraná - PR
236	Afastamento Portaria n 454 art. 2 Único	CHC da Universidade Federal do Paraná - PR

236	Afastamento Portaria n 454 art. 2 Único	CHC da Universidade Federal do Paraná - PR
236	Afastamento Portaria n 454 art. 2 Único	CHC da Universidade Federal do Paraná - PR
236	Afastamento Portaria n 454 art. 2 Único	CHC da Universidade Federal do Paraná - PR
236	Afastamento Portaria n 454 art. 2 Único	CHC da Universidade Federal do Paraná - PR
236	Afastamento Portaria n 454 art. 2 Único	CHC da Universidade Federal do Paraná - PR
236	Afastamento Portaria n 454 art. 2 Único	CHC da Universidade Federal do Paraná - PR
236	Afastamento Portaria n 454 art. 2 Único	CHC da Universidade Federal do Paraná - PR
236	Afastamento Portaria n 454 art. 2 Único	CHC da Universidade Federal do Paraná - PR
236	Afastamento Portaria n 454 art. 2 Único	CHC da Universidade Federal do Paraná - PR
236	Afastamento Portaria n 454 art. 2 Único	CHC da Universidade Federal do Paraná - PR
236	Afastamento Portaria n 454 art. 2 Único	CHC da Universidade Federal do Paraná - PR
236	Afastamento Portaria n 454 art. 2 Único	CHC da Universidade Federal do Paraná - PR
236	Afastamento Portaria n 454 art. 2 Único	CHC da Universidade Federal do Paraná - PR
236	Afastamento Portaria n 454 art. 2 Único	CHC da Universidade Federal do Paraná - PR
236	Afastamento Portaria n 454 art. 2 Único	CHC da Universidade Federal do Paraná - PR
236	Afastamento Portaria n 454 art. 2 Único	CHC da Universidade Federal do Paraná - PR
236	Afastamento Portaria n 454 art. 2 Único	CHC da Universidade Federal do Paraná - PR
236	Afastamento Portaria n 454 art. 2 Único	CHC da Universidade Federal do Paraná - PR
236	Afastamento Portaria n 454 art. 2 Único	CHC da Universidade Federal do Paraná - PR
236	Afastamento Portaria n 454 art. 2 Único	CHC da Universidade Federal do Paraná - PR
236	Afastamento Portaria n 454 art. 2 Único	CHC da Universidade Federal do Paraná - PR
236	Afastamento Portaria n 454 art. 2 Único	CHC da Universidade Federal do Paraná - PR
236	Afastamento Portaria n 454 art. 2 Único	CHC da Universidade Federal do Paraná - PR
236	Afastamento Portaria n 454 art. 2 Único	CHC da Universidade Federal do Paraná - PR
236	Afastamento Portaria n 454 art. 2 Único	CHC da Universidade Federal do Paraná - PR
236	Afastamento Portaria n 454 art. 2 Único	CHC da Universidade Federal do Paraná - PR
236	Afastamento Portaria n 454 art. 2 Único	CHC da Universidade Federal do Paraná - PR
236	Afastamento Portaria n 454 art. 2 Único	CHC da Universidade Federal do Paraná - PR
236	Afastamento Portaria n 454 art. 2 Único	CHC da Universidade Federal do Paraná - PR
236	Afastamento Portaria n 454 art. 2 Único	CHC da Universidade Federal do Paraná - PR
236	Afastamento Portaria n 454 art. 2 Único	CHC da Universidade Federal do Paraná - PR
236	Afastamento Portaria n 454 art. 2 Único	CHC da Universidade Federal do Paraná - PR
236	Afastamento Portaria n 454 art. 2 Único	CHC da Universidade Federal do Paraná - PR
236	Afastamento Portaria n 454 art. 2 Único	CHC da Universidade Federal do Paraná - PR
236	Afastamento Portaria n 454 art. 2 Único	CHC da Universidade Federal do Paraná - PR
236	Afastamento Portaria n 454 art. 2 Único	CHC da Universidade Federal do Paraná - PR
236	Afastamento Portaria n 454 art. 2 Único	CHC da Universidade Federal do Paraná - PR
236	Afastamento Portaria n 454 art. 2 Único	CHC da Universidade Federal do Paraná - PR
236	Afastamento Portaria n 454 art. 2 Único	CHC da Universidade Federal do Paraná - PR
236	Afastamento Portaria n 454 art. 2 Único	CHC da Universidade Federal do Paraná - PR
236	Afastamento Portaria n 454 art. 2 Único	CHC da Universidade Federal do Paraná - PR
236	Afastamento Portaria n 454 art. 2 Único	CHC da Universidade Federal do Paraná - PR

236	Afastamento Portaria n 454 art. 2 Único	CHC da Universidade Federal do Paraná - PR
236	Afastamento Portaria n 454 art. 2 Único	CHC da Universidade Federal do Paraná - PR
236	Afastamento Portaria n 454 art. 2 Único	CHC da Universidade Federal do Paraná - PR
236	Afastamento Portaria n 454 art. 2 Único	CHC da Universidade Federal do Paraná - PR
236	Afastamento Portaria n 454 art. 2 Único	CHC da Universidade Federal do Paraná - PR
236	Afastamento Portaria n 454 art. 2 Único	CHC da Universidade Federal do Paraná - PR
236	Afastamento Portaria n 454 art. 2 Único	CHC da Universidade Federal do Paraná - PR
236	Afastamento Portaria n 454 art. 2 Único	CHC da Universidade Federal do Paraná - PR
236	Afastamento Portaria n 454 art. 2 Único	CHC da Universidade Federal do Paraná - PR
236	Afastamento Portaria n 454 art. 2 Único	CHC da Universidade Federal do Paraná - PR
236	Afastamento Portaria n 454 art. 2 Único	CHC da Universidade Federal do Paraná - PR
236	Afastamento Portaria n 454 art. 2 Único	CHC da Universidade Federal do Paraná - PR
236	Afastamento Portaria n 454 art. 2 Único	CHC da Universidade Federal do Paraná - PR
236	Afastamento Portaria n 454 art. 2 Único	CHC da Universidade Federal do Paraná - PR
236	Afastamento Portaria n 454 art. 2 Único	CHC da Universidade Federal do Paraná - PR
236	Afastamento Portaria n 454 art. 2 Único	CHC da Universidade Federal do Paraná - PR
236	Afastamento Portaria n 454 art. 2 Único	CHC da Universidade Federal do Paraná - PR
236	Afastamento Portaria n 454 art. 2 Único	CHC da Universidade Federal do Paraná - PR
236	Afastamento Portaria n 454 art. 2 Único	CHC da Universidade Federal do Paraná - PR
236	Afastamento Portaria n 454 art. 2 Único	CHC da Universidade Federal do Paraná - PR
236	Afastamento Portaria n 454 art. 2 Único	CHC da Universidade Federal do Paraná - PR
236	Afastamento Portaria n 454 art. 2 Único	CHC da Universidade Federal do Paraná - PR
236	Afastamento Portaria n 454 art. 2 Único	CHC da Universidade Federal do Paraná - PR
236	Afastamento Portaria n 454 art. 2 Único	CHC da Universidade Federal do Paraná - PR
236	Afastamento Portaria n 454 art. 2 Único	CHC da Universidade Federal do Paraná - PR
236	Afastamento Portaria n 454 art. 2 Único	CHC da Universidade Federal do Paraná - PR
236	Afastamento Portaria n 454 art. 2 Único	CHC da Universidade Federal do Paraná - PR
236	Afastamento Portaria n 454 art. 2 Único	CHC da Universidade Federal do Paraná - PR
236	Afastamento Portaria n 454 art. 2 Único	CHC da Universidade Federal do Paraná - PR
236	Afastamento Portaria n 454 art. 2 Único	CHC da Universidade Federal do Paraná - PR
236	Afastamento Portaria n 454 art. 2 Único	CHC da Universidade Federal do Paraná - PR
236	Afastamento Portaria n 454 art. 2 Único	CHC da Universidade Federal do Paraná - PR
236	Afastamento Portaria n 454 art. 2 Único	CHC da Universidade Federal do Paraná - PR
236	Afastamento Portaria n 454 art. 2 Único	CHC da Universidade Federal do Paraná - PR
236	Afastamento Portaria n 454 art. 2 Único	CHC da Universidade Federal do Paraná - PR
236	Afastamento Portaria n 454 art. 2 Único	CHC da Universidade Federal do Paraná - PR
236	Afastamento Portaria n 454 art. 2 Único	CHC da Universidade Federal do Paraná - PR
236	Afastamento Portaria n 454 art. 2 Único	CHC da Universidade Federal do Paraná - PR
236	Afastamento Portaria n 454 art. 2 Único	CHC da Universidade Federal do Paraná - PR
236	Afastamento Portaria n 454 art. 2 Único	CHC da Universidade Federal do Paraná - PR
236	Afastamento Portaria n 454 art. 2 Único	CHC da Universidade Federal do Paraná - PR
236	Afastamento Portaria n 454 art. 2 Único	CHC da Universidade Federal do Paraná - PR
236	Afastamento Portaria n 454 art. 2 Único	CHC da Universidade Federal do Paraná - PR
236	Afastamento Portaria n 454 art. 2 Único	CHC da Universidade Federal do Paraná - PR
236	Afastamento Portaria n 454 art. 2 Único	CHC da Universidade Federal do Paraná - PR
236	Afastamento Portaria n 454 art. 2 Único	CHC da Universidade Federal do Paraná - PR
236	Afastamento Portaria n 454 art. 2 Único	CHC da Universidade Federal do Paraná - PR
236	Afastamento Portaria n 454 art. 2 Único	CHC da Universidade Federal do Paraná - PR

236	Afastamento Portaria n 454 art. 2 Único	CHC da Universidade Federal do Paraná - PR
236	Afastamento Portaria n 454 art. 2 Único	CHC da Universidade Federal do Paraná - PR
236	Afastamento Portaria n 454 art. 2 Único	CHC da Universidade Federal do Paraná - PR
236	Afastamento Portaria n 454 art. 2 Único	CHC da Universidade Federal do Paraná - PR
236	Afastamento Portaria n 454 art. 2 Único	CHC da Universidade Federal do Paraná - PR
236	Afastamento Portaria n 454 art. 2 Único	CHC da Universidade Federal do Paraná - PR
236	Afastamento Portaria n 454 art. 2 Único	CHC da Universidade Federal do Paraná - PR
236	Afastamento Portaria n 454 art. 2 Único	CHC da Universidade Federal do Paraná - PR
236	Afastamento Portaria n 454 art. 2 Único	CHC da Universidade Federal do Paraná - PR
236	Afastamento Portaria n 454 art. 2 Único	CHC da Universidade Federal do Paraná - PR
236	Afastamento Portaria n 454 art. 2 Único	CHC da Universidade Federal do Paraná - PR
236	Afastamento Portaria n 454 art. 2 Único	CHC da Universidade Federal do Paraná - PR
236	Afastamento Portaria n 454 art. 2 Único	CHC da Universidade Federal do Paraná - PR
236	Afastamento Portaria n 454 art. 2 Único	CHC da Universidade Federal do Paraná - PR
236	Afastamento Portaria n 454 art. 2 Único	CHC da Universidade Federal do Paraná - PR
236	Afastamento Portaria n 454 art. 2 Único	CHC da Universidade Federal do Paraná - PR
236	Afastamento Portaria n 454 art. 2 Único	CHC da Universidade Federal do Paraná - PR
236	Afastamento Portaria n 454 art. 2 Único	CHC da Universidade Federal do Paraná - PR
236	Afastamento Portaria n 454 art. 2 Único	CHC da Universidade Federal do Paraná - PR
236	Afastamento Portaria n 454 art. 2 Único	CHC da Universidade Federal do Paraná - PR
237	Trabalho Remoto Instrução Normativa DGP 03/2020 art 6, IX.	CHC da Universidade Federal do Paraná - PR
237	Trabalho Remoto Instrução Normativa DGP 03/2020 art 6, IX.	CHC da Universidade Federal do Paraná - PR
237	Trabalho Remoto Instrução Normativa DGP 03/2020 art 6, IX.	CHC da Universidade Federal do Paraná - PR
237	Trabalho Remoto Instrução Normativa DGP 03/2020 art 6, IX.	CHC da Universidade Federal do Paraná - PR
237	Trabalho Remoto Instrução Normativa DGP 03/2020 art 6, IX.	CHC da Universidade Federal do Paraná - PR
237	Trabalho Remoto Instrução Normativa DGP 03/2020 art 6, IX.	CHC da Universidade Federal do Paraná - PR
237	Trabalho Remoto Instrução Normativa DGP 03/2020 art 6, IX.	CHC da Universidade Federal do Paraná - PR
237	Trabalho Remoto Instrução Normativa DGP 03/2020 art 6, IX.	CHC da Universidade Federal do Paraná - PR
237	Trabalho Remoto Instrução Normativa DGP 03/2020 art 6, IX.	CHC da Universidade Federal do Paraná - PR
237	Trabalho Remoto Instrução Normativa DGP 03/2020 art 6, IX.	CHC da Universidade Federal do Paraná - PR
237	Trabalho Remoto Instrução Normativa DGP 03/2020 art 6, IX.	CHC da Universidade Federal do Paraná - PR
237	Trabalho Remoto Instrução Normativa DGP 03/2020 art 6, IX.	CHC da Universidade Federal do Paraná - PR
237	Trabalho Remoto Instrução Normativa DGP 03/2020 art 6, IX.	CHC da Universidade Federal do Paraná - PR
237	Trabalho Remoto Instrução Normativa DGP 03/2020 art 6, IX.	CHC da Universidade Federal do Paraná - PR
237	Trabalho Remoto Instrução Normativa DGP 03/2020 art 6, IX.	CHC da Universidade Federal do Paraná - PR
237	Trabalho Remoto Instrução Normativa DGP 03/2020 art 6, IX.	CHC da Universidade Federal do Paraná - PR
237	Trabalho Remoto Instrução Normativa DGP 03/2020 art 6, IX.	CHC da Universidade Federal do Paraná - PR
237	Trabalho Remoto Instrução Normativa DGP 03/2020 art 6, IX.	CHC da Universidade Federal do Paraná - PR

237	Trabalho Remoto Instrução Normativa DGP 03/2020 art 6, IX.	CHC da Universidade Federal do Paraná - PR
237	Trabalho Remoto Instrução Normativa DGP 03/2020 art 6, IX.	CHC da Universidade Federal do Paraná - PR
237	Trabalho Remoto Instrução Normativa DGP 03/2020 art 6, IX.	CHC da Universidade Federal do Paraná - PR
237	Trabalho Remoto Instrução Normativa DGP 03/2020 art 6, IX.	CHC da Universidade Federal do Paraná - PR
237	Trabalho Remoto Instrução Normativa DGP 03/2020 art 6, IX.	CHC da Universidade Federal do Paraná - PR
237	Trabalho Remoto Instrução Normativa DGP 03/2020 art 6, IX.	CHC da Universidade Federal do Paraná - PR
237	Trabalho Remoto Instrução Normativa DGP 03/2020 art 6, IX.	CHC da Universidade Federal do Paraná - PR
237	Trabalho Remoto Instrução Normativa DGP 03/2020 art 6, IX.	CHC da Universidade Federal do Paraná - PR
237	Trabalho Remoto Instrução Normativa DGP 03/2020 art 6, IX.	CHC da Universidade Federal do Paraná - PR
237	Trabalho Remoto Instrução Normativa DGP 03/2020 art 6, IX.	CHC da Universidade Federal do Paraná - PR
237	Trabalho Remoto Instrução Normativa DGP 03/2020 art 6, IX.	CHC da Universidade Federal do Paraná - PR
237	Trabalho Remoto Instrução Normativa DGP 03/2020 art 6, IX.	CHC da Universidade Federal do Paraná - PR
237	Trabalho Remoto Instrução Normativa DGP 03/2020 art 6, IX.	CHC da Universidade Federal do Paraná - PR
237	Trabalho Remoto Instrução Normativa DGP 03/2020 art 6, IX.	CHC da Universidade Federal do Paraná - PR
237	Trabalho Remoto Instrução Normativa DGP 03/2020 art 6, IX.	CHC da Universidade Federal do Paraná - PR
237	Trabalho Remoto Instrução Normativa DGP 03/2020 art 6, IX.	CHC da Universidade Federal do Paraná - PR
237	Trabalho Remoto Instrução Normativa DGP 03/2020 art 6, IX.	CHC da Universidade Federal do Paraná - PR
237	Trabalho Remoto Instrução Normativa DGP 03/2020 art 6, IX.	CHC da Universidade Federal do Paraná - PR
237	Trabalho Remoto Instrução Normativa DGP 03/2020 art 6, IX.	CHC da Universidade Federal do Paraná - PR
237	Trabalho Remoto Instrução Normativa DGP 03/2020 art 6, IX.	CHC da Universidade Federal do Paraná - PR
237	Trabalho Remoto Instrução Normativa DGP 03/2020 art 6, IX.	CHC da Universidade Federal do Paraná - PR
237	Trabalho Remoto Instrução Normativa DGP 03/2020 art 6, IX.	CHC da Universidade Federal do Paraná - PR
237	Trabalho Remoto Instrução Normativa DGP 03/2020 art 6, IX.	CHC da Universidade Federal do Paraná - PR
237	Trabalho Remoto Instrução Normativa DGP 03/2020 art 6, IX.	CHC da Universidade Federal do Paraná - PR
237	Trabalho Remoto Instrução Normativa DGP 03/2020 art 6, IX.	CHC da Universidade Federal do Paraná - PR
237	Trabalho Remoto Instrução Normativa DGP 03/2020 art 6, IX.	CHC da Universidade Federal do Paraná - PR
237	Trabalho Remoto Instrução Normativa DGP 03/2020 art 6, IX.	CHC da Universidade Federal do Paraná - PR
237	Trabalho Remoto Instrução Normativa DGP 03/2020 art 6, IX.	CHC da Universidade Federal do Paraná - PR
237	Trabalho Remoto Instrução Normativa DGP 03/2020 art 6, IX.	CHC da Universidade Federal do Paraná - PR
237	Trabalho Remoto Instrução Normativa DGP 03/2020 art 6, IX.	CHC da Universidade Federal do Paraná - PR
237	Trabalho Remoto Instrução Normativa DGP 03/2020 art 6, IX.	CHC da Universidade Federal do Paraná - PR
237	Trabalho Remoto Instrução Normativa DGP 03/2020 art 6, IX.	CHC da Universidade Federal do Paraná - PR

237	Trabalho Remoto Instrução Normativa DGP 03/2020 art 6, IX.	CHC da Universidade Federal do Paraná - PR
237	Trabalho Remoto Instrução Normativa DGP 03/2020 art 6, IX.	CHC da Universidade Federal do Paraná - PR
237	Trabalho Remoto Instrução Normativa DGP 03/2020 art 6, IX.	CHC da Universidade Federal do Paraná - PR
237	Trabalho Remoto Instrução Normativa DGP 03/2020 art 6, IX.	CHC da Universidade Federal do Paraná - PR
237	Trabalho Remoto Instrução Normativa DGP 03/2020 art 6, IX.	CHC da Universidade Federal do Paraná - PR
237	Trabalho Remoto Instrução Normativa DGP 03/2020 art 6, IX.	CHC da Universidade Federal do Paraná - PR
237	Trabalho Remoto Instrução Normativa DGP 03/2020 art 6, IX.	CHC da Universidade Federal do Paraná - PR
237	Trabalho Remoto Instrução Normativa DGP 03/2020 art 6, IX.	CHC da Universidade Federal do Paraná - PR
237	Trabalho Remoto Instrução Normativa DGP 03/2020 art 6, IX.	CHC da Universidade Federal do Paraná - PR
237	Trabalho Remoto Instrução Normativa DGP 03/2020 art 6, IX.	CHC da Universidade Federal do Paraná - PR
237	Trabalho Remoto Instrução Normativa DGP 03/2020 art 6, IX.	CHC da Universidade Federal do Paraná - PR
237	Trabalho Remoto Instrução Normativa DGP 03/2020 art 6, IX.	CHC da Universidade Federal do Paraná - PR
237	Trabalho Remoto Instrução Normativa DGP 03/2020 art 6, IX.	CHC da Universidade Federal do Paraná - PR
237	Trabalho Remoto Instrução Normativa DGP 03/2020 art 6, IX.	CHC da Universidade Federal do Paraná - PR
237	Trabalho Remoto Instrução Normativa DGP 03/2020 art 6, IX.	CHC da Universidade Federal do Paraná - PR
237	Trabalho Remoto Instrução Normativa DGP 03/2020 art 6, IX.	CHC da Universidade Federal do Paraná - PR
237	Trabalho Remoto Instrução Normativa DGP 03/2020 art 6, IX.	CHC da Universidade Federal do Paraná - PR
237	Trabalho Remoto Instrução Normativa DGP 03/2020 art 6, IX.	CHC da Universidade Federal do Paraná - PR
237	Trabalho Remoto Instrução Normativa DGP 03/2020 art 6, IX.	CHC da Universidade Federal do Paraná - PR
237	Trabalho Remoto Instrução Normativa DGP 03/2020 art 6, IX.	CHC da Universidade Federal do Paraná - PR
237	Trabalho Remoto Instrução Normativa DGP 03/2020 art 6, IX.	CHC da Universidade Federal do Paraná - PR
237	Trabalho Remoto Instrução Normativa DGP 03/2020 art 6, IX.	CHC da Universidade Federal do Paraná - PR
237	Trabalho Remoto Instrução Normativa DGP 03/2020 art 6, IX.	CHC da Universidade Federal do Paraná - PR
237	Trabalho Remoto Instrução Normativa DGP 03/2020 art 6, IX.	CHC da Universidade Federal do Paraná - PR
237	Trabalho Remoto Instrução Normativa DGP 03/2020 art 6, IX.	CHC da Universidade Federal do Paraná - PR
237	Trabalho Remoto Instrução Normativa DGP 03/2020 art 6, IX.	CHC da Universidade Federal do Paraná - PR
237	Trabalho Remoto Instrução Normativa DGP 03/2020 art 6, IX.	CHC da Universidade Federal do Paraná - PR
237	Trabalho Remoto Instrução Normativa DGP 03/2020 art 6, IX.	CHC da Universidade Federal do Paraná - PR
237	Trabalho Remoto Instrução Normativa DGP 03/2020 art 6, IX.	CHC da Universidade Federal do Paraná - PR
237	Trabalho Remoto Instrução Normativa DGP 03/2020 art 6, IX.	CHC da Universidade Federal do Paraná - PR
237	Trabalho Remoto Instrução Normativa DGP 03/2020 art 6, IX.	CHC da Universidade Federal do Paraná - PR
237	Trabalho Remoto Instrução Normativa DGP 03/2020 art 6, IX.	CHC da Universidade Federal do Paraná - PR

237	Trabalho Remoto Instrução Normativa DGP 03/2020 art 6, IX.	CHC da Universidade Federal do Paraná - PR
237	Trabalho Remoto Instrução Normativa DGP 03/2020 art 6, IX.	CHC da Universidade Federal do Paraná - PR
237	Trabalho Remoto Instrução Normativa DGP 03/2020 art 6, IX.	CHC da Universidade Federal do Paraná - PR
237	Trabalho Remoto Instrução Normativa DGP 03/2020 art 6, IX.	CHC da Universidade Federal do Paraná - PR
237	Trabalho Remoto Instrução Normativa DGP 03/2020 art 6, IX.	CHC da Universidade Federal do Paraná - PR
237	Trabalho Remoto Instrução Normativa DGP 03/2020 art 6, IX.	CHC da Universidade Federal do Paraná - PR
237	Trabalho Remoto Instrução Normativa DGP 03/2020 art 6, IX.	CHC da Universidade Federal do Paraná - PR
237	Trabalho Remoto Instrução Normativa DGP 03/2020 art 6, IX.	CHC da Universidade Federal do Paraná - PR
237	Trabalho Remoto Instrução Normativa DGP 03/2020 art 6, IX.	CHC da Universidade Federal do Paraná - PR
237	Trabalho Remoto Instrução Normativa DGP 03/2020 art 6, IX.	CHC da Universidade Federal do Paraná - PR
237	Trabalho Remoto Instrução Normativa DGP 03/2020 art 6, IX.	CHC da Universidade Federal do Paraná - PR
237	Trabalho Remoto Instrução Normativa DGP 03/2020 art 6, IX.	CHC da Universidade Federal do Paraná - PR
237	Trabalho Remoto Instrução Normativa DGP 03/2020 art 6, IX.	CHC da Universidade Federal do Paraná - PR
237	Trabalho Remoto Instrução Normativa DGP 03/2020 art 6, IX.	CHC da Universidade Federal do Paraná - PR
237	Trabalho Remoto Instrução Normativa DGP 03/2020 art 6, IX.	CHC da Universidade Federal do Paraná - PR
237	Trabalho Remoto Instrução Normativa DGP 03/2020 art 6, IX.	CHC da Universidade Federal do Paraná - PR
237	Trabalho Remoto Instrução Normativa DGP 03/2020 art 6, IX.	CHC da Universidade Federal do Paraná - PR
237	Trabalho Remoto Instrução Normativa DGP 03/2020 art 6, IX.	CHC da Universidade Federal do Paraná - PR
237	Trabalho Remoto Instrução Normativa DGP 03/2020 art 6, IX.	CHC da Universidade Federal do Paraná - PR
237	Trabalho Remoto Instrução Normativa DGP 03/2020 art 6, IX.	CHC da Universidade Federal do Paraná - PR
237	Trabalho Remoto Instrução Normativa DGP 03/2020 art 6, IX.	CHC da Universidade Federal do Paraná - PR
237	Trabalho Remoto Instrução Normativa DGP 03/2020 art 6, IX.	CHC da Universidade Federal do Paraná - PR
237	Trabalho Remoto Instrução Normativa DGP 03/2020 art 6, IX.	CHC da Universidade Federal do Paraná - PR
237	Trabalho Remoto Instrução Normativa DGP 03/2020 art 6, IX.	CHC da Universidade Federal do Paraná - PR
237	Trabalho Remoto Instrução Normativa DGP 03/2020 art 6, IX.	CHC da Universidade Federal do Paraná - PR
237	Trabalho Remoto Instrução Normativa DGP 03/2020 art 6, IX.	CHC da Universidade Federal do Paraná - PR
237	Trabalho Remoto Instrução Normativa DGP 03/2020 art 6, IX.	CHC da Universidade Federal do Paraná - PR
237	Trabalho Remoto Instrução Normativa DGP 03/2020 art 6, IX.	CHC da Universidade Federal do Paraná - PR
237	Trabalho Remoto Instrução Normativa DGP 03/2020 art 6, IX.	CHC da Universidade Federal do Paraná - PR
237	Trabalho Remoto Instrução Normativa DGP 03/2020 art 6, IX.	CHC da Universidade Federal do Paraná - PR
237	Trabalho Remoto Instrução Normativa DGP 03/2020 art 6, IX.	CHC da Universidade Federal do Paraná - PR
237	Trabalho Remoto Instrução Normativa DGP 03/2020 art 6, IX.	CHC da Universidade Federal do Paraná - PR

237	Trabalho Remoto Instrução Normativa DGP 03/2020 art 6, IX.	CHC da Universidade Federal do Paraná - PR
237	Trabalho Remoto Instrução Normativa DGP 03/2020 art 6, IX.	CHC da Universidade Federal do Paraná - PR
237	Trabalho Remoto Instrução Normativa DGP 03/2020 art 6, IX.	CHC da Universidade Federal do Paraná - PR
237	Trabalho Remoto Instrução Normativa DGP 03/2020 art 6, IX.	CHC da Universidade Federal do Paraná - PR
237	Trabalho Remoto Instrução Normativa DGP 03/2020 art 6, IX.	CHC da Universidade Federal do Paraná - PR
237	Trabalho Remoto Instrução Normativa DGP 03/2020 art 6, IX.	CHC da Universidade Federal do Paraná - PR
237	Trabalho Remoto Instrução Normativa DGP 03/2020 art 6, IX.	CHC da Universidade Federal do Paraná - PR
237	Trabalho Remoto Instrução Normativa DGP 03/2020 art 6, IX.	CHC da Universidade Federal do Paraná - PR
237	Trabalho Remoto Instrução Normativa DGP 03/2020 art 6, IX.	CHC da Universidade Federal do Paraná - PR
237	Trabalho Remoto Instrução Normativa DGP 03/2020 art 6, IX.	CHC da Universidade Federal do Paraná - PR
237	Trabalho Remoto Instrução Normativa DGP 03/2020 art 6, IX.	CHC da Universidade Federal do Paraná - PR
237	Trabalho Remoto Instrução Normativa DGP 03/2020 art 6, IX.	CHC da Universidade Federal do Paraná - PR
237	Trabalho Remoto Instrução Normativa DGP 03/2020 art 6, IX.	CHC da Universidade Federal do Paraná - PR
237	Trabalho Remoto Instrução Normativa DGP 03/2020 art 6, IX.	CHC da Universidade Federal do Paraná - PR
237	Trabalho Remoto Instrução Normativa DGP 03/2020 art 6, IX.	CHC da Universidade Federal do Paraná - PR
237	Trabalho Remoto Instrução Normativa DGP 03/2020 art 6, IX.	CHC da Universidade Federal do Paraná - PR
237	Trabalho Remoto Instrução Normativa DGP 03/2020 art 6, IX.	CHC da Universidade Federal do Paraná - PR
237	Trabalho Remoto Instrução Normativa DGP 03/2020 art 6, IX.	CHC da Universidade Federal do Paraná - PR
237	Trabalho Remoto Instrução Normativa DGP 03/2020 art 6, IX.	CHC da Universidade Federal do Paraná - PR
237	Trabalho Remoto Instrução Normativa DGP 03/2020 art 6, IX.	CHC da Universidade Federal do Paraná - PR
237	Trabalho Remoto Instrução Normativa DGP 03/2020 art 6, IX.	CHC da Universidade Federal do Paraná - PR
237	Trabalho Remoto Instrução Normativa DGP 03/2020 art 6, IX.	CHC da Universidade Federal do Paraná - PR
237	Trabalho Remoto Instrução Normativa DGP 03/2020 art 6, IX.	CHC da Universidade Federal do Paraná - PR
237	Trabalho Remoto Instrução Normativa DGP 03/2020 art 6, IX.	CHC da Universidade Federal do Paraná - PR
237	Trabalho Remoto Instrução Normativa DGP 03/2020 art 6, IX.	CHC da Universidade Federal do Paraná - PR
237	Trabalho Remoto Instrução Normativa DGP 03/2020 art 6, IX.	CHC da Universidade Federal do Paraná - PR
237	Trabalho Remoto Instrução Normativa DGP 03/2020 art 6, IX.	CHC da Universidade Federal do Paraná - PR
237	Trabalho Remoto Instrução Normativa DGP 03/2020 art 6, IX.	CHC da Universidade Federal do Paraná - PR
237	Trabalho Remoto Instrução Normativa DGP 03/2020 art 6, IX.	CHC da Universidade Federal do Paraná - PR
237	Trabalho Remoto Instrução Normativa DGP 03/2020 art 6, IX.	CHC da Universidade Federal do Paraná - PR
237	Trabalho Remoto Instrução Normativa DGP 03/2020 art 6, IX.	CHC da Universidade Federal do Paraná - PR
237	Trabalho Remoto Instrução Normativa DGP 03/2020 art 6, IX.	CHC da Universidade Federal do Paraná - PR

237	Trabalho Remoto Instrução Normativa DGP 03/2020 art 6, IX.	CHC da Universidade Federal do Paraná - PR
237	Trabalho Remoto Instrução Normativa DGP 03/2020 art 6, IX.	CHC da Universidade Federal do Paraná - PR
237	Trabalho Remoto Instrução Normativa DGP 03/2020 art 6, IX.	CHC da Universidade Federal do Paraná - PR
237	Trabalho Remoto Instrução Normativa DGP 03/2020 art 6, IX.	CHC da Universidade Federal do Paraná - PR
237	Trabalho Remoto Instrução Normativa DGP 03/2020 art 6, IX.	CHC da Universidade Federal do Paraná - PR
237	Trabalho Remoto Instrução Normativa DGP 03/2020 art 6, IX.	CHC da Universidade Federal do Paraná - PR
237	Trabalho Remoto Instrução Normativa DGP 03/2020 art 6, IX.	CHC da Universidade Federal do Paraná - PR
237	Trabalho Remoto Instrução Normativa DGP 03/2020 art 6, IX.	CHC da Universidade Federal do Paraná - PR
237	Trabalho Remoto Instrução Normativa DGP 03/2020 art 6, IX.	CHC da Universidade Federal do Paraná - PR
237	Trabalho Remoto Instrução Normativa DGP 03/2020 art 6, IX.	CHC da Universidade Federal do Paraná - PR
237	Trabalho Remoto Instrução Normativa DGP 03/2020 art 6, IX.	CHC da Universidade Federal do Paraná - PR
237	Trabalho Remoto Instrução Normativa DGP 03/2020 art 6, IX.	CHC da Universidade Federal do Paraná - PR
237	Trabalho Remoto Instrução Normativa DGP 03/2020 art 6, IX.	CHC da Universidade Federal do Paraná - PR
237	Trabalho Remoto Instrução Normativa DGP 03/2020 art 6, IX.	CHC da Universidade Federal do Paraná - PR
237	Trabalho Remoto Instrução Normativa DGP 03/2020 art 6, IX.	CHC da Universidade Federal do Paraná - PR
237	Trabalho Remoto Instrução Normativa DGP 03/2020 art 6, IX.	CHC da Universidade Federal do Paraná - PR
237	Trabalho Remoto Instrução Normativa DGP 03/2020 art 6, IX.	CHC da Universidade Federal do Paraná - PR
237	Trabalho Remoto Instrução Normativa DGP 03/2020 art 6, IX.	CHC da Universidade Federal do Paraná - PR
237	Trabalho Remoto Instrução Normativa DGP 03/2020 art 6, IX.	CHC da Universidade Federal do Paraná - PR
237	Trabalho Remoto Instrução Normativa DGP 03/2020 art 6, IX.	CHC da Universidade Federal do Paraná - PR
237	Trabalho Remoto Instrução Normativa DGP 03/2020 art 6, IX.	CHC da Universidade Federal do Paraná - PR
237	Trabalho Remoto Instrução Normativa DGP 03/2020 art 6, IX.	CHC da Universidade Federal do Paraná - PR
237	Trabalho Remoto Instrução Normativa DGP 03/2020 art 6, IX.	CHC da Universidade Federal do Paraná - PR
237	Trabalho Remoto Instrução Normativa DGP 03/2020 art 6, IX.	CHC da Universidade Federal do Paraná - PR
237	Trabalho Remoto Instrução Normativa DGP 03/2020 art 6, IX.	CHC da Universidade Federal do Paraná - PR
237	Trabalho Remoto Instrução Normativa DGP 03/2020 art 6, IX.	CHC da Universidade Federal do Paraná - PR
237	Trabalho Remoto Instrução Normativa DGP 03/2020 art 6, IX.	CHC da Universidade Federal do Paraná - PR
237	Trabalho Remoto Instrução Normativa DGP 03/2020 art 6, IX.	CHC da Universidade Federal do Paraná - PR
237	Trabalho Remoto Instrução Normativa DGP 03/2020 art 6, IX.	CHC da Universidade Federal do Paraná - PR
237	Trabalho Remoto Instrução Normativa DGP 03/2020 art 6, IX.	CHC da Universidade Federal do Paraná - PR
237	Trabalho Remoto Instrução Normativa DGP 03/2020 art 6, IX.	CHC da Universidade Federal do Paraná - PR
237	Trabalho Remoto Instrução Normativa DGP 03/2020 art 6, IX.	CHC da Universidade Federal do Paraná - PR

237	Trabalho Remoto Instrução Normativa DGP 03/2020 art 6, IX.	CHC da Universidade Federal do Paraná - PR
237	Trabalho Remoto Instrução Normativa DGP 03/2020 art 6, IX.	CHC da Universidade Federal do Paraná - PR
237	Trabalho Remoto Instrução Normativa DGP 03/2020 art 6, IX.	CHC da Universidade Federal do Paraná - PR
237	Trabalho Remoto Instrução Normativa DGP 03/2020 art 6, IX.	CHC da Universidade Federal do Paraná - PR
237	Trabalho Remoto Instrução Normativa DGP 03/2020 art 6, IX.	CHC da Universidade Federal do Paraná - PR
237	Trabalho Remoto Instrução Normativa DGP 03/2020 art 6, IX.	CHC da Universidade Federal do Paraná - PR
237	Trabalho Remoto Instrução Normativa DGP 03/2020 art 6, IX.	CHC da Universidade Federal do Paraná - PR
237	Trabalho Remoto Instrução Normativa DGP 03/2020 art 6, IX.	CHC da Universidade Federal do Paraná - PR
237	Trabalho Remoto Instrução Normativa DGP 03/2020 art 6, IX.	CHC da Universidade Federal do Paraná - PR
237	Trabalho Remoto Instrução Normativa DGP 03/2020 art 6, IX.	CHC da Universidade Federal do Paraná - PR
237	Trabalho Remoto Instrução Normativa DGP 03/2020 art 6, IX.	CHC da Universidade Federal do Paraná - PR
237	Trabalho Remoto Instrução Normativa DGP 03/2020 art 6, IX.	CHC da Universidade Federal do Paraná - PR
237	Trabalho Remoto Instrução Normativa DGP 03/2020 art 6, IX.	CHC da Universidade Federal do Paraná - PR
237	Trabalho Remoto Instrução Normativa DGP 03/2020 art 6, IX.	CHC da Universidade Federal do Paraná - PR
237	Trabalho Remoto Instrução Normativa DGP 03/2020 art 6, IX.	CHC da Universidade Federal do Paraná - PR
237	Trabalho Remoto Instrução Normativa DGP 03/2020 art 6, IX.	CHC da Universidade Federal do Paraná - PR
237	Trabalho Remoto Instrução Normativa DGP 03/2020 art 6, IX.	CHC da Universidade Federal do Paraná - PR
237	Trabalho Remoto Instrução Normativa DGP 03/2020 art 6, IX.	CHC da Universidade Federal do Paraná - PR
237	Trabalho Remoto Instrução Normativa DGP 03/2020 art 6, IX.	CHC da Universidade Federal do Paraná - PR
237	Trabalho Remoto Instrução Normativa DGP 03/2020 art 6, IX.	CHC da Universidade Federal do Paraná - PR
237	Trabalho Remoto Instrução Normativa DGP 03/2020 art 6, IX.	CHC da Universidade Federal do Paraná - PR
237	Trabalho Remoto Instrução Normativa DGP 03/2020 art 6, IX.	CHC da Universidade Federal do Paraná - PR
237	Trabalho Remoto Instrução Normativa DGP 03/2020 art 6, IX.	CHC da Universidade Federal do Paraná - PR
237	Trabalho Remoto Instrução Normativa DGP 03/2020 art 6, IX.	CHC da Universidade Federal do Paraná - PR
237	Trabalho Remoto Instrução Normativa DGP 03/2020 art 6, IX.	CHC da Universidade Federal do Paraná - PR
237	Trabalho Remoto Instrução Normativa DGP 03/2020 art 6, IX.	CHC da Universidade Federal do Paraná - PR
237	Trabalho Remoto Instrução Normativa DGP 03/2020 art 6, IX.	CHC da Universidade Federal do Paraná - PR
237	Trabalho Remoto Instrução Normativa DGP 03/2020 art 6, IX.	CHC da Universidade Federal do Paraná - PR
237	Trabalho Remoto Instrução Normativa DGP 03/2020 art 6, IX.	CHC da Universidade Federal do Paraná - PR
237	Trabalho Remoto Instrução Normativa DGP 03/2020 art 6, IX.	CHC da Universidade Federal do Paraná - PR
237	Trabalho Remoto Instrução Normativa DGP 03/2020 art 6, IX.	CHC da Universidade Federal do Paraná - PR
237	Trabalho Remoto Instrução Normativa DGP 03/2020 art 6, IX.	CHC da Universidade Federal do Paraná - PR

237	Trabalho Remoto Instrução Normativa DGP 03/2020 art 6, IX.	CHC da Universidade Federal do Paraná - PR
237	Trabalho Remoto Instrução Normativa DGP 03/2020 art 6, IX.	CHC da Universidade Federal do Paraná - PR
237	Trabalho Remoto Instrução Normativa DGP 03/2020 art 6, IX.	CHC da Universidade Federal do Paraná - PR
237	Trabalho Remoto Instrução Normativa DGP 03/2020 art 6, IX.	CHC da Universidade Federal do Paraná - PR
237	Trabalho Remoto Instrução Normativa DGP 03/2020 art 6, IX.	CHC da Universidade Federal do Paraná - PR
237	Trabalho Remoto Instrução Normativa DGP 03/2020 art 6, IX.	CHC da Universidade Federal do Paraná - PR
237	Trabalho Remoto Instrução Normativa DGP 03/2020 art 6, IX.	CHC da Universidade Federal do Paraná - PR
237	Trabalho Remoto Instrução Normativa DGP 03/2020 art 6, IX.	CHC da Universidade Federal do Paraná - PR
237	Trabalho Remoto Instrução Normativa DGP 03/2020 art 6, IX.	CHC da Universidade Federal do Paraná - PR
237	Trabalho Remoto Instrução Normativa DGP 03/2020 art 6, IX.	CHC da Universidade Federal do Paraná - PR
237	Trabalho Remoto Instrução Normativa DGP 03/2020 art 6, IX.	CHC da Universidade Federal do Paraná - PR
237	Trabalho Remoto Instrução Normativa DGP 03/2020 art 6, IX.	CHC da Universidade Federal do Paraná - PR
237	Trabalho Remoto Instrução Normativa DGP 03/2020 art 6, IX.	CHC da Universidade Federal do Paraná - PR
237	Trabalho Remoto Instrução Normativa DGP 03/2020 art 6, IX.	CHC da Universidade Federal do Paraná - PR
237	Trabalho Remoto Instrução Normativa DGP 03/2020 art 6, IX.	CHC da Universidade Federal do Paraná - PR
237	Trabalho Remoto Instrução Normativa DGP 03/2020 art 6, IX.	CHC da Universidade Federal do Paraná - PR
237	Trabalho Remoto Instrução Normativa DGP 03/2020 art 6, IX.	CHC da Universidade Federal do Paraná - PR
237	Trabalho Remoto Instrução Normativa DGP 03/2020 art 6, IX.	CHC da Universidade Federal do Paraná - PR
237	Trabalho Remoto Instrução Normativa DGP 03/2020 art 6, IX.	CHC da Universidade Federal do Paraná - PR
237	Trabalho Remoto Instrução Normativa DGP 03/2020 art 6, IX.	CHC da Universidade Federal do Paraná - PR
237	Trabalho Remoto Instrução Normativa DGP 03/2020 art 6, IX.	CHC da Universidade Federal do Paraná - PR
237	Trabalho Remoto Instrução Normativa DGP 03/2020 art 6, IX.	CHC da Universidade Federal do Paraná - PR
237	Trabalho Remoto Instrução Normativa DGP 03/2020 art 6, IX.	CHC da Universidade Federal do Paraná - PR
237	Trabalho Remoto Instrução Normativa DGP 03/2020 art 6, IX.	CHC da Universidade Federal do Paraná - PR
237	Trabalho Remoto Instrução Normativa DGP 03/2020 art 6, IX.	CHC da Universidade Federal do Paraná - PR
237	Trabalho Remoto Instrução Normativa DGP 03/2020 art 6, IX.	CHC da Universidade Federal do Paraná - PR
237	Trabalho Remoto Instrução Normativa DGP 03/2020 art 6, IX.	CHC da Universidade Federal do Paraná - PR
237	Trabalho Remoto Instrução Normativa DGP 03/2020 art 6, IX.	CHC da Universidade Federal do Paraná - PR
237	Trabalho Remoto Instrução Normativa DGP 03/2020 art 6, IX.	CHC da Universidade Federal do Paraná - PR
237	Trabalho Remoto Instrução Normativa DGP 03/2020 art 6, IX.	CHC da Universidade Federal do Paraná - PR
237	Trabalho Remoto Instrução Normativa DGP 03/2020 art 6, IX.	CHC da Universidade Federal do Paraná - PR
237	Trabalho Remoto Instrução Normativa DGP 03/2020 art 6, IX.	CHC da Universidade Federal do Paraná - PR

237	Trabalho Remoto Instrução Normativa DGP 03/2020 art 6, IX.	CHC da Universidade Federal do Paraná - PR
237	Trabalho Remoto Instrução Normativa DGP 03/2020 art 6, IX.	CHC da Universidade Federal do Paraná - PR
237	Trabalho Remoto Instrução Normativa DGP 03/2020 art 6, IX.	CHC da Universidade Federal do Paraná - PR
237	Trabalho Remoto Instrução Normativa DGP 03/2020 art 6, IX.	CHC da Universidade Federal do Paraná - PR
237	Trabalho Remoto Instrução Normativa DGP 03/2020 art 6, IX.	CHC da Universidade Federal do Paraná - PR
237	Trabalho Remoto Instrução Normativa DGP 03/2020 art 6, IX.	CHC da Universidade Federal do Paraná - PR
237	Trabalho Remoto Instrução Normativa DGP 03/2020 art 6, IX.	CHC da Universidade Federal do Paraná - PR
237	Trabalho Remoto Instrução Normativa DGP 03/2020 art 6, IX.	CHC da Universidade Federal do Paraná - PR
237	Trabalho Remoto Instrução Normativa DGP 03/2020 art 6, IX.	CHC da Universidade Federal do Paraná - PR
237	Trabalho Remoto Instrução Normativa DGP 03/2020 art 6, IX.	CHC da Universidade Federal do Paraná - PR
237	Trabalho Remoto Instrução Normativa DGP 03/2020 art 6, IX.	CHC da Universidade Federal do Paraná - PR
237	Trabalho Remoto Instrução Normativa DGP 03/2020 art 6, IX.	CHC da Universidade Federal do Paraná - PR
237	Trabalho Remoto Instrução Normativa DGP 03/2020 art 6, IX.	CHC da Universidade Federal do Paraná - PR
237	Trabalho Remoto Instrução Normativa DGP 03/2020 art 6, IX.	CHC da Universidade Federal do Paraná - PR
237	Trabalho Remoto Instrução Normativa DGP 03/2020 art 6, IX.	CHC da Universidade Federal do Paraná - PR
237	Trabalho Remoto Instrução Normativa DGP 03/2020 art 6, IX.	CHC da Universidade Federal do Paraná - PR
237	Trabalho Remoto Instrução Normativa DGP 03/2020 art 6, IX.	CHC da Universidade Federal do Paraná - PR
237	Trabalho Remoto Instrução Normativa DGP 03/2020 art 6, IX.	CHC da Universidade Federal do Paraná - PR
237	Trabalho Remoto Instrução Normativa DGP 03/2020 art 6, IX.	CHC da Universidade Federal do Paraná - PR
237	Trabalho Remoto Instrução Normativa DGP 03/2020 art 6, IX.	CHC da Universidade Federal do Paraná - PR
237	Trabalho Remoto Instrução Normativa DGP 03/2020 art 6, IX.	CHC da Universidade Federal do Paraná - PR
237	Trabalho Remoto Instrução Normativa DGP 03/2020 art 6, IX.	CHC da Universidade Federal do Paraná - PR
237	Trabalho Remoto Instrução Normativa DGP 03/2020 art 6, IX.	CHC da Universidade Federal do Paraná - PR
237	Trabalho Remoto Instrução Normativa DGP 03/2020 art 6, IX.	CHC da Universidade Federal do Paraná - PR
237	Trabalho Remoto Instrução Normativa DGP 03/2020 art 6, IX.	CHC da Universidade Federal do Paraná - PR
237	Trabalho Remoto Instrução Normativa DGP 03/2020 art 6, IX.	CHC da Universidade Federal do Paraná - PR
237	Trabalho Remoto Instrução Normativa DGP 03/2020 art 6, IX.	CHC da Universidade Federal do Paraná - PR
237	Trabalho Remoto Instrução Normativa DGP 03/2020 art 6, IX.	CHC da Universidade Federal do Paraná - PR
237	Trabalho Remoto Instrução Normativa DGP 03/2020 art 6, IX.	CHC da Universidade Federal do Paraná - PR
237	Trabalho Remoto Instrução Normativa DGP 03/2020 art 6, IX.	CHC da Universidade Federal do Paraná - PR
237	Trabalho Remoto Instrução Normativa DGP 03/2020 art 6, IX.	CHC da Universidade Federal do Paraná - PR
237	Trabalho Remoto Instrução Normativa DGP 03/2020 art 6, IX.	CHC da Universidade Federal do Paraná - PR

237	Trabalho Remoto Instrução Normativa DGP 03/2020 art 6, IX.	CHC da Universidade Federal do Paraná - PR
237	Trabalho Remoto Instrução Normativa DGP 03/2020 art 6, IX.	CHC da Universidade Federal do Paraná - PR
237	Trabalho Remoto Instrução Normativa DGP 03/2020 art 6, IX.	CHC da Universidade Federal do Paraná - PR
237	Trabalho Remoto Instrução Normativa DGP 03/2020 art 6, IX.	CHC da Universidade Federal do Paraná - PR
237	Trabalho Remoto Instrução Normativa DGP 03/2020 art 6, IX.	CHC da Universidade Federal do Paraná - PR
237	Trabalho Remoto Instrução Normativa DGP 03/2020 art 6, IX.	CHC da Universidade Federal do Paraná - PR
237	Trabalho Remoto Instrução Normativa DGP 03/2020 art 6, IX.	CHC da Universidade Federal do Paraná - PR
237	Trabalho Remoto Instrução Normativa DGP 03/2020 art 6, IX.	CHC da Universidade Federal do Paraná - PR
237	Trabalho Remoto Instrução Normativa DGP 03/2020 art 6, IX.	CHC da Universidade Federal do Paraná - PR
237	Trabalho Remoto Instrução Normativa DGP 03/2020 art 6, IX.	CHC da Universidade Federal do Paraná - PR
237	Trabalho Remoto Instrução Normativa DGP 03/2020 art 6, IX.	CHC da Universidade Federal do Paraná - PR
237	Trabalho Remoto Instrução Normativa DGP 03/2020 art 6, IX.	CHC da Universidade Federal do Paraná - PR
4	LICENÇA MATERNIDADE - 120 DIAS	CHU da Universidade Federal do Pará - UFPA - PA
4	LICENÇA MATERNIDADE - 120 DIAS	CHU da Universidade Federal do Pará - UFPA - PA
4	LICENÇA MATERNIDADE - 120 DIAS	CHU da Universidade Federal do Pará - UFPA - PA
4	LICENÇA MATERNIDADE - 120 DIAS	CHU da Universidade Federal do Pará - UFPA - PA
4	LICENÇA MATERNIDADE - 120 DIAS	CHU da Universidade Federal do Pará - UFPA - PA
4	LICENÇA MATERNIDADE - 120 DIAS	CHU da Universidade Federal do Pará - UFPA - PA
4	LICENÇA MATERNIDADE - 120 DIAS	CHU da Universidade Federal do Pará - UFPA - PA
4	LICENÇA MATERNIDADE - 120 DIAS	CHU da Universidade Federal do Pará - UFPA - PA
4	LICENÇA MATERNIDADE - 120 DIAS	CHU da Universidade Federal do Pará - UFPA - PA
4	LICENÇA MATERNIDADE - 120 DIAS	CHU da Universidade Federal do Pará - UFPA - PA
4	LICENÇA MATERNIDADE - 120 DIAS	CHU da Universidade Federal do Pará - UFPA - PA
4	LICENÇA MATERNIDADE - 120 DIAS	CHU da Universidade Federal do Pará - UFPA - PA
4	LICENÇA MATERNIDADE - 120 DIAS	CHU da Universidade Federal do Pará - UFPA - PA
4	LICENÇA MATERNIDADE - 120 DIAS	CHU da Universidade Federal do Pará - UFPA - PA
4	LICENÇA MATERNIDADE - 120 DIAS	CHU da Universidade Federal do Pará - UFPA - PA
4	LICENÇA MATERNIDADE - 120 DIAS	CHU da Universidade Federal do Pará - UFPA - PA
4	LICENÇA MATERNIDADE - 120 DIAS	CHU da Universidade Federal do Pará - UFPA - PA
4	LICENÇA MATERNIDADE - 120 DIAS	CHU da Universidade Federal do Pará - UFPA - PA
4	LICENÇA MATERNIDADE - 120 DIAS	CHU da Universidade Federal do Pará - UFPA - PA
4	LICENÇA MATERNIDADE - 120 DIAS	CHU da Universidade Federal do Pará - UFPA - PA
4	LICENÇA MATERNIDADE - 120 DIAS	CHU da Universidade Federal do Pará - UFPA - PA
4	LICENÇA MATERNIDADE - 120 DIAS	CHU da Universidade Federal do Pará - UFPA - PA
4	LICENÇA MATERNIDADE - 120 DIAS	CHU da Universidade Federal do Pará - UFPA - PA
4	LICENÇA MATERNIDADE - 120 DIAS	CHU da Universidade Federal do Pará - UFPA - PA
4	LICENÇA MATERNIDADE - 120 DIAS	CHU da Universidade Federal do Pará - UFPA - PA
4	LICENÇA MATERNIDADE - 120 DIAS	CHU da Universidade Federal do Pará - UFPA - PA
4	LICENÇA MATERNIDADE - 120 DIAS	CHU da Universidade Federal do Pará - UFPA - PA

5	LICENÇA POR MORTE DE FAMILIAR - CLT E REGULAMENTO DE PESSOAL	CHU da Universidade Federal do Pará - UFPA - PA
5	LICENÇA POR MORTE DE FAMILIAR - CLT E REGULAMENTO DE PESSOAL	CHU da Universidade Federal do Pará - UFPA - PA
5	LICENÇA POR MORTE DE FAMILIAR - CLT E REGULAMENTO DE PESSOAL	CHU da Universidade Federal do Pará - UFPA - PA
5	LICENÇA POR MORTE DE FAMILIAR - CLT E REGULAMENTO DE PESSOAL	CHU da Universidade Federal do Pará - UFPA - PA
5	LICENÇA POR MORTE DE FAMILIAR - CLT E REGULAMENTO DE PESSOAL	CHU da Universidade Federal do Pará - UFPA - PA
5	LICENÇA POR MORTE DE FAMILIAR - CLT E REGULAMENTO DE PESSOAL	CHU da Universidade Federal do Pará - UFPA - PA
5	LICENÇA POR MORTE DE FAMILIAR - CLT E REGULAMENTO DE PESSOAL	CHU da Universidade Federal do Pará - UFPA - PA
5	LICENÇA POR MORTE DE FAMILIAR - CLT E REGULAMENTO DE PESSOAL	CHU da Universidade Federal do Pará - UFPA - PA
5	LICENÇA POR MORTE DE FAMILIAR - CLT E REGULAMENTO DE PESSOAL	CHU da Universidade Federal do Pará - UFPA - PA
5	LICENÇA POR MORTE DE FAMILIAR - CLT E REGULAMENTO DE PESSOAL	CHU da Universidade Federal do Pará - UFPA - PA
5	LICENÇA POR MORTE DE FAMILIAR - CLT E REGULAMENTO DE PESSOAL	CHU da Universidade Federal do Pará - UFPA - PA
5	LICENÇA POR MORTE DE FAMILIAR - CLT E REGULAMENTO DE PESSOAL	CHU da Universidade Federal do Pará - UFPA - PA
5	LICENÇA POR MORTE DE FAMILIAR - CLT E REGULAMENTO DE PESSOAL	CHU da Universidade Federal do Pará - UFPA - PA
5	LICENÇA POR MORTE DE FAMILIAR - CLT E REGULAMENTO DE PESSOAL	CHU da Universidade Federal do Pará - UFPA - PA
5	LICENÇA POR MORTE DE FAMILIAR - CLT E REGULAMENTO DE PESSOAL	CHU da Universidade Federal do Pará - UFPA - PA
5	LICENÇA POR MORTE DE FAMILIAR - CLT E REGULAMENTO DE PESSOAL	CHU da Universidade Federal do Pará - UFPA - PA
5	LICENÇA POR MORTE DE FAMILIAR - CLT E REGULAMENTO DE PESSOAL	CHU da Universidade Federal do Pará - UFPA - PA
5	LICENÇA POR MORTE DE FAMILIAR - CLT E REGULAMENTO DE PESSOAL	CHU da Universidade Federal do Pará - UFPA - PA
5	LICENÇA POR MORTE DE FAMILIAR - CLT E REGULAMENTO DE PESSOAL	CHU da Universidade Federal do Pará - UFPA - PA
5	LICENÇA POR MORTE DE FAMILIAR - CLT E REGULAMENTO DE PESSOAL	CHU da Universidade Federal do Pará - UFPA - PA
5	LICENÇA POR MORTE DE FAMILIAR - CLT E REGULAMENTO DE PESSOAL	CHU da Universidade Federal do Pará - UFPA - PA
5	LICENÇA POR MORTE DE FAMILIAR - CLT E REGULAMENTO DE PESSOAL	CHU da Universidade Federal do Pará - UFPA - PA
5	LICENÇA POR MORTE DE FAMILIAR - CLT E REGULAMENTO DE PESSOAL	CHU da Universidade Federal do Pará - UFPA - PA
5	LICENÇA POR MORTE DE FAMILIAR - CLT E REGULAMENTO DE PESSOAL	CHU da Universidade Federal do Pará - UFPA - PA
5	LICENÇA POR MORTE DE FAMILIAR - CLT E REGULAMENTO DE PESSOAL	CHU da Universidade Federal do Pará - UFPA - PA
5	LICENÇA POR MORTE DE FAMILIAR - CLT E REGULAMENTO DE PESSOAL	CHU da Universidade Federal do Pará - UFPA - PA
12	LICENÇA MATERNIDADE (COMPLEMENTAR 60 DIAS)	CHU da Universidade Federal do Pará - UFPA - PA
12	LICENÇA MATERNIDADE (COMPLEMENTAR 60 DIAS)	CHU da Universidade Federal do Pará - UFPA - PA
12	LICENÇA MATERNIDADE (COMPLEMENTAR 60 DIAS)	CHU da Universidade Federal do Pará - UFPA - PA
12	LICENÇA MATERNIDADE (COMPLEMENTAR 60 DIAS)	CHU da Universidade Federal do Pará - UFPA - PA
12	LICENÇA MATERNIDADE (COMPLEMENTAR 60 DIAS)	CHU da Universidade Federal do Pará - UFPA - PA
12	LICENÇA MATERNIDADE (COMPLEMENTAR 60 DIAS)	CHU da Universidade Federal do Pará - UFPA - PA
12	LICENÇA MATERNIDADE (COMPLEMENTAR 60 DIAS)	CHU da Universidade Federal do Pará - UFPA - PA
12	LICENÇA MATERNIDADE (COMPLEMENTAR 60 DIAS)	CHU da Universidade Federal do Pará - UFPA - PA

12	LICENÇA MATERNIDADE (COMPLEMENTAR 60 DIAS)	CHU da Universidade Federal do Pará - UFPA - PA
12	LICENÇA MATERNIDADE (COMPLEMENTAR 60 DIAS)	CHU da Universidade Federal do Pará - UFPA - PA
12	LICENÇA MATERNIDADE (COMPLEMENTAR 60 DIAS)	CHU da Universidade Federal do Pará - UFPA - PA
12	LICENÇA MATERNIDADE (COMPLEMENTAR 60 DIAS)	CHU da Universidade Federal do Pará - UFPA - PA
12	LICENÇA MATERNIDADE (COMPLEMENTAR 60 DIAS)	CHU da Universidade Federal do Pará - UFPA - PA
12	LICENÇA MATERNIDADE (COMPLEMENTAR 60 DIAS)	CHU da Universidade Federal do Pará - UFPA - PA
12	LICENÇA MATERNIDADE (COMPLEMENTAR 60 DIAS)	CHU da Universidade Federal do Pará - UFPA - PA
12	LICENÇA MATERNIDADE (COMPLEMENTAR 60 DIAS)	CHU da Universidade Federal do Pará - UFPA - PA
12	LICENÇA MATERNIDADE (COMPLEMENTAR 60 DIAS)	CHU da Universidade Federal do Pará - UFPA - PA
12	LICENÇA MATERNIDADE (COMPLEMENTAR 60 DIAS)	CHU da Universidade Federal do Pará - UFPA - PA
14	LICENÇA GALA - CLT ART 473 II	CHU da Universidade Federal do Pará - UFPA - PA
14	LICENÇA GALA - CLT ART 473 II	CHU da Universidade Federal do Pará - UFPA - PA
14	LICENÇA GALA - CLT ART 473 II	CHU da Universidade Federal do Pará - UFPA - PA
14	LICENÇA GALA - CLT ART 473 II	CHU da Universidade Federal do Pará - UFPA - PA
14	LICENÇA GALA - CLT ART 473 II	CHU da Universidade Federal do Pará - UFPA - PA
23	CAPACITAÇAO/EVENTO POR INTERESSE DA EMPRESA - ATE 30 DIAS	CHU da Universidade Federal do Pará - UFPA - PA
23	CAPACITAÇAO/EVENTO POR INTERESSE DA EMPRESA - ATE 30 DIAS	CHU da Universidade Federal do Pará - UFPA - PA
23	CAPACITAÇAO/EVENTO POR INTERESSE DA EMPRESA - ATE 30 DIAS	CHU da Universidade Federal do Pará - UFPA - PA
23	CAPACITAÇAO/EVENTO POR INTERESSE DA EMPRESA - ATE 30 DIAS	CHU da Universidade Federal do Pará - UFPA - PA
23	CAPACITAÇAO/EVENTO POR INTERESSE DA EMPRESA - ATE 30 DIAS	CHU da Universidade Federal do Pará - UFPA - PA
23	CAPACITAÇAO/EVENTO POR INTERESSE DA EMPRESA - ATE 30 DIAS	CHU da Universidade Federal do Pará - UFPA - PA
23	CAPACITAÇAO/EVENTO POR INTERESSE DA EMPRESA - ATE 30 DIAS	CHU da Universidade Federal do Pará - UFPA - PA
23	CAPACITAÇAO/EVENTO POR INTERESSE DA EMPRESA - ATE 30 DIAS	CHU da Universidade Federal do Pará - UFPA - PA
23	CAPACITAÇAO/EVENTO POR INTERESSE DA EMPRESA - ATE 30 DIAS	CHU da Universidade Federal do Pará - UFPA - PA
23	CAPACITAÇAO/EVENTO POR INTERESSE DA EMPRESA - ATE 30 DIAS	CHU da Universidade Federal do Pará - UFPA - PA
23	CAPACITAÇAO/EVENTO POR INTERESSE DA EMPRESA - ATE 30 DIAS	CHU da Universidade Federal do Pará - UFPA - PA
23	CAPACITAÇAO/EVENTO POR INTERESSE DA EMPRESA - ATE 30 DIAS	CHU da Universidade Federal do Pará - UFPA - PA
23	CAPACITAÇAO/EVENTO POR INTERESSE DA EMPRESA - ATE 30 DIAS	CHU da Universidade Federal do Pará - UFPA - PA
23	CAPACITAÇAO/EVENTO POR INTERESSE DA EMPRESA - ATE 30 DIAS	CHU da Universidade Federal do Pará - UFPA - PA
23	CAPACITAÇAO/EVENTO POR INTERESSE DA EMPRESA - ATE 30 DIAS	CHU da Universidade Federal do Pará - UFPA - PA
23	CAPACITAÇAO/EVENTO POR INTERESSE DA EMPRESA - ATE 30 DIAS	CHU da Universidade Federal do Pará - UFPA - PA
23	CAPACITAÇAO/EVENTO POR INTERESSE DA EMPRESA - ATE 30 DIAS	CHU da Universidade Federal do Pará - UFPA - PA
23	CAPACITAÇAO/EVENTO POR INTERESSE DA EMPRESA - ATE 30 DIAS	CHU da Universidade Federal do Pará - UFPA - PA
23	CAPACITAÇAO/EVENTO POR INTERESSE DA EMPRESA - ATE 30 DIAS	CHU da Universidade Federal do Pará - UFPA - PA
23	CAPACITAÇAO/EVENTO POR INTERESSE DA EMPRESA - ATE 30 DIAS	CHU da Universidade Federal do Pará - UFPA - PA
23	CAPACITAÇAO/EVENTO POR INTERESSE DA EMPRESA - ATE 30 DIAS	CHU da Universidade Federal do Pará - UFPA - PA

23	CAPACITAÇAO/EVENTO POR INTERESSE DA EMPRESA - ATE 30 DIAS	CHU da Universidade Federal do Pará - UFPA - PA
23	CAPACITAÇAO/EVENTO POR INTERESSE DA EMPRESA - ATE 30 DIAS	CHU da Universidade Federal do Pará - UFPA - PA
23	CAPACITAÇAO/EVENTO POR INTERESSE DA EMPRESA - ATE 30 DIAS	CHU da Universidade Federal do Pará - UFPA - PA
23	CAPACITAÇAO/EVENTO POR INTERESSE DA EMPRESA - ATE 30 DIAS	CHU da Universidade Federal do Pará - UFPA - PA
23	CAPACITAÇAO/EVENTO POR INTERESSE DA EMPRESA - ATE 30 DIAS	CHU da Universidade Federal do Pará - UFPA - PA
23	CAPACITAÇAO/EVENTO POR INTERESSE DA EMPRESA - ATE 30 DIAS	CHU da Universidade Federal do Pará - UFPA - PA
23	CAPACITAÇAO/EVENTO POR INTERESSE DA EMPRESA - ATE 30 DIAS	CHU da Universidade Federal do Pará - UFPA - PA
23	CAPACITAÇAO/EVENTO POR INTERESSE DA EMPRESA - ATE 30 DIAS	CHU da Universidade Federal do Pará - UFPA - PA
23	CAPACITAÇAO/EVENTO POR INTERESSE DA EMPRESA - ATE 30 DIAS	CHU da Universidade Federal do Pará - UFPA - PA
23	CAPACITAÇAO/EVENTO POR INTERESSE DA EMPRESA - ATE 30 DIAS	CHU da Universidade Federal do Pará - UFPA - PA
23	CAPACITAÇAO/EVENTO POR INTERESSE DA EMPRESA - ATE 30 DIAS	CHU da Universidade Federal do Pará - UFPA - PA
23	CAPACITAÇAO/EVENTO POR INTERESSE DA EMPRESA - ATE 30 DIAS	CHU da Universidade Federal do Pará - UFPA - PA
23	CAPACITAÇAO/EVENTO POR INTERESSE DA EMPRESA - ATE 30 DIAS	CHU da Universidade Federal do Pará - UFPA - PA
23	CAPACITAÇAO/EVENTO POR INTERESSE DA EMPRESA - ATE 30 DIAS	CHU da Universidade Federal do Pará - UFPA - PA
23	CAPACITAÇAO/EVENTO POR INTERESSE DA EMPRESA - ATE 30 DIAS	CHU da Universidade Federal do Pará - UFPA - PA
23	CAPACITAÇAO/EVENTO POR INTERESSE DA EMPRESA - ATE 30 DIAS	CHU da Universidade Federal do Pará - UFPA - PA
23	CAPACITAÇAO/EVENTO POR INTERESSE DA EMPRESA - ATE 30 DIAS	CHU da Universidade Federal do Pará - UFPA - PA
23	CAPACITAÇAO/EVENTO POR INTERESSE DA EMPRESA - ATE 30 DIAS	CHU da Universidade Federal do Pará - UFPA - PA
23	CAPACITAÇAO/EVENTO POR INTERESSE DA EMPRESA - ATE 30 DIAS	CHU da Universidade Federal do Pará - UFPA - PA
23	CAPACITAÇAO/EVENTO POR INTERESSE DA EMPRESA - ATE 30 DIAS	CHU da Universidade Federal do Pará - UFPA - PA
31	LICENÇA ADOÇÃO - 120 DIAS - LEI 10.421/02	CHU da Universidade Federal do Pará - UFPA - PA
36	AUXILIO DOENÇA (INSS) - A PARTIR DO 16 DIA	CHU da Universidade Federal do Pará - UFPA - PA
36	AUXILIO DOENÇA (INSS) - A PARTIR DO 16 DIA	CHU da Universidade Federal do Pará - UFPA - PA
36	AUXILIO DOENÇA (INSS) - A PARTIR DO 16 DIA	CHU da Universidade Federal do Pará - UFPA - PA
36	AUXILIO DOENÇA (INSS) - A PARTIR DO 16 DIA	CHU da Universidade Federal do Pará - UFPA - PA
36	AUXILIO DOENÇA (INSS) - A PARTIR DO 16 DIA	CHU da Universidade Federal do Pará - UFPA - PA
36	AUXILIO DOENÇA (INSS) - A PARTIR DO 16 DIA	CHU da Universidade Federal do Pará - UFPA - PA
36	AUXILIO DOENÇA (INSS) - A PARTIR DO 16 DIA	CHU da Universidade Federal do Pará - UFPA - PA
36	AUXILIO DOENÇA (INSS) - A PARTIR DO 16 DIA	CHU da Universidade Federal do Pará - UFPA - PA
36	AUXILIO DOENÇA (INSS) - A PARTIR DO 16 DIA	CHU da Universidade Federal do Pará - UFPA - PA
36	AUXILIO DOENÇA (INSS) - A PARTIR DO 16 DIA	CHU da Universidade Federal do Pará - UFPA - PA
36	AUXILIO DOENÇA (INSS) - A PARTIR DO 16 DIA	CHU da Universidade Federal do Pará - UFPA - PA
36	AUXILIO DOENÇA (INSS) - A PARTIR DO 16 DIA	CHU da Universidade Federal do Pará - UFPA - PA
36	AUXILIO DOENÇA (INSS) - A PARTIR DO 16 DIA	CHU da Universidade Federal do Pará - UFPA - PA
36	AUXILIO DOENÇA (INSS) - A PARTIR DO 16 DIA	CHU da Universidade Federal do Pará - UFPA - PA
36	AUXILIO DOENÇA (INSS) - A PARTIR DO 16 DIA	CHU da Universidade Federal do Pará - UFPA - PA

36	AUXILIO DOENÇA (INSS) - A PARTIR DO 16 DIA	CHU da Universidade Federal do Pará - UFPA - PA
36	AUXILIO DOENÇA (INSS) - A PARTIR DO 16 DIA	CHU da Universidade Federal do Pará - UFPA - PA
36	AUXILIO DOENÇA (INSS) - A PARTIR DO 16 DIA	CHU da Universidade Federal do Pará - UFPA - PA
36	AUXILIO DOENÇA (INSS) - A PARTIR DO 16 DIA	CHU da Universidade Federal do Pará - UFPA - PA
36	AUXILIO DOENÇA (INSS) - A PARTIR DO 16 DIA	CHU da Universidade Federal do Pará - UFPA - PA
36	AUXILIO DOENÇA (INSS) - A PARTIR DO 16 DIA	CHU da Universidade Federal do Pará - UFPA - PA
36	AUXILIO DOENÇA (INSS) - A PARTIR DO 16 DIA	CHU da Universidade Federal do Pará - UFPA - PA
36	AUXILIO DOENÇA (INSS) - A PARTIR DO 16 DIA	CHU da Universidade Federal do Pará - UFPA - PA
36	AUXILIO DOENÇA (INSS) - A PARTIR DO 16 DIA	CHU da Universidade Federal do Pará - UFPA - PA
36	AUXILIO DOENÇA (INSS) - A PARTIR DO 16 DIA	CHU da Universidade Federal do Pará - UFPA - PA
36	AUXILIO DOENÇA (INSS) - A PARTIR DO 16 DIA	CHU da Universidade Federal do Pará - UFPA - PA
36	AUXILIO DOENÇA (INSS) - A PARTIR DO 16 DIA	CHU da Universidade Federal do Pará - UFPA - PA
36	AUXILIO DOENÇA (INSS) - A PARTIR DO 16 DIA	CHU da Universidade Federal do Pará - UFPA - PA
36	AUXILIO DOENÇA (INSS) - A PARTIR DO 16 DIA	CHU da Universidade Federal do Pará - UFPA - PA
36	AUXILIO DOENÇA (INSS) - A PARTIR DO 16 DIA	CHU da Universidade Federal do Pará - UFPA - PA
36	AUXILIO DOENÇA (INSS) - A PARTIR DO 16 DIA	CHU da Universidade Federal do Pará - UFPA - PA
36	AUXILIO DOENÇA (INSS) - A PARTIR DO 16 DIA	CHU da Universidade Federal do Pará - UFPA - PA
36	AUXILIO DOENÇA (INSS) - A PARTIR DO 16 DIA	CHU da Universidade Federal do Pará - UFPA - PA
36	AUXILIO DOENÇA (INSS) - A PARTIR DO 16 DIA	CHU da Universidade Federal do Pará - UFPA - PA
36	AUXILIO DOENÇA (INSS) - A PARTIR DO 16 DIA	CHU da Universidade Federal do Pará - UFPA - PA
36	AUXILIO DOENÇA (INSS) - A PARTIR DO 16 DIA	CHU da Universidade Federal do Pará - UFPA - PA
36	AUXILIO DOENÇA (INSS) - A PARTIR DO 16 DIA	CHU da Universidade Federal do Pará - UFPA - PA
36	AUXILIO DOENÇA (INSS) - A PARTIR DO 16 DIA	CHU da Universidade Federal do Pará - UFPA - PA
36	AUXILIO DOENÇA (INSS) - A PARTIR DO 16 DIA	CHU da Universidade Federal do Pará - UFPA - PA
42	DOAÇÃO DE SANGUE - REGULAMENTO DE PESSOAL ART 36 I	CHU da Universidade Federal do Pará - UFPA - PA
42	DOAÇÃO DE SANGUE - REGULAMENTO DE PESSOAL ART 36 I	CHU da Universidade Federal do Pará - UFPA - PA
42	DOAÇÃO DE SANGUE - REGULAMENTO DE PESSOAL ART 36 I	CHU da Universidade Federal do Pará - UFPA - PA
42	DOAÇÃO DE SANGUE - REGULAMENTO DE PESSOAL ART 36 I	CHU da Universidade Federal do Pará - UFPA - PA
42	DOAÇÃO DE SANGUE - REGULAMENTO DE PESSOAL ART 36 I	CHU da Universidade Federal do Pará - UFPA - PA
42	DOAÇÃO DE SANGUE - REGULAMENTO DE PESSOAL ART 36 I	CHU da Universidade Federal do Pará - UFPA - PA
42	DOAÇÃO DE SANGUE - REGULAMENTO DE PESSOAL ART 36 I	CHU da Universidade Federal do Pará - UFPA - PA
42	DOAÇÃO DE SANGUE - REGULAMENTO DE PESSOAL ART 36 I	CHU da Universidade Federal do Pará - UFPA - PA
42	DOAÇÃO DE SANGUE - REGULAMENTO DE PESSOAL ART 36 I	CHU da Universidade Federal do Pará - UFPA - PA
42	DOAÇÃO DE SANGUE - REGULAMENTO DE PESSOAL ART 36 I	CHU da Universidade Federal do Pará - UFPA - PA
42	DOAÇÃO DE SANGUE - REGULAMENTO DE PESSOAL ART 36 I	CHU da Universidade Federal do Pará - UFPA - PA
42	DOAÇÃO DE SANGUE - REGULAMENTO DE PESSOAL ART 36 I	CHU da Universidade Federal do Pará - UFPA - PA
42	DOAÇÃO DE SANGUE - REGULAMENTO DE PESSOAL ART 36 I	CHU da Universidade Federal do Pará - UFPA - PA
42	DOAÇÃO DE SANGUE - REGULAMENTO DE PESSOAL ART 36 I	CHU da Universidade Federal do Pará - UFPA - PA
42	DOAÇÃO DE SANGUE - REGULAMENTO DE PESSOAL ART 36 I	CHU da Universidade Federal do Pará - UFPA - PA
42	DOAÇÃO DE SANGUE - REGULAMENTO DE PESSOAL ART 36 I	CHU da Universidade Federal do Pará - UFPA - PA
45	FALTA INJUSTIFICADA (GERA DESCONTO NA FOLHA)	CHU da Universidade Federal do Pará - UFPA - PA
45	FALTA INJUSTIFICADA (GERA DESCONTO NA FOLHA)	CHU da Universidade Federal do Pará - UFPA - PA
45	FALTA INJUSTIFICADA (GERA DESCONTO NA FOLHA)	CHU da Universidade Federal do Pará - UFPA - PA

45	FALTA INJUSTIFICADA (GERA DESCONTO NA FOLHA)	CHU da Universidade Federal do Pará - UFPA - PA
45	FALTA INJUSTIFICADA (GERA DESCONTO NA FOLHA)	CHU da Universidade Federal do Pará - UFPA - PA
45	FALTA INJUSTIFICADA (GERA DESCONTO NA FOLHA)	CHU da Universidade Federal do Pará - UFPA - PA
45	FALTA INJUSTIFICADA (GERA DESCONTO NA FOLHA)	CHU da Universidade Federal do Pará - UFPA - PA
45	FALTA INJUSTIFICADA (GERA DESCONTO NA FOLHA)	CHU da Universidade Federal do Pará - UFPA - PA
45	FALTA INJUSTIFICADA (GERA DESCONTO NA FOLHA)	CHU da Universidade Federal do Pará - UFPA - PA
45	FALTA INJUSTIFICADA (GERA DESCONTO NA FOLHA)	CHU da Universidade Federal do Pará - UFPA - PA
45	FALTA INJUSTIFICADA (GERA DESCONTO NA FOLHA)	CHU da Universidade Federal do Pará - UFPA - PA
45	FALTA INJUSTIFICADA (GERA DESCONTO NA FOLHA)	CHU da Universidade Federal do Pará - UFPA - PA
45	FALTA INJUSTIFICADA (GERA DESCONTO NA FOLHA)	CHU da Universidade Federal do Pará - UFPA - PA
45	FALTA INJUSTIFICADA (GERA DESCONTO NA FOLHA)	CHU da Universidade Federal do Pará - UFPA - PA
45	FALTA INJUSTIFICADA (GERA DESCONTO NA FOLHA)	CHU da Universidade Federal do Pará - UFPA - PA
45	FALTA INJUSTIFICADA (GERA DESCONTO NA FOLHA)	CHU da Universidade Federal do Pará - UFPA - PA
45	FALTA INJUSTIFICADA (GERA DESCONTO NA FOLHA)	CHU da Universidade Federal do Pará - UFPA - PA
45	FALTA INJUSTIFICADA (GERA DESCONTO NA FOLHA)	CHU da Universidade Federal do Pará - UFPA - PA
45	FALTA INJUSTIFICADA (GERA DESCONTO NA FOLHA)	CHU da Universidade Federal do Pará - UFPA - PA
45	FALTA INJUSTIFICADA (GERA DESCONTO NA FOLHA)	CHU da Universidade Federal do Pará - UFPA - PA
45	FALTA INJUSTIFICADA (GERA DESCONTO NA FOLHA)	CHU da Universidade Federal do Pará - UFPA - PA
45	FALTA INJUSTIFICADA (GERA DESCONTO NA FOLHA)	CHU da Universidade Federal do Pará - UFPA - PA
45	FALTA INJUSTIFICADA (GERA DESCONTO NA FOLHA)	CHU da Universidade Federal do Pará - UFPA - PA
45	FALTA INJUSTIFICADA (GERA DESCONTO NA FOLHA)	CHU da Universidade Federal do Pará - UFPA - PA
45	FALTA INJUSTIFICADA (GERA DESCONTO NA FOLHA)	CHU da Universidade Federal do Pará - UFPA - PA
48	AFASTAMENTO ACIDENTE DO TRABALHO A PARTIR 16 DIA (INSS)	CHU da Universidade Federal do Pará - UFPA - PA
48	AFASTAMENTO ACIDENTE DO TRABALHO A PARTIR 16 DIA (INSS)	CHU da Universidade Federal do Pará - UFPA - PA
48	AFASTAMENTO ACIDENTE DO TRABALHO A PARTIR 16 DIA (INSS)	CHU da Universidade Federal do Pará - UFPA - PA
48	AFASTAMENTO ACIDENTE DO TRABALHO A PARTIR 16 DIA (INSS)	CHU da Universidade Federal do Pará - UFPA - PA
48	AFASTAMENTO ACIDENTE DO TRABALHO A PARTIR 16 DIA (INSS)	CHU da Universidade Federal do Pará - UFPA - PA
48	AFASTAMENTO ACIDENTE DO TRABALHO A PARTIR 16 DIA (INSS)	CHU da Universidade Federal do Pará - UFPA - PA
51	A SERVIÇO DA JUSTIÇA ELEITORAL - LEI 9.504 ART. 98	CHU da Universidade Federal do Pará - UFPA - PA
51	A SERVIÇO DA JUSTIÇA ELEITORAL - LEI 9.504 ART. 98	CHU da Universidade Federal do Pará - UFPA - PA
51	A SERVIÇO DA JUSTIÇA ELEITORAL - LEI 9.504 ART. 98	CHU da Universidade Federal do Pará - UFPA - PA
51	A SERVIÇO DA JUSTIÇA ELEITORAL - LEI 9.504 ART. 98	CHU da Universidade Federal do Pará - UFPA - PA
51	A SERVIÇO DA JUSTIÇA ELEITORAL - LEI 9.504 ART. 98	CHU da Universidade Federal do Pará - UFPA - PA
51	A SERVIÇO DA JUSTIÇA ELEITORAL - LEI 9.504 ART. 98	CHU da Universidade Federal do Pará - UFPA - PA
51	A SERVIÇO DA JUSTIÇA ELEITORAL - LEI 9.504 ART. 98	CHU da Universidade Federal do Pará - UFPA - PA

51	A SERVIÇO DA JUSTIÇA ELEITORAL - LEI 9.504 ART. 98	CHU da Universidade Federal do Pará - UFPA - PA
51	A SERVIÇO DA JUSTIÇA ELEITORAL - LEI 9.504 ART. 98	CHU da Universidade Federal do Pará - UFPA - PA
51	A SERVIÇO DA JUSTIÇA ELEITORAL - LEI 9.504 ART. 98	CHU da Universidade Federal do Pará - UFPA - PA
51	A SERVIÇO DA JUSTIÇA ELEITORAL - LEI 9.504 ART. 98	CHU da Universidade Federal do Pará - UFPA - PA
51	A SERVIÇO DA JUSTIÇA ELEITORAL - LEI 9.504 ART. 98	CHU da Universidade Federal do Pará - UFPA - PA
51	A SERVIÇO DA JUSTIÇA ELEITORAL - LEI 9.504 ART. 98	CHU da Universidade Federal do Pará - UFPA - PA
51	A SERVIÇO DA JUSTIÇA ELEITORAL - LEI 9.504 ART. 98	CHU da Universidade Federal do Pará - UFPA - PA
51	A SERVIÇO DA JUSTIÇA ELEITORAL - LEI 9.504 ART. 98	CHU da Universidade Federal do Pará - UFPA - PA
51	A SERVIÇO DA JUSTIÇA ELEITORAL - LEI 9.504 ART. 98	CHU da Universidade Federal do Pará - UFPA - PA
51	A SERVIÇO DA JUSTIÇA ELEITORAL - LEI 9.504 ART. 98	CHU da Universidade Federal do Pará - UFPA - PA
51	A SERVIÇO DA JUSTIÇA ELEITORAL - LEI 9.504 ART. 98	CHU da Universidade Federal do Pará - UFPA - PA
51	A SERVIÇO DA JUSTIÇA ELEITORAL - LEI 9.504 ART. 98	CHU da Universidade Federal do Pará - UFPA - PA
57	AFASTAMENTO POR ATESTADO MÉDICO - ATE 15 DIAS	CHU da Universidade Federal do Pará - UFPA - PA
57	AFASTAMENTO POR ATESTADO MÉDICO - ATE 15 DIAS	CHU da Universidade Federal do Pará - UFPA - PA
57	AFASTAMENTO POR ATESTADO MÉDICO - ATE 15 DIAS	CHU da Universidade Federal do Pará - UFPA - PA
57	AFASTAMENTO POR ATESTADO MÉDICO - ATE 15 DIAS	CHU da Universidade Federal do Pará - UFPA - PA
57	AFASTAMENTO POR ATESTADO MÉDICO - ATE 15 DIAS	CHU da Universidade Federal do Pará - UFPA - PA
57	AFASTAMENTO POR ATESTADO MÉDICO - ATE 15 DIAS	CHU da Universidade Federal do Pará - UFPA - PA
57	AFASTAMENTO POR ATESTADO MÉDICO - ATE 15 DIAS	CHU da Universidade Federal do Pará - UFPA - PA
57	AFASTAMENTO POR ATESTADO MÉDICO - ATE 15 DIAS	CHU da Universidade Federal do Pará - UFPA - PA
57	AFASTAMENTO POR ATESTADO MÉDICO - ATE 15 DIAS	CHU da Universidade Federal do Pará - UFPA - PA
57	AFASTAMENTO POR ATESTADO MÉDICO - ATE 15 DIAS	CHU da Universidade Federal do Pará - UFPA - PA
57	AFASTAMENTO POR ATESTADO MÉDICO - ATE 15 DIAS	CHU da Universidade Federal do Pará - UFPA - PA
57	AFASTAMENTO POR ATESTADO MÉDICO - ATE 15 DIAS	CHU da Universidade Federal do Pará - UFPA - PA
57	AFASTAMENTO POR ATESTADO MÉDICO - ATE 15 DIAS	CHU da Universidade Federal do Pará - UFPA - PA
57	AFASTAMENTO POR ATESTADO MÉDICO - ATE 15 DIAS	CHU da Universidade Federal do Pará - UFPA - PA
57	AFASTAMENTO POR ATESTADO MÉDICO - ATE 15 DIAS	CHU da Universidade Federal do Pará - UFPA - PA
57	AFASTAMENTO POR ATESTADO MÉDICO - ATE 15 DIAS	CHU da Universidade Federal do Pará - UFPA - PA
57	AFASTAMENTO POR ATESTADO MÉDICO - ATE 15 DIAS	CHU da Universidade Federal do Pará - UFPA - PA
57	AFASTAMENTO POR ATESTADO MÉDICO - ATE 15 DIAS	CHU da Universidade Federal do Pará - UFPA - PA
57	AFASTAMENTO POR ATESTADO MÉDICO - ATE 15 DIAS	CHU da Universidade Federal do Pará - UFPA - PA
57	AFASTAMENTO POR ATESTADO MÉDICO - ATE 15 DIAS	CHU da Universidade Federal do Pará - UFPA - PA
57	AFASTAMENTO POR ATESTADO MÉDICO - ATE 15 DIAS	CHU da Universidade Federal do Pará - UFPA - PA
57	AFASTAMENTO POR ATESTADO MÉDICO - ATE 15 DIAS	CHU da Universidade Federal do Pará - UFPA - PA
57	AFASTAMENTO POR ATESTADO MÉDICO - ATE 15 DIAS	CHU da Universidade Federal do Pará - UFPA - PA
57	AFASTAMENTO POR ATESTADO MÉDICO - ATE 15 DIAS	CHU da Universidade Federal do Pará - UFPA - PA
57	AFASTAMENTO POR ATESTADO MÉDICO - ATE 15 DIAS	CHU da Universidade Federal do Pará - UFPA - PA
57	AFASTAMENTO POR ATESTADO MÉDICO - ATE 15 DIAS	CHU da Universidade Federal do Pará - UFPA - PA
57	AFASTAMENTO POR ATESTADO MÉDICO - ATE 15 DIAS	CHU da Universidade Federal do Pará - UFPA - PA
57	AFASTAMENTO POR ATESTADO MÉDICO - ATE 15 DIAS	CHU da Universidade Federal do Pará - UFPA - PA
57	AFASTAMENTO POR ATESTADO MÉDICO - ATE 15 DIAS	CHU da Universidade Federal do Pará - UFPA - PA
57	AFASTAMENTO POR ATESTADO MÉDICO - ATE 15 DIAS	CHU da Universidade Federal do Pará - UFPA - PA
57	AFASTAMENTO POR ATESTADO MÉDICO - ATE 15 DIAS	CHU da Universidade Federal do Pará - UFPA - PA
57	AFASTAMENTO POR ATESTADO MÉDICO - ATE 15 DIAS	CHU da Universidade Federal do Pará - UFPA - PA
57	AFASTAMENTO POR ATESTADO MÉDICO - ATE 15 DIAS	CHU da Universidade Federal do Pará - UFPA - PA
57	AFASTAMENTO POR ATESTADO MÉDICO - ATE 15 DIAS	CHU da Universidade Federal do Pará - UFPA - PA
57	AFASTAMENTO POR ATESTADO MÉDICO - ATE 15 DIAS	CHU da Universidade Federal do Pará - UFPA - PA
57	AFASTAMENTO POR ATESTADO MÉDICO - ATE 15 DIAS	CHU da Universidade Federal do Pará - UFPA - PA

57	AFASTAMENTO POR ATESTADO MÉDICO - ATE 15 DIAS	CHU da Universidade Federal do Pará - UFPA - PA
57	AFASTAMENTO POR ATESTADO MÉDICO - ATE 15 DIAS	CHU da Universidade Federal do Pará - UFPA - PA
57	AFASTAMENTO POR ATESTADO MÉDICO - ATE 15 DIAS	CHU da Universidade Federal do Pará - UFPA - PA
57	AFASTAMENTO POR ATESTADO MÉDICO - ATE 15 DIAS	CHU da Universidade Federal do Pará - UFPA - PA
57	AFASTAMENTO POR ATESTADO MÉDICO - ATE 15 DIAS	CHU da Universidade Federal do Pará - UFPA - PA
57	AFASTAMENTO POR ATESTADO MÉDICO - ATE 15 DIAS	CHU da Universidade Federal do Pará - UFPA - PA
57	AFASTAMENTO POR ATESTADO MÉDICO - ATE 15 DIAS	CHU da Universidade Federal do Pará - UFPA - PA
57	AFASTAMENTO POR ATESTADO MÉDICO - ATE 15 DIAS	CHU da Universidade Federal do Pará - UFPA - PA
57	AFASTAMENTO POR ATESTADO MÉDICO - ATE 15 DIAS	CHU da Universidade Federal do Pará - UFPA - PA
57	AFASTAMENTO POR ATESTADO MÉDICO - ATE 15 DIAS	CHU da Universidade Federal do Pará - UFPA - PA
57	AFASTAMENTO POR ATESTADO MÉDICO - ATE 15 DIAS	CHU da Universidade Federal do Pará - UFPA - PA
57	AFASTAMENTO POR ATESTADO MÉDICO - ATE 15 DIAS	CHU da Universidade Federal do Pará - UFPA - PA
57	AFASTAMENTO POR ATESTADO MÉDICO - ATE 15 DIAS	CHU da Universidade Federal do Pará - UFPA - PA
57	AFASTAMENTO POR ATESTADO MÉDICO - ATE 15 DIAS	CHU da Universidade Federal do Pará - UFPA - PA
57	AFASTAMENTO POR ATESTADO MÉDICO - ATE 15 DIAS	CHU da Universidade Federal do Pará - UFPA - PA
57	AFASTAMENTO POR ATESTADO MÉDICO - ATE 15 DIAS	CHU da Universidade Federal do Pará - UFPA - PA
57	AFASTAMENTO POR ATESTADO MÉDICO - ATE 15 DIAS	CHU da Universidade Federal do Pará - UFPA - PA
57	AFASTAMENTO POR ATESTADO MÉDICO - ATE 15 DIAS	CHU da Universidade Federal do Pará - UFPA - PA
57	AFASTAMENTO POR ATESTADO MÉDICO - ATE 15 DIAS	CHU da Universidade Federal do Pará - UFPA - PA
57	AFASTAMENTO POR ATESTADO MÉDICO - ATE 15 DIAS	CHU da Universidade Federal do Pará - UFPA - PA
57	AFASTAMENTO POR ATESTADO MÉDICO - ATE 15 DIAS	CHU da Universidade Federal do Pará - UFPA - PA
57	AFASTAMENTO POR ATESTADO MÉDICO - ATE 15 DIAS	CHU da Universidade Federal do Pará - UFPA - PA
57	AFASTAMENTO POR ATESTADO MÉDICO - ATE 15 DIAS	CHU da Universidade Federal do Pará - UFPA - PA
57	AFASTAMENTO POR ATESTADO MÉDICO - ATE 15 DIAS	CHU da Universidade Federal do Pará - UFPA - PA
57	AFASTAMENTO POR ATESTADO MÉDICO - ATE 15 DIAS	CHU da Universidade Federal do Pará - UFPA - PA
57	AFASTAMENTO POR ATESTADO MÉDICO - ATE 15 DIAS	CHU da Universidade Federal do Pará - UFPA - PA
57	AFASTAMENTO POR ATESTADO MÉDICO - ATE 15 DIAS	CHU da Universidade Federal do Pará - UFPA - PA
57	AFASTAMENTO POR ATESTADO MÉDICO - ATE 15 DIAS	CHU da Universidade Federal do Pará - UFPA - PA
57	AFASTAMENTO POR ATESTADO MÉDICO - ATE 15 DIAS	CHU da Universidade Federal do Pará - UFPA - PA
57	AFASTAMENTO POR ATESTADO MÉDICO - ATE 15 DIAS	CHU da Universidade Federal do Pará - UFPA - PA
57	AFASTAMENTO POR ATESTADO MÉDICO - ATE 15 DIAS	CHU da Universidade Federal do Pará - UFPA - PA
57	AFASTAMENTO POR ATESTADO MÉDICO - ATE 15 DIAS	CHU da Universidade Federal do Pará - UFPA - PA
57	AFASTAMENTO POR ATESTADO MÉDICO - ATE 15 DIAS	CHU da Universidade Federal do Pará - UFPA - PA
57	AFASTAMENTO POR ATESTADO MÉDICO - ATE 15 DIAS	CHU da Universidade Federal do Pará - UFPA - PA
57	AFASTAMENTO POR ATESTADO MÉDICO - ATE 15 DIAS	CHU da Universidade Federal do Pará - UFPA - PA
57	AFASTAMENTO POR ATESTADO MÉDICO - ATE 15 DIAS	CHU da Universidade Federal do Pará - UFPA - PA
57	AFASTAMENTO POR ATESTADO MÉDICO - ATE 15 DIAS	CHU da Universidade Federal do Pará - UFPA - PA
57	AFASTAMENTO POR ATESTADO MÉDICO - ATE 15 DIAS	CHU da Universidade Federal do Pará - UFPA - PA
57	AFASTAMENTO POR ATESTADO MÉDICO - ATE 15 DIAS	CHU da Universidade Federal do Pará - UFPA - PA
57	AFASTAMENTO POR ATESTADO MÉDICO - ATE 15 DIAS	CHU da Universidade Federal do Pará - UFPA - PA
57	AFASTAMENTO POR ATESTADO MÉDICO - ATE 15 DIAS	CHU da Universidade Federal do Pará - UFPA - PA
57	AFASTAMENTO POR ATESTADO MÉDICO - ATE 15 DIAS	CHU da Universidade Federal do Pará - UFPA - PA
57	AFASTAMENTO POR ATESTADO MÉDICO - ATE 15 DIAS	CHU da Universidade Federal do Pará - UFPA - PA
57	AFASTAMENTO POR ATESTADO MÉDICO - ATE 15 DIAS	CHU da Universidade Federal do Pará - UFPA - PA
57	AFASTAMENTO POR ATESTADO MÉDICO - ATE 15 DIAS	CHU da Universidade Federal do Pará - UFPA - PA
57	AFASTAMENTO POR ATESTADO MÉDICO - ATE 15 DIAS	CHU da Universidade Federal do Pará - UFPA - PA
57	AFASTAMENTO POR ATESTADO MÉDICO - ATE 15 DIAS	CHU da Universidade Federal do Pará - UFPA - PA
57	AFASTAMENTO POR ATESTADO MÉDICO - ATE 15 DIAS	CHU da Universidade Federal do Pará - UFPA - PA

57	AFASTAMENTO POR ATESTADO MÉDICO - ATE 15 DIAS	CHU da Universidade Federal do Pará - UFPA - PA
57	AFASTAMENTO POR ATESTADO MÉDICO - ATE 15 DIAS	CHU da Universidade Federal do Pará - UFPA - PA
57	AFASTAMENTO POR ATESTADO MÉDICO - ATE 15 DIAS	CHU da Universidade Federal do Pará - UFPA - PA
57	AFASTAMENTO POR ATESTADO MÉDICO - ATE 15 DIAS	CHU da Universidade Federal do Pará - UFPA - PA
57	AFASTAMENTO POR ATESTADO MÉDICO - ATE 15 DIAS	CHU da Universidade Federal do Pará - UFPA - PA
57	AFASTAMENTO POR ATESTADO MÉDICO - ATE 15 DIAS	CHU da Universidade Federal do Pará - UFPA - PA
57	AFASTAMENTO POR ATESTADO MÉDICO - ATE 15 DIAS	CHU da Universidade Federal do Pará - UFPA - PA
57	AFASTAMENTO POR ATESTADO MÉDICO - ATE 15 DIAS	CHU da Universidade Federal do Pará - UFPA - PA
57	AFASTAMENTO POR ATESTADO MÉDICO - ATE 15 DIAS	CHU da Universidade Federal do Pará - UFPA - PA
57	AFASTAMENTO POR ATESTADO MÉDICO - ATE 15 DIAS	CHU da Universidade Federal do Pará - UFPA - PA
57	AFASTAMENTO POR ATESTADO MÉDICO - ATE 15 DIAS	CHU da Universidade Federal do Pará - UFPA - PA
57	AFASTAMENTO POR ATESTADO MÉDICO - ATE 15 DIAS	CHU da Universidade Federal do Pará - UFPA - PA
57	AFASTAMENTO POR ATESTADO MÉDICO - ATE 15 DIAS	CHU da Universidade Federal do Pará - UFPA - PA
57	AFASTAMENTO POR ATESTADO MÉDICO - ATE 15 DIAS	CHU da Universidade Federal do Pará - UFPA - PA
57	AFASTAMENTO POR ATESTADO MÉDICO - ATE 15 DIAS	CHU da Universidade Federal do Pará - UFPA - PA
57	AFASTAMENTO POR ATESTADO MÉDICO - ATE 15 DIAS	CHU da Universidade Federal do Pará - UFPA - PA
57	AFASTAMENTO POR ATESTADO MÉDICO - ATE 15 DIAS	CHU da Universidade Federal do Pará - UFPA - PA
57	AFASTAMENTO POR ATESTADO MÉDICO - ATE 15 DIAS	CHU da Universidade Federal do Pará - UFPA - PA
57	AFASTAMENTO POR ATESTADO MÉDICO - ATE 15 DIAS	CHU da Universidade Federal do Pará - UFPA - PA
57	AFASTAMENTO POR ATESTADO MÉDICO - ATE 15 DIAS	CHU da Universidade Federal do Pará - UFPA - PA
57	AFASTAMENTO POR ATESTADO MÉDICO - ATE 15 DIAS	CHU da Universidade Federal do Pará - UFPA - PA
57	AFASTAMENTO POR ATESTADO MÉDICO - ATE 15 DIAS	CHU da Universidade Federal do Pará - UFPA - PA
57	AFASTAMENTO POR ATESTADO MÉDICO - ATE 15 DIAS	CHU da Universidade Federal do Pará - UFPA - PA
57	AFASTAMENTO POR ATESTADO MÉDICO - ATE 15 DIAS	CHU da Universidade Federal do Pará - UFPA - PA
57	AFASTAMENTO POR ATESTADO MÉDICO - ATE 15 DIAS	CHU da Universidade Federal do Pará - UFPA - PA
57	AFASTAMENTO POR ATESTADO MÉDICO - ATE 15 DIAS	CHU da Universidade Federal do Pará - UFPA - PA
57	AFASTAMENTO POR ATESTADO MÉDICO - ATE 15 DIAS	CHU da Universidade Federal do Pará - UFPA - PA
57	AFASTAMENTO POR ATESTADO MÉDICO - ATE 15 DIAS	CHU da Universidade Federal do Pará - UFPA - PA
57	AFASTAMENTO POR ATESTADO MÉDICO - ATE 15 DIAS	CHU da Universidade Federal do Pará - UFPA - PA
57	AFASTAMENTO POR ATESTADO MÉDICO - ATE 15 DIAS	CHU da Universidade Federal do Pará - UFPA - PA
57	AFASTAMENTO POR ATESTADO MÉDICO - ATE 15 DIAS	CHU da Universidade Federal do Pará - UFPA - PA
57	AFASTAMENTO POR ATESTADO MÉDICO - ATE 15 DIAS	CHU da Universidade Federal do Pará - UFPA - PA
57	AFASTAMENTO POR ATESTADO MÉDICO - ATE 15 DIAS	CHU da Universidade Federal do Pará - UFPA - PA
57	AFASTAMENTO POR ATESTADO MÉDICO - ATE 15 DIAS	CHU da Universidade Federal do Pará - UFPA - PA
57	AFASTAMENTO POR ATESTADO MÉDICO - ATE 15 DIAS	CHU da Universidade Federal do Pará - UFPA - PA
57	AFASTAMENTO POR ATESTADO MÉDICO - ATE 15 DIAS	CHU da Universidade Federal do Pará - UFPA - PA
57	AFASTAMENTO POR ATESTADO MÉDICO - ATE 15 DIAS	CHU da Universidade Federal do Pará - UFPA - PA
57	AFASTAMENTO POR ATESTADO MÉDICO - ATE 15 DIAS	CHU da Universidade Federal do Pará - UFPA - PA
57	AFASTAMENTO POR ATESTADO MÉDICO - ATE 15 DIAS	CHU da Universidade Federal do Pará - UFPA - PA
57	AFASTAMENTO POR ATESTADO MÉDICO - ATE 15 DIAS	CHU da Universidade Federal do Pará - UFPA - PA
57	AFASTAMENTO POR ATESTADO MÉDICO - ATE 15 DIAS	CHU da Universidade Federal do Pará - UFPA - PA
57	AFASTAMENTO POR ATESTADO MÉDICO - ATE 15 DIAS	CHU da Universidade Federal do Pará - UFPA - PA
57	AFASTAMENTO POR ATESTADO MÉDICO - ATE 15 DIAS	CHU da Universidade Federal do Pará - UFPA - PA
57	AFASTAMENTO POR ATESTADO MÉDICO - ATE 15 DIAS	CHU da Universidade Federal do Pará - UFPA - PA
57	AFASTAMENTO POR ATESTADO MÉDICO - ATE 15 DIAS	CHU da Universidade Federal do Pará - UFPA - PA
57	AFASTAMENTO POR ATESTADO MÉDICO - ATE 15 DIAS	CHU da Universidade Federal do Pará - UFPA - PA
57	AFASTAMENTO POR ATESTADO MÉDICO - ATE 15 DIAS	CHU da Universidade Federal do Pará - UFPA - PA
57	AFASTAMENTO POR ATESTADO MÉDICO - ATE 15 DIAS	CHU da Universidade Federal do Pará - UFPA - PA

57	AFASTAMENTO POR ATESTADO MÉDICO - ATE 15 DIAS	CHU da Universidade Federal do Pará - UFPA - PA
57	AFASTAMENTO POR ATESTADO MÉDICO - ATE 15 DIAS	CHU da Universidade Federal do Pará - UFPA - PA
57	AFASTAMENTO POR ATESTADO MÉDICO - ATE 15 DIAS	CHU da Universidade Federal do Pará - UFPA - PA
57	AFASTAMENTO POR ATESTADO MÉDICO - ATE 15 DIAS	CHU da Universidade Federal do Pará - UFPA - PA
57	AFASTAMENTO POR ATESTADO MÉDICO - ATE 15 DIAS	CHU da Universidade Federal do Pará - UFPA - PA
57	AFASTAMENTO POR ATESTADO MÉDICO - ATE 15 DIAS	CHU da Universidade Federal do Pará - UFPA - PA
57	AFASTAMENTO POR ATESTADO MÉDICO - ATE 15 DIAS	CHU da Universidade Federal do Pará - UFPA - PA
57	AFASTAMENTO POR ATESTADO MÉDICO - ATE 15 DIAS	CHU da Universidade Federal do Pará - UFPA - PA
57	AFASTAMENTO POR ATESTADO MÉDICO - ATE 15 DIAS	CHU da Universidade Federal do Pará - UFPA - PA
57	AFASTAMENTO POR ATESTADO MÉDICO - ATE 15 DIAS	CHU da Universidade Federal do Pará - UFPA - PA
57	AFASTAMENTO POR ATESTADO MÉDICO - ATE 15 DIAS	CHU da Universidade Federal do Pará - UFPA - PA
57	AFASTAMENTO POR ATESTADO MÉDICO - ATE 15 DIAS	CHU da Universidade Federal do Pará - UFPA - PA
57	AFASTAMENTO POR ATESTADO MÉDICO - ATE 15 DIAS	CHU da Universidade Federal do Pará - UFPA - PA
57	AFASTAMENTO POR ATESTADO MÉDICO - ATE 15 DIAS	CHU da Universidade Federal do Pará - UFPA - PA
57	AFASTAMENTO POR ATESTADO MÉDICO - ATE 15 DIAS	CHU da Universidade Federal do Pará - UFPA - PA
57	AFASTAMENTO POR ATESTADO MÉDICO - ATE 15 DIAS	CHU da Universidade Federal do Pará - UFPA - PA
57	AFASTAMENTO POR ATESTADO MÉDICO - ATE 15 DIAS	CHU da Universidade Federal do Pará - UFPA - PA
57	AFASTAMENTO POR ATESTADO MÉDICO - ATE 15 DIAS	CHU da Universidade Federal do Pará - UFPA - PA
57	AFASTAMENTO POR ATESTADO MÉDICO - ATE 15 DIAS	CHU da Universidade Federal do Pará - UFPA - PA
57	AFASTAMENTO POR ATESTADO MÉDICO - ATE 15 DIAS	CHU da Universidade Federal do Pará - UFPA - PA
57	AFASTAMENTO POR ATESTADO MÉDICO - ATE 15 DIAS	CHU da Universidade Federal do Pará - UFPA - PA
57	AFASTAMENTO POR ATESTADO MÉDICO - ATE 15 DIAS	CHU da Universidade Federal do Pará - UFPA - PA
57	AFASTAMENTO POR ATESTADO MÉDICO - ATE 15 DIAS	CHU da Universidade Federal do Pará - UFPA - PA
57	AFASTAMENTO POR ATESTADO MÉDICO - ATE 15 DIAS	CHU da Universidade Federal do Pará - UFPA - PA
57	AFASTAMENTO POR ATESTADO MÉDICO - ATE 15 DIAS	CHU da Universidade Federal do Pará - UFPA - PA
57	AFASTAMENTO POR ATESTADO MÉDICO - ATE 15 DIAS	CHU da Universidade Federal do Pará - UFPA - PA
57	AFASTAMENTO POR ATESTADO MÉDICO - ATE 15 DIAS	CHU da Universidade Federal do Pará - UFPA - PA
57	AFASTAMENTO POR ATESTADO MÉDICO - ATE 15 DIAS	CHU da Universidade Federal do Pará - UFPA - PA
57	AFASTAMENTO POR ATESTADO MÉDICO - ATE 15 DIAS	CHU da Universidade Federal do Pará - UFPA - PA
57	AFASTAMENTO POR ATESTADO MÉDICO - ATE 15 DIAS	CHU da Universidade Federal do Pará - UFPA - PA
57	AFASTAMENTO POR ATESTADO MÉDICO - ATE 15 DIAS	CHU da Universidade Federal do Pará - UFPA - PA
57	AFASTAMENTO POR ATESTADO MÉDICO - ATE 15 DIAS	CHU da Universidade Federal do Pará - UFPA - PA

57	AFASTAMENTO POR ATESTADO MÉDICO - ATE 15 DIAS	CHU da Universidade Federal do Pará - UFPA - PA
57	AFASTAMENTO POR ATESTADO MÉDICO - ATE 15 DIAS	CHU da Universidade Federal do Pará - UFPA - PA
57	AFASTAMENTO POR ATESTADO MÉDICO - ATE 15 DIAS	CHU da Universidade Federal do Pará - UFPA - PA
57	AFASTAMENTO POR ATESTADO MÉDICO - ATE 15 DIAS	CHU da Universidade Federal do Pará - UFPA - PA
57	AFASTAMENTO POR ATESTADO MÉDICO - ATE 15 DIAS	CHU da Universidade Federal do Pará - UFPA - PA
57	AFASTAMENTO POR ATESTADO MÉDICO - ATE 15 DIAS	CHU da Universidade Federal do Pará - UFPA - PA
57	AFASTAMENTO POR ATESTADO MÉDICO - ATE 15 DIAS	CHU da Universidade Federal do Pará - UFPA - PA
57	AFASTAMENTO POR ATESTADO MÉDICO - ATE 15 DIAS	CHU da Universidade Federal do Pará - UFPA - PA
57	AFASTAMENTO POR ATESTADO MÉDICO - ATE 15 DIAS	CHU da Universidade Federal do Pará - UFPA - PA
57	AFASTAMENTO POR ATESTADO MÉDICO - ATE 15 DIAS	CHU da Universidade Federal do Pará - UFPA - PA
57	AFASTAMENTO POR ATESTADO MÉDICO - ATE 15 DIAS	CHU da Universidade Federal do Pará - UFPA - PA
57	AFASTAMENTO POR ATESTADO MÉDICO - ATE 15 DIAS	CHU da Universidade Federal do Pará - UFPA - PA
57	AFASTAMENTO POR ATESTADO MÉDICO - ATE 15 DIAS	CHU da Universidade Federal do Pará - UFPA - PA
57	AFASTAMENTO POR ATESTADO MÉDICO - ATE 15 DIAS	CHU da Universidade Federal do Pará - UFPA - PA
57	AFASTAMENTO POR ATESTADO MÉDICO - ATE 15 DIAS	CHU da Universidade Federal do Pará - UFPA - PA
57	AFASTAMENTO POR ATESTADO MÉDICO - ATE 15 DIAS	CHU da Universidade Federal do Pará - UFPA - PA
57	AFASTAMENTO POR ATESTADO MÉDICO - ATE 15 DIAS	CHU da Universidade Federal do Pará - UFPA - PA
57	AFASTAMENTO POR ATESTADO MÉDICO - ATE 15 DIAS	CHU da Universidade Federal do Pará - UFPA - PA
57	AFASTAMENTO POR ATESTADO MÉDICO - ATE 15 DIAS	CHU da Universidade Federal do Pará - UFPA - PA
57	AFASTAMENTO POR ATESTADO MÉDICO - ATE 15 DIAS	CHU da Universidade Federal do Pará - UFPA - PA
57	AFASTAMENTO POR ATESTADO MÉDICO - ATE 15 DIAS	CHU da Universidade Federal do Pará - UFPA - PA
57	AFASTAMENTO POR ATESTADO MÉDICO - ATE 15 DIAS	CHU da Universidade Federal do Pará - UFPA - PA
57	AFASTAMENTO POR ATESTADO MÉDICO - ATE 15 DIAS	CHU da Universidade Federal do Pará - UFPA - PA
57	AFASTAMENTO POR ATESTADO MÉDICO - ATE 15 DIAS	CHU da Universidade Federal do Pará - UFPA - PA
57	AFASTAMENTO POR ATESTADO MÉDICO - ATE 15 DIAS	CHU da Universidade Federal do Pará - UFPA - PA
57	AFASTAMENTO POR ATESTADO MÉDICO - ATE 15 DIAS	CHU da Universidade Federal do Pará - UFPA - PA
57	AFASTAMENTO POR ATESTADO MÉDICO - ATE 15 DIAS	CHU da Universidade Federal do Pará - UFPA - PA
57	AFASTAMENTO POR ATESTADO MÉDICO - ATE 15 DIAS	CHU da Universidade Federal do Pará - UFPA - PA
57	AFASTAMENTO POR ATESTADO MÉDICO - ATE 15 DIAS	CHU da Universidade Federal do Pará - UFPA - PA
57	AFASTAMENTO POR ATESTADO MÉDICO - ATE 15 DIAS	CHU da Universidade Federal do Pará - UFPA - PA
57	AFASTAMENTO POR ATESTADO MÉDICO - ATE 15 DIAS	CHU da Universidade Federal do Pará - UFPA - PA
57	AFASTAMENTO POR ATESTADO MÉDICO - ATE 15 DIAS	CHU da Universidade Federal do Pará - UFPA - PA
57	AFASTAMENTO POR ATESTADO MÉDICO - ATE 15 DIAS	CHU da Universidade Federal do Pará - UFPA - PA

57	AFASTAMENTO POR ATESTADO MÉDICO - ATE 15 DIAS	CHU da Universidade Federal do Pará - UFPA - PA
57	AFASTAMENTO POR ATESTADO MÉDICO - ATE 15 DIAS	CHU da Universidade Federal do Pará - UFPA - PA
57	AFASTAMENTO POR ATESTADO MÉDICO - ATE 15 DIAS	CHU da Universidade Federal do Pará - UFPA - PA
57	AFASTAMENTO POR ATESTADO MÉDICO - ATE 15 DIAS	CHU da Universidade Federal do Pará - UFPA - PA
57	AFASTAMENTO POR ATESTADO MÉDICO - ATE 15 DIAS	CHU da Universidade Federal do Pará - UFPA - PA
57	AFASTAMENTO POR ATESTADO MÉDICO - ATE 15 DIAS	CHU da Universidade Federal do Pará - UFPA - PA
57	AFASTAMENTO POR ATESTADO MÉDICO - ATE 15 DIAS	CHU da Universidade Federal do Pará - UFPA - PA
57	AFASTAMENTO POR ATESTADO MÉDICO - ATE 15 DIAS	CHU da Universidade Federal do Pará - UFPA - PA
57	AFASTAMENTO POR ATESTADO MÉDICO - ATE 15 DIAS	CHU da Universidade Federal do Pará - UFPA - PA
57	AFASTAMENTO POR ATESTADO MÉDICO - ATE 15 DIAS	CHU da Universidade Federal do Pará - UFPA - PA
57	AFASTAMENTO POR ATESTADO MÉDICO - ATE 15 DIAS	CHU da Universidade Federal do Pará - UFPA - PA
57	AFASTAMENTO POR ATESTADO MÉDICO - ATE 15 DIAS	CHU da Universidade Federal do Pará - UFPA - PA
57	AFASTAMENTO POR ATESTADO MÉDICO - ATE 15 DIAS	CHU da Universidade Federal do Pará - UFPA - PA
57	AFASTAMENTO POR ATESTADO MÉDICO - ATE 15 DIAS	CHU da Universidade Federal do Pará - UFPA - PA
57	AFASTAMENTO POR ATESTADO MÉDICO - ATE 15 DIAS	CHU da Universidade Federal do Pará - UFPA - PA
57	AFASTAMENTO POR ATESTADO MÉDICO - ATE 15 DIAS	CHU da Universidade Federal do Pará - UFPA - PA
57	AFASTAMENTO POR ATESTADO MÉDICO - ATE 15 DIAS	CHU da Universidade Federal do Pará - UFPA - PA
57	AFASTAMENTO POR ATESTADO MÉDICO - ATE 15 DIAS	CHU da Universidade Federal do Pará - UFPA - PA
57	AFASTAMENTO POR ATESTADO MÉDICO - ATE 15 DIAS	CHU da Universidade Federal do Pará - UFPA - PA
57	AFASTAMENTO POR ATESTADO MÉDICO - ATE 15 DIAS	CHU da Universidade Federal do Pará - UFPA - PA
57	AFASTAMENTO POR ATESTADO MÉDICO - ATE 15 DIAS	CHU da Universidade Federal do Pará - UFPA - PA
57	AFASTAMENTO POR ATESTADO MÉDICO - ATE 15 DIAS	CHU da Universidade Federal do Pará - UFPA - PA
57	AFASTAMENTO POR ATESTADO MÉDICO - ATE 15 DIAS	CHU da Universidade Federal do Pará - UFPA - PA
57	AFASTAMENTO POR ATESTADO MÉDICO - ATE 15 DIAS	CHU da Universidade Federal do Pará - UFPA - PA
57	AFASTAMENTO POR ATESTADO MÉDICO - ATE 15 DIAS	CHU da Universidade Federal do Pará - UFPA - PA
57	AFASTAMENTO POR ATESTADO MÉDICO - ATE 15 DIAS	CHU da Universidade Federal do Pará - UFPA - PA
57	AFASTAMENTO POR ATESTADO MÉDICO - ATE 15 DIAS	CHU da Universidade Federal do Pará - UFPA - PA
57	AFASTAMENTO POR ATESTADO MÉDICO - ATE 15 DIAS	CHU da Universidade Federal do Pará - UFPA - PA
57	AFASTAMENTO POR ATESTADO MÉDICO - ATE 15 DIAS	CHU da Universidade Federal do Pará - UFPA - PA
57	AFASTAMENTO POR ATESTADO MÉDICO - ATE 15 DIAS	CHU da Universidade Federal do Pará - UFPA - PA
57	AFASTAMENTO POR ATESTADO MÉDICO - ATE 15 DIAS	CHU da Universidade Federal do Pará - UFPA - PA
57	AFASTAMENTO POR ATESTADO MÉDICO - ATE 15 DIAS	CHU da Universidade Federal do Pará - UFPA - PA

57	AFASTAMENTO POR ATESTADO MÉDICO - ATE 15 DIAS	CHU da Universidade Federal do Pará - UFPA - PA
57	AFASTAMENTO POR ATESTADO MÉDICO - ATE 15 DIAS	CHU da Universidade Federal do Pará - UFPA - PA
57	AFASTAMENTO POR ATESTADO MÉDICO - ATE 15 DIAS	CHU da Universidade Federal do Pará - UFPA - PA
57	AFASTAMENTO POR ATESTADO MÉDICO - ATE 15 DIAS	CHU da Universidade Federal do Pará - UFPA - PA
57	AFASTAMENTO POR ATESTADO MÉDICO - ATE 15 DIAS	CHU da Universidade Federal do Pará - UFPA - PA
57	AFASTAMENTO POR ATESTADO MÉDICO - ATE 15 DIAS	CHU da Universidade Federal do Pará - UFPA - PA
57	AFASTAMENTO POR ATESTADO MÉDICO - ATE 15 DIAS	CHU da Universidade Federal do Pará - UFPA - PA
57	AFASTAMENTO POR ATESTADO MÉDICO - ATE 15 DIAS	CHU da Universidade Federal do Pará - UFPA - PA
57	AFASTAMENTO POR ATESTADO MÉDICO - ATE 15 DIAS	CHU da Universidade Federal do Pará - UFPA - PA
57	AFASTAMENTO POR ATESTADO MÉDICO - ATE 15 DIAS	CHU da Universidade Federal do Pará - UFPA - PA
57	AFASTAMENTO POR ATESTADO MÉDICO - ATE 15 DIAS	CHU da Universidade Federal do Pará - UFPA - PA
57	AFASTAMENTO POR ATESTADO MÉDICO - ATE 15 DIAS	CHU da Universidade Federal do Pará - UFPA - PA
57	AFASTAMENTO POR ATESTADO MÉDICO - ATE 15 DIAS	CHU da Universidade Federal do Pará - UFPA - PA
57	AFASTAMENTO POR ATESTADO MÉDICO - ATE 15 DIAS	CHU da Universidade Federal do Pará - UFPA - PA
57	AFASTAMENTO POR ATESTADO MÉDICO - ATE 15 DIAS	CHU da Universidade Federal do Pará - UFPA - PA
57	AFASTAMENTO POR ATESTADO MÉDICO - ATE 15 DIAS	CHU da Universidade Federal do Pará - UFPA - PA
57	AFASTAMENTO POR ATESTADO MÉDICO - ATE 15 DIAS	CHU da Universidade Federal do Pará - UFPA - PA
57	AFASTAMENTO POR ATESTADO MÉDICO - ATE 15 DIAS	CHU da Universidade Federal do Pará - UFPA - PA
57	AFASTAMENTO POR ATESTADO MÉDICO - ATE 15 DIAS	CHU da Universidade Federal do Pará - UFPA - PA
57	AFASTAMENTO POR ATESTADO MÉDICO - ATE 15 DIAS	CHU da Universidade Federal do Pará - UFPA - PA
57	AFASTAMENTO POR ATESTADO MÉDICO - ATE 15 DIAS	CHU da Universidade Federal do Pará - UFPA - PA
57	AFASTAMENTO POR ATESTADO MÉDICO - ATE 15 DIAS	CHU da Universidade Federal do Pará - UFPA - PA
57	AFASTAMENTO POR ATESTADO MÉDICO - ATE 15 DIAS	CHU da Universidade Federal do Pará - UFPA - PA
57	AFASTAMENTO POR ATESTADO MÉDICO - ATE 15 DIAS	CHU da Universidade Federal do Pará - UFPA - PA
57	AFASTAMENTO POR ATESTADO MÉDICO - ATE 15 DIAS	CHU da Universidade Federal do Pará - UFPA - PA
57	AFASTAMENTO POR ATESTADO MÉDICO - ATE 15 DIAS	CHU da Universidade Federal do Pará - UFPA - PA
57	AFASTAMENTO POR ATESTADO MÉDICO - ATE 15 DIAS	CHU da Universidade Federal do Pará - UFPA - PA
57	AFASTAMENTO POR ATESTADO MÉDICO - ATE 15 DIAS	CHU da Universidade Federal do Pará - UFPA - PA
57	AFASTAMENTO POR ATESTADO MÉDICO - ATE 15 DIAS	CHU da Universidade Federal do Pará - UFPA - PA
57	AFASTAMENTO POR ATESTADO MÉDICO - ATE 15 DIAS	CHU da Universidade Federal do Pará - UFPA - PA
57	AFASTAMENTO POR ATESTADO MÉDICO - ATE 15 DIAS	CHU da Universidade Federal do Pará - UFPA - PA
57	AFASTAMENTO POR ATESTADO MÉDICO - ATE 15 DIAS	CHU da Universidade Federal do Pará - UFPA - PA
57	AFASTAMENTO POR ATESTADO MÉDICO - ATE 15 DIAS	CHU da Universidade Federal do Pará - UFPA - PA
57	AFASTAMENTO POR ATESTADO MÉDICO - ATE 15 DIAS	CHU da Universidade Federal do Pará - UFPA - PA
57	AFASTAMENTO POR ATESTADO MÉDICO - ATE 15 DIAS	CHU da Universidade Federal do Pará - UFPA - PA
57	AFASTAMENTO POR ATESTADO MÉDICO - ATE 15 DIAS	CHU da Universidade Federal do Pará - UFPA - PA
57	AFASTAMENTO POR ATESTADO MÉDICO - ATE 15 DIAS	CHU da Universidade Federal do Pará - UFPA - PA
57	AFASTAMENTO POR ATESTADO MÉDICO - ATE 15 DIAS	CHU da Universidade Federal do Pará - UFPA - PA
57	AFASTAMENTO POR ATESTADO MÉDICO - ATE 15 DIAS	CHU da Universidade Federal do Pará - UFPA - PA
57	AFASTAMENTO POR ATESTADO MÉDICO - ATE 15 DIAS	CHU da Universidade Federal do Pará - UFPA - PA
57	AFASTAMENTO POR ATESTADO MÉDICO - ATE 15 DIAS	CHU da Universidade Federal do Pará - UFPA - PA

57	AFASTAMENTO POR ATESTADO MÉDICO - ATE 15 DIAS	CHU da Universidade Federal do Pará - UFPA - PA
57	AFASTAMENTO POR ATESTADO MÉDICO - ATE 15 DIAS	CHU da Universidade Federal do Pará - UFPA - PA
57	AFASTAMENTO POR ATESTADO MÉDICO - ATE 15 DIAS	CHU da Universidade Federal do Pará - UFPA - PA
57	AFASTAMENTO POR ATESTADO MÉDICO - ATE 15 DIAS	CHU da Universidade Federal do Pará - UFPA - PA
57	AFASTAMENTO POR ATESTADO MÉDICO - ATE 15 DIAS	CHU da Universidade Federal do Pará - UFPA - PA
57	AFASTAMENTO POR ATESTADO MÉDICO - ATE 15 DIAS	CHU da Universidade Federal do Pará - UFPA - PA
57	AFASTAMENTO POR ATESTADO MÉDICO - ATE 15 DIAS	CHU da Universidade Federal do Pará - UFPA - PA
57	AFASTAMENTO POR ATESTADO MÉDICO - ATE 15 DIAS	CHU da Universidade Federal do Pará - UFPA - PA
57	AFASTAMENTO POR ATESTADO MÉDICO - ATE 15 DIAS	CHU da Universidade Federal do Pará - UFPA - PA
57	AFASTAMENTO POR ATESTADO MÉDICO - ATE 15 DIAS	CHU da Universidade Federal do Pará - UFPA - PA
57	AFASTAMENTO POR ATESTADO MÉDICO - ATE 15 DIAS	CHU da Universidade Federal do Pará - UFPA - PA
57	AFASTAMENTO POR ATESTADO MÉDICO - ATE 15 DIAS	CHU da Universidade Federal do Pará - UFPA - PA
57	AFASTAMENTO POR ATESTADO MÉDICO - ATE 15 DIAS	CHU da Universidade Federal do Pará - UFPA - PA
57	AFASTAMENTO POR ATESTADO MÉDICO - ATE 15 DIAS	CHU da Universidade Federal do Pará - UFPA - PA
57	AFASTAMENTO POR ATESTADO MÉDICO - ATE 15 DIAS	CHU da Universidade Federal do Pará - UFPA - PA
57	AFASTAMENTO POR ATESTADO MÉDICO - ATE 15 DIAS	CHU da Universidade Federal do Pará - UFPA - PA
57	AFASTAMENTO POR ATESTADO MÉDICO - ATE 15 DIAS	CHU da Universidade Federal do Pará - UFPA - PA
57	AFASTAMENTO POR ATESTADO MÉDICO - ATE 15 DIAS	CHU da Universidade Federal do Pará - UFPA - PA
57	AFASTAMENTO POR ATESTADO MÉDICO - ATE 15 DIAS	CHU da Universidade Federal do Pará - UFPA - PA
57	AFASTAMENTO POR ATESTADO MÉDICO - ATE 15 DIAS	CHU da Universidade Federal do Pará - UFPA - PA
57	AFASTAMENTO POR ATESTADO MÉDICO - ATE 15 DIAS	CHU da Universidade Federal do Pará - UFPA - PA
57	AFASTAMENTO POR ATESTADO MÉDICO - ATE 15 DIAS	CHU da Universidade Federal do Pará - UFPA - PA
57	AFASTAMENTO POR ATESTADO MÉDICO - ATE 15 DIAS	CHU da Universidade Federal do Pará - UFPA - PA
57	AFASTAMENTO POR ATESTADO MÉDICO - ATE 15 DIAS	CHU da Universidade Federal do Pará - UFPA - PA
57	AFASTAMENTO POR ATESTADO MÉDICO - ATE 15 DIAS	CHU da Universidade Federal do Pará - UFPA - PA
57	AFASTAMENTO POR ATESTADO MÉDICO - ATE 15 DIAS	CHU da Universidade Federal do Pará - UFPA - PA
57	AFASTAMENTO POR ATESTADO MÉDICO - ATE 15 DIAS	CHU da Universidade Federal do Pará - UFPA - PA
57	AFASTAMENTO POR ATESTADO MÉDICO - ATE 15 DIAS	CHU da Universidade Federal do Pará - UFPA - PA
57	AFASTAMENTO POR ATESTADO MÉDICO - ATE 15 DIAS	CHU da Universidade Federal do Pará - UFPA - PA
57	AFASTAMENTO POR ATESTADO MÉDICO - ATE 15 DIAS	CHU da Universidade Federal do Pará - UFPA - PA
57	AFASTAMENTO POR ATESTADO MÉDICO - ATE 15 DIAS	CHU da Universidade Federal do Pará - UFPA - PA
57	AFASTAMENTO POR ATESTADO MÉDICO - ATE 15 DIAS	CHU da Universidade Federal do Pará - UFPA - PA
57	AFASTAMENTO POR ATESTADO MÉDICO - ATE 15 DIAS	CHU da Universidade Federal do Pará - UFPA - PA
57	AFASTAMENTO POR ATESTADO MÉDICO - ATE 15 DIAS	CHU da Universidade Federal do Pará - UFPA - PA
57	AFASTAMENTO POR ATESTADO MÉDICO - ATE 15 DIAS	CHU da Universidade Federal do Pará - UFPA - PA
57	AFASTAMENTO POR ATESTADO MÉDICO - ATE 15 DIAS	CHU da Universidade Federal do Pará - UFPA - PA
57	AFASTAMENTO POR ATESTADO MÉDICO - ATE 15 DIAS	CHU da Universidade Federal do Pará - UFPA - PA
57	AFASTAMENTO POR ATESTADO MÉDICO - ATE 15 DIAS	CHU da Universidade Federal do Pará - UFPA - PA
57	AFASTAMENTO POR ATESTADO MÉDICO - ATE 15 DIAS	CHU da Universidade Federal do Pará - UFPA - PA
57	AFASTAMENTO POR ATESTADO MÉDICO - ATE 15 DIAS	CHU da Universidade Federal do Pará - UFPA - PA
57	AFASTAMENTO POR ATESTADO MÉDICO - ATE 15 DIAS	CHU da Universidade Federal do Pará - UFPA - PA
57	AFASTAMENTO POR ATESTADO MÉDICO - ATE 15 DIAS	CHU da Universidade Federal do Pará - UFPA - PA
57	AFASTAMENTO POR ATESTADO MÉDICO - ATE 15 DIAS	CHU da Universidade Federal do Pará - UFPA - PA
57	AFASTAMENTO POR ATESTADO MÉDICO - ATE 15 DIAS	CHU da Universidade Federal do Pará - UFPA - PA
57	AFASTAMENTO POR ATESTADO MÉDICO - ATE 15 DIAS	CHU da Universidade Federal do Pará - UFPA - PA
57	AFASTAMENTO POR ATESTADO MÉDICO - ATE 15 DIAS	CHU da Universidade Federal do Pará - UFPA - PA
57	AFASTAMENTO POR ATESTADO MÉDICO - ATE 15 DIAS	CHU da Universidade Federal do Pará - UFPA - PA
57	AFASTAMENTO POR ATESTADO MÉDICO - ATE 15 DIAS	CHU da Universidade Federal do Pará - UFPA - PA

57	AFASTAMENTO POR ATESTADO MÉDICO - ATE 15 DIAS	CHU da Universidade Federal do Pará - UFPA - PA
57	AFASTAMENTO POR ATESTADO MÉDICO - ATE 15 DIAS	CHU da Universidade Federal do Pará - UFPA - PA
57	AFASTAMENTO POR ATESTADO MÉDICO - ATE 15 DIAS	CHU da Universidade Federal do Pará - UFPA - PA
57	AFASTAMENTO POR ATESTADO MÉDICO - ATE 15 DIAS	CHU da Universidade Federal do Pará - UFPA - PA
57	AFASTAMENTO POR ATESTADO MÉDICO - ATE 15 DIAS	CHU da Universidade Federal do Pará - UFPA - PA
57	AFASTAMENTO POR ATESTADO MÉDICO - ATE 15 DIAS	CHU da Universidade Federal do Pará - UFPA - PA
57	AFASTAMENTO POR ATESTADO MÉDICO - ATE 15 DIAS	CHU da Universidade Federal do Pará - UFPA - PA
57	AFASTAMENTO POR ATESTADO MÉDICO - ATE 15 DIAS	CHU da Universidade Federal do Pará - UFPA - PA
57	AFASTAMENTO POR ATESTADO MÉDICO - ATE 15 DIAS	CHU da Universidade Federal do Pará - UFPA - PA
57	AFASTAMENTO POR ATESTADO MÉDICO - ATE 15 DIAS	CHU da Universidade Federal do Pará - UFPA - PA
57	AFASTAMENTO POR ATESTADO MÉDICO - ATE 15 DIAS	CHU da Universidade Federal do Pará - UFPA - PA
57	AFASTAMENTO POR ATESTADO MÉDICO - ATE 15 DIAS	CHU da Universidade Federal do Pará - UFPA - PA
57	AFASTAMENTO POR ATESTADO MÉDICO - ATE 15 DIAS	CHU da Universidade Federal do Pará - UFPA - PA
57	AFASTAMENTO POR ATESTADO MÉDICO - ATE 15 DIAS	CHU da Universidade Federal do Pará - UFPA - PA
57	AFASTAMENTO POR ATESTADO MÉDICO - ATE 15 DIAS	CHU da Universidade Federal do Pará - UFPA - PA
57	AFASTAMENTO POR ATESTADO MÉDICO - ATE 15 DIAS	CHU da Universidade Federal do Pará - UFPA - PA
57	AFASTAMENTO POR ATESTADO MÉDICO - ATE 15 DIAS	CHU da Universidade Federal do Pará - UFPA - PA
57	AFASTAMENTO POR ATESTADO MÉDICO - ATE 15 DIAS	CHU da Universidade Federal do Pará - UFPA - PA
57	AFASTAMENTO POR ATESTADO MÉDICO - ATE 15 DIAS	CHU da Universidade Federal do Pará - UFPA - PA
57	AFASTAMENTO POR ATESTADO MÉDICO - ATE 15 DIAS	CHU da Universidade Federal do Pará - UFPA - PA
57	AFASTAMENTO POR ATESTADO MÉDICO - ATE 15 DIAS	CHU da Universidade Federal do Pará - UFPA - PA
57	AFASTAMENTO POR ATESTADO MÉDICO - ATE 15 DIAS	CHU da Universidade Federal do Pará - UFPA - PA
57	AFASTAMENTO POR ATESTADO MÉDICO - ATE 15 DIAS	CHU da Universidade Federal do Pará - UFPA - PA
57	AFASTAMENTO POR ATESTADO MÉDICO - ATE 15 DIAS	CHU da Universidade Federal do Pará - UFPA - PA
57	AFASTAMENTO POR ATESTADO MÉDICO - ATE 15 DIAS	CHU da Universidade Federal do Pará - UFPA - PA
57	AFASTAMENTO POR ATESTADO MÉDICO - ATE 15 DIAS	CHU da Universidade Federal do Pará - UFPA - PA
57	AFASTAMENTO POR ATESTADO MÉDICO - ATE 15 DIAS	CHU da Universidade Federal do Pará - UFPA - PA
57	AFASTAMENTO POR ATESTADO MÉDICO - ATE 15 DIAS	CHU da Universidade Federal do Pará - UFPA - PA
57	AFASTAMENTO POR ATESTADO MÉDICO - ATE 15 DIAS	CHU da Universidade Federal do Pará - UFPA - PA
57	AFASTAMENTO POR ATESTADO MÉDICO - ATE 15 DIAS	CHU da Universidade Federal do Pará - UFPA - PA
57	AFASTAMENTO POR ATESTADO MÉDICO - ATE 15 DIAS	CHU da Universidade Federal do Pará - UFPA - PA
57	AFASTAMENTO POR ATESTADO MÉDICO - ATE 15 DIAS	CHU da Universidade Federal do Pará - UFPA - PA
57	AFASTAMENTO POR ATESTADO MÉDICO - ATE 15 DIAS	CHU da Universidade Federal do Pará - UFPA - PA
57	AFASTAMENTO POR ATESTADO MÉDICO - ATE 15 DIAS	CHU da Universidade Federal do Pará - UFPA - PA
57	AFASTAMENTO POR ATESTADO MÉDICO - ATE 15 DIAS	CHU da Universidade Federal do Pará - UFPA - PA
57	AFASTAMENTO POR ATESTADO MÉDICO - ATE 15 DIAS	CHU da Universidade Federal do Pará - UFPA - PA
57	AFASTAMENTO POR ATESTADO MÉDICO - ATE 15 DIAS	CHU da Universidade Federal do Pará - UFPA - PA
57	AFASTAMENTO POR ATESTADO MÉDICO - ATE 15 DIAS	CHU da Universidade Federal do Pará - UFPA - PA
57	AFASTAMENTO POR ATESTADO MÉDICO - ATE 15 DIAS	CHU da Universidade Federal do Pará - UFPA - PA
57	AFASTAMENTO POR ATESTADO MÉDICO - ATE 15 DIAS	CHU da Universidade Federal do Pará - UFPA - PA
57	AFASTAMENTO POR ATESTADO MÉDICO - ATE 15 DIAS	CHU da Universidade Federal do Pará - UFPA - PA
57	AFASTAMENTO POR ATESTADO MÉDICO - ATE 15 DIAS	CHU da Universidade Federal do Pará - UFPA - PA
57	AFASTAMENTO POR ATESTADO MÉDICO - ATE 15 DIAS	CHU da Universidade Federal do Pará - UFPA - PA
57	AFASTAMENTO POR ATESTADO MÉDICO - ATE 15 DIAS	CHU da Universidade Federal do Pará - UFPA - PA
57	AFASTAMENTO POR ATESTADO MÉDICO - ATE 15 DIAS	CHU da Universidade Federal do Pará - UFPA - PA
57	AFASTAMENTO POR ATESTADO MÉDICO - ATE 15 DIAS	CHU da Universidade Federal do Pará - UFPA - PA
57	AFASTAMENTO POR ATESTADO MÉDICO - ATE 15 DIAS	CHU da Universidade Federal do Pará - UFPA - PA
57	AFASTAMENTO POR ATESTADO MÉDICO - ATE 15 DIAS	CHU da Universidade Federal do Pará - UFPA - PA

57	AFASTAMENTO POR ATESTADO MÉDICO - ATE 15 DIAS	CHU da Universidade Federal do Pará - UFPA - PA
57	AFASTAMENTO POR ATESTADO MÉDICO - ATE 15 DIAS	CHU da Universidade Federal do Pará - UFPA - PA
57	AFASTAMENTO POR ATESTADO MÉDICO - ATE 15 DIAS	CHU da Universidade Federal do Pará - UFPA - PA
57	AFASTAMENTO POR ATESTADO MÉDICO - ATE 15 DIAS	CHU da Universidade Federal do Pará - UFPA - PA
57	AFASTAMENTO POR ATESTADO MÉDICO - ATE 15 DIAS	CHU da Universidade Federal do Pará - UFPA - PA
57	AFASTAMENTO POR ATESTADO MÉDICO - ATE 15 DIAS	CHU da Universidade Federal do Pará - UFPA - PA
57	AFASTAMENTO POR ATESTADO MÉDICO - ATE 15 DIAS	CHU da Universidade Federal do Pará - UFPA - PA
57	AFASTAMENTO POR ATESTADO MÉDICO - ATE 15 DIAS	CHU da Universidade Federal do Pará - UFPA - PA
57	AFASTAMENTO POR ATESTADO MÉDICO - ATE 15 DIAS	CHU da Universidade Federal do Pará - UFPA - PA
57	AFASTAMENTO POR ATESTADO MÉDICO - ATE 15 DIAS	CHU da Universidade Federal do Pará - UFPA - PA
57	AFASTAMENTO POR ATESTADO MÉDICO - ATE 15 DIAS	CHU da Universidade Federal do Pará - UFPA - PA
57	AFASTAMENTO POR ATESTADO MÉDICO - ATE 15 DIAS	CHU da Universidade Federal do Pará - UFPA - PA
57	AFASTAMENTO POR ATESTADO MÉDICO - ATE 15 DIAS	CHU da Universidade Federal do Pará - UFPA - PA
57	AFASTAMENTO POR ATESTADO MÉDICO - ATE 15 DIAS	CHU da Universidade Federal do Pará - UFPA - PA
57	AFASTAMENTO POR ATESTADO MÉDICO - ATE 15 DIAS	CHU da Universidade Federal do Pará - UFPA - PA
57	AFASTAMENTO POR ATESTADO MÉDICO - ATE 15 DIAS	CHU da Universidade Federal do Pará - UFPA - PA
57	AFASTAMENTO POR ATESTADO MÉDICO - ATE 15 DIAS	CHU da Universidade Federal do Pará - UFPA - PA
57	AFASTAMENTO POR ATESTADO MÉDICO - ATE 15 DIAS	CHU da Universidade Federal do Pará - UFPA - PA
57	AFASTAMENTO POR ATESTADO MÉDICO - ATE 15 DIAS	CHU da Universidade Federal do Pará - UFPA - PA
57	AFASTAMENTO POR ATESTADO MÉDICO - ATE 15 DIAS	CHU da Universidade Federal do Pará - UFPA - PA
57	AFASTAMENTO POR ATESTADO MÉDICO - ATE 15 DIAS	CHU da Universidade Federal do Pará - UFPA - PA
57	AFASTAMENTO POR ATESTADO MÉDICO - ATE 15 DIAS	CHU da Universidade Federal do Pará - UFPA - PA
57	AFASTAMENTO POR ATESTADO MÉDICO - ATE 15 DIAS	CHU da Universidade Federal do Pará - UFPA - PA
57	AFASTAMENTO POR ATESTADO MÉDICO - ATE 15 DIAS	CHU da Universidade Federal do Pará - UFPA - PA
57	AFASTAMENTO POR ATESTADO MÉDICO - ATE 15 DIAS	CHU da Universidade Federal do Pará - UFPA - PA
57	AFASTAMENTO POR ATESTADO MÉDICO - ATE 15 DIAS	CHU da Universidade Federal do Pará - UFPA - PA
57	AFASTAMENTO POR ATESTADO MÉDICO - ATE 15 DIAS	CHU da Universidade Federal do Pará - UFPA - PA
57	AFASTAMENTO POR ATESTADO MÉDICO - ATE 15 DIAS	CHU da Universidade Federal do Pará - UFPA - PA
57	AFASTAMENTO POR ATESTADO MÉDICO - ATE 15 DIAS	CHU da Universidade Federal do Pará - UFPA - PA
57	AFASTAMENTO POR ATESTADO MÉDICO - ATE 15 DIAS	CHU da Universidade Federal do Pará - UFPA - PA
57	AFASTAMENTO POR ATESTADO MÉDICO - ATE 15 DIAS	CHU da Universidade Federal do Pará - UFPA - PA
57	AFASTAMENTO POR ATESTADO MÉDICO - ATE 15 DIAS	CHU da Universidade Federal do Pará - UFPA - PA
57	AFASTAMENTO POR ATESTADO MÉDICO - ATE 15 DIAS	CHU da Universidade Federal do Pará - UFPA - PA
57	AFASTAMENTO POR ATESTADO MÉDICO - ATE 15 DIAS	CHU da Universidade Federal do Pará - UFPA - PA
57	AFASTAMENTO POR ATESTADO MÉDICO - ATE 15 DIAS	CHU da Universidade Federal do Pará - UFPA - PA
57	AFASTAMENTO POR ATESTADO MÉDICO - ATE 15 DIAS	CHU da Universidade Federal do Pará - UFPA - PA
57	AFASTAMENTO POR ATESTADO MÉDICO - ATE 15 DIAS	CHU da Universidade Federal do Pará - UFPA - PA
57	AFASTAMENTO POR ATESTADO MÉDICO - ATE 15 DIAS	CHU da Universidade Federal do Pará - UFPA - PA
57	AFASTAMENTO POR ATESTADO MÉDICO - ATE 15 DIAS	CHU da Universidade Federal do Pará - UFPA - PA
57	AFASTAMENTO POR ATESTADO MÉDICO - ATE 15 DIAS	CHU da Universidade Federal do Pará - UFPA - PA
57	AFASTAMENTO POR ATESTADO MÉDICO - ATE 15 DIAS	CHU da Universidade Federal do Pará - UFPA - PA
57	AFASTAMENTO POR ATESTADO MÉDICO - ATE 15 DIAS	CHU da Universidade Federal do Pará - UFPA - PA
57	AFASTAMENTO POR ATESTADO MÉDICO - ATE 15 DIAS	CHU da Universidade Federal do Pará - UFPA - PA
57	AFASTAMENTO POR ATESTADO MÉDICO - ATE 15 DIAS	CHU da Universidade Federal do Pará - UFPA - PA
57	AFASTAMENTO POR ATESTADO MÉDICO - ATE 15 DIAS	CHU da Universidade Federal do Pará - UFPA - PA
57	AFASTAMENTO POR ATESTADO MÉDICO - ATE 15 DIAS	CHU da Universidade Federal do Pará - UFPA - PA
57	AFASTAMENTO POR ATESTADO MÉDICO - ATE 15 DIAS	CHU da Universidade Federal do Pará - UFPA - PA
57	AFASTAMENTO POR ATESTADO MÉDICO - ATE 15 DIAS	CHU da Universidade Federal do Pará - UFPA - PA

57	AFASTAMENTO POR ATESTADO MÉDICO - ATE 15 DIAS	CHU da Universidade Federal do Pará - UFPA - PA
57	AFASTAMENTO POR ATESTADO MÉDICO - ATE 15 DIAS	CHU da Universidade Federal do Pará - UFPA - PA
57	AFASTAMENTO POR ATESTADO MÉDICO - ATE 15 DIAS	CHU da Universidade Federal do Pará - UFPA - PA
57	AFASTAMENTO POR ATESTADO MÉDICO - ATE 15 DIAS	CHU da Universidade Federal do Pará - UFPA - PA
57	AFASTAMENTO POR ATESTADO MÉDICO - ATE 15 DIAS	CHU da Universidade Federal do Pará - UFPA - PA
57	AFASTAMENTO POR ATESTADO MÉDICO - ATE 15 DIAS	CHU da Universidade Federal do Pará - UFPA - PA
57	AFASTAMENTO POR ATESTADO MÉDICO - ATE 15 DIAS	CHU da Universidade Federal do Pará - UFPA - PA
57	AFASTAMENTO POR ATESTADO MÉDICO - ATE 15 DIAS	CHU da Universidade Federal do Pará - UFPA - PA
57	AFASTAMENTO POR ATESTADO MÉDICO - ATE 15 DIAS	CHU da Universidade Federal do Pará - UFPA - PA
57	AFASTAMENTO POR ATESTADO MÉDICO - ATE 15 DIAS	CHU da Universidade Federal do Pará - UFPA - PA
57	AFASTAMENTO POR ATESTADO MÉDICO - ATE 15 DIAS	CHU da Universidade Federal do Pará - UFPA - PA
57	AFASTAMENTO POR ATESTADO MÉDICO - ATE 15 DIAS	CHU da Universidade Federal do Pará - UFPA - PA
57	AFASTAMENTO POR ATESTADO MÉDICO - ATE 15 DIAS	CHU da Universidade Federal do Pará - UFPA - PA
57	AFASTAMENTO POR ATESTADO MÉDICO - ATE 15 DIAS	CHU da Universidade Federal do Pará - UFPA - PA
57	AFASTAMENTO POR ATESTADO MÉDICO - ATE 15 DIAS	CHU da Universidade Federal do Pará - UFPA - PA
57	AFASTAMENTO POR ATESTADO MÉDICO - ATE 15 DIAS	CHU da Universidade Federal do Pará - UFPA - PA
57	AFASTAMENTO POR ATESTADO MÉDICO - ATE 15 DIAS	CHU da Universidade Federal do Pará - UFPA - PA
57	AFASTAMENTO POR ATESTADO MÉDICO - ATE 15 DIAS	CHU da Universidade Federal do Pará - UFPA - PA
57	AFASTAMENTO POR ATESTADO MÉDICO - ATE 15 DIAS	CHU da Universidade Federal do Pará - UFPA - PA
57	AFASTAMENTO POR ATESTADO MÉDICO - ATE 15 DIAS	CHU da Universidade Federal do Pará - UFPA - PA
57	AFASTAMENTO POR ATESTADO MÉDICO - ATE 15 DIAS	CHU da Universidade Federal do Pará - UFPA - PA
57	AFASTAMENTO POR ATESTADO MÉDICO - ATE 15 DIAS	CHU da Universidade Federal do Pará - UFPA - PA
57	AFASTAMENTO POR ATESTADO MÉDICO - ATE 15 DIAS	CHU da Universidade Federal do Pará - UFPA - PA
57	AFASTAMENTO POR ATESTADO MÉDICO - ATE 15 DIAS	CHU da Universidade Federal do Pará - UFPA - PA
57	AFASTAMENTO POR ATESTADO MÉDICO - ATE 15 DIAS	CHU da Universidade Federal do Pará - UFPA - PA
57	AFASTAMENTO POR ATESTADO MÉDICO - ATE 15 DIAS	CHU da Universidade Federal do Pará - UFPA - PA
57	AFASTAMENTO POR ATESTADO MÉDICO - ATE 15 DIAS	CHU da Universidade Federal do Pará - UFPA - PA
57	AFASTAMENTO POR ATESTADO MÉDICO - ATE 15 DIAS	CHU da Universidade Federal do Pará - UFPA - PA
57	AFASTAMENTO POR ATESTADO MÉDICO - ATE 15 DIAS	CHU da Universidade Federal do Pará - UFPA - PA
57	AFASTAMENTO POR ATESTADO MÉDICO - ATE 15 DIAS	CHU da Universidade Federal do Pará - UFPA - PA
57	AFASTAMENTO POR ATESTADO MÉDICO - ATE 15 DIAS	CHU da Universidade Federal do Pará - UFPA - PA
57	AFASTAMENTO POR ATESTADO MÉDICO - ATE 15 DIAS	CHU da Universidade Federal do Pará - UFPA - PA
57	AFASTAMENTO POR ATESTADO MÉDICO - ATE 15 DIAS	CHU da Universidade Federal do Pará - UFPA - PA
57	AFASTAMENTO POR ATESTADO MÉDICO - ATE 15 DIAS	CHU da Universidade Federal do Pará - UFPA - PA
57	AFASTAMENTO POR ATESTADO MÉDICO - ATE 15 DIAS	CHU da Universidade Federal do Pará - UFPA - PA

57	AFASTAMENTO POR ATESTADO MÉDICO - ATE 15 DIAS	CHU da Universidade Federal do Pará - UFPA - PA
57	AFASTAMENTO POR ATESTADO MÉDICO - ATE 15 DIAS	CHU da Universidade Federal do Pará - UFPA - PA
57	AFASTAMENTO POR ATESTADO MÉDICO - ATE 15 DIAS	CHU da Universidade Federal do Pará - UFPA - PA
57	AFASTAMENTO POR ATESTADO MÉDICO - ATE 15 DIAS	CHU da Universidade Federal do Pará - UFPA - PA
57	AFASTAMENTO POR ATESTADO MÉDICO - ATE 15 DIAS	CHU da Universidade Federal do Pará - UFPA - PA
57	AFASTAMENTO POR ATESTADO MÉDICO - ATE 15 DIAS	CHU da Universidade Federal do Pará - UFPA - PA
57	AFASTAMENTO POR ATESTADO MÉDICO - ATE 15 DIAS	CHU da Universidade Federal do Pará - UFPA - PA
57	AFASTAMENTO POR ATESTADO MÉDICO - ATE 15 DIAS	CHU da Universidade Federal do Pará - UFPA - PA
57	AFASTAMENTO POR ATESTADO MÉDICO - ATE 15 DIAS	CHU da Universidade Federal do Pará - UFPA - PA
57	AFASTAMENTO POR ATESTADO MÉDICO - ATE 15 DIAS	CHU da Universidade Federal do Pará - UFPA - PA
57	AFASTAMENTO POR ATESTADO MÉDICO - ATE 15 DIAS	CHU da Universidade Federal do Pará - UFPA - PA
57	AFASTAMENTO POR ATESTADO MÉDICO - ATE 15 DIAS	CHU da Universidade Federal do Pará - UFPA - PA
57	AFASTAMENTO POR ATESTADO MÉDICO - ATE 15 DIAS	CHU da Universidade Federal do Pará - UFPA - PA
57	AFASTAMENTO POR ATESTADO MÉDICO - ATE 15 DIAS	CHU da Universidade Federal do Pará - UFPA - PA
57	AFASTAMENTO POR ATESTADO MÉDICO - ATE 15 DIAS	CHU da Universidade Federal do Pará - UFPA - PA
57	AFASTAMENTO POR ATESTADO MÉDICO - ATE 15 DIAS	CHU da Universidade Federal do Pará - UFPA - PA
57	AFASTAMENTO POR ATESTADO MÉDICO - ATE 15 DIAS	CHU da Universidade Federal do Pará - UFPA - PA
57	AFASTAMENTO POR ATESTADO MÉDICO - ATE 15 DIAS	CHU da Universidade Federal do Pará - UFPA - PA
57	AFASTAMENTO POR ATESTADO MÉDICO - ATE 15 DIAS	CHU da Universidade Federal do Pará - UFPA - PA
57	AFASTAMENTO POR ATESTADO MÉDICO - ATE 15 DIAS	CHU da Universidade Federal do Pará - UFPA - PA
57	AFASTAMENTO POR ATESTADO MÉDICO - ATE 15 DIAS	CHU da Universidade Federal do Pará - UFPA - PA
57	AFASTAMENTO POR ATESTADO MÉDICO - ATE 15 DIAS	CHU da Universidade Federal do Pará - UFPA - PA
57	AFASTAMENTO POR ATESTADO MÉDICO - ATE 15 DIAS	CHU da Universidade Federal do Pará - UFPA - PA
57	AFASTAMENTO POR ATESTADO MÉDICO - ATE 15 DIAS	CHU da Universidade Federal do Pará - UFPA - PA
57	AFASTAMENTO POR ATESTADO MÉDICO - ATE 15 DIAS	CHU da Universidade Federal do Pará - UFPA - PA
57	AFASTAMENTO POR ATESTADO MÉDICO - ATE 15 DIAS	CHU da Universidade Federal do Pará - UFPA - PA
57	AFASTAMENTO POR ATESTADO MÉDICO - ATE 15 DIAS	CHU da Universidade Federal do Pará - UFPA - PA
57	AFASTAMENTO POR ATESTADO MÉDICO - ATE 15 DIAS	CHU da Universidade Federal do Pará - UFPA - PA
57	AFASTAMENTO POR ATESTADO MÉDICO - ATE 15 DIAS	CHU da Universidade Federal do Pará - UFPA - PA
57	AFASTAMENTO POR ATESTADO MÉDICO - ATE 15 DIAS	CHU da Universidade Federal do Pará - UFPA - PA
57	AFASTAMENTO POR ATESTADO MÉDICO - ATE 15 DIAS	CHU da Universidade Federal do Pará - UFPA - PA
57	AFASTAMENTO POR ATESTADO MÉDICO - ATE 15 DIAS	CHU da Universidade Federal do Pará - UFPA - PA

57	AFASTAMENTO POR ATESTADO MÉDICO - ATE 15 DIAS	CHU da Universidade Federal do Pará - UFPA - PA
57	AFASTAMENTO POR ATESTADO MÉDICO - ATE 15 DIAS	CHU da Universidade Federal do Pará - UFPA - PA
57	AFASTAMENTO POR ATESTADO MÉDICO - ATE 15 DIAS	CHU da Universidade Federal do Pará - UFPA - PA
57	AFASTAMENTO POR ATESTADO MÉDICO - ATE 15 DIAS	CHU da Universidade Federal do Pará - UFPA - PA
57	AFASTAMENTO POR ATESTADO MÉDICO - ATE 15 DIAS	CHU da Universidade Federal do Pará - UFPA - PA
57	AFASTAMENTO POR ATESTADO MÉDICO - ATE 15 DIAS	CHU da Universidade Federal do Pará - UFPA - PA
57	AFASTAMENTO POR ATESTADO MÉDICO - ATE 15 DIAS	CHU da Universidade Federal do Pará - UFPA - PA
57	AFASTAMENTO POR ATESTADO MÉDICO - ATE 15 DIAS	CHU da Universidade Federal do Pará - UFPA - PA
57	AFASTAMENTO POR ATESTADO MÉDICO - ATE 15 DIAS	CHU da Universidade Federal do Pará - UFPA - PA
57	AFASTAMENTO POR ATESTADO MÉDICO - ATE 15 DIAS	CHU da Universidade Federal do Pará - UFPA - PA
57	AFASTAMENTO POR ATESTADO MÉDICO - ATE 15 DIAS	CHU da Universidade Federal do Pará - UFPA - PA
57	AFASTAMENTO POR ATESTADO MÉDICO - ATE 15 DIAS	CHU da Universidade Federal do Pará - UFPA - PA
57	AFASTAMENTO POR ATESTADO MÉDICO - ATE 15 DIAS	CHU da Universidade Federal do Pará - UFPA - PA
57	AFASTAMENTO POR ATESTADO MÉDICO - ATE 15 DIAS	CHU da Universidade Federal do Pará - UFPA - PA
57	AFASTAMENTO POR ATESTADO MÉDICO - ATE 15 DIAS	CHU da Universidade Federal do Pará - UFPA - PA
57	AFASTAMENTO POR ATESTADO MÉDICO - ATE 15 DIAS	CHU da Universidade Federal do Pará - UFPA - PA
57	AFASTAMENTO POR ATESTADO MÉDICO - ATE 15 DIAS	CHU da Universidade Federal do Pará - UFPA - PA
57	AFASTAMENTO POR ATESTADO MÉDICO - ATE 15 DIAS	CHU da Universidade Federal do Pará - UFPA - PA
57	AFASTAMENTO POR ATESTADO MÉDICO - ATE 15 DIAS	CHU da Universidade Federal do Pará - UFPA - PA
57	AFASTAMENTO POR ATESTADO MÉDICO - ATE 15 DIAS	CHU da Universidade Federal do Pará - UFPA - PA
57	AFASTAMENTO POR ATESTADO MÉDICO - ATE 15 DIAS	CHU da Universidade Federal do Pará - UFPA - PA
57	AFASTAMENTO POR ATESTADO MÉDICO - ATE 15 DIAS	CHU da Universidade Federal do Pará - UFPA - PA
57	AFASTAMENTO POR ATESTADO MÉDICO - ATE 15 DIAS	CHU da Universidade Federal do Pará - UFPA - PA
57	AFASTAMENTO POR ATESTADO MÉDICO - ATE 15 DIAS	CHU da Universidade Federal do Pará - UFPA - PA
57	AFASTAMENTO POR ATESTADO MÉDICO - ATE 15 DIAS	CHU da Universidade Federal do Pará - UFPA - PA
57	AFASTAMENTO POR ATESTADO MÉDICO - ATE 15 DIAS	CHU da Universidade Federal do Pará - UFPA - PA
57	AFASTAMENTO POR ATESTADO MÉDICO - ATE 15 DIAS	CHU da Universidade Federal do Pará - UFPA - PA
57	AFASTAMENTO POR ATESTADO MÉDICO - ATE 15 DIAS	CHU da Universidade Federal do Pará - UFPA - PA
57	AFASTAMENTO POR ATESTADO MÉDICO - ATE 15 DIAS	CHU da Universidade Federal do Pará - UFPA - PA
57	AFASTAMENTO POR ATESTADO MÉDICO - ATE 15 DIAS	CHU da Universidade Federal do Pará - UFPA - PA
57	AFASTAMENTO POR ATESTADO MÉDICO - ATE 15 DIAS	CHU da Universidade Federal do Pará - UFPA - PA
57	AFASTAMENTO POR ATESTADO MÉDICO - ATE 15 DIAS	CHU da Universidade Federal do Pará - UFPA - PA
57	AFASTAMENTO POR ATESTADO MÉDICO - ATE 15 DIAS	CHU da Universidade Federal do Pará - UFPA - PA
57	AFASTAMENTO POR ATESTADO MÉDICO - ATE 15 DIAS	CHU da Universidade Federal do Pará - UFPA - PA
57	AFASTAMENTO POR ATESTADO MÉDICO - ATE 15 DIAS	CHU da Universidade Federal do Pará - UFPA - PA
57	AFASTAMENTO POR ATESTADO MÉDICO - ATE 15 DIAS	CHU da Universidade Federal do Pará - UFPA - PA
57	AFASTAMENTO POR ATESTADO MÉDICO - ATE 15 DIAS	CHU da Universidade Federal do Pará - UFPA - PA
57	AFASTAMENTO POR ATESTADO MÉDICO - ATE 15 DIAS	CHU da Universidade Federal do Pará - UFPA - PA
57	AFASTAMENTO POR ATESTADO MÉDICO - ATE 15 DIAS	CHU da Universidade Federal do Pará - UFPA - PA
57	AFASTAMENTO POR ATESTADO MÉDICO - ATE 15 DIAS	CHU da Universidade Federal do Pará - UFPA - PA
57	AFASTAMENTO POR ATESTADO MÉDICO - ATE 15 DIAS	CHU da Universidade Federal do Pará - UFPA - PA
57	AFASTAMENTO POR ATESTADO MÉDICO - ATE 15 DIAS	CHU da Universidade Federal do Pará - UFPA - PA

57	AFASTAMENTO POR ATESTADO MÉDICO - ATE 15 DIAS	CHU da Universidade Federal do Pará - UFPA - PA
57	AFASTAMENTO POR ATESTADO MÉDICO - ATE 15 DIAS	CHU da Universidade Federal do Pará - UFPA - PA
57	AFASTAMENTO POR ATESTADO MÉDICO - ATE 15 DIAS	CHU da Universidade Federal do Pará - UFPA - PA
57	AFASTAMENTO POR ATESTADO MÉDICO - ATE 15 DIAS	CHU da Universidade Federal do Pará - UFPA - PA
57	AFASTAMENTO POR ATESTADO MÉDICO - ATE 15 DIAS	CHU da Universidade Federal do Pará - UFPA - PA
57	AFASTAMENTO POR ATESTADO MÉDICO - ATE 15 DIAS	CHU da Universidade Federal do Pará - UFPA - PA
57	AFASTAMENTO POR ATESTADO MÉDICO - ATE 15 DIAS	CHU da Universidade Federal do Pará - UFPA - PA
57	AFASTAMENTO POR ATESTADO MÉDICO - ATE 15 DIAS	CHU da Universidade Federal do Pará - UFPA - PA
57	AFASTAMENTO POR ATESTADO MÉDICO - ATE 15 DIAS	CHU da Universidade Federal do Pará - UFPA - PA
57	AFASTAMENTO POR ATESTADO MÉDICO - ATE 15 DIAS	CHU da Universidade Federal do Pará - UFPA - PA
57	AFASTAMENTO POR ATESTADO MÉDICO - ATE 15 DIAS	CHU da Universidade Federal do Pará - UFPA - PA
57	AFASTAMENTO POR ATESTADO MÉDICO - ATE 15 DIAS	CHU da Universidade Federal do Pará - UFPA - PA
57	AFASTAMENTO POR ATESTADO MÉDICO - ATE 15 DIAS	CHU da Universidade Federal do Pará - UFPA - PA
57	AFASTAMENTO POR ATESTADO MÉDICO - ATE 15 DIAS	CHU da Universidade Federal do Pará - UFPA - PA
57	AFASTAMENTO POR ATESTADO MÉDICO - ATE 15 DIAS	CHU da Universidade Federal do Pará - UFPA - PA
57	AFASTAMENTO POR ATESTADO MÉDICO - ATE 15 DIAS	CHU da Universidade Federal do Pará - UFPA - PA
57	AFASTAMENTO POR ATESTADO MÉDICO - ATE 15 DIAS	CHU da Universidade Federal do Pará - UFPA - PA
57	AFASTAMENTO POR ATESTADO MÉDICO - ATE 15 DIAS	CHU da Universidade Federal do Pará - UFPA - PA
57	AFASTAMENTO POR ATESTADO MÉDICO - ATE 15 DIAS	CHU da Universidade Federal do Pará - UFPA - PA
57	AFASTAMENTO POR ATESTADO MÉDICO - ATE 15 DIAS	CHU da Universidade Federal do Pará - UFPA - PA
57	AFASTAMENTO POR ATESTADO MÉDICO - ATE 15 DIAS	CHU da Universidade Federal do Pará - UFPA - PA
57	AFASTAMENTO POR ATESTADO MÉDICO - ATE 15 DIAS	CHU da Universidade Federal do Pará - UFPA - PA
57	AFASTAMENTO POR ATESTADO MÉDICO - ATE 15 DIAS	CHU da Universidade Federal do Pará - UFPA - PA
57	AFASTAMENTO POR ATESTADO MÉDICO - ATE 15 DIAS	CHU da Universidade Federal do Pará - UFPA - PA
57	AFASTAMENTO POR ATESTADO MÉDICO - ATE 15 DIAS	CHU da Universidade Federal do Pará - UFPA - PA
57	AFASTAMENTO POR ATESTADO MÉDICO - ATE 15 DIAS	CHU da Universidade Federal do Pará - UFPA - PA
57	AFASTAMENTO POR ATESTADO MÉDICO - ATE 15 DIAS	CHU da Universidade Federal do Pará - UFPA - PA
57	AFASTAMENTO POR ATESTADO MÉDICO - ATE 15 DIAS	CHU da Universidade Federal do Pará - UFPA - PA
57	AFASTAMENTO POR ATESTADO MÉDICO - ATE 15 DIAS	CHU da Universidade Federal do Pará - UFPA - PA
57	AFASTAMENTO POR ATESTADO MÉDICO - ATE 15 DIAS	CHU da Universidade Federal do Pará - UFPA - PA
57	AFASTAMENTO POR ATESTADO MÉDICO - ATE 15 DIAS	CHU da Universidade Federal do Pará - UFPA - PA
57	AFASTAMENTO POR ATESTADO MÉDICO - ATE 15 DIAS	CHU da Universidade Federal do Pará - UFPA - PA
57	AFASTAMENTO POR ATESTADO MÉDICO - ATE 15 DIAS	CHU da Universidade Federal do Pará - UFPA - PA
57	AFASTAMENTO POR ATESTADO MÉDICO - ATE 15 DIAS	CHU da Universidade Federal do Pará - UFPA - PA
57	AFASTAMENTO POR ATESTADO MÉDICO - ATE 15 DIAS	CHU da Universidade Federal do Pará - UFPA - PA
57	AFASTAMENTO POR ATESTADO MÉDICO - ATE 15 DIAS	CHU da Universidade Federal do Pará - UFPA - PA

57	AFASTAMENTO POR ATESTADO MÉDICO - ATE 15 DIAS	CHU da Universidade Federal do Pará - UFPA - PA
57	AFASTAMENTO POR ATESTADO MÉDICO - ATE 15 DIAS	CHU da Universidade Federal do Pará - UFPA - PA
57	AFASTAMENTO POR ATESTADO MÉDICO - ATE 15 DIAS	CHU da Universidade Federal do Pará - UFPA - PA
57	AFASTAMENTO POR ATESTADO MÉDICO - ATE 15 DIAS	CHU da Universidade Federal do Pará - UFPA - PA
57	AFASTAMENTO POR ATESTADO MÉDICO - ATE 15 DIAS	CHU da Universidade Federal do Pará - UFPA - PA
57	AFASTAMENTO POR ATESTADO MÉDICO - ATE 15 DIAS	CHU da Universidade Federal do Pará - UFPA - PA
57	AFASTAMENTO POR ATESTADO MÉDICO - ATE 15 DIAS	CHU da Universidade Federal do Pará - UFPA - PA
57	AFASTAMENTO POR ATESTADO MÉDICO - ATE 15 DIAS	CHU da Universidade Federal do Pará - UFPA - PA
57	AFASTAMENTO POR ATESTADO MÉDICO - ATE 15 DIAS	CHU da Universidade Federal do Pará - UFPA - PA
57	AFASTAMENTO POR ATESTADO MÉDICO - ATE 15 DIAS	CHU da Universidade Federal do Pará - UFPA - PA
57	AFASTAMENTO POR ATESTADO MÉDICO - ATE 15 DIAS	CHU da Universidade Federal do Pará - UFPA - PA
57	AFASTAMENTO POR ATESTADO MÉDICO - ATE 15 DIAS	CHU da Universidade Federal do Pará - UFPA - PA
57	AFASTAMENTO POR ATESTADO MÉDICO - ATE 15 DIAS	CHU da Universidade Federal do Pará - UFPA - PA
57	AFASTAMENTO POR ATESTADO MÉDICO - ATE 15 DIAS	CHU da Universidade Federal do Pará - UFPA - PA
57	AFASTAMENTO POR ATESTADO MÉDICO - ATE 15 DIAS	CHU da Universidade Federal do Pará - UFPA - PA
57	AFASTAMENTO POR ATESTADO MÉDICO - ATE 15 DIAS	CHU da Universidade Federal do Pará - UFPA - PA
57	AFASTAMENTO POR ATESTADO MÉDICO - ATE 15 DIAS	CHU da Universidade Federal do Pará - UFPA - PA
57	AFASTAMENTO POR ATESTADO MÉDICO - ATE 15 DIAS	CHU da Universidade Federal do Pará - UFPA - PA
57	AFASTAMENTO POR ATESTADO MÉDICO - ATE 15 DIAS	CHU da Universidade Federal do Pará - UFPA - PA
57	AFASTAMENTO POR ATESTADO MÉDICO - ATE 15 DIAS	CHU da Universidade Federal do Pará - UFPA - PA
57	AFASTAMENTO POR ATESTADO MÉDICO - ATE 15 DIAS	CHU da Universidade Federal do Pará - UFPA - PA
57	AFASTAMENTO POR ATESTADO MÉDICO - ATE 15 DIAS	CHU da Universidade Federal do Pará - UFPA - PA
57	AFASTAMENTO POR ATESTADO MÉDICO - ATE 15 DIAS	CHU da Universidade Federal do Pará - UFPA - PA
57	AFASTAMENTO POR ATESTADO MÉDICO - ATE 15 DIAS	CHU da Universidade Federal do Pará - UFPA - PA
57	AFASTAMENTO POR ATESTADO MÉDICO - ATE 15 DIAS	CHU da Universidade Federal do Pará - UFPA - PA
57	AFASTAMENTO POR ATESTADO MÉDICO - ATE 15 DIAS	CHU da Universidade Federal do Pará - UFPA - PA
57	AFASTAMENTO POR ATESTADO MÉDICO - ATE 15 DIAS	CHU da Universidade Federal do Pará - UFPA - PA
57	AFASTAMENTO POR ATESTADO MÉDICO - ATE 15 DIAS	CHU da Universidade Federal do Pará - UFPA - PA
57	AFASTAMENTO POR ATESTADO MÉDICO - ATE 15 DIAS	CHU da Universidade Federal do Pará - UFPA - PA
57	AFASTAMENTO POR ATESTADO MÉDICO - ATE 15 DIAS	CHU da Universidade Federal do Pará - UFPA - PA
57	AFASTAMENTO POR ATESTADO MÉDICO - ATE 15 DIAS	CHU da Universidade Federal do Pará - UFPA - PA
57	AFASTAMENTO POR ATESTADO MÉDICO - ATE 15 DIAS	CHU da Universidade Federal do Pará - UFPA - PA

57	AFASTAMENTO POR ATESTADO MÉDICO - ATE 15 DIAS	CHU da Universidade Federal do Pará - UFPA - PA
57	AFASTAMENTO POR ATESTADO MÉDICO - ATE 15 DIAS	CHU da Universidade Federal do Pará - UFPA - PA
57	AFASTAMENTO POR ATESTADO MÉDICO - ATE 15 DIAS	CHU da Universidade Federal do Pará - UFPA - PA
57	AFASTAMENTO POR ATESTADO MÉDICO - ATE 15 DIAS	CHU da Universidade Federal do Pará - UFPA - PA
57	AFASTAMENTO POR ATESTADO MÉDICO - ATE 15 DIAS	CHU da Universidade Federal do Pará - UFPA - PA
57	AFASTAMENTO POR ATESTADO MÉDICO - ATE 15 DIAS	CHU da Universidade Federal do Pará - UFPA - PA
57	AFASTAMENTO POR ATESTADO MÉDICO - ATE 15 DIAS	CHU da Universidade Federal do Pará - UFPA - PA
57	AFASTAMENTO POR ATESTADO MÉDICO - ATE 15 DIAS	CHU da Universidade Federal do Pará - UFPA - PA
57	AFASTAMENTO POR ATESTADO MÉDICO - ATE 15 DIAS	CHU da Universidade Federal do Pará - UFPA - PA
57	AFASTAMENTO POR ATESTADO MÉDICO - ATE 15 DIAS	CHU da Universidade Federal do Pará - UFPA - PA
57	AFASTAMENTO POR ATESTADO MÉDICO - ATE 15 DIAS	CHU da Universidade Federal do Pará - UFPA - PA
57	AFASTAMENTO POR ATESTADO MÉDICO - ATE 15 DIAS	CHU da Universidade Federal do Pará - UFPA - PA
57	AFASTAMENTO POR ATESTADO MÉDICO - ATE 15 DIAS	CHU da Universidade Federal do Pará - UFPA - PA
57	AFASTAMENTO POR ATESTADO MÉDICO - ATE 15 DIAS	CHU da Universidade Federal do Pará - UFPA - PA
57	AFASTAMENTO POR ATESTADO MÉDICO - ATE 15 DIAS	CHU da Universidade Federal do Pará - UFPA - PA
57	AFASTAMENTO POR ATESTADO MÉDICO - ATE 15 DIAS	CHU da Universidade Federal do Pará - UFPA - PA
57	AFASTAMENTO POR ATESTADO MÉDICO - ATE 15 DIAS	CHU da Universidade Federal do Pará - UFPA - PA
57	AFASTAMENTO POR ATESTADO MÉDICO - ATE 15 DIAS	CHU da Universidade Federal do Pará - UFPA - PA
57	AFASTAMENTO POR ATESTADO MÉDICO - ATE 15 DIAS	CHU da Universidade Federal do Pará - UFPA - PA
57	AFASTAMENTO POR ATESTADO MÉDICO - ATE 15 DIAS	CHU da Universidade Federal do Pará - UFPA - PA
57	AFASTAMENTO POR ATESTADO MÉDICO - ATE 15 DIAS	CHU da Universidade Federal do Pará - UFPA - PA
57	AFASTAMENTO POR ATESTADO MÉDICO - ATE 15 DIAS	CHU da Universidade Federal do Pará - UFPA - PA
57	AFASTAMENTO POR ATESTADO MÉDICO - ATE 15 DIAS	CHU da Universidade Federal do Pará - UFPA - PA
57	AFASTAMENTO POR ATESTADO MÉDICO - ATE 15 DIAS	CHU da Universidade Federal do Pará - UFPA - PA
57	AFASTAMENTO POR ATESTADO MÉDICO - ATE 15 DIAS	CHU da Universidade Federal do Pará - UFPA - PA
57	AFASTAMENTO POR ATESTADO MÉDICO - ATE 15 DIAS	CHU da Universidade Federal do Pará - UFPA - PA
57	AFASTAMENTO POR ATESTADO MÉDICO - ATE 15 DIAS	CHU da Universidade Federal do Pará - UFPA - PA
57	AFASTAMENTO POR ATESTADO MÉDICO - ATE 15 DIAS	CHU da Universidade Federal do Pará - UFPA - PA
57	AFASTAMENTO POR ATESTADO MÉDICO - ATE 15 DIAS	CHU da Universidade Federal do Pará - UFPA - PA
57	AFASTAMENTO POR ATESTADO MÉDICO - ATE 15 DIAS	CHU da Universidade Federal do Pará - UFPA - PA
57	AFASTAMENTO POR ATESTADO MÉDICO - ATE 15 DIAS	CHU da Universidade Federal do Pará - UFPA - PA
57	AFASTAMENTO POR ATESTADO MÉDICO - ATE 15 DIAS	CHU da Universidade Federal do Pará - UFPA - PA
57	AFASTAMENTO POR ATESTADO MÉDICO - ATE 15 DIAS	CHU da Universidade Federal do Pará - UFPA - PA
57	AFASTAMENTO POR ATESTADO MÉDICO - ATE 15 DIAS	CHU da Universidade Federal do Pará - UFPA - PA
57	AFASTAMENTO POR ATESTADO MÉDICO - ATE 15 DIAS	CHU da Universidade Federal do Pará - UFPA - PA
57	AFASTAMENTO POR ATESTADO MÉDICO - ATE 15 DIAS	CHU da Universidade Federal do Pará - UFPA - PA
57	AFASTAMENTO POR ATESTADO MÉDICO - ATE 15 DIAS	CHU da Universidade Federal do Pará - UFPA - PA
57	AFASTAMENTO POR ATESTADO MÉDICO - ATE 15 DIAS	CHU da Universidade Federal do Pará - UFPA - PA
57	AFASTAMENTO POR ATESTADO MÉDICO - ATE 15 DIAS	CHU da Universidade Federal do Pará - UFPA - PA
57	AFASTAMENTO POR ATESTADO MÉDICO - ATE 15 DIAS	CHU da Universidade Federal do Pará - UFPA - PA
57	AFASTAMENTO POR ATESTADO MÉDICO - ATE 15 DIAS	CHU da Universidade Federal do Pará - UFPA - PA
57	AFASTAMENTO POR ATESTADO MÉDICO - ATE 15 DIAS	CHU da Universidade Federal do Pará - UFPA - PA
57	AFASTAMENTO POR ATESTADO MÉDICO - ATE 15 DIAS	CHU da Universidade Federal do Pará - UFPA - PA
57	AFASTAMENTO POR ATESTADO MÉDICO - ATE 15 DIAS	CHU da Universidade Federal do Pará - UFPA - PA

57	AFASTAMENTO POR ATESTADO MÉDICO - ATE 15 DIAS	CHU da Universidade Federal do Pará - UFPA - PA
57	AFASTAMENTO POR ATESTADO MÉDICO - ATE 15 DIAS	CHU da Universidade Federal do Pará - UFPA - PA
57	AFASTAMENTO POR ATESTADO MÉDICO - ATE 15 DIAS	CHU da Universidade Federal do Pará - UFPA - PA
57	AFASTAMENTO POR ATESTADO MÉDICO - ATE 15 DIAS	CHU da Universidade Federal do Pará - UFPA - PA
57	AFASTAMENTO POR ATESTADO MÉDICO - ATE 15 DIAS	CHU da Universidade Federal do Pará - UFPA - PA
57	AFASTAMENTO POR ATESTADO MÉDICO - ATE 15 DIAS	CHU da Universidade Federal do Pará - UFPA - PA
57	AFASTAMENTO POR ATESTADO MÉDICO - ATE 15 DIAS	CHU da Universidade Federal do Pará - UFPA - PA
57	AFASTAMENTO POR ATESTADO MÉDICO - ATE 15 DIAS	CHU da Universidade Federal do Pará - UFPA - PA
57	AFASTAMENTO POR ATESTADO MÉDICO - ATE 15 DIAS	CHU da Universidade Federal do Pará - UFPA - PA
57	AFASTAMENTO POR ATESTADO MÉDICO - ATE 15 DIAS	CHU da Universidade Federal do Pará - UFPA - PA
57	AFASTAMENTO POR ATESTADO MÉDICO - ATE 15 DIAS	CHU da Universidade Federal do Pará - UFPA - PA
57	AFASTAMENTO POR ATESTADO MÉDICO - ATE 15 DIAS	CHU da Universidade Federal do Pará - UFPA - PA
57	AFASTAMENTO POR ATESTADO MÉDICO - ATE 15 DIAS	CHU da Universidade Federal do Pará - UFPA - PA
57	AFASTAMENTO POR ATESTADO MÉDICO - ATE 15 DIAS	CHU da Universidade Federal do Pará - UFPA - PA
57	AFASTAMENTO POR ATESTADO MÉDICO - ATE 15 DIAS	CHU da Universidade Federal do Pará - UFPA - PA
57	AFASTAMENTO POR ATESTADO MÉDICO - ATE 15 DIAS	CHU da Universidade Federal do Pará - UFPA - PA
57	AFASTAMENTO POR ATESTADO MÉDICO - ATE 15 DIAS	CHU da Universidade Federal do Pará - UFPA - PA
57	AFASTAMENTO POR ATESTADO MÉDICO - ATE 15 DIAS	CHU da Universidade Federal do Pará - UFPA - PA
57	AFASTAMENTO POR ATESTADO MÉDICO - ATE 15 DIAS	CHU da Universidade Federal do Pará - UFPA - PA
57	AFASTAMENTO POR ATESTADO MÉDICO - ATE 15 DIAS	CHU da Universidade Federal do Pará - UFPA - PA
57	AFASTAMENTO POR ATESTADO MÉDICO - ATE 15 DIAS	CHU da Universidade Federal do Pará - UFPA - PA
57	AFASTAMENTO POR ATESTADO MÉDICO - ATE 15 DIAS	CHU da Universidade Federal do Pará - UFPA - PA
57	AFASTAMENTO POR ATESTADO MÉDICO - ATE 15 DIAS	CHU da Universidade Federal do Pará - UFPA - PA
57	AFASTAMENTO POR ATESTADO MÉDICO - ATE 15 DIAS	CHU da Universidade Federal do Pará - UFPA - PA
57	AFASTAMENTO POR ATESTADO MÉDICO - ATE 15 DIAS	CHU da Universidade Federal do Pará - UFPA - PA
57	AFASTAMENTO POR ATESTADO MÉDICO - ATE 15 DIAS	CHU da Universidade Federal do Pará - UFPA - PA
57	AFASTAMENTO POR ATESTADO MÉDICO - ATE 15 DIAS	CHU da Universidade Federal do Pará - UFPA - PA
57	AFASTAMENTO POR ATESTADO MÉDICO - ATE 15 DIAS	CHU da Universidade Federal do Pará - UFPA - PA
57	AFASTAMENTO POR ATESTADO MÉDICO - ATE 15 DIAS	CHU da Universidade Federal do Pará - UFPA - PA
57	AFASTAMENTO POR ATESTADO MÉDICO - ATE 15 DIAS	CHU da Universidade Federal do Pará - UFPA - PA
57	AFASTAMENTO POR ATESTADO MÉDICO - ATE 15 DIAS	CHU da Universidade Federal do Pará - UFPA - PA
57	AFASTAMENTO POR ATESTADO MÉDICO - ATE 15 DIAS	CHU da Universidade Federal do Pará - UFPA - PA
57	AFASTAMENTO POR ATESTADO MÉDICO - ATE 15 DIAS	CHU da Universidade Federal do Pará - UFPA - PA
57	AFASTAMENTO POR ATESTADO MÉDICO - ATE 15 DIAS	CHU da Universidade Federal do Pará - UFPA - PA
57	AFASTAMENTO POR ATESTADO MÉDICO - ATE 15 DIAS	CHU da Universidade Federal do Pará - UFPA - PA
57	AFASTAMENTO POR ATESTADO MÉDICO - ATE 15 DIAS	CHU da Universidade Federal do Pará - UFPA - PA
57	AFASTAMENTO POR ATESTADO MÉDICO - ATE 15 DIAS	CHU da Universidade Federal do Pará - UFPA - PA
57	AFASTAMENTO POR ATESTADO MÉDICO - ATE 15 DIAS	CHU da Universidade Federal do Pará - UFPA - PA
57	AFASTAMENTO POR ATESTADO MÉDICO - ATE 15 DIAS	CHU da Universidade Federal do Pará - UFPA - PA
57	AFASTAMENTO POR ATESTADO MÉDICO - ATE 15 DIAS	CHU da Universidade Federal do Pará - UFPA - PA
57	AFASTAMENTO POR ATESTADO MÉDICO - ATE 15 DIAS	CHU da Universidade Federal do Pará - UFPA - PA
57	AFASTAMENTO POR ATESTADO MÉDICO - ATE 15 DIAS	CHU da Universidade Federal do Pará - UFPA - PA
57	AFASTAMENTO POR ATESTADO MÉDICO - ATE 15 DIAS	CHU da Universidade Federal do Pará - UFPA - PA
57	AFASTAMENTO POR ATESTADO MÉDICO - ATE 15 DIAS	CHU da Universidade Federal do Pará - UFPA - PA
57	AFASTAMENTO POR ATESTADO MÉDICO - ATE 15 DIAS	CHU da Universidade Federal do Pará - UFPA - PA
57	AFASTAMENTO POR ATESTADO MÉDICO - ATE 15 DIAS	CHU da Universidade Federal do Pará - UFPA - PA
57	AFASTAMENTO POR ATESTADO MÉDICO - ATE 15 DIAS	CHU da Universidade Federal do Pará - UFPA - PA
57	AFASTAMENTO POR ATESTADO MÉDICO - ATE 15 DIAS	CHU da Universidade Federal do Pará - UFPA - PA

57	AFASTAMENTO POR ATESTADO MÉDICO - ATE 15 DIAS	CHU da Universidade Federal do Pará - UFPA - PA
57	AFASTAMENTO POR ATESTADO MÉDICO - ATE 15 DIAS	CHU da Universidade Federal do Pará - UFPA - PA
57	AFASTAMENTO POR ATESTADO MÉDICO - ATE 15 DIAS	CHU da Universidade Federal do Pará - UFPA - PA
57	AFASTAMENTO POR ATESTADO MÉDICO - ATE 15 DIAS	CHU da Universidade Federal do Pará - UFPA - PA
57	AFASTAMENTO POR ATESTADO MÉDICO - ATE 15 DIAS	CHU da Universidade Federal do Pará - UFPA - PA
57	AFASTAMENTO POR ATESTADO MÉDICO - ATE 15 DIAS	CHU da Universidade Federal do Pará - UFPA - PA
57	AFASTAMENTO POR ATESTADO MÉDICO - ATE 15 DIAS	CHU da Universidade Federal do Pará - UFPA - PA
57	AFASTAMENTO POR ATESTADO MÉDICO - ATE 15 DIAS	CHU da Universidade Federal do Pará - UFPA - PA
57	AFASTAMENTO POR ATESTADO MÉDICO - ATE 15 DIAS	CHU da Universidade Federal do Pará - UFPA - PA
57	AFASTAMENTO POR ATESTADO MÉDICO - ATE 15 DIAS	CHU da Universidade Federal do Pará - UFPA - PA
57	AFASTAMENTO POR ATESTADO MÉDICO - ATE 15 DIAS	CHU da Universidade Federal do Pará - UFPA - PA
57	AFASTAMENTO POR ATESTADO MÉDICO - ATE 15 DIAS	CHU da Universidade Federal do Pará - UFPA - PA
57	AFASTAMENTO POR ATESTADO MÉDICO - ATE 15 DIAS	CHU da Universidade Federal do Pará - UFPA - PA
57	AFASTAMENTO POR ATESTADO MÉDICO - ATE 15 DIAS	CHU da Universidade Federal do Pará - UFPA - PA
57	AFASTAMENTO POR ATESTADO MÉDICO - ATE 15 DIAS	CHU da Universidade Federal do Pará - UFPA - PA
57	AFASTAMENTO POR ATESTADO MÉDICO - ATE 15 DIAS	CHU da Universidade Federal do Pará - UFPA - PA
57	AFASTAMENTO POR ATESTADO MÉDICO - ATE 15 DIAS	CHU da Universidade Federal do Pará - UFPA - PA
57	AFASTAMENTO POR ATESTADO MÉDICO - ATE 15 DIAS	CHU da Universidade Federal do Pará - UFPA - PA
57	AFASTAMENTO POR ATESTADO MÉDICO - ATE 15 DIAS	CHU da Universidade Federal do Pará - UFPA - PA
57	AFASTAMENTO POR ATESTADO MÉDICO - ATE 15 DIAS	CHU da Universidade Federal do Pará - UFPA - PA
57	AFASTAMENTO POR ATESTADO MÉDICO - ATE 15 DIAS	CHU da Universidade Federal do Pará - UFPA - PA
57	AFASTAMENTO POR ATESTADO MÉDICO - ATE 15 DIAS	CHU da Universidade Federal do Pará - UFPA - PA
57	AFASTAMENTO POR ATESTADO MÉDICO - ATE 15 DIAS	CHU da Universidade Federal do Pará - UFPA - PA
57	AFASTAMENTO POR ATESTADO MÉDICO - ATE 15 DIAS	CHU da Universidade Federal do Pará - UFPA - PA
57	AFASTAMENTO POR ATESTADO MÉDICO - ATE 15 DIAS	CHU da Universidade Federal do Pará - UFPA - PA
57	AFASTAMENTO POR ATESTADO MÉDICO - ATE 15 DIAS	CHU da Universidade Federal do Pará - UFPA - PA
57	AFASTAMENTO POR ATESTADO MÉDICO - ATE 15 DIAS	CHU da Universidade Federal do Pará - UFPA - PA
57	AFASTAMENTO POR ATESTADO MÉDICO - ATE 15 DIAS	CHU da Universidade Federal do Pará - UFPA - PA
57	AFASTAMENTO POR ATESTADO MÉDICO - ATE 15 DIAS	CHU da Universidade Federal do Pará - UFPA - PA
57	AFASTAMENTO POR ATESTADO MÉDICO - ATE 15 DIAS	CHU da Universidade Federal do Pará - UFPA - PA
57	AFASTAMENTO POR ATESTADO MÉDICO - ATE 15 DIAS	CHU da Universidade Federal do Pará - UFPA - PA
57	AFASTAMENTO POR ATESTADO MÉDICO - ATE 15 DIAS	CHU da Universidade Federal do Pará - UFPA - PA
57	AFASTAMENTO POR ATESTADO MÉDICO - ATE 15 DIAS	CHU da Universidade Federal do Pará - UFPA - PA
57	AFASTAMENTO POR ATESTADO MÉDICO - ATE 15 DIAS	CHU da Universidade Federal do Pará - UFPA - PA
57	AFASTAMENTO POR ATESTADO MÉDICO - ATE 15 DIAS	CHU da Universidade Federal do Pará - UFPA - PA
57	AFASTAMENTO POR ATESTADO MÉDICO - ATE 15 DIAS	CHU da Universidade Federal do Pará - UFPA - PA
57	AFASTAMENTO POR ATESTADO MÉDICO - ATE 15 DIAS	CHU da Universidade Federal do Pará - UFPA - PA
57	AFASTAMENTO POR ATESTADO MÉDICO - ATE 15 DIAS	CHU da Universidade Federal do Pará - UFPA - PA
57	AFASTAMENTO POR ATESTADO MÉDICO - ATE 15 DIAS	CHU da Universidade Federal do Pará - UFPA - PA
57	AFASTAMENTO POR ATESTADO MÉDICO - ATE 15 DIAS	CHU da Universidade Federal do Pará - UFPA - PA
57	AFASTAMENTO POR ATESTADO MÉDICO - ATE 15 DIAS	CHU da Universidade Federal do Pará - UFPA - PA
57	AFASTAMENTO POR ATESTADO MÉDICO - ATE 15 DIAS	CHU da Universidade Federal do Pará - UFPA - PA
57	AFASTAMENTO POR ATESTADO MÉDICO - ATE 15 DIAS	CHU da Universidade Federal do Pará - UFPA - PA
57	AFASTAMENTO POR ATESTADO MÉDICO - ATE 15 DIAS	CHU da Universidade Federal do Pará - UFPA - PA

57	AFASTAMENTO POR ATESTADO MÉDICO - ATE 15 DIAS	CHU da Universidade Federal do Pará - UFPA - PA
57	AFASTAMENTO POR ATESTADO MÉDICO - ATE 15 DIAS	CHU da Universidade Federal do Pará - UFPA - PA
57	AFASTAMENTO POR ATESTADO MÉDICO - ATE 15 DIAS	CHU da Universidade Federal do Pará - UFPA - PA
57	AFASTAMENTO POR ATESTADO MÉDICO - ATE 15 DIAS	CHU da Universidade Federal do Pará - UFPA - PA
57	AFASTAMENTO POR ATESTADO MÉDICO - ATE 15 DIAS	CHU da Universidade Federal do Pará - UFPA - PA
57	AFASTAMENTO POR ATESTADO MÉDICO - ATE 15 DIAS	CHU da Universidade Federal do Pará - UFPA - PA
57	AFASTAMENTO POR ATESTADO MÉDICO - ATE 15 DIAS	CHU da Universidade Federal do Pará - UFPA - PA
57	AFASTAMENTO POR ATESTADO MÉDICO - ATE 15 DIAS	CHU da Universidade Federal do Pará - UFPA - PA
57	AFASTAMENTO POR ATESTADO MÉDICO - ATE 15 DIAS	CHU da Universidade Federal do Pará - UFPA - PA
57	AFASTAMENTO POR ATESTADO MÉDICO - ATE 15 DIAS	CHU da Universidade Federal do Pará - UFPA - PA
57	AFASTAMENTO POR ATESTADO MÉDICO - ATE 15 DIAS	CHU da Universidade Federal do Pará - UFPA - PA
57	AFASTAMENTO POR ATESTADO MÉDICO - ATE 15 DIAS	CHU da Universidade Federal do Pará - UFPA - PA
57	AFASTAMENTO POR ATESTADO MÉDICO - ATE 15 DIAS	CHU da Universidade Federal do Pará - UFPA - PA
57	AFASTAMENTO POR ATESTADO MÉDICO - ATE 15 DIAS	CHU da Universidade Federal do Pará - UFPA - PA
57	AFASTAMENTO POR ATESTADO MÉDICO - ATE 15 DIAS	CHU da Universidade Federal do Pará - UFPA - PA
57	AFASTAMENTO POR ATESTADO MÉDICO - ATE 15 DIAS	CHU da Universidade Federal do Pará - UFPA - PA
57	AFASTAMENTO POR ATESTADO MÉDICO - ATE 15 DIAS	CHU da Universidade Federal do Pará - UFPA - PA
57	AFASTAMENTO POR ATESTADO MÉDICO - ATE 15 DIAS	CHU da Universidade Federal do Pará - UFPA - PA
57	AFASTAMENTO POR ATESTADO MÉDICO - ATE 15 DIAS	CHU da Universidade Federal do Pará - UFPA - PA
57	AFASTAMENTO POR ATESTADO MÉDICO - ATE 15 DIAS	CHU da Universidade Federal do Pará - UFPA - PA
57	AFASTAMENTO POR ATESTADO MÉDICO - ATE 15 DIAS	CHU da Universidade Federal do Pará - UFPA - PA
57	AFASTAMENTO POR ATESTADO MÉDICO - ATE 15 DIAS	CHU da Universidade Federal do Pará - UFPA - PA
57	AFASTAMENTO POR ATESTADO MÉDICO - ATE 15 DIAS	CHU da Universidade Federal do Pará - UFPA - PA
57	AFASTAMENTO POR ATESTADO MÉDICO - ATE 15 DIAS	CHU da Universidade Federal do Pará - UFPA - PA
57	AFASTAMENTO POR ATESTADO MÉDICO - ATE 15 DIAS	CHU da Universidade Federal do Pará - UFPA - PA
57	AFASTAMENTO POR ATESTADO MÉDICO - ATE 15 DIAS	CHU da Universidade Federal do Pará - UFPA - PA
57	AFASTAMENTO POR ATESTADO MÉDICO - ATE 15 DIAS	CHU da Universidade Federal do Pará - UFPA - PA
57	AFASTAMENTO POR ATESTADO MÉDICO - ATE 15 DIAS	CHU da Universidade Federal do Pará - UFPA - PA
57	AFASTAMENTO POR ATESTADO MÉDICO - ATE 15 DIAS	CHU da Universidade Federal do Pará - UFPA - PA
57	AFASTAMENTO POR ATESTADO MÉDICO - ATE 15 DIAS	CHU da Universidade Federal do Pará - UFPA - PA
57	AFASTAMENTO POR ATESTADO MÉDICO - ATE 15 DIAS	CHU da Universidade Federal do Pará - UFPA - PA
57	AFASTAMENTO POR ATESTADO MÉDICO - ATE 15 DIAS	CHU da Universidade Federal do Pará - UFPA - PA
57	AFASTAMENTO POR ATESTADO MÉDICO - ATE 15 DIAS	CHU da Universidade Federal do Pará - UFPA - PA
57	AFASTAMENTO POR ATESTADO MÉDICO - ATE 15 DIAS	CHU da Universidade Federal do Pará - UFPA - PA
57	AFASTAMENTO POR ATESTADO MÉDICO - ATE 15 DIAS	CHU da Universidade Federal do Pará - UFPA - PA
57	AFASTAMENTO POR ATESTADO MÉDICO - ATE 15 DIAS	CHU da Universidade Federal do Pará - UFPA - PA
57	AFASTAMENTO POR ATESTADO MÉDICO - ATE 15 DIAS	CHU da Universidade Federal do Pará - UFPA - PA
57	AFASTAMENTO POR ATESTADO MÉDICO - ATE 15 DIAS	CHU da Universidade Federal do Pará - UFPA - PA
57	AFASTAMENTO POR ATESTADO MÉDICO - ATE 15 DIAS	CHU da Universidade Federal do Pará - UFPA - PA
57	AFASTAMENTO POR ATESTADO MÉDICO - ATE 15 DIAS	CHU da Universidade Federal do Pará - UFPA - PA
57	AFASTAMENTO POR ATESTADO MÉDICO - ATE 15 DIAS	CHU da Universidade Federal do Pará - UFPA - PA
57	AFASTAMENTO POR ATESTADO MÉDICO - ATE 15 DIAS	CHU da Universidade Federal do Pará - UFPA - PA
57	AFASTAMENTO POR ATESTADO MÉDICO - ATE 15 DIAS	CHU da Universidade Federal do Pará - UFPA - PA
57	AFASTAMENTO POR ATESTADO MÉDICO - ATE 15 DIAS	CHU da Universidade Federal do Pará - UFPA - PA
57	AFASTAMENTO POR ATESTADO MÉDICO - ATE 15 DIAS	CHU da Universidade Federal do Pará - UFPA - PA
57	AFASTAMENTO POR ATESTADO MÉDICO - ATE 15 DIAS	CHU da Universidade Federal do Pará - UFPA - PA
57	AFASTAMENTO POR ATESTADO MÉDICO - ATE 15 DIAS	CHU da Universidade Federal do Pará - UFPA - PA
57	AFASTAMENTO POR ATESTADO MÉDICO - ATE 15 DIAS	CHU da Universidade Federal do Pará - UFPA - PA

57	AFASTAMENTO POR ATESTADO MÉDICO - ATE 15 DIAS	CHU da Universidade Federal do Pará - UFPA - PA
57	AFASTAMENTO POR ATESTADO MÉDICO - ATE 15 DIAS	CHU da Universidade Federal do Pará - UFPA - PA
57	AFASTAMENTO POR ATESTADO MÉDICO - ATE 15 DIAS	CHU da Universidade Federal do Pará - UFPA - PA
57	AFASTAMENTO POR ATESTADO MÉDICO - ATE 15 DIAS	CHU da Universidade Federal do Pará - UFPA - PA
57	AFASTAMENTO POR ATESTADO MÉDICO - ATE 15 DIAS	CHU da Universidade Federal do Pará - UFPA - PA
57	AFASTAMENTO POR ATESTADO MÉDICO - ATE 15 DIAS	CHU da Universidade Federal do Pará - UFPA - PA
57	AFASTAMENTO POR ATESTADO MÉDICO - ATE 15 DIAS	CHU da Universidade Federal do Pará - UFPA - PA
57	AFASTAMENTO POR ATESTADO MÉDICO - ATE 15 DIAS	CHU da Universidade Federal do Pará - UFPA - PA
57	AFASTAMENTO POR ATESTADO MÉDICO - ATE 15 DIAS	CHU da Universidade Federal do Pará - UFPA - PA
57	AFASTAMENTO POR ATESTADO MÉDICO - ATE 15 DIAS	CHU da Universidade Federal do Pará - UFPA - PA
57	AFASTAMENTO POR ATESTADO MÉDICO - ATE 15 DIAS	CHU da Universidade Federal do Pará - UFPA - PA
57	AFASTAMENTO POR ATESTADO MÉDICO - ATE 15 DIAS	CHU da Universidade Federal do Pará - UFPA - PA
57	AFASTAMENTO POR ATESTADO MÉDICO - ATE 15 DIAS	CHU da Universidade Federal do Pará - UFPA - PA
57	AFASTAMENTO POR ATESTADO MÉDICO - ATE 15 DIAS	CHU da Universidade Federal do Pará - UFPA - PA
57	AFASTAMENTO POR ATESTADO MÉDICO - ATE 15 DIAS	CHU da Universidade Federal do Pará - UFPA - PA
57	AFASTAMENTO POR ATESTADO MÉDICO - ATE 15 DIAS	CHU da Universidade Federal do Pará - UFPA - PA
57	AFASTAMENTO POR ATESTADO MÉDICO - ATE 15 DIAS	CHU da Universidade Federal do Pará - UFPA - PA
57	AFASTAMENTO POR ATESTADO MÉDICO - ATE 15 DIAS	CHU da Universidade Federal do Pará - UFPA - PA
57	AFASTAMENTO POR ATESTADO MÉDICO - ATE 15 DIAS	CHU da Universidade Federal do Pará - UFPA - PA
57	AFASTAMENTO POR ATESTADO MÉDICO - ATE 15 DIAS	CHU da Universidade Federal do Pará - UFPA - PA
57	AFASTAMENTO POR ATESTADO MÉDICO - ATE 15 DIAS	CHU da Universidade Federal do Pará - UFPA - PA
57	AFASTAMENTO POR ATESTADO MÉDICO - ATE 15 DIAS	CHU da Universidade Federal do Pará - UFPA - PA
57	AFASTAMENTO POR ATESTADO MÉDICO - ATE 15 DIAS	CHU da Universidade Federal do Pará - UFPA - PA
57	AFASTAMENTO POR ATESTADO MÉDICO - ATE 15 DIAS	CHU da Universidade Federal do Pará - UFPA - PA
57	AFASTAMENTO POR ATESTADO MÉDICO - ATE 15 DIAS	CHU da Universidade Federal do Pará - UFPA - PA
57	AFASTAMENTO POR ATESTADO MÉDICO - ATE 15 DIAS	CHU da Universidade Federal do Pará - UFPA - PA
57	AFASTAMENTO POR ATESTADO MÉDICO - ATE 15 DIAS	CHU da Universidade Federal do Pará - UFPA - PA
57	AFASTAMENTO POR ATESTADO MÉDICO - ATE 15 DIAS	CHU da Universidade Federal do Pará - UFPA - PA
57	AFASTAMENTO POR ATESTADO MÉDICO - ATE 15 DIAS	CHU da Universidade Federal do Pará - UFPA - PA
57	AFASTAMENTO POR ATESTADO MÉDICO - ATE 15 DIAS	CHU da Universidade Federal do Pará - UFPA - PA
57	AFASTAMENTO POR ATESTADO MÉDICO - ATE 15 DIAS	CHU da Universidade Federal do Pará - UFPA - PA
57	AFASTAMENTO POR ATESTADO MÉDICO - ATE 15 DIAS	CHU da Universidade Federal do Pará - UFPA - PA
57	AFASTAMENTO POR ATESTADO MÉDICO - ATE 15 DIAS	CHU da Universidade Federal do Pará - UFPA - PA
57	AFASTAMENTO POR ATESTADO MÉDICO - ATE 15 DIAS	CHU da Universidade Federal do Pará - UFPA - PA
57	AFASTAMENTO POR ATESTADO MÉDICO - ATE 15 DIAS	CHU da Universidade Federal do Pará - UFPA - PA
57	AFASTAMENTO POR ATESTADO MÉDICO - ATE 15 DIAS	CHU da Universidade Federal do Pará - UFPA - PA
57	AFASTAMENTO POR ATESTADO MÉDICO - ATE 15 DIAS	CHU da Universidade Federal do Pará - UFPA - PA
57	AFASTAMENTO POR ATESTADO MÉDICO - ATE 15 DIAS	CHU da Universidade Federal do Pará - UFPA - PA
57	AFASTAMENTO POR ATESTADO MÉDICO - ATE 15 DIAS	CHU da Universidade Federal do Pará - UFPA - PA
57	AFASTAMENTO POR ATESTADO MÉDICO - ATE 15 DIAS	CHU da Universidade Federal do Pará - UFPA - PA

57	AFASTAMENTO POR ATESTADO MÉDICO - ATE 15 DIAS	CHU da Universidade Federal do Pará - UFPA - PA
57	AFASTAMENTO POR ATESTADO MÉDICO - ATE 15 DIAS	CHU da Universidade Federal do Pará - UFPA - PA
57	AFASTAMENTO POR ATESTADO MÉDICO - ATE 15 DIAS	CHU da Universidade Federal do Pará - UFPA - PA
57	AFASTAMENTO POR ATESTADO MÉDICO - ATE 15 DIAS	CHU da Universidade Federal do Pará - UFPA - PA
57	AFASTAMENTO POR ATESTADO MÉDICO - ATE 15 DIAS	CHU da Universidade Federal do Pará - UFPA - PA
57	AFASTAMENTO POR ATESTADO MÉDICO - ATE 15 DIAS	CHU da Universidade Federal do Pará - UFPA - PA
57	AFASTAMENTO POR ATESTADO MÉDICO - ATE 15 DIAS	CHU da Universidade Federal do Pará - UFPA - PA
57	AFASTAMENTO POR ATESTADO MÉDICO - ATE 15 DIAS	CHU da Universidade Federal do Pará - UFPA - PA
57	AFASTAMENTO POR ATESTADO MÉDICO - ATE 15 DIAS	CHU da Universidade Federal do Pará - UFPA - PA
57	AFASTAMENTO POR ATESTADO MÉDICO - ATE 15 DIAS	CHU da Universidade Federal do Pará - UFPA - PA
57	AFASTAMENTO POR ATESTADO MÉDICO - ATE 15 DIAS	CHU da Universidade Federal do Pará - UFPA - PA
57	AFASTAMENTO POR ATESTADO MÉDICO - ATE 15 DIAS	CHU da Universidade Federal do Pará - UFPA - PA
57	AFASTAMENTO POR ATESTADO MÉDICO - ATE 15 DIAS	CHU da Universidade Federal do Pará - UFPA - PA
57	AFASTAMENTO POR ATESTADO MÉDICO - ATE 15 DIAS	CHU da Universidade Federal do Pará - UFPA - PA
57	AFASTAMENTO POR ATESTADO MÉDICO - ATE 15 DIAS	CHU da Universidade Federal do Pará - UFPA - PA
57	AFASTAMENTO POR ATESTADO MÉDICO - ATE 15 DIAS	CHU da Universidade Federal do Pará - UFPA - PA
57	AFASTAMENTO POR ATESTADO MÉDICO - ATE 15 DIAS	CHU da Universidade Federal do Pará - UFPA - PA
57	AFASTAMENTO POR ATESTADO MÉDICO - ATE 15 DIAS	CHU da Universidade Federal do Pará - UFPA - PA
57	AFASTAMENTO POR ATESTADO MÉDICO - ATE 15 DIAS	CHU da Universidade Federal do Pará - UFPA - PA
57	AFASTAMENTO POR ATESTADO MÉDICO - ATE 15 DIAS	CHU da Universidade Federal do Pará - UFPA - PA
57	AFASTAMENTO POR ATESTADO MÉDICO - ATE 15 DIAS	CHU da Universidade Federal do Pará - UFPA - PA
57	AFASTAMENTO POR ATESTADO MÉDICO - ATE 15 DIAS	CHU da Universidade Federal do Pará - UFPA - PA
57	AFASTAMENTO POR ATESTADO MÉDICO - ATE 15 DIAS	CHU da Universidade Federal do Pará - UFPA - PA
57	AFASTAMENTO POR ATESTADO MÉDICO - ATE 15 DIAS	CHU da Universidade Federal do Pará - UFPA - PA
57	AFASTAMENTO POR ATESTADO MÉDICO - ATE 15 DIAS	CHU da Universidade Federal do Pará - UFPA - PA
57	AFASTAMENTO POR ATESTADO MÉDICO - ATE 15 DIAS	CHU da Universidade Federal do Pará - UFPA - PA
57	AFASTAMENTO POR ATESTADO MÉDICO - ATE 15 DIAS	CHU da Universidade Federal do Pará - UFPA - PA
57	AFASTAMENTO POR ATESTADO MÉDICO - ATE 15 DIAS	CHU da Universidade Federal do Pará - UFPA - PA
57	AFASTAMENTO POR ATESTADO MÉDICO - ATE 15 DIAS	CHU da Universidade Federal do Pará - UFPA - PA
57	AFASTAMENTO POR ATESTADO MÉDICO - ATE 15 DIAS	CHU da Universidade Federal do Pará - UFPA - PA
57	AFASTAMENTO POR ATESTADO MÉDICO - ATE 15 DIAS	CHU da Universidade Federal do Pará - UFPA - PA
57	AFASTAMENTO POR ATESTADO MÉDICO - ATE 15 DIAS	CHU da Universidade Federal do Pará - UFPA - PA
57	AFASTAMENTO POR ATESTADO MÉDICO - ATE 15 DIAS	CHU da Universidade Federal do Pará - UFPA - PA
57	AFASTAMENTO POR ATESTADO MÉDICO - ATE 15 DIAS	CHU da Universidade Federal do Pará - UFPA - PA
57	AFASTAMENTO POR ATESTADO MÉDICO - ATE 15 DIAS	CHU da Universidade Federal do Pará - UFPA - PA
57	AFASTAMENTO POR ATESTADO MÉDICO - ATE 15 DIAS	CHU da Universidade Federal do Pará - UFPA - PA
57	AFASTAMENTO POR ATESTADO MÉDICO - ATE 15 DIAS	CHU da Universidade Federal do Pará - UFPA - PA
57	AFASTAMENTO POR ATESTADO MÉDICO - ATE 15 DIAS	CHU da Universidade Federal do Pará - UFPA - PA
57	AFASTAMENTO POR ATESTADO MÉDICO - ATE 15 DIAS	CHU da Universidade Federal do Pará - UFPA - PA
57	AFASTAMENTO POR ATESTADO MÉDICO - ATE 15 DIAS	CHU da Universidade Federal do Pará - UFPA - PA
57	AFASTAMENTO POR ATESTADO MÉDICO - ATE 15 DIAS	CHU da Universidade Federal do Pará - UFPA - PA
58	AFASTAMENTO POR ATESTADO ODONTOLOGICO - ATE 15 DIAS	CHU da Universidade Federal do Pará - UFPA - PA
58	AFASTAMENTO POR ATESTADO ODONTOLOGICO - ATE 15 DIAS	CHU da Universidade Federal do Pará - UFPA - PA
58	AFASTAMENTO POR ATESTADO ODONTOLOGICO - ATE 15 DIAS	CHU da Universidade Federal do Pará - UFPA - PA
58	AFASTAMENTO POR ATESTADO ODONTOLOGICO - ATE 15 DIAS	CHU da Universidade Federal do Pará - UFPA - PA

58	AFASTAMENTO POR ATESTADO ODONTOLOGICO - ATE 15 DIAS	CHU da Universidade Federal do Pará - UFPA - PA
58	AFASTAMENTO POR ATESTADO ODONTOLOGICO - ATE 15 DIAS	CHU da Universidade Federal do Pará - UFPA - PA
58	AFASTAMENTO POR ATESTADO ODONTOLOGICO - ATE 15 DIAS	CHU da Universidade Federal do Pará - UFPA - PA
58	AFASTAMENTO POR ATESTADO ODONTOLOGICO - ATE 15 DIAS	CHU da Universidade Federal do Pará - UFPA - PA
58	AFASTAMENTO POR ATESTADO ODONTOLOGICO - ATE 15 DIAS	CHU da Universidade Federal do Pará - UFPA - PA
58	AFASTAMENTO POR ATESTADO ODONTOLOGICO - ATE 15 DIAS	CHU da Universidade Federal do Pará - UFPA - PA
58	AFASTAMENTO POR ATESTADO ODONTOLOGICO - ATE 15 DIAS	CHU da Universidade Federal do Pará - UFPA - PA
58	AFASTAMENTO POR ATESTADO ODONTOLOGICO - ATE 15 DIAS	CHU da Universidade Federal do Pará - UFPA - PA
58	AFASTAMENTO POR ATESTADO ODONTOLOGICO - ATE 15 DIAS	CHU da Universidade Federal do Pará - UFPA - PA
58	AFASTAMENTO POR ATESTADO ODONTOLOGICO - ATE 15 DIAS	CHU da Universidade Federal do Pará - UFPA - PA
58	AFASTAMENTO POR ATESTADO ODONTOLOGICO - ATE 15 DIAS	CHU da Universidade Federal do Pará - UFPA - PA
58	AFASTAMENTO POR ATESTADO ODONTOLOGICO - ATE 15 DIAS	CHU da Universidade Federal do Pará - UFPA - PA
58	AFASTAMENTO POR ATESTADO ODONTOLOGICO - ATE 15 DIAS	CHU da Universidade Federal do Pará - UFPA - PA
58	AFASTAMENTO POR ATESTADO ODONTOLOGICO - ATE 15 DIAS	CHU da Universidade Federal do Pará - UFPA - PA
58	AFASTAMENTO POR ATESTADO ODONTOLOGICO - ATE 15 DIAS	CHU da Universidade Federal do Pará - UFPA - PA
58	AFASTAMENTO POR ATESTADO ODONTOLOGICO - ATE 15 DIAS	CHU da Universidade Federal do Pará - UFPA - PA
58	AFASTAMENTO POR ATESTADO ODONTOLOGICO - ATE 15 DIAS	CHU da Universidade Federal do Pará - UFPA - PA
58	AFASTAMENTO POR ATESTADO ODONTOLOGICO - ATE 15 DIAS	CHU da Universidade Federal do Pará - UFPA - PA
58	AFASTAMENTO POR ATESTADO ODONTOLOGICO - ATE 15 DIAS	CHU da Universidade Federal do Pará - UFPA - PA
62	LICENÇA PATERNIDADE CELETISTA - Até 5 DIAS - ADCT CF ART 10	CHU da Universidade Federal do Pará - UFPA - PA
62	LICENÇA PATERNIDADE CELETISTA - Até 5 DIAS - ADCT CF ART 10	CHU da Universidade Federal do Pará - UFPA - PA
62	LICENÇA PATERNIDADE CELETISTA - Até 5 DIAS - ADCT CF ART 10	CHU da Universidade Federal do Pará - UFPA - PA
62	LICENÇA PATERNIDADE CELETISTA - Até 5 DIAS - ADCT CF ART 10	CHU da Universidade Federal do Pará - UFPA - PA
62	LICENÇA PATERNIDADE CELETISTA - Até 5 DIAS - ADCT CF ART 10	CHU da Universidade Federal do Pará - UFPA - PA
62	LICENÇA PATERNIDADE CELETISTA - Até 5 DIAS - ADCT CF ART 10	CHU da Universidade Federal do Pará - UFPA - PA
62	LICENÇA PATERNIDADE CELETISTA - Até 5 DIAS - ADCT CF ART 10	CHU da Universidade Federal do Pará - UFPA - PA
68	COMPARECIMENTO A JUIZO (JURI, INQUERITO POLICIAL, PROC. JUDICIAL) - CLT ART. 473 VIII	CHU da Universidade Federal do Pará - UFPA - PA
68	COMPARECIMENTO A JUIZO (JURI, INQUERITO POLICIAL, PROC. JUDICIAL) - CLT ART. 473 VIII	CHU da Universidade Federal do Pará - UFPA - PA
68	COMPARECIMENTO A JUIZO (JURI, INQUERITO POLICIAL, PROC. JUDICIAL) - CLT ART. 473 VIII	CHU da Universidade Federal do Pará - UFPA - PA
68	COMPARECIMENTO A JUIZO (JURI, INQUERITO POLICIAL, PROC. JUDICIAL) - CLT ART. 473 VIII	CHU da Universidade Federal do Pará - UFPA - PA
69	ABONO (ACT)	CHU da Universidade Federal do Pará - UFPA - PA

69	ABONO (ACT)	CHU da Universidade Federal do Pará - UFPA - PA
69	ABONO (ACT)	CHU da Universidade Federal do Pará - UFPA - PA
69	ABONO (ACT)	CHU da Universidade Federal do Pará - UFPA - PA
69	ABONO (ACT)	CHU da Universidade Federal do Pará - UFPA - PA
69	ABONO (ACT)	CHU da Universidade Federal do Pará - UFPA - PA
69	ABONO (ACT)	CHU da Universidade Federal do Pará - UFPA - PA
69	ABONO (ACT)	CHU da Universidade Federal do Pará - UFPA - PA
69	ABONO (ACT)	CHU da Universidade Federal do Pará - UFPA - PA
69	ABONO (ACT)	CHU da Universidade Federal do Pará - UFPA - PA
69	ABONO (ACT)	CHU da Universidade Federal do Pará - UFPA - PA
69	ABONO (ACT)	CHU da Universidade Federal do Pará - UFPA - PA
69	ABONO (ACT)	CHU da Universidade Federal do Pará - UFPA - PA
69	ABONO (ACT)	CHU da Universidade Federal do Pará - UFPA - PA
69	ABONO (ACT)	CHU da Universidade Federal do Pará - UFPA - PA
69	ABONO (ACT)	CHU da Universidade Federal do Pará - UFPA - PA
69	ABONO (ACT)	CHU da Universidade Federal do Pará - UFPA - PA
69	ABONO (ACT)	CHU da Universidade Federal do Pará - UFPA - PA
69	ABONO (ACT)	CHU da Universidade Federal do Pará - UFPA - PA
69	ABONO (ACT)	CHU da Universidade Federal do Pará - UFPA - PA
69	ABONO (ACT)	CHU da Universidade Federal do Pará - UFPA - PA
69	ABONO (ACT)	CHU da Universidade Federal do Pará - UFPA - PA
69	ABONO (ACT)	CHU da Universidade Federal do Pará - UFPA - PA
69	ABONO (ACT)	CHU da Universidade Federal do Pará - UFPA - PA
69	ABONO (ACT)	CHU da Universidade Federal do Pará - UFPA - PA
69	ABONO (ACT)	CHU da Universidade Federal do Pará - UFPA - PA
69	ABONO (ACT)	CHU da Universidade Federal do Pará - UFPA - PA
69	ABONO (ACT)	CHU da Universidade Federal do Pará - UFPA - PA
69	ABONO (ACT)	CHU da Universidade Federal do Pará - UFPA - PA
69	ABONO (ACT)	CHU da Universidade Federal do Pará - UFPA - PA
69	ABONO (ACT)	CHU da Universidade Federal do Pará - UFPA - PA
69	ABONO (ACT)	CHU da Universidade Federal do Pará - UFPA - PA
69	ABONO (ACT)	CHU da Universidade Federal do Pará - UFPA - PA
69	ABONO (ACT)	CHU da Universidade Federal do Pará - UFPA - PA
69	ABONO (ACT)	CHU da Universidade Federal do Pará - UFPA - PA
69	ABONO (ACT)	CHU da Universidade Federal do Pará - UFPA - PA
69	ABONO (ACT)	CHU da Universidade Federal do Pará - UFPA - PA
69	ABONO (ACT)	CHU da Universidade Federal do Pará - UFPA - PA
69	ABONO (ACT)	CHU da Universidade Federal do Pará - UFPA - PA
69	ABONO (ACT)	CHU da Universidade Federal do Pará - UFPA - PA
69	ABONO (ACT)	CHU da Universidade Federal do Pará - UFPA - PA
69	ABONO (ACT)	CHU da Universidade Federal do Pará - UFPA - PA
69	ABONO (ACT)	CHU da Universidade Federal do Pará - UFPA - PA
69	ABONO (ACT)	CHU da Universidade Federal do Pará - UFPA - PA
69	ABONO (ACT)	CHU da Universidade Federal do Pará - UFPA - PA
69	ABONO (ACT)	CHU da Universidade Federal do Pará - UFPA - PA
69	ABONO (ACT)	CHU da Universidade Federal do Pará - UFPA - PA
69	ABONO (ACT)	CHU da Universidade Federal do Pará - UFPA - PA
69	ABONO (ACT)	CHU da Universidade Federal do Pará - UFPA - PA

69	ABONO (ACT)	CHU da Universidade Federal do Pará - UFPA - PA
69	ABONO (ACT)	CHU da Universidade Federal do Pará - UFPA - PA
69	ABONO (ACT)	CHU da Universidade Federal do Pará - UFPA - PA
69	ABONO (ACT)	CHU da Universidade Federal do Pará - UFPA - PA
69	ABONO (ACT)	CHU da Universidade Federal do Pará - UFPA - PA
69	ABONO (ACT)	CHU da Universidade Federal do Pará - UFPA - PA
69	ABONO (ACT)	CHU da Universidade Federal do Pará - UFPA - PA
69	ABONO (ACT)	CHU da Universidade Federal do Pará - UFPA - PA
69	ABONO (ACT)	CHU da Universidade Federal do Pará - UFPA - PA
69	ABONO (ACT)	CHU da Universidade Federal do Pará - UFPA - PA
69	ABONO (ACT)	CHU da Universidade Federal do Pará - UFPA - PA
69	ABONO (ACT)	CHU da Universidade Federal do Pará - UFPA - PA
69	ABONO (ACT)	CHU da Universidade Federal do Pará - UFPA - PA
69	ABONO (ACT)	CHU da Universidade Federal do Pará - UFPA - PA
69	ABONO (ACT)	CHU da Universidade Federal do Pará - UFPA - PA
69	ABONO (ACT)	CHU da Universidade Federal do Pará - UFPA - PA
69	ABONO (ACT)	CHU da Universidade Federal do Pará - UFPA - PA
69	ABONO (ACT)	CHU da Universidade Federal do Pará - UFPA - PA
69	ABONO (ACT)	CHU da Universidade Federal do Pará - UFPA - PA
69	ABONO (ACT)	CHU da Universidade Federal do Pará - UFPA - PA
69	ABONO (ACT)	CHU da Universidade Federal do Pará - UFPA - PA
69	ABONO (ACT)	CHU da Universidade Federal do Pará - UFPA - PA
69	ABONO (ACT)	CHU da Universidade Federal do Pará - UFPA - PA
69	ABONO (ACT)	CHU da Universidade Federal do Pará - UFPA - PA
69	ABONO (ACT)	CHU da Universidade Federal do Pará - UFPA - PA
69	ABONO (ACT)	CHU da Universidade Federal do Pará - UFPA - PA
69	ABONO (ACT)	CHU da Universidade Federal do Pará - UFPA - PA
69	ABONO (ACT)	CHU da Universidade Federal do Pará - UFPA - PA
69	ABONO (ACT)	CHU da Universidade Federal do Pará - UFPA - PA
69	ABONO (ACT)	CHU da Universidade Federal do Pará - UFPA - PA
69	ABONO (ACT)	CHU da Universidade Federal do Pará - UFPA - PA
69	ABONO (ACT)	CHU da Universidade Federal do Pará - UFPA - PA
69	ABONO (ACT)	CHU da Universidade Federal do Pará - UFPA - PA
69	ABONO (ACT)	CHU da Universidade Federal do Pará - UFPA - PA
69	ABONO (ACT)	CHU da Universidade Federal do Pará - UFPA - PA
69	ABONO (ACT)	CHU da Universidade Federal do Pará - UFPA - PA
69	ABONO (ACT)	CHU da Universidade Federal do Pará - UFPA - PA
69	ABONO (ACT)	CHU da Universidade Federal do Pará - UFPA - PA
69	ABONO (ACT)	CHU da Universidade Federal do Pará - UFPA - PA
69	ABONO (ACT)	CHU da Universidade Federal do Pará - UFPA - PA
69	ABONO (ACT)	CHU da Universidade Federal do Pará - UFPA - PA
69	ABONO (ACT)	CHU da Universidade Federal do Pará - UFPA - PA
69	ABONO (ACT)	CHU da Universidade Federal do Pará - UFPA - PA
69	ABONO (ACT)	CHU da Universidade Federal do Pará - UFPA - PA
69	ABONO (ACT)	CHU da Universidade Federal do Pará - UFPA - PA

69	ABONO (ACT)	CHU da Universidade Federal do Pará - UFPA - PA
69	ABONO (ACT)	CHU da Universidade Federal do Pará - UFPA - PA
69	ABONO (ACT)	CHU da Universidade Federal do Pará - UFPA - PA
69	ABONO (ACT)	CHU da Universidade Federal do Pará - UFPA - PA
69	ABONO (ACT)	CHU da Universidade Federal do Pará - UFPA - PA
69	ABONO (ACT)	CHU da Universidade Federal do Pará - UFPA - PA
69	ABONO (ACT)	CHU da Universidade Federal do Pará - UFPA - PA
69	ABONO (ACT)	CHU da Universidade Federal do Pará - UFPA - PA
69	ABONO (ACT)	CHU da Universidade Federal do Pará - UFPA - PA
69	ABONO (ACT)	CHU da Universidade Federal do Pará - UFPA - PA
69	ABONO (ACT)	CHU da Universidade Federal do Pará - UFPA - PA
69	ABONO (ACT)	CHU da Universidade Federal do Pará - UFPA - PA
69	ABONO (ACT)	CHU da Universidade Federal do Pará - UFPA - PA
69	ABONO (ACT)	CHU da Universidade Federal do Pará - UFPA - PA
69	ABONO (ACT)	CHU da Universidade Federal do Pará - UFPA - PA
69	ABONO (ACT)	CHU da Universidade Federal do Pará - UFPA - PA
69	ABONO (ACT)	CHU da Universidade Federal do Pará - UFPA - PA
69	ABONO (ACT)	CHU da Universidade Federal do Pará - UFPA - PA
69	ABONO (ACT)	CHU da Universidade Federal do Pará - UFPA - PA
69	ABONO (ACT)	CHU da Universidade Federal do Pará - UFPA - PA
69	ABONO (ACT)	CHU da Universidade Federal do Pará - UFPA - PA
69	ABONO (ACT)	CHU da Universidade Federal do Pará - UFPA - PA
69	ABONO (ACT)	CHU da Universidade Federal do Pará - UFPA - PA
69	ABONO (ACT)	CHU da Universidade Federal do Pará - UFPA - PA
69	ABONO (ACT)	CHU da Universidade Federal do Pará - UFPA - PA
69	ABONO (ACT)	CHU da Universidade Federal do Pará - UFPA - PA
69	ABONO (ACT)	CHU da Universidade Federal do Pará - UFPA - PA
69	ABONO (ACT)	CHU da Universidade Federal do Pará - UFPA - PA
69	ABONO (ACT)	CHU da Universidade Federal do Pará - UFPA - PA
69	ABONO (ACT)	CHU da Universidade Federal do Pará - UFPA - PA
69	ABONO (ACT)	CHU da Universidade Federal do Pará - UFPA - PA
69	ABONO (ACT)	CHU da Universidade Federal do Pará - UFPA - PA

69	ABONO (ACT)	CHU da Universidade Federal do Pará - UFPA - PA
69	ABONO (ACT)	CHU da Universidade Federal do Pará - UFPA - PA
69	ABONO (ACT)	CHU da Universidade Federal do Pará - UFPA - PA
69	ABONO (ACT)	CHU da Universidade Federal do Pará - UFPA - PA
69	ABONO (ACT)	CHU da Universidade Federal do Pará - UFPA - PA
69	ABONO (ACT)	CHU da Universidade Federal do Pará - UFPA - PA
69	ABONO (ACT)	CHU da Universidade Federal do Pará - UFPA - PA
69	ABONO (ACT)	CHU da Universidade Federal do Pará - UFPA - PA
69	ABONO (ACT)	CHU da Universidade Federal do Pará - UFPA - PA
69	ABONO (ACT)	CHU da Universidade Federal do Pará - UFPA - PA
69	ABONO (ACT)	CHU da Universidade Federal do Pará - UFPA - PA
69	ABONO (ACT)	CHU da Universidade Federal do Pará - UFPA - PA
69	ABONO (ACT)	CHU da Universidade Federal do Pará - UFPA - PA
69	ABONO (ACT)	CHU da Universidade Federal do Pará - UFPA - PA
69	ABONO (ACT)	CHU da Universidade Federal do Pará - UFPA - PA
69	ABONO (ACT)	CHU da Universidade Federal do Pará - UFPA - PA
69	ABONO (ACT)	CHU da Universidade Federal do Pará - UFPA - PA
69	ABONO (ACT)	CHU da Universidade Federal do Pará - UFPA - PA
69	ABONO (ACT)	CHU da Universidade Federal do Pará - UFPA - PA
69	ABONO (ACT)	CHU da Universidade Federal do Pará - UFPA - PA
69	ABONO (ACT)	CHU da Universidade Federal do Pará - UFPA - PA
69	ABONO (ACT)	CHU da Universidade Federal do Pará - UFPA - PA
69	ABONO (ACT)	CHU da Universidade Federal do Pará - UFPA - PA
69	ABONO (ACT)	CHU da Universidade Federal do Pará - UFPA - PA
69	ABONO (ACT)	CHU da Universidade Federal do Pará - UFPA - PA
69	ABONO (ACT)	CHU da Universidade Federal do Pará - UFPA - PA
69	ABONO (ACT)	CHU da Universidade Federal do Pará - UFPA - PA
69	ABONO (ACT)	CHU da Universidade Federal do Pará - UFPA - PA
69	ABONO (ACT)	CHU da Universidade Federal do Pará - UFPA - PA
69	ABONO (ACT)	CHU da Universidade Federal do Pará - UFPA - PA
69	ABONO (ACT)	CHU da Universidade Federal do Pará - UFPA - PA
69	ABONO (ACT)	CHU da Universidade Federal do Pará - UFPA - PA
69	ABONO (ACT)	CHU da Universidade Federal do Pará - UFPA - PA

69	ABONO (ACT)	CHU da Universidade Federal do Pará - UFPA - PA
69	ABONO (ACT)	CHU da Universidade Federal do Pará - UFPA - PA
69	ABONO (ACT)	CHU da Universidade Federal do Pará - UFPA - PA
69	ABONO (ACT)	CHU da Universidade Federal do Pará - UFPA - PA
69	ABONO (ACT)	CHU da Universidade Federal do Pará - UFPA - PA
69	ABONO (ACT)	CHU da Universidade Federal do Pará - UFPA - PA
69	ABONO (ACT)	CHU da Universidade Federal do Pará - UFPA - PA
69	ABONO (ACT)	CHU da Universidade Federal do Pará - UFPA - PA
69	ABONO (ACT)	CHU da Universidade Federal do Pará - UFPA - PA
69	ABONO (ACT)	CHU da Universidade Federal do Pará - UFPA - PA
69	ABONO (ACT)	CHU da Universidade Federal do Pará - UFPA - PA
69	ABONO (ACT)	CHU da Universidade Federal do Pará - UFPA - PA
69	ABONO (ACT)	CHU da Universidade Federal do Pará - UFPA - PA
69	ABONO (ACT)	CHU da Universidade Federal do Pará - UFPA - PA
69	ABONO (ACT)	CHU da Universidade Federal do Pará - UFPA - PA
69	ABONO (ACT)	CHU da Universidade Federal do Pará - UFPA - PA
69	ABONO (ACT)	CHU da Universidade Federal do Pará - UFPA - PA
69	ABONO (ACT)	CHU da Universidade Federal do Pará - UFPA - PA
69	ABONO (ACT)	CHU da Universidade Federal do Pará - UFPA - PA
69	ABONO (ACT)	CHU da Universidade Federal do Pará - UFPA - PA
69	ABONO (ACT)	CHU da Universidade Federal do Pará - UFPA - PA
69	ABONO (ACT)	CHU da Universidade Federal do Pará - UFPA - PA
69	ABONO (ACT)	CHU da Universidade Federal do Pará - UFPA - PA
69	ABONO (ACT)	CHU da Universidade Federal do Pará - UFPA - PA
69	ABONO (ACT)	CHU da Universidade Federal do Pará - UFPA - PA
69	ABONO (ACT)	CHU da Universidade Federal do Pará - UFPA - PA
69	ABONO (ACT)	CHU da Universidade Federal do Pará - UFPA - PA
69	ABONO (ACT)	CHU da Universidade Federal do Pará - UFPA - PA
69	ABONO (ACT)	CHU da Universidade Federal do Pará - UFPA - PA
69	ABONO (ACT)	CHU da Universidade Federal do Pará - UFPA - PA
69	ABONO (ACT)	CHU da Universidade Federal do Pará - UFPA - PA
69	ABONO (ACT)	CHU da Universidade Federal do Pará - UFPA - PA
69	ABONO (ACT)	CHU da Universidade Federal do Pará - UFPA - PA
69	ABONO (ACT)	CHU da Universidade Federal do Pará - UFPA - PA
69	ABONO (ACT)	CHU da Universidade Federal do Pará - UFPA - PA
69	ABONO (ACT)	CHU da Universidade Federal do Pará - UFPA - PA

69	ABONO (ACT)	CHU da Universidade Federal do Pará - UFPA - PA
69	ABONO (ACT)	CHU da Universidade Federal do Pará - UFPA - PA
69	ABONO (ACT)	CHU da Universidade Federal do Pará - UFPA - PA
69	ABONO (ACT)	CHU da Universidade Federal do Pará - UFPA - PA
69	ABONO (ACT)	CHU da Universidade Federal do Pará - UFPA - PA
69	ABONO (ACT)	CHU da Universidade Federal do Pará - UFPA - PA
69	ABONO (ACT)	CHU da Universidade Federal do Pará - UFPA - PA
69	ABONO (ACT)	CHU da Universidade Federal do Pará - UFPA - PA
69	ABONO (ACT)	CHU da Universidade Federal do Pará - UFPA - PA
69	ABONO (ACT)	CHU da Universidade Federal do Pará - UFPA - PA
69	ABONO (ACT)	CHU da Universidade Federal do Pará - UFPA - PA
69	ABONO (ACT)	CHU da Universidade Federal do Pará - UFPA - PA
69	ABONO (ACT)	CHU da Universidade Federal do Pará - UFPA - PA
69	ABONO (ACT)	CHU da Universidade Federal do Pará - UFPA - PA
69	ABONO (ACT)	CHU da Universidade Federal do Pará - UFPA - PA
69	ABONO (ACT)	CHU da Universidade Federal do Pará - UFPA - PA
69	ABONO (ACT)	CHU da Universidade Federal do Pará - UFPA - PA
69	ABONO (ACT)	CHU da Universidade Federal do Pará - UFPA - PA
69	ABONO (ACT)	CHU da Universidade Federal do Pará - UFPA - PA
69	ABONO (ACT)	CHU da Universidade Federal do Pará - UFPA - PA
69	ABONO (ACT)	CHU da Universidade Federal do Pará - UFPA - PA
69	ABONO (ACT)	CHU da Universidade Federal do Pará - UFPA - PA
69	ABONO (ACT)	CHU da Universidade Federal do Pará - UFPA - PA
69	ABONO (ACT)	CHU da Universidade Federal do Pará - UFPA - PA
69	ABONO (ACT)	CHU da Universidade Federal do Pará - UFPA - PA
69	ABONO (ACT)	CHU da Universidade Federal do Pará - UFPA - PA
69	ABONO (ACT)	CHU da Universidade Federal do Pará - UFPA - PA
69	ABONO (ACT)	CHU da Universidade Federal do Pará - UFPA - PA
69	ABONO (ACT)	CHU da Universidade Federal do Pará - UFPA - PA
69	ABONO (ACT)	CHU da Universidade Federal do Pará - UFPA - PA
69	ABONO (ACT)	CHU da Universidade Federal do Pará - UFPA - PA
69	ABONO (ACT)	CHU da Universidade Federal do Pará - UFPA - PA
69	ABONO (ACT)	CHU da Universidade Federal do Pará - UFPA - PA
69	ABONO (ACT)	CHU da Universidade Federal do Pará - UFPA - PA
69	ABONO (ACT)	CHU da Universidade Federal do Pará - UFPA - PA
69	ABONO (ACT)	CHU da Universidade Federal do Pará - UFPA - PA
69	ABONO (ACT)	CHU da Universidade Federal do Pará - UFPA - PA
69	ABONO (ACT)	CHU da Universidade Federal do Pará - UFPA - PA
69	ABONO (ACT)	CHU da Universidade Federal do Pará - UFPA - PA
69	ABONO (ACT)	CHU da Universidade Federal do Pará - UFPA - PA
69	ABONO (ACT)	CHU da Universidade Federal do Pará - UFPA - PA
69	ABONO (ACT)	CHU da Universidade Federal do Pará - UFPA - PA
69	ABONO (ACT)	CHU da Universidade Federal do Pará - UFPA - PA
69	ABONO (ACT)	CHU da Universidade Federal do Pará - UFPA - PA
69	ABONO (ACT)	CHU da Universidade Federal do Pará - UFPA - PA
69	ABONO (ACT)	CHU da Universidade Federal do Pará - UFPA - PA
69	ABONO (ACT)	CHU da Universidade Federal do Pará - UFPA - PA
69	ABONO (ACT)	CHU da Universidade Federal do Pará - UFPA - PA

69	ABONO (ACT)	CHU da Universidade Federal do Pará - UFPA - PA
69	ABONO (ACT)	CHU da Universidade Federal do Pará - UFPA - PA
69	ABONO (ACT)	CHU da Universidade Federal do Pará - UFPA - PA
69	ABONO (ACT)	CHU da Universidade Federal do Pará - UFPA - PA
69	ABONO (ACT)	CHU da Universidade Federal do Pará - UFPA - PA
69	ABONO (ACT)	CHU da Universidade Federal do Pará - UFPA - PA
69	ABONO (ACT)	CHU da Universidade Federal do Pará - UFPA - PA
69	ABONO (ACT)	CHU da Universidade Federal do Pará - UFPA - PA
69	ABONO (ACT)	CHU da Universidade Federal do Pará - UFPA - PA
69	ABONO (ACT)	CHU da Universidade Federal do Pará - UFPA - PA
69	ABONO (ACT)	CHU da Universidade Federal do Pará - UFPA - PA
69	ABONO (ACT)	CHU da Universidade Federal do Pará - UFPA - PA
69	ABONO (ACT)	CHU da Universidade Federal do Pará - UFPA - PA
69	ABONO (ACT)	CHU da Universidade Federal do Pará - UFPA - PA
69	ABONO (ACT)	CHU da Universidade Federal do Pará - UFPA - PA
69	ABONO (ACT)	CHU da Universidade Federal do Pará - UFPA - PA
69	ABONO (ACT)	CHU da Universidade Federal do Pará - UFPA - PA
69	ABONO (ACT)	CHU da Universidade Federal do Pará - UFPA - PA
69	ABONO (ACT)	CHU da Universidade Federal do Pará - UFPA - PA
69	ABONO (ACT)	CHU da Universidade Federal do Pará - UFPA - PA
69	ABONO (ACT)	CHU da Universidade Federal do Pará - UFPA - PA
69	ABONO (ACT)	CHU da Universidade Federal do Pará - UFPA - PA
69	ABONO (ACT)	CHU da Universidade Federal do Pará - UFPA - PA
69	ABONO (ACT)	CHU da Universidade Federal do Pará - UFPA - PA
69	ABONO (ACT)	CHU da Universidade Federal do Pará - UFPA - PA
69	ABONO (ACT)	CHU da Universidade Federal do Pará - UFPA - PA
69	ABONO (ACT)	CHU da Universidade Federal do Pará - UFPA - PA
69	ABONO (ACT)	CHU da Universidade Federal do Pará - UFPA - PA
69	ABONO (ACT)	CHU da Universidade Federal do Pará - UFPA - PA
69	ABONO (ACT)	CHU da Universidade Federal do Pará - UFPA - PA
69	ABONO (ACT)	CHU da Universidade Federal do Pará - UFPA - PA
69	ABONO (ACT)	CHU da Universidade Federal do Pará - UFPA - PA
69	ABONO (ACT)	CHU da Universidade Federal do Pará - UFPA - PA
69	ABONO (ACT)	CHU da Universidade Federal do Pará - UFPA - PA
69	ABONO (ACT)	CHU da Universidade Federal do Pará - UFPA - PA
69	ABONO (ACT)	CHU da Universidade Federal do Pará - UFPA - PA
69	ABONO (ACT)	CHU da Universidade Federal do Pará - UFPA - PA
69	ABONO (ACT)	CHU da Universidade Federal do Pará - UFPA - PA
69	ABONO (ACT)	CHU da Universidade Federal do Pará - UFPA - PA
69	ABONO (ACT)	CHU da Universidade Federal do Pará - UFPA - PA
69	ABONO (ACT)	CHU da Universidade Federal do Pará - UFPA - PA
69	ABONO (ACT)	CHU da Universidade Federal do Pará - UFPA - PA
69	ABONO (ACT)	CHU da Universidade Federal do Pará - UFPA - PA
69	ABONO (ACT)	CHU da Universidade Federal do Pará - UFPA - PA
69	ABONO (ACT)	CHU da Universidade Federal do Pará - UFPA - PA
69	ABONO (ACT)	CHU da Universidade Federal do Pará - UFPA - PA
69	ABONO (ACT)	CHU da Universidade Federal do Pará - UFPA - PA
69	ABONO (ACT)	CHU da Universidade Federal do Pará - UFPA - PA

69	ABONO (ACT)	CHU da Universidade Federal do Pará - UFPA - PA
69	ABONO (ACT)	CHU da Universidade Federal do Pará - UFPA - PA
69	ABONO (ACT)	CHU da Universidade Federal do Pará - UFPA - PA
69	ABONO (ACT)	CHU da Universidade Federal do Pará - UFPA - PA
69	ABONO (ACT)	CHU da Universidade Federal do Pará - UFPA - PA
69	ABONO (ACT)	CHU da Universidade Federal do Pará - UFPA - PA
69	ABONO (ACT)	CHU da Universidade Federal do Pará - UFPA - PA
69	ABONO (ACT)	CHU da Universidade Federal do Pará - UFPA - PA
69	ABONO (ACT)	CHU da Universidade Federal do Pará - UFPA - PA
69	ABONO (ACT)	CHU da Universidade Federal do Pará - UFPA - PA
69	ABONO (ACT)	CHU da Universidade Federal do Pará - UFPA - PA
69	ABONO (ACT)	CHU da Universidade Federal do Pará - UFPA - PA
69	ABONO (ACT)	CHU da Universidade Federal do Pará - UFPA - PA
69	ABONO (ACT)	CHU da Universidade Federal do Pará - UFPA - PA
69	ABONO (ACT)	CHU da Universidade Federal do Pará - UFPA - PA
69	ABONO (ACT)	CHU da Universidade Federal do Pará - UFPA - PA
69	ABONO (ACT)	CHU da Universidade Federal do Pará - UFPA - PA
69	ABONO (ACT)	CHU da Universidade Federal do Pará - UFPA - PA
69	ABONO (ACT)	CHU da Universidade Federal do Pará - UFPA - PA
69	ABONO (ACT)	CHU da Universidade Federal do Pará - UFPA - PA
69	ABONO (ACT)	CHU da Universidade Federal do Pará - UFPA - PA
69	ABONO (ACT)	CHU da Universidade Federal do Pará - UFPA - PA
69	ABONO (ACT)	CHU da Universidade Federal do Pará - UFPA - PA
69	ABONO (ACT)	CHU da Universidade Federal do Pará - UFPA - PA
69	ABONO (ACT)	CHU da Universidade Federal do Pará - UFPA - PA
69	ABONO (ACT)	CHU da Universidade Federal do Pará - UFPA - PA
69	ABONO (ACT)	CHU da Universidade Federal do Pará - UFPA - PA
69	ABONO (ACT)	CHU da Universidade Federal do Pará - UFPA - PA
69	ABONO (ACT)	CHU da Universidade Federal do Pará - UFPA - PA
69	ABONO (ACT)	CHU da Universidade Federal do Pará - UFPA - PA
69	ABONO (ACT)	CHU da Universidade Federal do Pará - UFPA - PA
69	ABONO (ACT)	CHU da Universidade Federal do Pará - UFPA - PA
69	ABONO (ACT)	CHU da Universidade Federal do Pará - UFPA - PA
69	ABONO (ACT)	CHU da Universidade Federal do Pará - UFPA - PA
69	ABONO (ACT)	CHU da Universidade Federal do Pará - UFPA - PA
69	ABONO (ACT)	CHU da Universidade Federal do Pará - UFPA - PA
69	ABONO (ACT)	CHU da Universidade Federal do Pará - UFPA - PA
69	ABONO (ACT)	CHU da Universidade Federal do Pará - UFPA - PA
69	ABONO (ACT)	CHU da Universidade Federal do Pará - UFPA - PA
69	ABONO (ACT)	CHU da Universidade Federal do Pará - UFPA - PA
69	ABONO (ACT)	CHU da Universidade Federal do Pará - UFPA - PA
69	ABONO (ACT)	CHU da Universidade Federal do Pará - UFPA - PA
69	ABONO (ACT)	CHU da Universidade Federal do Pará - UFPA - PA

69	ABONO (ACT)	CHU da Universidade Federal do Pará - UFPA - PA
69	ABONO (ACT)	CHU da Universidade Federal do Pará - UFPA - PA
69	ABONO (ACT)	CHU da Universidade Federal do Pará - UFPA - PA
69	ABONO (ACT)	CHU da Universidade Federal do Pará - UFPA - PA
69	ABONO (ACT)	CHU da Universidade Federal do Pará - UFPA - PA
69	ABONO (ACT)	CHU da Universidade Federal do Pará - UFPA - PA
69	ABONO (ACT)	CHU da Universidade Federal do Pará - UFPA - PA
69	ABONO (ACT)	CHU da Universidade Federal do Pará - UFPA - PA
69	ABONO (ACT)	CHU da Universidade Federal do Pará - UFPA - PA
69	ABONO (ACT)	CHU da Universidade Federal do Pará - UFPA - PA
69	ABONO (ACT)	CHU da Universidade Federal do Pará - UFPA - PA
69	ABONO (ACT)	CHU da Universidade Federal do Pará - UFPA - PA
69	ABONO (ACT)	CHU da Universidade Federal do Pará - UFPA - PA
69	ABONO (ACT)	CHU da Universidade Federal do Pará - UFPA - PA
69	ABONO (ACT)	CHU da Universidade Federal do Pará - UFPA - PA
69	ABONO (ACT)	CHU da Universidade Federal do Pará - UFPA - PA
69	ABONO (ACT)	CHU da Universidade Federal do Pará - UFPA - PA
69	ABONO (ACT)	CHU da Universidade Federal do Pará - UFPA - PA
69	ABONO (ACT)	CHU da Universidade Federal do Pará - UFPA - PA
69	ABONO (ACT)	CHU da Universidade Federal do Pará - UFPA - PA
69	ABONO (ACT)	CHU da Universidade Federal do Pará - UFPA - PA
69	ABONO (ACT)	CHU da Universidade Federal do Pará - UFPA - PA
69	ABONO (ACT)	CHU da Universidade Federal do Pará - UFPA - PA
69	ABONO (ACT)	CHU da Universidade Federal do Pará - UFPA - PA
69	ABONO (ACT)	CHU da Universidade Federal do Pará - UFPA - PA
69	ABONO (ACT)	CHU da Universidade Federal do Pará - UFPA - PA
69	ABONO (ACT)	CHU da Universidade Federal do Pará - UFPA - PA
69	ABONO (ACT)	CHU da Universidade Federal do Pará - UFPA - PA
69	ABONO (ACT)	CHU da Universidade Federal do Pará - UFPA - PA
69	ABONO (ACT)	CHU da Universidade Federal do Pará - UFPA - PA
69	ABONO (ACT)	CHU da Universidade Federal do Pará - UFPA - PA
69	ABONO (ACT)	CHU da Universidade Federal do Pará - UFPA - PA

69	ABONO (ACT)	CHU da Universidade Federal do Pará - UFPA - PA
69	ABONO (ACT)	CHU da Universidade Federal do Pará - UFPA - PA
69	ABONO (ACT)	CHU da Universidade Federal do Pará - UFPA - PA
69	ABONO (ACT)	CHU da Universidade Federal do Pará - UFPA - PA
69	ABONO (ACT)	CHU da Universidade Federal do Pará - UFPA - PA
69	ABONO (ACT)	CHU da Universidade Federal do Pará - UFPA - PA
69	ABONO (ACT)	CHU da Universidade Federal do Pará - UFPA - PA
69	ABONO (ACT)	CHU da Universidade Federal do Pará - UFPA - PA
69	ABONO (ACT)	CHU da Universidade Federal do Pará - UFPA - PA
69	ABONO (ACT)	CHU da Universidade Federal do Pará - UFPA - PA
69	ABONO (ACT)	CHU da Universidade Federal do Pará - UFPA - PA
69	ABONO (ACT)	CHU da Universidade Federal do Pará - UFPA - PA
69	ABONO (ACT)	CHU da Universidade Federal do Pará - UFPA - PA
69	ABONO (ACT)	CHU da Universidade Federal do Pará - UFPA - PA
69	ABONO (ACT)	CHU da Universidade Federal do Pará - UFPA - PA
69	ABONO (ACT)	CHU da Universidade Federal do Pará - UFPA - PA
69	ABONO (ACT)	CHU da Universidade Federal do Pará - UFPA - PA
69	ABONO (ACT)	CHU da Universidade Federal do Pará - UFPA - PA
69	ABONO (ACT)	CHU da Universidade Federal do Pará - UFPA - PA
69	ABONO (ACT)	CHU da Universidade Federal do Pará - UFPA - PA
69	ABONO (ACT)	CHU da Universidade Federal do Pará - UFPA - PA
69	ABONO (ACT)	CHU da Universidade Federal do Pará - UFPA - PA
69	ABONO (ACT)	CHU da Universidade Federal do Pará - UFPA - PA
69	ABONO (ACT)	CHU da Universidade Federal do Pará - UFPA - PA
69	ABONO (ACT)	CHU da Universidade Federal do Pará - UFPA - PA
69	ABONO (ACT)	CHU da Universidade Federal do Pará - UFPA - PA
69	ABONO (ACT)	CHU da Universidade Federal do Pará - UFPA - PA
69	ABONO (ACT)	CHU da Universidade Federal do Pará - UFPA - PA
69	ABONO (ACT)	CHU da Universidade Federal do Pará - UFPA - PA
69	ABONO (ACT)	CHU da Universidade Federal do Pará - UFPA - PA
69	ABONO (ACT)	CHU da Universidade Federal do Pará - UFPA - PA
69	ABONO (ACT)	CHU da Universidade Federal do Pará - UFPA - PA
69	ABONO (ACT)	CHU da Universidade Federal do Pará - UFPA - PA
69	ABONO (ACT)	CHU da Universidade Federal do Pará - UFPA - PA
69	ABONO (ACT)	CHU da Universidade Federal do Pará - UFPA - PA
69	ABONO (ACT)	CHU da Universidade Federal do Pará - UFPA - PA
69	ABONO (ACT)	CHU da Universidade Federal do Pará - UFPA - PA
69	ABONO (ACT)	CHU da Universidade Federal do Pará - UFPA - PA
69	ABONO (ACT)	CHU da Universidade Federal do Pará - UFPA - PA
69	ABONO (ACT)	CHU da Universidade Federal do Pará - UFPA - PA
69	ABONO (ACT)	CHU da Universidade Federal do Pará - UFPA - PA
69	ABONO (ACT)	CHU da Universidade Federal do Pará - UFPA - PA
69	ABONO (ACT)	CHU da Universidade Federal do Pará - UFPA - PA
69	ABONO (ACT)	CHU da Universidade Federal do Pará - UFPA - PA

69	ABONO (ACT)	CHU da Universidade Federal do Pará - UFPA - PA
69	ABONO (ACT)	CHU da Universidade Federal do Pará - UFPA - PA
69	ABONO (ACT)	CHU da Universidade Federal do Pará - UFPA - PA
69	ABONO (ACT)	CHU da Universidade Federal do Pará - UFPA - PA
69	ABONO (ACT)	CHU da Universidade Federal do Pará - UFPA - PA
69	ABONO (ACT)	CHU da Universidade Federal do Pará - UFPA - PA
69	ABONO (ACT)	CHU da Universidade Federal do Pará - UFPA - PA
69	ABONO (ACT)	CHU da Universidade Federal do Pará - UFPA - PA
69	ABONO (ACT)	CHU da Universidade Federal do Pará - UFPA - PA
69	ABONO (ACT)	CHU da Universidade Federal do Pará - UFPA - PA
69	ABONO (ACT)	CHU da Universidade Federal do Pará - UFPA - PA
69	ABONO (ACT)	CHU da Universidade Federal do Pará - UFPA - PA
69	ABONO (ACT)	CHU da Universidade Federal do Pará - UFPA - PA
69	ABONO (ACT)	CHU da Universidade Federal do Pará - UFPA - PA
69	ABONO (ACT)	CHU da Universidade Federal do Pará - UFPA - PA
69	ABONO (ACT)	CHU da Universidade Federal do Pará - UFPA - PA
69	ABONO (ACT)	CHU da Universidade Federal do Pará - UFPA - PA
69	ABONO (ACT)	CHU da Universidade Federal do Pará - UFPA - PA
69	ABONO (ACT)	CHU da Universidade Federal do Pará - UFPA - PA
69	ABONO (ACT)	CHU da Universidade Federal do Pará - UFPA - PA
69	ABONO (ACT)	CHU da Universidade Federal do Pará - UFPA - PA
69	ABONO (ACT)	CHU da Universidade Federal do Pará - UFPA - PA
69	ABONO (ACT)	CHU da Universidade Federal do Pará - UFPA - PA
69	ABONO (ACT)	CHU da Universidade Federal do Pará - UFPA - PA
69	ABONO (ACT)	CHU da Universidade Federal do Pará - UFPA - PA
69	ABONO (ACT)	CHU da Universidade Federal do Pará - UFPA - PA
69	ABONO (ACT)	CHU da Universidade Federal do Pará - UFPA - PA
69	ABONO (ACT)	CHU da Universidade Federal do Pará - UFPA - PA
69	ABONO (ACT)	CHU da Universidade Federal do Pará - UFPA - PA
69	ABONO (ACT)	CHU da Universidade Federal do Pará - UFPA - PA
69	ABONO (ACT)	CHU da Universidade Federal do Pará - UFPA - PA
69	ABONO (ACT)	CHU da Universidade Federal do Pará - UFPA - PA
69	ABONO (ACT)	CHU da Universidade Federal do Pará - UFPA - PA
69	ABONO (ACT)	CHU da Universidade Federal do Pará - UFPA - PA

69	ABONO (ACT)	CHU da Universidade Federal do Pará - UFPA - PA
69	ABONO (ACT)	CHU da Universidade Federal do Pará - UFPA - PA
69	ABONO (ACT)	CHU da Universidade Federal do Pará - UFPA - PA
69	ABONO (ACT)	CHU da Universidade Federal do Pará - UFPA - PA
69	ABONO (ACT)	CHU da Universidade Federal do Pará - UFPA - PA
69	ABONO (ACT)	CHU da Universidade Federal do Pará - UFPA - PA
69	ABONO (ACT)	CHU da Universidade Federal do Pará - UFPA - PA
69	ABONO (ACT)	CHU da Universidade Federal do Pará - UFPA - PA
69	ABONO (ACT)	CHU da Universidade Federal do Pará - UFPA - PA
69	ABONO (ACT)	CHU da Universidade Federal do Pará - UFPA - PA
69	ABONO (ACT)	CHU da Universidade Federal do Pará - UFPA - PA
69	ABONO (ACT)	CHU da Universidade Federal do Pará - UFPA - PA
69	ABONO (ACT)	CHU da Universidade Federal do Pará - UFPA - PA
69	ABONO (ACT)	CHU da Universidade Federal do Pará - UFPA - PA
69	ABONO (ACT)	CHU da Universidade Federal do Pará - UFPA - PA
69	ABONO (ACT)	CHU da Universidade Federal do Pará - UFPA - PA
69	ABONO (ACT)	CHU da Universidade Federal do Pará - UFPA - PA
69	ABONO (ACT)	CHU da Universidade Federal do Pará - UFPA - PA
69	ABONO (ACT)	CHU da Universidade Federal do Pará - UFPA - PA
69	ABONO (ACT)	CHU da Universidade Federal do Pará - UFPA - PA
69	ABONO (ACT)	CHU da Universidade Federal do Pará - UFPA - PA
69	ABONO (ACT)	CHU da Universidade Federal do Pará - UFPA - PA
69	ABONO (ACT)	CHU da Universidade Federal do Pará - UFPA - PA
69	ABONO (ACT)	CHU da Universidade Federal do Pará - UFPA - PA
69	ABONO (ACT)	CHU da Universidade Federal do Pará - UFPA - PA
69	ABONO (ACT)	CHU da Universidade Federal do Pará - UFPA - PA
69	ABONO (ACT)	CHU da Universidade Federal do Pará - UFPA - PA
69	ABONO (ACT)	CHU da Universidade Federal do Pará - UFPA - PA
69	ABONO (ACT)	CHU da Universidade Federal do Pará - UFPA - PA
69	ABONO (ACT)	CHU da Universidade Federal do Pará - UFPA - PA
69	ABONO (ACT)	CHU da Universidade Federal do Pará - UFPA - PA
69	ABONO (ACT)	CHU da Universidade Federal do Pará - UFPA - PA
69	ABONO (ACT)	CHU da Universidade Federal do Pará - UFPA - PA
69	ABONO (ACT)	CHU da Universidade Federal do Pará - UFPA - PA
69	ABONO (ACT)	CHU da Universidade Federal do Pará - UFPA - PA
69	ABONO (ACT)	CHU da Universidade Federal do Pará - UFPA - PA
69	ABONO (ACT)	CHU da Universidade Federal do Pará - UFPA - PA
69	ABONO (ACT)	CHU da Universidade Federal do Pará - UFPA - PA
69	ABONO (ACT)	CHU da Universidade Federal do Pará - UFPA - PA
69	ABONO (ACT)	CHU da Universidade Federal do Pará - UFPA - PA
69	ABONO (ACT)	CHU da Universidade Federal do Pará - UFPA - PA
69	ABONO (ACT)	CHU da Universidade Federal do Pará - UFPA - PA
69	ABONO (ACT)	CHU da Universidade Federal do Pará - UFPA - PA
69	ABONO (ACT)	CHU da Universidade Federal do Pará - UFPA - PA
69	ABONO (ACT)	CHU da Universidade Federal do Pará - UFPA - PA
69	ABONO (ACT)	CHU da Universidade Federal do Pará - UFPA - PA

69	ABONO (ACT)	CHU da Universidade Federal do Pará - UFPA - PA
69	ABONO (ACT)	CHU da Universidade Federal do Pará - UFPA - PA
69	ABONO (ACT)	CHU da Universidade Federal do Pará - UFPA - PA
69	ABONO (ACT)	CHU da Universidade Federal do Pará - UFPA - PA
69	ABONO (ACT)	CHU da Universidade Federal do Pará - UFPA - PA
69	ABONO (ACT)	CHU da Universidade Federal do Pará - UFPA - PA
69	ABONO (ACT)	CHU da Universidade Federal do Pará - UFPA - PA
69	ABONO (ACT)	CHU da Universidade Federal do Pará - UFPA - PA
69	ABONO (ACT)	CHU da Universidade Federal do Pará - UFPA - PA
69	ABONO (ACT)	CHU da Universidade Federal do Pará - UFPA - PA
69	ABONO (ACT)	CHU da Universidade Federal do Pará - UFPA - PA
69	ABONO (ACT)	CHU da Universidade Federal do Pará - UFPA - PA
69	ABONO (ACT)	CHU da Universidade Federal do Pará - UFPA - PA
69	ABONO (ACT)	CHU da Universidade Federal do Pará - UFPA - PA
69	ABONO (ACT)	CHU da Universidade Federal do Pará - UFPA - PA
69	ABONO (ACT)	CHU da Universidade Federal do Pará - UFPA - PA
69	ABONO (ACT)	CHU da Universidade Federal do Pará - UFPA - PA
69	ABONO (ACT)	CHU da Universidade Federal do Pará - UFPA - PA
69	ABONO (ACT)	CHU da Universidade Federal do Pará - UFPA - PA
69	ABONO (ACT)	CHU da Universidade Federal do Pará - UFPA - PA
69	ABONO (ACT)	CHU da Universidade Federal do Pará - UFPA - PA
69	ABONO (ACT)	CHU da Universidade Federal do Pará - UFPA - PA
69	ABONO (ACT)	CHU da Universidade Federal do Pará - UFPA - PA
69	ABONO (ACT)	CHU da Universidade Federal do Pará - UFPA - PA
69	ABONO (ACT)	CHU da Universidade Federal do Pará - UFPA - PA
69	ABONO (ACT)	CHU da Universidade Federal do Pará - UFPA - PA
69	ABONO (ACT)	CHU da Universidade Federal do Pará - UFPA - PA
69	ABONO (ACT)	CHU da Universidade Federal do Pará - UFPA - PA
69	ABONO (ACT)	CHU da Universidade Federal do Pará - UFPA - PA
69	ABONO (ACT)	CHU da Universidade Federal do Pará - UFPA - PA
69	ABONO (ACT)	CHU da Universidade Federal do Pará - UFPA - PA
69	ABONO (ACT)	CHU da Universidade Federal do Pará - UFPA - PA
69	ABONO (ACT)	CHU da Universidade Federal do Pará - UFPA - PA
69	ABONO (ACT)	CHU da Universidade Federal do Pará - UFPA - PA
69	ABONO (ACT)	CHU da Universidade Federal do Pará - UFPA - PA
69	ABONO (ACT)	CHU da Universidade Federal do Pará - UFPA - PA
69	ABONO (ACT)	CHU da Universidade Federal do Pará - UFPA - PA
69	ABONO (ACT)	CHU da Universidade Federal do Pará - UFPA - PA
69	ABONO (ACT)	CHU da Universidade Federal do Pará - UFPA - PA
69	ABONO (ACT)	CHU da Universidade Federal do Pará - UFPA - PA
69	ABONO (ACT)	CHU da Universidade Federal do Pará - UFPA - PA
69	ABONO (ACT)	CHU da Universidade Federal do Pará - UFPA - PA
69	ABONO (ACT)	CHU da Universidade Federal do Pará - UFPA - PA
69	ABONO (ACT)	CHU da Universidade Federal do Pará - UFPA - PA
69	ABONO (ACT)	CHU da Universidade Federal do Pará - UFPA - PA
69	ABONO (ACT)	CHU da Universidade Federal do Pará - UFPA - PA
69	ABONO (ACT)	CHU da Universidade Federal do Pará - UFPA - PA
69	ABONO (ACT)	CHU da Universidade Federal do Pará - UFPA - PA

69	ABONO (ACT)	CHU da Universidade Federal do Pará - UFPA - PA
69	ABONO (ACT)	CHU da Universidade Federal do Pará - UFPA - PA
69	ABONO (ACT)	CHU da Universidade Federal do Pará - UFPA - PA
69	ABONO (ACT)	CHU da Universidade Federal do Pará - UFPA - PA
69	ABONO (ACT)	CHU da Universidade Federal do Pará - UFPA - PA
69	ABONO (ACT)	CHU da Universidade Federal do Pará - UFPA - PA
69	ABONO (ACT)	CHU da Universidade Federal do Pará - UFPA - PA
69	ABONO (ACT)	CHU da Universidade Federal do Pará - UFPA - PA
69	ABONO (ACT)	CHU da Universidade Federal do Pará - UFPA - PA
69	ABONO (ACT)	CHU da Universidade Federal do Pará - UFPA - PA
69	ABONO (ACT)	CHU da Universidade Federal do Pará - UFPA - PA
69	ABONO (ACT)	CHU da Universidade Federal do Pará - UFPA - PA
69	ABONO (ACT)	CHU da Universidade Federal do Pará - UFPA - PA
69	ABONO (ACT)	CHU da Universidade Federal do Pará - UFPA - PA
69	ABONO (ACT)	CHU da Universidade Federal do Pará - UFPA - PA
69	ABONO (ACT)	CHU da Universidade Federal do Pará - UFPA - PA
69	ABONO (ACT)	CHU da Universidade Federal do Pará - UFPA - PA
69	ABONO (ACT)	CHU da Universidade Federal do Pará - UFPA - PA
69	ABONO (ACT)	CHU da Universidade Federal do Pará - UFPA - PA
69	ABONO (ACT)	CHU da Universidade Federal do Pará - UFPA - PA
69	ABONO (ACT)	CHU da Universidade Federal do Pará - UFPA - PA
69	ABONO (ACT)	CHU da Universidade Federal do Pará - UFPA - PA
69	ABONO (ACT)	CHU da Universidade Federal do Pará - UFPA - PA
69	ABONO (ACT)	CHU da Universidade Federal do Pará - UFPA - PA
69	ABONO (ACT)	CHU da Universidade Federal do Pará - UFPA - PA
69	ABONO (ACT)	CHU da Universidade Federal do Pará - UFPA - PA
69	ABONO (ACT)	CHU da Universidade Federal do Pará - UFPA - PA
69	ABONO (ACT)	CHU da Universidade Federal do Pará - UFPA - PA
69	ABONO (ACT)	CHU da Universidade Federal do Pará - UFPA - PA
69	ABONO (ACT)	CHU da Universidade Federal do Pará - UFPA - PA
69	ABONO (ACT)	CHU da Universidade Federal do Pará - UFPA - PA
69	ABONO (ACT)	CHU da Universidade Federal do Pará - UFPA - PA
69	ABONO (ACT)	CHU da Universidade Federal do Pará - UFPA - PA
69	ABONO (ACT)	CHU da Universidade Federal do Pará - UFPA - PA
69	ABONO (ACT)	CHU da Universidade Federal do Pará - UFPA - PA
69	ABONO (ACT)	CHU da Universidade Federal do Pará - UFPA - PA
69	ABONO (ACT)	CHU da Universidade Federal do Pará - UFPA - PA
69	ABONO (ACT)	CHU da Universidade Federal do Pará - UFPA - PA
69	ABONO (ACT)	CHU da Universidade Federal do Pará - UFPA - PA
69	ABONO (ACT)	CHU da Universidade Federal do Pará - UFPA - PA
69	ABONO (ACT)	CHU da Universidade Federal do Pará - UFPA - PA
69	ABONO (ACT)	CHU da Universidade Federal do Pará - UFPA - PA
69	ABONO (ACT)	CHU da Universidade Federal do Pará - UFPA - PA
69	ABONO (ACT)	CHU da Universidade Federal do Pará - UFPA - PA
69	ABONO (ACT)	CHU da Universidade Federal do Pará - UFPA - PA

69	ABONO (ACT)	CHU da Universidade Federal do Pará - UFPA - PA
69	ABONO (ACT)	CHU da Universidade Federal do Pará - UFPA - PA
69	ABONO (ACT)	CHU da Universidade Federal do Pará - UFPA - PA
69	ABONO (ACT)	CHU da Universidade Federal do Pará - UFPA - PA
69	ABONO (ACT)	CHU da Universidade Federal do Pará - UFPA - PA
69	ABONO (ACT)	CHU da Universidade Federal do Pará - UFPA - PA
69	ABONO (ACT)	CHU da Universidade Federal do Pará - UFPA - PA
69	ABONO (ACT)	CHU da Universidade Federal do Pará - UFPA - PA
69	ABONO (ACT)	CHU da Universidade Federal do Pará - UFPA - PA
69	ABONO (ACT)	CHU da Universidade Federal do Pará - UFPA - PA
69	ABONO (ACT)	CHU da Universidade Federal do Pará - UFPA - PA
69	ABONO (ACT)	CHU da Universidade Federal do Pará - UFPA - PA
69	ABONO (ACT)	CHU da Universidade Federal do Pará - UFPA - PA
69	ABONO (ACT)	CHU da Universidade Federal do Pará - UFPA - PA
69	ABONO (ACT)	CHU da Universidade Federal do Pará - UFPA - PA
69	ABONO (ACT)	CHU da Universidade Federal do Pará - UFPA - PA
69	ABONO (ACT)	CHU da Universidade Federal do Pará - UFPA - PA
69	ABONO (ACT)	CHU da Universidade Federal do Pará - UFPA - PA
69	ABONO (ACT)	CHU da Universidade Federal do Pará - UFPA - PA
69	ABONO (ACT)	CHU da Universidade Federal do Pará - UFPA - PA
69	ABONO (ACT)	CHU da Universidade Federal do Pará - UFPA - PA
69	ABONO (ACT)	CHU da Universidade Federal do Pará - UFPA - PA
69	ABONO (ACT)	CHU da Universidade Federal do Pará - UFPA - PA
69	ABONO (ACT)	CHU da Universidade Federal do Pará - UFPA - PA
69	ABONO (ACT)	CHU da Universidade Federal do Pará - UFPA - PA
69	ABONO (ACT)	CHU da Universidade Federal do Pará - UFPA - PA
69	ABONO (ACT)	CHU da Universidade Federal do Pará - UFPA - PA
69	ABONO (ACT)	CHU da Universidade Federal do Pará - UFPA - PA
69	ABONO (ACT)	CHU da Universidade Federal do Pará - UFPA - PA
69	ABONO (ACT)	CHU da Universidade Federal do Pará - UFPA - PA
69	ABONO (ACT)	CHU da Universidade Federal do Pará - UFPA - PA
69	ABONO (ACT)	CHU da Universidade Federal do Pará - UFPA - PA
69	ABONO (ACT)	CHU da Universidade Federal do Pará - UFPA - PA
69	ABONO (ACT)	CHU da Universidade Federal do Pará - UFPA - PA
69	ABONO (ACT)	CHU da Universidade Federal do Pará - UFPA - PA
69	ABONO (ACT)	CHU da Universidade Federal do Pará - UFPA - PA
69	ABONO (ACT)	CHU da Universidade Federal do Pará - UFPA - PA
69	ABONO (ACT)	CHU da Universidade Federal do Pará - UFPA - PA
69	ABONO (ACT)	CHU da Universidade Federal do Pará - UFPA - PA
69	ABONO (ACT)	CHU da Universidade Federal do Pará - UFPA - PA
69	ABONO (ACT)	CHU da Universidade Federal do Pará - UFPA - PA
69	ABONO (ACT)	CHU da Universidade Federal do Pará - UFPA - PA
69	ABONO (ACT)	CHU da Universidade Federal do Pará - UFPA - PA

69	ABONO (ACT)	CHU da Universidade Federal do Pará - UFPA - PA
69	ABONO (ACT)	CHU da Universidade Federal do Pará - UFPA - PA
69	ABONO (ACT)	CHU da Universidade Federal do Pará - UFPA - PA
69	ABONO (ACT)	CHU da Universidade Federal do Pará - UFPA - PA
69	ABONO (ACT)	CHU da Universidade Federal do Pará - UFPA - PA
69	ABONO (ACT)	CHU da Universidade Federal do Pará - UFPA - PA
69	ABONO (ACT)	CHU da Universidade Federal do Pará - UFPA - PA
69	ABONO (ACT)	CHU da Universidade Federal do Pará - UFPA - PA
69	ABONO (ACT)	CHU da Universidade Federal do Pará - UFPA - PA
69	ABONO (ACT)	CHU da Universidade Federal do Pará - UFPA - PA
69	ABONO (ACT)	CHU da Universidade Federal do Pará - UFPA - PA
69	ABONO (ACT)	CHU da Universidade Federal do Pará - UFPA - PA
69	ABONO (ACT)	CHU da Universidade Federal do Pará - UFPA - PA
69	ABONO (ACT)	CHU da Universidade Federal do Pará - UFPA - PA
69	ABONO (ACT)	CHU da Universidade Federal do Pará - UFPA - PA
69	ABONO (ACT)	CHU da Universidade Federal do Pará - UFPA - PA
69	ABONO (ACT)	CHU da Universidade Federal do Pará - UFPA - PA
69	ABONO (ACT)	CHU da Universidade Federal do Pará - UFPA - PA
69	ABONO (ACT)	CHU da Universidade Federal do Pará - UFPA - PA
69	ABONO (ACT)	CHU da Universidade Federal do Pará - UFPA - PA
69	ABONO (ACT)	CHU da Universidade Federal do Pará - UFPA - PA
69	ABONO (ACT)	CHU da Universidade Federal do Pará - UFPA - PA
69	ABONO (ACT)	CHU da Universidade Federal do Pará - UFPA - PA
69	ABONO (ACT)	CHU da Universidade Federal do Pará - UFPA - PA
69	ABONO (ACT)	CHU da Universidade Federal do Pará - UFPA - PA
69	ABONO (ACT)	CHU da Universidade Federal do Pará - UFPA - PA
69	ABONO (ACT)	CHU da Universidade Federal do Pará - UFPA - PA
69	ABONO (ACT)	CHU da Universidade Federal do Pará - UFPA - PA
69	ABONO (ACT)	CHU da Universidade Federal do Pará - UFPA - PA
69	ABONO (ACT)	CHU da Universidade Federal do Pará - UFPA - PA
69	ABONO (ACT)	CHU da Universidade Federal do Pará - UFPA - PA
69	ABONO (ACT)	CHU da Universidade Federal do Pará - UFPA - PA
69	ABONO (ACT)	CHU da Universidade Federal do Pará - UFPA - PA
69	ABONO (ACT)	CHU da Universidade Federal do Pará - UFPA - PA
69	ABONO (ACT)	CHU da Universidade Federal do Pará - UFPA - PA
69	ABONO (ACT)	CHU da Universidade Federal do Pará - UFPA - PA
69	ABONO (ACT)	CHU da Universidade Federal do Pará - UFPA - PA
69	ABONO (ACT)	CHU da Universidade Federal do Pará - UFPA - PA
69	ABONO (ACT)	CHU da Universidade Federal do Pará - UFPA - PA
69	ABONO (ACT)	CHU da Universidade Federal do Pará - UFPA - PA
69	ABONO (ACT)	CHU da Universidade Federal do Pará - UFPA - PA
69	ABONO (ACT)	CHU da Universidade Federal do Pará - UFPA - PA
69	ABONO (ACT)	CHU da Universidade Federal do Pará - UFPA - PA
69	ABONO (ACT)	CHU da Universidade Federal do Pará - UFPA - PA
70	AUSÊNCIA COM COMPENSAÇÃO	CHU da Universidade Federal do Pará - UFPA - PA
70	AUSÊNCIA COM COMPENSAÇÃO	CHU da Universidade Federal do Pará - UFPA - PA
70	AUSÊNCIA COM COMPENSAÇÃO	CHU da Universidade Federal do Pará - UFPA - PA
70	AUSÊNCIA COM COMPENSAÇÃO	CHU da Universidade Federal do Pará - UFPA - PA

70	AUSÊNCIA COM COMPENSAÇÃO	CHU da Universidade Federal do Pará - UFPA - PA
70	AUSÊNCIA COM COMPENSAÇÃO	CHU da Universidade Federal do Pará - UFPA - PA
70	AUSÊNCIA COM COMPENSAÇÃO	CHU da Universidade Federal do Pará - UFPA - PA
70	AUSÊNCIA COM COMPENSAÇÃO	CHU da Universidade Federal do Pará - UFPA - PA
70	AUSÊNCIA COM COMPENSAÇÃO	CHU da Universidade Federal do Pará - UFPA - PA
70	AUSÊNCIA COM COMPENSAÇÃO	CHU da Universidade Federal do Pará - UFPA - PA
70	AUSÊNCIA COM COMPENSAÇÃO	CHU da Universidade Federal do Pará - UFPA - PA
70	AUSÊNCIA COM COMPENSAÇÃO	CHU da Universidade Federal do Pará - UFPA - PA
70	AUSÊNCIA COM COMPENSAÇÃO	CHU da Universidade Federal do Pará - UFPA - PA
70	AUSÊNCIA COM COMPENSAÇÃO	CHU da Universidade Federal do Pará - UFPA - PA
70	AUSÊNCIA COM COMPENSAÇÃO	CHU da Universidade Federal do Pará - UFPA - PA
70	AUSÊNCIA COM COMPENSAÇÃO	CHU da Universidade Federal do Pará - UFPA - PA
70	AUSÊNCIA COM COMPENSAÇÃO	CHU da Universidade Federal do Pará - UFPA - PA
70	AUSÊNCIA COM COMPENSAÇÃO	CHU da Universidade Federal do Pará - UFPA - PA
70	AUSÊNCIA COM COMPENSAÇÃO	CHU da Universidade Federal do Pará - UFPA - PA
70	AUSÊNCIA COM COMPENSAÇÃO	CHU da Universidade Federal do Pará - UFPA - PA
70	AUSÊNCIA COM COMPENSAÇÃO	CHU da Universidade Federal do Pará - UFPA - PA
70	AUSÊNCIA COM COMPENSAÇÃO	CHU da Universidade Federal do Pará - UFPA - PA
70	AUSÊNCIA COM COMPENSAÇÃO	CHU da Universidade Federal do Pará - UFPA - PA
70	AUSÊNCIA COM COMPENSAÇÃO	CHU da Universidade Federal do Pará - UFPA - PA
70	AUSÊNCIA COM COMPENSAÇÃO	CHU da Universidade Federal do Pará - UFPA - PA
70	AUSÊNCIA COM COMPENSAÇÃO	CHU da Universidade Federal do Pará - UFPA - PA
70	AUSÊNCIA COM COMPENSAÇÃO	CHU da Universidade Federal do Pará - UFPA - PA
70	AUSÊNCIA COM COMPENSAÇÃO	CHU da Universidade Federal do Pará - UFPA - PA
70	AUSÊNCIA COM COMPENSAÇÃO	CHU da Universidade Federal do Pará - UFPA - PA
70	AUSÊNCIA COM COMPENSAÇÃO	CHU da Universidade Federal do Pará - UFPA - PA
70	AUSÊNCIA COM COMPENSAÇÃO	CHU da Universidade Federal do Pará - UFPA - PA
70	AUSÊNCIA COM COMPENSAÇÃO	CHU da Universidade Federal do Pará - UFPA - PA
70	AUSÊNCIA COM COMPENSAÇÃO	CHU da Universidade Federal do Pará - UFPA - PA
70	AUSÊNCIA COM COMPENSAÇÃO	CHU da Universidade Federal do Pará - UFPA - PA
70	AUSÊNCIA COM COMPENSAÇÃO	CHU da Universidade Federal do Pará - UFPA - PA
70	AUSÊNCIA COM COMPENSAÇÃO	CHU da Universidade Federal do Pará - UFPA - PA
70	AUSÊNCIA COM COMPENSAÇÃO	CHU da Universidade Federal do Pará - UFPA - PA
70	AUSÊNCIA COM COMPENSAÇÃO	CHU da Universidade Federal do Pará - UFPA - PA
70	AUSÊNCIA COM COMPENSAÇÃO	CHU da Universidade Federal do Pará - UFPA - PA
70	AUSÊNCIA COM COMPENSAÇÃO	CHU da Universidade Federal do Pará - UFPA - PA
70	AUSÊNCIA COM COMPENSAÇÃO	CHU da Universidade Federal do Pará - UFPA - PA
70	AUSÊNCIA COM COMPENSAÇÃO	CHU da Universidade Federal do Pará - UFPA - PA
70	AUSÊNCIA COM COMPENSAÇÃO	CHU da Universidade Federal do Pará - UFPA - PA
70	AUSÊNCIA COM COMPENSAÇÃO	CHU da Universidade Federal do Pará - UFPA - PA
70	AUSÊNCIA COM COMPENSAÇÃO	CHU da Universidade Federal do Pará - UFPA - PA
70	AUSÊNCIA COM COMPENSAÇÃO	CHU da Universidade Federal do Pará - UFPA - PA
70	AUSÊNCIA COM COMPENSAÇÃO	CHU da Universidade Federal do Pará - UFPA - PA
70	AUSÊNCIA COM COMPENSAÇÃO	CHU da Universidade Federal do Pará - UFPA - PA
70	AUSÊNCIA COM COMPENSAÇÃO	CHU da Universidade Federal do Pará - UFPA - PA
70	AUSÊNCIA COM COMPENSAÇÃO	CHU da Universidade Federal do Pará - UFPA - PA
70	AUSÊNCIA COM COMPENSAÇÃO	CHU da Universidade Federal do Pará - UFPA - PA
70	AUSÊNCIA COM COMPENSAÇÃO	CHU da Universidade Federal do Pará - UFPA - PA

70	AUSÊNCIA COM COMPENSAÇÃO	CHU da Universidade Federal do Pará - UFPA - PA
70	AUSÊNCIA COM COMPENSAÇÃO	CHU da Universidade Federal do Pará - UFPA - PA
70	AUSÊNCIA COM COMPENSAÇÃO	CHU da Universidade Federal do Pará - UFPA - PA
70	AUSÊNCIA COM COMPENSAÇÃO	CHU da Universidade Federal do Pará - UFPA - PA
70	AUSÊNCIA COM COMPENSAÇÃO	CHU da Universidade Federal do Pará - UFPA - PA
70	AUSÊNCIA COM COMPENSAÇÃO	CHU da Universidade Federal do Pará - UFPA - PA
70	AUSÊNCIA COM COMPENSAÇÃO	CHU da Universidade Federal do Pará - UFPA - PA
70	AUSÊNCIA COM COMPENSAÇÃO	CHU da Universidade Federal do Pará - UFPA - PA
70	AUSÊNCIA COM COMPENSAÇÃO	CHU da Universidade Federal do Pará - UFPA - PA
70	AUSÊNCIA COM COMPENSAÇÃO	CHU da Universidade Federal do Pará - UFPA - PA
70	AUSÊNCIA COM COMPENSAÇÃO	CHU da Universidade Federal do Pará - UFPA - PA
70	AUSÊNCIA COM COMPENSAÇÃO	CHU da Universidade Federal do Pará - UFPA - PA
70	AUSÊNCIA COM COMPENSAÇÃO	CHU da Universidade Federal do Pará - UFPA - PA
70	AUSÊNCIA COM COMPENSAÇÃO	CHU da Universidade Federal do Pará - UFPA - PA
70	AUSÊNCIA COM COMPENSAÇÃO	CHU da Universidade Federal do Pará - UFPA - PA
70	AUSÊNCIA COM COMPENSAÇÃO	CHU da Universidade Federal do Pará - UFPA - PA
70	AUSÊNCIA COM COMPENSAÇÃO	CHU da Universidade Federal do Pará - UFPA - PA
70	AUSÊNCIA COM COMPENSAÇÃO	CHU da Universidade Federal do Pará - UFPA - PA
70	AUSÊNCIA COM COMPENSAÇÃO	CHU da Universidade Federal do Pará - UFPA - PA
70	AUSÊNCIA COM COMPENSAÇÃO	CHU da Universidade Federal do Pará - UFPA - PA
70	AUSÊNCIA COM COMPENSAÇÃO	CHU da Universidade Federal do Pará - UFPA - PA
70	AUSÊNCIA COM COMPENSAÇÃO	CHU da Universidade Federal do Pará - UFPA - PA
70	AUSÊNCIA COM COMPENSAÇÃO	CHU da Universidade Federal do Pará - UFPA - PA
70	AUSÊNCIA COM COMPENSAÇÃO	CHU da Universidade Federal do Pará - UFPA - PA
70	AUSÊNCIA COM COMPENSAÇÃO	CHU da Universidade Federal do Pará - UFPA - PA
70	AUSÊNCIA COM COMPENSAÇÃO	CHU da Universidade Federal do Pará - UFPA - PA
70	AUSÊNCIA COM COMPENSAÇÃO	CHU da Universidade Federal do Pará - UFPA - PA
70	AUSÊNCIA COM COMPENSAÇÃO	CHU da Universidade Federal do Pará - UFPA - PA
70	AUSÊNCIA COM COMPENSAÇÃO	CHU da Universidade Federal do Pará - UFPA - PA
70	AUSÊNCIA COM COMPENSAÇÃO	CHU da Universidade Federal do Pará - UFPA - PA
70	AUSÊNCIA COM COMPENSAÇÃO	CHU da Universidade Federal do Pará - UFPA - PA
70	AUSÊNCIA COM COMPENSAÇÃO	CHU da Universidade Federal do Pará - UFPA - PA
70	AUSÊNCIA COM COMPENSAÇÃO	CHU da Universidade Federal do Pará - UFPA - PA
70	AUSÊNCIA COM COMPENSAÇÃO	CHU da Universidade Federal do Pará - UFPA - PA
70	AUSÊNCIA COM COMPENSAÇÃO	CHU da Universidade Federal do Pará - UFPA - PA
70	AUSÊNCIA COM COMPENSAÇÃO	CHU da Universidade Federal do Pará - UFPA - PA
70	AUSÊNCIA COM COMPENSAÇÃO	CHU da Universidade Federal do Pará - UFPA - PA
70	AUSÊNCIA COM COMPENSAÇÃO	CHU da Universidade Federal do Pará - UFPA - PA
70	AUSÊNCIA COM COMPENSAÇÃO	CHU da Universidade Federal do Pará - UFPA - PA
70	AUSÊNCIA COM COMPENSAÇÃO	CHU da Universidade Federal do Pará - UFPA - PA
70	AUSÊNCIA COM COMPENSAÇÃO	CHU da Universidade Federal do Pará - UFPA - PA
70	AUSÊNCIA COM COMPENSAÇÃO	CHU da Universidade Federal do Pará - UFPA - PA
70	AUSÊNCIA COM COMPENSAÇÃO	CHU da Universidade Federal do Pará - UFPA - PA
70	AUSÊNCIA COM COMPENSAÇÃO	CHU da Universidade Federal do Pará - UFPA - PA
70	AUSÊNCIA COM COMPENSAÇÃO	CHU da Universidade Federal do Pará - UFPA - PA
70	AUSÊNCIA COM COMPENSAÇÃO	CHU da Universidade Federal do Pará - UFPA - PA
70	AUSÊNCIA COM COMPENSAÇÃO	CHU da Universidade Federal do Pará - UFPA - PA
70	AUSÊNCIA COM COMPENSAÇÃO	CHU da Universidade Federal do Pará - UFPA - PA

70	AUSÊNCIA COM COMPENSAÇÃO	CHU da Universidade Federal do Pará - UFPA - PA
70	AUSÊNCIA COM COMPENSAÇÃO	CHU da Universidade Federal do Pará - UFPA - PA
70	AUSÊNCIA COM COMPENSAÇÃO	CHU da Universidade Federal do Pará - UFPA - PA
70	AUSÊNCIA COM COMPENSAÇÃO	CHU da Universidade Federal do Pará - UFPA - PA
70	AUSÊNCIA COM COMPENSAÇÃO	CHU da Universidade Federal do Pará - UFPA - PA
70	AUSÊNCIA COM COMPENSAÇÃO	CHU da Universidade Federal do Pará - UFPA - PA
70	AUSÊNCIA COM COMPENSAÇÃO	CHU da Universidade Federal do Pará - UFPA - PA
70	AUSÊNCIA COM COMPENSAÇÃO	CHU da Universidade Federal do Pará - UFPA - PA
70	AUSÊNCIA COM COMPENSAÇÃO	CHU da Universidade Federal do Pará - UFPA - PA
70	AUSÊNCIA COM COMPENSAÇÃO	CHU da Universidade Federal do Pará - UFPA - PA
70	AUSÊNCIA COM COMPENSAÇÃO	CHU da Universidade Federal do Pará - UFPA - PA
70	AUSÊNCIA COM COMPENSAÇÃO	CHU da Universidade Federal do Pará - UFPA - PA
70	AUSÊNCIA COM COMPENSAÇÃO	CHU da Universidade Federal do Pará - UFPA - PA
70	AUSÊNCIA COM COMPENSAÇÃO	CHU da Universidade Federal do Pará - UFPA - PA
70	AUSÊNCIA COM COMPENSAÇÃO	CHU da Universidade Federal do Pará - UFPA - PA
70	AUSÊNCIA COM COMPENSAÇÃO	CHU da Universidade Federal do Pará - UFPA - PA
70	AUSÊNCIA COM COMPENSAÇÃO	CHU da Universidade Federal do Pará - UFPA - PA
70	AUSÊNCIA COM COMPENSAÇÃO	CHU da Universidade Federal do Pará - UFPA - PA
70	AUSÊNCIA COM COMPENSAÇÃO	CHU da Universidade Federal do Pará - UFPA - PA
70	AUSÊNCIA COM COMPENSAÇÃO	CHU da Universidade Federal do Pará - UFPA - PA
70	AUSÊNCIA COM COMPENSAÇÃO	CHU da Universidade Federal do Pará - UFPA - PA
70	AUSÊNCIA COM COMPENSAÇÃO	CHU da Universidade Federal do Pará - UFPA - PA
70	AUSÊNCIA COM COMPENSAÇÃO	CHU da Universidade Federal do Pará - UFPA - PA
70	AUSÊNCIA COM COMPENSAÇÃO	CHU da Universidade Federal do Pará - UFPA - PA
70	AUSÊNCIA COM COMPENSAÇÃO	CHU da Universidade Federal do Pará - UFPA - PA
70	AUSÊNCIA COM COMPENSAÇÃO	CHU da Universidade Federal do Pará - UFPA - PA
70	AUSÊNCIA COM COMPENSAÇÃO	CHU da Universidade Federal do Pará - UFPA - PA
70	AUSÊNCIA COM COMPENSAÇÃO	CHU da Universidade Federal do Pará - UFPA - PA
70	AUSÊNCIA COM COMPENSAÇÃO	CHU da Universidade Federal do Pará - UFPA - PA
70	AUSÊNCIA COM COMPENSAÇÃO	CHU da Universidade Federal do Pará - UFPA - PA
70	AUSÊNCIA COM COMPENSAÇÃO	CHU da Universidade Federal do Pará - UFPA - PA
70	AUSÊNCIA COM COMPENSAÇÃO	CHU da Universidade Federal do Pará - UFPA - PA
70	AUSÊNCIA COM COMPENSAÇÃO	CHU da Universidade Federal do Pará - UFPA - PA
70	AUSÊNCIA COM COMPENSAÇÃO	CHU da Universidade Federal do Pará - UFPA - PA
70	AUSÊNCIA COM COMPENSAÇÃO	CHU da Universidade Federal do Pará - UFPA - PA
70	AUSÊNCIA COM COMPENSAÇÃO	CHU da Universidade Federal do Pará - UFPA - PA
70	AUSÊNCIA COM COMPENSAÇÃO	CHU da Universidade Federal do Pará - UFPA - PA
70	AUSÊNCIA COM COMPENSAÇÃO	CHU da Universidade Federal do Pará - UFPA - PA
70	AUSÊNCIA COM COMPENSAÇÃO	CHU da Universidade Federal do Pará - UFPA - PA
70	AUSÊNCIA COM COMPENSAÇÃO	CHU da Universidade Federal do Pará - UFPA - PA
70	AUSÊNCIA COM COMPENSAÇÃO	CHU da Universidade Federal do Pará - UFPA - PA
70	AUSÊNCIA COM COMPENSAÇÃO	CHU da Universidade Federal do Pará - UFPA - PA
70	AUSÊNCIA COM COMPENSAÇÃO	CHU da Universidade Federal do Pará - UFPA - PA
70	AUSÊNCIA COM COMPENSAÇÃO	CHU da Universidade Federal do Pará - UFPA - PA
70	AUSÊNCIA COM COMPENSAÇÃO	CHU da Universidade Federal do Pará - UFPA - PA

70	AUSÊNCIA COM COMPENSAÇÃO	CHU da Universidade Federal do Pará - UFPA - PA
70	AUSÊNCIA COM COMPENSAÇÃO	CHU da Universidade Federal do Pará - UFPA - PA
70	AUSÊNCIA COM COMPENSAÇÃO	CHU da Universidade Federal do Pará - UFPA - PA
70	AUSÊNCIA COM COMPENSAÇÃO	CHU da Universidade Federal do Pará - UFPA - PA
70	AUSÊNCIA COM COMPENSAÇÃO	CHU da Universidade Federal do Pará - UFPA - PA
70	AUSÊNCIA COM COMPENSAÇÃO	CHU da Universidade Federal do Pará - UFPA - PA
70	AUSÊNCIA COM COMPENSAÇÃO	CHU da Universidade Federal do Pará - UFPA - PA
70	AUSÊNCIA COM COMPENSAÇÃO	CHU da Universidade Federal do Pará - UFPA - PA
70	AUSÊNCIA COM COMPENSAÇÃO	CHU da Universidade Federal do Pará - UFPA - PA
70	AUSÊNCIA COM COMPENSAÇÃO	CHU da Universidade Federal do Pará - UFPA - PA
70	AUSÊNCIA COM COMPENSAÇÃO	CHU da Universidade Federal do Pará - UFPA - PA
70	AUSÊNCIA COM COMPENSAÇÃO	CHU da Universidade Federal do Pará - UFPA - PA
70	AUSÊNCIA COM COMPENSAÇÃO	CHU da Universidade Federal do Pará - UFPA - PA
70	AUSÊNCIA COM COMPENSAÇÃO	CHU da Universidade Federal do Pará - UFPA - PA
70	AUSÊNCIA COM COMPENSAÇÃO	CHU da Universidade Federal do Pará - UFPA - PA
70	AUSÊNCIA COM COMPENSAÇÃO	CHU da Universidade Federal do Pará - UFPA - PA
70	AUSÊNCIA COM COMPENSAÇÃO	CHU da Universidade Federal do Pará - UFPA - PA
70	AUSÊNCIA COM COMPENSAÇÃO	CHU da Universidade Federal do Pará - UFPA - PA
70	AUSÊNCIA COM COMPENSAÇÃO	CHU da Universidade Federal do Pará - UFPA - PA
70	AUSÊNCIA COM COMPENSAÇÃO	CHU da Universidade Federal do Pará - UFPA - PA
70	AUSÊNCIA COM COMPENSAÇÃO	CHU da Universidade Federal do Pará - UFPA - PA
70	AUSÊNCIA COM COMPENSAÇÃO	CHU da Universidade Federal do Pará - UFPA - PA
70	AUSÊNCIA COM COMPENSAÇÃO	CHU da Universidade Federal do Pará - UFPA - PA
70	AUSÊNCIA COM COMPENSAÇÃO	CHU da Universidade Federal do Pará - UFPA - PA
70	AUSÊNCIA COM COMPENSAÇÃO	CHU da Universidade Federal do Pará - UFPA - PA
70	AUSÊNCIA COM COMPENSAÇÃO	CHU da Universidade Federal do Pará - UFPA - PA
70	AUSÊNCIA COM COMPENSAÇÃO	CHU da Universidade Federal do Pará - UFPA - PA
70	AUSÊNCIA COM COMPENSAÇÃO	CHU da Universidade Federal do Pará - UFPA - PA
70	AUSÊNCIA COM COMPENSAÇÃO	CHU da Universidade Federal do Pará - UFPA - PA
70	AUSÊNCIA COM COMPENSAÇÃO	CHU da Universidade Federal do Pará - UFPA - PA
70	AUSÊNCIA COM COMPENSAÇÃO	CHU da Universidade Federal do Pará - UFPA - PA
70	AUSÊNCIA COM COMPENSAÇÃO	CHU da Universidade Federal do Pará - UFPA - PA
70	AUSÊNCIA COM COMPENSAÇÃO	CHU da Universidade Federal do Pará - UFPA - PA
70	AUSÊNCIA COM COMPENSAÇÃO	CHU da Universidade Federal do Pará - UFPA - PA
70	AUSÊNCIA COM COMPENSAÇÃO	CHU da Universidade Federal do Pará - UFPA - PA
70	AUSÊNCIA COM COMPENSAÇÃO	CHU da Universidade Federal do Pará - UFPA - PA
70	AUSÊNCIA COM COMPENSAÇÃO	CHU da Universidade Federal do Pará - UFPA - PA
70	AUSÊNCIA COM COMPENSAÇÃO	CHU da Universidade Federal do Pará - UFPA - PA
70	AUSÊNCIA COM COMPENSAÇÃO	CHU da Universidade Federal do Pará - UFPA - PA
70	AUSÊNCIA COM COMPENSAÇÃO	CHU da Universidade Federal do Pará - UFPA - PA
70	AUSÊNCIA COM COMPENSAÇÃO	CHU da Universidade Federal do Pará - UFPA - PA

70	AUSÊNCIA COM COMPENSAÇÃO	CHU da Universidade Federal do Pará - UFPA - PA
70	AUSÊNCIA COM COMPENSAÇÃO	CHU da Universidade Federal do Pará - UFPA - PA
70	AUSÊNCIA COM COMPENSAÇÃO	CHU da Universidade Federal do Pará - UFPA - PA
70	AUSÊNCIA COM COMPENSAÇÃO	CHU da Universidade Federal do Pará - UFPA - PA
70	AUSÊNCIA COM COMPENSAÇÃO	CHU da Universidade Federal do Pará - UFPA - PA
70	AUSÊNCIA COM COMPENSAÇÃO	CHU da Universidade Federal do Pará - UFPA - PA
70	AUSÊNCIA COM COMPENSAÇÃO	CHU da Universidade Federal do Pará - UFPA - PA
70	AUSÊNCIA COM COMPENSAÇÃO	CHU da Universidade Federal do Pará - UFPA - PA
70	AUSÊNCIA COM COMPENSAÇÃO	CHU da Universidade Federal do Pará - UFPA - PA
70	AUSÊNCIA COM COMPENSAÇÃO	CHU da Universidade Federal do Pará - UFPA - PA
70	AUSÊNCIA COM COMPENSAÇÃO	CHU da Universidade Federal do Pará - UFPA - PA
70	AUSÊNCIA COM COMPENSAÇÃO	CHU da Universidade Federal do Pará - UFPA - PA
70	AUSÊNCIA COM COMPENSAÇÃO	CHU da Universidade Federal do Pará - UFPA - PA
70	AUSÊNCIA COM COMPENSAÇÃO	CHU da Universidade Federal do Pará - UFPA - PA
70	AUSÊNCIA COM COMPENSAÇÃO	CHU da Universidade Federal do Pará - UFPA - PA
70	AUSÊNCIA COM COMPENSAÇÃO	CHU da Universidade Federal do Pará - UFPA - PA
70	AUSÊNCIA COM COMPENSAÇÃO	CHU da Universidade Federal do Pará - UFPA - PA
70	AUSÊNCIA COM COMPENSAÇÃO	CHU da Universidade Federal do Pará - UFPA - PA
70	AUSÊNCIA COM COMPENSAÇÃO	CHU da Universidade Federal do Pará - UFPA - PA
70	AUSÊNCIA COM COMPENSAÇÃO	CHU da Universidade Federal do Pará - UFPA - PA
70	AUSÊNCIA COM COMPENSAÇÃO	CHU da Universidade Federal do Pará - UFPA - PA
70	AUSÊNCIA COM COMPENSAÇÃO	CHU da Universidade Federal do Pará - UFPA - PA
70	AUSÊNCIA COM COMPENSAÇÃO	CHU da Universidade Federal do Pará - UFPA - PA
70	AUSÊNCIA COM COMPENSAÇÃO	CHU da Universidade Federal do Pará - UFPA - PA
70	AUSÊNCIA COM COMPENSAÇÃO	CHU da Universidade Federal do Pará - UFPA - PA
70	AUSÊNCIA COM COMPENSAÇÃO	CHU da Universidade Federal do Pará - UFPA - PA
70	AUSÊNCIA COM COMPENSAÇÃO	CHU da Universidade Federal do Pará - UFPA - PA
70	AUSÊNCIA COM COMPENSAÇÃO	CHU da Universidade Federal do Pará - UFPA - PA
70	AUSÊNCIA COM COMPENSAÇÃO	CHU da Universidade Federal do Pará - UFPA - PA
70	AUSÊNCIA COM COMPENSAÇÃO	CHU da Universidade Federal do Pará - UFPA - PA
70	AUSÊNCIA COM COMPENSAÇÃO	CHU da Universidade Federal do Pará - UFPA - PA
70	AUSÊNCIA COM COMPENSAÇÃO	CHU da Universidade Federal do Pará - UFPA - PA
70	AUSÊNCIA COM COMPENSAÇÃO	CHU da Universidade Federal do Pará - UFPA - PA
70	AUSÊNCIA COM COMPENSAÇÃO	CHU da Universidade Federal do Pará - UFPA - PA
71	Repouso Remunerado - Artigo 395 CLT - 14 dias	CHU da Universidade Federal do Pará - UFPA - PA
71	Repouso Remunerado - Artigo 395 CLT - 14 dias	CHU da Universidade Federal do Pará - UFPA - PA
71	Repouso Remunerado - Artigo 395 CLT - 14 dias	CHU da Universidade Federal do Pará - UFPA - PA
71	Repouso Remunerado - Artigo 395 CLT - 14 dias	CHU da Universidade Federal do Pará - UFPA - PA
75	LICENÇA PATERNIDADE CELETISTA - 20 Dias (c/ prorrogação) - LEI 11.770/08 ART 1 II	CHU da Universidade Federal do Pará - UFPA - PA
75	LICENÇA PATERNIDADE CELETISTA - 20 Dias (c/ prorrogação) - LEI 11.770/08 ART 1 II	CHU da Universidade Federal do Pará - UFPA - PA
75	LICENÇA PATERNIDADE CELETISTA - 20 Dias (c/ prorrogação) - LEI 11.770/08 ART 1 II	CHU da Universidade Federal do Pará - UFPA - PA
75	LICENÇA PATERNIDADE CELETISTA - 20 Dias (c/ prorrogação) - LEI 11.770/08 ART 1 II	CHU da Universidade Federal do Pará - UFPA - PA
75	LICENÇA PATERNIDADE CELETISTA - 20 Dias (c/ prorrogação) - LEI 11.770/08 ART 1 II	CHU da Universidade Federal do Pará - UFPA - PA
75	LICENÇA PATERNIDADE CELETISTA - 20 Dias (c/ prorrogação) - LEI 11.770/08 ART 1 II	CHU da Universidade Federal do Pará - UFPA - PA

97	RECESSO FINAL DE ANO	CHU da Universidade Federal do Pará - UFPA - PA
97	RECESSO FINAL DE ANO	CHU da Universidade Federal do Pará - UFPA - PA
97	RECESSO FINAL DE ANO	CHU da Universidade Federal do Pará - UFPA - PA
97	RECESSO FINAL DE ANO	CHU da Universidade Federal do Pará - UFPA - PA
97	RECESSO FINAL DE ANO	CHU da Universidade Federal do Pará - UFPA - PA
97	RECESSO FINAL DE ANO	CHU da Universidade Federal do Pará - UFPA - PA
97	RECESSO FINAL DE ANO	CHU da Universidade Federal do Pará - UFPA - PA
97	RECESSO FINAL DE ANO	CHU da Universidade Federal do Pará - UFPA - PA
97	RECESSO FINAL DE ANO	CHU da Universidade Federal do Pará - UFPA - PA
97	RECESSO FINAL DE ANO	CHU da Universidade Federal do Pará - UFPA - PA
97	RECESSO FINAL DE ANO	CHU da Universidade Federal do Pará - UFPA - PA
97	RECESSO FINAL DE ANO	CHU da Universidade Federal do Pará - UFPA - PA
97	RECESSO FINAL DE ANO	CHU da Universidade Federal do Pará - UFPA - PA
97	RECESSO FINAL DE ANO	CHU da Universidade Federal do Pará - UFPA - PA
97	RECESSO FINAL DE ANO	CHU da Universidade Federal do Pará - UFPA - PA
97	RECESSO FINAL DE ANO	CHU da Universidade Federal do Pará - UFPA - PA
97	RECESSO FINAL DE ANO	CHU da Universidade Federal do Pará - UFPA - PA
97	RECESSO FINAL DE ANO	CHU da Universidade Federal do Pará - UFPA - PA
97	RECESSO FINAL DE ANO	CHU da Universidade Federal do Pará - UFPA - PA
97	RECESSO FINAL DE ANO	CHU da Universidade Federal do Pará - UFPA - PA
97	RECESSO FINAL DE ANO	CHU da Universidade Federal do Pará - UFPA - PA
97	RECESSO FINAL DE ANO	CHU da Universidade Federal do Pará - UFPA - PA
97	RECESSO FINAL DE ANO	CHU da Universidade Federal do Pará - UFPA - PA
97	RECESSO FINAL DE ANO	CHU da Universidade Federal do Pará - UFPA - PA
97	RECESSO FINAL DE ANO	CHU da Universidade Federal do Pará - UFPA - PA
97	RECESSO FINAL DE ANO	CHU da Universidade Federal do Pará - UFPA - PA
97	RECESSO FINAL DE ANO	CHU da Universidade Federal do Pará - UFPA - PA
97	RECESSO FINAL DE ANO	CHU da Universidade Federal do Pará - UFPA - PA
97	RECESSO FINAL DE ANO	CHU da Universidade Federal do Pará - UFPA - PA
97	RECESSO FINAL DE ANO	CHU da Universidade Federal do Pará - UFPA - PA
97	RECESSO FINAL DE ANO	CHU da Universidade Federal do Pará - UFPA - PA
97	RECESSO FINAL DE ANO	CHU da Universidade Federal do Pará - UFPA - PA
97	RECESSO FINAL DE ANO	CHU da Universidade Federal do Pará - UFPA - PA
97	RECESSO FINAL DE ANO	CHU da Universidade Federal do Pará - UFPA - PA
97	RECESSO FINAL DE ANO	CHU da Universidade Federal do Pará - UFPA - PA
97	RECESSO FINAL DE ANO	CHU da Universidade Federal do Pará - UFPA - PA
97	RECESSO FINAL DE ANO	CHU da Universidade Federal do Pará - UFPA - PA
97	RECESSO FINAL DE ANO	CHU da Universidade Federal do Pará - UFPA - PA
97	RECESSO FINAL DE ANO	CHU da Universidade Federal do Pará - UFPA - PA
97	RECESSO FINAL DE ANO	CHU da Universidade Federal do Pará - UFPA - PA
97	RECESSO FINAL DE ANO	CHU da Universidade Federal do Pará - UFPA - PA
97	RECESSO FINAL DE ANO	CHU da Universidade Federal do Pará - UFPA - PA
97	RECESSO FINAL DE ANO	CHU da Universidade Federal do Pará - UFPA - PA
97	RECESSO FINAL DE ANO	CHU da Universidade Federal do Pará - UFPA - PA
97	RECESSO FINAL DE ANO	CHU da Universidade Federal do Pará - UFPA - PA
97	RECESSO FINAL DE ANO	CHU da Universidade Federal do Pará - UFPA - PA

97	RECESSO FINAL DE ANO	CHU da Universidade Federal do Pará - UFPA - PA
97	RECESSO FINAL DE ANO	CHU da Universidade Federal do Pará - UFPA - PA
97	RECESSO FINAL DE ANO	CHU da Universidade Federal do Pará - UFPA - PA
97	RECESSO FINAL DE ANO	CHU da Universidade Federal do Pará - UFPA - PA
97	RECESSO FINAL DE ANO	CHU da Universidade Federal do Pará - UFPA - PA
97	RECESSO FINAL DE ANO	CHU da Universidade Federal do Pará - UFPA - PA
97	RECESSO FINAL DE ANO	CHU da Universidade Federal do Pará - UFPA - PA
97	RECESSO FINAL DE ANO	CHU da Universidade Federal do Pará - UFPA - PA
97	RECESSO FINAL DE ANO	CHU da Universidade Federal do Pará - UFPA - PA
97	RECESSO FINAL DE ANO	CHU da Universidade Federal do Pará - UFPA - PA
97	RECESSO FINAL DE ANO	CHU da Universidade Federal do Pará - UFPA - PA
97	RECESSO FINAL DE ANO	CHU da Universidade Federal do Pará - UFPA - PA
97	RECESSO FINAL DE ANO	CHU da Universidade Federal do Pará - UFPA - PA
97	RECESSO FINAL DE ANO	CHU da Universidade Federal do Pará - UFPA - PA
97	RECESSO FINAL DE ANO	CHU da Universidade Federal do Pará - UFPA - PA
97	RECESSO FINAL DE ANO	CHU da Universidade Federal do Pará - UFPA - PA
97	RECESSO FINAL DE ANO	CHU da Universidade Federal do Pará - UFPA - PA
97	RECESSO FINAL DE ANO	CHU da Universidade Federal do Pará - UFPA - PA
97	RECESSO FINAL DE ANO	CHU da Universidade Federal do Pará - UFPA - PA
97	RECESSO FINAL DE ANO	CHU da Universidade Federal do Pará - UFPA - PA
98	DESLOCAMENTO PARA NOVA UNIDADE (TRANSITO) - NORMA OPERACIONAL DGP N 01/2017	CHU da Universidade Federal do Pará - UFPA - PA
102	ACOMPANHAMENTO DE ESPOSA GESTANTE - CLT ART. 473 X	CHU da Universidade Federal do Pará - UFPA - PA
102	ACOMPANHAMENTO DE ESPOSA GESTANTE - CLT ART. 473 X	CHU da Universidade Federal do Pará - UFPA - PA
104	Acompanhamento filho menor 6 anos - Art. 473, XI, CLT	CHU da Universidade Federal do Pará - UFPA - PA
104	Acompanhamento filho menor 6 anos - Art. 473, XI, CLT	CHU da Universidade Federal do Pará - UFPA - PA
104	Acompanhamento filho menor 6 anos - Art. 473, XI, CLT	CHU da Universidade Federal do Pará - UFPA - PA
104	Acompanhamento filho menor 6 anos - Art. 473, XI, CLT	CHU da Universidade Federal do Pará - UFPA - PA
104	Acompanhamento filho menor 6 anos - Art. 473, XI, CLT	CHU da Universidade Federal do Pará - UFPA - PA
104	Acompanhamento filho menor 6 anos - Art. 473, XI, CLT	CHU da Universidade Federal do Pará - UFPA - PA
104	Acompanhamento filho menor 6 anos - Art. 473, XI, CLT	CHU da Universidade Federal do Pará - UFPA - PA
104	Acompanhamento filho menor 6 anos - Art. 473, XI, CLT	CHU da Universidade Federal do Pará - UFPA - PA
104	Acompanhamento filho menor 6 anos - Art. 473, XI, CLT	CHU da Universidade Federal do Pará - UFPA - PA
104	Acompanhamento filho menor 6 anos - Art. 473, XI, CLT	CHU da Universidade Federal do Pará - UFPA - PA
105	LICENÇA ADOÇAO (PRORROGAÇAO 60 DIAS) - LEI 11.770/08 ART 1 2	CHU da Universidade Federal do Pará - UFPA - PA
224	REALIZAÇÃO DE EXAME PREVENTIVO DE CÂNCER. LEI 13.767	CHU da Universidade Federal do Pará - UFPA - PA
227	ATIVIDADE EXTERNA (ESPII COVID19)	CHU da Universidade Federal do Pará - UFPA - PA
227	ATIVIDADE EXTERNA (ESPII COVID19)	CHU da Universidade Federal do Pará - UFPA - PA
227	ATIVIDADE EXTERNA (ESPII COVID19)	CHU da Universidade Federal do Pará - UFPA - PA
227	ATIVIDADE EXTERNA (ESPII COVID19)	CHU da Universidade Federal do Pará - UFPA - PA
227	ATIVIDADE EXTERNA (ESPII COVID19)	CHU da Universidade Federal do Pará - UFPA - PA
227	ATIVIDADE EXTERNA (ESPII COVID19)	CHU da Universidade Federal do Pará - UFPA - PA
227	ATIVIDADE EXTERNA (ESPII COVID19)	CHU da Universidade Federal do Pará - UFPA - PA
227	ATIVIDADE EXTERNA (ESPII COVID19)	CHU da Universidade Federal do Pará - UFPA - PA

227	ATIVIDADE EXTERNA (ESPII COVID19)	CHU da Universidade Federal do Pará - UFPA - PA
227	ATIVIDADE EXTERNA (ESPII COVID19)	CHU da Universidade Federal do Pará - UFPA - PA
227	ATIVIDADE EXTERNA (ESPII COVID19)	CHU da Universidade Federal do Pará - UFPA - PA
227	ATIVIDADE EXTERNA (ESPII COVID19)	CHU da Universidade Federal do Pará - UFPA - PA
227	ATIVIDADE EXTERNA (ESPII COVID19)	CHU da Universidade Federal do Pará - UFPA - PA
227	ATIVIDADE EXTERNA (ESPII COVID19)	CHU da Universidade Federal do Pará - UFPA - PA
227	ATIVIDADE EXTERNA (ESPII COVID19)	CHU da Universidade Federal do Pará - UFPA - PA
227	ATIVIDADE EXTERNA (ESPII COVID19)	CHU da Universidade Federal do Pará - UFPA - PA
227	ATIVIDADE EXTERNA (ESPII COVID19)	CHU da Universidade Federal do Pará - UFPA - PA
227	ATIVIDADE EXTERNA (ESPII COVID19)	CHU da Universidade Federal do Pará - UFPA - PA
227	ATIVIDADE EXTERNA (ESPII COVID19)	CHU da Universidade Federal do Pará - UFPA - PA
227	ATIVIDADE EXTERNA (ESPII COVID19)	CHU da Universidade Federal do Pará - UFPA - PA
227	ATIVIDADE EXTERNA (ESPII COVID19)	CHU da Universidade Federal do Pará - UFPA - PA
227	ATIVIDADE EXTERNA (ESPII COVID19)	CHU da Universidade Federal do Pará - UFPA - PA
228	ATIVIDADE EXTERNA (ESPII COVID19) - 7 Dias. DESATIVADO DE ACORDO COM A IN 02 - DGP de 26/03/2020.	CHU da Universidade Federal do Pará - UFPA - PA
228	ATIVIDADE EXTERNA (ESPII COVID19) - 7 Dias. DESATIVADO DE ACORDO COM A IN 02 - DGP de 26/03/2020.	CHU da Universidade Federal do Pará - UFPA - PA
228	ATIVIDADE EXTERNA (ESPII COVID19) - 7 Dias. DESATIVADO DE ACORDO COM A IN 02 - DGP de 26/03/2020.	CHU da Universidade Federal do Pará - UFPA - PA
228	ATIVIDADE EXTERNA (ESPII COVID19) - 7 Dias. DESATIVADO DE ACORDO COM A IN 02 - DGP de 26/03/2020.	CHU da Universidade Federal do Pará - UFPA - PA
229	ATIVIDADE EXTERNA (ESPII COVID19) - 14 dias.	CHU da Universidade Federal do Pará - UFPA - PA
229	ATIVIDADE EXTERNA (ESPII COVID19) - 14 dias.	CHU da Universidade Federal do Pará - UFPA - PA
229	ATIVIDADE EXTERNA (ESPII COVID19) - 14 dias.	CHU da Universidade Federal do Pará - UFPA - PA
229	ATIVIDADE EXTERNA (ESPII COVID19) - 14 dias.	CHU da Universidade Federal do Pará - UFPA - PA
229	ATIVIDADE EXTERNA (ESPII COVID19) - 14 dias.	CHU da Universidade Federal do Pará - UFPA - PA
229	ATIVIDADE EXTERNA (ESPII COVID19) - 14 dias.	CHU da Universidade Federal do Pará - UFPA - PA
229	ATIVIDADE EXTERNA (ESPII COVID19) - 14 dias.	CHU da Universidade Federal do Pará - UFPA - PA
229	ATIVIDADE EXTERNA (ESPII COVID19) - 14 dias.	CHU da Universidade Federal do Pará - UFPA - PA
230	AFASTAMENTO (COVID 19)14 dias. DESATIVADO DE ACORDO COM A IN 02 - DGP de 26/03/2020.	CHU da Universidade Federal do Pará - UFPA - PA
230	AFASTAMENTO (COVID 19)14 dias. DESATIVADO DE ACORDO COM A IN 02 - DGP de 26/03/2020.	CHU da Universidade Federal do Pará - UFPA - PA
231	Estado gripal (autodeclaratório) COVID-19. DESATIVADO DE ACORDO COM A IN 02 - DGP de 26/03/2020.	CHU da Universidade Federal do Pará - UFPA - PA
233	ATIVIDADE EXTERNA ESPII - RODÍZIO	CHU da Universidade Federal do Pará - UFPA - PA
233	ATIVIDADE EXTERNA ESPII - RODÍZIO	CHU da Universidade Federal do Pará - UFPA - PA
233	ATIVIDADE EXTERNA ESPII - RODÍZIO	CHU da Universidade Federal do Pará - UFPA - PA
233	ATIVIDADE EXTERNA ESPII - RODÍZIO	CHU da Universidade Federal do Pará - UFPA - PA
233	ATIVIDADE EXTERNA ESPII - RODÍZIO	CHU da Universidade Federal do Pará - UFPA - PA
233	ATIVIDADE EXTERNA ESPII - RODÍZIO	CHU da Universidade Federal do Pará - UFPA - PA
233	ATIVIDADE EXTERNA ESPII - RODÍZIO	CHU da Universidade Federal do Pará - UFPA - PA
233	ATIVIDADE EXTERNA ESPII - RODÍZIO	CHU da Universidade Federal do Pará - UFPA - PA
233	ATIVIDADE EXTERNA ESPII - RODÍZIO	CHU da Universidade Federal do Pará - UFPA - PA
233	ATIVIDADE EXTERNA ESPII - RODÍZIO	CHU da Universidade Federal do Pará - UFPA - PA
233	ATIVIDADE EXTERNA ESPII - RODÍZIO	CHU da Universidade Federal do Pará - UFPA - PA
233	ATIVIDADE EXTERNA ESPII - RODÍZIO	CHU da Universidade Federal do Pará - UFPA - PA
233	ATIVIDADE EXTERNA ESPII - RODÍZIO	CHU da Universidade Federal do Pará - UFPA - PA
233	ATIVIDADE EXTERNA ESPII - RODÍZIO	CHU da Universidade Federal do Pará - UFPA - PA

233	ATIVIDADE EXTERNA ESPII - RODÍZIO	CHU da Universidade Federal do Pará - UFPA - PA
233	ATIVIDADE EXTERNA ESPII - RODÍZIO	CHU da Universidade Federal do Pará - UFPA - PA
233	ATIVIDADE EXTERNA ESPII - RODÍZIO	CHU da Universidade Federal do Pará - UFPA - PA
233	ATIVIDADE EXTERNA ESPII - RODÍZIO	CHU da Universidade Federal do Pará - UFPA - PA
233	ATIVIDADE EXTERNA ESPII - RODÍZIO	CHU da Universidade Federal do Pará - UFPA - PA
233	ATIVIDADE EXTERNA ESPII - RODÍZIO	CHU da Universidade Federal do Pará - UFPA - PA
233	ATIVIDADE EXTERNA ESPII - RODÍZIO	CHU da Universidade Federal do Pará - UFPA - PA
233	ATIVIDADE EXTERNA ESPII - RODÍZIO	CHU da Universidade Federal do Pará - UFPA - PA
233	ATIVIDADE EXTERNA ESPII - RODÍZIO	CHU da Universidade Federal do Pará - UFPA - PA
233	ATIVIDADE EXTERNA ESPII - RODÍZIO	CHU da Universidade Federal do Pará - UFPA - PA
233	ATIVIDADE EXTERNA ESPII - RODÍZIO	CHU da Universidade Federal do Pará - UFPA - PA
233	ATIVIDADE EXTERNA ESPII - RODÍZIO	CHU da Universidade Federal do Pará - UFPA - PA
233	ATIVIDADE EXTERNA ESPII - RODÍZIO	CHU da Universidade Federal do Pará - UFPA - PA
233	ATIVIDADE EXTERNA ESPII - RODÍZIO	CHU da Universidade Federal do Pará - UFPA - PA
233	ATIVIDADE EXTERNA ESPII - RODÍZIO	CHU da Universidade Federal do Pará - UFPA - PA
233	ATIVIDADE EXTERNA ESPII - RODÍZIO	CHU da Universidade Federal do Pará - UFPA - PA
233	ATIVIDADE EXTERNA ESPII - RODÍZIO	CHU da Universidade Federal do Pará - UFPA - PA
233	ATIVIDADE EXTERNA ESPII - RODÍZIO	CHU da Universidade Federal do Pará - UFPA - PA
233	ATIVIDADE EXTERNA ESPII - RODÍZIO	CHU da Universidade Federal do Pará - UFPA - PA
233	ATIVIDADE EXTERNA ESPII - RODÍZIO	CHU da Universidade Federal do Pará - UFPA - PA
233	ATIVIDADE EXTERNA ESPII - RODÍZIO	CHU da Universidade Federal do Pará - UFPA - PA
233	ATIVIDADE EXTERNA ESPII - RODÍZIO	CHU da Universidade Federal do Pará - UFPA - PA
233	ATIVIDADE EXTERNA ESPII - RODÍZIO	CHU da Universidade Federal do Pará - UFPA - PA
233	ATIVIDADE EXTERNA ESPII - RODÍZIO	CHU da Universidade Federal do Pará - UFPA - PA
233	ATIVIDADE EXTERNA ESPII - RODÍZIO	CHU da Universidade Federal do Pará - UFPA - PA
233	ATIVIDADE EXTERNA ESPII - RODÍZIO	CHU da Universidade Federal do Pará - UFPA - PA
233	ATIVIDADE EXTERNA ESPII - RODÍZIO	CHU da Universidade Federal do Pará - UFPA - PA
233	ATIVIDADE EXTERNA ESPII - RODÍZIO	CHU da Universidade Federal do Pará - UFPA - PA
233	ATIVIDADE EXTERNA ESPII - RODÍZIO	CHU da Universidade Federal do Pará - UFPA - PA
233	ATIVIDADE EXTERNA ESPII - RODÍZIO	CHU da Universidade Federal do Pará - UFPA - PA
233	ATIVIDADE EXTERNA ESPII - RODÍZIO	CHU da Universidade Federal do Pará - UFPA - PA
233	ATIVIDADE EXTERNA ESPII - RODÍZIO	CHU da Universidade Federal do Pará - UFPA - PA
233	ATIVIDADE EXTERNA ESPII - RODÍZIO	CHU da Universidade Federal do Pará - UFPA - PA
233	ATIVIDADE EXTERNA ESPII - RODÍZIO	CHU da Universidade Federal do Pará - UFPA - PA
233	ATIVIDADE EXTERNA ESPII - RODÍZIO	CHU da Universidade Federal do Pará - UFPA - PA
233	ATIVIDADE EXTERNA ESPII - RODÍZIO	CHU da Universidade Federal do Pará - UFPA - PA
233	ATIVIDADE EXTERNA ESPII - RODÍZIO	CHU da Universidade Federal do Pará - UFPA - PA
233	ATIVIDADE EXTERNA ESPII - RODÍZIO	CHU da Universidade Federal do Pará - UFPA - PA
233	ATIVIDADE EXTERNA ESPII - RODÍZIO	CHU da Universidade Federal do Pará - UFPA - PA
233	ATIVIDADE EXTERNA ESPII - RODÍZIO	CHU da Universidade Federal do Pará - UFPA - PA
233	ATIVIDADE EXTERNA ESPII - RODÍZIO	CHU da Universidade Federal do Pará - UFPA - PA
233	ATIVIDADE EXTERNA ESPII - RODÍZIO	CHU da Universidade Federal do Pará - UFPA - PA
233	ATIVIDADE EXTERNA ESPII - RODÍZIO	CHU da Universidade Federal do Pará - UFPA - PA
233	ATIVIDADE EXTERNA ESPII - RODÍZIO	CHU da Universidade Federal do Pará - UFPA - PA
233	ATIVIDADE EXTERNA ESPII - RODÍZIO	CHU da Universidade Federal do Pará - UFPA - PA
233	ATIVIDADE EXTERNA ESPII - RODÍZIO	CHU da Universidade Federal do Pará - UFPA - PA
233	ATIVIDADE EXTERNA ESPII - RODÍZIO	CHU da Universidade Federal do Pará - UFPA - PA
233	ATIVIDADE EXTERNA ESPII - RODÍZIO	CHU da Universidade Federal do Pará - UFPA - PA

233	ATIVIDADE EXTERNA ESPII - RODÍZIO	CHU da Universidade Federal do Pará - UFPA - PA
233	ATIVIDADE EXTERNA ESPII - RODÍZIO	CHU da Universidade Federal do Pará - UFPA - PA
233	ATIVIDADE EXTERNA ESPII - RODÍZIO	CHU da Universidade Federal do Pará - UFPA - PA
233	ATIVIDADE EXTERNA ESPII - RODÍZIO	CHU da Universidade Federal do Pará - UFPA - PA
233	ATIVIDADE EXTERNA ESPII - RODÍZIO	CHU da Universidade Federal do Pará - UFPA - PA
233	ATIVIDADE EXTERNA ESPII - RODÍZIO	CHU da Universidade Federal do Pará - UFPA - PA
233	ATIVIDADE EXTERNA ESPII - RODÍZIO	CHU da Universidade Federal do Pará - UFPA - PA
233	ATIVIDADE EXTERNA ESPII - RODÍZIO	CHU da Universidade Federal do Pará - UFPA - PA
233	ATIVIDADE EXTERNA ESPII - RODÍZIO	CHU da Universidade Federal do Pará - UFPA - PA
233	ATIVIDADE EXTERNA ESPII - RODÍZIO	CHU da Universidade Federal do Pará - UFPA - PA
233	ATIVIDADE EXTERNA ESPII - RODÍZIO	CHU da Universidade Federal do Pará - UFPA - PA
233	ATIVIDADE EXTERNA ESPII - RODÍZIO	CHU da Universidade Federal do Pará - UFPA - PA
233	ATIVIDADE EXTERNA ESPII - RODÍZIO	CHU da Universidade Federal do Pará - UFPA - PA
233	ATIVIDADE EXTERNA ESPII - RODÍZIO	CHU da Universidade Federal do Pará - UFPA - PA
233	ATIVIDADE EXTERNA ESPII - RODÍZIO	CHU da Universidade Federal do Pará - UFPA - PA
233	ATIVIDADE EXTERNA ESPII - RODÍZIO	CHU da Universidade Federal do Pará - UFPA - PA
233	ATIVIDADE EXTERNA ESPII - RODÍZIO	CHU da Universidade Federal do Pará - UFPA - PA
233	ATIVIDADE EXTERNA ESPII - RODÍZIO	CHU da Universidade Federal do Pará - UFPA - PA
233	ATIVIDADE EXTERNA ESPII - RODÍZIO	CHU da Universidade Federal do Pará - UFPA - PA
233	ATIVIDADE EXTERNA ESPII - RODÍZIO	CHU da Universidade Federal do Pará - UFPA - PA
233	ATIVIDADE EXTERNA ESPII - RODÍZIO	CHU da Universidade Federal do Pará - UFPA - PA
233	ATIVIDADE EXTERNA ESPII - RODÍZIO	CHU da Universidade Federal do Pará - UFPA - PA
233	ATIVIDADE EXTERNA ESPII - RODÍZIO	CHU da Universidade Federal do Pará - UFPA - PA
233	ATIVIDADE EXTERNA ESPII - RODÍZIO	CHU da Universidade Federal do Pará - UFPA - PA
233	ATIVIDADE EXTERNA ESPII - RODÍZIO	CHU da Universidade Federal do Pará - UFPA - PA
233	ATIVIDADE EXTERNA ESPII - RODÍZIO	CHU da Universidade Federal do Pará - UFPA - PA
233	ATIVIDADE EXTERNA ESPII - RODÍZIO	CHU da Universidade Federal do Pará - UFPA - PA
233	ATIVIDADE EXTERNA ESPII - RODÍZIO	CHU da Universidade Federal do Pará - UFPA - PA
233	ATIVIDADE EXTERNA ESPII - RODÍZIO	CHU da Universidade Federal do Pará - UFPA - PA
233	ATIVIDADE EXTERNA ESPII - RODÍZIO	CHU da Universidade Federal do Pará - UFPA - PA
233	ATIVIDADE EXTERNA ESPII - RODÍZIO	CHU da Universidade Federal do Pará - UFPA - PA
233	ATIVIDADE EXTERNA ESPII - RODÍZIO	CHU da Universidade Federal do Pará - UFPA - PA
233	ATIVIDADE EXTERNA ESPII - RODÍZIO	CHU da Universidade Federal do Pará - UFPA - PA
233	ATIVIDADE EXTERNA ESPII - RODÍZIO	CHU da Universidade Federal do Pará - UFPA - PA
233	ATIVIDADE EXTERNA ESPII - RODÍZIO	CHU da Universidade Federal do Pará - UFPA - PA
233	ATIVIDADE EXTERNA ESPII - RODÍZIO	CHU da Universidade Federal do Pará - UFPA - PA
233	ATIVIDADE EXTERNA ESPII - RODÍZIO	CHU da Universidade Federal do Pará - UFPA - PA
233	ATIVIDADE EXTERNA ESPII - RODÍZIO	CHU da Universidade Federal do Pará - UFPA - PA
233	ATIVIDADE EXTERNA ESPII - RODÍZIO	CHU da Universidade Federal do Pará - UFPA - PA
233	ATIVIDADE EXTERNA ESPII - RODÍZIO	CHU da Universidade Federal do Pará - UFPA - PA
233	ATIVIDADE EXTERNA ESPII - RODÍZIO	CHU da Universidade Federal do Pará - UFPA - PA
233	ATIVIDADE EXTERNA ESPII - RODÍZIO	CHU da Universidade Federal do Pará - UFPA - PA
233	ATIVIDADE EXTERNA ESPII - RODÍZIO	CHU da Universidade Federal do Pará - UFPA - PA
233	ATIVIDADE EXTERNA ESPII - RODÍZIO	CHU da Universidade Federal do Pará - UFPA - PA
233	ATIVIDADE EXTERNA ESPII - RODÍZIO	CHU da Universidade Federal do Pará - UFPA - PA
233	ATIVIDADE EXTERNA ESPII - RODÍZIO	CHU da Universidade Federal do Pará - UFPA - PA
233	ATIVIDADE EXTERNA ESPII - RODÍZIO	CHU da Universidade Federal do Pará - UFPA - PA
233	ATIVIDADE EXTERNA ESPII - RODÍZIO	CHU da Universidade Federal do Pará - UFPA - PA

233	ATIVIDADE EXTERNA ESPII - RODÍZIO	CHU da Universidade Federal do Pará - UFPA - PA
233	ATIVIDADE EXTERNA ESPII - RODÍZIO	CHU da Universidade Federal do Pará - UFPA - PA
233	ATIVIDADE EXTERNA ESPII - RODÍZIO	CHU da Universidade Federal do Pará - UFPA - PA
233	ATIVIDADE EXTERNA ESPII - RODÍZIO	CHU da Universidade Federal do Pará - UFPA - PA
233	ATIVIDADE EXTERNA ESPII - RODÍZIO	CHU da Universidade Federal do Pará - UFPA - PA
233	ATIVIDADE EXTERNA ESPII - RODÍZIO	CHU da Universidade Federal do Pará - UFPA - PA
233	ATIVIDADE EXTERNA ESPII - RODÍZIO	CHU da Universidade Federal do Pará - UFPA - PA
233	ATIVIDADE EXTERNA ESPII - RODÍZIO	CHU da Universidade Federal do Pará - UFPA - PA
233	ATIVIDADE EXTERNA ESPII - RODÍZIO	CHU da Universidade Federal do Pará - UFPA - PA
233	ATIVIDADE EXTERNA ESPII - RODÍZIO	CHU da Universidade Federal do Pará - UFPA - PA
233	ATIVIDADE EXTERNA ESPII - RODÍZIO	CHU da Universidade Federal do Pará - UFPA - PA
233	ATIVIDADE EXTERNA ESPII - RODÍZIO	CHU da Universidade Federal do Pará - UFPA - PA
233	ATIVIDADE EXTERNA ESPII - RODÍZIO	CHU da Universidade Federal do Pará - UFPA - PA
233	ATIVIDADE EXTERNA ESPII - RODÍZIO	CHU da Universidade Federal do Pará - UFPA - PA
233	ATIVIDADE EXTERNA ESPII - RODÍZIO	CHU da Universidade Federal do Pará - UFPA - PA
233	ATIVIDADE EXTERNA ESPII - RODÍZIO	CHU da Universidade Federal do Pará - UFPA - PA
233	ATIVIDADE EXTERNA ESPII - RODÍZIO	CHU da Universidade Federal do Pará - UFPA - PA
233	ATIVIDADE EXTERNA ESPII - RODÍZIO	CHU da Universidade Federal do Pará - UFPA - PA
233	ATIVIDADE EXTERNA ESPII - RODÍZIO	CHU da Universidade Federal do Pará - UFPA - PA
233	ATIVIDADE EXTERNA ESPII - RODÍZIO	CHU da Universidade Federal do Pará - UFPA - PA
233	ATIVIDADE EXTERNA ESPII - RODÍZIO	CHU da Universidade Federal do Pará - UFPA - PA
233	ATIVIDADE EXTERNA ESPII - RODÍZIO	CHU da Universidade Federal do Pará - UFPA - PA
233	ATIVIDADE EXTERNA ESPII - RODÍZIO	CHU da Universidade Federal do Pará - UFPA - PA
233	ATIVIDADE EXTERNA ESPII - RODÍZIO	CHU da Universidade Federal do Pará - UFPA - PA
233	ATIVIDADE EXTERNA ESPII - RODÍZIO	CHU da Universidade Federal do Pará - UFPA - PA
233	ATIVIDADE EXTERNA ESPII - RODÍZIO	CHU da Universidade Federal do Pará - UFPA - PA
233	ATIVIDADE EXTERNA ESPII - RODÍZIO	CHU da Universidade Federal do Pará - UFPA - PA
233	ATIVIDADE EXTERNA ESPII - RODÍZIO	CHU da Universidade Federal do Pará - UFPA - PA
233	ATIVIDADE EXTERNA ESPII - RODÍZIO	CHU da Universidade Federal do Pará - UFPA - PA
233	ATIVIDADE EXTERNA ESPII - RODÍZIO	CHU da Universidade Federal do Pará - UFPA - PA
233	ATIVIDADE EXTERNA ESPII - RODÍZIO	CHU da Universidade Federal do Pará - UFPA - PA
233	ATIVIDADE EXTERNA ESPII - RODÍZIO	CHU da Universidade Federal do Pará - UFPA - PA
233	ATIVIDADE EXTERNA ESPII - RODÍZIO	CHU da Universidade Federal do Pará - UFPA - PA
233	ATIVIDADE EXTERNA ESPII - RODÍZIO	CHU da Universidade Federal do Pará - UFPA - PA
233	ATIVIDADE EXTERNA ESPII - RODÍZIO	CHU da Universidade Federal do Pará - UFPA - PA
233	ATIVIDADE EXTERNA ESPII - RODÍZIO	CHU da Universidade Federal do Pará - UFPA - PA
233	ATIVIDADE EXTERNA ESPII - RODÍZIO	CHU da Universidade Federal do Pará - UFPA - PA
233	ATIVIDADE EXTERNA ESPII - RODÍZIO	CHU da Universidade Federal do Pará - UFPA - PA
233	ATIVIDADE EXTERNA ESPII - RODÍZIO	CHU da Universidade Federal do Pará - UFPA - PA
233	ATIVIDADE EXTERNA ESPII - RODÍZIO	CHU da Universidade Federal do Pará - UFPA - PA
233	ATIVIDADE EXTERNA ESPII - RODÍZIO	CHU da Universidade Federal do Pará - UFPA - PA
233	ATIVIDADE EXTERNA ESPII - RODÍZIO	CHU da Universidade Federal do Pará - UFPA - PA
233	ATIVIDADE EXTERNA ESPII - RODÍZIO	CHU da Universidade Federal do Pará - UFPA - PA
233	ATIVIDADE EXTERNA ESPII - RODÍZIO	CHU da Universidade Federal do Pará - UFPA - PA
233	ATIVIDADE EXTERNA ESPII - RODÍZIO	CHU da Universidade Federal do Pará - UFPA - PA
233	ATIVIDADE EXTERNA ESPII - RODÍZIO	CHU da Universidade Federal do Pará - UFPA - PA
233	ATIVIDADE EXTERNA ESPII - RODÍZIO	CHU da Universidade Federal do Pará - UFPA - PA
233	ATIVIDADE EXTERNA ESPII - RODÍZIO	CHU da Universidade Federal do Pará - UFPA - PA

233	ATIVIDADE EXTERNA ESPII - RODÍZIO	CHU da Universidade Federal do Pará - UFPA - PA
233	ATIVIDADE EXTERNA ESPII - RODÍZIO	CHU da Universidade Federal do Pará - UFPA - PA
233	ATIVIDADE EXTERNA ESPII - RODÍZIO	CHU da Universidade Federal do Pará - UFPA - PA
233	ATIVIDADE EXTERNA ESPII - RODÍZIO	CHU da Universidade Federal do Pará - UFPA - PA
233	ATIVIDADE EXTERNA ESPII - RODÍZIO	CHU da Universidade Federal do Pará - UFPA - PA
233	ATIVIDADE EXTERNA ESPII - RODÍZIO	CHU da Universidade Federal do Pará - UFPA - PA
233	ATIVIDADE EXTERNA ESPII - RODÍZIO	CHU da Universidade Federal do Pará - UFPA - PA
233	ATIVIDADE EXTERNA ESPII - RODÍZIO	CHU da Universidade Federal do Pará - UFPA - PA
233	ATIVIDADE EXTERNA ESPII - RODÍZIO	CHU da Universidade Federal do Pará - UFPA - PA
233	ATIVIDADE EXTERNA ESPII - RODÍZIO	CHU da Universidade Federal do Pará - UFPA - PA
233	ATIVIDADE EXTERNA ESPII - RODÍZIO	CHU da Universidade Federal do Pará - UFPA - PA
233	ATIVIDADE EXTERNA ESPII - RODÍZIO	CHU da Universidade Federal do Pará - UFPA - PA
233	ATIVIDADE EXTERNA ESPII - RODÍZIO	CHU da Universidade Federal do Pará - UFPA - PA
233	ATIVIDADE EXTERNA ESPII - RODÍZIO	CHU da Universidade Federal do Pará - UFPA - PA
233	ATIVIDADE EXTERNA ESPII - RODÍZIO	CHU da Universidade Federal do Pará - UFPA - PA
233	ATIVIDADE EXTERNA ESPII - RODÍZIO	CHU da Universidade Federal do Pará - UFPA - PA
233	ATIVIDADE EXTERNA ESPII - RODÍZIO	CHU da Universidade Federal do Pará - UFPA - PA
233	ATIVIDADE EXTERNA ESPII - RODÍZIO	CHU da Universidade Federal do Pará - UFPA - PA
233	ATIVIDADE EXTERNA ESPII - RODÍZIO	CHU da Universidade Federal do Pará - UFPA - PA
233	ATIVIDADE EXTERNA ESPII - RODÍZIO	CHU da Universidade Federal do Pará - UFPA - PA
233	ATIVIDADE EXTERNA ESPII - RODÍZIO	CHU da Universidade Federal do Pará - UFPA - PA
233	ATIVIDADE EXTERNA ESPII - RODÍZIO	CHU da Universidade Federal do Pará - UFPA - PA
233	ATIVIDADE EXTERNA ESPII - RODÍZIO	CHU da Universidade Federal do Pará - UFPA - PA
233	ATIVIDADE EXTERNA ESPII - RODÍZIO	CHU da Universidade Federal do Pará - UFPA - PA
233	ATIVIDADE EXTERNA ESPII - RODÍZIO	CHU da Universidade Federal do Pará - UFPA - PA
233	ATIVIDADE EXTERNA ESPII - RODÍZIO	CHU da Universidade Federal do Pará - UFPA - PA
233	ATIVIDADE EXTERNA ESPII - RODÍZIO	CHU da Universidade Federal do Pará - UFPA - PA
233	ATIVIDADE EXTERNA ESPII - RODÍZIO	CHU da Universidade Federal do Pará - UFPA - PA
233	ATIVIDADE EXTERNA ESPII - RODÍZIO	CHU da Universidade Federal do Pará - UFPA - PA
233	ATIVIDADE EXTERNA ESPII - RODÍZIO	CHU da Universidade Federal do Pará - UFPA - PA
233	ATIVIDADE EXTERNA ESPII - RODÍZIO	CHU da Universidade Federal do Pará - UFPA - PA
233	ATIVIDADE EXTERNA ESPII - RODÍZIO	CHU da Universidade Federal do Pará - UFPA - PA
233	ATIVIDADE EXTERNA ESPII - RODÍZIO	CHU da Universidade Federal do Pará - UFPA - PA
233	ATIVIDADE EXTERNA ESPII - RODÍZIO	CHU da Universidade Federal do Pará - UFPA - PA
233	ATIVIDADE EXTERNA ESPII - RODÍZIO	CHU da Universidade Federal do Pará - UFPA - PA
233	ATIVIDADE EXTERNA ESPII - RODÍZIO	CHU da Universidade Federal do Pará - UFPA - PA
233	ATIVIDADE EXTERNA ESPII - RODÍZIO	CHU da Universidade Federal do Pará - UFPA - PA
233	ATIVIDADE EXTERNA ESPII - RODÍZIO	CHU da Universidade Federal do Pará - UFPA - PA
233	ATIVIDADE EXTERNA ESPII - RODÍZIO	CHU da Universidade Federal do Pará - UFPA - PA
233	ATIVIDADE EXTERNA ESPII - RODÍZIO	CHU da Universidade Federal do Pará - UFPA - PA
233	ATIVIDADE EXTERNA ESPII - RODÍZIO	CHU da Universidade Federal do Pará - UFPA - PA
233	ATIVIDADE EXTERNA ESPII - RODÍZIO	CHU da Universidade Federal do Pará - UFPA - PA
233	ATIVIDADE EXTERNA ESPII - RODÍZIO	CHU da Universidade Federal do Pará - UFPA - PA
233	ATIVIDADE EXTERNA ESPII - RODÍZIO	CHU da Universidade Federal do Pará - UFPA - PA
233	ATIVIDADE EXTERNA ESPII - RODÍZIO	CHU da Universidade Federal do Pará - UFPA - PA
233	ATIVIDADE EXTERNA ESPII - RODÍZIO	CHU da Universidade Federal do Pará - UFPA - PA
233	ATIVIDADE EXTERNA ESPII - RODÍZIO	CHU da Universidade Federal do Pará - UFPA - PA
233	ATIVIDADE EXTERNA ESPII - RODÍZIO	CHU da Universidade Federal do Pará - UFPA - PA

233	ATIVIDADE EXTERNA ESPII - RODÍZIO	CHU da Universidade Federal do Pará - UFPA - PA
233	ATIVIDADE EXTERNA ESPII - RODÍZIO	CHU da Universidade Federal do Pará - UFPA - PA
233	ATIVIDADE EXTERNA ESPII - RODÍZIO	CHU da Universidade Federal do Pará - UFPA - PA
233	ATIVIDADE EXTERNA ESPII - RODÍZIO	CHU da Universidade Federal do Pará - UFPA - PA
233	ATIVIDADE EXTERNA ESPII - RODÍZIO	CHU da Universidade Federal do Pará - UFPA - PA
233	ATIVIDADE EXTERNA ESPII - RODÍZIO	CHU da Universidade Federal do Pará - UFPA - PA
233	ATIVIDADE EXTERNA ESPII - RODÍZIO	CHU da Universidade Federal do Pará - UFPA - PA
233	ATIVIDADE EXTERNA ESPII - RODÍZIO	CHU da Universidade Federal do Pará - UFPA - PA
233	ATIVIDADE EXTERNA ESPII - RODÍZIO	CHU da Universidade Federal do Pará - UFPA - PA
233	ATIVIDADE EXTERNA ESPII - RODÍZIO	CHU da Universidade Federal do Pará - UFPA - PA
233	ATIVIDADE EXTERNA ESPII - RODÍZIO	CHU da Universidade Federal do Pará - UFPA - PA
233	ATIVIDADE EXTERNA ESPII - RODÍZIO	CHU da Universidade Federal do Pará - UFPA - PA
233	ATIVIDADE EXTERNA ESPII - RODÍZIO	CHU da Universidade Federal do Pará - UFPA - PA
233	ATIVIDADE EXTERNA ESPII - RODÍZIO	CHU da Universidade Federal do Pará - UFPA - PA
233	ATIVIDADE EXTERNA ESPII - RODÍZIO	CHU da Universidade Federal do Pará - UFPA - PA
233	ATIVIDADE EXTERNA ESPII - RODÍZIO	CHU da Universidade Federal do Pará - UFPA - PA
233	ATIVIDADE EXTERNA ESPII - RODÍZIO	CHU da Universidade Federal do Pará - UFPA - PA
233	ATIVIDADE EXTERNA ESPII - RODÍZIO	CHU da Universidade Federal do Pará - UFPA - PA
233	ATIVIDADE EXTERNA ESPII - RODÍZIO	CHU da Universidade Federal do Pará - UFPA - PA
233	ATIVIDADE EXTERNA ESPII - RODÍZIO	CHU da Universidade Federal do Pará - UFPA - PA
233	ATIVIDADE EXTERNA ESPII - RODÍZIO	CHU da Universidade Federal do Pará - UFPA - PA
233	ATIVIDADE EXTERNA ESPII - RODÍZIO	CHU da Universidade Federal do Pará - UFPA - PA
233	ATIVIDADE EXTERNA ESPII - RODÍZIO	CHU da Universidade Federal do Pará - UFPA - PA
233	ATIVIDADE EXTERNA ESPII - RODÍZIO	CHU da Universidade Federal do Pará - UFPA - PA
233	ATIVIDADE EXTERNA ESPII - RODÍZIO	CHU da Universidade Federal do Pará - UFPA - PA
233	ATIVIDADE EXTERNA ESPII - RODÍZIO	CHU da Universidade Federal do Pará - UFPA - PA
233	ATIVIDADE EXTERNA ESPII - RODÍZIO	CHU da Universidade Federal do Pará - UFPA - PA
233	ATIVIDADE EXTERNA ESPII - RODÍZIO	CHU da Universidade Federal do Pará - UFPA - PA
233	ATIVIDADE EXTERNA ESPII - RODÍZIO	CHU da Universidade Federal do Pará - UFPA - PA
233	ATIVIDADE EXTERNA ESPII - RODÍZIO	CHU da Universidade Federal do Pará - UFPA - PA
233	ATIVIDADE EXTERNA ESPII - RODÍZIO	CHU da Universidade Federal do Pará - UFPA - PA
233	ATIVIDADE EXTERNA ESPII - RODÍZIO	CHU da Universidade Federal do Pará - UFPA - PA
233	ATIVIDADE EXTERNA ESPII - RODÍZIO	CHU da Universidade Federal do Pará - UFPA - PA
233	ATIVIDADE EXTERNA ESPII - RODÍZIO	CHU da Universidade Federal do Pará - UFPA - PA
233	ATIVIDADE EXTERNA ESPII - RODÍZIO	CHU da Universidade Federal do Pará - UFPA - PA
233	ATIVIDADE EXTERNA ESPII - RODÍZIO	CHU da Universidade Federal do Pará - UFPA - PA
233	ATIVIDADE EXTERNA ESPII - RODÍZIO	CHU da Universidade Federal do Pará - UFPA - PA
233	ATIVIDADE EXTERNA ESPII - RODÍZIO	CHU da Universidade Federal do Pará - UFPA - PA
233	ATIVIDADE EXTERNA ESPII - RODÍZIO	CHU da Universidade Federal do Pará - UFPA - PA
233	ATIVIDADE EXTERNA ESPII - RODÍZIO	CHU da Universidade Federal do Pará - UFPA - PA
233	ATIVIDADE EXTERNA ESPII - RODÍZIO	CHU da Universidade Federal do Pará - UFPA - PA
233	ATIVIDADE EXTERNA ESPII - RODÍZIO	CHU da Universidade Federal do Pará - UFPA - PA
233	ATIVIDADE EXTERNA ESPII - RODÍZIO	CHU da Universidade Federal do Pará - UFPA - PA
233	ATIVIDADE EXTERNA ESPII - RODÍZIO	CHU da Universidade Federal do Pará - UFPA - PA
233	ATIVIDADE EXTERNA ESPII - RODÍZIO	CHU da Universidade Federal do Pará - UFPA - PA
233	ATIVIDADE EXTERNA ESPII - RODÍZIO	CHU da Universidade Federal do Pará - UFPA - PA
233	ATIVIDADE EXTERNA ESPII - RODÍZIO	CHU da Universidade Federal do Pará - UFPA - PA
233	ATIVIDADE EXTERNA ESPII - RODÍZIO	CHU da Universidade Federal do Pará - UFPA - PA

233	ATIVIDADE EXTERNA ESPII - RODÍZIO	CHU da Universidade Federal do Pará - UFPA - PA
233	ATIVIDADE EXTERNA ESPII - RODÍZIO	CHU da Universidade Federal do Pará - UFPA - PA
233	ATIVIDADE EXTERNA ESPII - RODÍZIO	CHU da Universidade Federal do Pará - UFPA - PA
233	ATIVIDADE EXTERNA ESPII - RODÍZIO	CHU da Universidade Federal do Pará - UFPA - PA
233	ATIVIDADE EXTERNA ESPII - RODÍZIO	CHU da Universidade Federal do Pará - UFPA - PA
233	ATIVIDADE EXTERNA ESPII - RODÍZIO	CHU da Universidade Federal do Pará - UFPA - PA
233	ATIVIDADE EXTERNA ESPII - RODÍZIO	CHU da Universidade Federal do Pará - UFPA - PA
233	ATIVIDADE EXTERNA ESPII - RODÍZIO	CHU da Universidade Federal do Pará - UFPA - PA
233	ATIVIDADE EXTERNA ESPII - RODÍZIO	CHU da Universidade Federal do Pará - UFPA - PA
233	ATIVIDADE EXTERNA ESPII - RODÍZIO	CHU da Universidade Federal do Pará - UFPA - PA
233	ATIVIDADE EXTERNA ESPII - RODÍZIO	CHU da Universidade Federal do Pará - UFPA - PA
233	ATIVIDADE EXTERNA ESPII - RODÍZIO	CHU da Universidade Federal do Pará - UFPA - PA
233	ATIVIDADE EXTERNA ESPII - RODÍZIO	CHU da Universidade Federal do Pará - UFPA - PA
233	ATIVIDADE EXTERNA ESPII - RODÍZIO	CHU da Universidade Federal do Pará - UFPA - PA
233	ATIVIDADE EXTERNA ESPII - RODÍZIO	CHU da Universidade Federal do Pará - UFPA - PA
233	ATIVIDADE EXTERNA ESPII - RODÍZIO	CHU da Universidade Federal do Pará - UFPA - PA
233	ATIVIDADE EXTERNA ESPII - RODÍZIO	CHU da Universidade Federal do Pará - UFPA - PA
233	ATIVIDADE EXTERNA ESPII - RODÍZIO	CHU da Universidade Federal do Pará - UFPA - PA
233	ATIVIDADE EXTERNA ESPII - RODÍZIO	CHU da Universidade Federal do Pará - UFPA - PA
233	ATIVIDADE EXTERNA ESPII - RODÍZIO	CHU da Universidade Federal do Pará - UFPA - PA
233	ATIVIDADE EXTERNA ESPII - RODÍZIO	CHU da Universidade Federal do Pará - UFPA - PA
233	ATIVIDADE EXTERNA ESPII - RODÍZIO	CHU da Universidade Federal do Pará - UFPA - PA
233	ATIVIDADE EXTERNA ESPII - RODÍZIO	CHU da Universidade Federal do Pará - UFPA - PA
233	ATIVIDADE EXTERNA ESPII - RODÍZIO	CHU da Universidade Federal do Pará - UFPA - PA
233	ATIVIDADE EXTERNA ESPII - RODÍZIO	CHU da Universidade Federal do Pará - UFPA - PA
233	ATIVIDADE EXTERNA ESPII - RODÍZIO	CHU da Universidade Federal do Pará - UFPA - PA
233	ATIVIDADE EXTERNA ESPII - RODÍZIO	CHU da Universidade Federal do Pará - UFPA - PA
233	ATIVIDADE EXTERNA ESPII - RODÍZIO	CHU da Universidade Federal do Pará - UFPA - PA
233	ATIVIDADE EXTERNA ESPII - RODÍZIO	CHU da Universidade Federal do Pará - UFPA - PA
233	ATIVIDADE EXTERNA ESPII - RODÍZIO	CHU da Universidade Federal do Pará - UFPA - PA
233	ATIVIDADE EXTERNA ESPII - RODÍZIO	CHU da Universidade Federal do Pará - UFPA - PA
233	ATIVIDADE EXTERNA ESPII - RODÍZIO	CHU da Universidade Federal do Pará - UFPA - PA
233	ATIVIDADE EXTERNA ESPII - RODÍZIO	CHU da Universidade Federal do Pará - UFPA - PA
233	ATIVIDADE EXTERNA ESPII - RODÍZIO	CHU da Universidade Federal do Pará - UFPA - PA
233	ATIVIDADE EXTERNA ESPII - RODÍZIO	CHU da Universidade Federal do Pará - UFPA - PA
233	ATIVIDADE EXTERNA ESPII - RODÍZIO	CHU da Universidade Federal do Pará - UFPA - PA
233	ATIVIDADE EXTERNA ESPII - RODÍZIO	CHU da Universidade Federal do Pará - UFPA - PA
233	ATIVIDADE EXTERNA ESPII - RODÍZIO	CHU da Universidade Federal do Pará - UFPA - PA
233	ATIVIDADE EXTERNA ESPII - RODÍZIO	CHU da Universidade Federal do Pará - UFPA - PA
233	ATIVIDADE EXTERNA ESPII - RODÍZIO	CHU da Universidade Federal do Pará - UFPA - PA
233	ATIVIDADE EXTERNA ESPII - RODÍZIO	CHU da Universidade Federal do Pará - UFPA - PA
233	ATIVIDADE EXTERNA ESPII - RODÍZIO	CHU da Universidade Federal do Pará - UFPA - PA
233	ATIVIDADE EXTERNA ESPII - RODÍZIO	CHU da Universidade Federal do Pará - UFPA - PA
233	ATIVIDADE EXTERNA ESPII - RODÍZIO	CHU da Universidade Federal do Pará - UFPA - PA
233	ATIVIDADE EXTERNA ESPII - RODÍZIO	CHU da Universidade Federal do Pará - UFPA - PA
233	ATIVIDADE EXTERNA ESPII - RODÍZIO	CHU da Universidade Federal do Pará - UFPA - PA
233	ATIVIDADE EXTERNA ESPII - RODÍZIO	CHU da Universidade Federal do Pará - UFPA - PA
233	ATIVIDADE EXTERNA ESPII - RODÍZIO	CHU da Universidade Federal do Pará - UFPA - PA

233	ATIVIDADE EXTERNA ESPII - RODÍZIO	CHU da Universidade Federal do Pará - UFPA - PA
233	ATIVIDADE EXTERNA ESPII - RODÍZIO	CHU da Universidade Federal do Pará - UFPA - PA
233	ATIVIDADE EXTERNA ESPII - RODÍZIO	CHU da Universidade Federal do Pará - UFPA - PA
233	ATIVIDADE EXTERNA ESPII - RODÍZIO	CHU da Universidade Federal do Pará - UFPA - PA
233	ATIVIDADE EXTERNA ESPII - RODÍZIO	CHU da Universidade Federal do Pará - UFPA - PA
233	ATIVIDADE EXTERNA ESPII - RODÍZIO	CHU da Universidade Federal do Pará - UFPA - PA
233	ATIVIDADE EXTERNA ESPII - RODÍZIO	CHU da Universidade Federal do Pará - UFPA - PA
233	ATIVIDADE EXTERNA ESPII - RODÍZIO	CHU da Universidade Federal do Pará - UFPA - PA
233	ATIVIDADE EXTERNA ESPII - RODÍZIO	CHU da Universidade Federal do Pará - UFPA - PA
233	ATIVIDADE EXTERNA ESPII - RODÍZIO	CHU da Universidade Federal do Pará - UFPA - PA
233	ATIVIDADE EXTERNA ESPII - RODÍZIO	CHU da Universidade Federal do Pará - UFPA - PA
233	ATIVIDADE EXTERNA ESPII - RODÍZIO	CHU da Universidade Federal do Pará - UFPA - PA
233	ATIVIDADE EXTERNA ESPII - RODÍZIO	CHU da Universidade Federal do Pará - UFPA - PA
233	ATIVIDADE EXTERNA ESPII - RODÍZIO	CHU da Universidade Federal do Pará - UFPA - PA
233	ATIVIDADE EXTERNA ESPII - RODÍZIO	CHU da Universidade Federal do Pará - UFPA - PA
233	ATIVIDADE EXTERNA ESPII - RODÍZIO	CHU da Universidade Federal do Pará - UFPA - PA
233	ATIVIDADE EXTERNA ESPII - RODÍZIO	CHU da Universidade Federal do Pará - UFPA - PA
233	ATIVIDADE EXTERNA ESPII - RODÍZIO	CHU da Universidade Federal do Pará - UFPA - PA
233	ATIVIDADE EXTERNA ESPII - RODÍZIO	CHU da Universidade Federal do Pará - UFPA - PA
233	ATIVIDADE EXTERNA ESPII - RODÍZIO	CHU da Universidade Federal do Pará - UFPA - PA
233	ATIVIDADE EXTERNA ESPII - RODÍZIO	CHU da Universidade Federal do Pará - UFPA - PA
233	ATIVIDADE EXTERNA ESPII - RODÍZIO	CHU da Universidade Federal do Pará - UFPA - PA
233	ATIVIDADE EXTERNA ESPII - RODÍZIO	CHU da Universidade Federal do Pará - UFPA - PA
233	ATIVIDADE EXTERNA ESPII - RODÍZIO	CHU da Universidade Federal do Pará - UFPA - PA
233	ATIVIDADE EXTERNA ESPII - RODÍZIO	CHU da Universidade Federal do Pará - UFPA - PA
233	ATIVIDADE EXTERNA ESPII - RODÍZIO	CHU da Universidade Federal do Pará - UFPA - PA
233	ATIVIDADE EXTERNA ESPII - RODÍZIO	CHU da Universidade Federal do Pará - UFPA - PA
233	ATIVIDADE EXTERNA ESPII - RODÍZIO	CHU da Universidade Federal do Pará - UFPA - PA
233	ATIVIDADE EXTERNA ESPII - RODÍZIO	CHU da Universidade Federal do Pará - UFPA - PA
233	ATIVIDADE EXTERNA ESPII - RODÍZIO	CHU da Universidade Federal do Pará - UFPA - PA
233	ATIVIDADE EXTERNA ESPII - RODÍZIO	CHU da Universidade Federal do Pará - UFPA - PA
233	ATIVIDADE EXTERNA ESPII - RODÍZIO	CHU da Universidade Federal do Pará - UFPA - PA
233	ATIVIDADE EXTERNA ESPII - RODÍZIO	CHU da Universidade Federal do Pará - UFPA - PA
233	ATIVIDADE EXTERNA ESPII - RODÍZIO	CHU da Universidade Federal do Pará - UFPA - PA
233	ATIVIDADE EXTERNA ESPII - RODÍZIO	CHU da Universidade Federal do Pará - UFPA - PA
233	ATIVIDADE EXTERNA ESPII - RODÍZIO	CHU da Universidade Federal do Pará - UFPA - PA
233	ATIVIDADE EXTERNA ESPII - RODÍZIO	CHU da Universidade Federal do Pará - UFPA - PA
233	ATIVIDADE EXTERNA ESPII - RODÍZIO	CHU da Universidade Federal do Pará - UFPA - PA
233	ATIVIDADE EXTERNA ESPII - RODÍZIO	CHU da Universidade Federal do Pará - UFPA - PA
233	ATIVIDADE EXTERNA ESPII - RODÍZIO	CHU da Universidade Federal do Pará - UFPA - PA
233	ATIVIDADE EXTERNA ESPII - RODÍZIO	CHU da Universidade Federal do Pará - UFPA - PA
233	ATIVIDADE EXTERNA ESPII - RODÍZIO	CHU da Universidade Federal do Pará - UFPA - PA
233	ATIVIDADE EXTERNA ESPII - RODÍZIO	CHU da Universidade Federal do Pará - UFPA - PA
233	ATIVIDADE EXTERNA ESPII - RODÍZIO	CHU da Universidade Federal do Pará - UFPA - PA
233	ATIVIDADE EXTERNA ESPII - RODÍZIO	CHU da Universidade Federal do Pará - UFPA - PA
233	ATIVIDADE EXTERNA ESPII - RODÍZIO	CHU da Universidade Federal do Pará - UFPA - PA
233	ATIVIDADE EXTERNA ESPII - RODÍZIO	CHU da Universidade Federal do Pará - UFPA - PA
233	ATIVIDADE EXTERNA ESPII - RODÍZIO	CHU da Universidade Federal do Pará - UFPA - PA

233	ATIVIDADE EXTERNA ESPII - RODÍZIO	CHU da Universidade Federal do Pará - UFPA - PA
233	ATIVIDADE EXTERNA ESPII - RODÍZIO	CHU da Universidade Federal do Pará - UFPA - PA
233	ATIVIDADE EXTERNA ESPII - RODÍZIO	CHU da Universidade Federal do Pará - UFPA - PA
233	ATIVIDADE EXTERNA ESPII - RODÍZIO	CHU da Universidade Federal do Pará - UFPA - PA
233	ATIVIDADE EXTERNA ESPII - RODÍZIO	CHU da Universidade Federal do Pará - UFPA - PA
233	ATIVIDADE EXTERNA ESPII - RODÍZIO	CHU da Universidade Federal do Pará - UFPA - PA
233	ATIVIDADE EXTERNA ESPII - RODÍZIO	CHU da Universidade Federal do Pará - UFPA - PA
233	ATIVIDADE EXTERNA ESPII - RODÍZIO	CHU da Universidade Federal do Pará - UFPA - PA
233	ATIVIDADE EXTERNA ESPII - RODÍZIO	CHU da Universidade Federal do Pará - UFPA - PA
233	ATIVIDADE EXTERNA ESPII - RODÍZIO	CHU da Universidade Federal do Pará - UFPA - PA
233	ATIVIDADE EXTERNA ESPII - RODÍZIO	CHU da Universidade Federal do Pará - UFPA - PA
233	ATIVIDADE EXTERNA ESPII - RODÍZIO	CHU da Universidade Federal do Pará - UFPA - PA
233	ATIVIDADE EXTERNA ESPII - RODÍZIO	CHU da Universidade Federal do Pará - UFPA - PA
233	ATIVIDADE EXTERNA ESPII - RODÍZIO	CHU da Universidade Federal do Pará - UFPA - PA
233	ATIVIDADE EXTERNA ESPII - RODÍZIO	CHU da Universidade Federal do Pará - UFPA - PA
233	ATIVIDADE EXTERNA ESPII - RODÍZIO	CHU da Universidade Federal do Pará - UFPA - PA
233	ATIVIDADE EXTERNA ESPII - RODÍZIO	CHU da Universidade Federal do Pará - UFPA - PA
233	ATIVIDADE EXTERNA ESPII - RODÍZIO	CHU da Universidade Federal do Pará - UFPA - PA
233	ATIVIDADE EXTERNA ESPII - RODÍZIO	CHU da Universidade Federal do Pará - UFPA - PA
233	ATIVIDADE EXTERNA ESPII - RODÍZIO	CHU da Universidade Federal do Pará - UFPA - PA
233	ATIVIDADE EXTERNA ESPII - RODÍZIO	CHU da Universidade Federal do Pará - UFPA - PA
233	ATIVIDADE EXTERNA ESPII - RODÍZIO	CHU da Universidade Federal do Pará - UFPA - PA
233	ATIVIDADE EXTERNA ESPII - RODÍZIO	CHU da Universidade Federal do Pará - UFPA - PA
233	ATIVIDADE EXTERNA ESPII - RODÍZIO	CHU da Universidade Federal do Pará - UFPA - PA
233	ATIVIDADE EXTERNA ESPII - RODÍZIO	CHU da Universidade Federal do Pará - UFPA - PA
233	ATIVIDADE EXTERNA ESPII - RODÍZIO	CHU da Universidade Federal do Pará - UFPA - PA
233	ATIVIDADE EXTERNA ESPII - RODÍZIO	CHU da Universidade Federal do Pará - UFPA - PA
233	ATIVIDADE EXTERNA ESPII - RODÍZIO	CHU da Universidade Federal do Pará - UFPA - PA
233	ATIVIDADE EXTERNA ESPII - RODÍZIO	CHU da Universidade Federal do Pará - UFPA - PA
233	ATIVIDADE EXTERNA ESPII - RODÍZIO	CHU da Universidade Federal do Pará - UFPA - PA
233	ATIVIDADE EXTERNA ESPII - RODÍZIO	CHU da Universidade Federal do Pará - UFPA - PA
233	ATIVIDADE EXTERNA ESPII - RODÍZIO	CHU da Universidade Federal do Pará - UFPA - PA
233	ATIVIDADE EXTERNA ESPII - RODÍZIO	CHU da Universidade Federal do Pará - UFPA - PA
233	ATIVIDADE EXTERNA ESPII - RODÍZIO	CHU da Universidade Federal do Pará - UFPA - PA
233	ATIVIDADE EXTERNA ESPII - RODÍZIO	CHU da Universidade Federal do Pará - UFPA - PA
233	ATIVIDADE EXTERNA ESPII - RODÍZIO	CHU da Universidade Federal do Pará - UFPA - PA
233	ATIVIDADE EXTERNA ESPII - RODÍZIO	CHU da Universidade Federal do Pará - UFPA - PA
233	ATIVIDADE EXTERNA ESPII - RODÍZIO	CHU da Universidade Federal do Pará - UFPA - PA
233	ATIVIDADE EXTERNA ESPII - RODÍZIO	CHU da Universidade Federal do Pará - UFPA - PA
233	ATIVIDADE EXTERNA ESPII - RODÍZIO	CHU da Universidade Federal do Pará - UFPA - PA
233	ATIVIDADE EXTERNA ESPII - RODÍZIO	CHU da Universidade Federal do Pará - UFPA - PA
233	ATIVIDADE EXTERNA ESPII - RODÍZIO	CHU da Universidade Federal do Pará - UFPA - PA
233	ATIVIDADE EXTERNA ESPII - RODÍZIO	CHU da Universidade Federal do Pará - UFPA - PA
233	ATIVIDADE EXTERNA ESPII - RODÍZIO	CHU da Universidade Federal do Pará - UFPA - PA
233	ATIVIDADE EXTERNA ESPII - RODÍZIO	CHU da Universidade Federal do Pará - UFPA - PA
233	ATIVIDADE EXTERNA ESPII - RODÍZIO	CHU da Universidade Federal do Pará - UFPA - PA
233	ATIVIDADE EXTERNA ESPII - RODÍZIO	CHU da Universidade Federal do Pará - UFPA - PA
233	ATIVIDADE EXTERNA ESPII - RODÍZIO	CHU da Universidade Federal do Pará - UFPA - PA

233	ATIVIDADE EXTERNA ESPII - RODÍZIO	CHU da Universidade Federal do Pará - UFPA - PA
233	ATIVIDADE EXTERNA ESPII - RODÍZIO	CHU da Universidade Federal do Pará - UFPA - PA
233	ATIVIDADE EXTERNA ESPII - RODÍZIO	CHU da Universidade Federal do Pará - UFPA - PA
233	ATIVIDADE EXTERNA ESPII - RODÍZIO	CHU da Universidade Federal do Pará - UFPA - PA
233	ATIVIDADE EXTERNA ESPII - RODÍZIO	CHU da Universidade Federal do Pará - UFPA - PA
233	ATIVIDADE EXTERNA ESPII - RODÍZIO	CHU da Universidade Federal do Pará - UFPA - PA
233	ATIVIDADE EXTERNA ESPII - RODÍZIO	CHU da Universidade Federal do Pará - UFPA - PA
233	ATIVIDADE EXTERNA ESPII - RODÍZIO	CHU da Universidade Federal do Pará - UFPA - PA
233	ATIVIDADE EXTERNA ESPII - RODÍZIO	CHU da Universidade Federal do Pará - UFPA - PA
233	ATIVIDADE EXTERNA ESPII - RODÍZIO	CHU da Universidade Federal do Pará - UFPA - PA
233	ATIVIDADE EXTERNA ESPII - RODÍZIO	CHU da Universidade Federal do Pará - UFPA - PA
233	ATIVIDADE EXTERNA ESPII - RODÍZIO	CHU da Universidade Federal do Pará - UFPA - PA
233	ATIVIDADE EXTERNA ESPII - RODÍZIO	CHU da Universidade Federal do Pará - UFPA - PA
233	ATIVIDADE EXTERNA ESPII - RODÍZIO	CHU da Universidade Federal do Pará - UFPA - PA
233	ATIVIDADE EXTERNA ESPII - RODÍZIO	CHU da Universidade Federal do Pará - UFPA - PA
233	ATIVIDADE EXTERNA ESPII - RODÍZIO	CHU da Universidade Federal do Pará - UFPA - PA
233	ATIVIDADE EXTERNA ESPII - RODÍZIO	CHU da Universidade Federal do Pará - UFPA - PA
233	ATIVIDADE EXTERNA ESPII - RODÍZIO	CHU da Universidade Federal do Pará - UFPA - PA
233	ATIVIDADE EXTERNA ESPII - RODÍZIO	CHU da Universidade Federal do Pará - UFPA - PA
233	ATIVIDADE EXTERNA ESPII - RODÍZIO	CHU da Universidade Federal do Pará - UFPA - PA
233	ATIVIDADE EXTERNA ESPII - RODÍZIO	CHU da Universidade Federal do Pará - UFPA - PA
233	ATIVIDADE EXTERNA ESPII - RODÍZIO	CHU da Universidade Federal do Pará - UFPA - PA
233	ATIVIDADE EXTERNA ESPII - RODÍZIO	CHU da Universidade Federal do Pará - UFPA - PA
233	ATIVIDADE EXTERNA ESPII - RODÍZIO	CHU da Universidade Federal do Pará - UFPA - PA
233	ATIVIDADE EXTERNA ESPII - RODÍZIO	CHU da Universidade Federal do Pará - UFPA - PA
233	ATIVIDADE EXTERNA ESPII - RODÍZIO	CHU da Universidade Federal do Pará - UFPA - PA
233	ATIVIDADE EXTERNA ESPII - RODÍZIO	CHU da Universidade Federal do Pará - UFPA - PA
233	ATIVIDADE EXTERNA ESPII - RODÍZIO	CHU da Universidade Federal do Pará - UFPA - PA
233	ATIVIDADE EXTERNA ESPII - RODÍZIO	CHU da Universidade Federal do Pará - UFPA - PA
233	ATIVIDADE EXTERNA ESPII - RODÍZIO	CHU da Universidade Federal do Pará - UFPA - PA
233	ATIVIDADE EXTERNA ESPII - RODÍZIO	CHU da Universidade Federal do Pará - UFPA - PA
233	ATIVIDADE EXTERNA ESPII - RODÍZIO	CHU da Universidade Federal do Pará - UFPA - PA
233	ATIVIDADE EXTERNA ESPII - RODÍZIO	CHU da Universidade Federal do Pará - UFPA - PA
233	ATIVIDADE EXTERNA ESPII - RODÍZIO	CHU da Universidade Federal do Pará - UFPA - PA
233	ATIVIDADE EXTERNA ESPII - RODÍZIO	CHU da Universidade Federal do Pará - UFPA - PA
233	ATIVIDADE EXTERNA ESPII - RODÍZIO	CHU da Universidade Federal do Pará - UFPA - PA
233	ATIVIDADE EXTERNA ESPII - RODÍZIO	CHU da Universidade Federal do Pará - UFPA - PA
233	ATIVIDADE EXTERNA ESPII - RODÍZIO	CHU da Universidade Federal do Pará - UFPA - PA
233	ATIVIDADE EXTERNA ESPII - RODÍZIO	CHU da Universidade Federal do Pará - UFPA - PA

233	ATIVIDADE EXTERNA ESPII - RODÍZIO	CHU da Universidade Federal do Pará - UFPA - PA
233	ATIVIDADE EXTERNA ESPII - RODÍZIO	CHU da Universidade Federal do Pará - UFPA - PA
233	ATIVIDADE EXTERNA ESPII - RODÍZIO	CHU da Universidade Federal do Pará - UFPA - PA
233	ATIVIDADE EXTERNA ESPII - RODÍZIO	CHU da Universidade Federal do Pará - UFPA - PA
233	ATIVIDADE EXTERNA ESPII - RODÍZIO	CHU da Universidade Federal do Pará - UFPA - PA
233	ATIVIDADE EXTERNA ESPII - RODÍZIO	CHU da Universidade Federal do Pará - UFPA - PA
233	ATIVIDADE EXTERNA ESPII - RODÍZIO	CHU da Universidade Federal do Pará - UFPA - PA
233	ATIVIDADE EXTERNA ESPII - RODÍZIO	CHU da Universidade Federal do Pará - UFPA - PA
233	ATIVIDADE EXTERNA ESPII - RODÍZIO	CHU da Universidade Federal do Pará - UFPA - PA
233	ATIVIDADE EXTERNA ESPII - RODÍZIO	CHU da Universidade Federal do Pará - UFPA - PA
233	ATIVIDADE EXTERNA ESPII - RODÍZIO	CHU da Universidade Federal do Pará - UFPA - PA
233	ATIVIDADE EXTERNA ESPII - RODÍZIO	CHU da Universidade Federal do Pará - UFPA - PA
233	ATIVIDADE EXTERNA ESPII - RODÍZIO	CHU da Universidade Federal do Pará - UFPA - PA
233	ATIVIDADE EXTERNA ESPII - RODÍZIO	CHU da Universidade Federal do Pará - UFPA - PA
233	ATIVIDADE EXTERNA ESPII - RODÍZIO	CHU da Universidade Federal do Pará - UFPA - PA
233	ATIVIDADE EXTERNA ESPII - RODÍZIO	CHU da Universidade Federal do Pará - UFPA - PA
233	ATIVIDADE EXTERNA ESPII - RODÍZIO	CHU da Universidade Federal do Pará - UFPA - PA
233	ATIVIDADE EXTERNA ESPII - RODÍZIO	CHU da Universidade Federal do Pará - UFPA - PA
233	ATIVIDADE EXTERNA ESPII - RODÍZIO	CHU da Universidade Federal do Pará - UFPA - PA
233	ATIVIDADE EXTERNA ESPII - RODÍZIO	CHU da Universidade Federal do Pará - UFPA - PA
233	ATIVIDADE EXTERNA ESPII - RODÍZIO	CHU da Universidade Federal do Pará - UFPA - PA
233	ATIVIDADE EXTERNA ESPII - RODÍZIO	CHU da Universidade Federal do Pará - UFPA - PA
233	ATIVIDADE EXTERNA ESPII - RODÍZIO	CHU da Universidade Federal do Pará - UFPA - PA
233	ATIVIDADE EXTERNA ESPII - RODÍZIO	CHU da Universidade Federal do Pará - UFPA - PA
233	ATIVIDADE EXTERNA ESPII - RODÍZIO	CHU da Universidade Federal do Pará - UFPA - PA
233	ATIVIDADE EXTERNA ESPII - RODÍZIO	CHU da Universidade Federal do Pará - UFPA - PA
233	ATIVIDADE EXTERNA ESPII - RODÍZIO	CHU da Universidade Federal do Pará - UFPA - PA
233	ATIVIDADE EXTERNA ESPII - RODÍZIO	CHU da Universidade Federal do Pará - UFPA - PA
233	ATIVIDADE EXTERNA ESPII - RODÍZIO	CHU da Universidade Federal do Pará - UFPA - PA
233	ATIVIDADE EXTERNA ESPII - RODÍZIO	CHU da Universidade Federal do Pará - UFPA - PA
233	ATIVIDADE EXTERNA ESPII - RODÍZIO	CHU da Universidade Federal do Pará - UFPA - PA
233	ATIVIDADE EXTERNA ESPII - RODÍZIO	CHU da Universidade Federal do Pará - UFPA - PA
233	ATIVIDADE EXTERNA ESPII - RODÍZIO	CHU da Universidade Federal do Pará - UFPA - PA
233	ATIVIDADE EXTERNA ESPII - RODÍZIO	CHU da Universidade Federal do Pará - UFPA - PA
233	ATIVIDADE EXTERNA ESPII - RODÍZIO	CHU da Universidade Federal do Pará - UFPA - PA
233	ATIVIDADE EXTERNA ESPII - RODÍZIO	CHU da Universidade Federal do Pará - UFPA - PA
233	ATIVIDADE EXTERNA ESPII - RODÍZIO	CHU da Universidade Federal do Pará - UFPA - PA
233	ATIVIDADE EXTERNA ESPII - RODÍZIO	CHU da Universidade Federal do Pará - UFPA - PA
233	ATIVIDADE EXTERNA ESPII - RODÍZIO	CHU da Universidade Federal do Pará - UFPA - PA
233	ATIVIDADE EXTERNA ESPII - RODÍZIO	CHU da Universidade Federal do Pará - UFPA - PA
233	ATIVIDADE EXTERNA ESPII - RODÍZIO	CHU da Universidade Federal do Pará - UFPA - PA
233	ATIVIDADE EXTERNA ESPII - RODÍZIO	CHU da Universidade Federal do Pará - UFPA - PA

233	ATIVIDADE EXTERNA ESPII - RODÍZIO	CHU da Universidade Federal do Pará - UFPA - PA
233	ATIVIDADE EXTERNA ESPII - RODÍZIO	CHU da Universidade Federal do Pará - UFPA - PA
233	ATIVIDADE EXTERNA ESPII - RODÍZIO	CHU da Universidade Federal do Pará - UFPA - PA
233	ATIVIDADE EXTERNA ESPII - RODÍZIO	CHU da Universidade Federal do Pará - UFPA - PA
233	ATIVIDADE EXTERNA ESPII - RODÍZIO	CHU da Universidade Federal do Pará - UFPA - PA
233	ATIVIDADE EXTERNA ESPII - RODÍZIO	CHU da Universidade Federal do Pará - UFPA - PA
233	ATIVIDADE EXTERNA ESPII - RODÍZIO	CHU da Universidade Federal do Pará - UFPA - PA
233	ATIVIDADE EXTERNA ESPII - RODÍZIO	CHU da Universidade Federal do Pará - UFPA - PA
233	ATIVIDADE EXTERNA ESPII - RODÍZIO	CHU da Universidade Federal do Pará - UFPA - PA
233	ATIVIDADE EXTERNA ESPII - RODÍZIO	CHU da Universidade Federal do Pará - UFPA - PA
233	ATIVIDADE EXTERNA ESPII - RODÍZIO	CHU da Universidade Federal do Pará - UFPA - PA
233	ATIVIDADE EXTERNA ESPII - RODÍZIO	CHU da Universidade Federal do Pará - UFPA - PA
233	ATIVIDADE EXTERNA ESPII - RODÍZIO	CHU da Universidade Federal do Pará - UFPA - PA
233	ATIVIDADE EXTERNA ESPII - RODÍZIO	CHU da Universidade Federal do Pará - UFPA - PA
233	ATIVIDADE EXTERNA ESPII - RODÍZIO	CHU da Universidade Federal do Pará - UFPA - PA
233	ATIVIDADE EXTERNA ESPII - RODÍZIO	CHU da Universidade Federal do Pará - UFPA - PA
233	ATIVIDADE EXTERNA ESPII - RODÍZIO	CHU da Universidade Federal do Pará - UFPA - PA
233	ATIVIDADE EXTERNA ESPII - RODÍZIO	CHU da Universidade Federal do Pará - UFPA - PA
233	ATIVIDADE EXTERNA ESPII - RODÍZIO	CHU da Universidade Federal do Pará - UFPA - PA
233	ATIVIDADE EXTERNA ESPII - RODÍZIO	CHU da Universidade Federal do Pará - UFPA - PA
233	ATIVIDADE EXTERNA ESPII - RODÍZIO	CHU da Universidade Federal do Pará - UFPA - PA
233	ATIVIDADE EXTERNA ESPII - RODÍZIO	CHU da Universidade Federal do Pará - UFPA - PA
233	ATIVIDADE EXTERNA ESPII - RODÍZIO	CHU da Universidade Federal do Pará - UFPA - PA
233	ATIVIDADE EXTERNA ESPII - RODÍZIO	CHU da Universidade Federal do Pará - UFPA - PA
233	ATIVIDADE EXTERNA ESPII - RODÍZIO	CHU da Universidade Federal do Pará - UFPA - PA
233	ATIVIDADE EXTERNA ESPII - RODÍZIO	CHU da Universidade Federal do Pará - UFPA - PA
233	ATIVIDADE EXTERNA ESPII - RODÍZIO	CHU da Universidade Federal do Pará - UFPA - PA
233	ATIVIDADE EXTERNA ESPII - RODÍZIO	CHU da Universidade Federal do Pará - UFPA - PA
233	ATIVIDADE EXTERNA ESPII - RODÍZIO	CHU da Universidade Federal do Pará - UFPA - PA
233	ATIVIDADE EXTERNA ESPII - RODÍZIO	CHU da Universidade Federal do Pará - UFPA - PA
233	ATIVIDADE EXTERNA ESPII - RODÍZIO	CHU da Universidade Federal do Pará - UFPA - PA
233	ATIVIDADE EXTERNA ESPII - RODÍZIO	CHU da Universidade Federal do Pará - UFPA - PA
233	ATIVIDADE EXTERNA ESPII - RODÍZIO	CHU da Universidade Federal do Pará - UFPA - PA
233	ATIVIDADE EXTERNA ESPII - RODÍZIO	CHU da Universidade Federal do Pará - UFPA - PA
233	ATIVIDADE EXTERNA ESPII - RODÍZIO	CHU da Universidade Federal do Pará - UFPA - PA
233	ATIVIDADE EXTERNA ESPII - RODÍZIO	CHU da Universidade Federal do Pará - UFPA - PA
233	ATIVIDADE EXTERNA ESPII - RODÍZIO	CHU da Universidade Federal do Pará - UFPA - PA
233	ATIVIDADE EXTERNA ESPII - RODÍZIO	CHU da Universidade Federal do Pará - UFPA - PA
233	ATIVIDADE EXTERNA ESPII - RODÍZIO	CHU da Universidade Federal do Pará - UFPA - PA
233	ATIVIDADE EXTERNA ESPII - RODÍZIO	CHU da Universidade Federal do Pará - UFPA - PA
233	ATIVIDADE EXTERNA ESPII - RODÍZIO	CHU da Universidade Federal do Pará - UFPA - PA
233	ATIVIDADE EXTERNA ESPII - RODÍZIO	CHU da Universidade Federal do Pará - UFPA - PA
233	ATIVIDADE EXTERNA ESPII - RODÍZIO	CHU da Universidade Federal do Pará - UFPA - PA
233	ATIVIDADE EXTERNA ESPII - RODÍZIO	CHU da Universidade Federal do Pará - UFPA - PA
233	ATIVIDADE EXTERNA ESPII - RODÍZIO	CHU da Universidade Federal do Pará - UFPA - PA
233	ATIVIDADE EXTERNA ESPII - RODÍZIO	CHU da Universidade Federal do Pará - UFPA - PA
233	ATIVIDADE EXTERNA ESPII - RODÍZIO	CHU da Universidade Federal do Pará - UFPA - PA
233	ATIVIDADE EXTERNA ESPII - RODÍZIO	CHU da Universidade Federal do Pará - UFPA - PA

233	ATIVIDADE EXTERNA ESPII - RODÍZIO	CHU da Universidade Federal do Pará - UFPA - PA
233	ATIVIDADE EXTERNA ESPII - RODÍZIO	CHU da Universidade Federal do Pará - UFPA - PA
233	ATIVIDADE EXTERNA ESPII - RODÍZIO	CHU da Universidade Federal do Pará - UFPA - PA
233	ATIVIDADE EXTERNA ESPII - RODÍZIO	CHU da Universidade Federal do Pará - UFPA - PA
233	ATIVIDADE EXTERNA ESPII - RODÍZIO	CHU da Universidade Federal do Pará - UFPA - PA
233	ATIVIDADE EXTERNA ESPII - RODÍZIO	CHU da Universidade Federal do Pará - UFPA - PA
233	ATIVIDADE EXTERNA ESPII - RODÍZIO	CHU da Universidade Federal do Pará - UFPA - PA
233	ATIVIDADE EXTERNA ESPII - RODÍZIO	CHU da Universidade Federal do Pará - UFPA - PA
233	ATIVIDADE EXTERNA ESPII - RODÍZIO	CHU da Universidade Federal do Pará - UFPA - PA
233	ATIVIDADE EXTERNA ESPII - RODÍZIO	CHU da Universidade Federal do Pará - UFPA - PA
233	ATIVIDADE EXTERNA ESPII - RODÍZIO	CHU da Universidade Federal do Pará - UFPA - PA
233	ATIVIDADE EXTERNA ESPII - RODÍZIO	CHU da Universidade Federal do Pará - UFPA - PA
233	ATIVIDADE EXTERNA ESPII - RODÍZIO	CHU da Universidade Federal do Pará - UFPA - PA
233	ATIVIDADE EXTERNA ESPII - RODÍZIO	CHU da Universidade Federal do Pará - UFPA - PA
233	ATIVIDADE EXTERNA ESPII - RODÍZIO	CHU da Universidade Federal do Pará - UFPA - PA
233	ATIVIDADE EXTERNA ESPII - RODÍZIO	CHU da Universidade Federal do Pará - UFPA - PA
233	ATIVIDADE EXTERNA ESPII - RODÍZIO	CHU da Universidade Federal do Pará - UFPA - PA
233	ATIVIDADE EXTERNA ESPII - RODÍZIO	CHU da Universidade Federal do Pará - UFPA - PA
233	ATIVIDADE EXTERNA ESPII - RODÍZIO	CHU da Universidade Federal do Pará - UFPA - PA
233	ATIVIDADE EXTERNA ESPII - RODÍZIO	CHU da Universidade Federal do Pará - UFPA - PA
233	ATIVIDADE EXTERNA ESPII - RODÍZIO	CHU da Universidade Federal do Pará - UFPA - PA
233	ATIVIDADE EXTERNA ESPII - RODÍZIO	CHU da Universidade Federal do Pará - UFPA - PA
233	ATIVIDADE EXTERNA ESPII - RODÍZIO	CHU da Universidade Federal do Pará - UFPA - PA
233	ATIVIDADE EXTERNA ESPII - RODÍZIO	CHU da Universidade Federal do Pará - UFPA - PA
233	ATIVIDADE EXTERNA ESPII - RODÍZIO	CHU da Universidade Federal do Pará - UFPA - PA
233	ATIVIDADE EXTERNA ESPII - RODÍZIO	CHU da Universidade Federal do Pará - UFPA - PA
233	ATIVIDADE EXTERNA ESPII - RODÍZIO	CHU da Universidade Federal do Pará - UFPA - PA
233	ATIVIDADE EXTERNA ESPII - RODÍZIO	CHU da Universidade Federal do Pará - UFPA - PA
233	ATIVIDADE EXTERNA ESPII - RODÍZIO	CHU da Universidade Federal do Pará - UFPA - PA
233	ATIVIDADE EXTERNA ESPII - RODÍZIO	CHU da Universidade Federal do Pará - UFPA - PA
233	ATIVIDADE EXTERNA ESPII - RODÍZIO	CHU da Universidade Federal do Pará - UFPA - PA
233	ATIVIDADE EXTERNA ESPII - RODÍZIO	CHU da Universidade Federal do Pará - UFPA - PA
233	ATIVIDADE EXTERNA ESPII - RODÍZIO	CHU da Universidade Federal do Pará - UFPA - PA
233	ATIVIDADE EXTERNA ESPII - RODÍZIO	CHU da Universidade Federal do Pará - UFPA - PA
233	ATIVIDADE EXTERNA ESPII - RODÍZIO	CHU da Universidade Federal do Pará - UFPA - PA
233	ATIVIDADE EXTERNA ESPII - RODÍZIO	CHU da Universidade Federal do Pará - UFPA - PA
233	ATIVIDADE EXTERNA ESPII - RODÍZIO	CHU da Universidade Federal do Pará - UFPA - PA
233	ATIVIDADE EXTERNA ESPII - RODÍZIO	CHU da Universidade Federal do Pará - UFPA - PA
233	ATIVIDADE EXTERNA ESPII - RODÍZIO	CHU da Universidade Federal do Pará - UFPA - PA
233	ATIVIDADE EXTERNA ESPII - RODÍZIO	CHU da Universidade Federal do Pará - UFPA - PA
233	ATIVIDADE EXTERNA ESPII - RODÍZIO	CHU da Universidade Federal do Pará - UFPA - PA
233	ATIVIDADE EXTERNA ESPII - RODÍZIO	CHU da Universidade Federal do Pará - UFPA - PA
233	ATIVIDADE EXTERNA ESPII - RODÍZIO	CHU da Universidade Federal do Pará - UFPA - PA
233	ATIVIDADE EXTERNA ESPII - RODÍZIO	CHU da Universidade Federal do Pará - UFPA - PA
233	ATIVIDADE EXTERNA ESPII - RODÍZIO	CHU da Universidade Federal do Pará - UFPA - PA
233	ATIVIDADE EXTERNA ESPII - RODÍZIO	CHU da Universidade Federal do Pará - UFPA - PA
233	ATIVIDADE EXTERNA ESPII - RODÍZIO	CHU da Universidade Federal do Pará - UFPA - PA
233	ATIVIDADE EXTERNA ESPII - RODÍZIO	CHU da Universidade Federal do Pará - UFPA - PA

233	ATIVIDADE EXTERNA ESPII - RODÍZIO	CHU da Universidade Federal do Pará - UFPA - PA
233	ATIVIDADE EXTERNA ESPII - RODÍZIO	CHU da Universidade Federal do Pará - UFPA - PA
233	ATIVIDADE EXTERNA ESPII - RODÍZIO	CHU da Universidade Federal do Pará - UFPA - PA
233	ATIVIDADE EXTERNA ESPII - RODÍZIO	CHU da Universidade Federal do Pará - UFPA - PA
233	ATIVIDADE EXTERNA ESPII - RODÍZIO	CHU da Universidade Federal do Pará - UFPA - PA
233	ATIVIDADE EXTERNA ESPII - RODÍZIO	CHU da Universidade Federal do Pará - UFPA - PA
233	ATIVIDADE EXTERNA ESPII - RODÍZIO	CHU da Universidade Federal do Pará - UFPA - PA
233	ATIVIDADE EXTERNA ESPII - RODÍZIO	CHU da Universidade Federal do Pará - UFPA - PA
233	ATIVIDADE EXTERNA ESPII - RODÍZIO	CHU da Universidade Federal do Pará - UFPA - PA
233	ATIVIDADE EXTERNA ESPII - RODÍZIO	CHU da Universidade Federal do Pará - UFPA - PA
233	ATIVIDADE EXTERNA ESPII - RODÍZIO	CHU da Universidade Federal do Pará - UFPA - PA
233	ATIVIDADE EXTERNA ESPII - RODÍZIO	CHU da Universidade Federal do Pará - UFPA - PA
233	ATIVIDADE EXTERNA ESPII - RODÍZIO	CHU da Universidade Federal do Pará - UFPA - PA
233	ATIVIDADE EXTERNA ESPII - RODÍZIO	CHU da Universidade Federal do Pará - UFPA - PA
233	ATIVIDADE EXTERNA ESPII - RODÍZIO	CHU da Universidade Federal do Pará - UFPA - PA
233	ATIVIDADE EXTERNA ESPII - RODÍZIO	CHU da Universidade Federal do Pará - UFPA - PA
233	ATIVIDADE EXTERNA ESPII - RODÍZIO	CHU da Universidade Federal do Pará - UFPA - PA
233	ATIVIDADE EXTERNA ESPII - RODÍZIO	CHU da Universidade Federal do Pará - UFPA - PA
233	ATIVIDADE EXTERNA ESPII - RODÍZIO	CHU da Universidade Federal do Pará - UFPA - PA
233	ATIVIDADE EXTERNA ESPII - RODÍZIO	CHU da Universidade Federal do Pará - UFPA - PA
233	ATIVIDADE EXTERNA ESPII - RODÍZIO	CHU da Universidade Federal do Pará - UFPA - PA
233	ATIVIDADE EXTERNA ESPII - RODÍZIO	CHU da Universidade Federal do Pará - UFPA - PA
233	ATIVIDADE EXTERNA ESPII - RODÍZIO	CHU da Universidade Federal do Pará - UFPA - PA
233	ATIVIDADE EXTERNA ESPII - RODÍZIO	CHU da Universidade Federal do Pará - UFPA - PA
233	ATIVIDADE EXTERNA ESPII - RODÍZIO	CHU da Universidade Federal do Pará - UFPA - PA
233	ATIVIDADE EXTERNA ESPII - RODÍZIO	CHU da Universidade Federal do Pará - UFPA - PA
233	ATIVIDADE EXTERNA ESPII - RODÍZIO	CHU da Universidade Federal do Pará - UFPA - PA
233	ATIVIDADE EXTERNA ESPII - RODÍZIO	CHU da Universidade Federal do Pará - UFPA - PA
233	ATIVIDADE EXTERNA ESPII - RODÍZIO	CHU da Universidade Federal do Pará - UFPA - PA
233	ATIVIDADE EXTERNA ESPII - RODÍZIO	CHU da Universidade Federal do Pará - UFPA - PA
233	ATIVIDADE EXTERNA ESPII - RODÍZIO	CHU da Universidade Federal do Pará - UFPA - PA
233	ATIVIDADE EXTERNA ESPII - RODÍZIO	CHU da Universidade Federal do Pará - UFPA - PA
233	ATIVIDADE EXTERNA ESPII - RODÍZIO	CHU da Universidade Federal do Pará - UFPA - PA
233	ATIVIDADE EXTERNA ESPII - RODÍZIO	CHU da Universidade Federal do Pará - UFPA - PA
233	ATIVIDADE EXTERNA ESPII - RODÍZIO	CHU da Universidade Federal do Pará - UFPA - PA
233	ATIVIDADE EXTERNA ESPII - RODÍZIO	CHU da Universidade Federal do Pará - UFPA - PA
233	ATIVIDADE EXTERNA ESPII - RODÍZIO	CHU da Universidade Federal do Pará - UFPA - PA
233	ATIVIDADE EXTERNA ESPII - RODÍZIO	CHU da Universidade Federal do Pará - UFPA - PA
233	ATIVIDADE EXTERNA ESPII - RODÍZIO	CHU da Universidade Federal do Pará - UFPA - PA
233	ATIVIDADE EXTERNA ESPII - RODÍZIO	CHU da Universidade Federal do Pará - UFPA - PA
233	ATIVIDADE EXTERNA ESPII - RODÍZIO	CHU da Universidade Federal do Pará - UFPA - PA
233	ATIVIDADE EXTERNA ESPII - RODÍZIO	CHU da Universidade Federal do Pará - UFPA - PA
233	ATIVIDADE EXTERNA ESPII - RODÍZIO	CHU da Universidade Federal do Pará - UFPA - PA
233	ATIVIDADE EXTERNA ESPII - RODÍZIO	CHU da Universidade Federal do Pará - UFPA - PA
233	ATIVIDADE EXTERNA ESPII - RODÍZIO	CHU da Universidade Federal do Pará - UFPA - PA
233	ATIVIDADE EXTERNA ESPII - RODÍZIO	CHU da Universidade Federal do Pará - UFPA - PA
233	ATIVIDADE EXTERNA ESPII - RODÍZIO	CHU da Universidade Federal do Pará - UFPA - PA
233	ATIVIDADE EXTERNA ESPII - RODÍZIO	CHU da Universidade Federal do Pará - UFPA - PA

233	ATIVIDADE EXTERNA ESPII - RODÍZIO	CHU da Universidade Federal do Pará - UFPA - PA
233	ATIVIDADE EXTERNA ESPII - RODÍZIO	CHU da Universidade Federal do Pará - UFPA - PA
233	ATIVIDADE EXTERNA ESPII - RODÍZIO	CHU da Universidade Federal do Pará - UFPA - PA
233	ATIVIDADE EXTERNA ESPII - RODÍZIO	CHU da Universidade Federal do Pará - UFPA - PA
233	ATIVIDADE EXTERNA ESPII - RODÍZIO	CHU da Universidade Federal do Pará - UFPA - PA
233	ATIVIDADE EXTERNA ESPII - RODÍZIO	CHU da Universidade Federal do Pará - UFPA - PA
233	ATIVIDADE EXTERNA ESPII - RODÍZIO	CHU da Universidade Federal do Pará - UFPA - PA
233	ATIVIDADE EXTERNA ESPII - RODÍZIO	CHU da Universidade Federal do Pará - UFPA - PA
233	ATIVIDADE EXTERNA ESPII - RODÍZIO	CHU da Universidade Federal do Pará - UFPA - PA
233	ATIVIDADE EXTERNA ESPII - RODÍZIO	CHU da Universidade Federal do Pará - UFPA - PA
233	ATIVIDADE EXTERNA ESPII - RODÍZIO	CHU da Universidade Federal do Pará - UFPA - PA
233	ATIVIDADE EXTERNA ESPII - RODÍZIO	CHU da Universidade Federal do Pará - UFPA - PA
233	ATIVIDADE EXTERNA ESPII - RODÍZIO	CHU da Universidade Federal do Pará - UFPA - PA
233	ATIVIDADE EXTERNA ESPII - RODÍZIO	CHU da Universidade Federal do Pará - UFPA - PA
233	ATIVIDADE EXTERNA ESPII - RODÍZIO	CHU da Universidade Federal do Pará - UFPA - PA
233	ATIVIDADE EXTERNA ESPII - RODÍZIO	CHU da Universidade Federal do Pará - UFPA - PA
233	ATIVIDADE EXTERNA ESPII - RODÍZIO	CHU da Universidade Federal do Pará - UFPA - PA
233	ATIVIDADE EXTERNA ESPII - RODÍZIO	CHU da Universidade Federal do Pará - UFPA - PA
233	ATIVIDADE EXTERNA ESPII - RODÍZIO	CHU da Universidade Federal do Pará - UFPA - PA
233	ATIVIDADE EXTERNA ESPII - RODÍZIO	CHU da Universidade Federal do Pará - UFPA - PA
233	ATIVIDADE EXTERNA ESPII - RODÍZIO	CHU da Universidade Federal do Pará - UFPA - PA
233	ATIVIDADE EXTERNA ESPII - RODÍZIO	CHU da Universidade Federal do Pará - UFPA - PA
233	ATIVIDADE EXTERNA ESPII - RODÍZIO	CHU da Universidade Federal do Pará - UFPA - PA
233	ATIVIDADE EXTERNA ESPII - RODÍZIO	CHU da Universidade Federal do Pará - UFPA - PA
233	ATIVIDADE EXTERNA ESPII - RODÍZIO	CHU da Universidade Federal do Pará - UFPA - PA
233	ATIVIDADE EXTERNA ESPII - RODÍZIO	CHU da Universidade Federal do Pará - UFPA - PA
233	ATIVIDADE EXTERNA ESPII - RODÍZIO	CHU da Universidade Federal do Pará - UFPA - PA
233	ATIVIDADE EXTERNA ESPII - RODÍZIO	CHU da Universidade Federal do Pará - UFPA - PA
233	ATIVIDADE EXTERNA ESPII - RODÍZIO	CHU da Universidade Federal do Pará - UFPA - PA
233	ATIVIDADE EXTERNA ESPII - RODÍZIO	CHU da Universidade Federal do Pará - UFPA - PA
233	ATIVIDADE EXTERNA ESPII - RODÍZIO	CHU da Universidade Federal do Pará - UFPA - PA
233	ATIVIDADE EXTERNA ESPII - RODÍZIO	CHU da Universidade Federal do Pará - UFPA - PA
233	ATIVIDADE EXTERNA ESPII - RODÍZIO	CHU da Universidade Federal do Pará - UFPA - PA
233	ATIVIDADE EXTERNA ESPII - RODÍZIO	CHU da Universidade Federal do Pará - UFPA - PA
233	ATIVIDADE EXTERNA ESPII - RODÍZIO	CHU da Universidade Federal do Pará - UFPA - PA
233	ATIVIDADE EXTERNA ESPII - RODÍZIO	CHU da Universidade Federal do Pará - UFPA - PA
233	ATIVIDADE EXTERNA ESPII - RODÍZIO	CHU da Universidade Federal do Pará - UFPA - PA
233	ATIVIDADE EXTERNA ESPII - RODÍZIO	CHU da Universidade Federal do Pará - UFPA - PA
233	ATIVIDADE EXTERNA ESPII - RODÍZIO	CHU da Universidade Federal do Pará - UFPA - PA
233	ATIVIDADE EXTERNA ESPII - RODÍZIO	CHU da Universidade Federal do Pará - UFPA - PA
233	ATIVIDADE EXTERNA ESPII - RODÍZIO	CHU da Universidade Federal do Pará - UFPA - PA
233	ATIVIDADE EXTERNA ESPII - RODÍZIO	CHU da Universidade Federal do Pará - UFPA - PA
233	ATIVIDADE EXTERNA ESPII - RODÍZIO	CHU da Universidade Federal do Pará - UFPA - PA
233	ATIVIDADE EXTERNA ESPII - RODÍZIO	CHU da Universidade Federal do Pará - UFPA - PA
233	ATIVIDADE EXTERNA ESPII - RODÍZIO	CHU da Universidade Federal do Pará - UFPA - PA
233	ATIVIDADE EXTERNA ESPII - RODÍZIO	CHU da Universidade Federal do Pará - UFPA - PA
233	ATIVIDADE EXTERNA ESPII - RODÍZIO	CHU da Universidade Federal do Pará - UFPA - PA
233	ATIVIDADE EXTERNA ESPII - RODÍZIO	CHU da Universidade Federal do Pará - UFPA - PA

233	ATIVIDADE EXTERNA ESPII - RODÍZIO	CHU da Universidade Federal do Pará - UFPA - PA
233	ATIVIDADE EXTERNA ESPII - RODÍZIO	CHU da Universidade Federal do Pará - UFPA - PA
233	ATIVIDADE EXTERNA ESPII - RODÍZIO	CHU da Universidade Federal do Pará - UFPA - PA
233	ATIVIDADE EXTERNA ESPII - RODÍZIO	CHU da Universidade Federal do Pará - UFPA - PA
233	ATIVIDADE EXTERNA ESPII - RODÍZIO	CHU da Universidade Federal do Pará - UFPA - PA
233	ATIVIDADE EXTERNA ESPII - RODÍZIO	CHU da Universidade Federal do Pará - UFPA - PA
233	ATIVIDADE EXTERNA ESPII - RODÍZIO	CHU da Universidade Federal do Pará - UFPA - PA
233	ATIVIDADE EXTERNA ESPII - RODÍZIO	CHU da Universidade Federal do Pará - UFPA - PA
233	ATIVIDADE EXTERNA ESPII - RODÍZIO	CHU da Universidade Federal do Pará - UFPA - PA
233	ATIVIDADE EXTERNA ESPII - RODÍZIO	CHU da Universidade Federal do Pará - UFPA - PA
233	ATIVIDADE EXTERNA ESPII - RODÍZIO	CHU da Universidade Federal do Pará - UFPA - PA
233	ATIVIDADE EXTERNA ESPII - RODÍZIO	CHU da Universidade Federal do Pará - UFPA - PA
233	ATIVIDADE EXTERNA ESPII - RODÍZIO	CHU da Universidade Federal do Pará - UFPA - PA
233	ATIVIDADE EXTERNA ESPII - RODÍZIO	CHU da Universidade Federal do Pará - UFPA - PA
233	ATIVIDADE EXTERNA ESPII - RODÍZIO	CHU da Universidade Federal do Pará - UFPA - PA
233	ATIVIDADE EXTERNA ESPII - RODÍZIO	CHU da Universidade Federal do Pará - UFPA - PA
233	ATIVIDADE EXTERNA ESPII - RODÍZIO	CHU da Universidade Federal do Pará - UFPA - PA
233	ATIVIDADE EXTERNA ESPII - RODÍZIO	CHU da Universidade Federal do Pará - UFPA - PA
233	ATIVIDADE EXTERNA ESPII - RODÍZIO	CHU da Universidade Federal do Pará - UFPA - PA
233	ATIVIDADE EXTERNA ESPII - RODÍZIO	CHU da Universidade Federal do Pará - UFPA - PA
233	ATIVIDADE EXTERNA ESPII - RODÍZIO	CHU da Universidade Federal do Pará - UFPA - PA
233	ATIVIDADE EXTERNA ESPII - RODÍZIO	CHU da Universidade Federal do Pará - UFPA - PA
233	ATIVIDADE EXTERNA ESPII - RODÍZIO	CHU da Universidade Federal do Pará - UFPA - PA
233	ATIVIDADE EXTERNA ESPII - RODÍZIO	CHU da Universidade Federal do Pará - UFPA - PA
233	ATIVIDADE EXTERNA ESPII - RODÍZIO	CHU da Universidade Federal do Pará - UFPA - PA
233	ATIVIDADE EXTERNA ESPII - RODÍZIO	CHU da Universidade Federal do Pará - UFPA - PA
233	ATIVIDADE EXTERNA ESPII - RODÍZIO	CHU da Universidade Federal do Pará - UFPA - PA
233	ATIVIDADE EXTERNA ESPII - RODÍZIO	CHU da Universidade Federal do Pará - UFPA - PA
233	ATIVIDADE EXTERNA ESPII - RODÍZIO	CHU da Universidade Federal do Pará - UFPA - PA
233	ATIVIDADE EXTERNA ESPII - RODÍZIO	CHU da Universidade Federal do Pará - UFPA - PA
233	ATIVIDADE EXTERNA ESPII - RODÍZIO	CHU da Universidade Federal do Pará - UFPA - PA
233	ATIVIDADE EXTERNA ESPII - RODÍZIO	CHU da Universidade Federal do Pará - UFPA - PA
233	ATIVIDADE EXTERNA ESPII - RODÍZIO	CHU da Universidade Federal do Pará - UFPA - PA
233	ATIVIDADE EXTERNA ESPII - RODÍZIO	CHU da Universidade Federal do Pará - UFPA - PA
233	ATIVIDADE EXTERNA ESPII - RODÍZIO	CHU da Universidade Federal do Pará - UFPA - PA
233	ATIVIDADE EXTERNA ESPII - RODÍZIO	CHU da Universidade Federal do Pará - UFPA - PA
233	ATIVIDADE EXTERNA ESPII - RODÍZIO	CHU da Universidade Federal do Pará - UFPA - PA
233	ATIVIDADE EXTERNA ESPII - RODÍZIO	CHU da Universidade Federal do Pará - UFPA - PA
233	ATIVIDADE EXTERNA ESPII - RODÍZIO	CHU da Universidade Federal do Pará - UFPA - PA
233	ATIVIDADE EXTERNA ESPII - RODÍZIO	CHU da Universidade Federal do Pará - UFPA - PA
233	ATIVIDADE EXTERNA ESPII - RODÍZIO	CHU da Universidade Federal do Pará - UFPA - PA
233	ATIVIDADE EXTERNA ESPII - RODÍZIO	CHU da Universidade Federal do Pará - UFPA - PA
233	ATIVIDADE EXTERNA ESPII - RODÍZIO	CHU da Universidade Federal do Pará - UFPA - PA
233	ATIVIDADE EXTERNA ESPII - RODÍZIO	CHU da Universidade Federal do Pará - UFPA - PA

233	ATIVIDADE EXTERNA ESPII - RODÍZIO	CHU da Universidade Federal do Pará - UFPA - PA
233	ATIVIDADE EXTERNA ESPII - RODÍZIO	CHU da Universidade Federal do Pará - UFPA - PA
233	ATIVIDADE EXTERNA ESPII - RODÍZIO	CHU da Universidade Federal do Pará - UFPA - PA
233	ATIVIDADE EXTERNA ESPII - RODÍZIO	CHU da Universidade Federal do Pará - UFPA - PA
233	ATIVIDADE EXTERNA ESPII - RODÍZIO	CHU da Universidade Federal do Pará - UFPA - PA
233	ATIVIDADE EXTERNA ESPII - RODÍZIO	CHU da Universidade Federal do Pará - UFPA - PA
233	ATIVIDADE EXTERNA ESPII - RODÍZIO	CHU da Universidade Federal do Pará - UFPA - PA
233	ATIVIDADE EXTERNA ESPII - RODÍZIO	CHU da Universidade Federal do Pará - UFPA - PA
233	ATIVIDADE EXTERNA ESPII - RODÍZIO	CHU da Universidade Federal do Pará - UFPA - PA
233	ATIVIDADE EXTERNA ESPII - RODÍZIO	CHU da Universidade Federal do Pará - UFPA - PA
233	ATIVIDADE EXTERNA ESPII - RODÍZIO	CHU da Universidade Federal do Pará - UFPA - PA
233	ATIVIDADE EXTERNA ESPII - RODÍZIO	CHU da Universidade Federal do Pará - UFPA - PA
233	ATIVIDADE EXTERNA ESPII - RODÍZIO	CHU da Universidade Federal do Pará - UFPA - PA
233	ATIVIDADE EXTERNA ESPII - RODÍZIO	CHU da Universidade Federal do Pará - UFPA - PA
233	ATIVIDADE EXTERNA ESPII - RODÍZIO	CHU da Universidade Federal do Pará - UFPA - PA
233	ATIVIDADE EXTERNA ESPII - RODÍZIO	CHU da Universidade Federal do Pará - UFPA - PA
233	ATIVIDADE EXTERNA ESPII - RODÍZIO	CHU da Universidade Federal do Pará - UFPA - PA
233	ATIVIDADE EXTERNA ESPII - RODÍZIO	CHU da Universidade Federal do Pará - UFPA - PA
233	ATIVIDADE EXTERNA ESPII - RODÍZIO	CHU da Universidade Federal do Pará - UFPA - PA
233	ATIVIDADE EXTERNA ESPII - RODÍZIO	CHU da Universidade Federal do Pará - UFPA - PA
233	ATIVIDADE EXTERNA ESPII - RODÍZIO	CHU da Universidade Federal do Pará - UFPA - PA
233	ATIVIDADE EXTERNA ESPII - RODÍZIO	CHU da Universidade Federal do Pará - UFPA - PA
233	ATIVIDADE EXTERNA ESPII - RODÍZIO	CHU da Universidade Federal do Pará - UFPA - PA
233	ATIVIDADE EXTERNA ESPII - RODÍZIO	CHU da Universidade Federal do Pará - UFPA - PA
233	ATIVIDADE EXTERNA ESPII - RODÍZIO	CHU da Universidade Federal do Pará - UFPA - PA
233	ATIVIDADE EXTERNA ESPII - RODÍZIO	CHU da Universidade Federal do Pará - UFPA - PA
233	ATIVIDADE EXTERNA ESPII - RODÍZIO	CHU da Universidade Federal do Pará - UFPA - PA
233	ATIVIDADE EXTERNA ESPII - RODÍZIO	CHU da Universidade Federal do Pará - UFPA - PA
233	ATIVIDADE EXTERNA ESPII - RODÍZIO	CHU da Universidade Federal do Pará - UFPA - PA
233	ATIVIDADE EXTERNA ESPII - RODÍZIO	CHU da Universidade Federal do Pará - UFPA - PA
233	ATIVIDADE EXTERNA ESPII - RODÍZIO	CHU da Universidade Federal do Pará - UFPA - PA
233	ATIVIDADE EXTERNA ESPII - RODÍZIO	CHU da Universidade Federal do Pará - UFPA - PA

233	ATIVIDADE EXTERNA ESPII - RODÍZIO	CHU da Universidade Federal do Pará - UFPA - PA
233	ATIVIDADE EXTERNA ESPII - RODÍZIO	CHU da Universidade Federal do Pará - UFPA - PA
233	ATIVIDADE EXTERNA ESPII - RODÍZIO	CHU da Universidade Federal do Pará - UFPA - PA
233	ATIVIDADE EXTERNA ESPII - RODÍZIO	CHU da Universidade Federal do Pará - UFPA - PA
233	ATIVIDADE EXTERNA ESPII - RODÍZIO	CHU da Universidade Federal do Pará - UFPA - PA
233	ATIVIDADE EXTERNA ESPII - RODÍZIO	CHU da Universidade Federal do Pará - UFPA - PA
233	ATIVIDADE EXTERNA ESPII - RODÍZIO	CHU da Universidade Federal do Pará - UFPA - PA
233	ATIVIDADE EXTERNA ESPII - RODÍZIO	CHU da Universidade Federal do Pará - UFPA - PA
233	ATIVIDADE EXTERNA ESPII - RODÍZIO	CHU da Universidade Federal do Pará - UFPA - PA
233	ATIVIDADE EXTERNA ESPII - RODÍZIO	CHU da Universidade Federal do Pará - UFPA - PA
233	ATIVIDADE EXTERNA ESPII - RODÍZIO	CHU da Universidade Federal do Pará - UFPA - PA
233	ATIVIDADE EXTERNA ESPII - RODÍZIO	CHU da Universidade Federal do Pará - UFPA - PA
233	ATIVIDADE EXTERNA ESPII - RODÍZIO	CHU da Universidade Federal do Pará - UFPA - PA
233	ATIVIDADE EXTERNA ESPII - RODÍZIO	CHU da Universidade Federal do Pará - UFPA - PA
233	ATIVIDADE EXTERNA ESPII - RODÍZIO	CHU da Universidade Federal do Pará - UFPA - PA
233	ATIVIDADE EXTERNA ESPII - RODÍZIO	CHU da Universidade Federal do Pará - UFPA - PA
233	ATIVIDADE EXTERNA ESPII - RODÍZIO	CHU da Universidade Federal do Pará - UFPA - PA
233	ATIVIDADE EXTERNA ESPII - RODÍZIO	CHU da Universidade Federal do Pará - UFPA - PA
233	ATIVIDADE EXTERNA ESPII - RODÍZIO	CHU da Universidade Federal do Pará - UFPA - PA
233	ATIVIDADE EXTERNA ESPII - RODÍZIO	CHU da Universidade Federal do Pará - UFPA - PA
233	ATIVIDADE EXTERNA ESPII - RODÍZIO	CHU da Universidade Federal do Pará - UFPA - PA
233	ATIVIDADE EXTERNA ESPII - RODÍZIO	CHU da Universidade Federal do Pará - UFPA - PA
233	ATIVIDADE EXTERNA ESPII - RODÍZIO	CHU da Universidade Federal do Pará - UFPA - PA
233	ATIVIDADE EXTERNA ESPII - RODÍZIO	CHU da Universidade Federal do Pará - UFPA - PA
233	ATIVIDADE EXTERNA ESPII - RODÍZIO	CHU da Universidade Federal do Pará - UFPA - PA
233	ATIVIDADE EXTERNA ESPII - RODÍZIO	CHU da Universidade Federal do Pará - UFPA - PA
233	ATIVIDADE EXTERNA ESPII - RODÍZIO	CHU da Universidade Federal do Pará - UFPA - PA
233	ATIVIDADE EXTERNA ESPII - RODÍZIO	CHU da Universidade Federal do Pará - UFPA - PA
233	ATIVIDADE EXTERNA ESPII - RODÍZIO	CHU da Universidade Federal do Pará - UFPA - PA
233	ATIVIDADE EXTERNA ESPII - RODÍZIO	CHU da Universidade Federal do Pará - UFPA - PA
233	ATIVIDADE EXTERNA ESPII - RODÍZIO	CHU da Universidade Federal do Pará - UFPA - PA
233	ATIVIDADE EXTERNA ESPII - RODÍZIO	CHU da Universidade Federal do Pará - UFPA - PA
233	ATIVIDADE EXTERNA ESPII - RODÍZIO	CHU da Universidade Federal do Pará - UFPA - PA
233	ATIVIDADE EXTERNA ESPII - RODÍZIO	CHU da Universidade Federal do Pará - UFPA - PA
233	ATIVIDADE EXTERNA ESPII - RODÍZIO	CHU da Universidade Federal do Pará - UFPA - PA
233	ATIVIDADE EXTERNA ESPII - RODÍZIO	CHU da Universidade Federal do Pará - UFPA - PA
233	ATIVIDADE EXTERNA ESPII - RODÍZIO	CHU da Universidade Federal do Pará - UFPA - PA
233	ATIVIDADE EXTERNA ESPII - RODÍZIO	CHU da Universidade Federal do Pará - UFPA - PA

233	ATIVIDADE EXTERNA ESPII - RODÍZIO	CHU da Universidade Federal do Pará - UFPA - PA
233	ATIVIDADE EXTERNA ESPII - RODÍZIO	CHU da Universidade Federal do Pará - UFPA - PA
235	Afastamento Portaria n 454 art 3	CHU da Universidade Federal do Pará - UFPA - PA
235	Afastamento Portaria n 454 art 3	CHU da Universidade Federal do Pará - UFPA - PA
235	Afastamento Portaria n 454 art 3	CHU da Universidade Federal do Pará - UFPA - PA
235	Afastamento Portaria n 454 art 3	CHU da Universidade Federal do Pará - UFPA - PA
235	Afastamento Portaria n 454 art 3	CHU da Universidade Federal do Pará - UFPA - PA
235	Afastamento Portaria n 454 art 3	CHU da Universidade Federal do Pará - UFPA - PA
235	Afastamento Portaria n 454 art 3	CHU da Universidade Federal do Pará - UFPA - PA
235	Afastamento Portaria n 454 art 3	CHU da Universidade Federal do Pará - UFPA - PA
235	Afastamento Portaria n 454 art 3	CHU da Universidade Federal do Pará - UFPA - PA
235	Afastamento Portaria n 454 art 3	CHU da Universidade Federal do Pará - UFPA - PA
235	Afastamento Portaria n 454 art 3	CHU da Universidade Federal do Pará - UFPA - PA
235	Afastamento Portaria n 454 art 3	CHU da Universidade Federal do Pará - UFPA - PA
235	Afastamento Portaria n 454 art 3	CHU da Universidade Federal do Pará - UFPA - PA
235	Afastamento Portaria n 454 art 3	CHU da Universidade Federal do Pará - UFPA - PA
235	Afastamento Portaria n 454 art 3	CHU da Universidade Federal do Pará - UFPA - PA
235	Afastamento Portaria n 454 art 3	CHU da Universidade Federal do Pará - UFPA - PA
235	Afastamento Portaria n 454 art 3	CHU da Universidade Federal do Pará - UFPA - PA
235	Afastamento Portaria n 454 art 3	CHU da Universidade Federal do Pará - UFPA - PA
235	Afastamento Portaria n 454 art 3	CHU da Universidade Federal do Pará - UFPA - PA
235	Afastamento Portaria n 454 art 3	CHU da Universidade Federal do Pará - UFPA - PA
235	Afastamento Portaria n 454 art 3	CHU da Universidade Federal do Pará - UFPA - PA
235	Afastamento Portaria n 454 art 3	CHU da Universidade Federal do Pará - UFPA - PA
235	Afastamento Portaria n 454 art 3	CHU da Universidade Federal do Pará - UFPA - PA
235	Afastamento Portaria n 454 art 3	CHU da Universidade Federal do Pará - UFPA - PA
235	Afastamento Portaria n 454 art 3	CHU da Universidade Federal do Pará - UFPA - PA
235	Afastamento Portaria n 454 art 3	CHU da Universidade Federal do Pará - UFPA - PA
235	Afastamento Portaria n 454 art 3	CHU da Universidade Federal do Pará - UFPA - PA
235	Afastamento Portaria n 454 art 3	CHU da Universidade Federal do Pará - UFPA - PA
235	Afastamento Portaria n 454 art 3	CHU da Universidade Federal do Pará - UFPA - PA
235	Afastamento Portaria n 454 art 3	CHU da Universidade Federal do Pará - UFPA - PA
235	Afastamento Portaria n 454 art 3	CHU da Universidade Federal do Pará - UFPA - PA
235	Afastamento Portaria n 454 art 3	CHU da Universidade Federal do Pará - UFPA - PA
235	Afastamento Portaria n 454 art 3	CHU da Universidade Federal do Pará - UFPA - PA
235	Afastamento Portaria n 454 art 3	CHU da Universidade Federal do Pará - UFPA - PA
235	Afastamento Portaria n 454 art 3	CHU da Universidade Federal do Pará - UFPA - PA
235	Afastamento Portaria n 454 art 3	CHU da Universidade Federal do Pará - UFPA - PA
235	Afastamento Portaria n 454 art 3	CHU da Universidade Federal do Pará - UFPA - PA
235	Afastamento Portaria n 454 art 3	CHU da Universidade Federal do Pará - UFPA - PA
235	Afastamento Portaria n 454 art 3	CHU da Universidade Federal do Pará - UFPA - PA
235	Afastamento Portaria n 454 art 3	CHU da Universidade Federal do Pará - UFPA - PA

235	Afastamento Portaria n 454 art 3	CHU da Universidade Federal do Pará - UFPA - PA
235	Afastamento Portaria n 454 art 3	CHU da Universidade Federal do Pará - UFPA - PA
235	Afastamento Portaria n 454 art 3	CHU da Universidade Federal do Pará - UFPA - PA
235	Afastamento Portaria n 454 art 3	CHU da Universidade Federal do Pará - UFPA - PA
235	Afastamento Portaria n 454 art 3	CHU da Universidade Federal do Pará - UFPA - PA
235	Afastamento Portaria n 454 art 3	CHU da Universidade Federal do Pará - UFPA - PA
235	Afastamento Portaria n 454 art 3	CHU da Universidade Federal do Pará - UFPA - PA
235	Afastamento Portaria n 454 art 3	CHU da Universidade Federal do Pará - UFPA - PA
235	Afastamento Portaria n 454 art 3	CHU da Universidade Federal do Pará - UFPA - PA
235	Afastamento Portaria n 454 art 3	CHU da Universidade Federal do Pará - UFPA - PA
235	Afastamento Portaria n 454 art 3	CHU da Universidade Federal do Pará - UFPA - PA
235	Afastamento Portaria n 454 art 3	CHU da Universidade Federal do Pará - UFPA - PA
235	Afastamento Portaria n 454 art 3	CHU da Universidade Federal do Pará - UFPA - PA
235	Afastamento Portaria n 454 art 3	CHU da Universidade Federal do Pará - UFPA - PA
235	Afastamento Portaria n 454 art 3	CHU da Universidade Federal do Pará - UFPA - PA
235	Afastamento Portaria n 454 art 3	CHU da Universidade Federal do Pará - UFPA - PA
235	Afastamento Portaria n 454 art 3	CHU da Universidade Federal do Pará - UFPA - PA
235	Afastamento Portaria n 454 art 3	CHU da Universidade Federal do Pará - UFPA - PA
235	Afastamento Portaria n 454 art 3	CHU da Universidade Federal do Pará - UFPA - PA
235	Afastamento Portaria n 454 art 3	CHU da Universidade Federal do Pará - UFPA - PA
235	Afastamento Portaria n 454 art 3	CHU da Universidade Federal do Pará - UFPA - PA
235	Afastamento Portaria n 454 art 3	CHU da Universidade Federal do Pará - UFPA - PA
235	Afastamento Portaria n 454 art 3	CHU da Universidade Federal do Pará - UFPA - PA
235	Afastamento Portaria n 454 art 3	CHU da Universidade Federal do Pará - UFPA - PA
235	Afastamento Portaria n 454 art 3	CHU da Universidade Federal do Pará - UFPA - PA
235	Afastamento Portaria n 454 art 3	CHU da Universidade Federal do Pará - UFPA - PA
235	Afastamento Portaria n 454 art 3	CHU da Universidade Federal do Pará - UFPA - PA
235	Afastamento Portaria n 454 art 3	CHU da Universidade Federal do Pará - UFPA - PA
235	Afastamento Portaria n 454 art 3	CHU da Universidade Federal do Pará - UFPA - PA
235	Afastamento Portaria n 454 art 3	CHU da Universidade Federal do Pará - UFPA - PA
235	Afastamento Portaria n 454 art 3	CHU da Universidade Federal do Pará - UFPA - PA
235	Afastamento Portaria n 454 art 3	CHU da Universidade Federal do Pará - UFPA - PA
235	Afastamento Portaria n 454 art 3	CHU da Universidade Federal do Pará - UFPA - PA

235	Afastamento Portaria n 454 art 3	CHU da Universidade Federal do Pará - UFPA - PA
235	Afastamento Portaria n 454 art 3	CHU da Universidade Federal do Pará - UFPA - PA
235	Afastamento Portaria n 454 art 3	CHU da Universidade Federal do Pará - UFPA - PA
235	Afastamento Portaria n 454 art 3	CHU da Universidade Federal do Pará - UFPA - PA
235	Afastamento Portaria n 454 art 3	CHU da Universidade Federal do Pará - UFPA - PA
235	Afastamento Portaria n 454 art 3	CHU da Universidade Federal do Pará - UFPA - PA
235	Afastamento Portaria n 454 art 3	CHU da Universidade Federal do Pará - UFPA - PA
235	Afastamento Portaria n 454 art 3	CHU da Universidade Federal do Pará - UFPA - PA
235	Afastamento Portaria n 454 art 3	CHU da Universidade Federal do Pará - UFPA - PA
235	Afastamento Portaria n 454 art 3	CHU da Universidade Federal do Pará - UFPA - PA
235	Afastamento Portaria n 454 art 3	CHU da Universidade Federal do Pará - UFPA - PA
235	Afastamento Portaria n 454 art 3	CHU da Universidade Federal do Pará - UFPA - PA
235	Afastamento Portaria n 454 art 3	CHU da Universidade Federal do Pará - UFPA - PA
235	Afastamento Portaria n 454 art 3	CHU da Universidade Federal do Pará - UFPA - PA
235	Afastamento Portaria n 454 art 3	CHU da Universidade Federal do Pará - UFPA - PA
235	Afastamento Portaria n 454 art 3	CHU da Universidade Federal do Pará - UFPA - PA
235	Afastamento Portaria n 454 art 3	CHU da Universidade Federal do Pará - UFPA - PA
235	Afastamento Portaria n 454 art 3	CHU da Universidade Federal do Pará - UFPA - PA
235	Afastamento Portaria n 454 art 3	CHU da Universidade Federal do Pará - UFPA - PA
235	Afastamento Portaria n 454 art 3	CHU da Universidade Federal do Pará - UFPA - PA
235	Afastamento Portaria n 454 art 3	CHU da Universidade Federal do Pará - UFPA - PA
235	Afastamento Portaria n 454 art 3	CHU da Universidade Federal do Pará - UFPA - PA
235	Afastamento Portaria n 454 art 3	CHU da Universidade Federal do Pará - UFPA - PA
235	Afastamento Portaria n 454 art 3	CHU da Universidade Federal do Pará - UFPA - PA
235	Afastamento Portaria n 454 art 3	CHU da Universidade Federal do Pará - UFPA - PA
235	Afastamento Portaria n 454 art 3	CHU da Universidade Federal do Pará - UFPA - PA
235	Afastamento Portaria n 454 art 3	CHU da Universidade Federal do Pará - UFPA - PA
235	Afastamento Portaria n 454 art 3	CHU da Universidade Federal do Pará - UFPA - PA
235	Afastamento Portaria n 454 art 3	CHU da Universidade Federal do Pará - UFPA - PA
235	Afastamento Portaria n 454 art 3	CHU da Universidade Federal do Pará - UFPA - PA
235	Afastamento Portaria n 454 art 3	CHU da Universidade Federal do Pará - UFPA - PA
235	Afastamento Portaria n 454 art 3	CHU da Universidade Federal do Pará - UFPA - PA
235	Afastamento Portaria n 454 art 3	CHU da Universidade Federal do Pará - UFPA - PA
235	Afastamento Portaria n 454 art 3	CHU da Universidade Federal do Pará - UFPA - PA
235	Afastamento Portaria n 454 art 3	CHU da Universidade Federal do Pará - UFPA - PA
235	Afastamento Portaria n 454 art 3	CHU da Universidade Federal do Pará - UFPA - PA
235	Afastamento Portaria n 454 art 3	CHU da Universidade Federal do Pará - UFPA - PA
235	Afastamento Portaria n 454 art 3	CHU da Universidade Federal do Pará - UFPA - PA
235	Afastamento Portaria n 454 art 3	CHU da Universidade Federal do Pará - UFPA - PA
235	Afastamento Portaria n 454 art 3	CHU da Universidade Federal do Pará - UFPA - PA
235	Afastamento Portaria n 454 art 3	CHU da Universidade Federal do Pará - UFPA - PA
235	Afastamento Portaria n 454 art 3	CHU da Universidade Federal do Pará - UFPA - PA
235	Afastamento Portaria n 454 art 3	CHU da Universidade Federal do Pará - UFPA - PA
235	Afastamento Portaria n 454 art 3	CHU da Universidade Federal do Pará - UFPA - PA
235	Afastamento Portaria n 454 art 3	CHU da Universidade Federal do Pará - UFPA - PA

235	Afastamento Portaria n 454 art 3	CHU da Universidade Federal do Pará - UFPA - PA
235	Afastamento Portaria n 454 art 3	CHU da Universidade Federal do Pará - UFPA - PA
235	Afastamento Portaria n 454 art 3	CHU da Universidade Federal do Pará - UFPA - PA
235	Afastamento Portaria n 454 art 3	CHU da Universidade Federal do Pará - UFPA - PA
235	Afastamento Portaria n 454 art 3	CHU da Universidade Federal do Pará - UFPA - PA
235	Afastamento Portaria n 454 art 3	CHU da Universidade Federal do Pará - UFPA - PA
235	Afastamento Portaria n 454 art 3	CHU da Universidade Federal do Pará - UFPA - PA
235	Afastamento Portaria n 454 art 3	CHU da Universidade Federal do Pará - UFPA - PA
235	Afastamento Portaria n 454 art 3	CHU da Universidade Federal do Pará - UFPA - PA
235	Afastamento Portaria n 454 art 3	CHU da Universidade Federal do Pará - UFPA - PA
235	Afastamento Portaria n 454 art 3	CHU da Universidade Federal do Pará - UFPA - PA
235	Afastamento Portaria n 454 art 3	CHU da Universidade Federal do Pará - UFPA - PA
235	Afastamento Portaria n 454 art 3	CHU da Universidade Federal do Pará - UFPA - PA
235	Afastamento Portaria n 454 art 3	CHU da Universidade Federal do Pará - UFPA - PA
235	Afastamento Portaria n 454 art 3	CHU da Universidade Federal do Pará - UFPA - PA
235	Afastamento Portaria n 454 art 3	CHU da Universidade Federal do Pará - UFPA - PA
235	Afastamento Portaria n 454 art 3	CHU da Universidade Federal do Pará - UFPA - PA
235	Afastamento Portaria n 454 art 3	CHU da Universidade Federal do Pará - UFPA - PA
235	Afastamento Portaria n 454 art 3	CHU da Universidade Federal do Pará - UFPA - PA
235	Afastamento Portaria n 454 art 3	CHU da Universidade Federal do Pará - UFPA - PA
235	Afastamento Portaria n 454 art 3	CHU da Universidade Federal do Pará - UFPA - PA
235	Afastamento Portaria n 454 art 3	CHU da Universidade Federal do Pará - UFPA - PA
235	Afastamento Portaria n 454 art 3	CHU da Universidade Federal do Pará - UFPA - PA
235	Afastamento Portaria n 454 art 3	CHU da Universidade Federal do Pará - UFPA - PA
235	Afastamento Portaria n 454 art 3	CHU da Universidade Federal do Pará - UFPA - PA
235	Afastamento Portaria n 454 art 3	CHU da Universidade Federal do Pará - UFPA - PA
235	Afastamento Portaria n 454 art 3	CHU da Universidade Federal do Pará - UFPA - PA
235	Afastamento Portaria n 454 art 3	CHU da Universidade Federal do Pará - UFPA - PA
235	Afastamento Portaria n 454 art 3	CHU da Universidade Federal do Pará - UFPA - PA
235	Afastamento Portaria n 454 art 3	CHU da Universidade Federal do Pará - UFPA - PA
235	Afastamento Portaria n 454 art 3	CHU da Universidade Federal do Pará - UFPA - PA
235	Afastamento Portaria n 454 art 3	CHU da Universidade Federal do Pará - UFPA - PA
235	Afastamento Portaria n 454 art 3	CHU da Universidade Federal do Pará - UFPA - PA
235	Afastamento Portaria n 454 art 3	CHU da Universidade Federal do Pará - UFPA - PA
235	Afastamento Portaria n 454 art 3	CHU da Universidade Federal do Pará - UFPA - PA
235	Afastamento Portaria n 454 art 3	CHU da Universidade Federal do Pará - UFPA - PA
235	Afastamento Portaria n 454 art 3	CHU da Universidade Federal do Pará - UFPA - PA

235	Afastamento Portaria n 454 art 3	CHU da Universidade Federal do Pará - UFPA - PA
235	Afastamento Portaria n 454 art 3	CHU da Universidade Federal do Pará - UFPA - PA
235	Afastamento Portaria n 454 art 3	CHU da Universidade Federal do Pará - UFPA - PA
235	Afastamento Portaria n 454 art 3	CHU da Universidade Federal do Pará - UFPA - PA
235	Afastamento Portaria n 454 art 3	CHU da Universidade Federal do Pará - UFPA - PA
235	Afastamento Portaria n 454 art 3	CHU da Universidade Federal do Pará - UFPA - PA
235	Afastamento Portaria n 454 art 3	CHU da Universidade Federal do Pará - UFPA - PA
235	Afastamento Portaria n 454 art 3	CHU da Universidade Federal do Pará - UFPA - PA
235	Afastamento Portaria n 454 art 3	CHU da Universidade Federal do Pará - UFPA - PA
235	Afastamento Portaria n 454 art 3	CHU da Universidade Federal do Pará - UFPA - PA
235	Afastamento Portaria n 454 art 3	CHU da Universidade Federal do Pará - UFPA - PA
235	Afastamento Portaria n 454 art 3	CHU da Universidade Federal do Pará - UFPA - PA
235	Afastamento Portaria n 454 art 3	CHU da Universidade Federal do Pará - UFPA - PA
235	Afastamento Portaria n 454 art 3	CHU da Universidade Federal do Pará - UFPA - PA
235	Afastamento Portaria n 454 art 3	CHU da Universidade Federal do Pará - UFPA - PA
235	Afastamento Portaria n 454 art 3	CHU da Universidade Federal do Pará - UFPA - PA
235	Afastamento Portaria n 454 art 3	CHU da Universidade Federal do Pará - UFPA - PA
235	Afastamento Portaria n 454 art 3	CHU da Universidade Federal do Pará - UFPA - PA
235	Afastamento Portaria n 454 art 3	CHU da Universidade Federal do Pará - UFPA - PA
235	Afastamento Portaria n 454 art 3	CHU da Universidade Federal do Pará - UFPA - PA
235	Afastamento Portaria n 454 art 3	CHU da Universidade Federal do Pará - UFPA - PA
235	Afastamento Portaria n 454 art 3	CHU da Universidade Federal do Pará - UFPA - PA
235	Afastamento Portaria n 454 art 3	CHU da Universidade Federal do Pará - UFPA - PA
235	Afastamento Portaria n 454 art 3	CHU da Universidade Federal do Pará - UFPA - PA
235	Afastamento Portaria n 454 art 3	CHU da Universidade Federal do Pará - UFPA - PA
235	Afastamento Portaria n 454 art 3	CHU da Universidade Federal do Pará - UFPA - PA
235	Afastamento Portaria n 454 art 3	CHU da Universidade Federal do Pará - UFPA - PA
235	Afastamento Portaria n 454 art 3	CHU da Universidade Federal do Pará - UFPA - PA
235	Afastamento Portaria n 454 art 3	CHU da Universidade Federal do Pará - UFPA - PA
235	Afastamento Portaria n 454 art 3	CHU da Universidade Federal do Pará - UFPA - PA
235	Afastamento Portaria n 454 art 3	CHU da Universidade Federal do Pará - UFPA - PA
235	Afastamento Portaria n 454 art 3	CHU da Universidade Federal do Pará - UFPA - PA
235	Afastamento Portaria n 454 art 3	CHU da Universidade Federal do Pará - UFPA - PA
235	Afastamento Portaria n 454 art 3	CHU da Universidade Federal do Pará - UFPA - PA
235	Afastamento Portaria n 454 art 3	CHU da Universidade Federal do Pará - UFPA - PA

235	Afastamento Portaria n 454 art 3	CHU da Universidade Federal do Pará - UFPA - PA
235	Afastamento Portaria n 454 art 3	CHU da Universidade Federal do Pará - UFPA - PA
235	Afastamento Portaria n 454 art 3	CHU da Universidade Federal do Pará - UFPA - PA
235	Afastamento Portaria n 454 art 3	CHU da Universidade Federal do Pará - UFPA - PA
235	Afastamento Portaria n 454 art 3	CHU da Universidade Federal do Pará - UFPA - PA
235	Afastamento Portaria n 454 art 3	CHU da Universidade Federal do Pará - UFPA - PA
235	Afastamento Portaria n 454 art 3	CHU da Universidade Federal do Pará - UFPA - PA
235	Afastamento Portaria n 454 art 3	CHU da Universidade Federal do Pará - UFPA - PA
235	Afastamento Portaria n 454 art 3	CHU da Universidade Federal do Pará - UFPA - PA
235	Afastamento Portaria n 454 art 3	CHU da Universidade Federal do Pará - UFPA - PA
235	Afastamento Portaria n 454 art 3	CHU da Universidade Federal do Pará - UFPA - PA
235	Afastamento Portaria n 454 art 3	CHU da Universidade Federal do Pará - UFPA - PA
235	Afastamento Portaria n 454 art 3	CHU da Universidade Federal do Pará - UFPA - PA
235	Afastamento Portaria n 454 art 3	CHU da Universidade Federal do Pará - UFPA - PA
235	Afastamento Portaria n 454 art 3	CHU da Universidade Federal do Pará - UFPA - PA
235	Afastamento Portaria n 454 art 3	CHU da Universidade Federal do Pará - UFPA - PA
235	Afastamento Portaria n 454 art 3	CHU da Universidade Federal do Pará - UFPA - PA
235	Afastamento Portaria n 454 art 3	CHU da Universidade Federal do Pará - UFPA - PA
235	Afastamento Portaria n 454 art 3	CHU da Universidade Federal do Pará - UFPA - PA
235	Afastamento Portaria n 454 art 3	CHU da Universidade Federal do Pará - UFPA - PA
235	Afastamento Portaria n 454 art 3	CHU da Universidade Federal do Pará - UFPA - PA
235	Afastamento Portaria n 454 art 3	CHU da Universidade Federal do Pará - UFPA - PA
235	Afastamento Portaria n 454 art 3	CHU da Universidade Federal do Pará - UFPA - PA
235	Afastamento Portaria n 454 art 3	CHU da Universidade Federal do Pará - UFPA - PA
235	Afastamento Portaria n 454 art 3	CHU da Universidade Federal do Pará - UFPA - PA
235	Afastamento Portaria n 454 art 3	CHU da Universidade Federal do Pará - UFPA - PA
235	Afastamento Portaria n 454 art 3	CHU da Universidade Federal do Pará - UFPA - PA
235	Afastamento Portaria n 454 art 3	CHU da Universidade Federal do Pará - UFPA - PA
235	Afastamento Portaria n 454 art 3	CHU da Universidade Federal do Pará - UFPA - PA
235	Afastamento Portaria n 454 art 3	CHU da Universidade Federal do Pará - UFPA - PA
235	Afastamento Portaria n 454 art 3	CHU da Universidade Federal do Pará - UFPA - PA
235	Afastamento Portaria n 454 art 3	CHU da Universidade Federal do Pará - UFPA - PA
235	Afastamento Portaria n 454 art 3	CHU da Universidade Federal do Pará - UFPA - PA
235	Afastamento Portaria n 454 art 3	CHU da Universidade Federal do Pará - UFPA - PA
235	Afastamento Portaria n 454 art 3	CHU da Universidade Federal do Pará - UFPA - PA
235	Afastamento Portaria n 454 art 3	CHU da Universidade Federal do Pará - UFPA - PA
235	Afastamento Portaria n 454 art 3	CHU da Universidade Federal do Pará - UFPA - PA
235	Afastamento Portaria n 454 art 3	CHU da Universidade Federal do Pará - UFPA - PA
235	Afastamento Portaria n 454 art 3	CHU da Universidade Federal do Pará - UFPA - PA
235	Afastamento Portaria n 454 art 3	CHU da Universidade Federal do Pará - UFPA - PA
235	Afastamento Portaria n 454 art 3	CHU da Universidade Federal do Pará - UFPA - PA
235	Afastamento Portaria n 454 art 3	CHU da Universidade Federal do Pará - UFPA - PA
235	Afastamento Portaria n 454 art 3	CHU da Universidade Federal do Pará - UFPA - PA
235	Afastamento Portaria n 454 art 3	CHU da Universidade Federal do Pará - UFPA - PA
235	Afastamento Portaria n 454 art 3	CHU da Universidade Federal do Pará - UFPA - PA
235	Afastamento Portaria n 454 art 3	CHU da Universidade Federal do Pará - UFPA - PA

235	Afastamento Portaria n 454 art 3	CHU da Universidade Federal do Pará - UFPA - PA
235	Afastamento Portaria n 454 art 3	CHU da Universidade Federal do Pará - UFPA - PA
235	Afastamento Portaria n 454 art 3	CHU da Universidade Federal do Pará - UFPA - PA
235	Afastamento Portaria n 454 art 3	CHU da Universidade Federal do Pará - UFPA - PA
235	Afastamento Portaria n 454 art 3	CHU da Universidade Federal do Pará - UFPA - PA
235	Afastamento Portaria n 454 art 3	CHU da Universidade Federal do Pará - UFPA - PA
235	Afastamento Portaria n 454 art 3	CHU da Universidade Federal do Pará - UFPA - PA
235	Afastamento Portaria n 454 art 3	CHU da Universidade Federal do Pará - UFPA - PA
235	Afastamento Portaria n 454 art 3	CHU da Universidade Federal do Pará - UFPA - PA
235	Afastamento Portaria n 454 art 3	CHU da Universidade Federal do Pará - UFPA - PA
235	Afastamento Portaria n 454 art 3	CHU da Universidade Federal do Pará - UFPA - PA
235	Afastamento Portaria n 454 art 3	CHU da Universidade Federal do Pará - UFPA - PA
235	Afastamento Portaria n 454 art 3	CHU da Universidade Federal do Pará - UFPA - PA
235	Afastamento Portaria n 454 art 3	CHU da Universidade Federal do Pará - UFPA - PA
235	Afastamento Portaria n 454 art 3	CHU da Universidade Federal do Pará - UFPA - PA
235	Afastamento Portaria n 454 art 3	CHU da Universidade Federal do Pará - UFPA - PA
235	Afastamento Portaria n 454 art 3	CHU da Universidade Federal do Pará - UFPA - PA
235	Afastamento Portaria n 454 art 3	CHU da Universidade Federal do Pará - UFPA - PA
235	Afastamento Portaria n 454 art 3	CHU da Universidade Federal do Pará - UFPA - PA
235	Afastamento Portaria n 454 art 3	CHU da Universidade Federal do Pará - UFPA - PA
235	Afastamento Portaria n 454 art 3	CHU da Universidade Federal do Pará - UFPA - PA
235	Afastamento Portaria n 454 art 3	CHU da Universidade Federal do Pará - UFPA - PA
235	Afastamento Portaria n 454 art 3	CHU da Universidade Federal do Pará - UFPA - PA
235	Afastamento Portaria n 454 art 3	CHU da Universidade Federal do Pará - UFPA - PA
235	Afastamento Portaria n 454 art 3	CHU da Universidade Federal do Pará - UFPA - PA
235	Afastamento Portaria n 454 art 3	CHU da Universidade Federal do Pará - UFPA - PA
235	Afastamento Portaria n 454 art 3	CHU da Universidade Federal do Pará - UFPA - PA
236	Afastamento Portaria n 454 art. 2 Único	CHU da Universidade Federal do Pará - UFPA - PA
236	Afastamento Portaria n 454 art. 2 Único	CHU da Universidade Federal do Pará - UFPA - PA
236	Afastamento Portaria n 454 art. 2 Único	CHU da Universidade Federal do Pará - UFPA - PA
236	Afastamento Portaria n 454 art. 2 Único	CHU da Universidade Federal do Pará - UFPA - PA
236	Afastamento Portaria n 454 art. 2 Único	CHU da Universidade Federal do Pará - UFPA - PA
236	Afastamento Portaria n 454 art. 2 Único	CHU da Universidade Federal do Pará - UFPA - PA
236	Afastamento Portaria n 454 art. 2 Único	CHU da Universidade Federal do Pará - UFPA - PA
236	Afastamento Portaria n 454 art. 2 Único	CHU da Universidade Federal do Pará - UFPA - PA
236	Afastamento Portaria n 454 art. 2 Único	CHU da Universidade Federal do Pará - UFPA - PA
236	Afastamento Portaria n 454 art. 2 Único	CHU da Universidade Federal do Pará - UFPA - PA
236	Afastamento Portaria n 454 art. 2 Único	CHU da Universidade Federal do Pará - UFPA - PA
236	Afastamento Portaria n 454 art. 2 Único	CHU da Universidade Federal do Pará - UFPA - PA
236	Afastamento Portaria n 454 art. 2 Único	CHU da Universidade Federal do Pará - UFPA - PA
236	Afastamento Portaria n 454 art. 2 Único	CHU da Universidade Federal do Pará - UFPA - PA
236	Afastamento Portaria n 454 art. 2 Único	CHU da Universidade Federal do Pará - UFPA - PA
236	Afastamento Portaria n 454 art. 2 Único	CHU da Universidade Federal do Pará - UFPA - PA
236	Afastamento Portaria n 454 art. 2 Único	CHU da Universidade Federal do Pará - UFPA - PA

236	Afastamento Portaria n 454 art. 2 Único	CHU da Universidade Federal do Pará - UFPA - PA
236	Afastamento Portaria n 454 art. 2 Único	CHU da Universidade Federal do Pará - UFPA - PA
236	Afastamento Portaria n 454 art. 2 Único	CHU da Universidade Federal do Pará - UFPA - PA
236	Afastamento Portaria n 454 art. 2 Único	CHU da Universidade Federal do Pará - UFPA - PA
236	Afastamento Portaria n 454 art. 2 Único	CHU da Universidade Federal do Pará - UFPA - PA
236	Afastamento Portaria n 454 art. 2 Único	CHU da Universidade Federal do Pará - UFPA - PA
236	Afastamento Portaria n 454 art. 2 Único	CHU da Universidade Federal do Pará - UFPA - PA
236	Afastamento Portaria n 454 art. 2 Único	CHU da Universidade Federal do Pará - UFPA - PA
236	Afastamento Portaria n 454 art. 2 Único	CHU da Universidade Federal do Pará - UFPA - PA
236	Afastamento Portaria n 454 art. 2 Único	CHU da Universidade Federal do Pará - UFPA - PA
236	Afastamento Portaria n 454 art. 2 Único	CHU da Universidade Federal do Pará - UFPA - PA
236	Afastamento Portaria n 454 art. 2 Único	CHU da Universidade Federal do Pará - UFPA - PA
236	Afastamento Portaria n 454 art. 2 Único	CHU da Universidade Federal do Pará - UFPA - PA
236	Afastamento Portaria n 454 art. 2 Único	CHU da Universidade Federal do Pará - UFPA - PA
236	Afastamento Portaria n 454 art. 2 Único	CHU da Universidade Federal do Pará - UFPA - PA
236	Afastamento Portaria n 454 art. 2 Único	CHU da Universidade Federal do Pará - UFPA - PA
236	Afastamento Portaria n 454 art. 2 Único	CHU da Universidade Federal do Pará - UFPA - PA
236	Afastamento Portaria n 454 art. 2 Único	CHU da Universidade Federal do Pará - UFPA - PA
236	Afastamento Portaria n 454 art. 2 Único	CHU da Universidade Federal do Pará - UFPA - PA
236	Afastamento Portaria n 454 art. 2 Único	CHU da Universidade Federal do Pará - UFPA - PA
236	Afastamento Portaria n 454 art. 2 Único	CHU da Universidade Federal do Pará - UFPA - PA
236	Afastamento Portaria n 454 art. 2 Único	CHU da Universidade Federal do Pará - UFPA - PA
236	Afastamento Portaria n 454 art. 2 Único	CHU da Universidade Federal do Pará - UFPA - PA
236	Afastamento Portaria n 454 art. 2 Único	CHU da Universidade Federal do Pará - UFPA - PA
236	Afastamento Portaria n 454 art. 2 Único	CHU da Universidade Federal do Pará - UFPA - PA
236	Afastamento Portaria n 454 art. 2 Único	CHU da Universidade Federal do Pará - UFPA - PA
236	Afastamento Portaria n 454 art. 2 Único	CHU da Universidade Federal do Pará - UFPA - PA
236	Afastamento Portaria n 454 art. 2 Único	CHU da Universidade Federal do Pará - UFPA - PA
236	Afastamento Portaria n 454 art. 2 Único	CHU da Universidade Federal do Pará - UFPA - PA
236	Afastamento Portaria n 454 art. 2 Único	CHU da Universidade Federal do Pará - UFPA - PA
236	Afastamento Portaria n 454 art. 2 Único	CHU da Universidade Federal do Pará - UFPA - PA
236	Afastamento Portaria n 454 art. 2 Único	CHU da Universidade Federal do Pará - UFPA - PA
236	Afastamento Portaria n 454 art. 2 Único	CHU da Universidade Federal do Pará - UFPA - PA
236	Afastamento Portaria n 454 art. 2 Único	CHU da Universidade Federal do Pará - UFPA - PA
236	Afastamento Portaria n 454 art. 2 Único	CHU da Universidade Federal do Pará - UFPA - PA
236	Afastamento Portaria n 454 art. 2 Único	CHU da Universidade Federal do Pará - UFPA - PA
236	Afastamento Portaria n 454 art. 2 Único	CHU da Universidade Federal do Pará - UFPA - PA
236	Afastamento Portaria n 454 art. 2 Único	CHU da Universidade Federal do Pará - UFPA - PA
236	Afastamento Portaria n 454 art. 2 Único	CHU da Universidade Federal do Pará - UFPA - PA

236	Afastamento Portaria n 454 art. 2 Único	CHU da Universidade Federal do Pará - UFPA - PA
236	Afastamento Portaria n 454 art. 2 Único	CHU da Universidade Federal do Pará - UFPA - PA
236	Afastamento Portaria n 454 art. 2 Único	CHU da Universidade Federal do Pará - UFPA - PA
236	Afastamento Portaria n 454 art. 2 Único	CHU da Universidade Federal do Pará - UFPA - PA
236	Afastamento Portaria n 454 art. 2 Único	CHU da Universidade Federal do Pará - UFPA - PA
236	Afastamento Portaria n 454 art. 2 Único	CHU da Universidade Federal do Pará - UFPA - PA
236	Afastamento Portaria n 454 art. 2 Único	CHU da Universidade Federal do Pará - UFPA - PA
236	Afastamento Portaria n 454 art. 2 Único	CHU da Universidade Federal do Pará - UFPA - PA
236	Afastamento Portaria n 454 art. 2 Único	CHU da Universidade Federal do Pará - UFPA - PA
236	Afastamento Portaria n 454 art. 2 Único	CHU da Universidade Federal do Pará - UFPA - PA
236	Afastamento Portaria n 454 art. 2 Único	CHU da Universidade Federal do Pará - UFPA - PA
236	Afastamento Portaria n 454 art. 2 Único	CHU da Universidade Federal do Pará - UFPA - PA
236	Afastamento Portaria n 454 art. 2 Único	CHU da Universidade Federal do Pará - UFPA - PA
236	Afastamento Portaria n 454 art. 2 Único	CHU da Universidade Federal do Pará - UFPA - PA
236	Afastamento Portaria n 454 art. 2 Único	CHU da Universidade Federal do Pará - UFPA - PA
236	Afastamento Portaria n 454 art. 2 Único	CHU da Universidade Federal do Pará - UFPA - PA
236	Afastamento Portaria n 454 art. 2 Único	CHU da Universidade Federal do Pará - UFPA - PA
236	Afastamento Portaria n 454 art. 2 Único	CHU da Universidade Federal do Pará - UFPA - PA
236	Afastamento Portaria n 454 art. 2 Único	CHU da Universidade Federal do Pará - UFPA - PA
236	Afastamento Portaria n 454 art. 2 Único	CHU da Universidade Federal do Pará - UFPA - PA
236	Afastamento Portaria n 454 art. 2 Único	CHU da Universidade Federal do Pará - UFPA - PA
236	Afastamento Portaria n 454 art. 2 Único	CHU da Universidade Federal do Pará - UFPA - PA
237	Trabalho Remoto Instrução Normativa DGP 03/2020 art 6, IX.	CHU da Universidade Federal do Pará - UFPA - PA
237	Trabalho Remoto Instrução Normativa DGP 03/2020 art 6, IX.	CHU da Universidade Federal do Pará - UFPA - PA
237	Trabalho Remoto Instrução Normativa DGP 03/2020 art 6, IX.	CHU da Universidade Federal do Pará - UFPA - PA
237	Trabalho Remoto Instrução Normativa DGP 03/2020 art 6, IX.	CHU da Universidade Federal do Pará - UFPA - PA
237	Trabalho Remoto Instrução Normativa DGP 03/2020 art 6, IX.	CHU da Universidade Federal do Pará - UFPA - PA
237	Trabalho Remoto Instrução Normativa DGP 03/2020 art 6, IX.	CHU da Universidade Federal do Pará - UFPA - PA
237	Trabalho Remoto Instrução Normativa DGP 03/2020 art 6, IX.	CHU da Universidade Federal do Pará - UFPA - PA
237	Trabalho Remoto Instrução Normativa DGP 03/2020 art 6, IX.	CHU da Universidade Federal do Pará - UFPA - PA
237	Trabalho Remoto Instrução Normativa DGP 03/2020 art 6, IX.	CHU da Universidade Federal do Pará - UFPA - PA
237	Trabalho Remoto Instrução Normativa DGP 03/2020 art 6, IX.	CHU da Universidade Federal do Pará - UFPA - PA
237	Trabalho Remoto Instrução Normativa DGP 03/2020 art 6, IX.	CHU da Universidade Federal do Pará - UFPA - PA
237	Trabalho Remoto Instrução Normativa DGP 03/2020 art 6, IX.	CHU da Universidade Federal do Pará - UFPA - PA
237	Trabalho Remoto Instrução Normativa DGP 03/2020 art 6, IX.	CHU da Universidade Federal do Pará - UFPA - PA
237	Trabalho Remoto Instrução Normativa DGP 03/2020 art 6, IX.	CHU da Universidade Federal do Pará - UFPA - PA
237	Trabalho Remoto Instrução Normativa DGP 03/2020 art 6, IX.	CHU da Universidade Federal do Pará - UFPA - PA

237	Trabalho Remoto Instrução Normativa DGP 03/2020 art 6, IX.	CHU da Universidade Federal do Pará - UFPA - PA
237	Trabalho Remoto Instrução Normativa DGP 03/2020 art 6, IX.	CHU da Universidade Federal do Pará - UFPA - PA
237	Trabalho Remoto Instrução Normativa DGP 03/2020 art 6, IX.	CHU da Universidade Federal do Pará - UFPA - PA
237	Trabalho Remoto Instrução Normativa DGP 03/2020 art 6, IX.	CHU da Universidade Federal do Pará - UFPA - PA
237	Trabalho Remoto Instrução Normativa DGP 03/2020 art 6, IX.	CHU da Universidade Federal do Pará - UFPA - PA
237	Trabalho Remoto Instrução Normativa DGP 03/2020 art 6, IX.	CHU da Universidade Federal do Pará - UFPA - PA
237	Trabalho Remoto Instrução Normativa DGP 03/2020 art 6, IX.	CHU da Universidade Federal do Pará - UFPA - PA
237	Trabalho Remoto Instrução Normativa DGP 03/2020 art 6, IX.	CHU da Universidade Federal do Pará - UFPA - PA
237	Trabalho Remoto Instrução Normativa DGP 03/2020 art 6, IX.	CHU da Universidade Federal do Pará - UFPA - PA
237	Trabalho Remoto Instrução Normativa DGP 03/2020 art 6, IX.	CHU da Universidade Federal do Pará - UFPA - PA
237	Trabalho Remoto Instrução Normativa DGP 03/2020 art 6, IX.	CHU da Universidade Federal do Pará - UFPA - PA
237	Trabalho Remoto Instrução Normativa DGP 03/2020 art 6, IX.	CHU da Universidade Federal do Pará - UFPA - PA
237	Trabalho Remoto Instrução Normativa DGP 03/2020 art 6, IX.	CHU da Universidade Federal do Pará - UFPA - PA
237	Trabalho Remoto Instrução Normativa DGP 03/2020 art 6, IX.	CHU da Universidade Federal do Pará - UFPA - PA
237	Trabalho Remoto Instrução Normativa DGP 03/2020 art 6, IX.	CHU da Universidade Federal do Pará - UFPA - PA
237	Trabalho Remoto Instrução Normativa DGP 03/2020 art 6, IX.	CHU da Universidade Federal do Pará - UFPA - PA
237	Trabalho Remoto Instrução Normativa DGP 03/2020 art 6, IX.	CHU da Universidade Federal do Pará - UFPA - PA
237	Trabalho Remoto Instrução Normativa DGP 03/2020 art 6, IX.	CHU da Universidade Federal do Pará - UFPA - PA
237	Trabalho Remoto Instrução Normativa DGP 03/2020 art 6, IX.	CHU da Universidade Federal do Pará - UFPA - PA
237	Trabalho Remoto Instrução Normativa DGP 03/2020 art 6, IX.	CHU da Universidade Federal do Pará - UFPA - PA
237	Trabalho Remoto Instrução Normativa DGP 03/2020 art 6, IX.	CHU da Universidade Federal do Pará - UFPA - PA
237	Trabalho Remoto Instrução Normativa DGP 03/2020 art 6, IX.	CHU da Universidade Federal do Pará - UFPA - PA
237	Trabalho Remoto Instrução Normativa DGP 03/2020 art 6, IX.	CHU da Universidade Federal do Pará - UFPA - PA
237	Trabalho Remoto Instrução Normativa DGP 03/2020 art 6, IX.	CHU da Universidade Federal do Pará - UFPA - PA
237	Trabalho Remoto Instrução Normativa DGP 03/2020 art 6, IX.	CHU da Universidade Federal do Pará - UFPA - PA
237	Trabalho Remoto Instrução Normativa DGP 03/2020 art 6, IX.	CHU da Universidade Federal do Pará - UFPA - PA
237	Trabalho Remoto Instrução Normativa DGP 03/2020 art 6, IX.	CHU da Universidade Federal do Pará - UFPA - PA
237	Trabalho Remoto Instrução Normativa DGP 03/2020 art 6, IX.	CHU da Universidade Federal do Pará - UFPA - PA
237	Trabalho Remoto Instrução Normativa DGP 03/2020 art 6, IX.	CHU da Universidade Federal do Pará - UFPA - PA
237	Trabalho Remoto Instrução Normativa DGP 03/2020 art 6, IX.	CHU da Universidade Federal do Pará - UFPA - PA
237	Trabalho Remoto Instrução Normativa DGP 03/2020 art 6, IX.	CHU da Universidade Federal do Pará - UFPA - PA
237	Trabalho Remoto Instrução Normativa DGP 03/2020 art 6, IX.	CHU da Universidade Federal do Pará - UFPA - PA

237	Trabalho Remoto Instrução Normativa DGP 03/2020 art 6, IX.	CHU da Universidade Federal do Pará - UFPA - PA
237	Trabalho Remoto Instrução Normativa DGP 03/2020 art 6, IX.	CHU da Universidade Federal do Pará - UFPA - PA
237	Trabalho Remoto Instrução Normativa DGP 03/2020 art 6, IX.	CHU da Universidade Federal do Pará - UFPA - PA
237	Trabalho Remoto Instrução Normativa DGP 03/2020 art 6, IX.	CHU da Universidade Federal do Pará - UFPA - PA
237	Trabalho Remoto Instrução Normativa DGP 03/2020 art 6, IX.	CHU da Universidade Federal do Pará - UFPA - PA
237	Trabalho Remoto Instrução Normativa DGP 03/2020 art 6, IX.	CHU da Universidade Federal do Pará - UFPA - PA
237	Trabalho Remoto Instrução Normativa DGP 03/2020 art 6, IX.	CHU da Universidade Federal do Pará - UFPA - PA
237	Trabalho Remoto Instrução Normativa DGP 03/2020 art 6, IX.	CHU da Universidade Federal do Pará - UFPA - PA
237	Trabalho Remoto Instrução Normativa DGP 03/2020 art 6, IX.	CHU da Universidade Federal do Pará - UFPA - PA
237	Trabalho Remoto Instrução Normativa DGP 03/2020 art 6, IX.	CHU da Universidade Federal do Pará - UFPA - PA
237	Trabalho Remoto Instrução Normativa DGP 03/2020 art 6, IX.	CHU da Universidade Federal do Pará - UFPA - PA
237	Trabalho Remoto Instrução Normativa DGP 03/2020 art 6, IX.	CHU da Universidade Federal do Pará - UFPA - PA
237	Trabalho Remoto Instrução Normativa DGP 03/2020 art 6, IX.	CHU da Universidade Federal do Pará - UFPA - PA
237	Trabalho Remoto Instrução Normativa DGP 03/2020 art 6, IX.	CHU da Universidade Federal do Pará - UFPA - PA
237	Trabalho Remoto Instrução Normativa DGP 03/2020 art 6, IX.	CHU da Universidade Federal do Pará - UFPA - PA
237	Trabalho Remoto Instrução Normativa DGP 03/2020 art 6, IX.	CHU da Universidade Federal do Pará - UFPA - PA
237	Trabalho Remoto Instrução Normativa DGP 03/2020 art 6, IX.	CHU da Universidade Federal do Pará - UFPA - PA
237	Trabalho Remoto Instrução Normativa DGP 03/2020 art 6, IX.	CHU da Universidade Federal do Pará - UFPA - PA
237	Trabalho Remoto Instrução Normativa DGP 03/2020 art 6, IX.	CHU da Universidade Federal do Pará - UFPA - PA
237	Trabalho Remoto Instrução Normativa DGP 03/2020 art 6, IX.	CHU da Universidade Federal do Pará - UFPA - PA
237	Trabalho Remoto Instrução Normativa DGP 03/2020 art 6, IX.	CHU da Universidade Federal do Pará - UFPA - PA
237	Trabalho Remoto Instrução Normativa DGP 03/2020 art 6, IX.	CHU da Universidade Federal do Pará - UFPA - PA
237	Trabalho Remoto Instrução Normativa DGP 03/2020 art 6, IX.	CHU da Universidade Federal do Pará - UFPA - PA
237	Trabalho Remoto Instrução Normativa DGP 03/2020 art 6, IX.	CHU da Universidade Federal do Pará - UFPA - PA
237	Trabalho Remoto Instrução Normativa DGP 03/2020 art 6, IX.	CHU da Universidade Federal do Pará - UFPA - PA
237	Trabalho Remoto Instrução Normativa DGP 03/2020 art 6, IX.	CHU da Universidade Federal do Pará - UFPA - PA
237	Trabalho Remoto Instrução Normativa DGP 03/2020 art 6, IX.	CHU da Universidade Federal do Pará - UFPA - PA
237	Trabalho Remoto Instrução Normativa DGP 03/2020 art 6, IX.	CHU da Universidade Federal do Pará - UFPA - PA
237	Trabalho Remoto Instrução Normativa DGP 03/2020 art 6, IX.	CHU da Universidade Federal do Pará - UFPA - PA
237	Trabalho Remoto Instrução Normativa DGP 03/2020 art 6, IX.	CHU da Universidade Federal do Pará - UFPA - PA
237	Trabalho Remoto Instrução Normativa DGP 03/2020 art 6, IX.	CHU da Universidade Federal do Pará - UFPA - PA
237	Trabalho Remoto Instrução Normativa DGP 03/2020 art 6, IX.	CHU da Universidade Federal do Pará - UFPA - PA

237	Trabalho Remoto Instrução Normativa DGP 03/2020 art 6, IX.	CHU da Universidade Federal do Pará - UFPA - PA
237	Trabalho Remoto Instrução Normativa DGP 03/2020 art 6, IX.	CHU da Universidade Federal do Pará - UFPA - PA
237	Trabalho Remoto Instrução Normativa DGP 03/2020 art 6, IX.	CHU da Universidade Federal do Pará - UFPA - PA
237	Trabalho Remoto Instrução Normativa DGP 03/2020 art 6, IX.	CHU da Universidade Federal do Pará - UFPA - PA
237	Trabalho Remoto Instrução Normativa DGP 03/2020 art 6, IX.	CHU da Universidade Federal do Pará - UFPA - PA
238	Afastamento Intrução Normativa DGP 03/2020. Art 4 2	CHU da Universidade Federal do Pará - UFPA - PA
238	Afastamento Intrução Normativa DGP 03/2020. Art 4 2	CHU da Universidade Federal do Pará - UFPA - PA
238	Afastamento Intrução Normativa DGP 03/2020. Art 4 2	CHU da Universidade Federal do Pará - UFPA - PA
238	Afastamento Intrução Normativa DGP 03/2020. Art 4 2	CHU da Universidade Federal do Pará - UFPA - PA
238	Afastamento Intrução Normativa DGP 03/2020. Art 4 2	CHU da Universidade Federal do Pará - UFPA - PA
238	Afastamento Intrução Normativa DGP 03/2020. Art 4 2	CHU da Universidade Federal do Pará - UFPA - PA
238	Afastamento Intrução Normativa DGP 03/2020. Art 4 2	CHU da Universidade Federal do Pará - UFPA - PA
238	Afastamento Intrução Normativa DGP 03/2020. Art 4 2	CHU da Universidade Federal do Pará - UFPA - PA
238	Afastamento Intrução Normativa DGP 03/2020. Art 4 2	CHU da Universidade Federal do Pará - UFPA - PA
238	Afastamento Intrução Normativa DGP 03/2020. Art 4 2	CHU da Universidade Federal do Pará - UFPA - PA
238	Afastamento Intrução Normativa DGP 03/2020. Art 4 2	CHU da Universidade Federal do Pará - UFPA - PA
238	Afastamento Intrução Normativa DGP 03/2020. Art 4 2	CHU da Universidade Federal do Pará - UFPA - PA
238	Afastamento Intrução Normativa DGP 03/2020. Art 4 2	CHU da Universidade Federal do Pará - UFPA - PA
238	Afastamento Intrução Normativa DGP 03/2020. Art 4 2	CHU da Universidade Federal do Pará - UFPA - PA
238	Afastamento Intrução Normativa DGP 03/2020. Art 4 2	CHU da Universidade Federal do Pará - UFPA - PA
238	Afastamento Intrução Normativa DGP 03/2020. Art 4 2	CHU da Universidade Federal do Pará - UFPA - PA
238	Afastamento Intrução Normativa DGP 03/2020. Art 4 2	CHU da Universidade Federal do Pará - UFPA - PA
238	Afastamento Intrução Normativa DGP 03/2020. Art 4 2	CHU da Universidade Federal do Pará - UFPA - PA
238	Afastamento Intrução Normativa DGP 03/2020. Art 4 2	CHU da Universidade Federal do Pará - UFPA - PA
238	Afastamento Intrução Normativa DGP 03/2020. Art 4 2	CHU da Universidade Federal do Pará - UFPA - PA
238	Afastamento Intrução Normativa DGP 03/2020. Art 4 2	CHU da Universidade Federal do Pará - UFPA - PA
238	Afastamento Intrução Normativa DGP 03/2020. Art 4 2	CHU da Universidade Federal do Pará - UFPA - PA
238	Afastamento Intrução Normativa DGP 03/2020. Art 4 2	CHU da Universidade Federal do Pará - UFPA - PA
238	Afastamento Intrução Normativa DGP 03/2020. Art 4 2	CHU da Universidade Federal do Pará - UFPA - PA
238	Afastamento Intrução Normativa DGP 03/2020. Art 4 2	CHU da Universidade Federal do Pará - UFPA - PA
238	Afastamento Intrução Normativa DGP 03/2020. Art 4 2	CHU da Universidade Federal do Pará - UFPA - PA
238	Afastamento Intrução Normativa DGP 03/2020. Art 4 2	CHU da Universidade Federal do Pará - UFPA - PA
238	Afastamento Intrução Normativa DGP 03/2020. Art 4 2	CHU da Universidade Federal do Pará - UFPA - PA
238	Afastamento Intrução Normativa DGP 03/2020. Art 4 2	CHU da Universidade Federal do Pará - UFPA - PA
238	Afastamento Intrução Normativa DGP 03/2020. Art 4 2	CHU da Universidade Federal do Pará - UFPA - PA
238	Afastamento Intrução Normativa DGP 03/2020. Art 4 2	CHU da Universidade Federal do Pará - UFPA - PA
238	Afastamento Intrução Normativa DGP 03/2020. Art 4 2	CHU da Universidade Federal do Pará - UFPA - PA
238	Afastamento Intrução Normativa DGP 03/2020. Art 4 2	CHU da Universidade Federal do Pará - UFPA - PA
238	Afastamento Intrução Normativa DGP 03/2020. Art 4 2	CHU da Universidade Federal do Pará - UFPA - PA
238	Afastamento Intrução Normativa DGP 03/2020. Art 4 2	CHU da Universidade Federal do Pará - UFPA - PA
238	Afastamento Intrução Normativa DGP 03/2020. Art 4 2	CHU da Universidade Federal do Pará - UFPA - PA

238	Afastamento Intrução Normativa DGP 03/2020. Art 4 2	CHU da Universidade Federal do Pará - UFPA - PA
238	Afastamento Intrução Normativa DGP 03/2020. Art 4 2	CHU da Universidade Federal do Pará - UFPA - PA
238	Afastamento Intrução Normativa DGP 03/2020. Art 4 2	CHU da Universidade Federal do Pará - UFPA - PA
238	Afastamento Intrução Normativa DGP 03/2020. Art 4 2	CHU da Universidade Federal do Pará - UFPA - PA
238	Afastamento Intrução Normativa DGP 03/2020. Art 4 2	CHU da Universidade Federal do Pará - UFPA - PA
238	Afastamento Intrução Normativa DGP 03/2020. Art 4 2	CHU da Universidade Federal do Pará - UFPA - PA
3	LICENÇA PATERNIDADE - RJU - 20 DIAS - LEI 8.112/90 E DECRETO 8737/16	HC da Universidade Federal de Goiás - GO
4	LICENÇA MATERNIDADE - 120 DIAS	HC da Universidade Federal de Goiás - GO
4	LICENÇA MATERNIDADE - 120 DIAS	HC da Universidade Federal de Goiás - GO
4	LICENÇA MATERNIDADE - 120 DIAS	HC da Universidade Federal de Goiás - GO
4	LICENÇA MATERNIDADE - 120 DIAS	HC da Universidade Federal de Goiás - GO
4	LICENÇA MATERNIDADE - 120 DIAS	HC da Universidade Federal de Goiás - GO
4	LICENÇA MATERNIDADE - 120 DIAS	HC da Universidade Federal de Goiás - GO
4	LICENÇA MATERNIDADE - 120 DIAS	HC da Universidade Federal de Goiás - GO
4	LICENÇA MATERNIDADE - 120 DIAS	HC da Universidade Federal de Goiás - GO
4	LICENÇA MATERNIDADE - 120 DIAS	HC da Universidade Federal de Goiás - GO
4	LICENÇA MATERNIDADE - 120 DIAS	HC da Universidade Federal de Goiás - GO
4	LICENÇA MATERNIDADE - 120 DIAS	HC da Universidade Federal de Goiás - GO
4	LICENÇA MATERNIDADE - 120 DIAS	HC da Universidade Federal de Goiás - GO
4	LICENÇA MATERNIDADE - 120 DIAS	HC da Universidade Federal de Goiás - GO
4	LICENÇA MATERNIDADE - 120 DIAS	HC da Universidade Federal de Goiás - GO
4	LICENÇA MATERNIDADE - 120 DIAS	HC da Universidade Federal de Goiás - GO
5	LICENÇA POR MORTE DE FAMILIAR - CLT E REGULAMENTO DE PESSOAL	HC da Universidade Federal de Goiás - GO
5	LICENÇA POR MORTE DE FAMILIAR - CLT E REGULAMENTO DE PESSOAL	HC da Universidade Federal de Goiás - GO
5	LICENÇA POR MORTE DE FAMILIAR - CLT E REGULAMENTO DE PESSOAL	HC da Universidade Federal de Goiás - GO
5	LICENÇA POR MORTE DE FAMILIAR - CLT E REGULAMENTO DE PESSOAL	HC da Universidade Federal de Goiás - GO
5	LICENÇA POR MORTE DE FAMILIAR - CLT E REGULAMENTO DE PESSOAL	HC da Universidade Federal de Goiás - GO
5	LICENÇA POR MORTE DE FAMILIAR - CLT E REGULAMENTO DE PESSOAL	HC da Universidade Federal de Goiás - GO
5	LICENÇA POR MORTE DE FAMILIAR - CLT E REGULAMENTO DE PESSOAL	HC da Universidade Federal de Goiás - GO
5	LICENÇA POR MORTE DE FAMILIAR - CLT E REGULAMENTO DE PESSOAL	HC da Universidade Federal de Goiás - GO
5	LICENÇA POR MORTE DE FAMILIAR - CLT E REGULAMENTO DE PESSOAL	HC da Universidade Federal de Goiás - GO
5	LICENÇA POR MORTE DE FAMILIAR - CLT E REGULAMENTO DE PESSOAL	HC da Universidade Federal de Goiás - GO
5	LICENÇA POR MORTE DE FAMILIAR - CLT E REGULAMENTO DE PESSOAL	HC da Universidade Federal de Goiás - GO
5	LICENÇA POR MORTE DE FAMILIAR - CLT E REGULAMENTO DE PESSOAL	HC da Universidade Federal de Goiás - GO
5	LICENÇA POR MORTE DE FAMILIAR - CLT E REGULAMENTO DE PESSOAL	HC da Universidade Federal de Goiás - GO
5	LICENÇA POR MORTE DE FAMILIAR - CLT E REGULAMENTO DE PESSOAL	HC da Universidade Federal de Goiás - GO
5	LICENÇA POR MORTE DE FAMILIAR - CLT E REGULAMENTO DE PESSOAL	HC da Universidade Federal de Goiás - GO
5	LICENÇA POR MORTE DE FAMILIAR - CLT E REGULAMENTO DE PESSOAL	HC da Universidade Federal de Goiás - GO

5	LICENÇA POR MORTE DE FAMILIAR - CLT E REGULAMENTO DE PESSOAL	HC da Universidade Federal de Goiás - GO
5	LICENÇA POR MORTE DE FAMILIAR - CLT E REGULAMENTO DE PESSOAL	HC da Universidade Federal de Goiás - GO
5	LICENÇA POR MORTE DE FAMILIAR - CLT E REGULAMENTO DE PESSOAL	HC da Universidade Federal de Goiás - GO
5	LICENÇA POR MORTE DE FAMILIAR - CLT E REGULAMENTO DE PESSOAL	HC da Universidade Federal de Goiás - GO
6	LICENÇA INTERESSE PARTICULAR - REGULAMENTO DE PESSOAL ART 35 VII	HC da Universidade Federal de Goiás - GO
6	LICENÇA INTERESSE PARTICULAR - REGULAMENTO DE PESSOAL ART 35 VII	HC da Universidade Federal de Goiás - GO
6	LICENÇA INTERESSE PARTICULAR - REGULAMENTO DE PESSOAL ART 35 VII	HC da Universidade Federal de Goiás - GO
6	LICENÇA INTERESSE PARTICULAR - REGULAMENTO DE PESSOAL ART 35 VII	HC da Universidade Federal de Goiás - GO
12	LICENÇA MATERNIDADE (COMPLEMENTAR 60 DIAS)	HC da Universidade Federal de Goiás - GO
12	LICENÇA MATERNIDADE (COMPLEMENTAR 60 DIAS)	HC da Universidade Federal de Goiás - GO
12	LICENÇA MATERNIDADE (COMPLEMENTAR 60 DIAS)	HC da Universidade Federal de Goiás - GO
12	LICENÇA MATERNIDADE (COMPLEMENTAR 60 DIAS)	HC da Universidade Federal de Goiás - GO
12	LICENÇA MATERNIDADE (COMPLEMENTAR 60 DIAS)	HC da Universidade Federal de Goiás - GO
12	LICENÇA MATERNIDADE (COMPLEMENTAR 60 DIAS)	HC da Universidade Federal de Goiás - GO
12	LICENÇA MATERNIDADE (COMPLEMENTAR 60 DIAS)	HC da Universidade Federal de Goiás - GO
12	LICENÇA MATERNIDADE (COMPLEMENTAR 60 DIAS)	HC da Universidade Federal de Goiás - GO
12	LICENÇA MATERNIDADE (COMPLEMENTAR 60 DIAS)	HC da Universidade Federal de Goiás - GO
12	LICENÇA MATERNIDADE (COMPLEMENTAR 60 DIAS)	HC da Universidade Federal de Goiás - GO
12	LICENÇA MATERNIDADE (COMPLEMENTAR 60 DIAS)	HC da Universidade Federal de Goiás - GO
12	LICENÇA MATERNIDADE (COMPLEMENTAR 60 DIAS)	HC da Universidade Federal de Goiás - GO
12	LICENÇA MATERNIDADE (COMPLEMENTAR 60 DIAS)	HC da Universidade Federal de Goiás - GO
12	LICENÇA MATERNIDADE (COMPLEMENTAR 60 DIAS)	HC da Universidade Federal de Goiás - GO
12	LICENÇA MATERNIDADE (COMPLEMENTAR 60 DIAS)	HC da Universidade Federal de Goiás - GO
14	LICENÇA GALA - CLT ART 473 II	HC da Universidade Federal de Goiás - GO
14	LICENÇA GALA - CLT ART 473 II	HC da Universidade Federal de Goiás - GO
14	LICENÇA GALA - CLT ART 473 II	HC da Universidade Federal de Goiás - GO
14	LICENÇA GALA - CLT ART 473 II	HC da Universidade Federal de Goiás - GO
14	LICENÇA GALA - CLT ART 473 II	HC da Universidade Federal de Goiás - GO
14	LICENÇA GALA - CLT ART 473 II	HC da Universidade Federal de Goiás - GO
14	LICENÇA GALA - CLT ART 473 II	HC da Universidade Federal de Goiás - GO
14	LICENÇA GALA - CLT ART 473 II	HC da Universidade Federal de Goiás - GO
23	CAPACITAÇAO/EVENTO POR INTERESSE DA EMPRESA - ATE 30 DIAS	HC da Universidade Federal de Goiás - GO
23	CAPACITAÇAO/EVENTO POR INTERESSE DA EMPRESA - ATE 30 DIAS	HC da Universidade Federal de Goiás - GO
23	CAPACITAÇAO/EVENTO POR INTERESSE DA EMPRESA - ATE 30 DIAS	HC da Universidade Federal de Goiás - GO
23	CAPACITAÇAO/EVENTO POR INTERESSE DA EMPRESA - ATE 30 DIAS	HC da Universidade Federal de Goiás - GO
23	CAPACITAÇAO/EVENTO POR INTERESSE DA EMPRESA - ATE 30 DIAS	HC da Universidade Federal de Goiás - GO
23	CAPACITAÇAO/EVENTO POR INTERESSE DA EMPRESA - ATE 30 DIAS	HC da Universidade Federal de Goiás - GO
23	CAPACITAÇAO/EVENTO POR INTERESSE DA EMPRESA - ATE 30 DIAS	HC da Universidade Federal de Goiás - GO

23	CAPACITAÇAO/EVENTO POR INTERESSE DA EMPRESA - ATE 30 DIAS	HC da Universidade Federal de Goiás - GO
23	CAPACITAÇAO/EVENTO POR INTERESSE DA EMPRESA - ATE 30 DIAS	HC da Universidade Federal de Goiás - GO
23	CAPACITAÇAO/EVENTO POR INTERESSE DA EMPRESA - ATE 30 DIAS	HC da Universidade Federal de Goiás - GO
23	CAPACITAÇAO/EVENTO POR INTERESSE DA EMPRESA - ATE 30 DIAS	HC da Universidade Federal de Goiás - GO
23	CAPACITAÇAO/EVENTO POR INTERESSE DA EMPRESA - ATE 30 DIAS	HC da Universidade Federal de Goiás - GO
23	CAPACITAÇAO/EVENTO POR INTERESSE DA EMPRESA - ATE 30 DIAS	HC da Universidade Federal de Goiás - GO
23	CAPACITAÇAO/EVENTO POR INTERESSE DA EMPRESA - ATE 30 DIAS	HC da Universidade Federal de Goiás - GO
23	CAPACITAÇAO/EVENTO POR INTERESSE DA EMPRESA - ATE 30 DIAS	HC da Universidade Federal de Goiás - GO
23	CAPACITAÇAO/EVENTO POR INTERESSE DA EMPRESA - ATE 30 DIAS	HC da Universidade Federal de Goiás - GO
23	CAPACITAÇAO/EVENTO POR INTERESSE DA EMPRESA - ATE 30 DIAS	HC da Universidade Federal de Goiás - GO
23	CAPACITAÇAO/EVENTO POR INTERESSE DA EMPRESA - ATE 30 DIAS	HC da Universidade Federal de Goiás - GO
23	CAPACITAÇAO/EVENTO POR INTERESSE DA EMPRESA - ATE 30 DIAS	HC da Universidade Federal de Goiás - GO
23	CAPACITAÇAO/EVENTO POR INTERESSE DA EMPRESA - ATE 30 DIAS	HC da Universidade Federal de Goiás - GO
23	CAPACITAÇAO/EVENTO POR INTERESSE DA EMPRESA - ATE 30 DIAS	HC da Universidade Federal de Goiás - GO
23	CAPACITAÇAO/EVENTO POR INTERESSE DA EMPRESA - ATE 30 DIAS	HC da Universidade Federal de Goiás - GO
23	CAPACITAÇAO/EVENTO POR INTERESSE DA EMPRESA - ATE 30 DIAS	HC da Universidade Federal de Goiás - GO
23	CAPACITAÇAO/EVENTO POR INTERESSE DA EMPRESA - ATE 30 DIAS	HC da Universidade Federal de Goiás - GO
23	CAPACITAÇAO/EVENTO POR INTERESSE DA EMPRESA - ATE 30 DIAS	HC da Universidade Federal de Goiás - GO
23	CAPACITAÇAO/EVENTO POR INTERESSE DA EMPRESA - ATE 30 DIAS	HC da Universidade Federal de Goiás - GO
23	CAPACITAÇAO/EVENTO POR INTERESSE DA EMPRESA - ATE 30 DIAS	HC da Universidade Federal de Goiás - GO
23	CAPACITAÇAO/EVENTO POR INTERESSE DA EMPRESA - ATE 30 DIAS	HC da Universidade Federal de Goiás - GO
23	CAPACITAÇAO/EVENTO POR INTERESSE DA EMPRESA - ATE 30 DIAS	HC da Universidade Federal de Goiás - GO
23	CAPACITAÇAO/EVENTO POR INTERESSE DA EMPRESA - ATE 30 DIAS	HC da Universidade Federal de Goiás - GO
23	CAPACITAÇAO/EVENTO POR INTERESSE DA EMPRESA - ATE 30 DIAS	HC da Universidade Federal de Goiás - GO
23	CAPACITAÇAO/EVENTO POR INTERESSE DA EMPRESA - ATE 30 DIAS	HC da Universidade Federal de Goiás - GO
23	CAPACITAÇAO/EVENTO POR INTERESSE DA EMPRESA - ATE 30 DIAS	HC da Universidade Federal de Goiás - GO
23	CAPACITAÇAO/EVENTO POR INTERESSE DA EMPRESA - ATE 30 DIAS	HC da Universidade Federal de Goiás - GO
23	CAPACITAÇAO/EVENTO POR INTERESSE DA EMPRESA - ATE 30 DIAS	HC da Universidade Federal de Goiás - GO
23	CAPACITAÇAO/EVENTO POR INTERESSE DA EMPRESA - ATE 30 DIAS	HC da Universidade Federal de Goiás - GO
23	CAPACITAÇAO/EVENTO POR INTERESSE DA EMPRESA - ATE 30 DIAS	HC da Universidade Federal de Goiás - GO
23	CAPACITAÇAO/EVENTO POR INTERESSE DA EMPRESA - ATE 30 DIAS	HC da Universidade Federal de Goiás - GO
23	CAPACITAÇAO/EVENTO POR INTERESSE DA EMPRESA - ATE 30 DIAS	HC da Universidade Federal de Goiás - GO

23	CAPACITAÇAO/EVENTO POR INTERESSE DA EMPRESA - ATE 30 DIAS	HC da Universidade Federal de Goiás - GO
23	CAPACITAÇAO/EVENTO POR INTERESSE DA EMPRESA - ATE 30 DIAS	HC da Universidade Federal de Goiás - GO
23	CAPACITAÇAO/EVENTO POR INTERESSE DA EMPRESA - ATE 30 DIAS	HC da Universidade Federal de Goiás - GO
23	CAPACITAÇAO/EVENTO POR INTERESSE DA EMPRESA - ATE 30 DIAS	HC da Universidade Federal de Goiás - GO
23	CAPACITAÇAO/EVENTO POR INTERESSE DA EMPRESA - ATE 30 DIAS	HC da Universidade Federal de Goiás - GO
23	CAPACITAÇAO/EVENTO POR INTERESSE DA EMPRESA - ATE 30 DIAS	HC da Universidade Federal de Goiás - GO
23	CAPACITAÇAO/EVENTO POR INTERESSE DA EMPRESA - ATE 30 DIAS	HC da Universidade Federal de Goiás - GO
23	CAPACITAÇAO/EVENTO POR INTERESSE DA EMPRESA - ATE 30 DIAS	HC da Universidade Federal de Goiás - GO
23	CAPACITAÇAO/EVENTO POR INTERESSE DA EMPRESA - ATE 30 DIAS	HC da Universidade Federal de Goiás - GO
23	CAPACITAÇAO/EVENTO POR INTERESSE DA EMPRESA - ATE 30 DIAS	HC da Universidade Federal de Goiás - GO
23	CAPACITAÇAO/EVENTO POR INTERESSE DA EMPRESA - ATE 30 DIAS	HC da Universidade Federal de Goiás - GO
23	CAPACITAÇAO/EVENTO POR INTERESSE DA EMPRESA - ATE 30 DIAS	HC da Universidade Federal de Goiás - GO
23	CAPACITAÇAO/EVENTO POR INTERESSE DA EMPRESA - ATE 30 DIAS	HC da Universidade Federal de Goiás - GO
23	CAPACITAÇAO/EVENTO POR INTERESSE DA EMPRESA - ATE 30 DIAS	HC da Universidade Federal de Goiás - GO
36	AUXILIO DOENÇA (INSS) - A PARTIR DO 16 DIA	HC da Universidade Federal de Goiás - GO
36	AUXILIO DOENÇA (INSS) - A PARTIR DO 16 DIA	HC da Universidade Federal de Goiás - GO
36	AUXILIO DOENÇA (INSS) - A PARTIR DO 16 DIA	HC da Universidade Federal de Goiás - GO
36	AUXILIO DOENÇA (INSS) - A PARTIR DO 16 DIA	HC da Universidade Federal de Goiás - GO
36	AUXILIO DOENÇA (INSS) - A PARTIR DO 16 DIA	HC da Universidade Federal de Goiás - GO
36	AUXILIO DOENÇA (INSS) - A PARTIR DO 16 DIA	HC da Universidade Federal de Goiás - GO
36	AUXILIO DOENÇA (INSS) - A PARTIR DO 16 DIA	HC da Universidade Federal de Goiás - GO
36	AUXILIO DOENÇA (INSS) - A PARTIR DO 16 DIA	HC da Universidade Federal de Goiás - GO
36	AUXILIO DOENÇA (INSS) - A PARTIR DO 16 DIA	HC da Universidade Federal de Goiás - GO
36	AUXILIO DOENÇA (INSS) - A PARTIR DO 16 DIA	HC da Universidade Federal de Goiás - GO
36	AUXILIO DOENÇA (INSS) - A PARTIR DO 16 DIA	HC da Universidade Federal de Goiás - GO
36	AUXILIO DOENÇA (INSS) - A PARTIR DO 16 DIA	HC da Universidade Federal de Goiás - GO
36	AUXILIO DOENÇA (INSS) - A PARTIR DO 16 DIA	HC da Universidade Federal de Goiás - GO
36	AUXILIO DOENÇA (INSS) - A PARTIR DO 16 DIA	HC da Universidade Federal de Goiás - GO
36	AUXILIO DOENÇA (INSS) - A PARTIR DO 16 DIA	HC da Universidade Federal de Goiás - GO
36	AUXILIO DOENÇA (INSS) - A PARTIR DO 16 DIA	HC da Universidade Federal de Goiás - GO
36	AUXILIO DOENÇA (INSS) - A PARTIR DO 16 DIA	HC da Universidade Federal de Goiás - GO
36	AUXILIO DOENÇA (INSS) - A PARTIR DO 16 DIA	HC da Universidade Federal de Goiás - GO
36	AUXILIO DOENÇA (INSS) - A PARTIR DO 16 DIA	HC da Universidade Federal de Goiás - GO
36	AUXILIO DOENÇA (INSS) - A PARTIR DO 16 DIA	HC da Universidade Federal de Goiás - GO
36	AUXILIO DOENÇA (INSS) - A PARTIR DO 16 DIA	HC da Universidade Federal de Goiás - GO
36	AUXILIO DOENÇA (INSS) - A PARTIR DO 16 DIA	HC da Universidade Federal de Goiás - GO
36	AUXILIO DOENÇA (INSS) - A PARTIR DO 16 DIA	HC da Universidade Federal de Goiás - GO
36	AUXILIO DOENÇA (INSS) - A PARTIR DO 16 DIA	HC da Universidade Federal de Goiás - GO
36	AUXILIO DOENÇA (INSS) - A PARTIR DO 16 DIA	HC da Universidade Federal de Goiás - GO

36	AUXILIO DOENÇA (INSS) - A PARTIR DO 16 DIA	HC da Universidade Federal de Goiás - GO
36	AUXILIO DOENÇA (INSS) - A PARTIR DO 16 DIA	HC da Universidade Federal de Goiás - GO
36	AUXILIO DOENÇA (INSS) - A PARTIR DO 16 DIA	HC da Universidade Federal de Goiás - GO
36	AUXILIO DOENÇA (INSS) - A PARTIR DO 16 DIA	HC da Universidade Federal de Goiás - GO
36	AUXILIO DOENÇA (INSS) - A PARTIR DO 16 DIA	HC da Universidade Federal de Goiás - GO
36	AUXILIO DOENÇA (INSS) - A PARTIR DO 16 DIA	HC da Universidade Federal de Goiás - GO
36	AUXILIO DOENÇA (INSS) - A PARTIR DO 16 DIA	HC da Universidade Federal de Goiás - GO
36	AUXILIO DOENÇA (INSS) - A PARTIR DO 16 DIA	HC da Universidade Federal de Goiás - GO
36	AUXILIO DOENÇA (INSS) - A PARTIR DO 16 DIA	HC da Universidade Federal de Goiás - GO
36	AUXILIO DOENÇA (INSS) - A PARTIR DO 16 DIA	HC da Universidade Federal de Goiás - GO
36	AUXILIO DOENÇA (INSS) - A PARTIR DO 16 DIA	HC da Universidade Federal de Goiás - GO
36	AUXILIO DOENÇA (INSS) - A PARTIR DO 16 DIA	HC da Universidade Federal de Goiás - GO
42	DOAÇÃO DE SANGUE - REGULAMENTO DE PESSOAL ART 36 I	HC da Universidade Federal de Goiás - GO
42	DOAÇÃO DE SANGUE - REGULAMENTO DE PESSOAL ART 36 I	HC da Universidade Federal de Goiás - GO
42	DOAÇÃO DE SANGUE - REGULAMENTO DE PESSOAL ART 36 I	HC da Universidade Federal de Goiás - GO
42	DOAÇÃO DE SANGUE - REGULAMENTO DE PESSOAL ART 36 I	HC da Universidade Federal de Goiás - GO
42	DOAÇÃO DE SANGUE - REGULAMENTO DE PESSOAL ART 36 I	HC da Universidade Federal de Goiás - GO
42	DOAÇÃO DE SANGUE - REGULAMENTO DE PESSOAL ART 36 I	HC da Universidade Federal de Goiás - GO
42	DOAÇÃO DE SANGUE - REGULAMENTO DE PESSOAL ART 36 I	HC da Universidade Federal de Goiás - GO
42	DOAÇÃO DE SANGUE - REGULAMENTO DE PESSOAL ART 36 I	HC da Universidade Federal de Goiás - GO
42	DOAÇÃO DE SANGUE - REGULAMENTO DE PESSOAL ART 36 I	HC da Universidade Federal de Goiás - GO
42	DOAÇÃO DE SANGUE - REGULAMENTO DE PESSOAL ART 36 I	HC da Universidade Federal de Goiás - GO
42	DOAÇÃO DE SANGUE - REGULAMENTO DE PESSOAL ART 36 I	HC da Universidade Federal de Goiás - GO
42	DOAÇÃO DE SANGUE - REGULAMENTO DE PESSOAL ART 36 I	HC da Universidade Federal de Goiás - GO
42	DOAÇÃO DE SANGUE - REGULAMENTO DE PESSOAL ART 36 I	HC da Universidade Federal de Goiás - GO
42	DOAÇÃO DE SANGUE - REGULAMENTO DE PESSOAL ART 36 I	HC da Universidade Federal de Goiás - GO
42	DOAÇÃO DE SANGUE - REGULAMENTO DE PESSOAL ART 36 I	HC da Universidade Federal de Goiás - GO
42	DOAÇÃO DE SANGUE - REGULAMENTO DE PESSOAL ART 36 I	HC da Universidade Federal de Goiás - GO
42	DOAÇÃO DE SANGUE - REGULAMENTO DE PESSOAL ART 36 I	HC da Universidade Federal de Goiás - GO
42	DOAÇÃO DE SANGUE - REGULAMENTO DE PESSOAL ART 36 I	HC da Universidade Federal de Goiás - GO
42	DOAÇÃO DE SANGUE - REGULAMENTO DE PESSOAL ART 36 I	HC da Universidade Federal de Goiás - GO
42	DOAÇÃO DE SANGUE - REGULAMENTO DE PESSOAL ART 36 I	HC da Universidade Federal de Goiás - GO
42	DOAÇÃO DE SANGUE - REGULAMENTO DE PESSOAL ART 36 I	HC da Universidade Federal de Goiás - GO
42	DOAÇÃO DE SANGUE - REGULAMENTO DE PESSOAL ART 36 I	HC da Universidade Federal de Goiás - GO
42	DOAÇÃO DE SANGUE - REGULAMENTO DE PESSOAL ART 36 I	HC da Universidade Federal de Goiás - GO
42	DOAÇÃO DE SANGUE - REGULAMENTO DE PESSOAL ART 36 I	HC da Universidade Federal de Goiás - GO
45	FALTA INJUSTIFICADA (GERA DESCONTO NA FOLHA)	HC da Universidade Federal de Goiás - GO
45	FALTA INJUSTIFICADA (GERA DESCONTO NA FOLHA)	HC da Universidade Federal de Goiás - GO
45	FALTA INJUSTIFICADA (GERA DESCONTO NA FOLHA)	HC da Universidade Federal de Goiás - GO
45	FALTA INJUSTIFICADA (GERA DESCONTO NA FOLHA)	HC da Universidade Federal de Goiás - GO
45	FALTA INJUSTIFICADA (GERA DESCONTO NA FOLHA)	HC da Universidade Federal de Goiás - GO
45	FALTA INJUSTIFICADA (GERA DESCONTO NA FOLHA)	HC da Universidade Federal de Goiás - GO
45	FALTA INJUSTIFICADA (GERA DESCONTO NA FOLHA)	HC da Universidade Federal de Goiás - GO
45	FALTA INJUSTIFICADA (GERA DESCONTO NA FOLHA)	HC da Universidade Federal de Goiás - GO

45	FALTA INJUSTIFICADA (GERA DESCONTO NA FOLHA)	HC da Universidade Federal de Goiás - GO
45	FALTA INJUSTIFICADA (GERA DESCONTO NA FOLHA)	HC da Universidade Federal de Goiás - GO
45	FALTA INJUSTIFICADA (GERA DESCONTO NA FOLHA)	HC da Universidade Federal de Goiás - GO
45	FALTA INJUSTIFICADA (GERA DESCONTO NA FOLHA)	HC da Universidade Federal de Goiás - GO
45	FALTA INJUSTIFICADA (GERA DESCONTO NA FOLHA)	HC da Universidade Federal de Goiás - GO
45	FALTA INJUSTIFICADA (GERA DESCONTO NA FOLHA)	HC da Universidade Federal de Goiás - GO
45	FALTA INJUSTIFICADA (GERA DESCONTO NA FOLHA)	HC da Universidade Federal de Goiás - GO
45	FALTA INJUSTIFICADA (GERA DESCONTO NA FOLHA)	HC da Universidade Federal de Goiás - GO
45	FALTA INJUSTIFICADA (GERA DESCONTO NA FOLHA)	HC da Universidade Federal de Goiás - GO
45	FALTA INJUSTIFICADA (GERA DESCONTO NA FOLHA)	HC da Universidade Federal de Goiás - GO
45	FALTA INJUSTIFICADA (GERA DESCONTO NA FOLHA)	HC da Universidade Federal de Goiás - GO
45	FALTA INJUSTIFICADA (GERA DESCONTO NA FOLHA)	HC da Universidade Federal de Goiás - GO
45	FALTA INJUSTIFICADA (GERA DESCONTO NA FOLHA)	HC da Universidade Federal de Goiás - GO
45	FALTA INJUSTIFICADA (GERA DESCONTO NA FOLHA)	HC da Universidade Federal de Goiás - GO
45	FALTA INJUSTIFICADA (GERA DESCONTO NA FOLHA)	HC da Universidade Federal de Goiás - GO
45	FALTA INJUSTIFICADA (GERA DESCONTO NA FOLHA)	HC da Universidade Federal de Goiás - GO
45	FALTA INJUSTIFICADA (GERA DESCONTO NA FOLHA)	HC da Universidade Federal de Goiás - GO
45	FALTA INJUSTIFICADA (GERA DESCONTO NA FOLHA)	HC da Universidade Federal de Goiás - GO
45	FALTA INJUSTIFICADA (GERA DESCONTO NA FOLHA)	HC da Universidade Federal de Goiás - GO
45	FALTA INJUSTIFICADA (GERA DESCONTO NA FOLHA)	HC da Universidade Federal de Goiás - GO
45	FALTA INJUSTIFICADA (GERA DESCONTO NA FOLHA)	HC da Universidade Federal de Goiás - GO
45	FALTA INJUSTIFICADA (GERA DESCONTO NA FOLHA)	HC da Universidade Federal de Goiás - GO
45	FALTA INJUSTIFICADA (GERA DESCONTO NA FOLHA)	HC da Universidade Federal de Goiás - GO
45	FALTA INJUSTIFICADA (GERA DESCONTO NA FOLHA)	HC da Universidade Federal de Goiás - GO
45	FALTA INJUSTIFICADA (GERA DESCONTO NA FOLHA)	HC da Universidade Federal de Goiás - GO
45	FALTA INJUSTIFICADA (GERA DESCONTO NA FOLHA)	HC da Universidade Federal de Goiás - GO
45	FALTA INJUSTIFICADA (GERA DESCONTO NA FOLHA)	HC da Universidade Federal de Goiás - GO
45	FALTA INJUSTIFICADA (GERA DESCONTO NA FOLHA)	HC da Universidade Federal de Goiás - GO
45	FALTA INJUSTIFICADA (GERA DESCONTO NA FOLHA)	HC da Universidade Federal de Goiás - GO
45	FALTA INJUSTIFICADA (GERA DESCONTO NA FOLHA)	HC da Universidade Federal de Goiás - GO
45	FALTA INJUSTIFICADA (GERA DESCONTO NA FOLHA)	HC da Universidade Federal de Goiás - GO
51	A SERVIÇO DA JUSTIÇA ELEITORAL - LEI 9.504 ART. 98	HC da Universidade Federal de Goiás - GO
51	A SERVIÇO DA JUSTIÇA ELEITORAL - LEI 9.504 ART. 98	HC da Universidade Federal de Goiás - GO
51	A SERVIÇO DA JUSTIÇA ELEITORAL - LEI 9.504 ART. 98	HC da Universidade Federal de Goiás - GO
51	A SERVIÇO DA JUSTIÇA ELEITORAL - LEI 9.504 ART. 98	HC da Universidade Federal de Goiás - GO
51	A SERVIÇO DA JUSTIÇA ELEITORAL - LEI 9.504 ART. 98	HC da Universidade Federal de Goiás - GO
51	A SERVIÇO DA JUSTIÇA ELEITORAL - LEI 9.504 ART. 98	HC da Universidade Federal de Goiás - GO
51	A SERVIÇO DA JUSTIÇA ELEITORAL - LEI 9.504 ART. 98	HC da Universidade Federal de Goiás - GO
51	A SERVIÇO DA JUSTIÇA ELEITORAL - LEI 9.504 ART. 98	HC da Universidade Federal de Goiás - GO
51	A SERVIÇO DA JUSTIÇA ELEITORAL - LEI 9.504 ART. 98	HC da Universidade Federal de Goiás - GO
51	A SERVIÇO DA JUSTIÇA ELEITORAL - LEI 9.504 ART. 98	HC da Universidade Federal de Goiás - GO
51	A SERVIÇO DA JUSTIÇA ELEITORAL - LEI 9.504 ART. 98	HC da Universidade Federal de Goiás - GO
51	A SERVIÇO DA JUSTIÇA ELEITORAL - LEI 9.504 ART. 98	HC da Universidade Federal de Goiás - GO
51	A SERVIÇO DA JUSTIÇA ELEITORAL - LEI 9.504 ART. 98	HC da Universidade Federal de Goiás - GO
51	A SERVIÇO DA JUSTIÇA ELEITORAL - LEI 9.504 ART. 98	HC da Universidade Federal de Goiás - GO
51	A SERVIÇO DA JUSTIÇA ELEITORAL - LEI 9.504 ART. 98	HC da Universidade Federal de Goiás - GO

57	AFASTAMENTO POR ATESTADO MÉDICO - ATE 15 DIAS	HC da Universidade Federal de Goiás - GO
57	AFASTAMENTO POR ATESTADO MÉDICO - ATE 15 DIAS	HC da Universidade Federal de Goiás - GO
57	AFASTAMENTO POR ATESTADO MÉDICO - ATE 15 DIAS	HC da Universidade Federal de Goiás - GO
57	AFASTAMENTO POR ATESTADO MÉDICO - ATE 15 DIAS	HC da Universidade Federal de Goiás - GO
57	AFASTAMENTO POR ATESTADO MÉDICO - ATE 15 DIAS	HC da Universidade Federal de Goiás - GO
57	AFASTAMENTO POR ATESTADO MÉDICO - ATE 15 DIAS	HC da Universidade Federal de Goiás - GO
57	AFASTAMENTO POR ATESTADO MÉDICO - ATE 15 DIAS	HC da Universidade Federal de Goiás - GO
57	AFASTAMENTO POR ATESTADO MÉDICO - ATE 15 DIAS	HC da Universidade Federal de Goiás - GO
57	AFASTAMENTO POR ATESTADO MÉDICO - ATE 15 DIAS	HC da Universidade Federal de Goiás - GO
57	AFASTAMENTO POR ATESTADO MÉDICO - ATE 15 DIAS	HC da Universidade Federal de Goiás - GO
57	AFASTAMENTO POR ATESTADO MÉDICO - ATE 15 DIAS	HC da Universidade Federal de Goiás - GO
57	AFASTAMENTO POR ATESTADO MÉDICO - ATE 15 DIAS	HC da Universidade Federal de Goiás - GO
57	AFASTAMENTO POR ATESTADO MÉDICO - ATE 15 DIAS	HC da Universidade Federal de Goiás - GO
57	AFASTAMENTO POR ATESTADO MÉDICO - ATE 15 DIAS	HC da Universidade Federal de Goiás - GO
57	AFASTAMENTO POR ATESTADO MÉDICO - ATE 15 DIAS	HC da Universidade Federal de Goiás - GO
57	AFASTAMENTO POR ATESTADO MÉDICO - ATE 15 DIAS	HC da Universidade Federal de Goiás - GO
57	AFASTAMENTO POR ATESTADO MÉDICO - ATE 15 DIAS	HC da Universidade Federal de Goiás - GO
57	AFASTAMENTO POR ATESTADO MÉDICO - ATE 15 DIAS	HC da Universidade Federal de Goiás - GO
57	AFASTAMENTO POR ATESTADO MÉDICO - ATE 15 DIAS	HC da Universidade Federal de Goiás - GO
57	AFASTAMENTO POR ATESTADO MÉDICO - ATE 15 DIAS	HC da Universidade Federal de Goiás - GO
57	AFASTAMENTO POR ATESTADO MÉDICO - ATE 15 DIAS	HC da Universidade Federal de Goiás - GO
57	AFASTAMENTO POR ATESTADO MÉDICO - ATE 15 DIAS	HC da Universidade Federal de Goiás - GO
57	AFASTAMENTO POR ATESTADO MÉDICO - ATE 15 DIAS	HC da Universidade Federal de Goiás - GO
57	AFASTAMENTO POR ATESTADO MÉDICO - ATE 15 DIAS	HC da Universidade Federal de Goiás - GO
57	AFASTAMENTO POR ATESTADO MÉDICO - ATE 15 DIAS	HC da Universidade Federal de Goiás - GO
57	AFASTAMENTO POR ATESTADO MÉDICO - ATE 15 DIAS	HC da Universidade Federal de Goiás - GO
57	AFASTAMENTO POR ATESTADO MÉDICO - ATE 15 DIAS	HC da Universidade Federal de Goiás - GO
57	AFASTAMENTO POR ATESTADO MÉDICO - ATE 15 DIAS	HC da Universidade Federal de Goiás - GO
57	AFASTAMENTO POR ATESTADO MÉDICO - ATE 15 DIAS	HC da Universidade Federal de Goiás - GO
57	AFASTAMENTO POR ATESTADO MÉDICO - ATE 15 DIAS	HC da Universidade Federal de Goiás - GO
57	AFASTAMENTO POR ATESTADO MÉDICO - ATE 15 DIAS	HC da Universidade Federal de Goiás - GO
57	AFASTAMENTO POR ATESTADO MÉDICO - ATE 15 DIAS	HC da Universidade Federal de Goiás - GO
57	AFASTAMENTO POR ATESTADO MÉDICO - ATE 15 DIAS	HC da Universidade Federal de Goiás - GO
57	AFASTAMENTO POR ATESTADO MÉDICO - ATE 15 DIAS	HC da Universidade Federal de Goiás - GO
57	AFASTAMENTO POR ATESTADO MÉDICO - ATE 15 DIAS	HC da Universidade Federal de Goiás - GO
57	AFASTAMENTO POR ATESTADO MÉDICO - ATE 15 DIAS	HC da Universidade Federal de Goiás - GO
57	AFASTAMENTO POR ATESTADO MÉDICO - ATE 15 DIAS	HC da Universidade Federal de Goiás - GO
57	AFASTAMENTO POR ATESTADO MÉDICO - ATE 15 DIAS	HC da Universidade Federal de Goiás - GO
57	AFASTAMENTO POR ATESTADO MÉDICO - ATE 15 DIAS	HC da Universidade Federal de Goiás - GO
57	AFASTAMENTO POR ATESTADO MÉDICO - ATE 15 DIAS	HC da Universidade Federal de Goiás - GO
57	AFASTAMENTO POR ATESTADO MÉDICO - ATE 15 DIAS	HC da Universidade Federal de Goiás - GO
57	AFASTAMENTO POR ATESTADO MÉDICO - ATE 15 DIAS	HC da Universidade Federal de Goiás - GO
57	AFASTAMENTO POR ATESTADO MÉDICO - ATE 15 DIAS	HC da Universidade Federal de Goiás - GO
57	AFASTAMENTO POR ATESTADO MÉDICO - ATE 15 DIAS	HC da Universidade Federal de Goiás - GO

57	AFASTAMENTO POR ATESTADO MÉDICO - ATE 15 DIAS	HC da Universidade Federal de Goiás - GO
57	AFASTAMENTO POR ATESTADO MÉDICO - ATE 15 DIAS	HC da Universidade Federal de Goiás - GO
57	AFASTAMENTO POR ATESTADO MÉDICO - ATE 15 DIAS	HC da Universidade Federal de Goiás - GO
57	AFASTAMENTO POR ATESTADO MÉDICO - ATE 15 DIAS	HC da Universidade Federal de Goiás - GO
57	AFASTAMENTO POR ATESTADO MÉDICO - ATE 15 DIAS	HC da Universidade Federal de Goiás - GO
57	AFASTAMENTO POR ATESTADO MÉDICO - ATE 15 DIAS	HC da Universidade Federal de Goiás - GO
57	AFASTAMENTO POR ATESTADO MÉDICO - ATE 15 DIAS	HC da Universidade Federal de Goiás - GO
57	AFASTAMENTO POR ATESTADO MÉDICO - ATE 15 DIAS	HC da Universidade Federal de Goiás - GO
57	AFASTAMENTO POR ATESTADO MÉDICO - ATE 15 DIAS	HC da Universidade Federal de Goiás - GO
57	AFASTAMENTO POR ATESTADO MÉDICO - ATE 15 DIAS	HC da Universidade Federal de Goiás - GO
57	AFASTAMENTO POR ATESTADO MÉDICO - ATE 15 DIAS	HC da Universidade Federal de Goiás - GO
57	AFASTAMENTO POR ATESTADO MÉDICO - ATE 15 DIAS	HC da Universidade Federal de Goiás - GO
57	AFASTAMENTO POR ATESTADO MÉDICO - ATE 15 DIAS	HC da Universidade Federal de Goiás - GO
57	AFASTAMENTO POR ATESTADO MÉDICO - ATE 15 DIAS	HC da Universidade Federal de Goiás - GO
57	AFASTAMENTO POR ATESTADO MÉDICO - ATE 15 DIAS	HC da Universidade Federal de Goiás - GO
57	AFASTAMENTO POR ATESTADO MÉDICO - ATE 15 DIAS	HC da Universidade Federal de Goiás - GO
57	AFASTAMENTO POR ATESTADO MÉDICO - ATE 15 DIAS	HC da Universidade Federal de Goiás - GO
57	AFASTAMENTO POR ATESTADO MÉDICO - ATE 15 DIAS	HC da Universidade Federal de Goiás - GO
57	AFASTAMENTO POR ATESTADO MÉDICO - ATE 15 DIAS	HC da Universidade Federal de Goiás - GO
57	AFASTAMENTO POR ATESTADO MÉDICO - ATE 15 DIAS	HC da Universidade Federal de Goiás - GO
57	AFASTAMENTO POR ATESTADO MÉDICO - ATE 15 DIAS	HC da Universidade Federal de Goiás - GO
57	AFASTAMENTO POR ATESTADO MÉDICO - ATE 15 DIAS	HC da Universidade Federal de Goiás - GO
57	AFASTAMENTO POR ATESTADO MÉDICO - ATE 15 DIAS	HC da Universidade Federal de Goiás - GO
57	AFASTAMENTO POR ATESTADO MÉDICO - ATE 15 DIAS	HC da Universidade Federal de Goiás - GO
57	AFASTAMENTO POR ATESTADO MÉDICO - ATE 15 DIAS	HC da Universidade Federal de Goiás - GO
57	AFASTAMENTO POR ATESTADO MÉDICO - ATE 15 DIAS	HC da Universidade Federal de Goiás - GO
57	AFASTAMENTO POR ATESTADO MÉDICO - ATE 15 DIAS	HC da Universidade Federal de Goiás - GO
57	AFASTAMENTO POR ATESTADO MÉDICO - ATE 15 DIAS	HC da Universidade Federal de Goiás - GO
57	AFASTAMENTO POR ATESTADO MÉDICO - ATE 15 DIAS	HC da Universidade Federal de Goiás - GO
57	AFASTAMENTO POR ATESTADO MÉDICO - ATE 15 DIAS	HC da Universidade Federal de Goiás - GO
57	AFASTAMENTO POR ATESTADO MÉDICO - ATE 15 DIAS	HC da Universidade Federal de Goiás - GO
57	AFASTAMENTO POR ATESTADO MÉDICO - ATE 15 DIAS	HC da Universidade Federal de Goiás - GO
57	AFASTAMENTO POR ATESTADO MÉDICO - ATE 15 DIAS	HC da Universidade Federal de Goiás - GO
57	AFASTAMENTO POR ATESTADO MÉDICO - ATE 15 DIAS	HC da Universidade Federal de Goiás - GO
57	AFASTAMENTO POR ATESTADO MÉDICO - ATE 15 DIAS	HC da Universidade Federal de Goiás - GO
57	AFASTAMENTO POR ATESTADO MÉDICO - ATE 15 DIAS	HC da Universidade Federal de Goiás - GO
57	AFASTAMENTO POR ATESTADO MÉDICO - ATE 15 DIAS	HC da Universidade Federal de Goiás - GO
57	AFASTAMENTO POR ATESTADO MÉDICO - ATE 15 DIAS	HC da Universidade Federal de Goiás - GO
57	AFASTAMENTO POR ATESTADO MÉDICO - ATE 15 DIAS	HC da Universidade Federal de Goiás - GO
57	AFASTAMENTO POR ATESTADO MÉDICO - ATE 15 DIAS	HC da Universidade Federal de Goiás - GO
57	AFASTAMENTO POR ATESTADO MÉDICO - ATE 15 DIAS	HC da Universidade Federal de Goiás - GO
57	AFASTAMENTO POR ATESTADO MÉDICO - ATE 15 DIAS	HC da Universidade Federal de Goiás - GO
57	AFASTAMENTO POR ATESTADO MÉDICO - ATE 15 DIAS	HC da Universidade Federal de Goiás - GO
57	AFASTAMENTO POR ATESTADO MÉDICO - ATE 15 DIAS	HC da Universidade Federal de Goiás - GO
57	AFASTAMENTO POR ATESTADO MÉDICO - ATE 15 DIAS	HC da Universidade Federal de Goiás - GO
57	AFASTAMENTO POR ATESTADO MÉDICO - ATE 15 DIAS	HC da Universidade Federal de Goiás - GO
57	AFASTAMENTO POR ATESTADO MÉDICO - ATE 15 DIAS	HC da Universidade Federal de Goiás - GO
57	AFASTAMENTO POR ATESTADO MÉDICO - ATE 15 DIAS	HC da Universidade Federal de Goiás - GO

57	AFASTAMENTO POR ATESTADO MÉDICO - ATE 15 DIAS	HC da Universidade Federal de Goiás - GO
57	AFASTAMENTO POR ATESTADO MÉDICO - ATE 15 DIAS	HC da Universidade Federal de Goiás - GO
57	AFASTAMENTO POR ATESTADO MÉDICO - ATE 15 DIAS	HC da Universidade Federal de Goiás - GO
57	AFASTAMENTO POR ATESTADO MÉDICO - ATE 15 DIAS	HC da Universidade Federal de Goiás - GO
57	AFASTAMENTO POR ATESTADO MÉDICO - ATE 15 DIAS	HC da Universidade Federal de Goiás - GO
57	AFASTAMENTO POR ATESTADO MÉDICO - ATE 15 DIAS	HC da Universidade Federal de Goiás - GO
57	AFASTAMENTO POR ATESTADO MÉDICO - ATE 15 DIAS	HC da Universidade Federal de Goiás - GO
57	AFASTAMENTO POR ATESTADO MÉDICO - ATE 15 DIAS	HC da Universidade Federal de Goiás - GO
57	AFASTAMENTO POR ATESTADO MÉDICO - ATE 15 DIAS	HC da Universidade Federal de Goiás - GO
57	AFASTAMENTO POR ATESTADO MÉDICO - ATE 15 DIAS	HC da Universidade Federal de Goiás - GO
57	AFASTAMENTO POR ATESTADO MÉDICO - ATE 15 DIAS	HC da Universidade Federal de Goiás - GO
57	AFASTAMENTO POR ATESTADO MÉDICO - ATE 15 DIAS	HC da Universidade Federal de Goiás - GO
57	AFASTAMENTO POR ATESTADO MÉDICO - ATE 15 DIAS	HC da Universidade Federal de Goiás - GO
57	AFASTAMENTO POR ATESTADO MÉDICO - ATE 15 DIAS	HC da Universidade Federal de Goiás - GO
57	AFASTAMENTO POR ATESTADO MÉDICO - ATE 15 DIAS	HC da Universidade Federal de Goiás - GO
57	AFASTAMENTO POR ATESTADO MÉDICO - ATE 15 DIAS	HC da Universidade Federal de Goiás - GO
57	AFASTAMENTO POR ATESTADO MÉDICO - ATE 15 DIAS	HC da Universidade Federal de Goiás - GO
57	AFASTAMENTO POR ATESTADO MÉDICO - ATE 15 DIAS	HC da Universidade Federal de Goiás - GO
57	AFASTAMENTO POR ATESTADO MÉDICO - ATE 15 DIAS	HC da Universidade Federal de Goiás - GO
57	AFASTAMENTO POR ATESTADO MÉDICO - ATE 15 DIAS	HC da Universidade Federal de Goiás - GO
57	AFASTAMENTO POR ATESTADO MÉDICO - ATE 15 DIAS	HC da Universidade Federal de Goiás - GO
57	AFASTAMENTO POR ATESTADO MÉDICO - ATE 15 DIAS	HC da Universidade Federal de Goiás - GO
57	AFASTAMENTO POR ATESTADO MÉDICO - ATE 15 DIAS	HC da Universidade Federal de Goiás - GO
57	AFASTAMENTO POR ATESTADO MÉDICO - ATE 15 DIAS	HC da Universidade Federal de Goiás - GO
57	AFASTAMENTO POR ATESTADO MÉDICO - ATE 15 DIAS	HC da Universidade Federal de Goiás - GO
57	AFASTAMENTO POR ATESTADO MÉDICO - ATE 15 DIAS	HC da Universidade Federal de Goiás - GO
57	AFASTAMENTO POR ATESTADO MÉDICO - ATE 15 DIAS	HC da Universidade Federal de Goiás - GO
57	AFASTAMENTO POR ATESTADO MÉDICO - ATE 15 DIAS	HC da Universidade Federal de Goiás - GO
57	AFASTAMENTO POR ATESTADO MÉDICO - ATE 15 DIAS	HC da Universidade Federal de Goiás - GO
57	AFASTAMENTO POR ATESTADO MÉDICO - ATE 15 DIAS	HC da Universidade Federal de Goiás - GO
57	AFASTAMENTO POR ATESTADO MÉDICO - ATE 15 DIAS	HC da Universidade Federal de Goiás - GO
57	AFASTAMENTO POR ATESTADO MÉDICO - ATE 15 DIAS	HC da Universidade Federal de Goiás - GO
57	AFASTAMENTO POR ATESTADO MÉDICO - ATE 15 DIAS	HC da Universidade Federal de Goiás - GO
57	AFASTAMENTO POR ATESTADO MÉDICO - ATE 15 DIAS	HC da Universidade Federal de Goiás - GO
57	AFASTAMENTO POR ATESTADO MÉDICO - ATE 15 DIAS	HC da Universidade Federal de Goiás - GO
57	AFASTAMENTO POR ATESTADO MÉDICO - ATE 15 DIAS	HC da Universidade Federal de Goiás - GO
57	AFASTAMENTO POR ATESTADO MÉDICO - ATE 15 DIAS	HC da Universidade Federal de Goiás - GO
57	AFASTAMENTO POR ATESTADO MÉDICO - ATE 15 DIAS	HC da Universidade Federal de Goiás - GO
57	AFASTAMENTO POR ATESTADO MÉDICO - ATE 15 DIAS	HC da Universidade Federal de Goiás - GO
57	AFASTAMENTO POR ATESTADO MÉDICO - ATE 15 DIAS	HC da Universidade Federal de Goiás - GO
57	AFASTAMENTO POR ATESTADO MÉDICO - ATE 15 DIAS	HC da Universidade Federal de Goiás - GO
57	AFASTAMENTO POR ATESTADO MÉDICO - ATE 15 DIAS	HC da Universidade Federal de Goiás - GO
57	AFASTAMENTO POR ATESTADO MÉDICO - ATE 15 DIAS	HC da Universidade Federal de Goiás - GO
57	AFASTAMENTO POR ATESTADO MÉDICO - ATE 15 DIAS	HC da Universidade Federal de Goiás - GO

57	AFASTAMENTO POR ATESTADO MÉDICO - ATE 15 DIAS	HC da Universidade Federal de Goiás - GO
57	AFASTAMENTO POR ATESTADO MÉDICO - ATE 15 DIAS	HC da Universidade Federal de Goiás - GO
57	AFASTAMENTO POR ATESTADO MÉDICO - ATE 15 DIAS	HC da Universidade Federal de Goiás - GO
57	AFASTAMENTO POR ATESTADO MÉDICO - ATE 15 DIAS	HC da Universidade Federal de Goiás - GO
57	AFASTAMENTO POR ATESTADO MÉDICO - ATE 15 DIAS	HC da Universidade Federal de Goiás - GO
57	AFASTAMENTO POR ATESTADO MÉDICO - ATE 15 DIAS	HC da Universidade Federal de Goiás - GO
57	AFASTAMENTO POR ATESTADO MÉDICO - ATE 15 DIAS	HC da Universidade Federal de Goiás - GO
57	AFASTAMENTO POR ATESTADO MÉDICO - ATE 15 DIAS	HC da Universidade Federal de Goiás - GO
57	AFASTAMENTO POR ATESTADO MÉDICO - ATE 15 DIAS	HC da Universidade Federal de Goiás - GO
57	AFASTAMENTO POR ATESTADO MÉDICO - ATE 15 DIAS	HC da Universidade Federal de Goiás - GO
57	AFASTAMENTO POR ATESTADO MÉDICO - ATE 15 DIAS	HC da Universidade Federal de Goiás - GO
57	AFASTAMENTO POR ATESTADO MÉDICO - ATE 15 DIAS	HC da Universidade Federal de Goiás - GO
57	AFASTAMENTO POR ATESTADO MÉDICO - ATE 15 DIAS	HC da Universidade Federal de Goiás - GO
57	AFASTAMENTO POR ATESTADO MÉDICO - ATE 15 DIAS	HC da Universidade Federal de Goiás - GO
57	AFASTAMENTO POR ATESTADO MÉDICO - ATE 15 DIAS	HC da Universidade Federal de Goiás - GO
57	AFASTAMENTO POR ATESTADO MÉDICO - ATE 15 DIAS	HC da Universidade Federal de Goiás - GO
57	AFASTAMENTO POR ATESTADO MÉDICO - ATE 15 DIAS	HC da Universidade Federal de Goiás - GO
57	AFASTAMENTO POR ATESTADO MÉDICO - ATE 15 DIAS	HC da Universidade Federal de Goiás - GO
57	AFASTAMENTO POR ATESTADO MÉDICO - ATE 15 DIAS	HC da Universidade Federal de Goiás - GO
57	AFASTAMENTO POR ATESTADO MÉDICO - ATE 15 DIAS	HC da Universidade Federal de Goiás - GO
57	AFASTAMENTO POR ATESTADO MÉDICO - ATE 15 DIAS	HC da Universidade Federal de Goiás - GO
57	AFASTAMENTO POR ATESTADO MÉDICO - ATE 15 DIAS	HC da Universidade Federal de Goiás - GO
57	AFASTAMENTO POR ATESTADO MÉDICO - ATE 15 DIAS	HC da Universidade Federal de Goiás - GO
57	AFASTAMENTO POR ATESTADO MÉDICO - ATE 15 DIAS	HC da Universidade Federal de Goiás - GO
57	AFASTAMENTO POR ATESTADO MÉDICO - ATE 15 DIAS	HC da Universidade Federal de Goiás - GO
57	AFASTAMENTO POR ATESTADO MÉDICO - ATE 15 DIAS	HC da Universidade Federal de Goiás - GO
57	AFASTAMENTO POR ATESTADO MÉDICO - ATE 15 DIAS	HC da Universidade Federal de Goiás - GO
57	AFASTAMENTO POR ATESTADO MÉDICO - ATE 15 DIAS	HC da Universidade Federal de Goiás - GO
57	AFASTAMENTO POR ATESTADO MÉDICO - ATE 15 DIAS	HC da Universidade Federal de Goiás - GO
57	AFASTAMENTO POR ATESTADO MÉDICO - ATE 15 DIAS	HC da Universidade Federal de Goiás - GO
57	AFASTAMENTO POR ATESTADO MÉDICO - ATE 15 DIAS	HC da Universidade Federal de Goiás - GO
57	AFASTAMENTO POR ATESTADO MÉDICO - ATE 15 DIAS	HC da Universidade Federal de Goiás - GO
57	AFASTAMENTO POR ATESTADO MÉDICO - ATE 15 DIAS	HC da Universidade Federal de Goiás - GO
57	AFASTAMENTO POR ATESTADO MÉDICO - ATE 15 DIAS	HC da Universidade Federal de Goiás - GO
57	AFASTAMENTO POR ATESTADO MÉDICO - ATE 15 DIAS	HC da Universidade Federal de Goiás - GO
57	AFASTAMENTO POR ATESTADO MÉDICO - ATE 15 DIAS	HC da Universidade Federal de Goiás - GO
57	AFASTAMENTO POR ATESTADO MÉDICO - ATE 15 DIAS	HC da Universidade Federal de Goiás - GO
57	AFASTAMENTO POR ATESTADO MÉDICO - ATE 15 DIAS	HC da Universidade Federal de Goiás - GO
57	AFASTAMENTO POR ATESTADO MÉDICO - ATE 15 DIAS	HC da Universidade Federal de Goiás - GO
57	AFASTAMENTO POR ATESTADO MÉDICO - ATE 15 DIAS	HC da Universidade Federal de Goiás - GO
57	AFASTAMENTO POR ATESTADO MÉDICO - ATE 15 DIAS	HC da Universidade Federal de Goiás - GO
57	AFASTAMENTO POR ATESTADO MÉDICO - ATE 15 DIAS	HC da Universidade Federal de Goiás - GO
57	AFASTAMENTO POR ATESTADO MÉDICO - ATE 15 DIAS	HC da Universidade Federal de Goiás - GO
57	AFASTAMENTO POR ATESTADO MÉDICO - ATE 15 DIAS	HC da Universidade Federal de Goiás - GO
57	AFASTAMENTO POR ATESTADO MÉDICO - ATE 15 DIAS	HC da Universidade Federal de Goiás - GO
57	AFASTAMENTO POR ATESTADO MÉDICO - ATE 15 DIAS	HC da Universidade Federal de Goiás - GO
57	AFASTAMENTO POR ATESTADO MÉDICO - ATE 15 DIAS	HC da Universidade Federal de Goiás - GO

57	AFASTAMENTO POR ATESTADO MÉDICO - ATE 15 DIAS	HC da Universidade Federal de Goiás - GO
57	AFASTAMENTO POR ATESTADO MÉDICO - ATE 15 DIAS	HC da Universidade Federal de Goiás - GO
57	AFASTAMENTO POR ATESTADO MÉDICO - ATE 15 DIAS	HC da Universidade Federal de Goiás - GO
57	AFASTAMENTO POR ATESTADO MÉDICO - ATE 15 DIAS	HC da Universidade Federal de Goiás - GO
57	AFASTAMENTO POR ATESTADO MÉDICO - ATE 15 DIAS	HC da Universidade Federal de Goiás - GO
57	AFASTAMENTO POR ATESTADO MÉDICO - ATE 15 DIAS	HC da Universidade Federal de Goiás - GO
57	AFASTAMENTO POR ATESTADO MÉDICO - ATE 15 DIAS	HC da Universidade Federal de Goiás - GO
57	AFASTAMENTO POR ATESTADO MÉDICO - ATE 15 DIAS	HC da Universidade Federal de Goiás - GO
57	AFASTAMENTO POR ATESTADO MÉDICO - ATE 15 DIAS	HC da Universidade Federal de Goiás - GO
57	AFASTAMENTO POR ATESTADO MÉDICO - ATE 15 DIAS	HC da Universidade Federal de Goiás - GO
57	AFASTAMENTO POR ATESTADO MÉDICO - ATE 15 DIAS	HC da Universidade Federal de Goiás - GO
57	AFASTAMENTO POR ATESTADO MÉDICO - ATE 15 DIAS	HC da Universidade Federal de Goiás - GO
57	AFASTAMENTO POR ATESTADO MÉDICO - ATE 15 DIAS	HC da Universidade Federal de Goiás - GO
57	AFASTAMENTO POR ATESTADO MÉDICO - ATE 15 DIAS	HC da Universidade Federal de Goiás - GO
57	AFASTAMENTO POR ATESTADO MÉDICO - ATE 15 DIAS	HC da Universidade Federal de Goiás - GO
57	AFASTAMENTO POR ATESTADO MÉDICO - ATE 15 DIAS	HC da Universidade Federal de Goiás - GO
57	AFASTAMENTO POR ATESTADO MÉDICO - ATE 15 DIAS	HC da Universidade Federal de Goiás - GO
57	AFASTAMENTO POR ATESTADO MÉDICO - ATE 15 DIAS	HC da Universidade Federal de Goiás - GO
57	AFASTAMENTO POR ATESTADO MÉDICO - ATE 15 DIAS	HC da Universidade Federal de Goiás - GO
57	AFASTAMENTO POR ATESTADO MÉDICO - ATE 15 DIAS	HC da Universidade Federal de Goiás - GO
57	AFASTAMENTO POR ATESTADO MÉDICO - ATE 15 DIAS	HC da Universidade Federal de Goiás - GO
57	AFASTAMENTO POR ATESTADO MÉDICO - ATE 15 DIAS	HC da Universidade Federal de Goiás - GO
57	AFASTAMENTO POR ATESTADO MÉDICO - ATE 15 DIAS	HC da Universidade Federal de Goiás - GO
57	AFASTAMENTO POR ATESTADO MÉDICO - ATE 15 DIAS	HC da Universidade Federal de Goiás - GO
57	AFASTAMENTO POR ATESTADO MÉDICO - ATE 15 DIAS	HC da Universidade Federal de Goiás - GO
57	AFASTAMENTO POR ATESTADO MÉDICO - ATE 15 DIAS	HC da Universidade Federal de Goiás - GO
57	AFASTAMENTO POR ATESTADO MÉDICO - ATE 15 DIAS	HC da Universidade Federal de Goiás - GO
57	AFASTAMENTO POR ATESTADO MÉDICO - ATE 15 DIAS	HC da Universidade Federal de Goiás - GO
57	AFASTAMENTO POR ATESTADO MÉDICO - ATE 15 DIAS	HC da Universidade Federal de Goiás - GO
57	AFASTAMENTO POR ATESTADO MÉDICO - ATE 15 DIAS	HC da Universidade Federal de Goiás - GO
57	AFASTAMENTO POR ATESTADO MÉDICO - ATE 15 DIAS	HC da Universidade Federal de Goiás - GO
57	AFASTAMENTO POR ATESTADO MÉDICO - ATE 15 DIAS	HC da Universidade Federal de Goiás - GO

57	AFASTAMENTO POR ATESTADO MÉDICO - ATE 15 DIAS	HC da Universidade Federal de Goiás - GO
57	AFASTAMENTO POR ATESTADO MÉDICO - ATE 15 DIAS	HC da Universidade Federal de Goiás - GO
57	AFASTAMENTO POR ATESTADO MÉDICO - ATE 15 DIAS	HC da Universidade Federal de Goiás - GO
57	AFASTAMENTO POR ATESTADO MÉDICO - ATE 15 DIAS	HC da Universidade Federal de Goiás - GO
57	AFASTAMENTO POR ATESTADO MÉDICO - ATE 15 DIAS	HC da Universidade Federal de Goiás - GO
57	AFASTAMENTO POR ATESTADO MÉDICO - ATE 15 DIAS	HC da Universidade Federal de Goiás - GO
57	AFASTAMENTO POR ATESTADO MÉDICO - ATE 15 DIAS	HC da Universidade Federal de Goiás - GO
57	AFASTAMENTO POR ATESTADO MÉDICO - ATE 15 DIAS	HC da Universidade Federal de Goiás - GO
57	AFASTAMENTO POR ATESTADO MÉDICO - ATE 15 DIAS	HC da Universidade Federal de Goiás - GO
57	AFASTAMENTO POR ATESTADO MÉDICO - ATE 15 DIAS	HC da Universidade Federal de Goiás - GO
57	AFASTAMENTO POR ATESTADO MÉDICO - ATE 15 DIAS	HC da Universidade Federal de Goiás - GO
57	AFASTAMENTO POR ATESTADO MÉDICO - ATE 15 DIAS	HC da Universidade Federal de Goiás - GO
57	AFASTAMENTO POR ATESTADO MÉDICO - ATE 15 DIAS	HC da Universidade Federal de Goiás - GO
57	AFASTAMENTO POR ATESTADO MÉDICO - ATE 15 DIAS	HC da Universidade Federal de Goiás - GO
57	AFASTAMENTO POR ATESTADO MÉDICO - ATE 15 DIAS	HC da Universidade Federal de Goiás - GO
57	AFASTAMENTO POR ATESTADO MÉDICO - ATE 15 DIAS	HC da Universidade Federal de Goiás - GO
57	AFASTAMENTO POR ATESTADO MÉDICO - ATE 15 DIAS	HC da Universidade Federal de Goiás - GO
57	AFASTAMENTO POR ATESTADO MÉDICO - ATE 15 DIAS	HC da Universidade Federal de Goiás - GO
57	AFASTAMENTO POR ATESTADO MÉDICO - ATE 15 DIAS	HC da Universidade Federal de Goiás - GO
57	AFASTAMENTO POR ATESTADO MÉDICO - ATE 15 DIAS	HC da Universidade Federal de Goiás - GO
57	AFASTAMENTO POR ATESTADO MÉDICO - ATE 15 DIAS	HC da Universidade Federal de Goiás - GO
57	AFASTAMENTO POR ATESTADO MÉDICO - ATE 15 DIAS	HC da Universidade Federal de Goiás - GO
57	AFASTAMENTO POR ATESTADO MÉDICO - ATE 15 DIAS	HC da Universidade Federal de Goiás - GO
57	AFASTAMENTO POR ATESTADO MÉDICO - ATE 15 DIAS	HC da Universidade Federal de Goiás - GO
57	AFASTAMENTO POR ATESTADO MÉDICO - ATE 15 DIAS	HC da Universidade Federal de Goiás - GO
57	AFASTAMENTO POR ATESTADO MÉDICO - ATE 15 DIAS	HC da Universidade Federal de Goiás - GO
57	AFASTAMENTO POR ATESTADO MÉDICO - ATE 15 DIAS	HC da Universidade Federal de Goiás - GO
57	AFASTAMENTO POR ATESTADO MÉDICO - ATE 15 DIAS	HC da Universidade Federal de Goiás - GO
57	AFASTAMENTO POR ATESTADO MÉDICO - ATE 15 DIAS	HC da Universidade Federal de Goiás - GO
57	AFASTAMENTO POR ATESTADO MÉDICO - ATE 15 DIAS	HC da Universidade Federal de Goiás - GO
57	AFASTAMENTO POR ATESTADO MÉDICO - ATE 15 DIAS	HC da Universidade Federal de Goiás - GO
57	AFASTAMENTO POR ATESTADO MÉDICO - ATE 15 DIAS	HC da Universidade Federal de Goiás - GO
57	AFASTAMENTO POR ATESTADO MÉDICO - ATE 15 DIAS	HC da Universidade Federal de Goiás - GO
57	AFASTAMENTO POR ATESTADO MÉDICO - ATE 15 DIAS	HC da Universidade Federal de Goiás - GO
57	AFASTAMENTO POR ATESTADO MÉDICO - ATE 15 DIAS	HC da Universidade Federal de Goiás - GO
57	AFASTAMENTO POR ATESTADO MÉDICO - ATE 15 DIAS	HC da Universidade Federal de Goiás - GO
57	AFASTAMENTO POR ATESTADO MÉDICO - ATE 15 DIAS	HC da Universidade Federal de Goiás - GO
57	AFASTAMENTO POR ATESTADO MÉDICO - ATE 15 DIAS	HC da Universidade Federal de Goiás - GO
57	AFASTAMENTO POR ATESTADO MÉDICO - ATE 15 DIAS	HC da Universidade Federal de Goiás - GO
57	AFASTAMENTO POR ATESTADO MÉDICO - ATE 15 DIAS	HC da Universidade Federal de Goiás - GO
57	AFASTAMENTO POR ATESTADO MÉDICO - ATE 15 DIAS	HC da Universidade Federal de Goiás - GO

57	AFASTAMENTO POR ATESTADO MÉDICO - ATE 15 DIAS	HC da Universidade Federal de Goiás - GO
57	AFASTAMENTO POR ATESTADO MÉDICO - ATE 15 DIAS	HC da Universidade Federal de Goiás - GO
57	AFASTAMENTO POR ATESTADO MÉDICO - ATE 15 DIAS	HC da Universidade Federal de Goiás - GO
57	AFASTAMENTO POR ATESTADO MÉDICO - ATE 15 DIAS	HC da Universidade Federal de Goiás - GO
57	AFASTAMENTO POR ATESTADO MÉDICO - ATE 15 DIAS	HC da Universidade Federal de Goiás - GO
57	AFASTAMENTO POR ATESTADO MÉDICO - ATE 15 DIAS	HC da Universidade Federal de Goiás - GO
57	AFASTAMENTO POR ATESTADO MÉDICO - ATE 15 DIAS	HC da Universidade Federal de Goiás - GO
57	AFASTAMENTO POR ATESTADO MÉDICO - ATE 15 DIAS	HC da Universidade Federal de Goiás - GO
57	AFASTAMENTO POR ATESTADO MÉDICO - ATE 15 DIAS	HC da Universidade Federal de Goiás - GO
57	AFASTAMENTO POR ATESTADO MÉDICO - ATE 15 DIAS	HC da Universidade Federal de Goiás - GO
57	AFASTAMENTO POR ATESTADO MÉDICO - ATE 15 DIAS	HC da Universidade Federal de Goiás - GO
57	AFASTAMENTO POR ATESTADO MÉDICO - ATE 15 DIAS	HC da Universidade Federal de Goiás - GO
57	AFASTAMENTO POR ATESTADO MÉDICO - ATE 15 DIAS	HC da Universidade Federal de Goiás - GO
57	AFASTAMENTO POR ATESTADO MÉDICO - ATE 15 DIAS	HC da Universidade Federal de Goiás - GO
57	AFASTAMENTO POR ATESTADO MÉDICO - ATE 15 DIAS	HC da Universidade Federal de Goiás - GO
57	AFASTAMENTO POR ATESTADO MÉDICO - ATE 15 DIAS	HC da Universidade Federal de Goiás - GO
57	AFASTAMENTO POR ATESTADO MÉDICO - ATE 15 DIAS	HC da Universidade Federal de Goiás - GO
57	AFASTAMENTO POR ATESTADO MÉDICO - ATE 15 DIAS	HC da Universidade Federal de Goiás - GO
57	AFASTAMENTO POR ATESTADO MÉDICO - ATE 15 DIAS	HC da Universidade Federal de Goiás - GO
57	AFASTAMENTO POR ATESTADO MÉDICO - ATE 15 DIAS	HC da Universidade Federal de Goiás - GO
57	AFASTAMENTO POR ATESTADO MÉDICO - ATE 15 DIAS	HC da Universidade Federal de Goiás - GO
57	AFASTAMENTO POR ATESTADO MÉDICO - ATE 15 DIAS	HC da Universidade Federal de Goiás - GO
57	AFASTAMENTO POR ATESTADO MÉDICO - ATE 15 DIAS	HC da Universidade Federal de Goiás - GO
57	AFASTAMENTO POR ATESTADO MÉDICO - ATE 15 DIAS	HC da Universidade Federal de Goiás - GO
57	AFASTAMENTO POR ATESTADO MÉDICO - ATE 15 DIAS	HC da Universidade Federal de Goiás - GO
57	AFASTAMENTO POR ATESTADO MÉDICO - ATE 15 DIAS	HC da Universidade Federal de Goiás - GO
57	AFASTAMENTO POR ATESTADO MÉDICO - ATE 15 DIAS	HC da Universidade Federal de Goiás - GO
57	AFASTAMENTO POR ATESTADO MÉDICO - ATE 15 DIAS	HC da Universidade Federal de Goiás - GO
57	AFASTAMENTO POR ATESTADO MÉDICO - ATE 15 DIAS	HC da Universidade Federal de Goiás - GO
57	AFASTAMENTO POR ATESTADO MÉDICO - ATE 15 DIAS	HC da Universidade Federal de Goiás - GO
57	AFASTAMENTO POR ATESTADO MÉDICO - ATE 15 DIAS	HC da Universidade Federal de Goiás - GO
57	AFASTAMENTO POR ATESTADO MÉDICO - ATE 15 DIAS	HC da Universidade Federal de Goiás - GO
57	AFASTAMENTO POR ATESTADO MÉDICO - ATE 15 DIAS	HC da Universidade Federal de Goiás - GO
57	AFASTAMENTO POR ATESTADO MÉDICO - ATE 15 DIAS	HC da Universidade Federal de Goiás - GO
57	AFASTAMENTO POR ATESTADO MÉDICO - ATE 15 DIAS	HC da Universidade Federal de Goiás - GO
57	AFASTAMENTO POR ATESTADO MÉDICO - ATE 15 DIAS	HC da Universidade Federal de Goiás - GO
57	AFASTAMENTO POR ATESTADO MÉDICO - ATE 15 DIAS	HC da Universidade Federal de Goiás - GO
57	AFASTAMENTO POR ATESTADO MÉDICO - ATE 15 DIAS	HC da Universidade Federal de Goiás - GO
57	AFASTAMENTO POR ATESTADO MÉDICO - ATE 15 DIAS	HC da Universidade Federal de Goiás - GO
57	AFASTAMENTO POR ATESTADO MÉDICO - ATE 15 DIAS	HC da Universidade Federal de Goiás - GO
57	AFASTAMENTO POR ATESTADO MÉDICO - ATE 15 DIAS	HC da Universidade Federal de Goiás - GO
57	AFASTAMENTO POR ATESTADO MÉDICO - ATE 15 DIAS	HC da Universidade Federal de Goiás - GO
57	AFASTAMENTO POR ATESTADO MÉDICO - ATE 15 DIAS	HC da Universidade Federal de Goiás - GO
57	AFASTAMENTO POR ATESTADO MÉDICO - ATE 15 DIAS	HC da Universidade Federal de Goiás - GO
57	AFASTAMENTO POR ATESTADO MÉDICO - ATE 15 DIAS	HC da Universidade Federal de Goiás - GO
57	AFASTAMENTO POR ATESTADO MÉDICO - ATE 15 DIAS	HC da Universidade Federal de Goiás - GO
57	AFASTAMENTO POR ATESTADO MÉDICO - ATE 15 DIAS	HC da Universidade Federal de Goiás - GO
57	AFASTAMENTO POR ATESTADO MÉDICO - ATE 15 DIAS	HC da Universidade Federal de Goiás - GO

57	AFASTAMENTO POR ATESTADO MÉDICO - ATE 15 DIAS	HC da Universidade Federal de Goiás - GO
57	AFASTAMENTO POR ATESTADO MÉDICO - ATE 15 DIAS	HC da Universidade Federal de Goiás - GO
57	AFASTAMENTO POR ATESTADO MÉDICO - ATE 15 DIAS	HC da Universidade Federal de Goiás - GO
57	AFASTAMENTO POR ATESTADO MÉDICO - ATE 15 DIAS	HC da Universidade Federal de Goiás - GO
57	AFASTAMENTO POR ATESTADO MÉDICO - ATE 15 DIAS	HC da Universidade Federal de Goiás - GO
57	AFASTAMENTO POR ATESTADO MÉDICO - ATE 15 DIAS	HC da Universidade Federal de Goiás - GO
57	AFASTAMENTO POR ATESTADO MÉDICO - ATE 15 DIAS	HC da Universidade Federal de Goiás - GO
57	AFASTAMENTO POR ATESTADO MÉDICO - ATE 15 DIAS	HC da Universidade Federal de Goiás - GO
57	AFASTAMENTO POR ATESTADO MÉDICO - ATE 15 DIAS	HC da Universidade Federal de Goiás - GO
57	AFASTAMENTO POR ATESTADO MÉDICO - ATE 15 DIAS	HC da Universidade Federal de Goiás - GO
57	AFASTAMENTO POR ATESTADO MÉDICO - ATE 15 DIAS	HC da Universidade Federal de Goiás - GO
57	AFASTAMENTO POR ATESTADO MÉDICO - ATE 15 DIAS	HC da Universidade Federal de Goiás - GO
57	AFASTAMENTO POR ATESTADO MÉDICO - ATE 15 DIAS	HC da Universidade Federal de Goiás - GO
57	AFASTAMENTO POR ATESTADO MÉDICO - ATE 15 DIAS	HC da Universidade Federal de Goiás - GO
57	AFASTAMENTO POR ATESTADO MÉDICO - ATE 15 DIAS	HC da Universidade Federal de Goiás - GO
57	AFASTAMENTO POR ATESTADO MÉDICO - ATE 15 DIAS	HC da Universidade Federal de Goiás - GO
57	AFASTAMENTO POR ATESTADO MÉDICO - ATE 15 DIAS	HC da Universidade Federal de Goiás - GO
57	AFASTAMENTO POR ATESTADO MÉDICO - ATE 15 DIAS	HC da Universidade Federal de Goiás - GO
57	AFASTAMENTO POR ATESTADO MÉDICO - ATE 15 DIAS	HC da Universidade Federal de Goiás - GO
57	AFASTAMENTO POR ATESTADO MÉDICO - ATE 15 DIAS	HC da Universidade Federal de Goiás - GO
57	AFASTAMENTO POR ATESTADO MÉDICO - ATE 15 DIAS	HC da Universidade Federal de Goiás - GO
57	AFASTAMENTO POR ATESTADO MÉDICO - ATE 15 DIAS	HC da Universidade Federal de Goiás - GO
57	AFASTAMENTO POR ATESTADO MÉDICO - ATE 15 DIAS	HC da Universidade Federal de Goiás - GO
57	AFASTAMENTO POR ATESTADO MÉDICO - ATE 15 DIAS	HC da Universidade Federal de Goiás - GO
57	AFASTAMENTO POR ATESTADO MÉDICO - ATE 15 DIAS	HC da Universidade Federal de Goiás - GO
57	AFASTAMENTO POR ATESTADO MÉDICO - ATE 15 DIAS	HC da Universidade Federal de Goiás - GO
57	AFASTAMENTO POR ATESTADO MÉDICO - ATE 15 DIAS	HC da Universidade Federal de Goiás - GO
57	AFASTAMENTO POR ATESTADO MÉDICO - ATE 15 DIAS	HC da Universidade Federal de Goiás - GO
57	AFASTAMENTO POR ATESTADO MÉDICO - ATE 15 DIAS	HC da Universidade Federal de Goiás - GO
57	AFASTAMENTO POR ATESTADO MÉDICO - ATE 15 DIAS	HC da Universidade Federal de Goiás - GO
57	AFASTAMENTO POR ATESTADO MÉDICO - ATE 15 DIAS	HC da Universidade Federal de Goiás - GO
57	AFASTAMENTO POR ATESTADO MÉDICO - ATE 15 DIAS	HC da Universidade Federal de Goiás - GO
57	AFASTAMENTO POR ATESTADO MÉDICO - ATE 15 DIAS	HC da Universidade Federal de Goiás - GO
57	AFASTAMENTO POR ATESTADO MÉDICO - ATE 15 DIAS	HC da Universidade Federal de Goiás - GO
57	AFASTAMENTO POR ATESTADO MÉDICO - ATE 15 DIAS	HC da Universidade Federal de Goiás - GO
57	AFASTAMENTO POR ATESTADO MÉDICO - ATE 15 DIAS	HC da Universidade Federal de Goiás - GO
57	AFASTAMENTO POR ATESTADO MÉDICO - ATE 15 DIAS	HC da Universidade Federal de Goiás - GO
57	AFASTAMENTO POR ATESTADO MÉDICO - ATE 15 DIAS	HC da Universidade Federal de Goiás - GO
57	AFASTAMENTO POR ATESTADO MÉDICO - ATE 15 DIAS	HC da Universidade Federal de Goiás - GO
57	AFASTAMENTO POR ATESTADO MÉDICO - ATE 15 DIAS	HC da Universidade Federal de Goiás - GO
57	AFASTAMENTO POR ATESTADO MÉDICO - ATE 15 DIAS	HC da Universidade Federal de Goiás - GO
57	AFASTAMENTO POR ATESTADO MÉDICO - ATE 15 DIAS	HC da Universidade Federal de Goiás - GO
57	AFASTAMENTO POR ATESTADO MÉDICO - ATE 15 DIAS	HC da Universidade Federal de Goiás - GO
57	AFASTAMENTO POR ATESTADO MÉDICO - ATE 15 DIAS	HC da Universidade Federal de Goiás - GO
57	AFASTAMENTO POR ATESTADO MÉDICO - ATE 15 DIAS	HC da Universidade Federal de Goiás - GO
57	AFASTAMENTO POR ATESTADO MÉDICO - ATE 15 DIAS	HC da Universidade Federal de Goiás - GO
57	AFASTAMENTO POR ATESTADO MÉDICO - ATE 15 DIAS	HC da Universidade Federal de Goiás - GO
57	AFASTAMENTO POR ATESTADO MÉDICO - ATE 15 DIAS	HC da Universidade Federal de Goiás - GO

57	AFASTAMENTO POR ATESTADO MÉDICO - ATE 15 DIAS	HC da Universidade Federal de Goiás - GO
57	AFASTAMENTO POR ATESTADO MÉDICO - ATE 15 DIAS	HC da Universidade Federal de Goiás - GO
57	AFASTAMENTO POR ATESTADO MÉDICO - ATE 15 DIAS	HC da Universidade Federal de Goiás - GO
57	AFASTAMENTO POR ATESTADO MÉDICO - ATE 15 DIAS	HC da Universidade Federal de Goiás - GO
57	AFASTAMENTO POR ATESTADO MÉDICO - ATE 15 DIAS	HC da Universidade Federal de Goiás - GO
57	AFASTAMENTO POR ATESTADO MÉDICO - ATE 15 DIAS	HC da Universidade Federal de Goiás - GO
57	AFASTAMENTO POR ATESTADO MÉDICO - ATE 15 DIAS	HC da Universidade Federal de Goiás - GO
57	AFASTAMENTO POR ATESTADO MÉDICO - ATE 15 DIAS	HC da Universidade Federal de Goiás - GO
57	AFASTAMENTO POR ATESTADO MÉDICO - ATE 15 DIAS	HC da Universidade Federal de Goiás - GO
57	AFASTAMENTO POR ATESTADO MÉDICO - ATE 15 DIAS	HC da Universidade Federal de Goiás - GO
57	AFASTAMENTO POR ATESTADO MÉDICO - ATE 15 DIAS	HC da Universidade Federal de Goiás - GO
57	AFASTAMENTO POR ATESTADO MÉDICO - ATE 15 DIAS	HC da Universidade Federal de Goiás - GO
57	AFASTAMENTO POR ATESTADO MÉDICO - ATE 15 DIAS	HC da Universidade Federal de Goiás - GO
57	AFASTAMENTO POR ATESTADO MÉDICO - ATE 15 DIAS	HC da Universidade Federal de Goiás - GO
57	AFASTAMENTO POR ATESTADO MÉDICO - ATE 15 DIAS	HC da Universidade Federal de Goiás - GO
57	AFASTAMENTO POR ATESTADO MÉDICO - ATE 15 DIAS	HC da Universidade Federal de Goiás - GO
57	AFASTAMENTO POR ATESTADO MÉDICO - ATE 15 DIAS	HC da Universidade Federal de Goiás - GO
57	AFASTAMENTO POR ATESTADO MÉDICO - ATE 15 DIAS	HC da Universidade Federal de Goiás - GO
57	AFASTAMENTO POR ATESTADO MÉDICO - ATE 15 DIAS	HC da Universidade Federal de Goiás - GO
57	AFASTAMENTO POR ATESTADO MÉDICO - ATE 15 DIAS	HC da Universidade Federal de Goiás - GO
57	AFASTAMENTO POR ATESTADO MÉDICO - ATE 15 DIAS	HC da Universidade Federal de Goiás - GO
57	AFASTAMENTO POR ATESTADO MÉDICO - ATE 15 DIAS	HC da Universidade Federal de Goiás - GO
57	AFASTAMENTO POR ATESTADO MÉDICO - ATE 15 DIAS	HC da Universidade Federal de Goiás - GO
57	AFASTAMENTO POR ATESTADO MÉDICO - ATE 15 DIAS	HC da Universidade Federal de Goiás - GO
57	AFASTAMENTO POR ATESTADO MÉDICO - ATE 15 DIAS	HC da Universidade Federal de Goiás - GO
57	AFASTAMENTO POR ATESTADO MÉDICO - ATE 15 DIAS	HC da Universidade Federal de Goiás - GO
57	AFASTAMENTO POR ATESTADO MÉDICO - ATE 15 DIAS	HC da Universidade Federal de Goiás - GO
57	AFASTAMENTO POR ATESTADO MÉDICO - ATE 15 DIAS	HC da Universidade Federal de Goiás - GO
57	AFASTAMENTO POR ATESTADO MÉDICO - ATE 15 DIAS	HC da Universidade Federal de Goiás - GO
57	AFASTAMENTO POR ATESTADO MÉDICO - ATE 15 DIAS	HC da Universidade Federal de Goiás - GO
57	AFASTAMENTO POR ATESTADO MÉDICO - ATE 15 DIAS	HC da Universidade Federal de Goiás - GO
57	AFASTAMENTO POR ATESTADO MÉDICO - ATE 15 DIAS	HC da Universidade Federal de Goiás - GO
57	AFASTAMENTO POR ATESTADO MÉDICO - ATE 15 DIAS	HC da Universidade Federal de Goiás - GO
57	AFASTAMENTO POR ATESTADO MÉDICO - ATE 15 DIAS	HC da Universidade Federal de Goiás - GO
57	AFASTAMENTO POR ATESTADO MÉDICO - ATE 15 DIAS	HC da Universidade Federal de Goiás - GO
57	AFASTAMENTO POR ATESTADO MÉDICO - ATE 15 DIAS	HC da Universidade Federal de Goiás - GO
57	AFASTAMENTO POR ATESTADO MÉDICO - ATE 15 DIAS	HC da Universidade Federal de Goiás - GO
57	AFASTAMENTO POR ATESTADO MÉDICO - ATE 15 DIAS	HC da Universidade Federal de Goiás - GO
57	AFASTAMENTO POR ATESTADO MÉDICO - ATE 15 DIAS	HC da Universidade Federal de Goiás - GO
57	AFASTAMENTO POR ATESTADO MÉDICO - ATE 15 DIAS	HC da Universidade Federal de Goiás - GO
57	AFASTAMENTO POR ATESTADO MÉDICO - ATE 15 DIAS	HC da Universidade Federal de Goiás - GO
57	AFASTAMENTO POR ATESTADO MÉDICO - ATE 15 DIAS	HC da Universidade Federal de Goiás - GO
57	AFASTAMENTO POR ATESTADO MÉDICO - ATE 15 DIAS	HC da Universidade Federal de Goiás - GO

57	AFASTAMENTO POR ATESTADO MÉDICO - ATE 15 DIAS	HC da Universidade Federal de Goiás - GO
57	AFASTAMENTO POR ATESTADO MÉDICO - ATE 15 DIAS	HC da Universidade Federal de Goiás - GO
57	AFASTAMENTO POR ATESTADO MÉDICO - ATE 15 DIAS	HC da Universidade Federal de Goiás - GO
57	AFASTAMENTO POR ATESTADO MÉDICO - ATE 15 DIAS	HC da Universidade Federal de Goiás - GO
57	AFASTAMENTO POR ATESTADO MÉDICO - ATE 15 DIAS	HC da Universidade Federal de Goiás - GO
57	AFASTAMENTO POR ATESTADO MÉDICO - ATE 15 DIAS	HC da Universidade Federal de Goiás - GO
57	AFASTAMENTO POR ATESTADO MÉDICO - ATE 15 DIAS	HC da Universidade Federal de Goiás - GO
57	AFASTAMENTO POR ATESTADO MÉDICO - ATE 15 DIAS	HC da Universidade Federal de Goiás - GO
57	AFASTAMENTO POR ATESTADO MÉDICO - ATE 15 DIAS	HC da Universidade Federal de Goiás - GO
57	AFASTAMENTO POR ATESTADO MÉDICO - ATE 15 DIAS	HC da Universidade Federal de Goiás - GO
57	AFASTAMENTO POR ATESTADO MÉDICO - ATE 15 DIAS	HC da Universidade Federal de Goiás - GO
57	AFASTAMENTO POR ATESTADO MÉDICO - ATE 15 DIAS	HC da Universidade Federal de Goiás - GO
57	AFASTAMENTO POR ATESTADO MÉDICO - ATE 15 DIAS	HC da Universidade Federal de Goiás - GO
57	AFASTAMENTO POR ATESTADO MÉDICO - ATE 15 DIAS	HC da Universidade Federal de Goiás - GO
57	AFASTAMENTO POR ATESTADO MÉDICO - ATE 15 DIAS	HC da Universidade Federal de Goiás - GO
57	AFASTAMENTO POR ATESTADO MÉDICO - ATE 15 DIAS	HC da Universidade Federal de Goiás - GO
57	AFASTAMENTO POR ATESTADO MÉDICO - ATE 15 DIAS	HC da Universidade Federal de Goiás - GO
57	AFASTAMENTO POR ATESTADO MÉDICO - ATE 15 DIAS	HC da Universidade Federal de Goiás - GO
57	AFASTAMENTO POR ATESTADO MÉDICO - ATE 15 DIAS	HC da Universidade Federal de Goiás - GO
57	AFASTAMENTO POR ATESTADO MÉDICO - ATE 15 DIAS	HC da Universidade Federal de Goiás - GO
57	AFASTAMENTO POR ATESTADO MÉDICO - ATE 15 DIAS	HC da Universidade Federal de Goiás - GO
57	AFASTAMENTO POR ATESTADO MÉDICO - ATE 15 DIAS	HC da Universidade Federal de Goiás - GO
57	AFASTAMENTO POR ATESTADO MÉDICO - ATE 15 DIAS	HC da Universidade Federal de Goiás - GO
57	AFASTAMENTO POR ATESTADO MÉDICO - ATE 15 DIAS	HC da Universidade Federal de Goiás - GO
57	AFASTAMENTO POR ATESTADO MÉDICO - ATE 15 DIAS	HC da Universidade Federal de Goiás - GO
57	AFASTAMENTO POR ATESTADO MÉDICO - ATE 15 DIAS	HC da Universidade Federal de Goiás - GO
57	AFASTAMENTO POR ATESTADO MÉDICO - ATE 15 DIAS	HC da Universidade Federal de Goiás - GO
57	AFASTAMENTO POR ATESTADO MÉDICO - ATE 15 DIAS	HC da Universidade Federal de Goiás - GO
57	AFASTAMENTO POR ATESTADO MÉDICO - ATE 15 DIAS	HC da Universidade Federal de Goiás - GO
57	AFASTAMENTO POR ATESTADO MÉDICO - ATE 15 DIAS	HC da Universidade Federal de Goiás - GO
57	AFASTAMENTO POR ATESTADO MÉDICO - ATE 15 DIAS	HC da Universidade Federal de Goiás - GO
57	AFASTAMENTO POR ATESTADO MÉDICO - ATE 15 DIAS	HC da Universidade Federal de Goiás - GO
57	AFASTAMENTO POR ATESTADO MÉDICO - ATE 15 DIAS	HC da Universidade Federal de Goiás - GO
57	AFASTAMENTO POR ATESTADO MÉDICO - ATE 15 DIAS	HC da Universidade Federal de Goiás - GO

57	AFASTAMENTO POR ATESTADO MÉDICO - ATE 15 DIAS	HC da Universidade Federal de Goiás - GO
57	AFASTAMENTO POR ATESTADO MÉDICO - ATE 15 DIAS	HC da Universidade Federal de Goiás - GO
57	AFASTAMENTO POR ATESTADO MÉDICO - ATE 15 DIAS	HC da Universidade Federal de Goiás - GO
57	AFASTAMENTO POR ATESTADO MÉDICO - ATE 15 DIAS	HC da Universidade Federal de Goiás - GO
57	AFASTAMENTO POR ATESTADO MÉDICO - ATE 15 DIAS	HC da Universidade Federal de Goiás - GO
57	AFASTAMENTO POR ATESTADO MÉDICO - ATE 15 DIAS	HC da Universidade Federal de Goiás - GO
57	AFASTAMENTO POR ATESTADO MÉDICO - ATE 15 DIAS	HC da Universidade Federal de Goiás - GO
57	AFASTAMENTO POR ATESTADO MÉDICO - ATE 15 DIAS	HC da Universidade Federal de Goiás - GO
57	AFASTAMENTO POR ATESTADO MÉDICO - ATE 15 DIAS	HC da Universidade Federal de Goiás - GO
57	AFASTAMENTO POR ATESTADO MÉDICO - ATE 15 DIAS	HC da Universidade Federal de Goiás - GO
57	AFASTAMENTO POR ATESTADO MÉDICO - ATE 15 DIAS	HC da Universidade Federal de Goiás - GO
57	AFASTAMENTO POR ATESTADO MÉDICO - ATE 15 DIAS	HC da Universidade Federal de Goiás - GO
57	AFASTAMENTO POR ATESTADO MÉDICO - ATE 15 DIAS	HC da Universidade Federal de Goiás - GO
57	AFASTAMENTO POR ATESTADO MÉDICO - ATE 15 DIAS	HC da Universidade Federal de Goiás - GO
57	AFASTAMENTO POR ATESTADO MÉDICO - ATE 15 DIAS	HC da Universidade Federal de Goiás - GO
57	AFASTAMENTO POR ATESTADO MÉDICO - ATE 15 DIAS	HC da Universidade Federal de Goiás - GO
57	AFASTAMENTO POR ATESTADO MÉDICO - ATE 15 DIAS	HC da Universidade Federal de Goiás - GO
57	AFASTAMENTO POR ATESTADO MÉDICO - ATE 15 DIAS	HC da Universidade Federal de Goiás - GO
57	AFASTAMENTO POR ATESTADO MÉDICO - ATE 15 DIAS	HC da Universidade Federal de Goiás - GO
57	AFASTAMENTO POR ATESTADO MÉDICO - ATE 15 DIAS	HC da Universidade Federal de Goiás - GO
57	AFASTAMENTO POR ATESTADO MÉDICO - ATE 15 DIAS	HC da Universidade Federal de Goiás - GO
57	AFASTAMENTO POR ATESTADO MÉDICO - ATE 15 DIAS	HC da Universidade Federal de Goiás - GO
57	AFASTAMENTO POR ATESTADO MÉDICO - ATE 15 DIAS	HC da Universidade Federal de Goiás - GO
57	AFASTAMENTO POR ATESTADO MÉDICO - ATE 15 DIAS	HC da Universidade Federal de Goiás - GO
57	AFASTAMENTO POR ATESTADO MÉDICO - ATE 15 DIAS	HC da Universidade Federal de Goiás - GO
57	AFASTAMENTO POR ATESTADO MÉDICO - ATE 15 DIAS	HC da Universidade Federal de Goiás - GO
57	AFASTAMENTO POR ATESTADO MÉDICO - ATE 15 DIAS	HC da Universidade Federal de Goiás - GO
57	AFASTAMENTO POR ATESTADO MÉDICO - ATE 15 DIAS	HC da Universidade Federal de Goiás - GO
57	AFASTAMENTO POR ATESTADO MÉDICO - ATE 15 DIAS	HC da Universidade Federal de Goiás - GO
57	AFASTAMENTO POR ATESTADO MÉDICO - ATE 15 DIAS	HC da Universidade Federal de Goiás - GO
57	AFASTAMENTO POR ATESTADO MÉDICO - ATE 15 DIAS	HC da Universidade Federal de Goiás - GO
57	AFASTAMENTO POR ATESTADO MÉDICO - ATE 15 DIAS	HC da Universidade Federal de Goiás - GO
57	AFASTAMENTO POR ATESTADO MÉDICO - ATE 15 DIAS	HC da Universidade Federal de Goiás - GO
57	AFASTAMENTO POR ATESTADO MÉDICO - ATE 15 DIAS	HC da Universidade Federal de Goiás - GO
57	AFASTAMENTO POR ATESTADO MÉDICO - ATE 15 DIAS	HC da Universidade Federal de Goiás - GO
57	AFASTAMENTO POR ATESTADO MÉDICO - ATE 15 DIAS	HC da Universidade Federal de Goiás - GO
57	AFASTAMENTO POR ATESTADO MÉDICO - ATE 15 DIAS	HC da Universidade Federal de Goiás - GO
57	AFASTAMENTO POR ATESTADO MÉDICO - ATE 15 DIAS	HC da Universidade Federal de Goiás - GO
57	AFASTAMENTO POR ATESTADO MÉDICO - ATE 15 DIAS	HC da Universidade Federal de Goiás - GO
57	AFASTAMENTO POR ATESTADO MÉDICO - ATE 15 DIAS	HC da Universidade Federal de Goiás - GO
57	AFASTAMENTO POR ATESTADO MÉDICO - ATE 15 DIAS	HC da Universidade Federal de Goiás - GO
57	AFASTAMENTO POR ATESTADO MÉDICO - ATE 15 DIAS	HC da Universidade Federal de Goiás - GO
57	AFASTAMENTO POR ATESTADO MÉDICO - ATE 15 DIAS	HC da Universidade Federal de Goiás - GO
57	AFASTAMENTO POR ATESTADO MÉDICO - ATE 15 DIAS	HC da Universidade Federal de Goiás - GO
57	AFASTAMENTO POR ATESTADO MÉDICO - ATE 15 DIAS	HC da Universidade Federal de Goiás - GO
57	AFASTAMENTO POR ATESTADO MÉDICO - ATE 15 DIAS	HC da Universidade Federal de Goiás - GO
57	AFASTAMENTO POR ATESTADO MÉDICO - ATE 15 DIAS	HC da Universidade Federal de Goiás - GO
57	AFASTAMENTO POR ATESTADO MÉDICO - ATE 15 DIAS	HC da Universidade Federal de Goiás - GO

57	AFASTAMENTO POR ATESTADO MÉDICO - ATE 15 DIAS	HC da Universidade Federal de Goiás - GO
57	AFASTAMENTO POR ATESTADO MÉDICO - ATE 15 DIAS	HC da Universidade Federal de Goiás - GO
57	AFASTAMENTO POR ATESTADO MÉDICO - ATE 15 DIAS	HC da Universidade Federal de Goiás - GO
57	AFASTAMENTO POR ATESTADO MÉDICO - ATE 15 DIAS	HC da Universidade Federal de Goiás - GO
57	AFASTAMENTO POR ATESTADO MÉDICO - ATE 15 DIAS	HC da Universidade Federal de Goiás - GO
57	AFASTAMENTO POR ATESTADO MÉDICO - ATE 15 DIAS	HC da Universidade Federal de Goiás - GO
57	AFASTAMENTO POR ATESTADO MÉDICO - ATE 15 DIAS	HC da Universidade Federal de Goiás - GO
57	AFASTAMENTO POR ATESTADO MÉDICO - ATE 15 DIAS	HC da Universidade Federal de Goiás - GO
57	AFASTAMENTO POR ATESTADO MÉDICO - ATE 15 DIAS	HC da Universidade Federal de Goiás - GO
57	AFASTAMENTO POR ATESTADO MÉDICO - ATE 15 DIAS	HC da Universidade Federal de Goiás - GO
57	AFASTAMENTO POR ATESTADO MÉDICO - ATE 15 DIAS	HC da Universidade Federal de Goiás - GO
57	AFASTAMENTO POR ATESTADO MÉDICO - ATE 15 DIAS	HC da Universidade Federal de Goiás - GO
57	AFASTAMENTO POR ATESTADO MÉDICO - ATE 15 DIAS	HC da Universidade Federal de Goiás - GO
57	AFASTAMENTO POR ATESTADO MÉDICO - ATE 15 DIAS	HC da Universidade Federal de Goiás - GO
57	AFASTAMENTO POR ATESTADO MÉDICO - ATE 15 DIAS	HC da Universidade Federal de Goiás - GO
57	AFASTAMENTO POR ATESTADO MÉDICO - ATE 15 DIAS	HC da Universidade Federal de Goiás - GO
57	AFASTAMENTO POR ATESTADO MÉDICO - ATE 15 DIAS	HC da Universidade Federal de Goiás - GO
57	AFASTAMENTO POR ATESTADO MÉDICO - ATE 15 DIAS	HC da Universidade Federal de Goiás - GO
57	AFASTAMENTO POR ATESTADO MÉDICO - ATE 15 DIAS	HC da Universidade Federal de Goiás - GO
57	AFASTAMENTO POR ATESTADO MÉDICO - ATE 15 DIAS	HC da Universidade Federal de Goiás - GO
57	AFASTAMENTO POR ATESTADO MÉDICO - ATE 15 DIAS	HC da Universidade Federal de Goiás - GO
57	AFASTAMENTO POR ATESTADO MÉDICO - ATE 15 DIAS	HC da Universidade Federal de Goiás - GO
57	AFASTAMENTO POR ATESTADO MÉDICO - ATE 15 DIAS	HC da Universidade Federal de Goiás - GO
57	AFASTAMENTO POR ATESTADO MÉDICO - ATE 15 DIAS	HC da Universidade Federal de Goiás - GO
57	AFASTAMENTO POR ATESTADO MÉDICO - ATE 15 DIAS	HC da Universidade Federal de Goiás - GO
57	AFASTAMENTO POR ATESTADO MÉDICO - ATE 15 DIAS	HC da Universidade Federal de Goiás - GO
57	AFASTAMENTO POR ATESTADO MÉDICO - ATE 15 DIAS	HC da Universidade Federal de Goiás - GO
57	AFASTAMENTO POR ATESTADO MÉDICO - ATE 15 DIAS	HC da Universidade Federal de Goiás - GO
57	AFASTAMENTO POR ATESTADO MÉDICO - ATE 15 DIAS	HC da Universidade Federal de Goiás - GO
57	AFASTAMENTO POR ATESTADO MÉDICO - ATE 15 DIAS	HC da Universidade Federal de Goiás - GO
57	AFASTAMENTO POR ATESTADO MÉDICO - ATE 15 DIAS	HC da Universidade Federal de Goiás - GO
57	AFASTAMENTO POR ATESTADO MÉDICO - ATE 15 DIAS	HC da Universidade Federal de Goiás - GO
57	AFASTAMENTO POR ATESTADO MÉDICO - ATE 15 DIAS	HC da Universidade Federal de Goiás - GO
57	AFASTAMENTO POR ATESTADO MÉDICO - ATE 15 DIAS	HC da Universidade Federal de Goiás - GO
57	AFASTAMENTO POR ATESTADO MÉDICO - ATE 15 DIAS	HC da Universidade Federal de Goiás - GO
57	AFASTAMENTO POR ATESTADO MÉDICO - ATE 15 DIAS	HC da Universidade Federal de Goiás - GO
57	AFASTAMENTO POR ATESTADO MÉDICO - ATE 15 DIAS	HC da Universidade Federal de Goiás - GO
57	AFASTAMENTO POR ATESTADO MÉDICO - ATE 15 DIAS	HC da Universidade Federal de Goiás - GO
57	AFASTAMENTO POR ATESTADO MÉDICO - ATE 15 DIAS	HC da Universidade Federal de Goiás - GO
57	AFASTAMENTO POR ATESTADO MÉDICO - ATE 15 DIAS	HC da Universidade Federal de Goiás - GO
57	AFASTAMENTO POR ATESTADO MÉDICO - ATE 15 DIAS	HC da Universidade Federal de Goiás - GO
57	AFASTAMENTO POR ATESTADO MÉDICO - ATE 15 DIAS	HC da Universidade Federal de Goiás - GO
57	AFASTAMENTO POR ATESTADO MÉDICO - ATE 15 DIAS	HC da Universidade Federal de Goiás - GO
57	AFASTAMENTO POR ATESTADO MÉDICO - ATE 15 DIAS	HC da Universidade Federal de Goiás - GO
57	AFASTAMENTO POR ATESTADO MÉDICO - ATE 15 DIAS	HC da Universidade Federal de Goiás - GO
57	AFASTAMENTO POR ATESTADO MÉDICO - ATE 15 DIAS	HC da Universidade Federal de Goiás - GO
57	AFASTAMENTO POR ATESTADO MÉDICO - ATE 15 DIAS	HC da Universidade Federal de Goiás - GO
57	AFASTAMENTO POR ATESTADO MÉDICO - ATE 15 DIAS	HC da Universidade Federal de Goiás - GO

57	AFASTAMENTO POR ATESTADO MÉDICO - ATE 15 DIAS	HC da Universidade Federal de Goiás - GO
57	AFASTAMENTO POR ATESTADO MÉDICO - ATE 15 DIAS	HC da Universidade Federal de Goiás - GO
57	AFASTAMENTO POR ATESTADO MÉDICO - ATE 15 DIAS	HC da Universidade Federal de Goiás - GO
57	AFASTAMENTO POR ATESTADO MÉDICO - ATE 15 DIAS	HC da Universidade Federal de Goiás - GO
57	AFASTAMENTO POR ATESTADO MÉDICO - ATE 15 DIAS	HC da Universidade Federal de Goiás - GO
57	AFASTAMENTO POR ATESTADO MÉDICO - ATE 15 DIAS	HC da Universidade Federal de Goiás - GO
57	AFASTAMENTO POR ATESTADO MÉDICO - ATE 15 DIAS	HC da Universidade Federal de Goiás - GO
57	AFASTAMENTO POR ATESTADO MÉDICO - ATE 15 DIAS	HC da Universidade Federal de Goiás - GO
57	AFASTAMENTO POR ATESTADO MÉDICO - ATE 15 DIAS	HC da Universidade Federal de Goiás - GO
57	AFASTAMENTO POR ATESTADO MÉDICO - ATE 15 DIAS	HC da Universidade Federal de Goiás - GO
57	AFASTAMENTO POR ATESTADO MÉDICO - ATE 15 DIAS	HC da Universidade Federal de Goiás - GO
57	AFASTAMENTO POR ATESTADO MÉDICO - ATE 15 DIAS	HC da Universidade Federal de Goiás - GO
57	AFASTAMENTO POR ATESTADO MÉDICO - ATE 15 DIAS	HC da Universidade Federal de Goiás - GO
57	AFASTAMENTO POR ATESTADO MÉDICO - ATE 15 DIAS	HC da Universidade Federal de Goiás - GO
57	AFASTAMENTO POR ATESTADO MÉDICO - ATE 15 DIAS	HC da Universidade Federal de Goiás - GO
57	AFASTAMENTO POR ATESTADO MÉDICO - ATE 15 DIAS	HC da Universidade Federal de Goiás - GO
57	AFASTAMENTO POR ATESTADO MÉDICO - ATE 15 DIAS	HC da Universidade Federal de Goiás - GO
57	AFASTAMENTO POR ATESTADO MÉDICO - ATE 15 DIAS	HC da Universidade Federal de Goiás - GO
57	AFASTAMENTO POR ATESTADO MÉDICO - ATE 15 DIAS	HC da Universidade Federal de Goiás - GO
57	AFASTAMENTO POR ATESTADO MÉDICO - ATE 15 DIAS	HC da Universidade Federal de Goiás - GO
57	AFASTAMENTO POR ATESTADO MÉDICO - ATE 15 DIAS	HC da Universidade Federal de Goiás - GO
57	AFASTAMENTO POR ATESTADO MÉDICO - ATE 15 DIAS	HC da Universidade Federal de Goiás - GO
57	AFASTAMENTO POR ATESTADO MÉDICO - ATE 15 DIAS	HC da Universidade Federal de Goiás - GO
57	AFASTAMENTO POR ATESTADO MÉDICO - ATE 15 DIAS	HC da Universidade Federal de Goiás - GO
57	AFASTAMENTO POR ATESTADO MÉDICO - ATE 15 DIAS	HC da Universidade Federal de Goiás - GO
57	AFASTAMENTO POR ATESTADO MÉDICO - ATE 15 DIAS	HC da Universidade Federal de Goiás - GO
57	AFASTAMENTO POR ATESTADO MÉDICO - ATE 15 DIAS	HC da Universidade Federal de Goiás - GO
57	AFASTAMENTO POR ATESTADO MÉDICO - ATE 15 DIAS	HC da Universidade Federal de Goiás - GO
57	AFASTAMENTO POR ATESTADO MÉDICO - ATE 15 DIAS	HC da Universidade Federal de Goiás - GO
57	AFASTAMENTO POR ATESTADO MÉDICO - ATE 15 DIAS	HC da Universidade Federal de Goiás - GO
57	AFASTAMENTO POR ATESTADO MÉDICO - ATE 15 DIAS	HC da Universidade Federal de Goiás - GO
57	AFASTAMENTO POR ATESTADO MÉDICO - ATE 15 DIAS	HC da Universidade Federal de Goiás - GO
57	AFASTAMENTO POR ATESTADO MÉDICO - ATE 15 DIAS	HC da Universidade Federal de Goiás - GO
57	AFASTAMENTO POR ATESTADO MÉDICO - ATE 15 DIAS	HC da Universidade Federal de Goiás - GO
57	AFASTAMENTO POR ATESTADO MÉDICO - ATE 15 DIAS	HC da Universidade Federal de Goiás - GO
57	AFASTAMENTO POR ATESTADO MÉDICO - ATE 15 DIAS	HC da Universidade Federal de Goiás - GO
57	AFASTAMENTO POR ATESTADO MÉDICO - ATE 15 DIAS	HC da Universidade Federal de Goiás - GO
57	AFASTAMENTO POR ATESTADO MÉDICO - ATE 15 DIAS	HC da Universidade Federal de Goiás - GO
57	AFASTAMENTO POR ATESTADO MÉDICO - ATE 15 DIAS	HC da Universidade Federal de Goiás - GO
57	AFASTAMENTO POR ATESTADO MÉDICO - ATE 15 DIAS	HC da Universidade Federal de Goiás - GO
57	AFASTAMENTO POR ATESTADO MÉDICO - ATE 15 DIAS	HC da Universidade Federal de Goiás - GO
57	AFASTAMENTO POR ATESTADO MÉDICO - ATE 15 DIAS	HC da Universidade Federal de Goiás - GO
57	AFASTAMENTO POR ATESTADO MÉDICO - ATE 15 DIAS	HC da Universidade Federal de Goiás - GO
57	AFASTAMENTO POR ATESTADO MÉDICO - ATE 15 DIAS	HC da Universidade Federal de Goiás - GO
57	AFASTAMENTO POR ATESTADO MÉDICO - ATE 15 DIAS	HC da Universidade Federal de Goiás - GO
57	AFASTAMENTO POR ATESTADO MÉDICO - ATE 15 DIAS	HC da Universidade Federal de Goiás - GO
57	AFASTAMENTO POR ATESTADO MÉDICO - ATE 15 DIAS	HC da Universidade Federal de Goiás - GO
57	AFASTAMENTO POR ATESTADO MÉDICO - ATE 15 DIAS	HC da Universidade Federal de Goiás - GO

57	AFASTAMENTO POR ATESTADO MÉDICO - ATE 15 DIAS	HC da Universidade Federal de Goiás - GO
57	AFASTAMENTO POR ATESTADO MÉDICO - ATE 15 DIAS	HC da Universidade Federal de Goiás - GO
57	AFASTAMENTO POR ATESTADO MÉDICO - ATE 15 DIAS	HC da Universidade Federal de Goiás - GO
57	AFASTAMENTO POR ATESTADO MÉDICO - ATE 15 DIAS	HC da Universidade Federal de Goiás - GO
57	AFASTAMENTO POR ATESTADO MÉDICO - ATE 15 DIAS	HC da Universidade Federal de Goiás - GO
57	AFASTAMENTO POR ATESTADO MÉDICO - ATE 15 DIAS	HC da Universidade Federal de Goiás - GO
57	AFASTAMENTO POR ATESTADO MÉDICO - ATE 15 DIAS	HC da Universidade Federal de Goiás - GO
57	AFASTAMENTO POR ATESTADO MÉDICO - ATE 15 DIAS	HC da Universidade Federal de Goiás - GO
57	AFASTAMENTO POR ATESTADO MÉDICO - ATE 15 DIAS	HC da Universidade Federal de Goiás - GO
57	AFASTAMENTO POR ATESTADO MÉDICO - ATE 15 DIAS	HC da Universidade Federal de Goiás - GO
57	AFASTAMENTO POR ATESTADO MÉDICO - ATE 15 DIAS	HC da Universidade Federal de Goiás - GO
57	AFASTAMENTO POR ATESTADO MÉDICO - ATE 15 DIAS	HC da Universidade Federal de Goiás - GO
57	AFASTAMENTO POR ATESTADO MÉDICO - ATE 15 DIAS	HC da Universidade Federal de Goiás - GO
57	AFASTAMENTO POR ATESTADO MÉDICO - ATE 15 DIAS	HC da Universidade Federal de Goiás - GO
57	AFASTAMENTO POR ATESTADO MÉDICO - ATE 15 DIAS	HC da Universidade Federal de Goiás - GO
57	AFASTAMENTO POR ATESTADO MÉDICO - ATE 15 DIAS	HC da Universidade Federal de Goiás - GO
57	AFASTAMENTO POR ATESTADO MÉDICO - ATE 15 DIAS	HC da Universidade Federal de Goiás - GO
57	AFASTAMENTO POR ATESTADO MÉDICO - ATE 15 DIAS	HC da Universidade Federal de Goiás - GO
57	AFASTAMENTO POR ATESTADO MÉDICO - ATE 15 DIAS	HC da Universidade Federal de Goiás - GO
57	AFASTAMENTO POR ATESTADO MÉDICO - ATE 15 DIAS	HC da Universidade Federal de Goiás - GO
57	AFASTAMENTO POR ATESTADO MÉDICO - ATE 15 DIAS	HC da Universidade Federal de Goiás - GO
57	AFASTAMENTO POR ATESTADO MÉDICO - ATE 15 DIAS	HC da Universidade Federal de Goiás - GO
57	AFASTAMENTO POR ATESTADO MÉDICO - ATE 15 DIAS	HC da Universidade Federal de Goiás - GO
57	AFASTAMENTO POR ATESTADO MÉDICO - ATE 15 DIAS	HC da Universidade Federal de Goiás - GO
57	AFASTAMENTO POR ATESTADO MÉDICO - ATE 15 DIAS	HC da Universidade Federal de Goiás - GO
58	AFASTAMENTO POR ATESTADO ODONTOLOGICO - ATE 15 DIAS	HC da Universidade Federal de Goiás - GO
58	AFASTAMENTO POR ATESTADO ODONTOLOGICO - ATE 15 DIAS	HC da Universidade Federal de Goiás - GO
58	AFASTAMENTO POR ATESTADO ODONTOLOGICO - ATE 15 DIAS	HC da Universidade Federal de Goiás - GO
58	AFASTAMENTO POR ATESTADO ODONTOLOGICO - ATE 15 DIAS	HC da Universidade Federal de Goiás - GO
58	AFASTAMENTO POR ATESTADO ODONTOLOGICO - ATE 15 DIAS	HC da Universidade Federal de Goiás - GO
58	AFASTAMENTO POR ATESTADO ODONTOLOGICO - ATE 15 DIAS	HC da Universidade Federal de Goiás - GO
58	AFASTAMENTO POR ATESTADO ODONTOLOGICO - ATE 15 DIAS	HC da Universidade Federal de Goiás - GO
58	AFASTAMENTO POR ATESTADO ODONTOLOGICO - ATE 15 DIAS	HC da Universidade Federal de Goiás - GO
58	AFASTAMENTO POR ATESTADO ODONTOLOGICO - ATE 15 DIAS	HC da Universidade Federal de Goiás - GO
58	AFASTAMENTO POR ATESTADO ODONTOLOGICO - ATE 15 DIAS	HC da Universidade Federal de Goiás - GO
58	AFASTAMENTO POR ATESTADO ODONTOLOGICO - ATE 15 DIAS	HC da Universidade Federal de Goiás - GO
58	AFASTAMENTO POR ATESTADO ODONTOLOGICO - ATE 15 DIAS	HC da Universidade Federal de Goiás - GO
58	AFASTAMENTO POR ATESTADO ODONTOLOGICO - ATE 15 DIAS	HC da Universidade Federal de Goiás - GO
58	AFASTAMENTO POR ATESTADO ODONTOLOGICO - ATE 15 DIAS	HC da Universidade Federal de Goiás - GO
58	AFASTAMENTO POR ATESTADO ODONTOLOGICO - ATE 15 DIAS	HC da Universidade Federal de Goiás - GO

58	AFASTAMENTO POR ATESTADO ODONTOLOGICO - ATE 15 DIAS	HC da Universidade Federal de Goiás - GO
58	AFASTAMENTO POR ATESTADO ODONTOLOGICO - ATE 15 DIAS	HC da Universidade Federal de Goiás - GO
58	AFASTAMENTO POR ATESTADO ODONTOLOGICO - ATE 15 DIAS	HC da Universidade Federal de Goiás - GO
58	AFASTAMENTO POR ATESTADO ODONTOLOGICO - ATE 15 DIAS	HC da Universidade Federal de Goiás - GO
58	AFASTAMENTO POR ATESTADO ODONTOLOGICO - ATE 15 DIAS	HC da Universidade Federal de Goiás - GO
58	AFASTAMENTO POR ATESTADO ODONTOLOGICO - ATE 15 DIAS	HC da Universidade Federal de Goiás - GO
58	AFASTAMENTO POR ATESTADO ODONTOLOGICO - ATE 15 DIAS	HC da Universidade Federal de Goiás - GO
58	AFASTAMENTO POR ATESTADO ODONTOLOGICO - ATE 15 DIAS	HC da Universidade Federal de Goiás - GO
58	AFASTAMENTO POR ATESTADO ODONTOLOGICO - ATE 15 DIAS	HC da Universidade Federal de Goiás - GO
58	AFASTAMENTO POR ATESTADO ODONTOLOGICO - ATE 15 DIAS	HC da Universidade Federal de Goiás - GO
58	AFASTAMENTO POR ATESTADO ODONTOLOGICO - ATE 15 DIAS	HC da Universidade Federal de Goiás - GO
58	AFASTAMENTO POR ATESTADO ODONTOLOGICO - ATE 15 DIAS	HC da Universidade Federal de Goiás - GO
58	AFASTAMENTO POR ATESTADO ODONTOLOGICO - ATE 15 DIAS	HC da Universidade Federal de Goiás - GO
58	AFASTAMENTO POR ATESTADO ODONTOLOGICO - ATE 15 DIAS	HC da Universidade Federal de Goiás - GO
62	LICENÇA PATERNIDADE CELETISTA - Até 5 DIAS - ADCT CF ART 10	HC da Universidade Federal de Goiás - GO
62	LICENÇA PATERNIDADE CELETISTA - Até 5 DIAS - ADCT CF ART 10	HC da Universidade Federal de Goiás - GO
62	LICENÇA PATERNIDADE CELETISTA - Até 5 DIAS - ADCT CF ART 10	HC da Universidade Federal de Goiás - GO
62	LICENÇA PATERNIDADE CELETISTA - Até 5 DIAS - ADCT CF ART 10	HC da Universidade Federal de Goiás - GO
62	LICENÇA PATERNIDADE CELETISTA - Até 5 DIAS - ADCT CF ART 10	HC da Universidade Federal de Goiás - GO
62	LICENÇA PATERNIDADE CELETISTA - Até 5 DIAS - ADCT CF ART 10	HC da Universidade Federal de Goiás - GO
62	LICENÇA PATERNIDADE CELETISTA - Até 5 DIAS - ADCT CF ART 10	HC da Universidade Federal de Goiás - GO
62	LICENÇA PATERNIDADE CELETISTA - Até 5 DIAS - ADCT CF ART 10	HC da Universidade Federal de Goiás - GO
62	LICENÇA PATERNIDADE CELETISTA - Até 5 DIAS - ADCT CF ART 10	HC da Universidade Federal de Goiás - GO
62	LICENÇA PATERNIDADE CELETISTA - Até 5 DIAS - ADCT CF ART 10	HC da Universidade Federal de Goiás - GO
68	COMPARECIMENTO A JUIZO (JURI, INQUERITO POLICIAL, PROC. JUDICIAL) - CLT ART. 473 VIII	HC da Universidade Federal de Goiás - GO
68	COMPARECIMENTO A JUIZO (JURI, INQUERITO POLICIAL, PROC. JUDICIAL) - CLT ART. 473 VIII	HC da Universidade Federal de Goiás - GO
68	COMPARECIMENTO A JUIZO (JURI, INQUERITO POLICIAL, PROC. JUDICIAL) - CLT ART. 473 VIII	HC da Universidade Federal de Goiás - GO
69	ABONO (ACT)	HC da Universidade Federal de Goiás - GO
69	ABONO (ACT)	HC da Universidade Federal de Goiás - GO
69	ABONO (ACT)	HC da Universidade Federal de Goiás - GO
69	ABONO (ACT)	HC da Universidade Federal de Goiás - GO
69	ABONO (ACT)	HC da Universidade Federal de Goiás - GO
69	ABONO (ACT)	HC da Universidade Federal de Goiás - GO
69	ABONO (ACT)	HC da Universidade Federal de Goiás - GO

69	ABONO (ACT)	HC da Universidade Federal de Goiás - GO
69	ABONO (ACT)	HC da Universidade Federal de Goiás - GO
69	ABONO (ACT)	HC da Universidade Federal de Goiás - GO
69	ABONO (ACT)	HC da Universidade Federal de Goiás - GO
69	ABONO (ACT)	HC da Universidade Federal de Goiás - GO
69	ABONO (ACT)	HC da Universidade Federal de Goiás - GO
69	ABONO (ACT)	HC da Universidade Federal de Goiás - GO
69	ABONO (ACT)	HC da Universidade Federal de Goiás - GO
69	ABONO (ACT)	HC da Universidade Federal de Goiás - GO
69	ABONO (ACT)	HC da Universidade Federal de Goiás - GO
69	ABONO (ACT)	HC da Universidade Federal de Goiás - GO
69	ABONO (ACT)	HC da Universidade Federal de Goiás - GO
69	ABONO (ACT)	HC da Universidade Federal de Goiás - GO
69	ABONO (ACT)	HC da Universidade Federal de Goiás - GO
69	ABONO (ACT)	HC da Universidade Federal de Goiás - GO
69	ABONO (ACT)	HC da Universidade Federal de Goiás - GO
69	ABONO (ACT)	HC da Universidade Federal de Goiás - GO
69	ABONO (ACT)	HC da Universidade Federal de Goiás - GO
69	ABONO (ACT)	HC da Universidade Federal de Goiás - GO
69	ABONO (ACT)	HC da Universidade Federal de Goiás - GO
69	ABONO (ACT)	HC da Universidade Federal de Goiás - GO
69	ABONO (ACT)	HC da Universidade Federal de Goiás - GO
69	ABONO (ACT)	HC da Universidade Federal de Goiás - GO
69	ABONO (ACT)	HC da Universidade Federal de Goiás - GO
69	ABONO (ACT)	HC da Universidade Federal de Goiás - GO
69	ABONO (ACT)	HC da Universidade Federal de Goiás - GO
69	ABONO (ACT)	HC da Universidade Federal de Goiás - GO
69	ABONO (ACT)	HC da Universidade Federal de Goiás - GO
69	ABONO (ACT)	HC da Universidade Federal de Goiás - GO
69	ABONO (ACT)	HC da Universidade Federal de Goiás - GO
69	ABONO (ACT)	HC da Universidade Federal de Goiás - GO
69	ABONO (ACT)	HC da Universidade Federal de Goiás - GO
69	ABONO (ACT)	HC da Universidade Federal de Goiás - GO
69	ABONO (ACT)	HC da Universidade Federal de Goiás - GO
69	ABONO (ACT)	HC da Universidade Federal de Goiás - GO
69	ABONO (ACT)	HC da Universidade Federal de Goiás - GO
69	ABONO (ACT)	HC da Universidade Federal de Goiás - GO
69	ABONO (ACT)	HC da Universidade Federal de Goiás - GO
69	ABONO (ACT)	HC da Universidade Federal de Goiás - GO
69	ABONO (ACT)	HC da Universidade Federal de Goiás - GO
69	ABONO (ACT)	HC da Universidade Federal de Goiás - GO
69	ABONO (ACT)	HC da Universidade Federal de Goiás - GO
69	ABONO (ACT)	HC da Universidade Federal de Goiás - GO
69	ABONO (ACT)	HC da Universidade Federal de Goiás - GO
69	ABONO (ACT)	HC da Universidade Federal de Goiás - GO
69	ABONO (ACT)	HC da Universidade Federal de Goiás - GO

69	ABONO (ACT)	HC da Universidade Federal de Goiás - GO
69	ABONO (ACT)	HC da Universidade Federal de Goiás - GO
69	ABONO (ACT)	HC da Universidade Federal de Goiás - GO
69	ABONO (ACT)	HC da Universidade Federal de Goiás - GO
69	ABONO (ACT)	HC da Universidade Federal de Goiás - GO
69	ABONO (ACT)	HC da Universidade Federal de Goiás - GO
69	ABONO (ACT)	HC da Universidade Federal de Goiás - GO
69	ABONO (ACT)	HC da Universidade Federal de Goiás - GO
69	ABONO (ACT)	HC da Universidade Federal de Goiás - GO
69	ABONO (ACT)	HC da Universidade Federal de Goiás - GO
69	ABONO (ACT)	HC da Universidade Federal de Goiás - GO
69	ABONO (ACT)	HC da Universidade Federal de Goiás - GO
69	ABONO (ACT)	HC da Universidade Federal de Goiás - GO
69	ABONO (ACT)	HC da Universidade Federal de Goiás - GO
69	ABONO (ACT)	HC da Universidade Federal de Goiás - GO
69	ABONO (ACT)	HC da Universidade Federal de Goiás - GO
69	ABONO (ACT)	HC da Universidade Federal de Goiás - GO
69	ABONO (ACT)	HC da Universidade Federal de Goiás - GO
69	ABONO (ACT)	HC da Universidade Federal de Goiás - GO
69	ABONO (ACT)	HC da Universidade Federal de Goiás - GO
69	ABONO (ACT)	HC da Universidade Federal de Goiás - GO
69	ABONO (ACT)	HC da Universidade Federal de Goiás - GO
69	ABONO (ACT)	HC da Universidade Federal de Goiás - GO
69	ABONO (ACT)	HC da Universidade Federal de Goiás - GO
69	ABONO (ACT)	HC da Universidade Federal de Goiás - GO
69	ABONO (ACT)	HC da Universidade Federal de Goiás - GO
69	ABONO (ACT)	HC da Universidade Federal de Goiás - GO
69	ABONO (ACT)	HC da Universidade Federal de Goiás - GO
69	ABONO (ACT)	HC da Universidade Federal de Goiás - GO
69	ABONO (ACT)	HC da Universidade Federal de Goiás - GO
69	ABONO (ACT)	HC da Universidade Federal de Goiás - GO
69	ABONO (ACT)	HC da Universidade Federal de Goiás - GO
69	ABONO (ACT)	HC da Universidade Federal de Goiás - GO
69	ABONO (ACT)	HC da Universidade Federal de Goiás - GO
69	ABONO (ACT)	HC da Universidade Federal de Goiás - GO
69	ABONO (ACT)	HC da Universidade Federal de Goiás - GO
69	ABONO (ACT)	HC da Universidade Federal de Goiás - GO
69	ABONO (ACT)	HC da Universidade Federal de Goiás - GO
69	ABONO (ACT)	HC da Universidade Federal de Goiás - GO
69	ABONO (ACT)	HC da Universidade Federal de Goiás - GO
69	ABONO (ACT)	HC da Universidade Federal de Goiás - GO
69	ABONO (ACT)	HC da Universidade Federal de Goiás - GO
69	ABONO (ACT)	HC da Universidade Federal de Goiás - GO
69	ABONO (ACT)	HC da Universidade Federal de Goiás - GO
69	ABONO (ACT)	HC da Universidade Federal de Goiás - GO

69	ABONO (ACT)	HC da Universidade Federal de Goiás - GO
69	ABONO (ACT)	HC da Universidade Federal de Goiás - GO
69	ABONO (ACT)	HC da Universidade Federal de Goiás - GO
69	ABONO (ACT)	HC da Universidade Federal de Goiás - GO
69	ABONO (ACT)	HC da Universidade Federal de Goiás - GO
69	ABONO (ACT)	HC da Universidade Federal de Goiás - GO
69	ABONO (ACT)	HC da Universidade Federal de Goiás - GO
69	ABONO (ACT)	HC da Universidade Federal de Goiás - GO
69	ABONO (ACT)	HC da Universidade Federal de Goiás - GO
69	ABONO (ACT)	HC da Universidade Federal de Goiás - GO
69	ABONO (ACT)	HC da Universidade Federal de Goiás - GO
69	ABONO (ACT)	HC da Universidade Federal de Goiás - GO
69	ABONO (ACT)	HC da Universidade Federal de Goiás - GO
69	ABONO (ACT)	HC da Universidade Federal de Goiás - GO
69	ABONO (ACT)	HC da Universidade Federal de Goiás - GO
69	ABONO (ACT)	HC da Universidade Federal de Goiás - GO
69	ABONO (ACT)	HC da Universidade Federal de Goiás - GO
69	ABONO (ACT)	HC da Universidade Federal de Goiás - GO
69	ABONO (ACT)	HC da Universidade Federal de Goiás - GO
69	ABONO (ACT)	HC da Universidade Federal de Goiás - GO
69	ABONO (ACT)	HC da Universidade Federal de Goiás - GO
69	ABONO (ACT)	HC da Universidade Federal de Goiás - GO
69	ABONO (ACT)	HC da Universidade Federal de Goiás - GO
69	ABONO (ACT)	HC da Universidade Federal de Goiás - GO
69	ABONO (ACT)	HC da Universidade Federal de Goiás - GO
69	ABONO (ACT)	HC da Universidade Federal de Goiás - GO
69	ABONO (ACT)	HC da Universidade Federal de Goiás - GO
69	ABONO (ACT)	HC da Universidade Federal de Goiás - GO
69	ABONO (ACT)	HC da Universidade Federal de Goiás - GO
69	ABONO (ACT)	HC da Universidade Federal de Goiás - GO
69	ABONO (ACT)	HC da Universidade Federal de Goiás - GO
69	ABONO (ACT)	HC da Universidade Federal de Goiás - GO
69	ABONO (ACT)	HC da Universidade Federal de Goiás - GO
69	ABONO (ACT)	HC da Universidade Federal de Goiás - GO
69	ABONO (ACT)	HC da Universidade Federal de Goiás - GO
69	ABONO (ACT)	HC da Universidade Federal de Goiás - GO
69	ABONO (ACT)	HC da Universidade Federal de Goiás - GO
69	ABONO (ACT)	HC da Universidade Federal de Goiás - GO
69	ABONO (ACT)	HC da Universidade Federal de Goiás - GO
69	ABONO (ACT)	HC da Universidade Federal de Goiás - GO
69	ABONO (ACT)	HC da Universidade Federal de Goiás - GO
69	ABONO (ACT)	HC da Universidade Federal de Goiás - GO
69	ABONO (ACT)	HC da Universidade Federal de Goiás - GO
69	ABONO (ACT)	HC da Universidade Federal de Goiás - GO

69	ABONO (ACT)	HC da Universidade Federal de Goiás - GO
69	ABONO (ACT)	HC da Universidade Federal de Goiás - GO
69	ABONO (ACT)	HC da Universidade Federal de Goiás - GO
69	ABONO (ACT)	HC da Universidade Federal de Goiás - GO
69	ABONO (ACT)	HC da Universidade Federal de Goiás - GO
69	ABONO (ACT)	HC da Universidade Federal de Goiás - GO
69	ABONO (ACT)	HC da Universidade Federal de Goiás - GO
69	ABONO (ACT)	HC da Universidade Federal de Goiás - GO
69	ABONO (ACT)	HC da Universidade Federal de Goiás - GO
69	ABONO (ACT)	HC da Universidade Federal de Goiás - GO
69	ABONO (ACT)	HC da Universidade Federal de Goiás - GO
69	ABONO (ACT)	HC da Universidade Federal de Goiás - GO
69	ABONO (ACT)	HC da Universidade Federal de Goiás - GO
69	ABONO (ACT)	HC da Universidade Federal de Goiás - GO
69	ABONO (ACT)	HC da Universidade Federal de Goiás - GO
69	ABONO (ACT)	HC da Universidade Federal de Goiás - GO
69	ABONO (ACT)	HC da Universidade Federal de Goiás - GO
69	ABONO (ACT)	HC da Universidade Federal de Goiás - GO
69	ABONO (ACT)	HC da Universidade Federal de Goiás - GO
69	ABONO (ACT)	HC da Universidade Federal de Goiás - GO
69	ABONO (ACT)	HC da Universidade Federal de Goiás - GO
69	ABONO (ACT)	HC da Universidade Federal de Goiás - GO
69	ABONO (ACT)	HC da Universidade Federal de Goiás - GO
69	ABONO (ACT)	HC da Universidade Federal de Goiás - GO
69	ABONO (ACT)	HC da Universidade Federal de Goiás - GO
69	ABONO (ACT)	HC da Universidade Federal de Goiás - GO
69	ABONO (ACT)	HC da Universidade Federal de Goiás - GO
69	ABONO (ACT)	HC da Universidade Federal de Goiás - GO
69	ABONO (ACT)	HC da Universidade Federal de Goiás - GO
69	ABONO (ACT)	HC da Universidade Federal de Goiás - GO
69	ABONO (ACT)	HC da Universidade Federal de Goiás - GO
69	ABONO (ACT)	HC da Universidade Federal de Goiás - GO
69	ABONO (ACT)	HC da Universidade Federal de Goiás - GO
69	ABONO (ACT)	HC da Universidade Federal de Goiás - GO
69	ABONO (ACT)	HC da Universidade Federal de Goiás - GO
69	ABONO (ACT)	HC da Universidade Federal de Goiás - GO
69	ABONO (ACT)	HC da Universidade Federal de Goiás - GO
69	ABONO (ACT)	HC da Universidade Federal de Goiás - GO
69	ABONO (ACT)	HC da Universidade Federal de Goiás - GO
69	ABONO (ACT)	HC da Universidade Federal de Goiás - GO
69	ABONO (ACT)	HC da Universidade Federal de Goiás - GO
69	ABONO (ACT)	HC da Universidade Federal de Goiás - GO
69	ABONO (ACT)	HC da Universidade Federal de Goiás - GO

69	ABONO (ACT)	HC da Universidade Federal de Goiás - GO
69	ABONO (ACT)	HC da Universidade Federal de Goiás - GO
69	ABONO (ACT)	HC da Universidade Federal de Goiás - GO
69	ABONO (ACT)	HC da Universidade Federal de Goiás - GO
69	ABONO (ACT)	HC da Universidade Federal de Goiás - GO
69	ABONO (ACT)	HC da Universidade Federal de Goiás - GO
69	ABONO (ACT)	HC da Universidade Federal de Goiás - GO
69	ABONO (ACT)	HC da Universidade Federal de Goiás - GO
69	ABONO (ACT)	HC da Universidade Federal de Goiás - GO
69	ABONO (ACT)	HC da Universidade Federal de Goiás - GO
69	ABONO (ACT)	HC da Universidade Federal de Goiás - GO
69	ABONO (ACT)	HC da Universidade Federal de Goiás - GO
69	ABONO (ACT)	HC da Universidade Federal de Goiás - GO
69	ABONO (ACT)	HC da Universidade Federal de Goiás - GO
69	ABONO (ACT)	HC da Universidade Federal de Goiás - GO
69	ABONO (ACT)	HC da Universidade Federal de Goiás - GO
69	ABONO (ACT)	HC da Universidade Federal de Goiás - GO
69	ABONO (ACT)	HC da Universidade Federal de Goiás - GO
69	ABONO (ACT)	HC da Universidade Federal de Goiás - GO
69	ABONO (ACT)	HC da Universidade Federal de Goiás - GO
69	ABONO (ACT)	HC da Universidade Federal de Goiás - GO
69	ABONO (ACT)	HC da Universidade Federal de Goiás - GO
69	ABONO (ACT)	HC da Universidade Federal de Goiás - GO
69	ABONO (ACT)	HC da Universidade Federal de Goiás - GO
69	ABONO (ACT)	HC da Universidade Federal de Goiás - GO
69	ABONO (ACT)	HC da Universidade Federal de Goiás - GO
69	ABONO (ACT)	HC da Universidade Federal de Goiás - GO
69	ABONO (ACT)	HC da Universidade Federal de Goiás - GO
69	ABONO (ACT)	HC da Universidade Federal de Goiás - GO
69	ABONO (ACT)	HC da Universidade Federal de Goiás - GO
69	ABONO (ACT)	HC da Universidade Federal de Goiás - GO
69	ABONO (ACT)	HC da Universidade Federal de Goiás - GO
69	ABONO (ACT)	HC da Universidade Federal de Goiás - GO

69	ABONO (ACT)	HC da Universidade Federal de Goiás - GO
69	ABONO (ACT)	HC da Universidade Federal de Goiás - GO
69	ABONO (ACT)	HC da Universidade Federal de Goiás - GO
69	ABONO (ACT)	HC da Universidade Federal de Goiás - GO
69	ABONO (ACT)	HC da Universidade Federal de Goiás - GO
69	ABONO (ACT)	HC da Universidade Federal de Goiás - GO
69	ABONO (ACT)	HC da Universidade Federal de Goiás - GO
69	ABONO (ACT)	HC da Universidade Federal de Goiás - GO
69	ABONO (ACT)	HC da Universidade Federal de Goiás - GO
69	ABONO (ACT)	HC da Universidade Federal de Goiás - GO
69	ABONO (ACT)	HC da Universidade Federal de Goiás - GO
69	ABONO (ACT)	HC da Universidade Federal de Goiás - GO
69	ABONO (ACT)	HC da Universidade Federal de Goiás - GO
69	ABONO (ACT)	HC da Universidade Federal de Goiás - GO
69	ABONO (ACT)	HC da Universidade Federal de Goiás - GO
69	ABONO (ACT)	HC da Universidade Federal de Goiás - GO
69	ABONO (ACT)	HC da Universidade Federal de Goiás - GO
69	ABONO (ACT)	HC da Universidade Federal de Goiás - GO
69	ABONO (ACT)	HC da Universidade Federal de Goiás - GO
69	ABONO (ACT)	HC da Universidade Federal de Goiás - GO
69	ABONO (ACT)	HC da Universidade Federal de Goiás - GO
69	ABONO (ACT)	HC da Universidade Federal de Goiás - GO
69	ABONO (ACT)	HC da Universidade Federal de Goiás - GO
69	ABONO (ACT)	HC da Universidade Federal de Goiás - GO
69	ABONO (ACT)	HC da Universidade Federal de Goiás - GO
69	ABONO (ACT)	HC da Universidade Federal de Goiás - GO
69	ABONO (ACT)	HC da Universidade Federal de Goiás - GO
69	ABONO (ACT)	HC da Universidade Federal de Goiás - GO
69	ABONO (ACT)	HC da Universidade Federal de Goiás - GO
69	ABONO (ACT)	HC da Universidade Federal de Goiás - GO
69	ABONO (ACT)	HC da Universidade Federal de Goiás - GO
69	ABONO (ACT)	HC da Universidade Federal de Goiás - GO
69	ABONO (ACT)	HC da Universidade Federal de Goiás - GO
69	ABONO (ACT)	HC da Universidade Federal de Goiás - GO
69	ABONO (ACT)	HC da Universidade Federal de Goiás - GO
69	ABONO (ACT)	HC da Universidade Federal de Goiás - GO
69	ABONO (ACT)	HC da Universidade Federal de Goiás - GO
69	ABONO (ACT)	HC da Universidade Federal de Goiás - GO
69	ABONO (ACT)	HC da Universidade Federal de Goiás - GO
69	ABONO (ACT)	HC da Universidade Federal de Goiás - GO
69	ABONO (ACT)	HC da Universidade Federal de Goiás - GO
69	ABONO (ACT)	HC da Universidade Federal de Goiás - GO
69	ABONO (ACT)	HC da Universidade Federal de Goiás - GO
69	ABONO (ACT)	HC da Universidade Federal de Goiás - GO
69	ABONO (ACT)	HC da Universidade Federal de Goiás - GO
69	ABONO (ACT)	HC da Universidade Federal de Goiás - GO
69	ABONO (ACT)	HC da Universidade Federal de Goiás - GO
69	ABONO (ACT)	HC da Universidade Federal de Goiás - GO

69	ABONO (ACT)	HC da Universidade Federal de Goiás - GO
69	ABONO (ACT)	HC da Universidade Federal de Goiás - GO
69	ABONO (ACT)	HC da Universidade Federal de Goiás - GO
69	ABONO (ACT)	HC da Universidade Federal de Goiás - GO
69	ABONO (ACT)	HC da Universidade Federal de Goiás - GO
69	ABONO (ACT)	HC da Universidade Federal de Goiás - GO
69	ABONO (ACT)	HC da Universidade Federal de Goiás - GO
69	ABONO (ACT)	HC da Universidade Federal de Goiás - GO
69	ABONO (ACT)	HC da Universidade Federal de Goiás - GO
69	ABONO (ACT)	HC da Universidade Federal de Goiás - GO
69	ABONO (ACT)	HC da Universidade Federal de Goiás - GO
69	ABONO (ACT)	HC da Universidade Federal de Goiás - GO
69	ABONO (ACT)	HC da Universidade Federal de Goiás - GO
69	ABONO (ACT)	HC da Universidade Federal de Goiás - GO
69	ABONO (ACT)	HC da Universidade Federal de Goiás - GO
69	ABONO (ACT)	HC da Universidade Federal de Goiás - GO
69	ABONO (ACT)	HC da Universidade Federal de Goiás - GO
69	ABONO (ACT)	HC da Universidade Federal de Goiás - GO
69	ABONO (ACT)	HC da Universidade Federal de Goiás - GO
69	ABONO (ACT)	HC da Universidade Federal de Goiás - GO
69	ABONO (ACT)	HC da Universidade Federal de Goiás - GO
69	ABONO (ACT)	HC da Universidade Federal de Goiás - GO
69	ABONO (ACT)	HC da Universidade Federal de Goiás - GO
69	ABONO (ACT)	HC da Universidade Federal de Goiás - GO
69	ABONO (ACT)	HC da Universidade Federal de Goiás - GO
69	ABONO (ACT)	HC da Universidade Federal de Goiás - GO
69	ABONO (ACT)	HC da Universidade Federal de Goiás - GO
69	ABONO (ACT)	HC da Universidade Federal de Goiás - GO
69	ABONO (ACT)	HC da Universidade Federal de Goiás - GO
69	ABONO (ACT)	HC da Universidade Federal de Goiás - GO
69	ABONO (ACT)	HC da Universidade Federal de Goiás - GO
69	ABONO (ACT)	HC da Universidade Federal de Goiás - GO
69	ABONO (ACT)	HC da Universidade Federal de Goiás - GO
69	ABONO (ACT)	HC da Universidade Federal de Goiás - GO
69	ABONO (ACT)	HC da Universidade Federal de Goiás - GO
69	ABONO (ACT)	HC da Universidade Federal de Goiás - GO
69	ABONO (ACT)	HC da Universidade Federal de Goiás - GO
69	ABONO (ACT)	HC da Universidade Federal de Goiás - GO
69	ABONO (ACT)	HC da Universidade Federal de Goiás - GO
69	ABONO (ACT)	HC da Universidade Federal de Goiás - GO
69	ABONO (ACT)	HC da Universidade Federal de Goiás - GO
69	ABONO (ACT)	HC da Universidade Federal de Goiás - GO
69	ABONO (ACT)	HC da Universidade Federal de Goiás - GO
69	ABONO (ACT)	HC da Universidade Federal de Goiás - GO
69	ABONO (ACT)	HC da Universidade Federal de Goiás - GO
69	ABONO (ACT)	HC da Universidade Federal de Goiás - GO
69	ABONO (ACT)	HC da Universidade Federal de Goiás - GO
69	ABONO (ACT)	HC da Universidade Federal de Goiás - GO

69	ABONO (ACT)	HC da Universidade Federal de Goiás - GO
69	ABONO (ACT)	HC da Universidade Federal de Goiás - GO
69	ABONO (ACT)	HC da Universidade Federal de Goiás - GO
69	ABONO (ACT)	HC da Universidade Federal de Goiás - GO
69	ABONO (ACT)	HC da Universidade Federal de Goiás - GO
69	ABONO (ACT)	HC da Universidade Federal de Goiás - GO
69	ABONO (ACT)	HC da Universidade Federal de Goiás - GO
69	ABONO (ACT)	HC da Universidade Federal de Goiás - GO
69	ABONO (ACT)	HC da Universidade Federal de Goiás - GO
69	ABONO (ACT)	HC da Universidade Federal de Goiás - GO
69	ABONO (ACT)	HC da Universidade Federal de Goiás - GO
69	ABONO (ACT)	HC da Universidade Federal de Goiás - GO
69	ABONO (ACT)	HC da Universidade Federal de Goiás - GO
69	ABONO (ACT)	HC da Universidade Federal de Goiás - GO
69	ABONO (ACT)	HC da Universidade Federal de Goiás - GO
69	ABONO (ACT)	HC da Universidade Federal de Goiás - GO
69	ABONO (ACT)	HC da Universidade Federal de Goiás - GO
69	ABONO (ACT)	HC da Universidade Federal de Goiás - GO
69	ABONO (ACT)	HC da Universidade Federal de Goiás - GO
69	ABONO (ACT)	HC da Universidade Federal de Goiás - GO
69	ABONO (ACT)	HC da Universidade Federal de Goiás - GO
69	ABONO (ACT)	HC da Universidade Federal de Goiás - GO
69	ABONO (ACT)	HC da Universidade Federal de Goiás - GO
69	ABONO (ACT)	HC da Universidade Federal de Goiás - GO
69	ABONO (ACT)	HC da Universidade Federal de Goiás - GO
69	ABONO (ACT)	HC da Universidade Federal de Goiás - GO
69	ABONO (ACT)	HC da Universidade Federal de Goiás - GO
69	ABONO (ACT)	HC da Universidade Federal de Goiás - GO
69	ABONO (ACT)	HC da Universidade Federal de Goiás - GO
69	ABONO (ACT)	HC da Universidade Federal de Goiás - GO
69	ABONO (ACT)	HC da Universidade Federal de Goiás - GO
69	ABONO (ACT)	HC da Universidade Federal de Goiás - GO
69	ABONO (ACT)	HC da Universidade Federal de Goiás - GO
69	ABONO (ACT)	HC da Universidade Federal de Goiás - GO
69	ABONO (ACT)	HC da Universidade Federal de Goiás - GO
69	ABONO (ACT)	HC da Universidade Federal de Goiás - GO
69	ABONO (ACT)	HC da Universidade Federal de Goiás - GO
69	ABONO (ACT)	HC da Universidade Federal de Goiás - GO
69	ABONO (ACT)	HC da Universidade Federal de Goiás - GO
69	ABONO (ACT)	HC da Universidade Federal de Goiás - GO
69	ABONO (ACT)	HC da Universidade Federal de Goiás - GO
69	ABONO (ACT)	HC da Universidade Federal de Goiás - GO
69	ABONO (ACT)	HC da Universidade Federal de Goiás - GO
69	ABONO (ACT)	HC da Universidade Federal de Goiás - GO
69	ABONO (ACT)	HC da Universidade Federal de Goiás - GO
69	ABONO (ACT)	HC da Universidade Federal de Goiás - GO
69	ABONO (ACT)	HC da Universidade Federal de Goiás - GO
69	ABONO (ACT)	HC da Universidade Federal de Goiás - GO

69	ABONO (ACT)	HC da Universidade Federal de Goiás - GO
69	ABONO (ACT)	HC da Universidade Federal de Goiás - GO
69	ABONO (ACT)	HC da Universidade Federal de Goiás - GO
69	ABONO (ACT)	HC da Universidade Federal de Goiás - GO
69	ABONO (ACT)	HC da Universidade Federal de Goiás - GO
69	ABONO (ACT)	HC da Universidade Federal de Goiás - GO
69	ABONO (ACT)	HC da Universidade Federal de Goiás - GO
69	ABONO (ACT)	HC da Universidade Federal de Goiás - GO
69	ABONO (ACT)	HC da Universidade Federal de Goiás - GO
69	ABONO (ACT)	HC da Universidade Federal de Goiás - GO
69	ABONO (ACT)	HC da Universidade Federal de Goiás - GO
69	ABONO (ACT)	HC da Universidade Federal de Goiás - GO
69	ABONO (ACT)	HC da Universidade Federal de Goiás - GO
69	ABONO (ACT)	HC da Universidade Federal de Goiás - GO
69	ABONO (ACT)	HC da Universidade Federal de Goiás - GO
69	ABONO (ACT)	HC da Universidade Federal de Goiás - GO
69	ABONO (ACT)	HC da Universidade Federal de Goiás - GO
69	ABONO (ACT)	HC da Universidade Federal de Goiás - GO
69	ABONO (ACT)	HC da Universidade Federal de Goiás - GO
69	ABONO (ACT)	HC da Universidade Federal de Goiás - GO
69	ABONO (ACT)	HC da Universidade Federal de Goiás - GO
69	ABONO (ACT)	HC da Universidade Federal de Goiás - GO
69	ABONO (ACT)	HC da Universidade Federal de Goiás - GO
69	ABONO (ACT)	HC da Universidade Federal de Goiás - GO
69	ABONO (ACT)	HC da Universidade Federal de Goiás - GO
69	ABONO (ACT)	HC da Universidade Federal de Goiás - GO
69	ABONO (ACT)	HC da Universidade Federal de Goiás - GO
69	ABONO (ACT)	HC da Universidade Federal de Goiás - GO
69	ABONO (ACT)	HC da Universidade Federal de Goiás - GO
69	ABONO (ACT)	HC da Universidade Federal de Goiás - GO
69	ABONO (ACT)	HC da Universidade Federal de Goiás - GO
69	ABONO (ACT)	HC da Universidade Federal de Goiás - GO
69	ABONO (ACT)	HC da Universidade Federal de Goiás - GO
69	ABONO (ACT)	HC da Universidade Federal de Goiás - GO
69	ABONO (ACT)	HC da Universidade Federal de Goiás - GO
69	ABONO (ACT)	HC da Universidade Federal de Goiás - GO
69	ABONO (ACT)	HC da Universidade Federal de Goiás - GO
69	ABONO (ACT)	HC da Universidade Federal de Goiás - GO
69	ABONO (ACT)	HC da Universidade Federal de Goiás - GO
69	ABONO (ACT)	HC da Universidade Federal de Goiás - GO
69	ABONO (ACT)	HC da Universidade Federal de Goiás - GO

69	ABONO (ACT)	HC da Universidade Federal de Goiás - GO
69	ABONO (ACT)	HC da Universidade Federal de Goiás - GO
69	ABONO (ACT)	HC da Universidade Federal de Goiás - GO
69	ABONO (ACT)	HC da Universidade Federal de Goiás - GO
69	ABONO (ACT)	HC da Universidade Federal de Goiás - GO
69	ABONO (ACT)	HC da Universidade Federal de Goiás - GO
69	ABONO (ACT)	HC da Universidade Federal de Goiás - GO
69	ABONO (ACT)	HC da Universidade Federal de Goiás - GO
69	ABONO (ACT)	HC da Universidade Federal de Goiás - GO
69	ABONO (ACT)	HC da Universidade Federal de Goiás - GO
69	ABONO (ACT)	HC da Universidade Federal de Goiás - GO
69	ABONO (ACT)	HC da Universidade Federal de Goiás - GO
69	ABONO (ACT)	HC da Universidade Federal de Goiás - GO
69	ABONO (ACT)	HC da Universidade Federal de Goiás - GO
69	ABONO (ACT)	HC da Universidade Federal de Goiás - GO
69	ABONO (ACT)	HC da Universidade Federal de Goiás - GO
69	ABONO (ACT)	HC da Universidade Federal de Goiás - GO
69	ABONO (ACT)	HC da Universidade Federal de Goiás - GO
69	ABONO (ACT)	HC da Universidade Federal de Goiás - GO
69	ABONO (ACT)	HC da Universidade Federal de Goiás - GO
69	ABONO (ACT)	HC da Universidade Federal de Goiás - GO
69	ABONO (ACT)	HC da Universidade Federal de Goiás - GO
69	ABONO (ACT)	HC da Universidade Federal de Goiás - GO
69	ABONO (ACT)	HC da Universidade Federal de Goiás - GO
69	ABONO (ACT)	HC da Universidade Federal de Goiás - GO
69	ABONO (ACT)	HC da Universidade Federal de Goiás - GO
69	ABONO (ACT)	HC da Universidade Federal de Goiás - GO
69	ABONO (ACT)	HC da Universidade Federal de Goiás - GO
69	ABONO (ACT)	HC da Universidade Federal de Goiás - GO
69	ABONO (ACT)	HC da Universidade Federal de Goiás - GO
69	ABONO (ACT)	HC da Universidade Federal de Goiás - GO
69	ABONO (ACT)	HC da Universidade Federal de Goiás - GO
69	ABONO (ACT)	HC da Universidade Federal de Goiás - GO
69	ABONO (ACT)	HC da Universidade Federal de Goiás - GO
69	ABONO (ACT)	HC da Universidade Federal de Goiás - GO
69	ABONO (ACT)	HC da Universidade Federal de Goiás - GO
69	ABONO (ACT)	HC da Universidade Federal de Goiás - GO
69	ABONO (ACT)	HC da Universidade Federal de Goiás - GO

69	ABONO (ACT)	HC da Universidade Federal de Goiás - GO
69	ABONO (ACT)	HC da Universidade Federal de Goiás - GO
69	ABONO (ACT)	HC da Universidade Federal de Goiás - GO
69	ABONO (ACT)	HC da Universidade Federal de Goiás - GO
69	ABONO (ACT)	HC da Universidade Federal de Goiás - GO
69	ABONO (ACT)	HC da Universidade Federal de Goiás - GO
69	ABONO (ACT)	HC da Universidade Federal de Goiás - GO
69	ABONO (ACT)	HC da Universidade Federal de Goiás - GO
69	ABONO (ACT)	HC da Universidade Federal de Goiás - GO
69	ABONO (ACT)	HC da Universidade Federal de Goiás - GO
69	ABONO (ACT)	HC da Universidade Federal de Goiás - GO
69	ABONO (ACT)	HC da Universidade Federal de Goiás - GO
69	ABONO (ACT)	HC da Universidade Federal de Goiás - GO
69	ABONO (ACT)	HC da Universidade Federal de Goiás - GO
69	ABONO (ACT)	HC da Universidade Federal de Goiás - GO
69	ABONO (ACT)	HC da Universidade Federal de Goiás - GO
69	ABONO (ACT)	HC da Universidade Federal de Goiás - GO
69	ABONO (ACT)	HC da Universidade Federal de Goiás - GO
69	ABONO (ACT)	HC da Universidade Federal de Goiás - GO
69	ABONO (ACT)	HC da Universidade Federal de Goiás - GO
69	ABONO (ACT)	HC da Universidade Federal de Goiás - GO
69	ABONO (ACT)	HC da Universidade Federal de Goiás - GO
69	ABONO (ACT)	HC da Universidade Federal de Goiás - GO
69	ABONO (ACT)	HC da Universidade Federal de Goiás - GO
69	ABONO (ACT)	HC da Universidade Federal de Goiás - GO
69	ABONO (ACT)	HC da Universidade Federal de Goiás - GO
69	ABONO (ACT)	HC da Universidade Federal de Goiás - GO
69	ABONO (ACT)	HC da Universidade Federal de Goiás - GO
69	ABONO (ACT)	HC da Universidade Federal de Goiás - GO
69	ABONO (ACT)	HC da Universidade Federal de Goiás - GO
69	ABONO (ACT)	HC da Universidade Federal de Goiás - GO
69	ABONO (ACT)	HC da Universidade Federal de Goiás - GO
69	ABONO (ACT)	HC da Universidade Federal de Goiás - GO
69	ABONO (ACT)	HC da Universidade Federal de Goiás - GO
69	ABONO (ACT)	HC da Universidade Federal de Goiás - GO
69	ABONO (ACT)	HC da Universidade Federal de Goiás - GO
69	ABONO (ACT)	HC da Universidade Federal de Goiás - GO
69	ABONO (ACT)	HC da Universidade Federal de Goiás - GO
69	ABONO (ACT)	HC da Universidade Federal de Goiás - GO
69	ABONO (ACT)	HC da Universidade Federal de Goiás - GO
69	ABONO (ACT)	HC da Universidade Federal de Goiás - GO
69	ABONO (ACT)	HC da Universidade Federal de Goiás - GO
69	ABONO (ACT)	HC da Universidade Federal de Goiás - GO
69	ABONO (ACT)	HC da Universidade Federal de Goiás - GO
69	ABONO (ACT)	HC da Universidade Federal de Goiás - GO
69	ABONO (ACT)	HC da Universidade Federal de Goiás - GO
69	ABONO (ACT)	HC da Universidade Federal de Goiás - GO
69	ABONO (ACT)	HC da Universidade Federal de Goiás - GO

69	ABONO (ACT)	HC da Universidade Federal de Goiás - GO
69	ABONO (ACT)	HC da Universidade Federal de Goiás - GO
69	ABONO (ACT)	HC da Universidade Federal de Goiás - GO
69	ABONO (ACT)	HC da Universidade Federal de Goiás - GO
69	ABONO (ACT)	HC da Universidade Federal de Goiás - GO
69	ABONO (ACT)	HC da Universidade Federal de Goiás - GO
69	ABONO (ACT)	HC da Universidade Federal de Goiás - GO
69	ABONO (ACT)	HC da Universidade Federal de Goiás - GO
69	ABONO (ACT)	HC da Universidade Federal de Goiás - GO
69	ABONO (ACT)	HC da Universidade Federal de Goiás - GO
69	ABONO (ACT)	HC da Universidade Federal de Goiás - GO
69	ABONO (ACT)	HC da Universidade Federal de Goiás - GO
69	ABONO (ACT)	HC da Universidade Federal de Goiás - GO
69	ABONO (ACT)	HC da Universidade Federal de Goiás - GO
69	ABONO (ACT)	HC da Universidade Federal de Goiás - GO
69	ABONO (ACT)	HC da Universidade Federal de Goiás - GO
69	ABONO (ACT)	HC da Universidade Federal de Goiás - GO
69	ABONO (ACT)	HC da Universidade Federal de Goiás - GO
69	ABONO (ACT)	HC da Universidade Federal de Goiás - GO
69	ABONO (ACT)	HC da Universidade Federal de Goiás - GO
69	ABONO (ACT)	HC da Universidade Federal de Goiás - GO
69	ABONO (ACT)	HC da Universidade Federal de Goiás - GO
69	ABONO (ACT)	HC da Universidade Federal de Goiás - GO
69	ABONO (ACT)	HC da Universidade Federal de Goiás - GO
69	ABONO (ACT)	HC da Universidade Federal de Goiás - GO
69	ABONO (ACT)	HC da Universidade Federal de Goiás - GO
69	ABONO (ACT)	HC da Universidade Federal de Goiás - GO
69	ABONO (ACT)	HC da Universidade Federal de Goiás - GO
69	ABONO (ACT)	HC da Universidade Federal de Goiás - GO
69	ABONO (ACT)	HC da Universidade Federal de Goiás - GO
69	ABONO (ACT)	HC da Universidade Federal de Goiás - GO
69	ABONO (ACT)	HC da Universidade Federal de Goiás - GO
69	ABONO (ACT)	HC da Universidade Federal de Goiás - GO
69	ABONO (ACT)	HC da Universidade Federal de Goiás - GO
69	ABONO (ACT)	HC da Universidade Federal de Goiás - GO
69	ABONO (ACT)	HC da Universidade Federal de Goiás - GO
69	ABONO (ACT)	HC da Universidade Federal de Goiás - GO
69	ABONO (ACT)	HC da Universidade Federal de Goiás - GO
69	ABONO (ACT)	HC da Universidade Federal de Goiás - GO
69	ABONO (ACT)	HC da Universidade Federal de Goiás - GO
69	ABONO (ACT)	HC da Universidade Federal de Goiás - GO
69	ABONO (ACT)	HC da Universidade Federal de Goiás - GO
69	ABONO (ACT)	HC da Universidade Federal de Goiás - GO
69	ABONO (ACT)	HC da Universidade Federal de Goiás - GO
69	ABONO (ACT)	HC da Universidade Federal de Goiás - GO
69	ABONO (ACT)	HC da Universidade Federal de Goiás - GO
69	ABONO (ACT)	HC da Universidade Federal de Goiás - GO
69	ABONO (ACT)	HC da Universidade Federal de Goiás - GO

69	ABONO (ACT)	HC da Universidade Federal de Goiás - GO
69	ABONO (ACT)	HC da Universidade Federal de Goiás - GO
69	ABONO (ACT)	HC da Universidade Federal de Goiás - GO
69	ABONO (ACT)	HC da Universidade Federal de Goiás - GO
69	ABONO (ACT)	HC da Universidade Federal de Goiás - GO
69	ABONO (ACT)	HC da Universidade Federal de Goiás - GO
69	ABONO (ACT)	HC da Universidade Federal de Goiás - GO
69	ABONO (ACT)	HC da Universidade Federal de Goiás - GO
69	ABONO (ACT)	HC da Universidade Federal de Goiás - GO
69	ABONO (ACT)	HC da Universidade Federal de Goiás - GO
69	ABONO (ACT)	HC da Universidade Federal de Goiás - GO
69	ABONO (ACT)	HC da Universidade Federal de Goiás - GO
69	ABONO (ACT)	HC da Universidade Federal de Goiás - GO
69	ABONO (ACT)	HC da Universidade Federal de Goiás - GO
69	ABONO (ACT)	HC da Universidade Federal de Goiás - GO
69	ABONO (ACT)	HC da Universidade Federal de Goiás - GO
69	ABONO (ACT)	HC da Universidade Federal de Goiás - GO
69	ABONO (ACT)	HC da Universidade Federal de Goiás - GO
69	ABONO (ACT)	HC da Universidade Federal de Goiás - GO
69	ABONO (ACT)	HC da Universidade Federal de Goiás - GO
69	ABONO (ACT)	HC da Universidade Federal de Goiás - GO
69	ABONO (ACT)	HC da Universidade Federal de Goiás - GO
69	ABONO (ACT)	HC da Universidade Federal de Goiás - GO
69	ABONO (ACT)	HC da Universidade Federal de Goiás - GO
69	ABONO (ACT)	HC da Universidade Federal de Goiás - GO
69	ABONO (ACT)	HC da Universidade Federal de Goiás - GO
69	ABONO (ACT)	HC da Universidade Federal de Goiás - GO
69	ABONO (ACT)	HC da Universidade Federal de Goiás - GO
69	ABONO (ACT)	HC da Universidade Federal de Goiás - GO
69	ABONO (ACT)	HC da Universidade Federal de Goiás - GO
69	ABONO (ACT)	HC da Universidade Federal de Goiás - GO
69	ABONO (ACT)	HC da Universidade Federal de Goiás - GO
69	ABONO (ACT)	HC da Universidade Federal de Goiás - GO
70	AUSÊNCIA COM COMPENSAÇÃO	HC da Universidade Federal de Goiás - GO
70	AUSÊNCIA COM COMPENSAÇÃO	HC da Universidade Federal de Goiás - GO
70	AUSÊNCIA COM COMPENSAÇÃO	HC da Universidade Federal de Goiás - GO
70	AUSÊNCIA COM COMPENSAÇÃO	HC da Universidade Federal de Goiás - GO
70	AUSÊNCIA COM COMPENSAÇÃO	HC da Universidade Federal de Goiás - GO
70	AUSÊNCIA COM COMPENSAÇÃO	HC da Universidade Federal de Goiás - GO
70	AUSÊNCIA COM COMPENSAÇÃO	HC da Universidade Federal de Goiás - GO
70	AUSÊNCIA COM COMPENSAÇÃO	HC da Universidade Federal de Goiás - GO
70	AUSÊNCIA COM COMPENSAÇÃO	HC da Universidade Federal de Goiás - GO
70	AUSÊNCIA COM COMPENSAÇÃO	HC da Universidade Federal de Goiás - GO
70	AUSÊNCIA COM COMPENSAÇÃO	HC da Universidade Federal de Goiás - GO
70	AUSÊNCIA COM COMPENSAÇÃO	HC da Universidade Federal de Goiás - GO
70	AUSÊNCIA COM COMPENSAÇÃO	HC da Universidade Federal de Goiás - GO
70	AUSÊNCIA COM COMPENSAÇÃO	HC da Universidade Federal de Goiás - GO
70	AUSÊNCIA COM COMPENSAÇÃO	HC da Universidade Federal de Goiás - GO

70	AUSÊNCIA COM COMPENSAÇÃO	HC da Universidade Federal de Goiás - GO
70	AUSÊNCIA COM COMPENSAÇÃO	HC da Universidade Federal de Goiás - GO
75	LICENÇA PATERNIDADE CELETISTA - 20 Dias (c/ prorrogação) - LEI 11.770/08 ART 1 II	HC da Universidade Federal de Goiás - GO
75	LICENÇA PATERNIDADE CELETISTA - 20 Dias (c/ prorrogação) - LEI 11.770/08 ART 1 II	HC da Universidade Federal de Goiás - GO
75	LICENÇA PATERNIDADE CELETISTA - 20 Dias (c/ prorrogação) - LEI 11.770/08 ART 1 II	HC da Universidade Federal de Goiás - GO
75	LICENÇA PATERNIDADE CELETISTA - 20 Dias (c/ prorrogação) - LEI 11.770/08 ART 1 II	HC da Universidade Federal de Goiás - GO
75	LICENÇA PATERNIDADE CELETISTA - 20 Dias (c/ prorrogação) - LEI 11.770/08 ART 1 II	HC da Universidade Federal de Goiás - GO
75	LICENÇA PATERNIDADE CELETISTA - 20 Dias (c/ prorrogação) - LEI 11.770/08 ART 1 II	HC da Universidade Federal de Goiás - GO
75	LICENÇA PATERNIDADE CELETISTA - 20 Dias (c/ prorrogação) - LEI 11.770/08 ART 1 II	HC da Universidade Federal de Goiás - GO
75	LICENÇA PATERNIDADE CELETISTA - 20 Dias (c/ prorrogação) - LEI 11.770/08 ART 1 II	HC da Universidade Federal de Goiás - GO
75	LICENÇA PATERNIDADE CELETISTA - 20 Dias (c/ prorrogação) - LEI 11.770/08 ART 1 II	HC da Universidade Federal de Goiás - GO
75	LICENÇA PATERNIDADE CELETISTA - 20 Dias (c/ prorrogação) - LEI 11.770/08 ART 1 II	HC da Universidade Federal de Goiás - GO
75	LICENÇA PATERNIDADE CELETISTA - 20 Dias (c/ prorrogação) - LEI 11.770/08 ART 1 II	HC da Universidade Federal de Goiás - GO
75	LICENÇA PATERNIDADE CELETISTA - 20 Dias (c/ prorrogação) - LEI 11.770/08 ART 1 II	HC da Universidade Federal de Goiás - GO
75	LICENÇA PATERNIDADE CELETISTA - 20 Dias (c/ prorrogação) - LEI 11.770/08 ART 1 II	HC da Universidade Federal de Goiás - GO
97	RECESSO FINAL DE ANO	HC da Universidade Federal de Goiás - GO
97	RECESSO FINAL DE ANO	HC da Universidade Federal de Goiás - GO
97	RECESSO FINAL DE ANO	HC da Universidade Federal de Goiás - GO
97	RECESSO FINAL DE ANO	HC da Universidade Federal de Goiás - GO
97	RECESSO FINAL DE ANO	HC da Universidade Federal de Goiás - GO
97	RECESSO FINAL DE ANO	HC da Universidade Federal de Goiás - GO
97	RECESSO FINAL DE ANO	HC da Universidade Federal de Goiás - GO
97	RECESSO FINAL DE ANO	HC da Universidade Federal de Goiás - GO
97	RECESSO FINAL DE ANO	HC da Universidade Federal de Goiás - GO
97	RECESSO FINAL DE ANO	HC da Universidade Federal de Goiás - GO
97	RECESSO FINAL DE ANO	HC da Universidade Federal de Goiás - GO
97	RECESSO FINAL DE ANO	HC da Universidade Federal de Goiás - GO
97	RECESSO FINAL DE ANO	HC da Universidade Federal de Goiás - GO
97	RECESSO FINAL DE ANO	HC da Universidade Federal de Goiás - GO
97	RECESSO FINAL DE ANO	HC da Universidade Federal de Goiás - GO
97	RECESSO FINAL DE ANO	HC da Universidade Federal de Goiás - GO
97	RECESSO FINAL DE ANO	HC da Universidade Federal de Goiás - GO
97	RECESSO FINAL DE ANO	HC da Universidade Federal de Goiás - GO
97	RECESSO FINAL DE ANO	HC da Universidade Federal de Goiás - GO
97	RECESSO FINAL DE ANO	HC da Universidade Federal de Goiás - GO
97	RECESSO FINAL DE ANO	HC da Universidade Federal de Goiás - GO
97	RECESSO FINAL DE ANO	HC da Universidade Federal de Goiás - GO
97	RECESSO FINAL DE ANO	HC da Universidade Federal de Goiás - GO
97	RECESSO FINAL DE ANO	HC da Universidade Federal de Goiás - GO
97	RECESSO FINAL DE ANO	HC da Universidade Federal de Goiás - GO
97	RECESSO FINAL DE ANO	HC da Universidade Federal de Goiás - GO

97	RECESSO FINAL DE ANO	HC da Universidade Federal de Goiás - GO
97	RECESSO FINAL DE ANO	HC da Universidade Federal de Goiás - GO
97	RECESSO FINAL DE ANO	HC da Universidade Federal de Goiás - GO
97	RECESSO FINAL DE ANO	HC da Universidade Federal de Goiás - GO
97	RECESSO FINAL DE ANO	HC da Universidade Federal de Goiás - GO
97	RECESSO FINAL DE ANO	HC da Universidade Federal de Goiás - GO
97	RECESSO FINAL DE ANO	HC da Universidade Federal de Goiás - GO
97	RECESSO FINAL DE ANO	HC da Universidade Federal de Goiás - GO
97	RECESSO FINAL DE ANO	HC da Universidade Federal de Goiás - GO
97	RECESSO FINAL DE ANO	HC da Universidade Federal de Goiás - GO
97	RECESSO FINAL DE ANO	HC da Universidade Federal de Goiás - GO
97	RECESSO FINAL DE ANO	HC da Universidade Federal de Goiás - GO
97	RECESSO FINAL DE ANO	HC da Universidade Federal de Goiás - GO
97	RECESSO FINAL DE ANO	HC da Universidade Federal de Goiás - GO
97	RECESSO FINAL DE ANO	HC da Universidade Federal de Goiás - GO
97	RECESSO FINAL DE ANO	HC da Universidade Federal de Goiás - GO
97	RECESSO FINAL DE ANO	HC da Universidade Federal de Goiás - GO
97	RECESSO FINAL DE ANO	HC da Universidade Federal de Goiás - GO
97	RECESSO FINAL DE ANO	HC da Universidade Federal de Goiás - GO
97	RECESSO FINAL DE ANO	HC da Universidade Federal de Goiás - GO
97	RECESSO FINAL DE ANO	HC da Universidade Federal de Goiás - GO
97	RECESSO FINAL DE ANO	HC da Universidade Federal de Goiás - GO
97	RECESSO FINAL DE ANO	HC da Universidade Federal de Goiás - GO
97	RECESSO FINAL DE ANO	HC da Universidade Federal de Goiás - GO
97	RECESSO FINAL DE ANO	HC da Universidade Federal de Goiás - GO
97	RECESSO FINAL DE ANO	HC da Universidade Federal de Goiás - GO
97	RECESSO FINAL DE ANO	HC da Universidade Federal de Goiás - GO
97	RECESSO FINAL DE ANO	HC da Universidade Federal de Goiás - GO
97	RECESSO FINAL DE ANO	HC da Universidade Federal de Goiás - GO
97	RECESSO FINAL DE ANO	HC da Universidade Federal de Goiás - GO
97	RECESSO FINAL DE ANO	HC da Universidade Federal de Goiás - GO
97	RECESSO FINAL DE ANO	HC da Universidade Federal de Goiás - GO
97	RECESSO FINAL DE ANO	HC da Universidade Federal de Goiás - GO
97	RECESSO FINAL DE ANO	HC da Universidade Federal de Goiás - GO
97	RECESSO FINAL DE ANO	HC da Universidade Federal de Goiás - GO
97	RECESSO FINAL DE ANO	HC da Universidade Federal de Goiás - GO
97	RECESSO FINAL DE ANO	HC da Universidade Federal de Goiás - GO
97	RECESSO FINAL DE ANO	HC da Universidade Federal de Goiás - GO
97	RECESSO FINAL DE ANO	HC da Universidade Federal de Goiás - GO
97	RECESSO FINAL DE ANO	HC da Universidade Federal de Goiás - GO
97	RECESSO FINAL DE ANO	HC da Universidade Federal de Goiás - GO
97	RECESSO FINAL DE ANO	HC da Universidade Federal de Goiás - GO

97	RECESSO FINAL DE ANO	HC da Universidade Federal de Goiás - GO
97	RECESSO FINAL DE ANO	HC da Universidade Federal de Goiás - GO
97	RECESSO FINAL DE ANO	HC da Universidade Federal de Goiás - GO
97	RECESSO FINAL DE ANO	HC da Universidade Federal de Goiás - GO
97	RECESSO FINAL DE ANO	HC da Universidade Federal de Goiás - GO
97	RECESSO FINAL DE ANO	HC da Universidade Federal de Goiás - GO
97	RECESSO FINAL DE ANO	HC da Universidade Federal de Goiás - GO
97	RECESSO FINAL DE ANO	HC da Universidade Federal de Goiás - GO
97	RECESSO FINAL DE ANO	HC da Universidade Federal de Goiás - GO
97	RECESSO FINAL DE ANO	HC da Universidade Federal de Goiás - GO
97	RECESSO FINAL DE ANO	HC da Universidade Federal de Goiás - GO
97	RECESSO FINAL DE ANO	HC da Universidade Federal de Goiás - GO
97	RECESSO FINAL DE ANO	HC da Universidade Federal de Goiás - GO
97	RECESSO FINAL DE ANO	HC da Universidade Federal de Goiás - GO
97	RECESSO FINAL DE ANO	HC da Universidade Federal de Goiás - GO
97	RECESSO FINAL DE ANO	HC da Universidade Federal de Goiás - GO
97	RECESSO FINAL DE ANO	HC da Universidade Federal de Goiás - GO
97	RECESSO FINAL DE ANO	HC da Universidade Federal de Goiás - GO
97	RECESSO FINAL DE ANO	HC da Universidade Federal de Goiás - GO
97	RECESSO FINAL DE ANO	HC da Universidade Federal de Goiás - GO
97	RECESSO FINAL DE ANO	HC da Universidade Federal de Goiás - GO
97	RECESSO FINAL DE ANO	HC da Universidade Federal de Goiás - GO
98	DESLOCAMENTO PARA NOVA UNIDADE (TRANSITO) - NORMA OPERACIONAL DGP N 01/2017	HC da Universidade Federal de Goiás - GO
98	DESLOCAMENTO PARA NOVA UNIDADE (TRANSITO) - NORMA OPERACIONAL DGP N 01/2017	HC da Universidade Federal de Goiás - GO
98	DESLOCAMENTO PARA NOVA UNIDADE (TRANSITO) - NORMA OPERACIONAL DGP N 01/2017	HC da Universidade Federal de Goiás - GO
98	DESLOCAMENTO PARA NOVA UNIDADE (TRANSITO) - NORMA OPERACIONAL DGP N 01/2017	HC da Universidade Federal de Goiás - GO
98	DESLOCAMENTO PARA NOVA UNIDADE (TRANSITO) - NORMA OPERACIONAL DGP N 01/2017	HC da Universidade Federal de Goiás - GO
98	DESLOCAMENTO PARA NOVA UNIDADE (TRANSITO) - NORMA OPERACIONAL DGP N 01/2017	HC da Universidade Federal de Goiás - GO
107	LICENÇA MATERNIDADE/ADOÇÃO - RJU	HC da Universidade Federal de Goiás - GO
221	Afastamento Saúde Ocupacional	HC da Universidade Federal de Goiás - GO
221	Afastamento Saúde Ocupacional	HC da Universidade Federal de Goiás - GO
227	ATIVIDADE EXTERNA (ESPII COVID19)	HC da Universidade Federal de Goiás - GO
227	ATIVIDADE EXTERNA (ESPII COVID19)	HC da Universidade Federal de Goiás - GO
227	ATIVIDADE EXTERNA (ESPII COVID19)	HC da Universidade Federal de Goiás - GO
227	ATIVIDADE EXTERNA (ESPII COVID19)	HC da Universidade Federal de Goiás - GO
227	ATIVIDADE EXTERNA (ESPII COVID19)	HC da Universidade Federal de Goiás - GO
227	ATIVIDADE EXTERNA (ESPII COVID19)	HC da Universidade Federal de Goiás - GO
227	ATIVIDADE EXTERNA (ESPII COVID19)	HC da Universidade Federal de Goiás - GO
227	ATIVIDADE EXTERNA (ESPII COVID19)	HC da Universidade Federal de Goiás - GO
227	ATIVIDADE EXTERNA (ESPII COVID19)	HC da Universidade Federal de Goiás - GO
227	ATIVIDADE EXTERNA (ESPII COVID19)	HC da Universidade Federal de Goiás - GO

227	ATIVIDADE EXTERNA (ESPII COVID19)	HC da Universidade Federal de Goiás - GO
227	ATIVIDADE EXTERNA (ESPII COVID19)	HC da Universidade Federal de Goiás - GO
227	ATIVIDADE EXTERNA (ESPII COVID19)	HC da Universidade Federal de Goiás - GO
227	ATIVIDADE EXTERNA (ESPII COVID19)	HC da Universidade Federal de Goiás - GO
227	ATIVIDADE EXTERNA (ESPII COVID19)	HC da Universidade Federal de Goiás - GO
227	ATIVIDADE EXTERNA (ESPII COVID19)	HC da Universidade Federal de Goiás - GO
227	ATIVIDADE EXTERNA (ESPII COVID19)	HC da Universidade Federal de Goiás - GO
227	ATIVIDADE EXTERNA (ESPII COVID19)	HC da Universidade Federal de Goiás - GO
227	ATIVIDADE EXTERNA (ESPII COVID19)	HC da Universidade Federal de Goiás - GO
227	ATIVIDADE EXTERNA (ESPII COVID19)	HC da Universidade Federal de Goiás - GO
227	ATIVIDADE EXTERNA (ESPII COVID19)	HC da Universidade Federal de Goiás - GO
227	ATIVIDADE EXTERNA (ESPII COVID19)	HC da Universidade Federal de Goiás - GO
227	ATIVIDADE EXTERNA (ESPII COVID19)	HC da Universidade Federal de Goiás - GO
228	ATIVIDADE EXTERNA (ESPII COVID19) - 7 Dias. DESATIVADO DE ACORDO COM A IN 02 - DGP de 26/03/2020.	HC da Universidade Federal de Goiás - GO
229	ATIVIDADE EXTERNA (ESPII COVID19) - 14 dias.	HC da Universidade Federal de Goiás - GO
229	ATIVIDADE EXTERNA (ESPII COVID19) - 14 dias.	HC da Universidade Federal de Goiás - GO
231	Estado gripal (autodeclaratório) COVID-19. DESATIVADO DE ACORDO COM A IN 02 - DGP de 26/03/2020.	HC da Universidade Federal de Goiás - GO
232	ABONO ADMINISTRATIVO - COVID 19.	HC da Universidade Federal de Goiás - GO
232	ABONO ADMINISTRATIVO - COVID 19.	HC da Universidade Federal de Goiás - GO
232	ABONO ADMINISTRATIVO - COVID 19.	HC da Universidade Federal de Goiás - GO
232	ABONO ADMINISTRATIVO - COVID 19.	HC da Universidade Federal de Goiás - GO
232	ABONO ADMINISTRATIVO - COVID 19.	HC da Universidade Federal de Goiás - GO
232	ABONO ADMINISTRATIVO - COVID 19.	HC da Universidade Federal de Goiás - GO
232	ABONO ADMINISTRATIVO - COVID 19.	HC da Universidade Federal de Goiás - GO
232	ABONO ADMINISTRATIVO - COVID 19.	HC da Universidade Federal de Goiás - GO
232	ABONO ADMINISTRATIVO - COVID 19.	HC da Universidade Federal de Goiás - GO
232	ABONO ADMINISTRATIVO - COVID 19.	HC da Universidade Federal de Goiás - GO
232	ABONO ADMINISTRATIVO - COVID 19.	HC da Universidade Federal de Goiás - GO
232	ABONO ADMINISTRATIVO - COVID 19.	HC da Universidade Federal de Goiás - GO
232	ABONO ADMINISTRATIVO - COVID 19.	HC da Universidade Federal de Goiás - GO
232	ABONO ADMINISTRATIVO - COVID 19.	HC da Universidade Federal de Goiás - GO
232	ABONO ADMINISTRATIVO - COVID 19.	HC da Universidade Federal de Goiás - GO
233	ATIVIDADE EXTERNA ESPII - RODÍZIO	HC da Universidade Federal de Goiás - GO
233	ATIVIDADE EXTERNA ESPII - RODÍZIO	HC da Universidade Federal de Goiás - GO
233	ATIVIDADE EXTERNA ESPII - RODÍZIO	HC da Universidade Federal de Goiás - GO
233	ATIVIDADE EXTERNA ESPII - RODÍZIO	HC da Universidade Federal de Goiás - GO
233	ATIVIDADE EXTERNA ESPII - RODÍZIO	HC da Universidade Federal de Goiás - GO
233	ATIVIDADE EXTERNA ESPII - RODÍZIO	HC da Universidade Federal de Goiás - GO
233	ATIVIDADE EXTERNA ESPII - RODÍZIO	HC da Universidade Federal de Goiás - GO
233	ATIVIDADE EXTERNA ESPII - RODÍZIO	HC da Universidade Federal de Goiás - GO
233	ATIVIDADE EXTERNA ESPII - RODÍZIO	HC da Universidade Federal de Goiás - GO
233	ATIVIDADE EXTERNA ESPII - RODÍZIO	HC da Universidade Federal de Goiás - GO

233	ATIVIDADE EXTERNA ESPII - RODÍZIO	HC da Universidade Federal de Goiás - GO
233	ATIVIDADE EXTERNA ESPII - RODÍZIO	HC da Universidade Federal de Goiás - GO
233	ATIVIDADE EXTERNA ESPII - RODÍZIO	HC da Universidade Federal de Goiás - GO
233	ATIVIDADE EXTERNA ESPII - RODÍZIO	HC da Universidade Federal de Goiás - GO
233	ATIVIDADE EXTERNA ESPII - RODÍZIO	HC da Universidade Federal de Goiás - GO
233	ATIVIDADE EXTERNA ESPII - RODÍZIO	HC da Universidade Federal de Goiás - GO
233	ATIVIDADE EXTERNA ESPII - RODÍZIO	HC da Universidade Federal de Goiás - GO
233	ATIVIDADE EXTERNA ESPII - RODÍZIO	HC da Universidade Federal de Goiás - GO
233	ATIVIDADE EXTERNA ESPII - RODÍZIO	HC da Universidade Federal de Goiás - GO
233	ATIVIDADE EXTERNA ESPII - RODÍZIO	HC da Universidade Federal de Goiás - GO
233	ATIVIDADE EXTERNA ESPII - RODÍZIO	HC da Universidade Federal de Goiás - GO
233	ATIVIDADE EXTERNA ESPII - RODÍZIO	HC da Universidade Federal de Goiás - GO
233	ATIVIDADE EXTERNA ESPII - RODÍZIO	HC da Universidade Federal de Goiás - GO
233	ATIVIDADE EXTERNA ESPII - RODÍZIO	HC da Universidade Federal de Goiás - GO
233	ATIVIDADE EXTERNA ESPII - RODÍZIO	HC da Universidade Federal de Goiás - GO
233	ATIVIDADE EXTERNA ESPII - RODÍZIO	HC da Universidade Federal de Goiás - GO
233	ATIVIDADE EXTERNA ESPII - RODÍZIO	HC da Universidade Federal de Goiás - GO
233	ATIVIDADE EXTERNA ESPII - RODÍZIO	HC da Universidade Federal de Goiás - GO
233	ATIVIDADE EXTERNA ESPII - RODÍZIO	HC da Universidade Federal de Goiás - GO
233	ATIVIDADE EXTERNA ESPII - RODÍZIO	HC da Universidade Federal de Goiás - GO
233	ATIVIDADE EXTERNA ESPII - RODÍZIO	HC da Universidade Federal de Goiás - GO
233	ATIVIDADE EXTERNA ESPII - RODÍZIO	HC da Universidade Federal de Goiás - GO
233	ATIVIDADE EXTERNA ESPII - RODÍZIO	HC da Universidade Federal de Goiás - GO
233	ATIVIDADE EXTERNA ESPII - RODÍZIO	HC da Universidade Federal de Goiás - GO
233	ATIVIDADE EXTERNA ESPII - RODÍZIO	HC da Universidade Federal de Goiás - GO
233	ATIVIDADE EXTERNA ESPII - RODÍZIO	HC da Universidade Federal de Goiás - GO
233	ATIVIDADE EXTERNA ESPII - RODÍZIO	HC da Universidade Federal de Goiás - GO
233	ATIVIDADE EXTERNA ESPII - RODÍZIO	HC da Universidade Federal de Goiás - GO
233	ATIVIDADE EXTERNA ESPII - RODÍZIO	HC da Universidade Federal de Goiás - GO
233	ATIVIDADE EXTERNA ESPII - RODÍZIO	HC da Universidade Federal de Goiás - GO
233	ATIVIDADE EXTERNA ESPII - RODÍZIO	HC da Universidade Federal de Goiás - GO
233	ATIVIDADE EXTERNA ESPII - RODÍZIO	HC da Universidade Federal de Goiás - GO
233	ATIVIDADE EXTERNA ESPII - RODÍZIO	HC da Universidade Federal de Goiás - GO
233	ATIVIDADE EXTERNA ESPII - RODÍZIO	HC da Universidade Federal de Goiás - GO
233	ATIVIDADE EXTERNA ESPII - RODÍZIO	HC da Universidade Federal de Goiás - GO
233	ATIVIDADE EXTERNA ESPII - RODÍZIO	HC da Universidade Federal de Goiás - GO
233	ATIVIDADE EXTERNA ESPII - RODÍZIO	HC da Universidade Federal de Goiás - GO
233	ATIVIDADE EXTERNA ESPII - RODÍZIO	HC da Universidade Federal de Goiás - GO
233	ATIVIDADE EXTERNA ESPII - RODÍZIO	HC da Universidade Federal de Goiás - GO
233	ATIVIDADE EXTERNA ESPII - RODÍZIO	HC da Universidade Federal de Goiás - GO
233	ATIVIDADE EXTERNA ESPII - RODÍZIO	HC da Universidade Federal de Goiás - GO
233	ATIVIDADE EXTERNA ESPII - RODÍZIO	HC da Universidade Federal de Goiás - GO
233	ATIVIDADE EXTERNA ESPII - RODÍZIO	HC da Universidade Federal de Goiás - GO
233	ATIVIDADE EXTERNA ESPII - RODÍZIO	HC da Universidade Federal de Goiás - GO
233	ATIVIDADE EXTERNA ESPII - RODÍZIO	HC da Universidade Federal de Goiás - GO
233	ATIVIDADE EXTERNA ESPII - RODÍZIO	HC da Universidade Federal de Goiás - GO
233	ATIVIDADE EXTERNA ESPII - RODÍZIO	HC da Universidade Federal de Goiás - GO
233	ATIVIDADE EXTERNA ESPII - RODÍZIO	HC da Universidade Federal de Goiás - GO

233	ATIVIDADE EXTERNA ESPII - RODÍZIO	HC da Universidade Federal de Goiás - GO
233	ATIVIDADE EXTERNA ESPII - RODÍZIO	HC da Universidade Federal de Goiás - GO
233	ATIVIDADE EXTERNA ESPII - RODÍZIO	HC da Universidade Federal de Goiás - GO
233	ATIVIDADE EXTERNA ESPII - RODÍZIO	HC da Universidade Federal de Goiás - GO
233	ATIVIDADE EXTERNA ESPII - RODÍZIO	HC da Universidade Federal de Goiás - GO
233	ATIVIDADE EXTERNA ESPII - RODÍZIO	HC da Universidade Federal de Goiás - GO
233	ATIVIDADE EXTERNA ESPII - RODÍZIO	HC da Universidade Federal de Goiás - GO
233	ATIVIDADE EXTERNA ESPII - RODÍZIO	HC da Universidade Federal de Goiás - GO
233	ATIVIDADE EXTERNA ESPII - RODÍZIO	HC da Universidade Federal de Goiás - GO
233	ATIVIDADE EXTERNA ESPII - RODÍZIO	HC da Universidade Federal de Goiás - GO
233	ATIVIDADE EXTERNA ESPII - RODÍZIO	HC da Universidade Federal de Goiás - GO
233	ATIVIDADE EXTERNA ESPII - RODÍZIO	HC da Universidade Federal de Goiás - GO
233	ATIVIDADE EXTERNA ESPII - RODÍZIO	HC da Universidade Federal de Goiás - GO
233	ATIVIDADE EXTERNA ESPII - RODÍZIO	HC da Universidade Federal de Goiás - GO
233	ATIVIDADE EXTERNA ESPII - RODÍZIO	HC da Universidade Federal de Goiás - GO
233	ATIVIDADE EXTERNA ESPII - RODÍZIO	HC da Universidade Federal de Goiás - GO
233	ATIVIDADE EXTERNA ESPII - RODÍZIO	HC da Universidade Federal de Goiás - GO
233	ATIVIDADE EXTERNA ESPII - RODÍZIO	HC da Universidade Federal de Goiás - GO
233	ATIVIDADE EXTERNA ESPII - RODÍZIO	HC da Universidade Federal de Goiás - GO
233	ATIVIDADE EXTERNA ESPII - RODÍZIO	HC da Universidade Federal de Goiás - GO
233	ATIVIDADE EXTERNA ESPII - RODÍZIO	HC da Universidade Federal de Goiás - GO
233	ATIVIDADE EXTERNA ESPII - RODÍZIO	HC da Universidade Federal de Goiás - GO
233	ATIVIDADE EXTERNA ESPII - RODÍZIO	HC da Universidade Federal de Goiás - GO
233	ATIVIDADE EXTERNA ESPII - RODÍZIO	HC da Universidade Federal de Goiás - GO
233	ATIVIDADE EXTERNA ESPII - RODÍZIO	HC da Universidade Federal de Goiás - GO
233	ATIVIDADE EXTERNA ESPII - RODÍZIO	HC da Universidade Federal de Goiás - GO
233	ATIVIDADE EXTERNA ESPII - RODÍZIO	HC da Universidade Federal de Goiás - GO
233	ATIVIDADE EXTERNA ESPII - RODÍZIO	HC da Universidade Federal de Goiás - GO
233	ATIVIDADE EXTERNA ESPII - RODÍZIO	HC da Universidade Federal de Goiás - GO
233	ATIVIDADE EXTERNA ESPII - RODÍZIO	HC da Universidade Federal de Goiás - GO
233	ATIVIDADE EXTERNA ESPII - RODÍZIO	HC da Universidade Federal de Goiás - GO
233	ATIVIDADE EXTERNA ESPII - RODÍZIO	HC da Universidade Federal de Goiás - GO
233	ATIVIDADE EXTERNA ESPII - RODÍZIO	HC da Universidade Federal de Goiás - GO
233	ATIVIDADE EXTERNA ESPII - RODÍZIO	HC da Universidade Federal de Goiás - GO
233	ATIVIDADE EXTERNA ESPII - RODÍZIO	HC da Universidade Federal de Goiás - GO
233	ATIVIDADE EXTERNA ESPII - RODÍZIO	HC da Universidade Federal de Goiás - GO
233	ATIVIDADE EXTERNA ESPII - RODÍZIO	HC da Universidade Federal de Goiás - GO
233	ATIVIDADE EXTERNA ESPII - RODÍZIO	HC da Universidade Federal de Goiás - GO
233	ATIVIDADE EXTERNA ESPII - RODÍZIO	HC da Universidade Federal de Goiás - GO
233	ATIVIDADE EXTERNA ESPII - RODÍZIO	HC da Universidade Federal de Goiás - GO
233	ATIVIDADE EXTERNA ESPII - RODÍZIO	HC da Universidade Federal de Goiás - GO
233	ATIVIDADE EXTERNA ESPII - RODÍZIO	HC da Universidade Federal de Goiás - GO
233	ATIVIDADE EXTERNA ESPII - RODÍZIO	HC da Universidade Federal de Goiás - GO
233	ATIVIDADE EXTERNA ESPII - RODÍZIO	HC da Universidade Federal de Goiás - GO
233	ATIVIDADE EXTERNA ESPII - RODÍZIO	HC da Universidade Federal de Goiás - GO
233	ATIVIDADE EXTERNA ESPII - RODÍZIO	HC da Universidade Federal de Goiás - GO
233	ATIVIDADE EXTERNA ESPII - RODÍZIO	HC da Universidade Federal de Goiás - GO
233	ATIVIDADE EXTERNA ESPII - RODÍZIO	HC da Universidade Federal de Goiás - GO

233	ATIVIDADE EXTERNA ESPII - RODÍZIO	HC da Universidade Federal de Goiás - GO
233	ATIVIDADE EXTERNA ESPII - RODÍZIO	HC da Universidade Federal de Goiás - GO
233	ATIVIDADE EXTERNA ESPII - RODÍZIO	HC da Universidade Federal de Goiás - GO
233	ATIVIDADE EXTERNA ESPII - RODÍZIO	HC da Universidade Federal de Goiás - GO
233	ATIVIDADE EXTERNA ESPII - RODÍZIO	HC da Universidade Federal de Goiás - GO
233	ATIVIDADE EXTERNA ESPII - RODÍZIO	HC da Universidade Federal de Goiás - GO
233	ATIVIDADE EXTERNA ESPII - RODÍZIO	HC da Universidade Federal de Goiás - GO
233	ATIVIDADE EXTERNA ESPII - RODÍZIO	HC da Universidade Federal de Goiás - GO
233	ATIVIDADE EXTERNA ESPII - RODÍZIO	HC da Universidade Federal de Goiás - GO
233	ATIVIDADE EXTERNA ESPII - RODÍZIO	HC da Universidade Federal de Goiás - GO
233	ATIVIDADE EXTERNA ESPII - RODÍZIO	HC da Universidade Federal de Goiás - GO
233	ATIVIDADE EXTERNA ESPII - RODÍZIO	HC da Universidade Federal de Goiás - GO
233	ATIVIDADE EXTERNA ESPII - RODÍZIO	HC da Universidade Federal de Goiás - GO
233	ATIVIDADE EXTERNA ESPII - RODÍZIO	HC da Universidade Federal de Goiás - GO
233	ATIVIDADE EXTERNA ESPII - RODÍZIO	HC da Universidade Federal de Goiás - GO
233	ATIVIDADE EXTERNA ESPII - RODÍZIO	HC da Universidade Federal de Goiás - GO
233	ATIVIDADE EXTERNA ESPII - RODÍZIO	HC da Universidade Federal de Goiás - GO
233	ATIVIDADE EXTERNA ESPII - RODÍZIO	HC da Universidade Federal de Goiás - GO
233	ATIVIDADE EXTERNA ESPII - RODÍZIO	HC da Universidade Federal de Goiás - GO
233	ATIVIDADE EXTERNA ESPII - RODÍZIO	HC da Universidade Federal de Goiás - GO
233	ATIVIDADE EXTERNA ESPII - RODÍZIO	HC da Universidade Federal de Goiás - GO
233	ATIVIDADE EXTERNA ESPII - RODÍZIO	HC da Universidade Federal de Goiás - GO
233	ATIVIDADE EXTERNA ESPII - RODÍZIO	HC da Universidade Federal de Goiás - GO
233	ATIVIDADE EXTERNA ESPII - RODÍZIO	HC da Universidade Federal de Goiás - GO
233	ATIVIDADE EXTERNA ESPII - RODÍZIO	HC da Universidade Federal de Goiás - GO
233	ATIVIDADE EXTERNA ESPII - RODÍZIO	HC da Universidade Federal de Goiás - GO
233	ATIVIDADE EXTERNA ESPII - RODÍZIO	HC da Universidade Federal de Goiás - GO
233	ATIVIDADE EXTERNA ESPII - RODÍZIO	HC da Universidade Federal de Goiás - GO
233	ATIVIDADE EXTERNA ESPII - RODÍZIO	HC da Universidade Federal de Goiás - GO
233	ATIVIDADE EXTERNA ESPII - RODÍZIO	HC da Universidade Federal de Goiás - GO
233	ATIVIDADE EXTERNA ESPII - RODÍZIO	HC da Universidade Federal de Goiás - GO
233	ATIVIDADE EXTERNA ESPII - RODÍZIO	HC da Universidade Federal de Goiás - GO
233	ATIVIDADE EXTERNA ESPII - RODÍZIO	HC da Universidade Federal de Goiás - GO
233	ATIVIDADE EXTERNA ESPII - RODÍZIO	HC da Universidade Federal de Goiás - GO
233	ATIVIDADE EXTERNA ESPII - RODÍZIO	HC da Universidade Federal de Goiás - GO
233	ATIVIDADE EXTERNA ESPII - RODÍZIO	HC da Universidade Federal de Goiás - GO
233	ATIVIDADE EXTERNA ESPII - RODÍZIO	HC da Universidade Federal de Goiás - GO
233	ATIVIDADE EXTERNA ESPII - RODÍZIO	HC da Universidade Federal de Goiás - GO
233	ATIVIDADE EXTERNA ESPII - RODÍZIO	HC da Universidade Federal de Goiás - GO
233	ATIVIDADE EXTERNA ESPII - RODÍZIO	HC da Universidade Federal de Goiás - GO
233	ATIVIDADE EXTERNA ESPII - RODÍZIO	HC da Universidade Federal de Goiás - GO
233	ATIVIDADE EXTERNA ESPII - RODÍZIO	HC da Universidade Federal de Goiás - GO
233	ATIVIDADE EXTERNA ESPII - RODÍZIO	HC da Universidade Federal de Goiás - GO
233	ATIVIDADE EXTERNA ESPII - RODÍZIO	HC da Universidade Federal de Goiás - GO
233	ATIVIDADE EXTERNA ESPII - RODÍZIO	HC da Universidade Federal de Goiás - GO
233	ATIVIDADE EXTERNA ESPII - RODÍZIO	HC da Universidade Federal de Goiás - GO
233	ATIVIDADE EXTERNA ESPII - RODÍZIO	HC da Universidade Federal de Goiás - GO
233	ATIVIDADE EXTERNA ESPII - RODÍZIO	HC da Universidade Federal de Goiás - GO

233	ATIVIDADE EXTERNA ESPII - RODÍZIO	HC da Universidade Federal de Goiás - GO
233	ATIVIDADE EXTERNA ESPII - RODÍZIO	HC da Universidade Federal de Goiás - GO
233	ATIVIDADE EXTERNA ESPII - RODÍZIO	HC da Universidade Federal de Goiás - GO
233	ATIVIDADE EXTERNA ESPII - RODÍZIO	HC da Universidade Federal de Goiás - GO
233	ATIVIDADE EXTERNA ESPII - RODÍZIO	HC da Universidade Federal de Goiás - GO
233	ATIVIDADE EXTERNA ESPII - RODÍZIO	HC da Universidade Federal de Goiás - GO
233	ATIVIDADE EXTERNA ESPII - RODÍZIO	HC da Universidade Federal de Goiás - GO
233	ATIVIDADE EXTERNA ESPII - RODÍZIO	HC da Universidade Federal de Goiás - GO
233	ATIVIDADE EXTERNA ESPII - RODÍZIO	HC da Universidade Federal de Goiás - GO
233	ATIVIDADE EXTERNA ESPII - RODÍZIO	HC da Universidade Federal de Goiás - GO
233	ATIVIDADE EXTERNA ESPII - RODÍZIO	HC da Universidade Federal de Goiás - GO
233	ATIVIDADE EXTERNA ESPII - RODÍZIO	HC da Universidade Federal de Goiás - GO
233	ATIVIDADE EXTERNA ESPII - RODÍZIO	HC da Universidade Federal de Goiás - GO
233	ATIVIDADE EXTERNA ESPII - RODÍZIO	HC da Universidade Federal de Goiás - GO
233	ATIVIDADE EXTERNA ESPII - RODÍZIO	HC da Universidade Federal de Goiás - GO
233	ATIVIDADE EXTERNA ESPII - RODÍZIO	HC da Universidade Federal de Goiás - GO
233	ATIVIDADE EXTERNA ESPII - RODÍZIO	HC da Universidade Federal de Goiás - GO
233	ATIVIDADE EXTERNA ESPII - RODÍZIO	HC da Universidade Federal de Goiás - GO
233	ATIVIDADE EXTERNA ESPII - RODÍZIO	HC da Universidade Federal de Goiás - GO
233	ATIVIDADE EXTERNA ESPII - RODÍZIO	HC da Universidade Federal de Goiás - GO
233	ATIVIDADE EXTERNA ESPII - RODÍZIO	HC da Universidade Federal de Goiás - GO
233	ATIVIDADE EXTERNA ESPII - RODÍZIO	HC da Universidade Federal de Goiás - GO
233	ATIVIDADE EXTERNA ESPII - RODÍZIO	HC da Universidade Federal de Goiás - GO
233	ATIVIDADE EXTERNA ESPII - RODÍZIO	HC da Universidade Federal de Goiás - GO
233	ATIVIDADE EXTERNA ESPII - RODÍZIO	HC da Universidade Federal de Goiás - GO
233	ATIVIDADE EXTERNA ESPII - RODÍZIO	HC da Universidade Federal de Goiás - GO
233	ATIVIDADE EXTERNA ESPII - RODÍZIO	HC da Universidade Federal de Goiás - GO
233	ATIVIDADE EXTERNA ESPII - RODÍZIO	HC da Universidade Federal de Goiás - GO
233	ATIVIDADE EXTERNA ESPII - RODÍZIO	HC da Universidade Federal de Goiás - GO
233	ATIVIDADE EXTERNA ESPII - RODÍZIO	HC da Universidade Federal de Goiás - GO
233	ATIVIDADE EXTERNA ESPII - RODÍZIO	HC da Universidade Federal de Goiás - GO
233	ATIVIDADE EXTERNA ESPII - RODÍZIO	HC da Universidade Federal de Goiás - GO
233	ATIVIDADE EXTERNA ESPII - RODÍZIO	HC da Universidade Federal de Goiás - GO
233	ATIVIDADE EXTERNA ESPII - RODÍZIO	HC da Universidade Federal de Goiás - GO
233	ATIVIDADE EXTERNA ESPII - RODÍZIO	HC da Universidade Federal de Goiás - GO
233	ATIVIDADE EXTERNA ESPII - RODÍZIO	HC da Universidade Federal de Goiás - GO
233	ATIVIDADE EXTERNA ESPII - RODÍZIO	HC da Universidade Federal de Goiás - GO
233	ATIVIDADE EXTERNA ESPII - RODÍZIO	HC da Universidade Federal de Goiás - GO
233	ATIVIDADE EXTERNA ESPII - RODÍZIO	HC da Universidade Federal de Goiás - GO
233	ATIVIDADE EXTERNA ESPII - RODÍZIO	HC da Universidade Federal de Goiás - GO
233	ATIVIDADE EXTERNA ESPII - RODÍZIO	HC da Universidade Federal de Goiás - GO
233	ATIVIDADE EXTERNA ESPII - RODÍZIO	HC da Universidade Federal de Goiás - GO
233	ATIVIDADE EXTERNA ESPII - RODÍZIO	HC da Universidade Federal de Goiás - GO
233	ATIVIDADE EXTERNA ESPII - RODÍZIO	HC da Universidade Federal de Goiás - GO
233	ATIVIDADE EXTERNA ESPII - RODÍZIO	HC da Universidade Federal de Goiás - GO
233	ATIVIDADE EXTERNA ESPII - RODÍZIO	HC da Universidade Federal de Goiás - GO
233	ATIVIDADE EXTERNA ESPII - RODÍZIO	HC da Universidade Federal de Goiás - GO
233	ATIVIDADE EXTERNA ESPII - RODÍZIO	HC da Universidade Federal de Goiás - GO

233	ATIVIDADE EXTERNA ESPII - RODÍZIO	HC da Universidade Federal de Goiás - GO
233	ATIVIDADE EXTERNA ESPII - RODÍZIO	HC da Universidade Federal de Goiás - GO
233	ATIVIDADE EXTERNA ESPII - RODÍZIO	HC da Universidade Federal de Goiás - GO
233	ATIVIDADE EXTERNA ESPII - RODÍZIO	HC da Universidade Federal de Goiás - GO
233	ATIVIDADE EXTERNA ESPII - RODÍZIO	HC da Universidade Federal de Goiás - GO
233	ATIVIDADE EXTERNA ESPII - RODÍZIO	HC da Universidade Federal de Goiás - GO
233	ATIVIDADE EXTERNA ESPII - RODÍZIO	HC da Universidade Federal de Goiás - GO
233	ATIVIDADE EXTERNA ESPII - RODÍZIO	HC da Universidade Federal de Goiás - GO
233	ATIVIDADE EXTERNA ESPII - RODÍZIO	HC da Universidade Federal de Goiás - GO
233	ATIVIDADE EXTERNA ESPII - RODÍZIO	HC da Universidade Federal de Goiás - GO
233	ATIVIDADE EXTERNA ESPII - RODÍZIO	HC da Universidade Federal de Goiás - GO
233	ATIVIDADE EXTERNA ESPII - RODÍZIO	HC da Universidade Federal de Goiás - GO
233	ATIVIDADE EXTERNA ESPII - RODÍZIO	HC da Universidade Federal de Goiás - GO
233	ATIVIDADE EXTERNA ESPII - RODÍZIO	HC da Universidade Federal de Goiás - GO
233	ATIVIDADE EXTERNA ESPII - RODÍZIO	HC da Universidade Federal de Goiás - GO
233	ATIVIDADE EXTERNA ESPII - RODÍZIO	HC da Universidade Federal de Goiás - GO
233	ATIVIDADE EXTERNA ESPII - RODÍZIO	HC da Universidade Federal de Goiás - GO
233	ATIVIDADE EXTERNA ESPII - RODÍZIO	HC da Universidade Federal de Goiás - GO
233	ATIVIDADE EXTERNA ESPII - RODÍZIO	HC da Universidade Federal de Goiás - GO
233	ATIVIDADE EXTERNA ESPII - RODÍZIO	HC da Universidade Federal de Goiás - GO
233	ATIVIDADE EXTERNA ESPII - RODÍZIO	HC da Universidade Federal de Goiás - GO
233	ATIVIDADE EXTERNA ESPII - RODÍZIO	HC da Universidade Federal de Goiás - GO
233	ATIVIDADE EXTERNA ESPII - RODÍZIO	HC da Universidade Federal de Goiás - GO
233	ATIVIDADE EXTERNA ESPII - RODÍZIO	HC da Universidade Federal de Goiás - GO
233	ATIVIDADE EXTERNA ESPII - RODÍZIO	HC da Universidade Federal de Goiás - GO
233	ATIVIDADE EXTERNA ESPII - RODÍZIO	HC da Universidade Federal de Goiás - GO
233	ATIVIDADE EXTERNA ESPII - RODÍZIO	HC da Universidade Federal de Goiás - GO
233	ATIVIDADE EXTERNA ESPII - RODÍZIO	HC da Universidade Federal de Goiás - GO
233	ATIVIDADE EXTERNA ESPII - RODÍZIO	HC da Universidade Federal de Goiás - GO
233	ATIVIDADE EXTERNA ESPII - RODÍZIO	HC da Universidade Federal de Goiás - GO
233	ATIVIDADE EXTERNA ESPII - RODÍZIO	HC da Universidade Federal de Goiás - GO
233	ATIVIDADE EXTERNA ESPII - RODÍZIO	HC da Universidade Federal de Goiás - GO
233	ATIVIDADE EXTERNA ESPII - RODÍZIO	HC da Universidade Federal de Goiás - GO
233	ATIVIDADE EXTERNA ESPII - RODÍZIO	HC da Universidade Federal de Goiás - GO
233	ATIVIDADE EXTERNA ESPII - RODÍZIO	HC da Universidade Federal de Goiás - GO
233	ATIVIDADE EXTERNA ESPII - RODÍZIO	HC da Universidade Federal de Goiás - GO
233	ATIVIDADE EXTERNA ESPII - RODÍZIO	HC da Universidade Federal de Goiás - GO
233	ATIVIDADE EXTERNA ESPII - RODÍZIO	HC da Universidade Federal de Goiás - GO
233	ATIVIDADE EXTERNA ESPII - RODÍZIO	HC da Universidade Federal de Goiás - GO
233	ATIVIDADE EXTERNA ESPII - RODÍZIO	HC da Universidade Federal de Goiás - GO
233	ATIVIDADE EXTERNA ESPII - RODÍZIO	HC da Universidade Federal de Goiás - GO
233	ATIVIDADE EXTERNA ESPII - RODÍZIO	HC da Universidade Federal de Goiás - GO
233	ATIVIDADE EXTERNA ESPII - RODÍZIO	HC da Universidade Federal de Goiás - GO
233	ATIVIDADE EXTERNA ESPII - RODÍZIO	HC da Universidade Federal de Goiás - GO
233	ATIVIDADE EXTERNA ESPII - RODÍZIO	HC da Universidade Federal de Goiás - GO
233	ATIVIDADE EXTERNA ESPII - RODÍZIO	HC da Universidade Federal de Goiás - GO
233	ATIVIDADE EXTERNA ESPII - RODÍZIO	HC da Universidade Federal de Goiás - GO
233	ATIVIDADE EXTERNA ESPII - RODÍZIO	HC da Universidade Federal de Goiás - GO

233	ATIVIDADE EXTERNA ESPII - RODÍZIO	HC da Universidade Federal de Goiás - GO
233	ATIVIDADE EXTERNA ESPII - RODÍZIO	HC da Universidade Federal de Goiás - GO
233	ATIVIDADE EXTERNA ESPII - RODÍZIO	HC da Universidade Federal de Goiás - GO
233	ATIVIDADE EXTERNA ESPII - RODÍZIO	HC da Universidade Federal de Goiás - GO
233	ATIVIDADE EXTERNA ESPII - RODÍZIO	HC da Universidade Federal de Goiás - GO
233	ATIVIDADE EXTERNA ESPII - RODÍZIO	HC da Universidade Federal de Goiás - GO
233	ATIVIDADE EXTERNA ESPII - RODÍZIO	HC da Universidade Federal de Goiás - GO
233	ATIVIDADE EXTERNA ESPII - RODÍZIO	HC da Universidade Federal de Goiás - GO
233	ATIVIDADE EXTERNA ESPII - RODÍZIO	HC da Universidade Federal de Goiás - GO
233	ATIVIDADE EXTERNA ESPII - RODÍZIO	HC da Universidade Federal de Goiás - GO
233	ATIVIDADE EXTERNA ESPII - RODÍZIO	HC da Universidade Federal de Goiás - GO
233	ATIVIDADE EXTERNA ESPII - RODÍZIO	HC da Universidade Federal de Goiás - GO
233	ATIVIDADE EXTERNA ESPII - RODÍZIO	HC da Universidade Federal de Goiás - GO
233	ATIVIDADE EXTERNA ESPII - RODÍZIO	HC da Universidade Federal de Goiás - GO
233	ATIVIDADE EXTERNA ESPII - RODÍZIO	HC da Universidade Federal de Goiás - GO
233	ATIVIDADE EXTERNA ESPII - RODÍZIO	HC da Universidade Federal de Goiás - GO
233	ATIVIDADE EXTERNA ESPII - RODÍZIO	HC da Universidade Federal de Goiás - GO
233	ATIVIDADE EXTERNA ESPII - RODÍZIO	HC da Universidade Federal de Goiás - GO
233	ATIVIDADE EXTERNA ESPII - RODÍZIO	HC da Universidade Federal de Goiás - GO
233	ATIVIDADE EXTERNA ESPII - RODÍZIO	HC da Universidade Federal de Goiás - GO
233	ATIVIDADE EXTERNA ESPII - RODÍZIO	HC da Universidade Federal de Goiás - GO
233	ATIVIDADE EXTERNA ESPII - RODÍZIO	HC da Universidade Federal de Goiás - GO
233	ATIVIDADE EXTERNA ESPII - RODÍZIO	HC da Universidade Federal de Goiás - GO
233	ATIVIDADE EXTERNA ESPII - RODÍZIO	HC da Universidade Federal de Goiás - GO
233	ATIVIDADE EXTERNA ESPII - RODÍZIO	HC da Universidade Federal de Goiás - GO
233	ATIVIDADE EXTERNA ESPII - RODÍZIO	HC da Universidade Federal de Goiás - GO
233	ATIVIDADE EXTERNA ESPII - RODÍZIO	HC da Universidade Federal de Goiás - GO
233	ATIVIDADE EXTERNA ESPII - RODÍZIO	HC da Universidade Federal de Goiás - GO
233	ATIVIDADE EXTERNA ESPII - RODÍZIO	HC da Universidade Federal de Goiás - GO
233	ATIVIDADE EXTERNA ESPII - RODÍZIO	HC da Universidade Federal de Goiás - GO
233	ATIVIDADE EXTERNA ESPII - RODÍZIO	HC da Universidade Federal de Goiás - GO
233	ATIVIDADE EXTERNA ESPII - RODÍZIO	HC da Universidade Federal de Goiás - GO
233	ATIVIDADE EXTERNA ESPII - RODÍZIO	HC da Universidade Federal de Goiás - GO
233	ATIVIDADE EXTERNA ESPII - RODÍZIO	HC da Universidade Federal de Goiás - GO
233	ATIVIDADE EXTERNA ESPII - RODÍZIO	HC da Universidade Federal de Goiás - GO
233	ATIVIDADE EXTERNA ESPII - RODÍZIO	HC da Universidade Federal de Goiás - GO
233	ATIVIDADE EXTERNA ESPII - RODÍZIO	HC da Universidade Federal de Goiás - GO
233	ATIVIDADE EXTERNA ESPII - RODÍZIO	HC da Universidade Federal de Goiás - GO
233	ATIVIDADE EXTERNA ESPII - RODÍZIO	HC da Universidade Federal de Goiás - GO
233	ATIVIDADE EXTERNA ESPII - RODÍZIO	HC da Universidade Federal de Goiás - GO
233	ATIVIDADE EXTERNA ESPII - RODÍZIO	HC da Universidade Federal de Goiás - GO
233	ATIVIDADE EXTERNA ESPII - RODÍZIO	HC da Universidade Federal de Goiás - GO
233	ATIVIDADE EXTERNA ESPII - RODÍZIO	HC da Universidade Federal de Goiás - GO

233	ATIVIDADE EXTERNA ESPII - RODÍZIO	HC da Universidade Federal de Goiás - GO
233	ATIVIDADE EXTERNA ESPII - RODÍZIO	HC da Universidade Federal de Goiás - GO
233	ATIVIDADE EXTERNA ESPII - RODÍZIO	HC da Universidade Federal de Goiás - GO
233	ATIVIDADE EXTERNA ESPII - RODÍZIO	HC da Universidade Federal de Goiás - GO
233	ATIVIDADE EXTERNA ESPII - RODÍZIO	HC da Universidade Federal de Goiás - GO
233	ATIVIDADE EXTERNA ESPII - RODÍZIO	HC da Universidade Federal de Goiás - GO
233	ATIVIDADE EXTERNA ESPII - RODÍZIO	HC da Universidade Federal de Goiás - GO
233	ATIVIDADE EXTERNA ESPII - RODÍZIO	HC da Universidade Federal de Goiás - GO
233	ATIVIDADE EXTERNA ESPII - RODÍZIO	HC da Universidade Federal de Goiás - GO
233	ATIVIDADE EXTERNA ESPII - RODÍZIO	HC da Universidade Federal de Goiás - GO
233	ATIVIDADE EXTERNA ESPII - RODÍZIO	HC da Universidade Federal de Goiás - GO
233	ATIVIDADE EXTERNA ESPII - RODÍZIO	HC da Universidade Federal de Goiás - GO
233	ATIVIDADE EXTERNA ESPII - RODÍZIO	HC da Universidade Federal de Goiás - GO
233	ATIVIDADE EXTERNA ESPII - RODÍZIO	HC da Universidade Federal de Goiás - GO
233	ATIVIDADE EXTERNA ESPII - RODÍZIO	HC da Universidade Federal de Goiás - GO
233	ATIVIDADE EXTERNA ESPII - RODÍZIO	HC da Universidade Federal de Goiás - GO
233	ATIVIDADE EXTERNA ESPII - RODÍZIO	HC da Universidade Federal de Goiás - GO
233	ATIVIDADE EXTERNA ESPII - RODÍZIO	HC da Universidade Federal de Goiás - GO
233	ATIVIDADE EXTERNA ESPII - RODÍZIO	HC da Universidade Federal de Goiás - GO
233	ATIVIDADE EXTERNA ESPII - RODÍZIO	HC da Universidade Federal de Goiás - GO
233	ATIVIDADE EXTERNA ESPII - RODÍZIO	HC da Universidade Federal de Goiás - GO
233	ATIVIDADE EXTERNA ESPII - RODÍZIO	HC da Universidade Federal de Goiás - GO
233	ATIVIDADE EXTERNA ESPII - RODÍZIO	HC da Universidade Federal de Goiás - GO
233	ATIVIDADE EXTERNA ESPII - RODÍZIO	HC da Universidade Federal de Goiás - GO
233	ATIVIDADE EXTERNA ESPII - RODÍZIO	HC da Universidade Federal de Goiás - GO
233	ATIVIDADE EXTERNA ESPII - RODÍZIO	HC da Universidade Federal de Goiás - GO
233	ATIVIDADE EXTERNA ESPII - RODÍZIO	HC da Universidade Federal de Goiás - GO
233	ATIVIDADE EXTERNA ESPII - RODÍZIO	HC da Universidade Federal de Goiás - GO
233	ATIVIDADE EXTERNA ESPII - RODÍZIO	HC da Universidade Federal de Goiás - GO
233	ATIVIDADE EXTERNA ESPII - RODÍZIO	HC da Universidade Federal de Goiás - GO
233	ATIVIDADE EXTERNA ESPII - RODÍZIO	HC da Universidade Federal de Goiás - GO
233	ATIVIDADE EXTERNA ESPII - RODÍZIO	HC da Universidade Federal de Goiás - GO
233	ATIVIDADE EXTERNA ESPII - RODÍZIO	HC da Universidade Federal de Goiás - GO
233	ATIVIDADE EXTERNA ESPII - RODÍZIO	HC da Universidade Federal de Goiás - GO
233	ATIVIDADE EXTERNA ESPII - RODÍZIO	HC da Universidade Federal de Goiás - GO
233	ATIVIDADE EXTERNA ESPII - RODÍZIO	HC da Universidade Federal de Goiás - GO
233	ATIVIDADE EXTERNA ESPII - RODÍZIO	HC da Universidade Federal de Goiás - GO
233	ATIVIDADE EXTERNA ESPII - RODÍZIO	HC da Universidade Federal de Goiás - GO
233	ATIVIDADE EXTERNA ESPII - RODÍZIO	HC da Universidade Federal de Goiás - GO
233	ATIVIDADE EXTERNA ESPII - RODÍZIO	HC da Universidade Federal de Goiás - GO
233	ATIVIDADE EXTERNA ESPII - RODÍZIO	HC da Universidade Federal de Goiás - GO
233	ATIVIDADE EXTERNA ESPII - RODÍZIO	HC da Universidade Federal de Goiás - GO
233	ATIVIDADE EXTERNA ESPII - RODÍZIO	HC da Universidade Federal de Goiás - GO
233	ATIVIDADE EXTERNA ESPII - RODÍZIO	HC da Universidade Federal de Goiás - GO
233	ATIVIDADE EXTERNA ESPII - RODÍZIO	HC da Universidade Federal de Goiás - GO
233	ATIVIDADE EXTERNA ESPII - RODÍZIO	HC da Universidade Federal de Goiás - GO
233	ATIVIDADE EXTERNA ESPII - RODÍZIO	HC da Universidade Federal de Goiás - GO
233	ATIVIDADE EXTERNA ESPII - RODÍZIO	HC da Universidade Federal de Goiás - GO

233	ATIVIDADE EXTERNA ESPII - RODÍZIO	HC da Universidade Federal de Goiás - GO
233	ATIVIDADE EXTERNA ESPII - RODÍZIO	HC da Universidade Federal de Goiás - GO
233	ATIVIDADE EXTERNA ESPII - RODÍZIO	HC da Universidade Federal de Goiás - GO
233	ATIVIDADE EXTERNA ESPII - RODÍZIO	HC da Universidade Federal de Goiás - GO
233	ATIVIDADE EXTERNA ESPII - RODÍZIO	HC da Universidade Federal de Goiás - GO
233	ATIVIDADE EXTERNA ESPII - RODÍZIO	HC da Universidade Federal de Goiás - GO
233	ATIVIDADE EXTERNA ESPII - RODÍZIO	HC da Universidade Federal de Goiás - GO
233	ATIVIDADE EXTERNA ESPII - RODÍZIO	HC da Universidade Federal de Goiás - GO
233	ATIVIDADE EXTERNA ESPII - RODÍZIO	HC da Universidade Federal de Goiás - GO
233	ATIVIDADE EXTERNA ESPII - RODÍZIO	HC da Universidade Federal de Goiás - GO
233	ATIVIDADE EXTERNA ESPII - RODÍZIO	HC da Universidade Federal de Goiás - GO
233	ATIVIDADE EXTERNA ESPII - RODÍZIO	HC da Universidade Federal de Goiás - GO
233	ATIVIDADE EXTERNA ESPII - RODÍZIO	HC da Universidade Federal de Goiás - GO
233	ATIVIDADE EXTERNA ESPII - RODÍZIO	HC da Universidade Federal de Goiás - GO
233	ATIVIDADE EXTERNA ESPII - RODÍZIO	HC da Universidade Federal de Goiás - GO
233	ATIVIDADE EXTERNA ESPII - RODÍZIO	HC da Universidade Federal de Goiás - GO
233	ATIVIDADE EXTERNA ESPII - RODÍZIO	HC da Universidade Federal de Goiás - GO
233	ATIVIDADE EXTERNA ESPII - RODÍZIO	HC da Universidade Federal de Goiás - GO
233	ATIVIDADE EXTERNA ESPII - RODÍZIO	HC da Universidade Federal de Goiás - GO
233	ATIVIDADE EXTERNA ESPII - RODÍZIO	HC da Universidade Federal de Goiás - GO
233	ATIVIDADE EXTERNA ESPII - RODÍZIO	HC da Universidade Federal de Goiás - GO
233	ATIVIDADE EXTERNA ESPII - RODÍZIO	HC da Universidade Federal de Goiás - GO
233	ATIVIDADE EXTERNA ESPII - RODÍZIO	HC da Universidade Federal de Goiás - GO
233	ATIVIDADE EXTERNA ESPII - RODÍZIO	HC da Universidade Federal de Goiás - GO
233	ATIVIDADE EXTERNA ESPII - RODÍZIO	HC da Universidade Federal de Goiás - GO
233	ATIVIDADE EXTERNA ESPII - RODÍZIO	HC da Universidade Federal de Goiás - GO
233	ATIVIDADE EXTERNA ESPII - RODÍZIO	HC da Universidade Federal de Goiás - GO
233	ATIVIDADE EXTERNA ESPII - RODÍZIO	HC da Universidade Federal de Goiás - GO
233	ATIVIDADE EXTERNA ESPII - RODÍZIO	HC da Universidade Federal de Goiás - GO
233	ATIVIDADE EXTERNA ESPII - RODÍZIO	HC da Universidade Federal de Goiás - GO
233	ATIVIDADE EXTERNA ESPII - RODÍZIO	HC da Universidade Federal de Goiás - GO
233	ATIVIDADE EXTERNA ESPII - RODÍZIO	HC da Universidade Federal de Goiás - GO
233	ATIVIDADE EXTERNA ESPII - RODÍZIO	HC da Universidade Federal de Goiás - GO
233	ATIVIDADE EXTERNA ESPII - RODÍZIO	HC da Universidade Federal de Goiás - GO
233	ATIVIDADE EXTERNA ESPII - RODÍZIO	HC da Universidade Federal de Goiás - GO
233	ATIVIDADE EXTERNA ESPII - RODÍZIO	HC da Universidade Federal de Goiás - GO
233	ATIVIDADE EXTERNA ESPII - RODÍZIO	HC da Universidade Federal de Goiás - GO
233	ATIVIDADE EXTERNA ESPII - RODÍZIO	HC da Universidade Federal de Goiás - GO
233	ATIVIDADE EXTERNA ESPII - RODÍZIO	HC da Universidade Federal de Goiás - GO
233	ATIVIDADE EXTERNA ESPII - RODÍZIO	HC da Universidade Federal de Goiás - GO
233	ATIVIDADE EXTERNA ESPII - RODÍZIO	HC da Universidade Federal de Goiás - GO
233	ATIVIDADE EXTERNA ESPII - RODÍZIO	HC da Universidade Federal de Goiás - GO
233	ATIVIDADE EXTERNA ESPII - RODÍZIO	HC da Universidade Federal de Goiás - GO
233	ATIVIDADE EXTERNA ESPII - RODÍZIO	HC da Universidade Federal de Goiás - GO
233	ATIVIDADE EXTERNA ESPII - RODÍZIO	HC da Universidade Federal de Goiás - GO
233	ATIVIDADE EXTERNA ESPII - RODÍZIO	HC da Universidade Federal de Goiás - GO
233	ATIVIDADE EXTERNA ESPII - RODÍZIO	HC da Universidade Federal de Goiás - GO
233	ATIVIDADE EXTERNA ESPII - RODÍZIO	HC da Universidade Federal de Goiás - GO

233	ATIVIDADE EXTERNA ESPII - RODÍZIO	HC da Universidade Federal de Goiás - GO
233	ATIVIDADE EXTERNA ESPII - RODÍZIO	HC da Universidade Federal de Goiás - GO
233	ATIVIDADE EXTERNA ESPII - RODÍZIO	HC da Universidade Federal de Goiás - GO
233	ATIVIDADE EXTERNA ESPII - RODÍZIO	HC da Universidade Federal de Goiás - GO
233	ATIVIDADE EXTERNA ESPII - RODÍZIO	HC da Universidade Federal de Goiás - GO
233	ATIVIDADE EXTERNA ESPII - RODÍZIO	HC da Universidade Federal de Goiás - GO
233	ATIVIDADE EXTERNA ESPII - RODÍZIO	HC da Universidade Federal de Goiás - GO
233	ATIVIDADE EXTERNA ESPII - RODÍZIO	HC da Universidade Federal de Goiás - GO
233	ATIVIDADE EXTERNA ESPII - RODÍZIO	HC da Universidade Federal de Goiás - GO
233	ATIVIDADE EXTERNA ESPII - RODÍZIO	HC da Universidade Federal de Goiás - GO
233	ATIVIDADE EXTERNA ESPII - RODÍZIO	HC da Universidade Federal de Goiás - GO
233	ATIVIDADE EXTERNA ESPII - RODÍZIO	HC da Universidade Federal de Goiás - GO
233	ATIVIDADE EXTERNA ESPII - RODÍZIO	HC da Universidade Federal de Goiás - GO
233	ATIVIDADE EXTERNA ESPII - RODÍZIO	HC da Universidade Federal de Goiás - GO
233	ATIVIDADE EXTERNA ESPII - RODÍZIO	HC da Universidade Federal de Goiás - GO
233	ATIVIDADE EXTERNA ESPII - RODÍZIO	HC da Universidade Federal de Goiás - GO
233	ATIVIDADE EXTERNA ESPII - RODÍZIO	HC da Universidade Federal de Goiás - GO
233	ATIVIDADE EXTERNA ESPII - RODÍZIO	HC da Universidade Federal de Goiás - GO
233	ATIVIDADE EXTERNA ESPII - RODÍZIO	HC da Universidade Federal de Goiás - GO
233	ATIVIDADE EXTERNA ESPII - RODÍZIO	HC da Universidade Federal de Goiás - GO
233	ATIVIDADE EXTERNA ESPII - RODÍZIO	HC da Universidade Federal de Goiás - GO
233	ATIVIDADE EXTERNA ESPII - RODÍZIO	HC da Universidade Federal de Goiás - GO
233	ATIVIDADE EXTERNA ESPII - RODÍZIO	HC da Universidade Federal de Goiás - GO
233	ATIVIDADE EXTERNA ESPII - RODÍZIO	HC da Universidade Federal de Goiás - GO
233	ATIVIDADE EXTERNA ESPII - RODÍZIO	HC da Universidade Federal de Goiás - GO
233	ATIVIDADE EXTERNA ESPII - RODÍZIO	HC da Universidade Federal de Goiás - GO
233	ATIVIDADE EXTERNA ESPII - RODÍZIO	HC da Universidade Federal de Goiás - GO
233	ATIVIDADE EXTERNA ESPII - RODÍZIO	HC da Universidade Federal de Goiás - GO
233	ATIVIDADE EXTERNA ESPII - RODÍZIO	HC da Universidade Federal de Goiás - GO
233	ATIVIDADE EXTERNA ESPII - RODÍZIO	HC da Universidade Federal de Goiás - GO
233	ATIVIDADE EXTERNA ESPII - RODÍZIO	HC da Universidade Federal de Goiás - GO
233	ATIVIDADE EXTERNA ESPII - RODÍZIO	HC da Universidade Federal de Goiás - GO
233	ATIVIDADE EXTERNA ESPII - RODÍZIO	HC da Universidade Federal de Goiás - GO
233	ATIVIDADE EXTERNA ESPII - RODÍZIO	HC da Universidade Federal de Goiás - GO
233	ATIVIDADE EXTERNA ESPII - RODÍZIO	HC da Universidade Federal de Goiás - GO
233	ATIVIDADE EXTERNA ESPII - RODÍZIO	HC da Universidade Federal de Goiás - GO
233	ATIVIDADE EXTERNA ESPII - RODÍZIO	HC da Universidade Federal de Goiás - GO
233	ATIVIDADE EXTERNA ESPII - RODÍZIO	HC da Universidade Federal de Goiás - GO
233	ATIVIDADE EXTERNA ESPII - RODÍZIO	HC da Universidade Federal de Goiás - GO
233	ATIVIDADE EXTERNA ESPII - RODÍZIO	HC da Universidade Federal de Goiás - GO
233	ATIVIDADE EXTERNA ESPII - RODÍZIO	HC da Universidade Federal de Goiás - GO
233	ATIVIDADE EXTERNA ESPII - RODÍZIO	HC da Universidade Federal de Goiás - GO
233	ATIVIDADE EXTERNA ESPII - RODÍZIO	HC da Universidade Federal de Goiás - GO
233	ATIVIDADE EXTERNA ESPII - RODÍZIO	HC da Universidade Federal de Goiás - GO
233	ATIVIDADE EXTERNA ESPII - RODÍZIO	HC da Universidade Federal de Goiás - GO
233	ATIVIDADE EXTERNA ESPII - RODÍZIO	HC da Universidade Federal de Goiás - GO
233	ATIVIDADE EXTERNA ESPII - RODÍZIO	HC da Universidade Federal de Goiás - GO
233	ATIVIDADE EXTERNA ESPII - RODÍZIO	HC da Universidade Federal de Goiás - GO

233	ATIVIDADE EXTERNA ESPII - RODÍZIO	HC da Universidade Federal de Goiás - GO
233	ATIVIDADE EXTERNA ESPII - RODÍZIO	HC da Universidade Federal de Goiás - GO
233	ATIVIDADE EXTERNA ESPII - RODÍZIO	HC da Universidade Federal de Goiás - GO
233	ATIVIDADE EXTERNA ESPII - RODÍZIO	HC da Universidade Federal de Goiás - GO
233	ATIVIDADE EXTERNA ESPII - RODÍZIO	HC da Universidade Federal de Goiás - GO
233	ATIVIDADE EXTERNA ESPII - RODÍZIO	HC da Universidade Federal de Goiás - GO
233	ATIVIDADE EXTERNA ESPII - RODÍZIO	HC da Universidade Federal de Goiás - GO
233	ATIVIDADE EXTERNA ESPII - RODÍZIO	HC da Universidade Federal de Goiás - GO
233	ATIVIDADE EXTERNA ESPII - RODÍZIO	HC da Universidade Federal de Goiás - GO
233	ATIVIDADE EXTERNA ESPII - RODÍZIO	HC da Universidade Federal de Goiás - GO
233	ATIVIDADE EXTERNA ESPII - RODÍZIO	HC da Universidade Federal de Goiás - GO
233	ATIVIDADE EXTERNA ESPII - RODÍZIO	HC da Universidade Federal de Goiás - GO
233	ATIVIDADE EXTERNA ESPII - RODÍZIO	HC da Universidade Federal de Goiás - GO
233	ATIVIDADE EXTERNA ESPII - RODÍZIO	HC da Universidade Federal de Goiás - GO
233	ATIVIDADE EXTERNA ESPII - RODÍZIO	HC da Universidade Federal de Goiás - GO
233	ATIVIDADE EXTERNA ESPII - RODÍZIO	HC da Universidade Federal de Goiás - GO
233	ATIVIDADE EXTERNA ESPII - RODÍZIO	HC da Universidade Federal de Goiás - GO
233	ATIVIDADE EXTERNA ESPII - RODÍZIO	HC da Universidade Federal de Goiás - GO
233	ATIVIDADE EXTERNA ESPII - RODÍZIO	HC da Universidade Federal de Goiás - GO
233	ATIVIDADE EXTERNA ESPII - RODÍZIO	HC da Universidade Federal de Goiás - GO
233	ATIVIDADE EXTERNA ESPII - RODÍZIO	HC da Universidade Federal de Goiás - GO
233	ATIVIDADE EXTERNA ESPII - RODÍZIO	HC da Universidade Federal de Goiás - GO
233	ATIVIDADE EXTERNA ESPII - RODÍZIO	HC da Universidade Federal de Goiás - GO
233	ATIVIDADE EXTERNA ESPII - RODÍZIO	HC da Universidade Federal de Goiás - GO
233	ATIVIDADE EXTERNA ESPII - RODÍZIO	HC da Universidade Federal de Goiás - GO
233	ATIVIDADE EXTERNA ESPII - RODÍZIO	HC da Universidade Federal de Goiás - GO
233	ATIVIDADE EXTERNA ESPII - RODÍZIO	HC da Universidade Federal de Goiás - GO
233	ATIVIDADE EXTERNA ESPII - RODÍZIO	HC da Universidade Federal de Goiás - GO
233	ATIVIDADE EXTERNA ESPII - RODÍZIO	HC da Universidade Federal de Goiás - GO
233	ATIVIDADE EXTERNA ESPII - RODÍZIO	HC da Universidade Federal de Goiás - GO
233	ATIVIDADE EXTERNA ESPII - RODÍZIO	HC da Universidade Federal de Goiás - GO
233	ATIVIDADE EXTERNA ESPII - RODÍZIO	HC da Universidade Federal de Goiás - GO
233	ATIVIDADE EXTERNA ESPII - RODÍZIO	HC da Universidade Federal de Goiás - GO
233	ATIVIDADE EXTERNA ESPII - RODÍZIO	HC da Universidade Federal de Goiás - GO
233	ATIVIDADE EXTERNA ESPII - RODÍZIO	HC da Universidade Federal de Goiás - GO
233	ATIVIDADE EXTERNA ESPII - RODÍZIO	HC da Universidade Federal de Goiás - GO
233	ATIVIDADE EXTERNA ESPII - RODÍZIO	HC da Universidade Federal de Goiás - GO
233	ATIVIDADE EXTERNA ESPII - RODÍZIO	HC da Universidade Federal de Goiás - GO
233	ATIVIDADE EXTERNA ESPII - RODÍZIO	HC da Universidade Federal de Goiás - GO
233	ATIVIDADE EXTERNA ESPII - RODÍZIO	HC da Universidade Federal de Goiás - GO
233	ATIVIDADE EXTERNA ESPII - RODÍZIO	HC da Universidade Federal de Goiás - GO
233	ATIVIDADE EXTERNA ESPII - RODÍZIO	HC da Universidade Federal de Goiás - GO
233	ATIVIDADE EXTERNA ESPII - RODÍZIO	HC da Universidade Federal de Goiás - GO
233	ATIVIDADE EXTERNA ESPII - RODÍZIO	HC da Universidade Federal de Goiás - GO
233	ATIVIDADE EXTERNA ESPII - RODÍZIO	HC da Universidade Federal de Goiás - GO
233	ATIVIDADE EXTERNA ESPII - RODÍZIO	HC da Universidade Federal de Goiás - GO
233	ATIVIDADE EXTERNA ESPII - RODÍZIO	HC da Universidade Federal de Goiás - GO
233	ATIVIDADE EXTERNA ESPII - RODÍZIO	HC da Universidade Federal de Goiás - GO

233	ATIVIDADE EXTERNA ESPII - RODÍZIO	HC da Universidade Federal de Goiás - GO
233	ATIVIDADE EXTERNA ESPII - RODÍZIO	HC da Universidade Federal de Goiás - GO
233	ATIVIDADE EXTERNA ESPII - RODÍZIO	HC da Universidade Federal de Goiás - GO
233	ATIVIDADE EXTERNA ESPII - RODÍZIO	HC da Universidade Federal de Goiás - GO
233	ATIVIDADE EXTERNA ESPII - RODÍZIO	HC da Universidade Federal de Goiás - GO
233	ATIVIDADE EXTERNA ESPII - RODÍZIO	HC da Universidade Federal de Goiás - GO
233	ATIVIDADE EXTERNA ESPII - RODÍZIO	HC da Universidade Federal de Goiás - GO
233	ATIVIDADE EXTERNA ESPII - RODÍZIO	HC da Universidade Federal de Goiás - GO
233	ATIVIDADE EXTERNA ESPII - RODÍZIO	HC da Universidade Federal de Goiás - GO
233	ATIVIDADE EXTERNA ESPII - RODÍZIO	HC da Universidade Federal de Goiás - GO
233	ATIVIDADE EXTERNA ESPII - RODÍZIO	HC da Universidade Federal de Goiás - GO
233	ATIVIDADE EXTERNA ESPII - RODÍZIO	HC da Universidade Federal de Goiás - GO
233	ATIVIDADE EXTERNA ESPII - RODÍZIO	HC da Universidade Federal de Goiás - GO
233	ATIVIDADE EXTERNA ESPII - RODÍZIO	HC da Universidade Federal de Goiás - GO
233	ATIVIDADE EXTERNA ESPII - RODÍZIO	HC da Universidade Federal de Goiás - GO
233	ATIVIDADE EXTERNA ESPII - RODÍZIO	HC da Universidade Federal de Goiás - GO
233	ATIVIDADE EXTERNA ESPII - RODÍZIO	HC da Universidade Federal de Goiás - GO
233	ATIVIDADE EXTERNA ESPII - RODÍZIO	HC da Universidade Federal de Goiás - GO
233	ATIVIDADE EXTERNA ESPII - RODÍZIO	HC da Universidade Federal de Goiás - GO
233	ATIVIDADE EXTERNA ESPII - RODÍZIO	HC da Universidade Federal de Goiás - GO
233	ATIVIDADE EXTERNA ESPII - RODÍZIO	HC da Universidade Federal de Goiás - GO
233	ATIVIDADE EXTERNA ESPII - RODÍZIO	HC da Universidade Federal de Goiás - GO
233	ATIVIDADE EXTERNA ESPII - RODÍZIO	HC da Universidade Federal de Goiás - GO
233	ATIVIDADE EXTERNA ESPII - RODÍZIO	HC da Universidade Federal de Goiás - GO
233	ATIVIDADE EXTERNA ESPII - RODÍZIO	HC da Universidade Federal de Goiás - GO
233	ATIVIDADE EXTERNA ESPII - RODÍZIO	HC da Universidade Federal de Goiás - GO
233	ATIVIDADE EXTERNA ESPII - RODÍZIO	HC da Universidade Federal de Goiás - GO
233	ATIVIDADE EXTERNA ESPII - RODÍZIO	HC da Universidade Federal de Goiás - GO
233	ATIVIDADE EXTERNA ESPII - RODÍZIO	HC da Universidade Federal de Goiás - GO
233	ATIVIDADE EXTERNA ESPII - RODÍZIO	HC da Universidade Federal de Goiás - GO
233	ATIVIDADE EXTERNA ESPII - RODÍZIO	HC da Universidade Federal de Goiás - GO
233	ATIVIDADE EXTERNA ESPII - RODÍZIO	HC da Universidade Federal de Goiás - GO
233	ATIVIDADE EXTERNA ESPII - RODÍZIO	HC da Universidade Federal de Goiás - GO
233	ATIVIDADE EXTERNA ESPII - RODÍZIO	HC da Universidade Federal de Goiás - GO
233	ATIVIDADE EXTERNA ESPII - RODÍZIO	HC da Universidade Federal de Goiás - GO
233	ATIVIDADE EXTERNA ESPII - RODÍZIO	HC da Universidade Federal de Goiás - GO
233	ATIVIDADE EXTERNA ESPII - RODÍZIO	HC da Universidade Federal de Goiás - GO
233	ATIVIDADE EXTERNA ESPII - RODÍZIO	HC da Universidade Federal de Goiás - GO
233	ATIVIDADE EXTERNA ESPII - RODÍZIO	HC da Universidade Federal de Goiás - GO
233	ATIVIDADE EXTERNA ESPII - RODÍZIO	HC da Universidade Federal de Goiás - GO
233	ATIVIDADE EXTERNA ESPII - RODÍZIO	HC da Universidade Federal de Goiás - GO
233	ATIVIDADE EXTERNA ESPII - RODÍZIO	HC da Universidade Federal de Goiás - GO
233	ATIVIDADE EXTERNA ESPII - RODÍZIO	HC da Universidade Federal de Goiás - GO
233	ATIVIDADE EXTERNA ESPII - RODÍZIO	HC da Universidade Federal de Goiás - GO
233	ATIVIDADE EXTERNA ESPII - RODÍZIO	HC da Universidade Federal de Goiás - GO
233	ATIVIDADE EXTERNA ESPII - RODÍZIO	HC da Universidade Federal de Goiás - GO
233	ATIVIDADE EXTERNA ESPII - RODÍZIO	HC da Universidade Federal de Goiás - GO
233	ATIVIDADE EXTERNA ESPII - RODÍZIO	HC da Universidade Federal de Goiás - GO

233	ATIVIDADE EXTERNA ESPII - RODÍZIO	HC da Universidade Federal de Goiás - GO
233	ATIVIDADE EXTERNA ESPII - RODÍZIO	HC da Universidade Federal de Goiás - GO
233	ATIVIDADE EXTERNA ESPII - RODÍZIO	HC da Universidade Federal de Goiás - GO
233	ATIVIDADE EXTERNA ESPII - RODÍZIO	HC da Universidade Federal de Goiás - GO
233	ATIVIDADE EXTERNA ESPII - RODÍZIO	HC da Universidade Federal de Goiás - GO
233	ATIVIDADE EXTERNA ESPII - RODÍZIO	HC da Universidade Federal de Goiás - GO
233	ATIVIDADE EXTERNA ESPII - RODÍZIO	HC da Universidade Federal de Goiás - GO
233	ATIVIDADE EXTERNA ESPII - RODÍZIO	HC da Universidade Federal de Goiás - GO
233	ATIVIDADE EXTERNA ESPII - RODÍZIO	HC da Universidade Federal de Goiás - GO
233	ATIVIDADE EXTERNA ESPII - RODÍZIO	HC da Universidade Federal de Goiás - GO
233	ATIVIDADE EXTERNA ESPII - RODÍZIO	HC da Universidade Federal de Goiás - GO
233	ATIVIDADE EXTERNA ESPII - RODÍZIO	HC da Universidade Federal de Goiás - GO
233	ATIVIDADE EXTERNA ESPII - RODÍZIO	HC da Universidade Federal de Goiás - GO
233	ATIVIDADE EXTERNA ESPII - RODÍZIO	HC da Universidade Federal de Goiás - GO
233	ATIVIDADE EXTERNA ESPII - RODÍZIO	HC da Universidade Federal de Goiás - GO
233	ATIVIDADE EXTERNA ESPII - RODÍZIO	HC da Universidade Federal de Goiás - GO
233	ATIVIDADE EXTERNA ESPII - RODÍZIO	HC da Universidade Federal de Goiás - GO
233	ATIVIDADE EXTERNA ESPII - RODÍZIO	HC da Universidade Federal de Goiás - GO
233	ATIVIDADE EXTERNA ESPII - RODÍZIO	HC da Universidade Federal de Goiás - GO
233	ATIVIDADE EXTERNA ESPII - RODÍZIO	HC da Universidade Federal de Goiás - GO
233	ATIVIDADE EXTERNA ESPII - RODÍZIO	HC da Universidade Federal de Goiás - GO
233	ATIVIDADE EXTERNA ESPII - RODÍZIO	HC da Universidade Federal de Goiás - GO
233	ATIVIDADE EXTERNA ESPII - RODÍZIO	HC da Universidade Federal de Goiás - GO
233	ATIVIDADE EXTERNA ESPII - RODÍZIO	HC da Universidade Federal de Goiás - GO
233	ATIVIDADE EXTERNA ESPII - RODÍZIO	HC da Universidade Federal de Goiás - GO
233	ATIVIDADE EXTERNA ESPII - RODÍZIO	HC da Universidade Federal de Goiás - GO
233	ATIVIDADE EXTERNA ESPII - RODÍZIO	HC da Universidade Federal de Goiás - GO
233	ATIVIDADE EXTERNA ESPII - RODÍZIO	HC da Universidade Federal de Goiás - GO
233	ATIVIDADE EXTERNA ESPII - RODÍZIO	HC da Universidade Federal de Goiás - GO
233	ATIVIDADE EXTERNA ESPII - RODÍZIO	HC da Universidade Federal de Goiás - GO
233	ATIVIDADE EXTERNA ESPII - RODÍZIO	HC da Universidade Federal de Goiás - GO
233	ATIVIDADE EXTERNA ESPII - RODÍZIO	HC da Universidade Federal de Goiás - GO
233	ATIVIDADE EXTERNA ESPII - RODÍZIO	HC da Universidade Federal de Goiás - GO
233	ATIVIDADE EXTERNA ESPII - RODÍZIO	HC da Universidade Federal de Goiás - GO
233	ATIVIDADE EXTERNA ESPII - RODÍZIO	HC da Universidade Federal de Goiás - GO
233	ATIVIDADE EXTERNA ESPII - RODÍZIO	HC da Universidade Federal de Goiás - GO
233	ATIVIDADE EXTERNA ESPII - RODÍZIO	HC da Universidade Federal de Goiás - GO
233	ATIVIDADE EXTERNA ESPII - RODÍZIO	HC da Universidade Federal de Goiás - GO
233	ATIVIDADE EXTERNA ESPII - RODÍZIO	HC da Universidade Federal de Goiás - GO
233	ATIVIDADE EXTERNA ESPII - RODÍZIO	HC da Universidade Federal de Goiás - GO
233	ATIVIDADE EXTERNA ESPII - RODÍZIO	HC da Universidade Federal de Goiás - GO
233	ATIVIDADE EXTERNA ESPII - RODÍZIO	HC da Universidade Federal de Goiás - GO
233	ATIVIDADE EXTERNA ESPII - RODÍZIO	HC da Universidade Federal de Goiás - GO
233	ATIVIDADE EXTERNA ESPII - RODÍZIO	HC da Universidade Federal de Goiás - GO
233	ATIVIDADE EXTERNA ESPII - RODÍZIO	HC da Universidade Federal de Goiás - GO
233	ATIVIDADE EXTERNA ESPII - RODÍZIO	HC da Universidade Federal de Goiás - GO
233	ATIVIDADE EXTERNA ESPII - RODÍZIO	HC da Universidade Federal de Goiás - GO
233	ATIVIDADE EXTERNA ESPII - RODÍZIO	HC da Universidade Federal de Goiás - GO

233	ATIVIDADE EXTERNA ESPII - RODÍZIO	HC da Universidade Federal de Goiás - GO
233	ATIVIDADE EXTERNA ESPII - RODÍZIO	HC da Universidade Federal de Goiás - GO
233	ATIVIDADE EXTERNA ESPII - RODÍZIO	HC da Universidade Federal de Goiás - GO
233	ATIVIDADE EXTERNA ESPII - RODÍZIO	HC da Universidade Federal de Goiás - GO
233	ATIVIDADE EXTERNA ESPII - RODÍZIO	HC da Universidade Federal de Goiás - GO
233	ATIVIDADE EXTERNA ESPII - RODÍZIO	HC da Universidade Federal de Goiás - GO
233	ATIVIDADE EXTERNA ESPII - RODÍZIO	HC da Universidade Federal de Goiás - GO
233	ATIVIDADE EXTERNA ESPII - RODÍZIO	HC da Universidade Federal de Goiás - GO
233	ATIVIDADE EXTERNA ESPII - RODÍZIO	HC da Universidade Federal de Goiás - GO
233	ATIVIDADE EXTERNA ESPII - RODÍZIO	HC da Universidade Federal de Goiás - GO
233	ATIVIDADE EXTERNA ESPII - RODÍZIO	HC da Universidade Federal de Goiás - GO
233	ATIVIDADE EXTERNA ESPII - RODÍZIO	HC da Universidade Federal de Goiás - GO
233	ATIVIDADE EXTERNA ESPII - RODÍZIO	HC da Universidade Federal de Goiás - GO
233	ATIVIDADE EXTERNA ESPII - RODÍZIO	HC da Universidade Federal de Goiás - GO
233	ATIVIDADE EXTERNA ESPII - RODÍZIO	HC da Universidade Federal de Goiás - GO
233	ATIVIDADE EXTERNA ESPII - RODÍZIO	HC da Universidade Federal de Goiás - GO
233	ATIVIDADE EXTERNA ESPII - RODÍZIO	HC da Universidade Federal de Goiás - GO
233	ATIVIDADE EXTERNA ESPII - RODÍZIO	HC da Universidade Federal de Goiás - GO
233	ATIVIDADE EXTERNA ESPII - RODÍZIO	HC da Universidade Federal de Goiás - GO
233	ATIVIDADE EXTERNA ESPII - RODÍZIO	HC da Universidade Federal de Goiás - GO
233	ATIVIDADE EXTERNA ESPII - RODÍZIO	HC da Universidade Federal de Goiás - GO
233	ATIVIDADE EXTERNA ESPII - RODÍZIO	HC da Universidade Federal de Goiás - GO
233	ATIVIDADE EXTERNA ESPII - RODÍZIO	HC da Universidade Federal de Goiás - GO
233	ATIVIDADE EXTERNA ESPII - RODÍZIO	HC da Universidade Federal de Goiás - GO
233	ATIVIDADE EXTERNA ESPII - RODÍZIO	HC da Universidade Federal de Goiás - GO
233	ATIVIDADE EXTERNA ESPII - RODÍZIO	HC da Universidade Federal de Goiás - GO
233	ATIVIDADE EXTERNA ESPII - RODÍZIO	HC da Universidade Federal de Goiás - GO
233	ATIVIDADE EXTERNA ESPII - RODÍZIO	HC da Universidade Federal de Goiás - GO
233	ATIVIDADE EXTERNA ESPII - RODÍZIO	HC da Universidade Federal de Goiás - GO
233	ATIVIDADE EXTERNA ESPII - RODÍZIO	HC da Universidade Federal de Goiás - GO
233	ATIVIDADE EXTERNA ESPII - RODÍZIO	HC da Universidade Federal de Goiás - GO
233	ATIVIDADE EXTERNA ESPII - RODÍZIO	HC da Universidade Federal de Goiás - GO
233	ATIVIDADE EXTERNA ESPII - RODÍZIO	HC da Universidade Federal de Goiás - GO
233	ATIVIDADE EXTERNA ESPII - RODÍZIO	HC da Universidade Federal de Goiás - GO
233	ATIVIDADE EXTERNA ESPII - RODÍZIO	HC da Universidade Federal de Goiás - GO
233	ATIVIDADE EXTERNA ESPII - RODÍZIO	HC da Universidade Federal de Goiás - GO
233	ATIVIDADE EXTERNA ESPII - RODÍZIO	HC da Universidade Federal de Goiás - GO
233	ATIVIDADE EXTERNA ESPII - RODÍZIO	HC da Universidade Federal de Goiás - GO
233	ATIVIDADE EXTERNA ESPII - RODÍZIO	HC da Universidade Federal de Goiás - GO
233	ATIVIDADE EXTERNA ESPII - RODÍZIO	HC da Universidade Federal de Goiás - GO
233	ATIVIDADE EXTERNA ESPII - RODÍZIO	HC da Universidade Federal de Goiás - GO
233	ATIVIDADE EXTERNA ESPII - RODÍZIO	HC da Universidade Federal de Goiás - GO
233	ATIVIDADE EXTERNA ESPII - RODÍZIO	HC da Universidade Federal de Goiás - GO
233	ATIVIDADE EXTERNA ESPII - RODÍZIO	HC da Universidade Federal de Goiás - GO
233	ATIVIDADE EXTERNA ESPII - RODÍZIO	HC da Universidade Federal de Goiás - GO
233	ATIVIDADE EXTERNA ESPII - RODÍZIO	HC da Universidade Federal de Goiás - GO
233	ATIVIDADE EXTERNA ESPII - RODÍZIO	HC da Universidade Federal de Goiás - GO
233	ATIVIDADE EXTERNA ESPII - RODÍZIO	HC da Universidade Federal de Goiás - GO

233	ATIVIDADE EXTERNA ESPII - RODÍZIO	HC da Universidade Federal de Goiás - GO
233	ATIVIDADE EXTERNA ESPII - RODÍZIO	HC da Universidade Federal de Goiás - GO
233	ATIVIDADE EXTERNA ESPII - RODÍZIO	HC da Universidade Federal de Goiás - GO
233	ATIVIDADE EXTERNA ESPII - RODÍZIO	HC da Universidade Federal de Goiás - GO
233	ATIVIDADE EXTERNA ESPII - RODÍZIO	HC da Universidade Federal de Goiás - GO
233	ATIVIDADE EXTERNA ESPII - RODÍZIO	HC da Universidade Federal de Goiás - GO
233	ATIVIDADE EXTERNA ESPII - RODÍZIO	HC da Universidade Federal de Goiás - GO
233	ATIVIDADE EXTERNA ESPII - RODÍZIO	HC da Universidade Federal de Goiás - GO
233	ATIVIDADE EXTERNA ESPII - RODÍZIO	HC da Universidade Federal de Goiás - GO
233	ATIVIDADE EXTERNA ESPII - RODÍZIO	HC da Universidade Federal de Goiás - GO
233	ATIVIDADE EXTERNA ESPII - RODÍZIO	HC da Universidade Federal de Goiás - GO
233	ATIVIDADE EXTERNA ESPII - RODÍZIO	HC da Universidade Federal de Goiás - GO
233	ATIVIDADE EXTERNA ESPII - RODÍZIO	HC da Universidade Federal de Goiás - GO
233	ATIVIDADE EXTERNA ESPII - RODÍZIO	HC da Universidade Federal de Goiás - GO
233	ATIVIDADE EXTERNA ESPII - RODÍZIO	HC da Universidade Federal de Goiás - GO
233	ATIVIDADE EXTERNA ESPII - RODÍZIO	HC da Universidade Federal de Goiás - GO
233	ATIVIDADE EXTERNA ESPII - RODÍZIO	HC da Universidade Federal de Goiás - GO
233	ATIVIDADE EXTERNA ESPII - RODÍZIO	HC da Universidade Federal de Goiás - GO
233	ATIVIDADE EXTERNA ESPII - RODÍZIO	HC da Universidade Federal de Goiás - GO
233	ATIVIDADE EXTERNA ESPII - RODÍZIO	HC da Universidade Federal de Goiás - GO
233	ATIVIDADE EXTERNA ESPII - RODÍZIO	HC da Universidade Federal de Goiás - GO
233	ATIVIDADE EXTERNA ESPII - RODÍZIO	HC da Universidade Federal de Goiás - GO
233	ATIVIDADE EXTERNA ESPII - RODÍZIO	HC da Universidade Federal de Goiás - GO
233	ATIVIDADE EXTERNA ESPII - RODÍZIO	HC da Universidade Federal de Goiás - GO
233	ATIVIDADE EXTERNA ESPII - RODÍZIO	HC da Universidade Federal de Goiás - GO
233	ATIVIDADE EXTERNA ESPII - RODÍZIO	HC da Universidade Federal de Goiás - GO
233	ATIVIDADE EXTERNA ESPII - RODÍZIO	HC da Universidade Federal de Goiás - GO
233	ATIVIDADE EXTERNA ESPII - RODÍZIO	HC da Universidade Federal de Goiás - GO
233	ATIVIDADE EXTERNA ESPII - RODÍZIO	HC da Universidade Federal de Goiás - GO
233	ATIVIDADE EXTERNA ESPII - RODÍZIO	HC da Universidade Federal de Goiás - GO
233	ATIVIDADE EXTERNA ESPII - RODÍZIO	HC da Universidade Federal de Goiás - GO
233	ATIVIDADE EXTERNA ESPII - RODÍZIO	HC da Universidade Federal de Goiás - GO
233	ATIVIDADE EXTERNA ESPII - RODÍZIO	HC da Universidade Federal de Goiás - GO
233	ATIVIDADE EXTERNA ESPII - RODÍZIO	HC da Universidade Federal de Goiás - GO
233	ATIVIDADE EXTERNA ESPII - RODÍZIO	HC da Universidade Federal de Goiás - GO
233	ATIVIDADE EXTERNA ESPII - RODÍZIO	HC da Universidade Federal de Goiás - GO
233	ATIVIDADE EXTERNA ESPII - RODÍZIO	HC da Universidade Federal de Goiás - GO
233	ATIVIDADE EXTERNA ESPII - RODÍZIO	HC da Universidade Federal de Goiás - GO
233	ATIVIDADE EXTERNA ESPII - RODÍZIO	HC da Universidade Federal de Goiás - GO
233	ATIVIDADE EXTERNA ESPII - RODÍZIO	HC da Universidade Federal de Goiás - GO
233	ATIVIDADE EXTERNA ESPII - RODÍZIO	HC da Universidade Federal de Goiás - GO
233	ATIVIDADE EXTERNA ESPII - RODÍZIO	HC da Universidade Federal de Goiás - GO
233	ATIVIDADE EXTERNA ESPII - RODÍZIO	HC da Universidade Federal de Goiás - GO
233	ATIVIDADE EXTERNA ESPII - RODÍZIO	HC da Universidade Federal de Goiás - GO
233	ATIVIDADE EXTERNA ESPII - RODÍZIO	HC da Universidade Federal de Goiás - GO
233	ATIVIDADE EXTERNA ESPII - RODÍZIO	HC da Universidade Federal de Goiás - GO
233	ATIVIDADE EXTERNA ESPII - RODÍZIO	HC da Universidade Federal de Goiás - GO
233	ATIVIDADE EXTERNA ESPII - RODÍZIO	HC da Universidade Federal de Goiás - GO

233	ATIVIDADE EXTERNA ESPII - RODÍZIO	HC da Universidade Federal de Goiás - GO
233	ATIVIDADE EXTERNA ESPII - RODÍZIO	HC da Universidade Federal de Goiás - GO
233	ATIVIDADE EXTERNA ESPII - RODÍZIO	HC da Universidade Federal de Goiás - GO
233	ATIVIDADE EXTERNA ESPII - RODÍZIO	HC da Universidade Federal de Goiás - GO
233	ATIVIDADE EXTERNA ESPII - RODÍZIO	HC da Universidade Federal de Goiás - GO
233	ATIVIDADE EXTERNA ESPII - RODÍZIO	HC da Universidade Federal de Goiás - GO
233	ATIVIDADE EXTERNA ESPII - RODÍZIO	HC da Universidade Federal de Goiás - GO
233	ATIVIDADE EXTERNA ESPII - RODÍZIO	HC da Universidade Federal de Goiás - GO
233	ATIVIDADE EXTERNA ESPII - RODÍZIO	HC da Universidade Federal de Goiás - GO
233	ATIVIDADE EXTERNA ESPII - RODÍZIO	HC da Universidade Federal de Goiás - GO
233	ATIVIDADE EXTERNA ESPII - RODÍZIO	HC da Universidade Federal de Goiás - GO
233	ATIVIDADE EXTERNA ESPII - RODÍZIO	HC da Universidade Federal de Goiás - GO
233	ATIVIDADE EXTERNA ESPII - RODÍZIO	HC da Universidade Federal de Goiás - GO
233	ATIVIDADE EXTERNA ESPII - RODÍZIO	HC da Universidade Federal de Goiás - GO
233	ATIVIDADE EXTERNA ESPII - RODÍZIO	HC da Universidade Federal de Goiás - GO
233	ATIVIDADE EXTERNA ESPII - RODÍZIO	HC da Universidade Federal de Goiás - GO
233	ATIVIDADE EXTERNA ESPII - RODÍZIO	HC da Universidade Federal de Goiás - GO
233	ATIVIDADE EXTERNA ESPII - RODÍZIO	HC da Universidade Federal de Goiás - GO
233	ATIVIDADE EXTERNA ESPII - RODÍZIO	HC da Universidade Federal de Goiás - GO
233	ATIVIDADE EXTERNA ESPII - RODÍZIO	HC da Universidade Federal de Goiás - GO
233	ATIVIDADE EXTERNA ESPII - RODÍZIO	HC da Universidade Federal de Goiás - GO
233	ATIVIDADE EXTERNA ESPII - RODÍZIO	HC da Universidade Federal de Goiás - GO
233	ATIVIDADE EXTERNA ESPII - RODÍZIO	HC da Universidade Federal de Goiás - GO
233	ATIVIDADE EXTERNA ESPII - RODÍZIO	HC da Universidade Federal de Goiás - GO
233	ATIVIDADE EXTERNA ESPII - RODÍZIO	HC da Universidade Federal de Goiás - GO
233	ATIVIDADE EXTERNA ESPII - RODÍZIO	HC da Universidade Federal de Goiás - GO
233	ATIVIDADE EXTERNA ESPII - RODÍZIO	HC da Universidade Federal de Goiás - GO
233	ATIVIDADE EXTERNA ESPII - RODÍZIO	HC da Universidade Federal de Goiás - GO
233	ATIVIDADE EXTERNA ESPII - RODÍZIO	HC da Universidade Federal de Goiás - GO
233	ATIVIDADE EXTERNA ESPII - RODÍZIO	HC da Universidade Federal de Goiás - GO
233	ATIVIDADE EXTERNA ESPII - RODÍZIO	HC da Universidade Federal de Goiás - GO
233	ATIVIDADE EXTERNA ESPII - RODÍZIO	HC da Universidade Federal de Goiás - GO
233	ATIVIDADE EXTERNA ESPII - RODÍZIO	HC da Universidade Federal de Goiás - GO
233	ATIVIDADE EXTERNA ESPII - RODÍZIO	HC da Universidade Federal de Goiás - GO
233	ATIVIDADE EXTERNA ESPII - RODÍZIO	HC da Universidade Federal de Goiás - GO
233	ATIVIDADE EXTERNA ESPII - RODÍZIO	HC da Universidade Federal de Goiás - GO
233	ATIVIDADE EXTERNA ESPII - RODÍZIO	HC da Universidade Federal de Goiás - GO
233	ATIVIDADE EXTERNA ESPII - RODÍZIO	HC da Universidade Federal de Goiás - GO
233	ATIVIDADE EXTERNA ESPII - RODÍZIO	HC da Universidade Federal de Goiás - GO
233	ATIVIDADE EXTERNA ESPII - RODÍZIO	HC da Universidade Federal de Goiás - GO
233	ATIVIDADE EXTERNA ESPII - RODÍZIO	HC da Universidade Federal de Goiás - GO
233	ATIVIDADE EXTERNA ESPII - RODÍZIO	HC da Universidade Federal de Goiás - GO
233	ATIVIDADE EXTERNA ESPII - RODÍZIO	HC da Universidade Federal de Goiás - GO
233	ATIVIDADE EXTERNA ESPII - RODÍZIO	HC da Universidade Federal de Goiás - GO
233	ATIVIDADE EXTERNA ESPII - RODÍZIO	HC da Universidade Federal de Goiás - GO
233	ATIVIDADE EXTERNA ESPII - RODÍZIO	HC da Universidade Federal de Goiás - GO
233	ATIVIDADE EXTERNA ESPII - RODÍZIO	HC da Universidade Federal de Goiás - GO
233	ATIVIDADE EXTERNA ESPII - RODÍZIO	HC da Universidade Federal de Goiás - GO

233	ATIVIDADE EXTERNA ESPII - RODÍZIO	HC da Universidade Federal de Goiás - GO
233	ATIVIDADE EXTERNA ESPII - RODÍZIO	HC da Universidade Federal de Goiás - GO
233	ATIVIDADE EXTERNA ESPII - RODÍZIO	HC da Universidade Federal de Goiás - GO
233	ATIVIDADE EXTERNA ESPII - RODÍZIO	HC da Universidade Federal de Goiás - GO
233	ATIVIDADE EXTERNA ESPII - RODÍZIO	HC da Universidade Federal de Goiás - GO
233	ATIVIDADE EXTERNA ESPII - RODÍZIO	HC da Universidade Federal de Goiás - GO
233	ATIVIDADE EXTERNA ESPII - RODÍZIO	HC da Universidade Federal de Goiás - GO
233	ATIVIDADE EXTERNA ESPII - RODÍZIO	HC da Universidade Federal de Goiás - GO
233	ATIVIDADE EXTERNA ESPII - RODÍZIO	HC da Universidade Federal de Goiás - GO
233	ATIVIDADE EXTERNA ESPII - RODÍZIO	HC da Universidade Federal de Goiás - GO
233	ATIVIDADE EXTERNA ESPII - RODÍZIO	HC da Universidade Federal de Goiás - GO
233	ATIVIDADE EXTERNA ESPII - RODÍZIO	HC da Universidade Federal de Goiás - GO
233	ATIVIDADE EXTERNA ESPII - RODÍZIO	HC da Universidade Federal de Goiás - GO
233	ATIVIDADE EXTERNA ESPII - RODÍZIO	HC da Universidade Federal de Goiás - GO
233	ATIVIDADE EXTERNA ESPII - RODÍZIO	HC da Universidade Federal de Goiás - GO
233	ATIVIDADE EXTERNA ESPII - RODÍZIO	HC da Universidade Federal de Goiás - GO
233	ATIVIDADE EXTERNA ESPII - RODÍZIO	HC da Universidade Federal de Goiás - GO
233	ATIVIDADE EXTERNA ESPII - RODÍZIO	HC da Universidade Federal de Goiás - GO
233	ATIVIDADE EXTERNA ESPII - RODÍZIO	HC da Universidade Federal de Goiás - GO
233	ATIVIDADE EXTERNA ESPII - RODÍZIO	HC da Universidade Federal de Goiás - GO
233	ATIVIDADE EXTERNA ESPII - RODÍZIO	HC da Universidade Federal de Goiás - GO
233	ATIVIDADE EXTERNA ESPII - RODÍZIO	HC da Universidade Federal de Goiás - GO
233	ATIVIDADE EXTERNA ESPII - RODÍZIO	HC da Universidade Federal de Goiás - GO
233	ATIVIDADE EXTERNA ESPII - RODÍZIO	HC da Universidade Federal de Goiás - GO
233	ATIVIDADE EXTERNA ESPII - RODÍZIO	HC da Universidade Federal de Goiás - GO
233	ATIVIDADE EXTERNA ESPII - RODÍZIO	HC da Universidade Federal de Goiás - GO
233	ATIVIDADE EXTERNA ESPII - RODÍZIO	HC da Universidade Federal de Goiás - GO
233	ATIVIDADE EXTERNA ESPII - RODÍZIO	HC da Universidade Federal de Goiás - GO
233	ATIVIDADE EXTERNA ESPII - RODÍZIO	HC da Universidade Federal de Goiás - GO
233	ATIVIDADE EXTERNA ESPII - RODÍZIO	HC da Universidade Federal de Goiás - GO
233	ATIVIDADE EXTERNA ESPII - RODÍZIO	HC da Universidade Federal de Goiás - GO
233	ATIVIDADE EXTERNA ESPII - RODÍZIO	HC da Universidade Federal de Goiás - GO
233	ATIVIDADE EXTERNA ESPII - RODÍZIO	HC da Universidade Federal de Goiás - GO
233	ATIVIDADE EXTERNA ESPII - RODÍZIO	HC da Universidade Federal de Goiás - GO
233	ATIVIDADE EXTERNA ESPII - RODÍZIO	HC da Universidade Federal de Goiás - GO
233	ATIVIDADE EXTERNA ESPII - RODÍZIO	HC da Universidade Federal de Goiás - GO
233	ATIVIDADE EXTERNA ESPII - RODÍZIO	HC da Universidade Federal de Goiás - GO
233	ATIVIDADE EXTERNA ESPII - RODÍZIO	HC da Universidade Federal de Goiás - GO
233	ATIVIDADE EXTERNA ESPII - RODÍZIO	HC da Universidade Federal de Goiás - GO
233	ATIVIDADE EXTERNA ESPII - RODÍZIO	HC da Universidade Federal de Goiás - GO
233	ATIVIDADE EXTERNA ESPII - RODÍZIO	HC da Universidade Federal de Goiás - GO
233	ATIVIDADE EXTERNA ESPII - RODÍZIO	HC da Universidade Federal de Goiás - GO
233	ATIVIDADE EXTERNA ESPII - RODÍZIO	HC da Universidade Federal de Goiás - GO
233	ATIVIDADE EXTERNA ESPII - RODÍZIO	HC da Universidade Federal de Goiás - GO
233	ATIVIDADE EXTERNA ESPII - RODÍZIO	HC da Universidade Federal de Goiás - GO
233	ATIVIDADE EXTERNA ESPII - RODÍZIO	HC da Universidade Federal de Goiás - GO
233	ATIVIDADE EXTERNA ESPII - RODÍZIO	HC da Universidade Federal de Goiás - GO
233	ATIVIDADE EXTERNA ESPII - RODÍZIO	HC da Universidade Federal de Goiás - GO

233	ATIVIDADE EXTERNA ESPII - RODÍZIO	HC da Universidade Federal de Goiás - GO
233	ATIVIDADE EXTERNA ESPII - RODÍZIO	HC da Universidade Federal de Goiás - GO
233	ATIVIDADE EXTERNA ESPII - RODÍZIO	HC da Universidade Federal de Goiás - GO
233	ATIVIDADE EXTERNA ESPII - RODÍZIO	HC da Universidade Federal de Goiás - GO
233	ATIVIDADE EXTERNA ESPII - RODÍZIO	HC da Universidade Federal de Goiás - GO
233	ATIVIDADE EXTERNA ESPII - RODÍZIO	HC da Universidade Federal de Goiás - GO
233	ATIVIDADE EXTERNA ESPII - RODÍZIO	HC da Universidade Federal de Goiás - GO
233	ATIVIDADE EXTERNA ESPII - RODÍZIO	HC da Universidade Federal de Goiás - GO
233	ATIVIDADE EXTERNA ESPII - RODÍZIO	HC da Universidade Federal de Goiás - GO
233	ATIVIDADE EXTERNA ESPII - RODÍZIO	HC da Universidade Federal de Goiás - GO
233	ATIVIDADE EXTERNA ESPII - RODÍZIO	HC da Universidade Federal de Goiás - GO
233	ATIVIDADE EXTERNA ESPII - RODÍZIO	HC da Universidade Federal de Goiás - GO
233	ATIVIDADE EXTERNA ESPII - RODÍZIO	HC da Universidade Federal de Goiás - GO
233	ATIVIDADE EXTERNA ESPII - RODÍZIO	HC da Universidade Federal de Goiás - GO
233	ATIVIDADE EXTERNA ESPII - RODÍZIO	HC da Universidade Federal de Goiás - GO
233	ATIVIDADE EXTERNA ESPII - RODÍZIO	HC da Universidade Federal de Goiás - GO
233	ATIVIDADE EXTERNA ESPII - RODÍZIO	HC da Universidade Federal de Goiás - GO
233	ATIVIDADE EXTERNA ESPII - RODÍZIO	HC da Universidade Federal de Goiás - GO
233	ATIVIDADE EXTERNA ESPII - RODÍZIO	HC da Universidade Federal de Goiás - GO
233	ATIVIDADE EXTERNA ESPII - RODÍZIO	HC da Universidade Federal de Goiás - GO
233	ATIVIDADE EXTERNA ESPII - RODÍZIO	HC da Universidade Federal de Goiás - GO
233	ATIVIDADE EXTERNA ESPII - RODÍZIO	HC da Universidade Federal de Goiás - GO
233	ATIVIDADE EXTERNA ESPII - RODÍZIO	HC da Universidade Federal de Goiás - GO
233	ATIVIDADE EXTERNA ESPII - RODÍZIO	HC da Universidade Federal de Goiás - GO
233	ATIVIDADE EXTERNA ESPII - RODÍZIO	HC da Universidade Federal de Goiás - GO
233	ATIVIDADE EXTERNA ESPII - RODÍZIO	HC da Universidade Federal de Goiás - GO
233	ATIVIDADE EXTERNA ESPII - RODÍZIO	HC da Universidade Federal de Goiás - GO
233	ATIVIDADE EXTERNA ESPII - RODÍZIO	HC da Universidade Federal de Goiás - GO
233	ATIVIDADE EXTERNA ESPII - RODÍZIO	HC da Universidade Federal de Goiás - GO
233	ATIVIDADE EXTERNA ESPII - RODÍZIO	HC da Universidade Federal de Goiás - GO
233	ATIVIDADE EXTERNA ESPII - RODÍZIO	HC da Universidade Federal de Goiás - GO
233	ATIVIDADE EXTERNA ESPII - RODÍZIO	HC da Universidade Federal de Goiás - GO
233	ATIVIDADE EXTERNA ESPII - RODÍZIO	HC da Universidade Federal de Goiás - GO
233	ATIVIDADE EXTERNA ESPII - RODÍZIO	HC da Universidade Federal de Goiás - GO
233	ATIVIDADE EXTERNA ESPII - RODÍZIO	HC da Universidade Federal de Goiás - GO
233	ATIVIDADE EXTERNA ESPII - RODÍZIO	HC da Universidade Federal de Goiás - GO
233	ATIVIDADE EXTERNA ESPII - RODÍZIO	HC da Universidade Federal de Goiás - GO
233	ATIVIDADE EXTERNA ESPII - RODÍZIO	HC da Universidade Federal de Goiás - GO
233	ATIVIDADE EXTERNA ESPII - RODÍZIO	HC da Universidade Federal de Goiás - GO
233	ATIVIDADE EXTERNA ESPII - RODÍZIO	HC da Universidade Federal de Goiás - GO
233	ATIVIDADE EXTERNA ESPII - RODÍZIO	HC da Universidade Federal de Goiás - GO
233	ATIVIDADE EXTERNA ESPII - RODÍZIO	HC da Universidade Federal de Goiás - GO
233	ATIVIDADE EXTERNA ESPII - RODÍZIO	HC da Universidade Federal de Goiás - GO
233	ATIVIDADE EXTERNA ESPII - RODÍZIO	HC da Universidade Federal de Goiás - GO
233	ATIVIDADE EXTERNA ESPII - RODÍZIO	HC da Universidade Federal de Goiás - GO
233	ATIVIDADE EXTERNA ESPII - RODÍZIO	HC da Universidade Federal de Goiás - GO
233	ATIVIDADE EXTERNA ESPII - RODÍZIO	HC da Universidade Federal de Goiás - GO
233	ATIVIDADE EXTERNA ESPII - RODÍZIO	HC da Universidade Federal de Goiás - GO

233	ATIVIDADE EXTERNA ESPII - RODÍZIO	HC da Universidade Federal de Goiás - GO
233	ATIVIDADE EXTERNA ESPII - RODÍZIO	HC da Universidade Federal de Goiás - GO
233	ATIVIDADE EXTERNA ESPII - RODÍZIO	HC da Universidade Federal de Goiás - GO
233	ATIVIDADE EXTERNA ESPII - RODÍZIO	HC da Universidade Federal de Goiás - GO
233	ATIVIDADE EXTERNA ESPII - RODÍZIO	HC da Universidade Federal de Goiás - GO
233	ATIVIDADE EXTERNA ESPII - RODÍZIO	HC da Universidade Federal de Goiás - GO
233	ATIVIDADE EXTERNA ESPII - RODÍZIO	HC da Universidade Federal de Goiás - GO
233	ATIVIDADE EXTERNA ESPII - RODÍZIO	HC da Universidade Federal de Goiás - GO
233	ATIVIDADE EXTERNA ESPII - RODÍZIO	HC da Universidade Federal de Goiás - GO
233	ATIVIDADE EXTERNA ESPII - RODÍZIO	HC da Universidade Federal de Goiás - GO
233	ATIVIDADE EXTERNA ESPII - RODÍZIO	HC da Universidade Federal de Goiás - GO
233	ATIVIDADE EXTERNA ESPII - RODÍZIO	HC da Universidade Federal de Goiás - GO
233	ATIVIDADE EXTERNA ESPII - RODÍZIO	HC da Universidade Federal de Goiás - GO
233	ATIVIDADE EXTERNA ESPII - RODÍZIO	HC da Universidade Federal de Goiás - GO
233	ATIVIDADE EXTERNA ESPII - RODÍZIO	HC da Universidade Federal de Goiás - GO
233	ATIVIDADE EXTERNA ESPII - RODÍZIO	HC da Universidade Federal de Goiás - GO
233	ATIVIDADE EXTERNA ESPII - RODÍZIO	HC da Universidade Federal de Goiás - GO
233	ATIVIDADE EXTERNA ESPII - RODÍZIO	HC da Universidade Federal de Goiás - GO
233	ATIVIDADE EXTERNA ESPII - RODÍZIO	HC da Universidade Federal de Goiás - GO
233	ATIVIDADE EXTERNA ESPII - RODÍZIO	HC da Universidade Federal de Goiás - GO
233	ATIVIDADE EXTERNA ESPII - RODÍZIO	HC da Universidade Federal de Goiás - GO
233	ATIVIDADE EXTERNA ESPII - RODÍZIO	HC da Universidade Federal de Goiás - GO
233	ATIVIDADE EXTERNA ESPII - RODÍZIO	HC da Universidade Federal de Goiás - GO
233	ATIVIDADE EXTERNA ESPII - RODÍZIO	HC da Universidade Federal de Goiás - GO
233	ATIVIDADE EXTERNA ESPII - RODÍZIO	HC da Universidade Federal de Goiás - GO
233	ATIVIDADE EXTERNA ESPII - RODÍZIO	HC da Universidade Federal de Goiás - GO
233	ATIVIDADE EXTERNA ESPII - RODÍZIO	HC da Universidade Federal de Goiás - GO
233	ATIVIDADE EXTERNA ESPII - RODÍZIO	HC da Universidade Federal de Goiás - GO
233	ATIVIDADE EXTERNA ESPII - RODÍZIO	HC da Universidade Federal de Goiás - GO
233	ATIVIDADE EXTERNA ESPII - RODÍZIO	HC da Universidade Federal de Goiás - GO
233	ATIVIDADE EXTERNA ESPII - RODÍZIO	HC da Universidade Federal de Goiás - GO
233	ATIVIDADE EXTERNA ESPII - RODÍZIO	HC da Universidade Federal de Goiás - GO
233	ATIVIDADE EXTERNA ESPII - RODÍZIO	HC da Universidade Federal de Goiás - GO
233	ATIVIDADE EXTERNA ESPII - RODÍZIO	HC da Universidade Federal de Goiás - GO
233	ATIVIDADE EXTERNA ESPII - RODÍZIO	HC da Universidade Federal de Goiás - GO
233	ATIVIDADE EXTERNA ESPII - RODÍZIO	HC da Universidade Federal de Goiás - GO
233	ATIVIDADE EXTERNA ESPII - RODÍZIO	HC da Universidade Federal de Goiás - GO
233	ATIVIDADE EXTERNA ESPII - RODÍZIO	HC da Universidade Federal de Goiás - GO
233	ATIVIDADE EXTERNA ESPII - RODÍZIO	HC da Universidade Federal de Goiás - GO
233	ATIVIDADE EXTERNA ESPII - RODÍZIO	HC da Universidade Federal de Goiás - GO
233	ATIVIDADE EXTERNA ESPII - RODÍZIO	HC da Universidade Federal de Goiás - GO
233	ATIVIDADE EXTERNA ESPII - RODÍZIO	HC da Universidade Federal de Goiás - GO
233	ATIVIDADE EXTERNA ESPII - RODÍZIO	HC da Universidade Federal de Goiás - GO
233	ATIVIDADE EXTERNA ESPII - RODÍZIO	HC da Universidade Federal de Goiás - GO
233	ATIVIDADE EXTERNA ESPII - RODÍZIO	HC da Universidade Federal de Goiás - GO
233	ATIVIDADE EXTERNA ESPII - RODÍZIO	HC da Universidade Federal de Goiás - GO
233	ATIVIDADE EXTERNA ESPII - RODÍZIO	HC da Universidade Federal de Goiás - GO
233	ATIVIDADE EXTERNA ESPII - RODÍZIO	HC da Universidade Federal de Goiás - GO

233	ATIVIDADE EXTERNA ESPII - RODÍZIO	HC da Universidade Federal de Goiás - GO
233	ATIVIDADE EXTERNA ESPII - RODÍZIO	HC da Universidade Federal de Goiás - GO
233	ATIVIDADE EXTERNA ESPII - RODÍZIO	HC da Universidade Federal de Goiás - GO
233	ATIVIDADE EXTERNA ESPII - RODÍZIO	HC da Universidade Federal de Goiás - GO
233	ATIVIDADE EXTERNA ESPII - RODÍZIO	HC da Universidade Federal de Goiás - GO
233	ATIVIDADE EXTERNA ESPII - RODÍZIO	HC da Universidade Federal de Goiás - GO
233	ATIVIDADE EXTERNA ESPII - RODÍZIO	HC da Universidade Federal de Goiás - GO
233	ATIVIDADE EXTERNA ESPII - RODÍZIO	HC da Universidade Federal de Goiás - GO
233	ATIVIDADE EXTERNA ESPII - RODÍZIO	HC da Universidade Federal de Goiás - GO
233	ATIVIDADE EXTERNA ESPII - RODÍZIO	HC da Universidade Federal de Goiás - GO
233	ATIVIDADE EXTERNA ESPII - RODÍZIO	HC da Universidade Federal de Goiás - GO
233	ATIVIDADE EXTERNA ESPII - RODÍZIO	HC da Universidade Federal de Goiás - GO
233	ATIVIDADE EXTERNA ESPII - RODÍZIO	HC da Universidade Federal de Goiás - GO
233	ATIVIDADE EXTERNA ESPII - RODÍZIO	HC da Universidade Federal de Goiás - GO
233	ATIVIDADE EXTERNA ESPII - RODÍZIO	HC da Universidade Federal de Goiás - GO
233	ATIVIDADE EXTERNA ESPII - RODÍZIO	HC da Universidade Federal de Goiás - GO
233	ATIVIDADE EXTERNA ESPII - RODÍZIO	HC da Universidade Federal de Goiás - GO
233	ATIVIDADE EXTERNA ESPII - RODÍZIO	HC da Universidade Federal de Goiás - GO
233	ATIVIDADE EXTERNA ESPII - RODÍZIO	HC da Universidade Federal de Goiás - GO
233	ATIVIDADE EXTERNA ESPII - RODÍZIO	HC da Universidade Federal de Goiás - GO
233	ATIVIDADE EXTERNA ESPII - RODÍZIO	HC da Universidade Federal de Goiás - GO
233	ATIVIDADE EXTERNA ESPII - RODÍZIO	HC da Universidade Federal de Goiás - GO
233	ATIVIDADE EXTERNA ESPII - RODÍZIO	HC da Universidade Federal de Goiás - GO
233	ATIVIDADE EXTERNA ESPII - RODÍZIO	HC da Universidade Federal de Goiás - GO
233	ATIVIDADE EXTERNA ESPII - RODÍZIO	HC da Universidade Federal de Goiás - GO
233	ATIVIDADE EXTERNA ESPII - RODÍZIO	HC da Universidade Federal de Goiás - GO
233	ATIVIDADE EXTERNA ESPII - RODÍZIO	HC da Universidade Federal de Goiás - GO
233	ATIVIDADE EXTERNA ESPII - RODÍZIO	HC da Universidade Federal de Goiás - GO
233	ATIVIDADE EXTERNA ESPII - RODÍZIO	HC da Universidade Federal de Goiás - GO
233	ATIVIDADE EXTERNA ESPII - RODÍZIO	HC da Universidade Federal de Goiás - GO
233	ATIVIDADE EXTERNA ESPII - RODÍZIO	HC da Universidade Federal de Goiás - GO
233	ATIVIDADE EXTERNA ESPII - RODÍZIO	HC da Universidade Federal de Goiás - GO
233	ATIVIDADE EXTERNA ESPII - RODÍZIO	HC da Universidade Federal de Goiás - GO
233	ATIVIDADE EXTERNA ESPII - RODÍZIO	HC da Universidade Federal de Goiás - GO
233	ATIVIDADE EXTERNA ESPII - RODÍZIO	HC da Universidade Federal de Goiás - GO
233	ATIVIDADE EXTERNA ESPII - RODÍZIO	HC da Universidade Federal de Goiás - GO
233	ATIVIDADE EXTERNA ESPII - RODÍZIO	HC da Universidade Federal de Goiás - GO
233	ATIVIDADE EXTERNA ESPII - RODÍZIO	HC da Universidade Federal de Goiás - GO
233	ATIVIDADE EXTERNA ESPII - RODÍZIO	HC da Universidade Federal de Goiás - GO
233	ATIVIDADE EXTERNA ESPII - RODÍZIO	HC da Universidade Federal de Goiás - GO
233	ATIVIDADE EXTERNA ESPII - RODÍZIO	HC da Universidade Federal de Goiás - GO
233	ATIVIDADE EXTERNA ESPII - RODÍZIO	HC da Universidade Federal de Goiás - GO
233	ATIVIDADE EXTERNA ESPII - RODÍZIO	HC da Universidade Federal de Goiás - GO
233	ATIVIDADE EXTERNA ESPII - RODÍZIO	HC da Universidade Federal de Goiás - GO
233	ATIVIDADE EXTERNA ESPII - RODÍZIO	HC da Universidade Federal de Goiás - GO
233	ATIVIDADE EXTERNA ESPII - RODÍZIO	HC da Universidade Federal de Goiás - GO
233	ATIVIDADE EXTERNA ESPII - RODÍZIO	HC da Universidade Federal de Goiás - GO
233	ATIVIDADE EXTERNA ESPII - RODÍZIO	HC da Universidade Federal de Goiás - GO

233	ATIVIDADE EXTERNA ESPII - RODÍZIO	HC da Universidade Federal de Goiás - GO
233	ATIVIDADE EXTERNA ESPII - RODÍZIO	HC da Universidade Federal de Goiás - GO
233	ATIVIDADE EXTERNA ESPII - RODÍZIO	HC da Universidade Federal de Goiás - GO
233	ATIVIDADE EXTERNA ESPII - RODÍZIO	HC da Universidade Federal de Goiás - GO
233	ATIVIDADE EXTERNA ESPII - RODÍZIO	HC da Universidade Federal de Goiás - GO
233	ATIVIDADE EXTERNA ESPII - RODÍZIO	HC da Universidade Federal de Goiás - GO
233	ATIVIDADE EXTERNA ESPII - RODÍZIO	HC da Universidade Federal de Goiás - GO
233	ATIVIDADE EXTERNA ESPII - RODÍZIO	HC da Universidade Federal de Goiás - GO
233	ATIVIDADE EXTERNA ESPII - RODÍZIO	HC da Universidade Federal de Goiás - GO
233	ATIVIDADE EXTERNA ESPII - RODÍZIO	HC da Universidade Federal de Goiás - GO
233	ATIVIDADE EXTERNA ESPII - RODÍZIO	HC da Universidade Federal de Goiás - GO
233	ATIVIDADE EXTERNA ESPII - RODÍZIO	HC da Universidade Federal de Goiás - GO
233	ATIVIDADE EXTERNA ESPII - RODÍZIO	HC da Universidade Federal de Goiás - GO
233	ATIVIDADE EXTERNA ESPII - RODÍZIO	HC da Universidade Federal de Goiás - GO
233	ATIVIDADE EXTERNA ESPII - RODÍZIO	HC da Universidade Federal de Goiás - GO
233	ATIVIDADE EXTERNA ESPII - RODÍZIO	HC da Universidade Federal de Goiás - GO
233	ATIVIDADE EXTERNA ESPII - RODÍZIO	HC da Universidade Federal de Goiás - GO
233	ATIVIDADE EXTERNA ESPII - RODÍZIO	HC da Universidade Federal de Goiás - GO
233	ATIVIDADE EXTERNA ESPII - RODÍZIO	HC da Universidade Federal de Goiás - GO
233	ATIVIDADE EXTERNA ESPII - RODÍZIO	HC da Universidade Federal de Goiás - GO
233	ATIVIDADE EXTERNA ESPII - RODÍZIO	HC da Universidade Federal de Goiás - GO
233	ATIVIDADE EXTERNA ESPII - RODÍZIO	HC da Universidade Federal de Goiás - GO
233	ATIVIDADE EXTERNA ESPII - RODÍZIO	HC da Universidade Federal de Goiás - GO
233	ATIVIDADE EXTERNA ESPII - RODÍZIO	HC da Universidade Federal de Goiás - GO
233	ATIVIDADE EXTERNA ESPII - RODÍZIO	HC da Universidade Federal de Goiás - GO
233	ATIVIDADE EXTERNA ESPII - RODÍZIO	HC da Universidade Federal de Goiás - GO
233	ATIVIDADE EXTERNA ESPII - RODÍZIO	HC da Universidade Federal de Goiás - GO
233	ATIVIDADE EXTERNA ESPII - RODÍZIO	HC da Universidade Federal de Goiás - GO
233	ATIVIDADE EXTERNA ESPII - RODÍZIO	HC da Universidade Federal de Goiás - GO
233	ATIVIDADE EXTERNA ESPII - RODÍZIO	HC da Universidade Federal de Goiás - GO
233	ATIVIDADE EXTERNA ESPII - RODÍZIO	HC da Universidade Federal de Goiás - GO
233	ATIVIDADE EXTERNA ESPII - RODÍZIO	HC da Universidade Federal de Goiás - GO
233	ATIVIDADE EXTERNA ESPII - RODÍZIO	HC da Universidade Federal de Goiás - GO
233	ATIVIDADE EXTERNA ESPII - RODÍZIO	HC da Universidade Federal de Goiás - GO
233	ATIVIDADE EXTERNA ESPII - RODÍZIO	HC da Universidade Federal de Goiás - GO
233	ATIVIDADE EXTERNA ESPII - RODÍZIO	HC da Universidade Federal de Goiás - GO
233	ATIVIDADE EXTERNA ESPII - RODÍZIO	HC da Universidade Federal de Goiás - GO
233	ATIVIDADE EXTERNA ESPII - RODÍZIO	HC da Universidade Federal de Goiás - GO
233	ATIVIDADE EXTERNA ESPII - RODÍZIO	HC da Universidade Federal de Goiás - GO

233	ATIVIDADE EXTERNA ESPII - RODÍZIO	HC da Universidade Federal de Goiás - GO
233	ATIVIDADE EXTERNA ESPII - RODÍZIO	HC da Universidade Federal de Goiás - GO
233	ATIVIDADE EXTERNA ESPII - RODÍZIO	HC da Universidade Federal de Goiás - GO
233	ATIVIDADE EXTERNA ESPII - RODÍZIO	HC da Universidade Federal de Goiás - GO
233	ATIVIDADE EXTERNA ESPII - RODÍZIO	HC da Universidade Federal de Goiás - GO
233	ATIVIDADE EXTERNA ESPII - RODÍZIO	HC da Universidade Federal de Goiás - GO
233	ATIVIDADE EXTERNA ESPII - RODÍZIO	HC da Universidade Federal de Goiás - GO
233	ATIVIDADE EXTERNA ESPII - RODÍZIO	HC da Universidade Federal de Goiás - GO
233	ATIVIDADE EXTERNA ESPII - RODÍZIO	HC da Universidade Federal de Goiás - GO
233	ATIVIDADE EXTERNA ESPII - RODÍZIO	HC da Universidade Federal de Goiás - GO
233	ATIVIDADE EXTERNA ESPII - RODÍZIO	HC da Universidade Federal de Goiás - GO
233	ATIVIDADE EXTERNA ESPII - RODÍZIO	HC da Universidade Federal de Goiás - GO
233	ATIVIDADE EXTERNA ESPII - RODÍZIO	HC da Universidade Federal de Goiás - GO
233	ATIVIDADE EXTERNA ESPII - RODÍZIO	HC da Universidade Federal de Goiás - GO
233	ATIVIDADE EXTERNA ESPII - RODÍZIO	HC da Universidade Federal de Goiás - GO
233	ATIVIDADE EXTERNA ESPII - RODÍZIO	HC da Universidade Federal de Goiás - GO
233	ATIVIDADE EXTERNA ESPII - RODÍZIO	HC da Universidade Federal de Goiás - GO
233	ATIVIDADE EXTERNA ESPII - RODÍZIO	HC da Universidade Federal de Goiás - GO
233	ATIVIDADE EXTERNA ESPII - RODÍZIO	HC da Universidade Federal de Goiás - GO
233	ATIVIDADE EXTERNA ESPII - RODÍZIO	HC da Universidade Federal de Goiás - GO
233	ATIVIDADE EXTERNA ESPII - RODÍZIO	HC da Universidade Federal de Goiás - GO
233	ATIVIDADE EXTERNA ESPII - RODÍZIO	HC da Universidade Federal de Goiás - GO
233	ATIVIDADE EXTERNA ESPII - RODÍZIO	HC da Universidade Federal de Goiás - GO
233	ATIVIDADE EXTERNA ESPII - RODÍZIO	HC da Universidade Federal de Goiás - GO
233	ATIVIDADE EXTERNA ESPII - RODÍZIO	HC da Universidade Federal de Goiás - GO
233	ATIVIDADE EXTERNA ESPII - RODÍZIO	HC da Universidade Federal de Goiás - GO
233	ATIVIDADE EXTERNA ESPII - RODÍZIO	HC da Universidade Federal de Goiás - GO
233	ATIVIDADE EXTERNA ESPII - RODÍZIO	HC da Universidade Federal de Goiás - GO
233	ATIVIDADE EXTERNA ESPII - RODÍZIO	HC da Universidade Federal de Goiás - GO
233	ATIVIDADE EXTERNA ESPII - RODÍZIO	HC da Universidade Federal de Goiás - GO
233	ATIVIDADE EXTERNA ESPII - RODÍZIO	HC da Universidade Federal de Goiás - GO
233	ATIVIDADE EXTERNA ESPII - RODÍZIO	HC da Universidade Federal de Goiás - GO
233	ATIVIDADE EXTERNA ESPII - RODÍZIO	HC da Universidade Federal de Goiás - GO
233	ATIVIDADE EXTERNA ESPII - RODÍZIO	HC da Universidade Federal de Goiás - GO
233	ATIVIDADE EXTERNA ESPII - RODÍZIO	HC da Universidade Federal de Goiás - GO
233	ATIVIDADE EXTERNA ESPII - RODÍZIO	HC da Universidade Federal de Goiás - GO
233	ATIVIDADE EXTERNA ESPII - RODÍZIO	HC da Universidade Federal de Goiás - GO
233	ATIVIDADE EXTERNA ESPII - RODÍZIO	HC da Universidade Federal de Goiás - GO
233	ATIVIDADE EXTERNA ESPII - RODÍZIO	HC da Universidade Federal de Goiás - GO
233	ATIVIDADE EXTERNA ESPII - RODÍZIO	HC da Universidade Federal de Goiás - GO
233	ATIVIDADE EXTERNA ESPII - RODÍZIO	HC da Universidade Federal de Goiás - GO
233	ATIVIDADE EXTERNA ESPII - RODÍZIO	HC da Universidade Federal de Goiás - GO
233	ATIVIDADE EXTERNA ESPII - RODÍZIO	HC da Universidade Federal de Goiás - GO
233	ATIVIDADE EXTERNA ESPII - RODÍZIO	HC da Universidade Federal de Goiás - GO
233	ATIVIDADE EXTERNA ESPII - RODÍZIO	HC da Universidade Federal de Goiás - GO
233	ATIVIDADE EXTERNA ESPII - RODÍZIO	HC da Universidade Federal de Goiás - GO
233	ATIVIDADE EXTERNA ESPII - RODÍZIO	HC da Universidade Federal de Goiás - GO

233	ATIVIDADE EXTERNA ESPII - RODÍZIO	HC da Universidade Federal de Goiás - GO
233	ATIVIDADE EXTERNA ESPII - RODÍZIO	HC da Universidade Federal de Goiás - GO
233	ATIVIDADE EXTERNA ESPII - RODÍZIO	HC da Universidade Federal de Goiás - GO
233	ATIVIDADE EXTERNA ESPII - RODÍZIO	HC da Universidade Federal de Goiás - GO
233	ATIVIDADE EXTERNA ESPII - RODÍZIO	HC da Universidade Federal de Goiás - GO
233	ATIVIDADE EXTERNA ESPII - RODÍZIO	HC da Universidade Federal de Goiás - GO
233	ATIVIDADE EXTERNA ESPII - RODÍZIO	HC da Universidade Federal de Goiás - GO
233	ATIVIDADE EXTERNA ESPII - RODÍZIO	HC da Universidade Federal de Goiás - GO
233	ATIVIDADE EXTERNA ESPII - RODÍZIO	HC da Universidade Federal de Goiás - GO
233	ATIVIDADE EXTERNA ESPII - RODÍZIO	HC da Universidade Federal de Goiás - GO
233	ATIVIDADE EXTERNA ESPII - RODÍZIO	HC da Universidade Federal de Goiás - GO
233	ATIVIDADE EXTERNA ESPII - RODÍZIO	HC da Universidade Federal de Goiás - GO
233	ATIVIDADE EXTERNA ESPII - RODÍZIO	HC da Universidade Federal de Goiás - GO
233	ATIVIDADE EXTERNA ESPII - RODÍZIO	HC da Universidade Federal de Goiás - GO
233	ATIVIDADE EXTERNA ESPII - RODÍZIO	HC da Universidade Federal de Goiás - GO
233	ATIVIDADE EXTERNA ESPII - RODÍZIO	HC da Universidade Federal de Goiás - GO
233	ATIVIDADE EXTERNA ESPII - RODÍZIO	HC da Universidade Federal de Goiás - GO
233	ATIVIDADE EXTERNA ESPII - RODÍZIO	HC da Universidade Federal de Goiás - GO
233	ATIVIDADE EXTERNA ESPII - RODÍZIO	HC da Universidade Federal de Goiás - GO
233	ATIVIDADE EXTERNA ESPII - RODÍZIO	HC da Universidade Federal de Goiás - GO
233	ATIVIDADE EXTERNA ESPII - RODÍZIO	HC da Universidade Federal de Goiás - GO
233	ATIVIDADE EXTERNA ESPII - RODÍZIO	HC da Universidade Federal de Goiás - GO
233	ATIVIDADE EXTERNA ESPII - RODÍZIO	HC da Universidade Federal de Goiás - GO
233	ATIVIDADE EXTERNA ESPII - RODÍZIO	HC da Universidade Federal de Goiás - GO
233	ATIVIDADE EXTERNA ESPII - RODÍZIO	HC da Universidade Federal de Goiás - GO
233	ATIVIDADE EXTERNA ESPII - RODÍZIO	HC da Universidade Federal de Goiás - GO
233	ATIVIDADE EXTERNA ESPII - RODÍZIO	HC da Universidade Federal de Goiás - GO
233	ATIVIDADE EXTERNA ESPII - RODÍZIO	HC da Universidade Federal de Goiás - GO
233	ATIVIDADE EXTERNA ESPII - RODÍZIO	HC da Universidade Federal de Goiás - GO
233	ATIVIDADE EXTERNA ESPII - RODÍZIO	HC da Universidade Federal de Goiás - GO
233	ATIVIDADE EXTERNA ESPII - RODÍZIO	HC da Universidade Federal de Goiás - GO
233	ATIVIDADE EXTERNA ESPII - RODÍZIO	HC da Universidade Federal de Goiás - GO
233	ATIVIDADE EXTERNA ESPII - RODÍZIO	HC da Universidade Federal de Goiás - GO
233	ATIVIDADE EXTERNA ESPII - RODÍZIO	HC da Universidade Federal de Goiás - GO
233	ATIVIDADE EXTERNA ESPII - RODÍZIO	HC da Universidade Federal de Goiás - GO
233	ATIVIDADE EXTERNA ESPII - RODÍZIO	HC da Universidade Federal de Goiás - GO

235	Afastamento Portaria n 454 art 3	HC da Universidade Federal de Goiás - GO
235	Afastamento Portaria n 454 art 3	HC da Universidade Federal de Goiás - GO
235	Afastamento Portaria n 454 art 3	HC da Universidade Federal de Goiás - GO
235	Afastamento Portaria n 454 art 3	HC da Universidade Federal de Goiás - GO
235	Afastamento Portaria n 454 art 3	HC da Universidade Federal de Goiás - GO
235	Afastamento Portaria n 454 art 3	HC da Universidade Federal de Goiás - GO
235	Afastamento Portaria n 454 art 3	HC da Universidade Federal de Goiás - GO
235	Afastamento Portaria n 454 art 3	HC da Universidade Federal de Goiás - GO
235	Afastamento Portaria n 454 art 3	HC da Universidade Federal de Goiás - GO
235	Afastamento Portaria n 454 art 3	HC da Universidade Federal de Goiás - GO
235	Afastamento Portaria n 454 art 3	HC da Universidade Federal de Goiás - GO
235	Afastamento Portaria n 454 art 3	HC da Universidade Federal de Goiás - GO
235	Afastamento Portaria n 454 art 3	HC da Universidade Federal de Goiás - GO
235	Afastamento Portaria n 454 art 3	HC da Universidade Federal de Goiás - GO
235	Afastamento Portaria n 454 art 3	HC da Universidade Federal de Goiás - GO
235	Afastamento Portaria n 454 art 3	HC da Universidade Federal de Goiás - GO
235	Afastamento Portaria n 454 art 3	HC da Universidade Federal de Goiás - GO
235	Afastamento Portaria n 454 art 3	HC da Universidade Federal de Goiás - GO
235	Afastamento Portaria n 454 art 3	HC da Universidade Federal de Goiás - GO
235	Afastamento Portaria n 454 art 3	HC da Universidade Federal de Goiás - GO
235	Afastamento Portaria n 454 art 3	HC da Universidade Federal de Goiás - GO
235	Afastamento Portaria n 454 art 3	HC da Universidade Federal de Goiás - GO
235	Afastamento Portaria n 454 art 3	HC da Universidade Federal de Goiás - GO
235	Afastamento Portaria n 454 art 3	HC da Universidade Federal de Goiás - GO
235	Afastamento Portaria n 454 art 3	HC da Universidade Federal de Goiás - GO
235	Afastamento Portaria n 454 art 3	HC da Universidade Federal de Goiás - GO
235	Afastamento Portaria n 454 art 3	HC da Universidade Federal de Goiás - GO
235	Afastamento Portaria n 454 art 3	HC da Universidade Federal de Goiás - GO
235	Afastamento Portaria n 454 art 3	HC da Universidade Federal de Goiás - GO
235	Afastamento Portaria n 454 art 3	HC da Universidade Federal de Goiás - GO
235	Afastamento Portaria n 454 art 3	HC da Universidade Federal de Goiás - GO
235	Afastamento Portaria n 454 art 3	HC da Universidade Federal de Goiás - GO
235	Afastamento Portaria n 454 art 3	HC da Universidade Federal de Goiás - GO
235	Afastamento Portaria n 454 art 3	HC da Universidade Federal de Goiás - GO
235	Afastamento Portaria n 454 art 3	HC da Universidade Federal de Goiás - GO
235	Afastamento Portaria n 454 art 3	HC da Universidade Federal de Goiás - GO
235	Afastamento Portaria n 454 art 3	HC da Universidade Federal de Goiás - GO
235	Afastamento Portaria n 454 art 3	HC da Universidade Federal de Goiás - GO
235	Afastamento Portaria n 454 art 3	HC da Universidade Federal de Goiás - GO
235	Afastamento Portaria n 454 art 3	HC da Universidade Federal de Goiás - GO
235	Afastamento Portaria n 454 art 3	HC da Universidade Federal de Goiás - GO

235	Afastamento Portaria n 454 art 3	HC da Universidade Federal de Goiás - GO
235	Afastamento Portaria n 454 art 3	HC da Universidade Federal de Goiás - GO
235	Afastamento Portaria n 454 art 3	HC da Universidade Federal de Goiás - GO
235	Afastamento Portaria n 454 art 3	HC da Universidade Federal de Goiás - GO
235	Afastamento Portaria n 454 art 3	HC da Universidade Federal de Goiás - GO
235	Afastamento Portaria n 454 art 3	HC da Universidade Federal de Goiás - GO
235	Afastamento Portaria n 454 art 3	HC da Universidade Federal de Goiás - GO
235	Afastamento Portaria n 454 art 3	HC da Universidade Federal de Goiás - GO
235	Afastamento Portaria n 454 art 3	HC da Universidade Federal de Goiás - GO
235	Afastamento Portaria n 454 art 3	HC da Universidade Federal de Goiás - GO
235	Afastamento Portaria n 454 art 3	HC da Universidade Federal de Goiás - GO
235	Afastamento Portaria n 454 art 3	HC da Universidade Federal de Goiás - GO
235	Afastamento Portaria n 454 art 3	HC da Universidade Federal de Goiás - GO
235	Afastamento Portaria n 454 art 3	HC da Universidade Federal de Goiás - GO
235	Afastamento Portaria n 454 art 3	HC da Universidade Federal de Goiás - GO
235	Afastamento Portaria n 454 art 3	HC da Universidade Federal de Goiás - GO
235	Afastamento Portaria n 454 art 3	HC da Universidade Federal de Goiás - GO
235	Afastamento Portaria n 454 art 3	HC da Universidade Federal de Goiás - GO
235	Afastamento Portaria n 454 art 3	HC da Universidade Federal de Goiás - GO
235	Afastamento Portaria n 454 art 3	HC da Universidade Federal de Goiás - GO
235	Afastamento Portaria n 454 art 3	HC da Universidade Federal de Goiás - GO
235	Afastamento Portaria n 454 art 3	HC da Universidade Federal de Goiás - GO
235	Afastamento Portaria n 454 art 3	HC da Universidade Federal de Goiás - GO
235	Afastamento Portaria n 454 art 3	HC da Universidade Federal de Goiás - GO
235	Afastamento Portaria n 454 art 3	HC da Universidade Federal de Goiás - GO
235	Afastamento Portaria n 454 art 3	HC da Universidade Federal de Goiás - GO
235	Afastamento Portaria n 454 art 3	HC da Universidade Federal de Goiás - GO
235	Afastamento Portaria n 454 art 3	HC da Universidade Federal de Goiás - GO
235	Afastamento Portaria n 454 art 3	HC da Universidade Federal de Goiás - GO
235	Afastamento Portaria n 454 art 3	HC da Universidade Federal de Goiás - GO
235	Afastamento Portaria n 454 art 3	HC da Universidade Federal de Goiás - GO
235	Afastamento Portaria n 454 art 3	HC da Universidade Federal de Goiás - GO
235	Afastamento Portaria n 454 art 3	HC da Universidade Federal de Goiás - GO
235	Afastamento Portaria n 454 art 3	HC da Universidade Federal de Goiás - GO
235	Afastamento Portaria n 454 art 3	HC da Universidade Federal de Goiás - GO
235	Afastamento Portaria n 454 art 3	HC da Universidade Federal de Goiás - GO
235	Afastamento Portaria n 454 art 3	HC da Universidade Federal de Goiás - GO
235	Afastamento Portaria n 454 art 3	HC da Universidade Federal de Goiás - GO
235	Afastamento Portaria n 454 art 3	HC da Universidade Federal de Goiás - GO
235	Afastamento Portaria n 454 art 3	HC da Universidade Federal de Goiás - GO
235	Afastamento Portaria n 454 art 3	HC da Universidade Federal de Goiás - GO
235	Afastamento Portaria n 454 art 3	HC da Universidade Federal de Goiás - GO
235	Afastamento Portaria n 454 art 3	HC da Universidade Federal de Goiás - GO
235	Afastamento Portaria n 454 art 3	HC da Universidade Federal de Goiás - GO
235	Afastamento Portaria n 454 art 3	HC da Universidade Federal de Goiás - GO

235	Afastamento Portaria n 454 art 3	HC da Universidade Federal de Goiás - GO
235	Afastamento Portaria n 454 art 3	HC da Universidade Federal de Goiás - GO
235	Afastamento Portaria n 454 art 3	HC da Universidade Federal de Goiás - GO
235	Afastamento Portaria n 454 art 3	HC da Universidade Federal de Goiás - GO
235	Afastamento Portaria n 454 art 3	HC da Universidade Federal de Goiás - GO
235	Afastamento Portaria n 454 art 3	HC da Universidade Federal de Goiás - GO
235	Afastamento Portaria n 454 art 3	HC da Universidade Federal de Goiás - GO
236	Afastamento Portaria n 454 art. 2 Único	HC da Universidade Federal de Goiás - GO
236	Afastamento Portaria n 454 art. 2 Único	HC da Universidade Federal de Goiás - GO
236	Afastamento Portaria n 454 art. 2 Único	HC da Universidade Federal de Goiás - GO
236	Afastamento Portaria n 454 art. 2 Único	HC da Universidade Federal de Goiás - GO
236	Afastamento Portaria n 454 art. 2 Único	HC da Universidade Federal de Goiás - GO
236	Afastamento Portaria n 454 art. 2 Único	HC da Universidade Federal de Goiás - GO
236	Afastamento Portaria n 454 art. 2 Único	HC da Universidade Federal de Goiás - GO
236	Afastamento Portaria n 454 art. 2 Único	HC da Universidade Federal de Goiás - GO
236	Afastamento Portaria n 454 art. 2 Único	HC da Universidade Federal de Goiás - GO
236	Afastamento Portaria n 454 art. 2 Único	HC da Universidade Federal de Goiás - GO
236	Afastamento Portaria n 454 art. 2 Único	HC da Universidade Federal de Goiás - GO
236	Afastamento Portaria n 454 art. 2 Único	HC da Universidade Federal de Goiás - GO
236	Afastamento Portaria n 454 art. 2 Único	HC da Universidade Federal de Goiás - GO
236	Afastamento Portaria n 454 art. 2 Único	HC da Universidade Federal de Goiás - GO
236	Afastamento Portaria n 454 art. 2 Único	HC da Universidade Federal de Goiás - GO
236	Afastamento Portaria n 454 art. 2 Único	HC da Universidade Federal de Goiás - GO
236	Afastamento Portaria n 454 art. 2 Único	HC da Universidade Federal de Goiás - GO
236	Afastamento Portaria n 454 art. 2 Único	HC da Universidade Federal de Goiás - GO
236	Afastamento Portaria n 454 art. 2 Único	HC da Universidade Federal de Goiás - GO
236	Afastamento Portaria n 454 art. 2 Único	HC da Universidade Federal de Goiás - GO
236	Afastamento Portaria n 454 art. 2 Único	HC da Universidade Federal de Goiás - GO
236	Afastamento Portaria n 454 art. 2 Único	HC da Universidade Federal de Goiás - GO
236	Afastamento Portaria n 454 art. 2 Único	HC da Universidade Federal de Goiás - GO
236	Afastamento Portaria n 454 art. 2 Único	HC da Universidade Federal de Goiás - GO
236	Afastamento Portaria n 454 art. 2 Único	HC da Universidade Federal de Goiás - GO
236	Afastamento Portaria n 454 art. 2 Único	HC da Universidade Federal de Goiás - GO
236	Afastamento Portaria n 454 art. 2 Único	HC da Universidade Federal de Goiás - GO
236	Afastamento Portaria n 454 art. 2 Único	HC da Universidade Federal de Goiás - GO
236	Afastamento Portaria n 454 art. 2 Único	HC da Universidade Federal de Goiás - GO
236	Afastamento Portaria n 454 art. 2 Único	HC da Universidade Federal de Goiás - GO
236	Afastamento Portaria n 454 art. 2 Único	HC da Universidade Federal de Goiás - GO
236	Afastamento Portaria n 454 art. 2 Único	HC da Universidade Federal de Goiás - GO
236	Afastamento Portaria n 454 art. 2 Único	HC da Universidade Federal de Goiás - GO
236	Afastamento Portaria n 454 art. 2 Único	HC da Universidade Federal de Goiás - GO
236	Afastamento Portaria n 454 art. 2 Único	HC da Universidade Federal de Goiás - GO
236	Afastamento Portaria n 454 art. 2 Único	HC da Universidade Federal de Goiás - GO
236	Afastamento Portaria n 454 art. 2 Único	HC da Universidade Federal de Goiás - GO
236	Afastamento Portaria n 454 art. 2 Único	HC da Universidade Federal de Goiás - GO
236	Afastamento Portaria n 454 art. 2 Único	HC da Universidade Federal de Goiás - GO

236	Afastamento Portaria n 454 art. 2 Único	HC da Universidade Federal de Goiás - GO
236	Afastamento Portaria n 454 art. 2 Único	HC da Universidade Federal de Goiás - GO
236	Afastamento Portaria n 454 art. 2 Único	HC da Universidade Federal de Goiás - GO
236	Afastamento Portaria n 454 art. 2 Único	HC da Universidade Federal de Goiás - GO
236	Afastamento Portaria n 454 art. 2 Único	HC da Universidade Federal de Goiás - GO
236	Afastamento Portaria n 454 art. 2 Único	HC da Universidade Federal de Goiás - GO
236	Afastamento Portaria n 454 art. 2 Único	HC da Universidade Federal de Goiás - GO
236	Afastamento Portaria n 454 art. 2 Único	HC da Universidade Federal de Goiás - GO
236	Afastamento Portaria n 454 art. 2 Único	HC da Universidade Federal de Goiás - GO
236	Afastamento Portaria n 454 art. 2 Único	HC da Universidade Federal de Goiás - GO
236	Afastamento Portaria n 454 art. 2 Único	HC da Universidade Federal de Goiás - GO
236	Afastamento Portaria n 454 art. 2 Único	HC da Universidade Federal de Goiás - GO
236	Afastamento Portaria n 454 art. 2 Único	HC da Universidade Federal de Goiás - GO
236	Afastamento Portaria n 454 art. 2 Único	HC da Universidade Federal de Goiás - GO
236	Afastamento Portaria n 454 art. 2 Único	HC da Universidade Federal de Goiás - GO
236	Afastamento Portaria n 454 art. 2 Único	HC da Universidade Federal de Goiás - GO
236	Afastamento Portaria n 454 art. 2 Único	HC da Universidade Federal de Goiás - GO
236	Afastamento Portaria n 454 art. 2 Único	HC da Universidade Federal de Goiás - GO
236	Afastamento Portaria n 454 art. 2 Único	HC da Universidade Federal de Goiás - GO
236	Afastamento Portaria n 454 art. 2 Único	HC da Universidade Federal de Goiás - GO
236	Afastamento Portaria n 454 art. 2 Único	HC da Universidade Federal de Goiás - GO
236	Afastamento Portaria n 454 art. 2 Único	HC da Universidade Federal de Goiás - GO
236	Afastamento Portaria n 454 art. 2 Único	HC da Universidade Federal de Goiás - GO
236	Afastamento Portaria n 454 art. 2 Único	HC da Universidade Federal de Goiás - GO
236	Afastamento Portaria n 454 art. 2 Único	HC da Universidade Federal de Goiás - GO
236	Afastamento Portaria n 454 art. 2 Único	HC da Universidade Federal de Goiás - GO
236	Afastamento Portaria n 454 art. 2 Único	HC da Universidade Federal de Goiás - GO
236	Afastamento Portaria n 454 art. 2 Único	HC da Universidade Federal de Goiás - GO
236	Afastamento Portaria n 454 art. 2 Único	HC da Universidade Federal de Goiás - GO
236	Afastamento Portaria n 454 art. 2 Único	HC da Universidade Federal de Goiás - GO
236	Afastamento Portaria n 454 art. 2 Único	HC da Universidade Federal de Goiás - GO
236	Afastamento Portaria n 454 art. 2 Único	HC da Universidade Federal de Goiás - GO
236	Afastamento Portaria n 454 art. 2 Único	HC da Universidade Federal de Goiás - GO
236	Afastamento Portaria n 454 art. 2 Único	HC da Universidade Federal de Goiás - GO
236	Afastamento Portaria n 454 art. 2 Único	HC da Universidade Federal de Goiás - GO
236	Afastamento Portaria n 454 art. 2 Único	HC da Universidade Federal de Goiás - GO
236	Afastamento Portaria n 454 art. 2 Único	HC da Universidade Federal de Goiás - GO
236	Afastamento Portaria n 454 art. 2 Único	HC da Universidade Federal de Goiás - GO
236	Afastamento Portaria n 454 art. 2 Único	HC da Universidade Federal de Goiás - GO
236	Afastamento Portaria n 454 art. 2 Único	HC da Universidade Federal de Goiás - GO

236	Afastamento Portaria n 454 art. 2 Único	HC da Universidade Federal de Goiás - GO
236	Afastamento Portaria n 454 art. 2 Único	HC da Universidade Federal de Goiás - GO
236	Afastamento Portaria n 454 art. 2 Único	HC da Universidade Federal de Goiás - GO
236	Afastamento Portaria n 454 art. 2 Único	HC da Universidade Federal de Goiás - GO
236	Afastamento Portaria n 454 art. 2 Único	HC da Universidade Federal de Goiás - GO
236	Afastamento Portaria n 454 art. 2 Único	HC da Universidade Federal de Goiás - GO
236	Afastamento Portaria n 454 art. 2 Único	HC da Universidade Federal de Goiás - GO
236	Afastamento Portaria n 454 art. 2 Único	HC da Universidade Federal de Goiás - GO
236	Afastamento Portaria n 454 art. 2 Único	HC da Universidade Federal de Goiás - GO
236	Afastamento Portaria n 454 art. 2 Único	HC da Universidade Federal de Goiás - GO
236	Afastamento Portaria n 454 art. 2 Único	HC da Universidade Federal de Goiás - GO
236	Afastamento Portaria n 454 art. 2 Único	HC da Universidade Federal de Goiás - GO
236	Afastamento Portaria n 454 art. 2 Único	HC da Universidade Federal de Goiás - GO
236	Afastamento Portaria n 454 art. 2 Único	HC da Universidade Federal de Goiás - GO
236	Afastamento Portaria n 454 art. 2 Único	HC da Universidade Federal de Goiás - GO
236	Afastamento Portaria n 454 art. 2 Único	HC da Universidade Federal de Goiás - GO
236	Afastamento Portaria n 454 art. 2 Único	HC da Universidade Federal de Goiás - GO
236	Afastamento Portaria n 454 art. 2 Único	HC da Universidade Federal de Goiás - GO
236	Afastamento Portaria n 454 art. 2 Único	HC da Universidade Federal de Goiás - GO
236	Afastamento Portaria n 454 art. 2 Único	HC da Universidade Federal de Goiás - GO
236	Afastamento Portaria n 454 art. 2 Único	HC da Universidade Federal de Goiás - GO
236	Afastamento Portaria n 454 art. 2 Único	HC da Universidade Federal de Goiás - GO
236	Afastamento Portaria n 454 art. 2 Único	HC da Universidade Federal de Goiás - GO
236	Afastamento Portaria n 454 art. 2 Único	HC da Universidade Federal de Goiás - GO
236	Afastamento Portaria n 454 art. 2 Único	HC da Universidade Federal de Goiás - GO
236	Afastamento Portaria n 454 art. 2 Único	HC da Universidade Federal de Goiás - GO
236	Afastamento Portaria n 454 art. 2 Único	HC da Universidade Federal de Goiás - GO
236	Afastamento Portaria n 454 art. 2 Único	HC da Universidade Federal de Goiás - GO
236	Afastamento Portaria n 454 art. 2 Único	HC da Universidade Federal de Goiás - GO
236	Afastamento Portaria n 454 art. 2 Único	HC da Universidade Federal de Goiás - GO
236	Afastamento Portaria n 454 art. 2 Único	HC da Universidade Federal de Goiás - GO
236	Afastamento Portaria n 454 art. 2 Único	HC da Universidade Federal de Goiás - GO
236	Afastamento Portaria n 454 art. 2 Único	HC da Universidade Federal de Goiás - GO
236	Afastamento Portaria n 454 art. 2 Único	HC da Universidade Federal de Goiás - GO
236	Afastamento Portaria n 454 art. 2 Único	HC da Universidade Federal de Goiás - GO
236	Afastamento Portaria n 454 art. 2 Único	HC da Universidade Federal de Goiás - GO
236	Afastamento Portaria n 454 art. 2 Único	HC da Universidade Federal de Goiás - GO
236	Afastamento Portaria n 454 art. 2 Único	HC da Universidade Federal de Goiás - GO
236	Afastamento Portaria n 454 art. 2 Único	HC da Universidade Federal de Goiás - GO
236	Afastamento Portaria n 454 art. 2 Único	HC da Universidade Federal de Goiás - GO
236	Afastamento Portaria n 454 art. 2 Único	HC da Universidade Federal de Goiás - GO
236	Afastamento Portaria n 454 art. 2 Único	HC da Universidade Federal de Goiás - GO
236	Afastamento Portaria n 454 art. 2 Único	HC da Universidade Federal de Goiás - GO
236	Afastamento Portaria n 454 art. 2 Único	HC da Universidade Federal de Goiás - GO
236	Afastamento Portaria n 454 art. 2 Único	HC da Universidade Federal de Goiás - GO
236	Afastamento Portaria n 454 art. 2 Único	HC da Universidade Federal de Goiás - GO
236	Afastamento Portaria n 454 art. 2 Único	HC da Universidade Federal de Goiás - GO
236	Afastamento Portaria n 454 art. 2 Único	HC da Universidade Federal de Goiás - GO

236	Afastamento Portaria n 454 art. 2 Único	HC da Universidade Federal de Goiás - GO
236	Afastamento Portaria n 454 art. 2 Único	HC da Universidade Federal de Goiás - GO
236	Afastamento Portaria n 454 art. 2 Único	HC da Universidade Federal de Goiás - GO
236	Afastamento Portaria n 454 art. 2 Único	HC da Universidade Federal de Goiás - GO
236	Afastamento Portaria n 454 art. 2 Único	HC da Universidade Federal de Goiás - GO
236	Afastamento Portaria n 454 art. 2 Único	HC da Universidade Federal de Goiás - GO
236	Afastamento Portaria n 454 art. 2 Único	HC da Universidade Federal de Goiás - GO
236	Afastamento Portaria n 454 art. 2 Único	HC da Universidade Federal de Goiás - GO
236	Afastamento Portaria n 454 art. 2 Único	HC da Universidade Federal de Goiás - GO
236	Afastamento Portaria n 454 art. 2 Único	HC da Universidade Federal de Goiás - GO
236	Afastamento Portaria n 454 art. 2 Único	HC da Universidade Federal de Goiás - GO
236	Afastamento Portaria n 454 art. 2 Único	HC da Universidade Federal de Goiás - GO
236	Afastamento Portaria n 454 art. 2 Único	HC da Universidade Federal de Goiás - GO
236	Afastamento Portaria n 454 art. 2 Único	HC da Universidade Federal de Goiás - GO
236	Afastamento Portaria n 454 art. 2 Único	HC da Universidade Federal de Goiás - GO
236	Afastamento Portaria n 454 art. 2 Único	HC da Universidade Federal de Goiás - GO
236	Afastamento Portaria n 454 art. 2 Único	HC da Universidade Federal de Goiás - GO
236	Afastamento Portaria n 454 art. 2 Único	HC da Universidade Federal de Goiás - GO
236	Afastamento Portaria n 454 art. 2 Único	HC da Universidade Federal de Goiás - GO
236	Afastamento Portaria n 454 art. 2 Único	HC da Universidade Federal de Goiás - GO
236	Afastamento Portaria n 454 art. 2 Único	HC da Universidade Federal de Goiás - GO
236	Afastamento Portaria n 454 art. 2 Único	HC da Universidade Federal de Goiás - GO
236	Afastamento Portaria n 454 art. 2 Único	HC da Universidade Federal de Goiás - GO
236	Afastamento Portaria n 454 art. 2 Único	HC da Universidade Federal de Goiás - GO
236	Afastamento Portaria n 454 art. 2 Único	HC da Universidade Federal de Goiás - GO
236	Afastamento Portaria n 454 art. 2 Único	HC da Universidade Federal de Goiás - GO
236	Afastamento Portaria n 454 art. 2 Único	HC da Universidade Federal de Goiás - GO
236	Afastamento Portaria n 454 art. 2 Único	HC da Universidade Federal de Goiás - GO
236	Afastamento Portaria n 454 art. 2 Único	HC da Universidade Federal de Goiás - GO
236	Afastamento Portaria n 454 art. 2 Único	HC da Universidade Federal de Goiás - GO
236	Afastamento Portaria n 454 art. 2 Único	HC da Universidade Federal de Goiás - GO
236	Afastamento Portaria n 454 art. 2 Único	HC da Universidade Federal de Goiás - GO
236	Afastamento Portaria n 454 art. 2 Único	HC da Universidade Federal de Goiás - GO
236	Afastamento Portaria n 454 art. 2 Único	HC da Universidade Federal de Goiás - GO
236	Afastamento Portaria n 454 art. 2 Único	HC da Universidade Federal de Goiás - GO
236	Afastamento Portaria n 454 art. 2 Único	HC da Universidade Federal de Goiás - GO
236	Afastamento Portaria n 454 art. 2 Único	HC da Universidade Federal de Goiás - GO
236	Afastamento Portaria n 454 art. 2 Único	HC da Universidade Federal de Goiás - GO
236	Afastamento Portaria n 454 art. 2 Único	HC da Universidade Federal de Goiás - GO
236	Afastamento Portaria n 454 art. 2 Único	HC da Universidade Federal de Goiás - GO
236	Afastamento Portaria n 454 art. 2 Único	HC da Universidade Federal de Goiás - GO
236	Afastamento Portaria n 454 art. 2 Único	HC da Universidade Federal de Goiás - GO
236	Afastamento Portaria n 454 art. 2 Único	HC da Universidade Federal de Goiás - GO
236	Afastamento Portaria n 454 art. 2 Único	HC da Universidade Federal de Goiás - GO
236	Afastamento Portaria n 454 art. 2 Único	HC da Universidade Federal de Goiás - GO
236	Afastamento Portaria n 454 art. 2 Único	HC da Universidade Federal de Goiás - GO
236	Afastamento Portaria n 454 art. 2 Único	HC da Universidade Federal de Goiás - GO
236	Afastamento Portaria n 454 art. 2 Único	HC da Universidade Federal de Goiás - GO

236	Afastamento Portaria n 454 art. 2 Único	HC da Universidade Federal de Goiás - GO
236	Afastamento Portaria n 454 art. 2 Único	HC da Universidade Federal de Goiás - GO
236	Afastamento Portaria n 454 art. 2 Único	HC da Universidade Federal de Goiás - GO
236	Afastamento Portaria n 454 art. 2 Único	HC da Universidade Federal de Goiás - GO
236	Afastamento Portaria n 454 art. 2 Único	HC da Universidade Federal de Goiás - GO
236	Afastamento Portaria n 454 art. 2 Único	HC da Universidade Federal de Goiás - GO
237	Trabalho Remoto Instrução Normativa DGP 03/2020 art 6, IX.	HC da Universidade Federal de Goiás - GO
237	Trabalho Remoto Instrução Normativa DGP 03/2020 art 6, IX.	HC da Universidade Federal de Goiás - GO
237	Trabalho Remoto Instrução Normativa DGP 03/2020 art 6, IX.	HC da Universidade Federal de Goiás - GO
237	Trabalho Remoto Instrução Normativa DGP 03/2020 art 6, IX.	HC da Universidade Federal de Goiás - GO
237	Trabalho Remoto Instrução Normativa DGP 03/2020 art 6, IX.	HC da Universidade Federal de Goiás - GO
237	Trabalho Remoto Instrução Normativa DGP 03/2020 art 6, IX.	HC da Universidade Federal de Goiás - GO
237	Trabalho Remoto Instrução Normativa DGP 03/2020 art 6, IX.	HC da Universidade Federal de Goiás - GO
237	Trabalho Remoto Instrução Normativa DGP 03/2020 art 6, IX.	HC da Universidade Federal de Goiás - GO
237	Trabalho Remoto Instrução Normativa DGP 03/2020 art 6, IX.	HC da Universidade Federal de Goiás - GO
237	Trabalho Remoto Instrução Normativa DGP 03/2020 art 6, IX.	HC da Universidade Federal de Goiás - GO
237	Trabalho Remoto Instrução Normativa DGP 03/2020 art 6, IX.	HC da Universidade Federal de Goiás - GO
237	Trabalho Remoto Instrução Normativa DGP 03/2020 art 6, IX.	HC da Universidade Federal de Goiás - GO
237	Trabalho Remoto Instrução Normativa DGP 03/2020 art 6, IX.	HC da Universidade Federal de Goiás - GO
237	Trabalho Remoto Instrução Normativa DGP 03/2020 art 6, IX.	HC da Universidade Federal de Goiás - GO
237	Trabalho Remoto Instrução Normativa DGP 03/2020 art 6, IX.	HC da Universidade Federal de Goiás - GO
237	Trabalho Remoto Instrução Normativa DGP 03/2020 art 6, IX.	HC da Universidade Federal de Goiás - GO
237	Trabalho Remoto Instrução Normativa DGP 03/2020 art 6, IX.	HC da Universidade Federal de Goiás - GO
237	Trabalho Remoto Instrução Normativa DGP 03/2020 art 6, IX.	HC da Universidade Federal de Goiás - GO
237	Trabalho Remoto Instrução Normativa DGP 03/2020 art 6, IX.	HC da Universidade Federal de Goiás - GO
237	Trabalho Remoto Instrução Normativa DGP 03/2020 art 6, IX.	HC da Universidade Federal de Goiás - GO
237	Trabalho Remoto Instrução Normativa DGP 03/2020 art 6, IX.	HC da Universidade Federal de Goiás - GO
237	Trabalho Remoto Instrução Normativa DGP 03/2020 art 6, IX.	HC da Universidade Federal de Goiás - GO
237	Trabalho Remoto Instrução Normativa DGP 03/2020 art 6, IX.	HC da Universidade Federal de Goiás - GO
237	Trabalho Remoto Instrução Normativa DGP 03/2020 art 6, IX.	HC da Universidade Federal de Goiás - GO
237	Trabalho Remoto Instrução Normativa DGP 03/2020 art 6, IX.	HC da Universidade Federal de Goiás - GO
237	Trabalho Remoto Instrução Normativa DGP 03/2020 art 6, IX.	HC da Universidade Federal de Goiás - GO
237	Trabalho Remoto Instrução Normativa DGP 03/2020 art 6, IX.	HC da Universidade Federal de Goiás - GO
237	Trabalho Remoto Instrução Normativa DGP 03/2020 art 6, IX.	HC da Universidade Federal de Goiás - GO

237	Trabalho Remoto Instrução Normativa DGP 03/2020 art 6, IX.	HC da Universidade Federal de Goiás - GO
237	Trabalho Remoto Instrução Normativa DGP 03/2020 art 6, IX.	HC da Universidade Federal de Goiás - GO
237	Trabalho Remoto Instrução Normativa DGP 03/2020 art 6, IX.	HC da Universidade Federal de Goiás - GO
237	Trabalho Remoto Instrução Normativa DGP 03/2020 art 6, IX.	HC da Universidade Federal de Goiás - GO
237	Trabalho Remoto Instrução Normativa DGP 03/2020 art 6, IX.	HC da Universidade Federal de Goiás - GO
237	Trabalho Remoto Instrução Normativa DGP 03/2020 art 6, IX.	HC da Universidade Federal de Goiás - GO
237	Trabalho Remoto Instrução Normativa DGP 03/2020 art 6, IX.	HC da Universidade Federal de Goiás - GO
237	Trabalho Remoto Instrução Normativa DGP 03/2020 art 6, IX.	HC da Universidade Federal de Goiás - GO
237	Trabalho Remoto Instrução Normativa DGP 03/2020 art 6, IX.	HC da Universidade Federal de Goiás - GO
237	Trabalho Remoto Instrução Normativa DGP 03/2020 art 6, IX.	HC da Universidade Federal de Goiás - GO
237	Trabalho Remoto Instrução Normativa DGP 03/2020 art 6, IX.	HC da Universidade Federal de Goiás - GO
238	Afastamento Intrução Normativa DGP 03/2020. Art 4 2	HC da Universidade Federal de Goiás - GO
3	LICENÇA PATERNIDADE - RJU - 20 DIAS - LEI 8.112/90 E DECRETO 8737/16	HC da Universidade Federal de Minas Gerais - MG
4	LICENÇA MATERNIDADE - 120 DIAS	HC da Universidade Federal de Minas Gerais - MG
4	LICENÇA MATERNIDADE - 120 DIAS	HC da Universidade Federal de Minas Gerais - MG
4	LICENÇA MATERNIDADE - 120 DIAS	HC da Universidade Federal de Minas Gerais - MG
4	LICENÇA MATERNIDADE - 120 DIAS	HC da Universidade Federal de Minas Gerais - MG
4	LICENÇA MATERNIDADE - 120 DIAS	HC da Universidade Federal de Minas Gerais - MG
4	LICENÇA MATERNIDADE - 120 DIAS	HC da Universidade Federal de Minas Gerais - MG
4	LICENÇA MATERNIDADE - 120 DIAS	HC da Universidade Federal de Minas Gerais - MG
4	LICENÇA MATERNIDADE - 120 DIAS	HC da Universidade Federal de Minas Gerais - MG
4	LICENÇA MATERNIDADE - 120 DIAS	HC da Universidade Federal de Minas Gerais - MG
4	LICENÇA MATERNIDADE - 120 DIAS	HC da Universidade Federal de Minas Gerais - MG
4	LICENÇA MATERNIDADE - 120 DIAS	HC da Universidade Federal de Minas Gerais - MG
4	LICENÇA MATERNIDADE - 120 DIAS	HC da Universidade Federal de Minas Gerais - MG
4	LICENÇA MATERNIDADE - 120 DIAS	HC da Universidade Federal de Minas Gerais - MG
4	LICENÇA MATERNIDADE - 120 DIAS	HC da Universidade Federal de Minas Gerais - MG
4	LICENÇA MATERNIDADE - 120 DIAS	HC da Universidade Federal de Minas Gerais - MG
4	LICENÇA MATERNIDADE - 120 DIAS	HC da Universidade Federal de Minas Gerais - MG
4	LICENÇA MATERNIDADE - 120 DIAS	HC da Universidade Federal de Minas Gerais - MG
4	LICENÇA MATERNIDADE - 120 DIAS	HC da Universidade Federal de Minas Gerais - MG
4	LICENÇA MATERNIDADE - 120 DIAS	HC da Universidade Federal de Minas Gerais - MG
4	LICENÇA MATERNIDADE - 120 DIAS	HC da Universidade Federal de Minas Gerais - MG
4	LICENÇA MATERNIDADE - 120 DIAS	HC da Universidade Federal de Minas Gerais - MG
4	LICENÇA MATERNIDADE - 120 DIAS	HC da Universidade Federal de Minas Gerais - MG
4	LICENÇA MATERNIDADE - 120 DIAS	HC da Universidade Federal de Minas Gerais - MG
4	LICENÇA MATERNIDADE - 120 DIAS	HC da Universidade Federal de Minas Gerais - MG
4	LICENÇA MATERNIDADE - 120 DIAS	HC da Universidade Federal de Minas Gerais - MG
4	LICENÇA MATERNIDADE - 120 DIAS	HC da Universidade Federal de Minas Gerais - MG
4	LICENÇA MATERNIDADE - 120 DIAS	HC da Universidade Federal de Minas Gerais - MG
4	LICENÇA MATERNIDADE - 120 DIAS	HC da Universidade Federal de Minas Gerais - MG

4	LICENÇA MATERNIDADE - 120 DIAS	HC da Universidade Federal de Minas Gerais - MG
4	LICENÇA MATERNIDADE - 120 DIAS	HC da Universidade Federal de Minas Gerais - MG
4	LICENÇA MATERNIDADE - 120 DIAS	HC da Universidade Federal de Minas Gerais - MG
4	LICENÇA MATERNIDADE - 120 DIAS	HC da Universidade Federal de Minas Gerais - MG
4	LICENÇA MATERNIDADE - 120 DIAS	HC da Universidade Federal de Minas Gerais - MG
4	LICENÇA MATERNIDADE - 120 DIAS	HC da Universidade Federal de Minas Gerais - MG
4	LICENÇA MATERNIDADE - 120 DIAS	HC da Universidade Federal de Minas Gerais - MG
4	LICENÇA MATERNIDADE - 120 DIAS	HC da Universidade Federal de Minas Gerais - MG
4	LICENÇA MATERNIDADE - 120 DIAS	HC da Universidade Federal de Minas Gerais - MG
4	LICENÇA MATERNIDADE - 120 DIAS	HC da Universidade Federal de Minas Gerais - MG
4	LICENÇA MATERNIDADE - 120 DIAS	HC da Universidade Federal de Minas Gerais - MG
4	LICENÇA MATERNIDADE - 120 DIAS	HC da Universidade Federal de Minas Gerais - MG
4	LICENÇA MATERNIDADE - 120 DIAS	HC da Universidade Federal de Minas Gerais - MG
4	LICENÇA MATERNIDADE - 120 DIAS	HC da Universidade Federal de Minas Gerais - MG
4	LICENÇA MATERNIDADE - 120 DIAS	HC da Universidade Federal de Minas Gerais - MG
4	LICENÇA MATERNIDADE - 120 DIAS	HC da Universidade Federal de Minas Gerais - MG
4	LICENÇA MATERNIDADE - 120 DIAS	HC da Universidade Federal de Minas Gerais - MG
4	LICENÇA MATERNIDADE - 120 DIAS	HC da Universidade Federal de Minas Gerais - MG
4	LICENÇA MATERNIDADE - 120 DIAS	HC da Universidade Federal de Minas Gerais - MG
4	LICENÇA MATERNIDADE - 120 DIAS	HC da Universidade Federal de Minas Gerais - MG
4	LICENÇA MATERNIDADE - 120 DIAS	HC da Universidade Federal de Minas Gerais - MG
4	LICENÇA MATERNIDADE - 120 DIAS	HC da Universidade Federal de Minas Gerais - MG
4	LICENÇA MATERNIDADE - 120 DIAS	HC da Universidade Federal de Minas Gerais - MG
4	LICENÇA MATERNIDADE - 120 DIAS	HC da Universidade Federal de Minas Gerais - MG
4	LICENÇA MATERNIDADE - 120 DIAS	HC da Universidade Federal de Minas Gerais - MG
4	LICENÇA MATERNIDADE - 120 DIAS	HC da Universidade Federal de Minas Gerais - MG
4	LICENÇA MATERNIDADE - 120 DIAS	HC da Universidade Federal de Minas Gerais - MG
4	LICENÇA MATERNIDADE - 120 DIAS	HC da Universidade Federal de Minas Gerais - MG
4	LICENÇA MATERNIDADE - 120 DIAS	HC da Universidade Federal de Minas Gerais - MG
4	LICENÇA MATERNIDADE - 120 DIAS	HC da Universidade Federal de Minas Gerais - MG
4	LICENÇA MATERNIDADE - 120 DIAS	HC da Universidade Federal de Minas Gerais - MG
4	LICENÇA MATERNIDADE - 120 DIAS	HC da Universidade Federal de Minas Gerais - MG
4	LICENÇA MATERNIDADE - 120 DIAS	HC da Universidade Federal de Minas Gerais - MG
4	LICENÇA MATERNIDADE - 120 DIAS	HC da Universidade Federal de Minas Gerais - MG
4	LICENÇA MATERNIDADE - 120 DIAS	HC da Universidade Federal de Minas Gerais - MG
4	LICENÇA MATERNIDADE - 120 DIAS	HC da Universidade Federal de Minas Gerais - MG
4	LICENÇA MATERNIDADE - 120 DIAS	HC da Universidade Federal de Minas Gerais - MG
4	LICENÇA MATERNIDADE - 120 DIAS	HC da Universidade Federal de Minas Gerais - MG
4	LICENÇA MATERNIDADE - 120 DIAS	HC da Universidade Federal de Minas Gerais - MG

4	LICENÇA MATERNIDADE - 120 DIAS	HC da Universidade Federal de Minas Gerais - MG
4	LICENÇA MATERNIDADE - 120 DIAS	HC da Universidade Federal de Minas Gerais - MG
4	LICENÇA MATERNIDADE - 120 DIAS	HC da Universidade Federal de Minas Gerais - MG
4	LICENÇA MATERNIDADE - 120 DIAS	HC da Universidade Federal de Minas Gerais - MG
4	LICENÇA MATERNIDADE - 120 DIAS	HC da Universidade Federal de Minas Gerais - MG
4	LICENÇA MATERNIDADE - 120 DIAS	HC da Universidade Federal de Minas Gerais - MG
4	LICENÇA MATERNIDADE - 120 DIAS	HC da Universidade Federal de Minas Gerais - MG
4	LICENÇA MATERNIDADE - 120 DIAS	HC da Universidade Federal de Minas Gerais - MG
4	LICENÇA MATERNIDADE - 120 DIAS	HC da Universidade Federal de Minas Gerais - MG
4	LICENÇA MATERNIDADE - 120 DIAS	HC da Universidade Federal de Minas Gerais - MG
4	LICENÇA MATERNIDADE - 120 DIAS	HC da Universidade Federal de Minas Gerais - MG
4	LICENÇA MATERNIDADE - 120 DIAS	HC da Universidade Federal de Minas Gerais - MG
4	LICENÇA MATERNIDADE - 120 DIAS	HC da Universidade Federal de Minas Gerais - MG
4	LICENÇA MATERNIDADE - 120 DIAS	HC da Universidade Federal de Minas Gerais - MG
4	LICENÇA MATERNIDADE - 120 DIAS	HC da Universidade Federal de Minas Gerais - MG
4	LICENÇA MATERNIDADE - 120 DIAS	HC da Universidade Federal de Minas Gerais - MG
4	LICENÇA MATERNIDADE - 120 DIAS	HC da Universidade Federal de Minas Gerais - MG
4	LICENÇA MATERNIDADE - 120 DIAS	HC da Universidade Federal de Minas Gerais - MG
4	LICENÇA MATERNIDADE - 120 DIAS	HC da Universidade Federal de Minas Gerais - MG
4	LICENÇA MATERNIDADE - 120 DIAS	HC da Universidade Federal de Minas Gerais - MG
4	LICENÇA MATERNIDADE - 120 DIAS	HC da Universidade Federal de Minas Gerais - MG
4	LICENÇA MATERNIDADE - 120 DIAS	HC da Universidade Federal de Minas Gerais - MG
5	LICENÇA POR MORTE DE FAMILIAR - CLT E REGULAMENTO DE PESSOAL	HC da Universidade Federal de Minas Gerais - MG
5	LICENÇA POR MORTE DE FAMILIAR - CLT E REGULAMENTO DE PESSOAL	HC da Universidade Federal de Minas Gerais - MG
5	LICENÇA POR MORTE DE FAMILIAR - CLT E REGULAMENTO DE PESSOAL	HC da Universidade Federal de Minas Gerais - MG
5	LICENÇA POR MORTE DE FAMILIAR - CLT E REGULAMENTO DE PESSOAL	HC da Universidade Federal de Minas Gerais - MG
5	LICENÇA POR MORTE DE FAMILIAR - CLT E REGULAMENTO DE PESSOAL	HC da Universidade Federal de Minas Gerais - MG
5	LICENÇA POR MORTE DE FAMILIAR - CLT E REGULAMENTO DE PESSOAL	HC da Universidade Federal de Minas Gerais - MG
5	LICENÇA POR MORTE DE FAMILIAR - CLT E REGULAMENTO DE PESSOAL	HC da Universidade Federal de Minas Gerais - MG
5	LICENÇA POR MORTE DE FAMILIAR - CLT E REGULAMENTO DE PESSOAL	HC da Universidade Federal de Minas Gerais - MG
5	LICENÇA POR MORTE DE FAMILIAR - CLT E REGULAMENTO DE PESSOAL	HC da Universidade Federal de Minas Gerais - MG
5	LICENÇA POR MORTE DE FAMILIAR - CLT E REGULAMENTO DE PESSOAL	HC da Universidade Federal de Minas Gerais - MG
5	LICENÇA POR MORTE DE FAMILIAR - CLT E REGULAMENTO DE PESSOAL	HC da Universidade Federal de Minas Gerais - MG
5	LICENÇA POR MORTE DE FAMILIAR - CLT E REGULAMENTO DE PESSOAL	HC da Universidade Federal de Minas Gerais - MG
5	LICENÇA POR MORTE DE FAMILIAR - CLT E REGULAMENTO DE PESSOAL	HC da Universidade Federal de Minas Gerais - MG
5	LICENÇA POR MORTE DE FAMILIAR - CLT E REGULAMENTO DE PESSOAL	HC da Universidade Federal de Minas Gerais - MG
5	LICENÇA POR MORTE DE FAMILIAR - CLT E REGULAMENTO DE PESSOAL	HC da Universidade Federal de Minas Gerais - MG

5	LICENÇA POR MORTE DE FAMILIAR - CLT E REGULAMENTO DE PESSOAL	HC da Universidade Federal de Minas Gerais - MG
5	LICENÇA POR MORTE DE FAMILIAR - CLT E REGULAMENTO DE PESSOAL	HC da Universidade Federal de Minas Gerais - MG
5	LICENÇA POR MORTE DE FAMILIAR - CLT E REGULAMENTO DE PESSOAL	HC da Universidade Federal de Minas Gerais - MG
5	LICENÇA POR MORTE DE FAMILIAR - CLT E REGULAMENTO DE PESSOAL	HC da Universidade Federal de Minas Gerais - MG
5	LICENÇA POR MORTE DE FAMILIAR - CLT E REGULAMENTO DE PESSOAL	HC da Universidade Federal de Minas Gerais - MG
5	LICENÇA POR MORTE DE FAMILIAR - CLT E REGULAMENTO DE PESSOAL	HC da Universidade Federal de Minas Gerais - MG
5	LICENÇA POR MORTE DE FAMILIAR - CLT E REGULAMENTO DE PESSOAL	HC da Universidade Federal de Minas Gerais - MG
5	LICENÇA POR MORTE DE FAMILIAR - CLT E REGULAMENTO DE PESSOAL	HC da Universidade Federal de Minas Gerais - MG
5	LICENÇA POR MORTE DE FAMILIAR - CLT E REGULAMENTO DE PESSOAL	HC da Universidade Federal de Minas Gerais - MG
5	LICENÇA POR MORTE DE FAMILIAR - CLT E REGULAMENTO DE PESSOAL	HC da Universidade Federal de Minas Gerais - MG
5	LICENÇA POR MORTE DE FAMILIAR - CLT E REGULAMENTO DE PESSOAL	HC da Universidade Federal de Minas Gerais - MG
5	LICENÇA POR MORTE DE FAMILIAR - CLT E REGULAMENTO DE PESSOAL	HC da Universidade Federal de Minas Gerais - MG
5	LICENÇA POR MORTE DE FAMILIAR - CLT E REGULAMENTO DE PESSOAL	HC da Universidade Federal de Minas Gerais - MG
5	LICENÇA POR MORTE DE FAMILIAR - CLT E REGULAMENTO DE PESSOAL	HC da Universidade Federal de Minas Gerais - MG
5	LICENÇA POR MORTE DE FAMILIAR - CLT E REGULAMENTO DE PESSOAL	HC da Universidade Federal de Minas Gerais - MG
5	LICENÇA POR MORTE DE FAMILIAR - CLT E REGULAMENTO DE PESSOAL	HC da Universidade Federal de Minas Gerais - MG
5	LICENÇA POR MORTE DE FAMILIAR - CLT E REGULAMENTO DE PESSOAL	HC da Universidade Federal de Minas Gerais - MG
5	LICENÇA POR MORTE DE FAMILIAR - CLT E REGULAMENTO DE PESSOAL	HC da Universidade Federal de Minas Gerais - MG
5	LICENÇA POR MORTE DE FAMILIAR - CLT E REGULAMENTO DE PESSOAL	HC da Universidade Federal de Minas Gerais - MG
5	LICENÇA POR MORTE DE FAMILIAR - CLT E REGULAMENTO DE PESSOAL	HC da Universidade Federal de Minas Gerais - MG
5	LICENÇA POR MORTE DE FAMILIAR - CLT E REGULAMENTO DE PESSOAL	HC da Universidade Federal de Minas Gerais - MG
5	LICENÇA POR MORTE DE FAMILIAR - CLT E REGULAMENTO DE PESSOAL	HC da Universidade Federal de Minas Gerais - MG
5	LICENÇA POR MORTE DE FAMILIAR - CLT E REGULAMENTO DE PESSOAL	HC da Universidade Federal de Minas Gerais - MG
5	LICENÇA POR MORTE DE FAMILIAR - CLT E REGULAMENTO DE PESSOAL	HC da Universidade Federal de Minas Gerais - MG
5	LICENÇA POR MORTE DE FAMILIAR - CLT E REGULAMENTO DE PESSOAL	HC da Universidade Federal de Minas Gerais - MG
5	LICENÇA POR MORTE DE FAMILIAR - CLT E REGULAMENTO DE PESSOAL	HC da Universidade Federal de Minas Gerais - MG
5	LICENÇA POR MORTE DE FAMILIAR - CLT E REGULAMENTO DE PESSOAL	HC da Universidade Federal de Minas Gerais - MG
5	LICENÇA POR MORTE DE FAMILIAR - CLT E REGULAMENTO DE PESSOAL	HC da Universidade Federal de Minas Gerais - MG
5	LICENÇA POR MORTE DE FAMILIAR - CLT E REGULAMENTO DE PESSOAL	HC da Universidade Federal de Minas Gerais - MG
7	ALISTAMENTO ELEITORAL - CLT ART 473 V	HC da Universidade Federal de Minas Gerais - MG
7	ALISTAMENTO ELEITORAL - CLT ART 473 V	HC da Universidade Federal de Minas Gerais - MG
7	ALISTAMENTO ELEITORAL - CLT ART 473 V	HC da Universidade Federal de Minas Gerais - MG
7	ALISTAMENTO ELEITORAL - CLT ART 473 V	HC da Universidade Federal de Minas Gerais - MG

7	ALISTAMENTO ELEITORAL - CLT ART 473 V	HC da Universidade Federal de Minas Gerais - MG
7	ALISTAMENTO ELEITORAL - CLT ART 473 V	HC da Universidade Federal de Minas Gerais - MG
7	ALISTAMENTO ELEITORAL - CLT ART 473 V	HC da Universidade Federal de Minas Gerais - MG
7	ALISTAMENTO ELEITORAL - CLT ART 473 V	HC da Universidade Federal de Minas Gerais - MG
7	ALISTAMENTO ELEITORAL - CLT ART 473 V	HC da Universidade Federal de Minas Gerais - MG
7	ALISTAMENTO ELEITORAL - CLT ART 473 V	HC da Universidade Federal de Minas Gerais - MG
7	ALISTAMENTO ELEITORAL - CLT ART 473 V	HC da Universidade Federal de Minas Gerais - MG
7	ALISTAMENTO ELEITORAL - CLT ART 473 V	HC da Universidade Federal de Minas Gerais - MG
7	ALISTAMENTO ELEITORAL - CLT ART 473 V	HC da Universidade Federal de Minas Gerais - MG
7	ALISTAMENTO ELEITORAL - CLT ART 473 V	HC da Universidade Federal de Minas Gerais - MG
7	ALISTAMENTO ELEITORAL - CLT ART 473 V	HC da Universidade Federal de Minas Gerais - MG
7	ALISTAMENTO ELEITORAL - CLT ART 473 V	HC da Universidade Federal de Minas Gerais - MG
7	ALISTAMENTO ELEITORAL - CLT ART 473 V	HC da Universidade Federal de Minas Gerais - MG
7	ALISTAMENTO ELEITORAL - CLT ART 473 V	HC da Universidade Federal de Minas Gerais - MG
7	ALISTAMENTO ELEITORAL - CLT ART 473 V	HC da Universidade Federal de Minas Gerais - MG
7	ALISTAMENTO ELEITORAL - CLT ART 473 V	HC da Universidade Federal de Minas Gerais - MG
7	ALISTAMENTO ELEITORAL - CLT ART 473 V	HC da Universidade Federal de Minas Gerais - MG
7	ALISTAMENTO ELEITORAL - CLT ART 473 V	HC da Universidade Federal de Minas Gerais - MG
7	ALISTAMENTO ELEITORAL - CLT ART 473 V	HC da Universidade Federal de Minas Gerais - MG
7	ALISTAMENTO ELEITORAL - CLT ART 473 V	HC da Universidade Federal de Minas Gerais - MG
7	ALISTAMENTO ELEITORAL - CLT ART 473 V	HC da Universidade Federal de Minas Gerais - MG
7	ALISTAMENTO ELEITORAL - CLT ART 473 V	HC da Universidade Federal de Minas Gerais - MG
7	ALISTAMENTO ELEITORAL - CLT ART 473 V	HC da Universidade Federal de Minas Gerais - MG
12	LICENÇA MATERNIDADE (COMPLEMENTAR 60 DIAS)	HC da Universidade Federal de Minas Gerais - MG
12	LICENÇA MATERNIDADE (COMPLEMENTAR 60 DIAS)	HC da Universidade Federal de Minas Gerais - MG
12	LICENÇA MATERNIDADE (COMPLEMENTAR 60 DIAS)	HC da Universidade Federal de Minas Gerais - MG
12	LICENÇA MATERNIDADE (COMPLEMENTAR 60 DIAS)	HC da Universidade Federal de Minas Gerais - MG
12	LICENÇA MATERNIDADE (COMPLEMENTAR 60 DIAS)	HC da Universidade Federal de Minas Gerais - MG
12	LICENÇA MATERNIDADE (COMPLEMENTAR 60 DIAS)	HC da Universidade Federal de Minas Gerais - MG
12	LICENÇA MATERNIDADE (COMPLEMENTAR 60 DIAS)	HC da Universidade Federal de Minas Gerais - MG
12	LICENÇA MATERNIDADE (COMPLEMENTAR 60 DIAS)	HC da Universidade Federal de Minas Gerais - MG
12	LICENÇA MATERNIDADE (COMPLEMENTAR 60 DIAS)	HC da Universidade Federal de Minas Gerais - MG
12	LICENÇA MATERNIDADE (COMPLEMENTAR 60 DIAS)	HC da Universidade Federal de Minas Gerais - MG
12	LICENÇA MATERNIDADE (COMPLEMENTAR 60 DIAS)	HC da Universidade Federal de Minas Gerais - MG
12	LICENÇA MATERNIDADE (COMPLEMENTAR 60 DIAS)	HC da Universidade Federal de Minas Gerais - MG
12	LICENÇA MATERNIDADE (COMPLEMENTAR 60 DIAS)	HC da Universidade Federal de Minas Gerais - MG
12	LICENÇA MATERNIDADE (COMPLEMENTAR 60 DIAS)	HC da Universidade Federal de Minas Gerais - MG
12	LICENÇA MATERNIDADE (COMPLEMENTAR 60 DIAS)	HC da Universidade Federal de Minas Gerais - MG
12	LICENÇA MATERNIDADE (COMPLEMENTAR 60 DIAS)	HC da Universidade Federal de Minas Gerais - MG
12	LICENÇA MATERNIDADE (COMPLEMENTAR 60 DIAS)	HC da Universidade Federal de Minas Gerais - MG
12	LICENÇA MATERNIDADE (COMPLEMENTAR 60 DIAS)	HC da Universidade Federal de Minas Gerais - MG
12	LICENÇA MATERNIDADE (COMPLEMENTAR 60 DIAS)	HC da Universidade Federal de Minas Gerais - MG
12	LICENÇA MATERNIDADE (COMPLEMENTAR 60 DIAS)	HC da Universidade Federal de Minas Gerais - MG
12	LICENÇA MATERNIDADE (COMPLEMENTAR 60 DIAS)	HC da Universidade Federal de Minas Gerais - MG

12	LICENÇA MATERNIDADE (COMPLEMENTAR 60 DIAS)	HC da Universidade Federal de Minas Gerais - MG
12	LICENÇA MATERNIDADE (COMPLEMENTAR 60 DIAS)	HC da Universidade Federal de Minas Gerais - MG
12	LICENÇA MATERNIDADE (COMPLEMENTAR 60 DIAS)	HC da Universidade Federal de Minas Gerais - MG
12	LICENÇA MATERNIDADE (COMPLEMENTAR 60 DIAS)	HC da Universidade Federal de Minas Gerais - MG
12	LICENÇA MATERNIDADE (COMPLEMENTAR 60 DIAS)	HC da Universidade Federal de Minas Gerais - MG
12	LICENÇA MATERNIDADE (COMPLEMENTAR 60 DIAS)	HC da Universidade Federal de Minas Gerais - MG
12	LICENÇA MATERNIDADE (COMPLEMENTAR 60 DIAS)	HC da Universidade Federal de Minas Gerais - MG
12	LICENÇA MATERNIDADE (COMPLEMENTAR 60 DIAS)	HC da Universidade Federal de Minas Gerais - MG
12	LICENÇA MATERNIDADE (COMPLEMENTAR 60 DIAS)	HC da Universidade Federal de Minas Gerais - MG
12	LICENÇA MATERNIDADE (COMPLEMENTAR 60 DIAS)	HC da Universidade Federal de Minas Gerais - MG
12	LICENÇA MATERNIDADE (COMPLEMENTAR 60 DIAS)	HC da Universidade Federal de Minas Gerais - MG
12	LICENÇA MATERNIDADE (COMPLEMENTAR 60 DIAS)	HC da Universidade Federal de Minas Gerais - MG
12	LICENÇA MATERNIDADE (COMPLEMENTAR 60 DIAS)	HC da Universidade Federal de Minas Gerais - MG
12	LICENÇA MATERNIDADE (COMPLEMENTAR 60 DIAS)	HC da Universidade Federal de Minas Gerais - MG
12	LICENÇA MATERNIDADE (COMPLEMENTAR 60 DIAS)	HC da Universidade Federal de Minas Gerais - MG
12	LICENÇA MATERNIDADE (COMPLEMENTAR 60 DIAS)	HC da Universidade Federal de Minas Gerais - MG
12	LICENÇA MATERNIDADE (COMPLEMENTAR 60 DIAS)	HC da Universidade Federal de Minas Gerais - MG
12	LICENÇA MATERNIDADE (COMPLEMENTAR 60 DIAS)	HC da Universidade Federal de Minas Gerais - MG
12	LICENÇA MATERNIDADE (COMPLEMENTAR 60 DIAS)	HC da Universidade Federal de Minas Gerais - MG
12	LICENÇA MATERNIDADE (COMPLEMENTAR 60 DIAS)	HC da Universidade Federal de Minas Gerais - MG
12	LICENÇA MATERNIDADE (COMPLEMENTAR 60 DIAS)	HC da Universidade Federal de Minas Gerais - MG
12	LICENÇA MATERNIDADE (COMPLEMENTAR 60 DIAS)	HC da Universidade Federal de Minas Gerais - MG
12	LICENÇA MATERNIDADE (COMPLEMENTAR 60 DIAS)	HC da Universidade Federal de Minas Gerais - MG
12	LICENÇA MATERNIDADE (COMPLEMENTAR 60 DIAS)	HC da Universidade Federal de Minas Gerais - MG
12	LICENÇA MATERNIDADE (COMPLEMENTAR 60 DIAS)	HC da Universidade Federal de Minas Gerais - MG
12	LICENÇA MATERNIDADE (COMPLEMENTAR 60 DIAS)	HC da Universidade Federal de Minas Gerais - MG
12	LICENÇA MATERNIDADE (COMPLEMENTAR 60 DIAS)	HC da Universidade Federal de Minas Gerais - MG
12	LICENÇA MATERNIDADE (COMPLEMENTAR 60 DIAS)	HC da Universidade Federal de Minas Gerais - MG
12	LICENÇA MATERNIDADE (COMPLEMENTAR 60 DIAS)	HC da Universidade Federal de Minas Gerais - MG
12	LICENÇA MATERNIDADE (COMPLEMENTAR 60 DIAS)	HC da Universidade Federal de Minas Gerais - MG
12	LICENÇA MATERNIDADE (COMPLEMENTAR 60 DIAS)	HC da Universidade Federal de Minas Gerais - MG
12	LICENÇA MATERNIDADE (COMPLEMENTAR 60 DIAS)	HC da Universidade Federal de Minas Gerais - MG
12	LICENÇA MATERNIDADE (COMPLEMENTAR 60 DIAS)	HC da Universidade Federal de Minas Gerais - MG
12	LICENÇA MATERNIDADE (COMPLEMENTAR 60 DIAS)	HC da Universidade Federal de Minas Gerais - MG
12	LICENÇA MATERNIDADE (COMPLEMENTAR 60 DIAS)	HC da Universidade Federal de Minas Gerais - MG
12	LICENÇA MATERNIDADE (COMPLEMENTAR 60 DIAS)	HC da Universidade Federal de Minas Gerais - MG
12	LICENÇA MATERNIDADE (COMPLEMENTAR 60 DIAS)	HC da Universidade Federal de Minas Gerais - MG
12	LICENÇA MATERNIDADE (COMPLEMENTAR 60 DIAS)	HC da Universidade Federal de Minas Gerais - MG

12	LICENÇA MATERNIDADE (COMPLEMENTAR 60 DIAS)	HC da Universidade Federal de Minas Gerais - MG
12	LICENÇA MATERNIDADE (COMPLEMENTAR 60 DIAS)	HC da Universidade Federal de Minas Gerais - MG
12	LICENÇA MATERNIDADE (COMPLEMENTAR 60 DIAS)	HC da Universidade Federal de Minas Gerais - MG
12	LICENÇA MATERNIDADE (COMPLEMENTAR 60 DIAS)	HC da Universidade Federal de Minas Gerais - MG
12	LICENÇA MATERNIDADE (COMPLEMENTAR 60 DIAS)	HC da Universidade Federal de Minas Gerais - MG
[truncated: 602,622 more chars]
