# Supplementary material for: Impact of COVID-19 on the mental health of public university hospital workers in Brazil: A cohort-based analysis of 32,691 workers
Source: PLoS One. 2022 Jun 16;17(6):e0269318. doi: 10.1371/journal.pone.0269318 (PMC9202958; doi:10.1371/journal.pone.0269318)
Supplement: S2 Data — (ZIP) [file Raw study data2019 .pdf › Raw study data2019 .pdf]

	Sexo	Tipo Servidor
	F	Empregado Efetivo
	F	Empregado Efetivo
	F	Empregado Efetivo
	F	Empregado Efetivo
	F	Empregado Efetivo
	F	Empregado Efetivo
	F	Empregado Efetivo
	F	Empregado Efetivo
	F	Empregado Efetivo
	F	Empregado Efetivo
	F	Empregado Efetivo
	F	Empregado Efetivo
	F	Empregado Efetivo
	F	Empregado Efetivo
	F	Empregado Efetivo
	F	Empregado Efetivo
	F	Empregado Efetivo
	F	Empregado Efetivo
	F	Empregado Efetivo
	F	Empregado Efetivo
	F	Empregado Efetivo
	F	Empregado Efetivo
	F	Empregado Efetivo
	F	Empregado Efetivo
	F	Empregado Efetivo
	F	Empregado Efetivo
	F	Empregado Efetivo
	F	Empregado Efetivo
	F	Empregado Efetivo
	F	Empregado Efetivo
	F	Empregado Efetivo
	F	Empregado Efetivo
	F	Empregado Efetivo
	F	Empregado Efetivo
	F	Empregado Efetivo
	F	Empregado Efetivo
	F	Empregado Efetivo
	F	Empregado Efetivo
	F	Empregado Efetivo
	F	Empregado Efetivo
	F	Empregado Efetivo
	F	Empregado Efetivo
	F	Empregado Efetivo
	F	Empregado Efetivo
	F	Empregado Efetivo
	F	Empregado Efetivo

	F	Empregado Efetivo
	F	Empregado Efetivo
	F	Empregado Efetivo
	F	Empregado Efetivo
	F	Empregado Efetivo
	F	Empregado Efetivo
	F	Empregado Efetivo
	F	Empregado Efetivo
	F	Empregado Efetivo
	F	Empregado Efetivo
	F	Empregado Efetivo
	F	Empregado Efetivo
	F	Empregado Efetivo
	F	Empregado Efetivo
	F	Empregado Cedido
	F	Empregado Efetivo
	F	Empregado Efetivo
	F	Empregado Efetivo
	F	Empregado Efetivo
	F	Empregado Efetivo
	F	Empregado Efetivo
	F	Empregado Efetivo
	F	Empregado Efetivo
	F	Empregado Efetivo
	F	Empregado Efetivo
	F	Empregado Efetivo
	F	Empregado Efetivo
	F	Empregado Efetivo
	F	Empregado Efetivo
	F	Empregado Efetivo
	F	Empregado Efetivo
	F	Empregado Efetivo
	F	Empregado Efetivo
	F	Empregado Efetivo
	F	Empregado Efetivo
	F	Empregado Efetivo
	F	Empregado Efetivo
	F	Empregado Efetivo
	F	Empregado Efetivo
	F	Empregado Efetivo
	F	Empregado Efetivo
	F	Empregado Efetivo
	F	Empregado Efetivo
	F	Empregado Efetivo

	F	Empregado Efetivo
	F	Empregado Efetivo
	F	Empregado Efetivo
	F	Empregado Efetivo
	F	Empregado Efetivo
	F	Empregado Efetivo
	F	Empregado Efetivo
	F	Empregado Efetivo
	M	Empregado Efetivo
	M	Empregado Efetivo
	M	Empregado Efetivo
	F	Empregado Efetivo
	F	Empregado Efetivo
	F	Empregado Efetivo
	M	Empregado Efetivo
	F	Empregado Efetivo
	F	Empregado Efetivo
	F	Empregado Efetivo
	F	Empregado Efetivo
	F	Empregado Efetivo
	F	Empregado Efetivo
	F	Empregado Efetivo
	F	Empregado Efetivo
	F	Empregado Efetivo
	F	Empregado Efetivo
	F	Empregado Efetivo
	F	Empregado Efetivo
	F	Empregado Efetivo
	F	Empregado Efetivo
	F	Empregado Efetivo
	M	Empregado Efetivo
	M	Empregado Efetivo

	F	Empregado Efetivo
	M	Empregado Efetivo
	M	Empregado Efetivo
	F	Empregado Efetivo
	F	Empregado Efetivo
	F	Empregado Efetivo
	F	Empregado Efetivo
	F	Empregado Efetivo
	F	Empregado Efetivo
	F	Empregado Efetivo
	F	Empregado Efetivo
	F	Empregado Efetivo
	F	Empregado Efetivo
	F	Empregado Efetivo
	M	Empregado Efetivo
	F	Empregado Efetivo
	F	Empregado Efetivo
	F	Empregado Efetivo
	F	Empregado Efetivo
	F	Empregado Efetivo
	F	Empregado Efetivo
	F	Empregado Efetivo
	F	Empregado Efetivo
	M	Empregado Efetivo
	M	Empregado Efetivo
	M	Empregado Efetivo
	M	Empregado Efetivo
	F	Empregado Efetivo
	F	Empregado Efetivo
	M	Empregado Efetivo
	F	Empregado Efetivo
	F	Empregado Efetivo

	M	Empregado Efetivo
	M	Empregado Efetivo
	F	Empregado Efetivo
	F	Empregado Efetivo
	M	Empregado Temporário
	F	Empregado Efetivo
	F	Empregado Efetivo
	F	Empregado Efetivo
	F	Empregado Efetivo
	F	Empregado Efetivo
	F	Empregado Efetivo
	F	Empregado Efetivo
	F	Empregado Efetivo
	F	Empregado Efetivo
	M	Empregado Efetivo
	M	Empregado Efetivo
	F	Empregado Efetivo
	F	Empregado Efetivo
	F	Empregado Efetivo
	M	Empregado Efetivo
	M	Empregado Efetivo
	M	Empregado Efetivo
	F	Empregado Efetivo
	F	Empregado Efetivo
	F	Empregado Efetivo
	F	Empregado Efetivo
	F	Empregado Efetivo
	F	Empregado Efetivo
	F	Empregado Efetivo
	F	Empregado Efetivo
	F	Empregado Efetivo
	F	Empregado Efetivo
	F	Empregado Efetivo
	F	Empregado Efetivo
	F	Empregado Efetivo
	F	Empregado Efetivo

	F	Empregado Efetivo
	F	Empregado Efetivo
	F	Empregado Efetivo
	F	Empregado Efetivo
	F	Empregado Efetivo
	F	Empregado Efetivo
	F	Empregado Efetivo
	F	Empregado Efetivo
	F	Empregado Efetivo
	F	Empregado Efetivo
	F	Empregado Efetivo
	F	Empregado Efetivo
	F	Empregado Efetivo
	F	Empregado Efetivo
	F	Empregado Efetivo
	F	Empregado Efetivo
	F	Empregado Efetivo
	F	Empregado Efetivo
	F	Empregado Efetivo
	F	Empregado Efetivo
	F	Empregado Efetivo
	F	Empregado Efetivo
	F	Empregado Efetivo
	F	Empregado Efetivo
	F	Empregado Efetivo
	F	Empregado Efetivo
	F	Empregado Efetivo
	F	Empregado Efetivo
	F	Empregado Efetivo
	F	Empregado Efetivo
	F	Empregado Efetivo
	F	Empregado Efetivo
	F	Empregado Efetivo
	F	Empregado Efetivo
	F	Empregado Efetivo
	F	Empregado Efetivo
	F	Empregado Efetivo
	F	Empregado Efetivo
	F	Empregado Efetivo
	F	Empregado Cedido
	F	Empregado Efetivo
	F	Empregado Efetivo
	F	Empregado Efetivo
	F	Empregado Efetivo
	F	Empregado Efetivo
	F	Empregado Efetivo
	F	Empregado Efetivo
	F	Empregado Efetivo

	F	Empregado Efetivo
	F	Empregado Efetivo
	F	Empregado Efetivo
	F	Empregado Efetivo
	F	Empregado Efetivo
	F	Empregado Efetivo
	F	Empregado Efetivo
	F	Empregado Efetivo
	F	Empregado Efetivo
	F	Empregado Efetivo
	F	Empregado Efetivo
	F	Empregado Efetivo
	F	Empregado Efetivo
	F	Empregado Efetivo
	F	Empregado Efetivo
	F	Empregado Efetivo
	F	Empregado Efetivo
	F	Empregado Efetivo
	F	Empregado Efetivo
	F	Empregado Efetivo
	M	Empregado Efetivo
	F	Empregado Efetivo
	F	Empregado Efetivo
	F	Empregado Efetivo
	F	Empregado Efetivo
	M	Empregado Efetivo
	F	Empregado Efetivo
	M	Empregado Efetivo
	M	Empregado Efetivo
	F	Empregado Efetivo
	F	Empregado Efetivo
	F	Empregado Efetivo
	M	Empregado Efetivo
	F	Empregado Efetivo
	F	Empregado Efetivo
	F	Empregado Efetivo
	F	Empregado Efetivo
	F	Empregado Efetivo
	F	Empregado Efetivo
	F	Empregado Efetivo
	F	Empregado Efetivo

	F	Empregado Efetivo
	F	Empregado Efetivo
	F	Empregado Efetivo
	F	Empregado Efetivo
	M	Empregado Efetivo
	F	Empregado Efetivo
	F	Empregado Efetivo
	F	Empregado Efetivo
	F	Empregado Efetivo
	F	Empregado Efetivo
	F	Empregado Efetivo
	F	Empregado Efetivo
	F	Empregado Efetivo
	F	Empregado Efetivo
	F	Empregado Efetivo
	F	Empregado Efetivo
	F	Empregado Efetivo
	F	Empregado Efetivo
	F	Empregado Efetivo
	F	Empregado Efetivo
	F	Empregado Efetivo
	F	Empregado Efetivo
	F	Empregado Efetivo
	F	Empregado Efetivo
	F	Empregado Efetivo
	F	Empregado Efetivo
	F	Empregado Efetivo
	F	Empregado Efetivo
	F	Empregado Efetivo
	M	Empregado Efetivo
	M	Empregado Efetivo
	F	Empregado Efetivo

	M	Empregado Efetivo
	F	Cedido - RJU
	F	Cedido - RJU
	F	Cedido - RJU
	F	Cedido - RJU
	F	Cedido - RJU
	F	Cedido - RJU
	F	Empregado Efetivo
	F	Empregado Efetivo
	F	Empregado Efetivo
	F	Empregado Efetivo
	F	Empregado Efetivo
	F	Empregado Efetivo
	F	Empregado Efetivo
	F	Empregado Efetivo
	F	Empregado Efetivo
	F	Empregado Efetivo
	F	Empregado Efetivo
	F	Empregado Efetivo
	F	Empregado Efetivo
	F	Empregado Efetivo
	F	Empregado Efetivo
	F	Empregado Efetivo
	F	Empregado Efetivo
	F	Empregado Efetivo
	F	Empregado Efetivo
	F	Empregado Efetivo
	F	Empregado Efetivo
	M	Empregado Efetivo
	F	Empregado Efetivo
	F	Empregado Efetivo
	F	Empregado Efetivo

	F	Empregado Efetivo
	F	Empregado Efetivo
	F	Empregado Efetivo
	F	Empregado Efetivo
	F	Empregado Efetivo
	F	Empregado Efetivo
	F	Empregado Efetivo
	F	Empregado Efetivo
	F	Empregado Efetivo
	F	Empregado Efetivo
	F	Empregado Efetivo
	F	Empregado Efetivo
	F	Empregado Efetivo
	F	Empregado Efetivo
	F	Empregado Efetivo
	F	Empregado Efetivo
	F	Empregado Efetivo
	F	Empregado Efetivo
	F	Empregado Efetivo
	F	Empregado Efetivo
	F	Empregado Efetivo
	F	Empregado Efetivo
	F	Empregado Efetivo
	F	Empregado Efetivo
	F	Empregado Efetivo
	F	Empregado Efetivo
	F	Empregado Efetivo
	F	Empregado Efetivo
	F	Empregado Efetivo
	F	Empregado Efetivo
	F	Empregado Efetivo
	F	Empregado Efetivo

	F	Empregado Efetivo
	F	Empregado Efetivo
	F	Empregado Efetivo
	F	Empregado Efetivo
	F	Empregado Efetivo
	F	Empregado Efetivo
	F	Empregado Efetivo
	F	Empregado Efetivo
	F	Empregado Efetivo
	F	Empregado Efetivo
	F	Empregado Efetivo
	F	Empregado Efetivo
	F	Empregado Efetivo
	F	Empregado Efetivo
	F	Empregado Efetivo
	F	Empregado Efetivo
	F	Empregado Efetivo
	F	Empregado Efetivo
	F	Empregado Efetivo
	F	Empregado Efetivo
	F	Empregado Efetivo
	F	Empregado Efetivo
	F	Empregado Efetivo
	F	Empregado Efetivo
	F	Empregado Efetivo
	F	Empregado Efetivo
	F	Empregado Efetivo
	F	Empregado Efetivo
	F	Empregado Efetivo
	F	Empregado Efetivo
	F	Empregado Efetivo
	F	Empregado Efetivo

	F	Empregado Efetivo
	F	Empregado Efetivo
	F	Empregado Efetivo
	F	Empregado Efetivo
	F	Empregado Efetivo
	F	Empregado Efetivo
	F	Empregado Efetivo
	F	Empregado Efetivo
	F	Empregado Efetivo
	F	Empregado Efetivo
	F	Empregado Efetivo
	F	Empregado Efetivo
	F	Cedido - RJU
	F	Empregado Efetivo
	F	Empregado Efetivo
	F	Empregado Efetivo
	F	Empregado Efetivo
	M	Empregado Efetivo
	F	Empregado Efetivo
	F	Empregado Efetivo
	F	Empregado Efetivo
	F	Empregado Efetivo
	F	Empregado Efetivo
	F	Empregado Efetivo
	F	Empregado Efetivo
	F	Empregado Efetivo
	M	Empregado Efetivo
	F	Empregado Efetivo
	F	Empregado Efetivo
	F	Empregado Efetivo
	F	Empregado Efetivo
	F	Empregado Efetivo

	F	Empregado Efetivo
	F	Empregado Efetivo
	F	Empregado Efetivo
	F	Empregado Efetivo
	M	Empregado Efetivo
	M	Empregado Efetivo
	M	Empregado Efetivo
	F	Empregado Efetivo
	F	Empregado Efetivo
	F	Empregado Efetivo
	M	Empregado Efetivo
	M	Empregado Efetivo
	F	Empregado Efetivo
	F	Empregado Efetivo
	M	Empregado Efetivo
	M	Empregado Efetivo
	M	Empregado Efetivo
	M	Empregado Efetivo
	F	Empregado Efetivo
	F	Empregado Efetivo
	F	Empregado Efetivo
	F	Empregado Efetivo
	F	Empregado Efetivo
	F	Empregado Efetivo
	F	Empregado Efetivo
	F	Empregado Efetivo
	F	Empregado Efetivo
	F	Empregado Efetivo
	F	Empregado Efetivo
	M	Empregado Efetivo
	M	Empregado Efetivo
	M	Empregado Efetivo

	F	Empregado Efetivo
	M	Empregado Efetivo
	F	Empregado Efetivo
	F	Empregado Efetivo
	M	Empregado Efetivo
	F	Empregado Efetivo
	F	Empregado Efetivo
	F	Empregado Efetivo
	M	Empregado Efetivo
	F	Empregado Efetivo
	M	Empregado Efetivo
	F	Empregado Efetivo
	F	Empregado Efetivo
	F	Empregado Efetivo
	F	Empregado Efetivo
	F	Empregado Efetivo
	F	Empregado Efetivo
	F	Empregado Efetivo
	M	Empregado Efetivo
	M	Empregado Efetivo
	F	Empregado Efetivo
	F	Empregado Efetivo
	M	Empregado Efetivo
	M	Empregado Efetivo
	M	Empregado Efetivo
	M	Empregado Efetivo
	M	Empregado Efetivo
	M	Empregado Efetivo
	M	Empregado Efetivo
	M	Empregado Efetivo
	M	Empregado Efetivo
	M	Empregado Efetivo

	F	Empregado Efetivo
	F	Empregado Efetivo
	F	Empregado Efetivo
	M	Empregado Efetivo
	F	Empregado Efetivo
	F	Empregado Efetivo
	F	Empregado Efetivo
	F	Empregado Efetivo
	F	Empregado Efetivo
	F	Empregado Efetivo
	F	Empregado Efetivo
	F	Empregado Efetivo
	F	Empregado Efetivo
	F	Empregado Efetivo
	F	Empregado Efetivo
	F	Empregado Efetivo
	F	Empregado Efetivo
	F	Empregado Efetivo
	F	Empregado Efetivo
	F	Empregado Efetivo
	F	Empregado Efetivo
	F	Empregado Efetivo
	F	Empregado Efetivo
	F	Empregado Efetivo
	F	Empregado Efetivo
	F	Empregado Efetivo
	F	Empregado Efetivo
	F	Empregado Efetivo
	F	Empregado Efetivo
	F	Empregado Efetivo
	F	Empregado Efetivo
	F	Empregado Efetivo

	F	Empregado Efetivo
	F	Empregado Efetivo
	F	Empregado Efetivo
	F	Empregado Efetivo
	F	Empregado Efetivo
	F	Empregado Efetivo
	F	Empregado Efetivo
	F	Empregado Efetivo
	F	Empregado Efetivo
	F	Empregado Efetivo
	F	Empregado Efetivo
	F	Empregado Efetivo
	F	Empregado Efetivo
	F	Empregado Efetivo
	F	Empregado Efetivo
	F	Empregado Efetivo
	F	Empregado Efetivo
	F	Empregado Efetivo
	F	Empregado Efetivo
	F	Empregado Efetivo
	F	Empregado Efetivo
	F	Empregado Efetivo
	F	Empregado Efetivo
	F	Empregado Efetivo
	F	Empregado Efetivo
	F	Empregado Efetivo
	F	Empregado Efetivo
	F	Empregado Efetivo
	F	Empregado Efetivo
	F	Empregado Efetivo
	F	Empregado Efetivo
	F	Empregado Efetivo

	F	Empregado Efetivo
	F	Empregado Efetivo
	F	Empregado Efetivo
	F	Empregado Efetivo
	F	Empregado Efetivo
	F	Empregado Efetivo
	F	Empregado Efetivo
	F	Empregado Efetivo
	F	Empregado Efetivo
	F	Empregado Efetivo
	M	Empregado Efetivo
	F	Empregado Efetivo
	M	Empregado Efetivo
	F	Empregado Efetivo
	F	Empregado Efetivo
	F	Empregado Efetivo
	M	Empregado Efetivo
	M	Empregado Efetivo
	M	Empregado Efetivo
	F	Empregado Efetivo
	F	Empregado Efetivo
	F	Empregado Efetivo
	M	Empregado Efetivo
	F	Empregado Efetivo
	F	Empregado Efetivo
	M	Empregado Efetivo
	F	Empregado Efetivo
	M	Empregado Efetivo
	F	Empregado Efetivo
	F	Empregado Efetivo
	F	Empregado Efetivo
	M	Empregado Efetivo

	F	Empregado Efetivo
	F	Empregado Efetivo
	F	Empregado Efetivo
	F	Empregado Efetivo
	F	Empregado Efetivo
	M	Empregado Efetivo
	F	Empregado Efetivo
	F	Empregado Efetivo
	F	Empregado Efetivo
	F	Empregado Efetivo
	F	Empregado Efetivo
	F	Empregado Efetivo
	F	Empregado Efetivo
	F	Empregado Efetivo
	F	Empregado Efetivo
	F	Cedido - RJU
	F	Empregado Efetivo
	F	Empregado Efetivo
	M	Empregado Efetivo
	M	Empregado Efetivo
	M	Empregado Efetivo
	F	Empregado Efetivo
	M	Empregado Efetivo
	F	Empregado Efetivo
	M	Empregado Efetivo
	F	Empregado Efetivo
	M	Empregado Efetivo
	F	Empregado Efetivo
	M	Empregado Efetivo
	M	Empregado Efetivo
	M	Empregado Efetivo
	M	Empregado Efetivo

	F	Empregado Efetivo
	M	Empregado Efetivo
	F	Empregado Efetivo
	M	Empregado Efetivo
	F	Empregado Efetivo
	M	Empregado Efetivo
	F	Empregado Efetivo
	M	Empregado Efetivo
	M	Empregado Efetivo
	M	Empregado Efetivo
	F	Empregado Efetivo
	M	Empregado Efetivo
	M	Empregado Efetivo
	M	Empregado Efetivo
	M	Empregado Efetivo
	M	Empregado Efetivo
	F	Empregado Efetivo
	M	Empregado Efetivo
	M	Empregado Efetivo
	M	Empregado Efetivo
	M	Empregado Efetivo
	F	Empregado Efetivo
	F	Empregado Efetivo
	F	Empregado Efetivo
	F	Empregado Efetivo
	F	Empregado Efetivo
	F	Empregado Efetivo
	F	Empregado Efetivo
	F	Empregado Efetivo
	F	Empregado Efetivo
	F	Empregado Efetivo
	F	Empregado Efetivo

	F	Empregado Efetivo
	F	Empregado Efetivo
	F	Empregado Efetivo
	F	Empregado Efetivo
	F	Empregado Efetivo
	M	Empregado Efetivo
	F	Empregado Efetivo
	F	Empregado Efetivo
	F	Empregado Efetivo
	F	Empregado Efetivo
	F	Empregado Efetivo
	F	Empregado Efetivo
	F	Empregado Efetivo
	F	Empregado Efetivo
	F	Empregado Efetivo
	F	Empregado Efetivo
	F	Empregado Efetivo
	F	Empregado Efetivo
	F	Empregado Efetivo
	F	Empregado Efetivo
	F	Empregado Efetivo
	F	Empregado Efetivo
	F	Empregado Efetivo
	F	Empregado Efetivo
	F	Empregado Efetivo
	F	Empregado Efetivo
	F	Empregado Efetivo
	F	Empregado Efetivo
	F	Empregado Efetivo
	F	Empregado Efetivo
	F	Empregado Efetivo
	F	Empregado Efetivo
	F	Empregado Efetivo
	F	Empregado Efetivo
	F	Empregado Efetivo
	F	Empregado Efetivo
	F	Empregado Efetivo
	F	Empregado Efetivo
	F	Empregado Efetivo
	F	Empregado Efetivo
	F	Empregado Efetivo
	F	Empregado Efetivo
	F	Empregado Efetivo
	M	Empregado Efetivo
	F	Empregado Efetivo

	F	Empregado Efetivo
	F	Empregado Efetivo
	F	Empregado Efetivo
	F	Empregado Efetivo
	F	Empregado Efetivo
	F	Empregado Efetivo
	F	Empregado Efetivo
	F	Empregado Efetivo
	F	Empregado Efetivo
	F	Empregado Efetivo
	F	Empregado Efetivo
	F	Empregado Efetivo
	F	Empregado Efetivo
	F	Empregado Efetivo
	F	Empregado Efetivo
	F	Empregado Efetivo
	M	Empregado Efetivo
	F	Empregado Efetivo
	F	Empregado Efetivo
	F	Empregado Efetivo
	M	Empregado Efetivo
	F	Empregado Efetivo
	F	Empregado Efetivo
	F	Empregado Efetivo
	F	Empregado Efetivo
	F	Empregado Efetivo
	M	Empregado Efetivo
	F	Empregado Efetivo
	F	Empregado Efetivo
	F	Empregado Efetivo
	F	Empregado Efetivo
	F	Empregado Efetivo
	M	Empregado Efetivo
	F	Empregado Efetivo
	F	Empregado Efetivo
	M	Empregado Efetivo
	F	Empregado Efetivo
	M	Empregado Efetivo
	F	Empregado Efetivo
	F	Empregado Efetivo
	F	Empregado Efetivo
	F	Empregado Efetivo
	F	Empregado Efetivo
	F	Empregado Efetivo

	F	Empregado Efetivo
	F	Empregado Efetivo
	M	Empregado Efetivo
	M	Empregado Efetivo
	F	Empregado Efetivo
	M	Empregado Efetivo
	F	Empregado Efetivo
	F	Empregado Efetivo
	F	Empregado Efetivo
	F	Empregado Efetivo
	M	Empregado Efetivo
	F	Empregado Efetivo
	M	Empregado Efetivo
	M	Empregado Efetivo
	M	Empregado Efetivo
	M	Empregado Efetivo
	F	Empregado Efetivo
	M	Empregado Efetivo
	M	Empregado Efetivo
	M	Empregado Efetivo
	M	Empregado Efetivo
	M	Empregado Efetivo
	M	Empregado Efetivo
	M	Empregado Efetivo
	F	Empregado Efetivo
	M	Empregado Efetivo
	M	Empregado Efetivo
	F	Empregado Efetivo
	M	Empregado Efetivo
	M	Empregado Efetivo
	M	Empregado Efetivo
	M	Empregado Efetivo
	F	Empregado Efetivo
	F	Empregado Efetivo
	M	Empregado Efetivo
	F	Empregado Efetivo
	M	Empregado Efetivo
	M	Empregado Efetivo
	M	Empregado Efetivo
	F	Empregado Efetivo
	F	Empregado Efetivo
	F	Empregado Efetivo
	F	Empregado Efetivo

	M	Empregado Efetivo
	M	Empregado Efetivo
	M	Empregado Efetivo
	F	Empregado Efetivo
	M	Empregado Efetivo
	M	Empregado Efetivo
	F	Empregado Efetivo
	F	Empregado Efetivo
	F	Empregado Efetivo
	M	Empregado Efetivo
	F	Empregado Efetivo
	F	Empregado Efetivo
	F	Empregado Efetivo
	F	Empregado Efetivo
	F	Empregado Efetivo
	F	Empregado Efetivo
	M	Empregado Efetivo
	F	Empregado Efetivo
	F	Empregado Efetivo
	F	Empregado Efetivo
	M	Empregado Efetivo
	F	Empregado Efetivo
	F	Empregado Efetivo
	F	Empregado Efetivo
	F	Empregado Efetivo
	F	Empregado Efetivo
	F	Empregado Efetivo
	F	Empregado Efetivo
	F	Empregado Efetivo
	F	Empregado Efetivo
	F	Empregado Efetivo
	F	Empregado Efetivo
	F	Empregado Efetivo
	F	Empregado Efetivo
	F	Empregado Efetivo
	F	Empregado Efetivo
	F	Empregado Efetivo
	F	Empregado Efetivo
	F	Empregado Efetivo
	F	Empregado Efetivo
	F	Empregado Efetivo
	F	Empregado Efetivo
	F	Empregado Efetivo
	F	Empregado Efetivo
	F	Empregado Efetivo
	F	Empregado Efetivo
	F	Empregado Efetivo
	F	Empregado Efetivo

	F	Empregado Efetivo
	F	Empregado Efetivo
	F	Empregado Efetivo
	F	Empregado Efetivo
	F	Empregado Efetivo
	F	Empregado Efetivo
	F	Empregado Efetivo
	F	Empregado Efetivo
	F	Empregado Efetivo
	F	Empregado Efetivo
	F	Empregado Efetivo
	F	Empregado Efetivo
	F	Empregado Efetivo
	F	Empregado Efetivo
	F	Empregado Efetivo
	F	Empregado Efetivo
	F	Empregado Efetivo
	M	Empregado Efetivo
	M	Empregado Efetivo
	M	Empregado Efetivo
	M	Empregado Efetivo
	F	Empregado Efetivo
	M	Empregado Efetivo
	F	Empregado Efetivo
	F	Empregado Efetivo
	F	Empregado Efetivo
	F	Empregado Efetivo
	F	Empregado Efetivo
	F	Empregado Efetivo
	F	Empregado Efetivo
	F	Empregado Efetivo
	F	Empregado Efetivo
	M	Empregado Efetivo
	F	Empregado Efetivo
	F	Empregado Efetivo
	M	Empregado Efetivo
	M	Empregado Efetivo
	M	Empregado Efetivo
	F	Empregado Efetivo
	F	Empregado Efetivo
	M	Empregado Efetivo
	F	Empregado Efetivo
	F	Empregado Efetivo
	M	Empregado Efetivo
	F	Empregado Efetivo
	F	Empregado Efetivo
	F	Empregado Efetivo
	F	Empregado Efetivo

	F	Empregado Efetivo
	F	Empregado Efetivo
	F	Empregado Efetivo
	F	Empregado Efetivo
	M	Empregado Efetivo
	F	Empregado Efetivo
	F	Empregado Efetivo
	F	Empregado Efetivo
	F	Empregado Efetivo
	F	Empregado Efetivo
	F	Empregado Efetivo
	F	Empregado Efetivo
	F	Empregado Efetivo
	F	Empregado Efetivo
	F	Empregado Efetivo
	F	Empregado Efetivo
	F	Empregado Efetivo
	F	Empregado Efetivo
	F	Empregado Efetivo
	M	Empregado Efetivo
	F	Empregado Efetivo
	F	Empregado Efetivo
	F	Empregado Efetivo
	M	Empregado Efetivo
	F	Empregado Efetivo
	F	Empregado Efetivo
	M	Empregado Efetivo
	F	Empregado Efetivo
	M	Empregado Efetivo
	F	Empregado Efetivo
	F	Empregado Efetivo
	F	Empregado Efetivo
	F	Empregado Efetivo
	F	Empregado Efetivo
	F	Empregado Efetivo
	F	Empregado Efetivo
	F	Empregado Efetivo
	M	Empregado Efetivo
	M	Empregado Efetivo
	M	Empregado Efetivo
	M	Empregado Efetivo
	M	Empregado Efetivo
	F	Empregado Efetivo
	M	Empregado Efetivo
	F	Empregado Efetivo
	F	Empregado Efetivo
	F	Empregado Efetivo
	F	Empregado Efetivo

	F	Empregado Efetivo
	F	Empregado Efetivo
	F	Empregado Efetivo
	M	Empregado Efetivo
	M	Empregado Efetivo
	F	Empregado Efetivo
	F	Empregado Efetivo
	F	Empregado Efetivo
	F	Empregado Efetivo
	F	Empregado Efetivo
	M	Empregado Efetivo
	F	Empregado Efetivo
	M	Empregado Efetivo
	M	Empregado Efetivo
	M	Empregado Efetivo
	F	Empregado Efetivo
	F	Empregado Efetivo
	F	Empregado Efetivo
	M	Empregado Efetivo
	F	Empregado Efetivo
	M	Empregado Efetivo
	F	Empregado Efetivo
	M	Empregado Efetivo
	M	Empregado Efetivo
	F	Empregado Efetivo
	F	Empregado Efetivo
	F	Empregado Efetivo
	M	Empregado Efetivo
	M	Empregado Efetivo
	M	Empregado Efetivo
	M	Empregado Efetivo
	M	Empregado Efetivo
	M	Empregado Efetivo
	F	Empregado Efetivo
	M	Empregado Efetivo
	M	Empregado Efetivo
	F	Empregado Efetivo
	M	Empregado Efetivo
	F	Empregado Efetivo
	M	Empregado Efetivo
	F	Empregado Efetivo
	F	Empregado Efetivo
	M	Empregado Efetivo
	M	Empregado Efetivo
	M	Empregado Efetivo

	M	Empregado Efetivo
	M	Empregado Efetivo
	M	Empregado Efetivo
	M	Empregado Efetivo
	M	Empregado Efetivo
	M	Empregado Efetivo
	M	Empregado Efetivo
	M	Empregado Efetivo
	M	Empregado Efetivo
	M	Empregado Efetivo
	M	Empregado Efetivo
	M	Empregado Efetivo
	M	Empregado Efetivo
	M	Empregado Efetivo
	M	Empregado Efetivo
	M	Empregado Efetivo
	M	Empregado Efetivo
	M	Empregado Efetivo
	M	Empregado Efetivo
	M	Empregado Efetivo
	M	Empregado Efetivo
	M	Empregado Efetivo
	M	Empregado Efetivo
	M	Empregado Efetivo
	M	Empregado Efetivo
	M	Empregado Efetivo
	M	Empregado Efetivo
	M	Empregado Efetivo
	M	Empregado Efetivo
	F	Empregado Efetivo
	M	Empregado Efetivo
	M	Empregado Efetivo

	M	Empregado Efetivo
	M	Empregado Efetivo
	M	Empregado Efetivo
	M	Empregado Efetivo
	M	Empregado Efetivo
	M	Empregado Efetivo
	F	Empregado Efetivo
	F	Empregado Efetivo
	M	Empregado Efetivo
	M	Empregado Efetivo
	M	Empregado Efetivo
	F	Empregado Efetivo
	F	Empregado Efetivo
	M	Empregado Efetivo
	M	Empregado Efetivo
	M	Empregado Efetivo
	M	Empregado Efetivo
	M	Empregado Efetivo
	M	Empregado Efetivo
	M	Empregado Efetivo
	M	Empregado Efetivo
	M	Empregado Efetivo
	M	Empregado Efetivo
	M	Empregado Efetivo
	M	Empregado Efetivo
	M	Empregado Efetivo
	M	Empregado Efetivo
	M	Empregado Efetivo
	M	Empregado Efetivo
	M	Empregado Efetivo
	M	Empregado Efetivo
	M	Empregado Efetivo
	M	Empregado Efetivo
	M	Empregado Efetivo
	M	Empregado Efetivo
	M	Empregado Efetivo
	M	Empregado Efetivo
	M	Empregado Efetivo
	M	Empregado Efetivo
	M	Empregado Efetivo
	M	Empregado Efetivo

	M	Empregado Efetivo
	M	Empregado Efetivo
	M	Empregado Efetivo
	M	Empregado Efetivo
	M	Empregado Efetivo
	M	Empregado Efetivo
	M	Empregado Efetivo
	M	Empregado Efetivo
	M	Empregado Efetivo
	M	Empregado Efetivo
	M	Empregado Efetivo
	M	Empregado Efetivo
	M	Empregado Efetivo
	M	Empregado Efetivo
	F	Empregado Efetivo
	M	Empregado Efetivo
	M	Empregado Efetivo
	M	Empregado Efetivo
	M	Empregado Efetivo
	M	Empregado Efetivo
	F	Empregado Efetivo
	M	Empregado Efetivo
	M	Empregado Efetivo
	F	Empregado Efetivo
	M	Empregado Efetivo
	F	Empregado Efetivo
	F	Empregado Efetivo
	F	Empregado Efetivo
	F	Empregado Efetivo
	F	Empregado Efetivo
	F	Empregado Efetivo
	F	Empregado Efetivo
	M	Empregado Efetivo
	F	Empregado Efetivo
	M	Empregado Efetivo
	F	Empregado Efetivo
	M	Empregado Efetivo
	M	Empregado Efetivo
	M	Empregado Efetivo
	F	Empregado Efetivo
	M	Empregado Efetivo
	M	Empregado Efetivo
	M	Empregado Efetivo
	M	Empregado Efetivo
	M	Empregado Efetivo

	M	Empregado Efetivo
	M	Empregado Efetivo
	M	Empregado Efetivo
	M	Empregado Efetivo
	M	Empregado Efetivo
	F	Empregado Efetivo
	F	Empregado Efetivo
	M	Empregado Efetivo
	M	Empregado Efetivo
	M	Empregado Efetivo
	M	Empregado Efetivo
	M	Empregado Efetivo
	M	Empregado Efetivo
	M	Empregado Efetivo
	F	Empregado Efetivo
	F	Empregado Efetivo
	M	Empregado Efetivo
	M	Empregado Temporário
	F	Empregado Efetivo
	F	Empregado Efetivo
	F	Empregado Efetivo
	F	Empregado Efetivo
	F	Empregado Efetivo
	F	Empregado Efetivo
	F	Empregado Efetivo
	F	Cedido - RJU
	F	Cedido - RJU
	F	Empregado Efetivo
	F	Empregado Efetivo
	F	Empregado Efetivo
	F	Empregado Efetivo
	F	Empregado Efetivo
	F	Empregado Efetivo
	F	Empregado Efetivo
	F	Empregado Efetivo
	F	Empregado Efetivo
	F	Empregado Efetivo
	F	Empregado Efetivo
	F	Empregado Efetivo
	F	Empregado Efetivo
	F	Empregado Efetivo
	M	Empregado Efetivo
	M	Empregado Efetivo
	F	Empregado Efetivo
	F	Empregado Efetivo
	F	Empregado Efetivo
	F	Empregado Efetivo

	F	Empregado Efetivo
	F	Empregado Efetivo
	F	Empregado Efetivo
	F	Empregado Efetivo
	F	Empregado Efetivo
	F	Empregado Efetivo
	F	Empregado Efetivo
	F	Empregado Efetivo
	F	Empregado Efetivo
	M	Empregado Efetivo
	F	Empregado Efetivo
	M	Empregado Efetivo
	F	Empregado Efetivo
	F	Empregado Efetivo
	F	Empregado Efetivo
	F	Empregado Efetivo
	F	Empregado Efetivo
	F	Empregado Efetivo
	F	Empregado Efetivo
	F	Empregado Efetivo
	F	Empregado Efetivo
	F	Empregado Efetivo
	F	Empregado Efetivo
	F	Empregado Efetivo
	F	Empregado Efetivo
	F	Empregado Efetivo
	F	Empregado Efetivo
	F	Empregado Efetivo
	F	Empregado Efetivo
	F	Empregado Efetivo
	M	Empregado Efetivo
	F	Empregado Efetivo
	F	Empregado Efetivo
	F	Empregado Efetivo
	F	Empregado Efetivo
	F	Empregado Efetivo
	F	Empregado Efetivo
	F	Empregado Efetivo
	F	Empregado Efetivo
	F	Empregado Efetivo
	F	Empregado Efetivo
	F	Empregado Efetivo
	F	Empregado Efetivo
	F	Empregado Efetivo
	F	Empregado Efetivo
	F	Empregado Efetivo
	F	Empregado Efetivo
	F	Empregado Efetivo

	F	Empregado Efetivo
	F	Empregado Efetivo
	F	Empregado Efetivo
	F	Empregado Efetivo
	F	Empregado Efetivo
	F	Empregado Efetivo
	F	Empregado Efetivo
	F	Empregado Efetivo
	F	Empregado Efetivo
	F	Empregado Efetivo
	F	Empregado Efetivo
	F	Empregado Efetivo
	F	Empregado Efetivo
	F	Empregado Efetivo
	F	Empregado Efetivo
	F	Empregado Efetivo
	F	Empregado Efetivo
	F	Empregado Efetivo
	F	Empregado Efetivo
	F	Empregado Efetivo
	F	Empregado Efetivo
	F	Empregado Efetivo
	F	Empregado Efetivo
	F	Empregado Efetivo
	F	Empregado Efetivo
	F	Empregado Efetivo
	F	Empregado Efetivo
	F	Empregado Efetivo
	F	Empregado Efetivo
	F	Empregado Efetivo
	F	Empregado Efetivo
	F	Empregado Efetivo
	F	Empregado Efetivo
	F	Empregado Efetivo
	F	Empregado Efetivo
	F	Empregado Efetivo
	F	Empregado Efetivo
	F	Empregado Efetivo
	F	Empregado Efetivo
	F	Empregado Efetivo
	F	Empregado Efetivo
	F	Empregado Efetivo
	F	Empregado Efetivo
	F	Empregado Efetivo
	F	Empregado Efetivo
	F	Empregado Efetivo
	F	Empregado Efetivo
	F	Empregado Efetivo

	F	Empregado Efetivo
	F	Empregado Efetivo
	F	Empregado Efetivo
	F	Empregado Efetivo
	M	Empregado Efetivo
	F	Empregado Efetivo
	F	Empregado Efetivo
	F	Empregado Efetivo
	F	Empregado Efetivo
	F	Empregado Efetivo
	F	Empregado Efetivo
	F	Empregado Efetivo
	F	Empregado Efetivo
	M	Empregado Efetivo
	F	Empregado Efetivo
	F	Empregado Efetivo
	F	Empregado Efetivo
	F	Empregado Efetivo
	M	Empregado Efetivo
	M	Empregado Efetivo
	F	Empregado Efetivo
	F	Empregado Efetivo
	F	Empregado Efetivo
	F	Empregado Efetivo
	M	Empregado Efetivo
	M	Empregado Efetivo
	M	Empregado Efetivo
	M	Empregado Efetivo
	F	Empregado Efetivo
	F	Empregado Efetivo
	F	Empregado Efetivo
	M	Empregado Efetivo
	M	Empregado Efetivo
	M	Empregado Efetivo
	M	Empregado Efetivo
	M	Empregado Efetivo
	F	Empregado Efetivo
	F	Empregado Efetivo
	F	Empregado Efetivo
	F	Empregado Efetivo
	F	Empregado Efetivo
	F	Empregado Efetivo
	F	Empregado Efetivo
	F	Empregado Efetivo
	F	Empregado Efetivo
	F	Empregado Efetivo

	F	Empregado Efetivo
	F	Empregado Efetivo
	M	Empregado Efetivo
	F	Empregado Efetivo
	F	Empregado Efetivo
	M	Empregado Efetivo
	M	Empregado Efetivo
	M	Empregado Efetivo
	M	Empregado Efetivo
	F	Empregado Efetivo
	F	Empregado Efetivo
	M	Empregado Efetivo
	M	Empregado Efetivo
	F	Empregado Efetivo
	F	Empregado Efetivo
	F	Empregado Efetivo
	F	Empregado Efetivo
	F	Empregado Efetivo
	F	Empregado Efetivo
	F	Empregado Efetivo
	F	Empregado Efetivo
	F	Empregado Efetivo
	M	Empregado Efetivo
	F	Empregado Efetivo
	F	Empregado Efetivo
	F	Empregado Efetivo
	F	Empregado Efetivo
	F	Empregado Efetivo
	F	Empregado Efetivo
	F	Empregado Efetivo
	F	Empregado Efetivo
	F	Empregado Efetivo
	F	Empregado Efetivo
	F	Empregado Efetivo
	F	Empregado Efetivo
	F	Empregado Efetivo
	F	Empregado Efetivo
	F	Empregado Efetivo
	M	Empregado Efetivo
	M	Empregado Efetivo
	F	Empregado Efetivo
	M	Empregado Efetivo

	F	Empregado Efetivo
	F	Empregado Efetivo
	F	Empregado Efetivo
	M	Empregado Efetivo
	M	Empregado Efetivo
	F	Empregado Efetivo
	F	Empregado Efetivo
	F	Empregado Efetivo
	F	Empregado Efetivo
	F	Empregado Efetivo
	F	Empregado Efetivo
	F	Empregado Efetivo
	F	Empregado Efetivo
	F	Empregado Efetivo
	F	Empregado Efetivo
	M	Empregado Efetivo
	F	Empregado Efetivo
	M	Empregado Efetivo
	F	Empregado Efetivo
	F	Empregado Efetivo
	F	Empregado Efetivo
	F	Empregado Efetivo
	F	Empregado Efetivo
	F	Empregado Efetivo
	F	Empregado Efetivo
	F	Empregado Efetivo
	F	Empregado Efetivo
	F	Empregado Efetivo
	F	Empregado Efetivo
	F	Empregado Efetivo
	F	Empregado Efetivo
	F	Empregado Efetivo
	F	Empregado Efetivo
	M	Empregado Efetivo
	F	Empregado Efetivo
	M	Empregado Efetivo
	M	Empregado Efetivo
	F	Empregado Efetivo
	M	Empregado Efetivo
	F	Empregado Efetivo
	F	Empregado Efetivo
	M	Empregado Efetivo
	M	Empregado Efetivo
	M	Empregado Efetivo
	M	Empregado Efetivo
	M	Empregado Efetivo
	F	Empregado Efetivo
	M	Empregado Efetivo

	M	Empregado Efetivo
	F	Empregado Efetivo
	M	Empregado Efetivo
	M	Empregado Efetivo
	M	Empregado Efetivo
	F	Empregado Efetivo
	F	Empregado Efetivo
	F	Empregado Efetivo
	F	Empregado Efetivo
	F	Empregado Efetivo
	F	Empregado Efetivo
	F	Empregado Efetivo
	F	Empregado Efetivo
	F	Empregado Efetivo
	F	Empregado Efetivo
	F	Empregado Efetivo
	F	Empregado Efetivo
	F	Empregado Efetivo
	F	Empregado Efetivo
	F	Empregado Efetivo
	F	Empregado Efetivo
	F	Empregado Efetivo
	F	Empregado Efetivo
	F	Empregado Efetivo
	F	Empregado Efetivo
	F	Empregado Efetivo
	M	Empregado Efetivo
	F	Empregado Efetivo
	F	Empregado Efetivo
	F	Empregado Efetivo
	F	Empregado Efetivo
	F	Empregado Efetivo
	F	Empregado Efetivo
	F	Empregado Efetivo
	F	Empregado Efetivo
	F	Empregado Efetivo
	F	Empregado Efetivo
	F	Empregado Efetivo
	F	Empregado Efetivo
	M	Empregado Efetivo
	M	Empregado Efetivo
	M	Empregado Efetivo
	F	Empregado Efetivo
	F	Empregado Efetivo
	F	Empregado Efetivo
	F	Empregado Efetivo
	F	Empregado Efetivo
	F	Empregado Efetivo

	F	Empregado Efetivo
	F	Empregado Efetivo
	M	Empregado Efetivo
	M	Empregado Efetivo
	M	Empregado Efetivo
	M	Empregado Efetivo
	M	Empregado Efetivo
	M	Empregado Efetivo
	M	Empregado Efetivo
	F	Empregado Efetivo
	F	Empregado Efetivo
	F	Empregado Efetivo
	F	Empregado Efetivo
	F	Empregado Efetivo
	M	Empregado Efetivo
	M	Empregado Efetivo
	M	Empregado Efetivo
	M	Empregado Efetivo
	M	Empregado Efetivo
	M	Empregado Efetivo
	M	Empregado Efetivo
	M	Empregado Efetivo
	M	Empregado Efetivo
	M	Empregado Efetivo
	M	Empregado Efetivo
	M	Empregado Efetivo
	M	Empregado Efetivo
	M	Empregado Efetivo
	M	Empregado Efetivo
	F	Empregado Efetivo
	M	Empregado Efetivo
	M	Empregado Efetivo
	M	Empregado Efetivo
	F	Empregado Efetivo
	F	Empregado Efetivo

	F	Empregado Efetivo
	F	Empregado Efetivo
	F	Empregado Efetivo
	F	Empregado Efetivo
	M	Empregado Efetivo
	M	Empregado Efetivo
	F	Empregado Efetivo
	F	Empregado Efetivo
	F	Empregado Efetivo
	F	Empregado Efetivo
	F	Empregado Efetivo
	F	Empregado Efetivo
	F	Empregado Efetivo
	F	Empregado Efetivo
	M	Empregado Efetivo
	F	Empregado Efetivo
	F	Empregado Efetivo
	M	Empregado Efetivo
	M	Empregado Efetivo
	F	Empregado Efetivo
	M	Empregado Efetivo
	M	Empregado Efetivo
	F	Empregado Efetivo
	F	Empregado Efetivo
	M	Empregado Efetivo
	F	Empregado Efetivo
	F	Empregado Efetivo
	F	Empregado Efetivo
	M	Empregado Efetivo
	M	Empregado Efetivo
	M	Empregado Efetivo
	M	Empregado Efetivo
	M	Empregado Efetivo
	M	Empregado Efetivo
	M	Empregado Efetivo
	F	Empregado Efetivo
	F	Empregado Efetivo
	M	Empregado Efetivo
	F	Empregado Efetivo
	F	Empregado Efetivo

	F	Empregado Efetivo
	F	Empregado Efetivo
	F	Empregado Efetivo
	F	Empregado Efetivo
	F	Empregado Efetivo
	F	Empregado Efetivo
	F	Empregado Efetivo
	M	Empregado Efetivo
	M	Empregado Efetivo
	M	Empregado Efetivo
	M	Empregado Efetivo
	M	Empregado Efetivo
	M	Empregado Efetivo
	M	Empregado Efetivo
	M	Empregado Efetivo
	F	Empregado Efetivo
	F	Empregado Efetivo
	F	Empregado Efetivo
	F	Empregado Efetivo
	F	Empregado Efetivo
	F	Empregado Efetivo
	F	Empregado Efetivo
	F	Empregado Efetivo
	F	Empregado Efetivo
	F	Empregado Efetivo
	M	Empregado Efetivo
	M	Empregado Efetivo
	F	Empregado Efetivo
	F	Empregado Efetivo
	F	Empregado Efetivo
	F	Empregado Efetivo
	M	Empregado Efetivo
	F	Empregado Efetivo
	F	Empregado Efetivo
	M	Empregado Efetivo
	M	Empregado Efetivo
	F	Empregado Efetivo
	F	Empregado Efetivo

	M	Empregado Efetivo
	F	Empregado Efetivo
	F	Empregado Efetivo
	F	Empregado Efetivo
	F	Empregado Efetivo
	F	Empregado Efetivo
	F	Empregado Efetivo
	F	Empregado Efetivo
	F	Empregado Efetivo
	F	Empregado Efetivo
	F	Empregado Efetivo
	F	Empregado Efetivo
	F	Empregado Efetivo
	F	Empregado Efetivo
	F	Empregado Efetivo
	F	Empregado Efetivo
	F	Empregado Efetivo
	F	Empregado Efetivo
	M	Empregado Efetivo
	M	Empregado Efetivo
	F	Empregado Efetivo
	F	Empregado Efetivo
	F	Empregado Efetivo
	F	Empregado Efetivo
	F	Empregado Efetivo
	F	Empregado Efetivo
	F	Empregado Efetivo
	F	Empregado Efetivo
	F	Empregado Efetivo
	F	Empregado Efetivo
	F	Empregado Efetivo
	F	Empregado Efetivo
	F	Empregado Efetivo
	F	Empregado Efetivo
	F	Empregado Efetivo
	F	Empregado Efetivo
	F	Empregado Efetivo
	F	Empregado Efetivo
	F	Empregado Efetivo
	F	Empregado Efetivo
	F	Empregado Efetivo
	F	Empregado Efetivo
	F	Empregado Efetivo
	F	Empregado Efetivo
	F	Empregado Efetivo
	F	Empregado Efetivo

	F	Empregado Efetivo
	M	Empregado Efetivo
	M	Empregado Efetivo
	M	Empregado Efetivo
	M	Empregado Efetivo
	M	Empregado Efetivo
	M	Empregado Efetivo
	M	Empregado Efetivo
	F	Empregado Efetivo
	M	Empregado Efetivo
	F	Empregado Efetivo
	F	Empregado Efetivo
	F	Empregado Efetivo
	F	Empregado Efetivo
	F	Empregado Efetivo
	M	Empregado Efetivo
	M	Empregado Efetivo
	M	Empregado Efetivo
	M	Empregado Efetivo
	M	Empregado Efetivo
	M	Empregado Efetivo
	F	Empregado Efetivo
	F	Empregado Efetivo
	F	Empregado Efetivo
	F	Empregado Efetivo
	F	Empregado Efetivo
	F	Empregado Efetivo
	F	Empregado Efetivo
	F	Empregado Efetivo
	F	Empregado Efetivo
	F	Empregado Efetivo
	F	Empregado Efetivo
	F	Empregado Efetivo
	F	Empregado Efetivo
	F	Empregado Efetivo
	F	Empregado Efetivo
	M	Empregado Efetivo
	F	Empregado Efetivo
	M	Empregado Efetivo
	M	Empregado Efetivo
	F	Empregado Efetivo
	F	Empregado Efetivo

	F	Empregado Efetivo
	F	Empregado Efetivo
	F	Empregado Efetivo
	F	Empregado Efetivo
	F	Empregado Efetivo
	F	Empregado Efetivo
	F	Empregado Efetivo
	F	Empregado Efetivo
	F	Empregado Efetivo
	F	Empregado Efetivo
	F	Empregado Efetivo
	F	Empregado Efetivo
	F	Empregado Efetivo
	M	Empregado Efetivo
	F	Empregado Efetivo
	F	Empregado Efetivo
	F	Empregado Efetivo
	F	Empregado Efetivo
	F	Empregado Efetivo
	F	Empregado Efetivo
	M	Empregado Efetivo
	F	Empregado Efetivo
	F	Empregado Efetivo
	F	Empregado Efetivo
	F	Empregado Efetivo
	F	Empregado Efetivo
	F	Empregado Efetivo
	F	Empregado Efetivo
	F	Empregado Efetivo
	F	Empregado Efetivo
	F	Empregado Efetivo
	F	Empregado Efetivo
	F	Empregado Efetivo
	F	Empregado Efetivo
	M	Empregado Efetivo
	F	Empregado Efetivo
	F	Empregado Efetivo
	M	Empregado Efetivo
	F	Empregado Efetivo
	F	Empregado Efetivo
	F	Empregado Efetivo
	M	Empregado Efetivo
	F	Empregado Efetivo
	F	Empregado Efetivo
	F	Empregado Efetivo
	F	Empregado Efetivo
	F	Empregado Efetivo
	F	Empregado Efetivo

	F	Empregado Efetivo
	F	Empregado Efetivo
	F	Empregado Efetivo
	F	Empregado Efetivo
	F	Empregado Efetivo
	F	Empregado Efetivo
	F	Empregado Efetivo
	F	Empregado Efetivo
	F	Empregado Efetivo
	F	Empregado Efetivo
	F	Empregado Efetivo
	F	Empregado Efetivo
	F	Empregado Efetivo
	F	Empregado Efetivo
	F	Empregado Efetivo
	F	Empregado Efetivo
	F	Empregado Efetivo
	F	Empregado Efetivo
	F	Empregado Efetivo
	M	Empregado Efetivo
	F	Empregado Efetivo
	F	Empregado Efetivo
	F	Empregado Efetivo
	F	Empregado Efetivo
	F	Empregado Efetivo
	F	Empregado Efetivo
	F	Empregado Efetivo
	F	Empregado Efetivo
	F	Empregado Efetivo
	M	Empregado Efetivo
	F	Empregado Efetivo
	F	Empregado Efetivo
	F	Empregado Efetivo
	F	Empregado Efetivo
	F	Empregado Efetivo
	M	Empregado Efetivo
	F	Empregado Efetivo
	F	Empregado Efetivo
	F	Empregado Efetivo
	M	Empregado Efetivo
	M	Empregado Efetivo
	F	Empregado Efetivo
	M	Empregado Efetivo
	F	Empregado Efetivo
	F	Empregado Efetivo
	F	Empregado Efetivo
	F	Empregado Efetivo
	F	Empregado Efetivo

	M	Empregado Efetivo
	F	Empregado Efetivo
	F	Empregado Efetivo
	F	Empregado Efetivo
	F	Empregado Efetivo
	F	Empregado Efetivo
	F	Empregado Efetivo
	M	Empregado Efetivo
	F	Empregado Efetivo
	F	Empregado Efetivo
	F	Empregado Efetivo
	F	Empregado Efetivo
	F	Empregado Efetivo
	M	Empregado Efetivo
	M	Empregado Efetivo
	M	Empregado Efetivo
	F	Empregado Efetivo
	F	Empregado Efetivo
	F	Empregado Efetivo
	F	Empregado Efetivo
	F	Empregado Efetivo
	F	Empregado Efetivo
	M	Empregado Efetivo
	F	Empregado Efetivo
	F	Empregado Efetivo
	M	Empregado Efetivo
	F	Empregado Efetivo
	F	Empregado Efetivo
	F	Empregado Efetivo
	F	Empregado Efetivo
	F	Empregado Efetivo
	F	Empregado Efetivo
	F	Empregado Efetivo
	F	Empregado Efetivo
	F	Empregado Efetivo
	F	Empregado Efetivo
	F	Empregado Efetivo
	F	Empregado Efetivo
	M	Empregado Efetivo
	M	Empregado Efetivo
	F	Empregado Efetivo
	M	Empregado Efetivo
	F	Empregado Efetivo
	F	Empregado Efetivo
	F	Empregado Efetivo
	F	Empregado Efetivo
	F	Empregado Efetivo
	F	Empregado Efetivo

	F	Empregado Efetivo
	F	Empregado Efetivo
	F	Empregado Efetivo
	M	Empregado Efetivo
	F	Empregado Efetivo
	F	Empregado Efetivo
	F	Empregado Efetivo
	F	Empregado Efetivo
	F	Empregado Efetivo
	F	Empregado Efetivo
	F	Empregado Efetivo
	F	Empregado Efetivo
	F	Empregado Efetivo
	F	Empregado Efetivo
	F	Empregado Efetivo
	F	Empregado Efetivo
	F	Empregado Efetivo
	F	Empregado Efetivo
	F	Empregado Efetivo
	F	Empregado Efetivo
	F	Empregado Efetivo
	F	Empregado Efetivo
	F	Empregado Efetivo
	F	Empregado Efetivo
	F	Empregado Efetivo
	F	Empregado Efetivo
	F	Empregado Efetivo
	F	Empregado Efetivo
	F	Empregado Efetivo
	F	Empregado Efetivo
	F	Empregado Efetivo
	F	Empregado Efetivo
	F	Empregado Efetivo
	F	Empregado Efetivo
	F	Empregado Efetivo
	F	Empregado Efetivo
	F	Empregado Efetivo
	F	Empregado Efetivo
	F	Empregado Efetivo
	M	Empregado Efetivo
	F	Empregado Efetivo
	F	Empregado Efetivo
	F	Empregado Efetivo
	F	Empregado Efetivo
	F	Empregado Efetivo
	F	Empregado Efetivo
	F	Empregado Efetivo
	F	Empregado Efetivo

	M	Empregado Efetivo
	F	Empregado Efetivo
	F	Empregado Efetivo
	F	Empregado Efetivo
	F	Empregado Efetivo
	F	Empregado Efetivo
	M	Empregado Efetivo
	F	Empregado Efetivo
	F	Empregado Efetivo
	F	Empregado Efetivo
	F	Empregado Efetivo
	F	Empregado Efetivo
	F	Empregado Efetivo
	F	Empregado Efetivo
	F	Empregado Efetivo
	F	Empregado Efetivo
	F	Empregado Efetivo
	F	Empregado Efetivo
	F	Empregado Efetivo
	F	Empregado Efetivo
	F	Empregado Efetivo
	F	Empregado Efetivo
	F	Empregado Efetivo
	F	Empregado Efetivo
	F	Empregado Efetivo
	F	Empregado Efetivo
	F	Empregado Efetivo
	M	Empregado Efetivo
	F	Empregado Efetivo
	M	Empregado Efetivo
	F	Empregado Efetivo
	F	Empregado Efetivo
	F	Empregado Efetivo
	F	Empregado Efetivo
	F	Empregado Efetivo
	F	Empregado Efetivo
	F	Empregado Efetivo
	F	Empregado Efetivo
	M	Empregado Efetivo
	F	Empregado Efetivo
	F	Empregado Efetivo
	M	Empregado Efetivo
	F	Empregado Efetivo
	F	Empregado Efetivo
	F	Empregado Efetivo
	F	Empregado Efetivo
	F	Empregado Efetivo
	M	Empregado Efetivo

	F	Empregado Efetivo
	M	Empregado Efetivo
	F	Empregado Efetivo
	F	Empregado Efetivo
	F	Empregado Efetivo
	F	Empregado Efetivo
	F	Empregado Efetivo
	F	Empregado Efetivo
	F	Empregado Efetivo
	F	Empregado Efetivo
	F	Empregado Efetivo
	F	Empregado Efetivo
	F	Empregado Efetivo
	F	Empregado Efetivo
	F	Empregado Efetivo
	F	Empregado Efetivo
	F	Empregado Efetivo
	F	Empregado Efetivo
	F	Empregado Efetivo
	F	Empregado Efetivo
	F	Empregado Efetivo
	F	Empregado Efetivo
	F	Empregado Efetivo
	F	Empregado Efetivo
	F	Empregado Efetivo
	F	Empregado Efetivo
	F	Empregado Efetivo
	F	Empregado Efetivo
	M	Empregado Efetivo
	F	Empregado Efetivo
	M	Empregado Efetivo
	F	Empregado Efetivo
	F	Empregado Efetivo
	F	Empregado Efetivo
	F	Empregado Efetivo
	F	Empregado Efetivo
	F	Empregado Efetivo
	F	Empregado Efetivo
	F	Empregado Efetivo
	F	Empregado Efetivo
	F	Empregado Efetivo
	M	Empregado Efetivo
	F	Empregado Efetivo
	F	Empregado Efetivo
	F	Empregado Efetivo
	F	Empregado Efetivo
	F	Empregado Efetivo
	F	Empregado Efetivo

	M	Empregado Efetivo
	F	Empregado Efetivo
	F	Empregado Efetivo
	F	Empregado Efetivo
	F	Empregado Efetivo
	F	Empregado Efetivo
	F	Empregado Efetivo
	F	Empregado Efetivo
	F	Empregado Efetivo
	F	Empregado Efetivo
	F	Empregado Efetivo
	F	Empregado Efetivo
	F	Empregado Efetivo
	F	Empregado Efetivo
	F	Empregado Efetivo
	F	Empregado Efetivo
	F	Empregado Efetivo
	F	Empregado Efetivo
	F	Empregado Efetivo
	F	Empregado Efetivo
	F	Empregado Efetivo
	M	Empregado Efetivo
	F	Empregado Efetivo
	F	Empregado Efetivo
	F	Empregado Efetivo
	F	Empregado Efetivo
	F	Empregado Efetivo
	F	Empregado Efetivo
	F	Empregado Efetivo
	F	Empregado Efetivo
	M	Empregado Efetivo
	F	Empregado Efetivo
	F	Empregado Efetivo
	F	Empregado Efetivo
	F	Empregado Efetivo
	F	Empregado Efetivo
	F	Empregado Efetivo
	F	Empregado Efetivo
	F	Empregado Efetivo
	F	Empregado Efetivo
	F	Empregado Efetivo
	F	Empregado Efetivo
	F	Empregado Efetivo
	M	Empregado Efetivo
	M	Empregado Efetivo
	F	Empregado Efetivo
	F	Empregado Efetivo
	F	Empregado Efetivo

	F	Empregado Efetivo
	F	Empregado Efetivo
	M	Empregado Efetivo
	F	Empregado Efetivo
	F	Empregado Efetivo
	F	Empregado Efetivo
	F	Empregado Efetivo
	F	Empregado Efetivo
	F	Empregado Efetivo
	F	Empregado Efetivo
	F	Empregado Efetivo
	F	Empregado Efetivo
	F	Empregado Efetivo
	F	Empregado Efetivo
	F	Empregado Efetivo
	F	Empregado Efetivo
	F	Empregado Efetivo
	F	Empregado Efetivo
	F	Empregado Efetivo
	F	Empregado Efetivo
	F	Empregado Efetivo
	F	Empregado Efetivo
	F	Empregado Efetivo
	F	Empregado Efetivo
	F	Empregado Efetivo
	F	Empregado Efetivo
	F	Empregado Efetivo
	F	Empregado Efetivo
	F	Empregado Efetivo
	F	Empregado Efetivo
	F	Empregado Efetivo
	F	Empregado Efetivo
	F	Empregado Efetivo
	F	Empregado Efetivo
	F	Empregado Efetivo
	F	Empregado Efetivo
	F	Empregado Efetivo
	F	Empregado Efetivo
	F	Empregado Efetivo
	F	Empregado Efetivo
	F	Empregado Efetivo
	F	Empregado Efetivo
	F	Empregado Efetivo
	F	Empregado Efetivo
	F	Empregado Efetivo
	F	Empregado Efetivo
	F	Empregado Efetivo
	F	Empregado Efetivo

	F	Empregado Efetivo
	F	Empregado Efetivo
	M	Empregado Efetivo
	F	Empregado Efetivo
	F	Empregado Efetivo
	F	Empregado Efetivo
	F	Empregado Efetivo
	F	Empregado Efetivo
	F	Empregado Efetivo
	F	Empregado Efetivo
	F	Empregado Efetivo
	F	Empregado Efetivo
	F	Empregado Efetivo
	F	Empregado Efetivo
	F	Empregado Efetivo
	F	Empregado Efetivo
	F	Empregado Efetivo
	F	Empregado Efetivo
	F	Empregado Efetivo
	F	Empregado Efetivo
	F	Empregado Efetivo
	F	Empregado Efetivo
	F	Empregado Efetivo
	F	Empregado Efetivo
	F	Empregado Efetivo
	F	Empregado Efetivo
	F	Empregado Efetivo
	F	Empregado Efetivo
	F	Empregado Efetivo
	F	Empregado Efetivo
	F	Empregado Efetivo
	F	Empregado Efetivo
	F	Empregado Efetivo
	F	Empregado Efetivo
	F	Empregado Efetivo
	M	Empregado Efetivo
	F	Empregado Efetivo
	F	Empregado Efetivo
	F	Empregado Efetivo
	F	Empregado Efetivo
	F	Empregado Efetivo
	F	Empregado Efetivo
	F	Empregado Efetivo
	F	Empregado Efetivo
	F	Empregado Efetivo
	F	Empregado Efetivo
	F	Empregado Efetivo
	F	Empregado Efetivo

	F	Empregado Efetivo
	F	Empregado Efetivo
	F	Empregado Efetivo
	F	Empregado Efetivo
	F	Empregado Efetivo
	F	Empregado Efetivo
	F	Empregado Efetivo
	F	Empregado Efetivo
	F	Empregado Efetivo
	F	Empregado Efetivo
	F	Empregado Efetivo
	F	Empregado Efetivo
	F	Empregado Efetivo
	F	Empregado Efetivo
	F	Empregado Efetivo
	F	Empregado Efetivo
	F	Empregado Efetivo
	F	Empregado Efetivo
	F	Empregado Efetivo
	F	Empregado Efetivo
	F	Empregado Efetivo
	F	Empregado Efetivo
	F	Empregado Efetivo
	F	Empregado Efetivo
	F	Empregado Efetivo
	F	Empregado Efetivo
	F	Empregado Efetivo
	F	Empregado Efetivo
	F	Empregado Efetivo
	F	Empregado Efetivo
	F	Empregado Efetivo
	M	Empregado Efetivo
	F	Empregado Efetivo
	F	Empregado Efetivo
	M	Empregado Efetivo
	F	Empregado Efetivo
	F	Empregado Efetivo
	F	Empregado Efetivo
	F	Empregado Efetivo
	F	Empregado Efetivo
	F	Empregado Efetivo
	F	Empregado Efetivo
	F	Empregado Efetivo
	F	Empregado Efetivo
	F	Empregado Efetivo
	F	Empregado Efetivo
	F	Empregado Efetivo
	F	Empregado Efetivo

	F	Empregado Efetivo
	F	Empregado Efetivo
	F	Empregado Efetivo
	F	Empregado Efetivo
	F	Empregado Efetivo
	F	Empregado Efetivo
	F	Empregado Efetivo
	F	Empregado Efetivo
	F	Empregado Efetivo
	M	Empregado Efetivo
	F	Empregado Efetivo
	F	Empregado Efetivo
	F	Empregado Efetivo
	F	Empregado Efetivo
	F	Empregado Efetivo
	F	Empregado Efetivo
	F	Empregado Efetivo
	F	Empregado Efetivo
	F	Empregado Efetivo
	F	Empregado Efetivo
	F	Empregado Efetivo
	F	Empregado Efetivo
	F	Empregado Efetivo
	F	Empregado Efetivo
	F	Empregado Efetivo
	F	Empregado Efetivo
	F	Empregado Efetivo
	F	Empregado Efetivo
	F	Empregado Efetivo
	F	Empregado Efetivo
	F	Empregado Efetivo
	F	Empregado Efetivo
	F	Empregado Efetivo
	F	Empregado Efetivo
	F	Empregado Efetivo
	M	Empregado Efetivo
	F	Empregado Efetivo
	F	Empregado Efetivo
	F	Empregado Efetivo
	F	Empregado Efetivo
	F	Empregado Efetivo
	F	Empregado Efetivo
	F	Empregado Efetivo
	F	Empregado Efetivo
	F	Empregado Efetivo
	M	Empregado Efetivo
	F	Empregado Efetivo
	F	Empregado Efetivo

	F	Empregado Efetivo
	F	Empregado Efetivo
	M	Empregado Efetivo
	F	Empregado Efetivo
	F	Empregado Efetivo
	F	Empregado Efetivo
	F	Empregado Efetivo
	F	Empregado Efetivo
	F	Empregado Efetivo
	F	Empregado Efetivo
	F	Empregado Efetivo
	F	Empregado Efetivo
	F	Empregado Efetivo
	F	Empregado Efetivo
	F	Empregado Efetivo
	F	Empregado Efetivo
	F	Empregado Efetivo
	F	Empregado Efetivo
	F	Empregado Efetivo
	F	Empregado Efetivo
	F	Empregado Efetivo
	F	Empregado Efetivo
	F	Empregado Efetivo
	F	Empregado Efetivo
	F	Empregado Efetivo
	F	Empregado Efetivo
	F	Empregado Efetivo
	F	Empregado Efetivo
	F	Empregado Efetivo
	F	Empregado Efetivo
	M	Empregado Efetivo
	F	Empregado Efetivo
	M	Empregado Efetivo
	F	Empregado Efetivo
	F	Empregado Efetivo
	F	Empregado Efetivo
	F	Empregado Efetivo
	F	Empregado Efetivo
	F	Empregado Efetivo
	F	Empregado Efetivo
	F	Empregado Efetivo
	F	Empregado Efetivo
	F	Empregado Efetivo
	F	Empregado Efetivo
	F	Empregado Efetivo
	F	Empregado Efetivo
	F	Empregado Efetivo
	F	Empregado Efetivo

	F	Empregado Efetivo
	F	Empregado Efetivo
	F	Empregado Efetivo
	F	Empregado Efetivo
	F	Empregado Efetivo
	F	Empregado Efetivo
	F	Empregado Efetivo
	F	Empregado Efetivo
	F	Empregado Efetivo
	F	Empregado Efetivo
	F	Empregado Efetivo
	F	Empregado Efetivo
	F	Empregado Efetivo
	F	Empregado Efetivo
	F	Empregado Efetivo
	F	Empregado Efetivo
	F	Empregado Efetivo
	F	Empregado Efetivo
	F	Empregado Efetivo
	F	Empregado Efetivo
	F	Empregado Efetivo
	F	Empregado Efetivo
	F	Empregado Efetivo
	F	Empregado Efetivo
	F	Empregado Efetivo
	F	Empregado Efetivo
	F	Empregado Efetivo
	F	Empregado Efetivo
	F	Empregado Efetivo
	F	Empregado Efetivo
	F	Empregado Efetivo
	F	Empregado Efetivo
	F	Empregado Efetivo
	F	Empregado Efetivo
	F	Empregado Efetivo
	F	Empregado Efetivo
	F	Empregado Efetivo
	F	Empregado Efetivo
	F	Empregado Efetivo
	F	Empregado Efetivo
	M	Empregado Efetivo
	F	Empregado Efetivo
	F	Empregado Efetivo
	F	Empregado Efetivo
	F	Empregado Efetivo
	F	Empregado Efetivo
	F	Empregado Efetivo
	F	Empregado Efetivo

	F	Empregado Efetivo
	F	Empregado Efetivo
	F	Empregado Efetivo
	F	Empregado Efetivo
	F	Empregado Efetivo
	F	Empregado Efetivo
	F	Empregado Efetivo
	F	Empregado Efetivo
	F	Empregado Efetivo
	F	Empregado Efetivo
	F	Empregado Efetivo
	F	Empregado Efetivo
	F	Empregado Efetivo
	F	Empregado Efetivo
	F	Empregado Efetivo
	F	Empregado Efetivo
	F	Empregado Efetivo
	F	Empregado Efetivo
	F	Empregado Efetivo
	F	Empregado Efetivo
	F	Empregado Efetivo
	M	Empregado Efetivo
	F	Empregado Efetivo
	F	Empregado Efetivo
	F	Empregado Efetivo
	F	Empregado Efetivo
	F	Empregado Efetivo
	F	Empregado Efetivo
	M	Empregado Efetivo
	F	Empregado Efetivo
	F	Empregado Efetivo
	F	Empregado Efetivo
	F	Empregado Efetivo
	F	Empregado Efetivo
	F	Empregado Efetivo
	F	Empregado Efetivo
	F	Empregado Efetivo
	F	Empregado Efetivo
	F	Empregado Efetivo
	F	Empregado Efetivo
	F	Empregado Efetivo
	F	Empregado Efetivo
	F	Empregado Efetivo
	F	Empregado Efetivo
	F	Empregado Efetivo
	F	Empregado Efetivo
	F	Empregado Efetivo
	F	Empregado Efetivo

	F	Empregado Efetivo
	F	Empregado Efetivo
	F	Empregado Efetivo
	F	Empregado Efetivo
	F	Empregado Efetivo
	F	Empregado Efetivo
	F	Empregado Efetivo
	F	Empregado Efetivo
	F	Empregado Efetivo
	F	Empregado Efetivo
	F	Empregado Efetivo
	M	Empregado Efetivo
	F	Empregado Efetivo
	F	Empregado Efetivo
	M	Empregado Efetivo
	F	Empregado Efetivo
	F	Empregado Efetivo
	F	Empregado Efetivo
	F	Empregado Efetivo
	F	Empregado Efetivo
	F	Empregado Efetivo
	F	Empregado Efetivo
	F	Empregado Efetivo
	F	Empregado Efetivo
	F	Empregado Efetivo
	F	Empregado Efetivo
	F	Empregado Efetivo
	F	Empregado Efetivo
	F	Empregado Efetivo
	F	Empregado Efetivo
	F	Empregado Efetivo
	F	Empregado Efetivo
	F	Empregado Efetivo
	F	Empregado Efetivo
	F	Empregado Efetivo
	F	Empregado Efetivo
	F	Empregado Efetivo
	F	Empregado Efetivo
	F	Empregado Efetivo
	F	Empregado Efetivo
	F	Empregado Efetivo
	F	Empregado Efetivo
	F	Empregado Efetivo
	F	Empregado Efetivo
	F	Empregado Efetivo
	F	Empregado Efetivo
	F	Empregado Efetivo
	F	Empregado Efetivo

	F	Empregado Efetivo
	F	Empregado Efetivo
	F	Empregado Efetivo
	F	Empregado Efetivo
	F	Empregado Efetivo
	F	Empregado Efetivo
	F	Empregado Efetivo
	F	Empregado Efetivo
	F	Empregado Efetivo
	F	Empregado Efetivo
	F	Empregado Efetivo
	F	Empregado Efetivo
	F	Empregado Efetivo
	F	Empregado Efetivo
	F	Empregado Efetivo
	F	Empregado Efetivo
	F	Empregado Efetivo
	F	Empregado Efetivo
	F	Empregado Efetivo
	F	Empregado Efetivo
	F	Empregado Efetivo
	F	Empregado Efetivo
	F	Empregado Efetivo
	F	Empregado Efetivo
	F	Empregado Efetivo
	F	Empregado Efetivo
	F	Empregado Efetivo
	F	Empregado Efetivo
	F	Empregado Efetivo
	F	Empregado Efetivo
	F	Empregado Efetivo
	F	Empregado Efetivo
	F	Empregado Efetivo
	F	Empregado Efetivo
	F	Empregado Efetivo
	F	Empregado Efetivo
	F	Empregado Efetivo
	F	Empregado Efetivo
	M	Empregado Efetivo
	F	Empregado Efetivo
	F	Empregado Efetivo
	F	Empregado Efetivo
	F	Empregado Efetivo
	F	Empregado Efetivo
	F	Empregado Efetivo
	F	Empregado Efetivo
	F	Empregado Efetivo
	F	Empregado Efetivo

	F	Empregado Efetivo
	F	Empregado Efetivo
	F	Empregado Efetivo
	F	Empregado Efetivo
	F	Empregado Efetivo
	M	Empregado Efetivo
	F	Empregado Efetivo
	F	Empregado Efetivo
	F	Empregado Efetivo
	F	Empregado Efetivo
	F	Empregado Efetivo
	F	Empregado Efetivo
	F	Empregado Efetivo
	F	Empregado Efetivo
	F	Empregado Efetivo
	F	Empregado Efetivo
	F	Empregado Efetivo
	F	Empregado Efetivo
	F	Empregado Efetivo
	F	Empregado Efetivo
	F	Empregado Efetivo
	F	Empregado Efetivo
	F	Empregado Efetivo
	F	Empregado Efetivo
	F	Empregado Efetivo
	F	Empregado Efetivo
	F	Empregado Efetivo
	F	Empregado Efetivo
	F	Empregado Efetivo
	F	Empregado Efetivo
	F	Empregado Efetivo
	F	Empregado Efetivo
	F	Empregado Efetivo
	F	Empregado Efetivo
	F	Empregado Efetivo
	F	Empregado Efetivo
	F	Empregado Efetivo
	F	Empregado Efetivo
	F	Empregado Efetivo
	M	Empregado Efetivo
	F	Empregado Efetivo
	F	Empregado Efetivo
	F	Empregado Efetivo
	F	Empregado Efetivo
	F	Empregado Efetivo
	F	Empregado Efetivo
	F	Empregado Efetivo
	F	Empregado Efetivo

	F	Empregado Efetivo
	F	Empregado Efetivo
	F	Empregado Efetivo
	F	Empregado Efetivo
	F	Empregado Efetivo
	F	Empregado Efetivo
	F	Empregado Efetivo
	F	Empregado Efetivo
	M	Empregado Efetivo
	F	Empregado Efetivo
	F	Empregado Efetivo
	F	Empregado Efetivo
	F	Empregado Efetivo
	F	Empregado Efetivo
	F	Empregado Efetivo
	M	Empregado Efetivo
	F	Empregado Efetivo
	F	Empregado Efetivo
	F	Empregado Efetivo
	F	Empregado Efetivo
	F	Empregado Efetivo
	F	Empregado Efetivo
	F	Empregado Efetivo
	F	Empregado Efetivo
	F	Empregado Efetivo
	F	Empregado Efetivo
	F	Empregado Efetivo
	F	Empregado Efetivo
	F	Empregado Efetivo
	F	Empregado Efetivo
	F	Empregado Efetivo
	F	Empregado Efetivo
	F	Empregado Efetivo
	F	Empregado Efetivo
	F	Empregado Efetivo
	F	Empregado Efetivo
	F	Empregado Efetivo
	F	Empregado Efetivo
	F	Empregado Efetivo
	F	Empregado Efetivo
	F	Empregado Efetivo
	F	Empregado Efetivo
	F	Empregado Efetivo
	F	Empregado Efetivo
	M	Empregado Efetivo
	M	Empregado Efetivo
	F	Empregado Efetivo
	F	Empregado Efetivo

	F	Empregado Efetivo
	F	Empregado Efetivo
	F	Empregado Efetivo
	F	Empregado Efetivo
	F	Empregado Efetivo
	F	Empregado Efetivo
	F	Empregado Efetivo
	F	Empregado Efetivo
	F	Empregado Efetivo
	F	Empregado Efetivo
	M	Empregado Efetivo
	F	Empregado Efetivo
	F	Empregado Efetivo
	F	Empregado Efetivo
	F	Empregado Efetivo
	F	Empregado Efetivo
	F	Empregado Efetivo
	F	Empregado Efetivo
	F	Empregado Efetivo
	F	Empregado Efetivo
	F	Empregado Efetivo
	F	Empregado Efetivo
	F	Empregado Efetivo
	F	Empregado Efetivo
	F	Empregado Efetivo
	F	Empregado Efetivo
	M	Empregado Efetivo
	F	Empregado Efetivo
	M	Empregado Efetivo
	F	Empregado Efetivo
	F	Empregado Efetivo
	F	Empregado Efetivo
	F	Empregado Efetivo
	F	Empregado Efetivo
	F	Empregado Efetivo
	F	Empregado Efetivo
	F	Empregado Efetivo
	F	Empregado Efetivo
	F	Empregado Efetivo
	F	Empregado Efetivo
	F	Empregado Efetivo
	F	Empregado Efetivo
	F	Empregado Efetivo
	F	Empregado Efetivo
	F	Empregado Efetivo
	F	Empregado Efetivo
	F	Empregado Efetivo
	F	Empregado Efetivo

	F	Empregado Efetivo
	F	Empregado Efetivo
	F	Empregado Efetivo
	F	Empregado Efetivo
	M	Empregado Efetivo
	M	Empregado Efetivo
	M	Empregado Efetivo
	F	Empregado Efetivo
	M	Empregado Efetivo
	F	Empregado Efetivo
	M	Empregado Efetivo
	F	Empregado Efetivo
	M	Empregado Efetivo
	F	Empregado Efetivo
	F	Empregado Efetivo
	M	Empregado Efetivo
	M	Empregado Efetivo
	M	Empregado Efetivo
	M	Empregado Efetivo
	M	Empregado Efetivo
	F	Empregado Efetivo
	M	Empregado Efetivo
	F	Empregado Efetivo
	M	Empregado Efetivo
	F	Empregado Efetivo
	F	Empregado Efetivo
	F	Empregado Efetivo
	M	Empregado Efetivo
	F	Empregado Efetivo
	F	Empregado Efetivo
	F	Empregado Efetivo
	F	Empregado Efetivo
	F	Empregado Efetivo
	F	Empregado Efetivo
	F	Empregado Efetivo
	F	Empregado Efetivo
	F	Empregado Efetivo
	F	Empregado Efetivo
	F	Empregado Efetivo
	F	Empregado Efetivo
	F	Empregado Efetivo
	F	Empregado Efetivo
	F	Empregado Efetivo
	F	Empregado Efetivo
	M	Empregado Efetivo
	M	Empregado Efetivo
	F	Empregado Efetivo
	F	Empregado Efetivo

	F	Empregado Efetivo
	M	Empregado Efetivo
	F	Empregado Efetivo
	F	Empregado Efetivo
	F	Empregado Efetivo
	M	Empregado Efetivo
	F	Empregado Efetivo
	M	Empregado Efetivo
	M	Empregado Efetivo
	F	Empregado Efetivo
	M	Empregado Efetivo
	M	Empregado Efetivo
	F	Empregado Efetivo
	M	Empregado Efetivo
	F	Empregado Efetivo
	F	Empregado Efetivo
	M	Empregado Efetivo
	M	Empregado Efetivo
	M	Empregado Efetivo
	F	Empregado Efetivo
	M	Empregado Efetivo
	M	Empregado Efetivo
	M	Empregado Efetivo
	F	Empregado Efetivo
	M	Empregado Efetivo
	M	Empregado Efetivo
	M	Empregado Efetivo
	M	Empregado Efetivo
	M	Empregado Efetivo
	M	Empregado Efetivo
	M	Empregado Efetivo
	M	Empregado Efetivo
	M	Empregado Efetivo
	M	Empregado Efetivo
	F	Empregado Efetivo
	F	Empregado Efetivo
	F	Empregado Efetivo
	F	Empregado Efetivo
	F	Empregado Efetivo
	F	Empregado Efetivo
	F	Empregado Efetivo
	F	Empregado Efetivo
	F	Empregado Efetivo

	F	Empregado Efetivo
	F	Empregado Efetivo
	F	Empregado Efetivo
	F	Empregado Efetivo
	F	Empregado Efetivo
	M	Empregado Efetivo
	F	Empregado Efetivo
	F	Empregado Efetivo
	F	Empregado Efetivo
	F	Empregado Efetivo
	F	Empregado Efetivo
	F	Empregado Efetivo
	F	Empregado Efetivo
	F	Empregado Efetivo
	M	Empregado Efetivo
	F	Empregado Efetivo
	F	Empregado Efetivo
	M	Empregado Efetivo
	F	Empregado Efetivo
	F	Empregado Efetivo
	F	Empregado Efetivo
	M	Empregado Efetivo
	F	Empregado Efetivo
	F	Empregado Efetivo
	M	Empregado Efetivo
	F	Empregado Efetivo
	F	Empregado Efetivo
	F	Empregado Efetivo
	F	Empregado Efetivo
	F	Empregado Efetivo
	F	Empregado Efetivo
	F	Empregado Efetivo
	F	Empregado Efetivo
	M	Empregado Efetivo
	F	Empregado Efetivo
	F	Empregado Efetivo
	F	Empregado Efetivo
	F	Empregado Efetivo
	F	Empregado Efetivo
	F	Empregado Efetivo
	F	Empregado Efetivo
	M	Empregado Efetivo
	F	Empregado Efetivo
	F	Empregado Efetivo
	F	Empregado Efetivo
	F	Empregado Efetivo
	F	Empregado Efetivo
	F	Empregado Efetivo

	F	Empregado Efetivo
	F	Empregado Efetivo
	F	Empregado Efetivo
	F	Empregado Efetivo
	F	Empregado Efetivo
	F	Empregado Efetivo
	F	Empregado Efetivo
	M	Empregado Efetivo
	F	Empregado Efetivo
	F	Empregado Efetivo
	F	Empregado Efetivo
	F	Empregado Efetivo
	F	Empregado Efetivo
	F	Empregado Efetivo
	F	Empregado Efetivo
	F	Empregado Efetivo
	F	Empregado Efetivo
	F	Empregado Efetivo
	F	Empregado Efetivo
	F	Empregado Efetivo
	F	Empregado Efetivo
	F	Empregado Efetivo
	F	Empregado Efetivo
	F	Empregado Efetivo
	F	Empregado Efetivo
	F	Empregado Efetivo
	F	Empregado Efetivo
	F	Empregado Efetivo
	F	Empregado Efetivo
	F	Empregado Efetivo
	F	Empregado Efetivo
	F	Empregado Efetivo
	F	Empregado Efetivo
	F	Empregado Efetivo
	F	Empregado Efetivo
	F	Empregado Efetivo
	F	Empregado Efetivo
	F	Empregado Efetivo
	M	Empregado Efetivo
	F	Empregado Efetivo
	F	Empregado Efetivo
	F	Empregado Efetivo
	F	Empregado Efetivo
	F	Empregado Efetivo

	M	Empregado Efetivo
	F	Empregado Efetivo
	F	Empregado Efetivo
	F	Empregado Efetivo
	M	Empregado Efetivo
	F	Empregado Efetivo
	F	Empregado Efetivo
	F	Empregado Efetivo
	F	Empregado Efetivo
	F	Empregado Efetivo
	F	Empregado Efetivo
	F	Empregado Efetivo
	F	Empregado Efetivo
	F	Empregado Efetivo
	F	Empregado Efetivo
	F	Empregado Efetivo
	F	Empregado Efetivo
	F	Empregado Efetivo
	F	Empregado Efetivo
	F	Empregado Efetivo
	F	Empregado Efetivo
	F	Empregado Efetivo
	F	Empregado Efetivo
	F	Empregado Efetivo
	F	Empregado Efetivo
	F	Empregado Efetivo
	F	Empregado Efetivo
	F	Empregado Efetivo
	F	Empregado Efetivo
	F	Empregado Efetivo
	F	Empregado Efetivo
	F	Empregado Efetivo
	F	Empregado Efetivo
	F	Empregado Efetivo
	F	Empregado Efetivo
	F	Empregado Efetivo
	F	Empregado Efetivo
	F	Empregado Efetivo
	F	Empregado Efetivo
	F	Empregado Efetivo
	F	Empregado Efetivo
	F	Empregado Efetivo
	F	Empregado Efetivo
	F	Empregado Efetivo
	F	Empregado Efetivo
	F	Empregado Efetivo
	F	Empregado Efetivo

	M	Empregado Efetivo
	F	Empregado Efetivo
	F	Empregado Efetivo
	F	Empregado Efetivo
	F	Empregado Efetivo
	F	Empregado Efetivo
	F	Empregado Efetivo
	F	Empregado Efetivo
	F	Empregado Efetivo
	F	Empregado Efetivo
	F	Empregado Efetivo
	F	Empregado Efetivo
	F	Empregado Efetivo
	F	Empregado Efetivo
	F	Empregado Efetivo
	F	Empregado Efetivo
	F	Empregado Efetivo
	F	Empregado Efetivo
	F	Empregado Efetivo
	F	Empregado Efetivo
	F	Empregado Efetivo
	F	Empregado Efetivo
	F	Empregado Efetivo
	F	Empregado Efetivo
	F	Empregado Efetivo
	F	Empregado Efetivo
	F	Empregado Efetivo
	F	Empregado Efetivo
	F	Empregado Efetivo
	F	Empregado Efetivo
	F	Empregado Efetivo
	F	Empregado Efetivo
	F	Empregado Efetivo
	F	Empregado Efetivo
	M	Empregado Efetivo
	F	Empregado Efetivo
	F	Empregado Efetivo
	F	Empregado Efetivo
	F	Empregado Efetivo
	F	Empregado Efetivo
	F	Empregado Efetivo
	F	Empregado Efetivo
	F	Empregado Efetivo
	F	Empregado Efetivo
	F	Empregado Efetivo
	F	Empregado Efetivo
	M	Empregado Efetivo
	F	Empregado Efetivo

	F	Empregado Efetivo
	F	Empregado Efetivo
	F	Empregado Efetivo
	F	Empregado Efetivo
	F	Empregado Efetivo
	F	Empregado Efetivo
	M	Empregado Efetivo
	F	Empregado Efetivo
	F	Empregado Efetivo
	F	Empregado Efetivo
	F	Empregado Efetivo
	F	Empregado Efetivo
	M	Empregado Efetivo
	F	Empregado Efetivo
	F	Empregado Efetivo
	F	Empregado Efetivo
	F	Empregado Efetivo
	F	Empregado Efetivo
	F	Empregado Efetivo
	M	Empregado Efetivo
	F	Empregado Efetivo
	F	Empregado Efetivo
	F	Empregado Efetivo
	F	Empregado Efetivo
	F	Empregado Efetivo
	F	Empregado Efetivo
	M	Empregado Efetivo
	F	Empregado Efetivo
	M	Empregado Efetivo
	F	Empregado Efetivo
	M	Empregado Efetivo
	F	Empregado Efetivo
	F	Empregado Efetivo
	F	Empregado Efetivo
	F	Empregado Efetivo
	F	Empregado Efetivo
	F	Empregado Efetivo
	F	Empregado Efetivo
	F	Empregado Efetivo
	F	Empregado Efetivo
	F	Empregado Efetivo
	F	Empregado Efetivo
	F	Empregado Efetivo
	F	Empregado Efetivo
	F	Empregado Efetivo
	M	Empregado Efetivo

	F	Empregado Efetivo
	F	Empregado Efetivo
	F	Empregado Efetivo
	F	Empregado Efetivo
	F	Empregado Efetivo
	F	Empregado Efetivo
	F	Empregado Efetivo
	F	Empregado Efetivo
	F	Empregado Efetivo
	F	Empregado Efetivo
	F	Empregado Efetivo
	F	Empregado Efetivo
	F	Empregado Efetivo
	F	Empregado Efetivo
	F	Empregado Efetivo
	M	Empregado Efetivo
	F	Empregado Efetivo
	M	Empregado Efetivo
	M	Empregado Efetivo
	F	Empregado Efetivo
	F	Empregado Efetivo
	F	Empregado Efetivo
	F	Empregado Efetivo
	F	Empregado Efetivo
	F	Empregado Efetivo
	F	Empregado Efetivo
	F	Empregado Efetivo
	F	Empregado Efetivo
	F	Empregado Efetivo
	F	Empregado Efetivo
	F	Empregado Efetivo
	F	Empregado Efetivo
	F	Empregado Efetivo
	F	Empregado Efetivo
	M	Empregado Efetivo
	F	Empregado Efetivo
	F	Empregado Efetivo
	M	Empregado Efetivo
	F	Empregado Efetivo
	F	Empregado Efetivo
	F	Empregado Efetivo
	M	Empregado Efetivo

	F	Empregado Efetivo
	F	Empregado Efetivo
	F	Empregado Efetivo
	F	Empregado Efetivo
	F	Empregado Efetivo
	F	Empregado Efetivo
	F	Empregado Efetivo
	M	Empregado Efetivo
	F	Empregado Efetivo
	M	Empregado Efetivo
	F	Empregado Efetivo
	F	Empregado Efetivo
	F	Empregado Efetivo
	F	Empregado Efetivo
	F	Empregado Efetivo
	F	Empregado Efetivo
	F	Empregado Efetivo
	F	Empregado Efetivo
	F	Empregado Efetivo
	F	Empregado Efetivo
	F	Empregado Efetivo
	F	Empregado Efetivo
	F	Empregado Efetivo
	F	Empregado Efetivo
	M	Empregado Efetivo
	F	Empregado Efetivo
	F	Empregado Efetivo
	F	Empregado Efetivo
	F	Empregado Efetivo
	F	Empregado Efetivo
	F	Empregado Efetivo
	F	Empregado Efetivo
	F	Empregado Efetivo
	F	Empregado Efetivo
	F	Empregado Efetivo
	F	Empregado Efetivo
	F	Empregado Efetivo
	M	Empregado Efetivo
	F	Empregado Efetivo
	F	Empregado Efetivo
	F	Empregado Efetivo
	F	Empregado Efetivo
	F	Empregado Efetivo
	F	Empregado Efetivo
	F	Empregado Efetivo
	F	Empregado Efetivo
	F	Empregado Efetivo
	F	Empregado Efetivo

	F	Empregado Efetivo
	F	Empregado Efetivo
	F	Empregado Efetivo
	F	Empregado Efetivo
	F	Empregado Efetivo
	F	Empregado Efetivo
	F	Empregado Efetivo
	F	Empregado Efetivo
	F	Empregado Efetivo
	F	Empregado Efetivo
	F	Empregado Efetivo
	F	Empregado Efetivo
	F	Empregado Efetivo
	F	Empregado Efetivo
	F	Empregado Efetivo
	F	Empregado Efetivo
	M	Empregado Efetivo
	F	Empregado Efetivo
	F	Empregado Efetivo
	F	Empregado Efetivo
	F	Empregado Efetivo
	F	Empregado Efetivo
	F	Empregado Efetivo
	F	Empregado Efetivo
	F	Empregado Efetivo
	F	Empregado Efetivo
	F	Empregado Efetivo
	F	Empregado Efetivo
	M	Empregado Efetivo
	M	Empregado Efetivo
	F	Empregado Efetivo
	F	Empregado Efetivo
	M	Empregado Efetivo
	F	Empregado Efetivo
	M	Empregado Efetivo
	M	Empregado Efetivo
	F	Empregado Efetivo
	F	Empregado Efetivo
	F	Empregado Efetivo
	F	Empregado Efetivo
	F	Empregado Efetivo
	M	Empregado Efetivo
	F	Empregado Efetivo
	F	Empregado Efetivo
	F	Empregado Efetivo
	F	Empregado Efetivo
	F	Empregado Efetivo
	F	Empregado Efetivo

	M	Empregado Efetivo
	F	Empregado Efetivo
	F	Empregado Efetivo
	F	Empregado Efetivo
	F	Empregado Efetivo
	F	Empregado Efetivo
	F	Empregado Efetivo
	F	Empregado Efetivo
	F	Empregado Efetivo
	F	Empregado Efetivo
	F	Empregado Efetivo
	F	Empregado Efetivo
	F	Empregado Efetivo
	F	Empregado Efetivo
	F	Empregado Efetivo
	F	Empregado Efetivo
	F	Empregado Efetivo
	F	Empregado Efetivo
	F	Empregado Efetivo
	F	Empregado Efetivo
	F	Empregado Efetivo
	F	Empregado Efetivo
	F	Empregado Efetivo
	F	Empregado Efetivo
	F	Empregado Efetivo
	F	Empregado Efetivo
	F	Empregado Efetivo
	F	Empregado Efetivo
	F	Empregado Efetivo
	F	Empregado Efetivo
	F	Empregado Efetivo
	F	Empregado Efetivo
	F	Empregado Efetivo
	F	Empregado Efetivo
	F	Empregado Efetivo
	F	Empregado Efetivo
	F	Empregado Efetivo
	F	Empregado Efetivo
	F	Empregado Efetivo
	F	Empregado Efetivo
	F	Empregado Efetivo
	F	Empregado Efetivo
	F	Empregado Efetivo
	F	Empregado Efetivo
	F	Empregado Efetivo
	M	Empregado Efetivo
	F	Empregado Efetivo
	F	Empregado Efetivo

	F	Empregado Efetivo
	M	Empregado Efetivo
	M	Empregado Efetivo
	F	Empregado Efetivo
	F	Empregado Efetivo
	F	Empregado Efetivo
	F	Empregado Efetivo
	F	Empregado Efetivo
	F	Empregado Efetivo
	F	Empregado Efetivo
	F	Empregado Efetivo
	F	Empregado Efetivo
	F	Empregado Efetivo
	F	Empregado Efetivo
	F	Empregado Efetivo
	F	Empregado Efetivo
	F	Empregado Efetivo
	F	Empregado Efetivo
	F	Empregado Efetivo
	M	Empregado Efetivo
	F	Empregado Efetivo
	F	Empregado Efetivo
	F	Empregado Efetivo
	F	Empregado Efetivo
	F	Empregado Efetivo
	F	Empregado Efetivo
	F	Empregado Efetivo
	F	Empregado Efetivo
	M	Empregado Efetivo
	F	Empregado Efetivo
	F	Empregado Efetivo
	F	Empregado Efetivo
	F	Empregado Efetivo
	F	Empregado Efetivo
	F	Empregado Efetivo
	F	Empregado Efetivo
	F	Empregado Efetivo
	F	Empregado Efetivo
	F	Empregado Efetivo
	F	Empregado Efetivo
	M	Empregado Efetivo
	F	Empregado Efetivo
	F	Empregado Efetivo
	F	Empregado Efetivo
	F	Empregado Efetivo
	F	Empregado Efetivo
	F	Empregado Efetivo
	F	Empregado Efetivo

	F	Empregado Efetivo
	F	Empregado Efetivo
	F	Empregado Efetivo
	F	Empregado Efetivo
	F	Empregado Efetivo
	F	Empregado Efetivo
	F	Empregado Efetivo
	M	Empregado Efetivo
	F	Empregado Efetivo
	F	Empregado Efetivo
	F	Empregado Efetivo
	F	Empregado Efetivo
	M	Empregado Efetivo
	F	Empregado Efetivo
	F	Empregado Efetivo
	F	Empregado Efetivo
	F	Empregado Efetivo
	F	Empregado Efetivo
	F	Empregado Efetivo
	F	Empregado Efetivo
	M	Empregado Efetivo
	F	Empregado Efetivo
	F	Empregado Efetivo
	F	Empregado Efetivo
	F	Empregado Efetivo
	F	Empregado Efetivo
	F	Empregado Efetivo
	F	Empregado Efetivo
	F	Empregado Efetivo
	F	Empregado Efetivo
	M	Empregado Efetivo
	F	Empregado Efetivo
	F	Empregado Efetivo
	F	Empregado Efetivo
	F	Empregado Efetivo
	F	Empregado Efetivo
	F	Empregado Efetivo
	F	Empregado Efetivo
	F	Empregado Efetivo
	F	Empregado Efetivo
	F	Empregado Efetivo
	F	Empregado Efetivo
	F	Empregado Efetivo
	F	Empregado Efetivo
	M	Empregado Efetivo
	F	Empregado Efetivo
	F	Empregado Efetivo
	F	Empregado Efetivo

	F	Empregado Efetivo
	M	Empregado Efetivo
	F	Empregado Efetivo
	M	Empregado Efetivo
	F	Empregado Efetivo
	F	Empregado Efetivo
	F	Empregado Efetivo
	F	Empregado Efetivo
	F	Empregado Efetivo
	F	Empregado Efetivo
	F	Empregado Efetivo
	F	Empregado Efetivo
	F	Empregado Efetivo
	F	Empregado Efetivo
	F	Empregado Efetivo
	F	Empregado Efetivo
	F	Empregado Efetivo
	F	Empregado Efetivo
	F	Empregado Efetivo
	F	Empregado Efetivo
	F	Empregado Efetivo
	M	Empregado Efetivo
	F	Empregado Efetivo
	F	Empregado Efetivo
	M	Empregado Efetivo
	F	Empregado Efetivo
	M	Empregado Efetivo
	F	Empregado Efetivo
	F	Empregado Efetivo
	F	Empregado Efetivo
	F	Empregado Efetivo
	F	Empregado Efetivo
	F	Empregado Efetivo
	M	Empregado Efetivo
	F	Empregado Efetivo
	M	Empregado Efetivo
	F	Empregado Efetivo
	F	Empregado Efetivo
	F	Empregado Efetivo
	F	Empregado Efetivo
	F	Empregado Efetivo
	F	Empregado Efetivo
	F	Empregado Efetivo
	F	Empregado Efetivo
	M	Empregado Efetivo
	F	Empregado Efetivo
	F	Empregado Efetivo
	F	Empregado Efetivo

	M	Empregado Efetivo
	F	Empregado Efetivo
	F	Empregado Efetivo
	F	Empregado Efetivo
	F	Empregado Efetivo
	F	Empregado Efetivo
	F	Empregado Efetivo
	F	Empregado Efetivo
	F	Empregado Efetivo
	F	Empregado Efetivo
	F	Empregado Efetivo
	F	Empregado Efetivo
	F	Empregado Efetivo
	F	Empregado Efetivo
	M	Empregado Efetivo
	F	Empregado Efetivo
	F	Empregado Efetivo
	F	Empregado Efetivo
	F	Empregado Efetivo
	F	Empregado Efetivo
	F	Empregado Efetivo
	M	Empregado Efetivo
	F	Empregado Efetivo
	F	Empregado Efetivo
	M	Empregado Efetivo
	F	Empregado Efetivo
	F	Empregado Efetivo
	F	Empregado Efetivo
	M	Empregado Efetivo
	M	Empregado Efetivo
	F	Empregado Efetivo
	F	Empregado Efetivo
	F	Empregado Efetivo
	F	Empregado Efetivo
	M	Empregado Efetivo
	F	Empregado Efetivo
	M	Empregado Efetivo
	F	Empregado Efetivo
	F	Empregado Efetivo
	F	Empregado Efetivo
	F	Empregado Efetivo
	F	Empregado Efetivo
	F	Empregado Efetivo
	F	Empregado Efetivo
	F	Empregado Efetivo
	F	Empregado Efetivo
	F	Empregado Efetivo
	M	Empregado Efetivo

	M	Empregado Efetivo
	F	Empregado Efetivo
	F	Empregado Efetivo
	M	Empregado Efetivo
	M	Empregado Efetivo
	F	Empregado Efetivo
	F	Empregado Efetivo
	F	Empregado Efetivo
	M	Empregado Efetivo
	M	Empregado Efetivo
	F	Empregado Efetivo
	F	Empregado Efetivo
	F	Empregado Efetivo
	F	Empregado Efetivo
	F	Empregado Efetivo
	F	Empregado Efetivo
	F	Empregado Efetivo
	F	Empregado Efetivo
	M	Empregado Efetivo
	F	Empregado Efetivo
	F	Empregado Efetivo
	F	Empregado Efetivo
	F	Empregado Efetivo
	F	Empregado Efetivo
	F	Empregado Efetivo
	M	Empregado Efetivo
	F	Empregado Efetivo
	F	Empregado Efetivo
	F	Empregado Efetivo
	F	Empregado Efetivo
	M	Empregado Efetivo
	F	Empregado Efetivo
	F	Empregado Efetivo
	F	Empregado Efetivo
	F	Empregado Efetivo
	F	Empregado Efetivo
	F	Empregado Efetivo
	F	Empregado Efetivo
	F	Empregado Efetivo
	F	Empregado Efetivo
	F	Empregado Efetivo
	F	Empregado Efetivo
	F	Empregado Efetivo
	F	Empregado Efetivo

	M	Empregado Efetivo
	F	Empregado Efetivo
	F	Empregado Efetivo
	F	Empregado Efetivo
	F	Empregado Efetivo
	F	Empregado Efetivo
	F	Empregado Efetivo
	F	Empregado Efetivo
	M	Empregado Efetivo
	F	Empregado Efetivo
	F	Empregado Efetivo
	F	Empregado Efetivo
	F	Empregado Efetivo
	F	Empregado Efetivo
	F	Empregado Efetivo
	F	Empregado Efetivo
	F	Empregado Efetivo
	M	Empregado Efetivo
	F	Empregado Efetivo
	F	Empregado Efetivo
	F	Empregado Efetivo
	F	Empregado Efetivo
	F	Empregado Efetivo
	M	Empregado Efetivo
	M	Empregado Efetivo
	F	Empregado Efetivo
	F	Empregado Efetivo
	M	Empregado Efetivo
	M	Empregado Efetivo
	F	Empregado Efetivo
	F	Empregado Efetivo
	F	Empregado Efetivo
	F	Empregado Efetivo
	F	Empregado Efetivo
	F	Empregado Efetivo
	F	Empregado Efetivo
	M	Empregado Efetivo
	F	Empregado Efetivo
	F	Empregado Efetivo
	F	Empregado Efetivo

	F	Empregado Efetivo
	F	Empregado Efetivo
	F	Empregado Efetivo
	F	Empregado Efetivo
	F	Empregado Efetivo
	F	Empregado Efetivo
	M	Empregado Efetivo
	M	Empregado Efetivo
	M	Empregado Efetivo
	M	Empregado Efetivo
	M	Empregado Efetivo
	F	Empregado Efetivo
	M	Empregado Efetivo
	M	Empregado Efetivo
	F	Empregado Efetivo
	F	Empregado Efetivo
	M	Empregado Efetivo
	F	Empregado Efetivo
	F	Empregado Efetivo
	M	Empregado Efetivo
	F	Empregado Efetivo
	M	Empregado Efetivo
	M	Empregado Efetivo
	M	Empregado Efetivo
	M	Empregado Efetivo
	M	Empregado Efetivo
	F	Empregado Efetivo
	M	Empregado Efetivo
	F	Empregado Efetivo
	M	Empregado Efetivo
	M	Empregado Efetivo
	F	Empregado Efetivo
	F	Empregado Efetivo
	M	Empregado Efetivo
	M	Empregado Efetivo
	M	Empregado Efetivo
	M	Empregado Efetivo
	F	Empregado Efetivo
	M	Empregado Efetivo
	F	Empregado Efetivo
	M	Empregado Efetivo
	M	Empregado Efetivo
	F	Empregado Efetivo
	F	Empregado Efetivo

	F	Empregado Efetivo
	M	Empregado Efetivo
	F	Empregado Efetivo
	F	Empregado Efetivo
	F	Empregado Efetivo
	M	Empregado Efetivo
	F	Empregado Efetivo
	F	Empregado Efetivo
	M	Empregado Efetivo
	M	Empregado Efetivo
	F	Empregado Efetivo
	F	Empregado Efetivo
	F	Empregado Efetivo
	F	Empregado Efetivo
	M	Empregado Efetivo
	M	Empregado Efetivo
	F	Empregado Efetivo
	F	Empregado Efetivo
	M	Empregado Efetivo
	F	Empregado Efetivo
	F	Empregado Efetivo
	F	Empregado Efetivo
	F	Empregado Efetivo
	M	Empregado Efetivo
	F	Empregado Efetivo
	F	Empregado Efetivo
	F	Empregado Efetivo
	M	Empregado Efetivo
	F	Empregado Efetivo
	F	Empregado Efetivo
	F	Empregado Efetivo
	F	Empregado Efetivo
	M	Empregado Efetivo
	F	Empregado Efetivo
	F	Empregado Efetivo
	F	Empregado Efetivo
	F	Empregado Efetivo
	F	Empregado Efetivo
	F	Empregado Efetivo
	F	Empregado Efetivo
	F	Empregado Efetivo
	F	Empregado Efetivo
	M	Empregado Efetivo
	F	Empregado Efetivo

	F	Empregado Efetivo
	F	Empregado Efetivo
	F	Empregado Efetivo
	M	Empregado Efetivo
	F	Empregado Efetivo
	F	Empregado Efetivo
	F	Empregado Efetivo
	F	Empregado Efetivo
	F	Empregado Efetivo
	M	Empregado Efetivo
	F	Empregado Efetivo
	F	Empregado Efetivo
	M	Empregado Efetivo
	F	Empregado Efetivo
	M	Empregado Efetivo
	M	Empregado Efetivo
	F	Empregado Efetivo
	F	Empregado Efetivo
	F	Empregado Efetivo
	F	Empregado Efetivo
	F	Empregado Efetivo
	F	Empregado Efetivo
	F	Empregado Efetivo
	F	Empregado Efetivo
	F	Empregado Efetivo
	F	Empregado Efetivo
	M	Empregado Efetivo
	F	Empregado Efetivo
	F	Empregado Efetivo
	M	Empregado Efetivo
	F	Empregado Efetivo
	M	Empregado Efetivo
	M	Empregado Efetivo
	M	Empregado Efetivo
	M	Empregado Efetivo
	F	Empregado Efetivo
	M	Empregado Efetivo
	M	Empregado Efetivo
	M	Empregado Efetivo
	M	Empregado Efetivo
	M	Empregado Efetivo

	M	Empregado Efetivo
	M	Empregado Efetivo
	M	Empregado Efetivo
	M	Empregado Efetivo
	M	Empregado Efetivo
	M	Empregado Efetivo
	F	Empregado Efetivo
	M	Empregado Efetivo
	M	Empregado Efetivo
	M	Empregado Efetivo
	M	Empregado Efetivo
	M	Empregado Efetivo
	F	Empregado Efetivo
	M	Empregado Efetivo
	F	Empregado Efetivo
	F	Empregado Efetivo
	F	Empregado Efetivo
	F	Empregado Efetivo
	F	Empregado Efetivo
	F	Empregado Efetivo
	F	Empregado Efetivo
	F	Empregado Efetivo
	F	Empregado Efetivo
	F	Empregado Efetivo
	M	Empregado Efetivo
	M	Empregado Efetivo
	F	Empregado Efetivo
	M	Empregado Efetivo
	M	Empregado Efetivo
	F	Empregado Efetivo
	F	Empregado Efetivo
	F	Empregado Efetivo
	M	Empregado Efetivo
	F	Empregado Efetivo
	F	Empregado Efetivo
	F	Empregado Efetivo
	F	Empregado Efetivo

	F	Empregado Efetivo
	F	Empregado Efetivo
	F	Empregado Efetivo
	F	Empregado Efetivo
	F	Empregado Efetivo
	F	Empregado Efetivo
	F	Empregado Efetivo
	F	Empregado Efetivo
	F	Empregado Efetivo
	F	Empregado Efetivo
	F	Empregado Efetivo
	F	Empregado Efetivo
	F	Empregado Efetivo
	F	Empregado Efetivo
	F	Empregado Efetivo
	F	Empregado Efetivo
	F	Empregado Efetivo
	F	Empregado Efetivo
	M	Empregado Efetivo
	F	Empregado Efetivo
	F	Empregado Efetivo
	F	Empregado Efetivo
	F	Empregado Efetivo
	F	Empregado Efetivo
	F	Empregado Efetivo
	M	Empregado Efetivo
	F	Empregado Efetivo
	M	Empregado Efetivo
	M	Empregado Efetivo
	M	Empregado Efetivo
	F	Empregado Efetivo
	M	Empregado Efetivo
	F	Empregado Efetivo
	F	Empregado Efetivo
	M	Empregado Efetivo
	F	Empregado Efetivo
	F	Empregado Efetivo
	F	Empregado Efetivo
	M	Empregado Efetivo

	F	Empregado Efetivo
	M	Empregado Efetivo
	M	Empregado Efetivo
	F	Empregado Efetivo
	F	Empregado Efetivo
	M	Empregado Efetivo
	F	Empregado Efetivo
	F	Empregado Efetivo
	F	Empregado Efetivo
	M	Empregado Efetivo
	M	Empregado Efetivo
	F	Empregado Efetivo
	M	Empregado Efetivo
	F	Empregado Efetivo
	M	Empregado Efetivo
	F	Empregado Efetivo
	F	Empregado Efetivo
	M	Empregado Efetivo
	M	Empregado Efetivo
	F	Empregado Efetivo
	M	Empregado Efetivo
	F	Empregado Efetivo
	F	Empregado Efetivo
	M	Empregado Efetivo
	M	Empregado Efetivo
	F	Empregado Efetivo
	F	Empregado Efetivo
	F	Empregado Efetivo
	M	Empregado Efetivo
	M	Empregado Efetivo
	M	Empregado Efetivo
	F	Empregado Efetivo
	F	Empregado Efetivo
	F	Empregado Efetivo
	F	Empregado Efetivo
	F	Empregado Efetivo
	M	Empregado Efetivo
	F	Empregado Efetivo
	M	Empregado Efetivo
	M	Empregado Efetivo
	M	Empregado Efetivo
	F	Empregado Efetivo
	F	Empregado Efetivo
	F	Empregado Efetivo
	F	Empregado Efetivo

	F	Empregado Efetivo
	F	Empregado Efetivo
	F	Empregado Efetivo
	F	Empregado Efetivo
	F	Empregado Efetivo
	F	Empregado Efetivo
	M	Empregado Efetivo
	F	Empregado Efetivo
	F	Empregado Efetivo
	F	Empregado Efetivo
	F	Empregado Efetivo
	F	Empregado Efetivo
	F	Empregado Efetivo
	F	Empregado Efetivo
	F	Empregado Efetivo
	F	Empregado Efetivo
	F	Empregado Efetivo
	F	Empregado Efetivo
	F	Empregado Efetivo
	M	Empregado Efetivo
	F	Empregado Efetivo
	F	Empregado Efetivo
	F	Empregado Efetivo
	F	Empregado Efetivo
	F	Empregado Efetivo
	F	Empregado Efetivo
	F	Empregado Efetivo
	F	Empregado Efetivo
	M	Empregado Efetivo
	F	Empregado Efetivo
	F	Empregado Efetivo
	F	Empregado Efetivo
	F	Empregado Efetivo
	F	Empregado Efetivo
	F	Empregado Efetivo
	F	Empregado Efetivo
	F	Empregado Efetivo
	F	Empregado Efetivo
	F	Empregado Efetivo
	F	Empregado Efetivo
	M	Empregado Efetivo
	F	Empregado Efetivo
	F	Empregado Efetivo
	F	Empregado Efetivo
	F	Empregado Efetivo
	F	Empregado Efetivo
	F	Empregado Efetivo
	M	Empregado Efetivo

	F	Empregado Efetivo
	F	Empregado Efetivo
	F	Empregado Efetivo
	F	Empregado Efetivo
	F	Empregado Efetivo
	M	Empregado Efetivo
	F	Empregado Efetivo
	M	Empregado Efetivo
	F	Empregado Efetivo
	F	Empregado Efetivo
	F	Empregado Efetivo
	F	Empregado Efetivo
	F	Empregado Efetivo
	F	Empregado Efetivo
	F	Empregado Efetivo
	M	Empregado Efetivo
	F	Empregado Efetivo
	F	Empregado Efetivo
	F	Empregado Efetivo
	M	Empregado Efetivo
	F	Empregado Efetivo
	F	Empregado Efetivo
	F	Empregado Efetivo
	F	Empregado Efetivo
	F	Empregado Efetivo
	F	Empregado Efetivo
	F	Empregado Efetivo
	F	Empregado Efetivo
	F	Empregado Efetivo
	M	Empregado Efetivo
	F	Empregado Efetivo
	F	Empregado Efetivo
	F	Empregado Efetivo
	F	Empregado Efetivo
	F	Empregado Efetivo
	M	Empregado Efetivo
	F	Empregado Efetivo
	F	Empregado Efetivo
	F	Empregado Efetivo
	F	Empregado Efetivo
	F	Empregado Efetivo
	F	Empregado Efetivo

	F	Empregado Efetivo
	F	Empregado Efetivo
	F	Empregado Efetivo
	F	Empregado Efetivo
	F	Empregado Efetivo
	F	Empregado Efetivo
	F	Empregado Efetivo
	F	Empregado Efetivo
	F	Empregado Efetivo
	F	Empregado Efetivo
	F	Empregado Efetivo
	F	Empregado Efetivo
	F	Empregado Efetivo
	M	Empregado Efetivo
	F	Empregado Efetivo
	F	Empregado Efetivo
	F	Empregado Efetivo
	F	Empregado Efetivo
	F	Empregado Efetivo
	F	Empregado Efetivo
	F	Empregado Efetivo
	F	Empregado Efetivo
	F	Empregado Efetivo
	M	Empregado Efetivo
	F	Empregado Efetivo
	F	Empregado Efetivo
	F	Empregado Efetivo
	F	Empregado Efetivo
	F	Empregado Efetivo
	F	Empregado Efetivo
	F	Empregado Efetivo
	F	Empregado Efetivo

	F	Empregado Efetivo
	F	Empregado Efetivo
	M	Empregado Efetivo
	F	Empregado Efetivo
	F	Empregado Efetivo
	F	Empregado Efetivo
	F	Empregado Efetivo
	F	Empregado Efetivo
	M	Empregado Efetivo
	F	Empregado Efetivo
	F	Empregado Efetivo
	F	Empregado Efetivo
	F	Empregado Efetivo
	F	Empregado Efetivo
	F	Empregado Efetivo
	F	Empregado Efetivo
	F	Empregado Efetivo
	F	Empregado Efetivo
	F	Empregado Efetivo
	F	Empregado Efetivo
	F	Empregado Efetivo
	F	Empregado Efetivo
	F	Empregado Efetivo
	M	Empregado Efetivo
	F	Empregado Efetivo
	F	Empregado Efetivo
	F	Empregado Efetivo
	F	Empregado Efetivo
	F	Empregado Efetivo
	F	Empregado Efetivo
	F	Empregado Efetivo
	F	Empregado Efetivo
	M	Empregado Efetivo
	F	Empregado Efetivo
	M	Empregado Efetivo
	M	Empregado Efetivo
	F	Empregado Efetivo
	F	Empregado Efetivo

	F	Empregado Efetivo
	F	Empregado Efetivo
	M	Empregado Efetivo
	M	Empregado Efetivo
	M	Empregado Efetivo
	M	Empregado Efetivo
	F	Empregado Efetivo
	M	Empregado Efetivo
	F	Empregado Efetivo
	M	Empregado Efetivo
	F	Empregado Efetivo
	F	Empregado Efetivo
	F	Empregado Efetivo
	F	Empregado Efetivo
	M	Empregado Efetivo
	F	Empregado Efetivo
	M	Empregado Efetivo
	F	Empregado Efetivo
	M	Empregado Efetivo
	F	Empregado Efetivo
	F	Empregado Efetivo
	F	Empregado Efetivo
	F	Empregado Efetivo
	M	Empregado Efetivo
	F	Empregado Efetivo
	M	Empregado Efetivo
	F	Empregado Efetivo
	M	Empregado Efetivo
	F	Empregado Efetivo
	F	Empregado Efetivo
	F	Empregado Efetivo
	F	Empregado Efetivo
	F	Empregado Efetivo
	M	Empregado Efetivo

	F	Empregado Efetivo
	F	Empregado Efetivo
	F	Empregado Efetivo
	F	Empregado Efetivo
	F	Empregado Efetivo
	F	Empregado Efetivo
	M	Empregado Efetivo
	F	Empregado Efetivo
	F	Empregado Efetivo
	F	Empregado Efetivo
	M	Empregado Efetivo
	F	Empregado Efetivo
	M	Empregado Efetivo
	F	Empregado Efetivo
	F	Empregado Efetivo
	F	Empregado Efetivo
	F	Empregado Efetivo
	F	Empregado Efetivo
	F	Empregado Efetivo
	F	Empregado Efetivo
	F	Empregado Efetivo
	F	Empregado Efetivo
	F	Empregado Efetivo
	F	Empregado Efetivo
	F	Empregado Efetivo
	F	Empregado Efetivo
	F	Empregado Efetivo
	F	Empregado Efetivo
	M	Empregado Efetivo
	F	Empregado Efetivo
	F	Empregado Efetivo
	F	Empregado Efetivo
	F	Empregado Efetivo
	F	Empregado Efetivo
	F	Empregado Efetivo
	F	Empregado Efetivo
	F	Empregado Efetivo
	M	Empregado Efetivo
	F	Empregado Efetivo
	F	Empregado Efetivo
	F	Empregado Efetivo
	F	Empregado Efetivo
	F	Empregado Efetivo
	F	Empregado Efetivo
	F	Empregado Efetivo
	F	Empregado Efetivo
	F	Empregado Efetivo
	F	Empregado Efetivo

	F	Empregado Efetivo
	F	Empregado Efetivo
	F	Empregado Efetivo
	F	Empregado Efetivo
	F	Empregado Efetivo
	F	Empregado Efetivo
	F	Empregado Efetivo
	M	Empregado Efetivo
	F	Empregado Efetivo
	F	Empregado Efetivo
	F	Empregado Efetivo
	F	Empregado Efetivo
	F	Empregado Efetivo
	F	Empregado Efetivo
	F	Empregado Efetivo
	F	Empregado Efetivo
	F	Empregado Efetivo
	F	Empregado Efetivo
	F	Empregado Efetivo
	F	Empregado Efetivo
	F	Empregado Efetivo
	F	Empregado Efetivo
	F	Empregado Efetivo
	F	Empregado Efetivo
	F	Empregado Efetivo
	F	Empregado Efetivo
	F	Empregado Efetivo
	F	Empregado Efetivo
	F	Empregado Efetivo
	F	Empregado Efetivo
	F	Empregado Efetivo
	F	Empregado Efetivo
	F	Empregado Efetivo
	F	Empregado Efetivo
	F	Empregado Efetivo
	F	Empregado Efetivo
	F	Empregado Efetivo
	F	Empregado Efetivo
	F	Empregado Efetivo
	F	Empregado Efetivo
	F	Empregado Efetivo
	F	Empregado Efetivo
	F	Empregado Efetivo
	F	Empregado Efetivo
	F	Empregado Efetivo
	F	Empregado Efetivo
	F	Empregado Efetivo
	F	Empregado Efetivo

	F	Empregado Efetivo
	F	Empregado Efetivo
	F	Empregado Efetivo
	F	Empregado Efetivo
	F	Empregado Efetivo
	F	Empregado Efetivo
	F	Empregado Efetivo
	F	Empregado Efetivo
	F	Empregado Efetivo
	F	Empregado Efetivo
	F	Empregado Efetivo
	F	Empregado Efetivo
	F	Empregado Efetivo
	F	Empregado Efetivo
	F	Empregado Efetivo
	F	Empregado Efetivo
	F	Empregado Efetivo
	F	Empregado Efetivo
	M	Empregado Efetivo
	M	Empregado Efetivo
	F	Empregado Efetivo
	F	Empregado Efetivo
	F	Empregado Efetivo
	F	Empregado Efetivo
	F	Empregado Efetivo
	F	Empregado Efetivo
	F	Empregado Efetivo
	F	Empregado Efetivo
	F	Empregado Efetivo
	F	Empregado Efetivo
	F	Empregado Efetivo
	F	Empregado Efetivo
	F	Empregado Efetivo
	F	Empregado Efetivo
	F	Empregado Efetivo
	F	Empregado Efetivo
	F	Empregado Efetivo
	M	Empregado Efetivo
	F	Empregado Efetivo
	M	Empregado Efetivo
	F	Empregado Efetivo
	F	Empregado Efetivo
	M	Empregado Efetivo
	M	Empregado Efetivo
	F	Empregado Efetivo
	M	Empregado Efetivo

	F	Empregado Efetivo
	M	Empregado Efetivo
	F	Empregado Efetivo
	F	Empregado Efetivo
	M	Empregado Efetivo
	F	Empregado Efetivo
	F	Empregado Efetivo
	F	Empregado Efetivo
	F	Empregado Efetivo
	M	Empregado Efetivo
	F	Empregado Efetivo
	M	Empregado Efetivo
	F	Empregado Efetivo
	M	Empregado Efetivo
	M	Empregado Efetivo
	F	Empregado Efetivo
	F	Empregado Efetivo
	M	Empregado Efetivo
	F	Empregado Efetivo
	F	Empregado Efetivo
	F	Empregado Efetivo
	M	Empregado Efetivo
	F	Empregado Efetivo
	M	Empregado Efetivo
	M	Empregado Efetivo
	F	Empregado Efetivo
	F	Empregado Efetivo
	F	Empregado Efetivo
	M	Empregado Efetivo
	M	Empregado Efetivo
	M	Empregado Efetivo
	M	Empregado Efetivo

	F	Empregado Efetivo
	F	Empregado Efetivo
	M	Empregado Efetivo
	F	Empregado Efetivo
	F	Empregado Efetivo
	F	Empregado Efetivo
	M	Empregado Efetivo
	M	Empregado Efetivo
	M	Empregado Efetivo
	F	Empregado Efetivo
	M	Empregado Efetivo
	F	Empregado Efetivo
	F	Empregado Efetivo
	F	Empregado Efetivo
	F	Empregado Efetivo
	F	Empregado Efetivo
	F	Empregado Efetivo
	M	Empregado Efetivo
	F	Empregado Efetivo
	F	Empregado Efetivo
	F	Empregado Efetivo
	F	Empregado Efetivo
	M	Empregado Efetivo
	M	Empregado Efetivo
	F	Empregado Efetivo
	M	Empregado Efetivo
	F	Empregado Efetivo
	M	Empregado Efetivo
	M	Empregado Efetivo
	F	Empregado Efetivo
	M	Empregado Efetivo
	M	Empregado Efetivo
	M	Empregado Efetivo
	M	Empregado Efetivo
	M	Empregado Efetivo
	M	Empregado Efetivo
	F	Empregado Efetivo
	M	Empregado Efetivo
	F	Empregado Efetivo
	M	Empregado Efetivo
	F	Empregado Efetivo
	F	Empregado Efetivo

	M	Empregado Temporário
	M	Empregado Efetivo
	F	Empregado Efetivo
	F	Empregado Efetivo
	M	Empregado Efetivo
	F	Empregado Efetivo
	F	Empregado Efetivo
	M	Empregado Efetivo
	M	Empregado Efetivo
	F	Empregado Efetivo
	F	Empregado Efetivo
	F	Empregado Efetivo
	F	Empregado Efetivo
	F	Empregado Efetivo
	F	Empregado Efetivo
	F	Empregado Efetivo
	F	Empregado Efetivo
	F	Empregado Efetivo
	F	Empregado Efetivo
	F	Empregado Efetivo
	F	Empregado Efetivo
	F	Empregado Efetivo
	F	Empregado Efetivo
	F	Empregado Efetivo
	F	Empregado Efetivo
	F	Empregado Efetivo
	F	Empregado Efetivo
	M	Empregado Efetivo
	M	Empregado Efetivo
	F	Empregado Efetivo
	F	Empregado Efetivo
	F	Empregado Efetivo
	F	Empregado Efetivo
	F	Empregado Efetivo

	M	Empregado Efetivo
	F	Empregado Efetivo
	F	Empregado Efetivo
	F	Empregado Efetivo
	F	Empregado Efetivo
	F	Empregado Efetivo
	F	Empregado Efetivo
	F	Empregado Efetivo
	F	Empregado Efetivo
	F	Empregado Efetivo
	F	Empregado Efetivo
	F	Empregado Efetivo
	F	Empregado Efetivo
	F	Empregado Efetivo
	F	Empregado Efetivo
	F	Empregado Efetivo
	F	Empregado Efetivo
	F	Empregado Efetivo
	M	Empregado Efetivo
	F	Empregado Efetivo
	F	Empregado Efetivo
	F	Empregado Efetivo
	F	Empregado Efetivo
	F	Empregado Efetivo
	F	Empregado Efetivo
	F	Empregado Efetivo
	F	Empregado Efetivo
	F	Empregado Efetivo
	F	Empregado Efetivo
	M	Empregado Efetivo
	F	Empregado Efetivo
	F	Empregado Efetivo

	F	Empregado Efetivo
	F	Empregado Efetivo
	F	Empregado Efetivo
	F	Empregado Efetivo
	F	Empregado Efetivo
	F	Empregado Efetivo
	F	Empregado Efetivo
	F	Empregado Efetivo
	M	Empregado Efetivo
	F	Empregado Efetivo
	M	Empregado Efetivo
	F	Empregado Efetivo
	F	Empregado Efetivo
	F	Empregado Efetivo
	F	Empregado Efetivo
	F	Empregado Efetivo
	F	Empregado Efetivo
	F	Empregado Efetivo
	F	Empregado Efetivo
	F	Empregado Efetivo
	F	Empregado Efetivo
	F	Empregado Efetivo
	F	Empregado Efetivo
	F	Empregado Efetivo
	F	Empregado Efetivo
	F	Empregado Efetivo
	F	Empregado Efetivo
	F	Empregado Efetivo
	F	Empregado Efetivo
	F	Empregado Efetivo
	F	Empregado Efetivo
	F	Empregado Efetivo
	F	Empregado Efetivo
	F	Empregado Efetivo
	F	Empregado Efetivo
	F	Empregado Efetivo
	F	Empregado Efetivo
	F	Empregado Efetivo

	F	Empregado Efetivo
	F	Empregado Efetivo
	F	Empregado Efetivo
	F	Empregado Efetivo
	F	Empregado Efetivo
	F	Empregado Efetivo
	F	Empregado Efetivo
	F	Empregado Efetivo
	F	Empregado Efetivo
	F	Empregado Efetivo
	F	Empregado Efetivo
	F	Empregado Efetivo
	F	Empregado Efetivo
	F	Empregado Efetivo
	F	Empregado Efetivo
	F	Empregado Efetivo
	F	Empregado Efetivo
	F	Empregado Efetivo
	F	Empregado Efetivo
	F	Empregado Efetivo
	F	Empregado Efetivo
	F	Empregado Efetivo
	F	Empregado Efetivo
	F	Empregado Efetivo
	F	Empregado Efetivo
	F	Empregado Efetivo
	F	Empregado Efetivo
	F	Empregado Efetivo
	F	Empregado Efetivo
	F	Empregado Efetivo
	F	Empregado Efetivo
	F	Empregado Efetivo

	F	Empregado Efetivo
	F	Empregado Efetivo
	M	Empregado Efetivo
	F	Empregado Efetivo
	M	Empregado Efetivo
	M	Empregado Efetivo
	F	Empregado Efetivo
	F	Empregado Efetivo
	F	Empregado Efetivo
	F	Empregado Efetivo
	F	Empregado Efetivo
	F	Empregado Efetivo
	F	Empregado Efetivo
	F	Empregado Efetivo
	F	Empregado Efetivo
	M	Empregado Efetivo
	M	Empregado Efetivo
	M	Empregado Efetivo
	M	Empregado Efetivo
	M	Empregado Efetivo
	M	Empregado Efetivo
	M	Empregado Efetivo
	M	Empregado Efetivo
	M	Empregado Efetivo
	M	Empregado Efetivo
	M	Empregado Efetivo
	M	Empregado Efetivo
	M	Empregado Efetivo
	M	Empregado Efetivo
	M	Empregado Efetivo
	M	Empregado Efetivo
	M	Empregado Efetivo

	M	Empregado Efetivo
	M	Empregado Efetivo
	M	Empregado Efetivo
	M	Empregado Efetivo
	M	Empregado Efetivo
	M	Empregado Temporário
	M	Empregado Efetivo
	F	Empregado Efetivo
	M	Empregado Efetivo
	F	Empregado Efetivo
	M	Empregado Efetivo
	M	Empregado Efetivo
	M	Empregado Efetivo
	F	Empregado Efetivo
	F	Empregado Efetivo
	F	Empregado Efetivo
	F	Empregado Efetivo
	F	Empregado Efetivo
	F	Empregado Efetivo
	F	Empregado Efetivo
	F	Empregado Efetivo
	F	Empregado Efetivo
	F	Empregado Efetivo
	F	Empregado Efetivo
	F	Empregado Efetivo
	M	Empregado Efetivo
	F	Empregado Efetivo
	F	Empregado Efetivo
	M	Empregado Efetivo
	F	Empregado Efetivo
	F	Empregado Efetivo
	F	Empregado Efetivo
	F	Empregado Efetivo
	M	Empregado Efetivo
	F	Empregado Efetivo
	F	Empregado Efetivo
	F	Empregado Efetivo
	F	Empregado Efetivo

	F	Empregado Efetivo
	F	Empregado Efetivo
	F	Empregado Efetivo
	F	Empregado Efetivo
	F	Empregado Efetivo
	F	Empregado Efetivo
	F	Empregado Efetivo
	F	Empregado Efetivo
	M	Empregado Efetivo
	F	Empregado Efetivo
	F	Empregado Efetivo
	F	Empregado Efetivo
	F	Empregado Efetivo
	F	Empregado Efetivo
	F	Empregado Efetivo
	F	Empregado Efetivo
	F	Empregado Efetivo
	M	Empregado Efetivo
	M	Empregado Efetivo
	M	Empregado Efetivo
	F	Empregado Efetivo
	M	Empregado Efetivo
	F	Empregado Efetivo
	F	Empregado Efetivo
	F	Empregado Efetivo
	F	Empregado Efetivo
	M	Empregado Efetivo
	F	Empregado Efetivo
	F	Empregado Efetivo
	M	Empregado Efetivo
	F	Empregado Efetivo
	M	Empregado Efetivo

	M	Empregado Efetivo
	M	Empregado Efetivo
	F	Empregado Efetivo
	F	Empregado Efetivo
	F	Empregado Efetivo
	F	Empregado Efetivo
	M	Empregado Efetivo
	F	Empregado Efetivo
	F	Empregado Efetivo
	M	Empregado Efetivo
	F	Empregado Efetivo
	F	Empregado Efetivo
	F	Empregado Efetivo
	F	Empregado Efetivo
	F	Empregado Efetivo
	M	Empregado Efetivo
	F	Empregado Efetivo
	F	Empregado Efetivo
	F	Empregado Efetivo
	F	Empregado Efetivo
	F	Empregado Efetivo
	F	Empregado Efetivo
	F	Empregado Efetivo
	F	Empregado Efetivo
	F	Empregado Efetivo
	F	Empregado Efetivo
	F	Empregado Efetivo
	M	Empregado Efetivo
	M	Empregado Efetivo
	F	Empregado Efetivo
	F	Empregado Efetivo
	M	Empregado Efetivo
	M	Empregado Efetivo
	F	Empregado Efetivo
	F	Empregado Efetivo
	F	Empregado Efetivo
	F	Empregado Efetivo
	F	Empregado Efetivo
	F	Empregado Efetivo
	F	Empregado Efetivo
	F	Empregado Efetivo
	M	Empregado Efetivo
	F	Empregado Efetivo
	F	Empregado Efetivo

	F	Empregado Efetivo
	F	Empregado Efetivo
	M	Empregado Efetivo
	M	Empregado Efetivo
	F	Empregado Efetivo
	F	Empregado Efetivo
	F	Empregado Efetivo
	F	Empregado Efetivo
	M	Empregado Efetivo
	M	Empregado Efetivo
	F	Empregado Efetivo
	F	Empregado Efetivo
	F	Empregado Efetivo
	F	Empregado Efetivo
	M	Empregado Efetivo
	F	Empregado Efetivo
	F	Empregado Efetivo
	F	Empregado Efetivo
	F	Empregado Efetivo
	F	Empregado Efetivo
	M	Empregado Efetivo
	M	Empregado Efetivo
	F	Empregado Efetivo
	M	Empregado Efetivo
	F	Empregado Efetivo
	F	Empregado Efetivo
	M	Empregado Efetivo
	F	Empregado Efetivo
	F	Empregado Efetivo
	F	Empregado Efetivo
	M	Empregado Efetivo
	F	Empregado Efetivo
	F	Empregado Efetivo
	F	Empregado Efetivo
	M	Empregado Efetivo
	F	Empregado Efetivo
	M	Empregado Efetivo
	F	Empregado Efetivo
	F	Empregado Efetivo
	F	Empregado Efetivo
	F	Empregado Efetivo
	F	Empregado Efetivo
	F	Empregado Efetivo
	F	Empregado Efetivo
	F	Empregado Efetivo
	F	Empregado Efetivo
	F	Empregado Efetivo
	F	Empregado Efetivo

	F	Empregado Efetivo
	F	Empregado Efetivo
	F	Empregado Efetivo
	M	Empregado Efetivo
	F	Empregado Efetivo
	F	Empregado Efetivo
	M	Empregado Efetivo
	F	Empregado Efetivo
	F	Empregado Efetivo
	F	Empregado Efetivo
	M	Empregado Efetivo
	F	Empregado Efetivo
	M	Empregado Efetivo
	F	Empregado Efetivo
	F	Empregado Efetivo
	M	Empregado Efetivo
	F	Empregado Efetivo
	M	Empregado Efetivo
	F	Empregado Efetivo
	M	Empregado Efetivo
	F	Empregado Efetivo
	F	Empregado Efetivo
	F	Empregado Efetivo
	F	Empregado Efetivo
	F	Empregado Efetivo
	F	Empregado Efetivo
	M	Empregado Efetivo
	F	Empregado Efetivo
	M	Empregado Efetivo
	F	Empregado Efetivo
	M	Empregado Efetivo
	F	Empregado Efetivo
	F	Empregado Efetivo
	F	Empregado Efetivo
	M	Empregado Efetivo
	F	Empregado Efetivo
	F	Empregado Efetivo
	F	Empregado Efetivo
	F	Empregado Efetivo
	M	Empregado Efetivo
	F	Empregado Efetivo
	F	Empregado Efetivo
	F	Empregado Efetivo
	F	Empregado Efetivo
	M	Empregado Efetivo
	F	Empregado Efetivo
	F	Empregado Efetivo
	F	Empregado Efetivo

	F	Empregado Efetivo
	F	Empregado Efetivo
	M	Empregado Efetivo
	F	Empregado Efetivo
	F	Empregado Efetivo
	F	Empregado Efetivo
	F	Empregado Efetivo
	M	Empregado Efetivo
	F	Empregado Efetivo
	F	Empregado Efetivo
	F	Empregado Efetivo
	M	Empregado Efetivo
	F	Empregado Efetivo
	F	Empregado Efetivo
	F	Empregado Efetivo
	F	Empregado Efetivo
	F	Empregado Efetivo
	F	Empregado Efetivo
	F	Empregado Efetivo
	F	Empregado Efetivo
	F	Empregado Efetivo
	F	Empregado Efetivo
	F	Empregado Efetivo
	F	Empregado Efetivo
	M	Empregado Efetivo
	F	Empregado Efetivo
	F	Empregado Efetivo
	F	Empregado Efetivo
	M	Empregado Efetivo
	F	Empregado Efetivo
	M	Empregado Efetivo
	F	Empregado Efetivo
	F	Empregado Efetivo
	F	Empregado Efetivo
	F	Empregado Efetivo
	F	Empregado Efetivo
	F	Empregado Efetivo
	F	Empregado Efetivo
	F	Empregado Efetivo
	F	Empregado Efetivo
	F	Empregado Efetivo
	F	Empregado Efetivo
	M	Empregado Efetivo
	F	Empregado Efetivo
	F	Empregado Efetivo
	F	Empregado Efetivo
	F	Empregado Efetivo
	M	Empregado Efetivo

	F	Empregado Efetivo
	M	Empregado Efetivo
	F	Empregado Efetivo
	F	Empregado Efetivo
	F	Empregado Efetivo
	F	Empregado Efetivo
	M	Empregado Efetivo
	F	Empregado Efetivo
	F	Empregado Efetivo
	F	Empregado Efetivo
	F	Empregado Efetivo
	F	Empregado Efetivo
	F	Empregado Efetivo
	F	Empregado Efetivo
	F	Empregado Efetivo
	F	Empregado Efetivo
	F	Empregado Efetivo
	F	Empregado Efetivo
	F	Empregado Efetivo
	F	Empregado Efetivo
	F	Empregado Efetivo
	M	Empregado Efetivo
	F	Empregado Efetivo
	F	Empregado Efetivo
	F	Empregado Efetivo
	F	Empregado Efetivo
	F	Empregado Efetivo
	F	Empregado Efetivo
	F	Empregado Efetivo
	F	Empregado Efetivo
	M	Empregado Efetivo
	F	Empregado Efetivo
	F	Empregado Efetivo
	F	Empregado Efetivo
	F	Empregado Efetivo
	F	Empregado Efetivo
	F	Empregado Efetivo
	F	Empregado Efetivo
	F	Empregado Efetivo
	M	Empregado Efetivo
	M	Empregado Efetivo
	F	Empregado Efetivo
	F	Empregado Efetivo
	F	Empregado Efetivo
	F	Empregado Efetivo
	F	Empregado Efetivo
	M	Empregado Efetivo
	F	Empregado Efetivo

	F	Empregado Efetivo
	F	Empregado Efetivo
	F	Empregado Efetivo
	F	Empregado Efetivo
	F	Empregado Efetivo
	F	Empregado Efetivo
	F	Empregado Efetivo
	F	Empregado Efetivo
	F	Empregado Efetivo
	F	Empregado Efetivo
	F	Empregado Efetivo
	F	Empregado Efetivo
	F	Empregado Efetivo
	F	Empregado Efetivo
	F	Empregado Efetivo
	F	Empregado Efetivo
	F	Empregado Efetivo
	F	Empregado Efetivo
	F	Empregado Efetivo
	F	Empregado Efetivo
	F	Empregado Efetivo
	F	Empregado Efetivo
	F	Empregado Efetivo
	F	Empregado Efetivo
	F	Empregado Efetivo
	F	Empregado Efetivo
	F	Empregado Efetivo
	F	Empregado Efetivo
	M	Empregado Efetivo
	F	Empregado Efetivo
	F	Empregado Efetivo
	F	Empregado Efetivo
	F	Empregado Efetivo
	F	Empregado Efetivo
	F	Empregado Efetivo
	F	Empregado Efetivo
	F	Empregado Efetivo
	F	Empregado Efetivo
	F	Empregado Efetivo
	M	Empregado Efetivo
	F	Empregado Efetivo
	F	Empregado Efetivo
	F	Empregado Efetivo
	F	Empregado Efetivo
	F	Empregado Efetivo
	F	Empregado Efetivo
	F	Empregado Efetivo
	F	Empregado Efetivo

	F	Empregado Efetivo
	F	Empregado Efetivo
	F	Empregado Efetivo
	F	Empregado Efetivo
	F	Empregado Efetivo
	F	Empregado Efetivo
	F	Empregado Efetivo
	F	Empregado Efetivo
	F	Empregado Efetivo
	F	Empregado Efetivo
	F	Empregado Efetivo
	F	Empregado Efetivo
	F	Empregado Efetivo
	F	Empregado Efetivo
	F	Empregado Efetivo
	F	Empregado Efetivo
	F	Empregado Efetivo
	F	Empregado Efetivo
	F	Empregado Efetivo
	F	Empregado Efetivo
	F	Empregado Efetivo
	F	Empregado Efetivo
	F	Empregado Efetivo
	F	Empregado Efetivo
	F	Empregado Efetivo
	F	Empregado Efetivo
	F	Empregado Efetivo
	F	Empregado Efetivo
	F	Empregado Efetivo
	F	Empregado Efetivo
	M	Empregado Efetivo
	F	Empregado Efetivo
	F	Empregado Efetivo
	F	Empregado Efetivo
	F	Empregado Efetivo
	F	Empregado Efetivo
	F	Empregado Efetivo
	F	Empregado Efetivo
	F	Empregado Efetivo
	F	Empregado Efetivo
	F	Empregado Efetivo
	F	Empregado Efetivo
	F	Empregado Efetivo
	F	Empregado Efetivo
	F	Empregado Efetivo
	F	Empregado Efetivo
	F	Empregado Efetivo
	F	Empregado Efetivo

	F	Empregado Efetivo
	F	Empregado Efetivo
	F	Empregado Efetivo
	F	Empregado Efetivo
	F	Empregado Efetivo
	F	Empregado Efetivo
	F	Empregado Efetivo
	F	Empregado Efetivo
	M	Empregado Efetivo
	F	Empregado Efetivo
	F	Empregado Efetivo
	F	Empregado Efetivo
	F	Empregado Efetivo
	F	Empregado Efetivo
	F	Empregado Efetivo
	F	Empregado Efetivo
	F	Empregado Efetivo
	F	Empregado Efetivo
	F	Empregado Efetivo
	F	Empregado Efetivo
	F	Empregado Efetivo
	F	Empregado Efetivo
	F	Empregado Efetivo
	F	Empregado Efetivo
	F	Empregado Efetivo
	F	Empregado Efetivo
	F	Empregado Efetivo
	F	Empregado Efetivo
	F	Empregado Efetivo
	F	Empregado Efetivo
	F	Empregado Efetivo
	F	Empregado Efetivo
	F	Empregado Efetivo
	F	Empregado Efetivo
	F	Empregado Efetivo
	F	Empregado Efetivo
	F	Empregado Efetivo
	F	Empregado Efetivo
	F	Empregado Efetivo
	F	Empregado Efetivo
	F	Empregado Efetivo
	F	Empregado Efetivo
	M	Empregado Efetivo
	F	Empregado Efetivo
	F	Empregado Efetivo
	F	Empregado Efetivo
	F	Empregado Efetivo
	F	Empregado Efetivo

	F	Empregado Efetivo
	F	Empregado Efetivo
	F	Empregado Efetivo
	F	Empregado Efetivo
	F	Empregado Efetivo
	F	Empregado Efetivo
	F	Empregado Efetivo
	F	Empregado Efetivo
	F	Empregado Efetivo
	F	Empregado Efetivo
	F	Empregado Efetivo
	F	Empregado Efetivo
	F	Empregado Efetivo
	F	Empregado Efetivo
	F	Empregado Efetivo
	F	Empregado Efetivo
	F	Empregado Efetivo
	F	Empregado Efetivo
	F	Empregado Efetivo
	F	Empregado Efetivo
	F	Empregado Efetivo
	F	Empregado Efetivo
	F	Empregado Efetivo
	F	Empregado Efetivo
	F	Empregado Efetivo
	M	Empregado Efetivo
	F	Empregado Efetivo
	F	Empregado Efetivo
	F	Empregado Efetivo
	F	Empregado Efetivo
	F	Empregado Efetivo
	F	Empregado Efetivo
	F	Empregado Efetivo
	F	Empregado Efetivo
	M	Empregado Efetivo
	M	Empregado Efetivo
	F	Empregado Efetivo
	F	Empregado Efetivo
	F	Empregado Efetivo
	F	Empregado Efetivo
	F	Empregado Efetivo
	F	Empregado Efetivo
	F	Empregado Efetivo
	F	Empregado Efetivo
	F	Empregado Efetivo
	F	Empregado Efetivo
	F	Empregado Efetivo
	F	Empregado Efetivo

	F	Empregado Efetivo
	M	Empregado Efetivo
	F	Empregado Efetivo
	F	Empregado Efetivo
	F	Empregado Efetivo
	F	Empregado Efetivo
	F	Empregado Efetivo
	F	Empregado Efetivo
	F	Empregado Efetivo
	F	Empregado Efetivo
	F	Empregado Efetivo
	F	Empregado Efetivo
	F	Empregado Efetivo
	F	Empregado Efetivo
	F	Empregado Efetivo
	F	Empregado Efetivo
	F	Empregado Efetivo
	F	Empregado Efetivo
	F	Empregado Efetivo
	M	Empregado Efetivo
	F	Empregado Efetivo
	F	Empregado Efetivo
	F	Empregado Efetivo
	M	Empregado Efetivo
	F	Empregado Efetivo
	F	Empregado Efetivo
	F	Empregado Efetivo
	F	Empregado Efetivo
	M	Empregado Efetivo
	F	Empregado Efetivo
	M	Empregado Efetivo
	M	Empregado Efetivo
	F	Empregado Efetivo
	F	Empregado Efetivo
	M	Empregado Efetivo
	F	Empregado Efetivo
	F	Empregado Efetivo
	F	Empregado Efetivo
	F	Empregado Efetivo
	F	Empregado Efetivo
	F	Empregado Efetivo
	F	Empregado Efetivo
	F	Empregado Efetivo
	F	Empregado Efetivo
	F	Empregado Efetivo
	F	Empregado Efetivo
	F	Empregado Efetivo
	F	Empregado Efetivo

	F	Empregado Efetivo
	F	Empregado Efetivo
	F	Empregado Efetivo
	F	Empregado Efetivo
	F	Empregado Efetivo
	F	Empregado Efetivo
	F	Empregado Efetivo
	F	Empregado Efetivo
	M	Empregado Efetivo
	F	Empregado Efetivo
	F	Empregado Efetivo
	F	Empregado Efetivo
	F	Empregado Efetivo
	F	Empregado Efetivo
	F	Empregado Efetivo
	F	Empregado Efetivo
	F	Empregado Efetivo
	F	Empregado Efetivo
	F	Empregado Efetivo
	F	Empregado Efetivo
	F	Empregado Efetivo
	F	Empregado Efetivo
	F	Empregado Efetivo
	F	Empregado Efetivo
	F	Empregado Efetivo
	F	Empregado Efetivo
	F	Empregado Efetivo
	F	Empregado Efetivo
	F	Empregado Efetivo
	F	Empregado Efetivo
	F	Empregado Efetivo
	F	Empregado Efetivo
	F	Empregado Efetivo
	F	Empregado Efetivo
	F	Empregado Efetivo
	F	Empregado Efetivo
	F	Empregado Efetivo
	F	Empregado Efetivo
	F	Empregado Efetivo
	F	Empregado Efetivo
	F	Empregado Efetivo
	F	Empregado Efetivo
	F	Empregado Efetivo
	F	Empregado Efetivo
	F	Empregado Efetivo
	F	Empregado Efetivo
	F	Empregado Efetivo
	F	Empregado Efetivo

	F	Empregado Efetivo
	F	Empregado Efetivo
	F	Empregado Efetivo
	F	Empregado Efetivo
	F	Empregado Efetivo
	F	Empregado Efetivo
	F	Empregado Efetivo
	F	Empregado Efetivo
	M	Empregado Efetivo
	F	Empregado Efetivo
	F	Empregado Efetivo
	F	Empregado Efetivo
	F	Empregado Efetivo
	F	Empregado Efetivo
	F	Empregado Efetivo
	F	Empregado Efetivo
	F	Empregado Efetivo
	F	Empregado Efetivo
	F	Empregado Efetivo
	F	Empregado Efetivo
	F	Empregado Efetivo
	F	Empregado Efetivo
	F	Empregado Efetivo
	F	Empregado Efetivo
	F	Empregado Efetivo
	F	Empregado Efetivo
	F	Empregado Efetivo
	F	Empregado Efetivo
	F	Empregado Efetivo
	F	Empregado Efetivo
	F	Empregado Efetivo
	M	Empregado Efetivo
	F	Empregado Efetivo
	F	Empregado Efetivo
	F	Empregado Efetivo
	F	Empregado Efetivo
	F	Empregado Efetivo
	F	Empregado Efetivo
	F	Empregado Efetivo
	F	Empregado Efetivo
	F	Empregado Efetivo
	F	Empregado Efetivo
	F	Empregado Efetivo
	F	Empregado Efetivo
	F	Empregado Efetivo
	F	Empregado Efetivo
	F	Empregado Efetivo
	M	Empregado Efetivo

	F	Empregado Efetivo
	F	Empregado Efetivo
	F	Empregado Efetivo
	F	Empregado Efetivo
	F	Empregado Efetivo
	F	Empregado Efetivo
	F	Empregado Efetivo
	M	Empregado Efetivo
	F	Empregado Efetivo
	F	Empregado Efetivo
	F	Empregado Efetivo
	F	Empregado Efetivo
	F	Empregado Efetivo
	F	Empregado Efetivo
	F	Empregado Efetivo
	F	Empregado Efetivo
	F	Empregado Efetivo
	F	Empregado Efetivo
	F	Empregado Efetivo
	F	Empregado Efetivo
	F	Empregado Efetivo
	F	Empregado Efetivo
	F	Empregado Efetivo
	F	Empregado Efetivo
	F	Empregado Efetivo
	F	Empregado Efetivo
	F	Empregado Efetivo
	M	Empregado Efetivo
	M	Empregado Efetivo
	M	Empregado Efetivo
	M	Empregado Efetivo
	M	Empregado Efetivo
	M	Empregado Efetivo
	M	Empregado Efetivo
	M	Empregado Efetivo
	M	Empregado Efetivo
	F	Empregado Efetivo
	M	Empregado Efetivo
	M	Empregado Efetivo
	M	Empregado Efetivo
	F	Empregado Efetivo
	M	Empregado Efetivo
	M	Empregado Efetivo
	M	Empregado Efetivo
	M	Empregado Efetivo
	M	Empregado Efetivo
	F	Empregado Efetivo
	M	Empregado Efetivo

	F	Empregado Efetivo
	M	Empregado Efetivo
	F	Empregado Efetivo
	F	Empregado Efetivo
	M	Empregado Efetivo
	M	Empregado Efetivo
	F	Empregado Efetivo
	M	Empregado Efetivo
	M	Empregado Efetivo
	F	Empregado Efetivo
	M	Empregado Efetivo
	M	Empregado Efetivo
	M	Empregado Efetivo
	M	Empregado Efetivo
	F	Empregado Efetivo
	M	Empregado Efetivo
	F	Empregado Efetivo
	F	Empregado Efetivo
	F	Empregado Efetivo
	F	Empregado Efetivo
	F	Empregado Efetivo
	F	Empregado Efetivo
	F	Empregado Efetivo
	F	Empregado Efetivo
	F	Empregado Efetivo
	F	Empregado Efetivo
	F	Empregado Efetivo
	F	Empregado Efetivo
	M	Empregado Efetivo
	M	Empregado Efetivo
	M	Empregado Efetivo
	M	Empregado Efetivo
	F	Empregado Efetivo
	F	Empregado Efetivo
	M	Empregado Efetivo
	M	Empregado Efetivo
	M	Empregado Efetivo
	M	Empregado Efetivo
	F	Empregado Efetivo
	M	Empregado Efetivo
	F	Empregado Efetivo
	F	Empregado Efetivo
	F	Empregado Efetivo
	F	Empregado Efetivo
	M	Empregado Efetivo
	M	Empregado Efetivo
	M	Empregado Efetivo
	F	Empregado Efetivo

	M	Empregado Efetivo
	M	Empregado Efetivo
	F	Empregado Efetivo
	F	Empregado Efetivo
	F	Empregado Efetivo
	M	Empregado Efetivo
	M	Empregado Efetivo
	F	Empregado Efetivo
	M	Empregado Efetivo
	F	Empregado Efetivo
	F	Empregado Efetivo
	F	Empregado Efetivo
	M	Empregado Efetivo
	F	Empregado Efetivo
	M	Empregado Efetivo
	M	Empregado Efetivo
	M	Empregado Efetivo
	M	Empregado Efetivo
	M	Empregado Efetivo
	M	Empregado Efetivo
	M	Empregado Efetivo
	F	Empregado Efetivo
	M	Empregado Efetivo
	M	Empregado Efetivo
	F	Empregado Efetivo
	F	Empregado Efetivo
	F	Empregado Efetivo
	F	Empregado Efetivo
	F	Empregado Efetivo
	M	Empregado Efetivo
	M	Empregado Efetivo
	F	Empregado Efetivo
	F	Empregado Efetivo
	F	Empregado Efetivo
	F	Empregado Efetivo
	M	Empregado Efetivo
	F	Empregado Efetivo
	F	Empregado Efetivo
	F	Empregado Efetivo
	F	Empregado Efetivo
	F	Empregado Efetivo
	F	Empregado Efetivo
	F	Empregado Efetivo
	F	Empregado Efetivo
	F	Empregado Efetivo
	F	Empregado Efetivo

	F	Empregado Efetivo
	F	Empregado Efetivo
	F	Empregado Efetivo
	F	Empregado Efetivo
	F	Empregado Efetivo
	F	Empregado Efetivo
	F	Empregado Efetivo
	F	Empregado Efetivo
	F	Empregado Efetivo
	F	Empregado Efetivo
	F	Empregado Efetivo
	F	Empregado Efetivo
	F	Empregado Efetivo
	F	Empregado Efetivo
	F	Empregado Efetivo
	F	Empregado Efetivo
	F	Empregado Efetivo
	M	Empregado Efetivo
	F	Empregado Efetivo
	F	Empregado Efetivo
	M	Empregado Efetivo
	F	Empregado Efetivo
	F	Empregado Efetivo
	F	Empregado Efetivo
	F	Empregado Efetivo
	F	Empregado Efetivo
	F	Empregado Efetivo
	M	Empregado Efetivo
	F	Empregado Efetivo
	F	Empregado Efetivo
	M	Empregado Efetivo
	F	Empregado Efetivo
	F	Empregado Efetivo
	M	Empregado Efetivo
	F	Empregado Efetivo
	F	Empregado Efetivo
	F	Empregado Efetivo
	F	Empregado Efetivo
	F	Empregado Efetivo
	F	Empregado Efetivo
	F	Empregado Efetivo

	F	Empregado Efetivo
	F	Empregado Efetivo
	F	Empregado Efetivo
	F	Empregado Efetivo
	F	Empregado Efetivo
	M	Empregado Efetivo
	F	Empregado Efetivo
	F	Empregado Efetivo
	F	Empregado Efetivo
	F	Empregado Efetivo
	F	Empregado Efetivo
	F	Empregado Efetivo
	F	Empregado Efetivo
	F	Empregado Efetivo
	F	Empregado Efetivo
	F	Empregado Efetivo
	F	Empregado Efetivo
	F	Empregado Efetivo
	F	Empregado Efetivo
	F	Empregado Efetivo
	F	Empregado Efetivo
	F	Empregado Efetivo
	F	Empregado Efetivo
	M	Empregado Efetivo
	F	Empregado Efetivo
	F	Empregado Efetivo
	F	Empregado Efetivo
	F	Empregado Efetivo
	F	Empregado Efetivo
	F	Empregado Efetivo
	F	Empregado Efetivo
	F	Empregado Efetivo
	F	Empregado Efetivo
	F	Empregado Efetivo
	F	Empregado Efetivo
	F	Empregado Efetivo
	F	Empregado Efetivo
	F	Empregado Efetivo
	M	Empregado Efetivo
	F	Empregado Efetivo
	F	Empregado Efetivo
	M	Empregado Efetivo
	F	Empregado Efetivo
	M	Empregado Efetivo
	F	Empregado Efetivo
	F	Empregado Efetivo
	M	Empregado Efetivo
	M	Empregado Efetivo

	F	Empregado Efetivo
	M	Empregado Efetivo
	M	Empregado Efetivo
	F	Empregado Efetivo
	F	Empregado Efetivo
	M	Empregado Efetivo
	F	Empregado Efetivo
	M	Empregado Efetivo
	F	Empregado Efetivo
	M	Empregado Efetivo
	M	Empregado Efetivo
	F	Empregado Efetivo
	F	Empregado Efetivo
	F	Empregado Efetivo
	F	Empregado Efetivo
	F	Empregado Efetivo
	F	Empregado Efetivo
	F	Empregado Efetivo
	F	Empregado Efetivo
	M	Empregado Efetivo
	M	Empregado Efetivo
	F	Empregado Efetivo
	M	Empregado Efetivo
	F	Empregado Efetivo
	M	Empregado Efetivo
	F	Empregado Efetivo
	F	Empregado Efetivo
	F	Empregado Efetivo
	F	Empregado Efetivo
	F	Empregado Efetivo
	F	Empregado Efetivo
	F	Empregado Efetivo
	F	Empregado Efetivo
	F	Empregado Efetivo
	F	Empregado Efetivo
	F	Empregado Efetivo
	F	Empregado Efetivo
	F	Empregado Efetivo
	F	Empregado Efetivo
	F	Empregado Efetivo
	F	Empregado Efetivo

	F	Empregado Efetivo
	F	Empregado Efetivo
	F	Empregado Efetivo
	F	Empregado Efetivo
	F	Empregado Efetivo
	F	Empregado Efetivo
	F	Empregado Efetivo
	F	Empregado Efetivo
	F	Empregado Efetivo
	F	Empregado Efetivo
	F	Empregado Efetivo
	F	Empregado Efetivo
	F	Empregado Efetivo
	M	Empregado Efetivo
	F	Empregado Efetivo
	F	Empregado Efetivo
	M	Empregado Efetivo
	F	Empregado Efetivo
	F	Empregado Efetivo
	F	Empregado Efetivo
	F	Empregado Efetivo
	F	Empregado Efetivo
	F	Empregado Efetivo
	F	Empregado Efetivo
	F	Empregado Efetivo
	F	Empregado Efetivo
	F	Empregado Efetivo
	F	Empregado Efetivo
	F	Empregado Efetivo
	F	Empregado Efetivo
	F	Empregado Efetivo
	F	Empregado Efetivo
	F	Empregado Efetivo
	F	Empregado Efetivo
	F	Empregado Efetivo
	F	Empregado Efetivo
	F	Empregado Efetivo
	F	Empregado Efetivo
	M	Empregado Efetivo
	M	Empregado Efetivo
	F	Empregado Efetivo
	F	Empregado Efetivo
	F	Empregado Efetivo
	M	Empregado Efetivo
	M	Empregado Efetivo
	F	Empregado Efetivo
	F	Empregado Efetivo
	F	Empregado Efetivo

	F	Empregado Efetivo
	F	Empregado Efetivo
	F	Empregado Efetivo
	F	Empregado Efetivo
	F	Empregado Efetivo
	F	Empregado Efetivo
	F	Empregado Efetivo
	F	Empregado Efetivo
	F	Empregado Efetivo
	F	Empregado Efetivo
	F	Empregado Efetivo
	F	Empregado Efetivo
	F	Empregado Efetivo
	F	Empregado Efetivo
	F	Empregado Efetivo
	F	Empregado Efetivo
	F	Empregado Efetivo
	F	Empregado Efetivo
	F	Empregado Efetivo
	F	Empregado Efetivo
	F	Empregado Efetivo
	M	Empregado Efetivo
	F	Empregado Efetivo
	F	Empregado Efetivo
	F	Empregado Efetivo
	F	Empregado Efetivo
	F	Empregado Efetivo
	F	Empregado Efetivo
	F	Empregado Efetivo
	F	Empregado Efetivo
	F	Empregado Efetivo
	F	Empregado Efetivo
	F	Empregado Efetivo
	F	Empregado Efetivo
	F	Empregado Efetivo
	M	Empregado Efetivo
	F	Empregado Efetivo
	F	Empregado Efetivo
	M	Empregado Efetivo
	F	Empregado Efetivo
	F	Empregado Efetivo
	F	Empregado Efetivo
	F	Empregado Efetivo
	F	Empregado Efetivo
	F	Empregado Efetivo
	F	Empregado Efetivo
	F	Empregado Efetivo
	F	Empregado Efetivo

	F	Empregado Efetivo
	F	Empregado Efetivo
	F	Empregado Efetivo
	F	Empregado Efetivo
	F	Empregado Efetivo
	F	Empregado Efetivo
	F	Empregado Efetivo
	F	Empregado Efetivo
	F	Empregado Efetivo
	F	Empregado Efetivo
	F	Empregado Efetivo
	M	Empregado Efetivo
	F	Empregado Efetivo
	F	Empregado Efetivo
	F	Empregado Efetivo
	M	Empregado Efetivo
	F	Empregado Efetivo
	F	Empregado Efetivo
	F	Empregado Efetivo
	M	Empregado Efetivo
	F	Empregado Efetivo
	F	Empregado Efetivo
	M	Empregado Efetivo
	F	Empregado Efetivo
	F	Empregado Efetivo
	F	Empregado Efetivo
	F	Empregado Efetivo
	M	Empregado Efetivo
	F	Empregado Efetivo
	F	Empregado Efetivo
	M	Empregado Efetivo
	F	Empregado Efetivo
	F	Empregado Efetivo
	F	Empregado Efetivo
	F	Empregado Efetivo
	F	Empregado Efetivo
	F	Empregado Efetivo
	F	Empregado Efetivo
	M	Empregado Efetivo
	M	Empregado Efetivo
	M	Empregado Efetivo
	F	Empregado Efetivo
	F	Empregado Efetivo
	M	Empregado Efetivo
	F	Empregado Efetivo
	F	Empregado Efetivo
	F	Empregado Efetivo
	F	Empregado Efetivo

	M	Empregado Efetivo
	M	Empregado Efetivo
	M	Empregado Efetivo
	F	Empregado Efetivo
	F	Empregado Efetivo
	F	Empregado Efetivo
	F	Empregado Efetivo
	F	Empregado Efetivo
	F	Empregado Efetivo
	M	Empregado Efetivo
	M	Empregado Efetivo
	M	Empregado Efetivo
	F	Empregado Efetivo
	F	Empregado Efetivo
	F	Empregado Efetivo
	F	Empregado Efetivo
	F	Empregado Efetivo
	F	Empregado Efetivo
	F	Empregado Efetivo
	M	Empregado Efetivo
	F	Empregado Efetivo
	F	Empregado Efetivo
	M	Empregado Efetivo
	M	Empregado Efetivo
	F	Empregado Efetivo
	M	Empregado Efetivo
	F	Empregado Efetivo
	M	Empregado Efetivo
	F	Empregado Efetivo
	F	Empregado Efetivo
	M	Empregado Efetivo
	F	Empregado Efetivo
	F	Empregado Efetivo
	F	Empregado Efetivo
	M	Empregado Efetivo
	M	Empregado Efetivo
	F	Empregado Efetivo
	F	Empregado Efetivo
	M	Empregado Efetivo
	F	Empregado Efetivo
	F	Empregado Efetivo
	M	Empregado Efetivo
	M	Empregado Efetivo
	F	Empregado Efetivo
	F	Empregado Efetivo
	F	Empregado Efetivo
	M	Empregado Efetivo
	M	Empregado Efetivo

	M	Empregado Efetivo
	M	Empregado Efetivo
	F	Empregado Efetivo
	M	Empregado Efetivo
	F	Empregado Efetivo
	M	Empregado Efetivo
	M	Empregado Efetivo
	M	Empregado Efetivo
	M	Empregado Efetivo
	M	Empregado Efetivo
	F	Empregado Efetivo
	F	Empregado Efetivo
	M	Empregado Efetivo
	M	Empregado Efetivo
	F	Empregado Efetivo
	M	Empregado Efetivo
	F	Empregado Efetivo
	M	Empregado Efetivo
	F	Empregado Efetivo
	F	Empregado Efetivo
	F	Empregado Efetivo
	M	Empregado Efetivo
	F	Empregado Efetivo
	M	Empregado Efetivo
	M	Empregado Efetivo
	M	Empregado Efetivo
	M	Empregado Efetivo
	M	Empregado Efetivo
	F	Empregado Efetivo
	F	Empregado Efetivo
	F	Empregado Efetivo
	M	Empregado Efetivo
	F	Empregado Efetivo
	F	Empregado Efetivo
	M	Empregado Efetivo
	M	Empregado Efetivo
	F	Empregado Efetivo
	F	Empregado Efetivo
	F	Empregado Efetivo
	F	Empregado Efetivo
	F	Empregado Efetivo
	F	Empregado Efetivo
	F	Empregado Efetivo
	F	Empregado Efetivo
	F	Empregado Efetivo
	F	Empregado Efetivo
	F	Empregado Efetivo
	M	Empregado Efetivo

	F	Empregado Efetivo
	F	Empregado Efetivo
	F	Empregado Efetivo
	F	Empregado Efetivo
	F	Empregado Efetivo
	F	Empregado Efetivo
	F	Empregado Efetivo
	F	Empregado Efetivo
	F	Empregado Efetivo
	M	Empregado Efetivo
	F	Empregado Efetivo
	M	Empregado Efetivo
	M	Empregado Efetivo
	F	Empregado Efetivo
	F	Empregado Efetivo
	F	Empregado Efetivo
	F	Empregado Efetivo
	F	Empregado Efetivo
	F	Empregado Efetivo
	F	Empregado Efetivo
	M	Empregado Efetivo
	F	Empregado Efetivo
	F	Empregado Efetivo
	M	Empregado Efetivo
	F	Empregado Efetivo
	F	Empregado Efetivo
	F	Empregado Efetivo
	M	Empregado Efetivo
	F	Empregado Efetivo
	F	Empregado Efetivo
	F	Empregado Efetivo
	F	Empregado Efetivo
	M	Empregado Efetivo
	M	Empregado Efetivo
	M	Empregado Efetivo
	F	Empregado Efetivo
	F	Empregado Efetivo
	F	Empregado Efetivo

	F	Empregado Efetivo
	F	Empregado Efetivo
	M	Empregado Efetivo
	M	Empregado Efetivo
	F	Empregado Efetivo
	F	Empregado Efetivo
	M	Empregado Efetivo
	F	Empregado Efetivo
	F	Empregado Efetivo
	F	Empregado Efetivo
	M	Empregado Efetivo
	M	Empregado Efetivo
	F	Empregado Efetivo
	F	Empregado Efetivo
	M	Empregado Efetivo
	F	Empregado Efetivo
	F	Empregado Efetivo
	M	Empregado Efetivo
	F	Empregado Efetivo
	F	Empregado Efetivo
	M	Empregado Efetivo
	F	Empregado Efetivo
	M	Empregado Efetivo
	F	Empregado Efetivo
	F	Empregado Efetivo
	F	Empregado Efetivo
	F	Empregado Efetivo
	F	Empregado Efetivo
	F	Empregado Efetivo
	F	Empregado Efetivo
	F	Empregado Efetivo
	F	Empregado Efetivo
	F	Empregado Efetivo
	M	Empregado Efetivo
	F	Empregado Efetivo
	M	Empregado Efetivo
	M	Empregado Efetivo

	F	Empregado Efetivo
	F	Empregado Efetivo
	M	Empregado Efetivo
	M	Empregado Efetivo
	M	Empregado Efetivo
	M	Empregado Efetivo
	M	Empregado Efetivo
	M	Empregado Efetivo
	F	Empregado Efetivo
	M	Empregado Efetivo
	M	Empregado Efetivo
	M	Empregado Efetivo
	M	Empregado Efetivo
	M	Empregado Efetivo
	M	Empregado Efetivo
	M	Empregado Efetivo
	M	Empregado Efetivo
	M	Empregado Efetivo
	M	Empregado Efetivo
	M	Empregado Efetivo
	F	Empregado Efetivo
	M	Empregado Efetivo
	M	Empregado Efetivo
	M	Empregado Efetivo
	M	Empregado Efetivo
	M	Empregado Efetivo
	M	Empregado Efetivo
	F	Empregado Efetivo
	M	Empregado Efetivo
	M	Empregado Efetivo
	M	Empregado Efetivo
	M	Empregado Efetivo
	M	Empregado Efetivo
	M	Empregado Efetivo
	M	Empregado Efetivo
	M	Empregado Efetivo
	F	Empregado Efetivo
	F	Empregado Efetivo
	F	Empregado Efetivo
	M	Empregado Efetivo
	F	Empregado Efetivo
	M	Empregado Efetivo
	F	Empregado Efetivo
	M	Empregado Efetivo
	M	Empregado Efetivo

	M	Empregado Efetivo
	M	Empregado Efetivo
	M	Empregado Efetivo
	M	Empregado Efetivo
	M	Empregado Efetivo
	F	Empregado Efetivo
	F	Empregado Efetivo
	F	Empregado Efetivo
	M	Empregado Efetivo
	F	Empregado Efetivo
	F	Empregado Efetivo
	F	Empregado Efetivo
	F	Empregado Efetivo
	F	Empregado Efetivo
	M	Empregado Efetivo
	F	Empregado Efetivo
	F	Empregado Efetivo
	F	Empregado Efetivo
	F	Empregado Efetivo
	F	Empregado Efetivo
	F	Empregado Efetivo
	F	Empregado Efetivo
	M	Empregado Efetivo
	M	Empregado Efetivo
	M	Empregado Efetivo
	M	Empregado Efetivo
	F	Empregado Efetivo
	M	Empregado Efetivo
	M	Empregado Efetivo
	F	Empregado Efetivo
	F	Empregado Efetivo
	F	Empregado Efetivo
	F	Empregado Efetivo
	F	Empregado Efetivo
	M	Empregado Efetivo
	M	Empregado Efetivo
	M	Empregado Efetivo
	M	Empregado Efetivo
	M	Empregado Efetivo
	M	Empregado Efetivo
	F	Empregado Efetivo
	M	Empregado Efetivo

	M	Empregado Efetivo
	M	Empregado Efetivo
	M	Empregado Efetivo
	M	Empregado Efetivo
	F	Empregado Efetivo
	F	Empregado Efetivo
	M	Empregado Efetivo
	F	Empregado Efetivo
	F	Empregado Efetivo
	M	Empregado Efetivo
	F	Empregado Efetivo
	F	Empregado Efetivo
	M	Empregado Efetivo
	M	Empregado Efetivo
	M	Empregado Efetivo
	F	Empregado Efetivo
	F	Empregado Efetivo
	F	Empregado Efetivo
	F	Empregado Efetivo
	F	Empregado Efetivo
	F	Empregado Efetivo
	F	Empregado Efetivo
	F	Empregado Efetivo
	M	Empregado Efetivo
	F	Empregado Efetivo
	F	Empregado Efetivo
	M	Empregado Efetivo
	M	Empregado Efetivo
	F	Empregado Efetivo
	F	Empregado Efetivo
	F	Empregado Efetivo
	M	Empregado Efetivo
	M	Empregado Efetivo
	M	Empregado Efetivo
	F	Empregado Efetivo
	M	Empregado Efetivo
	M	Empregado Efetivo
	F	Empregado Efetivo
	M	Empregado Efetivo
	F	Empregado Efetivo

	M	Empregado Efetivo
	F	Empregado Efetivo
	F	Empregado Efetivo
	F	Empregado Efetivo
	M	Empregado Efetivo
	F	Empregado Efetivo
	M	Empregado Efetivo
	M	Empregado Efetivo
	M	Empregado Efetivo
	F	Empregado Efetivo
	M	Empregado Efetivo
	M	Empregado Efetivo
	M	Empregado Efetivo
	F	Empregado Efetivo
	F	Empregado Efetivo
	M	Empregado Efetivo
	F	Empregado Efetivo
	F	Empregado Efetivo
	F	Empregado Efetivo
	F	Empregado Efetivo
	F	Empregado Efetivo
	M	Empregado Efetivo
	F	Empregado Efetivo
	F	Empregado Efetivo
	F	Empregado Efetivo
	M	Empregado Efetivo
	M	Empregado Efetivo
	M	Empregado Efetivo
	F	Empregado Efetivo
	M	Empregado Efetivo
	M	Empregado Efetivo
	F	Empregado Efetivo
	M	Empregado Efetivo
	F	Empregado Efetivo
	F	Empregado Efetivo
	F	Empregado Efetivo
	F	Empregado Efetivo
	F	Empregado Efetivo
	M	Empregado Efetivo
	M	Empregado Efetivo
	F	Empregado Efetivo
	F	Empregado Efetivo
	M	Empregado Efetivo
	M	Empregado Efetivo

	F	Empregado Efetivo
	F	Empregado Efetivo
	M	Empregado Efetivo
	F	Empregado Efetivo
	F	Empregado Efetivo
	M	Empregado Efetivo
	F	Empregado Efetivo
	F	Empregado Efetivo
	F	Empregado Efetivo
	F	Empregado Efetivo
	M	Empregado Efetivo
	F	Empregado Efetivo
	M	Empregado Efetivo
	F	Empregado Efetivo
	F	Empregado Efetivo
	F	Empregado Efetivo
	F	Empregado Efetivo
	F	Empregado Efetivo
	F	Empregado Efetivo
	F	Empregado Efetivo
	F	Empregado Efetivo
	F	Empregado Efetivo
	F	Empregado Efetivo
	F	Empregado Efetivo
	F	Empregado Efetivo
	F	Empregado Efetivo
	F	Empregado Efetivo
	F	Empregado Efetivo
	F	Empregado Efetivo
	F	Empregado Efetivo
	F	Empregado Efetivo
	F	Empregado Efetivo
	F	Empregado Efetivo
	F	Empregado Efetivo
	M	Empregado Efetivo
	F	Empregado Efetivo
	F	Empregado Efetivo
	F	Empregado Efetivo
	F	Empregado Efetivo
	M	Empregado Efetivo
	F	Empregado Efetivo
	F	Empregado Efetivo
	F	Empregado Efetivo
	F	Empregado Efetivo
	F	Empregado Efetivo
	F	Empregado Efetivo

	F	Empregado Efetivo
	M	Empregado Efetivo
	M	Empregado Efetivo
	F	Empregado Efetivo
	F	Empregado Efetivo
	M	Empregado Efetivo
	F	Empregado Efetivo
	F	Empregado Efetivo
	F	Empregado Efetivo
	F	Empregado Efetivo
	F	Empregado Efetivo
	F	Empregado Efetivo
	F	Empregado Efetivo
	M	Empregado Efetivo
	M	Empregado Efetivo
	F	Empregado Efetivo
	M	Empregado Efetivo
	F	Empregado Efetivo
	F	Empregado Efetivo
	F	Empregado Efetivo
	F	Empregado Efetivo
	F	Empregado Efetivo
	F	Empregado Efetivo
	F	Empregado Efetivo
	F	Empregado Efetivo
	M	Empregado Efetivo
	F	Empregado Efetivo
	F	Empregado Efetivo
	M	Empregado Efetivo
	M	Empregado Efetivo
	F	Empregado Efetivo
	F	Empregado Efetivo
	M	Empregado Efetivo
	F	Empregado Efetivo
	M	Empregado Efetivo

	F	Empregado Efetivo
	F	Empregado Efetivo
	F	Empregado Efetivo
	F	Empregado Efetivo
	F	Empregado Efetivo
	F	Empregado Efetivo
	F	Empregado Efetivo
	M	Empregado Efetivo
	F	Empregado Efetivo
	F	Empregado Efetivo
	M	Empregado Efetivo
	F	Empregado Efetivo
	M	Empregado Efetivo
	F	Empregado Efetivo
	F	Empregado Efetivo
	M	Empregado Efetivo
	F	Empregado Efetivo
	F	Empregado Efetivo
	F	Empregado Efetivo
	F	Empregado Efetivo
	M	Empregado Efetivo
	F	Empregado Efetivo
	F	Empregado Efetivo
	F	Empregado Efetivo
	M	Empregado Efetivo
	F	Empregado Efetivo
	F	Empregado Efetivo
	F	Empregado Efetivo
	M	Empregado Efetivo
	M	Empregado Efetivo
	M	Empregado Efetivo
	F	Empregado Efetivo
	M	Empregado Efetivo
	F	Empregado Efetivo

	F	Empregado Efetivo
	M	Empregado Efetivo
	M	Empregado Efetivo
	F	Empregado Efetivo
	F	Empregado Efetivo
	F	Empregado Efetivo
	M	Empregado Efetivo
	F	Empregado Efetivo
	M	Empregado Efetivo
	F	Empregado Efetivo
	M	Empregado Efetivo
	F	Empregado Efetivo
	M	Empregado Efetivo
	F	Empregado Efetivo
	F	Empregado Efetivo
	M	Empregado Efetivo
	M	Empregado Efetivo
	M	Empregado Efetivo
	F	Empregado Efetivo
	M	Empregado Efetivo
	M	Empregado Efetivo
	F	Empregado Efetivo
	F	Empregado Efetivo
	F	Empregado Efetivo
	F	Empregado Efetivo
	F	Empregado Efetivo
	F	Empregado Efetivo
	F	Empregado Efetivo
	F	Empregado Efetivo
	F	Empregado Efetivo
	F	Empregado Efetivo
	M	Empregado Efetivo
	F	Empregado Efetivo
	F	Empregado Efetivo
	M	Empregado Efetivo
	M	Empregado Efetivo
	M	Empregado Efetivo
	F	Empregado Efetivo
	F	Empregado Efetivo
	F	Empregado Efetivo
	F	Empregado Efetivo
	F	Empregado Efetivo

	F	Empregado Efetivo
	F	Empregado Efetivo
	F	Empregado Efetivo
	F	Empregado Efetivo
	F	Empregado Efetivo
	M	Empregado Efetivo
	F	Empregado Efetivo
	F	Empregado Efetivo
	F	Empregado Efetivo
	M	Empregado Efetivo
	F	Empregado Efetivo
	F	Empregado Efetivo
	F	Empregado Efetivo
	M	Empregado Efetivo
	F	Empregado Efetivo
	M	Empregado Efetivo
	F	Empregado Efetivo
	F	Empregado Efetivo
	M	Empregado Efetivo
	F	Empregado Efetivo
	F	Empregado Efetivo
	F	Empregado Efetivo
	F	Empregado Efetivo
	F	Empregado Efetivo
	F	Empregado Efetivo
	F	Empregado Efetivo
	F	Empregado Efetivo
	F	Empregado Efetivo
	F	Empregado Efetivo
	F	Empregado Efetivo
	F	Empregado Efetivo
	F	Empregado Efetivo
	F	Empregado Efetivo
	F	Empregado Efetivo
	F	Empregado Efetivo
	F	Empregado Efetivo
	F	Empregado Efetivo
	F	Empregado Efetivo
	F	Empregado Efetivo
	F	Empregado Efetivo
	F	Empregado Efetivo
	F	Empregado Efetivo
	F	Empregado Efetivo
	F	Empregado Efetivo
	F	Empregado Efetivo
	F	Empregado Efetivo
	F	Empregado Efetivo
	F	Empregado Efetivo

	F	Empregado Efetivo
	F	Empregado Efetivo
	F	Empregado Efetivo
	F	Empregado Efetivo
	F	Empregado Efetivo
	F	Empregado Efetivo
	F	Empregado Efetivo
	F	Empregado Efetivo
	M	Empregado Efetivo
	F	Empregado Efetivo
	F	Empregado Efetivo
	F	Empregado Efetivo
	F	Empregado Efetivo
	M	Empregado Efetivo
	F	Empregado Efetivo
	F	Empregado Efetivo
	F	Empregado Efetivo
	F	Empregado Efetivo
	F	Empregado Efetivo
	F	Empregado Efetivo
	M	Empregado Efetivo
	F	Empregado Efetivo
	F	Empregado Efetivo
	F	Empregado Efetivo
	F	Empregado Efetivo
	F	Empregado Efetivo
	F	Empregado Efetivo
	F	Empregado Efetivo
	F	Empregado Efetivo
	F	Empregado Efetivo
	F	Empregado Efetivo
	F	Empregado Efetivo
	F	Empregado Efetivo
	F	Empregado Efetivo
	F	Empregado Efetivo
	F	Empregado Efetivo
	F	Empregado Efetivo
	F	Empregado Efetivo
	F	Empregado Efetivo
	F	Empregado Efetivo
	F	Empregado Efetivo
	F	Empregado Efetivo
	F	Empregado Efetivo
	F	Empregado Efetivo
	F	Empregado Efetivo
	F	Empregado Efetivo
	F	Empregado Efetivo

	F	Empregado Efetivo
	F	Empregado Efetivo
	F	Empregado Efetivo
	M	Empregado Efetivo
	M	Empregado Efetivo
	F	Empregado Efetivo
	M	Empregado Efetivo
	M	Empregado Efetivo
	F	Empregado Efetivo
	M	Empregado Efetivo
	M	Empregado Efetivo
	F	Empregado Efetivo
	F	Empregado Efetivo
	F	Empregado Efetivo
	F	Empregado Efetivo
	F	Empregado Efetivo
	F	Empregado Efetivo
	M	Empregado Efetivo
	M	Empregado Efetivo
	F	Empregado Efetivo
	F	Empregado Efetivo
	M	Empregado Efetivo
	M	Empregado Efetivo
	F	Empregado Efetivo
	M	Empregado Efetivo
	F	Empregado Efetivo
	F	Empregado Efetivo
	M	Empregado Efetivo
	M	Empregado Efetivo
	M	Empregado Efetivo
	M	Empregado Efetivo
	M	Empregado Efetivo
	F	Empregado Efetivo
	M	Empregado Efetivo
	M	Empregado Efetivo
	M	Empregado Efetivo
	F	Empregado Efetivo
	M	Empregado Efetivo
	F	Empregado Efetivo
	M	Empregado Efetivo
	M	Empregado Efetivo

	M	Empregado Efetivo
	M	Empregado Efetivo
	M	Empregado Efetivo
	F	Empregado Efetivo
	M	Empregado Efetivo
	M	Empregado Efetivo
	M	Empregado Efetivo
	M	Empregado Efetivo
	F	Empregado Efetivo
	M	Empregado Efetivo
	F	Empregado Efetivo
	M	Empregado Efetivo
	F	Empregado Efetivo
	M	Empregado Efetivo
	M	Empregado Efetivo
	F	Empregado Efetivo
	M	Empregado Efetivo
	F	Empregado Efetivo
	M	Empregado Efetivo
	M	Empregado Efetivo
	F	Empregado Efetivo
	M	Empregado Efetivo
	F	Empregado Efetivo
	M	Empregado Efetivo
	M	Empregado Efetivo
	M	Empregado Efetivo
	F	Empregado Efetivo
	M	Empregado Efetivo
	F	Empregado Efetivo
	F	Empregado Efetivo
	M	Empregado Efetivo
	F	Empregado Efetivo
	F	Empregado Efetivo
	F	Empregado Efetivo
	F	Empregado Efetivo
	F	Empregado Efetivo
	F	Empregado Efetivo
	F	Empregado Efetivo
	F	Empregado Efetivo
	F	Empregado Efetivo
	M	Empregado Efetivo
	F	Empregado Efetivo

	F	Empregado Efetivo
	F	Empregado Efetivo
	F	Empregado Efetivo
	F	Empregado Efetivo
	M	Empregado Efetivo
	F	Empregado Efetivo
	F	Empregado Efetivo
	F	Empregado Efetivo
	F	Empregado Efetivo
	F	Empregado Efetivo
	M	Empregado Efetivo
	F	Empregado Efetivo
	F	Empregado Efetivo
	F	Empregado Efetivo
	F	Empregado Efetivo
	F	Empregado Efetivo
	F	Empregado Efetivo
	F	Empregado Efetivo
	F	Empregado Efetivo
	F	Empregado Efetivo
	F	Empregado Efetivo
	F	Empregado Efetivo
	F	Empregado Efetivo
	F	Empregado Efetivo
	M	Empregado Efetivo
	F	Empregado Efetivo
	F	Empregado Efetivo
	F	Empregado Efetivo
	F	Empregado Efetivo
	F	Empregado Efetivo
	M	Empregado Efetivo
	M	Empregado Efetivo

	M	Empregado Efetivo
	F	Empregado Efetivo
	F	Empregado Efetivo
	F	Empregado Efetivo
	F	Empregado Efetivo
	F	Empregado Efetivo
	F	Empregado Efetivo
	F	Empregado Efetivo
	F	Empregado Efetivo
	F	Empregado Efetivo
	F	Empregado Efetivo
	F	Empregado Efetivo
	F	Empregado Efetivo
	F	Empregado Efetivo
	F	Empregado Efetivo
	F	Empregado Efetivo
	F	Empregado Efetivo
	F	Empregado Efetivo
	M	Empregado Efetivo
	F	Empregado Efetivo
	F	Empregado Efetivo
	M	Empregado Efetivo
	F	Empregado Efetivo
	F	Empregado Efetivo
	F	Empregado Efetivo
	F	Empregado Efetivo
	F	Empregado Efetivo
	F	Empregado Efetivo
	F	Empregado Efetivo
	F	Empregado Efetivo
	F	Empregado Efetivo
	F	Empregado Efetivo
	F	Empregado Efetivo
	F	Empregado Efetivo
	F	Empregado Efetivo
	F	Empregado Efetivo
	F	Empregado Efetivo
	F	Empregado Efetivo
	F	Empregado Efetivo
	F	Empregado Efetivo
	F	Empregado Efetivo
	F	Empregado Efetivo
	F	Empregado Efetivo
	F	Empregado Efetivo
	F	Empregado Efetivo
	F	Empregado Efetivo
	F	Empregado Efetivo

	F	Empregado Efetivo
	F	Empregado Efetivo
	F	Empregado Efetivo
	F	Empregado Efetivo
	F	Empregado Efetivo
	F	Empregado Efetivo
	F	Empregado Efetivo
	F	Empregado Efetivo
	F	Empregado Efetivo
	F	Empregado Efetivo
	F	Empregado Efetivo
	F	Empregado Efetivo
	F	Empregado Efetivo
	F	Empregado Efetivo
	F	Empregado Efetivo
	F	Empregado Efetivo
	F	Empregado Efetivo
	F	Empregado Efetivo
	F	Empregado Efetivo
	F	Empregado Efetivo
	F	Empregado Efetivo
	F	Empregado Efetivo
	F	Empregado Efetivo
	F	Empregado Efetivo
	F	Empregado Efetivo
	F	Empregado Efetivo
	F	Empregado Efetivo
	F	Empregado Efetivo
	F	Empregado Efetivo
	F	Empregado Efetivo
	F	Empregado Efetivo
	F	Empregado Efetivo
	F	Empregado Efetivo
	F	Empregado Efetivo
	F	Empregado Efetivo
	F	Empregado Efetivo
	F	Empregado Efetivo
	F	Empregado Efetivo
	F	Empregado Efetivo
	F	Empregado Efetivo
	F	Empregado Efetivo
	F	Empregado Efetivo
	F	Empregado Efetivo
	F	Empregado Efetivo
	F	Empregado Efetivo
	F	Empregado Efetivo
	F	Empregado Efetivo
	F	Empregado Efetivo

	F	Empregado Efetivo
	F	Empregado Efetivo
	F	Empregado Efetivo
	F	Empregado Efetivo
	F	Empregado Efetivo
	F	Empregado Efetivo
	F	Empregado Efetivo
	F	Empregado Efetivo
	F	Empregado Efetivo
	F	Empregado Efetivo
	F	Empregado Efetivo
	M	Empregado Efetivo
	F	Empregado Efetivo
	F	Empregado Efetivo
	F	Empregado Efetivo
	F	Empregado Efetivo
	F	Empregado Efetivo
	F	Empregado Efetivo
	F	Empregado Efetivo
	F	Empregado Efetivo
	M	Empregado Efetivo
	M	Empregado Efetivo
	M	Empregado Efetivo
	M	Empregado Efetivo
	M	Empregado Efetivo
	M	Empregado Efetivo
	M	Empregado Efetivo
	M	Empregado Efetivo
	F	Empregado Efetivo
	F	Empregado Efetivo
	M	Empregado Efetivo
	F	Empregado Efetivo
	F	Empregado Efetivo
	F	Empregado Efetivo
	M	Empregado Efetivo
	F	Empregado Efetivo
	M	Empregado Efetivo
	F	Empregado Efetivo
	F	Empregado Efetivo
	F	Empregado Efetivo
	F	Empregado Efetivo
	F	Empregado Efetivo
	F	Empregado Efetivo
	F	Empregado Efetivo
	F	Empregado Efetivo
	F	Empregado Efetivo
	F	Empregado Efetivo

	F	Empregado Efetivo
	F	Empregado Efetivo
	F	Empregado Efetivo
	M	Empregado Efetivo
	F	Empregado Efetivo
	M	Empregado Efetivo
	F	Empregado Efetivo
	F	Empregado Efetivo
	F	Empregado Efetivo
	F	Empregado Efetivo
	F	Empregado Efetivo
	F	Empregado Efetivo
	F	Empregado Efetivo
	F	Empregado Efetivo
	F	Empregado Efetivo
	F	Empregado Efetivo
	F	Empregado Efetivo
	F	Empregado Efetivo
	F	Empregado Efetivo
	M	Empregado Efetivo
	F	Empregado Efetivo
	F	Empregado Efetivo
	F	Empregado Efetivo
	F	Empregado Efetivo
	F	Empregado Efetivo
	M	Empregado Efetivo
	F	Empregado Efetivo
	F	Empregado Efetivo
	F	Empregado Efetivo
	F	Empregado Efetivo
	F	Empregado Efetivo
	F	Empregado Efetivo
	F	Empregado Efetivo
	F	Empregado Efetivo
	F	Empregado Efetivo
	M	Empregado Efetivo
	M	Empregado Efetivo
	M	Empregado Efetivo
	M	Empregado Efetivo
	F	Empregado Efetivo
	F	Empregado Efetivo
	F	Empregado Efetivo
	F	Empregado Efetivo
	F	Empregado Efetivo
	F	Empregado Efetivo
	M	Empregado Efetivo

	M	Empregado Efetivo
	F	Empregado Efetivo
	M	Empregado Efetivo
	F	Empregado Efetivo
	F	Empregado Efetivo
	F	Empregado Efetivo
	F	Empregado Efetivo
	F	Empregado Efetivo
	M	Empregado Efetivo
	M	Empregado Efetivo
	M	Empregado Efetivo
	F	Empregado Efetivo
	F	Empregado Efetivo
	F	Empregado Efetivo
	F	Empregado Efetivo
	M	Empregado Efetivo
	M	Empregado Efetivo
	M	Empregado Efetivo
	M	Empregado Efetivo
	M	Empregado Efetivo
	M	Empregado Efetivo
	F	Empregado Efetivo
	M	Empregado Efetivo
	F	Empregado Efetivo
	F	Empregado Efetivo
	F	Empregado Efetivo
	F	Empregado Efetivo
	F	Empregado Efetivo
	F	Empregado Efetivo
	F	Empregado Efetivo
	F	Empregado Efetivo
	F	Empregado Efetivo
	M	Empregado Efetivo
	M	Empregado Efetivo
	F	Empregado Efetivo
	M	Empregado Efetivo
	M	Empregado Efetivo
	F	Empregado Efetivo
	M	Empregado Efetivo
	F	Empregado Efetivo
	F	Empregado Efetivo
	M	Empregado Efetivo
	F	Empregado Efetivo
	F	Empregado Efetivo
	M	Empregado Efetivo
	M	Empregado Efetivo
	M	Empregado Efetivo
	F	Empregado Efetivo

	F	Empregado Efetivo
	F	Empregado Efetivo
	F	Empregado Efetivo
	M	Empregado Efetivo
	M	Empregado Efetivo
	F	Empregado Efetivo
	F	Empregado Efetivo
	F	Empregado Efetivo
	F	Empregado Efetivo
	F	Empregado Efetivo
	F	Empregado Efetivo
	M	Empregado Efetivo
	M	Empregado Efetivo
	F	Empregado Efetivo
	F	Empregado Efetivo
	M	Empregado Efetivo
	F	Empregado Efetivo
	F	Empregado Efetivo
	F	Empregado Efetivo
	F	Empregado Efetivo
	M	Empregado Efetivo
	F	Empregado Efetivo
	M	Empregado Efetivo
	F	Empregado Efetivo
	M	Empregado Efetivo
	F	Empregado Efetivo
	M	Empregado Efetivo
	M	Empregado Efetivo
	F	Empregado Efetivo
	F	Empregado Efetivo
	M	Empregado Efetivo
	F	Empregado Efetivo
	M	Empregado Efetivo
	F	Empregado Efetivo
	F	Empregado Efetivo
	F	Empregado Efetivo
	F	Empregado Efetivo
	F	Empregado Efetivo
	F	Empregado Efetivo
	F	Empregado Efetivo
	M	Empregado Efetivo
	M	Empregado Efetivo
	M	Empregado Efetivo
	M	Empregado Efetivo
	M	Empregado Efetivo

	M	Empregado Efetivo
	F	Empregado Efetivo
	F	Empregado Efetivo
	M	Empregado Efetivo
	F	Empregado Efetivo
	F	Empregado Efetivo
	M	Empregado Efetivo
	F	Empregado Efetivo
	F	Empregado Efetivo
	F	Empregado Efetivo
	F	Empregado Efetivo
	F	Empregado Efetivo
	F	Empregado Efetivo
	F	Empregado Efetivo
	M	Empregado Efetivo
	F	Empregado Efetivo
	M	Empregado Efetivo
	F	Empregado Efetivo
	F	Empregado Efetivo
	F	Empregado Efetivo
	F	Empregado Efetivo
	F	Empregado Efetivo
	M	Empregado Efetivo
	F	Empregado Efetivo
	F	Empregado Efetivo
	F	Empregado Efetivo
	M	Empregado Efetivo
	F	Empregado Efetivo
	F	Empregado Efetivo
	M	Empregado Efetivo
	F	Empregado Efetivo
	F	Empregado Efetivo
	F	Empregado Efetivo
	F	Empregado Efetivo
	M	Empregado Efetivo
	M	Empregado Efetivo
	M	Empregado Efetivo
	M	Empregado Efetivo

	M	Empregado Efetivo
	M	Empregado Efetivo
	M	Empregado Efetivo
	M	Empregado Efetivo
	M	Empregado Efetivo
	M	Empregado Efetivo
	M	Empregado Efetivo
	M	Empregado Efetivo
	M	Empregado Efetivo
	M	Empregado Efetivo
	M	Empregado Efetivo
	M	Empregado Efetivo
	M	Empregado Efetivo
	F	Empregado Efetivo
	F	Empregado Efetivo
	M	Empregado Efetivo
	F	Empregado Efetivo
	M	Empregado Efetivo
	M	Empregado Efetivo
	F	Empregado Efetivo
	F	Empregado Efetivo
	M	Empregado Efetivo
	F	Empregado Efetivo
	F	Empregado Efetivo
	M	Empregado Efetivo
	F	Empregado Efetivo
	F	Empregado Efetivo
	F	Empregado Efetivo
	F	Empregado Efetivo
	F	Empregado Efetivo
	F	Empregado Efetivo
	F	Empregado Efetivo
	F	Empregado Efetivo
	F	Empregado Efetivo
	F	Empregado Efetivo
	F	Empregado Efetivo
	F	Empregado Efetivo
	F	Empregado Efetivo

	M	Empregado Efetivo
	M	Empregado Efetivo
	M	Empregado Efetivo
	F	Empregado Efetivo
	F	Empregado Efetivo
	M	Empregado Efetivo
	F	Empregado Efetivo
	M	Empregado Efetivo
	F	Empregado Efetivo
	F	Empregado Efetivo
	F	Empregado Efetivo
	F	Empregado Efetivo
	F	Empregado Efetivo
	F	Empregado Efetivo
	F	Empregado Efetivo
	F	Empregado Efetivo
	F	Empregado Efetivo
	F	Empregado Efetivo
	M	Empregado Efetivo
	F	Empregado Efetivo
	F	Empregado Efetivo
	F	Empregado Efetivo
	M	Empregado Efetivo
	M	Empregado Efetivo
	M	Empregado Efetivo
	M	Empregado Efetivo
	M	Empregado Efetivo
	F	Empregado Efetivo
	F	Empregado Efetivo
	F	Empregado Efetivo
	F	Empregado Efetivo
	F	Empregado Efetivo
	F	Empregado Efetivo
	F	Empregado Efetivo
	F	Empregado Efetivo
	F	Empregado Efetivo
	F	Empregado Efetivo
	F	Empregado Efetivo
	F	Empregado Efetivo
	F	Empregado Efetivo
	F	Empregado Efetivo
	F	Empregado Efetivo

	F	Empregado Efetivo
	F	Empregado Efetivo
	F	Empregado Efetivo
	F	Empregado Efetivo
	F	Empregado Efetivo
	F	Empregado Efetivo
	F	Empregado Efetivo
	F	Empregado Efetivo
	F	Empregado Efetivo
	F	Empregado Efetivo
	F	Empregado Efetivo
	F	Empregado Efetivo
	F	Empregado Efetivo
	F	Empregado Efetivo
	F	Empregado Efetivo
	F	Empregado Efetivo
	M	Empregado Efetivo
	F	Empregado Efetivo
	F	Empregado Efetivo
	F	Empregado Efetivo
	F	Empregado Efetivo
	F	Empregado Efetivo
	F	Empregado Efetivo
	F	Empregado Efetivo
	F	Empregado Efetivo
	F	Empregado Efetivo
	F	Empregado Efetivo
	F	Empregado Efetivo
	M	Empregado Efetivo
	M	Empregado Efetivo
	F	Empregado Efetivo
	M	Empregado Efetivo
	M	Empregado Efetivo
	M	Empregado Efetivo
	F	Empregado Efetivo
	M	Empregado Efetivo
	M	Empregado Efetivo
	M	Empregado Efetivo
	M	Empregado Efetivo
	M	Empregado Efetivo
	M	Empregado Efetivo
	F	Empregado Efetivo
	M	Empregado Efetivo

	F	Empregado Efetivo
	M	Empregado Efetivo
	M	Empregado Efetivo
	M	Empregado Efetivo
	F	Empregado Efetivo
	M	Empregado Efetivo
	M	Empregado Efetivo
	F	Empregado Efetivo
	M	Empregado Efetivo
	F	Empregado Efetivo
	M	Empregado Efetivo
	F	Empregado Efetivo
	F	Empregado Efetivo
	F	Empregado Efetivo
	M	Empregado Efetivo
	M	Empregado Efetivo
	F	Empregado Efetivo
	M	Empregado Efetivo
	M	Empregado Efetivo
	M	Empregado Efetivo
	M	Empregado Efetivo
	M	Empregado Efetivo
	M	Empregado Efetivo
	M	Empregado Efetivo
	M	Empregado Efetivo
	M	Empregado Efetivo
	M	Empregado Efetivo
	M	Empregado Efetivo
	F	Empregado Efetivo
	F	Empregado Efetivo
	F	Empregado Efetivo
	F	Empregado Efetivo
	F	Empregado Efetivo
	F	Empregado Efetivo
	F	Empregado Efetivo
	F	Empregado Efetivo
	F	Empregado Efetivo
	F	Empregado Efetivo
	F	Empregado Efetivo
	F	Empregado Efetivo
	F	Empregado Efetivo
	F	Empregado Efetivo

	F	Empregado Efetivo
	F	Empregado Efetivo
	F	Empregado Efetivo
	F	Empregado Efetivo
	F	Empregado Efetivo
	F	Empregado Efetivo
	F	Empregado Efetivo
	F	Empregado Efetivo
	F	Empregado Efetivo
	F	Empregado Efetivo
	F	Empregado Efetivo
	F	Empregado Efetivo
	F	Empregado Efetivo
	F	Empregado Efetivo
	F	Empregado Efetivo
	F	Empregado Efetivo
	F	Empregado Efetivo
	F	Empregado Efetivo
	F	Empregado Efetivo
	F	Empregado Efetivo
	F	Empregado Efetivo
	F	Empregado Efetivo
	F	Empregado Efetivo
	F	Empregado Efetivo
	F	Empregado Efetivo
	F	Empregado Efetivo
	F	Empregado Efetivo
	F	Empregado Efetivo
	F	Empregado Efetivo
	F	Empregado Efetivo
	F	Empregado Efetivo
	F	Empregado Efetivo
	F	Empregado Efetivo
	F	Empregado Efetivo
	F	Empregado Efetivo
	F	Empregado Efetivo
	F	Empregado Efetivo
	F	Empregado Efetivo
	F	Empregado Efetivo
	F	Empregado Efetivo
	F	Empregado Efetivo
	F	Empregado Efetivo
	F	Empregado Efetivo
	F	Empregado Efetivo
	F	Empregado Efetivo
	F	Empregado Efetivo
	F	Empregado Efetivo
	F	Empregado Efetivo

	F	Empregado Efetivo
	F	Empregado Efetivo
	F	Empregado Efetivo
	F	Empregado Efetivo
	F	Empregado Efetivo
	F	Empregado Efetivo
	F	Empregado Efetivo
	F	Empregado Efetivo
	F	Empregado Efetivo
	F	Empregado Efetivo
	F	Empregado Efetivo
	F	Empregado Efetivo
	F	Empregado Efetivo
	F	Empregado Efetivo
	F	Empregado Efetivo
	F	Empregado Efetivo
	F	Empregado Efetivo
	F	Empregado Efetivo
	F	Empregado Efetivo
	F	Empregado Efetivo
	F	Empregado Efetivo
	F	Empregado Efetivo
	F	Empregado Efetivo
	F	Empregado Efetivo
	F	Empregado Efetivo
	F	Empregado Efetivo
	F	Empregado Efetivo
	F	Empregado Efetivo
	F	Empregado Efetivo
	F	Empregado Efetivo
	F	Empregado Efetivo
	F	Empregado Efetivo
	F	Empregado Efetivo
	F	Empregado Efetivo
	F	Empregado Efetivo
	F	Empregado Efetivo
	F	Empregado Efetivo
	F	Empregado Efetivo
	F	Empregado Efetivo
	F	Empregado Efetivo
	F	Empregado Efetivo
	F	Empregado Efetivo
	F	Empregado Efetivo
	F	Empregado Efetivo
	F	Empregado Efetivo
	F	Empregado Efetivo
	F	Empregado Efetivo
	F	Empregado Efetivo

	F	Empregado Efetivo
	F	Empregado Efetivo
	F	Empregado Efetivo
	F	Empregado Efetivo
	F	Empregado Efetivo
	F	Empregado Efetivo
	F	Empregado Efetivo
	F	Empregado Efetivo
	F	Empregado Efetivo
	F	Empregado Efetivo
	F	Empregado Efetivo
	F	Empregado Efetivo
	F	Empregado Efetivo
	F	Empregado Efetivo
	F	Empregado Efetivo
	F	Empregado Efetivo
	F	Empregado Efetivo
	F	Empregado Efetivo
	F	Empregado Efetivo
	F	Empregado Efetivo
	F	Empregado Efetivo
	F	Empregado Efetivo
	F	Empregado Efetivo
	F	Empregado Efetivo
	F	Empregado Efetivo
	F	Empregado Efetivo
	F	Empregado Efetivo
	F	Empregado Efetivo
	F	Empregado Efetivo
	F	Empregado Efetivo
	F	Empregado Efetivo
	F	Empregado Efetivo
	F	Empregado Efetivo
	F	Empregado Efetivo
	F	Empregado Efetivo
	F	Empregado Efetivo
	F	Empregado Efetivo
	F	Empregado Efetivo
	F	Empregado Efetivo
	F	Empregado Efetivo
	F	Empregado Efetivo
	F	Empregado Efetivo
	F	Empregado Efetivo
	F	Empregado Efetivo
	F	Empregado Efetivo
	F	Empregado Efetivo
	F	Empregado Efetivo
	F	Empregado Efetivo

	F	Empregado Efetivo
	F	Empregado Efetivo
	F	Empregado Efetivo
	F	Empregado Efetivo
	F	Empregado Efetivo
	F	Empregado Efetivo
	F	Empregado Efetivo
	F	Empregado Efetivo
	F	Empregado Efetivo
	F	Empregado Efetivo
	F	Empregado Efetivo
	F	Empregado Efetivo
	F	Empregado Efetivo
	F	Empregado Efetivo
	F	Empregado Efetivo
	F	Empregado Efetivo
	F	Empregado Efetivo
	F	Empregado Efetivo
	F	Empregado Efetivo
	F	Empregado Efetivo
	F	Empregado Efetivo
	F	Empregado Efetivo
	F	Empregado Efetivo
	F	Empregado Efetivo
	F	Empregado Efetivo
	F	Empregado Efetivo
	F	Empregado Efetivo
	F	Empregado Efetivo
	F	Empregado Efetivo
	F	Empregado Efetivo
	F	Empregado Efetivo
	F	Empregado Efetivo
	F	Empregado Efetivo
	F	Empregado Efetivo
	F	Empregado Efetivo
	F	Empregado Efetivo
	F	Empregado Efetivo
	F	Empregado Efetivo
	F	Empregado Efetivo
	F	Empregado Efetivo
	F	Empregado Efetivo
	F	Empregado Efetivo
	F	Empregado Efetivo
	F	Empregado Efetivo
	F	Empregado Efetivo
	F	Empregado Efetivo
	F	Empregado Efetivo

	F	Empregado Efetivo
	F	Empregado Efetivo
	F	Empregado Efetivo
	F	Empregado Efetivo
	F	Empregado Efetivo
	F	Empregado Efetivo
	F	Empregado Efetivo
	F	Empregado Efetivo
	F	Empregado Efetivo
	F	Empregado Efetivo
	F	Empregado Efetivo
	F	Empregado Efetivo
	F	Empregado Efetivo
	F	Empregado Efetivo
	F	Empregado Efetivo
	F	Empregado Efetivo
	F	Empregado Efetivo
	F	Empregado Efetivo
	F	Empregado Efetivo
	F	Empregado Efetivo
	F	Empregado Efetivo
	F	Empregado Efetivo
	F	Empregado Efetivo
	F	Empregado Efetivo
	F	Empregado Efetivo
	F	Empregado Efetivo
	F	Empregado Efetivo
	F	Empregado Efetivo
	F	Empregado Efetivo
	F	Empregado Efetivo
	F	Empregado Efetivo
	F	Empregado Efetivo
	F	Empregado Efetivo
	F	Empregado Efetivo
	F	Empregado Efetivo
	F	Empregado Efetivo
	F	Empregado Efetivo
	F	Empregado Efetivo
	F	Empregado Efetivo
	F	Empregado Efetivo
	F	Empregado Efetivo
	F	Empregado Efetivo
	F	Empregado Efetivo
	F	Empregado Efetivo

	F	Empregado Efetivo
	F	Empregado Efetivo
	F	Empregado Efetivo
	F	Empregado Efetivo
	F	Empregado Efetivo
	F	Empregado Efetivo
	F	Empregado Efetivo
	F	Empregado Efetivo
	F	Empregado Efetivo
	F	Empregado Efetivo
	F	Empregado Efetivo
	F	Empregado Efetivo
	F	Empregado Efetivo
	F	Empregado Efetivo
	F	Empregado Efetivo
	F	Empregado Efetivo
	F	Empregado Efetivo
	F	Empregado Efetivo
	F	Empregado Efetivo
	F	Empregado Efetivo
	M	Empregado Efetivo
	M	Empregado Efetivo
	M	Empregado Efetivo
	M	Empregado Efetivo
	M	Empregado Efetivo
	M	Empregado Efetivo
	M	Empregado Efetivo
	M	Empregado Efetivo
	M	Empregado Efetivo
	M	Empregado Efetivo
	M	Empregado Efetivo
	M	Empregado Efetivo
	M	Empregado Efetivo
	M	Empregado Efetivo
	M	Empregado Efetivo
	M	Empregado Efetivo

	M	Empregado Efetivo
	M	Empregado Efetivo
	M	Empregado Efetivo
	M	Empregado Efetivo
	M	Empregado Efetivo
	M	Empregado Efetivo
	M	Empregado Temporário
	M	Empregado Efetivo
	F	Empregado Efetivo
	F	Empregado Efetivo
	F	Empregado Efetivo
	F	Empregado Efetivo
	F	Empregado Efetivo
	F	Empregado Efetivo
	M	Empregado Efetivo
	F	Empregado Efetivo
	F	Empregado Efetivo
	F	Empregado Efetivo
	F	Empregado Efetivo
	F	Empregado Efetivo
	F	Empregado Efetivo
	M	Empregado Efetivo
	M	Empregado Efetivo
	M	Empregado Efetivo
	M	Empregado Efetivo
	M	Empregado Efetivo
	F	Empregado Efetivo
	M	Empregado Efetivo
	M	Empregado Efetivo
	F	Empregado Efetivo
	F	Empregado Efetivo
	F	Empregado Efetivo
	F	Empregado Efetivo
	M	Empregado Efetivo
	F	Empregado Efetivo
	F	Empregado Efetivo
	F	Empregado Efetivo
	M	Empregado Efetivo
	F	Empregado Efetivo

	F	Empregado Efetivo
	F	Empregado Efetivo
	M	Empregado Efetivo
	F	Empregado Efetivo
	F	Empregado Efetivo
	F	Empregado Efetivo
	F	Empregado Efetivo
	F	Empregado Efetivo
	F	Empregado Efetivo
	F	Empregado Efetivo
	M	Empregado Efetivo
	M	Empregado Efetivo
	F	Empregado Efetivo
	F	Empregado Efetivo
	F	Empregado Efetivo
	F	Empregado Efetivo
	M	Empregado Efetivo
	M	Empregado Efetivo
	M	Empregado Efetivo
	M	Empregado Efetivo
	F	Empregado Efetivo
	F	Empregado Efetivo
	F	Empregado Efetivo
	F	Empregado Efetivo
	F	Empregado Efetivo
	F	Empregado Efetivo
	F	Empregado Efetivo
	F	Empregado Efetivo
	F	Empregado Efetivo
	F	Empregado Efetivo
	F	Empregado Efetivo
	F	Empregado Efetivo
	F	Empregado Efetivo
	F	Empregado Efetivo
	F	Empregado Efetivo

	F	Empregado Efetivo
	F	Empregado Efetivo
	F	Empregado Efetivo
	F	Empregado Efetivo
	F	Empregado Efetivo
	F	Empregado Efetivo
	F	Empregado Efetivo
	F	Empregado Efetivo
	F	Empregado Efetivo
	F	Empregado Efetivo
	F	Empregado Efetivo
	F	Empregado Efetivo
	F	Empregado Efetivo
	F	Empregado Efetivo
	F	Empregado Efetivo
	F	Empregado Efetivo
	F	Empregado Efetivo
	F	Empregado Efetivo
	F	Empregado Efetivo
	F	Empregado Efetivo
	F	Empregado Efetivo
	F	Empregado Efetivo
	F	Empregado Efetivo
	F	Empregado Efetivo
	F	Empregado Efetivo
	F	Empregado Efetivo
	F	Empregado Efetivo
	F	Empregado Efetivo
	F	Empregado Efetivo
	F	Empregado Efetivo
	F	Empregado Efetivo
	F	Empregado Efetivo

	F	Empregado Efetivo
	F	Empregado Efetivo
	F	Empregado Efetivo
	F	Empregado Efetivo
	F	Empregado Efetivo
	F	Empregado Efetivo
	F	Empregado Efetivo
	F	Empregado Efetivo
	F	Empregado Efetivo
	F	Empregado Efetivo
	F	Empregado Efetivo
	F	Empregado Efetivo
	F	Empregado Efetivo
	F	Empregado Efetivo
	F	Empregado Efetivo
	F	Empregado Efetivo
	F	Empregado Efetivo
	F	Empregado Efetivo
	F	Empregado Efetivo
	F	Empregado Efetivo
	F	Empregado Efetivo
	F	Empregado Efetivo
	M	Empregado Efetivo
	F	Empregado Efetivo
	M	Empregado Efetivo
	F	Empregado Efetivo
	F	Empregado Efetivo
	F	Empregado Efetivo
	F	Empregado Efetivo
	F	Empregado Efetivo
	F	Empregado Efetivo
	F	Empregado Efetivo
	F	Empregado Efetivo
	F	Empregado Efetivo

	F	Empregado Efetivo
	F	Empregado Efetivo
	F	Empregado Efetivo
	F	Empregado Efetivo
	F	Empregado Efetivo
	F	Empregado Efetivo
	F	Empregado Efetivo
	F	Empregado Efetivo
	F	Empregado Efetivo
	F	Empregado Efetivo
	F	Empregado Efetivo
	F	Empregado Efetivo
	F	Empregado Efetivo
	F	Empregado Efetivo
	F	Empregado Efetivo
	F	Empregado Efetivo
	F	Empregado Efetivo
	F	Empregado Efetivo
	F	Empregado Efetivo
	F	Empregado Efetivo
	F	Empregado Efetivo
	F	Empregado Efetivo
	F	Empregado Efetivo
	F	Empregado Efetivo
	F	Empregado Efetivo
	F	Empregado Efetivo
	F	Empregado Efetivo
	F	Empregado Efetivo
	F	Empregado Efetivo
	F	Empregado Efetivo
	F	Empregado Efetivo
	F	Empregado Efetivo
	F	Empregado Efetivo
	F	Empregado Efetivo
	F	Empregado Efetivo
	F	Empregado Efetivo
	F	Empregado Efetivo
	F	Empregado Efetivo
	F	Empregado Efetivo
	F	Empregado Efetivo
	F	Empregado Efetivo
	F	Empregado Efetivo
	F	Empregado Efetivo
	F	Empregado Efetivo
	F	Empregado Efetivo
	F	Empregado Efetivo
	F	Empregado Efetivo

	F	Empregado Efetivo
	F	Empregado Efetivo
	F	Empregado Efetivo
	F	Empregado Efetivo
	F	Empregado Efetivo
	F	Empregado Efetivo
	F	Empregado Efetivo
	F	Empregado Efetivo
	F	Comissionado
	M	Empregado Efetivo
	M	Empregado Efetivo
	M	Empregado Efetivo
	F	Empregado Efetivo
	F	Empregado Efetivo
	F	Empregado Efetivo
	F	Empregado Efetivo
	F	Empregado Efetivo
	F	Empregado Efetivo
	F	Empregado Efetivo
	F	Empregado Efetivo
	M	Empregado Efetivo
	F	Empregado Efetivo
	F	Empregado Efetivo
	F	Empregado Efetivo
	F	Empregado Efetivo
	F	Empregado Efetivo
	F	Empregado Efetivo
	F	Empregado Efetivo
	F	Empregado Efetivo
	M	Empregado Efetivo
	F	Empregado Efetivo
	M	Empregado Efetivo
	F	Empregado Efetivo

	F	Empregado Efetivo
	F	Empregado Efetivo
	F	Empregado Efetivo
	M	Empregado Efetivo
	F	Empregado Efetivo
	F	Empregado Efetivo
	F	Empregado Efetivo
	M	Empregado Efetivo
	M	Empregado Efetivo
	M	Empregado Efetivo
	F	Empregado Efetivo
	F	Empregado Efetivo
	F	Empregado Efetivo
	F	Empregado Efetivo
	F	Empregado Efetivo
	F	Empregado Efetivo
	F	Empregado Efetivo
	F	Empregado Efetivo
	F	Empregado Efetivo
	F	Empregado Efetivo
	F	Empregado Efetivo
	F	Empregado Efetivo
	F	Empregado Efetivo
	F	Empregado Efetivo
	F	Empregado Efetivo
	F	Empregado Efetivo
	F	Empregado Efetivo
	F	Empregado Efetivo
	F	Empregado Efetivo
	F	Empregado Efetivo
	F	Empregado Efetivo
	F	Empregado Efetivo
	F	Empregado Efetivo
	F	Empregado Efetivo
	F	Empregado Efetivo
	F	Empregado Efetivo
	F	Empregado Efetivo
	F	Empregado Efetivo
	F	Empregado Efetivo
	F	Empregado Efetivo
	F	Empregado Efetivo
	F	Empregado Efetivo
	F	Empregado Efetivo

	F	Empregado Efetivo
	F	Empregado Efetivo
	F	Empregado Efetivo
	F	Empregado Efetivo
	F	Empregado Efetivo
	F	Empregado Efetivo
	F	Empregado Efetivo
	F	Empregado Efetivo
	F	Empregado Efetivo
	F	Empregado Efetivo
	F	Empregado Efetivo
	F	Empregado Efetivo
	F	Empregado Efetivo
	F	Empregado Efetivo
	M	Empregado Efetivo
	F	Empregado Efetivo
	M	Empregado Efetivo
	F	Empregado Efetivo
	F	Empregado Efetivo
	F	Empregado Efetivo
	F	Empregado Efetivo
	F	Empregado Efetivo
	F	Empregado Efetivo
	F	Empregado Efetivo
	F	Empregado Efetivo
	F	Empregado Efetivo
	F	Empregado Efetivo
	M	Empregado Efetivo
	F	Empregado Efetivo
	M	Empregado Efetivo
	F	Empregado Efetivo
	F	Empregado Efetivo
	M	Empregado Efetivo
	F	Empregado Efetivo
	F	Empregado Efetivo
	F	Empregado Efetivo
	F	Empregado Efetivo
	F	Empregado Efetivo
	F	Empregado Efetivo
	F	Empregado Efetivo
	F	Empregado Efetivo
	F	Empregado Efetivo

	F	Empregado Efetivo
	F	Empregado Efetivo
	F	Empregado Efetivo
	F	Empregado Efetivo
	F	Empregado Efetivo
	F	Empregado Efetivo
	F	Empregado Efetivo
	F	Empregado Efetivo
	F	Empregado Efetivo
	F	Empregado Efetivo
	F	Empregado Efetivo
	F	Empregado Efetivo
	F	Empregado Efetivo
	M	Empregado Efetivo
	F	Empregado Efetivo
	M	Empregado Efetivo
	F	Empregado Efetivo
	M	Empregado Efetivo
	F	Empregado Efetivo
	F	Empregado Efetivo
	F	Empregado Efetivo
	F	Empregado Efetivo
	F	Empregado Efetivo
	M	Empregado Efetivo
	F	Empregado Efetivo
	M	Empregado Efetivo
	F	Empregado Efetivo
	F	Empregado Efetivo
	F	Empregado Efetivo
	F	Empregado Efetivo
	F	Empregado Efetivo
	M	Empregado Efetivo
	F	Empregado Efetivo
	M	Empregado Efetivo
	F	Empregado Efetivo
	M	Empregado Efetivo
	F	Empregado Efetivo
	F	Empregado Efetivo
	F	Empregado Efetivo
	F	Empregado Efetivo
	F	Empregado Efetivo
	M	Empregado Efetivo
	F	Empregado Efetivo
	F	Empregado Efetivo

	F	Empregado Efetivo
	F	Empregado Efetivo
	F	Empregado Efetivo
	F	Empregado Efetivo
	F	Empregado Efetivo
	F	Empregado Efetivo
	M	Empregado Efetivo
	M	Empregado Efetivo
	M	Empregado Efetivo
	F	Empregado Efetivo
	F	Empregado Efetivo
	F	Empregado Efetivo
	F	Empregado Efetivo
	F	Empregado Efetivo
	F	Empregado Efetivo
	F	Empregado Efetivo
	M	Empregado Efetivo
	M	Empregado Efetivo
	M	Empregado Efetivo
	M	Empregado Efetivo
	M	Empregado Efetivo
	F	Empregado Efetivo
	M	Empregado Efetivo
	F	Empregado Efetivo
	F	Empregado Efetivo
	M	Empregado Efetivo
	F	Empregado Efetivo
	F	Empregado Efetivo
	M	Empregado Efetivo
	F	Empregado Efetivo
	F	Empregado Efetivo
	F	Empregado Efetivo
	F	Empregado Efetivo
	F	Empregado Efetivo
	F	Empregado Efetivo
	F	Empregado Efetivo
	M	Empregado Efetivo
	F	Empregado Efetivo
	F	Empregado Efetivo
	F	Empregado Efetivo
	F	Empregado Efetivo
	F	Empregado Efetivo
	F	Empregado Efetivo
	F	Empregado Efetivo
	F	Empregado Efetivo
	F	Empregado Efetivo
	F	Empregado Efetivo

	F	Empregado Efetivo
	F	Empregado Efetivo
	F	Empregado Efetivo
	F	Empregado Efetivo
	F	Empregado Efetivo
	F	Empregado Efetivo
	F	Empregado Efetivo
	F	Empregado Efetivo
	F	Empregado Efetivo
	F	Empregado Efetivo
	F	Empregado Efetivo
	M	Empregado Efetivo
	M	Empregado Efetivo
	M	Empregado Efetivo
	F	Empregado Efetivo
	F	Empregado Efetivo
	F	Empregado Efetivo
	F	Empregado Efetivo
	F	Empregado Efetivo
	F	Empregado Efetivo
	F	Empregado Efetivo
	F	Empregado Efetivo
	M	Empregado Efetivo
	M	Empregado Efetivo
	F	Empregado Efetivo
	F	Empregado Efetivo
	M	Empregado Efetivo
	F	Empregado Efetivo
	F	Empregado Efetivo
	F	Empregado Efetivo
	F	Empregado Efetivo
	F	Empregado Efetivo
	M	Empregado Efetivo
	F	Empregado Efetivo
	F	Empregado Efetivo
	F	Empregado Efetivo
	F	Empregado Efetivo
	M	Empregado Efetivo
	M	Empregado Efetivo
	M	Empregado Efetivo
	M	Empregado Efetivo
	M	Empregado Efetivo
	M	Empregado Efetivo
	M	Empregado Efetivo
	M	Empregado Efetivo

	M	Empregado Efetivo
	M	Empregado Efetivo
	M	Empregado Efetivo
	M	Empregado Efetivo
	M	Empregado Efetivo
	M	Empregado Efetivo
	M	Empregado Efetivo
	M	Empregado Efetivo
	M	Empregado Efetivo
	M	Empregado Efetivo
	M	Empregado Efetivo
	M	Empregado Efetivo
	M	Empregado Efetivo
	M	Empregado Efetivo
	M	Empregado Efetivo
	M	Empregado Efetivo
	M	Empregado Efetivo
	M	Empregado Efetivo
	F	Empregado Efetivo
	M	Empregado Efetivo
	F	Empregado Efetivo
	F	Empregado Efetivo
	M	Empregado Efetivo
	M	Empregado Efetivo
	F	Empregado Efetivo
	M	Empregado Efetivo
	F	Empregado Efetivo
	M	Empregado Efetivo
	M	Empregado Efetivo
	F	Empregado Efetivo
	M	Empregado Efetivo
	F	Empregado Efetivo
	F	Empregado Efetivo
	F	Empregado Efetivo
	F	Empregado Efetivo
	F	Empregado Efetivo
	F	Empregado Efetivo
	F	Empregado Efetivo
	F	Empregado Efetivo
	F	Empregado Efetivo
	F	Empregado Efetivo
	F	Empregado Efetivo
	M	Empregado Efetivo
	F	Empregado Efetivo
	F	Empregado Efetivo
	F	Empregado Efetivo
	F	Empregado Efetivo
	F	Empregado Efetivo

	F	Empregado Efetivo
	F	Empregado Efetivo
	M	Empregado Efetivo
	F	Empregado Efetivo
	F	Empregado Efetivo
	F	Empregado Efetivo
	F	Empregado Efetivo
	F	Empregado Efetivo
	M	Empregado Efetivo
	M	Empregado Efetivo
	M	Empregado Efetivo
	M	Empregado Efetivo
	M	Empregado Efetivo
	F	Empregado Efetivo
	F	Empregado Efetivo
	F	Empregado Efetivo
	F	Empregado Efetivo
	M	Empregado Efetivo
	F	Empregado Efetivo
	F	Empregado Efetivo
	F	Empregado Efetivo
	F	Empregado Efetivo
	F	Empregado Efetivo
	M	Empregado Efetivo
	F	Empregado Efetivo
	M	Empregado Efetivo
	F	Empregado Efetivo
	F	Empregado Efetivo
	F	Empregado Efetivo
	F	Empregado Efetivo
	F	Empregado Efetivo
	F	Empregado Efetivo
	F	Empregado Efetivo
	F	Empregado Efetivo
	F	Empregado Efetivo
	F	Empregado Efetivo
	F	Empregado Efetivo
	F	Empregado Efetivo
	F	Empregado Efetivo
	F	Empregado Efetivo
	M	Empregado Efetivo
	F	Empregado Efetivo
	F	Empregado Efetivo
	F	Empregado Efetivo
	F	Empregado Efetivo
	F	Empregado Efetivo
	F	Empregado Efetivo

	F	Empregado Efetivo
	F	Empregado Efetivo
	F	Empregado Efetivo
	F	Empregado Efetivo
	F	Empregado Efetivo
	F	Empregado Efetivo
	F	Empregado Efetivo
	F	Empregado Efetivo
	F	Empregado Efetivo
	F	Empregado Efetivo
	M	Empregado Efetivo
	M	Empregado Efetivo
	F	Empregado Efetivo
	F	Empregado Efetivo
	F	Empregado Efetivo
	F	Empregado Efetivo
	F	Empregado Efetivo
	F	Empregado Efetivo
	F	Empregado Efetivo
	F	Empregado Efetivo
	F	Empregado Efetivo
	F	Empregado Efetivo
	F	Empregado Efetivo
	F	Empregado Efetivo
	M	Empregado Efetivo
	M	Empregado Efetivo
	M	Empregado Efetivo
	M	Empregado Efetivo
	M	Empregado Efetivo
	M	Empregado Efetivo
	M	Empregado Efetivo
	M	Empregado Efetivo
	F	Empregado Efetivo
	M	Empregado Efetivo
	M	Empregado Efetivo
	M	Empregado Efetivo
	M	Empregado Efetivo
	M	Empregado Efetivo
	F	Empregado Efetivo
	M	Empregado Efetivo
	M	Empregado Efetivo
	M	Empregado Efetivo
	F	Empregado Efetivo
	M	Empregado Efetivo
	M	Empregado Efetivo
	M	Empregado Efetivo
	M	Empregado Efetivo
	F	Empregado Efetivo

	F	Empregado Efetivo
	F	Empregado Efetivo
	F	Empregado Efetivo
	F	Empregado Efetivo
	F	Empregado Efetivo
	M	Empregado Efetivo
	M	Empregado Efetivo
	F	Empregado Efetivo
	F	Empregado Efetivo
	F	Empregado Efetivo
	F	Empregado Efetivo
	F	Empregado Efetivo
	F	Empregado Efetivo
	F	Empregado Efetivo
	F	Empregado Efetivo
	M	Empregado Efetivo
	F	Empregado Efetivo
	F	Empregado Efetivo
	F	Empregado Efetivo
	F	Empregado Efetivo
	F	Empregado Efetivo
	F	Empregado Efetivo
	F	Empregado Efetivo
	F	Empregado Efetivo
	F	Empregado Efetivo
	F	Empregado Efetivo
	F	Empregado Efetivo
	F	Empregado Efetivo
	F	Empregado Efetivo
	F	Empregado Efetivo
	F	Empregado Efetivo
	F	Empregado Efetivo
	F	Empregado Efetivo
	F	Empregado Efetivo
	F	Empregado Efetivo
	F	Empregado Efetivo
	F	Empregado Efetivo
	F	Empregado Efetivo
	F	Empregado Efetivo
	F	Empregado Efetivo
	F	Empregado Efetivo
	F	Empregado Efetivo
	F	Empregado Efetivo
	F	Empregado Efetivo
	F	Empregado Efetivo

	F	Empregado Efetivo
	F	Empregado Efetivo
	F	Empregado Efetivo
	F	Empregado Efetivo
	F	Empregado Efetivo
	M	Empregado Efetivo
	F	Empregado Efetivo
	F	Empregado Efetivo
	F	Empregado Efetivo
	F	Empregado Efetivo
	F	Empregado Efetivo
	F	Empregado Efetivo
	F	Empregado Efetivo
	F	Empregado Efetivo
	F	Empregado Efetivo
	F	Empregado Efetivo
	F	Empregado Efetivo
	F	Empregado Efetivo
	F	Empregado Efetivo
	F	Empregado Efetivo
	F	Empregado Efetivo
	F	Empregado Efetivo
	M	Empregado Efetivo
	M	Empregado Efetivo
	F	Empregado Efetivo
	M	Empregado Efetivo
	F	Empregado Efetivo
	M	Empregado Efetivo
	M	Empregado Efetivo
	M	Empregado Efetivo
	F	Empregado Efetivo
	M	Empregado Efetivo
	F	Empregado Efetivo
	F	Empregado Efetivo
	M	Empregado Efetivo
	F	Empregado Efetivo
	M	Empregado Efetivo
	F	Empregado Efetivo
	M	Empregado Efetivo
	F	Empregado Efetivo
	F	Empregado Efetivo
	F	Empregado Efetivo
	F	Empregado Efetivo
	F	Empregado Efetivo
	F	Empregado Efetivo
	F	Empregado Efetivo
	F	Empregado Efetivo
	F	Empregado Efetivo

	F	Empregado Efetivo
	M	Empregado Efetivo
	F	Empregado Efetivo
	F	Empregado Efetivo
	F	Empregado Efetivo
	F	Empregado Efetivo
	M	Empregado Efetivo
	F	Empregado Efetivo
	F	Empregado Efetivo
	F	Empregado Efetivo
	F	Empregado Efetivo
	F	Empregado Efetivo
	F	Empregado Efetivo
	F	Empregado Efetivo
	F	Empregado Efetivo
	F	Empregado Efetivo
	F	Empregado Efetivo
	F	Empregado Efetivo
	F	Empregado Efetivo
	F	Empregado Efetivo
	F	Empregado Efetivo
	F	Empregado Efetivo
	F	Empregado Efetivo
	F	Empregado Efetivo
	F	Empregado Efetivo
	F	Empregado Efetivo
	F	Empregado Efetivo
	M	Empregado Efetivo
	F	Empregado Efetivo
	F	Empregado Efetivo
	F	Empregado Efetivo
	F	Empregado Efetivo
	F	Empregado Efetivo
	F	Empregado Efetivo
	F	Empregado Efetivo
	F	Empregado Efetivo
	M	Empregado Efetivo
	F	Empregado Efetivo
	M	Empregado Efetivo
	F	Empregado Efetivo
	F	Empregado Efetivo
	F	Empregado Efetivo
	F	Empregado Efetivo
	F	Empregado Efetivo
	F	Empregado Efetivo
	F	Empregado Efetivo
	F	Empregado Efetivo
	M	Empregado Efetivo

	M	Empregado Efetivo
	M	Empregado Efetivo
	F	Empregado Efetivo
	F	Empregado Efetivo
	F	Empregado Efetivo
	F	Empregado Efetivo
	F	Empregado Efetivo
	F	Empregado Efetivo
	M	Empregado Efetivo
	F	Empregado Efetivo
	M	Empregado Efetivo
	F	Empregado Efetivo
	F	Empregado Efetivo
	F	Empregado Efetivo
	F	Empregado Efetivo
	F	Empregado Efetivo
	M	Empregado Efetivo
	M	Empregado Efetivo
	F	Empregado Efetivo
	F	Empregado Efetivo
	F	Empregado Efetivo
	F	Empregado Efetivo
	F	Empregado Efetivo
	M	Empregado Efetivo
	M	Empregado Efetivo
	F	Empregado Efetivo
	M	Empregado Efetivo
	F	Empregado Efetivo
	F	Empregado Efetivo
	F	Empregado Efetivo
	F	Empregado Efetivo
	F	Empregado Efetivo
	F	Empregado Efetivo
	F	Empregado Efetivo
	F	Empregado Efetivo
	F	Empregado Efetivo
	F	Empregado Efetivo
	F	Empregado Efetivo
	F	Empregado Efetivo
	F	Empregado Efetivo
	F	Empregado Efetivo
	F	Empregado Efetivo
	M	Empregado Efetivo
	F	Empregado Efetivo
	F	Empregado Efetivo
	F	Comissionado
	F	Empregado Efetivo
	F	Empregado Efetivo

	M	Empregado Efetivo
	M	Empregado Efetivo
	F	Empregado Efetivo
	F	Empregado Efetivo
	F	Empregado Efetivo
	M	Empregado Efetivo
	M	Empregado Efetivo
	F	Empregado Efetivo
	M	Empregado Efetivo
	M	Empregado Efetivo
	M	Empregado Efetivo
	M	Empregado Efetivo
	M	Empregado Efetivo
	M	Empregado Efetivo
	M	Empregado Efetivo
	M	Empregado Efetivo
	M	Empregado Efetivo
	M	Empregado Efetivo
	M	Empregado Efetivo
	M	Empregado Efetivo
	M	Empregado Efetivo
	M	Empregado Efetivo
	M	Empregado Efetivo
	M	Empregado Efetivo
	M	Empregado Efetivo
	M	Empregado Efetivo
	M	Empregado Efetivo
	M	Empregado Efetivo
	M	Empregado Efetivo
	M	Empregado Efetivo
	M	Empregado Efetivo
	M	Empregado Efetivo
	M	Empregado Efetivo
	M	Empregado Efetivo
	M	Empregado Efetivo
	M	Empregado Efetivo
	M	Empregado Efetivo
	M	Empregado Efetivo
	M	Empregado Efetivo
	M	Empregado Efetivo
	M	Empregado Efetivo
	M	Empregado Efetivo
	M	Empregado Efetivo
	M	Empregado Efetivo
	M	Empregado Efetivo
	M	Empregado Efetivo
	M	Empregado Efetivo
	M	Empregado Efetivo

	M	Empregado Efetivo
	M	Empregado Efetivo
	M	Empregado Efetivo
	F	Empregado Efetivo
	F	Empregado Efetivo
	F	Empregado Efetivo
	F	Empregado Efetivo
	F	Empregado Efetivo
	F	Empregado Efetivo
	F	Empregado Efetivo
	F	Empregado Efetivo
	F	Empregado Efetivo
	M	Empregado Efetivo
	M	Empregado Efetivo
	M	Empregado Efetivo
	M	Empregado Efetivo
	F	Empregado Efetivo
	F	Empregado Efetivo
	M	Empregado Efetivo
	F	Empregado Efetivo
	F	Empregado Efetivo
	F	Empregado Efetivo
	F	Empregado Efetivo
	M	Empregado Efetivo
	F	Empregado Efetivo
	M	Empregado Efetivo
	F	Empregado Efetivo
	F	Empregado Efetivo
	F	Empregado Efetivo
	F	Empregado Efetivo
	F	Empregado Efetivo
	F	Empregado Efetivo
	F	Empregado Efetivo
	F	Empregado Efetivo
	F	Empregado Efetivo
	F	Empregado Efetivo
	F	Empregado Efetivo
	F	Empregado Efetivo
	F	Empregado Efetivo
	F	Empregado Efetivo
	F	Empregado Efetivo
	F	Empregado Efetivo
	F	Empregado Efetivo
	F	Empregado Efetivo
	F	Empregado Efetivo
	F	Empregado Efetivo
	F	Empregado Efetivo
	F	Empregado Efetivo

	F	Empregado Efetivo
	M	Empregado Efetivo
	M	Empregado Efetivo
	M	Empregado Efetivo
	M	Empregado Efetivo
	F	Empregado Efetivo
	M	Empregado Efetivo
	F	Empregado Efetivo
	F	Cedido - RJU
	F	Empregado Efetivo
	F	Empregado Efetivo
	F	Empregado Efetivo
	F	Empregado Efetivo
	F	Empregado Efetivo
	M	Empregado Efetivo
	F	Empregado Efetivo
	F	Empregado Efetivo
	F	Empregado Efetivo
	F	Empregado Efetivo
	F	Empregado Efetivo
	F	Empregado Efetivo
	F	Empregado Efetivo
	F	Empregado Efetivo
	F	Empregado Efetivo
	F	Empregado Efetivo
	F	Empregado Efetivo
	M	Empregado Efetivo
	F	Empregado Efetivo
	M	Empregado Efetivo
	F	Empregado Efetivo
	M	Empregado Efetivo
	M	Empregado Efetivo
	M	Empregado Efetivo
	F	Empregado Efetivo
	F	Empregado Efetivo
	F	Empregado Efetivo
	F	Empregado Efetivo
	F	Empregado Efetivo
	F	Empregado Efetivo
	F	Empregado Efetivo
	M	Empregado Efetivo
	F	Empregado Efetivo
	F	Empregado Efetivo
	F	Empregado Efetivo
	F	Empregado Efetivo
	F	Empregado Efetivo
	F	Empregado Efetivo
	F	Empregado Efetivo

	F	Empregado Efetivo
	F	Empregado Efetivo
	F	Empregado Efetivo
	F	Empregado Efetivo
	F	Empregado Efetivo
	F	Empregado Efetivo
	M	Empregado Efetivo
	F	Empregado Efetivo
	F	Empregado Efetivo
	M	Empregado Efetivo
	F	Empregado Efetivo
	F	Empregado Efetivo
	F	Empregado Efetivo
	F	Empregado Efetivo
	F	Empregado Efetivo
	M	Empregado Efetivo
	M	Empregado Efetivo
	F	Empregado Efetivo
	F	Empregado Efetivo
	M	Empregado Efetivo
	M	Empregado Efetivo
	F	Empregado Efetivo
	F	Empregado Efetivo
	F	Empregado Efetivo
	F	Empregado Efetivo
	M	Empregado Efetivo
	F	Empregado Efetivo
	M	Empregado Efetivo
	F	Empregado Efetivo
	M	Empregado Efetivo
	F	Empregado Efetivo
	M	Empregado Efetivo
	M	Empregado Efetivo
	F	Empregado Efetivo
	F	Empregado Efetivo
	F	Empregado Efetivo
	F	Empregado Efetivo
	F	Empregado Efetivo
	F	Empregado Efetivo
	F	Empregado Efetivo
	F	Empregado Efetivo
	F	Empregado Efetivo
	F	Empregado Efetivo
	F	Empregado Efetivo
	F	Empregado Efetivo
	F	Empregado Efetivo
	F	Empregado Efetivo
	F	Empregado Efetivo

	F	Empregado Efetivo
	F	Empregado Efetivo
	F	Empregado Efetivo
	F	Empregado Efetivo
	F	Empregado Efetivo
	F	Empregado Efetivo
	M	Empregado Efetivo
	F	Empregado Efetivo
	M	Empregado Efetivo
	M	Empregado Efetivo
	F	Empregado Efetivo
	F	Empregado Efetivo
	F	Empregado Efetivo
	F	Empregado Efetivo
	F	Empregado Efetivo
	F	Empregado Efetivo
	F	Empregado Efetivo
	F	Empregado Efetivo
	F	Empregado Efetivo
	F	Empregado Efetivo
	F	Empregado Efetivo
	F	Empregado Efetivo
	F	Empregado Efetivo
	F	Empregado Efetivo
	F	Empregado Efetivo
	F	Empregado Efetivo
	F	Empregado Efetivo
	F	Empregado Efetivo
	F	Empregado Efetivo
	F	Empregado Efetivo
	F	Empregado Efetivo
	M	Empregado Efetivo
	M	Empregado Efetivo
	M	Empregado Efetivo
	F	Empregado Efetivo
	F	Empregado Efetivo
	F	Empregado Efetivo
	F	Empregado Efetivo
	F	Empregado Efetivo
	F	Empregado Efetivo
	M	Empregado Efetivo
	M	Empregado Efetivo
	F	Empregado Efetivo
	F	Empregado Efetivo
	F	Empregado Efetivo
	F	Empregado Efetivo
	F	Empregado Efetivo
	F	Empregado Efetivo

	F	Empregado Efetivo
	F	Empregado Efetivo
	F	Empregado Efetivo
	M	Empregado Efetivo
	F	Empregado Efetivo
	F	Empregado Efetivo
	F	Empregado Efetivo
	F	Empregado Efetivo
	F	Empregado Efetivo
	F	Empregado Efetivo
	F	Empregado Efetivo
	F	Empregado Efetivo
	M	Empregado Efetivo
	F	Empregado Efetivo
	F	Empregado Efetivo
	F	Empregado Efetivo
	F	Empregado Efetivo
	F	Empregado Efetivo
	F	Empregado Efetivo
	F	Empregado Efetivo
	F	Empregado Efetivo
	F	Empregado Efetivo
	F	Empregado Efetivo
	F	Empregado Efetivo
	F	Empregado Efetivo
	F	Empregado Efetivo
	F	Empregado Efetivo
	F	Empregado Efetivo
	F	Empregado Efetivo
	F	Empregado Efetivo
	F	Empregado Efetivo
	F	Empregado Efetivo
	F	Empregado Efetivo
	F	Empregado Efetivo
	F	Empregado Efetivo
	F	Empregado Efetivo
	F	Empregado Efetivo
	F	Empregado Efetivo
	M	Empregado Efetivo
	F	Empregado Efetivo
	F	Empregado Efetivo
	M	Empregado Efetivo
	F	Empregado Efetivo
	F	Empregado Efetivo
	F	Empregado Efetivo
	F	Empregado Efetivo
	M	Empregado Efetivo
	M	Empregado Efetivo

	F	Empregado Efetivo
	F	Empregado Efetivo
	M	Empregado Efetivo
	M	Empregado Efetivo
	F	Empregado Efetivo
	F	Empregado Efetivo
	F	Empregado Efetivo
	M	Empregado Efetivo
	F	Empregado Efetivo
	F	Empregado Efetivo
	F	Empregado Efetivo
	F	Empregado Efetivo
	F	Empregado Efetivo
	F	Empregado Efetivo
	M	Empregado Efetivo
	F	Empregado Efetivo
	F	Empregado Efetivo
	M	Empregado Efetivo
	F	Empregado Efetivo
	F	Empregado Efetivo
	F	Empregado Efetivo
	F	Empregado Efetivo
	M	Empregado Efetivo
	F	Empregado Efetivo
	F	Empregado Efetivo
	F	Empregado Efetivo
	F	Empregado Efetivo
	F	Empregado Efetivo
	F	Empregado Efetivo
	F	Empregado Efetivo
	F	Empregado Efetivo
	F	Empregado Efetivo
	F	Empregado Efetivo
	F	Empregado Efetivo
	F	Empregado Efetivo
	F	Empregado Efetivo
	F	Empregado Efetivo
	F	Empregado Efetivo
	F	Empregado Efetivo
	F	Empregado Efetivo
	F	Empregado Efetivo
	F	Empregado Efetivo
	F	Empregado Efetivo
	F	Empregado Efetivo
	F	Empregado Efetivo
	M	Empregado Efetivo
	F	Empregado Efetivo
	F	Empregado Efetivo

	F	Empregado Efetivo
	M	Empregado Efetivo
	F	Empregado Efetivo
	F	Empregado Efetivo
	F	Empregado Efetivo
	F	Empregado Efetivo
	F	Empregado Efetivo
	F	Empregado Efetivo
	F	Empregado Efetivo
	F	Empregado Efetivo
	F	Empregado Efetivo
	F	Empregado Efetivo
	F	Empregado Efetivo
	M	Empregado Efetivo
	F	Empregado Efetivo
	F	Empregado Efetivo
	F	Empregado Efetivo
	M	Empregado Efetivo
	F	Empregado Efetivo
	F	Empregado Efetivo
	F	Empregado Efetivo
	F	Empregado Efetivo
	F	Empregado Efetivo
	F	Empregado Efetivo
	F	Empregado Efetivo
	F	Empregado Efetivo
	F	Empregado Efetivo
	F	Empregado Efetivo
	F	Empregado Efetivo
	M	Empregado Efetivo
	M	Empregado Efetivo
	F	Empregado Efetivo
	M	Empregado Efetivo
	F	Empregado Efetivo
	M	Empregado Efetivo
	F	Empregado Efetivo
	F	Empregado Efetivo
	F	Empregado Efetivo
	F	Empregado Efetivo
	F	Empregado Efetivo
	F	Empregado Efetivo
	F	Empregado Efetivo
	F	Empregado Efetivo
	F	Empregado Efetivo
	F	Empregado Efetivo
	F	Empregado Efetivo
	F	Empregado Efetivo
	F	Empregado Efetivo

	F	Empregado Efetivo
	F	Empregado Efetivo
	F	Empregado Efetivo
	F	Empregado Efetivo
	F	Empregado Efetivo
	F	Empregado Efetivo
	F	Empregado Efetivo
	F	Empregado Efetivo
	M	Empregado Efetivo
	F	Empregado Efetivo
	F	Empregado Efetivo
	F	Empregado Efetivo
	F	Empregado Efetivo
	F	Empregado Efetivo
	F	Empregado Efetivo
	F	Empregado Efetivo
	M	Empregado Efetivo
	F	Empregado Efetivo
	F	Empregado Efetivo
	F	Empregado Efetivo
	F	Empregado Efetivo
	M	Empregado Efetivo
	F	Empregado Efetivo
	M	Empregado Efetivo
	F	Empregado Efetivo
	F	Empregado Efetivo
	F	Empregado Efetivo
	F	Empregado Efetivo
	F	Empregado Efetivo
	F	Empregado Efetivo
	F	Empregado Efetivo
	F	Empregado Efetivo
	F	Empregado Efetivo
	M	Empregado Efetivo
	M	Empregado Efetivo
	F	Empregado Efetivo
	F	Empregado Efetivo
	F	Empregado Efetivo
	M	Empregado Efetivo
	F	Empregado Efetivo
	F	Empregado Efetivo
	F	Empregado Efetivo
	F	Empregado Efetivo
	F	Empregado Efetivo
	M	Empregado Efetivo
	F	Empregado Efetivo
	F	Empregado Efetivo
	F	Empregado Efetivo

	F	Empregado Efetivo
	F	Empregado Efetivo
	F	Empregado Efetivo
	F	Empregado Efetivo
	F	Empregado Efetivo
	F	Empregado Efetivo
	F	Empregado Efetivo
	F	Empregado Efetivo
	M	Empregado Efetivo
	F	Empregado Efetivo
	F	Empregado Efetivo
	F	Empregado Efetivo
	F	Empregado Efetivo
	F	Empregado Efetivo
	F	Empregado Efetivo
	M	Empregado Efetivo
	F	Empregado Efetivo
	M	Empregado Efetivo
	F	Empregado Efetivo
	F	Empregado Efetivo
	F	Empregado Efetivo
	F	Empregado Efetivo
	F	Empregado Efetivo
	F	Empregado Efetivo
	F	Empregado Efetivo
	F	Empregado Efetivo
	F	Empregado Efetivo
	F	Empregado Efetivo
	F	Empregado Efetivo
	F	Empregado Efetivo
	F	Empregado Efetivo
	F	Empregado Efetivo
	F	Empregado Efetivo
	F	Empregado Efetivo
	F	Empregado Efetivo
	F	Empregado Efetivo
	F	Empregado Efetivo
	F	Empregado Efetivo
	F	Empregado Efetivo
	M	Empregado Efetivo
	F	Empregado Efetivo
	F	Empregado Efetivo
	F	Empregado Efetivo
	F	Empregado Efetivo
	F	Empregado Efetivo
	M	Empregado Efetivo
	F	Empregado Efetivo
	F	Empregado Efetivo

	F	Empregado Efetivo
	M	Empregado Efetivo
	F	Empregado Efetivo
	F	Empregado Efetivo
	F	Empregado Efetivo
	F	Empregado Efetivo
	F	Empregado Efetivo
	F	Empregado Efetivo
	F	Empregado Efetivo
	F	Empregado Efetivo
	F	Empregado Efetivo
	F	Empregado Efetivo
	F	Empregado Efetivo
	F	Empregado Efetivo
	F	Empregado Efetivo
	F	Empregado Efetivo
	F	Empregado Efetivo
	F	Empregado Efetivo
	M	Empregado Efetivo
	F	Empregado Efetivo
	F	Empregado Efetivo
	F	Empregado Efetivo
	F	Empregado Efetivo
	F	Empregado Efetivo
	M	Empregado Efetivo
	F	Empregado Efetivo
	F	Empregado Efetivo
	M	Empregado Efetivo
	M	Empregado Efetivo
	F	Empregado Efetivo
	F	Empregado Efetivo
	F	Empregado Efetivo
	F	Empregado Efetivo
	F	Empregado Efetivo
	F	Empregado Efetivo
	F	Empregado Efetivo
	F	Empregado Efetivo
	F	Empregado Efetivo
	M	Empregado Efetivo
	F	Empregado Efetivo
	F	Empregado Efetivo
	M	Empregado Efetivo
	F	Empregado Efetivo
	M	Empregado Efetivo
	F	Empregado Efetivo
	F	Empregado Efetivo
	F	Empregado Efetivo
	F	Empregado Efetivo

	F	Empregado Efetivo
	F	Empregado Efetivo
	F	Empregado Efetivo
	F	Empregado Efetivo
	M	Empregado Efetivo
	F	Empregado Efetivo
	F	Empregado Efetivo
	M	Empregado Efetivo
	F	Empregado Efetivo
	F	Empregado Efetivo
	F	Empregado Efetivo
	F	Empregado Efetivo
	F	Empregado Efetivo
	F	Empregado Efetivo
	F	Empregado Efetivo
	F	Empregado Efetivo
	F	Empregado Efetivo
	F	Empregado Efetivo
	F	Empregado Efetivo
	F	Empregado Efetivo
	F	Empregado Efetivo
	F	Empregado Efetivo
	F	Empregado Efetivo
	F	Empregado Efetivo
	F	Empregado Efetivo
	F	Empregado Efetivo
	F	Empregado Efetivo
	F	Empregado Efetivo
	M	Empregado Efetivo
	F	Empregado Efetivo
	F	Empregado Efetivo
	F	Empregado Efetivo
	F	Empregado Efetivo
	F	Empregado Efetivo
	F	Empregado Efetivo
	F	Empregado Efetivo
	F	Empregado Efetivo
	F	Empregado Efetivo
	M	Empregado Efetivo
	F	Empregado Efetivo
	F	Empregado Efetivo
	M	Empregado Efetivo
	F	Empregado Efetivo
	F	Empregado Efetivo
	F	Empregado Efetivo
	F	Empregado Efetivo
	F	Empregado Efetivo
	F	Empregado Efetivo

	F	Empregado Efetivo
	F	Empregado Efetivo
	M	Empregado Efetivo
	M	Empregado Efetivo
	M	Empregado Efetivo
	F	Empregado Efetivo
	F	Empregado Efetivo
	F	Empregado Efetivo
	F	Empregado Efetivo
	M	Empregado Efetivo
	F	Empregado Efetivo
	M	Empregado Efetivo
	F	Empregado Efetivo
	F	Empregado Efetivo
	F	Empregado Efetivo
	F	Empregado Efetivo
	F	Empregado Efetivo
	F	Empregado Efetivo
	M	Empregado Efetivo
	F	Empregado Efetivo
	F	Empregado Efetivo
	F	Empregado Efetivo
	M	Empregado Efetivo
	F	Empregado Efetivo
	M	Empregado Efetivo
	M	Empregado Efetivo
	F	Empregado Efetivo
	F	Empregado Efetivo
	F	Empregado Efetivo
	F	Empregado Efetivo
	F	Empregado Efetivo
	F	Empregado Efetivo
	M	Empregado Efetivo
	F	Empregado Efetivo
	F	Empregado Efetivo
	F	Empregado Efetivo
	F	Empregado Efetivo
	F	Empregado Efetivo
	F	Empregado Efetivo
	M	Empregado Efetivo
	F	Empregado Efetivo
	F	Empregado Efetivo
	F	Empregado Efetivo
	F	Empregado Efetivo
	F	Empregado Efetivo
	F	Empregado Efetivo
	F	Empregado Efetivo
	F	Empregado Efetivo

	F	Empregado Efetivo
	F	Empregado Efetivo
	F	Empregado Efetivo
	F	Empregado Efetivo
	F	Empregado Efetivo
	F	Empregado Efetivo
	F	Empregado Efetivo
	F	Empregado Efetivo
	F	Empregado Efetivo
	F	Empregado Efetivo
	F	Empregado Efetivo
	F	Empregado Efetivo
	F	Empregado Efetivo
	F	Empregado Efetivo
	F	Empregado Efetivo
	F	Empregado Efetivo
	F	Empregado Efetivo
	F	Empregado Efetivo
	F	Empregado Efetivo
	F	Empregado Efetivo
	F	Empregado Efetivo
	F	Empregado Efetivo
	F	Empregado Efetivo
	F	Empregado Efetivo
	M	Empregado Efetivo
	F	Empregado Efetivo
	F	Empregado Efetivo
	F	Empregado Efetivo
	F	Empregado Efetivo
	F	Empregado Efetivo
	F	Empregado Efetivo
	F	Empregado Efetivo
	F	Empregado Efetivo
	F	Empregado Efetivo
	M	Empregado Efetivo
	F	Empregado Efetivo
	F	Empregado Efetivo
	F	Empregado Efetivo
	F	Empregado Efetivo
	F	Empregado Efetivo
	F	Empregado Efetivo
	F	Empregado Efetivo
	F	Empregado Efetivo
	F	Empregado Efetivo
	F	Empregado Efetivo
	F	Empregado Efetivo
	F	Empregado Efetivo
	F	Empregado Efetivo

	F	Empregado Efetivo
	F	Empregado Efetivo
	F	Empregado Efetivo
	F	Empregado Efetivo
	F	Empregado Efetivo
	F	Empregado Efetivo
	F	Empregado Efetivo
	M	Empregado Efetivo
	M	Empregado Efetivo
	F	Empregado Efetivo
	M	Empregado Efetivo
	M	Empregado Efetivo
	M	Empregado Efetivo
	F	Empregado Efetivo
	M	Empregado Efetivo
	M	Empregado Efetivo
	M	Empregado Efetivo
	M	Empregado Efetivo
	M	Empregado Efetivo
	F	Empregado Efetivo
	M	Empregado Efetivo
	F	Empregado Efetivo
	M	Empregado Efetivo
	F	Empregado Efetivo
	M	Empregado Efetivo
	M	Empregado Efetivo
	M	Empregado Efetivo
	F	Empregado Efetivo
	M	Empregado Efetivo
	M	Empregado Efetivo
	M	Empregado Efetivo
	M	Empregado Efetivo
	M	Empregado Efetivo
	F	Empregado Efetivo
	F	Empregado Efetivo
	F	Empregado Efetivo
	F	Empregado Efetivo
	F	Empregado Efetivo
	F	Empregado Efetivo
	F	Empregado Efetivo
	F	Empregado Efetivo
	F	Empregado Efetivo
	F	Empregado Efetivo
	F	Empregado Efetivo
	F	Empregado Efetivo
	F	Empregado Efetivo
	F	Empregado Efetivo
	F	Empregado Efetivo

	F	Empregado Efetivo
	F	Empregado Efetivo
	F	Empregado Efetivo
	F	Empregado Efetivo
	F	Empregado Efetivo
	F	Empregado Efetivo
	F	Empregado Efetivo
	F	Empregado Efetivo
	F	Empregado Efetivo
	F	Empregado Efetivo
	F	Empregado Efetivo
	F	Empregado Efetivo
	F	Empregado Efetivo
	F	Empregado Efetivo
	F	Empregado Efetivo
	F	Empregado Efetivo
	F	Empregado Efetivo
	F	Empregado Efetivo
	F	Empregado Efetivo
	F	Empregado Efetivo
	F	Empregado Efetivo
	F	Empregado Efetivo
	F	Empregado Efetivo
	F	Empregado Efetivo
	F	Empregado Efetivo
	F	Empregado Efetivo
	F	Empregado Efetivo
	F	Empregado Efetivo
	F	Empregado Efetivo
	F	Empregado Efetivo
	F	Empregado Efetivo
	F	Empregado Efetivo
	F	Empregado Efetivo
	F	Empregado Efetivo
	F	Empregado Efetivo
	F	Empregado Efetivo
	F	Empregado Efetivo
	M	Empregado Efetivo
	F	Empregado Efetivo
	F	Empregado Efetivo
	F	Empregado Efetivo
	M	Empregado Efetivo
	F	Empregado Efetivo
	F	Empregado Efetivo
	F	Empregado Efetivo
	M	Empregado Efetivo
	M	Empregado Efetivo
	F	Empregado Efetivo

	M	Empregado Efetivo
	F	Empregado Efetivo
	F	Empregado Efetivo
	F	Empregado Efetivo
	F	Empregado Efetivo
	M	Empregado Efetivo
	F	Empregado Efetivo
	F	Empregado Efetivo
	M	Empregado Efetivo
	F	Empregado Efetivo
	M	Empregado Efetivo
	F	Empregado Efetivo
	M	Empregado Efetivo
	F	Empregado Efetivo
	F	Empregado Efetivo
	F	Empregado Efetivo
	M	Empregado Efetivo
	F	Empregado Efetivo
	F	Empregado Efetivo
	F	Empregado Efetivo
	M	Empregado Efetivo
	F	Empregado Efetivo
	F	Empregado Efetivo
	F	Empregado Efetivo
	F	Empregado Efetivo
	M	Empregado Efetivo
	F	Empregado Efetivo
	F	Empregado Efetivo
	F	Empregado Efetivo
	M	Empregado Efetivo
	F	Empregado Efetivo
	M	Empregado Efetivo
	F	Empregado Efetivo
	F	Empregado Efetivo
	F	Empregado Efetivo
	M	Empregado Efetivo
	F	Empregado Efetivo
	F	Empregado Efetivo
	M	Empregado Efetivo
	F	Empregado Efetivo
	F	Empregado Efetivo
	M	Empregado Efetivo
	M	Empregado Efetivo
	F	Empregado Efetivo
	F	Empregado Efetivo
	F	Empregado Efetivo
	F	Empregado Efetivo
	F	Empregado Efetivo

	F	Empregado Efetivo
	F	Empregado Efetivo
	F	Empregado Efetivo
	M	Empregado Efetivo
	F	Empregado Efetivo
	F	Empregado Efetivo
	F	Empregado Efetivo
	F	Empregado Efetivo
	F	Empregado Efetivo
	F	Empregado Efetivo
	F	Empregado Efetivo
	F	Empregado Efetivo
	M	Empregado Efetivo
	F	Empregado Efetivo
	F	Empregado Efetivo
	F	Empregado Efetivo
	F	Empregado Efetivo
	F	Empregado Efetivo
	F	Empregado Efetivo
	M	Empregado Efetivo
	F	Empregado Efetivo
	F	Empregado Efetivo
	M	Empregado Efetivo
	F	Empregado Efetivo
	F	Empregado Efetivo
	F	Empregado Efetivo
	M	Empregado Efetivo
	M	Empregado Efetivo
	F	Empregado Efetivo
	F	Empregado Efetivo
	F	Empregado Efetivo
	F	Empregado Efetivo
	F	Empregado Efetivo
	F	Empregado Efetivo
	F	Empregado Efetivo
	M	Empregado Efetivo
	M	Empregado Efetivo
	F	Empregado Efetivo
	F	Empregado Efetivo
	F	Empregado Efetivo
	F	Empregado Efetivo
	F	Empregado Efetivo
	F	Empregado Efetivo
	F	Empregado Efetivo
	F	Empregado Efetivo
	F	Empregado Efetivo
	F	Empregado Efetivo
	F	Empregado Efetivo

	M	Empregado Efetivo
	M	Empregado Efetivo
	F	Empregado Efetivo
	F	Empregado Efetivo
	F	Empregado Efetivo
	F	Empregado Efetivo
	M	Empregado Efetivo
	F	Empregado Efetivo
	F	Empregado Efetivo
	F	Empregado Efetivo
	F	Empregado Efetivo
	M	Empregado Efetivo
	M	Empregado Efetivo
	M	Empregado Efetivo
	M	Empregado Efetivo
	M	Empregado Efetivo
	F	Empregado Efetivo
	F	Empregado Efetivo
	F	Empregado Efetivo
	F	Empregado Efetivo
	F	Empregado Efetivo
	F	Empregado Efetivo
	F	Empregado Efetivo
	F	Empregado Efetivo
	F	Empregado Efetivo
	F	Empregado Efetivo
	F	Empregado Efetivo
	F	Empregado Efetivo
	F	Empregado Efetivo
	F	Empregado Efetivo
	F	Empregado Efetivo
	F	Empregado Efetivo
	F	Empregado Efetivo
	F	Empregado Efetivo
	F	Empregado Efetivo
	F	Empregado Efetivo
	F	Empregado Efetivo
	F	Empregado Efetivo
	F	Empregado Efetivo
	F	Empregado Efetivo
	F	Empregado Efetivo
	F	Empregado Efetivo
	F	Empregado Efetivo
	F	Empregado Efetivo
	F	Empregado Efetivo
	F	Empregado Efetivo
	F	Empregado Efetivo
	F	Empregado Efetivo

	F	Empregado Efetivo
	F	Empregado Efetivo
	F	Empregado Efetivo
	F	Empregado Efetivo
	F	Empregado Efetivo
	F	Empregado Efetivo
	F	Empregado Efetivo
	F	Empregado Efetivo
	F	Empregado Efetivo
	F	Empregado Efetivo
	F	Empregado Efetivo
	F	Empregado Efetivo
	F	Empregado Efetivo
	F	Empregado Efetivo
	F	Empregado Efetivo
	F	Empregado Efetivo
	F	Empregado Efetivo
	F	Empregado Efetivo
	F	Empregado Efetivo
	F	Empregado Efetivo
	F	Empregado Efetivo
	M	Empregado Efetivo
	M	Empregado Efetivo
	F	Empregado Efetivo
	F	Empregado Efetivo
	F	Empregado Efetivo
	F	Empregado Efetivo
	F	Empregado Efetivo
	F	Empregado Efetivo
	F	Empregado Efetivo
	F	Empregado Efetivo
	F	Empregado Efetivo
	F	Empregado Efetivo
	F	Empregado Efetivo
	F	Empregado Efetivo
	M	Empregado Efetivo
	F	Empregado Efetivo
	F	Empregado Efetivo
	F	Empregado Efetivo
	M	Empregado Efetivo
	F	Empregado Efetivo
	F	Empregado Efetivo
	F	Empregado Efetivo
	F	Empregado Efetivo
	F	Empregado Efetivo
	F	Empregado Efetivo
	F	Empregado Efetivo
	F	Empregado Efetivo

	F	Empregado Efetivo
	F	Empregado Efetivo
	F	Empregado Efetivo
	F	Empregado Efetivo
	F	Empregado Efetivo
	F	Empregado Efetivo
	F	Empregado Efetivo
	F	Empregado Efetivo
	F	Empregado Efetivo
	F	Empregado Efetivo
	F	Empregado Efetivo
	F	Empregado Efetivo
	F	Empregado Efetivo
	F	Empregado Efetivo
	F	Empregado Efetivo
	F	Empregado Efetivo
	M	Empregado Efetivo
	F	Empregado Efetivo
	F	Empregado Efetivo
	F	Empregado Efetivo
	F	Empregado Efetivo
	F	Empregado Efetivo
	F	Empregado Efetivo
	F	Empregado Efetivo
	F	Empregado Efetivo
	F	Empregado Efetivo
	F	Empregado Efetivo
	F	Empregado Efetivo
	F	Empregado Efetivo
	F	Empregado Efetivo
	F	Empregado Efetivo
	F	Empregado Efetivo
	F	Empregado Efetivo
	F	Empregado Efetivo
	F	Empregado Efetivo
	F	Empregado Efetivo
	M	Empregado Efetivo
	F	Empregado Efetivo
	F	Empregado Efetivo
	F	Empregado Efetivo
	F	Empregado Efetivo
	F	Empregado Efetivo
	F	Empregado Efetivo
	F	Empregado Efetivo
	F	Empregado Efetivo
	F	Empregado Efetivo
	F	Empregado Efetivo
	F	Empregado Efetivo

	F	Empregado Efetivo
	F	Empregado Efetivo
	F	Empregado Efetivo
	F	Empregado Efetivo
	F	Empregado Efetivo
	F	Empregado Efetivo
	F	Empregado Efetivo
	F	Empregado Efetivo
	F	Empregado Efetivo
	F	Empregado Efetivo
	F	Empregado Efetivo
	F	Empregado Efetivo
	M	Empregado Efetivo
	F	Empregado Efetivo
	F	Empregado Efetivo
	F	Empregado Efetivo
	F	Empregado Efetivo
	F	Empregado Efetivo
	F	Empregado Efetivo
	F	Empregado Efetivo
	F	Empregado Efetivo
	F	Empregado Efetivo
	F	Empregado Efetivo
	F	Empregado Efetivo
	M	Empregado Efetivo
	F	Empregado Efetivo
	F	Empregado Efetivo
	M	Empregado Efetivo
	F	Empregado Efetivo
	F	Empregado Efetivo
	F	Empregado Efetivo
	F	Empregado Efetivo
	F	Empregado Efetivo
	F	Empregado Efetivo
	F	Empregado Efetivo
	F	Empregado Efetivo
	F	Empregado Efetivo
	F	Empregado Efetivo
	F	Empregado Efetivo
	M	Empregado Efetivo
	F	Empregado Efetivo
	F	Empregado Efetivo
	F	Empregado Efetivo
	F	Empregado Efetivo
	F	Empregado Efetivo
	F	Empregado Efetivo
	F	Empregado Efetivo
	F	Empregado Efetivo

	F	Empregado Efetivo
	F	Empregado Efetivo
	F	Empregado Efetivo
	F	Empregado Efetivo
	F	Empregado Efetivo
	M	Empregado Efetivo
	F	Empregado Efetivo
	M	Empregado Efetivo
	F	Empregado Efetivo
	F	Empregado Efetivo
	F	Empregado Efetivo
	F	Empregado Efetivo
	F	Empregado Efetivo
	F	Empregado Efetivo
	M	Empregado Efetivo
	F	Empregado Efetivo
	M	Empregado Efetivo
	F	Empregado Efetivo
	F	Empregado Efetivo
	F	Empregado Efetivo
	F	Empregado Efetivo
	F	Empregado Efetivo
	F	Empregado Efetivo
	M	Empregado Efetivo
	F	Empregado Efetivo
	M	Empregado Efetivo
	F	Empregado Efetivo
	M	Empregado Efetivo
	M	Empregado Efetivo
	F	Empregado Efetivo
	F	Empregado Efetivo
	F	Empregado Efetivo
	F	Empregado Efetivo
	F	Empregado Efetivo
	F	Empregado Efetivo
	F	Empregado Efetivo
	M	Empregado Efetivo

	F	Empregado Efetivo
	M	Empregado Efetivo
	M	Empregado Efetivo
	F	Empregado Efetivo
	F	Empregado Efetivo
	F	Empregado Efetivo
	M	Empregado Efetivo
	M	Empregado Efetivo
	F	Empregado Efetivo
	F	Empregado Efetivo
	F	Empregado Efetivo
	M	Empregado Efetivo
	F	Empregado Efetivo
	F	Empregado Efetivo
	F	Empregado Efetivo
	M	Empregado Efetivo
	M	Empregado Efetivo
	M	Empregado Efetivo
	F	Empregado Efetivo
	M	Empregado Efetivo
	F	Empregado Efetivo
	F	Empregado Efetivo
	M	Empregado Efetivo
	F	Empregado Efetivo
	M	Empregado Efetivo
	F	Empregado Efetivo
	M	Empregado Efetivo
	F	Empregado Efetivo
	F	Empregado Efetivo
	F	Empregado Efetivo
	F	Empregado Efetivo
	F	Empregado Efetivo

	F	Empregado Efetivo
	F	Empregado Efetivo
	M	Empregado Efetivo
	F	Empregado Efetivo
	F	Empregado Efetivo
	F	Empregado Efetivo
	F	Empregado Efetivo
	F	Empregado Efetivo
	F	Empregado Efetivo
	M	Empregado Efetivo
	M	Empregado Efetivo
	F	Empregado Efetivo
	F	Empregado Efetivo
	F	Empregado Efetivo
	M	Empregado Efetivo
	F	Empregado Efetivo
	M	Empregado Efetivo
	M	Empregado Efetivo
	F	Empregado Efetivo
	F	Empregado Efetivo
	M	Empregado Efetivo
	F	Empregado Efetivo
	F	Empregado Efetivo
	M	Empregado Efetivo
	M	Empregado Efetivo
	F	Empregado Efetivo
	M	Empregado Efetivo
	F	Empregado Efetivo
	F	Empregado Efetivo
	F	Empregado Efetivo
	F	Empregado Efetivo
	F	Empregado Efetivo

	F	Empregado Efetivo
	F	Empregado Efetivo
	F	Empregado Efetivo
	M	Empregado Efetivo
	F	Empregado Efetivo
	F	Empregado Efetivo
	F	Empregado Efetivo
	F	Empregado Efetivo
	F	Empregado Efetivo
	F	Empregado Efetivo
	F	Empregado Efetivo
	F	Empregado Efetivo
	M	Empregado Efetivo
	F	Empregado Efetivo
	F	Empregado Efetivo
	F	Empregado Efetivo
	F	Empregado Efetivo
	F	Empregado Efetivo
	F	Empregado Efetivo
	F	Empregado Efetivo
	F	Empregado Efetivo
	F	Empregado Efetivo
	F	Empregado Efetivo
	F	Empregado Efetivo
	F	Empregado Efetivo
	F	Empregado Efetivo
	F	Empregado Efetivo
	M	Empregado Efetivo
	F	Empregado Efetivo
	F	Empregado Efetivo
	F	Empregado Efetivo
	F	Empregado Efetivo
	F	Empregado Efetivo
	F	Empregado Efetivo
	F	Empregado Efetivo
	F	Empregado Efetivo
	M	Empregado Efetivo
	F	Empregado Efetivo
	F	Empregado Efetivo

	F	Empregado Efetivo
	F	Empregado Efetivo
	F	Empregado Efetivo
	F	Empregado Efetivo
	F	Empregado Efetivo
	M	Empregado Efetivo
	F	Empregado Efetivo
	F	Empregado Efetivo
	F	Empregado Efetivo
	F	Empregado Efetivo
	F	Empregado Efetivo
	M	Empregado Efetivo
	F	Empregado Efetivo
	F	Empregado Efetivo
	F	Empregado Efetivo
	F	Empregado Efetivo
	F	Empregado Efetivo
	F	Empregado Efetivo
	F	Empregado Efetivo
	M	Empregado Efetivo
	F	Empregado Efetivo
	F	Empregado Efetivo
	F	Empregado Efetivo
	F	Empregado Efetivo
	F	Empregado Efetivo
	F	Empregado Efetivo
	F	Empregado Efetivo
	F	Empregado Efetivo
	F	Empregado Efetivo
	F	Empregado Efetivo
	F	Empregado Efetivo
	M	Empregado Efetivo
	F	Empregado Efetivo
	F	Empregado Efetivo
	F	Empregado Efetivo
	F	Empregado Efetivo
	F	Empregado Efetivo
	M	Empregado Efetivo
	F	Empregado Efetivo
	M	Empregado Efetivo
	M	Empregado Efetivo
	F	Empregado Efetivo
	F	Empregado Efetivo
	M	Empregado Efetivo
	M	Empregado Efetivo
	F	Empregado Efetivo
	F	Empregado Efetivo
	F	Empregado Efetivo

	F	Empregado Efetivo
	F	Empregado Efetivo
	F	Empregado Efetivo
	F	Empregado Efetivo
	F	Empregado Efetivo
	F	Empregado Efetivo
	F	Empregado Efetivo
	F	Empregado Efetivo
	F	Empregado Efetivo
	F	Empregado Efetivo
	F	Empregado Efetivo
	M	Empregado Efetivo
	F	Empregado Efetivo
	F	Empregado Efetivo
	F	Empregado Efetivo
	F	Empregado Efetivo
	F	Empregado Efetivo
	F	Empregado Efetivo
	F	Empregado Efetivo
	F	Empregado Efetivo
	M	Empregado Efetivo
	F	Empregado Efetivo
	M	Empregado Efetivo
	F	Empregado Efetivo
	F	Empregado Efetivo
	F	Empregado Efetivo
	F	Empregado Efetivo
	M	Empregado Efetivo
	F	Empregado Efetivo
	F	Empregado Efetivo
	F	Empregado Efetivo
	F	Empregado Efetivo
	M	Empregado Efetivo
	F	Empregado Efetivo
	F	Empregado Efetivo
	F	Empregado Efetivo
	F	Empregado Efetivo
	F	Empregado Efetivo
	F	Empregado Efetivo
	F	Empregado Efetivo
	M	Empregado Efetivo
	M	Empregado Efetivo
	F	Empregado Efetivo
	F	Empregado Efetivo
	M	Empregado Efetivo
	F	Empregado Efetivo
	F	Empregado Efetivo
	F	Empregado Efetivo

	M	Empregado Efetivo
	F	Empregado Efetivo
	F	Empregado Efetivo
	F	Empregado Efetivo
	F	Empregado Efetivo
	F	Empregado Efetivo
	F	Empregado Efetivo
	F	Empregado Efetivo
	M	Empregado Efetivo
	F	Empregado Efetivo
	M	Empregado Efetivo
	F	Empregado Efetivo
	F	Empregado Efetivo
	F	Empregado Efetivo
	F	Empregado Efetivo
	M	Empregado Efetivo
	F	Empregado Efetivo
	F	Empregado Efetivo
	F	Empregado Efetivo
	F	Empregado Efetivo
	F	Empregado Efetivo
	F	Empregado Efetivo
	F	Empregado Efetivo
	F	Empregado Efetivo
	F	Empregado Efetivo
	M	Empregado Efetivo
	F	Empregado Efetivo
	F	Empregado Efetivo
	F	Empregado Efetivo
	F	Empregado Efetivo
	M	Empregado Efetivo
	M	Empregado Efetivo
	M	Empregado Efetivo
	F	Empregado Efetivo
	F	Empregado Efetivo
	F	Empregado Efetivo
	F	Empregado Efetivo
	M	Empregado Efetivo
	F	Empregado Efetivo
	F	Empregado Efetivo
	F	Empregado Efetivo
	F	Empregado Efetivo
	F	Empregado Efetivo
	F	Empregado Efetivo
	F	Empregado Efetivo
	F	Empregado Efetivo
	F	Empregado Efetivo
	F	Empregado Efetivo

	F	Empregado Efetivo
	F	Empregado Efetivo
	F	Empregado Efetivo
	F	Empregado Efetivo
	M	Empregado Efetivo
	F	Empregado Efetivo
	M	Empregado Efetivo
	F	Empregado Efetivo
	F	Empregado Efetivo
	F	Empregado Efetivo
	F	Empregado Efetivo
	F	Empregado Efetivo
	F	Empregado Efetivo
	F	Empregado Efetivo
	F	Empregado Efetivo
	F	Empregado Efetivo
	F	Empregado Efetivo
	F	Empregado Efetivo
	F	Empregado Efetivo
	F	Empregado Efetivo
	F	Empregado Efetivo
	M	Empregado Efetivo
	M	Empregado Efetivo
	F	Empregado Efetivo
	F	Empregado Efetivo
	F	Empregado Efetivo
	M	Empregado Efetivo
	F	Empregado Efetivo
	M	Empregado Efetivo
	F	Empregado Efetivo
	F	Empregado Efetivo
	F	Empregado Efetivo
	F	Empregado Efetivo
	F	Empregado Efetivo
	M	Empregado Efetivo
	F	Empregado Efetivo
	F	Empregado Efetivo
	M	Empregado Efetivo
	F	Empregado Efetivo
	M	Empregado Efetivo
	F	Empregado Efetivo
	F	Empregado Efetivo
	F	Empregado Efetivo
	F	Empregado Efetivo
	F	Empregado Efetivo
	F	Empregado Efetivo
	F	Empregado Efetivo
	F	Empregado Efetivo

	F	Empregado Efetivo
	F	Empregado Efetivo
	F	Empregado Efetivo
	F	Empregado Efetivo
	F	Empregado Efetivo
	F	Empregado Efetivo
	F	Empregado Efetivo
	F	Empregado Efetivo
	F	Empregado Efetivo
	F	Empregado Efetivo
	F	Empregado Efetivo
	F	Empregado Efetivo
	F	Empregado Efetivo
	F	Empregado Efetivo
	F	Empregado Efetivo
	F	Empregado Efetivo
	M	Empregado Efetivo
	F	Empregado Efetivo
	F	Empregado Efetivo
	F	Empregado Efetivo
	F	Empregado Efetivo
	F	Empregado Efetivo
	F	Empregado Efetivo
	M	Empregado Efetivo
	F	Empregado Efetivo
	F	Empregado Efetivo
	F	Empregado Efetivo
	F	Empregado Efetivo
	M	Empregado Efetivo
	F	Empregado Efetivo
	F	Empregado Efetivo
	F	Empregado Efetivo
	F	Empregado Efetivo
	M	Empregado Efetivo
	F	Empregado Efetivo
	F	Empregado Efetivo
	F	Empregado Efetivo
	F	Empregado Efetivo
	F	Empregado Efetivo
	F	Empregado Efetivo
	F	Empregado Efetivo
	M	Empregado Efetivo
	F	Empregado Efetivo
	F	Empregado Efetivo
	F	Empregado Efetivo
	M	Empregado Efetivo
	F	Empregado Efetivo
	F	Empregado Efetivo

	M	Empregado Efetivo
	F	Empregado Efetivo
	F	Empregado Efetivo
	M	Empregado Efetivo
	F	Empregado Efetivo
	M	Empregado Efetivo
	F	Empregado Efetivo
	F	Empregado Efetivo
	F	Empregado Efetivo
	M	Empregado Efetivo
	F	Empregado Efetivo
	F	Empregado Efetivo
	F	Empregado Efetivo
	M	Empregado Efetivo
	M	Empregado Efetivo
	M	Empregado Efetivo
	F	Empregado Efetivo
	F	Empregado Efetivo
	M	Empregado Efetivo
	M	Empregado Efetivo
	F	Empregado Efetivo
	F	Empregado Efetivo
	F	Empregado Efetivo
	F	Empregado Efetivo
	F	Empregado Efetivo
	F	Empregado Efetivo
	F	Empregado Efetivo
	F	Empregado Efetivo
	F	Empregado Efetivo
	F	Empregado Efetivo
	M	Empregado Efetivo
	F	Empregado Efetivo
	M	Empregado Efetivo
	F	Empregado Efetivo
	F	Empregado Efetivo
	F	Empregado Efetivo
	F	Empregado Efetivo
	F	Empregado Efetivo
	F	Empregado Efetivo
	F	Empregado Efetivo
	M	Empregado Efetivo
	F	Empregado Efetivo
	F	Empregado Efetivo
	F	Empregado Efetivo
	F	Empregado Efetivo
	F	Empregado Efetivo
	F	Empregado Efetivo
	M	Empregado Efetivo

	F	Empregado Efetivo
	F	Empregado Efetivo
	F	Empregado Efetivo
	F	Empregado Efetivo
	F	Empregado Efetivo
	M	Empregado Efetivo
	F	Empregado Efetivo
	F	Empregado Efetivo
	F	Empregado Efetivo
	F	Empregado Efetivo
	F	Empregado Efetivo
	F	Empregado Efetivo
	F	Empregado Efetivo
	F	Empregado Efetivo
	F	Empregado Efetivo
	F	Empregado Efetivo
	M	Empregado Efetivo
	F	Empregado Efetivo
	F	Empregado Efetivo
	M	Empregado Efetivo
	F	Empregado Efetivo
	F	Empregado Efetivo
	F	Empregado Efetivo
	F	Empregado Efetivo
	F	Empregado Efetivo
	F	Empregado Efetivo
	F	Empregado Efetivo
	F	Empregado Efetivo
	F	Empregado Efetivo
	F	Empregado Efetivo
	F	Empregado Efetivo
	F	Empregado Efetivo
	F	Empregado Efetivo
	F	Empregado Efetivo
	F	Empregado Efetivo
	F	Empregado Efetivo
	F	Empregado Efetivo
	F	Empregado Efetivo
	F	Empregado Efetivo
	F	Empregado Efetivo
	F	Empregado Efetivo
	F	Empregado Efetivo
	F	Empregado Efetivo
	F	Empregado Efetivo
	F	Empregado Efetivo
	F	Empregado Efetivo
	F	Empregado Efetivo
	F	Empregado Efetivo

	F	Empregado Efetivo
	F	Empregado Efetivo
	F	Empregado Efetivo
	F	Empregado Efetivo
	F	Empregado Efetivo
	M	Empregado Efetivo
	F	Empregado Efetivo
	F	Empregado Efetivo
	F	Empregado Efetivo
	F	Empregado Efetivo
	F	Empregado Efetivo
	F	Empregado Efetivo
	M	Empregado Efetivo
	F	Empregado Efetivo
	F	Empregado Efetivo
	F	Empregado Efetivo
	F	Empregado Efetivo
	F	Empregado Efetivo
	F	Empregado Efetivo
	F	Empregado Efetivo
	F	Empregado Efetivo
	F	Empregado Efetivo
	F	Empregado Efetivo
	F	Empregado Efetivo
	F	Empregado Efetivo
	F	Empregado Efetivo
	F	Empregado Efetivo
	F	Empregado Efetivo
	F	Empregado Efetivo
	F	Empregado Efetivo
	F	Empregado Efetivo
	M	Empregado Efetivo
	F	Empregado Efetivo
	F	Empregado Efetivo
	F	Empregado Efetivo
	F	Empregado Efetivo
	F	Empregado Efetivo
	F	Empregado Efetivo
	F	Empregado Efetivo
	F	Empregado Efetivo
	F	Empregado Efetivo
	F	Empregado Efetivo
	F	Empregado Efetivo
	F	Empregado Efetivo
	F	Empregado Efetivo
	F	Empregado Efetivo
	F	Empregado Efetivo
	F	Empregado Efetivo

	F	Empregado Efetivo
	F	Empregado Efetivo
	F	Empregado Efetivo
	F	Empregado Efetivo
	F	Empregado Efetivo
	F	Empregado Efetivo
	F	Empregado Efetivo
	F	Empregado Efetivo
	F	Empregado Efetivo
	F	Empregado Efetivo
	F	Empregado Efetivo
	F	Empregado Efetivo
	F	Empregado Efetivo
	F	Empregado Efetivo
	F	Empregado Efetivo
	F	Empregado Efetivo
	F	Empregado Efetivo
	F	Empregado Efetivo
	F	Empregado Efetivo
	F	Empregado Efetivo
	F	Empregado Efetivo
	F	Empregado Efetivo
	F	Empregado Efetivo
	F	Empregado Efetivo
	M	Empregado Efetivo
	F	Empregado Efetivo
	M	Empregado Efetivo
	F	Empregado Efetivo
	F	Empregado Efetivo
	F	Empregado Efetivo
	F	Empregado Efetivo
	F	Empregado Efetivo
	F	Empregado Efetivo
	F	Empregado Efetivo
	F	Empregado Efetivo
	F	Empregado Efetivo
	F	Empregado Efetivo
	F	Empregado Efetivo
	F	Empregado Efetivo
	F	Empregado Efetivo
	F	Empregado Efetivo
	F	Empregado Efetivo
	F	Empregado Efetivo
	F	Empregado Efetivo
	F	Empregado Efetivo
	F	Empregado Efetivo
	F	Empregado Efetivo
	F	Empregado Efetivo

	F	Empregado Efetivo
	M	Empregado Efetivo
	F	Empregado Efetivo
	F	Empregado Efetivo
	F	Empregado Efetivo
	F	Empregado Efetivo
	F	Empregado Efetivo
	F	Empregado Efetivo
	F	Empregado Efetivo
	F	Empregado Efetivo
	F	Empregado Efetivo
	F	Empregado Efetivo
	F	Empregado Efetivo
	F	Empregado Efetivo
	F	Empregado Efetivo
	F	Empregado Efetivo
	F	Empregado Efetivo
	F	Empregado Efetivo
	F	Empregado Efetivo
	F	Empregado Efetivo
	F	Empregado Efetivo
	F	Empregado Efetivo
	M	Empregado Efetivo
	M	Empregado Efetivo
	F	Empregado Efetivo
	F	Empregado Efetivo
	F	Empregado Efetivo
	F	Empregado Efetivo
	F	Empregado Efetivo
	M	Empregado Efetivo
	F	Empregado Efetivo
	F	Empregado Efetivo
	F	Empregado Efetivo
	F	Empregado Efetivo
	F	Empregado Efetivo
	F	Empregado Efetivo
	M	Empregado Efetivo
	M	Empregado Efetivo
	F	Empregado Efetivo
	F	Empregado Efetivo
	M	Empregado Efetivo
	F	Empregado Efetivo
	F	Empregado Efetivo
	F	Empregado Efetivo
	F	Empregado Efetivo
	M	Empregado Efetivo
	F	Empregado Efetivo
	F	Empregado Efetivo

	F	Empregado Efetivo
	M	Empregado Efetivo
	F	Empregado Efetivo
	F	Empregado Efetivo
	M	Empregado Efetivo
	F	Empregado Efetivo
	F	Empregado Efetivo
	F	Empregado Efetivo
	F	Empregado Efetivo
	M	Empregado Efetivo
	F	Empregado Efetivo
	M	Empregado Efetivo
	F	Empregado Efetivo
	F	Empregado Efetivo
	F	Empregado Efetivo
	F	Empregado Efetivo
	F	Empregado Efetivo
	F	Empregado Efetivo
	F	Empregado Efetivo
	F	Empregado Efetivo
	M	Empregado Efetivo
	F	Empregado Efetivo
	F	Empregado Efetivo
	F	Empregado Efetivo
	F	Empregado Efetivo
	F	Empregado Efetivo
	F	Empregado Efetivo
	M	Empregado Efetivo
	M	Empregado Efetivo
	M	Empregado Efetivo
	M	Empregado Efetivo
	M	Empregado Efetivo
	M	Empregado Efetivo
	F	Empregado Efetivo
	F	Empregado Efetivo
	F	Empregado Efetivo
	F	Empregado Efetivo
	F	Empregado Efetivo
	F	Empregado Efetivo

	F	Empregado Efetivo
	F	Empregado Efetivo
	F	Empregado Efetivo
	M	Empregado Efetivo
	M	Empregado Efetivo
	M	Empregado Efetivo
	F	Empregado Efetivo
	F	Empregado Efetivo
	F	Empregado Efetivo
	F	Empregado Efetivo
	F	Empregado Efetivo
	F	Empregado Efetivo
	F	Empregado Efetivo
	F	Empregado Efetivo
	F	Empregado Efetivo
	F	Empregado Efetivo
	F	Empregado Efetivo
	F	Empregado Efetivo
	F	Empregado Efetivo
	M	Empregado Efetivo
	M	Empregado Efetivo
	F	Empregado Efetivo
	F	Empregado Efetivo
	F	Empregado Efetivo
	F	Empregado Efetivo
	F	Empregado Efetivo
	F	Empregado Efetivo
	F	Empregado Efetivo
	F	Empregado Efetivo
	F	Empregado Efetivo
	F	Empregado Efetivo
	M	Empregado Efetivo

	M	Empregado Efetivo
	M	Empregado Efetivo
	F	Empregado Efetivo
	F	Empregado Efetivo
	M	Empregado Efetivo
	F	Empregado Efetivo
	F	Empregado Efetivo
	F	Empregado Efetivo
	M	Empregado Efetivo
	F	Empregado Efetivo
	F	Empregado Efetivo
	F	Empregado Efetivo
	M	Empregado Efetivo
	F	Empregado Efetivo
	F	Empregado Efetivo
	F	Empregado Efetivo
	M	Empregado Efetivo
	F	Empregado Efetivo
	F	Empregado Efetivo
	F	Empregado Efetivo
	F	Empregado Efetivo
	F	Empregado Efetivo
	F	Empregado Efetivo
	F	Empregado Efetivo
	F	Empregado Efetivo
	F	Empregado Efetivo
	F	Empregado Efetivo
	F	Empregado Efetivo
	F	Empregado Efetivo
	F	Empregado Efetivo
	F	Empregado Efetivo
	F	Empregado Efetivo

	F	Empregado Efetivo
	M	Empregado Efetivo
	M	Empregado Efetivo
	M	Empregado Efetivo
	M	Empregado Efetivo
	M	Empregado Efetivo
	M	Empregado Efetivo
	F	Empregado Efetivo
	M	Empregado Efetivo
	M	Empregado Efetivo
	F	Empregado Efetivo
	F	Empregado Efetivo
	F	Empregado Efetivo
	M	Empregado Efetivo
	F	Empregado Efetivo
	F	Empregado Efetivo
	F	Empregado Efetivo
	F	Empregado Efetivo
	M	Empregado Efetivo
	M	Empregado Efetivo
	F	Empregado Efetivo
	F	Empregado Efetivo
	F	Empregado Efetivo
	F	Empregado Efetivo
	F	Empregado Efetivo
	F	Empregado Efetivo
	M	Empregado Efetivo
	F	Empregado Efetivo
	F	Empregado Efetivo
	F	Empregado Efetivo
	F	Empregado Efetivo
	F	Empregado Efetivo
	F	Empregado Efetivo
	F	Empregado Efetivo
	F	Empregado Efetivo
	F	Empregado Efetivo
	F	Empregado Efetivo
	F	Empregado Efetivo
	F	Empregado Efetivo
	F	Empregado Efetivo
	F	Empregado Efetivo
	M	Empregado Efetivo
	M	Empregado Efetivo
	F	Empregado Efetivo

	F	Empregado Efetivo
	F	Empregado Efetivo
	F	Empregado Efetivo
	F	Empregado Efetivo
	F	Empregado Efetivo
	F	Empregado Efetivo
	F	Empregado Efetivo
	F	Empregado Efetivo
	F	Empregado Efetivo
	F	Empregado Efetivo
	F	Empregado Efetivo
	F	Empregado Efetivo
	F	Empregado Efetivo
	F	Empregado Efetivo
	F	Empregado Efetivo
	F	Empregado Efetivo
	F	Empregado Efetivo
	F	Empregado Efetivo
	F	Empregado Efetivo
	F	Empregado Efetivo
	F	Empregado Efetivo
	F	Empregado Efetivo
	F	Empregado Efetivo
	F	Empregado Efetivo
	M	Empregado Efetivo
	M	Empregado Efetivo
	F	Empregado Efetivo
	F	Empregado Efetivo
	F	Empregado Efetivo
	F	Empregado Efetivo
	F	Empregado Efetivo
	F	Empregado Efetivo
	F	Empregado Efetivo
	F	Empregado Efetivo
	F	Empregado Efetivo
	F	Empregado Efetivo
	F	Empregado Efetivo
	F	Empregado Efetivo
	F	Empregado Efetivo
	F	Empregado Efetivo
	F	Empregado Efetivo
	F	Empregado Efetivo
	F	Empregado Efetivo
	F	Empregado Efetivo
	F	Empregado Efetivo
	F	Empregado Efetivo
	F	Empregado Efetivo
	F	Empregado Efetivo

	M	Empregado Efetivo
	F	Empregado Efetivo
	M	Empregado Efetivo
	F	Empregado Efetivo
	F	Empregado Efetivo
	F	Empregado Efetivo
	F	Empregado Efetivo
	F	Empregado Efetivo
	M	Empregado Efetivo
	M	Empregado Efetivo
	M	Empregado Efetivo
	M	Empregado Efetivo
	M	Empregado Efetivo
	M	Empregado Efetivo
	M	Empregado Efetivo
	M	Empregado Efetivo
	M	Empregado Efetivo
	M	Empregado Efetivo
	M	Empregado Efetivo
	F	Empregado Efetivo
	F	Empregado Efetivo
	M	Empregado Efetivo
	M	Empregado Efetivo
	M	Empregado Efetivo
	M	Empregado Efetivo
	M	Empregado Efetivo
	M	Empregado Efetivo
	M	Empregado Efetivo
	F	Empregado Efetivo
	F	Empregado Efetivo
	F	Empregado Efetivo
	F	Empregado Efetivo
	M	Empregado Efetivo
	M	Empregado Efetivo
	F	Empregado Efetivo
	M	Empregado Efetivo
	M	Empregado Efetivo
	F	Empregado Efetivo
	F	Empregado Efetivo
	F	Empregado Efetivo
	F	Empregado Efetivo
	M	Empregado Efetivo
	F	Empregado Efetivo
	F	Empregado Efetivo
	F	Empregado Efetivo
	M	Empregado Efetivo
	F	Empregado Efetivo
	F	Empregado Efetivo

	M	Empregado Efetivo
	M	Empregado Efetivo
	F	Empregado Efetivo
	M	Empregado Efetivo
	M	Empregado Efetivo
	M	Empregado Efetivo
	M	Empregado Efetivo
	F	Empregado Efetivo
	F	Empregado Efetivo
	F	Empregado Efetivo
	F	Empregado Efetivo
	F	Empregado Efetivo
	F	Empregado Efetivo
	M	Empregado Efetivo
	M	Empregado Efetivo
	F	Empregado Efetivo
	F	Empregado Efetivo
	F	Empregado Efetivo
	M	Empregado Efetivo
	F	Empregado Efetivo
	F	Empregado Efetivo
	F	Empregado Efetivo
	F	Empregado Efetivo
	M	Empregado Efetivo
	F	Empregado Efetivo
	M	Empregado Efetivo
	F	Empregado Efetivo
	F	Empregado Efetivo
	F	Empregado Efetivo
	F	Empregado Efetivo
	M	Empregado Efetivo
	M	Empregado Efetivo
	F	Empregado Efetivo
	M	Empregado Efetivo
	F	Empregado Efetivo
	M	Empregado Efetivo
	F	Empregado Efetivo
	M	Empregado Efetivo
	F	Empregado Efetivo
	M	Empregado Efetivo
	M	Empregado Efetivo
	F	Empregado Efetivo
	M	Empregado Efetivo
	F	Empregado Efetivo
	M	Empregado Efetivo
	M	Empregado Efetivo
	M	Empregado Efetivo
	M	Empregado Efetivo

	F	Empregado Efetivo
	M	Empregado Efetivo
	F	Empregado Efetivo
	M	Empregado Efetivo
	F	Empregado Efetivo
	F	Empregado Efetivo
	M	Empregado Efetivo
	F	Empregado Efetivo
	F	Empregado Efetivo
	F	Empregado Efetivo
	F	Empregado Efetivo
	F	Empregado Efetivo
	M	Empregado Efetivo
	F	Empregado Efetivo
	M	Empregado Efetivo
	F	Empregado Efetivo
	F	Empregado Efetivo
	M	Empregado Efetivo
	M	Empregado Efetivo
	F	Empregado Efetivo
	M	Empregado Efetivo
	F	Empregado Efetivo
	M	Empregado Efetivo
	F	Empregado Efetivo
	M	Empregado Efetivo
	M	Empregado Efetivo
	F	Empregado Efetivo
	F	Empregado Efetivo
	M	Empregado Efetivo
	F	Empregado Efetivo
	F	Empregado Efetivo
	F	Empregado Efetivo
	F	Empregado Efetivo
	M	Empregado Efetivo
	F	Empregado Efetivo
	M	Empregado Efetivo
	M	Empregado Efetivo
	M	Empregado Efetivo
	F	Empregado Efetivo
	F	Empregado Efetivo
	F	Empregado Efetivo
	M	Empregado Efetivo
	F	Empregado Efetivo
	F	Empregado Efetivo
	M	Empregado Efetivo
	M	Empregado Efetivo
	F	Empregado Efetivo
	M	Empregado Efetivo

	M	Empregado Efetivo
	F	Empregado Efetivo
	M	Empregado Efetivo
	M	Empregado Efetivo
	M	Empregado Efetivo
	F	Empregado Efetivo
	M	Empregado Efetivo
	F	Empregado Efetivo
	F	Empregado Efetivo
	F	Empregado Efetivo
	M	Empregado Efetivo
	M	Empregado Efetivo
	F	Empregado Efetivo
	F	Empregado Efetivo
	F	Empregado Efetivo
	F	Empregado Efetivo
	F	Empregado Efetivo
	F	Empregado Efetivo
	F	Empregado Efetivo
	M	Empregado Efetivo
	F	Empregado Efetivo
	F	Empregado Efetivo
	F	Empregado Efetivo
	M	Empregado Efetivo
	F	Empregado Efetivo
	F	Empregado Efetivo
	F	Empregado Efetivo
	M	Empregado Efetivo
	F	Empregado Efetivo
	F	Empregado Efetivo
	M	Empregado Efetivo
	M	Empregado Efetivo
	F	Empregado Efetivo
	M	Empregado Efetivo
	M	Empregado Efetivo
	F	Empregado Efetivo
	F	Empregado Efetivo
	F	Empregado Efetivo
	F	Empregado Efetivo
	M	Empregado Efetivo
	M	Empregado Efetivo
	F	Empregado Efetivo
	M	Empregado Efetivo
	M	Empregado Efetivo
	F	Empregado Efetivo
	M	Empregado Efetivo
	F	Empregado Efetivo
	F	Empregado Efetivo

	M	Empregado Efetivo
	M	Empregado Efetivo
	M	Empregado Efetivo
	F	Empregado Efetivo
	F	Empregado Efetivo
	F	Empregado Efetivo
	F	Empregado Efetivo
	F	Empregado Efetivo
	F	Empregado Efetivo
	F	Empregado Efetivo
	M	Empregado Efetivo
	F	Empregado Efetivo
	F	Empregado Efetivo
	F	Empregado Efetivo
	F	Empregado Efetivo
	F	Empregado Efetivo
	F	Empregado Efetivo
	F	Empregado Efetivo
	F	Empregado Efetivo
	F	Empregado Efetivo
	F	Empregado Efetivo
	M	Empregado Efetivo
	F	Empregado Efetivo
	M	Empregado Efetivo
	F	Empregado Efetivo
	F	Empregado Efetivo
	F	Empregado Efetivo
	F	Empregado Efetivo
	F	Empregado Efetivo
	M	Empregado Efetivo
	F	Empregado Efetivo
	M	Empregado Efetivo
	F	Empregado Efetivo
	F	Empregado Efetivo
	F	Empregado Efetivo
	F	Empregado Efetivo
	F	Empregado Efetivo
	F	Empregado Efetivo
	M	Empregado Efetivo
	F	Empregado Efetivo
	M	Empregado Efetivo
	M	Empregado Efetivo
	F	Empregado Efetivo
	M	Empregado Efetivo
	F	Empregado Efetivo
	F	Empregado Efetivo
	M	Empregado Efetivo
	F	Empregado Efetivo

	M	Empregado Efetivo
	F	Empregado Efetivo
	F	Empregado Efetivo
	M	Empregado Efetivo
	F	Empregado Efetivo
	F	Empregado Efetivo
	F	Empregado Efetivo
	F	Empregado Efetivo
	F	Empregado Efetivo
	F	Empregado Efetivo
	F	Empregado Efetivo
	F	Empregado Efetivo
	F	Empregado Efetivo
	F	Empregado Efetivo
	F	Empregado Efetivo
	M	Empregado Efetivo
	F	Empregado Efetivo
	F	Empregado Efetivo
	F	Empregado Efetivo
	M	Empregado Efetivo
	F	Empregado Efetivo
	M	Empregado Efetivo
	F	Empregado Efetivo
	F	Empregado Efetivo
	M	Empregado Efetivo
	F	Empregado Efetivo
	F	Empregado Efetivo
	F	Empregado Efetivo
	F	Empregado Efetivo
	F	Empregado Efetivo
	F	Empregado Efetivo
	F	Empregado Efetivo
	F	Empregado Efetivo
	F	Empregado Efetivo
	F	Empregado Efetivo
	F	Empregado Efetivo
	F	Empregado Efetivo
	F	Empregado Efetivo
	F	Empregado Efetivo
	F	Empregado Efetivo
	F	Empregado Efetivo
	F	Empregado Efetivo
	F	Empregado Efetivo
	F	Empregado Efetivo
	M	Empregado Efetivo
	M	Empregado Efetivo
	F	Empregado Efetivo
	F	Empregado Efetivo

	F	Empregado Efetivo
	F	Empregado Efetivo
	F	Empregado Efetivo
	M	Empregado Efetivo
	F	Empregado Efetivo
	F	Empregado Efetivo
	F	Empregado Efetivo
	F	Empregado Efetivo
	F	Empregado Efetivo
	F	Empregado Efetivo
	F	Empregado Efetivo
	M	Empregado Efetivo
	M	Empregado Efetivo
	F	Empregado Efetivo
	F	Empregado Efetivo
	F	Empregado Efetivo
	M	Empregado Efetivo
	F	Empregado Efetivo
	M	Empregado Efetivo
	F	Empregado Efetivo
	F	Empregado Efetivo
	F	Empregado Efetivo
	M	Empregado Efetivo
	M	Empregado Efetivo
	F	Empregado Efetivo
	M	Empregado Efetivo
	F	Empregado Efetivo
	F	Empregado Efetivo
	F	Empregado Efetivo
	F	Empregado Efetivo
	F	Empregado Efetivo
	F	Empregado Efetivo
	F	Empregado Efetivo
	F	Empregado Efetivo
	F	Empregado Efetivo
	F	Empregado Efetivo
	F	Empregado Efetivo
	M	Empregado Efetivo
	F	Empregado Efetivo
	M	Empregado Efetivo
	F	Empregado Efetivo
	F	Empregado Efetivo
	F	Empregado Efetivo
	F	Empregado Efetivo
	M	Empregado Efetivo
	F	Empregado Efetivo
	M	Empregado Efetivo
	F	Empregado Efetivo

	M	Empregado Efetivo
	F	Empregado Efetivo
	F	Empregado Efetivo
	F	Empregado Efetivo
	F	Empregado Efetivo
	M	Empregado Efetivo
	M	Empregado Efetivo
	M	Empregado Efetivo
	M	Empregado Efetivo
	M	Empregado Efetivo
	F	Empregado Efetivo
	M	Empregado Efetivo
	F	Empregado Efetivo
	M	Empregado Efetivo
	F	Empregado Efetivo
	F	Empregado Efetivo
	M	Empregado Efetivo
	F	Empregado Efetivo
	M	Empregado Efetivo
	F	Empregado Efetivo
	F	Empregado Efetivo
	F	Empregado Efetivo
	F	Empregado Efetivo
	M	Empregado Efetivo
	M	Empregado Efetivo
	F	Empregado Efetivo
	F	Empregado Efetivo
	M	Empregado Efetivo
	F	Empregado Efetivo
	M	Empregado Efetivo
	F	Empregado Efetivo
	M	Empregado Efetivo
	F	Empregado Efetivo
	F	Empregado Efetivo
	F	Empregado Efetivo
	M	Empregado Efetivo
	M	Empregado Efetivo
	F	Empregado Efetivo
	F	Empregado Efetivo
	F	Empregado Efetivo
	M	Empregado Efetivo
	M	Empregado Efetivo
	F	Empregado Efetivo
	M	Empregado Efetivo
	F	Empregado Efetivo
	F	Empregado Efetivo
	M	Empregado Efetivo
	F	Empregado Efetivo

	F	Empregado Efetivo
	F	Empregado Efetivo
	F	Empregado Efetivo
	F	Empregado Efetivo
	F	Empregado Efetivo
	F	Empregado Efetivo
	M	Empregado Efetivo
	M	Empregado Efetivo
	M	Empregado Efetivo
	F	Empregado Efetivo
	F	Empregado Efetivo
	F	Empregado Efetivo
	M	Empregado Efetivo
	F	Empregado Efetivo
	M	Empregado Efetivo
	F	Empregado Efetivo
	M	Empregado Efetivo
	M	Empregado Efetivo
	M	Empregado Efetivo
	F	Empregado Efetivo
	F	Empregado Efetivo
	M	Empregado Efetivo
	M	Empregado Efetivo
	M	Empregado Efetivo
	F	Empregado Efetivo
	F	Empregado Efetivo
	F	Empregado Efetivo
	F	Empregado Efetivo
	M	Empregado Efetivo
	M	Empregado Efetivo
	F	Empregado Efetivo
	M	Empregado Efetivo
	F	Empregado Efetivo
	F	Empregado Efetivo
	F	Empregado Efetivo
	F	Empregado Efetivo
	F	Empregado Efetivo
	M	Empregado Efetivo
	M	Empregado Efetivo
	F	Empregado Efetivo
	F	Empregado Efetivo
	F	Empregado Efetivo
	F	Empregado Efetivo
	M	Empregado Efetivo
	F	Empregado Efetivo
	F	Empregado Efetivo
	F	Empregado Efetivo
	M	Empregado Efetivo

	F	Empregado Efetivo
	F	Empregado Efetivo
	F	Empregado Efetivo
	F	Empregado Efetivo
	M	Empregado Efetivo
	F	Empregado Efetivo
	F	Empregado Efetivo
	F	Empregado Efetivo
	M	Empregado Efetivo
	M	Empregado Efetivo
	M	Empregado Efetivo
	M	Empregado Efetivo
	F	Empregado Efetivo
	F	Empregado Efetivo
	F	Empregado Efetivo
	F	Empregado Efetivo
	F	Empregado Efetivo
	M	Empregado Efetivo
	M	Empregado Efetivo
	F	Empregado Efetivo
	F	Empregado Efetivo
	F	Empregado Efetivo
	F	Empregado Efetivo
	F	Empregado Efetivo
	F	Empregado Efetivo
	F	Empregado Efetivo
	F	Empregado Efetivo
	F	Empregado Efetivo
	F	Empregado Efetivo
	M	Empregado Efetivo
	F	Empregado Efetivo
	F	Empregado Efetivo
	F	Empregado Efetivo
	F	Empregado Efetivo
	F	Empregado Efetivo
	F	Empregado Efetivo
	F	Empregado Efetivo
	F	Empregado Efetivo
	F	Empregado Efetivo
	F	Empregado Efetivo
	F	Empregado Efetivo
	F	Empregado Efetivo
	F	Empregado Efetivo
	F	Empregado Efetivo
	F	Empregado Efetivo
	F	Empregado Efetivo
	F	Empregado Efetivo
	M	Empregado Efetivo

	F	Empregado Efetivo
	F	Empregado Efetivo
	M	Empregado Efetivo
	F	Empregado Efetivo
	F	Empregado Efetivo
	F	Empregado Efetivo
	F	Empregado Efetivo
	F	Empregado Efetivo
	F	Empregado Efetivo
	M	Empregado Efetivo
	F	Empregado Efetivo
	M	Empregado Efetivo
	M	Empregado Efetivo
	M	Empregado Efetivo
	M	Empregado Efetivo
	M	Empregado Efetivo
	F	Empregado Efetivo
	M	Empregado Efetivo
	M	Empregado Efetivo
	F	Empregado Efetivo
	F	Empregado Efetivo
	F	Empregado Efetivo
	M	Empregado Efetivo
	M	Empregado Efetivo
	M	Empregado Efetivo
	M	Empregado Efetivo
	F	Empregado Efetivo
	M	Empregado Efetivo
	M	Empregado Efetivo
	M	Empregado Efetivo
	F	Empregado Efetivo
	F	Empregado Efetivo
	F	Empregado Efetivo
	F	Empregado Efetivo
	M	Empregado Efetivo
	M	Empregado Efetivo
	M	Empregado Efetivo
	M	Empregado Efetivo
	F	Empregado Efetivo
	M	Empregado Efetivo
	F	Empregado Efetivo
	M	Empregado Efetivo
	M	Empregado Efetivo
	F	Empregado Efetivo
	M	Empregado Efetivo
	F	Empregado Efetivo
	F	Empregado Efetivo
	F	Empregado Efetivo

	F	Empregado Efetivo
	F	Empregado Efetivo
	F	Empregado Efetivo
	F	Empregado Efetivo
	F	Empregado Efetivo
	F	Empregado Efetivo
	F	Empregado Efetivo
	F	Empregado Efetivo
	F	Empregado Efetivo
	M	Empregado Efetivo
	F	Empregado Efetivo
	M	Empregado Efetivo
	F	Empregado Efetivo
	F	Empregado Efetivo
	F	Empregado Efetivo
	F	Empregado Efetivo
	F	Empregado Efetivo
	F	Empregado Efetivo
	F	Empregado Efetivo
	F	Empregado Efetivo
	F	Empregado Efetivo
	F	Empregado Efetivo
	F	Empregado Efetivo
	F	Empregado Efetivo
	F	Empregado Efetivo
	F	Empregado Efetivo
	F	Empregado Efetivo
	F	Empregado Efetivo
	F	Empregado Efetivo
	F	Empregado Efetivo
	F	Empregado Efetivo
	F	Empregado Efetivo
	F	Empregado Efetivo
	F	Empregado Efetivo
	F	Empregado Efetivo
	F	Empregado Efetivo
	F	Empregado Efetivo
	F	Empregado Efetivo
	F	Empregado Efetivo
	F	Empregado Efetivo
	F	Empregado Efetivo
	F	Empregado Efetivo
	F	Empregado Efetivo
	F	Empregado Efetivo
	F	Empregado Efetivo
	F	Empregado Efetivo
	F	Empregado Efetivo
	F	Empregado Efetivo

	F	Empregado Efetivo
	M	Empregado Efetivo
	F	Empregado Efetivo
	F	Empregado Efetivo
	F	Empregado Efetivo
	F	Empregado Efetivo
	F	Empregado Efetivo
	F	Empregado Efetivo
	F	Empregado Efetivo
	F	Empregado Efetivo
	F	Empregado Efetivo
	F	Empregado Efetivo
	F	Empregado Efetivo
	M	Empregado Efetivo
	F	Empregado Efetivo
	F	Empregado Efetivo
	F	Empregado Efetivo
	F	Empregado Efetivo
	F	Empregado Efetivo
	F	Empregado Efetivo
	F	Empregado Efetivo
	F	Empregado Efetivo
	F	Empregado Efetivo
	F	Empregado Efetivo
	F	Empregado Efetivo
	F	Empregado Efetivo
	F	Empregado Efetivo
	F	Empregado Efetivo
	F	Empregado Efetivo
	F	Empregado Efetivo
	F	Empregado Efetivo
	F	Empregado Efetivo
	F	Empregado Efetivo
	F	Empregado Efetivo
	F	Empregado Efetivo
	M	Empregado Efetivo
	F	Empregado Efetivo
	F	Empregado Efetivo
	F	Empregado Efetivo
	M	Empregado Efetivo
	F	Empregado Efetivo
	M	Empregado Efetivo
	F	Empregado Efetivo
	F	Empregado Efetivo
	F	Empregado Efetivo
	F	Empregado Efetivo
	F	Empregado Efetivo
	F	Empregado Efetivo

	F	Empregado Efetivo
	F	Empregado Efetivo
	F	Empregado Efetivo
	M	Empregado Efetivo
	F	Empregado Efetivo
	F	Empregado Efetivo
	M	Empregado Efetivo
	F	Empregado Efetivo
	F	Empregado Efetivo
	F	Empregado Efetivo
	F	Empregado Efetivo
	F	Empregado Efetivo
	F	Empregado Efetivo
	F	Empregado Efetivo
	F	Empregado Efetivo
	F	Empregado Efetivo
	F	Empregado Efetivo
	M	Empregado Efetivo
	F	Empregado Efetivo
	F	Empregado Efetivo
	F	Empregado Efetivo
	F	Empregado Efetivo
	M	Empregado Efetivo
	F	Empregado Efetivo
	F	Empregado Efetivo
	F	Empregado Efetivo
	F	Empregado Efetivo
	F	Empregado Efetivo
	F	Empregado Efetivo
	F	Empregado Efetivo
	F	Empregado Efetivo
	F	Empregado Efetivo
	F	Empregado Efetivo
	M	Empregado Efetivo
	F	Empregado Efetivo
	F	Empregado Efetivo
	F	Empregado Efetivo
	F	Empregado Efetivo
	F	Empregado Efetivo
	F	Empregado Efetivo
	M	Empregado Efetivo
	F	Empregado Efetivo
	F	Empregado Efetivo
	F	Empregado Efetivo
	M	Empregado Efetivo
	M	Empregado Efetivo
	F	Empregado Efetivo
	F	Empregado Efetivo

	F	Empregado Efetivo
	F	Empregado Efetivo
	F	Empregado Efetivo
	F	Empregado Efetivo
	M	Empregado Efetivo
	F	Empregado Efetivo
	M	Empregado Efetivo
	F	Empregado Efetivo
	M	Empregado Efetivo
	F	Empregado Efetivo
	F	Empregado Efetivo
	M	Empregado Efetivo
	F	Empregado Efetivo
	M	Empregado Efetivo
	M	Empregado Efetivo
	F	Empregado Efetivo
	F	Empregado Efetivo
	F	Empregado Efetivo
	F	Empregado Efetivo
	M	Empregado Efetivo
	F	Empregado Efetivo
	F	Empregado Efetivo
	M	Empregado Efetivo
	F	Empregado Efetivo
	F	Empregado Efetivo
	F	Empregado Efetivo
	F	Empregado Efetivo
	F	Empregado Efetivo
	F	Empregado Efetivo
	F	Empregado Efetivo
	M	Empregado Efetivo
	F	Empregado Efetivo
	F	Empregado Efetivo
	F	Empregado Efetivo
	F	Empregado Efetivo
	F	Empregado Efetivo
	F	Empregado Efetivo
	F	Empregado Efetivo
	F	Empregado Efetivo
	F	Empregado Efetivo
	F	Empregado Efetivo
	F	Empregado Efetivo
	M	Empregado Efetivo
	F	Empregado Efetivo
	F	Empregado Efetivo
	F	Empregado Efetivo
	F	Empregado Efetivo
	F	Empregado Efetivo

	F	Empregado Efetivo
	M	Empregado Efetivo
	F	Empregado Efetivo
	M	Empregado Efetivo
	M	Empregado Efetivo
	F	Empregado Efetivo
	F	Empregado Efetivo
	F	Empregado Efetivo
	F	Empregado Efetivo
	F	Empregado Efetivo
	F	Empregado Efetivo
	F	Empregado Efetivo
	M	Empregado Efetivo
	F	Empregado Efetivo
	F	Empregado Efetivo
	F	Empregado Efetivo
	F	Empregado Efetivo
	M	Empregado Efetivo
	F	Empregado Efetivo
	F	Empregado Efetivo
	M	Empregado Efetivo
	F	Empregado Efetivo
	F	Empregado Efetivo
	M	Empregado Efetivo
	M	Empregado Efetivo
	F	Empregado Efetivo
	F	Empregado Efetivo
	F	Empregado Efetivo
	F	Empregado Efetivo
	F	Empregado Efetivo
	F	Empregado Efetivo
	F	Empregado Efetivo
	F	Empregado Efetivo
	M	Empregado Efetivo
	F	Empregado Efetivo
	F	Empregado Efetivo
	F	Empregado Efetivo

	F	Empregado Efetivo
	F	Empregado Efetivo
	M	Empregado Efetivo
	F	Empregado Efetivo
	F	Empregado Efetivo
	F	Empregado Efetivo
	F	Empregado Efetivo
	M	Empregado Efetivo
	M	Empregado Efetivo
	M	Empregado Efetivo
	F	Empregado Efetivo
	F	Empregado Efetivo
	M	Empregado Efetivo
	F	Empregado Efetivo
	F	Empregado Efetivo
	F	Empregado Efetivo
	F	Empregado Efetivo
	F	Empregado Efetivo
	M	Empregado Efetivo
	F	Empregado Efetivo
	F	Empregado Efetivo
	F	Empregado Efetivo
	F	Empregado Efetivo
	F	Empregado Efetivo
	F	Empregado Efetivo
	F	Empregado Efetivo
	F	Empregado Efetivo
	M	Empregado Efetivo
	M	Empregado Efetivo
	M	Empregado Efetivo
	F	Empregado Efetivo
	F	Empregado Efetivo
	F	Empregado Efetivo
	F	Empregado Efetivo
	F	Empregado Efetivo
	F	Empregado Efetivo
	M	Empregado Efetivo
	M	Empregado Efetivo
	M	Empregado Efetivo
	M	Empregado Efetivo
	F	Empregado Efetivo
	F	Empregado Efetivo
	M	Empregado Efetivo

	F	Empregado Efetivo
	M	Empregado Efetivo
	M	Empregado Efetivo
	M	Empregado Efetivo
	F	Empregado Efetivo
	M	Empregado Efetivo
	F	Empregado Efetivo
	F	Empregado Efetivo
	F	Empregado Efetivo
	M	Empregado Efetivo
	M	Empregado Efetivo
	F	Empregado Efetivo
	M	Empregado Efetivo
	M	Empregado Efetivo
	F	Empregado Efetivo
	M	Empregado Efetivo
	M	Empregado Efetivo
	F	Empregado Efetivo
	M	Empregado Efetivo
	F	Empregado Efetivo
	M	Empregado Efetivo
	M	Empregado Efetivo
	F	Empregado Efetivo
	F	Empregado Efetivo
	F	Empregado Efetivo
	M	Empregado Efetivo
	M	Empregado Efetivo
	F	Empregado Efetivo
	F	Empregado Efetivo
	F	Empregado Efetivo
	F	Empregado Efetivo
	M	Empregado Efetivo
	F	Empregado Efetivo
	F	Empregado Efetivo
	F	Empregado Efetivo
	F	Empregado Efetivo
	F	Empregado Efetivo
	M	Empregado Efetivo
	M	Empregado Efetivo
	F	Empregado Efetivo
	M	Empregado Efetivo
	F	Empregado Efetivo
	F	Empregado Efetivo
	F	Empregado Efetivo
	M	Empregado Efetivo
	F	Empregado Efetivo
	F	Empregado Efetivo
	F	Empregado Efetivo

	F	Empregado Efetivo
	M	Empregado Efetivo
	F	Empregado Efetivo
	F	Empregado Efetivo
	F	Empregado Efetivo
	F	Empregado Efetivo
	F	Empregado Efetivo
	F	Empregado Efetivo
	F	Empregado Efetivo
	F	Empregado Efetivo
	F	Empregado Efetivo
	F	Empregado Efetivo
	F	Empregado Efetivo
	F	Empregado Efetivo
	F	Empregado Efetivo
	F	Empregado Efetivo
	M	Empregado Efetivo
	M	Empregado Efetivo
	M	Empregado Efetivo
	M	Empregado Efetivo
	M	Empregado Efetivo
	F	Empregado Efetivo
	F	Empregado Efetivo
	M	Empregado Efetivo
	F	Empregado Efetivo
	F	Empregado Efetivo
	F	Empregado Efetivo
	F	Empregado Efetivo
	F	Empregado Efetivo
	F	Empregado Efetivo
	F	Empregado Efetivo
	F	Empregado Efetivo
	F	Empregado Efetivo
	F	Empregado Efetivo
	F	Empregado Efetivo
	M	Empregado Efetivo
	F	Empregado Efetivo
	F	Empregado Efetivo
	M	Empregado Efetivo
	M	Empregado Efetivo
	F	Empregado Efetivo
	F	Empregado Efetivo
	F	Empregado Efetivo
	F	Empregado Efetivo
	F	Empregado Efetivo
	F	Empregado Efetivo
	F	Empregado Efetivo
	F	Empregado Efetivo

	F	Empregado Efetivo
	F	Empregado Efetivo
	F	Empregado Efetivo
	F	Empregado Efetivo
	F	Empregado Efetivo
	F	Empregado Efetivo
	F	Empregado Efetivo
	M	Empregado Efetivo
	F	Empregado Efetivo
	M	Empregado Efetivo
	F	Empregado Efetivo
	F	Empregado Efetivo
	M	Empregado Efetivo
	M	Empregado Efetivo
	M	Empregado Efetivo
	M	Empregado Efetivo
	M	Empregado Efetivo
	M	Empregado Efetivo
	M	Empregado Efetivo
	F	Empregado Efetivo
	F	Empregado Efetivo
	F	Empregado Efetivo
	F	Empregado Efetivo
	F	Empregado Efetivo
	F	Empregado Efetivo
	F	Empregado Efetivo
	M	Empregado Efetivo
	F	Empregado Efetivo
	M	Empregado Efetivo
	M	Empregado Efetivo
	F	Empregado Efetivo
	F	Empregado Efetivo
	F	Empregado Efetivo
	F	Empregado Efetivo
	M	Empregado Efetivo
	M	Empregado Efetivo
	M	Empregado Efetivo
	M	Empregado Efetivo
	F	Empregado Efetivo
	M	Empregado Efetivo
	M	Empregado Efetivo
	F	Empregado Efetivo
	F	Empregado Efetivo
	M	Empregado Efetivo
	F	Empregado Efetivo
	F	Empregado Efetivo
	F	Empregado Efetivo
	F	Empregado Efetivo

	M	Empregado Efetivo
	F	Empregado Efetivo
	F	Empregado Efetivo
	F	Empregado Efetivo
	F	Empregado Efetivo
	M	Empregado Efetivo
	M	Empregado Efetivo
	F	Empregado Efetivo
	F	Empregado Efetivo
	F	Empregado Efetivo
	F	Empregado Efetivo
	F	Empregado Efetivo
	F	Empregado Efetivo
	F	Empregado Efetivo
	F	Empregado Efetivo
	F	Empregado Efetivo
	F	Empregado Efetivo
	F	Empregado Efetivo
	F	Empregado Efetivo
	F	Empregado Efetivo
	F	Empregado Efetivo
	F	Empregado Efetivo
	F	Empregado Efetivo
	F	Empregado Efetivo
	F	Empregado Efetivo
	F	Empregado Efetivo
	F	Empregado Efetivo
	F	Empregado Efetivo
	F	Empregado Efetivo
	F	Empregado Efetivo
	F	Empregado Efetivo
	F	Empregado Efetivo
	F	Empregado Efetivo
	F	Empregado Efetivo
	F	Empregado Efetivo
	F	Empregado Efetivo
	F	Empregado Efetivo
	F	Empregado Efetivo
	F	Empregado Efetivo
	M	Empregado Efetivo
	M	Empregado Efetivo
	F	Empregado Efetivo
	F	Empregado Efetivo
	F	Empregado Efetivo
	M	Empregado Efetivo
	F	Empregado Efetivo
	F	Empregado Efetivo
	F	Empregado Efetivo

	F	Empregado Efetivo
	F	Empregado Efetivo
	F	Empregado Efetivo
	F	Empregado Efetivo
	F	Empregado Efetivo
	F	Empregado Efetivo
	M	Empregado Efetivo
	F	Empregado Efetivo
	F	Empregado Efetivo
	F	Empregado Efetivo
	F	Empregado Efetivo
	F	Empregado Efetivo
	F	Empregado Efetivo
	M	Empregado Efetivo
	F	Empregado Efetivo
	F	Empregado Efetivo
	F	Empregado Efetivo
	F	Empregado Efetivo
	F	Empregado Efetivo
	F	Empregado Efetivo
	F	Empregado Efetivo
	F	Empregado Efetivo
	F	Empregado Efetivo
	F	Empregado Efetivo
	F	Empregado Efetivo
	M	Empregado Efetivo
	F	Empregado Efetivo
	F	Empregado Efetivo
	F	Empregado Efetivo
	F	Empregado Efetivo
	F	Empregado Efetivo
	F	Empregado Efetivo
	F	Empregado Efetivo
	F	Empregado Efetivo
	M	Empregado Efetivo
	F	Empregado Efetivo
	F	Empregado Efetivo
	M	Empregado Efetivo
	M	Empregado Efetivo
	M	Empregado Efetivo
	M	Empregado Efetivo
	M	Empregado Efetivo
	M	Empregado Efetivo
	M	Empregado Efetivo
	M	Empregado Efetivo
	M	Empregado Efetivo
	M	Empregado Efetivo
	M	Empregado Efetivo

	F	Empregado Efetivo
	F	Empregado Efetivo
	F	Empregado Efetivo
	F	Empregado Efetivo
	F	Empregado Efetivo
	F	Empregado Efetivo
	F	Empregado Efetivo
	F	Empregado Efetivo
	F	Empregado Efetivo
	F	Empregado Efetivo
	F	Empregado Efetivo
	F	Empregado Efetivo
	F	Empregado Efetivo
	F	Empregado Efetivo
	F	Empregado Efetivo
	F	Empregado Efetivo
	F	Empregado Efetivo
	F	Empregado Efetivo
	M	Empregado Efetivo
	M	Empregado Efetivo
	M	Empregado Efetivo
	M	Empregado Efetivo
	M	Empregado Efetivo
	M	Empregado Efetivo
	M	Empregado Efetivo
	F	Empregado Efetivo
	M	Empregado Efetivo
	M	Empregado Efetivo
	M	Empregado Efetivo
	M	Empregado Efetivo
	F	Empregado Efetivo
	F	Empregado Efetivo
	F	Empregado Efetivo
	F	Empregado Efetivo
	F	Empregado Efetivo
	F	Empregado Efetivo
	F	Empregado Efetivo
	F	Empregado Efetivo
	F	Empregado Efetivo
	F	Empregado Efetivo
	F	Empregado Efetivo
	F	Empregado Efetivo

	F	Empregado Efetivo
	F	Empregado Efetivo
	F	Empregado Efetivo
	F	Empregado Efetivo
	F	Empregado Efetivo
	F	Cedido - RJU
	F	Cedido - RJU
	F	Cedido - RJU
	M	Empregado Efetivo
	F	Empregado Efetivo
	F	Empregado Efetivo
	F	Empregado Efetivo
	F	Empregado Efetivo
	F	Empregado Efetivo
	F	Empregado Efetivo
	F	Empregado Efetivo
	F	Empregado Efetivo
	F	Empregado Efetivo
	F	Empregado Efetivo
	F	Empregado Efetivo
	F	Empregado Efetivo
	F	Empregado Efetivo
	F	Empregado Efetivo
	F	Empregado Efetivo
	F	Empregado Efetivo
	F	Empregado Efetivo
	F	Empregado Efetivo
	F	Empregado Efetivo
	F	Empregado Efetivo
	F	Empregado Efetivo
	F	Empregado Efetivo
	F	Empregado Efetivo
	F	Empregado Efetivo
	F	Empregado Efetivo
	F	Empregado Efetivo
	F	Empregado Efetivo
	F	Empregado Efetivo
	F	Empregado Efetivo

	F	Empregado Efetivo
	F	Empregado Efetivo
	F	Empregado Efetivo
	F	Empregado Efetivo
	M	Cedido - RJU
	F	Empregado Efetivo
	F	Empregado Efetivo
	M	Empregado Efetivo
	F	Empregado Efetivo
	F	Empregado Efetivo
	M	Empregado Efetivo
	M	Empregado Efetivo
	F	Empregado Efetivo
	M	Empregado Efetivo
	F	Empregado Efetivo
	F	Empregado Efetivo
	M	Empregado Efetivo
	F	Empregado Efetivo
	F	Empregado Efetivo
	F	Empregado Efetivo
	F	Empregado Efetivo
	F	Empregado Efetivo
	F	Empregado Efetivo
	F	Empregado Efetivo
	F	Empregado Efetivo
	F	Empregado Efetivo
	F	Empregado Efetivo
	F	Empregado Efetivo
	F	Empregado Efetivo
	F	Empregado Efetivo
	F	Empregado Efetivo
	F	Empregado Efetivo
	F	Empregado Efetivo
	F	Empregado Efetivo
	F	Empregado Efetivo
	F	Empregado Efetivo
	M	Empregado Efetivo
	F	Empregado Efetivo

	F	Empregado Efetivo
	M	Empregado Efetivo
	F	Empregado Efetivo
	F	Empregado Efetivo
	M	Empregado Efetivo
	F	Empregado Efetivo
	F	Empregado Efetivo
	M	Empregado Efetivo
	F	Empregado Efetivo
	M	Empregado Efetivo
	F	Empregado Efetivo
	F	Empregado Efetivo
	F	Empregado Efetivo
	F	Empregado Efetivo
	M	Empregado Efetivo
	F	Empregado Efetivo
	F	Empregado Efetivo
	F	Empregado Efetivo
	F	Empregado Efetivo
	F	Empregado Efetivo
	F	Empregado Efetivo
	F	Empregado Efetivo
	F	Empregado Efetivo
	F	Empregado Efetivo
	F	Empregado Efetivo
	F	Empregado Efetivo
	F	Empregado Efetivo
	M	Empregado Efetivo
	M	Empregado Efetivo
	F	Empregado Efetivo
	F	Empregado Efetivo
	F	Empregado Efetivo
	F	Empregado Efetivo

	F	Empregado Efetivo
	F	Empregado Efetivo
	F	Empregado Efetivo
	F	Empregado Efetivo
	M	Empregado Efetivo
	M	Empregado Efetivo
	F	Empregado Efetivo
	F	Empregado Efetivo
	F	Empregado Efetivo
	F	Empregado Efetivo
	F	Empregado Efetivo
	F	Empregado Efetivo
	M	Empregado Efetivo
	F	Empregado Efetivo
	F	Empregado Efetivo
	F	Empregado Efetivo
	F	Empregado Efetivo
	F	Empregado Efetivo
	F	Empregado Efetivo
	M	Empregado Efetivo
	F	Empregado Efetivo
	F	Empregado Efetivo
	F	Empregado Efetivo
	F	Empregado Efetivo
	M	Empregado Efetivo
	M	Empregado Efetivo
	F	Empregado Efetivo
	M	Empregado Efetivo
	M	Empregado Efetivo
	F	Empregado Efetivo
	F	Empregado Efetivo
	F	Empregado Efetivo

	F	Empregado Efetivo
	F	Empregado Efetivo
	M	Empregado Efetivo
	F	Empregado Efetivo
	F	Empregado Efetivo
	F	Empregado Efetivo
	F	Empregado Efetivo
	F	Empregado Efetivo
	F	Empregado Efetivo
	F	Empregado Efetivo
	F	Empregado Efetivo
	F	Empregado Efetivo
	M	Empregado Efetivo
	F	Empregado Efetivo
	M	Empregado Efetivo
	F	Empregado Efetivo
	F	Comissionado
	M	Empregado Efetivo
	M	Empregado Efetivo
	M	Empregado Efetivo
	M	Empregado Efetivo
	F	Empregado Efetivo
	M	Empregado Efetivo
	M	Empregado Efetivo
	F	Empregado Efetivo
	M	Empregado Efetivo
	F	Empregado Efetivo
	M	Empregado Efetivo
	F	Empregado Efetivo
	F	Empregado Efetivo
	F	Empregado Efetivo
	F	Empregado Efetivo
	M	Empregado Efetivo
	F	Empregado Efetivo
	F	Empregado Efetivo
	M	Empregado Efetivo
	M	Empregado Efetivo
	M	Empregado Efetivo

	M	Empregado Efetivo
	M	Empregado Efetivo
	F	Empregado Efetivo
	M	Empregado Efetivo
	F	Empregado Efetivo
	F	Empregado Efetivo
	M	Empregado Efetivo
	F	Empregado Efetivo
	F	Empregado Efetivo
	M	Empregado Efetivo
	M	Empregado Efetivo
	M	Empregado Efetivo
	M	Empregado Efetivo
	M	Empregado Efetivo
	M	Empregado Efetivo
	M	Empregado Efetivo
	M	Empregado Efetivo
	M	Empregado Efetivo
	M	Empregado Efetivo
	M	Empregado Efetivo
	M	Empregado Efetivo
	F	Empregado Efetivo
	F	Empregado Efetivo
	F	Empregado Efetivo
	F	Empregado Efetivo
	F	Empregado Efetivo
	M	Empregado Efetivo
	F	Empregado Efetivo
	F	Empregado Efetivo
	M	Empregado Efetivo
	F	Empregado Efetivo
	F	Empregado Efetivo
	F	Empregado Efetivo
	F	Empregado Efetivo
	F	Empregado Efetivo
	F	Empregado Efetivo
	F	Empregado Efetivo
	F	Empregado Efetivo
	F	Empregado Efetivo
	F	Empregado Efetivo
	F	Empregado Efetivo
	F	Empregado Efetivo
	M	Empregado Efetivo
	F	Empregado Efetivo

	M	Empregado Efetivo
	F	Empregado Efetivo
	F	Empregado Efetivo
	M	Empregado Efetivo
	F	Empregado Efetivo
	F	Empregado Efetivo
	F	Empregado Efetivo
	F	Empregado Efetivo
	F	Empregado Efetivo
	F	Empregado Efetivo
	M	Empregado Efetivo
	F	Empregado Efetivo
	M	Empregado Efetivo
	M	Empregado Efetivo
	F	Empregado Efetivo
	F	Empregado Efetivo
	F	Empregado Efetivo
	M	Empregado Efetivo
	M	Empregado Efetivo
	F	Empregado Efetivo
	F	Empregado Efetivo
	F	Empregado Efetivo
	M	Empregado Efetivo
	M	Empregado Efetivo
	M	Empregado Efetivo
	M	Empregado Efetivo
	M	Empregado Efetivo
	F	Empregado Efetivo
	F	Empregado Efetivo
	F	Empregado Efetivo
	F	Empregado Efetivo
	F	Empregado Efetivo
	F	Empregado Efetivo
	M	Empregado Efetivo
	M	Empregado Efetivo
	M	Empregado Efetivo
	F	Empregado Efetivo
	F	Empregado Efetivo
	F	Empregado Efetivo
	F	Empregado Efetivo
	F	Empregado Efetivo
	F	Empregado Efetivo
	F	Empregado Efetivo
	M	Empregado Efetivo
	F	Empregado Efetivo
	M	Empregado Efetivo

	F	Empregado Efetivo
	F	Empregado Efetivo
	F	Empregado Efetivo
	F	Empregado Efetivo
	F	Empregado Efetivo
	F	Empregado Efetivo
	M	Empregado Efetivo
	F	Empregado Efetivo
	F	Empregado Efetivo
	F	Empregado Efetivo
	F	Empregado Efetivo
	F	Empregado Efetivo
	F	Empregado Efetivo
	F	Empregado Efetivo
	F	Empregado Efetivo
	F	Empregado Efetivo
	F	Empregado Efetivo
	F	Empregado Efetivo
	F	Empregado Efetivo
	F	Empregado Efetivo
	M	Empregado Efetivo
	F	Empregado Efetivo
	F	Empregado Efetivo
	M	Empregado Efetivo
	F	Empregado Efetivo
	M	Empregado Efetivo
	M	Empregado Efetivo
	F	Empregado Efetivo
	F	Empregado Efetivo
	F	Empregado Efetivo
	F	Empregado Efetivo
	F	Empregado Efetivo
	F	Empregado Efetivo
	F	Empregado Efetivo
	F	Empregado Efetivo
	F	Empregado Efetivo
	F	Comissionado
	F	Empregado Efetivo
	F	Empregado Efetivo
	F	Empregado Efetivo
	F	Empregado Efetivo
	F	Empregado Efetivo
	F	Empregado Efetivo
	F	Empregado Efetivo
	F	Empregado Efetivo
	F	Empregado Efetivo
	F	Empregado Efetivo
	F	Empregado Efetivo

	M	Empregado Efetivo
	M	Empregado Efetivo
	M	Empregado Efetivo
	M	Empregado Efetivo
	M	Empregado Efetivo
	M	Empregado Efetivo
	F	Empregado Efetivo
	F	Empregado Efetivo
	F	Empregado Efetivo
	M	Empregado Efetivo
	M	Empregado Efetivo
	F	Empregado Efetivo
	F	Empregado Efetivo
	M	Empregado Efetivo
	M	Empregado Efetivo
	F	Empregado Efetivo
	M	Cedido - RJU
	F	Empregado Efetivo
	M	Empregado Efetivo
	M	Empregado Efetivo
	M	Empregado Efetivo
	M	Empregado Efetivo
	F	Empregado Efetivo
	F	Empregado Efetivo
	F	Empregado Efetivo
	F	Empregado Efetivo
	F	Empregado Efetivo
	F	Empregado Efetivo
	F	Empregado Efetivo
	F	Empregado Efetivo
	M	Empregado Efetivo
	M	Empregado Efetivo
	M	Empregado Efetivo
	M	Empregado Efetivo
	F	Empregado Efetivo
	F	Empregado Efetivo
	F	Empregado Efetivo
	F	Empregado Efetivo
	F	Empregado Efetivo
	F	Empregado Efetivo
	F	Empregado Efetivo
	F	Empregado Efetivo
	M	Empregado Efetivo
	M	Empregado Efetivo
	M	Empregado Efetivo
	M	Empregado Efetivo
	M	Empregado Efetivo

	M	Empregado Efetivo
	F	Empregado Efetivo
	F	Empregado Efetivo
	F	Empregado Efetivo
	F	Empregado Efetivo
	F	Empregado Efetivo
	F	Empregado Efetivo
	F	Empregado Efetivo
	F	Empregado Efetivo
	F	Empregado Efetivo
	F	Empregado Efetivo
	F	Empregado Efetivo
	F	Empregado Efetivo
	F	Empregado Efetivo
	F	Empregado Efetivo
	F	Empregado Efetivo
	F	Empregado Efetivo
	F	Empregado Efetivo
	F	Empregado Efetivo
	F	Empregado Efetivo
	F	Empregado Efetivo
	F	Empregado Efetivo
	F	Empregado Efetivo
	F	Empregado Efetivo
	F	Empregado Efetivo
	F	Empregado Efetivo
	F	Empregado Efetivo
	F	Empregado Efetivo
	F	Empregado Efetivo
	F	Empregado Efetivo
	F	Empregado Efetivo
	F	Empregado Efetivo

	F	Empregado Efetivo
	F	Empregado Efetivo
	F	Empregado Efetivo
	F	Empregado Efetivo
	F	Empregado Efetivo
	F	Empregado Efetivo
	F	Empregado Efetivo
	F	Empregado Efetivo
	F	Empregado Efetivo
	F	Empregado Efetivo
	F	Empregado Efetivo
	F	Empregado Efetivo
	F	Empregado Efetivo
	F	Empregado Efetivo
	F	Empregado Efetivo
	F	Empregado Efetivo
	F	Empregado Efetivo
	F	Empregado Efetivo
	F	Empregado Efetivo
	F	Empregado Efetivo
	F	Empregado Efetivo
	F	Empregado Efetivo
	F	Empregado Efetivo
	F	Empregado Efetivo
	F	Empregado Efetivo
	F	Empregado Efetivo
	M	Empregado Efetivo
	F	Empregado Efetivo
	F	Empregado Efetivo
	F	Empregado Efetivo
	F	Empregado Efetivo
	F	Empregado Efetivo
	F	Empregado Efetivo
	F	Empregado Efetivo
	F	Empregado Efetivo

	F	Empregado Efetivo
	F	Empregado Efetivo
	F	Empregado Efetivo
	F	Empregado Efetivo
	F	Empregado Efetivo
	F	Empregado Efetivo
	F	Empregado Efetivo
	M	Empregado Efetivo
	F	Empregado Efetivo
	F	Empregado Efetivo
	F	Empregado Efetivo
	F	Empregado Efetivo
	M	Empregado Efetivo
	M	Empregado Efetivo
	F	Empregado Efetivo
	F	Empregado Efetivo
	F	Empregado Efetivo
	F	Empregado Efetivo
	F	Empregado Efetivo
	F	Empregado Efetivo
	F	Empregado Efetivo
	M	Empregado Efetivo
	F	Empregado Efetivo
	F	Empregado Efetivo
	M	Empregado Efetivo
	F	Empregado Efetivo
	F	Empregado Efetivo
	M	Empregado Efetivo
	F	Empregado Efetivo
	F	Empregado Efetivo
	M	Empregado Efetivo
	M	Empregado Efetivo

	F	Empregado Efetivo
	F	Empregado Efetivo
	F	Empregado Efetivo
	F	Empregado Efetivo
	F	Empregado Efetivo
	F	Empregado Efetivo
	M	Empregado Efetivo
	F	Empregado Efetivo
	F	Empregado Efetivo
	F	Empregado Efetivo
	F	Empregado Efetivo
	F	Empregado Efetivo
	M	Empregado Efetivo
	F	Empregado Efetivo
	M	Empregado Efetivo
	M	Empregado Efetivo
	F	Empregado Efetivo
	M	Empregado Efetivo
	M	Empregado Efetivo
	M	Empregado Efetivo
	M	Empregado Efetivo
	F	Empregado Efetivo
	F	Empregado Efetivo
	F	Empregado Efetivo
	M	Empregado Efetivo
	M	Empregado Efetivo
	F	Empregado Efetivo
	F	Empregado Efetivo
	M	Empregado Efetivo
	F	Empregado Efetivo
	F	Empregado Efetivo
	F	Empregado Efetivo
	F	Empregado Efetivo
	M	Empregado Efetivo
	M	Empregado Efetivo
	F	Empregado Efetivo
	F	Empregado Efetivo
	F	Empregado Efetivo
	F	Empregado Efetivo
	F	Empregado Efetivo
	F	Empregado Efetivo
	F	Empregado Efetivo
	M	Empregado Efetivo
	F	Empregado Efetivo

	M	Empregado Efetivo
	M	Empregado Efetivo
	F	Empregado Efetivo
	F	Empregado Efetivo
	F	Empregado Efetivo
	M	Empregado Efetivo
	F	Empregado Efetivo
	M	Empregado Efetivo
	F	Empregado Efetivo
	F	Empregado Efetivo
	M	Empregado Efetivo
	F	Empregado Efetivo
	F	Empregado Efetivo
	F	Empregado Efetivo
	M	Empregado Efetivo
	M	Empregado Efetivo
	M	Empregado Efetivo
	F	Empregado Efetivo
	F	Empregado Efetivo
	F	Empregado Efetivo
	F	Empregado Efetivo
	F	Empregado Efetivo
	M	Empregado Efetivo
	F	Empregado Efetivo
	F	Empregado Efetivo
	F	Empregado Efetivo
	F	Empregado Efetivo
	F	Empregado Efetivo
	F	Empregado Efetivo
	M	Empregado Efetivo
	F	Empregado Efetivo
	F	Empregado Efetivo
	F	Empregado Efetivo
	F	Empregado Efetivo
	M	Empregado Efetivo
	F	Empregado Efetivo
	F	Empregado Efetivo
	F	Empregado Efetivo
	F	Empregado Efetivo
	F	Empregado Efetivo
	F	Empregado Efetivo
	F	Empregado Efetivo
	F	Empregado Efetivo
	F	Empregado Efetivo
	F	Empregado Efetivo
	F	Empregado Efetivo
	M	Empregado Efetivo
	F	Empregado Efetivo

	F	Empregado Efetivo
	F	Empregado Efetivo
	F	Empregado Efetivo
	F	Empregado Efetivo
	F	Empregado Efetivo
	F	Empregado Efetivo
	F	Empregado Efetivo
	F	Empregado Efetivo
	F	Empregado Efetivo
	F	Empregado Efetivo
	M	Empregado Efetivo
	F	Empregado Efetivo
	F	Empregado Efetivo
	F	Empregado Efetivo
	F	Empregado Efetivo
	F	Empregado Efetivo
	F	Empregado Efetivo
	F	Empregado Efetivo
	F	Empregado Efetivo
	F	Empregado Efetivo
	F	Empregado Efetivo
	M	Empregado Efetivo
	F	Empregado Efetivo
	F	Empregado Efetivo
	F	Empregado Efetivo
	M	Empregado Efetivo
	F	Empregado Efetivo
	F	Empregado Efetivo
	F	Empregado Efetivo
	F	Empregado Efetivo
	F	Empregado Efetivo
	F	Empregado Efetivo
	F	Empregado Efetivo
	F	Empregado Efetivo
	F	Empregado Efetivo
	M	Empregado Efetivo
	M	Empregado Efetivo
	F	Empregado Efetivo
	F	Empregado Efetivo
	F	Empregado Efetivo
	F	Empregado Efetivo
	F	Empregado Efetivo
	F	Empregado Efetivo
	F	Empregado Efetivo
	M	Empregado Efetivo
	F	Empregado Efetivo

	F	Empregado Efetivo
	F	Empregado Efetivo
	F	Empregado Efetivo
	F	Empregado Efetivo
	F	Empregado Efetivo
	F	Empregado Efetivo
	F	Empregado Efetivo
	M	Empregado Efetivo
	M	Empregado Efetivo
	F	Empregado Efetivo
	M	Empregado Efetivo
	F	Empregado Efetivo
	M	Empregado Efetivo
	M	Empregado Efetivo
	F	Empregado Efetivo
	F	Empregado Efetivo
	M	Empregado Efetivo
	F	Empregado Efetivo
	M	Empregado Efetivo
	M	Empregado Efetivo
	M	Empregado Efetivo
	M	Empregado Efetivo
	M	Empregado Efetivo
	M	Empregado Efetivo
	M	Empregado Efetivo
	M	Empregado Efetivo
	M	Empregado Efetivo
	M	Empregado Efetivo
	M	Empregado Efetivo
	M	Empregado Efetivo
	M	Empregado Efetivo
	M	Empregado Efetivo

	M	Empregado Efetivo
	M	Empregado Efetivo
	M	Empregado Efetivo
	M	Empregado Efetivo
	F	Empregado Efetivo
	F	Empregado Efetivo
	M	Empregado Efetivo
	F	Empregado Efetivo
	M	Empregado Efetivo
	F	Empregado Efetivo
	F	Empregado Efetivo
	M	Empregado Efetivo
	F	Empregado Efetivo
	M	Empregado Efetivo
	F	Empregado Efetivo
	M	Empregado Efetivo
	M	Empregado Efetivo
	M	Empregado Efetivo
	F	Empregado Efetivo
	F	Empregado Efetivo
	F	Empregado Efetivo
	M	Empregado Efetivo
	F	Empregado Efetivo
	F	Empregado Efetivo
	F	Empregado Efetivo
	F	Empregado Efetivo
	F	Empregado Efetivo
	M	Empregado Efetivo
	M	Empregado Efetivo
	F	Empregado Efetivo
	F	Empregado Efetivo
	F	Empregado Efetivo
	M	Empregado Efetivo
	F	Empregado Efetivo
	F	Empregado Efetivo
	F	Empregado Efetivo
	F	Empregado Efetivo
	M	Empregado Efetivo
	F	Empregado Efetivo
	M	Empregado Efetivo

	F	Empregado Efetivo
	M	Empregado Efetivo
	M	Empregado Efetivo
	F	Empregado Efetivo
	F	Empregado Efetivo
	F	Empregado Efetivo
	F	Empregado Efetivo
	M	Empregado Efetivo
	F	Empregado Efetivo
	M	Empregado Efetivo
	M	Empregado Efetivo
	F	Empregado Efetivo
	F	Empregado Efetivo
	F	Empregado Efetivo
	F	Empregado Efetivo
	M	Empregado Efetivo
	M	Empregado Efetivo
	F	Empregado Efetivo
	F	Empregado Efetivo
	M	Empregado Efetivo
	F	Empregado Efetivo
	F	Empregado Efetivo
	M	Empregado Efetivo
	F	Empregado Efetivo
	M	Empregado Efetivo
	F	Empregado Efetivo
	F	Empregado Efetivo
	F	Empregado Efetivo
	F	Empregado Efetivo
	F	Empregado Efetivo
	F	Empregado Efetivo
	F	Empregado Efetivo
	F	Empregado Efetivo
	F	Empregado Efetivo
	F	Empregado Efetivo
	F	Empregado Efetivo
	F	Empregado Efetivo
	M	Empregado Efetivo

	F	Empregado Efetivo
	F	Empregado Efetivo
	F	Empregado Efetivo
	M	Empregado Efetivo
	F	Empregado Efetivo
	F	Empregado Efetivo
	M	Empregado Efetivo
	M	Empregado Efetivo
	M	Empregado Efetivo
	F	Empregado Efetivo
	M	Empregado Efetivo
	F	Empregado Efetivo
	F	Empregado Efetivo
	F	Empregado Efetivo
	F	Empregado Efetivo
	M	Empregado Efetivo
	F	Empregado Efetivo
	M	Empregado Efetivo
	F	Empregado Efetivo
	F	Empregado Efetivo
	F	Empregado Efetivo
	F	Empregado Efetivo
	F	Empregado Efetivo
	F	Empregado Efetivo
	F	Empregado Efetivo
	M	Empregado Efetivo
	F	Empregado Efetivo
	F	Empregado Efetivo
	F	Empregado Efetivo
	F	Empregado Efetivo
	F	Empregado Efetivo
	F	Empregado Efetivo
	M	Empregado Efetivo
	F	Empregado Efetivo
	M	Empregado Efetivo
	F	Empregado Efetivo

	M	Empregado Efetivo
	F	Empregado Efetivo
	F	Empregado Efetivo
	F	Empregado Efetivo
	F	Empregado Efetivo
	F	Empregado Efetivo
	F	Empregado Efetivo
	F	Empregado Efetivo
	F	Empregado Efetivo
	F	Empregado Efetivo
	F	Empregado Efetivo
	F	Empregado Efetivo
	F	Empregado Efetivo
	F	Empregado Efetivo
	M	Empregado Efetivo
	F	Empregado Efetivo
	M	Empregado Efetivo
	M	Empregado Efetivo
	F	Empregado Efetivo
	M	Empregado Efetivo
	M	Empregado Efetivo
	F	Empregado Efetivo
	F	Empregado Efetivo
	F	Empregado Efetivo
	F	Empregado Efetivo
	M	Empregado Efetivo
	F	Empregado Efetivo
	F	Empregado Efetivo
	F	Empregado Efetivo
	F	Empregado Efetivo
	F	Empregado Efetivo
	F	Empregado Efetivo
	F	Empregado Efetivo
	F	Empregado Efetivo
	F	Empregado Efetivo
	F	Empregado Efetivo
	F	Empregado Efetivo
	F	Empregado Efetivo
	M	Empregado Efetivo
	F	Empregado Efetivo
	F	Empregado Efetivo
	F	Empregado Efetivo
	F	Empregado Efetivo
	M	Empregado Efetivo
	F	Empregado Efetivo
	F	Empregado Efetivo
	F	Empregado Efetivo

	F	Empregado Efetivo
	F	Empregado Efetivo
	F	Empregado Efetivo
	F	Empregado Efetivo
	M	Empregado Efetivo
	M	Empregado Efetivo
	F	Empregado Efetivo
	F	Empregado Efetivo
	F	Empregado Efetivo
	F	Empregado Efetivo
	F	Empregado Efetivo
	F	Empregado Efetivo
	F	Empregado Efetivo
	M	Empregado Efetivo
	F	Empregado Efetivo
	M	Empregado Efetivo
	F	Empregado Efetivo
	F	Empregado Efetivo
	F	Empregado Efetivo
	F	Empregado Efetivo
	F	Empregado Efetivo
	F	Empregado Efetivo
	F	Empregado Efetivo
	F	Empregado Efetivo
	F	Empregado Efetivo
	F	Empregado Efetivo
	F	Empregado Efetivo
	M	Cedido - RJU
	F	Empregado Efetivo
	F	Empregado Efetivo
	F	Empregado Efetivo
	F	Empregado Efetivo
	F	Empregado Efetivo
	F	Empregado Efetivo
	F	Empregado Efetivo
	F	Empregado Efetivo
	F	Empregado Efetivo
	F	Empregado Efetivo
	F	Empregado Efetivo
	F	Empregado Efetivo
	F	Empregado Efetivo
	F	Empregado Efetivo
	F	Empregado Efetivo

	F	Empregado Efetivo
	F	Empregado Efetivo
	F	Empregado Efetivo
	F	Empregado Efetivo
	F	Empregado Efetivo
	F	Empregado Efetivo
	F	Empregado Efetivo
	F	Empregado Efetivo
	F	Empregado Efetivo
	F	Empregado Efetivo
	F	Empregado Efetivo
	F	Empregado Efetivo
	F	Empregado Efetivo
	M	Empregado Efetivo
	F	Empregado Efetivo
	F	Empregado Efetivo
	F	Empregado Efetivo
	F	Empregado Efetivo
	M	Empregado Efetivo
	F	Empregado Efetivo
	F	Empregado Efetivo
	F	Empregado Efetivo
	F	Empregado Efetivo
	F	Empregado Efetivo
	M	Empregado Efetivo
	F	Empregado Efetivo
	F	Empregado Efetivo
	F	Empregado Efetivo
	M	Empregado Efetivo
	F	Empregado Efetivo
	M	Empregado Efetivo
	F	Empregado Efetivo
	F	Empregado Efetivo
	M	Empregado Efetivo
	M	Empregado Efetivo
	F	Empregado Efetivo
	M	Empregado Efetivo
	F	Empregado Efetivo
	F	Empregado Efetivo
	F	Empregado Efetivo
	M	Empregado Efetivo
	F	Empregado Efetivo
	F	Empregado Efetivo
	F	Empregado Efetivo
	F	Empregado Efetivo
	F	Empregado Efetivo
	F	Empregado Efetivo
	F	Empregado Efetivo

	F	Empregado Efetivo
	F	Empregado Efetivo
	F	Empregado Efetivo
	F	Empregado Efetivo
	F	Empregado Efetivo
	F	Empregado Efetivo
	F	Empregado Efetivo
	F	Empregado Efetivo
	F	Empregado Efetivo
	F	Empregado Efetivo
	F	Empregado Efetivo
	F	Empregado Efetivo
	F	Empregado Efetivo
	F	Empregado Efetivo
	M	Empregado Efetivo
	F	Empregado Efetivo
	F	Empregado Efetivo
	F	Empregado Efetivo
	F	Empregado Efetivo
	F	Empregado Efetivo
	M	Empregado Efetivo
	F	Empregado Efetivo
	M	Empregado Efetivo
	F	Empregado Efetivo
	F	Empregado Efetivo
	M	Empregado Efetivo
	F	Empregado Efetivo
	F	Empregado Efetivo
	M	Empregado Efetivo
	F	Empregado Efetivo
	F	Empregado Efetivo
	F	Empregado Efetivo
	F	Empregado Efetivo
	F	Empregado Efetivo
	F	Empregado Efetivo
	F	Empregado Efetivo
	F	Empregado Efetivo
	F	Empregado Efetivo
	F	Empregado Efetivo
	M	Empregado Efetivo
	M	Empregado Efetivo
	F	Empregado Efetivo

	M	Empregado Efetivo
	M	Empregado Efetivo
	M	Empregado Efetivo
	M	Empregado Efetivo
	F	Empregado Efetivo
	M	Empregado Efetivo
	M	Empregado Efetivo
	M	Empregado Efetivo
	M	Empregado Efetivo
	F	Empregado Efetivo
	F	Empregado Efetivo
	F	Empregado Efetivo
	M	Empregado Efetivo
	F	Empregado Efetivo
	F	Empregado Efetivo
	F	Empregado Efetivo
	F	Empregado Efetivo
	F	Empregado Efetivo
	F	Empregado Efetivo
	F	Empregado Efetivo
	M	Empregado Efetivo
	F	Empregado Efetivo
	M	Empregado Efetivo
	F	Empregado Efetivo
	F	Empregado Efetivo
	F	Empregado Efetivo
	F	Empregado Efetivo
	F	Empregado Efetivo
	M	Empregado Efetivo
	M	Empregado Efetivo
	F	Empregado Efetivo
	M	Empregado Efetivo
	F	Empregado Efetivo
	F	Empregado Efetivo
	M	Empregado Efetivo
	M	Empregado Efetivo
	M	Empregado Efetivo
	M	Empregado Efetivo
	M	Empregado Efetivo
	M	Empregado Efetivo

	M	Empregado Efetivo
	M	Empregado Efetivo
	M	Empregado Efetivo
	F	Empregado Efetivo
	F	Empregado Efetivo
	F	Empregado Efetivo
	F	Empregado Efetivo
	F	Empregado Efetivo
	M	Empregado Efetivo
	M	Empregado Efetivo
	F	Empregado Efetivo
	F	Empregado Efetivo
	F	Empregado Efetivo
	F	Empregado Efetivo
	F	Empregado Efetivo
	F	Empregado Efetivo
	F	Empregado Efetivo
	F	Empregado Efetivo
	F	Empregado Efetivo
	F	Empregado Efetivo
	F	Empregado Efetivo
	F	Empregado Efetivo
	F	Empregado Efetivo
	F	Empregado Efetivo
	F	Empregado Efetivo
	F	Empregado Efetivo
	F	Empregado Efetivo
	F	Empregado Efetivo
	F	Empregado Efetivo
	F	Empregado Efetivo
	F	Empregado Efetivo
	F	Empregado Efetivo
	F	Empregado Efetivo
	F	Empregado Efetivo
	F	Empregado Efetivo
	F	Empregado Efetivo

	F	Empregado Efetivo
	F	Empregado Efetivo
	M	Empregado Efetivo
	F	Empregado Efetivo
	F	Empregado Efetivo
	F	Empregado Efetivo
	F	Empregado Efetivo
	F	Empregado Efetivo
	F	Empregado Efetivo
	M	Empregado Efetivo
	F	Empregado Efetivo
	F	Empregado Efetivo
	F	Empregado Efetivo
	F	Empregado Efetivo
	F	Empregado Efetivo
	F	Empregado Efetivo
	F	Empregado Efetivo
	F	Empregado Efetivo
	F	Empregado Efetivo
	F	Empregado Efetivo
	F	Empregado Efetivo
	F	Empregado Efetivo
	F	Empregado Efetivo
	M	Empregado Efetivo
	F	Empregado Efetivo
	F	Empregado Efetivo
	F	Empregado Efetivo
	F	Empregado Efetivo
	F	Empregado Efetivo
	M	Empregado Efetivo
	M	Empregado Efetivo
	F	Empregado Efetivo

	F	Empregado Efetivo
	F	Empregado Efetivo
	F	Empregado Efetivo
	M	Empregado Efetivo
	F	Empregado Efetivo
	M	Empregado Efetivo
	F	Empregado Efetivo
	F	Empregado Efetivo
	F	Empregado Efetivo
	F	Empregado Efetivo
	F	Empregado Efetivo
	F	Empregado Efetivo
	F	Empregado Efetivo
	F	Empregado Efetivo
	F	Empregado Efetivo
	F	Empregado Efetivo
	F	Empregado Efetivo
	F	Empregado Efetivo
	F	Empregado Efetivo
	F	Empregado Efetivo
	F	Empregado Efetivo
	F	Empregado Efetivo
	M	Empregado Efetivo
	F	Empregado Efetivo
	M	Empregado Efetivo
	F	Empregado Efetivo
	F	Empregado Efetivo
	M	Empregado Efetivo
	M	Empregado Efetivo
	M	Empregado Efetivo
	F	Empregado Efetivo
	F	Empregado Efetivo
	M	Empregado Efetivo
	F	Empregado Efetivo
	F	Empregado Efetivo
	F	Empregado Efetivo
	F	Empregado Efetivo
	F	Empregado Efetivo

	F	Empregado Efetivo
	F	Empregado Efetivo
	F	Empregado Efetivo
	M	Empregado Efetivo
	F	Empregado Efetivo
	M	Empregado Efetivo
	F	Empregado Efetivo
	M	Empregado Efetivo
	F	Empregado Efetivo
	F	Empregado Efetivo
	F	Empregado Efetivo
	F	Empregado Efetivo
	M	Empregado Efetivo
	F	Empregado Efetivo
	F	Empregado Efetivo
	F	Empregado Efetivo
	F	Empregado Efetivo
	F	Empregado Efetivo
	M	Empregado Efetivo
	F	Empregado Efetivo
	F	Empregado Efetivo
	M	Empregado Efetivo
	F	Empregado Efetivo
	F	Empregado Efetivo
	F	Empregado Efetivo
	F	Empregado Efetivo
	F	Empregado Efetivo
	M	Empregado Efetivo
	F	Empregado Efetivo
	M	Empregado Efetivo
	F	Empregado Efetivo
	F	Empregado Efetivo
	F	Empregado Efetivo
	F	Empregado Efetivo
	M	Empregado Efetivo
	M	Empregado Efetivo
	M	Empregado Efetivo
	M	Empregado Efetivo
	M	Empregado Efetivo
	F	Empregado Efetivo
	F	Empregado Efetivo
	F	Empregado Efetivo
	F	Empregado Efetivo
	F	Empregado Efetivo
	F	Empregado Efetivo
	M	Empregado Efetivo
	F	Empregado Efetivo
	M	Empregado Efetivo

	F	Empregado Efetivo
	M	Empregado Efetivo
	F	Empregado Efetivo
	M	Empregado Efetivo
	F	Empregado Efetivo
	F	Empregado Efetivo
	F	Empregado Efetivo
	F	Empregado Efetivo
	F	Empregado Efetivo
	F	Empregado Efetivo
	F	Empregado Efetivo
	F	Empregado Efetivo
	F	Empregado Efetivo
	F	Empregado Efetivo
	F	Empregado Efetivo
	F	Empregado Efetivo
	F	Empregado Efetivo
	F	Empregado Efetivo
	M	Empregado Efetivo
	M	Empregado Efetivo
	F	Empregado Efetivo
	F	Empregado Efetivo
	F	Empregado Efetivo
	F	Empregado Efetivo
	F	Empregado Efetivo
	F	Empregado Efetivo
	M	Empregado Efetivo
	M	Empregado Efetivo
	M	Empregado Efetivo
	F	Empregado Efetivo
	F	Empregado Efetivo
	M	Empregado Efetivo
	M	Empregado Efetivo
	M	Empregado Efetivo
	F	Empregado Efetivo
	M	Empregado Efetivo
	M	Empregado Efetivo
	M	Empregado Efetivo

	M	Empregado Efetivo
	F	Empregado Efetivo
	F	Empregado Efetivo
	F	Empregado Efetivo
	F	Empregado Efetivo
	F	Empregado Efetivo
	F	Empregado Efetivo
	M	Empregado Efetivo
	F	Empregado Efetivo
	F	Empregado Efetivo
	F	Empregado Efetivo
	F	Empregado Efetivo
	M	Empregado Efetivo
	M	Empregado Efetivo
	F	Empregado Efetivo
	F	Empregado Efetivo
	M	Empregado Efetivo
	M	Empregado Efetivo
	F	Empregado Efetivo
	F	Empregado Efetivo
	F	Empregado Efetivo
	F	Empregado Efetivo
	F	Empregado Efetivo
	M	Empregado Efetivo
	F	Empregado Efetivo
	M	Empregado Efetivo
	M	Empregado Efetivo
	M	Empregado Efetivo
	F	Empregado Efetivo
	M	Empregado Efetivo
	M	Empregado Efetivo
	M	Empregado Efetivo
	F	Empregado Efetivo
	F	Empregado Efetivo
	F	Empregado Efetivo
	F	Empregado Efetivo
	M	Empregado Efetivo
	M	Empregado Efetivo
	F	Empregado Efetivo

	F	Empregado Efetivo
	F	Empregado Efetivo
	F	Empregado Efetivo
	F	Empregado Efetivo
	F	Empregado Efetivo
	F	Empregado Efetivo
	M	Empregado Efetivo
	F	Empregado Efetivo
	M	Empregado Efetivo
	F	Empregado Efetivo
	F	Empregado Efetivo
	F	Empregado Efetivo
	F	Empregado Efetivo
	M	Empregado Efetivo
	F	Empregado Efetivo
	M	Empregado Efetivo
	F	Empregado Efetivo
	M	Empregado Efetivo
	M	Empregado Efetivo
	F	Empregado Efetivo
	M	Empregado Efetivo
	M	Empregado Efetivo
	F	Empregado Efetivo
	F	Empregado Efetivo
	F	Empregado Efetivo
	M	Empregado Efetivo
	M	Empregado Efetivo
	F	Empregado Efetivo
	F	Empregado Efetivo
	F	Empregado Efetivo
	F	Empregado Efetivo
	F	Empregado Efetivo
	F	Empregado Efetivo
	F	Empregado Efetivo

	F	Empregado Efetivo
	F	Empregado Efetivo
	F	Empregado Efetivo
	M	Empregado Efetivo
	F	Empregado Efetivo
	M	Empregado Efetivo
	F	Empregado Efetivo
	F	Empregado Efetivo
	M	Empregado Efetivo
	M	Empregado Efetivo
	F	Empregado Efetivo
	F	Empregado Efetivo
	F	Empregado Efetivo
	F	Empregado Efetivo
	F	Empregado Efetivo
	M	Empregado Efetivo
	M	Empregado Efetivo
	F	Empregado Efetivo
	F	Empregado Efetivo
	F	Empregado Efetivo
	F	Empregado Efetivo
	F	Empregado Efetivo
	F	Empregado Efetivo
	F	Empregado Efetivo
	M	Empregado Efetivo
	F	Empregado Efetivo
	F	Empregado Efetivo
	F	Empregado Efetivo
	F	Empregado Efetivo
	F	Empregado Efetivo
	F	Empregado Efetivo
	F	Empregado Efetivo
	F	Empregado Efetivo
	F	Empregado Efetivo
	F	Empregado Efetivo
	F	Empregado Efetivo
	F	Empregado Efetivo
	F	Empregado Efetivo
	F	Empregado Efetivo
	M	Empregado Efetivo
	M	Empregado Efetivo
	F	Empregado Efetivo

	F	Empregado Efetivo
	F	Empregado Efetivo
	F	Empregado Efetivo
	F	Empregado Efetivo
	F	Empregado Efetivo
	F	Empregado Efetivo
	F	Empregado Efetivo
	M	Empregado Efetivo
	F	Empregado Efetivo
	F	Empregado Efetivo
	F	Empregado Efetivo
	F	Empregado Efetivo
	F	Empregado Efetivo
	F	Empregado Efetivo
	F	Empregado Efetivo
	M	Empregado Efetivo
	F	Empregado Efetivo
	F	Empregado Efetivo
	F	Empregado Efetivo
	F	Empregado Efetivo
	F	Empregado Efetivo
	F	Empregado Efetivo
	F	Empregado Efetivo
	F	Empregado Efetivo
	F	Empregado Efetivo
	F	Empregado Efetivo
	M	Empregado Efetivo
	F	Empregado Efetivo
	M	Empregado Efetivo
	M	Empregado Efetivo
	M	Empregado Efetivo
	F	Empregado Efetivo
	M	Empregado Efetivo
	F	Empregado Efetivo
	F	Empregado Efetivo
	F	Empregado Efetivo
	M	Empregado Efetivo
	M	Empregado Efetivo
	M	Empregado Efetivo
	F	Empregado Efetivo
	F	Empregado Efetivo
	F	Empregado Efetivo
	M	Empregado Efetivo
	M	Empregado Efetivo
	F	Empregado Efetivo
	F	Empregado Efetivo
	M	Empregado Efetivo
	M	Empregado Efetivo

	M	Empregado Efetivo
	M	Empregado Efetivo
	F	Empregado Efetivo
	F	Empregado Efetivo
	M	Empregado Efetivo
	F	Empregado Efetivo
	F	Empregado Efetivo
	F	Empregado Efetivo
	M	Empregado Efetivo
	M	Empregado Efetivo
	F	Empregado Efetivo
	F	Empregado Efetivo
	F	Empregado Efetivo
	F	Empregado Efetivo
	F	Empregado Efetivo
	M	Empregado Efetivo
	M	Empregado Efetivo
	M	Empregado Efetivo
	M	Empregado Efetivo
	F	Empregado Efetivo
	F	Empregado Efetivo
	F	Empregado Efetivo
	F	Empregado Efetivo
	F	Empregado Efetivo
	F	Empregado Efetivo
	F	Empregado Efetivo
	F	Empregado Efetivo
	F	Empregado Efetivo
	F	Empregado Efetivo
	M	Empregado Efetivo
	M	Empregado Efetivo
	M	Empregado Efetivo
	F	Empregado Efetivo
	F	Empregado Efetivo
	F	Empregado Efetivo
	F	Empregado Efetivo
	F	Empregado Efetivo
	F	Empregado Efetivo
	F	Empregado Efetivo
	F	Empregado Efetivo
	F	Empregado Efetivo
	F	Empregado Efetivo

	F	Empregado Efetivo
	F	Empregado Efetivo
	F	Empregado Efetivo
	F	Empregado Efetivo
	F	Empregado Efetivo
	F	Empregado Efetivo
	F	Empregado Efetivo
	F	Empregado Efetivo
	F	Empregado Efetivo
	M	Empregado Efetivo
	F	Empregado Efetivo
	F	Empregado Efetivo
	F	Empregado Efetivo
	F	Empregado Efetivo
	F	Empregado Efetivo
	M	Empregado Efetivo
	F	Empregado Efetivo
	M	Empregado Efetivo
	F	Empregado Efetivo
	F	Empregado Efetivo
	F	Empregado Efetivo
	M	Empregado Efetivo
	F	Empregado Efetivo
	F	Empregado Efetivo
	F	Empregado Efetivo
	F	Empregado Efetivo
	F	Empregado Efetivo
	F	Empregado Efetivo
	F	Empregado Efetivo
	F	Empregado Efetivo
	F	Empregado Efetivo
	F	Empregado Efetivo
	F	Empregado Efetivo
	F	Empregado Efetivo
	M	Empregado Efetivo
	F	Empregado Efetivo
	M	Empregado Efetivo
	M	Empregado Efetivo
	F	Empregado Efetivo
	F	Empregado Efetivo
	F	Empregado Efetivo
	M	Empregado Efetivo
	F	Empregado Efetivo
	F	Empregado Efetivo
	F	Empregado Efetivo

	F	Empregado Efetivo
	F	Empregado Efetivo
	F	Empregado Efetivo
	F	Empregado Efetivo
	F	Empregado Efetivo
	F	Empregado Efetivo
	F	Empregado Efetivo
	F	Empregado Efetivo
	F	Empregado Efetivo
	F	Empregado Efetivo
	M	Empregado Efetivo
	M	Empregado Efetivo
	F	Empregado Efetivo
	F	Empregado Efetivo
	F	Empregado Efetivo
	F	Empregado Efetivo
	F	Empregado Efetivo
	M	Empregado Efetivo
	M	Empregado Efetivo
	F	Empregado Efetivo
	F	Empregado Efetivo
	M	Empregado Efetivo
	F	Empregado Efetivo
	M	Empregado Efetivo
	F	Empregado Efetivo
	F	Empregado Efetivo
	M	Empregado Efetivo
	M	Empregado Efetivo
	M	Empregado Efetivo
	F	Empregado Efetivo
	F	Empregado Efetivo
	F	Empregado Efetivo
	M	Empregado Efetivo
	F	Empregado Efetivo
	F	Empregado Efetivo
	F	Empregado Efetivo
	F	Empregado Efetivo
	F	Empregado Efetivo
	F	Empregado Efetivo
	F	Empregado Efetivo
	F	Empregado Efetivo
	F	Empregado Efetivo
	F	Empregado Efetivo
	F	Empregado Efetivo
	F	Empregado Efetivo
	F	Empregado Efetivo
	F	Empregado Efetivo

	F	Empregado Efetivo
	M	Empregado Efetivo
	F	Empregado Efetivo
	F	Empregado Efetivo
	M	Empregado Efetivo
	F	Empregado Efetivo
	F	Empregado Efetivo
	F	Empregado Efetivo
	F	Empregado Efetivo
	F	Empregado Efetivo
	F	Empregado Efetivo
	F	Empregado Efetivo
	F	Empregado Efetivo
	M	Empregado Efetivo
	M	Empregado Efetivo
	M	Empregado Efetivo
	M	Empregado Efetivo
	M	Empregado Efetivo
	F	Empregado Efetivo
	M	Empregado Efetivo
	F	Empregado Efetivo
	F	Empregado Efetivo
	F	Empregado Efetivo
	F	Empregado Efetivo
	F	Empregado Efetivo
	F	Empregado Efetivo
	F	Empregado Efetivo
	F	Empregado Efetivo
	F	Empregado Efetivo
	F	Empregado Efetivo
	F	Empregado Efetivo
	F	Empregado Efetivo
	F	Empregado Efetivo
	F	Empregado Efetivo
	F	Empregado Efetivo
	F	Empregado Efetivo
	F	Empregado Efetivo
	F	Empregado Efetivo
	F	Empregado Efetivo
	F	Empregado Efetivo
	F	Empregado Efetivo

	F	Empregado Efetivo
	F	Empregado Efetivo
	F	Empregado Efetivo
	F	Empregado Efetivo
	F	Empregado Efetivo
	F	Empregado Efetivo
	F	Empregado Efetivo
	F	Empregado Efetivo
	F	Empregado Efetivo
	F	Empregado Efetivo
	F	Empregado Efetivo
	F	Empregado Efetivo
	F	Empregado Efetivo
	M	Empregado Efetivo
	F	Empregado Efetivo
	F	Empregado Efetivo
	F	Empregado Efetivo
	F	Empregado Efetivo
	M	Empregado Efetivo
	F	Empregado Efetivo
	F	Empregado Efetivo
	F	Empregado Efetivo
	F	Empregado Efetivo
	F	Empregado Efetivo
	F	Empregado Efetivo
	F	Empregado Efetivo
	F	Empregado Efetivo
	F	Empregado Efetivo
	F	Empregado Efetivo
	F	Empregado Efetivo
	F	Empregado Efetivo
	F	Empregado Efetivo
	F	Empregado Efetivo
	F	Empregado Efetivo
	F	Empregado Efetivo
	F	Empregado Efetivo
	F	Empregado Efetivo
	F	Empregado Efetivo

	F	Empregado Efetivo
	F	Empregado Efetivo
	F	Empregado Efetivo
	F	Empregado Efetivo
	F	Cedido - RJU
	M	Cedido - RJU
	F	Empregado Efetivo
	M	Empregado Efetivo
	F	Empregado Efetivo
	F	Empregado Efetivo
	F	Empregado Efetivo
	M	Empregado Efetivo
	F	Empregado Efetivo
	F	Empregado Efetivo
	F	Empregado Efetivo
	F	Empregado Efetivo
	F	Empregado Efetivo
	M	Empregado Efetivo
	M	Empregado Efetivo
	M	Empregado Efetivo
	F	Empregado Efetivo
	F	Empregado Efetivo
	F	Empregado Efetivo
	M	Empregado Efetivo
	F	Empregado Efetivo
	F	Empregado Efetivo
	M	Empregado Efetivo
	F	Empregado Efetivo
	M	Empregado Efetivo
	F	Empregado Efetivo
	F	Empregado Efetivo
	F	Empregado Efetivo
	F	Empregado Efetivo
	F	Empregado Efetivo
	F	Empregado Efetivo
	F	Empregado Efetivo
	M	Empregado Efetivo
	F	Empregado Efetivo
	F	Empregado Efetivo
	F	Empregado Efetivo
	M	Empregado Efetivo
	F	Empregado Efetivo
	M	Empregado Efetivo

	M	Empregado Efetivo
	M	Empregado Efetivo
	F	Empregado Efetivo
	F	Empregado Efetivo
	M	Empregado Efetivo
	M	Empregado Efetivo
	F	Empregado Efetivo
	M	Empregado Efetivo
	M	Empregado Efetivo
	M	Empregado Efetivo
	M	Empregado Efetivo
	M	Empregado Efetivo
	F	Empregado Efetivo
	F	Empregado Efetivo
	F	Empregado Efetivo
	F	Empregado Efetivo
	M	Empregado Efetivo
	F	Empregado Efetivo
	F	Empregado Efetivo
	F	Empregado Efetivo
	F	Empregado Efetivo
	F	Empregado Efetivo
	M	Empregado Efetivo
	M	Empregado Efetivo
	F	Empregado Efetivo
	F	Empregado Efetivo
	F	Empregado Efetivo
	F	Empregado Efetivo
	F	Empregado Efetivo
	F	Empregado Efetivo
	F	Empregado Efetivo
	F	Empregado Efetivo
	F	Empregado Efetivo
	F	Empregado Efetivo
	F	Empregado Efetivo
	F	Empregado Efetivo
	F	Empregado Efetivo
	F	Empregado Efetivo
	F	Empregado Efetivo
	F	Empregado Efetivo
	F	Empregado Efetivo
	F	Empregado Efetivo
	F	Empregado Efetivo
	F	Empregado Efetivo
	F	Empregado Efetivo

	F	Empregado Efetivo
	M	Empregado Efetivo
	M	Empregado Efetivo
	F	Empregado Efetivo
	F	Empregado Efetivo
	F	Empregado Efetivo
	F	Empregado Efetivo
	F	Empregado Efetivo
	F	Empregado Efetivo
	F	Empregado Efetivo
	F	Empregado Efetivo
	M	Empregado Efetivo
	F	Empregado Efetivo
	M	Empregado Efetivo
	M	Empregado Efetivo
	M	Empregado Efetivo
	M	Empregado Efetivo
	M	Empregado Efetivo
	M	Empregado Efetivo
	F	Empregado Efetivo
	M	Empregado Efetivo
	M	Empregado Efetivo
	M	Empregado Efetivo
	M	Empregado Efetivo
	M	Empregado Efetivo
	M	Empregado Efetivo
	M	Empregado Efetivo
	F	Empregado Efetivo
	F	Empregado Efetivo
	F	Empregado Efetivo
	F	Empregado Efetivo
	F	Empregado Efetivo
	F	Empregado Efetivo
	F	Empregado Efetivo
	M	Empregado Efetivo
	M	Empregado Efetivo
	M	Empregado Efetivo

	M	Empregado Efetivo
	F	Empregado Efetivo
	F	Empregado Efetivo
	M	Empregado Efetivo
	F	Empregado Efetivo
	F	Empregado Efetivo
	F	Empregado Efetivo
	F	Empregado Efetivo
	F	Empregado Efetivo
	M	Empregado Efetivo
	F	Empregado Efetivo
	F	Empregado Efetivo
	F	Empregado Efetivo
	F	Empregado Efetivo
	F	Empregado Efetivo
	F	Empregado Efetivo
	F	Empregado Efetivo
	F	Empregado Efetivo
	F	Empregado Efetivo
	F	Empregado Efetivo
	M	Empregado Efetivo
	F	Empregado Efetivo
	F	Empregado Efetivo
	M	Empregado Efetivo
	M	Empregado Efetivo
	F	Empregado Efetivo
	F	Empregado Efetivo
	F	Empregado Efetivo
	F	Empregado Efetivo
	F	Empregado Efetivo
	F	Empregado Efetivo
	F	Empregado Efetivo
	F	Empregado Efetivo
	M	Empregado Efetivo
	M	Empregado Efetivo
	M	Empregado Efetivo
	M	Empregado Efetivo
	M	Empregado Efetivo
	M	Empregado Efetivo
	M	Empregado Efetivo
	M	Empregado Efetivo
	M	Empregado Efetivo
	M	Empregado Efetivo
	M	Empregado Efetivo
	M	Empregado Efetivo
	M	Empregado Efetivo
	M	Empregado Efetivo
	M	Empregado Efetivo

	M	Empregado Efetivo
	M	Empregado Efetivo
	M	Empregado Efetivo
	M	Empregado Efetivo
	M	Empregado Efetivo
	M	Empregado Efetivo
	M	Empregado Efetivo
	M	Empregado Efetivo
	M	Empregado Efetivo
	M	Empregado Efetivo
	M	Empregado Efetivo
	M	Empregado Efetivo
	M	Empregado Efetivo
	M	Empregado Efetivo
	F	Empregado Efetivo
	F	Empregado Efetivo
	F	Empregado Efetivo
	F	Empregado Efetivo
	F	Empregado Efetivo
	F	Empregado Efetivo
	F	Empregado Efetivo
	F	Empregado Efetivo
	F	Empregado Efetivo
	F	Empregado Efetivo
	F	Empregado Efetivo
	F	Empregado Efetivo
	F	Empregado Efetivo
	F	Empregado Efetivo
	F	Empregado Efetivo
	F	Empregado Efetivo
	F	Empregado Efetivo
	F	Empregado Efetivo
	F	Empregado Efetivo
	F	Empregado Efetivo
	F	Empregado Efetivo
	F	Empregado Efetivo
	M	Empregado Efetivo
	F	Empregado Efetivo
	F	Empregado Efetivo
	F	Empregado Efetivo
	M	Empregado Efetivo
	F	Empregado Efetivo
	F	Empregado Efetivo
	F	Empregado Efetivo
	F	Empregado Efetivo
	F	Empregado Efetivo
	F	Empregado Efetivo
	M	Empregado Efetivo

	F	Empregado Efetivo
	F	Empregado Efetivo
	F	Empregado Efetivo
	F	Empregado Efetivo
	F	Empregado Efetivo
	F	Empregado Efetivo
	F	Empregado Efetivo
	F	Empregado Efetivo
	F	Empregado Efetivo
	F	Empregado Efetivo
	M	Empregado Efetivo
	M	Empregado Efetivo
	M	Empregado Efetivo
	M	Empregado Efetivo
	F	Empregado Efetivo
	F	Empregado Efetivo
	F	Empregado Efetivo
	F	Empregado Efetivo
	F	Empregado Efetivo
	F	Empregado Efetivo
	F	Empregado Efetivo
	F	Empregado Efetivo
	F	Empregado Efetivo
	M	Empregado Efetivo
	F	Empregado Efetivo
	F	Empregado Efetivo
	F	Empregado Efetivo
	F	Empregado Efetivo
	F	Empregado Efetivo
	F	Empregado Efetivo
	F	Empregado Efetivo
	F	Empregado Efetivo
	F	Empregado Efetivo
	F	Empregado Efetivo
	F	Empregado Efetivo
	M	Empregado Efetivo
	M	Empregado Efetivo
	F	Empregado Efetivo
	F	Empregado Efetivo
	F	Empregado Efetivo
	M	Empregado Efetivo
	F	Empregado Efetivo

	F	Empregado Efetivo
	F	Empregado Efetivo
	F	Empregado Efetivo
	M	Empregado Efetivo
	F	Empregado Efetivo
	F	Empregado Efetivo
	F	Empregado Efetivo
	F	Empregado Efetivo
	F	Empregado Efetivo
	F	Empregado Efetivo
	M	Empregado Efetivo
	F	Empregado Efetivo
	F	Empregado Efetivo
	M	Empregado Efetivo
	M	Empregado Efetivo
	F	Empregado Efetivo
	M	Empregado Efetivo
	F	Empregado Efetivo
	M	Empregado Efetivo
	M	Empregado Efetivo
	F	Empregado Efetivo
	F	Empregado Efetivo
	F	Empregado Efetivo
	F	Empregado Efetivo
	M	Empregado Efetivo
	F	Empregado Efetivo
	F	Empregado Efetivo
	F	Empregado Efetivo
	F	Empregado Efetivo
	F	Empregado Efetivo
	F	Empregado Efetivo
	F	Empregado Efetivo
	F	Empregado Efetivo
	F	Empregado Efetivo
	F	Empregado Efetivo
	F	Empregado Efetivo
	F	Empregado Efetivo
	F	Empregado Efetivo
	F	Empregado Efetivo
	M	Empregado Efetivo
	F	Empregado Efetivo
	F	Empregado Efetivo
	F	Empregado Efetivo

	F	Empregado Efetivo
	M	Empregado Efetivo
	M	Empregado Efetivo
	F	Empregado Efetivo
	M	Empregado Efetivo
	F	Empregado Efetivo
	F	Empregado Efetivo
	M	Empregado Efetivo
	M	Empregado Efetivo
	M	Empregado Efetivo
	M	Empregado Efetivo
	F	Empregado Efetivo
	F	Empregado Efetivo
	M	Empregado Efetivo
	F	Empregado Efetivo
	F	Empregado Efetivo
	F	Empregado Efetivo
	F	Empregado Efetivo
	F	Empregado Efetivo
	F	Empregado Efetivo
	F	Empregado Efetivo
	F	Empregado Efetivo
	F	Empregado Efetivo
	F	Empregado Efetivo
	F	Empregado Efetivo
	F	Empregado Efetivo
	M	Empregado Efetivo
	M	Empregado Efetivo
	M	Empregado Efetivo
	M	Empregado Efetivo
	M	Empregado Efetivo
	M	Empregado Efetivo
	F	Empregado Efetivo
	M	Empregado Efetivo
	M	Empregado Efetivo

	F	Empregado Efetivo
	M	Empregado Efetivo
	F	Empregado Efetivo
	M	Empregado Efetivo
	M	Empregado Efetivo
	F	Empregado Efetivo
	F	Empregado Efetivo
	M	Empregado Efetivo
	F	Empregado Efetivo
	M	Empregado Efetivo
	M	Empregado Efetivo
	F	Empregado Efetivo
	F	Empregado Efetivo
	F	Empregado Efetivo
	F	Empregado Efetivo
	M	Empregado Efetivo
	M	Empregado Efetivo
	F	Empregado Efetivo
	F	Empregado Efetivo
	F	Empregado Efetivo
	F	Empregado Efetivo
	M	Empregado Efetivo
	F	Empregado Efetivo
	F	Empregado Efetivo
	F	Empregado Efetivo
	M	Empregado Efetivo
	F	Empregado Efetivo
	F	Empregado Efetivo
	F	Empregado Efetivo
	F	Empregado Efetivo
	F	Empregado Efetivo
	F	Empregado Efetivo
	F	Empregado Efetivo
	F	Empregado Efetivo
	M	Empregado Efetivo
	F	Empregado Efetivo
	F	Empregado Efetivo
	F	Empregado Efetivo
	F	Empregado Efetivo
	F	Empregado Efetivo
	F	Empregado Efetivo
	F	Empregado Efetivo
	F	Empregado Efetivo
	F	Empregado Efetivo
	F	Empregado Efetivo
	F	Empregado Efetivo
	F	Empregado Efetivo
	F	Empregado Efetivo

	F	Empregado Efetivo
	F	Empregado Efetivo
	F	Empregado Efetivo
	F	Empregado Efetivo
	F	Empregado Efetivo
	F	Empregado Efetivo
	F	Empregado Efetivo
	F	Empregado Efetivo
	F	Empregado Efetivo
	F	Empregado Efetivo
	F	Empregado Efetivo
	F	Empregado Efetivo
	F	Empregado Efetivo
	F	Empregado Efetivo
	F	Empregado Efetivo
	F	Empregado Efetivo
	F	Empregado Efetivo
	F	Empregado Efetivo
	F	Empregado Efetivo
	F	Empregado Efetivo
	F	Empregado Efetivo
	F	Empregado Efetivo
	F	Empregado Efetivo
	F	Empregado Efetivo
	F	Empregado Efetivo
	F	Empregado Efetivo
	F	Empregado Efetivo
	F	Empregado Efetivo
	F	Empregado Efetivo
	F	Empregado Efetivo
	F	Empregado Efetivo
	F	Empregado Efetivo
	F	Empregado Efetivo
	F	Empregado Efetivo
	F	Empregado Efetivo
	F	Empregado Efetivo
	M	Empregado Efetivo
	F	Empregado Efetivo
	F	Empregado Efetivo
	F	Empregado Efetivo
	F	Empregado Efetivo
	F	Empregado Efetivo
	F	Empregado Efetivo
	F	Empregado Efetivo
	F	Empregado Efetivo
	F	Empregado Efetivo
	F	Empregado Efetivo
	F	Empregado Efetivo

	F	Empregado Efetivo
	F	Empregado Efetivo
	F	Empregado Efetivo
	F	Empregado Efetivo
	F	Empregado Efetivo
	F	Empregado Efetivo
	F	Empregado Efetivo
	F	Empregado Efetivo
	F	Empregado Efetivo
	F	Empregado Efetivo
	F	Empregado Efetivo
	F	Empregado Efetivo
	F	Empregado Efetivo
	F	Empregado Efetivo
	F	Empregado Efetivo
	F	Empregado Efetivo
	F	Empregado Efetivo
	F	Empregado Efetivo
	M	Empregado Efetivo
	F	Empregado Efetivo
	F	Empregado Efetivo
	F	Empregado Efetivo
	F	Empregado Efetivo
	F	Empregado Efetivo
	F	Empregado Efetivo
	F	Empregado Efetivo
	F	Empregado Efetivo
	F	Empregado Efetivo
	F	Empregado Efetivo
	F	Empregado Efetivo
	F	Empregado Efetivo
	F	Empregado Efetivo
	F	Empregado Efetivo
	F	Empregado Efetivo
	F	Empregado Efetivo
	F	Empregado Efetivo
	M	Empregado Efetivo
	F	Empregado Efetivo
	M	Empregado Efetivo
	M	Empregado Efetivo
	F	Empregado Efetivo

	F	Empregado Efetivo
	M	Empregado Efetivo
	F	Empregado Efetivo
	F	Empregado Efetivo
	M	Empregado Efetivo
	M	Empregado Efetivo
	M	Empregado Efetivo
	M	Empregado Efetivo
	F	Empregado Efetivo
	F	Empregado Efetivo
	F	Empregado Efetivo
	F	Empregado Efetivo
	F	Empregado Efetivo
	F	Empregado Efetivo
	F	Empregado Efetivo
	F	Empregado Efetivo
	F	Empregado Efetivo
	F	Empregado Efetivo
	F	Empregado Efetivo
	F	Empregado Efetivo
	M	Empregado Efetivo
	F	Empregado Efetivo
	F	Empregado Efetivo
	F	Empregado Efetivo
	F	Empregado Efetivo
	F	Empregado Efetivo
	M	Empregado Efetivo
	M	Empregado Efetivo
	M	Empregado Efetivo
	M	Empregado Efetivo
	F	Empregado Efetivo
	F	Empregado Efetivo
	F	Empregado Efetivo
	F	Empregado Efetivo
	F	Empregado Efetivo
	M	Empregado Efetivo
	F	Empregado Efetivo

	F	Empregado Efetivo
	M	Empregado Efetivo
	F	Empregado Efetivo
	F	Empregado Efetivo
	F	Empregado Efetivo
	M	Empregado Efetivo
	F	Empregado Efetivo
	M	Empregado Efetivo
	F	Empregado Efetivo
	F	Empregado Efetivo
	F	Empregado Efetivo
	F	Empregado Efetivo
	F	Empregado Efetivo
	F	Empregado Efetivo
	M	Empregado Efetivo
	F	Empregado Efetivo
	F	Empregado Efetivo
	F	Empregado Efetivo
	F	Empregado Efetivo
	F	Empregado Efetivo
	M	Empregado Efetivo
	F	Empregado Efetivo
	F	Empregado Efetivo
	F	Empregado Efetivo
	F	Empregado Efetivo
	F	Empregado Efetivo
	F	Empregado Efetivo
	F	Empregado Efetivo
	F	Empregado Efetivo
	F	Empregado Efetivo
	F	Empregado Efetivo
	F	Empregado Efetivo
	F	Empregado Efetivo
	F	Empregado Efetivo
	F	Empregado Efetivo
	F	Empregado Efetivo
	F	Empregado Efetivo
	F	Empregado Efetivo
	F	Empregado Efetivo
	F	Empregado Efetivo
	F	Empregado Efetivo
	F	Empregado Efetivo
	F	Empregado Efetivo
	F	Empregado Efetivo
	F	Empregado Efetivo
	F	Empregado Efetivo
	F	Empregado Efetivo
	F	Empregado Efetivo

	F	Empregado Efetivo
	M	Empregado Efetivo
	F	Empregado Efetivo
	F	Empregado Efetivo
	F	Empregado Efetivo
	M	Empregado Efetivo
	F	Empregado Efetivo
	M	Empregado Efetivo
	F	Empregado Efetivo
	M	Empregado Efetivo
	F	Empregado Efetivo
	F	Empregado Efetivo
	F	Empregado Efetivo
	F	Empregado Efetivo
	F	Empregado Efetivo
	M	Empregado Efetivo
	F	Empregado Efetivo
	F	Empregado Efetivo
	F	Empregado Efetivo
	F	Empregado Efetivo
	F	Empregado Efetivo
	F	Empregado Efetivo
	F	Empregado Efetivo
	F	Empregado Efetivo
	F	Empregado Efetivo
	F	Empregado Efetivo
	F	Empregado Efetivo
	F	Empregado Efetivo
	F	Empregado Efetivo
	F	Empregado Efetivo
	F	Empregado Efetivo
	F	Empregado Efetivo
	M	Empregado Efetivo
	M	Empregado Efetivo
	F	Empregado Efetivo
	M	Empregado Efetivo
	F	Empregado Efetivo
	F	Empregado Efetivo
	M	Empregado Efetivo
	M	Empregado Efetivo
	F	Empregado Efetivo
	M	Empregado Efetivo
	F	Empregado Efetivo
	F	Empregado Efetivo
	F	Empregado Efetivo
	F	Empregado Efetivo
	F	Empregado Efetivo
	F	Empregado Efetivo

	F	Empregado Efetivo
	F	Empregado Efetivo
	M	Empregado Efetivo
	F	Empregado Efetivo
	F	Empregado Efetivo
	F	Empregado Efetivo
	M	Empregado Efetivo
	F	Empregado Efetivo
	F	Empregado Efetivo
	F	Empregado Efetivo
	F	Empregado Efetivo
	F	Empregado Efetivo
	F	Empregado Efetivo
	F	Empregado Efetivo
	F	Empregado Efetivo
	F	Empregado Efetivo
	F	Empregado Efetivo
	F	Empregado Efetivo
	F	Empregado Efetivo
	M	Empregado Efetivo
	F	Empregado Efetivo
	F	Empregado Efetivo
	M	Empregado Efetivo
	M	Empregado Efetivo
	F	Empregado Efetivo
	F	Empregado Efetivo
	F	Empregado Efetivo
	F	Empregado Efetivo
	M	Empregado Efetivo
	M	Empregado Efetivo
	M	Empregado Efetivo
	F	Empregado Efetivo
	M	Empregado Efetivo
	M	Empregado Efetivo
	M	Empregado Efetivo
	F	Empregado Efetivo
	F	Empregado Efetivo
	F	Empregado Efetivo
	F	Empregado Efetivo
	F	Empregado Efetivo
	F	Empregado Efetivo
	F	Empregado Efetivo
	F	Empregado Efetivo
	F	Empregado Efetivo
	F	Empregado Efetivo
	F	Empregado Efetivo
	M	Empregado Efetivo
	M	Empregado Efetivo

	M	Cedido - RJU
	F	Empregado Efetivo
	M	Empregado Efetivo
	M	Empregado Efetivo
	M	Empregado Efetivo
	F	Empregado Efetivo
	F	Empregado Efetivo
	F	Empregado Efetivo
	F	Empregado Efetivo
	F	Empregado Efetivo
	F	Empregado Efetivo
	F	Empregado Efetivo
	F	Empregado Efetivo
	F	Empregado Efetivo
	F	Empregado Efetivo
	F	Empregado Efetivo
	F	Empregado Efetivo
	F	Empregado Efetivo
	F	Empregado Efetivo
	F	Empregado Efetivo
	F	Empregado Efetivo
	M	Empregado Efetivo
	M	Empregado Efetivo
	M	Empregado Efetivo
	F	Empregado Efetivo
	M	Empregado Efetivo
	M	Empregado Efetivo
	M	Empregado Efetivo
	M	Empregado Efetivo
	M	Empregado Efetivo
	M	Empregado Efetivo
	M	Empregado Efetivo

	M	Empregado Efetivo
	M	Empregado Efetivo
	M	Empregado Efetivo
	M	Empregado Efetivo
	M	Empregado Efetivo
	M	Empregado Efetivo
	M	Empregado Efetivo
	M	Empregado Efetivo
	F	Empregado Efetivo
	F	Empregado Efetivo
	F	Empregado Efetivo
	F	Empregado Efetivo
	M	Empregado Efetivo
	M	Empregado Efetivo
	F	Empregado Efetivo
	F	Empregado Efetivo
	F	Empregado Efetivo
	F	Empregado Efetivo
	F	Empregado Efetivo
	F	Empregado Efetivo
	F	Empregado Efetivo
	F	Empregado Efetivo
	F	Empregado Efetivo
	F	Empregado Efetivo
	M	Empregado Efetivo
	M	Empregado Efetivo
	M	Empregado Efetivo
	M	Empregado Efetivo
	M	Empregado Efetivo
	M	Empregado Efetivo
	M	Empregado Efetivo
	M	Empregado Efetivo
	F	Empregado Efetivo
	F	Empregado Efetivo
	M	Empregado Efetivo
	M	Empregado Efetivo
	M	Empregado Efetivo
	M	Empregado Efetivo
	F	Empregado Efetivo
	F	Empregado Efetivo
	F	Empregado Efetivo
	F	Empregado Efetivo

	F	Empregado Efetivo
	M	Empregado Efetivo
	F	Empregado Efetivo
	F	Empregado Efetivo
	F	Empregado Efetivo
	F	Empregado Efetivo
	F	Empregado Efetivo
	F	Empregado Efetivo
	M	Empregado Efetivo
	F	Empregado Efetivo
	F	Empregado Efetivo
	F	Empregado Efetivo
	F	Empregado Efetivo
	F	Empregado Efetivo
	M	Empregado Efetivo
	F	Empregado Efetivo
	F	Empregado Efetivo
	F	Empregado Efetivo
	F	Empregado Efetivo
	F	Empregado Efetivo
	M	Empregado Efetivo
	F	Empregado Efetivo
	F	Empregado Efetivo
	M	Empregado Efetivo
	F	Empregado Efetivo
	F	Empregado Efetivo
	M	Empregado Efetivo
	M	Empregado Efetivo
	F	Empregado Efetivo
	F	Empregado Efetivo
	M	Empregado Efetivo
	M	Empregado Efetivo
	F	Empregado Efetivo
	F	Empregado Efetivo
	F	Empregado Efetivo
	F	Empregado Efetivo
	F	Empregado Efetivo
	F	Empregado Efetivo
	F	Empregado Efetivo
	F	Empregado Efetivo
	M	Empregado Efetivo
	M	Empregado Efetivo
	F	Empregado Efetivo

	M	Empregado Efetivo
	F	Empregado Efetivo
	M	Empregado Efetivo
	M	Empregado Efetivo
	F	Empregado Efetivo
	F	Empregado Efetivo
	M	Empregado Efetivo
	M	Empregado Efetivo
	M	Empregado Efetivo
	F	Empregado Efetivo
	F	Empregado Efetivo
	M	Empregado Efetivo
	M	Empregado Efetivo
	F	Empregado Efetivo
	F	Empregado Efetivo
	M	Empregado Efetivo
	M	Empregado Efetivo
	M	Empregado Efetivo
	M	Empregado Efetivo
	M	Empregado Efetivo
	F	Empregado Efetivo
	M	Empregado Efetivo
	F	Empregado Efetivo
	M	Empregado Efetivo
	M	Empregado Efetivo
	F	Empregado Efetivo
	F	Empregado Efetivo
	M	Empregado Efetivo
	F	Empregado Efetivo
	M	Empregado Efetivo
	F	Empregado Efetivo
	F	Empregado Efetivo
	M	Empregado Efetivo
	M	Empregado Efetivo
	F	Empregado Efetivo
	F	Empregado Efetivo
	F	Empregado Efetivo
	M	Empregado Efetivo
	F	Empregado Efetivo
	M	Empregado Efetivo
	M	Empregado Efetivo
	F	Empregado Efetivo
	M	Empregado Efetivo

	F	Empregado Efetivo
	F	Empregado Efetivo
	F	Empregado Efetivo
	F	Empregado Efetivo
	M	Empregado Efetivo
	F	Empregado Efetivo
	F	Empregado Efetivo
	F	Empregado Efetivo
	F	Empregado Efetivo
	F	Empregado Efetivo
	F	Empregado Efetivo
	M	Empregado Efetivo
	F	Empregado Efetivo
	M	Empregado Efetivo
	F	Empregado Efetivo
	F	Empregado Efetivo
	F	Empregado Efetivo
	F	Empregado Efetivo
	F	Empregado Efetivo
	F	Empregado Efetivo
	F	Empregado Efetivo
	F	Empregado Efetivo
	F	Empregado Efetivo
	F	Empregado Efetivo
	M	Empregado Efetivo
	M	Empregado Efetivo
	M	Empregado Efetivo
	F	Empregado Efetivo
	F	Empregado Efetivo
	M	Empregado Efetivo
	M	Empregado Efetivo
	M	Empregado Efetivo
	M	Empregado Efetivo
	F	Empregado Efetivo
	F	Empregado Efetivo
	M	Empregado Efetivo

	F	Empregado Efetivo
	M	Empregado Efetivo
	M	Empregado Efetivo
	F	Empregado Efetivo
	F	Empregado Efetivo
	M	Empregado Efetivo
	F	Empregado Efetivo
	F	Empregado Efetivo
	M	Empregado Efetivo
	F	Empregado Efetivo
	M	Empregado Efetivo
	F	Empregado Efetivo
	F	Empregado Efetivo
	F	Empregado Efetivo
	F	Empregado Efetivo
	M	Empregado Efetivo
	F	Empregado Efetivo
	F	Empregado Efetivo
	F	Empregado Efetivo
	M	Empregado Efetivo
	M	Empregado Efetivo
	F	Empregado Efetivo
	F	Empregado Efetivo
	M	Empregado Efetivo
	F	Empregado Efetivo
	F	Empregado Efetivo
	F	Empregado Efetivo
	F	Empregado Efetivo
	F	Empregado Efetivo
	M	Empregado Efetivo
	F	Empregado Efetivo
	M	Empregado Efetivo
	F	Empregado Efetivo
	F	Empregado Efetivo
	F	Empregado Efetivo
	F	Empregado Efetivo
	F	Empregado Efetivo
	F	Empregado Efetivo
	F	Empregado Efetivo
	F	Empregado Efetivo
	F	Empregado Efetivo
	F	Empregado Efetivo
	F	Empregado Efetivo
	F	Empregado Efetivo
	F	Empregado Efetivo
	F	Empregado Efetivo
	F	Empregado Efetivo
	F	Empregado Efetivo

	F	Empregado Efetivo
	F	Empregado Efetivo
	F	Empregado Efetivo
	F	Empregado Efetivo
	F	Empregado Efetivo
	F	Empregado Efetivo
	F	Empregado Efetivo
	F	Empregado Efetivo
	F	Empregado Efetivo
	F	Empregado Efetivo
	F	Empregado Efetivo
	F	Empregado Efetivo
	F	Empregado Efetivo
	F	Empregado Efetivo
	F	Empregado Efetivo
	F	Empregado Efetivo
	F	Empregado Efetivo
	F	Empregado Efetivo
	F	Empregado Efetivo
	F	Empregado Efetivo
	F	Empregado Efetivo
	M	Empregado Efetivo
	F	Empregado Efetivo
	M	Empregado Efetivo
	F	Empregado Efetivo
	F	Empregado Efetivo
	F	Empregado Efetivo
	F	Empregado Efetivo
	F	Empregado Efetivo
	F	Empregado Efetivo
	F	Empregado Efetivo
	F	Empregado Efetivo
	F	Empregado Efetivo
	F	Empregado Efetivo
	F	Empregado Efetivo
	F	Empregado Efetivo
	F	Empregado Efetivo
	F	Empregado Efetivo
	F	Empregado Efetivo
	F	Empregado Efetivo
	F	Empregado Efetivo
	F	Empregado Efetivo
	F	Empregado Efetivo

	F	Empregado Efetivo
	F	Empregado Efetivo
	F	Empregado Efetivo
	F	Empregado Efetivo
	F	Empregado Efetivo
	F	Empregado Efetivo
	F	Empregado Efetivo
	F	Empregado Efetivo
	F	Empregado Efetivo
	M	Empregado Efetivo
	F	Empregado Efetivo
	F	Empregado Efetivo
	M	Empregado Efetivo
	F	Empregado Efetivo
	M	Empregado Efetivo
	F	Empregado Efetivo
	M	Empregado Efetivo
	F	Empregado Efetivo
	F	Empregado Efetivo
	F	Empregado Efetivo
	M	Empregado Efetivo
	F	Empregado Efetivo
	F	Empregado Efetivo
	F	Empregado Efetivo
	F	Empregado Efetivo
	M	Empregado Efetivo
	F	Empregado Efetivo
	F	Empregado Efetivo
	F	Empregado Efetivo
	F	Empregado Efetivo
	F	Empregado Efetivo
	F	Empregado Efetivo
	F	Empregado Efetivo
	F	Empregado Efetivo
	F	Empregado Efetivo
	F	Empregado Efetivo
	F	Empregado Efetivo
	F	Empregado Efetivo
	F	Empregado Efetivo
	F	Empregado Efetivo
	F	Empregado Efetivo
	F	Empregado Efetivo
	F	Empregado Efetivo
	F	Empregado Efetivo
	M	Empregado Efetivo
	F	Empregado Efetivo

	F	Empregado Efetivo
	F	Empregado Efetivo
	F	Empregado Efetivo
	F	Empregado Efetivo
	F	Empregado Efetivo
	F	Empregado Efetivo
	M	Empregado Efetivo
	M	Empregado Efetivo
	F	Empregado Efetivo
	F	Empregado Efetivo
	F	Empregado Efetivo
	F	Empregado Efetivo
	M	Empregado Efetivo
	M	Empregado Efetivo
	M	Empregado Efetivo
	M	Empregado Efetivo
	M	Empregado Efetivo
	M	Empregado Efetivo
	M	Empregado Efetivo
	M	Empregado Efetivo
	M	Empregado Efetivo
	M	Empregado Efetivo
	M	Empregado Efetivo
	F	Empregado Efetivo
	F	Empregado Efetivo
	F	Empregado Efetivo
	F	Empregado Efetivo
	F	Empregado Efetivo
	F	Empregado Efetivo
	F	Empregado Efetivo
	F	Empregado Efetivo
	F	Empregado Efetivo
	F	Empregado Efetivo
	F	Empregado Efetivo
	F	Empregado Efetivo
	M	Empregado Efetivo
	F	Empregado Efetivo
	M	Empregado Efetivo
	F	Empregado Efetivo
	F	Empregado Efetivo
	F	Empregado Efetivo
	F	Empregado Efetivo
	F	Empregado Efetivo
	F	Empregado Efetivo
	F	Empregado Efetivo
	F	Empregado Efetivo
	F	Empregado Efetivo

	F	Empregado Efetivo
	M	Empregado Efetivo
	M	Empregado Efetivo
	M	Empregado Efetivo
	M	Empregado Efetivo
	M	Empregado Efetivo
	M	Empregado Efetivo
	M	Empregado Efetivo
	M	Empregado Efetivo
	M	Empregado Efetivo
	M	Empregado Efetivo
	M	Empregado Efetivo
	M	Cedido - RJU
	F	Empregado Efetivo
	F	Empregado Efetivo
	M	Empregado Efetivo
	F	Empregado Efetivo
	F	Empregado Efetivo
	F	Empregado Efetivo
	M	Empregado Efetivo
	F	Empregado Efetivo
	M	Empregado Efetivo
	M	Empregado Efetivo
	M	Empregado Efetivo
	M	Empregado Efetivo
	M	Empregado Efetivo
	F	Empregado Efetivo
	M	Empregado Efetivo
	M	Empregado Efetivo
	F	Empregado Efetivo
	M	Empregado Efetivo
	F	Empregado Efetivo
	F	Empregado Efetivo
	F	Empregado Efetivo
	F	Empregado Efetivo
	M	Empregado Efetivo
	M	Empregado Efetivo
	F	Empregado Efetivo
	F	Empregado Efetivo
	F	Empregado Efetivo
	M	Empregado Efetivo
	F	Empregado Efetivo

	F	Empregado Efetivo
	F	Empregado Efetivo
	F	Empregado Efetivo
	M	Empregado Efetivo
	F	Empregado Efetivo
	M	Empregado Efetivo
	F	Empregado Efetivo
	M	Empregado Efetivo
	F	Empregado Efetivo
	F	Empregado Efetivo
	F	Empregado Efetivo
	M	Empregado Efetivo
	F	Empregado Efetivo
	F	Empregado Efetivo
	F	Empregado Efetivo
	F	Empregado Efetivo
	M	Empregado Efetivo
	F	Empregado Efetivo
	F	Empregado Efetivo
	F	Empregado Efetivo
	M	Empregado Efetivo
	F	Empregado Efetivo
	F	Empregado Efetivo
	F	Empregado Efetivo
	M	Empregado Efetivo
	F	Empregado Efetivo
	M	Empregado Efetivo
	F	Empregado Efetivo
	F	Empregado Efetivo
	F	Empregado Efetivo
	F	Empregado Efetivo
	F	Empregado Efetivo
	F	Empregado Efetivo
	F	Empregado Efetivo
	F	Empregado Efetivo
	F	Empregado Efetivo
	F	Empregado Efetivo
	F	Empregado Efetivo
	F	Empregado Efetivo
	M	Empregado Efetivo
	M	Empregado Efetivo
	F	Empregado Efetivo
	F	Empregado Efetivo
	M	Empregado Efetivo

	F	Empregado Efetivo
	M	Empregado Efetivo
	M	Empregado Efetivo
	F	Empregado Efetivo
	M	Empregado Efetivo
	F	Empregado Efetivo
	F	Empregado Efetivo
	F	Empregado Efetivo
	F	Empregado Efetivo
	F	Empregado Efetivo
	F	Empregado Efetivo
	F	Empregado Efetivo
	F	Empregado Efetivo
	F	Empregado Efetivo
	F	Empregado Efetivo
	F	Empregado Efetivo
	F	Empregado Efetivo
	F	Empregado Efetivo
	F	Empregado Efetivo
	F	Empregado Efetivo
	F	Empregado Efetivo
	F	Empregado Efetivo
	F	Empregado Efetivo
	F	Empregado Efetivo
	F	Empregado Efetivo
	F	Empregado Efetivo
	F	Empregado Efetivo
	F	Empregado Efetivo
	F	Empregado Efetivo
	F	Empregado Efetivo
	F	Empregado Efetivo
	F	Empregado Efetivo
	F	Empregado Efetivo
	F	Empregado Efetivo
	F	Empregado Efetivo
	F	Empregado Efetivo
	F	Empregado Efetivo
	F	Empregado Efetivo
	F	Empregado Efetivo
	F	Empregado Efetivo
	F	Empregado Efetivo

	F	Empregado Efetivo
	F	Empregado Efetivo
	F	Empregado Efetivo
	F	Empregado Efetivo
	F	Empregado Efetivo
	F	Empregado Efetivo
	F	Empregado Efetivo
	F	Empregado Efetivo
	F	Empregado Efetivo
	F	Empregado Efetivo
	F	Empregado Efetivo
	F	Empregado Efetivo
	F	Empregado Efetivo
	F	Empregado Efetivo
	F	Empregado Efetivo
	F	Empregado Efetivo
	F	Empregado Efetivo
	F	Empregado Efetivo
	F	Empregado Efetivo
	F	Empregado Efetivo
	F	Empregado Efetivo
	F	Empregado Efetivo
	F	Empregado Efetivo
	F	Empregado Efetivo
	F	Empregado Efetivo
	F	Empregado Efetivo
	F	Empregado Efetivo
	F	Empregado Efetivo
	F	Empregado Efetivo
	F	Empregado Efetivo
	F	Empregado Efetivo
	F	Empregado Efetivo
	F	Empregado Efetivo
	F	Empregado Efetivo
	F	Empregado Efetivo
	F	Empregado Efetivo

	F	Empregado Efetivo
	F	Empregado Efetivo
	F	Empregado Efetivo
	F	Empregado Efetivo
	M	Empregado Efetivo
	M	Empregado Efetivo
	M	Empregado Efetivo
	F	Empregado Efetivo
	F	Empregado Efetivo
	F	Empregado Efetivo
	F	Cedido - RJU
	F	Empregado Efetivo
	M	Empregado Efetivo
	F	Empregado Efetivo
	M	Empregado Efetivo
	F	Empregado Efetivo
	F	Empregado Efetivo
	F	Empregado Efetivo
	F	Empregado Efetivo
	F	Empregado Efetivo
	F	Empregado Efetivo
	F	Empregado Efetivo
	F	Empregado Efetivo
	F	Empregado Efetivo
	F	Empregado Efetivo
	M	Empregado Efetivo
	F	Empregado Efetivo
	F	Empregado Efetivo
	F	Empregado Efetivo
	F	Empregado Efetivo
	F	Empregado Efetivo
	F	Empregado Efetivo
	F	Empregado Efetivo

	F	Empregado Efetivo
	F	Empregado Efetivo
	F	Empregado Efetivo
	F	Empregado Efetivo
	F	Empregado Efetivo
	F	Empregado Efetivo
	F	Empregado Efetivo
	F	Empregado Efetivo
	F	Empregado Efetivo
	F	Empregado Efetivo
	F	Empregado Efetivo
	F	Empregado Efetivo
	F	Empregado Efetivo
	F	Empregado Efetivo
	F	Empregado Efetivo
	F	Empregado Efetivo
	F	Empregado Efetivo
	F	Empregado Efetivo
	F	Empregado Efetivo
	F	Empregado Efetivo
	F	Empregado Efetivo
	F	Empregado Efetivo
	F	Empregado Efetivo
	F	Empregado Efetivo
	F	Empregado Efetivo
	F	Empregado Efetivo
	F	Empregado Efetivo
	F	Empregado Efetivo
	F	Empregado Efetivo
	F	Empregado Efetivo
	F	Empregado Efetivo
	F	Empregado Efetivo
	F	Empregado Efetivo
	F	Empregado Efetivo
	F	Empregado Efetivo
	F	Empregado Efetivo
	F	Empregado Efetivo
	F	Empregado Efetivo
	F	Empregado Efetivo

	F	Empregado Efetivo
	F	Empregado Efetivo
	F	Empregado Efetivo
	F	Empregado Efetivo
	F	Empregado Efetivo
	F	Empregado Efetivo
	F	Empregado Efetivo
	F	Empregado Efetivo
	F	Empregado Efetivo
	F	Empregado Efetivo
	F	Empregado Efetivo
	F	Empregado Efetivo
	F	Empregado Efetivo
	F	Empregado Efetivo
	F	Empregado Efetivo
	F	Empregado Efetivo
	F	Empregado Efetivo
	F	Empregado Efetivo
	F	Empregado Efetivo
	F	Empregado Efetivo
	M	Empregado Efetivo
	F	Empregado Efetivo
	F	Empregado Efetivo
	F	Empregado Efetivo
	F	Empregado Efetivo
	F	Empregado Efetivo
	F	Empregado Efetivo
	F	Empregado Efetivo
	M	Empregado Efetivo
	F	Empregado Efetivo
	F	Empregado Efetivo
	F	Empregado Efetivo
	M	Empregado Efetivo
	F	Empregado Efetivo
	M	Empregado Efetivo
	F	Empregado Efetivo
	F	Empregado Efetivo
	F	Empregado Efetivo
	F	Empregado Efetivo

	F	Empregado Efetivo
	M	Empregado Efetivo
	F	Empregado Efetivo
	M	Empregado Efetivo
	F	Empregado Efetivo
	F	Empregado Efetivo
	F	Empregado Efetivo
	F	Empregado Efetivo
	F	Empregado Efetivo
	M	Empregado Efetivo
	F	Empregado Efetivo
	F	Empregado Efetivo
	F	Empregado Efetivo
	F	Empregado Efetivo
	M	Empregado Efetivo
	M	Empregado Efetivo
	F	Empregado Efetivo
	F	Empregado Efetivo
	M	Empregado Efetivo
	F	Empregado Efetivo
	F	Empregado Efetivo
	M	Empregado Efetivo
	F	Empregado Efetivo
	F	Empregado Efetivo
	F	Empregado Efetivo
	F	Empregado Efetivo
	F	Empregado Efetivo
	M	Empregado Efetivo
	F	Empregado Efetivo
	F	Empregado Efetivo
	F	Empregado Efetivo
	F	Empregado Efetivo
	F	Empregado Efetivo
	F	Empregado Efetivo
	F	Empregado Efetivo
	F	Empregado Efetivo

	F	Empregado Efetivo
	F	Empregado Efetivo
	F	Empregado Efetivo
	F	Empregado Efetivo
	F	Empregado Efetivo
	M	Empregado Efetivo
	F	Empregado Efetivo
	F	Empregado Efetivo
	F	Empregado Efetivo
	F	Empregado Efetivo
	F	Empregado Efetivo
	F	Empregado Efetivo
	M	Empregado Efetivo
	F	Empregado Efetivo
	F	Empregado Efetivo
	M	Empregado Efetivo
	M	Empregado Efetivo
	M	Empregado Efetivo
	M	Empregado Efetivo
	M	Empregado Efetivo
	M	Empregado Efetivo
	F	Empregado Efetivo
	F	Empregado Efetivo
	F	Empregado Efetivo
	F	Empregado Efetivo
	F	Empregado Efetivo
	F	Empregado Efetivo
	F	Empregado Efetivo
	M	Empregado Efetivo
	F	Empregado Efetivo
	F	Empregado Efetivo
	F	Empregado Efetivo
	F	Empregado Efetivo
	M	Empregado Efetivo
	F	Empregado Efetivo
	F	Empregado Efetivo
	F	Empregado Efetivo
	F	Empregado Efetivo
	M	Empregado Efetivo

	F	Empregado Efetivo
	F	Empregado Efetivo
	F	Empregado Efetivo
	F	Empregado Efetivo
	F	Empregado Efetivo
	M	Empregado Efetivo
	F	Empregado Efetivo
	M	Empregado Efetivo
	M	Empregado Efetivo
	F	Empregado Efetivo
	F	Empregado Efetivo
	F	Empregado Efetivo
	M	Empregado Efetivo
	M	Empregado Efetivo
	F	Empregado Efetivo
	M	Empregado Efetivo
	F	Empregado Efetivo
	F	Empregado Efetivo
	F	Empregado Efetivo
	F	Empregado Efetivo
	M	Empregado Efetivo
	F	Empregado Efetivo
	F	Empregado Efetivo
	F	Empregado Efetivo
	F	Empregado Efetivo
	F	Empregado Efetivo
	M	Empregado Efetivo
	M	Empregado Efetivo
	M	Empregado Efetivo
	F	Empregado Efetivo
	F	Empregado Efetivo
	F	Empregado Efetivo
	F	Empregado Efetivo
	F	Empregado Efetivo
	F	Empregado Efetivo
	M	Empregado Efetivo

	F	Empregado Efetivo
	F	Empregado Efetivo
	M	Empregado Efetivo
	F	Empregado Efetivo
	F	Empregado Efetivo
	M	Empregado Efetivo
	F	Empregado Efetivo
	F	Empregado Efetivo
	F	Empregado Efetivo
	F	Empregado Efetivo
	F	Empregado Efetivo
	F	Empregado Efetivo
	M	Empregado Efetivo
	F	Empregado Efetivo
	F	Empregado Efetivo
	M	Empregado Efetivo
	F	Empregado Efetivo
	F	Empregado Efetivo
	F	Empregado Efetivo
	F	Empregado Efetivo
	F	Empregado Efetivo
	F	Empregado Efetivo
	M	Empregado Efetivo
	F	Empregado Efetivo
	M	Empregado Efetivo
	F	Empregado Efetivo
	M	Empregado Efetivo
	F	Empregado Efetivo
	M	Empregado Efetivo
	M	Empregado Efetivo
	F	Empregado Efetivo
	M	Empregado Efetivo
	M	Empregado Efetivo
	M	Empregado Efetivo
	M	Empregado Efetivo
	F	Empregado Efetivo
	M	Empregado Efetivo
	M	Empregado Efetivo
	F	Empregado Efetivo
	F	Empregado Efetivo
	M	Empregado Efetivo
	M	Empregado Efetivo
	M	Empregado Efetivo
	F	Empregado Efetivo
	F	Empregado Efetivo
	F	Empregado Efetivo
	F	Empregado Efetivo
	M	Empregado Efetivo

	F	Empregado Efetivo
	F	Empregado Efetivo
	F	Empregado Efetivo
	F	Empregado Efetivo
	F	Empregado Efetivo
	F	Empregado Efetivo
	F	Empregado Efetivo
	F	Empregado Efetivo
	F	Empregado Efetivo
	F	Empregado Efetivo
	F	Empregado Efetivo
	F	Empregado Efetivo
	F	Empregado Efetivo
	F	Empregado Efetivo
	F	Empregado Efetivo
	F	Empregado Efetivo
	F	Empregado Efetivo
	F	Empregado Efetivo
	F	Empregado Efetivo
	F	Empregado Efetivo
	F	Empregado Efetivo
	F	Empregado Efetivo
	F	Empregado Efetivo
	M	Empregado Efetivo
	M	Empregado Efetivo
	M	Empregado Efetivo
	M	Empregado Efetivo
	M	Empregado Efetivo
	M	Empregado Efetivo
	F	Empregado Efetivo
	F	Empregado Efetivo
	M	Empregado Efetivo
	F	Empregado Efetivo
	F	Empregado Efetivo
	F	Empregado Efetivo
	F	Empregado Efetivo
	F	Empregado Efetivo
	F	Empregado Efetivo
	F	Empregado Efetivo
	F	Empregado Efetivo
	F	Empregado Efetivo
	F	Empregado Efetivo
	F	Empregado Efetivo
	F	Empregado Efetivo

	M	Empregado Efetivo
	F	Cedido - RJU
	M	Empregado Efetivo
	F	Empregado Efetivo
	F	Empregado Efetivo
	M	Empregado Efetivo
	M	Empregado Efetivo
	F	Empregado Efetivo
	F	Empregado Efetivo
	F	Empregado Efetivo
	F	Empregado Efetivo
	F	Empregado Efetivo
	F	Empregado Efetivo
	F	Empregado Efetivo
	F	Empregado Efetivo
	F	Empregado Efetivo
	F	Empregado Efetivo
	F	Empregado Efetivo
	F	Empregado Efetivo
	F	Empregado Efetivo
	F	Empregado Efetivo
	F	Empregado Efetivo
	F	Empregado Efetivo
	F	Empregado Efetivo
	F	Empregado Efetivo
	F	Empregado Efetivo
	F	Empregado Efetivo
	F	Empregado Efetivo
	F	Empregado Efetivo
	F	Empregado Efetivo
	F	Empregado Efetivo
	F	Empregado Efetivo
	F	Empregado Efetivo
	F	Empregado Efetivo
	F	Empregado Efetivo
	F	Empregado Efetivo
	F	Empregado Efetivo
	F	Empregado Efetivo
	F	Empregado Efetivo
	F	Empregado Efetivo
	F	Empregado Efetivo
	F	Empregado Efetivo
	F	Empregado Efetivo
	M	Empregado Efetivo

	M	Empregado Efetivo
	M	Empregado Efetivo
	M	Empregado Efetivo
	F	Empregado Efetivo
	F	Empregado Efetivo
	F	Empregado Efetivo
	F	Empregado Efetivo
	F	Empregado Efetivo
	F	Empregado Efetivo
	F	Empregado Efetivo
	F	Empregado Efetivo
	F	Empregado Efetivo
	F	Empregado Efetivo
	F	Empregado Efetivo
	F	Empregado Efetivo
	F	Empregado Efetivo
	F	Empregado Efetivo
	F	Empregado Efetivo
	F	Empregado Efetivo
	F	Empregado Efetivo
	F	Empregado Efetivo
	F	Empregado Efetivo
	F	Empregado Efetivo
	F	Empregado Efetivo
	F	Empregado Efetivo
	F	Empregado Efetivo
	F	Empregado Efetivo
	F	Empregado Efetivo
	F	Empregado Efetivo
	F	Empregado Efetivo
	F	Empregado Efetivo
	F	Empregado Efetivo
	F	Empregado Efetivo
	F	Empregado Efetivo
	F	Empregado Efetivo
	F	Empregado Efetivo
	F	Empregado Efetivo
	F	Empregado Efetivo
	F	Empregado Efetivo
	F	Empregado Efetivo
	F	Empregado Efetivo
	F	Empregado Efetivo
	F	Empregado Efetivo
	F	Empregado Efetivo
	F	Empregado Efetivo
	M	Empregado Efetivo
	M	Empregado Efetivo
	F	Empregado Efetivo

	F	Empregado Efetivo
	F	Empregado Efetivo
	M	Empregado Efetivo
	F	Empregado Efetivo
	M	Empregado Efetivo
	F	Empregado Efetivo
	F	Empregado Efetivo
	M	Empregado Efetivo
	F	Empregado Efetivo
	F	Empregado Efetivo
	F	Empregado Efetivo
	M	Empregado Efetivo
	F	Empregado Efetivo
	F	Empregado Efetivo
	F	Empregado Efetivo
	F	Empregado Efetivo
	M	Empregado Efetivo
	M	Empregado Efetivo
	F	Empregado Efetivo
	F	Empregado Efetivo
	F	Empregado Efetivo
	F	Empregado Efetivo
	F	Empregado Efetivo
	F	Empregado Efetivo
	F	Empregado Efetivo
	F	Empregado Efetivo
	F	Empregado Efetivo
	F	Empregado Efetivo
	F	Empregado Efetivo
	F	Empregado Efetivo
	M	Empregado Efetivo
	F	Empregado Efetivo
	F	Empregado Efetivo
	F	Empregado Efetivo
	F	Empregado Efetivo
	F	Empregado Efetivo
	F	Empregado Efetivo
	F	Empregado Efetivo
	F	Empregado Efetivo
	F	Empregado Efetivo
	F	Empregado Efetivo
	F	Empregado Efetivo
	F	Empregado Efetivo
	F	Empregado Efetivo
	F	Empregado Efetivo
	M	Empregado Efetivo
	F	Empregado Efetivo
	F	Empregado Efetivo

	F	Empregado Efetivo
	F	Empregado Efetivo
	F	Empregado Efetivo
	M	Empregado Efetivo
	F	Empregado Efetivo
	M	Empregado Efetivo
	F	Empregado Efetivo
	F	Empregado Efetivo
	F	Empregado Efetivo
	F	Empregado Efetivo
	F	Empregado Efetivo
	M	Empregado Efetivo
	F	Empregado Efetivo
	F	Empregado Efetivo
	F	Empregado Efetivo
	M	Empregado Efetivo
	F	Empregado Efetivo
	M	Empregado Efetivo
	F	Empregado Efetivo
	F	Empregado Efetivo
	F	Empregado Efetivo
	F	Empregado Efetivo
	F	Empregado Efetivo
	M	Empregado Efetivo
	F	Empregado Efetivo
	F	Empregado Efetivo
	F	Empregado Efetivo
	M	Empregado Efetivo
	F	Empregado Efetivo
	F	Empregado Efetivo
	F	Empregado Efetivo
	F	Empregado Efetivo
	F	Empregado Efetivo
	F	Empregado Efetivo
	F	Empregado Efetivo
	F	Empregado Efetivo
	F	Empregado Efetivo
	F	Empregado Efetivo
	F	Empregado Efetivo
	F	Empregado Efetivo
	F	Empregado Efetivo
	F	Empregado Efetivo
	F	Empregado Efetivo
	F	Empregado Efetivo
	F	Empregado Efetivo
	F	Empregado Efetivo
	M	Empregado Efetivo
	F	Empregado Efetivo

	M	Empregado Efetivo
	F	Empregado Efetivo
	F	Empregado Efetivo
	F	Empregado Efetivo
	M	Empregado Efetivo
	F	Empregado Efetivo
	F	Empregado Efetivo
	F	Empregado Efetivo
	F	Empregado Efetivo
	F	Empregado Efetivo
	F	Empregado Efetivo
	F	Empregado Efetivo
	M	Empregado Efetivo
	F	Empregado Efetivo
	F	Empregado Efetivo
	F	Empregado Efetivo
	F	Empregado Efetivo
	M	Empregado Efetivo
	F	Empregado Efetivo
	F	Empregado Efetivo
	M	Empregado Efetivo
	F	Empregado Efetivo
	F	Empregado Efetivo
	M	Empregado Efetivo
	F	Empregado Efetivo
	F	Empregado Efetivo
	F	Empregado Efetivo
	F	Empregado Efetivo
	F	Empregado Efetivo
	F	Empregado Efetivo
	F	Empregado Efetivo
	F	Empregado Efetivo
	F	Empregado Efetivo
	F	Empregado Efetivo
	F	Empregado Efetivo
	F	Empregado Efetivo
	F	Empregado Efetivo
	F	Empregado Efetivo
	M	Empregado Efetivo
	F	Empregado Efetivo
	F	Empregado Efetivo
	F	Empregado Efetivo
	F	Empregado Efetivo
	F	Empregado Efetivo
	F	Empregado Efetivo
	F	Empregado Efetivo
	F	Empregado Efetivo
	F	Empregado Efetivo

	F	Empregado Efetivo
	F	Empregado Efetivo
	F	Empregado Efetivo
	F	Empregado Efetivo
	F	Empregado Efetivo
	F	Empregado Efetivo
	F	Empregado Efetivo
	F	Empregado Efetivo
	F	Empregado Efetivo
	F	Empregado Efetivo
	F	Empregado Efetivo
	M	Empregado Efetivo
	M	Empregado Efetivo
	M	Empregado Efetivo
	F	Empregado Efetivo
	M	Empregado Efetivo
	F	Empregado Efetivo
	F	Empregado Efetivo
	F	Empregado Efetivo
	F	Empregado Efetivo
	M	Empregado Efetivo
	F	Empregado Efetivo
	F	Empregado Efetivo
	F	Empregado Efetivo
	F	Empregado Efetivo
	F	Empregado Efetivo
	M	Empregado Efetivo
	M	Empregado Efetivo
	F	Empregado Efetivo
	F	Empregado Efetivo
	F	Empregado Efetivo
	F	Empregado Efetivo
	M	Empregado Efetivo
	F	Empregado Efetivo
	M	Empregado Efetivo
	F	Empregado Efetivo
	F	Empregado Efetivo
	M	Empregado Efetivo
	F	Empregado Efetivo
	F	Empregado Efetivo
	F	Empregado Efetivo
	M	Empregado Efetivo
	F	Empregado Efetivo
	M	Empregado Efetivo
	F	Empregado Efetivo
	F	Empregado Efetivo
	F	Empregado Efetivo
	F	Empregado Efetivo

	F	Empregado Efetivo
	F	Empregado Efetivo
	M	Empregado Efetivo
	F	Empregado Efetivo
	F	Empregado Efetivo
	F	Empregado Efetivo
	M	Empregado Efetivo
	F	Empregado Efetivo
	M	Empregado Efetivo
	F	Empregado Efetivo
	F	Empregado Efetivo
	F	Empregado Efetivo
	F	Empregado Efetivo
	F	Empregado Efetivo
	M	Empregado Efetivo
	F	Empregado Efetivo
	F	Empregado Efetivo
	F	Empregado Efetivo
	F	Empregado Efetivo
	F	Empregado Efetivo
	F	Empregado Efetivo
	F	Empregado Efetivo
	F	Empregado Efetivo
	F	Empregado Efetivo
	F	Empregado Efetivo
	F	Empregado Efetivo
	F	Empregado Efetivo
	M	Empregado Efetivo
	M	Empregado Efetivo
	F	Empregado Efetivo
	M	Empregado Efetivo
	M	Empregado Efetivo
	M	Empregado Efetivo
	F	Empregado Efetivo
	M	Empregado Efetivo
	F	Empregado Efetivo
	M	Empregado Efetivo
	F	Empregado Efetivo
	F	Empregado Efetivo

	F	Empregado Efetivo
	F	Empregado Efetivo
	F	Empregado Efetivo
	F	Empregado Efetivo
	F	Empregado Efetivo
	F	Empregado Efetivo
	F	Empregado Efetivo
	F	Empregado Efetivo
	F	Empregado Efetivo
	F	Empregado Efetivo
	F	Empregado Efetivo
	F	Empregado Efetivo
	F	Empregado Efetivo
	F	Empregado Efetivo
	F	Empregado Efetivo
	M	Empregado Efetivo
	F	Empregado Efetivo
	F	Empregado Efetivo
	F	Empregado Efetivo
	F	Empregado Efetivo
	F	Empregado Efetivo
	F	Empregado Efetivo
	M	Empregado Efetivo
	F	Empregado Efetivo
	F	Empregado Efetivo
	F	Empregado Efetivo
	F	Empregado Efetivo
	F	Empregado Efetivo
	M	Empregado Efetivo
	F	Empregado Efetivo
	F	Empregado Efetivo
	M	Empregado Efetivo
	M	Empregado Efetivo
	M	Empregado Efetivo
	F	Empregado Efetivo
	F	Empregado Efetivo
	F	Empregado Efetivo
	F	Empregado Efetivo
	M	Empregado Efetivo
	F	Empregado Efetivo
	F	Empregado Efetivo
	F	Empregado Efetivo
	F	Empregado Efetivo
	F	Empregado Efetivo
	M	Empregado Efetivo
	F	Empregado Efetivo
	F	Empregado Efetivo
	F	Empregado Efetivo

	M	Empregado Efetivo
	F	Empregado Efetivo
	F	Empregado Efetivo
	F	Empregado Efetivo
	F	Empregado Efetivo
	F	Empregado Efetivo
	F	Empregado Efetivo
	F	Empregado Efetivo
	F	Empregado Efetivo
	F	Empregado Efetivo
	F	Empregado Efetivo
	F	Empregado Efetivo
	F	Empregado Efetivo
	F	Empregado Efetivo
	F	Empregado Efetivo
	F	Empregado Efetivo
	F	Empregado Efetivo
	F	Empregado Efetivo
	F	Empregado Efetivo
	F	Empregado Efetivo
	F	Empregado Efetivo
	F	Empregado Efetivo
	F	Empregado Efetivo
	F	Empregado Efetivo
	F	Empregado Efetivo
	F	Empregado Efetivo
	F	Empregado Efetivo
	F	Empregado Efetivo
	F	Empregado Efetivo
	F	Empregado Efetivo
	F	Empregado Efetivo
	F	Empregado Efetivo
	M	Empregado Efetivo
	F	Empregado Efetivo
	F	Empregado Efetivo
	F	Empregado Efetivo
	F	Empregado Efetivo
	F	Empregado Efetivo
	F	Empregado Efetivo
	F	Empregado Efetivo
	F	Empregado Efetivo
	F	Empregado Efetivo
	M	Empregado Efetivo
	F	Empregado Efetivo
	F	Empregado Efetivo
	M	Empregado Efetivo
	F	Empregado Efetivo
	F	Empregado Efetivo

	M	Empregado Efetivo
	F	Empregado Efetivo
	F	Empregado Efetivo
	F	Empregado Efetivo
	F	Empregado Efetivo
	F	Empregado Efetivo
	F	Empregado Efetivo
	F	Empregado Efetivo
	F	Empregado Efetivo
	F	Empregado Efetivo
	F	Empregado Efetivo
	F	Empregado Efetivo
	F	Empregado Efetivo
	F	Empregado Efetivo
	F	Empregado Efetivo
	F	Empregado Efetivo
	F	Empregado Efetivo
	F	Empregado Efetivo
	M	Empregado Efetivo
	F	Empregado Efetivo
	F	Empregado Efetivo
	F	Empregado Efetivo
	F	Empregado Efetivo
	F	Empregado Efetivo
	F	Empregado Efetivo
	F	Empregado Efetivo
	F	Empregado Efetivo
	F	Empregado Efetivo
	F	Empregado Efetivo
	F	Empregado Efetivo
	F	Empregado Efetivo
	M	Empregado Efetivo
	F	Empregado Efetivo
	M	Empregado Efetivo
	F	Empregado Efetivo
	F	Empregado Efetivo
	F	Empregado Efetivo
	F	Empregado Efetivo
	F	Empregado Efetivo
	F	Empregado Efetivo
	M	Empregado Efetivo
	F	Empregado Efetivo
	F	Empregado Efetivo
	M	Empregado Efetivo
	M	Empregado Efetivo
	F	Empregado Efetivo
	F	Empregado Efetivo
	F	Empregado Efetivo

	F	Empregado Efetivo
	F	Empregado Efetivo
	M	Empregado Efetivo
	F	Empregado Efetivo
	F	Empregado Efetivo
	F	Empregado Efetivo
	F	Empregado Efetivo
	F	Empregado Efetivo
	F	Empregado Efetivo
	F	Empregado Efetivo
	M	Empregado Efetivo
	F	Empregado Efetivo
	F	Empregado Efetivo
	M	Empregado Efetivo
	F	Empregado Efetivo
	F	Empregado Efetivo
	F	Empregado Efetivo
	F	Empregado Efetivo
	M	Empregado Efetivo
	M	Empregado Efetivo
	M	Empregado Efetivo
	M	Empregado Efetivo
	F	Empregado Efetivo
	M	Empregado Efetivo
	F	Empregado Efetivo
	F	Empregado Efetivo
	F	Empregado Efetivo
	F	Empregado Efetivo
	F	Empregado Efetivo
	F	Empregado Efetivo
	F	Empregado Efetivo
	F	Empregado Efetivo
	F	Empregado Efetivo
	F	Empregado Efetivo
	F	Empregado Efetivo
	F	Empregado Efetivo
	F	Empregado Efetivo
	F	Empregado Efetivo
	F	Empregado Efetivo
	F	Empregado Efetivo
	F	Empregado Efetivo
	F	Empregado Efetivo
	F	Empregado Efetivo
	M	Empregado Efetivo
	F	Empregado Efetivo
	F	Empregado Efetivo
	F	Empregado Efetivo
	F	Empregado Efetivo

	F	Empregado Efetivo
	F	Empregado Efetivo
	F	Empregado Efetivo
	F	Empregado Efetivo
	F	Empregado Efetivo
	F	Empregado Efetivo
	F	Empregado Efetivo
	F	Empregado Efetivo
	F	Empregado Efetivo
	F	Empregado Efetivo
	F	Empregado Efetivo
	F	Empregado Efetivo
	F	Empregado Efetivo
	F	Empregado Efetivo
	F	Empregado Efetivo
	F	Empregado Efetivo
	F	Empregado Efetivo
	M	Empregado Efetivo
	F	Empregado Efetivo
	F	Empregado Efetivo
	F	Empregado Efetivo
	F	Empregado Efetivo
	F	Empregado Efetivo
	M	Empregado Efetivo
	F	Empregado Efetivo
	F	Empregado Efetivo
	F	Empregado Efetivo
	M	Empregado Efetivo
	F	Empregado Efetivo
	F	Empregado Efetivo
	F	Empregado Efetivo
	M	Empregado Efetivo
	F	Empregado Efetivo
	F	Empregado Efetivo
	F	Empregado Efetivo
	F	Empregado Efetivo
	F	Empregado Efetivo
	F	Empregado Efetivo
	M	Empregado Efetivo
	F	Empregado Efetivo
	M	Empregado Efetivo
	F	Empregado Efetivo
	F	Empregado Efetivo
	F	Empregado Efetivo
	F	Empregado Efetivo
	F	Empregado Efetivo
	F	Empregado Efetivo
	F	Empregado Efetivo

	M	Empregado Efetivo
	F	Empregado Efetivo
	F	Empregado Efetivo
	F	Empregado Efetivo
	F	Empregado Efetivo
	F	Empregado Efetivo
	F	Empregado Efetivo
	M	Empregado Efetivo
	F	Empregado Efetivo
	F	Empregado Efetivo
	F	Empregado Efetivo
	M	Empregado Efetivo
	F	Empregado Efetivo
	F	Empregado Efetivo
	F	Empregado Efetivo
	M	Empregado Efetivo
	F	Empregado Efetivo
	F	Empregado Efetivo
	F	Empregado Efetivo
	F	Empregado Efetivo
	M	Empregado Efetivo
	F	Empregado Efetivo
	M	Empregado Efetivo
	F	Empregado Efetivo
	F	Empregado Efetivo
	F	Empregado Efetivo
	F	Empregado Efetivo
	M	Empregado Efetivo
	F	Empregado Efetivo
	F	Empregado Efetivo
	F	Empregado Efetivo
	M	Empregado Efetivo
	F	Empregado Efetivo
	F	Empregado Efetivo
	M	Empregado Efetivo
	F	Empregado Efetivo
	F	Empregado Efetivo
	F	Empregado Efetivo
	F	Empregado Efetivo
	F	Empregado Efetivo
	F	Empregado Efetivo
	M	Empregado Efetivo
	F	Empregado Efetivo
	F	Empregado Efetivo
	F	Empregado Efetivo
	F	Empregado Efetivo
	M	Empregado Efetivo
	F	Empregado Efetivo

	F	Empregado Efetivo
	F	Empregado Efetivo
	M	Empregado Efetivo
	F	Empregado Efetivo
	M	Empregado Efetivo
	F	Empregado Efetivo
	F	Empregado Efetivo
	F	Empregado Efetivo
	F	Empregado Efetivo
	F	Empregado Efetivo
	F	Empregado Efetivo
	F	Empregado Efetivo
	F	Empregado Efetivo
	F	Empregado Efetivo
	F	Empregado Efetivo
	F	Empregado Efetivo
	F	Empregado Efetivo
	F	Empregado Efetivo
	F	Empregado Efetivo
	F	Empregado Efetivo
	F	Empregado Efetivo
	F	Empregado Efetivo
	F	Empregado Efetivo
	F	Empregado Efetivo
	F	Empregado Efetivo
	F	Empregado Efetivo
	F	Empregado Efetivo
	F	Empregado Efetivo
	M	Empregado Efetivo
	F	Empregado Efetivo
	F	Empregado Efetivo
	M	Empregado Efetivo
	M	Empregado Efetivo
	F	Empregado Efetivo
	F	Empregado Efetivo
	M	Empregado Efetivo
	F	Empregado Efetivo
	F	Empregado Efetivo
	F	Empregado Efetivo
	F	Empregado Efetivo
	F	Empregado Efetivo
	F	Empregado Efetivo
	F	Empregado Efetivo
	F	Empregado Efetivo
	F	Empregado Efetivo
	F	Empregado Efetivo
	F	Empregado Efetivo
	F	Empregado Efetivo

	F	Empregado Efetivo
	F	Empregado Efetivo
	F	Empregado Efetivo
	F	Empregado Efetivo
	F	Empregado Efetivo
	F	Empregado Efetivo
	F	Empregado Efetivo
	M	Empregado Efetivo
	F	Empregado Efetivo
	F	Empregado Efetivo
	F	Empregado Efetivo
	F	Empregado Efetivo
	F	Empregado Efetivo
	F	Empregado Efetivo
	F	Empregado Efetivo
	F	Empregado Efetivo
	M	Empregado Efetivo
	F	Empregado Efetivo
	F	Empregado Efetivo
	F	Empregado Efetivo
	F	Empregado Efetivo
	F	Empregado Efetivo
	F	Empregado Efetivo
	F	Empregado Efetivo
	F	Empregado Efetivo
	F	Empregado Efetivo
	F	Empregado Efetivo
	M	Empregado Efetivo
	F	Empregado Efetivo
	M	Empregado Efetivo
	F	Empregado Efetivo
	F	Empregado Efetivo
	F	Empregado Efetivo
	F	Empregado Efetivo
	F	Empregado Efetivo
	F	Empregado Efetivo
	F	Empregado Efetivo

	F	Empregado Efetivo
	F	Empregado Efetivo
	F	Empregado Efetivo
	F	Empregado Efetivo
	F	Empregado Efetivo
	F	Empregado Efetivo
	F	Empregado Efetivo
	F	Empregado Efetivo
	F	Empregado Efetivo
	F	Empregado Efetivo
	F	Empregado Efetivo
	F	Empregado Efetivo
	F	Empregado Efetivo
	F	Empregado Efetivo
	F	Empregado Efetivo
	F	Empregado Efetivo
	F	Empregado Efetivo
	F	Empregado Efetivo
	F	Empregado Efetivo
	F	Empregado Efetivo
	M	Empregado Efetivo
	F	Empregado Efetivo
	M	Empregado Efetivo
	F	Empregado Efetivo
	M	Empregado Efetivo
	F	Empregado Efetivo
	M	Empregado Efetivo
	F	Empregado Efetivo
	F	Empregado Efetivo
	F	Empregado Efetivo
	F	Empregado Efetivo
	F	Empregado Efetivo

	F	Empregado Efetivo
	F	Empregado Efetivo
	F	Empregado Efetivo
	F	Empregado Efetivo
	M	Empregado Efetivo
	F	Empregado Efetivo
	F	Empregado Efetivo
	F	Empregado Efetivo
	F	Empregado Efetivo
	F	Empregado Efetivo
	F	Empregado Efetivo
	F	Empregado Efetivo
	F	Empregado Efetivo
	F	Empregado Efetivo
	F	Empregado Efetivo
	F	Empregado Efetivo
	F	Empregado Efetivo
	F	Empregado Efetivo
	F	Empregado Efetivo
	F	Empregado Efetivo
	F	Empregado Efetivo
	F	Empregado Efetivo
	F	Empregado Efetivo
	F	Empregado Efetivo
	M	Empregado Efetivo
	F	Empregado Efetivo
	F	Empregado Efetivo
	F	Empregado Efetivo
	F	Empregado Efetivo
	F	Empregado Efetivo
	F	Empregado Efetivo
	M	Empregado Efetivo

	F	Empregado Efetivo
	F	Empregado Efetivo
	M	Empregado Efetivo
	F	Empregado Efetivo
	F	Empregado Efetivo
	F	Empregado Efetivo
	F	Empregado Efetivo
	F	Empregado Efetivo
	F	Empregado Efetivo
	F	Empregado Efetivo
	F	Empregado Efetivo
	F	Empregado Efetivo
	F	Empregado Efetivo
	M	Empregado Efetivo
	F	Empregado Efetivo
	F	Empregado Efetivo
	F	Empregado Efetivo
	M	Empregado Efetivo
	F	Empregado Efetivo
	M	Empregado Efetivo
	F	Empregado Efetivo
	F	Empregado Efetivo
	F	Empregado Efetivo
	F	Empregado Efetivo
	F	Empregado Efetivo
	F	Empregado Efetivo
	F	Empregado Efetivo
	F	Empregado Efetivo
	F	Empregado Efetivo
	M	Empregado Efetivo
	F	Empregado Efetivo
	F	Empregado Efetivo

	F	Empregado Efetivo
	F	Empregado Efetivo
	M	Empregado Efetivo
	M	Empregado Efetivo
	F	Empregado Efetivo
	F	Empregado Efetivo
	F	Empregado Efetivo
	F	Empregado Efetivo
	F	Empregado Efetivo
	F	Empregado Efetivo
	F	Empregado Efetivo
	F	Empregado Efetivo
	M	Empregado Efetivo
	F	Empregado Efetivo
	F	Empregado Efetivo
	F	Empregado Efetivo
	F	Empregado Efetivo
	F	Empregado Efetivo
	F	Empregado Efetivo
	F	Empregado Efetivo
	F	Empregado Efetivo
	F	Empregado Efetivo
	F	Empregado Efetivo
	F	Empregado Efetivo
	F	Empregado Efetivo
	F	Empregado Efetivo
	F	Empregado Efetivo
	F	Empregado Efetivo
	F	Empregado Efetivo
	F	Empregado Efetivo
	F	Empregado Efetivo
	F	Empregado Efetivo

	F	Empregado Efetivo
	F	Empregado Efetivo
	F	Empregado Efetivo
	F	Empregado Efetivo
	F	Empregado Efetivo
	F	Empregado Efetivo
	F	Empregado Efetivo
	F	Empregado Efetivo
	F	Empregado Efetivo
	F	Empregado Efetivo
	F	Empregado Efetivo
	F	Empregado Efetivo
	M	Empregado Efetivo
	F	Empregado Efetivo
	F	Empregado Efetivo
	F	Empregado Efetivo
	M	Empregado Efetivo
	F	Empregado Efetivo
	F	Empregado Efetivo
	F	Empregado Efetivo
	F	Empregado Efetivo
	M	Empregado Efetivo
	F	Empregado Efetivo
	F	Empregado Efetivo
	F	Empregado Efetivo
	M	Empregado Efetivo
	F	Empregado Efetivo
	M	Empregado Efetivo
	M	Empregado Efetivo
	F	Empregado Efetivo
	F	Empregado Efetivo
	M	Empregado Efetivo
	M	Empregado Efetivo
	F	Empregado Efetivo
	F	Empregado Efetivo
	F	Empregado Efetivo

	F	Empregado Efetivo
	F	Empregado Efetivo
	M	Empregado Efetivo
	F	Empregado Efetivo
	M	Empregado Efetivo
	F	Empregado Efetivo
	M	Empregado Efetivo
	F	Empregado Efetivo
	M	Empregado Efetivo
	M	Empregado Efetivo
	F	Empregado Efetivo
	F	Empregado Efetivo
	M	Empregado Efetivo
	F	Empregado Efetivo
	F	Empregado Efetivo
	M	Empregado Efetivo
	M	Empregado Efetivo
	F	Empregado Efetivo
	F	Empregado Efetivo
	F	Empregado Efetivo
	F	Empregado Efetivo
	F	Empregado Efetivo
	F	Empregado Efetivo
	F	Empregado Efetivo
	F	Empregado Efetivo
	M	Empregado Efetivo
	F	Empregado Efetivo
	F	Empregado Efetivo
	F	Empregado Efetivo
	F	Empregado Efetivo
	F	Empregado Efetivo
	F	Empregado Efetivo
	M	Empregado Efetivo
	F	Empregado Efetivo
	F	Empregado Efetivo
	F	Empregado Efetivo
	F	Empregado Efetivo
	M	Empregado Efetivo
	M	Empregado Efetivo
	F	Empregado Efetivo
	M	Empregado Efetivo
	F	Empregado Efetivo
	M	Empregado Efetivo
	M	Empregado Efetivo
	F	Empregado Efetivo
	F	Empregado Efetivo
	F	Empregado Efetivo
	F	Empregado Efetivo

	F	Empregado Efetivo
	F	Empregado Efetivo
	M	Empregado Efetivo
	F	Empregado Efetivo
	F	Empregado Efetivo
	M	Empregado Efetivo
	F	Empregado Efetivo
	F	Empregado Efetivo
	F	Empregado Efetivo
	F	Empregado Efetivo
	F	Empregado Efetivo
	F	Empregado Efetivo
	F	Empregado Efetivo
	F	Empregado Efetivo
	F	Empregado Efetivo
	F	Empregado Efetivo
	F	Empregado Efetivo
	F	Empregado Efetivo
	F	Empregado Efetivo
	M	Empregado Efetivo
	F	Empregado Efetivo
	F	Empregado Efetivo
	F	Empregado Efetivo
	F	Empregado Efetivo
	F	Empregado Efetivo
	F	Empregado Efetivo
	F	Empregado Efetivo
	M	Empregado Efetivo
	F	Empregado Efetivo
	F	Empregado Efetivo
	F	Empregado Efetivo
	F	Empregado Efetivo

	M	Empregado Efetivo
	F	Empregado Efetivo
	F	Empregado Efetivo
	F	Empregado Efetivo
	F	Empregado Efetivo
	M	Empregado Efetivo
	M	Empregado Efetivo
	F	Empregado Efetivo
	F	Empregado Efetivo
	F	Empregado Efetivo
	F	Empregado Efetivo
	F	Empregado Efetivo
	M	Empregado Efetivo
	F	Empregado Efetivo
	F	Empregado Efetivo
	F	Empregado Efetivo
	F	Empregado Efetivo
	F	Empregado Efetivo
	F	Empregado Efetivo
	F	Empregado Efetivo
	F	Empregado Efetivo
	F	Empregado Efetivo
	F	Empregado Efetivo
	F	Empregado Efetivo
	F	Empregado Efetivo
	F	Empregado Efetivo
	F	Empregado Efetivo
	F	Empregado Efetivo
	F	Empregado Efetivo
	F	Empregado Efetivo
	F	Empregado Efetivo
	F	Empregado Efetivo

	F	Empregado Efetivo
	F	Empregado Efetivo
	F	Empregado Efetivo
	F	Empregado Efetivo
	F	Empregado Efetivo
	F	Empregado Efetivo
	F	Empregado Efetivo
	F	Empregado Efetivo
	F	Empregado Efetivo
	F	Empregado Efetivo
	F	Empregado Efetivo
	F	Empregado Efetivo
	F	Empregado Efetivo
	F	Empregado Efetivo
	F	Empregado Efetivo
	F	Empregado Efetivo
	M	Empregado Efetivo
	M	Empregado Efetivo
	M	Empregado Efetivo
	M	Empregado Efetivo
	M	Empregado Efetivo
	M	Empregado Efetivo
	M	Empregado Efetivo
	M	Empregado Efetivo
	F	Empregado Efetivo
	F	Empregado Efetivo
	F	Empregado Efetivo
	F	Empregado Efetivo
	F	Empregado Efetivo
	F	Empregado Efetivo
	F	Empregado Efetivo
	F	Empregado Efetivo
	F	Empregado Efetivo
	F	Empregado Efetivo
	F	Empregado Efetivo
	F	Empregado Efetivo
	F	Empregado Efetivo
	F	Empregado Efetivo
	F	Empregado Efetivo
	F	Empregado Efetivo

	M	Empregado Efetivo
	F	Empregado Efetivo
	F	Empregado Efetivo
	F	Empregado Efetivo
	F	Empregado Efetivo
	F	Empregado Efetivo
	F	Empregado Efetivo
	F	Empregado Efetivo
	F	Empregado Efetivo
	F	Empregado Efetivo
	F	Empregado Efetivo
	F	Empregado Efetivo
	F	Empregado Efetivo
	F	Empregado Efetivo
	F	Empregado Efetivo
	F	Empregado Efetivo
	F	Empregado Efetivo
	F	Empregado Efetivo
	F	Empregado Efetivo
	F	Empregado Efetivo
	F	Empregado Efetivo
	F	Empregado Efetivo
	F	Empregado Efetivo
	F	Empregado Efetivo
	F	Empregado Efetivo
	F	Empregado Efetivo
	F	Empregado Efetivo
	F	Empregado Efetivo
	F	Empregado Efetivo
	F	Empregado Efetivo
	F	Empregado Efetivo
	F	Empregado Efetivo

	F	Empregado Efetivo
	F	Empregado Efetivo
	F	Empregado Efetivo
	F	Empregado Efetivo
	F	Empregado Efetivo
	F	Empregado Efetivo
	F	Empregado Efetivo
	F	Empregado Efetivo
	F	Empregado Efetivo
	F	Empregado Efetivo
	F	Empregado Efetivo
	F	Empregado Efetivo
	F	Empregado Efetivo
	F	Empregado Efetivo
	F	Empregado Efetivo
	F	Empregado Efetivo
	F	Empregado Efetivo
	F	Empregado Efetivo
	F	Empregado Efetivo
	F	Empregado Efetivo
	F	Empregado Efetivo
	F	Empregado Efetivo
	F	Empregado Efetivo
	F	Empregado Efetivo
	F	Empregado Efetivo
	F	Empregado Efetivo
	F	Empregado Efetivo
	F	Empregado Efetivo
	F	Empregado Efetivo
	F	Empregado Efetivo
	F	Empregado Efetivo
	F	Empregado Efetivo

	F	Empregado Efetivo
	F	Empregado Efetivo
	F	Empregado Efetivo
	F	Empregado Efetivo
	F	Empregado Efetivo
	F	Empregado Efetivo
	F	Empregado Efetivo
	F	Empregado Efetivo
	F	Empregado Efetivo
	F	Empregado Efetivo
	F	Empregado Efetivo
	F	Empregado Efetivo
	F	Empregado Efetivo
	F	Empregado Efetivo
	F	Empregado Efetivo
	F	Empregado Efetivo
	F	Empregado Efetivo
	F	Empregado Efetivo
	F	Empregado Efetivo
	F	Empregado Efetivo
	F	Empregado Efetivo
	F	Empregado Efetivo
	M	Empregado Efetivo
	F	Empregado Efetivo
	F	Empregado Efetivo
	F	Empregado Efetivo
	F	Empregado Efetivo
	F	Empregado Efetivo
	F	Empregado Efetivo
	F	Empregado Efetivo
	F	Empregado Efetivo
	F	Empregado Efetivo

	F	Empregado Efetivo
	F	Empregado Efetivo
	F	Empregado Efetivo
	F	Empregado Efetivo
	F	Empregado Efetivo
	F	Empregado Efetivo
	F	Empregado Efetivo
	F	Empregado Efetivo
	F	Empregado Efetivo
	F	Empregado Efetivo
	F	Empregado Efetivo
	M	Empregado Efetivo
	F	Empregado Efetivo
	F	Empregado Efetivo
	F	Empregado Efetivo
	F	Empregado Efetivo
	F	Empregado Efetivo
	M	Empregado Efetivo
	F	Empregado Efetivo
	F	Empregado Efetivo
	F	Empregado Efetivo
	F	Empregado Efetivo
	F	Empregado Efetivo
	F	Empregado Efetivo
	F	Empregado Efetivo
	F	Empregado Efetivo
	F	Empregado Efetivo
	F	Empregado Efetivo
	F	Empregado Efetivo
	F	Empregado Efetivo
	F	Empregado Efetivo
	F	Empregado Efetivo

	F	Empregado Efetivo
	F	Empregado Efetivo
	F	Empregado Efetivo
	F	Empregado Efetivo
	F	Empregado Efetivo
	F	Empregado Efetivo
	F	Empregado Efetivo
	F	Empregado Efetivo
	F	Empregado Efetivo
	F	Empregado Efetivo
	F	Empregado Efetivo
	F	Empregado Efetivo
	F	Empregado Efetivo
	F	Empregado Efetivo
	F	Empregado Efetivo
	F	Empregado Efetivo
	F	Empregado Efetivo
	F	Empregado Efetivo
	F	Empregado Efetivo
	F	Empregado Efetivo
	F	Empregado Efetivo
	F	Empregado Efetivo
	F	Empregado Efetivo
	F	Empregado Efetivo
	F	Empregado Efetivo
	F	Empregado Efetivo
	F	Empregado Efetivo
	F	Empregado Efetivo
	F	Empregado Efetivo
	F	Empregado Efetivo
	F	Empregado Efetivo
	F	Empregado Efetivo
	F	Empregado Efetivo
	F	Empregado Efetivo
	F	Empregado Efetivo

	F	Empregado Efetivo
	F	Empregado Efetivo
	F	Empregado Efetivo
	F	Empregado Efetivo
	F	Empregado Efetivo
	F	Empregado Efetivo
	F	Empregado Efetivo
	F	Empregado Efetivo
	F	Empregado Efetivo
	F	Empregado Efetivo
	F	Empregado Efetivo
	F	Empregado Efetivo
	F	Empregado Efetivo
	F	Empregado Efetivo
	F	Empregado Efetivo
	F	Empregado Efetivo
	F	Empregado Efetivo
	F	Empregado Efetivo
	F	Empregado Efetivo
	F	Empregado Efetivo
	F	Empregado Efetivo
	F	Empregado Efetivo
	F	Empregado Efetivo
	F	Empregado Efetivo
	F	Empregado Efetivo
	F	Empregado Efetivo
	F	Empregado Efetivo
	F	Empregado Efetivo
	F	Empregado Efetivo
	F	Empregado Efetivo
	M	Empregado Efetivo
	F	Empregado Efetivo
	M	Empregado Efetivo
	F	Empregado Efetivo
	F	Empregado Efetivo
	F	Empregado Efetivo
	M	Empregado Efetivo
	F	Empregado Efetivo
	F	Empregado Efetivo
	F	Empregado Efetivo
	F	Empregado Efetivo
	F	Empregado Efetivo
	M	Empregado Efetivo
	F	Empregado Efetivo
	F	Empregado Efetivo
	F	Empregado Efetivo
	F	Empregado Efetivo
	F	Empregado Efetivo

	F	Empregado Efetivo
	F	Empregado Efetivo
	F	Empregado Efetivo
	F	Empregado Efetivo
	F	Empregado Efetivo
	F	Empregado Efetivo
	F	Empregado Efetivo
	F	Empregado Efetivo
	F	Empregado Efetivo
	F	Empregado Efetivo
	F	Empregado Efetivo
	F	Empregado Efetivo
	F	Empregado Efetivo
	F	Empregado Efetivo
	F	Empregado Efetivo
	F	Empregado Efetivo
	F	Empregado Efetivo
	F	Empregado Efetivo
	F	Empregado Efetivo
	F	Empregado Efetivo
	F	Empregado Efetivo
	F	Empregado Efetivo
	F	Empregado Efetivo
	F	Empregado Efetivo
	M	Empregado Efetivo
	M	Empregado Efetivo
	F	Empregado Efetivo
	F	Empregado Efetivo
	M	Empregado Efetivo
	F	Empregado Efetivo
	F	Empregado Efetivo
	F	Empregado Efetivo
	M	Empregado Efetivo
	F	Empregado Efetivo
	F	Empregado Efetivo
	F	Empregado Efetivo
	F	Empregado Efetivo
	F	Empregado Efetivo
	F	Empregado Efetivo
	F	Empregado Efetivo
	F	Empregado Efetivo
	F	Empregado Efetivo
	F	Empregado Efetivo
	F	Empregado Efetivo
	F	Empregado Efetivo
	F	Empregado Efetivo
	F	Empregado Efetivo
	F	Empregado Efetivo

	F	Empregado Efetivo
	F	Empregado Efetivo
	F	Empregado Efetivo
	F	Empregado Efetivo
	F	Empregado Efetivo
	F	Empregado Efetivo
	F	Empregado Efetivo
	F	Empregado Efetivo
	F	Empregado Efetivo
	F	Empregado Efetivo
	F	Empregado Efetivo
	F	Empregado Efetivo
	F	Empregado Efetivo
	F	Empregado Efetivo
	F	Empregado Efetivo
	F	Empregado Efetivo
	F	Empregado Efetivo
	F	Empregado Efetivo
	F	Empregado Efetivo
	F	Empregado Efetivo
	F	Empregado Efetivo
	F	Empregado Efetivo
	F	Empregado Efetivo
	M	Empregado Efetivo
	M	Empregado Efetivo
	F	Empregado Efetivo
	M	Empregado Efetivo
	F	Empregado Efetivo
	F	Empregado Efetivo
	M	Empregado Efetivo
	F	Empregado Efetivo
	F	Empregado Efetivo
	F	Empregado Efetivo
	F	Empregado Efetivo
	F	Empregado Efetivo
	F	Empregado Efetivo
	F	Empregado Efetivo
	F	Empregado Efetivo
	F	Empregado Efetivo
	F	Empregado Efetivo
	F	Empregado Efetivo
	F	Empregado Efetivo
	F	Empregado Efetivo
	F	Empregado Efetivo
	F	Empregado Efetivo
	M	Empregado Efetivo
	F	Empregado Efetivo
	F	Empregado Efetivo

	M	Empregado Efetivo
	F	Empregado Efetivo
	F	Empregado Efetivo
	F	Empregado Efetivo
	F	Empregado Efetivo
	M	Empregado Efetivo
	F	Empregado Efetivo
	F	Empregado Efetivo
	F	Empregado Efetivo
	F	Empregado Efetivo
	F	Empregado Efetivo
	F	Empregado Efetivo
	F	Empregado Efetivo
	F	Empregado Efetivo
	F	Empregado Efetivo
	F	Empregado Efetivo
	F	Empregado Efetivo
	F	Empregado Efetivo
	F	Empregado Efetivo
	F	Empregado Efetivo
	F	Empregado Efetivo
	F	Empregado Efetivo
	F	Empregado Efetivo
	F	Empregado Efetivo
	F	Empregado Efetivo
	F	Empregado Efetivo
	F	Empregado Efetivo
	F	Empregado Efetivo
	F	Empregado Efetivo
	F	Empregado Efetivo
	F	Empregado Efetivo
	F	Empregado Efetivo
	F	Empregado Efetivo
	F	Empregado Efetivo
	F	Empregado Efetivo
	F	Empregado Efetivo
	F	Empregado Efetivo
	F	Empregado Efetivo
	F	Empregado Efetivo
	F	Empregado Efetivo
	F	Empregado Efetivo
	F	Empregado Efetivo
	F	Empregado Efetivo
	F	Empregado Efetivo
	F	Empregado Efetivo

	F	Empregado Efetivo
	F	Empregado Efetivo
	F	Empregado Efetivo
	F	Empregado Efetivo
	F	Empregado Efetivo
	M	Empregado Efetivo
	F	Empregado Efetivo
	M	Empregado Efetivo
	F	Empregado Efetivo
	F	Empregado Efetivo
	F	Empregado Efetivo
	F	Empregado Efetivo
	F	Empregado Efetivo
	F	Empregado Efetivo
	F	Empregado Efetivo
	F	Empregado Efetivo
	F	Empregado Efetivo
	F	Empregado Efetivo
	F	Empregado Efetivo
	F	Empregado Efetivo
	F	Empregado Efetivo
	F	Empregado Efetivo
	F	Empregado Efetivo
	F	Empregado Efetivo
	F	Empregado Efetivo
	F	Empregado Efetivo
	F	Empregado Efetivo
	F	Empregado Efetivo
	F	Empregado Efetivo
	F	Empregado Efetivo
	F	Empregado Efetivo
	F	Empregado Efetivo
	F	Empregado Efetivo
	F	Empregado Efetivo
	F	Empregado Efetivo
	F	Empregado Efetivo
	F	Empregado Efetivo
	F	Empregado Efetivo
	F	Empregado Efetivo
	F	Empregado Efetivo
	F	Empregado Efetivo

	F	Empregado Efetivo
	F	Empregado Efetivo
	F	Empregado Efetivo
	F	Empregado Efetivo
	M	Empregado Efetivo
	F	Empregado Efetivo
	M	Empregado Efetivo
	F	Empregado Efetivo
	F	Empregado Efetivo
	F	Empregado Efetivo
	F	Empregado Efetivo
	F	Empregado Efetivo
	F	Empregado Efetivo
	F	Empregado Efetivo
	F	Empregado Efetivo
	M	Empregado Efetivo
	F	Empregado Efetivo
	F	Empregado Efetivo
	F	Empregado Efetivo
	F	Empregado Efetivo
	F	Empregado Efetivo
	F	Empregado Efetivo
	F	Empregado Efetivo
	F	Empregado Efetivo
	F	Empregado Efetivo
	F	Empregado Efetivo
	F	Empregado Efetivo
	F	Empregado Efetivo
	F	Empregado Efetivo
	M	Empregado Efetivo
	F	Empregado Efetivo
	F	Empregado Efetivo

	F	Empregado Efetivo
	F	Empregado Efetivo
	F	Empregado Efetivo
	F	Empregado Efetivo
	F	Empregado Efetivo
	F	Empregado Efetivo
	F	Empregado Efetivo
	F	Empregado Efetivo
	F	Empregado Efetivo
	F	Empregado Efetivo
	F	Empregado Efetivo
	F	Empregado Efetivo
	F	Empregado Efetivo
	F	Empregado Efetivo
	F	Empregado Efetivo
	F	Empregado Efetivo
	F	Empregado Efetivo
	F	Empregado Efetivo
	M	Empregado Efetivo
	F	Empregado Efetivo
	M	Empregado Efetivo
	F	Empregado Efetivo
	F	Empregado Efetivo
	F	Empregado Efetivo
	F	Empregado Efetivo
	M	Empregado Efetivo
	F	Empregado Efetivo
	F	Empregado Efetivo
	F	Empregado Efetivo
	F	Empregado Efetivo
	F	Empregado Efetivo
	F	Empregado Efetivo

	F	Empregado Efetivo
	F	Empregado Efetivo
	F	Empregado Efetivo
	F	Empregado Efetivo
	F	Empregado Efetivo
	F	Empregado Efetivo
	F	Empregado Efetivo
	F	Empregado Efetivo
	F	Empregado Efetivo
	F	Empregado Efetivo
	F	Empregado Efetivo
	F	Empregado Efetivo
	F	Empregado Efetivo
	M	Empregado Efetivo
	F	Empregado Efetivo
	M	Empregado Efetivo
	F	Empregado Efetivo
	F	Empregado Efetivo
	F	Empregado Efetivo
	F	Empregado Efetivo
	F	Empregado Efetivo
	F	Empregado Efetivo
	F	Empregado Efetivo
	F	Empregado Efetivo
	M	Empregado Efetivo
	F	Empregado Efetivo
	M	Empregado Efetivo
	F	Empregado Efetivo
	F	Empregado Efetivo
	F	Empregado Efetivo
	F	Empregado Efetivo
	F	Empregado Efetivo

	F	Empregado Efetivo
	F	Empregado Efetivo
	F	Empregado Efetivo
	F	Empregado Efetivo
	M	Empregado Efetivo
	M	Empregado Efetivo
	F	Empregado Efetivo
	F	Empregado Efetivo
	F	Empregado Efetivo
	F	Empregado Efetivo
	F	Empregado Efetivo
	M	Empregado Efetivo
	F	Empregado Efetivo
	F	Empregado Efetivo
	F	Empregado Efetivo
	F	Empregado Efetivo
	M	Empregado Efetivo
	F	Empregado Efetivo
	M	Empregado Efetivo
	F	Empregado Efetivo
	M	Empregado Efetivo
	F	Empregado Efetivo
	F	Empregado Efetivo
	M	Empregado Efetivo
	F	Empregado Efetivo
	F	Empregado Efetivo
	F	Empregado Efetivo
	F	Empregado Efetivo
	F	Empregado Efetivo
	F	Empregado Efetivo
	F	Empregado Efetivo
	F	Empregado Efetivo

	F	Empregado Efetivo
	F	Empregado Efetivo
	F	Empregado Efetivo
	F	Empregado Efetivo
	F	Empregado Efetivo
	M	Empregado Efetivo
	F	Empregado Efetivo
	F	Empregado Efetivo
	M	Empregado Efetivo
	F	Empregado Efetivo
	F	Empregado Efetivo
	F	Empregado Efetivo
	F	Empregado Efetivo
	F	Empregado Efetivo
	F	Empregado Efetivo
	F	Empregado Efetivo
	F	Empregado Efetivo
	F	Empregado Efetivo
	F	Empregado Efetivo
	F	Empregado Efetivo
	M	Empregado Efetivo
	F	Empregado Efetivo
	F	Empregado Efetivo
	F	Empregado Efetivo
	F	Empregado Efetivo
	F	Empregado Efetivo
	F	Empregado Efetivo
	F	Empregado Efetivo
	M	Empregado Efetivo
	M	Empregado Efetivo
	F	Empregado Efetivo
	F	Empregado Efetivo

	M	Empregado Efetivo
	F	Empregado Efetivo
	F	Empregado Efetivo
	F	Empregado Efetivo
	M	Empregado Efetivo
	F	Empregado Efetivo
	M	Empregado Efetivo
	F	Empregado Efetivo
	F	Empregado Efetivo
	F	Empregado Efetivo
	F	Empregado Efetivo
	F	Empregado Efetivo
	F	Empregado Efetivo
	F	Empregado Efetivo
	F	Empregado Efetivo
	M	Empregado Efetivo
	M	Empregado Efetivo
	F	Empregado Efetivo
	M	Empregado Efetivo
	M	Empregado Efetivo
	F	Empregado Efetivo
	F	Empregado Efetivo
	F	Empregado Efetivo
	F	Empregado Efetivo
	F	Empregado Efetivo
	F	Empregado Efetivo
	F	Empregado Efetivo
	F	Empregado Efetivo
	M	Empregado Efetivo
	F	Empregado Efetivo
	F	Empregado Efetivo
	F	Empregado Efetivo

	F	Empregado Efetivo
	F	Empregado Efetivo
	F	Empregado Efetivo
	M	Empregado Efetivo
	M	Empregado Efetivo
	M	Empregado Efetivo
	M	Empregado Efetivo
	F	Empregado Efetivo
	M	Empregado Efetivo
	F	Empregado Efetivo
	F	Empregado Efetivo
	M	Empregado Efetivo
	F	Empregado Efetivo
	M	Empregado Efetivo
	M	Empregado Efetivo
	F	Empregado Efetivo
	F	Empregado Efetivo
	F	Empregado Efetivo
	F	Empregado Efetivo
	F	Empregado Efetivo
	F	Empregado Efetivo
	M	Empregado Efetivo
	F	Empregado Efetivo
	F	Empregado Efetivo
	F	Empregado Efetivo
	F	Empregado Efetivo
	F	Empregado Efetivo
	M	Empregado Efetivo
	F	Empregado Efetivo
	F	Empregado Efetivo
	F	Empregado Efetivo
	F	Empregado Efetivo

	F	Empregado Efetivo
	F	Empregado Efetivo
	F	Empregado Efetivo
	F	Empregado Efetivo
	M	Empregado Efetivo
	F	Empregado Efetivo
	F	Empregado Efetivo
	M	Empregado Efetivo
	M	Empregado Efetivo
	M	Empregado Efetivo
	F	Empregado Efetivo
	M	Empregado Efetivo
	F	Empregado Efetivo
	F	Empregado Efetivo
	F	Empregado Efetivo
	M	Empregado Efetivo
	F	Empregado Efetivo
	F	Empregado Efetivo
	F	Empregado Efetivo
	M	Empregado Efetivo
	F	Empregado Efetivo
	F	Empregado Efetivo
	F	Empregado Efetivo
	F	Empregado Efetivo
	F	Empregado Efetivo
	F	Empregado Efetivo
	F	Empregado Efetivo
	F	Empregado Efetivo
	F	Empregado Efetivo
	F	Empregado Efetivo
	F	Empregado Efetivo
	F	Empregado Efetivo
	F	Empregado Efetivo
	F	Empregado Efetivo
	F	Empregado Efetivo

	F	Empregado Efetivo
	F	Empregado Efetivo
	M	Empregado Efetivo
	F	Empregado Efetivo
	F	Empregado Efetivo
	F	Empregado Efetivo
	F	Empregado Efetivo
	F	Empregado Efetivo
	F	Empregado Efetivo
	F	Empregado Efetivo
	F	Empregado Efetivo
	F	Empregado Efetivo
	F	Empregado Efetivo
	F	Empregado Efetivo
	F	Empregado Efetivo
	F	Empregado Efetivo
	F	Empregado Efetivo
	F	Empregado Efetivo
	F	Empregado Efetivo
	F	Empregado Efetivo
	F	Empregado Efetivo
	F	Empregado Efetivo
	F	Empregado Efetivo
	F	Empregado Efetivo
	F	Empregado Efetivo
	F	Empregado Efetivo
	F	Empregado Efetivo
	F	Empregado Efetivo
	F	Empregado Efetivo
	F	Empregado Efetivo
	F	Empregado Efetivo
	F	Empregado Efetivo
	F	Empregado Efetivo
	F	Empregado Efetivo
	F	Empregado Efetivo
	F	Empregado Efetivo
	F	Empregado Efetivo
	F	Empregado Efetivo
	F	Empregado Efetivo
	F	Empregado Efetivo
	M	Empregado Efetivo
	F	Empregado Efetivo
	F	Empregado Efetivo
	F	Empregado Efetivo
	F	Empregado Efetivo
	F	Empregado Efetivo
	F	Empregado Efetivo
	F	Empregado Efetivo

	F	Empregado Efetivo
	F	Empregado Efetivo
	F	Empregado Efetivo
	F	Empregado Efetivo
	F	Empregado Efetivo
	F	Empregado Efetivo
	F	Empregado Efetivo
	F	Empregado Efetivo
	F	Empregado Efetivo
	F	Empregado Efetivo
	F	Empregado Efetivo
	F	Empregado Efetivo
	F	Empregado Efetivo
	F	Empregado Efetivo
	F	Empregado Efetivo
	F	Empregado Efetivo
	F	Empregado Efetivo
	F	Empregado Efetivo
	F	Empregado Efetivo
	F	Empregado Efetivo
	F	Empregado Efetivo
	F	Empregado Efetivo
	M	Empregado Efetivo
	F	Empregado Efetivo
	F	Empregado Efetivo
	F	Empregado Efetivo
	F	Empregado Efetivo
	F	Empregado Efetivo
	F	Empregado Efetivo
	F	Empregado Efetivo
	F	Empregado Efetivo
	F	Empregado Efetivo
	F	Empregado Efetivo
	F	Empregado Efetivo
	F	Empregado Efetivo
	F	Empregado Efetivo
	F	Empregado Efetivo
	F	Empregado Efetivo
	F	Empregado Efetivo
	M	Empregado Efetivo
	F	Empregado Efetivo
	F	Empregado Efetivo
	F	Empregado Efetivo
	F	Empregado Efetivo
	F	Empregado Efetivo
	F	Empregado Efetivo
	F	Empregado Efetivo
	F	Empregado Efetivo

	F	Empregado Efetivo
	F	Empregado Efetivo
	F	Empregado Efetivo
	F	Empregado Efetivo
	F	Empregado Efetivo
	F	Empregado Efetivo
	F	Empregado Efetivo
	F	Empregado Efetivo
	F	Empregado Efetivo
	F	Empregado Efetivo
	F	Empregado Efetivo
	F	Empregado Efetivo
	F	Empregado Efetivo
	F	Empregado Efetivo
	M	Empregado Efetivo
	M	Empregado Efetivo
	M	Empregado Efetivo
	M	Empregado Efetivo
	F	Empregado Efetivo
	M	Empregado Efetivo
	M	Empregado Efetivo
	F	Empregado Efetivo
	M	Empregado Efetivo
	M	Empregado Efetivo
	F	Empregado Efetivo
	F	Empregado Efetivo
	M	Empregado Efetivo
	M	Empregado Efetivo
	F	Empregado Efetivo
	M	Empregado Efetivo
	F	Empregado Efetivo
	F	Empregado Efetivo
	F	Empregado Efetivo
	F	Empregado Efetivo
	F	Empregado Efetivo
	M	Empregado Efetivo
	M	Empregado Efetivo
	M	Empregado Efetivo
	F	Empregado Efetivo

	F	Empregado Efetivo
	F	Empregado Efetivo
	F	Empregado Efetivo
	F	Empregado Efetivo
	F	Empregado Efetivo
	F	Empregado Efetivo
	F	Empregado Efetivo
	F	Empregado Efetivo
	F	Empregado Efetivo
	F	Empregado Efetivo
	F	Empregado Efetivo
	F	Empregado Efetivo
	F	Empregado Efetivo
	M	Empregado Efetivo
	M	Empregado Efetivo
	F	Empregado Efetivo
	F	Empregado Efetivo
	F	Empregado Efetivo
	F	Empregado Efetivo
	F	Empregado Efetivo
	F	Empregado Efetivo
	F	Empregado Efetivo
	F	Empregado Efetivo
	F	Empregado Efetivo
	F	Empregado Efetivo
	F	Empregado Efetivo
	F	Empregado Efetivo
	M	Empregado Efetivo
	M	Empregado Efetivo
	M	Empregado Efetivo
	F	Empregado Efetivo
	F	Empregado Efetivo

	F	Empregado Efetivo
	F	Empregado Efetivo
	F	Empregado Efetivo
	F	Empregado Efetivo
	F	Empregado Efetivo
	F	Empregado Efetivo
	F	Empregado Efetivo
	F	Empregado Efetivo
	F	Empregado Efetivo
	F	Empregado Efetivo
	F	Empregado Efetivo
	F	Empregado Efetivo
	F	Empregado Efetivo
	F	Empregado Efetivo
	F	Empregado Efetivo
	F	Empregado Efetivo
	F	Empregado Efetivo
	F	Empregado Efetivo
	F	Empregado Efetivo
	F	Empregado Efetivo
	F	Empregado Efetivo
	F	Empregado Efetivo
	F	Empregado Efetivo
	F	Empregado Efetivo
	F	Empregado Efetivo
	F	Empregado Efetivo
	F	Empregado Efetivo
	F	Empregado Efetivo
	F	Empregado Efetivo
	F	Empregado Efetivo
	F	Empregado Efetivo
	F	Empregado Efetivo

	F	Empregado Efetivo
	F	Empregado Efetivo
	F	Empregado Efetivo
	M	Empregado Efetivo
	M	Empregado Efetivo
	F	Empregado Efetivo
	M	Empregado Efetivo
	M	Empregado Efetivo
	M	Empregado Efetivo
	M	Empregado Efetivo
	M	Empregado Efetivo
	M	Empregado Efetivo
	M	Empregado Efetivo
	M	Empregado Efetivo
	M	Empregado Efetivo
	M	Empregado Efetivo
	M	Empregado Efetivo
	M	Empregado Efetivo
	M	Empregado Efetivo
	M	Empregado Efetivo
	M	Empregado Efetivo
	M	Empregado Efetivo
	M	Empregado Efetivo
	M	Empregado Efetivo
	M	Empregado Efetivo
	M	Empregado Efetivo
	M	Empregado Efetivo
	M	Empregado Efetivo
	M	Empregado Efetivo
	M	Empregado Efetivo
	M	Empregado Efetivo
	M	Empregado Efetivo

	M	Cedido - RJU
	M	Cedido - RJU
	M	Cedido - RJU
	F	Empregado Efetivo
	M	Empregado Efetivo
	M	Empregado Efetivo
	M	Empregado Efetivo
	M	Empregado Efetivo
	M	Empregado Efetivo
	M	Empregado Efetivo
	M	Empregado Efetivo
	M	Empregado Efetivo
	F	Empregado Efetivo
	F	Empregado Efetivo
	F	Empregado Efetivo
	M	Empregado Efetivo
	M	Empregado Efetivo
	F	Empregado Efetivo
	F	Empregado Efetivo
	M	Empregado Efetivo
	M	Empregado Efetivo
	M	Empregado Efetivo
	M	Empregado Efetivo
	F	Empregado Efetivo
	M	Empregado Efetivo
	F	Empregado Efetivo
	M	Empregado Efetivo
	F	Empregado Efetivo
	F	Empregado Efetivo
	F	Empregado Efetivo
	F	Empregado Efetivo
	M	Empregado Efetivo
	M	Empregado Efetivo
	F	Empregado Efetivo
	F	Empregado Efetivo
	F	Empregado Efetivo
	F	Empregado Efetivo
	F	Empregado Efetivo
	F	Empregado Efetivo

	F	Empregado Efetivo
	F	Empregado Efetivo
	F	Empregado Efetivo
	F	Empregado Efetivo
	F	Empregado Efetivo
	F	Empregado Efetivo
	F	Empregado Efetivo
	F	Empregado Efetivo
	F	Empregado Efetivo
	F	Empregado Efetivo
	F	Empregado Efetivo
	F	Empregado Efetivo
	F	Empregado Efetivo
	F	Empregado Efetivo
	F	Empregado Efetivo
	F	Empregado Efetivo
	F	Empregado Efetivo
	F	Empregado Efetivo
	F	Empregado Efetivo
	F	Empregado Efetivo
	F	Empregado Efetivo
	F	Empregado Efetivo
	F	Empregado Efetivo
	F	Empregado Efetivo
	F	Empregado Efetivo
	F	Empregado Efetivo
	F	Empregado Efetivo
	F	Empregado Efetivo
	F	Empregado Efetivo
	F	Empregado Efetivo
	F	Empregado Efetivo
	F	Empregado Efetivo
	F	Empregado Efetivo
	F	Empregado Efetivo
	F	Empregado Efetivo
	F	Empregado Efetivo
	F	Empregado Efetivo
	F	Empregado Efetivo
	F	Empregado Efetivo
	F	Empregado Efetivo
	F	Empregado Efetivo

	F	Empregado Efetivo
	F	Empregado Efetivo
	F	Empregado Efetivo
	F	Empregado Efetivo
	F	Empregado Efetivo
	F	Empregado Efetivo
	F	Empregado Efetivo
	F	Empregado Efetivo
	F	Empregado Efetivo
	F	Empregado Efetivo
	F	Empregado Efetivo
	F	Empregado Efetivo
	F	Empregado Efetivo
	F	Empregado Efetivo
	F	Empregado Efetivo
	F	Empregado Efetivo
	F	Empregado Efetivo
	F	Empregado Efetivo
	F	Empregado Efetivo
	F	Empregado Efetivo
	F	Empregado Efetivo
	F	Empregado Efetivo
	F	Empregado Efetivo
	F	Empregado Efetivo
	F	Empregado Efetivo
	F	Empregado Efetivo
	F	Empregado Efetivo
	F	Empregado Efetivo
	F	Empregado Efetivo
	F	Empregado Efetivo
	F	Empregado Efetivo
	F	Empregado Efetivo
	F	Empregado Efetivo
	F	Empregado Efetivo
	F	Empregado Efetivo
	F	Empregado Efetivo
	F	Empregado Efetivo

	F	Empregado Efetivo
	F	Empregado Efetivo
	F	Empregado Efetivo
	F	Empregado Efetivo
	F	Empregado Efetivo
	F	Empregado Efetivo
	F	Empregado Efetivo
	F	Empregado Efetivo
	F	Empregado Efetivo
	F	Empregado Efetivo
	F	Empregado Efetivo
	F	Empregado Efetivo
	F	Empregado Efetivo
	F	Empregado Efetivo
	F	Empregado Efetivo
	F	Empregado Efetivo
	F	Empregado Efetivo
	M	Empregado Efetivo
	M	Empregado Efetivo
	M	Empregado Efetivo
	M	Empregado Efetivo
	M	Empregado Efetivo
	M	Empregado Efetivo
	M	Empregado Efetivo
	M	Empregado Efetivo
	M	Empregado Efetivo
	M	Empregado Efetivo
	M	Empregado Efetivo
	M	Empregado Efetivo
	M	Empregado Efetivo
	M	Empregado Efetivo
	M	Empregado Efetivo
	M	Empregado Efetivo
	M	Empregado Efetivo
	M	Empregado Efetivo
	M	Empregado Efetivo

	M	Empregado Efetivo
	M	Empregado Efetivo
	M	Empregado Efetivo
	M	Empregado Efetivo
	M	Empregado Efetivo
	M	Empregado Efetivo
	M	Empregado Efetivo
	M	Empregado Efetivo
	F	Empregado Efetivo
	M	Empregado Efetivo
	M	Empregado Efetivo
	M	Empregado Efetivo
	F	Empregado Efetivo
	F	Empregado Efetivo
	F	Empregado Efetivo
	F	Empregado Efetivo
	F	Empregado Efetivo
	F	Empregado Efetivo
	F	Empregado Efetivo
	F	Empregado Efetivo
	F	Empregado Efetivo
	F	Empregado Efetivo
	F	Empregado Efetivo
	F	Empregado Efetivo
	F	Empregado Efetivo
	F	Empregado Efetivo
	F	Empregado Efetivo
	F	Empregado Efetivo
	F	Empregado Efetivo
	F	Empregado Efetivo
	M	Empregado Efetivo
	F	Empregado Efetivo
	F	Empregado Efetivo
	F	Empregado Efetivo
	F	Empregado Efetivo
	F	Empregado Efetivo
	F	Empregado Efetivo
	F	Empregado Efetivo
	F	Empregado Efetivo
	F	Empregado Efetivo

	F	Empregado Efetivo
	F	Empregado Efetivo
	M	Empregado Efetivo
	F	Empregado Efetivo
	M	Empregado Efetivo
	F	Empregado Efetivo
	F	Empregado Efetivo
	F	Empregado Efetivo
	F	Empregado Efetivo
	F	Empregado Efetivo
	F	Empregado Efetivo
	F	Empregado Efetivo
	F	Empregado Efetivo
	F	Empregado Efetivo
	M	Empregado Efetivo
	F	Empregado Efetivo
	F	Empregado Efetivo
	F	Empregado Efetivo
	F	Empregado Efetivo
	F	Empregado Efetivo
	F	Empregado Efetivo
	F	Empregado Efetivo
	F	Empregado Efetivo
	F	Empregado Efetivo
	F	Empregado Efetivo
	F	Empregado Efetivo
	F	Empregado Efetivo
	F	Empregado Efetivo
	M	Empregado Efetivo
	F	Empregado Efetivo
	F	Empregado Efetivo
	F	Empregado Efetivo
	F	Empregado Efetivo
	M	Empregado Efetivo
	F	Empregado Efetivo
	F	Cedido - RJU

	F	Cedido - RJU
	F	Cedido - RJU
	F	Cedido - RJU
	F	Cedido - RJU
	F	Cedido - RJU
	F	Cedido - RJU
	F	Cedido - RJU
	F	Cedido - RJU
	M	Cedido - RJU
	F	Empregado Efetivo
	F	Empregado Efetivo
	F	Empregado Efetivo
	F	Empregado Efetivo
	F	Empregado Efetivo
	F	Empregado Efetivo
	F	Empregado Efetivo
	F	Empregado Efetivo
	F	Empregado Efetivo
	F	Empregado Efetivo
	F	Empregado Efetivo
	F	Empregado Efetivo
	F	Empregado Efetivo
	F	Empregado Efetivo
	F	Empregado Efetivo
	F	Empregado Efetivo
	F	Empregado Efetivo
	F	Empregado Efetivo
	F	Empregado Efetivo
	F	Empregado Efetivo
	F	Empregado Efetivo
	F	Empregado Efetivo
	F	Empregado Efetivo
	F	Empregado Efetivo
	F	Empregado Efetivo
	F	Empregado Efetivo

	F	Empregado Efetivo
	F	Empregado Efetivo
	F	Empregado Efetivo
	F	Empregado Efetivo
	F	Empregado Efetivo
	F	Empregado Efetivo
	F	Empregado Efetivo
	F	Empregado Efetivo
	F	Empregado Efetivo
	F	Empregado Efetivo
	F	Empregado Efetivo
	F	Empregado Efetivo
	F	Empregado Efetivo
	F	Empregado Efetivo
	F	Empregado Efetivo
	F	Empregado Efetivo
	F	Empregado Efetivo
	F	Empregado Efetivo
	F	Empregado Efetivo
	F	Empregado Efetivo
	F	Empregado Efetivo
	F	Empregado Efetivo
	F	Empregado Efetivo
	F	Empregado Efetivo
	F	Empregado Efetivo
	F	Empregado Efetivo
	F	Empregado Efetivo
	F	Empregado Efetivo
	F	Empregado Efetivo
	F	Empregado Efetivo
	F	Empregado Efetivo
	F	Empregado Efetivo
	F	Empregado Efetivo
	F	Empregado Efetivo
	F	Empregado Efetivo
	F	Empregado Efetivo
	F	Empregado Efetivo
	F	Empregado Efetivo
	F	Empregado Efetivo
	F	Empregado Efetivo
	F	Empregado Efetivo

	F	Empregado Efetivo
	F	Empregado Efetivo
	F	Empregado Efetivo
	F	Empregado Efetivo
	F	Empregado Efetivo
	M	Empregado Efetivo
	M	Empregado Efetivo
	F	Empregado Efetivo
	F	Empregado Efetivo
	F	Empregado Efetivo
	F	Empregado Efetivo
	F	Empregado Efetivo
	F	Empregado Efetivo
	F	Empregado Efetivo
	F	Empregado Efetivo
	F	Empregado Efetivo
	M	Empregado Efetivo
	F	Empregado Efetivo
	M	Empregado Efetivo
	M	Empregado Efetivo
	F	Empregado Efetivo
	F	Empregado Efetivo
	F	Empregado Efetivo
	F	Empregado Efetivo
	F	Empregado Efetivo
	M	Empregado Efetivo
	F	Empregado Efetivo
	F	Empregado Efetivo
	F	Empregado Efetivo
	F	Empregado Efetivo
	F	Empregado Efetivo
	F	Empregado Efetivo

	M	Empregado Efetivo
	F	Empregado Efetivo
	F	Empregado Efetivo
	F	Empregado Efetivo
	F	Empregado Efetivo
	F	Empregado Efetivo
	F	Empregado Efetivo
	F	Empregado Efetivo
	F	Empregado Efetivo
	F	Empregado Efetivo
	F	Empregado Efetivo
	F	Empregado Efetivo
	F	Empregado Efetivo
	F	Empregado Efetivo
	F	Empregado Efetivo
	F	Empregado Efetivo
	F	Empregado Efetivo
	F	Empregado Efetivo
	F	Empregado Efetivo
	F	Empregado Efetivo
	F	Empregado Efetivo
	F	Empregado Efetivo
	F	Empregado Efetivo
	F	Empregado Efetivo
	F	Empregado Efetivo
	F	Empregado Efetivo
	F	Empregado Efetivo
	F	Empregado Efetivo
	F	Empregado Efetivo
	F	Empregado Efetivo
	F	Empregado Efetivo
	F	Empregado Efetivo
	F	Empregado Efetivo
	F	Empregado Efetivo
	F	Empregado Efetivo
	F	Empregado Efetivo
	F	Empregado Efetivo
	F	Empregado Efetivo
	F	Empregado Efetivo
	F	Empregado Efetivo

	F	Empregado Efetivo
	F	Empregado Efetivo
	F	Empregado Efetivo
	F	Empregado Efetivo
	F	Empregado Efetivo
	F	Empregado Efetivo
	F	Empregado Efetivo
	F	Empregado Efetivo
	F	Empregado Efetivo
	F	Empregado Efetivo
	F	Empregado Efetivo
	F	Empregado Efetivo
	F	Empregado Efetivo
	F	Empregado Efetivo
	F	Empregado Efetivo
	F	Empregado Efetivo
	F	Empregado Efetivo
	F	Empregado Efetivo
	F	Empregado Efetivo
	F	Empregado Efetivo
	F	Empregado Efetivo
	F	Empregado Efetivo
	F	Empregado Efetivo
	F	Empregado Efetivo
	F	Empregado Efetivo
	F	Empregado Efetivo
	F	Empregado Efetivo
	M	Empregado Efetivo
	M	Empregado Efetivo
	M	Empregado Efetivo
	M	Empregado Efetivo
	M	Empregado Efetivo
	M	Empregado Efetivo
	M	Empregado Efetivo
	F	Empregado Efetivo
	F	Empregado Efetivo
	F	Empregado Efetivo
	F	Empregado Efetivo
	M	Cedido - RJU
	F	Empregado Efetivo
	F	Empregado Efetivo
	F	Empregado Efetivo
	F	Empregado Efetivo

	F	Empregado Efetivo
	F	Empregado Efetivo
	F	Empregado Efetivo
	F	Cedido - RJU
	M	Empregado Efetivo
	F	Empregado Efetivo
	F	Empregado Efetivo
	F	Empregado Efetivo
	F	Empregado Efetivo
	F	Empregado Efetivo
	F	Empregado Efetivo
	F	Empregado Efetivo
	F	Empregado Efetivo
	F	Empregado Efetivo
	F	Empregado Efetivo
	F	Empregado Efetivo
	F	Empregado Efetivo
	M	Empregado Efetivo
	F	Empregado Efetivo
	F	Empregado Efetivo
	M	Empregado Efetivo
	M	Empregado Efetivo
	F	Empregado Efetivo
	F	Empregado Efetivo
	M	Empregado Efetivo
	M	Empregado Efetivo
	M	Empregado Efetivo
	M	Empregado Efetivo
	F	Empregado Efetivo
	F	Empregado Efetivo
	F	Empregado Efetivo
	F	Empregado Efetivo

	F	Empregado Efetivo
	F	Empregado Efetivo
	F	Empregado Efetivo
	M	Empregado Efetivo
	F	Empregado Efetivo
	F	Empregado Efetivo
	F	Empregado Efetivo
	M	Empregado Efetivo
	F	Empregado Efetivo
	F	Empregado Efetivo
	F	Empregado Efetivo
	F	Empregado Efetivo
	M	Empregado Efetivo
	F	Empregado Efetivo
	F	Empregado Efetivo
	F	Empregado Efetivo
	F	Empregado Efetivo
	F	Empregado Efetivo
	F	Empregado Efetivo
	M	Cedido - RJU
	M	Empregado Efetivo
	M	Empregado Efetivo
	M	Empregado Efetivo
	M	Empregado Efetivo
	F	Empregado Efetivo
	F	Empregado Efetivo
	F	Empregado Efetivo
	F	Empregado Efetivo
	F	Empregado Efetivo
	F	Empregado Efetivo
	F	Empregado Efetivo
	F	Empregado Efetivo

	F	Empregado Efetivo
	M	Empregado Efetivo
	M	Empregado Efetivo
	F	Empregado Efetivo
	M	Empregado Efetivo
	F	Empregado Efetivo
	M	Cedido - RJU
	M	Empregado Efetivo
	F	Empregado Efetivo
	F	Empregado Efetivo
	M	Empregado Efetivo
	F	Empregado Efetivo
	F	Empregado Efetivo
	F	Empregado Efetivo
	F	Empregado Efetivo
	F	Empregado Efetivo
	M	Empregado Efetivo
	F	Empregado Efetivo
	M	Empregado Efetivo
	F	Empregado Efetivo
	F	Empregado Efetivo
	F	Empregado Efetivo
	F	Empregado Efetivo
	M	Empregado Efetivo
	M	Empregado Efetivo
	F	Empregado Efetivo
	F	Empregado Efetivo
	F	Empregado Efetivo
	F	Empregado Efetivo
	F	Cedido - RJU
	M	Empregado Efetivo
	M	Empregado Efetivo

	F	Empregado Efetivo
	F	Empregado Efetivo
	F	Empregado Efetivo
	F	Empregado Efetivo
	F	Empregado Efetivo
	F	Empregado Efetivo
	F	Empregado Efetivo
	F	Empregado Efetivo
	F	Empregado Efetivo
	M	Empregado Efetivo
	F	Empregado Efetivo
	F	Empregado Efetivo
	M	Empregado Efetivo
	F	Empregado Efetivo
	M	Empregado Efetivo
	F	Empregado Efetivo
	M	Empregado Efetivo
	F	Empregado Efetivo
	M	Empregado Efetivo
	F	Empregado Efetivo
	F	Empregado Efetivo
	F	Empregado Efetivo
	F	Empregado Efetivo
	F	Empregado Efetivo
	F	Empregado Efetivo
	F	Empregado Efetivo
	F	Empregado Efetivo
	F	Empregado Efetivo
	F	Empregado Efetivo
	F	Empregado Efetivo
	F	Empregado Efetivo
	F	Empregado Efetivo
	F	Empregado Efetivo
	F	Empregado Efetivo
	F	Empregado Efetivo

	F	Empregado Efetivo
	M	Empregado Efetivo
	F	Empregado Efetivo
	F	Empregado Efetivo
	F	Empregado Efetivo
	F	Empregado Efetivo
	M	Empregado Efetivo
	F	Empregado Efetivo
	F	Empregado Efetivo
	F	Empregado Efetivo
	M	Empregado Efetivo
	M	Empregado Efetivo
	M	Empregado Efetivo
	F	Empregado Efetivo
	F	Empregado Efetivo
	F	Empregado Efetivo
	F	Empregado Efetivo
	M	Empregado Efetivo
	F	Empregado Efetivo
	F	Empregado Efetivo
	F	Empregado Efetivo
	F	Empregado Efetivo
	F	Empregado Efetivo
	F	Empregado Efetivo
	F	Empregado Efetivo
	F	Empregado Efetivo
	F	Empregado Efetivo
	F	Empregado Efetivo
	F	Empregado Efetivo
	M	Empregado Efetivo
	F	Empregado Efetivo
	M	Empregado Efetivo
	F	Empregado Efetivo
	M	Empregado Efetivo
	F	Empregado Efetivo
	F	Empregado Efetivo
	M	Empregado Efetivo
	M	Empregado Efetivo
	M	Empregado Efetivo
	F	Empregado Efetivo
	F	Empregado Efetivo
	M	Empregado Efetivo
	M	Empregado Efetivo

	F	Empregado Efetivo
	M	Empregado Efetivo
	M	Empregado Efetivo
	F	Empregado Efetivo
	F	Empregado Efetivo
	F	Empregado Efetivo
	F	Empregado Efetivo
	F	Empregado Efetivo
	F	Empregado Efetivo
	M	Empregado Efetivo
	M	Empregado Efetivo
	M	Empregado Efetivo
	M	Empregado Efetivo
	F	Empregado Efetivo
	F	Empregado Efetivo
	M	Empregado Efetivo
	M	Empregado Efetivo
	M	Empregado Efetivo
	M	Empregado Efetivo
	M	Empregado Efetivo
	M	Empregado Efetivo
	M	Empregado Efetivo
	M	Empregado Efetivo
	F	Empregado Efetivo
	F	Empregado Efetivo
	F	Empregado Efetivo
	F	Empregado Efetivo
	F	Empregado Efetivo
	F	Empregado Efetivo
	F	Empregado Efetivo
	F	Empregado Efetivo
	F	Empregado Efetivo
	F	Empregado Efetivo
	F	Empregado Efetivo
	F	Empregado Efetivo
	F	Empregado Efetivo
	F	Empregado Efetivo
	F	Empregado Efetivo
	F	Empregado Efetivo
	F	Empregado Efetivo
	M	Empregado Efetivo
	F	Empregado Efetivo
	F	Empregado Efetivo
	F	Empregado Efetivo
	F	Empregado Efetivo
	F	Empregado Efetivo

	F	Empregado Efetivo
	F	Empregado Efetivo
	F	Empregado Efetivo
	F	Empregado Efetivo
	F	Empregado Efetivo
	F	Empregado Efetivo
	F	Empregado Efetivo
	F	Empregado Efetivo
	F	Empregado Efetivo
	F	Empregado Efetivo
	F	Empregado Efetivo
	F	Empregado Efetivo
	F	Empregado Efetivo
	F	Empregado Efetivo
	F	Empregado Efetivo
	F	Empregado Efetivo
	F	Empregado Efetivo
	F	Empregado Efetivo
	F	Empregado Efetivo
	F	Empregado Efetivo
	F	Empregado Efetivo
	F	Empregado Efetivo
	F	Empregado Efetivo
	F	Empregado Efetivo
	F	Empregado Efetivo
	F	Empregado Efetivo
	F	Empregado Efetivo
	F	Empregado Efetivo
	F	Empregado Efetivo
	F	Empregado Efetivo
	F	Empregado Efetivo
	F	Empregado Efetivo
	F	Empregado Efetivo
	F	Empregado Efetivo
	F	Empregado Efetivo
	F	Empregado Efetivo
	F	Empregado Efetivo
	F	Empregado Efetivo
	M	Empregado Efetivo
	F	Empregado Efetivo
	F	Empregado Efetivo
	M	Empregado Efetivo
	M	Empregado Efetivo
	M	Empregado Efetivo
	M	Empregado Efetivo

	M	Empregado Efetivo
	F	Empregado Efetivo
	F	Empregado Efetivo
	F	Empregado Efetivo
	F	Empregado Efetivo
	F	Empregado Efetivo
	F	Empregado Efetivo
	F	Empregado Efetivo
	F	Empregado Efetivo
	F	Empregado Efetivo
	F	Empregado Efetivo
	F	Empregado Efetivo
	F	Empregado Efetivo
	F	Empregado Efetivo
	F	Empregado Efetivo
	F	Empregado Efetivo
	F	Empregado Efetivo
	F	Empregado Efetivo
	F	Empregado Efetivo
	F	Empregado Efetivo
	F	Empregado Efetivo
	F	Empregado Efetivo
	F	Empregado Efetivo
	F	Empregado Efetivo
	F	Empregado Efetivo
	M	Empregado Efetivo
	M	Empregado Efetivo
	M	Empregado Efetivo
	M	Empregado Efetivo
	F	Empregado Efetivo
	F	Empregado Efetivo
	F	Empregado Efetivo
	F	Empregado Efetivo
	F	Empregado Efetivo
	F	Empregado Efetivo
	F	Empregado Efetivo
	F	Empregado Efetivo
	F	Empregado Efetivo
	F	Empregado Efetivo
	F	Empregado Efetivo
	F	Empregado Efetivo
	F	Empregado Efetivo
	F	Cedido - RJU
	F	Empregado Efetivo
	F	Empregado Efetivo
	F	Empregado Efetivo

	F	Empregado Efetivo
	F	Empregado Efetivo
	F	Empregado Efetivo
	F	Empregado Efetivo
	F	Empregado Efetivo
	F	Empregado Efetivo
	F	Empregado Efetivo
	F	Empregado Efetivo
	F	Empregado Efetivo
	F	Empregado Efetivo
	F	Empregado Efetivo
	F	Empregado Efetivo
	F	Empregado Efetivo
	F	Empregado Efetivo
	F	Empregado Efetivo
	M	Empregado Efetivo
	F	Empregado Efetivo
	F	Empregado Efetivo
	F	Empregado Efetivo
	F	Empregado Efetivo
	F	Empregado Efetivo
	F	Empregado Efetivo
	M	Empregado Efetivo
	F	Empregado Efetivo
	M	Empregado Efetivo
	F	Empregado Efetivo
	F	Empregado Efetivo
	F	Empregado Efetivo
	F	Empregado Efetivo
	F	Empregado Efetivo
	F	Empregado Efetivo
	F	Empregado Efetivo
	F	Empregado Efetivo
	F	Empregado Efetivo
	F	Empregado Efetivo
	F	Empregado Efetivo
	F	Empregado Efetivo
	F	Empregado Efetivo

	F	Empregado Efetivo
	F	Empregado Efetivo
	F	Empregado Efetivo
	F	Empregado Efetivo
	F	Empregado Efetivo
	F	Empregado Efetivo
	F	Empregado Efetivo
	F	Empregado Efetivo
	F	Empregado Efetivo
	F	Empregado Efetivo
	F	Empregado Efetivo
	F	Empregado Efetivo
	F	Empregado Efetivo
	F	Empregado Efetivo
	F	Empregado Efetivo
	F	Empregado Efetivo
	F	Empregado Efetivo
	F	Empregado Efetivo
	F	Empregado Efetivo
	M	Cedido - RJU
	F	Empregado Efetivo
	F	Empregado Efetivo
	F	Empregado Efetivo
	F	Empregado Efetivo
	F	Empregado Efetivo
	F	Empregado Efetivo
	F	Empregado Efetivo
	F	Empregado Efetivo
	F	Empregado Efetivo
	F	Empregado Efetivo
	F	Empregado Efetivo
	F	Empregado Efetivo
	F	Empregado Efetivo
	F	Empregado Efetivo
	F	Empregado Efetivo
	F	Empregado Efetivo
	F	Empregado Efetivo
	F	Empregado Efetivo
	F	Empregado Efetivo
	F	Empregado Efetivo
	F	Empregado Efetivo
	F	Empregado Efetivo
	F	Empregado Efetivo
	F	Empregado Efetivo
	F	Empregado Efetivo
	F	Empregado Efetivo
	F	Empregado Efetivo
	F	Empregado Efetivo

	F	Empregado Efetivo
	F	Empregado Efetivo
	F	Empregado Efetivo
	F	Empregado Efetivo
	F	Empregado Efetivo
	F	Empregado Efetivo
	F	Empregado Efetivo
	F	Empregado Efetivo
	F	Empregado Efetivo
	F	Empregado Efetivo
	F	Empregado Efetivo
	F	Empregado Efetivo
	F	Empregado Efetivo
	F	Empregado Efetivo
	F	Empregado Efetivo
	F	Empregado Efetivo
	M	Empregado Efetivo
	F	Empregado Efetivo
	F	Empregado Efetivo
	F	Empregado Efetivo
	F	Empregado Efetivo
	F	Empregado Efetivo
	F	Empregado Efetivo
	F	Empregado Efetivo
	F	Empregado Efetivo
	F	Empregado Efetivo
	F	Empregado Efetivo
	F	Empregado Efetivo
	F	Empregado Efetivo
	F	Empregado Efetivo
	F	Empregado Efetivo
	M	Empregado Efetivo
	M	Empregado Efetivo
	F	Empregado Efetivo
	F	Empregado Efetivo
	F	Empregado Efetivo
	F	Empregado Efetivo
	F	Empregado Efetivo
	F	Empregado Efetivo
	M	Empregado Efetivo
	F	Empregado Efetivo
	F	Empregado Efetivo
	F	Empregado Efetivo
	F	Empregado Efetivo
	F	Empregado Efetivo
	F	Empregado Efetivo
	F	Empregado Efetivo
	F	Empregado Efetivo

	F	Empregado Efetivo
	F	Empregado Efetivo
	F	Empregado Efetivo
	F	Empregado Efetivo
	F	Empregado Efetivo
	F	Empregado Efetivo
	F	Empregado Efetivo
	F	Empregado Efetivo
	F	Empregado Efetivo
	F	Empregado Efetivo
	F	Empregado Efetivo
	F	Empregado Efetivo
	F	Empregado Efetivo
	F	Empregado Efetivo
	F	Empregado Efetivo
	F	Empregado Efetivo
	F	Empregado Efetivo
	F	Empregado Efetivo
	F	Empregado Efetivo
	F	Empregado Efetivo
	F	Empregado Efetivo
	F	Empregado Efetivo
	F	Empregado Efetivo
	F	Empregado Efetivo
	F	Empregado Efetivo
	F	Empregado Efetivo
	F	Empregado Efetivo
	F	Empregado Efetivo
	M	Empregado Efetivo
	F	Empregado Efetivo
	F	Empregado Efetivo
	F	Empregado Efetivo
	F	Empregado Efetivo
	F	Empregado Efetivo
	F	Empregado Efetivo
	F	Empregado Efetivo
	F	Empregado Efetivo
	F	Empregado Efetivo
	F	Empregado Efetivo
	F	Empregado Efetivo
	F	Empregado Efetivo
	F	Empregado Efetivo
	F	Empregado Efetivo
	F	Empregado Efetivo
	F	Empregado Efetivo
	F	Empregado Efetivo
	F	Empregado Efetivo
	F	Empregado Efetivo

	F	Empregado Efetivo
	M	Empregado Efetivo
	F	Empregado Efetivo
	F	Empregado Efetivo
	F	Empregado Efetivo
	F	Empregado Efetivo
	F	Empregado Efetivo
	F	Empregado Efetivo
	F	Empregado Efetivo
	F	Empregado Efetivo
	M	Empregado Efetivo
	F	Empregado Efetivo
	F	Empregado Efetivo
	F	Empregado Efetivo
	M	Empregado Efetivo
	F	Empregado Efetivo
	M	Empregado Efetivo
	F	Empregado Efetivo
	F	Empregado Efetivo
	F	Empregado Efetivo
	F	Empregado Efetivo
	F	Empregado Efetivo
	F	Empregado Efetivo
	F	Empregado Efetivo
	F	Empregado Efetivo
	F	Empregado Efetivo
	F	Empregado Efetivo
	M	Empregado Efetivo
	F	Empregado Efetivo
	F	Empregado Efetivo
	F	Empregado Efetivo
	F	Empregado Efetivo
	F	Empregado Efetivo
	F	Empregado Efetivo
	F	Empregado Efetivo
	F	Empregado Efetivo
	F	Empregado Efetivo
	F	Empregado Efetivo
	F	Empregado Efetivo
	F	Empregado Efetivo
	F	Empregado Efetivo
	F	Empregado Efetivo
	F	Empregado Efetivo
	F	Empregado Efetivo

	F	Empregado Efetivo
	F	Empregado Efetivo
	F	Empregado Efetivo
	F	Empregado Efetivo
	F	Empregado Efetivo
	F	Empregado Efetivo
	F	Empregado Efetivo
	F	Empregado Efetivo
	F	Empregado Efetivo
	F	Empregado Efetivo
	F	Empregado Efetivo
	F	Empregado Efetivo
	F	Empregado Efetivo
	F	Empregado Efetivo
	F	Empregado Efetivo
	F	Empregado Efetivo
	F	Empregado Efetivo
	F	Empregado Efetivo
	F	Empregado Efetivo
	M	Empregado Efetivo
	M	Empregado Efetivo
	F	Empregado Efetivo
	F	Empregado Efetivo
	F	Empregado Efetivo
	F	Empregado Efetivo
	M	Empregado Efetivo
	F	Empregado Efetivo
	F	Empregado Efetivo
	F	Empregado Efetivo
	F	Empregado Efetivo
	F	Empregado Efetivo
	F	Empregado Efetivo
	F	Empregado Efetivo
	F	Empregado Efetivo
	M	Empregado Efetivo
	F	Empregado Efetivo
	M	Empregado Efetivo
	M	Empregado Efetivo

	F	Empregado Efetivo
	F	Empregado Efetivo
	F	Empregado Efetivo
	F	Empregado Efetivo
	F	Empregado Efetivo
	M	Empregado Efetivo
	F	Empregado Efetivo
	M	Empregado Efetivo
	F	Empregado Efetivo
	M	Empregado Efetivo
	F	Empregado Efetivo
	F	Empregado Efetivo
	F	Empregado Efetivo
	F	Empregado Efetivo
	F	Empregado Efetivo
	F	Empregado Efetivo
	F	Empregado Efetivo
	M	Empregado Efetivo
	M	Empregado Efetivo
	F	Empregado Efetivo
	F	Empregado Efetivo
	F	Empregado Efetivo
	F	Empregado Efetivo
	F	Empregado Efetivo
	F	Empregado Efetivo
	F	Empregado Efetivo
	F	Empregado Efetivo
	F	Empregado Efetivo
	F	Empregado Efetivo
	M	Empregado Efetivo
	F	Empregado Efetivo
	F	Empregado Efetivo
	F	Empregado Efetivo
	F	Empregado Efetivo
	M	Empregado Efetivo
	F	Empregado Efetivo
	F	Empregado Efetivo
	F	Empregado Efetivo
	F	Empregado Efetivo
	M	Empregado Efetivo

	F	Empregado Efetivo
	F	Empregado Efetivo
	F	Empregado Efetivo
	F	Empregado Efetivo
	M	Empregado Efetivo
	M	Empregado Efetivo
	F	Empregado Efetivo
	M	Empregado Efetivo
	F	Empregado Efetivo
	F	Empregado Efetivo
	F	Empregado Efetivo
	F	Empregado Efetivo
	F	Empregado Efetivo
	F	Empregado Efetivo
	F	Empregado Efetivo
	F	Empregado Efetivo
	F	Empregado Efetivo
	F	Empregado Efetivo
	F	Empregado Efetivo
	F	Empregado Efetivo
	F	Empregado Efetivo
	F	Empregado Efetivo
	F	Empregado Efetivo
	F	Empregado Efetivo
	F	Empregado Efetivo
	F	Empregado Efetivo
	F	Empregado Efetivo
	F	Empregado Efetivo
	F	Empregado Efetivo
	F	Empregado Efetivo
	F	Empregado Efetivo
	F	Empregado Efetivo
	F	Empregado Efetivo
	F	Empregado Efetivo
	F	Empregado Efetivo
	F	Empregado Efetivo
	F	Empregado Efetivo
	F	Empregado Efetivo
	M	Empregado Efetivo
	F	Empregado Efetivo
	F	Empregado Efetivo
	F	Empregado Efetivo
	F	Empregado Efetivo
	F	Empregado Efetivo

	F	Empregado Efetivo
	F	Empregado Efetivo
	F	Empregado Efetivo
	F	Empregado Efetivo
	F	Empregado Efetivo
	F	Empregado Efetivo
	F	Empregado Efetivo
	F	Empregado Efetivo
	F	Empregado Efetivo
	M	Empregado Efetivo
	F	Empregado Efetivo
	F	Empregado Efetivo
	F	Empregado Efetivo
	F	Empregado Efetivo
	F	Empregado Efetivo
	F	Empregado Efetivo
	M	Empregado Efetivo
	M	Empregado Efetivo
	F	Empregado Efetivo
	F	Empregado Efetivo
	F	Empregado Efetivo
	F	Empregado Efetivo
	F	Empregado Efetivo
	M	Empregado Efetivo
	F	Empregado Efetivo
	F	Empregado Efetivo
	F	Empregado Efetivo
	F	Empregado Efetivo
	F	Empregado Efetivo
	F	Empregado Efetivo
	F	Empregado Efetivo
	F	Empregado Efetivo
	F	Empregado Efetivo
	F	Empregado Efetivo
	F	Empregado Efetivo
	F	Empregado Efetivo
	F	Empregado Efetivo
	M	Empregado Efetivo
	M	Empregado Efetivo
	M	Empregado Efetivo
	F	Empregado Efetivo
	M	Empregado Efetivo
	F	Empregado Efetivo
	M	Empregado Efetivo
	F	Empregado Efetivo
	M	Empregado Efetivo
	M	Empregado Efetivo
	F	Empregado Efetivo

	F	Empregado Efetivo
	M	Empregado Efetivo
	F	Empregado Efetivo
	F	Empregado Efetivo
	F	Empregado Efetivo
	M	Empregado Efetivo
	F	Empregado Efetivo
	F	Empregado Efetivo
	F	Empregado Efetivo
	F	Empregado Efetivo
	F	Empregado Efetivo
	F	Empregado Efetivo
	F	Empregado Efetivo
	F	Empregado Efetivo
	F	Empregado Efetivo
	F	Empregado Efetivo
	F	Empregado Efetivo
	F	Empregado Efetivo
	F	Empregado Efetivo
	F	Empregado Efetivo
	F	Empregado Efetivo
	F	Empregado Efetivo
	F	Empregado Efetivo
	F	Empregado Efetivo
	F	Empregado Efetivo
	F	Empregado Efetivo
	F	Empregado Efetivo
	M	Empregado Efetivo
	M	Empregado Efetivo
	M	Empregado Efetivo
	M	Empregado Efetivo
	M	Empregado Efetivo
	M	Empregado Efetivo
	F	Empregado Efetivo
	F	Empregado Efetivo
	F	Empregado Efetivo
	M	Empregado Efetivo
	F	Empregado Efetivo
	M	Empregado Efetivo
	M	Empregado Efetivo
	M	Empregado Efetivo
	M	Empregado Efetivo
	F	Empregado Efetivo
	M	Empregado Efetivo
	F	Empregado Efetivo
	F	Empregado Efetivo
	F	Empregado Efetivo
	M	Empregado Efetivo

	F	Empregado Efetivo
	F	Empregado Efetivo
	F	Empregado Efetivo
	F	Empregado Efetivo
	F	Empregado Efetivo
	F	Empregado Efetivo
	M	Empregado Efetivo
	M	Empregado Efetivo
	F	Empregado Efetivo
	M	Empregado Efetivo
	F	Empregado Efetivo
	M	Empregado Efetivo
	F	Empregado Efetivo
	F	Empregado Efetivo
	M	Empregado Efetivo
	F	Empregado Efetivo
	F	Empregado Efetivo
	F	Empregado Efetivo
	F	Empregado Efetivo
	F	Empregado Efetivo
	F	Empregado Efetivo
	M	Empregado Efetivo
	F	Empregado Efetivo
	F	Empregado Efetivo
	M	Empregado Efetivo
	F	Empregado Efetivo
	F	Empregado Efetivo
	M	Empregado Efetivo
	M	Empregado Efetivo
	F	Empregado Efetivo
	F	Empregado Efetivo
	F	Empregado Efetivo
	F	Empregado Efetivo
	F	Empregado Efetivo
	F	Empregado Efetivo
	F	Empregado Efetivo
	F	Empregado Efetivo
	M	Empregado Efetivo
	F	Empregado Efetivo
	F	Empregado Efetivo
	F	Empregado Efetivo
	F	Empregado Efetivo
	F	Empregado Efetivo
	F	Empregado Efetivo
	F	Empregado Efetivo
	F	Empregado Efetivo
	F	Empregado Efetivo
	F	Empregado Efetivo

	F	Empregado Efetivo
	F	Empregado Efetivo
	M	Empregado Efetivo
	F	Empregado Efetivo
	F	Empregado Efetivo
	M	Empregado Efetivo
	M	Empregado Efetivo
	F	Empregado Efetivo
	F	Empregado Efetivo
	M	Empregado Efetivo
	F	Empregado Efetivo
	F	Empregado Efetivo
	M	Empregado Efetivo
	F	Empregado Efetivo
	F	Empregado Efetivo
	F	Empregado Efetivo
	M	Empregado Efetivo
	F	Empregado Efetivo
	F	Empregado Efetivo
	F	Empregado Efetivo
	F	Empregado Efetivo
	F	Empregado Efetivo
	F	Empregado Efetivo
	F	Empregado Efetivo
	F	Empregado Efetivo
	F	Empregado Efetivo
	F	Empregado Efetivo
	M	Empregado Efetivo
	F	Empregado Efetivo
	F	Empregado Efetivo
	F	Empregado Efetivo
	F	Empregado Efetivo
	F	Empregado Efetivo
	F	Empregado Efetivo
	F	Empregado Efetivo
	F	Empregado Efetivo
	F	Empregado Efetivo
	F	Empregado Efetivo
	F	Empregado Efetivo
	F	Empregado Efetivo
	M	Empregado Efetivo
	F	Empregado Efetivo
	F	Empregado Efetivo
	F	Empregado Efetivo
	F	Empregado Efetivo
	F	Empregado Efetivo
	F	Empregado Efetivo
	F	Empregado Efetivo

	M	Empregado Efetivo
	F	Empregado Efetivo
	F	Empregado Efetivo
	F	Empregado Efetivo
	F	Empregado Efetivo
	F	Empregado Efetivo
	F	Empregado Efetivo
	F	Empregado Efetivo
	F	Empregado Efetivo
	F	Empregado Efetivo
	F	Empregado Efetivo
	F	Empregado Efetivo
	F	Empregado Efetivo
	F	Empregado Efetivo
	F	Empregado Efetivo
	F	Empregado Efetivo
	F	Empregado Efetivo
	F	Empregado Efetivo
	F	Empregado Efetivo
	M	Empregado Efetivo
	F	Empregado Efetivo
	M	Empregado Efetivo
	F	Empregado Efetivo
	F	Empregado Efetivo
	F	Empregado Efetivo
	F	Empregado Efetivo
	F	Empregado Efetivo
	F	Empregado Efetivo
	M	Empregado Efetivo
	F	Empregado Efetivo
	M	Empregado Efetivo
	F	Empregado Efetivo
	F	Empregado Efetivo
	F	Empregado Efetivo
	M	Empregado Efetivo
	F	Empregado Efetivo
	M	Empregado Efetivo
	F	Empregado Efetivo
	F	Empregado Efetivo
	F	Empregado Efetivo
	M	Empregado Efetivo
	F	Empregado Efetivo
	F	Empregado Efetivo
	F	Empregado Efetivo
	F	Empregado Efetivo

	F	Empregado Efetivo
	M	Empregado Efetivo
	F	Empregado Efetivo
	F	Empregado Efetivo
	F	Empregado Efetivo
	F	Empregado Efetivo
	F	Empregado Efetivo
	F	Empregado Efetivo
	F	Empregado Efetivo
	M	Empregado Efetivo
	F	Empregado Efetivo
	F	Empregado Efetivo
	F	Empregado Efetivo
	M	Empregado Efetivo
	F	Empregado Efetivo
	F	Empregado Efetivo
	F	Empregado Efetivo
	F	Empregado Efetivo
	M	Empregado Efetivo
	F	Empregado Efetivo
	F	Empregado Efetivo
	F	Empregado Efetivo
	F	Empregado Efetivo
	M	Empregado Efetivo
	M	Empregado Efetivo
	F	Empregado Efetivo
	F	Empregado Efetivo
	F	Empregado Efetivo
	F	Empregado Efetivo
	F	Empregado Efetivo
	M	Empregado Efetivo
	F	Empregado Efetivo

	F	Empregado Efetivo
	M	Empregado Efetivo
	F	Empregado Efetivo
	F	Empregado Efetivo
	M	Empregado Efetivo
	F	Empregado Efetivo
	M	Empregado Efetivo
	M	Empregado Efetivo
	F	Empregado Efetivo
	F	Empregado Efetivo
	F	Empregado Efetivo
	F	Empregado Efetivo
	F	Empregado Efetivo
	M	Empregado Efetivo
	F	Empregado Efetivo
	F	Empregado Efetivo
	F	Empregado Efetivo
	F	Empregado Efetivo
	F	Empregado Efetivo
	F	Empregado Efetivo
	F	Empregado Efetivo
	F	Empregado Efetivo
	F	Empregado Efetivo
	F	Empregado Efetivo
	F	Empregado Efetivo
	F	Empregado Efetivo
	F	Empregado Efetivo
	F	Empregado Efetivo
	F	Empregado Efetivo
	F	Empregado Efetivo
	F	Empregado Efetivo
	F	Empregado Efetivo

	F	Empregado Efetivo
	F	Empregado Efetivo
	F	Empregado Efetivo
	F	Empregado Efetivo
	F	Empregado Efetivo
	F	Empregado Efetivo
	F	Empregado Efetivo
	F	Empregado Efetivo
	F	Empregado Efetivo
	F	Empregado Efetivo
	F	Empregado Efetivo
	F	Empregado Efetivo
	F	Empregado Efetivo
	M	Empregado Efetivo
	F	Empregado Efetivo
	F	Empregado Efetivo
	M	Empregado Efetivo
	F	Empregado Efetivo
	F	Empregado Efetivo
	F	Empregado Efetivo
	F	Empregado Efetivo
	F	Empregado Efetivo
	F	Empregado Efetivo
	F	Empregado Efetivo
	F	Empregado Efetivo
	F	Empregado Efetivo
	F	Empregado Efetivo
	F	Empregado Efetivo
	M	Empregado Efetivo
	F	Empregado Efetivo
	F	Empregado Efetivo
	F	Empregado Efetivo
	F	Empregado Efetivo
	F	Empregado Efetivo
	M	Empregado Efetivo

	M	Empregado Efetivo
	F	Empregado Efetivo
	F	Empregado Efetivo
	F	Empregado Efetivo
	F	Empregado Efetivo
	M	Empregado Efetivo
	F	Empregado Efetivo
	F	Empregado Efetivo
	F	Empregado Efetivo
	F	Empregado Efetivo
	F	Empregado Efetivo
	F	Empregado Efetivo
	M	Empregado Efetivo
	M	Empregado Efetivo
	M	Empregado Efetivo
	M	Empregado Efetivo
	M	Empregado Efetivo
	F	Empregado Efetivo
	F	Empregado Efetivo
	F	Empregado Efetivo
	F	Empregado Efetivo
	F	Empregado Efetivo
	F	Empregado Efetivo
	F	Empregado Efetivo
	F	Empregado Efetivo
	F	Empregado Efetivo
	F	Empregado Efetivo
	F	Empregado Efetivo
	F	Empregado Efetivo
	F	Empregado Efetivo
	F	Empregado Efetivo
	M	Empregado Efetivo
	M	Empregado Efetivo
	M	Empregado Efetivo
	M	Empregado Efetivo
	F	Empregado Efetivo
	F	Empregado Efetivo
	F	Empregado Efetivo
	F	Empregado Efetivo
	F	Empregado Efetivo
	F	Empregado Efetivo
	F	Empregado Efetivo
	F	Empregado Efetivo
	F	Empregado Efetivo
	F	Empregado Efetivo
	F	Empregado Efetivo
	F	Empregado Efetivo
	F	Empregado Efetivo

	F	Empregado Efetivo
	F	Empregado Efetivo
	F	Empregado Efetivo
	F	Empregado Efetivo
	F	Empregado Efetivo
	F	Empregado Efetivo
	F	Empregado Efetivo
	F	Empregado Efetivo
	F	Empregado Efetivo
	F	Empregado Efetivo
	M	Empregado Efetivo
	M	Empregado Efetivo
	M	Empregado Efetivo
	M	Empregado Efetivo
	M	Empregado Efetivo
	F	Empregado Efetivo
	F	Empregado Efetivo
	F	Empregado Efetivo
	F	Empregado Efetivo
	M	Empregado Efetivo
	F	Empregado Efetivo
	F	Empregado Efetivo
	F	Empregado Efetivo
	M	Empregado Efetivo
	F	Empregado Efetivo
	F	Empregado Efetivo
	F	Empregado Efetivo
	M	Empregado Efetivo
	M	Empregado Efetivo
	F	Empregado Efetivo
	F	Empregado Efetivo
	F	Empregado Efetivo
	F	Empregado Efetivo
	F	Empregado Efetivo
	F	Empregado Efetivo
	F	Empregado Efetivo
	M	Empregado Efetivo
	F	Empregado Efetivo
	F	Empregado Efetivo
	F	Empregado Efetivo
	M	Empregado Efetivo
	F	Empregado Efetivo
	F	Empregado Efetivo
	F	Empregado Efetivo
	F	Empregado Efetivo
	F	Empregado Efetivo
	F	Empregado Efetivo
	F	Empregado Efetivo

	F	Empregado Efetivo
	F	Empregado Efetivo
	F	Empregado Efetivo
	F	Empregado Efetivo
	F	Empregado Efetivo
	F	Empregado Efetivo
	F	Empregado Efetivo
	F	Empregado Efetivo
	F	Empregado Efetivo
	F	Empregado Efetivo
	F	Empregado Efetivo
	F	Empregado Efetivo
	M	Empregado Efetivo
	F	Empregado Efetivo
	F	Empregado Efetivo
	F	Empregado Efetivo
	F	Empregado Efetivo
	F	Empregado Efetivo
	F	Empregado Efetivo
	F	Empregado Efetivo
	M	Empregado Efetivo
	F	Empregado Efetivo
	F	Empregado Efetivo
	F	Empregado Efetivo
	F	Empregado Efetivo
	F	Empregado Efetivo
	F	Empregado Efetivo
	F	Empregado Efetivo
	F	Empregado Efetivo
	F	Empregado Efetivo
	F	Empregado Efetivo
	F	Empregado Efetivo

	F	Empregado Efetivo
	F	Empregado Efetivo
	F	Empregado Efetivo
	F	Empregado Efetivo
	F	Empregado Efetivo
	M	Empregado Efetivo
	F	Empregado Efetivo
	F	Empregado Efetivo
	M	Empregado Efetivo
	F	Empregado Efetivo
	F	Empregado Efetivo
	F	Empregado Efetivo
	F	Empregado Efetivo
	F	Empregado Efetivo
	F	Empregado Efetivo
	F	Empregado Efetivo
	F	Empregado Efetivo
	F	Empregado Efetivo
	F	Empregado Efetivo
	F	Empregado Efetivo
	F	Empregado Efetivo
	F	Empregado Efetivo
	F	Empregado Efetivo
	F	Empregado Efetivo
	F	Empregado Efetivo
	F	Empregado Efetivo
	F	Empregado Efetivo
	F	Empregado Efetivo
	F	Empregado Efetivo
	F	Empregado Efetivo
	F	Empregado Efetivo
	F	Empregado Efetivo
	M	Empregado Efetivo
	F	Empregado Efetivo
	F	Empregado Efetivo
	F	Empregado Efetivo
	F	Empregado Efetivo
	F	Empregado Efetivo
	F	Empregado Efetivo
	F	Empregado Efetivo
	F	Empregado Efetivo
	F	Empregado Efetivo
	F	Empregado Efetivo

	M	Empregado Efetivo
	F	Empregado Efetivo
	M	Empregado Efetivo
	F	Empregado Efetivo
	F	Empregado Efetivo
	F	Empregado Efetivo
	F	Empregado Efetivo
	F	Empregado Efetivo
	F	Empregado Efetivo
	F	Empregado Efetivo
	F	Empregado Efetivo
	F	Empregado Efetivo
	F	Empregado Efetivo
	F	Empregado Efetivo
	F	Empregado Efetivo
	F	Empregado Efetivo
	F	Empregado Efetivo
	F	Empregado Efetivo
	F	Empregado Efetivo
	F	Empregado Efetivo
	F	Empregado Efetivo
	F	Empregado Efetivo
	F	Empregado Efetivo
	F	Empregado Efetivo
	F	Empregado Efetivo
	F	Empregado Efetivo
	F	Empregado Efetivo
	F	Empregado Efetivo
	F	Empregado Efetivo
	F	Empregado Efetivo
	F	Empregado Efetivo
	F	Empregado Efetivo
	F	Empregado Efetivo
	F	Empregado Efetivo
	F	Empregado Efetivo
	F	Empregado Efetivo
	M	Empregado Efetivo
	M	Empregado Efetivo
	F	Empregado Efetivo
	F	Empregado Efetivo
	F	Empregado Efetivo
	F	Empregado Efetivo
	F	Empregado Efetivo
	F	Empregado Efetivo
	F	Empregado Efetivo
	F	Empregado Efetivo
	F	Empregado Efetivo
	F	Empregado Efetivo

	F	Empregado Efetivo
	F	Empregado Efetivo
	F	Empregado Efetivo
	F	Empregado Efetivo
	M	Empregado Efetivo
	M	Empregado Efetivo
	F	Empregado Efetivo
	F	Empregado Efetivo
	F	Empregado Efetivo
	F	Empregado Efetivo
	F	Empregado Efetivo
	F	Empregado Efetivo
	F	Empregado Efetivo
	M	Empregado Efetivo
	M	Empregado Efetivo
	F	Empregado Efetivo
	F	Empregado Efetivo
	F	Empregado Efetivo
	F	Empregado Efetivo
	M	Empregado Efetivo
	F	Empregado Efetivo
	F	Empregado Efetivo
	F	Empregado Efetivo
	M	Empregado Efetivo
	F	Empregado Efetivo
	F	Empregado Efetivo
	F	Empregado Efetivo
	F	Empregado Efetivo
	M	Empregado Efetivo
	F	Empregado Efetivo
	F	Empregado Efetivo
	F	Empregado Efetivo
	M	Empregado Efetivo
	F	Empregado Efetivo
	F	Empregado Efetivo
	F	Empregado Efetivo
	F	Empregado Efetivo
	F	Empregado Efetivo
	F	Empregado Efetivo
	F	Empregado Efetivo
	F	Empregado Efetivo
	F	Empregado Efetivo
	F	Empregado Efetivo
	M	Empregado Efetivo
	F	Empregado Efetivo
	F	Empregado Efetivo
	M	Empregado Efetivo
	F	Empregado Efetivo

	F	Empregado Efetivo
	F	Empregado Efetivo
	F	Empregado Efetivo
	M	Empregado Efetivo
	M	Empregado Efetivo
	F	Empregado Efetivo
	F	Empregado Efetivo
	F	Empregado Efetivo
	F	Empregado Efetivo
	F	Empregado Efetivo
	F	Empregado Efetivo
	M	Empregado Efetivo
	F	Empregado Efetivo
	M	Empregado Efetivo
	M	Empregado Efetivo
	M	Empregado Efetivo
	F	Empregado Efetivo
	F	Empregado Efetivo
	F	Empregado Efetivo
	M	Empregado Efetivo
	M	Empregado Efetivo
	F	Empregado Efetivo
	F	Empregado Efetivo
	F	Empregado Efetivo
	M	Empregado Efetivo
	F	Empregado Efetivo
	F	Empregado Efetivo
	F	Empregado Efetivo
	M	Empregado Efetivo
	M	Empregado Efetivo
	M	Empregado Efetivo
	F	Empregado Efetivo
	F	Empregado Efetivo
	F	Empregado Efetivo
	F	Empregado Efetivo
	M	Empregado Efetivo
	M	Empregado Efetivo
	F	Empregado Efetivo

	F	Empregado Efetivo
	M	Empregado Efetivo
	F	Empregado Efetivo
	F	Empregado Efetivo
	F	Empregado Efetivo
	M	Empregado Efetivo
	F	Empregado Efetivo
	F	Empregado Efetivo
	F	Empregado Efetivo
	M	Empregado Efetivo
	M	Empregado Efetivo
	F	Empregado Efetivo
	F	Empregado Efetivo
	F	Empregado Efetivo
	F	Empregado Efetivo
	F	Empregado Efetivo
	M	Empregado Efetivo
	F	Empregado Efetivo
	F	Empregado Efetivo
	F	Empregado Efetivo
	M	Empregado Efetivo
	M	Empregado Efetivo
	M	Empregado Efetivo
	M	Empregado Efetivo
	M	Empregado Efetivo
	M	Empregado Efetivo
	M	Empregado Efetivo
	F	Empregado Efetivo
	M	Empregado Efetivo
	F	Empregado Efetivo
	M	Empregado Efetivo
	M	Empregado Efetivo
	M	Empregado Efetivo
	M	Empregado Efetivo

	M	Empregado Efetivo
	M	Empregado Efetivo
	M	Empregado Efetivo
	M	Empregado Efetivo
	M	Empregado Efetivo
	M	Empregado Efetivo
	F	Empregado Efetivo
	F	Empregado Efetivo
	M	Empregado Efetivo
	M	Empregado Efetivo
	M	Cedido - RJU
	F	Empregado Efetivo
	F	Empregado Efetivo
	M	Empregado Efetivo
	M	Empregado Efetivo
	M	Empregado Efetivo
	M	Empregado Efetivo
	M	Empregado Efetivo
	M	Empregado Efetivo
	F	Empregado Efetivo
	M	Empregado Efetivo
	F	Empregado Efetivo
	M	Empregado Efetivo
	F	Empregado Efetivo
	M	Empregado Efetivo
	F	Empregado Efetivo
	F	Empregado Efetivo
	F	Empregado Efetivo
	F	Empregado Efetivo
	M	Empregado Efetivo
	F	Empregado Efetivo
	F	Empregado Efetivo
	F	Empregado Efetivo
	F	Empregado Efetivo
	F	Empregado Efetivo
	F	Empregado Efetivo
	M	Empregado Efetivo
	F	Empregado Efetivo

	F	Empregado Efetivo
	M	Empregado Efetivo
	M	Empregado Efetivo
	F	Empregado Efetivo
	F	Empregado Efetivo
	F	Empregado Efetivo
	M	Empregado Efetivo
	M	Empregado Efetivo
	M	Empregado Efetivo
	F	Empregado Efetivo
	F	Empregado Efetivo
	M	Empregado Efetivo
	F	Empregado Efetivo
	F	Empregado Efetivo
	M	Empregado Efetivo
	M	Empregado Efetivo
	M	Empregado Efetivo
	F	Empregado Efetivo
	F	Empregado Efetivo
	M	Empregado Efetivo
	F	Empregado Efetivo
	F	Empregado Efetivo
	F	Empregado Efetivo
	F	Empregado Efetivo
	F	Empregado Efetivo
	F	Empregado Efetivo
	M	Empregado Efetivo
	F	Empregado Efetivo
	F	Empregado Efetivo
	F	Empregado Efetivo
	F	Empregado Efetivo
	F	Empregado Efetivo
	M	Empregado Efetivo
	F	Empregado Efetivo

	F	Empregado Efetivo
	F	Empregado Efetivo
	F	Empregado Efetivo
	F	Empregado Efetivo
	F	Empregado Efetivo
	F	Empregado Efetivo
	F	Empregado Efetivo
	F	Empregado Efetivo
	M	Empregado Efetivo
	F	Empregado Efetivo
	F	Empregado Efetivo
	F	Empregado Efetivo
	F	Empregado Efetivo
	F	Empregado Efetivo
	F	Empregado Efetivo
	F	Empregado Efetivo
	F	Empregado Efetivo
	M	Empregado Efetivo
	M	Empregado Efetivo
	F	Empregado Efetivo
	M	Empregado Efetivo
	M	Empregado Efetivo
	F	Empregado Efetivo
	F	Empregado Efetivo
	M	Empregado Efetivo
	M	Empregado Efetivo
	M	Empregado Efetivo
	F	Empregado Efetivo
	M	Empregado Efetivo
	M	Empregado Efetivo
	F	Empregado Efetivo
	F	Empregado Efetivo
	F	Empregado Efetivo
	F	Empregado Efetivo
	F	Empregado Efetivo
	F	Empregado Efetivo
	F	Empregado Efetivo
	F	Empregado Efetivo
	F	Empregado Efetivo
	F	Empregado Efetivo
	F	Empregado Efetivo
	F	Empregado Efetivo
	F	Empregado Efetivo
	F	Empregado Efetivo
	F	Empregado Efetivo
	F	Empregado Efetivo
	F	Empregado Efetivo

	F	Empregado Efetivo
	F	Empregado Efetivo
	F	Empregado Efetivo
	F	Empregado Efetivo
	F	Empregado Efetivo
	F	Empregado Efetivo
	F	Empregado Efetivo
	F	Empregado Efetivo
	F	Empregado Efetivo
	F	Empregado Efetivo
	M	Empregado Efetivo
	F	Empregado Efetivo
	F	Empregado Efetivo
	M	Empregado Efetivo
	F	Empregado Efetivo
	F	Empregado Efetivo
	F	Empregado Efetivo
	F	Empregado Efetivo
	F	Empregado Efetivo
	F	Empregado Efetivo
	F	Empregado Efetivo
	F	Empregado Efetivo
	F	Empregado Efetivo
	F	Empregado Efetivo
	M	Empregado Efetivo
	F	Empregado Efetivo
	M	Empregado Efetivo
	F	Empregado Efetivo
	F	Empregado Efetivo
	F	Empregado Efetivo
	F	Empregado Efetivo
	F	Empregado Efetivo
	F	Empregado Efetivo
	F	Empregado Efetivo
	F	Empregado Efetivo
	F	Empregado Efetivo
	F	Empregado Efetivo
	F	Empregado Efetivo
	F	Empregado Efetivo
	F	Empregado Efetivo
	F	Empregado Efetivo
	F	Empregado Efetivo
	F	Empregado Efetivo
	F	Empregado Efetivo
	F	Empregado Efetivo
	F	Empregado Efetivo
	M	Empregado Efetivo
	F	Empregado Efetivo

	F	Empregado Efetivo
	F	Empregado Efetivo
	F	Empregado Efetivo
	F	Empregado Efetivo
	F	Empregado Efetivo
	F	Empregado Efetivo
	F	Empregado Efetivo
	F	Empregado Efetivo
	F	Empregado Efetivo
	F	Empregado Efetivo
	F	Empregado Efetivo
	F	Empregado Efetivo
	M	Empregado Efetivo
	M	Empregado Efetivo
	M	Empregado Efetivo
	F	Empregado Efetivo
	M	Empregado Efetivo
	M	Empregado Efetivo
	M	Empregado Efetivo
	F	Empregado Efetivo
	F	Empregado Efetivo
	F	Empregado Efetivo
	F	Empregado Efetivo
	F	Empregado Efetivo
	F	Empregado Efetivo
	F	Empregado Efetivo
	F	Empregado Efetivo
	F	Empregado Efetivo
	M	Empregado Efetivo
	F	Empregado Efetivo
	F	Empregado Efetivo
	F	Empregado Efetivo
	F	Empregado Efetivo
	F	Empregado Efetivo
	F	Empregado Efetivo
	M	Empregado Efetivo
	M	Empregado Efetivo
	F	Empregado Efetivo
	M	Empregado Efetivo
	F	Empregado Efetivo
	F	Empregado Efetivo
	F	Empregado Efetivo
	F	Empregado Efetivo
	F	Empregado Efetivo
	M	Empregado Efetivo
	M	Empregado Efetivo

	F	Empregado Efetivo
	M	Empregado Efetivo
	F	Empregado Efetivo
	F	Empregado Efetivo
	M	Empregado Efetivo
	M	Empregado Efetivo
	F	Empregado Efetivo
	F	Empregado Efetivo
	M	Empregado Efetivo
	F	Empregado Efetivo
	F	Empregado Efetivo
	F	Empregado Efetivo
	F	Empregado Efetivo
	M	Empregado Efetivo
	M	Empregado Efetivo
	F	Empregado Efetivo
	M	Empregado Efetivo
	M	Empregado Efetivo
	F	Empregado Efetivo
	F	Empregado Efetivo
	F	Empregado Efetivo
	F	Empregado Efetivo
	F	Empregado Efetivo
	M	Empregado Efetivo
	M	Empregado Efetivo
	F	Empregado Efetivo
	F	Empregado Efetivo
	F	Empregado Efetivo
	M	Empregado Efetivo
	F	Empregado Efetivo
	F	Empregado Efetivo
	M	Empregado Efetivo
	F	Empregado Efetivo
	M	Empregado Efetivo
	M	Empregado Efetivo
	F	Empregado Efetivo
	M	Empregado Efetivo
	F	Empregado Efetivo
	F	Empregado Efetivo
	F	Empregado Efetivo
	M	Empregado Efetivo
	F	Empregado Efetivo
	M	Empregado Efetivo
	F	Empregado Efetivo
	M	Empregado Efetivo
	F	Empregado Efetivo
	F	Empregado Efetivo
	F	Empregado Efetivo

	F	Empregado Efetivo
	M	Empregado Efetivo
	F	Empregado Efetivo
	F	Empregado Efetivo
	F	Empregado Efetivo
	F	Empregado Efetivo
	F	Empregado Efetivo
	F	Empregado Efetivo
	M	Empregado Efetivo
	F	Empregado Efetivo
	F	Empregado Efetivo
	F	Empregado Efetivo
	F	Empregado Efetivo
	F	Empregado Efetivo
	F	Empregado Efetivo
	F	Empregado Efetivo
	F	Empregado Efetivo
	F	Empregado Efetivo
	F	Empregado Efetivo
	M	Empregado Efetivo
	F	Empregado Efetivo
	M	Empregado Efetivo
	F	Empregado Efetivo
	F	Empregado Efetivo
	F	Empregado Efetivo
	F	Empregado Efetivo
	F	Empregado Efetivo
	F	Empregado Efetivo
	F	Empregado Efetivo
	F	Empregado Efetivo
	F	Empregado Efetivo
	F	Empregado Efetivo
	F	Empregado Efetivo
	F	Empregado Efetivo
	F	Empregado Efetivo
	F	Empregado Efetivo
	F	Empregado Efetivo
	F	Empregado Efetivo
	F	Empregado Efetivo
	F	Empregado Efetivo
	F	Empregado Efetivo
	F	Empregado Efetivo
	F	Empregado Efetivo
	M	Empregado Efetivo
	F	Empregado Efetivo
	F	Empregado Efetivo
	M	Empregado Efetivo

	M	Empregado Efetivo
	F	Empregado Efetivo
	F	Empregado Efetivo
	M	Empregado Efetivo
	F	Empregado Efetivo
	F	Empregado Efetivo
	F	Empregado Efetivo
	F	Empregado Efetivo
	F	Empregado Efetivo
	F	Empregado Efetivo
	M	Empregado Efetivo
	M	Empregado Efetivo
	M	Empregado Efetivo
	F	Empregado Efetivo
	F	Empregado Efetivo
	M	Empregado Efetivo
	M	Empregado Efetivo
	F	Empregado Efetivo
	F	Empregado Efetivo
	F	Empregado Efetivo
	F	Empregado Efetivo
	F	Empregado Efetivo
	F	Empregado Efetivo
	M	Empregado Efetivo
	M	Empregado Efetivo
	M	Empregado Efetivo
	F	Empregado Efetivo
	F	Empregado Efetivo
	M	Empregado Efetivo
	F	Empregado Efetivo
	F	Empregado Efetivo
	M	Empregado Efetivo
	M	Empregado Efetivo
	F	Empregado Efetivo
	M	Empregado Efetivo
	F	Empregado Efetivo
	M	Empregado Efetivo
	F	Empregado Efetivo
	F	Empregado Efetivo
	F	Empregado Efetivo
	M	Empregado Efetivo
	M	Empregado Efetivo
	F	Empregado Efetivo
	F	Empregado Efetivo
	F	Empregado Efetivo
	F	Empregado Efetivo
	M	Empregado Efetivo
	F	Empregado Efetivo

	M	Empregado Efetivo
	F	Empregado Efetivo
	F	Empregado Efetivo
	M	Empregado Efetivo
	F	Empregado Efetivo
	M	Empregado Efetivo
	F	Empregado Efetivo
	F	Empregado Efetivo
	F	Empregado Efetivo
	F	Empregado Efetivo
	F	Empregado Efetivo
	F	Empregado Efetivo
	F	Empregado Efetivo
	F	Empregado Efetivo
	M	Empregado Efetivo
	F	Empregado Efetivo
	F	Empregado Efetivo
	F	Empregado Efetivo
	M	Empregado Efetivo
	F	Empregado Efetivo
	F	Empregado Efetivo
	F	Empregado Efetivo
	M	Empregado Efetivo
	F	Empregado Efetivo
	F	Empregado Efetivo
	M	Empregado Efetivo
	M	Empregado Efetivo
	F	Empregado Efetivo
	F	Empregado Efetivo
	F	Empregado Efetivo
	F	Empregado Efetivo
	F	Empregado Efetivo
	F	Empregado Efetivo
	F	Empregado Efetivo
	M	Empregado Efetivo
	F	Empregado Efetivo
	M	Empregado Efetivo
	F	Empregado Efetivo
	M	Empregado Efetivo
	M	Empregado Efetivo
	F	Empregado Efetivo
	M	Empregado Efetivo
	F	Empregado Efetivo
	F	Empregado Efetivo
	F	Empregado Efetivo
	F	Empregado Efetivo
	F	Empregado Efetivo
	F	Empregado Efetivo

	F	Empregado Efetivo
	M	Empregado Efetivo
	F	Empregado Efetivo
	F	Empregado Efetivo
	F	Empregado Efetivo
	F	Empregado Efetivo
	F	Empregado Efetivo
	F	Empregado Efetivo
	F	Empregado Efetivo
	F	Empregado Efetivo
	F	Empregado Efetivo
	F	Empregado Efetivo
	F	Empregado Efetivo
	F	Empregado Efetivo
	M	Empregado Efetivo
	M	Empregado Efetivo
	F	Empregado Efetivo
	F	Empregado Efetivo
	M	Empregado Efetivo
	F	Empregado Efetivo
	F	Empregado Efetivo
	F	Empregado Efetivo
	M	Empregado Efetivo
	F	Empregado Efetivo
	F	Empregado Efetivo
	M	Empregado Efetivo
	F	Empregado Efetivo
	F	Empregado Efetivo
	F	Empregado Efetivo
	F	Empregado Efetivo
	F	Empregado Efetivo
	F	Empregado Efetivo
	F	Empregado Efetivo
	F	Empregado Efetivo
	M	Empregado Efetivo
	F	Empregado Efetivo
	F	Empregado Efetivo
	F	Empregado Efetivo
	F	Empregado Efetivo
	F	Empregado Efetivo
	F	Empregado Efetivo
	F	Empregado Efetivo
	F	Empregado Efetivo
	F	Empregado Efetivo
	M	Empregado Efetivo
	F	Empregado Efetivo
	M	Empregado Efetivo
	F	Empregado Efetivo

	F	Empregado Efetivo
	F	Empregado Efetivo
	M	Empregado Efetivo
	M	Empregado Efetivo
	M	Empregado Efetivo
	F	Empregado Efetivo
	M	Empregado Efetivo
	F	Empregado Efetivo
	M	Empregado Efetivo
	F	Empregado Efetivo
	F	Empregado Efetivo
	F	Empregado Efetivo
	F	Empregado Efetivo
	F	Empregado Efetivo
	F	Empregado Efetivo
	F	Empregado Efetivo
	F	Empregado Efetivo
	F	Empregado Efetivo
	F	Empregado Efetivo
	F	Empregado Efetivo
	F	Empregado Efetivo
	F	Empregado Efetivo
	M	Empregado Efetivo
	F	Empregado Efetivo
	M	Empregado Efetivo
	F	Empregado Efetivo
	F	Empregado Efetivo
	F	Empregado Efetivo
	F	Empregado Efetivo
	F	Empregado Efetivo
	F	Empregado Efetivo
	F	Empregado Efetivo
	F	Empregado Efetivo
	F	Empregado Efetivo
	F	Empregado Efetivo
	F	Empregado Efetivo
	F	Empregado Efetivo
	F	Empregado Efetivo

	F	Empregado Efetivo
	F	Empregado Efetivo
	F	Empregado Efetivo
	F	Empregado Efetivo
	F	Empregado Efetivo
	F	Empregado Efetivo
	F	Empregado Efetivo
	M	Empregado Efetivo
	F	Empregado Efetivo
	F	Empregado Efetivo
	F	Empregado Efetivo
	F	Empregado Efetivo
	F	Empregado Efetivo
	F	Empregado Efetivo
	F	Empregado Efetivo
	F	Empregado Efetivo
	F	Empregado Efetivo
	F	Empregado Efetivo
	F	Empregado Efetivo
	F	Empregado Efetivo
	F	Empregado Efetivo
	F	Empregado Efetivo
	F	Empregado Efetivo
	F	Empregado Efetivo
	F	Empregado Efetivo
	F	Empregado Efetivo
	F	Empregado Efetivo
	F	Empregado Efetivo
	F	Empregado Efetivo
	F	Empregado Efetivo
	F	Empregado Efetivo
	F	Empregado Efetivo

	F	Empregado Efetivo
	F	Empregado Efetivo
	F	Empregado Efetivo
	M	Empregado Efetivo
	F	Empregado Efetivo
	F	Empregado Efetivo
	F	Empregado Efetivo
	M	Empregado Efetivo
	F	Empregado Efetivo
	F	Empregado Efetivo
	M	Empregado Efetivo
	F	Empregado Efetivo
	F	Empregado Efetivo
	F	Empregado Efetivo
	M	Empregado Efetivo
	F	Empregado Efetivo
	F	Empregado Efetivo
	F	Empregado Efetivo
	F	Empregado Efetivo
	F	Empregado Efetivo
	M	Empregado Efetivo
	M	Empregado Efetivo
	F	Empregado Efetivo
	F	Empregado Efetivo
	F	Empregado Efetivo
	M	Empregado Efetivo
	F	Empregado Efetivo
	F	Empregado Efetivo
	F	Empregado Efetivo
	M	Empregado Efetivo
	M	Empregado Efetivo
	M	Empregado Efetivo
	M	Empregado Efetivo
	F	Empregado Efetivo
	F	Empregado Efetivo
	F	Empregado Efetivo
	M	Empregado Efetivo
	M	Empregado Efetivo
	F	Empregado Efetivo
	F	Empregado Efetivo
	F	Empregado Efetivo
	F	Empregado Efetivo
	F	Empregado Efetivo
	F	Empregado Efetivo

	F	Empregado Efetivo
	F	Empregado Efetivo
	F	Empregado Efetivo
	F	Empregado Efetivo
	F	Empregado Efetivo
	M	Empregado Efetivo
	M	Empregado Efetivo
	F	Empregado Efetivo
	F	Empregado Efetivo
	F	Empregado Efetivo
	M	Empregado Efetivo
	M	Empregado Efetivo
	F	Empregado Efetivo
	F	Empregado Efetivo
	F	Empregado Efetivo
	F	Empregado Efetivo
	F	Empregado Efetivo
	F	Empregado Efetivo
	F	Empregado Efetivo
	F	Empregado Efetivo
	F	Empregado Efetivo
	F	Empregado Efetivo
	F	Empregado Efetivo
	F	Empregado Efetivo
	F	Empregado Efetivo
	F	Empregado Efetivo
	F	Empregado Efetivo
	F	Empregado Efetivo
	F	Empregado Efetivo
	F	Empregado Efetivo
	F	Empregado Efetivo
	M	Empregado Efetivo
	F	Empregado Efetivo
	M	Empregado Efetivo
	F	Empregado Efetivo
	F	Empregado Efetivo
	F	Empregado Efetivo
	F	Empregado Efetivo
	M	Empregado Efetivo
	F	Empregado Efetivo
	F	Empregado Efetivo

	F	Empregado Efetivo
	F	Empregado Efetivo
	F	Empregado Efetivo
	M	Empregado Efetivo
	M	Empregado Efetivo
	F	Empregado Efetivo
	F	Empregado Efetivo
	F	Empregado Efetivo
	F	Empregado Efetivo
	F	Empregado Efetivo
	M	Empregado Efetivo
	F	Empregado Efetivo
	F	Empregado Efetivo
	F	Empregado Efetivo
	F	Empregado Efetivo
	F	Empregado Efetivo
	F	Empregado Efetivo
	F	Empregado Efetivo
	F	Empregado Efetivo
	F	Empregado Efetivo
	M	Empregado Efetivo
	M	Empregado Efetivo
	F	Empregado Efetivo
	F	Empregado Efetivo
	F	Empregado Efetivo
	F	Empregado Efetivo
	F	Empregado Efetivo
	F	Empregado Efetivo
	M	Empregado Efetivo
	M	Empregado Efetivo
	F	Empregado Efetivo
	M	Empregado Efetivo
	F	Empregado Efetivo
	F	Empregado Efetivo
	F	Empregado Efetivo
	F	Empregado Efetivo

	F	Empregado Efetivo
	F	Empregado Efetivo
	F	Empregado Efetivo
	F	Empregado Efetivo
	F	Empregado Efetivo
	F	Empregado Efetivo
	F	Empregado Efetivo
	F	Empregado Efetivo
	F	Empregado Efetivo
	M	Empregado Efetivo
	F	Empregado Efetivo
	M	Empregado Efetivo
	F	Empregado Efetivo
	F	Empregado Efetivo
	F	Empregado Efetivo
	M	Empregado Efetivo
	F	Empregado Efetivo
	M	Empregado Efetivo
	M	Empregado Efetivo
	F	Empregado Efetivo
	F	Empregado Efetivo
	F	Empregado Efetivo
	M	Empregado Efetivo
	F	Empregado Efetivo
	F	Empregado Efetivo
	M	Empregado Efetivo
	F	Empregado Efetivo
	M	Empregado Efetivo
	M	Empregado Efetivo
	F	Empregado Efetivo
	F	Empregado Efetivo
	M	Empregado Efetivo
	M	Empregado Efetivo
	F	Empregado Efetivo
	F	Empregado Efetivo
	F	Empregado Efetivo
	F	Empregado Efetivo
	F	Empregado Efetivo
	F	Empregado Efetivo
	F	Empregado Efetivo
	F	Empregado Efetivo
	F	Empregado Efetivo
	F	Empregado Efetivo
	F	Empregado Efetivo
	M	Empregado Efetivo
	F	Empregado Efetivo
	F	Empregado Efetivo
	F	Empregado Efetivo

	F	Empregado Efetivo
	F	Empregado Efetivo
	M	Empregado Efetivo
	F	Empregado Efetivo
	F	Empregado Efetivo
	M	Empregado Efetivo
	M	Empregado Efetivo
	F	Empregado Efetivo
	F	Empregado Efetivo
	F	Empregado Efetivo
	F	Empregado Efetivo
	F	Empregado Efetivo
	F	Empregado Efetivo
	F	Empregado Efetivo
	F	Empregado Efetivo
	F	Empregado Efetivo
	F	Empregado Efetivo
	F	Empregado Efetivo
	F	Empregado Efetivo
	F	Empregado Efetivo
	F	Empregado Efetivo
	F	Empregado Efetivo
	F	Empregado Efetivo
	F	Empregado Efetivo
	F	Empregado Efetivo
	F	Empregado Efetivo
	M	Empregado Efetivo
	M	Empregado Efetivo
	M	Empregado Efetivo
	F	Empregado Efetivo
	F	Empregado Efetivo
	F	Empregado Efetivo
	F	Empregado Efetivo
	F	Empregado Efetivo
	M	Empregado Efetivo
	F	Empregado Efetivo
	F	Empregado Efetivo
	F	Empregado Efetivo
	F	Empregado Efetivo
	M	Empregado Efetivo
	M	Empregado Efetivo
	M	Empregado Efetivo
	M	Empregado Efetivo
	F	Empregado Efetivo
	F	Empregado Efetivo
	F	Empregado Efetivo
	F	Empregado Efetivo
	F	Empregado Efetivo

	F	Empregado Efetivo
	F	Empregado Efetivo
	M	Empregado Efetivo
	F	Empregado Efetivo
	F	Empregado Efetivo
	F	Empregado Efetivo
	F	Empregado Efetivo
	F	Empregado Efetivo
	F	Empregado Efetivo
	F	Empregado Efetivo
	F	Empregado Efetivo
	F	Empregado Efetivo
	F	Empregado Efetivo
	F	Empregado Efetivo
	M	Empregado Efetivo
	F	Empregado Efetivo
	F	Empregado Efetivo
	F	Empregado Efetivo
	F	Empregado Efetivo
	F	Empregado Efetivo
	M	Empregado Efetivo
	F	Empregado Efetivo
	F	Empregado Efetivo
	F	Empregado Efetivo
	M	Empregado Efetivo
	F	Empregado Efetivo
	F	Empregado Efetivo
	F	Empregado Efetivo
	F	Empregado Efetivo
	M	Empregado Efetivo
	M	Empregado Efetivo
	F	Empregado Efetivo
	F	Empregado Efetivo
	M	Empregado Efetivo
	F	Empregado Efetivo
	F	Empregado Efetivo
	F	Empregado Efetivo
	F	Empregado Efetivo
	F	Empregado Efetivo
	F	Empregado Efetivo
	F	Empregado Efetivo
	F	Empregado Efetivo
	F	Empregado Efetivo
	F	Empregado Efetivo
	M	Empregado Efetivo
	F	Empregado Efetivo
	F	Empregado Efetivo
	F	Empregado Efetivo

	F	Empregado Efetivo
	F	Empregado Efetivo
	F	Empregado Efetivo
	F	Empregado Efetivo
	F	Empregado Efetivo
	F	Empregado Efetivo
	F	Empregado Efetivo
	F	Empregado Efetivo
	F	Empregado Efetivo
	M	Empregado Efetivo
	M	Empregado Efetivo
	F	Empregado Efetivo
	F	Empregado Efetivo
	F	Empregado Efetivo
	M	Empregado Efetivo
	F	Empregado Efetivo
	F	Empregado Efetivo
	F	Empregado Efetivo
	F	Empregado Efetivo
	F	Empregado Efetivo
	F	Empregado Efetivo
	M	Empregado Efetivo
	F	Empregado Efetivo
	F	Empregado Efetivo
	F	Empregado Efetivo
	F	Empregado Efetivo
	F	Empregado Efetivo
	F	Empregado Efetivo
	F	Empregado Efetivo
	F	Empregado Efetivo
	F	Empregado Efetivo
	F	Empregado Efetivo
	F	Empregado Efetivo
	F	Empregado Efetivo
	F	Empregado Efetivo
	F	Empregado Efetivo
	F	Empregado Efetivo
	F	Empregado Efetivo
	F	Empregado Efetivo
	F	Empregado Efetivo
	F	Empregado Efetivo
	F	Empregado Efetivo
	F	Empregado Efetivo
	F	Empregado Efetivo
	F	Empregado Efetivo
	F	Empregado Efetivo

	F	Empregado Efetivo
	F	Empregado Efetivo
	F	Empregado Efetivo
	F	Empregado Efetivo
	F	Empregado Efetivo
	F	Empregado Efetivo
	F	Empregado Efetivo
	F	Empregado Efetivo
	F	Empregado Efetivo
	F	Empregado Efetivo
	F	Empregado Efetivo
	F	Empregado Efetivo
	F	Empregado Efetivo
	F	Empregado Efetivo
	F	Empregado Efetivo
	F	Empregado Efetivo
	F	Empregado Efetivo
	F	Empregado Efetivo
	F	Empregado Efetivo
	F	Empregado Efetivo
	F	Empregado Efetivo
	F	Empregado Efetivo
	F	Empregado Efetivo
	F	Empregado Efetivo
	F	Empregado Efetivo
	F	Empregado Efetivo
	F	Empregado Efetivo
	F	Empregado Efetivo
	F	Empregado Efetivo
	F	Empregado Efetivo
	F	Empregado Efetivo
	F	Empregado Efetivo
	F	Empregado Efetivo
	F	Empregado Efetivo
	F	Empregado Efetivo
	F	Empregado Efetivo
	F	Empregado Efetivo
	F	Empregado Efetivo
	F	Empregado Efetivo
	F	Empregado Efetivo
	F	Empregado Efetivo
	F	Empregado Efetivo
	F	Empregado Efetivo

	F	Empregado Efetivo
	F	Empregado Efetivo
	F	Empregado Efetivo
	F	Empregado Efetivo
	F	Empregado Efetivo
	F	Empregado Efetivo
	F	Empregado Efetivo
	F	Empregado Efetivo
	F	Empregado Efetivo
	F	Empregado Efetivo
	F	Empregado Efetivo
	F	Empregado Efetivo
	F	Empregado Efetivo
	F	Empregado Efetivo
	F	Empregado Efetivo
	F	Empregado Efetivo
	F	Empregado Efetivo
	F	Empregado Efetivo
	F	Empregado Efetivo
	F	Empregado Efetivo
	F	Empregado Efetivo
	F	Empregado Efetivo
	F	Empregado Efetivo
	F	Empregado Efetivo
	F	Empregado Efetivo
	F	Empregado Efetivo
	F	Empregado Efetivo
	F	Empregado Efetivo
	F	Empregado Efetivo
	F	Empregado Efetivo
	F	Empregado Efetivo
	F	Empregado Efetivo
	F	Empregado Efetivo
	F	Empregado Efetivo
	F	Empregado Efetivo
	F	Empregado Efetivo
	F	Empregado Efetivo
	F	Empregado Efetivo
	F	Empregado Efetivo
	F	Empregado Efetivo
	F	Empregado Efetivo
	F	Empregado Efetivo
	F	Empregado Efetivo
	F	Empregado Efetivo
	F	Empregado Efetivo

	F	Empregado Efetivo
	F	Empregado Efetivo
	F	Empregado Efetivo
	F	Empregado Efetivo
	F	Empregado Efetivo
	F	Empregado Efetivo
	F	Empregado Efetivo
	F	Empregado Efetivo
	F	Empregado Efetivo
	F	Empregado Efetivo
	F	Empregado Efetivo
	F	Empregado Efetivo
	F	Empregado Efetivo
	F	Empregado Efetivo
	F	Empregado Efetivo
	F	Empregado Efetivo
	F	Empregado Efetivo
	F	Empregado Efetivo
	F	Empregado Efetivo
	F	Empregado Efetivo
	F	Empregado Efetivo
	F	Empregado Efetivo
	F	Empregado Efetivo
	F	Empregado Efetivo
	F	Empregado Efetivo
	F	Empregado Efetivo
	M	Empregado Efetivo
	M	Empregado Efetivo
	M	Empregado Efetivo
	M	Empregado Efetivo
	M	Empregado Efetivo
	M	Empregado Efetivo
	M	Empregado Efetivo
	M	Empregado Efetivo
	M	Empregado Efetivo
	F	Empregado Efetivo
	F	Empregado Efetivo
	F	Empregado Efetivo
	F	Empregado Efetivo
	M	Empregado Efetivo
	F	Empregado Efetivo
	F	Empregado Efetivo
	F	Cedido - RJU

	M	Cedido - RJU
	F	Empregado Efetivo
	F	Empregado Efetivo
	M	Empregado Efetivo
	F	Cedido - RJU
	F	Cedido - RJU
	F	Empregado Efetivo
	F	Empregado Efetivo
	M	Empregado Efetivo
	F	Empregado Efetivo
	F	Empregado Efetivo
	F	Empregado Efetivo
	F	Empregado Efetivo
	M	Empregado Efetivo
	F	Empregado Efetivo
	F	Empregado Efetivo
	F	Empregado Efetivo
	M	Empregado Efetivo
	F	Empregado Efetivo
	F	Cedido - RJU
	F	Empregado Efetivo
	F	Empregado Efetivo
	F	Empregado Efetivo
	F	Empregado Efetivo
	F	Empregado Efetivo
	F	Empregado Efetivo
	M	Empregado Efetivo
	M	Empregado Efetivo
	F	Cedido - RJU
	F	Empregado Efetivo
	F	Empregado Efetivo
	F	Empregado Efetivo
	F	Empregado Efetivo
	F	Empregado Efetivo
	F	Empregado Efetivo
	M	Empregado Efetivo
	M	Empregado Efetivo
	F	Empregado Efetivo
	M	Cedido - RJU
	F	Empregado Efetivo
	F	Empregado Efetivo
	F	Empregado Efetivo
	M	Cedido - RJU
	F	Cedido - RJU
	M	Empregado Efetivo
	M	Empregado Efetivo

	M	Empregado Efetivo
	F	Cedido - RJU
	F	Cedido - RJU
	M	Empregado Efetivo
	F	Cedido - RJU
	F	Empregado Efetivo
	F	Empregado Efetivo
	M	Empregado Efetivo
	F	Empregado Efetivo
	F	Empregado Efetivo
	F	Empregado Efetivo
	F	Empregado Efetivo
	F	Empregado Efetivo
	F	Empregado Efetivo
	F	Empregado Efetivo
	F	Empregado Efetivo
	F	Empregado Efetivo
	F	Empregado Efetivo
	F	Empregado Efetivo
	F	Empregado Efetivo
	F	Cedido - RJU
	F	Empregado Efetivo
	F	Empregado Efetivo
	F	Empregado Efetivo
	F	Empregado Efetivo
	M	Empregado Efetivo
	F	Empregado Efetivo
	F	Empregado Efetivo
	M	Empregado Efetivo
	F	Empregado Efetivo
	F	Empregado Efetivo
	M	Empregado Efetivo
	F	Empregado Efetivo
	F	Empregado Efetivo
	M	Empregado Efetivo
	F	Empregado Efetivo
	M	Empregado Efetivo
	F	Empregado Efetivo
	M	Empregado Efetivo

	F	Empregado Efetivo
	M	Empregado Efetivo
	F	Empregado Efetivo
	F	Empregado Efetivo
	F	Empregado Efetivo
	F	Empregado Efetivo
	F	Empregado Efetivo
	F	Empregado Efetivo
	F	Empregado Efetivo
	F	Empregado Efetivo
	F	Empregado Efetivo
	F	Empregado Efetivo
	F	Empregado Efetivo
	F	Empregado Efetivo
	F	Empregado Efetivo
	F	Empregado Efetivo
	F	Empregado Efetivo
	F	Empregado Efetivo
	F	Empregado Efetivo
	F	Empregado Efetivo
	F	Empregado Efetivo
	F	Empregado Efetivo
	F	Empregado Efetivo
	F	Empregado Efetivo
	F	Empregado Efetivo
	F	Empregado Efetivo
	F	Empregado Efetivo
	F	Empregado Efetivo
	F	Empregado Efetivo
	F	Empregado Efetivo
	F	Empregado Efetivo
	F	Empregado Efetivo

	F	Empregado Efetivo
	F	Empregado Efetivo
	F	Empregado Efetivo
	F	Empregado Efetivo
	F	Empregado Efetivo
	F	Empregado Efetivo
	F	Empregado Efetivo
	F	Empregado Efetivo
	F	Empregado Efetivo
	F	Empregado Efetivo
	F	Empregado Efetivo
	F	Empregado Efetivo
	F	Empregado Efetivo
	F	Empregado Efetivo
	F	Empregado Efetivo
	F	Empregado Efetivo
	F	Empregado Efetivo
	F	Empregado Efetivo
	F	Empregado Efetivo
	F	Empregado Efetivo
	F	Empregado Efetivo
	F	Empregado Efetivo
	F	Empregado Efetivo
	F	Empregado Efetivo
	F	Empregado Efetivo
	F	Empregado Efetivo
	F	Empregado Efetivo
	F	Empregado Efetivo
	F	Empregado Efetivo
	F	Empregado Efetivo
	F	Empregado Efetivo
	M	Empregado Efetivo
	M	Empregado Efetivo
	F	Empregado Efetivo
	F	Empregado Efetivo
	M	Empregado Efetivo
	F	Empregado Efetivo
	M	Empregado Efetivo
	F	Empregado Efetivo
	F	Empregado Efetivo
	F	Empregado Efetivo

	F	Empregado Efetivo
	F	Empregado Efetivo
	F	Empregado Efetivo
	F	Empregado Efetivo
	F	Empregado Efetivo
	F	Empregado Efetivo
	M	Empregado Efetivo
	M	Cedido - RJU
	F	Empregado Efetivo
	F	Empregado Efetivo
	F	Empregado Efetivo
	F	Empregado Efetivo
	F	Empregado Efetivo
	F	Empregado Efetivo
	F	Empregado Efetivo
	F	Empregado Efetivo
	F	Empregado Efetivo
	F	Empregado Efetivo
	F	Empregado Efetivo
	F	Empregado Efetivo
	F	Empregado Efetivo
	F	Empregado Efetivo
	F	Empregado Efetivo
	F	Empregado Efetivo
	F	Empregado Efetivo
	F	Empregado Efetivo
	F	Empregado Efetivo
	F	Empregado Efetivo
	F	Empregado Efetivo
	F	Empregado Efetivo
	F	Empregado Efetivo
	F	Empregado Efetivo
	F	Empregado Efetivo
	F	Empregado Efetivo
	F	Empregado Efetivo
	F	Empregado Efetivo
	F	Empregado Efetivo
	F	Empregado Efetivo
	F	Empregado Efetivo

	F	Empregado Efetivo
	F	Empregado Efetivo
	F	Empregado Efetivo
	F	Empregado Efetivo
	F	Empregado Efetivo
	M	Empregado Efetivo
	F	Empregado Efetivo
	F	Empregado Efetivo
	F	Empregado Efetivo
	F	Empregado Efetivo
	F	Empregado Efetivo
	F	Empregado Efetivo
	F	Empregado Efetivo
	F	Empregado Efetivo
	F	Empregado Efetivo
	F	Empregado Efetivo
	M	Empregado Efetivo
	M	Empregado Efetivo
	M	Empregado Efetivo
	M	Empregado Efetivo
	M	Empregado Efetivo
	M	Empregado Efetivo
	M	Empregado Efetivo
	F	Empregado Efetivo
	F	Cedido - RJU
	F	Empregado Efetivo
	M	Empregado Efetivo
	M	Empregado Efetivo
	F	Empregado Efetivo
	M	Empregado Efetivo
	M	Empregado Efetivo
	F	Empregado Efetivo
	F	Empregado Efetivo
	M	Empregado Efetivo
	M	Empregado Efetivo
	M	Empregado Efetivo

	F	Empregado Efetivo
	F	Empregado Efetivo
	F	Empregado Efetivo
	F	Empregado Efetivo
	F	Empregado Efetivo
	F	Empregado Efetivo
	F	Empregado Efetivo
	F	Empregado Efetivo
	M	Empregado Efetivo
	M	Empregado Efetivo
	F	Empregado Efetivo
	F	Empregado Efetivo
	F	Empregado Efetivo
	F	Empregado Efetivo
	F	Empregado Efetivo
	M	Empregado Efetivo
	M	Empregado Efetivo
	M	Empregado Efetivo
	F	Empregado Efetivo
	F	Empregado Efetivo
	F	Empregado Efetivo
	F	Empregado Efetivo
	F	Empregado Efetivo
	F	Empregado Efetivo
	M	Empregado Efetivo
	M	Empregado Efetivo
	F	Empregado Efetivo
	F	Empregado Efetivo
	F	Empregado Efetivo
	M	Empregado Efetivo
	F	Empregado Efetivo
	M	Empregado Efetivo
	F	Empregado Efetivo
	F	Empregado Efetivo
	F	Empregado Efetivo
	F	Empregado Efetivo
	M	Empregado Efetivo
	M	Empregado Efetivo
	M	Empregado Efetivo
	F	Empregado Efetivo
	F	Empregado Efetivo

	F	Empregado Efetivo
	F	Empregado Efetivo
	F	Empregado Efetivo
	M	Empregado Efetivo
	F	Empregado Efetivo
	F	Empregado Efetivo
	M	Empregado Efetivo
	F	Empregado Efetivo
	M	Empregado Efetivo
	F	Empregado Efetivo
	F	Empregado Efetivo
	F	Empregado Efetivo
	F	Empregado Efetivo
	F	Empregado Efetivo
	F	Empregado Efetivo
	F	Empregado Efetivo
	F	Empregado Efetivo
	F	Empregado Efetivo
	F	Empregado Efetivo
	F	Empregado Efetivo
	F	Empregado Efetivo
	F	Empregado Efetivo
	F	Empregado Efetivo
	F	Empregado Efetivo
	F	Empregado Efetivo
	F	Empregado Efetivo
	F	Empregado Efetivo
	F	Empregado Efetivo
	F	Empregado Efetivo
	F	Empregado Efetivo
	F	Empregado Efetivo
	F	Empregado Efetivo
	F	Empregado Efetivo
	F	Empregado Efetivo
	F	Empregado Efetivo
	F	Empregado Efetivo
	F	Empregado Efetivo
	M	Empregado Efetivo
	F	Empregado Efetivo
	F	Empregado Efetivo
	M	Empregado Efetivo
	F	Empregado Efetivo
	M	Empregado Efetivo
	F	Empregado Efetivo
	F	Empregado Efetivo
	F	Empregado Efetivo
	M	Empregado Efetivo

	M	Empregado Efetivo
	F	Empregado Efetivo
	F	Empregado Efetivo
	F	Empregado Efetivo
	F	Empregado Efetivo
	M	Empregado Efetivo
	F	Empregado Efetivo
	M	Empregado Efetivo
	F	Empregado Efetivo
	M	Empregado Efetivo
	M	Empregado Efetivo
	M	Empregado Efetivo
	F	Empregado Efetivo
	F	Empregado Efetivo
	M	Empregado Efetivo
	M	Empregado Efetivo
	F	Empregado Efetivo
	F	Empregado Efetivo
	F	Empregado Efetivo
	F	Empregado Efetivo
	F	Empregado Efetivo
	F	Empregado Efetivo
	F	Empregado Efetivo
	F	Empregado Efetivo
	F	Empregado Efetivo
	F	Empregado Efetivo
	F	Empregado Efetivo
	F	Empregado Efetivo
	F	Empregado Efetivo
	F	Empregado Efetivo
	M	Empregado Efetivo
	M	Empregado Efetivo
	F	Empregado Efetivo
	F	Empregado Efetivo
	F	Empregado Efetivo
	F	Empregado Efetivo
	F	Empregado Efetivo
	F	Empregado Efetivo
	F	Empregado Efetivo
	F	Empregado Efetivo
	F	Empregado Efetivo
	F	Empregado Efetivo
	M	Empregado Efetivo
	M	Empregado Efetivo
	F	Empregado Efetivo
	F	Empregado Efetivo

	F	Empregado Efetivo
	F	Empregado Efetivo
	F	Empregado Efetivo
	F	Empregado Efetivo
	M	Empregado Efetivo
	F	Empregado Efetivo
	F	Empregado Efetivo
	F	Empregado Efetivo
	F	Empregado Efetivo
	M	Empregado Efetivo
	F	Empregado Efetivo
	M	Empregado Efetivo
	F	Empregado Efetivo
	F	Empregado Efetivo
	F	Empregado Efetivo
	F	Empregado Efetivo
	F	Empregado Efetivo
	F	Empregado Efetivo
	F	Empregado Efetivo
	F	Empregado Efetivo
	F	Empregado Efetivo
	F	Empregado Efetivo
	F	Empregado Efetivo
	F	Empregado Efetivo
	F	Empregado Efetivo
	F	Empregado Efetivo
	F	Empregado Efetivo
	F	Empregado Efetivo
	F	Empregado Efetivo
	F	Empregado Efetivo
	F	Empregado Efetivo
	F	Empregado Efetivo
	F	Empregado Efetivo
	F	Empregado Efetivo
	M	Empregado Efetivo
	F	Empregado Efetivo
	M	Empregado Efetivo
	M	Empregado Efetivo
	F	Empregado Efetivo
	M	Empregado Efetivo
	F	Empregado Efetivo
	F	Empregado Efetivo
	F	Empregado Efetivo
	M	Empregado Efetivo
	M	Empregado Efetivo
	F	Empregado Efetivo
	F	Empregado Efetivo

	M	Empregado Efetivo
	M	Empregado Efetivo
	F	Empregado Efetivo
	F	Empregado Efetivo
	F	Empregado Efetivo
	F	Empregado Efetivo
	F	Empregado Efetivo
	F	Empregado Efetivo
	F	Empregado Efetivo
	F	Empregado Efetivo
	F	Empregado Efetivo
	F	Empregado Efetivo
	F	Empregado Efetivo
	F	Empregado Efetivo
	F	Empregado Efetivo
	F	Empregado Efetivo
	F	Empregado Efetivo
	F	Empregado Efetivo
	F	Empregado Efetivo
	F	Empregado Efetivo
	F	Empregado Efetivo
	F	Empregado Efetivo
	F	Empregado Efetivo
	F	Empregado Efetivo
	F	Empregado Efetivo
	F	Empregado Efetivo
	F	Empregado Efetivo
	F	Empregado Efetivo
	F	Empregado Efetivo
	F	Empregado Efetivo
	F	Empregado Efetivo
	F	Empregado Efetivo
	F	Empregado Efetivo
	F	Empregado Efetivo
	M	Empregado Efetivo
	F	Empregado Efetivo
	F	Empregado Efetivo
	F	Empregado Efetivo
	F	Empregado Efetivo
	F	Empregado Efetivo
	F	Empregado Efetivo
	F	Empregado Efetivo
	F	Empregado Efetivo
	F	Empregado Efetivo
	F	Empregado Efetivo
	F	Empregado Efetivo
	F	Empregado Efetivo
	F	Empregado Efetivo

	M	Empregado Efetivo
	M	Empregado Efetivo
	F	Empregado Efetivo
	F	Empregado Efetivo
	M	Empregado Efetivo
	F	Empregado Efetivo
	F	Empregado Efetivo
	F	Empregado Efetivo
	F	Empregado Efetivo
	F	Empregado Efetivo
	F	Empregado Efetivo
	F	Empregado Efetivo
	F	Empregado Efetivo
	F	Empregado Efetivo
	M	Empregado Efetivo
	F	Empregado Efetivo
	F	Empregado Efetivo
	F	Empregado Efetivo
	F	Empregado Efetivo
	F	Empregado Efetivo
	F	Empregado Efetivo
	F	Empregado Efetivo
	F	Empregado Efetivo
	F	Empregado Efetivo
	F	Empregado Efetivo
	F	Empregado Efetivo
	F	Empregado Efetivo
	F	Empregado Efetivo
	F	Empregado Efetivo
	F	Empregado Efetivo
	F	Empregado Efetivo
	F	Empregado Efetivo
	F	Empregado Efetivo
	F	Empregado Efetivo
	F	Empregado Efetivo
	F	Empregado Efetivo
	F	Empregado Efetivo
	F	Empregado Efetivo
	F	Empregado Efetivo
	F	Empregado Efetivo
	F	Empregado Efetivo
	F	Empregado Efetivo
	F	Empregado Efetivo
	F	Empregado Efetivo
	F	Empregado Efetivo
	F	Empregado Efetivo

	F	Empregado Efetivo
	F	Empregado Efetivo
	F	Empregado Efetivo
	F	Empregado Efetivo
	F	Empregado Efetivo
	F	Empregado Efetivo
	F	Empregado Efetivo
	F	Empregado Efetivo
	F	Empregado Efetivo
	F	Empregado Efetivo
	F	Empregado Efetivo
	F	Empregado Efetivo
	F	Empregado Efetivo
	F	Empregado Efetivo
	F	Empregado Efetivo
	F	Empregado Efetivo
	M	Empregado Efetivo
	F	Empregado Efetivo
	F	Empregado Efetivo
	M	Empregado Efetivo
	M	Empregado Efetivo
	M	Empregado Efetivo
	F	Empregado Efetivo
	M	Empregado Efetivo
	M	Empregado Efetivo
	F	Empregado Efetivo
	F	Empregado Efetivo
	F	Empregado Efetivo
	F	Empregado Efetivo
	F	Empregado Efetivo
	F	Empregado Efetivo
	F	Empregado Efetivo
	F	Empregado Efetivo
	F	Empregado Efetivo
	F	Empregado Efetivo
	F	Empregado Efetivo
	F	Empregado Efetivo
	F	Empregado Efetivo
	F	Empregado Efetivo
	F	Empregado Efetivo
	F	Empregado Efetivo
	F	Empregado Efetivo
	F	Empregado Efetivo
	F	Empregado Efetivo
	F	Empregado Efetivo
	F	Empregado Efetivo

	M	Empregado Efetivo
	M	Empregado Efetivo
	F	Empregado Efetivo
	F	Empregado Efetivo
	F	Empregado Efetivo
	F	Empregado Efetivo
	F	Empregado Efetivo
	F	Empregado Efetivo
	F	Empregado Efetivo
	F	Empregado Efetivo
	M	Empregado Efetivo
	F	Empregado Efetivo
	F	Empregado Efetivo
	M	Empregado Efetivo
	F	Empregado Efetivo
	F	Empregado Efetivo
	F	Empregado Efetivo
	F	Empregado Efetivo
	F	Empregado Efetivo
	F	Empregado Efetivo
	F	Empregado Efetivo
	M	Empregado Efetivo
	M	Empregado Efetivo
	F	Empregado Efetivo
	M	Empregado Efetivo
	F	Empregado Efetivo
	M	Empregado Efetivo
	F	Empregado Efetivo
	F	Empregado Efetivo
	M	Empregado Efetivo
	M	Empregado Efetivo
	F	Empregado Efetivo
	F	Empregado Efetivo
	M	Empregado Efetivo
	F	Empregado Efetivo
	F	Empregado Efetivo
	M	Empregado Efetivo
	M	Empregado Efetivo
	M	Empregado Efetivo
	F	Empregado Efetivo
	F	Empregado Efetivo
	F	Empregado Efetivo
	F	Empregado Efetivo
	M	Empregado Efetivo
	F	Empregado Efetivo
	F	Empregado Efetivo
	F	Empregado Efetivo
	F	Empregado Efetivo

	M	Empregado Efetivo
	F	Empregado Efetivo
	F	Empregado Efetivo
	M	Empregado Efetivo
	F	Empregado Efetivo
	M	Empregado Efetivo
	F	Empregado Efetivo
	F	Empregado Efetivo
	F	Empregado Efetivo
	F	Empregado Efetivo
	F	Empregado Efetivo
	F	Empregado Efetivo
	M	Empregado Efetivo
	F	Empregado Efetivo
	F	Empregado Efetivo
	F	Empregado Efetivo
	F	Empregado Efetivo
	F	Empregado Efetivo
	F	Empregado Efetivo
	F	Empregado Efetivo
	F	Empregado Efetivo
	F	Empregado Efetivo
	M	Empregado Efetivo
	M	Empregado Efetivo
	F	Empregado Efetivo
	F	Empregado Efetivo
	F	Empregado Efetivo
	F	Empregado Efetivo
	F	Empregado Efetivo
	F	Empregado Efetivo
	F	Empregado Efetivo
	F	Empregado Efetivo
	M	Empregado Efetivo
	F	Empregado Efetivo
	F	Empregado Efetivo
	F	Empregado Efetivo
	F	Empregado Efetivo
	F	Empregado Efetivo
	F	Empregado Efetivo
	F	Empregado Efetivo
	F	Empregado Efetivo
	F	Empregado Efetivo
	M	Empregado Efetivo
	F	Empregado Efetivo
	M	Empregado Efetivo
	M	Empregado Efetivo
	M	Empregado Efetivo
	F	Empregado Efetivo

	F	Empregado Efetivo
	F	Empregado Efetivo
	F	Empregado Efetivo
	F	Empregado Efetivo
	M	Empregado Efetivo
	F	Empregado Efetivo
	M	Empregado Efetivo
	F	Empregado Efetivo
	M	Empregado Efetivo
	F	Empregado Efetivo
	F	Empregado Efetivo
	F	Empregado Efetivo
	F	Empregado Efetivo
	F	Empregado Efetivo
	F	Empregado Efetivo
	F	Empregado Efetivo
	F	Empregado Efetivo
	F	Empregado Efetivo
	F	Empregado Efetivo
	F	Empregado Efetivo
	F	Empregado Efetivo
	F	Empregado Efetivo
	F	Empregado Efetivo
	F	Empregado Efetivo
	F	Empregado Efetivo
	F	Empregado Efetivo
	F	Empregado Efetivo
	F	Empregado Efetivo
	M	Empregado Efetivo
	F	Empregado Efetivo
	F	Empregado Efetivo
	F	Empregado Efetivo
	F	Empregado Efetivo
	F	Empregado Efetivo
	F	Empregado Efetivo
	F	Empregado Efetivo
	F	Empregado Efetivo
	F	Empregado Efetivo
	F	Empregado Efetivo
	F	Empregado Efetivo
	F	Empregado Efetivo
	F	Empregado Efetivo
	F	Empregado Efetivo
	F	Empregado Efetivo
	F	Empregado Efetivo

	F	Empregado Efetivo
	F	Empregado Efetivo
	F	Empregado Efetivo
	F	Empregado Efetivo
	F	Empregado Efetivo
	F	Empregado Efetivo
	F	Empregado Efetivo
	F	Empregado Efetivo
	F	Empregado Efetivo
	F	Empregado Efetivo
	F	Empregado Efetivo
	F	Empregado Efetivo
	F	Empregado Efetivo
	F	Empregado Efetivo
	F	Empregado Efetivo
	F	Empregado Efetivo
	F	Empregado Efetivo
	F	Empregado Efetivo
	F	Empregado Efetivo
	F	Empregado Efetivo
	F	Empregado Efetivo
	F	Empregado Efetivo
	F	Empregado Efetivo
	F	Empregado Efetivo
	F	Empregado Efetivo
	F	Empregado Efetivo
	F	Empregado Efetivo
	F	Empregado Efetivo
	F	Empregado Efetivo
	F	Empregado Efetivo
	F	Empregado Efetivo
	F	Empregado Efetivo
	F	Empregado Efetivo
	F	Empregado Efetivo
	F	Empregado Efetivo

	F	Empregado Efetivo
	F	Empregado Efetivo
	F	Empregado Efetivo
	F	Empregado Efetivo
	F	Empregado Efetivo
	F	Empregado Efetivo
	F	Empregado Efetivo
	F	Empregado Efetivo
	F	Empregado Efetivo
	F	Empregado Efetivo
	F	Empregado Efetivo
	F	Empregado Efetivo
	F	Empregado Efetivo
	F	Empregado Efetivo
	F	Empregado Efetivo
	F	Empregado Efetivo
	F	Empregado Efetivo
	F	Empregado Efetivo
	F	Empregado Efetivo
	F	Empregado Efetivo
	F	Empregado Efetivo
	F	Empregado Efetivo
	F	Empregado Efetivo
	F	Empregado Efetivo
	M	Empregado Efetivo
	F	Empregado Efetivo
	F	Empregado Efetivo
	F	Empregado Efetivo
	F	Empregado Efetivo
	F	Empregado Efetivo
	F	Empregado Efetivo
	F	Empregado Efetivo
	F	Empregado Efetivo
	F	Empregado Efetivo
	F	Empregado Efetivo
	F	Empregado Efetivo
	F	Empregado Efetivo
	F	Empregado Efetivo
	F	Empregado Efetivo
	M	Empregado Efetivo
	F	Empregado Efetivo
	M	Empregado Efetivo
	F	Empregado Efetivo
	F	Empregado Efetivo
	F	Empregado Efetivo
	F	Empregado Efetivo
	F	Empregado Efetivo
	F	Empregado Efetivo

	F	Empregado Efetivo
	F	Empregado Efetivo
	M	Empregado Efetivo
	F	Empregado Efetivo
	F	Empregado Efetivo
	F	Empregado Efetivo
	F	Empregado Efetivo
	F	Empregado Efetivo
	F	Empregado Efetivo
	M	Empregado Efetivo
	F	Empregado Efetivo
	F	Empregado Efetivo
	M	Empregado Efetivo
	F	Empregado Efetivo
	F	Empregado Efetivo
	F	Empregado Efetivo
	F	Empregado Efetivo
	F	Empregado Efetivo
	F	Empregado Efetivo
	F	Empregado Efetivo
	F	Empregado Efetivo
	F	Empregado Efetivo
	F	Empregado Efetivo
	F	Empregado Efetivo
	F	Empregado Efetivo
	F	Empregado Efetivo
	F	Empregado Efetivo
	F	Empregado Efetivo
	F	Empregado Efetivo
	F	Empregado Efetivo
	F	Empregado Efetivo
	M	Empregado Efetivo
	F	Empregado Efetivo
	F	Empregado Efetivo
	F	Empregado Efetivo
	M	Empregado Efetivo
	F	Empregado Efetivo
	F	Empregado Efetivo
	F	Empregado Efetivo
	F	Empregado Efetivo
	F	Empregado Efetivo
	F	Empregado Efetivo
	F	Empregado Efetivo
	F	Empregado Efetivo
	F	Empregado Efetivo
	F	Empregado Efetivo
	F	Empregado Efetivo
	F	Empregado Efetivo

	F	Empregado Efetivo
	F	Empregado Efetivo
	F	Empregado Efetivo
	M	Empregado Efetivo
	M	Empregado Efetivo
	F	Empregado Efetivo
	F	Empregado Efetivo
	F	Empregado Efetivo
	M	Empregado Efetivo
	F	Empregado Efetivo
	F	Empregado Efetivo
	F	Empregado Efetivo
	M	Empregado Efetivo
	F	Empregado Efetivo
	F	Empregado Efetivo
	F	Empregado Efetivo
	F	Empregado Efetivo
	F	Empregado Efetivo
	F	Empregado Efetivo
	F	Empregado Efetivo
	F	Empregado Efetivo
	F	Empregado Efetivo
	F	Empregado Efetivo
	F	Empregado Efetivo
	F	Empregado Efetivo
	M	Empregado Efetivo
	F	Empregado Efetivo
	F	Empregado Efetivo
	F	Empregado Efetivo
	F	Empregado Efetivo
	F	Empregado Efetivo
	F	Empregado Efetivo
	M	Empregado Efetivo
	M	Empregado Efetivo
	F	Empregado Efetivo
	F	Empregado Efetivo
	F	Empregado Efetivo
	F	Empregado Efetivo
	F	Empregado Efetivo
	F	Empregado Efetivo
	F	Empregado Efetivo
	F	Empregado Efetivo
	F	Empregado Efetivo
	F	Empregado Efetivo
	F	Empregado Efetivo
	F	Empregado Efetivo
	F	Empregado Efetivo
	F	Empregado Efetivo

	F	Empregado Efetivo
	F	Empregado Efetivo
	F	Empregado Efetivo
	F	Empregado Efetivo
	F	Empregado Efetivo
	F	Empregado Efetivo
	F	Empregado Efetivo
	F	Empregado Efetivo
	F	Empregado Efetivo
	M	Empregado Efetivo
	F	Empregado Efetivo
	F	Empregado Efetivo
	F	Empregado Efetivo
	F	Empregado Efetivo
	M	Empregado Efetivo
	F	Empregado Efetivo
	M	Empregado Efetivo
	M	Empregado Efetivo
	M	Empregado Efetivo
	F	Empregado Efetivo
	M	Empregado Efetivo
	F	Empregado Efetivo
	F	Empregado Efetivo
	M	Empregado Efetivo
	F	Empregado Efetivo
	F	Empregado Efetivo
	F	Empregado Efetivo
	F	Empregado Efetivo
	M	Empregado Efetivo
	F	Empregado Efetivo
	F	Empregado Efetivo
	F	Empregado Efetivo
	F	Empregado Efetivo
	M	Empregado Efetivo
	M	Empregado Efetivo
	M	Empregado Efetivo
	F	Empregado Efetivo
	F	Empregado Efetivo
	M	Empregado Efetivo
	M	Empregado Efetivo
	M	Empregado Efetivo
	M	Empregado Efetivo
	M	Empregado Efetivo
	F	Empregado Efetivo
	F	Empregado Efetivo
	F	Empregado Efetivo
	F	Empregado Efetivo
	F	Empregado Efetivo

	F	Empregado Efetivo
	F	Empregado Efetivo
	F	Empregado Efetivo
	F	Empregado Efetivo
	M	Empregado Efetivo
	F	Empregado Efetivo
	F	Empregado Efetivo
	F	Empregado Efetivo
	M	Empregado Efetivo
	F	Empregado Efetivo
	M	Empregado Efetivo
	F	Empregado Efetivo
	F	Empregado Efetivo
	F	Empregado Efetivo
	M	Empregado Efetivo
	F	Empregado Efetivo
	F	Empregado Efetivo
	M	Empregado Efetivo
	F	Empregado Efetivo
	F	Empregado Efetivo
	M	Empregado Efetivo
	F	Empregado Efetivo
	F	Empregado Efetivo
	F	Empregado Efetivo
	M	Empregado Efetivo
	F	Empregado Efetivo
	M	Empregado Efetivo
	M	Empregado Efetivo
	F	Empregado Efetivo
	M	Empregado Efetivo
	F	Empregado Efetivo
	M	Empregado Efetivo
	F	Empregado Efetivo
	F	Empregado Efetivo
	F	Empregado Efetivo
	F	Empregado Efetivo
	F	Empregado Efetivo
	M	Empregado Efetivo
	F	Empregado Efetivo
	F	Empregado Efetivo
	F	Empregado Efetivo
	F	Empregado Efetivo
	F	Empregado Efetivo
	F	Empregado Efetivo
	M	Empregado Efetivo
	F	Empregado Efetivo
	M	Empregado Efetivo
	M	Empregado Efetivo

	M	Empregado Efetivo
	F	Empregado Efetivo
	F	Empregado Efetivo
	F	Empregado Efetivo
	F	Empregado Efetivo
	M	Cedido - RJU
	F	Empregado Efetivo
	M	Empregado Efetivo
	M	Empregado Efetivo
	M	Empregado Efetivo
	F	Empregado Efetivo
	F	Empregado Efetivo
	M	Empregado Efetivo
	F	Empregado Efetivo
	M	Empregado Efetivo
	M	Empregado Efetivo
	F	Empregado Efetivo
	F	Empregado Efetivo
	F	Empregado Efetivo
	M	Empregado Efetivo
	F	Empregado Efetivo
	F	Empregado Efetivo
	F	Empregado Efetivo
	F	Empregado Efetivo
	F	Empregado Efetivo
	F	Empregado Efetivo
	M	Empregado Efetivo
	M	Empregado Efetivo
	M	Empregado Efetivo
	M	Empregado Efetivo
	F	Empregado Efetivo
	F	Empregado Efetivo
	F	Empregado Efetivo
	F	Empregado Efetivo
	M	Empregado Efetivo
	M	Empregado Efetivo
	M	Empregado Efetivo
	F	Empregado Efetivo
	F	Empregado Efetivo
	F	Empregado Efetivo
	F	Empregado Efetivo
	F	Empregado Efetivo
	M	Empregado Efetivo
	F	Empregado Efetivo
	M	Empregado Efetivo
	M	Empregado Efetivo
	M	Empregado Efetivo
	F	Empregado Efetivo

	M	Empregado Efetivo
	F	Empregado Efetivo
	M	Empregado Efetivo
	F	Empregado Efetivo
	M	Empregado Efetivo
	M	Empregado Efetivo
	M	Empregado Efetivo
	M	Empregado Efetivo
	M	Empregado Efetivo
	M	Empregado Efetivo
	F	Empregado Efetivo
	F	Empregado Efetivo
	M	Empregado Efetivo
	F	Empregado Efetivo
	F	Empregado Efetivo
	F	Empregado Efetivo
	F	Empregado Efetivo
	F	Empregado Efetivo
	F	Empregado Efetivo
	F	Empregado Efetivo
	F	Empregado Efetivo
	F	Empregado Efetivo
	M	Empregado Efetivo
	F	Empregado Efetivo
	M	Empregado Efetivo
	M	Empregado Efetivo
	F	Empregado Efetivo
	M	Empregado Efetivo
	F	Empregado Efetivo
	F	Empregado Efetivo
	F	Empregado Efetivo
	F	Empregado Efetivo
	F	Empregado Efetivo
	F	Empregado Efetivo
	F	Empregado Efetivo
	F	Empregado Efetivo
	F	Empregado Efetivo
	F	Empregado Efetivo
	F	Empregado Efetivo
	F	Empregado Efetivo
	F	Empregado Efetivo
	M	Empregado Efetivo
	F	Empregado Efetivo
	F	Empregado Efetivo
	F	Empregado Efetivo
	F	Empregado Efetivo
	M	Empregado Efetivo
	M	Empregado Efetivo

	F	Empregado Efetivo
	F	Empregado Efetivo
	F	Empregado Efetivo
	F	Empregado Efetivo
	F	Empregado Efetivo
	F	Empregado Efetivo
	M	Empregado Efetivo
	M	Empregado Efetivo
	F	Empregado Efetivo
	F	Empregado Efetivo
	M	Empregado Efetivo
	F	Empregado Efetivo
	M	Empregado Efetivo
	F	Empregado Efetivo
	F	Empregado Efetivo
	M	Empregado Efetivo
	M	Empregado Efetivo
	F	Empregado Efetivo
	F	Empregado Efetivo
	F	Empregado Efetivo
	M	Empregado Efetivo
	M	Empregado Efetivo
	F	Empregado Efetivo
	F	Empregado Efetivo
	F	Empregado Efetivo
	F	Empregado Efetivo
	F	Empregado Efetivo
	F	Empregado Efetivo
	F	Empregado Efetivo
	F	Empregado Efetivo
	F	Empregado Efetivo
	F	Empregado Efetivo
	F	Empregado Efetivo
	F	Empregado Efetivo
	M	Empregado Efetivo
	F	Empregado Efetivo
	F	Empregado Efetivo
	F	Empregado Efetivo
	F	Empregado Efetivo
	F	Empregado Efetivo
	F	Empregado Efetivo

	F	Empregado Efetivo
	F	Empregado Efetivo
	F	Empregado Efetivo
	F	Empregado Efetivo
	F	Empregado Efetivo
	F	Empregado Efetivo
	F	Empregado Efetivo
	F	Empregado Efetivo
	F	Empregado Efetivo
	F	Empregado Efetivo
	F	Empregado Efetivo
	F	Empregado Efetivo
	F	Empregado Efetivo
	F	Empregado Efetivo
	F	Empregado Efetivo
	F	Empregado Efetivo
	M	Empregado Efetivo
	F	Empregado Efetivo
	F	Empregado Efetivo
	F	Empregado Efetivo
	F	Empregado Efetivo
	F	Empregado Efetivo
	F	Empregado Efetivo
	F	Cedido - RJU
	F	Empregado Efetivo
	F	Empregado Efetivo
	F	Empregado Efetivo
	F	Empregado Efetivo
	F	Empregado Efetivo
	F	Empregado Efetivo
	F	Empregado Efetivo
	F	Empregado Efetivo
	F	Empregado Efetivo
	F	Empregado Efetivo
	F	Empregado Efetivo
	F	Empregado Efetivo
	M	Empregado Efetivo
	F	Empregado Efetivo
	F	Empregado Efetivo
	F	Empregado Efetivo
	F	Empregado Efetivo
	F	Empregado Efetivo
	F	Empregado Efetivo
	F	Empregado Efetivo
	F	Empregado Efetivo
	F	Empregado Efetivo
	F	Empregado Efetivo
	F	Empregado Efetivo

	F	Empregado Efetivo
	F	Empregado Efetivo
	F	Empregado Efetivo
	F	Empregado Efetivo
	F	Empregado Efetivo
	F	Empregado Efetivo
	F	Empregado Efetivo
	F	Empregado Efetivo
	F	Empregado Efetivo
	F	Empregado Efetivo
	F	Empregado Efetivo
	F	Empregado Efetivo
	F	Empregado Efetivo
	F	Empregado Efetivo
	F	Empregado Efetivo
	F	Empregado Efetivo
	F	Empregado Efetivo
	M	Empregado Efetivo
	M	Empregado Efetivo
	F	Empregado Efetivo
	F	Empregado Efetivo
	F	Empregado Efetivo
	F	Empregado Efetivo
	F	Empregado Efetivo
	F	Empregado Efetivo
	F	Empregado Efetivo
	F	Empregado Efetivo
	F	Empregado Efetivo
	F	Empregado Efetivo
	F	Empregado Efetivo
	M	Empregado Efetivo
	F	Empregado Efetivo
	F	Empregado Efetivo
	F	Empregado Efetivo
	F	Empregado Efetivo
	F	Empregado Efetivo
	F	Empregado Efetivo
	F	Empregado Efetivo
	F	Empregado Efetivo
	F	Empregado Efetivo
	F	Empregado Efetivo
	F	Empregado Efetivo
	F	Empregado Efetivo
	F	Empregado Efetivo
	F	Empregado Efetivo
	M	Empregado Efetivo
	F	Empregado Efetivo

	F	Empregado Efetivo
	F	Empregado Efetivo
	F	Empregado Efetivo
	F	Empregado Efetivo
	F	Empregado Efetivo
	F	Empregado Efetivo
	F	Empregado Efetivo
	F	Empregado Efetivo
	F	Empregado Efetivo
	F	Empregado Efetivo
	F	Empregado Efetivo
	F	Empregado Efetivo
	F	Empregado Efetivo
	F	Empregado Efetivo
	F	Empregado Efetivo
	F	Empregado Efetivo
	F	Empregado Efetivo
	F	Empregado Efetivo
	F	Empregado Efetivo
	F	Empregado Efetivo
	F	Empregado Efetivo
	F	Empregado Efetivo
	F	Empregado Efetivo
	F	Empregado Efetivo
	M	Empregado Efetivo
	F	Empregado Efetivo
	F	Empregado Efetivo
	F	Empregado Efetivo
	F	Empregado Efetivo
	F	Empregado Efetivo
	F	Empregado Efetivo
	F	Empregado Efetivo
	F	Empregado Efetivo
	F	Empregado Efetivo
	F	Empregado Efetivo
	F	Empregado Efetivo
	F	Empregado Efetivo
	F	Empregado Efetivo
	F	Empregado Efetivo
	F	Empregado Efetivo
	F	Empregado Efetivo
	F	Empregado Efetivo
	F	Empregado Efetivo
	F	Empregado Efetivo
	F	Empregado Efetivo
	F	Empregado Efetivo
	F	Empregado Efetivo
	F	Empregado Efetivo

	F	Empregado Efetivo
	F	Empregado Efetivo
	F	Empregado Efetivo
	F	Empregado Efetivo
	F	Empregado Efetivo
	F	Empregado Efetivo
	F	Empregado Efetivo
	F	Empregado Efetivo
	F	Empregado Efetivo
	M	Empregado Efetivo
	M	Empregado Efetivo
	M	Empregado Efetivo
	M	Empregado Efetivo
	M	Empregado Efetivo
	M	Empregado Efetivo
	M	Empregado Efetivo
	M	Empregado Efetivo
	F	Empregado Efetivo
	M	Empregado Efetivo
	M	Empregado Efetivo
	M	Empregado Efetivo
	M	Empregado Efetivo
	F	Empregado Efetivo
	F	Empregado Efetivo
	F	Empregado Efetivo
	F	Empregado Efetivo
	M	Empregado Efetivo
	F	Empregado Efetivo
	F	Empregado Efetivo
	M	Empregado Efetivo
	M	Empregado Efetivo
	F	Empregado Efetivo
	M	Empregado Efetivo
	F	Empregado Efetivo
	F	Empregado Efetivo
	F	Empregado Efetivo
	M	Empregado Efetivo
	M	Empregado Efetivo
	F	Empregado Efetivo

	M	Empregado Efetivo
	F	Empregado Efetivo
	F	Empregado Efetivo
	F	Empregado Efetivo
	M	Empregado Efetivo
	F	Empregado Efetivo
	M	Empregado Efetivo
	M	Empregado Efetivo
	F	Empregado Efetivo
	F	Empregado Efetivo
	F	Empregado Efetivo
	F	Empregado Efetivo
	F	Empregado Efetivo
	F	Empregado Efetivo
	F	Empregado Efetivo
	F	Empregado Efetivo
	F	Empregado Efetivo
	F	Empregado Efetivo
	F	Empregado Efetivo
	M	Empregado Efetivo
	F	Empregado Efetivo
	F	Empregado Efetivo
	F	Empregado Efetivo
	M	Empregado Efetivo
	F	Empregado Efetivo
	F	Empregado Efetivo
	F	Empregado Efetivo
	F	Empregado Efetivo
	F	Empregado Efetivo
	M	Empregado Efetivo
	F	Empregado Efetivo
	F	Empregado Efetivo
	F	Empregado Efetivo
	F	Empregado Efetivo
	M	Empregado Efetivo
	F	Empregado Efetivo
	F	Empregado Efetivo
	F	Empregado Efetivo
	F	Empregado Efetivo
	F	Empregado Efetivo
	F	Empregado Efetivo
	F	Empregado Efetivo
	F	Empregado Efetivo
	F	Empregado Efetivo
	M	Empregado Efetivo
	F	Empregado Efetivo
	F	Empregado Efetivo
	F	Empregado Efetivo

	F	Empregado Efetivo
	F	Empregado Efetivo
	F	Empregado Efetivo
	F	Empregado Efetivo
	F	Empregado Efetivo
	F	Empregado Efetivo
	F	Empregado Efetivo
	F	Empregado Efetivo
	M	Empregado Efetivo
	F	Empregado Efetivo
	M	Empregado Efetivo
	F	Empregado Efetivo
	M	Empregado Efetivo
	F	Empregado Efetivo
	F	Empregado Efetivo
	F	Empregado Efetivo
	F	Empregado Efetivo
	F	Empregado Efetivo
	F	Empregado Efetivo
	F	Empregado Efetivo
	F	Empregado Efetivo
	F	Empregado Efetivo
	F	Empregado Efetivo
	F	Empregado Efetivo
	F	Empregado Efetivo
	F	Empregado Efetivo
	F	Empregado Efetivo
	F	Empregado Efetivo
	F	Empregado Efetivo
	F	Empregado Efetivo
	F	Empregado Efetivo
	F	Empregado Efetivo
	F	Empregado Efetivo
	F	Empregado Efetivo
	F	Empregado Efetivo
	F	Empregado Efetivo
	F	Empregado Efetivo
	F	Empregado Efetivo
	F	Empregado Efetivo
	F	Empregado Efetivo
	F	Empregado Efetivo
	F	Empregado Efetivo
	F	Empregado Efetivo
	F	Empregado Efetivo
	F	Empregado Efetivo
	F	Empregado Efetivo
	F	Empregado Efetivo
	F	Empregado Efetivo

	F	Empregado Efetivo
	M	Empregado Efetivo
	F	Empregado Efetivo
	F	Empregado Efetivo
	F	Empregado Efetivo
	F	Empregado Efetivo
	F	Empregado Efetivo
	F	Empregado Efetivo
	F	Empregado Efetivo
	F	Empregado Efetivo
	F	Empregado Efetivo
	F	Empregado Efetivo
	F	Empregado Efetivo
	F	Empregado Efetivo
	F	Empregado Efetivo
	F	Empregado Efetivo
	F	Empregado Efetivo
	F	Empregado Efetivo
	F	Empregado Efetivo
	F	Empregado Efetivo
	F	Empregado Efetivo
	F	Empregado Efetivo
	F	Empregado Efetivo
	F	Empregado Efetivo
	F	Empregado Efetivo
	F	Empregado Efetivo
	F	Empregado Efetivo
	F	Empregado Efetivo
	F	Empregado Efetivo
	F	Empregado Efetivo
	F	Empregado Efetivo
	F	Empregado Efetivo
	F	Empregado Efetivo
	F	Empregado Efetivo
	F	Empregado Efetivo
	F	Empregado Efetivo
	F	Empregado Efetivo
	F	Empregado Efetivo
	F	Empregado Efetivo
	F	Empregado Efetivo
	F	Empregado Efetivo
	F	Empregado Efetivo
	F	Empregado Efetivo
	M	Empregado Efetivo
	F	Empregado Efetivo
	M	Empregado Efetivo

	F	Empregado Efetivo
	F	Empregado Efetivo
	F	Empregado Efetivo
	F	Empregado Efetivo
	F	Empregado Efetivo
	F	Empregado Efetivo
	F	Empregado Efetivo
	F	Empregado Efetivo
	F	Empregado Efetivo
	F	Empregado Efetivo
	F	Empregado Efetivo
	F	Empregado Efetivo
	F	Empregado Efetivo
	F	Empregado Efetivo
	F	Empregado Efetivo
	M	Empregado Efetivo
	F	Empregado Efetivo
	F	Empregado Efetivo
	F	Empregado Efetivo
	M	Empregado Efetivo
	M	Empregado Efetivo
	M	Empregado Efetivo
	F	Empregado Efetivo
	M	Empregado Efetivo
	M	Empregado Efetivo
	F	Empregado Efetivo
	M	Empregado Efetivo
	M	Empregado Efetivo
	F	Empregado Efetivo
	F	Empregado Efetivo
	M	Empregado Efetivo
	M	Empregado Efetivo

	F	Empregado Efetivo
	F	Empregado Efetivo
	F	Empregado Efetivo
	M	Empregado Efetivo
	F	Empregado Efetivo
	F	Empregado Efetivo
	F	Empregado Efetivo
	F	Empregado Efetivo
	F	Empregado Efetivo
	F	Empregado Efetivo
	F	Empregado Efetivo
	F	Empregado Efetivo
	F	Empregado Efetivo
	F	Empregado Efetivo
	M	Empregado Efetivo
	M	Empregado Efetivo
	M	Empregado Efetivo
	M	Empregado Efetivo
	M	Empregado Efetivo
	M	Empregado Efetivo
	M	Empregado Efetivo
	F	Empregado Efetivo
	F	Empregado Efetivo
	F	Empregado Efetivo
	F	Empregado Efetivo
	F	Empregado Efetivo
	F	Empregado Efetivo
	F	Empregado Efetivo
	M	Empregado Efetivo
	F	Empregado Efetivo
	F	Empregado Efetivo
	F	Empregado Efetivo
	F	Empregado Efetivo

	F	Empregado Efetivo
	F	Empregado Efetivo
	M	Empregado Efetivo
	F	Empregado Efetivo
	F	Empregado Efetivo
	F	Empregado Efetivo
	M	Empregado Efetivo
	M	Empregado Efetivo
	F	Empregado Efetivo
	F	Empregado Efetivo
	F	Empregado Efetivo
	M	Empregado Efetivo
	F	Empregado Efetivo
	M	Empregado Efetivo
	F	Empregado Efetivo
	F	Empregado Efetivo
	M	Empregado Efetivo
	M	Empregado Efetivo
	F	Empregado Efetivo
	F	Empregado Efetivo
	F	Empregado Efetivo
	F	Empregado Efetivo
	F	Empregado Efetivo
	F	Empregado Efetivo
	F	Empregado Efetivo
	F	Empregado Efetivo
	F	Empregado Efetivo
	F	Empregado Efetivo
	F	Empregado Efetivo
	F	Empregado Efetivo
	F	Empregado Efetivo
	F	Empregado Efetivo
	F	Empregado Efetivo
	F	Empregado Efetivo
	F	Empregado Efetivo
	F	Empregado Efetivo
	F	Empregado Efetivo
	F	Empregado Efetivo
	M	Empregado Efetivo
	M	Empregado Efetivo
	F	Empregado Efetivo
	M	Empregado Efetivo
	M	Empregado Efetivo
	F	Empregado Efetivo
	F	Empregado Efetivo
	F	Empregado Efetivo
	F	Empregado Efetivo

	M	Empregado Efetivo
	M	Empregado Efetivo
	F	Empregado Efetivo
	F	Empregado Efetivo
	F	Empregado Efetivo
	F	Empregado Efetivo
	F	Empregado Efetivo
	M	Empregado Efetivo
	M	Empregado Efetivo
	F	Empregado Efetivo
	M	Empregado Efetivo
	F	Empregado Efetivo
	M	Empregado Efetivo
	M	Empregado Efetivo
	F	Empregado Efetivo
	F	Empregado Efetivo
	M	Empregado Efetivo
	M	Empregado Efetivo
	F	Empregado Efetivo
	F	Empregado Efetivo
	F	Empregado Efetivo
	F	Empregado Efetivo
	F	Empregado Efetivo
	F	Empregado Efetivo
	F	Empregado Efetivo
	F	Empregado Efetivo
	F	Empregado Efetivo
	F	Empregado Efetivo
	F	Empregado Efetivo
	F	Empregado Efetivo
	F	Empregado Efetivo
	F	Empregado Efetivo
	F	Empregado Efetivo
	F	Empregado Efetivo
	F	Empregado Efetivo
	F	Empregado Efetivo
	F	Empregado Efetivo
	M	Empregado Efetivo

	F	Empregado Efetivo
	F	Empregado Efetivo
	F	Empregado Efetivo
	F	Empregado Efetivo
	F	Empregado Efetivo
	F	Empregado Efetivo
	M	Empregado Efetivo
	F	Empregado Efetivo
	M	Empregado Efetivo
	M	Empregado Efetivo
	M	Empregado Efetivo
	F	Empregado Efetivo
	F	Empregado Efetivo
	M	Empregado Efetivo
	F	Empregado Efetivo
	M	Empregado Efetivo
	F	Empregado Efetivo
	M	Empregado Efetivo
	F	Empregado Efetivo
	M	Empregado Efetivo
	M	Empregado Efetivo
	F	Empregado Efetivo
	M	Empregado Efetivo
	F	Empregado Efetivo
	F	Empregado Efetivo
	F	Empregado Efetivo
	F	Empregado Efetivo
	F	Empregado Efetivo
	M	Empregado Efetivo
	F	Empregado Efetivo
	F	Empregado Efetivo
	F	Empregado Efetivo
	F	Empregado Efetivo
	M	Empregado Efetivo
	M	Empregado Efetivo
	F	Empregado Efetivo
	M	Empregado Efetivo
	M	Empregado Efetivo
	F	Empregado Efetivo
	M	Empregado Efetivo
	M	Empregado Efetivo
	M	Empregado Efetivo
	F	Empregado Efetivo
	F	Empregado Efetivo
	F	Empregado Efetivo
	M	Empregado Efetivo
	F	Empregado Efetivo
	F	Empregado Efetivo

	F	Empregado Efetivo
	F	Empregado Efetivo
	F	Empregado Efetivo
	F	Empregado Efetivo
	F	Empregado Efetivo
	F	Empregado Efetivo
	F	Empregado Efetivo
	F	Empregado Efetivo
	F	Empregado Efetivo
	F	Empregado Efetivo
	M	Empregado Efetivo
	F	Empregado Efetivo
	F	Empregado Efetivo
	M	Empregado Efetivo
	M	Empregado Efetivo
	F	Empregado Efetivo
	F	Empregado Efetivo
	F	Empregado Efetivo
	F	Empregado Efetivo
	M	Empregado Efetivo
	F	Empregado Efetivo
	M	Empregado Efetivo
	F	Empregado Efetivo
	M	Empregado Efetivo
	F	Empregado Efetivo
	F	Empregado Efetivo
	F	Empregado Efetivo
	F	Empregado Efetivo
	M	Empregado Efetivo
	F	Empregado Efetivo
	F	Empregado Efetivo
	F	Empregado Efetivo
	F	Empregado Efetivo
	F	Empregado Efetivo
	M	Empregado Efetivo
	M	Empregado Efetivo

	M	Empregado Efetivo
	F	Empregado Efetivo
	F	Empregado Efetivo
	F	Empregado Efetivo
	M	Empregado Efetivo
	F	Empregado Efetivo
	F	Empregado Efetivo
	F	Empregado Efetivo
	F	Empregado Efetivo
	F	Empregado Efetivo
	F	Empregado Efetivo
	F	Empregado Efetivo
	F	Empregado Efetivo
	F	Empregado Efetivo
	F	Empregado Efetivo
	F	Empregado Efetivo
	F	Empregado Efetivo
	F	Empregado Efetivo
	F	Empregado Efetivo
	M	Empregado Efetivo
	F	Empregado Efetivo
	M	Empregado Efetivo
	M	Empregado Efetivo
	M	Empregado Efetivo
	F	Empregado Efetivo
	F	Empregado Efetivo
	F	Empregado Efetivo
	M	Empregado Efetivo
	F	Empregado Efetivo
	F	Empregado Efetivo
	F	Empregado Efetivo
	F	Empregado Efetivo
	F	Empregado Efetivo
	F	Empregado Efetivo
	F	Empregado Efetivo
	F	Empregado Efetivo
	F	Empregado Efetivo
	F	Empregado Efetivo
	F	Empregado Efetivo
	F	Empregado Efetivo
	F	Empregado Efetivo
	M	Empregado Efetivo
	F	Empregado Efetivo
	M	Empregado Efetivo
	M	Empregado Efetivo
	M	Empregado Efetivo
	F	Empregado Efetivo

	F	Empregado Efetivo
	F	Empregado Efetivo
	F	Empregado Efetivo
	M	Empregado Efetivo
	F	Empregado Efetivo
	M	Empregado Efetivo
	M	Empregado Efetivo
	F	Empregado Efetivo
	M	Empregado Efetivo
	M	Empregado Efetivo
	F	Empregado Efetivo
	F	Empregado Efetivo
	M	Empregado Efetivo
	F	Empregado Efetivo
	F	Empregado Efetivo
	F	Empregado Efetivo
	M	Empregado Efetivo
	F	Empregado Efetivo
	M	Empregado Efetivo
	F	Empregado Efetivo
	F	Empregado Efetivo
	F	Empregado Efetivo
	F	Empregado Efetivo
	F	Empregado Efetivo
	F	Empregado Efetivo
	F	Empregado Efetivo
	F	Empregado Efetivo
	F	Empregado Efetivo
	F	Empregado Efetivo
	F	Empregado Efetivo
	F	Empregado Efetivo
	F	Empregado Efetivo
	F	Empregado Efetivo
	F	Empregado Efetivo
	F	Empregado Efetivo
	F	Empregado Efetivo
	M	Empregado Efetivo
	F	Empregado Efetivo
	F	Empregado Efetivo
	F	Empregado Efetivo
	M	Empregado Efetivo
	F	Empregado Efetivo
	F	Empregado Efetivo
	F	Empregado Efetivo

	F	Empregado Efetivo
	F	Empregado Efetivo
	F	Empregado Efetivo
	F	Empregado Efetivo
	F	Empregado Efetivo
	F	Empregado Efetivo
	M	Empregado Efetivo
	F	Empregado Efetivo
	F	Empregado Efetivo
	F	Empregado Efetivo
	F	Empregado Efetivo
	F	Empregado Efetivo
	M	Empregado Efetivo
	M	Empregado Efetivo
	M	Empregado Efetivo
	M	Empregado Efetivo
	F	Empregado Efetivo
	F	Empregado Efetivo
	F	Empregado Efetivo
	M	Empregado Efetivo
	M	Empregado Efetivo
	F	Empregado Efetivo
	F	Empregado Efetivo
	F	Empregado Efetivo
	F	Empregado Efetivo
	F	Empregado Efetivo
	F	Empregado Efetivo
	F	Empregado Efetivo
	F	Empregado Efetivo
	F	Empregado Efetivo
	F	Empregado Efetivo
	M	Empregado Efetivo
	F	Empregado Efetivo
	F	Empregado Efetivo
	F	Empregado Efetivo
	F	Empregado Efetivo
	F	Empregado Efetivo
	F	Empregado Efetivo
	F	Empregado Efetivo
	F	Empregado Efetivo
	F	Empregado Efetivo
	F	Empregado Efetivo
	F	Empregado Efetivo
	F	Empregado Efetivo
	F	Empregado Efetivo
	F	Empregado Efetivo

	F	Empregado Efetivo
	F	Empregado Efetivo
	F	Empregado Efetivo
	F	Empregado Efetivo
	F	Empregado Efetivo
	F	Empregado Efetivo
	M	Empregado Efetivo
	M	Empregado Efetivo
	M	Empregado Efetivo
	M	Empregado Efetivo
	M	Empregado Efetivo
	M	Empregado Efetivo
	M	Empregado Efetivo
	M	Empregado Efetivo
	M	Empregado Efetivo
	F	Empregado Efetivo
	F	Empregado Efetivo
	F	Empregado Efetivo
	F	Empregado Efetivo
	M	Empregado Efetivo
	F	Empregado Efetivo
	F	Empregado Efetivo
	F	Empregado Efetivo
	M	Cedido - RJU
	F	Empregado Efetivo
	F	Empregado Efetivo
	M	Empregado Efetivo
	M	Empregado Efetivo
	F	Empregado Efetivo
	F	Empregado Efetivo
	F	Empregado Efetivo
	M	Empregado Efetivo
	F	Empregado Efetivo
	M	Empregado Efetivo
	F	Empregado Efetivo
	F	Empregado Efetivo
	F	Empregado Efetivo
	F	Empregado Efetivo
	M	Empregado Efetivo
	F	Empregado Efetivo
	F	Empregado Efetivo
	M	Empregado Efetivo

	M	Empregado Efetivo
	F	Empregado Efetivo
	F	Empregado Efetivo
	F	Empregado Efetivo
	M	Empregado Efetivo
	F	Empregado Efetivo
	F	Empregado Efetivo
	F	Empregado Efetivo
	F	Cedido - RJU
	M	Empregado Efetivo
	F	Cedido - RJU
	F	Empregado Efetivo
	M	Empregado Efetivo
	F	Empregado Efetivo
	F	Empregado Efetivo
	F	Empregado Efetivo
	M	Empregado Efetivo
	F	Empregado Efetivo
	F	Empregado Efetivo
	F	Empregado Efetivo
	F	Cedido - RJU
	F	Cedido - RJU
	F	Empregado Efetivo
	F	Empregado Efetivo
	F	Empregado Efetivo
	F	Empregado Efetivo
	F	Empregado Efetivo
	F	Empregado Efetivo
	F	Empregado Efetivo
	F	Empregado Efetivo
	F	Empregado Efetivo
	F	Empregado Efetivo
	F	Empregado Efetivo
	F	Empregado Efetivo
	F	Empregado Efetivo
	F	Empregado Efetivo
	F	Empregado Efetivo
	F	Empregado Efetivo
	F	Empregado Efetivo
	F	Empregado Efetivo
	F	Empregado Efetivo
	F	Empregado Efetivo
	F	Empregado Efetivo

	F	Empregado Efetivo
	F	Empregado Efetivo
	F	Empregado Efetivo
	F	Empregado Efetivo
	F	Empregado Efetivo
	F	Empregado Efetivo
	F	Empregado Efetivo
	F	Empregado Efetivo
	F	Empregado Efetivo
	F	Empregado Efetivo
	F	Empregado Efetivo
	F	Empregado Efetivo
	F	Empregado Efetivo
	M	Empregado Efetivo
	F	Empregado Efetivo
	M	Empregado Efetivo
	M	Empregado Efetivo
	F	Empregado Efetivo
	F	Empregado Efetivo
	F	Empregado Efetivo
	M	Empregado Efetivo
	F	Empregado Efetivo
	M	Empregado Efetivo
	F	Empregado Efetivo
	F	Empregado Efetivo
	F	Empregado Efetivo
	F	Empregado Efetivo
	F	Empregado Efetivo
	M	Cedido - RJU
	F	Empregado Efetivo
	F	Empregado Efetivo
	F	Empregado Efetivo
	F	Empregado Efetivo
	F	Empregado Efetivo

	F	Empregado Efetivo
	F	Empregado Efetivo
	F	Empregado Efetivo
	F	Empregado Efetivo
	F	Empregado Efetivo
	F	Empregado Efetivo
	F	Empregado Efetivo
	F	Empregado Efetivo
	F	Empregado Efetivo
	F	Empregado Efetivo
	F	Empregado Efetivo
	F	Empregado Efetivo
	F	Empregado Efetivo
	F	Empregado Efetivo
	F	Empregado Efetivo
	F	Empregado Efetivo
	F	Empregado Efetivo
	F	Empregado Efetivo
	F	Empregado Efetivo
	F	Empregado Efetivo
	F	Empregado Efetivo
	F	Empregado Efetivo
	F	Empregado Efetivo
	F	Empregado Efetivo
	F	Empregado Efetivo
	F	Empregado Efetivo
	F	Empregado Efetivo
	F	Empregado Efetivo
	F	Empregado Efetivo
	F	Empregado Efetivo
	F	Empregado Efetivo
	F	Empregado Efetivo
	F	Empregado Efetivo
	F	Empregado Efetivo
	F	Empregado Efetivo
	F	Empregado Efetivo
	F	Empregado Efetivo
	F	Empregado Efetivo
	M	Empregado Efetivo
	F	Empregado Efetivo

	M	Empregado Efetivo
	M	Empregado Efetivo
	M	Empregado Efetivo
	M	Empregado Efetivo
	F	Empregado Efetivo
	F	Empregado Efetivo
	F	Empregado Efetivo
	M	Empregado Efetivo
	M	Empregado Efetivo
	M	Empregado Efetivo
	M	Empregado Efetivo
	M	Empregado Efetivo
	M	Empregado Efetivo
	M	Empregado Efetivo
	M	Empregado Efetivo
	M	Empregado Efetivo
	M	Empregado Efetivo
	M	Empregado Efetivo
	M	Empregado Efetivo
	M	Empregado Efetivo
	M	Empregado Efetivo
	M	Empregado Efetivo
	M	Empregado Efetivo
	F	Empregado Efetivo
	F	Empregado Efetivo
	F	Empregado Efetivo
	F	Empregado Efetivo
	M	Empregado Efetivo
	M	Empregado Efetivo
	M	Empregado Efetivo
	M	Empregado Efetivo
	M	Empregado Efetivo
	M	Empregado Efetivo
	F	Empregado Efetivo

	M	Empregado Efetivo
	M	Empregado Efetivo
	M	Empregado Efetivo
	M	Empregado Efetivo
	M	Empregado Efetivo
	M	Empregado Efetivo
	M	Empregado Efetivo
	M	Empregado Efetivo
	M	Empregado Efetivo
	M	Empregado Efetivo
	F	Empregado Efetivo
	F	Empregado Efetivo
	F	Empregado Efetivo
	F	Empregado Efetivo
	F	Empregado Efetivo
	F	Empregado Efetivo
	F	Empregado Efetivo
	F	Empregado Efetivo
	F	Empregado Efetivo
	F	Empregado Efetivo
	F	Empregado Efetivo
	M	Empregado Efetivo
	M	Empregado Efetivo
	M	Empregado Efetivo
	M	Empregado Efetivo
	F	Empregado Efetivo
	F	Empregado Efetivo
	F	Empregado Efetivo
	F	Empregado Efetivo
	F	Empregado Efetivo
	F	Empregado Efetivo
	F	Empregado Efetivo
	F	Empregado Efetivo
	F	Empregado Efetivo
	F	Empregado Efetivo
	F	Empregado Efetivo
	F	Empregado Efetivo

	M	Empregado Efetivo
	M	Empregado Efetivo
	M	Empregado Efetivo
	M	Empregado Efetivo
	F	Empregado Efetivo
	F	Empregado Efetivo
	M	Empregado Efetivo
	M	Empregado Efetivo
	M	Empregado Efetivo
	M	Empregado Efetivo
	F	Empregado Efetivo
	F	Empregado Efetivo
	F	Empregado Efetivo
	M	Empregado Efetivo
	F	Empregado Efetivo
	F	Empregado Efetivo
	F	Empregado Efetivo
	F	Empregado Efetivo
	F	Empregado Efetivo
	F	Empregado Efetivo
	F	Empregado Efetivo
	F	Empregado Efetivo
	F	Empregado Efetivo
	F	Empregado Efetivo
	F	Empregado Efetivo
	F	Empregado Efetivo
	F	Empregado Efetivo
	F	Empregado Efetivo
	F	Empregado Efetivo
	M	Empregado Efetivo
	M	Empregado Efetivo
	M	Empregado Efetivo
	F	Empregado Efetivo
	M	Empregado Efetivo
	F	Empregado Efetivo
	F	Empregado Efetivo
	F	Empregado Efetivo
	M	Empregado Efetivo
	M	Empregado Efetivo
	M	Empregado Efetivo
	M	Empregado Efetivo
	M	Empregado Efetivo

	M	Empregado Efetivo
	F	Empregado Efetivo
	M	Empregado Efetivo
	F	Empregado Efetivo
	M	Empregado Efetivo
	F	Empregado Efetivo
	F	Empregado Efetivo
	M	Empregado Efetivo
	F	Empregado Efetivo
	F	Empregado Efetivo
	F	Empregado Efetivo
	F	Empregado Efetivo
	F	Empregado Efetivo
	F	Empregado Efetivo
	F	Empregado Efetivo
	F	Empregado Efetivo
	F	Empregado Efetivo
	F	Empregado Efetivo
	F	Empregado Efetivo
	F	Empregado Efetivo
	F	Empregado Efetivo
	F	Empregado Efetivo
	F	Empregado Efetivo
	F	Empregado Efetivo
	F	Empregado Efetivo
	F	Empregado Efetivo
	F	Empregado Efetivo
	F	Empregado Efetivo
	M	Empregado Efetivo
	M	Empregado Efetivo
	M	Empregado Efetivo
	F	Empregado Efetivo
	F	Empregado Efetivo
	F	Empregado Efetivo
	F	Empregado Efetivo
	F	Empregado Efetivo
	F	Empregado Efetivo
	F	Empregado Efetivo
	F	Empregado Efetivo
	M	Empregado Efetivo
	M	Empregado Efetivo
	M	Empregado Efetivo

	M	Empregado Efetivo
	F	Empregado Efetivo
	F	Empregado Efetivo
	F	Empregado Efetivo
	F	Empregado Efetivo
	F	Empregado Efetivo
	F	Empregado Efetivo
	F	Empregado Efetivo
	F	Empregado Efetivo
	M	Empregado Efetivo
	M	Empregado Efetivo
	M	Empregado Efetivo
	M	Empregado Efetivo
	F	Empregado Efetivo
	F	Empregado Efetivo
	M	Empregado Efetivo
	M	Empregado Efetivo
	M	Empregado Efetivo
	M	Empregado Efetivo
	M	Empregado Efetivo
	M	Empregado Efetivo
	F	Empregado Efetivo
	F	Empregado Efetivo
	F	Empregado Efetivo
	F	Empregado Efetivo
	M	Empregado Efetivo
	F	Empregado Efetivo
	F	Empregado Efetivo
	F	Empregado Efetivo
	F	Empregado Efetivo
	F	Empregado Efetivo
	F	Empregado Efetivo
	F	Empregado Efetivo
	F	Empregado Efetivo
	F	Empregado Efetivo
	F	Empregado Efetivo
	F	Empregado Efetivo
	F	Empregado Efetivo
	F	Empregado Efetivo
	F	Empregado Efetivo
	F	Empregado Efetivo
	F	Empregado Efetivo

	F	Empregado Efetivo
	F	Empregado Efetivo
	F	Empregado Efetivo
	F	Empregado Efetivo
	F	Empregado Efetivo
	F	Empregado Efetivo
	F	Empregado Efetivo
	F	Empregado Efetivo
	F	Empregado Efetivo
	F	Empregado Efetivo
	F	Empregado Efetivo
	F	Empregado Efetivo
	F	Empregado Efetivo
	F	Empregado Efetivo
	F	Empregado Efetivo
	F	Empregado Efetivo
	F	Empregado Efetivo
	M	Empregado Efetivo
	M	Empregado Efetivo
	M	Empregado Efetivo
	M	Empregado Efetivo
	M	Empregado Efetivo
	M	Empregado Efetivo
	M	Empregado Efetivo
	M	Empregado Efetivo
	M	Empregado Efetivo
	F	Empregado Efetivo
	M	Empregado Efetivo
	M	Empregado Efetivo
	F	Empregado Efetivo
	F	Empregado Efetivo
	F	Empregado Efetivo
	F	Empregado Efetivo
	F	Empregado Efetivo
	F	Empregado Efetivo

	F	Empregado Efetivo
	F	Empregado Efetivo
	F	Empregado Efetivo
	F	Empregado Efetivo
	F	Empregado Efetivo
	F	Empregado Efetivo
	M	Empregado Efetivo
	M	Empregado Efetivo
	F	Empregado Efetivo
	F	Empregado Efetivo
	F	Empregado Efetivo
	F	Empregado Efetivo
	F	Empregado Efetivo
	F	Empregado Efetivo
	F	Empregado Efetivo
	F	Empregado Efetivo
	M	Empregado Efetivo
	F	Empregado Efetivo
	F	Empregado Efetivo
	F	Empregado Efetivo
	F	Empregado Efetivo
	M	Empregado Efetivo
	F	Empregado Efetivo
	F	Empregado Efetivo
	F	Empregado Efetivo
	F	Empregado Efetivo
	F	Empregado Efetivo
	F	Empregado Efetivo
	F	Empregado Efetivo
	F	Empregado Efetivo
	F	Empregado Efetivo
	F	Empregado Efetivo
	F	Empregado Efetivo
	F	Empregado Efetivo
	F	Empregado Efetivo
	F	Empregado Efetivo
	F	Empregado Efetivo
	F	Empregado Efetivo
	F	Empregado Efetivo
	F	Empregado Efetivo
	F	Empregado Efetivo
	F	Empregado Efetivo
	F	Empregado Efetivo
	F	Empregado Efetivo
	F	Empregado Efetivo
	M	Empregado Efetivo
	F	Empregado Efetivo
	F	Empregado Efetivo

	F	Empregado Efetivo
	F	Empregado Efetivo
	F	Empregado Efetivo
	F	Empregado Efetivo
	F	Empregado Efetivo
	F	Empregado Efetivo
	F	Empregado Efetivo
	F	Empregado Efetivo
	F	Empregado Efetivo
	F	Empregado Efetivo
	F	Empregado Efetivo
	F	Empregado Efetivo
	M	Empregado Efetivo
	F	Empregado Efetivo
	F	Empregado Efetivo
	F	Empregado Efetivo
	F	Empregado Efetivo
	F	Empregado Efetivo
	F	Empregado Efetivo
	F	Empregado Efetivo
	F	Empregado Efetivo
	F	Empregado Efetivo
	M	Empregado Efetivo
	M	Empregado Efetivo
	M	Empregado Efetivo
	F	Empregado Efetivo
	F	Empregado Efetivo
	F	Empregado Efetivo
	F	Empregado Efetivo
	F	Empregado Efetivo
	F	Empregado Efetivo
	F	Empregado Efetivo
	F	Empregado Efetivo
	F	Empregado Efetivo
	M	Empregado Efetivo
	M	Empregado Efetivo
	M	Empregado Efetivo
	F	Empregado Efetivo
	F	Empregado Efetivo
	F	Empregado Efetivo
	F	Empregado Efetivo
	F	Empregado Efetivo
	F	Empregado Efetivo
	F	Empregado Efetivo
	M	Empregado Efetivo
	F	Empregado Efetivo

	F	Empregado Efetivo
	F	Empregado Efetivo
	F	Empregado Efetivo
	F	Empregado Efetivo
	F	Empregado Efetivo
	F	Empregado Efetivo
	F	Empregado Efetivo
	F	Empregado Efetivo
	F	Empregado Efetivo
	F	Empregado Efetivo
	F	Empregado Efetivo
	F	Empregado Efetivo
	F	Empregado Efetivo
	M	Empregado Efetivo
	M	Empregado Efetivo
	F	Empregado Efetivo
	F	Empregado Efetivo
	F	Empregado Efetivo
	F	Empregado Efetivo
	F	Empregado Efetivo
	F	Empregado Efetivo
	F	Empregado Efetivo
	F	Empregado Efetivo
	F	Empregado Efetivo
	F	Empregado Efetivo
	F	Empregado Efetivo
	F	Empregado Efetivo
	F	Empregado Efetivo
	F	Empregado Efetivo
	F	Empregado Efetivo
	F	Empregado Efetivo
	F	Empregado Efetivo
	F	Empregado Efetivo
	F	Empregado Efetivo
	F	Empregado Efetivo
	F	Empregado Efetivo
	M	Empregado Efetivo
	M	Empregado Efetivo
	M	Empregado Efetivo
	M	Empregado Efetivo
	F	Empregado Efetivo
	M	Empregado Efetivo
	F	Empregado Efetivo
	M	Empregado Efetivo
	M	Empregado Efetivo

	M	Empregado Efetivo
	M	Empregado Efetivo
	F	Empregado Efetivo
	F	Empregado Efetivo
	F	Empregado Efetivo
	M	Empregado Efetivo
	F	Empregado Efetivo
	F	Empregado Efetivo
	F	Empregado Efetivo
	F	Empregado Efetivo
	M	Empregado Efetivo
	M	Empregado Efetivo
	F	Empregado Efetivo
	M	Empregado Efetivo
	M	Empregado Efetivo
	F	Empregado Efetivo
	M	Empregado Efetivo
	M	Empregado Efetivo
	F	Empregado Efetivo
	M	Empregado Efetivo
	F	Empregado Efetivo
	F	Empregado Efetivo
	F	Empregado Efetivo
	F	Empregado Efetivo
	F	Empregado Efetivo
	F	Empregado Efetivo
	M	Empregado Efetivo
	F	Empregado Efetivo
	M	Empregado Efetivo
	F	Empregado Efetivo
	F	Empregado Efetivo
	F	Empregado Efetivo
	M	Empregado Efetivo
	F	Empregado Efetivo
	M	Empregado Efetivo
	F	Empregado Efetivo
	F	Empregado Efetivo
	F	Empregado Efetivo
	F	Empregado Efetivo
	F	Empregado Efetivo
	F	Empregado Efetivo
	F	Empregado Efetivo
	F	Empregado Efetivo
	M	Empregado Efetivo
	F	Empregado Efetivo
	F	Empregado Efetivo
	F	Empregado Efetivo
	F	Empregado Efetivo

	F	Empregado Efetivo
	F	Empregado Efetivo
	F	Empregado Efetivo
	F	Empregado Efetivo
	F	Empregado Efetivo
	M	Empregado Efetivo
	F	Empregado Efetivo
	M	Empregado Efetivo
	F	Empregado Efetivo
	M	Empregado Efetivo
	F	Empregado Efetivo
	F	Empregado Efetivo
	M	Empregado Efetivo
	F	Empregado Efetivo
	F	Empregado Efetivo
	F	Empregado Efetivo
	F	Empregado Efetivo
	M	Empregado Efetivo
	M	Empregado Efetivo
	M	Empregado Efetivo
	F	Empregado Efetivo
	M	Empregado Efetivo
	M	Empregado Efetivo
	F	Empregado Efetivo
	F	Empregado Efetivo
	F	Empregado Efetivo
	F	Empregado Efetivo
	F	Empregado Efetivo
	M	Empregado Efetivo
	F	Empregado Efetivo
	F	Empregado Efetivo
	M	Empregado Efetivo
	F	Empregado Efetivo
	M	Empregado Efetivo
	F	Empregado Efetivo
	F	Empregado Efetivo
	F	Empregado Efetivo
	F	Empregado Efetivo
	F	Empregado Efetivo
	M	Empregado Efetivo
	F	Empregado Efetivo
	F	Empregado Efetivo
	M	Empregado Efetivo
	F	Empregado Efetivo
	F	Empregado Efetivo
	M	Empregado Efetivo
	F	Empregado Efetivo
	F	Empregado Efetivo

	F	Empregado Efetivo
	M	Empregado Efetivo
	M	Empregado Efetivo
	M	Empregado Efetivo
	M	Empregado Efetivo
	F	Empregado Efetivo
	F	Empregado Efetivo
	F	Empregado Efetivo
	F	Empregado Efetivo
	F	Empregado Efetivo
	F	Empregado Efetivo
	M	Empregado Efetivo
	M	Empregado Efetivo
	F	Empregado Efetivo
	F	Empregado Efetivo
	M	Empregado Efetivo
	F	Empregado Efetivo
	F	Empregado Efetivo
	F	Empregado Efetivo
	F	Empregado Efetivo
	F	Empregado Efetivo
	F	Empregado Efetivo
	F	Empregado Efetivo
	F	Empregado Efetivo
	F	Empregado Efetivo
	F	Empregado Efetivo
	M	Empregado Efetivo
	F	Empregado Efetivo
	F	Empregado Efetivo
	F	Empregado Efetivo
	F	Empregado Efetivo
	F	Empregado Efetivo
	F	Empregado Efetivo
	F	Empregado Efetivo
	F	Empregado Efetivo
	F	Empregado Efetivo
	F	Empregado Efetivo
	F	Empregado Efetivo
	F	Empregado Efetivo
	F	Empregado Efetivo
	F	Empregado Efetivo
	F	Empregado Efetivo

	F	Empregado Efetivo
	F	Empregado Efetivo
	F	Empregado Efetivo
	F	Empregado Efetivo
	F	Empregado Efetivo
	F	Empregado Efetivo
	F	Empregado Efetivo
	F	Empregado Efetivo
	F	Empregado Efetivo
	F	Empregado Efetivo
	F	Empregado Efetivo
	F	Empregado Efetivo
	F	Empregado Efetivo
	F	Empregado Efetivo
	F	Empregado Efetivo
	F	Empregado Efetivo
	F	Empregado Efetivo
	F	Empregado Efetivo
	F	Empregado Efetivo
	M	Empregado Efetivo
	F	Empregado Efetivo
	F	Empregado Efetivo
	F	Empregado Efetivo
	F	Empregado Efetivo
	F	Empregado Efetivo
	F	Empregado Efetivo
	F	Empregado Efetivo
	F	Empregado Efetivo
	F	Empregado Efetivo
	F	Empregado Efetivo
	F	Empregado Efetivo
	F	Empregado Efetivo
	F	Empregado Efetivo
	F	Empregado Efetivo
	F	Empregado Efetivo
	F	Empregado Efetivo
	F	Empregado Efetivo
	F	Empregado Efetivo

	F	Empregado Efetivo
	F	Empregado Efetivo
	M	Empregado Efetivo
	F	Empregado Efetivo
	M	Empregado Efetivo
	F	Empregado Efetivo
	M	Empregado Efetivo
	M	Empregado Efetivo
	F	Empregado Efetivo
	F	Empregado Efetivo
	F	Empregado Efetivo
	F	Empregado Efetivo
	F	Empregado Efetivo
	F	Empregado Efetivo
	F	Empregado Efetivo
	F	Empregado Efetivo
	F	Empregado Efetivo
	F	Empregado Efetivo
	F	Empregado Efetivo
	F	Empregado Efetivo
	F	Empregado Efetivo
	F	Empregado Efetivo
	F	Empregado Efetivo
	M	Empregado Efetivo
	F	Empregado Efetivo
	M	Empregado Efetivo
	F	Empregado Efetivo
	F	Empregado Efetivo
	F	Empregado Efetivo
	F	Empregado Efetivo
	F	Empregado Efetivo
	F	Empregado Efetivo

	F	Empregado Efetivo
	F	Empregado Efetivo
	F	Empregado Efetivo
	F	Empregado Efetivo
	F	Empregado Efetivo
	F	Empregado Efetivo
	F	Empregado Efetivo
	M	Empregado Efetivo
	M	Empregado Efetivo
	M	Empregado Efetivo
	F	Empregado Efetivo
	F	Empregado Efetivo
	M	Empregado Efetivo
	F	Empregado Efetivo
	F	Empregado Efetivo
	F	Empregado Efetivo
	M	Empregado Efetivo
	F	Empregado Efetivo
	F	Empregado Efetivo
	F	Empregado Efetivo
	F	Empregado Efetivo
	F	Empregado Efetivo
	F	Empregado Efetivo
	F	Empregado Efetivo
	F	Empregado Efetivo
	M	Empregado Efetivo
	F	Empregado Efetivo
	F	Empregado Efetivo
	M	Empregado Efetivo
	M	Empregado Efetivo
	F	Empregado Efetivo
	F	Empregado Efetivo

	M	Empregado Efetivo
	F	Empregado Efetivo
	F	Empregado Efetivo
	F	Empregado Efetivo
	M	Empregado Efetivo
	F	Empregado Efetivo
	F	Empregado Efetivo
	F	Empregado Efetivo
	F	Empregado Efetivo
	F	Empregado Efetivo
	F	Empregado Efetivo
	F	Empregado Efetivo
	F	Empregado Efetivo
	F	Empregado Efetivo
	F	Empregado Efetivo
	F	Empregado Efetivo
	F	Empregado Efetivo
	M	Empregado Efetivo
	F	Empregado Efetivo
	F	Empregado Efetivo
	F	Empregado Efetivo
	F	Empregado Efetivo
	F	Empregado Efetivo
	F	Empregado Efetivo
	F	Empregado Efetivo
	F	Empregado Efetivo
	F	Empregado Efetivo
	F	Empregado Efetivo
	M	Empregado Efetivo
	F	Empregado Efetivo
	M	Empregado Efetivo
	M	Empregado Efetivo
	M	Empregado Efetivo
	F	Empregado Efetivo
	F	Empregado Efetivo
	F	Empregado Efetivo
	M	Empregado Efetivo
	F	Empregado Efetivo
	F	Empregado Efetivo
	M	Empregado Efetivo
	F	Empregado Efetivo
	F	Empregado Efetivo
	F	Empregado Efetivo
	M	Empregado Efetivo
	F	Empregado Efetivo
	F	Empregado Efetivo
	M	Empregado Efetivo
	F	Empregado Efetivo

	F	Empregado Efetivo
	F	Empregado Efetivo
	F	Empregado Efetivo
	F	Empregado Efetivo
	M	Empregado Efetivo
	M	Empregado Efetivo
	F	Empregado Efetivo
	F	Empregado Efetivo
	F	Empregado Efetivo
	F	Empregado Efetivo
	F	Empregado Efetivo
	M	Empregado Efetivo
	M	Empregado Efetivo
	M	Empregado Efetivo
	M	Empregado Efetivo
	M	Empregado Efetivo
	M	Empregado Efetivo
	F	Empregado Efetivo
	M	Empregado Efetivo
	F	Empregado Efetivo
	F	Empregado Efetivo
	F	Empregado Efetivo
	F	Empregado Efetivo
	M	Empregado Efetivo
	F	Empregado Efetivo
	F	Empregado Efetivo
	F	Empregado Efetivo
	F	Empregado Efetivo
	F	Empregado Efetivo
	M	Empregado Efetivo
	M	Empregado Efetivo
	M	Empregado Efetivo
	F	Empregado Efetivo
	F	Empregado Efetivo
	F	Empregado Efetivo
	F	Empregado Efetivo
	F	Empregado Efetivo
	F	Empregado Efetivo

	F	Empregado Efetivo
	F	Empregado Efetivo
	F	Empregado Efetivo
	F	Empregado Efetivo
	F	Empregado Efetivo
	F	Empregado Efetivo
	F	Empregado Efetivo
	F	Empregado Efetivo
	F	Empregado Efetivo
	F	Empregado Efetivo
	F	Empregado Efetivo
	F	Empregado Efetivo
	F	Empregado Efetivo
	F	Empregado Efetivo
	F	Empregado Efetivo
	F	Empregado Efetivo
	F	Empregado Efetivo
	F	Empregado Efetivo
	F	Empregado Efetivo
	F	Empregado Efetivo
	F	Empregado Efetivo
	F	Empregado Efetivo
	F	Empregado Efetivo
	F	Empregado Efetivo
	F	Empregado Efetivo
	F	Empregado Efetivo
	F	Empregado Efetivo
	F	Empregado Efetivo
	F	Empregado Efetivo
	F	Empregado Efetivo
	F	Empregado Efetivo
	F	Empregado Efetivo

	F	Empregado Efetivo
	F	Empregado Efetivo
	F	Empregado Efetivo
	F	Empregado Efetivo
	F	Empregado Efetivo
	F	Empregado Efetivo
	F	Empregado Efetivo
	F	Empregado Efetivo
	F	Empregado Efetivo
	F	Empregado Efetivo
	F	Empregado Efetivo
	F	Empregado Efetivo
	F	Empregado Efetivo
	F	Empregado Efetivo
	F	Empregado Efetivo
	F	Empregado Efetivo
	F	Empregado Efetivo
	F	Empregado Efetivo
	M	Empregado Efetivo
	F	Empregado Efetivo
	F	Empregado Efetivo
	F	Empregado Efetivo
	F	Empregado Efetivo
	F	Empregado Efetivo
	M	Empregado Efetivo
	F	Empregado Efetivo
	M	Empregado Efetivo
	M	Empregado Efetivo
	F	Empregado Efetivo
	M	Empregado Efetivo
	F	Empregado Efetivo
	M	Empregado Efetivo
	F	Empregado Efetivo
	F	Empregado Efetivo
	F	Empregado Efetivo
	M	Empregado Efetivo

	F	Empregado Efetivo
	F	Empregado Efetivo
	F	Empregado Efetivo
	F	Empregado Efetivo
	F	Empregado Efetivo
	M	Empregado Efetivo
	F	Empregado Efetivo
	F	Empregado Efetivo
	M	Empregado Efetivo
	F	Empregado Efetivo
	F	Empregado Efetivo
	F	Empregado Efetivo
	F	Empregado Efetivo
	F	Empregado Efetivo
	F	Empregado Efetivo
	F	Empregado Efetivo
	F	Empregado Efetivo
	F	Empregado Efetivo
	F	Empregado Efetivo
	F	Empregado Efetivo
	F	Empregado Efetivo
	F	Empregado Efetivo
	F	Empregado Efetivo
	F	Empregado Efetivo
	F	Empregado Efetivo
	F	Empregado Efetivo
	F	Empregado Efetivo
	F	Empregado Efetivo
	F	Empregado Efetivo
	F	Empregado Efetivo
	F	Empregado Efetivo
	F	Empregado Efetivo
	F	Empregado Efetivo
	F	Empregado Efetivo
	F	Empregado Efetivo
	F	Empregado Efetivo
	M	Empregado Efetivo
	F	Empregado Efetivo
	M	Empregado Efetivo
	M	Empregado Efetivo

	F	Empregado Efetivo
	F	Empregado Efetivo
	M	Empregado Efetivo
	F	Empregado Efetivo
	F	Empregado Efetivo
	F	Empregado Efetivo
	M	Empregado Efetivo
	F	Empregado Efetivo
	F	Empregado Efetivo
	F	Empregado Efetivo
	F	Empregado Efetivo
	F	Empregado Efetivo
	F	Empregado Efetivo
	F	Empregado Efetivo
	F	Empregado Efetivo
	M	Empregado Efetivo
	F	Empregado Efetivo
	F	Empregado Efetivo
	F	Empregado Efetivo
	F	Empregado Efetivo
	F	Empregado Efetivo
	F	Empregado Efetivo
	F	Empregado Efetivo
	F	Empregado Efetivo
	F	Empregado Efetivo
	F	Empregado Efetivo
	F	Empregado Efetivo
	F	Empregado Efetivo
	F	Empregado Efetivo
	F	Empregado Efetivo
	F	Empregado Efetivo
	F	Empregado Efetivo
	F	Empregado Efetivo
	F	Empregado Efetivo
	F	Empregado Efetivo
	F	Empregado Efetivo
	F	Empregado Efetivo
	F	Empregado Efetivo
	F	Empregado Efetivo

	F	Empregado Efetivo
	F	Empregado Efetivo
	F	Empregado Efetivo
	F	Empregado Efetivo
	F	Empregado Efetivo
	F	Empregado Efetivo
	F	Empregado Efetivo
	F	Empregado Efetivo
	F	Empregado Efetivo
	F	Empregado Efetivo
	F	Empregado Efetivo
	F	Empregado Efetivo
	F	Empregado Efetivo
	F	Empregado Efetivo
	F	Empregado Efetivo
	F	Empregado Efetivo
	F	Empregado Efetivo
	F	Empregado Efetivo
	F	Empregado Efetivo
	F	Empregado Efetivo
	F	Empregado Efetivo
	F	Empregado Efetivo
	F	Empregado Efetivo
	F	Empregado Efetivo
	F	Empregado Efetivo
	F	Empregado Efetivo
	F	Empregado Efetivo
	F	Empregado Efetivo
	F	Empregado Efetivo
	F	Empregado Efetivo
	F	Empregado Efetivo
	F	Empregado Efetivo
	F	Empregado Efetivo
	F	Empregado Efetivo
	F	Empregado Efetivo
	M	Empregado Efetivo
	F	Empregado Efetivo
	F	Empregado Efetivo
	F	Empregado Efetivo
	F	Empregado Efetivo

	F	Empregado Efetivo
	F	Empregado Efetivo
	F	Empregado Efetivo
	F	Empregado Efetivo
	F	Empregado Efetivo
	F	Empregado Efetivo
	F	Empregado Efetivo
	F	Empregado Efetivo
	M	Empregado Efetivo
	M	Empregado Efetivo
	F	Empregado Efetivo
	F	Empregado Efetivo
	M	Empregado Efetivo
	F	Empregado Efetivo
	F	Empregado Efetivo
	M	Empregado Efetivo
	M	Empregado Efetivo
	M	Empregado Efetivo
	F	Empregado Efetivo
	F	Empregado Efetivo
	F	Empregado Efetivo
	F	Empregado Efetivo
	F	Empregado Efetivo
	F	Empregado Efetivo
	F	Empregado Efetivo
	F	Empregado Efetivo
	F	Empregado Efetivo
	M	Empregado Efetivo
	F	Empregado Efetivo
	M	Empregado Efetivo
	F	Empregado Efetivo
	F	Empregado Efetivo
	M	Empregado Efetivo
	F	Empregado Efetivo
	F	Empregado Efetivo
	M	Empregado Efetivo
	F	Empregado Efetivo
	F	Empregado Efetivo
	M	Empregado Efetivo
	F	Empregado Efetivo
	F	Empregado Efetivo
	F	Empregado Efetivo
	F	Empregado Efetivo
	M	Empregado Efetivo
	F	Empregado Efetivo

	F	Empregado Efetivo
	F	Empregado Efetivo
	F	Empregado Efetivo
	F	Empregado Efetivo
	F	Empregado Efetivo
	F	Empregado Efetivo
	F	Empregado Efetivo
	F	Empregado Efetivo
	F	Empregado Efetivo
	F	Empregado Efetivo
	F	Empregado Efetivo
	F	Empregado Efetivo
	F	Empregado Efetivo
	F	Empregado Efetivo
	F	Empregado Efetivo
	M	Empregado Efetivo
	F	Empregado Efetivo
	F	Empregado Efetivo
	F	Empregado Efetivo
	F	Empregado Efetivo
	F	Empregado Efetivo
	F	Empregado Efetivo
	F	Empregado Efetivo
	F	Empregado Efetivo
	F	Empregado Efetivo
	F	Empregado Efetivo
	F	Empregado Efetivo
	F	Empregado Efetivo
	M	Empregado Efetivo
	M	Empregado Efetivo
	F	Empregado Efetivo
	M	Empregado Efetivo
	M	Empregado Efetivo
	F	Empregado Efetivo
	F	Empregado Efetivo
	F	Empregado Efetivo
	F	Empregado Efetivo
	F	Empregado Efetivo
	F	Empregado Efetivo
	M	Empregado Efetivo
	M	Empregado Efetivo

	F	Empregado Efetivo
	F	Empregado Efetivo
	F	Empregado Efetivo
	F	Empregado Efetivo
	F	Empregado Efetivo
	F	Empregado Efetivo
	F	Empregado Efetivo
	F	Empregado Efetivo
	F	Empregado Efetivo
	F	Empregado Efetivo
	F	Empregado Efetivo
	F	Empregado Efetivo
	F	Empregado Efetivo
	F	Empregado Efetivo
	F	Empregado Efetivo
	F	Empregado Efetivo
	F	Empregado Efetivo
	F	Empregado Efetivo
	F	Empregado Efetivo
	F	Empregado Efetivo
	F	Empregado Efetivo
	F	Empregado Efetivo
	F	Empregado Efetivo
	F	Empregado Efetivo
	F	Empregado Efetivo
	F	Empregado Efetivo
	F	Empregado Efetivo
	F	Empregado Efetivo
	F	Empregado Efetivo
	F	Empregado Efetivo
	F	Empregado Efetivo
	F	Empregado Efetivo

	M	Empregado Efetivo
	M	Empregado Efetivo
	M	Empregado Efetivo
	M	Empregado Efetivo
	M	Empregado Efetivo
	M	Empregado Efetivo
	M	Empregado Efetivo
	F	Empregado Efetivo
	F	Empregado Efetivo
	F	Empregado Efetivo
	M	Empregado Efetivo
	F	Empregado Efetivo
	M	Empregado Efetivo
	F	Empregado Efetivo
	M	Empregado Efetivo
	M	Empregado Efetivo
	F	Empregado Efetivo
	F	Empregado Efetivo
	F	Empregado Efetivo
	M	Empregado Efetivo
	M	Empregado Efetivo
	F	Empregado Efetivo
	F	Empregado Efetivo
	F	Empregado Efetivo
	F	Empregado Efetivo
	F	Empregado Efetivo
	F	Empregado Efetivo
	F	Empregado Efetivo
	M	Empregado Efetivo
	F	Empregado Efetivo
	F	Empregado Efetivo
	F	Empregado Efetivo

	F	Empregado Efetivo
	F	Empregado Efetivo
	F	Empregado Efetivo
	F	Empregado Efetivo
	F	Empregado Efetivo
	F	Empregado Efetivo
	M	Empregado Efetivo
	M	Empregado Efetivo
	M	Empregado Efetivo
	M	Empregado Efetivo
	F	Empregado Efetivo
	F	Empregado Efetivo
	F	Empregado Efetivo
	F	Empregado Efetivo
	F	Empregado Efetivo
	F	Empregado Efetivo
	F	Empregado Efetivo
	F	Empregado Efetivo
	F	Empregado Efetivo
	F	Empregado Efetivo
	F	Empregado Efetivo
	F	Empregado Efetivo
	F	Empregado Efetivo
	F	Empregado Efetivo
	F	Empregado Efetivo
	F	Empregado Efetivo
	M	Empregado Efetivo
	F	Empregado Efetivo
	F	Empregado Efetivo
	M	Empregado Efetivo
	F	Empregado Efetivo
	F	Empregado Efetivo

	M	Empregado Efetivo
	M	Empregado Efetivo
	F	Empregado Efetivo
	M	Empregado Efetivo
	M	Empregado Efetivo
	F	Empregado Efetivo
	F	Empregado Efetivo
	F	Empregado Efetivo
	M	Empregado Efetivo
	M	Empregado Efetivo
	M	Empregado Efetivo
	F	Empregado Efetivo
	F	Empregado Efetivo
	F	Empregado Efetivo
	F	Empregado Efetivo
	F	Empregado Efetivo
	M	Empregado Efetivo
	M	Empregado Efetivo
	F	Empregado Efetivo
	F	Empregado Efetivo
	M	Empregado Efetivo
	F	Empregado Efetivo
	F	Empregado Efetivo
	F	Empregado Efetivo
	F	Empregado Efetivo
	F	Empregado Efetivo
	F	Empregado Efetivo
	F	Empregado Efetivo
	F	Empregado Efetivo
	M	Empregado Efetivo
	F	Empregado Efetivo
	F	Empregado Efetivo
	F	Empregado Efetivo
	F	Empregado Efetivo
	F	Empregado Efetivo
	F	Empregado Efetivo
	F	Empregado Efetivo
	F	Empregado Efetivo
	F	Empregado Efetivo
	M	Empregado Efetivo
	F	Empregado Efetivo

	F	Empregado Efetivo
	F	Empregado Efetivo
	F	Empregado Efetivo
	F	Empregado Efetivo
	F	Empregado Efetivo
	F	Empregado Efetivo
	M	Empregado Efetivo
	F	Empregado Efetivo
	F	Empregado Efetivo
	M	Empregado Efetivo
	F	Empregado Efetivo
	F	Empregado Efetivo
	F	Empregado Efetivo
	F	Empregado Efetivo
	F	Empregado Efetivo
	F	Empregado Efetivo
	F	Empregado Efetivo
	F	Empregado Efetivo
	F	Empregado Efetivo
	F	Empregado Efetivo
	M	Empregado Efetivo
	F	Empregado Efetivo
	F	Empregado Efetivo
	F	Empregado Efetivo
	F	Empregado Efetivo
	M	Empregado Efetivo
	F	Empregado Efetivo
	M	Empregado Efetivo
	M	Empregado Efetivo
	F	Empregado Efetivo
	F	Empregado Efetivo
	M	Empregado Efetivo
	F	Empregado Efetivo
	F	Empregado Efetivo
	M	Empregado Efetivo
	M	Empregado Efetivo
	F	Empregado Efetivo
	M	Empregado Efetivo
	M	Empregado Efetivo
	M	Empregado Efetivo
	F	Empregado Efetivo
	F	Empregado Efetivo
	F	Empregado Efetivo
	F	Empregado Efetivo
	F	Empregado Efetivo
	F	Empregado Efetivo
	F	Empregado Efetivo
	F	Empregado Efetivo

	F	Empregado Efetivo
	F	Empregado Efetivo
	F	Empregado Efetivo
	F	Empregado Efetivo
	F	Empregado Efetivo
	F	Empregado Efetivo
	F	Empregado Efetivo
	F	Empregado Efetivo
	F	Empregado Efetivo
	M	Empregado Efetivo
	F	Empregado Efetivo
	M	Empregado Efetivo
	F	Empregado Efetivo
	F	Empregado Efetivo
	F	Empregado Efetivo
	F	Empregado Efetivo
	M	Empregado Efetivo
	F	Empregado Efetivo
	M	Empregado Efetivo
	M	Empregado Efetivo
	M	Empregado Efetivo
	M	Empregado Efetivo
	F	Empregado Efetivo
	F	Empregado Efetivo
	F	Empregado Efetivo
	F	Empregado Efetivo
	M	Empregado Efetivo
	F	Empregado Efetivo
	F	Empregado Efetivo
	F	Empregado Efetivo
	M	Empregado Efetivo
	M	Empregado Efetivo
	F	Empregado Efetivo
	F	Empregado Efetivo
	F	Empregado Efetivo
	F	Empregado Efetivo
	F	Empregado Efetivo
	F	Empregado Efetivo
	F	Empregado Efetivo
	F	Empregado Efetivo
	F	Empregado Efetivo

	F	Empregado Efetivo
	F	Empregado Efetivo
	F	Empregado Efetivo
	F	Empregado Efetivo
	F	Empregado Efetivo
	F	Empregado Efetivo
	F	Empregado Efetivo
	F	Empregado Efetivo
	F	Empregado Efetivo
	F	Empregado Efetivo
	F	Empregado Efetivo
	F	Empregado Efetivo
	M	Empregado Efetivo
	F	Empregado Efetivo
	F	Empregado Efetivo
	F	Empregado Efetivo
	F	Empregado Efetivo
	F	Empregado Efetivo
	M	Empregado Efetivo
	F	Empregado Efetivo
	M	Empregado Efetivo
	M	Empregado Efetivo
	F	Empregado Efetivo
	F	Empregado Efetivo
	F	Empregado Efetivo
	F	Empregado Efetivo
	F	Empregado Efetivo
	M	Empregado Efetivo
	F	Empregado Efetivo
	F	Empregado Efetivo
	F	Empregado Efetivo
	F	Empregado Efetivo
	M	Empregado Efetivo
	F	Empregado Efetivo
	F	Empregado Efetivo
	M	Empregado Efetivo
	M	Empregado Efetivo

	F	Empregado Efetivo
	M	Empregado Efetivo
	F	Empregado Efetivo
	M	Empregado Efetivo
	F	Empregado Efetivo
	M	Empregado Efetivo
	F	Empregado Efetivo
	M	Empregado Efetivo
	F	Empregado Efetivo
	F	Empregado Efetivo
	F	Empregado Efetivo
	F	Empregado Efetivo
	F	Empregado Efetivo
	F	Empregado Efetivo
	F	Empregado Efetivo
	F	Empregado Efetivo
	M	Empregado Efetivo
	F	Empregado Efetivo
	F	Empregado Efetivo
	F	Empregado Efetivo
	F	Empregado Efetivo
	F	Empregado Efetivo
	M	Empregado Efetivo
	F	Empregado Efetivo
	F	Empregado Efetivo
	M	Empregado Efetivo
	M	Empregado Efetivo
	F	Empregado Efetivo
	F	Empregado Efetivo
	F	Empregado Efetivo
	M	Empregado Efetivo
	M	Empregado Efetivo
	F	Empregado Efetivo
	M	Empregado Efetivo
	F	Empregado Efetivo
	F	Empregado Efetivo
	M	Empregado Efetivo
	F	Empregado Efetivo
	M	Empregado Efetivo
	F	Empregado Efetivo
	M	Empregado Efetivo
	F	Empregado Efetivo
	F	Empregado Efetivo
	M	Empregado Efetivo
	M	Empregado Efetivo
	F	Empregado Efetivo
	F	Empregado Efetivo
	F	Empregado Efetivo

	M	Empregado Efetivo
	M	Empregado Efetivo
	F	Empregado Efetivo
	F	Empregado Efetivo
	F	Empregado Efetivo
	F	Empregado Efetivo
	F	Empregado Efetivo
	F	Empregado Efetivo
	M	Empregado Efetivo
	F	Empregado Efetivo
	F	Empregado Efetivo
	F	Empregado Efetivo
	M	Empregado Efetivo
	F	Empregado Efetivo
	F	Empregado Efetivo
	F	Empregado Efetivo
	F	Empregado Efetivo
	F	Empregado Efetivo
	F	Empregado Efetivo
	M	Empregado Efetivo
	M	Empregado Efetivo
	F	Empregado Efetivo
	M	Empregado Efetivo
	F	Empregado Efetivo
	M	Empregado Efetivo
	M	Empregado Efetivo
	M	Empregado Efetivo
	M	Empregado Efetivo
	F	Empregado Efetivo
	M	Empregado Efetivo
	F	Empregado Efetivo
	F	Empregado Efetivo
	F	Empregado Efetivo
	F	Empregado Efetivo
	M	Empregado Efetivo
	F	Empregado Efetivo
	F	Empregado Efetivo
	M	Empregado Efetivo
	F	Empregado Efetivo
	M	Empregado Efetivo
	F	Empregado Efetivo
	F	Empregado Efetivo
	M	Empregado Efetivo
	F	Empregado Efetivo
	M	Empregado Efetivo
	M	Empregado Efetivo
	M	Empregado Efetivo
	M	Empregado Efetivo

	F	Empregado Efetivo
	F	Empregado Efetivo
	F	Empregado Efetivo
	F	Empregado Efetivo
	F	Empregado Efetivo
	F	Empregado Efetivo
	F	Empregado Efetivo
	M	Empregado Efetivo
	M	Empregado Efetivo
	F	Empregado Efetivo
	F	Empregado Efetivo
	M	Empregado Efetivo
	M	Empregado Efetivo
	M	Empregado Efetivo
	F	Empregado Efetivo
	F	Empregado Efetivo
	M	Empregado Efetivo
	F	Empregado Efetivo
	F	Empregado Efetivo
	F	Empregado Efetivo
	F	Empregado Efetivo
	F	Empregado Efetivo
	F	Empregado Efetivo
	F	Empregado Efetivo
	F	Empregado Efetivo
	F	Empregado Efetivo
	M	Empregado Efetivo
	F	Empregado Efetivo
	F	Empregado Efetivo
	F	Empregado Efetivo
	F	Empregado Efetivo
	F	Empregado Efetivo
	M	Empregado Efetivo
	F	Empregado Efetivo
	F	Empregado Efetivo
	F	Empregado Efetivo
	F	Empregado Efetivo
	F	Empregado Efetivo
	F	Empregado Efetivo
	F	Empregado Efetivo
	F	Empregado Efetivo
	F	Empregado Efetivo
	M	Empregado Efetivo
	M	Empregado Efetivo
	F	Empregado Efetivo
	F	Empregado Efetivo
	F	Empregado Efetivo
	F	Empregado Efetivo

	M	Empregado Efetivo
	F	Empregado Efetivo
	F	Empregado Efetivo
	F	Empregado Efetivo
	M	Empregado Efetivo
	M	Empregado Efetivo
	F	Empregado Efetivo
	F	Empregado Efetivo
	F	Empregado Efetivo
	M	Empregado Efetivo
	M	Empregado Efetivo
	M	Empregado Efetivo
	F	Empregado Efetivo
	F	Empregado Efetivo
	F	Empregado Efetivo
	F	Empregado Efetivo
	F	Empregado Efetivo
	F	Empregado Efetivo
	F	Empregado Efetivo
	F	Empregado Efetivo
	F	Empregado Efetivo
	F	Empregado Efetivo
	M	Empregado Efetivo
	F	Empregado Efetivo
	F	Empregado Efetivo
	F	Empregado Efetivo
	F	Empregado Efetivo
	F	Empregado Efetivo
	F	Empregado Efetivo
	M	Empregado Efetivo
	F	Empregado Efetivo
	F	Empregado Efetivo
	F	Empregado Efetivo
	F	Empregado Efetivo
	F	Empregado Efetivo
	F	Empregado Efetivo

	F	Empregado Efetivo
	F	Empregado Efetivo
	F	Empregado Efetivo
	M	Empregado Efetivo
	F	Empregado Efetivo
	M	Empregado Efetivo
	M	Empregado Efetivo
	F	Empregado Efetivo
	F	Empregado Efetivo
	M	Empregado Efetivo
	F	Empregado Efetivo
	F	Empregado Efetivo
	F	Empregado Efetivo
	F	Empregado Efetivo
	F	Empregado Efetivo
	F	Empregado Efetivo
	F	Empregado Efetivo
	M	Empregado Efetivo
	F	Empregado Efetivo
	F	Empregado Efetivo
	F	Empregado Efetivo
	F	Empregado Efetivo
	F	Empregado Efetivo
	M	Empregado Efetivo
	F	Empregado Efetivo
	F	Empregado Efetivo
	F	Empregado Efetivo
	F	Empregado Efetivo
	F	Empregado Efetivo
	F	Empregado Efetivo
	M	Empregado Efetivo
	F	Empregado Efetivo
	F	Empregado Efetivo
	F	Empregado Efetivo
	F	Empregado Efetivo
	F	Empregado Efetivo
	F	Empregado Efetivo
	M	Empregado Efetivo
	F	Empregado Efetivo
	F	Empregado Efetivo
	M	Empregado Efetivo
	F	Empregado Efetivo

	F	Empregado Efetivo
	F	Empregado Efetivo
	F	Empregado Efetivo
	F	Empregado Efetivo
	F	Empregado Efetivo
	M	Empregado Efetivo
	F	Empregado Efetivo
	F	Empregado Efetivo
	M	Empregado Efetivo
	M	Empregado Efetivo
	M	Empregado Efetivo
	F	Empregado Efetivo
	M	Empregado Efetivo
	F	Empregado Efetivo
	M	Empregado Efetivo
	F	Empregado Efetivo
	F	Empregado Efetivo
	F	Empregado Efetivo
	F	Empregado Efetivo
	F	Empregado Efetivo
	M	Empregado Efetivo
	M	Empregado Efetivo
	M	Empregado Efetivo
	F	Empregado Efetivo
	M	Empregado Efetivo
	F	Empregado Efetivo
	F	Empregado Efetivo
	F	Empregado Efetivo
	M	Empregado Efetivo
	F	Empregado Efetivo
	F	Empregado Efetivo
	M	Empregado Efetivo
	M	Empregado Efetivo
	M	Empregado Efetivo
	M	Empregado Efetivo
	F	Empregado Efetivo
	F	Empregado Efetivo
	M	Empregado Efetivo
	F	Empregado Efetivo
	M	Empregado Efetivo
	M	Empregado Efetivo
	F	Empregado Efetivo
	F	Empregado Efetivo

	M	Empregado Efetivo
	M	Empregado Efetivo
	F	Empregado Efetivo
	M	Empregado Efetivo
	F	Empregado Efetivo
	F	Empregado Efetivo
	F	Empregado Efetivo
	F	Empregado Efetivo
	M	Empregado Efetivo
	M	Empregado Efetivo
	F	Empregado Efetivo
	F	Empregado Efetivo
	M	Empregado Efetivo
	M	Empregado Efetivo
	F	Empregado Efetivo
	M	Empregado Efetivo
	F	Empregado Efetivo
	F	Empregado Efetivo
	F	Empregado Efetivo
	M	Empregado Efetivo
	F	Empregado Efetivo
	M	Empregado Efetivo
	M	Empregado Efetivo
	M	Empregado Efetivo
	F	Empregado Efetivo
	M	Empregado Efetivo
	F	Empregado Efetivo
	F	Empregado Efetivo
	F	Empregado Efetivo
	M	Empregado Efetivo
	M	Empregado Efetivo
	F	Empregado Efetivo
	F	Empregado Efetivo
	F	Empregado Efetivo
	F	Empregado Efetivo
	F	Empregado Efetivo
	F	Empregado Efetivo
	F	Empregado Efetivo
	F	Empregado Efetivo
	F	Empregado Efetivo
	F	Empregado Efetivo
	F	Empregado Efetivo

	F	Empregado Efetivo
	F	Empregado Efetivo
	F	Empregado Efetivo
	F	Empregado Efetivo
	F	Empregado Efetivo
	F	Empregado Efetivo
	F	Empregado Efetivo
	F	Empregado Efetivo
	F	Empregado Efetivo
	F	Empregado Efetivo
	F	Empregado Efetivo
	F	Empregado Efetivo
	F	Empregado Efetivo
	F	Empregado Efetivo
	F	Empregado Efetivo
	F	Empregado Efetivo
	M	Empregado Efetivo
	F	Empregado Efetivo
	F	Empregado Efetivo
	F	Empregado Efetivo
	F	Empregado Efetivo
	F	Empregado Efetivo
	F	Empregado Efetivo
	F	Empregado Efetivo
	F	Empregado Efetivo
	F	Empregado Efetivo
	F	Empregado Efetivo
	F	Empregado Efetivo
	F	Empregado Efetivo
	F	Empregado Efetivo
	F	Empregado Efetivo
	F	Empregado Efetivo

	F	Empregado Efetivo
	F	Empregado Efetivo
	F	Empregado Efetivo
	F	Empregado Efetivo
	F	Empregado Efetivo
	F	Empregado Efetivo
	F	Empregado Efetivo
	F	Empregado Efetivo
	F	Empregado Efetivo
	M	Empregado Efetivo
	F	Empregado Efetivo
	F	Empregado Efetivo
	M	Empregado Efetivo
	F	Empregado Efetivo
	F	Empregado Efetivo
	M	Empregado Efetivo
	F	Empregado Efetivo
	F	Empregado Efetivo
	F	Empregado Efetivo
	F	Empregado Efetivo
	F	Empregado Efetivo
	F	Empregado Efetivo
	F	Empregado Efetivo
	F	Empregado Efetivo
	F	Empregado Efetivo
	M	Empregado Efetivo
	M	Empregado Efetivo
	F	Empregado Efetivo
	F	Empregado Efetivo
	F	Empregado Efetivo
	F	Empregado Efetivo
	F	Empregado Efetivo

	F	Empregado Efetivo
	M	Empregado Efetivo
	F	Empregado Efetivo
	F	Empregado Efetivo
	F	Empregado Efetivo
	F	Empregado Efetivo
	F	Empregado Efetivo
	F	Empregado Efetivo
	F	Empregado Efetivo
	M	Empregado Efetivo
	F	Empregado Efetivo
	F	Empregado Efetivo
	M	Empregado Efetivo
	F	Empregado Efetivo
	F	Empregado Efetivo
	F	Empregado Efetivo
	M	Empregado Efetivo
	M	Empregado Efetivo
	M	Empregado Efetivo
	F	Empregado Efetivo
	M	Empregado Efetivo
	M	Empregado Efetivo
	F	Empregado Efetivo
	F	Empregado Efetivo
	M	Empregado Efetivo
	F	Empregado Efetivo
	F	Empregado Efetivo
	M	Empregado Efetivo
	F	Empregado Efetivo
	M	Empregado Efetivo
	F	Empregado Efetivo
	F	Empregado Efetivo
	M	Empregado Efetivo
	F	Empregado Efetivo
	F	Empregado Efetivo
	M	Empregado Efetivo
	F	Empregado Efetivo
	M	Empregado Efetivo
	M	Empregado Efetivo
	M	Empregado Efetivo

	F	Empregado Efetivo
	F	Empregado Efetivo
	F	Empregado Efetivo
	F	Empregado Efetivo
	F	Empregado Efetivo
	F	Empregado Efetivo
	F	Empregado Efetivo
	F	Empregado Efetivo
	M	Empregado Efetivo
	F	Empregado Efetivo
	M	Empregado Efetivo
	F	Empregado Efetivo
	F	Empregado Efetivo
	F	Empregado Efetivo
	M	Empregado Efetivo
	F	Empregado Efetivo
	F	Empregado Efetivo
	F	Empregado Efetivo
	F	Empregado Efetivo
	F	Empregado Efetivo
	F	Empregado Efetivo
	F	Empregado Efetivo
	F	Empregado Efetivo
	M	Empregado Efetivo
	F	Empregado Efetivo
	M	Empregado Efetivo
	F	Empregado Efetivo
	M	Empregado Efetivo
	M	Empregado Efetivo
	F	Empregado Efetivo
	F	Empregado Efetivo
	F	Empregado Efetivo
	F	Empregado Efetivo
	F	Empregado Efetivo
	F	Empregado Efetivo
	F	Empregado Efetivo
	F	Empregado Efetivo
	F	Empregado Efetivo
	F	Empregado Efetivo
	F	Empregado Efetivo

	M	Empregado Efetivo
	F	Empregado Efetivo
	M	Empregado Efetivo
	F	Empregado Efetivo
	F	Empregado Efetivo
	F	Empregado Efetivo
	F	Empregado Efetivo
	F	Empregado Efetivo
	F	Empregado Efetivo
	F	Empregado Efetivo
	F	Empregado Efetivo
	F	Empregado Efetivo
	F	Empregado Efetivo
	F	Empregado Efetivo
	F	Empregado Efetivo
	F	Empregado Efetivo
	M	Empregado Efetivo
	M	Empregado Efetivo
	F	Empregado Efetivo
	F	Empregado Efetivo
	F	Empregado Efetivo
	F	Empregado Efetivo
	F	Empregado Efetivo
	F	Empregado Efetivo
	F	Empregado Efetivo
	F	Empregado Efetivo
	F	Empregado Efetivo
	F	Empregado Efetivo
	F	Empregado Efetivo
	F	Empregado Efetivo
	F	Empregado Efetivo
	F	Empregado Efetivo
	F	Empregado Efetivo
	F	Empregado Efetivo
	F	Empregado Efetivo
	M	Empregado Efetivo
	M	Empregado Efetivo

	F	Empregado Efetivo
	M	Empregado Efetivo
	F	Empregado Efetivo
	M	Empregado Efetivo
	M	Empregado Efetivo
	F	Empregado Efetivo
	M	Empregado Efetivo
	F	Empregado Efetivo
	F	Empregado Efetivo
	M	Empregado Efetivo
	F	Empregado Efetivo
	F	Empregado Efetivo
	F	Empregado Efetivo
	F	Empregado Efetivo
	F	Empregado Efetivo
	F	Empregado Efetivo
	M	Empregado Efetivo
	M	Empregado Efetivo
	F	Empregado Efetivo
	F	Empregado Efetivo
	M	Empregado Efetivo
	F	Empregado Efetivo
	M	Empregado Efetivo
	F	Empregado Efetivo
	M	Empregado Efetivo
	F	Empregado Efetivo
	F	Empregado Efetivo
	F	Empregado Efetivo
	F	Empregado Efetivo
	F	Empregado Efetivo
	F	Empregado Efetivo
	M	Empregado Efetivo
	M	Empregado Efetivo
	F	Empregado Efetivo
	F	Empregado Efetivo
	M	Empregado Efetivo
	F	Empregado Efetivo
	F	Empregado Efetivo

	F	Empregado Efetivo
	F	Empregado Efetivo
	M	Empregado Efetivo
	F	Empregado Efetivo
	F	Empregado Efetivo
	F	Empregado Efetivo
	F	Empregado Efetivo
	F	Empregado Efetivo
	F	Empregado Efetivo
	M	Empregado Efetivo
	M	Empregado Efetivo
	M	Empregado Efetivo
	F	Empregado Efetivo
	F	Empregado Efetivo
	F	Empregado Efetivo
	M	Empregado Efetivo
	F	Empregado Efetivo
	M	Empregado Efetivo
	F	Empregado Efetivo
	F	Empregado Efetivo
	F	Empregado Efetivo
	F	Empregado Efetivo
	F	Empregado Efetivo
	F	Empregado Efetivo
	F	Empregado Efetivo
	F	Empregado Efetivo
	F	Empregado Efetivo
	F	Empregado Efetivo
	F	Empregado Efetivo
	F	Empregado Efetivo
	F	Empregado Efetivo
	F	Empregado Efetivo
	F	Empregado Efetivo
	F	Empregado Efetivo
	F	Empregado Efetivo
	M	Empregado Efetivo
	F	Empregado Efetivo
	F	Empregado Efetivo
	F	Empregado Efetivo
	F	Empregado Efetivo
	F	Empregado Efetivo
	F	Empregado Efetivo
	F	Empregado Efetivo
	F	Empregado Efetivo
	F	Empregado Efetivo
	F	Empregado Efetivo
	F	Empregado Efetivo
	M	Empregado Efetivo

	F	Empregado Efetivo
	F	Empregado Efetivo
	F	Empregado Efetivo
	F	Empregado Efetivo
	M	Empregado Efetivo
	F	Empregado Efetivo
	F	Empregado Efetivo
	F	Empregado Efetivo
	F	Empregado Efetivo
	F	Empregado Efetivo
	F	Empregado Efetivo
	F	Empregado Efetivo
	F	Empregado Efetivo
	F	Empregado Efetivo
	F	Empregado Efetivo
	F	Empregado Efetivo
	F	Empregado Efetivo
	F	Empregado Efetivo
	F	Empregado Efetivo
	F	Empregado Efetivo
	F	Empregado Efetivo
	F	Empregado Efetivo
	F	Empregado Efetivo
	M	Empregado Efetivo
	M	Empregado Efetivo
	M	Empregado Efetivo
	M	Empregado Efetivo
	F	Empregado Efetivo
	F	Empregado Efetivo
	F	Empregado Efetivo
	F	Empregado Efetivo
	F	Empregado Efetivo
	F	Empregado Efetivo
	F	Empregado Efetivo
	F	Empregado Efetivo
	F	Empregado Efetivo
	F	Empregado Efetivo
	F	Empregado Efetivo
	F	Empregado Efetivo
	F	Empregado Efetivo
	F	Empregado Efetivo
	F	Empregado Efetivo
	F	Empregado Efetivo
	F	Empregado Efetivo
	F	Empregado Efetivo
	F	Empregado Efetivo
	F	Empregado Efetivo
	M	Empregado Efetivo

	M	Empregado Efetivo
	F	Empregado Efetivo
	F	Empregado Efetivo
	M	Empregado Efetivo
	F	Empregado Efetivo
	F	Empregado Efetivo
	F	Empregado Efetivo
	M	Empregado Efetivo
	F	Empregado Efetivo
	F	Empregado Efetivo
	F	Empregado Efetivo
	F	Empregado Efetivo
	F	Empregado Efetivo
	M	Empregado Efetivo
	F	Empregado Efetivo
	F	Empregado Efetivo
	F	Empregado Efetivo
	M	Empregado Efetivo
	F	Empregado Efetivo
	F	Empregado Efetivo
	F	Empregado Efetivo
	M	Empregado Efetivo
	M	Empregado Efetivo
	F	Empregado Efetivo
	F	Empregado Efetivo
	F	Empregado Efetivo
	F	Empregado Efetivo
	F	Empregado Efetivo
	F	Empregado Efetivo
	M	Empregado Efetivo
	F	Empregado Efetivo
	M	Empregado Efetivo
	F	Empregado Efetivo
	M	Empregado Efetivo
	F	Empregado Efetivo
	F	Empregado Efetivo
	M	Empregado Efetivo
	F	Empregado Efetivo
	M	Empregado Efetivo
	M	Empregado Efetivo
	F	Empregado Efetivo
	F	Empregado Efetivo
	F	Empregado Efetivo
	F	Empregado Efetivo
	F	Empregado Efetivo
	F	Empregado Efetivo
	F	Empregado Efetivo
	F	Empregado Efetivo

	F	Empregado Efetivo
	M	Empregado Efetivo
	F	Empregado Efetivo
	F	Empregado Efetivo
	F	Empregado Efetivo
	F	Empregado Efetivo
	F	Empregado Efetivo
	F	Empregado Efetivo
	F	Empregado Efetivo
	F	Empregado Efetivo
	F	Empregado Efetivo
	M	Empregado Efetivo
	F	Empregado Efetivo
	F	Empregado Efetivo
	M	Empregado Efetivo
	M	Empregado Efetivo
	F	Empregado Efetivo
	F	Empregado Efetivo
	F	Empregado Efetivo
	M	Empregado Efetivo
	M	Empregado Efetivo
	F	Empregado Efetivo
	F	Empregado Efetivo
	M	Empregado Efetivo
	F	Empregado Efetivo
	M	Empregado Efetivo
	F	Empregado Efetivo
	M	Empregado Efetivo
	M	Empregado Efetivo
	F	Empregado Efetivo
	M	Empregado Efetivo
	M	Empregado Efetivo
	F	Empregado Efetivo
	F	Empregado Efetivo
	F	Empregado Efetivo
	F	Empregado Efetivo
	M	Empregado Efetivo
	F	Empregado Efetivo
	M	Empregado Efetivo
	M	Empregado Efetivo
	F	Empregado Efetivo
	F	Empregado Efetivo
	F	Empregado Efetivo
	F	Empregado Efetivo
	F	Empregado Efetivo
	M	Empregado Efetivo
	F	Empregado Efetivo
	M	Empregado Efetivo

	F	Empregado Efetivo
	F	Empregado Efetivo
	M	Empregado Efetivo
	M	Empregado Efetivo
	F	Empregado Efetivo
	F	Empregado Efetivo
	F	Empregado Efetivo
	M	Empregado Efetivo
	F	Empregado Efetivo
	M	Empregado Efetivo
	F	Empregado Efetivo
	F	Empregado Efetivo
	F	Empregado Efetivo
	F	Empregado Efetivo
	F	Empregado Efetivo
	F	Empregado Efetivo
	F	Empregado Efetivo
	M	Empregado Efetivo
	M	Empregado Efetivo
	F	Empregado Efetivo
	F	Empregado Efetivo
	F	Empregado Efetivo
	F	Empregado Efetivo
	F	Empregado Efetivo
	F	Empregado Efetivo
	M	Empregado Efetivo
	F	Empregado Efetivo
	F	Empregado Efetivo
	F	Empregado Efetivo
	F	Empregado Efetivo
	F	Empregado Efetivo
	F	Empregado Efetivo
	F	Empregado Efetivo
	F	Empregado Efetivo
	F	Empregado Efetivo
	F	Empregado Efetivo
	F	Empregado Efetivo
	M	Empregado Efetivo
	M	Empregado Efetivo
	M	Empregado Efetivo
	M	Empregado Efetivo
	F	Empregado Efetivo
	F	Empregado Efetivo
	M	Empregado Efetivo
	F	Empregado Efetivo
	M	Empregado Efetivo
	F	Empregado Efetivo

	F	Empregado Efetivo
	F	Empregado Efetivo
	F	Empregado Efetivo
	F	Empregado Efetivo
	F	Empregado Efetivo
	F	Empregado Efetivo
	F	Empregado Efetivo
	F	Empregado Efetivo
	F	Empregado Efetivo
	F	Empregado Efetivo
	F	Empregado Efetivo
	F	Empregado Efetivo
	F	Empregado Efetivo
	F	Empregado Efetivo
	F	Empregado Efetivo
	F	Empregado Efetivo
	F	Empregado Efetivo
	F	Empregado Efetivo
	F	Empregado Efetivo
	F	Empregado Efetivo
	F	Empregado Efetivo
	F	Empregado Efetivo
	F	Empregado Efetivo
	F	Empregado Efetivo
	F	Empregado Efetivo
	F	Empregado Efetivo
	F	Empregado Efetivo
	F	Empregado Efetivo
	F	Empregado Efetivo
	F	Empregado Efetivo
	F	Empregado Efetivo
	F	Empregado Efetivo
	F	Empregado Efetivo
	F	Empregado Efetivo
	F	Empregado Efetivo
	F	Empregado Efetivo
	F	Empregado Efetivo
	M	Empregado Efetivo
	M	Empregado Efetivo
	F	Empregado Efetivo
	F	Empregado Efetivo
	F	Empregado Efetivo
	F	Empregado Efetivo
	F	Empregado Efetivo
	F	Empregado Efetivo

	F	Empregado Efetivo
	M	Empregado Efetivo
	F	Empregado Efetivo
	F	Empregado Efetivo
	F	Empregado Efetivo
	F	Empregado Efetivo
	F	Empregado Efetivo
	F	Empregado Efetivo
	F	Empregado Efetivo
	F	Empregado Efetivo
	F	Empregado Efetivo
	M	Empregado Efetivo
	M	Empregado Efetivo
	F	Empregado Efetivo
	F	Empregado Efetivo
	F	Empregado Efetivo
	F	Empregado Efetivo
	F	Empregado Efetivo
	F	Empregado Efetivo
	F	Empregado Efetivo
	F	Empregado Efetivo
	F	Empregado Efetivo
	F	Empregado Efetivo
	M	Empregado Efetivo
	M	Empregado Efetivo
	F	Empregado Efetivo
	F	Empregado Efetivo
	F	Empregado Efetivo
	F	Empregado Efetivo
	F	Empregado Efetivo
	F	Empregado Efetivo
	F	Empregado Efetivo
	F	Empregado Efetivo
	F	Empregado Efetivo
	F	Empregado Efetivo
	F	Empregado Efetivo
	F	Empregado Efetivo
	M	Empregado Efetivo
	F	Empregado Efetivo
	F	Empregado Efetivo

	F	Empregado Efetivo
	F	Empregado Efetivo
	F	Empregado Efetivo
	F	Empregado Efetivo
	F	Empregado Efetivo
	F	Empregado Efetivo
	F	Empregado Efetivo
	F	Empregado Efetivo
	F	Empregado Efetivo
	F	Empregado Efetivo
	F	Empregado Efetivo
	F	Empregado Efetivo
	M	Empregado Efetivo
	F	Empregado Efetivo
	F	Empregado Efetivo
	F	Empregado Efetivo
	M	Empregado Efetivo
	F	Empregado Efetivo
	F	Empregado Efetivo
	M	Empregado Efetivo
	F	Empregado Efetivo
	F	Empregado Efetivo
	F	Empregado Efetivo
	F	Empregado Efetivo
	M	Empregado Efetivo
	M	Empregado Efetivo
	F	Empregado Efetivo
	F	Empregado Efetivo
	F	Empregado Efetivo
	F	Empregado Efetivo
	F	Empregado Efetivo
	F	Empregado Efetivo

	F	Empregado Efetivo
	F	Empregado Efetivo
	F	Empregado Efetivo
	F	Empregado Efetivo
	F	Empregado Efetivo
	F	Empregado Efetivo
	M	Empregado Efetivo
	F	Empregado Efetivo
	F	Empregado Efetivo
	F	Empregado Efetivo
	F	Empregado Efetivo
	F	Empregado Efetivo
	F	Empregado Efetivo
	M	Empregado Efetivo
	F	Empregado Efetivo
	F	Empregado Efetivo
	M	Empregado Efetivo
	M	Empregado Efetivo
	M	Empregado Efetivo
	F	Empregado Efetivo
	F	Empregado Efetivo
	M	Empregado Efetivo
	F	Empregado Efetivo
	M	Empregado Efetivo
	F	Empregado Efetivo
	F	Empregado Efetivo
	F	Empregado Efetivo
	F	Empregado Efetivo
	F	Empregado Efetivo
	F	Empregado Efetivo
	F	Empregado Efetivo
	F	Empregado Efetivo
	F	Empregado Efetivo
	F	Empregado Efetivo
	F	Empregado Efetivo
	F	Empregado Efetivo
	F	Empregado Efetivo
	F	Empregado Efetivo

	F	Empregado Efetivo
	F	Empregado Efetivo
	F	Empregado Efetivo
	F	Empregado Efetivo
	F	Empregado Efetivo
	F	Empregado Efetivo
	F	Empregado Efetivo
	F	Empregado Efetivo
	F	Empregado Efetivo
	F	Empregado Efetivo
	F	Empregado Efetivo
	F	Empregado Efetivo
	F	Empregado Efetivo
	F	Empregado Efetivo
	F	Empregado Efetivo
	F	Empregado Efetivo
	M	Empregado Efetivo
	F	Empregado Efetivo
	F	Empregado Efetivo
	M	Empregado Efetivo
	F	Empregado Efetivo
	F	Empregado Efetivo
	M	Empregado Efetivo
	F	Empregado Efetivo
	F	Empregado Efetivo
	F	Empregado Efetivo
	F	Empregado Efetivo
	F	Empregado Efetivo
	F	Empregado Efetivo
	F	Empregado Efetivo
	F	Empregado Efetivo
	F	Empregado Efetivo
	F	Empregado Efetivo
	M	Empregado Efetivo
	F	Empregado Efetivo
	F	Empregado Efetivo
	F	Empregado Efetivo
	F	Empregado Efetivo
	F	Empregado Efetivo
	M	Empregado Efetivo
	M	Empregado Efetivo
	F	Empregado Efetivo
	F	Empregado Efetivo
	F	Empregado Efetivo
	F	Empregado Efetivo
	F	Empregado Efetivo
	M	Empregado Efetivo

	F	Empregado Efetivo
	F	Empregado Efetivo
	F	Empregado Efetivo
	F	Empregado Efetivo
	F	Empregado Efetivo
	F	Empregado Efetivo
	F	Empregado Efetivo
	F	Empregado Efetivo
	M	Empregado Efetivo
	F	Empregado Efetivo
	F	Empregado Efetivo
	F	Empregado Efetivo
	F	Empregado Efetivo
	F	Empregado Efetivo
	F	Empregado Efetivo
	M	Empregado Efetivo
	F	Empregado Efetivo
	F	Empregado Efetivo
	F	Empregado Efetivo
	F	Empregado Efetivo
	F	Empregado Efetivo
	M	Empregado Efetivo
	F	Empregado Efetivo
	F	Empregado Efetivo
	F	Empregado Efetivo
	F	Empregado Efetivo
	F	Empregado Efetivo
	F	Empregado Efetivo
	F	Empregado Efetivo
	F	Empregado Efetivo
	F	Empregado Efetivo
	F	Empregado Efetivo
	F	Empregado Efetivo
	F	Empregado Efetivo
	F	Empregado Efetivo
	F	Empregado Efetivo
	F	Empregado Efetivo
	M	Empregado Efetivo
	F	Empregado Efetivo
	F	Empregado Efetivo
	F	Empregado Efetivo
	F	Empregado Efetivo
	F	Empregado Efetivo
	F	Empregado Efetivo
	F	Empregado Efetivo
	F	Empregado Efetivo
	F	Empregado Efetivo
	F	Empregado Efetivo

	F	Empregado Efetivo
	M	Empregado Efetivo
	F	Empregado Efetivo
	F	Empregado Efetivo
	F	Empregado Efetivo
	F	Empregado Efetivo
	F	Empregado Efetivo
	M	Empregado Efetivo
	F	Empregado Efetivo
	F	Empregado Efetivo
	F	Empregado Efetivo
	F	Empregado Efetivo
	F	Empregado Efetivo
	F	Empregado Efetivo
	F	Empregado Efetivo
	F	Empregado Efetivo
	F	Empregado Efetivo
	F	Empregado Efetivo
	F	Empregado Efetivo
	F	Empregado Efetivo
	F	Empregado Efetivo
	F	Empregado Efetivo
	F	Empregado Efetivo
	F	Empregado Efetivo
	F	Empregado Efetivo
	F	Empregado Efetivo
	F	Empregado Efetivo
	F	Empregado Efetivo
	F	Empregado Efetivo
	F	Empregado Efetivo
	F	Empregado Efetivo
	F	Empregado Efetivo
	F	Empregado Efetivo
	F	Empregado Efetivo
	F	Empregado Efetivo
	F	Empregado Efetivo
	F	Empregado Efetivo
	F	Empregado Efetivo
	F	Empregado Efetivo
	F	Empregado Efetivo
	F	Empregado Efetivo
	F	Empregado Efetivo
	F	Empregado Efetivo

	F	Empregado Efetivo
	F	Empregado Efetivo
	F	Empregado Efetivo
	F	Empregado Efetivo
	F	Empregado Efetivo
	F	Empregado Efetivo
	F	Empregado Efetivo
	F	Empregado Efetivo
	F	Empregado Efetivo
	F	Empregado Efetivo
	M	Empregado Efetivo
	M	Empregado Efetivo
	M	Empregado Efetivo
	M	Empregado Efetivo
	M	Empregado Efetivo
	M	Empregado Efetivo
	M	Empregado Efetivo
	F	Empregado Efetivo
	F	Empregado Efetivo
	F	Empregado Efetivo
	F	Empregado Efetivo
	M	Empregado Efetivo
	F	Empregado Efetivo
	F	Empregado Efetivo
	F	Empregado Efetivo
	F	Empregado Efetivo
	F	Empregado Efetivo
	F	Empregado Efetivo
	F	Empregado Efetivo
	F	Empregado Efetivo
	F	Empregado Efetivo
	M	Empregado Efetivo
	M	Empregado Efetivo
	F	Empregado Efetivo
	F	Empregado Efetivo
	F	Empregado Efetivo
	F	Empregado Efetivo
	F	Empregado Efetivo
	F	Empregado Efetivo

	F	Empregado Efetivo
	F	Empregado Efetivo
	F	Empregado Efetivo
	F	Empregado Efetivo
	F	Empregado Efetivo
	F	Empregado Efetivo
	F	Empregado Efetivo
	F	Empregado Efetivo
	M	Empregado Efetivo
	F	Empregado Efetivo
	F	Empregado Efetivo
	M	Empregado Efetivo
	F	Empregado Efetivo
	F	Empregado Efetivo
	M	Empregado Efetivo
	F	Empregado Efetivo
	M	Empregado Efetivo
	M	Empregado Efetivo
	M	Empregado Efetivo
	M	Empregado Efetivo
	F	Empregado Efetivo
	F	Empregado Efetivo
	F	Empregado Efetivo
	M	Empregado Efetivo
	M	Empregado Efetivo
	F	Empregado Efetivo
	F	Empregado Efetivo
	F	Empregado Efetivo
	F	Empregado Efetivo
	F	Empregado Efetivo
	M	Empregado Efetivo
	M	Empregado Efetivo
	M	Empregado Efetivo
	M	Empregado Efetivo
	M	Empregado Efetivo
	F	Empregado Efetivo
	M	Empregado Efetivo
	M	Empregado Efetivo
	M	Empregado Efetivo
	F	Empregado Efetivo
	F	Empregado Efetivo

	F	Empregado Efetivo
	F	Empregado Efetivo
	F	Empregado Efetivo
	M	Empregado Efetivo
	M	Empregado Efetivo
	F	Empregado Efetivo
	F	Empregado Efetivo
	M	Empregado Efetivo
	F	Empregado Efetivo
	F	Empregado Efetivo
	F	Empregado Efetivo
	F	Cedido - RJU
	F	Cedido - RJU
	F	Cedido - RJU
	F	Cedido - RJU
	F	Cedido - RJU
	F	Cedido - RJU
	F	Cedido - RJU
	F	Empregado Efetivo
	F	Empregado Efetivo
	F	Empregado Efetivo
	F	Empregado Efetivo
	F	Empregado Efetivo
	M	Empregado Efetivo
	F	Empregado Efetivo
	F	Empregado Efetivo
	F	Empregado Efetivo
	F	Empregado Efetivo
	F	Empregado Efetivo
	F	Empregado Efetivo
	F	Empregado Efetivo
	F	Empregado Efetivo
	F	Empregado Efetivo
	F	Empregado Efetivo
	F	Empregado Efetivo
	F	Empregado Efetivo

	F	Empregado Efetivo
	F	Empregado Efetivo
	F	Empregado Efetivo
	F	Empregado Efetivo
	F	Empregado Efetivo
	F	Empregado Efetivo
	F	Empregado Efetivo
	F	Empregado Efetivo
	M	Empregado Efetivo
	F	Empregado Efetivo
	F	Empregado Efetivo
	F	Empregado Efetivo
	F	Empregado Efetivo
	F	Empregado Efetivo
	F	Empregado Efetivo
	F	Empregado Efetivo
	F	Empregado Efetivo
	F	Empregado Efetivo
	F	Empregado Efetivo
	F	Empregado Efetivo
	F	Empregado Efetivo
	M	Empregado Efetivo
	M	Empregado Efetivo
	F	Empregado Efetivo
	F	Empregado Efetivo
	M	Empregado Efetivo
	M	Empregado Efetivo
	F	Empregado Efetivo
	F	Empregado Efetivo
	F	Empregado Efetivo
	F	Empregado Efetivo
	F	Empregado Efetivo
	F	Empregado Efetivo
	F	Empregado Efetivo
	F	Empregado Efetivo
	F	Empregado Efetivo

	F	Empregado Efetivo
	M	Empregado Efetivo
	M	Empregado Efetivo
	F	Empregado Efetivo
	M	Empregado Efetivo
	M	Empregado Efetivo
	F	Empregado Efetivo
	M	Empregado Efetivo
	F	Empregado Efetivo
	F	Empregado Efetivo
	F	Empregado Efetivo
	F	Empregado Efetivo
	M	Empregado Efetivo
	M	Empregado Efetivo
	F	Empregado Efetivo
	F	Empregado Efetivo
	F	Empregado Efetivo
	F	Empregado Efetivo
	F	Empregado Efetivo
	F	Empregado Efetivo
	F	Empregado Efetivo
	F	Cedido - RJU
	F	Empregado Efetivo
	M	Empregado Efetivo
	M	Empregado Efetivo
	F	Empregado Efetivo
	M	Empregado Efetivo
	M	Empregado Efetivo
	M	Empregado Efetivo
	M	Empregado Efetivo
	F	Empregado Efetivo
	F	Empregado Efetivo
	F	Empregado Efetivo
	F	Empregado Efetivo

	M	Empregado Efetivo
	F	Empregado Efetivo
	F	Empregado Efetivo
	M	Empregado Efetivo
	M	Empregado Efetivo
	M	Empregado Efetivo
	M	Empregado Efetivo
	F	Empregado Efetivo
	F	Empregado Efetivo
	M	Empregado Efetivo
	F	Empregado Efetivo
	F	Empregado Efetivo
	F	Empregado Efetivo
	F	Empregado Efetivo
	F	Empregado Efetivo
	F	Empregado Efetivo
	F	Empregado Efetivo
	F	Empregado Efetivo
	F	Empregado Efetivo
	F	Empregado Efetivo
	F	Empregado Efetivo
	F	Empregado Efetivo
	F	Empregado Efetivo
	F	Empregado Efetivo
	F	Empregado Efetivo
	F	Empregado Efetivo
	F	Empregado Efetivo
	F	Empregado Efetivo
	F	Empregado Efetivo
	M	Empregado Efetivo
	M	Empregado Efetivo
	M	Empregado Efetivo

	M	Empregado Efetivo
	M	Empregado Efetivo
	M	Empregado Efetivo
	M	Empregado Efetivo
	F	Empregado Efetivo
	M	Empregado Efetivo
	F	Empregado Efetivo
	M	Empregado Efetivo
	M	Empregado Efetivo
	M	Empregado Efetivo
	F	Empregado Efetivo
	F	Empregado Efetivo
	F	Empregado Efetivo
	M	Empregado Efetivo
	M	Empregado Efetivo
	F	Empregado Efetivo
	F	Empregado Efetivo
	F	Empregado Efetivo
	F	Empregado Efetivo
	F	Empregado Efetivo
	M	Empregado Efetivo
	F	Empregado Efetivo
	F	Empregado Efetivo
	F	Empregado Efetivo
	F	Empregado Efetivo
	F	Empregado Efetivo
	M	Empregado Efetivo
	M	Empregado Efetivo
	M	Empregado Efetivo
	F	Empregado Efetivo
	F	Empregado Efetivo
	M	Empregado Efetivo
	M	Empregado Efetivo
	F	Empregado Efetivo
	F	Empregado Efetivo
	F	Empregado Efetivo
	M	Empregado Efetivo
	M	Empregado Efetivo

	M	Empregado Efetivo
	M	Empregado Efetivo
	M	Empregado Efetivo
	M	Empregado Efetivo
	M	Empregado Efetivo
	F	Empregado Efetivo
	F	Empregado Efetivo
	M	Empregado Efetivo
	F	Empregado Efetivo
	F	Empregado Efetivo
	M	Empregado Efetivo
	F	Cedido - RJU
	F	Empregado Efetivo
	F	Empregado Efetivo
	F	Empregado Efetivo
	F	Empregado Efetivo
	F	Empregado Efetivo
	F	Empregado Efetivo
	F	Empregado Efetivo
	F	Empregado Efetivo
	F	Empregado Efetivo
	F	Empregado Efetivo
	F	Empregado Efetivo
	F	Empregado Efetivo
	M	Empregado Efetivo
	M	Empregado Efetivo
	M	Empregado Efetivo
	M	Empregado Efetivo
	F	Empregado Efetivo
	F	Empregado Efetivo
	F	Empregado Efetivo
	F	Empregado Efetivo
	F	Empregado Efetivo
	F	Empregado Efetivo
	M	Empregado Efetivo
	F	Empregado Efetivo
	F	Cedido - RJU
	F	Cedido - RJU
	F	Cedido - RJU
	F	Cedido - RJU
	F	Empregado Efetivo
	F	Cedido - RJU
	F	Empregado Efetivo
	F	Empregado Efetivo
	F	Cedido - RJU

	F	Cedido - RJU
	F	Empregado Efetivo
	F	Empregado Efetivo
	F	Empregado Efetivo
	F	Empregado Efetivo
	F	Empregado Efetivo
	F	Empregado Efetivo
	F	Empregado Efetivo
	F	Empregado Efetivo
	F	Empregado Efetivo
	F	Empregado Efetivo
	F	Empregado Efetivo
	F	Empregado Efetivo
	F	Empregado Efetivo
	F	Empregado Efetivo
	F	Empregado Efetivo
	M	Empregado Efetivo
	F	Empregado Efetivo
	F	Empregado Efetivo
	F	Empregado Efetivo
	F	Empregado Efetivo
	F	Empregado Efetivo
	F	Empregado Efetivo
	F	Empregado Efetivo
	F	Empregado Efetivo
	F	Empregado Efetivo
	F	Empregado Efetivo
	F	Empregado Efetivo
	M	Empregado Efetivo
	F	Empregado Efetivo
	F	Empregado Efetivo
	F	Empregado Efetivo
	F	Empregado Efetivo
	M	Empregado Efetivo
	F	Empregado Efetivo
	F	Empregado Efetivo
	F	Empregado Efetivo
	F	Empregado Efetivo
	F	Empregado Efetivo
	F	Empregado Efetivo
	F	Empregado Efetivo
	F	Empregado Efetivo
	M	Empregado Efetivo
	M	Empregado Efetivo
	M	Empregado Efetivo
	M	Empregado Efetivo
	M	Empregado Efetivo
	M	Empregado Efetivo

	F	Empregado Efetivo
	F	Empregado Efetivo
	F	Empregado Efetivo
	M	Empregado Efetivo
	F	Empregado Efetivo
	F	Empregado Efetivo
	F	Empregado Efetivo
	F	Empregado Efetivo
	F	Empregado Efetivo
	F	Empregado Efetivo
	F	Empregado Efetivo
	F	Empregado Efetivo
	F	Empregado Efetivo
	F	Empregado Efetivo
	F	Empregado Efetivo
	F	Empregado Efetivo
	F	Empregado Efetivo
	F	Empregado Efetivo
	F	Empregado Efetivo
	F	Empregado Efetivo
	F	Empregado Efetivo
	M	Empregado Efetivo
	M	Empregado Efetivo
	M	Empregado Efetivo
	M	Empregado Efetivo
	F	Empregado Efetivo
	F	Empregado Efetivo
	F	Empregado Efetivo
	F	Empregado Efetivo
	F	Empregado Efetivo
	F	Empregado Efetivo
	F	Empregado Efetivo
	F	Empregado Efetivo
	F	Empregado Efetivo
	M	Empregado Efetivo
	M	Empregado Efetivo
	F	Empregado Efetivo
	F	Empregado Efetivo

	F	Empregado Efetivo
	F	Empregado Efetivo
	F	Empregado Efetivo
	F	Empregado Efetivo
	F	Empregado Efetivo
	F	Empregado Efetivo
	F	Empregado Efetivo
	F	Empregado Efetivo
	F	Empregado Efetivo
	F	Empregado Efetivo
	F	Empregado Efetivo
	F	Empregado Efetivo
	F	Empregado Efetivo
	F	Empregado Efetivo
	F	Cedido - RJU
	F	Empregado Efetivo
	F	Empregado Efetivo
	F	Empregado Efetivo
	F	Empregado Efetivo
	F	Empregado Efetivo
	M	Empregado Efetivo
	F	Empregado Efetivo
	F	Empregado Efetivo
	M	Empregado Efetivo
	F	Empregado Efetivo
	M	Empregado Efetivo
	F	Empregado Efetivo
	F	Empregado Efetivo
	F	Empregado Efetivo
	F	Empregado Efetivo
	F	Empregado Efetivo
	F	Empregado Efetivo
	M	Empregado Efetivo
	F	Empregado Efetivo
	F	Empregado Efetivo
	M	Empregado Efetivo
	F	Empregado Efetivo
	F	Empregado Efetivo
	M	Empregado Efetivo
	F	Empregado Efetivo
	F	Empregado Efetivo
	F	Empregado Efetivo
	F	Empregado Efetivo
	M	Empregado Efetivo
	F	Empregado Efetivo
	F	Empregado Efetivo
	F	Empregado Efetivo
	M	Empregado Efetivo

	M	Empregado Efetivo
	M	Empregado Efetivo
	M	Empregado Efetivo
	M	Empregado Efetivo
	M	Empregado Efetivo
	M	Empregado Efetivo
	M	Empregado Efetivo
	M	Empregado Efetivo
	F	Empregado Efetivo
	F	Empregado Efetivo
	F	Empregado Efetivo
	F	Empregado Efetivo
	F	Empregado Efetivo
	F	Empregado Efetivo
	F	Empregado Efetivo
	F	Empregado Efetivo
	M	Empregado Efetivo
	M	Empregado Efetivo
	M	Empregado Efetivo
	M	Empregado Efetivo
	F	Empregado Efetivo
	F	Empregado Efetivo
	M	Empregado Efetivo
	M	Empregado Efetivo
	F	Empregado Efetivo
	F	Empregado Efetivo
	F	Empregado Efetivo
	F	Empregado Efetivo
	F	Empregado Efetivo
	F	Empregado Efetivo
	F	Empregado Efetivo
	F	Empregado Efetivo
	F	Empregado Efetivo
	F	Empregado Efetivo
	F	Empregado Efetivo
	F	Empregado Efetivo
	F	Empregado Efetivo
	F	Empregado Efetivo
	F	Empregado Efetivo
	F	Cedido - RJU
	F	Empregado Efetivo
	F	Empregado Efetivo
	F	Empregado Efetivo
	F	Empregado Efetivo
	F	Empregado Efetivo
	F	Empregado Efetivo
	F	Empregado Efetivo
	F	Empregado Efetivo

	F	Empregado Efetivo
	F	Empregado Efetivo
	F	Empregado Efetivo
	F	Empregado Efetivo
	F	Empregado Efetivo
	F	Empregado Efetivo
	M	Empregado Efetivo
	F	Empregado Efetivo
	F	Empregado Efetivo
	F	Empregado Efetivo
	F	Empregado Efetivo
	F	Empregado Efetivo
	F	Empregado Efetivo
	F	Empregado Efetivo
	F	Empregado Efetivo
	F	Empregado Efetivo
	F	Empregado Efetivo
	F	Empregado Efetivo
	F	Empregado Efetivo
	F	Empregado Efetivo
	F	Empregado Efetivo
	F	Empregado Efetivo
	F	Empregado Efetivo
	F	Empregado Efetivo
	F	Empregado Efetivo
	F	Empregado Efetivo
	F	Empregado Efetivo
	F	Empregado Efetivo
	F	Empregado Efetivo
	F	Empregado Efetivo
	M	Empregado Efetivo
	F	Empregado Efetivo
	F	Empregado Efetivo
	F	Empregado Efetivo
	F	Empregado Efetivo
	F	Empregado Efetivo
	F	Empregado Efetivo
	F	Empregado Efetivo
	F	Empregado Efetivo
	F	Empregado Efetivo
	F	Empregado Efetivo
	F	Empregado Efetivo
	F	Empregado Efetivo
	F	Empregado Efetivo
	F	Empregado Efetivo
	F	Empregado Efetivo
	F	Empregado Efetivo
	F	Empregado Efetivo

	F	Empregado Efetivo
	F	Empregado Efetivo
	F	Empregado Efetivo
	F	Empregado Efetivo
	M	Empregado Efetivo
	F	Empregado Efetivo
	F	Empregado Efetivo
	F	Empregado Efetivo
	F	Empregado Efetivo
	M	Empregado Efetivo
	F	Empregado Efetivo
	F	Empregado Efetivo
	F	Empregado Efetivo
	F	Empregado Efetivo
	F	Empregado Efetivo
	F	Empregado Efetivo
	F	Empregado Efetivo
	F	Empregado Efetivo
	F	Empregado Efetivo
	F	Empregado Efetivo
	F	Empregado Efetivo
	F	Empregado Efetivo
	F	Empregado Efetivo
	F	Empregado Efetivo
	F	Empregado Efetivo
	F	Empregado Efetivo
	F	Empregado Efetivo
	F	Empregado Efetivo
	F	Empregado Efetivo
	F	Empregado Efetivo
	F	Empregado Efetivo
	M	Empregado Efetivo
	F	Empregado Efetivo
	M	Empregado Efetivo
	M	Empregado Efetivo
	M	Empregado Efetivo
	M	Empregado Efetivo
	M	Empregado Efetivo
	M	Empregado Efetivo
	F	Empregado Efetivo
	M	Empregado Efetivo
	F	Empregado Efetivo
	M	Empregado Efetivo
	M	Empregado Efetivo
	M	Empregado Efetivo
	M	Empregado Efetivo
	M	Empregado Efetivo
	F	Empregado Efetivo

	F	Empregado Efetivo
	F	Empregado Efetivo
	M	Empregado Efetivo
	M	Empregado Efetivo
	M	Empregado Efetivo
	F	Empregado Efetivo
	F	Empregado Efetivo
	F	Empregado Efetivo
	F	Empregado Efetivo
	F	Empregado Efetivo
	M	Empregado Efetivo
	M	Empregado Efetivo
	M	Empregado Efetivo
	F	Empregado Efetivo
	F	Empregado Efetivo
	F	Empregado Efetivo
	F	Empregado Efetivo
	F	Empregado Efetivo
	F	Empregado Efetivo
	F	Empregado Efetivo
	F	Empregado Efetivo
	F	Empregado Efetivo
	F	Empregado Efetivo
	F	Empregado Efetivo
	F	Empregado Efetivo
	M	Empregado Efetivo
	M	Empregado Efetivo
	F	Empregado Efetivo
	F	Empregado Efetivo
	F	Empregado Efetivo
	M	Empregado Efetivo
	M	Empregado Efetivo
	F	Empregado Efetivo
	M	Empregado Efetivo
	M	Empregado Efetivo
	M	Empregado Efetivo
	F	Empregado Efetivo
	F	Empregado Efetivo
	M	Empregado Efetivo
	F	Empregado Efetivo
	M	Empregado Efetivo
	F	Empregado Efetivo
	F	Empregado Efetivo
	F	Empregado Efetivo
	F	Empregado Efetivo
	M	Empregado Efetivo
	F	Empregado Efetivo

	F	Empregado Efetivo
	F	Empregado Efetivo
	F	Empregado Efetivo
	F	Empregado Efetivo
	F	Empregado Efetivo
	F	Empregado Efetivo
	F	Empregado Efetivo
	F	Empregado Efetivo
	F	Empregado Efetivo
	F	Empregado Efetivo
	F	Empregado Efetivo
	F	Empregado Efetivo
	F	Empregado Efetivo
	F	Empregado Efetivo
	F	Empregado Efetivo
	F	Empregado Efetivo
	M	Empregado Efetivo
	F	Empregado Efetivo
	F	Empregado Efetivo
	M	Empregado Efetivo
	F	Empregado Efetivo
	F	Empregado Efetivo
	F	Empregado Efetivo
	F	Empregado Efetivo
	F	Empregado Efetivo
	F	Empregado Efetivo
	F	Empregado Efetivo
	F	Empregado Efetivo
	F	Empregado Efetivo
	F	Empregado Efetivo
	F	Empregado Efetivo
	F	Empregado Efetivo

	F	Empregado Efetivo
	M	Empregado Efetivo
	F	Empregado Efetivo
	F	Empregado Efetivo
	F	Empregado Efetivo
	M	Empregado Efetivo
	F	Empregado Efetivo
	F	Empregado Efetivo
	F	Empregado Efetivo
	F	Empregado Efetivo
	M	Empregado Efetivo
	F	Empregado Efetivo
	M	Empregado Efetivo
	F	Empregado Efetivo
	F	Empregado Efetivo
	F	Empregado Efetivo
	F	Empregado Efetivo
	F	Empregado Efetivo
	F	Empregado Efetivo
	F	Empregado Efetivo
	F	Empregado Efetivo
	F	Empregado Efetivo
	F	Empregado Efetivo
	M	Empregado Efetivo
	F	Empregado Efetivo
	F	Empregado Efetivo
	M	Empregado Efetivo
	F	Empregado Efetivo
	F	Empregado Efetivo
	F	Empregado Efetivo
	F	Empregado Efetivo
	F	Empregado Efetivo

	F	Empregado Efetivo
	M	Empregado Efetivo
	F	Empregado Efetivo
	F	Empregado Efetivo
	F	Empregado Efetivo
	M	Empregado Efetivo
	F	Empregado Efetivo
	F	Empregado Efetivo
	M	Empregado Efetivo
	F	Empregado Efetivo
	F	Empregado Efetivo
	F	Empregado Efetivo
	F	Empregado Efetivo
	F	Empregado Efetivo
	F	Empregado Efetivo
	F	Empregado Efetivo
	M	Empregado Efetivo
	F	Empregado Efetivo
	M	Empregado Efetivo
	F	Empregado Efetivo
	F	Empregado Efetivo
	F	Empregado Efetivo
	F	Empregado Efetivo
	F	Empregado Efetivo
	M	Empregado Efetivo
	F	Empregado Efetivo
	F	Empregado Efetivo
	M	Empregado Efetivo
	M	Empregado Efetivo
	F	Empregado Efetivo
	F	Empregado Efetivo
	F	Empregado Efetivo

	F	Empregado Efetivo
	M	Empregado Efetivo
	F	Empregado Efetivo
	F	Empregado Efetivo
	F	Empregado Efetivo
	F	Empregado Efetivo
	F	Empregado Efetivo
	F	Empregado Efetivo
	F	Empregado Efetivo
	F	Empregado Efetivo
	F	Empregado Efetivo
	F	Empregado Efetivo
	F	Empregado Efetivo
	F	Empregado Efetivo
	F	Empregado Efetivo
	F	Empregado Efetivo
	M	Empregado Efetivo
	F	Empregado Efetivo
	F	Empregado Efetivo
	F	Empregado Efetivo
	F	Empregado Efetivo
	M	Empregado Efetivo
	F	Empregado Efetivo
	F	Empregado Efetivo
	F	Empregado Efetivo
	F	Empregado Efetivo
	M	Empregado Efetivo
	F	Empregado Efetivo
	F	Empregado Efetivo
	F	Empregado Efetivo
	F	Empregado Efetivo
	M	Empregado Efetivo

	F	Empregado Efetivo
	M	Empregado Efetivo
	F	Empregado Efetivo
	F	Empregado Efetivo
	F	Empregado Efetivo
	F	Empregado Efetivo
	M	Empregado Efetivo
	F	Empregado Efetivo
	F	Empregado Efetivo
	F	Empregado Efetivo
	F	Empregado Efetivo
	F	Empregado Efetivo
	M	Empregado Efetivo
	F	Empregado Efetivo
	F	Empregado Efetivo
	F	Empregado Efetivo
	M	Empregado Efetivo
	M	Empregado Efetivo
	M	Empregado Efetivo
	F	Empregado Efetivo
	F	Empregado Efetivo
	F	Empregado Efetivo
	F	Empregado Efetivo
	F	Empregado Efetivo
	F	Empregado Efetivo
	F	Empregado Efetivo
	F	Empregado Efetivo
	F	Empregado Efetivo
	F	Empregado Efetivo
	F	Empregado Efetivo
	F	Empregado Efetivo
	F	Empregado Efetivo

	F	Empregado Efetivo
	F	Empregado Efetivo
	F	Empregado Efetivo
	F	Empregado Efetivo
	F	Empregado Efetivo
	F	Empregado Efetivo
	F	Empregado Efetivo
	F	Empregado Efetivo
	M	Empregado Efetivo
	F	Empregado Efetivo
	F	Empregado Efetivo
	F	Empregado Efetivo
	F	Empregado Efetivo
	F	Empregado Efetivo
	F	Empregado Efetivo
	F	Empregado Efetivo
	M	Empregado Efetivo
	F	Empregado Efetivo
	F	Empregado Efetivo
	M	Empregado Efetivo
	F	Empregado Efetivo
	F	Empregado Efetivo
	F	Empregado Efetivo
	F	Empregado Efetivo
	M	Empregado Efetivo
	F	Empregado Efetivo
	F	Empregado Efetivo
	F	Empregado Efetivo
	F	Empregado Efetivo
	F	Empregado Efetivo
	F	Empregado Efetivo
	M	Empregado Efetivo

	F	Empregado Efetivo
	M	Empregado Efetivo
	F	Empregado Efetivo
	F	Empregado Efetivo
	F	Empregado Efetivo
	F	Empregado Efetivo
	F	Empregado Efetivo
	F	Empregado Efetivo
	F	Empregado Efetivo
	F	Empregado Efetivo
	F	Empregado Efetivo
	F	Empregado Efetivo
	F	Empregado Efetivo
	F	Empregado Efetivo
	F	Empregado Efetivo
	F	Empregado Efetivo
	F	Empregado Efetivo
	F	Empregado Efetivo
	F	Empregado Efetivo
	F	Empregado Efetivo
	F	Empregado Efetivo
	F	Empregado Efetivo
	F	Empregado Efetivo
	F	Empregado Efetivo
	F	Empregado Efetivo
	F	Empregado Efetivo
	F	Empregado Efetivo
	F	Empregado Efetivo
	F	Empregado Efetivo
	F	Empregado Efetivo
	F	Empregado Efetivo
	F	Empregado Efetivo
	F	Empregado Efetivo
	F	Empregado Efetivo
	F	Empregado Efetivo
	F	Empregado Efetivo
	F	Empregado Efetivo
	F	Empregado Efetivo

	F	Empregado Efetivo
	F	Empregado Efetivo
	F	Empregado Efetivo
	F	Empregado Efetivo
	F	Empregado Efetivo
	F	Empregado Efetivo
	F	Empregado Efetivo
	F	Empregado Efetivo
	F	Empregado Efetivo
	F	Empregado Efetivo
	F	Empregado Efetivo
	F	Empregado Efetivo
	F	Empregado Efetivo
	F	Empregado Efetivo
	F	Empregado Efetivo
	F	Empregado Efetivo
	F	Empregado Efetivo
	F	Empregado Efetivo
	F	Empregado Efetivo
	F	Empregado Efetivo
	F	Empregado Efetivo
	F	Empregado Efetivo
	F	Empregado Efetivo
	F	Empregado Efetivo
	F	Empregado Efetivo
	F	Empregado Efetivo
	F	Empregado Efetivo
	F	Empregado Efetivo
	M	Empregado Efetivo
	F	Empregado Efetivo
	F	Empregado Efetivo
	F	Empregado Efetivo
	F	Empregado Efetivo
	F	Empregado Efetivo
	F	Empregado Efetivo
	F	Empregado Efetivo
	F	Empregado Efetivo
	F	Empregado Efetivo
	F	Empregado Efetivo
	F	Empregado Efetivo
	F	Empregado Efetivo
	F	Empregado Efetivo
	F	Empregado Efetivo
	F	Empregado Efetivo
	F	Empregado Efetivo
	F	Empregado Efetivo
	M	Empregado Efetivo
	F	Empregado Efetivo

	F	Empregado Efetivo
	F	Empregado Efetivo
	F	Empregado Efetivo
	M	Empregado Efetivo
	F	Empregado Efetivo
	M	Empregado Efetivo
	M	Empregado Efetivo
	M	Empregado Efetivo
	M	Empregado Efetivo
	M	Empregado Efetivo
	M	Empregado Efetivo
	M	Empregado Efetivo
	M	Empregado Efetivo
	M	Empregado Efetivo
	M	Empregado Efetivo
	M	Empregado Efetivo
	M	Empregado Efetivo
	F	Empregado Efetivo
	M	Empregado Efetivo
	M	Empregado Efetivo
	M	Empregado Efetivo
	M	Empregado Efetivo
	F	Empregado Efetivo
	M	Empregado Efetivo
	F	Empregado Efetivo
	F	Empregado Efetivo
	M	Empregado Efetivo
	M	Empregado Efetivo
	M	Empregado Efetivo
	M	Empregado Efetivo
	F	Empregado Efetivo
	F	Empregado Efetivo
	F	Empregado Efetivo
	F	Empregado Efetivo
	F	Empregado Efetivo
	F	Empregado Efetivo
	F	Empregado Efetivo
	F	Empregado Efetivo
	F	Empregado Efetivo
	F	Empregado Efetivo

	F	Empregado Efetivo
	F	Empregado Efetivo
	F	Empregado Efetivo
	F	Empregado Efetivo
	F	Empregado Efetivo
	M	Empregado Efetivo
	F	Empregado Efetivo
	F	Empregado Efetivo
	F	Empregado Efetivo
	F	Empregado Efetivo
	M	Empregado Efetivo
	F	Empregado Efetivo
	M	Empregado Efetivo
	M	Empregado Efetivo
	M	Cedido - RJU
	M	Empregado Efetivo
	F	Empregado Efetivo
	F	Empregado Efetivo
	F	Empregado Efetivo
	F	Empregado Efetivo
	F	Empregado Efetivo
	F	Empregado Efetivo
	F	Empregado Efetivo
	F	Empregado Efetivo
	F	Empregado Efetivo
	F	Empregado Efetivo
	F	Empregado Efetivo
	F	Empregado Efetivo
	M	Empregado Efetivo
	F	Empregado Efetivo
	M	Empregado Efetivo
	F	Empregado Efetivo
	F	Empregado Efetivo
	M	Empregado Efetivo
	F	Empregado Efetivo
	M	Empregado Efetivo
	F	Empregado Efetivo
	F	Empregado Efetivo

	M	Empregado Efetivo
	M	Empregado Efetivo
	F	Empregado Efetivo
	F	Empregado Efetivo
	F	Empregado Efetivo
	F	Empregado Efetivo
	F	Empregado Efetivo
	F	Empregado Efetivo
	F	Empregado Efetivo
	F	Empregado Efetivo
	M	Empregado Efetivo
	M	Empregado Efetivo
	M	Empregado Efetivo
	M	Empregado Efetivo
	M	Empregado Efetivo
	M	Empregado Efetivo
	F	Empregado Efetivo
	M	Empregado Efetivo
	M	Empregado Efetivo
	M	Empregado Efetivo
	M	Empregado Efetivo
	M	Empregado Efetivo
	M	Empregado Efetivo
	M	Empregado Efetivo
	M	Empregado Efetivo
	F	Empregado Efetivo
	M	Empregado Efetivo
	M	Empregado Efetivo
	M	Empregado Efetivo
	F	Empregado Efetivo
	M	Empregado Efetivo
	M	Empregado Efetivo
	M	Empregado Efetivo
	M	Empregado Efetivo
	M	Empregado Efetivo
	M	Empregado Efetivo
	M	Empregado Efetivo
	M	Empregado Efetivo
	M	Empregado Efetivo
	M	Empregado Efetivo
	M	Empregado Efetivo
	M	Empregado Efetivo
	M	Empregado Efetivo
	M	Empregado Efetivo
	M	Empregado Efetivo
	M	Empregado Efetivo
	F	Empregado Efetivo
	F	Empregado Efetivo

	F	Empregado Efetivo
	M	Empregado Efetivo
	M	Empregado Efetivo
	M	Empregado Efetivo
	F	Empregado Efetivo
	M	Empregado Efetivo
	F	Empregado Efetivo
	M	Empregado Efetivo
	M	Empregado Efetivo
	F	Empregado Efetivo
	M	Empregado Efetivo
	M	Empregado Efetivo
	M	Empregado Efetivo
	F	Empregado Efetivo
	M	Empregado Efetivo
	M	Empregado Efetivo
	M	Empregado Efetivo
	M	Empregado Efetivo
	M	Empregado Efetivo
	F	Empregado Efetivo
	F	Empregado Efetivo
	F	Empregado Efetivo
	F	Empregado Efetivo
	F	Empregado Efetivo
	F	Empregado Efetivo
	F	Empregado Efetivo
	F	Empregado Efetivo
	F	Empregado Efetivo
	F	Empregado Efetivo
	F	Empregado Efetivo
	F	Empregado Efetivo
	F	Empregado Efetivo
	F	Empregado Efetivo
	M	Empregado Efetivo
	F	Empregado Efetivo
	M	Empregado Efetivo
	F	Empregado Efetivo
	F	Empregado Efetivo
	F	Empregado Efetivo
	F	Empregado Efetivo
	F	Empregado Efetivo
	F	Empregado Efetivo
	F	Empregado Efetivo
	F	Empregado Efetivo
	F	Empregado Efetivo

	F	Empregado Efetivo
	M	Empregado Efetivo
	M	Empregado Efetivo
	F	Empregado Efetivo
	F	Empregado Efetivo
	F	Empregado Efetivo
	M	Empregado Efetivo
	F	Empregado Efetivo
	F	Empregado Efetivo
	F	Empregado Efetivo
	F	Empregado Efetivo
	F	Empregado Efetivo
	F	Empregado Efetivo
	F	Empregado Efetivo
	F	Empregado Efetivo
	F	Empregado Efetivo
	F	Empregado Efetivo
	F	Empregado Efetivo
	M	Empregado Efetivo
	F	Empregado Efetivo
	M	Empregado Efetivo
	F	Empregado Efetivo
	F	Empregado Efetivo
	F	Empregado Efetivo
	M	Empregado Efetivo
	F	Empregado Efetivo
	F	Empregado Efetivo
	F	Empregado Efetivo
	F	Empregado Efetivo
	F	Empregado Efetivo
	F	Empregado Efetivo
	F	Empregado Efetivo
	F	Empregado Efetivo
	F	Empregado Efetivo
	F	Empregado Efetivo
	F	Empregado Efetivo
	F	Empregado Efetivo
	F	Empregado Efetivo
	F	Empregado Efetivo
	M	Empregado Efetivo
	F	Empregado Efetivo
	F	Empregado Efetivo
	F	Empregado Efetivo
	F	Empregado Efetivo
	F	Empregado Efetivo
	F	Empregado Efetivo
	F	Empregado Efetivo
	F	Empregado Efetivo

	F	Empregado Efetivo
	F	Empregado Efetivo
	F	Empregado Efetivo
	F	Empregado Efetivo
	F	Empregado Efetivo
	F	Empregado Efetivo
	F	Empregado Efetivo
	F	Empregado Efetivo
	F	Empregado Efetivo
	M	Empregado Efetivo
	M	Empregado Efetivo
	F	Empregado Efetivo
	F	Empregado Efetivo
	F	Empregado Efetivo
	F	Empregado Efetivo
	M	Empregado Efetivo
	M	Empregado Efetivo
	F	Empregado Efetivo
	F	Empregado Efetivo
	M	Empregado Efetivo
	F	Empregado Efetivo
	M	Empregado Efetivo
	F	Empregado Efetivo
	F	Empregado Efetivo
	F	Empregado Efetivo
	F	Empregado Efetivo
	F	Empregado Efetivo
	M	Empregado Efetivo
	F	Empregado Efetivo
	F	Empregado Efetivo
	F	Empregado Efetivo
	F	Empregado Efetivo
	F	Empregado Efetivo
	F	Empregado Efetivo
	F	Empregado Efetivo
	F	Empregado Efetivo
	M	Empregado Efetivo
	F	Empregado Efetivo
	F	Empregado Efetivo
	M	Empregado Efetivo
	F	Empregado Efetivo
	M	Empregado Efetivo
	M	Empregado Efetivo
	M	Empregado Efetivo
	M	Empregado Efetivo
	M	Empregado Efetivo
	M	Empregado Efetivo
	M	Empregado Efetivo

	M	Empregado Efetivo
	M	Empregado Efetivo
	M	Empregado Efetivo
	M	Empregado Efetivo
	M	Empregado Efetivo
	M	Empregado Efetivo
	M	Empregado Efetivo
	M	Empregado Efetivo
	M	Empregado Efetivo
	F	Empregado Efetivo
	F	Empregado Efetivo
	M	Empregado Efetivo
	M	Empregado Efetivo
	M	Empregado Efetivo
	F	Empregado Efetivo
	M	Empregado Efetivo
	M	Empregado Efetivo
	F	Empregado Efetivo
	M	Empregado Efetivo
	F	Empregado Efetivo
	M	Empregado Efetivo
	F	Empregado Efetivo
	M	Empregado Efetivo
	M	Empregado Efetivo
	M	Empregado Efetivo
	F	Empregado Efetivo
	F	Empregado Efetivo
	F	Empregado Efetivo
	F	Empregado Efetivo
	F	Empregado Efetivo
	M	Empregado Efetivo
	F	Empregado Efetivo
	F	Empregado Efetivo
	M	Empregado Efetivo
	F	Empregado Efetivo
	F	Empregado Efetivo
	F	Empregado Efetivo
	F	Empregado Efetivo
	F	Empregado Efetivo
	F	Empregado Efetivo
	F	Empregado Efetivo
	F	Empregado Efetivo
	F	Empregado Efetivo
	M	Empregado Efetivo
	F	Empregado Efetivo
	F	Empregado Efetivo
	F	Empregado Efetivo
	F	Empregado Efetivo

	F	Empregado Efetivo
	F	Empregado Efetivo
	F	Empregado Efetivo
	F	Empregado Efetivo
	F	Empregado Efetivo
	F	Empregado Efetivo
	M	Empregado Efetivo
	F	Empregado Efetivo
	M	Empregado Efetivo
	F	Empregado Efetivo
	M	Empregado Efetivo
	M	Empregado Efetivo
	M	Empregado Efetivo
	M	Empregado Efetivo
	M	Empregado Efetivo
	M	Empregado Efetivo
	M	Empregado Efetivo
	F	Empregado Efetivo
	F	Empregado Efetivo
	F	Empregado Efetivo
	F	Empregado Efetivo
	F	Empregado Efetivo
	F	Empregado Efetivo
	F	Empregado Efetivo
	F	Empregado Efetivo
	F	Empregado Efetivo
	F	Empregado Efetivo
	F	Empregado Efetivo
	F	Empregado Efetivo
	F	Empregado Efetivo
	F	Empregado Efetivo
	M	Empregado Efetivo
	M	Empregado Efetivo
	F	Empregado Efetivo
	F	Empregado Efetivo
	F	Empregado Efetivo
	F	Empregado Efetivo
	F	Empregado Efetivo

	F	Empregado Efetivo
	F	Empregado Efetivo
	F	Empregado Efetivo
	F	Empregado Efetivo
	F	Empregado Efetivo
	F	Empregado Efetivo
	F	Empregado Efetivo
	F	Empregado Efetivo
	F	Empregado Efetivo
	F	Empregado Efetivo
	F	Empregado Efetivo
	F	Empregado Efetivo
	F	Empregado Efetivo
	F	Empregado Efetivo
	F	Empregado Efetivo
	M	Empregado Efetivo
	F	Empregado Efetivo
	M	Empregado Efetivo
	F	Empregado Efetivo
	F	Empregado Efetivo
	F	Empregado Efetivo
	F	Empregado Efetivo
	F	Empregado Efetivo
	M	Empregado Efetivo
	F	Empregado Efetivo
	F	Empregado Efetivo
	F	Empregado Efetivo
	F	Empregado Efetivo
	F	Empregado Efetivo
	F	Empregado Efetivo
	F	Empregado Efetivo
	F	Empregado Efetivo

	F	Empregado Efetivo
	F	Empregado Efetivo
	F	Empregado Efetivo
	M	Empregado Efetivo
	F	Empregado Efetivo
	F	Empregado Efetivo
	M	Empregado Efetivo
	F	Empregado Efetivo
	F	Empregado Efetivo
	F	Empregado Efetivo
	F	Empregado Efetivo
	F	Empregado Efetivo
	M	Empregado Efetivo
	M	Empregado Efetivo
	F	Empregado Efetivo
	F	Empregado Efetivo
	F	Empregado Efetivo
	F	Empregado Efetivo
	F	Empregado Efetivo
	F	Empregado Efetivo
	M	Empregado Efetivo
	F	Empregado Efetivo
	M	Empregado Efetivo
	F	Empregado Efetivo
	F	Empregado Efetivo
	F	Empregado Efetivo
	F	Empregado Efetivo
	F	Empregado Efetivo
	F	Empregado Efetivo
	F	Empregado Efetivo
	F	Empregado Efetivo
	F	Empregado Efetivo

	M	Empregado Efetivo
	F	Empregado Efetivo
	M	Empregado Efetivo
	F	Empregado Efetivo
	F	Empregado Efetivo
	F	Empregado Efetivo
	F	Empregado Efetivo
	F	Empregado Efetivo
	M	Empregado Efetivo
	F	Empregado Efetivo
	F	Empregado Efetivo
	F	Empregado Efetivo
	F	Empregado Efetivo
	F	Empregado Efetivo
	F	Empregado Efetivo
	F	Empregado Efetivo
	F	Empregado Efetivo
	F	Empregado Efetivo
	F	Empregado Efetivo
	M	Empregado Efetivo
	F	Empregado Efetivo
	M	Empregado Efetivo
	F	Empregado Efetivo
	F	Empregado Efetivo
	F	Empregado Efetivo
	F	Empregado Efetivo
	M	Empregado Efetivo
	F	Empregado Efetivo
	M	Empregado Efetivo
	F	Empregado Efetivo
	F	Empregado Efetivo
	F	Empregado Efetivo

	F	Empregado Efetivo
	M	Empregado Efetivo
	M	Empregado Efetivo
	F	Empregado Efetivo
	F	Empregado Efetivo
	F	Empregado Efetivo
	F	Empregado Efetivo
	M	Empregado Efetivo
	F	Empregado Efetivo
	M	Empregado Efetivo
	F	Empregado Efetivo
	F	Empregado Efetivo
	F	Empregado Efetivo
	F	Empregado Efetivo
	F	Empregado Efetivo
	F	Empregado Efetivo
	M	Empregado Efetivo
	M	Empregado Efetivo
	F	Empregado Efetivo
	F	Empregado Efetivo
	F	Empregado Efetivo
	F	Empregado Efetivo
	F	Empregado Efetivo
	F	Empregado Efetivo
	F	Empregado Efetivo
	F	Empregado Efetivo
	F	Empregado Efetivo
	F	Empregado Efetivo
	F	Empregado Efetivo
	F	Empregado Efetivo
	M	Empregado Efetivo
	F	Empregado Efetivo

	M	Empregado Efetivo
	F	Empregado Efetivo
	F	Empregado Efetivo
	M	Empregado Efetivo
	M	Empregado Efetivo
	M	Empregado Efetivo
	F	Empregado Efetivo
	F	Empregado Efetivo
	F	Empregado Efetivo
	F	Empregado Efetivo
	F	Empregado Efetivo
	F	Empregado Efetivo
	F	Empregado Efetivo
	F	Empregado Efetivo
	F	Empregado Efetivo
	F	Empregado Efetivo
	F	Empregado Efetivo
	F	Empregado Efetivo
	F	Empregado Efetivo
	F	Empregado Efetivo
	F	Empregado Efetivo
	F	Empregado Efetivo
	F	Empregado Efetivo
	F	Empregado Efetivo
	F	Empregado Efetivo
	F	Empregado Efetivo
	F	Empregado Efetivo
	F	Empregado Efetivo
	M	Empregado Efetivo
	F	Empregado Efetivo
	M	Empregado Efetivo
	F	Empregado Efetivo

	F	Empregado Efetivo
	F	Empregado Efetivo
	M	Empregado Efetivo
	F	Empregado Efetivo
	F	Empregado Efetivo
	F	Empregado Efetivo
	F	Empregado Efetivo
	F	Empregado Efetivo
	F	Empregado Efetivo
	F	Empregado Efetivo
	F	Empregado Efetivo
	F	Empregado Efetivo
	F	Empregado Efetivo
	F	Empregado Efetivo
	F	Empregado Efetivo
	M	Empregado Efetivo
	F	Empregado Efetivo
	F	Empregado Efetivo
	F	Empregado Efetivo
	M	Empregado Efetivo
	F	Empregado Efetivo
	M	Empregado Efetivo
	M	Empregado Efetivo
	F	Empregado Efetivo
	F	Empregado Efetivo
	F	Empregado Efetivo
	F	Empregado Efetivo
	M	Empregado Efetivo
	F	Empregado Efetivo
	F	Empregado Efetivo
	F	Empregado Efetivo
	F	Empregado Efetivo

	F	Empregado Efetivo
	F	Empregado Efetivo
	F	Empregado Efetivo
	F	Empregado Efetivo
	M	Empregado Efetivo
	F	Empregado Efetivo
	M	Empregado Efetivo
	F	Empregado Efetivo
	F	Empregado Efetivo
	F	Empregado Efetivo
	F	Empregado Efetivo
	F	Empregado Efetivo
	F	Empregado Efetivo
	F	Empregado Efetivo
	F	Empregado Efetivo
	M	Empregado Efetivo
	F	Empregado Efetivo
	M	Empregado Efetivo
	F	Empregado Efetivo
	F	Empregado Efetivo
	M	Empregado Efetivo
	F	Empregado Efetivo
	F	Empregado Efetivo
	F	Empregado Efetivo
	M	Empregado Efetivo
	F	Empregado Efetivo
	M	Empregado Efetivo
	F	Empregado Efetivo
	M	Empregado Efetivo
	M	Empregado Efetivo
	M	Empregado Efetivo
	F	Empregado Efetivo

	F	Empregado Efetivo
	F	Empregado Efetivo
	F	Empregado Efetivo
	F	Empregado Efetivo
	F	Empregado Efetivo
	F	Empregado Efetivo
	F	Empregado Efetivo
	M	Empregado Efetivo
	F	Empregado Efetivo
	F	Empregado Efetivo
	F	Empregado Efetivo
	F	Empregado Efetivo
	F	Empregado Efetivo
	F	Empregado Efetivo
	M	Empregado Efetivo
	F	Empregado Efetivo
	F	Empregado Efetivo
	F	Empregado Efetivo
	F	Empregado Efetivo
	F	Empregado Efetivo
	F	Empregado Efetivo
	F	Empregado Efetivo
	F	Empregado Efetivo
	F	Empregado Efetivo
	F	Empregado Efetivo
	F	Empregado Efetivo
	F	Empregado Efetivo
	F	Empregado Efetivo
	F	Empregado Efetivo
	F	Empregado Efetivo
	F	Empregado Efetivo
	F	Empregado Efetivo

	F	Empregado Efetivo
	F	Empregado Efetivo
	F	Empregado Efetivo
	F	Empregado Efetivo
	F	Empregado Efetivo
	F	Empregado Efetivo
	F	Empregado Efetivo
	F	Empregado Efetivo
	F	Empregado Efetivo
	M	Empregado Efetivo
	M	Empregado Efetivo
	M	Empregado Efetivo
	F	Empregado Efetivo
	F	Empregado Efetivo
	M	Empregado Efetivo
	F	Empregado Efetivo
	F	Empregado Efetivo
	F	Empregado Efetivo
	M	Empregado Efetivo
	F	Empregado Efetivo
	F	Empregado Efetivo
	M	Empregado Efetivo
	F	Empregado Efetivo
	F	Empregado Efetivo
	F	Empregado Efetivo
	M	Empregado Efetivo
	F	Empregado Efetivo
	F	Empregado Efetivo
	F	Empregado Efetivo
	M	Empregado Efetivo
	M	Empregado Efetivo
	F	Empregado Efetivo
	F	Empregado Efetivo
	F	Empregado Efetivo
	F	Empregado Efetivo

	M	Empregado Efetivo
	M	Empregado Efetivo
	M	Empregado Efetivo
	F	Empregado Efetivo
	F	Empregado Efetivo
	F	Empregado Efetivo
	F	Empregado Efetivo
	F	Empregado Efetivo
	F	Empregado Efetivo
	F	Empregado Efetivo
	F	Empregado Efetivo
	F	Empregado Efetivo
	F	Empregado Efetivo
	F	Empregado Efetivo
	F	Empregado Efetivo
	M	Empregado Efetivo
	M	Empregado Efetivo
	M	Empregado Efetivo
	F	Empregado Efetivo
	F	Empregado Efetivo
	F	Empregado Efetivo
	F	Empregado Efetivo
	F	Empregado Efetivo
	F	Empregado Efetivo
	F	Empregado Efetivo
	F	Empregado Efetivo
	M	Empregado Efetivo
	F	Empregado Efetivo
	F	Empregado Efetivo
	M	Empregado Efetivo
	F	Empregado Efetivo
	F	Empregado Efetivo
	F	Empregado Efetivo
	M	Empregado Efetivo
	M	Empregado Efetivo
	F	Empregado Efetivo
	F	Empregado Efetivo
	F	Empregado Efetivo
	F	Empregado Efetivo
	F	Empregado Efetivo

	F	Empregado Efetivo
	F	Empregado Efetivo
	F	Empregado Efetivo
	F	Empregado Efetivo
	F	Empregado Efetivo
	F	Empregado Efetivo
	F	Empregado Efetivo
	F	Empregado Efetivo
	F	Empregado Efetivo
	F	Empregado Efetivo
	F	Empregado Efetivo
	F	Empregado Efetivo
	F	Empregado Efetivo
	F	Empregado Efetivo
	F	Empregado Efetivo
	F	Empregado Efetivo
	F	Empregado Efetivo
	M	Empregado Efetivo
	F	Empregado Efetivo
	F	Empregado Efetivo
	M	Empregado Efetivo
	M	Empregado Efetivo
	M	Empregado Efetivo
	M	Empregado Efetivo
	M	Empregado Efetivo
	M	Empregado Efetivo
	F	Empregado Efetivo
	M	Empregado Efetivo
	M	Empregado Efetivo
	F	Empregado Efetivo
	M	Empregado Efetivo
	M	Empregado Efetivo
	M	Empregado Efetivo
	M	Empregado Efetivo
	M	Empregado Efetivo
	M	Empregado Efetivo
	M	Empregado Efetivo
	M	Empregado Efetivo
	M	Empregado Efetivo
	M	Empregado Efetivo
	M	Empregado Efetivo
	M	Empregado Efetivo
	M	Empregado Efetivo
	M	Empregado Efetivo
	M	Empregado Efetivo
	F	Empregado Efetivo
	M	Empregado Efetivo
	M	Empregado Efetivo

	M	Empregado Efetivo
	M	Empregado Efetivo
	M	Empregado Efetivo
	M	Empregado Efetivo
	M	Empregado Efetivo
	M	Empregado Efetivo
	M	Empregado Efetivo
	F	Empregado Efetivo
	F	Empregado Efetivo
	M	Empregado Efetivo
	F	Empregado Efetivo
	F	Empregado Efetivo
	F	Empregado Efetivo
	F	Empregado Efetivo
	F	Empregado Efetivo
	F	Empregado Efetivo
	F	Empregado Efetivo
	F	Empregado Efetivo
	F	Empregado Efetivo
	F	Empregado Efetivo
	F	Empregado Efetivo
	F	Empregado Efetivo
	F	Empregado Efetivo
	F	Empregado Efetivo
	M	Empregado Efetivo
	F	Empregado Efetivo
	F	Empregado Efetivo
	F	Empregado Efetivo
	F	Empregado Efetivo
	F	Empregado Efetivo
	F	Empregado Efetivo
	F	Empregado Efetivo
	M	Empregado Efetivo
	M	Empregado Efetivo
	M	Empregado Efetivo
	F	Empregado Efetivo
	M	Empregado Efetivo
	M	Empregado Efetivo
	M	Empregado Efetivo
	M	Empregado Efetivo
	F	Empregado Efetivo
	F	Empregado Efetivo
	F	Empregado Efetivo
	M	Empregado Efetivo
	M	Empregado Efetivo
	M	Empregado Efetivo

	F	Empregado Efetivo
	F	Empregado Efetivo
	F	Empregado Efetivo
	M	Empregado Efetivo
	F	Empregado Efetivo
	F	Empregado Efetivo
	F	Empregado Efetivo
	M	Empregado Efetivo
	F	Empregado Efetivo
	F	Empregado Efetivo
	M	Empregado Efetivo
	M	Empregado Efetivo
	M	Empregado Efetivo
	F	Empregado Efetivo
	F	Empregado Efetivo
	F	Empregado Efetivo
	F	Empregado Efetivo
	F	Empregado Efetivo
	F	Empregado Efetivo
	F	Empregado Efetivo
	F	Empregado Efetivo
	F	Empregado Efetivo
	F	Empregado Efetivo
	M	Empregado Efetivo
	F	Empregado Efetivo
	M	Empregado Efetivo
	F	Empregado Efetivo
	F	Empregado Efetivo
	F	Empregado Efetivo
	F	Empregado Efetivo
	F	Empregado Efetivo
	F	Empregado Efetivo
	M	Empregado Efetivo
	M	Empregado Efetivo
	F	Empregado Efetivo
	M	Empregado Efetivo
	M	Empregado Efetivo
	M	Empregado Efetivo
	F	Empregado Efetivo
	F	Empregado Efetivo
	F	Empregado Efetivo
	M	Empregado Efetivo
	M	Empregado Efetivo
	M	Empregado Efetivo
	F	Empregado Efetivo
	F	Empregado Efetivo
	F	Empregado Efetivo
	F	Empregado Efetivo

	F	Empregado Efetivo
	F	Empregado Efetivo
	M	Empregado Efetivo
	F	Empregado Efetivo
	F	Empregado Efetivo
	F	Empregado Efetivo
	M	Empregado Efetivo
	F	Empregado Efetivo
	F	Empregado Efetivo
	M	Empregado Efetivo
	M	Empregado Efetivo
	M	Empregado Efetivo
	F	Empregado Efetivo
	M	Empregado Efetivo
	F	Empregado Efetivo
	F	Empregado Efetivo
	F	Empregado Efetivo
	F	Empregado Efetivo
	F	Empregado Efetivo
	F	Empregado Efetivo
	F	Empregado Efetivo
	F	Empregado Efetivo
	F	Empregado Efetivo
	F	Empregado Efetivo
	F	Empregado Efetivo
	M	Empregado Efetivo
	F	Empregado Efetivo
	F	Empregado Efetivo
	F	Empregado Efetivo
	M	Empregado Efetivo
	F	Empregado Efetivo
	F	Empregado Efetivo
	F	Empregado Efetivo
	F	Empregado Efetivo

	M	Empregado Efetivo
	F	Empregado Efetivo
	F	Empregado Efetivo
	F	Empregado Efetivo
	F	Empregado Efetivo
	F	Empregado Efetivo
	F	Empregado Efetivo
	F	Empregado Efetivo
	M	Empregado Efetivo
	F	Empregado Efetivo
	F	Empregado Efetivo
	F	Empregado Efetivo
	F	Empregado Efetivo
	M	Empregado Efetivo
	F	Empregado Efetivo
	F	Empregado Efetivo
	F	Empregado Efetivo
	M	Empregado Efetivo
	F	Empregado Efetivo
	F	Empregado Efetivo
	F	Empregado Efetivo
	F	Empregado Efetivo
	F	Empregado Efetivo
	F	Empregado Efetivo
	F	Empregado Efetivo
	M	Empregado Efetivo
	F	Empregado Efetivo
	M	Empregado Efetivo
	F	Empregado Efetivo
	F	Empregado Efetivo
	F	Empregado Efetivo
	M	Empregado Efetivo

	M	Empregado Efetivo
	M	Empregado Efetivo
	F	Empregado Efetivo
	F	Empregado Efetivo
	F	Empregado Efetivo
	F	Empregado Efetivo
	F	Empregado Efetivo
	F	Empregado Efetivo
	F	Empregado Efetivo
	F	Empregado Efetivo
	F	Empregado Efetivo
	F	Empregado Efetivo
	F	Empregado Efetivo
	F	Empregado Efetivo
	F	Empregado Efetivo
	F	Empregado Efetivo
	F	Empregado Efetivo
	F	Empregado Efetivo
	F	Empregado Efetivo
	F	Empregado Efetivo
	M	Empregado Efetivo
	F	Empregado Efetivo
	F	Empregado Efetivo
	F	Empregado Efetivo
	F	Empregado Efetivo
	F	Empregado Efetivo
	F	Empregado Efetivo
	F	Empregado Efetivo
	M	Empregado Efetivo
	F	Empregado Efetivo
	F	Empregado Efetivo
	M	Empregado Efetivo

	F	Empregado Efetivo
	F	Empregado Efetivo
	F	Empregado Efetivo
	F	Empregado Efetivo
	F	Empregado Efetivo
	F	Empregado Efetivo
	F	Empregado Efetivo
	M	Empregado Efetivo
	F	Empregado Efetivo
	F	Empregado Efetivo
	M	Empregado Efetivo
	F	Empregado Efetivo
	F	Empregado Efetivo
	F	Empregado Efetivo
	F	Empregado Efetivo
	F	Empregado Efetivo
	M	Empregado Efetivo
	M	Empregado Efetivo
	F	Empregado Efetivo
	F	Empregado Efetivo
	F	Empregado Efetivo
	F	Empregado Efetivo
	M	Empregado Efetivo
	F	Empregado Efetivo
	M	Empregado Efetivo
	F	Empregado Efetivo
	F	Empregado Efetivo
	F	Empregado Efetivo
	M	Empregado Efetivo
	F	Empregado Efetivo
	F	Empregado Efetivo
	M	Empregado Efetivo

	F	Empregado Efetivo
	F	Empregado Efetivo
	F	Empregado Efetivo
	M	Empregado Efetivo
	F	Empregado Efetivo
	F	Empregado Efetivo
	M	Empregado Efetivo
	F	Empregado Efetivo
	F	Empregado Efetivo
	F	Empregado Efetivo
	F	Empregado Efetivo
	F	Empregado Efetivo
	F	Empregado Efetivo
	F	Empregado Efetivo
	F	Empregado Efetivo
	F	Empregado Efetivo
	F	Empregado Efetivo
	M	Empregado Efetivo
	F	Empregado Efetivo
	M	Empregado Efetivo
	F	Empregado Efetivo
	F	Empregado Efetivo
	F	Empregado Efetivo
	F	Empregado Efetivo
	F	Empregado Efetivo
	F	Empregado Efetivo
	F	Empregado Efetivo
	F	Empregado Efetivo
	F	Empregado Efetivo
	F	Empregado Efetivo
	F	Empregado Efetivo
	F	Empregado Efetivo

	F	Empregado Efetivo
	F	Empregado Efetivo
	M	Empregado Efetivo
	F	Empregado Efetivo
	F	Empregado Efetivo
	F	Empregado Efetivo
	F	Empregado Efetivo
	F	Empregado Efetivo
	M	Empregado Efetivo
	F	Empregado Efetivo
	F	Empregado Efetivo
	F	Empregado Efetivo
	F	Empregado Efetivo
	F	Empregado Efetivo
	F	Empregado Efetivo
	F	Empregado Efetivo
	M	Empregado Efetivo
	F	Empregado Efetivo
	F	Empregado Efetivo
	F	Empregado Efetivo
	F	Empregado Efetivo
	M	Empregado Efetivo
	F	Empregado Efetivo
	F	Empregado Efetivo
	F	Empregado Efetivo
	F	Empregado Efetivo
	F	Empregado Efetivo
	F	Empregado Efetivo
	M	Empregado Efetivo
	F	Empregado Efetivo
	F	Empregado Efetivo
	F	Empregado Efetivo

	M	Empregado Efetivo
	F	Empregado Efetivo
	F	Empregado Efetivo
	F	Empregado Efetivo
	F	Empregado Efetivo
	F	Empregado Efetivo
	F	Empregado Efetivo
	F	Empregado Efetivo
	F	Empregado Efetivo
	F	Empregado Efetivo
	F	Empregado Efetivo
	F	Empregado Efetivo
	F	Empregado Efetivo
	F	Empregado Efetivo
	M	Empregado Efetivo
	F	Empregado Efetivo
	F	Empregado Efetivo
	F	Empregado Efetivo
	F	Empregado Efetivo
	F	Empregado Efetivo
	M	Empregado Efetivo
	F	Empregado Efetivo
	F	Empregado Efetivo
	F	Empregado Efetivo
	F	Empregado Efetivo
	M	Empregado Efetivo
	F	Empregado Efetivo
	F	Empregado Efetivo
	F	Empregado Efetivo
	F	Empregado Efetivo
	M	Empregado Efetivo
	F	Empregado Efetivo

	F	Empregado Efetivo
	F	Empregado Efetivo
	M	Empregado Efetivo
	F	Empregado Efetivo
	F	Empregado Efetivo
	F	Empregado Efetivo
	F	Empregado Efetivo
	M	Empregado Efetivo
	F	Empregado Efetivo
	M	Empregado Efetivo
	F	Empregado Efetivo
	M	Empregado Efetivo
	F	Empregado Efetivo
	F	Empregado Efetivo
	F	Empregado Efetivo
	F	Empregado Efetivo
	M	Empregado Efetivo
	F	Empregado Efetivo
	F	Empregado Efetivo
	F	Empregado Efetivo
	F	Empregado Efetivo
	F	Empregado Efetivo
	F	Empregado Efetivo
	M	Empregado Efetivo
	F	Empregado Efetivo
	F	Empregado Efetivo
	F	Empregado Efetivo
	F	Empregado Efetivo
	M	Empregado Efetivo
	M	Empregado Efetivo
	F	Empregado Efetivo
	F	Empregado Efetivo

	M	Empregado Efetivo
	F	Empregado Efetivo
	F	Empregado Efetivo
	F	Empregado Efetivo
	M	Empregado Efetivo
	F	Empregado Efetivo
	F	Empregado Efetivo
	F	Empregado Efetivo
	M	Empregado Efetivo
	F	Empregado Efetivo
	F	Empregado Efetivo
	F	Empregado Efetivo
	F	Empregado Efetivo
	F	Empregado Efetivo
	F	Empregado Efetivo
	F	Empregado Efetivo
	F	Empregado Efetivo
	F	Empregado Efetivo
	F	Empregado Efetivo
	F	Empregado Efetivo
	F	Empregado Efetivo
	F	Empregado Efetivo
	F	Empregado Efetivo
	F	Empregado Efetivo
	F	Empregado Efetivo
	F	Empregado Efetivo
	M	Empregado Efetivo
	F	Empregado Efetivo
	F	Empregado Efetivo
	M	Empregado Efetivo
	F	Empregado Efetivo
	F	Empregado Efetivo
	F	Empregado Efetivo
	F	Empregado Efetivo
	F	Empregado Efetivo
	F	Empregado Efetivo
	F	Empregado Efetivo
	F	Empregado Efetivo

	F	Empregado Efetivo
	F	Empregado Efetivo
	F	Empregado Efetivo
	M	Empregado Efetivo
	M	Empregado Efetivo
	M	Empregado Efetivo
	F	Empregado Efetivo
	F	Empregado Efetivo
	F	Empregado Efetivo
	F	Empregado Efetivo
	F	Empregado Efetivo
	F	Empregado Efetivo
	F	Empregado Efetivo
	F	Empregado Efetivo
	F	Empregado Efetivo
	F	Empregado Efetivo
	F	Empregado Efetivo
	F	Empregado Efetivo
	F	Empregado Efetivo
	F	Empregado Efetivo
	F	Empregado Efetivo
	F	Empregado Efetivo
	F	Empregado Efetivo
	F	Empregado Efetivo
	F	Empregado Efetivo
	F	Empregado Efetivo
	F	Empregado Efetivo
	F	Empregado Efetivo
	F	Empregado Efetivo
	F	Empregado Efetivo
	F	Empregado Efetivo
	F	Empregado Efetivo
	F	Empregado Efetivo
	F	Empregado Efetivo
	F	Empregado Efetivo
	F	Empregado Efetivo
	F	Empregado Efetivo
	F	Empregado Efetivo
	F	Empregado Efetivo
	F	Empregado Efetivo
	F	Empregado Efetivo
	F	Empregado Efetivo
	F	Empregado Efetivo
	F	Empregado Efetivo
	F	Empregado Efetivo
	F	Empregado Efetivo

	F	Empregado Efetivo
	M	Empregado Efetivo
	M	Empregado Efetivo
	M	Empregado Efetivo
	M	Empregado Efetivo
	M	Empregado Efetivo
	M	Empregado Efetivo
	M	Empregado Efetivo
	M	Empregado Efetivo
	M	Empregado Efetivo
	M	Empregado Efetivo
	M	Empregado Efetivo
	M	Empregado Efetivo
	M	Empregado Efetivo
	M	Empregado Efetivo
	M	Empregado Efetivo
	F	Cedido - RJU
	F	Cedido - RJU
	M	Empregado Efetivo
	M	Empregado Efetivo
	F	Empregado Efetivo
	M	Empregado Efetivo
	M	Empregado Efetivo
	F	Empregado Efetivo
	M	Cedido - RJU
	M	Empregado Efetivo
	F	Empregado Efetivo
	M	Cedido - RJU
	F	Empregado Efetivo
	M	Empregado Efetivo
	M	Empregado Efetivo
	F	Empregado Efetivo
	F	Empregado Efetivo
	M	Empregado Efetivo
	F	Empregado Efetivo
	F	Empregado Efetivo
	F	Empregado Efetivo
	F	Empregado Efetivo
	F	Empregado Efetivo

	F	Cedido - RJU
	F	Empregado Efetivo
	M	Empregado Efetivo
	F	Empregado Efetivo
	F	Empregado Efetivo
	F	Empregado Efetivo
	F	Empregado Efetivo
	F	Empregado Efetivo
	F	Empregado Efetivo
	F	Empregado Efetivo
	F	Empregado Efetivo
	M	Empregado Efetivo
	M	Empregado Efetivo
	M	Empregado Efetivo
	F	Empregado Efetivo
	F	Empregado Efetivo
	M	Empregado Efetivo
	F	Empregado Efetivo
	F	Empregado Efetivo
	F	Empregado Efetivo
	F	Empregado Efetivo
	M	Empregado Efetivo
	F	Empregado Efetivo
	F	Empregado Efetivo
	M	Empregado Efetivo
	F	Empregado Efetivo
	F	Empregado Efetivo
	F	Empregado Efetivo
	F	Empregado Efetivo
	F	Empregado Efetivo
	F	Empregado Efetivo
	F	Empregado Efetivo
	F	Empregado Efetivo
	F	Empregado Efetivo
	F	Empregado Efetivo
	F	Empregado Efetivo
	F	Empregado Efetivo
	F	Empregado Efetivo

	F	Empregado Efetivo
	F	Empregado Efetivo
	F	Empregado Efetivo
	F	Empregado Efetivo
	F	Empregado Efetivo
	F	Empregado Efetivo
	F	Empregado Efetivo
	F	Empregado Efetivo
	F	Empregado Efetivo
	M	Empregado Efetivo
	F	Cedido - RJU
	M	Empregado Efetivo
	F	Empregado Efetivo
	F	Empregado Efetivo
	F	Empregado Efetivo
	F	Empregado Efetivo
	F	Empregado Efetivo
	F	Empregado Efetivo
	F	Empregado Efetivo
	F	Empregado Efetivo
	M	Cedido - CLT
	F	Empregado Efetivo
	F	Empregado Efetivo
	F	Empregado Movimentado - Po/MP n 193/2018
	F	Empregado Efetivo
	F	Empregado Efetivo
	F	Empregado Efetivo
	M	Empregado Efetivo
	M	Empregado Efetivo
	F	Empregado Efetivo
	F	Empregado Efetivo
	F	Empregado Efetivo
	M	Empregado Efetivo
	F	Empregado Efetivo
	F	Empregado Temporário
	F	Empregado Efetivo
	F	Empregado Movimentado - Po/MP n 193/2018
	F	Empregado Efetivo
	F	Empregado Efetivo
	F	Empregado Efetivo
	M	Empregado Efetivo

	F	Empregado Efetivo
	F	Empregado Efetivo
	F	Empregado Efetivo
	F	Empregado Efetivo
	F	Empregado Efetivo
	F	Empregado Efetivo
	M	Empregado Efetivo
	F	Empregado Efetivo
	F	Empregado Efetivo
	M	Empregado Efetivo
	F	Empregado Efetivo
	F	Empregado Efetivo
	F	Empregado Efetivo
	M	Empregado Efetivo
	M	Empregado Efetivo
	F	Empregado Efetivo
	F	Empregado Efetivo
	F	Empregado Efetivo
	F	Empregado Efetivo
	M	Empregado Efetivo
	M	Empregado Efetivo
	F	Empregado Efetivo
	F	Empregado Efetivo
	F	Empregado Efetivo
	F	Empregado Efetivo
	F	Empregado Efetivo
	F	Empregado Efetivo
	M	Empregado Efetivo
	M	Empregado Efetivo
	F	Empregado Efetivo
	M	Cedido - RJU
	M	Cedido - RJU
	M	Cedido - RJU
	M	Cedido - RJU
	F	Cedido - RJU
	F	Empregado Efetivo
	F	Empregado Efetivo
	M	Empregado Efetivo
	F	Empregado Efetivo
	F	Empregado Efetivo
	F	Empregado Efetivo
	F	Empregado Efetivo
	M	Empregado Efetivo
	M	Empregado Efetivo
	M	Empregado Efetivo
	M	Empregado Efetivo

	M	Empregado Efetivo
	M	Empregado Efetivo
	M	Empregado Efetivo
	M	Empregado Efetivo
	F	Empregado Efetivo
	M	Empregado Efetivo
	F	Empregado Efetivo
	F	Empregado Efetivo
	F	Empregado Efetivo
	F	Empregado Efetivo
	F	Empregado Efetivo
	M	Empregado Efetivo
	F	Empregado Efetivo
	M	Empregado Efetivo
	F	Empregado Efetivo
	F	Empregado Efetivo
	F	Empregado Efetivo
	F	Empregado Efetivo
	M	Empregado Efetivo
	M	Empregado Efetivo
	F	Empregado Efetivo
	M	Empregado Efetivo
	M	Empregado Efetivo
	M	Empregado Efetivo
	M	Empregado Efetivo
	F	Empregado Efetivo
	F	Empregado Efetivo
	F	Empregado Efetivo
	F	Empregado Efetivo
	F	Empregado Efetivo
	F	Empregado Efetivo
	M	Empregado Efetivo
	M	Empregado Efetivo
	M	Empregado Efetivo
	F	Empregado Efetivo
	F	Empregado Efetivo
	F	Empregado Efetivo
	M	Empregado Efetivo
	F	Empregado Efetivo
	M	Empregado Efetivo
	F	Empregado Efetivo
	F	Empregado Efetivo
	M	Empregado Efetivo
	F	Empregado Efetivo
	F	Empregado Efetivo
	M	Empregado Efetivo

	F	Empregado Efetivo
	F	Empregado Efetivo
	M	Empregado Temporário
	M	Empregado Temporário
	F	Empregado Efetivo
	F	Empregado Efetivo
	F	Empregado Efetivo
	M	Empregado Temporário
	F	Empregado Efetivo
	M	Empregado Temporário
	M	Empregado Efetivo
	M	Empregado Temporário
	F	Empregado Efetivo
	F	Empregado Efetivo
	F	Empregado Efetivo
	F	Empregado Efetivo
	F	Empregado Efetivo
	F	Empregado Efetivo
	F	Empregado Efetivo
	F	Empregado Efetivo
	F	Empregado Efetivo
	F	Empregado Efetivo
	F	Empregado Efetivo
	F	Empregado Efetivo
	F	Empregado Efetivo
	F	Empregado Efetivo
	F	Empregado Efetivo
	F	Empregado Efetivo
	F	Empregado Efetivo
	F	Empregado Efetivo
	F	Empregado Efetivo
	F	Empregado Efetivo
	F	Empregado Temporário
	M	Empregado Efetivo
	M	Empregado Efetivo
	F	Empregado Efetivo
	F	Empregado Temporário
	F	Empregado Efetivo
	F	Empregado Efetivo
	F	Empregado Efetivo
	M	Empregado Efetivo
	F	Empregado Temporário
	F	Empregado Efetivo
	F	Empregado Temporário
	F	Empregado Efetivo
	F	Empregado Efetivo
	F	Empregado Temporário
	F	Empregado Efetivo

	F	Empregado Efetivo
	F	Empregado Efetivo
	F	Empregado Efetivo
	F	Empregado Efetivo
	F	Empregado Efetivo
	F	Empregado Efetivo
	F	Empregado Efetivo
	F	Empregado Efetivo
	M	Empregado Efetivo
	M	Empregado Efetivo
	M	Empregado Efetivo
	F	Empregado Efetivo
	M	Empregado Efetivo
	M	Empregado Efetivo
	F	Empregado Efetivo
	M	Empregado Efetivo
	F	Empregado Efetivo
	F	Empregado Efetivo
	F	Empregado Efetivo
	F	Empregado Efetivo
	M	Empregado Efetivo
	M	Empregado Efetivo
	M	Empregado Efetivo
	F	Empregado Efetivo
	F	Empregado Efetivo
	F	Empregado Efetivo
	F	Empregado Efetivo
	F	Empregado Efetivo
	F	Empregado Efetivo
	F	Empregado Efetivo
	F	Empregado Efetivo
	F	Empregado Efetivo
	F	Empregado Efetivo
	F	Empregado Efetivo
	F	Empregado Efetivo
	M	Cedido - RJU
	F	Cedido - RJU
	F	Cedido - RJU
	F	Empregado Efetivo
	M	Empregado Efetivo
	M	Empregado Efetivo
	F	Empregado Efetivo
	M	Empregado Efetivo
	M	Empregado Efetivo
	M	Empregado Efetivo
	M	Empregado Efetivo
	M	Empregado Efetivo
	M	Empregado Efetivo

	F	Empregado Efetivo
	F	Empregado Efetivo
	F	Empregado Efetivo
	F	Empregado Efetivo
	F	Empregado Efetivo
	M	Empregado Efetivo
	F	Empregado Efetivo
	F	Empregado Efetivo
	M	Empregado Efetivo
	F	Empregado Efetivo
	M	Empregado Efetivo
	F	Empregado Efetivo
	M	Empregado Efetivo
	F	Empregado Efetivo
	F	Empregado Efetivo
	F	Empregado Efetivo
	F	Empregado Efetivo
	F	Empregado Efetivo
	F	Empregado Efetivo
	F	Empregado Efetivo
	M	Empregado Efetivo
	M	Empregado Efetivo
	F	Empregado Efetivo
	F	Empregado Efetivo
	M	Empregado Efetivo
	F	Empregado Efetivo
	F	Empregado Efetivo
	F	Empregado Efetivo
	M	Empregado Efetivo
	M	Empregado Efetivo
	M	Empregado Efetivo
	M	Empregado Efetivo
	F	Empregado Efetivo
	F	Empregado Efetivo
	M	Empregado Efetivo
	F	Empregado Efetivo
	F	Empregado Efetivo
	M	Empregado Efetivo
	F	Empregado Efetivo
	F	Empregado Efetivo
	M	Empregado Efetivo
	F	Empregado Efetivo
	F	Empregado Efetivo
	F	Empregado Efetivo
	F	Empregado Efetivo
	F	Empregado Efetivo
	F	Empregado Efetivo
	F	Empregado Efetivo

	M	Empregado Efetivo
	F	Empregado Efetivo
	F	Empregado Efetivo
	F	Empregado Efetivo
	F	Empregado Efetivo
	M	Empregado Efetivo
	F	Empregado Efetivo
	F	Empregado Efetivo
	F	Empregado Efetivo
	F	Empregado Efetivo
	F	Empregado Efetivo
	F	Empregado Efetivo
	F	Empregado Efetivo
	F	Empregado Efetivo
	F	Empregado Efetivo
	F	Empregado Efetivo
	M	Empregado Efetivo
	F	Empregado Efetivo
	F	Empregado Efetivo
	M	Empregado Efetivo
	M	Empregado Efetivo
	M	Empregado Efetivo
	F	Empregado Efetivo
	F	Empregado Efetivo
	M	Empregado Efetivo
	M	Empregado Efetivo
	M	Empregado Efetivo
	F	Empregado Efetivo
	F	Empregado Efetivo
	F	Empregado Efetivo
	F	Empregado Efetivo
	F	Empregado Efetivo
	F	Empregado Efetivo
	F	Empregado Efetivo
	F	Empregado Efetivo
	F	Empregado Efetivo
	M	Empregado Efetivo
	F	Empregado Efetivo
	F	Empregado Efetivo
	F	Empregado Efetivo
	F	Empregado Efetivo
	F	Empregado Efetivo
	F	Empregado Efetivo
	F	Empregado Efetivo
	M	Empregado Efetivo

	F	Empregado Efetivo
	F	Empregado Efetivo
	F	Empregado Efetivo
	F	Empregado Efetivo
	F	Empregado Efetivo
	F	Empregado Efetivo
	F	Empregado Efetivo
	F	Empregado Efetivo
	F	Empregado Efetivo
	F	Empregado Efetivo
	M	Empregado Efetivo
	F	Empregado Efetivo
	M	Empregado Efetivo
	F	Empregado Efetivo
	F	Empregado Efetivo
	F	Empregado Efetivo
	F	Empregado Efetivo
	M	Empregado Efetivo
	M	Empregado Efetivo
	F	Empregado Efetivo
	M	Empregado Efetivo
	F	Empregado Efetivo
	F	Empregado Efetivo
	F	Empregado Efetivo
	F	Empregado Efetivo
	F	Empregado Efetivo
	M	Empregado Efetivo
	F	Empregado Efetivo
	F	Empregado Efetivo
	F	Empregado Efetivo
	F	Empregado Efetivo
	F	Empregado Efetivo
	F	Empregado Efetivo
	F	Empregado Efetivo
	F	Empregado Efetivo
	M	Empregado Efetivo
	F	Empregado Efetivo
	F	Empregado Efetivo
	F	Empregado Efetivo
	F	Empregado Efetivo
	F	Empregado Efetivo
	F	Empregado Efetivo
	F	Empregado Efetivo
	F	Empregado Efetivo
	F	Empregado Efetivo
	F	Empregado Efetivo
	F	Empregado Efetivo
	F	Empregado Efetivo

	F	Empregado Efetivo
	F	Empregado Efetivo
	F	Empregado Efetivo
	F	Empregado Efetivo
	F	Empregado Efetivo
	F	Empregado Efetivo
	F	Empregado Efetivo
	M	Empregado Efetivo
	F	Empregado Efetivo
	M	Empregado Efetivo
	F	Empregado Efetivo
	F	Empregado Efetivo
	M	Empregado Efetivo
	F	Empregado Efetivo
	F	Empregado Efetivo
	F	Empregado Efetivo
	F	Empregado Efetivo
	F	Empregado Efetivo
	F	Empregado Efetivo
	F	Empregado Efetivo
	F	Empregado Efetivo
	F	Empregado Efetivo
	F	Empregado Efetivo
	F	Empregado Efetivo
	F	Empregado Efetivo
	F	Empregado Efetivo
	M	Empregado Efetivo
	F	Empregado Efetivo
	F	Empregado Efetivo
	F	Empregado Efetivo
	F	Empregado Efetivo
	F	Empregado Efetivo
	F	Empregado Efetivo
	F	Empregado Efetivo
	F	Empregado Efetivo
	F	Empregado Efetivo
	F	Empregado Efetivo
	M	Empregado Efetivo
	M	Empregado Efetivo
	F	Empregado Efetivo

	F	Empregado Efetivo
	F	Empregado Efetivo
	F	Empregado Efetivo
	F	Empregado Efetivo
	F	Empregado Efetivo
	F	Empregado Efetivo
	F	Empregado Efetivo
	M	Empregado Efetivo
	F	Empregado Efetivo
	F	Empregado Efetivo
	M	Empregado Efetivo
	M	Empregado Efetivo
	F	Empregado Efetivo
	F	Empregado Efetivo
	F	Empregado Efetivo
	M	Empregado Efetivo
	M	Empregado Efetivo
	M	Empregado Efetivo
	M	Empregado Efetivo
	M	Empregado Efetivo
	M	Empregado Efetivo
	M	Empregado Efetivo
	M	Empregado Efetivo
	M	Empregado Efetivo
	F	Empregado Efetivo
	M	Empregado Efetivo
	F	Empregado Efetivo
	F	Empregado Efetivo
	F	Empregado Efetivo
	M	Empregado Efetivo
	F	Empregado Efetivo
	F	Empregado Efetivo
	F	Empregado Efetivo
	M	Empregado Efetivo

	M	Empregado Efetivo
	F	Empregado Efetivo
	M	Empregado Efetivo
	M	Empregado Efetivo
	F	Empregado Efetivo
	F	Empregado Efetivo
	F	Empregado Efetivo
	F	Empregado Efetivo
	F	Empregado Efetivo
	F	Empregado Efetivo
	F	Empregado Efetivo
	F	Empregado Efetivo
	F	Empregado Efetivo
	F	Empregado Efetivo
	F	Empregado Efetivo
	M	Empregado Efetivo
	F	Empregado Efetivo
	F	Empregado Efetivo
	M	Empregado Efetivo
	F	Empregado Efetivo
	M	Empregado Efetivo
	F	Empregado Efetivo
	F	Empregado Efetivo
	M	Empregado Efetivo
	M	Empregado Efetivo
	F	Empregado Efetivo
	F	Empregado Efetivo
	F	Empregado Efetivo
	F	Empregado Efetivo
	F	Empregado Efetivo
	F	Empregado Efetivo
	F	Empregado Efetivo
	F	Empregado Efetivo
	F	Empregado Efetivo
	F	Empregado Efetivo
	F	Empregado Efetivo
	F	Empregado Efetivo
	F	Empregado Efetivo
	M	Empregado Efetivo
	F	Empregado Efetivo
	M	Empregado Efetivo
	M	Empregado Efetivo
	F	Empregado Efetivo
	M	Empregado Efetivo
	M	Empregado Efetivo
	F	Empregado Efetivo
	M	Empregado Efetivo
	F	Empregado Efetivo

	M	Empregado Efetivo
	F	Empregado Efetivo
	M	Empregado Efetivo
	F	Empregado Efetivo
	F	Empregado Efetivo
	F	Empregado Efetivo
	M	Empregado Efetivo
	M	Empregado Efetivo
	M	Empregado Efetivo
	M	Empregado Efetivo
	M	Empregado Efetivo
	M	Empregado Efetivo
	M	Empregado Efetivo
	M	Empregado Efetivo
	F	Empregado Efetivo
	M	Empregado Efetivo
	F	Empregado Efetivo
	M	Empregado Efetivo
	M	Empregado Efetivo
	F	Empregado Efetivo
	M	Empregado Efetivo
	F	Empregado Efetivo
	F	Empregado Efetivo
	M	Empregado Efetivo
	M	Empregado Efetivo
	M	Empregado Efetivo
	F	Empregado Efetivo
	F	Empregado Efetivo
	F	Empregado Efetivo
	F	Empregado Efetivo
	F	Empregado Efetivo
	F	Empregado Efetivo
	F	Empregado Efetivo
	F	Empregado Efetivo
	M	Empregado Efetivo
	F	Empregado Efetivo
	F	Empregado Efetivo
	F	Empregado Efetivo
	F	Empregado Efetivo
	F	Empregado Efetivo
	F	Empregado Efetivo
	F	Empregado Efetivo
	F	Empregado Efetivo
	F	Empregado Efetivo
	M	Empregado Efetivo
	F	Empregado Efetivo
	M	Empregado Efetivo
	M	Empregado Efetivo

	M	Empregado Efetivo
	F	Empregado Efetivo
	M	Empregado Efetivo
	M	Empregado Efetivo
	M	Empregado Efetivo
	F	Empregado Efetivo
	M	Empregado Efetivo
	F	Empregado Efetivo
	F	Empregado Efetivo
	M	Empregado Efetivo
	F	Empregado Efetivo
	F	Empregado Efetivo
	M	Empregado Efetivo
	F	Empregado Efetivo
	M	Empregado Efetivo
	F	Empregado Efetivo
	F	Empregado Efetivo
	F	Empregado Efetivo
	F	Empregado Efetivo
	F	Empregado Efetivo
	M	Empregado Efetivo
	F	Empregado Efetivo
	F	Empregado Efetivo
	F	Empregado Efetivo
	M	Empregado Efetivo
	F	Empregado Efetivo
	M	Empregado Efetivo
	F	Empregado Efetivo
	F	Empregado Efetivo
	M	Empregado Efetivo
	M	Empregado Efetivo
	M	Empregado Efetivo
	F	Empregado Efetivo
	M	Empregado Efetivo
	F	Empregado Efetivo
	F	Empregado Efetivo
	F	Empregado Efetivo
	F	Empregado Efetivo
	F	Empregado Efetivo
	F	Empregado Efetivo
	F	Empregado Efetivo
	M	Empregado Efetivo
	F	Empregado Efetivo
	F	Empregado Efetivo
	F	Empregado Efetivo
	F	Empregado Efetivo
	F	Empregado Efetivo
	F	Empregado Efetivo

	F	Empregado Efetivo
	M	Empregado Efetivo
	F	Empregado Temporário
	F	Empregado Efetivo
	F	Empregado Efetivo
	F	Empregado Efetivo
	F	Empregado Efetivo
	F	Empregado Efetivo
	F	Empregado Efetivo
	F	Empregado Efetivo
	F	Empregado Temporário
	F	Empregado Efetivo
	F	Empregado Efetivo
	M	Empregado Efetivo
	M	Empregado Efetivo
	M	Empregado Efetivo
	M	Empregado Efetivo
	M	Empregado Efetivo
	M	Empregado Efetivo
	M	Empregado Efetivo
	F	Empregado Efetivo
	M	Empregado Efetivo
	F	Empregado Efetivo
	M	Empregado Efetivo
	F	Cedido - RJU
	M	Empregado Efetivo
	F	Cedido - RJU
	F	Empregado Efetivo
	F	Empregado Efetivo
	F	Empregado Efetivo
	F	Empregado Efetivo
	F	Empregado Efetivo
	F	Empregado Efetivo
	F	Empregado Efetivo
	F	Empregado Efetivo
	F	Empregado Efetivo
	F	Empregado Efetivo
	F	Empregado Efetivo

	F	Empregado Efetivo
	F	Empregado Efetivo
	F	Empregado Efetivo
	F	Empregado Efetivo
	F	Empregado Efetivo
	F	Empregado Efetivo
	F	Empregado Efetivo
	F	Empregado Efetivo
	F	Empregado Efetivo
	F	Empregado Efetivo
	F	Empregado Efetivo
	M	Empregado Efetivo
	F	Empregado Efetivo
	F	Empregado Efetivo
	F	Empregado Efetivo
	F	Empregado Efetivo
	F	Empregado Efetivo
	M	Empregado Efetivo
	F	Empregado Efetivo
	F	Empregado Efetivo
	F	Empregado Efetivo
	F	Empregado Efetivo
	F	Empregado Efetivo
	F	Empregado Efetivo
	F	Empregado Efetivo
	F	Empregado Efetivo
	F	Empregado Efetivo
	F	Empregado Efetivo
	M	Empregado Efetivo
	F	Empregado Efetivo
	M	Empregado Efetivo
	M	Empregado Efetivo
	M	Empregado Efetivo
	F	Empregado Efetivo
	M	Empregado Efetivo
	F	Empregado Efetivo
	F	Empregado Efetivo
	M	Empregado Efetivo
	M	Empregado Efetivo
	M	Empregado Efetivo

	M	Empregado Efetivo
	F	Empregado Efetivo
	F	Empregado Efetivo
	F	Empregado Efetivo
	F	Empregado Efetivo
	F	Empregado Efetivo
	F	Empregado Efetivo
	F	Empregado Efetivo
	F	Empregado Efetivo
	M	Empregado Efetivo
	F	Empregado Efetivo
	M	Empregado Efetivo
	F	Empregado Efetivo
	F	Empregado Efetivo
	F	Empregado Efetivo
	F	Empregado Efetivo
	F	Empregado Efetivo
	F	Empregado Efetivo
	F	Empregado Efetivo
	F	Empregado Efetivo
	F	Empregado Efetivo
	F	Empregado Efetivo
	F	Empregado Efetivo
	F	Empregado Efetivo
	F	Empregado Efetivo
	M	Empregado Efetivo
	F	Empregado Efetivo
	F	Empregado Efetivo
	F	Empregado Efetivo
	F	Empregado Efetivo
	F	Empregado Efetivo
	F	Empregado Efetivo
	F	Empregado Efetivo
	M	Empregado Efetivo
	F	Empregado Efetivo
	M	Empregado Efetivo
	M	Empregado Efetivo
	M	Empregado Efetivo
	M	Empregado Efetivo

	F	Empregado Efetivo
	F	Empregado Efetivo
	F	Empregado Efetivo
	F	Empregado Efetivo
	F	Empregado Efetivo
	F	Empregado Efetivo
	F	Empregado Efetivo
	F	Empregado Efetivo
	F	Empregado Efetivo
	F	Empregado Efetivo
	F	Empregado Efetivo
	F	Empregado Efetivo
	F	Empregado Efetivo
	F	Empregado Efetivo
	F	Empregado Efetivo
	F	Empregado Efetivo
	M	Empregado Efetivo
	M	Empregado Efetivo
	M	Empregado Efetivo
	F	Empregado Efetivo
	F	Empregado Efetivo
	F	Empregado Efetivo
	F	Empregado Efetivo
	F	Empregado Efetivo
	F	Empregado Efetivo
	M	Empregado Efetivo
	M	Empregado Efetivo
	F	Empregado Efetivo
	F	Empregado Efetivo
	F	Empregado Efetivo
	F	Empregado Efetivo
	F	Empregado Efetivo
	F	Empregado Efetivo
	F	Empregado Efetivo
	F	Empregado Efetivo
	F	Empregado Efetivo
	F	Empregado Efetivo
	F	Empregado Efetivo
	F	Empregado Efetivo
	F	Empregado Efetivo
	F	Empregado Efetivo
	F	Empregado Efetivo
	F	Empregado Efetivo
	M	Empregado Efetivo

	F	Empregado Efetivo
	F	Empregado Efetivo
	M	Empregado Efetivo
	M	Empregado Efetivo
	M	Empregado Efetivo
	M	Empregado Efetivo
	M	Empregado Efetivo
	F	Empregado Efetivo
	M	Empregado Efetivo
	M	Empregado Efetivo
	F	Empregado Efetivo
	F	Empregado Efetivo
	F	Empregado Efetivo
	F	Empregado Efetivo
	F	Empregado Efetivo
	F	Empregado Efetivo
	F	Empregado Efetivo
	F	Empregado Efetivo
	F	Empregado Efetivo
	F	Empregado Efetivo
	M	Empregado Efetivo
	M	Empregado Efetivo
	M	Empregado Efetivo
	M	Empregado Efetivo
	M	Empregado Efetivo
	M	Empregado Efetivo
	M	Empregado Efetivo
	F	Empregado Efetivo
	F	Empregado Efetivo
	M	Empregado Efetivo
	M	Empregado Efetivo
	F	Empregado Efetivo
	F	Empregado Efetivo
	F	Empregado Efetivo
	F	Empregado Efetivo
	F	Empregado Efetivo
	F	Empregado Efetivo
	F	Empregado Efetivo
	F	Empregado Efetivo
	F	Empregado Efetivo
	F	Empregado Efetivo

	F	Empregado Efetivo
	F	Empregado Efetivo
	F	Empregado Efetivo
	F	Empregado Efetivo
	F	Empregado Efetivo
	F	Empregado Efetivo
	F	Empregado Efetivo
	F	Empregado Efetivo
	F	Empregado Efetivo
	F	Empregado Efetivo
	F	Empregado Efetivo
	F	Empregado Efetivo
	F	Empregado Efetivo
	F	Empregado Efetivo
	F	Empregado Efetivo
	M	Empregado Efetivo
	F	Empregado Efetivo
	F	Empregado Efetivo
	F	Empregado Efetivo
	F	Empregado Efetivo
	F	Empregado Efetivo
	F	Empregado Efetivo
	F	Empregado Efetivo
	F	Empregado Efetivo
	F	Empregado Efetivo
	M	Empregado Efetivo
	F	Empregado Efetivo
	F	Empregado Efetivo
	M	Empregado Efetivo
	F	Empregado Efetivo
	F	Empregado Efetivo
	M	Empregado Efetivo
	F	Empregado Efetivo
	F	Empregado Efetivo
	F	Empregado Efetivo
	F	Empregado Efetivo
	F	Empregado Efetivo
	M	Empregado Efetivo
	F	Empregado Efetivo
	F	Empregado Efetivo
	F	Empregado Efetivo
	F	Empregado Efetivo

	F	Empregado Efetivo
	F	Empregado Efetivo
	M	Empregado Efetivo
	F	Empregado Efetivo
	F	Empregado Efetivo
	F	Empregado Efetivo
	F	Empregado Efetivo
	F	Empregado Efetivo
	M	Empregado Efetivo
	M	Empregado Efetivo
	M	Empregado Efetivo
	F	Empregado Efetivo
	M	Empregado Efetivo
	M	Empregado Efetivo
	M	Empregado Efetivo
	F	Empregado Efetivo
	F	Empregado Efetivo
	F	Empregado Efetivo
	M	Empregado Efetivo
	F	Empregado Efetivo
	F	Empregado Efetivo
	F	Empregado Efetivo
	M	Empregado Efetivo
	F	Empregado Efetivo
	M	Empregado Efetivo
	F	Empregado Efetivo
	F	Empregado Efetivo
	F	Empregado Efetivo
	F	Empregado Efetivo
	F	Empregado Efetivo
	M	Empregado Efetivo
	F	Empregado Efetivo
	F	Empregado Efetivo
	F	Empregado Efetivo
	F	Empregado Efetivo
	F	Empregado Efetivo
	F	Empregado Efetivo
	F	Empregado Efetivo
	F	Empregado Efetivo
	F	Empregado Efetivo
	F	Empregado Efetivo
	F	Empregado Efetivo
	F	Empregado Efetivo
	F	Empregado Efetivo
	F	Empregado Efetivo
	F	Empregado Efetivo
	F	Empregado Efetivo
	F	Empregado Efetivo

	F	Empregado Efetivo
	F	Empregado Efetivo
	F	Empregado Efetivo
	F	Empregado Efetivo
	M	Empregado Efetivo
	F	Empregado Efetivo
	M	Empregado Efetivo
	F	Empregado Efetivo
	F	Empregado Efetivo
	F	Empregado Efetivo
	F	Empregado Efetivo
	F	Empregado Efetivo
	F	Empregado Efetivo
	F	Empregado Efetivo
	F	Empregado Efetivo
	F	Empregado Efetivo
	F	Empregado Efetivo
	F	Empregado Efetivo
	F	Empregado Efetivo
	F	Empregado Efetivo
	F	Empregado Efetivo
	F	Empregado Efetivo
	F	Empregado Efetivo
	F	Empregado Efetivo
	F	Empregado Efetivo
	F	Empregado Efetivo
	M	Empregado Efetivo
	M	Empregado Efetivo
	F	Empregado Efetivo
	F	Empregado Efetivo
	F	Empregado Efetivo
	F	Empregado Efetivo
	F	Empregado Efetivo
	M	Empregado Efetivo
	F	Empregado Efetivo
	M	Empregado Efetivo
	F	Empregado Efetivo
	F	Empregado Efetivo
	F	Empregado Efetivo
	M	Empregado Efetivo
	M	Empregado Efetivo
	F	Empregado Efetivo
	F	Empregado Efetivo
	F	Empregado Efetivo
	F	Empregado Efetivo

	F	Empregado Efetivo
	F	Empregado Efetivo
	F	Empregado Efetivo
	M	Empregado Efetivo
	F	Empregado Efetivo
	F	Empregado Efetivo
	F	Empregado Efetivo
	F	Empregado Efetivo
	F	Empregado Efetivo
	F	Empregado Efetivo
	F	Empregado Efetivo
	F	Empregado Efetivo
	M	Empregado Efetivo
	F	Empregado Efetivo
	F	Empregado Efetivo
	F	Empregado Efetivo
	F	Empregado Efetivo
	M	Empregado Efetivo
	M	Empregado Efetivo
	M	Empregado Efetivo
	F	Empregado Efetivo
	F	Empregado Efetivo
	F	Empregado Efetivo
	F	Empregado Efetivo
	F	Empregado Efetivo
	F	Empregado Efetivo
	F	Empregado Efetivo
	F	Empregado Efetivo
	F	Empregado Efetivo
	F	Empregado Efetivo
	F	Empregado Efetivo
	F	Empregado Efetivo
	F	Empregado Efetivo
	M	Empregado Efetivo
	F	Empregado Efetivo
	F	Empregado Efetivo
	F	Empregado Efetivo
	F	Empregado Efetivo
	F	Empregado Efetivo

	F	Empregado Efetivo
	F	Empregado Efetivo
	F	Empregado Efetivo
	F	Empregado Efetivo
	F	Empregado Efetivo
	F	Empregado Efetivo
	F	Empregado Efetivo
	F	Empregado Efetivo
	F	Empregado Efetivo
	F	Empregado Efetivo
	F	Empregado Efetivo
	M	Empregado Efetivo
	M	Empregado Efetivo
	M	Empregado Efetivo
	F	Empregado Efetivo
	M	Empregado Efetivo
	F	Empregado Efetivo
	M	Empregado Efetivo
	F	Empregado Efetivo
	F	Empregado Efetivo
	F	Empregado Efetivo
	M	Empregado Efetivo
	M	Empregado Efetivo
	M	Empregado Efetivo
	F	Empregado Efetivo
	F	Empregado Efetivo
	F	Empregado Efetivo
	F	Empregado Efetivo
	F	Empregado Efetivo
	F	Empregado Efetivo
	F	Empregado Efetivo
	F	Empregado Efetivo
	F	Empregado Efetivo
	M	Empregado Efetivo
	F	Empregado Efetivo
	F	Empregado Efetivo
	F	Empregado Efetivo
	F	Empregado Efetivo
	F	Empregado Efetivo
	F	Empregado Efetivo
	F	Empregado Efetivo
	M	Empregado Efetivo
	M	Empregado Efetivo

	M	Empregado Efetivo
	M	Empregado Efetivo
	M	Empregado Efetivo
	F	Empregado Efetivo
	M	Empregado Efetivo
	F	Empregado Efetivo
	F	Empregado Efetivo
	M	Empregado Efetivo
	F	Empregado Efetivo
	M	Empregado Efetivo
	F	Empregado Efetivo
	F	Empregado Efetivo
	F	Empregado Efetivo
	M	Empregado Efetivo
	F	Empregado Efetivo
	F	Empregado Efetivo
	M	Empregado Efetivo
	F	Empregado Efetivo
	F	Empregado Efetivo
	F	Empregado Efetivo
	M	Empregado Efetivo
	F	Empregado Efetivo
	F	Empregado Efetivo
	F	Empregado Efetivo
	M	Empregado Efetivo
	F	Empregado Efetivo
	F	Empregado Efetivo
	F	Empregado Efetivo
	F	Empregado Efetivo
	F	Empregado Efetivo
	F	Empregado Efetivo
	M	Empregado Efetivo
	F	Empregado Efetivo
	F	Empregado Efetivo
	F	Empregado Efetivo
	F	Empregado Efetivo
	F	Empregado Efetivo
	F	Empregado Efetivo
	F	Empregado Efetivo
	F	Empregado Efetivo
	F	Empregado Efetivo
	F	Empregado Efetivo
	F	Empregado Efetivo
	M	Empregado Efetivo
	F	Empregado Efetivo
	F	Empregado Efetivo
	M	Empregado Efetivo
	F	Empregado Efetivo

	M	Empregado Efetivo
	F	Empregado Efetivo
	F	Empregado Efetivo
	F	Empregado Efetivo
	F	Empregado Efetivo
	F	Empregado Efetivo
	F	Empregado Efetivo
	F	Empregado Efetivo
	F	Empregado Efetivo
	F	Empregado Efetivo
	F	Empregado Efetivo
	F	Empregado Efetivo
	F	Empregado Efetivo
	F	Empregado Efetivo
	F	Empregado Efetivo
	F	Empregado Efetivo
	F	Empregado Efetivo
	M	Empregado Efetivo
	F	Empregado Efetivo
	F	Empregado Efetivo
	F	Empregado Efetivo
	F	Empregado Efetivo
	M	Empregado Efetivo
	M	Empregado Efetivo
	M	Empregado Efetivo
	M	Empregado Efetivo
	F	Empregado Efetivo
	F	Empregado Efetivo
	M	Empregado Efetivo
	M	Empregado Efetivo
	M	Empregado Efetivo
	F	Empregado Efetivo
	F	Empregado Efetivo
	M	Empregado Efetivo
	M	Empregado Efetivo
	M	Empregado Efetivo
	F	Empregado Efetivo

	F	Empregado Efetivo
	M	Empregado Efetivo
	M	Empregado Efetivo
	F	Empregado Efetivo
	F	Empregado Efetivo
	F	Empregado Efetivo
	F	Empregado Efetivo
	M	Empregado Efetivo
	F	Empregado Efetivo
	F	Empregado Efetivo
	F	Empregado Efetivo
	F	Empregado Efetivo
	F	Empregado Efetivo
	M	Empregado Efetivo
	F	Empregado Efetivo
	F	Empregado Efetivo
	M	Empregado Efetivo
	M	Empregado Efetivo
	F	Empregado Efetivo
	F	Empregado Efetivo
	M	Empregado Efetivo
	M	Empregado Efetivo
	F	Empregado Efetivo
	F	Empregado Efetivo
	F	Empregado Efetivo
	F	Empregado Efetivo
	M	Empregado Efetivo
	M	Empregado Efetivo
	M	Empregado Efetivo
	M	Empregado Efetivo
	F	Empregado Efetivo
	F	Empregado Efetivo
	F	Empregado Efetivo
	F	Empregado Efetivo
	F	Empregado Efetivo
	F	Empregado Efetivo
	F	Empregado Efetivo
	F	Empregado Efetivo
	F	Empregado Efetivo
	F	Empregado Efetivo

	F	Empregado Efetivo
	F	Empregado Efetivo
	F	Empregado Efetivo
	F	Empregado Efetivo
	F	Empregado Efetivo
	F	Empregado Efetivo
	F	Empregado Efetivo
	F	Empregado Efetivo
	F	Empregado Efetivo
	F	Empregado Efetivo
	F	Empregado Efetivo
	F	Empregado Efetivo
	F	Empregado Efetivo
	F	Empregado Efetivo
	F	Empregado Efetivo
	M	Empregado Efetivo
	M	Empregado Efetivo
	F	Empregado Efetivo
	F	Empregado Efetivo
	F	Empregado Efetivo
	F	Empregado Efetivo
	M	Empregado Efetivo
	F	Empregado Efetivo
	F	Empregado Efetivo
	F	Empregado Efetivo
	F	Empregado Efetivo
	F	Empregado Efetivo
	F	Empregado Efetivo
	F	Empregado Efetivo
	F	Empregado Efetivo
	F	Empregado Efetivo
	F	Empregado Efetivo
	F	Empregado Efetivo
	M	Empregado Efetivo
	F	Empregado Efetivo
	M	Empregado Efetivo
	F	Empregado Efetivo
	M	Empregado Efetivo
	F	Empregado Efetivo
	F	Empregado Efetivo
	F	Empregado Efetivo

	M	Empregado Efetivo
	F	Empregado Efetivo
	M	Empregado Efetivo
	M	Empregado Efetivo
	M	Empregado Efetivo
	M	Empregado Efetivo
	M	Empregado Efetivo
	M	Empregado Efetivo
	M	Empregado Efetivo
	F	Empregado Efetivo
	M	Empregado Efetivo
	F	Empregado Efetivo
	F	Empregado Efetivo
	F	Empregado Efetivo
	M	Empregado Efetivo
	M	Empregado Efetivo
	M	Empregado Efetivo
	M	Empregado Efetivo
	M	Empregado Efetivo
	M	Empregado Efetivo
	M	Empregado Efetivo
	M	Empregado Efetivo
	F	Empregado Efetivo
	M	Empregado Efetivo
	F	Empregado Efetivo
	M	Empregado Efetivo
	F	Empregado Efetivo
	M	Empregado Efetivo
	F	Empregado Efetivo
	M	Empregado Efetivo
	F	Empregado Efetivo
	F	Empregado Efetivo
	F	Empregado Efetivo
	M	Empregado Efetivo
	F	Empregado Efetivo
	M	Empregado Efetivo
	M	Empregado Efetivo
	M	Empregado Efetivo
	F	Empregado Efetivo
	M	Empregado Efetivo
	M	Empregado Efetivo
	M	Empregado Efetivo
	F	Empregado Efetivo
	M	Empregado Efetivo
	M	Empregado Efetivo
	M	Empregado Efetivo
	M	Empregado Efetivo
	M	Empregado Efetivo

	M	Empregado Efetivo
	M	Empregado Efetivo
	F	Empregado Efetivo
	M	Empregado Efetivo
	F	Empregado Efetivo
	F	Empregado Efetivo
	M	Empregado Efetivo
	M	Empregado Efetivo
	M	Empregado Efetivo
	M	Empregado Efetivo
	M	Empregado Efetivo
	F	Empregado Efetivo
	M	Empregado Efetivo
	M	Empregado Efetivo
	M	Empregado Efetivo
	F	Empregado Efetivo
	F	Empregado Efetivo
	M	Empregado Efetivo
	F	Empregado Efetivo
	F	Empregado Efetivo
	F	Empregado Efetivo
	F	Empregado Efetivo
	F	Empregado Efetivo
	F	Empregado Efetivo
	F	Empregado Efetivo
	F	Empregado Efetivo
	F	Empregado Efetivo
	F	Empregado Efetivo
	M	Empregado Efetivo
	M	Empregado Efetivo
	F	Empregado Efetivo
	F	Empregado Efetivo
	F	Empregado Efetivo
	F	Empregado Efetivo
	F	Empregado Efetivo
	F	Empregado Efetivo
	F	Empregado Efetivo
	M	Empregado Efetivo
	F	Empregado Efetivo
	M	Empregado Efetivo
	F	Empregado Efetivo
	M	Empregado Efetivo
	F	Empregado Efetivo
	F	Empregado Efetivo
	F	Empregado Efetivo
	M	Empregado Efetivo
	F	Empregado Efetivo
	F	Empregado Efetivo

	M	Empregado Efetivo
	M	Empregado Efetivo
	F	Empregado Efetivo
	F	Empregado Efetivo
	F	Empregado Efetivo
	F	Empregado Efetivo
	F	Empregado Efetivo
	F	Empregado Efetivo
	F	Empregado Efetivo
	F	Empregado Efetivo
	M	Empregado Efetivo
	F	Empregado Efetivo
	F	Empregado Efetivo
	F	Empregado Efetivo
	F	Empregado Efetivo
	F	Empregado Efetivo
	F	Empregado Efetivo
	F	Empregado Efetivo
	F	Empregado Efetivo
	F	Empregado Efetivo
	F	Empregado Efetivo
	F	Empregado Efetivo
	M	Empregado Efetivo
	F	Empregado Efetivo
	F	Empregado Efetivo
	F	Empregado Efetivo
	F	Empregado Efetivo
	F	Empregado Efetivo
	F	Empregado Efetivo
	F	Empregado Efetivo
	F	Empregado Efetivo
	F	Empregado Efetivo
	F	Empregado Efetivo
	F	Empregado Efetivo
	F	Empregado Efetivo
	F	Empregado Efetivo
	F	Empregado Efetivo
	F	Empregado Efetivo
	F	Empregado Efetivo
	M	Empregado Efetivo
	F	Empregado Efetivo
	M	Empregado Efetivo

	M	Empregado Efetivo
	F	Empregado Efetivo
	M	Empregado Efetivo
	F	Empregado Efetivo
	F	Empregado Efetivo
	F	Empregado Efetivo
	F	Empregado Efetivo
	F	Empregado Efetivo
	F	Empregado Efetivo
	F	Empregado Efetivo
	F	Empregado Efetivo
	F	Empregado Efetivo
	F	Empregado Efetivo
	F	Empregado Efetivo
	F	Empregado Efetivo
	F	Empregado Efetivo
	M	Empregado Efetivo
	F	Empregado Efetivo
	F	Empregado Efetivo
	F	Empregado Efetivo
	M	Empregado Efetivo
	F	Empregado Efetivo
	M	Empregado Efetivo
	F	Empregado Efetivo
	M	Empregado Efetivo
	M	Empregado Efetivo
	M	Empregado Efetivo
	F	Empregado Efetivo
	M	Empregado Efetivo
	M	Empregado Efetivo
	F	Empregado Efetivo
	M	Empregado Efetivo
	M	Empregado Efetivo
	M	Empregado Efetivo
	F	Empregado Efetivo
	F	Empregado Efetivo
	F	Empregado Efetivo
	F	Empregado Efetivo
	F	Empregado Efetivo

	F	Empregado Efetivo
	F	Empregado Efetivo
	F	Empregado Efetivo
	F	Empregado Efetivo
	F	Empregado Efetivo
	F	Empregado Efetivo
	F	Empregado Efetivo
	F	Empregado Efetivo
	F	Empregado Efetivo
	F	Empregado Efetivo
	F	Empregado Efetivo
	F	Empregado Efetivo
	F	Empregado Efetivo
	F	Empregado Efetivo
	F	Empregado Efetivo
	F	Empregado Efetivo
	F	Empregado Efetivo
	M	Empregado Efetivo
	F	Empregado Efetivo
	F	Empregado Efetivo
	F	Empregado Efetivo
	F	Empregado Efetivo
	M	Empregado Efetivo
	F	Empregado Efetivo
	F	Empregado Efetivo
	F	Empregado Efetivo
	M	Empregado Efetivo
	F	Empregado Efetivo
	F	Empregado Efetivo
	F	Empregado Efetivo
	F	Empregado Efetivo
	F	Empregado Efetivo
	F	Empregado Efetivo
	F	Empregado Efetivo
	F	Empregado Efetivo
	F	Empregado Efetivo
	F	Empregado Efetivo
	M	Empregado Efetivo
	M	Empregado Efetivo
	M	Empregado Efetivo
	M	Empregado Efetivo
	F	Empregado Efetivo
	F	Empregado Efetivo
	F	Empregado Efetivo
	F	Empregado Efetivo
	F	Empregado Efetivo

	F	Empregado Efetivo
	M	Empregado Efetivo
	F	Empregado Efetivo
	F	Empregado Efetivo
	F	Empregado Efetivo
	M	Empregado Efetivo
	M	Empregado Efetivo
	F	Empregado Efetivo
	F	Empregado Efetivo
	M	Empregado Efetivo
	F	Empregado Efetivo
	F	Empregado Efetivo
	F	Empregado Efetivo
	M	Empregado Efetivo
	F	Empregado Efetivo
	F	Empregado Efetivo
	F	Empregado Efetivo
	F	Empregado Efetivo
	F	Empregado Efetivo
	F	Empregado Efetivo
	F	Empregado Efetivo
	F	Empregado Efetivo
	F	Empregado Efetivo
	F	Empregado Efetivo
	M	Empregado Efetivo
	M	Empregado Efetivo
	F	Empregado Efetivo
	F	Empregado Efetivo
	F	Empregado Efetivo
	F	Empregado Efetivo
	F	Empregado Efetivo
	F	Empregado Efetivo
	F	Empregado Efetivo
	M	Empregado Efetivo
	M	Empregado Efetivo
	F	Empregado Efetivo
	F	Empregado Efetivo
	F	Empregado Efetivo
	F	Empregado Efetivo
	M	Empregado Efetivo
	F	Empregado Efetivo
	F	Empregado Efetivo
	M	Empregado Efetivo
	M	Empregado Efetivo
	M	Empregado Efetivo
	F	Empregado Efetivo
	F	Empregado Efetivo
	F	Empregado Efetivo

	F	Empregado Efetivo
	M	Empregado Efetivo
	F	Empregado Efetivo
	F	Empregado Efetivo
	F	Empregado Efetivo
	F	Empregado Efetivo
	F	Empregado Efetivo
	M	Empregado Efetivo
	F	Empregado Efetivo
	M	Empregado Efetivo
	F	Empregado Efetivo
	F	Empregado Efetivo
	F	Empregado Efetivo
	F	Empregado Efetivo
	F	Empregado Efetivo
	M	Empregado Efetivo
	F	Empregado Efetivo
	F	Empregado Efetivo
	F	Empregado Efetivo
	F	Empregado Efetivo
	M	Empregado Efetivo
	F	Empregado Efetivo
	M	Empregado Efetivo
	F	Empregado Efetivo
	M	Empregado Efetivo
	M	Empregado Efetivo
	F	Empregado Efetivo
	M	Empregado Efetivo
	M	Empregado Efetivo
	M	Empregado Efetivo
	M	Empregado Efetivo
	F	Empregado Efetivo
	M	Empregado Efetivo
	F	Empregado Efetivo
	M	Empregado Efetivo
	M	Empregado Efetivo
	M	Empregado Efetivo
	F	Empregado Efetivo
	F	Empregado Efetivo
	F	Empregado Efetivo
	F	Empregado Efetivo
	F	Empregado Efetivo
	M	Empregado Efetivo
	M	Empregado Efetivo
	M	Empregado Efetivo
	M	Empregado Efetivo
	F	Empregado Efetivo

	F	Empregado Efetivo
	F	Empregado Efetivo
	M	Empregado Efetivo
	F	Empregado Efetivo
	F	Empregado Efetivo
	F	Empregado Efetivo
	F	Empregado Efetivo
	F	Empregado Efetivo
	F	Empregado Efetivo
	F	Empregado Efetivo
	F	Empregado Efetivo
	F	Empregado Efetivo
	F	Empregado Efetivo
	F	Empregado Efetivo
	F	Empregado Efetivo
	F	Empregado Efetivo
	F	Empregado Efetivo
	F	Empregado Efetivo
	M	Empregado Efetivo
	M	Empregado Efetivo
	M	Empregado Efetivo
	M	Empregado Efetivo
	F	Empregado Efetivo
	F	Empregado Efetivo
	M	Empregado Efetivo
	F	Empregado Efetivo
	F	Empregado Efetivo
	F	Empregado Efetivo
	F	Empregado Efetivo
	M	Empregado Efetivo
	F	Empregado Efetivo
	F	Empregado Efetivo
	F	Empregado Efetivo
	F	Empregado Efetivo
	F	Empregado Efetivo
	F	Empregado Efetivo
	F	Empregado Efetivo

	M	Empregado Efetivo
	F	Empregado Efetivo
	F	Empregado Efetivo
	F	Empregado Efetivo
	F	Empregado Efetivo
	F	Empregado Efetivo
	F	Empregado Efetivo
	F	Empregado Efetivo
	F	Empregado Efetivo
	F	Empregado Efetivo
	F	Empregado Efetivo
	F	Empregado Efetivo
	F	Empregado Efetivo
	F	Empregado Efetivo
	F	Empregado Efetivo
	F	Empregado Efetivo
	F	Empregado Efetivo
	F	Empregado Efetivo
	F	Empregado Efetivo
	F	Empregado Efetivo
	F	Empregado Efetivo
	F	Empregado Efetivo
	F	Empregado Efetivo
	F	Empregado Efetivo
	F	Empregado Efetivo
	F	Empregado Efetivo
	F	Empregado Efetivo
	M	Empregado Efetivo
	F	Empregado Efetivo
	F	Empregado Efetivo
	M	Empregado Efetivo
	F	Empregado Efetivo
	F	Empregado Efetivo
	M	Empregado Efetivo
	M	Empregado Efetivo
	M	Empregado Efetivo
	F	Empregado Efetivo
	M	Empregado Efetivo
	F	Empregado Efetivo

	F	Empregado Efetivo
	F	Empregado Efetivo
	F	Empregado Efetivo
	F	Empregado Efetivo
	F	Empregado Efetivo
	F	Empregado Efetivo
	F	Empregado Efetivo
	F	Empregado Efetivo
	F	Empregado Efetivo
	F	Empregado Efetivo
	F	Empregado Efetivo
	F	Empregado Efetivo
	F	Empregado Efetivo
	F	Empregado Efetivo
	F	Empregado Efetivo
	F	Empregado Efetivo
	F	Empregado Efetivo
	F	Empregado Efetivo
	F	Empregado Efetivo
	F	Empregado Efetivo
	M	Empregado Efetivo
	M	Empregado Efetivo
	F	Empregado Efetivo
	F	Empregado Efetivo
	F	Empregado Efetivo
	F	Empregado Efetivo
	F	Empregado Efetivo
	F	Empregado Efetivo
	F	Empregado Efetivo
	F	Empregado Efetivo
	F	Empregado Efetivo
	M	Empregado Efetivo
	F	Empregado Efetivo
	F	Empregado Efetivo
	M	Cedido - RJU
	M	Empregado Efetivo
	M	Empregado Efetivo
	F	Empregado Efetivo
	M	Empregado Efetivo

	F	Empregado Efetivo
	F	Empregado Efetivo
	F	Empregado Efetivo
	M	Empregado Efetivo
	F	Empregado Efetivo
	F	Empregado Efetivo
	F	Empregado Efetivo
	F	Empregado Efetivo
	F	Empregado Efetivo
	F	Empregado Efetivo
	F	Empregado Efetivo
	F	Empregado Efetivo
	F	Empregado Efetivo
	F	Empregado Efetivo
	F	Empregado Efetivo
	F	Empregado Efetivo
	F	Empregado Efetivo
	F	Empregado Efetivo
	F	Empregado Efetivo
	F	Empregado Efetivo
	F	Empregado Efetivo
	F	Empregado Efetivo
	F	Empregado Efetivo
	M	Empregado Efetivo
	M	Empregado Efetivo
	M	Empregado Efetivo
	M	Empregado Efetivo
	F	Empregado Efetivo
	M	Empregado Efetivo
	F	Empregado Efetivo
	F	Empregado Efetivo
	F	Empregado Efetivo

	F	Empregado Efetivo
	F	Empregado Efetivo
	F	Empregado Efetivo
	F	Empregado Efetivo
	F	Empregado Efetivo
	F	Empregado Efetivo
	F	Empregado Efetivo
	F	Empregado Efetivo
	F	Empregado Efetivo
	F	Empregado Efetivo
	M	Empregado Efetivo
	M	Empregado Efetivo
	M	Empregado Efetivo
	F	Empregado Efetivo
	M	Empregado Efetivo
	F	Empregado Efetivo
	F	Empregado Efetivo
	F	Empregado Efetivo
	F	Empregado Efetivo
	F	Empregado Efetivo
	F	Empregado Efetivo
	F	Empregado Efetivo
	F	Empregado Efetivo
	F	Empregado Efetivo
	F	Empregado Efetivo
	M	Empregado Efetivo
	M	Empregado Efetivo
	M	Empregado Efetivo
	F	Empregado Efetivo
	M	Empregado Efetivo
	M	Empregado Efetivo
	F	Empregado Efetivo
	F	Empregado Efetivo
	F	Empregado Efetivo
	F	Empregado Efetivo
	M	Empregado Efetivo
	F	Empregado Efetivo
	F	Empregado Efetivo
	M	Empregado Efetivo
	M	Empregado Efetivo
	F	Empregado Efetivo

	M	Empregado Efetivo
	F	Empregado Efetivo
	F	Empregado Efetivo
	M	Empregado Efetivo
	F	Empregado Efetivo
	F	Empregado Efetivo
	F	Empregado Efetivo
	F	Empregado Efetivo
	F	Empregado Efetivo
	F	Empregado Efetivo
	M	Empregado Efetivo
	M	Empregado Efetivo
	M	Empregado Efetivo
	M	Empregado Efetivo
	M	Empregado Efetivo
	M	Empregado Efetivo
	M	Empregado Efetivo
	M	Empregado Efetivo
	F	Empregado Efetivo
	M	Empregado Efetivo
	F	Empregado Efetivo
	F	Empregado Efetivo
	F	Empregado Efetivo
	F	Empregado Efetivo
	F	Empregado Efetivo
	F	Empregado Efetivo
	F	Empregado Efetivo
	F	Empregado Efetivo
	F	Empregado Efetivo
	F	Empregado Efetivo
	F	Empregado Efetivo
	F	Empregado Efetivo
	F	Empregado Efetivo
	F	Empregado Efetivo
	F	Empregado Efetivo
	M	Empregado Efetivo
	F	Empregado Efetivo
	F	Empregado Efetivo
	M	Empregado Efetivo
	F	Empregado Efetivo
	F	Empregado Efetivo
	F	Empregado Efetivo
	M	Empregado Efetivo
	F	Empregado Efetivo
	F	Empregado Efetivo
	M	Empregado Efetivo
	F	Empregado Efetivo

	F	Empregado Efetivo
	F	Empregado Efetivo
	F	Empregado Efetivo
	F	Empregado Efetivo
	F	Empregado Efetivo
	F	Empregado Efetivo
	F	Empregado Efetivo
	F	Empregado Efetivo
	F	Empregado Efetivo
	F	Empregado Efetivo
	F	Empregado Efetivo
	F	Empregado Efetivo
	F	Empregado Efetivo
	F	Empregado Efetivo
	F	Empregado Efetivo
	F	Empregado Efetivo
	F	Empregado Efetivo
	F	Empregado Efetivo
	F	Empregado Efetivo
	F	Empregado Efetivo
	F	Empregado Efetivo
	F	Empregado Efetivo
	F	Empregado Efetivo
	F	Empregado Efetivo
	F	Empregado Efetivo
	F	Empregado Efetivo
	F	Empregado Efetivo
	M	Empregado Efetivo
	F	Empregado Efetivo
	F	Empregado Efetivo
	F	Empregado Efetivo
	F	Empregado Efetivo
	F	Empregado Efetivo
	F	Empregado Efetivo
	F	Empregado Efetivo
	F	Empregado Efetivo
	F	Empregado Efetivo
	M	Empregado Efetivo
	F	Empregado Efetivo
	M	Empregado Efetivo
	M	Empregado Efetivo
	M	Empregado Efetivo
	M	Empregado Efetivo
	M	Empregado Efetivo
	M	Empregado Efetivo
	M	Empregado Efetivo

	F	Empregado Efetivo
	M	Empregado Efetivo
	M	Empregado Efetivo
	M	Empregado Efetivo
	M	Empregado Efetivo
	M	Empregado Efetivo
	M	Empregado Efetivo
	M	Empregado Efetivo
	F	Empregado Efetivo
	M	Empregado Efetivo
	F	Empregado Efetivo
	F	Empregado Efetivo
	M	Empregado Efetivo
	M	Empregado Efetivo
	M	Empregado Efetivo
	M	Empregado Efetivo
	M	Empregado Efetivo
	M	Empregado Efetivo
	M	Empregado Efetivo
	M	Empregado Efetivo
	M	Empregado Efetivo
	M	Empregado Efetivo
	F	Empregado Efetivo
	F	Empregado Efetivo
	F	Empregado Efetivo
	F	Empregado Efetivo
	F	Empregado Efetivo
	F	Empregado Efetivo
	F	Empregado Efetivo
	F	Empregado Efetivo
	F	Empregado Efetivo
	F	Empregado Efetivo
	F	Empregado Efetivo
	F	Empregado Efetivo
	F	Empregado Efetivo
	F	Empregado Efetivo
	F	Empregado Efetivo
	F	Empregado Efetivo
	F	Empregado Efetivo
	F	Empregado Efetivo
	F	Empregado Efetivo
	F	Empregado Efetivo
	F	Empregado Efetivo
	F	Empregado Efetivo
	F	Empregado Efetivo
	F	Empregado Efetivo

	F	Empregado Efetivo
	F	Empregado Efetivo
	F	Empregado Efetivo
	F	Empregado Efetivo
	F	Empregado Efetivo
	F	Empregado Efetivo
	M	Empregado Efetivo
	M	Empregado Efetivo
	F	Cedido - RJU
	M	Empregado Efetivo
	F	Empregado Efetivo
	F	Empregado Efetivo
	M	Empregado Efetivo
	F	Empregado Efetivo
	M	Empregado Efetivo
	F	Empregado Efetivo
	F	Empregado Efetivo
	M	Empregado Efetivo
	M	Empregado Efetivo
	F	Empregado Efetivo
	F	Empregado Efetivo
	F	Empregado Efetivo
	F	Empregado Efetivo
	F	Empregado Efetivo
	F	Empregado Efetivo
	M	Empregado Efetivo
	F	Empregado Efetivo
	F	Empregado Efetivo
	F	Empregado Efetivo
	F	Empregado Efetivo
	F	Empregado Efetivo
	F	Empregado Efetivo
	M	Empregado Efetivo
	F	Empregado Efetivo
	F	Empregado Efetivo
	F	Empregado Efetivo
	F	Empregado Efetivo
	F	Empregado Efetivo
	M	Empregado Efetivo
	F	Empregado Efetivo
	M	Empregado Efetivo
	F	Empregado Efetivo
	F	Empregado Efetivo
	F	Empregado Efetivo
	F	Empregado Efetivo
	M	Empregado Efetivo
	F	Empregado Efetivo

	F	Empregado Efetivo
	M	Empregado Efetivo
	F	Empregado Efetivo
	M	Empregado Efetivo
	F	Empregado Efetivo
	F	Empregado Efetivo
	M	Empregado Efetivo
	F	Empregado Efetivo
	F	Empregado Efetivo
	F	Empregado Efetivo
	F	Empregado Efetivo
	M	Cedido - RJU
	F	Empregado Efetivo
	F	Empregado Efetivo
	F	Empregado Efetivo
	M	Empregado Efetivo
	F	Empregado Efetivo
	F	Empregado Efetivo
	F	Empregado Efetivo
	F	Empregado Efetivo
	F	Empregado Efetivo
	M	Empregado Efetivo
	M	Empregado Efetivo
	M	Empregado Efetivo
	M	Empregado Efetivo
	M	Empregado Efetivo
	M	Empregado Efetivo
	M	Empregado Efetivo
	M	Empregado Efetivo
	M	Empregado Efetivo
	M	Empregado Efetivo
	M	Empregado Efetivo
	M	Empregado Efetivo
	M	Empregado Efetivo
	M	Empregado Efetivo
	F	Empregado Efetivo
	F	Empregado Efetivo
	M	Empregado Efetivo
	F	Empregado Efetivo
	F	Empregado Efetivo
	M	Empregado Efetivo
	F	Empregado Efetivo
	F	Empregado Efetivo
	F	Empregado Efetivo
	F	Empregado Efetivo
	F	Empregado Efetivo
	M	Empregado Efetivo
	F	Empregado Efetivo

	F	Empregado Efetivo
	F	Empregado Efetivo
	F	Empregado Efetivo
	M	Empregado Efetivo
	F	Empregado Efetivo
	F	Empregado Efetivo
	F	Empregado Efetivo
	F	Empregado Efetivo
	F	Empregado Efetivo
	M	Empregado Efetivo
	F	Empregado Efetivo
	M	Empregado Efetivo
	M	Empregado Efetivo
	F	Empregado Efetivo
	F	Empregado Efetivo
	F	Empregado Efetivo
	F	Empregado Efetivo
	F	Empregado Efetivo
	F	Empregado Efetivo
	F	Empregado Efetivo
	F	Empregado Efetivo
	F	Empregado Efetivo
	F	Empregado Efetivo
	F	Empregado Efetivo
	F	Empregado Efetivo
	F	Empregado Efetivo
	F	Empregado Efetivo
	F	Empregado Efetivo
	F	Empregado Efetivo
	F	Empregado Efetivo
	F	Empregado Efetivo
	F	Empregado Efetivo
	F	Empregado Efetivo
	F	Empregado Efetivo
	F	Empregado Efetivo
	F	Empregado Efetivo
	F	Empregado Efetivo
	F	Empregado Efetivo
	F	Empregado Efetivo
	F	Empregado Efetivo
	M	Empregado Efetivo
	F	Empregado Efetivo
	F	Empregado Efetivo
	F	Empregado Efetivo
	F	Empregado Efetivo
	F	Empregado Efetivo
	F	Empregado Efetivo
	F	Empregado Efetivo

	F	Empregado Efetivo
	F	Empregado Efetivo
	F	Empregado Efetivo
	F	Empregado Efetivo
	F	Empregado Efetivo
	F	Empregado Efetivo
	F	Empregado Efetivo
	F	Empregado Efetivo
	F	Empregado Efetivo
	F	Empregado Efetivo
	F	Empregado Efetivo
	F	Empregado Efetivo
	F	Empregado Efetivo
	F	Empregado Efetivo
	F	Empregado Efetivo
	F	Empregado Efetivo
	F	Empregado Efetivo
	F	Empregado Efetivo
	F	Empregado Efetivo
	F	Empregado Efetivo
	F	Empregado Efetivo
	M	Empregado Efetivo
	F	Empregado Efetivo
	F	Empregado Efetivo
	F	Empregado Efetivo
	F	Empregado Efetivo
	F	Empregado Efetivo
	F	Empregado Efetivo
	F	Empregado Efetivo
	F	Empregado Efetivo
	F	Empregado Efetivo
	M	Empregado Efetivo
	F	Empregado Efetivo
	M	Empregado Efetivo
	F	Empregado Efetivo
	F	Empregado Efetivo
	F	Empregado Efetivo
	F	Empregado Efetivo
	F	Empregado Efetivo
	F	Empregado Efetivo
	F	Empregado Efetivo
	F	Empregado Efetivo
	M	Empregado Efetivo
	F	Empregado Efetivo
	M	Empregado Efetivo
	F	Empregado Efetivo
	M	Empregado Efetivo
	F	Empregado Efetivo

	F	Empregado Efetivo
	M	Empregado Efetivo
	F	Empregado Efetivo
	F	Empregado Efetivo
	F	Empregado Efetivo
	M	Empregado Efetivo
	M	Empregado Efetivo
	F	Empregado Efetivo
	F	Empregado Efetivo
	F	Empregado Efetivo
	F	Empregado Efetivo
	F	Empregado Efetivo
	M	Empregado Efetivo
	F	Empregado Efetivo
	F	Empregado Efetivo
	F	Empregado Efetivo
	F	Empregado Efetivo
	F	Empregado Efetivo
	M	Empregado Efetivo
	F	Empregado Efetivo
	F	Empregado Efetivo
	F	Empregado Efetivo
	F	Empregado Efetivo
	F	Empregado Efetivo
	F	Empregado Efetivo
	M	Empregado Efetivo
	F	Empregado Efetivo
	M	Empregado Efetivo
	F	Empregado Efetivo
	M	Empregado Efetivo
	F	Empregado Efetivo
	M	Empregado Efetivo
	F	Empregado Efetivo
	F	Empregado Efetivo
	M	Empregado Efetivo
	M	Empregado Efetivo
	F	Empregado Efetivo
	F	Empregado Efetivo
	M	Empregado Efetivo
	M	Empregado Efetivo
	F	Empregado Efetivo
	F	Empregado Efetivo
	F	Empregado Efetivo
	F	Empregado Efetivo
	F	Empregado Efetivo
	F	Empregado Efetivo
	F	Empregado Efetivo
	M	Empregado Efetivo

	F	Empregado Efetivo
	F	Empregado Efetivo
	F	Empregado Efetivo
	M	Empregado Efetivo
	M	Empregado Efetivo
	F	Empregado Efetivo
	F	Empregado Efetivo
	M	Empregado Efetivo
	F	Empregado Efetivo
	F	Empregado Efetivo
	F	Empregado Efetivo
	F	Empregado Efetivo
	F	Empregado Efetivo
	F	Empregado Efetivo
	F	Empregado Efetivo
	F	Empregado Efetivo
	F	Empregado Efetivo
	F	Empregado Efetivo
	F	Empregado Efetivo
	F	Empregado Efetivo
	F	Empregado Efetivo
	F	Empregado Efetivo
	F	Empregado Efetivo
	F	Empregado Efetivo
	F	Empregado Efetivo
	F	Empregado Efetivo
	F	Empregado Efetivo
	M	Empregado Efetivo
	F	Empregado Efetivo
	F	Empregado Efetivo
	F	Empregado Efetivo
	F	Empregado Efetivo
	F	Empregado Efetivo
	F	Empregado Efetivo
	F	Empregado Efetivo
	F	Empregado Efetivo
	F	Empregado Efetivo
	F	Empregado Efetivo
	F	Empregado Efetivo
	F	Empregado Efetivo
	F	Empregado Efetivo
	M	Empregado Efetivo
	F	Empregado Efetivo
	F	Empregado Efetivo
	F	Empregado Efetivo
	F	Empregado Efetivo
	F	Empregado Efetivo
	F	Empregado Efetivo

	F	Empregado Efetivo
	F	Empregado Efetivo
	F	Empregado Efetivo
	F	Empregado Efetivo
	F	Empregado Efetivo
	F	Empregado Efetivo
	F	Empregado Efetivo
	F	Empregado Efetivo
	M	Empregado Efetivo
	F	Empregado Efetivo
	M	Empregado Efetivo
	F	Empregado Efetivo
	F	Empregado Efetivo
	F	Empregado Efetivo
	F	Empregado Efetivo
	F	Empregado Efetivo
	F	Empregado Efetivo
	M	Empregado Efetivo
	M	Empregado Efetivo
	F	Empregado Efetivo
	F	Empregado Efetivo
	F	Empregado Efetivo
	F	Empregado Efetivo
	M	Empregado Efetivo
	F	Empregado Efetivo
	F	Empregado Efetivo
	F	Empregado Efetivo
	F	Empregado Efetivo
	M	Empregado Efetivo
	M	Empregado Efetivo
	M	Empregado Efetivo
	M	Empregado Efetivo
	F	Empregado Efetivo
	M	Empregado Efetivo
	M	Empregado Efetivo
	F	Empregado Efetivo
	F	Empregado Efetivo
	F	Empregado Efetivo
	F	Empregado Efetivo
	F	Empregado Efetivo
	F	Empregado Efetivo
	M	Empregado Efetivo
	F	Empregado Efetivo
	F	Empregado Efetivo
	F	Empregado Efetivo

	F	Empregado Efetivo
	M	Empregado Efetivo
	M	Empregado Efetivo
	F	Empregado Efetivo
	F	Empregado Efetivo
	F	Empregado Efetivo
	F	Empregado Efetivo
	F	Empregado Efetivo
	F	Empregado Efetivo
	F	Empregado Efetivo
	F	Empregado Efetivo
	F	Empregado Efetivo
	F	Empregado Efetivo
	F	Empregado Efetivo
	M	Empregado Efetivo
	F	Empregado Efetivo
	F	Empregado Efetivo
	F	Empregado Efetivo
	F	Empregado Efetivo
	F	Empregado Efetivo
	F	Empregado Efetivo
	F	Empregado Efetivo
	F	Empregado Efetivo
	F	Empregado Efetivo
	M	Empregado Efetivo
	F	Empregado Efetivo
	F	Empregado Efetivo
	F	Empregado Efetivo
	F	Empregado Efetivo
	F	Empregado Efetivo
	F	Empregado Efetivo
	F	Empregado Efetivo
	F	Empregado Efetivo
	F	Empregado Efetivo
	M	Empregado Efetivo
	M	Empregado Efetivo
	F	Empregado Efetivo
	M	Empregado Efetivo
	M	Empregado Efetivo
	M	Empregado Efetivo
	F	Empregado Efetivo
	M	Empregado Efetivo
	M	Empregado Efetivo
	M	Empregado Efetivo

	M	Empregado Efetivo
	M	Empregado Efetivo
	M	Empregado Efetivo
	F	Empregado Efetivo
	M	Empregado Efetivo
	F	Empregado Efetivo
	M	Empregado Efetivo
	M	Empregado Efetivo
	F	Empregado Efetivo
	M	Empregado Efetivo
	F	Empregado Efetivo
	F	Empregado Efetivo
	F	Empregado Efetivo
	F	Empregado Efetivo
	F	Empregado Efetivo
	F	Empregado Efetivo
	M	Empregado Efetivo
	M	Empregado Efetivo
	F	Empregado Efetivo
	F	Empregado Efetivo
	M	Empregado Efetivo
	M	Empregado Efetivo
	M	Empregado Efetivo
	M	Empregado Efetivo
	M	Empregado Efetivo
	F	Empregado Efetivo
	M	Empregado Efetivo
	F	Empregado Efetivo
	F	Empregado Efetivo
	M	Empregado Efetivo
	F	Empregado Efetivo
	F	Empregado Efetivo
	M	Empregado Efetivo
	F	Empregado Efetivo
	M	Empregado Efetivo
	M	Empregado Efetivo
	M	Empregado Efetivo
	M	Empregado Efetivo
	M	Empregado Efetivo
	M	Empregado Efetivo
	M	Empregado Efetivo
	F	Empregado Efetivo
	F	Empregado Efetivo

	F	Empregado Efetivo
	F	Empregado Efetivo
	F	Empregado Efetivo
	F	Empregado Efetivo
	F	Empregado Efetivo
	F	Empregado Efetivo
	F	Empregado Efetivo
	F	Empregado Efetivo
	M	Empregado Efetivo
	F	Empregado Efetivo
	F	Empregado Efetivo
	F	Empregado Efetivo
	F	Empregado Efetivo
	F	Empregado Efetivo
	M	Empregado Efetivo
	M	Empregado Efetivo
	M	Empregado Efetivo
	M	Empregado Efetivo
	M	Empregado Efetivo
	M	Empregado Efetivo
	M	Empregado Efetivo
	M	Empregado Efetivo
	M	Empregado Efetivo
	M	Empregado Efetivo
	M	Empregado Efetivo
	M	Empregado Efetivo
	F	Empregado Efetivo
	F	Empregado Efetivo
	F	Empregado Efetivo
	F	Empregado Efetivo
	M	Empregado Efetivo
	M	Empregado Efetivo

	F	Empregado Efetivo
	F	Empregado Efetivo
	F	Empregado Efetivo
	F	Empregado Efetivo
	M	Empregado Efetivo
	F	Empregado Efetivo
	M	Empregado Efetivo
	F	Empregado Efetivo
	M	Empregado Efetivo
	M	Empregado Efetivo
	F	Empregado Efetivo
	M	Empregado Efetivo
	M	Empregado Efetivo
	F	Empregado Efetivo
	F	Empregado Efetivo
	F	Empregado Efetivo
	F	Empregado Efetivo
	M	Empregado Efetivo
	F	Empregado Efetivo
	M	Empregado Efetivo
	M	Empregado Efetivo
	M	Empregado Efetivo
	F	Empregado Efetivo
	F	Empregado Efetivo
	M	Empregado Efetivo
	F	Empregado Efetivo
	F	Empregado Efetivo
	F	Empregado Efetivo
	F	Empregado Efetivo
	M	Empregado Efetivo
	M	Empregado Efetivo
	M	Empregado Efetivo
	F	Empregado Efetivo
	F	Empregado Efetivo
	M	Empregado Efetivo
	M	Empregado Efetivo
	F	Empregado Efetivo
	M	Empregado Efetivo
	F	Empregado Efetivo
	F	Empregado Efetivo
	F	Empregado Efetivo
	M	Empregado Efetivo

	F	Empregado Efetivo
	M	Empregado Efetivo
	F	Empregado Efetivo
	M	Empregado Efetivo
	F	Empregado Efetivo
	F	Empregado Efetivo
	F	Empregado Efetivo
	F	Empregado Efetivo
	F	Empregado Efetivo
	F	Empregado Efetivo
	M	Empregado Efetivo
	F	Empregado Efetivo
	F	Empregado Efetivo
	F	Empregado Efetivo
	F	Empregado Efetivo
	M	Empregado Efetivo
	F	Empregado Efetivo
	M	Empregado Efetivo
	F	Empregado Efetivo
	F	Empregado Efetivo
	F	Empregado Efetivo
	F	Empregado Efetivo
	F	Empregado Efetivo
	M	Empregado Efetivo
	F	Empregado Efetivo
	F	Empregado Efetivo
	M	Empregado Efetivo
	M	Empregado Efetivo
	F	Empregado Efetivo
	M	Empregado Efetivo
	M	Empregado Efetivo
	M	Empregado Efetivo
	F	Empregado Efetivo
	M	Empregado Efetivo
	M	Empregado Efetivo
	F	Empregado Efetivo
	M	Empregado Efetivo
	F	Empregado Efetivo
	F	Empregado Efetivo
	M	Empregado Efetivo
	M	Empregado Efetivo
	M	Empregado Efetivo
	M	Empregado Efetivo
	M	Empregado Efetivo
	M	Empregado Efetivo
	F	Empregado Efetivo
	M	Empregado Efetivo
	F	Empregado Efetivo

	M	Empregado Efetivo
	M	Empregado Efetivo
	M	Empregado Efetivo
	F	Empregado Efetivo
	F	Empregado Efetivo
	M	Empregado Efetivo
	M	Empregado Efetivo
	F	Empregado Efetivo
	M	Empregado Efetivo
	F	Empregado Efetivo
	F	Empregado Efetivo
	F	Empregado Efetivo
	F	Empregado Efetivo
	F	Empregado Efetivo
	F	Empregado Efetivo
	M	Empregado Efetivo
	F	Empregado Efetivo
	F	Empregado Efetivo
	M	Empregado Efetivo
	F	Empregado Efetivo
	M	Empregado Efetivo
	F	Empregado Efetivo
	M	Empregado Efetivo
	M	Empregado Efetivo
	F	Empregado Efetivo
	M	Empregado Efetivo
	F	Empregado Efetivo
	F	Empregado Efetivo
	F	Empregado Efetivo
	F	Empregado Efetivo
	M	Empregado Efetivo
	M	Empregado Efetivo
	M	Empregado Efetivo
	F	Empregado Efetivo
	F	Empregado Efetivo
	F	Empregado Efetivo
	F	Empregado Efetivo
	F	Empregado Efetivo
	F	Empregado Efetivo
	F	Empregado Efetivo
	F	Empregado Efetivo
	M	Empregado Efetivo
	M	Empregado Efetivo
	F	Empregado Efetivo
	M	Empregado Efetivo
	F	Empregado Efetivo
	F	Empregado Efetivo
	M	Empregado Efetivo

	M	Empregado Efetivo
	F	Empregado Efetivo
	M	Empregado Efetivo
	F	Empregado Efetivo
	F	Empregado Efetivo
	F	Empregado Efetivo
	F	Empregado Efetivo
	F	Empregado Efetivo
	F	Empregado Efetivo
	F	Empregado Efetivo
	F	Empregado Efetivo
	F	Empregado Efetivo
	F	Empregado Efetivo
	F	Empregado Efetivo
	F	Empregado Efetivo
	F	Empregado Efetivo
	F	Empregado Efetivo
	F	Empregado Efetivo
	F	Empregado Efetivo
	F	Empregado Efetivo
	F	Empregado Efetivo
	F	Empregado Efetivo
	M	Empregado Efetivo
	F	Empregado Efetivo
	M	Empregado Efetivo
	F	Empregado Efetivo
	M	Empregado Efetivo
	F	Empregado Efetivo
	F	Empregado Efetivo
	M	Empregado Efetivo
	F	Empregado Efetivo
	F	Empregado Efetivo
	F	Empregado Efetivo
	F	Empregado Efetivo
	F	Empregado Efetivo
	F	Empregado Efetivo
	F	Empregado Efetivo
	F	Empregado Efetivo
	F	Empregado Efetivo
	F	Empregado Efetivo
	M	Empregado Efetivo
	F	Empregado Efetivo
	F	Empregado Efetivo
	F	Empregado Efetivo
	F	Empregado Efetivo
	M	Empregado Efetivo
	F	Empregado Efetivo
	F	Empregado Efetivo

	M	Empregado Efetivo
	F	Empregado Efetivo
	F	Empregado Efetivo
	F	Empregado Efetivo
	F	Empregado Efetivo
	F	Empregado Efetivo
	M	Empregado Efetivo
	F	Empregado Efetivo
	F	Empregado Efetivo
	F	Empregado Efetivo
	F	Empregado Efetivo
	M	Empregado Efetivo
	F	Empregado Efetivo
	F	Empregado Efetivo
	F	Empregado Efetivo
	F	Empregado Efetivo
	F	Empregado Efetivo
	F	Empregado Efetivo
	F	Empregado Efetivo
	F	Empregado Efetivo
	F	Empregado Efetivo
	F	Empregado Efetivo
	F	Empregado Efetivo
	F	Empregado Efetivo
	M	Empregado Efetivo
	F	Empregado Efetivo
	M	Empregado Efetivo
	M	Empregado Efetivo
	F	Empregado Efetivo
	M	Empregado Efetivo
	M	Empregado Efetivo
	M	Empregado Efetivo
	M	Empregado Efetivo
	F	Empregado Efetivo
	F	Empregado Efetivo
	F	Empregado Efetivo
	F	Empregado Efetivo
	F	Empregado Efetivo
	F	Empregado Efetivo
	F	Empregado Efetivo
	F	Empregado Efetivo
	F	Empregado Efetivo
	F	Empregado Efetivo
	F	Empregado Efetivo
	F	Empregado Efetivo
	M	Empregado Efetivo
	F	Empregado Efetivo
	F	Empregado Efetivo

	F	Empregado Efetivo
	F	Empregado Efetivo
	F	Empregado Efetivo
	F	Empregado Efetivo
	F	Empregado Efetivo
	M	Empregado Efetivo
	F	Empregado Efetivo
	F	Empregado Efetivo
	F	Empregado Efetivo
	M	Empregado Efetivo
	F	Empregado Efetivo
	F	Empregado Efetivo
	F	Empregado Efetivo
	F	Empregado Efetivo
	F	Empregado Efetivo
	M	Empregado Efetivo
	F	Empregado Efetivo
	M	Empregado Efetivo
	F	Empregado Efetivo
	F	Empregado Efetivo
	F	Empregado Efetivo
	F	Empregado Efetivo
	F	Empregado Efetivo
	F	Empregado Efetivo
	F	Empregado Efetivo
	M	Empregado Efetivo
	F	Empregado Efetivo
	M	Empregado Efetivo
	F	Empregado Efetivo
	F	Empregado Efetivo
	M	Empregado Efetivo
	F	Empregado Efetivo
	F	Empregado Efetivo
	M	Empregado Efetivo
	F	Empregado Efetivo
	F	Empregado Efetivo
	F	Empregado Efetivo
	F	Empregado Efetivo
	F	Empregado Efetivo
	F	Empregado Efetivo
	F	Empregado Efetivo
	F	Empregado Efetivo
	F	Empregado Efetivo
	F	Empregado Efetivo
	F	Empregado Efetivo
	M	Empregado Efetivo
	F	Empregado Efetivo
	F	Empregado Efetivo

	F	Empregado Efetivo
	F	Empregado Efetivo
	F	Empregado Efetivo
	F	Empregado Efetivo
	F	Empregado Efetivo
	F	Empregado Efetivo
	F	Empregado Efetivo
	M	Empregado Efetivo
	F	Empregado Efetivo
	M	Empregado Efetivo
	F	Empregado Efetivo
	M	Empregado Efetivo
	M	Empregado Efetivo
	M	Empregado Efetivo
	F	Empregado Efetivo
	M	Empregado Efetivo
	M	Empregado Efetivo
	M	Empregado Efetivo
	M	Empregado Efetivo
	F	Empregado Efetivo
	M	Empregado Efetivo
	F	Empregado Efetivo
	M	Empregado Efetivo
	M	Empregado Efetivo
	M	Empregado Efetivo
	M	Empregado Efetivo
	M	Empregado Efetivo
	M	Empregado Efetivo
	F	Empregado Efetivo
	M	Empregado Efetivo
	F	Empregado Efetivo
	F	Empregado Efetivo
	F	Empregado Efetivo
	F	Empregado Efetivo
	M	Empregado Efetivo
	F	Empregado Efetivo
	F	Empregado Efetivo
	F	Empregado Efetivo
	F	Empregado Efetivo
	F	Empregado Efetivo
	M	Empregado Efetivo

	M	Empregado Efetivo
	M	Empregado Efetivo
	F	Empregado Efetivo
	F	Empregado Efetivo
	M	Empregado Efetivo
	M	Empregado Efetivo
	M	Empregado Efetivo
	M	Empregado Efetivo
	M	Empregado Efetivo
	M	Empregado Efetivo
	M	Empregado Efetivo
	F	Empregado Efetivo
	M	Empregado Efetivo
	M	Empregado Efetivo
	F	Empregado Efetivo
	F	Empregado Efetivo
	F	Empregado Efetivo
	M	Empregado Efetivo
	F	Empregado Efetivo
	M	Empregado Efetivo
	F	Empregado Efetivo
	M	Empregado Efetivo
	F	Empregado Efetivo
	M	Empregado Efetivo
	F	Empregado Efetivo
	M	Empregado Efetivo
	M	Empregado Efetivo
	M	Empregado Efetivo
	F	Empregado Efetivo
	F	Empregado Efetivo
	F	Empregado Efetivo
	F	Empregado Efetivo
	F	Empregado Efetivo
	F	Empregado Efetivo
	F	Empregado Efetivo
	M	Empregado Efetivo
	F	Empregado Efetivo
	F	Empregado Efetivo
	F	Empregado Efetivo
	M	Empregado Efetivo
	F	Empregado Efetivo
	M	Empregado Efetivo

	F	Empregado Efetivo
	F	Empregado Efetivo
	M	Empregado Efetivo
	M	Empregado Efetivo
	M	Empregado Efetivo
	M	Empregado Efetivo
	F	Empregado Efetivo
	F	Empregado Efetivo
	F	Empregado Efetivo
	M	Empregado Efetivo
	M	Empregado Efetivo
	M	Empregado Efetivo
	M	Empregado Efetivo
	M	Empregado Efetivo
	M	Empregado Efetivo
	M	Empregado Efetivo
	F	Empregado Efetivo
	M	Empregado Efetivo
	M	Empregado Efetivo
	F	Empregado Efetivo
	M	Empregado Efetivo
	M	Empregado Efetivo
	M	Empregado Efetivo
	M	Empregado Efetivo
	F	Empregado Efetivo
	F	Empregado Efetivo
	F	Empregado Efetivo
	F	Empregado Efetivo
	M	Empregado Efetivo
	M	Empregado Efetivo
	M	Empregado Efetivo
	F	Empregado Efetivo
	F	Empregado Efetivo
	F	Empregado Efetivo
	F	Empregado Efetivo
	F	Empregado Efetivo
	F	Empregado Efetivo
	F	Empregado Efetivo
	F	Empregado Efetivo
	F	Empregado Efetivo
	F	Empregado Efetivo
	F	Empregado Efetivo
	F	Empregado Efetivo
	F	Empregado Efetivo
	F	Empregado Efetivo
	F	Empregado Efetivo
	F	Empregado Efetivo
	F	Empregado Efetivo

	F	Empregado Efetivo
	F	Empregado Efetivo
	F	Empregado Efetivo
	F	Empregado Efetivo
	F	Empregado Efetivo
	M	Empregado Efetivo
	M	Empregado Efetivo
	F	Empregado Efetivo
	F	Empregado Efetivo
	F	Empregado Efetivo
	F	Empregado Efetivo
	F	Empregado Efetivo
	M	Empregado Efetivo
	F	Empregado Efetivo
	M	Empregado Efetivo
	F	Empregado Efetivo
	F	Empregado Efetivo
	M	Empregado Efetivo
	M	Empregado Efetivo
	M	Empregado Efetivo
	F	Empregado Efetivo
	M	Empregado Efetivo
	F	Empregado Efetivo
	F	Empregado Efetivo
	F	Empregado Efetivo
	F	Empregado Efetivo
	F	Empregado Efetivo
	F	Empregado Efetivo
	F	Empregado Efetivo
	M	Empregado Efetivo
	F	Empregado Efetivo
	F	Empregado Efetivo
	F	Empregado Efetivo
	F	Empregado Efetivo
	M	Empregado Efetivo
	F	Empregado Efetivo
	F	Empregado Efetivo
	F	Empregado Efetivo
	F	Empregado Efetivo
	F	Empregado Efetivo
	M	Empregado Efetivo
	F	Empregado Efetivo
	F	Empregado Efetivo
	M	Empregado Efetivo
	M	Empregado Efetivo
	M	Empregado Efetivo
	M	Empregado Efetivo
	F	Empregado Efetivo

	F	Empregado Efetivo
	M	Empregado Efetivo
	F	Empregado Efetivo
	F	Empregado Efetivo
	M	Empregado Efetivo
	F	Empregado Efetivo
	F	Empregado Efetivo
	M	Empregado Efetivo
	M	Empregado Efetivo
	F	Empregado Efetivo
	M	Empregado Efetivo
	F	Empregado Efetivo
	M	Empregado Efetivo
	F	Empregado Efetivo
	F	Empregado Efetivo
	F	Empregado Efetivo
	M	Empregado Efetivo
	F	Empregado Efetivo
	M	Empregado Efetivo
	M	Empregado Efetivo
	F	Empregado Efetivo
	M	Empregado Efetivo
	F	Empregado Efetivo
	M	Empregado Efetivo
	M	Empregado Efetivo
	F	Empregado Efetivo
	F	Empregado Efetivo
	F	Empregado Efetivo
	F	Empregado Efetivo
	F	Empregado Efetivo
	F	Empregado Efetivo
	F	Empregado Efetivo
	F	Empregado Efetivo
	F	Empregado Efetivo
	F	Empregado Efetivo
	F	Empregado Efetivo
	F	Empregado Efetivo
	F	Empregado Efetivo
	M	Empregado Efetivo
	M	Empregado Efetivo
	F	Empregado Efetivo
	F	Empregado Efetivo
	F	Empregado Efetivo
	F	Empregado Efetivo
	F	Empregado Efetivo
	M	Empregado Efetivo
	M	Empregado Efetivo
	F	Empregado Efetivo

	F	Empregado Efetivo
	F	Empregado Efetivo
	M	Empregado Efetivo
	F	Empregado Efetivo
	F	Empregado Efetivo
	F	Empregado Efetivo
	F	Empregado Efetivo
	F	Empregado Efetivo
	F	Empregado Efetivo
	F	Empregado Efetivo
	F	Empregado Efetivo
	F	Empregado Efetivo
	F	Empregado Efetivo
	M	Empregado Efetivo
	F	Empregado Efetivo
	F	Empregado Efetivo
	F	Empregado Efetivo
	F	Empregado Efetivo
	F	Empregado Efetivo
	F	Empregado Efetivo
	F	Empregado Efetivo
	M	Empregado Efetivo
	F	Empregado Efetivo
	F	Empregado Efetivo
	F	Empregado Efetivo
	F	Empregado Efetivo
	F	Empregado Efetivo
	F	Empregado Efetivo
	M	Empregado Efetivo
	F	Empregado Efetivo
	F	Empregado Efetivo
	F	Empregado Efetivo
	F	Empregado Efetivo
	M	Empregado Efetivo
	F	Empregado Efetivo
	F	Empregado Efetivo
	F	Empregado Efetivo
	F	Empregado Efetivo
	M	Empregado Efetivo
	M	Empregado Efetivo
	M	Empregado Efetivo
	F	Empregado Efetivo
	M	Empregado Efetivo
	M	Empregado Efetivo
	M	Empregado Efetivo
	F	Empregado Efetivo
	F	Empregado Efetivo

	F	Empregado Efetivo
	M	Empregado Efetivo
	M	Empregado Efetivo
	F	Empregado Efetivo
	M	Empregado Efetivo
	F	Empregado Efetivo
	M	Empregado Efetivo
	F	Empregado Efetivo
	F	Empregado Efetivo
	F	Empregado Efetivo
	F	Empregado Efetivo
	F	Empregado Efetivo
	F	Empregado Efetivo
	F	Empregado Efetivo
	F	Empregado Efetivo
	M	Empregado Efetivo
	F	Empregado Efetivo
	F	Empregado Efetivo
	F	Empregado Efetivo
	F	Empregado Efetivo
	F	Empregado Efetivo
	M	Empregado Efetivo
	M	Empregado Efetivo
	M	Empregado Efetivo
	M	Empregado Efetivo
	M	Empregado Efetivo
	M	Empregado Efetivo
	M	Empregado Efetivo
	M	Empregado Efetivo
	M	Empregado Efetivo
	M	Empregado Efetivo
	F	Empregado Efetivo
	M	Empregado Efetivo
	F	Empregado Efetivo
	F	Empregado Efetivo
	F	Empregado Efetivo
	M	Empregado Efetivo
	M	Empregado Efetivo
	F	Empregado Efetivo
	F	Empregado Efetivo
	F	Empregado Efetivo
	F	Empregado Efetivo
	F	Empregado Efetivo
	F	Empregado Efetivo
	F	Empregado Efetivo
	F	Empregado Efetivo
	F	Empregado Efetivo

	F	Empregado Efetivo
	F	Empregado Efetivo
	F	Empregado Efetivo
	F	Empregado Efetivo
	F	Empregado Efetivo
	F	Empregado Efetivo
	F	Empregado Efetivo
	F	Empregado Efetivo
	F	Empregado Efetivo
	F	Empregado Efetivo
	F	Empregado Efetivo
	F	Empregado Efetivo
	F	Empregado Efetivo
	F	Empregado Efetivo
	F	Empregado Efetivo
	F	Empregado Efetivo
	F	Empregado Efetivo
	F	Empregado Efetivo
	F	Empregado Efetivo
	F	Empregado Efetivo
	F	Empregado Efetivo
	F	Empregado Efetivo
	F	Empregado Efetivo
	F	Empregado Efetivo
	F	Empregado Efetivo
	F	Empregado Efetivo
	F	Empregado Efetivo
	F	Empregado Efetivo
	F	Empregado Efetivo
	F	Empregado Efetivo
	F	Empregado Efetivo
	F	Empregado Efetivo
	F	Empregado Efetivo
	F	Empregado Efetivo
	M	Empregado Efetivo
	M	Empregado Efetivo
	M	Empregado Efetivo
	M	Empregado Efetivo
	M	Empregado Efetivo
	M	Empregado Efetivo
	M	Empregado Efetivo
	M	Cedido - RJU
	F	Empregado Efetivo
	M	Cedido - RJU

	M	Cedido - RJU
	M	Empregado Efetivo
	F	Empregado Efetivo
	F	Empregado Efetivo
	F	Cedido - RJU
	F	Empregado Efetivo
	F	Empregado Efetivo
	F	Empregado Efetivo
	F	Empregado Efetivo
	M	Empregado Efetivo
	F	Empregado Efetivo
	M	Empregado Efetivo
	M	Empregado Efetivo
	F	Empregado Efetivo
	M	Cedido - RJU
	M	Empregado Efetivo
	F	Empregado Efetivo
	M	Empregado Efetivo
	F	Empregado Efetivo
	M	Empregado Efetivo
	M	Empregado Efetivo
	F	Empregado Efetivo
	F	Empregado Efetivo
	M	Empregado Efetivo
	M	Empregado Efetivo
	F	Empregado Efetivo
	F	Empregado Efetivo
	M	Empregado Efetivo
	M	Empregado Efetivo
	M	Empregado Efetivo
	M	Empregado Efetivo
	M	Empregado Efetivo
	M	Empregado Efetivo
	M	Empregado Efetivo
	M	Empregado Efetivo
	F	Empregado Efetivo
	M	Empregado Efetivo
	F	Empregado Efetivo
	M	Empregado Efetivo
	F	Empregado Efetivo
	M	Empregado Efetivo

	F	Empregado Efetivo
	M	Empregado Efetivo
	F	Empregado Efetivo
	F	Empregado Efetivo
	F	Empregado Efetivo
	F	Empregado Efetivo
	F	Empregado Efetivo
	F	Empregado Efetivo
	F	Empregado Efetivo
	F	Empregado Efetivo
	F	Empregado Efetivo
	F	Empregado Efetivo
	F	Empregado Efetivo
	F	Empregado Efetivo
	F	Empregado Efetivo
	F	Empregado Efetivo
	F	Empregado Efetivo
	F	Empregado Efetivo
	F	Empregado Efetivo
	F	Empregado Efetivo
	F	Empregado Efetivo
	M	Empregado Efetivo
	F	Empregado Efetivo
	F	Empregado Efetivo
	F	Empregado Efetivo
	F	Empregado Efetivo
	F	Empregado Efetivo
	F	Empregado Efetivo
	F	Empregado Efetivo
	F	Empregado Efetivo
	F	Empregado Efetivo
	F	Empregado Efetivo
	F	Empregado Efetivo
	F	Empregado Efetivo
	F	Empregado Efetivo
	F	Empregado Efetivo
	F	Empregado Efetivo
	F	Empregado Efetivo
	F	Empregado Efetivo
	F	Empregado Efetivo
	F	Empregado Efetivo
	F	Empregado Efetivo
	M	Empregado Efetivo

	M	Empregado Efetivo
	F	Empregado Efetivo
	F	Empregado Efetivo
	F	Empregado Efetivo
	F	Empregado Efetivo
	F	Empregado Efetivo
	F	Empregado Efetivo
	F	Empregado Efetivo
	F	Empregado Efetivo
	F	Empregado Efetivo
	M	Empregado Efetivo
	F	Empregado Efetivo
	M	Empregado Efetivo
	F	Empregado Efetivo
	F	Empregado Efetivo
	F	Empregado Efetivo
	F	Empregado Efetivo
	F	Empregado Efetivo
	F	Empregado Efetivo
	F	Empregado Efetivo
	F	Empregado Efetivo
	F	Empregado Efetivo
	F	Empregado Efetivo
	F	Empregado Efetivo
	F	Empregado Efetivo
	F	Empregado Efetivo
	F	Empregado Efetivo
	F	Empregado Efetivo
	F	Empregado Efetivo
	F	Empregado Efetivo
	F	Empregado Efetivo
	F	Empregado Efetivo
	M	Empregado Efetivo
	F	Empregado Efetivo
	F	Empregado Efetivo
	M	Empregado Efetivo
	M	Empregado Efetivo
	M	Empregado Efetivo
	M	Empregado Efetivo
	F	Empregado Efetivo
	F	Empregado Efetivo
	F	Empregado Efetivo
	F	Empregado Efetivo
	F	Empregado Efetivo
	F	Empregado Efetivo
	M	Empregado Efetivo

	F	Empregado Efetivo
	F	Empregado Efetivo
	F	Empregado Efetivo
	M	Empregado Efetivo
	F	Empregado Efetivo
	F	Empregado Efetivo
	M	Empregado Efetivo
	M	Empregado Efetivo
	F	Empregado Efetivo
	F	Empregado Efetivo
	F	Empregado Efetivo
	F	Empregado Efetivo
	F	Empregado Efetivo
	F	Empregado Efetivo
	F	Empregado Efetivo
	F	Empregado Efetivo
	F	Empregado Efetivo
	F	Empregado Efetivo
	M	Empregado Efetivo
	F	Empregado Efetivo
	M	Empregado Efetivo
	F	Empregado Efetivo
	F	Empregado Efetivo
	F	Empregado Efetivo
	F	Empregado Efetivo
	F	Empregado Efetivo
	F	Empregado Efetivo
	F	Empregado Efetivo
	F	Empregado Efetivo
	M	Empregado Efetivo
	F	Empregado Efetivo
	F	Empregado Efetivo
	F	Empregado Efetivo
	F	Empregado Efetivo
	F	Empregado Efetivo
	F	Empregado Efetivo
	F	Empregado Efetivo
	F	Empregado Efetivo
	F	Empregado Efetivo
	M	Empregado Efetivo
	F	Empregado Efetivo
	F	Empregado Efetivo
	F	Empregado Efetivo
	M	Empregado Efetivo
	M	Empregado Efetivo
	F	Empregado Efetivo
	M	Empregado Efetivo
	M	Empregado Efetivo

	F	Empregado Efetivo
	M	Empregado Efetivo
	F	Empregado Efetivo
	M	Empregado Efetivo
	F	Empregado Efetivo
	F	Empregado Efetivo
	F	Empregado Efetivo
	M	Empregado Efetivo
	F	Empregado Efetivo
	F	Empregado Efetivo
	F	Empregado Efetivo
	F	Empregado Efetivo
	F	Empregado Efetivo
	F	Empregado Efetivo
	F	Empregado Efetivo
	F	Empregado Efetivo
	F	Empregado Efetivo
	F	Empregado Efetivo
	F	Empregado Efetivo
	F	Empregado Efetivo
	M	Empregado Efetivo
	F	Empregado Efetivo
	F	Empregado Efetivo
	F	Empregado Efetivo
	F	Empregado Efetivo
	F	Empregado Efetivo
	M	Empregado Efetivo
	F	Empregado Efetivo
	F	Empregado Efetivo
	M	Empregado Efetivo
	F	Empregado Efetivo
	F	Empregado Efetivo
	F	Empregado Efetivo
	F	Empregado Efetivo
	F	Empregado Efetivo
	F	Empregado Efetivo
	F	Empregado Efetivo
	M	Empregado Efetivo
	F	Empregado Efetivo
	M	Empregado Efetivo
	M	Empregado Efetivo
	F	Empregado Efetivo
	M	Empregado Efetivo
	F	Empregado Efetivo
	F	Empregado Efetivo
	F	Empregado Efetivo
	F	Empregado Efetivo
	F	Empregado Efetivo

	F	Empregado Efetivo
	M	Empregado Efetivo
	F	Empregado Efetivo
	F	Empregado Efetivo
	F	Empregado Efetivo
	F	Empregado Efetivo
	M	Empregado Efetivo
	F	Empregado Efetivo
	F	Empregado Efetivo
	F	Empregado Efetivo
	F	Empregado Efetivo
	F	Empregado Efetivo
	F	Empregado Efetivo
	F	Empregado Efetivo
	F	Empregado Efetivo
	F	Empregado Efetivo
	F	Empregado Efetivo
	F	Empregado Efetivo
	M	Empregado Efetivo
	M	Empregado Efetivo
	M	Empregado Efetivo
	F	Empregado Efetivo
	F	Empregado Efetivo
	M	Empregado Efetivo
	F	Empregado Efetivo
	F	Empregado Efetivo
	F	Empregado Efetivo
	F	Empregado Efetivo
	F	Empregado Efetivo
	F	Empregado Efetivo
	F	Empregado Efetivo
	M	Empregado Efetivo
	F	Empregado Efetivo
	M	Empregado Efetivo
	F	Empregado Efetivo
	F	Empregado Efetivo
	F	Empregado Efetivo
	F	Empregado Efetivo
	M	Empregado Efetivo
	F	Empregado Efetivo
	F	Empregado Efetivo
	F	Empregado Efetivo
	F	Empregado Efetivo
	F	Empregado Efetivo
	F	Empregado Efetivo
	F	Empregado Efetivo
	M	Empregado Efetivo
	F	Empregado Efetivo

	F	Empregado Efetivo
	M	Empregado Efetivo
	F	Empregado Efetivo
	F	Empregado Efetivo
	F	Empregado Efetivo
	M	Empregado Efetivo
	F	Empregado Efetivo
	F	Empregado Efetivo
	F	Empregado Efetivo
	M	Empregado Efetivo
	F	Empregado Efetivo
	M	Empregado Efetivo
	M	Empregado Efetivo
	M	Empregado Efetivo
	M	Empregado Efetivo
	F	Empregado Efetivo
	F	Empregado Efetivo
	F	Empregado Efetivo
	F	Empregado Efetivo
	M	Empregado Efetivo
	M	Empregado Efetivo
	F	Empregado Efetivo
	F	Empregado Efetivo
	F	Empregado Efetivo
	F	Empregado Efetivo
	F	Empregado Efetivo
	F	Empregado Efetivo
	F	Empregado Efetivo
	F	Empregado Efetivo
	F	Empregado Efetivo
	F	Empregado Efetivo
	F	Empregado Efetivo
	F	Empregado Efetivo
	F	Empregado Efetivo
	F	Empregado Efetivo
	F	Empregado Efetivo
	M	Empregado Efetivo
	M	Empregado Efetivo
	F	Empregado Efetivo
	F	Empregado Efetivo

	F	Empregado Efetivo
	F	Empregado Efetivo
	F	Empregado Efetivo
	F	Empregado Efetivo
	F	Empregado Efetivo
	F	Empregado Efetivo
	F	Empregado Efetivo
	M	Empregado Efetivo
	M	Empregado Efetivo
	F	Empregado Efetivo
	F	Empregado Efetivo
	M	Empregado Efetivo
	F	Empregado Efetivo
	M	Empregado Efetivo
	F	Empregado Efetivo
	F	Empregado Efetivo
	F	Empregado Efetivo
	M	Empregado Efetivo
	F	Empregado Efetivo
	F	Empregado Efetivo
	M	Empregado Efetivo
	F	Empregado Efetivo
	M	Empregado Efetivo
	M	Empregado Efetivo
	F	Empregado Efetivo
	M	Empregado Efetivo
	F	Empregado Efetivo
	F	Empregado Efetivo
	M	Empregado Efetivo
	F	Empregado Efetivo
	F	Empregado Efetivo
	F	Empregado Efetivo
	F	Empregado Efetivo
	F	Empregado Efetivo
	F	Empregado Efetivo
	F	Empregado Efetivo
	F	Empregado Efetivo
	F	Empregado Efetivo
	F	Empregado Efetivo
	F	Empregado Efetivo
	F	Empregado Efetivo
	F	Empregado Efetivo

	F	Empregado Efetivo
	F	Empregado Efetivo
	F	Empregado Efetivo
	F	Empregado Efetivo
	F	Empregado Efetivo
	F	Empregado Efetivo
	F	Empregado Efetivo
	F	Empregado Efetivo
	F	Empregado Efetivo
	F	Empregado Efetivo
	F	Empregado Efetivo
	F	Empregado Efetivo
	F	Empregado Efetivo
	F	Empregado Efetivo
	F	Empregado Efetivo
	F	Empregado Efetivo
	F	Empregado Efetivo
	F	Empregado Efetivo
	F	Empregado Efetivo
	F	Empregado Efetivo
	F	Empregado Efetivo
	F	Empregado Efetivo
	F	Empregado Efetivo
	F	Empregado Efetivo
	F	Empregado Efetivo
	F	Empregado Efetivo
	F	Empregado Efetivo
	F	Empregado Efetivo
	F	Empregado Efetivo
	F	Empregado Efetivo
	F	Empregado Efetivo
	F	Empregado Efetivo
	F	Empregado Efetivo
	F	Empregado Efetivo
	F	Empregado Efetivo
	F	Empregado Efetivo
	F	Empregado Efetivo
	F	Empregado Efetivo
	F	Empregado Efetivo
	F	Empregado Efetivo
	F	Empregado Efetivo
	F	Empregado Efetivo
	F	Empregado Efetivo

	F	Empregado Efetivo
	F	Empregado Efetivo
	F	Empregado Efetivo
	F	Empregado Efetivo
	F	Empregado Efetivo
	F	Empregado Efetivo
	F	Empregado Efetivo
	F	Empregado Efetivo
	F	Empregado Efetivo
	F	Empregado Efetivo
	F	Empregado Efetivo
	F	Empregado Efetivo
	F	Empregado Efetivo
	F	Empregado Efetivo
	F	Empregado Efetivo
	F	Empregado Efetivo
	F	Empregado Efetivo
	F	Empregado Efetivo
	F	Empregado Efetivo
	F	Empregado Efetivo
	F	Empregado Efetivo
	F	Empregado Efetivo
	M	Empregado Efetivo
	F	Empregado Efetivo
	F	Empregado Efetivo
	F	Empregado Efetivo
	F	Empregado Efetivo
	F	Empregado Efetivo
	F	Empregado Efetivo
	F	Empregado Efetivo
	F	Empregado Efetivo
	F	Empregado Efetivo
	M	Empregado Efetivo
	F	Empregado Efetivo
	F	Empregado Efetivo

	M	Empregado Efetivo
	F	Empregado Efetivo
	M	Empregado Efetivo
	F	Empregado Efetivo
	F	Empregado Efetivo
	M	Empregado Efetivo
	M	Empregado Efetivo
	F	Empregado Efetivo
	F	Empregado Efetivo
	F	Empregado Efetivo
	F	Empregado Efetivo
	F	Empregado Efetivo
	F	Empregado Efetivo
	F	Empregado Efetivo
	F	Empregado Efetivo
	F	Empregado Efetivo
	F	Empregado Efetivo
	F	Empregado Efetivo
	F	Empregado Efetivo
	F	Empregado Efetivo
	F	Empregado Efetivo
	F	Empregado Efetivo
	F	Empregado Efetivo
	M	Empregado Efetivo
	F	Empregado Efetivo
	F	Empregado Efetivo
	F	Empregado Efetivo
	F	Empregado Efetivo
	F	Empregado Efetivo
	F	Empregado Efetivo
	F	Empregado Efetivo
	F	Empregado Efetivo
	F	Empregado Efetivo

	F	Empregado Efetivo
	F	Empregado Efetivo
	F	Empregado Efetivo
	F	Empregado Efetivo
	F	Empregado Efetivo
	F	Empregado Efetivo
	F	Empregado Efetivo
	F	Empregado Efetivo
	F	Empregado Efetivo
	F	Empregado Efetivo
	F	Empregado Efetivo
	F	Empregado Efetivo
	F	Empregado Efetivo
	F	Empregado Efetivo
	F	Empregado Efetivo
	F	Empregado Efetivo
	F	Empregado Efetivo
	F	Empregado Efetivo
	F	Empregado Efetivo
	F	Empregado Efetivo
	F	Empregado Efetivo
	F	Empregado Efetivo
	F	Empregado Efetivo
	F	Empregado Efetivo
	F	Empregado Efetivo
	F	Empregado Efetivo
	F	Empregado Efetivo
	F	Empregado Efetivo
	F	Empregado Efetivo
	F	Empregado Efetivo
	F	Empregado Efetivo
	F	Empregado Efetivo
	F	Empregado Efetivo
	F	Cedido - RJU
	F	Empregado Efetivo
	F	Empregado Efetivo
	F	Empregado Efetivo
	F	Empregado Efetivo
	F	Empregado Efetivo
	M	Empregado Efetivo
	F	Empregado Efetivo

	F	Empregado Efetivo
	F	Empregado Efetivo
	F	Empregado Efetivo
	M	Empregado Efetivo
	M	Empregado Efetivo
	F	Empregado Efetivo
	F	Empregado Efetivo
	F	Empregado Efetivo
	F	Empregado Efetivo
	F	Empregado Efetivo
	F	Empregado Efetivo
	M	Cedido - RJU
	M	Cedido - RJU
	M	Cedido - RJU
	M	Empregado Efetivo
	F	Empregado Efetivo
	F	Empregado Efetivo
	F	Cedido - RJU
	M	Empregado Efetivo
	F	Empregado Efetivo
	F	Empregado Efetivo
	M	Empregado Efetivo
	F	Empregado Efetivo
	F	Empregado Efetivo
	F	Empregado Efetivo
	F	Empregado Efetivo
	F	Cedido - RJU
	F	Empregado Efetivo
	F	Empregado Efetivo
	F	Empregado Efetivo
	F	Empregado Efetivo
	F	Empregado Efetivo
	F	Empregado Efetivo
	F	Empregado Efetivo

	F	Empregado Efetivo
	M	Empregado Efetivo
	F	Empregado Efetivo
	F	Empregado Efetivo
	M	Empregado Efetivo
	F	Empregado Efetivo
	F	Empregado Efetivo
	F	Empregado Efetivo
	F	Empregado Efetivo
	F	Empregado Efetivo
	F	Empregado Efetivo
	F	Empregado Efetivo
	F	Empregado Efetivo
	F	Cedido - RJU
	M	Empregado Efetivo
	M	Empregado Efetivo
	M	Empregado Efetivo
	M	Empregado Efetivo
	M	Empregado Efetivo
	M	Empregado Efetivo
	M	Empregado Efetivo
	F	Empregado Efetivo
	M	Empregado Efetivo
	F	Empregado Efetivo
	M	Empregado Efetivo
	M	Empregado Efetivo
	F	Empregado Efetivo
	F	Empregado Efetivo
	F	Empregado Efetivo
	F	Empregado Efetivo
	F	Empregado Efetivo
	F	Empregado Efetivo

	F	Empregado Efetivo
	F	Empregado Efetivo
	M	Empregado Efetivo
	M	Empregado Efetivo
	F	Empregado Efetivo
	M	Empregado Efetivo
	F	Empregado Efetivo
	F	Empregado Efetivo
	F	Empregado Efetivo
	F	Empregado Efetivo
	M	Empregado Efetivo
	M	Empregado Efetivo
	M	Empregado Efetivo
	F	Empregado Efetivo
	M	Empregado Efetivo
	M	Empregado Efetivo
	M	Empregado Efetivo
	M	Empregado Efetivo
	F	Cedido - RJU
	F	Empregado Efetivo
	F	Empregado Efetivo
	F	Empregado Efetivo
	M	Empregado Efetivo
	F	Empregado Efetivo
	F	Empregado Efetivo
	F	Empregado Efetivo
	F	Empregado Efetivo
	F	Empregado Efetivo
	M	Empregado Efetivo
	M	Empregado Efetivo
	F	Empregado Efetivo
	F	Empregado Efetivo

	F	Empregado Efetivo
	F	Empregado Efetivo
	F	Empregado Efetivo
	F	Empregado Efetivo
	F	Empregado Efetivo
	F	Empregado Efetivo
	F	Empregado Efetivo
	F	Empregado Efetivo
	F	Empregado Efetivo
	F	Empregado Efetivo
	F	Empregado Efetivo
	F	Empregado Efetivo
	M	Empregado Efetivo
	F	Empregado Efetivo
	M	Empregado Efetivo
	F	Empregado Efetivo
	F	Empregado Efetivo
	F	Empregado Efetivo
	M	Empregado Efetivo
	F	Empregado Efetivo
	F	Empregado Efetivo
	F	Empregado Efetivo
	F	Empregado Efetivo
	F	Empregado Efetivo
	F	Empregado Efetivo
	F	Empregado Efetivo
	M	Empregado Efetivo
	F	Empregado Efetivo
	F	Empregado Efetivo
	F	Empregado Efetivo
	M	Empregado Efetivo
	F	Empregado Efetivo

	F	Empregado Efetivo
	F	Empregado Efetivo
	M	Empregado Cedido
	F	Empregado Efetivo
	F	Empregado Efetivo
	F	Empregado Efetivo
	F	Empregado Efetivo
	F	Empregado Efetivo
	F	Empregado Efetivo
	F	Empregado Efetivo
	F	Empregado Efetivo
	F	Empregado Efetivo
	F	Empregado Efetivo
	F	Empregado Efetivo
	F	Empregado Efetivo
	F	Empregado Efetivo
	F	Empregado Efetivo
	M	Empregado Efetivo
	M	Empregado Efetivo
	F	Empregado Efetivo
	F	Empregado Efetivo
	F	Empregado Efetivo
	F	Empregado Efetivo
	F	Empregado Efetivo
	F	Empregado Efetivo
	F	Empregado Efetivo
	F	Empregado Efetivo
	M	Empregado Efetivo
	M	Empregado Efetivo
	F	Empregado Efetivo
	F	Empregado Efetivo
	F	Empregado Efetivo
	M	Empregado Efetivo
	F	Empregado Efetivo
	F	Empregado Efetivo
	F	Empregado Efetivo
	F	Empregado Efetivo
	F	Empregado Efetivo
	M	Empregado Efetivo
	F	Empregado Efetivo
	M	Empregado Efetivo
	F	Empregado Efetivo
	F	Empregado Efetivo

	F	Empregado Efetivo
	M	Empregado Efetivo
	F	Empregado Efetivo
	M	Empregado Efetivo
	F	Empregado Efetivo
	F	Empregado Efetivo
	F	Empregado Efetivo
	M	Empregado Efetivo
	F	Empregado Efetivo
	F	Empregado Efetivo
	F	Empregado Efetivo
	M	Empregado Efetivo
	F	Empregado Efetivo
	F	Empregado Efetivo
	M	Empregado Efetivo
	M	Empregado Efetivo
	F	Empregado Efetivo
	M	Empregado Efetivo
	F	Empregado Efetivo
	F	Empregado Efetivo
	F	Empregado Efetivo
	F	Empregado Efetivo
	F	Empregado Efetivo
	F	Empregado Efetivo
	F	Empregado Efetivo
	F	Empregado Efetivo
	F	Empregado Efetivo
	F	Empregado Efetivo
	F	Empregado Efetivo
	F	Empregado Efetivo
	F	Empregado Efetivo
	F	Empregado Efetivo
	F	Empregado Efetivo
	F	Empregado Efetivo
	F	Empregado Efetivo
	M	Empregado Efetivo
	M	Empregado Efetivo
	F	Empregado Efetivo
	M	Empregado Efetivo
	F	Empregado Efetivo

	F	Empregado Efetivo
	F	Empregado Efetivo
	F	Empregado Efetivo
	M	Empregado Efetivo
	F	Empregado Efetivo
	F	Empregado Efetivo
	F	Empregado Efetivo
	F	Empregado Efetivo
	F	Empregado Efetivo
	F	Empregado Efetivo
	F	Empregado Efetivo
	F	Empregado Efetivo
	F	Empregado Efetivo
	M	Empregado Efetivo
	M	Empregado Efetivo
	F	Empregado Efetivo
	F	Empregado Efetivo
	F	Empregado Efetivo
	F	Empregado Efetivo
	F	Empregado Efetivo
	M	Empregado Efetivo
	F	Empregado Efetivo
	F	Empregado Efetivo
	F	Empregado Efetivo
	F	Empregado Efetivo
	F	Empregado Efetivo
	F	Empregado Efetivo
	F	Empregado Efetivo
	F	Empregado Efetivo
	F	Empregado Efetivo
	F	Empregado Efetivo
	F	Empregado Efetivo
	F	Empregado Efetivo
	F	Empregado Efetivo
	F	Empregado Efetivo
	M	Empregado Efetivo
	F	Empregado Efetivo
	F	Empregado Efetivo
	M	Empregado Efetivo
	F	Empregado Efetivo
	M	Empregado Efetivo
	M	Empregado Efetivo
	M	Empregado Efetivo
	F	Empregado Efetivo
	F	Empregado Efetivo
	M	Empregado Efetivo
	F	Empregado Efetivo
	M	Empregado Efetivo

	F	Empregado Efetivo
	F	Empregado Efetivo
	F	Empregado Efetivo
	M	Empregado Efetivo
	F	Empregado Efetivo
	F	Empregado Efetivo
	F	Empregado Efetivo
	F	Empregado Efetivo
	F	Empregado Efetivo
	F	Empregado Efetivo
	M	Empregado Efetivo
	M	Empregado Efetivo
	M	Empregado Efetivo
	M	Empregado Efetivo
	F	Empregado Efetivo
	M	Empregado Efetivo
	M	Empregado Efetivo
	F	Empregado Efetivo
	M	Empregado Efetivo
	F	Empregado Efetivo
	F	Empregado Efetivo
	F	Empregado Efetivo
	F	Empregado Efetivo
	M	Empregado Efetivo
	M	Empregado Efetivo
	F	Empregado Efetivo
	F	Empregado Efetivo
	F	Empregado Efetivo
	M	Empregado Efetivo
	M	Empregado Efetivo
	M	Empregado Efetivo
	M	Empregado Efetivo
	M	Empregado Efetivo
	M	Empregado Efetivo
	M	Empregado Efetivo
	F	Empregado Efetivo
	M	Empregado Efetivo
	M	Empregado Efetivo
	F	Empregado Efetivo
	F	Empregado Efetivo
	F	Empregado Efetivo
	M	Empregado Efetivo
	M	Empregado Efetivo
	M	Empregado Efetivo
	M	Empregado Efetivo

	F	Empregado Efetivo
	F	Empregado Efetivo
	F	Empregado Efetivo
	F	Empregado Efetivo
	M	Empregado Efetivo
	F	Empregado Efetivo
	M	Empregado Efetivo
	M	Empregado Efetivo
	F	Empregado Efetivo
	M	Empregado Efetivo
	F	Empregado Efetivo
	M	Empregado Efetivo
	M	Empregado Efetivo
	F	Empregado Efetivo
	F	Empregado Efetivo
	F	Empregado Efetivo
	F	Empregado Efetivo
	F	Empregado Efetivo
	F	Empregado Efetivo
	F	Empregado Efetivo
	F	Empregado Efetivo
	F	Empregado Efetivo
	F	Empregado Efetivo
	M	Empregado Efetivo
	M	Empregado Efetivo
	M	Empregado Efetivo
	M	Empregado Efetivo
	F	Empregado Efetivo
	F	Empregado Efetivo
	F	Empregado Efetivo
	F	Empregado Efetivo
	F	Empregado Efetivo
	F	Empregado Efetivo
	F	Empregado Efetivo
	F	Empregado Efetivo

	F	Empregado Efetivo
	F	Empregado Efetivo
	F	Empregado Efetivo
	F	Empregado Efetivo
	F	Empregado Efetivo
	F	Empregado Efetivo
	F	Empregado Efetivo
	F	Empregado Efetivo
	F	Empregado Efetivo
	F	Empregado Efetivo
	F	Empregado Efetivo
	F	Empregado Efetivo
	F	Empregado Efetivo
	F	Empregado Efetivo
	F	Empregado Efetivo
	F	Empregado Efetivo
	M	Empregado Efetivo
	F	Empregado Efetivo
	M	Empregado Efetivo
	F	Empregado Efetivo
	F	Empregado Efetivo
	F	Empregado Efetivo
	F	Empregado Efetivo
	F	Empregado Efetivo
	F	Empregado Efetivo
	M	Empregado Efetivo
	F	Empregado Efetivo
	F	Empregado Efetivo
	M	Empregado Efetivo
	M	Empregado Efetivo
	F	Empregado Efetivo
	F	Empregado Efetivo
	F	Empregado Efetivo
	F	Empregado Efetivo
	F	Empregado Efetivo
	F	Empregado Efetivo
	F	Empregado Efetivo
	F	Empregado Efetivo
	M	Empregado Efetivo

	F	Empregado Efetivo
	F	Empregado Efetivo
	M	Empregado Efetivo
	F	Empregado Efetivo
	F	Empregado Efetivo
	F	Empregado Efetivo
	F	Empregado Efetivo
	F	Empregado Efetivo
	F	Empregado Efetivo
	F	Empregado Efetivo
	F	Empregado Efetivo
	F	Empregado Efetivo
	M	Empregado Efetivo
	F	Empregado Efetivo
	M	Empregado Efetivo
	F	Empregado Efetivo
	F	Empregado Efetivo
	F	Empregado Efetivo
	F	Empregado Efetivo
	M	Empregado Efetivo
	F	Empregado Efetivo
	F	Empregado Efetivo
	F	Empregado Efetivo
	F	Empregado Efetivo
	F	Empregado Efetivo
	F	Empregado Efetivo
	F	Empregado Efetivo
	M	Empregado Efetivo
	M	Empregado Efetivo
	M	Empregado Efetivo
	M	Empregado Efetivo
	M	Empregado Efetivo
	M	Empregado Efetivo
	M	Empregado Efetivo
	F	Empregado Efetivo
	F	Empregado Efetivo
	F	Empregado Efetivo
	F	Empregado Efetivo
	F	Empregado Efetivo
	F	Empregado Efetivo
	F	Empregado Efetivo
	F	Empregado Efetivo
	F	Empregado Efetivo
	M	Empregado Efetivo
	F	Empregado Efetivo
	F	Empregado Efetivo

	F	Empregado Efetivo
	F	Empregado Efetivo
	F	Empregado Efetivo
	F	Empregado Efetivo
	F	Empregado Efetivo
	F	Empregado Efetivo
	F	Empregado Efetivo
	F	Empregado Efetivo
	F	Cedido - RJU
	F	Cedido - RJU
	M	Empregado Efetivo
	M	Empregado Efetivo
	M	Empregado Efetivo
	M	Empregado Cedido
	M	Empregado Cedido
	M	Empregado Efetivo
	M	Empregado Efetivo
	M	Empregado Efetivo
	M	Empregado Efetivo
	M	Empregado Efetivo
	M	Empregado Efetivo
	M	Empregado Efetivo
	M	Empregado Efetivo
	F	Empregado Efetivo
	F	Empregado Efetivo
	F	Empregado Efetivo
	F	Empregado Efetivo
	M	Empregado Efetivo
	M	Empregado Efetivo
	F	Empregado Efetivo
	F	Empregado Efetivo
	F	Empregado Efetivo
	F	Empregado Efetivo
	F	Empregado Efetivo
	M	Empregado Efetivo
	F	Empregado Efetivo
	F	Empregado Efetivo
	M	Empregado Efetivo
	F	Empregado Efetivo
	F	Empregado Efetivo
	F	Empregado Efetivo
	F	Empregado Efetivo
	M	Empregado Efetivo
	F	Empregado Efetivo
	F	Empregado Efetivo

	F	Empregado Efetivo
	M	Empregado Efetivo
	M	Empregado Efetivo
	M	Empregado Efetivo
	M	Empregado Efetivo
	F	Empregado Efetivo
	M	Empregado Efetivo
	M	Empregado Efetivo
	F	Empregado Efetivo
	F	Empregado Efetivo
	M	Empregado Efetivo
	F	Empregado Efetivo
	M	Empregado Efetivo
	M	Empregado Efetivo
	M	Empregado Efetivo
	F	Empregado Efetivo
	M	Empregado Efetivo
	F	Empregado Efetivo
	F	Empregado Efetivo
	F	Empregado Efetivo
	M	Empregado Efetivo
	F	Empregado Efetivo
	F	Empregado Efetivo
	F	Empregado Efetivo
	M	Empregado Efetivo
	F	Empregado Efetivo
	M	Empregado Efetivo
	F	Empregado Efetivo
	M	Empregado Efetivo
	F	Empregado Efetivo
	F	Empregado Efetivo
	M	Empregado Efetivo
	F	Empregado Efetivo
	M	Empregado Efetivo
	F	Empregado Efetivo
	M	Empregado Efetivo
	M	Empregado Efetivo
	M	Empregado Efetivo
	F	Empregado Efetivo
	F	Empregado Efetivo
	F	Empregado Efetivo
	M	Empregado Efetivo
	F	Empregado Efetivo
	F	Empregado Efetivo
	M	Empregado Efetivo
	F	Empregado Efetivo
	M	Empregado Efetivo
	M	Empregado Efetivo

	M	Empregado Efetivo
	F	Empregado Efetivo
	F	Empregado Efetivo
	F	Empregado Efetivo
	M	Empregado Efetivo
	M	Empregado Efetivo
	F	Empregado Efetivo
	F	Empregado Efetivo
	F	Empregado Efetivo
	F	Empregado Efetivo
	F	Empregado Efetivo
	F	Empregado Efetivo
	F	Empregado Efetivo
	M	Empregado Efetivo
	F	Empregado Efetivo
	F	Empregado Efetivo
	F	Empregado Efetivo
	M	Empregado Efetivo
	F	Empregado Efetivo
	F	Empregado Efetivo
	F	Empregado Efetivo
	F	Empregado Efetivo
	F	Empregado Efetivo
	F	Empregado Efetivo
	F	Empregado Efetivo
	F	Empregado Efetivo
	F	Empregado Efetivo
	F	Empregado Efetivo
	M	Empregado Efetivo
	F	Empregado Efetivo
	F	Empregado Efetivo
	F	Empregado Efetivo
	F	Empregado Efetivo
	F	Empregado Efetivo
	F	Empregado Efetivo
	F	Empregado Efetivo
	F	Empregado Efetivo
	F	Empregado Efetivo
	F	Empregado Efetivo
	M	Empregado Efetivo
	F	Empregado Efetivo
	F	Empregado Efetivo
	F	Empregado Efetivo
	M	Empregado Efetivo
	F	Empregado Efetivo
	F	Empregado Efetivo
	F	Empregado Efetivo

	F	Empregado Efetivo
	M	Empregado Efetivo
	F	Empregado Efetivo
	F	Empregado Efetivo
	F	Empregado Efetivo
	F	Empregado Efetivo
	F	Empregado Efetivo
	F	Empregado Efetivo
	F	Empregado Efetivo
	F	Empregado Efetivo
	F	Empregado Efetivo
	F	Empregado Efetivo
	F	Empregado Efetivo
	F	Empregado Efetivo
	F	Empregado Efetivo
	F	Empregado Efetivo
	F	Empregado Efetivo
	F	Empregado Efetivo
	F	Empregado Efetivo
	F	Empregado Efetivo
	F	Empregado Efetivo
	M	Empregado Efetivo
	F	Empregado Efetivo
	F	Empregado Efetivo
	F	Empregado Efetivo
	F	Empregado Efetivo
	F	Empregado Efetivo
	F	Empregado Efetivo
	F	Empregado Efetivo
	F	Empregado Efetivo
	F	Empregado Efetivo
	F	Empregado Efetivo
	F	Empregado Efetivo
	F	Empregado Efetivo
	F	Empregado Efetivo
	F	Empregado Efetivo
	F	Empregado Efetivo
	F	Empregado Efetivo
	F	Empregado Efetivo
	F	Empregado Efetivo
	F	Empregado Efetivo
	F	Empregado Efetivo
	F	Empregado Efetivo
	F	Empregado Efetivo
	F	Empregado Efetivo
	F	Empregado Efetivo
	F	Empregado Efetivo
	F	Empregado Efetivo

	F	Empregado Efetivo
	F	Empregado Efetivo
	M	Empregado Efetivo
	F	Empregado Efetivo
	M	Empregado Efetivo
	F	Empregado Efetivo
	F	Empregado Efetivo
	F	Empregado Efetivo
	F	Empregado Efetivo
	F	Empregado Efetivo
	F	Empregado Efetivo
	F	Empregado Efetivo
	F	Empregado Efetivo
	F	Empregado Efetivo
	F	Empregado Efetivo
	F	Empregado Efetivo
	F	Empregado Efetivo
	F	Empregado Efetivo
	F	Empregado Efetivo
	F	Empregado Efetivo
	F	Empregado Efetivo
	M	Empregado Efetivo
	F	Empregado Efetivo
	F	Empregado Efetivo
	F	Empregado Efetivo
	F	Empregado Efetivo
	M	Empregado Efetivo
	F	Empregado Efetivo
	F	Empregado Efetivo
	F	Empregado Efetivo
	F	Empregado Efetivo
	F	Empregado Efetivo
	F	Empregado Efetivo
	F	Empregado Efetivo
	F	Empregado Efetivo
	M	Empregado Efetivo
	F	Empregado Efetivo
	F	Empregado Efetivo
	F	Empregado Efetivo
	F	Empregado Efetivo
	F	Empregado Efetivo
	F	Empregado Efetivo
	F	Empregado Efetivo
	F	Empregado Efetivo
	F	Empregado Efetivo

	F	Empregado Efetivo
	F	Empregado Efetivo
	M	Empregado Efetivo
	F	Empregado Efetivo
	F	Empregado Efetivo
	M	Empregado Efetivo
	F	Empregado Efetivo
	F	Empregado Efetivo
	F	Empregado Efetivo
	F	Empregado Efetivo
	F	Empregado Efetivo
	F	Empregado Efetivo
	F	Empregado Efetivo
	F	Empregado Efetivo
	F	Empregado Efetivo
	F	Empregado Efetivo
	F	Empregado Efetivo
	F	Empregado Efetivo
	F	Empregado Efetivo
	F	Empregado Efetivo
	F	Empregado Efetivo
	F	Empregado Efetivo
	M	Empregado Efetivo
	F	Empregado Efetivo
	F	Empregado Efetivo
	F	Empregado Efetivo
	F	Empregado Efetivo
	F	Empregado Efetivo
	M	Empregado Efetivo
	F	Empregado Efetivo
	F	Empregado Efetivo
	F	Empregado Efetivo

	F	Empregado Efetivo
	F	Empregado Efetivo
	F	Empregado Efetivo
	F	Empregado Efetivo
	F	Empregado Efetivo
	F	Empregado Efetivo
	F	Empregado Efetivo
	F	Empregado Efetivo
	F	Empregado Efetivo
	F	Empregado Efetivo
	F	Empregado Efetivo
	F	Empregado Efetivo
	F	Empregado Efetivo
	F	Empregado Efetivo
	F	Empregado Efetivo
	F	Empregado Efetivo
	F	Empregado Efetivo
	F	Empregado Efetivo
	F	Empregado Efetivo
	F	Empregado Efetivo
	M	Empregado Efetivo
	F	Empregado Efetivo
	F	Empregado Efetivo
	F	Empregado Efetivo
	F	Empregado Efetivo
	F	Empregado Efetivo
	F	Empregado Efetivo
	F	Empregado Efetivo
	F	Empregado Efetivo
	F	Empregado Efetivo
	F	Empregado Efetivo
	M	Empregado Efetivo
	M	Empregado Efetivo
	M	Empregado Efetivo
	F	Empregado Efetivo
	F	Empregado Efetivo
	F	Empregado Efetivo
	F	Empregado Efetivo
	F	Empregado Efetivo
	F	Empregado Efetivo
	F	Empregado Efetivo
	F	Empregado Efetivo
	M	Empregado Efetivo
	F	Empregado Efetivo
	F	Empregado Efetivo
	M	Empregado Efetivo
	F	Empregado Efetivo
	F	Empregado Efetivo

	F	Empregado Efetivo
	F	Empregado Efetivo
	F	Empregado Efetivo
	F	Empregado Efetivo
	F	Empregado Efetivo
	M	Empregado Efetivo
	F	Empregado Efetivo
	F	Empregado Efetivo
	F	Empregado Efetivo
	F	Empregado Efetivo
	F	Empregado Efetivo
	F	Empregado Efetivo
	F	Empregado Efetivo
	F	Empregado Efetivo
	F	Empregado Efetivo
	M	Empregado Efetivo
	M	Empregado Efetivo
	F	Empregado Efetivo
	F	Empregado Efetivo
	M	Empregado Efetivo
	F	Empregado Efetivo
	F	Empregado Efetivo
	F	Empregado Efetivo
	F	Empregado Efetivo
	M	Empregado Efetivo
	F	Empregado Efetivo
	F	Empregado Efetivo
	F	Empregado Efetivo
	F	Empregado Efetivo
	F	Empregado Efetivo
	F	Empregado Efetivo
	F	Empregado Efetivo
	F	Empregado Efetivo
	F	Empregado Efetivo
	F	Empregado Efetivo
	M	Empregado Efetivo
	F	Empregado Efetivo
	F	Empregado Efetivo
	F	Empregado Efetivo
	F	Empregado Efetivo
	F	Empregado Efetivo
	F	Empregado Efetivo
	M	Empregado Efetivo
	F	Empregado Efetivo
	M	Empregado Efetivo
	F	Empregado Efetivo
	M	Empregado Efetivo
	F	Empregado Efetivo

	F	Empregado Efetivo
	F	Empregado Efetivo
	F	Empregado Efetivo
	F	Empregado Efetivo
	F	Empregado Efetivo
	F	Empregado Efetivo
	F	Empregado Efetivo
	M	Empregado Efetivo
	M	Empregado Efetivo
	F	Empregado Efetivo
	F	Empregado Efetivo
	F	Empregado Efetivo
	F	Empregado Efetivo
	F	Empregado Efetivo
	F	Empregado Efetivo
	F	Empregado Efetivo
	F	Empregado Efetivo
	F	Empregado Efetivo
	F	Empregado Efetivo
	F	Empregado Efetivo
	F	Empregado Efetivo
	F	Empregado Efetivo
	F	Empregado Efetivo
	F	Empregado Efetivo
	M	Empregado Efetivo
	F	Empregado Efetivo
	F	Empregado Efetivo
	F	Empregado Efetivo
	M	Empregado Efetivo
	F	Empregado Efetivo
	M	Empregado Efetivo
	F	Empregado Efetivo
	F	Empregado Efetivo
	F	Empregado Efetivo
	F	Empregado Efetivo
	F	Empregado Efetivo
	F	Empregado Efetivo
	M	Empregado Efetivo
	F	Empregado Efetivo
	F	Empregado Efetivo
	M	Empregado Efetivo
	F	Empregado Efetivo
	F	Empregado Efetivo
	F	Empregado Efetivo
	F	Empregado Efetivo
	F	Empregado Efetivo
	F	Empregado Efetivo
	M	Empregado Efetivo

	F	Empregado Efetivo
	F	Empregado Efetivo
	F	Empregado Efetivo
	M	Empregado Efetivo
	F	Empregado Efetivo
	F	Empregado Efetivo
	M	Empregado Efetivo
	M	Empregado Efetivo
	M	Empregado Efetivo
	F	Empregado Efetivo
	F	Empregado Efetivo
	F	Empregado Efetivo
	M	Empregado Efetivo
	F	Empregado Efetivo
	F	Empregado Efetivo
	F	Empregado Efetivo
	F	Empregado Efetivo
	F	Empregado Efetivo
	F	Empregado Efetivo
	F	Empregado Efetivo
	F	Empregado Efetivo
	F	Empregado Efetivo
	F	Empregado Efetivo
	F	Empregado Efetivo
	F	Empregado Efetivo
	F	Empregado Efetivo
	F	Empregado Efetivo
	F	Empregado Efetivo
	F	Empregado Efetivo
	F	Empregado Efetivo
	F	Empregado Efetivo
	M	Empregado Efetivo
	F	Empregado Efetivo
	F	Empregado Efetivo
	F	Empregado Efetivo
	F	Empregado Efetivo
	F	Empregado Efetivo
	F	Empregado Efetivo
	F	Empregado Efetivo
	F	Empregado Efetivo
	F	Empregado Efetivo
	F	Empregado Efetivo
	F	Empregado Efetivo
	F	Empregado Efetivo
	F	Empregado Efetivo
	F	Empregado Efetivo
	F	Empregado Efetivo
	F	Empregado Efetivo

	F	Empregado Efetivo
	F	Empregado Efetivo
	M	Empregado Efetivo
	F	Empregado Efetivo
	F	Empregado Efetivo
	F	Empregado Efetivo
	F	Empregado Efetivo
	F	Empregado Efetivo
	F	Empregado Efetivo
	F	Empregado Efetivo
	F	Empregado Efetivo
	M	Empregado Efetivo
	F	Empregado Efetivo
	F	Empregado Efetivo
	F	Empregado Efetivo
	F	Empregado Efetivo
	F	Empregado Efetivo
	F	Empregado Efetivo
	F	Empregado Efetivo
	F	Empregado Efetivo
	F	Empregado Efetivo
	M	Empregado Efetivo
	F	Empregado Efetivo
	M	Empregado Efetivo
	F	Empregado Efetivo
	F	Empregado Efetivo
	F	Empregado Efetivo
	F	Empregado Efetivo
	F	Empregado Efetivo
	F	Empregado Efetivo
	M	Empregado Efetivo
	F	Empregado Efetivo
	F	Empregado Efetivo
	F	Empregado Efetivo
	F	Empregado Efetivo
	F	Empregado Efetivo
	M	Empregado Efetivo

	F	Empregado Efetivo
	F	Empregado Efetivo
	F	Empregado Efetivo
	F	Empregado Efetivo
	F	Empregado Efetivo
	F	Empregado Efetivo
	F	Empregado Efetivo
	F	Empregado Efetivo
	F	Empregado Efetivo
	F	Empregado Efetivo
	F	Empregado Efetivo
	M	Empregado Efetivo
	F	Empregado Efetivo
	F	Empregado Efetivo
	F	Empregado Efetivo
	F	Empregado Efetivo
	F	Empregado Efetivo
	F	Empregado Efetivo
	M	Empregado Efetivo
	M	Empregado Efetivo
	M	Empregado Efetivo
	M	Empregado Efetivo
	M	Empregado Efetivo
	M	Empregado Efetivo
	F	Empregado Efetivo
	F	Empregado Efetivo
	M	Empregado Efetivo
	F	Empregado Efetivo
	F	Empregado Efetivo
	M	Empregado Efetivo
	M	Empregado Efetivo
	F	Empregado Efetivo

	F	Empregado Efetivo
	F	Empregado Efetivo
	F	Empregado Efetivo
	F	Empregado Efetivo
	M	Empregado Efetivo
	F	Empregado Efetivo
	F	Empregado Efetivo
	F	Empregado Efetivo
	F	Empregado Efetivo
	M	Empregado Efetivo
	F	Empregado Efetivo
	F	Empregado Efetivo
	F	Empregado Efetivo
	F	Empregado Efetivo
	F	Empregado Efetivo
	M	Empregado Efetivo
	F	Empregado Efetivo
	F	Empregado Efetivo
	M	Empregado Efetivo
	F	Empregado Efetivo
	F	Empregado Efetivo
	F	Empregado Efetivo
	F	Empregado Efetivo
	M	Empregado Efetivo
	M	Empregado Efetivo
	F	Empregado Efetivo
	M	Empregado Efetivo
	M	Empregado Efetivo
	M	Empregado Efetivo
	F	Empregado Efetivo
	F	Empregado Efetivo
	F	Empregado Efetivo

	F	Empregado Efetivo
	M	Empregado Efetivo
	F	Empregado Efetivo
	F	Empregado Efetivo
	F	Empregado Efetivo
	F	Empregado Efetivo
	M	Empregado Efetivo
	M	Empregado Efetivo
	M	Empregado Efetivo
	M	Empregado Efetivo
	F	Empregado Efetivo
	F	Empregado Efetivo
	F	Empregado Efetivo
	F	Empregado Efetivo
	F	Empregado Efetivo
	M	Empregado Efetivo
	M	Empregado Efetivo
	M	Empregado Efetivo
	M	Empregado Efetivo
	M	Empregado Efetivo
	M	Empregado Efetivo
	F	Empregado Efetivo
	F	Empregado Efetivo
	F	Empregado Efetivo
	F	Empregado Efetivo
	F	Cedido - RJU
	F	Empregado Efetivo
	F	Empregado Efetivo
	F	Empregado Efetivo
	F	Empregado Efetivo
	F	Empregado Efetivo
	F	Empregado Efetivo
	F	Empregado Efetivo

	F	Empregado Efetivo
	F	Empregado Efetivo
	F	Empregado Efetivo
	F	Empregado Efetivo
	F	Empregado Efetivo
	F	Empregado Efetivo
	F	Empregado Efetivo
	F	Empregado Efetivo
	M	Empregado Efetivo
	F	Empregado Efetivo
	F	Empregado Efetivo
	F	Empregado Efetivo
	F	Empregado Efetivo
	F	Empregado Efetivo
	F	Empregado Efetivo
	F	Empregado Efetivo
	M	Empregado Efetivo
	F	Empregado Efetivo
	F	Empregado Efetivo
	F	Empregado Efetivo
	F	Empregado Efetivo
	F	Empregado Efetivo
	F	Empregado Efetivo
	F	Empregado Efetivo
	F	Empregado Efetivo
	F	Empregado Efetivo
	F	Empregado Efetivo
	F	Empregado Efetivo
	F	Empregado Efetivo
	M	Empregado Efetivo
	F	Empregado Efetivo
	F	Empregado Efetivo
	M	Empregado Efetivo
	M	Empregado Efetivo
	F	Empregado Efetivo
	F	Empregado Efetivo
	M	Empregado Efetivo
	M	Empregado Efetivo

	M	Empregado Efetivo
	M	Empregado Efetivo
	F	Empregado Efetivo
	M	Empregado Efetivo
	F	Empregado Efetivo
	F	Empregado Efetivo
	F	Empregado Efetivo
	F	Empregado Efetivo
	M	Cedido - RJU
	F	Empregado Efetivo
	F	Empregado Efetivo
	M	Empregado Efetivo
	F	Empregado Efetivo
	M	Cedido - RJU
	M	Empregado Efetivo
	F	Empregado Efetivo
	F	Empregado Efetivo
	F	Empregado Efetivo
	F	Empregado Efetivo
	F	Empregado Efetivo
	F	Empregado Efetivo
	M	Empregado Efetivo
	F	Empregado Efetivo
	F	Empregado Efetivo
	F	Empregado Efetivo
	F	Empregado Efetivo
	F	Empregado Efetivo
	F	Empregado Efetivo
	F	Empregado Efetivo
	F	Empregado Efetivo
	M	Empregado Efetivo
	F	Empregado Efetivo
	F	Empregado Efetivo
	F	Empregado Efetivo
	F	Empregado Efetivo
	F	Empregado Efetivo
	F	Empregado Efetivo
	F	Empregado Efetivo
	F	Empregado Efetivo
	F	Empregado Efetivo
	F	Empregado Efetivo
	F	Empregado Efetivo
	F	Empregado Efetivo
	F	Empregado Efetivo
	F	Empregado Efetivo
	F	Empregado Efetivo
	F	Empregado Efetivo
	M	Empregado Efetivo
	F	Empregado Efetivo

	F	Empregado Efetivo
	F	Empregado Efetivo
	F	Empregado Efetivo
	F	Empregado Efetivo
	M	Empregado Efetivo
	M	Empregado Efetivo
	F	Empregado Efetivo
	F	Empregado Efetivo
	F	Empregado Efetivo
	F	Empregado Efetivo
	M	Empregado Efetivo
	M	Empregado Efetivo
	F	Empregado Efetivo
	F	Empregado Efetivo
	F	Empregado Efetivo
	M	Empregado Efetivo
	F	Empregado Efetivo
	F	Empregado Efetivo
	F	Empregado Efetivo
	F	Empregado Efetivo
	F	Empregado Efetivo
	F	Empregado Efetivo
	F	Empregado Efetivo
	F	Empregado Efetivo
	F	Empregado Efetivo
	F	Empregado Efetivo
	F	Empregado Efetivo
	F	Empregado Efetivo
	F	Empregado Efetivo
	F	Empregado Efetivo
	F	Empregado Efetivo
	F	Empregado Efetivo
	F	Empregado Efetivo
	F	Empregado Efetivo
	M	Empregado Efetivo
	M	Empregado Efetivo
	M	Empregado Efetivo
	M	Empregado Efetivo
	M	Empregado Efetivo
	M	Empregado Efetivo
	M	Empregado Efetivo
	M	Empregado Efetivo
	M	Empregado Efetivo
	M	Empregado Efetivo
	F	Empregado Efetivo
	F	Empregado Efetivo
	F	Empregado Efetivo
	F	Empregado Efetivo

	F	Empregado Efetivo
	F	Empregado Efetivo
	F	Empregado Efetivo
	F	Empregado Efetivo
	F	Empregado Efetivo
	F	Empregado Efetivo
	F	Empregado Efetivo
	F	Empregado Efetivo
	F	Empregado Efetivo
	F	Empregado Efetivo
	F	Empregado Efetivo
	F	Empregado Efetivo
	F	Empregado Efetivo
	F	Empregado Efetivo
	F	Empregado Efetivo
	F	Empregado Efetivo
	F	Empregado Efetivo
	F	Empregado Efetivo
	F	Empregado Efetivo
	F	Empregado Efetivo
	F	Empregado Efetivo
	F	Empregado Efetivo
	F	Empregado Efetivo
	F	Empregado Efetivo
	F	Empregado Efetivo
	F	Empregado Efetivo
	F	Empregado Efetivo
	F	Empregado Efetivo
	F	Empregado Efetivo
	F	Empregado Efetivo
	F	Empregado Efetivo
	F	Empregado Efetivo
	F	Empregado Efetivo
	F	Empregado Efetivo
	F	Empregado Efetivo
	F	Empregado Efetivo
	F	Empregado Efetivo
	F	Empregado Efetivo
	F	Empregado Efetivo
	F	Empregado Efetivo
	F	Empregado Efetivo
	F	Empregado Efetivo
	F	Empregado Efetivo
	F	Empregado Efetivo
	F	Empregado Efetivo
	F	Empregado Efetivo
	F	Empregado Efetivo
	F	Empregado Efetivo

	F	Empregado Efetivo
	F	Empregado Efetivo
	F	Empregado Efetivo
	F	Empregado Efetivo
	F	Empregado Efetivo
	F	Empregado Efetivo
	F	Empregado Efetivo
	F	Empregado Efetivo
	F	Empregado Efetivo
	F	Empregado Efetivo
	F	Empregado Efetivo
	F	Empregado Efetivo
	F	Empregado Efetivo
	F	Empregado Efetivo
	F	Empregado Efetivo
	F	Empregado Efetivo
	F	Empregado Efetivo
	F	Empregado Efetivo
	F	Empregado Efetivo
	F	Empregado Efetivo
	F	Empregado Efetivo
	F	Empregado Efetivo
	F	Empregado Efetivo
	M	Empregado Efetivo
	F	Empregado Efetivo
	F	Empregado Efetivo
	F	Empregado Efetivo
	F	Empregado Efetivo
	F	Empregado Efetivo
	F	Empregado Efetivo
	F	Empregado Efetivo
	F	Empregado Efetivo
	F	Empregado Efetivo
	F	Empregado Efetivo
	F	Empregado Efetivo
	F	Empregado Efetivo
	F	Empregado Efetivo
	F	Empregado Efetivo
	F	Empregado Efetivo
	F	Empregado Efetivo
	F	Empregado Efetivo
	F	Empregado Efetivo
	F	Empregado Efetivo
	F	Empregado Efetivo
	F	Empregado Efetivo
	F	Empregado Efetivo
	F	Empregado Efetivo
	F	Empregado Efetivo

	F	Empregado Efetivo
	M	Empregado Efetivo
	M	Empregado Efetivo
	M	Empregado Efetivo
	F	Empregado Efetivo
	F	Empregado Efetivo
	F	Empregado Efetivo
	F	Empregado Efetivo
	F	Empregado Efetivo
	F	Empregado Efetivo
	F	Empregado Efetivo
	M	Empregado Efetivo
	M	Empregado Efetivo
	F	Empregado Efetivo
	F	Empregado Efetivo
	F	Empregado Efetivo
	M	Empregado Efetivo
	F	Empregado Efetivo
	F	Empregado Efetivo
	F	Empregado Efetivo
	F	Empregado Efetivo
	F	Empregado Efetivo
	F	Empregado Efetivo
	F	Empregado Efetivo
	F	Empregado Efetivo
	F	Empregado Efetivo
	F	Empregado Efetivo
	F	Empregado Efetivo
	F	Empregado Efetivo
	F	Empregado Efetivo
	F	Empregado Efetivo
	F	Empregado Efetivo
	F	Empregado Efetivo
	M	Empregado Efetivo
	F	Empregado Efetivo
	F	Empregado Efetivo
	F	Empregado Efetivo
	F	Empregado Efetivo
	F	Empregado Efetivo
	F	Empregado Efetivo

	F	Empregado Efetivo
	F	Empregado Efetivo
	F	Empregado Efetivo
	F	Empregado Efetivo
	F	Empregado Efetivo
	F	Empregado Efetivo
	F	Empregado Efetivo
	F	Empregado Efetivo
	F	Empregado Efetivo
	F	Empregado Efetivo
	F	Empregado Efetivo
	F	Empregado Efetivo
	M	Empregado Efetivo
	F	Empregado Efetivo
	F	Empregado Efetivo
	F	Empregado Efetivo
	F	Empregado Efetivo
	M	Empregado Efetivo
	F	Empregado Efetivo
	F	Empregado Efetivo
	F	Empregado Efetivo
	F	Empregado Efetivo
	F	Empregado Efetivo
	F	Empregado Efetivo
	F	Empregado Efetivo
	F	Empregado Efetivo
	F	Empregado Efetivo
	F	Empregado Efetivo
	F	Empregado Efetivo
	F	Empregado Efetivo
	F	Empregado Efetivo
	F	Empregado Efetivo

	F	Empregado Efetivo
	F	Empregado Efetivo
	F	Empregado Efetivo
	F	Empregado Efetivo
	M	Empregado Efetivo
	F	Empregado Efetivo
	F	Empregado Efetivo
	F	Empregado Efetivo
	F	Empregado Efetivo
	F	Empregado Efetivo
	F	Empregado Efetivo
	F	Empregado Efetivo
	F	Empregado Efetivo
	F	Empregado Efetivo
	F	Empregado Efetivo
	F	Empregado Efetivo
	F	Empregado Efetivo
	F	Empregado Efetivo
	F	Empregado Efetivo
	F	Empregado Efetivo
	F	Empregado Efetivo
	F	Empregado Efetivo
	F	Empregado Efetivo
	F	Empregado Efetivo
	F	Empregado Efetivo
	F	Empregado Efetivo
	F	Empregado Efetivo
	F	Empregado Efetivo
	F	Empregado Efetivo
	F	Empregado Efetivo
	F	Empregado Efetivo
	F	Empregado Efetivo

	F	Empregado Efetivo
	F	Empregado Efetivo
	F	Empregado Efetivo
	F	Empregado Efetivo
	F	Empregado Efetivo
	F	Empregado Efetivo
	F	Empregado Efetivo
	F	Empregado Efetivo
	F	Empregado Efetivo
	F	Empregado Efetivo
	F	Empregado Efetivo
	F	Empregado Efetivo
	F	Empregado Efetivo
	F	Empregado Efetivo
	F	Empregado Efetivo
	F	Empregado Efetivo
	F	Empregado Efetivo
	F	Empregado Efetivo
	F	Empregado Efetivo
	F	Empregado Efetivo
	F	Empregado Efetivo
	F	Empregado Efetivo
	F	Empregado Efetivo
	F	Empregado Efetivo
	F	Empregado Efetivo
	F	Empregado Efetivo
	F	Empregado Efetivo
	F	Empregado Efetivo
	F	Empregado Efetivo
	F	Empregado Efetivo
	F	Empregado Efetivo
	F	Empregado Efetivo
	F	Empregado Efetivo

	F	Empregado Efetivo
	F	Empregado Efetivo
	F	Empregado Efetivo
	F	Empregado Efetivo
	F	Empregado Efetivo
	M	Empregado Efetivo
	F	Empregado Efetivo
	M	Empregado Efetivo
	F	Empregado Efetivo
	M	Empregado Efetivo
	F	Empregado Efetivo
	F	Empregado Efetivo
	M	Empregado Efetivo
	F	Empregado Efetivo
	F	Empregado Efetivo
	F	Empregado Efetivo
	F	Empregado Efetivo
	F	Empregado Efetivo
	F	Empregado Efetivo
	F	Empregado Efetivo
	F	Empregado Efetivo
	F	Empregado Efetivo
	F	Empregado Efetivo
	F	Empregado Efetivo
	M	Empregado Efetivo
	F	Empregado Efetivo
	F	Empregado Efetivo
	F	Empregado Efetivo
	F	Empregado Efetivo
	F	Empregado Efetivo
	F	Empregado Efetivo
	F	Empregado Efetivo
	F	Empregado Efetivo
	F	Empregado Efetivo
	F	Empregado Efetivo
	F	Empregado Efetivo
	F	Empregado Efetivo
	F	Empregado Efetivo
	F	Empregado Efetivo
	F	Empregado Efetivo
	F	Empregado Efetivo
	F	Empregado Efetivo
	F	Empregado Efetivo
	F	Empregado Efetivo
	M	Empregado Efetivo
	F	Empregado Efetivo
	F	Empregado Efetivo
	F	Empregado Efetivo

	F	Empregado Efetivo
	F	Empregado Efetivo
	F	Empregado Efetivo
	F	Empregado Efetivo
	F	Empregado Efetivo
	F	Empregado Efetivo
	F	Empregado Efetivo
	F	Empregado Efetivo
	F	Empregado Efetivo
	F	Empregado Efetivo
	F	Empregado Efetivo
	F	Empregado Efetivo
	F	Empregado Efetivo
	F	Empregado Efetivo
	F	Empregado Efetivo
	F	Empregado Efetivo
	F	Empregado Efetivo
	F	Empregado Efetivo
	F	Empregado Efetivo
	F	Empregado Efetivo
	F	Empregado Efetivo
	F	Empregado Efetivo
	F	Empregado Efetivo
	F	Empregado Efetivo
	F	Empregado Efetivo
	F	Empregado Efetivo
	F	Empregado Efetivo
	M	Empregado Efetivo
	M	Empregado Efetivo
	F	Empregado Efetivo
	F	Empregado Efetivo
	F	Empregado Efetivo
	F	Empregado Efetivo
	F	Empregado Efetivo
	F	Empregado Efetivo
	F	Empregado Efetivo
	F	Empregado Efetivo
	F	Empregado Efetivo
	F	Empregado Efetivo
	M	Empregado Efetivo
	F	Empregado Efetivo
	M	Empregado Efetivo
	M	Empregado Efetivo
	M	Empregado Efetivo
	M	Empregado Efetivo
	M	Empregado Efetivo
	M	Empregado Efetivo
	F	Empregado Efetivo

	F	Empregado Efetivo
	F	Empregado Efetivo
	F	Empregado Efetivo
	F	Empregado Efetivo
	F	Empregado Efetivo
	F	Empregado Efetivo
	M	Empregado Efetivo
	F	Empregado Efetivo
	M	Empregado Efetivo
	F	Comissionado
	M	Empregado Efetivo
	F	Empregado Efetivo
	M	Empregado Efetivo
	F	Empregado Efetivo
	F	Empregado Efetivo
	F	Empregado Efetivo
	F	Empregado Efetivo
	F	Empregado Efetivo
	F	Empregado Efetivo
	F	Empregado Efetivo
	M	Empregado Efetivo
	M	Empregado Efetivo
	M	Empregado Efetivo
	F	Empregado Efetivo
	F	Empregado Efetivo
	M	Empregado Efetivo
	M	Empregado Efetivo
	F	Empregado Efetivo
	F	Empregado Efetivo
	F	Empregado Efetivo
	M	Empregado Efetivo
	F	Empregado Efetivo
	M	Empregado Efetivo
	F	Empregado Efetivo
	F	Empregado Efetivo

	M	Empregado Efetivo
	F	Empregado Efetivo
	M	Empregado Efetivo
	F	Empregado Efetivo
	F	Empregado Efetivo
	M	Empregado Efetivo
	F	Empregado Efetivo
	M	Empregado Efetivo
	F	Empregado Efetivo
	F	Empregado Efetivo
	F	Empregado Efetivo
	F	Empregado Efetivo
	F	Empregado Efetivo
	M	Empregado Efetivo
	F	Empregado Efetivo
	F	Empregado Efetivo
	M	Empregado Efetivo
	M	Empregado Efetivo
	F	Empregado Efetivo
	F	Empregado Efetivo
	F	Empregado Efetivo
	F	Empregado Efetivo
	F	Empregado Efetivo
	F	Empregado Efetivo
	F	Empregado Efetivo
	F	Empregado Efetivo
	F	Empregado Efetivo
	F	Empregado Efetivo
	M	Empregado Efetivo
	M	Empregado Efetivo
	M	Empregado Efetivo
	M	Empregado Efetivo

	M	Empregado Efetivo
	M	Empregado Efetivo
	M	Empregado Efetivo
	M	Empregado Efetivo
	M	Empregado Efetivo
	M	Empregado Efetivo
	M	Empregado Efetivo
	M	Cedido - RJU
	M	Cedido - RJU
	F	Cedido - RJU
	M	Empregado Efetivo
	M	Empregado Efetivo
	M	Empregado Efetivo
	F	Empregado Efetivo
	F	Cedido - RJU
	M	Cedido - RJU
	F	Empregado Efetivo
	F	Empregado Efetivo
	F	Empregado Efetivo
	F	Empregado Efetivo
	M	Empregado Efetivo
	M	Empregado Efetivo
	F	Empregado Efetivo
	F	Empregado Efetivo
	F	Empregado Efetivo
	F	Empregado Efetivo
	F	Empregado Efetivo
	F	Empregado Efetivo
	F	Empregado Efetivo
	F	Empregado Efetivo
	M	Empregado Efetivo
	F	Empregado Efetivo
	F	Empregado Efetivo
	F	Empregado Efetivo
	F	Empregado Efetivo
	F	Empregado Efetivo
	F	Empregado Efetivo
	F	Empregado Efetivo
	F	Empregado Efetivo
	F	Empregado Efetivo
	F	Empregado Efetivo
	F	Empregado Efetivo

	M	Empregado Efetivo
	F	Empregado Efetivo
	F	Empregado Efetivo
	M	Empregado Efetivo
	M	Empregado Efetivo
	M	Empregado Efetivo
	M	Empregado Efetivo
	F	Empregado Efetivo
	F	Empregado Efetivo
	F	Empregado Efetivo
	M	Empregado Efetivo
	M	Empregado Efetivo
	M	Empregado Efetivo
	M	Empregado Efetivo
	F	Empregado Efetivo
	M	Empregado Efetivo
	M	Empregado Efetivo
	F	Empregado Efetivo
	F	Empregado Efetivo
	F	Empregado Efetivo
	F	Empregado Efetivo
	F	Empregado Efetivo
	F	Empregado Efetivo
	F	Empregado Efetivo
	M	Empregado Efetivo
	F	Empregado Efetivo
	M	Empregado Efetivo
	F	Empregado Efetivo
	M	Empregado Efetivo
	M	Empregado Efetivo
	F	Empregado Efetivo
	F	Empregado Efetivo
	F	Empregado Efetivo
	F	Empregado Efetivo
	F	Empregado Efetivo
	M	Empregado Efetivo
	M	Empregado Efetivo
	M	Empregado Efetivo
	F	Empregado Efetivo
	F	Empregado Efetivo
	F	Empregado Efetivo
	M	Empregado Efetivo
	F	Empregado Efetivo
	F	Empregado Efetivo
	M	Empregado Efetivo
	F	Empregado Efetivo
	F	Empregado Efetivo

	F	Empregado Efetivo
	M	Empregado Efetivo
	F	Empregado Efetivo
	F	Empregado Efetivo
	F	Empregado Efetivo
	F	Empregado Efetivo
	M	Empregado Efetivo
	F	Empregado Efetivo
	F	Empregado Efetivo
	M	Empregado Efetivo
	F	Empregado Efetivo
	F	Empregado Efetivo
	F	Empregado Efetivo
	F	Empregado Efetivo
	M	Empregado Efetivo
	F	Empregado Efetivo
	F	Empregado Efetivo
	F	Empregado Efetivo
	F	Empregado Efetivo
	M	Empregado Efetivo
	F	Empregado Efetivo
	F	Empregado Efetivo
	F	Empregado Efetivo
	F	Empregado Efetivo
	F	Empregado Efetivo
	M	Empregado Efetivo
	F	Empregado Efetivo
	F	Empregado Efetivo
	M	Empregado Efetivo
	F	Empregado Efetivo
	F	Empregado Efetivo
	M	Empregado Efetivo
	F	Empregado Efetivo
	M	Empregado Efetivo
	M	Empregado Efetivo
	F	Empregado Efetivo
	F	Empregado Efetivo
	F	Empregado Efetivo
	F	Empregado Efetivo
	F	Empregado Efetivo
	F	Empregado Efetivo
	M	Empregado Efetivo
	F	Empregado Efetivo
	M	Empregado Efetivo
	F	Empregado Efetivo
	M	Empregado Efetivo
	F	Empregado Efetivo
	F	Empregado Efetivo

	M	Empregado Efetivo
	F	Empregado Efetivo
	M	Empregado Efetivo
	M	Empregado Efetivo
	F	Empregado Efetivo
	M	Empregado Efetivo
	M	Empregado Efetivo
	F	Empregado Efetivo
	F	Empregado Efetivo
	M	Empregado Efetivo
	M	Empregado Efetivo
	F	Empregado Efetivo
	F	Empregado Efetivo
	F	Empregado Efetivo
	F	Empregado Efetivo
	F	Empregado Efetivo
	F	Empregado Efetivo
	M	Empregado Efetivo
	F	Empregado Efetivo
	M	Empregado Efetivo
	F	Empregado Efetivo
	F	Empregado Efetivo
	F	Empregado Efetivo
	F	Empregado Efetivo
	F	Empregado Efetivo
	F	Empregado Efetivo
	F	Empregado Efetivo
	F	Empregado Efetivo
	M	Empregado Efetivo
	M	Empregado Efetivo
	F	Empregado Efetivo
	F	Empregado Efetivo
	F	Empregado Efetivo
	F	Empregado Efetivo
	F	Empregado Efetivo
	F	Empregado Efetivo
	F	Empregado Efetivo
	F	Empregado Efetivo
	F	Empregado Efetivo
	F	Empregado Efetivo
	F	Empregado Efetivo
	F	Empregado Efetivo
	F	Empregado Efetivo
	F	Empregado Efetivo
	F	Empregado Efetivo
	F	Empregado Efetivo
	F	Empregado Efetivo
	F	Empregado Efetivo

	F	Empregado Efetivo
	F	Empregado Efetivo
	F	Empregado Efetivo
	F	Empregado Efetivo
	F	Empregado Efetivo
	F	Empregado Efetivo
	M	Empregado Efetivo
	F	Empregado Efetivo
	F	Empregado Efetivo
	F	Empregado Efetivo
	F	Empregado Efetivo
	F	Empregado Efetivo
	F	Empregado Efetivo
	F	Empregado Efetivo
	F	Empregado Efetivo
	F	Empregado Efetivo
	F	Empregado Efetivo
	F	Empregado Efetivo
	M	Empregado Efetivo
	F	Empregado Efetivo
	M	Empregado Efetivo
	F	Empregado Efetivo
	F	Empregado Efetivo
	F	Empregado Efetivo
	F	Empregado Efetivo
	F	Empregado Efetivo
	F	Empregado Efetivo
	F	Empregado Efetivo
	F	Empregado Efetivo
	F	Empregado Efetivo
	M	Empregado Efetivo
	F	Empregado Efetivo
	F	Empregado Efetivo
	F	Empregado Efetivo
	F	Empregado Efetivo
	F	Empregado Efetivo
	F	Empregado Efetivo
	F	Empregado Efetivo
	F	Empregado Efetivo
	F	Empregado Efetivo
	F	Empregado Efetivo
	F	Empregado Efetivo
	F	Empregado Efetivo
	M	Empregado Efetivo
	F	Empregado Efetivo
	F	Empregado Efetivo
	F	Empregado Efetivo
	F	Empregado Efetivo

	F	Empregado Efetivo
	F	Empregado Efetivo
	F	Empregado Efetivo
	F	Empregado Efetivo
	F	Empregado Efetivo
	F	Empregado Efetivo
	F	Empregado Efetivo
	F	Empregado Efetivo
	F	Empregado Efetivo
	F	Empregado Efetivo
	F	Empregado Efetivo
	F	Empregado Efetivo
	F	Empregado Efetivo
	F	Empregado Efetivo
	F	Empregado Efetivo
	F	Empregado Efetivo
	M	Empregado Efetivo
	F	Empregado Efetivo
	F	Empregado Efetivo
	F	Empregado Efetivo
	F	Empregado Efetivo
	M	Empregado Efetivo
	F	Empregado Efetivo
	F	Empregado Efetivo
	M	Empregado Efetivo
	F	Empregado Efetivo
	F	Empregado Efetivo
	F	Empregado Efetivo
	F	Empregado Efetivo
	F	Empregado Efetivo
	M	Empregado Efetivo
	F	Empregado Efetivo
	M	Empregado Efetivo
	F	Empregado Efetivo

	F	Empregado Efetivo
	F	Empregado Efetivo
	F	Empregado Efetivo
	F	Empregado Efetivo
	F	Empregado Efetivo
	F	Empregado Efetivo
	F	Empregado Efetivo
	F	Empregado Efetivo
	F	Empregado Efetivo
	F	Empregado Efetivo
	M	Empregado Efetivo
	F	Empregado Efetivo
	F	Empregado Efetivo
	M	Empregado Efetivo
	F	Empregado Efetivo
	M	Empregado Efetivo
	F	Empregado Efetivo
	F	Empregado Efetivo
	F	Empregado Efetivo
	F	Empregado Efetivo
	F	Empregado Efetivo
	M	Empregado Efetivo
	M	Empregado Efetivo
	F	Empregado Efetivo
	M	Empregado Efetivo
	M	Empregado Efetivo
	F	Empregado Efetivo
	F	Empregado Efetivo
	F	Empregado Efetivo
	F	Empregado Efetivo
	M	Empregado Efetivo
	F	Empregado Efetivo
	F	Empregado Efetivo
	F	Empregado Efetivo
	M	Empregado Efetivo
	M	Empregado Efetivo
	F	Empregado Efetivo
	F	Empregado Efetivo
	F	Empregado Efetivo
	M	Empregado Efetivo
	F	Empregado Efetivo
	F	Empregado Efetivo
	M	Empregado Efetivo
	F	Empregado Efetivo
	M	Empregado Efetivo

	F	Empregado Efetivo
	F	Empregado Efetivo
	F	Empregado Efetivo
	F	Empregado Efetivo
	M	Empregado Efetivo
	F	Empregado Efetivo
	F	Empregado Efetivo
	F	Empregado Efetivo
	F	Empregado Efetivo
	F	Empregado Efetivo
	M	Empregado Efetivo
	M	Empregado Efetivo
	M	Empregado Efetivo
	M	Empregado Efetivo
	F	Empregado Efetivo
	M	Empregado Efetivo
	M	Empregado Efetivo
	F	Empregado Efetivo
	M	Empregado Efetivo
	F	Empregado Efetivo
	M	Empregado Efetivo
	M	Empregado Efetivo
	M	Empregado Efetivo
	F	Empregado Efetivo
	M	Empregado Efetivo
	F	Empregado Efetivo
	M	Empregado Efetivo
	F	Empregado Efetivo
	F	Empregado Efetivo
	M	Empregado Efetivo
	F	Empregado Efetivo
	F	Empregado Efetivo
	M	Empregado Efetivo
	M	Empregado Efetivo
	M	Empregado Efetivo
	F	Empregado Efetivo
	M	Empregado Efetivo
	F	Empregado Efetivo
	F	Empregado Efetivo
	M	Empregado Efetivo
	M	Empregado Efetivo
	F	Empregado Efetivo
	F	Empregado Efetivo
	F	Empregado Efetivo
	M	Empregado Efetivo
	F	Empregado Efetivo
	F	Empregado Efetivo
	M	Empregado Efetivo

	F	Empregado Efetivo
	F	Empregado Efetivo
	F	Empregado Efetivo
	F	Empregado Efetivo
	M	Empregado Efetivo
	F	Empregado Efetivo
	F	Empregado Efetivo
	F	Empregado Efetivo
	F	Empregado Efetivo
	F	Empregado Efetivo
	F	Empregado Efetivo
	F	Empregado Efetivo
	F	Empregado Efetivo
	F	Empregado Efetivo
	M	Empregado Efetivo
	F	Empregado Efetivo
	F	Empregado Efetivo
	F	Empregado Efetivo
	M	Empregado Efetivo
	M	Empregado Efetivo
	F	Empregado Efetivo
	M	Empregado Efetivo
	F	Empregado Efetivo
	F	Empregado Efetivo
	F	Empregado Efetivo
	M	Empregado Efetivo
	M	Empregado Efetivo
	F	Empregado Efetivo
	F	Empregado Efetivo
	M	Empregado Efetivo
	F	Empregado Efetivo
	M	Empregado Efetivo
	F	Empregado Efetivo
	M	Empregado Efetivo
	F	Empregado Efetivo
	F	Empregado Efetivo
	M	Empregado Efetivo
	F	Empregado Efetivo
	M	Empregado Efetivo
	F	Empregado Efetivo
	F	Empregado Efetivo
	M	Empregado Efetivo
	F	Empregado Efetivo
	F	Empregado Efetivo
	F	Empregado Efetivo
	F	Empregado Efetivo
	F	Empregado Efetivo
	F	Empregado Efetivo

	M	Empregado Efetivo
	M	Empregado Efetivo
	F	Empregado Efetivo
	F	Empregado Efetivo
	F	Empregado Efetivo
	F	Empregado Efetivo
	F	Empregado Efetivo
	M	Empregado Efetivo
	M	Empregado Efetivo
	F	Empregado Efetivo
	F	Empregado Efetivo
	M	Empregado Efetivo
	F	Empregado Efetivo
	F	Empregado Efetivo
	F	Empregado Efetivo
	M	Empregado Efetivo
	F	Empregado Efetivo
	M	Empregado Efetivo
	F	Empregado Efetivo
	M	Empregado Efetivo
	M	Empregado Efetivo
	F	Empregado Efetivo
	F	Empregado Efetivo
	F	Empregado Efetivo
	F	Empregado Efetivo
	M	Empregado Efetivo
	M	Empregado Efetivo
	F	Empregado Efetivo
	F	Empregado Efetivo
	M	Empregado Efetivo
	F	Empregado Efetivo
	M	Empregado Efetivo
	F	Empregado Efetivo
	M	Empregado Efetivo
	M	Empregado Efetivo
	F	Empregado Efetivo
	F	Empregado Efetivo
	F	Empregado Efetivo
	M	Empregado Efetivo
	F	Empregado Efetivo
	F	Empregado Efetivo
	M	Empregado Efetivo
	F	Empregado Efetivo
	M	Empregado Efetivo
	F	Empregado Efetivo
	F	Empregado Efetivo
	M	Empregado Efetivo
	M	Empregado Efetivo

	M	Empregado Efetivo
	F	Empregado Efetivo
	F	Empregado Efetivo
	M	Empregado Efetivo
	F	Empregado Efetivo
	M	Empregado Efetivo
	M	Empregado Efetivo
	M	Empregado Efetivo
	F	Empregado Efetivo
	F	Empregado Efetivo
	F	Empregado Efetivo
	M	Empregado Efetivo
	M	Empregado Efetivo
	M	Empregado Efetivo
	M	Empregado Efetivo
	F	Empregado Efetivo
	M	Empregado Efetivo
	F	Empregado Efetivo
	F	Empregado Efetivo
	F	Empregado Efetivo
	F	Empregado Efetivo
	F	Empregado Efetivo
	F	Empregado Efetivo
	F	Empregado Efetivo
	F	Empregado Efetivo
	M	Empregado Efetivo
	F	Empregado Efetivo
	F	Empregado Efetivo
	F	Empregado Efetivo
	F	Empregado Efetivo
	F	Empregado Efetivo
	M	Empregado Efetivo
	F	Empregado Efetivo
	F	Empregado Efetivo
	M	Empregado Efetivo
	F	Empregado Efetivo
	F	Empregado Efetivo
	F	Empregado Efetivo
	F	Empregado Efetivo
	F	Empregado Efetivo
	F	Empregado Efetivo
	F	Empregado Efetivo
	F	Empregado Efetivo
	M	Empregado Efetivo
	M	Empregado Efetivo
	M	Empregado Efetivo
	M	Empregado Efetivo
	M	Empregado Efetivo

	F	Empregado Efetivo
	F	Empregado Efetivo
	F	Empregado Efetivo
	F	Empregado Efetivo
	F	Empregado Efetivo
	F	Empregado Efetivo
	F	Empregado Efetivo
	F	Empregado Efetivo
	M	Empregado Efetivo
	F	Empregado Efetivo
	F	Empregado Efetivo
	F	Empregado Efetivo
	F	Empregado Efetivo
	F	Empregado Efetivo
	F	Empregado Efetivo
	F	Empregado Efetivo
	F	Empregado Efetivo
	F	Empregado Efetivo
	F	Empregado Efetivo
	F	Empregado Efetivo
	F	Empregado Efetivo
	F	Empregado Efetivo
	F	Empregado Efetivo
	F	Empregado Efetivo
	F	Empregado Efetivo
	M	Empregado Efetivo
	F	Empregado Efetivo
	F	Empregado Efetivo
	F	Empregado Efetivo
	F	Empregado Efetivo
	F	Empregado Efetivo
	F	Empregado Efetivo
	F	Empregado Efetivo
	F	Empregado Efetivo

	F	Empregado Efetivo
	F	Empregado Efetivo
	F	Empregado Efetivo
	F	Empregado Efetivo
	F	Empregado Efetivo
	F	Empregado Efetivo
	F	Empregado Efetivo
	M	Empregado Efetivo
	M	Empregado Efetivo
	F	Empregado Efetivo
	M	Empregado Efetivo
	F	Empregado Efetivo
	F	Empregado Efetivo
	F	Empregado Efetivo
	F	Empregado Efetivo
	M	Empregado Efetivo
	F	Empregado Efetivo
	M	Empregado Efetivo
	F	Empregado Efetivo
	M	Empregado Efetivo
	F	Empregado Efetivo
	F	Empregado Efetivo
	F	Empregado Efetivo
	M	Empregado Efetivo
	M	Empregado Efetivo
	M	Empregado Efetivo
	M	Empregado Efetivo
	M	Empregado Efetivo
	F	Empregado Efetivo
	F	Empregado Efetivo
	M	Empregado Efetivo
	F	Empregado Efetivo

	F	Empregado Efetivo
	M	Empregado Efetivo
	M	Empregado Efetivo
	F	Empregado Efetivo
	F	Empregado Efetivo
	M	Empregado Efetivo
	F	Empregado Efetivo
	M	Empregado Efetivo
	F	Empregado Efetivo
	M	Empregado Efetivo
	F	Empregado Efetivo
	M	Empregado Efetivo
	F	Empregado Efetivo
	M	Empregado Efetivo
	F	Empregado Efetivo
	M	Empregado Efetivo
	F	Empregado Efetivo
	M	Empregado Efetivo
	F	Empregado Efetivo
	M	Empregado Efetivo
	M	Empregado Efetivo
	F	Empregado Efetivo
	M	Empregado Efetivo
	M	Empregado Efetivo
	M	Empregado Efetivo
	F	Empregado Efetivo
	M	Empregado Efetivo
	M	Empregado Efetivo
	M	Empregado Efetivo
	F	Empregado Efetivo
	M	Empregado Efetivo
	F	Empregado Efetivo

	M	Empregado Efetivo
	M	Empregado Efetivo
	M	Empregado Efetivo
	M	Empregado Efetivo
	M	Empregado Efetivo
	M	Empregado Efetivo
	F	Empregado Efetivo
	M	Empregado Efetivo
	M	Empregado Efetivo
	F	Empregado Efetivo
	F	Empregado Efetivo
	F	Empregado Efetivo
	F	Empregado Efetivo
	M	Empregado Efetivo
	M	Empregado Efetivo
	M	Empregado Efetivo
	F	Empregado Efetivo
	M	Empregado Efetivo
	F	Empregado Efetivo
	M	Empregado Efetivo
	M	Empregado Efetivo
	F	Empregado Efetivo
	F	Empregado Efetivo
	F	Empregado Efetivo
	F	Empregado Efetivo
	F	Empregado Efetivo
	F	Empregado Efetivo
	M	Empregado Efetivo
	F	Empregado Efetivo
	F	Empregado Efetivo
	M	Empregado Efetivo
	M	Empregado Efetivo
	F	Empregado Efetivo

	F	Empregado Efetivo
	M	Empregado Efetivo
	F	Empregado Efetivo
	F	Empregado Efetivo
	M	Empregado Efetivo
	F	Empregado Efetivo
	M	Empregado Efetivo
	F	Empregado Efetivo
	F	Empregado Efetivo
	F	Empregado Efetivo
	F	Empregado Efetivo
	F	Empregado Efetivo
	F	Empregado Efetivo
	F	Empregado Efetivo
	F	Empregado Efetivo
	F	Empregado Efetivo
	F	Empregado Efetivo
	M	Empregado Efetivo
	M	Empregado Efetivo
	M	Empregado Efetivo
	F	Empregado Efetivo
	F	Empregado Efetivo
	M	Empregado Efetivo
	M	Empregado Efetivo
	F	Empregado Efetivo
	M	Empregado Efetivo
	M	Empregado Efetivo
	F	Empregado Efetivo
	F	Empregado Efetivo
	F	Empregado Efetivo
	F	Empregado Efetivo
	F	Empregado Efetivo
	M	Empregado Efetivo
	M	Empregado Efetivo
	F	Empregado Efetivo
	F	Empregado Efetivo
	M	Empregado Efetivo
	M	Empregado Efetivo
	M	Empregado Efetivo
	F	Empregado Efetivo
	F	Empregado Efetivo
	M	Empregado Efetivo
	M	Empregado Efetivo
	F	Empregado Efetivo

	F	Empregado Efetivo
	M	Empregado Efetivo
	F	Empregado Efetivo
	F	Empregado Efetivo
	F	Empregado Efetivo
	F	Empregado Efetivo
	F	Empregado Efetivo
	F	Empregado Efetivo
	F	Empregado Efetivo
	F	Empregado Efetivo
	F	Empregado Efetivo
	F	Empregado Efetivo
	F	Empregado Efetivo
	F	Empregado Efetivo
	F	Empregado Efetivo
	F	Empregado Efetivo
	F	Empregado Efetivo
	F	Empregado Efetivo
	F	Empregado Efetivo
	F	Empregado Efetivo
	F	Empregado Efetivo
	M	Empregado Efetivo
	F	Empregado Efetivo
	F	Empregado Efetivo
	F	Empregado Efetivo
	F	Empregado Efetivo
	F	Empregado Efetivo
	F	Empregado Efetivo
	M	Empregado Efetivo
	M	Empregado Efetivo
	F	Empregado Efetivo
	F	Empregado Efetivo
	F	Empregado Efetivo
	F	Empregado Efetivo
	M	Empregado Efetivo
	M	Empregado Efetivo
	F	Empregado Efetivo
	F	Empregado Efetivo
	F	Empregado Efetivo
	F	Empregado Efetivo
	F	Empregado Efetivo
	F	Empregado Efetivo
	F	Empregado Efetivo
	F	Empregado Efetivo
	F	Empregado Efetivo
	F	Empregado Efetivo
	F	Empregado Efetivo
	F	Empregado Efetivo

	F	Empregado Efetivo
	F	Empregado Efetivo
	F	Empregado Efetivo
	F	Empregado Efetivo
	F	Empregado Efetivo
	F	Empregado Efetivo
	F	Empregado Efetivo
	F	Empregado Efetivo
	F	Empregado Efetivo
	F	Empregado Efetivo
	F	Empregado Efetivo
	F	Empregado Efetivo
	F	Empregado Efetivo
	F	Empregado Efetivo
	M	Empregado Efetivo
	F	Empregado Efetivo
	F	Empregado Efetivo
	F	Empregado Efetivo
	F	Empregado Efetivo
	F	Empregado Efetivo
	F	Empregado Efetivo
	F	Empregado Efetivo
	F	Empregado Efetivo
	F	Empregado Efetivo
	F	Empregado Efetivo
	F	Empregado Efetivo
	F	Empregado Efetivo
	F	Empregado Efetivo
	F	Empregado Efetivo
	F	Empregado Efetivo
	F	Empregado Efetivo
	F	Empregado Efetivo
	F	Empregado Efetivo
	F	Empregado Efetivo
	F	Empregado Efetivo
	F	Empregado Efetivo
	F	Empregado Efetivo
	F	Empregado Efetivo
	F	Empregado Efetivo
	F	Empregado Efetivo
	F	Empregado Efetivo
	F	Empregado Efetivo
	F	Empregado Efetivo
	F	Empregado Efetivo
	F	Empregado Efetivo
	F	Empregado Efetivo
	F	Empregado Efetivo
	F	Empregado Efetivo

	F	Empregado Efetivo
	F	Empregado Efetivo
	F	Empregado Efetivo
	F	Empregado Efetivo
	F	Empregado Efetivo
	F	Empregado Efetivo
	F	Empregado Efetivo
	F	Empregado Efetivo
	F	Empregado Efetivo
	F	Empregado Efetivo
	F	Empregado Efetivo
	F	Empregado Efetivo
	F	Empregado Efetivo
	F	Empregado Efetivo
	M	Empregado Efetivo
	F	Empregado Efetivo
	F	Empregado Efetivo
	F	Empregado Efetivo
	F	Empregado Efetivo
	F	Empregado Efetivo
	F	Empregado Efetivo
	F	Empregado Efetivo
	M	Empregado Efetivo
	M	Empregado Efetivo
	F	Empregado Efetivo
	M	Empregado Efetivo
	M	Empregado Efetivo
	F	Empregado Efetivo
	F	Empregado Efetivo
	F	Empregado Efetivo
	F	Empregado Efetivo
	M	Empregado Efetivo
	F	Empregado Efetivo
	M	Empregado Efetivo
	F	Empregado Efetivo
	F	Empregado Efetivo
	F	Empregado Efetivo
	F	Empregado Efetivo
	F	Empregado Efetivo
	F	Empregado Efetivo
	M	Empregado Efetivo
	M	Empregado Efetivo
	F	Empregado Efetivo
	F	Empregado Efetivo

	F	Empregado Efetivo
	F	Empregado Efetivo
	F	Empregado Efetivo
	F	Empregado Efetivo
	F	Empregado Efetivo
	F	Empregado Efetivo
	F	Empregado Efetivo
	F	Empregado Efetivo
	M	Empregado Efetivo
	F	Empregado Efetivo
	F	Empregado Efetivo
	F	Empregado Efetivo
	F	Empregado Efetivo
	M	Empregado Efetivo
	M	Empregado Efetivo
	F	Empregado Efetivo
	F	Empregado Efetivo
	F	Empregado Efetivo
	F	Empregado Efetivo
	F	Empregado Efetivo
	F	Empregado Efetivo
	F	Empregado Efetivo
	F	Empregado Efetivo
	F	Empregado Efetivo
	F	Empregado Efetivo
	F	Empregado Efetivo
	M	Empregado Efetivo
	F	Empregado Efetivo
	F	Empregado Efetivo
	F	Empregado Efetivo
	F	Empregado Efetivo
	F	Empregado Efetivo
	F	Empregado Efetivo

	F	Empregado Efetivo
	F	Empregado Efetivo
	F	Empregado Efetivo
	F	Empregado Efetivo
	F	Empregado Efetivo
	F	Empregado Efetivo
	F	Empregado Efetivo
	F	Empregado Efetivo
	F	Empregado Efetivo
	F	Empregado Efetivo
	F	Empregado Efetivo
	F	Empregado Efetivo
	F	Empregado Efetivo
	F	Empregado Efetivo
	F	Empregado Efetivo
	F	Empregado Efetivo
	F	Empregado Efetivo
	F	Empregado Efetivo
	F	Empregado Efetivo
	F	Empregado Efetivo
	F	Empregado Efetivo
	F	Empregado Efetivo
	F	Empregado Efetivo
	F	Empregado Efetivo
	M	Empregado Efetivo
	F	Empregado Efetivo
	F	Empregado Efetivo
	F	Empregado Efetivo
	M	Empregado Efetivo
	F	Empregado Efetivo
	M	Empregado Efetivo
	M	Empregado Efetivo

	F	Empregado Efetivo
	M	Empregado Efetivo
	F	Empregado Efetivo
	F	Empregado Efetivo
	F	Empregado Efetivo
	M	Empregado Efetivo
	M	Empregado Efetivo
	F	Empregado Efetivo
	F	Empregado Efetivo
	F	Empregado Efetivo
	M	Empregado Efetivo
	F	Empregado Efetivo
	F	Empregado Efetivo
	F	Empregado Efetivo
	F	Empregado Efetivo
	M	Empregado Efetivo
	M	Empregado Efetivo
	F	Empregado Efetivo
	F	Empregado Efetivo
	M	Empregado Efetivo
	M	Empregado Efetivo
	F	Empregado Efetivo
	F	Empregado Efetivo
	M	Empregado Efetivo
	M	Empregado Efetivo
	M	Empregado Efetivo
	F	Empregado Efetivo
	F	Empregado Efetivo
	F	Empregado Efetivo
	M	Empregado Efetivo
	F	Empregado Efetivo
	F	Empregado Efetivo
	M	Empregado Efetivo
	F	Empregado Efetivo
	M	Empregado Efetivo
	F	Empregado Efetivo
	F	Empregado Efetivo
	M	Empregado Efetivo
	F	Empregado Efetivo
	M	Empregado Efetivo
	F	Empregado Efetivo
	F	Empregado Efetivo

	F	Empregado Efetivo
	F	Empregado Efetivo
	F	Empregado Efetivo
	F	Empregado Efetivo
	M	Empregado Efetivo
	F	Empregado Efetivo
	F	Empregado Efetivo
	F	Empregado Efetivo
	F	Empregado Efetivo
	F	Empregado Efetivo
	F	Empregado Efetivo
	M	Empregado Efetivo
	F	Empregado Efetivo
	F	Empregado Efetivo
	F	Empregado Efetivo
	F	Empregado Efetivo
	F	Empregado Efetivo
	F	Empregado Efetivo
	F	Empregado Efetivo
	F	Empregado Efetivo
	F	Empregado Efetivo
	F	Empregado Efetivo
	F	Empregado Efetivo
	M	Empregado Efetivo
	F	Empregado Efetivo
	M	Empregado Efetivo
	M	Empregado Efetivo
	F	Empregado Efetivo
	F	Empregado Efetivo
	F	Empregado Efetivo
	M	Empregado Efetivo
	F	Empregado Efetivo
	F	Empregado Efetivo
	F	Empregado Efetivo
	M	Empregado Efetivo
	F	Empregado Efetivo
	F	Empregado Efetivo
	M	Empregado Efetivo
	F	Empregado Efetivo
	M	Empregado Efetivo
	F	Empregado Efetivo
	F	Empregado Efetivo
	F	Empregado Efetivo
	F	Empregado Efetivo
	F	Empregado Efetivo
	F	Empregado Efetivo
	F	Empregado Efetivo
	F	Empregado Efetivo

	F	Empregado Efetivo
	F	Empregado Efetivo
	F	Empregado Efetivo
	F	Empregado Efetivo
	M	Empregado Efetivo
	M	Empregado Efetivo
	M	Empregado Efetivo
	M	Empregado Efetivo
	F	Empregado Efetivo
	F	Empregado Efetivo
	F	Empregado Efetivo
	F	Empregado Efetivo
	F	Empregado Efetivo
	F	Empregado Efetivo
	F	Empregado Efetivo
	M	Empregado Efetivo
	M	Empregado Efetivo
	F	Empregado Efetivo
	F	Empregado Efetivo
	F	Empregado Efetivo
	F	Empregado Efetivo
	F	Empregado Efetivo
	F	Empregado Efetivo
	F	Empregado Efetivo
	F	Empregado Efetivo
	M	Empregado Efetivo
	F	Empregado Efetivo
	F	Empregado Efetivo
	F	Empregado Efetivo
	F	Empregado Efetivo
	F	Empregado Efetivo
	F	Empregado Efetivo
	F	Empregado Efetivo
	F	Empregado Efetivo
	F	Empregado Efetivo
	F	Empregado Efetivo
	F	Empregado Efetivo
	F	Empregado Efetivo
	F	Empregado Efetivo
	F	Empregado Efetivo
	F	Empregado Efetivo
	F	Empregado Efetivo
	F	Empregado Efetivo
	F	Empregado Efetivo
	F	Empregado Efetivo

	F	Empregado Efetivo
	F	Empregado Efetivo
	F	Empregado Efetivo
	F	Empregado Efetivo
	F	Empregado Efetivo
	F	Empregado Efetivo
	F	Empregado Efetivo
	F	Empregado Efetivo
	F	Empregado Efetivo
	F	Empregado Efetivo
	F	Empregado Efetivo
	F	Empregado Efetivo
	F	Empregado Efetivo
	F	Empregado Efetivo
	F	Empregado Efetivo
	F	Empregado Efetivo
	F	Empregado Efetivo
	F	Empregado Efetivo
	F	Empregado Efetivo
	F	Empregado Efetivo
	F	Empregado Efetivo
	F	Empregado Efetivo
	F	Empregado Efetivo
	F	Empregado Efetivo
	F	Empregado Efetivo
	F	Empregado Efetivo
	F	Empregado Efetivo
	F	Empregado Efetivo
	F	Empregado Efetivo
	F	Empregado Efetivo
	F	Empregado Efetivo
	F	Empregado Efetivo
	F	Empregado Efetivo
	F	Empregado Efetivo
	F	Empregado Efetivo
	F	Empregado Efetivo
	F	Empregado Efetivo
	F	Empregado Efetivo
	F	Empregado Efetivo
	F	Empregado Efetivo
	F	Empregado Efetivo

	F	Empregado Efetivo
	F	Empregado Efetivo
	F	Empregado Efetivo
	F	Empregado Efetivo
	F	Empregado Efetivo
	F	Empregado Efetivo
	F	Empregado Efetivo
	F	Empregado Efetivo
	F	Empregado Efetivo
	F	Empregado Efetivo
	F	Empregado Efetivo
	F	Empregado Efetivo
	F	Empregado Efetivo
	F	Empregado Efetivo
	M	Empregado Efetivo
	M	Empregado Efetivo
	M	Empregado Efetivo
	M	Empregado Efetivo
	M	Empregado Efetivo
	M	Empregado Efetivo
	M	Empregado Efetivo
	M	Empregado Efetivo
	M	Empregado Efetivo
	M	Empregado Efetivo
	M	Empregado Efetivo
	M	Empregado Efetivo
	M	Empregado Efetivo
	F	Empregado Efetivo
	M	Empregado Efetivo
	F	Empregado Efetivo
	F	Empregado Efetivo
	F	Empregado Efetivo
	F	Empregado Efetivo
	F	Empregado Efetivo
	F	Empregado Efetivo
	M	Empregado Efetivo
	M	Empregado Efetivo
	F	Empregado Efetivo
	M	Empregado Efetivo
	M	Empregado Efetivo
	F	Empregado Efetivo

	F	Empregado Efetivo
	M	Empregado Efetivo
	F	Empregado Efetivo
	M	Empregado Efetivo
	F	Empregado Efetivo
	M	Empregado Efetivo
	M	Empregado Efetivo
	F	Empregado Efetivo
	F	Empregado Efetivo
	F	Empregado Efetivo
	F	Empregado Efetivo
	F	Empregado Efetivo
	F	Empregado Efetivo
	M	Empregado Efetivo
	F	Empregado Efetivo
	F	Empregado Efetivo
	F	Empregado Efetivo
	F	Empregado Efetivo
	F	Empregado Efetivo
	F	Empregado Efetivo
	M	Empregado Efetivo
	M	Empregado Efetivo
	F	Empregado Efetivo
	F	Empregado Efetivo
	F	Empregado Efetivo
	F	Empregado Efetivo
	F	Empregado Efetivo
	F	Empregado Efetivo
	M	Empregado Efetivo
	F	Empregado Efetivo
	F	Empregado Efetivo
	M	Empregado Efetivo
	M	Empregado Efetivo
	M	Empregado Efetivo
	M	Empregado Efetivo
	M	Empregado Efetivo
	F	Empregado Efetivo
	F	Empregado Efetivo
	M	Empregado Efetivo
	F	Empregado Efetivo
	M	Empregado Efetivo
	F	Empregado Efetivo

	M	Empregado Efetivo
	M	Empregado Efetivo
	F	Empregado Efetivo
	F	Empregado Efetivo
	F	Empregado Efetivo
	F	Empregado Efetivo
	F	Empregado Efetivo
	F	Empregado Efetivo
	F	Empregado Efetivo
	F	Empregado Efetivo
	F	Empregado Efetivo
	M	Empregado Efetivo
	F	Empregado Efetivo
	F	Empregado Efetivo
	M	Empregado Efetivo
	F	Empregado Efetivo
	M	Empregado Efetivo
	F	Empregado Efetivo
	F	Empregado Efetivo
	M	Empregado Efetivo
	F	Empregado Efetivo
	M	Empregado Efetivo
	F	Empregado Efetivo
	F	Empregado Efetivo
	F	Empregado Efetivo
	F	Empregado Efetivo
	F	Empregado Efetivo
	F	Empregado Efetivo
	F	Empregado Efetivo
	F	Empregado Efetivo
	F	Empregado Efetivo
	F	Empregado Efetivo
	F	Empregado Efetivo
	F	Empregado Efetivo
	F	Empregado Efetivo
	F	Empregado Efetivo
	F	Empregado Efetivo
	F	Empregado Efetivo
	F	Empregado Efetivo
	F	Empregado Efetivo

	F	Empregado Efetivo
	F	Empregado Efetivo
	F	Empregado Efetivo
	F	Empregado Efetivo
	F	Empregado Efetivo
	F	Empregado Efetivo
	F	Empregado Efetivo
	F	Empregado Efetivo
	F	Empregado Efetivo
	F	Empregado Efetivo
	F	Empregado Efetivo
	M	Cedido - RJU
	F	Empregado Efetivo
	F	Empregado Efetivo
	F	Empregado Efetivo
	F	Empregado Temporário
	F	Empregado Efetivo
	F	Empregado Efetivo
	F	Empregado Efetivo
	F	Empregado Efetivo
	F	Empregado Efetivo
	F	Empregado Efetivo
	F	Empregado Efetivo
	F	Empregado Efetivo
	F	Empregado Efetivo
	F	Empregado Efetivo
	F	Empregado Efetivo
	F	Empregado Efetivo
	F	Empregado Efetivo
	F	Empregado Efetivo
	F	Empregado Efetivo
	F	Empregado Efetivo
	F	Empregado Efetivo
	F	Empregado Efetivo
	F	Empregado Efetivo
	F	Empregado Efetivo
	F	Empregado Efetivo
	F	Empregado Efetivo
	M	Empregado Efetivo

	M	Empregado Efetivo
	F	Empregado Efetivo
	F	Empregado Efetivo
	F	Empregado Efetivo
	F	Empregado Efetivo
	F	Empregado Efetivo
	M	Empregado Efetivo
	F	Empregado Efetivo
	F	Empregado Efetivo
	F	Empregado Efetivo
	F	Empregado Efetivo
	M	Empregado Efetivo
	F	Empregado Efetivo
	F	Empregado Efetivo
	M	Empregado Efetivo
	F	Empregado Efetivo
	M	Empregado Efetivo
	M	Empregado Efetivo
	F	Empregado Efetivo
	F	Empregado Efetivo
	F	Empregado Efetivo
	F	Empregado Efetivo
	F	Empregado Efetivo
	F	Empregado Efetivo
	F	Empregado Efetivo
	M	Empregado Efetivo
	M	Empregado Efetivo
	F	Empregado Efetivo
	F	Empregado Efetivo
	F	Empregado Efetivo
	F	Empregado Temporário
	F	Empregado Temporário
	F	Empregado Efetivo
	F	Empregado Efetivo

	F	Empregado Efetivo
	F	Empregado Efetivo
	F	Empregado Efetivo
	F	Empregado Efetivo
	F	Empregado Efetivo
	F	Empregado Efetivo
	F	Empregado Efetivo
	F	Empregado Efetivo
	F	Empregado Efetivo
	F	Empregado Efetivo
	F	Empregado Efetivo
	F	Empregado Efetivo
	F	Empregado Efetivo
	F	Empregado Efetivo
	F	Empregado Efetivo
	F	Empregado Efetivo
	F	Empregado Efetivo
	F	Empregado Efetivo
	M	Empregado Efetivo
	F	Empregado Efetivo
	M	Empregado Efetivo
	F	Empregado Efetivo
	M	Empregado Efetivo
	F	Empregado Efetivo
	F	Empregado Efetivo
	F	Empregado Efetivo
	F	Empregado Efetivo
	M	Cedido - RJU
	F	Empregado Efetivo
	M	Empregado Efetivo
	M	Empregado Efetivo
	M	Empregado Efetivo
	F	Empregado Efetivo
	M	Cedido - RJU
	F	Empregado Efetivo
	F	Empregado Efetivo
	M	Empregado Efetivo
	M	Empregado Efetivo

	F	Empregado Efetivo
	F	Empregado Efetivo
	M	Empregado Efetivo
	M	Empregado Efetivo
	F	Empregado Efetivo
	F	Empregado Efetivo
	F	Empregado Efetivo
	F	Empregado Efetivo
	M	Empregado Efetivo
	M	Empregado Efetivo
	F	Empregado Efetivo
	F	Empregado Efetivo
	F	Empregado Efetivo
	F	Empregado Efetivo
	M	Empregado Efetivo
	F	Empregado Efetivo
	M	Empregado Efetivo
	M	Empregado Efetivo
	F	Empregado Efetivo
	F	Empregado Efetivo
	F	Empregado Efetivo
	F	Empregado Efetivo
	F	Empregado Efetivo
	F	Empregado Efetivo
	F	Empregado Efetivo
	F	Empregado Efetivo
	F	Empregado Efetivo
	M	Empregado Efetivo
	F	Empregado Efetivo
	F	Empregado Efetivo
	F	Empregado Efetivo
	F	Empregado Efetivo

	M	Empregado Efetivo
	F	Empregado Efetivo
	F	Empregado Efetivo
	F	Empregado Efetivo
	F	Empregado Efetivo
	M	Empregado Efetivo
	F	Empregado Efetivo
	F	Empregado Efetivo
	F	Empregado Efetivo
	F	Empregado Efetivo
	M	Empregado Efetivo
	M	Empregado Efetivo
	M	Empregado Efetivo
	F	Empregado Efetivo
	F	Empregado Efetivo
	F	Empregado Efetivo
	F	Empregado Efetivo
	F	Empregado Efetivo
	F	Empregado Efetivo
	F	Empregado Efetivo
	F	Empregado Efetivo
	F	Empregado Efetivo
	F	Empregado Efetivo
	F	Empregado Efetivo
	F	Empregado Efetivo
	F	Empregado Efetivo
	F	Empregado Efetivo
	F	Empregado Efetivo
	M	Empregado Efetivo
	F	Empregado Efetivo
	F	Empregado Efetivo
	F	Empregado Efetivo
	F	Empregado Efetivo
	F	Empregado Efetivo
	F	Empregado Efetivo
	F	Empregado Efetivo
	F	Empregado Efetivo
	F	Empregado Efetivo
	F	Empregado Efetivo
	F	Empregado Efetivo
	F	Empregado Efetivo
	F	Empregado Efetivo
	F	Empregado Efetivo

	F	Empregado Efetivo
	F	Empregado Efetivo
	F	Empregado Efetivo
	F	Empregado Efetivo
	F	Empregado Efetivo
	F	Empregado Efetivo
	M	Empregado Efetivo
	M	Empregado Efetivo
	F	Empregado Efetivo
	M	Empregado Efetivo
	F	Empregado Efetivo
	M	Empregado Efetivo
	M	Empregado Efetivo
	M	Empregado Efetivo
	M	Empregado Efetivo
	M	Empregado Efetivo
	M	Empregado Efetivo
	F	Cedido - RJU
	F	Empregado Efetivo
	F	Empregado Efetivo
	F	Empregado Efetivo
	F	Empregado Efetivo
	F	Empregado Efetivo
	M	Empregado Efetivo
	F	Empregado Efetivo
	F	Empregado Efetivo
	F	Empregado Efetivo
	F	Empregado Efetivo
	F	Empregado Efetivo
	M	Empregado Efetivo
	M	Empregado Efetivo
	F	Empregado Efetivo
	M	Empregado Efetivo
	M	Empregado Efetivo
	M	Empregado Efetivo
	F	Empregado Efetivo
	F	Empregado Efetivo
	F	Empregado Efetivo
	F	Empregado Efetivo
	F	Cedido - RJU
	F	Empregado Efetivo
	M	Empregado Efetivo
	M	Empregado Efetivo
	F	Empregado Efetivo

	F	Empregado Efetivo
	F	Empregado Efetivo
	M	Cedido - RJU
	M	Empregado Efetivo
	M	Empregado Efetivo
	F	Cedido - RJU
	F	Empregado Efetivo
	F	Empregado Efetivo
	M	Empregado Efetivo
	F	Empregado Efetivo
	F	Empregado Efetivo
	F	Empregado Efetivo
	F	Empregado Efetivo
	F	Empregado Efetivo
	M	Empregado Efetivo
	F	Empregado Efetivo
	M	Empregado Efetivo
	F	Empregado Efetivo
	F	Empregado Efetivo
	M	Empregado Efetivo
	F	Empregado Efetivo
	F	Empregado Efetivo
	F	Empregado Efetivo
	F	Empregado Efetivo
	F	Empregado Efetivo
	F	Empregado Efetivo
	F	Empregado Efetivo
	F	Empregado Efetivo
	F	Empregado Efetivo
	F	Empregado Efetivo
	F	Empregado Efetivo
	F	Empregado Efetivo
	F	Empregado Efetivo
	F	Empregado Efetivo
	F	Empregado Efetivo
	F	Empregado Efetivo
	F	Empregado Efetivo
	F	Empregado Efetivo
	F	Empregado Efetivo
	F	Empregado Efetivo
	F	Empregado Efetivo
	F	Empregado Efetivo
	F	Empregado Efetivo
	F	Empregado Efetivo
	F	Empregado Efetivo
	F	Empregado Efetivo

	F	Empregado Efetivo
	F	Empregado Efetivo
	F	Empregado Efetivo
	F	Empregado Efetivo
	F	Empregado Efetivo
	F	Empregado Efetivo
	F	Empregado Efetivo
	F	Empregado Efetivo
	F	Empregado Efetivo
	F	Empregado Efetivo
	F	Empregado Efetivo
	F	Empregado Efetivo
	F	Empregado Efetivo
	F	Empregado Efetivo
	F	Empregado Efetivo
	F	Empregado Efetivo
	F	Empregado Efetivo
	F	Empregado Efetivo
	F	Empregado Efetivo
	F	Empregado Efetivo
	F	Empregado Efetivo
	M	Cedido - RJU
	M	Empregado Efetivo
	M	Empregado Efetivo
	M	Empregado Efetivo
	M	Empregado Efetivo
	F	Empregado Efetivo
	F	Empregado Efetivo
	F	Empregado Efetivo
	F	Empregado Efetivo
	F	Empregado Efetivo
	F	Empregado Efetivo
	F	Empregado Efetivo
	F	Empregado Efetivo
	F	Empregado Efetivo
	F	Empregado Efetivo
	F	Empregado Efetivo
	F	Empregado Efetivo
	F	Empregado Efetivo
	F	Empregado Efetivo
	M	Empregado Efetivo
	F	Empregado Efetivo

	F	Empregado Efetivo
	M	Empregado Efetivo
	M	Empregado Efetivo
	M	Empregado Efetivo
	F	Empregado Efetivo
	F	Empregado Efetivo
	F	Empregado Efetivo
	F	Cedido - RJU
	F	Cedido - RJU
	F	Cedido - RJU
	F	Empregado Efetivo
	F	Empregado Efetivo
	F	Empregado Efetivo
	F	Empregado Efetivo
	M	Empregado Efetivo
	M	Empregado Efetivo
	M	Empregado Efetivo
	M	Empregado Efetivo
	F	Empregado Efetivo
	M	Cedido - RJU
	M	Cedido - RJU
	F	Empregado Efetivo
	F	Empregado Efetivo
	F	Cedido - RJU
	F	Cedido - RJU
	F	Empregado Efetivo
	F	Empregado Efetivo
	F	Empregado Efetivo
	F	Cedido - RJU
	M	Empregado Efetivo
	F	Empregado Efetivo
	F	Empregado Efetivo
	F	Empregado Efetivo
	F	Empregado Efetivo
	F	Empregado Efetivo
	F	Empregado Efetivo
	F	Empregado Efetivo
	F	Empregado Efetivo
	F	Empregado Efetivo
	F	Empregado Efetivo
	F	Empregado Efetivo
	F	Empregado Efetivo
	F	Empregado Efetivo
	M	Empregado Efetivo

	F	Empregado Efetivo
	F	Empregado Efetivo
	F	Empregado Efetivo
	F	Empregado Efetivo
	F	Empregado Efetivo
	M	Empregado Efetivo
	F	Empregado Efetivo
	F	Empregado Efetivo
	F	Empregado Efetivo
	F	Empregado Efetivo
	F	Empregado Efetivo
	F	Empregado Efetivo
	F	Empregado Efetivo
	F	Empregado Efetivo
	M	Empregado Efetivo
	F	Empregado Efetivo
	M	Empregado Efetivo
	M	Empregado Efetivo
	F	Empregado Efetivo
	M	Empregado Efetivo
	F	Empregado Efetivo
	F	Empregado Efetivo
	F	Empregado Efetivo
	M	Empregado Efetivo
	F	Empregado Efetivo
	F	Empregado Efetivo
	M	Empregado Efetivo
	F	Empregado Efetivo
	M	Empregado Efetivo
	M	Empregado Efetivo
	M	Empregado Efetivo
	F	Empregado Efetivo
	F	Empregado Efetivo
	M	Empregado Efetivo
	M	Empregado Efetivo
	F	Empregado Efetivo
	F	Empregado Efetivo
	M	Empregado Efetivo
	M	Empregado Efetivo
	F	Empregado Efetivo
	F	Empregado Efetivo
	F	Empregado Efetivo
	F	Empregado Efetivo
	F	Empregado Efetivo
	F	Empregado Efetivo
	F	Empregado Efetivo
	F	Empregado Efetivo
	F	Empregado Efetivo

	F	Empregado Efetivo
	F	Empregado Efetivo
	F	Cedido - RJU
	M	Empregado Temporário
	F	Empregado Efetivo
	F	Empregado Efetivo
	F	Empregado Temporário
	F	Empregado Efetivo
	M	Empregado Efetivo
	M	Empregado Efetivo
	M	Empregado Efetivo
	M	Empregado Efetivo
	M	Empregado Efetivo
	M	Empregado Efetivo
	F	Empregado Efetivo
	M	Empregado Efetivo
	F	Empregado Efetivo
	F	Empregado Efetivo
	M	Empregado Efetivo
	M	Empregado Efetivo
	F	Empregado Efetivo
	M	Empregado Efetivo
	M	Empregado Efetivo
	M	Empregado Efetivo
	M	Empregado Efetivo
	M	Empregado Efetivo
	M	Empregado Efetivo
	M	Empregado Efetivo
	M	Empregado Efetivo
	M	Empregado Efetivo
	M	Empregado Efetivo
	M	Empregado Efetivo
	M	Empregado Efetivo
	F	Empregado Efetivo
	F	Empregado Efetivo
	F	Empregado Efetivo
	F	Empregado Efetivo
	F	Empregado Efetivo
	F	Empregado Efetivo
	F	Empregado Efetivo
	F	Empregado Efetivo
	F	Comissionado
	F	Comissionado
	F	Comissionado

	M	Empregado Efetivo
	M	Empregado Efetivo
	M	Empregado Efetivo
	M	Empregado Efetivo
	F	Empregado Efetivo
	F	Empregado Efetivo
	M	Empregado Efetivo
	M	Empregado Efetivo
	M	Empregado Efetivo
	M	Empregado Efetivo
	M	Empregado Efetivo
	M	Empregado Efetivo
	M	Empregado Efetivo
	F	Empregado Efetivo
	F	Empregado Efetivo
	F	Empregado Efetivo
	F	Empregado Efetivo
	F	Empregado Efetivo
	F	Empregado Efetivo
	F	Empregado Efetivo
	F	Empregado Efetivo
	F	Empregado Efetivo
	F	Empregado Efetivo
	F	Empregado Efetivo
	F	Empregado Efetivo
	F	Empregado Efetivo
	F	Empregado Efetivo
	F	Empregado Efetivo
	F	Empregado Efetivo
	M	Empregado Efetivo
	F	Empregado Efetivo
	F	Empregado Efetivo
	F	Empregado Efetivo
	F	Empregado Efetivo
	F	Empregado Efetivo
	F	Empregado Efetivo
	F	Empregado Efetivo

	F	Empregado Efetivo
	F	Empregado Efetivo
	F	Empregado Efetivo
	F	Empregado Efetivo
	F	Empregado Efetivo
	F	Empregado Efetivo
	F	Empregado Efetivo
	F	Empregado Efetivo
	F	Empregado Efetivo
	F	Empregado Efetivo
	F	Empregado Efetivo
	F	Empregado Efetivo
	F	Empregado Efetivo
	F	Empregado Efetivo
	F	Empregado Efetivo
	F	Empregado Efetivo
	F	Empregado Efetivo
	F	Empregado Efetivo
	F	Empregado Efetivo
	F	Empregado Efetivo
	F	Empregado Efetivo
	F	Empregado Efetivo
	F	Empregado Efetivo
	F	Empregado Efetivo
	F	Empregado Efetivo
	F	Empregado Efetivo
	F	Empregado Efetivo
	F	Empregado Efetivo
	F	Empregado Efetivo
	F	Empregado Efetivo
	F	Empregado Efetivo
	F	Empregado Efetivo

	F	Empregado Efetivo
	F	Empregado Efetivo
	F	Empregado Efetivo
	F	Empregado Efetivo
	F	Empregado Efetivo
	F	Empregado Efetivo
	F	Empregado Efetivo
	F	Empregado Efetivo
	F	Empregado Efetivo
	F	Empregado Efetivo
	M	Empregado Efetivo
	F	Empregado Efetivo
	F	Empregado Efetivo
	F	Empregado Efetivo
	F	Empregado Efetivo
	F	Empregado Efetivo
	F	Empregado Efetivo
	F	Empregado Efetivo
	F	Empregado Efetivo
	F	Empregado Efetivo
	M	Empregado Efetivo
	F	Empregado Efetivo
	F	Empregado Efetivo
	F	Empregado Efetivo
	F	Empregado Efetivo
	F	Empregado Efetivo
	F	Empregado Efetivo
	F	Empregado Efetivo
	F	Empregado Efetivo
	F	Empregado Efetivo
	F	Empregado Efetivo
	F	Empregado Efetivo
	F	Empregado Efetivo
	F	Empregado Efetivo
	F	Empregado Efetivo
	F	Empregado Efetivo
	F	Empregado Efetivo
	F	Empregado Efetivo
	M	Empregado Efetivo
	F	Empregado Efetivo

	F	Empregado Efetivo
	F	Empregado Efetivo
	F	Empregado Efetivo
	F	Empregado Efetivo
	F	Empregado Efetivo
	F	Empregado Efetivo
	M	Empregado Efetivo
	F	Empregado Efetivo
	F	Empregado Efetivo
	F	Empregado Efetivo
	F	Empregado Efetivo
	F	Empregado Efetivo
	F	Empregado Efetivo
	F	Empregado Efetivo
	F	Empregado Efetivo
	F	Empregado Efetivo
	F	Empregado Efetivo
	F	Empregado Efetivo
	F	Empregado Efetivo
	F	Empregado Efetivo
	F	Empregado Efetivo
	F	Empregado Efetivo
	F	Empregado Efetivo
	F	Empregado Efetivo
	F	Empregado Efetivo
	F	Empregado Efetivo
	F	Empregado Efetivo
	M	Empregado Efetivo
	F	Empregado Efetivo
	M	Empregado Efetivo
	F	Empregado Efetivo
	F	Empregado Efetivo
	F	Empregado Efetivo
	F	Empregado Efetivo
	F	Empregado Efetivo
	M	Empregado Efetivo
	F	Empregado Efetivo
	F	Empregado Efetivo
	M	Empregado Efetivo
	F	Empregado Efetivo
	F	Empregado Efetivo
	F	Empregado Efetivo
	F	Empregado Efetivo
	F	Empregado Efetivo
	F	Empregado Efetivo
	F	Empregado Efetivo
	F	Empregado Efetivo
	F	Empregado Efetivo

	F	Empregado Efetivo
	F	Empregado Efetivo
	F	Empregado Efetivo
	M	Empregado Efetivo
	M	Empregado Efetivo
	M	Empregado Efetivo
	F	Empregado Efetivo
	F	Empregado Efetivo
	F	Empregado Efetivo
	M	Empregado Efetivo
	M	Empregado Efetivo
	F	Empregado Efetivo
	F	Empregado Efetivo
	F	Empregado Efetivo
	F	Empregado Efetivo
	M	Empregado Efetivo
	F	Empregado Efetivo
	F	Empregado Efetivo
	F	Empregado Efetivo
	F	Empregado Efetivo
	F	Empregado Efetivo
	F	Empregado Efetivo
	F	Empregado Efetivo
	M	Empregado Efetivo
	F	Empregado Efetivo
	F	Empregado Efetivo
	F	Empregado Efetivo
	F	Empregado Efetivo
	M	Empregado Efetivo
	F	Empregado Efetivo
	M	Empregado Efetivo
	F	Empregado Efetivo
	F	Empregado Efetivo
	F	Empregado Efetivo
	M	Empregado Efetivo
	F	Empregado Efetivo
	F	Empregado Efetivo
	M	Empregado Efetivo
	F	Empregado Efetivo
	F	Empregado Efetivo
	M	Empregado Efetivo
	F	Empregado Efetivo
	F	Empregado Efetivo
	F	Empregado Efetivo
	F	Empregado Efetivo
	F	Empregado Efetivo
	F	Empregado Efetivo
	M	Empregado Efetivo

	F	Empregado Efetivo
	F	Empregado Efetivo
	F	Empregado Efetivo
	F	Empregado Efetivo
	F	Empregado Efetivo
	F	Empregado Efetivo
	F	Empregado Efetivo
	F	Empregado Efetivo
	F	Empregado Efetivo
	F	Empregado Efetivo
	F	Empregado Efetivo
	F	Empregado Efetivo
	F	Empregado Efetivo
	F	Empregado Efetivo
	F	Empregado Efetivo
	F	Empregado Efetivo
	F	Empregado Efetivo
	F	Empregado Efetivo
	F	Empregado Efetivo
	F	Empregado Efetivo
	F	Empregado Efetivo
	F	Empregado Efetivo
	F	Empregado Efetivo
	F	Empregado Efetivo
	F	Empregado Efetivo
	F	Empregado Efetivo
	F	Empregado Efetivo
	F	Empregado Efetivo
	F	Empregado Efetivo
	F	Empregado Efetivo
	F	Empregado Efetivo
	F	Empregado Efetivo
	F	Empregado Efetivo
	F	Empregado Efetivo
	F	Empregado Efetivo
	F	Empregado Efetivo
	F	Empregado Efetivo
	F	Empregado Efetivo
	F	Empregado Efetivo
	F	Empregado Efetivo
	F	Empregado Efetivo
	F	Empregado Efetivo
	F	Empregado Efetivo
	F	Empregado Efetivo
	F	Empregado Efetivo
	F	Empregado Efetivo
	M	Empregado Efetivo
	F	Empregado Efetivo

	F	Empregado Efetivo
	F	Empregado Efetivo
	F	Empregado Efetivo
	F	Empregado Efetivo
	F	Empregado Efetivo
	M	Empregado Efetivo
	F	Empregado Efetivo
	F	Empregado Efetivo
	F	Empregado Efetivo
	F	Empregado Efetivo
	F	Empregado Efetivo
	F	Empregado Efetivo
	F	Empregado Efetivo
	F	Empregado Efetivo
	M	Empregado Efetivo
	F	Empregado Efetivo
	M	Empregado Efetivo
	F	Empregado Efetivo
	F	Empregado Efetivo
	F	Empregado Efetivo
	F	Empregado Efetivo
	F	Empregado Efetivo
	F	Empregado Efetivo
	F	Empregado Efetivo
	F	Empregado Efetivo
	F	Empregado Efetivo
	F	Empregado Efetivo
	F	Empregado Efetivo
	F	Empregado Efetivo
	F	Empregado Efetivo
	F	Empregado Efetivo
	F	Empregado Efetivo
	M	Empregado Efetivo
	F	Empregado Efetivo
	F	Empregado Efetivo
	F	Empregado Efetivo
	F	Empregado Efetivo
	F	Empregado Efetivo
	F	Empregado Efetivo
	F	Empregado Efetivo
	F	Empregado Efetivo
	F	Empregado Efetivo
	F	Empregado Efetivo
	F	Empregado Efetivo
	F	Empregado Efetivo
	F	Empregado Efetivo
	F	Empregado Efetivo
	M	Empregado Efetivo

	F	Empregado Efetivo
	F	Empregado Efetivo
	M	Empregado Efetivo
	M	Empregado Efetivo
	F	Empregado Efetivo
	M	Empregado Efetivo
	M	Empregado Efetivo
	F	Empregado Efetivo
	F	Empregado Efetivo
	F	Empregado Efetivo
	M	Empregado Efetivo
	F	Empregado Efetivo
	F	Empregado Efetivo
	F	Empregado Efetivo
	F	Empregado Efetivo
	F	Empregado Efetivo
	F	Empregado Efetivo
	F	Empregado Efetivo
	F	Empregado Efetivo
	F	Empregado Efetivo
	F	Empregado Efetivo
	F	Empregado Efetivo
	F	Empregado Efetivo
	M	Empregado Efetivo
	M	Empregado Efetivo
	F	Empregado Efetivo
	F	Empregado Efetivo
	F	Empregado Efetivo
	F	Empregado Efetivo
	F	Empregado Efetivo
	M	Empregado Efetivo
	F	Empregado Efetivo
	F	Empregado Efetivo
	F	Empregado Efetivo
	F	Empregado Efetivo
	F	Empregado Efetivo
	F	Empregado Efetivo
	F	Empregado Efetivo
	M	Empregado Efetivo
	F	Empregado Efetivo
	F	Empregado Efetivo
	F	Empregado Efetivo
	F	Empregado Efetivo
	F	Empregado Efetivo
	F	Empregado Efetivo
	F	Empregado Efetivo
	F	Empregado Efetivo
	M	Empregado Efetivo

	F	Empregado Efetivo
	F	Empregado Efetivo
	M	Empregado Efetivo
	M	Empregado Efetivo
	M	Empregado Efetivo
	F	Empregado Efetivo
	M	Empregado Efetivo
	F	Empregado Efetivo
	M	Empregado Efetivo
	F	Empregado Efetivo
	F	Empregado Efetivo
	F	Empregado Efetivo
	F	Empregado Efetivo
	M	Empregado Efetivo
	F	Empregado Efetivo
	M	Empregado Efetivo
	F	Empregado Efetivo
	F	Empregado Efetivo
	F	Empregado Efetivo
	F	Empregado Efetivo
	F	Empregado Efetivo
	F	Empregado Efetivo
	F	Empregado Efetivo
	F	Empregado Efetivo
	F	Empregado Efetivo
	F	Empregado Efetivo
	F	Empregado Efetivo
	F	Empregado Efetivo
	F	Empregado Efetivo
	F	Empregado Efetivo
	F	Empregado Efetivo
	F	Empregado Efetivo

	M	Empregado Efetivo
	F	Empregado Efetivo
	F	Empregado Efetivo
	M	Empregado Efetivo
	F	Empregado Efetivo
	F	Empregado Efetivo
	F	Empregado Efetivo
	F	Empregado Efetivo
	F	Empregado Efetivo
	F	Empregado Efetivo
	M	Empregado Efetivo
	F	Empregado Efetivo
	F	Empregado Efetivo
	F	Empregado Efetivo
	F	Empregado Efetivo
	F	Empregado Efetivo
	F	Empregado Efetivo
	M	Empregado Efetivo
	F	Empregado Efetivo
	M	Empregado Efetivo
	F	Empregado Efetivo
	M	Empregado Efetivo
	F	Empregado Efetivo
	F	Empregado Efetivo
	F	Empregado Efetivo
	F	Empregado Efetivo
	F	Empregado Efetivo
	F	Empregado Efetivo
	M	Empregado Efetivo
	M	Empregado Efetivo
	M	Empregado Efetivo
	F	Empregado Efetivo
	F	Empregado Efetivo
	F	Empregado Efetivo

	F	Empregado Efetivo
	F	Empregado Efetivo
	F	Empregado Efetivo
	F	Empregado Efetivo
	F	Empregado Efetivo
	F	Empregado Efetivo
	F	Empregado Efetivo
	F	Empregado Efetivo
	F	Empregado Efetivo
	F	Empregado Efetivo
	F	Empregado Efetivo
	F	Empregado Efetivo
	F	Empregado Efetivo
	F	Empregado Efetivo
	F	Empregado Efetivo
	F	Empregado Efetivo
	F	Empregado Efetivo
	F	Empregado Efetivo
	F	Empregado Efetivo
	F	Empregado Efetivo
	F	Empregado Efetivo
	F	Empregado Efetivo
	F	Empregado Efetivo
	F	Empregado Efetivo
	F	Empregado Efetivo
	F	Empregado Efetivo
	F	Empregado Efetivo
	F	Empregado Efetivo
	F	Empregado Efetivo
	F	Empregado Efetivo
	F	Empregado Efetivo
	F	Empregado Efetivo
	F	Empregado Efetivo
	F	Empregado Efetivo
	F	Empregado Efetivo
	F	Empregado Efetivo
	F	Empregado Efetivo
	F	Empregado Efetivo
	F	Empregado Efetivo
	F	Empregado Efetivo
	F	Empregado Efetivo
	F	Empregado Efetivo
	F	Empregado Efetivo
	F	Empregado Efetivo
	F	Empregado Efetivo
	F	Empregado Efetivo
	F	Empregado Efetivo

	F	Empregado Efetivo
	F	Empregado Efetivo
	F	Empregado Efetivo
	F	Empregado Efetivo
	M	Empregado Efetivo
	F	Empregado Efetivo
	F	Empregado Efetivo
	F	Empregado Efetivo
	F	Empregado Efetivo
	F	Empregado Efetivo
	F	Empregado Efetivo
	F	Empregado Efetivo
	F	Empregado Efetivo
	F	Empregado Efetivo
	F	Empregado Efetivo
	F	Empregado Efetivo
	F	Empregado Efetivo
	F	Empregado Efetivo
	F	Empregado Efetivo
	F	Empregado Efetivo
	M	Empregado Efetivo
	F	Empregado Efetivo
	F	Empregado Efetivo
	M	Empregado Efetivo
	M	Empregado Efetivo
	M	Empregado Efetivo
	M	Empregado Efetivo
	M	Empregado Efetivo
	M	Empregado Efetivo
	F	Empregado Efetivo
	F	Empregado Efetivo
	M	Empregado Efetivo
	M	Empregado Efetivo
	M	Empregado Efetivo
	M	Empregado Efetivo
	F	Empregado Efetivo
	F	Empregado Efetivo
	M	Empregado Efetivo
	M	Empregado Efetivo
	M	Empregado Efetivo
	M	Empregado Efetivo
	F	Empregado Efetivo
	M	Empregado Efetivo

	M	Empregado Efetivo
	F	Empregado Efetivo
	F	Empregado Efetivo
	M	Empregado Efetivo
	M	Empregado Efetivo
	F	Empregado Efetivo
	F	Empregado Efetivo
	F	Empregado Efetivo
	F	Empregado Efetivo
	F	Empregado Efetivo
	F	Empregado Efetivo
	F	Empregado Efetivo
	F	Empregado Efetivo
	F	Empregado Efetivo
	F	Empregado Efetivo
	F	Empregado Efetivo
	M	Empregado Efetivo
	F	Empregado Efetivo
	F	Empregado Efetivo
	M	Empregado Efetivo
	F	Empregado Efetivo
	M	Empregado Efetivo
	F	Empregado Efetivo
	F	Empregado Efetivo
	F	Empregado Efetivo
	F	Empregado Efetivo
	M	Empregado Efetivo
	F	Empregado Efetivo
	F	Empregado Efetivo
	F	Empregado Efetivo
	F	Empregado Efetivo
	F	Empregado Efetivo
	F	Empregado Efetivo
	F	Empregado Efetivo
	F	Empregado Efetivo
	F	Empregado Efetivo
	M	Empregado Efetivo
	F	Empregado Efetivo
	M	Empregado Efetivo
	M	Empregado Efetivo
	F	Empregado Efetivo
	F	Empregado Efetivo
	M	Empregado Efetivo
	F	Empregado Efetivo
	F	Empregado Efetivo
	F	Empregado Efetivo
	M	Empregado Efetivo
	F	Empregado Efetivo

	F	Empregado Efetivo
	F	Empregado Efetivo
	F	Empregado Efetivo
	F	Empregado Efetivo
	F	Empregado Efetivo
	F	Empregado Efetivo
	F	Empregado Efetivo
	F	Empregado Efetivo
	F	Empregado Efetivo
	F	Empregado Efetivo
	F	Empregado Efetivo
	F	Empregado Efetivo
	F	Empregado Efetivo
	F	Empregado Efetivo
	F	Empregado Efetivo
	F	Empregado Efetivo
	F	Empregado Efetivo
	F	Empregado Efetivo
	F	Empregado Efetivo
	F	Empregado Efetivo
	F	Empregado Efetivo
	F	Empregado Efetivo
	F	Empregado Efetivo
	M	Empregado Efetivo
	M	Empregado Efetivo
	F	Empregado Efetivo
	F	Empregado Efetivo
	F	Empregado Efetivo
	F	Empregado Efetivo
	F	Empregado Efetivo
	F	Empregado Efetivo
	F	Empregado Efetivo
	F	Empregado Temporário
	F	Empregado Efetivo
	F	Empregado Efetivo
	F	Empregado Efetivo
	F	Empregado Efetivo
	M	Empregado Temporário
	F	Empregado Efetivo
	M	Empregado Efetivo
	F	Empregado Efetivo
	F	Empregado Efetivo
	F	Empregado Efetivo
	M	Empregado Efetivo
	F	Empregado Efetivo
	F	Empregado Efetivo
	F	Empregado Efetivo
	F	Empregado Temporário

	M	Empregado Temporário
	F	Empregado Efetivo
	F	Empregado Efetivo
	F	Empregado Efetivo
	M	Empregado Efetivo
	F	Empregado Efetivo
	F	Empregado Efetivo
	F	Empregado Efetivo
	F	Empregado Efetivo
	F	Empregado Efetivo
	F	Empregado Efetivo
	F	Empregado Efetivo
	M	Empregado Efetivo
	F	Empregado Efetivo
	F	Empregado Efetivo
	F	Empregado Efetivo
	F	Empregado Efetivo
	M	Empregado Efetivo
	F	Empregado Efetivo
	F	Empregado Efetivo
	M	Empregado Efetivo
	F	Empregado Efetivo
	F	Empregado Efetivo
	F	Empregado Efetivo
	F	Empregado Efetivo
	F	Empregado Efetivo
	F	Empregado Efetivo
	F	Empregado Efetivo
	F	Empregado Efetivo
	M	Empregado Efetivo
	F	Empregado Efetivo
	F	Empregado Efetivo
	M	Empregado Efetivo
	F	Empregado Efetivo
	F	Empregado Efetivo
	M	Empregado Efetivo
	M	Empregado Efetivo
	M	Empregado Efetivo
	M	Empregado Efetivo
	M	Empregado Efetivo
	M	Empregado Efetivo
	M	Empregado Efetivo
	F	Empregado Efetivo
	F	Empregado Efetivo
	F	Empregado Efetivo
	M	Empregado Efetivo
	M	Empregado Efetivo
	M	Empregado Efetivo

	M	Empregado Efetivo
	M	Empregado Efetivo
	M	Empregado Efetivo
	M	Empregado Efetivo
	M	Empregado Efetivo
	M	Empregado Efetivo
	F	Empregado Efetivo
	M	Empregado Efetivo
	M	Empregado Efetivo
	F	Empregado Efetivo
	F	Empregado Efetivo
	F	Empregado Efetivo
	F	Empregado Efetivo
	F	Empregado Efetivo
	M	Empregado Efetivo
	F	Empregado Efetivo
	F	Empregado Efetivo
	F	Empregado Efetivo
	F	Empregado Efetivo
	F	Empregado Efetivo
	F	Empregado Efetivo
	F	Empregado Efetivo
	F	Empregado Efetivo
	F	Empregado Efetivo
	F	Empregado Efetivo
	F	Empregado Efetivo
	F	Empregado Efetivo
	F	Empregado Efetivo
	F	Empregado Efetivo
	F	Empregado Efetivo
	F	Empregado Efetivo
	F	Empregado Efetivo
	F	Empregado Efetivo
	F	Empregado Efetivo
	F	Empregado Efetivo
	M	Empregado Efetivo
	F	Empregado Efetivo
	F	Empregado Efetivo
	F	Empregado Efetivo
	F	Empregado Efetivo
	F	Empregado Efetivo

	F	Empregado Efetivo
	F	Empregado Efetivo
	F	Empregado Efetivo
	F	Empregado Efetivo
	F	Empregado Efetivo
	F	Empregado Efetivo
	F	Empregado Efetivo
	F	Empregado Efetivo
	F	Empregado Efetivo
	F	Empregado Efetivo
	F	Empregado Efetivo
	M	Empregado Efetivo
	M	Empregado Efetivo
	F	Empregado Efetivo
	F	Empregado Efetivo
	F	Empregado Efetivo
	F	Empregado Efetivo
	F	Empregado Efetivo
	F	Empregado Efetivo
	M	Empregado Efetivo
	M	Empregado Efetivo
	M	Empregado Efetivo
	F	Empregado Efetivo
	F	Empregado Efetivo
	F	Empregado Efetivo
	F	Empregado Efetivo
	F	Empregado Efetivo
	M	Empregado Efetivo
	F	Empregado Efetivo
	M	Empregado Temporário
	M	Empregado Efetivo
	M	Empregado Efetivo

	M	Empregado Efetivo
	M	Empregado Efetivo
	M	Empregado Efetivo
	M	Empregado Efetivo
	F	Empregado Efetivo
	F	Empregado Efetivo
	F	Empregado Efetivo
	F	Empregado Efetivo
	F	Empregado Efetivo
	F	Empregado Efetivo
	F	Cedido - RJU
	F	Cedido - RJU
	F	Cedido - RJU
	F	Cedido - RJU
	F	Empregado Efetivo
	F	Empregado Efetivo
	M	Empregado Efetivo
	F	Empregado Efetivo
	M	Empregado Efetivo
	M	Empregado Efetivo
	F	Empregado Efetivo
	F	Empregado Efetivo
	F	Empregado Efetivo
	F	Empregado Efetivo
	F	Empregado Efetivo
	M	Empregado Efetivo
	M	Empregado Efetivo
	M	Empregado Efetivo
	M	Empregado Efetivo
	F	Empregado Efetivo
	F	Empregado Efetivo
	M	Empregado Efetivo
	F	Empregado Efetivo
	M	Empregado Efetivo
	M	Empregado Efetivo
	M	Empregado Efetivo
	M	Empregado Efetivo
	F	Empregado Temporário

	F	Empregado Temporário
	F	Empregado Temporário
	M	Empregado Efetivo
	F	Empregado Temporário
	F	Empregado Efetivo
	F	Empregado Temporário
	M	Empregado Temporário
	F	Empregado Temporário
	M	Empregado Temporário
	F	Empregado Efetivo
	F	Empregado Temporário
	M	Empregado Temporário
	M	Empregado Temporário
	M	Empregado Efetivo
	F	Empregado Efetivo
	F	Empregado Temporário
	F	Empregado Efetivo
	M	Empregado Efetivo
	M	Empregado Efetivo
	F	Empregado Efetivo
	M	Empregado Efetivo
	M	Empregado Efetivo
	M	Empregado Efetivo
	M	Empregado Efetivo
	M	Empregado Efetivo
	M	Empregado Efetivo
	F	Empregado Efetivo
	F	Empregado Efetivo
	F	Empregado Efetivo
	M	Empregado Efetivo
	M	Empregado Efetivo
	F	Empregado Efetivo
	F	Empregado Efetivo
	F	Empregado Efetivo
	F	Empregado Efetivo
	F	Empregado Efetivo
	F	Empregado Efetivo
	F	Empregado Efetivo
	F	Empregado Efetivo
	F	Empregado Efetivo

	F	Empregado Efetivo
	F	Empregado Efetivo
	F	Empregado Efetivo
	F	Empregado Efetivo
	F	Empregado Efetivo
	F	Empregado Efetivo
	F	Empregado Efetivo
	F	Empregado Efetivo
	F	Empregado Efetivo
	F	Empregado Efetivo
	F	Empregado Efetivo
	F	Empregado Efetivo
	F	Empregado Efetivo
	F	Empregado Efetivo
	F	Empregado Efetivo
	F	Empregado Efetivo
	F	Empregado Efetivo
	F	Empregado Efetivo
	F	Empregado Efetivo
	F	Empregado Efetivo
	F	Empregado Efetivo
	F	Empregado Efetivo
	F	Empregado Efetivo
	M	Empregado Efetivo
	F	Empregado Efetivo
	F	Empregado Efetivo
	F	Empregado Efetivo
	F	Empregado Efetivo
	F	Empregado Efetivo
	F	Empregado Efetivo
	F	Empregado Efetivo
	M	Empregado Efetivo
	F	Empregado Efetivo
	F	Empregado Efetivo
	F	Empregado Efetivo
	F	Empregado Efetivo
	F	Empregado Efetivo
	F	Empregado Efetivo
	F	Empregado Efetivo
	F	Empregado Efetivo
	F	Empregado Efetivo

	F	Empregado Efetivo
	F	Empregado Efetivo
	F	Empregado Efetivo
	F	Empregado Efetivo
	F	Empregado Efetivo
	M	Empregado Efetivo
	M	Empregado Efetivo
	F	Empregado Efetivo
	F	Empregado Efetivo
	F	Empregado Efetivo
	F	Empregado Efetivo
	F	Empregado Efetivo
	M	Empregado Efetivo
	F	Empregado Efetivo
	F	Empregado Efetivo
	F	Empregado Efetivo
	M	Empregado Efetivo
	F	Empregado Efetivo
	M	Empregado Efetivo
	F	Empregado Efetivo
	F	Empregado Efetivo
	M	Empregado Efetivo
	F	Empregado Efetivo
	F	Empregado Efetivo
	F	Empregado Efetivo
	F	Empregado Efetivo
	F	Empregado Efetivo
	F	Empregado Efetivo
	F	Empregado Efetivo
	F	Empregado Efetivo
	M	Empregado Efetivo
	F	Empregado Efetivo
	F	Empregado Efetivo
	F	Empregado Efetivo
	F	Empregado Efetivo
	F	Empregado Efetivo
	F	Empregado Efetivo
	F	Empregado Efetivo
	F	Empregado Efetivo
	F	Empregado Efetivo
	F	Empregado Efetivo
	F	Empregado Efetivo
	F	Empregado Efetivo
	F	Empregado Efetivo
	F	Empregado Efetivo
	M	Empregado Efetivo
	F	Empregado Efetivo
	F	Empregado Efetivo

	F	Empregado Efetivo
	F	Empregado Efetivo
	M	Empregado Efetivo
	F	Empregado Efetivo
	F	Empregado Efetivo
	F	Empregado Efetivo
	M	Empregado Efetivo
	M	Empregado Efetivo
	M	Empregado Efetivo
	M	Empregado Efetivo
	F	Empregado Efetivo
	F	Empregado Efetivo
	M	Empregado Efetivo
	F	Empregado Efetivo
	F	Empregado Efetivo
	M	Empregado Efetivo
	F	Empregado Efetivo
	M	Empregado Efetivo
	M	Empregado Efetivo
	M	Empregado Efetivo
	M	Empregado Efetivo
	F	Empregado Efetivo
	F	Empregado Efetivo
	F	Empregado Efetivo
	M	Empregado Efetivo
	F	Empregado Efetivo
	M	Empregado Efetivo
	F	Empregado Efetivo
	F	Empregado Efetivo
	F	Empregado Efetivo
	F	Empregado Efetivo
	F	Empregado Efetivo
	F	Empregado Efetivo
	M	Empregado Efetivo
	F	Empregado Efetivo
	F	Empregado Efetivo
	F	Empregado Efetivo
	F	Empregado Efetivo
	F	Empregado Efetivo
	F	Empregado Efetivo
	F	Empregado Efetivo
	F	Empregado Efetivo
	M	Empregado Efetivo
	F	Empregado Efetivo
	M	Empregado Efetivo
	F	Empregado Efetivo
	F	Empregado Efetivo
	F	Empregado Efetivo

	F	Empregado Efetivo
	F	Empregado Efetivo
	F	Empregado Efetivo
	F	Empregado Efetivo
	M	Empregado Efetivo
	F	Empregado Efetivo
	F	Empregado Efetivo
	F	Empregado Efetivo
	M	Empregado Efetivo
	F	Empregado Efetivo
	F	Empregado Efetivo
	M	Empregado Efetivo
	M	Empregado Efetivo
	F	Empregado Efetivo
	F	Empregado Efetivo
	F	Empregado Efetivo
	F	Empregado Efetivo
	F	Empregado Efetivo
	M	Empregado Efetivo
	F	Empregado Efetivo
	F	Empregado Efetivo
	F	Empregado Efetivo
	F	Empregado Efetivo
	M	Empregado Efetivo
	M	Empregado Efetivo
	M	Empregado Efetivo
	M	Empregado Efetivo
	M	Empregado Efetivo
	M	Empregado Efetivo
	F	Empregado Efetivo
	F	Empregado Efetivo
	F	Empregado Efetivo
	F	Empregado Efetivo
	M	Empregado Efetivo
	M	Empregado Efetivo
	M	Empregado Efetivo
	M	Empregado Efetivo
	M	Empregado Efetivo
	M	Empregado Efetivo
	M	Empregado Efetivo
	M	Empregado Efetivo
	F	Empregado Efetivo
	F	Empregado Efetivo
	M	Empregado Efetivo
	M	Empregado Efetivo
	F	Empregado Efetivo
	M	Empregado Efetivo
	F	Empregado Efetivo

	F	Empregado Efetivo
	F	Empregado Efetivo
	F	Empregado Efetivo
	F	Empregado Efetivo
	M	Empregado Efetivo
	F	Empregado Efetivo
	F	Empregado Efetivo
	F	Empregado Efetivo
	M	Empregado Efetivo
	M	Empregado Efetivo
	M	Empregado Efetivo
	F	Empregado Efetivo
	M	Empregado Efetivo
	F	Empregado Efetivo
	M	Empregado Efetivo
	F	Empregado Efetivo
	M	Empregado Efetivo
	F	Empregado Efetivo
	F	Empregado Efetivo
	M	Empregado Efetivo
	F	Empregado Efetivo
	F	Empregado Efetivo
	F	Empregado Efetivo
	F	Empregado Efetivo
	M	Empregado Efetivo
	F	Empregado Efetivo
	M	Empregado Efetivo
	F	Empregado Efetivo
	F	Empregado Efetivo
	M	Empregado Efetivo
	M	Empregado Efetivo
	F	Empregado Efetivo
	F	Empregado Efetivo
	M	Empregado Efetivo
	M	Empregado Efetivo
	F	Empregado Efetivo
	F	Empregado Efetivo
	F	Empregado Efetivo
	M	Empregado Efetivo
	F	Empregado Efetivo
	F	Empregado Efetivo

	F	Empregado Efetivo
	F	Empregado Efetivo
	F	Empregado Efetivo
	F	Empregado Efetivo
	F	Empregado Efetivo
	F	Empregado Efetivo
	M	Empregado Efetivo
	M	Empregado Efetivo
	F	Empregado Efetivo
	F	Empregado Efetivo
	M	Empregado Efetivo
	F	Empregado Efetivo
	M	Empregado Efetivo
	F	Empregado Efetivo
	F	Empregado Efetivo
	F	Empregado Efetivo
	F	Empregado Efetivo
	F	Empregado Efetivo
	M	Empregado Efetivo
	F	Empregado Efetivo
	F	Empregado Efetivo
	F	Empregado Efetivo
	F	Empregado Efetivo
	M	Empregado Efetivo
	M	Empregado Efetivo
	M	Empregado Efetivo
	F	Empregado Efetivo
	F	Empregado Efetivo
	M	Empregado Efetivo
	M	Empregado Efetivo
	F	Empregado Efetivo
	F	Empregado Efetivo
	F	Empregado Efetivo
	M	Empregado Efetivo
	F	Empregado Efetivo
	M	Empregado Efetivo
	M	Empregado Efetivo
	M	Empregado Efetivo
	M	Empregado Efetivo
	M	Empregado Efetivo
	M	Empregado Efetivo
	M	Empregado Efetivo
	M	Empregado Efetivo
	M	Empregado Efetivo

	M	Empregado Efetivo
	M	Empregado Efetivo
	M	Empregado Efetivo
	F	Empregado Efetivo
	M	Empregado Efetivo
	M	Empregado Efetivo
	M	Empregado Efetivo
	M	Empregado Efetivo
	M	Empregado Efetivo
	F	Empregado Efetivo
	F	Empregado Efetivo
	F	Empregado Efetivo
	M	Empregado Efetivo
	F	Empregado Efetivo
	M	Empregado Efetivo
	M	Empregado Efetivo
	M	Empregado Efetivo
	M	Empregado Efetivo
	F	Empregado Efetivo
	M	Empregado Efetivo
	F	Empregado Efetivo
	F	Empregado Efetivo
	F	Empregado Efetivo
	M	Empregado Efetivo
	M	Empregado Efetivo
	M	Empregado Efetivo
	F	Empregado Efetivo
	M	Empregado Efetivo
	F	Empregado Efetivo
	F	Empregado Efetivo
	F	Empregado Efetivo
	F	Empregado Efetivo
	F	Empregado Efetivo
	M	Empregado Efetivo
	F	Empregado Efetivo
	F	Empregado Efetivo
	F	Empregado Efetivo
	F	Empregado Efetivo
	F	Empregado Efetivo
	F	Empregado Efetivo
	F	Empregado Efetivo
	F	Empregado Efetivo
	F	Empregado Efetivo
	F	Empregado Efetivo
	F	Empregado Efetivo
	F	Empregado Efetivo
	F	Empregado Efetivo
	F	Empregado Efetivo

	M	Empregado Temporário
	F	Empregado Efetivo
	F	Empregado Efetivo
	F	Empregado Efetivo
	F	Empregado Efetivo
	F	Empregado Efetivo
	F	Empregado Efetivo
	M	Empregado Efetivo
	M	Empregado Temporário
	M	Empregado Efetivo
	F	Empregado Temporário
	F	Empregado Efetivo
	F	Empregado Efetivo
	M	Empregado Efetivo
	F	Empregado Efetivo
	F	Empregado Efetivo
	F	Empregado Efetivo
	F	Empregado Efetivo
	F	Empregado Efetivo
	F	Empregado Efetivo
	F	Empregado Efetivo
	F	Empregado Efetivo
	F	Empregado Efetivo
	F	Empregado Efetivo
	M	Empregado Efetivo
	F	Empregado Efetivo
	F	Empregado Efetivo
	M	Empregado Efetivo
	F	Empregado Efetivo
	M	Empregado Efetivo
	F	Empregado Efetivo
	F	Empregado Efetivo
	F	Empregado Efetivo
	F	Empregado Efetivo
	F	Empregado Efetivo
	F	Empregado Efetivo
	F	Empregado Efetivo
	F	Empregado Efetivo
	F	Empregado Efetivo
	F	Empregado Efetivo
	F	Empregado Efetivo
	M	Empregado Efetivo
	F	Empregado Efetivo
	F	Empregado Efetivo
	M	Empregado Efetivo
	F	Empregado Efetivo
	F	Empregado Efetivo
	F	Empregado Efetivo

	M	Empregado Efetivo
	M	Empregado Efetivo
	M	Empregado Efetivo
	M	Empregado Efetivo
	M	Empregado Efetivo
	F	Empregado Efetivo
	M	Empregado Temporário
	M	Empregado Efetivo
	M	Empregado Efetivo
	M	Empregado Temporário
	F	Empregado Efetivo
	M	Empregado Efetivo
	F	Empregado Efetivo
	F	Empregado Efetivo
	F	Empregado Efetivo
	M	Empregado Efetivo
	M	Empregado Efetivo
	F	Empregado Efetivo
	M	Empregado Efetivo
	F	Empregado Efetivo
	M	Empregado Efetivo
	F	Empregado Efetivo
	M	Empregado Efetivo
	M	Empregado Efetivo
	M	Empregado Efetivo
	M	Empregado Efetivo
	M	Empregado Efetivo
	F	Empregado Efetivo
	F	Empregado Efetivo
	M	Empregado Efetivo
	F	Empregado Efetivo
	F	Empregado Efetivo
	F	Empregado Efetivo
	F	Empregado Efetivo
	F	Empregado Efetivo
	M	Empregado Efetivo
	M	Empregado Efetivo
	F	Empregado Efetivo
	F	Empregado Efetivo
	F	Empregado Efetivo
	M	Empregado Efetivo
	F	Empregado Efetivo
	F	Empregado Efetivo
	F	Empregado Efetivo
	F	Empregado Efetivo
	M	Empregado Efetivo
	M	Empregado Efetivo
	F	Empregado Efetivo

	M	Empregado Temporário
	M	Empregado Efetivo
	M	Empregado Efetivo
	M	Empregado Efetivo
	M	Empregado Efetivo
	M	Empregado Efetivo
	M	Empregado Efetivo
	M	Empregado Efetivo
	M	Empregado Efetivo
	F	Empregado Efetivo
	F	Empregado Efetivo
	F	Empregado Efetivo
	M	Empregado Efetivo
	F	Empregado Efetivo
	F	Empregado Efetivo
	F	Empregado Efetivo
	M	Empregado Efetivo
	F	Cedido - RJU
	F	Cedido - RJU
	F	Cedido - RJU
	F	Cedido - RJU
	F	Cedido - RJU
	F	Cedido - RJU
	F	Cedido - RJU
	F	Cedido - RJU
	M	Cedido - RJU
	F	Cedido - RJU
	F	Cedido - RJU
	F	Cedido - RJU
	F	Cedido - RJU
	F	Empregado Efetivo
	F	Empregado Efetivo

	F	Empregado Efetivo
	F	Empregado Efetivo
	F	Empregado Efetivo
	F	Empregado Efetivo
	F	Empregado Efetivo
	F	Empregado Efetivo
	F	Empregado Efetivo
	F	Empregado Efetivo
	F	Empregado Efetivo
	F	Empregado Efetivo
	F	Empregado Efetivo
	F	Empregado Efetivo
	F	Empregado Efetivo
	F	Empregado Efetivo
	F	Empregado Efetivo
	F	Empregado Efetivo
	F	Empregado Efetivo
	F	Empregado Efetivo
	F	Empregado Efetivo
	F	Empregado Efetivo
	F	Empregado Efetivo
	F	Empregado Efetivo
	F	Empregado Efetivo
	F	Empregado Efetivo
	F	Empregado Efetivo
	F	Empregado Efetivo
	F	Empregado Efetivo
	F	Empregado Efetivo
	F	Empregado Efetivo
	F	Empregado Efetivo
	F	Empregado Efetivo
	F	Empregado Efetivo
	F	Empregado Efetivo
	F	Empregado Efetivo
	F	Empregado Efetivo
	F	Empregado Efetivo
	M	Empregado Efetivo
	M	Empregado Efetivo
	F	Empregado Efetivo
	F	Empregado Efetivo
	F	Empregado Efetivo

	F	Empregado Efetivo
	F	Empregado Efetivo
	F	Empregado Efetivo
	M	Empregado Efetivo
	M	Empregado Efetivo
	M	Empregado Efetivo
	M	Empregado Efetivo
	F	Empregado Efetivo
	F	Empregado Efetivo
	M	Empregado Efetivo
	F	Empregado Efetivo
	F	Empregado Efetivo
	F	Empregado Efetivo
	M	Empregado Efetivo
	M	Empregado Efetivo
	M	Empregado Efetivo
	F	Empregado Efetivo
	M	Empregado Efetivo
	F	Empregado Efetivo
	F	Empregado Efetivo
	F	Empregado Efetivo
	F	Empregado Efetivo
	F	Empregado Efetivo
	M	Empregado Efetivo
	F	Empregado Efetivo
	F	Empregado Efetivo
	F	Empregado Efetivo
	F	Empregado Efetivo
	F	Empregado Efetivo
	F	Empregado Efetivo
	F	Empregado Efetivo
	F	Empregado Efetivo
	F	Empregado Efetivo
	F	Empregado Efetivo
	F	Empregado Efetivo

	F	Empregado Efetivo
	F	Empregado Efetivo
	F	Empregado Efetivo
	F	Empregado Efetivo
	F	Empregado Efetivo
	F	Empregado Efetivo
	F	Empregado Efetivo
	F	Empregado Efetivo
	F	Empregado Efetivo
	F	Empregado Efetivo
	F	Empregado Efetivo
	F	Empregado Efetivo
	F	Empregado Efetivo
	F	Empregado Efetivo
	F	Empregado Efetivo
	F	Empregado Efetivo
	F	Empregado Efetivo
	F	Empregado Efetivo
	F	Empregado Efetivo
	F	Empregado Efetivo
	F	Empregado Efetivo
	F	Empregado Efetivo
	F	Empregado Efetivo
	F	Empregado Efetivo
	M	Empregado Efetivo
	M	Empregado Efetivo
	F	Empregado Efetivo
	F	Empregado Efetivo
	M	Empregado Efetivo
	F	Empregado Efetivo
	F	Empregado Efetivo
	F	Empregado Efetivo
	M	Empregado Efetivo
	M	Empregado Efetivo
	F	Empregado Efetivo
	M	Empregado Efetivo
	F	Empregado Efetivo
	M	Empregado Efetivo
	M	Empregado Efetivo
	F	Empregado Efetivo
	F	Empregado Efetivo
	F	Empregado Efetivo

	M	Empregado Efetivo
	F	Empregado Efetivo
	F	Empregado Efetivo
	F	Empregado Efetivo
	F	Empregado Efetivo
	M	Empregado Efetivo
	M	Empregado Efetivo
	F	Empregado Efetivo
	F	Empregado Efetivo
	F	Empregado Efetivo
	F	Empregado Efetivo
	F	Empregado Efetivo
	F	Empregado Efetivo
	F	Empregado Efetivo
	F	Empregado Efetivo
	F	Empregado Efetivo
	F	Empregado Efetivo
	M	Empregado Efetivo
	M	Empregado Efetivo
	M	Empregado Efetivo
	F	Empregado Efetivo
	F	Empregado Efetivo
	F	Empregado Efetivo
	F	Empregado Efetivo
	F	Empregado Efetivo
	F	Empregado Efetivo
	F	Empregado Efetivo
	F	Empregado Efetivo
	F	Empregado Efetivo
	F	Empregado Efetivo
	M	Empregado Efetivo
	F	Empregado Efetivo

	F	Empregado Efetivo
	F	Empregado Efetivo
	F	Empregado Efetivo
	M	Empregado Efetivo
	F	Empregado Efetivo
	F	Empregado Efetivo
	F	Empregado Efetivo
	F	Empregado Efetivo
	F	Empregado Efetivo
	M	Empregado Efetivo
	F	Empregado Efetivo
	F	Empregado Efetivo
	F	Empregado Efetivo
	F	Empregado Efetivo
	F	Empregado Efetivo
	F	Empregado Efetivo
	F	Empregado Efetivo
	M	Empregado Efetivo
	F	Empregado Efetivo
	F	Empregado Efetivo
	F	Empregado Efetivo
	F	Empregado Efetivo
	M	Empregado Efetivo
	F	Empregado Efetivo
	F	Empregado Efetivo
	F	Empregado Efetivo
	F	Empregado Efetivo
	F	Empregado Efetivo
	F	Empregado Efetivo
	F	Empregado Efetivo
	F	Empregado Efetivo
	F	Empregado Efetivo

	F	Empregado Efetivo
	F	Empregado Efetivo
	F	Empregado Efetivo
	F	Empregado Efetivo
	F	Empregado Efetivo
	F	Empregado Efetivo
	F	Empregado Efetivo
	F	Empregado Efetivo
	F	Empregado Efetivo
	F	Empregado Efetivo
	F	Empregado Efetivo
	F	Empregado Efetivo
	F	Empregado Efetivo
	F	Empregado Efetivo
	M	Empregado Efetivo
	M	Empregado Efetivo
	M	Empregado Efetivo
	M	Empregado Efetivo
	F	Empregado Efetivo
	F	Empregado Efetivo
	F	Empregado Efetivo
	F	Empregado Efetivo
	F	Empregado Efetivo
	F	Empregado Efetivo
	F	Cedido - RJU
	F	Empregado Efetivo
	F	Empregado Efetivo
	F	Empregado Efetivo
	F	Empregado Efetivo
	F	Empregado Efetivo
	F	Empregado Efetivo
	M	Empregado Efetivo

	M	Empregado Efetivo
	F	Empregado Efetivo
	M	Empregado Efetivo
	F	Empregado Efetivo
	F	Empregado Efetivo
	F	Empregado Efetivo
	F	Empregado Efetivo
	F	Empregado Efetivo
	F	Empregado Efetivo
	F	Empregado Efetivo
	F	Empregado Efetivo
	F	Empregado Efetivo
	F	Empregado Efetivo
	M	Empregado Efetivo
	M	Empregado Efetivo
	F	Empregado Efetivo
	F	Empregado Efetivo
	M	Empregado Efetivo
	F	Empregado Efetivo
	M	Empregado Efetivo
	M	Empregado Efetivo
	M	Empregado Efetivo
	F	Empregado Efetivo
	M	Empregado Efetivo
	F	Empregado Efetivo
	F	Empregado Efetivo
	M	Empregado Efetivo
	F	Empregado Efetivo
	F	Empregado Efetivo
	F	Empregado Efetivo
	M	Empregado Efetivo
	F	Empregado Efetivo

	F	Empregado Efetivo
	F	Empregado Efetivo
	M	Empregado Efetivo
	M	Empregado Efetivo
	F	Empregado Efetivo
	F	Empregado Efetivo
	F	Empregado Efetivo
	F	Empregado Efetivo
	F	Empregado Efetivo
	F	Empregado Efetivo
	M	Empregado Efetivo
	M	Empregado Efetivo
	M	Empregado Efetivo
	F	Empregado Efetivo
	F	Empregado Efetivo
	F	Empregado Efetivo
	F	Empregado Efetivo
	F	Empregado Efetivo
	F	Empregado Efetivo
	M	Empregado Efetivo
	F	Empregado Efetivo
	F	Empregado Efetivo
	F	Empregado Efetivo
	F	Empregado Efetivo
	F	Empregado Efetivo
	F	Empregado Efetivo
	F	Empregado Efetivo
	F	Empregado Efetivo
	F	Empregado Efetivo
	F	Empregado Efetivo
	F	Empregado Efetivo
	F	Empregado Efetivo
	F	Empregado Efetivo
	F	Empregado Efetivo
	F	Empregado Efetivo
	F	Empregado Efetivo
	F	Empregado Efetivo
	F	Empregado Efetivo

	F	Empregado Efetivo
	F	Empregado Efetivo
	F	Empregado Efetivo
	F	Empregado Efetivo
	M	Empregado Efetivo
	F	Empregado Efetivo
	F	Empregado Efetivo
	F	Empregado Efetivo
	F	Empregado Efetivo
	F	Empregado Efetivo
	F	Empregado Efetivo
	F	Empregado Efetivo
	F	Empregado Efetivo
	F	Empregado Efetivo
	M	Empregado Efetivo
	F	Empregado Efetivo
	F	Empregado Efetivo
	F	Empregado Efetivo
	M	Empregado Efetivo
	M	Empregado Efetivo
	M	Empregado Efetivo
	M	Empregado Efetivo
	F	Empregado Efetivo
	M	Empregado Efetivo
	F	Empregado Efetivo
	F	Empregado Efetivo
	F	Empregado Efetivo
	M	Empregado Efetivo
	M	Empregado Efetivo
	M	Empregado Efetivo
	F	Empregado Efetivo
	F	Empregado Efetivo
	M	Empregado Efetivo
	F	Empregado Efetivo
	F	Empregado Efetivo
	F	Empregado Efetivo
	F	Empregado Efetivo
	M	Empregado Efetivo
	M	Empregado Efetivo
	M	Empregado Efetivo
	F	Empregado Efetivo

	F	Empregado Efetivo
	F	Empregado Efetivo
	M	Empregado Efetivo
	M	Empregado Efetivo
	F	Empregado Efetivo
	F	Empregado Efetivo
	F	Empregado Efetivo
	F	Empregado Efetivo
	F	Empregado Efetivo
	F	Empregado Efetivo
	F	Empregado Efetivo
	F	Empregado Efetivo
	F	Empregado Efetivo
	M	Empregado Efetivo
	F	Empregado Efetivo
	F	Empregado Efetivo
	M	Empregado Efetivo
	F	Empregado Efetivo
	F	Empregado Efetivo
	F	Empregado Efetivo
	F	Empregado Efetivo
	F	Empregado Efetivo
	M	Empregado Efetivo
	M	Empregado Efetivo
	F	Empregado Efetivo
	F	Empregado Efetivo
	F	Empregado Efetivo
	F	Empregado Efetivo
	F	Empregado Efetivo
	F	Empregado Efetivo
	F	Empregado Efetivo
	M	Empregado Efetivo
	F	Empregado Efetivo
	F	Empregado Efetivo
	F	Empregado Efetivo
	F	Empregado Efetivo
	F	Empregado Efetivo
	F	Empregado Efetivo
	F	Empregado Efetivo
	F	Empregado Efetivo

	M	Empregado Efetivo
	M	Empregado Efetivo
	M	Empregado Efetivo
	F	Empregado Efetivo
	F	Empregado Efetivo
	F	Empregado Efetivo
	M	Empregado Efetivo
	F	Empregado Efetivo
	F	Empregado Efetivo
	M	Empregado Efetivo
	F	Empregado Efetivo
	F	Empregado Efetivo
	F	Empregado Efetivo
	F	Empregado Efetivo
	M	Empregado Efetivo
	F	Empregado Efetivo
	F	Empregado Efetivo
	F	Empregado Efetivo
	F	Empregado Efetivo
	F	Empregado Efetivo
	M	Empregado Efetivo
	M	Empregado Efetivo
	F	Empregado Efetivo
	F	Empregado Efetivo
	M	Empregado Efetivo
	M	Empregado Efetivo
	M	Empregado Efetivo
	M	Empregado Efetivo
	F	Empregado Efetivo
	F	Empregado Efetivo
	F	Empregado Efetivo
	F	Empregado Efetivo
	F	Empregado Efetivo
	F	Empregado Efetivo
	F	Empregado Efetivo
	F	Empregado Efetivo
	M	Empregado Efetivo
	M	Empregado Efetivo
	M	Empregado Efetivo
	F	Empregado Efetivo
	F	Empregado Efetivo
	M	Empregado Efetivo
	F	Empregado Efetivo
	M	Empregado Efetivo
	F	Empregado Efetivo
	M	Empregado Efetivo
	F	Empregado Efetivo
	F	Empregado Efetivo

	F	Empregado Efetivo
	F	Empregado Efetivo
	F	Empregado Efetivo
	F	Empregado Efetivo
	F	Empregado Efetivo
	F	Empregado Efetivo
	F	Empregado Efetivo
	F	Empregado Efetivo
	M	Empregado Efetivo
	M	Empregado Efetivo
	M	Empregado Efetivo
	M	Empregado Efetivo
	M	Empregado Efetivo
	M	Empregado Efetivo
	M	Empregado Efetivo
	M	Empregado Efetivo
	M	Empregado Efetivo
	F	Empregado Efetivo
	M	Empregado Efetivo
	M	Empregado Efetivo
	M	Empregado Efetivo
	F	Empregado Efetivo
	M	Empregado Efetivo
	F	Empregado Efetivo
	F	Empregado Efetivo
	F	Empregado Efetivo
	F	Empregado Efetivo
	F	Empregado Efetivo
	F	Empregado Efetivo
	F	Empregado Efetivo
	F	Empregado Efetivo
	F	Empregado Efetivo
	F	Empregado Efetivo
	F	Empregado Efetivo
	F	Empregado Efetivo
	F	Empregado Efetivo
	F	Empregado Efetivo
	F	Empregado Efetivo
	F	Empregado Efetivo
	F	Empregado Efetivo
	F	Empregado Efetivo
	F	Empregado Efetivo
	F	Empregado Efetivo
	F	Empregado Efetivo
	F	Empregado Efetivo
	F	Empregado Efetivo

	F	Empregado Efetivo
	M	Empregado Efetivo
	F	Empregado Efetivo
	F	Empregado Efetivo
	M	Empregado Efetivo
	M	Empregado Efetivo
	M	Empregado Efetivo
	F	Empregado Efetivo
	F	Empregado Efetivo
	F	Empregado Efetivo
	F	Empregado Efetivo
	F	Empregado Efetivo
	F	Empregado Efetivo
	F	Empregado Efetivo
	F	Empregado Efetivo
	F	Empregado Efetivo
	F	Empregado Efetivo
	F	Empregado Efetivo
	F	Empregado Efetivo
	F	Empregado Efetivo
	F	Empregado Efetivo
	F	Empregado Efetivo
	M	Empregado Efetivo
	M	Empregado Efetivo
	M	Empregado Efetivo
	M	Empregado Efetivo
	M	Empregado Efetivo
	M	Empregado Efetivo
	F	Empregado Efetivo
	F	Empregado Efetivo
	F	Empregado Efetivo
	F	Empregado Efetivo
	F	Empregado Efetivo
	F	Empregado Efetivo
	F	Empregado Efetivo
	F	Empregado Efetivo
	F	Empregado Efetivo
	F	Empregado Efetivo
	F	Empregado Efetivo
	F	Empregado Efetivo
	F	Empregado Efetivo
	F	Empregado Efetivo
	F	Empregado Efetivo
	F	Empregado Efetivo
	F	Empregado Efetivo

	F	Empregado Efetivo
	F	Empregado Efetivo
	F	Empregado Efetivo
	F	Empregado Efetivo
	F	Empregado Efetivo
	F	Empregado Efetivo
	M	Empregado Efetivo
	M	Empregado Efetivo
	F	Empregado Efetivo
	M	Empregado Efetivo
	M	Empregado Efetivo
	M	Empregado Efetivo
	M	Empregado Efetivo
	F	Empregado Efetivo
	M	Empregado Efetivo
	F	Empregado Efetivo
	F	Empregado Efetivo
	M	Empregado Efetivo
	F	Empregado Efetivo
	F	Empregado Efetivo
	F	Empregado Efetivo
	F	Empregado Efetivo
	M	Empregado Efetivo
	F	Empregado Efetivo
	M	Empregado Efetivo
	M	Empregado Efetivo
	F	Empregado Efetivo
	M	Empregado Efetivo
	F	Empregado Efetivo
	F	Empregado Efetivo
	M	Empregado Efetivo
	F	Empregado Efetivo
	M	Empregado Efetivo
	M	Empregado Efetivo
	F	Empregado Efetivo

	F	Empregado Efetivo
	M	Empregado Efetivo
	M	Empregado Efetivo
	F	Empregado Efetivo
	F	Empregado Efetivo
	M	Empregado Efetivo
	M	Empregado Efetivo
	M	Empregado Efetivo
	F	Empregado Efetivo
	F	Empregado Efetivo
	F	Empregado Efetivo
	F	Empregado Efetivo
	F	Empregado Efetivo
	F	Empregado Efetivo
	F	Empregado Efetivo
	F	Empregado Efetivo
	F	Empregado Efetivo
	F	Empregado Efetivo
	F	Empregado Efetivo
	M	Empregado Efetivo
	F	Empregado Efetivo
	F	Empregado Efetivo
	F	Empregado Efetivo
	F	Empregado Efetivo
	F	Empregado Efetivo
	M	Empregado Efetivo
	M	Empregado Efetivo
	F	Empregado Efetivo
	M	Empregado Efetivo
	M	Empregado Efetivo
	M	Empregado Efetivo
	F	Empregado Efetivo
	F	Empregado Efetivo
	M	Empregado Efetivo
	M	Empregado Efetivo
	M	Empregado Efetivo
	M	Empregado Efetivo
	F	Empregado Efetivo
	M	Empregado Efetivo
	M	Empregado Efetivo
	M	Empregado Efetivo
	F	Empregado Efetivo
	F	Empregado Efetivo
	F	Empregado Efetivo
	F	Empregado Efetivo
	F	Empregado Efetivo
	F	Empregado Efetivo

	F	Empregado Efetivo
	M	Empregado Efetivo
	F	Empregado Efetivo
	F	Empregado Efetivo
	F	Empregado Efetivo
	F	Empregado Efetivo
	M	Empregado Efetivo
	F	Empregado Efetivo
	F	Empregado Efetivo
	M	Empregado Efetivo
	M	Empregado Efetivo
	F	Empregado Efetivo
	F	Empregado Efetivo
	F	Empregado Efetivo
	F	Empregado Efetivo
	F	Empregado Efetivo
	M	Empregado Efetivo
	F	Empregado Efetivo
	F	Empregado Efetivo
	F	Empregado Efetivo
	F	Empregado Efetivo
	F	Empregado Efetivo
	F	Empregado Efetivo
	M	Empregado Efetivo
	F	Empregado Efetivo
	F	Empregado Efetivo
	M	Empregado Efetivo
	M	Empregado Efetivo
	F	Empregado Efetivo
	F	Empregado Efetivo
	F	Empregado Efetivo
	F	Empregado Efetivo
	F	Empregado Efetivo
	F	Empregado Efetivo
	F	Empregado Efetivo
	F	Empregado Efetivo
	M	Empregado Efetivo
	F	Empregado Efetivo
	F	Empregado Efetivo
	F	Empregado Efetivo
	F	Empregado Efetivo
	M	Empregado Efetivo
	F	Empregado Efetivo
	F	Empregado Efetivo
	F	Empregado Efetivo

	F	Empregado Efetivo
	M	Empregado Efetivo
	F	Empregado Efetivo
	F	Empregado Efetivo
	F	Empregado Efetivo
	F	Empregado Efetivo
	F	Empregado Efetivo
	F	Empregado Efetivo
	M	Empregado Efetivo
	F	Empregado Efetivo
	F	Empregado Efetivo
	F	Empregado Efetivo
	F	Empregado Efetivo
	M	Empregado Efetivo
	F	Empregado Efetivo
	F	Empregado Efetivo
	F	Empregado Efetivo
	F	Empregado Efetivo
	F	Empregado Efetivo
	F	Empregado Efetivo
	F	Empregado Efetivo
	F	Empregado Efetivo
	F	Empregado Efetivo
	M	Empregado Efetivo
	F	Empregado Efetivo
	F	Empregado Efetivo
	F	Empregado Efetivo
	F	Empregado Efetivo
	F	Empregado Efetivo
	F	Empregado Efetivo
	F	Empregado Efetivo
	F	Empregado Efetivo
	F	Empregado Efetivo
	F	Empregado Efetivo
	F	Empregado Efetivo
	F	Empregado Efetivo
	F	Empregado Efetivo
	F	Empregado Efetivo
	F	Empregado Efetivo
	M	Empregado Efetivo
	F	Empregado Efetivo
	M	Empregado Efetivo
	M	Empregado Efetivo
	F	Empregado Efetivo
	F	Empregado Efetivo
	M	Empregado Efetivo
	F	Empregado Efetivo
	F	Empregado Efetivo

	F	Empregado Efetivo
	F	Empregado Efetivo
	F	Empregado Efetivo
	F	Empregado Efetivo
	F	Empregado Efetivo
	F	Empregado Efetivo
	F	Empregado Efetivo
	F	Empregado Efetivo
	F	Empregado Efetivo
	F	Empregado Efetivo
	M	Empregado Efetivo
	F	Empregado Efetivo
	F	Empregado Efetivo
	F	Empregado Efetivo
	M	Empregado Efetivo
	F	Empregado Efetivo
	F	Empregado Efetivo
	F	Empregado Efetivo
	F	Empregado Efetivo
	F	Empregado Efetivo
	F	Empregado Efetivo
	F	Empregado Efetivo
	M	Empregado Efetivo
	F	Empregado Efetivo
	F	Empregado Efetivo
	F	Empregado Efetivo
	F	Empregado Efetivo
	M	Empregado Efetivo
	F	Empregado Efetivo
	F	Empregado Efetivo
	F	Empregado Efetivo
	F	Empregado Efetivo
	F	Empregado Efetivo
	F	Empregado Efetivo
	F	Empregado Efetivo
	F	Empregado Efetivo
	M	Empregado Efetivo
	F	Empregado Efetivo
	F	Empregado Efetivo
	F	Empregado Efetivo
	F	Empregado Efetivo
	F	Empregado Efetivo
	F	Empregado Efetivo
	F	Empregado Efetivo
	F	Empregado Efetivo
	F	Empregado Efetivo
	F	Empregado Efetivo
	F	Empregado Efetivo

	M	Empregado Efetivo
	F	Empregado Efetivo
	F	Empregado Efetivo
	F	Empregado Efetivo
	M	Empregado Efetivo
	F	Empregado Efetivo
	F	Empregado Efetivo
	M	Empregado Efetivo
	F	Empregado Efetivo
	F	Empregado Efetivo
	F	Empregado Efetivo
	F	Empregado Efetivo
	M	Empregado Efetivo
	M	Empregado Efetivo
	F	Empregado Efetivo
	M	Empregado Efetivo
	F	Empregado Efetivo
	F	Empregado Efetivo
	F	Empregado Efetivo
	M	Empregado Efetivo
	F	Empregado Efetivo
	F	Empregado Efetivo
	F	Empregado Efetivo
	F	Empregado Efetivo
	F	Empregado Efetivo
	F	Empregado Efetivo
	F	Empregado Efetivo
	F	Empregado Efetivo
	F	Empregado Efetivo
	F	Empregado Efetivo
	F	Empregado Efetivo
	F	Empregado Efetivo
	F	Empregado Efetivo
	M	Empregado Efetivo
	F	Empregado Efetivo
	M	Empregado Efetivo
	F	Empregado Efetivo
	M	Empregado Efetivo
	F	Empregado Efetivo
	M	Empregado Efetivo
	F	Empregado Efetivo
	F	Empregado Efetivo
	M	Empregado Efetivo
	F	Empregado Efetivo
	M	Empregado Efetivo
	F	Empregado Efetivo
	F	Empregado Efetivo
	F	Empregado Efetivo

	F	Empregado Efetivo
	F	Empregado Efetivo
	F	Empregado Efetivo
	M	Empregado Efetivo
	F	Empregado Efetivo
	M	Empregado Efetivo
	F	Empregado Efetivo
	F	Empregado Efetivo
	F	Empregado Efetivo
	F	Empregado Efetivo
	F	Empregado Efetivo
	F	Empregado Efetivo
	M	Cedido - RJU
	F	Empregado Efetivo
	F	Empregado Efetivo
	F	Empregado Efetivo
	F	Empregado Efetivo
	F	Empregado Efetivo
	F	Empregado Efetivo
	F	Empregado Efetivo
	F	Empregado Efetivo
	F	Empregado Efetivo
	M	Empregado Efetivo
	F	Empregado Efetivo
	M	Empregado Efetivo
	F	Empregado Efetivo
	M	Empregado Efetivo
	F	Empregado Efetivo
	M	Empregado Efetivo
	F	Empregado Efetivo
	M	Empregado Efetivo
	M	Empregado Efetivo
	M	Empregado Efetivo
	F	Empregado Efetivo
	M	Empregado Efetivo
	F	Empregado Efetivo
	F	Empregado Efetivo
	F	Empregado Efetivo
	F	Empregado Efetivo
	M	Empregado Efetivo
	F	Empregado Efetivo
	M	Empregado Efetivo
	M	Empregado Efetivo
	F	Empregado Efetivo
	F	Empregado Efetivo
	F	Empregado Efetivo
	M	Empregado Efetivo

	M	Empregado Efetivo
	M	Empregado Efetivo
	M	Empregado Efetivo
	M	Empregado Efetivo
	M	Empregado Efetivo
	M	Empregado Efetivo
	M	Empregado Efetivo
	M	Empregado Efetivo
	M	Empregado Efetivo
	F	Empregado Efetivo
	F	Empregado Efetivo
	F	Empregado Efetivo
	F	Empregado Efetivo
	F	Empregado Efetivo
	F	Empregado Efetivo
	F	Empregado Efetivo
	F	Empregado Efetivo
	F	Empregado Efetivo
	F	Empregado Efetivo
	F	Empregado Efetivo
	F	Empregado Efetivo
	F	Empregado Efetivo
	F	Empregado Efetivo
	F	Empregado Efetivo
	F	Empregado Efetivo
	F	Empregado Efetivo
	F	Empregado Efetivo
	F	Empregado Efetivo
	F	Empregado Efetivo
	F	Empregado Efetivo
	F	Empregado Efetivo
	F	Empregado Efetivo
	F	Empregado Efetivo
	F	Empregado Efetivo
	F	Empregado Efetivo
	F	Empregado Efetivo
	F	Empregado Efetivo
	F	Empregado Efetivo
	F	Empregado Efetivo
	F	Empregado Efetivo
	F	Empregado Efetivo
	F	Empregado Efetivo
	F	Empregado Efetivo
	F	Empregado Efetivo

	F	Empregado Efetivo
	F	Empregado Efetivo
	F	Empregado Efetivo
	F	Empregado Efetivo
	F	Empregado Efetivo
	F	Empregado Efetivo
	F	Empregado Efetivo
	F	Empregado Efetivo
	F	Empregado Efetivo
	F	Empregado Efetivo
	F	Empregado Efetivo
	F	Empregado Efetivo
	F	Empregado Efetivo
	F	Empregado Efetivo
	M	Empregado Efetivo
	F	Empregado Efetivo
	F	Empregado Efetivo
	M	Empregado Efetivo
	F	Empregado Efetivo
	F	Empregado Efetivo
	F	Empregado Efetivo
	F	Empregado Efetivo
	M	Empregado Efetivo
	F	Empregado Efetivo
	M	Empregado Efetivo
	F	Empregado Efetivo
	F	Empregado Efetivo
	F	Empregado Efetivo
	M	Empregado Efetivo
	F	Empregado Efetivo
	F	Empregado Efetivo
	M	Empregado Efetivo
	F	Empregado Efetivo
	M	Empregado Efetivo
	F	Empregado Efetivo
	F	Empregado Efetivo
	F	Empregado Efetivo
	M	Empregado Efetivo
	F	Empregado Efetivo
	F	Empregado Efetivo
	M	Empregado Efetivo

	F	Empregado Efetivo
	F	Empregado Efetivo
	F	Empregado Efetivo
	F	Empregado Efetivo
	F	Empregado Efetivo
	M	Empregado Efetivo
	F	Empregado Efetivo
	F	Empregado Efetivo
	F	Empregado Efetivo
	M	Empregado Efetivo
	M	Empregado Efetivo
	F	Empregado Efetivo
	F	Empregado Efetivo
	F	Empregado Efetivo
	F	Empregado Efetivo
	F	Empregado Efetivo
	F	Empregado Efetivo
	F	Empregado Efetivo
	F	Empregado Efetivo
	F	Empregado Efetivo
	F	Empregado Efetivo
	F	Empregado Efetivo
	F	Empregado Efetivo
	F	Empregado Efetivo
	M	Empregado Efetivo
	F	Empregado Efetivo
	M	Empregado Efetivo
	M	Empregado Efetivo
	F	Empregado Efetivo
	F	Empregado Efetivo
	F	Empregado Efetivo
	F	Empregado Efetivo
	F	Empregado Efetivo
	M	Empregado Efetivo
	F	Empregado Efetivo
	F	Empregado Efetivo
	F	Empregado Efetivo
	F	Empregado Efetivo
	F	Empregado Efetivo
	F	Empregado Efetivo
	F	Empregado Efetivo
	F	Empregado Efetivo
	F	Empregado Efetivo
	F	Empregado Efetivo

	F	Empregado Efetivo
	F	Empregado Efetivo
	F	Empregado Efetivo
	F	Empregado Efetivo
	F	Empregado Efetivo
	F	Empregado Efetivo
	F	Empregado Efetivo
	F	Empregado Efetivo
	F	Empregado Efetivo
	F	Empregado Efetivo
	F	Empregado Efetivo
	F	Empregado Efetivo
	F	Empregado Efetivo
	F	Empregado Efetivo
	F	Empregado Efetivo
	F	Empregado Efetivo
	F	Empregado Efetivo
	F	Empregado Efetivo
	F	Empregado Efetivo
	F	Empregado Efetivo
	F	Empregado Efetivo
	F	Empregado Efetivo
	F	Empregado Efetivo
	F	Empregado Efetivo
	F	Empregado Efetivo
	F	Empregado Efetivo
	F	Empregado Efetivo
	F	Empregado Efetivo
	F	Empregado Efetivo
	F	Empregado Efetivo
	F	Empregado Efetivo
	F	Empregado Efetivo

	M	Empregado Efetivo
	F	Empregado Efetivo
	F	Empregado Efetivo
	F	Empregado Efetivo
	F	Empregado Efetivo
	M	Empregado Efetivo
	F	Empregado Efetivo
	F	Empregado Efetivo
	F	Empregado Efetivo
	F	Empregado Efetivo
	F	Empregado Efetivo
	F	Empregado Efetivo
	M	Empregado Efetivo
	M	Empregado Efetivo
	F	Empregado Efetivo
	F	Empregado Efetivo
	F	Empregado Efetivo
	F	Empregado Efetivo
	F	Empregado Efetivo
	F	Empregado Efetivo
	F	Empregado Efetivo
	M	Empregado Efetivo
	M	Empregado Efetivo
	M	Empregado Efetivo
	F	Empregado Efetivo
	M	Empregado Efetivo
	F	Empregado Efetivo
	F	Empregado Efetivo
	F	Empregado Efetivo
	F	Empregado Efetivo
	F	Empregado Efetivo
	F	Empregado Efetivo
	F	Empregado Efetivo
	F	Empregado Efetivo
	F	Empregado Efetivo
	F	Empregado Efetivo
	F	Empregado Efetivo
	M	Empregado Efetivo
	M	Empregado Efetivo
	F	Empregado Efetivo
	F	Empregado Efetivo
	F	Empregado Efetivo

	F	Empregado Efetivo
	F	Empregado Efetivo
	F	Empregado Efetivo
	F	Empregado Efetivo
	F	Empregado Efetivo
	F	Empregado Efetivo
	F	Empregado Efetivo
	F	Empregado Efetivo
	F	Empregado Efetivo
	F	Empregado Efetivo
	F	Empregado Efetivo
	F	Empregado Efetivo
	F	Empregado Efetivo
	F	Empregado Efetivo
	M	Empregado Efetivo
	F	Empregado Efetivo
	F	Empregado Efetivo
	F	Empregado Efetivo
	F	Empregado Efetivo
	F	Empregado Efetivo
	F	Empregado Efetivo
	F	Empregado Efetivo
	F	Empregado Efetivo
	F	Empregado Efetivo
	F	Empregado Efetivo
	F	Empregado Efetivo
	F	Empregado Efetivo
	F	Empregado Efetivo
	F	Empregado Efetivo
	F	Empregado Efetivo
	F	Empregado Efetivo
	F	Empregado Efetivo
	F	Empregado Efetivo
	F	Empregado Efetivo
	F	Empregado Efetivo
	F	Empregado Efetivo
	F	Empregado Efetivo
	F	Empregado Efetivo
	F	Empregado Efetivo
	F	Empregado Efetivo
	M	Empregado Efetivo
	F	Empregado Efetivo
	F	Empregado Efetivo
	F	Empregado Efetivo
	M	Empregado Efetivo
	F	Empregado Efetivo
	F	Empregado Efetivo
	F	Empregado Efetivo
	F	Empregado Efetivo

	F	Empregado Efetivo
	M	Empregado Efetivo
	F	Empregado Efetivo
	F	Empregado Efetivo
	F	Empregado Efetivo
	F	Empregado Efetivo
	F	Empregado Efetivo
	F	Empregado Efetivo
	F	Empregado Efetivo
	F	Empregado Efetivo
	F	Empregado Efetivo
	F	Empregado Efetivo
	F	Empregado Efetivo
	F	Empregado Efetivo
	M	Empregado Efetivo
	M	Empregado Efetivo
	F	Empregado Efetivo
	F	Empregado Efetivo
	F	Empregado Efetivo
	F	Empregado Efetivo
	F	Empregado Efetivo
	F	Empregado Efetivo
	M	Empregado Efetivo
	F	Empregado Efetivo
	F	Empregado Efetivo
	F	Empregado Efetivo
	F	Empregado Efetivo
	F	Empregado Efetivo
	F	Empregado Efetivo
	F	Empregado Efetivo
	F	Empregado Efetivo
	F	Empregado Efetivo
	F	Empregado Efetivo
	F	Empregado Efetivo
	F	Empregado Efetivo
	F	Empregado Efetivo
	F	Empregado Efetivo
	F	Empregado Efetivo
	F	Empregado Efetivo
	F	Empregado Efetivo
	F	Empregado Efetivo
	F	Empregado Efetivo
	F	Empregado Efetivo
	F	Empregado Efetivo
	F	Empregado Efetivo
	F	Empregado Efetivo
	F	Empregado Efetivo
	F	Empregado Efetivo

	F	Empregado Efetivo
	F	Empregado Efetivo
	F	Empregado Efetivo
	F	Empregado Efetivo
	F	Empregado Efetivo
	F	Empregado Efetivo
	F	Empregado Efetivo
	F	Empregado Efetivo
	F	Empregado Efetivo
	M	Empregado Efetivo
	F	Empregado Efetivo
	F	Empregado Efetivo
	F	Empregado Efetivo
	M	Empregado Efetivo
	M	Empregado Efetivo
	F	Empregado Efetivo
	F	Empregado Efetivo
	F	Empregado Efetivo
	F	Empregado Efetivo
	F	Empregado Efetivo
	F	Empregado Efetivo
	F	Empregado Efetivo
	F	Empregado Efetivo
	F	Empregado Efetivo
	F	Empregado Efetivo
	F	Empregado Efetivo
	F	Empregado Efetivo
	F	Empregado Efetivo
	F	Empregado Efetivo
	F	Empregado Efetivo
	F	Empregado Efetivo
	F	Empregado Efetivo
	M	Empregado Efetivo
	F	Empregado Efetivo
	M	Empregado Efetivo
	F	Empregado Efetivo
	F	Empregado Efetivo
	M	Empregado Efetivo
	M	Empregado Efetivo
	M	Empregado Efetivo
	M	Empregado Efetivo
	M	Empregado Efetivo
	F	Empregado Efetivo
	F	Empregado Efetivo
	F	Empregado Efetivo
	F	Empregado Efetivo
	F	Empregado Efetivo
	F	Empregado Efetivo

	M	Empregado Efetivo
	M	Empregado Efetivo
	F	Empregado Efetivo
	F	Empregado Efetivo
	F	Empregado Efetivo
	F	Empregado Efetivo
	F	Empregado Efetivo
	F	Empregado Efetivo
	F	Empregado Efetivo
	F	Empregado Efetivo
	M	Empregado Efetivo
	M	Empregado Efetivo
	M	Empregado Efetivo
	F	Empregado Efetivo
	F	Empregado Efetivo
	F	Empregado Efetivo
	M	Empregado Efetivo
	F	Empregado Efetivo
	F	Empregado Efetivo
	F	Empregado Efetivo
	M	Empregado Efetivo
	F	Empregado Efetivo
	M	Empregado Efetivo
	M	Empregado Efetivo
	F	Empregado Efetivo
	M	Empregado Efetivo
	F	Empregado Efetivo
	F	Empregado Efetivo
	M	Empregado Efetivo
	F	Empregado Efetivo
	F	Empregado Efetivo
	M	Empregado Efetivo
	M	Empregado Efetivo
	M	Empregado Efetivo
	F	Empregado Efetivo
	F	Empregado Efetivo
	M	Empregado Efetivo
	M	Empregado Efetivo
	F	Empregado Efetivo
	M	Empregado Efetivo
	M	Empregado Efetivo
	M	Empregado Efetivo
	F	Empregado Efetivo

	M	Empregado Efetivo
	F	Empregado Efetivo
	M	Empregado Efetivo
	F	Empregado Efetivo
	F	Empregado Efetivo
	F	Empregado Efetivo
	F	Empregado Efetivo
	F	Empregado Efetivo
	F	Empregado Efetivo
	F	Empregado Efetivo
	F	Empregado Efetivo
	F	Empregado Efetivo
	M	Empregado Efetivo
	F	Empregado Efetivo
	M	Empregado Efetivo
	F	Empregado Efetivo
	F	Empregado Efetivo
	F	Empregado Efetivo
	M	Empregado Efetivo
	M	Empregado Efetivo
	F	Empregado Efetivo
	F	Empregado Efetivo
	F	Empregado Efetivo
	M	Empregado Efetivo
	M	Empregado Efetivo
	F	Empregado Efetivo
	F	Empregado Efetivo
	F	Empregado Efetivo
	F	Empregado Efetivo
	M	Empregado Efetivo
	M	Empregado Efetivo
	M	Empregado Efetivo

	F	Empregado Efetivo
	F	Empregado Efetivo
	F	Empregado Efetivo
	F	Empregado Efetivo
	F	Empregado Efetivo
	F	Empregado Efetivo
	F	Empregado Efetivo
	F	Empregado Efetivo
	M	Empregado Efetivo
	F	Empregado Efetivo
	F	Empregado Efetivo
	M	Empregado Efetivo
	F	Empregado Efetivo
	F	Empregado Efetivo
	F	Empregado Efetivo
	F	Empregado Efetivo
	M	Empregado Efetivo
	F	Empregado Efetivo
	F	Empregado Efetivo
	M	Empregado Efetivo
	M	Empregado Efetivo
	M	Empregado Efetivo
	M	Empregado Efetivo
	M	Empregado Efetivo
	F	Empregado Efetivo
	F	Empregado Efetivo
	F	Empregado Efetivo
	M	Empregado Efetivo
	M	Empregado Efetivo
	M	Empregado Efetivo
	M	Empregado Efetivo
	F	Empregado Efetivo

	M	Empregado Efetivo
	M	Empregado Efetivo
	F	Empregado Efetivo
	M	Empregado Efetivo
	M	Empregado Efetivo
	F	Empregado Efetivo
	F	Empregado Efetivo
	F	Empregado Efetivo
	M	Empregado Efetivo
	M	Empregado Efetivo
	M	Empregado Efetivo
	M	Empregado Efetivo
	F	Empregado Efetivo
	F	Empregado Efetivo
	F	Empregado Efetivo
	F	Empregado Efetivo
	F	Empregado Efetivo
	M	Empregado Efetivo
	F	Empregado Efetivo
	F	Empregado Efetivo
	F	Empregado Efetivo
	M	Empregado Efetivo
	M	Empregado Efetivo
	F	Empregado Efetivo
	M	Empregado Efetivo
	F	Empregado Efetivo
	F	Empregado Efetivo
	F	Empregado Efetivo
	F	Empregado Efetivo
	F	Empregado Efetivo
	F	Empregado Efetivo
	M	Empregado Efetivo
	M	Empregado Efetivo
	F	Empregado Efetivo
	F	Empregado Efetivo
	F	Empregado Efetivo
	F	Empregado Efetivo
	F	Empregado Efetivo
	M	Empregado Efetivo
	F	Empregado Efetivo
	F	Empregado Efetivo
	F	Empregado Efetivo
	F	Empregado Efetivo
	F	Empregado Efetivo
	F	Empregado Efetivo

	M	Empregado Efetivo
	F	Empregado Efetivo
	F	Empregado Efetivo
	M	Empregado Efetivo
	M	Empregado Efetivo
	F	Empregado Efetivo
	F	Empregado Efetivo
	F	Empregado Efetivo
	F	Empregado Efetivo
	M	Empregado Efetivo
	F	Empregado Efetivo
	F	Empregado Efetivo
	F	Empregado Efetivo
	M	Empregado Efetivo
	M	Empregado Efetivo
	F	Empregado Efetivo
	F	Empregado Efetivo
	F	Empregado Efetivo
	M	Empregado Efetivo
	M	Empregado Efetivo
	M	Empregado Efetivo
	F	Empregado Efetivo
	F	Empregado Efetivo
	F	Empregado Efetivo
	M	Empregado Efetivo
	F	Empregado Efetivo
	F	Empregado Efetivo
	F	Empregado Efetivo
	M	Empregado Efetivo
	F	Empregado Efetivo
	F	Empregado Efetivo
	F	Empregado Efetivo
	F	Empregado Efetivo
	F	Empregado Efetivo
	F	Empregado Efetivo
	F	Empregado Efetivo
	F	Empregado Efetivo
	F	Empregado Efetivo
	F	Empregado Efetivo
	F	Empregado Efetivo
	M	Empregado Efetivo
	F	Empregado Efetivo
	M	Empregado Efetivo
	F	Empregado Efetivo
	F	Empregado Efetivo
	M	Empregado Efetivo
	M	Empregado Efetivo
	F	Empregado Efetivo

	F	Empregado Efetivo
	M	Empregado Efetivo
	F	Empregado Efetivo
	F	Empregado Efetivo
	F	Empregado Efetivo
	F	Empregado Efetivo
	F	Empregado Efetivo
	F	Empregado Efetivo
	F	Empregado Efetivo
	F	Empregado Efetivo
	F	Empregado Efetivo
	F	Empregado Efetivo
	F	Empregado Efetivo
	F	Empregado Efetivo
	F	Empregado Efetivo
	M	Empregado Efetivo
	F	Empregado Efetivo
	F	Empregado Efetivo
	F	Empregado Efetivo
	F	Empregado Efetivo
	F	Empregado Efetivo
	F	Empregado Efetivo
	F	Empregado Efetivo
	F	Empregado Efetivo
	F	Empregado Efetivo
	F	Empregado Efetivo
	F	Empregado Efetivo
	F	Empregado Efetivo
	F	Empregado Efetivo
	F	Empregado Efetivo
	F	Empregado Efetivo
	F	Empregado Efetivo
	F	Empregado Efetivo
	F	Empregado Efetivo
	F	Empregado Efetivo
	F	Empregado Efetivo
	F	Empregado Efetivo
	F	Empregado Efetivo
	F	Empregado Efetivo
	F	Empregado Efetivo

	F	Empregado Efetivo
	F	Empregado Efetivo
	F	Empregado Efetivo
	F	Empregado Efetivo
	F	Empregado Efetivo
	F	Empregado Efetivo
	F	Empregado Efetivo
	F	Empregado Efetivo
	F	Empregado Efetivo
	F	Empregado Efetivo
	F	Empregado Efetivo
	F	Empregado Efetivo
	F	Empregado Efetivo
	F	Empregado Efetivo
	F	Empregado Efetivo
	F	Empregado Efetivo
	F	Empregado Efetivo
	M	Empregado Efetivo
	F	Empregado Efetivo
	M	Empregado Efetivo
	M	Empregado Efetivo
	M	Empregado Efetivo
	F	Empregado Efetivo
	F	Empregado Efetivo
	F	Empregado Efetivo
	F	Empregado Efetivo
	M	Empregado Efetivo
	F	Empregado Efetivo
	M	Empregado Efetivo
	M	Empregado Efetivo
	F	Empregado Efetivo
	F	Empregado Efetivo
	F	Empregado Efetivo
	F	Empregado Efetivo
	F	Empregado Efetivo
	M	Empregado Efetivo
	F	Empregado Efetivo
	M	Empregado Efetivo
	M	Empregado Efetivo
	F	Empregado Efetivo
	F	Empregado Efetivo
	F	Empregado Efetivo
	F	Empregado Efetivo
	F	Empregado Efetivo
	F	Empregado Efetivo
	F	Empregado Efetivo
	F	Empregado Efetivo
	F	Empregado Efetivo

	F	Empregado Efetivo
	F	Empregado Efetivo
	F	Empregado Efetivo
	F	Empregado Efetivo
	F	Empregado Efetivo
	F	Empregado Efetivo
	F	Empregado Efetivo
	F	Empregado Efetivo
	F	Empregado Efetivo
	F	Empregado Efetivo
	F	Empregado Efetivo
	F	Empregado Efetivo
	F	Empregado Efetivo
	F	Empregado Efetivo
	M	Empregado Efetivo
	F	Empregado Efetivo
	M	Empregado Efetivo
	F	Empregado Efetivo
	F	Empregado Efetivo
	F	Empregado Efetivo
	M	Empregado Efetivo
	F	Empregado Efetivo
	F	Empregado Efetivo
	F	Empregado Efetivo
	F	Empregado Efetivo
	F	Empregado Efetivo
	F	Empregado Efetivo
	F	Empregado Efetivo
	M	Empregado Efetivo
	M	Empregado Efetivo
	F	Empregado Efetivo
	M	Empregado Efetivo
	M	Empregado Efetivo
	M	Empregado Efetivo
	F	Empregado Efetivo
	F	Empregado Efetivo
	M	Empregado Efetivo
	F	Empregado Efetivo
	F	Empregado Efetivo
	M	Empregado Efetivo
	M	Empregado Efetivo
	F	Empregado Efetivo
	F	Empregado Efetivo
	F	Empregado Efetivo
	F	Empregado Efetivo
	F	Empregado Efetivo
	F	Empregado Efetivo
	F	Empregado Efetivo

	M	Empregado Efetivo
	F	Empregado Efetivo
	M	Empregado Efetivo
	F	Empregado Efetivo
	M	Empregado Efetivo
	M	Empregado Efetivo
	F	Empregado Efetivo
	F	Empregado Efetivo
	F	Empregado Efetivo
	F	Empregado Efetivo
	M	Empregado Efetivo
	F	Empregado Efetivo
	F	Empregado Efetivo
	F	Empregado Efetivo
	M	Empregado Efetivo
	F	Empregado Efetivo
	F	Empregado Efetivo
	F	Empregado Efetivo
	F	Empregado Efetivo
	F	Empregado Efetivo
	F	Empregado Efetivo
	F	Empregado Efetivo
	F	Empregado Efetivo
	M	Empregado Efetivo
	F	Empregado Efetivo
	F	Empregado Efetivo
	M	Empregado Efetivo
	F	Empregado Efetivo
	F	Empregado Efetivo
	F	Empregado Efetivo
	F	Empregado Efetivo
	M	Empregado Efetivo
	M	Empregado Efetivo
	F	Empregado Efetivo
	F	Empregado Efetivo
	F	Empregado Efetivo
	F	Empregado Efetivo
	F	Empregado Efetivo
	M	Empregado Efetivo
	F	Empregado Efetivo
	M	Empregado Efetivo
	M	Empregado Efetivo
	F	Empregado Efetivo
	F	Empregado Efetivo
	F	Empregado Efetivo
	F	Empregado Efetivo
	F	Empregado Efetivo
	F	Empregado Efetivo

	F	Empregado Efetivo
	M	Empregado Efetivo
	M	Empregado Efetivo
	F	Empregado Efetivo
	F	Empregado Efetivo
	M	Empregado Efetivo
	M	Empregado Efetivo
	F	Empregado Efetivo
	F	Empregado Efetivo
	F	Empregado Efetivo
	M	Empregado Efetivo
	F	Empregado Efetivo
	F	Empregado Efetivo
	F	Empregado Efetivo
	F	Empregado Efetivo
	F	Empregado Efetivo
	M	Empregado Efetivo
	M	Empregado Efetivo
	M	Empregado Efetivo
	F	Empregado Efetivo
	F	Empregado Efetivo
	F	Empregado Efetivo
	F	Empregado Efetivo
	F	Empregado Efetivo
	M	Empregado Efetivo
	M	Empregado Efetivo
	F	Empregado Efetivo
	F	Empregado Efetivo
	F	Empregado Efetivo
	F	Empregado Efetivo
	F	Empregado Efetivo
	F	Empregado Efetivo
	F	Empregado Efetivo

	F	Empregado Efetivo
	F	Empregado Efetivo
	F	Empregado Efetivo
	F	Empregado Efetivo
	M	Empregado Efetivo
	F	Empregado Efetivo
	M	Empregado Efetivo
	F	Empregado Efetivo
	F	Empregado Efetivo
	F	Empregado Efetivo
	F	Empregado Efetivo
	F	Empregado Efetivo
	F	Empregado Efetivo
	F	Empregado Efetivo
	M	Empregado Efetivo
	F	Empregado Efetivo
	F	Empregado Efetivo
	F	Empregado Efetivo
	F	Empregado Efetivo
	F	Empregado Efetivo
	F	Empregado Efetivo
	F	Empregado Efetivo
	M	Empregado Efetivo
	M	Empregado Efetivo
	M	Empregado Efetivo
	M	Empregado Efetivo
	M	Empregado Efetivo
	M	Empregado Efetivo
	M	Empregado Efetivo
	M	Empregado Efetivo
	M	Empregado Efetivo
	F	Cedido - RJU

	M	Cedido - RJU
	M	Cedido - RJU
	M	Empregado Efetivo
	F	Empregado Efetivo
	F	Empregado Efetivo
	F	Empregado Efetivo
	M	Empregado Efetivo
	M	Empregado Efetivo
	M	Empregado Efetivo
	M	Empregado Efetivo
	F	Empregado Efetivo
	M	Empregado Efetivo
	F	Empregado Efetivo
	F	Empregado Efetivo
	F	Empregado Efetivo
	M	Empregado Efetivo
	F	Empregado Efetivo
	F	Empregado Efetivo
	F	Empregado Efetivo
	M	Empregado Efetivo
	M	Empregado Efetivo
	M	Empregado Efetivo
	F	Empregado Efetivo
	F	Empregado Efetivo
	F	Empregado Efetivo
	F	Empregado Efetivo
	F	Empregado Efetivo
	M	Empregado Efetivo
	F	Empregado Efetivo
	M	Empregado Efetivo
	F	Empregado Efetivo
	M	Empregado Efetivo
	M	Empregado Efetivo
	F	Empregado Efetivo
	F	Empregado Efetivo
	M	Empregado Efetivo
	M	Empregado Efetivo
	F	Empregado Efetivo
	F	Empregado Efetivo

	F	Empregado Efetivo
	F	Empregado Efetivo
	F	Empregado Efetivo
	M	Empregado Efetivo
	F	Empregado Efetivo
	F	Empregado Efetivo
	M	Empregado Efetivo
	M	Empregado Efetivo
	F	Empregado Efetivo
	F	Empregado Efetivo
	M	Empregado Efetivo
	F	Empregado Efetivo
	F	Empregado Efetivo
	M	Empregado Efetivo
	F	Empregado Efetivo
	F	Empregado Efetivo
	F	Empregado Efetivo
	F	Empregado Efetivo
	F	Empregado Efetivo
	F	Empregado Efetivo
	F	Empregado Efetivo
	F	Empregado Efetivo
	M	Empregado Efetivo
	M	Empregado Efetivo
	M	Empregado Efetivo
	F	Empregado Efetivo
	F	Empregado Efetivo
	F	Empregado Efetivo
	F	Empregado Efetivo
	M	Empregado Efetivo
	M	Empregado Efetivo
	F	Empregado Efetivo
	F	Empregado Efetivo
	F	Empregado Efetivo
	F	Empregado Efetivo
	M	Empregado Efetivo
	M	Empregado Efetivo
	F	Empregado Efetivo
	M	Empregado Efetivo
	F	Empregado Efetivo
	M	Empregado Efetivo
	F	Empregado Efetivo
	F	Empregado Efetivo
	F	Empregado Efetivo
	F	Empregado Efetivo
	F	Empregado Efetivo
	F	Empregado Efetivo
	M	Empregado Efetivo

	F	Empregado Efetivo
	F	Empregado Efetivo
	F	Empregado Efetivo
	M	Empregado Efetivo
	F	Empregado Efetivo
	F	Empregado Efetivo
	F	Empregado Efetivo
	F	Empregado Efetivo
	F	Empregado Efetivo
	F	Empregado Efetivo
	F	Empregado Efetivo
	F	Empregado Efetivo
	F	Empregado Efetivo
	F	Empregado Efetivo
	M	Empregado Efetivo
	F	Empregado Efetivo
	F	Empregado Efetivo
	F	Empregado Efetivo
	F	Empregado Efetivo
	F	Empregado Efetivo
	F	Empregado Efetivo
	F	Empregado Efetivo
	F	Empregado Efetivo
	M	Empregado Efetivo
	F	Empregado Efetivo
	M	Empregado Efetivo
	F	Empregado Efetivo
	F	Empregado Efetivo
	F	Empregado Efetivo
	F	Empregado Efetivo
	M	Empregado Efetivo
	M	Empregado Efetivo
	F	Empregado Efetivo
	M	Empregado Efetivo
	F	Empregado Efetivo
	F	Empregado Efetivo
	F	Empregado Efetivo
	F	Empregado Efetivo
	M	Empregado Efetivo
	F	Empregado Efetivo
	F	Empregado Efetivo
	F	Empregado Efetivo
	F	Empregado Efetivo
	M	Empregado Efetivo
	F	Empregado Efetivo
	M	Empregado Efetivo
	F	Empregado Efetivo
	F	Empregado Efetivo

	M	Empregado Efetivo
	F	Empregado Efetivo
	F	Empregado Efetivo
	F	Empregado Efetivo
	F	Empregado Efetivo
	M	Empregado Efetivo
	F	Empregado Efetivo
	F	Empregado Efetivo
	M	Empregado Efetivo
	M	Empregado Efetivo
	M	Empregado Efetivo
	F	Empregado Efetivo
	F	Empregado Efetivo
	F	Empregado Efetivo
	F	Empregado Efetivo
	M	Empregado Efetivo
	F	Empregado Efetivo
	F	Empregado Efetivo
	F	Empregado Efetivo
	M	Empregado Efetivo
	F	Empregado Efetivo
	M	Empregado Efetivo
	F	Empregado Efetivo
	F	Empregado Efetivo
	M	Empregado Efetivo
	F	Empregado Efetivo
	F	Empregado Efetivo
	M	Empregado Efetivo
	F	Empregado Efetivo
	F	Empregado Efetivo
	F	Empregado Efetivo
	F	Empregado Efetivo
	M	Empregado Efetivo
	M	Empregado Efetivo
	F	Empregado Efetivo
	M	Empregado Efetivo
	F	Empregado Efetivo
	F	Empregado Efetivo
	F	Empregado Efetivo
	M	Empregado Efetivo
	F	Empregado Efetivo
	F	Empregado Efetivo
	F	Empregado Efetivo
	F	Empregado Efetivo
	F	Empregado Efetivo
	M	Empregado Efetivo
	F	Empregado Efetivo
	F	Empregado Efetivo

	F	Empregado Efetivo
	F	Empregado Efetivo
	F	Empregado Efetivo
	F	Empregado Efetivo
	F	Empregado Efetivo
	F	Empregado Efetivo
	F	Empregado Efetivo
	M	Empregado Efetivo
	M	Empregado Efetivo
	F	Empregado Efetivo
	F	Empregado Efetivo
	F	Empregado Efetivo
	F	Empregado Efetivo
	M	Empregado Efetivo
	F	Empregado Efetivo
	F	Empregado Efetivo
	M	Empregado Efetivo
	M	Empregado Efetivo
	F	Empregado Efetivo
	F	Empregado Efetivo
	F	Empregado Efetivo
	F	Empregado Efetivo
	F	Empregado Efetivo
	M	Empregado Efetivo
	F	Empregado Efetivo
	F	Empregado Efetivo
	F	Empregado Efetivo
	F	Empregado Efetivo
	M	Empregado Efetivo
	F	Empregado Efetivo
	F	Empregado Efetivo
	F	Empregado Efetivo
	M	Empregado Efetivo
	F	Empregado Efetivo
	M	Empregado Efetivo
	M	Empregado Efetivo
	M	Empregado Efetivo
	M	Empregado Efetivo
	F	Empregado Efetivo
	M	Empregado Efetivo
	F	Empregado Efetivo
	F	Empregado Efetivo
	M	Empregado Efetivo
	M	Empregado Efetivo
	M	Empregado Efetivo
	M	Empregado Efetivo
	M	Empregado Efetivo

	M	Empregado Efetivo
	F	Empregado Efetivo
	M	Empregado Efetivo
	M	Empregado Efetivo
	M	Empregado Efetivo
	M	Empregado Efetivo
	M	Empregado Efetivo
	M	Empregado Efetivo
	M	Empregado Efetivo
	F	Empregado Efetivo
	F	Empregado Efetivo
	F	Empregado Efetivo
	F	Empregado Efetivo
	F	Empregado Efetivo
	F	Empregado Efetivo
	M	Empregado Efetivo
	M	Empregado Efetivo
	M	Empregado Efetivo
	M	Empregado Efetivo
	M	Empregado Efetivo
	F	Empregado Efetivo
	F	Empregado Efetivo
	F	Empregado Efetivo
	F	Empregado Efetivo
	F	Empregado Efetivo
	F	Empregado Efetivo
	F	Empregado Efetivo
	F	Empregado Efetivo
	M	Empregado Efetivo
	F	Empregado Efetivo
	M	Empregado Efetivo
	M	Empregado Efetivo
	F	Empregado Efetivo
	F	Empregado Efetivo
	F	Empregado Efetivo
	F	Empregado Efetivo
	F	Empregado Efetivo
	F	Empregado Efetivo
	F	Empregado Efetivo
	F	Empregado Efetivo

	M	Empregado Efetivo
	M	Empregado Efetivo
	M	Empregado Efetivo
	M	Empregado Efetivo
	M	Empregado Efetivo
	F	Empregado Efetivo
	F	Empregado Efetivo
	M	Empregado Efetivo
	F	Empregado Efetivo
	F	Empregado Efetivo
	F	Empregado Efetivo
	M	Empregado Efetivo
	F	Empregado Efetivo
	F	Empregado Efetivo
	F	Empregado Efetivo
	F	Empregado Efetivo
	F	Empregado Efetivo
	M	Empregado Efetivo
	F	Empregado Efetivo
	F	Empregado Efetivo
	M	Empregado Efetivo
	M	Empregado Efetivo
	M	Empregado Efetivo
	F	Empregado Efetivo
	M	Empregado Efetivo
	F	Empregado Efetivo
	F	Empregado Efetivo
	F	Empregado Efetivo
	F	Empregado Efetivo
	F	Empregado Efetivo
	F	Empregado Efetivo
	F	Empregado Efetivo
	F	Empregado Efetivo
	F	Empregado Efetivo
	F	Empregado Efetivo
	F	Empregado Efetivo
	F	Empregado Efetivo
	F	Empregado Efetivo
	F	Empregado Efetivo
	F	Empregado Efetivo
	F	Empregado Efetivo
	F	Empregado Efetivo
	F	Empregado Efetivo
	F	Empregado Efetivo
	F	Empregado Efetivo
	F	Empregado Efetivo
	F	Empregado Efetivo
	F	Empregado Efetivo

	F	Empregado Efetivo
	F	Empregado Efetivo
	F	Empregado Efetivo
	F	Empregado Efetivo
	F	Empregado Efetivo
	F	Empregado Efetivo
	M	Empregado Efetivo
	F	Empregado Efetivo
	F	Empregado Efetivo
	M	Empregado Efetivo
	F	Empregado Efetivo
	F	Empregado Efetivo
	F	Empregado Efetivo
	F	Empregado Efetivo
	M	Empregado Efetivo
	F	Empregado Efetivo
	F	Empregado Efetivo
	M	Empregado Efetivo
	F	Empregado Efetivo
	F	Empregado Efetivo
	M	Empregado Efetivo
	F	Empregado Efetivo
	F	Empregado Efetivo
	F	Empregado Efetivo
	F	Empregado Efetivo
	F	Empregado Efetivo
	F	Empregado Efetivo
	F	Empregado Efetivo
	F	Empregado Efetivo
	F	Empregado Efetivo
	F	Empregado Efetivo
	F	Empregado Efetivo

	F	Empregado Efetivo
	F	Empregado Efetivo
	F	Empregado Efetivo
	F	Empregado Efetivo
	F	Empregado Efetivo
	F	Empregado Efetivo
	M	Empregado Efetivo
	F	Empregado Efetivo
	F	Empregado Efetivo
	F	Empregado Efetivo
	F	Empregado Efetivo
	F	Empregado Efetivo
	F	Empregado Efetivo
	F	Empregado Efetivo
	F	Empregado Efetivo
	F	Empregado Efetivo
	M	Empregado Efetivo
	F	Empregado Efetivo
	M	Empregado Efetivo
	F	Empregado Efetivo
	M	Empregado Efetivo
	F	Empregado Efetivo
	F	Empregado Efetivo
	F	Empregado Efetivo
	F	Empregado Efetivo
	F	Empregado Efetivo
	F	Empregado Efetivo
	F	Empregado Efetivo
	F	Empregado Efetivo
	F	Empregado Efetivo
	F	Empregado Efetivo
	M	Empregado Efetivo
	M	Empregado Efetivo
	M	Empregado Efetivo
	F	Empregado Efetivo
	F	Empregado Efetivo
	M	Empregado Efetivo
	F	Empregado Efetivo
	F	Empregado Efetivo
	F	Empregado Efetivo
	F	Empregado Efetivo
	M	Empregado Efetivo

	F	Empregado Efetivo
	F	Empregado Efetivo
	F	Empregado Efetivo
	F	Empregado Efetivo
	F	Empregado Efetivo
	F	Empregado Efetivo
	M	Empregado Efetivo
	M	Empregado Efetivo
	F	Empregado Efetivo
	F	Empregado Efetivo
	M	Empregado Efetivo
	F	Empregado Efetivo
	F	Empregado Efetivo
	F	Empregado Efetivo
	F	Empregado Efetivo
	M	Empregado Efetivo
	F	Empregado Efetivo
	F	Empregado Efetivo
	F	Empregado Efetivo
	F	Empregado Efetivo
	M	Empregado Efetivo
	F	Empregado Efetivo
	F	Empregado Efetivo
	F	Empregado Efetivo
	F	Empregado Efetivo
	F	Empregado Efetivo
	F	Empregado Efetivo
	F	Empregado Efetivo
	F	Empregado Efetivo
	F	Empregado Efetivo
	M	Empregado Efetivo
	F	Empregado Efetivo
	M	Empregado Efetivo
	F	Empregado Efetivo
	F	Empregado Efetivo
	F	Empregado Efetivo
	F	Empregado Efetivo
	F	Empregado Efetivo
	F	Empregado Efetivo
	F	Empregado Efetivo
	F	Empregado Efetivo
	M	Empregado Efetivo
	F	Empregado Efetivo
	M	Empregado Efetivo
	F	Empregado Efetivo
	F	Empregado Efetivo
	M	Empregado Efetivo
	F	Empregado Efetivo

	F	Empregado Efetivo
	F	Empregado Efetivo
	F	Empregado Efetivo
	F	Empregado Efetivo
	F	Empregado Efetivo
	F	Empregado Efetivo
	F	Empregado Efetivo
	F	Empregado Efetivo
	F	Empregado Efetivo
	F	Empregado Efetivo
	F	Empregado Efetivo
	F	Empregado Efetivo
	M	Empregado Efetivo
	M	Empregado Efetivo
	F	Empregado Efetivo
	F	Empregado Efetivo
	F	Empregado Efetivo
	F	Empregado Efetivo
	F	Empregado Efetivo
	F	Empregado Efetivo
	M	Empregado Efetivo
	F	Empregado Efetivo
	F	Empregado Efetivo
	F	Empregado Efetivo
	M	Empregado Efetivo
	F	Empregado Efetivo
	M	Empregado Efetivo
	M	Empregado Efetivo
	M	Empregado Efetivo
	M	Empregado Efetivo
	F	Empregado Efetivo
	M	Empregado Efetivo
	F	Empregado Efetivo
	M	Empregado Efetivo
	M	Empregado Efetivo
	F	Empregado Efetivo
	M	Empregado Efetivo
	M	Empregado Efetivo
	F	Empregado Efetivo
	M	Empregado Efetivo
	F	Empregado Efetivo
	M	Empregado Efetivo
	M	Empregado Efetivo
	F	Empregado Efetivo
	M	Empregado Efetivo
	F	Empregado Efetivo
	F	Empregado Efetivo
	F	Empregado Efetivo

	F	Empregado Efetivo
	F	Empregado Efetivo
	F	Empregado Efetivo
	M	Empregado Efetivo
	F	Empregado Efetivo
	M	Empregado Efetivo
	F	Empregado Efetivo
	M	Empregado Efetivo
	M	Empregado Efetivo
	M	Empregado Efetivo
	M	Empregado Efetivo
	F	Empregado Efetivo
	M	Empregado Efetivo
	M	Empregado Efetivo
	F	Empregado Efetivo
	F	Empregado Efetivo
	F	Empregado Efetivo
	M	Empregado Efetivo
	M	Empregado Efetivo
	F	Empregado Efetivo
	F	Empregado Efetivo
	F	Empregado Efetivo
	M	Empregado Efetivo
	F	Empregado Efetivo
	M	Empregado Efetivo
	M	Empregado Efetivo
	M	Empregado Efetivo
	M	Empregado Efetivo
	M	Empregado Efetivo
	M	Empregado Efetivo
	F	Empregado Efetivo
	F	Empregado Efetivo
	M	Empregado Efetivo
	M	Empregado Efetivo
	F	Empregado Efetivo
	M	Empregado Efetivo
	M	Empregado Efetivo
	M	Empregado Efetivo

	M	Empregado Efetivo
	M	Empregado Efetivo
	F	Empregado Efetivo
	M	Empregado Efetivo
	F	Empregado Efetivo
	F	Empregado Efetivo
	F	Empregado Efetivo
	M	Empregado Efetivo
	F	Empregado Efetivo
	F	Empregado Efetivo
	F	Empregado Efetivo
	F	Empregado Efetivo
	F	Empregado Efetivo
	F	Empregado Efetivo
	F	Empregado Efetivo
	M	Empregado Efetivo
	M	Empregado Efetivo
	M	Empregado Efetivo
	M	Empregado Efetivo
	F	Empregado Efetivo
	M	Empregado Efetivo
	F	Empregado Efetivo
	M	Empregado Efetivo
	M	Empregado Efetivo
	F	Empregado Efetivo
	F	Empregado Efetivo
	M	Empregado Efetivo
	F	Empregado Efetivo
	M	Empregado Efetivo
	M	Empregado Efetivo
	F	Empregado Efetivo
	F	Empregado Efetivo

	F	Empregado Efetivo
	M	Empregado Efetivo
	F	Empregado Efetivo
	M	Empregado Efetivo
	M	Empregado Efetivo
	F	Empregado Efetivo
	M	Empregado Efetivo
	F	Empregado Efetivo
	M	Empregado Efetivo
	M	Empregado Efetivo
	M	Empregado Efetivo
	M	Empregado Efetivo
	F	Empregado Efetivo
	M	Empregado Efetivo
	F	Empregado Efetivo
	F	Empregado Efetivo
	F	Empregado Efetivo
	M	Empregado Efetivo
	M	Empregado Efetivo
	F	Empregado Efetivo
	M	Empregado Efetivo
	F	Empregado Efetivo
	F	Empregado Efetivo
	M	Empregado Efetivo
	F	Empregado Efetivo
	F	Empregado Efetivo
	M	Empregado Efetivo
	M	Empregado Efetivo
	M	Empregado Efetivo
	F	Empregado Efetivo
	M	Empregado Efetivo
	M	Empregado Efetivo

	F	Empregado Efetivo
	F	Empregado Efetivo
	F	Empregado Efetivo
	F	Empregado Efetivo
	M	Empregado Efetivo
	F	Empregado Efetivo
	F	Empregado Efetivo
	M	Empregado Efetivo
	F	Empregado Efetivo
	F	Empregado Efetivo
	F	Empregado Efetivo
	M	Empregado Efetivo
	M	Empregado Efetivo
	M	Empregado Efetivo
	M	Empregado Efetivo
	F	Empregado Efetivo
	F	Empregado Efetivo
	F	Empregado Efetivo
	F	Empregado Efetivo
	F	Empregado Efetivo
	F	Empregado Efetivo
	M	Empregado Efetivo
	M	Empregado Efetivo
	F	Empregado Efetivo
	F	Empregado Efetivo
	F	Empregado Efetivo
	F	Empregado Efetivo
	F	Empregado Efetivo
	F	Empregado Efetivo
	F	Empregado Efetivo
	F	Empregado Efetivo
	M	Empregado Efetivo
	M	Empregado Efetivo
	M	Empregado Efetivo
	F	Empregado Efetivo
	F	Empregado Efetivo
	F	Empregado Efetivo
	F	Empregado Efetivo
	F	Empregado Efetivo
	F	Empregado Efetivo
	F	Empregado Efetivo
	F	Empregado Efetivo
	F	Empregado Efetivo
	F	Empregado Efetivo
	F	Empregado Efetivo
	F	Empregado Efetivo
	F	Empregado Efetivo

	F	Empregado Efetivo
	F	Empregado Efetivo
	F	Empregado Efetivo
	M	Empregado Efetivo
	F	Empregado Efetivo
	M	Empregado Efetivo
	F	Empregado Efetivo
	F	Empregado Efetivo
	M	Empregado Efetivo
	F	Empregado Efetivo
	M	Empregado Efetivo
	F	Empregado Efetivo
	M	Empregado Efetivo
	M	Empregado Efetivo
	M	Empregado Efetivo
	F	Empregado Efetivo
	F	Empregado Efetivo
	M	Empregado Efetivo
	F	Empregado Efetivo
	F	Empregado Efetivo
	F	Empregado Efetivo
	F	Empregado Efetivo
	F	Empregado Efetivo
	F	Empregado Efetivo
	F	Empregado Efetivo
	M	Empregado Efetivo
	F	Empregado Efetivo
	F	Empregado Efetivo
	M	Empregado Efetivo
	F	Empregado Efetivo
	M	Empregado Efetivo
	M	Empregado Efetivo
	F	Empregado Efetivo
	F	Empregado Efetivo
	M	Empregado Efetivo
	F	Empregado Efetivo
	M	Empregado Efetivo
	F	Empregado Efetivo
	F	Empregado Efetivo
	M	Empregado Efetivo
	F	Empregado Efetivo
	M	Empregado Efetivo
	F	Empregado Efetivo
	F	Empregado Efetivo
	M	Empregado Efetivo
	F	Empregado Efetivo
	F	Empregado Efetivo
	M	Empregado Efetivo

	M	Empregado Efetivo
	M	Empregado Efetivo
	M	Empregado Efetivo
	F	Empregado Efetivo
	F	Empregado Efetivo
	F	Empregado Efetivo
	F	Empregado Efetivo
	F	Empregado Efetivo
	F	Empregado Efetivo
	F	Empregado Efetivo
	F	Empregado Efetivo
	F	Empregado Efetivo
	F	Empregado Efetivo
	F	Empregado Efetivo
	F	Empregado Efetivo
	F	Empregado Efetivo
	M	Empregado Efetivo
	F	Empregado Efetivo
	M	Empregado Efetivo
	M	Empregado Efetivo
	F	Empregado Efetivo
	F	Empregado Efetivo
	M	Empregado Efetivo
	F	Empregado Efetivo
	M	Empregado Efetivo
	F	Empregado Efetivo
	F	Empregado Efetivo
	F	Empregado Efetivo
	F	Empregado Efetivo
	M	Empregado Efetivo
	F	Empregado Efetivo
	F	Empregado Efetivo
	F	Empregado Efetivo
	F	Empregado Efetivo
	F	Empregado Efetivo
	F	Empregado Efetivo
	F	Empregado Efetivo
	F	Empregado Efetivo
	F	Empregado Efetivo
	F	Empregado Efetivo
	F	Empregado Efetivo
	F	Empregado Efetivo
	M	Empregado Efetivo

	F	Empregado Efetivo
	F	Empregado Efetivo
	F	Empregado Efetivo
	F	Empregado Efetivo
	F	Empregado Efetivo
	F	Empregado Efetivo
	M	Empregado Efetivo
	F	Empregado Efetivo
	F	Empregado Efetivo
	F	Empregado Efetivo
	F	Empregado Efetivo
	F	Empregado Efetivo
	F	Empregado Efetivo
	F	Empregado Efetivo
	F	Empregado Efetivo
	F	Empregado Efetivo
	F	Empregado Efetivo
	F	Empregado Efetivo
	F	Empregado Efetivo
	F	Empregado Efetivo
	F	Empregado Efetivo
	F	Empregado Efetivo
	F	Empregado Efetivo
	F	Empregado Efetivo
	F	Empregado Efetivo
	F	Empregado Efetivo
	M	Empregado Efetivo
	F	Empregado Efetivo
	M	Empregado Efetivo
	F	Empregado Efetivo
	F	Empregado Efetivo
	F	Empregado Efetivo
	F	Empregado Efetivo
	F	Empregado Efetivo
	F	Empregado Efetivo
	M	Empregado Efetivo
	F	Empregado Efetivo
	F	Empregado Efetivo
	F	Empregado Efetivo
	F	Empregado Efetivo
	M	Empregado Efetivo
	F	Empregado Efetivo
	F	Empregado Efetivo
	F	Empregado Efetivo
	F	Empregado Efetivo
	M	Empregado Efetivo
	F	Empregado Efetivo
	F	Empregado Efetivo

	F	Empregado Efetivo
	M	Empregado Efetivo
	M	Empregado Efetivo
	M	Empregado Efetivo
	F	Empregado Efetivo
	F	Empregado Efetivo
	M	Empregado Efetivo
	F	Empregado Efetivo
	F	Empregado Efetivo
	F	Empregado Efetivo
	F	Empregado Efetivo
	F	Empregado Efetivo
	F	Empregado Efetivo
	M	Empregado Efetivo
	F	Empregado Efetivo
	F	Empregado Efetivo
	M	Empregado Efetivo
	M	Empregado Efetivo
	F	Empregado Efetivo
	F	Empregado Efetivo
	M	Empregado Efetivo
	M	Empregado Efetivo
	M	Empregado Efetivo
	F	Empregado Efetivo
	M	Empregado Efetivo
	M	Empregado Efetivo
	F	Empregado Efetivo
	M	Empregado Efetivo
	F	Empregado Efetivo
	F	Empregado Efetivo
	F	Empregado Efetivo
	M	Empregado Efetivo
	F	Empregado Efetivo
	F	Empregado Efetivo
	F	Empregado Efetivo
	M	Empregado Efetivo
	F	Empregado Efetivo
	F	Empregado Efetivo
	F	Empregado Efetivo
	F	Empregado Efetivo
	F	Empregado Efetivo
	F	Empregado Efetivo
	M	Empregado Efetivo
	M	Empregado Efetivo
	F	Empregado Efetivo
	M	Empregado Efetivo
	F	Empregado Efetivo
	F	Empregado Efetivo

	F	Empregado Efetivo
	F	Empregado Efetivo
	F	Empregado Efetivo
	F	Empregado Efetivo
	M	Empregado Efetivo
	F	Empregado Efetivo
	F	Empregado Efetivo
	M	Empregado Efetivo
	F	Empregado Efetivo
	F	Empregado Efetivo
	F	Empregado Efetivo
	M	Empregado Efetivo
	M	Empregado Efetivo
	F	Empregado Efetivo
	F	Empregado Efetivo
	F	Empregado Efetivo
	F	Empregado Efetivo
	M	Empregado Efetivo
	F	Empregado Efetivo
	F	Empregado Efetivo
	M	Empregado Efetivo
	F	Empregado Efetivo
	F	Empregado Efetivo
	F	Empregado Efetivo
	F	Empregado Efetivo
	F	Empregado Efetivo
	F	Empregado Efetivo
	F	Empregado Efetivo
	F	Empregado Efetivo
	F	Empregado Efetivo
	F	Empregado Efetivo
	F	Empregado Efetivo
	F	Empregado Efetivo
	F	Empregado Efetivo
	F	Empregado Efetivo
	F	Empregado Efetivo
	F	Empregado Efetivo
	F	Empregado Efetivo
	F	Empregado Efetivo
	F	Empregado Efetivo
	F	Empregado Efetivo
	M	Empregado Efetivo
	M	Empregado Efetivo
	F	Empregado Efetivo
	F	Empregado Efetivo
	F	Empregado Efetivo
	F	Empregado Efetivo
	F	Empregado Efetivo

	F	Empregado Efetivo
	F	Empregado Efetivo
	F	Empregado Efetivo
	M	Cedido - RJU
	F	Empregado Efetivo
	F	Empregado Efetivo
	F	Empregado Efetivo
	F	Empregado Efetivo
	F	Empregado Efetivo
	F	Empregado Efetivo
	F	Empregado Efetivo
	F	Empregado Efetivo
	F	Empregado Efetivo
	F	Empregado Efetivo
	F	Empregado Efetivo
	F	Empregado Efetivo
	F	Empregado Efetivo
	F	Empregado Efetivo
	M	Empregado Efetivo
	M	Empregado Efetivo
	M	Empregado Efetivo
	F	Empregado Efetivo
	F	Empregado Efetivo
	F	Empregado Efetivo
	F	Empregado Efetivo
	M	Empregado Efetivo
	F	Empregado Efetivo
	F	Empregado Efetivo
	F	Empregado Efetivo
	F	Empregado Efetivo
	F	Empregado Efetivo
	F	Empregado Efetivo
	F	Empregado Efetivo
	F	Empregado Efetivo
	F	Empregado Efetivo
	F	Empregado Efetivo
	F	Empregado Efetivo
	F	Empregado Efetivo
	F	Empregado Efetivo
	F	Empregado Efetivo
	F	Empregado Efetivo
	F	Empregado Efetivo
	F	Empregado Efetivo
	F	Empregado Efetivo
	F	Empregado Efetivo
	F	Empregado Efetivo

	F	Empregado Efetivo
	F	Empregado Efetivo
	F	Empregado Efetivo
	F	Empregado Efetivo
	F	Empregado Efetivo
	F	Empregado Efetivo
	F	Empregado Efetivo
	F	Empregado Efetivo
	F	Empregado Efetivo
	M	Empregado Efetivo
	F	Empregado Efetivo
	M	Empregado Efetivo
	F	Empregado Efetivo
	F	Empregado Efetivo
	F	Empregado Efetivo
	F	Empregado Efetivo
	F	Empregado Efetivo
	F	Empregado Efetivo
	F	Empregado Efetivo
	F	Empregado Efetivo
	F	Empregado Efetivo
	F	Empregado Efetivo
	F	Empregado Efetivo
	M	Empregado Efetivo
	F	Empregado Efetivo
	F	Empregado Efetivo
	F	Empregado Efetivo
	M	Empregado Efetivo
	M	Empregado Efetivo
	F	Empregado Efetivo
	F	Empregado Efetivo
	F	Empregado Efetivo
	F	Empregado Efetivo
	F	Empregado Efetivo
	F	Empregado Efetivo
	F	Empregado Efetivo
	F	Empregado Efetivo
	F	Empregado Efetivo
	F	Empregado Efetivo
	F	Empregado Efetivo
	M	Empregado Efetivo
	M	Empregado Efetivo
	M	Empregado Efetivo
	F	Empregado Efetivo

	F	Empregado Efetivo
	F	Empregado Efetivo
	F	Empregado Efetivo
	F	Empregado Efetivo
	F	Empregado Efetivo
	M	Empregado Efetivo
	M	Empregado Efetivo
	F	Empregado Efetivo
	F	Empregado Efetivo
	F	Empregado Efetivo
	F	Empregado Efetivo
	F	Empregado Efetivo
	F	Empregado Efetivo
	F	Empregado Efetivo
	F	Empregado Efetivo
	F	Empregado Efetivo
	F	Empregado Efetivo
	F	Empregado Efetivo
	F	Empregado Efetivo
	F	Empregado Efetivo
	F	Empregado Efetivo
	F	Empregado Efetivo
	F	Empregado Efetivo
	F	Empregado Efetivo
	F	Empregado Efetivo
	F	Empregado Efetivo
	F	Empregado Efetivo
	F	Empregado Efetivo
	F	Empregado Efetivo
	M	Empregado Efetivo
	F	Empregado Efetivo
	F	Empregado Efetivo
	F	Empregado Efetivo
	F	Empregado Efetivo
	F	Empregado Efetivo
	F	Empregado Efetivo
	M	Empregado Efetivo
	F	Empregado Efetivo
	F	Empregado Efetivo
	M	Empregado Efetivo
	M	Empregado Efetivo
	F	Empregado Efetivo
	F	Empregado Efetivo
	F	Empregado Efetivo
	F	Empregado Efetivo

	F	Empregado Efetivo
	F	Empregado Efetivo
	F	Empregado Efetivo
	F	Empregado Efetivo
	F	Empregado Efetivo
	F	Empregado Efetivo
	F	Empregado Efetivo
	F	Empregado Efetivo
	F	Empregado Efetivo
	F	Empregado Efetivo
	F	Empregado Efetivo
	F	Empregado Efetivo
	F	Empregado Efetivo
	F	Empregado Efetivo
	F	Empregado Efetivo
	F	Empregado Efetivo
	F	Empregado Efetivo
	F	Empregado Efetivo
	F	Empregado Efetivo
	F	Empregado Efetivo
	F	Empregado Efetivo
	F	Empregado Efetivo
	F	Empregado Efetivo
	F	Empregado Efetivo
	F	Empregado Efetivo
	F	Empregado Efetivo
	F	Empregado Efetivo
	F	Empregado Efetivo
	F	Empregado Efetivo
	F	Empregado Efetivo
	F	Empregado Efetivo
	F	Empregado Efetivo
	F	Empregado Efetivo
	F	Empregado Efetivo
	F	Empregado Efetivo
	F	Empregado Efetivo
	F	Empregado Efetivo
	F	Empregado Efetivo
	F	Empregado Efetivo
	F	Empregado Efetivo
	F	Empregado Efetivo
	F	Empregado Efetivo
	F	Empregado Efetivo

	F	Empregado Efetivo
	F	Empregado Efetivo
	F	Empregado Efetivo
	F	Empregado Efetivo
	F	Empregado Efetivo
	F	Empregado Efetivo
	F	Empregado Efetivo
	F	Empregado Efetivo
	F	Empregado Efetivo
	F	Empregado Efetivo
	F	Empregado Efetivo
	F	Empregado Efetivo
	F	Empregado Efetivo
	F	Empregado Efetivo
	F	Empregado Efetivo
	F	Empregado Efetivo
	F	Empregado Efetivo
	F	Empregado Efetivo
	F	Empregado Efetivo
	M	Empregado Efetivo
	M	Empregado Efetivo
	M	Empregado Efetivo
	M	Empregado Efetivo
	M	Empregado Efetivo
	M	Empregado Efetivo
	M	Empregado Efetivo
	M	Empregado Efetivo
	F	Empregado Efetivo
	M	Empregado Efetivo
	F	Empregado Efetivo
	F	Empregado Efetivo
	F	Empregado Efetivo
	F	Empregado Efetivo
	F	Empregado Efetivo
	F	Empregado Efetivo
	F	Empregado Efetivo
	F	Empregado Efetivo
	M	Empregado Efetivo
	F	Empregado Efetivo

	M	Empregado Efetivo
	F	Empregado Efetivo
	F	Empregado Efetivo
	M	Empregado Efetivo
	F	Empregado Efetivo
	F	Empregado Efetivo
	F	Empregado Efetivo
	F	Empregado Efetivo
	M	Empregado Efetivo
	M	Empregado Efetivo
	M	Empregado Efetivo
	M	Empregado Efetivo
	M	Empregado Efetivo
	F	Empregado Efetivo
	M	Empregado Efetivo
	M	Empregado Efetivo
	M	Empregado Efetivo
	F	Empregado Efetivo
	F	Empregado Efetivo
	F	Empregado Efetivo
	F	Empregado Efetivo
	F	Empregado Efetivo
	M	Empregado Efetivo
	F	Empregado Efetivo
	F	Empregado Efetivo
	F	Empregado Efetivo
	F	Empregado Efetivo
	F	Empregado Efetivo
	F	Empregado Efetivo
	M	Empregado Efetivo
	F	Empregado Efetivo
	F	Empregado Efetivo
	F	Empregado Efetivo
	M	Empregado Efetivo
	F	Empregado Efetivo
	F	Empregado Efetivo
	F	Empregado Efetivo
	F	Empregado Efetivo
	F	Empregado Efetivo
	F	Empregado Efetivo
	F	Empregado Efetivo
	F	Empregado Efetivo
	F	Empregado Efetivo
	F	Empregado Efetivo
	F	Empregado Efetivo
	F	Empregado Efetivo
	M	Empregado Efetivo

	F	Empregado Efetivo
	F	Empregado Efetivo
	M	Empregado Efetivo
	F	Empregado Efetivo
	F	Empregado Efetivo
	F	Empregado Efetivo
	F	Empregado Efetivo
	F	Empregado Efetivo
	F	Empregado Efetivo
	F	Empregado Efetivo
	F	Empregado Efetivo
	F	Empregado Efetivo
	F	Empregado Efetivo
	M	Empregado Efetivo
	F	Empregado Efetivo
	M	Empregado Efetivo
	F	Empregado Efetivo
	F	Empregado Efetivo
	F	Empregado Efetivo
	F	Empregado Efetivo
	M	Empregado Efetivo
	M	Empregado Efetivo
	F	Empregado Efetivo
	F	Empregado Efetivo
	F	Empregado Efetivo
	F	Empregado Efetivo
	F	Empregado Efetivo
	F	Empregado Efetivo
	F	Empregado Efetivo
	F	Empregado Efetivo
	F	Empregado Efetivo
	M	Empregado Efetivo
	F	Empregado Efetivo
	F	Empregado Efetivo
	F	Empregado Efetivo
	M	Empregado Efetivo
	M	Empregado Efetivo
	F	Empregado Efetivo
	M	Empregado Efetivo
	F	Empregado Efetivo
	F	Empregado Efetivo
	M	Empregado Efetivo
	M	Empregado Efetivo
	F	Empregado Efetivo
	F	Empregado Efetivo

	F	Empregado Efetivo
	M	Empregado Efetivo
	F	Empregado Efetivo
	F	Empregado Efetivo
	F	Empregado Efetivo
	F	Empregado Efetivo
	F	Empregado Efetivo
	F	Empregado Efetivo
	F	Empregado Efetivo
	F	Empregado Efetivo
	F	Empregado Efetivo
	F	Empregado Efetivo
	M	Empregado Efetivo
	M	Empregado Efetivo
	M	Empregado Efetivo
	F	Empregado Efetivo
	M	Empregado Efetivo
	F	Empregado Efetivo
	F	Empregado Efetivo
	F	Empregado Efetivo
	F	Empregado Efetivo
	M	Empregado Efetivo
	F	Empregado Efetivo
	F	Empregado Efetivo
	F	Empregado Efetivo
	F	Empregado Efetivo
	F	Empregado Efetivo
	M	Empregado Efetivo
	F	Empregado Efetivo
	F	Empregado Efetivo
	F	Empregado Efetivo
	F	Empregado Efetivo
	F	Empregado Efetivo
	F	Empregado Efetivo
	F	Empregado Efetivo
	F	Empregado Efetivo
	F	Empregado Efetivo
	F	Empregado Efetivo
	M	Empregado Efetivo
	M	Empregado Efetivo
	F	Empregado Efetivo
	M	Empregado Efetivo
	F	Empregado Efetivo
	F	Empregado Efetivo
	F	Empregado Efetivo
	F	Empregado Efetivo

	F	Empregado Efetivo
	F	Empregado Efetivo
	F	Empregado Efetivo
	F	Empregado Efetivo
	F	Empregado Efetivo
	F	Empregado Efetivo
	F	Empregado Efetivo
	M	Empregado Efetivo
	F	Empregado Efetivo
	F	Empregado Efetivo
	F	Empregado Efetivo
	F	Empregado Efetivo
	F	Empregado Efetivo
	F	Empregado Efetivo
	F	Empregado Efetivo
	F	Empregado Efetivo
	M	Empregado Efetivo
	M	Empregado Efetivo
	F	Cedido - RJU
	F	Cedido - RJU
	F	Cedido - RJU
	F	Cedido - RJU
	M	Cedido - RJU
	F	Cedido - RJU
	F	Cedido - RJU
	M	Cedido - RJU
	M	Cedido - RJU
	M	Cedido - RJU
	F	Empregado Efetivo
	F	Empregado Efetivo
	F	Empregado Efetivo
	F	Empregado Efetivo
	F	Empregado Efetivo

	F	Empregado Efetivo
	F	Empregado Efetivo
	F	Empregado Efetivo
	F	Empregado Efetivo
	F	Empregado Efetivo
	F	Empregado Efetivo
	M	Empregado Efetivo
	F	Empregado Efetivo
	F	Empregado Efetivo
	F	Empregado Efetivo
	F	Empregado Efetivo
	F	Empregado Efetivo
	F	Empregado Efetivo
	F	Empregado Efetivo
	F	Empregado Efetivo
	F	Empregado Efetivo
	F	Empregado Efetivo
	F	Empregado Efetivo
	F	Empregado Efetivo
	F	Empregado Efetivo
	F	Empregado Efetivo
	F	Empregado Efetivo
	M	Empregado Efetivo
	F	Empregado Efetivo
	F	Empregado Efetivo
	M	Empregado Efetivo
	F	Empregado Efetivo
	F	Empregado Efetivo
	F	Empregado Efetivo
	F	Empregado Efetivo
	F	Empregado Efetivo
	F	Empregado Efetivo
	F	Empregado Efetivo

	F	Empregado Efetivo
	F	Empregado Efetivo
	F	Empregado Efetivo
	F	Empregado Efetivo
	F	Empregado Efetivo
	F	Empregado Efetivo
	M	Empregado Efetivo
	F	Empregado Efetivo
	F	Empregado Efetivo
	F	Empregado Efetivo
	F	Empregado Efetivo
	F	Empregado Efetivo
	F	Empregado Efetivo
	F	Empregado Efetivo
	F	Empregado Efetivo
	F	Empregado Efetivo
	F	Empregado Efetivo
	M	Empregado Efetivo
	F	Empregado Efetivo
	F	Empregado Efetivo
	F	Empregado Efetivo
	F	Empregado Efetivo
	F	Empregado Efetivo
	F	Empregado Efetivo
	F	Empregado Efetivo
	F	Empregado Efetivo
	F	Empregado Efetivo
	F	Empregado Efetivo
	F	Empregado Efetivo
	F	Empregado Efetivo
	M	Empregado Efetivo
	M	Empregado Efetivo
	M	Empregado Efetivo
	F	Empregado Efetivo
	F	Empregado Efetivo

	F	Empregado Efetivo
	F	Empregado Efetivo
	F	Empregado Efetivo
	M	Empregado Efetivo
	M	Empregado Efetivo
	F	Empregado Efetivo
	M	Empregado Efetivo
	F	Empregado Efetivo
	F	Empregado Efetivo
	F	Empregado Efetivo
	F	Empregado Efetivo
	F	Empregado Efetivo
	F	Empregado Efetivo
	F	Empregado Efetivo
	M	Empregado Efetivo
	F	Empregado Efetivo
	F	Empregado Efetivo
	F	Empregado Efetivo
	M	Empregado Efetivo
	F	Empregado Efetivo
	F	Empregado Efetivo
	F	Empregado Efetivo
	M	Empregado Efetivo
	F	Empregado Efetivo
	M	Empregado Efetivo
	F	Empregado Efetivo
	F	Empregado Efetivo
	F	Empregado Efetivo
	F	Empregado Efetivo
	F	Empregado Efetivo
	M	Empregado Efetivo
	F	Empregado Efetivo

	F	Empregado Efetivo
	F	Empregado Efetivo
	F	Empregado Efetivo
	M	Empregado Efetivo
	F	Empregado Efetivo
	F	Empregado Efetivo
	M	Empregado Efetivo
	F	Empregado Efetivo
	M	Empregado Efetivo
	M	Empregado Efetivo
	M	Empregado Efetivo
	M	Empregado Efetivo
	M	Empregado Efetivo
	M	Empregado Efetivo
	M	Empregado Efetivo
	F	Empregado Efetivo
	F	Empregado Efetivo
	F	Empregado Efetivo
	F	Empregado Efetivo
	F	Empregado Efetivo
	M	Empregado Efetivo
	F	Empregado Efetivo
	F	Empregado Efetivo
	F	Empregado Efetivo
	F	Empregado Efetivo
	F	Empregado Efetivo
	F	Empregado Efetivo
	F	Empregado Efetivo
	F	Empregado Efetivo
	M	Empregado Efetivo
	M	Empregado Efetivo
	M	Empregado Efetivo

	F	Empregado Efetivo
	F	Empregado Efetivo
	F	Empregado Efetivo
	F	Empregado Efetivo
	F	Empregado Efetivo
	F	Empregado Efetivo
	F	Empregado Efetivo
	F	Empregado Efetivo
	F	Empregado Efetivo
	F	Empregado Efetivo
	F	Empregado Efetivo
	F	Empregado Efetivo
	F	Empregado Efetivo
	F	Empregado Efetivo
	F	Empregado Efetivo
	F	Empregado Efetivo
	F	Empregado Efetivo
	F	Empregado Efetivo
	F	Empregado Efetivo
	F	Empregado Efetivo
	F	Empregado Efetivo
	F	Empregado Efetivo
	F	Empregado Efetivo
	F	Empregado Efetivo
	F	Empregado Efetivo
	F	Empregado Efetivo
	F	Empregado Efetivo
	F	Empregado Efetivo
	F	Empregado Efetivo
	F	Empregado Efetivo
	F	Empregado Efetivo
	F	Empregado Efetivo

	F	Empregado Efetivo
	F	Empregado Efetivo
	F	Empregado Efetivo
	F	Empregado Efetivo
	F	Empregado Efetivo
	F	Empregado Efetivo
	F	Empregado Efetivo
	F	Empregado Efetivo
	F	Empregado Efetivo
	F	Empregado Efetivo
	F	Empregado Efetivo
	F	Empregado Efetivo
	F	Empregado Efetivo
	F	Empregado Efetivo
	F	Empregado Efetivo
	F	Empregado Efetivo
	F	Empregado Efetivo
	F	Empregado Efetivo
	M	Empregado Efetivo
	F	Empregado Efetivo
	F	Empregado Efetivo
	F	Empregado Efetivo
	M	Empregado Efetivo
	F	Empregado Efetivo
	F	Empregado Efetivo
	F	Empregado Efetivo
	F	Empregado Efetivo
	F	Empregado Efetivo
	F	Empregado Efetivo
	F	Empregado Efetivo
	F	Empregado Efetivo
	F	Empregado Efetivo

	F	Empregado Efetivo
	F	Empregado Efetivo
	F	Empregado Efetivo
	F	Empregado Efetivo
	F	Empregado Efetivo
	F	Empregado Efetivo
	F	Empregado Efetivo
	F	Empregado Efetivo
	F	Empregado Efetivo
	F	Empregado Efetivo
	F	Empregado Efetivo
	F	Empregado Efetivo
	F	Empregado Efetivo
	F	Empregado Efetivo
	F	Empregado Efetivo
	M	Empregado Efetivo
	F	Empregado Efetivo
	F	Empregado Efetivo
	M	Empregado Efetivo
	F	Empregado Efetivo
	F	Empregado Efetivo
	F	Empregado Efetivo
	F	Empregado Efetivo
	F	Empregado Efetivo
	F	Empregado Efetivo
	F	Empregado Efetivo
	F	Empregado Efetivo
	F	Empregado Efetivo
	F	Empregado Efetivo
	F	Empregado Efetivo
	F	Empregado Efetivo
	F	Empregado Efetivo

	F	Empregado Efetivo
	F	Empregado Efetivo
	M	Empregado Efetivo
	M	Empregado Efetivo
	F	Empregado Efetivo
	F	Empregado Efetivo
	F	Empregado Efetivo
	F	Empregado Efetivo
	F	Empregado Efetivo
	F	Empregado Efetivo
	F	Empregado Efetivo
	F	Empregado Efetivo
	M	Empregado Efetivo
	F	Empregado Efetivo
	F	Empregado Efetivo
	F	Empregado Efetivo
	F	Empregado Efetivo
	F	Empregado Efetivo
	F	Empregado Efetivo
	F	Empregado Efetivo
	F	Empregado Efetivo
	F	Empregado Efetivo
	F	Empregado Efetivo
	F	Empregado Efetivo
	F	Empregado Efetivo
	F	Empregado Efetivo
	F	Empregado Efetivo
	F	Empregado Efetivo
	M	Empregado Efetivo
	F	Empregado Efetivo
	F	Empregado Efetivo
	M	Empregado Efetivo

	F	Empregado Efetivo
	F	Empregado Efetivo
	F	Empregado Efetivo
	F	Empregado Efetivo
	F	Empregado Efetivo
	F	Empregado Efetivo
	F	Empregado Efetivo
	F	Empregado Efetivo
	F	Empregado Efetivo
	M	Empregado Efetivo
	F	Empregado Efetivo
	M	Empregado Efetivo
	M	Empregado Efetivo
	M	Empregado Efetivo
	M	Empregado Efetivo
	F	Empregado Efetivo
	M	Empregado Efetivo
	F	Empregado Efetivo
	F	Empregado Efetivo
	F	Empregado Efetivo
	F	Empregado Efetivo
	F	Empregado Efetivo
	F	Empregado Efetivo
	F	Empregado Efetivo
	F	Empregado Efetivo
	F	Empregado Efetivo
	F	Empregado Efetivo
	M	Empregado Efetivo
	M	Empregado Efetivo
	M	Empregado Efetivo
	F	Empregado Efetivo
	F	Empregado Efetivo

	F	Empregado Efetivo
	F	Empregado Efetivo
	F	Empregado Efetivo
	F	Empregado Efetivo
	F	Empregado Efetivo
	F	Empregado Efetivo
	F	Empregado Efetivo
	F	Empregado Efetivo
	F	Empregado Efetivo
	M	Empregado Efetivo
	F	Empregado Efetivo
	F	Empregado Efetivo
	M	Empregado Efetivo
	F	Empregado Efetivo
	F	Empregado Efetivo
	F	Empregado Efetivo
	M	Empregado Efetivo
	F	Empregado Efetivo
	F	Empregado Efetivo
	F	Empregado Efetivo
	M	Empregado Efetivo
	F	Empregado Efetivo
	M	Empregado Efetivo
	F	Empregado Efetivo
	F	Empregado Efetivo
	F	Empregado Efetivo
	F	Empregado Efetivo
	F	Empregado Efetivo
	F	Empregado Efetivo
	F	Empregado Efetivo
	F	Empregado Efetivo
	F	Empregado Efetivo
	F	Empregado Efetivo
	M	Empregado Efetivo
	M	Empregado Efetivo
	F	Empregado Efetivo
	F	Empregado Efetivo
	F	Empregado Efetivo
	F	Empregado Efetivo
	F	Empregado Efetivo
	M	Empregado Efetivo

	M	Empregado Efetivo
	M	Empregado Efetivo
	M	Empregado Efetivo
	F	Empregado Efetivo
	F	Empregado Efetivo
	F	Empregado Efetivo
	M	Empregado Efetivo
	F	Empregado Efetivo
	F	Empregado Efetivo
	F	Empregado Efetivo
	M	Empregado Efetivo
	F	Empregado Efetivo
	F	Empregado Efetivo
	F	Empregado Efetivo
	M	Empregado Efetivo
	F	Empregado Efetivo
	F	Empregado Efetivo
	F	Empregado Efetivo
	F	Empregado Efetivo
	F	Empregado Efetivo
	F	Empregado Efetivo
	F	Empregado Efetivo
	F	Empregado Efetivo
	F	Empregado Efetivo
	F	Empregado Efetivo
	M	Empregado Efetivo
	F	Empregado Efetivo
	F	Empregado Efetivo
	M	Empregado Efetivo
	M	Empregado Efetivo
	F	Empregado Efetivo
	F	Empregado Efetivo
	F	Empregado Efetivo
	F	Empregado Efetivo
	F	Empregado Efetivo
	F	Empregado Efetivo
	F	Empregado Efetivo
	F	Empregado Efetivo
	F	Empregado Efetivo
	F	Empregado Efetivo
	F	Empregado Efetivo
	F	Empregado Efetivo
	F	Empregado Efetivo
	F	Empregado Efetivo
	F	Empregado Efetivo
	F	Empregado Efetivo
	F	Empregado Efetivo

	F	Empregado Efetivo
	M	Empregado Efetivo
	F	Empregado Efetivo
	F	Empregado Efetivo
	M	Empregado Efetivo
	F	Empregado Efetivo
	M	Empregado Efetivo
	F	Empregado Efetivo
	M	Empregado Efetivo
	M	Empregado Efetivo
	M	Empregado Efetivo
	M	Empregado Efetivo
	M	Empregado Efetivo
	F	Empregado Efetivo
	F	Empregado Efetivo
	M	Empregado Efetivo
	M	Empregado Efetivo
	F	Empregado Efetivo
	F	Empregado Efetivo
	M	Empregado Efetivo
	F	Empregado Efetivo
	M	Empregado Efetivo
	F	Empregado Efetivo
	F	Empregado Efetivo
	F	Empregado Efetivo
	F	Empregado Efetivo
	F	Empregado Efetivo
	M	Empregado Efetivo
	F	Empregado Efetivo
	F	Empregado Efetivo
	F	Empregado Efetivo
	M	Empregado Efetivo
	F	Empregado Efetivo
	F	Empregado Efetivo
	F	Empregado Efetivo
	F	Empregado Efetivo
	F	Empregado Efetivo
	F	Empregado Efetivo
	F	Empregado Efetivo
	F	Empregado Efetivo
	F	Empregado Efetivo
	F	Empregado Efetivo

	F	Empregado Efetivo
	F	Empregado Efetivo
	F	Empregado Efetivo
	F	Empregado Efetivo
	F	Empregado Efetivo
	F	Empregado Efetivo
	F	Empregado Efetivo
	F	Empregado Efetivo
	F	Empregado Efetivo
	F	Empregado Efetivo
	F	Empregado Efetivo
	M	Empregado Efetivo
	F	Empregado Efetivo
	F	Empregado Efetivo
	F	Empregado Efetivo
	F	Empregado Efetivo
	F	Empregado Efetivo
	M	Empregado Efetivo
	F	Empregado Efetivo
	F	Empregado Efetivo
	F	Empregado Efetivo
	F	Empregado Efetivo
	F	Empregado Efetivo
	F	Empregado Efetivo
	F	Empregado Efetivo
	F	Empregado Efetivo
	F	Empregado Efetivo
	F	Empregado Efetivo
	F	Empregado Efetivo
	F	Empregado Efetivo
	F	Empregado Efetivo
	F	Empregado Efetivo
	F	Empregado Efetivo
	F	Empregado Efetivo
	F	Empregado Efetivo
	F	Empregado Efetivo
	F	Empregado Efetivo
	F	Empregado Efetivo
	F	Empregado Efetivo
	F	Empregado Efetivo
	F	Empregado Efetivo
	F	Empregado Efetivo
	F	Empregado Efetivo
	F	Empregado Efetivo

	F	Empregado Efetivo
	F	Empregado Efetivo
	F	Empregado Efetivo
	F	Empregado Efetivo
	F	Empregado Efetivo
	F	Empregado Efetivo
	F	Empregado Efetivo
	F	Empregado Efetivo
	F	Empregado Efetivo
	F	Empregado Efetivo
	F	Empregado Efetivo
	F	Empregado Efetivo
	F	Empregado Efetivo
	F	Empregado Efetivo
	F	Empregado Efetivo
	F	Empregado Efetivo
	F	Empregado Efetivo
	F	Empregado Efetivo
	M	Empregado Efetivo
	F	Empregado Efetivo
	F	Empregado Efetivo
	F	Empregado Efetivo
	M	Empregado Efetivo
	F	Empregado Efetivo
	F	Empregado Efetivo
	F	Empregado Efetivo
	F	Empregado Efetivo
	F	Empregado Efetivo
	F	Empregado Efetivo
	F	Empregado Efetivo
	F	Empregado Efetivo
	F	Empregado Efetivo
	F	Empregado Efetivo
	M	Empregado Efetivo
	F	Empregado Efetivo
	F	Empregado Efetivo
	F	Empregado Efetivo
	F	Empregado Efetivo
	F	Empregado Efetivo

	F	Empregado Efetivo
	F	Empregado Efetivo
	F	Empregado Efetivo
	F	Empregado Efetivo
	F	Empregado Efetivo
	M	Empregado Efetivo
	M	Empregado Efetivo
	F	Empregado Efetivo
	M	Empregado Efetivo
	F	Empregado Efetivo
	F	Empregado Efetivo
	F	Empregado Efetivo
	M	Empregado Efetivo
	F	Empregado Efetivo
	F	Empregado Efetivo
	F	Empregado Efetivo
	F	Empregado Efetivo
	F	Empregado Efetivo
	F	Empregado Efetivo
	F	Empregado Efetivo
	M	Empregado Efetivo
	M	Empregado Efetivo
	F	Empregado Efetivo
	F	Empregado Efetivo
	F	Empregado Efetivo
	M	Empregado Efetivo
	M	Empregado Efetivo
	F	Empregado Efetivo
	M	Empregado Efetivo
	F	Empregado Efetivo
	F	Empregado Efetivo
	F	Empregado Efetivo
	F	Empregado Efetivo
	M	Empregado Efetivo
	F	Empregado Efetivo
	F	Empregado Efetivo
	F	Empregado Efetivo
	F	Empregado Efetivo
	F	Empregado Efetivo
	M	Empregado Efetivo
	F	Empregado Efetivo
	F	Empregado Efetivo
	F	Empregado Efetivo
	F	Empregado Efetivo
	F	Empregado Efetivo
	F	Empregado Efetivo
	F	Empregado Efetivo
	F	Empregado Efetivo

	F	Empregado Efetivo
	F	Empregado Efetivo
	M	Empregado Efetivo
	F	Empregado Efetivo
	F	Empregado Efetivo
	F	Empregado Efetivo
	F	Empregado Efetivo
	M	Empregado Efetivo
	F	Empregado Efetivo
	F	Empregado Efetivo
	M	Empregado Efetivo
	F	Empregado Efetivo
	F	Empregado Efetivo
	F	Empregado Efetivo
	F	Empregado Efetivo
	F	Empregado Efetivo
	F	Empregado Efetivo
	F	Empregado Efetivo
	F	Empregado Efetivo
	F	Empregado Efetivo
	F	Empregado Efetivo
	F	Empregado Efetivo
	F	Empregado Efetivo
	F	Empregado Efetivo
	F	Empregado Efetivo
	M	Empregado Efetivo
	M	Empregado Efetivo
	F	Empregado Efetivo
	F	Empregado Efetivo
	F	Empregado Efetivo
	F	Empregado Efetivo
	F	Empregado Efetivo
	F	Empregado Efetivo
	M	Empregado Efetivo
	F	Empregado Efetivo
	F	Empregado Efetivo
	F	Empregado Efetivo
	F	Empregado Efetivo
	F	Empregado Efetivo
	F	Empregado Efetivo
	F	Empregado Efetivo
	F	Empregado Efetivo
	M	Empregado Efetivo

	F	Empregado Efetivo
	M	Empregado Efetivo
	M	Empregado Efetivo
	M	Empregado Efetivo
	M	Empregado Efetivo
	M	Empregado Efetivo
	M	Empregado Efetivo
	M	Empregado Efetivo
	M	Empregado Efetivo
	M	Empregado Efetivo
	M	Empregado Efetivo
	F	Empregado Efetivo
	F	Empregado Efetivo
	F	Empregado Efetivo
	F	Empregado Efetivo
	F	Empregado Efetivo
	F	Empregado Efetivo
	F	Empregado Efetivo
	F	Empregado Efetivo
	F	Empregado Efetivo
	F	Empregado Efetivo
	F	Empregado Efetivo
	F	Empregado Efetivo
	M	Empregado Efetivo
	M	Empregado Efetivo
	F	Empregado Efetivo
	M	Empregado Efetivo
	M	Empregado Efetivo
	F	Empregado Efetivo
	F	Empregado Efetivo
	F	Empregado Efetivo
	F	Empregado Efetivo
	F	Empregado Efetivo
	F	Empregado Efetivo
	F	Empregado Efetivo
	F	Empregado Efetivo
	F	Empregado Efetivo
	F	Empregado Efetivo
	M	Empregado Efetivo
	M	Empregado Efetivo
	M	Empregado Efetivo

	F	Empregado Efetivo
	M	Empregado Efetivo
	F	Empregado Efetivo
	F	Empregado Efetivo
	F	Empregado Efetivo
	M	Empregado Efetivo
	F	Empregado Efetivo
	M	Empregado Efetivo
	F	Empregado Efetivo
	M	Empregado Efetivo
	M	Empregado Efetivo
	F	Empregado Efetivo
	F	Empregado Efetivo
	F	Empregado Efetivo
	F	Empregado Efetivo
	F	Empregado Efetivo
	F	Empregado Efetivo
	F	Empregado Efetivo
	F	Empregado Efetivo
	F	Empregado Efetivo
	F	Empregado Efetivo
	F	Empregado Efetivo
	F	Empregado Efetivo
	F	Empregado Efetivo
	M	Empregado Efetivo
	M	Empregado Efetivo
	M	Empregado Efetivo
	M	Empregado Efetivo
	F	Empregado Efetivo
	M	Empregado Efetivo
	M	Empregado Efetivo
	M	Empregado Efetivo
	M	Empregado Efetivo
	M	Empregado Efetivo
	M	Empregado Efetivo
	F	Empregado Efetivo
	M	Empregado Efetivo
	M	Empregado Efetivo
	F	Empregado Efetivo
	F	Empregado Efetivo
	M	Empregado Efetivo
	F	Empregado Efetivo
	F	Empregado Efetivo

	M	Empregado Efetivo
	F	Empregado Efetivo
	F	Empregado Efetivo
	F	Empregado Efetivo
	F	Empregado Efetivo
	F	Empregado Efetivo
	F	Empregado Efetivo
	F	Empregado Efetivo
	F	Empregado Efetivo
	F	Empregado Efetivo
	F	Empregado Efetivo
	F	Empregado Efetivo
	F	Empregado Efetivo
	F	Empregado Efetivo
	F	Empregado Efetivo
	F	Empregado Efetivo
	F	Empregado Efetivo
	F	Empregado Efetivo
	F	Empregado Efetivo
	F	Empregado Efetivo
	F	Empregado Efetivo
	F	Empregado Efetivo
	F	Empregado Efetivo
	F	Empregado Efetivo
	F	Empregado Efetivo
	F	Empregado Efetivo
	F	Empregado Efetivo
	F	Empregado Efetivo
	F	Empregado Efetivo
	F	Empregado Efetivo
	M	Empregado Efetivo
	F	Empregado Efetivo
	M	Empregado Efetivo
	M	Empregado Efetivo
	F	Empregado Efetivo
	M	Empregado Efetivo
	M	Empregado Efetivo
	M	Empregado Efetivo
	F	Empregado Efetivo
	F	Empregado Efetivo
	F	Empregado Efetivo
	M	Empregado Efetivo
	M	Empregado Efetivo
	F	Empregado Efetivo
	F	Empregado Efetivo
	F	Empregado Efetivo
	F	Empregado Efetivo
	M	Empregado Efetivo

	F	Empregado Efetivo
	M	Empregado Efetivo
	M	Empregado Efetivo
	F	Empregado Efetivo
	M	Empregado Efetivo
	M	Empregado Efetivo
	M	Empregado Efetivo
	F	Empregado Efetivo
	F	Empregado Efetivo
	M	Empregado Efetivo
	F	Empregado Efetivo
	M	Empregado Efetivo
	M	Empregado Efetivo
	F	Empregado Efetivo
	M	Empregado Efetivo
	M	Empregado Efetivo
	F	Empregado Efetivo
	F	Empregado Efetivo
	F	Empregado Efetivo
	F	Empregado Efetivo
	F	Empregado Efetivo
	F	Empregado Efetivo
	F	Empregado Efetivo
	F	Empregado Efetivo
	F	Empregado Efetivo
	F	Empregado Efetivo
	F	Empregado Efetivo
	F	Empregado Efetivo
	F	Empregado Efetivo
	F	Empregado Efetivo
	F	Empregado Efetivo
	M	Empregado Efetivo
	F	Empregado Efetivo
	F	Empregado Efetivo
	F	Empregado Efetivo
	F	Empregado Efetivo
	F	Empregado Efetivo

	F	Empregado Efetivo
	F	Empregado Efetivo
	F	Empregado Efetivo
	F	Empregado Efetivo
	F	Empregado Efetivo
	F	Empregado Efetivo
	M	Empregado Efetivo
	M	Empregado Efetivo
	F	Empregado Efetivo
	F	Empregado Efetivo
	M	Empregado Efetivo
	F	Empregado Efetivo
	F	Empregado Efetivo
	F	Empregado Efetivo
	F	Empregado Efetivo
	F	Empregado Efetivo
	F	Empregado Efetivo
	M	Empregado Efetivo
	F	Empregado Efetivo
	F	Empregado Efetivo
	M	Empregado Efetivo
	F	Empregado Efetivo
	F	Empregado Efetivo
	F	Empregado Efetivo
	M	Empregado Efetivo
	F	Empregado Efetivo
	F	Empregado Efetivo
	F	Empregado Efetivo
	F	Empregado Efetivo
	M	Empregado Efetivo
	F	Empregado Efetivo
	M	Empregado Efetivo
	F	Empregado Efetivo
	F	Empregado Efetivo
	F	Empregado Efetivo
	F	Empregado Efetivo
	F	Empregado Efetivo
	F	Empregado Efetivo
	F	Empregado Efetivo
	F	Empregado Efetivo
	F	Empregado Efetivo
	F	Empregado Efetivo
	M	Empregado Efetivo
	F	Empregado Efetivo
	F	Empregado Efetivo

	M	Empregado Efetivo
	F	Empregado Efetivo
	M	Empregado Efetivo
	M	Empregado Efetivo
	F	Empregado Efetivo
	F	Empregado Efetivo
	F	Empregado Efetivo
	F	Empregado Efetivo
	F	Empregado Efetivo
	F	Empregado Efetivo
	F	Empregado Efetivo
	F	Empregado Efetivo
	F	Empregado Efetivo
	F	Empregado Efetivo
	M	Empregado Efetivo
	F	Empregado Efetivo
	M	Empregado Efetivo
	F	Empregado Efetivo
	M	Empregado Efetivo
	M	Empregado Efetivo
	F	Empregado Efetivo
	F	Empregado Efetivo
	F	Empregado Efetivo
	F	Empregado Efetivo
	F	Empregado Efetivo
	F	Empregado Efetivo
	F	Empregado Efetivo
	F	Empregado Efetivo
	F	Empregado Efetivo
	F	Empregado Efetivo
	F	Empregado Efetivo
	F	Empregado Efetivo
	M	Empregado Efetivo
	M	Empregado Efetivo
	F	Empregado Efetivo
	F	Empregado Efetivo
	M	Empregado Efetivo
	M	Empregado Efetivo
	M	Empregado Efetivo
	F	Empregado Efetivo
	F	Empregado Efetivo
	F	Empregado Efetivo
	M	Empregado Efetivo
	F	Empregado Efetivo
	F	Empregado Efetivo
	F	Empregado Efetivo
	F	Empregado Efetivo
	F	Empregado Efetivo

	F	Empregado Efetivo
	F	Empregado Efetivo
	F	Empregado Efetivo
	F	Empregado Efetivo
	F	Empregado Efetivo
	F	Empregado Efetivo
	F	Empregado Efetivo
	M	Empregado Efetivo
	F	Empregado Efetivo
	F	Empregado Efetivo
	M	Empregado Efetivo
	F	Empregado Efetivo
	F	Empregado Efetivo
	F	Empregado Efetivo
	F	Empregado Efetivo
	F	Empregado Efetivo
	M	Empregado Efetivo
	M	Empregado Efetivo
	F	Empregado Efetivo
	F	Empregado Efetivo
	F	Empregado Efetivo
	F	Empregado Efetivo
	F	Empregado Efetivo
	F	Empregado Efetivo
	F	Empregado Efetivo
	F	Empregado Efetivo
	F	Empregado Efetivo
	M	Empregado Efetivo
	M	Empregado Efetivo
	M	Empregado Efetivo
	M	Empregado Efetivo
	M	Empregado Efetivo
	M	Empregado Efetivo
	M	Empregado Efetivo
	M	Empregado Efetivo
	F	Empregado Efetivo
	F	Empregado Efetivo
	F	Empregado Efetivo
	F	Empregado Efetivo
	F	Empregado Efetivo
	F	Empregado Efetivo
	F	Empregado Efetivo
	F	Empregado Efetivo
	F	Empregado Efetivo
	F	Empregado Efetivo
	F	Empregado Efetivo

	M	Empregado Efetivo
	F	Empregado Efetivo
	F	Empregado Efetivo
	F	Empregado Efetivo
	F	Empregado Efetivo
	F	Empregado Efetivo
	F	Empregado Efetivo
	F	Empregado Efetivo
	F	Empregado Efetivo
	F	Empregado Efetivo
	F	Empregado Efetivo
	F	Empregado Efetivo
	F	Empregado Efetivo
	F	Empregado Efetivo
	F	Empregado Efetivo
	F	Empregado Efetivo
	F	Empregado Efetivo
	F	Empregado Efetivo
	F	Empregado Efetivo
	F	Empregado Efetivo
	F	Empregado Efetivo
	F	Empregado Efetivo
	F	Empregado Efetivo
	F	Empregado Efetivo
	F	Empregado Efetivo
	M	Empregado Efetivo
	M	Empregado Efetivo
	M	Empregado Efetivo
	M	Empregado Efetivo
	F	Empregado Efetivo
	M	Empregado Efetivo
	F	Empregado Efetivo
	F	Empregado Efetivo
	F	Empregado Efetivo
	F	Empregado Efetivo
	F	Empregado Efetivo
	F	Empregado Efetivo
	F	Empregado Efetivo
	M	Empregado Efetivo

	F	Empregado Efetivo
	F	Empregado Efetivo
	F	Empregado Efetivo
	M	Empregado Efetivo
	F	Empregado Efetivo
	F	Empregado Efetivo
	F	Empregado Efetivo
	M	Empregado Efetivo
	F	Empregado Efetivo
	F	Empregado Efetivo
	M	Cedido - RJU
	F	Cedido - RJU
	F	Cedido - RJU
	F	Cedido - RJU
	F	Empregado Efetivo
	F	Empregado Efetivo
	F	Empregado Efetivo
	F	Empregado Efetivo
	M	Cedido - RJU
	F	Empregado Efetivo
	F	Empregado Efetivo
	F	Empregado Efetivo
	F	Empregado Efetivo
	F	Empregado Efetivo
	F	Empregado Efetivo
	F	Empregado Efetivo
	F	Empregado Efetivo
	F	Empregado Efetivo
	F	Empregado Efetivo
	F	Empregado Efetivo
	F	Empregado Efetivo
	F	Empregado Efetivo
	F	Empregado Efetivo
	F	Empregado Efetivo
	F	Empregado Efetivo
	F	Empregado Efetivo
	F	Empregado Efetivo
	F	Empregado Efetivo
	F	Empregado Efetivo

	F	Empregado Efetivo
	F	Empregado Efetivo
	F	Empregado Efetivo
	F	Empregado Efetivo
	F	Empregado Efetivo
	F	Empregado Efetivo
	F	Empregado Efetivo
	F	Empregado Efetivo
	F	Empregado Efetivo
	F	Empregado Efetivo
	F	Empregado Efetivo
	F	Empregado Efetivo
	F	Empregado Efetivo
	F	Empregado Efetivo
	F	Empregado Efetivo
	F	Empregado Efetivo
	F	Empregado Efetivo
	M	Cedido - RJU
	F	Empregado Efetivo
	F	Empregado Efetivo
	F	Empregado Efetivo
	M	Empregado Efetivo
	F	Empregado Efetivo
	F	Empregado Efetivo
	F	Empregado Efetivo
	F	Empregado Efetivo
	F	Empregado Efetivo
	F	Empregado Efetivo
	F	Empregado Efetivo
	F	Empregado Efetivo
	M	Empregado Efetivo
	F	Empregado Efetivo
	M	Empregado Efetivo
	M	Empregado Efetivo
	F	Empregado Efetivo

	F	Empregado Efetivo
	F	Empregado Efetivo
	M	Empregado Efetivo
	F	Empregado Efetivo
	F	Empregado Efetivo
	F	Empregado Efetivo
	M	Empregado Efetivo
	F	Empregado Efetivo
	F	Empregado Efetivo
	F	Empregado Efetivo
	F	Empregado Efetivo
	F	Empregado Efetivo
	F	Empregado Efetivo
	F	Empregado Efetivo
	F	Empregado Efetivo
	F	Empregado Efetivo
	F	Empregado Efetivo
	F	Empregado Efetivo
	F	Empregado Efetivo
	F	Empregado Efetivo
	F	Empregado Efetivo
	F	Empregado Efetivo
	F	Empregado Efetivo
	F	Empregado Efetivo
	F	Empregado Efetivo
	F	Empregado Efetivo
	F	Empregado Efetivo
	F	Empregado Efetivo
	F	Empregado Efetivo
	F	Empregado Efetivo
	F	Empregado Efetivo
	F	Empregado Efetivo
	F	Empregado Efetivo
	F	Empregado Efetivo
	F	Empregado Efetivo
	F	Empregado Efetivo
	F	Empregado Efetivo
	F	Empregado Efetivo
	F	Empregado Efetivo

	M	Empregado Efetivo
	F	Empregado Efetivo
	F	Empregado Efetivo
	F	Empregado Efetivo
	F	Empregado Efetivo
	F	Empregado Efetivo
	F	Empregado Efetivo
	F	Empregado Efetivo
	F	Empregado Efetivo
	M	Empregado Efetivo
	M	Empregado Efetivo
	F	Empregado Efetivo
	M	Empregado Efetivo
	M	Empregado Efetivo
	F	Empregado Efetivo
	F	Empregado Efetivo
	F	Empregado Efetivo
	F	Empregado Efetivo
	F	Empregado Efetivo
	F	Empregado Efetivo
	F	Empregado Efetivo
	M	Empregado Efetivo
	F	Empregado Efetivo
	F	Empregado Efetivo
	F	Empregado Efetivo
	M	Empregado Efetivo
	F	Empregado Efetivo
	F	Empregado Efetivo
	M	Empregado Efetivo
	M	Empregado Efetivo
	M	Empregado Efetivo
	F	Empregado Efetivo
	M	Empregado Efetivo
	F	Empregado Efetivo

	F	Empregado Efetivo
	F	Empregado Efetivo
	F	Empregado Efetivo
	F	Empregado Efetivo
	F	Empregado Efetivo
	F	Empregado Efetivo
	F	Empregado Efetivo
	M	Empregado Efetivo
	M	Empregado Efetivo
	M	Empregado Efetivo
	F	Empregado Efetivo
	M	Empregado Efetivo
	F	Empregado Efetivo
	M	Empregado Efetivo
	F	Empregado Efetivo
	F	Empregado Efetivo
	F	Empregado Efetivo
	F	Empregado Efetivo
	F	Empregado Efetivo
	F	Empregado Efetivo
	F	Empregado Efetivo
	F	Empregado Efetivo
	M	Empregado Efetivo
	F	Empregado Efetivo
	F	Empregado Efetivo
	F	Empregado Efetivo
	F	Empregado Efetivo
	F	Empregado Efetivo
	F	Empregado Efetivo
	M	Empregado Efetivo
	F	Empregado Efetivo
	F	Empregado Efetivo

	F	Empregado Efetivo
	F	Empregado Efetivo
	M	Empregado Efetivo
	F	Empregado Efetivo
	F	Empregado Efetivo
	F	Empregado Efetivo
	F	Empregado Efetivo
	M	Empregado Efetivo
	M	Empregado Efetivo
	M	Empregado Efetivo
	F	Empregado Efetivo
	M	Empregado Efetivo
	M	Empregado Efetivo
	F	Empregado Efetivo
	F	Empregado Efetivo
	F	Empregado Efetivo
	F	Empregado Efetivo
	F	Empregado Efetivo
	F	Empregado Efetivo
	F	Empregado Efetivo
	F	Empregado Efetivo
	F	Empregado Efetivo
	F	Empregado Efetivo
	F	Empregado Efetivo
	F	Empregado Efetivo
	F	Empregado Efetivo
	F	Empregado Efetivo
	F	Empregado Efetivo
	F	Empregado Efetivo
	F	Empregado Efetivo
	F	Empregado Efetivo
	F	Empregado Efetivo

	F	Empregado Efetivo
	F	Empregado Efetivo
	F	Empregado Efetivo
	F	Empregado Efetivo
	F	Empregado Efetivo
	F	Empregado Efetivo
	F	Empregado Efetivo
	F	Empregado Efetivo
	F	Empregado Efetivo
	M	Empregado Efetivo
	F	Empregado Efetivo
	F	Empregado Efetivo
	F	Empregado Efetivo
	F	Empregado Efetivo
	M	Empregado Efetivo
	F	Empregado Efetivo
	F	Empregado Efetivo
	F	Empregado Efetivo
	M	Empregado Efetivo
	F	Empregado Efetivo
	F	Empregado Efetivo
	F	Empregado Efetivo
	F	Empregado Efetivo
	F	Empregado Efetivo
	F	Empregado Efetivo
	F	Empregado Efetivo
	F	Empregado Efetivo
	F	Empregado Efetivo
	F	Empregado Efetivo
	M	Empregado Efetivo
	F	Empregado Efetivo
	F	Empregado Efetivo

	F	Empregado Efetivo
	F	Empregado Efetivo
	F	Empregado Efetivo
	F	Empregado Efetivo
	F	Empregado Efetivo
	M	Empregado Efetivo
	F	Empregado Efetivo
	F	Empregado Efetivo
	F	Empregado Efetivo
	F	Empregado Efetivo
	F	Empregado Efetivo
	F	Empregado Efetivo
	F	Empregado Efetivo
	M	Empregado Efetivo
	F	Empregado Efetivo
	F	Empregado Efetivo
	M	Empregado Efetivo
	F	Empregado Efetivo
	F	Empregado Efetivo
	F	Empregado Efetivo
	F	Empregado Efetivo
	F	Empregado Efetivo
	F	Empregado Efetivo
	F	Empregado Efetivo
	F	Empregado Efetivo
	F	Empregado Efetivo
	F	Empregado Efetivo
	F	Empregado Efetivo
	M	Empregado Efetivo
	F	Empregado Efetivo
	F	Empregado Efetivo
	F	Empregado Efetivo

	F	Empregado Efetivo
	M	Empregado Efetivo
	M	Empregado Efetivo
	M	Empregado Efetivo
	M	Empregado Efetivo
	M	Empregado Efetivo
	M	Empregado Efetivo
	F	Empregado Efetivo
	M	Empregado Efetivo
	M	Empregado Efetivo
	M	Empregado Efetivo
	M	Empregado Efetivo
	M	Empregado Efetivo
	F	Empregado Efetivo
	F	Empregado Efetivo
	F	Empregado Efetivo
	M	Empregado Efetivo
	F	Empregado Efetivo
	F	Empregado Efetivo
	F	Empregado Efetivo
	F	Empregado Efetivo
	F	Empregado Efetivo
	F	Empregado Efetivo
	F	Empregado Efetivo
	F	Empregado Efetivo
	F	Empregado Efetivo
	F	Empregado Efetivo
	F	Empregado Efetivo
	F	Empregado Efetivo
	F	Empregado Efetivo
	F	Empregado Efetivo
	F	Empregado Efetivo
	F	Empregado Efetivo
	F	Empregado Efetivo
	F	Empregado Efetivo
	F	Empregado Efetivo
	M	Empregado Efetivo
	M	Empregado Efetivo
	M	Empregado Efetivo
	F	Empregado Efetivo

	F	Empregado Efetivo
	F	Empregado Efetivo
	M	Empregado Efetivo
	F	Empregado Efetivo
	F	Empregado Efetivo
	F	Empregado Efetivo
	F	Empregado Efetivo
	F	Empregado Efetivo
	F	Empregado Efetivo
	F	Empregado Efetivo
	F	Empregado Efetivo
	F	Empregado Efetivo
	F	Empregado Efetivo
	F	Empregado Efetivo
	F	Empregado Efetivo
	F	Empregado Efetivo
	F	Empregado Efetivo
	F	Empregado Efetivo
	F	Empregado Efetivo
	F	Empregado Efetivo
	F	Empregado Efetivo
	F	Empregado Efetivo
	F	Empregado Efetivo
	F	Empregado Efetivo
	F	Empregado Efetivo
	F	Empregado Efetivo
	M	Empregado Efetivo
	M	Empregado Efetivo
	M	Empregado Efetivo
	F	Empregado Efetivo
	F	Empregado Efetivo
	F	Empregado Efetivo
	F	Empregado Efetivo
	F	Empregado Efetivo
	F	Empregado Efetivo
	F	Empregado Efetivo
	F	Empregado Efetivo
	F	Empregado Efetivo
	F	Empregado Efetivo
	F	Empregado Efetivo
	F	Empregado Efetivo
	F	Empregado Efetivo
	F	Empregado Efetivo
	F	Empregado Efetivo

	F	Empregado Efetivo
	F	Empregado Efetivo
	F	Empregado Efetivo
	M	Empregado Efetivo
	M	Empregado Efetivo
	F	Empregado Efetivo
	F	Empregado Efetivo
	F	Empregado Efetivo
	F	Empregado Efetivo
	F	Empregado Efetivo
	F	Empregado Efetivo
	F	Empregado Efetivo
	F	Empregado Efetivo
	M	Empregado Efetivo
	F	Empregado Efetivo
	F	Empregado Efetivo
	M	Empregado Efetivo
	M	Empregado Efetivo
	M	Empregado Efetivo
	M	Empregado Efetivo
	M	Empregado Efetivo
	M	Empregado Efetivo
	F	Empregado Efetivo
	M	Empregado Efetivo
	M	Empregado Efetivo
	F	Empregado Efetivo
	F	Empregado Efetivo
	F	Empregado Efetivo
	M	Empregado Efetivo
	M	Empregado Efetivo
	M	Empregado Efetivo
	M	Empregado Efetivo
	M	Empregado Efetivo
	M	Empregado Efetivo
	M	Empregado Efetivo
	M	Empregado Efetivo
	M	Empregado Efetivo
	M	Empregado Efetivo
	M	Empregado Efetivo
	M	Empregado Efetivo
	M	Empregado Efetivo
	M	Empregado Efetivo
	M	Empregado Efetivo
	M	Empregado Efetivo

	M	Empregado Efetivo
	M	Empregado Efetivo
	M	Empregado Efetivo
	M	Empregado Efetivo
	M	Empregado Efetivo
	M	Empregado Efetivo
	M	Empregado Efetivo
	F	Empregado Efetivo
	M	Empregado Efetivo
	M	Empregado Efetivo
	M	Empregado Efetivo
	M	Empregado Efetivo
	M	Empregado Efetivo
	F	Empregado Efetivo
	F	Empregado Efetivo
	F	Empregado Efetivo
	M	Empregado Efetivo
	M	Empregado Efetivo
	F	Empregado Efetivo
	F	Empregado Efetivo
	F	Empregado Efetivo
	F	Empregado Efetivo
	F	Empregado Efetivo
	F	Empregado Efetivo
	M	Empregado Efetivo
	F	Empregado Efetivo
	F	Empregado Efetivo
	F	Empregado Efetivo
	F	Empregado Efetivo
	F	Empregado Efetivo
	F	Empregado Efetivo
	F	Empregado Efetivo
	M	Empregado Efetivo
	M	Empregado Efetivo
	F	Empregado Efetivo
	M	Empregado Efetivo
	M	Empregado Efetivo
	M	Empregado Efetivo
	F	Empregado Efetivo
	F	Empregado Efetivo
	F	Empregado Efetivo
	F	Empregado Efetivo
	F	Empregado Efetivo
	F	Empregado Efetivo

	F	Empregado Efetivo
	F	Empregado Efetivo
	M	Empregado Efetivo
	F	Empregado Efetivo
	F	Empregado Efetivo
	F	Empregado Efetivo
	M	Empregado Efetivo
	F	Empregado Efetivo
	F	Empregado Efetivo
	F	Empregado Efetivo
	F	Empregado Efetivo
	M	Empregado Efetivo
	F	Empregado Efetivo
	F	Empregado Efetivo
	M	Empregado Efetivo
	M	Empregado Efetivo
	F	Empregado Efetivo
	F	Empregado Efetivo
	F	Empregado Efetivo
	F	Empregado Efetivo
	F	Empregado Efetivo
	M	Empregado Efetivo
	M	Empregado Efetivo
	M	Empregado Efetivo
	M	Empregado Efetivo
	F	Empregado Efetivo
	F	Empregado Efetivo
	F	Empregado Efetivo
	F	Empregado Efetivo
	F	Empregado Efetivo
	M	Empregado Efetivo
	F	Empregado Efetivo
	F	Empregado Efetivo
	F	Empregado Efetivo
	F	Empregado Efetivo
	F	Empregado Efetivo
	F	Empregado Efetivo
	F	Empregado Efetivo
	M	Empregado Efetivo
	F	Empregado Efetivo
	F	Empregado Efetivo
	F	Empregado Efetivo
	F	Empregado Efetivo
	F	Empregado Efetivo

	F	Empregado Efetivo
	F	Empregado Efetivo
	F	Empregado Efetivo
	F	Empregado Efetivo
	F	Empregado Efetivo
	F	Empregado Efetivo
	F	Empregado Efetivo
	F	Empregado Efetivo
	F	Empregado Efetivo
	F	Empregado Efetivo
	F	Empregado Efetivo
	F	Empregado Efetivo
	F	Empregado Efetivo
	F	Empregado Efetivo
	F	Empregado Efetivo
	F	Empregado Efetivo
	F	Empregado Efetivo
	F	Empregado Efetivo
	F	Empregado Efetivo
	F	Empregado Efetivo
	F	Empregado Efetivo
	F	Empregado Efetivo
	F	Empregado Efetivo
	F	Empregado Efetivo
	F	Empregado Efetivo
	F	Empregado Efetivo
	F	Empregado Efetivo
	F	Empregado Efetivo
	F	Empregado Efetivo
	F	Empregado Efetivo
	F	Empregado Efetivo
	F	Empregado Efetivo
	F	Empregado Efetivo
	F	Empregado Efetivo
	F	Empregado Efetivo

	F	Empregado Efetivo
	F	Empregado Efetivo
	F	Empregado Efetivo
	F	Empregado Efetivo
	F	Empregado Efetivo
	F	Empregado Efetivo
	F	Empregado Efetivo
	F	Empregado Efetivo
	F	Empregado Efetivo
	F	Empregado Efetivo
	F	Empregado Efetivo
	F	Empregado Efetivo
	F	Empregado Efetivo
	F	Empregado Efetivo
	F	Empregado Efetivo
	F	Empregado Efetivo
	F	Empregado Efetivo
	F	Empregado Efetivo
	F	Empregado Efetivo
	F	Empregado Efetivo
	F	Empregado Efetivo
	F	Empregado Efetivo
	F	Empregado Efetivo
	F	Empregado Efetivo
	F	Empregado Efetivo
	F	Empregado Efetivo
	F	Empregado Efetivo
	F	Empregado Efetivo
	F	Empregado Efetivo
	F	Empregado Efetivo
	F	Empregado Efetivo
	F	Empregado Efetivo
	F	Empregado Efetivo
	F	Empregado Efetivo
	F	Empregado Efetivo
	F	Empregado Efetivo
	F	Empregado Efetivo
	F	Empregado Efetivo
	F	Empregado Efetivo
	F	Empregado Efetivo
	F	Empregado Efetivo
	F	Empregado Efetivo
	F	Empregado Efetivo
	F	Empregado Efetivo
	F	Empregado Efetivo
	F	Empregado Efetivo
	F	Empregado Efetivo
	F	Empregado Efetivo

	F	Empregado Efetivo
	F	Empregado Efetivo
	F	Empregado Efetivo
	F	Empregado Efetivo
	F	Empregado Efetivo
	F	Empregado Efetivo
	F	Empregado Efetivo
	F	Empregado Efetivo
	F	Empregado Efetivo
	F	Empregado Efetivo
	F	Empregado Efetivo
	F	Empregado Efetivo
	F	Empregado Efetivo
	F	Empregado Efetivo
	F	Empregado Efetivo
	F	Empregado Efetivo
	F	Empregado Efetivo
	F	Empregado Efetivo
	F	Empregado Efetivo
	F	Empregado Efetivo
	F	Empregado Efetivo
	F	Empregado Efetivo
	F	Empregado Efetivo
	F	Empregado Efetivo
	F	Empregado Efetivo
	F	Empregado Efetivo
	F	Empregado Efetivo
	F	Empregado Efetivo
	F	Empregado Efetivo
	F	Empregado Efetivo
	F	Empregado Efetivo
	M	Empregado Efetivo
	M	Empregado Efetivo
	M	Empregado Efetivo
	M	Empregado Efetivo
	F	Empregado Efetivo
	F	Empregado Efetivo
	F	Empregado Efetivo
	F	Empregado Efetivo
	F	Empregado Efetivo
	F	Empregado Efetivo
	F	Empregado Efetivo
	F	Empregado Efetivo
	F	Empregado Efetivo
	M	Empregado Efetivo
	M	Empregado Efetivo
	M	Empregado Efetivo
	F	Empregado Efetivo

	F	Empregado Efetivo
	F	Empregado Efetivo
	M	Empregado Efetivo
	F	Empregado Efetivo
	F	Empregado Efetivo
	F	Empregado Efetivo
	F	Empregado Efetivo
	F	Empregado Efetivo
	F	Empregado Efetivo
	F	Empregado Efetivo
	M	Empregado Efetivo
	F	Empregado Efetivo
	F	Empregado Efetivo
	F	Empregado Efetivo
	F	Empregado Efetivo
	F	Empregado Efetivo
	F	Empregado Efetivo
	F	Empregado Efetivo
	F	Empregado Efetivo
	M	Empregado Efetivo
	F	Empregado Efetivo
	F	Empregado Efetivo
	F	Empregado Efetivo
	F	Empregado Efetivo
	M	Empregado Efetivo
	F	Empregado Efetivo
	F	Empregado Efetivo
	F	Empregado Efetivo
	F	Empregado Efetivo
	F	Empregado Efetivo
	F	Empregado Efetivo
	F	Empregado Efetivo
	M	Empregado Efetivo
	F	Empregado Efetivo
	F	Empregado Efetivo
	F	Empregado Efetivo
	M	Empregado Efetivo
	F	Empregado Efetivo
	F	Empregado Efetivo
	F	Empregado Efetivo
	F	Empregado Efetivo
	F	Empregado Efetivo
	F	Empregado Efetivo
	F	Empregado Efetivo
	F	Empregado Efetivo
	F	Empregado Efetivo
	F	Empregado Efetivo
	F	Empregado Efetivo

	M	Empregado Efetivo
	F	Empregado Efetivo
	F	Empregado Efetivo
	F	Empregado Efetivo
	F	Empregado Efetivo
	F	Empregado Efetivo
	F	Empregado Efetivo
	F	Empregado Efetivo
	F	Empregado Efetivo
	F	Empregado Efetivo
	F	Empregado Efetivo
	F	Empregado Efetivo
	F	Empregado Efetivo
	F	Empregado Efetivo
	F	Empregado Efetivo
	F	Empregado Efetivo
	M	Empregado Efetivo
	F	Empregado Efetivo
	F	Empregado Efetivo
	F	Empregado Efetivo
	M	Empregado Efetivo
	F	Empregado Efetivo
	F	Empregado Efetivo
	F	Empregado Efetivo
	M	Empregado Efetivo
	F	Empregado Efetivo
	F	Empregado Efetivo
	F	Empregado Efetivo
	F	Empregado Efetivo
	F	Empregado Efetivo
	F	Empregado Efetivo
	F	Empregado Efetivo
	F	Empregado Efetivo
	F	Empregado Efetivo
	M	Empregado Efetivo
	M	Empregado Efetivo
	M	Empregado Efetivo
	F	Empregado Efetivo
	M	Empregado Efetivo
	F	Empregado Efetivo
	M	Empregado Efetivo
	F	Empregado Efetivo
	F	Empregado Efetivo
	F	Empregado Efetivo
	M	Empregado Efetivo
	M	Empregado Efetivo
	F	Empregado Efetivo
	F	Empregado Efetivo

	F	Empregado Efetivo
	M	Empregado Efetivo
	F	Empregado Efetivo
	F	Empregado Efetivo
	F	Empregado Efetivo
	F	Empregado Efetivo
	M	Empregado Efetivo
	F	Empregado Efetivo
	F	Empregado Efetivo
	F	Empregado Efetivo
	F	Empregado Efetivo
	F	Empregado Efetivo
	F	Empregado Efetivo
	M	Empregado Efetivo
	F	Empregado Efetivo
	F	Empregado Efetivo
	F	Empregado Efetivo
	F	Empregado Efetivo
	F	Empregado Efetivo
	F	Empregado Efetivo
	F	Empregado Efetivo
	F	Empregado Efetivo
	F	Empregado Efetivo
	F	Empregado Efetivo
	F	Empregado Efetivo
	M	Empregado Efetivo
	F	Empregado Efetivo
	F	Empregado Efetivo
	F	Empregado Efetivo
	F	Empregado Efetivo
	M	Empregado Efetivo
	F	Empregado Efetivo
	F	Empregado Efetivo
	F	Empregado Efetivo
	F	Empregado Efetivo
	M	Empregado Efetivo
	F	Empregado Efetivo
	F	Empregado Efetivo
	F	Empregado Efetivo
	F	Empregado Efetivo
	F	Empregado Efetivo
	F	Empregado Efetivo
	F	Empregado Efetivo
	F	Empregado Efetivo
	F	Empregado Efetivo
	F	Empregado Efetivo
	F	Empregado Efetivo
	F	Empregado Efetivo

	M	Empregado Efetivo
	M	Empregado Efetivo
	F	Empregado Efetivo
	M	Empregado Efetivo
	M	Empregado Efetivo
	F	Empregado Efetivo
	F	Empregado Efetivo
	F	Empregado Efetivo
	F	Empregado Efetivo
	F	Empregado Efetivo
	F	Empregado Efetivo
	F	Empregado Efetivo
	F	Empregado Efetivo
	F	Empregado Efetivo
	F	Empregado Efetivo
	F	Empregado Efetivo
	F	Empregado Efetivo
	F	Empregado Efetivo
	F	Empregado Efetivo
	F	Empregado Efetivo
	F	Empregado Efetivo
	F	Empregado Efetivo
	F	Empregado Efetivo
	F	Empregado Efetivo
	F	Empregado Efetivo
	F	Empregado Efetivo
	F	Empregado Efetivo
	F	Empregado Efetivo
	M	Empregado Efetivo
	M	Empregado Efetivo
	M	Empregado Efetivo
	F	Empregado Efetivo
	F	Empregado Efetivo

	F	Empregado Efetivo
	F	Empregado Efetivo
	F	Empregado Efetivo
	M	Empregado Efetivo
	F	Empregado Efetivo
	F	Empregado Efetivo
	F	Empregado Efetivo
	M	Empregado Efetivo
	F	Empregado Efetivo
	F	Empregado Efetivo
	F	Empregado Efetivo
	F	Empregado Efetivo
	F	Empregado Efetivo
	F	Empregado Efetivo
	F	Empregado Efetivo
	F	Empregado Efetivo
	M	Empregado Efetivo
	F	Empregado Efetivo
	M	Empregado Efetivo
	F	Empregado Efetivo
	F	Empregado Efetivo
	F	Empregado Efetivo
	M	Empregado Efetivo
	F	Empregado Efetivo
	F	Empregado Efetivo
	F	Empregado Efetivo
	F	Empregado Efetivo
	F	Empregado Efetivo
	F	Empregado Efetivo
	F	Empregado Efetivo
	M	Empregado Efetivo
	F	Empregado Efetivo

	M	Empregado Efetivo
	F	Empregado Efetivo
	F	Empregado Efetivo
	F	Empregado Efetivo
	F	Empregado Efetivo
	F	Empregado Efetivo
	M	Empregado Efetivo
	F	Empregado Efetivo
	F	Empregado Efetivo
	F	Empregado Efetivo
	F	Empregado Efetivo
	F	Empregado Efetivo
	F	Empregado Efetivo
	F	Empregado Efetivo
	F	Empregado Efetivo
	F	Empregado Efetivo
	F	Empregado Efetivo
	F	Empregado Efetivo
	F	Empregado Efetivo
	F	Empregado Efetivo
	F	Empregado Efetivo
	F	Empregado Efetivo
	F	Empregado Efetivo
	F	Empregado Efetivo
	F	Empregado Efetivo
	F	Empregado Efetivo
	F	Empregado Efetivo
	F	Empregado Efetivo
	M	Empregado Efetivo
	F	Empregado Efetivo
	F	Empregado Efetivo
	M	Empregado Efetivo

	M	Empregado Efetivo
	M	Empregado Efetivo
	M	Empregado Efetivo
	M	Empregado Efetivo
	F	Empregado Efetivo
	M	Empregado Efetivo
	M	Empregado Efetivo
	F	Empregado Efetivo
	M	Empregado Efetivo
	M	Empregado Efetivo
	M	Empregado Efetivo
	F	Empregado Efetivo
	F	Empregado Efetivo
	F	Empregado Efetivo
	F	Empregado Efetivo
	F	Empregado Efetivo
	F	Empregado Efetivo
	F	Empregado Efetivo
	F	Empregado Efetivo
	F	Empregado Efetivo
	F	Empregado Efetivo
	F	Empregado Efetivo
	F	Empregado Efetivo
	F	Empregado Efetivo
	F	Empregado Efetivo
	F	Empregado Efetivo
	F	Empregado Efetivo
	F	Empregado Efetivo
	F	Empregado Efetivo
	F	Empregado Efetivo
	F	Empregado Efetivo
	F	Empregado Efetivo
	F	Empregado Efetivo
	F	Empregado Efetivo
	F	Empregado Efetivo
	F	Empregado Efetivo
	F	Empregado Efetivo
	F	Empregado Efetivo
	F	Empregado Efetivo
	F	Empregado Efetivo
	F	Empregado Efetivo
	F	Empregado Efetivo
	F	Empregado Efetivo
	F	Empregado Efetivo
	F	Empregado Efetivo

	F	Empregado Efetivo
	F	Empregado Efetivo
	F	Empregado Efetivo
	F	Empregado Efetivo
	F	Empregado Efetivo
	F	Empregado Efetivo
	F	Empregado Efetivo
	F	Empregado Efetivo
	F	Empregado Efetivo
	F	Empregado Efetivo
	F	Empregado Efetivo
	F	Empregado Efetivo
	F	Empregado Efetivo
	F	Empregado Efetivo
	F	Empregado Efetivo
	F	Empregado Efetivo
	F	Empregado Efetivo
	M	Empregado Efetivo
	F	Empregado Efetivo
	F	Empregado Efetivo
	M	Empregado Efetivo
	F	Empregado Efetivo
	F	Empregado Efetivo
	M	Empregado Efetivo
	M	Empregado Efetivo
	F	Empregado Efetivo
	F	Empregado Efetivo
	F	Empregado Efetivo
	F	Empregado Efetivo
	F	Empregado Efetivo
	F	Empregado Efetivo
	F	Empregado Efetivo
	F	Empregado Efetivo
	M	Empregado Efetivo
	M	Empregado Efetivo
	M	Empregado Efetivo
	F	Empregado Efetivo
	F	Empregado Efetivo
	M	Empregado Efetivo
	M	Empregado Efetivo
	F	Empregado Efetivo
	F	Empregado Efetivo
	F	Empregado Efetivo
	F	Empregado Efetivo
	F	Empregado Efetivo
	F	Empregado Efetivo
	M	Empregado Efetivo

	F	Empregado Efetivo
	F	Empregado Efetivo
	M	Empregado Efetivo
	F	Empregado Efetivo
	M	Empregado Efetivo
	F	Empregado Efetivo
	F	Empregado Efetivo
	M	Empregado Efetivo
	F	Empregado Efetivo
	F	Empregado Efetivo
	M	Empregado Efetivo
	F	Empregado Efetivo
	F	Empregado Efetivo
	F	Empregado Efetivo
	F	Empregado Efetivo
	F	Empregado Efetivo
	M	Empregado Efetivo
	F	Empregado Efetivo
	F	Empregado Efetivo
	F	Empregado Efetivo
	F	Empregado Efetivo
	F	Empregado Efetivo
	F	Empregado Efetivo
	F	Empregado Efetivo
	F	Empregado Efetivo
	F	Empregado Efetivo
	F	Empregado Efetivo
	F	Empregado Efetivo
	M	Empregado Efetivo
	F	Empregado Efetivo
	F	Empregado Efetivo
	F	Empregado Efetivo
	M	Empregado Efetivo
	M	Empregado Efetivo
	M	Empregado Efetivo
	M	Empregado Efetivo
	F	Empregado Efetivo
	M	Empregado Efetivo
	F	Empregado Efetivo
	M	Empregado Efetivo

	M	Empregado Efetivo
	F	Empregado Efetivo
	F	Empregado Efetivo
	M	Empregado Efetivo
	F	Empregado Efetivo
	F	Empregado Efetivo
	F	Empregado Efetivo
	M	Empregado Efetivo
	F	Empregado Efetivo
	F	Empregado Efetivo
	F	Empregado Efetivo
	F	Empregado Efetivo
	F	Empregado Efetivo
	F	Empregado Efetivo
	F	Empregado Efetivo
	F	Empregado Efetivo
	M	Empregado Efetivo
	F	Empregado Efetivo
	F	Empregado Efetivo
	F	Empregado Efetivo
	M	Empregado Efetivo
	F	Empregado Efetivo
	F	Empregado Efetivo
	F	Empregado Efetivo
	F	Empregado Efetivo
	F	Empregado Efetivo
	F	Empregado Efetivo
	F	Empregado Efetivo
	F	Empregado Efetivo
	F	Empregado Efetivo
	F	Empregado Efetivo
	M	Empregado Efetivo
	F	Empregado Efetivo
	M	Empregado Efetivo
	F	Empregado Efetivo
	F	Empregado Efetivo
	M	Empregado Efetivo
	F	Empregado Efetivo
	F	Empregado Efetivo
	F	Empregado Efetivo
	F	Empregado Efetivo
	F	Empregado Efetivo
	F	Empregado Efetivo
	F	Empregado Efetivo
	M	Empregado Efetivo
	M	Empregado Efetivo
	M	Empregado Efetivo
	F	Empregado Efetivo

	F	Empregado Efetivo
	F	Empregado Efetivo
	M	Empregado Efetivo
	F	Empregado Efetivo
	F	Empregado Efetivo
	M	Empregado Efetivo
	F	Empregado Efetivo
	F	Empregado Efetivo
	M	Empregado Efetivo
	F	Empregado Efetivo
	F	Empregado Efetivo
	M	Empregado Efetivo
	F	Empregado Efetivo
	M	Empregado Efetivo
	F	Empregado Efetivo
	M	Empregado Efetivo
	M	Empregado Efetivo
	M	Empregado Efetivo
	M	Empregado Efetivo
	F	Empregado Efetivo
	F	Empregado Efetivo
	M	Empregado Efetivo
	M	Empregado Efetivo
	M	Empregado Efetivo
	F	Empregado Efetivo
	F	Empregado Efetivo
	F	Empregado Efetivo
	F	Empregado Efetivo
	F	Empregado Efetivo
	F	Empregado Efetivo
	F	Empregado Efetivo
	F	Empregado Efetivo
	F	Empregado Efetivo
	F	Empregado Efetivo
	F	Empregado Efetivo
	M	Empregado Efetivo
	F	Empregado Efetivo
	M	Empregado Efetivo
	F	Empregado Efetivo
	F	Empregado Efetivo

	M	Empregado Efetivo
	F	Empregado Efetivo
	F	Empregado Efetivo
	M	Empregado Efetivo
	M	Empregado Efetivo
	F	Empregado Efetivo
	F	Empregado Efetivo
	M	Empregado Efetivo
	F	Empregado Efetivo
	F	Empregado Efetivo
	M	Empregado Efetivo
	M	Empregado Efetivo
	M	Empregado Efetivo
	F	Empregado Efetivo
	F	Empregado Efetivo
	F	Empregado Efetivo
	F	Empregado Efetivo
	F	Empregado Efetivo
	F	Empregado Efetivo
	F	Empregado Efetivo
	F	Empregado Efetivo
	M	Empregado Efetivo
	M	Empregado Efetivo
	M	Empregado Efetivo
	M	Empregado Efetivo
	M	Empregado Efetivo
	M	Empregado Efetivo
	M	Empregado Efetivo
	M	Empregado Efetivo
	M	Empregado Efetivo
	M	Empregado Efetivo
	F	Empregado Efetivo

	M	Empregado Efetivo
	F	Empregado Efetivo
	M	Empregado Efetivo
	M	Empregado Efetivo
	M	Empregado Efetivo
	F	Empregado Efetivo
	F	Empregado Efetivo
	M	Empregado Efetivo
	F	Empregado Efetivo
	F	Empregado Efetivo
	F	Empregado Efetivo
	F	Empregado Efetivo
	F	Empregado Efetivo
	F	Empregado Efetivo
	F	Empregado Efetivo
	M	Empregado Efetivo
	M	Empregado Efetivo
	F	Empregado Efetivo
	M	Empregado Efetivo
	F	Empregado Efetivo
	F	Empregado Efetivo
	F	Empregado Efetivo
	M	Empregado Efetivo
	F	Empregado Efetivo
	F	Empregado Efetivo
	M	Empregado Efetivo
	F	Empregado Efetivo
	F	Empregado Efetivo
	M	Empregado Efetivo
	M	Empregado Efetivo
	M	Empregado Efetivo
	M	Empregado Efetivo
	F	Empregado Efetivo
	M	Empregado Efetivo
	M	Empregado Efetivo

	M	Empregado Efetivo
	M	Empregado Efetivo
	F	Empregado Efetivo
	F	Empregado Efetivo
	M	Empregado Efetivo
	M	Empregado Efetivo
	M	Empregado Efetivo
	M	Empregado Efetivo
	F	Empregado Efetivo
	M	Empregado Efetivo
	M	Empregado Efetivo
	M	Empregado Efetivo
	M	Empregado Efetivo
	M	Empregado Efetivo
	M	Empregado Efetivo
	M	Empregado Efetivo
	F	Empregado Efetivo
	F	Empregado Efetivo
	M	Empregado Efetivo
	M	Empregado Efetivo
	M	Empregado Efetivo
	M	Empregado Efetivo
	F	Empregado Efetivo
	M	Empregado Efetivo
	M	Empregado Efetivo
	F	Empregado Efetivo
	F	Empregado Efetivo
	F	Empregado Efetivo
	F	Empregado Efetivo
	F	Empregado Efetivo
	F	Empregado Efetivo
	M	Empregado Efetivo
	F	Empregado Efetivo
	F	Empregado Efetivo
	F	Empregado Efetivo
	F	Empregado Efetivo
	F	Empregado Efetivo
	F	Empregado Efetivo
	F	Empregado Efetivo

	F	Empregado Efetivo
	M	Empregado Efetivo
	F	Empregado Efetivo
	F	Empregado Efetivo
	F	Empregado Efetivo
	F	Empregado Efetivo
	M	Empregado Efetivo
	F	Empregado Efetivo
	F	Empregado Efetivo
	F	Empregado Efetivo
	F	Empregado Efetivo
	M	Empregado Efetivo
	F	Empregado Efetivo
	F	Empregado Efetivo
	F	Empregado Efetivo
	F	Empregado Efetivo
	F	Empregado Efetivo
	F	Empregado Efetivo
	F	Empregado Efetivo
	F	Empregado Efetivo
	M	Empregado Efetivo
	F	Empregado Efetivo
	F	Empregado Efetivo
	F	Empregado Efetivo
	F	Empregado Efetivo
	F	Empregado Efetivo
	F	Empregado Efetivo
	M	Empregado Efetivo
	F	Empregado Efetivo
	F	Empregado Efetivo
	F	Empregado Efetivo
	F	Empregado Efetivo
	F	Empregado Efetivo
	F	Empregado Efetivo
	F	Empregado Efetivo
	M	Empregado Efetivo
	F	Empregado Efetivo
	M	Empregado Efetivo
	F	Empregado Efetivo
	F	Empregado Efetivo

	F	Empregado Efetivo
	F	Empregado Efetivo
	M	Empregado Efetivo
	F	Empregado Efetivo
	F	Empregado Efetivo
	M	Empregado Efetivo
	F	Empregado Efetivo
	M	Empregado Efetivo
	F	Empregado Efetivo
	F	Empregado Efetivo
	M	Empregado Efetivo
	F	Empregado Efetivo
	F	Empregado Efetivo
	F	Empregado Efetivo
	M	Empregado Efetivo
	F	Empregado Efetivo
	M	Empregado Efetivo
	M	Empregado Efetivo
	F	Empregado Efetivo
	F	Empregado Efetivo
	F	Empregado Efetivo
	F	Empregado Efetivo
	F	Empregado Efetivo
	F	Empregado Efetivo
	F	Empregado Efetivo
	F	Empregado Efetivo
	F	Empregado Efetivo
	F	Empregado Efetivo
	M	Empregado Efetivo
	M	Empregado Efetivo
	F	Empregado Efetivo
	F	Empregado Efetivo
	F	Empregado Efetivo
	F	Empregado Efetivo
	F	Empregado Efetivo
	F	Empregado Efetivo
	M	Empregado Efetivo
	F	Empregado Efetivo
	M	Empregado Efetivo
	M	Empregado Efetivo
	F	Empregado Efetivo
	M	Empregado Efetivo
	F	Empregado Efetivo
	F	Empregado Efetivo
	F	Empregado Efetivo
	F	Empregado Efetivo
	M	Empregado Efetivo
	M	Empregado Efetivo

	F	Empregado Efetivo
	M	Empregado Efetivo
	F	Empregado Efetivo
	F	Empregado Efetivo
	F	Empregado Efetivo
	M	Empregado Efetivo
	F	Empregado Efetivo
	M	Empregado Efetivo
	M	Empregado Efetivo
	F	Empregado Efetivo
	F	Empregado Efetivo
	M	Empregado Efetivo
	F	Empregado Efetivo
	F	Empregado Efetivo
	F	Empregado Efetivo
	M	Empregado Efetivo
	F	Empregado Efetivo
	F	Empregado Efetivo
	F	Empregado Efetivo
	F	Empregado Efetivo
	F	Empregado Efetivo
	M	Empregado Efetivo
	F	Empregado Efetivo
	F	Empregado Efetivo
	M	Empregado Efetivo
	F	Empregado Efetivo
	F	Empregado Efetivo
	F	Empregado Efetivo
	M	Empregado Efetivo
	F	Empregado Efetivo
	F	Empregado Efetivo
	F	Empregado Efetivo
	M	Empregado Efetivo
	F	Empregado Efetivo
	F	Empregado Efetivo
	F	Empregado Efetivo
	F	Empregado Efetivo
	F	Empregado Efetivo
	F	Empregado Efetivo
	F	Empregado Efetivo
	F	Empregado Efetivo
	F	Empregado Efetivo
	F	Empregado Efetivo
	F	Empregado Efetivo
	F	Empregado Efetivo
	F	Empregado Efetivo
	F	Empregado Efetivo
	F	Empregado Efetivo

	F	Empregado Efetivo
	F	Empregado Efetivo
	F	Empregado Efetivo
	F	Empregado Efetivo
	F	Empregado Efetivo
	F	Empregado Efetivo
	F	Empregado Efetivo
	F	Empregado Efetivo
	F	Empregado Efetivo
	F	Empregado Efetivo
	M	Empregado Efetivo
	M	Empregado Efetivo
	M	Empregado Efetivo
	F	Empregado Efetivo
	F	Empregado Efetivo
	M	Empregado Efetivo
	M	Empregado Efetivo
	F	Empregado Efetivo
	F	Empregado Efetivo
	F	Empregado Efetivo
	F	Empregado Efetivo
	F	Empregado Efetivo
	F	Empregado Efetivo
	F	Empregado Efetivo
	F	Empregado Efetivo
	F	Empregado Efetivo
	M	Empregado Efetivo
	M	Empregado Efetivo
	F	Empregado Efetivo
	M	Empregado Efetivo
	F	Empregado Efetivo
	F	Empregado Efetivo
	F	Empregado Efetivo
	F	Empregado Efetivo
	F	Empregado Efetivo
	F	Empregado Efetivo
	M	Empregado Efetivo

	F	Empregado Efetivo
	F	Empregado Efetivo
	F	Empregado Efetivo
	F	Empregado Efetivo
	F	Empregado Efetivo
	M	Empregado Efetivo
	M	Empregado Efetivo
	F	Empregado Efetivo
	F	Empregado Efetivo
	F	Empregado Efetivo
	F	Empregado Efetivo
	M	Empregado Efetivo
	F	Empregado Efetivo
	F	Empregado Efetivo
	F	Empregado Efetivo
	F	Empregado Efetivo
	F	Empregado Efetivo
	M	Empregado Efetivo
	F	Empregado Efetivo
	F	Empregado Efetivo
	F	Empregado Efetivo
	F	Empregado Efetivo
	F	Empregado Efetivo
	F	Empregado Efetivo
	F	Empregado Efetivo
	F	Empregado Efetivo
	F	Empregado Efetivo
	F	Empregado Efetivo
	F	Empregado Efetivo
	F	Empregado Efetivo
	F	Empregado Efetivo
	F	Empregado Efetivo

	F	Empregado Efetivo
	F	Empregado Efetivo
	M	Empregado Efetivo
	F	Empregado Efetivo
	F	Empregado Efetivo
	F	Empregado Efetivo
	F	Empregado Efetivo
	F	Empregado Efetivo
	M	Empregado Efetivo
	M	Empregado Efetivo
	F	Empregado Efetivo
	F	Empregado Efetivo
	M	Empregado Efetivo
	F	Empregado Efetivo
	F	Empregado Efetivo
	F	Empregado Efetivo
	F	Empregado Efetivo
	F	Empregado Efetivo
	F	Empregado Efetivo
	F	Empregado Efetivo
	F	Empregado Efetivo
	F	Empregado Efetivo
	F	Empregado Efetivo
	F	Empregado Efetivo
	F	Empregado Efetivo
	F	Empregado Efetivo
	F	Empregado Efetivo
	F	Empregado Efetivo
	F	Empregado Efetivo
	F	Empregado Efetivo
	F	Empregado Efetivo
	M	Empregado Efetivo
	M	Empregado Efetivo
	M	Empregado Efetivo

	M	Empregado Efetivo
	M	Empregado Efetivo
	M	Empregado Efetivo
	F	Empregado Efetivo
	F	Empregado Efetivo
	F	Empregado Efetivo
	M	Empregado Efetivo
	M	Empregado Efetivo
	M	Empregado Efetivo
	M	Empregado Efetivo
	M	Empregado Efetivo
	M	Empregado Efetivo
	M	Empregado Efetivo
	M	Empregado Efetivo
	M	Empregado Efetivo
	M	Empregado Efetivo
	F	Empregado Efetivo
	M	Empregado Efetivo
	F	Empregado Efetivo
	F	Empregado Efetivo
	F	Empregado Efetivo
	F	Empregado Efetivo
	F	Empregado Efetivo
	F	Empregado Efetivo
	M	Empregado Efetivo
	F	Empregado Efetivo
	F	Empregado Efetivo
	F	Empregado Efetivo
	F	Empregado Efetivo
	F	Empregado Efetivo
	F	Empregado Efetivo
	F	Empregado Efetivo
	F	Empregado Efetivo
	F	Empregado Efetivo
	F	Empregado Efetivo
	F	Empregado Efetivo
	F	Empregado Efetivo
	F	Empregado Efetivo
	F	Empregado Efetivo
	F	Empregado Efetivo
	F	Empregado Efetivo
	F	Empregado Efetivo

	F	Empregado Efetivo
	F	Empregado Efetivo
	F	Empregado Efetivo
	F	Empregado Efetivo
	F	Empregado Efetivo
	F	Empregado Efetivo
	F	Empregado Efetivo
	M	Empregado Efetivo
	M	Empregado Efetivo
	F	Empregado Efetivo
	F	Empregado Efetivo
	M	Empregado Efetivo
	M	Empregado Efetivo
	F	Empregado Efetivo
	F	Empregado Efetivo
	F	Empregado Efetivo
	M	Empregado Efetivo
	F	Empregado Efetivo
	F	Empregado Efetivo
	F	Empregado Efetivo
	F	Empregado Efetivo
	F	Empregado Efetivo
	F	Empregado Efetivo
	F	Empregado Efetivo
	F	Empregado Efetivo
	F	Empregado Efetivo
	F	Empregado Efetivo
	F	Empregado Efetivo
	M	Empregado Efetivo
	F	Empregado Efetivo
	F	Empregado Efetivo
	F	Empregado Efetivo
	F	Empregado Efetivo
	F	Empregado Efetivo
	F	Empregado Efetivo
	F	Empregado Efetivo
	F	Empregado Efetivo
	M	Empregado Efetivo

	M	Empregado Efetivo
	M	Empregado Efetivo
	F	Empregado Efetivo
	M	Empregado Efetivo
	F	Empregado Efetivo
	M	Empregado Efetivo
	F	Empregado Efetivo
	M	Empregado Efetivo
	M	Empregado Efetivo
	F	Empregado Efetivo
	F	Empregado Efetivo
	F	Empregado Efetivo
	F	Empregado Efetivo
	F	Empregado Efetivo
	F	Empregado Efetivo
	M	Empregado Efetivo
	F	Empregado Efetivo
	M	Empregado Efetivo
	F	Empregado Efetivo
	M	Empregado Efetivo
	F	Empregado Efetivo
	M	Empregado Efetivo
	F	Empregado Efetivo
	F	Empregado Efetivo
	M	Empregado Efetivo
	F	Empregado Efetivo
	M	Empregado Efetivo
	F	Empregado Efetivo
	F	Empregado Efetivo
	M	Empregado Efetivo
	F	Empregado Efetivo
	F	Empregado Efetivo
	F	Empregado Efetivo
	M	Empregado Efetivo
	F	Empregado Efetivo
	M	Empregado Efetivo
	M	Empregado Efetivo
	M	Empregado Efetivo
	F	Empregado Efetivo
	F	Empregado Efetivo
	F	Empregado Efetivo
	F	Empregado Efetivo
	F	Empregado Efetivo
	F	Empregado Efetivo
	F	Empregado Efetivo
	F	Empregado Efetivo

	M	Empregado Efetivo
	M	Empregado Efetivo
	M	Empregado Efetivo
	M	Empregado Efetivo
	M	Empregado Efetivo
	M	Empregado Efetivo
	F	Empregado Efetivo
	F	Empregado Efetivo
	F	Empregado Efetivo
	F	Empregado Efetivo
	F	Empregado Efetivo
	F	Empregado Efetivo
	F	Empregado Efetivo
	F	Empregado Efetivo
	F	Empregado Efetivo
	F	Empregado Efetivo
	F	Empregado Efetivo
	F	Empregado Efetivo
	M	Empregado Efetivo
	F	Empregado Efetivo
	M	Empregado Efetivo
	F	Empregado Efetivo
	M	Empregado Efetivo
	M	Empregado Efetivo
	F	Empregado Efetivo
	F	Empregado Efetivo
	F	Empregado Efetivo
	F	Empregado Efetivo
	F	Empregado Efetivo
	F	Empregado Efetivo
	F	Empregado Efetivo
	M	Empregado Efetivo
	F	Empregado Efetivo
	F	Empregado Efetivo
	F	Empregado Efetivo
	F	Empregado Efetivo
	F	Empregado Efetivo
	F	Empregado Efetivo
	F	Empregado Efetivo
	F	Empregado Efetivo
	F	Empregado Efetivo
	M	Empregado Efetivo
	M	Empregado Efetivo
	M	Empregado Efetivo
	M	Empregado Efetivo
	F	Empregado Efetivo
	F	Empregado Efetivo
	M	Empregado Efetivo

	M	Empregado Efetivo
	F	Empregado Efetivo
	M	Empregado Efetivo
	M	Empregado Efetivo
	M	Empregado Efetivo
	F	Empregado Efetivo
	M	Empregado Efetivo
	M	Empregado Efetivo
	M	Empregado Efetivo
	M	Empregado Efetivo
	M	Empregado Efetivo
	M	Empregado Efetivo
	M	Empregado Efetivo
	F	Empregado Efetivo
	M	Empregado Efetivo
	M	Empregado Efetivo
	F	Empregado Efetivo
	M	Empregado Efetivo
	F	Empregado Efetivo
	F	Empregado Efetivo
	M	Empregado Efetivo
	M	Empregado Efetivo
	M	Empregado Efetivo
	M	Empregado Efetivo
	M	Empregado Efetivo
	F	Empregado Efetivo
	F	Empregado Efetivo
	F	Empregado Efetivo
	F	Empregado Efetivo
	F	Empregado Efetivo
	F	Empregado Efetivo
	F	Empregado Efetivo
	F	Empregado Efetivo
	F	Empregado Efetivo
	F	Empregado Efetivo
	F	Empregado Efetivo
	M	Empregado Efetivo
	F	Empregado Efetivo
	M	Empregado Efetivo
	F	Empregado Efetivo
	F	Empregado Efetivo
	F	Empregado Efetivo
	F	Empregado Efetivo
	F	Empregado Efetivo
	F	Empregado Efetivo
	F	Empregado Efetivo

	F	Empregado Efetivo
	F	Empregado Efetivo
	F	Empregado Efetivo
	F	Empregado Efetivo
	F	Empregado Efetivo
	F	Empregado Efetivo
	F	Empregado Efetivo
	F	Empregado Efetivo
	F	Empregado Efetivo
	M	Empregado Efetivo
	F	Empregado Efetivo
	M	Empregado Efetivo
	M	Empregado Efetivo
	M	Empregado Efetivo
	F	Empregado Efetivo
	M	Empregado Efetivo
	F	Empregado Efetivo
	F	Empregado Efetivo
	F	Empregado Efetivo
	F	Empregado Efetivo
	F	Empregado Efetivo
	F	Empregado Efetivo
	F	Empregado Efetivo
	F	Empregado Efetivo
	F	Empregado Efetivo
	F	Empregado Efetivo
	F	Empregado Efetivo
	F	Empregado Efetivo
	F	Empregado Efetivo
	F	Empregado Efetivo
	F	Empregado Efetivo
	F	Empregado Efetivo
	F	Empregado Efetivo
	M	Empregado Efetivo
	M	Empregado Efetivo
	F	Empregado Efetivo
	M	Empregado Efetivo
	M	Empregado Efetivo
	F	Empregado Efetivo
	F	Empregado Efetivo
	F	Empregado Efetivo
	F	Empregado Efetivo
	F	Empregado Efetivo
	M	Empregado Efetivo
	F	Empregado Efetivo

	F	Empregado Efetivo
	F	Empregado Efetivo
	F	Empregado Efetivo
	F	Empregado Efetivo
	F	Empregado Efetivo
	F	Empregado Efetivo
	F	Empregado Efetivo
	F	Empregado Efetivo
	F	Empregado Efetivo
	F	Empregado Efetivo
	M	Empregado Efetivo
	M	Empregado Efetivo
	M	Empregado Efetivo
	F	Empregado Efetivo
	M	Empregado Efetivo
	M	Empregado Efetivo
	M	Empregado Efetivo
	M	Empregado Efetivo
	F	Empregado Efetivo
	M	Empregado Efetivo
	M	Empregado Efetivo
	M	Empregado Efetivo
	F	Empregado Efetivo
	F	Empregado Efetivo
	F	Empregado Efetivo
	M	Empregado Efetivo
	F	Empregado Efetivo
	M	Empregado Efetivo
	M	Empregado Efetivo
	M	Empregado Efetivo
	F	Empregado Efetivo
	M	Empregado Efetivo
	F	Empregado Efetivo
	F	Empregado Efetivo
	F	Empregado Efetivo
	F	Empregado Efetivo
	F	Empregado Efetivo
	F	Empregado Efetivo
	F	Empregado Efetivo
	F	Empregado Efetivo
	F	Empregado Efetivo
	F	Empregado Efetivo
	F	Empregado Efetivo
	F	Empregado Efetivo
	F	Empregado Efetivo

	F	Empregado Efetivo
	F	Empregado Efetivo
	F	Empregado Efetivo
	F	Empregado Efetivo
	F	Empregado Efetivo
	F	Empregado Efetivo
	F	Empregado Efetivo
	F	Empregado Efetivo
	F	Empregado Efetivo
	F	Empregado Efetivo
	F	Empregado Efetivo
	F	Empregado Efetivo
	F	Empregado Efetivo
	F	Empregado Efetivo
	F	Empregado Efetivo
	F	Empregado Efetivo
	F	Empregado Efetivo
	F	Empregado Efetivo
	F	Empregado Efetivo
	F	Empregado Efetivo
	F	Empregado Efetivo
	F	Empregado Efetivo
	F	Empregado Efetivo
	F	Empregado Efetivo
	F	Empregado Efetivo
	F	Empregado Efetivo
	F	Empregado Efetivo
	F	Empregado Efetivo
	F	Empregado Efetivo
	F	Empregado Efetivo
	F	Empregado Efetivo
	F	Empregado Efetivo
	F	Empregado Efetivo
	F	Empregado Efetivo
	F	Empregado Efetivo
	F	Empregado Efetivo
	F	Empregado Efetivo
	F	Empregado Efetivo
	F	Empregado Efetivo
	F	Empregado Efetivo
	F	Empregado Efetivo
	F	Empregado Efetivo
	F	Empregado Efetivo
	F	Empregado Efetivo
	F	Empregado Efetivo
	F	Empregado Efetivo

	F	Empregado Efetivo
	F	Empregado Efetivo
	F	Empregado Efetivo
	F	Empregado Efetivo
	F	Empregado Efetivo
	F	Empregado Efetivo
	F	Empregado Efetivo
	F	Empregado Efetivo
	F	Empregado Efetivo
	F	Empregado Efetivo
	F	Empregado Efetivo
	F	Empregado Efetivo
	F	Empregado Efetivo
	F	Empregado Efetivo
	F	Empregado Efetivo
	F	Empregado Efetivo
	F	Empregado Efetivo
	F	Empregado Efetivo
	F	Empregado Efetivo
	F	Empregado Efetivo
	F	Empregado Efetivo
	F	Empregado Efetivo
	F	Empregado Efetivo
	F	Empregado Efetivo
	F	Empregado Efetivo
	F	Empregado Efetivo
	F	Empregado Efetivo
	F	Empregado Efetivo
	F	Empregado Efetivo
	F	Empregado Efetivo
	M	Empregado Efetivo
	M	Empregado Efetivo
	M	Empregado Efetivo
	M	Empregado Efetivo
	M	Empregado Efetivo
	M	Empregado Efetivo
	M	Empregado Efetivo
	M	Empregado Efetivo
	M	Empregado Efetivo
	M	Empregado Efetivo
	F	Empregado Efetivo
	F	Empregado Efetivo

	M	Empregado Efetivo
	M	Empregado Efetivo
	M	Cedido - RJU
	F	Empregado Efetivo
	F	Empregado Efetivo
	F	Empregado Efetivo
	M	Empregado Efetivo
	F	Empregado Efetivo
	M	Empregado Efetivo
	F	Empregado Efetivo
	M	Empregado Efetivo
	M	Empregado Efetivo
	M	Empregado Efetivo
	M	Empregado Efetivo
	M	Empregado Efetivo
	F	Empregado Efetivo
	F	Empregado Efetivo
	M	Empregado Efetivo
	M	Empregado Efetivo
	M	Empregado Efetivo
	M	Empregado Efetivo
	F	Empregado Efetivo
	M	Empregado Efetivo
	M	Empregado Efetivo
	F	Empregado Efetivo
	F	Empregado Efetivo
	F	Empregado Efetivo
	F	Empregado Efetivo
	M	Cedido - RJU
	F	Empregado Efetivo
	F	Empregado Efetivo
	M	Empregado Efetivo
	M	Empregado Efetivo
	F	Empregado Efetivo
	F	Empregado Efetivo
	F	Empregado Efetivo
	F	Empregado Efetivo
	F	Empregado Efetivo
	F	Empregado Efetivo
	F	Empregado Efetivo
	F	Empregado Efetivo
	F	Empregado Efetivo
	F	Empregado Efetivo
	F	Empregado Efetivo
	F	Empregado Efetivo

	M	Empregado Efetivo
	M	Empregado Efetivo
	M	Cedido - RJU
	F	Cedido - RJU
	F	Cedido - RJU
	F	Cedido - RJU
	F	Cedido - RJU
	F	Cedido - RJU
	F	Cedido - RJU
	F	Cedido - RJU
	F	Empregado Efetivo
	F	Empregado Efetivo
	F	Empregado Efetivo
	F	Empregado Efetivo
	F	Empregado Efetivo
	F	Comissionado
	F	Empregado Efetivo
	F	Empregado Efetivo
	F	Empregado Efetivo
	F	Empregado Efetivo
	F	Empregado Efetivo
	F	Empregado Efetivo
	F	Empregado Efetivo
	F	Empregado Efetivo
	F	Empregado Efetivo
	F	Empregado Efetivo
	F	Empregado Efetivo
	F	Empregado Efetivo
	F	Empregado Efetivo
	F	Empregado Efetivo
	F	Empregado Efetivo
	M	Empregado Efetivo
	F	Empregado Efetivo
	F	Empregado Efetivo
	F	Empregado Efetivo
	F	Empregado Efetivo
	F	Comissionado
	F	Empregado Efetivo

	F	Empregado Efetivo
	F	Empregado Efetivo
	F	Empregado Efetivo
	F	Empregado Efetivo
	F	Empregado Efetivo
	F	Empregado Efetivo
	F	Empregado Efetivo
	F	Empregado Efetivo
	F	Empregado Efetivo
	F	Empregado Efetivo
	F	Empregado Efetivo
	F	Empregado Efetivo
	F	Empregado Efetivo
	F	Empregado Efetivo
	F	Empregado Efetivo
	F	Empregado Efetivo
	F	Empregado Efetivo
	F	Empregado Efetivo
	M	Empregado Efetivo
	M	Empregado Efetivo
	M	Empregado Efetivo
	M	Empregado Efetivo
	M	Empregado Efetivo
	M	Empregado Efetivo
	M	Empregado Efetivo
	F	Empregado Efetivo
	F	Empregado Efetivo
	F	Empregado Efetivo
	M	Empregado Efetivo
	F	Empregado Efetivo
	M	Empregado Efetivo
	F	Empregado Efetivo
	F	Empregado Efetivo
	F	Empregado Efetivo
	F	Empregado Efetivo
	F	Empregado Efetivo

	F	Empregado Efetivo
	F	Empregado Efetivo
	F	Empregado Efetivo
	F	Empregado Efetivo
	F	Empregado Efetivo
	F	Empregado Efetivo
	F	Empregado Efetivo
	F	Empregado Efetivo
	M	Empregado Efetivo
	F	Empregado Efetivo
	F	Empregado Efetivo
	M	Empregado Efetivo
	F	Empregado Efetivo
	F	Empregado Efetivo
	F	Empregado Efetivo
	M	Empregado Efetivo
	F	Empregado Efetivo
	M	Empregado Efetivo
	M	Empregado Efetivo
	M	Empregado Efetivo
	F	Empregado Efetivo
	F	Empregado Efetivo
	M	Empregado Efetivo
	F	Empregado Efetivo
	M	Empregado Efetivo
	M	Empregado Efetivo
	F	Empregado Efetivo
	F	Empregado Efetivo
	F	Empregado Efetivo
	F	Empregado Efetivo
	F	Empregado Efetivo
	F	Empregado Efetivo

	F	Empregado Efetivo
	F	Empregado Efetivo
	F	Empregado Efetivo
	M	Empregado Efetivo
	F	Empregado Efetivo
	F	Empregado Efetivo
	F	Empregado Efetivo
	F	Empregado Efetivo
	F	Empregado Efetivo
	F	Empregado Efetivo
	F	Empregado Efetivo
	F	Empregado Efetivo
	F	Empregado Efetivo
	F	Empregado Efetivo
	F	Empregado Efetivo
	F	Empregado Efetivo
	F	Empregado Efetivo
	F	Empregado Efetivo
	F	Empregado Efetivo
	F	Empregado Efetivo
	F	Empregado Efetivo
	F	Empregado Efetivo
	F	Empregado Efetivo
	F	Empregado Efetivo
	F	Empregado Efetivo
	F	Empregado Efetivo
	F	Empregado Efetivo
	M	Empregado Efetivo
	M	Empregado Efetivo
	M	Empregado Efetivo
	F	Empregado Efetivo
	M	Empregado Efetivo
	M	Empregado Efetivo
	M	Empregado Efetivo
	M	Empregado Efetivo
	F	Empregado Efetivo
	F	Empregado Efetivo
	F	Empregado Efetivo
	M	Empregado Efetivo

	F	Empregado Efetivo
	F	Empregado Efetivo
	F	Empregado Efetivo
	F	Empregado Efetivo
	F	Empregado Efetivo
	M	Empregado Efetivo
	M	Empregado Efetivo
	M	Empregado Efetivo
	F	Empregado Efetivo
	F	Empregado Efetivo
	F	Empregado Efetivo
	F	Empregado Efetivo
	F	Empregado Efetivo
	F	Empregado Efetivo
	F	Empregado Efetivo
	F	Empregado Efetivo
	F	Empregado Efetivo
	F	Empregado Efetivo
	M	Empregado Efetivo
	M	Empregado Efetivo
	M	Empregado Efetivo
	M	Empregado Efetivo
	M	Empregado Efetivo
	M	Empregado Efetivo
	M	Empregado Efetivo
	M	Empregado Efetivo
	M	Empregado Efetivo
	F	Empregado Efetivo
	F	Empregado Efetivo
	F	Empregado Efetivo
	F	Empregado Efetivo
	M	Empregado Efetivo
	M	Empregado Efetivo
	F	Empregado Efetivo
	M	Empregado Efetivo
	M	Empregado Efetivo
	M	Empregado Efetivo
	F	Empregado Efetivo
	F	Empregado Efetivo
	F	Empregado Efetivo
	F	Empregado Efetivo

	F	Empregado Efetivo
	F	Empregado Efetivo
	F	Empregado Efetivo
	F	Empregado Efetivo
	F	Empregado Efetivo
	F	Empregado Efetivo
	F	Empregado Efetivo
	F	Empregado Efetivo
	M	Empregado Efetivo
	F	Empregado Efetivo
	F	Empregado Efetivo
	F	Empregado Efetivo
	F	Empregado Efetivo
	F	Empregado Efetivo
	F	Empregado Efetivo
	F	Empregado Efetivo
	F	Empregado Efetivo
	F	Empregado Efetivo
	F	Empregado Efetivo
	F	Empregado Efetivo
	F	Empregado Efetivo
	F	Empregado Efetivo
	F	Empregado Efetivo
	F	Empregado Efetivo
	F	Empregado Efetivo
	F	Empregado Efetivo
	F	Empregado Efetivo
	F	Empregado Efetivo
	F	Empregado Efetivo
	F	Empregado Efetivo
	F	Empregado Efetivo
	M	Empregado Efetivo
	F	Empregado Efetivo
	M	Empregado Efetivo
	F	Empregado Efetivo
	F	Empregado Efetivo
	F	Empregado Efetivo
	F	Empregado Efetivo
	F	Empregado Efetivo
	M	Empregado Efetivo
	F	Empregado Efetivo
	F	Empregado Efetivo
	F	Empregado Efetivo
	F	Empregado Efetivo
	F	Empregado Efetivo
	F	Empregado Efetivo

	F	Empregado Efetivo
	F	Empregado Efetivo
	F	Empregado Efetivo
	M	Empregado Efetivo
	M	Empregado Efetivo
	F	Empregado Efetivo
	F	Empregado Efetivo
	M	Empregado Efetivo
	F	Empregado Efetivo
	M	Empregado Efetivo
	F	Empregado Efetivo
	F	Empregado Efetivo
	F	Empregado Efetivo
	M	Empregado Efetivo
	F	Empregado Efetivo
	M	Empregado Efetivo
	F	Empregado Efetivo
	F	Empregado Efetivo
	F	Empregado Efetivo
	F	Empregado Efetivo
	F	Empregado Efetivo
	F	Empregado Efetivo
	F	Empregado Efetivo
	F	Empregado Efetivo
	M	Empregado Efetivo
	F	Empregado Efetivo
	F	Empregado Efetivo
	M	Empregado Efetivo
	F	Empregado Efetivo
	F	Empregado Efetivo
	M	Empregado Efetivo
	F	Empregado Efetivo
	F	Empregado Efetivo
	F	Empregado Efetivo
	F	Empregado Efetivo
	F	Empregado Efetivo
	F	Empregado Efetivo
	F	Empregado Efetivo

	M	Empregado Efetivo
	F	Empregado Efetivo
	F	Empregado Efetivo
	F	Empregado Efetivo
	F	Empregado Efetivo
	F	Empregado Efetivo
	F	Empregado Efetivo
	F	Empregado Efetivo
	F	Empregado Efetivo
	F	Empregado Efetivo
	F	Empregado Efetivo
	F	Empregado Efetivo
	F	Empregado Efetivo
	F	Empregado Efetivo
	F	Empregado Efetivo
	M	Empregado Efetivo
	F	Empregado Efetivo
	F	Empregado Efetivo
	F	Empregado Efetivo
	F	Empregado Efetivo
	F	Empregado Efetivo
	F	Empregado Efetivo
	F	Empregado Efetivo
	F	Empregado Efetivo
	F	Empregado Efetivo
	F	Empregado Efetivo
	F	Empregado Efetivo
	F	Empregado Efetivo
	F	Empregado Efetivo
	F	Empregado Efetivo
	F	Empregado Efetivo
	F	Empregado Efetivo

	F	Empregado Efetivo
	F	Empregado Efetivo
	F	Empregado Efetivo
	F	Empregado Efetivo
	F	Empregado Efetivo
	F	Empregado Efetivo
	F	Empregado Efetivo
	F	Empregado Efetivo
	F	Empregado Efetivo
	F	Empregado Efetivo
	F	Empregado Efetivo
	F	Empregado Efetivo
	F	Empregado Efetivo
	F	Empregado Efetivo
	F	Empregado Efetivo
	F	Empregado Efetivo
	F	Empregado Efetivo
	F	Empregado Efetivo
	F	Empregado Efetivo
	F	Empregado Efetivo
	F	Empregado Efetivo
	M	Empregado Efetivo
	M	Empregado Efetivo
	F	Empregado Efetivo
	F	Empregado Efetivo
	M	Empregado Efetivo
	F	Empregado Efetivo
	M	Empregado Efetivo
	F	Empregado Efetivo
	F	Empregado Efetivo
	F	Empregado Efetivo
	F	Empregado Efetivo

	F	Empregado Efetivo
	F	Empregado Efetivo
	F	Empregado Efetivo
	F	Empregado Efetivo
	F	Empregado Efetivo
	F	Empregado Efetivo
	F	Empregado Efetivo
	F	Empregado Efetivo
	F	Empregado Efetivo
	M	Empregado Efetivo
	F	Empregado Efetivo
	F	Empregado Efetivo
	M	Empregado Efetivo
	F	Empregado Efetivo
	F	Empregado Efetivo
	F	Empregado Efetivo
	M	Empregado Efetivo
	F	Empregado Efetivo
	F	Empregado Efetivo
	F	Empregado Efetivo
	F	Empregado Efetivo
	F	Empregado Efetivo
	F	Empregado Efetivo
	F	Empregado Efetivo
	F	Empregado Efetivo
	F	Empregado Efetivo
	F	Empregado Efetivo
	F	Empregado Efetivo
	F	Empregado Efetivo
	M	Empregado Efetivo
	F	Empregado Efetivo
	F	Empregado Efetivo
	F	Empregado Efetivo
	F	Cedido - RJU
	F	Empregado Efetivo
	M	Empregado Efetivo
	M	Empregado Efetivo
	M	Empregado Efetivo
	M	Empregado Efetivo
	M	Empregado Efetivo
	F	Empregado Efetivo
	F	Empregado Efetivo
	F	Empregado Efetivo
	M	Empregado Efetivo

	M	Empregado Efetivo
	F	Empregado Efetivo
	F	Empregado Efetivo
	F	Empregado Efetivo
	F	Empregado Efetivo
	F	Empregado Efetivo
	F	Empregado Efetivo
	F	Empregado Efetivo
	M	Empregado Efetivo
	F	Empregado Efetivo
	F	Empregado Efetivo
	F	Empregado Efetivo
	F	Empregado Efetivo
	F	Empregado Efetivo
	F	Empregado Efetivo
	F	Empregado Efetivo
	F	Empregado Efetivo
	F	Empregado Efetivo
	F	Empregado Efetivo
	F	Empregado Efetivo
	M	Empregado Efetivo
	F	Empregado Efetivo
	F	Empregado Efetivo
	F	Empregado Efetivo
	F	Empregado Efetivo
	F	Empregado Efetivo
	F	Empregado Efetivo
	F	Empregado Efetivo
	F	Empregado Efetivo
	M	Empregado Efetivo
	M	Empregado Efetivo
	M	Empregado Efetivo
	F	Empregado Efetivo
	F	Empregado Efetivo
	F	Empregado Efetivo
	F	Empregado Efetivo
	F	Empregado Efetivo
	M	Empregado Efetivo

	F	Empregado Efetivo
	M	Empregado Efetivo
	F	Empregado Efetivo
	M	Empregado Efetivo
	M	Empregado Efetivo
	M	Empregado Efetivo
	F	Empregado Efetivo
	F	Empregado Efetivo
	M	Empregado Efetivo
	M	Empregado Efetivo
	M	Empregado Efetivo
	F	Empregado Efetivo
	F	Empregado Efetivo
	M	Empregado Efetivo
	F	Empregado Efetivo
	F	Empregado Efetivo
	F	Empregado Efetivo
	F	Empregado Efetivo
	M	Empregado Efetivo
	F	Empregado Efetivo
	F	Empregado Efetivo
	F	Empregado Efetivo
	F	Empregado Efetivo
	F	Empregado Efetivo
	F	Empregado Efetivo
	F	Empregado Efetivo
	M	Empregado Efetivo
	M	Empregado Efetivo
	F	Empregado Efetivo
	F	Empregado Efetivo
	F	Empregado Efetivo
	F	Empregado Efetivo
	F	Empregado Efetivo
	F	Empregado Efetivo
	F	Empregado Efetivo
	F	Empregado Efetivo
	F	Empregado Efetivo
	F	Empregado Efetivo
	F	Empregado Efetivo
	F	Empregado Efetivo
	F	Empregado Efetivo
	F	Empregado Efetivo
	F	Empregado Efetivo
	F	Empregado Efetivo
	F	Empregado Efetivo

	F	Empregado Efetivo
	F	Empregado Efetivo
	F	Empregado Efetivo
	F	Empregado Efetivo
	F	Empregado Efetivo
	F	Empregado Efetivo
	F	Empregado Efetivo
	F	Empregado Efetivo
	M	Empregado Efetivo
	F	Empregado Efetivo
	F	Empregado Efetivo
	F	Empregado Efetivo
	F	Empregado Efetivo
	F	Empregado Efetivo
	F	Empregado Efetivo
	M	Empregado Efetivo
	F	Empregado Efetivo
	F	Empregado Efetivo
	F	Empregado Efetivo
	M	Empregado Efetivo
	M	Empregado Efetivo
	F	Empregado Efetivo
	F	Empregado Efetivo
	F	Empregado Efetivo
	M	Empregado Efetivo
	F	Empregado Efetivo
	F	Empregado Efetivo
	F	Empregado Efetivo
	M	Empregado Efetivo
	F	Empregado Efetivo
	F	Empregado Efetivo
	M	Empregado Efetivo
	F	Empregado Efetivo
	M	Empregado Efetivo
	F	Empregado Efetivo
	F	Empregado Efetivo
	M	Empregado Efetivo
	F	Empregado Efetivo
	F	Empregado Efetivo
	F	Empregado Efetivo
	M	Empregado Efetivo
	F	Empregado Efetivo
	F	Empregado Efetivo
	M	Empregado Efetivo
	F	Empregado Efetivo
	F	Empregado Efetivo
	M	Empregado Efetivo
	F	Empregado Efetivo

	M	Empregado Efetivo
	M	Empregado Efetivo
	F	Empregado Efetivo
	F	Empregado Efetivo
	F	Empregado Efetivo
	F	Empregado Efetivo
	F	Empregado Efetivo
	F	Empregado Efetivo
	F	Empregado Efetivo
	F	Empregado Efetivo
	F	Empregado Efetivo
	F	Empregado Efetivo
	F	Empregado Efetivo
	F	Empregado Efetivo
	F	Empregado Efetivo
	F	Empregado Efetivo
	F	Empregado Efetivo
	F	Empregado Efetivo
	F	Empregado Efetivo
	F	Empregado Efetivo
	F	Empregado Efetivo
	F	Empregado Efetivo
	F	Empregado Efetivo
	F	Empregado Efetivo
	F	Empregado Efetivo
	F	Empregado Efetivo
	F	Empregado Efetivo
	F	Empregado Efetivo
	F	Empregado Efetivo
	M	Empregado Efetivo
	F	Empregado Efetivo
	F	Empregado Efetivo

	F	Empregado Efetivo
	F	Empregado Efetivo
	F	Empregado Efetivo
	M	Empregado Efetivo
	F	Empregado Efetivo
	F	Empregado Efetivo
	F	Empregado Efetivo
	F	Empregado Efetivo
	F	Empregado Efetivo
	F	Empregado Efetivo
	F	Empregado Efetivo
	F	Empregado Efetivo
	F	Empregado Efetivo
	F	Empregado Efetivo
	F	Empregado Efetivo
	M	Empregado Efetivo
	F	Empregado Efetivo
	F	Empregado Efetivo
	F	Empregado Efetivo
	F	Empregado Efetivo
	F	Empregado Efetivo
	F	Empregado Efetivo
	F	Empregado Efetivo
	F	Empregado Efetivo
	F	Empregado Efetivo
	F	Empregado Efetivo
	F	Empregado Efetivo
	F	Empregado Efetivo
	F	Empregado Efetivo
	F	Empregado Efetivo
	F	Empregado Efetivo
	F	Empregado Efetivo

	F	Empregado Efetivo
	M	Empregado Efetivo
	M	Empregado Efetivo
	F	Empregado Efetivo
	F	Empregado Efetivo
	F	Empregado Efetivo
	F	Empregado Efetivo
	F	Empregado Efetivo
	F	Empregado Efetivo
	F	Empregado Efetivo
	F	Empregado Efetivo
	F	Empregado Efetivo
	F	Empregado Efetivo
	F	Empregado Efetivo
	F	Empregado Efetivo
	M	Empregado Efetivo
	M	Empregado Efetivo
	F	Empregado Efetivo
	M	Empregado Efetivo
	F	Empregado Efetivo
	F	Empregado Efetivo
	F	Empregado Efetivo
	F	Empregado Efetivo
	F	Empregado Efetivo
	M	Empregado Efetivo
	F	Empregado Efetivo
	F	Empregado Efetivo
	F	Empregado Efetivo
	F	Empregado Efetivo
	F	Empregado Efetivo
	F	Empregado Efetivo
	F	Empregado Efetivo
	F	Empregado Efetivo
	F	Empregado Efetivo
	M	Empregado Efetivo
	F	Empregado Efetivo
	M	Empregado Efetivo
	F	Empregado Efetivo
	F	Empregado Efetivo
	F	Empregado Efetivo
	F	Empregado Efetivo

	F	Empregado Efetivo
	F	Empregado Efetivo
	M	Empregado Efetivo
	F	Empregado Efetivo
	F	Empregado Efetivo
	F	Empregado Efetivo
	M	Empregado Efetivo
	F	Empregado Efetivo
	M	Empregado Efetivo
	M	Empregado Efetivo
	F	Empregado Efetivo
	F	Empregado Efetivo
	F	Empregado Efetivo
	M	Empregado Efetivo
	M	Empregado Efetivo
	M	Empregado Efetivo
	M	Empregado Efetivo
	F	Empregado Efetivo
	M	Empregado Efetivo
	M	Empregado Efetivo
	M	Empregado Efetivo
	F	Empregado Efetivo
	M	Empregado Efetivo
	M	Empregado Efetivo
	M	Empregado Efetivo
	F	Empregado Efetivo
	F	Empregado Efetivo
	M	Empregado Efetivo
	F	Empregado Efetivo
	F	Empregado Efetivo
	F	Empregado Efetivo
	M	Empregado Efetivo
	M	Empregado Efetivo
	M	Empregado Efetivo
	M	Empregado Efetivo
	F	Empregado Efetivo
	F	Empregado Efetivo

	F	Empregado Efetivo
	F	Empregado Efetivo
	F	Empregado Efetivo
	F	Empregado Efetivo
	F	Empregado Efetivo
	F	Empregado Efetivo
	M	Empregado Efetivo
	F	Empregado Efetivo
	M	Empregado Efetivo
	F	Empregado Efetivo
	M	Empregado Efetivo
	M	Empregado Efetivo
	F	Empregado Efetivo
	M	Empregado Efetivo
	M	Empregado Efetivo
	F	Empregado Efetivo
	F	Empregado Efetivo
	M	Empregado Efetivo
	F	Empregado Efetivo
	M	Empregado Efetivo
	F	Empregado Efetivo
	M	Empregado Efetivo
	F	Empregado Efetivo
	F	Empregado Efetivo
	F	Empregado Efetivo
	F	Empregado Efetivo
	F	Empregado Efetivo
	M	Empregado Efetivo
	M	Empregado Efetivo
	F	Empregado Efetivo
	M	Empregado Efetivo
	F	Empregado Efetivo
	F	Empregado Efetivo
	F	Empregado Efetivo
	F	Empregado Efetivo
	F	Empregado Efetivo
	F	Empregado Efetivo
	F	Empregado Efetivo
	F	Empregado Efetivo
	F	Empregado Efetivo
	M	Empregado Efetivo
	F	Empregado Efetivo
	M	Empregado Efetivo
	F	Empregado Efetivo
	F	Empregado Efetivo
	F	Empregado Efetivo
	F	Empregado Efetivo

	F	Empregado Efetivo
	F	Empregado Efetivo
	F	Empregado Efetivo
	F	Empregado Efetivo
	F	Empregado Efetivo
	M	Empregado Efetivo
	F	Empregado Efetivo
	F	Empregado Efetivo
	M	Empregado Efetivo
	F	Empregado Efetivo
	M	Empregado Efetivo
	F	Empregado Efetivo
	M	Empregado Efetivo
	F	Empregado Efetivo
	M	Empregado Efetivo
	F	Empregado Efetivo
	F	Empregado Efetivo
	M	Empregado Efetivo
	F	Empregado Efetivo
	F	Empregado Efetivo
	F	Empregado Efetivo
	F	Empregado Efetivo
	F	Empregado Efetivo
	M	Empregado Efetivo
	F	Empregado Efetivo
	F	Empregado Efetivo
	F	Empregado Efetivo
	F	Empregado Efetivo
	F	Empregado Efetivo
	F	Empregado Efetivo
	F	Empregado Efetivo
	F	Empregado Efetivo
	F	Empregado Efetivo
	F	Empregado Efetivo
	F	Empregado Efetivo
	F	Empregado Efetivo
	F	Empregado Efetivo
	F	Empregado Efetivo
	F	Empregado Efetivo
	M	Empregado Efetivo
	F	Empregado Efetivo
	F	Empregado Efetivo
	M	Empregado Efetivo
	M	Empregado Efetivo
	F	Empregado Efetivo
	F	Empregado Efetivo
	M	Empregado Efetivo
	F	Empregado Efetivo

	F	Empregado Efetivo
	F	Empregado Efetivo
	F	Empregado Efetivo
	F	Empregado Efetivo
	F	Empregado Efetivo
	F	Empregado Efetivo
	F	Empregado Efetivo
	F	Empregado Efetivo
	F	Empregado Efetivo
	F	Empregado Efetivo
	M	Empregado Efetivo
	M	Empregado Efetivo
	F	Empregado Efetivo
	M	Empregado Efetivo
	F	Empregado Efetivo
	F	Empregado Efetivo
	M	Empregado Efetivo
	F	Empregado Efetivo
	F	Empregado Efetivo
	F	Empregado Efetivo
	M	Empregado Efetivo
	F	Empregado Efetivo
	F	Empregado Efetivo
	F	Empregado Efetivo
	F	Empregado Efetivo
	F	Empregado Efetivo
	M	Empregado Efetivo
	F	Empregado Efetivo
	M	Empregado Efetivo
	F	Empregado Efetivo
	F	Empregado Efetivo
	F	Empregado Efetivo
	M	Empregado Efetivo
	F	Empregado Efetivo
	M	Empregado Efetivo
	F	Empregado Efetivo
	F	Empregado Efetivo
	M	Empregado Efetivo
	F	Empregado Efetivo
	F	Empregado Efetivo
	M	Empregado Efetivo
	F	Empregado Efetivo
	F	Empregado Efetivo
	F	Empregado Efetivo
	F	Empregado Efetivo
	M	Empregado Efetivo
	M	Empregado Efetivo
	F	Empregado Efetivo

	F	Empregado Efetivo
	F	Empregado Efetivo
	F	Empregado Efetivo
	F	Empregado Efetivo
	F	Empregado Efetivo
	F	Empregado Efetivo
	F	Empregado Efetivo
	F	Empregado Efetivo
	F	Empregado Efetivo
	F	Empregado Efetivo
	M	Empregado Efetivo
	F	Empregado Efetivo
	F	Empregado Efetivo
	M	Empregado Efetivo
	F	Empregado Efetivo
	F	Empregado Efetivo
	F	Empregado Efetivo
	F	Empregado Efetivo
	F	Empregado Efetivo
	F	Empregado Efetivo
	F	Empregado Efetivo
	F	Empregado Efetivo
	F	Empregado Efetivo
	F	Empregado Efetivo
	F	Empregado Efetivo
	F	Empregado Efetivo
	F	Empregado Efetivo
	F	Empregado Efetivo
	F	Empregado Efetivo
	F	Empregado Efetivo
	F	Empregado Efetivo
	F	Empregado Efetivo
	M	Empregado Efetivo
	F	Empregado Efetivo
	F	Empregado Efetivo
	F	Empregado Efetivo
	F	Empregado Efetivo
	F	Empregado Efetivo
	F	Empregado Efetivo
	F	Empregado Efetivo
	F	Empregado Efetivo
	F	Empregado Efetivo
	F	Empregado Efetivo
	F	Empregado Efetivo
	F	Empregado Efetivo
	F	Empregado Efetivo
	F	Empregado Efetivo
	F	Empregado Efetivo

	F	Empregado Efetivo
	F	Empregado Efetivo
	F	Empregado Efetivo
	F	Empregado Efetivo
	F	Empregado Efetivo
	F	Empregado Efetivo
	M	Empregado Efetivo
	F	Empregado Efetivo
	F	Empregado Efetivo
	M	Empregado Efetivo
	F	Empregado Efetivo
	F	Empregado Efetivo
	M	Empregado Efetivo
	F	Empregado Efetivo
	F	Empregado Efetivo
	F	Empregado Efetivo
	F	Empregado Efetivo
	M	Empregado Efetivo
	F	Empregado Efetivo
	F	Empregado Efetivo
	F	Empregado Efetivo
	M	Empregado Efetivo
	F	Empregado Efetivo
	M	Empregado Efetivo
	M	Empregado Efetivo
	M	Empregado Efetivo
	M	Empregado Efetivo
	F	Empregado Efetivo
	F	Empregado Efetivo
	F	Empregado Efetivo
	M	Empregado Efetivo
	F	Empregado Efetivo
	F	Empregado Efetivo
	M	Empregado Efetivo
	F	Empregado Efetivo
	F	Empregado Efetivo
	F	Empregado Efetivo
	F	Empregado Efetivo
	F	Empregado Efetivo
	F	Empregado Efetivo
	F	Empregado Efetivo
	F	Empregado Efetivo
	F	Empregado Efetivo
	M	Empregado Efetivo
	F	Empregado Efetivo
	F	Empregado Efetivo
	F	Empregado Efetivo
	F	Empregado Efetivo

	F	Empregado Efetivo
	F	Empregado Efetivo
	F	Empregado Efetivo
	F	Empregado Efetivo
	F	Empregado Efetivo
	F	Empregado Efetivo
	M	Empregado Efetivo
	F	Empregado Efetivo
	F	Empregado Efetivo
	F	Empregado Efetivo
	F	Empregado Efetivo
	F	Empregado Efetivo
	F	Empregado Efetivo
	F	Empregado Efetivo
	F	Empregado Efetivo
	F	Empregado Efetivo
	F	Empregado Efetivo
	F	Empregado Efetivo
	M	Empregado Efetivo
	F	Empregado Efetivo
	M	Empregado Efetivo
	F	Empregado Efetivo
	F	Empregado Efetivo
	F	Empregado Efetivo
	F	Empregado Efetivo
	F	Empregado Efetivo
	F	Empregado Efetivo
	F	Empregado Efetivo
	M	Empregado Efetivo
	M	Empregado Efetivo
	F	Empregado Efetivo
	F	Empregado Efetivo
	F	Empregado Efetivo
	M	Empregado Efetivo
	M	Empregado Efetivo
	F	Empregado Efetivo
	F	Empregado Efetivo
	M	Empregado Efetivo
	M	Empregado Efetivo
	F	Empregado Efetivo
	F	Empregado Efetivo
	M	Empregado Efetivo
	M	Empregado Efetivo
	M	Empregado Efetivo
	F	Empregado Efetivo
	M	Empregado Efetivo
	M	Empregado Efetivo
	M	Empregado Efetivo

	M	Empregado Efetivo
	F	Empregado Efetivo
	F	Empregado Efetivo
	F	Empregado Efetivo
	F	Empregado Efetivo
	F	Empregado Efetivo
	M	Empregado Efetivo
	F	Empregado Efetivo
	F	Empregado Efetivo
	M	Empregado Efetivo
	M	Empregado Efetivo
	F	Empregado Efetivo
	M	Empregado Efetivo
	F	Empregado Efetivo
	F	Empregado Efetivo
	F	Empregado Efetivo
	F	Empregado Efetivo
	F	Empregado Efetivo
	F	Empregado Efetivo
	F	Empregado Efetivo
	F	Empregado Efetivo
	M	Empregado Efetivo
	F	Empregado Efetivo
	F	Empregado Efetivo
	F	Empregado Efetivo
	F	Empregado Efetivo
	F	Empregado Efetivo
	F	Empregado Efetivo
	F	Empregado Efetivo
	F	Empregado Efetivo
	F	Empregado Efetivo
	F	Empregado Efetivo
	F	Empregado Efetivo
	F	Empregado Efetivo
	F	Empregado Efetivo
	F	Empregado Efetivo
	M	Empregado Efetivo
	F	Empregado Efetivo
	F	Empregado Efetivo
	F	Empregado Efetivo

	F	Empregado Efetivo
	F	Empregado Efetivo
	F	Empregado Efetivo
	F	Empregado Efetivo
	F	Empregado Efetivo
	F	Empregado Efetivo
	F	Empregado Efetivo
	F	Empregado Efetivo
	M	Empregado Efetivo
	F	Empregado Efetivo
	F	Empregado Efetivo
	F	Empregado Efetivo
	F	Empregado Efetivo
	F	Empregado Efetivo
	F	Empregado Efetivo
	F	Empregado Efetivo
	F	Empregado Efetivo
	F	Empregado Efetivo
	F	Empregado Efetivo
	F	Empregado Efetivo
	F	Empregado Efetivo
	F	Empregado Efetivo
	F	Empregado Efetivo
	F	Empregado Efetivo
	F	Empregado Efetivo
	F	Empregado Efetivo
	F	Empregado Efetivo
	F	Empregado Efetivo
	F	Empregado Efetivo
	F	Empregado Efetivo
	F	Empregado Efetivo
	F	Empregado Efetivo

	F	Empregado Efetivo
	F	Empregado Efetivo
	F	Empregado Efetivo
	M	Empregado Efetivo
	F	Empregado Efetivo
	F	Empregado Efetivo
	F	Empregado Efetivo
	F	Empregado Efetivo
	F	Empregado Efetivo
	F	Empregado Efetivo
	F	Empregado Efetivo
	F	Empregado Efetivo
	F	Empregado Efetivo
	F	Empregado Efetivo
	F	Empregado Efetivo
	F	Empregado Efetivo
	F	Empregado Efetivo
	F	Empregado Efetivo
	F	Empregado Efetivo
	F	Empregado Efetivo
	F	Empregado Efetivo
	F	Empregado Efetivo
	F	Empregado Efetivo
	F	Empregado Efetivo
	F	Empregado Efetivo
	F	Empregado Efetivo
	F	Empregado Efetivo
	M	Empregado Efetivo
	F	Empregado Efetivo
	F	Empregado Efetivo
	F	Empregado Efetivo
	F	Empregado Efetivo

	F	Empregado Efetivo
	F	Empregado Efetivo
	F	Empregado Efetivo
	F	Empregado Efetivo
	F	Empregado Efetivo
	F	Empregado Efetivo
	F	Empregado Efetivo
	F	Empregado Efetivo
	F	Empregado Efetivo
	F	Empregado Efetivo
	F	Empregado Efetivo
	F	Empregado Efetivo
	F	Empregado Efetivo
	F	Empregado Efetivo
	M	Empregado Efetivo
	F	Empregado Efetivo
	F	Empregado Efetivo
	F	Empregado Efetivo
	F	Empregado Efetivo
	F	Empregado Efetivo
	F	Empregado Efetivo
	F	Empregado Efetivo
	F	Empregado Efetivo
	F	Empregado Efetivo
	F	Empregado Efetivo
	F	Empregado Efetivo
	F	Empregado Efetivo
	F	Empregado Efetivo
	F	Empregado Efetivo
	F	Empregado Efetivo
	F	Empregado Efetivo
	F	Empregado Efetivo

	F	Empregado Efetivo
	F	Empregado Efetivo
	F	Empregado Efetivo
	F	Empregado Efetivo
	F	Empregado Efetivo
	F	Empregado Efetivo
	F	Empregado Efetivo
	F	Empregado Efetivo
	F	Empregado Efetivo
	F	Empregado Efetivo
	F	Empregado Efetivo
	F	Empregado Efetivo
	F	Empregado Efetivo
	F	Empregado Efetivo
	F	Empregado Efetivo
	F	Empregado Efetivo
	F	Empregado Efetivo
	F	Empregado Efetivo
	M	Empregado Efetivo
	F	Empregado Efetivo
	F	Empregado Efetivo
	F	Empregado Efetivo
	F	Empregado Efetivo
	F	Empregado Efetivo
	F	Empregado Efetivo
	F	Empregado Efetivo
	F	Empregado Efetivo
	F	Empregado Efetivo
	F	Empregado Efetivo
	M	Empregado Efetivo
	F	Empregado Efetivo
	F	Empregado Efetivo

	F	Empregado Efetivo
	F	Empregado Efetivo
	F	Empregado Efetivo
	F	Empregado Efetivo
	F	Empregado Efetivo
	F	Empregado Efetivo
	F	Empregado Efetivo
	M	Empregado Efetivo
	F	Empregado Efetivo
	F	Empregado Efetivo
	F	Empregado Efetivo
	F	Empregado Efetivo
	F	Empregado Efetivo
	M	Empregado Efetivo
	F	Empregado Efetivo
	F	Empregado Efetivo
	F	Empregado Efetivo
	F	Empregado Efetivo
	F	Empregado Efetivo
	M	Empregado Efetivo
	F	Empregado Efetivo
	F	Empregado Efetivo
	F	Empregado Efetivo
	F	Empregado Efetivo
	F	Empregado Efetivo
	F	Empregado Efetivo
	M	Empregado Efetivo
	F	Empregado Efetivo
	F	Empregado Efetivo
	F	Empregado Efetivo
	F	Empregado Efetivo
	M	Empregado Efetivo

	F	Empregado Efetivo
	F	Empregado Efetivo
	F	Empregado Efetivo
	F	Empregado Efetivo
	F	Empregado Efetivo
	F	Empregado Efetivo
	M	Empregado Efetivo
	F	Empregado Efetivo
	F	Empregado Efetivo
	F	Empregado Efetivo
	F	Empregado Efetivo
	F	Empregado Efetivo
	F	Empregado Efetivo
	F	Empregado Efetivo
	F	Empregado Efetivo
	F	Empregado Efetivo
	F	Empregado Efetivo
	F	Empregado Efetivo
	F	Empregado Efetivo
	F	Empregado Efetivo
	M	Empregado Efetivo
	F	Empregado Efetivo
	F	Empregado Efetivo
	F	Empregado Efetivo
	F	Empregado Efetivo
	F	Empregado Efetivo
	M	Empregado Efetivo
	F	Empregado Efetivo
	F	Empregado Efetivo
	M	Empregado Efetivo
	F	Empregado Efetivo
	F	Empregado Efetivo

	F	Empregado Efetivo
	F	Empregado Efetivo
	F	Empregado Efetivo
	F	Empregado Efetivo
	F	Empregado Efetivo
	F	Empregado Efetivo
	F	Empregado Efetivo
	F	Empregado Efetivo
	F	Empregado Efetivo
	F	Empregado Efetivo
	F	Empregado Efetivo
	F	Empregado Efetivo
	F	Empregado Efetivo
	F	Empregado Efetivo
	F	Empregado Efetivo
	F	Empregado Efetivo
	F	Empregado Efetivo
	F	Empregado Efetivo
	F	Empregado Efetivo
	F	Empregado Efetivo
	F	Empregado Efetivo
	F	Empregado Efetivo
	F	Empregado Efetivo
	F	Empregado Efetivo
	F	Empregado Efetivo
	F	Empregado Efetivo
	F	Empregado Efetivo
	F	Empregado Efetivo
	F	Empregado Efetivo
	F	Empregado Efetivo
	F	Empregado Efetivo
	F	Empregado Efetivo
	F	Empregado Efetivo

	F	Empregado Efetivo
	F	Empregado Efetivo
	F	Empregado Efetivo
	M	Empregado Efetivo
	M	Empregado Efetivo
	M	Empregado Efetivo
	M	Empregado Efetivo
	M	Empregado Efetivo
	M	Empregado Efetivo
	M	Empregado Efetivo
	M	Empregado Efetivo
	M	Empregado Efetivo
	M	Empregado Efetivo
	F	Empregado Efetivo
	F	Empregado Efetivo
	F	Empregado Efetivo
	F	Empregado Efetivo
	F	Empregado Efetivo
	M	Empregado Efetivo
	M	Empregado Efetivo
	F	Empregado Efetivo
	F	Empregado Efetivo
	F	Empregado Efetivo
	M	Empregado Efetivo
	F	Empregado Efetivo
	M	Empregado Efetivo
	F	Empregado Efetivo
	F	Empregado Efetivo
	M	Empregado Efetivo
	M	Empregado Efetivo
	F	Empregado Efetivo
	F	Empregado Efetivo
	F	Empregado Efetivo
	F	Empregado Efetivo
	F	Empregado Efetivo
	M	Empregado Efetivo
	F	Empregado Efetivo
	F	Empregado Efetivo
	M	Empregado Efetivo
	F	Empregado Efetivo
	F	Empregado Efetivo
	F	Empregado Efetivo
	M	Empregado Efetivo
	F	Empregado Efetivo
	F	Empregado Efetivo
	M	Empregado Efetivo
	F	Empregado Efetivo
	M	Empregado Efetivo

	F	Empregado Efetivo
	M	Empregado Efetivo
	M	Empregado Efetivo
	F	Empregado Efetivo
	M	Empregado Efetivo
	M	Empregado Efetivo
	F	Empregado Efetivo
	F	Empregado Efetivo
	F	Empregado Efetivo
	M	Empregado Efetivo
	F	Empregado Efetivo
	F	Empregado Efetivo
	F	Empregado Efetivo
	M	Empregado Efetivo
	M	Empregado Efetivo
	M	Empregado Efetivo
	M	Empregado Efetivo
	M	Empregado Efetivo
	F	Empregado Efetivo
	F	Empregado Efetivo
	F	Empregado Efetivo
	M	Empregado Efetivo
	M	Empregado Efetivo
	M	Empregado Efetivo
	F	Empregado Efetivo
	M	Empregado Efetivo
	F	Empregado Efetivo
	F	Empregado Efetivo
	F	Empregado Efetivo
	F	Empregado Efetivo
	M	Empregado Efetivo
	F	Empregado Efetivo
	F	Empregado Efetivo
	F	Empregado Efetivo
	F	Empregado Efetivo
	M	Empregado Efetivo
	F	Empregado Efetivo
	F	Empregado Efetivo
	M	Empregado Efetivo
	F	Empregado Efetivo
	F	Empregado Efetivo
	F	Empregado Efetivo
	F	Empregado Efetivo
	F	Empregado Efetivo

	F	Empregado Efetivo
	F	Empregado Efetivo
	F	Empregado Efetivo
	F	Empregado Efetivo
	F	Empregado Efetivo
	F	Empregado Efetivo
	F	Empregado Efetivo
	F	Empregado Efetivo
	M	Empregado Efetivo
	M	Empregado Efetivo
	M	Empregado Efetivo
	F	Empregado Efetivo
	M	Empregado Efetivo
	M	Empregado Efetivo
	M	Empregado Efetivo
	M	Empregado Efetivo
	M	Empregado Efetivo
	F	Empregado Efetivo
	F	Empregado Efetivo
	M	Empregado Efetivo
	F	Empregado Efetivo
	F	Empregado Efetivo
	F	Empregado Efetivo
	F	Empregado Efetivo
	F	Cedido - RJU
	F	Cedido - RJU
	M	Cedido - RJU
	F	Empregado Efetivo
	F	Empregado Efetivo
	F	Empregado Efetivo
	F	Empregado Efetivo
	F	Empregado Efetivo
	F	Empregado Efetivo
	F	Empregado Efetivo
	F	Empregado Efetivo
	F	Empregado Efetivo
	F	Empregado Efetivo
	F	Empregado Efetivo
	F	Empregado Efetivo
	F	Empregado Efetivo

	F	Empregado Efetivo
	F	Empregado Efetivo
	F	Empregado Efetivo
	F	Empregado Efetivo
	F	Empregado Efetivo
	F	Empregado Efetivo
	F	Empregado Efetivo
	F	Empregado Efetivo
	F	Empregado Efetivo
	F	Empregado Efetivo
	F	Empregado Efetivo
	F	Empregado Efetivo
	F	Empregado Efetivo
	F	Empregado Efetivo
	F	Empregado Efetivo
	F	Empregado Efetivo
	F	Empregado Efetivo
	F	Empregado Efetivo
	F	Empregado Efetivo
	F	Empregado Efetivo
	F	Empregado Efetivo
	F	Empregado Efetivo
	F	Empregado Efetivo
	F	Empregado Efetivo
	F	Empregado Efetivo
	F	Empregado Efetivo
	F	Empregado Efetivo
	F	Empregado Efetivo
	F	Empregado Efetivo
	F	Empregado Efetivo
	F	Empregado Efetivo
	F	Empregado Efetivo
	F	Empregado Efetivo
	F	Empregado Efetivo
	M	Empregado Efetivo
	F	Empregado Efetivo
	F	Empregado Efetivo
	F	Empregado Efetivo
	M	Empregado Efetivo
	F	Empregado Efetivo
	F	Empregado Efetivo
	F	Empregado Efetivo
	F	Empregado Efetivo

	F	Empregado Efetivo
	F	Empregado Efetivo
	F	Empregado Efetivo
	F	Empregado Efetivo
	F	Empregado Efetivo
	M	Empregado Efetivo
	F	Empregado Efetivo
	F	Empregado Efetivo
	F	Empregado Efetivo
	F	Empregado Efetivo
	F	Empregado Efetivo
	F	Empregado Efetivo
	M	Empregado Efetivo
	F	Empregado Efetivo
	F	Empregado Efetivo
	F	Empregado Efetivo
	M	Empregado Efetivo
	F	Empregado Efetivo
	F	Empregado Efetivo
	F	Empregado Efetivo
	F	Empregado Efetivo
	F	Empregado Efetivo
	M	Empregado Efetivo
	F	Empregado Efetivo
	F	Empregado Efetivo
	F	Empregado Efetivo
	F	Empregado Efetivo
	F	Empregado Efetivo
	F	Empregado Efetivo
	F	Empregado Efetivo
	F	Empregado Efetivo
	F	Empregado Efetivo
	F	Empregado Efetivo
	F	Empregado Efetivo
	F	Empregado Efetivo

	F	Empregado Efetivo
	F	Empregado Efetivo
	F	Empregado Efetivo
	F	Empregado Efetivo
	F	Empregado Efetivo
	F	Empregado Efetivo
	F	Empregado Efetivo
	F	Empregado Efetivo
	F	Empregado Efetivo
	F	Empregado Efetivo
	F	Empregado Efetivo
	F	Empregado Efetivo
	F	Empregado Efetivo
	F	Empregado Efetivo
	F	Empregado Efetivo
	F	Empregado Efetivo
	F	Empregado Efetivo
	F	Empregado Efetivo
	F	Empregado Efetivo
	F	Empregado Efetivo
	F	Empregado Efetivo
	F	Empregado Efetivo
	F	Empregado Efetivo
	F	Empregado Efetivo
	F	Empregado Efetivo
	F	Empregado Efetivo
	F	Empregado Efetivo
	M	Empregado Efetivo
	F	Empregado Efetivo
	F	Empregado Efetivo
	F	Empregado Efetivo
	M	Empregado Efetivo
	F	Empregado Efetivo
	F	Empregado Efetivo
	M	Empregado Efetivo
	F	Empregado Efetivo
	M	Empregado Efetivo
	M	Empregado Efetivo
	M	Empregado Efetivo
	M	Empregado Efetivo
	M	Empregado Efetivo
	M	Empregado Efetivo
	M	Empregado Efetivo

	M	Empregado Efetivo
	F	Empregado Efetivo
	F	Empregado Efetivo
	M	Empregado Efetivo
	M	Empregado Efetivo
	F	Empregado Efetivo
	F	Empregado Efetivo
	F	Empregado Efetivo
	F	Empregado Efetivo
	F	Empregado Efetivo
	F	Empregado Efetivo
	F	Empregado Efetivo
	F	Empregado Efetivo
	F	Empregado Efetivo
	F	Empregado Efetivo
	F	Empregado Efetivo
	F	Empregado Efetivo
	F	Empregado Efetivo
	F	Empregado Efetivo
	F	Empregado Efetivo
	F	Empregado Efetivo
	F	Empregado Efetivo
	F	Empregado Efetivo
	F	Empregado Efetivo
	F	Empregado Efetivo
	F	Empregado Efetivo
	M	Empregado Efetivo
	F	Empregado Efetivo
	F	Empregado Efetivo
	F	Empregado Efetivo
	F	Empregado Efetivo
	F	Empregado Efetivo
	F	Empregado Efetivo
	F	Empregado Efetivo
	F	Empregado Efetivo
	F	Empregado Efetivo
	F	Empregado Efetivo
	F	Empregado Efetivo
	F	Empregado Efetivo
	F	Empregado Efetivo
	F	Empregado Efetivo
	F	Empregado Efetivo
	F	Empregado Efetivo
	F	Empregado Efetivo
	F	Empregado Efetivo
	F	Empregado Efetivo

	M	Empregado Efetivo
	F	Empregado Efetivo
	F	Empregado Efetivo
	F	Empregado Efetivo
	F	Empregado Efetivo
	F	Empregado Efetivo
	F	Empregado Efetivo
	F	Empregado Efetivo
	F	Empregado Efetivo
	F	Empregado Efetivo
	F	Empregado Efetivo
	F	Empregado Efetivo
	F	Empregado Efetivo
	F	Empregado Efetivo
	F	Empregado Efetivo
	M	Empregado Efetivo
	M	Empregado Efetivo
	M	Empregado Efetivo
	F	Empregado Efetivo
	F	Empregado Efetivo
	F	Empregado Efetivo
	F	Empregado Efetivo
	M	Empregado Efetivo
	F	Empregado Efetivo
	F	Empregado Efetivo
	F	Empregado Efetivo
	F	Empregado Efetivo
	F	Empregado Efetivo
	F	Empregado Efetivo
	F	Empregado Efetivo
	F	Empregado Efetivo
	F	Empregado Efetivo
	F	Empregado Efetivo
	F	Empregado Efetivo
	F	Empregado Efetivo
	F	Empregado Efetivo
	F	Empregado Efetivo
	F	Empregado Efetivo
	F	Empregado Efetivo
	F	Empregado Efetivo
	F	Empregado Efetivo
	F	Empregado Efetivo
	F	Empregado Efetivo
	M	Empregado Efetivo

	F	Empregado Efetivo
	F	Empregado Efetivo
	M	Empregado Efetivo
	F	Empregado Efetivo
	F	Empregado Efetivo
	M	Empregado Efetivo
	F	Empregado Efetivo
	M	Empregado Efetivo
	F	Empregado Efetivo
	M	Empregado Efetivo
	F	Empregado Efetivo
	F	Empregado Efetivo
	F	Empregado Efetivo
	F	Empregado Efetivo
	M	Empregado Efetivo
	F	Empregado Efetivo
	F	Empregado Efetivo
	M	Empregado Efetivo
	F	Empregado Efetivo
	M	Empregado Efetivo
	F	Empregado Efetivo
	F	Empregado Efetivo
	M	Empregado Efetivo
	M	Empregado Efetivo
	M	Empregado Efetivo
	F	Empregado Efetivo
	F	Empregado Efetivo
	F	Empregado Efetivo
	F	Empregado Efetivo
	M	Empregado Efetivo
	M	Empregado Efetivo
	M	Empregado Efetivo
	M	Empregado Efetivo
	F	Empregado Efetivo
	F	Empregado Efetivo
	M	Empregado Efetivo
	M	Empregado Efetivo
	M	Empregado Efetivo
	M	Empregado Efetivo
	F	Empregado Efetivo
	F	Empregado Efetivo
	M	Empregado Efetivo
	F	Empregado Efetivo
	F	Empregado Efetivo
	M	Empregado Efetivo
	F	Empregado Efetivo
	F	Empregado Efetivo
	F	Empregado Efetivo

	F	Empregado Efetivo
	F	Empregado Efetivo
	F	Empregado Efetivo
	M	Empregado Efetivo
	M	Empregado Efetivo
	M	Empregado Efetivo
	F	Empregado Efetivo
	F	Empregado Efetivo
	M	Empregado Efetivo
	M	Empregado Efetivo
	F	Empregado Efetivo
	M	Empregado Efetivo
	M	Empregado Efetivo
	M	Empregado Efetivo
	F	Empregado Efetivo
	F	Empregado Efetivo
	M	Empregado Efetivo
	F	Empregado Efetivo
	F	Empregado Efetivo
	F	Empregado Efetivo
	F	Empregado Efetivo
	F	Empregado Efetivo
	F	Empregado Efetivo
	F	Empregado Efetivo
	M	Empregado Efetivo
	F	Empregado Efetivo
	F	Empregado Efetivo
	F	Empregado Efetivo
	M	Empregado Efetivo
	F	Empregado Efetivo
	F	Empregado Efetivo
	F	Empregado Efetivo
	F	Empregado Efetivo
	F	Empregado Efetivo
	M	Empregado Efetivo
	M	Empregado Efetivo
	M	Empregado Efetivo
	M	Empregado Efetivo
	F	Empregado Efetivo
	F	Empregado Efetivo
	F	Empregado Efetivo
	F	Empregado Efetivo
	F	Empregado Efetivo
	F	Empregado Efetivo
	F	Empregado Efetivo
	F	Empregado Efetivo
	F	Empregado Efetivo

	F	Empregado Efetivo
	F	Empregado Efetivo
	M	Empregado Efetivo
	F	Empregado Efetivo
	F	Empregado Efetivo
	M	Empregado Efetivo
	F	Empregado Efetivo
	F	Empregado Efetivo
	F	Empregado Efetivo
	M	Empregado Efetivo
	M	Empregado Efetivo
	F	Empregado Efetivo
	F	Empregado Efetivo
	F	Empregado Efetivo
	F	Empregado Efetivo
	F	Empregado Efetivo
	F	Empregado Efetivo
	F	Empregado Efetivo
	F	Empregado Efetivo
	F	Empregado Efetivo
	F	Empregado Efetivo
	F	Empregado Efetivo
	F	Empregado Efetivo
	F	Empregado Efetivo
	M	Empregado Efetivo
	M	Empregado Efetivo
	F	Empregado Efetivo
	F	Empregado Efetivo
	F	Empregado Efetivo
	F	Empregado Efetivo
	F	Empregado Efetivo
	F	Empregado Efetivo
	M	Empregado Efetivo
	F	Empregado Efetivo
	F	Empregado Efetivo
	F	Empregado Efetivo
	F	Empregado Efetivo
	F	Empregado Efetivo
	M	Empregado Efetivo
	F	Empregado Efetivo
	M	Empregado Efetivo
	F	Empregado Efetivo
	F	Empregado Efetivo
	F	Empregado Efetivo
	F	Empregado Efetivo
	F	Empregado Efetivo
	M	Empregado Efetivo
	F	Empregado Efetivo

	F	Empregado Efetivo
	F	Empregado Efetivo
	F	Empregado Efetivo
	F	Empregado Efetivo
	M	Empregado Efetivo
	M	Empregado Efetivo
	F	Empregado Efetivo
	M	Empregado Efetivo
	M	Empregado Efetivo
	F	Empregado Efetivo
	M	Empregado Efetivo
	M	Empregado Efetivo
	F	Empregado Efetivo
	F	Empregado Efetivo
	F	Empregado Efetivo
	F	Empregado Efetivo
	F	Empregado Efetivo
	M	Empregado Efetivo
	F	Empregado Efetivo
	F	Empregado Efetivo
	M	Empregado Efetivo
	M	Empregado Efetivo
	M	Empregado Efetivo
	F	Empregado Efetivo
	F	Empregado Efetivo
	F	Empregado Efetivo
	F	Empregado Efetivo
	F	Empregado Efetivo
	F	Empregado Efetivo
	F	Empregado Efetivo
	F	Empregado Efetivo
	F	Empregado Efetivo
	F	Empregado Efetivo
	M	Empregado Efetivo
	F	Empregado Efetivo
	M	Empregado Efetivo
	F	Empregado Efetivo
	F	Empregado Efetivo
	M	Empregado Efetivo
	F	Empregado Efetivo
	F	Empregado Efetivo
	F	Empregado Efetivo
	F	Empregado Efetivo
	F	Empregado Efetivo
	M	Empregado Efetivo
	F	Empregado Efetivo
	F	Empregado Efetivo
	M	Empregado Efetivo

	F	Empregado Efetivo
	F	Empregado Efetivo
	F	Empregado Efetivo
	F	Empregado Efetivo
	F	Empregado Efetivo
	M	Empregado Efetivo
	F	Empregado Efetivo
	F	Empregado Efetivo
	M	Empregado Efetivo
	M	Empregado Efetivo
	F	Empregado Efetivo
	F	Empregado Efetivo
	M	Empregado Efetivo
	F	Empregado Efetivo
	F	Empregado Efetivo
	F	Empregado Efetivo
	F	Empregado Efetivo
	F	Empregado Efetivo
	F	Empregado Efetivo
	F	Empregado Efetivo
	F	Empregado Efetivo
	F	Empregado Efetivo
	F	Empregado Efetivo
	M	Empregado Efetivo
	M	Empregado Efetivo
	F	Empregado Efetivo
	F	Empregado Efetivo
	F	Empregado Efetivo
	F	Empregado Efetivo
	M	Empregado Efetivo
	F	Empregado Efetivo
	M	Empregado Efetivo
	M	Empregado Efetivo
	F	Empregado Efetivo
	M	Empregado Efetivo
	M	Empregado Efetivo
	F	Empregado Efetivo
	F	Empregado Efetivo
	F	Empregado Efetivo
	F	Empregado Efetivo
	F	Empregado Efetivo
	F	Empregado Efetivo
	F	Empregado Efetivo
	M	Empregado Efetivo
	F	Empregado Efetivo
	F	Empregado Efetivo
	F	Empregado Efetivo
	F	Empregado Efetivo

	F	Empregado Efetivo
	M	Empregado Efetivo
	M	Empregado Efetivo
	M	Empregado Efetivo
	F	Empregado Efetivo
	F	Empregado Efetivo
	F	Empregado Efetivo
	M	Empregado Efetivo
	F	Empregado Efetivo
	M	Empregado Efetivo
	F	Empregado Efetivo
	M	Empregado Efetivo
	F	Empregado Efetivo
	F	Empregado Efetivo
	F	Empregado Efetivo
	F	Empregado Efetivo
	F	Empregado Efetivo
	F	Empregado Efetivo
	F	Empregado Efetivo
	F	Empregado Efetivo
	M	Empregado Efetivo
	M	Empregado Efetivo
	F	Empregado Efetivo
	F	Empregado Efetivo
	F	Empregado Efetivo
	F	Empregado Efetivo
	F	Empregado Efetivo
	M	Empregado Efetivo
	F	Empregado Efetivo
	F	Empregado Efetivo
	F	Empregado Efetivo
	M	Empregado Efetivo
	F	Empregado Efetivo
	F	Empregado Efetivo
	M	Empregado Efetivo
	F	Empregado Efetivo
	M	Empregado Efetivo
	F	Empregado Efetivo
	F	Empregado Efetivo
	F	Empregado Efetivo
	F	Empregado Efetivo
	M	Empregado Efetivo
	M	Empregado Efetivo
	F	Empregado Efetivo
	F	Empregado Efetivo
	F	Empregado Efetivo
	M	Empregado Efetivo
	F	Empregado Efetivo

	M	Empregado Efetivo
	M	Empregado Efetivo
	F	Empregado Efetivo
	M	Empregado Efetivo
	F	Empregado Efetivo
	M	Empregado Efetivo
	F	Empregado Efetivo
	M	Empregado Efetivo
	F	Empregado Efetivo
	F	Empregado Efetivo
	F	Empregado Efetivo
	F	Empregado Efetivo
	F	Empregado Efetivo
	F	Empregado Efetivo
	F	Empregado Efetivo
	F	Empregado Efetivo
	F	Empregado Efetivo
	F	Empregado Efetivo
	F	Empregado Efetivo
	F	Empregado Efetivo
	F	Empregado Efetivo
	F	Empregado Efetivo
	F	Empregado Efetivo
	F	Empregado Efetivo
	F	Empregado Efetivo
	F	Empregado Efetivo
	F	Empregado Efetivo
	F	Empregado Efetivo
	F	Empregado Efetivo
	F	Empregado Efetivo
	F	Empregado Efetivo
	F	Empregado Efetivo
	F	Empregado Efetivo
	F	Empregado Efetivo
	F	Empregado Efetivo
	F	Empregado Efetivo
	F	Empregado Efetivo
	F	Empregado Efetivo
	F	Empregado Efetivo
	F	Empregado Efetivo
	F	Empregado Efetivo
	F	Empregado Efetivo
	M	Empregado Efetivo
	M	Empregado Efetivo
	M	Empregado Efetivo
	M	Empregado Efetivo
	M	Empregado Efetivo
	M	Empregado Efetivo

	M	Empregado Efetivo
	M	Empregado Efetivo
	M	Empregado Efetivo
	M	Empregado Efetivo
	M	Empregado Efetivo
	M	Empregado Efetivo
	M	Empregado Efetivo
	F	Empregado Efetivo
	F	Empregado Efetivo
	M	Empregado Efetivo
	M	Empregado Efetivo
	F	Empregado Efetivo
	F	Empregado Efetivo
	M	Empregado Efetivo
	M	Empregado Efetivo
	F	Empregado Efetivo
	M	Empregado Efetivo
	F	Empregado Efetivo
	F	Empregado Efetivo
	M	Empregado Efetivo
	F	Empregado Efetivo
	M	Empregado Efetivo
	M	Empregado Efetivo
	M	Empregado Efetivo
	M	Empregado Efetivo
	M	Empregado Efetivo
	F	Empregado Efetivo
	F	Empregado Efetivo
	F	Empregado Efetivo
	F	Empregado Efetivo
	F	Empregado Efetivo
	F	Empregado Efetivo
	F	Empregado Efetivo
	F	Empregado Efetivo
	F	Empregado Efetivo
	F	Empregado Efetivo
	F	Empregado Efetivo
	F	Empregado Efetivo
	M	Empregado Efetivo
	M	Empregado Efetivo

	F	Empregado Efetivo
	F	Empregado Efetivo
	F	Empregado Efetivo
	F	Empregado Efetivo
	F	Empregado Efetivo
	F	Empregado Efetivo
	M	Empregado Efetivo
	F	Empregado Efetivo
	F	Empregado Efetivo
	F	Empregado Efetivo
	M	Empregado Efetivo
	F	Empregado Efetivo
	F	Empregado Efetivo
	F	Empregado Efetivo
	F	Empregado Efetivo
	F	Empregado Efetivo
	M	Empregado Efetivo
	F	Empregado Efetivo
	F	Empregado Efetivo
	M	Empregado Efetivo
	F	Empregado Efetivo
	F	Empregado Efetivo
	F	Empregado Efetivo
	F	Empregado Efetivo
	F	Empregado Efetivo
	F	Empregado Efetivo
	M	Empregado Efetivo
	M	Empregado Efetivo
	M	Empregado Efetivo
	M	Empregado Efetivo
	M	Empregado Efetivo
	M	Empregado Efetivo
	M	Empregado Efetivo
	M	Empregado Efetivo
	M	Empregado Efetivo
	F	Empregado Efetivo
	F	Empregado Efetivo
	F	Empregado Efetivo
	F	Empregado Efetivo
	F	Empregado Efetivo
	F	Empregado Efetivo
	F	Empregado Efetivo
	F	Empregado Efetivo
	F	Empregado Efetivo
	F	Empregado Efetivo
	F	Empregado Efetivo
	F	Empregado Efetivo

	F	Empregado Efetivo
	F	Empregado Efetivo
	F	Empregado Efetivo
	F	Empregado Efetivo
	F	Empregado Efetivo
	F	Empregado Efetivo
	F	Empregado Efetivo
	M	Empregado Efetivo
	F	Empregado Efetivo
	F	Empregado Efetivo
	F	Empregado Efetivo
	F	Empregado Efetivo
	F	Empregado Efetivo
	M	Empregado Efetivo
	F	Empregado Efetivo
	F	Empregado Efetivo
	F	Empregado Efetivo
	F	Empregado Efetivo
	F	Empregado Efetivo
	F	Empregado Efetivo
	F	Empregado Efetivo
	F	Empregado Efetivo
	M	Empregado Efetivo
	F	Empregado Efetivo
	F	Empregado Efetivo
	F	Empregado Efetivo
	M	Empregado Efetivo
	F	Empregado Efetivo
	F	Empregado Efetivo
	F	Empregado Efetivo
	F	Empregado Efetivo
	M	Empregado Efetivo
	F	Empregado Efetivo
	F	Empregado Efetivo
	F	Empregado Efetivo
	M	Empregado Efetivo
	F	Empregado Efetivo
	F	Empregado Efetivo
	F	Empregado Efetivo
	F	Empregado Efetivo
	F	Empregado Efetivo
	F	Empregado Efetivo
	F	Empregado Efetivo
	F	Empregado Efetivo
	F	Empregado Efetivo
	F	Empregado Efetivo
	F	Empregado Efetivo

	F	Empregado Efetivo
	F	Empregado Efetivo
	F	Empregado Efetivo
	F	Empregado Efetivo
	F	Empregado Efetivo
	F	Empregado Efetivo
	F	Empregado Efetivo
	F	Empregado Efetivo
	F	Empregado Efetivo
	F	Empregado Efetivo
	F	Empregado Efetivo
	F	Empregado Efetivo
	F	Empregado Efetivo
	F	Empregado Efetivo
	F	Empregado Efetivo
	F	Empregado Efetivo
	F	Empregado Efetivo
	F	Empregado Efetivo
	F	Empregado Efetivo
	F	Empregado Efetivo
	M	Empregado Efetivo
	F	Empregado Efetivo
	F	Empregado Efetivo
	F	Empregado Efetivo
	F	Empregado Efetivo
	F	Empregado Efetivo
	F	Empregado Efetivo
	F	Empregado Efetivo
	F	Empregado Efetivo
	F	Empregado Efetivo
	F	Empregado Efetivo
	F	Empregado Efetivo
	F	Empregado Efetivo
	F	Empregado Efetivo
	F	Empregado Efetivo
	M	Empregado Efetivo
	F	Empregado Efetivo
	F	Empregado Efetivo
	M	Empregado Efetivo
	F	Empregado Efetivo
	F	Empregado Efetivo
	M	Empregado Efetivo
	F	Empregado Efetivo
	M	Empregado Efetivo
	F	Empregado Efetivo
	F	Empregado Efetivo
	M	Empregado Efetivo
	F	Empregado Efetivo

	M	Empregado Efetivo
	F	Empregado Efetivo
	F	Empregado Efetivo
	F	Empregado Efetivo
	F	Empregado Efetivo
	F	Empregado Efetivo
	F	Empregado Efetivo
	F	Empregado Efetivo
	F	Empregado Efetivo
	F	Empregado Efetivo
	F	Empregado Efetivo
	F	Empregado Efetivo
	M	Empregado Efetivo
	F	Empregado Efetivo
	F	Empregado Efetivo
	F	Empregado Efetivo
	F	Empregado Efetivo
	F	Empregado Efetivo
	F	Empregado Efetivo
	F	Empregado Efetivo
	F	Empregado Efetivo
	F	Empregado Efetivo
	M	Empregado Efetivo
	F	Empregado Efetivo
	F	Empregado Efetivo
	F	Empregado Efetivo
	F	Empregado Efetivo
	F	Empregado Efetivo
	F	Empregado Efetivo
	F	Empregado Efetivo
	F	Empregado Efetivo
	F	Empregado Efetivo
	F	Empregado Efetivo
	F	Empregado Efetivo
	F	Empregado Efetivo
	F	Empregado Efetivo
	F	Empregado Efetivo
	M	Empregado Efetivo
	F	Empregado Efetivo
	F	Empregado Efetivo
	F	Empregado Efetivo
	F	Empregado Efetivo
	F	Empregado Efetivo
	F	Empregado Efetivo
	M	Empregado Efetivo
	F	Empregado Efetivo
	F	Empregado Efetivo
	F	Empregado Efetivo

	F	Empregado Efetivo
	F	Empregado Efetivo
	F	Empregado Efetivo
	M	Empregado Efetivo
	F	Empregado Efetivo
	F	Empregado Efetivo
	F	Empregado Efetivo
	M	Empregado Efetivo
	F	Empregado Efetivo
	F	Empregado Efetivo
	F	Empregado Efetivo
	F	Empregado Efetivo
	F	Empregado Efetivo
	F	Empregado Efetivo
	F	Empregado Efetivo
	F	Empregado Efetivo
	F	Empregado Efetivo
	F	Empregado Efetivo
	F	Empregado Efetivo
	F	Empregado Efetivo
	F	Empregado Efetivo
	F	Empregado Efetivo
	M	Empregado Efetivo
	F	Empregado Efetivo
	F	Empregado Efetivo
	F	Empregado Efetivo
	F	Empregado Efetivo
	F	Empregado Efetivo
	M	Empregado Efetivo
	F	Empregado Efetivo
	F	Empregado Efetivo
	F	Empregado Efetivo
	F	Empregado Efetivo
	F	Empregado Efetivo
	F	Empregado Efetivo
	F	Empregado Efetivo
	F	Empregado Efetivo
	F	Empregado Efetivo
	M	Empregado Efetivo
	M	Empregado Efetivo
	M	Empregado Efetivo
	F	Empregado Efetivo
	F	Empregado Efetivo
	F	Empregado Efetivo
	M	Empregado Efetivo
	F	Empregado Efetivo
	F	Empregado Efetivo
	F	Empregado Efetivo

	M	Empregado Efetivo
	F	Empregado Efetivo
	F	Empregado Efetivo
	F	Empregado Efetivo
	F	Empregado Efetivo
	F	Empregado Efetivo
	M	Empregado Efetivo
	F	Empregado Efetivo
	F	Empregado Efetivo
	F	Empregado Efetivo
	M	Empregado Efetivo
	F	Empregado Efetivo
	F	Empregado Efetivo
	F	Empregado Efetivo
	F	Empregado Efetivo
	F	Empregado Efetivo
	F	Empregado Efetivo
	F	Empregado Efetivo
	F	Empregado Efetivo
	F	Empregado Efetivo
	F	Empregado Efetivo
	F	Empregado Efetivo
	F	Empregado Efetivo
	F	Empregado Efetivo
	F	Empregado Efetivo
	F	Empregado Efetivo
	F	Empregado Efetivo
	F	Empregado Efetivo
	F	Empregado Efetivo
	F	Empregado Efetivo
	F	Empregado Efetivo
	M	Empregado Efetivo
	F	Empregado Efetivo
	F	Empregado Efetivo
	F	Empregado Efetivo
	F	Empregado Efetivo
	F	Empregado Efetivo
	M	Empregado Efetivo
	M	Empregado Efetivo
	F	Empregado Efetivo
	F	Empregado Efetivo
	F	Empregado Efetivo
	F	Empregado Efetivo
	F	Empregado Efetivo
	F	Empregado Efetivo
	F	Empregado Efetivo
	M	Empregado Efetivo
	F	Empregado Efetivo

	F	Empregado Efetivo
	F	Empregado Efetivo
	F	Empregado Efetivo
	F	Empregado Efetivo
	F	Empregado Efetivo
	F	Empregado Efetivo
	F	Empregado Efetivo
	F	Empregado Efetivo
	F	Empregado Efetivo
	F	Empregado Efetivo
	F	Empregado Efetivo
	F	Empregado Efetivo
	F	Empregado Efetivo
	M	Empregado Efetivo
	F	Empregado Efetivo
	F	Empregado Efetivo
	F	Empregado Efetivo
	F	Empregado Efetivo
	M	Empregado Efetivo
	M	Empregado Efetivo
	M	Empregado Efetivo
	F	Empregado Efetivo
	F	Empregado Efetivo
	F	Empregado Efetivo
	F	Empregado Efetivo
	F	Empregado Efetivo
	F	Empregado Efetivo
	F	Empregado Efetivo
	M	Empregado Efetivo
	F	Empregado Efetivo
	F	Empregado Efetivo
	F	Empregado Efetivo
	F	Empregado Efetivo
	M	Empregado Efetivo
	F	Empregado Efetivo
	F	Empregado Efetivo
	F	Empregado Efetivo
	F	Empregado Efetivo
	F	Empregado Efetivo
	F	Empregado Efetivo
	F	Empregado Efetivo
	F	Empregado Efetivo
	F	Empregado Efetivo
	F	Empregado Efetivo
	F	Empregado Efetivo
	F	Empregado Efetivo
	F	Empregado Efetivo
	F	Empregado Efetivo

	F	Empregado Efetivo
	F	Empregado Efetivo
	F	Empregado Efetivo
	M	Empregado Efetivo
	F	Empregado Efetivo
	F	Empregado Efetivo
	F	Empregado Efetivo
	F	Empregado Efetivo
	F	Empregado Efetivo
	F	Empregado Efetivo
	F	Empregado Efetivo
	F	Empregado Efetivo
	F	Empregado Efetivo
	F	Empregado Efetivo
	F	Empregado Efetivo
	M	Empregado Efetivo
	F	Empregado Efetivo
	F	Empregado Efetivo
	F	Empregado Efetivo
	F	Empregado Efetivo
	F	Empregado Efetivo
	M	Empregado Efetivo
	F	Empregado Efetivo
	F	Empregado Efetivo
	F	Empregado Efetivo
	F	Empregado Efetivo
	F	Empregado Efetivo
	F	Empregado Efetivo
	F	Empregado Efetivo
	F	Empregado Efetivo
	M	Empregado Efetivo
	F	Empregado Efetivo
	F	Empregado Efetivo
	M	Empregado Efetivo
	F	Empregado Efetivo
	F	Empregado Efetivo
	F	Empregado Efetivo
	F	Empregado Efetivo
	F	Empregado Efetivo
	F	Empregado Efetivo
	F	Empregado Efetivo
	M	Empregado Efetivo
	F	Empregado Efetivo
	M	Empregado Efetivo
	F	Empregado Efetivo
	F	Empregado Efetivo
	F	Empregado Efetivo
	F	Empregado Efetivo

	F	Empregado Efetivo
	F	Empregado Efetivo
	F	Empregado Efetivo
	M	Empregado Efetivo
	F	Empregado Efetivo
	F	Empregado Efetivo
	F	Empregado Efetivo
	M	Empregado Efetivo
	F	Empregado Efetivo
	F	Empregado Efetivo
	F	Empregado Efetivo
	M	Empregado Efetivo
	F	Empregado Efetivo
	F	Empregado Efetivo
	F	Empregado Efetivo
	M	Empregado Efetivo
	F	Empregado Efetivo
	F	Empregado Efetivo
	F	Empregado Efetivo
	F	Empregado Efetivo
	F	Empregado Efetivo
	M	Empregado Efetivo
	F	Empregado Efetivo
	F	Empregado Efetivo
	F	Empregado Efetivo
	F	Empregado Efetivo
	F	Empregado Efetivo
	F	Empregado Efetivo
	F	Empregado Efetivo
	M	Empregado Efetivo
	F	Empregado Efetivo
	F	Empregado Efetivo
	F	Empregado Efetivo
	F	Empregado Efetivo
	F	Empregado Efetivo
	F	Empregado Efetivo
	F	Empregado Efetivo
	F	Empregado Efetivo
	F	Empregado Efetivo
	F	Empregado Efetivo
	F	Empregado Efetivo
	M	Empregado Efetivo
	F	Empregado Efetivo
	F	Empregado Efetivo
	F	Empregado Efetivo
	F	Empregado Efetivo
	F	Empregado Efetivo
	F	Empregado Efetivo

	F	Empregado Efetivo
	F	Empregado Efetivo
	F	Empregado Efetivo
	F	Empregado Efetivo
	F	Empregado Efetivo
	F	Empregado Efetivo
	F	Empregado Efetivo
	F	Empregado Efetivo
	F	Empregado Efetivo
	M	Empregado Efetivo
	F	Empregado Efetivo
	F	Empregado Efetivo
	F	Empregado Efetivo
	M	Empregado Efetivo
	F	Empregado Efetivo
	M	Empregado Efetivo
	M	Empregado Efetivo
	F	Empregado Efetivo
	F	Empregado Efetivo
	M	Empregado Efetivo
	F	Empregado Efetivo
	F	Empregado Efetivo
	F	Empregado Efetivo
	M	Empregado Efetivo
	F	Empregado Efetivo
	M	Empregado Efetivo
	F	Empregado Efetivo
	F	Empregado Efetivo
	F	Empregado Efetivo
	F	Empregado Efetivo
	F	Empregado Efetivo
	F	Empregado Efetivo
	M	Empregado Efetivo
	F	Empregado Efetivo
	F	Empregado Efetivo
	F	Empregado Efetivo
	F	Empregado Efetivo
	F	Empregado Efetivo
	F	Empregado Efetivo
	F	Empregado Efetivo
	F	Empregado Efetivo
	F	Empregado Efetivo
	M	Empregado Efetivo
	F	Empregado Efetivo
	F	Empregado Efetivo
	F	Empregado Efetivo
	F	Empregado Efetivo
	F	Empregado Efetivo

	F	Empregado Efetivo
	M	Empregado Efetivo
	F	Empregado Efetivo
	F	Empregado Efetivo
	F	Empregado Efetivo
	F	Empregado Efetivo
	F	Empregado Efetivo
	F	Empregado Efetivo
	F	Empregado Efetivo
	F	Empregado Efetivo
	F	Empregado Efetivo
	F	Empregado Efetivo
	M	Empregado Efetivo
	F	Empregado Efetivo
	F	Empregado Efetivo
	F	Empregado Efetivo
	F	Empregado Efetivo
	F	Empregado Efetivo
	F	Empregado Efetivo
	F	Empregado Efetivo
	F	Empregado Efetivo
	F	Empregado Efetivo
	F	Empregado Efetivo
	M	Empregado Efetivo
	M	Empregado Efetivo
	M	Empregado Efetivo
	F	Empregado Efetivo
	F	Empregado Efetivo
	F	Empregado Efetivo
	F	Empregado Efetivo
	F	Empregado Efetivo
	F	Empregado Efetivo
	F	Empregado Efetivo
	F	Empregado Efetivo
	F	Empregado Efetivo
	F	Empregado Efetivo
	M	Empregado Efetivo
	F	Empregado Efetivo
	F	Empregado Efetivo
	F	Empregado Efetivo
	F	Empregado Efetivo
	F	Empregado Efetivo
	F	Empregado Efetivo
	F	Empregado Efetivo
	F	Empregado Efetivo
	F	Empregado Efetivo
	F	Empregado Efetivo
	F	Empregado Efetivo

	F	Empregado Efetivo
	F	Empregado Efetivo
	F	Empregado Efetivo
	F	Empregado Efetivo
	F	Empregado Efetivo
	F	Empregado Efetivo
	F	Empregado Efetivo
	F	Empregado Efetivo
	F	Empregado Efetivo
	M	Empregado Efetivo
	F	Empregado Efetivo
	F	Empregado Efetivo
	F	Empregado Efetivo
	F	Empregado Efetivo
	F	Empregado Efetivo
	F	Empregado Efetivo
	F	Empregado Efetivo
	F	Empregado Efetivo
	F	Empregado Efetivo
	F	Empregado Efetivo
	F	Empregado Efetivo
	F	Empregado Efetivo
	F	Empregado Efetivo
	M	Empregado Efetivo
	F	Empregado Efetivo
	M	Empregado Efetivo
	F	Empregado Efetivo
	F	Empregado Efetivo
	F	Empregado Efetivo
	F	Empregado Efetivo
	F	Empregado Efetivo
	M	Empregado Efetivo
	F	Empregado Efetivo
	F	Empregado Efetivo
	F	Empregado Efetivo
	F	Empregado Efetivo
	F	Empregado Efetivo
	F	Empregado Efetivo
	F	Empregado Efetivo
	F	Empregado Efetivo
	F	Empregado Efetivo
	F	Empregado Efetivo
	F	Empregado Efetivo
	F	Empregado Efetivo
	F	Empregado Efetivo
	F	Empregado Efetivo
	F	Empregado Efetivo
	F	Empregado Efetivo

	F	Empregado Efetivo
	F	Empregado Efetivo
	F	Empregado Efetivo
	F	Empregado Efetivo
	F	Empregado Efetivo
	M	Empregado Efetivo
	F	Empregado Efetivo
	F	Empregado Efetivo
	F	Empregado Efetivo
	F	Empregado Efetivo
	F	Empregado Efetivo
	F	Empregado Efetivo
	F	Empregado Efetivo
	F	Empregado Efetivo
	F	Empregado Efetivo
	F	Empregado Efetivo
	M	Empregado Efetivo
	F	Empregado Efetivo
	F	Empregado Efetivo
	F	Empregado Efetivo
	F	Empregado Efetivo
	F	Empregado Efetivo
	F	Empregado Efetivo
	M	Empregado Efetivo
	F	Empregado Efetivo
	F	Empregado Efetivo
	F	Empregado Efetivo
	F	Empregado Efetivo
	F	Empregado Efetivo
	M	Empregado Efetivo
	F	Empregado Efetivo
	F	Empregado Efetivo
	F	Empregado Efetivo
	F	Empregado Efetivo
	F	Empregado Efetivo
	F	Empregado Efetivo
	F	Empregado Efetivo
	F	Empregado Efetivo
	F	Empregado Efetivo
	F	Empregado Efetivo
	F	Empregado Efetivo
	F	Empregado Efetivo
	F	Empregado Efetivo
	F	Empregado Efetivo
	F	Empregado Efetivo
	F	Empregado Efetivo
	F	Empregado Efetivo
	F	Empregado Efetivo

	F	Empregado Efetivo
	M	Empregado Efetivo
	F	Empregado Efetivo
	F	Empregado Efetivo
	F	Empregado Efetivo
	F	Empregado Efetivo
	F	Empregado Efetivo
	F	Empregado Efetivo
	F	Empregado Efetivo
	F	Empregado Efetivo
	F	Empregado Efetivo
	F	Empregado Efetivo
	F	Empregado Efetivo
	F	Empregado Efetivo
	F	Empregado Efetivo
	M	Empregado Efetivo
	F	Empregado Efetivo
	F	Empregado Efetivo
	F	Empregado Efetivo
	F	Empregado Efetivo
	F	Empregado Efetivo
	F	Empregado Efetivo
	F	Empregado Efetivo
	M	Empregado Efetivo
	F	Empregado Efetivo
	F	Empregado Efetivo
	F	Empregado Efetivo
	M	Empregado Efetivo
	F	Empregado Efetivo
	F	Empregado Efetivo
	F	Empregado Efetivo
	F	Empregado Efetivo
	F	Empregado Efetivo
	M	Empregado Efetivo
	F	Empregado Efetivo
	F	Empregado Efetivo
	F	Empregado Efetivo
	F	Empregado Efetivo
	F	Empregado Efetivo
	F	Empregado Efetivo
	F	Empregado Efetivo
	F	Empregado Efetivo
	M	Empregado Efetivo
	F	Empregado Efetivo
	M	Empregado Efetivo
	F	Empregado Efetivo
	F	Empregado Efetivo
	F	Empregado Efetivo

	F	Empregado Efetivo
	F	Empregado Efetivo
	F	Empregado Efetivo
	F	Empregado Efetivo
	F	Empregado Efetivo
	F	Empregado Efetivo
	F	Empregado Efetivo
	F	Empregado Efetivo
	F	Empregado Efetivo
	M	Empregado Efetivo
	F	Empregado Efetivo
	F	Empregado Efetivo
	F	Empregado Efetivo
	F	Empregado Efetivo
	F	Empregado Efetivo
	M	Empregado Efetivo
	F	Empregado Efetivo
	F	Empregado Efetivo
	F	Empregado Efetivo
	M	Empregado Efetivo
	F	Empregado Efetivo
	F	Empregado Efetivo
	M	Empregado Efetivo
	F	Empregado Efetivo
	F	Empregado Efetivo
	F	Empregado Efetivo
	F	Empregado Efetivo
	F	Empregado Efetivo
	F	Empregado Efetivo
	F	Empregado Efetivo
	F	Empregado Efetivo
	F	Empregado Efetivo
	F	Empregado Efetivo
	F	Empregado Efetivo
	F	Empregado Efetivo
	F	Empregado Efetivo
	F	Empregado Efetivo
	M	Empregado Efetivo
	F	Empregado Efetivo
	F	Empregado Efetivo
	M	Empregado Efetivo
	F	Empregado Efetivo
	F	Empregado Efetivo
	M	Empregado Efetivo
	F	Empregado Efetivo
	F	Empregado Efetivo
	M	Empregado Efetivo
	F	Empregado Efetivo

	F	Empregado Efetivo
	F	Empregado Efetivo
	M	Empregado Efetivo
	F	Empregado Efetivo
	F	Empregado Efetivo
	F	Empregado Efetivo
	F	Empregado Efetivo
	F	Empregado Efetivo
	M	Empregado Efetivo
	F	Empregado Efetivo
	F	Empregado Efetivo
	F	Empregado Efetivo
	F	Empregado Efetivo
	F	Empregado Efetivo
	F	Empregado Efetivo
	F	Empregado Efetivo
	F	Empregado Efetivo
	F	Empregado Efetivo
	M	Empregado Efetivo
	F	Empregado Efetivo
	M	Empregado Efetivo
	F	Empregado Efetivo
	F	Empregado Efetivo
	F	Empregado Efetivo
	F	Empregado Efetivo
	F	Empregado Efetivo
	F	Empregado Efetivo
	F	Empregado Efetivo
	F	Empregado Efetivo
	F	Empregado Efetivo
	F	Empregado Efetivo
	M	Empregado Efetivo
	F	Empregado Efetivo
	F	Empregado Efetivo
	F	Empregado Efetivo
	F	Empregado Efetivo
	F	Empregado Efetivo
	F	Empregado Efetivo
	M	Empregado Efetivo
	F	Empregado Efetivo
	M	Empregado Efetivo
	F	Empregado Efetivo
	F	Empregado Efetivo
	F	Empregado Efetivo
	F	Empregado Efetivo
	F	Empregado Efetivo
	F	Empregado Efetivo
	F	Empregado Efetivo

	F	Empregado Efetivo
	M	Empregado Efetivo
	F	Empregado Efetivo
	F	Empregado Efetivo
	F	Empregado Efetivo
	F	Empregado Efetivo
	F	Empregado Efetivo
	F	Empregado Efetivo
	F	Empregado Efetivo
	M	Empregado Efetivo
	F	Empregado Efetivo
	F	Empregado Efetivo
	F	Empregado Efetivo
	F	Empregado Efetivo
	F	Empregado Efetivo
	F	Empregado Efetivo
	F	Empregado Efetivo
	F	Empregado Efetivo
	F	Empregado Efetivo
	F	Empregado Efetivo
	F	Empregado Efetivo
	F	Empregado Efetivo
	F	Empregado Efetivo
	F	Empregado Efetivo
	F	Empregado Efetivo
	F	Empregado Efetivo
	F	Empregado Efetivo
	M	Empregado Efetivo
	M	Empregado Efetivo
	F	Empregado Efetivo
	F	Empregado Efetivo
	F	Empregado Efetivo
	F	Empregado Efetivo
	M	Empregado Efetivo
	F	Empregado Efetivo
	F	Empregado Efetivo
	F	Empregado Efetivo
	F	Empregado Efetivo
	F	Empregado Efetivo
	F	Empregado Efetivo
	F	Empregado Efetivo
	M	Empregado Efetivo
	M	Empregado Efetivo
	F	Empregado Efetivo
	F	Empregado Efetivo
	F	Empregado Efetivo
	F	Empregado Efetivo
	F	Empregado Efetivo

	F	Empregado Efetivo
	F	Empregado Efetivo
	F	Empregado Efetivo
	F	Empregado Efetivo
	F	Empregado Efetivo
	F	Empregado Efetivo
	F	Empregado Efetivo
	F	Empregado Efetivo
	F	Empregado Efetivo
	F	Empregado Efetivo
	F	Empregado Efetivo
	F	Empregado Efetivo
	F	Empregado Efetivo
	F	Empregado Efetivo
	F	Empregado Efetivo
	F	Empregado Efetivo
	F	Empregado Efetivo
	F	Empregado Efetivo
	F	Empregado Efetivo
	F	Empregado Efetivo
	F	Empregado Efetivo
	F	Empregado Efetivo
	F	Empregado Efetivo
	F	Empregado Efetivo
	F	Empregado Efetivo
	F	Empregado Efetivo
	F	Empregado Efetivo
	F	Empregado Efetivo
	F	Empregado Efetivo
	F	Empregado Efetivo
	F	Empregado Efetivo
	F	Empregado Efetivo
	F	Empregado Efetivo
	F	Empregado Efetivo
	F	Empregado Efetivo
	F	Empregado Efetivo
	F	Empregado Efetivo
	F	Empregado Efetivo
	F	Empregado Efetivo
	F	Empregado Efetivo
	F	Empregado Efetivo
	F	Empregado Efetivo
	F	Empregado Efetivo
	F	Empregado Efetivo
	F	Empregado Efetivo
	F	Empregado Efetivo
	F	Empregado Efetivo
	F	Empregado Efetivo

	F	Empregado Efetivo
	F	Empregado Efetivo
	F	Empregado Efetivo
	F	Empregado Efetivo
	F	Empregado Efetivo
	F	Empregado Efetivo
	F	Empregado Efetivo
	F	Empregado Efetivo
	F	Empregado Efetivo
	F	Empregado Efetivo
	F	Empregado Efetivo
	M	Empregado Efetivo
	F	Empregado Efetivo
	F	Empregado Efetivo
	F	Empregado Efetivo
	F	Empregado Efetivo
	F	Empregado Efetivo
	F	Empregado Efetivo
	F	Empregado Efetivo
	F	Empregado Efetivo
	F	Empregado Efetivo
	F	Empregado Efetivo
	F	Empregado Efetivo
	F	Empregado Efetivo
	F	Empregado Efetivo
	F	Empregado Efetivo
	F	Empregado Efetivo
	F	Empregado Efetivo
	F	Empregado Efetivo
	F	Empregado Efetivo
	M	Empregado Efetivo
	F	Empregado Efetivo
	F	Empregado Efetivo
	F	Empregado Efetivo
	F	Empregado Efetivo
	F	Empregado Efetivo
	M	Empregado Efetivo
	F	Empregado Efetivo
	F	Empregado Efetivo
	F	Empregado Efetivo
	F	Empregado Efetivo
	F	Empregado Efetivo
	F	Empregado Efetivo
	F	Empregado Efetivo
	F	Empregado Efetivo
	F	Empregado Efetivo
	F	Empregado Efetivo
	F	Empregado Efetivo

	F	Empregado Efetivo
	M	Empregado Efetivo
	F	Empregado Efetivo
	M	Empregado Efetivo
	F	Empregado Efetivo
	F	Empregado Efetivo
	F	Empregado Efetivo
	F	Empregado Efetivo
	M	Empregado Efetivo
	F	Empregado Efetivo
	F	Empregado Efetivo
	F	Empregado Efetivo
	F	Empregado Efetivo
	F	Empregado Efetivo
	F	Empregado Efetivo
	F	Empregado Efetivo
	F	Empregado Efetivo
	F	Empregado Efetivo
	F	Empregado Efetivo
	F	Empregado Efetivo
	M	Empregado Efetivo
	M	Empregado Efetivo
	F	Empregado Efetivo
	F	Empregado Efetivo
	F	Empregado Efetivo
	F	Empregado Efetivo
	M	Empregado Efetivo
	M	Empregado Efetivo
	M	Empregado Efetivo
	M	Empregado Efetivo
	M	Empregado Efetivo
	M	Empregado Efetivo
	M	Empregado Efetivo
	M	Empregado Efetivo
	M	Empregado Efetivo
	M	Empregado Efetivo
	F	Empregado Efetivo
	M	Empregado Efetivo
	M	Empregado Efetivo
	F	Empregado Efetivo
	M	Empregado Efetivo
	F	Empregado Efetivo
	F	Empregado Efetivo
	M	Empregado Efetivo
	M	Empregado Efetivo
	F	Empregado Efetivo
	F	Empregado Efetivo
	M	Empregado Efetivo

	F	Empregado Efetivo
	F	Empregado Efetivo
	M	Empregado Efetivo
	F	Empregado Efetivo
	M	Empregado Efetivo
	M	Empregado Efetivo
	M	Empregado Efetivo
	M	Empregado Efetivo
	M	Empregado Efetivo
	M	Empregado Efetivo
	M	Empregado Efetivo
	F	Empregado Efetivo
	M	Empregado Efetivo
	M	Empregado Efetivo
	M	Empregado Efetivo
	M	Empregado Efetivo
	M	Empregado Efetivo
	M	Empregado Efetivo
	M	Empregado Efetivo
	M	Empregado Efetivo
	M	Empregado Efetivo
	M	Empregado Efetivo
	M	Empregado Efetivo
	M	Empregado Efetivo
	M	Empregado Efetivo
	M	Empregado Efetivo
	F	Empregado Efetivo
	M	Empregado Efetivo
	M	Empregado Efetivo
	M	Empregado Efetivo
	F	Empregado Efetivo
	F	Empregado Efetivo
	F	Empregado Efetivo
	F	Empregado Efetivo
	F	Empregado Efetivo
	F	Empregado Efetivo
	F	Empregado Efetivo
	F	Empregado Efetivo
	M	Empregado Efetivo
	M	Empregado Efetivo
	M	Empregado Efetivo
	F	Empregado Efetivo

	F	Empregado Efetivo
	M	Empregado Efetivo
	F	Empregado Efetivo
	F	Empregado Efetivo
	M	Empregado Efetivo
	M	Empregado Efetivo
	F	Empregado Efetivo
	F	Empregado Efetivo
	M	Empregado Efetivo
	F	Empregado Efetivo
	M	Empregado Efetivo
	M	Empregado Efetivo
	F	Empregado Efetivo
	M	Empregado Efetivo
	F	Empregado Efetivo
	F	Empregado Efetivo
	F	Empregado Efetivo
	F	Empregado Efetivo
	M	Empregado Efetivo
	F	Empregado Efetivo
	M	Empregado Efetivo
	M	Empregado Efetivo
	M	Empregado Efetivo
	F	Empregado Efetivo
	F	Empregado Efetivo
	F	Empregado Efetivo
	M	Empregado Efetivo
	F	Empregado Efetivo
	F	Empregado Efetivo
	F	Empregado Efetivo
	M	Empregado Efetivo
	F	Empregado Efetivo
	F	Empregado Efetivo
	F	Empregado Efetivo
	F	Empregado Efetivo
	F	Empregado Efetivo
	F	Empregado Efetivo
	M	Empregado Efetivo
	M	Empregado Efetivo
	F	Empregado Efetivo

	M	Empregado Efetivo
	F	Empregado Efetivo
	F	Empregado Efetivo
	F	Empregado Efetivo
	F	Empregado Efetivo
	F	Empregado Efetivo
	F	Empregado Efetivo
	F	Empregado Efetivo
	F	Empregado Efetivo
	M	Empregado Efetivo
	M	Empregado Efetivo
	M	Empregado Efetivo
	M	Empregado Efetivo
	M	Empregado Efetivo
	F	Empregado Efetivo
	F	Empregado Efetivo
	F	Empregado Efetivo
	F	Empregado Efetivo
	F	Empregado Efetivo
	F	Empregado Efetivo
	F	Empregado Efetivo
	F	Empregado Efetivo
	F	Empregado Efetivo
	F	Empregado Efetivo
	F	Empregado Efetivo
	F	Empregado Efetivo
	M	Empregado Efetivo
	F	Empregado Efetivo
	M	Empregado Efetivo
	F	Empregado Efetivo
	F	Empregado Efetivo
	F	Empregado Efetivo
	F	Empregado Efetivo
	F	Empregado Efetivo
	F	Empregado Efetivo
	F	Empregado Efetivo
	F	Empregado Efetivo
	F	Empregado Efetivo
	F	Empregado Efetivo
	F	Empregado Efetivo

	F	Empregado Efetivo
	F	Empregado Efetivo
	F	Empregado Efetivo
	M	Empregado Efetivo
	F	Empregado Efetivo
	M	Empregado Efetivo
	F	Empregado Efetivo
	F	Empregado Efetivo
	F	Empregado Efetivo
	F	Empregado Efetivo
	F	Empregado Efetivo
	F	Empregado Efetivo
	F	Empregado Efetivo
	F	Empregado Efetivo
	F	Empregado Efetivo
	F	Empregado Efetivo
	F	Empregado Efetivo
	F	Empregado Efetivo
	F	Empregado Efetivo
	F	Empregado Efetivo
	F	Empregado Efetivo
	F	Empregado Efetivo
	F	Empregado Efetivo
	F	Empregado Efetivo
	F	Empregado Efetivo
	F	Empregado Efetivo
	M	Empregado Efetivo
	F	Empregado Efetivo
	F	Empregado Efetivo
	F	Empregado Efetivo
	F	Empregado Efetivo
	F	Empregado Efetivo
	F	Empregado Efetivo
	F	Empregado Efetivo
	F	Empregado Efetivo
	F	Empregado Efetivo
	F	Empregado Efetivo
	F	Empregado Efetivo
	M	Empregado Efetivo
	M	Empregado Efetivo
	F	Empregado Efetivo
	F	Empregado Efetivo
	F	Empregado Efetivo
	F	Empregado Efetivo
	F	Empregado Efetivo
	F	Empregado Efetivo
	F	Empregado Efetivo
	F	Empregado Efetivo

	F	Empregado Efetivo
	F	Empregado Efetivo
	F	Empregado Efetivo
	F	Empregado Efetivo
	F	Empregado Efetivo
	F	Empregado Efetivo
	F	Empregado Efetivo
	F	Empregado Efetivo
	F	Empregado Efetivo
	F	Empregado Efetivo
	F	Empregado Efetivo
	F	Empregado Efetivo
	M	Empregado Efetivo
	F	Empregado Efetivo
	F	Empregado Efetivo
	F	Empregado Efetivo
	F	Empregado Efetivo
	F	Empregado Efetivo
	F	Empregado Efetivo
	F	Empregado Efetivo
	F	Empregado Efetivo
	F	Empregado Efetivo
	F	Empregado Efetivo
	F	Empregado Efetivo
	F	Empregado Efetivo
	F	Empregado Efetivo
	F	Empregado Efetivo
	F	Empregado Efetivo
	F	Empregado Efetivo
	F	Empregado Efetivo
	F	Empregado Efetivo
	M	Empregado Efetivo
	F	Empregado Efetivo
	M	Empregado Efetivo
	F	Empregado Efetivo
	F	Empregado Efetivo
	F	Empregado Efetivo
	F	Empregado Efetivo
	F	Empregado Efetivo
	F	Empregado Efetivo
	F	Empregado Efetivo
	F	Empregado Efetivo
	F	Empregado Efetivo
	M	Empregado Efetivo
	F	Empregado Efetivo
	F	Empregado Efetivo
	F	Empregado Efetivo
	F	Empregado Efetivo

	F	Empregado Efetivo
	F	Empregado Efetivo
	F	Empregado Efetivo
	F	Empregado Efetivo
	F	Empregado Efetivo
	F	Empregado Efetivo
	M	Empregado Efetivo
	F	Empregado Efetivo
	F	Empregado Efetivo
	M	Empregado Efetivo
	F	Empregado Efetivo
	F	Empregado Efetivo
	F	Empregado Efetivo
	F	Empregado Efetivo
	F	Empregado Efetivo
	F	Empregado Efetivo
	F	Empregado Efetivo
	F	Empregado Efetivo
	F	Empregado Efetivo
	F	Empregado Efetivo
	F	Empregado Efetivo
	F	Empregado Efetivo
	F	Empregado Efetivo
	F	Empregado Efetivo
	F	Empregado Efetivo
	F	Empregado Efetivo
	F	Empregado Efetivo
	F	Empregado Efetivo
	F	Empregado Efetivo
	F	Empregado Efetivo
	F	Empregado Efetivo
	F	Empregado Efetivo
	M	Empregado Efetivo
	F	Empregado Efetivo
	F	Empregado Efetivo
	F	Empregado Efetivo
	F	Empregado Efetivo
	F	Empregado Efetivo
	F	Empregado Efetivo
	F	Empregado Efetivo
	F	Empregado Efetivo
	F	Empregado Efetivo
	F	Empregado Efetivo
	F	Empregado Efetivo
	F	Empregado Efetivo
	F	Empregado Efetivo
	F	Empregado Efetivo
	F	Empregado Efetivo

	F	Empregado Efetivo
	F	Empregado Efetivo
	F	Empregado Efetivo
	F	Empregado Efetivo
	F	Empregado Efetivo
	F	Empregado Efetivo
	F	Empregado Efetivo
	F	Empregado Efetivo
	F	Empregado Efetivo
	F	Empregado Efetivo
	F	Empregado Efetivo
	F	Empregado Efetivo
	F	Empregado Efetivo
	F	Empregado Efetivo
	F	Empregado Efetivo
	F	Empregado Efetivo
	M	Empregado Efetivo
	F	Empregado Efetivo
	F	Empregado Efetivo
	F	Empregado Efetivo
	F	Empregado Efetivo
	F	Empregado Efetivo
	M	Empregado Efetivo
	F	Empregado Efetivo
	F	Empregado Efetivo
	F	Empregado Efetivo
	F	Empregado Efetivo
	F	Empregado Efetivo
	F	Empregado Efetivo
	F	Empregado Efetivo
	F	Empregado Efetivo
	F	Empregado Efetivo
	F	Empregado Efetivo
	F	Empregado Efetivo
	F	Empregado Efetivo
	F	Empregado Efetivo
	F	Empregado Efetivo
	F	Empregado Efetivo
	M	Empregado Efetivo
	F	Empregado Efetivo
	F	Empregado Efetivo
	M	Empregado Efetivo
	F	Empregado Efetivo
	F	Empregado Efetivo
	F	Empregado Efetivo
	F	Empregado Efetivo
	M	Empregado Efetivo
	F	Empregado Efetivo

	F	Empregado Efetivo
	F	Empregado Efetivo
	M	Empregado Efetivo
	F	Empregado Efetivo
	F	Empregado Efetivo
	F	Empregado Efetivo
	F	Empregado Efetivo
	M	Empregado Efetivo
	F	Empregado Efetivo
	F	Empregado Efetivo
	F	Empregado Efetivo
	M	Empregado Efetivo
	F	Empregado Efetivo
	F	Empregado Efetivo
	F	Empregado Efetivo
	F	Empregado Efetivo
	F	Empregado Efetivo
	F	Empregado Efetivo
	F	Empregado Efetivo
	M	Empregado Efetivo
	M	Empregado Efetivo
	M	Empregado Efetivo
	M	Empregado Efetivo
	M	Empregado Efetivo
	M	Empregado Efetivo
	M	Empregado Efetivo
	M	Empregado Efetivo
	M	Empregado Efetivo
	M	Empregado Efetivo
	M	Empregado Efetivo
	M	Empregado Efetivo
	M	Empregado Efetivo
	M	Empregado Efetivo
	M	Empregado Efetivo
	M	Empregado Efetivo
	F	Empregado Efetivo
	F	Empregado Efetivo
	M	Empregado Efetivo
	F	Empregado Efetivo
	F	Empregado Efetivo
	F	Empregado Efetivo
	F	Empregado Efetivo
	M	Empregado Efetivo
	M	Empregado Efetivo
	F	Empregado Efetivo
	M	Empregado Efetivo
	F	Empregado Efetivo
	F	Empregado Efetivo

	F	Empregado Efetivo
	F	Empregado Efetivo
	M	Empregado Efetivo
	M	Empregado Efetivo
	M	Empregado Efetivo
	F	Empregado Efetivo
	F	Empregado Efetivo
	F	Empregado Efetivo
	F	Empregado Efetivo
	F	Empregado Efetivo
	F	Empregado Efetivo
	F	Empregado Efetivo
	F	Empregado Efetivo
	F	Empregado Efetivo
	F	Empregado Efetivo
	F	Empregado Efetivo
	F	Empregado Efetivo
	F	Empregado Efetivo
	F	Empregado Efetivo
	F	Empregado Efetivo
	F	Empregado Efetivo
	F	Empregado Efetivo
	F	Empregado Efetivo
	F	Empregado Efetivo
	F	Empregado Efetivo
	F	Empregado Efetivo
	F	Empregado Efetivo
	F	Empregado Efetivo
	F	Empregado Efetivo
	F	Empregado Efetivo
	F	Empregado Efetivo
	F	Empregado Efetivo
	F	Empregado Efetivo
	M	Empregado Efetivo
	F	Empregado Efetivo
	F	Empregado Efetivo
	F	Empregado Efetivo
	F	Empregado Efetivo
	F	Empregado Efetivo
	F	Empregado Efetivo
	F	Empregado Efetivo
	M	Empregado Efetivo
	M	Empregado Efetivo
	F	Empregado Efetivo
	F	Empregado Efetivo
	M	Empregado Efetivo
	M	Empregado Efetivo

	M	Empregado Efetivo
	F	Empregado Efetivo
	M	Empregado Efetivo
	F	Empregado Efetivo
	M	Empregado Efetivo
	F	Empregado Efetivo
	F	Empregado Efetivo
	F	Empregado Efetivo
	F	Empregado Efetivo
	F	Empregado Efetivo
	F	Empregado Efetivo
	F	Empregado Efetivo
	M	Empregado Efetivo
	M	Empregado Efetivo
	M	Empregado Efetivo
	F	Empregado Efetivo
	F	Empregado Efetivo
	F	Empregado Efetivo
	F	Empregado Efetivo
	F	Empregado Efetivo
	F	Empregado Efetivo
	F	Empregado Efetivo
	F	Empregado Efetivo
	F	Empregado Efetivo
	F	Empregado Efetivo
	F	Empregado Efetivo
	F	Empregado Efetivo
	F	Empregado Efetivo
	M	Empregado Efetivo
	F	Empregado Efetivo
	F	Empregado Efetivo
	F	Empregado Efetivo
	M	Empregado Efetivo
	F	Empregado Efetivo
	F	Empregado Efetivo
	F	Empregado Efetivo
	F	Empregado Efetivo
	F	Empregado Efetivo
	F	Empregado Efetivo
	F	Empregado Efetivo
	F	Empregado Efetivo
	F	Empregado Efetivo
	F	Empregado Efetivo
	F	Empregado Efetivo
	F	Empregado Efetivo
	F	Empregado Efetivo
	F	Empregado Efetivo
	F	Empregado Efetivo

	F	Empregado Efetivo
	F	Empregado Efetivo
	F	Empregado Efetivo
	F	Empregado Efetivo
	F	Empregado Efetivo
	F	Empregado Efetivo
	F	Empregado Efetivo
	F	Empregado Efetivo
	F	Empregado Efetivo
	F	Empregado Efetivo
	F	Empregado Efetivo
	F	Empregado Efetivo
	F	Empregado Efetivo
	F	Empregado Efetivo
	F	Empregado Efetivo
	F	Empregado Efetivo
	F	Empregado Efetivo
	F	Empregado Efetivo
	F	Empregado Efetivo
	F	Empregado Efetivo
	F	Empregado Efetivo
	M	Empregado Efetivo
	F	Empregado Efetivo
	M	Empregado Efetivo
	M	Empregado Efetivo
	M	Empregado Efetivo
	F	Empregado Efetivo
	F	Empregado Efetivo
	F	Empregado Efetivo
	F	Empregado Efetivo
	F	Empregado Efetivo
	F	Empregado Efetivo
	F	Empregado Efetivo
	F	Empregado Efetivo
	F	Empregado Efetivo
	F	Empregado Efetivo
	F	Empregado Efetivo
	F	Empregado Efetivo
	M	Empregado Efetivo
	F	Empregado Efetivo
	F	Empregado Efetivo
	F	Empregado Efetivo
	F	Empregado Efetivo
	F	Empregado Efetivo
	F	Empregado Efetivo
	F	Empregado Efetivo
	F	Empregado Efetivo

	F	Empregado Efetivo
	F	Empregado Efetivo
	F	Empregado Efetivo
	F	Empregado Efetivo
	F	Empregado Efetivo
	F	Empregado Efetivo
	F	Empregado Efetivo
	M	Empregado Efetivo
	F	Empregado Efetivo
	F	Empregado Efetivo
	F	Empregado Efetivo
	F	Empregado Efetivo
	F	Empregado Efetivo
	F	Empregado Efetivo
	F	Empregado Efetivo
	F	Empregado Efetivo
	F	Empregado Efetivo
	F	Empregado Efetivo
	F	Empregado Efetivo
	F	Empregado Efetivo
	M	Empregado Efetivo
	M	Empregado Efetivo
	F	Empregado Efetivo
	F	Empregado Efetivo
	F	Empregado Efetivo
	F	Empregado Efetivo
	F	Empregado Efetivo
	F	Empregado Efetivo
	M	Empregado Efetivo
	F	Empregado Efetivo
	F	Empregado Efetivo
	F	Empregado Efetivo
	M	Empregado Efetivo
	F	Empregado Efetivo
	F	Empregado Efetivo
	M	Empregado Efetivo
	F	Empregado Efetivo
	F	Empregado Efetivo
	F	Empregado Efetivo
	F	Empregado Efetivo
	F	Empregado Efetivo
	F	Empregado Efetivo
	F	Empregado Efetivo
	M	Empregado Efetivo
	F	Empregado Efetivo
	F	Empregado Efetivo
	F	Empregado Efetivo
	F	Empregado Efetivo

	F	Empregado Efetivo
	F	Empregado Efetivo
	F	Empregado Efetivo
	F	Empregado Efetivo
	F	Empregado Efetivo
	M	Empregado Efetivo
	M	Empregado Efetivo
	F	Empregado Efetivo
	M	Empregado Efetivo
	M	Empregado Efetivo
	F	Empregado Efetivo
	F	Empregado Efetivo
	F	Empregado Efetivo
	F	Empregado Efetivo
	F	Empregado Efetivo
	F	Empregado Efetivo
	F	Empregado Efetivo
	F	Empregado Efetivo
	F	Empregado Efetivo
	F	Empregado Efetivo
	F	Empregado Efetivo
	M	Empregado Efetivo
	M	Empregado Efetivo
	F	Empregado Efetivo
	M	Empregado Efetivo
	F	Empregado Efetivo
	F	Empregado Efetivo
	F	Empregado Efetivo
	F	Empregado Efetivo
	F	Empregado Efetivo
	F	Empregado Efetivo
	F	Empregado Efetivo
	F	Empregado Efetivo
	M	Empregado Efetivo
	F	Empregado Efetivo
	M	Empregado Efetivo
	F	Empregado Efetivo
	F	Empregado Efetivo
	F	Empregado Efetivo
	F	Empregado Efetivo
	F	Empregado Efetivo
	F	Empregado Efetivo
	F	Empregado Efetivo
	F	Empregado Efetivo

	F	Empregado Efetivo
	F	Empregado Efetivo
	F	Empregado Efetivo
	F	Empregado Efetivo
	F	Empregado Efetivo
	F	Empregado Efetivo
	F	Empregado Efetivo
	F	Empregado Efetivo
	F	Empregado Efetivo
	F	Empregado Efetivo
	F	Empregado Efetivo
	F	Empregado Efetivo
	F	Empregado Efetivo
	F	Empregado Efetivo
	F	Empregado Efetivo
	M	Empregado Efetivo
	F	Empregado Efetivo
	F	Empregado Efetivo
	F	Empregado Efetivo
	F	Empregado Efetivo
	F	Empregado Efetivo
	F	Empregado Efetivo
	F	Empregado Efetivo
	F	Empregado Efetivo
	F	Empregado Efetivo
	F	Empregado Efetivo
	M	Empregado Efetivo
	M	Empregado Efetivo
	M	Empregado Efetivo
	M	Empregado Efetivo
	M	Empregado Efetivo
	M	Empregado Efetivo

	M	Empregado Efetivo
	M	Empregado Efetivo
	M	Empregado Efetivo
	M	Empregado Efetivo
	M	Empregado Efetivo
	F	Empregado Efetivo
	F	Empregado Efetivo
	F	Empregado Efetivo
	F	Empregado Efetivo
	F	Empregado Efetivo
	F	Empregado Efetivo
	F	Empregado Efetivo
	F	Empregado Efetivo
	M	Empregado Efetivo
	F	Empregado Efetivo
	M	Empregado Efetivo
	F	Empregado Efetivo
	F	Empregado Efetivo
	F	Empregado Efetivo
	F	Empregado Efetivo
	F	Empregado Efetivo
	F	Empregado Efetivo
	F	Empregado Efetivo
	F	Empregado Efetivo
	F	Empregado Efetivo
	F	Empregado Efetivo
	F	Empregado Efetivo
	F	Empregado Efetivo
	M	Empregado Efetivo
	F	Empregado Efetivo
	F	Empregado Efetivo
	F	Empregado Efetivo
	M	Empregado Efetivo
	F	Empregado Efetivo
	F	Empregado Efetivo
	F	Empregado Efetivo
	F	Empregado Efetivo
	F	Empregado Efetivo
	M	Empregado Efetivo
	F	Empregado Efetivo
	F	Empregado Efetivo

	F	Empregado Efetivo
	F	Empregado Efetivo
	F	Empregado Efetivo
	F	Empregado Efetivo
	F	Empregado Efetivo
	M	Empregado Efetivo
	F	Empregado Efetivo
	F	Empregado Efetivo
	F	Empregado Efetivo
	F	Empregado Efetivo
	F	Empregado Efetivo
	M	Empregado Efetivo
	F	Empregado Efetivo
	F	Empregado Efetivo
	F	Empregado Efetivo
	F	Empregado Efetivo
	F	Empregado Efetivo
	F	Empregado Efetivo
	F	Empregado Efetivo
	F	Empregado Efetivo
	F	Empregado Efetivo
	F	Empregado Efetivo
	F	Empregado Efetivo
	F	Empregado Efetivo
	F	Empregado Efetivo
	F	Empregado Efetivo
	F	Empregado Efetivo
	F	Empregado Efetivo
	F	Empregado Efetivo
	F	Empregado Efetivo
	F	Empregado Efetivo
	F	Empregado Efetivo
	F	Empregado Efetivo
	M	Empregado Efetivo
	F	Empregado Efetivo
	F	Empregado Efetivo
	F	Empregado Efetivo
	F	Empregado Efetivo
	F	Empregado Efetivo
	M	Empregado Efetivo
	F	Empregado Efetivo
	F	Empregado Efetivo
	M	Empregado Efetivo
	F	Empregado Efetivo
	F	Empregado Efetivo
	F	Empregado Efetivo
	F	Empregado Efetivo
	F	Empregado Efetivo

	M	Empregado Efetivo
	F	Empregado Efetivo
	F	Empregado Efetivo
	F	Empregado Efetivo
	F	Empregado Efetivo
	F	Empregado Efetivo
	F	Empregado Efetivo
	F	Empregado Efetivo
	F	Empregado Efetivo
	F	Empregado Efetivo
	F	Empregado Efetivo
	F	Empregado Efetivo
	F	Empregado Efetivo
	F	Empregado Efetivo
	F	Empregado Efetivo
	F	Empregado Efetivo
	F	Empregado Efetivo
	F	Empregado Efetivo
	F	Empregado Efetivo
	F	Empregado Efetivo
	F	Empregado Efetivo
	F	Empregado Efetivo
	F	Empregado Efetivo
	M	Empregado Efetivo
	M	Empregado Efetivo
	F	Empregado Efetivo
	F	Empregado Efetivo
	F	Empregado Efetivo
	F	Empregado Efetivo
	F	Empregado Efetivo
	F	Empregado Efetivo
	F	Empregado Efetivo
	F	Empregado Efetivo
	F	Empregado Efetivo
	F	Empregado Efetivo
	F	Empregado Efetivo
	F	Empregado Efetivo
	F	Empregado Efetivo
	M	Empregado Efetivo
	F	Empregado Efetivo
	F	Empregado Efetivo
	M	Empregado Efetivo
	F	Empregado Efetivo
	F	Empregado Efetivo
	F	Empregado Efetivo
	F	Empregado Efetivo
	M	Empregado Efetivo
	M	Empregado Efetivo

	F	Empregado Efetivo
	F	Empregado Efetivo
	F	Empregado Efetivo
	F	Empregado Efetivo
	F	Empregado Efetivo
	F	Empregado Efetivo
	M	Empregado Efetivo
	M	Empregado Efetivo
	F	Empregado Efetivo
	F	Empregado Efetivo
	F	Empregado Efetivo
	F	Empregado Efetivo
	F	Empregado Efetivo
	F	Empregado Efetivo
	M	Empregado Efetivo
	M	Empregado Efetivo
	M	Empregado Efetivo
	F	Empregado Efetivo
	F	Empregado Efetivo
	F	Empregado Efetivo
	F	Empregado Efetivo
	F	Empregado Efetivo
	F	Empregado Efetivo
	F	Empregado Efetivo
	F	Empregado Efetivo
	F	Empregado Efetivo
	F	Empregado Efetivo
	M	Empregado Efetivo
	F	Empregado Efetivo
	F	Empregado Efetivo
	F	Empregado Efetivo
	F	Empregado Efetivo
	F	Empregado Efetivo
	M	Empregado Efetivo
	F	Empregado Efetivo
	F	Empregado Efetivo
	M	Empregado Efetivo
	M	Empregado Efetivo
	F	Empregado Efetivo
	F	Empregado Efetivo
	F	Empregado Efetivo
	F	Empregado Efetivo
	F	Empregado Efetivo
	F	Empregado Efetivo
	F	Empregado Efetivo
	M	Empregado Efetivo
	M	Empregado Efetivo
	F	Empregado Efetivo

	F	Empregado Efetivo
	F	Empregado Efetivo
	F	Empregado Efetivo
	F	Empregado Efetivo
	M	Empregado Efetivo
	F	Empregado Efetivo
	F	Empregado Efetivo
	M	Empregado Efetivo
	F	Empregado Efetivo
	F	Empregado Efetivo
	F	Empregado Efetivo
	F	Empregado Efetivo
	F	Empregado Efetivo
	F	Empregado Efetivo
	F	Empregado Efetivo
	M	Empregado Efetivo
	F	Empregado Efetivo
	F	Empregado Efetivo
	F	Empregado Efetivo
	F	Empregado Efetivo
	F	Empregado Efetivo
	F	Empregado Efetivo
	F	Empregado Efetivo
	F	Empregado Efetivo
	F	Empregado Efetivo
	M	Empregado Efetivo
	F	Empregado Efetivo
	F	Empregado Efetivo
	F	Empregado Efetivo
	F	Empregado Efetivo
	F	Empregado Efetivo
	F	Empregado Efetivo
	M	Empregado Efetivo
	M	Empregado Efetivo
	F	Empregado Efetivo
	F	Empregado Efetivo
	F	Empregado Efetivo
	F	Empregado Efetivo
	F	Empregado Efetivo
	F	Empregado Efetivo
	M	Empregado Efetivo
	F	Empregado Efetivo
	F	Empregado Efetivo
	F	Empregado Efetivo
	F	Empregado Efetivo
	F	Empregado Efetivo
	F	Empregado Efetivo
	F	Empregado Efetivo

	F	Empregado Efetivo
	F	Empregado Efetivo
	F	Empregado Efetivo
	F	Empregado Efetivo
	F	Empregado Efetivo
	M	Empregado Efetivo
	M	Empregado Efetivo
	F	Empregado Efetivo
	F	Empregado Efetivo
	F	Empregado Efetivo
	M	Empregado Efetivo
	M	Empregado Efetivo
	F	Empregado Efetivo
	F	Empregado Efetivo
	F	Empregado Efetivo
	F	Empregado Efetivo
	M	Empregado Efetivo
	M	Empregado Efetivo
	M	Empregado Efetivo
	F	Empregado Efetivo
	F	Empregado Efetivo
	M	Empregado Efetivo
	F	Empregado Efetivo
	F	Empregado Efetivo
	F	Empregado Efetivo
	F	Empregado Efetivo
	F	Empregado Efetivo
	F	Empregado Efetivo
	F	Empregado Efetivo
	F	Empregado Efetivo
	F	Empregado Efetivo
	F	Empregado Efetivo
	F	Empregado Efetivo
	M	Empregado Efetivo
	F	Empregado Efetivo
	M	Empregado Efetivo
	F	Empregado Efetivo
	F	Empregado Efetivo
	F	Empregado Efetivo
	M	Empregado Efetivo
	F	Empregado Efetivo
	F	Empregado Efetivo
	F	Empregado Efetivo
	M	Empregado Efetivo
	F	Empregado Efetivo
	F	Empregado Efetivo
	M	Empregado Efetivo
	F	Empregado Efetivo

	F	Empregado Efetivo
	F	Empregado Efetivo
	F	Empregado Efetivo
	F	Empregado Efetivo
	F	Empregado Efetivo
	F	Empregado Efetivo
	F	Empregado Efetivo
	F	Empregado Efetivo
	M	Empregado Efetivo
	M	Empregado Efetivo
	M	Empregado Efetivo
	F	Empregado Efetivo
	M	Empregado Efetivo
	F	Empregado Efetivo
	F	Empregado Efetivo
	F	Empregado Efetivo
	F	Empregado Efetivo
	F	Empregado Efetivo
	M	Empregado Efetivo
	M	Empregado Efetivo
	F	Empregado Efetivo
	F	Empregado Efetivo
	F	Empregado Efetivo
	M	Empregado Efetivo
	M	Empregado Efetivo
	F	Empregado Efetivo
	F	Empregado Efetivo
	M	Empregado Efetivo
	F	Empregado Efetivo
	F	Empregado Efetivo
	F	Empregado Efetivo
	F	Empregado Efetivo
	M	Empregado Efetivo
	F	Empregado Efetivo
	M	Empregado Efetivo
	M	Empregado Efetivo
	F	Empregado Efetivo
	F	Empregado Efetivo
	F	Empregado Efetivo
	M	Empregado Efetivo
	F	Empregado Efetivo
	F	Empregado Efetivo
	M	Empregado Efetivo
	F	Empregado Efetivo
	F	Empregado Efetivo
	F	Empregado Efetivo
	F	Empregado Efetivo
	F	Empregado Efetivo

	M	Empregado Efetivo
	F	Empregado Efetivo
	M	Empregado Efetivo
	M	Empregado Efetivo
	F	Empregado Efetivo
	F	Empregado Efetivo
	M	Empregado Efetivo
	F	Empregado Efetivo
	F	Empregado Efetivo
	F	Empregado Efetivo
	F	Empregado Efetivo
	F	Empregado Efetivo
	F	Empregado Efetivo
	F	Empregado Efetivo
	M	Empregado Efetivo
	F	Empregado Efetivo
	F	Empregado Efetivo
	F	Empregado Efetivo
	M	Empregado Efetivo
	F	Empregado Efetivo
	M	Empregado Efetivo
	M	Empregado Efetivo
	M	Empregado Efetivo
	F	Empregado Efetivo
	F	Empregado Efetivo
	F	Empregado Efetivo
	F	Empregado Efetivo
	F	Empregado Efetivo
	F	Empregado Efetivo
	M	Empregado Efetivo
	M	Empregado Efetivo
	M	Empregado Efetivo
	M	Empregado Efetivo
	M	Empregado Efetivo
	M	Empregado Efetivo
	F	Empregado Efetivo
	F	Empregado Efetivo
	F	Empregado Efetivo
	F	Empregado Efetivo
	F	Empregado Efetivo
	M	Empregado Efetivo
	M	Empregado Efetivo
	F	Empregado Efetivo
	F	Empregado Efetivo
	F	Empregado Efetivo
	F	Empregado Efetivo
	F	Empregado Efetivo
	F	Empregado Efetivo

	F	Empregado Efetivo
	F	Empregado Efetivo
	F	Empregado Efetivo
	F	Empregado Efetivo
	F	Empregado Efetivo
	F	Empregado Efetivo
	F	Empregado Efetivo
	M	Empregado Efetivo
	F	Empregado Efetivo
	M	Empregado Efetivo
	F	Empregado Efetivo
	F	Empregado Efetivo
	F	Empregado Efetivo
	F	Empregado Efetivo
	M	Empregado Efetivo
	F	Empregado Efetivo
	F	Empregado Efetivo
	F	Empregado Efetivo
	F	Empregado Efetivo
	M	Empregado Efetivo
	M	Empregado Efetivo
	F	Empregado Efetivo
	F	Empregado Efetivo
	M	Empregado Efetivo
	M	Empregado Efetivo
	F	Empregado Efetivo
	F	Empregado Efetivo
	M	Empregado Efetivo
	F	Empregado Efetivo
	F	Empregado Efetivo
	F	Empregado Efetivo
	F	Empregado Efetivo
	F	Empregado Efetivo
	F	Empregado Efetivo
	F	Empregado Efetivo
	F	Empregado Efetivo
	F	Empregado Efetivo
	F	Empregado Efetivo
	F	Empregado Efetivo
	F	Empregado Efetivo
	F	Empregado Efetivo
	F	Empregado Efetivo
	M	Empregado Efetivo
	M	Empregado Efetivo
	F	Empregado Efetivo
	F	Empregado Efetivo
	F	Empregado Efetivo
	F	Empregado Efetivo

	F	Empregado Efetivo
	F	Empregado Efetivo
	F	Empregado Efetivo
	F	Empregado Efetivo
	F	Empregado Efetivo
	F	Empregado Efetivo
	F	Empregado Efetivo
	F	Empregado Efetivo
	F	Empregado Efetivo
	F	Empregado Efetivo
	F	Empregado Efetivo
	F	Empregado Efetivo
	F	Empregado Efetivo
	F	Empregado Efetivo
	F	Empregado Efetivo
	F	Empregado Efetivo
	F	Empregado Efetivo
	F	Empregado Efetivo
	F	Empregado Efetivo
	F	Empregado Efetivo
	F	Empregado Efetivo
	F	Empregado Efetivo
	F	Empregado Efetivo
	F	Empregado Efetivo
	F	Empregado Efetivo
	F	Empregado Efetivo
	F	Empregado Efetivo
	F	Empregado Efetivo
	F	Empregado Efetivo
	F	Empregado Efetivo
	F	Empregado Efetivo
	F	Empregado Efetivo
	F	Empregado Efetivo
	F	Empregado Efetivo
	F	Empregado Efetivo
	F	Empregado Efetivo
	F	Empregado Efetivo
	F	Empregado Efetivo
	F	Empregado Efetivo
	M	Empregado Efetivo
	M	Empregado Efetivo
	F	Empregado Efetivo
	F	Empregado Efetivo
	F	Empregado Efetivo
	F	Empregado Efetivo
	F	Empregado Efetivo
	F	Empregado Efetivo
	F	Empregado Efetivo

	F	Empregado Efetivo
	F	Empregado Efetivo
	F	Empregado Efetivo
	M	Empregado Efetivo
	F	Empregado Efetivo
	F	Empregado Efetivo
	F	Empregado Efetivo
	F	Empregado Efetivo
	F	Empregado Efetivo
	F	Empregado Efetivo
	F	Empregado Efetivo
	F	Empregado Efetivo
	F	Empregado Efetivo
	F	Empregado Efetivo
	F	Empregado Efetivo
	F	Empregado Efetivo
	F	Empregado Efetivo
	F	Empregado Efetivo
	F	Empregado Efetivo
	F	Empregado Efetivo
	F	Empregado Efetivo
	F	Empregado Efetivo
	F	Empregado Efetivo
	F	Empregado Efetivo
	F	Empregado Efetivo
	F	Empregado Efetivo
	F	Empregado Efetivo
	F	Empregado Efetivo
	F	Empregado Efetivo
	F	Empregado Efetivo
	M	Empregado Efetivo
	F	Empregado Efetivo
	M	Empregado Efetivo
	F	Empregado Efetivo
	F	Empregado Efetivo
	F	Empregado Efetivo
	F	Empregado Efetivo
	F	Empregado Efetivo
	F	Empregado Efetivo
	F	Empregado Efetivo
	M	Empregado Efetivo
	F	Empregado Efetivo
	F	Empregado Efetivo
	F	Empregado Efetivo
	F	Empregado Efetivo
	M	Empregado Efetivo
	M	Empregado Efetivo
	F	Empregado Efetivo

	F	Empregado Efetivo
	M	Empregado Efetivo
	M	Empregado Efetivo
	F	Empregado Efetivo
	F	Empregado Efetivo
	M	Empregado Efetivo
	F	Empregado Efetivo
	F	Empregado Efetivo
	M	Empregado Efetivo
	M	Empregado Efetivo
	M	Empregado Efetivo
	M	Empregado Efetivo
	M	Empregado Efetivo
	F	Empregado Efetivo
	F	Empregado Efetivo
	M	Empregado Efetivo
	M	Empregado Efetivo
	M	Empregado Efetivo
	M	Empregado Efetivo
	M	Empregado Efetivo
	F	Empregado Efetivo
	M	Empregado Efetivo
	F	Empregado Efetivo
	M	Empregado Efetivo
	F	Empregado Efetivo
	M	Empregado Efetivo
	F	Empregado Efetivo
	M	Empregado Efetivo
	M	Empregado Efetivo
	M	Empregado Efetivo
	M	Empregado Efetivo
	F	Empregado Efetivo
	M	Empregado Efetivo
	F	Empregado Efetivo
	M	Empregado Efetivo
	M	Empregado Efetivo
	M	Empregado Efetivo
	M	Empregado Efetivo
	M	Empregado Efetivo
	M	Empregado Efetivo
	M	Empregado Efetivo
	M	Empregado Efetivo
	M	Empregado Efetivo

	M	Empregado Efetivo
	M	Empregado Efetivo
	M	Empregado Efetivo
	M	Empregado Efetivo
	M	Empregado Efetivo
	M	Empregado Efetivo
	M	Empregado Efetivo
	M	Empregado Efetivo
	M	Empregado Efetivo
	F	Empregado Efetivo
	F	Empregado Efetivo
	F	Empregado Efetivo
	M	Empregado Efetivo
	F	Empregado Efetivo
	M	Empregado Efetivo
	F	Empregado Efetivo
	F	Empregado Efetivo
	F	Empregado Efetivo
	M	Empregado Efetivo
	M	Empregado Efetivo
	F	Empregado Efetivo
	F	Empregado Efetivo
	F	Empregado Efetivo
	F	Empregado Efetivo
	F	Empregado Efetivo
	M	Empregado Efetivo
	F	Empregado Efetivo
	M	Empregado Efetivo
	M	Empregado Efetivo
	M	Empregado Efetivo
	M	Empregado Efetivo
	F	Empregado Efetivo
	F	Empregado Efetivo
	F	Empregado Efetivo
	F	Empregado Efetivo
	F	Empregado Efetivo
	F	Empregado Efetivo
	F	Empregado Efetivo
	F	Empregado Efetivo
	F	Empregado Efetivo

	F	Empregado Efetivo
	F	Empregado Efetivo
	F	Empregado Efetivo
	F	Empregado Efetivo
	F	Empregado Efetivo
	F	Empregado Efetivo
	F	Empregado Efetivo
	F	Empregado Efetivo
	F	Empregado Efetivo
	F	Empregado Efetivo
	M	Empregado Efetivo
	F	Empregado Efetivo
	M	Empregado Efetivo
	F	Empregado Efetivo
	M	Empregado Efetivo
	M	Empregado Efetivo
	F	Empregado Efetivo
	M	Empregado Efetivo
	F	Empregado Efetivo
	F	Empregado Efetivo
	F	Empregado Efetivo
	M	Empregado Efetivo
	F	Empregado Efetivo
	F	Empregado Efetivo
	F	Empregado Efetivo
	F	Empregado Efetivo
	F	Empregado Efetivo
	M	Empregado Efetivo
	F	Empregado Efetivo
	F	Empregado Efetivo
	F	Empregado Efetivo
	F	Empregado Efetivo
	M	Empregado Efetivo
	F	Empregado Efetivo
	M	Empregado Efetivo
	M	Empregado Efetivo

	M	Empregado Efetivo
	F	Empregado Efetivo
	F	Empregado Efetivo
	F	Empregado Efetivo
	F	Empregado Efetivo
	F	Empregado Efetivo
	F	Empregado Efetivo
	F	Empregado Efetivo
	F	Empregado Efetivo
	F	Empregado Efetivo
	F	Empregado Efetivo
	F	Empregado Efetivo
	F	Empregado Efetivo
	F	Empregado Efetivo
	F	Empregado Efetivo
	F	Empregado Efetivo
	F	Empregado Efetivo
	F	Empregado Efetivo
	F	Empregado Efetivo
	F	Empregado Efetivo
	F	Empregado Efetivo
	F	Empregado Efetivo
	F	Empregado Efetivo
	F	Empregado Efetivo
	F	Empregado Efetivo
	F	Empregado Efetivo
	F	Empregado Efetivo
	F	Empregado Efetivo
	F	Empregado Efetivo
	F	Empregado Efetivo
	M	Empregado Efetivo
	F	Empregado Efetivo
	F	Empregado Efetivo
	F	Empregado Efetivo
	F	Empregado Efetivo
	F	Empregado Efetivo
	F	Empregado Efetivo
	F	Empregado Efetivo
	F	Empregado Efetivo
	F	Empregado Efetivo
	F	Empregado Efetivo
	M	Empregado Efetivo
	F	Empregado Efetivo
	F	Empregado Efetivo
	F	Empregado Efetivo
	M	Empregado Efetivo
	F	Empregado Efetivo
	F	Empregado Efetivo

	F	Empregado Efetivo
	F	Empregado Efetivo
	M	Empregado Efetivo
	F	Empregado Efetivo
	F	Empregado Efetivo
	F	Empregado Efetivo
	F	Empregado Efetivo
	M	Empregado Efetivo
	F	Empregado Efetivo
	F	Empregado Efetivo
	F	Empregado Efetivo
	F	Empregado Efetivo
	M	Empregado Efetivo
	M	Empregado Efetivo
	F	Empregado Efetivo
	F	Empregado Efetivo
	F	Empregado Efetivo
	F	Empregado Efetivo
	M	Empregado Efetivo
	F	Empregado Efetivo
	F	Empregado Efetivo
	F	Empregado Efetivo
	F	Empregado Efetivo
	F	Empregado Efetivo
	F	Empregado Efetivo
	M	Empregado Efetivo
	F	Empregado Efetivo
	F	Empregado Efetivo
	F	Empregado Efetivo
	F	Empregado Efetivo
	M	Empregado Efetivo
	F	Empregado Efetivo
	M	Empregado Efetivo
	F	Empregado Efetivo
	F	Empregado Efetivo
	F	Empregado Efetivo
	F	Empregado Efetivo
	M	Empregado Efetivo
	F	Empregado Efetivo
	F	Empregado Efetivo
	F	Empregado Efetivo
	F	Empregado Efetivo
	M	Empregado Efetivo
	F	Empregado Efetivo
	M	Empregado Efetivo
	F	Empregado Efetivo
	M	Empregado Efetivo
	F	Empregado Efetivo

	M	Empregado Efetivo
	M	Empregado Efetivo
	M	Empregado Efetivo
	M	Empregado Efetivo
	F	Empregado Efetivo
	F	Empregado Efetivo
	F	Empregado Efetivo
	F	Empregado Efetivo
	F	Empregado Efetivo
	F	Empregado Efetivo
	F	Empregado Efetivo
	F	Empregado Efetivo
	F	Empregado Efetivo
	F	Empregado Efetivo
	F	Empregado Efetivo
	F	Empregado Efetivo
	M	Empregado Efetivo
	F	Empregado Efetivo
	F	Empregado Efetivo
	F	Empregado Efetivo
	F	Empregado Efetivo
	F	Empregado Efetivo
	F	Empregado Efetivo
	F	Empregado Efetivo
	F	Empregado Efetivo
	F	Empregado Efetivo
	F	Empregado Efetivo
	M	Empregado Efetivo
	F	Empregado Efetivo
	F	Empregado Efetivo
	F	Empregado Efetivo
	F	Empregado Efetivo
	F	Empregado Efetivo
	F	Empregado Efetivo
	F	Empregado Efetivo
	F	Empregado Efetivo
	F	Empregado Efetivo
	M	Empregado Efetivo
	F	Empregado Efetivo
	M	Empregado Efetivo
	F	Empregado Efetivo
	F	Empregado Efetivo
	M	Empregado Efetivo
	F	Empregado Efetivo
	M	Empregado Efetivo
	F	Empregado Efetivo
	F	Empregado Efetivo
	F	Empregado Efetivo

	F	Empregado Efetivo
	F	Empregado Efetivo
	F	Empregado Efetivo
	M	Empregado Efetivo
	F	Empregado Efetivo
	F	Empregado Efetivo
	F	Empregado Efetivo
	F	Empregado Efetivo
	F	Empregado Efetivo
	M	Empregado Efetivo
	M	Empregado Efetivo
	M	Empregado Efetivo
	M	Empregado Efetivo
	M	Empregado Efetivo
	F	Empregado Efetivo
	M	Empregado Efetivo
	M	Empregado Efetivo
	F	Empregado Efetivo
	M	Empregado Efetivo
	M	Empregado Efetivo
	F	Empregado Efetivo
	F	Empregado Efetivo
	F	Empregado Efetivo
	M	Empregado Efetivo
	M	Empregado Efetivo
	M	Empregado Efetivo
	M	Empregado Efetivo
	M	Empregado Efetivo
	M	Empregado Efetivo
	F	Empregado Efetivo
	M	Empregado Efetivo
	M	Empregado Efetivo
	M	Empregado Efetivo
	M	Empregado Efetivo
	F	Empregado Efetivo
	F	Empregado Efetivo
	M	Empregado Efetivo
	M	Empregado Efetivo
	F	Empregado Efetivo
	F	Empregado Efetivo
	F	Empregado Efetivo
	F	Empregado Efetivo
	F	Empregado Efetivo
	F	Empregado Efetivo
	M	Empregado Efetivo
	M	Empregado Efetivo
	M	Empregado Efetivo
	F	Empregado Efetivo

	F	Empregado Efetivo
	F	Empregado Efetivo
	F	Empregado Efetivo
	F	Empregado Efetivo
	F	Empregado Efetivo
	F	Empregado Efetivo
	F	Empregado Efetivo
	F	Empregado Efetivo
	F	Empregado Efetivo
	M	Empregado Efetivo
	F	Empregado Efetivo
	M	Empregado Efetivo
	F	Empregado Efetivo
	M	Empregado Efetivo
	M	Empregado Efetivo
	F	Empregado Efetivo
	F	Empregado Efetivo
	F	Empregado Efetivo
	M	Empregado Efetivo
	F	Empregado Efetivo
	M	Empregado Efetivo
	F	Empregado Efetivo
	M	Empregado Efetivo
	F	Empregado Efetivo
	M	Empregado Efetivo
	F	Empregado Efetivo
	M	Empregado Efetivo
	F	Empregado Efetivo
	F	Empregado Efetivo
	F	Empregado Efetivo
	F	Empregado Efetivo
	M	Empregado Efetivo
	M	Empregado Efetivo
	F	Empregado Efetivo
	F	Empregado Efetivo
	F	Empregado Efetivo
	F	Empregado Efetivo
	F	Empregado Efetivo
	F	Empregado Efetivo
	F	Empregado Efetivo
	F	Empregado Efetivo
	M	Empregado Efetivo
	F	Empregado Efetivo
	F	Empregado Efetivo
	F	Empregado Efetivo
	M	Empregado Efetivo

	M	Empregado Efetivo
	M	Empregado Efetivo
	M	Empregado Efetivo
	M	Empregado Efetivo
	M	Empregado Efetivo
	M	Empregado Efetivo
	F	Empregado Efetivo
	F	Empregado Efetivo
	M	Empregado Efetivo
	M	Empregado Efetivo
	F	Empregado Efetivo
	F	Empregado Efetivo
	F	Empregado Efetivo
	F	Empregado Efetivo
	M	Empregado Efetivo
	M	Empregado Efetivo
	F	Empregado Efetivo
	F	Empregado Efetivo
	M	Empregado Efetivo
	F	Empregado Efetivo
	M	Empregado Efetivo
	F	Empregado Efetivo
	F	Empregado Efetivo
	M	Empregado Efetivo
	F	Empregado Efetivo
	F	Empregado Efetivo
	F	Empregado Efetivo
	M	Empregado Efetivo
	M	Empregado Efetivo
	F	Empregado Efetivo
	F	Empregado Efetivo
	F	Empregado Efetivo
	F	Empregado Efetivo
	F	Empregado Efetivo
	F	Empregado Efetivo
	F	Empregado Efetivo
	F	Empregado Efetivo
	F	Empregado Efetivo
	M	Empregado Efetivo
	M	Empregado Efetivo
	F	Empregado Efetivo
	M	Empregado Efetivo
	F	Empregado Efetivo
	F	Empregado Efetivo
	F	Empregado Efetivo
	F	Empregado Efetivo
	F	Empregado Efetivo

	F	Empregado Efetivo
	F	Empregado Efetivo
	F	Empregado Efetivo
	F	Empregado Efetivo
	F	Empregado Efetivo
	F	Empregado Efetivo
	F	Empregado Efetivo
	M	Empregado Efetivo
	F	Empregado Efetivo
	F	Empregado Efetivo
	F	Empregado Efetivo
	F	Empregado Efetivo
	M	Empregado Efetivo
	F	Empregado Efetivo
	M	Empregado Efetivo
	M	Empregado Efetivo
	M	Empregado Efetivo
	M	Empregado Efetivo
	F	Empregado Efetivo
	F	Empregado Efetivo
	F	Empregado Efetivo
	F	Empregado Efetivo
	M	Empregado Efetivo
	F	Empregado Efetivo
	F	Empregado Efetivo
	F	Empregado Efetivo
	M	Empregado Efetivo
	F	Empregado Efetivo
	F	Empregado Efetivo
	M	Empregado Efetivo
	F	Empregado Efetivo
	F	Empregado Efetivo
	M	Empregado Efetivo
	F	Empregado Efetivo
	F	Empregado Efetivo
	M	Empregado Efetivo
	F	Empregado Efetivo
	F	Empregado Efetivo
	F	Empregado Efetivo
	F	Empregado Efetivo
	F	Empregado Efetivo
	M	Empregado Efetivo
	F	Empregado Efetivo
	F	Empregado Efetivo
	M	Empregado Efetivo
	M	Empregado Efetivo
	F	Empregado Efetivo
	F	Empregado Efetivo

	M	Empregado Efetivo
	F	Empregado Efetivo
	M	Empregado Efetivo
	F	Empregado Efetivo
	F	Empregado Efetivo
	F	Empregado Efetivo
	F	Empregado Efetivo
	F	Empregado Efetivo
	M	Empregado Efetivo
	F	Empregado Efetivo
	F	Empregado Efetivo
	M	Empregado Efetivo
	F	Empregado Efetivo
	F	Empregado Efetivo
	F	Empregado Efetivo
	F	Empregado Efetivo
	F	Empregado Efetivo
	F	Empregado Efetivo
	F	Empregado Efetivo
	F	Empregado Efetivo
	M	Empregado Efetivo
	M	Empregado Efetivo
	M	Empregado Efetivo
	F	Empregado Efetivo
	F	Empregado Efetivo
	F	Empregado Efetivo
	F	Empregado Efetivo
	F	Empregado Efetivo
	M	Empregado Efetivo
	F	Empregado Efetivo
	F	Empregado Efetivo
	F	Empregado Efetivo
	F	Empregado Efetivo
	F	Empregado Efetivo
	F	Empregado Efetivo
	F	Empregado Efetivo
	F	Empregado Efetivo
	F	Empregado Efetivo
	F	Empregado Efetivo
	F	Empregado Efetivo
	F	Empregado Efetivo
	F	Empregado Efetivo
	F	Empregado Efetivo
	F	Empregado Efetivo
	F	Empregado Efetivo
	F	Empregado Efetivo
	F	Empregado Efetivo

	F	Empregado Efetivo
	F	Empregado Efetivo
	F	Empregado Efetivo
	F	Empregado Efetivo
	F	Empregado Efetivo
	F	Empregado Efetivo
	F	Empregado Efetivo
	F	Empregado Efetivo
	F	Empregado Efetivo
	F	Empregado Efetivo
	F	Empregado Efetivo
	F	Empregado Efetivo
	F	Empregado Efetivo
	F	Empregado Efetivo
	F	Empregado Efetivo
	F	Empregado Efetivo
	F	Empregado Efetivo
	F	Empregado Efetivo
	F	Empregado Efetivo
	F	Empregado Efetivo
	F	Empregado Efetivo
	F	Empregado Efetivo
	F	Empregado Efetivo
	F	Empregado Efetivo
	F	Empregado Efetivo
	F	Empregado Efetivo
	F	Empregado Efetivo
	F	Empregado Efetivo
	F	Empregado Efetivo
	F	Empregado Efetivo
	F	Empregado Efetivo
	F	Empregado Efetivo
	F	Empregado Efetivo
	F	Empregado Efetivo
	F	Empregado Efetivo
	F	Empregado Efetivo
	F	Empregado Efetivo
	F	Empregado Efetivo
	F	Empregado Efetivo
	F	Empregado Efetivo
	F	Empregado Efetivo
	F	Empregado Efetivo
	F	Empregado Efetivo
	F	Empregado Efetivo
	F	Empregado Efetivo
	F	Empregado Efetivo
	F	Empregado Efetivo
	F	Empregado Efetivo

	F	Empregado Efetivo
	F	Empregado Efetivo
	F	Empregado Efetivo
	F	Empregado Efetivo
	F	Empregado Efetivo
	F	Empregado Efetivo
	F	Empregado Efetivo
	F	Empregado Efetivo
	F	Empregado Efetivo
	F	Empregado Efetivo
	F	Empregado Efetivo
	F	Empregado Efetivo
	F	Empregado Efetivo
	F	Empregado Efetivo
	F	Empregado Efetivo
	F	Empregado Efetivo
	F	Empregado Efetivo
	F	Empregado Efetivo
	F	Empregado Efetivo
	F	Empregado Efetivo
	F	Empregado Efetivo
	F	Empregado Efetivo
	F	Empregado Efetivo
	F	Empregado Efetivo
	F	Empregado Efetivo
	F	Empregado Efetivo
	F	Empregado Efetivo
	F	Empregado Efetivo
	F	Empregado Efetivo
	F	Empregado Efetivo
	F	Empregado Efetivo
	F	Empregado Efetivo
	F	Empregado Efetivo
	F	Empregado Efetivo
	F	Empregado Efetivo
	F	Empregado Efetivo
	F	Empregado Efetivo
	F	Empregado Efetivo
	F	Empregado Efetivo
	F	Empregado Efetivo
	F	Empregado Efetivo
	F	Empregado Efetivo
	F	Empregado Efetivo
	F	Empregado Efetivo
	F	Empregado Efetivo
	F	Empregado Efetivo
	F	Empregado Efetivo
	F	Empregado Efetivo

	F	Empregado Efetivo
	F	Empregado Efetivo
	F	Empregado Efetivo
	F	Empregado Efetivo
	F	Empregado Efetivo
	F	Empregado Efetivo
	F	Empregado Efetivo
	F	Empregado Efetivo
	F	Empregado Efetivo
	F	Empregado Efetivo
	F	Empregado Efetivo
	F	Empregado Efetivo
	F	Empregado Efetivo
	F	Empregado Efetivo
	F	Empregado Efetivo
	F	Empregado Efetivo
	F	Empregado Efetivo
	F	Empregado Efetivo
	F	Empregado Efetivo
	F	Empregado Efetivo
	F	Empregado Efetivo
	F	Empregado Efetivo
	F	Empregado Efetivo
	F	Empregado Efetivo
	F	Empregado Efetivo
	F	Empregado Efetivo
	F	Empregado Efetivo
	F	Empregado Efetivo
	F	Empregado Efetivo
	F	Empregado Efetivo
	F	Empregado Efetivo
	F	Empregado Efetivo
	F	Empregado Efetivo
	F	Empregado Efetivo
	F	Empregado Efetivo
	F	Empregado Efetivo
	F	Empregado Efetivo
	F	Empregado Efetivo
	F	Empregado Efetivo
	F	Empregado Efetivo
	F	Empregado Efetivo
	F	Empregado Efetivo
	F	Empregado Efetivo
	F	Empregado Efetivo
	F	Empregado Efetivo

	F	Empregado Efetivo
	F	Empregado Efetivo
	F	Empregado Efetivo
	F	Empregado Efetivo
	F	Empregado Efetivo
	F	Empregado Efetivo
	F	Empregado Efetivo
	F	Empregado Efetivo
	F	Empregado Efetivo
	F	Empregado Efetivo
	F	Empregado Efetivo
	M	Empregado Efetivo
	M	Empregado Efetivo
	M	Empregado Efetivo
	M	Empregado Efetivo
	M	Empregado Efetivo
	M	Empregado Efetivo
	M	Empregado Efetivo
	M	Empregado Efetivo
	M	Empregado Efetivo
	M	Empregado Efetivo
	F	Empregado Efetivo
	F	Empregado Efetivo
	F	Empregado Efetivo
	M	Comissionado
	F	Empregado Efetivo
	F	Empregado Efetivo
	M	Empregado Efetivo
	F	Empregado Efetivo
	F	Empregado Efetivo
	F	Empregado Efetivo
	F	Empregado Efetivo
	F	Empregado Efetivo
	F	Empregado Efetivo
	F	Empregado Efetivo
	F	Empregado Efetivo
	M	Empregado Efetivo
	F	Empregado Efetivo
	F	Empregado Efetivo
	F	Empregado Efetivo
	F	Empregado Efetivo
	F	Empregado Efetivo
	F	Empregado Efetivo

	F	Empregado Efetivo
	F	Empregado Efetivo
	F	Empregado Efetivo
	M	Empregado Efetivo
	M	Empregado Efetivo
	F	Empregado Efetivo
	F	Empregado Efetivo
	F	Empregado Efetivo
	F	Empregado Efetivo
	F	Empregado Efetivo
	F	Empregado Efetivo
	F	Empregado Efetivo
	F	Empregado Efetivo
	F	Empregado Efetivo
	F	Empregado Efetivo
	F	Empregado Efetivo
	M	Empregado Efetivo
	M	Empregado Efetivo
	F	Empregado Efetivo
	F	Empregado Efetivo
	F	Empregado Efetivo
	F	Empregado Efetivo
	F	Empregado Efetivo
	F	Empregado Efetivo
	F	Empregado Efetivo
	F	Empregado Efetivo
	F	Empregado Efetivo
	F	Empregado Efetivo
	F	Empregado Efetivo
	F	Empregado Efetivo
	F	Empregado Efetivo
	M	Empregado Efetivo
	F	Empregado Efetivo
	F	Empregado Efetivo
	F	Empregado Efetivo
	F	Empregado Efetivo
	M	Empregado Efetivo
	F	Empregado Efetivo
	M	Empregado Efetivo
	M	Empregado Efetivo
	F	Empregado Efetivo
	F	Empregado Efetivo
	F	Empregado Efetivo
	F	Empregado Efetivo
	F	Empregado Efetivo
	F	Empregado Efetivo
	F	Empregado Efetivo
	F	Empregado Efetivo

	M	Empregado Efetivo
	F	Empregado Efetivo
	F	Empregado Efetivo
	F	Empregado Efetivo
	F	Empregado Efetivo
	F	Empregado Efetivo
	F	Empregado Efetivo
	M	Empregado Efetivo
	F	Empregado Efetivo
	F	Empregado Efetivo
	F	Empregado Efetivo
	M	Empregado Efetivo
	F	Empregado Efetivo
	M	Empregado Efetivo
	F	Empregado Efetivo
	F	Empregado Efetivo
	F	Empregado Efetivo
	F	Empregado Efetivo
	F	Empregado Efetivo
	F	Empregado Efetivo
	F	Empregado Efetivo
	F	Empregado Efetivo
	F	Empregado Efetivo
	F	Empregado Efetivo
	F	Empregado Efetivo
	F	Empregado Efetivo
	F	Empregado Efetivo
	F	Empregado Efetivo
	F	Empregado Efetivo
	F	Empregado Efetivo
	F	Empregado Efetivo
	F	Empregado Efetivo
	F	Empregado Efetivo
	M	Empregado Efetivo
	M	Empregado Efetivo
	F	Empregado Efetivo
	M	Empregado Efetivo
	M	Empregado Efetivo
	M	Empregado Efetivo
	F	Empregado Efetivo
	F	Empregado Efetivo
	F	Empregado Efetivo
	F	Empregado Efetivo
	F	Empregado Efetivo
	F	Empregado Efetivo
	F	Empregado Efetivo
	F	Empregado Efetivo

	F	Empregado Efetivo
	F	Empregado Efetivo
	F	Empregado Efetivo
	M	Empregado Efetivo
	F	Empregado Efetivo
	F	Empregado Efetivo
	F	Empregado Efetivo
	F	Empregado Efetivo
	F	Empregado Efetivo
	F	Empregado Efetivo
	M	Empregado Efetivo
	M	Empregado Efetivo
	F	Empregado Efetivo
	F	Empregado Efetivo
	F	Empregado Efetivo
	F	Empregado Efetivo
	F	Empregado Efetivo
	M	Empregado Efetivo
	M	Empregado Efetivo
	M	Empregado Efetivo
	F	Empregado Efetivo
	M	Empregado Efetivo
	F	Empregado Efetivo
	F	Empregado Efetivo
	M	Empregado Efetivo
	F	Empregado Efetivo
	M	Empregado Efetivo
	F	Empregado Efetivo
	F	Empregado Efetivo
	F	Empregado Efetivo
	M	Empregado Efetivo
	F	Empregado Efetivo
	M	Empregado Efetivo
	F	Empregado Efetivo
	M	Empregado Efetivo
	F	Empregado Efetivo
	M	Empregado Efetivo
	F	Empregado Efetivo
	F	Empregado Efetivo
	M	Empregado Efetivo
	F	Empregado Efetivo
	F	Empregado Efetivo
	F	Empregado Efetivo
	F	Empregado Efetivo
	F	Empregado Efetivo

	F	Empregado Efetivo
	F	Empregado Efetivo
	F	Empregado Efetivo
	F	Empregado Efetivo
	F	Empregado Efetivo
	F	Empregado Efetivo
	F	Empregado Efetivo
	F	Empregado Efetivo
	F	Empregado Efetivo
	M	Empregado Efetivo
	F	Empregado Efetivo
	F	Empregado Efetivo
	F	Empregado Efetivo
	F	Empregado Efetivo
	M	Empregado Efetivo
	F	Empregado Efetivo
	F	Empregado Efetivo
	F	Empregado Efetivo
	F	Empregado Efetivo
	F	Empregado Efetivo
	F	Empregado Efetivo
	F	Empregado Efetivo
	F	Empregado Efetivo
	F	Empregado Efetivo
	F	Empregado Efetivo
	M	Empregado Efetivo
	M	Empregado Efetivo
	M	Empregado Efetivo
	M	Empregado Efetivo
	M	Empregado Efetivo
	F	Empregado Efetivo
	M	Empregado Efetivo
	F	Empregado Efetivo
	M	Empregado Efetivo
	M	Empregado Efetivo
	F	Empregado Efetivo
	F	Empregado Efetivo
	F	Empregado Efetivo
	F	Empregado Efetivo
	F	Empregado Efetivo
	F	Empregado Efetivo
	F	Empregado Efetivo
	F	Empregado Efetivo
	M	Empregado Efetivo
	F	Empregado Efetivo
	F	Empregado Efetivo
	F	Empregado Efetivo
	M	Empregado Efetivo

	F	Empregado Efetivo
	F	Empregado Efetivo
	F	Empregado Efetivo
	M	Empregado Efetivo
	F	Empregado Efetivo
	F	Empregado Efetivo
	F	Empregado Efetivo
	M	Empregado Efetivo
	F	Empregado Efetivo
	F	Empregado Efetivo
	F	Empregado Efetivo
	F	Empregado Efetivo
	F	Empregado Efetivo
	F	Empregado Efetivo
	F	Empregado Efetivo
	M	Empregado Efetivo
	M	Empregado Efetivo
	F	Empregado Efetivo
	F	Empregado Efetivo
	F	Empregado Efetivo
	M	Empregado Efetivo
	F	Empregado Efetivo
	F	Empregado Efetivo
	F	Empregado Efetivo
	F	Empregado Efetivo
	F	Empregado Efetivo
	F	Empregado Efetivo
	F	Empregado Efetivo
	F	Empregado Efetivo
	M	Empregado Efetivo
	M	Empregado Efetivo
	M	Empregado Efetivo
	F	Empregado Efetivo
	F	Empregado Efetivo
	F	Empregado Efetivo
	F	Empregado Efetivo
	M	Empregado Efetivo
	F	Empregado Efetivo
	F	Empregado Efetivo
	M	Empregado Efetivo
	F	Empregado Efetivo
	F	Empregado Efetivo
	F	Empregado Efetivo
	M	Empregado Efetivo
	F	Empregado Efetivo

	F	Empregado Efetivo
	F	Empregado Efetivo
	F	Empregado Efetivo
	F	Empregado Efetivo
	F	Empregado Efetivo
	F	Empregado Efetivo
	M	Empregado Efetivo
	F	Empregado Efetivo
	F	Empregado Efetivo
	F	Empregado Efetivo
	F	Empregado Efetivo
	F	Empregado Efetivo
	F	Empregado Efetivo
	F	Empregado Efetivo
	F	Empregado Efetivo
	F	Empregado Efetivo
	M	Empregado Efetivo
	F	Empregado Efetivo
	F	Empregado Efetivo
	F	Empregado Efetivo
	F	Empregado Efetivo
	F	Empregado Efetivo
	F	Empregado Efetivo
	M	Empregado Efetivo
	F	Empregado Efetivo
	F	Empregado Efetivo
	F	Empregado Efetivo
	F	Empregado Efetivo
	F	Empregado Efetivo
	F	Empregado Efetivo
	F	Empregado Efetivo
	F	Empregado Efetivo
	F	Empregado Efetivo
	F	Empregado Efetivo
	F	Empregado Efetivo
	F	Empregado Efetivo
	F	Empregado Efetivo
	F	Empregado Efetivo
	F	Empregado Efetivo
	F	Empregado Efetivo
	F	Empregado Efetivo
	F	Empregado Efetivo
	F	Empregado Efetivo
	F	Empregado Efetivo
	F	Empregado Efetivo
	F	Empregado Efetivo

	F	Empregado Efetivo
	F	Empregado Efetivo
	F	Empregado Efetivo
	F	Empregado Efetivo
	F	Empregado Efetivo
	F	Empregado Efetivo
	F	Empregado Efetivo
	F	Empregado Efetivo
	F	Empregado Efetivo
	F	Empregado Efetivo
	F	Empregado Efetivo
	F	Empregado Efetivo
	F	Empregado Efetivo
	F	Empregado Efetivo
	F	Empregado Efetivo
	F	Empregado Efetivo
	F	Empregado Efetivo
	F	Empregado Efetivo
	F	Empregado Efetivo
	F	Empregado Efetivo
	F	Empregado Efetivo
	F	Empregado Efetivo
	F	Empregado Efetivo
	F	Empregado Efetivo
	F	Empregado Efetivo
	F	Empregado Efetivo
	F	Empregado Efetivo
	F	Empregado Efetivo
	F	Empregado Efetivo
	F	Empregado Efetivo
	F	Empregado Efetivo
	F	Empregado Efetivo
	F	Empregado Efetivo
	F	Empregado Efetivo
	F	Empregado Efetivo
	F	Empregado Efetivo
	F	Empregado Efetivo
	F	Empregado Efetivo
	F	Empregado Efetivo
	F	Empregado Efetivo
	F	Empregado Efetivo

	F	Empregado Efetivo
	F	Empregado Efetivo
	F	Empregado Efetivo
	F	Empregado Efetivo
	F	Empregado Efetivo
	F	Empregado Efetivo
	F	Empregado Efetivo
	F	Empregado Efetivo
	F	Empregado Efetivo
	F	Empregado Efetivo
	F	Empregado Efetivo
	F	Empregado Efetivo
	F	Empregado Efetivo
	F	Empregado Efetivo
	F	Empregado Efetivo
	F	Empregado Efetivo
	F	Empregado Efetivo
	F	Empregado Efetivo
	F	Empregado Efetivo
	F	Empregado Efetivo
	F	Empregado Efetivo
	F	Empregado Efetivo
	F	Empregado Efetivo
	F	Empregado Efetivo
	F	Empregado Efetivo
	F	Empregado Efetivo
	F	Empregado Efetivo
	F	Empregado Efetivo
	F	Empregado Efetivo
	F	Empregado Efetivo
	F	Empregado Efetivo
	M	Empregado Efetivo
	F	Empregado Efetivo
	M	Empregado Efetivo
	F	Empregado Efetivo
	F	Empregado Efetivo
	F	Empregado Efetivo
	F	Empregado Efetivo
	F	Empregado Efetivo
	M	Empregado Efetivo

	F	Empregado Efetivo
	F	Empregado Efetivo
	F	Empregado Efetivo
	F	Empregado Efetivo
	F	Empregado Efetivo
	F	Empregado Efetivo
	F	Empregado Efetivo
	F	Empregado Efetivo
	F	Empregado Efetivo
	F	Empregado Efetivo
	F	Empregado Efetivo
	F	Empregado Efetivo
	F	Empregado Efetivo
	F	Empregado Efetivo
	F	Empregado Efetivo
	F	Empregado Efetivo
	F	Empregado Efetivo
	F	Empregado Efetivo
	F	Empregado Efetivo
	F	Empregado Efetivo
	F	Empregado Efetivo
	F	Empregado Efetivo
	F	Empregado Efetivo
	F	Empregado Efetivo
	F	Empregado Efetivo
	F	Empregado Efetivo
	M	Empregado Efetivo
	F	Empregado Efetivo
	M	Empregado Efetivo
	F	Empregado Efetivo
	F	Empregado Efetivo
	F	Empregado Efetivo

	F	Empregado Efetivo
	F	Empregado Efetivo
	F	Empregado Efetivo
	F	Empregado Efetivo
	F	Empregado Efetivo
	F	Empregado Efetivo
	F	Empregado Efetivo
	F	Empregado Efetivo
	F	Empregado Efetivo
	F	Empregado Efetivo
	F	Empregado Efetivo
	F	Empregado Efetivo
	F	Empregado Efetivo
	F	Empregado Efetivo
	F	Empregado Efetivo
	F	Empregado Efetivo
	F	Empregado Efetivo
	F	Empregado Efetivo
	F	Empregado Efetivo
	F	Empregado Efetivo
	F	Empregado Efetivo
	F	Empregado Efetivo
	F	Empregado Efetivo
	F	Empregado Efetivo
	F	Empregado Efetivo
	F	Empregado Efetivo
	F	Empregado Efetivo
	F	Empregado Efetivo
	F	Empregado Efetivo
	F	Empregado Efetivo
	F	Empregado Efetivo
	F	Empregado Efetivo
	F	Empregado Efetivo
	F	Empregado Efetivo
	F	Empregado Efetivo
	F	Empregado Efetivo
	F	Empregado Efetivo
	F	Empregado Efetivo
	F	Empregado Efetivo
	F	Empregado Efetivo
	F	Empregado Efetivo
	F	Empregado Efetivo
	F	Empregado Efetivo

	F	Empregado Efetivo
	F	Empregado Efetivo
	F	Empregado Efetivo
	F	Empregado Efetivo
	F	Empregado Efetivo
	F	Empregado Efetivo
	F	Empregado Efetivo
	F	Empregado Efetivo
	F	Empregado Efetivo
	F	Empregado Efetivo
	F	Empregado Efetivo
	F	Empregado Efetivo
	F	Empregado Efetivo
	F	Empregado Efetivo
	F	Empregado Efetivo
	F	Empregado Efetivo
	F	Empregado Efetivo
	F	Empregado Efetivo
	F	Empregado Efetivo
	F	Empregado Efetivo
	F	Empregado Efetivo
	F	Empregado Efetivo
	M	Cedido - RJU
	F	Empregado Efetivo
	M	Empregado Efetivo
	F	Empregado Efetivo
	F	Empregado Efetivo
	F	Empregado Efetivo
	F	Empregado Efetivo
	F	Empregado Efetivo
	F	Empregado Efetivo
	F	Empregado Efetivo
	F	Empregado Efetivo
	F	Empregado Efetivo
	M	Empregado Efetivo
	F	Empregado Efetivo
	F	Empregado Efetivo
	M	Empregado Efetivo
	M	Empregado Efetivo
	M	Empregado Efetivo
	F	Empregado Efetivo
	F	Empregado Efetivo
	F	Empregado Efetivo
	F	Empregado Efetivo

	M	Empregado Efetivo
	M	Empregado Efetivo
	F	Empregado Efetivo
	M	Empregado Efetivo
	F	Empregado Efetivo
	F	Empregado Efetivo
	M	Empregado Efetivo
	F	Empregado Efetivo
	M	Comissionado
	M	Cedido - RJU
	F	Empregado Efetivo
	F	Empregado Efetivo
	F	Empregado Efetivo
	M	Empregado Efetivo
	F	Empregado Efetivo
	F	Empregado Efetivo
	F	Empregado Efetivo
	F	Empregado Efetivo
	F	Empregado Efetivo
	M	Empregado Efetivo
	M	Empregado Efetivo
	F	Empregado Efetivo
	M	Empregado Efetivo
	F	Empregado Efetivo
	M	Empregado Efetivo
	F	Empregado Efetivo
	F	Empregado Efetivo
	F	Empregado Efetivo
	F	Empregado Efetivo
	M	Empregado Efetivo
	M	Empregado Efetivo
	M	Empregado Efetivo
	M	Empregado Efetivo
	F	Empregado Efetivo

	F	Empregado Efetivo
	M	Empregado Efetivo
	F	Empregado Efetivo
	F	Empregado Efetivo
	F	Empregado Efetivo
	F	Empregado Efetivo
	M	Empregado Efetivo
	M	Empregado Efetivo
	F	Empregado Efetivo
	M	Empregado Efetivo
	F	Empregado Efetivo
	F	Empregado Efetivo
	M	Empregado Efetivo
	M	Empregado Efetivo
	M	Empregado Efetivo
	Rower	02/02/4748
	F	Empregado Efetivo
	F	Empregado Efetivo
	F	Empregado Efetivo
	F	Empregado Efetivo
	M	Empregado Efetivo
	F	Empregado Efetivo
	F	Empregado Efetivo
	F	Empregado Efetivo
	F	Empregado Efetivo
	F	Empregado Efetivo
	F	Empregado Efetivo
	F	Empregado Efetivo
	F	Empregado Efetivo
	F	Empregado Efetivo
	F	Empregado Efetivo
	F	Empregado Efetivo

	M	Empregado Efetivo
	M	Empregado Efetivo
	M	Empregado Efetivo
	F	Empregado Efetivo
	M	Empregado Efetivo
	F	Empregado Efetivo
	F	Empregado Efetivo
	F	Empregado Efetivo
	F	Empregado Efetivo
	F	Empregado Efetivo
	F	Empregado Efetivo
	F	Empregado Efetivo
	M	Empregado Efetivo
	F	Empregado Efetivo
	F	Empregado Efetivo
	M	Empregado Efetivo
	M	Empregado Efetivo
	F	Empregado Efetivo
	M	Empregado Efetivo
	F	Empregado Efetivo
	F	Empregado Efetivo
	M	Empregado Efetivo
	F	Empregado Efetivo
	F	Empregado Efetivo
	F	Empregado Efetivo
	F	Empregado Efetivo
	F	Empregado Efetivo
	M	Empregado Efetivo
	M	Empregado Efetivo
	F	Empregado Efetivo
	F	Empregado Efetivo
	M	Empregado Efetivo

	F	Empregado Efetivo
	M	Empregado Efetivo
	M	Empregado Efetivo
	M	Empregado Efetivo
	F	Empregado Efetivo
	F	Empregado Efetivo
	F	Empregado Efetivo
	M	Empregado Efetivo
	F	Empregado Efetivo
	M	Empregado Efetivo
	F	Empregado Efetivo
	F	Empregado Efetivo
	F	Empregado Efetivo
	F	Empregado Efetivo
	M	Empregado Efetivo
	F	Empregado Efetivo
	F	Empregado Efetivo
	F	Empregado Efetivo
	F	Empregado Efetivo
	M	Empregado Efetivo
	F	Empregado Efetivo
	F	Empregado Efetivo
	F	Empregado Efetivo
	F	Empregado Efetivo
	M	Empregado Efetivo
	F	Empregado Efetivo
	F	Empregado Efetivo
	F	Empregado Efetivo
	F	Empregado Efetivo
	F	Empregado Efetivo
	F	Empregado Efetivo
	F	Empregado Efetivo
	F	Empregado Efetivo
	M	Empregado Efetivo
	F	Empregado Efetivo
	F	Empregado Efetivo
	M	Empregado Efetivo
	F	Empregado Efetivo
	F	Empregado Efetivo
	F	Empregado Efetivo
	F	Empregado Efetivo
	F	Empregado Efetivo
	F	Empregado Efetivo
	F	Empregado Efetivo

	F	Empregado Efetivo
	F	Empregado Efetivo
	F	Empregado Efetivo
	F	Empregado Efetivo
	F	Empregado Efetivo
	F	Empregado Efetivo
	M	Empregado Efetivo
	M	Empregado Efetivo
	M	Empregado Efetivo
	F	Empregado Efetivo
	F	Empregado Efetivo
	F	Empregado Efetivo
	F	Empregado Efetivo
	M	Empregado Efetivo
	M	Empregado Efetivo
	M	Empregado Efetivo
	F	Empregado Efetivo
	F	Empregado Efetivo
	M	Empregado Efetivo
	F	Empregado Efetivo
	F	Empregado Efetivo
	F	Empregado Efetivo
	F	Empregado Efetivo
	F	Empregado Efetivo
	M	Empregado Efetivo
	M	Empregado Efetivo
	F	Empregado Efetivo
	F	Empregado Efetivo
	M	Empregado Efetivo
	F	Empregado Efetivo
	F	Empregado Efetivo
	M	Empregado Efetivo
	F	Empregado Efetivo
	M	Empregado Efetivo
	M	Empregado Efetivo
	F	Empregado Efetivo
	M	Empregado Efetivo
	M	Empregado Efetivo
	M	Empregado Efetivo
	F	Empregado Efetivo
	F	Empregado Efetivo
	M	Empregado Efetivo
	F	Empregado Efetivo
	F	Empregado Efetivo

	F	Empregado Efetivo
	F	Empregado Efetivo
	M	Empregado Efetivo
	F	Empregado Efetivo
	F	Empregado Efetivo
	M	Empregado Efetivo
	M	Empregado Efetivo
	M	Empregado Efetivo
	F	Empregado Efetivo
	M	Empregado Efetivo
	F	Empregado Efetivo
	F	Empregado Efetivo
	F	Empregado Efetivo
	F	Empregado Efetivo
	F	Empregado Efetivo
	F	Empregado Efetivo
	F	Empregado Efetivo
	F	Empregado Efetivo
	F	Empregado Efetivo
	F	Empregado Efetivo
	M	Empregado Efetivo
	F	Empregado Efetivo
	F	Empregado Efetivo
	F	Empregado Efetivo
	M	Empregado Efetivo
	F	Empregado Efetivo
	F	Empregado Efetivo
	F	Empregado Efetivo
	F	Empregado Efetivo
	F	Empregado Efetivo
	F	Empregado Efetivo
	F	Empregado Efetivo
	F	Empregado Efetivo
	F	Empregado Efetivo
	F	Empregado Efetivo
	F	Empregado Efetivo
	F	Empregado Efetivo
	F	Empregado Efetivo
	F	Empregado Efetivo
	F	Empregado Efetivo
	M	Empregado Efetivo
	F	Empregado Efetivo
	F	Empregado Efetivo
	F	Empregado Efetivo
	F	Empregado Efetivo
	F	Empregado Efetivo
	F	Empregado Efetivo
	F	Empregado Efetivo

	M	Empregado Efetivo
	F	Empregado Efetivo
	F	Empregado Efetivo
	F	Empregado Efetivo
	F	Empregado Efetivo
	F	Empregado Efetivo
	F	Empregado Efetivo
	F	Empregado Efetivo
	F	Empregado Efetivo
	F	Empregado Efetivo
	F	Empregado Efetivo
	F	Empregado Efetivo
	F	Empregado Efetivo
	M	Empregado Efetivo
	F	Empregado Efetivo
	F	Empregado Efetivo
	M	Empregado Efetivo
	F	Empregado Efetivo
	F	Empregado Efetivo
	F	Empregado Efetivo
	F	Empregado Efetivo
	F	Empregado Efetivo
	F	Empregado Efetivo
	F	Empregado Efetivo
	F	Empregado Efetivo
	F	Empregado Efetivo
	F	Empregado Efetivo
	F	Empregado Efetivo
	F	Empregado Efetivo
	F	Empregado Efetivo
	F	Empregado Efetivo
	F	Empregado Efetivo
	F	Empregado Efetivo
	F	Empregado Efetivo
	F	Empregado Efetivo
	F	Empregado Efetivo
	F	Empregado Efetivo
	F	Empregado Efetivo
	F	Empregado Efetivo
	F	Empregado Efetivo
	F	Empregado Efetivo
	F	Empregado Efetivo
	F	Empregado Efetivo
	F	Empregado Efetivo
	F	Empregado Efetivo
	F	Empregado Efetivo
	F	Empregado Efetivo
	F	Empregado Efetivo

	F	Empregado Efetivo
	F	Empregado Efetivo
	F	Empregado Efetivo
	F	Empregado Efetivo
	M	Empregado Efetivo
	M	Empregado Efetivo
	M	Empregado Efetivo
	M	Empregado Efetivo
	M	Empregado Efetivo
	M	Empregado Efetivo
	M	Empregado Efetivo
	M	Empregado Efetivo
	M	Empregado Efetivo
	M	Empregado Efetivo
	F	Empregado Efetivo
	F	Empregado Efetivo
	F	Empregado Efetivo
	F	Empregado Efetivo
	F	Empregado Efetivo
	F	Empregado Efetivo
	F	Empregado Efetivo
	F	Empregado Efetivo
	F	Empregado Efetivo
	F	Empregado Efetivo
	F	Empregado Efetivo
	F	Empregado Efetivo
	F	Empregado Efetivo
	F	Empregado Efetivo
	F	Empregado Efetivo
	F	Empregado Efetivo
	F	Empregado Efetivo
	F	Empregado Efetivo
	F	Empregado Efetivo
	F	Empregado Efetivo
	F	Empregado Efetivo
	F	Empregado Efetivo
	F	Empregado Efetivo
	F	Empregado Efetivo
	F	Empregado Efetivo
	F	Empregado Efetivo
	F	Empregado Efetivo
	M	Empregado Efetivo
	M	Empregado Efetivo
	M	Empregado Efetivo

	M	Empregado Efetivo
	F	Empregado Efetivo
	F	Empregado Efetivo
	F	Empregado Efetivo
	F	Empregado Efetivo
	F	Empregado Efetivo
	F	Empregado Efetivo
	F	Empregado Efetivo
	F	Empregado Efetivo
	M	Empregado Efetivo
	M	Empregado Efetivo
	F	Empregado Efetivo
	F	Empregado Efetivo
	M	Empregado Efetivo
	M	Empregado Efetivo
	F	Empregado Efetivo
	F	Empregado Efetivo
	F	Empregado Efetivo
	F	Empregado Efetivo
	F	Empregado Efetivo
	F	Empregado Efetivo
	F	Empregado Efetivo
	F	Empregado Efetivo
	F	Empregado Efetivo
	F	Empregado Efetivo
	F	Empregado Efetivo
	F	Empregado Efetivo
	F	Empregado Efetivo
	F	Empregado Efetivo
	F	Empregado Efetivo
	F	Empregado Efetivo
	F	Empregado Efetivo
	F	Empregado Efetivo
	F	Empregado Efetivo
	F	Empregado Efetivo
	F	Empregado Efetivo
	F	Empregado Efetivo
	F	Empregado Efetivo
	F	Empregado Efetivo
	F	Empregado Efetivo

	F	Empregado Efetivo
	F	Empregado Efetivo
	F	Empregado Efetivo
	F	Empregado Efetivo
	F	Empregado Efetivo
	F	Empregado Efetivo
	F	Empregado Efetivo
	F	Empregado Efetivo
	F	Empregado Efetivo
	F	Empregado Efetivo
	F	Empregado Efetivo
	F	Empregado Efetivo
	F	Empregado Efetivo
	F	Empregado Efetivo
	F	Empregado Efetivo
	F	Empregado Efetivo
	F	Empregado Efetivo
	F	Empregado Efetivo
	F	Empregado Efetivo
	F	Empregado Efetivo
	F	Empregado Efetivo
	F	Empregado Efetivo
	F	Empregado Efetivo
	M	Empregado Efetivo
	F	Empregado Efetivo
	F	Empregado Efetivo
	F	Empregado Efetivo
	M	Empregado Efetivo
	M	Empregado Efetivo
	F	Empregado Efetivo
	F	Empregado Efetivo
	F	Empregado Efetivo
	F	Empregado Efetivo
	F	Empregado Efetivo
	F	Empregado Efetivo
	F	Empregado Efetivo
	F	Empregado Efetivo
	F	Empregado Efetivo
	F	Empregado Efetivo
	F	Empregado Efetivo
	F	Empregado Efetivo
	F	Empregado Efetivo
	F	Empregado Efetivo
	F	Empregado Efetivo
	F	Empregado Efetivo
	F	Empregado Efetivo
	F	Empregado Efetivo

	F	Empregado Efetivo
	M	Empregado Efetivo
	M	Empregado Efetivo
	F	Empregado Efetivo
	M	Empregado Efetivo
	M	Empregado Efetivo
	M	Empregado Efetivo
	F	Empregado Efetivo
	F	Empregado Efetivo
	F	Empregado Efetivo
	F	Empregado Efetivo
	F	Empregado Efetivo
	F	Empregado Efetivo
	F	Empregado Efetivo
	M	Empregado Efetivo
	M	Empregado Efetivo
	M	Empregado Efetivo
	F	Empregado Efetivo
	F	Empregado Efetivo
	M	Empregado Efetivo
	M	Empregado Efetivo
	M	Empregado Efetivo
	F	Empregado Efetivo
	F	Empregado Efetivo
	M	Empregado Efetivo
	M	Empregado Efetivo
	M	Empregado Efetivo
	F	Empregado Efetivo
	F	Empregado Efetivo
	F	Empregado Efetivo
	F	Empregado Efetivo
	M	Empregado Efetivo
	M	Empregado Efetivo
	M	Empregado Efetivo
	F	Empregado Efetivo
	F	Empregado Efetivo
	F	Empregado Efetivo
	F	Empregado Efetivo
	F	Empregado Efetivo
	M	Empregado Efetivo
	M	Empregado Efetivo
	M	Empregado Efetivo
	F	Empregado Efetivo
	F	Empregado Efetivo
	F	Empregado Efetivo

	F	Empregado Efetivo
	F	Empregado Efetivo
	F	Empregado Efetivo
	F	Empregado Efetivo
	F	Empregado Efetivo
	F	Empregado Efetivo
	F	Empregado Efetivo
	F	Empregado Efetivo
	F	Empregado Efetivo
	F	Empregado Efetivo
	F	Empregado Efetivo
	F	Empregado Efetivo
	M	Empregado Efetivo
	F	Empregado Efetivo
	F	Empregado Efetivo
	F	Empregado Efetivo
	F	Empregado Efetivo
	F	Empregado Efetivo
	F	Empregado Efetivo
	F	Empregado Efetivo
	F	Empregado Efetivo
	F	Empregado Efetivo
	F	Empregado Efetivo
	F	Empregado Efetivo
	F	Empregado Efetivo
	M	Empregado Efetivo
	M	Empregado Efetivo
	F	Empregado Efetivo
	F	Empregado Efetivo
	F	Empregado Efetivo
	F	Empregado Efetivo
	F	Empregado Efetivo
	F	Empregado Efetivo
	M	Empregado Efetivo
	M	Empregado Efetivo
	M	Empregado Efetivo
	M	Empregado Efetivo
	F	Empregado Efetivo
	F	Empregado Efetivo
	F	Empregado Efetivo
	F	Empregado Efetivo
	F	Empregado Efetivo
	F	Empregado Efetivo

	F	Empregado Efetivo
	F	Empregado Efetivo
	F	Empregado Efetivo
	F	Empregado Efetivo
	F	Empregado Efetivo
	F	Empregado Efetivo
	F	Empregado Efetivo
	F	Empregado Efetivo
	F	Empregado Efetivo
	F	Empregado Efetivo
	F	Empregado Efetivo
	F	Empregado Efetivo
	F	Empregado Efetivo
	F	Empregado Efetivo
	F	Empregado Efetivo
	F	Empregado Efetivo
	F	Empregado Efetivo
	F	Empregado Efetivo
	F	Empregado Efetivo
	F	Empregado Efetivo
	M	Empregado Efetivo
	F	Empregado Efetivo
	F	Empregado Efetivo
	F	Empregado Efetivo
	M	Empregado Efetivo
	M	Empregado Efetivo
	F	Empregado Efetivo
	F	Empregado Efetivo
	F	Empregado Efetivo
	F	Empregado Efetivo
	F	Empregado Efetivo
	F	Empregado Efetivo
	F	Empregado Efetivo
	F	Empregado Efetivo
	F	Empregado Efetivo
	F	Empregado Efetivo
	F	Empregado Efetivo
	F	Empregado Efetivo
	F	Empregado Efetivo
	F	Empregado Efetivo
	F	Empregado Efetivo
	F	Empregado Efetivo
	F	Empregado Efetivo
	F	Empregado Efetivo
	F	Empregado Efetivo
	F	Empregado Efetivo
	F	Empregado Efetivo
	F	Empregado Efetivo

	F	Empregado Efetivo
	F	Empregado Efetivo
	F	Empregado Efetivo
	F	Empregado Efetivo
	F	Empregado Efetivo
	F	Empregado Efetivo
	F	Empregado Efetivo
	F	Empregado Efetivo
	F	Empregado Efetivo
	F	Empregado Efetivo
	F	Empregado Efetivo
	F	Empregado Efetivo
	F	Empregado Efetivo
	F	Empregado Efetivo
	F	Empregado Efetivo
	F	Empregado Efetivo
	F	Empregado Efetivo
	F	Empregado Efetivo
	F	Empregado Efetivo
	F	Empregado Efetivo
	F	Empregado Efetivo
	F	Empregado Efetivo
	F	Empregado Efetivo
	F	Empregado Efetivo
	F	Empregado Efetivo
	F	Empregado Efetivo
	F	Empregado Efetivo
	F	Empregado Efetivo
	F	Empregado Efetivo
	F	Empregado Efetivo
	F	Empregado Efetivo
	F	Empregado Efetivo
	F	Empregado Efetivo
	M	Empregado Efetivo
	M	Empregado Efetivo
	M	Empregado Efetivo
	M	Empregado Efetivo
	M	Empregado Efetivo
	M	Empregado Efetivo
	M	Empregado Efetivo
	M	Empregado Efetivo
	M	Empregado Efetivo
	F	Empregado Efetivo
	F	Empregado Efetivo
	M	Empregado Efetivo
	F	Empregado Efetivo
	M	Empregado Efetivo
	M	Empregado Efetivo

	M	Empregado Efetivo
	M	Empregado Efetivo
	M	Empregado Efetivo
	M	Empregado Efetivo
	F	Empregado Efetivo
	F	Empregado Efetivo
	F	Empregado Efetivo
	M	Empregado Efetivo
	M	Empregado Efetivo
	M	Empregado Efetivo
	F	Empregado Efetivo
	F	Empregado Efetivo
	M	Empregado Efetivo
	M	Empregado Efetivo
	F	Empregado Efetivo
	F	Empregado Efetivo
	F	Empregado Efetivo
	F	Empregado Efetivo
	F	Empregado Efetivo
	F	Empregado Efetivo
	F	Empregado Efetivo
	F	Empregado Efetivo
	F	Empregado Efetivo
	F	Empregado Efetivo
	F	Empregado Efetivo
	M	Empregado Efetivo
	F	Empregado Efetivo
	F	Empregado Efetivo
	F	Empregado Efetivo
	F	Empregado Efetivo
	F	Empregado Efetivo
	F	Empregado Efetivo
	F	Empregado Efetivo
	F	Empregado Efetivo
	F	Empregado Efetivo
	F	Empregado Efetivo
	F	Empregado Efetivo
	F	Empregado Efetivo

	F	Empregado Efetivo
	F	Empregado Efetivo
	F	Empregado Efetivo
	F	Empregado Efetivo
	F	Empregado Efetivo
	F	Empregado Efetivo
	F	Empregado Efetivo
	F	Empregado Efetivo
	M	Empregado Efetivo
	F	Empregado Efetivo
	M	Empregado Efetivo
	M	Empregado Efetivo
	F	Empregado Efetivo
	F	Empregado Efetivo
	F	Empregado Efetivo
	F	Empregado Efetivo
	F	Empregado Efetivo
	F	Empregado Efetivo
	F	Empregado Efetivo
	F	Empregado Efetivo
	F	Empregado Efetivo
	F	Empregado Efetivo
	F	Empregado Efetivo
	M	Empregado Efetivo
	F	Empregado Efetivo
	F	Empregado Efetivo
	M	Empregado Efetivo
	M	Empregado Efetivo
	F	Empregado Efetivo
	F	Empregado Efetivo
	M	Empregado Efetivo
	M	Empregado Efetivo
	F	Empregado Efetivo
	F	Empregado Efetivo
	F	Empregado Efetivo
	M	Empregado Efetivo
	F	Empregado Efetivo
	F	Empregado Efetivo
	F	Empregado Efetivo
	F	Empregado Efetivo
	F	Empregado Efetivo
	F	Empregado Efetivo
	M	Empregado Efetivo
	F	Empregado Efetivo
	F	Empregado Efetivo

	F	Empregado Efetivo
	F	Empregado Efetivo
	F	Empregado Efetivo
	F	Empregado Efetivo
	M	Empregado Efetivo
	F	Empregado Efetivo
	F	Empregado Efetivo
	F	Empregado Efetivo
	F	Empregado Efetivo
	F	Empregado Efetivo
	F	Empregado Efetivo
	F	Empregado Efetivo
	F	Empregado Efetivo
	F	Empregado Efetivo
	F	Empregado Efetivo
	F	Empregado Efetivo
	M	Empregado Efetivo
	F	Empregado Efetivo
	F	Empregado Efetivo
	F	Empregado Efetivo
	F	Empregado Efetivo
	F	Empregado Efetivo
	F	Empregado Efetivo
	F	Empregado Efetivo
	F	Empregado Efetivo
	F	Empregado Efetivo
	F	Empregado Efetivo
	F	Empregado Efetivo
	M	Empregado Efetivo
	F	Empregado Efetivo
	F	Empregado Efetivo
	F	Empregado Efetivo
	F	Empregado Efetivo
	F	Empregado Efetivo
	F	Empregado Efetivo
	F	Empregado Efetivo
	F	Empregado Efetivo
	F	Empregado Efetivo
	M	Empregado Efetivo
	F	Empregado Efetivo
	F	Empregado Efetivo
	F	Empregado Efetivo
	F	Empregado Efetivo
	F	Empregado Efetivo
	F	Empregado Efetivo
	F	Empregado Efetivo
	M	Empregado Efetivo
	F	Empregado Efetivo

	F	Empregado Efetivo
	F	Empregado Efetivo
	F	Empregado Efetivo
	F	Empregado Efetivo
	F	Empregado Efetivo
	F	Empregado Efetivo
	M	Empregado Efetivo
	F	Empregado Efetivo
	F	Empregado Efetivo
	F	Empregado Efetivo
	F	Empregado Efetivo
	F	Empregado Efetivo
	M	Empregado Efetivo
	M	Empregado Efetivo
	F	Empregado Efetivo
	F	Empregado Efetivo
	F	Empregado Efetivo
	F	Empregado Efetivo
	M	Empregado Efetivo
	F	Empregado Efetivo
	F	Empregado Efetivo
	F	Empregado Efetivo
	M	Empregado Efetivo
	F	Empregado Efetivo
	F	Empregado Efetivo
	F	Empregado Efetivo
	M	Empregado Efetivo
	F	Empregado Efetivo
	F	Empregado Efetivo
	F	Empregado Efetivo
	M	Empregado Efetivo
	F	Empregado Efetivo
	F	Empregado Efetivo
	F	Empregado Efetivo
	F	Empregado Efetivo
	F	Empregado Efetivo
	M	Empregado Efetivo
	F	Empregado Efetivo
	F	Empregado Efetivo
	F	Empregado Efetivo
	F	Empregado Efetivo
	F	Empregado Efetivo
	F	Empregado Efetivo
	F	Empregado Efetivo
	M	Empregado Efetivo
	F	Empregado Efetivo
	F	Empregado Efetivo
	F	Empregado Efetivo

	F	Empregado Efetivo
	F	Empregado Efetivo
	F	Empregado Efetivo
	F	Empregado Efetivo
	F	Empregado Efetivo
	M	Empregado Efetivo
	F	Empregado Efetivo
	F	Empregado Efetivo
	F	Empregado Efetivo
	M	Empregado Efetivo
	F	Empregado Efetivo
	F	Empregado Efetivo
	F	Empregado Efetivo
	F	Empregado Efetivo
	F	Empregado Efetivo
	F	Empregado Efetivo
	F	Empregado Efetivo
	F	Empregado Efetivo
	F	Empregado Efetivo
	F	Empregado Efetivo
	F	Empregado Efetivo
	F	Empregado Efetivo
	F	Empregado Efetivo
	F	Empregado Efetivo
	F	Empregado Efetivo
	F	Empregado Efetivo
	F	Empregado Efetivo
	F	Empregado Efetivo
	M	Empregado Efetivo
	F	Empregado Efetivo
	F	Empregado Efetivo
	M	Empregado Efetivo
	F	Empregado Efetivo
	F	Empregado Efetivo
	F	Empregado Efetivo
	F	Empregado Efetivo
	F	Empregado Efetivo
	M	Empregado Efetivo
	F	Empregado Efetivo
	F	Empregado Efetivo
	F	Empregado Efetivo
	F	Empregado Efetivo
	M	Empregado Efetivo
	F	Empregado Efetivo
	F	Empregado Efetivo
	M	Empregado Efetivo
	F	Empregado Efetivo
	F	Empregado Efetivo

	F	Empregado Efetivo
	F	Empregado Efetivo
	F	Empregado Efetivo
	F	Empregado Efetivo
	F	Empregado Efetivo
	F	Empregado Efetivo
	F	Empregado Efetivo
	M	Empregado Efetivo
	F	Empregado Efetivo
	F	Empregado Efetivo
	M	Empregado Efetivo
	F	Empregado Efetivo
	F	Empregado Efetivo
	F	Empregado Efetivo
	F	Empregado Efetivo
	F	Empregado Efetivo
	F	Empregado Efetivo
	F	Empregado Efetivo
	F	Empregado Efetivo
	F	Empregado Efetivo
	F	Empregado Efetivo
	F	Empregado Efetivo
	M	Empregado Efetivo
	F	Empregado Efetivo
	F	Empregado Efetivo
	F	Empregado Efetivo
	F	Empregado Efetivo
	F	Empregado Efetivo
	F	Empregado Efetivo
	M	Empregado Efetivo
	F	Empregado Efetivo
	F	Empregado Efetivo
	F	Empregado Efetivo
	F	Empregado Efetivo
	F	Empregado Efetivo
	F	Empregado Efetivo
	F	Empregado Efetivo
	F	Empregado Efetivo
	M	Empregado Efetivo
	F	Empregado Efetivo
	M	Empregado Efetivo
	F	Empregado Efetivo
	F	Empregado Efetivo
	F	Empregado Efetivo
	F	Empregado Efetivo
	F	Empregado Efetivo
	F	Empregado Efetivo
	F	Empregado Efetivo

	F	Empregado Efetivo
	M	Empregado Efetivo
	F	Empregado Efetivo
	F	Empregado Efetivo
	M	Empregado Efetivo
	F	Empregado Efetivo
	F	Empregado Efetivo
	F	Empregado Efetivo
	F	Empregado Efetivo
	F	Empregado Efetivo
	F	Empregado Efetivo
	M	Empregado Efetivo
	F	Empregado Efetivo
	F	Empregado Efetivo
	F	Empregado Efetivo
	F	Empregado Efetivo
	M	Empregado Efetivo
	F	Empregado Efetivo
	F	Empregado Efetivo
	F	Empregado Efetivo
	F	Empregado Efetivo
	F	Empregado Efetivo
	F	Empregado Efetivo
	F	Empregado Efetivo
	F	Empregado Efetivo
	F	Empregado Efetivo
	F	Empregado Efetivo
	F	Empregado Efetivo
	F	Empregado Efetivo
	F	Empregado Efetivo
	F	Empregado Efetivo
	F	Empregado Efetivo
	F	Empregado Efetivo
	F	Empregado Efetivo
	F	Empregado Efetivo
	M	Empregado Efetivo
	F	Empregado Efetivo
	F	Empregado Efetivo
	F	Empregado Efetivo
	F	Empregado Efetivo
	F	Empregado Efetivo
	F	Empregado Efetivo
	F	Empregado Efetivo
	F	Empregado Efetivo
	M	Empregado Efetivo
	M	Empregado Efetivo
	F	Empregado Efetivo
	F	Empregado Efetivo

	F	Empregado Efetivo
	F	Empregado Efetivo
	F	Empregado Efetivo
	F	Empregado Efetivo
	F	Empregado Efetivo
	F	Empregado Efetivo
	F	Empregado Efetivo
	M	Empregado Efetivo
	F	Empregado Efetivo
	F	Empregado Efetivo
	F	Empregado Efetivo
	F	Empregado Efetivo
	F	Empregado Efetivo
	F	Empregado Efetivo
	M	Empregado Efetivo
	F	Empregado Efetivo
	F	Empregado Efetivo
	F	Empregado Efetivo
	F	Empregado Efetivo
	F	Empregado Efetivo
	F	Empregado Efetivo
	F	Empregado Efetivo
	F	Empregado Efetivo
	F	Empregado Efetivo
	F	Empregado Efetivo
	M	Empregado Efetivo
	M	Empregado Efetivo
	M	Empregado Efetivo
	M	Empregado Efetivo
	F	Empregado Efetivo
	F	Empregado Efetivo
	M	Empregado Efetivo
	F	Empregado Efetivo
	F	Empregado Efetivo
	M	Empregado Efetivo
	F	Empregado Efetivo
	F	Empregado Efetivo
	F	Empregado Efetivo
	M	Empregado Efetivo
	F	Empregado Efetivo
	F	Empregado Efetivo
	F	Empregado Efetivo
	F	Empregado Efetivo
	F	Empregado Efetivo
	F	Empregado Efetivo
	M	Empregado Efetivo
	F	Empregado Efetivo
	F	Empregado Efetivo

	F	Empregado Efetivo
	F	Empregado Efetivo
	M	Empregado Efetivo
	F	Empregado Efetivo
	F	Empregado Efetivo
	F	Empregado Efetivo
	F	Empregado Efetivo
	F	Empregado Efetivo
	M	Empregado Efetivo
	F	Empregado Efetivo
	M	Empregado Efetivo
	F	Empregado Efetivo
	F	Empregado Efetivo
	F	Empregado Efetivo
	F	Empregado Efetivo
	F	Empregado Efetivo
	F	Empregado Efetivo
	F	Empregado Efetivo
	F	Empregado Efetivo
	F	Empregado Efetivo
	F	Empregado Efetivo
	F	Empregado Efetivo
	F	Empregado Efetivo
	F	Empregado Efetivo
	F	Empregado Efetivo
	F	Empregado Efetivo
	F	Empregado Efetivo
	F	Empregado Efetivo
	F	Empregado Efetivo
	F	Empregado Efetivo
	F	Empregado Efetivo
	F	Empregado Efetivo
	F	Empregado Efetivo
	F	Empregado Efetivo
	F	Empregado Efetivo
	F	Empregado Efetivo
	F	Empregado Efetivo
	F	Empregado Efetivo
	F	Empregado Efetivo
	F	Empregado Efetivo
	F	Empregado Efetivo
	F	Empregado Efetivo
	F	Empregado Efetivo
	F	Empregado Efetivo
	F	Empregado Efetivo
	F	Empregado Efetivo
	F	Empregado Efetivo
	F	Empregado Efetivo

	F	Empregado Efetivo
	F	Empregado Efetivo
	M	Empregado Efetivo
	F	Empregado Efetivo
	F	Empregado Efetivo
	F	Empregado Efetivo
	F	Empregado Efetivo
	M	Empregado Efetivo
	F	Empregado Efetivo
	F	Empregado Efetivo
	M	Empregado Efetivo
	F	Empregado Efetivo
	F	Empregado Efetivo
	F	Empregado Efetivo
	F	Empregado Efetivo
	F	Empregado Efetivo
	F	Empregado Efetivo
	F	Empregado Efetivo
	F	Empregado Efetivo
	F	Empregado Efetivo
	M	Empregado Efetivo
	F	Empregado Efetivo
	F	Empregado Efetivo
	F	Empregado Efetivo
	F	Empregado Efetivo
	F	Empregado Efetivo
	F	Empregado Efetivo
	F	Empregado Efetivo
	F	Empregado Efetivo
	F	Empregado Efetivo
	F	Empregado Efetivo
	M	Empregado Efetivo
	F	Empregado Efetivo
	F	Empregado Efetivo
	F	Empregado Efetivo
	F	Empregado Efetivo
	F	Empregado Efetivo
	F	Empregado Efetivo
	F	Empregado Efetivo
	F	Empregado Efetivo
	M	Empregado Efetivo
	F	Empregado Efetivo
	F	Empregado Efetivo
	F	Empregado Efetivo
	F	Empregado Efetivo
	F	Empregado Efetivo
	F	Empregado Efetivo
	F	Empregado Efetivo

	F	Empregado Efetivo
	F	Empregado Efetivo
	F	Empregado Efetivo
	F	Empregado Efetivo
	M	Empregado Efetivo
	F	Empregado Efetivo
	F	Empregado Efetivo
	F	Empregado Efetivo
	F	Empregado Efetivo
	F	Empregado Efetivo
	F	Empregado Efetivo
	F	Empregado Efetivo
	F	Empregado Efetivo
	F	Empregado Efetivo
	F	Empregado Efetivo
	F	Empregado Efetivo
	M	Empregado Efetivo
	F	Empregado Efetivo
	F	Empregado Efetivo
	F	Empregado Efetivo
	F	Empregado Efetivo
	F	Empregado Efetivo
	F	Empregado Efetivo
	M	Empregado Efetivo
	F	Empregado Efetivo
	F	Empregado Efetivo
	F	Empregado Efetivo
	F	Empregado Efetivo
	F	Empregado Efetivo
	M	Empregado Efetivo
	F	Empregado Efetivo
	F	Empregado Efetivo
	F	Empregado Efetivo
	F	Empregado Efetivo
	F	Empregado Efetivo
	F	Empregado Efetivo
	F	Empregado Efetivo
	F	Empregado Efetivo
	F	Empregado Efetivo
	F	Empregado Efetivo
	F	Empregado Efetivo
	F	Empregado Efetivo
	F	Empregado Efetivo
	F	Empregado Efetivo
	F	Empregado Efetivo
	F	Empregado Efetivo
	F	Empregado Efetivo
	F	Empregado Efetivo

	F	Empregado Efetivo
	F	Empregado Efetivo
	F	Empregado Efetivo
	F	Empregado Efetivo
	F	Empregado Efetivo
	F	Empregado Efetivo
	F	Empregado Efetivo
	F	Empregado Efetivo
	F	Empregado Efetivo
	F	Empregado Efetivo
	M	Empregado Efetivo
	F	Empregado Efetivo
	F	Empregado Efetivo
	F	Empregado Efetivo
	F	Empregado Efetivo
	F	Empregado Efetivo
	F	Empregado Efetivo
	F	Empregado Efetivo
	F	Empregado Efetivo
	F	Empregado Efetivo
	F	Empregado Efetivo
	F	Empregado Efetivo
	F	Empregado Efetivo
	F	Empregado Efetivo
	F	Empregado Efetivo
	F	Empregado Efetivo
	F	Empregado Efetivo
	F	Empregado Efetivo
	F	Empregado Efetivo
	F	Empregado Efetivo
	F	Empregado Efetivo
	M	Empregado Efetivo
	F	Empregado Efetivo
	F	Empregado Efetivo
	M	Empregado Efetivo
	F	Empregado Efetivo
	F	Empregado Efetivo
	F	Empregado Efetivo
	F	Empregado Efetivo
	F	Empregado Efetivo
	F	Empregado Efetivo
	F	Empregado Efetivo
	F	Empregado Efetivo
	F	Empregado Efetivo
	F	Empregado Efetivo
	F	Empregado Efetivo
	F	Empregado Efetivo
	F	Empregado Efetivo

	F	Empregado Efetivo
	F	Empregado Efetivo
	F	Empregado Efetivo
	F	Empregado Efetivo
	F	Empregado Efetivo
	F	Empregado Efetivo
	F	Empregado Efetivo
	F	Empregado Efetivo
	F	Empregado Efetivo
	F	Empregado Efetivo
	F	Empregado Efetivo
	F	Empregado Efetivo
	F	Empregado Efetivo
	F	Empregado Efetivo
	F	Empregado Efetivo
	F	Empregado Efetivo
	F	Empregado Efetivo
	F	Empregado Efetivo
	F	Empregado Efetivo
	F	Empregado Efetivo
	F	Empregado Efetivo
	F	Empregado Efetivo
	M	Empregado Efetivo
	F	Empregado Efetivo
	F	Empregado Efetivo
	F	Empregado Efetivo
	F	Empregado Efetivo
	F	Empregado Efetivo
	F	Empregado Efetivo
	F	Empregado Efetivo
	F	Empregado Efetivo
	F	Empregado Efetivo
	F	Empregado Efetivo
	F	Empregado Efetivo
	F	Empregado Efetivo
	F	Empregado Efetivo
	F	Empregado Efetivo
	F	Empregado Efetivo
	F	Empregado Efetivo
	F	Empregado Efetivo
	F	Empregado Efetivo
	F	Empregado Efetivo
	F	Empregado Efetivo
	F	Empregado Efetivo
	F	Empregado Efetivo
	M	Empregado Efetivo
	M	Empregado Efetivo
	F	Empregado Efetivo

	F	Empregado Efetivo
	F	Empregado Efetivo
	F	Empregado Efetivo
	M	Empregado Efetivo
	F	Empregado Efetivo
	F	Empregado Efetivo
	F	Empregado Efetivo
	F	Empregado Efetivo
	F	Empregado Efetivo
	F	Empregado Efetivo
	M	Empregado Efetivo
	F	Empregado Efetivo
	F	Empregado Efetivo
	M	Empregado Efetivo
	F	Empregado Efetivo
	F	Empregado Efetivo
	F	Empregado Efetivo
	F	Empregado Efetivo
	F	Empregado Efetivo
	F	Empregado Efetivo
	F	Empregado Efetivo
	F	Empregado Efetivo
	F	Empregado Efetivo
	F	Empregado Efetivo
	F	Empregado Efetivo
	F	Empregado Efetivo
	F	Empregado Efetivo
	M	Empregado Efetivo
	F	Empregado Efetivo
	F	Empregado Efetivo
	F	Empregado Efetivo
	F	Empregado Efetivo
	F	Empregado Efetivo
	F	Empregado Efetivo
	F	Empregado Efetivo
	F	Empregado Efetivo
	F	Empregado Efetivo
	F	Empregado Efetivo
	F	Empregado Efetivo
	F	Empregado Efetivo
	F	Empregado Efetivo
	F	Empregado Efetivo
	F	Empregado Efetivo
	F	Empregado Efetivo
	F	Empregado Efetivo
	F	Empregado Efetivo
	F	Empregado Efetivo
	F	Empregado Efetivo

	F	Empregado Efetivo
	F	Empregado Efetivo
	F	Empregado Efetivo
	M	Empregado Efetivo
	F	Empregado Efetivo
	F	Empregado Efetivo
	F	Empregado Efetivo
	F	Empregado Efetivo
	F	Empregado Efetivo
	F	Empregado Efetivo
	F	Empregado Efetivo
	F	Empregado Efetivo
	F	Empregado Efetivo
	M	Empregado Efetivo
	F	Empregado Efetivo
	F	Empregado Efetivo
	F	Empregado Efetivo
	F	Empregado Efetivo
	M	Empregado Efetivo
	F	Empregado Efetivo
	F	Empregado Efetivo
	F	Empregado Efetivo
	F	Empregado Efetivo
	F	Empregado Efetivo
	F	Empregado Efetivo
	F	Empregado Efetivo
	F	Empregado Efetivo
	F	Empregado Efetivo
	F	Empregado Efetivo
	F	Empregado Efetivo
	F	Empregado Efetivo
	M	Empregado Efetivo
	F	Empregado Efetivo
	F	Empregado Efetivo
	M	Empregado Efetivo
	F	Empregado Efetivo
	F	Empregado Efetivo
	F	Empregado Efetivo
	F	Empregado Efetivo
	F	Empregado Efetivo
	F	Empregado Efetivo
	F	Empregado Efetivo
	F	Empregado Efetivo
	F	Empregado Efetivo
	F	Empregado Efetivo
	F	Empregado Efetivo
	F	Empregado Efetivo
	F	Empregado Efetivo

	F	Empregado Efetivo
	F	Empregado Efetivo
	F	Empregado Efetivo
	F	Empregado Efetivo
	F	Empregado Efetivo
	M	Empregado Efetivo
	F	Empregado Efetivo
	F	Empregado Efetivo
	F	Empregado Efetivo
	F	Empregado Efetivo
	F	Empregado Efetivo
	F	Empregado Efetivo
	F	Empregado Efetivo
	F	Empregado Efetivo
	F	Empregado Efetivo
	F	Empregado Efetivo
	F	Empregado Efetivo
	F	Empregado Efetivo
	M	Empregado Efetivo
	F	Empregado Efetivo
	F	Empregado Efetivo
	F	Empregado Efetivo
	F	Empregado Efetivo
	F	Empregado Efetivo
	M	Empregado Efetivo
	F	Empregado Efetivo
	F	Empregado Efetivo
	M	Empregado Efetivo
	M	Empregado Efetivo
	F	Empregado Efetivo
	F	Empregado Efetivo
	F	Empregado Efetivo
	F	Empregado Efetivo
	F	Empregado Efetivo
	F	Empregado Efetivo
	M	Empregado Efetivo
	F	Empregado Efetivo
	F	Empregado Efetivo
	F	Empregado Efetivo
	F	Empregado Efetivo
	F	Empregado Efetivo
	M	Empregado Efetivo
	F	Empregado Efetivo
	F	Empregado Efetivo
	F	Empregado Efetivo
	F	Empregado Efetivo
	F	Empregado Efetivo
	F	Empregado Efetivo

	F	Empregado Efetivo
	F	Empregado Efetivo
	F	Empregado Efetivo
	F	Empregado Efetivo
	M	Empregado Efetivo
	F	Empregado Efetivo
	F	Empregado Efetivo
	M	Empregado Efetivo
	M	Empregado Efetivo
	F	Empregado Efetivo
	F	Empregado Efetivo
	F	Empregado Efetivo
	F	Empregado Efetivo
	M	Empregado Efetivo
	F	Empregado Efetivo
	F	Empregado Efetivo
	F	Empregado Efetivo
	F	Empregado Efetivo
	F	Empregado Efetivo
	F	Empregado Efetivo
	M	Empregado Efetivo
	M	Empregado Efetivo
	F	Empregado Efetivo
	F	Empregado Efetivo
	F	Empregado Efetivo
	F	Empregado Efetivo
	F	Empregado Efetivo
	F	Empregado Efetivo
	M	Empregado Efetivo
	F	Empregado Efetivo
	F	Empregado Efetivo
	F	Empregado Efetivo
	F	Empregado Efetivo
	M	Empregado Efetivo
	F	Empregado Efetivo
	M	Empregado Efetivo
	F	Empregado Efetivo
	F	Empregado Efetivo
	F	Empregado Efetivo
	F	Empregado Efetivo
	F	Empregado Efetivo
	F	Empregado Efetivo
	F	Empregado Efetivo
	F	Empregado Efetivo
	F	Empregado Efetivo
	F	Empregado Efetivo
	F	Empregado Efetivo
	F	Empregado Efetivo

	F	Empregado Efetivo
	F	Empregado Efetivo
	M	Empregado Efetivo
	F	Empregado Efetivo
	M	Empregado Efetivo
	M	Empregado Efetivo
	F	Empregado Efetivo
	F	Empregado Efetivo
	F	Empregado Efetivo
	F	Empregado Efetivo
	F	Empregado Efetivo
	F	Empregado Efetivo
	F	Empregado Efetivo
	F	Empregado Efetivo
	F	Empregado Efetivo
	M	Empregado Efetivo
	F	Empregado Efetivo
	F	Empregado Efetivo
	F	Empregado Efetivo
	F	Empregado Efetivo
	F	Empregado Efetivo
	F	Empregado Efetivo
	F	Empregado Efetivo
	F	Empregado Efetivo
	F	Empregado Efetivo
	F	Empregado Efetivo
	F	Empregado Efetivo
	F	Empregado Efetivo
	F	Empregado Efetivo
	F	Empregado Efetivo
	F	Empregado Efetivo
	F	Empregado Efetivo
	F	Empregado Efetivo
	F	Empregado Efetivo
	F	Empregado Efetivo
	F	Empregado Efetivo
	F	Empregado Efetivo
	F	Empregado Efetivo
	F	Empregado Efetivo
	F	Empregado Efetivo
	F	Empregado Efetivo
	F	Empregado Efetivo
	F	Empregado Efetivo

	F	Empregado Efetivo
	F	Empregado Efetivo
	F	Empregado Efetivo
	F	Empregado Efetivo
	F	Empregado Efetivo
	F	Empregado Efetivo
	F	Empregado Efetivo
	F	Empregado Efetivo
	F	Empregado Efetivo
	F	Empregado Efetivo
	F	Empregado Efetivo
	F	Empregado Efetivo
	M	Empregado Efetivo
	F	Empregado Efetivo
	F	Empregado Efetivo
	F	Empregado Efetivo
	M	Empregado Efetivo
	F	Empregado Efetivo
	F	Empregado Efetivo
	F	Empregado Efetivo
	F	Empregado Efetivo
	F	Empregado Efetivo
	F	Empregado Efetivo
	F	Empregado Efetivo
	F	Empregado Efetivo
	F	Empregado Efetivo
	F	Empregado Efetivo
	F	Empregado Efetivo
	F	Empregado Efetivo
	F	Empregado Efetivo
	M	Empregado Efetivo
	F	Empregado Efetivo

	M	Empregado Efetivo
	F	Empregado Efetivo
	F	Empregado Efetivo
	F	Empregado Efetivo
	M	Empregado Efetivo
	F	Empregado Efetivo
	F	Empregado Efetivo
	F	Empregado Efetivo
	F	Empregado Efetivo
	F	Empregado Efetivo
	F	Empregado Efetivo
	M	Empregado Efetivo
	F	Empregado Efetivo
	F	Empregado Efetivo
	F	Empregado Efetivo
	F	Empregado Efetivo
	F	Empregado Efetivo
	F	Empregado Efetivo
	F	Empregado Efetivo
	F	Empregado Efetivo
	F	Empregado Efetivo
	F	Empregado Efetivo
	F	Empregado Efetivo
	F	Empregado Efetivo
	F	Empregado Efetivo
	F	Empregado Efetivo
	F	Empregado Efetivo
	F	Empregado Efetivo
	F	Empregado Efetivo
	F	Empregado Efetivo
	F	Empregado Efetivo
	F	Empregado Efetivo

	M	Empregado Efetivo
	F	Empregado Efetivo
	F	Empregado Efetivo
	F	Empregado Efetivo
	F	Empregado Efetivo
	F	Empregado Efetivo
	F	Empregado Efetivo
	F	Empregado Efetivo
	F	Empregado Efetivo
	F	Empregado Efetivo
	F	Empregado Efetivo
	F	Empregado Efetivo
	F	Empregado Efetivo
	F	Empregado Efetivo
	F	Empregado Efetivo
	F	Empregado Efetivo
	F	Empregado Efetivo
	F	Empregado Efetivo
	F	Empregado Efetivo
	F	Empregado Efetivo
	F	Empregado Efetivo
	F	Empregado Efetivo
	F	Empregado Efetivo
	F	Empregado Efetivo
	F	Empregado Efetivo
	F	Empregado Efetivo
	F	Empregado Efetivo
	F	Empregado Efetivo
	F	Empregado Efetivo
	F	Empregado Efetivo
	F	Empregado Efetivo
	F	Empregado Efetivo
	F	Empregado Efetivo
	F	Empregado Efetivo
	F	Empregado Efetivo
	F	Empregado Efetivo
	F	Empregado Efetivo
	F	Empregado Efetivo
	F	Empregado Efetivo
	M	Empregado Efetivo
	F	Empregado Efetivo
	F	Empregado Efetivo
	M	Empregado Efetivo
	F	Empregado Efetivo
	F	Empregado Efetivo
	F	Empregado Efetivo

	F	Empregado Efetivo
	F	Empregado Efetivo
	F	Empregado Efetivo
	M	Empregado Efetivo
	F	Empregado Efetivo
	F	Empregado Efetivo
	F	Empregado Efetivo
	F	Empregado Efetivo
	F	Empregado Efetivo
	F	Empregado Efetivo
	F	Empregado Efetivo
	F	Empregado Efetivo
	F	Empregado Efetivo
	F	Empregado Efetivo
	F	Empregado Efetivo
	F	Empregado Efetivo
	F	Empregado Efetivo
	F	Empregado Efetivo
	F	Empregado Efetivo
	F	Empregado Efetivo
	F	Empregado Efetivo
	F	Empregado Efetivo
	F	Empregado Efetivo
	F	Empregado Efetivo
	F	Empregado Efetivo
	F	Empregado Efetivo
	F	Empregado Efetivo
	F	Empregado Efetivo
	F	Empregado Efetivo
	F	Empregado Efetivo
	F	Empregado Efetivo
	F	Empregado Efetivo
	F	Empregado Efetivo
	F	Empregado Efetivo
	F	Empregado Efetivo
	F	Empregado Efetivo
	F	Empregado Efetivo
	F	Empregado Efetivo
	F	Empregado Efetivo
	F	Empregado Efetivo
	F	Empregado Efetivo
	F	Empregado Efetivo
	F	Empregado Efetivo
	F	Empregado Efetivo
	F	Empregado Efetivo
	F	Empregado Efetivo
	F	Empregado Efetivo
	F	Empregado Efetivo

	F	Empregado Efetivo
	F	Empregado Efetivo
	F	Empregado Efetivo
	F	Empregado Efetivo
	M	Empregado Efetivo
	F	Empregado Efetivo
	F	Empregado Efetivo
	F	Empregado Efetivo
	F	Empregado Efetivo
	F	Empregado Efetivo
	F	Empregado Efetivo
	F	Empregado Efetivo
	F	Empregado Efetivo
	F	Empregado Efetivo
	F	Empregado Efetivo
	M	Empregado Efetivo
	F	Empregado Efetivo
	F	Empregado Efetivo
	F	Empregado Efetivo
	F	Empregado Efetivo
	F	Empregado Efetivo
	F	Empregado Efetivo
	F	Empregado Efetivo
	F	Empregado Efetivo
	F	Empregado Efetivo
	F	Empregado Efetivo
	F	Empregado Efetivo
	F	Empregado Efetivo
	F	Empregado Efetivo
	F	Empregado Efetivo
	F	Empregado Efetivo
	F	Empregado Efetivo
	F	Empregado Efetivo
	F	Empregado Efetivo
	F	Empregado Efetivo
	F	Empregado Efetivo
	F	Empregado Efetivo
	F	Empregado Efetivo
	M	Empregado Efetivo
	F	Empregado Efetivo
	F	Empregado Efetivo
	F	Empregado Efetivo
	F	Empregado Efetivo
	F	Empregado Efetivo
	F	Empregado Efetivo
	F	Empregado Efetivo
	F	Empregado Efetivo
	F	Empregado Efetivo

	F	Empregado Efetivo
	F	Empregado Efetivo
	F	Empregado Efetivo
	F	Empregado Efetivo
	F	Empregado Efetivo
	F	Empregado Efetivo
	F	Empregado Efetivo
	F	Empregado Efetivo
	F	Empregado Efetivo
	F	Empregado Efetivo
	F	Empregado Efetivo
	F	Empregado Efetivo
	F	Empregado Efetivo
	F	Empregado Efetivo
	F	Empregado Efetivo
	F	Empregado Efetivo
	F	Empregado Efetivo
	F	Empregado Efetivo
	M	Empregado Efetivo
	F	Empregado Efetivo
	F	Empregado Efetivo
	F	Empregado Efetivo
	F	Empregado Efetivo
	F	Empregado Efetivo
	F	Empregado Efetivo
	F	Empregado Efetivo
	F	Empregado Efetivo
	F	Empregado Efetivo
	F	Empregado Efetivo
	F	Empregado Efetivo
	F	Empregado Efetivo
	F	Empregado Efetivo
	F	Empregado Efetivo
	F	Empregado Efetivo
	F	Empregado Efetivo
	F	Empregado Efetivo
	F	Empregado Efetivo
	F	Empregado Efetivo
	F	Empregado Efetivo
	F	Empregado Efetivo
	F	Empregado Efetivo
	F	Empregado Efetivo
	F	Empregado Efetivo
	F	Empregado Efetivo
	F	Empregado Efetivo
	F	Empregado Efetivo
	F	Empregado Efetivo
	F	Empregado Efetivo

	F	Empregado Efetivo
	F	Empregado Efetivo
	F	Empregado Efetivo
	F	Empregado Efetivo
	F	Empregado Efetivo
	F	Empregado Efetivo
	F	Empregado Efetivo
	F	Empregado Efetivo
	F	Empregado Efetivo
	F	Empregado Efetivo
	F	Empregado Efetivo
	F	Empregado Efetivo
	F	Empregado Efetivo
	F	Empregado Efetivo
	F	Empregado Efetivo
	F	Empregado Efetivo
	F	Empregado Efetivo
	F	Empregado Efetivo
	F	Empregado Efetivo
	F	Empregado Efetivo
	F	Empregado Efetivo
	F	Empregado Efetivo
	F	Empregado Efetivo
	F	Empregado Efetivo
	F	Empregado Efetivo
	F	Empregado Efetivo
	F	Empregado Efetivo
	F	Empregado Efetivo
	F	Empregado Efetivo
	F	Empregado Efetivo
	F	Empregado Efetivo
	F	Empregado Efetivo
	F	Empregado Efetivo
	F	Empregado Efetivo
	F	Empregado Efetivo
	F	Empregado Efetivo
	F	Empregado Efetivo
	F	Empregado Efetivo
	F	Empregado Efetivo
	F	Empregado Efetivo
	F	Empregado Efetivo
	F	Empregado Efetivo
	M	Empregado Efetivo
	M	Empregado Efetivo
	F	Empregado Efetivo
	F	Empregado Efetivo
	F	Empregado Efetivo
	F	Empregado Efetivo

	F	Empregado Efetivo
	F	Empregado Efetivo
	F	Empregado Efetivo
	F	Empregado Efetivo
	F	Empregado Efetivo
	F	Empregado Efetivo
	F	Empregado Efetivo
	F	Empregado Efetivo
	F	Empregado Efetivo
	F	Empregado Efetivo
	F	Empregado Efetivo
	F	Empregado Efetivo
	M	Empregado Efetivo
	F	Empregado Efetivo
	F	Empregado Efetivo
	F	Empregado Efetivo
	F	Empregado Efetivo
	F	Empregado Efetivo
	F	Empregado Efetivo
	M	Empregado Efetivo
	M	Empregado Efetivo
	F	Empregado Efetivo
	F	Empregado Efetivo
	F	Empregado Efetivo
	F	Empregado Efetivo
	F	Empregado Efetivo
	F	Empregado Efetivo
	F	Empregado Efetivo
	F	Empregado Efetivo
	F	Empregado Efetivo
	F	Empregado Efetivo
	F	Empregado Efetivo
	F	Empregado Efetivo
	F	Empregado Efetivo
	F	Empregado Efetivo
	F	Empregado Efetivo
	M	Empregado Efetivo
	F	Empregado Efetivo
	M	Empregado Efetivo
	M	Empregado Efetivo
	M	Empregado Efetivo
	F	Empregado Efetivo
	F	Empregado Efetivo
	F	Empregado Efetivo
	F	Empregado Efetivo
	M	Empregado Efetivo
	F	Empregado Efetivo
	F	Empregado Efetivo

	F	Empregado Efetivo
	M	Empregado Efetivo
	F	Empregado Efetivo
	F	Empregado Efetivo
	F	Empregado Efetivo
	M	Empregado Efetivo
	M	Empregado Efetivo
	F	Empregado Efetivo
	F	Empregado Efetivo
	F	Empregado Efetivo
	F	Empregado Efetivo
	F	Empregado Efetivo
	F	Empregado Efetivo
	F	Empregado Efetivo
	F	Empregado Efetivo
	F	Empregado Efetivo
	F	Empregado Efetivo
	M	Empregado Efetivo
	F	Empregado Efetivo
	F	Empregado Efetivo
	F	Empregado Efetivo
	F	Empregado Efetivo
	F	Empregado Efetivo
	F	Empregado Efetivo
	M	Empregado Efetivo
	F	Empregado Efetivo
	M	Empregado Efetivo
	F	Empregado Efetivo
	F	Empregado Efetivo
	F	Empregado Efetivo
	F	Empregado Efetivo
	F	Empregado Efetivo
	M	Empregado Efetivo
	F	Empregado Efetivo
	F	Empregado Efetivo
	F	Empregado Efetivo
	M	Empregado Efetivo
	F	Empregado Efetivo
	F	Empregado Efetivo
	F	Empregado Efetivo
	M	Empregado Efetivo
	F	Empregado Efetivo
	M	Empregado Efetivo
	F	Empregado Efetivo
	F	Empregado Efetivo
	F	Empregado Efetivo
	M	Empregado Efetivo
	F	Empregado Efetivo

	F	Empregado Efetivo
	M	Empregado Efetivo
	F	Empregado Efetivo
	F	Empregado Efetivo
	F	Empregado Efetivo
	F	Empregado Efetivo
	F	Empregado Efetivo
	F	Empregado Efetivo
	F	Empregado Efetivo
	M	Empregado Efetivo
	F	Empregado Efetivo
	F	Empregado Efetivo
	F	Empregado Efetivo
	F	Empregado Efetivo
	F	Empregado Efetivo
	F	Empregado Efetivo
	F	Empregado Efetivo
	M	Empregado Efetivo
	F	Empregado Efetivo
	F	Empregado Efetivo
	F	Empregado Efetivo
	M	Empregado Efetivo
	F	Empregado Efetivo
	F	Empregado Efetivo
	M	Empregado Efetivo
	F	Empregado Efetivo
	F	Empregado Efetivo
	F	Empregado Efetivo
	F	Empregado Efetivo
	M	Empregado Efetivo
	M	Empregado Efetivo
	M	Empregado Efetivo
	F	Empregado Efetivo
	F	Empregado Efetivo
	F	Empregado Efetivo
	F	Empregado Efetivo
	F	Empregado Efetivo
	M	Empregado Efetivo
	F	Empregado Efetivo
	F	Empregado Efetivo
	F	Empregado Efetivo
	F	Empregado Efetivo
	F	Empregado Efetivo
	F	Empregado Efetivo
	M	Empregado Efetivo

	F	Empregado Efetivo
	M	Empregado Efetivo
	M	Empregado Efetivo
	M	Empregado Efetivo
	M	Empregado Efetivo
	M	Empregado Efetivo
	F	Empregado Efetivo
	M	Empregado Efetivo
	F	Empregado Efetivo
	M	Empregado Efetivo
	M	Empregado Efetivo
	M	Empregado Efetivo
	M	Empregado Efetivo
	F	Empregado Efetivo
	F	Empregado Efetivo
	F	Empregado Efetivo
	M	Empregado Efetivo
	M	Empregado Efetivo
	F	Empregado Efetivo
	F	Empregado Efetivo
	F	Empregado Efetivo
	F	Empregado Efetivo
	F	Empregado Efetivo
	F	Empregado Efetivo
	F	Empregado Efetivo
	F	Empregado Efetivo
	F	Empregado Efetivo
	F	Empregado Efetivo
	F	Empregado Efetivo
	F	Empregado Efetivo
	F	Empregado Efetivo
	F	Empregado Efetivo
	F	Empregado Efetivo
	M	Empregado Efetivo
	M	Empregado Efetivo
	F	Empregado Efetivo
	F	Empregado Efetivo
	F	Empregado Efetivo

	F	Empregado Efetivo
	F	Empregado Efetivo
	F	Empregado Efetivo
	F	Empregado Efetivo
	F	Empregado Efetivo
	F	Empregado Efetivo
	F	Empregado Efetivo
	M	Empregado Efetivo
	F	Empregado Efetivo
	F	Empregado Efetivo
	F	Empregado Efetivo
	F	Empregado Efetivo
	F	Empregado Efetivo
	F	Empregado Efetivo
	F	Empregado Efetivo
	F	Empregado Efetivo
	F	Empregado Efetivo
	F	Empregado Efetivo
	F	Empregado Efetivo
	F	Empregado Efetivo
	F	Empregado Efetivo
	F	Empregado Efetivo
	F	Empregado Efetivo
	F	Empregado Efetivo
	F	Empregado Efetivo
	F	Empregado Efetivo
	M	Empregado Efetivo
	F	Empregado Efetivo
	F	Empregado Efetivo
	F	Empregado Efetivo
	F	Empregado Efetivo
	F	Empregado Efetivo

	F	Empregado Efetivo
	F	Empregado Efetivo
	F	Empregado Efetivo
	F	Empregado Efetivo
	F	Empregado Efetivo
	F	Empregado Efetivo
	F	Empregado Efetivo
	F	Empregado Efetivo
	F	Empregado Efetivo
	M	Empregado Efetivo
	F	Empregado Efetivo
	F	Empregado Efetivo
	F	Empregado Efetivo
	F	Empregado Efetivo
	F	Empregado Efetivo
	F	Empregado Efetivo
	F	Empregado Efetivo
	F	Empregado Efetivo
	F	Empregado Efetivo
	F	Empregado Efetivo
	F	Empregado Efetivo
	F	Empregado Efetivo
	F	Empregado Efetivo
	F	Empregado Efetivo
	F	Empregado Efetivo
	F	Empregado Efetivo
	F	Empregado Efetivo
	F	Empregado Efetivo
	F	Empregado Efetivo
	M	Empregado Efetivo
	F	Empregado Efetivo
	F	Empregado Efetivo

	F	Empregado Efetivo
	F	Empregado Efetivo
	F	Empregado Efetivo
	F	Empregado Efetivo
	F	Empregado Efetivo
	F	Empregado Efetivo
	F	Empregado Efetivo
	F	Empregado Efetivo
	F	Empregado Efetivo
	M	Empregado Efetivo
	F	Empregado Efetivo
	M	Empregado Efetivo
	F	Empregado Efetivo
	F	Empregado Efetivo
	F	Empregado Efetivo
	F	Empregado Efetivo
	M	Empregado Efetivo
	F	Empregado Efetivo
	F	Empregado Efetivo
	F	Empregado Efetivo
	F	Empregado Efetivo
	F	Empregado Efetivo
	M	Empregado Efetivo
	F	Empregado Efetivo
	F	Empregado Efetivo
	M	Empregado Efetivo
	F	Empregado Efetivo
	M	Empregado Efetivo
	F	Empregado Efetivo
	F	Empregado Efetivo
	F	Empregado Efetivo
	F	Empregado Efetivo

	F	Empregado Efetivo
	M	Empregado Efetivo
	M	Empregado Efetivo
	F	Empregado Efetivo
	F	Empregado Efetivo
	F	Empregado Efetivo
	F	Empregado Efetivo
	F	Empregado Efetivo
	M	Empregado Efetivo
	F	Empregado Efetivo
	F	Empregado Efetivo
	F	Empregado Efetivo
	F	Empregado Efetivo
	M	Empregado Efetivo
	F	Empregado Efetivo
	F	Empregado Efetivo
	F	Empregado Efetivo
	F	Empregado Efetivo
	F	Empregado Efetivo
	F	Empregado Efetivo
	F	Empregado Efetivo
	F	Empregado Efetivo
	M	Empregado Efetivo
	M	Empregado Efetivo
	F	Empregado Efetivo
	F	Empregado Efetivo
	F	Empregado Efetivo
	M	Empregado Efetivo
	F	Empregado Efetivo
	M	Empregado Efetivo
	F	Empregado Efetivo
	M	Empregado Efetivo

	F	Empregado Efetivo
	F	Empregado Efetivo
	M	Empregado Efetivo
	F	Empregado Efetivo
	F	Empregado Efetivo
	F	Empregado Efetivo
	F	Empregado Efetivo
	M	Empregado Efetivo
	F	Empregado Efetivo
	M	Empregado Efetivo
	F	Empregado Efetivo
	F	Empregado Efetivo
	M	Empregado Efetivo
	F	Empregado Efetivo
	F	Empregado Efetivo
	F	Empregado Efetivo
	F	Empregado Efetivo
	F	Empregado Efetivo
	F	Empregado Efetivo
	F	Empregado Efetivo
	F	Empregado Efetivo
	F	Empregado Efetivo
	F	Empregado Efetivo
	M	Empregado Efetivo
	F	Empregado Efetivo
	F	Empregado Efetivo
	F	Empregado Efetivo
	F	Empregado Efetivo
	F	Empregado Efetivo
	F	Empregado Efetivo
	F	Empregado Efetivo
	F	Empregado Efetivo

	F	Empregado Efetivo
	F	Empregado Efetivo
	F	Empregado Efetivo
	M	Empregado Efetivo
	F	Empregado Efetivo
	F	Empregado Efetivo
	M	Empregado Efetivo
	F	Empregado Efetivo
	F	Empregado Efetivo
	F	Empregado Efetivo
	F	Empregado Efetivo
	F	Empregado Efetivo
	F	Empregado Efetivo
	F	Empregado Efetivo
	M	Empregado Efetivo
	F	Empregado Efetivo
	F	Empregado Efetivo
	F	Empregado Efetivo
	F	Empregado Efetivo
	F	Empregado Efetivo
	F	Empregado Efetivo
	M	Empregado Efetivo
	F	Empregado Efetivo
	F	Empregado Efetivo
	F	Empregado Efetivo
	F	Empregado Efetivo
	F	Empregado Efetivo
	F	Empregado Efetivo
	F	Empregado Efetivo
	F	Empregado Efetivo
	M	Empregado Efetivo
	F	Empregado Efetivo

	M	Empregado Efetivo
	F	Empregado Efetivo
	F	Empregado Efetivo
	M	Empregado Efetivo
	F	Empregado Efetivo
	F	Empregado Efetivo
	F	Empregado Efetivo
	F	Empregado Efetivo
	F	Empregado Efetivo
	F	Empregado Efetivo
	M	Empregado Efetivo
	F	Empregado Efetivo
	F	Empregado Efetivo
	F	Empregado Efetivo
	F	Empregado Efetivo
	F	Empregado Efetivo
	F	Empregado Efetivo
	M	Empregado Efetivo
	F	Empregado Efetivo
	F	Empregado Efetivo
	F	Empregado Efetivo
	F	Empregado Efetivo
	F	Empregado Efetivo
	F	Empregado Efetivo
	F	Empregado Efetivo
	M	Empregado Efetivo
	F	Empregado Efetivo
	M	Empregado Efetivo
	F	Empregado Efetivo
	F	Empregado Efetivo
	F	Cedido - RJU
	M	Empregado Efetivo

	F	Empregado Efetivo
	F	Empregado Efetivo
	F	Empregado Efetivo
	F	Empregado Efetivo
	F	Empregado Efetivo
	F	Empregado Efetivo
	F	Empregado Efetivo
	F	Empregado Efetivo
	F	Empregado Efetivo
	F	Empregado Efetivo
	F	Empregado Efetivo
	F	Empregado Efetivo
	F	Empregado Efetivo
	M	Empregado Efetivo
	F	Empregado Efetivo
	F	Empregado Efetivo
	F	Empregado Efetivo
	F	Empregado Efetivo
	F	Empregado Efetivo
	F	Empregado Efetivo
	F	Empregado Efetivo
	F	Empregado Efetivo
	F	Empregado Efetivo
	F	Empregado Efetivo
	F	Empregado Efetivo
	F	Empregado Efetivo
	F	Empregado Efetivo
	F	Empregado Efetivo
	F	Empregado Efetivo
	F	Empregado Efetivo
	M	Empregado Efetivo
	F	Empregado Efetivo
	F	Empregado Efetivo
	F	Empregado Efetivo
	F	Empregado Efetivo
	F	Empregado Efetivo
	F	Empregado Efetivo
	F	Empregado Efetivo
	F	Empregado Efetivo
	F	Empregado Efetivo
	F	Empregado Efetivo
	F	Empregado Efetivo
	F	Empregado Efetivo
	F	Empregado Efetivo
	F	Empregado Efetivo
	F	Empregado Efetivo
	F	Empregado Efetivo
	F	Empregado Efetivo

	F	Empregado Efetivo
	F	Empregado Efetivo
	F	Empregado Efetivo
	F	Empregado Efetivo
	F	Empregado Efetivo
	F	Empregado Efetivo
	F	Empregado Efetivo
	F	Empregado Efetivo
	F	Empregado Efetivo
	M	Empregado Efetivo
	F	Empregado Efetivo
	F	Empregado Efetivo
	F	Empregado Efetivo
	F	Empregado Efetivo
	F	Empregado Efetivo
	F	Empregado Efetivo
	F	Empregado Efetivo
	F	Empregado Efetivo
	F	Empregado Efetivo
	F	Empregado Efetivo
	F	Empregado Efetivo
	F	Empregado Efetivo
	F	Empregado Efetivo
	F	Empregado Efetivo
	M	Empregado Efetivo
	F	Empregado Efetivo
	F	Empregado Efetivo
	F	Empregado Efetivo
	F	Empregado Efetivo
	F	Empregado Efetivo
	F	Empregado Efetivo
	F	Empregado Efetivo
	F	Empregado Efetivo
	F	Empregado Efetivo
	F	Empregado Efetivo
	F	Empregado Efetivo
	F	Empregado Efetivo
	F	Empregado Efetivo
	F	Empregado Efetivo
	F	Empregado Efetivo
	F	Empregado Efetivo
	F	Empregado Efetivo
	F	Empregado Efetivo
	F	Empregado Efetivo
	F	Empregado Efetivo
	F	Empregado Efetivo
	F	Empregado Efetivo
	F	Empregado Efetivo

	F	Empregado Efetivo
	F	Empregado Efetivo
	F	Empregado Efetivo
	F	Empregado Efetivo
	F	Empregado Efetivo
	F	Empregado Efetivo
	F	Empregado Efetivo
	F	Empregado Efetivo
	F	Empregado Efetivo
	F	Empregado Efetivo
	F	Empregado Efetivo
	F	Empregado Efetivo
	F	Empregado Efetivo
	F	Empregado Efetivo
	F	Empregado Efetivo
	F	Empregado Efetivo
	F	Empregado Efetivo
	F	Empregado Efetivo
	F	Empregado Efetivo
	F	Empregado Efetivo
	F	Empregado Efetivo
	F	Empregado Efetivo
	M	Empregado Efetivo
	F	Empregado Efetivo
	F	Empregado Efetivo
	F	Empregado Efetivo
	F	Empregado Efetivo
	F	Empregado Efetivo
	M	Empregado Efetivo
	F	Empregado Efetivo
	F	Empregado Efetivo
	F	Empregado Efetivo
	F	Empregado Efetivo
	F	Empregado Efetivo
	F	Empregado Efetivo
	F	Empregado Efetivo
	F	Empregado Efetivo
	M	Empregado Efetivo
	F	Empregado Efetivo
	F	Empregado Efetivo
	F	Empregado Efetivo
	F	Empregado Efetivo
	M	Empregado Efetivo
	F	Empregado Efetivo
	F	Empregado Efetivo
	F	Empregado Efetivo
	F	Empregado Efetivo
	F	Empregado Efetivo

	F	Empregado Efetivo
	F	Empregado Efetivo
	F	Empregado Efetivo
	F	Empregado Efetivo
	F	Empregado Efetivo
	F	Empregado Efetivo
	F	Empregado Efetivo
	F	Empregado Efetivo
	F	Empregado Efetivo
	F	Empregado Efetivo
	F	Empregado Efetivo
	F	Empregado Efetivo
	F	Empregado Efetivo
	M	Empregado Efetivo
	M	Cedido - RJU
	F	Empregado Efetivo
	F	Empregado Efetivo
	F	Empregado Efetivo
	F	Empregado Efetivo
	M	Empregado Efetivo
	F	Empregado Efetivo
	F	Empregado Efetivo
	F	Empregado Efetivo
	F	Empregado Efetivo
	M	Empregado Efetivo
	F	Empregado Efetivo
	M	Empregado Efetivo
	F	Empregado Efetivo
	M	Empregado Efetivo
	F	Empregado Efetivo
	F	Empregado Efetivo
	F	Empregado Efetivo
	F	Empregado Efetivo
	F	Empregado Efetivo
	F	Empregado Efetivo
	F	Empregado Efetivo
	F	Empregado Efetivo
	M	Empregado Efetivo
	M	Empregado Efetivo
	F	Empregado Efetivo
	F	Empregado Efetivo
	F	Empregado Efetivo
	F	Empregado Efetivo
	F	Empregado Efetivo
	M	Empregado Efetivo
	F	Empregado Efetivo
	F	Empregado Efetivo
	M	Empregado Efetivo

	F	Empregado Efetivo
	F	Empregado Efetivo
	M	Empregado Efetivo
	F	Empregado Efetivo
	F	Empregado Efetivo
	F	Empregado Efetivo
	F	Empregado Efetivo
	F	Empregado Efetivo
	F	Empregado Efetivo
	F	Empregado Efetivo
	F	Empregado Efetivo
	F	Empregado Efetivo
	F	Empregado Efetivo
	F	Empregado Efetivo
	F	Empregado Efetivo
	M	Empregado Efetivo
	F	Empregado Efetivo
	M	Empregado Efetivo
	F	Empregado Efetivo
	F	Empregado Efetivo
	F	Empregado Efetivo
	F	Empregado Efetivo
	F	Empregado Efetivo
	F	Empregado Efetivo
	F	Empregado Efetivo
	F	Empregado Efetivo
	F	Empregado Efetivo
	M	Empregado Efetivo
	F	Empregado Efetivo
	F	Empregado Efetivo
	F	Empregado Efetivo
	F	Empregado Efetivo
	F	Empregado Efetivo
	F	Empregado Efetivo
	F	Empregado Efetivo
	F	Empregado Efetivo
	F	Empregado Efetivo
	F	Empregado Efetivo
	F	Empregado Efetivo
	F	Empregado Efetivo
	F	Empregado Efetivo
	M	Empregado Efetivo
	M	Empregado Efetivo
	F	Empregado Efetivo
	F	Empregado Efetivo
	F	Empregado Efetivo
	F	Empregado Efetivo
	F	Empregado Efetivo

	F	Empregado Efetivo
	F	Empregado Efetivo
	F	Empregado Efetivo
	F	Empregado Efetivo
	M	Empregado Efetivo
	F	Empregado Efetivo
	F	Empregado Efetivo
	F	Empregado Efetivo
	F	Empregado Efetivo
	F	Empregado Efetivo
	F	Empregado Efetivo
	F	Empregado Efetivo
	F	Empregado Efetivo
	F	Empregado Efetivo
	F	Empregado Efetivo
	F	Empregado Efetivo
	F	Empregado Efetivo
	F	Empregado Efetivo
	F	Empregado Efetivo
	F	Empregado Efetivo
	M	Empregado Efetivo
	F	Empregado Efetivo
	F	Empregado Efetivo
	F	Empregado Efetivo
	F	Empregado Efetivo
	F	Empregado Efetivo
	F	Empregado Efetivo
	M	Empregado Efetivo
	F	Empregado Efetivo
	F	Empregado Efetivo
	F	Empregado Efetivo
	F	Empregado Efetivo
	F	Empregado Efetivo
	F	Empregado Efetivo
	F	Empregado Efetivo
	F	Empregado Efetivo
	F	Empregado Efetivo
	F	Empregado Efetivo
	F	Empregado Efetivo
	F	Empregado Efetivo
	F	Empregado Efetivo
	F	Empregado Efetivo
	F	Empregado Efetivo
	F	Empregado Efetivo
	F	Empregado Efetivo
	F	Empregado Efetivo
	F	Empregado Efetivo
	F	Empregado Efetivo

	F	Empregado Efetivo
	F	Empregado Efetivo
	F	Empregado Efetivo
	F	Empregado Efetivo
	F	Empregado Efetivo
	F	Empregado Efetivo
	F	Empregado Efetivo
	F	Empregado Efetivo
	F	Empregado Efetivo
	F	Empregado Efetivo
	F	Empregado Efetivo
	F	Empregado Efetivo
	F	Empregado Efetivo
	F	Empregado Efetivo
	F	Empregado Efetivo
	F	Empregado Efetivo
	F	Empregado Efetivo
	F	Empregado Efetivo
	F	Empregado Efetivo
	F	Empregado Efetivo
	F	Empregado Efetivo
	F	Empregado Efetivo
	M	Empregado Efetivo
	F	Empregado Efetivo
	F	Empregado Efetivo
	F	Empregado Efetivo
	F	Empregado Efetivo
	F	Empregado Efetivo
	F	Empregado Efetivo
	M	Empregado Efetivo
	F	Empregado Efetivo
	F	Empregado Efetivo
	F	Empregado Efetivo
	F	Empregado Efetivo
	F	Empregado Efetivo
	F	Empregado Efetivo
	F	Empregado Efetivo
	F	Empregado Efetivo
	F	Empregado Efetivo
	F	Empregado Efetivo
	F	Empregado Efetivo
	F	Empregado Efetivo
	F	Empregado Efetivo
	F	Empregado Efetivo
	F	Empregado Efetivo
	F	Empregado Efetivo
	F	Empregado Efetivo
	F	Empregado Efetivo

	F	Empregado Efetivo
	F	Empregado Efetivo
	F	Empregado Efetivo
	M	Empregado Efetivo
	F	Empregado Efetivo
	F	Empregado Efetivo
	F	Empregado Efetivo
	F	Empregado Efetivo
	F	Empregado Efetivo
	F	Empregado Efetivo
	F	Empregado Efetivo
	F	Empregado Efetivo
	F	Empregado Efetivo
	F	Empregado Efetivo
	F	Empregado Efetivo
	F	Empregado Efetivo
	F	Empregado Efetivo
	F	Empregado Efetivo
	F	Empregado Efetivo
	F	Empregado Efetivo
	F	Empregado Efetivo
	F	Empregado Efetivo
	F	Empregado Efetivo
	F	Empregado Efetivo
	F	Empregado Efetivo
	F	Empregado Efetivo
	F	Empregado Efetivo
	F	Empregado Efetivo
	F	Empregado Efetivo
	F	Empregado Efetivo
	F	Empregado Efetivo
	F	Empregado Efetivo
	F	Empregado Efetivo
	M	Empregado Efetivo
	F	Empregado Efetivo
	F	Empregado Efetivo
	F	Empregado Efetivo
	F	Empregado Efetivo
	F	Empregado Efetivo
	F	Empregado Efetivo
	F	Empregado Efetivo
	F	Empregado Efetivo
	F	Empregado Efetivo
	F	Empregado Efetivo
	F	Empregado Efetivo
	F	Empregado Efetivo
	F	Empregado Efetivo
	F	Empregado Efetivo

	F	Empregado Efetivo
	F	Empregado Efetivo
	F	Empregado Efetivo
	F	Empregado Efetivo
	F	Empregado Efetivo
	F	Empregado Efetivo
	F	Empregado Efetivo
	F	Empregado Efetivo
	F	Empregado Efetivo
	F	Empregado Efetivo
	F	Empregado Efetivo
	M	Empregado Efetivo
	F	Empregado Efetivo
	F	Empregado Efetivo
	F	Empregado Efetivo
	F	Empregado Efetivo
	F	Empregado Efetivo
	F	Empregado Efetivo
	F	Empregado Efetivo
	F	Empregado Efetivo
	F	Empregado Efetivo
	F	Empregado Efetivo
	F	Empregado Efetivo
	F	Empregado Efetivo
	F	Empregado Efetivo
	F	Empregado Efetivo
	F	Empregado Efetivo
	F	Empregado Efetivo
	F	Empregado Efetivo
	F	Empregado Efetivo
	F	Empregado Efetivo
	F	Empregado Efetivo
	F	Empregado Efetivo
	F	Empregado Efetivo
	F	Empregado Efetivo
	F	Empregado Efetivo
	F	Empregado Efetivo
	F	Empregado Efetivo
	F	Empregado Efetivo
	F	Empregado Efetivo
	F	Empregado Efetivo
	F	Empregado Efetivo
	F	Empregado Efetivo
	F	Empregado Efetivo
	F	Empregado Efetivo
	F	Empregado Efetivo
	F	Empregado Efetivo
	F	Empregado Efetivo

	M	Empregado Efetivo
	F	Empregado Efetivo
	F	Empregado Efetivo
	F	Empregado Efetivo
	F	Empregado Efetivo
	F	Empregado Efetivo
	F	Empregado Efetivo
	F	Empregado Efetivo
	F	Empregado Efetivo
	F	Empregado Efetivo
	F	Empregado Efetivo
	F	Empregado Efetivo
	F	Empregado Efetivo
	F	Empregado Efetivo
	F	Empregado Efetivo
	F	Empregado Efetivo
	M	Empregado Efetivo
	F	Empregado Efetivo
	F	Empregado Efetivo
	M	Empregado Efetivo
	F	Empregado Efetivo
	F	Empregado Efetivo
	F	Empregado Efetivo
	M	Empregado Efetivo
	F	Empregado Efetivo
	F	Empregado Efetivo
	F	Empregado Efetivo
	F	Empregado Efetivo
	F	Empregado Efetivo
	F	Empregado Efetivo
	F	Empregado Efetivo
	F	Empregado Efetivo

	F	Empregado Efetivo
	F	Empregado Efetivo
	F	Empregado Efetivo
	F	Empregado Efetivo
	F	Empregado Efetivo
	M	Empregado Efetivo
	M	Empregado Efetivo
	F	Empregado Efetivo
	M	Empregado Efetivo
	F	Empregado Efetivo
	F	Empregado Efetivo
	F	Empregado Efetivo
	M	Empregado Efetivo
	F	Empregado Efetivo
	F	Empregado Efetivo
	F	Empregado Efetivo
	F	Empregado Efetivo
	F	Empregado Efetivo
	F	Empregado Efetivo
	F	Empregado Efetivo
	M	Empregado Efetivo
	F	Empregado Efetivo
	F	Empregado Efetivo
	F	Empregado Efetivo
	F	Empregado Efetivo
	F	Empregado Efetivo
	F	Empregado Efetivo
	F	Empregado Efetivo
	F	Empregado Efetivo
	F	Empregado Efetivo
	F	Empregado Efetivo
	F	Empregado Efetivo

	F	Empregado Efetivo
	F	Empregado Efetivo
	F	Empregado Efetivo
	M	Empregado Efetivo
	F	Empregado Efetivo
	F	Empregado Efetivo
	F	Empregado Efetivo
	F	Empregado Efetivo
	F	Empregado Efetivo
	F	Empregado Efetivo
	F	Empregado Efetivo
	F	Empregado Efetivo
	F	Empregado Efetivo
	F	Empregado Efetivo
	F	Empregado Efetivo
	F	Empregado Efetivo
	F	Empregado Efetivo
	F	Empregado Efetivo
	F	Empregado Efetivo
	F	Empregado Efetivo
	F	Empregado Efetivo
	F	Empregado Efetivo
	F	Empregado Efetivo
	M	Empregado Efetivo
	F	Empregado Efetivo
	F	Empregado Efetivo
	M	Empregado Efetivo
	F	Empregado Efetivo
	F	Empregado Efetivo
	M	Empregado Efetivo
	F	Empregado Efetivo
	F	Empregado Efetivo

	F	Empregado Efetivo
	F	Empregado Efetivo
	F	Empregado Efetivo
	F	Empregado Efetivo
	F	Empregado Efetivo
	F	Empregado Efetivo
	F	Empregado Efetivo
	F	Empregado Efetivo
	M	Empregado Efetivo
	M	Empregado Efetivo
	M	Empregado Efetivo
	F	Empregado Efetivo
	F	Empregado Efetivo
	F	Empregado Efetivo
	F	Empregado Efetivo
	F	Empregado Efetivo
	F	Empregado Efetivo
	F	Empregado Efetivo
	F	Empregado Efetivo
	F	Empregado Efetivo
	F	Empregado Efetivo
	F	Empregado Efetivo
	F	Empregado Efetivo
	F	Empregado Efetivo
	F	Empregado Efetivo
	F	Empregado Efetivo
	F	Empregado Efetivo
	F	Empregado Efetivo
	F	Empregado Efetivo
	M	Empregado Efetivo
	F	Empregado Efetivo
	M	Empregado Efetivo

	F	Empregado Efetivo
	F	Empregado Efetivo
	M	Empregado Efetivo
	F	Empregado Efetivo
	F	Empregado Efetivo
	F	Empregado Efetivo
	M	Empregado Efetivo
	F	Empregado Efetivo
	F	Empregado Efetivo
	F	Empregado Efetivo
	F	Empregado Efetivo
	F	Empregado Efetivo
	F	Empregado Efetivo
	F	Empregado Efetivo
	F	Empregado Efetivo
	F	Empregado Efetivo
	F	Empregado Efetivo
	F	Empregado Efetivo
	F	Empregado Efetivo
	F	Empregado Efetivo
	F	Empregado Efetivo
	M	Empregado Efetivo
	F	Empregado Efetivo
	F	Empregado Efetivo
	M	Empregado Efetivo
	M	Empregado Efetivo
	M	Empregado Efetivo
	M	Empregado Efetivo
	F	Empregado Efetivo
	F	Empregado Efetivo
	F	Empregado Efetivo
	F	Empregado Efetivo

	F	Empregado Efetivo
	F	Empregado Efetivo
	F	Empregado Efetivo
	F	Empregado Efetivo
	F	Empregado Efetivo
	F	Empregado Efetivo
	F	Empregado Efetivo
	F	Empregado Efetivo
	F	Empregado Efetivo
	M	Empregado Efetivo
	F	Empregado Efetivo
	F	Empregado Efetivo
	F	Empregado Efetivo
	F	Empregado Efetivo
	F	Empregado Efetivo
	F	Empregado Efetivo
	F	Empregado Efetivo
	F	Empregado Efetivo
	F	Empregado Efetivo
	F	Empregado Efetivo
	F	Empregado Efetivo
	M	Empregado Efetivo
	F	Empregado Efetivo
	F	Empregado Efetivo
	F	Empregado Efetivo
	F	Empregado Efetivo
	F	Empregado Efetivo
	F	Empregado Efetivo
	F	Empregado Efetivo
	F	Empregado Efetivo
	F	Empregado Efetivo
	F	Empregado Efetivo

	F	Empregado Efetivo
	F	Empregado Efetivo
	F	Empregado Efetivo
	F	Empregado Efetivo
	F	Empregado Efetivo
	F	Empregado Efetivo
	F	Empregado Efetivo
	F	Empregado Efetivo
	F	Empregado Efetivo
	M	Empregado Efetivo
	M	Empregado Efetivo
	M	Empregado Efetivo
	M	Empregado Efetivo
	M	Empregado Efetivo
	M	Empregado Efetivo
	M	Empregado Efetivo
	M	Empregado Efetivo
	M	Empregado Efetivo
	M	Empregado Efetivo
	M	Empregado Efetivo
	M	Empregado Efetivo
	M	Empregado Efetivo
	M	Empregado Efetivo
	F	Empregado Efetivo
	M	Empregado Efetivo
	M	Empregado Efetivo
	F	Empregado Efetivo
	M	Empregado Efetivo
	F	Empregado Efetivo
	F	Empregado Efetivo
	M	Empregado Efetivo
	F	Empregado Efetivo

	F	Empregado Efetivo
	F	Empregado Efetivo
	F	Empregado Efetivo
	F	Empregado Efetivo
	F	Empregado Efetivo
	F	Empregado Efetivo
	F	Empregado Efetivo
	F	Empregado Efetivo
	F	Empregado Efetivo
	F	Empregado Efetivo
	F	Empregado Efetivo
	F	Empregado Efetivo
	F	Empregado Efetivo
	F	Empregado Efetivo
	F	Empregado Efetivo
	F	Empregado Efetivo
	F	Empregado Efetivo
	F	Empregado Efetivo
	F	Empregado Efetivo
	F	Empregado Efetivo
	F	Empregado Efetivo
	F	Empregado Efetivo
	F	Empregado Efetivo
	F	Empregado Efetivo
	M	Empregado Efetivo
	F	Empregado Efetivo
	M	Empregado Efetivo
	F	Empregado Efetivo
	F	Empregado Efetivo
	F	Empregado Efetivo
	F	Empregado Efetivo
	F	Empregado Efetivo

	M	Empregado Efetivo
	M	Empregado Efetivo
	M	Empregado Efetivo
	F	Empregado Efetivo
	F	Empregado Efetivo
	F	Empregado Efetivo
	M	Empregado Efetivo
	F	Empregado Efetivo
	M	Empregado Efetivo
	M	Empregado Efetivo
	M	Empregado Efetivo
	M	Empregado Efetivo
	M	Empregado Efetivo
	F	Empregado Efetivo
	F	Empregado Efetivo
	F	Empregado Efetivo
	F	Empregado Efetivo
	F	Empregado Efetivo
	F	Empregado Efetivo
	F	Empregado Efetivo
	F	Empregado Efetivo
	F	Empregado Efetivo
	F	Empregado Efetivo
	F	Empregado Efetivo
	F	Empregado Efetivo
	F	Empregado Efetivo
	M	Empregado Efetivo
	F	Empregado Efetivo
	M	Empregado Efetivo
	M	Empregado Efetivo
	M	Empregado Efetivo
	M	Empregado Efetivo
	M	Empregado Efetivo
	F	Empregado Efetivo
	F	Empregado Efetivo
	F	Empregado Efetivo
	M	Empregado Efetivo
	F	Empregado Efetivo
	M	Empregado Efetivo
	M	Empregado Efetivo
	M	Empregado Efetivo
	M	Empregado Efetivo
	M	Empregado Efetivo
	M	Empregado Efetivo
	M	Empregado Efetivo
	M	Empregado Efetivo
	F	Empregado Efetivo
	M	Empregado Efetivo

	F	Empregado Efetivo
	F	Empregado Efetivo
	F	Empregado Efetivo
	M	Empregado Efetivo
	M	Empregado Efetivo
	M	Empregado Efetivo
	F	Empregado Efetivo
	M	Empregado Efetivo
	M	Empregado Efetivo
	M	Empregado Efetivo
	M	Empregado Efetivo
	F	Empregado Efetivo
	M	Empregado Efetivo
	M	Empregado Efetivo
	M	Empregado Efetivo
	M	Empregado Efetivo
	F	Empregado Efetivo
	F	Empregado Efetivo
	F	Empregado Efetivo
	M	Empregado Efetivo
	F	Empregado Efetivo
	F	Empregado Efetivo
	F	Empregado Efetivo
	F	Empregado Efetivo
	F	Empregado Efetivo
	F	Empregado Efetivo
	F	Empregado Efetivo
	F	Empregado Efetivo
	F	Empregado Efetivo
	F	Empregado Efetivo
	F	Empregado Efetivo
	F	Empregado Efetivo
	F	Empregado Efetivo
	F	Empregado Efetivo
	M	Empregado Efetivo
	M	Empregado Efetivo
	F	Empregado Efetivo
	F	Empregado Efetivo
	M	Empregado Efetivo
	F	Empregado Efetivo
	F	Empregado Efetivo
	F	Empregado Efetivo

	F	Empregado Efetivo
	F	Empregado Efetivo
	M	Empregado Efetivo
	M	Empregado Efetivo
	M	Empregado Efetivo
	F	Empregado Efetivo
	F	Empregado Efetivo
	F	Empregado Efetivo
	M	Empregado Efetivo
	F	Empregado Efetivo
	F	Empregado Efetivo
	M	Empregado Efetivo
	M	Empregado Efetivo
	M	Empregado Efetivo
	M	Empregado Efetivo
	M	Empregado Efetivo
	M	Empregado Efetivo
	M	Empregado Efetivo
	M	Empregado Efetivo
	M	Empregado Efetivo
	M	Empregado Efetivo
	F	Empregado Efetivo
	M	Empregado Efetivo
	M	Empregado Efetivo
	M	Empregado Efetivo
	M	Empregado Efetivo
	M	Empregado Efetivo
	M	Empregado Efetivo
	M	Empregado Efetivo
	M	Empregado Efetivo
	M	Empregado Efetivo
	M	Empregado Efetivo
	M	Empregado Efetivo
	M	Empregado Efetivo
	M	Empregado Efetivo
	M	Empregado Efetivo
	M	Empregado Efetivo
	M	Empregado Efetivo
	F	Empregado Efetivo
	M	Empregado Efetivo
	F	Empregado Efetivo
	M	Empregado Efetivo
	M	Empregado Efetivo
	F	Empregado Efetivo
	F	Empregado Efetivo
	F	Empregado Efetivo
	F	Empregado Efetivo
	F	Empregado Efetivo

	M	Empregado Efetivo
	F	Empregado Efetivo
	F	Empregado Efetivo
	M	Empregado Efetivo
	F	Empregado Efetivo
	F	Empregado Efetivo
	F	Empregado Efetivo
	F	Empregado Efetivo
	F	Empregado Efetivo
	F	Empregado Efetivo
	F	Empregado Efetivo
	F	Empregado Efetivo
	F	Empregado Efetivo
	F	Empregado Efetivo
	M	Empregado Efetivo
	F	Empregado Efetivo
	F	Empregado Efetivo
	F	Empregado Efetivo
	F	Empregado Efetivo
	F	Empregado Efetivo
	F	Empregado Efetivo
	F	Empregado Efetivo
	F	Empregado Efetivo
	F	Empregado Efetivo
	F	Empregado Efetivo
	F	Empregado Efetivo
	F	Empregado Efetivo
	F	Empregado Efetivo
	M	Empregado Efetivo
	F	Empregado Efetivo
	F	Empregado Efetivo
	F	Empregado Efetivo
	F	Empregado Efetivo
	F	Empregado Efetivo
	F	Empregado Efetivo
	F	Empregado Efetivo
	F	Empregado Efetivo
	F	Empregado Efetivo
	F	Empregado Efetivo
	F	Empregado Efetivo
	F	Empregado Efetivo
	F	Empregado Efetivo
	F	Empregado Efetivo
	F	Empregado Efetivo
	F	Empregado Efetivo
	F	Empregado Efetivo
	F	Empregado Efetivo
	F	Empregado Efetivo

	F	Empregado Efetivo
	F	Empregado Efetivo
	F	Empregado Efetivo
	F	Empregado Efetivo
	M	Empregado Efetivo
	F	Empregado Efetivo
	F	Empregado Efetivo
	F	Empregado Efetivo
	F	Empregado Efetivo
	F	Empregado Efetivo
	F	Empregado Efetivo
	F	Empregado Efetivo
	F	Empregado Efetivo
	F	Empregado Efetivo
	M	Empregado Efetivo
	M	Empregado Efetivo
	F	Empregado Efetivo
	F	Empregado Efetivo
	F	Empregado Efetivo
	F	Empregado Efetivo
	F	Empregado Efetivo
	M	Empregado Efetivo
	F	Empregado Efetivo
	F	Empregado Efetivo
	F	Empregado Efetivo
	F	Empregado Efetivo
	F	Empregado Efetivo
	F	Empregado Efetivo
	F	Empregado Efetivo
	M	Empregado Efetivo
	M	Empregado Efetivo
	F	Empregado Efetivo
	M	Empregado Efetivo
	M	Empregado Efetivo
	F	Empregado Efetivo
	F	Empregado Efetivo
	F	Empregado Efetivo
	M	Empregado Efetivo
	F	Empregado Efetivo
	F	Empregado Efetivo
	M	Empregado Efetivo
	F	Empregado Efetivo
	F	Empregado Efetivo
	F	Empregado Efetivo
	F	Empregado Efetivo
	F	Empregado Efetivo
	F	Empregado Efetivo
	F	Empregado Efetivo

	F	Empregado Efetivo
	M	Empregado Efetivo
	M	Empregado Efetivo
	F	Empregado Efetivo
	F	Empregado Efetivo
	F	Empregado Efetivo
	F	Empregado Efetivo
	M	Empregado Efetivo
	F	Empregado Efetivo
	F	Empregado Efetivo
	F	Empregado Efetivo
	M	Empregado Efetivo
	F	Empregado Efetivo
	F	Empregado Efetivo
	F	Empregado Efetivo
	F	Empregado Efetivo
	F	Empregado Efetivo
	M	Empregado Efetivo
	F	Empregado Efetivo
	F	Empregado Efetivo
	F	Empregado Efetivo
	F	Empregado Efetivo
	F	Empregado Efetivo
	F	Empregado Efetivo
	F	Empregado Efetivo
	F	Empregado Efetivo
	F	Empregado Efetivo
	F	Empregado Efetivo
	F	Empregado Efetivo
	F	Empregado Efetivo
	F	Empregado Efetivo
	M	Empregado Efetivo
	F	Empregado Efetivo
	M	Empregado Efetivo
	F	Empregado Efetivo
	F	Empregado Efetivo
	F	Empregado Efetivo
	M	Empregado Efetivo
	M	Empregado Efetivo
	F	Empregado Efetivo
	M	Empregado Efetivo
	F	Empregado Efetivo
	F	Empregado Efetivo
	F	Empregado Efetivo
	F	Empregado Efetivo
	F	Empregado Efetivo
	F	Empregado Efetivo
	F	Empregado Efetivo

	F	Empregado Efetivo
	F	Empregado Efetivo
	F	Empregado Efetivo
	F	Empregado Efetivo
	F	Empregado Efetivo
	F	Empregado Efetivo
	F	Empregado Efetivo
	F	Empregado Efetivo
	F	Empregado Efetivo
	F	Empregado Efetivo
	F	Empregado Efetivo
	M	Empregado Efetivo
	F	Empregado Efetivo
	F	Empregado Efetivo
	M	Empregado Efetivo
	M	Empregado Efetivo
	F	Empregado Efetivo
	F	Empregado Efetivo
	F	Empregado Efetivo
	F	Empregado Efetivo
	F	Empregado Efetivo
	M	Empregado Efetivo
	F	Empregado Efetivo
	F	Empregado Efetivo
	F	Empregado Efetivo
	F	Empregado Efetivo
	F	Empregado Efetivo
	F	Empregado Efetivo
	F	Empregado Efetivo
	M	Empregado Efetivo
	F	Empregado Efetivo
	M	Empregado Efetivo
	F	Empregado Efetivo
	F	Empregado Efetivo
	F	Empregado Efetivo
	F	Empregado Efetivo
	M	Empregado Efetivo
	M	Empregado Efetivo
	F	Empregado Efetivo
	F	Empregado Efetivo
	F	Empregado Efetivo
	F	Empregado Efetivo
	F	Empregado Efetivo
	F	Empregado Efetivo
	F	Empregado Efetivo
	F	Empregado Efetivo
	M	Empregado Efetivo
	F	Empregado Efetivo

	F	Empregado Efetivo
	F	Empregado Efetivo
	F	Empregado Efetivo
	M	Empregado Efetivo
	F	Empregado Efetivo
	F	Empregado Efetivo
	M	Empregado Efetivo
	M	Empregado Efetivo
	F	Empregado Efetivo
	F	Empregado Efetivo
	F	Empregado Efetivo
	F	Empregado Efetivo
	F	Empregado Efetivo
	F	Empregado Efetivo
	M	Empregado Efetivo
	F	Empregado Efetivo
	F	Empregado Efetivo
	F	Empregado Efetivo
	F	Empregado Efetivo
	F	Empregado Efetivo
	F	Empregado Efetivo
	F	Empregado Efetivo
	M	Empregado Efetivo
	F	Empregado Efetivo
	F	Empregado Efetivo
	M	Empregado Efetivo
	F	Empregado Efetivo
	M	Empregado Efetivo
	F	Empregado Efetivo
	M	Empregado Efetivo
	M	Empregado Efetivo
	F	Empregado Efetivo
	M	Empregado Efetivo
	F	Empregado Efetivo
	M	Empregado Efetivo
	F	Empregado Efetivo
	F	Empregado Efetivo
	F	Empregado Efetivo
	F	Empregado Efetivo
	F	Empregado Efetivo
	F	Empregado Efetivo
	F	Empregado Efetivo
	F	Empregado Efetivo
	F	Empregado Efetivo
	F	Empregado Efetivo
	F	Empregado Efetivo
	F	Empregado Efetivo
	F	Empregado Efetivo

	F	Empregado Efetivo
	F	Empregado Efetivo
	F	Empregado Efetivo
	F	Empregado Efetivo
	F	Empregado Efetivo
	F	Empregado Efetivo
	F	Empregado Efetivo
	F	Empregado Efetivo
	M	Empregado Efetivo
	F	Empregado Efetivo
	F	Empregado Efetivo
	M	Empregado Efetivo
	F	Empregado Efetivo
	F	Empregado Efetivo
	F	Empregado Efetivo
	F	Empregado Efetivo
	F	Empregado Efetivo
	F	Empregado Efetivo
	F	Empregado Efetivo
	F	Empregado Efetivo
	F	Empregado Efetivo
	F	Empregado Efetivo
	F	Empregado Efetivo
	F	Empregado Efetivo
	F	Empregado Efetivo
	F	Empregado Efetivo
	F	Empregado Efetivo
	M	Empregado Efetivo
	F	Empregado Efetivo
	F	Empregado Efetivo
	F	Empregado Efetivo
	F	Empregado Efetivo
	F	Empregado Efetivo
	F	Empregado Efetivo
	F	Empregado Efetivo
	F	Empregado Efetivo
	F	Empregado Efetivo
	F	Empregado Efetivo
	M	Empregado Efetivo
	F	Empregado Efetivo
	F	Empregado Efetivo
	M	Empregado Efetivo
	F	Empregado Efetivo
	F	Empregado Efetivo
	M	Empregado Efetivo
	F	Empregado Efetivo
	F	Empregado Efetivo
	F	Empregado Efetivo

	F	Empregado Efetivo
	F	Empregado Efetivo
	F	Empregado Efetivo
	F	Empregado Efetivo
	F	Empregado Efetivo
	F	Empregado Efetivo
	F	Empregado Efetivo
	F	Empregado Efetivo
	F	Empregado Efetivo
	F	Empregado Efetivo
	F	Empregado Efetivo
	F	Empregado Efetivo
	M	Empregado Efetivo
	F	Empregado Efetivo
	F	Empregado Efetivo
	F	Empregado Efetivo
	F	Empregado Efetivo
	F	Empregado Efetivo
	F	Empregado Efetivo
	F	Empregado Efetivo
	F	Empregado Efetivo
	F	Empregado Efetivo
	F	Empregado Efetivo
	M	Empregado Efetivo
	F	Empregado Efetivo
	F	Empregado Efetivo
	F	Empregado Efetivo
	M	Empregado Efetivo
	M	Empregado Efetivo
	F	Empregado Efetivo
	F	Empregado Efetivo
	F	Empregado Efetivo
	F	Empregado Efetivo
	M	Empregado Efetivo
	F	Empregado Efetivo
	F	Empregado Efetivo
	F	Empregado Efetivo
	F	Empregado Efetivo
	F	Empregado Efetivo
	F	Empregado Efetivo
	M	Empregado Efetivo
	F	Empregado Efetivo
	F	Empregado Efetivo
	F	Empregado Efetivo
	F	Empregado Efetivo
	F	Empregado Efetivo
	M	Empregado Efetivo
	M	Empregado Efetivo

	M	Empregado Efetivo
	F	Empregado Efetivo
	M	Empregado Efetivo
	F	Empregado Efetivo
	F	Empregado Efetivo
	M	Empregado Efetivo
	M	Empregado Efetivo
	F	Empregado Efetivo
	F	Empregado Efetivo
	M	Empregado Efetivo
	F	Empregado Efetivo
	F	Empregado Efetivo
	F	Empregado Efetivo
	F	Empregado Efetivo
	F	Empregado Efetivo
	F	Empregado Efetivo
	M	Empregado Efetivo
	F	Empregado Efetivo
	F	Empregado Efetivo
	M	Empregado Efetivo
	F	Empregado Efetivo
	F	Empregado Efetivo
	F	Empregado Efetivo
	F	Empregado Efetivo
	F	Empregado Efetivo
	F	Empregado Efetivo
	M	Empregado Efetivo
	F	Empregado Efetivo
	F	Empregado Efetivo
	F	Empregado Efetivo
	F	Empregado Efetivo
	F	Empregado Efetivo
	F	Empregado Efetivo
	F	Empregado Efetivo
	F	Empregado Efetivo
	F	Empregado Efetivo
	F	Empregado Efetivo
	F	Empregado Efetivo
	F	Empregado Efetivo
	F	Empregado Efetivo
	F	Empregado Efetivo
	M	Empregado Efetivo
	F	Empregado Efetivo
	F	Empregado Efetivo
	F	Empregado Efetivo
	F	Empregado Efetivo
	F	Empregado Efetivo
	F	Empregado Efetivo

	M	Empregado Efetivo
	F	Empregado Efetivo
	F	Empregado Efetivo
	F	Empregado Efetivo
	M	Empregado Efetivo
	M	Empregado Efetivo
	F	Empregado Efetivo
	F	Empregado Efetivo
	F	Empregado Efetivo
	F	Empregado Efetivo
	F	Empregado Efetivo
	F	Empregado Efetivo
	F	Empregado Efetivo
	M	Empregado Efetivo
	F	Empregado Efetivo
	F	Empregado Efetivo
	M	Empregado Efetivo
	M	Empregado Efetivo
	F	Empregado Efetivo
	F	Empregado Efetivo
	M	Empregado Efetivo
	F	Empregado Efetivo
	F	Empregado Efetivo
	F	Empregado Efetivo
	F	Empregado Efetivo
	F	Empregado Efetivo
	F	Empregado Efetivo
	F	Empregado Efetivo
	M	Empregado Efetivo
	F	Empregado Efetivo
	M	Empregado Efetivo
	F	Empregado Efetivo
	F	Empregado Efetivo
	F	Empregado Efetivo
	F	Empregado Efetivo
	F	Empregado Efetivo

	F	Empregado Efetivo
	F	Empregado Efetivo
	M	Empregado Efetivo
	F	Empregado Efetivo
	F	Empregado Efetivo
	M	Empregado Efetivo
	F	Empregado Efetivo
	M	Empregado Efetivo
	F	Empregado Efetivo
	F	Empregado Efetivo
	F	Empregado Efetivo
	F	Empregado Efetivo
	F	Empregado Efetivo
	F	Empregado Efetivo
	F	Empregado Efetivo
	F	Empregado Efetivo
	F	Empregado Efetivo
	F	Empregado Efetivo
	M	Empregado Efetivo
	M	Empregado Efetivo
	F	Empregado Efetivo
	F	Empregado Efetivo
	M	Empregado Efetivo
	M	Empregado Efetivo
	F	Empregado Efetivo
	F	Empregado Efetivo
	F	Empregado Efetivo
	F	Empregado Efetivo
	F	Empregado Efetivo
	F	Empregado Efetivo
	F	Empregado Efetivo
	F	Empregado Efetivo
	F	Empregado Efetivo
	F	Empregado Efetivo

	F	Empregado Efetivo
	F	Empregado Efetivo
	F	Empregado Efetivo
	F	Empregado Efetivo
	F	Empregado Efetivo
	F	Empregado Efetivo
	F	Empregado Efetivo
	F	Empregado Efetivo
	F	Empregado Efetivo
	F	Empregado Efetivo
	F	Empregado Efetivo
	F	Empregado Efetivo
	F	Empregado Efetivo
	F	Empregado Efetivo
	F	Empregado Efetivo
	F	Empregado Efetivo
	F	Empregado Efetivo
	F	Empregado Efetivo
	F	Empregado Efetivo
	M	Empregado Efetivo
	M	Empregado Efetivo
	F	Empregado Efetivo
	M	Empregado Efetivo
	F	Empregado Efetivo
	F	Empregado Efetivo
	M	Empregado Efetivo
	F	Empregado Efetivo
	F	Empregado Efetivo
	F	Empregado Efetivo
	M	Empregado Efetivo
	F	Empregado Efetivo
	F	Empregado Efetivo
	M	Empregado Efetivo
	F	Empregado Efetivo
	F	Empregado Efetivo
	F	Empregado Efetivo
	M	Empregado Efetivo
	F	Empregado Efetivo
	F	Empregado Efetivo
	F	Empregado Efetivo
	M	Empregado Efetivo
	F	Empregado Efetivo
	F	Empregado Efetivo
	F	Empregado Efetivo
	F	Empregado Efetivo
	F	Empregado Efetivo
	M	Empregado Efetivo
	F	Empregado Efetivo

	F	Empregado Efetivo
	F	Empregado Efetivo
	F	Empregado Efetivo
	M	Empregado Efetivo
	F	Empregado Efetivo
	F	Empregado Efetivo
	F	Empregado Efetivo
	F	Empregado Efetivo
	F	Empregado Efetivo
	F	Empregado Efetivo
	F	Empregado Efetivo
	F	Empregado Efetivo
	F	Empregado Efetivo
	M	Empregado Efetivo
	F	Empregado Efetivo
	F	Empregado Efetivo
	F	Empregado Efetivo
	M	Empregado Efetivo
	F	Empregado Efetivo
	F	Empregado Efetivo
	F	Empregado Efetivo
	M	Empregado Efetivo
	M	Empregado Efetivo
	F	Empregado Efetivo
	F	Empregado Efetivo
	F	Empregado Efetivo
	F	Empregado Efetivo
	F	Empregado Efetivo
	F	Empregado Efetivo
	F	Empregado Efetivo
	F	Empregado Efetivo
	F	Empregado Efetivo
	M	Empregado Efetivo
	F	Empregado Efetivo
	F	Empregado Efetivo
	M	Empregado Efetivo
	F	Empregado Efetivo
	F	Empregado Efetivo
	F	Empregado Efetivo
	M	Empregado Efetivo
	F	Empregado Efetivo
	F	Empregado Efetivo
	F	Empregado Efetivo
	F	Empregado Efetivo
	F	Empregado Efetivo
	F	Empregado Efetivo
	F	Empregado Efetivo
	M	Empregado Efetivo

	M	Empregado Efetivo
	F	Empregado Efetivo
	F	Empregado Efetivo
	F	Empregado Efetivo
	M	Empregado Efetivo
	F	Empregado Efetivo
	F	Empregado Efetivo
	F	Empregado Efetivo
	F	Empregado Efetivo
	M	Empregado Efetivo
	F	Empregado Efetivo
	M	Empregado Efetivo
	F	Empregado Efetivo
	F	Empregado Efetivo
	F	Empregado Efetivo
	F	Empregado Efetivo
	F	Empregado Efetivo
	M	Empregado Efetivo
	F	Empregado Efetivo
	F	Empregado Efetivo
	F	Empregado Efetivo
	F	Empregado Efetivo
	F	Empregado Efetivo
	F	Empregado Efetivo
	F	Empregado Efetivo
	F	Empregado Efetivo
	F	Empregado Efetivo
	F	Empregado Efetivo
	F	Empregado Efetivo
	M	Empregado Efetivo
	M	Empregado Efetivo
	M	Empregado Efetivo
	M	Empregado Efetivo
	F	Empregado Efetivo
	F	Empregado Efetivo
	M	Empregado Efetivo
	M	Empregado Efetivo
	M	Empregado Efetivo
	M	Empregado Efetivo
	M	Empregado Efetivo
	M	Empregado Efetivo
	M	Empregado Efetivo
	M	Empregado Efetivo
	M	Empregado Efetivo
	M	Empregado Efetivo
	M	Empregado Efetivo
	F	Empregado Efetivo
	M	Empregado Efetivo

	M	Empregado Efetivo
	M	Empregado Efetivo
	F	Empregado Efetivo
	M	Empregado Efetivo
	M	Empregado Efetivo
	M	Empregado Efetivo
	M	Empregado Efetivo
	M	Empregado Efetivo
	M	Empregado Efetivo
	F	Empregado Efetivo
	M	Empregado Efetivo
	F	Empregado Efetivo
	M	Empregado Efetivo
	F	Empregado Efetivo
	F	Empregado Efetivo
	M	Empregado Efetivo
	F	Empregado Efetivo
	F	Empregado Efetivo
	M	Empregado Efetivo
	F	Empregado Efetivo
	F	Empregado Efetivo
	F	Empregado Efetivo
	F	Empregado Efetivo
	F	Empregado Efetivo
	F	Empregado Efetivo
	M	Empregado Efetivo
	M	Empregado Efetivo
	M	Empregado Efetivo
	F	Empregado Efetivo
	M	Empregado Efetivo
	F	Empregado Efetivo
	F	Empregado Efetivo
	M	Empregado Efetivo
	F	Empregado Efetivo
	F	Empregado Efetivo
	M	Empregado Efetivo
	M	Empregado Efetivo
	M	Empregado Efetivo
	M	Empregado Efetivo
	F	Empregado Efetivo
	M	Empregado Efetivo
	F	Empregado Efetivo
	F	Empregado Efetivo
	M	Empregado Efetivo
	M	Empregado Efetivo
	M	Empregado Efetivo
	F	Empregado Efetivo
	M	Empregado Efetivo

	F	Empregado Efetivo
	F	Empregado Efetivo
	F	Empregado Efetivo
	M	Empregado Efetivo
	M	Empregado Efetivo
	M	Empregado Efetivo
	M	Empregado Efetivo
	M	Empregado Efetivo
	F	Empregado Efetivo
	F	Empregado Efetivo
	F	Empregado Efetivo
	F	Empregado Efetivo
	F	Empregado Efetivo
	F	Empregado Efetivo
	M	Empregado Efetivo
	M	Empregado Efetivo
	F	Empregado Efetivo
	F	Empregado Efetivo
	M	Empregado Efetivo
	F	Empregado Efetivo
	Rower	02/02/4748
	F	Empregado Efetivo
	F	Empregado Efetivo
	F	Empregado Efetivo
	F	Empregado Efetivo
	F	Empregado Efetivo
	F	Empregado Efetivo
	F	Empregado Efetivo
	F	Empregado Efetivo
	F	Empregado Efetivo
	F	Empregado Efetivo
	M	Empregado Efetivo
	F	Empregado Efetivo
	F	Empregado Efetivo
	F	Empregado Efetivo
	F	Empregado Efetivo
	F	Empregado Efetivo
	F	Empregado Efetivo
	F	Empregado Efetivo
	M	Empregado Efetivo
	F	Empregado Efetivo
	F	Empregado Efetivo
	M	Empregado Efetivo
	F	Empregado Efetivo
	F	Empregado Efetivo
	M	Empregado Efetivo
	F	Empregado Efetivo
	M	Empregado Efetivo

	F	Empregado Efetivo
	M	Empregado Efetivo
	M	Empregado Efetivo
	M	Empregado Efetivo
	M	Empregado Efetivo
	M	Empregado Efetivo
	F	Empregado Efetivo
	F	Empregado Efetivo
	F	Empregado Efetivo
	F	Empregado Efetivo
	M	Empregado Efetivo
	F	Empregado Efetivo
	F	Empregado Efetivo
	M	Empregado Efetivo
	M	Empregado Efetivo
	M	Empregado Efetivo
	F	Empregado Efetivo
	F	Empregado Efetivo
	M	Empregado Efetivo
	F	Empregado Efetivo
	F	Empregado Efetivo
	M	Empregado Efetivo
	M	Empregado Efetivo
	F	Empregado Efetivo
	M	Empregado Efetivo
	M	Empregado Efetivo
	M	Empregado Efetivo
	F	Empregado Efetivo
	M	Empregado Efetivo
	F	Empregado Efetivo
	M	Empregado Efetivo
	F	Empregado Efetivo
	F	Empregado Efetivo
	F	Empregado Efetivo
	F	Empregado Efetivo
	F	Empregado Efetivo
	F	Empregado Efetivo
	F	Empregado Efetivo
	F	Empregado Efetivo
	M	Empregado Efetivo
	F	Empregado Efetivo
	F	Empregado Efetivo
	M	Empregado Efetivo
	M	Empregado Efetivo
	F	Empregado Efetivo
	F	Empregado Efetivo
	M	Empregado Efetivo
	F	Empregado Efetivo

	F	Empregado Efetivo
	F	Empregado Efetivo
	F	Empregado Efetivo
	F	Empregado Efetivo
	F	Empregado Efetivo
	M	Empregado Efetivo
	M	Empregado Efetivo
	M	Empregado Efetivo
	M	Empregado Efetivo
	M	Empregado Efetivo
	F	Empregado Efetivo
	F	Empregado Efetivo
	F	Empregado Efetivo
	M	Empregado Efetivo
	M	Empregado Efetivo
	F	Empregado Efetivo
	F	Empregado Efetivo
	M	Empregado Efetivo
	F	Empregado Efetivo
	F	Empregado Efetivo
	F	Empregado Efetivo
	F	Empregado Efetivo
	M	Empregado Efetivo
	M	Empregado Efetivo
	F	Empregado Efetivo
	F	Empregado Efetivo
	F	Empregado Efetivo
	F	Empregado Efetivo
	M	Empregado Efetivo
	M	Empregado Efetivo
	M	Empregado Efetivo
	F	Empregado Efetivo
	F	Empregado Efetivo
	F	Empregado Efetivo
	F	Empregado Efetivo
	M	Empregado Efetivo
	F	Empregado Efetivo
	F	Empregado Efetivo
	F	Empregado Efetivo
	F	Empregado Efetivo

	M	Empregado Efetivo
	F	Empregado Efetivo
	M	Empregado Efetivo
	F	Empregado Efetivo
	F	Empregado Efetivo
	M	Empregado Efetivo
	F	Empregado Efetivo
	M	Empregado Efetivo
	F	Empregado Efetivo
	F	Empregado Efetivo
	F	Empregado Efetivo
	M	Empregado Efetivo
	M	Empregado Efetivo
	F	Empregado Efetivo
	F	Empregado Efetivo
	F	Empregado Efetivo
	F	Empregado Efetivo
	M	Empregado Efetivo
	M	Empregado Efetivo
	M	Empregado Efetivo
	F	Empregado Efetivo
	M	Empregado Efetivo
	M	Empregado Efetivo
	F	Empregado Efetivo
	M	Empregado Efetivo
	F	Empregado Efetivo
	F	Empregado Efetivo
	M	Empregado Efetivo
	F	Empregado Efetivo
	M	Empregado Efetivo
	F	Empregado Efetivo
	F	Empregado Efetivo
	F	Empregado Efetivo
	F	Empregado Efetivo
	F	Empregado Efetivo
	F	Empregado Efetivo

	F	Empregado Efetivo
	F	Empregado Efetivo
	F	Empregado Efetivo
	F	Empregado Efetivo
	M	Empregado Efetivo
	F	Empregado Efetivo
	F	Empregado Efetivo
	F	Empregado Efetivo
	M	Empregado Efetivo
	F	Empregado Efetivo
	F	Empregado Efetivo
	F	Empregado Efetivo
	F	Empregado Efetivo
	M	Empregado Efetivo
	F	Empregado Efetivo
	F	Empregado Efetivo
	F	Empregado Efetivo
	F	Empregado Efetivo
	F	Empregado Efetivo
	M	Empregado Efetivo
	F	Empregado Efetivo
	F	Empregado Efetivo
	F	Empregado Efetivo
	M	Empregado Efetivo
	F	Empregado Efetivo
	F	Empregado Efetivo
	F	Empregado Efetivo
	M	Empregado Efetivo
	F	Empregado Efetivo
	F	Empregado Efetivo
	M	Empregado Efetivo
	F	Empregado Efetivo
	M	Empregado Efetivo
	F	Empregado Efetivo

	F	Empregado Efetivo
	F	Empregado Efetivo
	M	Empregado Efetivo
	F	Empregado Efetivo
	M	Empregado Efetivo
	M	Empregado Efetivo
	F	Empregado Efetivo
	F	Empregado Efetivo
	F	Empregado Efetivo
	F	Empregado Efetivo
	F	Empregado Efetivo
	M	Empregado Efetivo
	F	Empregado Efetivo
	F	Empregado Efetivo
	F	Empregado Efetivo
	M	Empregado Efetivo
	M	Empregado Efetivo
	M	Empregado Efetivo
	F	Empregado Efetivo
	M	Empregado Efetivo
	F	Empregado Efetivo
	F	Empregado Efetivo
	F	Empregado Efetivo
	F	Empregado Efetivo
	M	Empregado Efetivo
	F	Empregado Efetivo
	F	Empregado Efetivo
	F	Empregado Efetivo
	M	Empregado Efetivo
	M	Empregado Efetivo
	F	Empregado Efetivo
	F	Empregado Efetivo

	F	Empregado Efetivo
	F	Empregado Efetivo
	F	Empregado Efetivo
	M	Empregado Efetivo
	M	Empregado Efetivo
	M	Empregado Efetivo
	M	Empregado Efetivo
	M	Empregado Efetivo
	F	Empregado Efetivo
	M	Empregado Efetivo
	M	Empregado Efetivo
	M	Empregado Efetivo
	F	Empregado Efetivo
	M	Empregado Efetivo
	F	Empregado Efetivo
	F	Empregado Efetivo
	F	Empregado Efetivo
	F	Empregado Efetivo
	M	Empregado Efetivo
	F	Empregado Efetivo
	F	Empregado Efetivo
	F	Empregado Efetivo
	F	Empregado Efetivo
	M	Empregado Efetivo
	F	Empregado Efetivo
	F	Empregado Efetivo
	F	Empregado Efetivo
	F	Empregado Efetivo
	F	Empregado Efetivo
	M	Empregado Efetivo
	F	Empregado Efetivo
	F	Empregado Efetivo
	F	Empregado Efetivo
	F	Empregado Efetivo
	F	Empregado Efetivo
	F	Empregado Efetivo
	M	Empregado Efetivo
	F	Empregado Efetivo
	F	Empregado Efetivo

	F	Empregado Efetivo
	F	Empregado Efetivo
	F	Empregado Efetivo
	F	Empregado Efetivo
	F	Empregado Efetivo
	F	Empregado Efetivo
	F	Empregado Efetivo
	F	Empregado Efetivo
	F	Empregado Efetivo
	F	Empregado Efetivo
	F	Empregado Efetivo
	F	Empregado Efetivo
	F	Empregado Efetivo
	F	Empregado Efetivo
	F	Empregado Efetivo
	F	Empregado Efetivo
	F	Empregado Efetivo
	F	Empregado Efetivo
	F	Empregado Efetivo
	F	Empregado Efetivo
	F	Empregado Efetivo
	F	Empregado Efetivo
	F	Empregado Efetivo
	F	Empregado Efetivo
	M	Empregado Efetivo
	F	Empregado Efetivo
	F	Empregado Efetivo
	F	Empregado Efetivo
	F	Empregado Efetivo
	F	Empregado Efetivo
	F	Empregado Efetivo
	F	Empregado Efetivo
	F	Empregado Efetivo
	F	Empregado Efetivo
	F	Empregado Efetivo
	M	Empregado Efetivo
	F	Empregado Efetivo
	F	Empregado Efetivo
	F	Empregado Efetivo
	F	Empregado Efetivo
	F	Empregado Efetivo
	F	Empregado Efetivo
	F	Empregado Efetivo
	F	Empregado Efetivo
	F	Empregado Efetivo
	F	Empregado Efetivo
	F	Empregado Efetivo
	F	Empregado Efetivo

	F	Empregado Efetivo
	F	Empregado Efetivo
	F	Empregado Efetivo
	F	Empregado Efetivo
	F	Empregado Efetivo
	F	Empregado Efetivo
	F	Empregado Efetivo
	M	Empregado Efetivo
	F	Empregado Efetivo
	F	Empregado Efetivo
	M	Empregado Efetivo
	F	Empregado Efetivo
	F	Empregado Efetivo
	M	Empregado Efetivo
	F	Empregado Efetivo
	F	Empregado Efetivo
	F	Empregado Efetivo
	F	Empregado Efetivo
	F	Empregado Efetivo
	F	Empregado Efetivo
	F	Empregado Efetivo
	F	Empregado Efetivo
	F	Empregado Efetivo
	M	Empregado Efetivo
	F	Empregado Efetivo
	F	Empregado Efetivo
	F	Empregado Efetivo
	F	Empregado Efetivo
	M	Empregado Efetivo
	M	Empregado Efetivo
	F	Empregado Efetivo
	F	Empregado Efetivo
	F	Empregado Efetivo
	F	Empregado Efetivo
	F	Empregado Efetivo
	M	Empregado Efetivo
	M	Empregado Efetivo
	M	Empregado Efetivo
	M	Empregado Efetivo
	F	Empregado Efetivo
	F	Empregado Efetivo
	F	Empregado Efetivo
	F	Empregado Efetivo
	M	Empregado Efetivo
	M	Empregado Efetivo
	F	Empregado Efetivo
	M	Empregado Efetivo
	M	Empregado Efetivo

	F	Empregado Efetivo
	M	Empregado Efetivo
	F	Empregado Efetivo
	F	Empregado Efetivo
	F	Empregado Efetivo
	M	Empregado Efetivo
	M	Empregado Efetivo
	M	Empregado Efetivo
	M	Empregado Efetivo
	F	Empregado Efetivo
	M	Empregado Efetivo
	F	Empregado Efetivo
	F	Empregado Efetivo
	M	Empregado Efetivo
	F	Empregado Efetivo
	F	Empregado Efetivo
	M	Empregado Efetivo
	F	Empregado Efetivo
	F	Empregado Efetivo
	M	Empregado Efetivo
	F	Empregado Efetivo
	F	Empregado Efetivo
	F	Empregado Efetivo
	M	Empregado Efetivo
	F	Empregado Efetivo
	F	Empregado Efetivo
	M	Empregado Efetivo
	M	Empregado Efetivo
	F	Empregado Efetivo
	F	Empregado Efetivo
	M	Empregado Efetivo
	F	Empregado Efetivo
	M	Empregado Efetivo
	F	Empregado Efetivo
	M	Empregado Efetivo
	F	Empregado Efetivo
	F	Empregado Efetivo
	F	Empregado Efetivo
	F	Empregado Efetivo
	F	Empregado Efetivo
	M	Empregado Efetivo
	F	Empregado Efetivo
	F	Empregado Efetivo
	F	Empregado Efetivo
	F	Empregado Efetivo
	M	Empregado Efetivo
	F	Empregado Efetivo
	M	Empregado Efetivo

	M	Empregado Efetivo
	M	Empregado Efetivo
	M	Empregado Efetivo
	M	Empregado Efetivo
	F	Empregado Efetivo
	M	Empregado Efetivo
	M	Empregado Efetivo
	M	Empregado Efetivo
	M	Empregado Efetivo
	M	Empregado Efetivo
	M	Empregado Efetivo
	M	Empregado Efetivo
	F	Empregado Efetivo
	F	Empregado Efetivo
	F	Empregado Efetivo
	F	Empregado Efetivo
	F	Empregado Efetivo
	M	Empregado Efetivo
	F	Empregado Efetivo
	F	Empregado Efetivo
	F	Empregado Efetivo
	M	Empregado Efetivo
	F	Empregado Efetivo
	F	Empregado Efetivo
	F	Empregado Efetivo
	F	Empregado Efetivo
	F	Empregado Efetivo
	F	Empregado Efetivo
	F	Empregado Efetivo
	F	Empregado Efetivo
	F	Empregado Efetivo
	F	Empregado Efetivo
	F	Empregado Efetivo
	F	Empregado Efetivo
	F	Empregado Efetivo
	F	Empregado Efetivo
	F	Empregado Efetivo
	F	Empregado Efetivo
	F	Empregado Efetivo
	F	Empregado Efetivo
	F	Empregado Efetivo
	F	Empregado Efetivo
	F	Empregado Efetivo
	F	Empregado Efetivo
	F	Empregado Efetivo
	M	Empregado Efetivo
	F	Empregado Efetivo
	F	Empregado Efetivo

	F	Empregado Efetivo
	F	Empregado Efetivo
	F	Empregado Efetivo
	F	Empregado Efetivo
	F	Empregado Efetivo
	F	Empregado Efetivo
	F	Empregado Efetivo
	F	Empregado Efetivo
	F	Empregado Efetivo
	F	Empregado Efetivo
	F	Empregado Efetivo
	F	Empregado Efetivo
	F	Empregado Efetivo
	M	Empregado Efetivo
	F	Empregado Efetivo
	F	Empregado Efetivo
	F	Empregado Efetivo
	F	Empregado Efetivo
	M	Empregado Efetivo
	F	Empregado Efetivo
	F	Empregado Efetivo
	F	Empregado Efetivo
	F	Empregado Efetivo
	F	Empregado Efetivo
	F	Empregado Efetivo
	F	Empregado Efetivo
	F	Empregado Efetivo
	F	Empregado Efetivo
	F	Empregado Efetivo
	F	Empregado Efetivo
	M	Empregado Efetivo
	F	Empregado Efetivo
	F	Empregado Efetivo
	F	Empregado Efetivo
	F	Empregado Efetivo
	M	Empregado Efetivo
	F	Empregado Efetivo
	F	Empregado Efetivo
	F	Empregado Efetivo
	M	Empregado Efetivo
	F	Empregado Efetivo
	F	Empregado Efetivo
	F	Empregado Efetivo
	F	Empregado Efetivo
	F	Empregado Efetivo
	F	Empregado Efetivo
	F	Empregado Efetivo
	M	Empregado Efetivo

	F	Empregado Efetivo
	F	Empregado Efetivo
	F	Empregado Efetivo
	F	Empregado Efetivo
	F	Empregado Efetivo
	F	Empregado Efetivo
	F	Empregado Efetivo
	F	Empregado Efetivo
	F	Empregado Efetivo
	F	Empregado Efetivo
	F	Empregado Efetivo
	M	Empregado Efetivo
	F	Empregado Efetivo
	F	Empregado Efetivo
	F	Empregado Efetivo
	F	Empregado Efetivo
	F	Empregado Efetivo
	F	Empregado Efetivo
	F	Empregado Efetivo
	F	Empregado Efetivo
	F	Empregado Efetivo
	F	Empregado Efetivo
	F	Empregado Efetivo
	M	Empregado Efetivo
	F	Empregado Efetivo
	M	Empregado Efetivo
	M	Empregado Efetivo
	F	Empregado Efetivo
	F	Empregado Efetivo
	M	Empregado Efetivo
	F	Empregado Efetivo
	F	Empregado Efetivo
	F	Empregado Efetivo
	F	Empregado Efetivo
	F	Empregado Efetivo
	M	Empregado Efetivo
	F	Empregado Efetivo
	F	Empregado Efetivo
	F	Empregado Efetivo
	F	Empregado Efetivo
	M	Empregado Efetivo
	M	Empregado Efetivo
	F	Empregado Efetivo
	F	Empregado Efetivo
	F	Empregado Efetivo
	F	Empregado Efetivo
	F	Empregado Efetivo
	M	Empregado Efetivo

	F	Empregado Efetivo
	F	Empregado Efetivo
	F	Empregado Efetivo
	F	Empregado Efetivo
	F	Empregado Efetivo
	F	Empregado Efetivo
	F	Empregado Efetivo
	M	Empregado Efetivo
	F	Empregado Efetivo
	F	Empregado Efetivo
	F	Empregado Efetivo
	F	Empregado Efetivo
	F	Empregado Efetivo
	F	Empregado Efetivo
	F	Empregado Efetivo
	F	Empregado Efetivo
	F	Empregado Efetivo
	F	Empregado Efetivo
	F	Empregado Efetivo
	F	Empregado Efetivo
	F	Empregado Efetivo
	F	Empregado Efetivo
	F	Empregado Efetivo
	F	Empregado Efetivo
	F	Empregado Efetivo
	F	Empregado Efetivo
	F	Empregado Efetivo
	F	Empregado Efetivo
	F	Empregado Efetivo
	F	Empregado Efetivo
	F	Empregado Efetivo
	F	Empregado Efetivo
	F	Empregado Efetivo
	F	Empregado Efetivo
	F	Empregado Efetivo
	F	Empregado Efetivo
	F	Empregado Efetivo
	F	Empregado Efetivo
	F	Empregado Efetivo
	F	Empregado Efetivo
	F	Empregado Efetivo
	F	Empregado Efetivo
	F	Empregado Efetivo
	F	Empregado Efetivo
	F	Empregado Efetivo
	F	Empregado Efetivo
	F	Empregado Efetivo
	F	Empregado Efetivo

	F	Empregado Efetivo
	F	Empregado Efetivo
	F	Empregado Efetivo
	F	Empregado Efetivo
	F	Empregado Efetivo
	M	Empregado Efetivo
	F	Empregado Efetivo
	F	Empregado Efetivo
	F	Empregado Efetivo
	F	Empregado Efetivo
	F	Empregado Efetivo
	F	Empregado Efetivo
	F	Empregado Efetivo
	F	Empregado Efetivo
	F	Empregado Efetivo
	F	Empregado Efetivo
	F	Empregado Efetivo
	F	Empregado Efetivo
	F	Empregado Efetivo
	F	Empregado Efetivo
	F	Empregado Efetivo
	F	Empregado Efetivo
	F	Empregado Efetivo
	F	Empregado Efetivo
	F	Empregado Efetivo
	F	Empregado Efetivo
	F	Empregado Efetivo
	F	Empregado Efetivo
	F	Empregado Efetivo
	F	Empregado Efetivo
	F	Empregado Efetivo
	F	Empregado Efetivo
	F	Empregado Efetivo
	F	Empregado Efetivo
	F	Empregado Efetivo
	F	Empregado Efetivo
	F	Empregado Efetivo
	F	Empregado Efetivo
	F	Empregado Efetivo
	F	Empregado Efetivo
	F	Empregado Efetivo
	F	Empregado Efetivo
	F	Empregado Efetivo
	F	Empregado Efetivo
	F	Empregado Efetivo
	F	Empregado Efetivo
	F	Empregado Efetivo
	F	Empregado Efetivo

	F	Empregado Efetivo
	F	Empregado Efetivo
	F	Empregado Efetivo
	F	Empregado Efetivo
	F	Empregado Efetivo
	F	Empregado Efetivo
	F	Empregado Efetivo
	F	Empregado Efetivo
	F	Empregado Efetivo
	F	Empregado Efetivo
	F	Empregado Efetivo
	F	Empregado Efetivo
	F	Empregado Efetivo
	F	Empregado Efetivo
	F	Empregado Efetivo
	F	Empregado Efetivo
	F	Empregado Efetivo
	F	Empregado Efetivo
	F	Empregado Efetivo
	F	Empregado Efetivo
	F	Empregado Efetivo
	F	Empregado Efetivo
	F	Empregado Efetivo
	F	Empregado Efetivo
	F	Empregado Efetivo
	F	Empregado Efetivo
	F	Empregado Efetivo
	F	Empregado Efetivo
	F	Empregado Efetivo
	F	Empregado Efetivo
	F	Empregado Efetivo
	F	Empregado Efetivo
	F	Empregado Efetivo
	F	Empregado Efetivo
	F	Empregado Efetivo
	F	Empregado Efetivo
	F	Empregado Efetivo
	F	Empregado Efetivo
	F	Empregado Efetivo
	F	Empregado Efetivo
	F	Empregado Efetivo

	F	Empregado Efetivo
	F	Empregado Efetivo
	F	Empregado Efetivo
	F	Empregado Efetivo
	F	Empregado Efetivo
	F	Empregado Efetivo
	F	Empregado Efetivo
	F	Empregado Efetivo
	F	Empregado Efetivo
	F	Empregado Efetivo
	F	Empregado Efetivo
	F	Empregado Efetivo
	F	Empregado Efetivo
	F	Empregado Efetivo
	F	Empregado Efetivo
	F	Empregado Efetivo
	F	Empregado Efetivo
	F	Empregado Efetivo
	F	Empregado Efetivo
	F	Empregado Efetivo
	F	Empregado Efetivo
	F	Empregado Efetivo
	F	Empregado Efetivo
	F	Empregado Efetivo
	F	Empregado Efetivo
	F	Empregado Efetivo
	F	Empregado Efetivo
	F	Empregado Efetivo
	F	Empregado Efetivo
	F	Empregado Efetivo
	F	Empregado Efetivo
	F	Empregado Efetivo
	F	Empregado Efetivo
	F	Empregado Efetivo
	F	Empregado Efetivo
	F	Empregado Efetivo
	F	Empregado Efetivo
	F	Empregado Efetivo
	F	Empregado Efetivo
	F	Empregado Efetivo
	F	Empregado Efetivo
	F	Empregado Efetivo

	F	Empregado Efetivo
	F	Empregado Efetivo
	F	Empregado Efetivo
	F	Empregado Efetivo
	F	Empregado Efetivo
	F	Empregado Efetivo
	F	Empregado Efetivo
	F	Empregado Efetivo
	F	Empregado Efetivo
	F	Empregado Efetivo
	F	Empregado Efetivo
	F	Empregado Efetivo
	F	Empregado Efetivo
	F	Empregado Efetivo
	F	Empregado Efetivo
	F	Empregado Efetivo
	F	Empregado Efetivo
	F	Empregado Efetivo
	F	Empregado Efetivo
	F	Empregado Efetivo
	F	Empregado Efetivo
	F	Empregado Efetivo
	F	Empregado Efetivo
	F	Empregado Efetivo
	F	Empregado Efetivo
	F	Empregado Efetivo
	F	Empregado Efetivo
	F	Empregado Efetivo
	F	Empregado Efetivo
	F	Empregado Efetivo
	F	Empregado Efetivo
	F	Empregado Efetivo
	F	Empregado Efetivo
	F	Empregado Efetivo
	F	Empregado Efetivo
	F	Empregado Efetivo
	F	Empregado Efetivo

	F	Empregado Efetivo
	F	Empregado Efetivo
	F	Empregado Efetivo
	F	Empregado Efetivo
	F	Empregado Efetivo
	F	Empregado Efetivo
	F	Empregado Efetivo
	F	Empregado Efetivo
	F	Empregado Efetivo
	F	Empregado Efetivo
	F	Empregado Efetivo
	F	Empregado Efetivo
	F	Empregado Efetivo
	F	Empregado Efetivo
	F	Empregado Efetivo
	F	Empregado Efetivo
	F	Empregado Efetivo
	F	Empregado Efetivo
	F	Empregado Efetivo
	F	Empregado Efetivo
	M	Empregado Efetivo
	M	Empregado Efetivo
	M	Empregado Efetivo
	M	Empregado Efetivo
	M	Empregado Efetivo
	M	Empregado Efetivo
	M	Empregado Efetivo
	M	Empregado Efetivo
	M	Empregado Efetivo
	M	Empregado Efetivo
	M	Empregado Efetivo
	M	Empregado Efetivo
	M	Empregado Efetivo
	M	Empregado Efetivo
	M	Empregado Efetivo
	F	Empregado Efetivo
	F	Empregado Efetivo
	F	Empregado Efetivo
	F	Empregado Efetivo
	F	Empregado Efetivo
	F	Cedido - RJU
	M	Empregado Efetivo

	M	Empregado Efetivo
	M	Empregado Efetivo
	M	Empregado Efetivo
	M	Empregado Efetivo
	M	Empregado Efetivo
	M	Empregado Efetivo
	M	Empregado Efetivo
	M	Empregado Efetivo
	F	Empregado Efetivo
	F	Empregado Efetivo
	F	Empregado Efetivo
	M	Empregado Efetivo
	M	Empregado Efetivo
	F	Empregado Efetivo
	M	Empregado Efetivo
	M	Empregado Efetivo
	M	Empregado Efetivo
	M	Empregado Efetivo
	M	Empregado Efetivo
	M	Empregado Efetivo
	F	Empregado Efetivo
	M	Empregado Efetivo
	F	Empregado Efetivo
	F	Empregado Efetivo
	M	Empregado Efetivo
	F	Empregado Efetivo
	F	Empregado Efetivo
	M	Empregado Efetivo
	F	Empregado Efetivo
	M	Empregado Efetivo
	F	Empregado Efetivo
	F	Empregado Efetivo
	F	Empregado Efetivo
	F	Empregado Efetivo
	M	Cedido - RJU
	F	Empregado Efetivo
	M	Empregado Efetivo
	F	Empregado Efetivo
	F	Empregado Efetivo
	M	Empregado Efetivo
	M	Empregado Efetivo
	F	Empregado Efetivo
	F	Empregado Efetivo
	M	Empregado Efetivo
	F	Empregado Efetivo
	M	Empregado Efetivo
	M	Empregado Efetivo

	M	Empregado Efetivo
	F	Empregado Efetivo
	F	Empregado Efetivo
	F	Empregado Efetivo
	M	Empregado Efetivo
	M	Empregado Efetivo
	M	Empregado Efetivo
	F	Empregado Efetivo
	F	Empregado Efetivo
	M	Empregado Efetivo
	F	Empregado Efetivo
	F	Empregado Efetivo
	F	Empregado Efetivo
	F	Empregado Efetivo
	F	Empregado Efetivo
	F	Empregado Efetivo
	M	Empregado Efetivo
	F	Empregado Efetivo
	F	Empregado Efetivo
	F	Empregado Efetivo
	F	Empregado Efetivo
	F	Empregado Efetivo
	F	Empregado Efetivo
	F	Empregado Efetivo
	F	Empregado Efetivo
	F	Empregado Efetivo
	F	Empregado Efetivo
	F	Empregado Efetivo
	F	Empregado Efetivo
	F	Empregado Efetivo
	F	Empregado Efetivo
	F	Empregado Efetivo
	F	Empregado Efetivo
	F	Empregado Efetivo
	F	Empregado Efetivo
	F	Empregado Efetivo
	F	Empregado Efetivo
	F	Empregado Efetivo
	F	Empregado Efetivo
	F	Empregado Efetivo
	F	Empregado Efetivo
	F	Empregado Efetivo

	F	Empregado Efetivo
	F	Empregado Efetivo
	F	Empregado Efetivo
	F	Empregado Efetivo
	F	Empregado Efetivo
	F	Empregado Efetivo
	M	Empregado Efetivo
	F	Empregado Efetivo
	F	Empregado Efetivo
	F	Empregado Efetivo
	F	Empregado Efetivo
	F	Empregado Efetivo
	F	Empregado Efetivo
	F	Empregado Efetivo
	M	Empregado Efetivo
	F	Empregado Efetivo
	F	Empregado Efetivo
	M	Empregado Efetivo
	F	Empregado Efetivo
	F	Empregado Efetivo
	F	Empregado Efetivo
	F	Empregado Efetivo
	F	Empregado Efetivo
	F	Empregado Efetivo
	F	Empregado Efetivo
	F	Empregado Efetivo
	F	Empregado Efetivo
	F	Empregado Efetivo
	F	Empregado Efetivo
	F	Empregado Efetivo
	F	Empregado Efetivo
	F	Empregado Efetivo
	F	Empregado Efetivo
	F	Empregado Efetivo
	M	Empregado Efetivo
	F	Empregado Efetivo
	F	Empregado Efetivo
	M	Empregado Efetivo
	M	Empregado Efetivo
	M	Empregado Efetivo
	F	Empregado Efetivo
	F	Empregado Efetivo
	F	Empregado Efetivo
	M	Empregado Efetivo

	F	Empregado Efetivo
	F	Empregado Efetivo
	F	Empregado Efetivo
	M	Empregado Efetivo
	F	Empregado Efetivo
	M	Empregado Efetivo
	M	Empregado Efetivo
	F	Empregado Efetivo
	F	Empregado Efetivo
	F	Empregado Efetivo
	M	Empregado Efetivo
	F	Empregado Efetivo
	F	Empregado Efetivo
	F	Empregado Efetivo
	F	Empregado Efetivo
	M	Empregado Efetivo
	F	Empregado Efetivo
	F	Empregado Efetivo
	F	Empregado Efetivo
	M	Empregado Efetivo
	F	Empregado Efetivo
	F	Empregado Efetivo
	F	Empregado Efetivo
	M	Empregado Efetivo
	F	Empregado Efetivo
	F	Empregado Efetivo
	F	Empregado Efetivo
	F	Empregado Efetivo
	F	Empregado Efetivo
	M	Empregado Efetivo
	F	Empregado Efetivo
	F	Empregado Efetivo
	F	Empregado Efetivo
	F	Empregado Efetivo
	F	Empregado Efetivo
	F	Empregado Efetivo
	F	Empregado Efetivo
	F	Empregado Efetivo
	F	Empregado Efetivo
	F	Empregado Efetivo

	M	Empregado Efetivo
	F	Empregado Efetivo
	F	Empregado Efetivo
	F	Empregado Efetivo
	F	Empregado Efetivo
	F	Empregado Efetivo
	M	Empregado Efetivo
	F	Empregado Efetivo
	F	Empregado Efetivo
	F	Empregado Efetivo
	F	Empregado Efetivo
	F	Empregado Efetivo
	M	Empregado Efetivo
	F	Empregado Efetivo
	F	Empregado Efetivo
	M	Empregado Efetivo
	F	Empregado Efetivo
	F	Empregado Efetivo
	F	Empregado Efetivo
	F	Empregado Efetivo
	F	Empregado Efetivo
	F	Empregado Efetivo
	F	Empregado Efetivo
	F	Empregado Efetivo
	F	Empregado Efetivo
	F	Empregado Efetivo
	F	Empregado Efetivo
	F	Empregado Efetivo
	F	Empregado Efetivo
	F	Empregado Efetivo
	F	Empregado Efetivo
	F	Empregado Efetivo
	F	Empregado Efetivo
	F	Empregado Efetivo
	F	Empregado Efetivo
	F	Empregado Efetivo
	F	Empregado Efetivo
	F	Empregado Efetivo
	F	Empregado Efetivo
	F	Empregado Efetivo
	F	Empregado Efetivo
	F	Empregado Efetivo
	F	Empregado Efetivo
	F	Empregado Efetivo
	F	Empregado Efetivo

	F	Empregado Efetivo
	F	Empregado Efetivo
	F	Empregado Efetivo
	F	Empregado Efetivo
	F	Empregado Efetivo
	F	Empregado Efetivo
	F	Empregado Efetivo
	F	Empregado Efetivo
	F	Empregado Efetivo
	F	Empregado Efetivo
	F	Empregado Efetivo
	F	Empregado Efetivo
	F	Empregado Efetivo
	F	Empregado Efetivo
	F	Empregado Efetivo
	F	Empregado Efetivo
	F	Empregado Efetivo
	F	Empregado Efetivo
	F	Empregado Efetivo
	F	Empregado Efetivo
	F	Empregado Efetivo
	F	Empregado Efetivo
	M	Empregado Efetivo
	M	Empregado Efetivo
	F	Empregado Efetivo
	F	Empregado Efetivo
	F	Empregado Efetivo
	F	Empregado Efetivo
	F	Empregado Efetivo
	M	Empregado Efetivo
	F	Empregado Efetivo
	F	Empregado Efetivo
	F	Empregado Efetivo
	F	Empregado Efetivo
	F	Empregado Efetivo
	F	Empregado Efetivo

	M	Empregado Efetivo
	M	Empregado Efetivo
	M	Empregado Efetivo
	F	Empregado Efetivo
	M	Empregado Efetivo
	F	Empregado Efetivo
	M	Empregado Efetivo
	M	Empregado Efetivo
	M	Empregado Efetivo
	M	Empregado Efetivo
	F	Empregado Efetivo
	F	Empregado Efetivo
	M	Empregado Efetivo
	M	Empregado Efetivo
	M	Empregado Efetivo
	F	Empregado Efetivo
	F	Empregado Efetivo
	F	Empregado Efetivo
	M	Empregado Efetivo
	M	Empregado Efetivo
	F	Empregado Efetivo
	F	Empregado Efetivo
	F	Empregado Efetivo
	F	Empregado Efetivo
	F	Empregado Efetivo
	F	Empregado Efetivo
	F	Empregado Efetivo
	F	Empregado Efetivo
	F	Empregado Efetivo
	F	Empregado Efetivo
	F	Empregado Efetivo
	F	Empregado Efetivo
	F	Empregado Efetivo
	F	Empregado Efetivo
	F	Empregado Efetivo
	F	Empregado Efetivo
	F	Empregado Efetivo

	F	Empregado Efetivo
	F	Empregado Efetivo
	F	Empregado Efetivo
	F	Empregado Efetivo
	F	Empregado Efetivo
	F	Empregado Efetivo
	F	Empregado Efetivo
	F	Empregado Efetivo
	F	Empregado Efetivo
	F	Empregado Efetivo
	F	Empregado Efetivo
	F	Empregado Efetivo
	F	Empregado Efetivo
	F	Empregado Efetivo
	F	Empregado Efetivo
	F	Empregado Efetivo
	F	Empregado Efetivo
	F	Empregado Efetivo
	F	Empregado Efetivo
	F	Empregado Efetivo
	F	Empregado Efetivo
	F	Empregado Efetivo
	F	Empregado Efetivo
	F	Empregado Efetivo
	F	Empregado Efetivo
	F	Empregado Efetivo
	F	Empregado Efetivo
	F	Empregado Efetivo
	F	Empregado Efetivo
	F	Empregado Efetivo
	F	Empregado Efetivo
	M	Empregado Efetivo
	F	Empregado Efetivo
	F	Empregado Efetivo
	F	Empregado Efetivo
	F	Empregado Efetivo
	F	Empregado Efetivo
	M	Empregado Efetivo
	M	Empregado Efetivo
	M	Empregado Efetivo
	F	Empregado Efetivo
	F	Empregado Efetivo
	F	Empregado Efetivo

	F	Empregado Efetivo
	F	Empregado Efetivo
	F	Empregado Efetivo
	F	Empregado Efetivo
	F	Empregado Efetivo
	F	Empregado Efetivo
	F	Empregado Efetivo
	F	Empregado Efetivo
	F	Empregado Efetivo
	F	Empregado Efetivo
	M	Empregado Efetivo
	M	Empregado Efetivo
	M	Empregado Efetivo
	F	Empregado Efetivo
	M	Empregado Efetivo
	F	Empregado Efetivo
	F	Empregado Efetivo
	F	Empregado Efetivo
	M	Empregado Efetivo
	F	Empregado Efetivo
	F	Empregado Efetivo
	F	Empregado Efetivo
	F	Empregado Efetivo
	F	Empregado Efetivo
	F	Empregado Efetivo
	M	Empregado Efetivo
	F	Empregado Efetivo
	M	Empregado Efetivo
	F	Empregado Efetivo
	F	Empregado Efetivo
	F	Empregado Efetivo
	F	Empregado Efetivo

	F	Empregado Efetivo
	F	Empregado Efetivo
	M	Empregado Efetivo
	M	Empregado Efetivo
	F	Empregado Efetivo
	F	Empregado Efetivo
	F	Empregado Efetivo
	F	Empregado Efetivo
	F	Empregado Efetivo
	F	Empregado Efetivo
	F	Empregado Efetivo
	F	Empregado Efetivo
	F	Empregado Efetivo
	F	Empregado Efetivo
	F	Empregado Efetivo
	F	Empregado Efetivo
	F	Empregado Efetivo
	F	Empregado Efetivo
	F	Empregado Efetivo
	F	Empregado Efetivo
	F	Empregado Efetivo
	M	Empregado Efetivo
	F	Empregado Efetivo
	F	Empregado Efetivo
	F	Empregado Efetivo
	M	Empregado Efetivo
	F	Empregado Efetivo
	F	Empregado Efetivo
	M	Empregado Efetivo
	F	Empregado Efetivo
	F	Empregado Efetivo
	M	Empregado Efetivo

	M	Empregado Efetivo
	M	Empregado Efetivo
	M	Empregado Efetivo
	M	Empregado Efetivo
	F	Empregado Efetivo
	F	Empregado Efetivo
	M	Empregado Efetivo
	M	Empregado Efetivo
	M	Empregado Efetivo
	F	Empregado Efetivo
	M	Empregado Efetivo
	M	Empregado Efetivo
	M	Empregado Efetivo
	M	Empregado Efetivo
	M	Empregado Efetivo
	M	Empregado Efetivo
	M	Empregado Efetivo
	M	Empregado Efetivo
	M	Empregado Efetivo
	M	Empregado Efetivo
	M	Empregado Efetivo
	M	Empregado Efetivo
	F	Empregado Efetivo
	F	Empregado Efetivo
	F	Empregado Efetivo
	F	Empregado Efetivo
	F	Empregado Efetivo
	F	Empregado Efetivo
	F	Empregado Efetivo
	F	Empregado Efetivo
	F	Empregado Efetivo
	F	Empregado Efetivo

	M	Empregado Efetivo
	M	Empregado Efetivo
	M	Empregado Efetivo
	F	Empregado Efetivo
	F	Empregado Efetivo
	F	Empregado Efetivo
	F	Empregado Efetivo
	F	Empregado Efetivo
	F	Empregado Efetivo
	M	Empregado Efetivo
	M	Empregado Efetivo
	M	Empregado Efetivo
	M	Empregado Efetivo
	F	Empregado Efetivo
	F	Empregado Efetivo
	F	Empregado Efetivo
	F	Empregado Efetivo
	F	Empregado Efetivo
	F	Empregado Efetivo
	M	Empregado Efetivo
	F	Empregado Efetivo
	F	Empregado Efetivo
	M	Empregado Efetivo
	F	Empregado Efetivo
	F	Empregado Efetivo
	F	Empregado Efetivo
	F	Empregado Cedido
	F	Empregado Efetivo
	F	Empregado Efetivo
	F	Empregado Efetivo
	M	Empregado Efetivo
	F	Empregado Efetivo
	F	Empregado Efetivo
	F	Empregado Efetivo
	F	Empregado Efetivo
	F	Empregado Efetivo
	F	Empregado Efetivo
	F	Empregado Efetivo
	M	Empregado Efetivo

	M	Empregado Efetivo
	M	Empregado Efetivo
	M	Empregado Efetivo
	F	Empregado Efetivo
	F	Empregado Efetivo
	F	Empregado Efetivo
	F	Empregado Efetivo
	M	Empregado Efetivo
	F	Empregado Efetivo
	F	Empregado Efetivo
	F	Empregado Efetivo
	F	Empregado Efetivo
	M	Empregado Efetivo
	F	Empregado Efetivo
	F	Empregado Efetivo
	F	Empregado Efetivo
	F	Empregado Efetivo
	F	Empregado Efetivo
	M	Empregado Efetivo
	F	Empregado Efetivo
	F	Empregado Efetivo
	F	Empregado Efetivo
	F	Empregado Efetivo
	F	Empregado Efetivo
	F	Empregado Efetivo
	M	Empregado Efetivo
	F	Empregado Efetivo
	M	Empregado Efetivo
	F	Empregado Efetivo
	F	Empregado Efetivo
	F	Empregado Efetivo
	F	Empregado Efetivo
	F	Empregado Efetivo
	F	Empregado Efetivo
	M	Empregado Efetivo
	F	Empregado Efetivo
	F	Empregado Efetivo
	F	Empregado Efetivo
	F	Empregado Efetivo
	F	Empregado Efetivo
	M	Empregado Efetivo
	F	Empregado Efetivo
	F	Empregado Efetivo

	F	Empregado Efetivo
	F	Empregado Efetivo
	F	Empregado Efetivo
	F	Empregado Efetivo
	F	Empregado Efetivo
	M	Empregado Efetivo
	F	Empregado Efetivo
	F	Empregado Efetivo
	F	Empregado Efetivo
	F	Empregado Efetivo
	F	Empregado Efetivo
	M	Empregado Efetivo
	F	Empregado Efetivo
	F	Empregado Efetivo
	F	Empregado Efetivo
	F	Empregado Efetivo
	F	Empregado Efetivo
	F	Empregado Efetivo
	F	Empregado Efetivo
	F	Empregado Efetivo
	F	Empregado Efetivo
	F	Empregado Efetivo
	F	Empregado Efetivo
	M	Empregado Efetivo
	F	Empregado Efetivo
	F	Empregado Efetivo
	F	Empregado Efetivo
	F	Empregado Efetivo
	M	Empregado Efetivo
	F	Empregado Efetivo
	F	Empregado Efetivo
	F	Empregado Efetivo
	F	Empregado Efetivo
	M	Empregado Efetivo
	F	Empregado Efetivo
	F	Empregado Efetivo
	F	Empregado Efetivo
	F	Empregado Efetivo
	F	Empregado Efetivo
	M	Empregado Efetivo
	F	Empregado Efetivo
	F	Empregado Efetivo
	F	Empregado Efetivo
	F	Empregado Efetivo
	F	Empregado Efetivo
	F	Empregado Efetivo
	M	Empregado Efetivo
	M	Empregado Efetivo

	M	Empregado Efetivo
	M	Empregado Efetivo
	M	Empregado Efetivo
	M	Empregado Efetivo
	F	Empregado Efetivo
	F	Empregado Efetivo
	M	Empregado Efetivo
	F	Empregado Efetivo
	F	Empregado Efetivo
	F	Empregado Efetivo
	F	Empregado Efetivo
	M	Empregado Efetivo
	F	Empregado Efetivo
	M	Empregado Efetivo
	F	Empregado Efetivo
	F	Empregado Efetivo
	F	Empregado Efetivo
	F	Empregado Efetivo
	F	Empregado Efetivo
	M	Empregado Efetivo
	F	Empregado Efetivo
	F	Empregado Efetivo
	M	Empregado Efetivo
	F	Empregado Efetivo
	M	Empregado Efetivo
	F	Empregado Efetivo
	F	Empregado Efetivo
	F	Empregado Efetivo
	F	Empregado Efetivo
	M	Empregado Efetivo
	F	Empregado Efetivo
	M	Empregado Efetivo
	F	Empregado Efetivo
	F	Empregado Efetivo
	F	Empregado Efetivo
	F	Empregado Efetivo
	F	Empregado Efetivo
	F	Empregado Efetivo
	F	Empregado Efetivo
	F	Empregado Efetivo
	F	Empregado Efetivo
	F	Empregado Efetivo
	M	Empregado Efetivo
	F	Empregado Efetivo

	F	Empregado Efetivo
	F	Empregado Efetivo
	M	Empregado Efetivo
	F	Empregado Efetivo
	M	Empregado Efetivo
	F	Empregado Efetivo
	F	Empregado Efetivo
	F	Empregado Efetivo
	F	Empregado Efetivo
	M	Empregado Efetivo
	F	Empregado Efetivo
	M	Empregado Efetivo
	F	Empregado Efetivo
	F	Empregado Efetivo
	F	Empregado Efetivo
	F	Empregado Efetivo
	F	Empregado Efetivo
	F	Empregado Efetivo
	F	Empregado Efetivo
	F	Empregado Efetivo
	F	Empregado Efetivo
	F	Empregado Efetivo
	M	Empregado Efetivo
	M	Empregado Efetivo
	F	Empregado Efetivo
	M	Empregado Efetivo
	F	Empregado Efetivo
	F	Empregado Efetivo
	M	Empregado Efetivo
	M	Empregado Efetivo
	M	Empregado Efetivo
	F	Empregado Efetivo
	F	Empregado Efetivo
	M	Empregado Efetivo
	F	Empregado Efetivo
	F	Empregado Efetivo
	F	Empregado Efetivo
	F	Empregado Efetivo
	F	Empregado Efetivo
	F	Empregado Efetivo
	F	Empregado Efetivo
	F	Empregado Efetivo
	F	Empregado Efetivo
	F	Empregado Efetivo
	M	Empregado Efetivo
	M	Empregado Efetivo
	F	Empregado Efetivo
	F	Empregado Efetivo

	F	Empregado Efetivo
	F	Empregado Efetivo
	F	Empregado Efetivo
	F	Empregado Efetivo
	M	Empregado Efetivo
	M	Empregado Efetivo
	F	Empregado Efetivo
	M	Empregado Efetivo
	F	Empregado Efetivo
	F	Empregado Efetivo
	M	Empregado Efetivo
	F	Empregado Efetivo
	M	Empregado Efetivo
	F	Empregado Efetivo
	F	Empregado Efetivo
	F	Empregado Efetivo
	M	Empregado Efetivo
	M	Empregado Efetivo
	F	Empregado Efetivo
	M	Empregado Efetivo
	F	Empregado Efetivo
	F	Empregado Efetivo
	F	Empregado Efetivo
	M	Empregado Efetivo
	M	Empregado Efetivo
	F	Empregado Efetivo
	F	Empregado Efetivo
	M	Empregado Efetivo
	M	Empregado Efetivo
	F	Empregado Efetivo
	F	Empregado Efetivo
	F	Empregado Efetivo
	F	Empregado Efetivo
	F	Empregado Efetivo
	M	Empregado Efetivo
	F	Empregado Efetivo
	F	Empregado Efetivo
	F	Empregado Efetivo
	M	Empregado Efetivo
	F	Empregado Efetivo
	F	Empregado Efetivo
	M	Empregado Efetivo
	M	Empregado Efetivo

	M	Empregado Efetivo
	F	Empregado Efetivo
	F	Empregado Efetivo
	M	Empregado Efetivo
	F	Empregado Efetivo
	M	Empregado Efetivo
	M	Empregado Efetivo
	M	Empregado Efetivo
	M	Empregado Efetivo
	M	Empregado Efetivo
	M	Empregado Efetivo
	F	Empregado Efetivo
	F	Empregado Efetivo
	F	Empregado Efetivo
	F	Empregado Efetivo
	F	Empregado Efetivo
	F	Empregado Efetivo
	M	Empregado Efetivo
	F	Empregado Efetivo
	F	Empregado Efetivo
	F	Empregado Efetivo
	F	Empregado Efetivo
	F	Empregado Efetivo
	F	Empregado Efetivo
	F	Empregado Efetivo
	F	Empregado Efetivo
	F	Empregado Efetivo
	F	Empregado Efetivo
	F	Empregado Efetivo
	F	Empregado Efetivo
	F	Empregado Efetivo
	F	Empregado Efetivo
	F	Empregado Efetivo
	F	Empregado Efetivo
	F	Empregado Efetivo
	F	Empregado Efetivo
	F	Empregado Efetivo
	F	Empregado Efetivo
	F	Empregado Efetivo
	F	Empregado Efetivo
	F	Empregado Efetivo
	F	Empregado Efetivo
	F	Empregado Efetivo
	F	Empregado Efetivo
	F	Empregado Efetivo
	F	Empregado Efetivo
	F	Empregado Efetivo
	F	Empregado Efetivo

	F	Empregado Efetivo
	F	Empregado Efetivo
	F	Empregado Efetivo
	F	Empregado Efetivo
	F	Empregado Efetivo
	F	Empregado Efetivo
	F	Empregado Efetivo
	F	Empregado Efetivo
	F	Empregado Efetivo
	F	Empregado Efetivo
	F	Empregado Efetivo
	F	Empregado Efetivo
	F	Empregado Efetivo
	F	Empregado Efetivo
	F	Empregado Efetivo
	F	Empregado Efetivo
	F	Empregado Efetivo
	F	Empregado Efetivo
	F	Empregado Efetivo
	F	Empregado Efetivo
	F	Empregado Efetivo
	F	Empregado Efetivo
	F	Empregado Efetivo
	F	Empregado Efetivo
	F	Empregado Efetivo
	F	Empregado Efetivo
	F	Empregado Efetivo
	F	Empregado Efetivo
	M	Empregado Efetivo
	M	Empregado Efetivo
	M	Empregado Efetivo
	M	Empregado Efetivo
	M	Empregado Efetivo
	M	Empregado Efetivo
	F	Empregado Efetivo
	M	Empregado Efetivo
	M	Empregado Efetivo
	F	Empregado Efetivo
	F	Empregado Efetivo
	M	Empregado Efetivo
	M	Empregado Efetivo
	M	Empregado Efetivo
	F	Empregado Efetivo
	M	Empregado Efetivo
	M	Empregado Efetivo
	M	Empregado Efetivo
	M	Empregado Efetivo
	M	Empregado Efetivo

	M	Empregado Efetivo
	M	Empregado Efetivo
	M	Empregado Efetivo
	M	Empregado Efetivo
	M	Empregado Efetivo
	F	Empregado Efetivo
	M	Empregado Efetivo
	F	Empregado Efetivo
	M	Empregado Efetivo
	M	Empregado Efetivo
	M	Empregado Efetivo
	F	Empregado Efetivo
	M	Empregado Efetivo
	M	Empregado Efetivo
	F	Empregado Efetivo
	F	Empregado Efetivo
	F	Empregado Efetivo
	F	Empregado Efetivo
	F	Empregado Efetivo
	F	Empregado Efetivo
	F	Empregado Efetivo
	F	Empregado Efetivo
	F	Empregado Efetivo
	F	Empregado Efetivo
	F	Empregado Efetivo
	F	Empregado Efetivo
	F	Empregado Efetivo
	F	Empregado Efetivo
	F	Empregado Efetivo
	F	Empregado Efetivo
	F	Empregado Efetivo
	F	Empregado Efetivo
	F	Empregado Efetivo
	F	Empregado Efetivo
	F	Empregado Efetivo
	M	Empregado Efetivo
	F	Empregado Efetivo
	F	Empregado Efetivo
	M	Empregado Efetivo
	M	Empregado Efetivo
	M	Empregado Efetivo
	M	Empregado Efetivo
	M	Empregado Efetivo
	M	Empregado Efetivo
	M	Empregado Efetivo
	M	Empregado Efetivo
	M	Empregado Efetivo

	M	Empregado Efetivo
	F	Empregado Efetivo
	F	Empregado Efetivo
	M	Empregado Efetivo
	F	Empregado Efetivo
	F	Empregado Efetivo
	F	Empregado Efetivo
	F	Empregado Efetivo
	F	Empregado Efetivo
	F	Empregado Efetivo
	F	Empregado Efetivo
	F	Empregado Efetivo
	F	Empregado Efetivo
	F	Empregado Efetivo
	F	Empregado Efetivo
	F	Empregado Efetivo
	F	Empregado Efetivo
	F	Empregado Efetivo
	F	Empregado Efetivo
	F	Empregado Efetivo
	F	Empregado Efetivo
	M	Empregado Efetivo
	M	Empregado Efetivo
	M	Empregado Efetivo
	M	Empregado Efetivo
	F	Empregado Efetivo
	F	Empregado Efetivo
	F	Empregado Efetivo
	F	Empregado Efetivo
	F	Empregado Efetivo
	F	Empregado Efetivo
	F	Empregado Efetivo
	M	Empregado Efetivo
	F	Empregado Efetivo
	F	Empregado Efetivo
	F	Cedido - RJU
	F	Empregado Efetivo
	F	Empregado Efetivo
	M	Empregado Efetivo
	F	Empregado Efetivo
	F	Empregado Efetivo
	M	Empregado Efetivo
	F	Empregado Efetivo

	M	Empregado Efetivo
	M	Empregado Efetivo
	F	Empregado Efetivo
	F	Empregado Efetivo
	F	Empregado Efetivo
	F	Empregado Efetivo
	F	Empregado Efetivo
	F	Empregado Efetivo
	M	Empregado Efetivo
	F	Empregado Efetivo
	F	Empregado Efetivo
	F	Empregado Efetivo
	F	Empregado Efetivo
	F	Empregado Efetivo
	F	Empregado Efetivo
	F	Empregado Efetivo
	M	Empregado Efetivo
	M	Empregado Efetivo
	F	Empregado Efetivo
	F	Empregado Efetivo
	F	Empregado Efetivo
	F	Empregado Efetivo
	F	Empregado Efetivo
	F	Empregado Efetivo
	F	Empregado Efetivo
	F	Empregado Efetivo
	M	Empregado Efetivo
	F	Empregado Efetivo
	F	Empregado Efetivo
	F	Empregado Efetivo
	F	Empregado Efetivo
	F	Empregado Efetivo
	M	Empregado Efetivo
	F	Empregado Efetivo
	F	Empregado Efetivo
	F	Empregado Efetivo
	F	Empregado Efetivo
	F	Empregado Efetivo
	F	Empregado Efetivo
	F	Empregado Efetivo
	F	Empregado Efetivo
	F	Empregado Efetivo
	F	Empregado Efetivo
	F	Empregado Efetivo
	F	Empregado Efetivo
	F	Empregado Efetivo
	F	Empregado Efetivo
	F	Empregado Efetivo

	M	Empregado Efetivo
	F	Empregado Efetivo
	F	Empregado Efetivo
	F	Empregado Efetivo
	F	Empregado Efetivo
	M	Empregado Efetivo
	F	Empregado Efetivo
	M	Empregado Efetivo
	M	Empregado Efetivo
	F	Empregado Efetivo
	M	Empregado Efetivo
	F	Empregado Efetivo
	F	Empregado Efetivo
	F	Empregado Efetivo
	F	Empregado Efetivo
	F	Empregado Efetivo
	M	Empregado Efetivo
	F	Empregado Efetivo
	F	Empregado Efetivo
	M	Empregado Efetivo
	F	Empregado Efetivo
	F	Empregado Efetivo
	F	Empregado Efetivo
	M	Empregado Efetivo
	F	Empregado Efetivo
	F	Empregado Efetivo
	F	Empregado Efetivo
	F	Empregado Efetivo
	F	Empregado Efetivo
	F	Empregado Efetivo
	F	Empregado Efetivo
	F	Empregado Efetivo
	F	Empregado Efetivo
	F	Empregado Efetivo
	F	Empregado Efetivo
	F	Empregado Efetivo
	F	Empregado Efetivo
	F	Empregado Efetivo
	F	Empregado Efetivo
	F	Empregado Efetivo
	M	Empregado Efetivo
	F	Empregado Efetivo
	F	Empregado Efetivo
	F	Empregado Efetivo
	F	Empregado Efetivo
	F	Empregado Efetivo
	F	Empregado Efetivo
	F	Empregado Efetivo

	F	Empregado Efetivo
	F	Empregado Efetivo
	F	Empregado Efetivo
	F	Empregado Efetivo
	F	Empregado Efetivo
	F	Empregado Efetivo
	F	Empregado Efetivo
	F	Empregado Efetivo
	F	Empregado Efetivo
	F	Empregado Efetivo
	F	Empregado Efetivo
	F	Empregado Efetivo
	F	Empregado Efetivo
	F	Empregado Efetivo
	F	Empregado Efetivo
	F	Empregado Efetivo
	M	Empregado Efetivo
	F	Empregado Efetivo
	F	Empregado Efetivo
	F	Empregado Efetivo
	F	Empregado Efetivo
	M	Empregado Efetivo
	M	Empregado Efetivo
	F	Empregado Efetivo
	M	Empregado Efetivo
	F	Empregado Efetivo
	M	Empregado Efetivo
	F	Empregado Efetivo
	M	Empregado Efetivo
	M	Empregado Efetivo
	F	Empregado Efetivo
	M	Empregado Efetivo
	F	Empregado Efetivo
	F	Empregado Efetivo
	F	Empregado Efetivo
	F	Empregado Efetivo
	M	Empregado Efetivo
	F	Empregado Efetivo
	F	Empregado Efetivo
	M	Empregado Efetivo
	F	Empregado Efetivo
	F	Empregado Efetivo
	F	Empregado Efetivo
	M	Empregado Efetivo

	F	Empregado Efetivo
	F	Empregado Efetivo
	F	Empregado Efetivo
	F	Empregado Efetivo
	F	Empregado Efetivo
	M	Empregado Efetivo
	F	Empregado Efetivo
	F	Empregado Efetivo
	F	Empregado Efetivo
	F	Empregado Efetivo
	M	Empregado Efetivo
	F	Empregado Efetivo
	F	Empregado Efetivo
	F	Empregado Efetivo
	F	Empregado Efetivo
	F	Empregado Efetivo
	M	Empregado Efetivo
	F	Empregado Efetivo
	F	Empregado Efetivo
	F	Empregado Efetivo
	M	Empregado Efetivo
	F	Empregado Efetivo
	F	Empregado Efetivo
	M	Empregado Efetivo
	M	Empregado Efetivo
	F	Empregado Efetivo
	M	Empregado Efetivo
	M	Empregado Efetivo
	F	Empregado Efetivo
	F	Empregado Efetivo
	F	Empregado Efetivo
	F	Empregado Efetivo
	F	Empregado Efetivo

	M	Empregado Efetivo
	M	Empregado Efetivo
	F	Empregado Efetivo
	M	Empregado Efetivo
	M	Empregado Efetivo
	M	Empregado Efetivo
	M	Empregado Efetivo
	M	Empregado Efetivo
	M	Empregado Efetivo
	F	Empregado Efetivo
	F	Empregado Efetivo
	F	Empregado Efetivo
	F	Empregado Efetivo
	F	Empregado Efetivo
	F	Empregado Efetivo
	F	Empregado Efetivo
	F	Empregado Efetivo
	F	Empregado Efetivo
	F	Empregado Efetivo
	F	Empregado Efetivo
	F	Empregado Efetivo
	F	Empregado Efetivo
	F	Empregado Efetivo
	F	Empregado Efetivo
	F	Empregado Efetivo
	F	Empregado Efetivo
	F	Empregado Efetivo
	F	Empregado Efetivo
	F	Empregado Efetivo
	F	Empregado Efetivo
	F	Empregado Efetivo
	F	Empregado Efetivo
	M	Empregado Efetivo
	F	Empregado Efetivo
	F	Empregado Efetivo
	M	Empregado Efetivo
	F	Empregado Efetivo
	F	Empregado Efetivo
	F	Empregado Efetivo
	M	Empregado Efetivo
	F	Empregado Efetivo
	M	Empregado Efetivo
	M	Empregado Efetivo
	F	Empregado Efetivo
	F	Empregado Efetivo
	M	Empregado Efetivo
	F	Empregado Efetivo
	F	Empregado Efetivo

	F	Empregado Efetivo
	F	Empregado Efetivo
	F	Empregado Efetivo
	M	Empregado Efetivo
	M	Empregado Efetivo
	F	Empregado Efetivo
	F	Empregado Efetivo
	F	Empregado Efetivo
	F	Empregado Efetivo
	F	Empregado Efetivo
	M	Empregado Efetivo
	M	Empregado Efetivo
	F	Empregado Efetivo
	F	Empregado Efetivo
	M	Empregado Efetivo
	F	Empregado Efetivo
	F	Empregado Efetivo
	M	Empregado Efetivo
	M	Empregado Efetivo
	F	Empregado Efetivo
	F	Empregado Efetivo
	F	Empregado Efetivo
	F	Empregado Efetivo
	F	Empregado Efetivo
	F	Empregado Efetivo
	F	Empregado Efetivo
	M	Empregado Efetivo
	F	Empregado Efetivo
	M	Empregado Efetivo
	F	Empregado Efetivo
	F	Empregado Efetivo
	M	Empregado Efetivo
	M	Empregado Efetivo
	M	Empregado Efetivo
	F	Empregado Efetivo
	M	Empregado Efetivo
	F	Empregado Efetivo
	F	Empregado Efetivo
	F	Empregado Efetivo
	F	Empregado Efetivo
	F	Empregado Efetivo
	F	Empregado Efetivo
	F	Empregado Efetivo
	M	Empregado Efetivo
	F	Empregado Efetivo
	M	Empregado Efetivo
	F	Empregado Efetivo
	F	Empregado Efetivo

	M	Empregado Efetivo
	M	Empregado Efetivo
	F	Empregado Efetivo
	F	Empregado Efetivo
	M	Empregado Efetivo
	M	Empregado Efetivo
	F	Empregado Efetivo
	F	Empregado Efetivo
	F	Empregado Efetivo
	F	Empregado Efetivo
	M	Empregado Efetivo
	F	Empregado Efetivo
	F	Empregado Efetivo
	F	Empregado Efetivo
	F	Empregado Efetivo
	F	Empregado Efetivo
	F	Empregado Efetivo
	F	Empregado Efetivo
	F	Empregado Efetivo
	F	Empregado Efetivo
	F	Empregado Efetivo
	F	Empregado Efetivo
	F	Empregado Efetivo
	M	Empregado Efetivo
	F	Empregado Efetivo
	F	Empregado Efetivo
	F	Empregado Efetivo
	M	Empregado Efetivo
	F	Empregado Efetivo
	F	Empregado Efetivo
	F	Empregado Efetivo
	M	Empregado Efetivo
	F	Empregado Efetivo
	M	Empregado Efetivo
	F	Empregado Efetivo
	M	Empregado Efetivo
	F	Empregado Efetivo
	F	Empregado Efetivo
	M	Empregado Efetivo
	F	Empregado Efetivo
	F	Empregado Efetivo
	F	Empregado Efetivo
	F	Empregado Efetivo
	M	Empregado Efetivo
	F	Empregado Efetivo
	F	Empregado Efetivo
	F	Empregado Efetivo
	F	Empregado Efetivo

	F	Empregado Efetivo
	F	Empregado Efetivo
	F	Empregado Efetivo
	F	Empregado Efetivo
	F	Empregado Efetivo
	F	Empregado Efetivo
	F	Empregado Efetivo
	F	Empregado Efetivo
	M	Empregado Efetivo
	M	Empregado Efetivo
	M	Empregado Efetivo
	M	Empregado Efetivo
	M	Empregado Efetivo
	F	Empregado Efetivo
	F	Empregado Efetivo
	F	Empregado Efetivo
	F	Empregado Efetivo
	F	Empregado Efetivo
	F	Empregado Efetivo
	F	Empregado Efetivo
	F	Empregado Efetivo
	F	Empregado Efetivo
	F	Empregado Efetivo
	M	Empregado Efetivo
	F	Empregado Efetivo
	M	Empregado Efetivo
	F	Empregado Efetivo
	M	Empregado Efetivo
	F	Empregado Efetivo
	F	Empregado Efetivo
	F	Empregado Efetivo
	F	Empregado Efetivo
	M	Empregado Efetivo
	F	Empregado Efetivo
	M	Empregado Efetivo
	F	Empregado Efetivo
	F	Empregado Efetivo
	F	Empregado Efetivo
	F	Empregado Efetivo
	F	Empregado Efetivo
	F	Empregado Efetivo
	F	Empregado Efetivo
	F	Empregado Efetivo
	F	Empregado Efetivo
	F	Empregado Efetivo
	F	Empregado Efetivo
	F	Empregado Efetivo
	F	Empregado Efetivo

	F	Empregado Efetivo
	F	Empregado Efetivo
	F	Empregado Efetivo
	F	Empregado Efetivo
	F	Empregado Efetivo
	F	Empregado Efetivo
	M	Empregado Efetivo
	F	Empregado Efetivo
	F	Empregado Efetivo
	F	Empregado Efetivo
	F	Empregado Efetivo
	F	Empregado Efetivo
	F	Empregado Efetivo
	F	Empregado Efetivo
	F	Empregado Efetivo
	F	Empregado Efetivo
	F	Empregado Efetivo
	F	Empregado Efetivo
	F	Empregado Efetivo
	F	Empregado Efetivo
	F	Empregado Efetivo
	F	Empregado Efetivo
	F	Empregado Efetivo
	F	Empregado Efetivo
	M	Empregado Efetivo
	F	Empregado Efetivo
	F	Empregado Efetivo
	F	Empregado Efetivo
	F	Empregado Efetivo
	F	Empregado Efetivo
	F	Empregado Efetivo
	F	Empregado Efetivo
	F	Empregado Efetivo
	F	Empregado Efetivo
	M	Empregado Efetivo
	F	Empregado Efetivo
	F	Empregado Efetivo
	M	Empregado Efetivo
	M	Empregado Efetivo
	F	Empregado Efetivo
	F	Empregado Efetivo
	M	Empregado Efetivo
	F	Empregado Efetivo
	M	Empregado Efetivo
	F	Empregado Efetivo
	F	Empregado Efetivo
	F	Empregado Efetivo
	F	Empregado Efetivo

	F	Empregado Efetivo
	M	Empregado Efetivo
	F	Empregado Efetivo
	M	Empregado Efetivo
	M	Empregado Efetivo
	M	Empregado Efetivo
	F	Empregado Efetivo
	F	Empregado Efetivo
	F	Empregado Efetivo
	F	Empregado Efetivo
	F	Empregado Efetivo
	F	Empregado Efetivo
	M	Empregado Efetivo
	F	Empregado Efetivo
	M	Empregado Efetivo
	F	Empregado Efetivo
	F	Empregado Efetivo
	F	Empregado Efetivo
	F	Empregado Efetivo
	F	Empregado Efetivo
	M	Empregado Efetivo
	M	Empregado Efetivo
	M	Empregado Efetivo
	M	Empregado Efetivo
	F	Empregado Efetivo
	M	Empregado Efetivo
	M	Empregado Efetivo
	F	Empregado Efetivo
	F	Empregado Efetivo
	M	Empregado Efetivo
	F	Empregado Efetivo
	F	Empregado Efetivo
	F	Empregado Efetivo
	M	Empregado Efetivo
	M	Empregado Efetivo
	F	Empregado Efetivo
	F	Empregado Efetivo
	M	Empregado Efetivo
	M	Empregado Efetivo
	M	Empregado Efetivo
	M	Empregado Efetivo
	M	Empregado Efetivo
	M	Empregado Efetivo
	M	Empregado Efetivo
	M	Empregado Efetivo
	M	Empregado Efetivo
	M	Empregado Efetivo
	M	Empregado Efetivo

	F	Empregado Efetivo
	F	Empregado Efetivo
	F	Empregado Efetivo
	F	Empregado Efetivo
	F	Empregado Efetivo
	F	Empregado Efetivo
	F	Empregado Efetivo
	F	Empregado Efetivo
	F	Empregado Efetivo
	F	Empregado Efetivo
	F	Empregado Efetivo
	F	Empregado Efetivo
	F	Empregado Efetivo
	F	Empregado Efetivo
	F	Empregado Efetivo
	F	Empregado Efetivo
	F	Empregado Efetivo
	F	Empregado Efetivo
	F	Empregado Efetivo
	F	Empregado Efetivo
	F	Empregado Efetivo
	F	Empregado Efetivo
	F	Empregado Efetivo
	F	Empregado Efetivo
	F	Empregado Efetivo
	F	Empregado Efetivo
	F	Empregado Efetivo
	M	Empregado Efetivo
	M	Empregado Efetivo
	M	Empregado Efetivo
	M	Empregado Efetivo
	F	Empregado Efetivo
	F	Empregado Efetivo
	F	Empregado Efetivo
	F	Empregado Efetivo
	F	Empregado Efetivo
	F	Empregado Efetivo
	F	Empregado Efetivo
	F	Empregado Efetivo
	F	Empregado Efetivo
	F	Empregado Efetivo
	F	Empregado Efetivo
	F	Empregado Efetivo
	F	Empregado Efetivo
	M	Empregado Efetivo
	F	Empregado Efetivo
	F	Empregado Efetivo
	M	Empregado Efetivo

	F	Empregado Efetivo
	F	Empregado Efetivo
	F	Empregado Efetivo
	F	Empregado Efetivo
	F	Empregado Efetivo
	F	Empregado Efetivo
	F	Empregado Efetivo
	F	Empregado Efetivo
	F	Empregado Efetivo
	F	Empregado Efetivo
	F	Empregado Efetivo
	F	Empregado Efetivo
	F	Empregado Efetivo
	F	Empregado Efetivo
	F	Empregado Efetivo
	F	Empregado Efetivo
	F	Empregado Efetivo
	F	Empregado Efetivo
	F	Empregado Efetivo
	F	Empregado Efetivo
	F	Empregado Efetivo
	F	Empregado Efetivo
	M	Empregado Efetivo
	M	Empregado Efetivo
	F	Empregado Efetivo
	F	Empregado Efetivo
	F	Empregado Efetivo
	F	Empregado Efetivo
	M	Empregado Efetivo
	F	Empregado Efetivo
	M	Empregado Efetivo
	F	Empregado Efetivo
	F	Empregado Efetivo
	M	Empregado Efetivo
	F	Empregado Efetivo
	F	Empregado Efetivo

	M	Empregado Efetivo
	F	Empregado Efetivo
	F	Empregado Efetivo
	F	Empregado Efetivo
	F	Empregado Efetivo
	F	Empregado Efetivo
	F	Empregado Efetivo
	F	Empregado Efetivo
	M	Empregado Efetivo
	M	Empregado Efetivo
	F	Empregado Efetivo
	F	Empregado Efetivo
	F	Empregado Efetivo
	M	Empregado Efetivo
	M	Empregado Efetivo
	F	Empregado Efetivo
	M	Empregado Efetivo
	F	Empregado Efetivo
	F	Empregado Efetivo
	M	Empregado Efetivo
	M	Empregado Efetivo
	F	Empregado Efetivo
	F	Empregado Efetivo
	F	Empregado Efetivo
	F	Empregado Efetivo
	F	Empregado Efetivo
	F	Empregado Efetivo
	F	Empregado Efetivo
	M	Empregado Efetivo
	F	Empregado Efetivo
	F	Empregado Efetivo
	F	Empregado Efetivo
	F	Empregado Efetivo
	F	Empregado Efetivo
	F	Empregado Efetivo
	F	Empregado Efetivo
	M	Empregado Efetivo
	F	Empregado Efetivo
	F	Empregado Efetivo
	F	Empregado Efetivo
	F	Empregado Efetivo
	F	Empregado Efetivo
	F	Empregado Efetivo
	F	Empregado Efetivo
	F	Empregado Efetivo
	F	Empregado Efetivo
	F	Empregado Efetivo
	F	Empregado Efetivo

	F	Empregado Efetivo
	F	Empregado Efetivo
	F	Empregado Efetivo
	F	Empregado Efetivo
	F	Empregado Efetivo
	F	Empregado Efetivo
	F	Empregado Efetivo
	F	Empregado Efetivo
	F	Empregado Efetivo
	F	Empregado Efetivo
	F	Empregado Efetivo
	F	Empregado Efetivo
	F	Empregado Efetivo
	F	Empregado Efetivo
	F	Empregado Efetivo
	F	Empregado Efetivo
	F	Empregado Efetivo
	F	Empregado Efetivo
	F	Empregado Efetivo
	F	Empregado Efetivo
	F	Empregado Efetivo
	F	Empregado Efetivo
	F	Empregado Efetivo
	F	Empregado Efetivo
	F	Empregado Efetivo
	F	Empregado Efetivo
	F	Empregado Efetivo
	F	Empregado Efetivo
	F	Empregado Efetivo
	F	Empregado Efetivo
	F	Empregado Efetivo
	F	Empregado Efetivo
	F	Empregado Efetivo
	F	Empregado Efetivo
	F	Empregado Efetivo
	F	Empregado Efetivo
	M	Empregado Efetivo
	F	Empregado Efetivo
	F	Empregado Efetivo
	F	Empregado Efetivo
	F	Empregado Efetivo
	F	Empregado Efetivo
	F	Empregado Efetivo
	F	Empregado Efetivo
	F	Empregado Efetivo
	F	Empregado Efetivo
	F	Empregado Efetivo

	F	Empregado Efetivo
	F	Empregado Efetivo
	F	Empregado Efetivo
	F	Empregado Efetivo
	F	Empregado Efetivo
	F	Empregado Efetivo
	F	Empregado Efetivo
	F	Empregado Efetivo
	F	Empregado Efetivo
	F	Empregado Efetivo
	F	Empregado Efetivo
	F	Empregado Efetivo
	F	Empregado Efetivo
	F	Empregado Efetivo
	F	Empregado Efetivo
	F	Empregado Efetivo
	F	Empregado Efetivo
	F	Empregado Efetivo
	F	Empregado Efetivo
	F	Empregado Efetivo
	F	Empregado Efetivo
	F	Empregado Efetivo
	F	Empregado Efetivo
	F	Empregado Efetivo
	F	Empregado Efetivo
	F	Empregado Efetivo
	F	Empregado Efetivo
	F	Empregado Efetivo
	F	Empregado Efetivo
	F	Empregado Efetivo
	F	Empregado Efetivo
	F	Empregado Efetivo
	F	Empregado Efetivo
	F	Empregado Efetivo
	M	Empregado Efetivo
	F	Empregado Efetivo
	F	Empregado Efetivo
	F	Empregado Efetivo
	F	Empregado Efetivo
	F	Empregado Efetivo
	F	Empregado Efetivo
	F	Empregado Efetivo
	F	Empregado Efetivo
	F	Empregado Efetivo
	F	Empregado Efetivo
	F	Empregado Efetivo
	F	Empregado Efetivo
	F	Empregado Efetivo

	F	Empregado Efetivo
	M	Empregado Efetivo
	F	Empregado Efetivo
	F	Empregado Efetivo
	F	Empregado Efetivo
	M	Empregado Efetivo
	F	Empregado Efetivo
	F	Empregado Efetivo
	F	Empregado Efetivo
	F	Empregado Efetivo
	F	Empregado Efetivo
	F	Empregado Efetivo
	F	Empregado Efetivo
	F	Empregado Efetivo
	F	Empregado Efetivo
	F	Empregado Efetivo
	F	Empregado Efetivo
	F	Empregado Efetivo
	F	Empregado Efetivo
	F	Empregado Efetivo
	F	Empregado Efetivo
	F	Empregado Efetivo
	F	Empregado Efetivo
	F	Empregado Efetivo
	F	Empregado Efetivo
	F	Empregado Efetivo
	F	Empregado Efetivo
	M	Empregado Efetivo
	F	Empregado Efetivo
	F	Empregado Efetivo
	F	Empregado Efetivo
	M	Empregado Efetivo
	F	Empregado Efetivo
	F	Empregado Efetivo
	F	Empregado Efetivo
	F	Empregado Efetivo
	M	Empregado Efetivo
	M	Empregado Efetivo
	F	Empregado Efetivo
	F	Empregado Efetivo
	F	Empregado Efetivo
	F	Empregado Efetivo
	F	Empregado Efetivo
	F	Empregado Efetivo
	F	Empregado Efetivo
	F	Empregado Efetivo
	M	Empregado Efetivo
	F	Empregado Efetivo

	F	Empregado Efetivo
	F	Empregado Efetivo
	F	Empregado Efetivo
	F	Empregado Efetivo
	F	Empregado Efetivo
	F	Empregado Efetivo
	F	Empregado Efetivo
	F	Empregado Efetivo
	F	Empregado Efetivo
	F	Empregado Efetivo
	F	Empregado Efetivo
	F	Empregado Efetivo
	F	Empregado Efetivo
	F	Empregado Efetivo
	F	Empregado Efetivo
	F	Empregado Efetivo
	F	Empregado Efetivo
	F	Empregado Efetivo
	F	Empregado Efetivo
	F	Empregado Efetivo
	F	Empregado Efetivo
	F	Empregado Efetivo
	F	Empregado Efetivo
	M	Empregado Efetivo
	F	Empregado Efetivo
	F	Empregado Efetivo
	F	Empregado Efetivo
	F	Empregado Efetivo
	F	Empregado Efetivo
	F	Empregado Efetivo
	F	Empregado Efetivo
	F	Empregado Efetivo
	F	Empregado Efetivo
	F	Empregado Efetivo
	F	Empregado Efetivo
	F	Empregado Efetivo
	F	Empregado Efetivo
	F	Empregado Efetivo
	F	Empregado Efetivo
	F	Empregado Efetivo
	F	Empregado Efetivo
	F	Empregado Efetivo
	F	Empregado Efetivo
	F	Empregado Efetivo
	F	Empregado Efetivo
	F	Empregado Efetivo
	F	Empregado Efetivo
	F	Empregado Efetivo

	F	Empregado Efetivo
	F	Empregado Efetivo
	F	Empregado Efetivo
	F	Empregado Efetivo
	F	Empregado Efetivo
	F	Empregado Efetivo
	F	Empregado Efetivo
	M	Empregado Efetivo
	F	Empregado Efetivo
	F	Empregado Efetivo
	F	Empregado Efetivo
	F	Empregado Efetivo
	F	Empregado Efetivo
	F	Empregado Efetivo
	F	Empregado Efetivo
	F	Empregado Efetivo
	F	Empregado Efetivo
	F	Empregado Efetivo
	F	Empregado Efetivo
	F	Empregado Efetivo
	F	Empregado Efetivo
	F	Empregado Efetivo
	F	Empregado Efetivo
	F	Empregado Efetivo
	F	Empregado Efetivo
	F	Empregado Efetivo
	F	Empregado Efetivo
	F	Empregado Efetivo
	F	Empregado Efetivo
	F	Empregado Efetivo
	F	Empregado Efetivo
	F	Empregado Efetivo
	F	Empregado Efetivo
	F	Empregado Efetivo
	F	Empregado Efetivo
	F	Empregado Efetivo
	F	Empregado Efetivo
	F	Empregado Efetivo
	F	Empregado Efetivo
	F	Empregado Efetivo
	F	Empregado Efetivo
	F	Empregado Efetivo
	F	Empregado Efetivo
	F	Empregado Efetivo
	M	Empregado Efetivo
	F	Empregado Efetivo
	F	Empregado Efetivo
	F	Empregado Efetivo

	F	Empregado Efetivo
	F	Empregado Efetivo
	F	Empregado Efetivo
	F	Empregado Efetivo
	F	Empregado Efetivo
	F	Empregado Efetivo
	F	Empregado Efetivo
	F	Empregado Efetivo
	F	Empregado Efetivo
	M	Empregado Efetivo
	F	Empregado Efetivo
	F	Empregado Efetivo
	F	Empregado Efetivo
	F	Empregado Efetivo
	F	Empregado Efetivo
	F	Empregado Efetivo
	F	Empregado Efetivo
	F	Empregado Efetivo
	F	Empregado Efetivo
	F	Empregado Efetivo
	F	Empregado Efetivo
	F	Empregado Efetivo
	F	Empregado Efetivo
	F	Empregado Efetivo
	F	Empregado Efetivo
	F	Empregado Efetivo
	F	Empregado Efetivo
	F	Empregado Efetivo
	F	Empregado Efetivo
	F	Empregado Efetivo
	F	Empregado Efetivo
	F	Empregado Efetivo
	F	Empregado Efetivo
	F	Empregado Efetivo
	F	Empregado Efetivo
	F	Empregado Efetivo
	F	Empregado Efetivo
	F	Empregado Efetivo
	F	Empregado Efetivo
	F	Empregado Efetivo
	F	Empregado Efetivo
	F	Empregado Efetivo
	F	Empregado Efetivo
	F	Empregado Efetivo
	F	Empregado Efetivo
	F	Empregado Efetivo
	F	Empregado Efetivo
	F	Empregado Efetivo

	F	Empregado Efetivo
	F	Empregado Efetivo
	F	Empregado Efetivo
	F	Empregado Efetivo
	F	Empregado Efetivo
	F	Empregado Efetivo
	F	Empregado Efetivo
	F	Empregado Efetivo
	F	Empregado Efetivo
	F	Empregado Efetivo
	F	Empregado Efetivo
	F	Empregado Efetivo
	F	Empregado Efetivo
	F	Empregado Efetivo
	F	Empregado Efetivo
	F	Empregado Efetivo
	F	Empregado Efetivo
	F	Empregado Efetivo
	F	Empregado Efetivo
	F	Empregado Efetivo
	F	Empregado Efetivo
	F	Empregado Efetivo
	F	Empregado Efetivo
	F	Empregado Efetivo
	M	Empregado Efetivo
	M	Empregado Efetivo
	F	Empregado Efetivo
	F	Empregado Efetivo
	F	Empregado Efetivo
	F	Empregado Efetivo
	F	Empregado Efetivo
	F	Empregado Efetivo
	F	Empregado Efetivo
	F	Empregado Efetivo
	F	Empregado Efetivo
	F	Empregado Efetivo
	F	Empregado Efetivo
	F	Empregado Efetivo
	M	Empregado Efetivo
	F	Empregado Efetivo
	F	Empregado Efetivo
	F	Empregado Efetivo
	F	Empregado Efetivo
	F	Empregado Efetivo
	F	Empregado Efetivo
	F	Empregado Efetivo
	F	Empregado Efetivo
	F	Empregado Efetivo

	F	Empregado Efetivo
	F	Empregado Efetivo
	M	Empregado Efetivo
	M	Empregado Efetivo
	F	Empregado Efetivo
	F	Empregado Efetivo
	F	Empregado Efetivo
	F	Empregado Efetivo
	M	Empregado Efetivo
	F	Empregado Efetivo
	F	Empregado Efetivo
	F	Empregado Efetivo
	F	Empregado Efetivo
	F	Empregado Efetivo
	F	Empregado Efetivo
	F	Empregado Efetivo
	F	Empregado Efetivo
	F	Empregado Efetivo
	F	Empregado Efetivo
	F	Empregado Efetivo
	F	Empregado Efetivo
	F	Empregado Efetivo
	F	Empregado Efetivo
	F	Empregado Efetivo
	F	Empregado Efetivo
	F	Empregado Efetivo
	F	Empregado Efetivo
	F	Empregado Efetivo
	F	Empregado Efetivo
	F	Empregado Efetivo
	F	Empregado Efetivo
	F	Empregado Efetivo
	F	Empregado Efetivo
	F	Empregado Efetivo
	F	Empregado Efetivo
	F	Empregado Efetivo
	F	Empregado Efetivo
	F	Empregado Efetivo
	F	Empregado Efetivo
	F	Empregado Efetivo
	M	Empregado Efetivo
	F	Empregado Efetivo
	F	Empregado Efetivo
	F	Empregado Efetivo
	F	Empregado Efetivo
	F	Empregado Efetivo
	F	Empregado Efetivo
	F	Empregado Efetivo

	F	Empregado Efetivo
	F	Empregado Efetivo
	F	Empregado Efetivo
	F	Empregado Efetivo
	F	Empregado Efetivo
	F	Empregado Efetivo
	F	Empregado Efetivo
	F	Empregado Efetivo
	F	Empregado Efetivo
	F	Empregado Efetivo
	F	Empregado Efetivo
	F	Empregado Efetivo
	F	Empregado Efetivo
	F	Empregado Efetivo
	F	Empregado Efetivo
	F	Empregado Efetivo
	F	Empregado Efetivo
	F	Empregado Efetivo
	F	Empregado Efetivo
	F	Empregado Efetivo
	F	Empregado Efetivo
	F	Empregado Efetivo
	F	Empregado Efetivo
	F	Empregado Efetivo
	F	Empregado Efetivo
	M	Empregado Efetivo
	F	Empregado Efetivo
	F	Empregado Efetivo
	F	Empregado Efetivo
	F	Empregado Efetivo
	F	Empregado Efetivo
	M	Empregado Efetivo
	M	Empregado Efetivo
	F	Empregado Efetivo
	F	Empregado Efetivo
	F	Empregado Efetivo
	F	Empregado Efetivo
	F	Empregado Efetivo
	F	Empregado Efetivo
	F	Empregado Efetivo
	F	Empregado Efetivo
	F	Empregado Efetivo
	F	Empregado Efetivo
	F	Empregado Efetivo
	F	Empregado Efetivo
	F	Empregado Efetivo
	F	Empregado Efetivo
	F	Empregado Efetivo

	F	Empregado Efetivo
	F	Empregado Efetivo
	F	Empregado Efetivo
	F	Empregado Efetivo
	F	Empregado Efetivo
	F	Empregado Efetivo
	F	Empregado Efetivo
	F	Empregado Efetivo
	F	Empregado Efetivo
	F	Empregado Efetivo
	F	Empregado Efetivo
	F	Empregado Efetivo
	F	Empregado Efetivo
	F	Empregado Efetivo
	F	Empregado Efetivo
	F	Empregado Efetivo
	F	Empregado Efetivo
	F	Empregado Efetivo
	F	Empregado Efetivo
	F	Empregado Efetivo
	F	Empregado Efetivo
	F	Empregado Efetivo
	M	Empregado Efetivo
	F	Empregado Efetivo
	F	Empregado Efetivo
	F	Empregado Efetivo
	F	Empregado Efetivo
	F	Empregado Efetivo
	M	Empregado Efetivo
	F	Empregado Efetivo
	F	Empregado Efetivo
	F	Empregado Efetivo
	F	Empregado Efetivo
	F	Empregado Efetivo
	F	Empregado Efetivo
	M	Empregado Efetivo
	F	Empregado Efetivo
	M	Empregado Efetivo
	F	Empregado Efetivo
	F	Empregado Efetivo
	F	Empregado Efetivo
	M	Empregado Efetivo
	F	Empregado Efetivo
	F	Empregado Efetivo
	F	Empregado Efetivo
	F	Empregado Efetivo
	F	Empregado Efetivo
	F	Empregado Efetivo

	F	Empregado Efetivo
	F	Empregado Efetivo
	F	Empregado Efetivo
	F	Empregado Efetivo
	F	Empregado Efetivo
	F	Empregado Efetivo
	F	Empregado Efetivo
	F	Empregado Efetivo
	F	Empregado Efetivo
	F	Empregado Efetivo
	F	Empregado Efetivo
	F	Empregado Efetivo
	F	Empregado Efetivo
	F	Empregado Efetivo
	F	Empregado Efetivo
	F	Empregado Efetivo
	F	Empregado Efetivo
	F	Empregado Efetivo
	F	Comissionado
	F	Empregado Efetivo
	F	Empregado Efetivo
	F	Empregado Efetivo
	F	Empregado Efetivo
	F	Empregado Efetivo
	F	Empregado Efetivo
	F	Empregado Efetivo
	F	Empregado Efetivo
	F	Empregado Efetivo
	F	Empregado Efetivo
	F	Empregado Efetivo
	F	Empregado Efetivo
	F	Empregado Efetivo
	M	Empregado Efetivo
	F	Empregado Efetivo
	F	Empregado Efetivo
	F	Empregado Efetivo
	M	Empregado Efetivo
	M	Empregado Efetivo
	F	Empregado Efetivo
	M	Empregado Efetivo
	F	Empregado Efetivo
	F	Empregado Efetivo
	F	Empregado Efetivo
	F	Empregado Efetivo
	F	Empregado Efetivo
	M	Empregado Efetivo
	F	Empregado Efetivo
	F	Empregado Efetivo

	F	Empregado Efetivo
	F	Empregado Efetivo
	F	Empregado Efetivo
	F	Empregado Efetivo
	F	Empregado Efetivo
	F	Empregado Efetivo
	F	Empregado Efetivo
	F	Empregado Efetivo
	F	Empregado Efetivo
	F	Empregado Efetivo
	F	Empregado Efetivo
	M	Empregado Efetivo
	F	Empregado Efetivo
	F	Empregado Efetivo
	F	Empregado Efetivo
	F	Empregado Efetivo
	F	Empregado Efetivo
	M	Empregado Efetivo
	F	Empregado Efetivo
	M	Empregado Efetivo
	F	Empregado Efetivo
	M	Empregado Efetivo
	F	Empregado Efetivo
	M	Empregado Efetivo
	M	Empregado Efetivo
	M	Empregado Efetivo
	F	Empregado Efetivo
	F	Empregado Efetivo
	F	Empregado Efetivo
	F	Empregado Efetivo
	M	Empregado Efetivo
	M	Empregado Efetivo
	M	Empregado Efetivo
	M	Empregado Efetivo
	F	Empregado Efetivo
	M	Empregado Efetivo
	F	Empregado Efetivo
	F	Empregado Efetivo
	F	Empregado Efetivo
	F	Empregado Efetivo
	F	Empregado Efetivo
	F	Empregado Efetivo
	F	Empregado Efetivo
	F	Empregado Efetivo
	M	Empregado Efetivo
	M	Empregado Efetivo

	F	Empregado Efetivo
	F	Empregado Efetivo
	F	Empregado Efetivo
	M	Empregado Efetivo
	F	Empregado Efetivo
	F	Empregado Efetivo
	F	Empregado Efetivo
	F	Empregado Efetivo
	M	Empregado Efetivo
	M	Empregado Efetivo
	F	Empregado Efetivo
	F	Empregado Efetivo
	F	Empregado Efetivo
	F	Empregado Efetivo
	F	Empregado Efetivo
	F	Empregado Efetivo
	F	Empregado Efetivo
	F	Empregado Efetivo
	F	Empregado Efetivo
	M	Empregado Efetivo
	F	Empregado Efetivo
	M	Empregado Efetivo
	M	Empregado Efetivo
	F	Empregado Efetivo
	F	Empregado Efetivo
	F	Empregado Efetivo
	F	Empregado Efetivo
	M	Empregado Efetivo
	M	Empregado Efetivo
	F	Empregado Efetivo
	F	Empregado Efetivo
	F	Empregado Efetivo
	F	Empregado Efetivo
	M	Empregado Efetivo
	F	Empregado Efetivo
	M	Empregado Efetivo
	M	Empregado Efetivo

	M	Empregado Efetivo
	M	Empregado Efetivo
	F	Empregado Efetivo
	F	Empregado Efetivo
	M	Empregado Efetivo
	F	Empregado Efetivo
	F	Empregado Efetivo
	F	Empregado Efetivo
	F	Empregado Efetivo
	F	Empregado Efetivo
	F	Empregado Efetivo
	M	Empregado Efetivo
	F	Empregado Efetivo
	M	Empregado Efetivo
	F	Empregado Efetivo
	M	Empregado Efetivo
	M	Empregado Efetivo
	F	Empregado Efetivo
	M	Empregado Efetivo
	F	Empregado Efetivo
	F	Empregado Efetivo
	F	Empregado Efetivo
	M	Empregado Efetivo
	F	Empregado Efetivo
	F	Empregado Efetivo
	M	Empregado Efetivo
	F	Empregado Efetivo
	F	Empregado Efetivo
	M	Empregado Efetivo
	M	Empregado Efetivo
	F	Empregado Efetivo
	F	Empregado Efetivo

	F	Empregado Efetivo
	F	Empregado Efetivo
	F	Empregado Efetivo
	M	Empregado Efetivo
	F	Empregado Efetivo
	M	Empregado Efetivo
	M	Empregado Efetivo
	F	Empregado Efetivo
	F	Empregado Efetivo
	F	Empregado Efetivo
	M	Empregado Efetivo
	F	Empregado Efetivo
	M	Empregado Efetivo
	M	Empregado Efetivo
	M	Empregado Efetivo
	F	Empregado Efetivo
	F	Empregado Efetivo
	F	Empregado Efetivo
	M	Empregado Efetivo
	F	Empregado Efetivo
	F	Empregado Efetivo
	F	Empregado Efetivo
	F	Empregado Efetivo
	F	Empregado Efetivo
	F	Empregado Efetivo
	F	Empregado Efetivo
	M	Empregado Efetivo
	F	Empregado Efetivo
	F	Empregado Efetivo
	M	Empregado Efetivo
	F	Empregado Efetivo
	F	Empregado Efetivo
	F	Empregado Efetivo
	F	Empregado Efetivo
	F	Empregado Efetivo
	F	Empregado Efetivo
	F	Empregado Efetivo
	F	Empregado Efetivo

	F	Empregado Efetivo
	F	Empregado Efetivo
	F	Empregado Efetivo
	F	Empregado Efetivo
	F	Empregado Efetivo
	F	Empregado Efetivo
	F	Empregado Efetivo
	F	Empregado Efetivo
	F	Empregado Efetivo
	F	Empregado Efetivo
	F	Empregado Efetivo
	F	Empregado Efetivo
	F	Empregado Efetivo
	F	Cedido - RJU
	F	Empregado Efetivo
	F	Empregado Efetivo
	F	Empregado Efetivo
	F	Empregado Efetivo
	F	Empregado Efetivo
	F	Empregado Efetivo
	F	Empregado Efetivo
	F	Empregado Efetivo
	F	Empregado Efetivo
	M	Empregado Efetivo
	F	Empregado Efetivo
	F	Empregado Efetivo
	F	Empregado Efetivo
	F	Empregado Efetivo
	F	Empregado Efetivo
	F	Empregado Efetivo
	F	Empregado Efetivo
	F	Empregado Efetivo
	F	Empregado Efetivo
	F	Empregado Efetivo
	M	Empregado Efetivo
	F	Empregado Efetivo

	M	Empregado Efetivo
	F	Empregado Efetivo
	F	Empregado Efetivo
	F	Empregado Efetivo
	M	Empregado Efetivo
	F	Empregado Efetivo
	M	Empregado Efetivo
	F	Empregado Efetivo
	F	Empregado Efetivo
	F	Empregado Efetivo
	F	Empregado Efetivo
	M	Empregado Efetivo
	F	Empregado Efetivo
	F	Empregado Efetivo
	F	Empregado Efetivo
	F	Empregado Efetivo
	M	Empregado Efetivo
	F	Empregado Efetivo
	F	Empregado Efetivo
	M	Empregado Efetivo
	F	Empregado Efetivo
	F	Empregado Efetivo
	M	Empregado Efetivo
	F	Empregado Efetivo
	F	Empregado Efetivo
	F	Empregado Efetivo
	F	Empregado Efetivo
	M	Empregado Efetivo
	F	Empregado Efetivo
	F	Empregado Efetivo
	F	Empregado Efetivo
	M	Empregado Efetivo

	M	Empregado Efetivo
	F	Empregado Efetivo
	M	Empregado Efetivo
	M	Empregado Efetivo
	F	Empregado Efetivo
	F	Empregado Efetivo
	F	Empregado Efetivo
	F	Empregado Efetivo
	F	Empregado Efetivo
	F	Empregado Efetivo
	F	Empregado Efetivo
	F	Empregado Efetivo
	F	Empregado Efetivo
	F	Empregado Efetivo
	F	Empregado Efetivo
	F	Empregado Efetivo
	F	Empregado Efetivo
	F	Empregado Efetivo
	F	Empregado Efetivo
	M	Empregado Efetivo
	M	Empregado Efetivo
	M	Empregado Efetivo
	M	Empregado Efetivo
	F	Empregado Efetivo
	F	Empregado Efetivo
	F	Empregado Efetivo
	F	Empregado Efetivo
	F	Empregado Efetivo
	F	Empregado Efetivo
	F	Empregado Efetivo
	F	Empregado Efetivo
	F	Empregado Efetivo
	F	Empregado Efetivo
	F	Empregado Efetivo
	F	Empregado Efetivo
	F	Empregado Efetivo
	F	Empregado Efetivo
	F	Empregado Efetivo
	F	Empregado Efetivo
	F	Empregado Efetivo
	F	Empregado Efetivo
	F	Empregado Efetivo
	F	Empregado Efetivo
	F	Empregado Efetivo
	F	Empregado Efetivo
	F	Empregado Efetivo
	F	Empregado Efetivo
	F	Empregado Efetivo

	M	Empregado Efetivo
	F	Empregado Efetivo
	F	Empregado Efetivo
	F	Empregado Efetivo
	F	Empregado Efetivo
	F	Empregado Efetivo
	F	Empregado Efetivo
	F	Empregado Efetivo
	F	Empregado Efetivo
	F	Empregado Efetivo
	F	Empregado Efetivo
	F	Empregado Efetivo
	F	Empregado Efetivo
	F	Empregado Efetivo
	F	Empregado Efetivo
	F	Empregado Efetivo
	F	Empregado Efetivo
	F	Empregado Efetivo
	F	Empregado Efetivo
	F	Empregado Efetivo
	F	Empregado Efetivo
	F	Empregado Efetivo
	F	Empregado Efetivo
	F	Empregado Efetivo
	F	Empregado Efetivo
	F	Empregado Efetivo
	F	Empregado Efetivo
	F	Empregado Efetivo
	F	Empregado Efetivo
	F	Empregado Efetivo
	F	Empregado Efetivo
	F	Empregado Efetivo
	F	Empregado Efetivo
	F	Empregado Efetivo
	F	Empregado Efetivo
	F	Empregado Efetivo
	F	Empregado Efetivo
	F	Empregado Efetivo
	F	Empregado Efetivo
	F	Empregado Efetivo
	F	Empregado Efetivo
	F	Empregado Efetivo
	F	Empregado Efetivo
	F	Empregado Efetivo
	F	Empregado Efetivo
	F	Empregado Efetivo
	F	Empregado Efetivo
	F	Empregado Efetivo

	F	Empregado Efetivo
	F	Empregado Efetivo
	F	Empregado Efetivo
	F	Empregado Efetivo
	M	Empregado Efetivo
	F	Empregado Efetivo
	F	Empregado Efetivo
	F	Empregado Efetivo
	F	Empregado Efetivo
	F	Empregado Efetivo
	F	Empregado Efetivo
	F	Empregado Efetivo
	F	Empregado Efetivo
	F	Empregado Efetivo
	F	Empregado Efetivo
	F	Empregado Efetivo
	F	Empregado Efetivo
	F	Empregado Efetivo
	F	Empregado Efetivo
	F	Empregado Efetivo
	F	Empregado Efetivo
	F	Empregado Efetivo
	M	Empregado Efetivo
	F	Empregado Efetivo
	F	Empregado Efetivo
	F	Empregado Efetivo
	F	Empregado Efetivo
	F	Empregado Efetivo
	M	Empregado Efetivo
	F	Empregado Efetivo
	M	Empregado Efetivo
	F	Empregado Efetivo
	F	Empregado Efetivo
	M	Empregado Efetivo
	F	Empregado Efetivo
	F	Empregado Efetivo
	F	Empregado Efetivo
	F	Empregado Efetivo
	F	Empregado Efetivo
	F	Empregado Efetivo
	F	Empregado Efetivo
	M	Empregado Efetivo
	F	Empregado Efetivo
	F	Empregado Efetivo
	M	Empregado Efetivo
	F	Empregado Efetivo
	F	Empregado Efetivo
	M	Empregado Efetivo

	F	Empregado Efetivo
	M	Empregado Efetivo
	F	Empregado Efetivo
	F	Empregado Efetivo
	F	Empregado Efetivo
	F	Empregado Efetivo
	M	Empregado Efetivo
	F	Empregado Efetivo
	F	Empregado Efetivo
	F	Empregado Efetivo
	F	Empregado Efetivo
	F	Empregado Efetivo
	F	Empregado Efetivo
	F	Empregado Efetivo
	M	Empregado Efetivo
	F	Empregado Efetivo
	F	Empregado Efetivo
	F	Empregado Efetivo
	F	Empregado Efetivo
	M	Empregado Efetivo
	F	Empregado Efetivo
	F	Empregado Efetivo
	F	Empregado Efetivo
	F	Empregado Efetivo
	F	Empregado Efetivo
	F	Empregado Efetivo
	F	Empregado Efetivo
	F	Empregado Efetivo
	F	Empregado Efetivo
	F	Empregado Efetivo
	F	Empregado Efetivo
	F	Empregado Efetivo
	F	Empregado Efetivo
	F	Empregado Efetivo
	F	Empregado Efetivo
	M	Empregado Efetivo
	M	Empregado Efetivo
	F	Empregado Efetivo
	M	Empregado Efetivo
	F	Empregado Efetivo
	F	Empregado Efetivo
	M	Empregado Efetivo
	F	Empregado Efetivo
	F	Empregado Efetivo
	F	Empregado Efetivo
	M	Empregado Efetivo
	F	Empregado Efetivo
	F	Empregado Efetivo

	F	Empregado Efetivo
	F	Empregado Efetivo
	F	Empregado Efetivo
	F	Empregado Efetivo
	F	Empregado Efetivo
	F	Empregado Efetivo
	F	Empregado Efetivo
	F	Empregado Efetivo
	F	Empregado Efetivo
	F	Empregado Efetivo
	F	Empregado Efetivo
	M	Empregado Efetivo
	F	Empregado Efetivo
	F	Empregado Efetivo
	M	Empregado Efetivo
	F	Empregado Efetivo
	F	Empregado Efetivo
	M	Empregado Efetivo
	M	Empregado Efetivo
	M	Empregado Efetivo
	F	Empregado Efetivo
	F	Empregado Efetivo
	F	Empregado Efetivo
	F	Empregado Efetivo
	F	Empregado Efetivo
	F	Empregado Efetivo
	F	Empregado Efetivo
	F	Empregado Efetivo
	F	Empregado Efetivo
	F	Empregado Efetivo
	F	Empregado Efetivo
	F	Empregado Efetivo
	M	Empregado Efetivo
	F	Empregado Efetivo
	F	Empregado Efetivo
	F	Empregado Efetivo
	F	Empregado Efetivo
	F	Empregado Efetivo
	F	Empregado Efetivo
	F	Empregado Efetivo
	F	Empregado Efetivo
	F	Empregado Efetivo
	F	Empregado Efetivo
	F	Empregado Efetivo
	F	Empregado Efetivo
	F	Empregado Efetivo
	F	Empregado Efetivo
	F	Empregado Efetivo

	F	Empregado Efetivo
	M	Empregado Efetivo
	F	Empregado Efetivo
	F	Empregado Efetivo
	F	Empregado Efetivo
	F	Empregado Efetivo
	F	Empregado Efetivo
	F	Empregado Efetivo
	F	Empregado Efetivo
	F	Empregado Efetivo
	F	Empregado Efetivo
	F	Empregado Efetivo
	F	Empregado Efetivo
	F	Empregado Efetivo
	F	Empregado Efetivo
	F	Empregado Efetivo
	F	Empregado Efetivo
	F	Empregado Efetivo
	M	Empregado Efetivo
	F	Empregado Efetivo
	F	Empregado Efetivo
	F	Empregado Efetivo
	M	Empregado Efetivo
	F	Empregado Efetivo
	F	Empregado Efetivo
	F	Empregado Efetivo
	F	Empregado Efetivo
	F	Empregado Efetivo
	F	Empregado Efetivo
	F	Empregado Efetivo
	F	Empregado Efetivo
	F	Empregado Efetivo
	F	Empregado Efetivo
	M	Empregado Efetivo
	F	Empregado Efetivo
	F	Empregado Efetivo
	F	Empregado Efetivo
	F	Empregado Efetivo
	F	Empregado Efetivo
	F	Empregado Efetivo
	F	Empregado Efetivo
	M	Empregado Efetivo
	F	Empregado Efetivo
	M	Empregado Efetivo
	M	Empregado Efetivo
	F	Empregado Efetivo
	F	Empregado Efetivo
	F	Empregado Efetivo

	F	Empregado Efetivo
	F	Empregado Efetivo
	F	Empregado Efetivo
	M	Empregado Efetivo
	F	Empregado Efetivo
	F	Empregado Efetivo
	F	Empregado Efetivo
	F	Empregado Efetivo
	F	Empregado Efetivo
	F	Empregado Efetivo
	M	Empregado Efetivo
	F	Empregado Efetivo
	F	Empregado Efetivo
	F	Empregado Efetivo
	M	Empregado Efetivo
	F	Empregado Efetivo
	F	Empregado Efetivo
	F	Empregado Efetivo
	F	Empregado Efetivo
	M	Empregado Efetivo
	F	Empregado Efetivo
	F	Empregado Efetivo
	F	Empregado Efetivo
	F	Empregado Efetivo
	F	Empregado Efetivo
	F	Empregado Efetivo
	M	Empregado Efetivo
	F	Empregado Efetivo
	F	Empregado Efetivo
	F	Empregado Efetivo
	F	Empregado Efetivo
	M	Empregado Efetivo
	F	Empregado Efetivo
	F	Empregado Efetivo
	F	Empregado Efetivo
	F	Empregado Efetivo
	F	Empregado Efetivo
	F	Empregado Efetivo
	F	Empregado Efetivo
	F	Empregado Efetivo
	F	Empregado Efetivo
	F	Empregado Efetivo
	M	Empregado Efetivo
	F	Empregado Efetivo
	F	Empregado Efetivo
	F	Empregado Efetivo
	F	Empregado Efetivo
	F	Empregado Efetivo

	F	Empregado Efetivo
	F	Empregado Efetivo
	M	Empregado Efetivo
	F	Empregado Efetivo
	F	Empregado Efetivo
	F	Empregado Efetivo
	F	Empregado Efetivo
	F	Empregado Efetivo
	F	Empregado Efetivo
	F	Empregado Efetivo
	F	Empregado Efetivo
	F	Empregado Efetivo
	F	Empregado Efetivo
	F	Empregado Efetivo
	F	Empregado Efetivo
	F	Empregado Efetivo
	F	Empregado Efetivo
	F	Empregado Efetivo
	F	Empregado Efetivo
	F	Empregado Efetivo
	F	Empregado Efetivo
	F	Empregado Efetivo
	M	Empregado Efetivo
	M	Empregado Efetivo
	F	Empregado Efetivo
	F	Empregado Efetivo
	F	Empregado Efetivo
	F	Empregado Efetivo
	F	Empregado Efetivo
	F	Empregado Efetivo
	F	Empregado Efetivo
	F	Empregado Efetivo
	F	Empregado Efetivo
	F	Empregado Efetivo
	F	Empregado Efetivo
	F	Empregado Efetivo
	F	Empregado Efetivo
	F	Empregado Efetivo
	F	Empregado Efetivo
	F	Empregado Efetivo
	F	Empregado Efetivo
	F	Empregado Efetivo
	F	Empregado Efetivo
	F	Empregado Efetivo
	F	Empregado Efetivo
	F	Empregado Efetivo
	M	Empregado Efetivo
	M	Empregado Efetivo

	F	Empregado Efetivo
	F	Empregado Efetivo
	M	Empregado Efetivo
	F	Empregado Efetivo
	M	Empregado Efetivo
	F	Empregado Efetivo
	F	Empregado Efetivo
	F	Empregado Efetivo
	M	Empregado Efetivo
	F	Empregado Efetivo
	M	Empregado Efetivo
	F	Empregado Efetivo
	F	Empregado Efetivo
	M	Empregado Efetivo
	M	Empregado Efetivo
	F	Empregado Efetivo
	M	Empregado Efetivo
	F	Empregado Efetivo
	F	Empregado Efetivo
	F	Empregado Efetivo
	F	Empregado Efetivo
	F	Empregado Efetivo
	F	Empregado Efetivo
	F	Empregado Efetivo
	F	Empregado Efetivo
	F	Empregado Efetivo
	F	Empregado Efetivo
	F	Empregado Efetivo
	F	Empregado Efetivo
	F	Empregado Efetivo
	F	Empregado Efetivo
	F	Empregado Efetivo
	M	Empregado Efetivo
	M	Empregado Efetivo
	F	Empregado Efetivo
	M	Empregado Efetivo
	M	Empregado Efetivo
	F	Empregado Efetivo
	F	Empregado Efetivo
	F	Empregado Efetivo
	F	Empregado Efetivo
	F	Empregado Efetivo
	F	Empregado Efetivo
	F	Empregado Efetivo
	F	Empregado Efetivo
	F	Empregado Efetivo
	F	Empregado Efetivo
	F	Empregado Efetivo

	F	Empregado Efetivo
	F	Empregado Efetivo
	F	Empregado Efetivo
	F	Empregado Efetivo
	F	Empregado Efetivo
	F	Empregado Efetivo
	M	Empregado Efetivo
	F	Empregado Efetivo
	F	Empregado Efetivo
	F	Empregado Efetivo
	F	Empregado Efetivo
	F	Empregado Efetivo
	F	Empregado Efetivo
	F	Empregado Efetivo
	F	Empregado Efetivo
	F	Empregado Efetivo
	F	Empregado Efetivo
	F	Empregado Efetivo
	F	Empregado Efetivo
	F	Empregado Efetivo
	F	Empregado Efetivo
	F	Empregado Efetivo
	F	Empregado Efetivo
	M	Empregado Efetivo
	F	Empregado Efetivo
	F	Empregado Efetivo
	F	Empregado Efetivo
	F	Empregado Efetivo
	F	Empregado Efetivo
	F	Empregado Efetivo
	F	Empregado Efetivo
	F	Empregado Efetivo
	F	Empregado Efetivo
	F	Empregado Efetivo
	F	Empregado Efetivo
	M	Empregado Efetivo
	F	Empregado Efetivo
	F	Empregado Efetivo
	M	Empregado Efetivo
	F	Empregado Efetivo
	F	Empregado Efetivo
	F	Empregado Efetivo
	F	Empregado Efetivo
	F	Empregado Efetivo
	F	Empregado Efetivo
	F	Empregado Efetivo
	F	Empregado Efetivo
	M	Empregado Efetivo

	F	Empregado Efetivo
	F	Empregado Efetivo
	F	Empregado Efetivo
	F	Empregado Efetivo
	F	Empregado Efetivo
	F	Empregado Efetivo
	F	Empregado Efetivo
	F	Empregado Efetivo
	M	Empregado Efetivo
	F	Empregado Efetivo
	M	Empregado Efetivo
	F	Empregado Efetivo
	F	Empregado Efetivo
	F	Empregado Efetivo
	F	Empregado Efetivo
	M	Empregado Efetivo
	F	Empregado Efetivo
	F	Empregado Efetivo
	F	Empregado Efetivo
	F	Empregado Efetivo
	F	Empregado Efetivo
	M	Empregado Efetivo
	F	Empregado Efetivo
	F	Empregado Efetivo
	F	Empregado Efetivo
	F	Empregado Efetivo
	F	Empregado Efetivo
	F	Empregado Efetivo
	F	Empregado Efetivo
	F	Empregado Efetivo
	F	Empregado Efetivo
	M	Empregado Efetivo
	F	Empregado Efetivo
	F	Empregado Efetivo
	F	Empregado Efetivo
	F	Empregado Efetivo
	F	Empregado Efetivo
	F	Empregado Efetivo
	F	Empregado Efetivo
	F	Empregado Efetivo
	M	Empregado Efetivo
	F	Empregado Efetivo
	F	Empregado Efetivo
	F	Empregado Efetivo
	F	Empregado Efetivo
	F	Empregado Efetivo
	M	Empregado Efetivo
	F	Empregado Efetivo

	F	Empregado Efetivo
	F	Empregado Efetivo
	F	Empregado Efetivo
	F	Empregado Efetivo
	F	Empregado Efetivo
	F	Empregado Efetivo
	F	Empregado Efetivo
	F	Empregado Efetivo
	F	Empregado Efetivo
	F	Empregado Efetivo
	F	Empregado Efetivo
	F	Empregado Efetivo
	F	Empregado Efetivo
	F	Empregado Efetivo
	F	Empregado Efetivo
	M	Empregado Efetivo
	F	Empregado Efetivo
	F	Empregado Efetivo
	F	Empregado Efetivo
	M	Empregado Efetivo
	F	Empregado Efetivo
	F	Empregado Efetivo
	F	Empregado Efetivo
	F	Empregado Efetivo
	F	Empregado Efetivo
	F	Empregado Efetivo
	F	Empregado Efetivo
	F	Empregado Efetivo
	F	Empregado Efetivo
	F	Empregado Efetivo
	F	Empregado Efetivo
	F	Empregado Efetivo
	F	Empregado Efetivo
	F	Empregado Efetivo
	F	Empregado Efetivo
	F	Empregado Efetivo
	F	Empregado Efetivo
	F	Empregado Efetivo
	F	Empregado Efetivo
	F	Empregado Efetivo
	F	Empregado Efetivo
	M	Empregado Efetivo
	F	Empregado Efetivo
	F	Empregado Efetivo
	F	Empregado Efetivo
	F	Empregado Efetivo
	F	Empregado Efetivo
	F	Empregado Efetivo

	F	Empregado Efetivo
	F	Empregado Efetivo
	F	Empregado Efetivo
	F	Empregado Efetivo
	M	Empregado Efetivo
	F	Empregado Efetivo
	F	Empregado Efetivo
	F	Empregado Efetivo
	F	Empregado Efetivo
	F	Empregado Efetivo
	F	Empregado Efetivo
	F	Empregado Efetivo
	F	Empregado Efetivo
	F	Empregado Efetivo
	F	Empregado Efetivo
	F	Empregado Efetivo
	F	Empregado Efetivo
	M	Empregado Efetivo
	F	Empregado Efetivo
	F	Empregado Efetivo
	F	Empregado Efetivo
	F	Empregado Efetivo
	F	Empregado Efetivo
	M	Empregado Efetivo
	M	Empregado Efetivo
	F	Empregado Efetivo
	F	Empregado Efetivo
	F	Empregado Efetivo
	F	Empregado Efetivo
	F	Empregado Efetivo
	F	Empregado Efetivo
	M	Empregado Efetivo
	M	Empregado Efetivo
	F	Empregado Efetivo
	F	Empregado Efetivo
	F	Empregado Efetivo
	F	Empregado Efetivo
	F	Empregado Efetivo
	F	Empregado Efetivo
	F	Empregado Efetivo
	F	Empregado Efetivo
	M	Empregado Efetivo
	M	Empregado Efetivo
	M	Empregado Efetivo
	M	Empregado Efetivo
	M	Empregado Efetivo
	F	Empregado Efetivo
	F	Empregado Efetivo

	F	Empregado Efetivo
	M	Empregado Efetivo
	M	Empregado Efetivo
	M	Empregado Efetivo
	M	Empregado Efetivo
	F	Empregado Efetivo
	F	Empregado Efetivo
	M	Empregado Efetivo
	M	Empregado Efetivo
	M	Empregado Efetivo
	M	Empregado Efetivo
	M	Empregado Efetivo
	M	Empregado Efetivo
	M	Empregado Efetivo
	M	Empregado Efetivo
	F	Empregado Efetivo
	F	Empregado Efetivo
	F	Empregado Efetivo
	M	Empregado Efetivo
	M	Empregado Efetivo
	M	Empregado Efetivo
	F	Empregado Efetivo
	M	Empregado Efetivo
	F	Empregado Efetivo
	M	Empregado Efetivo
	F	Empregado Efetivo
	M	Empregado Efetivo
	M	Empregado Efetivo
	M	Empregado Efetivo
	M	Empregado Efetivo
	F	Empregado Efetivo
	F	Empregado Efetivo
	F	Empregado Efetivo
	F	Empregado Efetivo
	F	Empregado Efetivo
	F	Empregado Efetivo
	F	Empregado Efetivo
	F	Empregado Efetivo
	F	Empregado Efetivo
	M	Empregado Efetivo
	M	Empregado Efetivo
	M	Empregado Efetivo
	M	Empregado Efetivo
	M	Empregado Efetivo
	M	Empregado Efetivo
	F	Empregado Efetivo
	M	Empregado Efetivo

	F	Empregado Efetivo
	F	Empregado Efetivo
	F	Empregado Efetivo
	F	Empregado Efetivo
	F	Empregado Efetivo
	M	Empregado Efetivo
	M	Empregado Efetivo
	F	Empregado Efetivo
	M	Empregado Efetivo
	F	Empregado Efetivo
	F	Empregado Efetivo
	F	Empregado Efetivo
	M	Empregado Efetivo
	F	Empregado Efetivo
	F	Empregado Efetivo
	F	Empregado Efetivo
	F	Empregado Efetivo
	F	Empregado Efetivo
	M	Empregado Efetivo
	F	Empregado Efetivo
	F	Empregado Efetivo
	F	Empregado Efetivo
	F	Empregado Efetivo
	F	Empregado Efetivo
	F	Empregado Efetivo
	F	Empregado Efetivo
	F	Empregado Efetivo
	M	Empregado Efetivo
	F	Empregado Efetivo
	F	Empregado Efetivo
	M	Empregado Efetivo
	M	Empregado Efetivo
	F	Empregado Efetivo
	M	Empregado Efetivo
	F	Empregado Efetivo
	F	Empregado Efetivo
	M	Empregado Efetivo
	F	Empregado Efetivo
	F	Empregado Efetivo
	M	Empregado Efetivo
	M	Empregado Efetivo
	F	Empregado Efetivo
	M	Empregado Efetivo
	F	Empregado Efetivo
	F	Empregado Efetivo
	M	Empregado Efetivo
	F	Empregado Efetivo
	F	Empregado Efetivo

	M	Empregado Efetivo
	M	Empregado Efetivo
	F	Empregado Efetivo
	F	Empregado Efetivo
	F	Empregado Efetivo
	F	Empregado Efetivo
	F	Empregado Efetivo
	F	Empregado Efetivo
	F	Empregado Efetivo
	F	Empregado Efetivo
	M	Empregado Efetivo
	M	Empregado Efetivo
	M	Empregado Efetivo
	F	Empregado Efetivo
	F	Empregado Efetivo
	M	Empregado Efetivo
	M	Empregado Efetivo
	F	Empregado Efetivo
	F	Empregado Efetivo
	F	Empregado Efetivo
	F	Empregado Efetivo
	F	Empregado Efetivo
	M	Empregado Efetivo
	F	Empregado Efetivo
	F	Empregado Efetivo
	F	Empregado Efetivo
	F	Empregado Efetivo
	F	Empregado Efetivo
	F	Empregado Efetivo
	F	Empregado Efetivo
	F	Empregado Efetivo
	F	Empregado Efetivo
	F	Empregado Efetivo
	F	Empregado Efetivo
	M	Empregado Efetivo
	F	Empregado Efetivo
	M	Empregado Efetivo
	M	Empregado Efetivo
	M	Empregado Efetivo
	F	Empregado Efetivo
	F	Empregado Efetivo
	F	Empregado Efetivo
	F	Empregado Efetivo
	F	Empregado Efetivo
	M	Empregado Efetivo
	F	Empregado Efetivo
	M	Empregado Efetivo
	F	Empregado Efetivo

	M	Empregado Efetivo
	M	Empregado Efetivo
	F	Empregado Efetivo
	F	Empregado Efetivo
	M	Empregado Efetivo
	F	Empregado Efetivo
	F	Empregado Efetivo
	F	Empregado Efetivo
	F	Empregado Efetivo
	F	Empregado Efetivo
	M	Empregado Efetivo
	F	Empregado Efetivo
	F	Empregado Efetivo
	M	Empregado Efetivo
	F	Empregado Efetivo
	F	Empregado Efetivo
	M	Empregado Efetivo
	M	Empregado Efetivo
	M	Empregado Efetivo
	M	Empregado Efetivo
	F	Empregado Efetivo
	M	Empregado Efetivo
	M	Empregado Efetivo
	F	Empregado Efetivo
	M	Empregado Efetivo
	F	Empregado Efetivo
	F	Empregado Efetivo
	F	Empregado Efetivo
	M	Empregado Efetivo
	F	Empregado Efetivo
	M	Empregado Efetivo
	F	Empregado Efetivo
	M	Empregado Efetivo
	M	Empregado Efetivo
	F	Empregado Efetivo
	F	Empregado Efetivo
	F	Empregado Efetivo
	F	Empregado Efetivo
	F	Empregado Efetivo
	F	Empregado Efetivo
	F	Empregado Efetivo
	F	Empregado Efetivo
	F	Empregado Efetivo
	F	Empregado Efetivo
	M	Empregado Efetivo
	F	Empregado Efetivo
	F	Empregado Efetivo
	F	Empregado Efetivo

	F	Empregado Efetivo
	F	Empregado Efetivo
	F	Empregado Efetivo
	F	Empregado Efetivo
	F	Empregado Efetivo
	F	Empregado Efetivo
	F	Empregado Efetivo
	M	Empregado Efetivo
	F	Empregado Efetivo
	F	Empregado Efetivo
	F	Empregado Efetivo
	F	Empregado Efetivo
	F	Empregado Efetivo
	F	Empregado Efetivo
	F	Empregado Efetivo
	F	Empregado Efetivo
	F	Empregado Efetivo
	F	Empregado Efetivo
	F	Empregado Efetivo
	F	Empregado Efetivo
	M	Empregado Efetivo
	M	Empregado Efetivo
	M	Empregado Efetivo
	F	Empregado Efetivo
	F	Empregado Efetivo
	F	Empregado Efetivo
	F	Empregado Efetivo
	M	Empregado Efetivo
	F	Empregado Efetivo
	F	Empregado Efetivo
	F	Empregado Efetivo
	F	Empregado Efetivo
	M	Empregado Efetivo
	F	Empregado Efetivo
	F	Empregado Efetivo
	F	Empregado Efetivo
	F	Empregado Efetivo
	F	Empregado Efetivo
	F	Empregado Efetivo
	F	Empregado Efetivo
	F	Empregado Efetivo
	F	Empregado Efetivo
	F	Empregado Efetivo
	F	Empregado Efetivo
	F	Empregado Efetivo
	F	Empregado Efetivo
	F	Empregado Efetivo
	M	Empregado Efetivo

	F	Empregado Efetivo
	M	Empregado Efetivo
	F	Empregado Efetivo
	F	Empregado Efetivo
	F	Empregado Efetivo
	M	Empregado Efetivo
	F	Empregado Efetivo
	F	Empregado Efetivo
	F	Empregado Efetivo
	F	Empregado Efetivo
	M	Empregado Efetivo
	F	Empregado Efetivo
	F	Empregado Efetivo
	F	Empregado Efetivo
	F	Empregado Efetivo
	F	Empregado Efetivo
	M	Empregado Efetivo
	F	Empregado Efetivo
	M	Empregado Efetivo
	F	Empregado Efetivo
	M	Empregado Efetivo
	F	Empregado Efetivo
	F	Empregado Efetivo
	F	Empregado Efetivo
	F	Empregado Efetivo
	F	Empregado Efetivo
	F	Empregado Efetivo
	F	Empregado Efetivo
	F	Empregado Efetivo
	M	Empregado Efetivo
	F	Empregado Efetivo
	M	Empregado Efetivo
	F	Empregado Efetivo
	M	Empregado Efetivo
	F	Empregado Efetivo
	F	Empregado Efetivo
	F	Empregado Efetivo
	F	Empregado Efetivo
	F	Empregado Efetivo
	F	Empregado Efetivo
	F	Empregado Efetivo
	F	Empregado Efetivo
	F	Empregado Efetivo

	F	Empregado Efetivo
	F	Empregado Efetivo
	F	Empregado Efetivo
	F	Empregado Efetivo
	F	Empregado Efetivo
	F	Empregado Efetivo
	F	Empregado Efetivo
	F	Empregado Efetivo
	F	Empregado Efetivo
	F	Empregado Efetivo
	F	Empregado Efetivo
	F	Empregado Efetivo
	F	Empregado Efetivo
	F	Empregado Efetivo
	F	Empregado Efetivo
	F	Empregado Efetivo
	F	Empregado Efetivo
	F	Empregado Efetivo
	F	Empregado Efetivo
	F	Empregado Efetivo
	F	Empregado Efetivo
	F	Empregado Efetivo
	F	Empregado Efetivo
	F	Empregado Efetivo
	F	Empregado Efetivo
	F	Empregado Efetivo
	F	Empregado Efetivo
	F	Empregado Efetivo
	F	Empregado Efetivo
	F	Empregado Efetivo
	F	Empregado Efetivo
	M	Empregado Efetivo

	F	Empregado Efetivo
	F	Empregado Efetivo
	F	Empregado Efetivo
	F	Empregado Efetivo
	M	Empregado Efetivo
	F	Empregado Efetivo
	F	Empregado Efetivo
	F	Empregado Efetivo
	F	Empregado Efetivo
	F	Empregado Efetivo
	F	Empregado Efetivo
	F	Empregado Efetivo
	F	Empregado Efetivo
	F	Empregado Efetivo
	F	Empregado Efetivo
	F	Empregado Efetivo
	F	Empregado Efetivo
	F	Empregado Efetivo
	F	Empregado Efetivo
	F	Empregado Efetivo
	F	Empregado Efetivo
	F	Empregado Efetivo
	F	Empregado Efetivo
	F	Empregado Efetivo
	F	Empregado Efetivo
	F	Empregado Efetivo
	F	Empregado Efetivo
	F	Empregado Efetivo
	F	Empregado Efetivo
	F	Empregado Efetivo
	F	Empregado Efetivo
	F	Empregado Efetivo
	F	Empregado Efetivo
	F	Empregado Efetivo
	F	Empregado Efetivo
	F	Empregado Efetivo

	F	Empregado Efetivo
	F	Empregado Efetivo
	F	Empregado Efetivo
	F	Empregado Efetivo
	F	Empregado Efetivo
	F	Empregado Efetivo
	F	Empregado Efetivo
	F	Empregado Efetivo
	F	Empregado Efetivo
	F	Empregado Efetivo
	F	Empregado Efetivo
	F	Empregado Efetivo
	F	Empregado Efetivo
	M	Empregado Efetivo
	F	Empregado Efetivo
	F	Empregado Efetivo
	F	Empregado Efetivo
	F	Empregado Efetivo
	F	Empregado Efetivo
	M	Empregado Efetivo
	F	Empregado Efetivo
	F	Empregado Efetivo
	F	Empregado Efetivo
	F	Empregado Efetivo
	F	Empregado Efetivo
	F	Empregado Efetivo
	F	Empregado Efetivo
	F	Empregado Efetivo
	F	Empregado Efetivo
	F	Empregado Efetivo
	F	Empregado Efetivo
	M	Empregado Efetivo
	F	Empregado Efetivo
	F	Empregado Efetivo
	F	Empregado Efetivo
	F	Empregado Efetivo
	F	Empregado Efetivo
	F	Empregado Efetivo
	F	Empregado Efetivo
	F	Empregado Efetivo
	F	Empregado Efetivo
	F	Empregado Efetivo
	F	Empregado Efetivo
	F	Empregado Efetivo
	F	Empregado Efetivo
	F	Empregado Efetivo
	F	Empregado Efetivo
	F	Empregado Efetivo

	F	Empregado Efetivo
	F	Empregado Efetivo
	F	Empregado Efetivo
	F	Empregado Efetivo
	F	Empregado Efetivo
	F	Empregado Efetivo
	F	Empregado Efetivo
	F	Empregado Efetivo
	F	Empregado Efetivo
	F	Empregado Efetivo
	F	Empregado Efetivo
	F	Empregado Efetivo
	F	Empregado Efetivo
	M	Empregado Efetivo
	F	Empregado Efetivo
	F	Empregado Efetivo
	F	Empregado Efetivo
	M	Empregado Efetivo
	F	Empregado Efetivo
	F	Empregado Efetivo
	M	Empregado Efetivo
	F	Empregado Efetivo
	F	Empregado Efetivo
	F	Empregado Efetivo
	F	Empregado Efetivo
	F	Empregado Efetivo
	M	Empregado Efetivo
	F	Empregado Efetivo
	M	Empregado Efetivo
	M	Empregado Efetivo
	F	Empregado Efetivo
	F	Empregado Efetivo
	M	Empregado Efetivo
	F	Empregado Efetivo
	M	Empregado Efetivo
	F	Empregado Efetivo
	F	Empregado Efetivo
	M	Empregado Efetivo
	M	Empregado Efetivo
	F	Empregado Efetivo
	F	Empregado Efetivo
	M	Empregado Efetivo
	F	Empregado Efetivo
	F	Empregado Efetivo
	F	Empregado Efetivo
	F	Empregado Efetivo
	F	Empregado Efetivo
	M	Empregado Efetivo

	F	Empregado Efetivo
	F	Empregado Efetivo
	F	Empregado Efetivo
	F	Empregado Efetivo
	M	Empregado Efetivo
	F	Empregado Efetivo
	M	Empregado Efetivo
	M	Empregado Efetivo
	M	Empregado Efetivo
	M	Empregado Efetivo
	M	Empregado Efetivo
	M	Empregado Efetivo
	M	Empregado Efetivo
	M	Empregado Efetivo
	M	Empregado Efetivo
	M	Empregado Efetivo
	M	Empregado Efetivo
	F	Empregado Efetivo
	F	Empregado Efetivo
	F	Empregado Efetivo
	F	Empregado Efetivo
	F	Empregado Efetivo
	F	Empregado Efetivo
	F	Empregado Efetivo
	F	Empregado Efetivo
	F	Empregado Efetivo
	F	Empregado Efetivo
	F	Empregado Efetivo
	F	Empregado Efetivo
	F	Empregado Efetivo
	M	Empregado Efetivo
	F	Empregado Efetivo
	M	Empregado Efetivo
	F	Empregado Efetivo
	F	Empregado Efetivo
	F	Empregado Efetivo
	F	Empregado Efetivo
	F	Empregado Efetivo
	F	Empregado Efetivo
	F	Empregado Efetivo
	M	Empregado Efetivo
	M	Empregado Efetivo
	F	Empregado Efetivo
	M	Empregado Efetivo
	F	Empregado Efetivo
	F	Empregado Efetivo
	F	Empregado Efetivo
	M	Empregado Efetivo

	F	Empregado Efetivo
	M	Empregado Efetivo
	M	Empregado Efetivo
	M	Empregado Efetivo
	M	Empregado Efetivo
	M	Empregado Efetivo
	M	Empregado Efetivo
	F	Empregado Efetivo
	M	Empregado Efetivo
	F	Empregado Efetivo
	F	Empregado Efetivo
	M	Empregado Efetivo
	M	Empregado Efetivo
	F	Empregado Efetivo
	M	Empregado Efetivo
	M	Empregado Efetivo
	M	Empregado Efetivo
	M	Empregado Efetivo
	M	Empregado Efetivo
	F	Empregado Efetivo
	M	Empregado Efetivo
	M	Empregado Efetivo
	M	Empregado Efetivo
	M	Empregado Efetivo
	M	Empregado Efetivo
	M	Empregado Efetivo
	F	Empregado Efetivo
	F	Empregado Efetivo
	F	Empregado Efetivo
	M	Empregado Efetivo
	F	Empregado Efetivo
	F	Empregado Efetivo
	F	Empregado Efetivo
	M	Empregado Efetivo
	F	Empregado Efetivo
	M	Empregado Efetivo
	F	Empregado Efetivo
	F	Empregado Efetivo
	F	Empregado Efetivo
	F	Empregado Efetivo
	F	Empregado Efetivo
	M	Empregado Efetivo
	F	Empregado Efetivo
	F	Empregado Efetivo
	F	Empregado Efetivo
	M	Empregado Efetivo
	F	Empregado Efetivo
	F	Empregado Efetivo

	M	Empregado Efetivo
	F	Empregado Efetivo
	F	Empregado Efetivo
	F	Empregado Efetivo
	F	Empregado Efetivo
	F	Empregado Efetivo
	F	Empregado Efetivo
	F	Empregado Efetivo
	F	Empregado Efetivo
	F	Empregado Efetivo
	M	Empregado Efetivo
	F	Empregado Efetivo
	M	Empregado Efetivo
	F	Empregado Efetivo
	F	Empregado Efetivo
	F	Empregado Efetivo
	F	Empregado Efetivo
	F	Empregado Efetivo
	F	Empregado Efetivo
	F	Empregado Efetivo
	F	Empregado Efetivo
	F	Empregado Efetivo
	F	Empregado Efetivo
	F	Empregado Efetivo
	M	Empregado Efetivo
	F	Empregado Efetivo
	F	Empregado Efetivo
	F	Empregado Efetivo
	F	Empregado Efetivo
	F	Empregado Efetivo
	F	Empregado Efetivo
	F	Empregado Efetivo
	M	Empregado Efetivo
	F	Empregado Efetivo
	M	Empregado Efetivo
	M	Empregado Efetivo
	M	Cedido - RJU
	F	Empregado Efetivo
	F	Empregado Efetivo
	F	Empregado Efetivo
	F	Empregado Efetivo
	F	Empregado Efetivo
	F	Empregado Efetivo
	F	Empregado Efetivo
	F	Empregado Efetivo
	F	Empregado Efetivo
	F	Empregado Efetivo
	F	Empregado Efetivo

	F	Empregado Efetivo
	F	Empregado Efetivo
	F	Empregado Efetivo
	F	Empregado Efetivo
	F	Empregado Efetivo
	F	Empregado Efetivo
	F	Empregado Efetivo
	F	Empregado Efetivo
	F	Empregado Efetivo
	F	Empregado Efetivo
	F	Empregado Efetivo
	F	Empregado Efetivo
	M	Empregado Efetivo
	F	Empregado Efetivo
	F	Empregado Efetivo
	F	Empregado Efetivo
	F	Empregado Efetivo
	F	Empregado Efetivo
	F	Empregado Efetivo
	F	Empregado Efetivo
	F	Empregado Efetivo
	F	Empregado Efetivo
	M	Empregado Efetivo
	F	Empregado Efetivo
	F	Empregado Efetivo
	F	Empregado Efetivo
	F	Empregado Efetivo
	F	Empregado Efetivo
	F	Empregado Efetivo
	F	Empregado Efetivo
	M	Empregado Efetivo
	F	Empregado Efetivo
	F	Empregado Efetivo
	F	Empregado Efetivo
	F	Empregado Efetivo
	F	Empregado Efetivo
	F	Empregado Efetivo
	F	Empregado Efetivo
	F	Empregado Efetivo
	F	Empregado Efetivo
	F	Empregado Efetivo
	M	Empregado Efetivo

	M	Empregado Efetivo
	M	Empregado Efetivo
	M	Empregado Efetivo
	M	Empregado Efetivo
	F	Empregado Efetivo
	F	Empregado Efetivo
	F	Empregado Efetivo
	F	Empregado Efetivo
	M	Empregado Efetivo
	F	Empregado Efetivo
	F	Empregado Efetivo
	F	Empregado Efetivo
	F	Empregado Efetivo
	M	Empregado Efetivo
	F	Empregado Efetivo
	M	Empregado Efetivo
	M	Empregado Efetivo
	F	Empregado Efetivo
	F	Empregado Efetivo
	F	Empregado Efetivo
	M	Empregado Efetivo
	M	Empregado Efetivo
	M	Empregado Efetivo
	F	Empregado Efetivo
	M	Empregado Efetivo
	F	Empregado Efetivo
	M	Empregado Efetivo
	F	Empregado Efetivo
	F	Empregado Efetivo
	M	Empregado Efetivo
	M	Empregado Efetivo
	F	Empregado Efetivo

	F	Empregado Efetivo
	F	Empregado Efetivo
	M	Empregado Efetivo
	F	Empregado Efetivo
	F	Empregado Efetivo
	F	Empregado Efetivo
	M	Empregado Efetivo
	F	Empregado Efetivo
	F	Empregado Efetivo
	F	Empregado Efetivo
	F	Empregado Efetivo
	F	Empregado Efetivo
	M	Empregado Efetivo
	M	Empregado Efetivo
	M	Empregado Efetivo
	M	Empregado Efetivo
	M	Empregado Efetivo
	M	Empregado Efetivo
	M	Empregado Efetivo
	M	Empregado Efetivo
	M	Empregado Efetivo
	M	Empregado Efetivo
	M	Empregado Efetivo
	M	Empregado Efetivo
	F	Empregado Efetivo
	F	Empregado Efetivo
	F	Empregado Efetivo
	F	Empregado Efetivo
	F	Empregado Efetivo
	F	Empregado Efetivo
	F	Empregado Efetivo
	F	Empregado Efetivo

	F	Empregado Efetivo
	F	Empregado Efetivo
	F	Empregado Efetivo
	F	Empregado Efetivo
	F	Empregado Efetivo
	F	Empregado Efetivo
	F	Empregado Efetivo
	F	Empregado Efetivo
	F	Empregado Efetivo
	M	Empregado Efetivo
	M	Empregado Efetivo
	F	Empregado Efetivo
	F	Empregado Efetivo
	F	Empregado Efetivo
	F	Empregado Efetivo
	F	Empregado Efetivo
	F	Empregado Efetivo
	F	Empregado Efetivo
	F	Empregado Efetivo
	F	Empregado Efetivo
	F	Empregado Efetivo
	F	Empregado Efetivo
	F	Empregado Efetivo
	M	Empregado Efetivo
	F	Empregado Efetivo
	M	Empregado Efetivo
	F	Empregado Efetivo
	F	Empregado Efetivo
	F	Empregado Efetivo
	F	Empregado Efetivo
	F	Empregado Efetivo
	F	Empregado Efetivo

	F	Empregado Efetivo
	F	Empregado Efetivo
	F	Empregado Efetivo
	F	Empregado Efetivo
	F	Empregado Efetivo
	F	Empregado Efetivo
	F	Empregado Efetivo
	M	Empregado Efetivo
	F	Empregado Efetivo
	M	Empregado Efetivo
	F	Empregado Efetivo
	F	Empregado Efetivo
	F	Empregado Efetivo
	F	Empregado Efetivo
	F	Empregado Efetivo
	F	Empregado Efetivo
	F	Empregado Efetivo
	F	Empregado Efetivo
	F	Empregado Efetivo
	F	Empregado Efetivo
	F	Empregado Efetivo
	F	Empregado Efetivo
	F	Empregado Efetivo
	F	Empregado Efetivo
	F	Empregado Efetivo
	F	Empregado Efetivo
	F	Empregado Efetivo
	F	Empregado Efetivo
	F	Empregado Efetivo
	M	Empregado Efetivo
	F	Empregado Efetivo
	M	Empregado Efetivo
	F	Empregado Efetivo
	F	Empregado Efetivo
	F	Empregado Efetivo
	M	Empregado Efetivo
	F	Empregado Efetivo
	F	Empregado Efetivo
	F	Empregado Efetivo
	F	Empregado Efetivo
	F	Empregado Efetivo

	M	Empregado Efetivo
	F	Empregado Efetivo
	F	Empregado Efetivo
	F	Empregado Efetivo
	F	Empregado Efetivo
	M	Empregado Efetivo
	F	Empregado Efetivo
	F	Empregado Efetivo
	M	Empregado Efetivo
	F	Empregado Efetivo
	F	Empregado Efetivo
	F	Empregado Efetivo
	F	Empregado Efetivo
	F	Empregado Efetivo
	F	Empregado Efetivo
	F	Empregado Efetivo
	F	Empregado Efetivo
	F	Empregado Efetivo
	F	Empregado Efetivo
	F	Empregado Efetivo
	F	Empregado Efetivo
	F	Empregado Efetivo
	F	Empregado Efetivo
	M	Empregado Efetivo
	F	Empregado Efetivo
	F	Empregado Efetivo
	F	Empregado Efetivo
	M	Empregado Efetivo
	F	Empregado Efetivo
	F	Empregado Efetivo
	F	Empregado Efetivo
	F	Empregado Efetivo
	F	Empregado Efetivo
	F	Empregado Efetivo
	F	Empregado Efetivo
	F	Empregado Efetivo
	F	Empregado Efetivo
	F	Empregado Efetivo
	F	Empregado Efetivo
	F	Empregado Efetivo
	F	Empregado Efetivo
	F	Empregado Efetivo
	M	Empregado Efetivo
	F	Empregado Efetivo
	F	Empregado Efetivo
	F	Empregado Efetivo
	F	Empregado Efetivo
	F	Empregado Efetivo

	F	Empregado Efetivo
	F	Empregado Efetivo
	F	Empregado Efetivo
	F	Empregado Efetivo
	F	Empregado Efetivo
	F	Empregado Efetivo
	F	Empregado Efetivo
	F	Empregado Efetivo
	M	Empregado Efetivo
	F	Empregado Efetivo
	F	Empregado Efetivo
	F	Empregado Efetivo
	F	Empregado Efetivo
	F	Empregado Efetivo
	F	Empregado Efetivo
	F	Empregado Efetivo
	F	Empregado Efetivo
	F	Empregado Efetivo
	F	Empregado Efetivo
	F	Empregado Efetivo
	F	Empregado Efetivo
	F	Empregado Efetivo
	F	Empregado Efetivo
	M	Empregado Efetivo
	F	Empregado Efetivo
	F	Empregado Efetivo
	F	Empregado Efetivo
	F	Empregado Efetivo
	F	Empregado Efetivo
	F	Empregado Efetivo
	F	Empregado Efetivo
	F	Empregado Efetivo
	F	Empregado Efetivo
	F	Empregado Efetivo
	M	Empregado Efetivo
	F	Empregado Efetivo
	F	Empregado Efetivo
	F	Empregado Efetivo
	F	Empregado Efetivo
	F	Empregado Efetivo
	F	Empregado Efetivo
	F	Empregado Efetivo
	F	Empregado Efetivo
	F	Empregado Efetivo
	M	Empregado Efetivo
	F	Empregado Efetivo
	F	Empregado Efetivo
	F	Empregado Efetivo

	F	Empregado Efetivo
	F	Empregado Efetivo
	M	Empregado Efetivo
	F	Empregado Efetivo
	F	Empregado Efetivo
	F	Empregado Efetivo
	F	Empregado Efetivo
	F	Empregado Efetivo
	F	Empregado Efetivo
	F	Empregado Efetivo
	M	Empregado Efetivo
	F	Empregado Efetivo
	F	Empregado Efetivo
	F	Empregado Efetivo
	F	Empregado Efetivo
	F	Empregado Efetivo
	F	Empregado Efetivo
	M	Empregado Efetivo
	F	Empregado Efetivo
	F	Empregado Efetivo
	F	Empregado Efetivo
	M	Empregado Efetivo
	F	Empregado Efetivo
	F	Empregado Efetivo
	F	Empregado Efetivo
	F	Empregado Efetivo
	F	Empregado Efetivo
	F	Empregado Efetivo
	F	Empregado Efetivo
	F	Empregado Efetivo
	F	Empregado Efetivo
	F	Empregado Efetivo
	M	Empregado Efetivo
	F	Empregado Efetivo
	F	Empregado Efetivo
	F	Empregado Efetivo
	F	Empregado Efetivo
	F	Empregado Efetivo
	F	Empregado Efetivo
	F	Empregado Efetivo
	F	Empregado Efetivo
	F	Empregado Efetivo
	F	Empregado Efetivo
	F	Empregado Efetivo
	F	Empregado Efetivo
	F	Empregado Efetivo
	F	Empregado Efetivo
	F	Empregado Efetivo
	F	Empregado Efetivo

	F	Empregado Efetivo
	F	Empregado Efetivo
	F	Empregado Efetivo
	M	Empregado Efetivo
	M	Empregado Efetivo
	F	Empregado Efetivo
	M	Empregado Efetivo
	F	Empregado Efetivo
	F	Empregado Efetivo
	F	Empregado Efetivo
	F	Empregado Efetivo
	F	Empregado Efetivo
	F	Empregado Efetivo
	F	Empregado Efetivo
	F	Empregado Efetivo
	F	Empregado Efetivo
	F	Empregado Efetivo
	M	Empregado Efetivo
	F	Empregado Efetivo
	F	Empregado Efetivo
	F	Empregado Efetivo
	F	Empregado Efetivo
	F	Empregado Efetivo
	F	Empregado Efetivo
	F	Empregado Efetivo
	M	Empregado Efetivo
	F	Empregado Efetivo
	M	Empregado Efetivo
	F	Empregado Efetivo
	M	Empregado Efetivo
	M	Empregado Efetivo
	F	Empregado Efetivo
	F	Empregado Efetivo
	F	Empregado Efetivo
	F	Empregado Efetivo
	F	Empregado Efetivo
	F	Empregado Efetivo
	M	Empregado Efetivo
	M	Empregado Efetivo
	F	Empregado Efetivo
	F	Empregado Efetivo
	F	Empregado Efetivo
	M	Empregado Efetivo

	F	Empregado Efetivo
	M	Empregado Efetivo
	F	Empregado Efetivo
	F	Empregado Efetivo
	M	Empregado Efetivo
	F	Empregado Efetivo
	F	Empregado Efetivo
	F	Empregado Efetivo
	M	Empregado Efetivo
	F	Empregado Efetivo
	M	Empregado Efetivo
	M	Empregado Efetivo
	F	Empregado Efetivo
	M	Empregado Efetivo
	F	Empregado Efetivo
	F	Empregado Efetivo
	M	Empregado Efetivo
	F	Empregado Efetivo
	F	Empregado Efetivo
	M	Empregado Efetivo
	F	Empregado Efetivo
	M	Empregado Efetivo
	F	Empregado Efetivo
	M	Empregado Efetivo
	M	Empregado Efetivo
	F	Empregado Efetivo
	F	Empregado Efetivo
	M	Empregado Efetivo
	M	Empregado Efetivo
	M	Empregado Efetivo
	F	Empregado Efetivo
	M	Empregado Efetivo
	F	Empregado Efetivo
	F	Empregado Efetivo

	F	Empregado Efetivo
	M	Empregado Efetivo
	F	Empregado Efetivo
	F	Empregado Efetivo
	F	Empregado Efetivo
	F	Empregado Efetivo
	F	Empregado Efetivo
	F	Empregado Efetivo
	F	Empregado Efetivo
	F	Empregado Efetivo
	F	Empregado Efetivo
	F	Empregado Efetivo
	F	Empregado Efetivo
	F	Empregado Efetivo
	F	Empregado Efetivo
	M	Empregado Efetivo
	F	Empregado Efetivo
	F	Empregado Efetivo
	F	Empregado Efetivo
	F	Empregado Efetivo
	F	Empregado Efetivo
	F	Empregado Efetivo
	F	Empregado Efetivo
	M	Empregado Efetivo
	M	Empregado Efetivo
	M	Empregado Efetivo
	F	Empregado Efetivo
	F	Empregado Efetivo
	M	Empregado Efetivo
	F	Empregado Efetivo
	M	Empregado Efetivo
	M	Empregado Efetivo
	F	Empregado Efetivo
	F	Empregado Efetivo
	F	Empregado Efetivo
	F	Empregado Efetivo
	F	Empregado Efetivo
	M	Empregado Efetivo
	M	Empregado Efetivo
	F	Empregado Efetivo
	F	Empregado Efetivo
	M	Empregado Efetivo
	F	Empregado Efetivo
	M	Empregado Efetivo
	F	Empregado Efetivo
	F	Empregado Efetivo
	M	Empregado Efetivo
	M	Empregado Efetivo

	F	Empregado Efetivo
	F	Empregado Efetivo
	M	Empregado Efetivo
	M	Empregado Efetivo
	M	Empregado Efetivo
	M	Empregado Efetivo
	M	Empregado Efetivo
	M	Empregado Efetivo
	M	Empregado Efetivo
	M	Empregado Efetivo
	M	Empregado Efetivo
	M	Empregado Efetivo
	M	Empregado Efetivo
	F	Empregado Efetivo
	F	Empregado Efetivo
	F	Empregado Efetivo
	F	Empregado Efetivo
	F	Empregado Efetivo
	F	Empregado Efetivo
	F	Empregado Efetivo
	F	Empregado Efetivo
	F	Empregado Efetivo
	F	Empregado Efetivo
	M	Empregado Efetivo
	M	Empregado Efetivo
	F	Empregado Efetivo
	F	Empregado Efetivo
	F	Empregado Efetivo
	F	Empregado Efetivo
	F	Empregado Efetivo
	F	Empregado Efetivo
	F	Empregado Efetivo
	F	Empregado Efetivo
	F	Empregado Efetivo
	M	Empregado Efetivo
	F	Empregado Efetivo
	F	Empregado Efetivo
	F	Empregado Efetivo
	F	Empregado Efetivo
	F	Empregado Efetivo
	M	Empregado Efetivo
	F	Empregado Efetivo
	F	Empregado Efetivo
	F	Empregado Efetivo
	F	Empregado Efetivo
	F	Empregado Efetivo
	F	Empregado Efetivo
	F	Empregado Efetivo

	F	Empregado Efetivo
	F	Empregado Efetivo
	F	Empregado Efetivo
	F	Empregado Efetivo
	F	Empregado Efetivo
	F	Empregado Efetivo
	F	Empregado Efetivo
	F	Empregado Efetivo
	F	Empregado Efetivo
	M	Empregado Efetivo
	F	Empregado Efetivo
	F	Empregado Efetivo
	F	Empregado Efetivo
	F	Empregado Efetivo
	F	Empregado Efetivo
	F	Empregado Efetivo
	M	Empregado Efetivo
	M	Empregado Efetivo
	F	Empregado Efetivo
	F	Empregado Efetivo
	M	Empregado Efetivo
	M	Empregado Efetivo
	M	Empregado Efetivo
	M	Empregado Efetivo
	M	Empregado Efetivo
	M	Empregado Efetivo
	M	Empregado Efetivo
	M	Empregado Efetivo
	F	Empregado Efetivo
	F	Empregado Efetivo
	M	Empregado Efetivo
	M	Empregado Efetivo
	M	Empregado Efetivo
	M	Empregado Efetivo
	M	Empregado Efetivo
	F	Empregado Efetivo
	M	Empregado Efetivo
	F	Empregado Efetivo
	F	Empregado Efetivo
	M	Empregado Efetivo
	F	Empregado Efetivo
	F	Empregado Efetivo
	M	Empregado Efetivo
	F	Empregado Efetivo
	F	Empregado Efetivo
	M	Empregado Efetivo
	F	Empregado Efetivo
	F	Empregado Efetivo

	F	Empregado Efetivo
	M	Empregado Efetivo
	F	Empregado Efetivo
	M	Empregado Efetivo
	F	Empregado Efetivo
	F	Empregado Efetivo
	F	Empregado Efetivo
	F	Empregado Efetivo
	F	Empregado Efetivo
	F	Empregado Efetivo
	M	Empregado Efetivo
	F	Empregado Efetivo
	F	Empregado Efetivo
	M	Empregado Efetivo
	F	Empregado Efetivo
	F	Empregado Efetivo
	F	Empregado Efetivo
	F	Empregado Efetivo
	M	Empregado Efetivo
	M	Empregado Efetivo
	M	Empregado Efetivo
	M	Empregado Efetivo
	M	Empregado Efetivo
	F	Empregado Efetivo
	M	Empregado Efetivo
	M	Empregado Efetivo
	F	Empregado Efetivo
	M	Empregado Efetivo
	F	Empregado Efetivo
	F	Empregado Efetivo
	M	Empregado Efetivo
	F	Empregado Efetivo
	F	Empregado Efetivo
	F	Empregado Efetivo
	M	Empregado Efetivo
	F	Empregado Efetivo
	M	Empregado Efetivo
	F	Empregado Efetivo
	F	Empregado Efetivo
	M	Empregado Efetivo
	F	Empregado Efetivo
	F	Empregado Efetivo
	F	Empregado Efetivo
	M	Empregado Efetivo
	F	Empregado Efetivo
	F	Empregado Efetivo
	F	Empregado Efetivo
	F	Empregado Efetivo

	F	Empregado Efetivo
	M	Empregado Efetivo
	F	Empregado Efetivo
	F	Empregado Efetivo
	F	Empregado Efetivo
	F	Empregado Efetivo
	M	Empregado Efetivo
	M	Empregado Efetivo
	F	Empregado Efetivo
	F	Empregado Efetivo
	M	Empregado Efetivo
	F	Empregado Efetivo
	F	Empregado Efetivo
	M	Empregado Efetivo
	F	Empregado Efetivo
	M	Empregado Efetivo
	F	Empregado Efetivo
	M	Empregado Efetivo
	M	Empregado Efetivo
	F	Empregado Efetivo
	F	Empregado Efetivo
	M	Empregado Efetivo
	F	Empregado Efetivo
	M	Empregado Efetivo
	F	Empregado Efetivo
	F	Empregado Efetivo
	F	Empregado Efetivo
	F	Empregado Efetivo
	F	Empregado Efetivo
	F	Empregado Efetivo
	M	Empregado Efetivo
	F	Empregado Efetivo
	F	Empregado Efetivo
	F	Empregado Efetivo
	F	Empregado Efetivo
	F	Empregado Efetivo
	F	Empregado Efetivo
	M	Empregado Efetivo
	M	Empregado Efetivo
	M	Empregado Efetivo
	M	Empregado Efetivo
	F	Empregado Efetivo
	F	Empregado Efetivo
	F	Empregado Efetivo
	F	Empregado Efetivo
	F	Empregado Efetivo
	F	Empregado Efetivo
	M	Empregado Efetivo

	M	Empregado Efetivo
	M	Empregado Efetivo
	M	Empregado Efetivo
	M	Empregado Efetivo
	F	Empregado Efetivo
	M	Empregado Efetivo
	F	Empregado Efetivo
	F	Empregado Efetivo
	M	Empregado Efetivo
	M	Empregado Efetivo
	M	Empregado Efetivo
	F	Empregado Efetivo
	F	Empregado Efetivo
	F	Empregado Efetivo
	F	Empregado Efetivo
	F	Empregado Efetivo
	F	Empregado Efetivo
	F	Empregado Efetivo
	F	Empregado Efetivo
	F	Empregado Efetivo
	F	Empregado Efetivo
	F	Empregado Efetivo
	F	Empregado Efetivo
	M	Empregado Efetivo
	M	Empregado Efetivo
	F	Empregado Efetivo
	M	Empregado Efetivo
	F	Empregado Efetivo
	M	Empregado Efetivo
	F	Empregado Efetivo
	M	Empregado Efetivo
	F	Empregado Efetivo
	M	Empregado Efetivo
	M	Empregado Efetivo
	F	Empregado Efetivo
	F	Empregado Efetivo
	F	Empregado Efetivo
	M	Empregado Efetivo
	F	Empregado Efetivo
	F	Empregado Efetivo
	M	Empregado Efetivo
	F	Empregado Efetivo
	M	Empregado Efetivo
	F	Empregado Efetivo
	F	Empregado Efetivo
	F	Empregado Efetivo
	M	Empregado Efetivo
	F	Empregado Efetivo

	M	Empregado Efetivo
	M	Empregado Efetivo
	F	Empregado Efetivo
	F	Empregado Efetivo
	F	Empregado Efetivo
	F	Empregado Efetivo
	F	Empregado Efetivo
	F	Empregado Efetivo
	F	Empregado Efetivo
	M	Empregado Efetivo
	F	Empregado Efetivo
	F	Empregado Efetivo
	F	Empregado Efetivo
	F	Empregado Efetivo
	F	Empregado Efetivo
	M	Empregado Efetivo
	M	Empregado Efetivo
	F	Empregado Efetivo
	M	Empregado Efetivo
	F	Empregado Efetivo
	M	Empregado Efetivo
	F	Empregado Efetivo
	F	Empregado Efetivo
	F	Empregado Efetivo
	F	Empregado Efetivo
	M	Empregado Efetivo
	F	Empregado Efetivo
	F	Empregado Efetivo
	F	Empregado Efetivo
	M	Empregado Efetivo
	F	Empregado Efetivo
	F	Empregado Efetivo
	M	Empregado Efetivo
	M	Empregado Efetivo
	F	Empregado Efetivo
	F	Empregado Efetivo
	M	Empregado Efetivo
	F	Empregado Efetivo
	F	Empregado Efetivo
	F	Empregado Efetivo
	F	Empregado Efetivo
	F	Empregado Efetivo
	F	Empregado Efetivo
	F	Empregado Efetivo
	M	Empregado Efetivo
	F	Empregado Efetivo
	M	Empregado Efetivo
	F	Empregado Efetivo

	M	Empregado Efetivo
	F	Empregado Efetivo
	F	Empregado Efetivo
	F	Empregado Efetivo
	F	Empregado Efetivo
	F	Empregado Efetivo
	F	Empregado Efetivo
	M	Empregado Efetivo
	F	Empregado Efetivo
	F	Empregado Efetivo
	F	Empregado Efetivo
	M	Empregado Efetivo
	F	Empregado Efetivo
	M	Empregado Efetivo
	M	Empregado Efetivo
	M	Empregado Efetivo
	F	Empregado Efetivo
	F	Empregado Efetivo
	M	Empregado Efetivo
	F	Empregado Efetivo
	F	Empregado Efetivo
	F	Empregado Efetivo
	F	Empregado Efetivo
	M	Empregado Efetivo
	F	Empregado Efetivo
	F	Empregado Efetivo
	F	Empregado Efetivo
	F	Empregado Efetivo
	F	Empregado Efetivo
	F	Empregado Efetivo
	F	Empregado Efetivo
	F	Empregado Efetivo
	F	Empregado Efetivo
	M	Empregado Efetivo
	F	Empregado Efetivo
	F	Empregado Efetivo
	M	Empregado Efetivo
	F	Empregado Efetivo

	F	Empregado Efetivo
	F	Empregado Efetivo
	M	Empregado Efetivo
	F	Empregado Efetivo
	F	Empregado Efetivo
	M	Empregado Efetivo
	M	Empregado Efetivo
	F	Empregado Efetivo
	F	Empregado Efetivo
	M	Empregado Efetivo
	M	Empregado Efetivo
	M	Empregado Efetivo
	F	Empregado Efetivo
	F	Empregado Efetivo
	F	Empregado Efetivo
	F	Empregado Efetivo
	F	Empregado Efetivo
	F	Empregado Efetivo
	M	Empregado Efetivo
	M	Empregado Efetivo
	F	Empregado Efetivo
	F	Empregado Efetivo
	F	Empregado Efetivo
	F	Empregado Efetivo
	F	Empregado Efetivo
	F	Empregado Efetivo
	F	Empregado Efetivo
	F	Empregado Efetivo
	F	Empregado Efetivo
	F	Empregado Efetivo
	F	Empregado Efetivo
	F	Empregado Efetivo
	M	Empregado Efetivo
	M	Empregado Efetivo
	M	Empregado Efetivo
	M	Empregado Efetivo
	M	Empregado Efetivo
	F	Empregado Efetivo
	F	Empregado Efetivo
	F	Empregado Efetivo
	F	Empregado Efetivo
	F	Empregado Efetivo
	F	Empregado Efetivo
	F	Empregado Efetivo
	F	Empregado Efetivo
	M	Empregado Efetivo
	F	Empregado Efetivo
	M	Empregado Efetivo

	M	Empregado Efetivo
	M	Empregado Efetivo
	F	Empregado Efetivo
	F	Empregado Efetivo
	M	Empregado Efetivo
	M	Empregado Efetivo
	F	Empregado Efetivo
	M	Empregado Efetivo
	M	Empregado Efetivo
	M	Empregado Efetivo
	M	Empregado Efetivo
	M	Empregado Efetivo
	M	Empregado Efetivo
	M	Empregado Efetivo
	M	Empregado Efetivo
	M	Empregado Efetivo
	F	Empregado Efetivo
	F	Empregado Efetivo
	F	Empregado Efetivo
	M	Empregado Efetivo
	F	Empregado Efetivo
	F	Empregado Efetivo
	F	Empregado Efetivo
	F	Empregado Efetivo
	F	Empregado Efetivo
	F	Empregado Efetivo
	F	Empregado Efetivo
	F	Empregado Efetivo
	F	Empregado Efetivo
	F	Empregado Efetivo
	F	Empregado Efetivo
	F	Empregado Efetivo
	F	Empregado Efetivo
	F	Empregado Efetivo
	F	Empregado Efetivo
	F	Empregado Efetivo
	F	Empregado Efetivo
	F	Empregado Efetivo
	M	Empregado Efetivo
	M	Empregado Efetivo
	M	Empregado Efetivo
	M	Empregado Efetivo
	F	Empregado Efetivo
	F	Empregado Efetivo
	F	Empregado Efetivo
	F	Empregado Efetivo
	F	Empregado Efetivo
	M	Empregado Efetivo

	F	Empregado Efetivo
	F	Empregado Efetivo
	F	Empregado Efetivo
	M	Empregado Efetivo
	F	Empregado Efetivo
	F	Empregado Efetivo
	F	Empregado Efetivo
	F	Empregado Efetivo
	F	Empregado Efetivo
	F	Empregado Efetivo
	F	Empregado Efetivo
	F	Empregado Efetivo
	F	Empregado Efetivo
	F	Empregado Efetivo
	F	Empregado Efetivo
	F	Empregado Efetivo
	F	Empregado Efetivo
	F	Empregado Efetivo
	F	Empregado Efetivo
	F	Empregado Efetivo
	F	Empregado Efetivo
	M	Empregado Efetivo
	M	Empregado Efetivo
	M	Empregado Efetivo
	M	Empregado Efetivo
	M	Empregado Efetivo
	F	Empregado Efetivo
	F	Empregado Efetivo
	F	Empregado Efetivo
	F	Empregado Efetivo
	F	Empregado Efetivo
	F	Empregado Efetivo
	F	Empregado Efetivo
	F	Empregado Efetivo
	F	Empregado Efetivo
	F	Empregado Efetivo
	F	Empregado Efetivo
	F	Empregado Efetivo
	F	Empregado Efetivo
	F	Empregado Efetivo
	F	Empregado Efetivo
	F	Empregado Efetivo
	F	Empregado Efetivo
	F	Empregado Efetivo
	F	Empregado Efetivo
	F	Empregado Efetivo
	F	Empregado Efetivo
	F	Empregado Efetivo

	F	Empregado Efetivo
	F	Empregado Efetivo
	F	Empregado Efetivo
	F	Empregado Efetivo
	F	Empregado Efetivo
	F	Empregado Efetivo
	F	Empregado Efetivo
	F	Empregado Efetivo
	F	Empregado Efetivo
	F	Empregado Efetivo
	F	Empregado Efetivo
	F	Empregado Efetivo
	F	Empregado Efetivo
	F	Empregado Efetivo
	F	Empregado Efetivo
	F	Empregado Efetivo
	F	Empregado Efetivo
	F	Empregado Efetivo
	F	Empregado Efetivo
	F	Empregado Efetivo
	F	Empregado Efetivo
	F	Empregado Efetivo
	F	Empregado Efetivo
	F	Empregado Efetivo
	F	Empregado Efetivo
	F	Empregado Efetivo
	F	Empregado Efetivo
	F	Empregado Efetivo
	F	Empregado Efetivo
	F	Empregado Efetivo
	F	Empregado Efetivo
	F	Empregado Efetivo
	F	Empregado Efetivo
	F	Empregado Efetivo
	F	Empregado Efetivo
	F	Empregado Efetivo
	F	Empregado Efetivo
	F	Empregado Efetivo
	F	Empregado Efetivo
	F	Empregado Efetivo
	F	Empregado Efetivo
	F	Empregado Efetivo
	F	Empregado Efetivo
	F	Empregado Efetivo
	F	Empregado Efetivo
	F	Empregado Efetivo
	F	Empregado Efetivo
	F	Empregado Efetivo

	F	Empregado Efetivo
	F	Empregado Efetivo
	F	Empregado Efetivo
	F	Empregado Efetivo
	F	Empregado Efetivo
	F	Empregado Efetivo
	F	Empregado Efetivo
	F	Empregado Efetivo
	F	Empregado Efetivo
	F	Empregado Efetivo
	F	Empregado Efetivo
	F	Empregado Efetivo
	F	Empregado Efetivo
	F	Empregado Efetivo
	F	Empregado Efetivo
	F	Empregado Efetivo
	F	Empregado Efetivo
	F	Empregado Efetivo
	F	Empregado Efetivo
	F	Empregado Efetivo
	F	Empregado Efetivo
	F	Empregado Efetivo
	F	Empregado Efetivo
	F	Empregado Efetivo
	F	Empregado Efetivo
	F	Empregado Efetivo
	F	Empregado Efetivo
	F	Empregado Efetivo
	F	Empregado Efetivo
	F	Empregado Efetivo
	F	Empregado Efetivo
	F	Empregado Efetivo
	F	Empregado Efetivo
	F	Empregado Efetivo
	F	Empregado Efetivo
	F	Empregado Efetivo
	F	Empregado Efetivo

	F	Empregado Efetivo
	F	Empregado Efetivo
	F	Empregado Efetivo
	F	Empregado Efetivo
	F	Empregado Efetivo
	F	Empregado Efetivo
	F	Empregado Efetivo
	F	Empregado Efetivo
	F	Empregado Efetivo
	F	Empregado Efetivo
	F	Empregado Efetivo
	F	Empregado Efetivo
	F	Empregado Efetivo
	F	Empregado Efetivo
	F	Empregado Efetivo
	F	Empregado Efetivo
	F	Empregado Efetivo
	F	Empregado Efetivo
	F	Empregado Efetivo
	F	Empregado Efetivo
	F	Empregado Efetivo
	F	Empregado Efetivo
	F	Empregado Efetivo
	F	Empregado Efetivo
	F	Empregado Efetivo
	F	Empregado Efetivo
	F	Empregado Efetivo
	F	Empregado Efetivo
	F	Empregado Efetivo
	F	Empregado Efetivo
	F	Empregado Efetivo
	F	Empregado Efetivo
	F	Empregado Efetivo
	F	Empregado Efetivo
	F	Empregado Efetivo
	F	Empregado Efetivo
	F	Empregado Efetivo
	F	Empregado Efetivo
	F	Empregado Efetivo
	F	Empregado Efetivo
	F	Empregado Efetivo
	F	Empregado Efetivo
	F	Empregado Efetivo
	F	Empregado Efetivo
	F	Empregado Efetivo
	F	Empregado Efetivo
	F	Empregado Efetivo

	F	Empregado Efetivo
	F	Empregado Efetivo
	F	Empregado Efetivo
	F	Empregado Efetivo
	F	Empregado Efetivo
	F	Empregado Efetivo
	F	Empregado Efetivo
	F	Empregado Efetivo
	F	Empregado Efetivo
	F	Empregado Efetivo
	F	Empregado Efetivo
	F	Empregado Efetivo
	F	Empregado Efetivo
	F	Empregado Efetivo
	F	Empregado Efetivo
	F	Empregado Efetivo
	F	Empregado Efetivo
	F	Empregado Efetivo
	F	Empregado Efetivo
	F	Empregado Efetivo
	F	Empregado Efetivo
	F	Empregado Efetivo
	F	Empregado Efetivo
	F	Empregado Efetivo
	F	Empregado Efetivo
	F	Empregado Efetivo
	F	Empregado Efetivo
	F	Empregado Efetivo
	F	Empregado Efetivo
	F	Empregado Efetivo
	F	Empregado Efetivo
	F	Empregado Efetivo
	F	Empregado Efetivo
	F	Empregado Efetivo
	F	Empregado Efetivo
	F	Empregado Efetivo
	F	Empregado Efetivo
	F	Empregado Efetivo
	F	Empregado Efetivo
	F	Empregado Efetivo
	F	Empregado Efetivo
	F	Empregado Efetivo
	F	Empregado Efetivo
	F	Empregado Efetivo
	F	Empregado Efetivo
	F	Empregado Efetivo
	F	Empregado Efetivo
	F	Empregado Efetivo

	F	Empregado Efetivo
	F	Empregado Efetivo
	F	Empregado Efetivo
	F	Empregado Efetivo
	F	Empregado Efetivo
	F	Empregado Efetivo
	F	Empregado Efetivo
	F	Empregado Efetivo
	F	Empregado Efetivo
	F	Empregado Efetivo
	F	Empregado Efetivo
	F	Empregado Efetivo
	F	Empregado Efetivo
	F	Empregado Efetivo
	F	Empregado Efetivo
	F	Empregado Efetivo
	F	Empregado Efetivo
	F	Empregado Efetivo
	F	Empregado Efetivo
	F	Empregado Efetivo
	F	Empregado Efetivo
	F	Empregado Efetivo
	F	Empregado Efetivo
	F	Empregado Efetivo
	F	Empregado Efetivo
	F	Empregado Efetivo
	F	Empregado Efetivo
	F	Empregado Efetivo
	F	Empregado Efetivo
	F	Empregado Efetivo
	F	Empregado Efetivo
	F	Empregado Efetivo
	F	Empregado Efetivo
	F	Empregado Efetivo
	F	Empregado Efetivo
	F	Empregado Efetivo
	F	Empregado Efetivo
	F	Empregado Efetivo
	F	Empregado Efetivo
	F	Empregado Efetivo
	F	Empregado Efetivo
	F	Empregado Efetivo
	F	Empregado Efetivo
	F	Empregado Efetivo
	F	Empregado Efetivo
	F	Empregado Efetivo
	F	Empregado Efetivo
	F	Empregado Efetivo

	F	Empregado Efetivo
	F	Empregado Efetivo
	F	Empregado Efetivo
	F	Empregado Efetivo
	F	Empregado Efetivo
	F	Empregado Efetivo
	F	Empregado Efetivo
	F	Empregado Efetivo
	F	Empregado Efetivo
	F	Empregado Efetivo
	F	Empregado Efetivo
	F	Empregado Efetivo
	F	Empregado Efetivo
	F	Empregado Efetivo
	F	Empregado Efetivo
	F	Empregado Efetivo
	F	Empregado Efetivo
	F	Empregado Efetivo
	F	Empregado Efetivo
	F	Empregado Efetivo
	F	Empregado Efetivo
	F	Empregado Efetivo
	F	Empregado Efetivo
	F	Empregado Efetivo
	F	Empregado Efetivo
	F	Empregado Efetivo
	F	Empregado Efetivo
	F	Empregado Efetivo
	F	Empregado Efetivo
	F	Empregado Efetivo
	F	Empregado Efetivo
	F	Empregado Efetivo
	F	Empregado Efetivo
	F	Empregado Efetivo
	F	Empregado Efetivo
	F	Empregado Efetivo
	F	Empregado Efetivo
	F	Empregado Efetivo
	M	Empregado Efetivo
	M	Empregado Efetivo
	M	Empregado Efetivo
	M	Empregado Efetivo
	M	Empregado Efetivo
	M	Empregado Efetivo

	M	Empregado Efetivo
	M	Empregado Efetivo
	F	Empregado Efetivo
	F	Empregado Efetivo
	F	Empregado Efetivo
	F	Empregado Efetivo
	F	Empregado Efetivo
	F	Empregado Efetivo
	M	Empregado Efetivo
	F	Empregado Efetivo
	M	Empregado Efetivo
	F	Empregado Efetivo
	F	Empregado Efetivo
	F	Empregado Efetivo
	M	Empregado Efetivo
	M	Empregado Efetivo
	M	Empregado Efetivo
	M	Empregado Efetivo
	M	Empregado Efetivo
	M	Empregado Efetivo
	M	Empregado Efetivo
	F	Empregado Efetivo
	M	Empregado Efetivo
	F	Empregado Efetivo
	F	Empregado Efetivo
	F	Empregado Efetivo
	F	Empregado Efetivo
	F	Empregado Efetivo
	F	Empregado Efetivo
	F	Empregado Efetivo
	F	Empregado Efetivo
	F	Empregado Efetivo
	F	Empregado Efetivo
	F	Empregado Efetivo
	F	Empregado Efetivo

	F	Empregado Efetivo
	F	Empregado Efetivo
	F	Empregado Efetivo
	F	Empregado Efetivo
	F	Empregado Efetivo
	F	Empregado Efetivo
	F	Empregado Efetivo
	F	Empregado Efetivo
	M	Empregado Efetivo
	F	Empregado Efetivo
	F	Empregado Efetivo
	F	Empregado Efetivo
	F	Empregado Efetivo
	F	Empregado Efetivo
	F	Empregado Efetivo
	F	Empregado Efetivo
	F	Empregado Efetivo
	F	Empregado Efetivo
	F	Empregado Efetivo
	F	Empregado Efetivo
	F	Empregado Efetivo
	F	Empregado Efetivo
	M	Empregado Efetivo
	F	Empregado Efetivo
	F	Empregado Efetivo
	M	Empregado Efetivo
	F	Empregado Efetivo
	M	Empregado Efetivo
	F	Empregado Efetivo
	F	Empregado Efetivo
	F	Empregado Efetivo
	F	Empregado Efetivo
	F	Empregado Efetivo
	F	Empregado Efetivo
	F	Empregado Efetivo
	F	Empregado Efetivo
	F	Empregado Efetivo
	F	Empregado Efetivo
	F	Empregado Efetivo
	F	Empregado Efetivo
	F	Empregado Efetivo
	F	Empregado Efetivo
	F	Empregado Efetivo
	F	Empregado Efetivo

	F	Empregado Efetivo
	F	Empregado Efetivo
	F	Empregado Efetivo
	M	Empregado Efetivo
	M	Empregado Efetivo
	F	Empregado Efetivo
	M	Empregado Efetivo
	F	Empregado Efetivo
	F	Empregado Efetivo
	F	Empregado Efetivo
	F	Empregado Efetivo
	F	Empregado Efetivo
	F	Empregado Efetivo
	F	Empregado Efetivo
	F	Empregado Efetivo
	F	Empregado Efetivo
	F	Empregado Efetivo
	F	Empregado Efetivo
	F	Empregado Efetivo
	F	Empregado Efetivo
	F	Empregado Efetivo
	F	Empregado Efetivo
	F	Empregado Efetivo
	F	Empregado Efetivo
	F	Empregado Efetivo
	F	Empregado Efetivo
	F	Empregado Efetivo
	F	Empregado Efetivo
	F	Empregado Efetivo
	F	Empregado Efetivo
	F	Empregado Efetivo
	F	Empregado Efetivo
	F	Empregado Efetivo
	F	Empregado Efetivo
	F	Empregado Efetivo
	F	Empregado Efetivo
	F	Empregado Efetivo
	F	Empregado Efetivo

	F	Empregado Efetivo
	F	Empregado Efetivo
	F	Empregado Efetivo
	F	Empregado Efetivo
	F	Empregado Efetivo
	F	Empregado Efetivo
	F	Empregado Efetivo
	F	Empregado Efetivo
	F	Empregado Efetivo
	F	Empregado Efetivo
	F	Empregado Efetivo
	F	Empregado Efetivo
	F	Empregado Efetivo
	F	Empregado Efetivo
	F	Empregado Efetivo
	M	Cedido - RJU
	F	Empregado Efetivo
	F	Empregado Efetivo
	F	Empregado Efetivo
	F	Empregado Efetivo
	F	Empregado Efetivo
	F	Empregado Efetivo
	F	Empregado Efetivo
	F	Empregado Efetivo
	F	Empregado Efetivo
	F	Empregado Efetivo
	F	Empregado Efetivo
	F	Empregado Efetivo
	F	Empregado Efetivo
	F	Empregado Efetivo
	F	Empregado Efetivo
	F	Empregado Efetivo
	F	Empregado Efetivo
	F	Empregado Efetivo
	F	Empregado Efetivo
	F	Empregado Efetivo
	F	Empregado Efetivo
	F	Empregado Efetivo
	F	Empregado Efetivo

	F	Empregado Efetivo
	F	Empregado Efetivo
	F	Empregado Efetivo
	F	Empregado Efetivo
	F	Empregado Efetivo
	F	Empregado Efetivo
	F	Empregado Efetivo
	F	Empregado Efetivo
	F	Empregado Efetivo
	F	Empregado Efetivo
	F	Empregado Efetivo
	F	Empregado Efetivo
	F	Empregado Efetivo
	F	Empregado Efetivo
	F	Empregado Efetivo
	F	Empregado Efetivo
	F	Empregado Efetivo
	F	Empregado Efetivo
	F	Empregado Efetivo
	F	Empregado Efetivo
	F	Empregado Efetivo
	F	Empregado Efetivo
	F	Empregado Efetivo
	F	Empregado Efetivo
	F	Empregado Efetivo
	F	Empregado Efetivo
	F	Empregado Efetivo
	F	Empregado Efetivo
	F	Empregado Efetivo
	F	Empregado Efetivo
	F	Empregado Efetivo
	F	Empregado Efetivo
	F	Empregado Efetivo
	F	Empregado Efetivo
	F	Empregado Efetivo
	F	Empregado Efetivo
	F	Empregado Efetivo
	F	Empregado Efetivo
	F	Empregado Efetivo
	F	Empregado Efetivo
	F	Empregado Efetivo
	F	Empregado Efetivo
	F	Empregado Efetivo

	F	Empregado Efetivo
	F	Empregado Efetivo
	F	Empregado Efetivo
	F	Empregado Efetivo
	F	Empregado Efetivo
	M	Empregado Efetivo
	M	Empregado Efetivo
	M	Empregado Efetivo
	M	Empregado Efetivo
	M	Empregado Efetivo
	F	Empregado Efetivo
	M	Empregado Efetivo
	F	Empregado Efetivo
	M	Empregado Efetivo
	M	Empregado Efetivo
	M	Empregado Efetivo
	F	Empregado Efetivo
	M	Empregado Efetivo
	F	Empregado Efetivo
	F	Empregado Efetivo
	F	Empregado Efetivo
	F	Empregado Efetivo
	F	Empregado Efetivo
	M	Empregado Efetivo
	M	Empregado Efetivo
	F	Empregado Efetivo
	M	Empregado Efetivo
	F	Empregado Efetivo
	F	Empregado Efetivo
	F	Empregado Efetivo
	F	Empregado Efetivo
	F	Empregado Efetivo

	M	Empregado Efetivo
	F	Empregado Efetivo
	M	Empregado Efetivo
	M	Empregado Efetivo
	M	Empregado Efetivo
	M	Empregado Efetivo
	F	Empregado Efetivo
	M	Empregado Efetivo
	M	Empregado Efetivo
	M	Empregado Efetivo
	M	Empregado Efetivo
	F	Empregado Efetivo
	F	Empregado Efetivo
	F	Empregado Efetivo
	F	Empregado Efetivo
	F	Empregado Efetivo
	F	Empregado Efetivo
	F	Empregado Efetivo
	F	Empregado Efetivo
	F	Empregado Efetivo
	F	Empregado Efetivo
	F	Empregado Efetivo
	F	Empregado Efetivo
	F	Empregado Efetivo
	F	Empregado Efetivo
	F	Empregado Efetivo
	F	Empregado Efetivo
	F	Empregado Efetivo
	F	Empregado Efetivo
	F	Empregado Efetivo
	F	Empregado Efetivo
	F	Empregado Efetivo
	F	Empregado Efetivo
	F	Empregado Efetivo
	F	Empregado Efetivo
	F	Empregado Efetivo
	F	Empregado Efetivo
	F	Empregado Efetivo
	F	Empregado Efetivo
	F	Empregado Efetivo
	F	Empregado Efetivo
	F	Empregado Efetivo

	F	Empregado Efetivo
	F	Empregado Efetivo
	F	Empregado Efetivo
	F	Empregado Efetivo
	F	Empregado Efetivo
	F	Empregado Efetivo
	F	Empregado Efetivo
	F	Empregado Efetivo
	F	Empregado Efetivo
	F	Empregado Efetivo
	F	Empregado Efetivo
	F	Empregado Efetivo
	F	Empregado Efetivo
	F	Empregado Efetivo
	F	Empregado Efetivo
	F	Empregado Efetivo
	F	Empregado Efetivo
	F	Empregado Efetivo
	F	Empregado Efetivo
	F	Empregado Efetivo
	F	Empregado Efetivo
	F	Empregado Efetivo
	M	Empregado Efetivo
	F	Empregado Efetivo
	M	Empregado Efetivo
	F	Empregado Efetivo
	F	Empregado Efetivo
	F	Empregado Efetivo
	F	Empregado Efetivo
	F	Empregado Efetivo
	F	Empregado Efetivo
	F	Empregado Efetivo
	F	Empregado Efetivo
	F	Empregado Efetivo
	F	Empregado Efetivo
	F	Empregado Efetivo
	F	Cedido - RJU
	F	Cedido - RJU
	F	Cedido - RJU
	F	Empregado Efetivo

	F	Empregado Efetivo
	F	Empregado Efetivo
	F	Empregado Efetivo
	F	Empregado Efetivo
	F	Empregado Efetivo
	F	Empregado Efetivo
	F	Empregado Efetivo
	F	Empregado Efetivo
	F	Empregado Efetivo
	M	Empregado Efetivo
	M	Empregado Efetivo
	M	Empregado Efetivo
	F	Empregado Efetivo
	F	Empregado Efetivo
	F	Empregado Efetivo
	F	Empregado Efetivo
	F	Empregado Efetivo
	F	Empregado Efetivo
	F	Empregado Efetivo
	F	Empregado Efetivo
	F	Empregado Efetivo
	F	Empregado Efetivo
	F	Empregado Efetivo
	F	Empregado Efetivo
	F	Empregado Efetivo
	F	Empregado Efetivo
	M	Empregado Efetivo
	F	Empregado Efetivo
	F	Empregado Efetivo
	F	Empregado Efetivo
	F	Empregado Efetivo
	M	Empregado Efetivo

	M	Empregado Efetivo
	M	Empregado Efetivo
	F	Empregado Efetivo
	F	Cedido - RJU
	F	Empregado Efetivo
	F	Empregado Efetivo
	F	Empregado Efetivo
	M	Empregado Efetivo
	M	Empregado Efetivo
	F	Empregado Efetivo
	F	Empregado Efetivo
	F	Empregado Efetivo
	F	Empregado Efetivo
	F	Empregado Efetivo
	F	Empregado Efetivo
	F	Empregado Efetivo
	F	Empregado Efetivo
	F	Empregado Efetivo
	F	Empregado Efetivo
	F	Empregado Efetivo
	F	Empregado Efetivo
	F	Empregado Efetivo
	F	Empregado Efetivo
	F	Empregado Efetivo
	F	Empregado Efetivo
	F	Cedido - RJU
	F	Empregado Efetivo
	F	Empregado Efetivo
	F	Empregado Efetivo
	F	Empregado Efetivo
	F	Empregado Efetivo
	F	Empregado Efetivo

	F	Empregado Efetivo
	F	Empregado Efetivo
	F	Empregado Efetivo
	F	Empregado Efetivo
	F	Empregado Efetivo
	F	Empregado Efetivo
	F	Empregado Efetivo
	F	Empregado Efetivo
	F	Empregado Efetivo
	F	Empregado Efetivo
	F	Empregado Efetivo
	F	Empregado Efetivo
	F	Empregado Efetivo
	M	Empregado Efetivo
	F	Empregado Efetivo
	F	Empregado Efetivo
	F	Empregado Efetivo
	F	Empregado Efetivo
	F	Empregado Efetivo
	F	Empregado Efetivo
	F	Empregado Efetivo
	F	Empregado Efetivo
	F	Empregado Efetivo
	M	Empregado Efetivo
	M	Empregado Efetivo
	F	Empregado Efetivo
	F	Empregado Efetivo
	F	Empregado Efetivo
	M	Empregado Efetivo
	F	Empregado Efetivo
	F	Empregado Efetivo
	F	Empregado Efetivo
	F	Empregado Efetivo
	F	Empregado Efetivo
	M	Empregado Efetivo
	M	Empregado Efetivo
	F	Empregado Efetivo
	F	Empregado Efetivo
	F	Empregado Efetivo
	F	Empregado Efetivo

	M	Empregado Efetivo
	F	Empregado Efetivo
	F	Empregado Efetivo
	F	Empregado Efetivo
	F	Empregado Efetivo
	M	Empregado Efetivo
	F	Empregado Efetivo
	M	Empregado Efetivo
	F	Empregado Efetivo
	F	Empregado Efetivo
	M	Empregado Efetivo
	F	Empregado Efetivo
	F	Empregado Efetivo
	F	Empregado Efetivo
	F	Empregado Efetivo
	M	Empregado Efetivo
	F	Empregado Efetivo
	M	Empregado Efetivo
	F	Empregado Efetivo
	F	Empregado Efetivo
	F	Empregado Efetivo
	F	Empregado Efetivo
	F	Empregado Efetivo
	F	Empregado Efetivo
	F	Empregado Efetivo
	F	Empregado Efetivo
	M	Empregado Efetivo
	F	Empregado Efetivo
	M	Empregado Efetivo
	M	Empregado Efetivo
	F	Empregado Efetivo
	M	Empregado Efetivo
	F	Empregado Efetivo
	F	Empregado Efetivo
	F	Empregado Efetivo
	F	Empregado Efetivo
	F	Empregado Efetivo
	F	Empregado Efetivo
	M	Empregado Efetivo
	M	Empregado Efetivo
	M	Empregado Efetivo
	F	Empregado Efetivo
	F	Empregado Efetivo
	F	Empregado Efetivo
	F	Empregado Efetivo
	F	Empregado Efetivo

	F	Empregado Efetivo
	F	Empregado Efetivo
	F	Empregado Efetivo
	F	Empregado Efetivo
	F	Empregado Efetivo
	M	Empregado Efetivo
	F	Empregado Efetivo
	F	Empregado Efetivo
	F	Empregado Efetivo
	F	Empregado Efetivo
	F	Empregado Efetivo
	F	Empregado Efetivo
	F	Empregado Efetivo
	M	Empregado Efetivo
	M	Empregado Efetivo
	M	Empregado Efetivo
	M	Empregado Efetivo
	F	Empregado Efetivo
	F	Empregado Efetivo
	M	Empregado Efetivo
	M	Empregado Efetivo
	F	Empregado Efetivo
	F	Empregado Efetivo
	M	Empregado Efetivo
	F	Empregado Efetivo
	M	Empregado Efetivo
	M	Empregado Efetivo
	F	Empregado Efetivo
	F	Empregado Efetivo
	M	Empregado Efetivo
	F	Empregado Efetivo
	F	Empregado Efetivo
	M	Empregado Efetivo
	F	Empregado Efetivo
	M	Empregado Efetivo
	M	Empregado Efetivo
	F	Empregado Efetivo
	M	Empregado Efetivo
	F	Empregado Efetivo
	F	Empregado Efetivo
	M	Empregado Efetivo
	F	Empregado Efetivo
	F	Empregado Efetivo
	F	Empregado Efetivo
	M	Empregado Efetivo
	M	Empregado Efetivo
	M	Empregado Efetivo
	F	Empregado Efetivo

	F	Empregado Efetivo
	F	Empregado Efetivo
	M	Empregado Efetivo
	F	Empregado Efetivo
	M	Empregado Efetivo
	F	Empregado Efetivo
	F	Empregado Efetivo
	F	Empregado Efetivo
	F	Empregado Efetivo
	M	Empregado Efetivo
	M	Empregado Efetivo
	F	Empregado Efetivo
	F	Empregado Efetivo
	M	Empregado Efetivo
	M	Empregado Efetivo
	M	Empregado Efetivo
	M	Empregado Efetivo
	M	Empregado Efetivo
	M	Empregado Efetivo
	F	Empregado Efetivo
	F	Empregado Efetivo
	M	Empregado Efetivo
	M	Empregado Efetivo
	M	Empregado Efetivo
	M	Empregado Efetivo
	M	Empregado Efetivo
	M	Empregado Efetivo
	M	Empregado Efetivo
	F	Empregado Efetivo
	F	Empregado Efetivo
	F	Empregado Efetivo
	F	Empregado Efetivo
	F	Empregado Efetivo
	F	Empregado Efetivo
	F	Empregado Efetivo
	F	Empregado Efetivo
	F	Empregado Efetivo
	F	Empregado Efetivo
	F	Empregado Efetivo
	F	Empregado Efetivo
	F	Empregado Efetivo
	F	Empregado Efetivo
	F	Empregado Efetivo

	F	Empregado Efetivo
	F	Empregado Efetivo
	M	Empregado Efetivo
	F	Empregado Efetivo
	F	Empregado Efetivo
	M	Empregado Efetivo
	F	Empregado Efetivo
	F	Empregado Efetivo
	F	Empregado Efetivo
	F	Empregado Efetivo
	F	Empregado Efetivo
	F	Empregado Efetivo
	F	Empregado Efetivo
	F	Empregado Efetivo
	F	Empregado Efetivo
	F	Empregado Efetivo
	F	Empregado Efetivo
	F	Empregado Efetivo
	F	Empregado Efetivo
	F	Empregado Efetivo
	F	Empregado Efetivo
	F	Empregado Efetivo
	F	Empregado Efetivo
	F	Empregado Efetivo
	F	Empregado Efetivo
	F	Empregado Efetivo
	F	Empregado Efetivo
	F	Empregado Efetivo
	F	Empregado Efetivo
	F	Empregado Efetivo
	F	Empregado Efetivo
	F	Empregado Efetivo
	F	Empregado Efetivo
	F	Empregado Efetivo
	F	Empregado Efetivo
	F	Empregado Efetivo
	F	Empregado Efetivo
	F	Empregado Efetivo
	F	Empregado Efetivo
	F	Empregado Efetivo
	F	Empregado Efetivo
	F	Empregado Efetivo
	F	Empregado Efetivo
	F	Empregado Efetivo
	F	Empregado Efetivo
	F	Empregado Efetivo
	F	Empregado Efetivo
	F	Empregado Efetivo

	F	Empregado Efetivo
	F	Empregado Efetivo
	F	Empregado Efetivo
	F	Empregado Efetivo
	F	Empregado Efetivo
	F	Empregado Efetivo
	F	Empregado Efetivo
	F	Empregado Efetivo
	F	Empregado Efetivo
	F	Empregado Efetivo
	F	Empregado Efetivo
	F	Empregado Efetivo
	F	Empregado Efetivo
	F	Empregado Efetivo
	F	Empregado Efetivo
	F	Empregado Efetivo
	F	Empregado Efetivo
	F	Empregado Efetivo
	F	Empregado Efetivo
	F	Empregado Efetivo
	F	Empregado Efetivo
	F	Empregado Efetivo
	F	Empregado Efetivo
	F	Empregado Efetivo
	F	Empregado Efetivo
	F	Empregado Efetivo
	F	Empregado Efetivo
	F	Empregado Efetivo
	F	Empregado Efetivo
	F	Empregado Efetivo
	F	Empregado Efetivo
	F	Empregado Efetivo
	F	Empregado Efetivo
	F	Empregado Efetivo
	F	Empregado Efetivo
	F	Empregado Efetivo
	M	Empregado Efetivo
	M	Empregado Efetivo
	F	Empregado Efetivo
	F	Empregado Efetivo
	F	Empregado Efetivo
	M	Empregado Efetivo
	F	Empregado Efetivo
	F	Empregado Efetivo
	F	Empregado Efetivo
	M	Empregado Efetivo
	M	Empregado Efetivo
	F	Empregado Efetivo

	F	Empregado Efetivo
	M	Empregado Efetivo
	M	Empregado Efetivo
	F	Empregado Efetivo
	F	Empregado Efetivo
	F	Empregado Efetivo
	F	Empregado Efetivo
	M	Empregado Efetivo
	M	Empregado Efetivo
	F	Empregado Efetivo
	F	Empregado Efetivo
	F	Empregado Efetivo
	F	Empregado Efetivo
	F	Empregado Efetivo
	F	Empregado Efetivo
	F	Empregado Efetivo
	F	Empregado Efetivo
	F	Empregado Efetivo
	F	Empregado Efetivo
	F	Empregado Efetivo
	F	Empregado Efetivo
	F	Empregado Efetivo
	M	Empregado Efetivo
	M	Empregado Efetivo
	F	Empregado Efetivo
	F	Empregado Efetivo
	F	Empregado Efetivo
	M	Empregado Efetivo
	F	Empregado Efetivo
	F	Empregado Efetivo
	F	Empregado Efetivo
	F	Empregado Efetivo
	M	Empregado Efetivo
	F	Empregado Efetivo
	F	Empregado Efetivo

	F	Empregado Efetivo
	F	Empregado Efetivo
	F	Empregado Efetivo
	F	Empregado Efetivo
	F	Empregado Efetivo
	M	Empregado Efetivo
	F	Empregado Efetivo
	F	Empregado Efetivo
	M	Empregado Efetivo
	M	Empregado Efetivo
	M	Empregado Efetivo
	M	Empregado Efetivo
	M	Empregado Efetivo
	F	Empregado Efetivo
	F	Empregado Efetivo
	F	Empregado Efetivo
	F	Empregado Efetivo
	F	Empregado Efetivo
	F	Empregado Efetivo
	F	Empregado Efetivo
	F	Empregado Efetivo
	F	Empregado Efetivo
	F	Empregado Efetivo
	F	Empregado Efetivo
	F	Empregado Efetivo
	M	Empregado Efetivo
	F	Empregado Efetivo
	F	Empregado Efetivo
	F	Empregado Efetivo
	F	Empregado Efetivo
	F	Empregado Efetivo
	F	Empregado Efetivo
	F	Empregado Efetivo
	F	Empregado Efetivo
	F	Empregado Efetivo
	F	Empregado Efetivo
	F	Empregado Efetivo
	F	Empregado Efetivo
	F	Empregado Efetivo

	F	Empregado Efetivo
	F	Empregado Efetivo
	M	Empregado Efetivo
	F	Empregado Efetivo
	F	Empregado Efetivo
	F	Empregado Efetivo
	F	Empregado Efetivo
	F	Empregado Efetivo
	F	Empregado Efetivo
	F	Empregado Efetivo
	F	Empregado Efetivo
	F	Empregado Efetivo
	F	Empregado Efetivo
	F	Empregado Efetivo
	M	Empregado Efetivo
	M	Empregado Efetivo
	M	Empregado Efetivo
	F	Empregado Efetivo
	F	Empregado Efetivo
	F	Empregado Efetivo
	M	Empregado Efetivo
	M	Empregado Efetivo
	M	Empregado Efetivo
	M	Empregado Efetivo
	M	Empregado Efetivo
	F	Empregado Efetivo
	F	Empregado Efetivo
	F	Empregado Efetivo
	F	Empregado Efetivo
	F	Empregado Efetivo
	F	Empregado Efetivo
	F	Empregado Efetivo
	F	Empregado Efetivo
	F	Empregado Efetivo
	M	Empregado Efetivo
	F	Empregado Efetivo
	M	Empregado Efetivo
	M	Empregado Efetivo
	F	Empregado Efetivo
	M	Empregado Efetivo
	M	Empregado Efetivo
	F	Empregado Efetivo
	F	Empregado Efetivo
	F	Empregado Efetivo
	F	Empregado Efetivo
	F	Empregado Efetivo
	F	Empregado Efetivo
	F	Empregado Efetivo

	F	Empregado Efetivo
	F	Empregado Efetivo
	F	Empregado Efetivo
	F	Empregado Efetivo
	F	Empregado Efetivo
	M	Empregado Efetivo
	M	Empregado Efetivo
	F	Empregado Efetivo
	M	Empregado Efetivo
	F	Empregado Efetivo
	M	Empregado Efetivo
	F	Empregado Efetivo
	F	Empregado Efetivo
	M	Empregado Efetivo
	F	Empregado Efetivo
	M	Empregado Efetivo
	M	Empregado Efetivo
	M	Empregado Efetivo
	F	Empregado Efetivo
	M	Empregado Efetivo
	M	Empregado Efetivo
	F	Empregado Efetivo
	F	Empregado Efetivo
	F	Empregado Efetivo
	F	Empregado Efetivo
	F	Empregado Efetivo
	F	Empregado Efetivo
	F	Empregado Efetivo
	F	Empregado Efetivo
	F	Empregado Efetivo
	F	Empregado Efetivo
	F	Empregado Efetivo
	F	Empregado Efetivo
	F	Empregado Efetivo
	F	Empregado Efetivo
	F	Empregado Efetivo
	F	Empregado Efetivo
	F	Empregado Efetivo
	F	Empregado Efetivo
	F	Empregado Efetivo
	F	Empregado Efetivo
	F	Empregado Efetivo
	F	Empregado Efetivo
	F	Empregado Efetivo
	F	Empregado Efetivo
	F	Empregado Efetivo
	F	Empregado Efetivo
	F	Empregado Efetivo

	F	Empregado Efetivo
	F	Empregado Efetivo
	M	Empregado Efetivo
	M	Empregado Efetivo
	M	Empregado Efetivo
	M	Empregado Efetivo
	M	Empregado Efetivo
	M	Empregado Efetivo
	F	Empregado Efetivo
	F	Empregado Efetivo
	F	Empregado Efetivo
	F	Empregado Efetivo
	F	Empregado Efetivo
	F	Empregado Efetivo
	F	Empregado Efetivo
	F	Empregado Efetivo
	F	Empregado Efetivo
	F	Empregado Efetivo
	F	Empregado Efetivo
	F	Empregado Efetivo
	F	Empregado Efetivo
	F	Empregado Efetivo
	F	Empregado Efetivo
	F	Empregado Efetivo
	M	Empregado Efetivo
	M	Empregado Efetivo
	M	Empregado Efetivo
	M	Empregado Efetivo
	F	Empregado Efetivo
	F	Empregado Efetivo
	F	Empregado Efetivo
	F	Empregado Efetivo
	F	Empregado Efetivo
	F	Empregado Efetivo
	F	Empregado Efetivo
	F	Empregado Efetivo
	F	Empregado Efetivo
	F	Empregado Efetivo
	F	Empregado Efetivo
	F	Empregado Efetivo
	F	Empregado Efetivo
	F	Empregado Efetivo
	F	Empregado Efetivo
	F	Empregado Efetivo
	F	Empregado Efetivo
	M	Empregado Efetivo

	M	Empregado Efetivo
	M	Empregado Efetivo
	M	Empregado Efetivo
	F	Empregado Efetivo
	F	Empregado Efetivo
	F	Empregado Efetivo
	F	Empregado Efetivo
	F	Empregado Efetivo
	F	Empregado Efetivo
	F	Empregado Efetivo
	F	Empregado Efetivo
	M	Empregado Efetivo
	M	Empregado Efetivo
	M	Empregado Efetivo
	M	Empregado Efetivo
	M	Empregado Efetivo
	M	Empregado Efetivo
	F	Empregado Efetivo
	F	Empregado Efetivo
	F	Empregado Efetivo
	F	Empregado Efetivo
	F	Empregado Efetivo
	F	Empregado Efetivo
	F	Empregado Efetivo
	F	Empregado Efetivo
	F	Empregado Efetivo
	F	Empregado Efetivo
	F	Empregado Efetivo
	F	Empregado Efetivo
	F	Empregado Efetivo
	F	Empregado Efetivo
	F	Empregado Efetivo
	F	Empregado Efetivo
	M	Empregado Efetivo
	F	Empregado Efetivo
	M	Empregado Efetivo
	F	Empregado Efetivo
	M	Empregado Efetivo
	M	Empregado Efetivo
	M	Empregado Efetivo
	M	Empregado Efetivo
	M	Empregado Efetivo
	F	Empregado Efetivo
	M	Empregado Efetivo
	F	Empregado Efetivo
	F	Empregado Efetivo
	F	Empregado Efetivo
	M	Empregado Efetivo

	F	Empregado Efetivo
	F	Empregado Efetivo
	F	Empregado Efetivo
	F	Comissionado
	F	Empregado Efetivo
	F	Empregado Efetivo
	M	Cedido - RJU
	F	Empregado Efetivo
	F	Empregado Efetivo
	F	Empregado Efetivo
	F	Empregado Efetivo
	F	Empregado Efetivo
	F	Empregado Efetivo
	F	Empregado Efetivo
	F	Empregado Efetivo
	M	Empregado Efetivo
	F	Empregado Efetivo
	F	Empregado Efetivo
	F	Empregado Efetivo
	M	Empregado Efetivo
	M	Empregado Efetivo
	F	Empregado Efetivo
	F	Empregado Efetivo
	F	Empregado Efetivo
	F	Empregado Efetivo
	M	Empregado Efetivo
	F	Empregado Efetivo
	F	Empregado Efetivo
	F	Empregado Efetivo
	M	Empregado Efetivo
	F	Empregado Efetivo
	F	Empregado Efetivo
	M	Empregado Efetivo
	M	Empregado Efetivo
	M	Empregado Efetivo
	F	Empregado Efetivo
	F	Empregado Efetivo
	M	Empregado Efetivo
	F	Empregado Efetivo
	M	Empregado Efetivo
	F	Empregado Efetivo
	F	Empregado Efetivo
	M	Empregado Efetivo
	F	Empregado Efetivo
	F	Empregado Efetivo
	M	Empregado Efetivo
	F	Empregado Efetivo

	F	Empregado Efetivo
	M	Empregado Efetivo
	M	Empregado Efetivo
	F	Empregado Efetivo
	F	Empregado Efetivo
	M	Empregado Movimentado - Po/MP n 193/2018
	F	Empregado Efetivo
	F	Empregado Efetivo
	F	Empregado Efetivo
	F	Empregado Efetivo
	F	Empregado Efetivo
	F	Empregado Efetivo
	F	Empregado Efetivo
	F	Empregado Efetivo
	F	Empregado Efetivo
	F	Empregado Efetivo
	F	Empregado Efetivo
	F	Empregado Efetivo
	M	Empregado Efetivo
	M	Empregado Efetivo
	F	Empregado Efetivo
	F	Empregado Efetivo
	F	Empregado Efetivo
	F	Empregado Efetivo
	F	Empregado Efetivo
	F	Empregado Efetivo
	F	Empregado Efetivo
	M	Empregado Efetivo
	M	Empregado Efetivo
	M	Empregado Efetivo
	M	Empregado Efetivo
	M	Empregado Efetivo
	M	Empregado Efetivo
	M	Empregado Efetivo
	M	Empregado Efetivo
	M	Empregado Efetivo
	M	Empregado Efetivo
	F	Empregado Efetivo
	M	Empregado Efetivo
	M	Empregado Efetivo
	F	Empregado Efetivo
	M	Empregado Efetivo
	F	Empregado Efetivo
	M	Empregado Efetivo
	M	Empregado Efetivo
	F	Empregado Efetivo
	F	Empregado Efetivo
	M	Empregado Efetivo

	M	Empregado Efetivo
	M	Empregado Efetivo
	F	Empregado Efetivo
	F	Empregado Efetivo
	F	Empregado Efetivo
	F	Empregado Efetivo
	F	Empregado Efetivo
	F	Empregado Efetivo
	F	Empregado Efetivo
	F	Empregado Efetivo
	F	Empregado Efetivo
	F	Empregado Efetivo
	F	Empregado Efetivo
	F	Empregado Efetivo
	F	Empregado Efetivo
	F	Empregado Efetivo
	F	Empregado Efetivo
	F	Empregado Efetivo
	F	Empregado Efetivo
	F	Empregado Efetivo
	F	Empregado Efetivo
	F	Empregado Efetivo
	F	Empregado Efetivo
	F	Empregado Efetivo
	F	Empregado Efetivo
	F	Empregado Efetivo
	F	Empregado Efetivo
	F	Empregado Efetivo
	F	Empregado Efetivo
	F	Empregado Efetivo
	F	Empregado Efetivo
	F	Empregado Efetivo
	F	Empregado Efetivo
	F	Empregado Efetivo
	F	Empregado Efetivo
	F	Empregado Efetivo
	F	Empregado Efetivo
	F	Empregado Efetivo
	F	Empregado Efetivo
	M	Empregado Efetivo
	F	Empregado Efetivo

	F	Empregado Efetivo
	F	Empregado Efetivo
	M	Empregado Efetivo
	F	Empregado Efetivo
	M	Empregado Efetivo
	M	Empregado Efetivo
	M	Empregado Efetivo
	M	Empregado Efetivo
	M	Empregado Efetivo
	M	Empregado Efetivo
	F	Empregado Efetivo
	M	Empregado Efetivo
	F	Empregado Efetivo
	F	Empregado Efetivo
	F	Empregado Efetivo
	M	Empregado Efetivo
	F	Empregado Efetivo
	F	Empregado Efetivo
	F	Empregado Efetivo
	M	Empregado Efetivo
	F	Empregado Efetivo
	M	Empregado Efetivo
	F	Empregado Efetivo
	M	Empregado Efetivo
	F	Empregado Efetivo
	F	Empregado Efetivo
	M	Empregado Efetivo
	F	Empregado Efetivo
	F	Empregado Efetivo
	F	Empregado Efetivo
	F	Empregado Efetivo
	F	Empregado Efetivo
	F	Empregado Efetivo
	F	Empregado Efetivo
	M	Empregado Efetivo
	F	Empregado Efetivo
	F	Empregado Efetivo
	F	Empregado Efetivo
	F	Empregado Efetivo
	F	Empregado Efetivo
	F	Empregado Efetivo
	F	Empregado Efetivo
	F	Empregado Efetivo
	F	Empregado Efetivo
	F	Empregado Efetivo
	F	Empregado Efetivo
	F	Empregado Efetivo
	F	Empregado Efetivo

	F	Empregado Efetivo
	F	Empregado Efetivo
	F	Empregado Efetivo
	F	Empregado Efetivo
	F	Empregado Efetivo
	M	Empregado Efetivo
	F	Empregado Efetivo
	M	Empregado Efetivo
	M	Empregado Efetivo
	F	Empregado Efetivo
	F	Empregado Efetivo
	F	Empregado Efetivo
	F	Empregado Efetivo
	F	Empregado Efetivo
	F	Empregado Efetivo
	F	Empregado Efetivo
	F	Empregado Efetivo
	F	Empregado Efetivo
	F	Empregado Efetivo
	F	Empregado Efetivo
	F	Empregado Efetivo
	F	Empregado Efetivo
	F	Empregado Efetivo
	F	Empregado Efetivo
	F	Empregado Efetivo
	M	Empregado Efetivo
	F	Empregado Efetivo
	F	Empregado Efetivo
	F	Empregado Efetivo
	F	Empregado Efetivo
	F	Empregado Efetivo
	M	Empregado Efetivo
	M	Empregado Efetivo
	F	Empregado Efetivo
	M	Empregado Efetivo
	F	Empregado Efetivo
	M	Empregado Efetivo
	F	Empregado Efetivo
	F	Empregado Efetivo
	F	Empregado Efetivo
	F	Empregado Efetivo
	F	Empregado Efetivo
	F	Empregado Efetivo
	F	Empregado Efetivo
	M	Empregado Efetivo
	F	Empregado Efetivo
	F	Empregado Efetivo
	F	Empregado Efetivo

	F	Empregado Efetivo
	F	Empregado Efetivo
	F	Empregado Efetivo
	F	Empregado Efetivo
	M	Empregado Efetivo
	M	Empregado Efetivo
	M	Empregado Efetivo
	F	Empregado Efetivo
	F	Empregado Efetivo
	F	Empregado Efetivo
	F	Empregado Efetivo
	F	Empregado Efetivo
	F	Empregado Efetivo
	F	Empregado Efetivo
	F	Empregado Efetivo
	F	Empregado Efetivo
	F	Empregado Efetivo
	F	Empregado Efetivo
	F	Empregado Efetivo
	F	Empregado Efetivo
	F	Empregado Efetivo
	F	Empregado Efetivo
	F	Empregado Efetivo
	F	Empregado Efetivo
	F	Empregado Efetivo
	F	Empregado Efetivo
	F	Empregado Efetivo
	F	Empregado Efetivo
	M	Empregado Efetivo
	F	Empregado Efetivo
	F	Empregado Efetivo
	F	Empregado Efetivo
	F	Empregado Efetivo
	F	Empregado Efetivo
	F	Empregado Efetivo
	F	Empregado Efetivo
	F	Empregado Efetivo
	M	Empregado Efetivo
	F	Empregado Efetivo
	F	Empregado Efetivo
	F	Empregado Efetivo
	F	Empregado Efetivo
	F	Empregado Efetivo
	F	Empregado Efetivo
	F	Empregado Efetivo
	F	Empregado Efetivo
	F	Empregado Efetivo
	F	Empregado Efetivo

	F	Empregado Efetivo
	F	Empregado Efetivo
	F	Empregado Efetivo
	F	Empregado Efetivo
	M	Empregado Efetivo
	F	Empregado Efetivo
	F	Empregado Efetivo
	F	Empregado Efetivo
	F	Empregado Efetivo
	F	Empregado Efetivo
	F	Empregado Efetivo
	F	Empregado Efetivo
	F	Empregado Efetivo
	F	Empregado Efetivo
	F	Empregado Efetivo
	F	Empregado Efetivo
	F	Empregado Efetivo
	F	Empregado Efetivo
	F	Empregado Efetivo
	F	Empregado Efetivo
	F	Empregado Efetivo
	F	Empregado Efetivo
	F	Empregado Efetivo
	F	Empregado Efetivo
	F	Empregado Efetivo
	F	Empregado Efetivo
	F	Empregado Efetivo
	F	Empregado Efetivo
	F	Empregado Efetivo
	F	Empregado Efetivo
	F	Empregado Efetivo
	F	Empregado Efetivo
	F	Empregado Efetivo
	F	Empregado Efetivo
	M	Empregado Efetivo
	F	Empregado Efetivo
	F	Empregado Efetivo
	F	Empregado Efetivo
	F	Empregado Efetivo
	F	Empregado Efetivo
	F	Empregado Efetivo
	F	Empregado Efetivo
	F	Empregado Efetivo
	F	Empregado Efetivo
	F	Empregado Efetivo
	F	Empregado Efetivo
	F	Empregado Efetivo
	F	Empregado Efetivo

	F	Empregado Efetivo
	F	Empregado Efetivo
	F	Empregado Efetivo
	F	Empregado Efetivo
	F	Empregado Efetivo
	F	Empregado Efetivo
	F	Empregado Efetivo
	F	Empregado Efetivo
	F	Empregado Efetivo
	F	Empregado Efetivo
	F	Empregado Efetivo
	F	Empregado Efetivo
	F	Empregado Efetivo
	M	Empregado Efetivo
	F	Empregado Efetivo
	F	Empregado Efetivo
	F	Empregado Efetivo
	F	Empregado Efetivo
	F	Empregado Efetivo
	M	Empregado Efetivo
	F	Empregado Efetivo
	F	Empregado Efetivo
	F	Empregado Efetivo
	F	Empregado Efetivo
	F	Empregado Efetivo
	F	Empregado Efetivo
	F	Empregado Efetivo
	F	Empregado Efetivo
	F	Empregado Efetivo
	F	Empregado Efetivo
	F	Empregado Efetivo
	F	Empregado Efetivo
	F	Empregado Efetivo
	F	Empregado Efetivo
	F	Empregado Efetivo
	F	Empregado Efetivo
	F	Empregado Efetivo
	F	Empregado Efetivo
	F	Empregado Efetivo
	M	Empregado Efetivo
	M	Empregado Efetivo
	M	Empregado Efetivo
	M	Empregado Efetivo
	M	Empregado Efetivo
	M	Empregado Efetivo
	F	Empregado Efetivo
	F	Empregado Efetivo
	F	Empregado Efetivo

	F	Empregado Efetivo
	F	Empregado Efetivo
	F	Empregado Efetivo
	F	Empregado Efetivo
	F	Empregado Efetivo
	F	Empregado Efetivo
	F	Empregado Efetivo
	F	Empregado Efetivo
	F	Empregado Efetivo
	F	Empregado Efetivo
	M	Empregado Efetivo
	F	Empregado Efetivo
	F	Empregado Efetivo
	F	Empregado Efetivo
	M	Empregado Efetivo
	F	Empregado Efetivo
	M	Empregado Efetivo
	M	Empregado Efetivo
	F	Empregado Efetivo
	M	Empregado Efetivo
	F	Empregado Efetivo
	F	Empregado Efetivo
	F	Empregado Efetivo
	M	Empregado Efetivo
	M	Empregado Efetivo
	F	Empregado Efetivo
	F	Empregado Efetivo
	F	Empregado Efetivo
	F	Empregado Efetivo
	M	Empregado Efetivo
	F	Empregado Efetivo
	F	Empregado Efetivo
	F	Empregado Efetivo
	F	Empregado Efetivo
	F	Empregado Efetivo
	F	Empregado Efetivo
	F	Empregado Efetivo
	F	Empregado Efetivo
	F	Empregado Efetivo
	F	Empregado Efetivo
	F	Empregado Efetivo
	F	Empregado Efetivo
	F	Empregado Efetivo
	M	Empregado Efetivo
	F	Empregado Efetivo
	F	Empregado Efetivo
	F	Empregado Efetivo
	F	Empregado Efetivo

	F	Empregado Efetivo
	F	Empregado Efetivo
	F	Empregado Efetivo
	F	Empregado Efetivo
	F	Empregado Efetivo
	F	Empregado Efetivo
	F	Empregado Efetivo
	F	Empregado Efetivo
	F	Empregado Efetivo
	F	Empregado Efetivo
	F	Empregado Efetivo
	M	Empregado Efetivo
	F	Empregado Efetivo
	F	Empregado Efetivo
	F	Empregado Efetivo
	M	Empregado Efetivo
	F	Empregado Efetivo
	M	Empregado Efetivo
	M	Empregado Efetivo
	F	Empregado Efetivo
	F	Empregado Efetivo
	F	Empregado Efetivo
	M	Empregado Efetivo
	F	Empregado Efetivo
	F	Empregado Efetivo
	F	Empregado Efetivo
	M	Empregado Efetivo
	M	Empregado Efetivo
	F	Empregado Efetivo
	F	Empregado Efetivo
	F	Empregado Efetivo
	F	Empregado Efetivo
	F	Empregado Efetivo
	F	Empregado Efetivo
	F	Empregado Efetivo
	F	Empregado Efetivo
	F	Empregado Efetivo
	M	Empregado Efetivo
	F	Empregado Efetivo
	F	Empregado Efetivo
	M	Empregado Efetivo
	F	Empregado Efetivo
	M	Empregado Efetivo
	F	Empregado Efetivo
	F	Empregado Efetivo
	F	Empregado Efetivo
	F	Empregado Efetivo
	F	Empregado Efetivo

	M	Empregado Efetivo
	F	Empregado Efetivo
	F	Empregado Efetivo
	M	Empregado Efetivo
	F	Empregado Efetivo
	F	Empregado Efetivo
	F	Empregado Efetivo
	M	Empregado Efetivo
	F	Empregado Efetivo
	F	Empregado Efetivo
	F	Empregado Efetivo
	F	Empregado Efetivo
	F	Empregado Efetivo
	F	Empregado Efetivo
	F	Empregado Efetivo
	F	Empregado Efetivo
	F	Empregado Efetivo
	F	Empregado Efetivo
	F	Empregado Efetivo
	F	Empregado Efetivo
	F	Empregado Efetivo
	F	Empregado Efetivo
	F	Empregado Efetivo
	F	Empregado Efetivo
	M	Empregado Efetivo
	F	Empregado Efetivo
	F	Empregado Efetivo
	F	Empregado Efetivo
	F	Empregado Efetivo
	F	Empregado Efetivo
	F	Empregado Efetivo
	F	Empregado Efetivo
	F	Empregado Efetivo
	F	Empregado Efetivo
	F	Empregado Efetivo
	F	Empregado Efetivo
	F	Empregado Efetivo
	M	Empregado Efetivo
	F	Empregado Efetivo
	F	Empregado Efetivo
	F	Empregado Efetivo
	F	Empregado Efetivo
	F	Empregado Efetivo
	F	Empregado Efetivo
	F	Empregado Efetivo
	M	Empregado Efetivo
	F	Empregado Efetivo
	F	Empregado Efetivo

	F	Empregado Efetivo
	F	Empregado Efetivo
	F	Empregado Efetivo
	F	Empregado Efetivo
	M	Empregado Efetivo
	F	Empregado Efetivo
	F	Empregado Efetivo
	F	Empregado Efetivo
	F	Empregado Efetivo
	F	Empregado Efetivo
	F	Empregado Efetivo
	F	Empregado Efetivo
	F	Empregado Efetivo
	F	Empregado Efetivo
	F	Empregado Efetivo
	F	Empregado Efetivo
	F	Empregado Efetivo
	F	Empregado Efetivo
	F	Empregado Efetivo
	F	Empregado Efetivo
	M	Empregado Efetivo
	F	Empregado Efetivo
	F	Empregado Efetivo
	F	Empregado Efetivo
	F	Empregado Efetivo
	M	Empregado Efetivo
	F	Empregado Efetivo
	M	Empregado Efetivo
	M	Empregado Efetivo
	F	Empregado Efetivo
	F	Empregado Efetivo
	F	Empregado Efetivo
	F	Empregado Efetivo
	F	Empregado Efetivo
	F	Empregado Efetivo
	M	Empregado Efetivo
	F	Empregado Efetivo
	M	Empregado Efetivo
	F	Empregado Efetivo
	F	Empregado Efetivo
	F	Empregado Efetivo
	F	Empregado Efetivo
	F	Empregado Efetivo
	F	Empregado Efetivo
	F	Empregado Efetivo
	F	Empregado Efetivo
	F	Empregado Efetivo
	F	Empregado Efetivo
	F	Empregado Efetivo

	F	Empregado Efetivo
	M	Empregado Efetivo
	F	Empregado Efetivo
	F	Empregado Efetivo
	F	Empregado Efetivo
	F	Empregado Efetivo
	F	Empregado Efetivo
	F	Empregado Efetivo
	F	Empregado Efetivo
	F	Empregado Efetivo
	M	Empregado Efetivo
	F	Empregado Efetivo
	F	Empregado Efetivo
	F	Empregado Efetivo
	F	Empregado Efetivo
	F	Empregado Efetivo
	F	Empregado Efetivo
	F	Empregado Efetivo
	F	Empregado Efetivo
	F	Empregado Efetivo
	F	Empregado Efetivo
	F	Empregado Efetivo
	F	Empregado Efetivo
	F	Empregado Efetivo
	F	Empregado Efetivo
	F	Empregado Efetivo
	F	Empregado Efetivo
	F	Empregado Efetivo
	F	Empregado Efetivo
	F	Empregado Efetivo
	M	Empregado Efetivo
	F	Empregado Efetivo
	F	Empregado Efetivo
	F	Empregado Efetivo
	F	Empregado Efetivo
	F	Empregado Efetivo
	F	Empregado Efetivo
	F	Empregado Efetivo
	F	Empregado Efetivo
	F	Empregado Efetivo
	M	Empregado Efetivo
	F	Empregado Efetivo
	M	Empregado Efetivo
	F	Empregado Efetivo
	M	Empregado Efetivo
	M	Empregado Efetivo
	F	Empregado Efetivo
	F	Empregado Efetivo

	M	Empregado Efetivo
	M	Empregado Efetivo
	F	Empregado Efetivo
	F	Empregado Efetivo
	F	Empregado Efetivo
	F	Empregado Efetivo
	F	Empregado Efetivo
	F	Empregado Efetivo
	M	Empregado Efetivo
	F	Empregado Efetivo
	F	Empregado Efetivo
	M	Empregado Efetivo
	F	Empregado Efetivo
	F	Empregado Efetivo
	M	Empregado Efetivo
	F	Empregado Efetivo
	M	Empregado Efetivo
	F	Empregado Efetivo
	F	Empregado Efetivo
	F	Empregado Efetivo
	M	Empregado Efetivo
	F	Empregado Efetivo
	M	Empregado Efetivo
	F	Empregado Efetivo
	M	Empregado Efetivo
	F	Empregado Efetivo
	M	Empregado Efetivo
	M	Empregado Efetivo
	M	Empregado Efetivo
	F	Empregado Efetivo
	F	Empregado Efetivo
	F	Empregado Efetivo
	F	Empregado Efetivo
	F	Empregado Efetivo
	F	Empregado Efetivo
	M	Empregado Efetivo
	F	Empregado Efetivo
	F	Empregado Efetivo
	F	Empregado Efetivo
	M	Empregado Efetivo
	F	Empregado Efetivo
	F	Empregado Efetivo
	F	Empregado Efetivo
	F	Empregado Efetivo
	F	Empregado Efetivo
	F	Empregado Efetivo
	F	Empregado Efetivo
	F	Empregado Efetivo

	F	Empregado Efetivo
	F	Empregado Efetivo
	F	Empregado Efetivo
	F	Empregado Efetivo
	F	Empregado Efetivo
	F	Empregado Efetivo
	F	Empregado Efetivo
	M	Empregado Efetivo
	M	Empregado Efetivo
	F	Empregado Efetivo
	F	Empregado Efetivo
	F	Empregado Efetivo
	F	Empregado Efetivo
	F	Empregado Efetivo
	F	Empregado Efetivo
	F	Empregado Efetivo
	F	Empregado Efetivo
	M	Empregado Efetivo
	F	Empregado Efetivo
	F	Empregado Efetivo
	F	Empregado Efetivo
	F	Empregado Efetivo
	F	Empregado Efetivo
	F	Empregado Efetivo
	M	Empregado Efetivo
	F	Empregado Efetivo
	F	Empregado Efetivo
	M	Empregado Efetivo
	M	Empregado Efetivo
	F	Empregado Efetivo
	F	Empregado Efetivo
	M	Empregado Efetivo
	F	Empregado Efetivo
	F	Empregado Efetivo
	F	Empregado Efetivo
	F	Empregado Efetivo
	F	Empregado Efetivo
	M	Empregado Efetivo
	F	Empregado Efetivo
	M	Empregado Efetivo
	F	Empregado Efetivo
	F	Empregado Efetivo
	M	Empregado Efetivo
	F	Empregado Efetivo
	F	Empregado Efetivo
	M	Empregado Efetivo
	M	Empregado Efetivo
	M	Empregado Efetivo

	F	Empregado Efetivo
	F	Empregado Efetivo
	F	Empregado Efetivo
	M	Empregado Efetivo
	F	Empregado Efetivo
	F	Empregado Efetivo
	F	Empregado Efetivo
	F	Empregado Efetivo
	M	Empregado Efetivo
	F	Empregado Efetivo
	F	Empregado Efetivo
	F	Empregado Efetivo
	M	Empregado Efetivo
	F	Empregado Efetivo
	F	Empregado Efetivo
	F	Empregado Efetivo
	M	Empregado Efetivo
	M	Empregado Efetivo
	M	Empregado Efetivo
	F	Empregado Efetivo
	F	Empregado Efetivo
	F	Empregado Efetivo
	F	Empregado Efetivo
	M	Empregado Efetivo
	F	Empregado Efetivo
	F	Empregado Efetivo
	F	Empregado Efetivo
	F	Empregado Efetivo
	M	Empregado Efetivo
	M	Empregado Efetivo
	M	Empregado Efetivo
	F	Empregado Efetivo
	F	Empregado Efetivo
	M	Empregado Efetivo
	F	Empregado Efetivo
	M	Empregado Efetivo
	F	Empregado Efetivo
	F	Empregado Efetivo
	M	Empregado Efetivo
	M	Empregado Efetivo
	F	Empregado Efetivo
	F	Empregado Efetivo
	F	Empregado Efetivo
	F	Empregado Efetivo
	F	Empregado Efetivo
	F	Empregado Efetivo
	M	Empregado Efetivo
	F	Empregado Efetivo

	F	Empregado Efetivo
	F	Empregado Efetivo
	F	Empregado Efetivo
	M	Empregado Efetivo
	F	Empregado Efetivo
	F	Empregado Efetivo
	F	Empregado Efetivo
	F	Empregado Efetivo
	F	Empregado Efetivo
	M	Empregado Efetivo
	F	Empregado Efetivo
	F	Empregado Efetivo
	F	Empregado Efetivo
	F	Empregado Efetivo
	F	Empregado Efetivo
	F	Empregado Efetivo
	M	Empregado Efetivo
	F	Empregado Efetivo
	F	Empregado Efetivo
	F	Empregado Efetivo
	F	Empregado Efetivo
	F	Empregado Efetivo
	F	Empregado Efetivo
	F	Empregado Efetivo
	M	Empregado Efetivo
	F	Empregado Efetivo
	F	Empregado Efetivo
	F	Empregado Efetivo
	F	Empregado Efetivo
	F	Empregado Efetivo
	F	Empregado Efetivo
	M	Empregado Efetivo
	F	Empregado Efetivo
	F	Empregado Efetivo
	M	Empregado Efetivo
	F	Empregado Efetivo
	F	Empregado Efetivo
	M	Empregado Efetivo
	F	Empregado Efetivo
	F	Empregado Efetivo
	F	Empregado Efetivo
	M	Empregado Efetivo
	F	Empregado Efetivo
	F	Empregado Efetivo
	M	Empregado Efetivo
	F	Empregado Efetivo
	F	Empregado Efetivo
	M	Empregado Efetivo

	F	Empregado Efetivo
	M	Empregado Efetivo
	F	Empregado Efetivo
	F	Empregado Efetivo
	F	Empregado Efetivo
	F	Empregado Efetivo
	F	Empregado Efetivo
	M	Empregado Efetivo
	F	Empregado Efetivo
	F	Empregado Efetivo
	M	Empregado Efetivo
	F	Empregado Efetivo
	F	Empregado Efetivo
	F	Empregado Efetivo
	M	Empregado Efetivo
	F	Empregado Efetivo
	M	Empregado Efetivo
	M	Empregado Efetivo
	F	Empregado Efetivo
	M	Empregado Efetivo
	F	Empregado Efetivo
	F	Empregado Efetivo
	M	Empregado Efetivo
	F	Empregado Efetivo
	M	Empregado Efetivo
	F	Empregado Efetivo
	F	Empregado Efetivo
	F	Empregado Efetivo
	F	Empregado Efetivo
	F	Empregado Efetivo
	F	Empregado Efetivo
	F	Empregado Efetivo
	F	Empregado Efetivo
	F	Empregado Efetivo
	F	Empregado Efetivo
	F	Empregado Efetivo
	F	Empregado Efetivo
	F	Empregado Efetivo
	F	Empregado Efetivo
	F	Empregado Efetivo
	F	Cedido - RJU
	F	Empregado Efetivo
	F	Empregado Efetivo
	F	Empregado Efetivo
	F	Empregado Efetivo
	F	Empregado Efetivo
	M	Empregado Efetivo
	M	Empregado Efetivo

	F	Empregado Efetivo
	F	Empregado Efetivo
	F	Empregado Efetivo
	F	Empregado Efetivo
	M	Empregado Efetivo
	F	Empregado Efetivo
	F	Empregado Efetivo
	F	Empregado Efetivo
	F	Empregado Efetivo
	F	Empregado Efetivo
	F	Empregado Efetivo
	F	Empregado Efetivo
	F	Empregado Efetivo
	F	Empregado Efetivo
	F	Empregado Efetivo
	F	Empregado Efetivo
	M	Empregado Efetivo
	F	Empregado Efetivo
	F	Empregado Efetivo
	F	Empregado Efetivo
	F	Empregado Efetivo
	F	Empregado Efetivo
	F	Empregado Efetivo
	F	Empregado Efetivo
	F	Empregado Efetivo
	F	Empregado Efetivo
	F	Empregado Efetivo
	F	Empregado Efetivo
	F	Empregado Efetivo
	F	Empregado Efetivo
	F	Empregado Efetivo
	F	Empregado Efetivo
	M	Empregado Efetivo
	F	Empregado Efetivo
	M	Empregado Efetivo
	F	Empregado Efetivo
	F	Empregado Efetivo
	F	Empregado Efetivo
	F	Empregado Efetivo
	F	Empregado Efetivo
	F	Empregado Efetivo
	M	Empregado Efetivo
	M	Empregado Efetivo
	F	Empregado Efetivo
	M	Empregado Efetivo
	F	Empregado Efetivo
	F	Empregado Efetivo
	F	Empregado Efetivo

	F	Empregado Efetivo
	F	Empregado Efetivo
	F	Empregado Efetivo
	F	Empregado Efetivo
	F	Empregado Efetivo
	F	Empregado Efetivo
	F	Empregado Efetivo
	M	Empregado Efetivo
	F	Empregado Efetivo
	F	Empregado Efetivo
	F	Empregado Efetivo
	F	Empregado Efetivo
	F	Empregado Efetivo
	F	Empregado Efetivo
	F	Empregado Efetivo
	F	Empregado Efetivo
	F	Empregado Efetivo
	F	Empregado Efetivo
	F	Empregado Efetivo
	F	Empregado Efetivo
	F	Empregado Efetivo
	F	Empregado Efetivo
	F	Empregado Efetivo
	F	Empregado Efetivo
	F	Empregado Efetivo
	F	Empregado Efetivo
	F	Empregado Efetivo
	F	Empregado Efetivo
	F	Empregado Efetivo
	F	Empregado Efetivo
	F	Empregado Efetivo
	M	Empregado Efetivo
	F	Empregado Efetivo
	F	Empregado Efetivo
	F	Empregado Efetivo
	F	Empregado Efetivo
	F	Empregado Efetivo
	F	Empregado Efetivo
	F	Empregado Efetivo
	F	Empregado Efetivo
	F	Empregado Efetivo
	F	Empregado Efetivo
	F	Empregado Efetivo
	F	Empregado Efetivo
	M	Empregado Efetivo
	M	Empregado Efetivo
	F	Empregado Efetivo
	F	Empregado Efetivo

	F	Empregado Efetivo
	F	Empregado Efetivo
	M	Empregado Efetivo
	F	Empregado Efetivo
	F	Empregado Efetivo
	F	Empregado Efetivo
	M	Empregado Efetivo
	M	Empregado Efetivo
	F	Empregado Efetivo
	F	Empregado Efetivo
	F	Empregado Efetivo
	F	Empregado Efetivo
	M	Empregado Efetivo
	M	Empregado Efetivo
	F	Empregado Efetivo
	F	Empregado Efetivo
	F	Empregado Efetivo
	M	Empregado Efetivo
	M	Empregado Efetivo
	F	Empregado Efetivo
	M	Empregado Efetivo
	F	Empregado Efetivo
	F	Empregado Efetivo
	F	Empregado Efetivo
	F	Empregado Efetivo
	F	Empregado Efetivo
	F	Empregado Efetivo
	F	Empregado Efetivo
	F	Empregado Efetivo
	F	Empregado Efetivo
	F	Empregado Efetivo
	M	Empregado Efetivo
	F	Empregado Efetivo
	M	Empregado Efetivo
	F	Empregado Efetivo
	F	Empregado Efetivo
	F	Empregado Efetivo
	F	Empregado Efetivo
	F	Empregado Efetivo
	F	Empregado Efetivo
	F	Empregado Efetivo
	F	Empregado Efetivo
	F	Empregado Efetivo
	F	Empregado Efetivo
	F	Empregado Efetivo
	F	Empregado Efetivo
	F	Empregado Efetivo
	F	Empregado Efetivo

	M	Empregado Efetivo
	M	Empregado Efetivo
	F	Empregado Efetivo
	F	Empregado Efetivo
	F	Empregado Efetivo
	M	Empregado Efetivo
	F	Empregado Efetivo
	F	Empregado Efetivo
	F	Empregado Efetivo
	M	Empregado Efetivo
	F	Empregado Efetivo
	F	Empregado Efetivo
	F	Empregado Efetivo
	F	Empregado Efetivo
	F	Empregado Efetivo
	F	Empregado Efetivo
	F	Empregado Efetivo
	F	Empregado Efetivo
	F	Empregado Efetivo
	F	Empregado Efetivo
	F	Empregado Efetivo
	F	Empregado Efetivo
	F	Empregado Efetivo
	F	Empregado Efetivo
	F	Empregado Efetivo
	F	Empregado Efetivo
	F	Empregado Efetivo
	F	Empregado Efetivo
	F	Empregado Efetivo
	F	Empregado Efetivo
	F	Empregado Efetivo
	F	Empregado Efetivo
	F	Empregado Efetivo
	F	Empregado Efetivo
	F	Empregado Efetivo
	F	Empregado Efetivo
	F	Empregado Efetivo
	F	Empregado Efetivo
	F	Empregado Efetivo
	F	Empregado Efetivo
	F	Empregado Efetivo
	F	Empregado Efetivo
	F	Empregado Efetivo
	F	Empregado Efetivo
	F	Empregado Efetivo
	F	Empregado Efetivo
	F	Empregado Efetivo
	F	Empregado Efetivo

	M	Empregado Efetivo
	F	Empregado Efetivo
	F	Empregado Efetivo
	F	Empregado Efetivo
	M	Empregado Efetivo
	F	Empregado Efetivo
	F	Empregado Efetivo
	F	Empregado Efetivo
	F	Empregado Efetivo
	F	Empregado Efetivo
	F	Empregado Efetivo
	F	Empregado Efetivo
	F	Empregado Efetivo
	F	Empregado Efetivo
	F	Empregado Efetivo
	F	Empregado Efetivo
	F	Empregado Efetivo
	M	Empregado Efetivo
	F	Empregado Efetivo
	F	Empregado Efetivo
	F	Empregado Efetivo
	F	Empregado Efetivo
	F	Empregado Efetivo
	F	Empregado Efetivo
	M	Empregado Efetivo
	F	Empregado Efetivo
	M	Empregado Efetivo
	F	Empregado Efetivo
	F	Empregado Efetivo
	F	Empregado Efetivo
	F	Empregado Efetivo
	F	Empregado Efetivo
	M	Empregado Efetivo
	F	Empregado Efetivo
	F	Empregado Efetivo
	M	Empregado Efetivo
	M	Empregado Efetivo
	F	Empregado Efetivo
	F	Empregado Efetivo
	F	Empregado Efetivo
	M	Empregado Efetivo
	F	Empregado Efetivo
	F	Empregado Efetivo
	F	Empregado Efetivo
	F	Empregado Efetivo
	F	Empregado Efetivo
	F	Empregado Efetivo
	M	Empregado Efetivo

	F	Empregado Efetivo
	F	Empregado Efetivo
	F	Empregado Efetivo
	M	Empregado Efetivo
	M	Empregado Efetivo
	M	Empregado Efetivo
	F	Empregado Efetivo
	M	Empregado Efetivo
	F	Empregado Efetivo
	F	Empregado Efetivo
	M	Empregado Efetivo
	F	Empregado Efetivo
	F	Empregado Efetivo
	F	Empregado Efetivo
	M	Empregado Efetivo
	M	Empregado Efetivo
	F	Empregado Efetivo
	F	Empregado Efetivo
	M	Empregado Efetivo
	M	Empregado Efetivo
	F	Empregado Efetivo
	F	Empregado Efetivo
	M	Empregado Efetivo
	M	Empregado Efetivo
	M	Empregado Efetivo
	F	Empregado Efetivo
	F	Empregado Efetivo
	M	Empregado Efetivo
	F	Empregado Efetivo
	F	Empregado Efetivo
	F	Empregado Efetivo
	M	Empregado Efetivo
	F	Empregado Efetivo
	M	Empregado Efetivo
	F	Empregado Efetivo
	M	Empregado Efetivo
	F	Empregado Efetivo
	F	Empregado Efetivo
	F	Empregado Efetivo
	F	Empregado Efetivo
	F	Empregado Efetivo
	M	Empregado Efetivo
	F	Empregado Efetivo
	F	Empregado Efetivo
	F	Empregado Efetivo
	F	Empregado Efetivo
	M	Empregado Efetivo
	F	Empregado Efetivo

	F	Empregado Efetivo
	F	Empregado Efetivo
	F	Empregado Efetivo
	F	Empregado Efetivo
	F	Empregado Efetivo
	M	Empregado Efetivo
	F	Empregado Efetivo
	F	Empregado Efetivo
	F	Empregado Efetivo
	F	Empregado Efetivo
	F	Empregado Efetivo
	F	Empregado Efetivo
	M	Empregado Efetivo
	F	Empregado Efetivo
	F	Empregado Efetivo
	M	Empregado Efetivo
	M	Empregado Efetivo
	M	Empregado Efetivo
	F	Empregado Efetivo
	F	Empregado Efetivo
	M	Empregado Efetivo
	F	Empregado Efetivo
	M	Empregado Efetivo
	M	Empregado Efetivo
	M	Empregado Efetivo
	F	Empregado Efetivo
	F	Empregado Efetivo
	F	Empregado Efetivo
	F	Empregado Efetivo
	F	Empregado Efetivo
	F	Empregado Efetivo
	F	Empregado Efetivo
	F	Empregado Efetivo
	F	Empregado Efetivo
	F	Empregado Efetivo
	F	Empregado Efetivo
	M	Empregado Efetivo
	F	Empregado Efetivo
	M	Empregado Efetivo
	F	Empregado Efetivo
	F	Empregado Efetivo
	F	Empregado Efetivo
	M	Empregado Efetivo
	F	Empregado Efetivo
	M	Empregado Efetivo
	M	Empregado Efetivo
	F	Empregado Efetivo
	F	Empregado Efetivo

	F	Empregado Efetivo
	F	Empregado Efetivo
	F	Empregado Efetivo
	F	Empregado Efetivo
	F	Empregado Efetivo
	F	Empregado Efetivo
	F	Empregado Efetivo
	F	Empregado Efetivo
	F	Empregado Efetivo
	F	Empregado Efetivo
	F	Empregado Efetivo
	M	Empregado Efetivo
	F	Empregado Efetivo
	F	Empregado Efetivo
	F	Empregado Efetivo
	F	Empregado Efetivo
	F	Empregado Efetivo
	F	Empregado Efetivo
	F	Empregado Efetivo
	M	Empregado Efetivo
	F	Empregado Efetivo
	F	Empregado Efetivo
	F	Empregado Efetivo
	F	Empregado Efetivo
	F	Empregado Efetivo
	F	Empregado Efetivo
	F	Empregado Efetivo
	M	Empregado Efetivo
	F	Empregado Efetivo
	M	Empregado Efetivo
	M	Empregado Efetivo
	F	Empregado Efetivo
	F	Empregado Efetivo
	M	Empregado Efetivo
	F	Empregado Efetivo
	F	Empregado Efetivo
	F	Empregado Efetivo
	F	Empregado Efetivo
	M	Empregado Efetivo
	M	Empregado Efetivo
	M	Empregado Efetivo
	M	Empregado Efetivo
	M	Empregado Efetivo
	F	Empregado Efetivo
	F	Empregado Efetivo
	M	Empregado Efetivo
	M	Empregado Efetivo
	M	Empregado Efetivo

	M	Empregado Efetivo
	M	Empregado Efetivo
	F	Empregado Efetivo
	F	Empregado Efetivo
	M	Empregado Efetivo
	F	Empregado Efetivo
	F	Empregado Efetivo
	F	Empregado Efetivo
	M	Empregado Efetivo
	F	Empregado Efetivo
	F	Empregado Efetivo
	F	Empregado Efetivo
	F	Empregado Efetivo
	F	Empregado Efetivo
	F	Empregado Efetivo
	F	Empregado Efetivo
	F	Empregado Efetivo
	F	Empregado Efetivo
	F	Empregado Efetivo
	F	Empregado Efetivo
	F	Empregado Efetivo
	F	Empregado Efetivo
	F	Empregado Efetivo
	F	Empregado Efetivo
	F	Empregado Efetivo
	F	Empregado Efetivo
	F	Empregado Efetivo
	F	Empregado Efetivo
	F	Empregado Efetivo
	F	Empregado Efetivo
	F	Empregado Efetivo
	F	Empregado Efetivo
	F	Empregado Efetivo
	F	Empregado Efetivo
	F	Empregado Efetivo
	F	Empregado Efetivo
	F	Empregado Efetivo
	F	Empregado Efetivo
	F	Empregado Efetivo
	F	Empregado Efetivo
	F	Empregado Efetivo
	F	Empregado Efetivo
	F	Empregado Efetivo
	F	Empregado Efetivo
	F	Empregado Efetivo
	F	Empregado Efetivo
	F	Empregado Efetivo
	F	Empregado Efetivo

	F	Empregado Efetivo
	F	Empregado Efetivo
	F	Empregado Efetivo
	F	Empregado Efetivo
	F	Empregado Efetivo
	F	Empregado Efetivo
	F	Empregado Efetivo
	F	Empregado Efetivo
	F	Empregado Efetivo
	F	Empregado Efetivo
	F	Empregado Efetivo
	F	Empregado Efetivo
	F	Empregado Efetivo
	F	Empregado Efetivo
	F	Empregado Efetivo
	F	Empregado Efetivo
	F	Empregado Efetivo
	F	Empregado Efetivo
	F	Empregado Efetivo
	F	Empregado Efetivo
	F	Empregado Efetivo
	F	Empregado Efetivo
	F	Empregado Efetivo
	M	Empregado Efetivo
	F	Empregado Efetivo
	F	Empregado Efetivo
	F	Empregado Efetivo
	F	Empregado Efetivo
	F	Empregado Efetivo
	F	Empregado Efetivo
	F	Empregado Efetivo
	F	Empregado Efetivo
	F	Empregado Efetivo
	M	Empregado Efetivo
	M	Empregado Efetivo
	M	Empregado Efetivo
	F	Empregado Efetivo
	F	Empregado Efetivo
	F	Empregado Efetivo
	F	Empregado Efetivo
	F	Empregado Efetivo
	F	Empregado Efetivo
	M	Empregado Efetivo
	F	Empregado Efetivo
	F	Empregado Efetivo
	F	Empregado Efetivo
	F	Empregado Efetivo
	F	Empregado Efetivo

	F	Empregado Efetivo
	F	Empregado Efetivo
	F	Empregado Efetivo
	F	Empregado Efetivo
	F	Empregado Efetivo
	F	Empregado Efetivo
	F	Empregado Efetivo
	F	Empregado Efetivo
	F	Empregado Efetivo
	F	Empregado Efetivo
	F	Empregado Efetivo
	F	Empregado Efetivo
	M	Empregado Efetivo
	M	Empregado Efetivo
	F	Empregado Efetivo
	F	Empregado Efetivo
	F	Empregado Efetivo
	M	Empregado Efetivo
	M	Empregado Efetivo
	M	Empregado Efetivo
	M	Empregado Efetivo
	F	Empregado Efetivo
	F	Empregado Efetivo
	F	Empregado Efetivo
	F	Empregado Efetivo
	M	Empregado Efetivo
	M	Empregado Efetivo
	F	Empregado Efetivo
	F	Empregado Efetivo
	F	Empregado Efetivo
	F	Empregado Efetivo
	F	Empregado Efetivo
	M	Empregado Efetivo
	F	Empregado Efetivo
	F	Empregado Efetivo
	F	Empregado Efetivo
	F	Empregado Efetivo
	F	Empregado Efetivo
	M	Empregado Efetivo
	F	Empregado Efetivo
	M	Empregado Efetivo

	F	Empregado Efetivo
	F	Empregado Efetivo
	F	Empregado Efetivo
	F	Empregado Efetivo
	F	Empregado Efetivo
	F	Empregado Efetivo
	F	Empregado Efetivo
	F	Empregado Efetivo
	F	Empregado Efetivo
	F	Empregado Efetivo
	F	Empregado Efetivo
	F	Empregado Efetivo
	F	Empregado Efetivo
	F	Empregado Efetivo
	F	Empregado Efetivo
	F	Empregado Efetivo
	M	Empregado Efetivo
	F	Empregado Efetivo
	F	Empregado Efetivo
	F	Empregado Efetivo
	F	Empregado Efetivo
	M	Empregado Efetivo
	F	Empregado Efetivo
	F	Empregado Efetivo
	F	Empregado Efetivo
	M	Empregado Efetivo
	F	Empregado Efetivo
	F	Empregado Efetivo
	F	Empregado Efetivo
	F	Empregado Efetivo
	F	Empregado Efetivo
	F	Empregado Efetivo

	F	Empregado Efetivo
	F	Empregado Efetivo
	F	Empregado Efetivo
	F	Empregado Efetivo
	F	Empregado Efetivo
	F	Empregado Efetivo
	M	Empregado Efetivo
	F	Empregado Efetivo
	F	Empregado Efetivo
	F	Empregado Efetivo
	F	Empregado Efetivo
	F	Empregado Efetivo
	F	Empregado Efetivo
	F	Empregado Efetivo
	F	Empregado Efetivo
	F	Empregado Efetivo
	F	Empregado Efetivo
	F	Empregado Efetivo
	M	Empregado Efetivo
	F	Empregado Efetivo
	M	Empregado Efetivo
	F	Empregado Efetivo
	F	Empregado Efetivo
	F	Empregado Efetivo
	M	Empregado Efetivo
	M	Empregado Efetivo
	F	Empregado Efetivo
	F	Empregado Efetivo
	F	Empregado Efetivo
	M	Empregado Efetivo
	F	Empregado Efetivo
	F	Empregado Efetivo

	F	Empregado Efetivo
	M	Empregado Efetivo
	F	Empregado Efetivo
	F	Empregado Efetivo
	F	Empregado Efetivo
	F	Empregado Efetivo
	F	Empregado Efetivo
	F	Empregado Efetivo
	F	Empregado Efetivo
	F	Empregado Efetivo
	F	Empregado Efetivo
	F	Empregado Efetivo
	M	Empregado Efetivo
	F	Empregado Efetivo
	F	Empregado Efetivo
	F	Empregado Efetivo
	F	Empregado Efetivo
	M	Empregado Efetivo
	F	Empregado Efetivo
	M	Empregado Efetivo
	F	Empregado Efetivo
	F	Empregado Efetivo
	F	Empregado Efetivo
	F	Empregado Efetivo
	F	Empregado Efetivo
	F	Empregado Efetivo
	F	Empregado Efetivo
	F	Empregado Efetivo
	M	Empregado Efetivo
	M	Empregado Efetivo
	F	Empregado Efetivo
	F	Empregado Efetivo
	F	Empregado Efetivo
	F	Empregado Efetivo
	M	Empregado Efetivo
	F	Empregado Efetivo
	F	Empregado Efetivo
	F	Empregado Efetivo

	F	Empregado Efetivo
	F	Empregado Efetivo
	F	Empregado Efetivo
	F	Empregado Efetivo
	F	Empregado Efetivo
	F	Empregado Efetivo
	F	Empregado Efetivo
	F	Empregado Efetivo
	F	Empregado Efetivo
	M	Empregado Efetivo
	F	Empregado Efetivo
	F	Empregado Efetivo
	F	Empregado Efetivo
	F	Empregado Efetivo
	F	Empregado Efetivo
	F	Empregado Efetivo
	F	Empregado Efetivo
	F	Empregado Efetivo
	F	Empregado Efetivo
	F	Empregado Efetivo
	F	Empregado Efetivo
	F	Empregado Efetivo
	F	Empregado Efetivo
	F	Empregado Efetivo
	F	Empregado Efetivo
	F	Empregado Efetivo
	F	Empregado Efetivo
	F	Empregado Efetivo
	F	Empregado Efetivo
	F	Empregado Efetivo
	F	Empregado Efetivo
	F	Empregado Efetivo
	F	Empregado Efetivo
	M	Empregado Efetivo
	F	Empregado Efetivo
	M	Empregado Efetivo
	F	Empregado Efetivo
	M	Empregado Efetivo
	M	Empregado Efetivo
	M	Empregado Efetivo
	F	Empregado Efetivo
	F	Empregado Efetivo
	F	Empregado Efetivo
	F	Empregado Efetivo
	F	Empregado Efetivo
	F	Empregado Efetivo
	M	Empregado Efetivo
	F	Empregado Efetivo

	F	Empregado Efetivo
	F	Empregado Efetivo
	M	Empregado Efetivo
	M	Empregado Efetivo
	M	Empregado Efetivo
	F	Empregado Efetivo
	F	Empregado Efetivo
	F	Empregado Efetivo
	F	Empregado Efetivo
	F	Empregado Efetivo
	F	Empregado Efetivo
	F	Empregado Efetivo
	F	Empregado Efetivo
	F	Empregado Efetivo
	F	Empregado Efetivo
	F	Empregado Efetivo
	F	Empregado Efetivo
	F	Empregado Efetivo
	F	Empregado Efetivo
	F	Empregado Efetivo
	F	Empregado Efetivo
	F	Empregado Efetivo
	F	Empregado Efetivo
	F	Empregado Efetivo
	F	Empregado Efetivo
	F	Empregado Efetivo
	F	Empregado Efetivo
	F	Empregado Efetivo
	F	Empregado Efetivo
	F	Empregado Efetivo
	F	Empregado Efetivo
	F	Empregado Efetivo
	F	Empregado Efetivo
	F	Empregado Efetivo
	F	Empregado Efetivo
	F	Empregado Efetivo
	F	Empregado Efetivo
	F	Empregado Efetivo
	F	Empregado Efetivo
	F	Empregado Efetivo
	F	Empregado Efetivo
	F	Empregado Efetivo
	F	Empregado Efetivo
	F	Empregado Efetivo
	F	Empregado Efetivo
	F	Empregado Efetivo
	F	Empregado Efetivo
	F	Empregado Efetivo

	F	Empregado Efetivo
	F	Empregado Efetivo
	F	Empregado Efetivo
	F	Empregado Efetivo
	F	Empregado Efetivo
	F	Empregado Efetivo
	F	Empregado Efetivo
	F	Empregado Efetivo
	F	Empregado Efetivo
	M	Empregado Efetivo
	M	Empregado Efetivo
	F	Empregado Efetivo
	F	Empregado Efetivo
	F	Empregado Efetivo
	F	Empregado Efetivo
	F	Empregado Efetivo
	F	Empregado Efetivo
	F	Empregado Efetivo
	F	Empregado Efetivo
	M	Empregado Efetivo
	M	Empregado Efetivo
	F	Empregado Efetivo
	F	Empregado Efetivo
	M	Empregado Efetivo
	F	Empregado Efetivo
	M	Empregado Efetivo
	F	Empregado Efetivo
	F	Empregado Efetivo
	F	Empregado Efetivo
	M	Empregado Efetivo
	F	Empregado Efetivo
	F	Empregado Efetivo
	F	Empregado Efetivo
	F	Empregado Efetivo
	F	Empregado Efetivo
	M	Empregado Efetivo
	F	Empregado Efetivo
	F	Empregado Efetivo
	F	Empregado Efetivo
	F	Empregado Efetivo
	F	Empregado Efetivo
	M	Empregado Efetivo
	F	Empregado Efetivo
	M	Empregado Efetivo
	F	Empregado Efetivo
	M	Empregado Efetivo
	F	Empregado Efetivo
	M	Empregado Efetivo

	F	Empregado Efetivo
	F	Empregado Efetivo
	F	Empregado Efetivo
	F	Empregado Efetivo
	F	Empregado Efetivo
	F	Empregado Efetivo
	F	Empregado Efetivo
	F	Empregado Efetivo
	M	Empregado Efetivo
	F	Empregado Efetivo
	F	Empregado Efetivo
	M	Empregado Efetivo
	F	Empregado Efetivo
	F	Empregado Efetivo
	M	Empregado Efetivo
	F	Empregado Efetivo
	F	Empregado Efetivo
	M	Empregado Efetivo
	F	Empregado Efetivo
	F	Empregado Efetivo
	F	Empregado Efetivo
	F	Empregado Efetivo
	F	Cedido - RJU
	F	Empregado Efetivo
	F	Empregado Efetivo
	F	Empregado Efetivo
	F	Empregado Efetivo
	M	Empregado Efetivo
	M	Empregado Efetivo
	M	Empregado Efetivo
	F	Empregado Efetivo
	F	Empregado Efetivo
	F	Empregado Efetivo
	F	Empregado Efetivo
	M	Empregado Efetivo
	F	Empregado Efetivo
	F	Empregado Efetivo
	M	Empregado Efetivo
	F	Empregado Efetivo
	M	Empregado Efetivo
	F	Empregado Efetivo
	F	Empregado Efetivo
	F	Empregado Efetivo
	F	Empregado Efetivo
	M	Empregado Efetivo
	M	Empregado Efetivo
	F	Empregado Efetivo
	M	Empregado Efetivo

	F	Empregado Efetivo
	F	Empregado Efetivo
	F	Empregado Efetivo
	F	Empregado Efetivo
	F	Empregado Efetivo
	F	Empregado Efetivo
	F	Empregado Efetivo
	F	Empregado Efetivo
	F	Empregado Efetivo
	F	Empregado Efetivo
	F	Empregado Efetivo
	F	Empregado Efetivo
	F	Empregado Efetivo
	F	Empregado Efetivo
	F	Empregado Temporário
	F	Empregado Efetivo
	F	Empregado Temporário
	M	Empregado Efetivo
	F	Empregado Efetivo
	F	Empregado Efetivo
	F	Empregado Efetivo
	F	Empregado Efetivo
	F	Empregado Efetivo
	F	Empregado Efetivo
	F	Empregado Efetivo
	M	Empregado Efetivo
	F	Empregado Efetivo
	F	Empregado Efetivo
	F	Empregado Efetivo
	F	Empregado Efetivo
	F	Empregado Efetivo
	F	Empregado Efetivo
	F	Empregado Efetivo
	F	Empregado Efetivo
	F	Empregado Efetivo
	F	Empregado Efetivo
	F	Empregado Efetivo
	F	Empregado Efetivo
	F	Empregado Efetivo
	M	Empregado Efetivo
	F	Empregado Efetivo
	F	Empregado Efetivo
	F	Empregado Efetivo
	F	Empregado Efetivo
	F	Empregado Efetivo
	F	Empregado Efetivo
	F	Empregado Efetivo
	F	Empregado Efetivo

	F	Empregado Efetivo
	F	Empregado Efetivo
	F	Empregado Efetivo
	F	Empregado Efetivo
	F	Empregado Efetivo
	F	Empregado Efetivo
	F	Empregado Efetivo
	F	Empregado Efetivo
	F	Empregado Efetivo
	F	Empregado Efetivo
	M	Empregado Efetivo
	F	Empregado Efetivo
	F	Empregado Efetivo
	F	Empregado Efetivo
	F	Empregado Efetivo
	F	Empregado Efetivo
	F	Empregado Efetivo
	F	Empregado Efetivo
	F	Empregado Efetivo
	F	Empregado Efetivo
	F	Empregado Efetivo
	F	Empregado Efetivo
	F	Empregado Efetivo
	F	Empregado Efetivo
	F	Empregado Efetivo
	M	Empregado Efetivo
	F	Empregado Efetivo
	F	Empregado Efetivo
	F	Empregado Efetivo
	F	Empregado Efetivo
	F	Empregado Efetivo
	F	Empregado Efetivo
	F	Empregado Efetivo
	F	Empregado Efetivo
	F	Empregado Efetivo
	F	Empregado Efetivo
	F	Empregado Efetivo
	F	Empregado Efetivo
	F	Empregado Efetivo
	F	Empregado Efetivo
	M	Empregado Efetivo
	F	Empregado Efetivo
	F	Empregado Efetivo
	F	Empregado Efetivo
	F	Empregado Efetivo
	F	Empregado Efetivo
	F	Empregado Efetivo
	F	Empregado Efetivo

	F	Empregado Efetivo
	F	Empregado Efetivo
	F	Empregado Efetivo
	F	Empregado Efetivo
	M	Empregado Efetivo
	F	Empregado Efetivo
	F	Empregado Efetivo
	F	Empregado Efetivo
	F	Empregado Efetivo
	F	Empregado Efetivo
	M	Empregado Efetivo
	F	Empregado Efetivo
	F	Empregado Efetivo
	M	Empregado Efetivo
	F	Empregado Efetivo
	F	Empregado Efetivo
	F	Empregado Efetivo
	F	Empregado Efetivo
	F	Empregado Efetivo
	F	Empregado Efetivo
	F	Empregado Efetivo
	F	Empregado Efetivo
	F	Empregado Efetivo
	M	Empregado Efetivo
	F	Empregado Efetivo
	F	Empregado Efetivo
	F	Empregado Efetivo
	F	Empregado Efetivo
	F	Empregado Efetivo
	F	Empregado Efetivo
	F	Empregado Efetivo
	M	Empregado Efetivo
	F	Empregado Efetivo
	F	Empregado Efetivo
	F	Empregado Efetivo
	F	Empregado Efetivo
	F	Empregado Efetivo
	F	Empregado Efetivo
	F	Empregado Efetivo
	F	Empregado Efetivo
	F	Empregado Efetivo
	F	Empregado Efetivo
	F	Empregado Efetivo
	F	Empregado Efetivo
	F	Empregado Efetivo
	F	Empregado Efetivo
	F	Empregado Efetivo
	F	Empregado Efetivo

	F	Empregado Efetivo
	F	Empregado Efetivo
	F	Empregado Efetivo
	F	Empregado Efetivo
	F	Empregado Efetivo
	F	Empregado Efetivo
	F	Empregado Efetivo
	M	Empregado Efetivo
	F	Empregado Efetivo
	F	Empregado Efetivo
	F	Empregado Efetivo
	F	Empregado Efetivo
	F	Empregado Efetivo
	F	Empregado Efetivo
	F	Empregado Efetivo
	M	Empregado Efetivo
	F	Empregado Efetivo
	F	Empregado Efetivo
	F	Empregado Efetivo
	F	Empregado Efetivo
	F	Empregado Efetivo
	F	Empregado Efetivo
	M	Empregado Efetivo
	M	Empregado Efetivo
	F	Empregado Efetivo
	F	Empregado Efetivo
	F	Empregado Efetivo
	F	Empregado Efetivo
	M	Empregado Efetivo
	F	Empregado Efetivo
	F	Empregado Efetivo
	F	Empregado Efetivo
	F	Empregado Efetivo
	F	Empregado Efetivo
	F	Empregado Efetivo
	F	Empregado Efetivo
	F	Empregado Efetivo
	F	Empregado Efetivo
	F	Empregado Efetivo
	F	Empregado Efetivo
	F	Empregado Efetivo
	F	Empregado Efetivo
	F	Empregado Efetivo
	F	Empregado Efetivo
	F	Empregado Efetivo
	F	Empregado Efetivo
	F	Empregado Efetivo
	F	Empregado Efetivo

	F	Empregado Efetivo
	F	Empregado Efetivo
	F	Empregado Efetivo
	F	Empregado Efetivo
	F	Empregado Efetivo
	F	Empregado Efetivo
	F	Empregado Efetivo
	F	Empregado Efetivo
	F	Empregado Efetivo
	F	Empregado Efetivo
	F	Empregado Efetivo
	F	Empregado Efetivo
	F	Empregado Efetivo
	F	Empregado Efetivo
	F	Empregado Efetivo
	M	Empregado Efetivo
	F	Empregado Efetivo
	F	Empregado Efetivo
	F	Empregado Efetivo
	F	Empregado Efetivo
	F	Empregado Efetivo
	F	Empregado Efetivo
	F	Empregado Efetivo
	F	Empregado Efetivo
	F	Empregado Efetivo
	F	Empregado Efetivo
	F	Empregado Efetivo
	F	Empregado Efetivo
	F	Empregado Efetivo
	F	Empregado Efetivo
	F	Empregado Efetivo
	F	Empregado Efetivo
	F	Empregado Efetivo
	F	Empregado Efetivo
	F	Empregado Efetivo
	F	Empregado Efetivo
	F	Empregado Efetivo
	F	Empregado Efetivo
	F	Empregado Efetivo
	F	Empregado Efetivo
	F	Empregado Efetivo
	F	Empregado Efetivo
	F	Empregado Efetivo
	F	Empregado Efetivo
	F	Empregado Efetivo
	F	Empregado Efetivo
	F	Empregado Efetivo
	F	Empregado Efetivo

	F	Empregado Efetivo
	F	Empregado Efetivo
	M	Empregado Efetivo
	F	Empregado Efetivo
	F	Empregado Efetivo
	F	Empregado Efetivo
	F	Empregado Efetivo
	F	Empregado Efetivo
	M	Empregado Efetivo
	F	Empregado Efetivo
	F	Empregado Efetivo
	F	Empregado Efetivo
	F	Empregado Efetivo
	F	Empregado Efetivo
	F	Empregado Efetivo
	F	Empregado Efetivo
	F	Empregado Efetivo
	F	Empregado Efetivo
	M	Empregado Efetivo
	F	Empregado Efetivo
	F	Empregado Efetivo
	F	Empregado Efetivo
	M	Empregado Efetivo
	F	Empregado Efetivo
	M	Empregado Efetivo
	F	Empregado Efetivo
	F	Empregado Efetivo
	F	Empregado Efetivo
	F	Empregado Efetivo
	F	Empregado Efetivo
	F	Empregado Efetivo
	F	Empregado Efetivo
	F	Empregado Efetivo
	F	Empregado Efetivo
	F	Empregado Efetivo
	F	Empregado Efetivo
	F	Empregado Efetivo
	F	Empregado Efetivo
	F	Empregado Efetivo
	F	Empregado Efetivo
	F	Empregado Efetivo
	F	Empregado Efetivo
	F	Empregado Efetivo
	F	Empregado Efetivo
	F	Empregado Efetivo
	F	Empregado Efetivo
	F	Empregado Efetivo
	F	Empregado Efetivo

	M	Empregado Efetivo
	F	Empregado Efetivo
	F	Empregado Efetivo
	F	Empregado Efetivo
	F	Empregado Efetivo
	F	Empregado Efetivo
	F	Empregado Efetivo
	F	Empregado Efetivo
	F	Empregado Efetivo
	F	Empregado Efetivo
	F	Empregado Efetivo
	F	Empregado Efetivo
	F	Empregado Efetivo
	F	Empregado Efetivo
	F	Empregado Efetivo
	F	Empregado Efetivo
	F	Empregado Efetivo
	F	Empregado Efetivo
	F	Empregado Efetivo
	F	Empregado Efetivo
	F	Empregado Efetivo
	F	Empregado Efetivo
	F	Empregado Efetivo
	F	Empregado Efetivo
	F	Empregado Efetivo
	M	Empregado Efetivo
	F	Empregado Efetivo
	F	Empregado Efetivo
	F	Empregado Efetivo
	F	Empregado Efetivo
	F	Empregado Efetivo
	F	Empregado Efetivo
	F	Empregado Efetivo
	F	Empregado Efetivo
	F	Empregado Efetivo
	F	Empregado Efetivo
	F	Empregado Efetivo
	F	Empregado Efetivo
	F	Empregado Efetivo
	F	Empregado Efetivo
	F	Empregado Efetivo
	F	Empregado Efetivo
	F	Empregado Efetivo
	F	Empregado Efetivo
	F	Empregado Efetivo
	F	Empregado Efetivo
	F	Empregado Efetivo
	F	Empregado Efetivo

	F	Empregado Efetivo
	F	Empregado Efetivo
	F	Empregado Efetivo
	M	Empregado Efetivo
	F	Empregado Efetivo
	F	Empregado Efetivo
	F	Empregado Efetivo
	F	Empregado Efetivo
	F	Empregado Efetivo
	M	Empregado Efetivo
	F	Empregado Efetivo
	F	Empregado Efetivo
	F	Empregado Efetivo
	F	Empregado Efetivo
	F	Empregado Efetivo
	F	Empregado Efetivo
	F	Empregado Efetivo
	M	Empregado Efetivo
	M	Empregado Efetivo
	F	Empregado Efetivo
	F	Empregado Efetivo
	F	Empregado Efetivo
	M	Empregado Efetivo
	F	Empregado Efetivo
	F	Empregado Efetivo
	F	Empregado Efetivo
	F	Empregado Efetivo
	F	Empregado Efetivo
	F	Empregado Efetivo
	F	Empregado Efetivo
	M	Empregado Efetivo
	F	Empregado Efetivo
	M	Empregado Efetivo
	F	Empregado Efetivo
	F	Empregado Efetivo
	F	Empregado Efetivo
	F	Empregado Efetivo
	F	Empregado Efetivo
	F	Empregado Efetivo
	F	Empregado Efetivo
	F	Empregado Efetivo
	F	Empregado Efetivo
	F	Empregado Efetivo
	F	Empregado Efetivo
	M	Empregado Efetivo
	F	Empregado Efetivo
	M	Empregado Efetivo
	F	Empregado Efetivo

	F	Empregado Efetivo
	F	Empregado Efetivo
	F	Empregado Efetivo
	F	Empregado Efetivo
	F	Empregado Efetivo
	F	Empregado Efetivo
	F	Empregado Efetivo
	M	Empregado Efetivo
	F	Empregado Efetivo
	F	Empregado Efetivo
	F	Empregado Efetivo
	F	Empregado Efetivo
	F	Empregado Efetivo
	F	Empregado Efetivo
	F	Empregado Efetivo
	F	Empregado Efetivo
	F	Empregado Efetivo
	F	Empregado Efetivo
	F	Empregado Efetivo
	F	Empregado Efetivo
	F	Empregado Efetivo
	F	Empregado Efetivo
	F	Empregado Efetivo
	F	Empregado Efetivo
	F	Empregado Efetivo
	F	Empregado Efetivo
	F	Empregado Efetivo
	M	Empregado Efetivo
	F	Empregado Efetivo
	M	Empregado Efetivo
	F	Empregado Efetivo
	F	Empregado Efetivo
	F	Empregado Efetivo
	M	Empregado Efetivo

	M	Empregado Efetivo
	M	Empregado Efetivo
	F	Empregado Efetivo
	M	Empregado Efetivo
	F	Empregado Efetivo
	M	Empregado Efetivo
	M	Empregado Efetivo
	F	Empregado Efetivo
	F	Empregado Efetivo
	F	Empregado Efetivo
	F	Empregado Efetivo
	F	Empregado Efetivo
	F	Empregado Efetivo
	F	Empregado Efetivo
	M	Empregado Efetivo
	M	Empregado Efetivo
	F	Empregado Efetivo
	F	Empregado Efetivo
	M	Empregado Efetivo
	F	Empregado Efetivo
	F	Empregado Efetivo
	M	Empregado Efetivo
	F	Empregado Efetivo
	F	Empregado Efetivo
	F	Empregado Efetivo
	M	Empregado Efetivo
	F	Empregado Efetivo
	F	Empregado Efetivo
	F	Empregado Efetivo
	F	Empregado Efetivo
	F	Empregado Efetivo
	F	Empregado Efetivo

	F	Empregado Efetivo
	F	Empregado Efetivo
	F	Empregado Efetivo
	F	Empregado Efetivo
	F	Empregado Efetivo
	F	Empregado Efetivo
	F	Empregado Efetivo
	F	Empregado Efetivo
	F	Empregado Efetivo
	F	Empregado Efetivo
	F	Empregado Efetivo
	F	Empregado Efetivo
	F	Empregado Efetivo
	F	Empregado Efetivo
	F	Empregado Efetivo
	F	Empregado Efetivo
	F	Empregado Efetivo
	F	Empregado Efetivo
	F	Empregado Efetivo
	F	Empregado Efetivo
	F	Empregado Efetivo
	F	Empregado Efetivo
	F	Empregado Efetivo
	F	Empregado Efetivo
	F	Empregado Efetivo
	F	Empregado Efetivo
	F	Empregado Efetivo
	F	Empregado Efetivo
	F	Empregado Efetivo
	F	Empregado Efetivo
	F	Empregado Efetivo
	F	Empregado Efetivo

	F	Empregado Efetivo
	F	Empregado Efetivo
	F	Empregado Efetivo
	M	Empregado Efetivo
	M	Empregado Efetivo
	M	Empregado Efetivo
	M	Empregado Efetivo
	M	Empregado Efetivo
	M	Empregado Efetivo
	M	Empregado Efetivo
	M	Empregado Efetivo
	M	Empregado Efetivo
	M	Empregado Efetivo
	M	Empregado Efetivo
	M	Empregado Efetivo
	M	Empregado Efetivo
	M	Empregado Efetivo
	M	Empregado Efetivo
	M	Empregado Efetivo
	F	Empregado Efetivo
	F	Empregado Efetivo
	F	Cedido - RJU
	M	Empregado Efetivo
	F	Empregado Efetivo
	M	Empregado Efetivo
	F	Empregado Efetivo
	M	Empregado Efetivo
	F	Empregado Efetivo
	M	Empregado Efetivo
	F	Empregado Efetivo
	F	Empregado Efetivo
	F	Empregado Efetivo
	F	Empregado Efetivo

	F	Empregado Efetivo
	F	Empregado Efetivo
	F	Empregado Efetivo
	F	Empregado Efetivo
	F	Empregado Efetivo
	F	Empregado Efetivo
	F	Empregado Efetivo
	F	Empregado Efetivo
	F	Empregado Efetivo
	F	Empregado Efetivo
	F	Empregado Efetivo
	M	Empregado Efetivo
	M	Empregado Efetivo
	M	Empregado Efetivo
	M	Empregado Efetivo
	M	Empregado Efetivo
	M	Empregado Efetivo
	M	Empregado Efetivo
	F	Empregado Efetivo
	F	Empregado Efetivo
	F	Empregado Efetivo
	F	Empregado Efetivo
	F	Empregado Efetivo
	M	Empregado Efetivo
	M	Empregado Efetivo
	F	Empregado Efetivo
	F	Empregado Efetivo
	F	Empregado Efetivo
	F	Empregado Efetivo
	F	Empregado Efetivo
	F	Empregado Efetivo
	F	Empregado Efetivo
	F	Empregado Efetivo
	M	Empregado Efetivo
	F	Empregado Efetivo

	F	Empregado Efetivo
	M	Empregado Efetivo
	F	Empregado Efetivo
	F	Empregado Efetivo
	M	Empregado Efetivo
	M	Empregado Efetivo
	M	Empregado Efetivo
	F	Empregado Efetivo
	M	Empregado Efetivo
	F	Empregado Efetivo
	F	Empregado Efetivo
	F	Empregado Efetivo
	F	Empregado Efetivo
	M	Empregado Efetivo
	F	Empregado Efetivo
	F	Empregado Efetivo
	F	Empregado Efetivo
	M	Empregado Efetivo
	F	Empregado Efetivo
	M	Empregado Efetivo
	M	Empregado Efetivo
	F	Empregado Efetivo
	F	Empregado Efetivo
	M	Empregado Efetivo
	M	Empregado Efetivo
	M	Empregado Efetivo
	F	Empregado Efetivo
	M	Empregado Efetivo
	F	Empregado Efetivo
	F	Empregado Efetivo
	F	Empregado Efetivo
	F	Empregado Efetivo
	F	Empregado Efetivo
	F	Empregado Efetivo
	M	Empregado Efetivo
	F	Empregado Efetivo
	F	Empregado Efetivo
	M	Empregado Efetivo
	F	Empregado Efetivo
	F	Empregado Efetivo
	F	Empregado Efetivo
	F	Empregado Efetivo
	M	Empregado Efetivo

	M	Empregado Efetivo
	F	Empregado Efetivo
	F	Empregado Efetivo
	F	Empregado Efetivo
	F	Empregado Efetivo
	M	Empregado Efetivo
	F	Empregado Efetivo
	F	Empregado Efetivo
	F	Empregado Efetivo
	M	Empregado Efetivo
	F	Empregado Efetivo
	M	Empregado Efetivo
	F	Empregado Efetivo
	F	Empregado Efetivo
	F	Empregado Efetivo
	M	Empregado Efetivo
	F	Empregado Efetivo
	F	Empregado Efetivo
	F	Empregado Efetivo
	F	Empregado Efetivo
	F	Empregado Efetivo
	F	Empregado Efetivo
	F	Empregado Efetivo
	F	Empregado Efetivo
	M	Empregado Efetivo
	M	Empregado Efetivo
	F	Empregado Efetivo
	F	Empregado Efetivo
	F	Empregado Efetivo
	M	Empregado Efetivo
	F	Empregado Efetivo
	F	Empregado Efetivo
	F	Empregado Efetivo
	F	Empregado Efetivo
	F	Empregado Efetivo
	F	Empregado Efetivo
	M	Empregado Efetivo
	M	Empregado Efetivo
	F	Empregado Efetivo
	M	Empregado Efetivo
	M	Empregado Efetivo
	M	Empregado Efetivo
	F	Empregado Efetivo
	M	Empregado Efetivo
	F	Empregado Efetivo
	M	Empregado Efetivo
	F	Empregado Efetivo
	M	Empregado Efetivo

	F	Empregado Efetivo
	M	Empregado Efetivo
	F	Empregado Efetivo
	M	Empregado Efetivo
	F	Empregado Efetivo
	F	Empregado Efetivo
	F	Empregado Efetivo
	F	Empregado Efetivo
	F	Empregado Efetivo
	M	Empregado Efetivo
	F	Empregado Efetivo
	F	Empregado Efetivo
	F	Empregado Efetivo
	F	Empregado Efetivo
	M	Empregado Efetivo
	F	Empregado Efetivo
	F	Empregado Efetivo
	M	Empregado Efetivo
	M	Empregado Efetivo
	F	Empregado Efetivo
	M	Empregado Efetivo
	M	Empregado Efetivo
	M	Empregado Efetivo
	F	Empregado Efetivo
	F	Empregado Efetivo
	M	Empregado Efetivo
	M	Empregado Efetivo
	F	Empregado Efetivo
	F	Empregado Efetivo
	F	Empregado Efetivo
	F	Empregado Efetivo
	F	Empregado Efetivo
	F	Empregado Efetivo
	F	Empregado Efetivo
	F	Empregado Efetivo
	F	Empregado Efetivo
	F	Empregado Efetivo
	F	Empregado Efetivo
	F	Empregado Efetivo
	F	Empregado Efetivo
	F	Empregado Efetivo
	F	Empregado Efetivo
	F	Empregado Efetivo
	F	Empregado Efetivo
	F	Empregado Efetivo
	F	Empregado Efetivo
	F	Empregado Efetivo
	F	Empregado Efetivo

	F	Empregado Efetivo
	F	Empregado Efetivo
	F	Empregado Efetivo
	F	Empregado Efetivo
	F	Empregado Efetivo
	F	Empregado Efetivo
	F	Empregado Efetivo
	F	Empregado Efetivo
	F	Empregado Efetivo
	M	Empregado Efetivo
	F	Empregado Efetivo
	F	Empregado Efetivo
	M	Empregado Efetivo
	M	Empregado Efetivo
	F	Empregado Efetivo
	F	Empregado Efetivo
	F	Empregado Efetivo
	F	Empregado Efetivo
	M	Empregado Efetivo
	F	Empregado Efetivo
	F	Empregado Efetivo
	F	Empregado Efetivo
	F	Empregado Efetivo
	F	Empregado Efetivo
	F	Empregado Efetivo
	F	Empregado Efetivo
	F	Empregado Efetivo
	F	Empregado Efetivo
	F	Empregado Efetivo
	F	Empregado Efetivo
	M	Empregado Efetivo
	F	Empregado Efetivo
	F	Empregado Efetivo
	F	Empregado Efetivo
	F	Empregado Efetivo
	F	Empregado Efetivo
	F	Empregado Efetivo
	F	Empregado Efetivo
	F	Empregado Efetivo
	F	Empregado Efetivo
	F	Empregado Efetivo
	F	Empregado Efetivo
	F	Empregado Efetivo
	F	Empregado Efetivo
	F	Empregado Efetivo
	F	Empregado Efetivo
	M	Empregado Efetivo
	F	Empregado Efetivo

	M	Empregado Efetivo
	F	Empregado Efetivo
	M	Empregado Efetivo
	F	Empregado Efetivo
	F	Empregado Efetivo
	M	Empregado Efetivo
	F	Empregado Efetivo
	F	Empregado Efetivo
	M	Empregado Efetivo
	F	Empregado Efetivo
	F	Empregado Efetivo
	M	Empregado Efetivo
	F	Empregado Efetivo
	M	Empregado Efetivo
	M	Empregado Efetivo
	F	Empregado Efetivo
	F	Empregado Efetivo
	F	Empregado Efetivo
	M	Empregado Efetivo
	F	Empregado Efetivo
	M	Empregado Efetivo
	F	Empregado Efetivo
	F	Empregado Efetivo
	F	Empregado Efetivo
	F	Empregado Efetivo
	M	Empregado Efetivo
	M	Empregado Efetivo
	F	Empregado Efetivo
	M	Empregado Efetivo
	F	Empregado Efetivo
	F	Empregado Efetivo
	M	Empregado Efetivo
	F	Empregado Efetivo
	M	Empregado Efetivo
	F	Empregado Efetivo
	F	Empregado Efetivo
	F	Empregado Efetivo
	F	Empregado Efetivo
	F	Empregado Efetivo
	F	Empregado Efetivo
	F	Empregado Efetivo
	F	Empregado Efetivo
	F	Empregado Efetivo
	F	Empregado Efetivo
	F	Empregado Efetivo
	F	Empregado Efetivo
	F	Empregado Efetivo
	F	Empregado Efetivo

	F	Empregado Efetivo
	M	Empregado Efetivo
	F	Empregado Efetivo
	F	Empregado Efetivo
	F	Empregado Efetivo
	M	Empregado Efetivo
	M	Empregado Efetivo
	M	Empregado Efetivo
	F	Empregado Efetivo
	F	Empregado Efetivo
	M	Empregado Efetivo
	F	Empregado Efetivo
	F	Empregado Efetivo
	F	Empregado Efetivo
	F	Empregado Efetivo
	F	Empregado Efetivo
	F	Empregado Efetivo
	F	Empregado Efetivo
	F	Empregado Efetivo
	F	Empregado Efetivo
	F	Empregado Efetivo
	M	Empregado Efetivo
	M	Empregado Efetivo
	F	Empregado Efetivo
	M	Empregado Efetivo
	F	Empregado Efetivo
	F	Empregado Efetivo
	F	Empregado Efetivo
	M	Empregado Efetivo
	F	Empregado Efetivo
	M	Empregado Efetivo
	F	Empregado Efetivo
	F	Empregado Efetivo
	M	Empregado Efetivo
	F	Empregado Efetivo
	M	Empregado Efetivo
	F	Empregado Efetivo
	F	Empregado Efetivo
	F	Empregado Efetivo
	M	Empregado Efetivo
	M	Empregado Efetivo
	F	Empregado Efetivo
	F	Empregado Efetivo
	F	Empregado Efetivo
	F	Empregado Efetivo
	M	Empregado Efetivo
	F	Empregado Efetivo
	M	Empregado Efetivo

	F	Empregado Efetivo
	M	Empregado Efetivo
	M	Empregado Efetivo
	M	Empregado Efetivo
	F	Empregado Efetivo
	M	Empregado Efetivo
	F	Empregado Efetivo
	F	Empregado Efetivo
	M	Empregado Efetivo
	F	Empregado Efetivo
	F	Empregado Efetivo
	F	Empregado Efetivo
	F	Empregado Efetivo
	F	Empregado Efetivo
	F	Empregado Efetivo
	F	Empregado Efetivo
	F	Empregado Efetivo
	F	Empregado Efetivo
	F	Empregado Efetivo
	F	Empregado Efetivo
	F	Empregado Efetivo
	F	Empregado Efetivo
	F	Empregado Efetivo
	F	Empregado Efetivo
	F	Empregado Efetivo
	F	Empregado Efetivo
	F	Empregado Efetivo
	F	Empregado Efetivo
	F	Empregado Efetivo
	M	Empregado Efetivo
	M	Empregado Efetivo
	M	Empregado Efetivo
	F	Empregado Efetivo
	M	Empregado Efetivo
	M	Empregado Efetivo
	M	Empregado Efetivo
	M	Empregado Efetivo
	F	Empregado Efetivo
	M	Empregado Efetivo
	F	Empregado Efetivo
	F	Empregado Efetivo
	F	Empregado Efetivo
	M	Empregado Efetivo
	F	Empregado Efetivo
	F	Empregado Efetivo
	F	Empregado Efetivo
	F	Empregado Efetivo
	F	Empregado Efetivo

	F	Empregado Efetivo
	M	Empregado Efetivo
	F	Empregado Efetivo
	F	Empregado Efetivo
	F	Empregado Efetivo
	F	Empregado Efetivo
	M	Empregado Efetivo
	F	Empregado Efetivo
	M	Empregado Efetivo
	F	Empregado Efetivo
	F	Empregado Efetivo
	F	Empregado Efetivo
	M	Empregado Efetivo
	M	Empregado Efetivo
	M	Empregado Efetivo
	F	Empregado Efetivo
	M	Empregado Efetivo
	F	Empregado Efetivo
	F	Empregado Efetivo
	F	Empregado Efetivo
	M	Empregado Efetivo
	M	Empregado Efetivo
	F	Empregado Efetivo
	F	Empregado Efetivo
	F	Empregado Efetivo
	M	Empregado Efetivo
	F	Empregado Efetivo
	F	Empregado Efetivo
	F	Empregado Efetivo
	F	Empregado Efetivo
	F	Empregado Efetivo
	M	Empregado Efetivo
	M	Empregado Efetivo
	M	Empregado Efetivo
	F	Empregado Efetivo
	F	Empregado Efetivo
	F	Empregado Efetivo
	M	Empregado Efetivo
	F	Empregado Efetivo
	F	Empregado Efetivo
	F	Empregado Efetivo
	F	Empregado Efetivo
	F	Empregado Efetivo
	F	Empregado Efetivo
	F	Empregado Efetivo
	F	Empregado Efetivo
	F	Empregado Efetivo
	F	Empregado Efetivo

	F	Empregado Efetivo
	M	Empregado Efetivo
	F	Empregado Efetivo
	F	Empregado Efetivo
	F	Empregado Efetivo
	F	Empregado Efetivo
	F	Empregado Efetivo
	F	Empregado Efetivo
	F	Empregado Efetivo
	M	Empregado Efetivo
	M	Empregado Efetivo
	F	Empregado Efetivo
	F	Empregado Efetivo
	F	Empregado Efetivo
	F	Empregado Efetivo
	F	Empregado Efetivo
	F	Empregado Efetivo
	F	Empregado Efetivo
	F	Empregado Efetivo
	F	Empregado Efetivo
	F	Empregado Efetivo
	F	Empregado Efetivo
	F	Empregado Efetivo
	F	Empregado Efetivo
	M	Empregado Efetivo
	F	Empregado Efetivo
	F	Empregado Efetivo
	F	Empregado Efetivo
	F	Empregado Efetivo
	F	Empregado Efetivo
	F	Empregado Efetivo
	F	Empregado Efetivo
	F	Empregado Efetivo
	F	Empregado Efetivo
	F	Empregado Efetivo
	F	Empregado Efetivo
	F	Empregado Efetivo
	F	Empregado Efetivo
	F	Empregado Efetivo
	M	Empregado Efetivo
	F	Empregado Efetivo
	F	Empregado Efetivo
	F	Empregado Efetivo
	F	Empregado Efetivo
	F	Empregado Efetivo
	F	Empregado Efetivo
	F	Empregado Efetivo
	F	Empregado Efetivo

	F	Empregado Efetivo
	M	Empregado Efetivo
	M	Empregado Efetivo
	F	Empregado Efetivo
	M	Empregado Efetivo
	F	Empregado Efetivo
	M	Empregado Efetivo
	M	Empregado Efetivo
	M	Empregado Efetivo
	F	Empregado Efetivo
	F	Empregado Efetivo
	F	Empregado Efetivo
	F	Empregado Efetivo
	F	Empregado Efetivo
	F	Empregado Efetivo
	F	Empregado Efetivo
	F	Empregado Efetivo
	M	Empregado Efetivo
	F	Empregado Efetivo
	M	Empregado Efetivo
	F	Empregado Efetivo
	M	Empregado Efetivo
	F	Empregado Efetivo
	F	Empregado Efetivo
	F	Empregado Efetivo
	F	Empregado Efetivo
	M	Empregado Efetivo
	F	Empregado Efetivo
	F	Empregado Efetivo
	F	Empregado Efetivo
	F	Empregado Efetivo
	M	Empregado Efetivo
	F	Empregado Efetivo
	F	Empregado Efetivo
	F	Empregado Efetivo
	F	Empregado Efetivo
	F	Empregado Efetivo
	F	Empregado Efetivo
	F	Empregado Efetivo
	M	Empregado Efetivo
	F	Empregado Efetivo
	M	Empregado Efetivo
	M	Empregado Efetivo
	F	Empregado Efetivo
	F	Empregado Efetivo
	F	Empregado Efetivo
	M	Empregado Efetivo
	M	Empregado Efetivo

	F	Empregado Efetivo
	F	Empregado Efetivo
	M	Empregado Efetivo
	M	Empregado Efetivo
	F	Empregado Efetivo
	F	Empregado Efetivo
	F	Empregado Efetivo
	F	Empregado Efetivo
	M	Empregado Efetivo
	F	Empregado Efetivo
	F	Empregado Efetivo
	F	Empregado Efetivo
	M	Empregado Efetivo
	F	Empregado Efetivo
	F	Empregado Efetivo
	M	Empregado Efetivo
	F	Empregado Efetivo
	F	Empregado Efetivo
	F	Empregado Efetivo
	F	Empregado Efetivo
	F	Empregado Efetivo
	F	Empregado Efetivo
	F	Empregado Efetivo
	F	Empregado Efetivo
	F	Empregado Efetivo
	M	Empregado Efetivo
	F	Empregado Efetivo
	M	Empregado Efetivo
	F	Empregado Efetivo
	F	Empregado Efetivo
	M	Empregado Efetivo
	F	Empregado Efetivo
	F	Empregado Efetivo
	F	Empregado Efetivo
	M	Empregado Efetivo
	F	Empregado Efetivo
	M	Empregado Efetivo
	M	Empregado Efetivo
	M	Empregado Efetivo
	M	Empregado Efetivo
	M	Empregado Efetivo
	M	Empregado Efetivo
	F	Empregado Efetivo
	M	Empregado Efetivo
	F	Empregado Efetivo
	F	Empregado Efetivo
	F	Empregado Efetivo
	M	Empregado Efetivo

	M	Empregado Efetivo
	F	Empregado Efetivo
	F	Empregado Efetivo
	M	Empregado Efetivo
	F	Empregado Efetivo
	M	Empregado Efetivo
	F	Empregado Efetivo
	M	Empregado Efetivo
	M	Empregado Efetivo
	M	Empregado Efetivo
	M	Empregado Efetivo
	F	Empregado Efetivo
	F	Empregado Efetivo
	M	Empregado Efetivo
	F	Empregado Efetivo
	F	Empregado Efetivo
	F	Empregado Efetivo
	F	Empregado Efetivo
	F	Empregado Efetivo
	F	Empregado Efetivo
	M	Empregado Efetivo
	F	Empregado Efetivo
	F	Empregado Efetivo
	F	Empregado Efetivo
	M	Empregado Efetivo
	F	Empregado Efetivo
	M	Empregado Efetivo
	M	Empregado Efetivo
	F	Empregado Efetivo
	M	Empregado Efetivo
	F	Empregado Efetivo
	F	Empregado Efetivo
	F	Empregado Efetivo
	F	Empregado Efetivo
	F	Empregado Efetivo
	F	Empregado Efetivo
	F	Empregado Efetivo
	F	Empregado Efetivo
	F	Empregado Efetivo
	F	Empregado Efetivo
	M	Empregado Efetivo
	M	Empregado Efetivo
	F	Empregado Efetivo
	M	Empregado Efetivo
	F	Empregado Efetivo
	M	Empregado Efetivo
	F	Empregado Efetivo
	M	Empregado Efetivo

	M	Empregado Efetivo
	M	Empregado Efetivo
	F	Empregado Efetivo
	M	Empregado Efetivo
	M	Empregado Efetivo
	M	Empregado Efetivo
	F	Empregado Efetivo
	F	Empregado Efetivo
	M	Empregado Efetivo
	M	Empregado Efetivo
	M	Empregado Efetivo
	M	Empregado Efetivo
	M	Empregado Efetivo
	M	Empregado Efetivo
	M	Empregado Efetivo
	M	Empregado Efetivo
	F	Empregado Efetivo
	F	Empregado Efetivo
	F	Empregado Efetivo
	M	Empregado Efetivo
	M	Empregado Efetivo
	M	Empregado Efetivo
	F	Empregado Efetivo
	F	Empregado Efetivo
	M	Empregado Efetivo
	F	Empregado Efetivo
	F	Empregado Efetivo
	F	Empregado Efetivo
	F	Empregado Efetivo
	M	Empregado Efetivo
	F	Empregado Efetivo
	F	Empregado Efetivo
	F	Empregado Efetivo
	F	Empregado Efetivo
	F	Empregado Efetivo
	F	Empregado Efetivo
	M	Empregado Efetivo
	M	Empregado Efetivo
	M	Empregado Efetivo
	F	Empregado Efetivo
	F	Empregado Efetivo
	F	Empregado Efetivo
	F	Empregado Efetivo
	F	Empregado Efetivo
	M	Empregado Efetivo
	M	Empregado Efetivo
	F	Empregado Efetivo
	F	Empregado Efetivo

	F	Empregado Efetivo
	F	Empregado Efetivo
	F	Empregado Efetivo
	F	Empregado Efetivo
	F	Empregado Efetivo
	F	Empregado Efetivo
	F	Empregado Efetivo
	F	Empregado Efetivo
	F	Empregado Efetivo
	F	Empregado Efetivo
	F	Empregado Efetivo
	F	Empregado Efetivo
	M	Empregado Efetivo
	M	Empregado Efetivo
	F	Empregado Efetivo
	F	Empregado Efetivo
	F	Empregado Efetivo
	F	Empregado Efetivo
	F	Empregado Efetivo
	F	Empregado Efetivo
	F	Empregado Efetivo
	M	Empregado Efetivo
	M	Empregado Efetivo
	M	Empregado Efetivo
	M	Empregado Efetivo
	F	Empregado Efetivo
	F	Empregado Efetivo
	F	Empregado Efetivo
	F	Empregado Efetivo
	F	Empregado Efetivo
	F	Empregado Efetivo
	F	Empregado Efetivo
	F	Empregado Efetivo
	M	Empregado Efetivo
	M	Empregado Efetivo
	F	Empregado Efetivo
	F	Empregado Efetivo
	F	Empregado Efetivo
	F	Empregado Efetivo
	F	Empregado Efetivo
	M	Empregado Efetivo
	F	Empregado Efetivo
	M	Empregado Efetivo
	M	Empregado Efetivo
	F	Empregado Efetivo
	F	Empregado Efetivo
	F	Empregado Efetivo
	F	Empregado Efetivo

	F	Empregado Efetivo
	F	Empregado Efetivo
	F	Empregado Efetivo
	F	Empregado Efetivo
	M	Empregado Efetivo
	F	Empregado Efetivo
	M	Empregado Efetivo
	F	Empregado Efetivo
	F	Empregado Efetivo
	F	Empregado Efetivo
	F	Empregado Efetivo
	F	Empregado Efetivo
	M	Empregado Efetivo
	M	Empregado Efetivo
	M	Empregado Efetivo
	F	Empregado Efetivo
	F	Empregado Efetivo
	M	Empregado Efetivo
	M	Empregado Efetivo
	M	Empregado Efetivo
	M	Empregado Efetivo
	F	Empregado Efetivo
	F	Empregado Efetivo
	F	Empregado Efetivo
	M	Empregado Efetivo
	M	Empregado Efetivo
	M	Empregado Efetivo
	F	Empregado Efetivo
	F	Empregado Efetivo
	M	Empregado Efetivo
	F	Empregado Efetivo
	F	Empregado Efetivo
	M	Empregado Efetivo
	M	Empregado Efetivo
	F	Empregado Efetivo
	M	Empregado Efetivo
	M	Empregado Efetivo
	F	Empregado Efetivo
	F	Empregado Efetivo
	F	Empregado Efetivo
	F	Empregado Efetivo
	F	Empregado Efetivo
	F	Empregado Efetivo
	M	Empregado Efetivo
	F	Empregado Efetivo
	F	Empregado Efetivo
	F	Empregado Efetivo
	F	Empregado Efetivo

	F	Empregado Efetivo
	F	Empregado Efetivo
	F	Empregado Efetivo
	F	Empregado Efetivo
	F	Empregado Efetivo
	F	Empregado Efetivo
	F	Empregado Efetivo
	F	Empregado Efetivo
	F	Empregado Efetivo
	M	Empregado Efetivo
	F	Empregado Efetivo
	F	Empregado Efetivo
	M	Empregado Efetivo
	M	Empregado Efetivo
	F	Empregado Efetivo
	F	Empregado Efetivo
	F	Empregado Efetivo
	F	Empregado Efetivo
	F	Empregado Efetivo
	F	Empregado Efetivo
	F	Empregado Efetivo
	F	Empregado Efetivo
	F	Empregado Efetivo
	F	Empregado Efetivo
	F	Empregado Efetivo
	F	Empregado Efetivo
	F	Empregado Efetivo
	F	Empregado Efetivo
	F	Empregado Efetivo
	F	Empregado Efetivo
	F	Empregado Efetivo
	M	Empregado Efetivo
	F	Empregado Efetivo
	M	Empregado Efetivo
	F	Empregado Efetivo
	F	Empregado Efetivo
	M	Empregado Efetivo
	F	Empregado Efetivo
	M	Empregado Efetivo
	F	Empregado Efetivo
	F	Empregado Efetivo
	M	Empregado Efetivo
	F	Empregado Efetivo
	F	Empregado Efetivo
	F	Empregado Efetivo
	M	Empregado Efetivo
	F	Empregado Efetivo
	F	Empregado Efetivo

	F	Empregado Efetivo
	M	Empregado Efetivo
	M	Empregado Efetivo
	M	Empregado Efetivo
	F	Empregado Efetivo
	F	Empregado Efetivo
	F	Empregado Efetivo
	F	Empregado Efetivo
	M	Empregado Efetivo
	M	Empregado Efetivo
	F	Empregado Efetivo
	F	Empregado Efetivo
	M	Empregado Efetivo
	F	Empregado Efetivo
	M	Empregado Efetivo
	F	Empregado Efetivo
	F	Empregado Efetivo
	M	Empregado Efetivo
	F	Empregado Efetivo
	F	Empregado Efetivo
	F	Empregado Efetivo
	F	Empregado Efetivo
	F	Empregado Efetivo
	F	Empregado Efetivo
	F	Empregado Efetivo
	F	Empregado Efetivo
	F	Empregado Efetivo
	F	Empregado Efetivo
	F	Empregado Efetivo
	F	Empregado Efetivo
	F	Empregado Efetivo
	F	Empregado Efetivo
	F	Empregado Efetivo
	F	Empregado Efetivo
	F	Empregado Efetivo
	F	Empregado Efetivo
	F	Empregado Efetivo
	F	Empregado Efetivo
	F	Empregado Efetivo

	M	Empregado Efetivo
	F	Empregado Efetivo
	F	Empregado Efetivo
	F	Empregado Efetivo
	F	Empregado Efetivo
	F	Empregado Efetivo
	F	Empregado Efetivo
	F	Empregado Efetivo
	F	Empregado Efetivo
	M	Empregado Efetivo
	M	Empregado Efetivo
	F	Empregado Efetivo
	F	Empregado Efetivo
	M	Empregado Efetivo
	F	Empregado Efetivo
	M	Empregado Efetivo
	M	Empregado Efetivo
	F	Empregado Efetivo
	M	Empregado Efetivo
	F	Empregado Efetivo
	F	Empregado Efetivo
	F	Empregado Efetivo
	M	Empregado Efetivo
	M	Empregado Efetivo
	M	Empregado Efetivo
	F	Empregado Efetivo
	F	Empregado Efetivo
	M	Empregado Efetivo
	F	Empregado Efetivo
	F	Empregado Efetivo
	F	Empregado Efetivo
	F	Empregado Efetivo
	F	Empregado Efetivo
	M	Empregado Efetivo
	F	Empregado Efetivo

	F	Empregado Efetivo
	F	Empregado Efetivo
	F	Empregado Efetivo
	F	Empregado Efetivo
	F	Empregado Efetivo
	F	Empregado Efetivo
	F	Empregado Efetivo
	F	Empregado Efetivo
	F	Empregado Efetivo
	M	Empregado Efetivo
	M	Empregado Efetivo
	F	Empregado Efetivo
	M	Empregado Efetivo
	F	Empregado Efetivo
	F	Empregado Efetivo
	F	Empregado Efetivo
	F	Empregado Efetivo
	F	Empregado Efetivo
	F	Empregado Efetivo
	F	Empregado Efetivo
	F	Empregado Efetivo
	F	Empregado Efetivo
	F	Empregado Efetivo
	M	Empregado Efetivo
	F	Empregado Efetivo
	F	Empregado Efetivo
	F	Empregado Efetivo
	M	Empregado Efetivo
	F	Empregado Efetivo
	F	Empregado Efetivo
	F	Empregado Efetivo
	F	Empregado Efetivo
	F	Empregado Efetivo
	M	Empregado Efetivo
	F	Empregado Efetivo
	F	Empregado Efetivo
	F	Empregado Efetivo
	M	Empregado Efetivo
	F	Empregado Efetivo
	F	Empregado Efetivo
	F	Empregado Efetivo
	F	Empregado Efetivo
	F	Empregado Efetivo
	F	Empregado Efetivo
	F	Empregado Efetivo
	F	Empregado Efetivo
	F	Empregado Efetivo
	M	Empregado Efetivo

	F	Empregado Efetivo
	F	Empregado Efetivo
	F	Empregado Efetivo
	F	Empregado Efetivo
	M	Empregado Efetivo
	M	Empregado Efetivo
	M	Empregado Efetivo
	F	Empregado Efetivo
	M	Empregado Efetivo
	M	Empregado Efetivo
	F	Empregado Efetivo
	F	Empregado Efetivo
	F	Empregado Efetivo
	F	Empregado Efetivo
	F	Empregado Efetivo
	M	Empregado Efetivo
	M	Empregado Efetivo
	F	Empregado Efetivo
	F	Empregado Efetivo
	F	Empregado Efetivo
	F	Empregado Efetivo
	F	Empregado Efetivo
	F	Empregado Efetivo
	F	Empregado Efetivo
	F	Empregado Efetivo
	F	Empregado Efetivo
	F	Empregado Efetivo
	F	Empregado Efetivo
	F	Empregado Efetivo
	F	Empregado Efetivo
	F	Empregado Efetivo
	M	Empregado Efetivo
	F	Empregado Efetivo
	F	Empregado Efetivo
	M	Empregado Efetivo
	F	Empregado Efetivo
	M	Empregado Efetivo
	F	Empregado Efetivo
	F	Empregado Efetivo
	F	Empregado Efetivo
	F	Empregado Efetivo
	F	Empregado Efetivo
	F	Empregado Efetivo
	F	Empregado Efetivo
	F	Empregado Efetivo
	F	Empregado Efetivo
	F	Empregado Efetivo
	M	Empregado Efetivo

	F	Empregado Efetivo
	F	Empregado Efetivo
	M	Empregado Efetivo
	F	Empregado Efetivo
	F	Empregado Efetivo
	M	Empregado Efetivo
	F	Empregado Efetivo
	F	Empregado Efetivo
	M	Empregado Efetivo
	F	Empregado Efetivo
	M	Empregado Efetivo
	M	Empregado Efetivo
	F	Empregado Efetivo
	F	Empregado Efetivo
	F	Empregado Efetivo
	F	Empregado Efetivo
	F	Empregado Efetivo
	F	Empregado Efetivo
	M	Empregado Efetivo
	F	Empregado Efetivo
	F	Empregado Efetivo
	F	Empregado Efetivo
	F	Empregado Efetivo
	F	Empregado Efetivo
	M	Empregado Efetivo
	M	Empregado Efetivo
	F	Empregado Efetivo
	F	Empregado Efetivo
	F	Empregado Efetivo
	F	Empregado Efetivo
	F	Empregado Efetivo
	F	Empregado Efetivo
	F	Empregado Efetivo
	F	Empregado Efetivo
	F	Empregado Efetivo
	F	Empregado Efetivo
	F	Empregado Efetivo
	F	Empregado Efetivo
	F	Empregado Efetivo
	F	Empregado Efetivo
	F	Empregado Efetivo
	M	Empregado Efetivo
	F	Empregado Efetivo
	M	Empregado Efetivo
	F	Empregado Efetivo
	F	Empregado Efetivo
	F	Empregado Efetivo
	F	Empregado Efetivo

	F	Empregado Efetivo
	F	Empregado Efetivo
	M	Empregado Efetivo
	F	Empregado Efetivo
	F	Empregado Efetivo
	F	Empregado Efetivo
	F	Empregado Efetivo
	F	Empregado Efetivo
	F	Empregado Efetivo
	F	Empregado Efetivo
	M	Empregado Efetivo
	M	Empregado Efetivo
	M	Empregado Efetivo
	M	Empregado Efetivo
	F	Empregado Efetivo
	F	Empregado Efetivo
	F	Empregado Efetivo
	F	Empregado Efetivo
	F	Empregado Efetivo
	F	Empregado Efetivo
	M	Empregado Efetivo
	F	Empregado Efetivo
	F	Empregado Efetivo
	F	Empregado Efetivo
	F	Empregado Efetivo
	F	Empregado Efetivo
	F	Empregado Efetivo
	F	Empregado Efetivo
	F	Empregado Efetivo
	F	Empregado Efetivo
	F	Empregado Efetivo
	F	Empregado Efetivo
	F	Empregado Efetivo
	F	Empregado Efetivo
	F	Empregado Efetivo
	F	Empregado Efetivo
	F	Empregado Efetivo
	F	Empregado Efetivo
	M	Empregado Efetivo
	M	Empregado Efetivo
	F	Empregado Efetivo
	F	Empregado Efetivo
	M	Empregado Efetivo
	F	Empregado Efetivo
	F	Empregado Efetivo
	F	Empregado Efetivo
	F	Empregado Efetivo
	F	Empregado Efetivo

	F	Empregado Efetivo
	M	Empregado Efetivo
	F	Empregado Efetivo
	F	Empregado Efetivo
	F	Empregado Efetivo
	F	Empregado Efetivo
	F	Empregado Efetivo
	F	Empregado Efetivo
	F	Empregado Efetivo
	F	Empregado Efetivo
	F	Empregado Efetivo
	F	Empregado Efetivo
	F	Empregado Efetivo
	F	Empregado Efetivo
	F	Empregado Efetivo
	F	Empregado Efetivo
	F	Empregado Efetivo
	F	Empregado Efetivo
	F	Empregado Efetivo
	F	Empregado Efetivo
	F	Empregado Efetivo
	F	Empregado Efetivo
	F	Empregado Efetivo
	M	Empregado Efetivo
	F	Empregado Efetivo
	F	Empregado Efetivo
	F	Empregado Efetivo
	F	Empregado Efetivo
	F	Empregado Efetivo
	F	Empregado Efetivo
	F	Empregado Efetivo
	F	Empregado Efetivo
	F	Empregado Efetivo
	F	Empregado Efetivo
	F	Empregado Efetivo
	F	Empregado Efetivo
	F	Empregado Efetivo
	F	Empregado Efetivo
	F	Empregado Efetivo
	M	Empregado Efetivo
	F	Empregado Efetivo
	F	Empregado Efetivo
	M	Empregado Efetivo
	F	Empregado Efetivo
	F	Empregado Efetivo
	F	Empregado Efetivo
	F	Empregado Efetivo
	M	Empregado Efetivo

	F	Empregado Efetivo
	F	Empregado Efetivo
	F	Empregado Efetivo
	M	Empregado Efetivo
	M	Empregado Efetivo
	F	Empregado Efetivo
	F	Empregado Efetivo
	F	Empregado Efetivo
	F	Empregado Efetivo
	F	Empregado Efetivo
	F	Empregado Efetivo
	F	Empregado Efetivo
	F	Empregado Efetivo
	F	Empregado Efetivo
	F	Empregado Efetivo
	F	Empregado Efetivo
	F	Empregado Efetivo
	F	Empregado Efetivo
	F	Empregado Efetivo
	F	Empregado Efetivo
	F	Empregado Efetivo
	F	Empregado Efetivo
	F	Empregado Efetivo
	F	Empregado Efetivo
	F	Empregado Efetivo
	F	Empregado Efetivo
	F	Empregado Efetivo
	F	Empregado Efetivo
	F	Empregado Efetivo
	F	Empregado Efetivo
	F	Empregado Efetivo
	F	Empregado Efetivo
	F	Empregado Efetivo
	F	Empregado Efetivo
	F	Empregado Efetivo
	F	Empregado Efetivo
	M	Empregado Efetivo
	F	Empregado Efetivo
	F	Empregado Efetivo
	F	Empregado Efetivo
	F	Empregado Efetivo
	F	Empregado Efetivo
	F	Empregado Efetivo
	F	Empregado Efetivo
	F	Empregado Efetivo
	F	Empregado Efetivo
	F	Empregado Efetivo
	F	Empregado Efetivo

	F	Empregado Efetivo
	F	Empregado Efetivo
	M	Empregado Efetivo
	F	Empregado Efetivo
	F	Empregado Efetivo
	F	Empregado Efetivo
	F	Empregado Efetivo
	F	Empregado Efetivo
	F	Empregado Efetivo
	F	Empregado Efetivo
	F	Empregado Efetivo
	F	Empregado Efetivo
	F	Empregado Efetivo
	F	Empregado Efetivo
	M	Empregado Efetivo
	F	Empregado Efetivo
	F	Empregado Efetivo
	F	Empregado Efetivo
	F	Empregado Efetivo
	F	Empregado Efetivo
	M	Empregado Efetivo
	F	Empregado Efetivo
	F	Empregado Efetivo
	M	Empregado Efetivo
	F	Empregado Efetivo
	F	Empregado Efetivo
	F	Empregado Efetivo
	F	Empregado Efetivo
	F	Empregado Efetivo
	F	Empregado Efetivo
	F	Empregado Efetivo
	F	Empregado Efetivo
	F	Empregado Efetivo
	F	Empregado Efetivo
	F	Empregado Efetivo
	M	Empregado Efetivo
	F	Empregado Efetivo
	F	Empregado Efetivo
	F	Empregado Efetivo
	F	Empregado Efetivo
	F	Empregado Efetivo
	F	Empregado Efetivo
	F	Empregado Efetivo
	F	Empregado Efetivo
	F	Empregado Efetivo
	F	Empregado Efetivo
	F	Empregado Efetivo
	F	Empregado Efetivo

	F	Empregado Efetivo
	F	Empregado Efetivo
	F	Empregado Efetivo
	F	Empregado Efetivo
	F	Empregado Efetivo
	F	Empregado Efetivo
	F	Empregado Efetivo
	F	Empregado Efetivo
	F	Empregado Efetivo
	F	Empregado Efetivo
	F	Empregado Efetivo
	F	Empregado Efetivo
	F	Empregado Efetivo
	F	Empregado Efetivo
	F	Empregado Efetivo
	M	Empregado Efetivo
	F	Empregado Efetivo
	F	Empregado Efetivo
	F	Empregado Efetivo
	F	Empregado Efetivo
	F	Empregado Efetivo
	F	Empregado Efetivo
	F	Empregado Efetivo
	F	Empregado Efetivo
	F	Empregado Efetivo
	F	Empregado Efetivo
	F	Empregado Efetivo
	F	Empregado Efetivo
	F	Empregado Efetivo
	F	Empregado Efetivo
	F	Empregado Efetivo
	F	Empregado Efetivo
	M	Empregado Efetivo
	F	Empregado Efetivo
	F	Empregado Efetivo
	M	Empregado Efetivo
	F	Empregado Efetivo
	F	Empregado Efetivo
	M	Empregado Efetivo
	M	Empregado Efetivo
	M	Empregado Efetivo
	F	Empregado Efetivo
	F	Empregado Efetivo
	F	Empregado Efetivo
	F	Empregado Efetivo
	M	Empregado Efetivo

	M	Empregado Efetivo
	M	Empregado Efetivo
	M	Empregado Efetivo
	M	Empregado Efetivo
	F	Empregado Efetivo
	M	Empregado Efetivo
	F	Empregado Efetivo
	F	Empregado Efetivo
	F	Empregado Efetivo
	F	Empregado Efetivo
	F	Empregado Efetivo
	F	Empregado Efetivo
	F	Empregado Efetivo
	F	Empregado Efetivo
	F	Empregado Efetivo
	F	Empregado Efetivo
	F	Empregado Efetivo
	F	Empregado Efetivo
	F	Empregado Efetivo
	M	Empregado Efetivo
	M	Empregado Efetivo
	F	Empregado Efetivo
	F	Empregado Efetivo
	M	Empregado Efetivo
	M	Empregado Efetivo
	F	Empregado Efetivo
	F	Empregado Efetivo
	F	Empregado Efetivo
	F	Empregado Efetivo
	F	Empregado Efetivo
	F	Empregado Efetivo
	M	Empregado Efetivo
	M	Empregado Efetivo
	F	Empregado Efetivo
	F	Empregado Efetivo
	F	Empregado Efetivo
	F	Empregado Efetivo
	F	Empregado Efetivo
	F	Empregado Efetivo
	M	Empregado Efetivo
	F	Empregado Efetivo
	F	Empregado Efetivo
	F	Empregado Efetivo
	F	Empregado Efetivo
	M	Empregado Efetivo
	F	Empregado Efetivo
	F	Empregado Efetivo
	F	Empregado Efetivo

	F	Empregado Efetivo
	F	Empregado Efetivo
	F	Empregado Efetivo
	F	Empregado Efetivo
	F	Empregado Efetivo
	F	Empregado Efetivo
	F	Empregado Efetivo
	F	Empregado Efetivo
	F	Empregado Efetivo
	M	Empregado Efetivo
	F	Empregado Efetivo
	F	Empregado Efetivo
	F	Empregado Efetivo
	F	Empregado Efetivo
	F	Empregado Efetivo
	F	Empregado Efetivo
	F	Empregado Efetivo
	M	Empregado Efetivo
	M	Empregado Efetivo
	M	Empregado Efetivo
	M	Empregado Efetivo
	M	Empregado Efetivo
	M	Empregado Efetivo
	M	Empregado Efetivo
	M	Empregado Efetivo
	F	Empregado Efetivo
	F	Empregado Efetivo
	M	Empregado Efetivo
	M	Empregado Efetivo
	F	Empregado Efetivo
	F	Empregado Efetivo
	F	Empregado Efetivo
	F	Empregado Efetivo
	F	Empregado Efetivo
	F	Empregado Efetivo
	F	Empregado Efetivo
	M	Empregado Efetivo
	F	Empregado Efetivo
	F	Empregado Efetivo
	M	Empregado Efetivo
	F	Empregado Efetivo
	M	Empregado Efetivo
	M	Empregado Efetivo
	F	Empregado Efetivo
	M	Empregado Efetivo
	M	Empregado Efetivo
	F	Empregado Efetivo
	F	Empregado Efetivo

	F	Empregado Efetivo
	M	Empregado Efetivo
	M	Empregado Efetivo
	F	Empregado Efetivo
	F	Empregado Efetivo
	F	Empregado Efetivo
	F	Empregado Efetivo
	M	Empregado Efetivo
	F	Empregado Efetivo
	F	Empregado Efetivo
	F	Empregado Efetivo
	F	Empregado Efetivo
	F	Empregado Efetivo
	F	Empregado Efetivo
	F	Empregado Efetivo
	F	Empregado Efetivo
	F	Empregado Efetivo
	F	Empregado Efetivo
	F	Empregado Efetivo
	F	Empregado Efetivo
	F	Empregado Efetivo
	F	Empregado Efetivo
	F	Empregado Efetivo
	M	Empregado Efetivo
	M	Empregado Efetivo
	M	Empregado Efetivo
	F	Empregado Efetivo
	F	Empregado Efetivo
	F	Empregado Efetivo
	F	Empregado Efetivo
	F	Empregado Efetivo
	F	Empregado Efetivo
	F	Empregado Efetivo
	F	Empregado Efetivo
	F	Empregado Efetivo
	F	Empregado Efetivo
	F	Empregado Efetivo
	F	Empregado Efetivo
	M	Empregado Efetivo

	F	Empregado Efetivo
	F	Empregado Efetivo
	F	Empregado Efetivo
	F	Empregado Efetivo
	F	Empregado Efetivo
	F	Empregado Efetivo
	F	Empregado Efetivo
	F	Empregado Efetivo
	F	Empregado Efetivo
	F	Empregado Efetivo
	F	Empregado Efetivo
	M	Empregado Efetivo
	F	Empregado Efetivo
	F	Empregado Efetivo
	F	Empregado Efetivo
	F	Empregado Efetivo
	M	Empregado Efetivo
	F	Empregado Efetivo
	F	Empregado Efetivo
	F	Empregado Efetivo
	F	Empregado Efetivo
	F	Empregado Efetivo
	F	Empregado Efetivo
	F	Empregado Efetivo
	M	Empregado Efetivo
	F	Empregado Efetivo
	F	Empregado Efetivo
	M	Empregado Efetivo
	F	Empregado Efetivo
	F	Empregado Efetivo
	F	Empregado Efetivo
	F	Empregado Efetivo
	F	Empregado Efetivo
	F	Empregado Efetivo
	F	Empregado Efetivo
	F	Empregado Efetivo
	F	Empregado Efetivo
	F	Empregado Efetivo
	F	Empregado Efetivo
	F	Empregado Efetivo
	F	Empregado Efetivo
	F	Empregado Efetivo
	F	Empregado Efetivo
	F	Empregado Efetivo
	F	Empregado Efetivo
	F	Empregado Efetivo
	F	Empregado Efetivo
	F	Empregado Efetivo

	F	Empregado Efetivo
	F	Empregado Efetivo
	F	Empregado Efetivo
	F	Empregado Efetivo
	F	Empregado Efetivo
	M	Empregado Efetivo
	M	Empregado Efetivo
	M	Empregado Efetivo
	M	Empregado Efetivo
	M	Empregado Efetivo
	M	Empregado Efetivo
	M	Empregado Efetivo
	F	Empregado Efetivo
	F	Empregado Efetivo
	F	Empregado Efetivo
	F	Empregado Efetivo
	F	Empregado Efetivo
	F	Empregado Efetivo
	F	Empregado Efetivo
	F	Empregado Efetivo
	F	Empregado Efetivo
	F	Empregado Efetivo
	F	Empregado Efetivo
	F	Empregado Efetivo
	F	Empregado Efetivo
	F	Empregado Efetivo
	F	Empregado Efetivo
	F	Empregado Efetivo
	F	Empregado Efetivo
	F	Empregado Efetivo
	F	Empregado Efetivo
	F	Empregado Efetivo
	F	Empregado Efetivo
	F	Empregado Efetivo
	F	Empregado Efetivo
	F	Empregado Efetivo
	F	Empregado Efetivo
	F	Empregado Efetivo
	F	Empregado Efetivo
	F	Empregado Efetivo
	F	Empregado Efetivo
	F	Empregado Efetivo
	F	Empregado Efetivo
	F	Empregado Efetivo
	F	Empregado Efetivo

	F	Empregado Efetivo
	F	Empregado Efetivo
	F	Empregado Efetivo
	F	Empregado Efetivo
	F	Empregado Efetivo
	F	Empregado Efetivo
	F	Empregado Efetivo
	F	Empregado Efetivo
	F	Empregado Efetivo
	F	Empregado Efetivo
	F	Empregado Efetivo
	F	Empregado Efetivo
	F	Empregado Efetivo
	F	Empregado Efetivo
	F	Empregado Efetivo
	F	Empregado Efetivo
	F	Empregado Efetivo
	F	Empregado Efetivo
	F	Empregado Efetivo
	F	Empregado Efetivo
	F	Empregado Efetivo
	F	Empregado Efetivo
	F	Empregado Efetivo
	F	Empregado Efetivo
	F	Empregado Efetivo
	F	Empregado Efetivo
	F	Empregado Efetivo
	F	Empregado Efetivo
	F	Empregado Efetivo
	F	Empregado Efetivo
	F	Empregado Efetivo
	F	Empregado Efetivo
	F	Empregado Efetivo
	F	Empregado Efetivo
	F	Empregado Efetivo
	F	Empregado Efetivo
	F	Empregado Efetivo
	F	Empregado Efetivo
	F	Empregado Efetivo
	F	Empregado Efetivo
	F	Empregado Efetivo
	F	Empregado Efetivo
	F	Empregado Efetivo
	F	Empregado Efetivo
	F	Empregado Efetivo
	F	Empregado Efetivo
	F	Empregado Efetivo

	F	Empregado Efetivo
	F	Empregado Efetivo
	F	Empregado Efetivo
	F	Empregado Efetivo
	F	Empregado Efetivo
	F	Empregado Efetivo
	F	Empregado Efetivo
	F	Empregado Efetivo
	F	Empregado Efetivo
	F	Empregado Efetivo
	F	Empregado Efetivo
	F	Empregado Efetivo
	F	Empregado Efetivo
	F	Empregado Efetivo
	F	Empregado Efetivo
	M	Empregado Efetivo
	M	Empregado Efetivo
	M	Empregado Efetivo
	M	Empregado Efetivo
	M	Empregado Efetivo
	M	Empregado Efetivo
	M	Empregado Efetivo
	M	Empregado Efetivo
	M	Empregado Efetivo
	M	Empregado Efetivo
	M	Empregado Efetivo
	M	Empregado Efetivo
	M	Empregado Efetivo
	M	Empregado Efetivo
	M	Empregado Efetivo
	M	Empregado Efetivo
	F	Empregado Efetivo
	F	Comissionado
	F	Cedido - RJU
	F	Empregado Efetivo
	M	Cedido - RJU
	M	Empregado Efetivo
	F	Empregado Efetivo

	M	Empregado Efetivo
	F	Empregado Efetivo
	M	Empregado Efetivo
	F	Empregado Efetivo
	F	Empregado Efetivo
	M	Empregado Efetivo
	M	Empregado Efetivo
	F	Cedido - RJU
	M	Empregado Efetivo
	M	Empregado Efetivo
	M	Empregado Efetivo
	F	Empregado Efetivo
	M	Empregado Efetivo
	M	Empregado Efetivo
	M	Empregado Efetivo
	F	Empregado Efetivo
	M	Cedido - RJU
	F	Empregado Efetivo
	M	Empregado Efetivo
	F	Empregado Efetivo
	F	Empregado Efetivo
	M	Empregado Efetivo
	F	Empregado Efetivo
	F	Empregado Efetivo
	M	Empregado Efetivo
	F	Empregado Efetivo
	F	Empregado Efetivo
	M	Empregado Efetivo
	M	Empregado Efetivo
	F	Empregado Efetivo
	F	Empregado Efetivo
	F	Empregado Efetivo
	M	Empregado Efetivo
	F	Empregado Efetivo
	F	Empregado Efetivo
	F	Empregado Efetivo
	F	Empregado Efetivo
	F	Empregado Efetivo
	F	Empregado Efetivo
	F	Empregado Efetivo
	F	Empregado Efetivo
	M	Empregado Efetivo
	F	Empregado Efetivo
	M	Empregado Efetivo
	F	Empregado Efetivo
	F	Empregado Efetivo
	F	Empregado Efetivo

	F	Empregado Efetivo
	F	Empregado Efetivo
	F	Empregado Efetivo
	F	Empregado Efetivo
	M	Empregado Efetivo
	F	Empregado Efetivo
	F	Empregado Efetivo
	M	Empregado Efetivo
	F	Empregado Efetivo
	F	Empregado Efetivo
	F	Empregado Efetivo
	F	Empregado Efetivo
	F	Empregado Efetivo
	F	Empregado Efetivo
	M	Empregado Efetivo
	F	Empregado Efetivo
	M	Empregado Efetivo
	F	Empregado Efetivo
	F	Empregado Efetivo
	F	Empregado Efetivo
	F	Empregado Efetivo
	F	Empregado Efetivo
	M	Empregado Efetivo
	M	Empregado Efetivo
	M	Empregado Efetivo
	M	Empregado Efetivo
	F	Empregado Efetivo
	F	Empregado Efetivo
	F	Empregado Efetivo
	F	Empregado Efetivo
	F	Empregado Efetivo
	M	Empregado Efetivo
	F	Empregado Efetivo
	F	Empregado Efetivo
	M	Empregado Efetivo
	F	Empregado Efetivo
	M	Empregado Efetivo
	M	Empregado Efetivo
	F	Empregado Efetivo
	F	Empregado Efetivo
	F	Empregado Efetivo
	F	Empregado Efetivo
	F	Empregado Efetivo
	F	Empregado Efetivo
	F	Empregado Efetivo
	F	Empregado Efetivo
	M	Empregado Efetivo
	M	Empregado Efetivo

	M	Empregado Efetivo
	F	Empregado Efetivo
	M	Empregado Efetivo
	F	Empregado Efetivo
	F	Empregado Efetivo
	F	Empregado Efetivo
	F	Empregado Efetivo
	F	Empregado Efetivo
	M	Empregado Efetivo
	F	Empregado Efetivo
	F	Empregado Efetivo
	F	Empregado Efetivo
	F	Empregado Efetivo
	M	Empregado Efetivo
	F	Empregado Efetivo
	F	Empregado Efetivo
	M	Empregado Efetivo
	M	Empregado Efetivo
	F	Empregado Efetivo
	M	Empregado Efetivo
	F	Empregado Efetivo
	M	Empregado Efetivo
	F	Empregado Efetivo
	M	Empregado Efetivo
	M	Empregado Efetivo
	F	Empregado Efetivo
	M	Empregado Efetivo
	F	Empregado Efetivo
	F	Empregado Efetivo
	M	Empregado Efetivo
	F	Empregado Efetivo
	F	Empregado Efetivo
	F	Empregado Efetivo
	F	Empregado Efetivo
	F	Empregado Efetivo
	F	Empregado Efetivo
	F	Empregado Efetivo
	F	Empregado Efetivo
	F	Empregado Efetivo
	F	Empregado Efetivo
	M	Empregado Efetivo
	F	Empregado Efetivo
	M	Empregado Efetivo
	M	Empregado Efetivo
	M	Empregado Efetivo
	F	Empregado Efetivo
	F	Empregado Efetivo
	F	Empregado Efetivo

	M	Empregado Efetivo
	F	Cedido - RJU
	M	Empregado Efetivo
	F	Empregado Efetivo
	M	Empregado Efetivo
	M	Empregado Efetivo
	F	Empregado Efetivo
	M	Empregado Efetivo
	M	Empregado Efetivo
	M	Empregado Efetivo
	M	Empregado Efetivo
	M	Empregado Efetivo
	F	Empregado Efetivo
	M	Empregado Efetivo
	F	Empregado Efetivo
	F	Empregado Efetivo
	F	Empregado Efetivo
	F	Empregado Efetivo
	F	Empregado Efetivo
	M	Empregado Efetivo
	F	Empregado Efetivo
	F	Empregado Efetivo
	F	Empregado Efetivo
	M	Empregado Efetivo
	F	Empregado Efetivo
	F	Empregado Efetivo
	F	Empregado Efetivo
	M	Empregado Efetivo
	F	Empregado Efetivo
	F	Empregado Efetivo
	F	Empregado Efetivo
	M	Empregado Efetivo
	F	Empregado Efetivo
	F	Empregado Efetivo
	M	Empregado Efetivo
	F	Empregado Efetivo
	M	Empregado Efetivo
	F	Empregado Efetivo
	F	Empregado Efetivo
	F	Empregado Efetivo
	F	Empregado Efetivo
	F	Empregado Efetivo
	F	Empregado Efetivo
	M	Empregado Efetivo
	F	Empregado Efetivo
	F	Empregado Efetivo
	F	Empregado Efetivo

	F	Empregado Efetivo
	M	Empregado Efetivo
	F	Empregado Efetivo
	F	Empregado Efetivo
	F	Empregado Efetivo
	F	Empregado Efetivo
	M	Empregado Efetivo
	M	Empregado Efetivo
	F	Empregado Efetivo
	F	Empregado Efetivo
	F	Empregado Efetivo
	M	Empregado Efetivo
	F	Empregado Efetivo
	M	Empregado Efetivo
	F	Empregado Efetivo
	F	Empregado Efetivo
	M	Empregado Efetivo
	F	Empregado Efetivo
	F	Empregado Efetivo
	M	Empregado Efetivo
	F	Empregado Efetivo
	F	Empregado Efetivo
	F	Empregado Efetivo
	F	Empregado Efetivo
	F	Empregado Efetivo
	F	Empregado Efetivo
	F	Empregado Efetivo
	F	Empregado Efetivo
	M	Empregado Efetivo
	F	Empregado Efetivo
	F	Empregado Efetivo
	F	Cedido - RJU
	F	Cedido - RJU
	F	Empregado Efetivo
	M	Empregado Efetivo
	F	Empregado Efetivo
	M	Empregado Efetivo
	F	Empregado Efetivo
	F	Empregado Efetivo

	F	Empregado Efetivo
	F	Empregado Efetivo
	F	Empregado Efetivo
	F	Empregado Efetivo
	F	Empregado Efetivo
	F	Empregado Efetivo
	F	Empregado Efetivo
	F	Empregado Efetivo
	F	Empregado Efetivo
	M	Cedido - RJU
	F	Empregado Efetivo
	F	Empregado Efetivo
	F	Empregado Efetivo
	F	Empregado Efetivo
	F	Empregado Efetivo
	F	Empregado Efetivo
	F	Empregado Efetivo
	F	Empregado Efetivo
	F	Empregado Efetivo
	F	Empregado Efetivo
	F	Empregado Efetivo
	F	Empregado Efetivo
	F	Empregado Efetivo
	F	Empregado Efetivo
	F	Empregado Efetivo
	F	Empregado Efetivo
	F	Empregado Efetivo
	F	Empregado Efetivo
	F	Empregado Efetivo
	F	Empregado Efetivo
	F	Empregado Efetivo
	F	Empregado Efetivo
	F	Empregado Efetivo
	F	Empregado Efetivo
	M	Empregado Efetivo
	M	Empregado Efetivo

	F	Empregado Efetivo
	F	Empregado Efetivo
	F	Empregado Efetivo
	F	Empregado Efetivo
	M	Empregado Efetivo
	F	Empregado Efetivo
	M	Empregado Efetivo
	M	Empregado Efetivo
	F	Empregado Efetivo
	F	Empregado Efetivo
	F	Empregado Efetivo
	F	Empregado Efetivo
	F	Empregado Efetivo
	F	Empregado Efetivo
	F	Empregado Efetivo
	F	Empregado Efetivo
	F	Empregado Efetivo
	F	Empregado Efetivo
	F	Empregado Efetivo
	F	Empregado Efetivo
	M	Empregado Efetivo
	M	Empregado Efetivo
	M	Empregado Efetivo
	F	Empregado Efetivo
	F	Empregado Efetivo
	F	Empregado Efetivo
	F	Empregado Efetivo
	F	Empregado Efetivo
	M	Empregado Efetivo
	F	Empregado Efetivo
	F	Empregado Efetivo
	M	Empregado Efetivo
	F	Empregado Efetivo
	F	Empregado Efetivo
	F	Empregado Efetivo
	M	Empregado Efetivo
	F	Empregado Efetivo

	F	Empregado Efetivo
	F	Empregado Efetivo
	M	Empregado Efetivo
	F	Empregado Efetivo
	M	Empregado Efetivo
	F	Empregado Efetivo
	F	Empregado Efetivo
	M	Empregado Efetivo
	F	Empregado Efetivo
	F	Empregado Efetivo
	F	Empregado Efetivo
	F	Empregado Efetivo
	F	Empregado Efetivo
	M	Empregado Efetivo
	M	Empregado Efetivo
	F	Cedido - RJU
	M	Empregado Efetivo
	M	Empregado Efetivo
	F	Empregado Efetivo
	M	Empregado Efetivo
	M	Empregado Efetivo
	F	Empregado Efetivo
	M	Empregado Efetivo
	M	Empregado Efetivo
	M	Empregado Efetivo
	F	Empregado Efetivo
	F	Empregado Efetivo
	F	Empregado Efetivo
	F	Empregado Efetivo
	F	Empregado Efetivo
	F	Empregado Efetivo
	F	Empregado Efetivo
	F	Empregado Efetivo
	F	Empregado Efetivo
	F	Empregado Efetivo
	M	Empregado Efetivo
	F	Empregado Efetivo
	M	Empregado Efetivo
	F	Empregado Efetivo
	F	Empregado Efetivo
	F	Empregado Efetivo
	F	Empregado Efetivo
	M	Empregado Efetivo

	M	Empregado Efetivo
	M	Empregado Efetivo
	M	Empregado Efetivo
	M	Empregado Efetivo
	M	Empregado Efetivo
	F	Empregado Efetivo
	F	Empregado Efetivo
	F	Empregado Efetivo
	F	Empregado Efetivo
	M	Empregado Efetivo
	M	Empregado Efetivo
	M	Empregado Efetivo
	M	Empregado Efetivo
	M	Empregado Efetivo
	F	Empregado Efetivo
	F	Empregado Efetivo
	F	Empregado Efetivo
	F	Empregado Efetivo
	F	Empregado Efetivo
	F	Empregado Efetivo
	F	Empregado Efetivo
	F	Empregado Efetivo
	F	Empregado Efetivo
	F	Empregado Efetivo
	F	Empregado Efetivo
	F	Empregado Efetivo
	F	Empregado Efetivo
	F	Empregado Efetivo
	F	Empregado Efetivo
	F	Empregado Efetivo
	F	Empregado Efetivo
	F	Empregado Efetivo
	M	Empregado Efetivo
	M	Empregado Efetivo
	M	Empregado Efetivo
	M	Empregado Efetivo
	F	Empregado Efetivo
	M	Empregado Efetivo
	M	Empregado Efetivo

	M	Empregado Efetivo
	F	Empregado Efetivo
	M	Empregado Efetivo
	F	Empregado Efetivo
	F	Empregado Efetivo
	F	Empregado Efetivo
	F	Empregado Efetivo
	F	Empregado Efetivo
	F	Empregado Efetivo
	M	Empregado Efetivo
	F	Empregado Efetivo
	F	Empregado Efetivo
	F	Empregado Efetivo
	F	Empregado Efetivo
	F	Empregado Efetivo
	F	Empregado Efetivo
	F	Empregado Efetivo
	F	Empregado Efetivo
	F	Empregado Efetivo
	F	Empregado Efetivo
	F	Empregado Efetivo
	F	Empregado Efetivo
	F	Empregado Efetivo
	M	Empregado Efetivo
	F	Empregado Efetivo
	F	Empregado Efetivo
	F	Empregado Efetivo
	F	Empregado Efetivo
	M	Empregado Efetivo
	F	Empregado Efetivo
	F	Empregado Efetivo
	F	Empregado Efetivo
	F	Empregado Efetivo
	F	Empregado Efetivo
	F	Empregado Efetivo
	F	Empregado Efetivo
	F	Empregado Efetivo
	F	Empregado Efetivo
	F	Empregado Efetivo
	F	Empregado Efetivo
	M	Empregado Efetivo
	M	Empregado Efetivo
	F	Empregado Efetivo
	M	Empregado Efetivo
	F	Empregado Efetivo
	M	Empregado Efetivo
	F	Empregado Efetivo
	F	Empregado Efetivo

	F	Empregado Efetivo
	F	Empregado Efetivo
	F	Empregado Efetivo
	F	Empregado Efetivo
	F	Empregado Efetivo
	F	Empregado Efetivo
	F	Empregado Efetivo
	F	Empregado Efetivo
	F	Empregado Efetivo
	F	Empregado Efetivo
	F	Empregado Efetivo
	M	Empregado Efetivo
	M	Empregado Efetivo
	F	Empregado Efetivo
	F	Empregado Efetivo
	M	Empregado Efetivo
	F	Empregado Efetivo
	F	Empregado Efetivo
	F	Empregado Efetivo
	F	Empregado Efetivo
	F	Empregado Efetivo
	F	Empregado Efetivo
	M	Empregado Efetivo
	M	Empregado Efetivo
	F	Empregado Efetivo
	F	Empregado Efetivo
	M	Empregado Efetivo
	M	Empregado Efetivo
	F	Empregado Efetivo
	M	Empregado Efetivo
	F	Empregado Efetivo
	F	Empregado Efetivo
	F	Empregado Efetivo
	F	Empregado Efetivo
	M	Empregado Efetivo
	F	Empregado Efetivo
	F	Empregado Efetivo
	F	Empregado Efetivo
	M	Empregado Efetivo
	M	Empregado Efetivo
	M	Empregado Efetivo
	F	Empregado Efetivo
	F	Empregado Efetivo
	F	Empregado Efetivo
	F	Empregado Efetivo
	F	Empregado Efetivo
	F	Empregado Efetivo

	F	Empregado Efetivo
	F	Empregado Efetivo
	F	Empregado Efetivo
	F	Empregado Efetivo
	F	Empregado Efetivo
	F	Empregado Efetivo
	F	Empregado Efetivo
	F	Empregado Efetivo
	F	Empregado Efetivo
	M	Empregado Efetivo
	M	Empregado Efetivo
	F	Empregado Efetivo
	F	Empregado Efetivo
	F	Empregado Efetivo
	M	Empregado Efetivo
	M	Empregado Efetivo
	F	Empregado Efetivo
	M	Empregado Efetivo
	F	Empregado Efetivo
	M	Empregado Efetivo
	M	Empregado Efetivo
	F	Empregado Efetivo
	F	Empregado Efetivo
	M	Empregado Efetivo
	M	Empregado Efetivo
	F	Empregado Efetivo
	F	Empregado Efetivo
	M	Empregado Efetivo
	F	Empregado Efetivo
	F	Empregado Efetivo
	F	Empregado Efetivo
	F	Empregado Efetivo
	F	Empregado Efetivo
	F	Empregado Efetivo
	F	Empregado Efetivo
	M	Empregado Efetivo
	M	Empregado Efetivo
	M	Empregado Efetivo
	F	Empregado Efetivo

	M	Empregado Efetivo
	F	Empregado Efetivo
	F	Empregado Efetivo
	F	Empregado Efetivo
	F	Empregado Efetivo
	M	Empregado Efetivo
	F	Empregado Efetivo
	M	Empregado Efetivo
	F	Empregado Efetivo
	F	Empregado Efetivo
	M	Empregado Efetivo
	F	Empregado Efetivo
	F	Empregado Efetivo
	F	Empregado Efetivo
	M	Empregado Efetivo
	M	Empregado Efetivo
	F	Empregado Efetivo
	M	Empregado Efetivo
	F	Empregado Efetivo
	F	Empregado Efetivo
	F	Empregado Efetivo
	F	Empregado Efetivo
	F	Empregado Efetivo
	F	Empregado Efetivo
	M	Empregado Efetivo
	F	Empregado Efetivo
	F	Empregado Efetivo
	M	Empregado Efetivo
	F	Empregado Efetivo
	F	Empregado Efetivo
	F	Empregado Efetivo
	F	Empregado Efetivo
	F	Empregado Efetivo
	F	Empregado Efetivo
	F	Empregado Efetivo
	F	Empregado Efetivo
	F	Empregado Efetivo
	M	Empregado Efetivo
	F	Empregado Efetivo
	F	Empregado Efetivo
	F	Empregado Efetivo
	F	Empregado Efetivo
	F	Empregado Efetivo
	F	Empregado Efetivo
	M	Empregado Efetivo
	F	Empregado Efetivo
	F	Empregado Efetivo
	F	Empregado Efetivo

	M	Empregado Efetivo
	F	Empregado Efetivo
	F	Empregado Efetivo
	F	Empregado Efetivo
	F	Empregado Efetivo
	F	Empregado Efetivo
	M	Empregado Efetivo
	F	Empregado Efetivo
	F	Empregado Efetivo
	F	Empregado Efetivo
	F	Empregado Efetivo
	F	Empregado Efetivo
	F	Empregado Efetivo
	F	Empregado Efetivo
	F	Empregado Efetivo
	F	Empregado Efetivo
	F	Empregado Efetivo
	F	Empregado Efetivo
	F	Empregado Efetivo
	M	Empregado Efetivo
	F	Empregado Efetivo
	M	Empregado Efetivo
	F	Empregado Efetivo
	F	Empregado Efetivo
	F	Empregado Efetivo
	M	Empregado Efetivo
	F	Empregado Efetivo
	M	Empregado Efetivo
	M	Empregado Efetivo
	F	Empregado Efetivo
	M	Empregado Efetivo
	M	Empregado Efetivo
	F	Empregado Efetivo
	M	Empregado Efetivo
	M	Empregado Efetivo
	F	Empregado Efetivo
	F	Empregado Efetivo
	F	Empregado Efetivo
	F	Empregado Efetivo
	F	Empregado Efetivo
	F	Empregado Efetivo
	F	Empregado Efetivo

	M	Empregado Efetivo
	M	Empregado Efetivo
	F	Empregado Efetivo
	F	Empregado Efetivo
	F	Empregado Efetivo
	F	Empregado Efetivo
	M	Empregado Efetivo
	F	Empregado Efetivo
	F	Empregado Efetivo
	F	Empregado Efetivo
	F	Empregado Efetivo
	F	Empregado Efetivo
	F	Empregado Efetivo
	F	Empregado Efetivo
	M	Empregado Efetivo
	M	Empregado Efetivo
	M	Empregado Efetivo
	F	Empregado Efetivo
	F	Empregado Efetivo
	F	Empregado Efetivo
	F	Empregado Efetivo
	F	Empregado Efetivo
	F	Empregado Efetivo
	M	Empregado Efetivo
	F	Empregado Efetivo
	F	Empregado Efetivo
	F	Empregado Efetivo
	F	Empregado Efetivo
	F	Empregado Efetivo
	F	Empregado Efetivo
	M	Empregado Efetivo
	F	Empregado Efetivo
	F	Empregado Efetivo
	F	Empregado Efetivo
	F	Empregado Efetivo
	M	Empregado Efetivo
	F	Empregado Efetivo
	F	Empregado Efetivo
	F	Empregado Efetivo
	F	Empregado Efetivo
	M	Empregado Efetivo
	F	Empregado Efetivo
	F	Empregado Efetivo
	F	Empregado Efetivo

	F	Empregado Efetivo
	M	Empregado Efetivo
	F	Empregado Efetivo
	F	Empregado Efetivo
	F	Empregado Efetivo
	M	Empregado Efetivo
	F	Empregado Efetivo
	F	Empregado Efetivo
	F	Empregado Efetivo
	F	Empregado Efetivo
	F	Empregado Efetivo
	F	Empregado Efetivo
	F	Empregado Efetivo
	F	Empregado Efetivo
	F	Empregado Efetivo
	F	Empregado Efetivo
	F	Empregado Efetivo
	F	Empregado Efetivo
	F	Empregado Efetivo
	M	Empregado Efetivo
	F	Empregado Efetivo
	F	Empregado Efetivo
	F	Empregado Efetivo
	M	Empregado Efetivo
	F	Empregado Efetivo
	M	Empregado Efetivo
	M	Empregado Efetivo
	F	Empregado Efetivo
	F	Empregado Efetivo
	F	Empregado Efetivo
	F	Empregado Efetivo
	M	Empregado Efetivo
	F	Empregado Efetivo
	F	Empregado Efetivo
	F	Empregado Efetivo
	F	Empregado Efetivo
	F	Empregado Efetivo
	F	Empregado Efetivo
	F	Empregado Efetivo
	M	Empregado Efetivo
	F	Empregado Efetivo
	F	Empregado Efetivo
	F	Empregado Efetivo
	F	Empregado Efetivo
	F	Empregado Efetivo
	F	Empregado Efetivo
	F	Empregado Efetivo
	F	Empregado Efetivo

	F	Empregado Efetivo
	M	Empregado Efetivo
	F	Empregado Efetivo
	M	Empregado Efetivo
	F	Empregado Efetivo
	F	Empregado Efetivo
	M	Empregado Efetivo
	F	Empregado Efetivo
	F	Empregado Efetivo
	F	Empregado Efetivo
	F	Empregado Efetivo
	M	Empregado Efetivo
	F	Empregado Efetivo
	M	Empregado Efetivo
	F	Empregado Efetivo
	M	Empregado Efetivo
	F	Empregado Efetivo
	F	Empregado Efetivo
	F	Empregado Efetivo
	F	Empregado Efetivo
	F	Empregado Efetivo
	F	Empregado Efetivo
	F	Empregado Efetivo
	F	Empregado Efetivo
	F	Empregado Efetivo
	F	Empregado Efetivo
	M	Empregado Efetivo
	F	Empregado Efetivo
	M	Empregado Efetivo
	F	Empregado Efetivo
	F	Empregado Efetivo
	F	Empregado Efetivo
	F	Empregado Efetivo
	F	Empregado Efetivo
	F	Empregado Efetivo
	F	Empregado Efetivo
	F	Empregado Efetivo
	F	Empregado Efetivo
	F	Empregado Efetivo
	F	Empregado Efetivo
	M	Empregado Efetivo
	F	Empregado Efetivo
	F	Empregado Efetivo
	F	Empregado Efetivo
	F	Empregado Efetivo
	F	Empregado Efetivo
	M	Empregado Efetivo
	F	Empregado Efetivo

	F	Empregado Efetivo
	F	Empregado Efetivo
	F	Empregado Efetivo
	M	Empregado Efetivo
	M	Empregado Efetivo
	M	Empregado Efetivo
	F	Empregado Efetivo
	M	Empregado Efetivo
	M	Empregado Efetivo
	F	Empregado Efetivo
	M	Empregado Efetivo
	M	Empregado Efetivo
	F	Empregado Efetivo
	M	Empregado Efetivo
	M	Empregado Efetivo
	F	Empregado Efetivo
	M	Empregado Efetivo
	M	Empregado Efetivo
	M	Empregado Efetivo
	M	Empregado Efetivo
	M	Empregado Efetivo
	F	Empregado Efetivo
	M	Empregado Efetivo
	M	Empregado Efetivo
	F	Empregado Efetivo
	F	Empregado Efetivo
	F	Empregado Efetivo
	F	Empregado Efetivo
	F	Empregado Efetivo
	F	Empregado Efetivo
	F	Empregado Efetivo
	M	Empregado Efetivo
	F	Empregado Efetivo
	M	Empregado Efetivo
	M	Empregado Efetivo
	M	Empregado Efetivo
	F	Empregado Efetivo

	F	Empregado Efetivo
	F	Empregado Efetivo
	F	Empregado Efetivo
	M	Empregado Efetivo
	M	Empregado Efetivo
	F	Empregado Efetivo
	F	Empregado Efetivo
	M	Empregado Efetivo
	F	Empregado Efetivo
	F	Empregado Efetivo
	M	Empregado Efetivo
	F	Empregado Efetivo
	F	Empregado Efetivo
	F	Empregado Efetivo
	F	Empregado Efetivo
	F	Empregado Efetivo
	F	Empregado Efetivo
	M	Empregado Efetivo
	F	Empregado Efetivo
	F	Empregado Efetivo
	F	Empregado Efetivo
	F	Empregado Efetivo
	F	Empregado Efetivo
	F	Empregado Efetivo
	F	Empregado Efetivo
	F	Empregado Efetivo
	F	Empregado Efetivo
	F	Empregado Efetivo
	F	Empregado Efetivo
	F	Empregado Efetivo
	F	Empregado Efetivo
	F	Empregado Efetivo
	F	Empregado Efetivo
	F	Empregado Efetivo
	F	Empregado Efetivo
	F	Empregado Efetivo
	F	Empregado Efetivo
	F	Empregado Efetivo
	F	Empregado Efetivo
	F	Empregado Efetivo
	M	Empregado Efetivo
	F	Empregado Efetivo
	F	Empregado Efetivo
	M	Empregado Efetivo

	M	Empregado Efetivo
	M	Empregado Efetivo
	F	Empregado Efetivo
	F	Empregado Efetivo
	F	Empregado Efetivo
	F	Empregado Efetivo
	F	Empregado Efetivo
	F	Empregado Efetivo
	M	Empregado Efetivo
	F	Empregado Efetivo
	F	Empregado Efetivo
	F	Empregado Efetivo
	M	Empregado Efetivo
	F	Empregado Efetivo
	M	Empregado Efetivo
	M	Empregado Efetivo
	F	Empregado Efetivo
	F	Empregado Efetivo
	M	Empregado Efetivo
	M	Empregado Efetivo
	F	Empregado Efetivo
	F	Empregado Efetivo
	M	Empregado Efetivo
	M	Empregado Efetivo
	F	Empregado Efetivo
	M	Empregado Efetivo
	F	Empregado Efetivo
	M	Empregado Efetivo
	M	Empregado Efetivo
	M	Empregado Efetivo
	M	Empregado Efetivo
	M	Empregado Efetivo
	M	Empregado Efetivo
	M	Empregado Efetivo
	M	Empregado Efetivo
	M	Empregado Efetivo
	M	Empregado Efetivo
	M	Empregado Efetivo
	F	Empregado Efetivo
	M	Empregado Efetivo
	M	Empregado Efetivo
	M	Empregado Efetivo
	F	Empregado Efetivo

	M	Empregado Efetivo
	F	Empregado Efetivo
	F	Empregado Efetivo
	F	Empregado Efetivo
	F	Empregado Efetivo
	M	Empregado Efetivo
	F	Empregado Efetivo
	F	Empregado Efetivo
	F	Empregado Efetivo
	F	Empregado Efetivo
	M	Empregado Efetivo
	M	Empregado Efetivo
	F	Empregado Efetivo
	F	Empregado Efetivo
	F	Empregado Efetivo
	M	Empregado Efetivo
	F	Empregado Efetivo
	F	Empregado Efetivo
	F	Empregado Efetivo
	F	Empregado Efetivo
	F	Empregado Efetivo
	M	Empregado Efetivo
	M	Empregado Efetivo
	M	Empregado Efetivo
	M	Empregado Efetivo
	M	Empregado Efetivo
	M	Empregado Efetivo
	F	Empregado Efetivo
	M	Empregado Efetivo
	F	Empregado Efetivo
	M	Empregado Efetivo
	F	Empregado Efetivo
	F	Empregado Efetivo
	M	Empregado Efetivo
	M	Empregado Efetivo
	F	Empregado Efetivo
	F	Empregado Efetivo
	F	Empregado Efetivo

	F	Empregado Efetivo
	M	Empregado Efetivo
	M	Empregado Efetivo
	M	Empregado Efetivo
	M	Empregado Efetivo
	F	Empregado Efetivo
	M	Empregado Efetivo
	F	Empregado Efetivo
	F	Empregado Efetivo
	M	Empregado Efetivo
	M	Empregado Efetivo
	F	Empregado Efetivo
	F	Empregado Efetivo
	F	Empregado Efetivo
	F	Empregado Efetivo
	F	Empregado Efetivo
	F	Empregado Efetivo
	F	Empregado Efetivo
	F	Empregado Efetivo
	F	Empregado Efetivo
	F	Empregado Efetivo
	F	Empregado Efetivo
	M	Empregado Efetivo
	F	Empregado Efetivo
	F	Empregado Efetivo
	F	Empregado Efetivo
	F	Empregado Efetivo
	F	Empregado Efetivo
	F	Empregado Efetivo
	F	Empregado Efetivo
	F	Empregado Efetivo
	F	Empregado Efetivo
	F	Empregado Efetivo
	F	Empregado Efetivo
	F	Empregado Efetivo
	F	Empregado Efetivo
	F	Empregado Efetivo
	F	Empregado Efetivo
	F	Empregado Efetivo
	M	Empregado Efetivo
	F	Empregado Efetivo
	F	Empregado Efetivo
	F	Empregado Efetivo
	F	Empregado Efetivo
	M	Empregado Efetivo
	F	Empregado Efetivo
	F	Empregado Efetivo
	M	Empregado Efetivo

	F	Empregado Efetivo
	F	Empregado Efetivo
	F	Empregado Efetivo
	M	Empregado Efetivo
	M	Empregado Efetivo
	F	Empregado Efetivo
	M	Empregado Efetivo
	F	Empregado Efetivo
	F	Empregado Efetivo
	M	Empregado Efetivo
	F	Empregado Efetivo
	F	Empregado Efetivo
	F	Empregado Efetivo
	F	Empregado Efetivo
	F	Empregado Efetivo
	F	Empregado Efetivo
	F	Empregado Efetivo
	F	Empregado Efetivo
	F	Empregado Efetivo
	M	Empregado Efetivo
	F	Empregado Efetivo
	M	Empregado Efetivo
	M	Empregado Efetivo
	M	Empregado Efetivo
	F	Empregado Efetivo
	M	Empregado Efetivo
	M	Empregado Efetivo
	F	Empregado Efetivo
	M	Empregado Efetivo
	M	Empregado Efetivo
	F	Empregado Efetivo
	M	Empregado Efetivo
	M	Empregado Efetivo
	F	Empregado Efetivo
	F	Empregado Efetivo
	F	Empregado Efetivo
	F	Empregado Efetivo
	F	Empregado Efetivo
	M	Empregado Efetivo
	F	Empregado Efetivo
	M	Empregado Efetivo
	F	Empregado Efetivo
	M	Empregado Efetivo

	F	Empregado Efetivo
	F	Empregado Efetivo
	F	Empregado Efetivo
	F	Empregado Efetivo
	F	Empregado Efetivo
	F	Empregado Efetivo
	F	Empregado Efetivo
	F	Empregado Efetivo
	F	Empregado Efetivo
	F	Empregado Efetivo
	F	Empregado Efetivo
	F	Empregado Efetivo
	M	Empregado Efetivo
	M	Empregado Efetivo
	M	Empregado Efetivo
	M	Empregado Efetivo
	F	Empregado Efetivo
	M	Empregado Efetivo
	F	Empregado Efetivo
	M	Empregado Efetivo
	F	Empregado Efetivo
	F	Empregado Efetivo
	F	Empregado Efetivo
	F	Empregado Efetivo
	F	Empregado Efetivo
	M	Empregado Efetivo
	M	Empregado Efetivo
	F	Empregado Efetivo
	M	Empregado Efetivo
	F	Empregado Efetivo
	M	Empregado Efetivo
	M	Empregado Efetivo
	M	Empregado Efetivo
	M	Empregado Efetivo
	M	Empregado Efetivo
	F	Empregado Efetivo
	M	Empregado Efetivo
	M	Empregado Efetivo
	F	Empregado Efetivo
	F	Empregado Efetivo
	F	Empregado Efetivo
	M	Empregado Efetivo
	F	Empregado Efetivo
	M	Empregado Efetivo
	F	Empregado Efetivo
	M	Empregado Efetivo
	F	Empregado Efetivo

	F	Empregado Efetivo
	F	Empregado Efetivo
	F	Empregado Efetivo
	M	Empregado Efetivo
	M	Empregado Efetivo
	F	Empregado Efetivo
	F	Empregado Efetivo
	F	Empregado Efetivo
	F	Empregado Efetivo
	M	Empregado Efetivo
	M	Empregado Efetivo
	F	Empregado Efetivo
	F	Empregado Efetivo
	F	Empregado Efetivo
	M	Empregado Efetivo
	F	Empregado Efetivo
	F	Empregado Efetivo
	F	Empregado Efetivo
	F	Empregado Efetivo
	F	Empregado Efetivo
	F	Empregado Efetivo
	F	Empregado Efetivo
	M	Empregado Efetivo
	M	Empregado Efetivo
	F	Empregado Efetivo
	M	Empregado Efetivo
	M	Empregado Efetivo
	F	Empregado Efetivo
	M	Empregado Efetivo
	M	Empregado Efetivo
	F	Empregado Efetivo
	F	Empregado Efetivo
	F	Empregado Efetivo
	M	Empregado Efetivo
	F	Empregado Efetivo
	M	Empregado Efetivo
	M	Empregado Efetivo
	F	Empregado Efetivo
	F	Empregado Efetivo
	F	Empregado Efetivo
	F	Empregado Efetivo
	M	Empregado Efetivo
	M	Empregado Efetivo
	F	Empregado Efetivo
	F	Empregado Efetivo
	F	Empregado Efetivo
	M	Empregado Efetivo
	F	Empregado Efetivo

	F	Empregado Efetivo
	F	Empregado Efetivo
	F	Empregado Efetivo
	F	Empregado Efetivo
	F	Empregado Efetivo
	F	Empregado Efetivo
	F	Empregado Efetivo
	F	Empregado Efetivo
	F	Empregado Efetivo
	F	Empregado Efetivo
	F	Empregado Efetivo
	F	Empregado Efetivo
	M	Empregado Efetivo
	F	Empregado Efetivo
	M	Empregado Efetivo
	M	Empregado Efetivo
	M	Empregado Efetivo
	F	Empregado Efetivo
	F	Empregado Efetivo
	M	Empregado Efetivo
	F	Empregado Efetivo
	F	Empregado Efetivo
	F	Empregado Efetivo
	F	Empregado Efetivo
	M	Empregado Efetivo
	M	Empregado Efetivo
	F	Empregado Efetivo
	F	Empregado Efetivo
	F	Empregado Efetivo
	M	Empregado Efetivo
	F	Empregado Efetivo
	F	Empregado Efetivo
	M	Empregado Efetivo
	F	Empregado Efetivo
	M	Empregado Efetivo
	F	Empregado Efetivo
	F	Empregado Efetivo
	M	Empregado Efetivo
	F	Empregado Efetivo
	F	Empregado Efetivo
	F	Empregado Efetivo
	F	Empregado Efetivo
	F	Empregado Efetivo
	F	Empregado Efetivo
	F	Empregado Efetivo
	F	Empregado Efetivo
	F	Empregado Efetivo
	M	Empregado Efetivo

	M	Empregado Efetivo
	M	Empregado Efetivo
	M	Empregado Efetivo
	F	Empregado Efetivo
	M	Empregado Efetivo
	F	Empregado Efetivo
	F	Empregado Efetivo
	F	Empregado Efetivo
	F	Empregado Efetivo
	F	Empregado Efetivo
	F	Empregado Efetivo
	F	Empregado Efetivo
	F	Empregado Efetivo
	M	Empregado Efetivo
	M	Empregado Efetivo
	F	Empregado Efetivo
	M	Empregado Efetivo
	M	Empregado Efetivo
	F	Empregado Efetivo
	M	Empregado Efetivo
	M	Empregado Efetivo
	M	Empregado Efetivo
	M	Empregado Efetivo
	M	Empregado Efetivo
	M	Empregado Efetivo
	M	Empregado Efetivo
	M	Empregado Efetivo
	M	Empregado Efetivo
	F	Empregado Efetivo
	F	Empregado Efetivo
	F	Empregado Efetivo
	F	Empregado Efetivo
	F	Empregado Efetivo
	F	Empregado Efetivo
	F	Empregado Efetivo
	F	Empregado Efetivo
	F	Empregado Efetivo
	F	Empregado Efetivo
	F	Empregado Efetivo
	M	Empregado Efetivo
	F	Empregado Efetivo
	F	Empregado Efetivo
	F	Empregado Efetivo

	F	Empregado Efetivo
	M	Empregado Efetivo
	M	Empregado Efetivo
	M	Empregado Efetivo
	M	Empregado Efetivo
	M	Empregado Efetivo
	M	Empregado Efetivo
	F	Empregado Efetivo
	F	Empregado Efetivo
	F	Empregado Efetivo
	M	Empregado Efetivo
	M	Empregado Efetivo
	M	Empregado Efetivo
	M	Empregado Efetivo
	M	Empregado Efetivo
	M	Empregado Efetivo
	M	Empregado Efetivo
	M	Empregado Efetivo
	F	Empregado Efetivo
	M	Empregado Efetivo
	F	Empregado Efetivo
	F	Empregado Efetivo
	F	Empregado Efetivo
	F	Empregado Efetivo
	F	Empregado Efetivo
	F	Empregado Efetivo
	F	Empregado Efetivo
	F	Empregado Efetivo
	F	Empregado Efetivo
	F	Empregado Efetivo
	M	Empregado Efetivo
	M	Empregado Efetivo
	M	Empregado Efetivo
	F	Empregado Efetivo
	F	Empregado Efetivo
	M	Empregado Efetivo
	F	Empregado Efetivo
	F	Empregado Efetivo
	M	Cedido - RJU
	F	Empregado Efetivo
	F	Empregado Efetivo
	M	Empregado Efetivo

	F	Empregado Efetivo
	M	Empregado Efetivo
	M	Empregado Efetivo
	F	Empregado Efetivo
	M	Empregado Efetivo
	F	Empregado Efetivo
	M	Empregado Efetivo
	M	Empregado Efetivo
	M	Empregado Efetivo
	F	Empregado Efetivo
	F	Empregado Efetivo
	F	Empregado Efetivo
	M	Empregado Efetivo
	M	Empregado Efetivo
	F	Empregado Efetivo
	M	Empregado Efetivo
	M	Empregado Efetivo
	F	Empregado Efetivo
	M	Empregado Efetivo
	F	Empregado Efetivo
	F	Empregado Efetivo
	F	Empregado Efetivo
	F	Empregado Efetivo
	F	Empregado Efetivo
	F	Empregado Efetivo
	M	Empregado Efetivo
	F	Empregado Efetivo
	F	Empregado Efetivo
	M	Empregado Efetivo
	M	Empregado Efetivo
	F	Empregado Efetivo
	M	Empregado Efetivo
	M	Empregado Efetivo
	M	Empregado Efetivo

	F	Empregado Efetivo
	F	Empregado Efetivo
	F	Empregado Efetivo
	F	Empregado Efetivo
	F	Empregado Efetivo
	F	Empregado Efetivo
	F	Empregado Efetivo
	F	Empregado Efetivo
	F	Empregado Efetivo
	F	Empregado Efetivo
	F	Empregado Efetivo
	F	Empregado Efetivo
	F	Empregado Efetivo
	F	Empregado Efetivo
	F	Empregado Efetivo
	F	Empregado Efetivo
	F	Empregado Efetivo
	F	Empregado Efetivo
	F	Empregado Efetivo
	F	Empregado Efetivo
	F	Empregado Efetivo
	F	Empregado Efetivo
	F	Empregado Efetivo
	F	Empregado Efetivo
	F	Empregado Efetivo
	F	Empregado Efetivo
	F	Empregado Efetivo
	F	Empregado Efetivo
	F	Empregado Efetivo
	F	Empregado Efetivo
	F	Empregado Efetivo
	F	Empregado Efetivo
	F	Empregado Efetivo
	F	Empregado Efetivo
	F	Empregado Efetivo
	M	Empregado Efetivo
	M	Empregado Efetivo
	F	Empregado Efetivo
	F	Empregado Efetivo
	F	Empregado Efetivo
	F	Empregado Efetivo

	F	Empregado Efetivo
	F	Empregado Efetivo
	M	Empregado Efetivo
	F	Empregado Efetivo
	M	Empregado Efetivo
	M	Empregado Efetivo
	F	Empregado Efetivo
	F	Empregado Efetivo
	F	Empregado Efetivo
	F	Empregado Efetivo
	F	Empregado Efetivo
	F	Empregado Efetivo
	F	Empregado Efetivo
	F	Empregado Efetivo
	F	Empregado Efetivo
	F	Empregado Efetivo
	F	Empregado Efetivo
	F	Empregado Efetivo
	F	Empregado Efetivo
	F	Empregado Efetivo
	F	Empregado Efetivo
	F	Empregado Efetivo
	F	Empregado Efetivo
	F	Empregado Efetivo
	F	Empregado Efetivo
	F	Empregado Efetivo
	F	Empregado Efetivo
	M	Empregado Efetivo
	M	Empregado Efetivo
	F	Empregado Efetivo
	F	Empregado Efetivo
	M	Empregado Efetivo
	F	Empregado Efetivo
	F	Empregado Efetivo
	M	Empregado Efetivo
	M	Empregado Efetivo
	M	Empregado Efetivo
	M	Empregado Efetivo
	F	Empregado Efetivo
	F	Empregado Efetivo

	F	Empregado Efetivo
	M	Empregado Efetivo
	M	Empregado Efetivo
	M	Empregado Efetivo
	M	Empregado Efetivo
	F	Empregado Efetivo
	F	Empregado Efetivo
	F	Empregado Efetivo
	M	Empregado Efetivo
	F	Empregado Efetivo
	F	Empregado Efetivo
	F	Empregado Efetivo
	F	Empregado Efetivo
	F	Empregado Efetivo
	F	Empregado Efetivo
	M	Empregado Efetivo
	F	Empregado Efetivo
	F	Empregado Efetivo
	F	Empregado Efetivo
	F	Empregado Efetivo
	M	Empregado Efetivo
	M	Empregado Efetivo
	F	Empregado Efetivo
	F	Empregado Efetivo
	F	Empregado Efetivo
	F	Empregado Efetivo
	F	Empregado Efetivo
	F	Empregado Efetivo
	F	Empregado Efetivo
	M	Empregado Efetivo
	M	Empregado Efetivo
	F	Empregado Efetivo

	M	Empregado Efetivo
	M	Empregado Efetivo
	M	Empregado Efetivo
	F	Empregado Efetivo
	F	Empregado Efetivo
	F	Empregado Efetivo
	F	Empregado Efetivo
	F	Empregado Efetivo
	F	Empregado Efetivo
	F	Empregado Efetivo
	F	Empregado Efetivo
	F	Empregado Efetivo
	F	Empregado Efetivo
	F	Empregado Efetivo
	F	Empregado Efetivo
	F	Empregado Efetivo
	F	Empregado Efetivo
	F	Empregado Efetivo
	F	Empregado Efetivo
	F	Empregado Efetivo
	F	Empregado Efetivo
	M	Empregado Efetivo
	F	Empregado Efetivo
	M	Empregado Efetivo
	F	Empregado Efetivo
	F	Empregado Efetivo
	M	Empregado Efetivo
	M	Empregado Efetivo
	F	Empregado Efetivo
	F	Empregado Efetivo
	F	Empregado Efetivo
	F	Empregado Efetivo
	M	Empregado Efetivo
	F	Empregado Efetivo
	F	Empregado Efetivo
	F	Empregado Efetivo
	F	Empregado Efetivo
	F	Empregado Efetivo
	F	Empregado Efetivo
	F	Empregado Efetivo
	F	Empregado Efetivo
	F	Empregado Efetivo
	F	Empregado Efetivo
	F	Empregado Efetivo
	F	Empregado Efetivo
	F	Empregado Efetivo

	F	Empregado Efetivo
	F	Empregado Efetivo
	F	Empregado Efetivo
	F	Empregado Efetivo
	F	Empregado Efetivo
	F	Empregado Efetivo
	F	Empregado Efetivo
	F	Empregado Efetivo
	F	Empregado Efetivo
	F	Empregado Efetivo
	F	Empregado Efetivo
	F	Empregado Efetivo
	F	Empregado Efetivo
	F	Empregado Efetivo
	F	Empregado Efetivo
	F	Empregado Efetivo
	F	Empregado Efetivo
	F	Empregado Efetivo
	F	Empregado Efetivo
	F	Empregado Efetivo
	F	Empregado Efetivo
	F	Empregado Efetivo
	F	Empregado Efetivo
	F	Empregado Efetivo
	F	Empregado Efetivo
	F	Empregado Efetivo
	F	Empregado Efetivo
	F	Empregado Efetivo
	F	Empregado Efetivo
	F	Empregado Efetivo
	F	Empregado Efetivo
	F	Empregado Efetivo
	F	Empregado Efetivo
	F	Empregado Efetivo
	F	Empregado Efetivo
	F	Empregado Efetivo
	F	Empregado Efetivo
	F	Empregado Efetivo
	M	Empregado Efetivo
	M	Empregado Efetivo
	M	Empregado Efetivo
	M	Empregado Efetivo
	F	Empregado Efetivo
	M	Empregado Efetivo
	M	Empregado Efetivo
	F	Empregado Efetivo
	M	Empregado Efetivo

	F	Empregado Efetivo
	F	Empregado Efetivo
	F	Empregado Efetivo
	F	Empregado Efetivo
	F	Empregado Efetivo
	M	Empregado Efetivo
	M	Empregado Efetivo
	M	Empregado Efetivo
	F	Empregado Efetivo
	F	Empregado Efetivo
	F	Empregado Efetivo
	M	Empregado Efetivo
	F	Empregado Efetivo
	F	Empregado Efetivo
	F	Empregado Efetivo
	M	Empregado Efetivo
	F	Empregado Efetivo
	M	Empregado Efetivo
	M	Empregado Efetivo
	M	Empregado Efetivo
	M	Empregado Efetivo
	M	Empregado Efetivo
	F	Empregado Efetivo
	F	Empregado Efetivo
	F	Empregado Efetivo
	F	Empregado Efetivo
	F	Empregado Efetivo
	F	Empregado Efetivo
	F	Empregado Efetivo
	F	Empregado Efetivo
	F	Empregado Efetivo
	F	Empregado Efetivo
	F	Empregado Efetivo
	F	Empregado Efetivo
	F	Empregado Efetivo
	F	Empregado Efetivo
	F	Cedido - RJU
	F	Empregado Efetivo
	F	Empregado Efetivo
	F	Empregado Efetivo
	F	Empregado Efetivo
	F	Empregado Efetivo
	F	Empregado Efetivo
	F	Empregado Efetivo
	F	Cedido - RJU
	F	Empregado Efetivo
	F	Empregado Efetivo

	F	Empregado Efetivo
	F	Empregado Efetivo
	F	Empregado Efetivo
	F	Empregado Efetivo
	F	Empregado Efetivo
	F	Empregado Efetivo
	F	Empregado Efetivo
	F	Empregado Efetivo
	F	Empregado Efetivo
	F	Empregado Efetivo
	F	Empregado Efetivo
	F	Empregado Efetivo
	M	Empregado Efetivo
	F	Empregado Efetivo
	F	Empregado Efetivo
	F	Empregado Efetivo
	F	Empregado Efetivo
	F	Empregado Efetivo
	F	Empregado Efetivo
	F	Empregado Efetivo
	F	Empregado Efetivo
	F	Empregado Efetivo
	M	Empregado Efetivo
	M	Empregado Efetivo
	F	Empregado Efetivo
	F	Empregado Efetivo
	F	Empregado Efetivo
	F	Empregado Efetivo
	F	Empregado Efetivo
	F	Empregado Efetivo
	F	Empregado Efetivo
	F	Empregado Efetivo
	F	Empregado Efetivo
	F	Empregado Efetivo
	F	Empregado Efetivo
	M	Empregado Efetivo
	F	Empregado Efetivo
	F	Empregado Efetivo
	F	Empregado Efetivo
	M	Empregado Efetivo
	F	Empregado Efetivo
	M	Empregado Efetivo
	F	Empregado Efetivo
	F	Empregado Efetivo
	F	Empregado Efetivo
	F	Empregado Efetivo
	M	Empregado Efetivo
	F	Empregado Efetivo

	F	Empregado Efetivo
	M	Empregado Efetivo
	F	Empregado Efetivo
	M	Empregado Efetivo
	M	Empregado Efetivo
	F	Empregado Efetivo
	M	Empregado Efetivo
	M	Empregado Efetivo
	F	Empregado Efetivo
	M	Empregado Efetivo
	F	Empregado Efetivo
	M	Empregado Efetivo
	F	Empregado Efetivo
	F	Empregado Efetivo
	M	Empregado Efetivo
	M	Empregado Efetivo
	F	Empregado Efetivo
	F	Empregado Efetivo
	F	Empregado Efetivo
	F	Empregado Efetivo
	M	Empregado Efetivo
	M	Empregado Efetivo
	M	Empregado Efetivo
	F	Empregado Efetivo
	F	Empregado Efetivo
	F	Empregado Efetivo
	F	Cedido - RJU
	F	Cedido - RJU
	F	Empregado Efetivo
	M	Empregado Efetivo
	F	Empregado Efetivo
	F	Empregado Efetivo
	F	Empregado Efetivo
	F	Empregado Efetivo
	F	Empregado Efetivo
	F	Empregado Efetivo
	M	Empregado Efetivo
	F	Empregado Efetivo
	F	Empregado Efetivo
	F	Empregado Efetivo
	F	Empregado Efetivo
	F	Empregado Efetivo
	F	Empregado Efetivo
	F	Empregado Efetivo
	M	Empregado Efetivo
	M	Empregado Efetivo
	M	Empregado Efetivo

	M	Empregado Efetivo
	F	Empregado Efetivo
	M	Empregado Efetivo
	F	Empregado Efetivo
	F	Empregado Efetivo
	F	Empregado Efetivo
	M	Empregado Efetivo
	M	Empregado Efetivo
	F	Empregado Efetivo
	F	Empregado Efetivo
	F	Empregado Efetivo
	F	Empregado Efetivo
	F	Empregado Efetivo
	F	Empregado Efetivo
	F	Empregado Efetivo
	F	Empregado Efetivo
	F	Empregado Efetivo
	M	Empregado Efetivo
	F	Empregado Efetivo
	M	Empregado Efetivo
	F	Empregado Efetivo
	M	Empregado Efetivo
	F	Empregado Efetivo
	F	Empregado Efetivo
	F	Empregado Efetivo
	M	Empregado Efetivo
	F	Empregado Efetivo
	F	Empregado Efetivo
	F	Empregado Efetivo
	F	Empregado Efetivo
	M	Empregado Efetivo
	F	Empregado Efetivo
	M	Empregado Efetivo
	F	Empregado Efetivo
	F	Empregado Efetivo
	F	Empregado Efetivo
	M	Empregado Efetivo
	F	Empregado Efetivo
	F	Empregado Efetivo
	F	Empregado Efetivo
	F	Empregado Efetivo
	M	Empregado Efetivo
	F	Empregado Efetivo
	F	Empregado Efetivo
	F	Empregado Efetivo
	F	Empregado Efetivo
	F	Empregado Efetivo

	M	Empregado Efetivo
	F	Empregado Efetivo
	F	Empregado Efetivo
	F	Empregado Efetivo
	M	Empregado Efetivo
	F	Empregado Efetivo
	M	Empregado Efetivo
	M	Empregado Efetivo
	F	Empregado Efetivo
	M	Cedido - RJU
	F	Empregado Efetivo
	F	Empregado Efetivo
	F	Empregado Efetivo
	F	Empregado Efetivo
	F	Empregado Efetivo
	F	Empregado Efetivo
	F	Cedido - RJU
	F	Empregado Efetivo
	F	Empregado Efetivo
	F	Empregado Efetivo
	F	Empregado Efetivo
	M	Empregado Efetivo
	F	Empregado Efetivo
	F	Empregado Efetivo
	F	Empregado Efetivo
	F	Empregado Efetivo
	F	Empregado Efetivo
	F	Empregado Efetivo
	F	Empregado Efetivo
	F	Empregado Efetivo
	F	Empregado Efetivo
	F	Empregado Efetivo
	F	Empregado Efetivo
	F	Empregado Efetivo
	F	Empregado Efetivo
	F	Empregado Efetivo
	F	Empregado Efetivo
	F	Empregado Efetivo
	F	Empregado Efetivo
	F	Empregado Efetivo

	F	Empregado Efetivo
	M	Empregado Efetivo
	M	Empregado Efetivo
	F	Empregado Efetivo
	F	Empregado Efetivo
	F	Empregado Efetivo
	M	Empregado Efetivo
	F	Empregado Efetivo
	F	Empregado Efetivo
	F	Empregado Efetivo
	M	Empregado Efetivo
	F	Empregado Efetivo
	F	Empregado Efetivo
	M	Empregado Efetivo
	F	Empregado Efetivo
	F	Empregado Efetivo
	F	Empregado Efetivo
	F	Empregado Efetivo
	F	Empregado Efetivo
	F	Empregado Efetivo
	F	Empregado Efetivo
	F	Empregado Efetivo
	F	Empregado Efetivo
	F	Empregado Efetivo
	F	Empregado Efetivo
	F	Empregado Efetivo
	F	Empregado Efetivo
	F	Empregado Efetivo
	F	Empregado Efetivo
	F	Empregado Efetivo
	F	Empregado Efetivo
	F	Empregado Efetivo
	F	Empregado Efetivo
	F	Empregado Efetivo
	F	Empregado Efetivo
	F	Empregado Efetivo
	F	Empregado Efetivo
	F	Empregado Efetivo
	F	Empregado Efetivo
	F	Empregado Efetivo
	F	Empregado Efetivo
	F	Empregado Efetivo
	F	Empregado Efetivo
	F	Empregado Efetivo
	F	Empregado Efetivo
	F	Empregado Efetivo
	F	Empregado Efetivo

	F	Empregado Efetivo
	F	Empregado Efetivo
	M	Empregado Efetivo
	F	Empregado Efetivo
	F	Empregado Efetivo
	F	Empregado Efetivo
	F	Empregado Efetivo
	F	Empregado Efetivo
	F	Empregado Efetivo
	M	Empregado Efetivo
	F	Empregado Efetivo
	F	Empregado Efetivo
	F	Empregado Efetivo
	F	Empregado Efetivo
	F	Empregado Efetivo
	F	Empregado Efetivo
	F	Empregado Efetivo
	F	Empregado Efetivo
	F	Empregado Efetivo
	F	Empregado Efetivo
	F	Empregado Efetivo
	M	Empregado Efetivo
	F	Empregado Efetivo
	F	Empregado Efetivo
	F	Empregado Efetivo
	F	Empregado Efetivo
	F	Empregado Efetivo
	F	Empregado Efetivo
	F	Empregado Efetivo
	F	Empregado Efetivo
	F	Empregado Efetivo
	F	Empregado Efetivo
	F	Empregado Efetivo
	F	Empregado Efetivo
	F	Empregado Efetivo
	M	Empregado Efetivo
	F	Empregado Efetivo
	M	Empregado Efetivo
	F	Empregado Efetivo
	F	Empregado Efetivo
	M	Empregado Efetivo
	F	Empregado Efetivo
	F	Empregado Efetivo
	F	Empregado Efetivo
	F	Empregado Efetivo
	F	Empregado Efetivo
	F	Empregado Efetivo
	F	Empregado Efetivo

	F	Empregado Efetivo
	F	Empregado Efetivo
	F	Empregado Efetivo
	F	Empregado Efetivo
	F	Empregado Efetivo
	F	Empregado Efetivo
	F	Empregado Efetivo
	F	Empregado Efetivo
	F	Empregado Efetivo
	M	Empregado Efetivo
	F	Empregado Efetivo
	F	Empregado Efetivo
	F	Empregado Efetivo
	F	Empregado Efetivo
	F	Empregado Efetivo
	F	Empregado Efetivo
	F	Empregado Efetivo
	M	Empregado Efetivo
	F	Empregado Efetivo
	F	Empregado Efetivo
	F	Empregado Efetivo
	F	Empregado Efetivo
	F	Empregado Efetivo
	F	Empregado Efetivo
	F	Empregado Efetivo
	F	Empregado Efetivo
	M	Empregado Efetivo
	M	Empregado Efetivo
	F	Empregado Efetivo
	F	Empregado Efetivo
	F	Empregado Efetivo
	F	Empregado Efetivo
	F	Empregado Efetivo
	F	Empregado Efetivo
	F	Empregado Efetivo
	F	Empregado Efetivo
	F	Empregado Efetivo
	F	Empregado Efetivo
	F	Empregado Efetivo
	F	Empregado Efetivo
	F	Empregado Efetivo
	F	Empregado Efetivo
	F	Empregado Efetivo
	F	Empregado Efetivo
	F	Empregado Efetivo
	M	Empregado Efetivo
	F	Empregado Efetivo
	F	Empregado Efetivo

	F	Empregado Efetivo
	F	Empregado Efetivo
	F	Empregado Efetivo
	F	Empregado Efetivo
	F	Empregado Efetivo
	F	Empregado Efetivo
	F	Empregado Efetivo
	F	Empregado Efetivo
	F	Empregado Efetivo
	F	Empregado Efetivo
	F	Empregado Efetivo
	F	Empregado Efetivo
	F	Empregado Efetivo
	F	Empregado Efetivo
	F	Empregado Efetivo
	F	Empregado Efetivo
	M	Empregado Efetivo
	F	Empregado Efetivo
	F	Empregado Efetivo
	F	Empregado Efetivo
	F	Empregado Efetivo
	F	Empregado Efetivo
	F	Empregado Efetivo
	F	Empregado Efetivo
	F	Empregado Efetivo
	F	Empregado Efetivo
	F	Empregado Efetivo
	F	Empregado Efetivo
	F	Empregado Efetivo
	F	Empregado Efetivo
	F	Empregado Efetivo
	F	Empregado Efetivo
	F	Empregado Efetivo
	F	Empregado Efetivo
	F	Empregado Efetivo
	F	Empregado Efetivo
	F	Empregado Efetivo
	F	Empregado Efetivo
	F	Empregado Efetivo
	F	Empregado Efetivo
	F	Empregado Efetivo
	F	Empregado Efetivo
	F	Empregado Efetivo
	F	Empregado Efetivo
	F	Empregado Efetivo
	F	Empregado Efetivo
	F	Empregado Efetivo

	M	Cedido - RJU
	F	Empregado Efetivo
	F	Empregado Efetivo
	F	Empregado Efetivo
	F	Empregado Efetivo
	F	Empregado Efetivo
	M	Empregado Efetivo
	M	Empregado Efetivo
	M	Empregado Efetivo
	F	Empregado Efetivo
	M	Empregado Efetivo
	M	Empregado Efetivo
	F	Empregado Efetivo
	F	Empregado Efetivo
	F	Empregado Efetivo
	F	Empregado Efetivo
	F	Empregado Efetivo
	F	Empregado Efetivo
	F	Empregado Efetivo
	F	Empregado Efetivo
	M	Empregado Efetivo
	F	Empregado Efetivo
	M	Empregado Efetivo
	F	Empregado Efetivo
	F	Empregado Efetivo
	F	Empregado Efetivo
	F	Empregado Efetivo
	F	Empregado Efetivo
	F	Empregado Efetivo
	F	Empregado Efetivo
	F	Empregado Efetivo
	F	Empregado Efetivo
	F	Empregado Efetivo
	F	Empregado Efetivo
	F	Empregado Efetivo
	M	Empregado Efetivo
	F	Empregado Efetivo
	F	Empregado Efetivo
	F	Empregado Efetivo

	F	Empregado Efetivo
	F	Empregado Efetivo
	F	Empregado Efetivo
	F	Empregado Efetivo
	F	Empregado Efetivo
	F	Empregado Efetivo
	F	Empregado Efetivo
	M	Empregado Efetivo
	F	Empregado Efetivo
	M	Empregado Efetivo
	F	Empregado Efetivo
	F	Empregado Efetivo
	M	Empregado Efetivo
	M	Empregado Efetivo
	M	Empregado Efetivo
	F	Empregado Efetivo
	M	Empregado Efetivo
	F	Empregado Efetivo
	M	Empregado Efetivo
	F	Empregado Efetivo
	F	Empregado Efetivo
	F	Empregado Efetivo
	M	Empregado Efetivo
	F	Empregado Efetivo
	M	Empregado Efetivo
	M	Empregado Efetivo
	M	Empregado Efetivo
	F	Empregado Efetivo
	M	Empregado Efetivo
	F	Empregado Efetivo
	M	Empregado Efetivo
	F	Empregado Efetivo
	F	Empregado Efetivo
	F	Empregado Efetivo
	M	Empregado Efetivo
	M	Empregado Efetivo
	M	Empregado Efetivo
	F	Empregado Efetivo
	F	Empregado Efetivo
	F	Empregado Efetivo
	M	Empregado Efetivo
	F	Empregado Efetivo

	F	Empregado Efetivo
	M	Empregado Efetivo
	M	Empregado Efetivo
	M	Empregado Efetivo
	M	Empregado Efetivo
	F	Empregado Efetivo
	F	Empregado Efetivo
	F	Empregado Efetivo
	F	Empregado Efetivo
	F	Empregado Efetivo
	F	Empregado Efetivo
	F	Empregado Efetivo
	F	Empregado Efetivo
	F	Empregado Efetivo
	M	Empregado Efetivo
	F	Empregado Efetivo
	M	Empregado Efetivo
	F	Empregado Efetivo
	M	Empregado Efetivo
	F	Empregado Efetivo
	F	Empregado Efetivo
	F	Empregado Efetivo
	M	Empregado Efetivo
	M	Empregado Efetivo
	M	Empregado Efetivo
	M	Empregado Efetivo
	M	Empregado Efetivo
	M	Empregado Efetivo
	M	Empregado Efetivo
	M	Empregado Efetivo
	M	Empregado Efetivo
	M	Empregado Efetivo

	M	Empregado Efetivo
	M	Empregado Efetivo
	M	Empregado Efetivo
	M	Empregado Efetivo
	F	Empregado Efetivo
	F	Empregado Efetivo
	M	Empregado Efetivo
	F	Empregado Efetivo
	F	Empregado Efetivo
	M	Empregado Efetivo
	M	Empregado Efetivo
	F	Empregado Efetivo
	F	Empregado Efetivo
	F	Empregado Efetivo
	F	Empregado Efetivo
	F	Empregado Efetivo
	F	Empregado Efetivo
	F	Empregado Efetivo
	F	Empregado Efetivo
	F	Empregado Efetivo
	F	Empregado Efetivo
	F	Empregado Efetivo
	F	Empregado Efetivo
	F	Empregado Efetivo
	F	Empregado Efetivo
	F	Empregado Efetivo
	F	Empregado Efetivo
	F	Empregado Efetivo
	F	Empregado Efetivo
	F	Empregado Efetivo
	F	Empregado Efetivo
	F	Empregado Efetivo
	F	Empregado Efetivo
	F	Empregado Efetivo
	F	Empregado Efetivo
	F	Empregado Efetivo
	F	Empregado Efetivo
	F	Empregado Efetivo
	F	Empregado Efetivo
	F	Empregado Efetivo
	F	Empregado Efetivo
	F	Empregado Efetivo

	F	Empregado Efetivo
	F	Empregado Efetivo
	F	Empregado Efetivo
	F	Empregado Efetivo
	F	Empregado Efetivo
	F	Empregado Efetivo
	F	Empregado Efetivo
	F	Empregado Efetivo
	F	Empregado Efetivo
	F	Empregado Efetivo
	F	Empregado Efetivo
	F	Empregado Efetivo
	F	Empregado Efetivo
	F	Empregado Efetivo
	F	Empregado Efetivo
	F	Empregado Efetivo
	F	Empregado Efetivo
	F	Empregado Efetivo
	F	Empregado Efetivo
	F	Empregado Efetivo
	F	Empregado Efetivo
	F	Empregado Efetivo
	F	Empregado Efetivo
	F	Empregado Efetivo
	F	Empregado Efetivo
	F	Empregado Efetivo
	F	Empregado Efetivo
	F	Empregado Efetivo
	F	Empregado Efetivo
	F	Empregado Efetivo
	F	Empregado Efetivo
	F	Empregado Efetivo
	F	Empregado Efetivo
	M	Empregado Efetivo
	M	Empregado Efetivo
	F	Empregado Efetivo
	F	Empregado Efetivo
	M	Empregado Efetivo
	M	Empregado Efetivo
	M	Empregado Efetivo
	F	Empregado Efetivo
	F	Empregado Efetivo
	M	Empregado Efetivo
	F	Empregado Efetivo
	M	Empregado Efetivo
	F	Empregado Efetivo
	M	Empregado Efetivo
	F	Empregado Efetivo

	M	Empregado Efetivo
	M	Empregado Efetivo
	M	Empregado Efetivo
	M	Empregado Efetivo
	M	Empregado Efetivo
	M	Empregado Efetivo
	M	Empregado Efetivo
	F	Empregado Efetivo
	M	Empregado Efetivo
	M	Empregado Efetivo
	M	Empregado Efetivo
	F	Empregado Efetivo
	F	Empregado Efetivo
	F	Empregado Efetivo
	M	Empregado Efetivo
	F	Empregado Efetivo
	F	Empregado Efetivo
	M	Empregado Efetivo
	F	Empregado Efetivo
	F	Empregado Efetivo
	F	Empregado Efetivo
	F	Empregado Efetivo
	M	Empregado Efetivo
	M	Empregado Efetivo
	M	Empregado Efetivo
	M	Empregado Efetivo
	F	Empregado Efetivo
	F	Empregado Efetivo
	F	Empregado Efetivo
	M	Empregado Efetivo
	M	Empregado Efetivo
	M	Empregado Efetivo
	M	Empregado Efetivo
	M	Empregado Efetivo
	M	Empregado Efetivo
	M	Empregado Efetivo
	M	Empregado Efetivo
	M	Empregado Efetivo
	M	Empregado Efetivo
	M	Empregado Efetivo
	M	Empregado Efetivo
	F	Empregado Efetivo
	F	Empregado Efetivo
	F	Empregado Efetivo
	M	Empregado Efetivo
	F	Empregado Efetivo
	F	Empregado Efetivo

	M	Empregado Efetivo
	M	Empregado Efetivo
	F	Empregado Efetivo
	F	Empregado Efetivo
	M	Empregado Efetivo
	F	Empregado Efetivo
	M	Empregado Efetivo
	M	Empregado Efetivo
	M	Empregado Efetivo
	F	Empregado Efetivo
	F	Empregado Efetivo
	M	Empregado Efetivo
	F	Empregado Efetivo
	F	Empregado Efetivo
	F	Empregado Efetivo
	M	Empregado Efetivo
	M	Empregado Efetivo
	F	Empregado Efetivo
	F	Empregado Efetivo
	F	Empregado Efetivo
	M	Empregado Efetivo
	F	Empregado Efetivo
	F	Empregado Efetivo
	F	Empregado Efetivo
	M	Empregado Efetivo
	M	Empregado Efetivo
	F	Empregado Efetivo
	F	Empregado Efetivo
	F	Empregado Efetivo
	F	Empregado Efetivo
	M	Empregado Efetivo
	M	Empregado Efetivo
	F	Empregado Efetivo
	F	Empregado Efetivo
	F	Empregado Efetivo
	F	Empregado Efetivo
	F	Empregado Efetivo
	F	Empregado Efetivo
	F	Empregado Efetivo
	F	Empregado Efetivo
	F	Empregado Efetivo
	F	Empregado Efetivo
	F	Empregado Efetivo
	M	Empregado Efetivo
	M	Empregado Efetivo
	F	Empregado Efetivo
	M	Empregado Efetivo
	F	Empregado Efetivo

	F	Empregado Efetivo
	F	Empregado Efetivo
	F	Empregado Efetivo
	M	Empregado Efetivo
	M	Empregado Efetivo
	F	Empregado Efetivo
	F	Empregado Efetivo
	M	Empregado Efetivo
	F	Empregado Efetivo
	F	Empregado Efetivo
	F	Empregado Efetivo
	F	Empregado Efetivo
	F	Empregado Efetivo
	M	Empregado Efetivo
	F	Empregado Efetivo
	F	Empregado Efetivo
	F	Empregado Efetivo
	F	Empregado Efetivo
	F	Empregado Efetivo
	F	Empregado Efetivo
	F	Empregado Efetivo
	M	Empregado Efetivo
	F	Empregado Efetivo
	M	Empregado Efetivo
	M	Empregado Efetivo
	F	Empregado Efetivo
	M	Empregado Efetivo
	F	Empregado Efetivo
	F	Empregado Efetivo
	F	Empregado Efetivo
	F	Empregado Efetivo
	F	Empregado Efetivo
	F	Empregado Efetivo
	F	Empregado Efetivo
	M	Empregado Efetivo
	F	Empregado Efetivo
	M	Empregado Efetivo
	M	Empregado Efetivo
	M	Empregado Efetivo
	F	Empregado Efetivo
	F	Empregado Efetivo
	F	Empregado Efetivo

	M	Empregado Efetivo
	F	Empregado Efetivo
	F	Empregado Efetivo
	F	Empregado Efetivo
	F	Empregado Efetivo
	F	Empregado Efetivo
	M	Empregado Efetivo
	F	Empregado Efetivo
	M	Empregado Efetivo
	F	Empregado Efetivo
	F	Empregado Efetivo
	M	Empregado Efetivo
	F	Empregado Efetivo
	M	Empregado Efetivo
	M	Empregado Efetivo
	F	Empregado Efetivo
	F	Empregado Efetivo
	F	Empregado Efetivo
	F	Empregado Efetivo
	F	Empregado Efetivo
	F	Empregado Efetivo
	F	Empregado Efetivo
	F	Empregado Efetivo
	F	Empregado Efetivo
	F	Empregado Efetivo
	F	Empregado Efetivo
	F	Empregado Efetivo
	F	Empregado Efetivo
	F	Empregado Efetivo
	F	Empregado Efetivo
	F	Empregado Efetivo
	F	Empregado Efetivo
	F	Empregado Efetivo
	F	Empregado Efetivo
	F	Empregado Efetivo
	F	Empregado Efetivo
	F	Empregado Efetivo
	F	Empregado Efetivo
	F	Empregado Efetivo
	F	Empregado Efetivo
	F	Empregado Efetivo
	F	Empregado Efetivo
	F	Empregado Efetivo
	F	Empregado Efetivo
	M	Empregado Efetivo
	F	Empregado Efetivo
	M	Empregado Efetivo

	M	Empregado Efetivo
	M	Empregado Efetivo
	F	Empregado Efetivo
	F	Empregado Efetivo
	F	Empregado Efetivo
	M	Empregado Efetivo
	F	Empregado Efetivo
	M	Empregado Efetivo
	F	Empregado Efetivo
	F	Empregado Efetivo
	F	Empregado Efetivo
	F	Empregado Efetivo
	M	Empregado Efetivo
	M	Empregado Efetivo
	F	Empregado Efetivo
	F	Empregado Efetivo
	M	Empregado Efetivo
	F	Empregado Efetivo
	F	Empregado Efetivo
	M	Empregado Efetivo
	F	Empregado Efetivo
	F	Empregado Efetivo
	F	Empregado Efetivo
	F	Empregado Efetivo
	F	Empregado Efetivo
	F	Empregado Efetivo
	F	Empregado Efetivo
	F	Empregado Efetivo
	F	Empregado Efetivo
	F	Empregado Efetivo
	M	Empregado Efetivo
	F	Empregado Efetivo
	F	Empregado Efetivo
	F	Empregado Efetivo
	F	Empregado Efetivo
	F	Empregado Efetivo
	M	Empregado Efetivo
	M	Empregado Efetivo
	F	Empregado Efetivo
	F	Empregado Efetivo
	F	Empregado Efetivo
	F	Empregado Efetivo
	F	Empregado Efetivo
	F	Empregado Efetivo
	F	Empregado Efetivo
	F	Empregado Efetivo
	F	Empregado Efetivo
	F	Empregado Efetivo

	F	Empregado Efetivo
	F	Empregado Efetivo
	F	Empregado Efetivo
	F	Empregado Efetivo
	F	Empregado Efetivo
	F	Empregado Efetivo
	F	Empregado Efetivo
	F	Empregado Efetivo
	F	Empregado Efetivo
	F	Empregado Efetivo
	F	Empregado Efetivo
	F	Empregado Efetivo
	M	Empregado Efetivo
	F	Empregado Efetivo
	F	Empregado Efetivo
	F	Empregado Efetivo
	F	Empregado Efetivo
	F	Empregado Efetivo
	F	Empregado Efetivo
	F	Empregado Efetivo
	F	Empregado Efetivo
	M	Empregado Efetivo
	M	Empregado Efetivo
	F	Empregado Efetivo
	F	Empregado Efetivo
	F	Empregado Efetivo
	M	Empregado Efetivo
	F	Empregado Efetivo
	F	Empregado Efetivo
	F	Empregado Efetivo
	M	Empregado Efetivo
	F	Empregado Efetivo
	F	Empregado Efetivo
	F	Empregado Efetivo
	F	Empregado Efetivo
	F	Empregado Efetivo
	F	Empregado Efetivo
	F	Empregado Efetivo
	F	Empregado Efetivo
	F	Empregado Efetivo
	F	Empregado Efetivo
	F	Empregado Efetivo
	F	Empregado Efetivo
	F	Empregado Efetivo
	F	Empregado Efetivo
	F	Empregado Efetivo
	F	Empregado Efetivo
	F	Empregado Efetivo

	F	Empregado Efetivo
	F	Empregado Efetivo
	F	Empregado Efetivo
	F	Empregado Efetivo
	F	Empregado Efetivo
	M	Empregado Efetivo
	F	Empregado Efetivo
	M	Empregado Efetivo
	F	Empregado Efetivo
	M	Empregado Efetivo
	M	Empregado Efetivo
	F	Empregado Efetivo
	M	Empregado Efetivo
	F	Empregado Efetivo
	F	Empregado Efetivo
	F	Empregado Efetivo
	F	Empregado Efetivo
	M	Empregado Efetivo
	F	Empregado Efetivo
	M	Empregado Efetivo
	F	Empregado Efetivo
	F	Empregado Efetivo
	M	Empregado Efetivo
	F	Empregado Efetivo
	F	Empregado Efetivo
	F	Empregado Efetivo
	F	Empregado Efetivo
	F	Empregado Efetivo
	F	Empregado Efetivo
	F	Empregado Efetivo
	F	Empregado Efetivo
	F	Empregado Efetivo
	F	Empregado Efetivo
	M	Empregado Efetivo
	M	Empregado Efetivo
	F	Empregado Efetivo
	F	Empregado Efetivo
	F	Empregado Efetivo
	F	Empregado Efetivo
	F	Empregado Efetivo
	M	Empregado Efetivo
	M	Empregado Efetivo
	M	Empregado Efetivo
	F	Empregado Efetivo
	F	Empregado Efetivo
	F	Empregado Efetivo
	F	Empregado Efetivo
	F	Empregado Efetivo

	F	Empregado Efetivo
	F	Empregado Efetivo
	F	Empregado Efetivo
	M	Empregado Efetivo
	M	Empregado Efetivo
	M	Empregado Efetivo
	F	Empregado Efetivo
	F	Empregado Efetivo
	F	Empregado Efetivo
	M	Empregado Efetivo
	F	Empregado Efetivo
	F	Empregado Efetivo
	F	Empregado Efetivo
	F	Empregado Efetivo
	M	Empregado Efetivo
	F	Empregado Efetivo
	F	Empregado Efetivo
	M	Empregado Efetivo
	F	Empregado Efetivo
	M	Empregado Efetivo
	F	Empregado Efetivo
	F	Empregado Efetivo
	F	Empregado Efetivo
	F	Empregado Efetivo
	F	Empregado Efetivo
	M	Empregado Efetivo
	M	Empregado Efetivo
	M	Empregado Efetivo
	F	Empregado Efetivo
	F	Empregado Efetivo
	M	Empregado Efetivo
	M	Empregado Efetivo
	F	Empregado Efetivo
	F	Empregado Efetivo
	F	Empregado Efetivo
	M	Empregado Efetivo
	F	Empregado Efetivo
	F	Empregado Efetivo
	F	Empregado Efetivo

	M	Empregado Efetivo
	M	Empregado Efetivo
	F	Empregado Efetivo
	F	Empregado Efetivo
	F	Empregado Efetivo
	F	Empregado Efetivo
	F	Empregado Efetivo
	F	Empregado Efetivo
	M	Empregado Efetivo
	M	Empregado Efetivo
	F	Empregado Efetivo
	F	Empregado Efetivo
	F	Empregado Efetivo
	F	Empregado Efetivo
	F	Empregado Efetivo
	F	Empregado Efetivo
	F	Empregado Efetivo
	M	Empregado Efetivo
	F	Empregado Efetivo
	F	Empregado Efetivo
	F	Empregado Efetivo
	F	Empregado Efetivo
	M	Empregado Efetivo
	F	Empregado Efetivo
	M	Empregado Efetivo
	F	Empregado Efetivo
	F	Empregado Efetivo
	F	Empregado Efetivo
	F	Empregado Efetivo
	F	Empregado Efetivo
	F	Empregado Efetivo
	M	Empregado Efetivo
	F	Empregado Efetivo
	F	Empregado Efetivo
	F	Empregado Efetivo
	M	Empregado Efetivo
	F	Empregado Efetivo
	F	Empregado Efetivo
	F	Empregado Efetivo
	F	Empregado Efetivo
	F	Empregado Efetivo
	F	Empregado Efetivo
	F	Empregado Efetivo
	F	Empregado Efetivo
	F	Empregado Efetivo
	F	Empregado Efetivo
	F	Empregado Efetivo

	M	Empregado Efetivo
	M	Empregado Efetivo
	M	Empregado Efetivo
	F	Empregado Efetivo
	F	Empregado Efetivo
	F	Empregado Efetivo
	F	Empregado Efetivo
	F	Empregado Efetivo
	F	Empregado Efetivo
	F	Empregado Efetivo
	F	Empregado Efetivo
	F	Empregado Efetivo
	F	Empregado Efetivo
	F	Empregado Efetivo
	F	Empregado Efetivo
	F	Empregado Efetivo
	F	Empregado Efetivo
	F	Empregado Efetivo
	F	Empregado Efetivo
	F	Empregado Efetivo
	F	Empregado Efetivo
	F	Empregado Efetivo
	F	Empregado Efetivo
	F	Empregado Efetivo
	F	Empregado Efetivo
	F	Empregado Efetivo
	F	Empregado Efetivo
	F	Empregado Efetivo
	F	Empregado Efetivo
	F	Empregado Efetivo
	F	Empregado Efetivo
	F	Empregado Efetivo
	F	Empregado Efetivo
	F	Empregado Efetivo
	M	Empregado Efetivo
	M	Empregado Efetivo
	M	Empregado Efetivo
	M	Empregado Efetivo
	M	Empregado Efetivo
	M	Empregado Efetivo
	M	Empregado Efetivo
	M	Empregado Efetivo

	M	Empregado Efetivo
	M	Empregado Efetivo
	M	Empregado Efetivo
	M	Empregado Efetivo
	M	Empregado Efetivo
	M	Empregado Efetivo
	F	Empregado Efetivo
	F	Cedido - RJU
	F	Cedido - RJU
	F	Cedido - RJU
	F	Cedido - RJU
	F	Empregado Efetivo
	F	Empregado Efetivo
	F	Empregado Efetivo
	M	Empregado Efetivo
	F	Empregado Efetivo
	F	Empregado Efetivo
	F	Empregado Efetivo
	F	Empregado Efetivo
	F	Empregado Efetivo
	F	Empregado Efetivo
	F	Empregado Efetivo
	F	Empregado Efetivo
	F	Empregado Efetivo
	F	Empregado Efetivo
	F	Empregado Efetivo
	F	Empregado Efetivo
	F	Empregado Efetivo
	F	Empregado Efetivo
	M	Empregado Efetivo
	M	Empregado Efetivo
	F	Empregado Efetivo
	M	Empregado Efetivo
	F	Empregado Efetivo
	F	Empregado Efetivo
	F	Empregado Efetivo
	F	Empregado Efetivo

	F	Empregado Efetivo
	F	Empregado Efetivo
	F	Empregado Efetivo
	F	Empregado Efetivo
	F	Empregado Efetivo
	F	Empregado Efetivo
	F	Empregado Efetivo
	F	Empregado Efetivo
	M	Cedido - RJU
	F	Empregado Efetivo
	M	Empregado Efetivo
	M	Empregado Efetivo
	F	Empregado Efetivo
	F	Empregado Efetivo
	M	Empregado Efetivo
	M	Empregado Efetivo
	F	Empregado Efetivo
	F	Empregado Efetivo
	F	Empregado Efetivo
	F	Empregado Efetivo
	F	Empregado Efetivo
	F	Empregado Efetivo
	F	Empregado Efetivo
	F	Empregado Efetivo
	M	Cedido - RJU
	M	Cedido - RJU
	F	Empregado Efetivo
	M	Empregado Efetivo
	M	Empregado Efetivo
	F	Empregado Efetivo
	F	Empregado Efetivo
	F	Empregado Efetivo
	M	Empregado Efetivo
	M	Empregado Efetivo
	F	Empregado Efetivo

	F	Empregado Efetivo
	F	Empregado Efetivo
	F	Empregado Efetivo
	F	Empregado Efetivo
	F	Empregado Efetivo
	F	Empregado Efetivo
	F	Empregado Efetivo
	M	Empregado Efetivo
	M	Empregado Efetivo
	M	Empregado Efetivo
	F	Empregado Efetivo
	M	Empregado Efetivo
	F	Cedido - RJU
	F	Empregado Efetivo
	M	Empregado Efetivo
	M	Empregado Efetivo
	M	Cedido - RJU
	M	Empregado Efetivo
	M	Empregado Efetivo
	F	Empregado Efetivo
	M	Empregado Efetivo
	F	Empregado Efetivo
	F	Empregado Efetivo
	F	Empregado Efetivo
	F	Empregado Efetivo
	F	Empregado Efetivo
	F	Empregado Efetivo
	F	Empregado Efetivo
	F	Empregado Efetivo
	F	Empregado Efetivo
	F	Empregado Efetivo
	F	Empregado Efetivo

	F	Empregado Efetivo
	F	Empregado Efetivo
	F	Empregado Efetivo
	F	Empregado Efetivo
	F	Empregado Efetivo
	F	Empregado Efetivo
	F	Empregado Efetivo
	F	Empregado Efetivo
	F	Empregado Efetivo
	F	Empregado Efetivo
	F	Empregado Efetivo
	M	Empregado Efetivo
	F	Empregado Efetivo
	M	Empregado Efetivo
	F	Empregado Efetivo
	M	Empregado Efetivo
	M	Empregado Efetivo
	M	Empregado Efetivo
	M	Empregado Efetivo
	M	Empregado Efetivo
	M	Empregado Efetivo
	M	Empregado Efetivo
	M	Empregado Efetivo
	M	Empregado Efetivo
	M	Empregado Efetivo
	M	Empregado Efetivo
	M	Empregado Efetivo
	M	Empregado Efetivo
	M	Empregado Efetivo
	F	Empregado Efetivo
	F	Empregado Efetivo
	F	Empregado Efetivo
	F	Empregado Efetivo
	F	Empregado Efetivo
	F	Empregado Efetivo
	M	Empregado Efetivo
	F	Empregado Efetivo
	F	Empregado Efetivo
	F	Empregado Efetivo

	F	Empregado Efetivo
	F	Empregado Efetivo
	F	Empregado Efetivo
	F	Empregado Efetivo
	F	Empregado Efetivo
	F	Empregado Efetivo
	F	Empregado Efetivo
	F	Empregado Efetivo
	F	Empregado Efetivo
	F	Empregado Efetivo
	F	Empregado Efetivo
	F	Empregado Efetivo
	F	Empregado Efetivo
	F	Empregado Efetivo
	F	Empregado Efetivo
	F	Empregado Efetivo
	F	Empregado Efetivo
	F	Empregado Efetivo
	F	Empregado Efetivo
	F	Empregado Efetivo
	F	Empregado Efetivo
	F	Empregado Efetivo
	F	Empregado Efetivo
	F	Empregado Efetivo
	F	Empregado Efetivo
	M	Empregado Efetivo
	F	Empregado Efetivo
	F	Empregado Efetivo
	F	Empregado Efetivo
	F	Empregado Efetivo
	M	Empregado Efetivo
	M	Empregado Efetivo
	M	Empregado Efetivo
	F	Empregado Efetivo
	F	Empregado Efetivo
	F	Empregado Efetivo
	M	Empregado Efetivo
	M	Empregado Efetivo
	M	Empregado Efetivo
	M	Empregado Efetivo
	M	Empregado Efetivo
	M	Empregado Efetivo
	M	Empregado Efetivo
	M	Empregado Efetivo
	M	Empregado Efetivo

	M	Empregado Efetivo
	M	Empregado Efetivo
	M	Empregado Efetivo
	F	Empregado Efetivo
	F	Empregado Efetivo
	F	Empregado Efetivo
	F	Empregado Efetivo
	F	Empregado Efetivo
	F	Empregado Efetivo
	F	Empregado Efetivo
	F	Empregado Efetivo
	F	Empregado Efetivo
	M	Empregado Efetivo
	F	Empregado Efetivo
	F	Empregado Efetivo
	M	Empregado Efetivo
	M	Empregado Efetivo
	F	Empregado Efetivo
	F	Empregado Efetivo
	F	Empregado Efetivo
	F	Empregado Efetivo
	F	Empregado Efetivo
	F	Empregado Efetivo
	F	Empregado Efetivo
	F	Empregado Efetivo
	F	Empregado Efetivo
	F	Empregado Efetivo
	F	Empregado Efetivo
	M	Empregado Efetivo
	F	Empregado Efetivo
	M	Empregado Efetivo
	M	Empregado Efetivo
	M	Empregado Efetivo
	F	Empregado Efetivo
	M	Empregado Efetivo
	M	Empregado Efetivo
	F	Empregado Efetivo
	F	Empregado Efetivo
	F	Empregado Efetivo
	F	Empregado Efetivo
	F	Empregado Efetivo
	F	Empregado Efetivo
	F	Empregado Efetivo
	F	Empregado Efetivo
	F	Empregado Efetivo
	F	Empregado Efetivo
	F	Empregado Efetivo
	F	Empregado Efetivo

	F	Empregado Efetivo
	F	Empregado Efetivo
	F	Empregado Efetivo
	F	Empregado Efetivo
	F	Empregado Efetivo
	F	Empregado Efetivo
	F	Empregado Efetivo
	F	Empregado Efetivo
	F	Empregado Efetivo
	F	Empregado Efetivo
	F	Empregado Efetivo
	F	Empregado Efetivo
	F	Empregado Efetivo
	M	Empregado Efetivo
	F	Empregado Efetivo
	F	Empregado Efetivo
	F	Empregado Efetivo
	F	Empregado Efetivo
	F	Empregado Efetivo
	F	Empregado Efetivo
	F	Empregado Efetivo
	F	Empregado Efetivo
	F	Empregado Efetivo
	M	Empregado Efetivo
	F	Empregado Efetivo
	F	Empregado Efetivo
	F	Empregado Efetivo
	F	Empregado Efetivo
	M	Empregado Efetivo
	F	Empregado Efetivo
	F	Empregado Efetivo
	F	Empregado Efetivo

	F	Empregado Efetivo
	M	Empregado Efetivo
	F	Empregado Efetivo
	F	Empregado Efetivo
	F	Empregado Efetivo
	F	Empregado Efetivo
	F	Empregado Efetivo
	F	Empregado Efetivo
	F	Empregado Efetivo
	F	Empregado Efetivo
	F	Empregado Efetivo
	F	Empregado Efetivo
	F	Empregado Efetivo
	F	Empregado Efetivo
	F	Empregado Efetivo
	F	Empregado Efetivo
	M	Empregado Efetivo
	F	Empregado Efetivo
	F	Empregado Efetivo
	F	Empregado Efetivo
	F	Empregado Efetivo
	F	Empregado Efetivo
	F	Empregado Efetivo
	F	Empregado Efetivo
	F	Empregado Efetivo
	F	Empregado Efetivo
	F	Empregado Efetivo
	M	Empregado Efetivo
	F	Empregado Efetivo
	F	Empregado Efetivo
	F	Empregado Efetivo
	F	Empregado Efetivo
	M	Empregado Efetivo
	F	Empregado Efetivo
	M	Empregado Efetivo
	F	Empregado Efetivo

	F	Empregado Efetivo
	F	Empregado Efetivo
	F	Empregado Efetivo
	F	Empregado Efetivo
	M	Empregado Efetivo
	F	Empregado Efetivo
	F	Empregado Efetivo
	F	Empregado Efetivo
	M	Empregado Efetivo
	F	Empregado Efetivo
	F	Empregado Efetivo
	F	Empregado Efetivo
	F	Empregado Efetivo
	F	Empregado Efetivo
	F	Empregado Efetivo
	F	Empregado Efetivo
	F	Empregado Efetivo
	F	Empregado Efetivo
	F	Empregado Efetivo
	F	Empregado Efetivo
	F	Empregado Efetivo
	F	Empregado Efetivo
	F	Empregado Efetivo
	F	Empregado Efetivo
	F	Empregado Efetivo
	F	Empregado Efetivo
	F	Empregado Efetivo
	F	Empregado Efetivo
	F	Empregado Efetivo
	M	Empregado Efetivo
	M	Empregado Efetivo
	F	Empregado Efetivo
	F	Empregado Efetivo
	F	Empregado Efetivo
	M	Empregado Efetivo
	F	Empregado Efetivo
	F	Empregado Efetivo
	F	Empregado Efetivo

	F	Empregado Efetivo
	M	Empregado Efetivo
	F	Empregado Efetivo
	F	Empregado Efetivo
	M	Empregado Efetivo
	F	Empregado Efetivo
	F	Empregado Efetivo
	F	Empregado Efetivo
	F	Empregado Efetivo
	M	Empregado Efetivo
	M	Empregado Efetivo
	F	Empregado Efetivo
	M	Empregado Efetivo
	F	Empregado Efetivo
	M	Empregado Efetivo
	F	Empregado Efetivo
	F	Empregado Efetivo
	F	Empregado Efetivo
	F	Empregado Efetivo
	F	Empregado Efetivo
	F	Empregado Efetivo
	M	Empregado Efetivo
	F	Empregado Efetivo
	M	Empregado Efetivo
	M	Empregado Efetivo
	M	Empregado Efetivo
	M	Empregado Efetivo
	F	Empregado Efetivo
	F	Empregado Efetivo
	M	Empregado Efetivo
	M	Empregado Efetivo
	F	Empregado Efetivo
	F	Empregado Efetivo
	M	Empregado Efetivo
	M	Empregado Efetivo
	M	Empregado Efetivo
	F	Empregado Efetivo
	M	Empregado Efetivo
	M	Empregado Efetivo
	F	Empregado Efetivo
	F	Empregado Efetivo
	F	Empregado Efetivo
	F	Empregado Efetivo
	F	Empregado Efetivo
	F	Empregado Efetivo
	F	Empregado Efetivo

	F	Empregado Efetivo
	F	Empregado Efetivo
	F	Empregado Efetivo
	F	Empregado Efetivo
	F	Empregado Efetivo
	F	Empregado Efetivo
	F	Empregado Efetivo
	F	Empregado Efetivo
	M	Empregado Efetivo
	F	Empregado Efetivo
	M	Empregado Efetivo
	M	Empregado Efetivo
	F	Empregado Efetivo
	F	Empregado Efetivo
	F	Empregado Efetivo
	F	Empregado Efetivo
	F	Empregado Efetivo
	F	Empregado Efetivo
	M	Empregado Efetivo
	F	Empregado Efetivo
	M	Empregado Efetivo
	M	Empregado Efetivo
	F	Empregado Efetivo
	F	Empregado Efetivo
	F	Empregado Efetivo
	F	Empregado Efetivo
	F	Empregado Efetivo
	F	Empregado Efetivo
	M	Empregado Efetivo
	F	Empregado Efetivo
	F	Empregado Efetivo
	M	Empregado Efetivo
	M	Empregado Efetivo
	M	Empregado Efetivo
	F	Empregado Efetivo
	F	Empregado Efetivo
	F	Empregado Efetivo
	F	Empregado Efetivo
	F	Empregado Efetivo
	F	Empregado Efetivo
	F	Empregado Efetivo
	M	Empregado Efetivo
	F	Empregado Efetivo
	F	Empregado Efetivo
	F	Empregado Efetivo
	F	Empregado Efetivo
	F	Empregado Efetivo
	M	Empregado Efetivo

	M	Empregado Efetivo
	F	Empregado Efetivo
	M	Empregado Efetivo
	F	Empregado Efetivo
	F	Empregado Efetivo
	F	Empregado Efetivo
	F	Empregado Efetivo
	F	Empregado Efetivo
	F	Empregado Efetivo
	F	Empregado Efetivo
	F	Empregado Efetivo
	F	Empregado Efetivo
	F	Empregado Efetivo
	F	Empregado Efetivo
	F	Empregado Efetivo
	F	Empregado Efetivo
	F	Empregado Efetivo
	F	Empregado Efetivo
	F	Empregado Efetivo
	F	Empregado Efetivo
	F	Empregado Efetivo
	F	Empregado Efetivo
	F	Empregado Efetivo
	F	Empregado Efetivo
	F	Empregado Efetivo
	F	Empregado Efetivo
	F	Empregado Efetivo
	F	Empregado Efetivo
	F	Empregado Efetivo
	F	Empregado Efetivo
	F	Empregado Efetivo
	F	Empregado Efetivo
	F	Empregado Efetivo
	F	Empregado Efetivo
	M	Empregado Efetivo
	F	Empregado Efetivo
	F	Empregado Efetivo
	F	Empregado Efetivo
	F	Empregado Efetivo
	F	Empregado Efetivo

	F	Empregado Efetivo
	F	Empregado Efetivo
	F	Empregado Efetivo
	F	Empregado Efetivo
	F	Empregado Efetivo
	F	Empregado Efetivo
	M	Empregado Efetivo
	F	Empregado Efetivo
	F	Empregado Efetivo
	F	Empregado Efetivo
	F	Empregado Efetivo
	F	Empregado Efetivo
	M	Empregado Efetivo
	F	Empregado Efetivo
	F	Empregado Efetivo
	M	Empregado Efetivo
	F	Empregado Efetivo
	F	Empregado Efetivo
	F	Empregado Efetivo
	F	Empregado Efetivo
	F	Empregado Efetivo
	F	Empregado Efetivo
	F	Empregado Efetivo
	F	Empregado Efetivo
	F	Empregado Efetivo
	F	Empregado Efetivo
	F	Empregado Efetivo
	F	Empregado Efetivo
	F	Empregado Efetivo
	M	Empregado Efetivo
	F	Empregado Efetivo
	F	Empregado Efetivo

	F	Empregado Efetivo
	F	Empregado Efetivo
	M	Empregado Efetivo
	F	Empregado Efetivo
	F	Empregado Efetivo
	F	Empregado Efetivo
	F	Empregado Efetivo
	F	Empregado Efetivo
	F	Empregado Efetivo
	M	Empregado Efetivo
	F	Empregado Efetivo
	F	Empregado Efetivo
	F	Empregado Efetivo
	F	Empregado Efetivo
	F	Empregado Efetivo
	F	Empregado Efetivo
	F	Empregado Efetivo
	F	Empregado Efetivo
	M	Empregado Efetivo
	F	Empregado Efetivo
	F	Empregado Efetivo
	F	Empregado Efetivo
	F	Empregado Efetivo
	F	Empregado Efetivo
	F	Empregado Efetivo
	F	Empregado Efetivo
	F	Empregado Efetivo
	F	Empregado Efetivo
	M	Empregado Efetivo
	F	Empregado Efetivo
	F	Empregado Efetivo
	F	Empregado Efetivo

	F	Empregado Efetivo
	M	Empregado Efetivo
	F	Empregado Efetivo
	F	Empregado Efetivo
	F	Empregado Efetivo
	F	Empregado Efetivo
	F	Empregado Efetivo
	F	Empregado Efetivo
	F	Empregado Efetivo
	F	Empregado Efetivo
	F	Empregado Efetivo
	F	Empregado Efetivo
	F	Empregado Efetivo
	M	Empregado Efetivo
	F	Empregado Efetivo
	F	Empregado Efetivo
	M	Empregado Efetivo
	F	Empregado Efetivo
	F	Empregado Efetivo
	F	Empregado Efetivo
	M	Empregado Efetivo
	F	Empregado Efetivo
	F	Empregado Efetivo
	M	Empregado Efetivo
	M	Empregado Efetivo
	F	Empregado Efetivo
	F	Empregado Efetivo
	F	Empregado Efetivo
	F	Empregado Efetivo
	M	Empregado Efetivo
	M	Empregado Efetivo
	F	Empregado Efetivo

	F	Empregado Efetivo
	F	Empregado Efetivo
	M	Empregado Efetivo
	F	Empregado Efetivo
	M	Empregado Efetivo
	F	Empregado Efetivo
	F	Empregado Efetivo
	F	Empregado Efetivo
	F	Empregado Efetivo
	F	Empregado Efetivo
	M	Empregado Efetivo
	M	Empregado Efetivo
	F	Empregado Efetivo
	M	Empregado Efetivo
	M	Empregado Efetivo
	M	Empregado Efetivo
	M	Empregado Efetivo
	F	Empregado Efetivo
	M	Empregado Efetivo
	F	Empregado Efetivo
	F	Empregado Efetivo
	F	Empregado Efetivo
	F	Empregado Efetivo
	F	Empregado Efetivo
	M	Empregado Efetivo
	M	Empregado Efetivo
	F	Empregado Efetivo
	F	Empregado Efetivo
	F	Empregado Efetivo
	F	Empregado Efetivo
	F	Empregado Efetivo
	F	Empregado Efetivo
	F	Empregado Efetivo
	F	Empregado Efetivo

	F	Empregado Efetivo
	F	Empregado Efetivo
	F	Empregado Efetivo
	F	Empregado Efetivo
	F	Empregado Efetivo
	F	Empregado Efetivo
	F	Empregado Efetivo
	F	Empregado Efetivo
	F	Empregado Efetivo
	M	Empregado Efetivo
	F	Empregado Efetivo
	F	Empregado Efetivo
	F	Empregado Efetivo
	F	Empregado Efetivo
	F	Empregado Efetivo
	F	Empregado Efetivo
	F	Empregado Efetivo
	F	Empregado Efetivo
	M	Empregado Efetivo
	M	Empregado Efetivo
	M	Empregado Efetivo
	F	Empregado Efetivo
	F	Empregado Efetivo
	F	Empregado Efetivo
	M	Empregado Efetivo
	F	Empregado Efetivo
	M	Empregado Efetivo
	F	Empregado Efetivo
	F	Empregado Efetivo
	F	Empregado Efetivo
	F	Empregado Efetivo
	F	Empregado Efetivo
	F	Empregado Efetivo
	M	Empregado Efetivo
	M	Empregado Efetivo
	F	Empregado Efetivo
	F	Empregado Efetivo
	M	Empregado Efetivo
	F	Empregado Efetivo
	F	Empregado Efetivo
	F	Empregado Efetivo
	F	Empregado Efetivo
	F	Empregado Efetivo
	F	Empregado Efetivo
	F	Empregado Efetivo
	F	Empregado Efetivo
	F	Empregado Efetivo
	F	Empregado Efetivo

	F	Empregado Efetivo
	F	Empregado Efetivo
	F	Empregado Efetivo
	F	Empregado Efetivo
	F	Empregado Efetivo
	F	Empregado Efetivo
	M	Empregado Efetivo
	M	Empregado Efetivo
	F	Empregado Efetivo
	F	Empregado Efetivo
	F	Empregado Efetivo
	F	Empregado Efetivo
	F	Empregado Efetivo
	F	Empregado Efetivo
	F	Empregado Efetivo
	F	Empregado Efetivo
	F	Empregado Efetivo
	F	Empregado Efetivo
	F	Empregado Efetivo
	F	Empregado Efetivo
	M	Empregado Efetivo
	M	Empregado Efetivo
	M	Empregado Efetivo
	M	Empregado Efetivo
	F	Empregado Efetivo
	F	Empregado Efetivo
	F	Empregado Efetivo
	F	Empregado Efetivo
	F	Empregado Efetivo
	F	Empregado Efetivo
	F	Empregado Efetivo
	F	Empregado Efetivo
	F	Empregado Efetivo
	M	Empregado Efetivo
	M	Empregado Efetivo
	F	Empregado Efetivo
	F	Empregado Efetivo
	F	Empregado Efetivo
	F	Empregado Efetivo
	F	Empregado Efetivo

	F	Empregado Efetivo
	F	Empregado Efetivo
	F	Empregado Efetivo
	F	Empregado Efetivo
	F	Empregado Efetivo
	F	Empregado Efetivo
	F	Empregado Efetivo
	F	Empregado Efetivo
	F	Empregado Efetivo
	F	Empregado Efetivo
	F	Empregado Efetivo
	F	Empregado Efetivo
	F	Empregado Efetivo
	F	Empregado Efetivo
	F	Empregado Efetivo
	F	Empregado Efetivo
	M	Empregado Efetivo
	M	Empregado Efetivo
	M	Empregado Efetivo
	F	Empregado Efetivo
	F	Empregado Efetivo
	F	Cedido - RJU
	F	Empregado Efetivo
	F	Empregado Efetivo
	F	Empregado Efetivo
	F	Empregado Efetivo
	F	Empregado Efetivo
	F	Empregado Efetivo
	F	Empregado Efetivo
	F	Empregado Efetivo
	F	Empregado Efetivo
	F	Empregado Efetivo
	F	Empregado Efetivo
	F	Empregado Efetivo
	F	Empregado Efetivo
	F	Empregado Efetivo
	F	Empregado Efetivo
	F	Empregado Efetivo
	F	Empregado Efetivo
	F	Empregado Efetivo
	F	Empregado Efetivo

	F	Empregado Efetivo
	F	Empregado Efetivo
	M	Empregado Efetivo
	F	Empregado Efetivo
	F	Empregado Efetivo
	F	Empregado Efetivo
	F	Empregado Efetivo
	F	Empregado Efetivo
	F	Empregado Efetivo
	F	Empregado Efetivo
	F	Empregado Efetivo
	M	Empregado Efetivo
	M	Empregado Efetivo
	M	Empregado Efetivo
	F	Empregado Efetivo
	M	Empregado Efetivo
	F	Empregado Efetivo
	F	Empregado Efetivo
	F	Empregado Efetivo
	F	Empregado Efetivo
	F	Empregado Efetivo
	F	Empregado Efetivo
	F	Empregado Efetivo
	F	Empregado Efetivo
	M	Empregado Efetivo
	M	Empregado Efetivo
	M	Empregado Efetivo
	M	Empregado Efetivo
	M	Empregado Efetivo
	M	Empregado Efetivo
	M	Empregado Efetivo
	M	Empregado Efetivo
	M	Empregado Efetivo
	M	Empregado Efetivo

	M	Empregado Efetivo
	M	Empregado Efetivo
	M	Empregado Efetivo
	F	Empregado Efetivo
	M	Empregado Efetivo
	M	Empregado Efetivo
	M	Empregado Efetivo
	F	Empregado Efetivo
	F	Empregado Efetivo
	F	Empregado Efetivo
	F	Empregado Efetivo
	F	Empregado Efetivo
	F	Empregado Efetivo
	F	Empregado Efetivo
	F	Empregado Efetivo
	M	Empregado Efetivo
	F	Empregado Efetivo
	F	Empregado Efetivo
	F	Empregado Efetivo
	M	Empregado Efetivo
	M	Empregado Efetivo
	F	Empregado Efetivo
	F	Empregado Efetivo
	M	Empregado Efetivo
	M	Empregado Efetivo
	M	Empregado Efetivo
	M	Empregado Efetivo
	M	Empregado Efetivo
	M	Empregado Efetivo
	M	Empregado Efetivo
	F	Empregado Efetivo
	F	Empregado Efetivo
	F	Empregado Efetivo
	M	Empregado Efetivo
	M	Empregado Efetivo
	F	Empregado Efetivo
	F	Empregado Efetivo
	M	Empregado Efetivo
	M	Empregado Efetivo

	F	Empregado Efetivo
	M	Empregado Efetivo
	M	Empregado Efetivo
	M	Empregado Efetivo
	M	Empregado Efetivo
	F	Empregado Efetivo
	M	Empregado Efetivo
	M	Empregado Efetivo
	F	Empregado Efetivo
	F	Empregado Efetivo
	F	Empregado Efetivo
	M	Empregado Efetivo
	F	Empregado Efetivo
	F	Empregado Efetivo
	F	Empregado Efetivo
	F	Empregado Efetivo
	F	Empregado Efetivo
	F	Empregado Efetivo
	F	Empregado Efetivo
	M	Empregado Efetivo
	F	Empregado Efetivo
	F	Empregado Efetivo
	F	Empregado Efetivo
	M	Empregado Efetivo
	M	Empregado Efetivo
	M	Empregado Efetivo
	M	Empregado Efetivo
	M	Empregado Efetivo
	M	Empregado Efetivo
	M	Empregado Efetivo
	M	Empregado Efetivo
	M	Empregado Efetivo
	F	Empregado Efetivo
	M	Empregado Efetivo
	M	Empregado Efetivo
	M	Empregado Efetivo
	F	Empregado Efetivo
	F	Empregado Efetivo
	F	Empregado Efetivo
	F	Empregado Efetivo
	F	Empregado Efetivo

	F	Empregado Efetivo
	F	Empregado Efetivo
	F	Empregado Efetivo
	F	Empregado Efetivo
	F	Empregado Efetivo
	F	Empregado Efetivo
	M	Empregado Efetivo
	M	Empregado Efetivo
	F	Empregado Efetivo
	F	Empregado Efetivo
	F	Empregado Efetivo
	F	Empregado Efetivo
	M	Empregado Efetivo
	M	Empregado Efetivo
	M	Empregado Efetivo
	M	Empregado Efetivo
	M	Empregado Efetivo
	M	Empregado Efetivo
	M	Empregado Efetivo
	F	Empregado Efetivo
	F	Empregado Efetivo
	F	Empregado Efetivo
	F	Empregado Efetivo
	F	Empregado Efetivo
	F	Empregado Efetivo
	F	Empregado Efetivo
	F	Empregado Efetivo
	F	Empregado Efetivo
	F	Empregado Efetivo
	F	Empregado Efetivo
	F	Empregado Efetivo
	F	Empregado Efetivo
	F	Empregado Efetivo
	F	Empregado Efetivo
	F	Empregado Efetivo
	F	Empregado Efetivo
	F	Empregado Efetivo
	M	Empregado Efetivo
	M	Empregado Efetivo
	M	Empregado Efetivo
	F	Empregado Efetivo
	M	Empregado Efetivo
	M	Empregado Efetivo
	F	Empregado Efetivo
	M	Empregado Efetivo
	F	Empregado Efetivo
	F	Empregado Efetivo
	F	Empregado Efetivo

	F	Empregado Efetivo
	F	Cedido - RJU
	M	Empregado Efetivo
	M	Empregado Efetivo
	F	Empregado Efetivo
	F	Empregado Efetivo
	F	Empregado Efetivo
	F	Empregado Efetivo
	F	Empregado Efetivo
	F	Empregado Efetivo
	F	Empregado Efetivo
	F	Empregado Efetivo
	F	Empregado Efetivo
	F	Empregado Efetivo
	F	Empregado Efetivo
	F	Empregado Efetivo
	F	Empregado Efetivo
	F	Empregado Efetivo
	F	Empregado Efetivo
	F	Empregado Efetivo
	F	Empregado Efetivo
	F	Empregado Efetivo
	F	Empregado Efetivo
	F	Empregado Efetivo
	F	Empregado Efetivo
	M	Empregado Efetivo
	M	Empregado Efetivo
	F	Empregado Efetivo
	F	Empregado Efetivo
	M	Empregado Efetivo
	F	Empregado Efetivo
	F	Empregado Efetivo
	F	Empregado Efetivo
	F	Empregado Efetivo
	M	Empregado Efetivo
	F	Empregado Efetivo
	M	Empregado Efetivo
	F	Empregado Efetivo
	M	Empregado Efetivo
	M	Empregado Efetivo
	F	Empregado Efetivo
	F	Empregado Efetivo
	F	Empregado Efetivo
	M	Empregado Efetivo
	F	Empregado Efetivo
	F	Empregado Efetivo

	F	Empregado Efetivo
	M	Empregado Efetivo
	M	Empregado Efetivo
	F	Empregado Efetivo
	F	Empregado Efetivo
	F	Empregado Efetivo
	F	Empregado Efetivo
	F	Empregado Efetivo
	F	Empregado Efetivo
	F	Empregado Efetivo
	F	Empregado Efetivo
	F	Empregado Efetivo
	F	Empregado Efetivo
	F	Comissionado
	F	Comissionado
	M	Empregado Efetivo
	M	Empregado Efetivo
	M	Empregado Efetivo
	F	Empregado Efetivo
	F	Empregado Efetivo
	F	Empregado Efetivo
	F	Empregado Efetivo
	F	Empregado Efetivo
	F	Empregado Efetivo
	F	Empregado Efetivo
	F	Empregado Efetivo
	F	Empregado Efetivo
	F	Empregado Efetivo
	F	Empregado Efetivo
	F	Empregado Efetivo
	M	Empregado Efetivo
	M	Empregado Efetivo
	M	Empregado Efetivo
	F	Empregado Efetivo
	M	Empregado Efetivo
	F	Empregado Efetivo
	F	Empregado Efetivo
	M	Empregado Efetivo
	M	Empregado Efetivo
	M	Empregado Efetivo
	M	Empregado Efetivo
	M	Empregado Efetivo
	F	Cedido - RJU
	F	Cedido - RJU
	M	Empregado Efetivo
	M	Empregado Efetivo
	M	Empregado Efetivo

	F	Empregado Efetivo
	M	Empregado Efetivo
	M	Empregado Efetivo
	F	Empregado Efetivo
	F	Empregado Efetivo
	F	Empregado Efetivo
	F	Empregado Efetivo
	F	Empregado Efetivo
	F	Empregado Efetivo
	F	Empregado Efetivo
	F	Empregado Efetivo
	F	Empregado Efetivo
	F	Empregado Efetivo
	F	Empregado Efetivo
	F	Empregado Efetivo
	F	Empregado Efetivo
	F	Empregado Efetivo
	F	Empregado Efetivo
	F	Empregado Efetivo
	F	Empregado Efetivo
	F	Empregado Efetivo
	F	Empregado Efetivo
	F	Empregado Efetivo
	F	Empregado Efetivo
	F	Empregado Efetivo
	M	Empregado Efetivo
	F	Empregado Efetivo
	F	Empregado Efetivo
	F	Empregado Efetivo
	F	Empregado Efetivo
	F	Empregado Efetivo
	F	Empregado Efetivo
	F	Empregado Efetivo
	M	Empregado Efetivo
	M	Empregado Efetivo
	F	Empregado Efetivo
	M	Empregado Efetivo
	M	Empregado Efetivo
	M	Empregado Efetivo
	M	Empregado Efetivo
	F	Empregado Efetivo
	F	Empregado Efetivo
	M	Empregado Efetivo
	F	Empregado Efetivo
	F	Empregado Efetivo

	F	Empregado Efetivo
	F	Empregado Efetivo
	F	Empregado Efetivo
	F	Empregado Efetivo
	M	Empregado Efetivo
	F	Empregado Efetivo
	M	Empregado Efetivo
	F	Empregado Efetivo
	M	Empregado Efetivo
	F	Empregado Efetivo
	F	Empregado Efetivo
	M	Empregado Efetivo
	F	Empregado Efetivo
	M	Empregado Efetivo
	F	Empregado Efetivo
	F	Empregado Efetivo
	M	Empregado Efetivo
	M	Empregado Efetivo
	F	Empregado Efetivo
	M	Empregado Efetivo
	F	Empregado Efetivo
	F	Empregado Efetivo
	F	Empregado Efetivo
	F	Empregado Efetivo
	F	Empregado Efetivo
	F	Empregado Efetivo
	M	Empregado Efetivo
	F	Empregado Efetivo
	F	Empregado Efetivo
	F	Empregado Efetivo
	F	Empregado Efetivo
	M	Empregado Efetivo
	F	Empregado Efetivo
	F	Empregado Efetivo
	F	Empregado Efetivo
	F	Empregado Efetivo
	F	Empregado Efetivo
	F	Empregado Efetivo
	F	Empregado Efetivo
	F	Empregado Efetivo
	M	Empregado Efetivo
	F	Empregado Efetivo
	F	Empregado Efetivo
	F	Empregado Efetivo
	F	Empregado Efetivo
	F	Empregado Efetivo
	F	Empregado Efetivo
	F	Empregado Efetivo

	M	Empregado Efetivo
	M	Empregado Efetivo
	F	Empregado Efetivo
	F	Empregado Efetivo
	F	Empregado Efetivo
	F	Empregado Efetivo
	F	Empregado Efetivo
	F	Empregado Efetivo
	M	Empregado Efetivo
	F	Empregado Efetivo
	F	Empregado Efetivo
	F	Empregado Efetivo
	F	Empregado Efetivo
	F	Empregado Efetivo
	F	Empregado Efetivo
	M	Empregado Efetivo
	F	Empregado Efetivo
	F	Empregado Efetivo
	F	Empregado Efetivo
	F	Empregado Efetivo
	F	Empregado Efetivo
	F	Empregado Efetivo
	F	Empregado Efetivo
	F	Empregado Efetivo
	F	Empregado Efetivo
	F	Empregado Efetivo
	F	Empregado Efetivo
	M	Empregado Efetivo
	F	Empregado Efetivo
	F	Empregado Efetivo
	F	Empregado Efetivo
	F	Empregado Efetivo
	F	Empregado Efetivo
	F	Empregado Efetivo
	F	Empregado Efetivo
	F	Empregado Efetivo
	F	Empregado Efetivo
	F	Empregado Efetivo
	F	Empregado Efetivo
	F	Empregado Efetivo
	F	Empregado Efetivo
	F	Empregado Efetivo
	F	Empregado Efetivo
	M	Empregado Efetivo
	M	Empregado Efetivo
	M	Empregado Efetivo
	F	Empregado Efetivo
	F	Empregado Efetivo

	M	Empregado Efetivo
	F	Empregado Efetivo
	F	Empregado Efetivo
	F	Empregado Efetivo
	F	Empregado Efetivo
	F	Empregado Efetivo
	F	Empregado Efetivo
	F	Empregado Efetivo
	F	Empregado Efetivo
	F	Empregado Efetivo
	F	Empregado Efetivo
	F	Empregado Efetivo
	M	Empregado Efetivo
	F	Empregado Efetivo
	F	Empregado Efetivo
	F	Empregado Efetivo
	F	Empregado Efetivo
	F	Empregado Efetivo
	M	Empregado Efetivo
	F	Empregado Efetivo
	F	Empregado Efetivo
	F	Empregado Efetivo
	F	Empregado Efetivo
	F	Empregado Efetivo
	F	Empregado Efetivo
	F	Empregado Efetivo
	F	Empregado Efetivo
	F	Empregado Efetivo
	F	Empregado Efetivo
	F	Empregado Efetivo
	F	Empregado Efetivo
	F	Empregado Efetivo
	M	Empregado Efetivo
	F	Empregado Efetivo
	F	Empregado Efetivo
	F	Empregado Efetivo
	F	Empregado Efetivo
	F	Empregado Efetivo
	M	Empregado Efetivo
	F	Empregado Efetivo
	F	Empregado Efetivo
	F	Empregado Efetivo
	M	Empregado Efetivo
	F	Empregado Efetivo

	F	Empregado Efetivo
	M	Empregado Efetivo
	F	Empregado Efetivo
	M	Empregado Efetivo
	F	Empregado Efetivo
	F	Empregado Efetivo
	M	Empregado Efetivo
	F	Empregado Efetivo
	M	Empregado Efetivo
	M	Empregado Efetivo
	F	Empregado Efetivo
	M	Empregado Efetivo
	F	Empregado Efetivo
	F	Empregado Efetivo
	F	Empregado Efetivo
	F	Empregado Efetivo
	M	Empregado Efetivo
	F	Empregado Efetivo
	M	Empregado Efetivo
	F	Empregado Efetivo
	M	Empregado Efetivo
	M	Empregado Efetivo
	F	Empregado Efetivo
	M	Empregado Efetivo
	M	Empregado Efetivo
	M	Empregado Efetivo
	M	Empregado Efetivo
	M	Empregado Efetivo
	F	Empregado Efetivo
	M	Empregado Efetivo
	F	Empregado Efetivo
	F	Empregado Efetivo

	F	Empregado Efetivo
	M	Empregado Efetivo
	M	Empregado Efetivo
	F	Empregado Efetivo
	F	Empregado Efetivo
	F	Empregado Efetivo
	M	Empregado Efetivo
	F	Empregado Efetivo
	M	Empregado Efetivo
	F	Empregado Efetivo
	M	Empregado Efetivo
	M	Empregado Efetivo
	F	Empregado Efetivo
	M	Empregado Efetivo
	F	Empregado Efetivo
	M	Empregado Efetivo
	F	Empregado Efetivo
	F	Empregado Efetivo
	M	Empregado Efetivo
	M	Empregado Efetivo
	F	Empregado Efetivo
	F	Empregado Efetivo
	M	Empregado Efetivo
	F	Empregado Efetivo
	F	Empregado Efetivo
	M	Empregado Efetivo
	F	Empregado Efetivo
	F	Empregado Efetivo
	F	Empregado Efetivo
	F	Empregado Efetivo
	M	Empregado Efetivo
	F	Empregado Efetivo
	F	Empregado Efetivo
	F	Empregado Efetivo
	M	Empregado Efetivo
	M	Empregado Efetivo

	F	Cedido - RJU
	F	Empregado Efetivo
	F	Empregado Efetivo
	M	Empregado Efetivo
	F	Empregado Efetivo
	F	Empregado Efetivo
	F	Empregado Efetivo
	F	Empregado Efetivo
	F	Empregado Efetivo
	F	Empregado Efetivo
	F	Empregado Efetivo
	F	Empregado Efetivo
	F	Empregado Efetivo
	F	Empregado Efetivo
	M	Empregado Efetivo
	M	Empregado Efetivo
	M	Empregado Efetivo
	F	Empregado Efetivo
	F	Empregado Efetivo
	M	Empregado Efetivo
	F	Empregado Efetivo
	F	Empregado Efetivo
	M	Empregado Efetivo
	M	Empregado Efetivo
	M	Empregado Efetivo
	M	Empregado Efetivo
	M	Empregado Efetivo
	F	Empregado Efetivo
	M	Empregado Efetivo
	M	Empregado Efetivo
	F	Empregado Efetivo
	M	Empregado Efetivo
	F	Empregado Efetivo
	M	Empregado Efetivo
	F	Empregado Efetivo
	M	Empregado Efetivo
	M	Empregado Efetivo
	M	Empregado Efetivo
	F	Empregado Efetivo
	M	Empregado Efetivo
	M	Empregado Efetivo
	F	Empregado Efetivo
	F	Empregado Efetivo
	F	Empregado Efetivo
	F	Empregado Efetivo
	F	Empregado Efetivo
	F	Empregado Efetivo
	F	Empregado Efetivo

	F	Empregado Efetivo
	F	Empregado Efetivo
	F	Empregado Efetivo
	F	Empregado Efetivo
	F	Empregado Efetivo
	F	Empregado Efetivo
	F	Empregado Efetivo
	F	Empregado Efetivo
	F	Empregado Efetivo
	F	Empregado Efetivo
	F	Empregado Efetivo
	F	Empregado Efetivo
	M	Empregado Efetivo
	F	Empregado Efetivo
	F	Empregado Efetivo
	M	Empregado Efetivo
	F	Empregado Efetivo
	F	Empregado Efetivo
	F	Empregado Efetivo
	F	Empregado Efetivo
	F	Empregado Efetivo
	F	Empregado Efetivo
	F	Empregado Efetivo
	M	Empregado Efetivo
	F	Empregado Efetivo
	F	Empregado Efetivo
	F	Empregado Efetivo
	F	Empregado Efetivo
	M	Empregado Efetivo
	F	Empregado Efetivo
	F	Empregado Efetivo
	M	Empregado Efetivo
	F	Empregado Efetivo
	F	Empregado Efetivo
	F	Empregado Efetivo
	F	Empregado Efetivo
	F	Empregado Efetivo
	F	Empregado Efetivo
	F	Empregado Efetivo
	F	Empregado Efetivo
	M	Empregado Efetivo
	F	Empregado Efetivo
	F	Empregado Efetivo
	M	Empregado Efetivo
	F	Empregado Efetivo
	F	Empregado Efetivo
	M	Empregado Efetivo
	F	Empregado Efetivo

	F	Empregado Efetivo
	F	Empregado Efetivo
	F	Empregado Efetivo
	F	Empregado Efetivo
	F	Empregado Efetivo
	F	Empregado Efetivo
	M	Empregado Efetivo
	F	Empregado Efetivo
	F	Empregado Efetivo
	F	Empregado Efetivo
	F	Empregado Efetivo
	F	Empregado Efetivo
	F	Empregado Efetivo
	F	Empregado Efetivo
	F	Empregado Efetivo
	M	Empregado Efetivo
	F	Empregado Efetivo
	F	Empregado Efetivo
	F	Empregado Efetivo
	F	Empregado Efetivo
	F	Empregado Efetivo
	F	Empregado Efetivo
	F	Empregado Efetivo
	F	Empregado Efetivo
	F	Empregado Efetivo
	F	Empregado Efetivo
	F	Empregado Efetivo
	F	Empregado Efetivo
	F	Empregado Efetivo
	F	Empregado Efetivo
	F	Empregado Efetivo
	F	Empregado Efetivo
	F	Empregado Efetivo
	F	Empregado Efetivo
	F	Empregado Efetivo
	F	Empregado Efetivo
	F	Empregado Efetivo
	F	Empregado Efetivo
	F	Empregado Efetivo
	F	Empregado Efetivo
	F	Empregado Efetivo
	F	Empregado Efetivo
	F	Empregado Efetivo
	F	Empregado Efetivo
	F	Empregado Efetivo
	F	Empregado Efetivo
	F	Empregado Efetivo
	F	Empregado Efetivo

	F	Empregado Efetivo
	F	Empregado Efetivo
	F	Empregado Efetivo
	F	Empregado Efetivo
	F	Empregado Efetivo
	F	Empregado Efetivo
	F	Empregado Efetivo
	F	Empregado Efetivo
	F	Empregado Efetivo
	M	Empregado Efetivo
	M	Empregado Efetivo
	F	Empregado Efetivo
	M	Empregado Efetivo
	F	Empregado Efetivo
	F	Empregado Efetivo
	F	Empregado Efetivo
	F	Empregado Efetivo
	M	Empregado Efetivo
	F	Empregado Efetivo
	F	Empregado Efetivo
	F	Empregado Efetivo
	F	Empregado Efetivo
	M	Empregado Efetivo
	M	Empregado Efetivo
	F	Cedido - RJU
	F	Empregado Efetivo
	F	Empregado Efetivo
	F	Empregado Efetivo
	F	Empregado Efetivo
	F	Empregado Efetivo
	F	Empregado Efetivo
	F	Empregado Efetivo
	F	Empregado Efetivo

	F	Empregado Efetivo
	F	Empregado Efetivo
	F	Empregado Efetivo
	F	Empregado Efetivo
	F	Empregado Efetivo
	F	Empregado Efetivo
	F	Empregado Efetivo
	F	Empregado Efetivo
	F	Empregado Efetivo
	F	Empregado Efetivo
	F	Empregado Efetivo
	M	Empregado Efetivo
	F	Empregado Efetivo
	M	Empregado Efetivo
	M	Empregado Efetivo
	F	Empregado Efetivo
	F	Empregado Efetivo
	F	Empregado Efetivo
	F	Empregado Efetivo
	F	Empregado Efetivo
	M	Empregado Efetivo
	M	Empregado Efetivo
	M	Empregado Efetivo
	F	Empregado Efetivo
	M	Empregado Efetivo
	M	Empregado Efetivo
	F	Empregado Efetivo
	F	Empregado Efetivo
	F	Empregado Efetivo
	M	Empregado Efetivo
	M	Empregado Efetivo
	M	Empregado Efetivo
	F	Empregado Efetivo
	M	Empregado Efetivo
	F	Empregado Efetivo
	M	Empregado Efetivo
	F	Empregado Efetivo
	M	Empregado Efetivo
	F	Empregado Efetivo
	M	Empregado Efetivo

	F	Empregado Efetivo
	M	Empregado Efetivo
	M	Empregado Efetivo
	M	Empregado Efetivo
	F	Empregado Efetivo
	M	Empregado Efetivo
	F	Empregado Efetivo
	F	Empregado Efetivo
	M	Empregado Efetivo
	F	Empregado Efetivo
	M	Empregado Efetivo
	F	Empregado Efetivo
	M	Empregado Efetivo
	F	Empregado Efetivo
	F	Empregado Efetivo
	F	Empregado Efetivo
	F	Empregado Efetivo
	F	Empregado Efetivo
	F	Empregado Efetivo
	F	Empregado Efetivo
	F	Empregado Efetivo
	F	Empregado Efetivo
	F	Empregado Efetivo
	F	Empregado Efetivo
	M	Empregado Efetivo
	M	Empregado Efetivo
	M	Empregado Efetivo
	F	Empregado Efetivo
	M	Empregado Efetivo
	M	Empregado Efetivo
	M	Empregado Efetivo
	M	Empregado Efetivo
	M	Empregado Efetivo
	M	Empregado Efetivo
	F	Empregado Efetivo
	F	Empregado Efetivo
	M	Empregado Efetivo
	F	Empregado Efetivo
	F	Empregado Efetivo
	F	Empregado Efetivo
	M	Empregado Efetivo
	M	Empregado Efetivo

	M	Empregado Efetivo
	F	Empregado Efetivo
	F	Empregado Efetivo
	M	Empregado Efetivo
	M	Empregado Efetivo
	F	Empregado Efetivo
	F	Empregado Efetivo
	F	Empregado Efetivo
	F	Empregado Efetivo
	M	Empregado Efetivo
	M	Empregado Efetivo
	M	Empregado Efetivo
	M	Empregado Efetivo
	F	Empregado Efetivo
	F	Empregado Efetivo
	F	Empregado Efetivo
	M	Empregado Efetivo
	F	Empregado Efetivo
	F	Empregado Efetivo
	M	Empregado Efetivo
	F	Empregado Efetivo
	F	Empregado Efetivo
	F	Empregado Efetivo
	F	Empregado Efetivo
	F	Empregado Efetivo
	M	Empregado Efetivo
	F	Empregado Efetivo
	F	Empregado Efetivo
	F	Empregado Efetivo
	F	Empregado Efetivo
	F	Empregado Efetivo
	M	Empregado Efetivo
	M	Empregado Efetivo
	F	Empregado Efetivo
	M	Empregado Efetivo
	F	Empregado Efetivo
	M	Empregado Efetivo
	M	Empregado Efetivo
	F	Empregado Efetivo
	M	Empregado Efetivo
	M	Empregado Efetivo
	F	Empregado Efetivo
	F	Empregado Efetivo
	F	Empregado Efetivo
	F	Empregado Efetivo
	M	Empregado Efetivo
	M	Empregado Efetivo
	M	Empregado Efetivo

	F	Empregado Efetivo
	M	Empregado Efetivo
	F	Empregado Efetivo
	F	Empregado Efetivo
	F	Empregado Efetivo
	F	Empregado Efetivo
	F	Empregado Efetivo
	M	Empregado Efetivo
	F	Empregado Efetivo
	F	Empregado Efetivo
	F	Empregado Efetivo
	F	Empregado Efetivo
	M	Empregado Efetivo
	M	Empregado Efetivo
	F	Empregado Efetivo
	M	Empregado Efetivo
	F	Empregado Efetivo
	M	Empregado Efetivo
	F	Empregado Efetivo
	M	Empregado Efetivo
	F	Empregado Efetivo
	M	Empregado Efetivo
	M	Empregado Efetivo
	F	Empregado Efetivo
	F	Empregado Efetivo
	F	Empregado Efetivo
	F	Empregado Efetivo
	F	Empregado Efetivo
	F	Empregado Efetivo
	F	Empregado Efetivo
	F	Empregado Efetivo
	F	Empregado Efetivo
	F	Empregado Efetivo
	F	Empregado Efetivo
	F	Empregado Efetivo
	F	Empregado Efetivo
	F	Empregado Efetivo
	M	Empregado Efetivo
	F	Empregado Efetivo
	M	Empregado Efetivo
	M	Empregado Efetivo
	M	Empregado Efetivo
	F	Empregado Efetivo
	F	Empregado Efetivo
	M	Empregado Efetivo
	M	Empregado Efetivo
	M	Empregado Efetivo
	F	Empregado Efetivo

	M	Empregado Efetivo
	M	Empregado Efetivo
	F	Empregado Efetivo
	M	Empregado Efetivo
	F	Empregado Efetivo
	M	Empregado Efetivo
	F	Empregado Efetivo
	M	Empregado Efetivo
	F	Empregado Efetivo
	M	Empregado Efetivo
	M	Empregado Efetivo
	M	Empregado Efetivo
	M	Empregado Efetivo
	M	Empregado Efetivo
	M	Empregado Efetivo
	M	Empregado Efetivo
	M	Empregado Efetivo
	F	Empregado Efetivo
	F	Empregado Efetivo
	F	Empregado Efetivo
	F	Empregado Efetivo
	F	Empregado Efetivo
	M	Empregado Efetivo
	F	Empregado Efetivo
	F	Empregado Efetivo
	F	Empregado Efetivo
	F	Empregado Efetivo
	F	Empregado Efetivo
	M	Empregado Efetivo
	M	Empregado Efetivo
	F	Empregado Efetivo
	F	Empregado Efetivo
	F	Empregado Efetivo
	M	Empregado Efetivo
	M	Empregado Efetivo
	M	Empregado Efetivo
	F	Empregado Efetivo
	F	Empregado Efetivo
	F	Empregado Efetivo
	M	Empregado Efetivo
	M	Empregado Efetivo
	F	Empregado Efetivo
	F	Empregado Efetivo
	F	Empregado Efetivo

	F	Empregado Efetivo
	F	Empregado Efetivo
	M	Empregado Efetivo
	M	Empregado Efetivo
	M	Empregado Efetivo
	F	Empregado Efetivo
	M	Empregado Efetivo
	F	Empregado Efetivo
	F	Empregado Efetivo
	F	Empregado Efetivo
	M	Empregado Efetivo
	F	Empregado Efetivo
	F	Empregado Efetivo
	M	Empregado Efetivo
	M	Empregado Efetivo
	M	Empregado Efetivo
	M	Empregado Efetivo
	F	Empregado Efetivo
	M	Empregado Efetivo
	F	Empregado Efetivo
	F	Empregado Efetivo
	F	Empregado Efetivo
	F	Empregado Efetivo
	M	Empregado Efetivo
	F	Empregado Efetivo
	F	Empregado Efetivo
	M	Empregado Efetivo
	M	Empregado Efetivo
	M	Empregado Efetivo
	M	Empregado Efetivo
	M	Empregado Efetivo
	M	Empregado Efetivo
	F	Empregado Efetivo
	F	Empregado Efetivo
	F	Empregado Efetivo
	F	Empregado Efetivo
	F	Empregado Efetivo
	F	Empregado Efetivo
	F	Empregado Efetivo
	F	Empregado Efetivo
	F	Empregado Efetivo
	F	Empregado Efetivo
	F	Empregado Efetivo
	F	Empregado Efetivo
	F	Empregado Efetivo
	F	Empregado Efetivo
	M	Empregado Efetivo
	F	Empregado Efetivo

	M	Empregado Efetivo
	F	Empregado Efetivo
	F	Empregado Efetivo
	F	Empregado Efetivo
	F	Empregado Efetivo
	M	Empregado Efetivo
	M	Empregado Efetivo
	F	Empregado Efetivo
	F	Empregado Efetivo
	F	Empregado Efetivo
	M	Empregado Efetivo
	M	Empregado Efetivo
	M	Empregado Efetivo
	M	Empregado Efetivo
	F	Empregado Efetivo
	F	Empregado Efetivo
	F	Empregado Efetivo
	M	Empregado Efetivo
	F	Empregado Efetivo
	F	Empregado Efetivo
	F	Empregado Efetivo
	F	Empregado Efetivo
	F	Empregado Efetivo
	F	Empregado Efetivo
	F	Empregado Efetivo
	F	Empregado Efetivo
	F	Empregado Efetivo
	M	Empregado Efetivo
	F	Empregado Efetivo
	M	Empregado Efetivo
	M	Empregado Efetivo
	M	Empregado Efetivo
	M	Empregado Efetivo
	M	Empregado Efetivo
	M	Empregado Efetivo
	M	Empregado Efetivo
	M	Empregado Efetivo
	M	Empregado Efetivo
	M	Empregado Efetivo
	F	Empregado Efetivo
	F	Empregado Efetivo
	M	Empregado Efetivo
	M	Empregado Efetivo
	F	Empregado Efetivo
	F	Empregado Efetivo

	M	Cedido - RJU
	F	Empregado Efetivo
	F	Empregado Efetivo
	F	Empregado Efetivo
	F	Empregado Efetivo
	F	Empregado Efetivo
	F	Empregado Efetivo
	F	Empregado Efetivo
	F	Empregado Efetivo
	F	Empregado Efetivo
	F	Empregado Efetivo
	F	Empregado Efetivo
	F	Empregado Efetivo
	M	Empregado Efetivo
	M	Empregado Efetivo
	F	Empregado Efetivo
	F	Empregado Efetivo
	F	Empregado Efetivo
	F	Empregado Efetivo
	F	Empregado Efetivo
	F	Empregado Efetivo
	F	Empregado Efetivo
	F	Empregado Efetivo
	F	Empregado Efetivo
	F	Empregado Efetivo
	F	Empregado Efetivo
	F	Empregado Efetivo
	F	Empregado Efetivo
	M	Empregado Efetivo
	M	Empregado Efetivo
	M	Empregado Efetivo
	M	Empregado Efetivo
	F	Empregado Efetivo
	F	Empregado Efetivo
	F	Empregado Efetivo
	F	Empregado Efetivo
	F	Empregado Efetivo
	M	Empregado Efetivo

	F	Empregado Efetivo
	F	Empregado Efetivo
	F	Empregado Efetivo
	M	Empregado Efetivo
	M	Empregado Efetivo
	M	Empregado Efetivo
	M	Empregado Efetivo
	M	Empregado Efetivo
	M	Empregado Efetivo
	M	Empregado Efetivo
	M	Empregado Efetivo
	F	Empregado Efetivo
	M	Empregado Efetivo
	M	Empregado Efetivo
	F	Empregado Efetivo
	F	Empregado Efetivo
	M	Empregado Efetivo
	F	Empregado Efetivo
	M	Empregado Efetivo
	F	Empregado Efetivo
	F	Empregado Efetivo
	M	Empregado Efetivo
	F	Empregado Efetivo
	F	Empregado Efetivo
	M	Empregado Efetivo
	M	Empregado Efetivo
	M	Empregado Efetivo
	M	Empregado Efetivo
	M	Empregado Efetivo
	M	Empregado Efetivo
	M	Empregado Efetivo
	M	Empregado Efetivo
	M	Empregado Efetivo
	M	Empregado Efetivo
	F	Empregado Efetivo
	F	Empregado Efetivo
	F	Empregado Efetivo
	F	Empregado Efetivo
	F	Empregado Efetivo
	F	Empregado Efetivo
	F	Empregado Efetivo
	F	Empregado Efetivo
	F	Empregado Efetivo

	F	Empregado Efetivo
	F	Empregado Efetivo
	F	Empregado Efetivo
	F	Empregado Efetivo
	F	Empregado Efetivo
	F	Empregado Efetivo
	F	Empregado Efetivo
	F	Empregado Efetivo
	F	Empregado Efetivo
	M	Empregado Efetivo
	F	Empregado Efetivo
	M	Empregado Efetivo
	F	Empregado Efetivo
	F	Empregado Efetivo
	M	Empregado Efetivo
	F	Empregado Efetivo
	M	Empregado Efetivo
	M	Empregado Efetivo
	M	Empregado Efetivo
	F	Empregado Efetivo
	M	Empregado Efetivo
	F	Cedido - RJU
	F	Cedido - RJU
	F	Cedido - RJU
	M	Empregado Efetivo
	F	Cedido - RJU
	F	Empregado Efetivo
	F	Empregado Efetivo
	F	Cedido - RJU
	M	Empregado Efetivo
	M	Empregado Efetivo
	F	Empregado Efetivo
	F	Empregado Efetivo
	F	Empregado Efetivo
	M	Empregado Efetivo
	F	Empregado Efetivo
	M	Empregado Efetivo
	F	Empregado Efetivo
	F	Empregado Efetivo
	M	Empregado Efetivo
	M	Empregado Efetivo
	F	Empregado Efetivo
	F	Empregado Efetivo
	F	Empregado Efetivo
	M	Empregado Efetivo
	F	Empregado Efetivo
	M	Empregado Efetivo

	F	Empregado Efetivo
	F	Empregado Efetivo
	F	Empregado Efetivo
	F	Empregado Efetivo
	F	Empregado Efetivo
	M	Empregado Efetivo
	F	Empregado Efetivo
	F	Empregado Efetivo
	M	Empregado Efetivo
	F	Empregado Efetivo
	F	Empregado Efetivo
	F	Empregado Efetivo
	F	Empregado Efetivo
	F	Empregado Efetivo
	F	Empregado Efetivo
	F	Empregado Efetivo
	F	Empregado Efetivo
	F	Empregado Efetivo
	F	Empregado Efetivo
	M	Empregado Efetivo
	M	Empregado Efetivo
	M	Cedido - RJU
	F	Empregado Efetivo
	F	Empregado Efetivo
	F	Empregado Efetivo
	M	Empregado Efetivo
	F	Empregado Efetivo
	F	Empregado Efetivo
	F	Empregado Efetivo
	F	Empregado Efetivo
	F	Empregado Efetivo
	F	Empregado Efetivo
	F	Empregado Efetivo
	F	Empregado Efetivo
	M	Empregado Efetivo
	M	Empregado Efetivo
	M	Empregado Efetivo
	M	Empregado Efetivo
	M	Empregado Efetivo
	M	Empregado Efetivo
	M	Empregado Efetivo
	F	Empregado Efetivo
	M	Empregado Efetivo
	M	Empregado Efetivo
	F	Empregado Efetivo

	F	Empregado Efetivo
	F	Empregado Efetivo
	F	Empregado Efetivo
	F	Empregado Efetivo
	F	Empregado Efetivo
	F	Empregado Efetivo
	F	Empregado Efetivo
	F	Empregado Efetivo
	F	Empregado Efetivo
	F	Empregado Efetivo
	F	Empregado Efetivo
	F	Empregado Efetivo
	F	Empregado Efetivo
	F	Empregado Efetivo
	F	Empregado Efetivo
	F	Empregado Efetivo
	F	Empregado Efetivo
	M	Empregado Efetivo
	F	Empregado Efetivo
	F	Empregado Efetivo
	F	Empregado Efetivo
	F	Empregado Efetivo
	F	Empregado Efetivo
	F	Empregado Efetivo
	F	Empregado Efetivo
	F	Empregado Efetivo
	F	Empregado Efetivo
	F	Empregado Efetivo
	F	Empregado Efetivo
	F	Empregado Efetivo
	F	Empregado Efetivo
	F	Empregado Efetivo
	F	Empregado Efetivo
	F	Empregado Efetivo
	F	Empregado Efetivo
	F	Empregado Efetivo
	F	Empregado Efetivo
	F	Empregado Efetivo
	F	Empregado Efetivo
	F	Empregado Efetivo
	F	Empregado Efetivo

	F	Empregado Efetivo
	F	Empregado Efetivo
	F	Empregado Efetivo
	M	Cedido - RJU
	F	Empregado Efetivo
	F	Empregado Efetivo
	F	Empregado Efetivo
	F	Empregado Efetivo
	F	Empregado Efetivo
	F	Empregado Efetivo
	F	Empregado Efetivo
	M	Empregado Efetivo
	F	Empregado Efetivo
	F	Empregado Efetivo
	F	Empregado Efetivo
	F	Empregado Efetivo
	F	Empregado Efetivo
	F	Empregado Efetivo
	F	Empregado Efetivo
	F	Empregado Efetivo
	F	Empregado Efetivo
	F	Empregado Efetivo
	F	Empregado Efetivo
	F	Empregado Efetivo
	F	Empregado Efetivo
	M	Empregado Efetivo
	M	Empregado Efetivo
	F	Empregado Efetivo
	F	Empregado Efetivo
	F	Empregado Efetivo
	F	Empregado Efetivo
	F	Empregado Efetivo
	F	Empregado Efetivo
	M	Empregado Efetivo
	M	Empregado Efetivo
	M	Empregado Efetivo
	F	Empregado Efetivo
	F	Empregado Efetivo
	F	Empregado Efetivo
	F	Empregado Efetivo
	M	Empregado Efetivo
	M	Empregado Efetivo
	F	Empregado Efetivo

	M	Empregado Efetivo
	M	Empregado Efetivo
	F	Empregado Efetivo
	F	Empregado Efetivo
	F	Empregado Efetivo
	F	Empregado Efetivo
	F	Empregado Efetivo
	F	Empregado Efetivo
	M	Empregado Efetivo
	F	Empregado Efetivo
	F	Empregado Efetivo
	M	Empregado Efetivo
	M	Empregado Efetivo
	F	Empregado Efetivo
	F	Empregado Efetivo
	F	Empregado Efetivo
	F	Empregado Efetivo
	F	Empregado Efetivo
	M	Empregado Efetivo
	M	Empregado Efetivo
	F	Empregado Efetivo
	M	Empregado Efetivo
	F	Empregado Efetivo
	M	Empregado Efetivo
	F	Empregado Efetivo
	M	Empregado Efetivo
	M	Empregado Efetivo
	M	Empregado Efetivo
	F	Empregado Efetivo
	F	Empregado Efetivo
	F	Empregado Efetivo
	M	Empregado Efetivo
	M	Empregado Efetivo
	M	Empregado Efetivo
	M	Empregado Efetivo
	M	Empregado Efetivo
	F	Empregado Efetivo
	F	Empregado Efetivo
	M	Empregado Efetivo
	F	Empregado Efetivo
	M	Empregado Efetivo
	M	Empregado Efetivo
	M	Empregado Efetivo
	M	Empregado Efetivo
	M	Empregado Efetivo

	M	Empregado Efetivo
	F	Empregado Efetivo
	F	Empregado Efetivo
	F	Empregado Efetivo
	F	Empregado Efetivo
	F	Empregado Efetivo
	F	Empregado Efetivo
	F	Empregado Efetivo
	F	Empregado Efetivo
	M	Empregado Efetivo
	M	Empregado Efetivo
	M	Empregado Efetivo
	F	Empregado Efetivo
	F	Empregado Efetivo
	M	Empregado Efetivo
	M	Empregado Efetivo
	M	Empregado Efetivo
	M	Empregado Efetivo
	M	Empregado Efetivo
	M	Empregado Efetivo
	M	Empregado Efetivo
	M	Empregado Efetivo
	M	Empregado Efetivo
	M	Empregado Efetivo
	M	Empregado Efetivo
	M	Empregado Efetivo
	M	Empregado Efetivo
	M	Empregado Efetivo
	M	Empregado Efetivo
	M	Empregado Efetivo
	M	Empregado Efetivo
	F	Empregado Efetivo
	F	Empregado Efetivo
	F	Empregado Efetivo
	F	Empregado Efetivo
	F	Empregado Efetivo
	F	Empregado Efetivo
	F	Empregado Efetivo
	F	Empregado Efetivo
	F	Empregado Efetivo

	F	Empregado Efetivo
	M	Empregado Efetivo
	M	Empregado Efetivo
	F	Empregado Efetivo
	M	Empregado Efetivo
	F	Empregado Efetivo
	F	Empregado Efetivo
	M	Empregado Efetivo
	F	Empregado Efetivo
	F	Empregado Efetivo
	F	Empregado Efetivo
	F	Empregado Efetivo
	F	Empregado Efetivo
	F	Empregado Efetivo
	M	Empregado Efetivo
	F	Empregado Efetivo
	F	Empregado Efetivo
	F	Empregado Efetivo
	F	Empregado Efetivo
	F	Empregado Efetivo
	F	Empregado Efetivo
	F	Empregado Efetivo
	F	Empregado Efetivo
	F	Empregado Efetivo
	F	Empregado Efetivo
	F	Empregado Efetivo
	M	Empregado Efetivo
	M	Empregado Efetivo
	M	Empregado Efetivo
	F	Empregado Efetivo
	F	Empregado Efetivo
	F	Empregado Efetivo
	F	Empregado Efetivo
	F	Empregado Efetivo
	F	Empregado Efetivo
	F	Empregado Efetivo
	F	Empregado Efetivo
	M	Empregado Efetivo
	F	Empregado Efetivo
	F	Empregado Efetivo
	F	Empregado Efetivo

	F	Empregado Efetivo
	F	Empregado Efetivo
	F	Empregado Efetivo
	F	Empregado Efetivo
	F	Empregado Efetivo
	F	Empregado Efetivo
	F	Empregado Efetivo
	F	Empregado Efetivo
	F	Empregado Efetivo
	M	Empregado Efetivo
	M	Empregado Efetivo
	F	Empregado Efetivo
	M	Empregado Efetivo
	M	Empregado Efetivo
	M	Empregado Efetivo
	F	Empregado Efetivo
	M	Empregado Efetivo
	F	Empregado Efetivo
	F	Empregado Efetivo
	F	Empregado Efetivo
	M	Empregado Efetivo
	F	Empregado Efetivo
	F	Empregado Efetivo
	F	Empregado Efetivo
	M	Empregado Efetivo
	F	Empregado Efetivo
	F	Empregado Efetivo
	F	Empregado Efetivo
	F	Empregado Efetivo
	F	Empregado Efetivo
	M	Empregado Efetivo
	F	Empregado Efetivo
	M	Empregado Efetivo
	F	Empregado Efetivo
	F	Empregado Efetivo
	F	Empregado Efetivo
	F	Empregado Efetivo
	M	Empregado Efetivo
	F	Empregado Efetivo
	F	Empregado Efetivo
	F	Empregado Efetivo
	M	Empregado Efetivo
	F	Empregado Efetivo
	M	Empregado Efetivo
	F	Empregado Efetivo
	F	Empregado Efetivo
	F	Empregado Efetivo
	F	Empregado Efetivo

	F	Empregado Efetivo
	M	Empregado Efetivo
	M	Empregado Efetivo
	F	Empregado Efetivo
	F	Empregado Efetivo
	F	Empregado Efetivo
	F	Empregado Efetivo
	F	Empregado Efetivo
	M	Empregado Efetivo
	F	Empregado Efetivo
	M	Empregado Efetivo
	F	Empregado Efetivo
	M	Empregado Efetivo
	F	Empregado Efetivo
	F	Empregado Efetivo
	F	Empregado Efetivo
	M	Empregado Efetivo
	F	Empregado Efetivo
	F	Empregado Efetivo
	F	Empregado Efetivo
	F	Empregado Efetivo
	M	Empregado Efetivo
	M	Empregado Efetivo
	F	Empregado Efetivo
	F	Empregado Efetivo
	F	Empregado Efetivo
	F	Empregado Efetivo
	F	Empregado Efetivo
	F	Empregado Efetivo
	F	Empregado Efetivo
	F	Empregado Efetivo
	F	Empregado Efetivo
	F	Empregado Efetivo
	F	Empregado Efetivo
	F	Empregado Efetivo
	F	Empregado Efetivo
	F	Empregado Efetivo
	F	Empregado Efetivo
	F	Empregado Efetivo
	F	Empregado Efetivo

	F	Empregado Efetivo
	F	Empregado Efetivo
	F	Empregado Efetivo
	F	Empregado Efetivo
	F	Empregado Efetivo
	F	Empregado Efetivo
	F	Empregado Efetivo
	F	Empregado Efetivo
	M	Empregado Efetivo
	M	Empregado Efetivo
	M	Empregado Efetivo
	M	Empregado Efetivo
	F	Empregado Efetivo
	F	Empregado Efetivo
	M	Empregado Efetivo
	F	Empregado Efetivo
	F	Empregado Efetivo
	F	Empregado Efetivo
	M	Empregado Efetivo
	F	Empregado Efetivo
	F	Empregado Efetivo
	F	Empregado Efetivo
	F	Empregado Efetivo
	F	Empregado Efetivo
	F	Empregado Efetivo
	F	Empregado Efetivo
	F	Empregado Efetivo
	F	Empregado Efetivo
	F	Empregado Efetivo
	F	Empregado Efetivo
	F	Empregado Efetivo
	F	Empregado Efetivo
	F	Empregado Efetivo
	F	Empregado Efetivo
	F	Empregado Efetivo
	F	Empregado Efetivo
	F	Empregado Efetivo
	F	Empregado Efetivo

	F	Empregado Efetivo
	F	Empregado Efetivo
	F	Empregado Efetivo
	F	Empregado Efetivo
	F	Empregado Efetivo
	F	Empregado Efetivo
	F	Empregado Efetivo
	F	Empregado Efetivo
	M	Empregado Efetivo
	F	Empregado Efetivo
	F	Empregado Efetivo
	M	Empregado Efetivo
	F	Empregado Efetivo
	F	Empregado Efetivo
	F	Empregado Efetivo
	F	Empregado Efetivo
	F	Empregado Efetivo
	F	Empregado Efetivo
	F	Empregado Efetivo
	F	Empregado Efetivo
	F	Empregado Efetivo
	F	Empregado Efetivo
	M	Empregado Efetivo
	M	Empregado Efetivo
	M	Empregado Efetivo
	M	Empregado Efetivo
	M	Empregado Efetivo
	M	Empregado Efetivo
	M	Empregado Efetivo
	F	Empregado Efetivo
	F	Empregado Efetivo
	F	Empregado Efetivo
	M	Empregado Efetivo
	M	Empregado Efetivo
	M	Empregado Efetivo

	M	Empregado Efetivo
	M	Empregado Efetivo
	F	Empregado Efetivo
	F	Empregado Efetivo
	F	Empregado Efetivo
	F	Empregado Efetivo
	M	Empregado Efetivo
	F	Empregado Efetivo
	M	Empregado Efetivo
	M	Empregado Efetivo
	M	Empregado Efetivo
	M	Empregado Efetivo
	M	Empregado Efetivo
	F	Empregado Efetivo
	F	Empregado Efetivo
	M	Empregado Efetivo
	F	Empregado Efetivo
	F	Empregado Efetivo
	M	Empregado Efetivo
	F	Empregado Efetivo
	F	Empregado Efetivo
	F	Empregado Efetivo
	F	Empregado Efetivo
	F	Empregado Efetivo
	F	Empregado Efetivo
	F	Empregado Efetivo
	F	Empregado Efetivo
	F	Empregado Efetivo
	F	Empregado Efetivo
	F	Empregado Efetivo
	F	Empregado Efetivo
	F	Empregado Efetivo
	F	Empregado Efetivo
	F	Empregado Efetivo
	F	Empregado Efetivo
	F	Empregado Efetivo

	F	Empregado Efetivo
	F	Empregado Efetivo
	M	Empregado Efetivo
	F	Empregado Efetivo
	M	Empregado Efetivo
	M	Empregado Efetivo
	M	Empregado Efetivo
	F	Empregado Efetivo
	F	Empregado Efetivo
	M	Empregado Efetivo
	F	Empregado Efetivo
	M	Empregado Efetivo
	F	Empregado Efetivo
	F	Empregado Efetivo
	F	Empregado Efetivo
	M	Empregado Efetivo
	F	Empregado Efetivo
	F	Empregado Efetivo
	M	Empregado Efetivo
	M	Empregado Efetivo
	F	Empregado Efetivo
	F	Empregado Efetivo
	M	Empregado Efetivo
	F	Empregado Efetivo
	M	Empregado Efetivo
	M	Empregado Efetivo
	M	Empregado Efetivo
	M	Empregado Efetivo
	M	Empregado Efetivo
	M	Empregado Efetivo
	M	Empregado Efetivo
	M	Empregado Efetivo
	F	Empregado Efetivo
	M	Empregado Efetivo
	M	Empregado Efetivo
	M	Empregado Efetivo
	M	Empregado Efetivo
	M	Empregado Efetivo
	M	Empregado Efetivo
	M	Empregado Efetivo
	M	Empregado Efetivo
	M	Empregado Efetivo
	M	Empregado Efetivo
	M	Empregado Efetivo
	M	Empregado Efetivo

	M	Empregado Efetivo
	M	Empregado Efetivo
	M	Empregado Efetivo
	M	Empregado Efetivo
	M	Empregado Efetivo
	F	Empregado Efetivo
	M	Empregado Efetivo
	M	Empregado Efetivo
	F	Empregado Efetivo
	F	Empregado Efetivo
	F	Empregado Efetivo
	F	Empregado Efetivo
	F	Empregado Efetivo
	F	Empregado Efetivo
	F	Empregado Efetivo
	F	Empregado Efetivo
	F	Empregado Efetivo
	F	Empregado Efetivo
	F	Empregado Efetivo
	F	Empregado Efetivo
	F	Empregado Efetivo
	F	Empregado Efetivo
	F	Empregado Efetivo
	F	Empregado Efetivo
	F	Empregado Efetivo
	M	Empregado Efetivo
	M	Empregado Efetivo
	M	Empregado Efetivo
	M	Empregado Efetivo
	F	Empregado Efetivo
	F	Empregado Efetivo
	F	Empregado Efetivo
	F	Empregado Efetivo
	M	Empregado Efetivo
	M	Empregado Efetivo
	M	Empregado Efetivo
	F	Empregado Efetivo
	F	Empregado Efetivo
	M	Empregado Efetivo
	M	Empregado Efetivo
	M	Empregado Efetivo
	M	Empregado Efetivo
	M	Empregado Efetivo
	M	Empregado Efetivo
	F	Empregado Efetivo
	F	Empregado Efetivo
	F	Empregado Efetivo

	F	Empregado Efetivo
	F	Empregado Efetivo
	F	Empregado Efetivo
	F	Empregado Efetivo
	F	Empregado Efetivo
	M	Empregado Efetivo
	M	Empregado Efetivo
	F	Empregado Efetivo
	F	Empregado Efetivo
	M	Empregado Efetivo
	M	Empregado Efetivo
	M	Empregado Efetivo
	M	Empregado Efetivo
	F	Empregado Efetivo
	F	Empregado Efetivo
	F	Empregado Efetivo
	M	Empregado Efetivo
	M	Empregado Efetivo
	F	Empregado Efetivo
	F	Empregado Efetivo
	F	Empregado Efetivo
	F	Empregado Efetivo
	F	Empregado Efetivo
	F	Empregado Efetivo
	F	Empregado Efetivo
	F	Empregado Efetivo
	F	Empregado Efetivo
	F	Empregado Efetivo
	F	Empregado Efetivo
	F	Empregado Efetivo
	F	Empregado Efetivo
	F	Empregado Efetivo
	F	Empregado Efetivo
	F	Empregado Efetivo
	F	Empregado Efetivo
	F	Empregado Efetivo
	F	Empregado Efetivo
	F	Empregado Efetivo
	M	Empregado Efetivo
	M	Empregado Efetivo
	M	Empregado Efetivo
	M	Empregado Efetivo

	F	Empregado Efetivo
	F	Empregado Efetivo
	F	Empregado Efetivo
	F	Empregado Efetivo
	F	Empregado Efetivo
	F	Empregado Efetivo
	F	Empregado Efetivo
	F	Empregado Efetivo
	F	Empregado Efetivo
	F	Empregado Efetivo
	F	Empregado Efetivo
	F	Empregado Efetivo
	F	Empregado Efetivo
	F	Empregado Efetivo
	F	Empregado Efetivo
	F	Empregado Efetivo
	F	Empregado Efetivo
	F	Empregado Efetivo
	F	Empregado Efetivo
	F	Empregado Efetivo
	F	Empregado Efetivo
	F	Empregado Efetivo
	M	Empregado Efetivo
	F	Empregado Efetivo
	F	Empregado Efetivo
	F	Empregado Efetivo
	F	Empregado Efetivo
	F	Empregado Efetivo
	F	Empregado Efetivo
	F	Empregado Efetivo
	F	Empregado Efetivo
	F	Empregado Efetivo
	F	Empregado Efetivo
	F	Empregado Efetivo
	F	Empregado Efetivo
	F	Empregado Efetivo
	F	Empregado Efetivo
	F	Empregado Efetivo
	M	Empregado Efetivo
	F	Empregado Efetivo
	F	Empregado Efetivo
	M	Empregado Efetivo

	F	Empregado Efetivo
	F	Empregado Efetivo
	F	Empregado Efetivo
	F	Empregado Efetivo
	M	Empregado Efetivo
	M	Empregado Efetivo
	F	Empregado Efetivo
	F	Empregado Efetivo
	F	Empregado Efetivo
	F	Empregado Efetivo
	F	Empregado Efetivo
	F	Empregado Efetivo
	F	Empregado Efetivo
	F	Empregado Efetivo
	F	Empregado Efetivo
	F	Empregado Efetivo
	F	Empregado Efetivo
	F	Empregado Efetivo
	F	Empregado Efetivo
	F	Empregado Efetivo
	F	Empregado Efetivo
	F	Empregado Efetivo
	F	Empregado Efetivo
	F	Empregado Efetivo
	F	Empregado Efetivo
	F	Empregado Efetivo
	M	Empregado Efetivo
	F	Empregado Efetivo
	F	Empregado Efetivo
	F	Empregado Efetivo
	M	Empregado Efetivo
	F	Empregado Efetivo
	F	Empregado Efetivo
	M	Empregado Efetivo
	F	Empregado Efetivo
	M	Empregado Efetivo
	M	Empregado Efetivo
	F	Empregado Efetivo
	M	Empregado Efetivo
	M	Empregado Efetivo
	F	Empregado Efetivo
	F	Empregado Efetivo
	M	Empregado Efetivo
	M	Empregado Efetivo
	M	Empregado Efetivo

	F	Empregado Efetivo
	M	Empregado Efetivo
	F	Empregado Efetivo
	M	Empregado Efetivo
	F	Empregado Efetivo
	F	Empregado Efetivo
	F	Empregado Efetivo
	F	Empregado Efetivo
	F	Empregado Efetivo
	F	Empregado Efetivo
	F	Empregado Efetivo
	F	Empregado Efetivo
	F	Empregado Efetivo
	F	Empregado Efetivo
	F	Empregado Efetivo
	F	Empregado Efetivo
	F	Empregado Efetivo
	F	Empregado Efetivo
	F	Empregado Efetivo
	F	Empregado Efetivo
	F	Empregado Efetivo
	F	Empregado Efetivo
	F	Empregado Efetivo
	F	Empregado Efetivo
	F	Empregado Efetivo
	F	Empregado Efetivo
	M	Empregado Efetivo
	F	Empregado Efetivo
	F	Empregado Efetivo
	F	Empregado Efetivo
	F	Empregado Efetivo
	M	Empregado Efetivo
	F	Empregado Efetivo
	F	Empregado Efetivo
	M	Empregado Efetivo
	F	Empregado Efetivo
	M	Empregado Efetivo
	F	Empregado Efetivo
	F	Empregado Efetivo
	F	Empregado Efetivo
	F	Empregado Efetivo
	F	Empregado Efetivo
	F	Empregado Efetivo
	F	Empregado Efetivo
	F	Empregado Efetivo
	F	Empregado Efetivo

	F	Empregado Efetivo
	F	Empregado Efetivo
	F	Empregado Efetivo
	M	Empregado Efetivo
	M	Empregado Efetivo
	M	Empregado Efetivo
	F	Empregado Efetivo
	F	Empregado Efetivo
	F	Empregado Efetivo
	F	Empregado Efetivo
	F	Empregado Efetivo
	F	Empregado Efetivo
	F	Empregado Efetivo
	F	Empregado Efetivo
	F	Empregado Efetivo
	F	Empregado Efetivo
	F	Empregado Efetivo
	F	Empregado Efetivo
	F	Empregado Efetivo
	F	Empregado Efetivo
	F	Empregado Efetivo
	M	Empregado Efetivo
	F	Empregado Efetivo
	F	Empregado Efetivo
	F	Empregado Efetivo
	M	Empregado Efetivo
	F	Empregado Efetivo
	F	Empregado Efetivo
	F	Empregado Efetivo
	F	Empregado Efetivo
	F	Empregado Efetivo
	F	Empregado Efetivo

	F	Empregado Efetivo
	F	Empregado Efetivo
	F	Empregado Efetivo
	F	Empregado Efetivo
	F	Empregado Efetivo
	F	Empregado Efetivo
	F	Empregado Efetivo
	F	Empregado Efetivo
	F	Empregado Efetivo
	F	Empregado Efetivo
	F	Empregado Efetivo
	F	Empregado Efetivo
	F	Empregado Efetivo
	F	Empregado Efetivo
	F	Empregado Efetivo
	F	Empregado Efetivo
	F	Empregado Efetivo
	F	Empregado Efetivo
	F	Empregado Efetivo
	F	Empregado Efetivo
	F	Empregado Efetivo
	F	Empregado Efetivo
	F	Empregado Efetivo
	F	Empregado Efetivo
	F	Empregado Efetivo
	F	Empregado Efetivo
	F	Empregado Efetivo
	F	Empregado Efetivo
	M	Empregado Efetivo
	F	Empregado Efetivo
	F	Empregado Efetivo
	F	Empregado Efetivo
	F	Empregado Efetivo
	M	Empregado Efetivo
	F	Empregado Efetivo
	M	Empregado Efetivo
	F	Empregado Efetivo

	F	Empregado Efetivo
	M	Empregado Efetivo
	F	Empregado Efetivo
	F	Empregado Efetivo
	F	Empregado Efetivo
	F	Empregado Efetivo
	F	Empregado Efetivo
	F	Empregado Efetivo
	F	Empregado Efetivo
	F	Empregado Efetivo
	F	Empregado Efetivo
	M	Empregado Efetivo
	F	Empregado Efetivo
	F	Empregado Efetivo
	F	Empregado Efetivo
	F	Empregado Efetivo
	F	Empregado Efetivo
	F	Empregado Efetivo
	M	Empregado Efetivo
	M	Empregado Efetivo
	M	Empregado Efetivo
	M	Empregado Efetivo
	M	Empregado Efetivo
	M	Empregado Efetivo
	M	Empregado Efetivo
	F	Empregado Efetivo
	F	Empregado Efetivo
	M	Empregado Efetivo
	F	Empregado Efetivo
	F	Empregado Efetivo
	F	Empregado Efetivo
	F	Empregado Efetivo
	F	Empregado Efetivo
	F	Empregado Efetivo
	F	Empregado Efetivo
	F	Empregado Efetivo
	F	Empregado Efetivo
	F	Empregado Efetivo
	M	Empregado Efetivo
	F	Empregado Efetivo
	M	Empregado Efetivo
	M	Empregado Efetivo
	F	Empregado Efetivo
	F	Empregado Efetivo
	F	Empregado Efetivo
	F	Empregado Efetivo
	F	Empregado Efetivo
	F	Empregado Efetivo

	F	Empregado Efetivo
	F	Empregado Efetivo
	F	Empregado Efetivo
	F	Empregado Efetivo
	F	Empregado Efetivo
	F	Empregado Efetivo
	F	Empregado Efetivo
	F	Empregado Efetivo
	F	Empregado Efetivo
	F	Empregado Efetivo
	F	Empregado Efetivo
	F	Empregado Efetivo
	F	Empregado Efetivo
	F	Empregado Efetivo
	F	Empregado Efetivo
	F	Empregado Efetivo
	F	Empregado Efetivo
	F	Empregado Efetivo
	M	Empregado Efetivo
	F	Empregado Efetivo
	F	Empregado Efetivo
	F	Empregado Efetivo
	F	Empregado Efetivo
	F	Empregado Efetivo
	F	Empregado Efetivo
	F	Empregado Efetivo
	F	Empregado Efetivo
	F	Empregado Efetivo
	F	Empregado Efetivo
	F	Empregado Efetivo
	F	Empregado Efetivo
	F	Empregado Efetivo
	F	Empregado Efetivo
	F	Empregado Efetivo
	F	Empregado Efetivo
	F	Empregado Efetivo
	F	Empregado Efetivo
	F	Empregado Efetivo
	F	Empregado Efetivo
	F	Empregado Efetivo
	F	Empregado Efetivo
	F	Empregado Efetivo
	F	Empregado Efetivo
	F	Empregado Efetivo
	F	Empregado Efetivo
	F	Empregado Efetivo
	F	Empregado Efetivo
	F	Empregado Efetivo

	F	Empregado Efetivo
	F	Empregado Efetivo
	F	Empregado Efetivo
	M	Empregado Efetivo
	F	Empregado Efetivo
	F	Empregado Efetivo
	F	Empregado Efetivo
	F	Empregado Efetivo
	F	Empregado Efetivo
	F	Empregado Efetivo
	F	Empregado Efetivo
	F	Empregado Efetivo
	M	Empregado Efetivo
	F	Empregado Efetivo
	F	Empregado Efetivo
	M	Empregado Efetivo
	M	Empregado Efetivo
	F	Empregado Efetivo
	F	Empregado Efetivo
	F	Empregado Efetivo
	M	Empregado Efetivo
	F	Empregado Efetivo
	F	Empregado Efetivo
	M	Empregado Efetivo
	F	Empregado Efetivo
	F	Empregado Efetivo
	M	Empregado Efetivo
	F	Empregado Efetivo
	F	Empregado Efetivo
	F	Empregado Efetivo
	F	Empregado Efetivo
	F	Empregado Efetivo
	M	Empregado Efetivo
	F	Empregado Efetivo
	M	Empregado Efetivo
	F	Empregado Efetivo

	F	Empregado Efetivo
	F	Empregado Efetivo
	F	Empregado Efetivo
	F	Empregado Efetivo
	F	Empregado Efetivo
	F	Empregado Efetivo
	F	Empregado Efetivo
	F	Empregado Efetivo
	M	Empregado Efetivo
	F	Empregado Efetivo
	F	Empregado Efetivo
	F	Empregado Efetivo
	F	Empregado Efetivo
	F	Empregado Efetivo
	F	Empregado Efetivo
	F	Empregado Efetivo
	M	Empregado Efetivo
	M	Empregado Efetivo
	F	Empregado Efetivo
	F	Empregado Efetivo
	F	Empregado Efetivo
	F	Empregado Efetivo
	F	Empregado Efetivo
	F	Empregado Efetivo
	F	Empregado Efetivo
	F	Empregado Efetivo
	F	Empregado Efetivo
	F	Empregado Efetivo
	F	Empregado Efetivo
	M	Empregado Efetivo
	F	Empregado Efetivo
	F	Empregado Efetivo
	F	Empregado Efetivo

	F	Empregado Efetivo
	F	Empregado Efetivo
	F	Empregado Efetivo
	M	Empregado Efetivo
	M	Empregado Efetivo
	F	Empregado Efetivo
	M	Empregado Efetivo
	M	Empregado Efetivo
	M	Empregado Efetivo
	M	Empregado Efetivo
	F	Empregado Efetivo
	M	Comissionado
	M	Empregado Efetivo
	M	Empregado Efetivo
	M	Empregado Efetivo
	M	Empregado Efetivo
	F	Empregado Efetivo
	M	Empregado Efetivo
	M	Empregado Efetivo
	F	Empregado Efetivo
	F	Empregado Efetivo
	F	Empregado Efetivo
	F	Empregado Efetivo
	F	Empregado Efetivo
	F	Empregado Efetivo
	F	Empregado Efetivo
	F	Empregado Efetivo
	F	Empregado Efetivo
	M	Empregado Efetivo
	M	Empregado Efetivo
	F	Empregado Efetivo
	F	Empregado Efetivo
	F	Empregado Efetivo
	F	Empregado Efetivo
	F	Empregado Efetivo
	F	Empregado Efetivo

	F	Empregado Efetivo
	M	Empregado Efetivo
	M	Empregado Efetivo
	M	Empregado Efetivo
	M	Empregado Efetivo
	M	Empregado Efetivo
	F	Empregado Efetivo
	F	Empregado Efetivo
	F	Empregado Efetivo
	F	Empregado Efetivo
	F	Empregado Efetivo
	M	Empregado Efetivo
	F	Empregado Efetivo
	F	Empregado Efetivo
	F	Empregado Efetivo
	F	Empregado Efetivo
	F	Empregado Efetivo
	F	Empregado Efetivo
	F	Empregado Efetivo
	F	Empregado Efetivo
	F	Empregado Efetivo
	F	Empregado Efetivo
	F	Empregado Efetivo
	M	Empregado Efetivo
	F	Empregado Efetivo
	M	Empregado Efetivo
	F	Empregado Efetivo
	F	Empregado Efetivo
	F	Empregado Efetivo
	F	Empregado Efetivo
	F	Empregado Efetivo
	F	Empregado Efetivo
	F	Empregado Efetivo
	F	Empregado Efetivo
	F	Empregado Efetivo
	F	Empregado Efetivo
	F	Empregado Efetivo
	F	Empregado Efetivo
	F	Empregado Efetivo
	F	Empregado Efetivo

	F	Empregado Efetivo
	F	Empregado Efetivo
	F	Empregado Efetivo
	F	Empregado Efetivo
	F	Empregado Efetivo
	F	Empregado Efetivo
	F	Empregado Efetivo
	F	Empregado Efetivo
	F	Empregado Efetivo
	F	Empregado Efetivo
	F	Empregado Efetivo
	F	Empregado Efetivo
	F	Empregado Efetivo
	F	Empregado Efetivo
	F	Empregado Efetivo
	F	Empregado Efetivo
	F	Empregado Efetivo
	M	Empregado Efetivo
	M	Empregado Efetivo
	M	Empregado Efetivo
	M	Empregado Efetivo
	F	Empregado Efetivo
	F	Empregado Efetivo
	F	Empregado Efetivo
	F	Empregado Efetivo
	F	Empregado Efetivo
	F	Empregado Efetivo
	F	Empregado Efetivo
	M	Empregado Efetivo
	M	Empregado Efetivo
	F	Empregado Efetivo
	F	Empregado Efetivo
	F	Empregado Efetivo
	F	Empregado Efetivo
	M	Empregado Efetivo
	F	Empregado Efetivo
	F	Empregado Efetivo
	M	Empregado Efetivo
	M	Empregado Efetivo
	F	Empregado Efetivo
	F	Empregado Efetivo
	F	Empregado Efetivo
	F	Empregado Efetivo
	F	Empregado Efetivo
	F	Empregado Efetivo
	F	Empregado Efetivo

	F	Empregado Efetivo
	M	Empregado Efetivo
	M	Empregado Efetivo
	M	Empregado Efetivo
	F	Empregado Efetivo
	F	Empregado Efetivo
	F	Empregado Efetivo
	F	Empregado Efetivo
	M	Empregado Efetivo
	F	Empregado Efetivo
	M	Empregado Efetivo
	F	Empregado Efetivo
	M	Empregado Efetivo
	M	Empregado Efetivo
	F	Empregado Efetivo
	F	Empregado Efetivo
	F	Empregado Efetivo
	M	Empregado Efetivo
	F	Empregado Efetivo
	F	Empregado Efetivo
	F	Empregado Efetivo
	F	Empregado Efetivo
	M	Empregado Efetivo
	M	Empregado Efetivo
	M	Empregado Efetivo
	M	Empregado Efetivo
	M	Empregado Efetivo
	F	Empregado Efetivo
	F	Empregado Efetivo
	F	Empregado Efetivo
	M	Empregado Efetivo
	F	Empregado Efetivo
	F	Empregado Efetivo
	F	Empregado Efetivo
	F	Empregado Efetivo
	F	Empregado Efetivo
	M	Empregado Efetivo
	F	Empregado Efetivo
	M	Empregado Efetivo
	M	Empregado Efetivo
	M	Empregado Efetivo

	M	Empregado Efetivo
	F	Empregado Efetivo
	F	Empregado Efetivo
	M	Empregado Efetivo
	F	Empregado Efetivo
	F	Empregado Efetivo
	M	Empregado Efetivo
	F	Empregado Efetivo
	M	Empregado Efetivo
	F	Empregado Efetivo
	M	Empregado Efetivo
	M	Empregado Efetivo
	F	Empregado Efetivo
	F	Empregado Efetivo
	F	Empregado Efetivo
	F	Empregado Efetivo
	F	Empregado Efetivo
	F	Empregado Efetivo
	F	Empregado Efetivo
	F	Empregado Efetivo
	F	Empregado Efetivo
	F	Empregado Efetivo
	F	Empregado Efetivo
	F	Empregado Efetivo
	F	Empregado Efetivo
	F	Empregado Efetivo
	F	Empregado Efetivo
	F	Empregado Efetivo
	F	Empregado Efetivo
	F	Empregado Efetivo
	F	Empregado Efetivo
	F	Empregado Efetivo

	F	Empregado Efetivo
	F	Empregado Efetivo
	F	Empregado Efetivo
	F	Empregado Efetivo
	F	Empregado Efetivo
	F	Empregado Efetivo
	F	Empregado Efetivo
	F	Empregado Efetivo
	F	Empregado Efetivo
	M	Empregado Efetivo
	M	Empregado Efetivo
	F	Empregado Efetivo
	F	Empregado Efetivo
	F	Empregado Efetivo
	F	Empregado Efetivo
	F	Empregado Efetivo
	F	Empregado Efetivo
	M	Empregado Efetivo
	M	Empregado Efetivo
	F	Empregado Efetivo
	M	Empregado Efetivo
	F	Empregado Efetivo
	F	Empregado Efetivo
	F	Empregado Efetivo
	F	Empregado Efetivo
	F	Empregado Efetivo
	M	Empregado Efetivo
	F	Empregado Efetivo
	F	Empregado Efetivo
	F	Empregado Efetivo
	F	Empregado Efetivo
	F	Empregado Efetivo
	F	Empregado Efetivo
	F	Empregado Efetivo
	F	Empregado Efetivo
	F	Empregado Efetivo
	F	Empregado Efetivo
	F	Empregado Efetivo
	F	Empregado Efetivo
	F	Empregado Efetivo
	M	Empregado Efetivo
	F	Empregado Efetivo
	M	Empregado Efetivo

	M	Empregado Efetivo
	M	Empregado Efetivo
	M	Empregado Efetivo
	F	Empregado Efetivo
	F	Empregado Efetivo
	F	Empregado Efetivo
	F	Empregado Efetivo
	F	Empregado Efetivo
	M	Empregado Efetivo
	F	Empregado Efetivo
	F	Empregado Efetivo
	F	Empregado Efetivo
	F	Empregado Efetivo
	F	Empregado Efetivo
	M	Empregado Efetivo
	F	Empregado Efetivo
	F	Empregado Efetivo
	M	Empregado Efetivo
	F	Empregado Efetivo
	F	Empregado Efetivo
	F	Empregado Efetivo
	M	Empregado Efetivo
	F	Empregado Efetivo
	F	Empregado Efetivo
	F	Empregado Efetivo
	F	Empregado Efetivo
	M	Empregado Efetivo
	M	Empregado Efetivo
	M	Empregado Efetivo
	F	Empregado Efetivo
	F	Empregado Efetivo
	F	Empregado Efetivo
	F	Empregado Efetivo
	F	Empregado Efetivo
	F	Empregado Efetivo
	F	Empregado Efetivo

	F	Empregado Efetivo
	F	Empregado Efetivo
	F	Empregado Efetivo
	M	Empregado Efetivo
	M	Empregado Efetivo
	F	Empregado Efetivo
	M	Empregado Efetivo
	F	Empregado Efetivo
	F	Empregado Efetivo
	F	Empregado Efetivo
	M	Comissionado
	M	Empregado Efetivo
	M	Empregado Efetivo
	M	Empregado Efetivo
	M	Empregado Efetivo
	M	Empregado Efetivo
	M	Empregado Efetivo
	M	Empregado Efetivo
	M	Empregado Efetivo
	M	Empregado Efetivo
	F	Empregado Efetivo
	F	Empregado Efetivo
	F	Empregado Efetivo
	M	Empregado Efetivo
	F	Empregado Efetivo
	F	Empregado Efetivo
	F	Empregado Efetivo
	F	Empregado Efetivo
	F	Empregado Efetivo
	F	Empregado Efetivo
	F	Empregado Efetivo
	F	Empregado Efetivo
	F	Cedido - RJU
	F	Empregado Efetivo
	F	Empregado Efetivo
	F	Empregado Efetivo
	F	Empregado Efetivo
	F	Empregado Efetivo
	F	Empregado Efetivo
	F	Empregado Efetivo

	F	Empregado Efetivo
	F	Empregado Efetivo
	F	Empregado Efetivo
	F	Empregado Efetivo
	F	Empregado Efetivo
	F	Empregado Efetivo
	F	Empregado Efetivo
	F	Empregado Efetivo
	F	Empregado Efetivo
	F	Empregado Efetivo
	F	Empregado Efetivo
	F	Empregado Efetivo
	F	Empregado Efetivo
	F	Empregado Efetivo
	F	Empregado Efetivo
	F	Empregado Efetivo
	F	Empregado Efetivo
	F	Empregado Efetivo
	F	Empregado Efetivo
	F	Empregado Efetivo
	F	Empregado Efetivo
	F	Empregado Efetivo
	F	Empregado Efetivo
	F	Empregado Efetivo
	F	Empregado Efetivo
	F	Empregado Efetivo
	F	Empregado Efetivo
	F	Empregado Efetivo
	F	Empregado Efetivo
	F	Empregado Efetivo
	F	Empregado Efetivo
	F	Empregado Efetivo
	F	Empregado Efetivo
	F	Empregado Efetivo
	F	Empregado Efetivo
	F	Empregado Efetivo
	F	Empregado Efetivo
	F	Empregado Efetivo
	F	Empregado Efetivo
	F	Empregado Efetivo
	F	Empregado Efetivo
	F	Empregado Efetivo
	F	Empregado Efetivo
	F	Empregado Efetivo
	F	Empregado Efetivo
	F	Empregado Efetivo
	F	Empregado Efetivo

	F	Empregado Efetivo
	F	Empregado Efetivo
	F	Empregado Efetivo
	F	Empregado Efetivo
	F	Empregado Efetivo
	F	Empregado Efetivo
	F	Empregado Efetivo
	F	Empregado Efetivo
	F	Empregado Efetivo
	F	Empregado Efetivo
	F	Empregado Efetivo
	F	Empregado Efetivo
	F	Empregado Efetivo
	F	Empregado Efetivo
	F	Empregado Efetivo
	F	Empregado Efetivo
	F	Empregado Efetivo
	F	Empregado Efetivo
	M	Empregado Efetivo
	F	Empregado Efetivo
	F	Empregado Efetivo
	F	Empregado Efetivo
	F	Empregado Efetivo
	F	Empregado Efetivo
	F	Empregado Efetivo
	F	Empregado Efetivo
	F	Empregado Efetivo
	M	Empregado Efetivo
	F	Empregado Efetivo
	F	Empregado Efetivo
	F	Empregado Efetivo
	F	Empregado Efetivo
	F	Empregado Efetivo
	M	Empregado Efetivo
	F	Empregado Efetivo
	M	Empregado Efetivo

	F	Empregado Efetivo
	M	Empregado Efetivo
	F	Empregado Efetivo
	F	Empregado Efetivo
	F	Empregado Efetivo
	F	Empregado Efetivo
	F	Empregado Efetivo
	F	Empregado Efetivo
	F	Empregado Efetivo
	F	Empregado Efetivo
	F	Empregado Efetivo
	F	Empregado Efetivo
	F	Empregado Efetivo
	F	Empregado Efetivo
	F	Empregado Efetivo
	F	Empregado Efetivo
	F	Empregado Efetivo
	F	Empregado Efetivo
	F	Empregado Efetivo
	F	Empregado Efetivo
	F	Empregado Efetivo
	F	Empregado Efetivo
	F	Empregado Efetivo
	F	Empregado Efetivo
	F	Empregado Efetivo
	F	Empregado Efetivo
	F	Empregado Efetivo
	F	Empregado Efetivo
	F	Empregado Efetivo
	F	Empregado Efetivo
	F	Empregado Efetivo
	F	Empregado Efetivo
	F	Empregado Efetivo
	F	Empregado Efetivo
	F	Empregado Efetivo
	F	Empregado Efetivo
	F	Empregado Efetivo
	F	Empregado Efetivo
	F	Empregado Efetivo

	F	Empregado Efetivo
	F	Empregado Efetivo
	F	Empregado Efetivo
	F	Empregado Efetivo
	F	Empregado Efetivo
	F	Empregado Efetivo
	F	Empregado Efetivo
	F	Empregado Efetivo
	F	Empregado Efetivo
	F	Empregado Efetivo
	F	Empregado Efetivo
	F	Empregado Efetivo
	F	Empregado Efetivo
	F	Empregado Efetivo
	F	Empregado Efetivo
	F	Empregado Efetivo
	F	Empregado Efetivo
	F	Empregado Efetivo
	M	Empregado Efetivo
	F	Empregado Efetivo
	M	Empregado Efetivo
	F	Empregado Efetivo
	F	Empregado Efetivo
	F	Empregado Efetivo
	F	Empregado Efetivo
	M	Empregado Efetivo
	F	Empregado Efetivo
	F	Empregado Efetivo
	F	Empregado Efetivo
	M	Empregado Efetivo
	F	Empregado Efetivo
	F	Empregado Efetivo
	F	Empregado Efetivo
	F	Empregado Efetivo
	F	Empregado Efetivo
	F	Empregado Efetivo
	F	Empregado Efetivo
	F	Empregado Efetivo
	M	Empregado Efetivo
	F	Empregado Efetivo
	F	Empregado Efetivo
	F	Empregado Efetivo

	F	Empregado Efetivo
	F	Empregado Efetivo
	F	Empregado Efetivo
	F	Empregado Efetivo
	F	Empregado Efetivo
	F	Empregado Efetivo
	F	Empregado Efetivo
	F	Empregado Efetivo
	F	Empregado Efetivo
	F	Empregado Efetivo
	M	Empregado Efetivo
	F	Empregado Efetivo
	F	Empregado Efetivo
	F	Empregado Efetivo
	F	Empregado Efetivo
	F	Empregado Efetivo
	F	Empregado Efetivo
	F	Empregado Efetivo
	F	Empregado Efetivo
	F	Empregado Efetivo
	F	Empregado Efetivo
	F	Empregado Efetivo
	F	Empregado Efetivo
	F	Empregado Efetivo
	F	Empregado Efetivo
	F	Empregado Efetivo
	F	Empregado Efetivo
	F	Empregado Efetivo
	F	Empregado Efetivo
	F	Empregado Efetivo
	M	Empregado Efetivo
	M	Empregado Efetivo

	M	Empregado Efetivo
	F	Empregado Efetivo
	M	Empregado Efetivo
	F	Empregado Efetivo
	M	Empregado Efetivo
	F	Empregado Efetivo
	F	Empregado Efetivo
	F	Empregado Efetivo
	F	Empregado Efetivo
	F	Empregado Efetivo
	F	Empregado Efetivo
	F	Empregado Efetivo
	M	Empregado Efetivo
	M	Empregado Efetivo
	F	Empregado Efetivo
	F	Empregado Efetivo
	F	Empregado Efetivo
	F	Empregado Efetivo
	F	Empregado Efetivo
	F	Empregado Efetivo
	F	Empregado Efetivo
	F	Empregado Efetivo
	F	Empregado Efetivo
	F	Empregado Efetivo
	M	Empregado Efetivo
	F	Empregado Efetivo
	F	Empregado Efetivo
	M	Empregado Efetivo
	F	Empregado Efetivo
	F	Empregado Efetivo
	F	Empregado Efetivo
	F	Empregado Efetivo

	F	Empregado Efetivo
	F	Empregado Efetivo
	F	Empregado Efetivo
	M	Empregado Efetivo
	F	Empregado Efetivo
	F	Empregado Efetivo
	F	Empregado Efetivo
	F	Empregado Efetivo
	F	Empregado Efetivo
	F	Empregado Efetivo
	F	Empregado Efetivo
	F	Empregado Efetivo
	F	Empregado Efetivo
	F	Empregado Efetivo
	F	Empregado Efetivo
	F	Empregado Efetivo
	F	Empregado Efetivo
	M	Empregado Efetivo
	M	Empregado Efetivo
	F	Empregado Efetivo
	F	Empregado Efetivo
	M	Empregado Efetivo
	M	Empregado Efetivo
	F	Empregado Efetivo
	F	Empregado Efetivo
	M	Empregado Efetivo
	F	Empregado Efetivo
	F	Empregado Efetivo
	F	Empregado Efetivo
	F	Empregado Efetivo
	F	Empregado Efetivo
	F	Empregado Efetivo

	M	Empregado Efetivo
	F	Empregado Efetivo
	F	Empregado Efetivo
	F	Empregado Efetivo
	F	Empregado Efetivo
	F	Empregado Efetivo
	F	Empregado Efetivo
	F	Empregado Efetivo
	F	Empregado Efetivo
	F	Empregado Efetivo
	F	Empregado Efetivo
	M	Empregado Efetivo
	F	Empregado Efetivo
	F	Empregado Efetivo
	F	Empregado Efetivo
	F	Empregado Efetivo
	F	Empregado Efetivo
	F	Empregado Efetivo
	F	Empregado Efetivo
	F	Empregado Efetivo
	F	Empregado Efetivo
	F	Empregado Efetivo
	F	Empregado Efetivo
	M	Empregado Efetivo
	F	Empregado Efetivo
	F	Empregado Efetivo
	F	Empregado Efetivo
	F	Empregado Efetivo
	F	Empregado Efetivo
	F	Empregado Efetivo
	F	Empregado Efetivo
	F	Empregado Efetivo
	F	Empregado Efetivo
	F	Empregado Efetivo
	F	Empregado Efetivo
	M	Empregado Efetivo
	F	Empregado Efetivo
	F	Empregado Efetivo
	M	Empregado Efetivo
	F	Empregado Efetivo
	F	Empregado Efetivo
	M	Empregado Efetivo

	M	Empregado Efetivo
	M	Empregado Efetivo
	M	Empregado Efetivo
	F	Empregado Efetivo
	F	Empregado Efetivo
	F	Empregado Efetivo
	M	Empregado Efetivo
	F	Empregado Efetivo
	F	Empregado Efetivo
	M	Empregado Efetivo
	F	Empregado Efetivo
	F	Empregado Efetivo
	F	Empregado Efetivo
	F	Empregado Efetivo
	F	Empregado Efetivo
	F	Empregado Efetivo
	M	Empregado Efetivo
	F	Empregado Efetivo
	F	Empregado Efetivo
	F	Empregado Efetivo
	F	Empregado Efetivo
	F	Empregado Efetivo
	F	Empregado Efetivo
	F	Empregado Efetivo
	F	Empregado Efetivo
	F	Empregado Efetivo
	F	Empregado Efetivo
	M	Empregado Efetivo
	F	Empregado Efetivo
	F	Empregado Efetivo
	M	Empregado Efetivo
	F	Empregado Efetivo
	F	Empregado Efetivo
	F	Empregado Efetivo
	F	Empregado Efetivo
	F	Empregado Efetivo
	M	Empregado Efetivo
	F	Empregado Efetivo
	F	Empregado Efetivo
	F	Empregado Efetivo
	F	Empregado Efetivo
	M	Empregado Efetivo
	F	Empregado Efetivo
	F	Empregado Efetivo
	M	Empregado Efetivo
	M	Empregado Efetivo
	F	Empregado Efetivo

	F	Empregado Efetivo
	F	Empregado Efetivo
	M	Empregado Efetivo
	M	Empregado Efetivo
	M	Empregado Efetivo
	M	Empregado Efetivo
	F	Empregado Efetivo
	M	Empregado Efetivo
	F	Empregado Efetivo
	M	Empregado Efetivo
	F	Empregado Efetivo
	M	Empregado Efetivo
	M	Empregado Efetivo
	M	Empregado Efetivo
	M	Empregado Efetivo
	M	Empregado Efetivo
	M	Empregado Efetivo
	M	Empregado Efetivo
	M	Empregado Efetivo
	M	Empregado Efetivo
	F	Empregado Efetivo
	M	Empregado Efetivo
	F	Empregado Efetivo
	F	Empregado Efetivo
	M	Empregado Efetivo
	F	Empregado Efetivo
	M	Empregado Efetivo
	M	Empregado Efetivo
	M	Empregado Efetivo
	F	Empregado Efetivo
	F	Empregado Efetivo
	F	Empregado Efetivo
	F	Empregado Efetivo
	F	Empregado Efetivo
	F	Empregado Efetivo
	F	Empregado Efetivo
	F	Empregado Efetivo
	F	Empregado Efetivo
	F	Empregado Efetivo
	F	Empregado Efetivo
	F	Empregado Efetivo
	F	Empregado Efetivo
	F	Empregado Efetivo
	F	Empregado Efetivo
	F	Empregado Efetivo
	F	Empregado Efetivo

	F	Empregado Efetivo
	M	Empregado Efetivo
	F	Empregado Efetivo
	F	Empregado Efetivo
	F	Empregado Efetivo
	F	Empregado Efetivo
	F	Empregado Efetivo
	M	Empregado Efetivo
	F	Empregado Efetivo
	F	Empregado Efetivo
	F	Empregado Efetivo
	F	Empregado Efetivo
	F	Empregado Efetivo
	F	Empregado Efetivo
	F	Empregado Efetivo
	F	Empregado Efetivo
	F	Empregado Efetivo
	F	Empregado Efetivo
	F	Empregado Efetivo
	F	Empregado Efetivo
	M	Empregado Efetivo
	M	Empregado Efetivo
	M	Empregado Efetivo
	M	Empregado Efetivo
	M	Empregado Efetivo
	F	Empregado Efetivo
	F	Empregado Efetivo
	F	Empregado Efetivo
	F	Empregado Efetivo
	F	Empregado Efetivo
	F	Empregado Efetivo
	F	Empregado Efetivo
	F	Empregado Efetivo
	F	Empregado Efetivo
	F	Empregado Efetivo
	F	Empregado Efetivo
	F	Empregado Efetivo
	F	Empregado Efetivo
	F	Empregado Efetivo
	F	Empregado Efetivo
	F	Empregado Efetivo
	F	Empregado Efetivo
	F	Empregado Efetivo
	F	Empregado Efetivo
	F	Empregado Efetivo
	F	Empregado Efetivo
	F	Empregado Efetivo
	F	Empregado Efetivo

	F	Empregado Efetivo
	F	Empregado Efetivo
	F	Empregado Efetivo
	F	Empregado Efetivo
	F	Empregado Efetivo
	F	Empregado Efetivo
	F	Empregado Efetivo
	F	Empregado Efetivo
	F	Empregado Efetivo
	F	Empregado Efetivo
	F	Empregado Efetivo
	F	Empregado Efetivo
	F	Empregado Efetivo
	F	Empregado Efetivo
	F	Empregado Efetivo
	F	Empregado Efetivo
	F	Empregado Efetivo
	F	Empregado Efetivo
	F	Empregado Efetivo
	F	Empregado Efetivo
	F	Empregado Efetivo
	F	Empregado Efetivo
	F	Empregado Efetivo
	F	Empregado Efetivo
	F	Empregado Efetivo
	F	Empregado Efetivo
	F	Empregado Efetivo
	F	Empregado Efetivo
	F	Empregado Efetivo
	F	Empregado Efetivo
	F	Empregado Efetivo
	F	Empregado Efetivo
	F	Empregado Efetivo
	F	Empregado Efetivo
	F	Empregado Efetivo
	F	Empregado Efetivo
	F	Empregado Efetivo
	F	Empregado Efetivo

	F	Empregado Efetivo
	F	Empregado Efetivo
	F	Empregado Efetivo
	F	Empregado Efetivo
	F	Empregado Efetivo
	F	Empregado Efetivo
	F	Empregado Efetivo
	F	Empregado Efetivo
	F	Empregado Efetivo
	F	Empregado Efetivo
	F	Empregado Efetivo
	F	Empregado Efetivo
	F	Empregado Efetivo
	F	Empregado Efetivo
	F	Empregado Efetivo
	F	Empregado Efetivo
	F	Empregado Efetivo
	F	Empregado Efetivo
	F	Empregado Efetivo
	F	Empregado Efetivo
	F	Empregado Efetivo
	M	Empregado Efetivo
	F	Empregado Efetivo
	F	Empregado Efetivo
	F	Empregado Efetivo
	F	Empregado Efetivo
	M	Empregado Efetivo
	F	Empregado Efetivo
	F	Empregado Efetivo
	F	Empregado Efetivo
	M	Empregado Efetivo
	F	Empregado Efetivo
	F	Empregado Efetivo
	F	Empregado Efetivo
	F	Empregado Efetivo
	F	Empregado Efetivo
	F	Empregado Efetivo
	F	Empregado Efetivo
	F	Empregado Efetivo
	F	Empregado Efetivo
	F	Empregado Efetivo
	M	Empregado Efetivo
	F	Empregado Efetivo
	F	Empregado Efetivo
	F	Empregado Efetivo
	F	Empregado Efetivo
	M	Empregado Efetivo
	M	Empregado Efetivo

	F	Empregado Efetivo
	F	Empregado Efetivo
	M	Empregado Efetivo
	M	Empregado Efetivo
	M	Empregado Efetivo
	F	Empregado Efetivo
	F	Empregado Efetivo
	F	Empregado Efetivo
	M	Empregado Efetivo
	M	Empregado Efetivo
	F	Empregado Efetivo
	F	Empregado Efetivo
	F	Empregado Efetivo
	M	Empregado Efetivo
	F	Empregado Efetivo
	F	Empregado Efetivo
	F	Empregado Efetivo
	F	Empregado Efetivo
	F	Empregado Efetivo
	F	Empregado Efetivo
	F	Empregado Efetivo
	F	Empregado Efetivo
	F	Empregado Efetivo
	M	Empregado Efetivo
	M	Empregado Efetivo
	M	Empregado Efetivo
	F	Empregado Efetivo
	F	Empregado Efetivo
	F	Empregado Efetivo
	F	Empregado Efetivo
	F	Empregado Efetivo
	M	Empregado Efetivo
	M	Empregado Efetivo
	F	Empregado Efetivo
	F	Empregado Efetivo
	F	Empregado Efetivo
	F	Empregado Efetivo
	F	Empregado Efetivo
	F	Empregado Efetivo
	F	Empregado Efetivo
	M	Empregado Efetivo
	F	Empregado Efetivo
	F	Empregado Efetivo
	F	Empregado Efetivo
	F	Empregado Efetivo
	F	Empregado Efetivo
	F	Empregado Efetivo
	M	Empregado Efetivo

	M	Empregado Efetivo
	F	Empregado Efetivo
	F	Empregado Efetivo
	F	Empregado Efetivo
	M	Empregado Efetivo
	M	Empregado Efetivo
	F	Empregado Efetivo
	F	Empregado Efetivo
	M	Empregado Efetivo
	F	Empregado Efetivo
	F	Empregado Efetivo
	F	Empregado Efetivo
	M	Empregado Efetivo
	F	Empregado Efetivo
	M	Empregado Efetivo
	F	Empregado Efetivo
	F	Empregado Efetivo
	F	Empregado Efetivo
	F	Empregado Efetivo
	F	Empregado Efetivo
	F	Empregado Efetivo
	F	Empregado Efetivo
	F	Empregado Efetivo
	F	Empregado Efetivo
	F	Empregado Efetivo
	F	Empregado Efetivo
	F	Empregado Efetivo
	M	Empregado Efetivo
	F	Empregado Efetivo
	F	Empregado Efetivo
	F	Empregado Efetivo
	F	Empregado Efetivo
	F	Empregado Efetivo
	F	Empregado Efetivo
	F	Empregado Efetivo
	M	Empregado Efetivo
	F	Empregado Efetivo
	F	Empregado Efetivo
	M	Empregado Efetivo
	F	Empregado Efetivo
	F	Empregado Efetivo
	F	Empregado Efetivo
	F	Empregado Efetivo
	F	Empregado Efetivo
	F	Empregado Efetivo
	F	Empregado Efetivo
	F	Empregado Efetivo
	F	Empregado Efetivo

	F	Empregado Efetivo
	F	Empregado Efetivo
	F	Empregado Efetivo
	M	Empregado Efetivo
	M	Empregado Efetivo
	M	Empregado Efetivo
	M	Empregado Efetivo
	M	Empregado Efetivo
	M	Empregado Efetivo
	M	Empregado Efetivo
	M	Empregado Efetivo
	M	Empregado Efetivo
	M	Empregado Efetivo
	M	Empregado Efetivo
	F	Empregado Efetivo
	M	Empregado Efetivo
	M	Empregado Efetivo
	M	Empregado Efetivo
	F	Empregado Efetivo
	F	Empregado Efetivo
	F	Empregado Efetivo
	F	Empregado Efetivo
	F	Empregado Efetivo
	F	Empregado Efetivo
	F	Empregado Efetivo
	F	Empregado Efetivo
	F	Empregado Efetivo
	F	Empregado Efetivo
	F	Empregado Efetivo
	F	Empregado Efetivo
	F	Empregado Efetivo
	F	Empregado Efetivo
	F	Empregado Efetivo
	F	Empregado Efetivo
	F	Empregado Efetivo
	F	Empregado Efetivo
	F	Empregado Efetivo
	F	Empregado Efetivo
	F	Empregado Efetivo
	F	Empregado Efetivo
	M	Empregado Efetivo
	M	Empregado Efetivo
	F	Empregado Efetivo
	F	Empregado Efetivo
	M	Empregado Efetivo
	M	Empregado Efetivo
	F	Empregado Efetivo

	F	Empregado Efetivo
	F	Empregado Efetivo
	F	Empregado Efetivo
	F	Empregado Efetivo
	F	Empregado Efetivo
	F	Empregado Efetivo
	F	Empregado Efetivo
	F	Empregado Efetivo
	F	Empregado Efetivo
	F	Empregado Efetivo
	F	Empregado Efetivo
	F	Empregado Efetivo
	F	Empregado Efetivo
	F	Empregado Efetivo
	F	Empregado Efetivo
	F	Empregado Efetivo
	F	Empregado Efetivo
	F	Empregado Efetivo
	F	Empregado Efetivo
	F	Empregado Efetivo
	F	Empregado Efetivo
	F	Empregado Efetivo
	F	Empregado Efetivo
	F	Empregado Efetivo
	F	Empregado Efetivo
	F	Empregado Efetivo
	F	Empregado Efetivo
	F	Empregado Efetivo
	F	Empregado Efetivo
	F	Empregado Efetivo
	M	Empregado Efetivo
	M	Empregado Efetivo
	M	Empregado Efetivo
	M	Empregado Efetivo
	F	Empregado Efetivo
	F	Empregado Efetivo
	F	Empregado Efetivo
	F	Empregado Efetivo
	F	Empregado Efetivo
	F	Empregado Efetivo
	F	Empregado Efetivo
	F	Empregado Efetivo
	F	Empregado Efetivo
	F	Empregado Efetivo
	F	Empregado Efetivo
	F	Empregado Efetivo
	F	Empregado Efetivo
	F	Empregado Efetivo

	F	Empregado Efetivo
	F	Empregado Efetivo
	F	Empregado Efetivo
	F	Empregado Efetivo
	F	Empregado Efetivo
	F	Empregado Efetivo
	F	Empregado Efetivo
	F	Empregado Efetivo
	F	Empregado Efetivo
	F	Empregado Efetivo
	F	Empregado Efetivo
	F	Empregado Efetivo
	F	Empregado Efetivo
	F	Empregado Efetivo
	F	Empregado Efetivo
	F	Empregado Efetivo
	F	Empregado Efetivo
	F	Empregado Efetivo
	F	Empregado Efetivo
	F	Empregado Efetivo
	F	Empregado Efetivo
	F	Empregado Efetivo
	F	Empregado Efetivo
	F	Empregado Efetivo
	F	Empregado Efetivo
	F	Empregado Efetivo
	F	Empregado Efetivo
	F	Empregado Efetivo
	F	Empregado Efetivo
	F	Empregado Efetivo
	F	Empregado Efetivo
	F	Empregado Efetivo
	F	Empregado Efetivo
	M	Empregado Efetivo
	M	Empregado Efetivo
	M	Empregado Efetivo
	F	Empregado Efetivo
	F	Empregado Efetivo
	F	Empregado Efetivo
	F	Empregado Efetivo
	F	Empregado Efetivo
	F	Empregado Efetivo
	F	Empregado Efetivo
	F	Empregado Efetivo
	F	Empregado Efetivo
	F	Empregado Efetivo
	F	Empregado Efetivo
	F	Empregado Efetivo

	F	Empregado Efetivo
	F	Empregado Efetivo
	F	Empregado Efetivo
	F	Empregado Efetivo
	F	Empregado Efetivo
	F	Empregado Efetivo
	F	Empregado Efetivo
	F	Empregado Efetivo
	F	Empregado Efetivo
	F	Empregado Efetivo
	F	Empregado Efetivo
	F	Empregado Efetivo
	F	Empregado Efetivo
	F	Empregado Efetivo
	F	Empregado Efetivo
	F	Empregado Efetivo
	F	Empregado Efetivo
	F	Empregado Efetivo
	M	Empregado Efetivo
	M	Empregado Efetivo
	M	Empregado Efetivo
	M	Empregado Efetivo
	M	Empregado Efetivo
	F	Empregado Efetivo
	F	Empregado Efetivo
	F	Empregado Efetivo
	F	Empregado Efetivo
	F	Empregado Efetivo
	F	Empregado Efetivo
	F	Empregado Efetivo
	F	Empregado Efetivo
	F	Empregado Efetivo
	F	Empregado Efetivo
	F	Empregado Efetivo
	F	Empregado Efetivo
	F	Empregado Efetivo
	F	Empregado Efetivo
	F	Empregado Efetivo
	F	Empregado Efetivo
	F	Empregado Efetivo
	F	Empregado Efetivo
	M	Empregado Efetivo
	F	Empregado Efetivo
	F	Empregado Efetivo
	F	Empregado Efetivo
	F	Empregado Efetivo
	F	Empregado Efetivo
	F	Empregado Efetivo

	F	Empregado Efetivo
	F	Empregado Efetivo
	F	Empregado Efetivo
	F	Empregado Efetivo
	F	Empregado Efetivo
	F	Empregado Efetivo
	F	Empregado Efetivo
	F	Empregado Efetivo
	F	Empregado Efetivo
	F	Empregado Efetivo
	F	Empregado Efetivo
	F	Empregado Efetivo
	F	Empregado Efetivo
	F	Empregado Efetivo
	F	Empregado Efetivo
	F	Empregado Efetivo
	F	Empregado Efetivo
	M	Empregado Efetivo
	F	Empregado Efetivo
	F	Empregado Efetivo
	F	Empregado Efetivo
	F	Empregado Efetivo
	F	Empregado Efetivo
	F	Empregado Efetivo
	F	Empregado Efetivo
	F	Empregado Efetivo
	F	Empregado Efetivo
	M	Empregado Efetivo
	F	Empregado Efetivo
	F	Empregado Efetivo
	M	Empregado Efetivo
	F	Empregado Efetivo
	F	Empregado Efetivo
	M	Empregado Efetivo
	M	Empregado Efetivo
	F	Empregado Efetivo
	F	Empregado Efetivo
	F	Empregado Efetivo
	F	Empregado Efetivo
	F	Empregado Efetivo
	F	Empregado Efetivo
	F	Empregado Efetivo
	F	Empregado Efetivo
	F	Empregado Efetivo
	F	Empregado Efetivo
	M	Empregado Efetivo
	F	Empregado Efetivo
	F	Empregado Efetivo

	F	Empregado Efetivo
	M	Empregado Efetivo
	F	Empregado Efetivo
	F	Empregado Efetivo
	F	Empregado Efetivo
	F	Empregado Efetivo
	F	Empregado Efetivo
	F	Empregado Efetivo
	F	Empregado Efetivo
	F	Empregado Efetivo
	F	Empregado Efetivo
	F	Empregado Efetivo
	F	Empregado Efetivo
	F	Empregado Efetivo
	M	Empregado Efetivo
	F	Empregado Efetivo
	F	Empregado Efetivo
	M	Empregado Efetivo
	F	Empregado Efetivo
	F	Empregado Efetivo
	F	Empregado Efetivo
	M	Empregado Efetivo
	F	Empregado Efetivo
	M	Empregado Efetivo
	F	Empregado Efetivo
	F	Empregado Efetivo
	F	Empregado Efetivo
	F	Empregado Efetivo
	F	Empregado Efetivo
	F	Empregado Efetivo
	M	Empregado Efetivo
	F	Empregado Efetivo
	M	Empregado Efetivo
	F	Empregado Efetivo
	F	Empregado Efetivo
	F	Empregado Efetivo
	M	Empregado Efetivo
	F	Empregado Efetivo
	F	Empregado Efetivo
	F	Empregado Efetivo
	F	Empregado Efetivo
	F	Empregado Efetivo
	M	Empregado Efetivo
	F	Empregado Efetivo
	F	Empregado Efetivo
	F	Empregado Efetivo
	M	Empregado Efetivo
	F	Empregado Efetivo

	F	Empregado Efetivo
	F	Empregado Efetivo
	F	Empregado Efetivo
	F	Empregado Efetivo
	M	Empregado Efetivo
	M	Cedido - RJU
	M	Empregado Efetivo
	M	Empregado Efetivo
	F	Cedido - RJU
	F	Cedido - RJU
	M	Cedido - RJU
	M	Empregado Efetivo
	F	Empregado Efetivo
	F	Empregado Efetivo
	F	Empregado Efetivo
	F	Empregado Efetivo
	F	Empregado Efetivo
	M	Empregado Efetivo
	F	Empregado Efetivo
	M	Empregado Efetivo
	M	Empregado Efetivo
	F	Empregado Efetivo
	M	Empregado Efetivo
	M	Empregado Efetivo
	F	Empregado Efetivo
	F	Empregado Efetivo
	M	Empregado Efetivo
	F	Empregado Efetivo
	F	Empregado Efetivo
	F	Empregado Efetivo
	F	Empregado Efetivo
	F	Empregado Efetivo
	M	Empregado Efetivo
	F	Empregado Efetivo
	F	Empregado Efetivo
	M	Empregado Efetivo
	M	Empregado Efetivo
	F	Empregado Efetivo
	M	Empregado Efetivo
	M	Empregado Efetivo
	M	Empregado Efetivo
	F	Empregado Efetivo
	F	Empregado Efetivo

	F	Empregado Efetivo
	F	Empregado Efetivo
	F	Empregado Efetivo
	F	Empregado Efetivo
	F	Empregado Efetivo
	M	Empregado Efetivo
	F	Empregado Efetivo
	F	Empregado Efetivo
	F	Empregado Efetivo
	F	Empregado Efetivo
	F	Empregado Efetivo
	F	Empregado Efetivo
	F	Empregado Efetivo
	F	Empregado Efetivo
	F	Empregado Efetivo
	F	Empregado Efetivo
	F	Empregado Efetivo
	M	Empregado Efetivo
	M	Empregado Efetivo
	F	Empregado Efetivo
	F	Empregado Efetivo
	F	Empregado Efetivo
	F	Empregado Efetivo
	F	Empregado Efetivo
	F	Empregado Efetivo
	F	Empregado Efetivo
	F	Empregado Efetivo
	F	Empregado Efetivo
	F	Empregado Efetivo
	F	Empregado Efetivo
	F	Empregado Efetivo
	F	Empregado Efetivo
	F	Empregado Efetivo
	F	Empregado Efetivo
	F	Empregado Efetivo
	F	Empregado Efetivo
	F	Empregado Efetivo
	F	Empregado Efetivo
	F	Empregado Efetivo
	F	Empregado Efetivo
	F	Empregado Efetivo
	F	Empregado Efetivo
	F	Empregado Efetivo
	F	Empregado Efetivo
	F	Empregado Efetivo
	F	Empregado Efetivo
	F	Empregado Efetivo

	F	Empregado Efetivo
	F	Empregado Efetivo
	F	Empregado Efetivo
	F	Empregado Efetivo
	F	Empregado Efetivo
	F	Empregado Efetivo
	F	Empregado Efetivo
	F	Empregado Efetivo
	F	Empregado Efetivo
	F	Empregado Efetivo
	F	Empregado Efetivo
	F	Empregado Efetivo
	F	Empregado Efetivo
	M	Empregado Efetivo
	F	Empregado Efetivo
	F	Empregado Efetivo
	F	Empregado Efetivo
	F	Empregado Efetivo
	F	Empregado Efetivo
	M	Empregado Efetivo
	F	Empregado Efetivo
	F	Empregado Efetivo
	M	Empregado Efetivo
	F	Empregado Efetivo
	F	Empregado Efetivo
	M	Empregado Efetivo
	F	Empregado Efetivo
	F	Empregado Efetivo
	F	Empregado Efetivo
	F	Empregado Efetivo
	F	Empregado Efetivo
	F	Empregado Efetivo
	F	Empregado Efetivo
	F	Empregado Efetivo
	F	Empregado Efetivo
	F	Empregado Efetivo
	F	Empregado Efetivo

	F	Empregado Efetivo
	F	Empregado Efetivo
	F	Empregado Efetivo
	F	Empregado Efetivo
	F	Empregado Efetivo
	F	Empregado Efetivo
	F	Empregado Efetivo
	F	Empregado Efetivo
	F	Empregado Efetivo
	F	Empregado Efetivo
	F	Empregado Efetivo
	F	Empregado Efetivo
	F	Empregado Efetivo
	F	Empregado Efetivo
	F	Empregado Efetivo
	F	Empregado Efetivo
	M	Empregado Efetivo
	F	Cedido - RJU
	M	Empregado Efetivo
	F	Empregado Efetivo
	F	Empregado Efetivo
	F	Empregado Efetivo
	F	Empregado Efetivo
	F	Empregado Efetivo
	F	Empregado Efetivo
	F	Empregado Efetivo
	F	Empregado Efetivo
	F	Empregado Efetivo
	F	Empregado Efetivo
	F	Empregado Efetivo
	F	Empregado Efetivo
	F	Empregado Efetivo
	F	Empregado Efetivo
	F	Empregado Efetivo
	F	Empregado Efetivo
	F	Empregado Efetivo
	F	Empregado Efetivo
	F	Empregado Efetivo
	F	Empregado Efetivo
	F	Empregado Efetivo
	F	Empregado Efetivo
	F	Empregado Efetivo
	F	Empregado Efetivo
	F	Empregado Efetivo
	F	Empregado Efetivo
	F	Empregado Efetivo
	F	Empregado Efetivo

	F	Empregado Efetivo
	M	Empregado Efetivo
	F	Empregado Efetivo
	F	Empregado Efetivo
	F	Empregado Efetivo
	F	Empregado Efetivo
	F	Empregado Efetivo
	F	Empregado Efetivo
	F	Empregado Efetivo
	F	Empregado Efetivo
	F	Empregado Efetivo
	M	Empregado Efetivo
	F	Empregado Efetivo
	F	Empregado Efetivo
	F	Empregado Efetivo
	F	Empregado Efetivo
	F	Empregado Efetivo
	F	Empregado Efetivo
	F	Empregado Efetivo
	F	Empregado Efetivo
	F	Empregado Efetivo
	M	Empregado Efetivo
	M	Empregado Efetivo
	F	Empregado Efetivo
	F	Empregado Efetivo
	F	Empregado Efetivo
	F	Empregado Efetivo
	F	Empregado Efetivo
	F	Empregado Efetivo
	F	Empregado Efetivo
	F	Empregado Efetivo
	F	Empregado Efetivo

	F	Empregado Efetivo
	F	Empregado Efetivo
	F	Empregado Efetivo
	F	Empregado Efetivo
	F	Empregado Efetivo
	F	Empregado Efetivo
	F	Empregado Efetivo
	M	Empregado Efetivo
	F	Empregado Efetivo
	F	Empregado Efetivo
	F	Empregado Efetivo
	F	Empregado Efetivo
	F	Empregado Efetivo
	F	Empregado Efetivo
	F	Empregado Efetivo
	F	Empregado Efetivo
	F	Empregado Efetivo
	F	Empregado Efetivo
	F	Empregado Efetivo
	F	Empregado Efetivo
	F	Empregado Efetivo
	F	Empregado Efetivo
	F	Empregado Efetivo
	F	Empregado Efetivo
	F	Empregado Efetivo
	F	Empregado Efetivo
	F	Empregado Efetivo
	F	Empregado Efetivo
	F	Empregado Efetivo
	F	Empregado Efetivo
	F	Empregado Efetivo
	F	Empregado Efetivo
	F	Empregado Efetivo
	F	Empregado Efetivo
	F	Empregado Efetivo
	F	Empregado Efetivo
	F	Empregado Efetivo
	F	Empregado Efetivo
	F	Empregado Efetivo
	F	Empregado Efetivo
	F	Empregado Efetivo

	F	Empregado Efetivo
	F	Empregado Efetivo
	F	Empregado Efetivo
	F	Empregado Efetivo
	M	Empregado Efetivo
	F	Empregado Efetivo
	F	Empregado Efetivo
	F	Empregado Efetivo
	F	Empregado Efetivo
	F	Empregado Efetivo
	F	Empregado Efetivo
	F	Empregado Efetivo
	F	Empregado Efetivo
	M	Empregado Efetivo
	F	Empregado Efetivo
	F	Empregado Efetivo
	F	Empregado Efetivo
	F	Empregado Efetivo
	F	Empregado Efetivo
	F	Empregado Efetivo
	F	Empregado Efetivo
	F	Empregado Efetivo
	M	Empregado Efetivo
	F	Empregado Efetivo
	F	Empregado Efetivo
	M	Empregado Efetivo
	F	Empregado Efetivo
	F	Empregado Efetivo
	F	Empregado Efetivo
	F	Empregado Efetivo
	F	Empregado Efetivo
	F	Empregado Efetivo
	F	Empregado Efetivo
	F	Empregado Efetivo
	M	Empregado Efetivo
	M	Empregado Efetivo
	F	Empregado Efetivo
	F	Empregado Efetivo
	F	Empregado Efetivo
	F	Empregado Efetivo
	F	Empregado Efetivo
	F	Empregado Efetivo
	M	Empregado Efetivo
	F	Empregado Efetivo
	F	Empregado Efetivo
	F	Empregado Efetivo
	F	Empregado Efetivo
	F	Empregado Efetivo

	F	Empregado Efetivo
	F	Empregado Efetivo
	F	Empregado Efetivo
	F	Empregado Efetivo
	F	Empregado Efetivo
	F	Empregado Efetivo
	M	Empregado Efetivo
	F	Empregado Efetivo
	F	Empregado Efetivo
	F	Empregado Efetivo
	F	Empregado Efetivo
	F	Empregado Efetivo
	F	Empregado Efetivo
	F	Empregado Efetivo
	M	Empregado Efetivo
	F	Empregado Efetivo
	F	Empregado Efetivo
	F	Empregado Efetivo
	F	Empregado Efetivo
	M	Empregado Efetivo
	F	Empregado Efetivo
	M	Empregado Efetivo
	F	Empregado Efetivo
	F	Empregado Efetivo
	F	Empregado Efetivo
	F	Empregado Efetivo
	F	Empregado Efetivo
	F	Empregado Efetivo
	M	Empregado Efetivo
	F	Empregado Efetivo
	F	Empregado Efetivo
	F	Empregado Efetivo
	F	Empregado Efetivo
	F	Empregado Efetivo
	F	Empregado Efetivo
	M	Empregado Efetivo
	F	Empregado Efetivo
	M	Empregado Efetivo
	F	Empregado Efetivo
	M	Empregado Efetivo
	F	Empregado Efetivo
	F	Empregado Efetivo
	F	Empregado Efetivo
	F	Empregado Efetivo
	F	Empregado Efetivo
	F	Empregado Efetivo
	M	Empregado Efetivo
	F	Empregado Efetivo

	F	Empregado Efetivo
	F	Empregado Efetivo
	F	Empregado Efetivo
	F	Empregado Efetivo
	F	Empregado Efetivo
	F	Empregado Efetivo
	M	Empregado Efetivo
	F	Empregado Efetivo
	F	Empregado Efetivo
	F	Empregado Efetivo
	F	Empregado Efetivo
	F	Empregado Efetivo
	M	Empregado Efetivo
	F	Empregado Efetivo
	F	Empregado Efetivo
	F	Empregado Efetivo
	F	Empregado Efetivo
	F	Empregado Efetivo
	F	Empregado Efetivo
	F	Empregado Efetivo
	M	Empregado Efetivo
	F	Empregado Efetivo
	F	Empregado Efetivo
	F	Empregado Efetivo
	F	Empregado Efetivo
	F	Empregado Efetivo
	F	Empregado Efetivo
	F	Empregado Efetivo
	F	Empregado Efetivo
	F	Empregado Efetivo
	F	Empregado Efetivo
	F	Empregado Efetivo
	F	Empregado Efetivo
	F	Empregado Efetivo
	F	Empregado Efetivo
	F	Empregado Efetivo
	M	Empregado Efetivo
	F	Empregado Efetivo
	F	Empregado Efetivo
	F	Empregado Efetivo
	F	Empregado Efetivo
	F	Empregado Efetivo
	F	Empregado Efetivo
	F	Empregado Efetivo
	F	Empregado Efetivo
	F	Empregado Efetivo
	M	Empregado Efetivo
	F	Empregado Efetivo

	M	Empregado Efetivo
	F	Empregado Efetivo
	F	Empregado Efetivo
	F	Empregado Efetivo
	M	Empregado Efetivo
	F	Empregado Efetivo
	F	Empregado Efetivo
	F	Empregado Efetivo
	F	Empregado Efetivo
	F	Empregado Efetivo
	F	Empregado Efetivo
	F	Empregado Efetivo
	F	Empregado Efetivo
	F	Empregado Efetivo
	F	Empregado Efetivo
	F	Empregado Efetivo
	M	Empregado Efetivo
	F	Empregado Efetivo
	F	Empregado Efetivo
	F	Empregado Efetivo
	F	Empregado Efetivo
	F	Empregado Efetivo
	F	Empregado Efetivo
	M	Empregado Efetivo
	F	Empregado Efetivo
	F	Empregado Efetivo
	F	Empregado Efetivo
	F	Empregado Efetivo
	F	Empregado Efetivo
	F	Empregado Efetivo
	F	Empregado Efetivo
	F	Empregado Efetivo
	F	Empregado Efetivo
	F	Empregado Efetivo
	F	Empregado Efetivo
	M	Empregado Efetivo
	F	Empregado Efetivo
	F	Empregado Efetivo
	F	Empregado Efetivo
	F	Empregado Efetivo
	F	Empregado Efetivo
	F	Empregado Efetivo
	F	Empregado Efetivo
	F	Empregado Efetivo
	F	Empregado Efetivo
	F	Empregado Efetivo
	F	Empregado Efetivo
	F	Empregado Efetivo

	F	Empregado Efetivo
	F	Empregado Efetivo
	F	Empregado Efetivo
	M	Empregado Efetivo
	F	Empregado Efetivo
	F	Empregado Efetivo
	M	Empregado Efetivo
	F	Empregado Efetivo
	F	Empregado Efetivo
	F	Empregado Efetivo
	F	Empregado Efetivo
	F	Empregado Efetivo
	F	Empregado Efetivo
	F	Empregado Efetivo
	F	Empregado Efetivo
	F	Empregado Efetivo
	F	Empregado Efetivo
	F	Empregado Efetivo
	F	Empregado Efetivo
	F	Empregado Efetivo
	M	Empregado Efetivo
	F	Empregado Efetivo
	F	Empregado Efetivo
	M	Empregado Efetivo
	F	Empregado Efetivo
	F	Empregado Efetivo
	F	Empregado Efetivo
	F	Empregado Efetivo
	F	Empregado Efetivo
	M	Empregado Efetivo
	F	Empregado Efetivo
	F	Empregado Efetivo
	F	Empregado Efetivo
	F	Empregado Efetivo
	F	Empregado Efetivo
	F	Empregado Efetivo
	F	Empregado Efetivo
	F	Empregado Efetivo
	F	Empregado Efetivo
	M	Empregado Efetivo
	F	Empregado Efetivo
	M	Empregado Efetivo
	F	Empregado Efetivo
	F	Empregado Efetivo
	F	Empregado Efetivo
	F	Empregado Efetivo
	F	Empregado Efetivo
	F	Empregado Efetivo

	F	Empregado Efetivo
	F	Empregado Efetivo
	F	Empregado Efetivo
	F	Empregado Efetivo
	F	Empregado Efetivo
	F	Empregado Efetivo
	F	Empregado Efetivo
	M	Empregado Efetivo
	F	Empregado Efetivo
	F	Empregado Efetivo
	F	Empregado Efetivo
	F	Empregado Efetivo
	F	Empregado Efetivo
	F	Empregado Efetivo
	M	Empregado Efetivo
	F	Empregado Efetivo
	F	Empregado Efetivo
	F	Empregado Efetivo
	F	Empregado Efetivo
	F	Empregado Efetivo
	F	Empregado Efetivo
	F	Empregado Efetivo
	F	Empregado Efetivo
	F	Empregado Efetivo
	F	Empregado Efetivo
	F	Empregado Efetivo
	M	Empregado Efetivo
	F	Empregado Efetivo
	F	Empregado Efetivo
	F	Empregado Efetivo
	F	Empregado Efetivo
	F	Empregado Efetivo
	F	Empregado Efetivo
	F	Empregado Efetivo
	F	Empregado Efetivo
	F	Empregado Efetivo
	F	Empregado Efetivo
	F	Empregado Efetivo
	F	Empregado Efetivo
	F	Empregado Efetivo
	M	Empregado Efetivo
	F	Empregado Efetivo
	F	Empregado Efetivo
	F	Empregado Efetivo
	F	Empregado Efetivo
	F	Empregado Efetivo
	F	Empregado Efetivo
	M	Empregado Efetivo

	F	Empregado Efetivo
	F	Empregado Efetivo
	F	Empregado Efetivo
	F	Empregado Efetivo
	F	Empregado Efetivo
	F	Empregado Efetivo
	F	Empregado Efetivo
	M	Empregado Efetivo
	M	Empregado Efetivo
	F	Empregado Efetivo
	F	Empregado Efetivo
	M	Empregado Efetivo
	F	Empregado Efetivo
	F	Empregado Efetivo
	F	Empregado Efetivo
	F	Empregado Efetivo
	F	Empregado Efetivo
	F	Empregado Efetivo
	M	Empregado Efetivo
	F	Empregado Efetivo
	M	Empregado Efetivo
	F	Empregado Efetivo
	F	Empregado Efetivo
	F	Empregado Efetivo
	F	Empregado Efetivo
	F	Empregado Efetivo
	F	Empregado Efetivo
	F	Empregado Efetivo
	F	Empregado Efetivo
	F	Empregado Efetivo
	F	Empregado Efetivo
	M	Cedido - RJU
	F	Empregado Efetivo
	F	Empregado Efetivo
	F	Empregado Efetivo
	F	Empregado Efetivo
	F	Empregado Efetivo
	F	Empregado Efetivo
	F	Empregado Efetivo
	F	Empregado Efetivo
	F	Empregado Efetivo
	F	Empregado Efetivo
	F	Empregado Efetivo
	F	Empregado Efetivo
	F	Empregado Efetivo
	F	Empregado Efetivo
	F	Empregado Efetivo
	F	Empregado Efetivo

	F	Empregado Efetivo
	F	Empregado Efetivo
	M	Empregado Efetivo
	M	Empregado Efetivo
	F	Empregado Efetivo
	M	Empregado Efetivo
	F	Empregado Efetivo
	M	Empregado Efetivo
	F	Empregado Efetivo
	F	Empregado Efetivo
	F	Empregado Efetivo
	M	Empregado Efetivo
	F	Empregado Efetivo
	F	Empregado Efetivo
	F	Empregado Efetivo
	F	Empregado Efetivo
	M	Empregado Efetivo
	F	Empregado Efetivo
	F	Empregado Efetivo
	F	Empregado Efetivo
	F	Empregado Efetivo
	F	Empregado Efetivo
	F	Empregado Efetivo
	F	Empregado Efetivo
	F	Empregado Efetivo
	F	Empregado Efetivo
	F	Empregado Efetivo
	F	Empregado Efetivo
	F	Empregado Efetivo
	F	Empregado Efetivo
	F	Empregado Efetivo
	F	Empregado Efetivo
	F	Empregado Efetivo
	F	Empregado Efetivo
	F	Empregado Efetivo
	F	Empregado Efetivo
	M	Empregado Efetivo
	F	Empregado Efetivo
	F	Empregado Efetivo
	F	Empregado Efetivo
	F	Empregado Efetivo
	F	Empregado Efetivo
	M	Empregado Efetivo
	F	Empregado Efetivo
	F	Empregado Efetivo
	F	Empregado Efetivo
	F	Empregado Efetivo
	F	Empregado Efetivo

	F	Empregado Efetivo
	F	Empregado Efetivo
	F	Empregado Efetivo
	F	Empregado Efetivo
	F	Empregado Efetivo
	F	Empregado Efetivo
	F	Empregado Efetivo
	F	Empregado Efetivo
	F	Empregado Efetivo
	F	Empregado Efetivo
	F	Empregado Efetivo
	F	Empregado Efetivo
	F	Empregado Efetivo
	M	Empregado Efetivo
	F	Empregado Efetivo
	F	Empregado Efetivo
	F	Empregado Efetivo
	F	Empregado Efetivo
	M	Empregado Efetivo
	F	Empregado Efetivo
	F	Empregado Efetivo
	M	Empregado Efetivo
	F	Empregado Efetivo
	F	Empregado Efetivo
	F	Empregado Efetivo
	F	Empregado Efetivo
	F	Empregado Efetivo
	F	Empregado Efetivo
	F	Empregado Efetivo
	F	Empregado Efetivo
	F	Empregado Efetivo
	F	Empregado Efetivo
	F	Empregado Efetivo
	F	Empregado Efetivo
	F	Empregado Efetivo
	F	Empregado Efetivo
	F	Empregado Efetivo
	F	Empregado Efetivo
	F	Empregado Efetivo
	M	Empregado Efetivo
	F	Empregado Efetivo
	F	Empregado Efetivo
	F	Empregado Efetivo
	M	Empregado Efetivo
	F	Empregado Efetivo
	F	Empregado Efetivo
	F	Empregado Efetivo
	M	Empregado Efetivo

	M	Empregado Efetivo
	M	Empregado Efetivo
	F	Empregado Efetivo
	F	Empregado Efetivo
	M	Empregado Efetivo
	M	Empregado Efetivo
	M	Empregado Efetivo
	F	Empregado Efetivo
	F	Empregado Efetivo
	M	Empregado Efetivo
	F	Empregado Efetivo
	F	Empregado Efetivo
	F	Empregado Efetivo
	M	Empregado Efetivo
	F	Empregado Efetivo
	F	Empregado Efetivo
	F	Empregado Efetivo
	F	Empregado Efetivo
	F	Empregado Efetivo
	F	Empregado Efetivo
	F	Empregado Efetivo
	F	Empregado Efetivo
	F	Empregado Efetivo
	F	Empregado Efetivo
	F	Empregado Efetivo
	F	Empregado Efetivo
	F	Empregado Efetivo
	F	Empregado Efetivo
	M	Empregado Efetivo
	F	Empregado Efetivo
	F	Empregado Efetivo
	F	Empregado Efetivo
	F	Empregado Efetivo
	F	Empregado Efetivo
	F	Empregado Efetivo
	F	Empregado Efetivo
	F	Empregado Efetivo
	M	Empregado Efetivo
	F	Empregado Efetivo
	F	Empregado Efetivo
	F	Empregado Efetivo
	F	Empregado Efetivo
	F	Empregado Efetivo
	F	Empregado Efetivo
	M	Empregado Efetivo
	M	Empregado Efetivo
	F	Empregado Efetivo
	F	Empregado Efetivo

	F	Empregado Efetivo
	M	Empregado Efetivo
	F	Empregado Efetivo
	F	Empregado Efetivo
	F	Empregado Efetivo
	F	Empregado Efetivo
	F	Empregado Efetivo
	F	Empregado Efetivo
	M	Empregado Efetivo
	F	Empregado Efetivo
	F	Empregado Efetivo
	F	Empregado Efetivo
	F	Empregado Efetivo
	F	Empregado Efetivo
	M	Empregado Efetivo
	F	Empregado Efetivo
	F	Empregado Efetivo
	F	Empregado Efetivo
	F	Empregado Efetivo
	F	Empregado Efetivo
	F	Empregado Efetivo
	F	Empregado Efetivo
	F	Empregado Efetivo
	F	Empregado Efetivo
	F	Empregado Efetivo
	F	Empregado Efetivo
	F	Empregado Efetivo
	F	Empregado Efetivo
	F	Empregado Efetivo
	F	Empregado Efetivo
	M	Empregado Efetivo
	F	Empregado Efetivo
	F	Empregado Efetivo
	F	Empregado Efetivo
	M	Empregado Efetivo
	M	Empregado Efetivo
	F	Empregado Efetivo
	F	Empregado Efetivo
	F	Empregado Efetivo
	M	Empregado Efetivo
	F	Empregado Efetivo
	M	Empregado Efetivo
	M	Empregado Efetivo
	F	Empregado Efetivo
	M	Empregado Efetivo
	M	Empregado Efetivo
	F	Empregado Efetivo
	F	Empregado Efetivo

	F	Empregado Efetivo
	F	Empregado Efetivo
	F	Empregado Efetivo
	F	Empregado Efetivo
	M	Empregado Efetivo
	M	Empregado Efetivo
	F	Empregado Efetivo
	F	Empregado Efetivo
	F	Empregado Efetivo
	F	Empregado Efetivo
	F	Empregado Efetivo
	F	Empregado Efetivo
	F	Empregado Efetivo
	F	Empregado Efetivo
	F	Empregado Efetivo
	M	Empregado Efetivo
	F	Empregado Efetivo
	F	Empregado Efetivo
	F	Empregado Efetivo
	F	Empregado Efetivo
	F	Empregado Efetivo
	F	Empregado Efetivo
	F	Empregado Efetivo
	M	Empregado Efetivo
	F	Empregado Efetivo
	F	Empregado Efetivo
	M	Empregado Efetivo
	M	Empregado Efetivo
	F	Empregado Efetivo
	M	Empregado Efetivo
	M	Empregado Efetivo
	F	Empregado Efetivo
	M	Empregado Efetivo
	F	Empregado Efetivo
	F	Empregado Efetivo
	F	Empregado Efetivo
	F	Empregado Efetivo
	M	Empregado Efetivo
	F	Empregado Efetivo
	M	Empregado Efetivo
	F	Empregado Efetivo
	M	Empregado Efetivo
	F	Empregado Efetivo
	F	Empregado Efetivo
	M	Empregado Efetivo
	M	Empregado Efetivo
	M	Empregado Efetivo
	F	Empregado Efetivo

	M	Empregado Efetivo
	M	Empregado Efetivo
	F	Empregado Efetivo
	F	Empregado Efetivo
	M	Empregado Efetivo
	M	Empregado Efetivo
	M	Empregado Efetivo
	F	Empregado Efetivo
	F	Empregado Efetivo
	F	Empregado Efetivo
	F	Empregado Efetivo
	F	Empregado Efetivo
	F	Empregado Efetivo
	F	Empregado Efetivo
	F	Empregado Efetivo
	F	Empregado Efetivo
	F	Empregado Efetivo
	F	Empregado Efetivo
	F	Empregado Efetivo
	F	Empregado Efetivo
	F	Empregado Efetivo
	F	Empregado Efetivo
	F	Empregado Efetivo
	F	Empregado Efetivo
	F	Empregado Efetivo
	F	Empregado Efetivo
	F	Empregado Efetivo
	F	Empregado Efetivo
	F	Empregado Efetivo
	F	Empregado Efetivo
	F	Empregado Efetivo
	F	Empregado Efetivo
	F	Empregado Efetivo
	F	Empregado Efetivo
	F	Empregado Efetivo
	F	Empregado Efetivo
	F	Empregado Efetivo
	M	Empregado Efetivo
	F	Empregado Efetivo
	F	Empregado Efetivo
	F	Empregado Efetivo
	F	Empregado Efetivo
	F	Empregado Efetivo
	F	Empregado Efetivo
	M	Empregado Efetivo
	M	Empregado Efetivo
	F	Empregado Efetivo
	M	Empregado Efetivo
	F	Empregado Efetivo

	M	Empregado Efetivo
	M	Empregado Efetivo
	F	Empregado Efetivo
	F	Empregado Efetivo
	F	Empregado Efetivo
	F	Empregado Efetivo
	F	Empregado Efetivo
	M	Empregado Efetivo
	F	Empregado Efetivo
	F	Empregado Efetivo
	M	Empregado Efetivo
	F	Empregado Efetivo
	M	Empregado Efetivo
	F	Empregado Efetivo
	F	Empregado Efetivo
	F	Empregado Efetivo
	F	Empregado Efetivo
	F	Empregado Efetivo
	F	Empregado Efetivo
	F	Empregado Efetivo
	F	Empregado Efetivo
	F	Empregado Efetivo
	F	Empregado Efetivo
	F	Empregado Efetivo
	F	Empregado Efetivo
	F	Empregado Efetivo
	F	Empregado Efetivo
	F	Empregado Efetivo
	F	Empregado Efetivo
	F	Empregado Efetivo
	M	Empregado Efetivo
	M	Empregado Efetivo
	M	Empregado Efetivo
	F	Empregado Efetivo
	F	Empregado Efetivo
	M	Empregado Efetivo
	M	Empregado Efetivo
	F	Empregado Efetivo
	F	Empregado Efetivo
	F	Empregado Efetivo
	F	Empregado Efetivo
	M	Empregado Efetivo
	F	Empregado Efetivo
	M	Empregado Efetivo
	M	Empregado Efetivo
	M	Empregado Efetivo

	F	Empregado Efetivo
	F	Empregado Efetivo
	F	Empregado Efetivo
	M	Empregado Efetivo
	F	Empregado Efetivo
	F	Empregado Efetivo
	F	Empregado Efetivo
	F	Empregado Efetivo
	M	Empregado Efetivo
	F	Empregado Efetivo
	F	Empregado Efetivo
	F	Empregado Efetivo
	M	Empregado Efetivo
	M	Empregado Efetivo
	F	Empregado Efetivo
	F	Empregado Efetivo
	F	Empregado Efetivo
	F	Empregado Efetivo
	F	Empregado Efetivo
	F	Empregado Efetivo
	M	Empregado Efetivo
	F	Empregado Efetivo
	F	Empregado Efetivo
	F	Empregado Efetivo
	M	Empregado Efetivo
	F	Empregado Efetivo
	F	Empregado Efetivo
	F	Empregado Efetivo
	F	Empregado Efetivo
	F	Empregado Efetivo
	F	Empregado Efetivo
	F	Empregado Efetivo
	M	Empregado Efetivo
	F	Empregado Efetivo
	M	Empregado Efetivo
	M	Empregado Efetivo
	F	Empregado Efetivo
	M	Empregado Efetivo
	F	Empregado Efetivo
	F	Empregado Efetivo
	M	Empregado Efetivo
	F	Empregado Efetivo
	F	Empregado Efetivo
	F	Empregado Efetivo
	M	Empregado Efetivo
	F	Empregado Efetivo
	F	Empregado Efetivo
	M	Empregado Efetivo

	M	Empregado Efetivo
	F	Empregado Efetivo
	F	Empregado Efetivo
	F	Empregado Efetivo
	F	Empregado Efetivo
	F	Empregado Efetivo
	F	Empregado Efetivo
	F	Empregado Efetivo
	F	Empregado Efetivo
	F	Empregado Efetivo
	F	Empregado Efetivo
	F	Empregado Efetivo
	M	Empregado Efetivo
	F	Empregado Efetivo
	F	Empregado Efetivo
	F	Empregado Efetivo
	F	Empregado Efetivo
	F	Empregado Efetivo
	F	Empregado Efetivo
	M	Empregado Efetivo
	F	Empregado Efetivo
	F	Empregado Efetivo
	M	Empregado Efetivo
	F	Empregado Efetivo
	F	Empregado Efetivo
	F	Empregado Efetivo
	F	Empregado Efetivo
	F	Empregado Efetivo
	F	Empregado Efetivo
	F	Empregado Efetivo
	F	Empregado Efetivo
	F	Empregado Efetivo
	F	Empregado Efetivo
	F	Empregado Efetivo
	F	Empregado Efetivo
	F	Empregado Efetivo
	M	Empregado Efetivo
	M	Empregado Efetivo
	F	Empregado Efetivo
	F	Empregado Efetivo
	M	Empregado Efetivo
	F	Empregado Efetivo
	F	Empregado Efetivo
	F	Empregado Efetivo
	M	Empregado Efetivo
	F	Empregado Efetivo
	M	Empregado Efetivo
	F	Empregado Efetivo

	M	Empregado Efetivo
	F	Empregado Efetivo
	M	Empregado Efetivo
	M	Empregado Efetivo
	F	Empregado Efetivo
	F	Empregado Efetivo
	F	Empregado Efetivo
	F	Empregado Efetivo
	F	Empregado Efetivo
	F	Empregado Efetivo
	M	Empregado Efetivo
	F	Empregado Efetivo
	F	Empregado Efetivo
	F	Empregado Efetivo
	F	Empregado Efetivo
	F	Empregado Efetivo
	F	Empregado Efetivo
	M	Empregado Efetivo
	M	Empregado Efetivo
	F	Empregado Efetivo
	F	Empregado Efetivo
	F	Empregado Efetivo
	F	Empregado Efetivo
	F	Empregado Efetivo
	F	Empregado Efetivo
	F	Empregado Efetivo
	F	Empregado Efetivo
	F	Empregado Efetivo
	F	Empregado Efetivo
	F	Empregado Efetivo
	M	Empregado Efetivo
	F	Empregado Efetivo
	F	Empregado Efetivo
	F	Empregado Efetivo
	F	Empregado Efetivo
	F	Empregado Efetivo
	F	Empregado Efetivo
	F	Empregado Efetivo
	F	Empregado Efetivo
	F	Empregado Efetivo
	F	Empregado Efetivo
	F	Empregado Efetivo
	F	Empregado Efetivo
	F	Empregado Efetivo
	F	Empregado Efetivo
	F	Empregado Efetivo
	F	Empregado Efetivo
	F	Empregado Efetivo

	F	Empregado Efetivo
	F	Empregado Efetivo
	F	Empregado Efetivo
	F	Empregado Efetivo
	F	Empregado Efetivo
	F	Empregado Efetivo
	F	Empregado Efetivo
	F	Empregado Efetivo
	F	Empregado Efetivo
	F	Empregado Efetivo
	M	Empregado Efetivo
	F	Empregado Efetivo
	F	Empregado Efetivo
	F	Empregado Efetivo
	F	Empregado Efetivo
	F	Empregado Efetivo
	F	Empregado Efetivo
	F	Empregado Efetivo
	F	Empregado Efetivo
	M	Empregado Efetivo
	F	Empregado Efetivo
	F	Empregado Efetivo
	F	Empregado Efetivo
	F	Empregado Efetivo
	F	Empregado Efetivo
	F	Empregado Efetivo
	F	Empregado Efetivo
	F	Empregado Efetivo
	M	Empregado Efetivo
	M	Empregado Efetivo
	F	Empregado Efetivo
	F	Empregado Efetivo
	F	Empregado Efetivo
	F	Empregado Efetivo
	F	Empregado Efetivo
	F	Empregado Efetivo
	M	Empregado Efetivo
	F	Empregado Efetivo
	F	Empregado Efetivo
	F	Empregado Efetivo
	M	Empregado Efetivo
	M	Empregado Efetivo
	M	Empregado Efetivo
	M	Empregado Efetivo
	F	Empregado Efetivo
	F	Empregado Efetivo
	F	Empregado Efetivo
	F	Empregado Efetivo

	F	Empregado Efetivo
	F	Empregado Efetivo
	F	Empregado Efetivo
	F	Empregado Efetivo
	F	Empregado Efetivo
	M	Empregado Efetivo
	F	Empregado Efetivo
	F	Empregado Efetivo
	M	Empregado Efetivo
	F	Empregado Efetivo
	M	Empregado Efetivo
	F	Empregado Efetivo
	F	Empregado Efetivo
	F	Empregado Efetivo
	F	Empregado Efetivo
	F	Empregado Efetivo
	F	Empregado Efetivo
	F	Empregado Efetivo
	F	Empregado Efetivo
	F	Empregado Efetivo
	F	Empregado Efetivo
	F	Empregado Efetivo
	F	Empregado Efetivo
	F	Empregado Efetivo
	F	Empregado Efetivo
	F	Empregado Efetivo
	F	Empregado Efetivo
	F	Empregado Efetivo
	F	Empregado Efetivo
	F	Empregado Efetivo
	F	Empregado Efetivo
	F	Empregado Efetivo
	M	Empregado Efetivo
	M	Empregado Efetivo
	F	Empregado Efetivo
	F	Empregado Efetivo
	F	Empregado Efetivo
	F	Empregado Efetivo
	F	Empregado Efetivo
	F	Empregado Efetivo
	F	Empregado Efetivo
	F	Empregado Efetivo
	F	Empregado Efetivo
	F	Empregado Efetivo
	M	Empregado Efetivo
	M	Empregado Efetivo
	M	Empregado Efetivo
	F	Empregado Efetivo

	F	Empregado Efetivo
	F	Empregado Efetivo
	F	Empregado Efetivo
	F	Empregado Efetivo
	F	Empregado Efetivo
	F	Empregado Efetivo
	F	Empregado Efetivo
	M	Empregado Efetivo
	F	Empregado Efetivo
	M	Empregado Efetivo
	M	Empregado Efetivo
	F	Empregado Efetivo
	F	Empregado Efetivo
	F	Empregado Efetivo
	F	Empregado Efetivo
	F	Empregado Efetivo
	M	Empregado Efetivo
	M	Empregado Efetivo
	F	Empregado Efetivo
	F	Empregado Efetivo
	F	Empregado Efetivo
	F	Empregado Efetivo
	F	Empregado Efetivo
	F	Empregado Efetivo
	F	Empregado Efetivo
	F	Empregado Efetivo
	F	Empregado Efetivo
	F	Empregado Efetivo
	F	Empregado Efetivo
	F	Empregado Efetivo
	F	Empregado Efetivo
	F	Empregado Efetivo
	F	Empregado Efetivo
	F	Empregado Efetivo
	M	Empregado Efetivo
	M	Empregado Efetivo
	F	Empregado Efetivo
	F	Empregado Efetivo
	F	Empregado Efetivo
	F	Empregado Efetivo
	F	Empregado Efetivo
	F	Empregado Efetivo
	F	Empregado Efetivo
	F	Empregado Efetivo
	F	Empregado Efetivo
	M	Empregado Efetivo
	M	Empregado Efetivo
	F	Empregado Efetivo

	F	Empregado Efetivo
	F	Empregado Efetivo
	F	Empregado Efetivo
	F	Empregado Efetivo
	F	Empregado Efetivo
	F	Empregado Efetivo
	F	Empregado Efetivo
	F	Empregado Efetivo
	F	Empregado Efetivo
	F	Empregado Efetivo
	F	Empregado Efetivo
	F	Empregado Efetivo
	F	Empregado Efetivo
	F	Empregado Efetivo
	F	Empregado Efetivo
	M	Empregado Efetivo
	F	Empregado Efetivo
	F	Empregado Efetivo
	F	Empregado Efetivo
	F	Empregado Efetivo
	F	Empregado Efetivo
	F	Empregado Efetivo
	F	Empregado Efetivo
	F	Empregado Efetivo
	F	Empregado Efetivo
	F	Empregado Efetivo
	F	Empregado Efetivo
	F	Empregado Efetivo
	F	Empregado Efetivo
	F	Empregado Efetivo
	F	Empregado Efetivo
	F	Empregado Efetivo
	F	Empregado Efetivo
	F	Empregado Efetivo
	F	Empregado Efetivo
	F	Empregado Efetivo
	F	Empregado Efetivo
	F	Empregado Efetivo
	F	Empregado Efetivo
	M	Empregado Efetivo
	F	Empregado Efetivo
	F	Empregado Efetivo
	F	Empregado Efetivo
	F	Empregado Efetivo
	F	Empregado Efetivo
	F	Empregado Efetivo
	F	Empregado Efetivo
	F	Empregado Efetivo

	F	Empregado Efetivo
	F	Empregado Efetivo
	F	Empregado Efetivo
	F	Empregado Efetivo
	F	Empregado Efetivo
	F	Empregado Efetivo
	F	Empregado Efetivo
	F	Empregado Efetivo
	F	Empregado Efetivo
	F	Empregado Efetivo
	F	Empregado Efetivo
	M	Empregado Efetivo
	F	Empregado Efetivo
	F	Empregado Efetivo
	F	Empregado Efetivo
	F	Empregado Efetivo
	F	Empregado Efetivo
	M	Empregado Efetivo
	F	Empregado Efetivo
	F	Empregado Efetivo
	F	Empregado Efetivo
	F	Empregado Efetivo
	F	Empregado Efetivo
	F	Empregado Efetivo
	F	Empregado Efetivo
	F	Empregado Efetivo
	M	Empregado Efetivo
	F	Empregado Efetivo
	F	Empregado Efetivo
	F	Empregado Efetivo
	M	Empregado Efetivo
	F	Empregado Efetivo
	F	Empregado Efetivo
	F	Empregado Efetivo
	M	Empregado Efetivo
	M	Empregado Efetivo
	F	Empregado Efetivo
	M	Empregado Efetivo
	F	Empregado Efetivo
	F	Empregado Efetivo
	F	Empregado Efetivo
	M	Empregado Efetivo
	F	Empregado Efetivo
	F	Empregado Efetivo
	M	Empregado Efetivo
	F	Empregado Efetivo
	F	Empregado Efetivo
	M	Empregado Efetivo

	F	Empregado Efetivo
	M	Empregado Efetivo
	F	Empregado Efetivo
	F	Empregado Efetivo
	F	Empregado Efetivo
	F	Empregado Efetivo
	F	Empregado Efetivo
	F	Empregado Efetivo
	F	Empregado Efetivo
	F	Empregado Efetivo
	F	Empregado Efetivo
	F	Empregado Efetivo
	F	Empregado Efetivo
	M	Empregado Efetivo
	M	Empregado Efetivo
	F	Empregado Efetivo
	F	Empregado Efetivo
	F	Empregado Efetivo
	F	Empregado Efetivo
	M	Empregado Efetivo
	M	Empregado Efetivo
	F	Empregado Efetivo
	F	Empregado Efetivo
	F	Empregado Efetivo
	F	Empregado Efetivo
	F	Empregado Efetivo
	F	Empregado Efetivo
	F	Empregado Efetivo
	F	Empregado Efetivo
	F	Empregado Efetivo
	F	Empregado Efetivo
	F	Empregado Efetivo
	F	Empregado Efetivo
	M	Empregado Efetivo
	F	Empregado Efetivo
	F	Empregado Efetivo
	F	Empregado Efetivo
	F	Empregado Efetivo
	M	Empregado Efetivo
	F	Empregado Efetivo
	F	Empregado Efetivo
	F	Empregado Efetivo
	F	Empregado Efetivo
	F	Empregado Efetivo
	F	Empregado Efetivo
	M	Empregado Efetivo
	F	Empregado Efetivo
	F	Empregado Efetivo

	M	Empregado Efetivo
	M	Empregado Efetivo
	M	Empregado Efetivo
	M	Empregado Efetivo
	F	Empregado Efetivo
	F	Empregado Efetivo
	F	Empregado Efetivo
	F	Empregado Efetivo
	F	Empregado Efetivo
	F	Empregado Efetivo
	F	Empregado Efetivo
	F	Empregado Efetivo
	F	Empregado Efetivo
	F	Empregado Efetivo
	F	Empregado Efetivo
	F	Empregado Efetivo
	F	Empregado Efetivo
	F	Empregado Efetivo
	F	Empregado Efetivo
	F	Empregado Efetivo
	F	Empregado Efetivo
	F	Empregado Efetivo
	F	Empregado Efetivo
	F	Empregado Efetivo
	F	Empregado Efetivo
	F	Empregado Efetivo
	F	Empregado Efetivo
	F	Empregado Efetivo
	F	Empregado Efetivo
	F	Empregado Efetivo
	F	Empregado Efetivo
	F	Empregado Efetivo
	F	Empregado Efetivo
	F	Empregado Efetivo
	F	Empregado Efetivo
	F	Empregado Efetivo
	F	Empregado Efetivo
	F	Empregado Efetivo
	F	Empregado Efetivo
	F	Empregado Efetivo
	F	Empregado Efetivo
	F	Empregado Efetivo
	F	Empregado Efetivo
	F	Empregado Efetivo
	F	Empregado Efetivo
	F	Empregado Efetivo
	F	Empregado Efetivo
	F	Empregado Efetivo

	F	Empregado Efetivo
	F	Empregado Efetivo
	F	Empregado Efetivo
	F	Empregado Efetivo
	F	Empregado Efetivo
	F	Empregado Efetivo
	F	Empregado Efetivo
	F	Empregado Efetivo
	F	Empregado Efetivo
	F	Empregado Efetivo
	F	Empregado Efetivo
	F	Empregado Efetivo
	F	Empregado Efetivo
	F	Empregado Efetivo
	F	Empregado Efetivo
	F	Empregado Efetivo
	F	Empregado Efetivo
	F	Empregado Efetivo
	F	Empregado Efetivo
	F	Empregado Efetivo
	F	Empregado Efetivo
	F	Empregado Efetivo
	F	Empregado Efetivo
	F	Empregado Efetivo
	F	Empregado Efetivo
	F	Empregado Efetivo
	F	Empregado Efetivo
	F	Empregado Efetivo
	F	Empregado Efetivo
	F	Empregado Efetivo
	F	Empregado Efetivo
	F	Empregado Efetivo
	F	Empregado Efetivo
	F	Empregado Efetivo
	F	Empregado Efetivo
	F	Empregado Efetivo
	F	Empregado Efetivo
	F	Empregado Efetivo
	F	Empregado Efetivo
	F	Empregado Efetivo
	F	Empregado Efetivo
	F	Empregado Efetivo
	F	Empregado Efetivo
	F	Empregado Efetivo
	F	Empregado Efetivo
	F	Empregado Efetivo
	F	Empregado Efetivo
	F	Empregado Efetivo

	F	Empregado Efetivo
	F	Empregado Efetivo
	F	Empregado Efetivo
	F	Empregado Efetivo
	F	Empregado Efetivo
	F	Empregado Efetivo
	F	Empregado Efetivo
	F	Empregado Efetivo
	F	Empregado Efetivo
	F	Empregado Efetivo
	F	Empregado Efetivo
	F	Empregado Efetivo
	M	Empregado Efetivo
	F	Empregado Efetivo
	F	Empregado Efetivo
	F	Empregado Efetivo
	F	Empregado Efetivo
	F	Empregado Efetivo
	F	Empregado Efetivo
	F	Empregado Efetivo
	M	Empregado Efetivo
	F	Empregado Efetivo
	F	Empregado Efetivo
	F	Empregado Efetivo
	F	Empregado Efetivo
	F	Empregado Efetivo
	F	Empregado Efetivo
	F	Empregado Efetivo
	F	Empregado Efetivo
	F	Empregado Efetivo
	F	Empregado Efetivo
	F	Empregado Efetivo
	F	Empregado Efetivo
	F	Empregado Efetivo
	F	Empregado Efetivo
	F	Empregado Efetivo
	F	Empregado Efetivo
	F	Empregado Efetivo
	F	Empregado Efetivo
	F	Empregado Efetivo
	F	Empregado Efetivo
	F	Empregado Efetivo
	F	Empregado Efetivo
	F	Empregado Efetivo
	F	Empregado Efetivo
	F	Empregado Efetivo
	F	Empregado Efetivo
	F	Empregado Efetivo

	M	Empregado Efetivo
	F	Empregado Efetivo
	F	Empregado Efetivo
	F	Empregado Efetivo
	F	Empregado Efetivo
	F	Empregado Efetivo
	F	Empregado Efetivo
	F	Empregado Efetivo
	F	Empregado Efetivo
	F	Empregado Efetivo
	M	Empregado Efetivo
	M	Empregado Efetivo
	M	Empregado Efetivo
	F	Empregado Efetivo
	F	Empregado Efetivo
	F	Empregado Efetivo
	F	Empregado Efetivo
	M	Empregado Efetivo
	F	Empregado Efetivo
	M	Empregado Efetivo
	M	Empregado Efetivo
	M	Empregado Efetivo
	M	Empregado Efetivo
	F	Empregado Efetivo
	M	Empregado Efetivo
	F	Empregado Efetivo
	F	Empregado Efetivo
	F	Empregado Efetivo
	F	Empregado Efetivo
	F	Empregado Efetivo
	F	Empregado Efetivo
	M	Empregado Efetivo
	M	Empregado Efetivo
	M	Empregado Efetivo
	M	Empregado Efetivo

	M	Empregado Efetivo
	M	Empregado Efetivo
	M	Empregado Efetivo
	M	Empregado Efetivo
	M	Empregado Efetivo
	M	Empregado Efetivo
	M	Empregado Efetivo
	M	Empregado Efetivo
	M	Empregado Efetivo
	F	Empregado Efetivo
	M	Empregado Efetivo
	M	Empregado Efetivo
	F	Empregado Efetivo
	F	Empregado Efetivo
	F	Empregado Efetivo
	M	Empregado Efetivo
	F	Empregado Efetivo
	F	Empregado Efetivo
	F	Empregado Efetivo
	M	Empregado Efetivo
	F	Empregado Efetivo
	F	Empregado Efetivo
	F	Empregado Efetivo
	F	Empregado Efetivo
	F	Empregado Efetivo
	M	Empregado Efetivo
	F	Empregado Efetivo
	F	Empregado Efetivo
	F	Empregado Efetivo
	F	Empregado Efetivo
	F	Empregado Efetivo
	F	Empregado Efetivo

	F	Empregado Efetivo
	F	Empregado Efetivo
	F	Empregado Efetivo
	F	Empregado Efetivo
	F	Empregado Efetivo
	M	Empregado Efetivo
	F	Empregado Efetivo
	F	Empregado Efetivo
	M	Empregado Efetivo
	F	Empregado Efetivo
	F	Empregado Efetivo
	F	Empregado Efetivo
	F	Empregado Efetivo
	M	Empregado Efetivo
	M	Empregado Efetivo
	M	Empregado Efetivo
	M	Empregado Efetivo
	M	Empregado Efetivo
	M	Empregado Efetivo
	M	Empregado Efetivo
	F	Empregado Efetivo
	M	Empregado Efetivo
	M	Empregado Efetivo
	F	Empregado Efetivo
	F	Empregado Efetivo
	F	Empregado Efetivo
	F	Empregado Efetivo
	F	Empregado Efetivo
	F	Empregado Efetivo
	F	Empregado Efetivo
	F	Empregado Efetivo
	F	Empregado Efetivo
	F	Empregado Efetivo
	F	Empregado Efetivo
	F	Empregado Efetivo
	M	Empregado Efetivo
	F	Empregado Efetivo

	F	Empregado Efetivo
	M	Empregado Efetivo
	M	Empregado Efetivo
	M	Empregado Efetivo
	F	Empregado Efetivo
	F	Empregado Efetivo
	F	Empregado Efetivo
	F	Empregado Efetivo
	M	Empregado Efetivo
	F	Empregado Efetivo
	F	Empregado Efetivo
	F	Empregado Efetivo
	M	Empregado Efetivo
	F	Empregado Efetivo
	M	Empregado Efetivo
	F	Empregado Efetivo
	F	Empregado Efetivo
	F	Empregado Efetivo
	F	Empregado Efetivo
	M	Empregado Efetivo
	F	Empregado Efetivo
	F	Empregado Efetivo
	F	Empregado Efetivo
	F	Empregado Efetivo
	M	Empregado Efetivo
	F	Empregado Efetivo
	M	Empregado Efetivo
	F	Empregado Efetivo
	F	Empregado Efetivo
	F	Empregado Efetivo
	M	Empregado Efetivo
	F	Empregado Efetivo
	M	Empregado Efetivo
	F	Empregado Efetivo
	F	Empregado Efetivo
	F	Empregado Efetivo
	F	Empregado Efetivo
	M	Empregado Efetivo
	M	Empregado Efetivo
	M	Empregado Efetivo
	M	Empregado Efetivo
	M	Empregado Efetivo
	F	Empregado Efetivo
	M	Empregado Efetivo
	M	Empregado Efetivo
	F	Empregado Efetivo
	M	Empregado Efetivo

	F	Empregado Efetivo
	F	Empregado Efetivo
	M	Empregado Efetivo
	F	Empregado Efetivo
	F	Empregado Efetivo
	M	Empregado Efetivo
	M	Empregado Efetivo
	M	Empregado Efetivo
	F	Empregado Efetivo
	F	Empregado Efetivo
	M	Empregado Efetivo
	M	Empregado Efetivo
	M	Empregado Efetivo
	M	Empregado Efetivo
	F	Empregado Efetivo
	F	Empregado Efetivo
	M	Empregado Efetivo
	F	Empregado Efetivo
	F	Empregado Efetivo
	M	Empregado Efetivo
	M	Empregado Efetivo
	M	Empregado Efetivo
	F	Empregado Efetivo
	M	Empregado Efetivo
	M	Empregado Efetivo
	F	Empregado Efetivo
	M	Empregado Efetivo
	M	Empregado Efetivo
	M	Empregado Efetivo
	M	Empregado Efetivo
	M	Empregado Efetivo
	F	Empregado Efetivo
	F	Empregado Efetivo
	F	Empregado Efetivo
	F	Empregado Efetivo
	F	Empregado Efetivo
	F	Empregado Efetivo
	F	Empregado Efetivo
	F	Empregado Efetivo
	F	Empregado Efetivo
	F	Empregado Efetivo
	F	Empregado Efetivo
	F	Empregado Efetivo
	F	Empregado Efetivo

	F	Empregado Efetivo
	F	Empregado Efetivo
	F	Empregado Efetivo
	F	Empregado Efetivo
	F	Empregado Efetivo
	F	Empregado Efetivo
	F	Empregado Efetivo
	F	Empregado Efetivo
	F	Empregado Efetivo
	F	Empregado Efetivo
	F	Empregado Efetivo
	F	Empregado Efetivo
	F	Empregado Efetivo
	F	Empregado Efetivo
	F	Empregado Efetivo
	F	Empregado Efetivo
	F	Empregado Efetivo
	F	Empregado Efetivo
	F	Empregado Efetivo
	F	Empregado Efetivo
	F	Empregado Efetivo
	F	Empregado Efetivo
	F	Empregado Efetivo
	F	Empregado Efetivo
	F	Empregado Efetivo
	F	Empregado Efetivo
	F	Empregado Efetivo
	F	Empregado Efetivo
	F	Empregado Efetivo
	F	Empregado Efetivo
	F	Empregado Efetivo
	F	Empregado Efetivo
	F	Empregado Efetivo
	F	Empregado Efetivo
	F	Empregado Efetivo
	F	Empregado Efetivo
	F	Empregado Efetivo

	F	Empregado Efetivo
	F	Empregado Efetivo
	F	Empregado Efetivo
	F	Empregado Efetivo
	M	Empregado Efetivo
	F	Empregado Efetivo
	F	Empregado Efetivo
	F	Empregado Efetivo
	F	Empregado Efetivo
	F	Empregado Efetivo
	F	Empregado Efetivo
	F	Empregado Efetivo
	F	Empregado Efetivo
	F	Empregado Efetivo
	F	Empregado Efetivo
	F	Empregado Efetivo
	F	Empregado Efetivo
	F	Empregado Efetivo
	F	Empregado Efetivo
	F	Empregado Efetivo
	F	Empregado Efetivo
	F	Empregado Efetivo
	F	Empregado Efetivo
	F	Empregado Efetivo
	F	Empregado Efetivo
	F	Empregado Efetivo
	F	Empregado Efetivo
	F	Empregado Efetivo
	F	Empregado Efetivo
	F	Empregado Efetivo
	F	Empregado Efetivo
	F	Empregado Efetivo
	F	Empregado Efetivo
	F	Empregado Efetivo
	F	Empregado Efetivo
	F	Empregado Efetivo
	F	Empregado Efetivo
	F	Empregado Efetivo
	F	Empregado Efetivo
	F	Empregado Efetivo
	F	Empregado Efetivo

	F	Empregado Efetivo
	F	Empregado Efetivo
	F	Empregado Efetivo
	F	Empregado Efetivo
	F	Empregado Efetivo
	F	Empregado Efetivo
	F	Empregado Efetivo
	F	Empregado Efetivo
	F	Empregado Efetivo
	F	Empregado Efetivo
	F	Empregado Efetivo
	F	Empregado Efetivo
	F	Empregado Efetivo
	F	Empregado Efetivo
	F	Empregado Efetivo
	F	Empregado Efetivo
	F	Empregado Efetivo
	F	Empregado Efetivo
	F	Empregado Efetivo
	F	Empregado Efetivo
	F	Empregado Efetivo
	F	Empregado Efetivo
	F	Empregado Efetivo
	F	Empregado Efetivo
	F	Empregado Efetivo
	F	Empregado Efetivo
	F	Empregado Efetivo
	F	Empregado Efetivo
	M	Empregado Efetivo
	M	Empregado Efetivo
	F	Empregado Efetivo
	F	Empregado Efetivo

	F	Empregado Efetivo
	F	Empregado Efetivo
	M	Empregado Efetivo
	F	Empregado Efetivo
	F	Empregado Efetivo
	F	Empregado Efetivo
	F	Empregado Efetivo
	F	Empregado Efetivo
	F	Empregado Efetivo
	M	Empregado Efetivo
	F	Empregado Efetivo
	F	Empregado Efetivo
	F	Empregado Efetivo
	F	Empregado Efetivo
	F	Empregado Efetivo
	F	Empregado Efetivo
	F	Empregado Efetivo
	F	Empregado Efetivo
	F	Empregado Efetivo
	F	Empregado Efetivo
	F	Empregado Efetivo
	F	Empregado Efetivo
	F	Empregado Efetivo
	F	Empregado Efetivo
	F	Empregado Efetivo
	F	Empregado Efetivo
	F	Empregado Efetivo
	F	Empregado Efetivo
	F	Empregado Efetivo
	F	Empregado Efetivo
	F	Empregado Efetivo
	F	Empregado Efetivo
	F	Empregado Efetivo
	F	Empregado Efetivo
	F	Empregado Efetivo
	F	Empregado Efetivo
	M	Empregado Efetivo
	F	Empregado Efetivo
	F	Empregado Efetivo
	M	Empregado Efetivo
	F	Empregado Efetivo
	M	Empregado Efetivo

	F	Empregado Efetivo
	F	Empregado Efetivo
	F	Empregado Efetivo
	M	Empregado Efetivo
	M	Empregado Efetivo
	F	Empregado Efetivo
	F	Empregado Efetivo
	F	Empregado Efetivo
	M	Empregado Efetivo
	F	Empregado Efetivo
	F	Empregado Efetivo
	F	Empregado Efetivo
	F	Empregado Efetivo
	M	Empregado Efetivo
	F	Empregado Efetivo
	F	Empregado Efetivo
	F	Empregado Efetivo
	M	Empregado Efetivo
	F	Empregado Efetivo
	F	Empregado Efetivo
	F	Empregado Efetivo
	F	Empregado Efetivo
	F	Empregado Efetivo
	F	Empregado Efetivo
	F	Empregado Efetivo
	M	Empregado Efetivo
	M	Empregado Efetivo
	F	Empregado Efetivo
	F	Empregado Efetivo
	F	Empregado Efetivo
	F	Empregado Efetivo
	F	Empregado Efetivo
	F	Empregado Efetivo
	M	Empregado Efetivo
	F	Empregado Efetivo
	M	Empregado Efetivo
	F	Empregado Efetivo
	F	Empregado Efetivo
	F	Empregado Efetivo
	F	Empregado Efetivo
	F	Empregado Efetivo
	F	Empregado Efetivo
	M	Empregado Efetivo
	M	Empregado Efetivo
	M	Empregado Efetivo
	F	Empregado Efetivo
	M	Empregado Efetivo
	M	Empregado Efetivo

	F	Empregado Efetivo
	M	Empregado Efetivo
	M	Empregado Efetivo
	M	Empregado Efetivo
	M	Empregado Efetivo
	F	Empregado Efetivo
	M	Empregado Efetivo
	M	Empregado Efetivo
	F	Empregado Efetivo
	M	Empregado Efetivo
	F	Empregado Efetivo
	M	Empregado Efetivo
	M	Empregado Efetivo
	M	Empregado Efetivo
	F	Empregado Efetivo
	M	Empregado Efetivo
	M	Empregado Efetivo
	F	Empregado Efetivo
	F	Empregado Efetivo
	F	Empregado Efetivo
	M	Empregado Efetivo
	F	Empregado Efetivo
	F	Empregado Efetivo
	M	Empregado Efetivo
	F	Empregado Efetivo
	M	Empregado Efetivo
	F	Empregado Efetivo
	F	Empregado Efetivo
	M	Empregado Efetivo
	M	Empregado Efetivo
	M	Empregado Efetivo
	M	Empregado Efetivo
	F	Empregado Efetivo
	F	Empregado Efetivo
	F	Empregado Efetivo
	F	Empregado Efetivo
	F	Empregado Efetivo
	F	Empregado Efetivo
	F	Empregado Efetivo
	F	Empregado Efetivo
	F	Empregado Efetivo
	F	Empregado Efetivo
	F	Empregado Efetivo
	F	Empregado Efetivo
	F	Empregado Efetivo
	F	Empregado Efetivo
	F	Empregado Efetivo
	F	Empregado Efetivo

	F	Empregado Efetivo
	F	Empregado Efetivo
	F	Empregado Efetivo
	F	Empregado Efetivo
	F	Empregado Efetivo
	F	Empregado Efetivo
	F	Empregado Efetivo
	F	Empregado Efetivo
	F	Empregado Efetivo
	F	Empregado Efetivo
	F	Empregado Efetivo
	F	Empregado Efetivo
	F	Empregado Efetivo
	F	Empregado Efetivo
	F	Empregado Efetivo
	F	Empregado Efetivo
	F	Empregado Efetivo
	F	Empregado Efetivo
	F	Empregado Efetivo
	F	Empregado Efetivo
	F	Empregado Efetivo
	F	Empregado Efetivo
	F	Empregado Efetivo
	F	Empregado Efetivo
	M	Empregado Efetivo
	F	Empregado Efetivo
	F	Empregado Efetivo
	F	Empregado Efetivo
	F	Empregado Efetivo
	M	Empregado Efetivo
	F	Empregado Efetivo
	F	Empregado Efetivo
	F	Empregado Efetivo
	F	Empregado Efetivo
	F	Empregado Efetivo
	F	Empregado Efetivo
	F	Empregado Efetivo
	F	Empregado Efetivo
	F	Empregado Efetivo
	F	Empregado Efetivo
	M	Empregado Efetivo
	F	Empregado Efetivo
	F	Empregado Efetivo
	F	Empregado Efetivo
	F	Empregado Efetivo
	F	Empregado Efetivo
	F	Empregado Efetivo
	F	Empregado Efetivo

	F	Empregado Efetivo
	F	Empregado Efetivo
	F	Empregado Efetivo
	F	Empregado Efetivo
	F	Empregado Efetivo
	F	Empregado Efetivo
	F	Empregado Efetivo
	F	Empregado Efetivo
	F	Empregado Efetivo
	F	Empregado Efetivo
	F	Empregado Efetivo
	F	Empregado Efetivo
	F	Empregado Efetivo
	F	Empregado Efetivo
	F	Empregado Efetivo
	F	Empregado Efetivo
	F	Empregado Efetivo
	F	Empregado Efetivo
	F	Empregado Efetivo
	F	Empregado Efetivo
	M	Empregado Efetivo
	F	Empregado Efetivo
	F	Empregado Efetivo
	F	Empregado Efetivo
	F	Empregado Efetivo
	F	Empregado Efetivo
	M	Empregado Efetivo
	M	Empregado Efetivo
	F	Empregado Efetivo
	F	Empregado Efetivo
	F	Empregado Efetivo
	F	Empregado Efetivo
	F	Empregado Efetivo
	F	Empregado Efetivo
	F	Empregado Efetivo
	F	Empregado Efetivo
	F	Empregado Efetivo
	F	Empregado Efetivo
	F	Empregado Efetivo
	F	Empregado Efetivo
	F	Empregado Efetivo
	F	Empregado Efetivo
	M	Empregado Efetivo
	F	Empregado Efetivo
	F	Empregado Efetivo
	M	Empregado Efetivo
	F	Empregado Efetivo
	F	Empregado Efetivo

	F	Empregado Efetivo
	F	Empregado Efetivo
	M	Empregado Efetivo
	F	Empregado Efetivo
	F	Empregado Efetivo
	F	Empregado Efetivo
	F	Empregado Efetivo
	F	Empregado Efetivo
	F	Empregado Efetivo
	F	Empregado Efetivo
	F	Empregado Efetivo
	F	Empregado Efetivo
	F	Empregado Efetivo
	M	Empregado Efetivo
	F	Empregado Efetivo
	F	Empregado Efetivo
	F	Empregado Efetivo
	F	Empregado Efetivo
	M	Empregado Efetivo
	F	Empregado Efetivo
	F	Empregado Efetivo
	F	Empregado Efetivo
	F	Empregado Efetivo
	F	Empregado Efetivo
	F	Empregado Efetivo
	F	Empregado Efetivo
	F	Empregado Efetivo
	F	Empregado Efetivo
	F	Empregado Efetivo
	F	Empregado Efetivo
	F	Empregado Efetivo
	M	Empregado Efetivo
	F	Empregado Efetivo
	F	Empregado Efetivo
	F	Empregado Efetivo
	F	Empregado Efetivo
	F	Empregado Efetivo
	F	Empregado Efetivo
	M	Empregado Efetivo
	M	Empregado Efetivo
	M	Empregado Efetivo
	F	Empregado Efetivo
	F	Empregado Efetivo
	F	Empregado Efetivo
	F	Empregado Efetivo
	F	Empregado Efetivo
	F	Empregado Efetivo
	F	Empregado Efetivo

	M	Empregado Efetivo
	M	Empregado Efetivo
	F	Empregado Efetivo
	F	Empregado Efetivo
	M	Empregado Efetivo
	F	Empregado Efetivo
	F	Empregado Efetivo
	F	Empregado Efetivo
	F	Empregado Efetivo
	F	Empregado Efetivo
	F	Empregado Efetivo
	F	Empregado Efetivo
	M	Empregado Efetivo
	F	Empregado Efetivo
	F	Empregado Efetivo
	F	Empregado Efetivo
	F	Empregado Efetivo
	F	Empregado Efetivo
	F	Empregado Efetivo
	F	Empregado Efetivo
	F	Empregado Efetivo
	F	Empregado Efetivo
	F	Empregado Efetivo
	F	Empregado Efetivo
	F	Empregado Efetivo
	F	Empregado Efetivo
	F	Empregado Efetivo
	F	Empregado Efetivo
	M	Empregado Efetivo
	F	Empregado Efetivo
	M	Empregado Efetivo
	F	Empregado Efetivo
	M	Empregado Efetivo
	F	Empregado Efetivo
	F	Empregado Efetivo
	F	Empregado Efetivo
	M	Empregado Efetivo
	F	Empregado Efetivo
	F	Empregado Efetivo
	M	Empregado Efetivo
	M	Empregado Efetivo
	F	Empregado Efetivo
	F	Empregado Efetivo
	M	Empregado Efetivo
	F	Empregado Efetivo
	F	Empregado Efetivo
	F	Empregado Efetivo
	F	Empregado Efetivo

	M	Empregado Efetivo
	M	Empregado Efetivo
	M	Empregado Efetivo
	F	Empregado Efetivo
	F	Empregado Efetivo
	F	Empregado Efetivo
	F	Empregado Efetivo
	F	Empregado Efetivo
	F	Empregado Efetivo
	F	Empregado Efetivo
	F	Empregado Efetivo
	F	Empregado Efetivo
	F	Empregado Efetivo
	F	Empregado Efetivo
	F	Empregado Efetivo
	F	Empregado Efetivo
	F	Empregado Efetivo
	M	Empregado Efetivo
	F	Empregado Efetivo
	F	Empregado Efetivo
	M	Empregado Efetivo
	F	Empregado Efetivo
	F	Empregado Efetivo
	M	Empregado Efetivo
	F	Empregado Efetivo
	M	Empregado Efetivo
	F	Empregado Efetivo
	F	Empregado Efetivo
	F	Empregado Efetivo
	F	Empregado Efetivo
	F	Empregado Efetivo
	F	Empregado Efetivo
	F	Empregado Efetivo
	M	Empregado Efetivo
	F	Empregado Efetivo
	F	Empregado Efetivo
	F	Empregado Efetivo
	F	Empregado Efetivo
	M	Empregado Efetivo
	F	Empregado Efetivo
	F	Empregado Efetivo
	F	Empregado Efetivo
	F	Empregado Efetivo
	F	Empregado Efetivo
	F	Empregado Efetivo
	M	Empregado Efetivo
	M	Empregado Efetivo
	F	Empregado Efetivo

	M	Empregado Efetivo
	F	Empregado Efetivo
	F	Empregado Efetivo
	F	Empregado Efetivo
	F	Empregado Efetivo
	F	Empregado Efetivo
	F	Empregado Efetivo
	F	Empregado Efetivo
	F	Empregado Efetivo
	M	Empregado Efetivo
	F	Empregado Efetivo
	M	Empregado Efetivo
	F	Empregado Efetivo
	F	Empregado Efetivo
	F	Empregado Efetivo
	M	Empregado Efetivo
	F	Empregado Efetivo
	F	Empregado Efetivo
	F	Empregado Efetivo
	F	Empregado Efetivo
	F	Empregado Efetivo
	M	Empregado Efetivo
	F	Empregado Efetivo
	F	Empregado Efetivo
	F	Empregado Efetivo
	M	Empregado Efetivo
	F	Empregado Efetivo
	F	Empregado Efetivo
	F	Empregado Efetivo
	M	Empregado Efetivo
	M	Empregado Efetivo
	F	Empregado Efetivo
	M	Empregado Efetivo
	F	Empregado Efetivo
	M	Empregado Efetivo
	F	Empregado Efetivo
	F	Empregado Efetivo
	F	Empregado Efetivo
	F	Empregado Efetivo
	F	Empregado Efetivo
	F	Empregado Efetivo
	F	Empregado Efetivo
	F	Empregado Efetivo
	F	Empregado Efetivo
	M	Empregado Efetivo
	F	Empregado Efetivo
	F	Empregado Efetivo
	F	Empregado Efetivo

	F	Empregado Efetivo
	F	Empregado Efetivo
	M	Empregado Efetivo
	F	Empregado Efetivo
	F	Empregado Efetivo
	F	Empregado Efetivo
	F	Empregado Efetivo
	F	Empregado Efetivo
	F	Empregado Efetivo
	F	Empregado Efetivo
	F	Empregado Efetivo
	F	Empregado Efetivo
	F	Empregado Efetivo
	F	Empregado Efetivo
	M	Empregado Efetivo
	F	Empregado Efetivo
	M	Empregado Efetivo
	F	Empregado Efetivo
	F	Empregado Efetivo
	F	Empregado Efetivo
	F	Empregado Efetivo
	M	Empregado Efetivo
	F	Empregado Efetivo
	F	Empregado Efetivo
	M	Empregado Efetivo
	F	Empregado Efetivo
	M	Empregado Efetivo
	M	Empregado Efetivo
	M	Empregado Efetivo
	M	Empregado Efetivo
	M	Empregado Efetivo
	M	Empregado Efetivo
	F	Empregado Efetivo
	M	Empregado Efetivo
	M	Empregado Efetivo
	F	Empregado Efetivo
	M	Empregado Efetivo
	M	Empregado Efetivo
	M	Empregado Efetivo
	M	Empregado Efetivo
	M	Empregado Efetivo
	M	Empregado Efetivo
	M	Empregado Efetivo
	M	Empregado Efetivo
	F	Empregado Efetivo
	M	Empregado Efetivo
	M	Empregado Efetivo
	M	Empregado Efetivo

	F	Empregado Efetivo
	M	Empregado Efetivo
	F	Empregado Efetivo
	F	Empregado Efetivo
	F	Empregado Efetivo
	F	Empregado Efetivo
	F	Empregado Efetivo
	M	Empregado Efetivo
	F	Empregado Efetivo
	F	Empregado Efetivo
	F	Empregado Efetivo
	M	Empregado Efetivo
	F	Empregado Efetivo
	F	Empregado Efetivo
	M	Empregado Efetivo
	F	Empregado Efetivo
	F	Empregado Efetivo
	F	Empregado Efetivo
	F	Empregado Efetivo
	F	Empregado Efetivo
	F	Empregado Efetivo
	F	Empregado Efetivo
	F	Empregado Efetivo
	F	Empregado Efetivo
	F	Empregado Efetivo
	F	Empregado Efetivo
	F	Empregado Efetivo
	F	Empregado Efetivo
	F	Empregado Efetivo
	F	Empregado Efetivo
	F	Empregado Efetivo
	F	Empregado Efetivo
	F	Empregado Efetivo
	F	Empregado Efetivo
	F	Empregado Efetivo
	F	Empregado Efetivo
	F	Empregado Efetivo
	F	Empregado Efetivo
	F	Empregado Efetivo
	F	Empregado Efetivo
	F	Empregado Efetivo
	F	Empregado Efetivo
	F	Empregado Efetivo
	M	Empregado Efetivo
	F	Empregado Efetivo
	F	Empregado Efetivo
	F	Empregado Efetivo
	F	Empregado Efetivo

	F	Empregado Efetivo
	F	Empregado Efetivo
	F	Empregado Efetivo
	F	Empregado Efetivo
	M	Empregado Efetivo
	F	Empregado Efetivo
	F	Empregado Efetivo
	F	Empregado Efetivo
	M	Empregado Efetivo
	F	Empregado Efetivo
	F	Empregado Efetivo
	F	Empregado Efetivo
	F	Empregado Efetivo
	F	Empregado Efetivo
	F	Empregado Efetivo
	F	Empregado Efetivo
	F	Empregado Efetivo
	F	Empregado Efetivo
	F	Empregado Efetivo
	M	Empregado Efetivo
	F	Empregado Efetivo
	M	Empregado Efetivo
	F	Empregado Efetivo
	M	Empregado Efetivo
	F	Empregado Efetivo
	F	Empregado Efetivo
	F	Empregado Efetivo
	F	Empregado Efetivo
	F	Empregado Efetivo
	F	Empregado Efetivo
	F	Empregado Efetivo
	F	Empregado Efetivo
	F	Empregado Efetivo
	M	Empregado Efetivo
	F	Empregado Efetivo
	M	Empregado Efetivo
	M	Empregado Efetivo
	F	Empregado Efetivo
	F	Empregado Efetivo
	F	Empregado Efetivo
	F	Empregado Efetivo
	F	Empregado Efetivo
	M	Empregado Efetivo
	M	Empregado Efetivo
	F	Empregado Efetivo
	F	Empregado Efetivo
	F	Empregado Efetivo

	F	Empregado Efetivo
	M	Empregado Efetivo
	M	Empregado Efetivo
	F	Empregado Efetivo
	F	Empregado Efetivo
	F	Empregado Efetivo
	F	Empregado Efetivo
	F	Empregado Efetivo
	F	Empregado Efetivo
	F	Empregado Efetivo
	F	Empregado Efetivo
	M	Empregado Efetivo
	M	Empregado Efetivo
	F	Empregado Efetivo
	F	Empregado Efetivo
	F	Empregado Efetivo
	F	Empregado Efetivo
	M	Empregado Efetivo
	F	Empregado Efetivo
	F	Empregado Efetivo
	F	Empregado Efetivo
	F	Empregado Efetivo
	M	Empregado Efetivo
	F	Empregado Efetivo
	F	Empregado Efetivo
	F	Empregado Efetivo
	F	Empregado Efetivo
	F	Empregado Efetivo
	F	Empregado Efetivo
	F	Empregado Efetivo
	F	Empregado Efetivo
	F	Empregado Efetivo
	F	Empregado Efetivo
	F	Empregado Efetivo
	F	Empregado Efetivo
	F	Empregado Efetivo
	F	Empregado Efetivo
	F	Empregado Efetivo
	F	Empregado Efetivo
	F	Empregado Efetivo
	F	Empregado Efetivo
	F	Empregado Efetivo
	F	Empregado Efetivo
	F	Empregado Efetivo
	F	Empregado Efetivo
	F	Empregado Efetivo
	F	Empregado Efetivo
	F	Empregado Efetivo

	F	Empregado Efetivo
	F	Empregado Efetivo
	F	Empregado Efetivo
	M	Empregado Efetivo
	F	Empregado Efetivo
	M	Empregado Efetivo
	F	Empregado Efetivo
	M	Empregado Efetivo
	F	Empregado Efetivo
	M	Empregado Efetivo
	F	Empregado Efetivo
	M	Empregado Efetivo
	F	Empregado Efetivo
	F	Empregado Efetivo
	M	Empregado Efetivo
	M	Empregado Efetivo
	F	Empregado Efetivo
	F	Empregado Efetivo
	F	Empregado Efetivo
	F	Empregado Efetivo
	M	Empregado Efetivo
	F	Empregado Efetivo
	F	Empregado Efetivo
	F	Empregado Efetivo
	F	Empregado Efetivo
	F	Empregado Efetivo
	F	Empregado Efetivo
	F	Empregado Efetivo
	F	Empregado Efetivo
	F	Empregado Efetivo
	F	Empregado Efetivo
	F	Empregado Efetivo
	F	Empregado Efetivo
	F	Empregado Efetivo
	F	Empregado Efetivo
	F	Empregado Efetivo
	M	Empregado Efetivo
	M	Empregado Efetivo
	M	Empregado Efetivo
	F	Empregado Efetivo
	M	Empregado Efetivo
	F	Empregado Efetivo
	F	Empregado Efetivo
	F	Empregado Efetivo
	F	Empregado Efetivo
	F	Empregado Efetivo

	F	Empregado Efetivo
	F	Empregado Efetivo
	F	Empregado Efetivo
	M	Empregado Efetivo
	M	Empregado Efetivo
	F	Empregado Efetivo
	F	Empregado Efetivo
	F	Empregado Efetivo
	M	Empregado Efetivo
	F	Empregado Efetivo
	F	Empregado Efetivo
	F	Empregado Efetivo
	F	Empregado Efetivo
	F	Empregado Efetivo
	F	Empregado Efetivo
	F	Empregado Efetivo
	F	Empregado Efetivo
	F	Empregado Efetivo
	F	Empregado Efetivo
	F	Empregado Efetivo
	F	Empregado Efetivo
	F	Empregado Efetivo
	F	Empregado Efetivo
	F	Empregado Efetivo
	F	Empregado Efetivo
	F	Empregado Efetivo
	F	Empregado Efetivo
	F	Empregado Efetivo
	F	Empregado Efetivo
	F	Empregado Efetivo
	F	Empregado Efetivo
	F	Empregado Efetivo
	F	Empregado Efetivo
	F	Empregado Efetivo
	F	Empregado Efetivo
	F	Empregado Efetivo
	F	Empregado Efetivo
	M	Empregado Efetivo
	M	Empregado Efetivo
	F	Empregado Efetivo
	F	Empregado Efetivo
	F	Empregado Efetivo
	F	Empregado Efetivo
	F	Empregado Efetivo
	F	Empregado Efetivo
	F	Empregado Efetivo
	M	Empregado Efetivo
	F	Empregado Efetivo

	F	Empregado Efetivo
	F	Empregado Efetivo
	F	Empregado Efetivo
	F	Empregado Efetivo
	F	Empregado Efetivo
	F	Empregado Efetivo
	F	Empregado Efetivo
	F	Empregado Efetivo
	M	Empregado Efetivo
	F	Empregado Efetivo
	F	Empregado Efetivo
	F	Empregado Efetivo
	F	Empregado Efetivo
	M	Empregado Efetivo
	F	Empregado Efetivo
	M	Empregado Efetivo
	M	Empregado Efetivo
	F	Empregado Efetivo
	F	Empregado Efetivo
	F	Empregado Efetivo
	F	Empregado Efetivo
	F	Empregado Efetivo
	F	Empregado Efetivo
	F	Empregado Efetivo
	F	Empregado Efetivo
	M	Empregado Efetivo
	F	Empregado Efetivo
	F	Empregado Efetivo
	F	Empregado Efetivo
	F	Empregado Efetivo
	F	Empregado Efetivo
	F	Empregado Efetivo
	M	Empregado Efetivo
	M	Empregado Efetivo
	F	Empregado Efetivo
	M	Empregado Efetivo
	M	Empregado Efetivo
	F	Empregado Efetivo
	F	Empregado Efetivo
	F	Empregado Efetivo
	F	Empregado Efetivo
	F	Empregado Efetivo
	F	Empregado Efetivo
	F	Empregado Efetivo
	F	Empregado Efetivo
	F	Empregado Efetivo
	F	Empregado Efetivo
	F	Empregado Efetivo

	M	Empregado Efetivo
	F	Empregado Efetivo
	F	Empregado Efetivo
	M	Empregado Efetivo
	F	Empregado Efetivo
	M	Empregado Efetivo
	F	Empregado Efetivo
	F	Empregado Efetivo
	F	Empregado Efetivo
	F	Empregado Efetivo
	F	Empregado Efetivo
	M	Empregado Efetivo
	F	Empregado Efetivo
	F	Empregado Efetivo
	F	Empregado Efetivo
	M	Empregado Efetivo
	M	Empregado Efetivo
	F	Empregado Efetivo
	F	Empregado Efetivo
	M	Empregado Efetivo
	M	Empregado Efetivo
	F	Empregado Efetivo
	F	Empregado Efetivo
	F	Empregado Efetivo
	M	Empregado Efetivo
	F	Empregado Efetivo
	F	Empregado Efetivo
	F	Empregado Efetivo
	F	Empregado Efetivo
	F	Empregado Efetivo
	M	Empregado Efetivo
	F	Empregado Efetivo
	F	Empregado Efetivo
	F	Empregado Efetivo
	F	Empregado Efetivo
	M	Empregado Efetivo
	F	Empregado Efetivo
	F	Empregado Efetivo
	F	Empregado Efetivo
	F	Empregado Efetivo
	F	Empregado Efetivo
	F	Empregado Efetivo
	F	Empregado Efetivo
	F	Empregado Efetivo
	F	Empregado Efetivo
	F	Empregado Efetivo
	F	Empregado Efetivo
	M	Empregado Efetivo

	F	Empregado Efetivo
	F	Empregado Efetivo
	F	Empregado Efetivo
	F	Empregado Efetivo
	M	Empregado Efetivo
	F	Empregado Efetivo
	M	Empregado Efetivo
	M	Empregado Efetivo
	F	Empregado Efetivo
	M	Empregado Efetivo
	F	Empregado Efetivo
	F	Empregado Efetivo
	F	Empregado Efetivo
	F	Empregado Efetivo
	F	Empregado Efetivo
	F	Empregado Efetivo
	F	Empregado Efetivo
	F	Empregado Efetivo
	F	Empregado Efetivo
	F	Empregado Efetivo
	F	Empregado Efetivo
	F	Empregado Efetivo
	F	Empregado Efetivo
	F	Empregado Efetivo
	F	Empregado Efetivo
	F	Empregado Efetivo
	F	Empregado Efetivo
	F	Empregado Efetivo
	F	Empregado Efetivo
	F	Empregado Efetivo
	F	Empregado Efetivo
	F	Empregado Efetivo
	F	Empregado Efetivo
	F	Empregado Efetivo
	F	Empregado Efetivo
	F	Empregado Efetivo
	F	Empregado Efetivo
	M	Empregado Efetivo
	F	Empregado Efetivo
	F	Empregado Efetivo
	F	Empregado Efetivo
	F	Empregado Efetivo
	F	Empregado Efetivo
	F	Empregado Efetivo
	F	Empregado Efetivo
	F	Empregado Efetivo
	M	Empregado Efetivo
	F	Empregado Efetivo

	F	Empregado Efetivo
	F	Empregado Efetivo
	F	Empregado Efetivo
	F	Empregado Efetivo
	F	Empregado Efetivo
	F	Empregado Efetivo
	F	Empregado Efetivo
	F	Empregado Efetivo
	F	Empregado Efetivo
	F	Empregado Efetivo
	F	Empregado Efetivo
	F	Empregado Efetivo
	F	Empregado Efetivo
	F	Empregado Efetivo
	F	Empregado Efetivo
	F	Empregado Efetivo
	F	Empregado Efetivo
	F	Empregado Efetivo
	F	Empregado Efetivo
	F	Empregado Efetivo
	F	Empregado Efetivo
	F	Empregado Efetivo
	M	Empregado Efetivo
	F	Empregado Efetivo
	F	Empregado Efetivo
	F	Empregado Efetivo
	F	Empregado Efetivo
	F	Empregado Efetivo
	F	Empregado Efetivo
	F	Empregado Efetivo
	F	Empregado Efetivo
	F	Empregado Efetivo
	F	Empregado Efetivo
	F	Empregado Efetivo
	F	Empregado Efetivo
	F	Empregado Efetivo
	M	Empregado Efetivo
	F	Empregado Efetivo
	F	Empregado Efetivo
	F	Empregado Efetivo
	F	Empregado Efetivo
	F	Empregado Efetivo
	F	Empregado Efetivo
	F	Empregado Efetivo
	F	Empregado Efetivo
	F	Empregado Efetivo
	F	Empregado Efetivo
	F	Empregado Efetivo

	F	Empregado Efetivo
	F	Empregado Efetivo
	F	Empregado Efetivo
	F	Empregado Efetivo
	F	Empregado Efetivo
	F	Empregado Efetivo
	F	Empregado Efetivo
	F	Empregado Efetivo
	F	Empregado Efetivo
	F	Empregado Efetivo
	F	Empregado Efetivo
	F	Empregado Efetivo
	F	Empregado Efetivo
	F	Empregado Efetivo
	F	Empregado Efetivo
	F	Empregado Efetivo
	F	Empregado Efetivo
	F	Empregado Efetivo
	F	Empregado Efetivo
	F	Empregado Efetivo
	F	Empregado Efetivo
	F	Empregado Efetivo
	M	Empregado Efetivo
	M	Empregado Efetivo
	F	Empregado Efetivo
	F	Empregado Efetivo
	F	Empregado Efetivo
	F	Empregado Efetivo
	F	Empregado Efetivo
	F	Empregado Efetivo
	F	Empregado Efetivo
	F	Empregado Efetivo
	F	Empregado Efetivo
	F	Empregado Efetivo
	F	Empregado Efetivo
	F	Empregado Efetivo
	F	Empregado Efetivo
	F	Empregado Efetivo
	F	Empregado Efetivo
	F	Empregado Efetivo
	F	Empregado Efetivo
	F	Empregado Efetivo
	F	Empregado Efetivo
	F	Empregado Efetivo
	F	Empregado Efetivo
	F	Empregado Efetivo
	M	Empregado Efetivo
	F	Empregado Efetivo

	M	Empregado Efetivo
	F	Empregado Efetivo
	F	Empregado Efetivo
	F	Empregado Efetivo
	F	Empregado Efetivo
	F	Empregado Efetivo
	M	Empregado Efetivo
	M	Empregado Efetivo
	F	Empregado Efetivo
	F	Empregado Efetivo
	F	Empregado Efetivo
	F	Empregado Efetivo
	F	Empregado Efetivo
	M	Empregado Efetivo
	M	Empregado Efetivo
	M	Empregado Efetivo
	M	Empregado Efetivo
	M	Empregado Efetivo
	F	Empregado Efetivo
	M	Empregado Efetivo
	M	Empregado Efetivo
	M	Empregado Efetivo
	F	Empregado Efetivo
	F	Empregado Efetivo
	M	Empregado Efetivo
	F	Empregado Efetivo
	F	Empregado Efetivo
	F	Empregado Efetivo
	F	Empregado Efetivo
	F	Empregado Efetivo
	F	Empregado Efetivo
	F	Empregado Efetivo
	F	Empregado Efetivo
	F	Empregado Efetivo
	F	Empregado Efetivo
	F	Empregado Efetivo
	F	Empregado Efetivo
	F	Empregado Efetivo
	F	Empregado Efetivo
	M	Empregado Efetivo
	F	Empregado Efetivo
	F	Empregado Efetivo
	F	Empregado Efetivo

	F	Empregado Efetivo
	F	Empregado Efetivo
	F	Empregado Efetivo
	M	Empregado Efetivo
	F	Empregado Efetivo
	F	Empregado Efetivo
	F	Empregado Efetivo
	F	Empregado Efetivo
	M	Empregado Efetivo
	F	Empregado Efetivo
	F	Empregado Efetivo
	F	Empregado Efetivo
	F	Empregado Efetivo
	F	Empregado Efetivo
	F	Empregado Efetivo
	M	Empregado Efetivo
	F	Empregado Efetivo
	F	Empregado Efetivo
	F	Empregado Efetivo
	F	Empregado Efetivo
	F	Empregado Efetivo
	F	Empregado Efetivo
	F	Empregado Efetivo
	F	Empregado Efetivo
	F	Empregado Efetivo
	M	Empregado Efetivo
	M	Empregado Efetivo
	F	Empregado Efetivo
	F	Empregado Efetivo
	M	Empregado Efetivo
	M	Empregado Efetivo
	M	Empregado Efetivo
	F	Empregado Efetivo
	F	Empregado Efetivo
	M	Empregado Efetivo
	M	Empregado Efetivo
	F	Empregado Efetivo
	F	Empregado Efetivo
	M	Empregado Efetivo
	M	Empregado Efetivo

	M	Empregado Efetivo
	M	Empregado Efetivo
	F	Empregado Efetivo
	F	Empregado Efetivo
	M	Empregado Efetivo
	M	Empregado Efetivo
	F	Empregado Efetivo
	M	Empregado Efetivo
	F	Empregado Efetivo
	F	Empregado Efetivo
	M	Empregado Efetivo
	F	Empregado Efetivo
	F	Empregado Efetivo
	M	Empregado Efetivo
	F	Empregado Efetivo
	F	Empregado Efetivo
	F	Empregado Efetivo
	F	Empregado Efetivo
	F	Empregado Efetivo
	F	Empregado Efetivo
	F	Empregado Efetivo
	F	Empregado Efetivo
	F	Empregado Efetivo
	F	Empregado Efetivo
	F	Empregado Efetivo
	F	Empregado Efetivo
	F	Empregado Efetivo
	F	Empregado Efetivo
	F	Empregado Efetivo
	F	Empregado Efetivo
	M	Empregado Efetivo
	M	Empregado Efetivo
	F	Empregado Efetivo
	F	Empregado Efetivo
	F	Empregado Efetivo
	F	Empregado Efetivo
	F	Empregado Efetivo
	F	Empregado Efetivo
	F	Empregado Efetivo
	F	Empregado Efetivo
	F	Empregado Efetivo
	M	Empregado Efetivo
	F	Empregado Efetivo
	F	Empregado Efetivo
	F	Empregado Efetivo
	F	Empregado Efetivo
	F	Empregado Efetivo
	F	Empregado Efetivo

	F	Empregado Efetivo
	F	Empregado Efetivo
	F	Empregado Efetivo
	F	Empregado Efetivo
	F	Empregado Efetivo
	F	Empregado Efetivo
	F	Empregado Efetivo
	F	Empregado Efetivo
	F	Empregado Efetivo
	F	Empregado Efetivo
	F	Empregado Efetivo
	F	Empregado Efetivo
	M	Empregado Efetivo
	F	Empregado Efetivo
	F	Empregado Efetivo
	F	Empregado Efetivo
	F	Empregado Efetivo
	F	Empregado Efetivo
	F	Empregado Efetivo
	M	Empregado Efetivo
	F	Empregado Efetivo
	F	Empregado Efetivo
	F	Empregado Efetivo
	F	Empregado Efetivo
	F	Empregado Efetivo
	F	Empregado Efetivo
	F	Empregado Efetivo
	F	Empregado Efetivo
	F	Empregado Efetivo
	F	Empregado Efetivo
	F	Empregado Efetivo
	F	Empregado Efetivo
	F	Empregado Efetivo
	F	Empregado Efetivo
	F	Empregado Efetivo
	F	Empregado Efetivo
	F	Empregado Efetivo
	F	Empregado Efetivo
	F	Empregado Efetivo
	F	Empregado Efetivo
	M	Empregado Efetivo
	F	Empregado Efetivo
	F	Empregado Efetivo
	F	Empregado Efetivo
	F	Empregado Efetivo
	F	Empregado Efetivo
	M	Empregado Efetivo
	M	Empregado Efetivo

	F	Empregado Efetivo
	F	Empregado Efetivo
	F	Empregado Efetivo
	F	Empregado Efetivo
	F	Empregado Efetivo
	F	Empregado Efetivo
	M	Empregado Efetivo
	M	Empregado Efetivo
	M	Empregado Efetivo
	F	Empregado Efetivo
	F	Empregado Efetivo
	F	Empregado Efetivo
	F	Empregado Efetivo
	F	Empregado Efetivo
	F	Empregado Efetivo
	F	Empregado Efetivo
	M	Empregado Efetivo
	M	Empregado Efetivo
	M	Empregado Efetivo
	F	Empregado Efetivo
	F	Empregado Efetivo
	M	Empregado Efetivo
	F	Empregado Efetivo
	F	Empregado Efetivo
	F	Empregado Efetivo
	F	Empregado Efetivo
	F	Empregado Efetivo
	F	Empregado Efetivo
	F	Empregado Efetivo
	F	Empregado Efetivo
	F	Empregado Efetivo
	F	Empregado Efetivo
	F	Empregado Efetivo
	F	Empregado Efetivo
	M	Empregado Efetivo
	M	Empregado Efetivo
	M	Empregado Efetivo
	F	Empregado Efetivo
	F	Empregado Efetivo
	F	Empregado Efetivo
	M	Empregado Efetivo
	F	Empregado Efetivo
	F	Empregado Efetivo
	F	Empregado Efetivo
	F	Empregado Efetivo
	F	Empregado Efetivo
	F	Empregado Efetivo

	F	Empregado Efetivo
	M	Empregado Efetivo
	F	Empregado Efetivo
	F	Empregado Efetivo
	M	Empregado Efetivo
	F	Empregado Efetivo
	F	Empregado Efetivo
	F	Empregado Efetivo
	F	Empregado Efetivo
	F	Empregado Efetivo
	F	Empregado Efetivo
	F	Empregado Efetivo
	F	Empregado Efetivo
	M	Empregado Efetivo
	F	Empregado Efetivo
	F	Empregado Efetivo
	F	Empregado Efetivo
	F	Empregado Efetivo
	M	Empregado Efetivo
	M	Empregado Efetivo
	F	Empregado Efetivo
	M	Empregado Efetivo
	F	Empregado Efetivo
	F	Empregado Efetivo
	M	Empregado Efetivo
	F	Empregado Efetivo
	F	Empregado Efetivo
	F	Empregado Efetivo
	F	Empregado Efetivo
	F	Empregado Efetivo
	F	Empregado Efetivo
	F	Empregado Efetivo
	F	Empregado Efetivo
	F	Empregado Efetivo
	F	Empregado Efetivo
	F	Empregado Efetivo
	F	Empregado Efetivo
	F	Empregado Efetivo
	F	Empregado Efetivo
	F	Empregado Efetivo
	F	Empregado Efetivo
	F	Empregado Efetivo
	F	Empregado Efetivo
	F	Empregado Efetivo
	F	Empregado Efetivo
	F	Empregado Efetivo
	F	Empregado Efetivo
	F	Empregado Efetivo

	F	Empregado Efetivo
	F	Empregado Efetivo
	F	Empregado Efetivo
	F	Empregado Efetivo
	M	Empregado Efetivo
	F	Empregado Efetivo
	F	Empregado Efetivo
	F	Empregado Efetivo
	F	Empregado Efetivo
	F	Empregado Efetivo
	F	Empregado Efetivo
	F	Empregado Efetivo
	F	Empregado Efetivo
	F	Empregado Efetivo
	F	Empregado Efetivo
	F	Empregado Efetivo
	F	Empregado Efetivo
	F	Empregado Efetivo
	F	Empregado Efetivo
	F	Empregado Efetivo
	F	Empregado Efetivo
	M	Empregado Efetivo
	F	Empregado Efetivo
	F	Empregado Efetivo
	M	Empregado Efetivo
	F	Empregado Efetivo
	F	Empregado Efetivo
	F	Empregado Efetivo
	F	Empregado Efetivo
	F	Empregado Efetivo
	F	Empregado Efetivo
	F	Empregado Efetivo
	F	Empregado Efetivo
	F	Empregado Efetivo
	F	Empregado Efetivo
	F	Empregado Efetivo
	F	Empregado Efetivo
	F	Empregado Efetivo
	F	Empregado Efetivo
	F	Empregado Efetivo
	F	Empregado Efetivo
	F	Empregado Efetivo
	F	Empregado Efetivo
	F	Empregado Efetivo
	F	Empregado Efetivo
	F	Empregado Efetivo
	F	Empregado Efetivo
	F	Empregado Efetivo

	F	Empregado Efetivo
	F	Empregado Efetivo
	F	Empregado Efetivo
	F	Empregado Efetivo
	F	Empregado Efetivo
	F	Empregado Efetivo
	F	Empregado Efetivo
	F	Empregado Efetivo
	F	Empregado Efetivo
	F	Empregado Efetivo
	F	Empregado Efetivo
	F	Empregado Efetivo
	F	Empregado Efetivo
	F	Empregado Efetivo
	F	Empregado Efetivo
	F	Empregado Efetivo
	F	Empregado Efetivo
	F	Empregado Efetivo
	F	Empregado Efetivo
	F	Empregado Efetivo
	F	Empregado Efetivo
	F	Empregado Efetivo
	F	Empregado Efetivo
	F	Empregado Efetivo
	F	Empregado Efetivo
	F	Empregado Efetivo
	F	Empregado Efetivo
	F	Empregado Efetivo
	F	Empregado Efetivo
	F	Empregado Efetivo
	F	Empregado Efetivo
	F	Empregado Efetivo
	F	Empregado Efetivo
	F	Empregado Efetivo
	F	Empregado Efetivo
	F	Empregado Efetivo
	F	Empregado Efetivo
	F	Empregado Efetivo
	F	Empregado Efetivo
	F	Empregado Efetivo
	F	Empregado Efetivo
	F	Empregado Efetivo
	F	Empregado Efetivo
	F	Empregado Efetivo
	F	Empregado Efetivo
	F	Empregado Efetivo
	F	Empregado Efetivo
	F	Empregado Efetivo

	F	Empregado Efetivo
	F	Empregado Efetivo
	F	Empregado Efetivo
	F	Empregado Efetivo
	F	Empregado Efetivo
	F	Empregado Efetivo
	F	Empregado Efetivo
	F	Empregado Efetivo
	F	Empregado Efetivo
	F	Empregado Efetivo
	F	Empregado Efetivo
	F	Empregado Efetivo
	F	Empregado Efetivo
	F	Empregado Efetivo
	F	Empregado Efetivo
	F	Empregado Efetivo
	F	Empregado Efetivo
	F	Empregado Efetivo
	F	Empregado Efetivo
	F	Empregado Efetivo
	F	Empregado Efetivo
	F	Empregado Efetivo
	F	Empregado Efetivo
	F	Empregado Efetivo
	F	Empregado Efetivo
	F	Empregado Efetivo
	F	Empregado Efetivo
	F	Empregado Efetivo
	F	Empregado Efetivo
	F	Empregado Efetivo
	F	Empregado Efetivo
	F	Empregado Efetivo
	F	Empregado Efetivo
	F	Empregado Efetivo
	F	Empregado Efetivo
	F	Empregado Efetivo
	F	Empregado Efetivo
	F	Empregado Efetivo
	F	Empregado Efetivo
	F	Empregado Efetivo
	F	Empregado Efetivo
	F	Empregado Efetivo
	F	Empregado Efetivo
	F	Empregado Efetivo
	F	Empregado Efetivo
	F	Empregado Efetivo
	F	Empregado Efetivo
	F	Empregado Efetivo

	F	Empregado Efetivo
	F	Empregado Efetivo
	F	Empregado Efetivo
	F	Empregado Efetivo
	F	Empregado Efetivo
	F	Empregado Efetivo
	F	Empregado Efetivo
	F	Empregado Efetivo
	F	Empregado Efetivo
	F	Empregado Efetivo
	F	Empregado Efetivo
	F	Empregado Efetivo
	F	Empregado Efetivo
	F	Empregado Efetivo
	F	Empregado Efetivo
	F	Empregado Efetivo
	F	Empregado Efetivo
	F	Empregado Efetivo
	F	Empregado Efetivo
	F	Empregado Efetivo
	M	Empregado Efetivo
	M	Empregado Efetivo
	M	Empregado Efetivo
	M	Empregado Efetivo
	M	Empregado Efetivo
	M	Empregado Efetivo
	M	Empregado Efetivo
	M	Empregado Efetivo
	M	Empregado Efetivo
	F	Empregado Efetivo
	F	Empregado Efetivo
	F	Empregado Efetivo
	F	Empregado Efetivo
	F	Empregado Efetivo
	F	Empregado Efetivo
	F	Empregado Efetivo
	M	Empregado Efetivo
	M	Empregado Efetivo
	F	Empregado Efetivo
	F	Empregado Efetivo
	M	Empregado Efetivo
	M	Empregado Efetivo
	F	Empregado Efetivo

	M	Empregado Efetivo
	F	Empregado Efetivo
	M	Empregado Efetivo
	M	Empregado Efetivo
	M	Empregado Efetivo
	M	Empregado Efetivo
	F	Empregado Efetivo
	F	Empregado Efetivo
	F	Cedido - RJU
	M	Empregado Efetivo
	M	Empregado Efetivo
	F	Empregado Efetivo
	M	Empregado Efetivo
	M	Empregado Efetivo
	M	Empregado Efetivo
	F	Empregado Efetivo
	F	Empregado Efetivo
	F	Empregado Efetivo
	F	Empregado Efetivo
	M	Empregado Efetivo
	F	Empregado Efetivo
	F	Empregado Efetivo
	M	Empregado Efetivo
	F	Empregado Efetivo
	F	Empregado Efetivo
	F	Empregado Efetivo
	F	Empregado Efetivo
	F	Empregado Efetivo
	F	Empregado Efetivo
	F	Empregado Efetivo
	F	Empregado Efetivo
	M	Empregado Efetivo
	F	Empregado Efetivo
	F	Empregado Efetivo
	F	Empregado Efetivo
	M	Empregado Efetivo
	F	Empregado Efetivo
	M	Empregado Efetivo
	M	Empregado Efetivo
	F	Empregado Efetivo
	F	Empregado Efetivo
	F	Empregado Efetivo
	F	Empregado Efetivo
	F	Empregado Efetivo

	F	Empregado Efetivo
	F	Empregado Efetivo
	F	Empregado Efetivo
	F	Empregado Efetivo
	F	Empregado Efetivo
	M	Empregado Efetivo
	F	Empregado Efetivo
	M	Empregado Efetivo
	F	Empregado Efetivo
	F	Empregado Efetivo
	M	Empregado Efetivo
	F	Empregado Efetivo
	F	Empregado Efetivo
	M	Empregado Efetivo
	M	Empregado Efetivo
	F	Empregado Efetivo
	F	Empregado Efetivo
	M	Empregado Efetivo
	F	Empregado Efetivo
	M	Empregado Efetivo
	M	Empregado Efetivo
	F	Empregado Efetivo
	F	Empregado Efetivo
	F	Empregado Efetivo
	F	Empregado Efetivo
	F	Empregado Efetivo
	F	Empregado Efetivo
	M	Empregado Efetivo
	M	Empregado Efetivo
	F	Empregado Efetivo
	F	Empregado Efetivo
	M	Empregado Efetivo
	F	Empregado Efetivo
	F	Empregado Efetivo
	F	Empregado Efetivo
	F	Empregado Efetivo
	F	Empregado Efetivo
	F	Empregado Efetivo
	F	Empregado Efetivo
	F	Empregado Efetivo
	F	Empregado Efetivo
	F	Empregado Efetivo
	F	Empregado Efetivo
	M	Empregado Efetivo
	F	Empregado Efetivo
	F	Empregado Efetivo
	M	Empregado Efetivo
	M	Empregado Efetivo

	M	Empregado Efetivo
	M	Empregado Efetivo
	F	Empregado Efetivo
	F	Empregado Efetivo
	F	Empregado Efetivo
	F	Empregado Efetivo
	F	Empregado Efetivo
	F	Empregado Efetivo
	F	Empregado Efetivo
	M	Empregado Efetivo
	F	Empregado Efetivo
	F	Empregado Efetivo
	F	Empregado Efetivo
	F	Empregado Efetivo
	M	Empregado Efetivo
	F	Empregado Efetivo
	F	Empregado Efetivo
	M	Empregado Efetivo
	F	Empregado Efetivo
	F	Empregado Efetivo
	F	Empregado Efetivo
	M	Empregado Efetivo
	F	Empregado Efetivo
	F	Empregado Efetivo
	F	Empregado Efetivo
	F	Empregado Efetivo
	M	Empregado Efetivo
	F	Empregado Efetivo
	M	Empregado Efetivo
	F	Empregado Efetivo
	F	Empregado Efetivo
	F	Empregado Efetivo
	F	Empregado Efetivo
	M	Empregado Efetivo
	M	Empregado Efetivo
	F	Empregado Efetivo
	F	Empregado Efetivo

	F	Empregado Efetivo
	F	Empregado Efetivo
	F	Empregado Efetivo
	F	Empregado Efetivo
	F	Empregado Efetivo
	F	Empregado Efetivo
	F	Empregado Efetivo
	F	Empregado Efetivo
	M	Empregado Efetivo
	F	Empregado Efetivo
	F	Empregado Efetivo
	F	Empregado Efetivo
	F	Empregado Efetivo
	F	Empregado Efetivo
	F	Empregado Efetivo
	F	Empregado Efetivo
	F	Empregado Efetivo
	F	Empregado Efetivo
	F	Empregado Efetivo
	F	Empregado Efetivo
	F	Empregado Efetivo
	F	Empregado Efetivo
	F	Empregado Efetivo
	F	Empregado Efetivo
	F	Empregado Efetivo
	F	Empregado Efetivo
	F	Empregado Efetivo
	F	Empregado Efetivo
	F	Empregado Efetivo
	F	Empregado Efetivo
	F	Empregado Efetivo
	F	Empregado Efetivo
	F	Empregado Efetivo
	F	Empregado Efetivo
	F	Empregado Efetivo
	F	Empregado Efetivo
	F	Empregado Efetivo
	F	Empregado Efetivo
	F	Empregado Efetivo

	F	Empregado Efetivo
	F	Empregado Efetivo
	F	Empregado Efetivo
	F	Empregado Efetivo
	F	Empregado Efetivo
	F	Empregado Efetivo
	F	Empregado Efetivo
	F	Empregado Efetivo
	F	Empregado Efetivo
	F	Empregado Efetivo
	M	Empregado Efetivo
	F	Empregado Efetivo
	M	Empregado Efetivo
	F	Empregado Efetivo
	F	Empregado Efetivo
	M	Empregado Efetivo
	M	Empregado Efetivo
	M	Empregado Efetivo
	M	Empregado Efetivo
	F	Empregado Efetivo
	F	Empregado Efetivo
	F	Empregado Efetivo
	F	Empregado Efetivo
	F	Empregado Efetivo
	F	Empregado Efetivo
	F	Empregado Efetivo
	F	Empregado Efetivo
	M	Empregado Efetivo
	F	Empregado Efetivo
	F	Empregado Efetivo
	M	Empregado Efetivo
	M	Empregado Efetivo
	F	Empregado Efetivo
	F	Empregado Efetivo
	M	Empregado Efetivo

	F	Empregado Efetivo
	M	Empregado Efetivo
	M	Empregado Efetivo
	F	Empregado Efetivo
	F	Empregado Efetivo
	M	Empregado Efetivo
	F	Empregado Efetivo
	M	Empregado Efetivo
	F	Empregado Efetivo
	M	Empregado Efetivo
	M	Empregado Efetivo
	F	Empregado Efetivo
	F	Empregado Efetivo
	F	Empregado Efetivo
	F	Empregado Efetivo
	F	Empregado Efetivo
	F	Empregado Efetivo
	F	Empregado Efetivo
	F	Empregado Efetivo
	F	Empregado Efetivo
	F	Empregado Efetivo
	F	Empregado Efetivo
	F	Empregado Efetivo
	F	Empregado Efetivo
	F	Empregado Efetivo
	F	Empregado Efetivo
	F	Empregado Efetivo
	F	Empregado Efetivo
	F	Empregado Efetivo
	F	Empregado Efetivo
	F	Empregado Efetivo
	F	Empregado Efetivo
	F	Empregado Efetivo
	F	Empregado Efetivo
	F	Empregado Efetivo
	F	Empregado Efetivo
	F	Empregado Efetivo
	F	Empregado Efetivo
	F	Empregado Efetivo

	F	Empregado Efetivo
	F	Empregado Efetivo
	M	Empregado Efetivo
	F	Empregado Efetivo
	M	Empregado Efetivo
	F	Empregado Efetivo
	M	Empregado Efetivo
	F	Empregado Efetivo
	F	Empregado Efetivo
	M	Empregado Efetivo
	M	Empregado Efetivo
	F	Empregado Efetivo
	F	Empregado Efetivo
	F	Empregado Efetivo
	F	Empregado Efetivo
	F	Empregado Efetivo
	F	Empregado Efetivo
	F	Cedido - RJU
	M	Empregado Efetivo
	M	Empregado Efetivo
	M	Empregado Efetivo
	M	Empregado Efetivo
	M	Empregado Efetivo
	M	Empregado Efetivo
	M	Empregado Efetivo
	M	Empregado Efetivo
	M	Empregado Efetivo
	M	Empregado Efetivo
	M	Empregado Efetivo
	M	Empregado Efetivo
	M	Empregado Efetivo
	M	Empregado Efetivo
	M	Empregado Efetivo
	M	Empregado Efetivo

	M	Empregado Efetivo
	M	Empregado Efetivo
	M	Empregado Efetivo
	M	Empregado Efetivo
	M	Empregado Efetivo
	M	Empregado Efetivo
	M	Empregado Efetivo
	M	Empregado Efetivo
	F	Empregado Efetivo
	F	Empregado Efetivo
	F	Empregado Efetivo
	F	Empregado Efetivo
	F	Empregado Efetivo
	F	Empregado Efetivo
	F	Empregado Efetivo
	F	Empregado Efetivo
	F	Empregado Efetivo
	F	Empregado Efetivo
	F	Empregado Efetivo
	F	Empregado Efetivo
	F	Empregado Efetivo
	F	Empregado Efetivo
	F	Empregado Efetivo
	F	Empregado Efetivo
	F	Empregado Efetivo
	F	Empregado Efetivo
	F	Empregado Efetivo
	F	Empregado Efetivo
	F	Empregado Efetivo
	F	Empregado Efetivo
	F	Empregado Efetivo
	F	Empregado Efetivo

	F	Empregado Efetivo
	F	Empregado Efetivo
	F	Empregado Efetivo
	F	Empregado Efetivo
	F	Empregado Efetivo
	F	Empregado Efetivo
	F	Empregado Efetivo
	F	Empregado Efetivo
	F	Empregado Efetivo
	F	Empregado Efetivo
	F	Empregado Efetivo
	F	Empregado Efetivo
	F	Empregado Efetivo
	F	Empregado Efetivo
	F	Empregado Efetivo
	F	Empregado Efetivo
	F	Empregado Efetivo
	F	Empregado Efetivo
	F	Empregado Efetivo
	F	Empregado Efetivo
	F	Empregado Efetivo
	F	Empregado Efetivo
	F	Empregado Efetivo
	F	Empregado Efetivo
	F	Empregado Efetivo
	F	Empregado Efetivo
	F	Empregado Efetivo
	F	Empregado Efetivo
	F	Empregado Efetivo
	M	Empregado Efetivo
	F	Empregado Efetivo
	F	Empregado Efetivo

	F	Empregado Efetivo
	F	Empregado Efetivo
	F	Empregado Efetivo
	F	Empregado Efetivo
	F	Empregado Efetivo
	M	Empregado Efetivo
	F	Empregado Efetivo
	F	Empregado Efetivo
	M	Empregado Efetivo
	F	Empregado Efetivo
	F	Empregado Efetivo
	F	Empregado Efetivo
	F	Empregado Efetivo
	F	Empregado Efetivo
	F	Empregado Efetivo
	M	Empregado Efetivo
	F	Empregado Efetivo
	F	Empregado Efetivo
	F	Empregado Efetivo
	F	Empregado Efetivo
	F	Empregado Efetivo
	F	Empregado Efetivo
	M	Empregado Efetivo
	F	Empregado Efetivo
	M	Empregado Efetivo
	F	Empregado Efetivo
	M	Empregado Efetivo
	M	Empregado Efetivo
	F	Empregado Efetivo
	M	Empregado Efetivo
	M	Empregado Efetivo
	F	Empregado Efetivo

	M	Empregado Efetivo
	M	Empregado Efetivo
	F	Empregado Efetivo
	F	Empregado Efetivo
	F	Empregado Efetivo
	F	Empregado Efetivo
	M	Empregado Efetivo
	F	Empregado Efetivo
	F	Empregado Efetivo
	F	Empregado Efetivo
	F	Empregado Efetivo
	F	Empregado Efetivo
	F	Empregado Efetivo
	F	Empregado Efetivo
	M	Empregado Efetivo
	F	Empregado Efetivo
	F	Empregado Efetivo
	F	Empregado Efetivo
	F	Empregado Efetivo
	F	Empregado Efetivo
	F	Empregado Efetivo
	F	Empregado Efetivo
	F	Empregado Efetivo
	F	Empregado Efetivo
	F	Empregado Efetivo
	F	Empregado Efetivo
	F	Empregado Efetivo
	F	Empregado Efetivo
	F	Empregado Efetivo
	F	Empregado Efetivo
	F	Empregado Efetivo
	F	Empregado Efetivo

	F	Empregado Efetivo
	F	Empregado Efetivo
	F	Empregado Efetivo
	F	Empregado Efetivo
	F	Empregado Efetivo
	F	Empregado Efetivo
	F	Empregado Efetivo
	F	Empregado Efetivo
	F	Empregado Efetivo
	F	Empregado Efetivo
	F	Empregado Efetivo
	F	Empregado Efetivo
	F	Empregado Efetivo
	F	Empregado Efetivo
	F	Empregado Efetivo
	F	Empregado Efetivo
	F	Empregado Efetivo
	F	Empregado Efetivo
	F	Empregado Efetivo
	F	Empregado Efetivo
	F	Empregado Efetivo
	F	Empregado Efetivo
	F	Empregado Efetivo
	F	Empregado Efetivo
	F	Empregado Efetivo
	F	Empregado Efetivo
	F	Empregado Efetivo
	F	Empregado Efetivo
	F	Empregado Efetivo
	F	Empregado Efetivo
	F	Empregado Efetivo
	F	Empregado Efetivo

	F	Empregado Efetivo
	F	Empregado Efetivo
	F	Empregado Efetivo
	F	Empregado Efetivo
	F	Empregado Efetivo
	F	Empregado Efetivo
	F	Empregado Efetivo
	F	Empregado Efetivo
	F	Empregado Efetivo
	F	Empregado Efetivo
	F	Empregado Efetivo
	F	Empregado Efetivo
	F	Empregado Efetivo
	F	Empregado Efetivo
	F	Empregado Efetivo
	F	Empregado Efetivo
	F	Empregado Efetivo
	F	Empregado Efetivo
	F	Empregado Efetivo
	F	Empregado Efetivo
	F	Empregado Efetivo
	F	Empregado Efetivo
	F	Empregado Efetivo
	F	Empregado Efetivo
	F	Empregado Efetivo
	F	Empregado Efetivo
	F	Empregado Efetivo
	F	Empregado Efetivo
	F	Empregado Efetivo
	F	Empregado Efetivo
	M	Empregado Efetivo
	F	Empregado Efetivo

	F	Empregado Efetivo
	F	Empregado Efetivo
	F	Empregado Efetivo
	F	Empregado Efetivo
	F	Empregado Efetivo
	F	Empregado Efetivo
	F	Empregado Efetivo
	F	Empregado Efetivo
	F	Empregado Efetivo
	F	Empregado Efetivo
	F	Empregado Efetivo
	F	Empregado Efetivo
	F	Empregado Efetivo
	F	Empregado Efetivo
	F	Empregado Efetivo
	F	Empregado Efetivo
	F	Empregado Efetivo
	F	Empregado Efetivo
	F	Empregado Efetivo
	F	Empregado Efetivo
	F	Empregado Efetivo
	F	Empregado Efetivo
	F	Empregado Efetivo
	F	Empregado Efetivo
	F	Empregado Efetivo
	F	Empregado Efetivo
	F	Empregado Efetivo
	F	Empregado Efetivo
	F	Empregado Efetivo
	F	Empregado Efetivo
	F	Empregado Efetivo
	F	Empregado Efetivo

	F	Empregado Efetivo
	M	Empregado Efetivo
	F	Empregado Efetivo
	F	Empregado Efetivo
	F	Empregado Efetivo
	M	Empregado Efetivo
	F	Empregado Efetivo
	F	Empregado Efetivo
	F	Empregado Efetivo
	F	Empregado Efetivo
	M	Empregado Efetivo
	F	Empregado Efetivo
	M	Empregado Efetivo
	F	Empregado Efetivo
	F	Empregado Efetivo
	F	Empregado Efetivo
	F	Empregado Efetivo
	F	Empregado Efetivo
	F	Empregado Efetivo
	F	Empregado Efetivo
	F	Empregado Efetivo
	F	Empregado Efetivo
	F	Empregado Efetivo
	F	Empregado Efetivo
	F	Empregado Efetivo
	F	Empregado Efetivo
	F	Empregado Efetivo
	F	Empregado Efetivo
	F	Empregado Efetivo
	F	Empregado Efetivo
	F	Empregado Efetivo
	F	Empregado Efetivo

	F	Empregado Efetivo
	F	Empregado Efetivo
	F	Empregado Efetivo
	F	Empregado Efetivo
	F	Empregado Efetivo
	F	Empregado Efetivo
	F	Empregado Efetivo
	F	Empregado Efetivo
	F	Empregado Efetivo
	F	Empregado Efetivo
	F	Empregado Efetivo
	F	Empregado Efetivo
	F	Empregado Efetivo
	F	Empregado Efetivo
	F	Empregado Efetivo
	F	Empregado Efetivo
	F	Empregado Efetivo
	F	Empregado Efetivo
	M	Empregado Efetivo
	F	Empregado Efetivo
	F	Empregado Efetivo
	F	Empregado Efetivo
	F	Empregado Efetivo
	F	Empregado Efetivo
	F	Empregado Efetivo
	F	Empregado Efetivo
	F	Empregado Efetivo
	F	Empregado Efetivo
	F	Empregado Efetivo
	F	Empregado Efetivo
	F	Empregado Efetivo
	F	Empregado Efetivo

	F	Empregado Efetivo
	F	Empregado Efetivo
	F	Empregado Efetivo
	F	Empregado Efetivo
	F	Empregado Efetivo
	F	Empregado Efetivo
	F	Empregado Efetivo
	F	Empregado Efetivo
	F	Empregado Efetivo
	F	Empregado Efetivo
	F	Empregado Efetivo
	F	Empregado Efetivo
	F	Empregado Efetivo
	F	Empregado Efetivo
	F	Empregado Efetivo
	F	Empregado Efetivo
	F	Empregado Efetivo
	F	Empregado Efetivo
	F	Empregado Efetivo
	F	Empregado Efetivo
	F	Empregado Efetivo
	F	Empregado Efetivo
	F	Empregado Efetivo
	F	Empregado Efetivo
	F	Empregado Efetivo
	F	Empregado Efetivo
	F	Empregado Efetivo
	F	Empregado Efetivo
	F	Empregado Efetivo
	F	Empregado Efetivo
	F	Empregado Efetivo
	F	Empregado Efetivo

	F	Empregado Efetivo
	F	Empregado Efetivo
	M	Empregado Efetivo
	M	Empregado Efetivo
	M	Empregado Efetivo
	M	Empregado Efetivo
	M	Empregado Efetivo
	M	Empregado Efetivo
	M	Empregado Efetivo
	F	Empregado Efetivo
	F	Empregado Efetivo
	F	Empregado Efetivo
	M	Empregado Efetivo
	M	Empregado Efetivo
	M	Empregado Efetivo
	F	Empregado Efetivo
	F	Empregado Efetivo
	F	Empregado Efetivo
	F	Empregado Efetivo
	F	Empregado Efetivo
	F	Empregado Efetivo
	F	Empregado Efetivo
	F	Empregado Efetivo
	F	Empregado Efetivo
	F	Empregado Efetivo
	F	Empregado Efetivo
	F	Empregado Efetivo
	F	Empregado Efetivo
	F	Empregado Efetivo
	F	Empregado Efetivo
	F	Empregado Efetivo
	F	Empregado Efetivo

	F	Empregado Efetivo
	F	Empregado Efetivo
	F	Empregado Efetivo
	F	Empregado Efetivo
	F	Empregado Efetivo
	M	Empregado Efetivo
	M	Empregado Efetivo
	M	Empregado Efetivo
	M	Empregado Efetivo
	M	Empregado Efetivo
	M	Empregado Efetivo
	F	Empregado Efetivo
	F	Empregado Efetivo
	F	Empregado Efetivo
	F	Empregado Efetivo
	F	Empregado Efetivo
	F	Empregado Efetivo
	F	Empregado Efetivo
	F	Empregado Efetivo
	F	Empregado Efetivo
	F	Empregado Efetivo
	F	Empregado Efetivo
	F	Empregado Efetivo
	F	Empregado Efetivo
	F	Empregado Efetivo
	F	Empregado Efetivo
	F	Empregado Efetivo
	F	Empregado Efetivo
	F	Empregado Efetivo
	M	Empregado Efetivo
	F	Empregado Efetivo
	F	Empregado Efetivo
	F	Empregado Efetivo
	F	Empregado Efetivo
	M	Empregado Efetivo
	M	Empregado Efetivo
	F	Empregado Efetivo
	F	Empregado Efetivo
	F	Empregado Efetivo
	F	Empregado Efetivo

	F	Empregado Efetivo
	F	Empregado Efetivo
	F	Empregado Efetivo
	F	Empregado Efetivo
	F	Empregado Efetivo
	M	Empregado Efetivo
	M	Empregado Efetivo
	F	Empregado Efetivo
	F	Empregado Efetivo
	F	Empregado Efetivo
	F	Empregado Efetivo
	F	Empregado Efetivo
	F	Empregado Efetivo
	F	Empregado Efetivo
	F	Empregado Efetivo
	M	Empregado Efetivo
	F	Empregado Efetivo
	F	Empregado Efetivo
	F	Empregado Efetivo
	M	Empregado Efetivo
	M	Empregado Efetivo
	F	Empregado Efetivo
	F	Empregado Efetivo
	F	Empregado Efetivo
	F	Empregado Efetivo
	F	Empregado Efetivo
	M	Empregado Efetivo
	F	Empregado Efetivo
	F	Empregado Efetivo
	M	Empregado Efetivo
	F	Empregado Efetivo
	F	Empregado Efetivo
	F	Empregado Efetivo
	M	Empregado Efetivo
	M	Empregado Efetivo
	M	Empregado Efetivo
	F	Empregado Efetivo
	M	Empregado Efetivo
	F	Empregado Efetivo
	F	Empregado Efetivo
	M	Empregado Efetivo

	F	Empregado Efetivo
	F	Empregado Efetivo
	F	Empregado Efetivo
	F	Empregado Efetivo
	F	Empregado Efetivo
	F	Empregado Efetivo
	F	Empregado Efetivo
	F	Empregado Efetivo
	F	Empregado Efetivo
	F	Empregado Efetivo
	M	Empregado Efetivo
	F	Empregado Efetivo
	F	Empregado Efetivo
	M	Empregado Efetivo
	M	Empregado Efetivo
	M	Empregado Efetivo
	F	Empregado Efetivo
	F	Empregado Efetivo
	M	Empregado Efetivo
	F	Empregado Efetivo
	F	Empregado Efetivo
	F	Empregado Efetivo
	F	Empregado Efetivo
	M	Empregado Efetivo
	F	Empregado Efetivo
	F	Empregado Efetivo
	M	Empregado Efetivo
	M	Empregado Efetivo
	M	Empregado Efetivo
	M	Empregado Efetivo
	M	Empregado Efetivo
	F	Empregado Efetivo
	F	Empregado Efetivo
	M	Empregado Efetivo
	M	Empregado Efetivo
	M	Empregado Efetivo
	M	Empregado Efetivo
	F	Empregado Efetivo
	M	Empregado Efetivo
	F	Empregado Efetivo
	M	Empregado Efetivo
	M	Empregado Efetivo
	M	Empregado Efetivo

	M	Empregado Efetivo
	M	Empregado Efetivo
	M	Empregado Efetivo
	M	Empregado Efetivo
	M	Empregado Efetivo
	M	Empregado Efetivo
	F	Empregado Efetivo
	M	Empregado Efetivo
	F	Empregado Efetivo
	F	Empregado Efetivo
	M	Empregado Efetivo
	F	Empregado Efetivo
	F	Empregado Efetivo
	F	Empregado Efetivo
	F	Empregado Efetivo
	F	Empregado Efetivo
	F	Empregado Efetivo
	F	Empregado Efetivo
	M	Empregado Efetivo
	F	Empregado Efetivo
	M	Empregado Efetivo
	M	Empregado Efetivo
	F	Empregado Efetivo
	F	Empregado Efetivo
	M	Empregado Efetivo
	M	Empregado Efetivo
	M	Empregado Efetivo
	M	Empregado Efetivo
	M	Empregado Efetivo
	M	Empregado Efetivo
	M	Empregado Efetivo
	M	Empregado Efetivo
	M	Empregado Efetivo
	M	Empregado Efetivo
	M	Empregado Efetivo
	M	Empregado Efetivo
	F	Empregado Efetivo
	M	Empregado Efetivo
	M	Empregado Efetivo
	M	Empregado Efetivo
	M	Empregado Efetivo
	M	Empregado Efetivo
	M	Empregado Efetivo
	M	Empregado Efetivo
	M	Empregado Efetivo
	M	Empregado Efetivo
	M	Empregado Efetivo
	M	Empregado Efetivo

	M	Empregado Efetivo
	M	Empregado Efetivo
	M	Empregado Efetivo
	M	Empregado Efetivo
	M	Empregado Efetivo
	M	Empregado Efetivo
	M	Empregado Efetivo
	M	Empregado Efetivo
	M	Empregado Efetivo
	M	Empregado Efetivo
	M	Empregado Efetivo
	M	Empregado Efetivo
	M	Empregado Efetivo
	M	Empregado Efetivo
	M	Empregado Efetivo
	M	Empregado Efetivo
	M	Empregado Efetivo
	M	Empregado Efetivo
	M	Empregado Efetivo
	M	Empregado Efetivo
	M	Empregado Efetivo
	M	Empregado Efetivo
	M	Empregado Efetivo
	M	Empregado Efetivo
	M	Empregado Efetivo
	M	Empregado Efetivo
	M	Empregado Efetivo
	M	Empregado Efetivo
	M	Empregado Efetivo
	M	Empregado Efetivo
	M	Empregado Efetivo
	M	Empregado Efetivo
	M	Empregado Efetivo
	M	Empregado Efetivo
	F	Empregado Efetivo
	F	Empregado Efetivo
	F	Empregado Efetivo
	F	Empregado Efetivo
	F	Empregado Efetivo
	M	Empregado Efetivo
	M	Empregado Efetivo
	M	Empregado Efetivo
	M	Empregado Efetivo
	M	Empregado Efetivo
	F	Empregado Efetivo
	F	Empregado Efetivo

	F	Empregado Efetivo
	M	Empregado Efetivo
	F	Empregado Efetivo
	F	Empregado Efetivo
	F	Empregado Efetivo
	F	Empregado Efetivo
	F	Empregado Efetivo
	F	Empregado Efetivo
	F	Empregado Efetivo
	F	Empregado Efetivo
	F	Empregado Efetivo
	F	Empregado Efetivo
	F	Empregado Efetivo
	F	Empregado Efetivo
	F	Empregado Efetivo
	F	Empregado Efetivo
	F	Empregado Efetivo
	F	Empregado Efetivo
	F	Empregado Efetivo
	F	Empregado Efetivo
	F	Empregado Efetivo
	F	Empregado Efetivo
	M	Empregado Efetivo
	F	Empregado Efetivo
	F	Empregado Efetivo
	F	Empregado Efetivo
	F	Empregado Efetivo
	F	Empregado Efetivo
	F	Empregado Efetivo
	F	Empregado Efetivo
	F	Empregado Efetivo
	F	Empregado Efetivo
	M	Empregado Efetivo
	M	Empregado Efetivo
	M	Empregado Efetivo
	M	Empregado Efetivo
	M	Empregado Efetivo
	M	Empregado Efetivo
	M	Empregado Efetivo
	M	Empregado Efetivo
	M	Empregado Efetivo
	M	Empregado Efetivo
	M	Empregado Efetivo
	F	Empregado Efetivo
	F	Empregado Efetivo
	F	Empregado Efetivo
	M	Empregado Efetivo
	F	Empregado Efetivo

	M	Empregado Efetivo
	M	Empregado Efetivo
	F	Empregado Efetivo
	F	Empregado Efetivo
	M	Empregado Efetivo
	M	Empregado Efetivo
	M	Empregado Efetivo
	M	Empregado Efetivo
	F	Empregado Efetivo
	M	Empregado Efetivo
	M	Empregado Efetivo
	M	Empregado Efetivo
	M	Empregado Efetivo
	M	Empregado Efetivo
	M	Empregado Efetivo
	F	Empregado Efetivo
	M	Empregado Efetivo
	M	Empregado Efetivo
	F	Empregado Efetivo
	F	Empregado Efetivo
	F	Empregado Efetivo
	F	Empregado Efetivo
	F	Empregado Efetivo
	F	Empregado Efetivo
	F	Empregado Efetivo
	F	Empregado Efetivo
	M	Empregado Efetivo
	M	Empregado Efetivo
	F	Empregado Efetivo
	F	Empregado Efetivo
	F	Empregado Efetivo
	F	Empregado Efetivo
	M	Empregado Efetivo
	M	Empregado Efetivo
	F	Empregado Efetivo
	M	Empregado Efetivo
	F	Empregado Efetivo
	F	Empregado Efetivo

	M	Empregado Efetivo
	M	Empregado Efetivo
	F	Empregado Efetivo
	F	Empregado Efetivo
	F	Empregado Efetivo
	F	Empregado Efetivo
	F	Empregado Efetivo
	M	Empregado Efetivo
	M	Empregado Efetivo
	M	Empregado Efetivo
	M	Empregado Efetivo
	M	Empregado Efetivo
	M	Empregado Efetivo
	M	Empregado Efetivo
	M	Empregado Efetivo
	M	Empregado Efetivo
	M	Empregado Efetivo
	F	Empregado Efetivo
	F	Empregado Efetivo
	F	Empregado Efetivo
	F	Empregado Efetivo
	F	Empregado Efetivo
	F	Empregado Efetivo
	M	Empregado Efetivo
	M	Empregado Efetivo
	M	Empregado Efetivo
	F	Empregado Efetivo
	F	Empregado Efetivo
	F	Empregado Efetivo
	F	Empregado Efetivo
	F	Empregado Efetivo
	M	Empregado Efetivo
	F	Empregado Efetivo
	F	Empregado Efetivo
	F	Empregado Efetivo
	F	Empregado Efetivo
	F	Empregado Efetivo
	F	Empregado Efetivo
	M	Empregado Efetivo
	F	Empregado Efetivo
	F	Empregado Efetivo
	F	Empregado Efetivo
	F	Empregado Efetivo
	F	Empregado Efetivo
	F	Empregado Efetivo
	F	Empregado Efetivo
	F	Empregado Efetivo
	F	Empregado Efetivo

	F	Empregado Efetivo
	F	Empregado Efetivo
	F	Empregado Efetivo
	F	Empregado Efetivo
	F	Empregado Efetivo
	F	Empregado Efetivo
	F	Empregado Efetivo
	F	Empregado Efetivo
	F	Empregado Efetivo
	F	Empregado Efetivo
	F	Empregado Efetivo
	F	Empregado Efetivo
	F	Empregado Efetivo
	F	Empregado Efetivo
	F	Empregado Efetivo
	F	Empregado Efetivo
	F	Empregado Efetivo
	M	Empregado Efetivo
	M	Empregado Efetivo
	M	Empregado Efetivo
	F	Empregado Efetivo
	F	Empregado Efetivo
	F	Empregado Efetivo
	F	Empregado Efetivo
	F	Empregado Efetivo
	F	Empregado Efetivo
	F	Empregado Efetivo
	F	Empregado Efetivo
	F	Empregado Efetivo
	M	Empregado Efetivo
	M	Empregado Efetivo
	M	Empregado Efetivo
	F	Empregado Efetivo
	F	Empregado Efetivo
	F	Empregado Efetivo
	M	Empregado Efetivo
	F	Empregado Efetivo
	F	Empregado Efetivo
	F	Empregado Efetivo
	F	Empregado Efetivo
	F	Empregado Efetivo
	M	Empregado Efetivo
	M	Empregado Efetivo

	M	Empregado Efetivo
	M	Empregado Efetivo
	M	Empregado Efetivo
	M	Empregado Efetivo
	M	Empregado Efetivo
	M	Empregado Efetivo
	M	Empregado Efetivo
	M	Empregado Efetivo
	M	Empregado Efetivo
	M	Empregado Efetivo
	M	Empregado Efetivo
	M	Empregado Efetivo
	M	Empregado Efetivo
	M	Empregado Efetivo
	M	Empregado Efetivo
	M	Empregado Efetivo
	M	Empregado Efetivo
	M	Empregado Efetivo
	M	Empregado Efetivo
	M	Empregado Efetivo
	M	Empregado Efetivo
	M	Empregado Efetivo
	F	Empregado Efetivo
	F	Empregado Efetivo
	F	Empregado Efetivo
	F	Empregado Efetivo
	F	Empregado Efetivo
	F	Empregado Efetivo
	F	Empregado Efetivo
	F	Empregado Efetivo
	F	Empregado Efetivo
	F	Empregado Efetivo
	F	Empregado Efetivo
	F	Empregado Efetivo
	F	Empregado Efetivo
	F	Empregado Efetivo
	F	Empregado Efetivo
	F	Empregado Efetivo
	F	Empregado Efetivo
	F	Empregado Efetivo
	F	Empregado Efetivo
	F	Empregado Efetivo
	F	Empregado Efetivo
	F	Empregado Efetivo
	F	Empregado Efetivo
	F	Empregado Efetivo
	F	Empregado Efetivo
	F	Empregado Efetivo

	F	Empregado Efetivo
	F	Empregado Efetivo
	F	Empregado Efetivo
	F	Empregado Efetivo
	F	Empregado Efetivo
	F	Empregado Efetivo
	F	Empregado Efetivo
	F	Empregado Efetivo
	F	Empregado Efetivo
	F	Empregado Efetivo
	F	Empregado Efetivo
	F	Empregado Efetivo
	F	Empregado Efetivo
	F	Empregado Efetivo
	F	Empregado Efetivo
	F	Empregado Efetivo
	F	Empregado Efetivo
	F	Empregado Efetivo
	F	Empregado Efetivo
	F	Empregado Efetivo
	F	Empregado Efetivo
	F	Empregado Efetivo
	F	Empregado Efetivo
	F	Empregado Efetivo
	F	Empregado Efetivo
	F	Empregado Efetivo
	F	Empregado Efetivo
	F	Empregado Efetivo
	F	Empregado Efetivo
	F	Empregado Efetivo
	F	Empregado Efetivo
	F	Empregado Efetivo
	F	Empregado Efetivo
	F	Empregado Efetivo
	F	Empregado Efetivo
	F	Empregado Efetivo
	F	Empregado Efetivo
	F	Empregado Efetivo
	F	Empregado Efetivo
	F	Empregado Efetivo
	F	Empregado Efetivo
	F	Empregado Efetivo
	F	Empregado Efetivo
	F	Empregado Efetivo
	F	Empregado Efetivo
	F	Empregado Efetivo
	F	Empregado Efetivo
	F	Empregado Efetivo

	F	Empregado Efetivo
	F	Empregado Efetivo
	F	Empregado Efetivo
	F	Empregado Efetivo
	F	Empregado Efetivo
	F	Empregado Efetivo
	F	Empregado Efetivo
	F	Empregado Efetivo
	F	Empregado Efetivo
	F	Empregado Efetivo
	F	Empregado Efetivo
	F	Empregado Efetivo
	F	Empregado Efetivo
	F	Empregado Efetivo
	F	Empregado Efetivo
	F	Empregado Efetivo
	F	Empregado Efetivo
	F	Empregado Efetivo
	F	Empregado Efetivo
	F	Empregado Efetivo
	F	Empregado Efetivo
	F	Empregado Efetivo
	F	Empregado Efetivo
	F	Empregado Efetivo
	F	Empregado Efetivo
	F	Empregado Efetivo
	F	Empregado Efetivo
	F	Empregado Efetivo
	F	Empregado Efetivo
	F	Empregado Efetivo
	F	Empregado Efetivo
	F	Empregado Efetivo
	F	Empregado Efetivo
	F	Empregado Efetivo
	F	Empregado Efetivo
	F	Empregado Efetivo
	F	Empregado Efetivo
	F	Empregado Efetivo
	F	Empregado Efetivo
	F	Empregado Efetivo
	F	Empregado Efetivo
	F	Empregado Efetivo
	M	Empregado Efetivo
	F	Empregado Efetivo
	F	Empregado Efetivo
	F	Empregado Efetivo
	F	Empregado Efetivo
	F	Empregado Efetivo

	F	Empregado Efetivo
	F	Empregado Efetivo
	F	Empregado Efetivo
	F	Empregado Efetivo
	F	Empregado Efetivo
	F	Empregado Efetivo
	F	Empregado Efetivo
	F	Empregado Efetivo
	F	Empregado Efetivo
	F	Empregado Efetivo
	F	Empregado Efetivo
	F	Empregado Efetivo
	F	Empregado Efetivo
	F	Empregado Efetivo
	M	Empregado Efetivo
	F	Empregado Efetivo
	F	Empregado Efetivo
	F	Empregado Efetivo
	F	Empregado Efetivo
	F	Empregado Efetivo
	F	Empregado Efetivo
	F	Empregado Efetivo
	F	Empregado Efetivo
	F	Empregado Efetivo
	F	Empregado Efetivo
	F	Empregado Efetivo
	F	Empregado Efetivo
	F	Empregado Efetivo
	F	Empregado Efetivo
	F	Empregado Efetivo
	F	Empregado Efetivo
	F	Empregado Efetivo
	F	Empregado Efetivo
	F	Empregado Efetivo
	F	Empregado Efetivo
	F	Empregado Efetivo
	F	Empregado Efetivo
	F	Empregado Efetivo
	F	Empregado Efetivo
	F	Empregado Efetivo
	F	Empregado Efetivo
	F	Empregado Efetivo
	F	Empregado Efetivo
	F	Empregado Efetivo
	F	Empregado Efetivo
	F	Empregado Efetivo
	F	Empregado Efetivo
	F	Empregado Efetivo

	F	Empregado Efetivo
	F	Empregado Efetivo
	F	Empregado Efetivo
	F	Empregado Efetivo
	F	Empregado Efetivo
	F	Empregado Efetivo
	F	Empregado Efetivo
	F	Empregado Efetivo
	F	Empregado Efetivo
	F	Empregado Efetivo
	M	Empregado Efetivo
	F	Empregado Efetivo
	F	Empregado Efetivo
	F	Empregado Efetivo
	F	Empregado Efetivo
	M	Empregado Efetivo
	M	Empregado Efetivo
	F	Empregado Efetivo
	F	Empregado Efetivo
	M	Empregado Efetivo
	M	Empregado Efetivo
	M	Empregado Efetivo
	F	Empregado Efetivo
	F	Empregado Efetivo
	M	Empregado Efetivo
	F	Empregado Efetivo
	F	Empregado Efetivo
	F	Empregado Efetivo
	M	Empregado Efetivo
	M	Empregado Efetivo
	F	Empregado Efetivo
	F	Empregado Efetivo
	F	Empregado Efetivo
	F	Empregado Efetivo
	M	Empregado Efetivo
	M	Empregado Efetivo
	F	Empregado Efetivo
	F	Empregado Efetivo
	F	Empregado Efetivo

	M	Empregado Efetivo
	F	Empregado Efetivo
	F	Empregado Efetivo
	F	Empregado Efetivo
	F	Empregado Efetivo
	F	Empregado Efetivo
	F	Empregado Efetivo
	F	Empregado Efetivo
	F	Empregado Efetivo
	F	Empregado Efetivo
	F	Empregado Efetivo
	F	Empregado Efetivo
	F	Empregado Efetivo
	M	Empregado Efetivo
	M	Empregado Efetivo
	F	Empregado Efetivo
	F	Empregado Efetivo
	F	Empregado Efetivo
	F	Empregado Efetivo
	M	Empregado Efetivo
	F	Empregado Efetivo
	F	Empregado Efetivo
	F	Empregado Efetivo
	F	Empregado Efetivo
	F	Empregado Efetivo
	F	Empregado Efetivo
	M	Empregado Efetivo
	F	Empregado Efetivo
	F	Empregado Efetivo
	F	Empregado Efetivo
	F	Empregado Efetivo
	F	Empregado Efetivo
	F	Empregado Efetivo
	F	Empregado Efetivo
	F	Empregado Efetivo
	F	Empregado Efetivo
	F	Empregado Efetivo
	F	Empregado Efetivo
	F	Empregado Efetivo
	F	Empregado Efetivo
	F	Empregado Efetivo
	F	Empregado Efetivo
	F	Empregado Efetivo
	F	Empregado Efetivo
	F	Empregado Efetivo
	F	Empregado Efetivo
	F	Empregado Efetivo
	M	Empregado Efetivo

	M	Empregado Efetivo
	M	Empregado Efetivo
	F	Empregado Efetivo
	F	Empregado Efetivo
	F	Empregado Efetivo
	F	Empregado Efetivo
	F	Empregado Efetivo
	F	Empregado Efetivo
	F	Empregado Efetivo
	F	Empregado Efetivo
	M	Empregado Efetivo
	F	Empregado Efetivo
	F	Empregado Efetivo
	F	Empregado Efetivo
	F	Empregado Efetivo
	F	Empregado Efetivo
	M	Empregado Efetivo
	F	Empregado Efetivo
	F	Empregado Efetivo
	F	Empregado Efetivo
	F	Empregado Efetivo
	F	Empregado Efetivo
	F	Empregado Efetivo
	M	Empregado Efetivo
	M	Empregado Efetivo
	F	Empregado Efetivo
	F	Empregado Efetivo
	F	Empregado Efetivo
	F	Empregado Efetivo
	F	Empregado Efetivo
	F	Empregado Efetivo
	M	Empregado Efetivo
	F	Empregado Efetivo
	F	Empregado Efetivo
	F	Empregado Efetivo
	F	Empregado Efetivo
	F	Empregado Efetivo
	F	Empregado Efetivo
	F	Empregado Efetivo
	F	Empregado Efetivo
	M	Empregado Efetivo
	F	Empregado Efetivo
	F	Empregado Efetivo
	F	Empregado Efetivo
	F	Empregado Efetivo
	F	Empregado Efetivo
	F	Empregado Efetivo
	F	Empregado Efetivo

	M	Empregado Efetivo
	F	Empregado Efetivo
	F	Empregado Efetivo
	F	Empregado Efetivo
	M	Empregado Efetivo
	F	Empregado Efetivo
	M	Empregado Efetivo
	F	Empregado Efetivo
	M	Empregado Efetivo
	M	Empregado Efetivo
	M	Empregado Efetivo
	F	Empregado Efetivo
	F	Empregado Efetivo
	M	Empregado Efetivo
	M	Empregado Efetivo
	M	Empregado Efetivo
	F	Empregado Efetivo
	M	Empregado Efetivo
	F	Empregado Efetivo
	M	Empregado Efetivo
	F	Empregado Efetivo
	F	Empregado Efetivo
	F	Empregado Efetivo
	F	Empregado Efetivo
	M	Empregado Efetivo
	F	Empregado Efetivo
	M	Empregado Efetivo
	M	Empregado Efetivo
	F	Empregado Efetivo
	F	Empregado Efetivo
	M	Empregado Efetivo
	F	Empregado Efetivo
	F	Empregado Efetivo
	F	Empregado Efetivo
	F	Empregado Efetivo
	F	Empregado Efetivo
	F	Empregado Efetivo
	F	Empregado Efetivo
	M	Empregado Efetivo
	F	Empregado Efetivo
	F	Empregado Efetivo
	M	Empregado Efetivo
	M	Empregado Efetivo
	M	Empregado Efetivo
	F	Empregado Efetivo
	F	Empregado Efetivo
	F	Empregado Efetivo
	F	Empregado Efetivo

	F	Empregado Efetivo
	F	Empregado Efetivo
	F	Empregado Efetivo
	F	Empregado Efetivo
	M	Empregado Efetivo
	M	Empregado Efetivo
	F	Empregado Efetivo
	M	Empregado Efetivo
	F	Empregado Efetivo
	F	Empregado Efetivo
	M	Empregado Efetivo
	F	Empregado Efetivo
	F	Empregado Efetivo
	M	Empregado Efetivo
	F	Empregado Efetivo
	M	Empregado Efetivo
	M	Empregado Efetivo
	F	Empregado Efetivo
	M	Empregado Efetivo
	M	Empregado Efetivo
	M	Empregado Efetivo
	M	Empregado Efetivo
	M	Empregado Efetivo
	F	Empregado Efetivo
	F	Empregado Efetivo
	F	Empregado Efetivo
	F	Empregado Efetivo
	M	Empregado Efetivo
	M	Empregado Efetivo
	F	Empregado Efetivo
	F	Empregado Efetivo
	M	Empregado Efetivo
	F	Empregado Efetivo
	F	Empregado Efetivo
	F	Empregado Efetivo
	M	Empregado Efetivo
	F	Empregado Efetivo
	F	Empregado Efetivo
	M	Empregado Efetivo
	F	Empregado Efetivo
	F	Empregado Efetivo
	F	Empregado Efetivo
	F	Empregado Efetivo

	F	Empregado Efetivo
	F	Empregado Efetivo
	F	Empregado Efetivo
	F	Empregado Efetivo
	F	Empregado Efetivo
	F	Empregado Efetivo
	F	Empregado Efetivo
	F	Empregado Efetivo
	F	Empregado Efetivo
	F	Empregado Efetivo
	F	Empregado Efetivo
	F	Empregado Efetivo
	F	Empregado Efetivo
	F	Empregado Efetivo
	F	Empregado Efetivo
	F	Empregado Efetivo
	F	Empregado Efetivo
	F	Empregado Efetivo
	F	Empregado Efetivo
	F	Empregado Efetivo
	F	Empregado Efetivo
	F	Empregado Efetivo
	F	Empregado Efetivo
	M	Empregado Efetivo
	F	Empregado Efetivo
	F	Empregado Efetivo
	F	Empregado Efetivo
	F	Empregado Efetivo
	F	Empregado Efetivo
	F	Empregado Efetivo
	F	Empregado Efetivo
	M	Empregado Efetivo

	F	Empregado Efetivo
	F	Empregado Efetivo
	F	Empregado Efetivo
	F	Empregado Efetivo
	F	Empregado Efetivo
	M	Empregado Efetivo
	F	Empregado Efetivo
	F	Empregado Efetivo
	F	Empregado Efetivo
	M	Empregado Efetivo
	M	Empregado Efetivo
	F	Empregado Efetivo
	F	Empregado Efetivo
	F	Empregado Efetivo
	F	Empregado Efetivo
	F	Empregado Efetivo
	F	Empregado Efetivo
	F	Empregado Efetivo
	F	Empregado Efetivo
	F	Empregado Efetivo
	F	Empregado Efetivo
	M	Empregado Efetivo
	F	Empregado Efetivo
	F	Empregado Efetivo
	F	Empregado Efetivo
	F	Empregado Efetivo
	F	Empregado Efetivo
	F	Empregado Efetivo
	M	Empregado Efetivo
	F	Empregado Efetivo
	M	Empregado Efetivo
	F	Empregado Efetivo

	F	Empregado Efetivo
	F	Empregado Efetivo
	M	Empregado Efetivo
	M	Empregado Efetivo
	M	Empregado Efetivo
	F	Empregado Efetivo
	F	Empregado Efetivo
	F	Empregado Efetivo
	F	Empregado Efetivo
	F	Empregado Efetivo
	F	Empregado Efetivo
	F	Empregado Efetivo
	F	Empregado Efetivo
	F	Empregado Efetivo
	F	Empregado Efetivo
	F	Empregado Efetivo
	F	Empregado Efetivo
	F	Empregado Efetivo
	F	Empregado Efetivo
	F	Empregado Efetivo
	M	Empregado Efetivo
	F	Empregado Efetivo
	F	Empregado Efetivo
	F	Empregado Efetivo
	M	Empregado Efetivo
	F	Empregado Efetivo
	F	Empregado Efetivo
	F	Empregado Efetivo
	F	Empregado Efetivo
	F	Empregado Efetivo
	F	Empregado Efetivo
	F	Empregado Efetivo
	F	Empregado Efetivo
	F	Empregado Efetivo
	F	Empregado Efetivo
	F	Empregado Efetivo
	F	Empregado Efetivo
	F	Empregado Efetivo
	F	Empregado Efetivo
	F	Empregado Efetivo
	F	Empregado Efetivo

	F	Empregado Efetivo
	F	Empregado Efetivo
	F	Empregado Efetivo
	M	Empregado Efetivo
	M	Empregado Efetivo
	F	Empregado Efetivo
	F	Empregado Efetivo
	F	Empregado Efetivo
	F	Empregado Efetivo
	F	Empregado Efetivo
	F	Empregado Efetivo
	F	Empregado Efetivo
	F	Empregado Efetivo
	F	Empregado Efetivo
	F	Empregado Efetivo
	F	Empregado Efetivo
	F	Empregado Efetivo
	F	Empregado Efetivo
	F	Empregado Efetivo
	F	Empregado Efetivo
	M	Empregado Efetivo
	M	Empregado Efetivo
	F	Empregado Efetivo
	M	Empregado Efetivo
	F	Empregado Efetivo
	F	Empregado Efetivo
	F	Empregado Efetivo
	F	Empregado Efetivo
	F	Empregado Efetivo
	F	Empregado Efetivo
	F	Empregado Efetivo
	F	Empregado Efetivo
	F	Empregado Efetivo
	M	Empregado Efetivo
	F	Empregado Efetivo
	F	Empregado Efetivo
	F	Empregado Efetivo
	F	Empregado Efetivo
	F	Empregado Efetivo
	M	Empregado Efetivo
	M	Empregado Efetivo
	M	Empregado Efetivo

	M	Empregado Efetivo
	F	Empregado Efetivo
	M	Empregado Efetivo
	M	Empregado Efetivo
	M	Empregado Efetivo
	F	Empregado Efetivo
	M	Empregado Efetivo
	M	Empregado Efetivo
	F	Empregado Efetivo
	M	Empregado Efetivo
	M	Empregado Efetivo
	F	Empregado Efetivo
	F	Empregado Efetivo
	F	Empregado Efetivo
	F	Empregado Efetivo
	F	Empregado Efetivo
	F	Empregado Efetivo
	F	Empregado Efetivo
	M	Empregado Efetivo
	F	Empregado Efetivo
	F	Empregado Efetivo
	F	Empregado Efetivo
	F	Empregado Efetivo
	M	Empregado Efetivo
	F	Empregado Efetivo
	F	Empregado Efetivo
	F	Empregado Efetivo
	F	Empregado Efetivo
	F	Empregado Efetivo
	F	Empregado Efetivo
	M	Empregado Efetivo
	F	Empregado Efetivo
	M	Empregado Efetivo
	F	Empregado Efetivo
	F	Empregado Efetivo
	M	Empregado Efetivo
	F	Empregado Efetivo
	F	Empregado Efetivo
	F	Empregado Efetivo

	F	Empregado Efetivo
	F	Empregado Efetivo
	F	Empregado Efetivo
	F	Empregado Efetivo
	F	Empregado Efetivo
	F	Empregado Efetivo
	F	Empregado Efetivo
	F	Empregado Efetivo
	F	Empregado Efetivo
	F	Empregado Efetivo
	M	Empregado Efetivo
	F	Empregado Efetivo
	F	Empregado Efetivo
	F	Empregado Efetivo
	F	Empregado Efetivo
	F	Empregado Efetivo
	F	Empregado Efetivo
	F	Empregado Efetivo
	F	Empregado Efetivo
	M	Empregado Efetivo
	F	Empregado Efetivo
	F	Empregado Efetivo
	M	Empregado Efetivo
	F	Empregado Efetivo
	F	Empregado Efetivo
	F	Empregado Efetivo
	M	Empregado Efetivo
	F	Empregado Efetivo
	F	Empregado Efetivo
	F	Empregado Efetivo
	F	Empregado Efetivo
	F	Empregado Efetivo
	F	Empregado Efetivo
	F	Empregado Efetivo

	F	Empregado Efetivo
	F	Empregado Efetivo
	M	Empregado Efetivo
	M	Empregado Efetivo
	M	Empregado Efetivo
	F	Empregado Efetivo
	M	Empregado Efetivo
	F	Empregado Efetivo
	F	Empregado Efetivo
	F	Empregado Efetivo
	F	Empregado Efetivo
	M	Empregado Efetivo
	F	Empregado Efetivo
	F	Empregado Efetivo
	F	Empregado Efetivo
	F	Empregado Efetivo
	F	Empregado Efetivo
	F	Empregado Efetivo
	F	Empregado Efetivo
	F	Empregado Efetivo
	M	Empregado Efetivo
	F	Empregado Efetivo
	F	Empregado Efetivo
	F	Empregado Efetivo
	F	Empregado Efetivo
	F	Empregado Efetivo
	F	Empregado Efetivo
	F	Empregado Efetivo
	F	Empregado Efetivo
	F	Empregado Efetivo
	F	Empregado Efetivo
	M	Empregado Efetivo
	F	Empregado Efetivo
	F	Empregado Efetivo
	F	Empregado Efetivo
	F	Empregado Efetivo
	F	Empregado Efetivo
	F	Empregado Efetivo
	F	Empregado Efetivo
	F	Empregado Efetivo
	F	Empregado Efetivo
	F	Empregado Efetivo
	F	Empregado Efetivo
	M	Empregado Efetivo
	F	Empregado Efetivo
	F	Empregado Efetivo
	F	Empregado Efetivo
	F	Empregado Efetivo

	M	Empregado Efetivo
	F	Empregado Efetivo
	F	Empregado Efetivo
	F	Empregado Efetivo
	F	Empregado Efetivo
	M	Empregado Efetivo
	F	Empregado Efetivo
	F	Empregado Efetivo
	F	Empregado Efetivo
	F	Empregado Efetivo
	F	Empregado Efetivo
	F	Empregado Efetivo
	F	Empregado Efetivo
	F	Empregado Efetivo
	F	Empregado Efetivo
	F	Empregado Efetivo
	F	Empregado Efetivo
	F	Empregado Efetivo
	F	Empregado Efetivo
	F	Empregado Efetivo
	F	Empregado Efetivo
	F	Empregado Efetivo
	F	Empregado Efetivo
	F	Empregado Efetivo
	F	Empregado Efetivo
	F	Empregado Efetivo
	F	Empregado Efetivo
	F	Empregado Efetivo
	F	Empregado Efetivo
	F	Empregado Efetivo
	F	Empregado Efetivo
	F	Empregado Efetivo
	F	Empregado Efetivo
	F	Empregado Efetivo
	F	Empregado Efetivo
	F	Empregado Efetivo
	F	Empregado Efetivo
	F	Empregado Efetivo
	F	Empregado Efetivo
	F	Empregado Efetivo
	F	Empregado Efetivo
	F	Empregado Efetivo
	F	Empregado Efetivo
	F	Empregado Efetivo
	F	Empregado Efetivo
	F	Empregado Efetivo
	F	Empregado Efetivo
	F	Empregado Efetivo

	F	Empregado Efetivo
	M	Empregado Efetivo
	F	Empregado Efetivo
	M	Empregado Efetivo
	M	Empregado Efetivo
	F	Empregado Efetivo
	F	Empregado Efetivo
	F	Empregado Efetivo
	F	Empregado Efetivo
	M	Empregado Efetivo
	F	Empregado Efetivo
	F	Empregado Efetivo
	M	Empregado Efetivo
	M	Empregado Efetivo
	M	Empregado Efetivo
	F	Empregado Efetivo
	M	Empregado Efetivo
	M	Empregado Efetivo
	M	Empregado Efetivo
	F	Empregado Efetivo
	F	Empregado Efetivo
	M	Empregado Efetivo
	M	Empregado Efetivo
	F	Empregado Efetivo
	F	Empregado Efetivo
	F	Empregado Efetivo
	F	Empregado Efetivo
	F	Empregado Efetivo
	M	Empregado Efetivo
	F	Empregado Efetivo
	M	Empregado Efetivo
	F	Empregado Efetivo
	M	Empregado Efetivo
	M	Empregado Efetivo
	M	Empregado Efetivo
	F	Empregado Efetivo
	F	Empregado Efetivo
	M	Empregado Efetivo
	F	Empregado Efetivo
	F	Empregado Efetivo
	F	Empregado Efetivo
	M	Empregado Efetivo
	F	Empregado Efetivo
	M	Empregado Efetivo
	F	Empregado Efetivo
	F	Empregado Efetivo
	M	Empregado Efetivo
	F	Empregado Efetivo

	F	Empregado Efetivo
	M	Empregado Efetivo
	F	Empregado Efetivo
	M	Empregado Efetivo
	M	Empregado Efetivo
	M	Empregado Efetivo
	F	Empregado Efetivo
	M	Empregado Efetivo
	F	Empregado Efetivo
	F	Empregado Efetivo
	F	Empregado Efetivo
	M	Empregado Efetivo
	M	Empregado Efetivo
	M	Empregado Efetivo
	M	Empregado Efetivo
	F	Empregado Efetivo
	F	Empregado Efetivo
	F	Empregado Efetivo
	M	Empregado Efetivo
	F	Empregado Efetivo
	F	Empregado Efetivo
	F	Empregado Efetivo
	M	Empregado Efetivo
	M	Empregado Efetivo
	F	Empregado Efetivo
	M	Empregado Efetivo
	F	Empregado Efetivo
	M	Empregado Efetivo
	M	Empregado Efetivo
	F	Empregado Efetivo
	M	Empregado Efetivo
	M	Empregado Efetivo
	M	Empregado Efetivo
	M	Empregado Efetivo
	F	Empregado Efetivo
	M	Empregado Efetivo
	M	Empregado Efetivo
	M	Empregado Efetivo
	F	Empregado Efetivo
	F	Empregado Efetivo
	M	Empregado Efetivo
	F	Empregado Efetivo
	F	Empregado Efetivo
	F	Empregado Efetivo
	F	Empregado Efetivo
	F	Empregado Efetivo
	F	Empregado Efetivo
	F	Empregado Efetivo

	M	Empregado Efetivo
	M	Empregado Efetivo
	F	Empregado Efetivo
	F	Empregado Efetivo
	F	Empregado Efetivo
	F	Empregado Efetivo
	F	Empregado Efetivo
	F	Empregado Efetivo
	F	Empregado Efetivo
	F	Empregado Efetivo
	F	Empregado Efetivo
	F	Empregado Efetivo
	F	Empregado Efetivo
	M	Empregado Efetivo
	F	Empregado Efetivo
	M	Empregado Efetivo
	F	Empregado Efetivo
	F	Empregado Efetivo
	F	Empregado Efetivo
	F	Empregado Efetivo
	M	Empregado Efetivo
	F	Empregado Efetivo
	M	Empregado Efetivo
	F	Empregado Efetivo
	F	Empregado Efetivo
	F	Empregado Efetivo
	F	Empregado Efetivo
	F	Empregado Efetivo
	M	Empregado Efetivo
	F	Empregado Efetivo
	F	Empregado Efetivo
	F	Empregado Efetivo
	F	Empregado Efetivo
	F	Empregado Efetivo
	M	Empregado Efetivo
	F	Empregado Efetivo
	F	Empregado Efetivo
	F	Empregado Efetivo
	F	Empregado Efetivo
	F	Empregado Efetivo
	F	Empregado Efetivo
	M	Empregado Efetivo
	M	Empregado Efetivo
	F	Empregado Efetivo
	M	Empregado Efetivo

	M	Empregado Efetivo
	M	Empregado Efetivo
	F	Empregado Efetivo
	F	Empregado Efetivo
	F	Empregado Efetivo
	F	Empregado Efetivo
	F	Empregado Efetivo
	F	Empregado Efetivo
	F	Empregado Efetivo
	F	Empregado Efetivo
	F	Empregado Efetivo
	F	Empregado Efetivo
	F	Empregado Efetivo
	F	Empregado Efetivo
	F	Empregado Efetivo
	F	Empregado Efetivo
	M	Empregado Efetivo
	F	Empregado Efetivo
	F	Empregado Efetivo
	F	Empregado Efetivo
	F	Empregado Efetivo
	F	Empregado Efetivo
	M	Empregado Efetivo
	M	Empregado Efetivo
	M	Empregado Efetivo
	M	Empregado Efetivo
	F	Empregado Efetivo
	F	Empregado Efetivo
	F	Empregado Efetivo
	F	Empregado Efetivo
	F	Empregado Efetivo
	F	Empregado Efetivo

	M	Empregado Efetivo
	F	Empregado Efetivo
	F	Empregado Efetivo
	F	Empregado Efetivo
	M	Empregado Efetivo
	M	Empregado Efetivo
	F	Empregado Efetivo
	M	Empregado Efetivo
	F	Empregado Efetivo
	F	Empregado Efetivo
	M	Empregado Efetivo
	M	Empregado Efetivo
	M	Empregado Efetivo
	M	Empregado Efetivo
	M	Empregado Efetivo
	M	Empregado Efetivo
	M	Empregado Efetivo
	M	Empregado Efetivo
	M	Empregado Efetivo
	M	Empregado Efetivo
	M	Empregado Efetivo
	M	Empregado Efetivo
	M	Empregado Efetivo
	M	Empregado Efetivo
	M	Empregado Efetivo
	M	Empregado Efetivo
	M	Empregado Efetivo
	F	Empregado Efetivo
	M	Empregado Efetivo
	M	Empregado Efetivo
	F	Empregado Efetivo
	M	Empregado Efetivo
	F	Empregado Efetivo

	F	Empregado Efetivo
	F	Empregado Efetivo
	F	Empregado Efetivo
	F	Empregado Efetivo
	F	Empregado Efetivo
	F	Empregado Efetivo
	F	Empregado Efetivo
	F	Empregado Efetivo
	F	Empregado Efetivo
	F	Empregado Efetivo
	F	Empregado Efetivo
	F	Empregado Efetivo
	M	Empregado Efetivo
	M	Empregado Efetivo
	F	Empregado Efetivo
	M	Empregado Efetivo
	M	Empregado Efetivo
	F	Empregado Efetivo
	M	Empregado Efetivo
	M	Empregado Efetivo
	F	Empregado Efetivo
	F	Empregado Efetivo
	F	Empregado Efetivo
	M	Empregado Efetivo
	F	Empregado Efetivo
	M	Empregado Efetivo
	M	Empregado Efetivo
	F	Empregado Efetivo
	M	Empregado Efetivo
	F	Empregado Efetivo
	F	Empregado Efetivo
	M	Empregado Efetivo

	M	Empregado Efetivo
	F	Empregado Efetivo
	M	Empregado Efetivo
	F	Empregado Efetivo
	F	Empregado Efetivo
	F	Empregado Efetivo
	F	Empregado Efetivo
	F	Empregado Efetivo
	F	Empregado Efetivo
	F	Empregado Efetivo
	M	Empregado Efetivo
	M	Empregado Efetivo
	F	Empregado Efetivo
	M	Empregado Efetivo
	M	Empregado Efetivo
	F	Empregado Efetivo
	F	Empregado Efetivo
	F	Empregado Efetivo
	F	Empregado Efetivo
	F	Empregado Efetivo
	F	Empregado Efetivo
	F	Empregado Efetivo
	F	Empregado Efetivo
	F	Empregado Efetivo
	F	Empregado Efetivo
	M	Empregado Efetivo
	M	Empregado Efetivo
	M	Empregado Efetivo
	F	Empregado Efetivo
	M	Empregado Efetivo
	F	Empregado Efetivo
	F	Empregado Efetivo
	F	Empregado Efetivo
	F	Empregado Efetivo
	M	Empregado Efetivo
	M	Empregado Efetivo
	M	Empregado Efetivo
	M	Empregado Efetivo
	M	Empregado Efetivo
	M	Empregado Efetivo
	M	Empregado Efetivo
	M	Empregado Efetivo
	F	Empregado Efetivo
	F	Empregado Efetivo

	F	Empregado Efetivo
	F	Empregado Efetivo
	F	Empregado Efetivo
	M	Empregado Efetivo
	F	Empregado Efetivo
	F	Empregado Efetivo
	F	Empregado Efetivo
	F	Empregado Efetivo
	F	Empregado Efetivo
	F	Empregado Efetivo
	F	Empregado Efetivo
	F	Empregado Efetivo
	F	Empregado Efetivo
	F	Empregado Efetivo
	F	Empregado Efetivo
	F	Empregado Efetivo
	F	Empregado Efetivo
	F	Empregado Efetivo
	F	Empregado Efetivo
	F	Empregado Efetivo
	F	Empregado Efetivo
	F	Empregado Efetivo
	F	Empregado Efetivo
	F	Empregado Efetivo
	F	Empregado Efetivo
	F	Empregado Efetivo
	F	Empregado Efetivo
	F	Empregado Efetivo
	F	Empregado Efetivo
	F	Empregado Efetivo
	F	Empregado Efetivo
	F	Empregado Efetivo
	F	Empregado Efetivo
	F	Empregado Efetivo
	F	Empregado Efetivo
	F	Empregado Efetivo
	F	Empregado Efetivo
	F	Empregado Efetivo
	F	Empregado Efetivo
	F	Empregado Efetivo
	F	Empregado Efetivo
	F	Empregado Efetivo
	F	Empregado Efetivo
	F	Empregado Efetivo
	F	Empregado Efetivo
	F	Empregado Efetivo
	F	Empregado Efetivo
	F	Empregado Efetivo

	F	Empregado Efetivo
	F	Empregado Efetivo
	F	Empregado Efetivo
	F	Empregado Efetivo
	F	Empregado Efetivo
	F	Empregado Efetivo
	F	Empregado Efetivo
	F	Empregado Efetivo
	F	Empregado Efetivo
	F	Empregado Efetivo
	F	Empregado Efetivo
	F	Empregado Efetivo
	F	Empregado Efetivo
	F	Empregado Efetivo
	F	Empregado Efetivo
	F	Empregado Efetivo
	F	Empregado Efetivo
	F	Empregado Efetivo
	F	Empregado Efetivo
	F	Empregado Efetivo
	F	Empregado Efetivo
	F	Empregado Efetivo
	F	Empregado Efetivo
	F	Empregado Efetivo
	F	Empregado Efetivo
	F	Empregado Efetivo
	F	Empregado Efetivo
	F	Empregado Efetivo
	F	Empregado Efetivo
	F	Empregado Efetivo
	F	Empregado Efetivo
	F	Empregado Efetivo
	F	Empregado Efetivo
	F	Empregado Efetivo
	F	Empregado Efetivo
	M	Empregado Efetivo
	F	Empregado Efetivo
	F	Empregado Efetivo
	F	Empregado Efetivo
	F	Empregado Efetivo
	F	Empregado Efetivo
	F	Empregado Efetivo
	F	Empregado Efetivo
	F	Empregado Efetivo
	F	Empregado Efetivo
	F	Empregado Efetivo
	F	Empregado Efetivo
	F	Empregado Efetivo

	F	Empregado Efetivo
	F	Empregado Efetivo
	F	Empregado Efetivo
	F	Empregado Efetivo
	F	Empregado Efetivo
	F	Empregado Efetivo
	M	Empregado Efetivo
	F	Empregado Efetivo
	F	Empregado Efetivo
	F	Empregado Efetivo
	M	Empregado Efetivo
	F	Empregado Efetivo
	F	Empregado Efetivo
	F	Empregado Efetivo
	M	Empregado Efetivo
	F	Empregado Efetivo
	F	Empregado Efetivo
	M	Empregado Efetivo
	F	Empregado Efetivo
	F	Empregado Efetivo
	F	Empregado Efetivo
	F	Empregado Efetivo
	F	Empregado Efetivo
	F	Empregado Efetivo
	F	Empregado Efetivo
	F	Empregado Efetivo
	F	Empregado Efetivo
	F	Empregado Efetivo
	F	Empregado Efetivo
	F	Empregado Efetivo
	F	Empregado Efetivo
	M	Empregado Efetivo
	M	Empregado Efetivo
	F	Empregado Efetivo
	M	Empregado Efetivo
	M	Empregado Efetivo
	F	Empregado Efetivo
	F	Empregado Efetivo
	F	Empregado Efetivo
	F	Empregado Efetivo
	F	Empregado Efetivo
	F	Empregado Efetivo
	M	Empregado Efetivo
	F	Empregado Efetivo

	M	Empregado Efetivo
	F	Empregado Efetivo
	F	Empregado Efetivo
	M	Empregado Efetivo
	F	Empregado Efetivo
	F	Empregado Efetivo
	F	Empregado Efetivo
	F	Empregado Efetivo
	F	Empregado Efetivo
	F	Empregado Efetivo
	F	Empregado Efetivo
	F	Empregado Efetivo
	M	Empregado Efetivo
	F	Empregado Efetivo
	M	Empregado Efetivo
	F	Empregado Efetivo
	M	Empregado Efetivo
	M	Empregado Efetivo
	F	Empregado Efetivo
	M	Empregado Efetivo
	F	Empregado Efetivo
	F	Empregado Efetivo
	F	Empregado Efetivo
	F	Empregado Efetivo
	F	Empregado Efetivo
	M	Empregado Efetivo
	F	Empregado Efetivo
	F	Empregado Efetivo
	F	Empregado Efetivo
	F	Empregado Efetivo
	F	Empregado Efetivo
	F	Empregado Efetivo
	F	Empregado Efetivo
	M	Empregado Efetivo
	F	Empregado Efetivo
	M	Empregado Efetivo
	F	Empregado Efetivo
	F	Empregado Efetivo
	F	Empregado Efetivo
	M	Empregado Efetivo
	F	Empregado Efetivo
	F	Empregado Efetivo
	M	Empregado Efetivo
	M	Empregado Efetivo
	F	Empregado Efetivo

	M	Empregado Efetivo
	M	Empregado Efetivo
	M	Empregado Efetivo
	F	Empregado Efetivo
	F	Empregado Efetivo
	F	Empregado Efetivo
	M	Empregado Efetivo
	M	Empregado Efetivo
	M	Empregado Efetivo
	M	Empregado Efetivo
	F	Empregado Efetivo
	F	Empregado Efetivo
	M	Empregado Efetivo
	M	Empregado Efetivo
	F	Empregado Efetivo
	M	Empregado Efetivo
	F	Empregado Efetivo
	F	Empregado Efetivo
	F	Empregado Efetivo
	F	Empregado Efetivo
	M	Empregado Efetivo
	F	Empregado Efetivo
	M	Empregado Efetivo
	M	Empregado Efetivo
	M	Empregado Efetivo
	M	Empregado Efetivo
	M	Empregado Efetivo
	M	Empregado Efetivo
	F	Empregado Efetivo
	F	Empregado Efetivo
	M	Empregado Efetivo
	F	Empregado Efetivo
	M	Empregado Efetivo
	F	Empregado Efetivo
	M	Empregado Efetivo
	M	Empregado Efetivo
	M	Empregado Efetivo
	M	Empregado Efetivo
	F	Empregado Efetivo
	M	Empregado Efetivo
	F	Empregado Efetivo
	F	Empregado Efetivo
	M	Empregado Efetivo
	M	Empregado Efetivo
	F	Empregado Efetivo
	M	Empregado Efetivo
	F	Empregado Efetivo
	F	Empregado Efetivo

	M	Empregado Efetivo
	M	Empregado Efetivo
	M	Empregado Efetivo
	M	Empregado Efetivo
	F	Empregado Efetivo
	F	Empregado Efetivo
	F	Empregado Efetivo
	M	Empregado Efetivo
	F	Empregado Efetivo
	F	Empregado Efetivo
	F	Empregado Efetivo
	F	Empregado Efetivo
	F	Empregado Efetivo
	F	Empregado Efetivo
	F	Empregado Efetivo
	M	Empregado Efetivo
	F	Empregado Efetivo
	M	Empregado Efetivo
	M	Empregado Efetivo
	F	Empregado Efetivo
	M	Empregado Efetivo
	M	Empregado Efetivo
	F	Empregado Efetivo
	M	Empregado Efetivo
	F	Empregado Efetivo
	F	Empregado Efetivo
	F	Empregado Efetivo
	M	Empregado Efetivo
	M	Empregado Efetivo
	F	Empregado Efetivo
	F	Empregado Efetivo
	F	Empregado Efetivo
	M	Empregado Efetivo
	F	Empregado Efetivo
	M	Empregado Efetivo
	F	Empregado Efetivo
	M	Empregado Efetivo
	F	Empregado Efetivo
	F	Empregado Efetivo
	F	Empregado Efetivo
	F	Empregado Efetivo
	M	Empregado Efetivo
	M	Empregado Efetivo
	F	Empregado Efetivo
	F	Empregado Efetivo
	F	Empregado Efetivo
	M	Empregado Efetivo
	M	Empregado Efetivo

	M	Empregado Efetivo
	F	Empregado Efetivo
	M	Empregado Efetivo
	M	Empregado Efetivo
	F	Empregado Efetivo
	F	Empregado Efetivo
	M	Empregado Efetivo
	F	Empregado Efetivo
	F	Empregado Efetivo
	F	Empregado Efetivo
	F	Empregado Efetivo
	M	Empregado Efetivo
	M	Empregado Efetivo
	F	Empregado Efetivo
	F	Empregado Efetivo
	F	Empregado Efetivo
	M	Empregado Efetivo
	F	Empregado Efetivo
	F	Empregado Efetivo
	F	Empregado Efetivo
	F	Empregado Efetivo
	F	Empregado Efetivo
	M	Empregado Efetivo
	M	Empregado Efetivo
	F	Empregado Efetivo
	F	Empregado Efetivo
	M	Empregado Efetivo
	M	Empregado Efetivo
	F	Empregado Efetivo
	F	Empregado Efetivo
	M	Empregado Efetivo
	F	Empregado Efetivo
	F	Empregado Efetivo
	F	Empregado Efetivo
	F	Empregado Efetivo
	M	Empregado Efetivo
	F	Empregado Efetivo
	M	Empregado Efetivo
	F	Empregado Efetivo
	F	Empregado Efetivo

	F	Empregado Efetivo
	F	Empregado Efetivo
	F	Empregado Efetivo
	F	Empregado Efetivo
	M	Empregado Efetivo
	F	Empregado Efetivo
	M	Empregado Efetivo
	M	Empregado Efetivo
	M	Empregado Efetivo
	M	Empregado Efetivo
	M	Empregado Efetivo
	M	Empregado Efetivo
	M	Empregado Efetivo
	M	Empregado Efetivo
	F	Empregado Efetivo
	M	Empregado Efetivo
	M	Empregado Efetivo
	M	Empregado Efetivo
	M	Empregado Efetivo
	M	Empregado Efetivo
	M	Empregado Efetivo
	F	Empregado Efetivo
	M	Empregado Efetivo
	M	Empregado Efetivo
	M	Empregado Efetivo
	M	Empregado Efetivo
	F	Empregado Efetivo
	M	Empregado Efetivo
	F	Empregado Efetivo
	F	Empregado Efetivo
	M	Empregado Efetivo
	F	Empregado Efetivo

	F	Empregado Efetivo
	F	Empregado Efetivo
	F	Empregado Efetivo
	M	Empregado Efetivo
	F	Empregado Efetivo
	F	Empregado Efetivo
	M	Empregado Efetivo
	M	Empregado Efetivo
	F	Empregado Efetivo
	F	Empregado Efetivo
	M	Empregado Efetivo
	M	Empregado Efetivo
	F	Empregado Efetivo
	F	Empregado Efetivo
	F	Empregado Efetivo
	F	Empregado Efetivo
	F	Empregado Efetivo
	F	Empregado Efetivo
	M	Empregado Efetivo
	F	Empregado Efetivo
	F	Empregado Efetivo
	F	Empregado Efetivo
	F	Empregado Efetivo
	F	Empregado Efetivo
	M	Empregado Efetivo
	M	Empregado Efetivo
	F	Empregado Efetivo
	F	Empregado Efetivo
	F	Empregado Efetivo
	M	Empregado Efetivo
	M	Empregado Efetivo
	F	Empregado Efetivo
	F	Empregado Efetivo
	F	Empregado Efetivo
	F	Empregado Efetivo
	F	Empregado Efetivo
	M	Empregado Efetivo
	F	Empregado Efetivo
	F	Empregado Efetivo
	M	Empregado Efetivo

	M	Empregado Efetivo
	M	Empregado Efetivo
	F	Empregado Efetivo
	F	Empregado Efetivo
	M	Empregado Efetivo
	F	Empregado Efetivo
	F	Empregado Efetivo
	M	Empregado Efetivo
	M	Empregado Efetivo
	F	Empregado Efetivo
	F	Empregado Efetivo
	F	Empregado Efetivo
	F	Empregado Efetivo
	F	Empregado Efetivo
	F	Empregado Efetivo
	M	Empregado Efetivo
	F	Empregado Efetivo
	F	Empregado Efetivo
	F	Empregado Efetivo
	M	Empregado Efetivo
	F	Empregado Efetivo
	F	Empregado Efetivo
	M	Empregado Efetivo
	F	Empregado Efetivo
	M	Empregado Efetivo
	F	Empregado Efetivo
	F	Empregado Efetivo
	F	Empregado Efetivo
	M	Empregado Efetivo
	M	Empregado Efetivo
	F	Empregado Efetivo
	M	Empregado Efetivo
	M	Empregado Efetivo
	F	Empregado Efetivo
	F	Empregado Efetivo
	F	Empregado Efetivo
	M	Empregado Efetivo
	F	Empregado Efetivo
	F	Empregado Efetivo
	F	Empregado Efetivo
	F	Empregado Efetivo
	F	Empregado Efetivo
	F	Empregado Efetivo
	M	Empregado Efetivo
	F	Empregado Efetivo

	F	Empregado Efetivo
	F	Empregado Efetivo
	M	Empregado Efetivo
	F	Empregado Efetivo
	F	Empregado Efetivo
	F	Empregado Efetivo
	F	Empregado Efetivo
	F	Empregado Efetivo
	M	Empregado Efetivo
	F	Empregado Efetivo
	F	Empregado Efetivo
	M	Empregado Efetivo
	F	Empregado Efetivo
	M	Empregado Efetivo
	F	Empregado Efetivo
	F	Empregado Efetivo
	F	Empregado Efetivo
	F	Empregado Efetivo
	F	Empregado Efetivo
	F	Empregado Efetivo
	F	Empregado Efetivo
	F	Empregado Efetivo
	F	Empregado Efetivo
	F	Empregado Efetivo
	F	Empregado Efetivo
	F	Empregado Efetivo
	F	Empregado Efetivo
	F	Empregado Efetivo
	F	Empregado Efetivo
	F	Empregado Efetivo
	F	Empregado Efetivo
	F	Empregado Efetivo
	M	Empregado Efetivo
	F	Empregado Efetivo
	M	Empregado Efetivo
	M	Empregado Efetivo
	F	Empregado Efetivo
	F	Empregado Efetivo
	F	Empregado Efetivo
	M	Empregado Efetivo
	M	Empregado Efetivo

	F	Empregado Efetivo
	M	Empregado Efetivo
	F	Empregado Efetivo
	F	Empregado Efetivo
	M	Empregado Efetivo
	F	Empregado Efetivo
	F	Empregado Efetivo
	F	Empregado Efetivo
	F	Empregado Efetivo
	M	Empregado Efetivo
	M	Empregado Efetivo
	F	Empregado Efetivo
	F	Empregado Efetivo
	F	Empregado Efetivo
	F	Empregado Efetivo
	F	Empregado Efetivo
	F	Empregado Efetivo
	F	Empregado Efetivo
	F	Empregado Efetivo
	F	Empregado Efetivo
	F	Empregado Efetivo
	F	Empregado Efetivo
	F	Empregado Efetivo
	F	Empregado Efetivo
	F	Empregado Efetivo
	M	Empregado Efetivo
	F	Empregado Efetivo
	M	Empregado Efetivo
	F	Empregado Efetivo
	F	Empregado Efetivo
	M	Empregado Efetivo
	F	Empregado Efetivo
	M	Empregado Efetivo
	M	Empregado Efetivo
	F	Empregado Efetivo
	M	Empregado Efetivo
	M	Empregado Efetivo
	M	Empregado Efetivo
	M	Empregado Efetivo
	M	Empregado Efetivo
	M	Empregado Efetivo
	M	Empregado Efetivo
	F	Empregado Efetivo
	M	Empregado Efetivo
	M	Empregado Efetivo
	F	Empregado Efetivo
	M	Empregado Efetivo
	M	Empregado Efetivo

	F	Empregado Efetivo
	F	Empregado Efetivo
	F	Empregado Efetivo
	F	Empregado Efetivo
	F	Empregado Efetivo
	F	Empregado Efetivo
	M	Empregado Efetivo
	M	Empregado Efetivo
	F	Empregado Efetivo
	F	Empregado Efetivo
	F	Empregado Efetivo
	F	Empregado Efetivo
	F	Empregado Efetivo
	F	Empregado Efetivo
	M	Empregado Efetivo
	F	Empregado Efetivo
	M	Empregado Efetivo
	F	Empregado Efetivo
	M	Empregado Efetivo
	F	Empregado Efetivo
	M	Empregado Efetivo
	F	Empregado Efetivo
	F	Empregado Efetivo
	F	Empregado Efetivo
	F	Empregado Efetivo
	F	Empregado Efetivo
	F	Empregado Efetivo
	M	Empregado Efetivo
	F	Empregado Efetivo
	F	Empregado Efetivo
	M	Empregado Efetivo
	M	Empregado Efetivo
	M	Empregado Efetivo
	M	Empregado Efetivo
	M	Empregado Efetivo
	F	Empregado Efetivo
	M	Empregado Efetivo
	M	Empregado Efetivo
	M	Empregado Efetivo
	F	Empregado Efetivo
	M	Empregado Efetivo
	F	Empregado Efetivo
	F	Empregado Efetivo
	M	Empregado Efetivo
	M	Empregado Efetivo
	M	Empregado Efetivo
	F	Empregado Efetivo
	F	Empregado Efetivo

	F	Empregado Efetivo
	F	Empregado Efetivo
	M	Empregado Efetivo
	F	Empregado Efetivo
	F	Empregado Efetivo
	F	Empregado Efetivo
	F	Empregado Efetivo
	F	Empregado Efetivo
	F	Empregado Efetivo
	M	Empregado Efetivo
	F	Empregado Efetivo
	M	Empregado Efetivo
	M	Empregado Efetivo
	F	Empregado Efetivo
	M	Empregado Efetivo
	M	Empregado Efetivo
	M	Empregado Efetivo
	F	Empregado Efetivo
	M	Empregado Efetivo
	F	Empregado Efetivo
	F	Empregado Efetivo
	F	Empregado Efetivo
	F	Empregado Efetivo
	F	Empregado Efetivo
	M	Empregado Efetivo
	M	Empregado Efetivo
	M	Empregado Efetivo
	M	Empregado Efetivo
	F	Empregado Efetivo
	F	Empregado Efetivo
	F	Empregado Efetivo
	F	Empregado Efetivo
	F	Empregado Efetivo
	M	Empregado Efetivo
	M	Empregado Efetivo
	F	Empregado Efetivo
	F	Empregado Efetivo
	M	Empregado Efetivo
	F	Empregado Efetivo
	F	Empregado Efetivo
	F	Empregado Efetivo
	F	Empregado Efetivo
	F	Empregado Efetivo
	F	Empregado Efetivo
	F	Empregado Efetivo
	F	Empregado Efetivo
	F	Empregado Efetivo
	M	Empregado Efetivo

	F	Empregado Efetivo
	F	Empregado Efetivo
	F	Empregado Efetivo
	F	Empregado Efetivo
	F	Empregado Efetivo
	M	Empregado Efetivo
	F	Empregado Efetivo
	M	Empregado Efetivo
	F	Empregado Efetivo
	M	Empregado Efetivo
	F	Empregado Efetivo
	F	Empregado Efetivo
	F	Empregado Efetivo
	F	Empregado Efetivo
	F	Empregado Efetivo
	F	Empregado Efetivo
	F	Empregado Efetivo
	M	Empregado Efetivo
	F	Empregado Efetivo
	F	Empregado Efetivo
	F	Empregado Efetivo
	F	Empregado Efetivo
	F	Empregado Efetivo
	F	Empregado Efetivo
	M	Empregado Efetivo
	M	Empregado Efetivo
	M	Empregado Efetivo
	F	Empregado Efetivo
	F	Empregado Efetivo
	F	Empregado Efetivo
	M	Empregado Efetivo
	F	Empregado Efetivo
	F	Empregado Efetivo
	F	Empregado Efetivo
	F	Empregado Efetivo
	M	Empregado Efetivo
	F	Empregado Efetivo
	F	Empregado Efetivo
	F	Empregado Efetivo
	F	Empregado Efetivo
	F	Empregado Efetivo
	F	Empregado Efetivo
	F	Empregado Efetivo
	F	Empregado Efetivo
	F	Empregado Efetivo
	F	Empregado Efetivo
	F	Empregado Efetivo

	F	Empregado Efetivo
	F	Empregado Efetivo
	F	Empregado Efetivo
	F	Empregado Efetivo
	F	Empregado Efetivo
	F	Empregado Efetivo
	M	Empregado Efetivo
	M	Empregado Efetivo
	M	Empregado Efetivo
	M	Empregado Efetivo
	M	Empregado Efetivo
	F	Empregado Efetivo
	F	Empregado Efetivo
	F	Empregado Efetivo
	F	Empregado Efetivo
	F	Empregado Efetivo
	F	Empregado Efetivo
	F	Empregado Efetivo
	F	Empregado Efetivo
	F	Empregado Efetivo
	F	Empregado Efetivo
	F	Empregado Efetivo
	F	Empregado Efetivo
	F	Empregado Efetivo
	F	Empregado Efetivo
	F	Empregado Efetivo
	F	Empregado Efetivo
	F	Empregado Efetivo
	F	Empregado Efetivo
	F	Empregado Efetivo
	F	Empregado Efetivo
	F	Empregado Efetivo
	F	Empregado Efetivo
	F	Empregado Efetivo
	F	Empregado Efetivo
	F	Empregado Efetivo
	F	Empregado Efetivo
	F	Empregado Efetivo
	F	Empregado Efetivo
	F	Empregado Efetivo
	F	Empregado Efetivo
	F	Empregado Efetivo
	F	Empregado Efetivo
	F	Empregado Efetivo
	F	Empregado Efetivo
	M	Empregado Efetivo
	F	Empregado Efetivo

	M	Empregado Efetivo
	M	Empregado Efetivo
	M	Empregado Efetivo
	M	Empregado Efetivo
	F	Empregado Efetivo
	M	Empregado Efetivo
	F	Empregado Efetivo
	M	Empregado Efetivo
	M	Empregado Efetivo
	M	Empregado Efetivo
	M	Empregado Efetivo
	F	Empregado Efetivo
	M	Empregado Efetivo
	F	Empregado Efetivo
	F	Empregado Efetivo
	F	Empregado Efetivo
	F	Empregado Efetivo
	F	Empregado Efetivo
	M	Empregado Efetivo
	F	Empregado Efetivo
	F	Empregado Efetivo
	F	Empregado Efetivo
	F	Empregado Efetivo
	F	Empregado Efetivo
	M	Empregado Efetivo
	F	Empregado Efetivo
	F	Empregado Efetivo
	M	Empregado Efetivo
	F	Empregado Efetivo
	F	Empregado Efetivo
	F	Empregado Efetivo
	F	Empregado Efetivo
	F	Empregado Efetivo
	F	Empregado Efetivo
	F	Empregado Efetivo
	M	Empregado Efetivo
	F	Empregado Efetivo
	F	Empregado Efetivo

	F	Empregado Efetivo
	F	Empregado Efetivo
	M	Empregado Efetivo
	M	Empregado Efetivo
	M	Empregado Efetivo
	F	Empregado Efetivo
	M	Empregado Efetivo
	F	Empregado Efetivo
	F	Empregado Efetivo
	M	Empregado Efetivo
	F	Empregado Efetivo
	F	Empregado Efetivo
	F	Empregado Efetivo
	F	Empregado Efetivo
	M	Empregado Efetivo
	M	Empregado Efetivo
	M	Empregado Efetivo
	M	Empregado Efetivo
	M	Empregado Efetivo
	M	Empregado Efetivo
	F	Empregado Efetivo
	F	Empregado Efetivo
	M	Empregado Efetivo
	F	Empregado Efetivo
	F	Empregado Efetivo
	M	Empregado Efetivo
	M	Empregado Efetivo
	F	Empregado Efetivo
	M	Empregado Efetivo
	F	Empregado Efetivo
	M	Empregado Efetivo
	M	Empregado Efetivo
	M	Empregado Efetivo
	M	Empregado Efetivo
	M	Empregado Efetivo
	F	Empregado Efetivo
	F	Empregado Efetivo
	M	Empregado Efetivo
	F	Empregado Efetivo
	F	Empregado Efetivo
	F	Empregado Efetivo
	F	Empregado Efetivo
	M	Empregado Efetivo
	F	Empregado Efetivo
	F	Empregado Efetivo
	M	Empregado Efetivo
	F	Empregado Efetivo
	F	Empregado Efetivo

	F	Empregado Efetivo
	M	Empregado Efetivo
	F	Empregado Efetivo
	F	Empregado Efetivo
	F	Empregado Efetivo
	M	Empregado Efetivo
	F	Empregado Efetivo
	F	Empregado Efetivo
	F	Empregado Efetivo
	F	Empregado Efetivo
	F	Empregado Efetivo
	F	Empregado Efetivo
	F	Empregado Efetivo
	F	Empregado Efetivo
	F	Empregado Efetivo
	F	Empregado Efetivo
	F	Empregado Efetivo
	M	Empregado Efetivo
	F	Empregado Efetivo
	F	Empregado Efetivo
	F	Empregado Efetivo
	F	Empregado Efetivo
	F	Empregado Efetivo
	F	Empregado Efetivo
	F	Empregado Efetivo
	F	Empregado Efetivo
	F	Empregado Efetivo
	F	Empregado Efetivo
	F	Empregado Efetivo
	F	Empregado Efetivo
	F	Empregado Efetivo
	M	Empregado Efetivo
	F	Empregado Efetivo
	M	Empregado Efetivo
	F	Empregado Efetivo
	F	Empregado Efetivo
	M	Empregado Efetivo
	M	Empregado Efetivo
	F	Empregado Efetivo
	M	Empregado Efetivo
	F	Empregado Efetivo
	F	Empregado Efetivo
	F	Empregado Efetivo
	F	Empregado Efetivo
	F	Empregado Efetivo
	F	Empregado Efetivo
	M	Empregado Efetivo
	F	Empregado Efetivo

	F	Empregado Efetivo
	F	Empregado Efetivo
	F	Empregado Efetivo
	F	Empregado Efetivo
	F	Empregado Efetivo
	F	Empregado Efetivo
	F	Empregado Efetivo
	F	Empregado Efetivo
	F	Empregado Efetivo
	F	Empregado Efetivo
	F	Empregado Efetivo
	F	Empregado Efetivo
	F	Empregado Efetivo
	F	Empregado Efetivo
	F	Empregado Efetivo
	F	Empregado Efetivo
	F	Empregado Efetivo
	F	Empregado Efetivo
	F	Empregado Efetivo
	F	Empregado Efetivo
	F	Empregado Efetivo
	F	Empregado Efetivo
	M	Empregado Efetivo
	F	Empregado Efetivo
	F	Empregado Efetivo
	F	Empregado Efetivo
	M	Empregado Efetivo
	F	Empregado Efetivo
	F	Empregado Efetivo
	F	Empregado Efetivo
	F	Empregado Efetivo
	F	Empregado Efetivo
	M	Empregado Efetivo
	F	Empregado Efetivo
	F	Empregado Efetivo
	F	Empregado Efetivo
	F	Empregado Efetivo
	M	Empregado Efetivo
	M	Empregado Efetivo
	M	Empregado Efetivo
	F	Empregado Efetivo
	F	Empregado Efetivo
	F	Empregado Efetivo
	F	Empregado Efetivo
	M	Empregado Efetivo
	F	Empregado Efetivo
	M	Empregado Efetivo
	M	Empregado Efetivo

	F	Empregado Efetivo
	F	Empregado Efetivo
	F	Empregado Efetivo
	F	Empregado Efetivo
	F	Empregado Efetivo
	F	Empregado Efetivo
	F	Empregado Efetivo
	F	Empregado Efetivo
	F	Empregado Efetivo
	F	Empregado Efetivo
	F	Empregado Efetivo
	F	Empregado Efetivo
	M	Empregado Efetivo
	M	Empregado Efetivo
	F	Empregado Efetivo
	M	Empregado Efetivo
	F	Empregado Efetivo
	F	Empregado Efetivo
	F	Empregado Efetivo
	F	Empregado Efetivo
	F	Empregado Efetivo
	F	Empregado Efetivo
	F	Empregado Efetivo
	F	Empregado Efetivo
	F	Empregado Efetivo
	F	Empregado Efetivo
	F	Empregado Efetivo
	F	Empregado Efetivo
	F	Empregado Efetivo
	F	Empregado Efetivo
	F	Empregado Efetivo
	F	Empregado Efetivo
	F	Empregado Efetivo
	F	Empregado Efetivo
	F	Empregado Efetivo
	F	Empregado Efetivo
	F	Empregado Efetivo
	F	Empregado Efetivo
	F	Empregado Efetivo
	F	Empregado Efetivo

	F	Empregado Efetivo
	F	Empregado Efetivo
	F	Empregado Efetivo
	F	Empregado Efetivo
	F	Empregado Efetivo
	M	Empregado Efetivo
	M	Empregado Efetivo
	M	Empregado Efetivo
	F	Empregado Efetivo
	F	Empregado Efetivo
	M	Empregado Efetivo
	M	Empregado Efetivo
	M	Empregado Efetivo
	F	Empregado Efetivo
	M	Empregado Efetivo
	F	Empregado Efetivo
	M	Empregado Efetivo
	F	Empregado Efetivo
	M	Empregado Efetivo
	M	Empregado Efetivo
	M	Empregado Efetivo
	M	Empregado Efetivo
	F	Empregado Efetivo
	F	Empregado Efetivo
	F	Empregado Efetivo
	F	Empregado Efetivo
	F	Empregado Efetivo
	F	Empregado Efetivo
	F	Empregado Efetivo
	F	Empregado Efetivo
	F	Empregado Efetivo
	F	Empregado Efetivo
	F	Empregado Efetivo
	M	Empregado Efetivo
	M	Empregado Efetivo
	F	Empregado Efetivo
	M	Empregado Efetivo

	M	Empregado Efetivo
	M	Empregado Efetivo
	F	Empregado Efetivo
	F	Empregado Efetivo
	F	Empregado Efetivo
	F	Empregado Efetivo
	M	Empregado Efetivo
	M	Empregado Efetivo
	M	Empregado Efetivo
	M	Empregado Efetivo
	F	Empregado Efetivo
	M	Empregado Efetivo
	F	Empregado Efetivo
	F	Empregado Efetivo
	F	Empregado Efetivo
	M	Empregado Efetivo
	M	Empregado Efetivo
	F	Empregado Efetivo
	F	Empregado Efetivo
	F	Empregado Efetivo
	M	Empregado Efetivo
	F	Empregado Efetivo
	M	Empregado Efetivo
	F	Empregado Efetivo
	F	Empregado Efetivo
	F	Empregado Efetivo
	F	Empregado Efetivo
	F	Empregado Efetivo
	M	Empregado Efetivo
	F	Empregado Efetivo
	F	Empregado Efetivo
	F	Empregado Efetivo
	F	Empregado Efetivo
	M	Empregado Efetivo
	F	Empregado Efetivo
	F	Empregado Efetivo
	M	Empregado Efetivo
	F	Empregado Efetivo
	M	Empregado Efetivo

	F	Empregado Efetivo
	F	Empregado Efetivo
	F	Empregado Efetivo
	F	Empregado Efetivo
	M	Empregado Efetivo
	F	Empregado Efetivo
	F	Empregado Efetivo
	F	Empregado Efetivo
	F	Empregado Efetivo
	F	Empregado Efetivo
	F	Empregado Efetivo
	F	Empregado Efetivo
	M	Empregado Efetivo
	F	Empregado Efetivo
	F	Empregado Efetivo
	F	Empregado Efetivo
	F	Empregado Efetivo
	M	Empregado Efetivo
	F	Empregado Efetivo
	F	Empregado Efetivo
	M	Empregado Efetivo
	F	Empregado Efetivo
	M	Empregado Efetivo
	M	Empregado Efetivo
	M	Empregado Efetivo
	M	Empregado Efetivo
	M	Empregado Efetivo
	M	Empregado Efetivo
	M	Empregado Efetivo
	F	Empregado Efetivo
	F	Empregado Efetivo
	F	Empregado Efetivo
	M	Empregado Efetivo
	M	Empregado Efetivo
	F	Empregado Efetivo
	F	Empregado Efetivo
	F	Empregado Efetivo
	M	Empregado Efetivo
	M	Empregado Efetivo
	M	Empregado Efetivo
	F	Empregado Efetivo
	F	Empregado Efetivo
	M	Empregado Efetivo
	M	Empregado Efetivo
	F	Empregado Efetivo
	M	Empregado Efetivo
	M	Empregado Efetivo

	M	Empregado Efetivo
	M	Empregado Efetivo
	M	Empregado Efetivo
	F	Empregado Efetivo
	M	Empregado Efetivo
	F	Empregado Efetivo
	M	Empregado Efetivo
	M	Empregado Efetivo
	F	Empregado Efetivo
	F	Empregado Efetivo
	F	Empregado Efetivo
	F	Empregado Efetivo
	M	Empregado Efetivo
	F	Empregado Efetivo
	M	Empregado Efetivo
	M	Empregado Efetivo
	F	Empregado Efetivo
	F	Empregado Efetivo
	F	Empregado Efetivo
	M	Empregado Efetivo
	M	Empregado Efetivo
	M	Empregado Efetivo
	F	Empregado Efetivo
	F	Empregado Efetivo
	F	Empregado Efetivo
	M	Empregado Efetivo
	F	Empregado Efetivo
	M	Empregado Efetivo
	F	Empregado Efetivo
	M	Empregado Efetivo
	M	Empregado Efetivo
	M	Empregado Efetivo
	F	Empregado Efetivo
	M	Empregado Efetivo
	F	Empregado Efetivo
	F	Empregado Efetivo
	M	Empregado Efetivo
	F	Empregado Efetivo
	M	Empregado Efetivo
	F	Empregado Efetivo
	F	Empregado Efetivo
	F	Empregado Efetivo
	F	Empregado Efetivo
	M	Empregado Efetivo
	M	Empregado Efetivo
	M	Empregado Efetivo
	F	Empregado Efetivo
	F	Empregado Efetivo

	F	Empregado Efetivo
	F	Empregado Efetivo
	F	Empregado Efetivo
	F	Empregado Efetivo
	M	Empregado Efetivo
	M	Empregado Efetivo
	M	Empregado Efetivo
	M	Empregado Efetivo
	F	Empregado Efetivo
	M	Empregado Efetivo
	F	Empregado Efetivo
	M	Empregado Efetivo
	F	Empregado Efetivo
	F	Empregado Efetivo
	F	Empregado Efetivo
	M	Empregado Efetivo
	M	Empregado Efetivo
	M	Empregado Efetivo
	F	Empregado Efetivo
	M	Empregado Efetivo
	M	Empregado Efetivo
	M	Empregado Efetivo
	M	Empregado Efetivo
	M	Empregado Efetivo
	M	Empregado Efetivo
	M	Empregado Efetivo
	M	Empregado Efetivo
	M	Empregado Efetivo
	M	Empregado Efetivo
	M	Empregado Efetivo
	M	Empregado Efetivo
	F	Empregado Efetivo
	M	Empregado Efetivo
	F	Empregado Efetivo
	F	Empregado Efetivo
	F	Empregado Efetivo
	M	Empregado Efetivo
	F	Empregado Efetivo
	F	Empregado Efetivo
	M	Empregado Efetivo
	F	Empregado Efetivo
	F	Empregado Efetivo
	M	Empregado Efetivo
	M	Empregado Efetivo
	M	Empregado Efetivo
	M	Empregado Efetivo
	M	Empregado Efetivo
	F	Empregado Efetivo

	M	Empregado Efetivo
	M	Empregado Efetivo
	M	Empregado Efetivo
	M	Empregado Efetivo
	M	Empregado Efetivo
	M	Empregado Efetivo
	F	Empregado Efetivo
	M	Empregado Efetivo
	F	Empregado Efetivo
	F	Empregado Efetivo
	M	Empregado Efetivo
	M	Empregado Efetivo
	F	Empregado Efetivo
	F	Empregado Efetivo
	F	Empregado Efetivo
	M	Empregado Efetivo
	M	Empregado Efetivo
	M	Empregado Efetivo
	F	Empregado Efetivo
	M	Empregado Efetivo
	F	Empregado Efetivo
	F	Empregado Efetivo
	F	Empregado Efetivo
	F	Empregado Efetivo
	M	Empregado Efetivo
	M	Empregado Efetivo
	M	Empregado Efetivo
	M	Empregado Efetivo
	M	Empregado Efetivo
	M	Empregado Efetivo
	M	Empregado Efetivo
	M	Empregado Efetivo
	M	Empregado Efetivo
	M	Empregado Efetivo
	M	Empregado Efetivo
	M	Empregado Efetivo
	M	Empregado Efetivo
	M	Empregado Efetivo
	M	Empregado Efetivo
	F	Empregado Efetivo
	F	Empregado Efetivo
	F	Empregado Efetivo
	F	Empregado Efetivo
	M	Empregado Efetivo
	F	Empregado Efetivo
	F	Empregado Efetivo
	F	Empregado Efetivo
	M	Empregado Efetivo

	F	Empregado Efetivo
	M	Empregado Efetivo
	F	Empregado Efetivo
	F	Empregado Efetivo
	F	Empregado Efetivo
	M	Empregado Efetivo
	F	Empregado Efetivo
	F	Empregado Efetivo
	F	Empregado Efetivo
	M	Empregado Efetivo
	F	Empregado Efetivo
	F	Empregado Efetivo
	F	Empregado Efetivo
	F	Empregado Efetivo
	F	Empregado Efetivo
	M	Empregado Efetivo
	F	Empregado Efetivo
	F	Empregado Efetivo
	F	Empregado Efetivo
	F	Empregado Efetivo
	F	Empregado Efetivo
	F	Empregado Efetivo
	F	Empregado Efetivo
	F	Empregado Efetivo
	M	Empregado Efetivo
	F	Empregado Efetivo
	F	Empregado Efetivo
	F	Empregado Efetivo
	F	Empregado Efetivo
	M	Empregado Efetivo
	F	Empregado Efetivo
	F	Empregado Efetivo
	F	Empregado Efetivo
	F	Empregado Efetivo
	F	Empregado Efetivo
	F	Empregado Efetivo
	F	Empregado Efetivo
	F	Empregado Efetivo
	F	Empregado Efetivo
	F	Empregado Efetivo
	F	Empregado Efetivo
	F	Empregado Efetivo
	F	Empregado Efetivo
	F	Empregado Efetivo
	F	Empregado Efetivo

	F	Empregado Efetivo
	F	Empregado Efetivo
	F	Empregado Efetivo
	F	Empregado Efetivo
	F	Empregado Efetivo
	F	Empregado Efetivo
	F	Empregado Efetivo
	F	Empregado Efetivo
	F	Empregado Efetivo
	F	Empregado Efetivo
	F	Empregado Efetivo
	F	Empregado Efetivo
	F	Empregado Efetivo
	F	Empregado Efetivo
	F	Empregado Efetivo
	F	Empregado Efetivo
	F	Empregado Efetivo
	F	Empregado Efetivo
	F	Empregado Efetivo
	F	Empregado Efetivo
	F	Empregado Efetivo
	F	Empregado Efetivo
	F	Empregado Efetivo
	F	Empregado Efetivo
	F	Empregado Efetivo
	F	Empregado Efetivo
	F	Empregado Efetivo
	F	Empregado Efetivo
	F	Empregado Efetivo
	F	Empregado Efetivo
	F	Empregado Efetivo
	F	Empregado Efetivo
	F	Empregado Efetivo
	F	Empregado Efetivo
	F	Empregado Efetivo
	F	Empregado Efetivo
	F	Empregado Efetivo
	F	Empregado Efetivo
	F	Empregado Efetivo
	F	Empregado Efetivo
	F	Empregado Efetivo
	F	Empregado Efetivo
	F	Empregado Efetivo

	F	Empregado Efetivo
	F	Empregado Efetivo
	F	Empregado Efetivo
	F	Empregado Efetivo
	F	Empregado Efetivo
	F	Empregado Efetivo
	F	Empregado Efetivo
	F	Empregado Efetivo
	F	Empregado Efetivo
	F	Empregado Efetivo
	F	Empregado Efetivo
	F	Empregado Efetivo
	F	Empregado Efetivo
	F	Empregado Efetivo
	F	Empregado Efetivo
	F	Empregado Efetivo
	F	Empregado Efetivo
	F	Empregado Efetivo
	F	Empregado Efetivo
	F	Empregado Efetivo
	F	Empregado Efetivo
	F	Empregado Efetivo
	F	Empregado Efetivo
	F	Empregado Efetivo
	F	Empregado Efetivo
	F	Empregado Efetivo
	M	Empregado Efetivo
	M	Empregado Efetivo
	M	Empregado Efetivo
	M	Empregado Efetivo
	M	Empregado Efetivo
	M	Empregado Efetivo
	M	Empregado Efetivo
	M	Empregado Efetivo
	M	Empregado Efetivo
	M	Empregado Efetivo
	M	Empregado Efetivo

	M	Empregado Efetivo
	M	Empregado Efetivo
	M	Empregado Efetivo
	F	Empregado Efetivo
	F	Empregado Efetivo
	F	Empregado Efetivo
	M	Empregado Efetivo
	F	Empregado Efetivo
	M	Empregado Efetivo
	M	Empregado Efetivo
[truncated: 484,581 more chars]
