# Supplementary material for: Impact of COVID-19 on the mental health of public university hospital workers in Brazil: A cohort-based analysis of 32,691 workers
Source: PLoS One. 2022 Jun 16;17(6):e0269318. doi: 10.1371/journal.pone.0269318 (PMC9202958; doi:10.1371/journal.pone.0269318)
Supplement: S4 File — (PDF) [file pone.0269318.s004.pdf]

Supporting Information Part 4

Gender differences as to proportions of individuals with work absences due to ALL CAUSES

```
* Generalized Estimating Equations.
GENLIN AbAC (REFERENCE=FIRST) BY AsgnGrp Sex (ORDER=DESCENDING)
  /MODEL AsgnGrp Sex AsgnGrp*Sex INTERCEPT=YES
 DISTRIBUTION=BINOMIAL LINK=LOGIT
  /CRITERIA METHOD=FISHER(1) SCALE=1 MAXITERATIONS=100 MAXSTEPHALVING=5 PCONVERGE=1E-006(ABSOLUTE)
    SINGULAR=1E-012 ANALYSISTYPE=3(WALD) CILEVEL=95 LIKELIHOOD=FULL
  /REPEATED SUBJECT=ID WITHINSUBJECT=AsgnGrp SORT=YES CORRTYPE=UNSTRUCTURED ADJUSTCORR=YES
    COVB=ROBUST MAXITERATIONS=100 PCONVERGE=1e-006(ABSOLUTE) UPDATECORR=1
  /MISSING CLASSMISSING=EXCLUDE
  /PRINT CPS DESCRIPTIVES MODELINFO FIT SUMMARY SOLUTION (EXPONENTIATED).
```

Generalized Linear Models

[DataSet2] C:\Users\trabalho\Documents\COVID-19\Centro Pesquisa HUB COVID19\BDForceII_AbsperInd_Vertic.sav

Model Information

Dependent Variable	Any work absence due to ALL CAUSES occurred in the period[a]
Probability Distribution	Binomial

Link Function		Logit
Subject Effect	1	ID
Within-Subject Effect	1	Exposure to the pandemia environment
Working Correlation Matrix Structure		Unstructured

a. The procedure models Yes as the response, treating No as the reference category.

Case Processing Summary

	N	Percent
Included	65382	100,0%
Excluded	0	0,0%
Total	65382	100,0%

Correlated Data Summary

Number of Levels	Subject Effect	ID	32691
	Within-Subject Effect	Exposure to the pandemia environment	2
Number of Subjects			32691
Number of Measurements per Subject	Minimum		2
	Maximum		2
Correlation Matrix Dimension			2

Categorical Variable Information

			N	Percent
Dependent Variable	Any work absence due to ALL CAUSES occurred in the period	No	34347	52,5%
		Yes	31035	47,5%
		Total	65382	100,0%
Factor	Exposure to the pandemia environment	Exposed (2020)	32691	50,0%
		Non-Exposed (2019)	32691	50,0%
		Total	65382	100,0%
	Gender	Male	19418	29,7%
		Female	45964	70,3%
		Total	65382	100,0%

Goodness of Fit[a]

	Value
Quasi Likelihood under Independence Model Criterion (QIC)[b]	88626,933
Corrected Quasi Likelihood under Independence Model Criterion (QICC)[b]	88626,933

Dependent Variable: Any work absence due to ALL CAUSES occurred in the period
Model: (Intercept), Exposure to the pandemia environment, Gender, Exposure to the pandemia environment * Gender[a]
a. Information criteria are in smaller-is-better form.
b. Computed using the full log quasi-likelihood function.

Parameter Estimates

Parameter	B	Std. Error	95% Wald Confidence Interval		Hypothesis Test			Exp(B)	95% Wald Confidence Interval for Exp(B)	
			Lower	Upper	Wald Chi-Square	df	Sig.		Lower	Upper
(Intercept)	,249	,0133	,223	,275	349,643	1	,000	1,282	1,249	1,316
[Exposure to the pandemia environment=1]	-,313	,0166	-,346	-,281	354,884	1	,000	,731	,708	,755
[Exposure to the pandemia environment=0]	0[a]	.	.	.	.	.	.	1	.	.
[Gender=1]	-,663	,0246	-,712	-,615	725,252	1	,000	,515	,491	,541
[Gender=0]	0[a]	.	.	.	.	.	.	1	.	.
[Exposure to the pandemia environment=1] * [Gender=1]	-,009	,0315	-,071	,053	,083	1	,773	,991	,932	1,054
[Exposure to the pandemia environment=1] * [Gender=0]	0[a]	.	.	.	.	.	.	1	.	.
[Exposure to the pandemia environment=0] * [Gender=1]	0[a]	.	.	.	.	.	.	1	.	.
[Exposure to the pandemia environment=0] * [Gender=0]	0[a]	.	.	.	.	.	.	1	.	.
(Scale)	1									

Dependent Variable: Any work absence due to ALL CAUSES occurred in the period

Model: (Intercept), Exposure to the pandemia environment, Gender, Exposure to the pandemia environment * Gender

a. Set to zero because this parameter is redundant.

Tests of Model Effects

Source	Type III

	Wald Chi-Square	df	Sig.
(Intercept)	619,880	1	,000
Exposure to the pandemia environment	407,937	1	,000
Gender	1180,095	1	,000
Exposure to the pandemia environment * Gender	,083	1	,773

Dependent Variable: Any work absence due to ALL CAUSES occurred in the period
Model: (Intercept), Exposure to the pandemia environment, Gender, Exposure to the pandemia environment * Gender

Gender differences as to proportions of individuals with work absences due to ADMINISTRATIVE CAUSES

```
* Generalized Estimating Equations.
GENLIN AbADM (REFERENCE=FIRST) BY AsgnGrp Sex (ORDER=DESCENDING)
  /MODEL AsgnGrp Sex INTERCEPT=YES
 DISTRIBUTION=BINOMIAL LINK=LOGIT
  /CRITERIA METHOD=FISHER(1) SCALE=1 MAXITERATIONS=100 MAXSTEPHALVING=5 PCONVERGE=1E-006(ABSOLUTE)
    SINGULAR=1E-012 ANALYSISTYPE=3(WALD) CILEVEL=95 LIKELIHOOD=FULL
  /REPEATED SUBJECT=ID WITHINSUBJECT=AsgnGrp SORT=YES CORRTYPE=UNSTRUCTURED ADJUSTCORR=YES
    COVB=ROBUST MAXITERATIONS=100 PCONVERGE=1e-006(ABSOLUTE) UPDATECORR=1
  /MISSING CLASSMISSING=EXCLUDE
  /PRINT CPS DESCRIPTIVES MODELINFO FIT SUMMARY SOLUTION (EXPONENTIATED).
```

Generalized Linear Models

Model Information

Dependent Variable		Any work absence due to ADMINISTRATIVE CAUSES occurred in the period[a]
Probability Distribution		Binomial
Link Function		Logit
Subject Effect	1	ID
Within-Subject Effect	1	Exposure to the pandemia environment
Working Correlation Matrix Structure		Unstructured

a. The procedure models Yes as the response, treating No as the reference category.

Case Processing Summary

	N	Percent
Included	65382	100,0%
Excluded	0	0,0%
Total	65382	100,0%

Correlated Data Summary

Number of Levels	Subject Effect	ID	32691
	Within-Subject Effect	Exposure to the pandemia environment	2
Number of Subjects			32691
Number of Measurements per Subject	Minimum		2
	Maximum		2
Correlation Matrix Dimension			2

Categorical Variable Information

			N	Percent
Dependent Variable	Any work absence due to ADMINISTRATIVE CAUSES occurred in the period	No	58184	89,0%
		Yes	7198	11,0%
		Total	65382	100,0%
Factor	Exposure to the pandemia environment	Exposed (2020)	32691	50,0%
		Non-Exposed (2019)	32691	50,0%
		Total	65382	100,0%
	Gender	Male	19418	29,7%
		Female	45964	70,3%
		Total	65382	100,0%

Goodness of Fit[a]

	Value
Quasi Likelihood under Independence Model Criterion (QIC)[b]	44983,494
Corrected Quasi Likelihood under Independence Model Criterion (QICC)[b]	44983,234

Dependent Variable: Any work absence due to ADMINISTRATIVE CAUSES occurred in the period

Model: (Intercept), Exposure to the pandemia environment, Gender[a]

a. Information criteria are in smaller-is-better form.

b. Computed using the full log quasi-likelihood function.

Tests of Model Effects

	Type III		
Source	Wald Chi-Square	df	Sig.
(Intercept)	20184,633	1	,000
Exposure to the pandemia environment	389,820	1	,000
Gender	12,238	1	,000

Dependent Variable: Any work absence due to ADMINISTRATIVE CAUSES occurred in the period

Model: (Intercept), Exposure to the pandemia environment, Gender

Parameter Estimates

Parameter	B	Std. Error	95% Wald Confidence Interval		Hypothesis Test			Exp(B)	95% Wald Confidence Interval for Exp(B)	
			Lower	Upper	Wald Chi-Square	df	Sig.		Lower	Upper
(Intercept)	-1,845	,0182	-1,881	-1,810	10251,117	1	,000	,158	,152	,164
[Exposure to the pandemia environment=1]	-,470	,0237	-,517	-,424	392,101	1	,000	,625	,596	,655
[Exposure to the pandemia environment=0]	0[a]	.	.	.	.	.	.	1	.	.
[Gender=1]	-,104	,0297	-,162	-,046	12,238	1	,000	,901	,851	,955
[Gender=0]	0[a]	.	.	.	.	.	.	1	.	.
(Scale)	1									

Dependent Variable: Any work absence due to ADMINISTRATIVE CAUSES occurred in the period

Model: (Intercept), Exposure to the pandemia environment, Gender

a. Set to zero because this parameter is redundant.

Gender differences as to proportions of individuals with work absences due to HEALTH-RELATED CAUSES

* Generalized Estimating Equations.

Supporting Information Part 4

```
GENLIN AbHRel (REFERENCE=FIRST) BY AsgnGrp Sex (ORDER=DESCENDING)
  /MODEL AsgnGrp Sex INTERCEPT=YES
 DISTRIBUTION=BINOMIAL LINK=LOGIT
  /CRITERIA METHOD=FISHER(1) SCALE=1 MAXITERATIONS=100 MAXSTEPHALVING=5 PCONVERGE=1E-006(ABSOLUTE)
    SINGULAR=1E-012 ANALYSISTYPE=3(WALD) CILEVEL=95 LIKELIHOOD=FULL
  /REPEATED SUBJECT=ID WITHINSUBJECT=AsgnGrp SORT=YES CORRTYPE=UNSTRUCTURED ADJUSTCORR=YES
    COVB=ROBUST MAXITERATIONS=100 PCONVERGE=1e-006(ABSOLUTE) UPDATECORR=1
  /MISSING CLASSMISSING=EXCLUDE
  /PRINT CPS DESCRIPTIVES MODELINFO FIT SUMMARY SOLUTION (EXPONENTIATED).
```

Generalized Linear Models

Model Information

Dependent Variable		Any work absence due to HEALTH-RELATED CAUSES occurred in the period[a]
Probability Distribution		Binomial
Link Function		Logit
Subject Effect	1	ID
Within-Subject Effect	1	Exposure to the pandemia environment
Working Correlation Matrix Structure		Unstructured

a. The procedure models Yes as the response, treating No as the reference category.

Supporting Information Part 4

Case Processing Summary

	N	Percent
Included	65382	100,0%
Excluded	0	0,0%
Total	65382	100,0%

Correlated Data Summary

Number of Levels	Subject Effect	ID	32691
	Within-Subject Effect	Exposure to the pandemia environment	2
Number of Subjects			32691
Number of Measurements per Subject	Minimum		2
	Maximum		2
Correlation Matrix Dimension			2

Categorical Variable Information

			N	Percent
Dependent Variable	Any work absence due to HEALTH-RELATED CAUSES occurred in the period	No	40969	62,7%
		Yes	24413	37,3%
		Total	65382	100,0%

Factor	Exposure to the pandemia environment	Exposed (2020)	32691	50,0%
		Non-Exposed (2019)	32691	50,0%
		Total	65382	100,0%
	Gender	Male	19418	29,7%
		Female	45964	70,3%
		Total	65382	100,0%

Goodness of Fit[a]

	Value
Quasi Likelihood under Independence Model Criterion (QIC)[b]	84266,543
Corrected Quasi Likelihood under Independence Model Criterion (QICC)[b]	84266,088

Dependent Variable: Any work absence due to HEALTH-RELATED CAUSES occurred in the period
Model: (Intercept), Exposure to the pandemia environment, Gender[a]
a. Information criteria are in smaller-is-better form.
b. Computed using the full log quasi-likelihood function.

Tests of Model Effects

Source	Type III		
	Wald Chi-Square	df	Sig.
(Intercept)	4389,335	1	,000
Exposure to the pandemia environment	388,268	1	,000
Gender	1417,060	1	,000

Dependent Variable: Any work absence due to HEALTH-RELATED CAUSES occurred in the period

Model: (Intercept), Exposure to the pandemia environment, Gender

Parameter Estimates

Parameter	B	Std. Error	95% Wald Confidence Interval Lower	95% Wald Confidence Interval Upper	Hypothesis Test Wald Chi-Square	Hypothesis Test df	Hypothesis Test Sig.	Exp(B)	95% Wald Confidence Interval for Exp(B) Lower	95% Wald Confidence Interval for Exp(B) Upper
(Intercept)	-,161	,0126	-,185	-,136	162,044	1	,000	,852	,831	,873
[Exposure to the pandemia environment=1]	-,285	,0145	-,313	-,256	388,268	1	,000	,752	,731	,774
[Exposure to the pandemia environment=0]	0[a]	.	.	.	.	.	.	1	.	.
[Gender=1]	-,796	,0212	-,838	-,755	1417,060	1	,000	,451	,433	,470
[Gender=0]	0[a]	.	.	.	.	.	.	1	.	.
(Scale)	1									

Dependent Variable: Any work absence due to HEALTH-RELATED CAUSES occurred in the period

Model: (Intercept), Exposure to the pandemia environment, Gender

a. Set to zero because this parameter is redundant.

Comparisons between professional categories

Custom Tables: comparisons between HCP categories

```
[DataSet2] C:\Users\User\Documents\Pesquisa\Force II\BDForceII_AbsperInd_Vertic.sav
```

	Exposure to the pandemia environment	
	Non-Exposed (2019)	Exposed (2020)

Supporting Information Part 4

				Count	Column N %	Count	Column N %
Healthcare provider category	Physicians	Any work absence due to ALL CAUSES occurred in the period	No	4265	62,1%	4803	69,9%
			Yes	2606	37,9%	2068	30,1%
			Total	6871	100,0%	6871	100,0%
		Any work absence due to ADMINISTRATIVE CAUSES occurred in the period	No	6025	87,7%	6561	95,5%
			Yes	846	12,3%	310	4,5%
			Total	6871	100,0%	6871	100,0%
		Any work absence due to HEALTH-RELATED CAUSES occurred in the period	No	5227	76,1%	5404	78,6%
			Yes	1644	23,9%	1467	21,4%
			Total	6871	100,0%	6871	100,0%
		Any work absence due to MENTAL DISEASE occurred in the period	No	6814	99,2%	6757	98,3%
			Yes	57	0,8%	114	1,7%
			Total	6871	100,0%	6871	100,0%
	Nurses	Any work absence due to ALL CAUSES occurred in the period	No	2226	40,0%	2668	47,9%
			Yes	3344	60,0%	2902	52,1%
			Total	5570	100,0%	5570	100,0%
		Any work absence due to ADMINISTRATIVE CAUSES occurred in the period	No	4803	86,2%	5068	91,0%
			Yes	767	13,8%	502	9,0%
			Total	5570	100,0%	5570	100,0%
		Any work absence due to HEALTH-RELATED CAUSES occurred in the period	No	2808	50,4%	3214	57,7%
			Yes	2762	49,6%	2356	42,3%
			Total	5570	100,0%	5570	100,0%
		Any work absence due to MENTAL DISEASE occurred in the period	No	5382	96,6%	5300	95,2%
			Yes	188	3,4%	270	4,8%
			Total	5570	100,0%	5570	100,0%

	Physiotherapists	Any work absence due to ALL CAUSES occurred in the period	No	454	44,6%	547	53,7%
			Yes	565	55,4%	472	46,3%
			Total	1019	100,0%	1019	100,0%
		Any work absence due to ADMINISTRATIVE CAUSES occurred in the period	No	903	88,6%	951	93,3%
			Yes	116	11,4%	68	6,7%
			Total	1019	100,0%	1019	100,0%
		Any work absence due to HEALTH-RELATED CAUSES occurred in the period	No	564	55,3%	649	63,7%
			Yes	455	44,7%	370	36,3%
			Total	1019	100,0%	1019	100,0%
		Any work absence due to MENTAL DISEASE occurred in the period	No	988	97,0%	973	95,5%
			Yes	31	3,0%	46	4,5%
			Total	1019	100,0%	1019	100,0%
	Nurse assistants	Any work absence due to ALL CAUSES occurred in the period	No	4331	42,2%	4711	45,9%
			Yes	5935	57,8%	5555	54,1%
			Total	10266	100,0%	10266	100,0%
		Any work absence due to ADMINISTRATIVE CAUSES occurred in the period	No	8990	87,6%	9116	88,8%
			Yes	1276	12,4%	1150	11,2%
			Total	10266	100,0%	10266	100,0%
		Any work absence due to HEALTH-RELATED CAUSES occurred in the period	No	5171	50,4%	5652	55,1%
			Yes	5095	49,6%	4614	44,9%
			Total	10266	100,0%	10266	100,0%
		Any work absence due to MENTAL DISEASE occurred in the period	No	9936	96,8%	9837	95,8%
			Yes	330	3,2%	429	4,2%
			Total	10266	100,0%	10266	100,0%

	Other HCP	Any work absence due to ALL CAUSES occurred in the period	No	1425	44,1%	1751	54,2%
			Yes	1805	55,9%	1479	45,8%
			Total	3230	100,0%	3230	100,0%
		Any work absence due to ADMINISTRATIVE CAUSES occurred in the period	No	2710	83,9%	2891	89,5%
			Yes	520	16,1%	339	10,5%
			Total	3230	100,0%	3230	100,0%
		Any work absence due to HEALTH-RELATED CAUSES occurred in the period	No	1807	55,9%	2092	64,8%
			Yes	1423	44,1%	1138	35,2%
			Total	3230	100,0%	3230	100,0%
		Any work absence due to MENTAL DISEASE occurred in the period	No	3151	97,6%	3106	96,2%
			Yes	79	2,4%	124	3,8%
			Total	3230	100,0%	3230	100,0%

Are the apparent differences regarding the physicians compared to other HCP, in terms of work absences, significant within each isolate period (2019 and 2020)? No repeated measures.

Generalized Linear Models

[DataSet1] C:\Users\User\Documents\Pesquisa\Force II\BDForceII_AbsperInd_Horiz.sav

Model Information

Dependent Variable	Any work absence due to ALL CAUSES occurred in the period - T2 (exposed)[a]
Probability Distribution	Binomial
Link Function	Logit

a. The procedure models Yes as the response, treating No as the reference category.

Case Processing Summary

	N	Percent
Included	26956	82,5%
Excluded	5735	17,5%
Total	32691	100,0%

Categorical Variable Information

			N	Percent
Dependent Variable	Any work absence due to ALL CAUSES occurred in the period - T2 (exposed)	No	14480	53,7%
		Yes	12476	46,3%
		Total	26956	100,0%

Supporting Information Part 4

Factor	Healthcare provider category	Other HCP	3230	12,0%
		Nurse assistants	10266	38,1%
		Physiotherapists	1019	3,8%
		Nurses	5570	20,7%
		Physicians	6871	25,5%
		Total	26956	100,0%
	Gender	Male	7108	26,4%
		Female	19848	73,6%
		Total	26956	100,0%

Goodness of Fit[a]

	Value	df	Value/df
Deviance	40,476	4	10,119
Scaled Deviance	40,476	4	
Pearson Chi-Square	40,366	4	10,091
Scaled Pearson Chi-Square	40,366	4	
Log Likelihood[b]	-59,590		
Akaike's Information Criterion (AIC)	131,181		
Finite Sample Corrected AIC (AICC)	131,184		
Bayesian Information Criterion (BIC)	180,392		
Consistent AIC (CAIC)	186,392		

Dependent Variable: Any work absence due to ALL CAUSES occurred in the period - T2

Model: (Intercept), Healthcare provider category, Gender[a]

a. Information criteria are in smaller-is-better form.

b. The full log likelihood function is displayed and used in computing information criteria.

Omnibus Test[a]

Likelihood Ratio Chi-Square	df	Sig.
1319,679	5	,000

Dependent Variable: Any work absence due to ALL CAUSES occurred in the period - T2

Model: (Intercept), Healthcare provider category, Gender[a]

a. Compares the fitted model against the intercept-only model.

Tests of Model Effects

Source	Type III Wald Chi-Square	df	Sig.
(Intercept)	254,526	1	,000
Healthcare provider category	735,725	4	,000
Gender	238,768	1	,000

Dependent Variable: Any work absence due to ALL CAUSES occurred in the period - T2

Model: (Intercept), Healthcare provider category, Gender

Parameter Estimates

Parameter	B	Std. Error	95% Wald Confidence Interval		Hypothesis Test			Exp(B)	95% Wald Confidence Interval for Exp(B)	
			Lower	Upper	Wald Chi-Square	df	Sig.		Lower	Upper
(Intercept)	-,640	,0292	-,698	-,583	480,633	1	,000	,527	,498	,558
[Healthcare provider category=5]	,615	,0444	,528	,702	191,633	1	,000	1,849	1,695	2,017
[Healthcare provider category=4]	,888	,0338	,822	,955	689,944	1	,000	2,431	2,275	2,598
[Healthcare provider category=3]	,595	,0687	,461	,730	75,153	1	,000	1,814	1,585	2,075
[Healthcare provider category=2]	,797	,0385	,722	,873	428,315	1	,000	2,219	2,058	2,393
[Healthcare provider category=1]	0[a]	.	.	.	.	.	.	1	.	.
[Gender=1]	-,464	,0300	-,523	-,405	238,768	1	,000	,629	,593	,667
[Gender=0]	0[a]	.	.	.	.	.	.	1	.	.
(Scale)	1[b]									

Dependent Variable: Any work absence due to ALL CAUSES occurred in the period - T2

Model: (Intercept), Healthcare provider category, Gender

a. Set to zero because this parameter is redundant.

b. Fixed at the displayed value.

```
* Generalized Linear Models.
GENLIN AbAC_T1 (REFERENCE=FIRST) BY HCPcateg Sex (ORDER=DESCENDING)
  /MODEL HCPcateg Sex INTERCEPT=YES
 DISTRIBUTION=BINOMIAL LINK=LOGIT
  /CRITERIA METHOD=FISHER(1) SCALE=1 COVB=MODEL MAXITERATIONS=100 MAXSTEPHALVING=5
```

```
    PCONVERGE=1E-006(ABSOLUTE) SINGULAR=1E-012 ANALYSISTYPE=3(WALD) CILEVEL=95 CITYPE=WALD
    LIKELIHOOD=FULL
  /MISSING CLASSMISSING=EXCLUDE
  /PRINT CPS DESCRIPTIVES MODELINFO FIT SUMMARY SOLUTION (EXPONENTIATED).
```

Generalized Linear Models

Model Information

Dependent Variable	Any work absence due to ALL CAUSES occurred in the period - T1 (non-exposed)[a]
Probability Distribution	Binomial
Link Function	Logit

a. The procedure models Yes as the response, treating No as the reference category.

Case Processing Summary

	N	Percent
Included	26956	82,5%
Excluded	5735	17,5%

Total	32691	100,0%

Categorical Variable Information

			N	Percent
Dependent Variable	Any work absence due to ALL CAUSES occurred in the period - T1 (non-exposed)	No	12701	47,1%
		Yes	14255	52,9%
		Total	26956	100,0%
Factor	Healthcare provider category	Other HCP	3230	12,0%
		Nurse assistants	10266	38,1%
		Physiotherapists	1019	3,8%
		Nurses	5570	20,7%
		Physicians	6871	25,5%
		Total	26956	100,0%
	Gender	Male	7108	26,4%
		Female	19848	73,6%
		Total	26956	100,0%

Goodness of Fit[a]

	Value	df	Value/df
Deviance	30,390	4	7,598
Scaled Deviance	30,390	4	
Pearson Chi-Square	30,337	4	7,584

Scaled Pearson Chi-Square	30,337	4	
Log Likelihood[b]	-54,659		
Akaike's Information Criterion (AIC)	121,317		
Finite Sample Corrected AIC (AICC)	121,320		
Bayesian Information Criterion (BIC)	170,529		
Consistent AIC (CAIC)	176,529		

Dependent Variable: Any work absence due to ALL CAUSES occurred in the period - T1

Model: (Intercept), Healthcare provider category, Gender[a]

a. Information criteria are in smaller-is-better form.

b. The full log likelihood function is displayed and used in computing information criteria.

Omnibus Test[a]

Likelihood Ratio Chi-Square	df	Sig.
1167,675	5	,000

Dependent Variable: Any work absence due to ALL CAUSES occurred in the period - T1

Model: (Intercept), Healthcare provider category, Gender[a]

a. Compares the fitted model against the intercept-only model.

Tests of Model Effects

Source	Type III		
	Wald Chi-Square	df	Sig.
(Intercept)	,702	1	,402
Healthcare provider category	548,234	4	,000
Gender	316,542	1	,000

Dependent Variable: Any work absence due to ALL CAUSES occurred in the period - T1

Model: (Intercept), Healthcare provider category, Gender

Supporting Information Part 4

Parameter Estimates

Parameter	B	Std. Error	95% Wald Confidence Interval		Hypothesis Test			Exp(B)	95% Wald Confidence Interval for Exp(B)	
			Lower	Upper	Wald Chi-Square	df	Sig.		Lower	Upper
(Intercept)	-,261	,0280	-,316	-,206	86,925	1	,000	,770	,729	,814
[Healthcare provider category=5]	,664	,0437	,579	,750	230,812	1	,000	1,943	1,783	2,117
[Healthcare provider category=4]	,672	,0329	,607	,736	416,761	1	,000	1,957	1,835	2,088
[Healthcare provider category=3]	,599	,0684	,465	,733	76,711	1	,000	1,821	1,592	2,082
[Healthcare provider category=2]	,753	,0380	,678	,827	393,161	1	,000	2,123	1,971	2,287
[Healthcare provider category=1]	0[a]	.	.	.	.	.	.	1	.	.
[Gender=1]	-,522	,0294	-,580	-,465	316,542	1	,000	,593	,560	,628
[Gender=0]	0[a]	.	.	.	.	.	.	1	.	.
(Scale)	1[b]									

Dependent Variable: Any work absence due to ALL CAUSES occurred in the period - T1

Model: (Intercept), Healthcare provider category, Gender

a. Set to zero because this parameter is redundant.

b. Fixed at the displayed value.

```
* Generalized Linear Models.
GENLIN AbHRel_T2 (REFERENCE=FIRST) BY HCPcateg Sex (ORDER=DESCENDING)
  /MODEL HCPcateg Sex INTERCEPT=YES
 DISTRIBUTION=BINOMIAL LINK=LOGIT
  /CRITERIA METHOD=FISHER(1) SCALE=1 COVB=MODEL MAXITERATIONS=100 MAXSTEPHALVING=5
    PCONVERGE=1E-006(ABSOLUTE) SINGULAR=1E-012 ANALYSISTYPE=3(WALD) CILEVEL=95 CITYPE=WALD
    LIKELIHOOD=FULL
  /MISSING CLASSMISSING=EXCLUDE
  /PRINT CPS DESCRIPTIVES MODELINFO FIT SUMMARY SOLUTION (EXPONENTIATED).
```

Generalized Linear Models

Model Information

Dependent Variable	Any work absence due to HEALTH-RELATED CAUSES occurred in the period - T2[a]
Probability Distribution	Binomial
Link Function	Logit

a. The procedure models Yes as the response, treating No as the reference category.

Case Processing Summary

	N	Percent
Included	26956	82,5%
Excluded	5735	17,5%
Total	32691	100,0%

Categorical Variable Information

			N	Percent
Dependent Variable	Any work absence due to HEALTH-RELATED CAUSES occurred in the period - T2	No	17011	63,1%
		Yes	9945	36,9%
		Total	26956	100,0%
Factor	Healthcare provider category	Other HCP	3230	12,0%
		Nurse assistants	10266	38,1%
		Physiotherapists	1019	3,8%
		Nurses	5570	20,7%
		Physicians	6871	25,5%
		Total	26956	100,0%
	Gender	Male	7108	26,4%
		Female	19848	73,6%
		Total	26956	100,0%

Goodness of Fit[a]

	Value	df	Value/df
Deviance	28,472	4	7,118
Scaled Deviance	28,472	4	
Pearson Chi-Square	28,399	4	7,100
Scaled Pearson Chi-Square	28,399	4	
Log Likelihood[b]	-53,060		
Akaike's Information Criterion (AIC)	118,121		

Finite Sample Corrected AIC (AICC)	118,124		
Bayesian Information Criterion (BIC)	167,333		
Consistent AIC (CAIC)	173,333		

Dependent Variable: Any work absence due to HEALTH-RELATED CAUSES occurred in the period - T2

Model: (Intercept), Healthcare provider category, Gender[a]

a. Information criteria are in smaller-is-better form.

b. The full log likelihood function is displayed and used in computing information criteria.

Omnibus Test[a]

Likelihood Ratio Chi-Square	df	Sig.
1367,899	5	,000

Dependent Variable: Any work absence due to HEALTH-RELATED CAUSES occurred in the period - T2

Model: (Intercept), Healthcare provider category, Gender[a]

a. Compares the fitted model against the intercept-only model.

Tests of Model Effects

Source	Type III		
	Wald Chi-Square	df	Sig.
(Intercept)	1367,021	1	,000
Healthcare provider category	755,294	4	,000
Gender	234,747	1	,000

Dependent Variable: Any work absence due to HEALTH-RELATED CAUSES occurred in the period - T2

Model: (Intercept), Healthcare provider category, Gender

Parameter Estimates

Parameter	B	Std. Error	95% Wald Confidence Interval		Hypothesis Test			Exp(B)	95% Wald Confidence Interval for Exp(B)	
			Lower	Upper	Wald Chi-Square	df	Sig.		Lower	Upper
(Intercept)	-1,095	,0321	-1,158	-1,032	1162,753	1	,000	,334	,314	,356
[Healthcare provider category=5]	,633	,0475	,540	,726	177,155	1	,000	1,883	1,715	2,066
[Healthcare provider category=4]	,977	,0364	,906	1,049	721,753	1	,000	2,658	2,475	2,854
[Healthcare provider category=3]	,638	,0721	,497	,779	78,318	1	,000	1,892	1,643	2,179
[Healthcare provider category=2]	,859	,0410	,778	,939	439,228	1	,000	2,360	2,178	2,558
[Healthcare provider category=1]	0[a]	.	.	.	.	.	.	1	.	.
[Gender=1]	-,492	,0321	-,555	-,429	234,747	1	,000	,611	,574	,651
[Gender=0]	0[a]	.	.	.	.	.	.	1	.	.
(Scale)	1[b]									

Dependent Variable: Any work absence due to HEALTH-RELATED CAUSES occurred in the period - T2

Model: (Intercept), Healthcare provider category, Gender

a. Set to zero because this parameter is redundant.

b. Fixed at the displayed value.

```
* Generalized Linear Models.
GENLIN AbHRel_T1 (REFERENCE=FIRST) BY HCPcateg Sex (ORDER=DESCENDING)
  /MODEL HCPcateg Sex INTERCEPT=YES
 DISTRIBUTION=BINOMIAL LINK=LOGIT
  /CRITERIA METHOD=FISHER(1) SCALE=1 COVB=MODEL MAXITERATIONS=100 MAXSTEPHALVING=5
    PCONVERGE=1E-006(ABSOLUTE) SINGULAR=1E-012 ANALYSISTYPE=3(WALD) CILEVEL=95 CITYPE=WALD
    LIKELIHOOD=FULL
  /MISSING CLASSMISSING=EXCLUDE
  /PRINT CPS DESCRIPTIVES MODELINFO FIT SUMMARY SOLUTION (EXPONENTIATED).
```

Generalized Linear Models

Model Information

Dependent Variable	Any work absence due to HEALTH-RELATED CAUSES occurred in the period - T1[a]
Probability Distribution	Binomial
Link Function	Logit

a. The procedure models Yes as the response, treating No as the reference category.

Case Processing Summary

	N	Percent
Included	26956	82,5%
Excluded	5735	17,5%
Total	32691	100,0%

Categorical Variable Information

			N	Percent
Dependent Variable	Any work absence due to HEALTH-RELATED CAUSES occurred in the period - T1	No	15577	57,8%
		Yes	11379	42,2%
		Total	26956	100,0%
Factor	Healthcare provider category	Other HCP	3230	12,0%
		Nurse assistants	10266	38,1%
		Physiotherapists	1019	3,8%
		Nurses	5570	20,7%
		Physicians	6871	25,5%
		Total	26956	100,0%
	Gender	Male	7108	26,4%
		Female	19848	73,6%

Total	26956	100,0%

Goodness of Fit[a]

	Value	df	Value/df
Deviance	38,075	4	9,519
Scaled Deviance	38,075	4	
Pearson Chi-Square	37,813	4	9,453
Scaled Pearson Chi-Square	37,813	4	
Log Likelihood[b]	-58,128		
Akaike's Information Criterion (AIC)	128,255		
Finite Sample Corrected AIC (AICC)	128,259		
Bayesian Information Criterion (BIC)	177,467		
Consistent AIC (CAIC)	183,467		

Dependent Variable: Any work absence due to HEALTH-RELATED CAUSES occurred in the period - T1

Model: (Intercept), Healthcare provider category, Gender[a]

a. Information criteria are in smaller-is-better form.

b. The full log likelihood function is displayed and used in computing information criteria.

Omnibus Test[a]

Likelihood Ratio Chi-Square	df	Sig.
1757,491	5	,000

Dependent Variable: Any work absence due to HEALTH-RELATED CAUSES occurred in the period - T1

Model: (Intercept), Healthcare provider category, Gender[a]

a. Compares the fitted model against the intercept-only model.

Tests of Model Effects

	Type III		
Source	Wald Chi-Square	df	Sig.
(Intercept)	642,068	1	,000
Healthcare provider category	884,279	4	,000
Gender	380,554	1	,000

Dependent Variable: Any work absence due to HEALTH-RELATED CAUSES occurred in the period - T1

Model: (Intercept), Healthcare provider category, Gender

Parameter Estimates

Parameter	B	Std. Error	95% Wald Confidence Interval Lower	95% Wald Confidence Interval Upper	Hypothesis Test Wald Chi-Square	Hypothesis Test df	Hypothesis Test Sig.	Exp(B)	95% Wald Confidence Interval for Exp(B) Lower	95% Wald Confidence Interval for Exp(B) Upper
(Intercept)	-,901	,0309	-,961	-,840	850,653	1	,000	,406	,382	,432
[Healthcare provider category=5]	,847	,0458	,758	,937	341,840	1	,000	2,333	2,133	2,553
[Healthcare provider category=4]	,993	,0354	,924	1,062	788,082	1	,000	2,699	2,518	2,893
[Healthcare provider category=3]	,818	,0698	,682	,955	137,461	1	,000	2,267	1,977	2,600
[Healthcare provider category=2]	,978	,0399	,900	1,056	601,225	1	,000	2,660	2,460	2,876
[Healthcare provider category=1]	0[a]	.	.	.	.	.	.	1	.	.
[Gender=1]	-,610	,0313	-,672	-,549	380,554	1	,000	,543	,511	,578
[Gender=0]	0[a]	.	.	.	.	.	.	1	.	.
(Scale)	1[b]									

Dependent Variable: Any work absence due to HEALTH-RELATED CAUSES occurred in the period - T1

Model: (Intercept), Healthcare provider category, Gender

a. Set to zero because this parameter is redundant.

b. Fixed at the displayed value.

```
* Generalized Linear Models.
GENLIN AbMnt_T2 (REFERENCE=FIRST) BY HCPcateg Sex (ORDER=DESCENDING)
  /MODEL HCPcateg Sex INTERCEPT=YES
 DISTRIBUTION=BINOMIAL LINK=LOGIT
  /CRITERIA METHOD=FISHER(1) SCALE=1 COVB=MODEL MAXITERATIONS=100 MAXSTEPHALVING=5
    PCONVERGE=1E-006(ABSOLUTE) SINGULAR=1E-012 ANALYSISTYPE=3(WALD) CILEVEL=95 CITYPE=WALD
    LIKELIHOOD=FULL
  /MISSING CLASSMISSING=EXCLUDE
  /PRINT CPS DESCRIPTIVES MODELINFO FIT SUMMARY SOLUTION (EXPONENTIATED).
```

Generalized Linear Models

Model Information

Dependent Variable	Any work absence due to MENTAL DISEASE occurred in the period - T2[a]
Probability Distribution	Binomial
Link Function	Logit

a. The procedure models Yes as the response, treating No as the reference category.

Case Processing Summary

	N	Percent
Included	26956	82,5%
Excluded	5735	17,5%
Total	32691	100,0%

Categorical Variable Information

	N	Percent

Dependent Variable	Any work absence due to MENTAL DISEASE occurred in the period - T2	No	25973	96,4%
		Yes	983	3,6%
		Total	26956	100,0%
Factor	Healthcare provider category	Other HCP	3230	12,0%
		Nurse assistants	10266	38,1%
		Physiotherapists	1019	3,8%
		Nurses	5570	20,7%
		Physicians	6871	25,5%
		Total	26956	100,0%
	Gender	Male	7108	26,4%
		Female	19848	73,6%
		Total	26956	100,0%

Goodness of Fit[a]

	Value	df	Value/df
Deviance	20,615	4	5,154
Scaled Deviance	20,615	4	
Pearson Chi-Square	17,639	4	4,410
Scaled Pearson Chi-Square	17,639	4	
Log Likelihood[b]	-38,556		
Akaike's Information Criterion (AIC)	89,111		
Finite Sample Corrected AIC (AICC)	89,115		

Bayesian Information Criterion (BIC)	138,323		
Consistent AIC (CAIC)	144,323		

Dependent Variable: Any work absence due to MENTAL DISEASE occurred in the period - T2

Model: (Intercept), Healthcare provider category, Gender[a]

a. Information criteria are in smaller-is-better form.

b. The full log likelihood function is displayed and used in computing information criteria.

Omnibus Test[a]

Likelihood Ratio Chi-Square	df	Sig.
172,312	5	,000

Dependent Variable: Any work absence due to MENTAL DISEASE occurred in the period - T2

Model: (Intercept), Healthcare provider category, Gender[a]

a. Compares the fitted model against the intercept-only model.

Tests of Model Effects

Source	Type III

	Wald Chi-Square	df	Sig.
(Intercept)	4293,340	1	,000
Healthcare provider category	72,671	4	,000
Gender	39,851	1	,000

Dependent Variable: Any work absence due to MENTAL DISEASE occurred in the period - T2

Model: (Intercept), Healthcare provider category, Gender

Parameter Estimates

			95% Wald Confidence Interval		Hypothesis Test				95% Wald Confidence Interval for Exp(B)	
Parameter	B	Std. Error	Lower	Upper	Wald Chi-Square	df	Sig.	Exp(B)	Lower	Upper
(Intercept)	-3,850	,0993	-4,045	-3,656	1503,719	1	,000	,021	,018	,026
[Healthcare provider category=5]	,784	,1321	,525	1,043	35,182	1	,000	2,189	1,690	2,836
[Healthcare provider category=4]	,803	,1085	,590	1,015	54,755	1	,000	2,232	1,804	2,760
[Healthcare provider category=3]	,907	,1790	,556	1,258	25,699	1	,000	2,477	1,744	3,518
[Healthcare provider category=2]	,948	,1152	,722	1,174	67,646	1	,000	2,580	2,059	3,234
[Healthcare provider category=1]	0[a]	.	.	.	.	.	.	1	.	.
[Gender=1]	-,595	,0943	-,780	-,410	39,851	1	,000	,552	,459	,663
[Gender=0]	0[a]	.	.	.	.	.	.	1	.	.
(Scale)	1[b]									

Dependent Variable: Any work absence due to MENTAL DISEASE occurred in the period - T2

Model: (Intercept), Healthcare provider category, Gender

a. Set to zero because this parameter is redundant.

b. Fixed at the displayed value.

```
* Generalized Linear Models.
GENLIN AbMnt_T1 (REFERENCE=FIRST) BY HCPcateg Sex (ORDER=DESCENDING)
  /MODEL HCPcateg Sex INTERCEPT=YES
 DISTRIBUTION=BINOMIAL LINK=LOGIT
  /CRITERIA METHOD=FISHER(1) SCALE=1 COVB=MODEL MAXITERATIONS=100 MAXSTEPHALVING=5
    PCONVERGE=1E-006(ABSOLUTE) SINGULAR=1E-012 ANALYSISTYPE=3(WALD) CILEVEL=95 CITYPE=WALD
    LIKELIHOOD=FULL
  /MISSING CLASSMISSING=EXCLUDE
  /PRINT CPS DESCRIPTIVES MODELINFO FIT SUMMARY SOLUTION (EXPONENTIATED).
```

Generalized Linear Models

Model Information

Dependent Variable	Any work absence due to MENTAL DISEASE occurred in the period - T1[a]
Probability Distribution	Binomial
Link Function	Logit

a. The procedure models Yes as the response, treating No as the reference category.

Case Processing Summary

	N	Percent
Included	26956	82,5%
Excluded	5735	17,5%
Total	32691	100,0%

Categorical Variable Information

			N	Percent
Dependent Variable	Any work absence due to MENTAL DISEASE occurred in the period - T1	No	26271	97,5%
		Yes	685	2,5%
		Total	26956	100,0%
Factor	Healthcare provider category	Other HCP	3230	12,0%
		Nurse assistants	10266	38,1%
		Physiotherapists	1019	3,8%
		Nurses	5570	20,7%
		Physicians	6871	25,5%
		Total	26956	100,0%
	Gender	Male	7108	26,4%
		Female	19848	73,6%
		Total	26956	100,0%

Goodness of Fit[a]

	Value	df	Value/df
Deviance	5,169	4	1,292
Scaled Deviance	5,169	4	
Pearson Chi-Square	5,009	4	1,252
Scaled Pearson Chi-Square	5,009	4	
Log Likelihood[b]	-29,525		
Akaike's Information Criterion (AIC)	71,050		
Finite Sample Corrected AIC (AICC)	71,053		
Bayesian Information Criterion (BIC)	120,262		
Consistent AIC (CAIC)	126,262		

Dependent Variable: Any work absence due to MENTAL DISEASE occurred in the period - T1

Model: (Intercept), Healthcare provider category, Gender[a]

a. Information criteria are in smaller-is-better form.

b. The full log likelihood function is displayed and used in computing information criteria.

Omnibus Test[a]

Likelihood Ratio Chi-Square	df	Sig.
167,372	5	,000

Dependent Variable: Any work absence due to MENTAL DISEASE occurred in the period - T1
Model: (Intercept), Healthcare provider category, Gender[a]
a. Compares the fitted model against the intercept-only model.

Tests of Model Effects

Source	Type III		
	Wald Chi-Square	df	Sig.
(Intercept)	3611,196	1	,000
Healthcare provider category	79,056	4	,000
Gender	22,429	1	,000

Dependent Variable: Any work absence due to MENTAL DISEASE occurred in the period - T1
Model: (Intercept), Healthcare provider category, Gender

Parameter Estimates

Parameter	B	Std. Error	95% Wald Confidence Interval		Hypothesis Test			Exp(B)	95% Wald Confidence Interval for Exp(B)	
			Lower	Upper	Wald Chi-Square	df	Sig.		Lower	Upper
(Intercept)	-4,572	,1383	-4,843	-4,301	1093,428	1	,000	,010	,008	,014
[Healthcare provider category=5]	1,027	,1757	,683	1,371	34,160	1	,000	2,792	1,979	3,940
[Healthcare provider category=4]	1,245	,1464	,958	1,532	72,324	1	,000	3,474	2,607	4,629
[Healthcare provider category=3]	1,210	,2268	,765	1,654	28,457	1	,000	3,353	2,150	5,230
[Healthcare provider category=2]	1,286	,1546	,983	1,589	69,221	1	,000	3,619	2,673	4,900
[Healthcare provider category=1]	0[a]	.	.	.	.	.	.	1	.	.
[Gender=1]	-,534	,1128	-,755	-,313	22,429	1	,000	,586	,470	,731
[Gender=0]	0[a]	.	.	.	.	.	.	1	.	.
(Scale)	1[b]									

Dependent Variable: Any work absence due to MENTAL DISEASE occurred in the period - T1

Model: (Intercept), Healthcare provider category, Gender

a. Set to zero because this parameter is redundant.

b. Fixed at the displayed value.

Are the apparent differences observed across the periods of pre-pandemia (2019) and pandemia (2020) regarding the proportions of individuals presenting different types of work absences still significant taking into consideration the observed differences among professional categories and gender?

```
* Generalized Estimating Equations.
GENLIN AbAC (REFERENCE=FIRST) BY AsgnGrp HCPcateg Sex (ORDER=DESCENDING)
  /MODEL AsgnGrp HCPcateg Sex INTERCEPT=YES
 DISTRIBUTION=BINOMIAL LINK=LOGIT
  /CRITERIA METHOD=FISHER(1) SCALE=1 MAXITERATIONS=100 MAXSTEPHALVING=5 PCONVERGE=1E-006(ABSOLUTE)
```

```
  SINGULAR=1E-012 ANALYSISTYPE=3(WALD) CILEVEL=95 LIKELIHOOD=FULL
/REPEATED SUBJECT=ID WITHINSUBJECT=AsgnGrp SORT=YES CORRTYPE=UNSTRUCTURED ADJUSTCORR=YES
  COVB=ROBUST MAXITERATIONS=100 PCONVERGE=1e-006(ABSOLUTE) UPDATECORR=1
/MISSING CLASSMISSING=EXCLUDE
/PRINT CPS DESCRIPTIVES MODELINFO FIT SUMMARY SOLUTION (EXPONENTIATED).
```

Generalized Linear Models

Model Information

Dependent Variable		Any work absence due to ALL CAUSES occurred in the period[a]
Probability Distribution		Binomial
Link Function		Logit
Subject Effect	1	ID
Within-Subject Effect	1	Exposure to the pandemia environment
Working Correlation Matrix Structure		Unstructured

a. The procedure models Yes as the response, treating No as the reference category.

Case Processing Summary

	N	Percent

Included	53912	82,5%
Excluded	11470	17,5%
Total	65382	100,0%

Correlated Data Summary

Number of Levels	Subject Effect	ID	26956
	Within-Subject Effect	Exposure to the pandemia environment	2
Number of Subjects			26956
Number of Measurements per Subject	Minimum		2
	Maximum		2
Correlation Matrix Dimension			2

Categorical Variable Information

			N	Percent
Dependent Variable	Any work absence due to ALL CAUSES occurred in the period	No	27181	50,4%
		Yes	26731	49,6%
		Total	53912	100,0%
Factor	Exposure to the pandemia environment	Exposed (2020)	26956	50,0%
		Non-Exposed (2019)	26956	50,0%
		Total	53912	100,0%
	Healthcare provider category	Other HCP	6460	12,0%

		Nurse assistants	20532	38,1%
		Physiotherapists	2038	3,8%
		Nurses	11140	20,7%
		Physicians	13742	25,5%
		Total	53912	100,0%
	Gender	Male	14216	26,4%
		Female	39696	73,6%
		Total	53912	100,0%

Goodness of Fit[a]

	Value
Quasi Likelihood under Independence Model Criterion (QIC)[b]	72063,421
Corrected Quasi Likelihood under Independence Model Criterion (QICC)[b]	72061,646

Dependent Variable: Any work absence due to ALL CAUSES occurred in the period

Model: (Intercept), Exposure to the pandemia environment, Healthcare provider category, Gender[a]

a. Information criteria are in smaller-is-better form.

b. Computed using the full log quasi-likelihood function.

Tests of Model Effects

Source	Type III		
	Wald Chi-Square	df	Sig.
(Intercept)	99,058	1	,000
Exposure to the pandemia environment	301,494	1	,000
Healthcare provider category	1078,491	4	,000
Gender	468,751	1	,000

Dependent Variable: Any work absence due to ALL CAUSES occurred in the period

Model: (Intercept), Exposure to the pandemia environment, Healthcare provider category, Gender

Parameter Estimates

Parameter	B	Std. Error	95% Wald Confidence Interval		Hypothesis Test			Exp(B)	95% Wald Confidence Interval for Exp(B)	
			Lower	Upper	Wald Chi-Square	df	Sig.		Lower	Upper
(Intercept)	-,310	,0231	-,355	-,265	181,102	1	,000	,733	,701	,767
[Exposure to the pandemia environment=1]	-,277	,0159	-,308	-,246	301,494	1	,000	,758	,735	,782
[Exposure to the pandemia environment=0]	0[a]	.	.	.	.	.	.	1	.	.
[Healthcare provider category=5]	,637	,0334	,571	,702	364,013	1	,000	1,890	1,771	2,018

[Healthcare provider category=4]	,779	,0255	,729	,829	931,393	1	,000	2,179	2,073	2,291
[Healthcare provider category=3]	,595	,0524	,492	,698	128,992	1	,000	1,813	1,636	2,009
[Healthcare provider category=2]	,772	,0292	,715	,829	697,433	1	,000	2,164	2,044	2,292
[Healthcare provider category=1]	0[a]	.	.	.	.	.	.	1	.	.
[Gender=1]	-,493	,0228	-,538	-,449	468,751	1	,000	,611	,584	,638
[Gender=0]	0[a]	.	.	.	.	.	.	1	.	.
(Scale)	1									

Dependent Variable: Any work absence due to ALL CAUSES occurred in the period

Model: (Intercept), Exposure to the pandemia environment, Healthcare provider category, Gender

a. Set to zero because this parameter is redundant.

Generalized Linear Models

Model Information

Dependent Variable		Any work absence due to HEALTH-RELATED CAUSES occurred in the period[a]
Probability Distribution		Binomial
Link Function		Logit
Subject Effect	1	ID

Supporting Information Part 4

Within-Subject Effect	1	Exposure to the pandemia environment
Working Correlation Matrix Structure		Unstructured

a. The procedure models Yes as the response, treating No as the reference category.

```
* Generalized Estimating Equations.
GENLIN AbHRel (REFERENCE=FIRST) BY AsgnGrp HCPcateg Sex (ORDER=DESCENDING)
  /MODEL AsgnGrp HCPcateg Sex INTERCEPT=YES
 DISTRIBUTION=BINOMIAL LINK=LOGIT
  /CRITERIA METHOD=FISHER(1) SCALE=1 MAXITERATIONS=100 MAXSTEPHALVING=5 PCONVERGE=1E-006(ABSOLUTE)
    SINGULAR=1E-012 ANALYSISTYPE=3(WALD) CILEVEL=95 LIKELIHOOD=FULL
  /REPEATED SUBJECT=ID WITHINSUBJECT=AsgnGrp SORT=YES CORRTYPE=UNSTRUCTURED ADJUSTCORR=YES
    COVB=ROBUST MAXITERATIONS=100 PCONVERGE=1e-006(ABSOLUTE) UPDATECORR=1
  /MISSING CLASSMISSING=EXCLUDE
  /PRINT CPS DESCRIPTIVES MODELINFO FIT SUMMARY SOLUTION (EXPONENTIATED).
```

Case Processing Summary

	N	Percent
Included	53912	82,5%
Excluded	11470	17,5%
Total	65382	100,0%

Correlated Data Summary

Number of Levels	Subject Effect	ID	26956

	Within-Subject Effect	Exposure to the pandemia environment	2
Number of Subjects			26956
Number of Measurements per Subject	Minimum		2
	Maximum		2
Correlation Matrix Dimension			2

Categorical Variable Information

			N	Percent
Dependent Variable	Any work absence due to HEALTH-RELATED CAUSES occurred in the period	No	32588	60,4%
		Yes	21324	39,6%
		Total	53912	100,0%
Factor	Exposure to the pandemia environment	Exposed (2020)	26956	50,0%
		Non-Exposed (2019)	26956	50,0%
		Total	53912	100,0%
	Healthcare provider category	Other HCP	6460	12,0%
		Nurse assistants	20532	38,1%
		Physiotherapists	2038	3,8%
		Nurses	11140	20,7%
		Physicians	13742	25,5%
		Total	53912	100,0%
	Gender	Male	14216	26,4%
		Female	39696	73,6%

Total	53912	100,0%

Goodness of Fit[a]

	Value
Quasi Likelihood under Independence Model Criterion (QIC)[b]	69123,422
Corrected Quasi Likelihood under Independence Model Criterion (QICC)[b]	69121,573

Dependent Variable: Any work absence due to HEALTH-RELATED CAUSES occurred in the period
Model: (Intercept), Exposure to the pandemia environment, Healthcare provider category, Gender[a]
a. Information criteria are in smaller-is-better form.
b. Computed using the full log quasi-likelihood function.

Tests of Model Effects

Source	Type III Wald Chi-Square	df	Sig.
(Intercept)	1676,559	1	,000

Exposure to the pandemia environment	209,671	1	,000
Healthcare provider category	1372,876	4	,000
Gender	508,863	1	,000

Dependent Variable: Any work absence due to HEALTH-RELATED CAUSES occurred in the period

Model: (Intercept), Exposure to the pandemia environment, Healthcare provider category, Gender

Parameter Estimates

			95% Wald Confidence Interval		Hypothesis Test				95% Wald Confidence Interval for Exp(B)	
Parameter	B	Std. Error	Lower	Upper	Wald Chi-Square	df	Sig.	Exp(B)	Lower	Upper
(Intercept)	-,882	,0253	-,932	-,833	1213,556	1	,000	,414	,394	,435
[Exposure to the pandemia environment=1]	-,236	,0163	-,267	-,204	209,671	1	,000	,790	,765	,816
[Exposure to the pandemia environment=0]	0[a]	.	.	.	.	.	.	1	.	.
[Healthcare provider category=5]	,745	,0356	,675	,814	438,316	1	,000	2,106	1,964	2,258
[Healthcare provider category=4]	,987	,0277	,933	1,041	1270,416	1	,000	2,684	2,542	2,833
[Healthcare provider category=3]	,732	,0542	,626	,838	182,236	1	,000	2,080	1,870	2,313
[Healthcare provider category=2]	,921	,0310	,860	,982	882,148	1	,000	2,512	2,364	2,669
[Healthcare provider category=1]	0[a]	.	.	.	.	.	.	1	.	.
[Gender=1]	-,554	,0245	-,602	-,505	508,863	1	,000	,575	,548	,603

Supporting Information Part 4

[Gender=0]	0[a]	.	.	.	.	.	.	1	.	.
(Scale)	1									

Dependent Variable: Any work absence due to HEALTH-RELATED CAUSES occurred in the period

Model: (Intercept), Exposure to the pandemia environment, Healthcare provider category, Gender

a. Set to zero because this parameter is redundant.

```
* Generalized Estimating Equations.
GENLIN AbMnt (REFERENCE=FIRST) BY AsgnGrp HCPcateg Sex (ORDER=DESCENDING)
  /MODEL AsgnGrp HCPcateg Sex INTERCEPT=YES
 DISTRIBUTION=BINOMIAL LINK=LOGIT
  /CRITERIA METHOD=FISHER(1) SCALE=1 MAXITERATIONS=100 MAXSTEPHALVING=5 PCONVERGE=1E-006(ABSOLUTE)
    SINGULAR=1E-012 ANALYSISTYPE=3(WALD) CILEVEL=95 LIKELIHOOD=FULL
  /REPEATED SUBJECT=ID WITHINSUBJECT=AsgnGrp SORT=YES CORRTYPE=UNSTRUCTURED ADJUSTCORR=YES
    COVB=ROBUST MAXITERATIONS=100 PCONVERGE=1e-006(ABSOLUTE) UPDATECORR=1
  /MISSING CLASSMISSING=EXCLUDE
  /PRINT CPS DESCRIPTIVES MODELINFO FIT SUMMARY SOLUTION (EXPONENTIATED).
```

Supporting Information. Continues in the next file.
