# Supplementary material for: Impact of COVID-19 on the mental health of public university hospital workers in Brazil: A cohort-based analysis of 32,691 workers
Source: PLoS One. 2022 Jun 16;17(6):e0269318. doi: 10.1371/journal.pone.0269318 (PMC9202958; doi:10.1371/journal.pone.0269318)
Supplement: S5 File — (PDF) [file pone.0269318.s005.pdf]

Supporting Information. Part5

# Generalized Linear Models

## Model Information

| | | |
|---|---|---|
| Dependent Variable | | Any work absence due to MENTAL DISEASE occurred in the period[a] |
| Probability Distribution | | Binomial |
| Link Function | | Logit |
| Subject Effect | 1 | ID |
| Within-Subject Effect | 1 | Exposure to the pandemia environment |
| Working Correlation Matrix Structure | | Unstructured |

a. The procedure models Yes as the response, treating No as the reference category.

## Case Processing Summary

| | N | Percent |
|---|---|---|
| Included | 53912 | 82,5% |
| Excluded | 11470 | 17,5% |
| Total | 65382 | 100,0% |

## Correlated Data Summary

| | | | |
|---|---|---|---|
| Number of Levels | Subject Effect | ID | 26956 |
| | Within-Subject Effect | Exposure to the pandemia environment | 2 |
| Number of Subjects | | | 26956 |

Supporting Information. Part5

| | | |
|---|---|---|
| Number of Measurements per Subject | Minimum | 2 |
| | Maximum | 2 |
| Correlation Matrix Dimension | | 2 |

**Categorical Variable Information**

| | | | N | Percent |
|---|---|---|---|---|
| Dependent Variable | Any work absence due to MENTAL DISEASE occurred in the period | No | 52244 | 96,9% |
| | | Yes | 1668 | 3,1% |
| | | Total | 53912 | 100,0% |
| Factor | Exposure to the pandemia environment | Exposed (2020) | 26956 | 50,0% |
| | | Non-Exposed (2019) | 26956 | 50,0% |
| | | Total | 53912 | 100,0% |
| | Healthcare provider category | Other HCP | 6460 | 12,0% |
| | | Nurse assistants | 20532 | 38,1% |
| | | Physiotherapists | 2038 | 3,8% |
| | | Nurses | 11140 | 20,7% |
| | | Physicians | 13742 | 25,5% |
| | | Total | 53912 | 100,0% |
| | Gender | Male | 14216 | 26,4% |
| | | Female | 39696 | 73,6% |
| | | Total | 53912 | 100,0% |

**Goodness of Fit[a]**

| | Value |
|---|---|
| Quasi Likelihood under Independence Model Criterion (QIC)[b] | 14506,229 |

| | |
|---|---|
| Corrected Quasi Likelihood under Independence Model Criterion (QICC)[b] | 14504,580 |

Dependent Variable: Any work absence due to MENTAL DISEASE occurred in the period
Model: (Intercept), Exposure to the pandemia environment, Healthcare provider category, Gender[a]
a. Information criteria are in smaller-is-better form.
b. Computed using the full log quasi-likelihood function.

## Tests of Model Effects

| Source | Type III | | |
|---|---|---|---|
| | Wald Chi-Square | df | Sig. |
| (Intercept) | 7205,049 | 1 | ,000 |
| Exposure to the pandemia environment | 65,221 | 1 | ,000 |
| Healthcare provider category | 124,516 | 4 | ,000 |
| Gender | 50,744 | 1 | ,000 |

Dependent Variable: Any work absence due to MENTAL DISEASE occurred in the period
Model: (Intercept), Exposure to the pandemia environment, Healthcare provider category, Gender

## Parameter Estimates

| Parameter | B | Std. Error | 95% Wald Confidence Interval | | Hypothesis Test | | | Exp(B) | 95% Wald Confidence Interval for Exp(B) | |
|---|---|---|---|---|---|---|---|---|---|---|
| | | | Lower | Upper | Wald Chi-Square | df | Sig. | | Lower | Upper |
| (Intercept) | -4,349 | ,0916 | -4,529 | -4,170 | 2254,693 | 1 | ,000 | ,013 | ,011 | ,015 |

| | | | | | | | | | | |
|---|---|---|---|---|---|---|---|---|---|---|
| [Exposure to the pandemia environment=1] | ,375 | ,0464 | ,284 | ,465 | 65,221 | 1 | ,000 | 1,454 | 1,328 | 1,593 |
| [Exposure to the pandemia environment=0] | 0[a] | . | . | . | . | . | . | 1 | . | . |
| [Healthcare provider category=5] | ,868 | ,1127 | ,647 | 1,089 | 59,247 | 1 | ,000 | 2,381 | 1,909 | 2,970 |
| [Healthcare provider category=4] | ,966 | ,0948 | ,780 | 1,152 | 103,855 | 1 | ,000 | 2,628 | 2,182 | 3,164 |
| [Healthcare provider category=3] | 1,014 | ,1511 | ,718 | 1,310 | 45,049 | 1 | ,000 | 2,756 | 2,050 | 3,706 |
| [Healthcare provider category=2] | 1,069 | ,1001 | ,873 | 1,265 | 114,061 | 1 | ,000 | 2,911 | 2,393 | 3,542 |
| [Healthcare provider category=1] | 0[a] | . | . | . | . | . | . | 1 | . | . |
| [Gender=1] | -,571 | ,0802 | -,728 | -,414 | 50,744 | 1 | ,000 | ,565 | ,483 | ,661 |
| [Gender=0] | 0[a] | . | . | . | . | . | . | 1 | . | . |
| (Scale) | 1 | | | | | | | | | |

Dependent Variable: Any work absence due to MENTAL DISEASE occurred in the period

Model: (Intercept), Exposure to the pandemia environment, Healthcare provider category, Gender

a. Set to zero because this parameter is redundant.

## Did the physicians themselves changed their profile regarding work absences across the periods of pre-pandemia (2019) and pandemia (2020)?

```
USE ALL.
COMPUTE filter_$=(HCPcateg = 1).
VARIABLE LABELS filter_$ 'HCPcateg = 1 (FILTER)'.
VALUE LABELS filter_$ 0 'Not Selected' 1 'Selected'.
FORMATS filter_$ (f1.0).
FILTER BY filter_$.
EXECUTE.
* Generalized Estimating Equations.
GENLIN AbAC (REFERENCE=FIRST) BY AsgnGrp Sex (ORDER=DESCENDING)
  /MODEL AsgnGrp Sex INTERCEPT=YES
 DISTRIBUTION=BINOMIAL LINK=LOGIT
  /CRITERIA METHOD=FISHER(1) SCALE=1 MAXITERATIONS=100 MAXSTEPHALVING=5 PCONVERGE=1E-006(ABSOLUTE)
    SINGULAR=1E-012 ANALYSISTYPE=3(WALD) CILEVEL=95 LIKELIHOOD=FULL
  /REPEATED SUBJECT=ID WITHINSUBJECT=AsgnGrp SORT=YES CORRTYPE=UNSTRUCTURED ADJUSTCORR=YES
    COVB=ROBUST MAXITERATIONS=100 PCONVERGE=1e-006(ABSOLUTE) UPDATECORR=1
```

```
  /MISSING CLASSMISSING=EXCLUDE
  /PRINT CPS DESCRIPTIVES MODELINFO FIT SUMMARY SOLUTION (EXPONENTIATED).
```

# Generalized Linear Models

### Model Information

| | | |
|---|---|---|
| Dependent Variable | | Any work absence due to ALL CAUSES occurred in the period[a] |
| Probability Distribution | | Binomial |
| Link Function | | Logit |
| Subject Effect | 1 | ID |
| Within-Subject Effect | 1 | Exposure to the pandemia environment |
| Working Correlation Matrix Structure | | Unstructured |

a. The procedure models Yes as the response, treating No as the reference category.

### Case Processing Summary

| | N | Percent |
|---|---|---|
| Included | 13742 | 100,0% |
| Excluded | 0 | 0,0% |
| Total | 13742 | 100,0% |

### Correlated Data Summary

Supporting Information. Part5

| | | | |
|---|---|---|---|
| Number of Levels | Subject Effect | ID | 6871 |
| | Within-Subject Effect | Exposure to the pandemia environment | 2 |
| Number of Subjects | | | 6871 |
| Number of Measurements per Subject | Minimum | | 2 |
| | Maximum | | 2 |
| Correlation Matrix Dimension | | | 2 |

**Categorical Variable Information**

| | | | N | Percent |
|---|---|---|---|---|
| Dependent Variable | Any work absence due to ALL CAUSES occurred in the period | No | 9068 | 66,0% |
| | | Yes | 4674 | 34,0% |
| | | Total | 13742 | 100,0% |
| Factor | Exposure to the pandemia environment | Exposed (2020) | 6871 | 50,0% |
| | | Non-Exposed (2019) | 6871 | 50,0% |
| | | Total | 13742 | 100,0% |
| | Gender | Male | 6310 | 45,9% |
| | | Female | 7432 | 54,1% |
| | | Total | 13742 | 100,0% |

**Goodness of Fit[a]**

| | Value |
|---|---|
| Quasi Likelihood under Independence Model Criterion (QIC)[b] | 17171,106 |

Supporting Information. Part5

| | |
|---|---|
| Corrected Quasi Likelihood under Independence Model Criterion (QICC)[b] | 17170,830 |

Dependent Variable: Any work absence due to ALL CAUSES occurred in the period
Model: (Intercept), Exposure to the pandemia environment, Gender[a]
a. Information criteria are in smaller-is-better form.
b. Computed using the full log quasi-likelihood function.

## Tests of Model Effects

| | Type III | | |
|---|---|---|---|
| Source | Wald Chi-Square | df | Sig. |
| (Intercept) | 1288,536 | 1 | ,000 |
| Exposure to the pandemia environment | 111,548 | 1 | ,000 |
| Gender | 309,243 | 1 | ,000 |

Dependent Variable: Any work absence due to ALL CAUSES occurred in the period
Model: (Intercept), Exposure to the pandemia environment, Gender

## Parameter Estimates

| | | | 95% Wald Confidence Interval | | Hypothesis Test | | | | 95% Wald Confidence Interval for Exp(B) | |
|---|---|---|---|---|---|---|---|---|---|---|
| Parameter | B | Std. Error | Lower | Upper | Wald Chi-Square | df | Sig. | Exp(B) | Lower | Upper |
| (Intercept) | -,186 | ,0301 | -,245 | -,127 | 38,172 | 1 | ,000 | ,830 | ,783 | ,881 |
| [Exposure to the pandemia environment=1] | -,359 | ,0340 | -,426 | -,293 | 111,548 | 1 | ,000 | ,698 | ,653 | ,746 |

| | | | | | | | | | | |
|---|---|---|---|---|---|---|---|---|---|---|
| [Exposure to the pandemia environment=0] | 0[a] | . | . | . | . | . | . | 1 | . | . |
| [Gender=1] | -,701 | ,0399 | -,779 | -,623 | 309,243 | 1 | ,000 | ,496 | ,459 | ,537 |
| [Gender=0] | 0[a] | . | . | . | . | . | . | 1 | . | . |
| (Scale) | 1 | | | | | | | | | |

Dependent Variable: Any work absence due to ALL CAUSES occurred in the period

Model: (Intercept), Exposure to the pandemia environment, Gender

a. Set to zero because this parameter is redundant.

```
* Generalized Estimating Equations.
GENLIN AbHRel (REFERENCE=FIRST) BY AsgnGrp Sex (ORDER=DESCENDING)
  /MODEL AsgnGrp Sex INTERCEPT=YES
 DISTRIBUTION=BINOMIAL LINK=LOGIT
  /CRITERIA METHOD=FISHER(1) SCALE=1 MAXITERATIONS=100 MAXSTEPHALVING=5 PCONVERGE=1E-006(ABSOLUTE)
    SINGULAR=1E-012 ANALYSISTYPE=3(WALD) CILEVEL=95 LIKELIHOOD=FULL
  /REPEATED SUBJECT=ID WITHINSUBJECT=AsgnGrp SORT=YES CORRTYPE=UNSTRUCTURED ADJUSTCORR=YES
    COVB=ROBUST MAXITERATIONS=100 PCONVERGE=1e-006(ABSOLUTE) UPDATECORR=1
  /MISSING CLASSMISSING=EXCLUDE
  /PRINT CPS DESCRIPTIVES MODELINFO FIT SUMMARY SOLUTION (EXPONENTIATED).
```

# Generalized Linear Models

**Model Information**

| | |
|---|---|
| Dependent Variable | Any work absence due to HEALTH-RELATED CAUSES occurred in the period[a] |
| Probability Distribution | Binomial |
| Link Function | Logit |

Supporting Information. Part5

| | | |
|---|---|---|
| Subject Effect | 1 | ID |
| Within-Subject Effect | 1 | Exposure to the pandemia environment |
| Working Correlation Matrix Structure | | Unstructured |

a. The procedure models Yes as the response, treating No as the reference category.

### Case Processing Summary

| | N | Percent |
|---|---|---|
| Included | 13742 | 100,0% |
| Excluded | 0 | 0,0% |
| Total | 13742 | 100,0% |

### Correlated Data Summary

| | | | |
|---|---|---|---|
| Number of Levels | Subject Effect | ID | 6871 |
| | Within-Subject Effect | Exposure to the pandemia environment | 2 |
| Number of Subjects | | | 6871 |
| Number of Measurements per Subject | Minimum | | 2 |
| | Maximum | | 2 |
| Correlation Matrix Dimension | | | 2 |

### Categorical Variable Information

| | | | N | Percent |
|---|---|---|---|---|
| Dependent Variable | Any work absence due to HEALTH-RELATED CAUSES occurred in the period | No | 10631 | 77,4% |
| | | Yes | 3111 | 22,6% |
| | | Total | 13742 | 100,0% |

Supporting Information. Part5

| | | | | |
|---|---|---|---|---|
| Factor | Exposure to the pandemia environment | Exposed (2020) | 6871 | 50,0% |
| | | Non-Exposed (2019) | 6871 | 50,0% |
| | | Total | 13742 | 100,0% |
| | Gender | Male | 6310 | 45,9% |
| | | Female | 7432 | 54,1% |
| | | Total | 13742 | 100,0% |

### Goodness of Fit[a]

| | Value |
|---|---|
| Quasi Likelihood under Independence Model Criterion (QIC)[b] | 14323,357 |
| Corrected Quasi Likelihood under Independence Model Criterion (QICC)[b] | 14323,057 |

Dependent Variable: Any work absence due to HEALTH-RELATED CAUSES occurred in the period

Model: (Intercept), Exposure to the pandemia environment, Gender[a]

a. Information criteria are in smaller-is-better form.

b. Computed using the full log quasi-likelihood function.

### Tests of Model Effects

| Source | Type III Wald Chi-Square | df | Sig. |
|---|---|---|---|
| (Intercept) | 3160,331 | 1 | ,000 |

Supporting Information. Part5

| | | | |
|---|---|---|---|
| Exposure to the pandemia environment | 15,702 | 1 | ,000 |
| Gender | 306,043 | 1 | ,000 |

Dependent Variable: Any work absence due to HEALTH-RELATED CAUSES occurred in the period

Model: (Intercept), Exposure to the pandemia environment, Gender

**Parameter Estimates**

| Parameter | B | Std. Error | 95% Wald Confidence Interval | | Hypothesis Test | | | Exp(B) | 95% Wald Confidence Interval for Exp(B) | |
|---|---|---|---|---|---|---|---|---|---|---|
| | | | Lower | Upper | Wald Chi-Square | df | Sig. | | Lower | Upper |
| (Intercept) | -,826 | ,0332 | -,891 | -,761 | 619,786 | 1 | ,000 | ,438 | ,410 | ,467 |
| [Exposure to the pandemia environment=1] | -,151 | ,0382 | -,226 | -,076 | 15,702 | 1 | ,000 | ,860 | ,798 | ,926 |
| [Exposure to the pandemia environment=0] | 0[a] | . | . | . | . | . | . | 1 | . | . |
| [Gender=1] | -,814 | ,0465 | -,905 | -,722 | 306,043 | 1 | ,000 | ,443 | ,405 | ,486 |
| [Gender=0] | 0[a] | . | . | . | . | . | . | 1 | . | . |
| (Scale) | 1 | | | | | | | | | |

Dependent Variable: Any work absence due to HEALTH-RELATED CAUSES occurred in the period

Model: (Intercept), Exposure to the pandemia environment, Gender

a. Set to zero because this parameter is redundant.

```
* Generalized Estimating Equations.
GENLIN AbMnt (REFERENCE=FIRST) BY AsgnGrp Sex (ORDER=DESCENDING)
  /MODEL AsgnGrp Sex INTERCEPT=YES
 DISTRIBUTION=BINOMIAL LINK=LOGIT
  /CRITERIA METHOD=FISHER(1) SCALE=1 MAXITERATIONS=100 MAXSTEPHALVING=5 PCONVERGE=1E-006(ABSOLUTE)
    SINGULAR=1E-012 ANALYSISTYPE=3(WALD) CILEVEL=95 LIKELIHOOD=FULL
  /REPEATED SUBJECT=ID WITHINSUBJECT=AsgnGrp SORT=YES CORRTYPE=UNSTRUCTURED ADJUSTCORR=YES
    COVB=ROBUST MAXITERATIONS=100 PCONVERGE=1e-006(ABSOLUTE) UPDATECORR=1
  /MISSING CLASSMISSING=EXCLUDE
  /PRINT CPS DESCRIPTIVES MODELINFO FIT SUMMARY SOLUTION (EXPONENTIATED).
```

Supporting Information. Part5

# Generalized Linear Models

### Model Information

| | | |
|---|---|---|
| Dependent Variable | | Any work absence due to MENTAL DISEASE occurred in the period[a] |
| Probability Distribution | | Binomial |
| Link Function | | Logit |
| Subject Effect | 1 | ID |
| Within-Subject Effect | 1 | Exposure to the pandemia environment |
| Working Correlation Matrix Structure | | Unstructured |

a. The procedure models Yes as the response, treating No as the reference category.

### Case Processing Summary

| | N | Percent |
|---|---|---|
| Included | 13742 | 100,0% |
| Excluded | 0 | 0,0% |
| Total | 13742 | 100,0% |

### Correlated Data Summary

Supporting Information. Part5

| | | | |
|---|---|---|---|
| Number of Levels | Subject Effect | ID | 6871 |
| | Within-Subject Effect | Exposure to the pandemia environment | 2 |
| Number of Subjects | | | 6871 |
| Number of Measurements per Subject | Minimum | | 2 |
| | Maximum | | 2 |
| Correlation Matrix Dimension | | | 2 |

### Categorical Variable Information

| | | | N | Percent |
|---|---|---|---|---|
| Dependent Variable | Any work absence due to MENTAL DISEASE occurred in the period | No | 13571 | 98,8% |
| | | Yes | 171 | 1,2% |
| | | Total | 13742 | 100,0% |
| Factor | Exposure to the pandemia environment | Exposed (2020) | 6871 | 50,0% |
| | | Non-Exposed (2019) | 6871 | 50,0% |
| | | Total | 13742 | 100,0% |
| | Gender | Male | 6310 | 45,9% |
| | | Female | 7432 | 54,1% |
| | | Total | 13742 | 100,0% |

### Goodness of Fit[a]

| | Value |
|---|---|
| Quasi Likelihood under Independence Model Criterion (QIC)[b] | 1780,015 |

| | |
|---|---|
| Corrected Quasi Likelihood under Independence Model Criterion (QICC)[b] | 1779,872 |

Dependent Variable: Any work absence due to MENTAL DISEASE occurred in the period
Model: (Intercept), Exposure to the pandemia environment, Gender[a]
a. Information criteria are in smaller-is-better form.
b. Computed using the full log quasi-likelihood function.

## Tests of Model Effects

| Source | Type III Wald Chi-Square | df | Sig. |
|---|---|---|---|
| (Intercept) | 2065,771 | 1 | ,000 |
| Exposure to the pandemia environment | 21,295 | 1 | ,000 |
| Gender | 36,296 | 1 | ,000 |

Dependent Variable: Any work absence due to MENTAL DISEASE occurred in the period
Model: (Intercept), Exposure to the pandemia environment, Gender

## Parameter Estimates

| | | | 95% Wald Confidence Interval | | Hypothesis Test | | | | 95% Wald Confidence Interval for Exp(B) | |
|---|---|---|---|---|---|---|---|---|---|---|
| Parameter | B | Std. Error | Lower | Upper | Wald Chi-Square | df | Sig. | Exp(B) | Lower | Upper |
| (Intercept) | -4,400 | ,1398 | -4,674 | -4,126 | 990,904 | 1 | ,000 | ,012 | ,009 | ,016 |
| [Exposure to the pandemia environment=1] | ,704 | ,1525 | ,405 | 1,003 | 21,295 | 1 | ,000 | 2,021 | 1,499 | 2,725 |

| | | | | | | | | | | |
|---|---|---|---|---|---|---|---|---|---|---|
| [Exposure to the pandemia environment=0] | 0[a] | . | . | . | . | . | . | 1 | . | . |
| [Gender=1] | -1,175 | ,1951 | -1,558 | -,793 | 36,296 | 1 | ,000 | ,309 | ,211 | ,452 |
| [Gender=0] | 0[a] | . | . | . | . | . | . | 1 | . | . |
| (Scale) | 1 | | | | | | | | | |

Dependent Variable: Any work absence due to MENTAL DISEASE occurred in the period

Model: (Intercept), Exposure to the pandemia environment, Gender

a. Set to zero because this parameter is redundant.

Did the cumulative incidence of COVID cases in the city of the professionals affect the profile of non-COVID Health-Related work absences among health-care providers in those locations?

## GGraph

Supporting Information. Part5

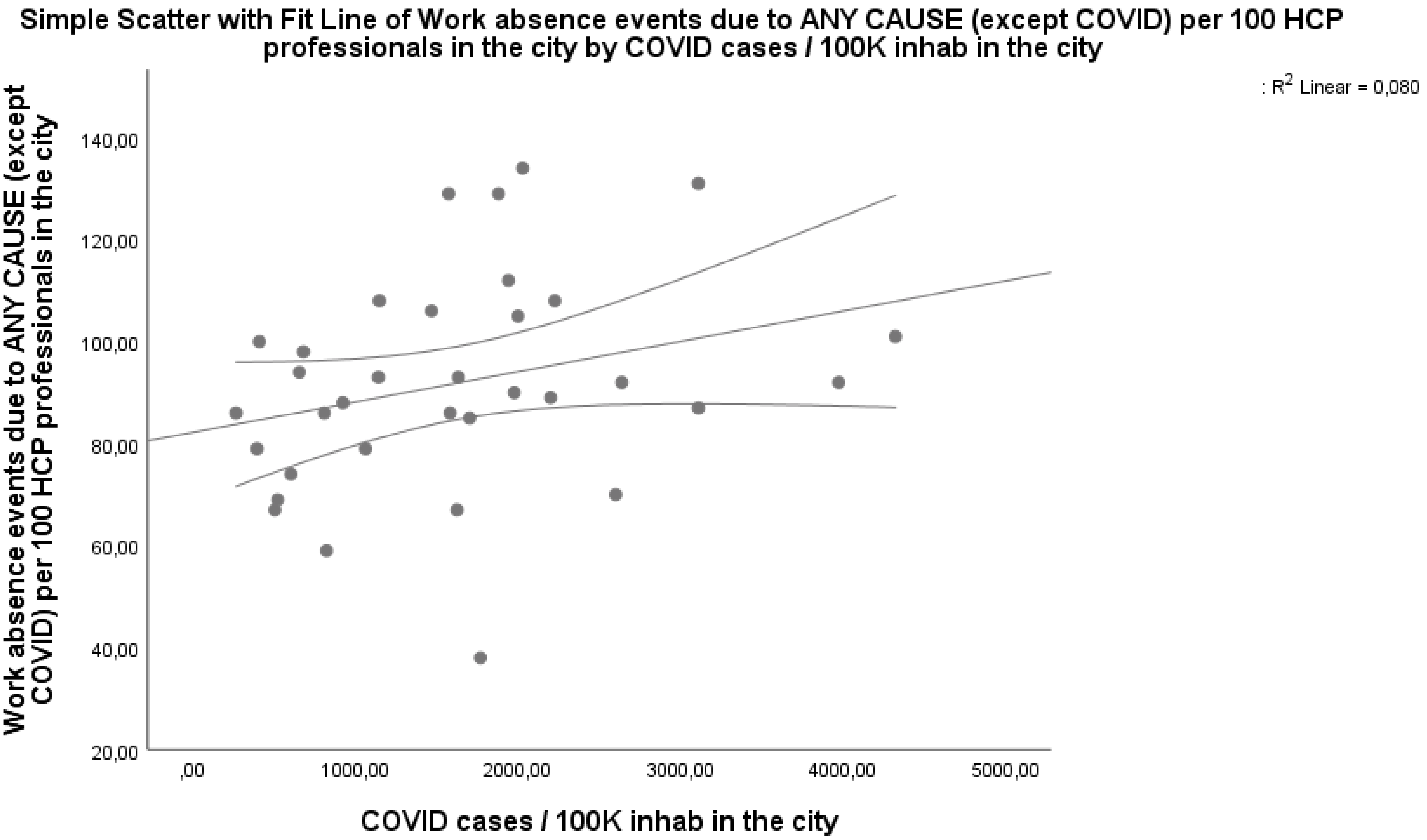


EXAMINE VARIABLES=CityCovCases100k AbAC_HCP_N

Supporting Information. Part5

```
  /PLOT HISTOGRAM NPPLOT
  /PERCENTILES(5,10,25,50,75,90,95) HAVERAGE
  /STATISTICS DESCRIPTIVES
  /CINTERVAL 95
  /MISSING PAIRWISE
  /NOTOTAL.
```

# Explore

## Case Processing Summary

| | Cases | | | | | |
|---|---|---|---|---|---|---|
| | Valid | | Missing | | Total | |
| | N | Percent | N | Percent | N | Percent |
| COVID cases / 100K inhab in the city | 34 | 100,0% | 0 | 0,0% | 34 | 100,0% |
| Work absence events due to ANY CAUSE (except COVID) per 100 HCP professionals in the city | 34 | 100,0% | 0 | 0,0% | 34 | 100,0% |

## Descriptives

| | | | Statistic | Std. Error |
|---|---|---|---|---|
| COVID cases / 100K inhab in the city | Mean | | 1624,7937 | 172,14783 |
| | 95% Confidence Interval for Mean | Lower Bound | 1274,5563 | |
| | | Upper Bound | 1975,0311 | |
| | 5% Trimmed Mean | | 1555,7030 | |
| | Median | | 1601,5129 | |

| | | | | |
|---|---|---|---|---|
| | Variance | | 1007585,741 | |
| | Std. Deviation | | 1003,78570 | |
| | Minimum | | 264,89 | |
| | Maximum | | 4318,79 | |
| | Range | | 4053,90 | |
| | Interquartile Range | | 1293,95 | |
| | Skewness | | ,890 | ,403 |
| | Kurtosis | | ,643 | ,788 |
| Work absence events due to ANY CAUSE (except COVID) per 100 HCP professionals in the city | Mean | | 91,8824 | 3,61068 |
| | 95% Confidence Interval for Mean | Lower Bound | 84,5364 | |
| | | Upper Bound | 99,2283 | |
| | 5% Trimmed Mean | | 92,1242 | |
| | Median | | 91,0000 | |
| | Variance | | 443,258 | |
| | Std. Deviation | | 21,05370 | |
| | Minimum | | 38,00 | |
| | Maximum | | 134,00 | |
| | Range | | 96,00 | |
| | Interquartile Range | | 26,25 | |
| | Skewness | | ,012 | ,403 |
| | Kurtosis | | ,536 | ,788 |

## Percentiles

| | | Percentiles | | | | | | |
|---|---|---|---|---|---|---|---|---|
| | | 5 | 10 | 25 | 50 | 75 | 90 | 95 |
| Weighted Average(Definition 1) | COVID cases / 100K inhab in the city | 361,5726 | 455,7713 | 775,1292 | 1601,5129 | 2069,0827 | 3107,3040 | 4057,3388 |

| | | | | | | | | |
|---|---|---|---|---|---|---|---|---|
| | Work absence events due to ANY CAUSE (except COVID) per 100 HCP professionals in the city | 53,7500 | 67,0000 | 79,0000 | 91,0000 | 105,2500 | 129,0000 | 131,7500 |
| Tukey's Hinges | COVID cases / 100K inhab in the city | | | 807,1431 | 1601,5129 | 2026,0278 | | |
| | Work absence events due to ANY CAUSE (except COVID) per 100 HCP professionals in the city | | | 79,0000 | 91,0000 | 105,0000 | | |

**Tests of Normality**

| | Kolmogorov-Smirnov[a] | | | Shapiro-Wilk | | |
|---|---|---|---|---|---|---|
| | Statistic | df | Sig. | Statistic | df | Sig. |
| COVID cases / 100K inhab in the city | ,109 | 34 | ,200* | ,930 | 34 | ,031 |
| Work absence events due to ANY CAUSE (except COVID) per 100 HCP professionals in the city | ,107 | 34 | ,200* | ,968 | 34 | ,409 |

*. This is a lower bound of the true significance.

a. Lilliefors Significance Correction

## COVID cases / 100K inhab in the city

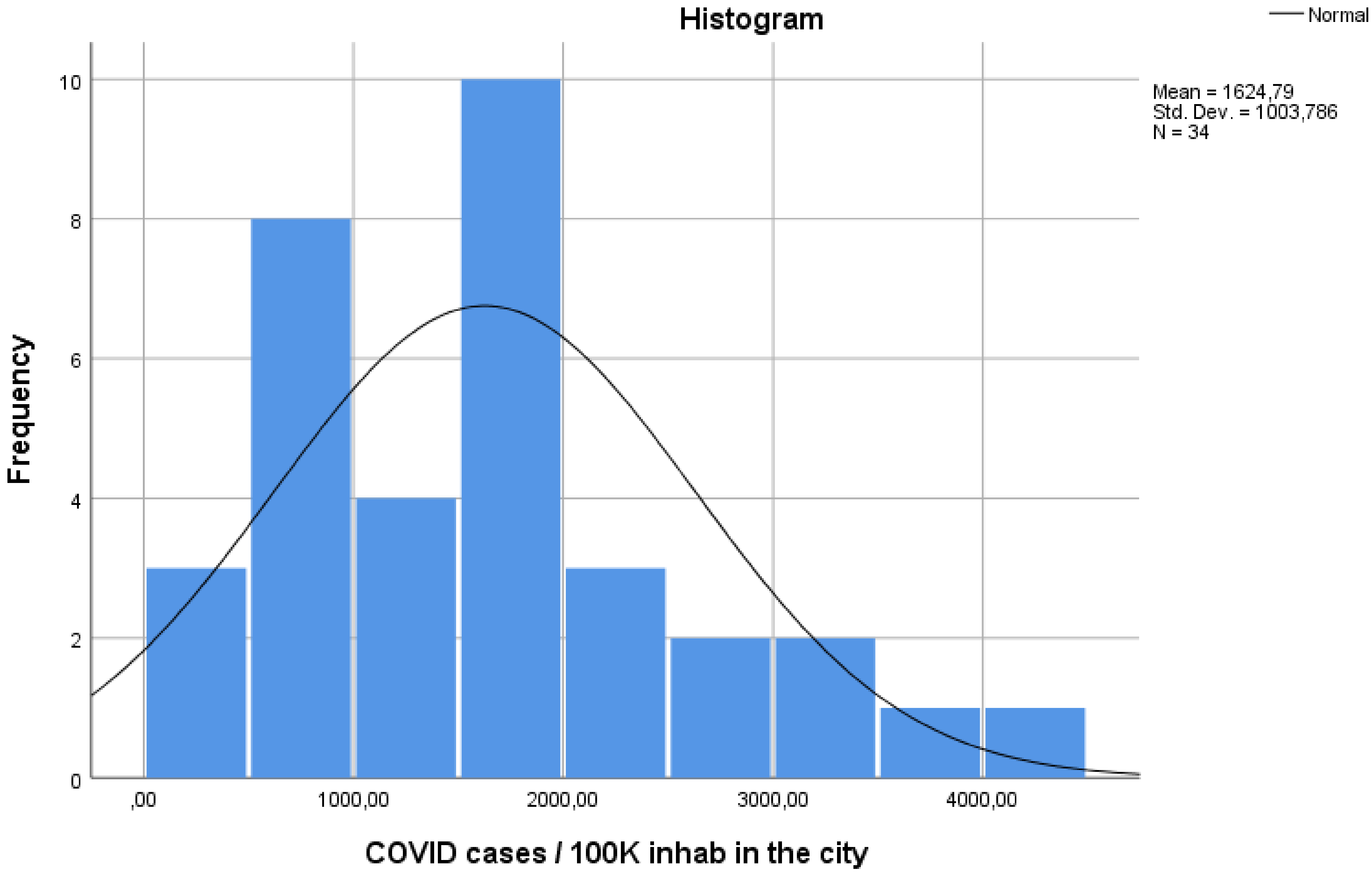
Histogram
Normal
Mean = 1624,79
Std. Dev. = 1003,786
N = 34
Frequency
0
2
4
6
8
10
,00
1000,00
2000,00
3000,00
4000,00
COVID cases / 100K inhab in the city

Supporting Information. Part5

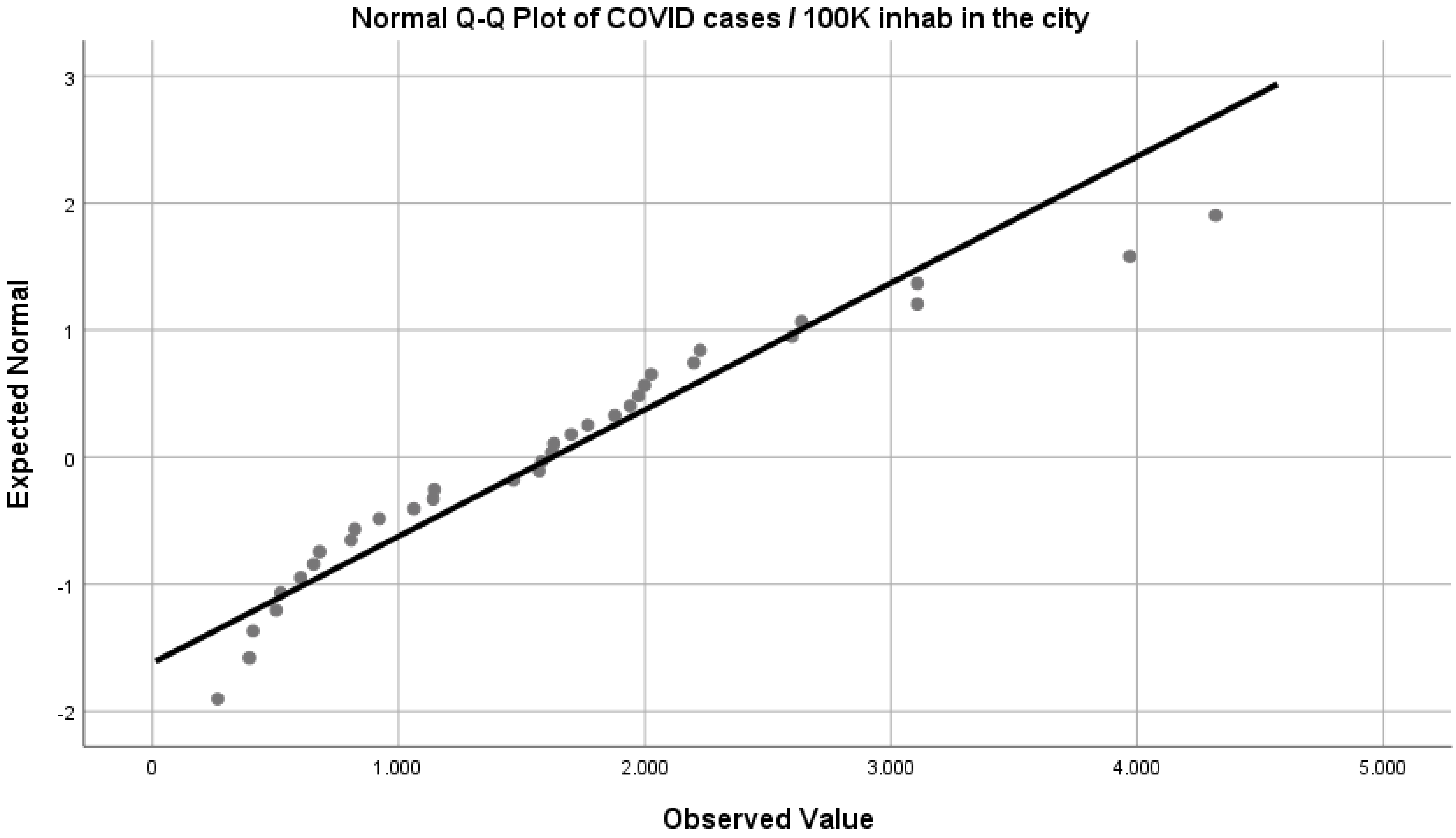

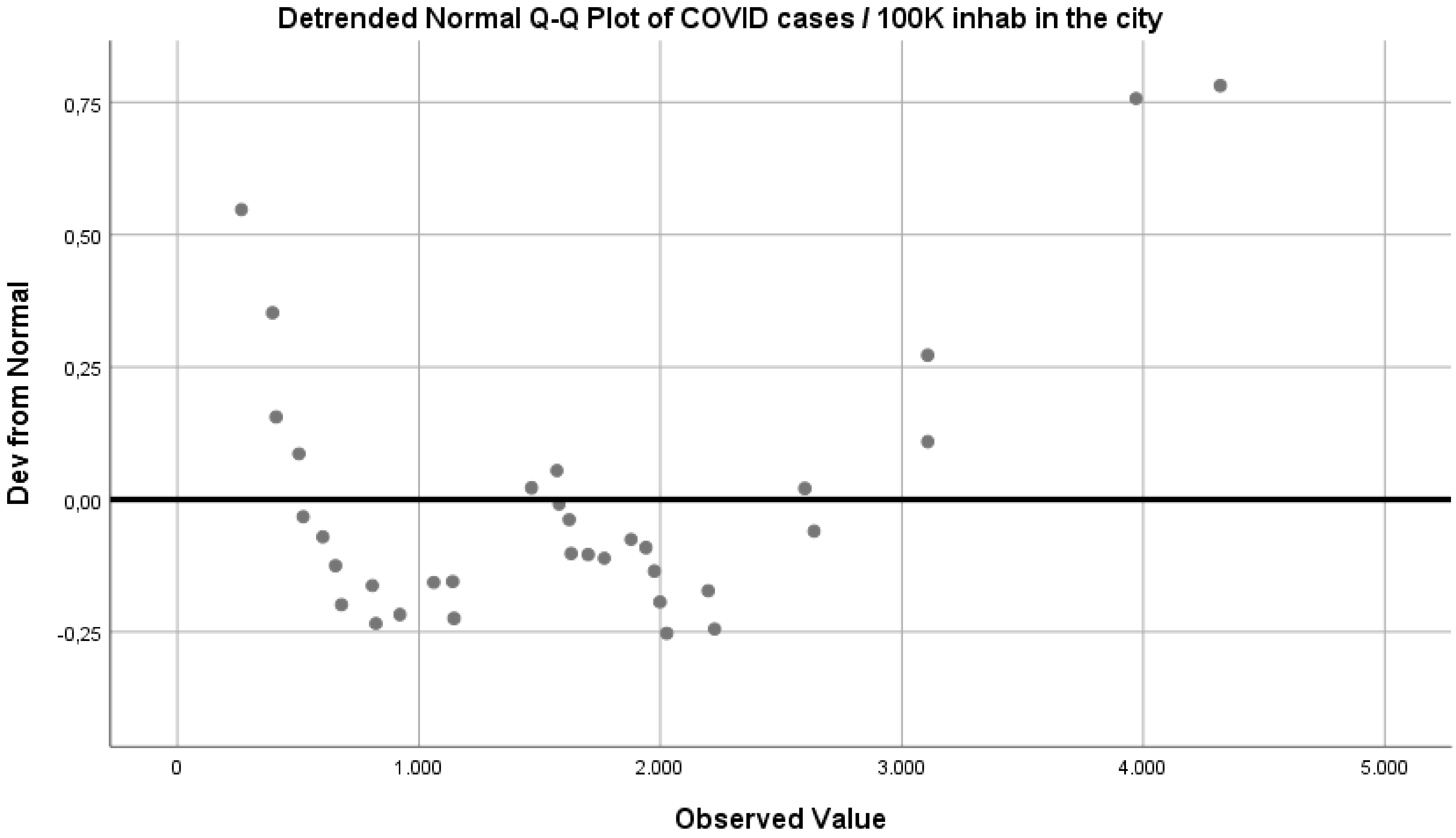
Detrended Normal Q-Q Plot of COVID cases / 100K inhab in the city
Dev from Normal
0,75
0,50
0,25
0,00
-0,25
0
1.000
2.000
3.000
4.000
5.000
Observed Value

## Work absence events due to ANY CAUSE (except COVID) per 100 HCP professionals in the city

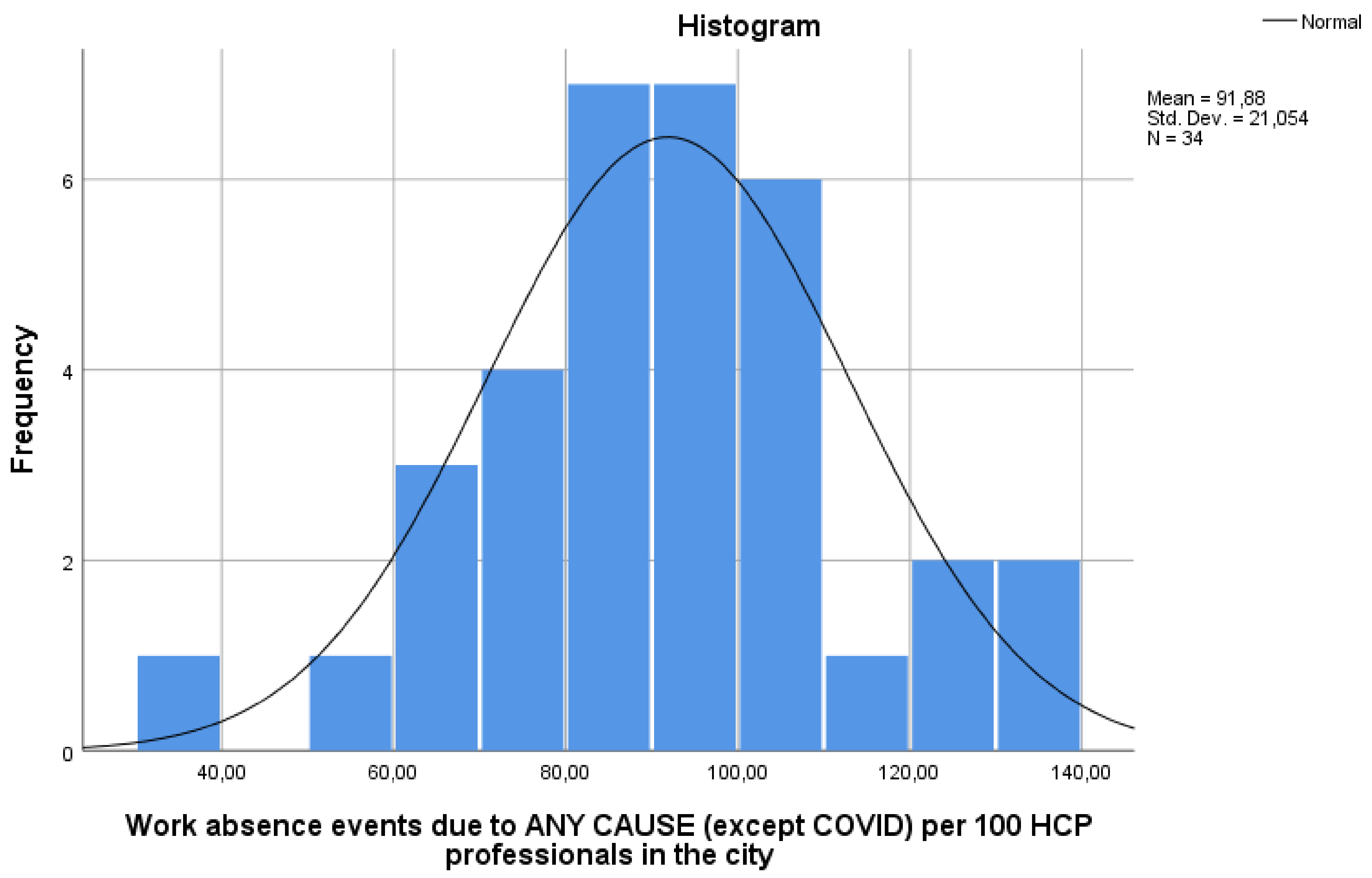
Histogram
Normal
Mean = 91,88
Std. Dev. = 21,054
N = 34
Frequency
0
2
4
6
40,00
60,00
80,00
100,00
120,00
140,00
Work absence events due to ANY CAUSE (except COVID) per 100 HCP professionals in the city

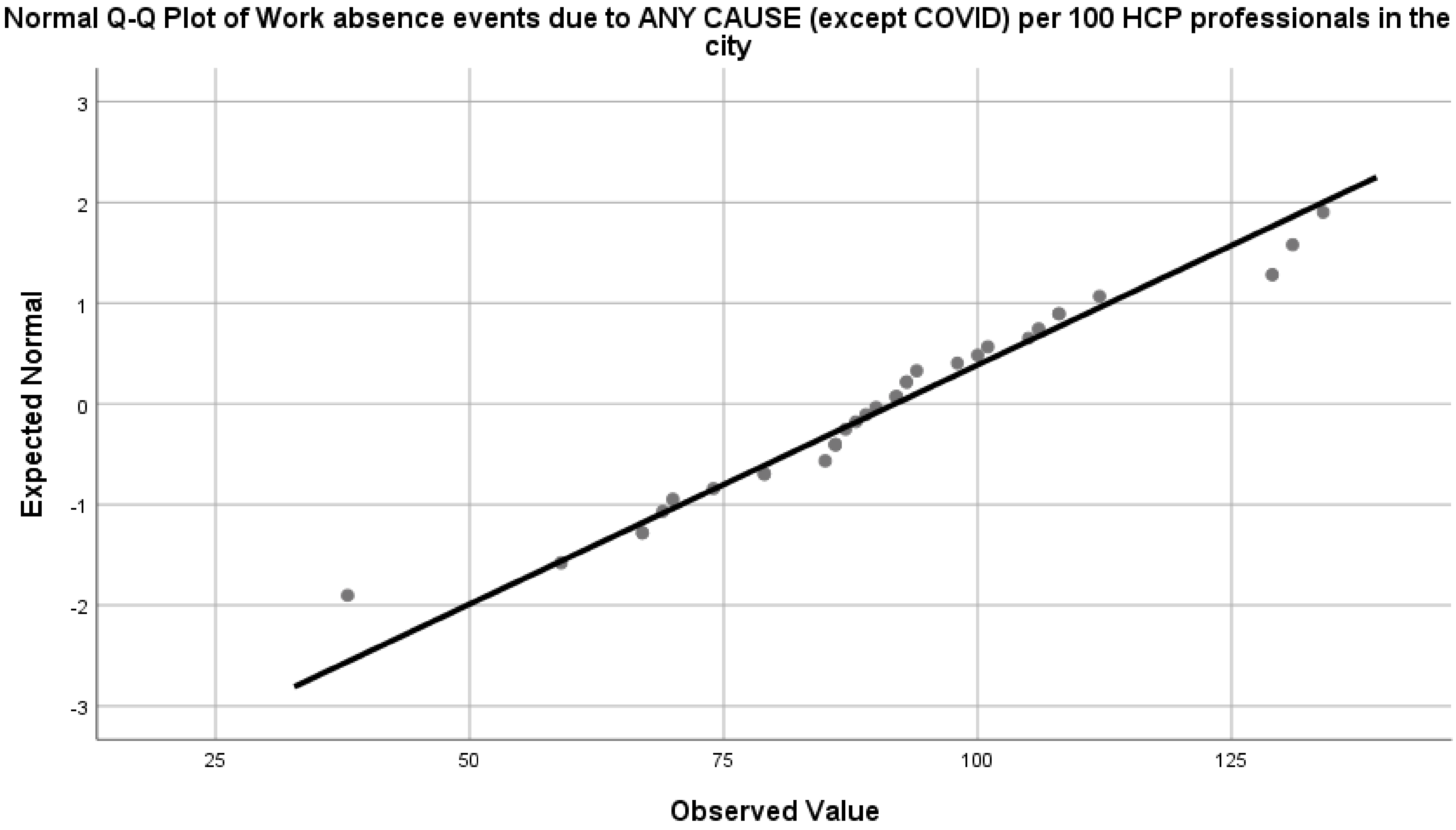
Normal Q-Q Plot of Work absence events due to ANY CAUSE (except COVID) per 100 HCP professionals in the city
Expected Normal
Observed Value
3
2
1
0
-1
-2
-3
25
50
75
100
125

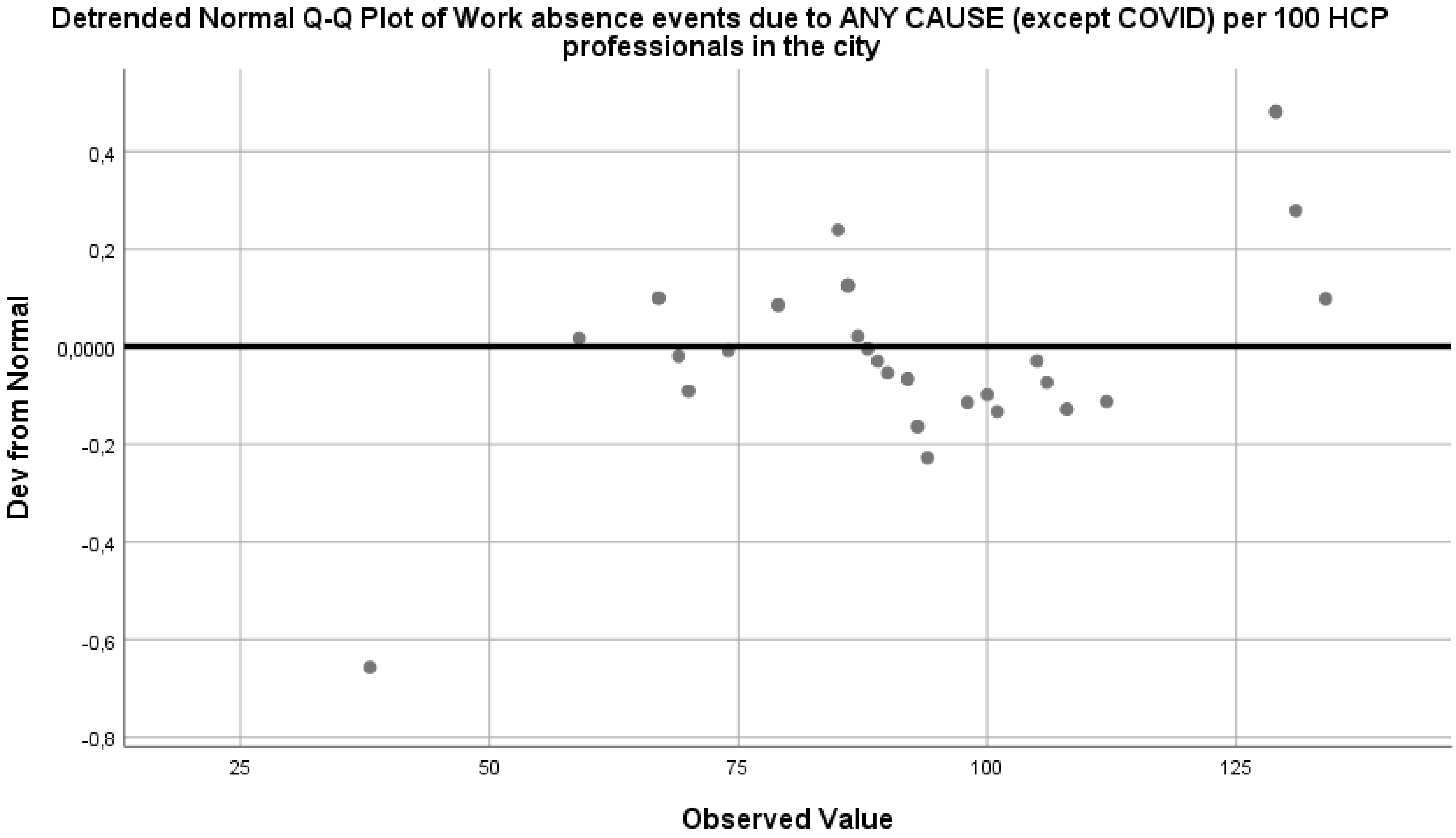


```
COMPUTE LogCovCases100k=LN(CityCovCases100k).
```

Supporting Information. Part5

```
VARIABLE LABELS  LogCovCases100k 'Log COVID cases / 100K inhab in the city'.
EXECUTE.
* Chart Builder.
GGRAPH
  /GRAPHDATASET NAME="graphdataset" VARIABLES=LogCovCases100k AbAC_HCP_N MISSING=LISTWISE
    REPORTMISSING=NO
  /GRAPHSPEC SOURCE=INLINE
  /FITLINE TOTAL=YES.
BEGIN GPL
  SOURCE: s=userSource(id("graphdataset"))
  DATA: LogCovCases100k=col(source(s), name("LogCovCases100k"))
  DATA: AbAC_HCP_N=col(source(s), name("AbAC_HCP_N"))
  GUIDE: axis(dim(1), label("Log COVID cases / 100K inhab in the city"))
  GUIDE: axis(dim(2), label("Work absence events due to ANY CAUSE (except COVID) per 100 HCP ",
    "professionals in the city"))
  GUIDE: text.title(label("Simple Scatter with Fit Line of Work absence events due to ANY CAUSE ",
    "(except COVID) per 100 HCP professionals in the city by Log COVID cases / 100K inhab in the city"))
  ELEMENT: point(position(LogCovCases100k*AbAC_HCP_N))
END GPL.
```

## GGraph

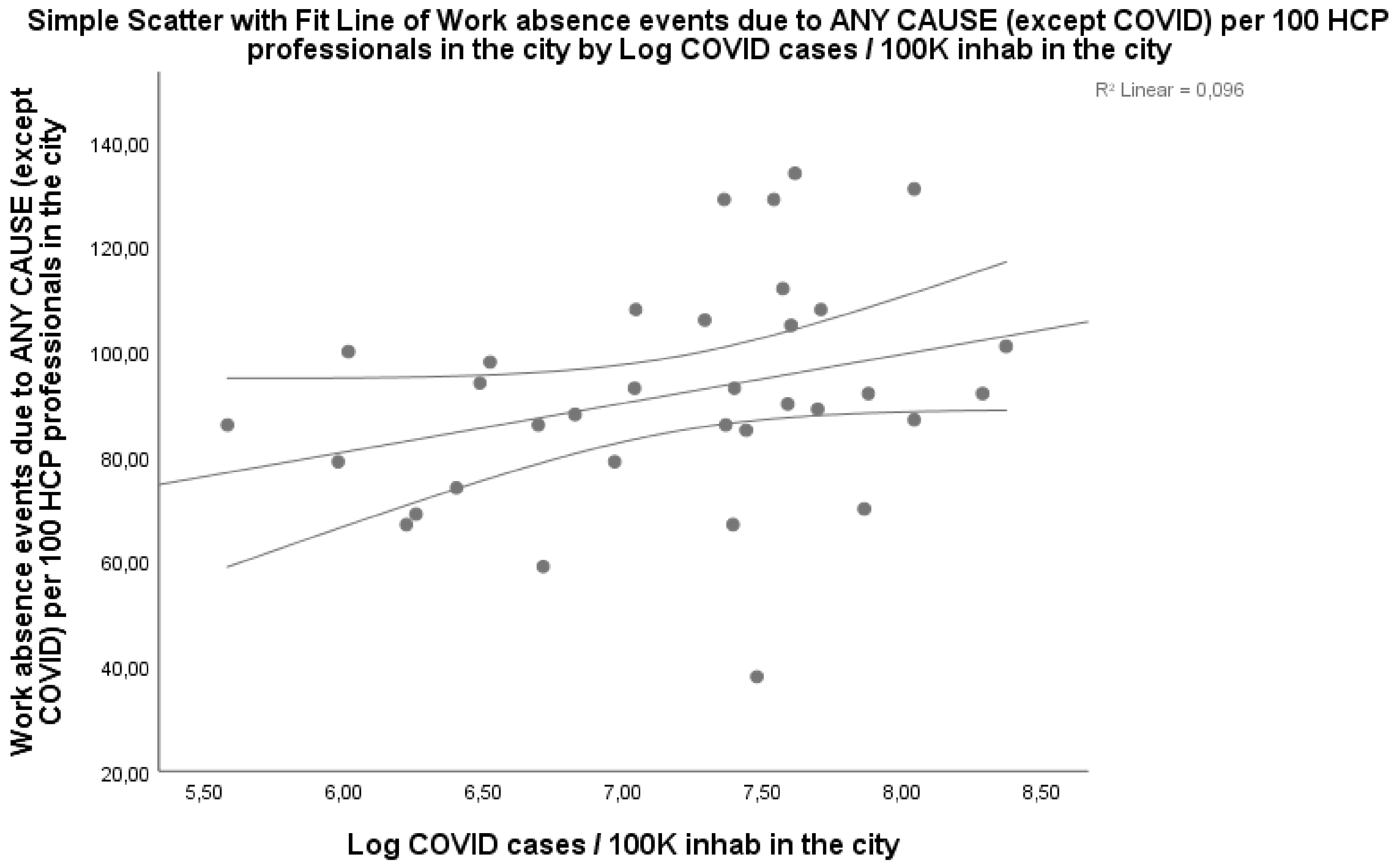
Simple Scatter with Fit Line of Work absence events due to ANY CAUSE (except COVID) per 100 HCP professionals in the city by Log COVID cases / 100K inhab in the city
R² Linear = 0,096
Work absence events due to ANY CAUSE (except COVID) per 100 HCP professionals in the city
140,00
120,00
100,00
80,00
60,00
40,00
20,00
5,50
6,00
6,50
7,00
7,50
8,00
8,50
Log COVID cases / 100K inhab in the city

NONPAR CORR

Supporting Information. Part5

```
/VARIABLES=CityCovCases100k AbAC_N AbAC_HCP_N AbHRel_N AbHRel_HCP_N AbMnt_N AbMnt_HCP_N
/PRINT=SPEARMAN TWOTAIL NOSIG
/MISSING=PAIRWISE.
```

# Nonparametric Correlations

**Correlations**

| | | | COVID cases / 100K inhab in the city | Work absence events due to ANY CAUSE (except COVID) per 100 professionals in the city | Work absence events due to ANY CAUSE (except COVID) per 100 HCP professionals in the city | Work absence events due to HEALTH-RELATED causes (except COVID) per 100 professionals in the city | Work absence events due to HEALTH-RELATED causes (except COVID) per 100 HCP professionals in the city | Work absence events due to MENTAL DISEASE per 100 professionals in the city | Work absence events due to MENTAL DISEASE per 100 HCP professionals in the city |
|---|---|---|---|---|---|---|---|---|---|
| Spearman's rho | COVID cases / 100K inhab in the city | Correlation Coefficient | 1,000 | ,349* | ,358* | ,301 | ,266 | ,219 | ,188 |
| | | Sig. (2-tailed) | . | ,043 | ,038 | ,084 | ,128 | ,214 | ,286 |
| | | N | 34 | 34 | 34 | 34 | 34 | 34 | 34 |
| | Work absence events due to ANY CAUSE (except COVID) per 100 professionals in the city | Correlation Coefficient | ,349* | 1,000 | ,954** | ,834** | ,830** | ,318 | ,291 |
| | | Sig. (2-tailed) | ,043 | . | ,000 | ,000 | ,000 | ,067 | ,095 |
| | | N | 34 | 34 | 34 | 34 | 34 | 34 | 34 |
| | Work absence events due to ANY CAUSE (except COVID) per 100 HCP professionals in the city | Correlation Coefficient | ,358* | ,954** | 1,000 | ,810** | ,834** | ,363* | ,359* |
| | | Sig. (2-tailed) | ,038 | ,000 | . | ,000 | ,000 | ,035 | ,037 |
| | | N | 34 | 34 | 34 | 34 | 34 | 34 | 34 |
| | Work absence events due to HEALTH-RELATED causes | Correlation Coefficient | ,301 | ,834** | ,810** | 1,000 | ,981** | ,414* | ,338 |
| | | Sig. (2-tailed) | ,084 | ,000 | ,000 | . | ,000 | ,015 | ,051 |

| | | | | | | | | |
|---|---|---|---|---|---|---|---|---|
| (except COVID) per 100 professionals in the city | N | 34 | 34 | 34 | 34 | 34 | 34 | 34 |
| Work absence events due to HEALTH-RELATED causes (except COVID) per 100 HCP professionals in the city | Correlation Coefficient | ,266 | ,830** | ,834** | ,981** | 1,000 | ,396* | ,337 |
| | Sig. (2-tailed) | ,128 | ,000 | ,000 | ,000 | . | ,020 | ,052 |
| | N | 34 | 34 | 34 | 34 | 34 | 34 | 34 |
| Work absence events due to MENTAL DISEASE per 100 professionals in the city | Correlation Coefficient | ,219 | ,318 | ,363* | ,414* | ,396* | 1,000 | ,974** |
| | Sig. (2-tailed) | ,214 | ,067 | ,035 | ,015 | ,020 | . | ,000 |
| | N | 34 | 34 | 34 | 34 | 34 | 34 | 34 |
| Work absence events due to MENTAL DISEASE per 100 HCP professionals in the city | Correlation Coefficient | ,188 | ,291 | ,359* | ,338 | ,337 | ,974** | 1,000 |
| | Sig. (2-tailed) | ,286 | ,095 | ,037 | ,051 | ,052 | ,000 | . |
| | N | 34 | 34 | 34 | 34 | 34 | 34 | 34 |

*. Correlation is significant at the 0.05 level (2-tailed).

**. Correlation is significant at the 0.01 level (2-tailed).

Graphs (Forrest Plots) summarizing the differences (Odds Ratios) between individuals, regarding several causes of work absences, found along the aforementioned analyses.

## Graph

Supporting Information. Part5

**Figure - Comparisons between exposed vs. non-exposed individuals (N=32961) regarding several causes of work absences (unadjusted)**

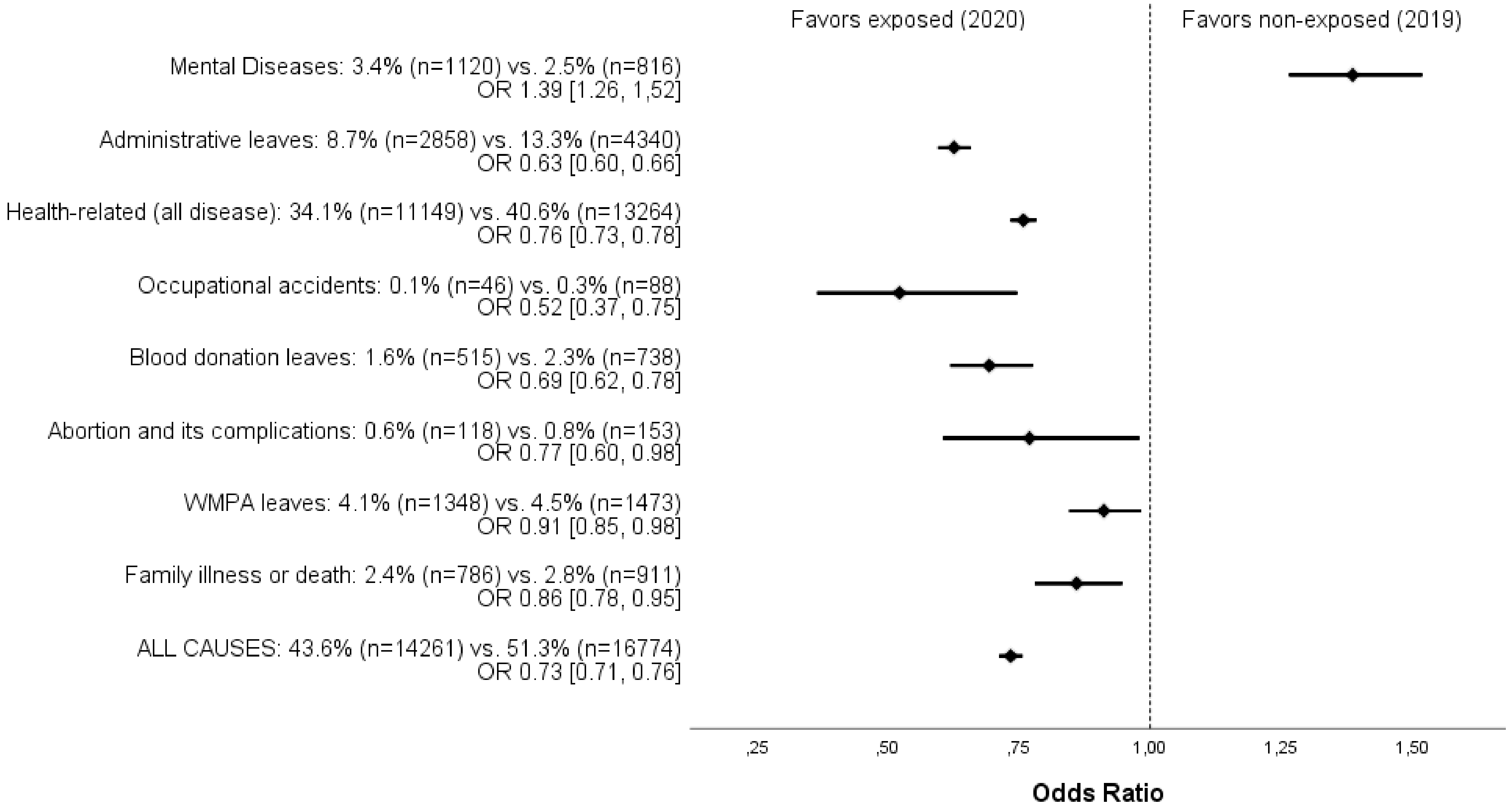

Supporting Information. Part5

## Graph

**Figure - Comparisons between exposed vs. non-exposed Healthcare Professionals (N=26956) regarding work absences due to All Causes, Health-Related Causes and Mental Diseases, adjusted for differences in HCP categories and gender**

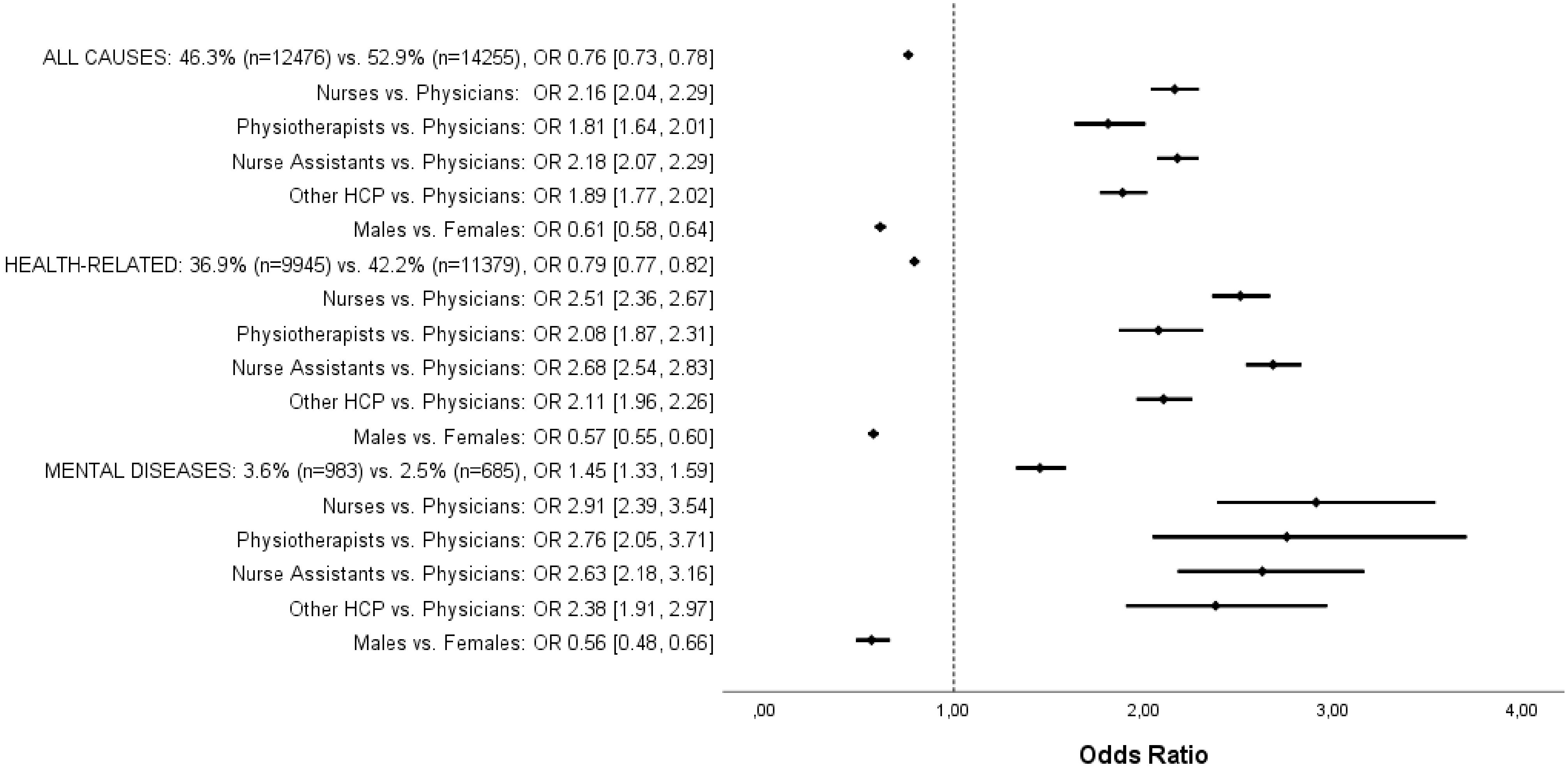

## Graph

Figure - Comparisons between exposed vs. non-exposed PHYSICIANS (N = 6871) regarding work absences due to All Causes, Health-Related Causes and Mental Diseases, adjusted for gender differences

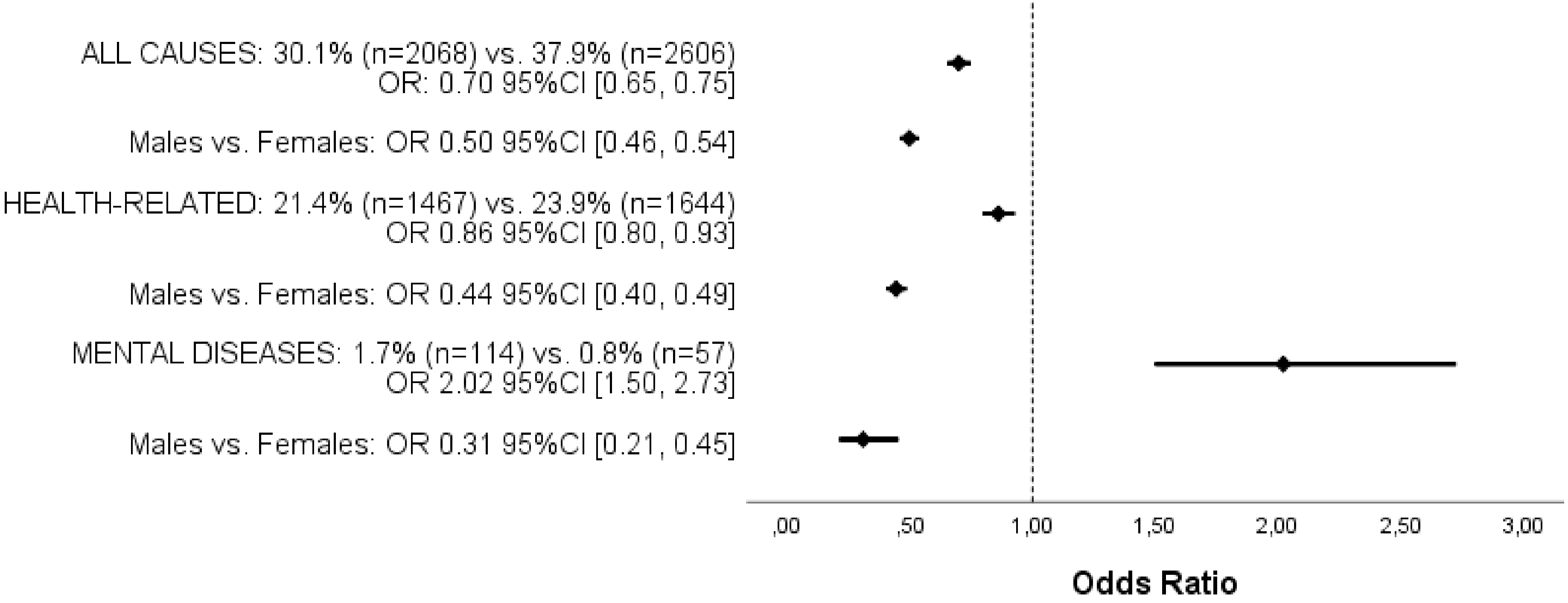

## Custom Tables

| | | | | Exposure to the pandemia environment | | | |
|---|---|---|---|---|---|---|---|
| | | | | Non-Exposed (2019) | | Exposed (2020) | |
| | | | | Count | Column N % | Count | Column N % |
| Healthcare provider category | Physicians | Any work absence due to ALL CAUSES occurred in the period | No | 4265 | 62,1% | 4803 | 69,9% |
| | | | Yes | 2606 | 37,9% | 2068 | 30,1% |
| | | | Total | 6871 | 100,0% | 6871 | 100,0% |
| | | Any work absence due to HEALTH-RELATED CAUSES occurred in the period | No | 5227 | 76,1% | 5404 | 78,6% |
| | | | Yes | 1644 | 23,9% | 1467 | 21,4% |
| | | | Total | 6871 | 100,0% | 6871 | 100,0% |
| | | Any work absence due to MENTAL DISEASE occurred in the period | No | 6814 | 99,2% | 6757 | 98,3% |
| | | | Yes | 57 | 0,8% | 114 | 1,7% |
| | | | Total | 6871 | 100,0% | 6871 | 100,0% |
| | Nurses | Any work absence due to ALL CAUSES occurred in the period | No | 2226 | 40,0% | 2668 | 47,9% |
| | | | Yes | 3344 | 60,0% | 2902 | 52,1% |
| | | | Total | 5570 | 100,0% | 5570 | 100,0% |
| | | Any work absence due to HEALTH-RELATED CAUSES occurred in the period | No | 2808 | 50,4% | 3214 | 57,7% |
| | | | Yes | 2762 | 49,6% | 2356 | 42,3% |
| | | | Total | 5570 | 100,0% | 5570 | 100,0% |
| | | Any work absence due to MENTAL DISEASE occurred in the period | No | 5382 | 96,6% | 5300 | 95,2% |
| | | | Yes | 188 | 3,4% | 270 | 4,8% |
| | | | Total | 5570 | 100,0% | 5570 | 100,0% |
| | Physiotherapists | Any work absence due to ALL CAUSES occurred in the period | No | 454 | 44,6% | 547 | 53,7% |
| | | | Yes | 565 | 55,4% | 472 | 46,3% |

| | | | | | | | |
|---|---|---|---|---|---|---|---|
| | | | Total | 1019 | 100,0% | 1019 | 100,0% |
| | | Any work absence due to HEALTH-RELATED CAUSES occurred in the period | No | 564 | 55,3% | 649 | 63,7% |
| | | | Yes | 455 | 44,7% | 370 | 36,3% |
| | | | Total | 1019 | 100,0% | 1019 | 100,0% |
| | | Any work absence due to MENTAL DISEASE occurred in the period | No | 988 | 97,0% | 973 | 95,5% |
| | | | Yes | 31 | 3,0% | 46 | 4,5% |
| | | | Total | 1019 | 100,0% | 1019 | 100,0% |
| | Nurse assistants | Any work absence due to ALL CAUSES occurred in the period | No | 4331 | 42,2% | 4711 | 45,9% |
| | | | Yes | 5935 | 57,8% | 5555 | 54,1% |
| | | | Total | 10266 | 100,0% | 10266 | 100,0% |
| | | Any work absence due to HEALTH-RELATED CAUSES occurred in the period | No | 5171 | 50,4% | 5652 | 55,1% |
| | | | Yes | 5095 | 49,6% | 4614 | 44,9% |
| | | | Total | 10266 | 100,0% | 10266 | 100,0% |
| | | Any work absence due to MENTAL DISEASE occurred in the period | No | 9936 | 96,8% | 9837 | 95,8% |
| | | | Yes | 330 | 3,2% | 429 | 4,2% |
| | | | Total | 10266 | 100,0% | 10266 | 100,0% |
| | Other HCP | Any work absence due to ALL CAUSES occurred in the period | No | 1425 | 44,1% | 1751 | 54,2% |
| | | | Yes | 1805 | 55,9% | 1479 | 45,8% |
| | | | Total | 3230 | 100,0% | 3230 | 100,0% |
| | | Any work absence due to HEALTH-RELATED CAUSES occurred in the period | No | 1807 | 55,9% | 2092 | 64,8% |
| | | | Yes | 1423 | 44,1% | 1138 | 35,2% |
| | | | Total | 3230 | 100,0% | 3230 | 100,0% |
| | | Any work absence due to MENTAL DISEASE occurred in the period | No | 3151 | 97,6% | 3106 | 96,2% |
| | | | Yes | 79 | 2,4% | 124 | 3,8% |
| | | | Total | 3230 | 100,0% | 3230 | 100,0% |
| | Total | Any work absence due to ALL CAUSES occurred in the period | No | 12701 | 47,1% | 14480 | 53,7% |
| | | | Yes | 14255 | 52,9% | 12476 | 46,3% |
| | | | Total | 26956 | 100,0% | 26956 | 100,0% |
| | | Any work absence due to HEALTH- | No | 15577 | 57,8% | 17011 | 63,1% |

Supporting Information. Part5

| | | | | | | |
|---|---|---|---|---|---|---|
| | RELATED CAUSES occurred in the period | Yes | 11379 | 42,2% | 9945 | 36,9% |
| | | Total | 26956 | 100,0% | 26956 | 100,0% |
| | Any work absence due to MENTAL DISEASE occurred in the period | No | 26271 | 97,5% | 25973 | 96,4% |
| | | Yes | 685 | 2,5% | 983 | 3,6% |
| | | Total | 26956 | 100,0% | 26956 | 100,0% |
