# Supplementary material for: Impact of COVID-19 on the mental health of public university hospital workers in Brazil: A cohort-based analysis of 32,691 workers
Source: PLoS One. 2022 Jun 16;17(6):e0269318. doi: 10.1371/journal.pone.0269318 (PMC9202958; doi:10.1371/journal.pone.0269318)
Supplement: Attachment — Submitted filename: PLOS one _brazil_MH_leaves.docx [file pone.0269318.s008.docx]

**Dear Rosemary Frey,	Date: 17^th^ November 2021**

**PLOS ONE**

Thanks for inviting me to review the paper titled “Impact of COVID-19 on the mental health of public university hospital workers in Brazil: a cohort-based analysis of 32,691 workers.”

Authors have conducted a meaningful study. There are some methodological issues and reporting of study findings.. After going through the paper in its entirety, hereby sharing my comments on paper.

**Abstract:** Comprehensive

**Main file**

**Introduction-**

The sentence-“adding to work-related stress that prior to the pandemic was at its highest level since 2014.[2]” needs simplification.

**Methodology-**

***Statistics:*** Though authors talk about using GEE and McNemar test, they have not be clearly shown in tables. I would suggest an opinion of biostatistician be taken for the same.

**Results:** Figure 2, please check the placement of favors pre-pandemic and pandemic. As per the figure, it seems that mental illness related leaves were higher in pre-pandemic period.

Figure 3 mentions log_rang in place of log-rank please correct it.

Tables: The tables has been represented as such it is produced by the SPSS. Authors need to present it properly.

Discussion: Discussion has been appropriate.

**Conclusion:** fine

**Reference:** many of the references missing doi. Please add them wherever indicated.

**Final comments:**

Overall, a well conducted study and written manuscript. However, minor changes can be made to improve the quality of the paper.

Regards,

Dr. Snehil Gupta

ORCID ID- 0000-0001-5498-2917

Publons ID: [ABH-4007-2020](https://publons.com/researcher/ABH-4007-2020/)
