# Supplementary material for: Impact of COVID-19 on the mental health of public university hospital workers in Brazil: A cohort-based analysis of 32,691 workers
Source: PLoS One. 2022 Jun 16;17(6):e0269318. doi: 10.1371/journal.pone.0269318 (PMC9202958; doi:10.1371/journal.pone.0269318)
Supplement: Attachment — Submitted filename: Response to reviewers.docx [file pone.0269318.s009.docx]

Dear Academic Editor and Reviewers

First, we would like to thank the opportunity to evaluate our work as well as the suggestions sent by the reviewers.

The following are the answers to the questions listed by the editors:

1. As for Figure 1, we chose to remove the figure.
2. As for the availability of the data, we inform you that the raw study data are available at Supporting Information.
3. As for the references, all were reviewed and all DOI were included as requested. There were changes in references 1, for greater ease of access to information and in reference 3, which was replaced. It was included reference 14, to support the argumentation.

The following are the answers to the questions listed by **Reviewer 1:**

***“Statistics: Though authors talk about using GEE and McNemar test, they have not be clearly shown in tables. I would suggest an opinion of biostatistician be taken for the same.”***

The data were analysed by a biostatistician. All bivariate (unadjusted) analyses comparing proportions between groups in the pre- and pandemic periods were performed using the McNemar test for repeated measurements, as indicated in the methodology. All multivariate (adjusted) analyses, because it is a design of repeated measures used GHG models, as indicated in the methodology. However, to make clearer the use of each method chosen in each case, we add the information in the notes of each figure.

***“Results: Figure 2, please check the placement of favors pre-pandemic and pandemic. As per the figure, it seems that mental illness related leaves were higher in pre-pandemic period.***

***Figure 3 mentions log_rang in place of log-rank please correct it.***

***Tables: The tables has been represented as such it is produced by the SPSS. Authors need to present it properly.”***

The suggestions regarding the correction of Figure 2, we chose to adjust the figure, which became figure 1. The subtitles of the figure were adjusted, previously written: "Favors during pandemic 2020 / Favors pre pandemic 2019", after the correction was written: "lower risk / increased risk". A footnote was included, with the statistical method used.

The orientation regarding figure 3 has been corrected. Previously was "log-rang", after correction the term used is "log-rank". A footnote has been added, with the statistical method used.

Table 1 was redone in order to meet the reviewer's suggestions, contemplating the instructions of the journal.

In figure 4, a footnote was included, with the statistical method used.

The S1Fig was included as Figure 2 in the article.

***“Reference: many of the references missing doi. Please add them wherever indicated.”***

All DOI were included in the references.

The following are the answers to the questions listed by **Reviewer 2**:

**“*Abstract is too lengthy. Need to shorten/condense a bit*.”**

The abstract was condensed to achieve the suggestion. It was already in the number of characters allowed (268 words), but additional reduction was made now with 249 words, as requested by the reviewer.

All spelling corrections were performed.

***“The discussion and conclusion sections contain some speculations re causes of the results which have little or no support in the study's results.”***

The text was reformulated, and reference 14 was included in order to support argumentation.

***“There needs to be a separate 'limitations of this study' section.”***

This section was included in the article.

We hope that the adjustments will meet the suggested recommendations and look forward to a conclusive response as soon as possible.

Respectfully

Prof. Licia Mota
