# Supplementary material for: Impact of COVID-19 on the mental health of public university hospital workers in Brazil: A cohort-based analysis of 32,691 workers
Source: PLoS One. 2022 Jun 16;17(6):e0269318. doi: 10.1371/journal.pone.0269318 (PMC9202958; doi:10.1371/journal.pone.0269318)
Supplement: Attachment — Submitted filename: Response to reviewers.docx [file pone.0269318.s010.docx]

Dear Editor and Reviewers,

About the comments listed by the reviewer 1:

Regarding the commentary about the McNemar test, we agree that the (categorical) nature of the variables would give consideration to a Chi-squared test. However, our research followed a repeated-measures design, meaning that every participant were assessed twice (before and after).

Because of that, a regular Pearson's Chi-squared test for independent samples would not apply. Instead, we used the McNemar test, which is one of the most used approaches to compare correlated proportions (in paired samples). [1] The McNemar test does follow a Chi-squared distribution with one degree of freedom (in 2x2 table), but focuses in the discordances between the repeated measures.

The support information is quoted in the manuscript in the Data sharing item.

Answering the comments listed by the reviewer 3:

Regarding grammatical correction and writing in English, a new revision was performed to make the text appropriate to the required standards. We have included the certificate of review and professional publishing attached.

Regarding the changes requested to comply to Plos One's submission guidelines, all references to funding were removed from the manuscript and the ethical statement was inserted at the beginning of the methods section.

We hope that the adjustments will meet the suggested recommendations and look forward to a conclusive response as soon as possible.

Respectfully

Prof. Licia Mota

[1] *McNemar, Quinn (18 de junho de 1947). «Note on the sampling error of the difference between correlated proportions or percentages». Psychometrika.* ***12*** *(2): 153–157.* [*PMID*](https://pt.wikipedia.org/wiki/PubMed_Identifier) [*20254758*](https://www.ncbi.nlm.nih.gov/pubmed/20254758)*.* [*doi*](https://pt.wikipedia.org/wiki/Digital_object_identifier)*:*[*10.1007/BF02295996*](https://dx.doi.org/10.1007%2FBF02295996)
